# Polymetallic nodules, sediments, and deep waters in the equatorial North Pacific exhibit highly diverse and distinct bacterial, archaeal, and microeukaryotic communities

**DOI:** 10.1002/mbo3.428

**Published:** 2016-11-21

**Authors:** Christine N. Shulse, Brianne Maillot, Craig R. Smith, Matthew J. Church

**Affiliations:** ^1^ Center for Microbial Oceanography: Research and Education (C‐MORE)University of Hawaii at ManoaHonoluluHIUSA; ^2^ Department of OceanographySchool of Ocean and Earth Science and TechnologyUniversity of Hawaii at ManoaHonoluluHIUSA; ^3^ Present address: Department of Energy Joint Genome InstituteWalnut CreekCAUSA.; ^4^ Present address: Flathead Lake Biological StationUniversity of MontanaPolsonCAUSA.

**Keywords:** 16S rRNA, 18S rRNA, diversity, microbial ecology, polymetallic nodules, sediment

## Abstract

Concentrated seabed deposits of polymetallic nodules, which are rich in economically valuable metals (e.g., copper, nickel, cobalt, manganese), occur over vast areas of the abyssal Pacific Ocean floor. Little is currently known about the diversity of microorganisms inhabiting abyssal habitats. In this study, sediment, nodule, and water column samples were collected from the Clarion‐Clipperton Zone of the Eastern North Pacific. The diversities of prokaryote and microeukaryote communities associated with these habitats were examined. Microbial community composition and diversity varied with habitat type, water column depth, and sediment horizon. *Thaumarchaeota* were relatively enriched in the sediments and nodules compared to the water column, whereas Gammaproteobacteria were the most abundant sequences associated with nodules. Among the Eukaryota, rRNA genes belonging to the *Cryptomonadales* were relatively most abundant among organisms associated with nodules, whereas rRNA gene sequences deriving from members of the *Alveolata* were relatively enriched in sediments and the water column. Nine operational taxonomic unit (OTU)s were identified that occur in all nodules in this dataset, as well as all nodules found in a study 3000–9000 km from our site. Microbial communities in the sediments had the highest diversity, followed by nodules, and then by the water column with <1/3 the number of OTUs as in the sediments.

## 1 Introduction

Polymetallic (i.e., manganese) nodules occur over vast areas of the abyssal ocean floor (Ghosh & Mukhopadhyay, 2000) and are enriched in commercially valuable minerals such as manganese (Mn), cobalt (Co), copper (Cu), nickel (Ni), and rare‐earth elements (Wegorzewski & Kuhn, 2014). Although nodules are estimated to have very slow growth rates of <1 nm year^−1^ (Kerr, 1984), rising global demand for these metals has renewed interests in commercial mining of deep‐sea nodule deposits. The Clarion‐Clipperton Zone (CCZ) in the Pacific Ocean is of particular interest for mining due to nodule concentrations up to 10–15 kg m^−2^ (Smith, Levin, Koslow, Tyler, & Glover, 2008). As of August 2015, the International Seabed Authority had granted 16 exploration licenses for nodule mining within the CCZ (Wedding et al., 2015). As currently conceived, mining operations would (1) remove nodules and seafloor sediments, and create near‐bottom sediment plumes that could spread over thousands of square kilometers, (2) pump nodules and associated sediments from the seabed to the ocean's surface, and (3) release mining tailings composed of nodule‐free sediments back into the water column, thereby potentially impacting much larger areas than due to the deep water plume (Rolinski, Segschneider, & Sundermann, 2001), and significantly disturbing large areas of the abyssal seafloor and overlying water column (Smith et al., 2008). To fully understand potential impacts on microbial processes that may be disturbed by mining activities, it is important to characterize the microbial communities of the abyssal seafloor and the overlying water column since both will likely be impacted by mining.

Although there is limited information on microbial communities inhabiting abyssal sediments in regions where polymetallic nodules are abundant, studies of abyssal sediments lacking nodules indicate that the resident prokaryotic communities are mainly composed of *Proteobacteria*,* Acidobacteria*,* Actinomycetes*,* Chloroflexi*,* Planctomycetes,* and *Crenarchaeota* (Durbin & Teske, 2010; Schauer, Bienhold, Ramette, & Harder, 2010), reviewed in Corinaldesi (2015). In the CCZ, the low particulate organic‐carbon flux from the overlying waters compared to continental margins results in oxygenated sediments to depths of 2–3 m (Mewes et al., 2014; Smith, De Leo, Bernardino, Sweetman, & Arbizu, 2008). The abyssal plain can be a relatively stable environment over a period of many years, and benthic production can be low as it depends on input of organic particles from the euphotic zone. The physical and chemical structure of polymetallic nodules potentially provides a unique niche for bacterial, archaeal, and microeukaryotic communities to colonize. Little is known about nodule formation, although it has been hypothesized to be an abiotic process (Kerr, 1984). However, a recent study posited a microbially mediated mechanism for nodule initiation because X‐ray and microscopy studies indicated high concentrations of bacteria in Mn‐rich micronodules (Wang, Schlossmacher, Wiens, Schroeder, & Mueller, 2009). Indeed, an early study of polymetallic nodules using light and scanning electron microscopy revealed biofilms and filamentous microorganisms associated with nodule surfaces (Burnett & Nealson, 1981). Three recent studies (Blothe et al., 2015; Tully & Heidelberg, 2013; Wu et al., 2013), relying on gene‐based surveys, identified unique bacterial and archaeal operational taxonomic unit (OTUs) specifically associated with nodules compared to surrounding sediments in the eastern North Pacific Subtropical Gyre (NPSG), the central South Pacific Gyre and the central and western NPSG. A fourth study investigated only bacterial diversity in the sediments of the CCZ but not from nodules (Wang et al., 2010). The nodule‐associated protistan community has thus far not been investigated beyond morphological identification of selected foraminifera (e.g., (Mullineaux, 1987; Veillette, Juniper, Gooday, & Sarrazin, 2007; Veillette, Sarrazin et al. 2007)).

**Figure 1:**
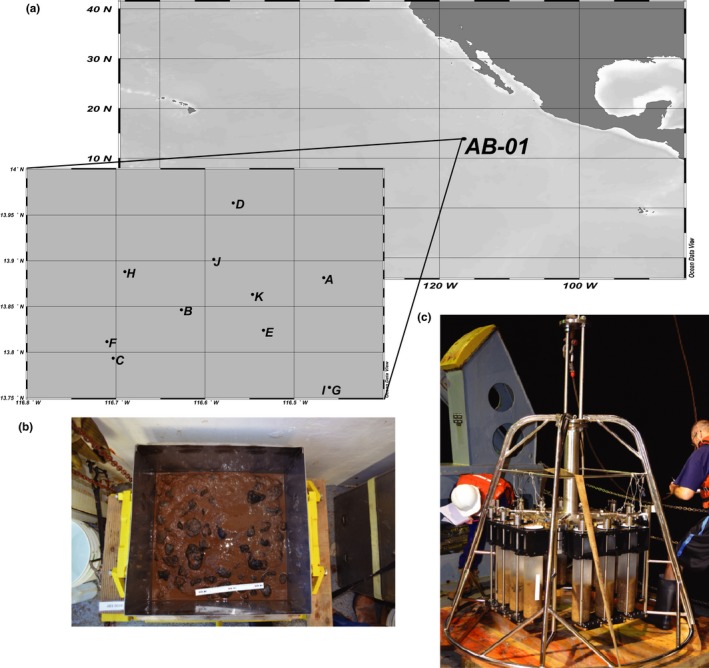
Study location and design. (a) Location of AB‐01 stratum within the Clarion‐Clipperton Zone of the Pacific Ocean, where sampling occurred. Locations of sampling stations are indicated by dots and labeled with letters as in Table 1. (b) Example of a boxcore from which nodules (black globular structure) were sampled. In this example, the topwater has been drained from the box corer; box is 50 cm on a side. (Photo credit: Craig Smith.) (c) Example of a megacoring device after recovery with core tubes containing abyssal sediments during AB‐01

Here we present findings of the bacterial, archaeal, and microeukaryotic communities associated with a polymetallic nodule field based on amplification and sequencing of rRNA genes. These analyses included 75 sediment samples, 24 water column samples, and 20 individual nodules from 11 stations randomly distributed over a 30 × 30 km stratum (AB‐01; Table 1, Figure 1a) within the United Kingdom's UK‐1 exploration claim area. Our results confirm that polymetallic nodules harbor diverse types of microorganisms that are distinct from both the surrounding sediments and overlying water, and that microbial diversity is substantially higher in the seafloor ecosystem than in the water column above. Our results reveal for the first time a diverse nodule eukaryotic community harboring OTUs assigned to the *Sar*,* Opisthokonta*, and *Cryptophyceae* lineages, as well as a core prokaryotic nodule community with OTUs found in nodules collected 3000 to >9000 km from our study site. These results suggest polymetallic nodules may harbor a persistent and stable microbiome.

**Table 1:** Sampling site locations in the UK‐1 claim area, dates, and depths for this study

Station	Location	Date (MM/DD/YY)	Station bottom depth (m)
A	(CT^a^) 13°52.90 N, 116°28.00 W	10/08/13	4113
B	(CT) 13°50.79 N, 116°37.59 W	10/11/13	4025
	(BC^b^) 13°50.99 N, 116°38.70 W	10/09/13	4108
	(MC^b^) 13°50.79 N, 116°27.59 W	10/10/13	4079
C	(BC) 13°47.60 N, 116°37.19 W	10/11/13	4081
	(MC) 13°47.62 N, 116°42.19 W	10/11/13	4078
D	(CT) 13°57.80 N, 116°34.10 W	10/13/13	4025
	(MC) 13°57.80 N, 116°34.09 W	10/13/13	4084
E	(BC) 13°49.45 N, 116°32.06 W	10/14/13	4054
	(MC) 13°49.45 N, 116°32.06 W	10/15/13	4054
F	(BC/MC)^c^ 13°48.70 N, 116°42.60 W	10/16/13	4076
G	(MC) 13°45.71 N, 116°27.60 W	10/18/13	4111
H	(BC/MC)^c^ 13°53.30 N, 116°41.40 W	10/19/13	4150
I	(BC) 13°45.00 N, 116°30.80 W	10/18/13	4036
	(MC) 13°45.70 N, 116°27.62 W	10/18/13	4111
J	(BC) 13°54.11 N, 116°35.40 W	10/21/13	4163
	(MC) 13°54.10 N, 116°35.40 W	10/21/13	4166
K	(BC) 13°51.78 N, 116°32.93 W	10/20/13	4050
	(MC) 13°51.80 N, 116°32.80 W	10/21/13	4053

a CT, niskin rosette on the conductivity‐temperature‐depth device.

b BC, Boxcore; MC, Megacore.

c Ship remained on station; boxcore and megacore deployments occurred sequentially.

## 2 Experimental Procedures

### 2.1 Sample collection

Abyssal sediments, polymetallic nodules, and seawater samples were collected as part of the MV1313 research cruise (October 2013) aboard the R/V *Melville* to a 30 × 30 km stratum centered at 13**°**49′N, 116°36′W within the UK‐1 claim area in the Clarion‐Clipperton Zone. Samples were collected from stations randomly located within this 30 × 30 km area, designated AB‐01 or Stratum A (Table 1, Figure 1); 11 stations were sampled for sediments and nodules and three stations for seawater. Seawater samples were collected from eight discrete depths within the water column (5, 150, 300, 500, 1000, 2000, 3000 m, and near‐bottom waters) using a conductivity‐temperature‐depth (CTD; SBE 911plus; Sea‐Bird Electronics) rosette sampler equipped with 24 10 L sampling bottles. The rosette sensor package also included a fluorometer (Seapoint Chlorophyll Fluorometer; Seapoint Sensors, Inc.) and dissolved oxygen (O_2_) sensor (SBE 43; Sea‐Bird Electronics). Seawater (2 L each from 5, 150, 300, and 500 m and 8 L from 1000, 2000, 3000 m, and near‐bottom waters) was subsampled from the rosette bottles into polycarbonate carboys (4.5 L carboys for 2 L samples and 10 L carboys for 8 L samples) and immediately filtered using a peristaltic pump onto in‐line 25 mm diameter, 0.2 μm pore‐sized, Supor filters. Filtration times varied from 40 min to 2.5 hr depending on the volume. Filters were flash‐frozen in liquid nitrogen and stored at −80°C until shore‐based laboratory processing. Water samples (1.5 ml) for subsequent flow cytometric analyses of picoplankton abundance were fixed with 0.22 μm‐filtered formaldehyde (2% final concentration), incubated at 4°C for 15 min, flash‐frozen in liquid nitrogen, and stored at −80°C until shore‐based analyses.

Nodules and sediments were aseptically sampled from 0.25 m^2^ box cores (nodules) or 80 cm^2^ megacore tubes (nodules and sediments) (see (Glover, Dahlgren, Wiklund, Mohrbeck, & Smith, 2016) for box‐coring and megacoring equipment and sampling protocols). Nodules were mostly found within the 0–5 cm fraction of the box core and megacore, although some were recovered from our maximum sampling depth of 10 cm. All nodules found in megacore tubes designated for microbiology (usually 2 per deployment) were collected, as were a random subset of nodules from boxcores. From this collection of megacore and boxcore nodules, a random subset were selected for subsequent DNA extraction, and amplification and sequencing of 16S and 18S rRNA genes. In total, extracts from 20 nodules were used for 16S rRNA amplification and sequencing, with 18 of these nodule‐derived DNA extracts also used for 18S rRNA gene amplification and sequencing. Subcores of sediments were obtained using sterile 20 mL syringes with the tip ends cutoff, in each of four sediment horizons: 0–5 cm below seafloor (cmbf), 5–6 cmbf, 6–8 cmbf, and 8–10 cmbf. Sediment subcores were stored in sterile Whirl‐Pak bags (Nasco, Fort Atkinson, Wisconsin) at −80°C. Nodules were rinsed with 0.2 μm‐filtered ambient bottom water to remove sediment adhering to the surface and stored whole in sterile Whirl‐Pak bags at −80°C.

### 2.2 DNA extraction

In the shore‐based laboratory, genomic DNA was extracted from seawater samples using a DNeasy Plant Mini Kit (Qiagen) following a modified protocol (Paerl, Foster, Jenkins, Montoya, & Zehr, 2008). Briefly, the filters were subjected to chemical and physical (bead‐beating step using both 0.1 and 0.5 mm beads) means for cell disruption. Total lysates were purified using the DNeasy Mini spin column procedure (Qiagen) following the manufacturer's recommendations.

Under sterile laboratory conditions, the polymetallic nodules were rinsed with 0.2 μm‐filtered, autoclaved, bottom (~4000 m) seawater, returned to Whirl‐Pak bags, and broken while still in the bag using an autoclaved mortar and pestle. Two ~500 mg pieces from the interior of each nodule were subjected to DNA extraction. Extraction of DNA from nodules and sediments was performed using the FastDNA Spin Kit for Soil (MP Biomedicals, USA) following the manufacturer's protocol, modified as follows: homogenization was performed in a Mini‐Beadbeater‐16 (Biospec Products, Bartlesville, Oklahoma) and centrifugation following homogenization was extended to 15 min. An extraction blank (FastDNA Spin Kit for Soil spin column with no sample added) was processed alongside samples. DNA concentrations were determined from 4 μl of each sample using the Qubit 2.0 Fluorometer and the Qubit dsDNA High Sensitivity Assay kit (Life Technologies). Extracts with DNA concentrations >0.1 ng/μl were purified and concentrated using the Zymo Clean & Concentrator‐5 (2:1 DNA Binding Buffer) kit with the resulting DNA eluted in sterile, DNase‐free water.

### 2.3 PCR amplification and Illumina sequencing of 16S and 18S rRNA genes

The V4 region of the 16S rRNA gene was amplified by the polymerase chain reaction (PCR) using the oligonucleotide primer pair 515f/806r, which include the Illumina flowcell adapter sequences and a sample‐specific barcode exactly as described in Caporaso et al., (2011, 2012). Of the 20 nodules sampled for 16S rRNA genes, most were sampled in duplicate (i.e.*,* two separate ~500 mg pieces from the same nodules were subjected to DNA extraction, PCR amplification, and sequencing). Initial 16S rRNA gene results from the duplicate samples generated from each nodule sample revealed a high degrees of similarity between profiles (Figure S3). To conserve the extracted genomic material, in most cases the duplicate extracts were pooled and only a single 18S rRNA gene amplification was performed from each nodule. The V9 region of the 18S rRNA gene was PCR amplified using the oligonucleotide primer pair 1391f/EukBr (Amaral‐Zettler, McCliment, Ducklow, & Huse, 2009) exactly as described in the Earth Microbiome Project 18S rRNA Amplification Protocol (Gilbert, Jansson, & Knight, 2014); this region (V9) of the 18S rRNA gene has previously been used in a large‐scale biodiversity assessment of marine eukaryotic communities (de Vargas et al., 2015). Sequencing of the 16S and 18S rRNA gene amplification products, including extraction blanks, was performed in separate runs on an Illumina MiSeq at the Hawaii Institute of Marine Biology Genetics Core Facility (Kaneohe, HI).

### 2.4 Bioinformatic analyses of sequences

Illumina paired‐end 16S rRNA gene reads were joined using the bioinformatic software fastq‐join (Aronesty, 2013) and sequences were processed, including an initial quality filtering and sequence sample‐mapping by barcode, using QIIME version 1.8.0 (Caporaso, Kuczynski, et al. 2010). Potentially chimeric sequences were identified using the UCHIME algorithm within the USEARCH package (Edgar, 2010) and removed from further analysis. Open reference‐based OTU picking was performed using the UCLUST algorithm (Edgar, 2010), one of the principal clustering algorithms in the QIIME package, at a 97% sequence similarity cutoff against the Greengenes rRNA gene database release 13_8 (DeSantis et al., 2006). OTUs that occurred as absolute singletons or were observed in the extraction and/or PCR blanks were filtered from the experimental samples. Taxonomy was assigned based on the Greengenes taxonomy (McDonald et al., 2012; Werner et al., 2012) using a UCLUST‐based consensus taxonomy assigner (Bokulich et al., 2015). A total of 13,835,715 high‐quality sequences were generated, with an average of 101,133 sequences/sample (minimum sequences/sample = 16,753; maximum sequences/sample = 236,702). These data were normalized to 16,000 reads/sample to account for uneven sampling depth using the script single_rarefaction.py, which randomly subsamples the input OTU table without replacement, and this normalized OTU table was used in subsequent analyses unless otherwise specified. The script summarize_otu_by_cat.py in the QIIME package was used to collapse this OTU table by sample type and/or depth when necessary. The only exceptions were the differential abundance analysis in which the full dataset was used, and alpha diversity analyses in which samples were collapsed by sample type (water column, nodules, or sediments) and the dataset was subsampled randomly multiple times at different depths, with a maximum depth of 2,401,000 sequences in order to take maximum advantage of this large dataset.

Illumina 5′ 18S rRNA gene reads were processed similarly to 16S rRNA reads, except reference‐based OTU picking was performed against the SILVA 119 rRNA gene database (Quast et al., 2013). Taxonomy was assigned based on the SILVA 119 taxonomy (Yilmaz et al., 2014) using BLAST (Altschul, Gish, Miller, Myers, & Lipman, 1990). The resulting OTUs were filtered to exclude 38,523 bacterial OTUs, 23,733 archaeal OTUs, and 3,126 OTUs that could not be identified at the domain level. A total of 54,819 Eukaryota OTUs comprised of 5,353,354 high‐quality sequences remained, with an average of 45,367 sequences/sample (minimum sequences/sample = 5450; maximum sequences/sample = 154,747). These data were normalized to 5,400 reads/sample to account for uneven sampling depth, and either this normalized OTU table, or a table normalized to relative abundance, was used in subsequent analyses, except the differential abundance analysis in which the full dataset was used and alpha diversity analyses in which samples were collapsed by sample type (water column, nodules, or sediments) and the dataset was subsampled randomly multiple times at different depths, with a maximum depth of 100,100 sequences. Joined, quality filtered 16S fastq files and 5′, quality filtered 18S fastq files have been deposited in the NCBI's Sequence Read Archive under BioProject ID PRJNA281530, SRA ID SRP057408.

The nodule prokaryotic core microbiome was computed using the script compute_core_microbiome.py within the QIIME package. The Wu et al. dataset was downloaded from NCBI and OTUs were picked and taxonomy assigned as described for our dataset. OTUs within our core microbiome that hit to Greengenes were compared to the newly created Wu et al. OTU table in order to identify reference‐based OTUs that were present in both datasets.

### 2.5 Statistical methodologies

Principal Coordinates Analysis (PCoA) was used to visualize patterns in microbial community structure based on sample type within the CCZ. Analysis of similarities (ANOSIM; (Chapman & Underwood, 1999) was performed on weighted UniFrac distance measurements of both 16S and 18S gene sequences, and implemented using the compare_categories.py script within the QIIME package. Briefly, UniFrac calculates a distance measure based on the fraction of branch length shared between two communities within a phylogenetic tree; weighted UniFrac additionally takes into account the differences in relative abundances of taxa within each community (Lozupone, Lladser, Knights, Stombaugh, & Knight, 2011). The prokaryotic phylogenetic tree used for UniFrac was built using FastTree (Price, Dehal, & Arkin, 2010) from representative sequences aligned with PyNAST (Caporaso, Desantis, et al., 2010), as implemented in the pick_open_reference_otus.py workflow, and is available as Figure S8; sequences which failed to align were omitted from both the tree and the OTU table. The eukaryotic phylogenetic tree was created similarly from representative sequences aligned with Infernal (Nawrocki, Kolbe, & Eddy, 2009) and is available as Figure S9. A heatmap (Figure 5) was created using the function heatmap.2 in the R package gplots (R Core Team, 2015; Warnes et al., 2016). A Bray–Curtis dissimilarity matrix was created from an OTU table containing the 10 most abundant OTUs in each habitat, average linkage hierarchical clustering was performed and a dendrogram was created using the R package *vegan* (Oksanen et al., 2016 *)*. Colors came from the R package RColorBrewer (Neuwirth, 2014). Average linkage hierarchical clustering was also done on the full dataset and the results were similar, that is, sediments, nodules, and the water column each formed groups (Figure S10). To create Figure 9, a differential analysis of count data using shrinkage estimation (DESeq2, (Love, Huber, & Anders, 2014)) was implemented on the full dataset (not rarefied) within the phyloseq package (McMurdie & Holmes, 2014). Differential OTUs which had a base mean of ≥100 (prokaryotes) or ≥10 (eukaryotes) were reported and visualized using the R package ggplot2 (Wickham, 2009).

### 2.6 Flow cytometric cell abundances

Seawater samples for flow cytometric analyses were thawed and 250 μl aliquots were transferred to 96‐well plates and stained with SYBR Green I (final concentration of 1X). Abundances of picoplanktonic cells were determined using an Attune Acoustic Focusing Cytometer (Life Technologies, Carlsbad, CA) at a flow rate of 100 μl min^−1^, using an excitation of 488 nm and detected using a 530/30 bandpass filter and side scatter.

### 2.7 Seawater nutrient and DOC concentrations

Seawater samples for subsequent analysis of phosphate (PO_4_^3‐^), nitrate plus nitrite (NO_3_^−^ + NO_2_^−^), silicate, and dissolved organic carbon (DOC) were collected into clean, acid washed 125 ml polyethylene bottles and frozen upright at −20°C. In the shore‐based laboratory, nutrient concentrations were measured colorimetrically using a Bran+Luebbe Autoanalyzer III (Karl et al., 2001). DOC analyses relied on high‐temperature combustion using a Shimadzu TOC‐V (DOM Analytical Lab, Santa Barbara, CA; (Carlson et al., 2010)).

## 3 Results and Discussion

### 3.1 Chemical characterization of UK‐1 claim area

Seawater nutrient concentrations and microbial cell abundances were sampled from vertical profiles of the water column overlying the abyssal seabed. Nutrient concentrations were typical for this region, with low (<0.2 μmol L^−1^) concentrations of nitrate + nitrite (N+N) in the near‐surface waters, increasing rapidly with depth, reaching concentrations (~45 μmol L^−1^) typical for the deep waters of the Eastern Tropical North Pacific (Garcia et al., 2014) (Table 2). One of the most prominent features was the presence of a large, relatively shallow, oxygen minimum zone (OMZ), where dissolved oxygen concentrations declined to <10 μmol L^−1^ (< 0.2 ml/L) between ~50 m to ~1000 m (Figure S1).

**Table 2:** Mean concentrations of seawater nutrients, DOC, and picoplankton cell abundances from those depths where seawater samples were collected for rRNA gene analyses

Depth (m)	PO_4_^3−^(μmol L^−1^)	N+N (μmol L^−1^)	SiO_4_ (μmol L^−1^)	DOC (μmol L^−1^)	Picoplankton abundances (cells ml^−1^)
5	0.18 ± 0.03	0.17 ± 0.01	1.89 ± 0.42	76.37 ± 0.54	5.68 × 10^5 ^± 8.13 × 10^4^
150	2.59 ± 0.02	26.29 ± 0.51	32.54 ± 0.50	46.35 ± 0.17	3.05 × 10^5^ ± 2.37 × 10^4^
300	2.82 ± 0.04	29.14 ± 0.69	41.51 ± 1.47	43.29 ± 0.71	1.55 × 10^5 ^± 6.89 × 10^3^
500	3.14 ± 0.00	37.03 ± 0.13	63.72 ± 0.31	39.98 ± 1.46	7.92 × 10^4^ ± 4.34 × 10^3^
1000	3.35 ± 0.01	46.63 ± 0.13	105.64 ± 0.03	39.06 ± 1.30	4.98 × 10^4^ ± 4.41 × 10^3^
2000	2.85 ± 0.02	41.46 ± 0.14	152.16 ± 0.50	37.49 ± 0.39	3.67 × 10^4^ ± 1.78 × 10^3^
3000	2.67 ± 0.01	39.34 ± 0.10	160.81 ± 0.12	37.08 ± 1.21	4.40 × 10^4^ ± 7.27 × 10^3^
Near‐bottom waters (4013 –4103)	2.59 ± 0.08	38.07 ± 1.35	156.98 ± 2.93	38.94 ± 0.64	3.86 × 10^4^ ± 7.77 × 10^2^

DOC, dissolved organic carbon; PO_4_^3–^, phosphate; N+N, nitrate+nitrite; SiO_4_, silicic acid.

Shown are mean ± SD concentrations of the four water column stations sampled in the UK‐1 claim region.

### 3.2 General characteristics of the UK‐1 rRNA gene phylogenetic profiles

In total, after rarefaction, 112,926 distinct prokaryotic OTUs (defined as 16S rRNA gene sequences sharing ≥97% sequence identity) were recovered from the three types of samples (nodules, sediment, and seawater), with 74% of these amplicons deriving from bacteria, 24% from archaea, and an additional 2% that could not be confidently assigned at the Domain level. Within this dataset, a large proportion of OTUs were taxonomically assigned to the *Proteobacteria* (45% of sequences), including *Gammaproteobacteria* (21%), *Alphaproteobacteria* (14%), *Deltaproteobacteria* (9%), and *Betaproteobacteria* (1%). OTUs taxonomically assigned to the *Thaumarchaeota* also comprised a relatively large proportion of this dataset (22% of the sequences). Among the eukaryote 18S rRNA genes, 26,011 distinct OTUs (defined as sequences sharing ≥97% sequence identity) were recovered from the three sample types. The greatest proportion (51%) of these eukaryotic sequences fell into OTUs assigned to the *Sar* (formal taxon name derived from *Stramenopiles*,* Alveolata*, and *Rhizaria*; (Adl et al., 2012)) supergroup, whereas lower proportions grouped among the *Opisthokonta* (14%), *Cryptophyceae* (14%), *Excavata* (8%), *Archaeplastida* (8%), and *Amoebozoa* (2%). *Sar* was the dominant supergroup in all three habitats (seawater, sediment, and nodules). However, the *Cryptomonadales*, an order within the *Cryptophyceae*, represented the greatest relative abundance (25%) of classified sequences on nodules, more than any individual order within the *Sar*. In contrast, in the water column and sediments, the lineage *Alveolata* within the *Sar* supergroup dominated relative sequence abundances (43% and 30%, respectively).

### 3.3 Seawater communities

**Figure 2:**
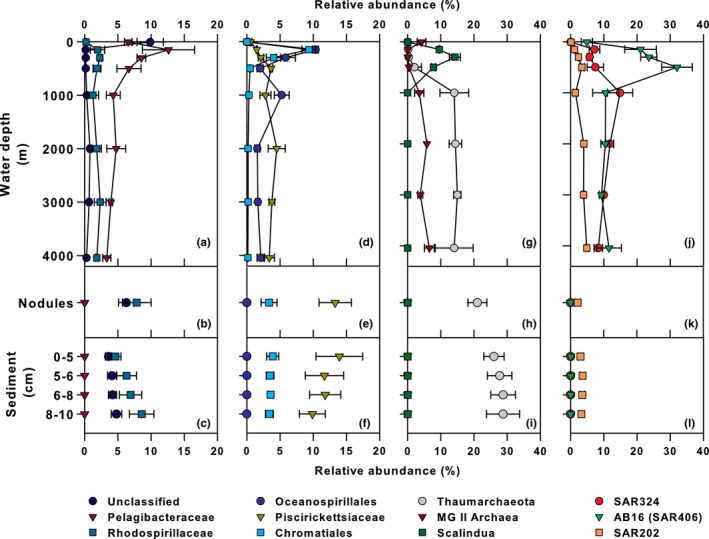
Vertical profiles depicting relative abundances of selected prokaryote rRNA gene lineages from the water column (a, d, g, & j), sediments (b, e, h, & k), and nodules (c, f, i, & l). Depicted are mean and standard deviation (error bars) of samples collected during AB‐01 from various locations in UK‐1 study region

The distributions and relative abundances of prokaryote 16S rRNA genes in the water column of this region appeared typical of open‐ocean habitats, with notable exceptions observed in the low oxygen waters of the mesopelagic zone (Figure S2). OTUs identified as belonging to the candidate phylum *Marine Group A* (MGA) (formerly known as SAR406; (Fuhrman & Davis, 1997)), specifically the AB16 gene lineage, were the dominant lineage within the OMZ, comprising relative abundances of 24% and 32% at 300 m and 500 m, respectively (Figure 2j, and S2a). There are currently no cultivated representatives of the MGA, but these organisms appear abundant and diverse in OMZ waters (Allers et al., 2013; Fuchs, Woebken, Zubkov, Burkill, & Amann, 2005; Stevens & Ulloa, 2008), and recent metagenomic analyses from seawater samples collected in the North Pacific suggest these organisms are involved in oxidation of various reduced sulfur substrates (Wright et al., 2014). OTUs clustering among the *Planctomycetes* were also enriched in the oxygen minimum zone (averaging 12% relative abundance across all stations investigated, Figure S2a), and these OTUs demonstrated low relative abundances (averaging 2%) at other depths. This enrichment in *Planctomycetes* OTUs was driven by sequences deriving from members of the ‘Candidatus *Scalindua*’ (Figure 2g), a genus capable of catalyzing anaerobic ammonium oxidation (anammox; (Kuypers et al., 2003)). The anammox reaction is known to be a major loss for fixed nitrogen in various regions of the ocean (Kuypers et al., 2005; Ulloa, Canfield, DeLong, Letelier, & Stewart, 2012).

**Figure 3:**
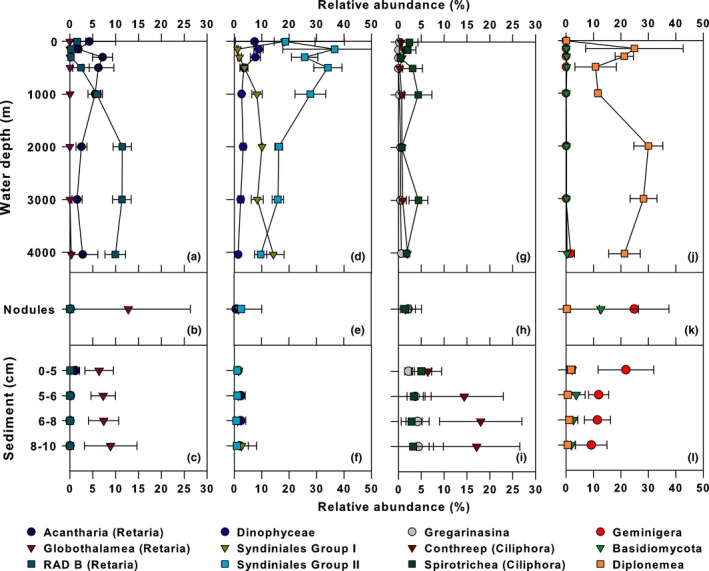
Vertical profiles depicting relative abundances of selected eukaryote rRNA gene lineages from the water column (a, d, g, & j), sediments (b, e, h, & k), and nodules (c, f, i, & l). Depicted are mean and standard deviation (error bars) of samples collected during AB‐01 from various locations in UK‐1 study region

The water column microeukaryote community was dominated by *Protalveolata* (*Sar*:* Alveolata*; 35%), *Discicristata* (*Excavata*:* Discoba*; 20%), and *Retaria* (*Sar*:* Rhizaria*; 15%); the relative abundances of these lineages varying with depth (Figure S2b). Nearly all the *Protalveolata* sequences clustered among the *Syndiniales*, a presumably exclusively endoparasitic group common in culture‐independent surveys of marine environments (Guillou et al., 2008; de Vargas et al., 2015). Group II *Syndiniales* dominated the anoxic and suboxic OMZ, whereas Groups I and II coexisted in the photic and oxygenated bathypelagic zones (Figure 3d). The *Discicristata* were solely *Euglenozoa*, mostly *Diplonemea*, a group of heterotrophic flagellates with major uncultured clades distributed throughout the deep‐sea (Lara, Moreira, Vereshchaka, & Lopez‐Garcia, 2009). This was reflected in our data, as the *Diplonemea* (*Discicristata*) were nearly absent from the euphotic zone samples and increased in relative abundance with depth (Figure 3j and S2b). The *Retaria* were represented by *Radiolaria*, mainly the *Acantharia* (4%), *Polycystinea* (6%), and members of radiolarian sequence group RAD B (5%). It has been hypothesized that one or more of these Radiolarian groups may serve as a host for the parasitic *Syndiniales* (Not, Gausling, Azam, Heidelberg, & Worden, 2007). Within our dataset, OTUs assigned to the *Acantharia* (Figure 3a) and *Polycystinea* (data not shown) had similar depth‐distribution patterns to OTUs assigned to *Syndinales* Group II (Figure 3d), a finding consistent with the Not et al. (2007) hypothesis.

### 3.4 Seabed‐associated communities

**Figure 4:**
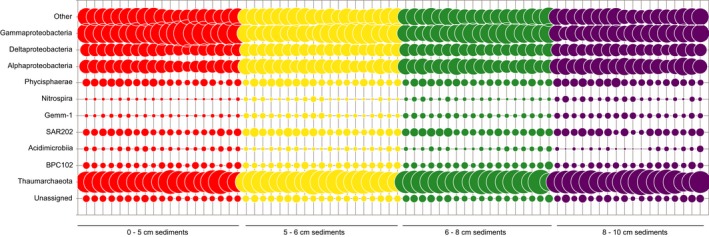
Major class‐level lineages of prokaryotic sediment taxa present at ≥2% relative rRNA gene abundances in at least two samples. Vertical lines represent the individual samples collected from each sediment horizon. Category “Other” represents all named taxa that did not reach the ≥2% relative abundance cutoff

**Figure 5:**
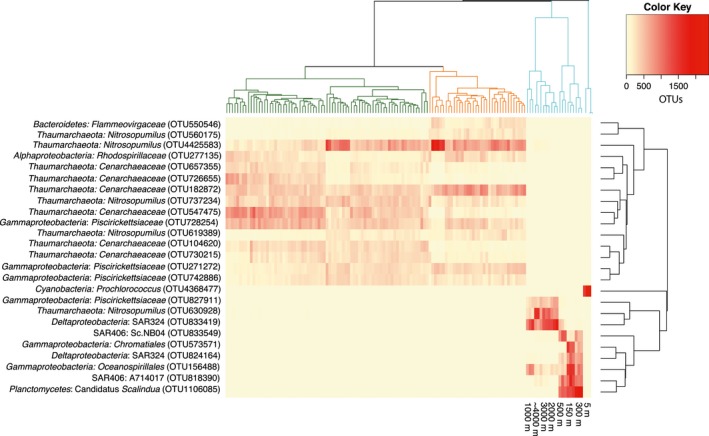
Heatmap of top 10 most abundant prokaryotic rRNA gene OTUs from each sample type. In some cases, the most abundant rRNA gene OTUs from sediments and nodules were the same, so total number of OTUs depicted is 25. Dendrograms were created via average linkage hierarchical clustering on a Bray–Curtis dissimilarity matrix of the selected dataset. Dendrogram on the *Y*‐axis is color‐coded by sample type as throughout the manuscript (blue = water, green = sediments, orange =  nodules). Dendrogram on the *X*‐axis clusters OTUs that occur most frequently together. Heatmap color represents the number of OTUs found in each sample after normalization to 16,000 reads/sample. OTU, operational taxonomic unit

The relative abundance of nodule‐associated 16S rRNA gene sequences was dominated by members of the *Gammaproteobacteria* (23%), *Thaumarchaeota* (21%), and *Alphaproteobacteria* (18%; Figure S3), consistent with previous studies examining nodule‐associated archaeal and bacterial communities (Tully & Heidelberg, 2013; Wu et al., 2013). The same three major groups of prokaryotes were also dominant in the sediment samples (Figure 4): *Thaumarchaeota* (28%), *Gammaproteobacteria* (20%), *Alphaproteobacteria* (12%). Within the *Thaumarchaeota*, the genus *Nitrosopumilus* was prevalent in both the sediments and nodules (12% and 10%, respectively). The most abundant OTUs classified as *Nitrosopumilus* in the sediments and nodules were distinct from the abundant *Nitrosopumilus* OTU found in the bottom waters, and indeed, throughout the bathypelagic zone in AB‐01 (Figure 5), implying niche specialization between the sediment/nodules and water column. The genus *Nitrosopumilus* contains the ammonia‐oxidizing chemolithoautotrophic archaeal nitrifiers (Konneke et al., 2005); the relatively large proportion of these archaea in sediment and nodule samples could reflect increased importance of reduced substrates such as ammonia in the benthic environment as compared with the water column (Orcutt, Sylvan, Knab, & Edwards, 2011). In addition to the *Thaumarchaeota*, the class *Nitrospira*, containing potential chemolithoautotrophs, comprised a minor but measurable (1%) portion of the sediment community (Figure. 4).

Among the *Gammaproteobacteria*, sequences clustering among unclassified genera within the family *Piscirickettsiaceae* demonstrated the greatest relative abundance in both the sediments and nodules (12% and 13% relative abundances, respectively), with relative abundances decreasing with depth in the sediment. The *Piscirickettsiaceae* are a family of aerobic, aquatic bacteria, and a recent study indicated these organisms were enriched in seawater microcosms treated with cadmium (Wang et al., 2015). Generalized resistance to metal toxicity may explain their relatively high abundances within the nodule field. Additionally, sequences classified as belonging to the order *Chromatiales* comprised 2–5% of the total 16S rRNA genes throughout the sediments. Previous studies in the central and western Pacific have recovered rRNA genes belonging to the *Chromatiales* from sediments and nodules (Wu et al., 2013), as well as sediments associated with cobalt‐rich sediment crusts (Liao et al., 2011).

The dominant *Alphaproteobacteria* in both nodules and sediments were an unclassified genus within the family *Rhodospirillaceae*, occurring at 8% and 7% relative abundance, respectively, which was not present in the water column (Figure 5). Although *Rhodospirillaceae* are often found in anaerobic environments, this family contains the genus *Magnetospirillum,* a microaerophilic heterotroph with relatives known from sediments previously collected in the Pacific Nodule Province (Xu, Wang, Meng, & Xiao, 2007).

All the major Eukaryotic supergroups were represented in the nodule and sediment datasets (Figure S4, 6 and 3b–l). On nodules, the groups *Geminigera* (25%; a genus of cryptophytes), *Fungi* (14%), and *Retaria* (13%; a clade within *Rhizaria*) demonstrated the greatest relative abundances (Fig. S4). *Fungi* were almost exclusively comprised of *Basidiomycota* (13% of total sequences), a broad phylum that contains both yeasts and filamentous fungi. Sequences clustering among the *Ciliophora* (20%), *Geminigera* (13%), and *Retaria* (13%) were abundant in the sediments, although the relative abundances of these organisms varied with depth (Figure 3c, i and l). *Ciliophora* are relatively well‐studied ciliated, heterotrophic eukaryotes (Pierce & Turner, 1992; Verni & Gualtieri, 1997); however, the ecology and biogeochemical role of deep‐sea groups of these organisms remains unknown. Within our dataset, the *Conthreep* and *Spirotrichea* (*Ciliophora*) lineages were most abundant, with *Conthreep* OTUs increasing below the 0–5 cm sediment horizon, whereas the *Spirotrichea* remained at low relative abundance throughout the sediment (Figure 3i). The 18S rRNA profiles in the both the sediments and nodules also contained OTUs from the *Archaeplastida* and the *Cryptophyceae*, two groups of organisms most often associated with plastid‐containing photosynthetic organisms. In total, the sediment and nodule communities contained 1002 and 629 *Archaeplastida* OTUs and 1327 and 759 *Cryptophyceae* OTUs, respectively. Such results are consistent with previous 18S rRNA gene surveys in deep waters of the Gulf of California, and the Arctic and Southern Oceans (Edgcomb, Kysela, Teske, Gomez, & Sogin, 2002; Pawlowski et al., 2011), a finding attributed to sinking of surface water organisms and subsequent preservation in the sediment (Pawlowski et al., 2011).

**Figure 6:**
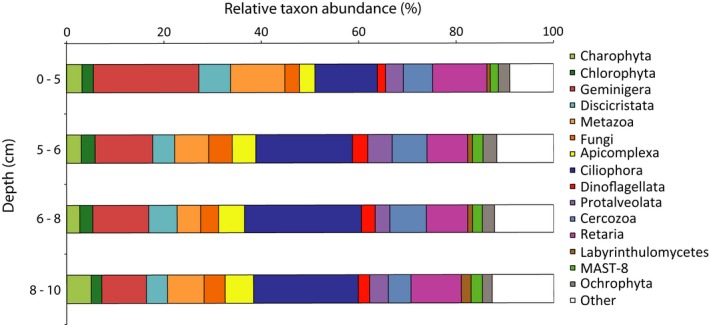
Eukaryotic sediment taxa from the 0–5 cm, 5–6 cm, 6–8 cm, and 8–10 cm horizons, averaged across all samples taken at a given depth; rRNA gene taxa present ≥2% relative abundance (on average) in at least one horizon are depicted. Category “Other” represents all named taxa that did not reach the ≥2% relative abundance cutoff

### 3.5 Alpha diversity of Clarion‐Clipperton Zone microbial communities

**Figure 7:**
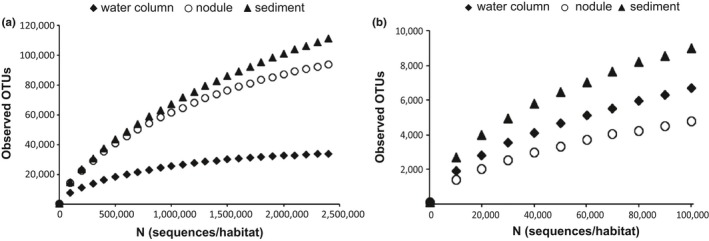
Rarefaction analyses of observed operational taxonomic units among nodule, sediment, and water column (a) prokaryotic and (b) eukaryotic communities

After pooling by habitat and rarefaction to 2,401,000 sequences per habitat in order to account for differences in sequencing depth while still utilizing a large portion of the dataset, 33,732 prokaryotic OTUs were found in the water column, 93,790 prokaryotic OTUs were found in the nodules, and 111,413 prokaryotic OTUs were found in the sediments (Table S1). Species accumulation curves indicate that OTUs were still accumulating in the sediments and nodules at this depth of sequencing, whereas sequence diversity in the water column appeared to plateau (Figure 7). Chao1 predicts a species richness of 35,083 prokaryotic OTUs in the water column, 118,552 prokaryotic OTUs in the nodules, and 184,335 prokaryotic OTUs in the sediments (Table S1). For eukaryotes, at a sampling depth of 100,100 sequences (pooled by habitat), the accumulation curves do not appear to approach an asymptote for nodules, the water column, or the sediments, indicating undersampling of all three habitats (Figure 7). There were 6,704 eukaryotic observed OTUs in the water column, 4,744 eukaryotic OTUs in the nodules, and 9004 eukaryotic OTUs in the sediments, with Chao1 richness estimates of 13,373, 8831, and 15,344 OTUs, respectively (Table S1).

Based on the three different measures of diversity assessed in this study (Chao1, exponential of Shannon's, and observed OTUs), nodule and sediment prokaryotic communities harbored greater alpha diversity than water column communities when pooled by habitat (Table S1). This held true when the alpha diversity of individual samples within these habitats were considered as well (rarefied to 16,000 sequences/sample, nonparametric *t*‐test, observed OTUs, *p* = .003 for both nodule versus water column and sediment vs. water column; Chao1, *p* = .003 for both nodule versus water column and sediment vs. water column; exponential of Shannon's, *p *= .003 for both nodules vs. water column and sediment vs. water column). Additionally, on average, prokaryotic sediment communities demonstrated greater alpha diversity than nodule communities (nonparametric *t*‐test, observed OTUs, *p* = .006).

### 3.6 Beta and gamma diversity of Clarion Clipperton Zone microbial communities

**Figure 8:**
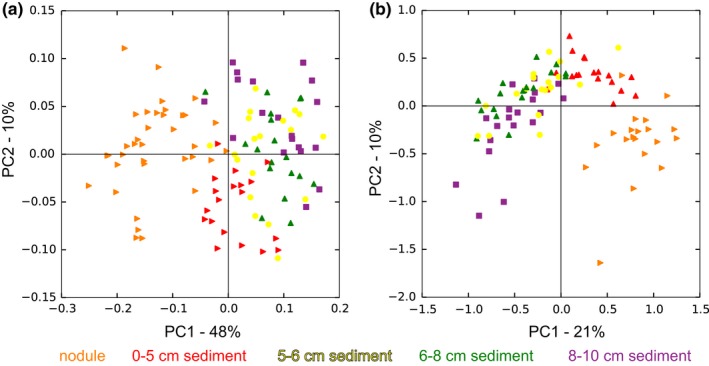
Principal Component Analysis (PCoA) plots based on weighted UniFrac distance measurements comparing: (a) prokaryotic communities; (b) eukaryotic communities associated with different layers of sediment and nodules in the study area

Principal Coordinates Analysis (PCoA) of weighted UniFrac (Lozupone & Knight, 2005) distances of the 16S and 18S rRNA gene amplicon communities, revealed that the seawater, sediments, and nodules each harbored distinct prokaryotic (Figure S5a) and eukaryotic (Figure S5b) assemblages and this was supported by ANOSIM (*R* statistic = .8203, *p* = .001 and *R* statistic = .7996, *p* = .001, respectively). PCoA analysis of the nodules and different sediments layers was conducted, and both nodules and 0–5 cm sediments harbored prokaryotic and eukaryotic communities distinct from each other and from the deeper sediments (Figure 8). Microbial prokaryote and eukaryote assemblages within the sediments were further shown to vary with depth via ANOSIM (*R* statistic = .3105, *p *= 0.001 and *R* statistic = .3357, *p *= 0.001, respectively). However, there were no significant differences in microbial community structure among the different stations sampled within the 30 × 30 km stratum of the study site (ANOSIM; prokaryotes: *R* statistic = .1126, *p* = .002; eukaryotes: *R* statistic = .0432, *p* = .079), suggesting that these assemblages are widespread within the ~900 km^2^ sampling area. Repeating the PCoA analyses with abundance weighted Jaccard distance matrices gave similar results (Figure S6 & S7).

### 3.7 Trends in microbes differentially represented in sediments and nodules

**Figure 9:**
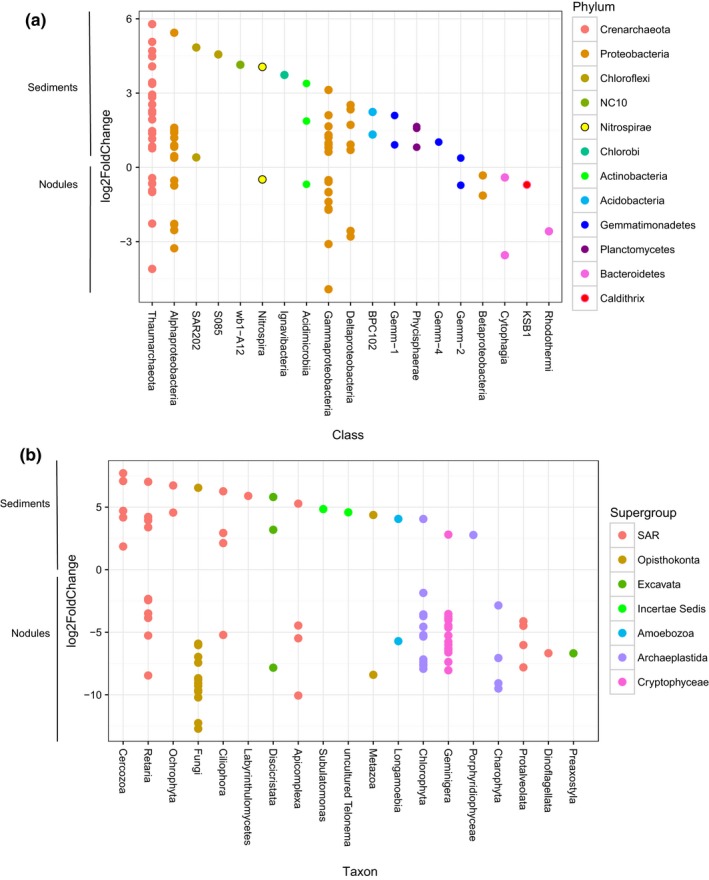
Plot depicting differentially abundant operational taxonomic units as the log2(fold change) of sediment versus nodule samples for (a) prokaryotic and (b) eukaryotic communities. OTUs that are more abundant in sediments are above the zero line, whereas those that are more abundant in nodules are below the zero line. OTUs are colored by phylum (prokaryotes) or supergroup (eukaryotes). OTU, operational taxonomic unit

A differential analysis of count data (Love et al., 2014) was performed to determine whether specific OTUs discriminated between nodules and sediment samples. Ninety‐three prokaryotic OTUs with assigned taxonomy were identified as differentiating the 75 sediment samples from the 36 nodule samples (Figure 9a). Ninety eukaryotic OTUs differentiated the 74 sediment samples from the 20 pooled nodule samples (Figure 9b). Although the prokaryotic OTUs that differentiated the sediment and nodule samples came from diverse phyla, some general trends emerged. All the differential *Chloroflexi* OTUs recovered (4 OTUs; 4% of differential OTUs) were more abundant in sediment samples than nodule samples (Figure 9a). This agrees with data from the German mining claim area, to the west of our study site, where *Chloroflexi* were found in sediments but were not associated with nodules (Blothe et al., 2015). Fifteen (16%) of the differentially abundant OTUs were *Alphaproteobacteria* (Figure 9a). Of those *Alphaproteobacteria* more highly represented on the nodules, two fell into the family Hyphomicrobiaceae within the *Rhizobiales,* a group that contains members known to be involved in manganese cycling (Larsen, Sly, & McEwan, 1999). The Alphaproteobacterial OTUs identified as more abundant in the sediments than nodules were either *Rhodospirillales* or unclassified beyond the class level; none of them were classified as *Rhizobiales*, potentially indicating a unique niche for *Rhizobiales* on the nodules. Five of the seven deltaproteobacterial OTUs were overrepresented in the sediments relative to the nodules. *Deltaproteobacteria* are ubiquitous members of the deep‐sea surface sediment community (Kouridaki, Polymenakou, Tselepides, Mandalakis, & Smith, 2010; Schauer et al., 2010), including within polymetallic nodule fields (Wang et al., 2010; Xu, Wang, Wang, & Xiao, 2005).

Eukaryotic communities in both the various sediment samples and on collected nodules demonstrated considerable sample‐to‐sample heterogeneity (Figure S4), but differential abundance analysis identified several taxa that appear to prefer either sediments or nodules (Figure 9b). For example, three of the four *Ciliophora* OTUs identified as differentially abundant were more abundant in the sediments, and differential OTUs from the clade *Cercozoa*, a diverse group of heterotrophic protists, were found solely in the sediments (Figure 9b). Fifteen (23%) of the OTUs that were more abundant on the nodules derived from the *Opisthokonta*, the supergroup containing both the *Fungi* and the *Metazoa* (Figure 9b). This included one metazoan OTU unique to this study classified as *Enoplea* (a nematode), and 14 fungal OTUs, three of which were related to the yeast‐like fungus *Pseudozyma*.

### 3.8 Comparisons to other polymetallic nodule datasets

In addition to describing the microbial communities associated with the sediments, nodules, and overlying waters, we compared our results to previously published studies from geographically diverse sites within the Pacific Ocean to place our observations in a basin‐scale context of known nodule‐associated microbial communities. The large number of nodules collected in this study allowed us to compute a “nodule core microbiome” for the AB‐01 region in order to compare it to nodules across the CCZ. In total, 196 prokaryotic OTUs were present in 100% of our nodules sampled; 168 of these fell into OTUs with representative sequences within the Greengenes database whereas 28 were novel (Table S2). In order to look at connectivity across the CCZ, we compared the 168 reference‐based OTUs to OTUs identified in the three nodules sampled from two sites at distances ~3000 km and >9000 km from AB‐01, within the North Pacific (Wu et al., 2013). Forty‐seven (28%) of these OTUs were also retrieved in at least one of the nodules studied by Wu et al.; nine of these OTUs were found in all three nodules studied by Wu et al. (Table 3). Many of these core OTUs fell into taxa that our differential abundance analyses revealed to prefer nodules rather than sediments, such as the *Cytophagia* and the *Rhizobiales* (Table 3, Figure 9a). These results indicate that certain stable and consistent associations between microbes and nodules exist over thousands of kilometers of abyssal ocean.

**Table 3:** Core nodule OTUs found in all nodules in this study and all nodules in Wu et al., at 3000 – 9000 km distance from the AB‐01 site

Greengenes OTU ID	Taxonomy
1054	*Archaea; Crenarchaeota; Thaumarchaeota; Cenarchaeales; Cenarchaeaceae*
182872	*Archaea; Crenarchaeota; Thaumarchaeota; Cenarchaeales; Cenarchaeaceae*
196754	*Archaea; Crenarchaeota; Thaumarchaeota; Cenarchaeales; Cenarchaeaceae*
540244	*Archaea; Crenarchaeota; Thaumarchaeota; Cenarchaeales; Cenarchaeaceae*
737234	*Archaea; Crenarchaeota; Thaumarchaeota; Cenarchaeales; Cenarchaeaceae; Nitrosopumilus*
550546	*Bacteria; Bacteroidetes; Cytophagia; Cytophagales; Flammeovirgaceae*
704361	*Bacteria; Planctomycetes; Phycisphaerae; C86*
567782	*Bacteria; Proteobacteria; Alphaproteobacteria*
260794	*Bacteria; Proteobacteria; Alphaproteobacteria; Rhizobiales; Hyphomicrobiaceae*

A recent study of the microbes associated with two polymetallic nodules in the east German license area, ~300 km from our study site, found that members of the gammaproteobacterial genera *Colwellia* and *Shewanella* made up 30–50% of the bacterial sequences (*n *=* *75) in clone libraries and 66–84% of gammaproteobacterial sequences derived from pyrosequencing (Blothe et al., 2015). Similarly, a study of three nodules collected in the South Pacific Gyre found that *Colwellia* comprised ~40–55% of the prokaryotic community on one of three nodules, but were absent or nearly absent (0.3%) on the other two nodules (Tully & Heidelberg, 2013). Our analysis of 20 nodules from 8 stations, sampled by two different apparatus (megacore and boxcore), recovered no sequences classified as *Colwellia* and a minute fraction (<0.0006%) classified as *Shewanella*. One possible explanation for the high amounts of *Colwellia* and *Shewanella* in the Tully and Heidelberg and Blothe et al. studies could be sediment contamination, since our methodology employed a stringent double wash of the exterior of the nodule samples prior to DNA extraction and amplification. However, we find vanishingly small amounts of *Colwellia* (0.00008%) and *Shewanella* (0.002%) even in our sediment samples. Blothe et al. considered this possibility as well and performed group‐specific PCR of sediment samples to ensure that the *Shewanella* signal they found truly came from the nodules rather than sediment contamination. Additionally, in the Tully and Heidelberg study, the *Colwellia* OTUs were predominantly retrieved from the inner portion of the nodule sample (42–55%) rather than the outer portion (6%). Hence, we suspect sediment contamination is likely not the explanation for differences between our study and these other studies. *Colwellia* and *Shewanella* are generally considered copiotriophs, demonstrating rapid growth rates and hence are found in regions where concentrations of organic and inorganic nutrients are elevated (Lauro et al., 2009), and *Colwellia* and *Shewanella* are both known to colonize marine invertebrates (Gillan, Speksnijder, Zwart, & De Ridder, 1998; Ivanova et al., 2004). The greater abundances of these *Alteromondales* in various nodules from other studies and complete absence in this study (as well as absence from individual nodules in the Tully and Heidelberg (2013) study) may reflect regional differences within the CCZ in the colonization of nodules by invertebrates and their associated microflora, or simply a high degree of endemism at the abyssal seafloor when examined over larger spatial scales (>300 km) (Bienhold, Zinger, Boetius, & Ramette, 2016). In addition, differences among these studies could reflect biases associated with the choice of PCR primers; for this study, the forward (515f) and reverse (805r) PCR primers we relied on for prokaryotic identification can yield single‐nucleotide mismatches to the 16S rRNA genes of members of the *Thaumarcheota* Marine Group I (Parada, Needham, & Fuhrman, 2015) and the SAR11 clade (Apprill, McNally, Parsons, & Weber, 2015), respectively. However, this would not explain the lack of the *Gammaproteobacteria Colwellia* and *Shewanella* associated with nodules sampled in our study. We further checked these primers using Silva TestPrime 1.0 and found that the primer pair 515f/805r had coverage of 92% within the *Shewanella* and 89% within the *Colwellia* when no mismatches were allowed. When one mismatch was allowed, this coverage rose to 96 and 94%, respectively. Therefore, it seems unlikely that the primers used for amplification in our study are biased against these genera. We utilized the same primers for all the samples collected for this study, facilitating comparative assessment of prokaryotic diversity across the different types of habitats (seawater, sediment, and nodules) and stations sampled. Finally, there may have been differences in the physical and/or chemical structure of the nodules within the German claim area and those nodules sampled from the UK claim area, which might promote differences in the microbiota observed between these studies.

## 4 Conclusions

We provide strong evidence supporting the growing view that polymetallic nodules, surrounding sediments, and the overlying water column constitute distinct microbial habitats with characteristic microbial assemblages. Microbial assemblages differ with depth into the sediment such that surface sediment removal and/or accelerated burial by resettling from a near‐bottom sediment plume are likely to fundamentally alter microbial community structure. Sediments and nodules are major reservoirs of microbial diversity distinct from even the deep water column, suggesting that large‐scale removal of nodules and sediments might alter local, and even regional, patterns of microbial diversity and ultimately modify specific ecosystem functions. Over the ~30 km scales of our study, each of these habitats appears to harbor similar microbial assemblages. Additional work is needed to determine if microbial assemblages in the sediments and nodules vary over the ~6 million square km expanse of the Clarion‐Clipperton Zone, as suggested by the lack of *Colwellia* and *Shewanella* sequences in our samples versus within the German claim area ~300 km away. However, our current study provides the first evidence of a widespread core nodule microbial community across large regions of the Pacific Ocean. Additionally, many of the prominent prokaryotic genera retrieved from the sediment and nodules in our study and others suggest an important role for chemoautotrophy within and above the nodule field. The energy sources sustaining such metabolisms remain unknown. Finally, future work is needed to understand the stability and resilience of these microbial ecosystems to perturbations such as those likely to result from nodule‐mining operations.

## Conflict of Interest

The authors declare no conflict of interest.
