# Supplementary material for: Polymetallic nodules, sediments, and deep waters in the equatorial North Pacific exhibit highly diverse and distinct bacterial, archaeal, and microeukaryotic communities
Source: Microbiologyopen. 2016 Nov 21;6(2):e00428. doi: 10.1002/mbo3.428 (PMC5387330; doi:10.1002/mbo3.428)
Supplement: Supplementary file 11 [file MBO3-6-na-s011.pdf]

Table S1 OTU and species richness estimates at sampling depths of 2,401,000 and 100,100 sequences for prokaryotes and eukaryotes, respectively.

	Observed OTUs	Chao1	Exponential of Shannon's
Water column (prokaryotes)	33,732	35,083	7435
Nodules (prokaryotes)	93,790	118,552	25,345
Sediment (prokaryotes)	111,413	184,335	24,443
Water column (eukaryotes)	6704	13,373	9566
Nodules (eukaryotes)	4744	8831	3508
Sediment (eukaryotes)	9004	15,344	22,692
