# Supplementary material for: Polymetallic nodules, sediments, and deep waters in the equatorial North Pacific exhibit highly diverse and distinct bacterial, archaeal, and microeukaryotic communities
Source: Microbiologyopen. 2016 Nov 21;6(2):e00428. doi: 10.1002/mbo3.428 (PMC5387330; doi:10.1002/mbo3.428)
Supplement: Supplementary file 12 [file MBO3-6-na-s012.pdf]

Table S2 Core prokaryotic OTUs found across 100% of nodule samples analyzed from AB01:

Greengenes ID	Taxonomic assignment
657355	[k__Archaea, p__Crenarchaeota, c__Thaumarchaeota, o__Cenarchaeales, f__Cenarchaeaceae, g__, s__]
741963	[k__Archaea, p__Crenarchaeota, c__Thaumarchaeota, o__Cenarchaeales, f__Cenarchaeaceae, g__, s__]
182872	[k__Archaea, p__Crenarchaeota, c__Thaumarchaeota, o__Cenarchaeales, f__Cenarchaeaceae, g__, s__]
776850	[k__Archaea, p__Crenarchaeota, c__Thaumarchaeota, o__Cenarchaeales, f__Cenarchaeaceae, g__, s__]
1054	[k__Archaea, p__Crenarchaeota, c__Thaumarchaeota, o__Cenarchaeales, f__Cenarchaeaceae, g__, s__]
730215	[k__Archaea, p__Crenarchaeota, c__Thaumarchaeota, o__Cenarchaeales, f__Cenarchaeaceae, g__, s__]
196754	[k__Archaea, p__Crenarchaeota, c__Thaumarchaeota, o__Cenarchaeales, f__Cenarchaeaceae, g__, s__]
104620	[k__Archaea, p__Crenarchaeota, c__Thaumarchaeota, o__Cenarchaeales, f__Cenarchaeaceae, g__, s__]
753582	[k__Archaea, p__Crenarchaeota, c__Thaumarchaeota, o__Cenarchaeales, f__Cenarchaeaceae, g__, s__]
1584736	[k__Archaea, p__Crenarchaeota, c__Thaumarchaeota, o__Cenarchaeales, f__Cenarchaeaceae, g__, s__]
New.ReferenceOTU1359	[k__Archaea, p__Crenarchaeota, c__Thaumarchaeota, o__Cenarchaeales, f__Cenarchaeaceae, g__, s__]
540244	[k__Archaea, p__Crenarchaeota, c__Thaumarchaeota, o__Cenarchaeales, f__Cenarchaeaceae, g__Nitrosopumilus, s__]
869040	[k__Archaea, p__Crenarchaeota, c__Thaumarchaeota, o__Cenarchaeales, f__Cenarchaeaceae, g__Nitrosopumilus, s__]
659087	[k__Archaea, p__Crenarchaeota, c__Thaumarchaeota, o__Cenarchaeales, f__Cenarchaeaceae, g__Nitrosopumilus, s__]
1087314	[k__Archaea, p__Crenarchaeota, c__Thaumarchaeota, o__Cenarchaeales, f__Cenarchaeaceae, g__Nitrosopumilus, s__]
4425583	[k__Archaea, p__Crenarchaeota, c__Thaumarchaeota, o__Cenarchaeales, f__Cenarchaeaceae, g__Nitrosopumilus, s__]
4369009	[k__Archaea, p__Crenarchaeota, c__Thaumarchaeota, o__Cenarchaeales, f__Cenarchaeaceae, g__Nitrosopumilus, s__]
619389	[k__Archaea, p__Crenarchaeota, c__Thaumarchaeota, o__Cenarchaeales, f__Cenarchaeaceae, g__Nitrosopumilus, s__]
553746	[k__Archaea, p__Crenarchaeota, c__Thaumarchaeota, o__Cenarchaeales, f__Cenarchaeaceae, g__Nitrosopumilus, s__]
348549	[k__Archaea, p__Crenarchaeota, c__Thaumarchaeota, o__Cenarchaeales, f__Cenarchaeaceae, g__Nitrosopumilus, s__]
737234	[k__Archaea, p__Crenarchaeota, c__Thaumarchaeota, o__Cenarchaeales, f__Cenarchaeaceae, g__Nitrosopumilus, s__]
560175	[k__Archaea, p__Crenarchaeota, c__Thaumarchaeota, o__Cenarchaeales, f__Cenarchaeaceae, g__Nitrosopumilus, s__]
New.ReferenceOTU254	[k__Archaea, p__Crenarchaeota, c__Thaumarchaeota, o__Cenarchaeales, f__Cenarchaeaceae, g__Nitrosopumilus, s__]
New.ReferenceOTU1600	[k__Archaea, p__Crenarchaeota, c__Thaumarchaeota, o__Cenarchaeales, f__Cenarchaeaceae, g__Nitrosopumilus, s__]
4352607	[k__Bacteria, p__[Caldithrix], c__KSB1, o__Ucn15732, f__, g__, s__]
262829	[k__Bacteria, p__[Caldithrix], c__KSB1, o__Ucn15732, f__, g__, s__]
272885	[k__Bacteria, p__Acidobacteria, c__[Chloracidobacteria], o__RB41, f__Ellin6075, g__, s__]
4037775	[k__Bacteria, p__Acidobacteria, c__Acidobacteria-6, o__BPC015, f__, g__, s__]
2449617	[k__Bacteria, p__Acidobacteria, c__BPC102, o__B110, f__, g__, s__]
4475363	[k__Bacteria, p__Acidobacteria, c__BPC102, o__B110, f__, g__, s__]
1045667	[k__Bacteria, p__Acidobacteria, c__BPC102, o__B110, f__, g__, s__]
New.ReferenceOTU1401	[k__Bacteria, p__Acidobacteria, c__BPC102, o__B110, f__, g__, s__]
203449	[k__Bacteria, p__Acidobacteria, c__DA052, o__E29, f__, g__, s__]

1110602	[k__Bacteria, p__Acidobacteria, c__Sva0725, o__Sva0725, f__, g__, s__]
138174	[k__Bacteria, p__Actinobacteria, c__Acidimicrobiia, o__Acidimicrobiales, f__koll13, g__, s__]
272708	[k__Bacteria, p__Actinobacteria, c__Acidimicrobiia, o__Acidimicrobiales, f__wb1_P06, g__, s__]
4348977	[k__Bacteria, p__Actinobacteria, c__Thermoleophilia, o__Solirubrobacterales, f__, g__, s__]
568999	[k__Bacteria, p__Bacteroidetes, c__[Rhodothermi], o__[Rhodothermales], f__Rhodothermaceae, g__, s__]
New.ReferenceOTU91	[k__Bacteria, p__Bacteroidetes, c__[Rhodothermi], o__[Rhodothermales], f__Rhodothermaceae, g__, s__]
539477	[k__Bacteria, p__Bacteroidetes, c__[Rhodothermi], o__[Rhodothermales], f__Rhodothermaceae, g__Rubricoccus, s__]
571975	[k__Bacteria, p__Bacteroidetes, c__Cytophagia, o__Cytophagales, f__Flammeovirgaceae, g__, s__]
550546	[k__Bacteria, p__Bacteroidetes, c__Cytophagia, o__Cytophagales, f__Flammeovirgaceae, g__, s__]
New.ReferenceOTU1947	[k__Bacteria, p__Bacteroidetes, c__Cytophagia, o__Cytophagales, f__Flammeovirgaceae, g__, s__]
246587	[k__Bacteria, p__Bacteroidetes, c__Flavobacteriia, o__Flavobacteriales, f__Flavobacteriaceae, g__, s__]
2582030	[k__Bacteria, p__Chloroflexi, c__SAR202, o__, f__, g__, s__]
576457	[k__Bacteria, p__Chloroflexi, c__SAR202, o__, f__, g__, s__]
663206	[k__Bacteria, p__Chloroflexi, c__SAR202, o__, f__, g__, s__]
2585229	[k__Bacteria, p__Chloroflexi, c__SAR202, o__, f__, g__, s__]
20758	[k__Bacteria, p__Chloroflexi, c__SAR202, o__, f__, g__, s__]
554951	[k__Bacteria, p__Chloroflexi, c__SAR202, o__, f__, g__, s__]
New.ReferenceOTU2440	[k__Bacteria, p__Chloroflexi, c__SAR202, o__, f__, g__, s__]
4417556	[k__Bacteria, p__Gemmatimonadetes, c__Gemm-1, o__, f__, g__, s__]
New.ReferenceOTU1911	[k__Bacteria, p__Gemmatimonadetes, c__Gemm-1, o__, f__, g__, s__]
New.ReferenceOTU1866	[k__Bacteria, p__Gemmatimonadetes, c__Gemm-1, o__, f__, g__, s__]
537373	[k__Bacteria, p__Gemmatimonadetes, c__Gemm-2, o__, f__, g__, s__]
358080	[k__Bacteria, p__Gemmatimonadetes, c__Gemm-2, o__, f__, g__, s__]
4256117	[k__Bacteria, p__Gemmatimonadetes, c__Gemm-2, o__, f__, g__, s__]
565523	[k__Bacteria, p__Gemmatimonadetes, c__Gemm-4, o__, f__, g__, s__]
244505	[k__Bacteria, p__Gemmatimonadetes, c__Gemm-4, o__, f__, g__, s__]
1591289	[k__Bacteria, p__Gemmatimonadetes, c__Gemm-4, o__, f__, g__, s__]
New.ReferenceOTU2053	[k__Bacteria, p__Gemmatimonadetes, c__Gemm-4, o__, f__, g__, s__]
1449	[k__Bacteria, p__Nitrospirae, c__Nitrospira, o__Nitrospirales, f__Nitrospiraceae, g__, s__]
4335834	[k__Bacteria, p__Nitrospirae, c__Nitrospira, o__Nitrospirales, f__Nitrospiraceae, g__, s__]
237590	[k__Bacteria, p__Nitrospirae, c__Nitrospira, o__Nitrospirales, f__Nitrospiraceae, g__, s__]
552101	[k__Bacteria, p__OP3, c__koll11, o__GIF10, f__kpj58rc, g__, s__]
New.ReferenceOTU1317	[k__Bacteria, p__Planctomycetes, c__BD7-11, o__, f__, g__, s__]
704361	[k__Bacteria, p__Planctomycetes, c__Phycisphaerae, o__C86, f__, g__, s__]
272431	[k__Bacteria, p__Planctomycetes, c__Phycisphaerae, o__Phycisphaerales, f__, g__, s__]

124913	[k__Bacteria, p__Planctomycetes, c__Phycisphaerae, o__Phycisphaerales, f__, g__, s__]
3211673	[k__Bacteria, p__Planctomycetes, c__Phycisphaerae, o__S-70, f__, g__, s__]
263866	[k__Bacteria, p__Planctomycetes, c__Planctomycetia, o__Pirellulales, f__Pirellulaceae, g__, s__]
364773	[k__Bacteria, p__Planctomycetes, c__Planctomycetia, o__Pirellulales, f__Pirellulaceae, g__, s__]
New.ReferenceOTU1438	[k__Bacteria, p__Planctomycetes, c__Planctomycetia, o__Pirellulales, f__Pirellulaceae, g__, s__]
4307148	[k__Bacteria, p__Planctomycetes, c__Planctomycetia, o__Pirellulales, f__Pirellulaceae, g__A17, s__]
719818	[k__Bacteria, p__Planctomycetes, c__Planctomycetia, o__Pirellulales, f__Pirellulaceae, g__A17, s__]
266612	[k__Bacteria, p__Proteobacteria, c__Alphaproteobacteria, o__, f__, g__, s__]
265780	[k__Bacteria, p__Proteobacteria, c__Alphaproteobacteria, o__, f__, g__, s__]
1123043	[k__Bacteria, p__Proteobacteria, c__Alphaproteobacteria, o__, f__, g__, s__]
138344	[k__Bacteria, p__Proteobacteria, c__Alphaproteobacteria, o__, f__, g__, s__]
567782	[k__Bacteria, p__Proteobacteria, c__Alphaproteobacteria, o__, f__, g__, s__]
95741	[k__Bacteria, p__Proteobacteria, c__Alphaproteobacteria, o__, f__, g__, s__]
3223711	[k__Bacteria, p__Proteobacteria, c__Alphaproteobacteria, o__, f__, g__, s__]
831594	[k__Bacteria, p__Proteobacteria, c__Alphaproteobacteria, o__, f__, g__, s__]
1083150	[k__Bacteria, p__Proteobacteria, c__Alphaproteobacteria, o__, f__, g__, s__]
718100	[k__Bacteria, p__Proteobacteria, c__Alphaproteobacteria, o__, f__, g__, s__]
565274	[k__Bacteria, p__Proteobacteria, c__Alphaproteobacteria, o__, f__, g__, s__]
543882	[k__Bacteria, p__Proteobacteria, c__Alphaproteobacteria, o__, f__, g__, s__]
819552	[k__Bacteria, p__Proteobacteria, c__Alphaproteobacteria, o__, f__, g__, s__]
4423336	[k__Bacteria, p__Proteobacteria, c__Alphaproteobacteria, o__, f__, g__, s__]
2644916	[k__Bacteria, p__Proteobacteria, c__Alphaproteobacteria, o__, f__, g__, s__]
583912	[k__Bacteria, p__Proteobacteria, c__Alphaproteobacteria, o__, f__, g__, s__]
New.ReferenceOTU1855	[k__Bacteria, p__Proteobacteria, c__Alphaproteobacteria, o__, f__, g__, s__]
New.ReferenceOTU2553	[k__Bacteria, p__Proteobacteria, c__Alphaproteobacteria, o__, f__, g__, s__]
4385354	[k__Bacteria, p__Proteobacteria, c__Alphaproteobacteria, o__Rhizobiales, f__, g__, s__]
4385422	[k__Bacteria, p__Proteobacteria, c__Alphaproteobacteria, o__Rhizobiales, f__Hyphomicrobiaceae, g__, s__]
272493	[k__Bacteria, p__Proteobacteria, c__Alphaproteobacteria, o__Rhizobiales, f__Hyphomicrobiaceae, g__, s__]
3768672	[k__Bacteria, p__Proteobacteria, c__Alphaproteobacteria, o__Rhizobiales, f__Hyphomicrobiaceae, g__, s__]
171619	[k__Bacteria, p__Proteobacteria, c__Alphaproteobacteria, o__Rhizobiales, f__Hyphomicrobiaceae, g__Hyphomicrobium, s__]
260794	[k__Bacteria, p__Proteobacteria, c__Alphaproteobacteria, o__Rhizobiales, f__Hyphomicrobiaceae, g__Hyphomicrobium, s__]
863772	[k__Bacteria, p__Proteobacteria, c__Alphaproteobacteria, o__Rhizobiales, f__Hyphomicrobiaceae, g__Hyphomicrobium, s__]
New.ReferenceOTU2844	[k__Bacteria, p__Proteobacteria, c__Alphaproteobacteria, o__Rhizobiales, f__Hyphomicrobiaceae]
5807	[k__Bacteria, p__Proteobacteria, c__Alphaproteobacteria, o__Rhodobacterales, f__Rhodobacteraceae, g__, s__]
4348883	[k__Bacteria, p__Proteobacteria, c__Alphaproteobacteria, o__Rhodospirillales, f__Rhodospirillaceae, g__, s__]

3708318	[k__Bacteria, p__Proteobacteria, c__Alphaproteobacteria, o__Rhodospirillales, f__Rhodospirillaceae, g__, s__]
277135	[k__Bacteria, p__Proteobacteria, c__Alphaproteobacteria, o__Rhodospirillales, f__Rhodospirillaceae, g__, s__]
734841	[k__Bacteria, p__Proteobacteria, c__Alphaproteobacteria, o__Rhodospirillales, f__Rhodospirillaceae, g__, s__]
1118195	[k__Bacteria, p__Proteobacteria, c__Alphaproteobacteria, o__Rhodospirillales, f__Rhodospirillaceae, g__, s__]
757981	[k__Bacteria, p__Proteobacteria, c__Alphaproteobacteria, o__Rhodospirillales, f__Rhodospirillaceae, g__, s__]
669611	[k__Bacteria, p__Proteobacteria, c__Alphaproteobacteria, o__Rhodospirillales, f__Rhodospirillaceae, g__, s__]
569704	[k__Bacteria, p__Proteobacteria, c__Alphaproteobacteria, o__Rhodospirillales, f__Rhodospirillaceae, g__, s__]
4423338	[k__Bacteria, p__Proteobacteria, c__Alphaproteobacteria, o__Rhodospirillales, f__Rhodospirillaceae, g__, s__]
569555	[k__Bacteria, p__Proteobacteria, c__Alphaproteobacteria, o__Rhodospirillales, f__Rhodospirillaceae, g__, s__]
4413241	[k__Bacteria, p__Proteobacteria, c__Alphaproteobacteria, o__Rhodospirillales, f__Rhodospirillaceae, g__, s__]
682480	[k__Bacteria, p__Proteobacteria, c__Alphaproteobacteria, o__Rhodospirillales, f__Rhodospirillaceae, g__, s__]
837201	[k__Bacteria, p__Proteobacteria, c__Alphaproteobacteria, o__Rhodospirillales, f__Rhodospirillaceae, g__, s__]
278297	[k__Bacteria, p__Proteobacteria, c__Alphaproteobacteria, o__Rhodospirillales, f__Rhodospirillaceae, g__, s__]
3582703	[k__Bacteria, p__Proteobacteria, c__Alphaproteobacteria, o__Rhodospirillales, f__Rhodospirillaceae, g__, s__]
200860	[k__Bacteria, p__Proteobacteria, c__Alphaproteobacteria, o__Rhodospirillales, f__Rhodospirillaceae, g__, s__]
693333	[k__Bacteria, p__Proteobacteria, c__Alphaproteobacteria, o__Rhodospirillales, f__Rhodospirillaceae, g__, s__]
2632957	[k__Bacteria, p__Proteobacteria, c__Alphaproteobacteria, o__Rhodospirillales, f__Rhodospirillaceae, g__, s__]
New.ReferenceOTU103	[k__Bacteria, p__Proteobacteria, c__Alphaproteobacteria, o__Rhodospirillales, f__Rhodospirillaceae, g__, s__]
New.ReferenceOTU1642	[k__Bacteria, p__Proteobacteria, c__Alphaproteobacteria, o__Rhodospirillales, f__Rhodospirillaceae, g__, s__]
New.ReferenceOTU1977	[k__Bacteria, p__Proteobacteria, c__Alphaproteobacteria, o__Rhodospirillales, f__Rhodospirillaceae, g__, s__]
New.ReferenceOTU87	[k__Bacteria, p__Proteobacteria, c__Alphaproteobacteria, o__Rhodospirillales, f__Rhodospirillaceae, g__, s__]
4395653	[k__Bacteria, p__Proteobacteria, c__Betaproteobacteria, o__Nitrosomonadales, f__Nitrosomonadaceae, g__, s__]
145637	[k__Bacteria, p__Proteobacteria, c__Betaproteobacteria, o__Nitrosomonadales, f__Nitrosomonadaceae, g__, s__]
New.ReferenceOTU1086	[k__Bacteria, p__Proteobacteria, c__Betaproteobacteria, o__Rhodocyclales, f__Rhodocyclaceae]
327296	[k__Bacteria, p__Proteobacteria, c__Deltaproteobacteria, o__, f__, g__, s__]
4380792	[k__Bacteria, p__Proteobacteria, c__Deltaproteobacteria, o__[Entotheonellales], f__[Entotheonellaceae], g__, s__]
274620	[k__Bacteria, p__Proteobacteria, c__Deltaproteobacteria, o__Desulfobacterales, f__Nitrospinaceae, g__Nitrospina, s__]
138264	[k__Bacteria, p__Proteobacteria, c__Deltaproteobacteria, o__Desulfobacterales, f__Nitrospinaceae, g__Nitrospina, s__]
299227	[k__Bacteria, p__Proteobacteria, c__Deltaproteobacteria, o__Desulfobacterales, f__Nitrospinaceae, g__Nitrospina, s__]
793842	[k__Bacteria, p__Proteobacteria, c__Deltaproteobacteria, o__Desulfobacterales, f__Nitrospinaceae, g__Nitrospina, s__]
792011	[k__Bacteria, p__Proteobacteria, c__Deltaproteobacteria, o__Myxococcales, f__, g__, s__]
572150	[k__Bacteria, p__Proteobacteria, c__Deltaproteobacteria, o__Myxococcales, f__, g__, s__]
New.ReferenceOTU1232	[k__Bacteria, p__Proteobacteria, c__Deltaproteobacteria, o__NB1-j, f__, g__, s__]
808856	[k__Bacteria, p__Proteobacteria, c__Deltaproteobacteria, o__NB1-j, f__JTB38, g__, s__]
659717	[k__Bacteria, p__Proteobacteria, c__Deltaproteobacteria, o__NB1-j, f__JTB38, g__, s__]

317719	[k__Bacteria, p__Proteobacteria, c__Deltaproteobacteria, o__NB1-j, f__JTB38, g__, s__]
659088	[k__Bacteria, p__Proteobacteria, c__Deltaproteobacteria, o__NB1-j, f__MND4, g__, s__]
New.ReferenceOTU2273	[k__Bacteria, p__Proteobacteria, c__Deltaproteobacteria, o__NB1-j, f__MND4, g__, s__]
New.ReferenceOTU2144	[k__Bacteria, p__Proteobacteria, c__Deltaproteobacteria, o__NB1-j, f__MND4, g__, s__]
New.ReferenceOTU263	[k__Bacteria, p__Proteobacteria, c__Deltaproteobacteria, o__NB1-j, f__MND4, g__, s__]
250443	[k__Bacteria, p__Proteobacteria, c__Deltaproteobacteria, o__NB1-j, f__NB1-i, g__, s__]
2495965	[k__Bacteria, p__Proteobacteria, c__Deltaproteobacteria, o__NB1-j, f__NB1-i, g__, s__]
4437142	[k__Bacteria, p__Proteobacteria, c__Deltaproteobacteria, o__NB1-j, f__NB1-i, g__, s__]
657368	[k__Bacteria, p__Proteobacteria, c__Deltaproteobacteria, o__NB1-j, f__NB1-i, g__, s__]
325676	[k__Bacteria, p__Proteobacteria, c__Deltaproteobacteria, o__NB1-j, f__NB1-i, g__, s__]
4484249	[k__Bacteria, p__Proteobacteria, c__Deltaproteobacteria, o__NB1-j, f__NB1-i, g__, s__]
1591296	[k__Bacteria, p__Proteobacteria, c__Deltaproteobacteria, o__Sva0853, f__JTB36, g__, s__]
3211674	[k__Bacteria, p__Proteobacteria, c__Deltaproteobacteria, o__Sva0853, f__JTB36, g__, s__]
4348974	[k__Bacteria, p__Proteobacteria, c__Deltaproteobacteria, o__Sva0853, f__JTB36, g__, s__]
4434771	[k__Bacteria, p__Proteobacteria, c__Deltaproteobacteria, o__Sva0853, f__JTB36, g__, s__]
New.ReferenceOTU337	[k__Bacteria, p__Proteobacteria, c__Deltaproteobacteria, o__Sva0853, f__JTB36, g__, s__]
4324265	[k__Bacteria, p__Proteobacteria, c__Deltaproteobacteria, o__Syntrophobacterales, f__Syntrophobacteraceae, g__, s__]
834257	[k__Bacteria, p__Proteobacteria, c__Deltaproteobacteria, o__Syntrophobacterales, f__Syntrophobacteraceae, g__, s__]
239869	[k__Bacteria, p__Proteobacteria, c__Gammaproteobacteria, o__, f__, g__, s__]
342284	[k__Bacteria, p__Proteobacteria, c__Gammaproteobacteria, o__[Marinicellales], f__[Marinicellaceae], g__, s__]
4414633	[k__Bacteria, p__Proteobacteria, c__Gammaproteobacteria, o__Alteromonadales, f__OM60, g__, s__]
4039575	[k__Bacteria, p__Proteobacteria, c__Gammaproteobacteria, o__Chromatiales, f__, g__, s__]
149753	[k__Bacteria, p__Proteobacteria, c__Gammaproteobacteria, o__Chromatiales, f__, g__, s__]
544130	[k__Bacteria, p__Proteobacteria, c__Gammaproteobacteria, o__Chromatiales, f__, g__, s__]
241556	[k__Bacteria, p__Proteobacteria, c__Gammaproteobacteria, o__Chromatiales, f__, g__, s__]
4480288	[k__Bacteria, p__Proteobacteria, c__Gammaproteobacteria, o__Chromatiales, f__, g__, s__]
718758	[k__Bacteria, p__Proteobacteria, c__Gammaproteobacteria, o__Chromatiales, f__, g__, s__]
832176	[k__Bacteria, p__Proteobacteria, c__Gammaproteobacteria, o__Chromatiales, f__, g__, s__]
1012494	[k__Bacteria, p__Proteobacteria, c__Gammaproteobacteria, o__Chromatiales, f__, g__, s__]
80943	[k__Bacteria, p__Proteobacteria, c__Gammaproteobacteria, o__Chromatiales, f__, g__, s__]
247507	[k__Bacteria, p__Proteobacteria, c__Gammaproteobacteria, o__Chromatiales, f__Ectothiorhodospiraceae, g__, s__]
4352040	[k__Bacteria, p__Proteobacteria, c__Gammaproteobacteria, o__HTCC2188, f__HTCC2089, g__, s__]
243868	[k__Bacteria, p__Proteobacteria, c__Gammaproteobacteria, o__HTCC2188, f__HTCC2089, g__, s__]
274135	[k__Bacteria, p__Proteobacteria, c__Gammaproteobacteria, o__Oceanospirillales, f__, g__, s__]
742886	[k__Bacteria, p__Proteobacteria, c__Gammaproteobacteria, o__Thiotrichales, f__Piscirickettsiaceae, g__, s__]

3726190	[k__Bacteria, p__Proteobacteria, c__Gammaproteobacteria, o__Thiotrichales, f__Piscirickettsiaceae, g__, s__]
264970	[k__Bacteria, p__Proteobacteria, c__Gammaproteobacteria, o__Thiotrichales, f__Piscirickettsiaceae, g__, s__]
4300016	[k__Bacteria, p__Proteobacteria, c__Gammaproteobacteria, o__Thiotrichales, f__Piscirickettsiaceae, g__, s__]
728254	[k__Bacteria, p__Proteobacteria, c__Gammaproteobacteria, o__Thiotrichales, f__Piscirickettsiaceae, g__, s__]
247606	[k__Bacteria, p__Proteobacteria, c__Gammaproteobacteria, o__Thiotrichales, f__Piscirickettsiaceae, g__, s__]
257985	[k__Bacteria, p__Proteobacteria, c__Gammaproteobacteria, o__Thiotrichales, f__Piscirickettsiaceae, g__, s__]
248278	[k__Bacteria, p__Proteobacteria, c__Gammaproteobacteria, o__Thiotrichales, f__Piscirickettsiaceae, g__, s__]
4041618	[k__Bacteria, p__Proteobacteria, c__Gammaproteobacteria, o__Thiotrichales, f__Piscirickettsiaceae, g__, s__]
303000	[k__Bacteria, p__Proteobacteria, c__Gammaproteobacteria, o__Thiotrichales, f__Piscirickettsiaceae, g__, s__]
90324	[k__Bacteria, p__Proteobacteria, c__Gammaproteobacteria, o__Thiotrichales, f__Piscirickettsiaceae, g__, s__]
271272	[k__Bacteria, p__Proteobacteria, c__Gammaproteobacteria, o__Thiotrichales, f__Piscirickettsiaceae, g__, s__]
4480536	[k__Bacteria, p__Proteobacteria, c__Gammaproteobacteria, o__Thiotrichales, f__Piscirickettsiaceae, g__, s__]
95014	[k__Bacteria, p__Proteobacteria, c__Gammaproteobacteria, o__Thiotrichales, f__Piscirickettsiaceae, g__, s__]
4296905	[k__Bacteria, p__Proteobacteria, c__Gammaproteobacteria, o__Thiotrichales, f__Piscirickettsiaceae, g__, s__]
265051	[k__Bacteria, p__Proteobacteria, c__Gammaproteobacteria, o__Thiotrichales, f__Piscirickettsiaceae, g__, s__]
295290	[k__Bacteria, p__Proteobacteria, c__Gammaproteobacteria, o__Thiotrichales, f__Piscirickettsiaceae, g__, s__]
724681	[k__Bacteria, p__Proteobacteria, c__Gammaproteobacteria, o__Thiotrichales, f__Piscirickettsiaceae, g__, s__]
239964	[k__Bacteria, p__Proteobacteria, c__Gammaproteobacteria, o__Thiotrichales, f__Piscirickettsiaceae, g__, s__]
New.ReferenceOTU527	[k__Bacteria, p__Proteobacteria, c__Gammaproteobacteria, o__Thiotrichales, f__Piscirickettsiaceae, g__, s__]
New.ReferenceOTU909	[k__Bacteria, p__Proteobacteria, c__Gammaproteobacteria, o__Thiotrichales, f__Piscirickettsiaceae, g__, s__]
263284	[k__Bacteria, p__SBR1093, c__, o__, f__, g__, s__]
203274	[k__Bacteria, p__SBR1093, c__VHS-B5-50, o__, f__, g__, s__]
New.ReferenceOTU2406	[Unassigned]
