# Supplementary material for: Polymetallic nodules, sediments, and deep waters in the equatorial North Pacific exhibit highly diverse and distinct bacterial, archaeal, and microeukaryotic communities
Source: Microbiologyopen. 2016 Nov 21;6(2):e00428. doi: 10.1002/mbo3.428 (PMC5387330; doi:10.1002/mbo3.428)
Supplement: Supplementary file 8 [file MBO3-6-na-s008.pdf]

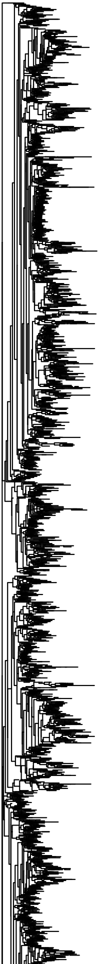

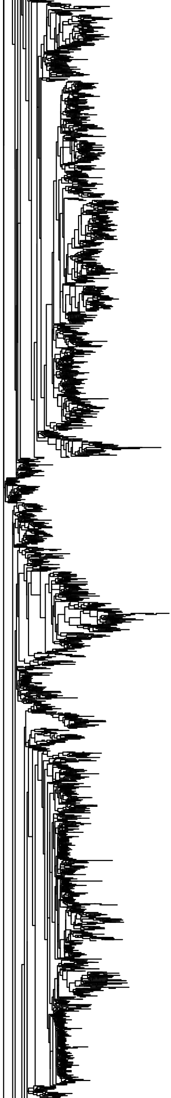

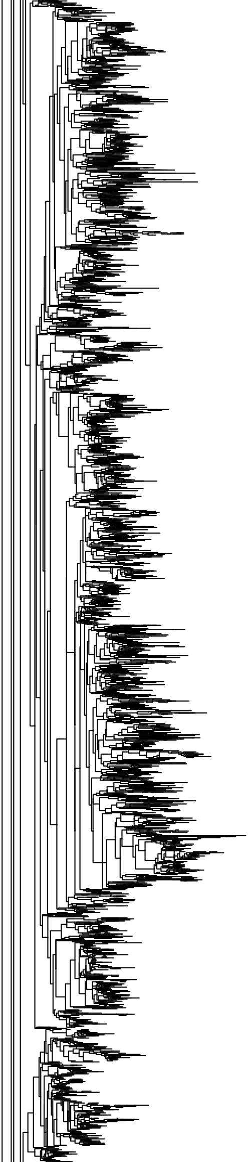

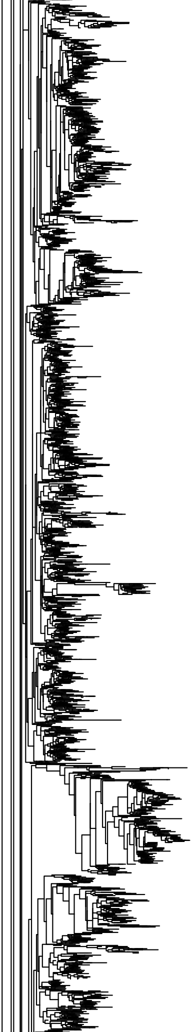

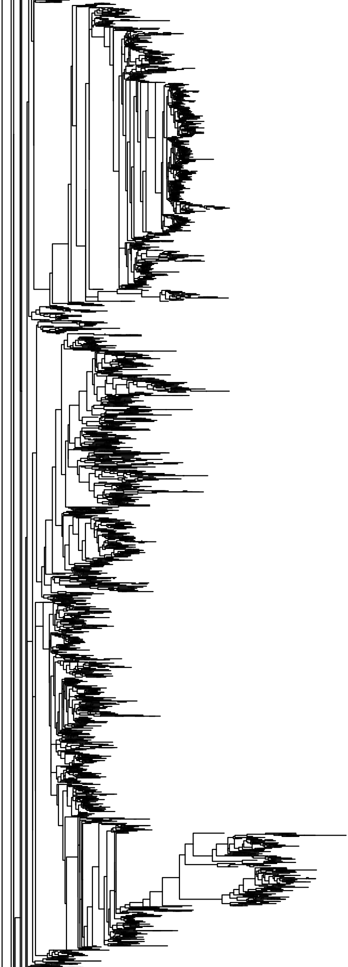

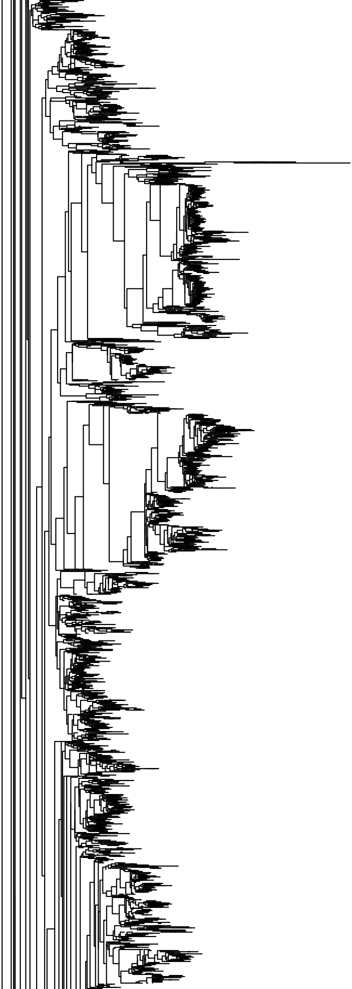

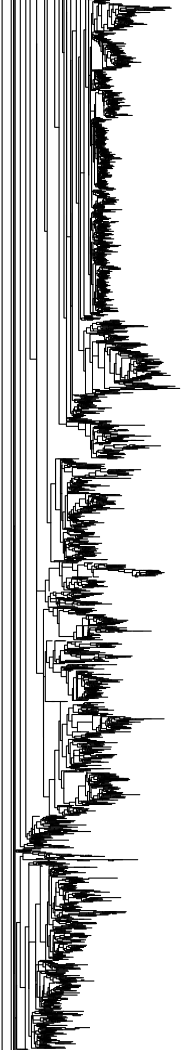

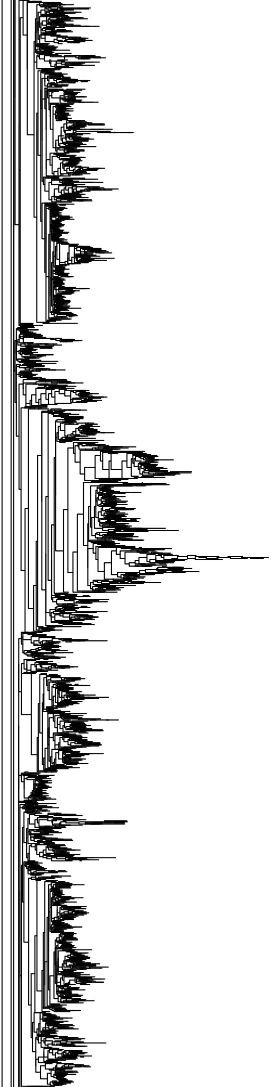

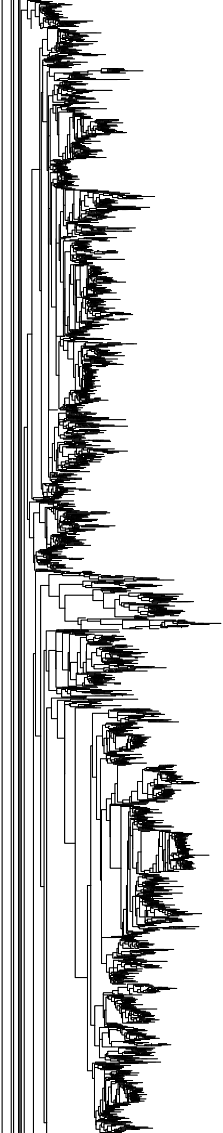

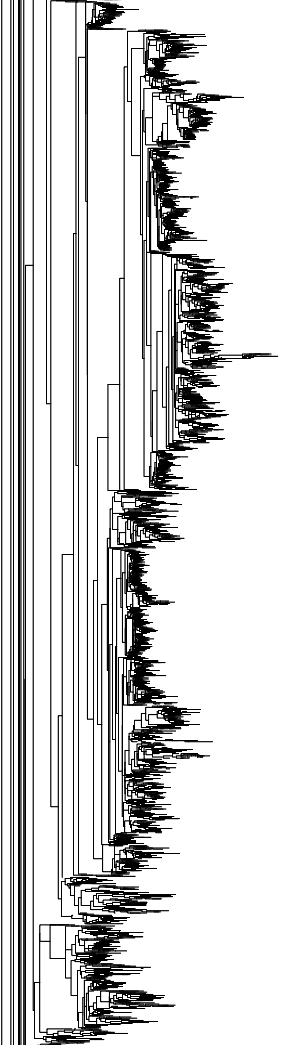

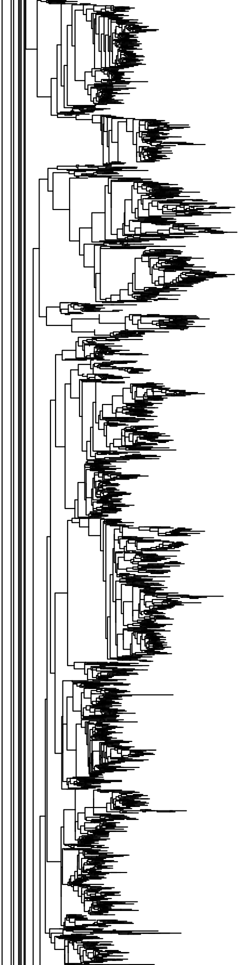

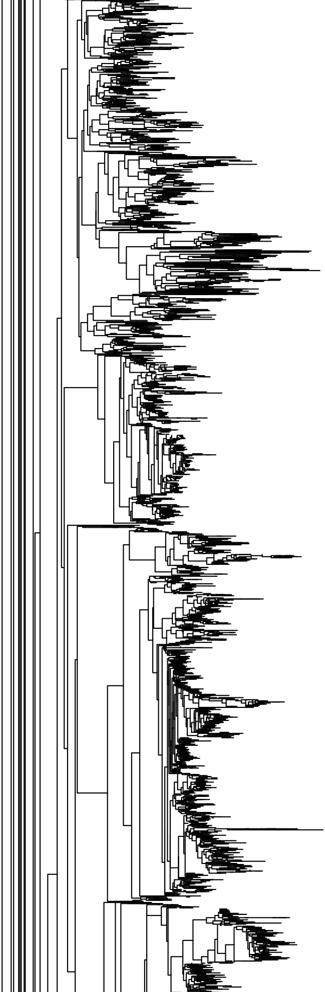

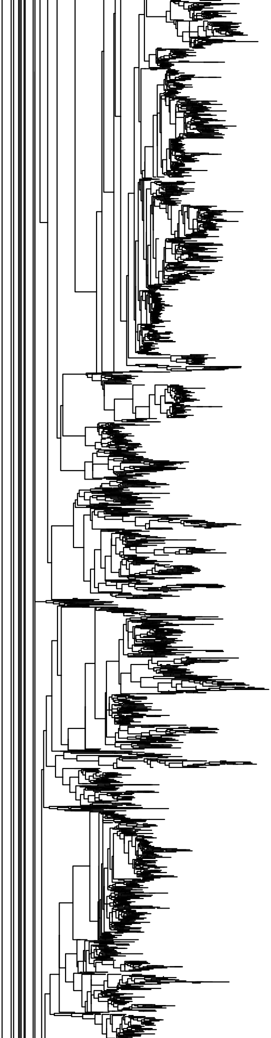

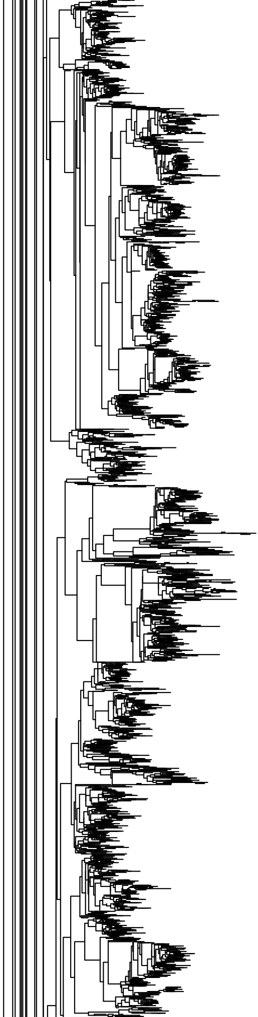

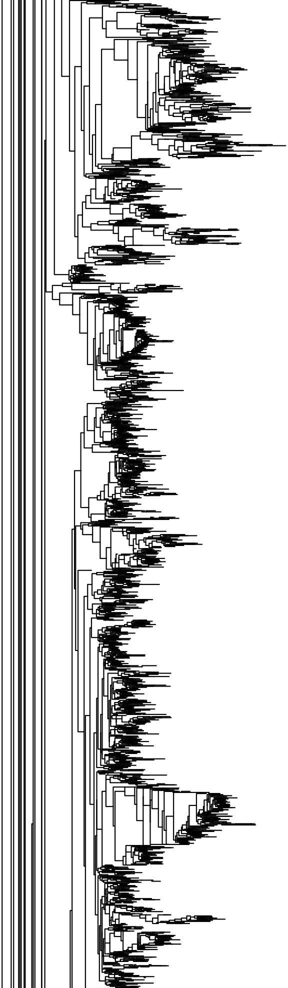

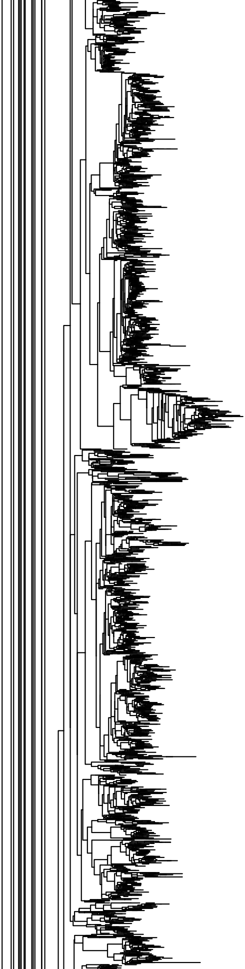

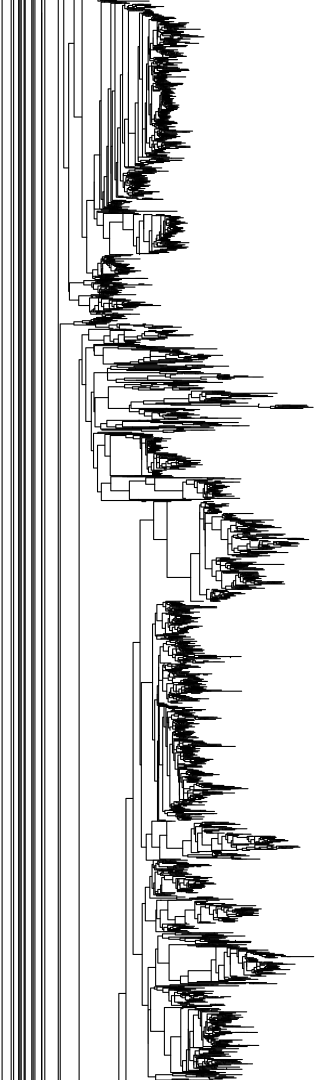

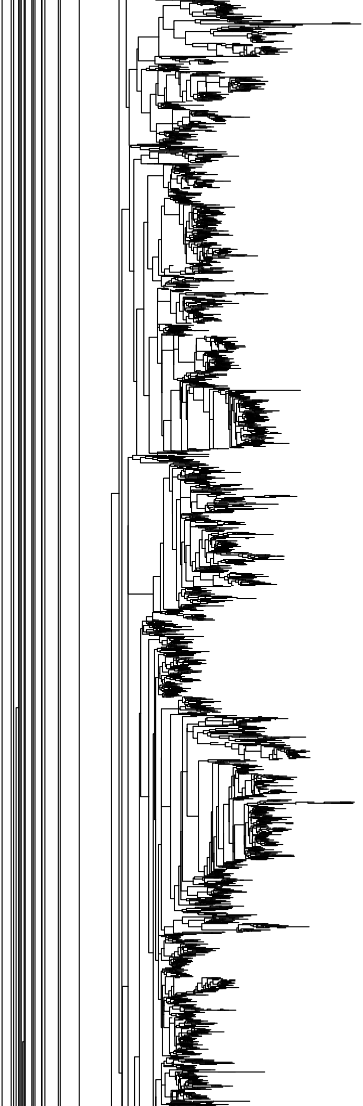

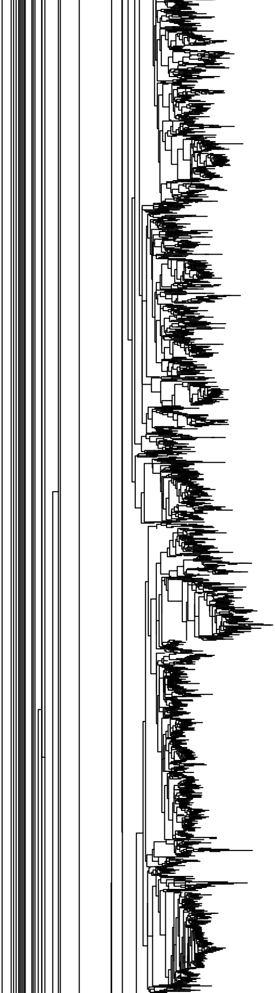

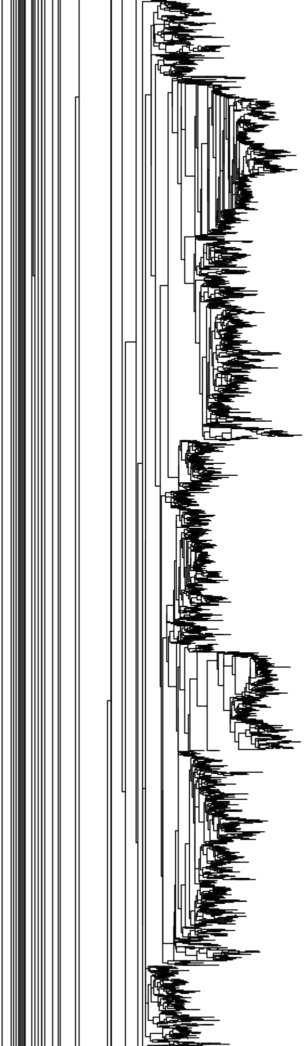

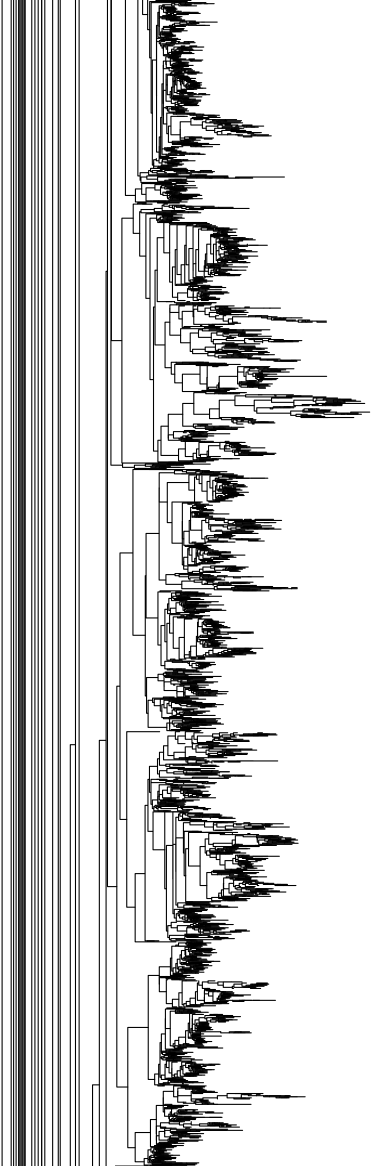

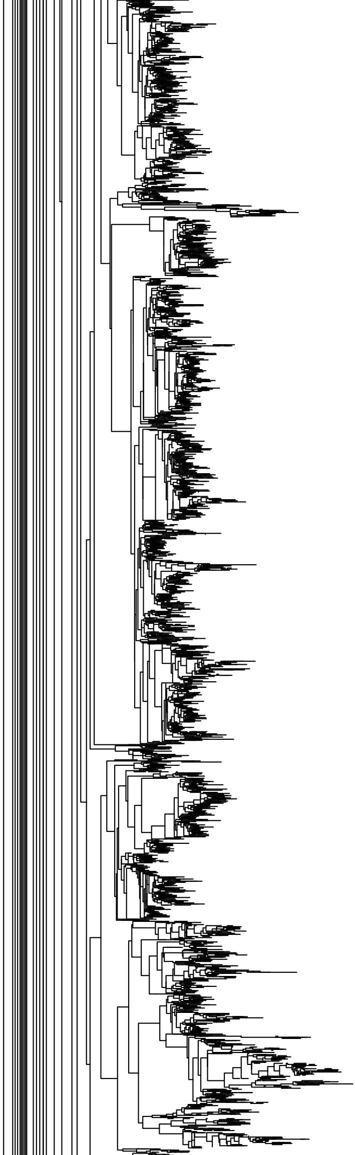

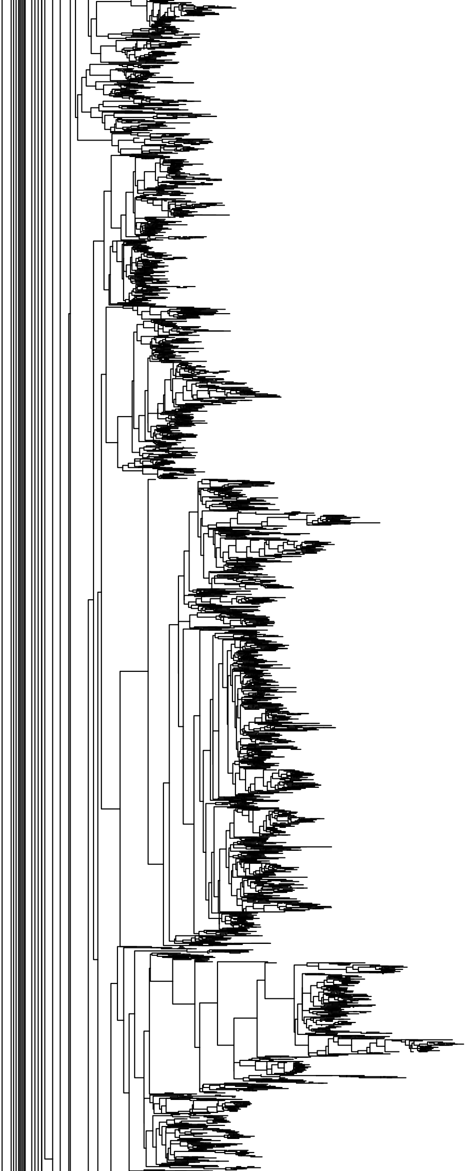

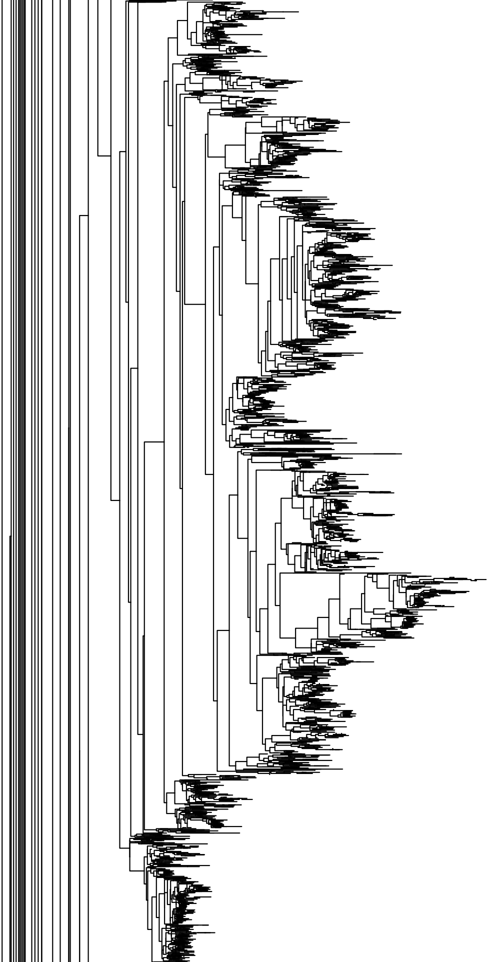

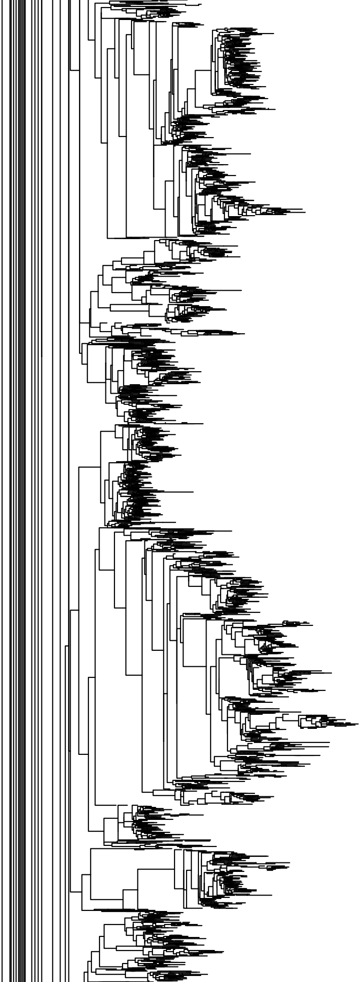

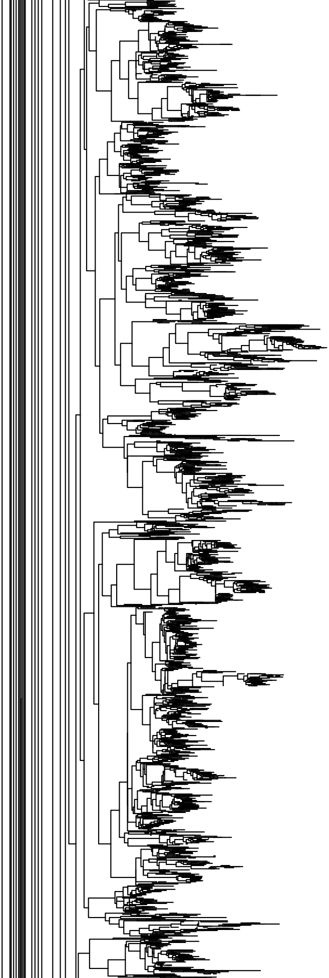

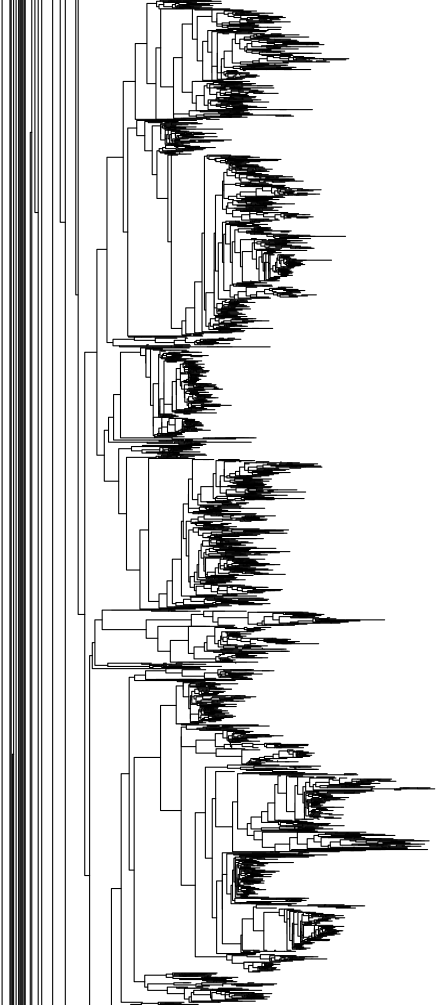

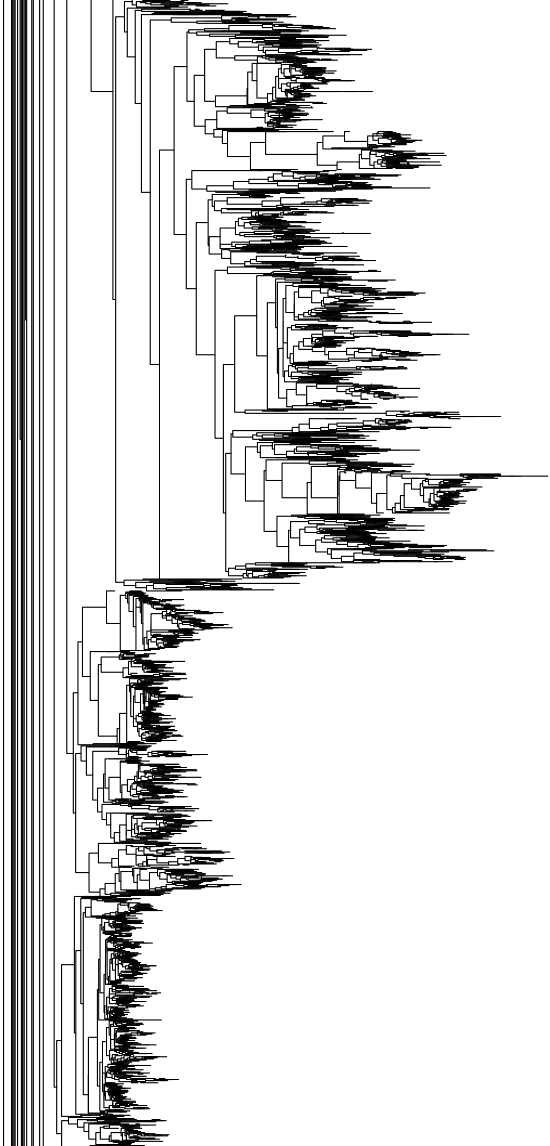

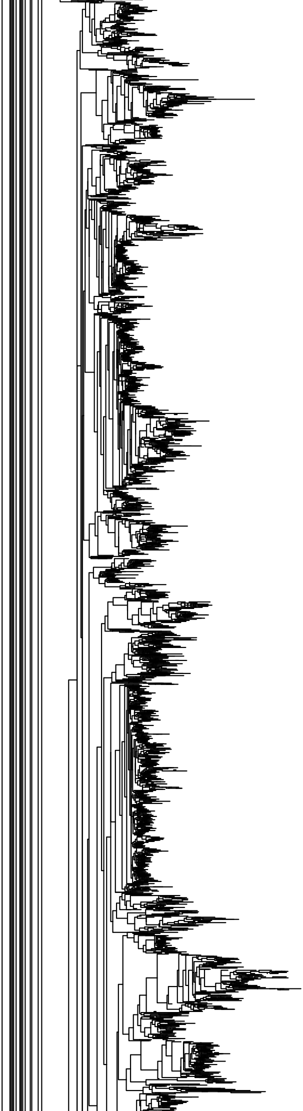

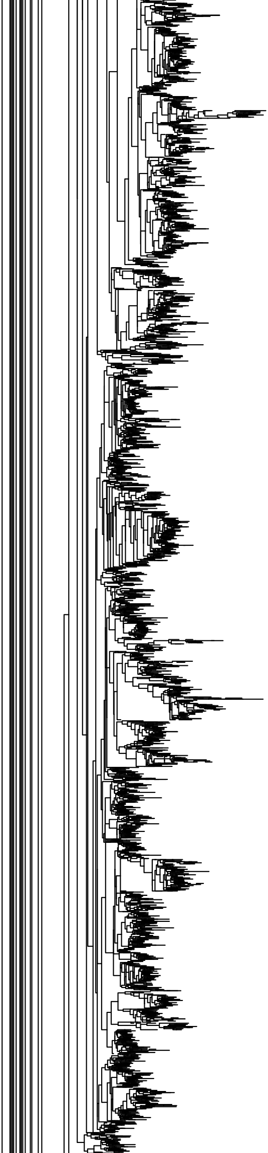

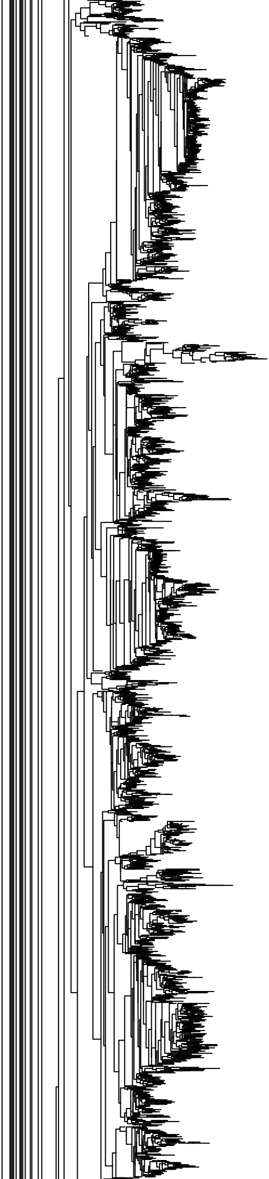

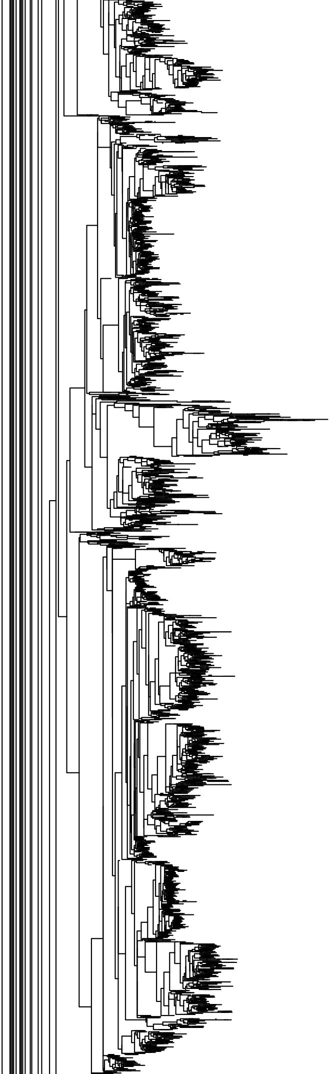

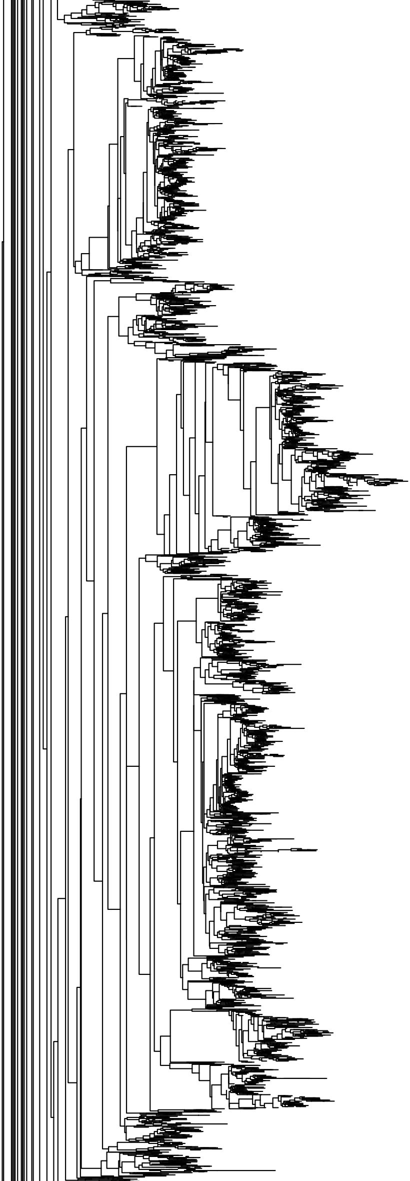

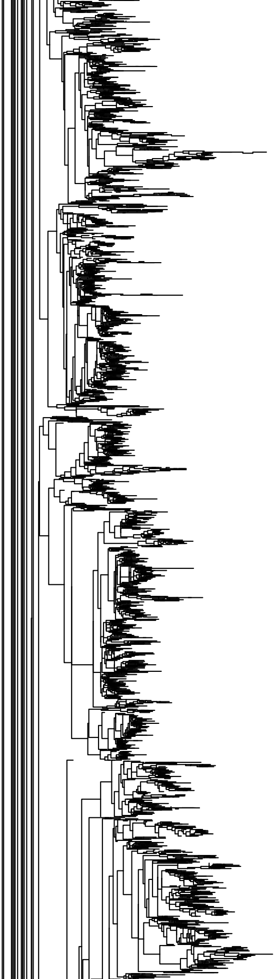

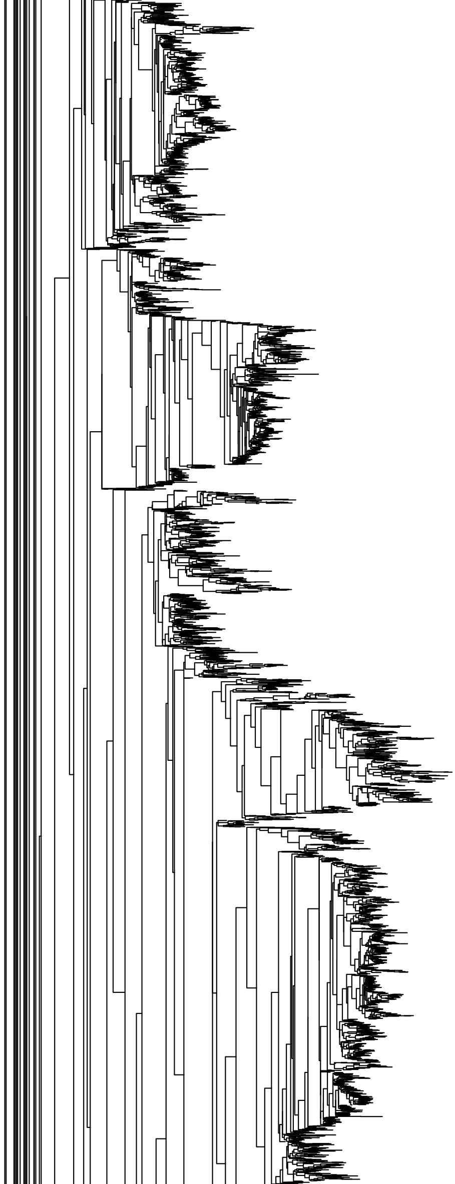

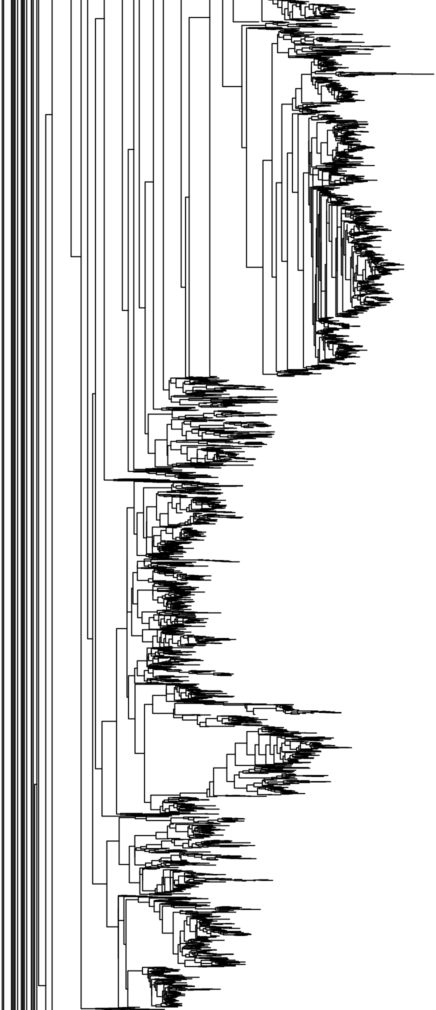

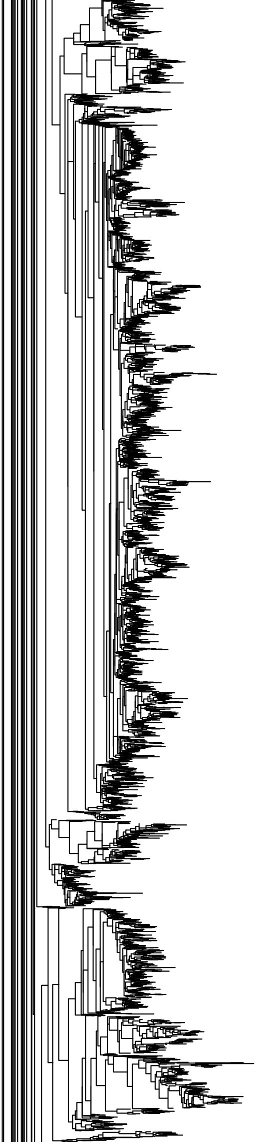

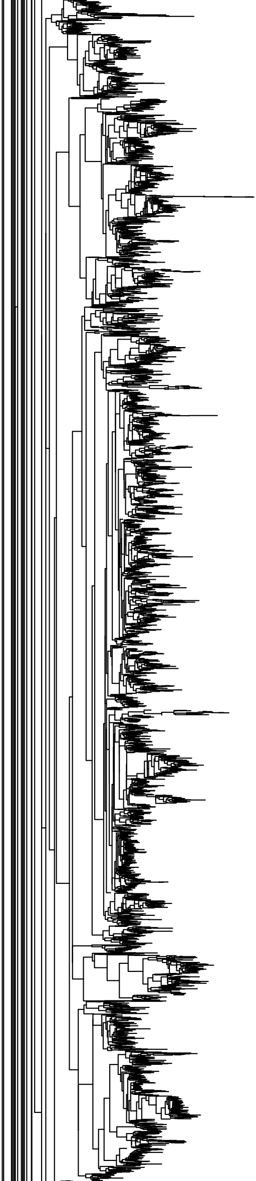

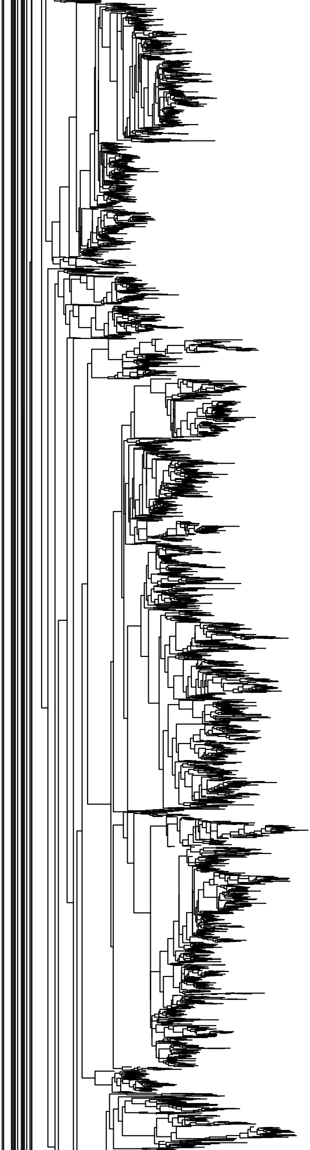

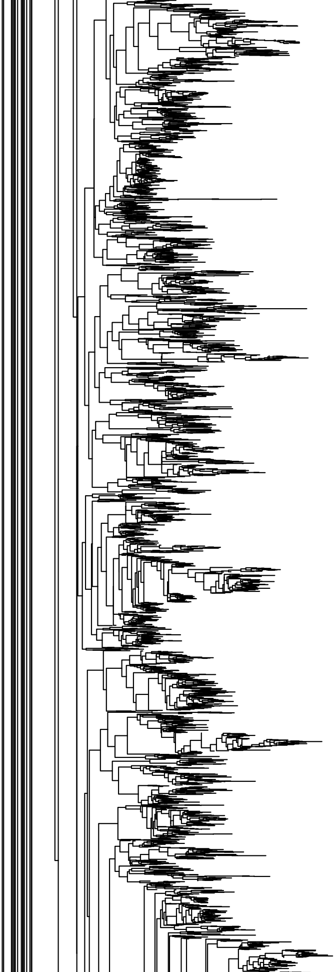

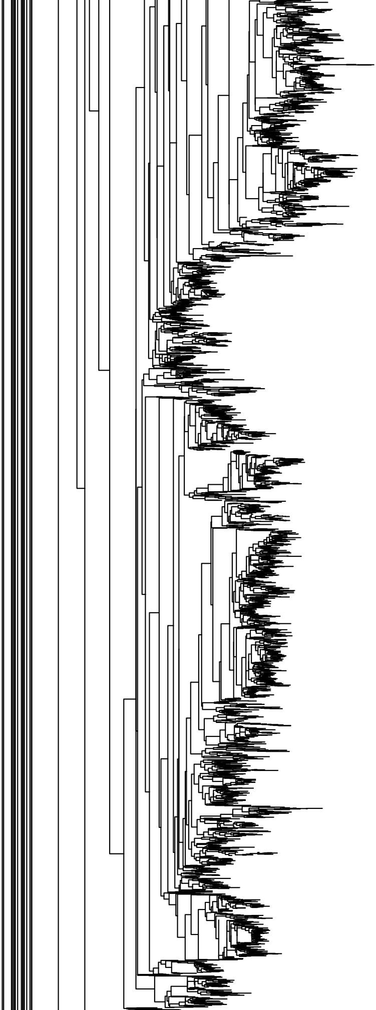

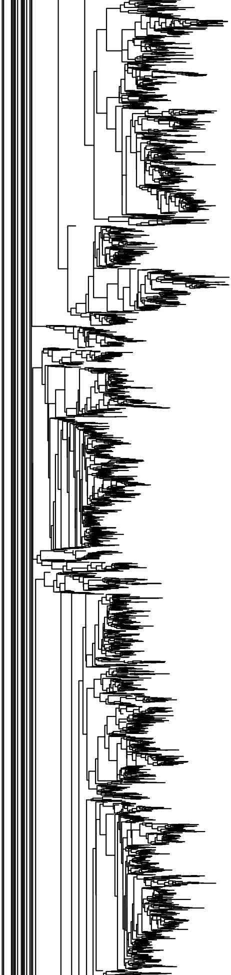

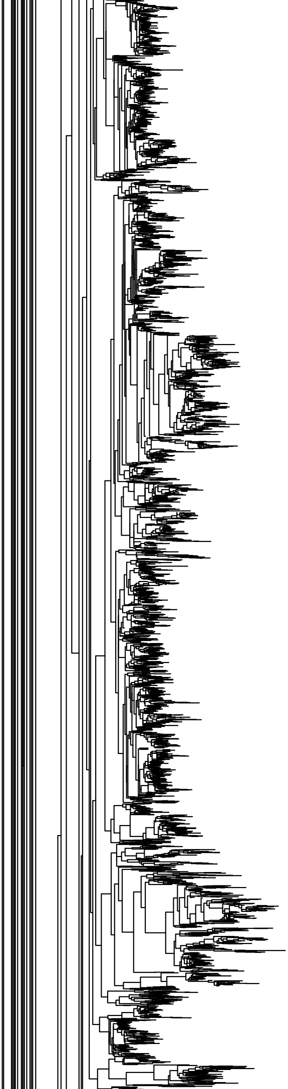

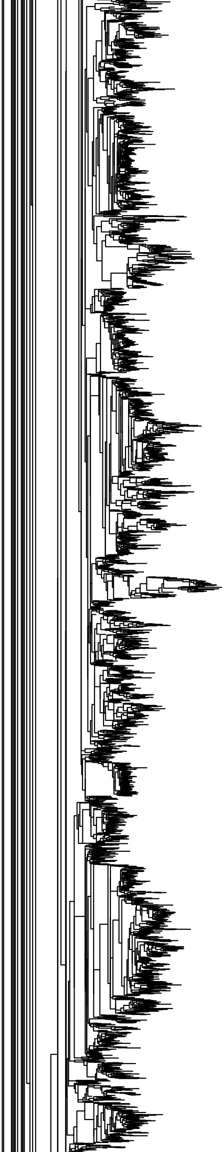

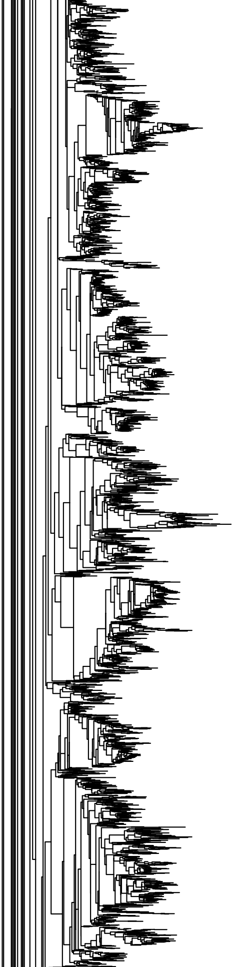

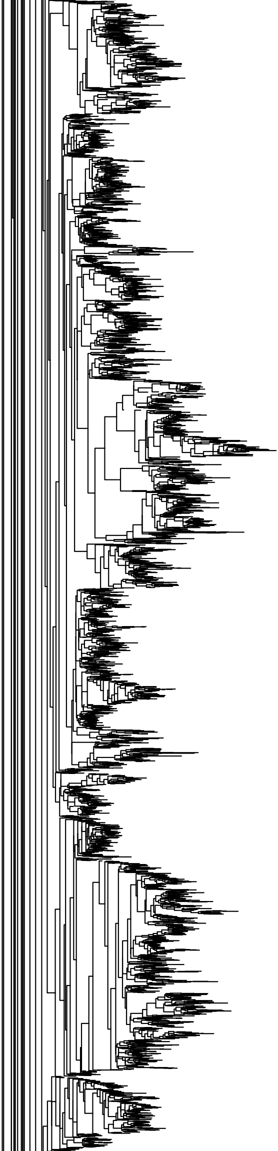

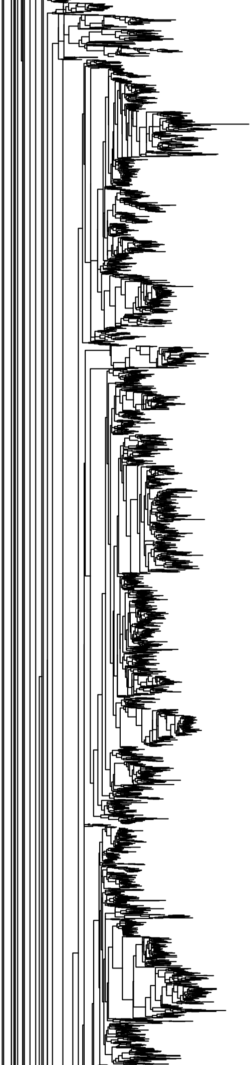

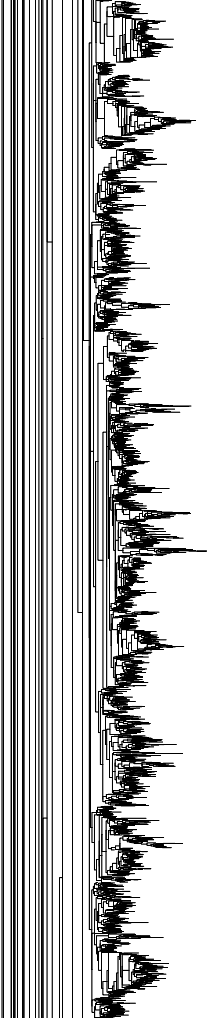

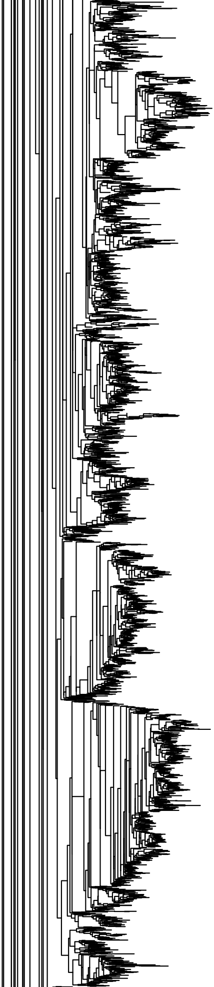

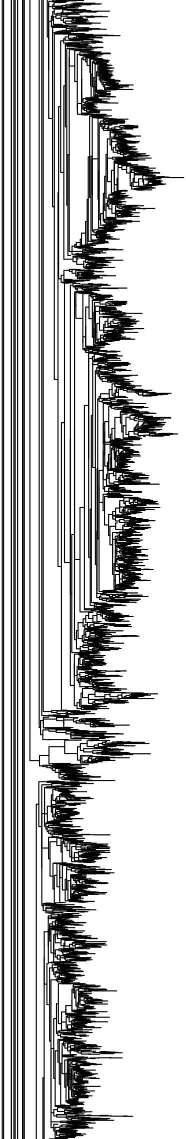

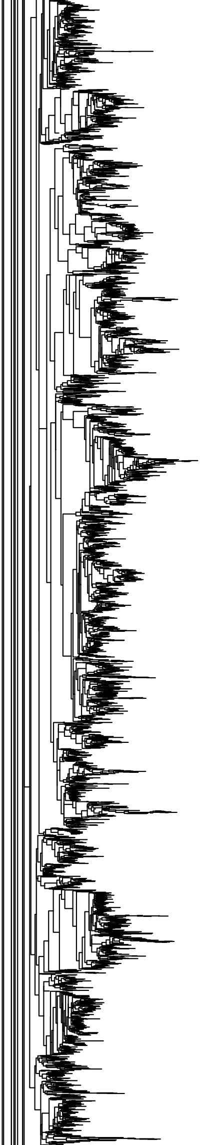

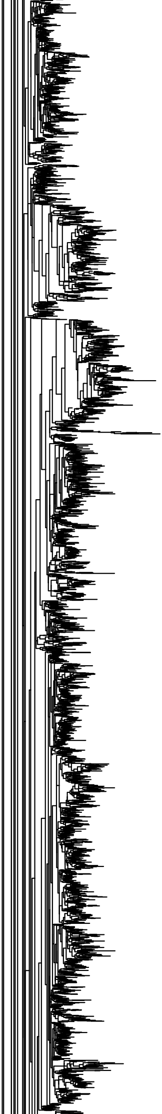

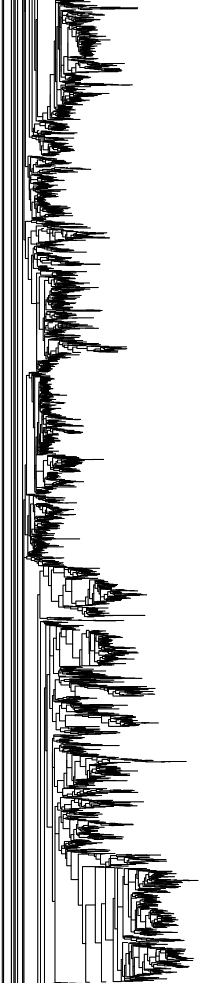

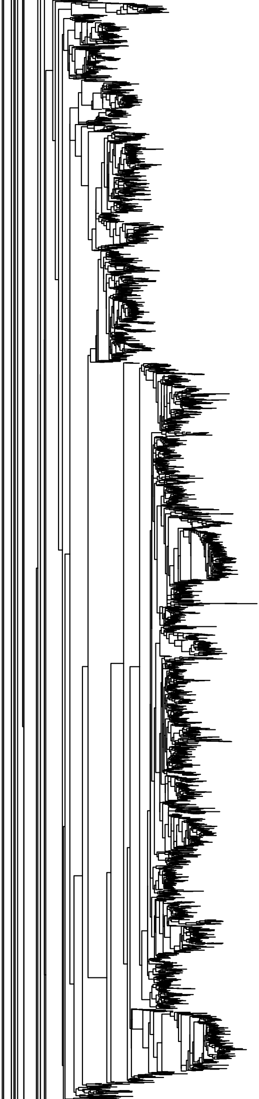

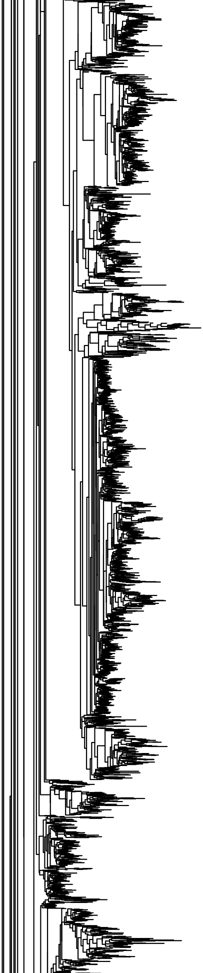

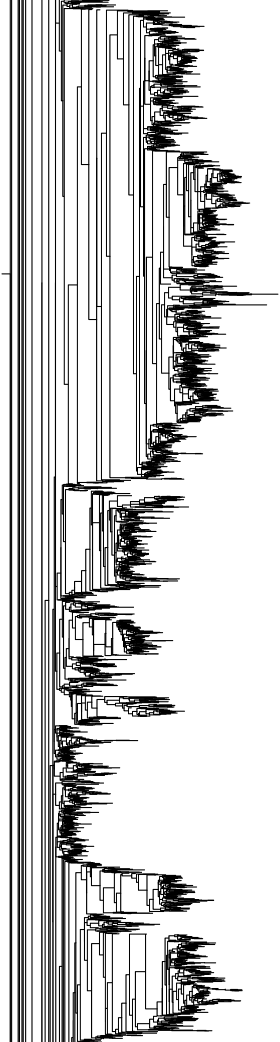

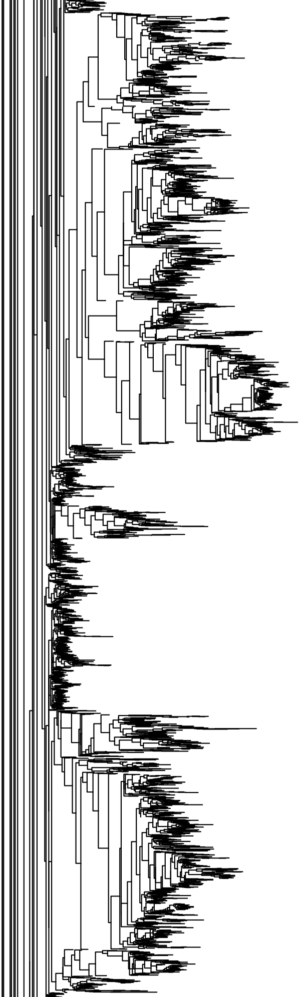

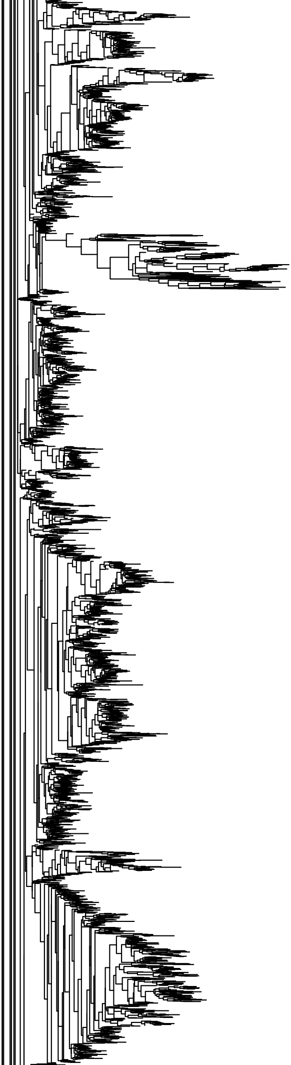

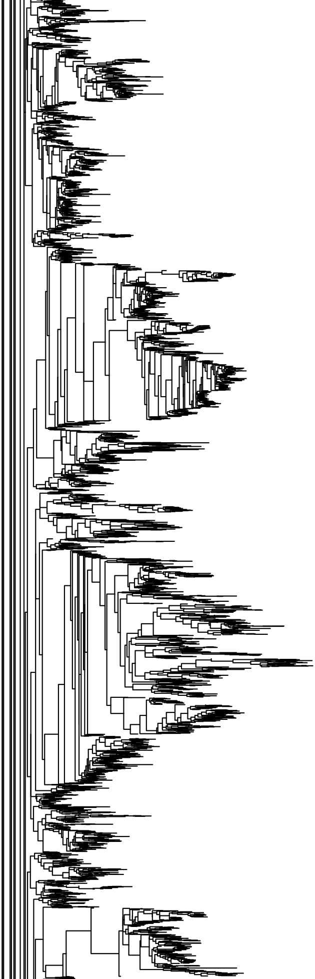

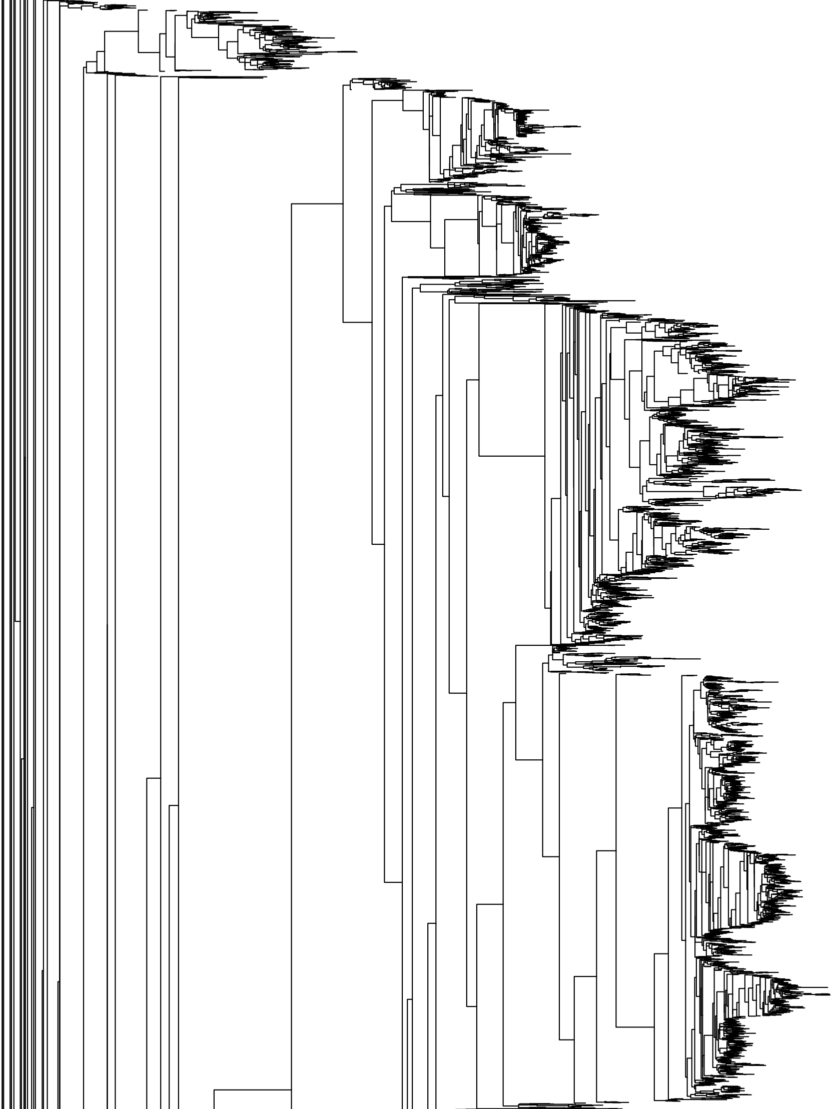

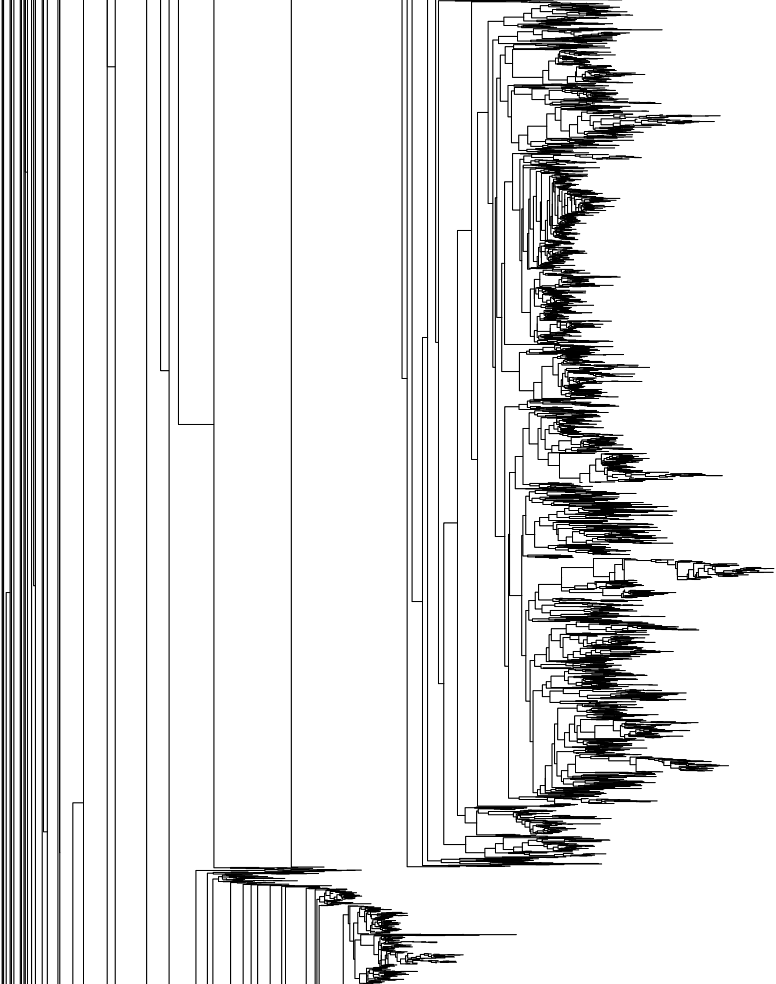

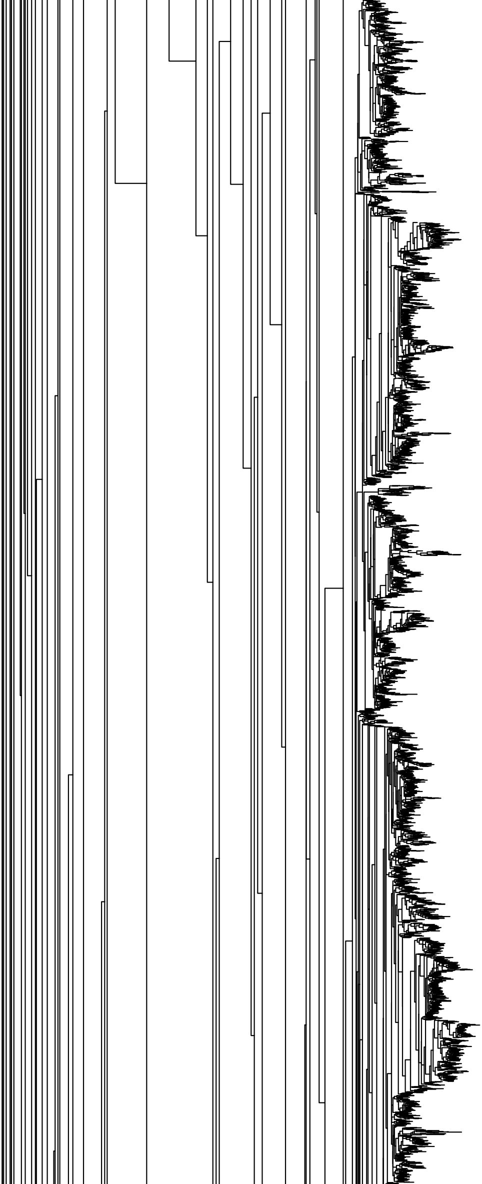

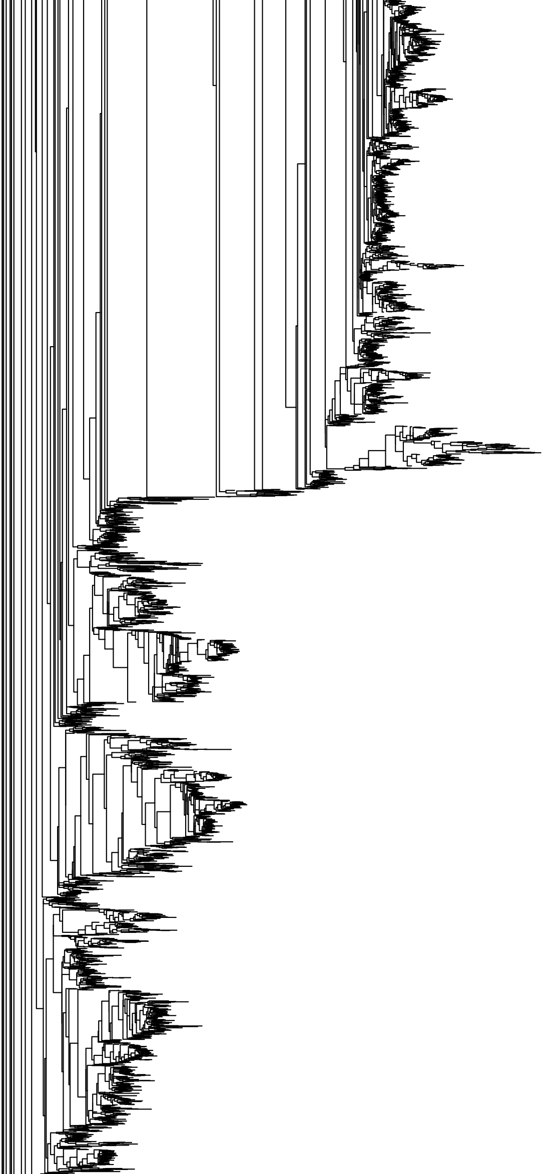

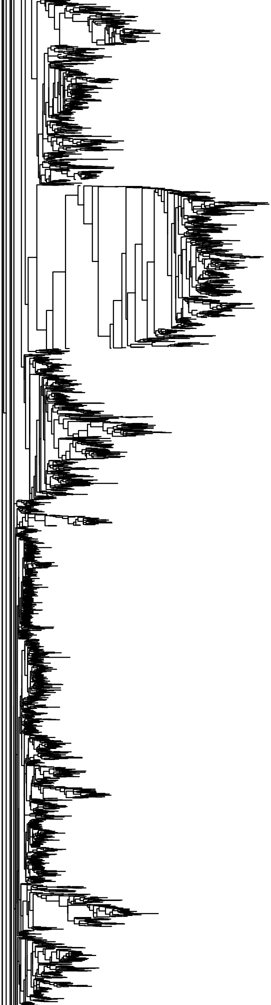

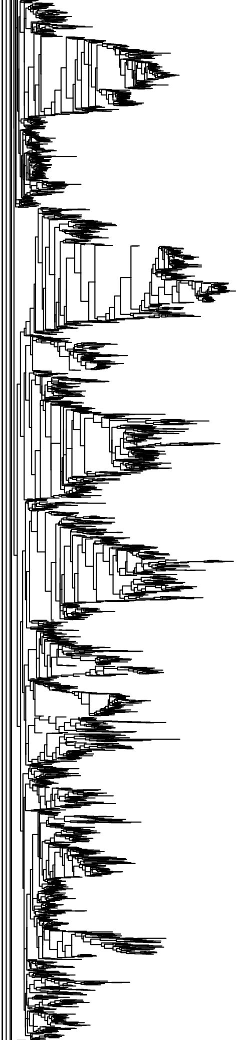

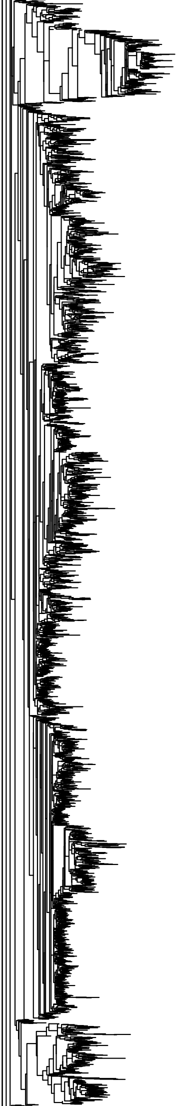

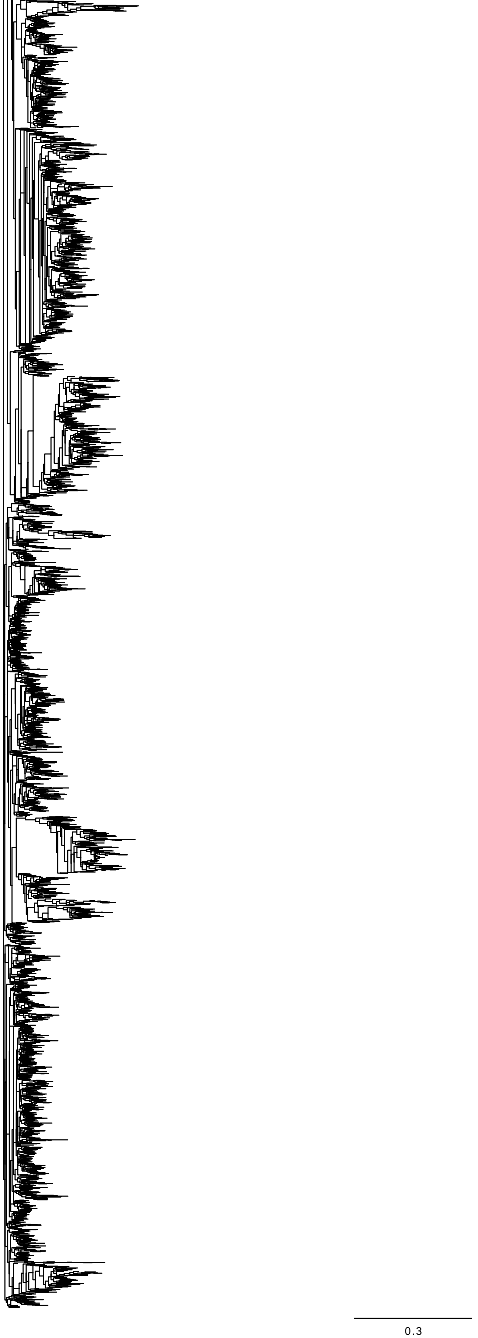
0.3
