# Supplementary material for: Polymetallic nodules, sediments, and deep waters in the equatorial North Pacific exhibit highly diverse and distinct bacterial, archaeal, and microeukaryotic communities
Source: Microbiologyopen. 2016 Nov 21;6(2):e00428. doi: 10.1002/mbo3.428 (PMC5387330; doi:10.1002/mbo3.428)
Supplement: Supplementary file 9 [file MBO3-6-na-s009.pdf]

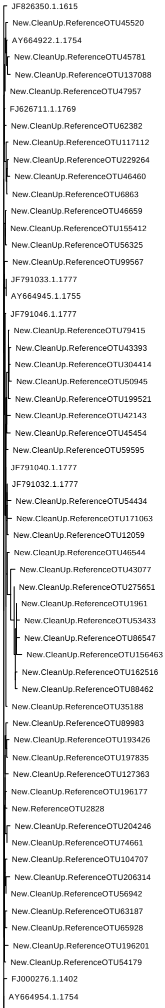
JF826350.1.1615
New.CleanUp.ReferenceOTU45520
AY664922.1.1754
New.CleanUp.ReferenceOTU45781
New.CleanUp.ReferenceOTU137088
New.CleanUp.ReferenceOTU47957
FJ626711.1.1769
New.CleanUp.ReferenceOTU62382
New.CleanUp.ReferenceOTU117112
New.CleanUp.ReferenceOTU229264
New.CleanUp.ReferenceOTU46460
New.CleanUp.ReferenceOTU6863
New.CleanUp.ReferenceOTU46659
New.CleanUp.ReferenceOTU155412
New.CleanUp.ReferenceOTU56325
New.CleanUp.ReferenceOTU99567
JF791033.1.1777
AY664945.1.1755
JF791046.1.1777
New.CleanUp.ReferenceOTU79415
New.CleanUp.ReferenceOTU43393
New.CleanUp.ReferenceOTU304414
New.CleanUp.ReferenceOTU50945
New.CleanUp.ReferenceOTU199521
New.CleanUp.ReferenceOTU42143
New.CleanUp.ReferenceOTU45454
New.CleanUp.ReferenceOTU59595
JF791040.1.1777
JF791032.1.1777
New.CleanUp.ReferenceOTU54434
New.CleanUp.ReferenceOTU171063
New.CleanUp.ReferenceOTU12059
New.CleanUp.ReferenceOTU46544
New.CleanUp.ReferenceOTU43077
New.CleanUp.ReferenceOTU275651
New.CleanUp.ReferenceOTU1961
New.CleanUp.ReferenceOTU53433
New.CleanUp.ReferenceOTU86547
New.CleanUp.ReferenceOTU156463
New.CleanUp.ReferenceOTU162516
New.CleanUp.ReferenceOTU88462
New.CleanUp.ReferenceOTU35188
New.CleanUp.ReferenceOTU89983
New.CleanUp.ReferenceOTU193426
New.CleanUp.ReferenceOTU197835
New.CleanUp.ReferenceOTU127363
New.CleanUp.ReferenceOTU196177
New.ReferenceOTU2828
New.CleanUp.ReferenceOTU204246
New.CleanUp.ReferenceOTU74661
New.CleanUp.ReferenceOTU104707
New.CleanUp.ReferenceOTU206314
New.CleanUp.ReferenceOTU56942
New.CleanUp.ReferenceOTU63187
New.CleanUp.ReferenceOTU65928
New.CleanUp.ReferenceOTU196201
New.CleanUp.ReferenceOTU54179
FJ000276.1.1402
AY664954.1.1754

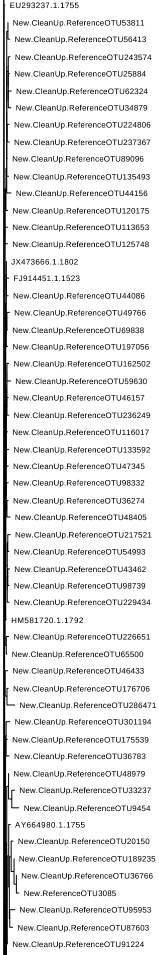
EU293237.1.1755
New.CleanUp.ReferenceOTU53811
New.CleanUp.ReferenceOTU56413
New.CleanUp.ReferenceOTU243574
New.CleanUp.ReferenceOTU25884
New.CleanUp.ReferenceOTU62324
New.CleanUp.ReferenceOTU34879
New.CleanUp.ReferenceOTU224806
New.CleanUp.ReferenceOTU237367
New.CleanUp.ReferenceOTU89096
New.CleanUp.ReferenceOTU135493
New.CleanUp.ReferenceOTU44156
New.CleanUp.ReferenceOTU120175
New.CleanUp.ReferenceOTU113653
New.CleanUp.ReferenceOTU125748
JX473666.1.1802
FJ914451.1.1523
New.CleanUp.ReferenceOTU44086
New.CleanUp.ReferenceOTU49766
New.CleanUp.ReferenceOTU69838
New.CleanUp.ReferenceOTU197056
New.CleanUp.ReferenceOTU162502
New.CleanUp.ReferenceOTU59630
New.CleanUp.ReferenceOTU46157
New.CleanUp.ReferenceOTU236249
New.CleanUp.ReferenceOTU116017
New.CleanUp.ReferenceOTU133592
New.CleanUp.ReferenceOTU47345
New.CleanUp.ReferenceOTU98332
New.CleanUp.ReferenceOTU36274
New.CleanUp.ReferenceOTU48405
New.CleanUp.ReferenceOTU217521
New.CleanUp.ReferenceOTU54993
New.CleanUp.ReferenceOTU43462
New.CleanUp.ReferenceOTU98739
New.CleanUp.ReferenceOTU229434
HM581720.1.1792
New.CleanUp.ReferenceOTU226651
New.CleanUp.ReferenceOTU65500
New.CleanUp.ReferenceOTU46433
New.CleanUp.ReferenceOTU176706
New.CleanUp.ReferenceOTU286471
New.CleanUp.ReferenceOTU301194
New.CleanUp.ReferenceOTU175539
New.CleanUp.ReferenceOTU36783
New.CleanUp.ReferenceOTU48979
New.CleanUp.ReferenceOTU33237
New.CleanUp.ReferenceOTU9454
AY664980.1.1755
New.CleanUp.ReferenceOTU20150
New.CleanUp.ReferenceOTU189235
New.CleanUp.ReferenceOTU36766
New.ReferenceOTU3085
New.CleanUp.ReferenceOTU95953
New.CleanUp.ReferenceOTU87603
New.CleanUp.ReferenceOTU91224

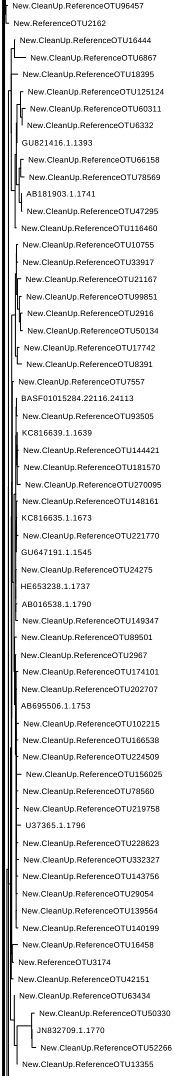
New.CleanUp.ReferenceOTU96457
New.ReferenceOTU2162
New.CleanUp.ReferenceOTU16444
New.CleanUp.ReferenceOTU6867
New.CleanUp.ReferenceOTU18395
New.CleanUp.ReferenceOTU125124
New.CleanUp.ReferenceOTU60311
New.CleanUp.ReferenceOTU6332
GU821416.1.1393
New.CleanUp.ReferenceOTU66158
New.CleanUp.ReferenceOTU78569
AB181903.1.1741
New.CleanUp.ReferenceOTU47295
New.CleanUp.ReferenceOTU116460
New.CleanUp.ReferenceOTU10755
New.CleanUp.ReferenceOTU33917
New.CleanUp.ReferenceOTU21167
New.CleanUp.ReferenceOTU99851
New.CleanUp.ReferenceOTU2916
New.CleanUp.ReferenceOTU50134
New.CleanUp.ReferenceOTU17742
New.CleanUp.ReferenceOTU8391
New.CleanUp.ReferenceOTU7557
BASF01015284.22116.24113
New.CleanUp.ReferenceOTU93505
KC816639.1.1639
New.CleanUp.ReferenceOTU144421
New.CleanUp.ReferenceOTU181570
New.CleanUp.ReferenceOTU270095
New.CleanUp.ReferenceOTU148161
KC816635.1.1673
New.CleanUp.ReferenceOTU221770
GU647191.1.1545
New.CleanUp.ReferenceOTU24275
HE653238.1.1737
AB016538.1.1790
New.CleanUp.ReferenceOTU149347
New.CleanUp.ReferenceOTU89501
New.CleanUp.ReferenceOTU2967
New.CleanUp.ReferenceOTU174101
New.CleanUp.ReferenceOTU202707
AB695506.1.1753
New.CleanUp.ReferenceOTU102215
New.CleanUp.ReferenceOTU166538
New.CleanUp.ReferenceOTU224509
New.CleanUp.ReferenceOTU156025
New.CleanUp.ReferenceOTU78560
New.CleanUp.ReferenceOTU219758
U37365.1.1796
New.CleanUp.ReferenceOTU228623
New.CleanUp.ReferenceOTU332327
New.CleanUp.ReferenceOTU143756
New.CleanUp.ReferenceOTU29054
New.CleanUp.ReferenceOTU139564
New.CleanUp.ReferenceOTU140199
New.CleanUp.ReferenceOTU16458
New.ReferenceOTU3174
New.CleanUp.ReferenceOTU42151
New.CleanUp.ReferenceOTU63434
New.CleanUp.ReferenceOTU50330
JN832709.1.1770
New.CleanUp.ReferenceOTU52266
New.CleanUp.ReferenceOTU13355

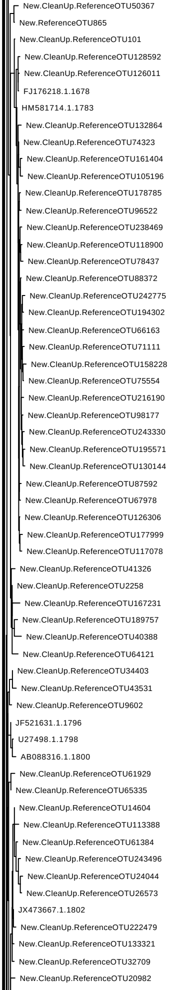
New.CleanUp.ReferenceOTU50367
New.ReferenceOTU865
New.CleanUp.ReferenceOTU101
New.CleanUp.ReferenceOTU128592
New.CleanUp.ReferenceOTU126011
FJ176218.1.1678
HM581714.1.1783
New.CleanUp.ReferenceOTU132864
New.CleanUp.ReferenceOTU74323
New.CleanUp.ReferenceOTU161404
New.CleanUp.ReferenceOTU105196
New.CleanUp.ReferenceOTU178785
New.CleanUp.ReferenceOTU96522
New.CleanUp.ReferenceOTU238469
New.CleanUp.ReferenceOTU118900
New.CleanUp.ReferenceOTU78437
New.CleanUp.ReferenceOTU88372
New.CleanUp.ReferenceOTU242775
New.CleanUp.ReferenceOTU194302
New.CleanUp.ReferenceOTU66163
New.CleanUp.ReferenceOTU71111
New.CleanUp.ReferenceOTU158228
New.CleanUp.ReferenceOTU75554
New.CleanUp.ReferenceOTU216190
New.CleanUp.ReferenceOTU98177
New.CleanUp.ReferenceOTU243330
New.CleanUp.ReferenceOTU195571
New.CleanUp.ReferenceOTU130144
New.CleanUp.ReferenceOTU87592
New.CleanUp.ReferenceOTU67978
New.CleanUp.ReferenceOTU126306
New.CleanUp.ReferenceOTU177999
New.CleanUp.ReferenceOTU117078
New.CleanUp.ReferenceOTU41326
New.CleanUp.ReferenceOTU2258
New.CleanUp.ReferenceOTU167231
New.CleanUp.ReferenceOTU189757
New.CleanUp.ReferenceOTU40388
New.CleanUp.ReferenceOTU64121
New.CleanUp.ReferenceOTU34403
New.CleanUp.ReferenceOTU43531
New.CleanUp.ReferenceOTU9602
JF521631.1.1796
U27498.1.1798
AB088316.1.1800
New.CleanUp.ReferenceOTU61929
New.CleanUp.ReferenceOTU65335
New.CleanUp.ReferenceOTU14604
New.CleanUp.ReferenceOTU113388
New.CleanUp.ReferenceOTU61384
New.CleanUp.ReferenceOTU243496
New.CleanUp.ReferenceOTU24044
New.CleanUp.ReferenceOTU26573
JX473667.1.1802
New.CleanUp.ReferenceOTU222479
New.CleanUp.ReferenceOTU133321
New.CleanUp.ReferenceOTU32709
New.CleanUp.ReferenceOTU20982

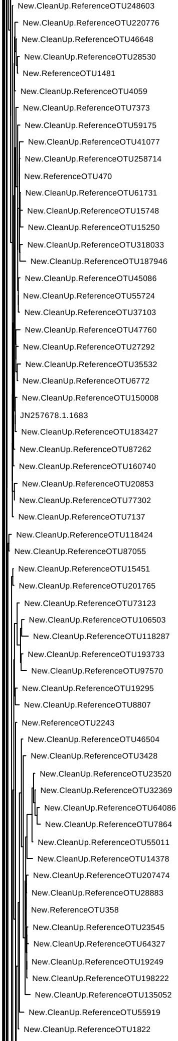
New.CleanUp.ReferenceOTU248603
New.CleanUp.ReferenceOTU220776
New.CleanUp.ReferenceOTU46648
New.CleanUp.ReferenceOTU28530
New.ReferenceOTU1481
New.CleanUp.ReferenceOTU4059
New.CleanUp.ReferenceOTU7373
New.CleanUp.ReferenceOTU59175
New.CleanUp.ReferenceOTU41077
New.CleanUp.ReferenceOTU258714
New.ReferenceOTU470
New.CleanUp.ReferenceOTU61731
New.CleanUp.ReferenceOTU15748
New.CleanUp.ReferenceOTU15250
New.CleanUp.ReferenceOTU318033
New.CleanUp.ReferenceOTU187946
New.CleanUp.ReferenceOTU45086
New.CleanUp.ReferenceOTU55724
New.CleanUp.ReferenceOTU37103
New.CleanUp.ReferenceOTU47760
New.CleanUp.ReferenceOTU27292
New.CleanUp.ReferenceOTU35532
New.CleanUp.ReferenceOTU6772
New.CleanUp.ReferenceOTU150008
JN257678.1.1683
New.CleanUp.ReferenceOTU183427
New.CleanUp.ReferenceOTU87262
New.CleanUp.ReferenceOTU160740
New.CleanUp.ReferenceOTU20853
New.CleanUp.ReferenceOTU77302
New.CleanUp.ReferenceOTU7137
New.CleanUp.ReferenceOTU118424
New.CleanUp.ReferenceOTU87055
New.CleanUp.ReferenceOTU15451
New.CleanUp.ReferenceOTU201765
New.CleanUp.ReferenceOTU73123
New.CleanUp.ReferenceOTU106503
New.CleanUp.ReferenceOTU118287
New.CleanUp.ReferenceOTU193733
New.CleanUp.ReferenceOTU97570
New.CleanUp.ReferenceOTU19295
New.CleanUp.ReferenceOTU8807
New.ReferenceOTU2243
New.CleanUp.ReferenceOTU46504
New.CleanUp.ReferenceOTU3428
New.CleanUp.ReferenceOTU23520
New.CleanUp.ReferenceOTU32369
New.CleanUp.ReferenceOTU64086
New.CleanUp.ReferenceOTU7864
New.CleanUp.ReferenceOTU55011
New.CleanUp.ReferenceOTU14378
New.CleanUp.ReferenceOTU207474
New.CleanUp.ReferenceOTU28883
New.ReferenceOTU358
New.CleanUp.ReferenceOTU23545
New.CleanUp.ReferenceOTU64327
New.CleanUp.ReferenceOTU19249
New.CleanUp.ReferenceOTU198222
New.CleanUp.ReferenceOTU135052
New.CleanUp.ReferenceOTU55919
New.CleanUp.ReferenceOTU1822

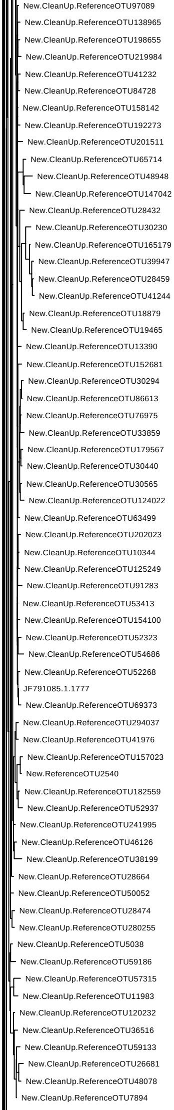
New.CleanUp.ReferenceOTU97089
New.CleanUp.ReferenceOTU138965
New.CleanUp.ReferenceOTU198655
New.CleanUp.ReferenceOTU219984
New.CleanUp.ReferenceOTU41232
New.CleanUp.ReferenceOTU84728
New.CleanUp.ReferenceOTU158142
New.CleanUp.ReferenceOTU192273
New.CleanUp.ReferenceOTU201511
New.CleanUp.ReferenceOTU65714
New.CleanUp.ReferenceOTU48948
New.CleanUp.ReferenceOTU147042
New.CleanUp.ReferenceOTU28432
New.CleanUp.ReferenceOTU30230
New.CleanUp.ReferenceOTU165179
New.CleanUp.ReferenceOTU39947
New.CleanUp.ReferenceOTU28459
New.CleanUp.ReferenceOTU41244
New.CleanUp.ReferenceOTU18879
New.CleanUp.ReferenceOTU19465
New.CleanUp.ReferenceOTU13390
New.CleanUp.ReferenceOTU152681
New.CleanUp.ReferenceOTU30294
New.CleanUp.ReferenceOTU86613
New.CleanUp.ReferenceOTU76975
New.CleanUp.ReferenceOTU33859
New.CleanUp.ReferenceOTU179567
New.CleanUp.ReferenceOTU30440
New.CleanUp.ReferenceOTU30565
New.CleanUp.ReferenceOTU124022
New.CleanUp.ReferenceOTU63499
New.CleanUp.ReferenceOTU202023
New.CleanUp.ReferenceOTU10344
New.CleanUp.ReferenceOTU125249
New.CleanUp.ReferenceOTU91283
New.CleanUp.ReferenceOTU53413
New.CleanUp.ReferenceOTU154100
New.CleanUp.ReferenceOTU52323
New.CleanUp.ReferenceOTU54686
New.CleanUp.ReferenceOTU52268
JF791085.1.1777
New.CleanUp.ReferenceOTU69373
New.CleanUp.ReferenceOTU294037
New.CleanUp.ReferenceOTU41976
New.CleanUp.ReferenceOTU157023
New.ReferenceOTU2540
New.CleanUp.ReferenceOTU182559
New.CleanUp.ReferenceOTU52937
New.CleanUp.ReferenceOTU241995
New.CleanUp.ReferenceOTU46126
New.CleanUp.ReferenceOTU38199
New.CleanUp.ReferenceOTU28664
New.CleanUp.ReferenceOTU50052
New.CleanUp.ReferenceOTU28474
New.CleanUp.ReferenceOTU280255
New.CleanUp.ReferenceOTU5038
New.CleanUp.ReferenceOTU59186
New.CleanUp.ReferenceOTU57315
New.CleanUp.ReferenceOTU11983
New.CleanUp.ReferenceOTU120232
New.CleanUp.ReferenceOTU36516
New.CleanUp.ReferenceOTU59133
New.CleanUp.ReferenceOTU26681
New.CleanUp.ReferenceOTU48078
New.CleanUp.ReferenceOTU7894

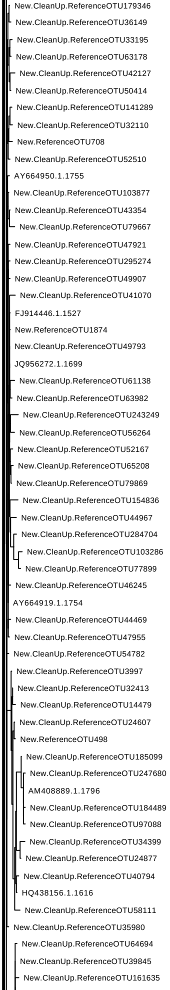
New.CleanUp.ReferenceOTU179346
New.CleanUp.ReferenceOTU36149
New.CleanUp.ReferenceOTU33195
New.CleanUp.ReferenceOTU63178
New.CleanUp.ReferenceOTU42127
New.CleanUp.ReferenceOTU50414
New.CleanUp.ReferenceOTU141289
New.CleanUp.ReferenceOTU32110
New.ReferenceOTU708
New.CleanUp.ReferenceOTU52510
AY664950.1.1755
New.CleanUp.ReferenceOTU103877
New.CleanUp.ReferenceOTU43354
New.CleanUp.ReferenceOTU79667
New.CleanUp.ReferenceOTU47921
New.CleanUp.ReferenceOTU295274
New.CleanUp.ReferenceOTU49907
New.CleanUp.ReferenceOTU41070
FJ914446.1.1527
New.ReferenceOTU1874
New.CleanUp.ReferenceOTU49793
JQ956272.1.1699
New.CleanUp.ReferenceOTU61138
New.CleanUp.ReferenceOTU63982
New.CleanUp.ReferenceOTU243249
New.CleanUp.ReferenceOTU56264
New.CleanUp.ReferenceOTU52167
New.CleanUp.ReferenceOTU65208
New.CleanUp.ReferenceOTU79869
New.CleanUp.ReferenceOTU154836
New.CleanUp.ReferenceOTU44967
New.CleanUp.ReferenceOTU284704
New.CleanUp.ReferenceOTU103286
New.CleanUp.ReferenceOTU77899
New.CleanUp.ReferenceOTU46245
AY664919.1.1754
New.CleanUp.ReferenceOTU44469
New.CleanUp.ReferenceOTU47955
New.CleanUp.ReferenceOTU54782
New.CleanUp.ReferenceOTU3997
New.CleanUp.ReferenceOTU32413
New.CleanUp.ReferenceOTU14479
New.CleanUp.ReferenceOTU24607
New.ReferenceOTU498
New.CleanUp.ReferenceOTU185099
New.CleanUp.ReferenceOTU247680
AM408889.1.1796
New.CleanUp.ReferenceOTU184489
New.CleanUp.ReferenceOTU97088
New.CleanUp.ReferenceOTU34399
New.CleanUp.ReferenceOTU24877
New.CleanUp.ReferenceOTU40794
HQ438156.1.1616
New.CleanUp.ReferenceOTU58111
New.CleanUp.ReferenceOTU35980
New.CleanUp.ReferenceOTU64694
New.CleanUp.ReferenceOTU39845
New.CleanUp.ReferenceOTU161635

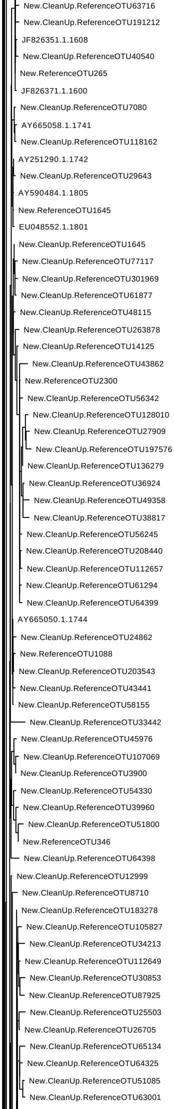
New.CleanUp.ReferenceOTU63716
New.CleanUp.ReferenceOTU191212
JF826351.1.1608
New.CleanUp.ReferenceOTU40540
New.ReferenceOTU265
JF826371.1.1600
New.CleanUp.ReferenceOTU7080
AY665058.1.1741
New.CleanUp.ReferenceOTU118162
AY251290.1.1742
New.CleanUp.ReferenceOTU29643
AY590484.1.1805
New.ReferenceOTU1645
EU048552.1.1801
New.CleanUp.ReferenceOTU1645
New.CleanUp.ReferenceOTU77117
New.CleanUp.ReferenceOTU301969
New.CleanUp.ReferenceOTU61877
New.CleanUp.ReferenceOTU48115
New.CleanUp.ReferenceOTU263878
New.CleanUp.ReferenceOTU14125
New.CleanUp.ReferenceOTU43862
New.ReferenceOTU2300
New.CleanUp.ReferenceOTU56342
New.CleanUp.ReferenceOTU128010
New.CleanUp.ReferenceOTU27909
New.CleanUp.ReferenceOTU197576
New.CleanUp.ReferenceOTU136279
New.CleanUp.ReferenceOTU36924
New.CleanUp.ReferenceOTU49358
New.CleanUp.ReferenceOTU38817
New.CleanUp.ReferenceOTU56245
New.CleanUp.ReferenceOTU208440
New.CleanUp.ReferenceOTU112657
New.CleanUp.ReferenceOTU61294
New.CleanUp.ReferenceOTU64399
AY665050.1.1744
New.CleanUp.ReferenceOTU24862
New.ReferenceOTU1088
New.CleanUp.ReferenceOTU203543
New.CleanUp.ReferenceOTU43441
New.CleanUp.ReferenceOTU58155
New.CleanUp.ReferenceOTU33442
New.CleanUp.ReferenceOTU45976
New.CleanUp.ReferenceOTU107069
New.CleanUp.ReferenceOTU3900
New.CleanUp.ReferenceOTU54330
New.CleanUp.ReferenceOTU39960
New.CleanUp.ReferenceOTU51800
New.ReferenceOTU346
New.CleanUp.ReferenceOTU64398
New.CleanUp.ReferenceOTU12999
New.CleanUp.ReferenceOTU8710
New.CleanUp.ReferenceOTU183278
New.CleanUp.ReferenceOTU105827
New.CleanUp.ReferenceOTU34213
New.CleanUp.ReferenceOTU112649
New.CleanUp.ReferenceOTU30853
New.CleanUp.ReferenceOTU87925
New.CleanUp.ReferenceOTU25503
New.CleanUp.ReferenceOTU26705
New.CleanUp.ReferenceOTU65134
New.CleanUp.ReferenceOTU64325
New.CleanUp.ReferenceOTU51085
New.CleanUp.ReferenceOTU63001

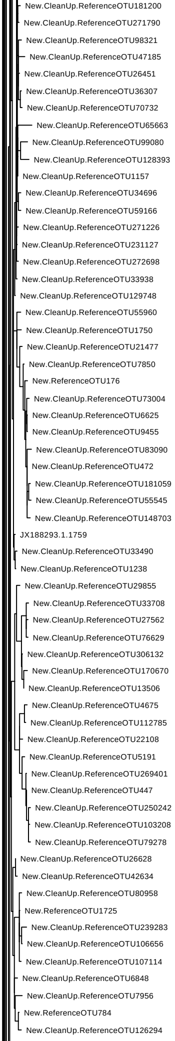
New.CleanUp.ReferenceOTU181200
New.CleanUp.ReferenceOTU271790
New.CleanUp.ReferenceOTU98321
New.CleanUp.ReferenceOTU47185
New.CleanUp.ReferenceOTU26451
New.CleanUp.ReferenceOTU36307
New.CleanUp.ReferenceOTU70732
New.CleanUp.ReferenceOTU65663
New.CleanUp.ReferenceOTU99080
New.CleanUp.ReferenceOTU128393
New.CleanUp.ReferenceOTU1157
New.CleanUp.ReferenceOTU34696
New.CleanUp.ReferenceOTU59166
New.CleanUp.ReferenceOTU271226
New.CleanUp.ReferenceOTU231127
New.CleanUp.ReferenceOTU272698
New.CleanUp.ReferenceOTU33938
New.CleanUp.ReferenceOTU129748
New.CleanUp.ReferenceOTU55960
New.CleanUp.ReferenceOTU1750
New.CleanUp.ReferenceOTU21477
New.CleanUp.ReferenceOTU7850
New.ReferenceOTU176
New.CleanUp.ReferenceOTU73004
New.CleanUp.ReferenceOTU6625
New.CleanUp.ReferenceOTU9455
New.CleanUp.ReferenceOTU83090
New.CleanUp.ReferenceOTU472
New.CleanUp.ReferenceOTU181059
New.CleanUp.ReferenceOTU55545
New.CleanUp.ReferenceOTU148703
JX188293.1.1759
New.CleanUp.ReferenceOTU33490
New.CleanUp.ReferenceOTU1238
New.CleanUp.ReferenceOTU29855
New.CleanUp.ReferenceOTU33708
New.CleanUp.ReferenceOTU27562
New.CleanUp.ReferenceOTU76629
New.CleanUp.ReferenceOTU306132
New.CleanUp.ReferenceOTU170670
New.CleanUp.ReferenceOTU13506
New.CleanUp.ReferenceOTU4675
New.CleanUp.ReferenceOTU112785
New.CleanUp.ReferenceOTU22108
New.CleanUp.ReferenceOTU5191
New.CleanUp.ReferenceOTU269401
New.CleanUp.ReferenceOTU447
New.CleanUp.ReferenceOTU250242
New.CleanUp.ReferenceOTU103208
New.CleanUp.ReferenceOTU79278
New.CleanUp.ReferenceOTU26628
New.CleanUp.ReferenceOTU42634
New.CleanUp.ReferenceOTU80958
New.ReferenceOTU1725
New.CleanUp.ReferenceOTU239283
New.CleanUp.ReferenceOTU106656
New.CleanUp.ReferenceOTU107114
New.CleanUp.ReferenceOTU6848
New.CleanUp.ReferenceOTU7956
New.ReferenceOTU784
New.CleanUp.ReferenceOTU126294

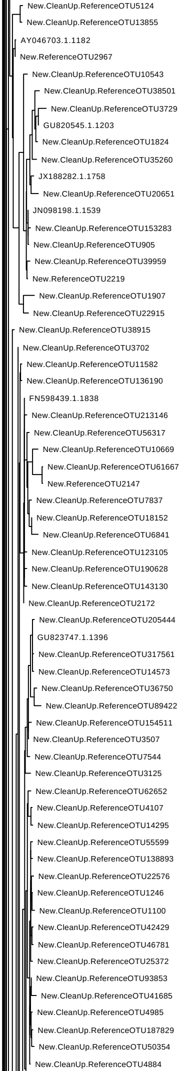
New.CleanUp.ReferenceOTU5124
New.CleanUp.ReferenceOTU13855
AY046703.1.1182
New.ReferenceOTU2967
New.CleanUp.ReferenceOTU10543
New.CleanUp.ReferenceOTU38501
New.CleanUp.ReferenceOTU3729
GU820545.1.1203
New.CleanUp.ReferenceOTU1824
New.CleanUp.ReferenceOTU35260
JX188282.1.1758
New.CleanUp.ReferenceOTU20651
JN098198.1.1539
New.CleanUp.ReferenceOTU153283
New.CleanUp.ReferenceOTU905
New.CleanUp.ReferenceOTU39959
New.ReferenceOTU2219
New.CleanUp.ReferenceOTU1907
New.CleanUp.ReferenceOTU22915
New.CleanUp.ReferenceOTU38915
New.CleanUp.ReferenceOTU3702
New.CleanUp.ReferenceOTU11582
New.CleanUp.ReferenceOTU136190
FN598439.1.1838
New.CleanUp.ReferenceOTU213146
New.CleanUp.ReferenceOTU56317
New.CleanUp.ReferenceOTU10669
New.CleanUp.ReferenceOTU61667
New.ReferenceOTU2147
New.CleanUp.ReferenceOTU7837
New.CleanUp.ReferenceOTU18152
New.CleanUp.ReferenceOTU6841
New.CleanUp.ReferenceOTU123105
New.CleanUp.ReferenceOTU190628
New.CleanUp.ReferenceOTU143130
New.CleanUp.ReferenceOTU2172
New.CleanUp.ReferenceOTU205444
GU823747.1.1396
New.CleanUp.ReferenceOTU317561
New.CleanUp.ReferenceOTU14573
New.CleanUp.ReferenceOTU36750
New.CleanUp.ReferenceOTU89422
New.CleanUp.ReferenceOTU154511
New.CleanUp.ReferenceOTU3507
New.CleanUp.ReferenceOTU7544
New.CleanUp.ReferenceOTU3125
New.CleanUp.ReferenceOTU62652
New.CleanUp.ReferenceOTU4107
New.CleanUp.ReferenceOTU14295
New.CleanUp.ReferenceOTU55599
New.CleanUp.ReferenceOTU138893
New.CleanUp.ReferenceOTU22576
New.CleanUp.ReferenceOTU1246
New.CleanUp.ReferenceOTU1100
New.CleanUp.ReferenceOTU42429
New.CleanUp.ReferenceOTU46781
New.CleanUp.ReferenceOTU25372
New.CleanUp.ReferenceOTU93853
New.CleanUp.ReferenceOTU41685
New.CleanUp.ReferenceOTU4985
New.CleanUp.ReferenceOTU187829
New.CleanUp.ReferenceOTU50354
New.CleanUp.ReferenceOTU4884

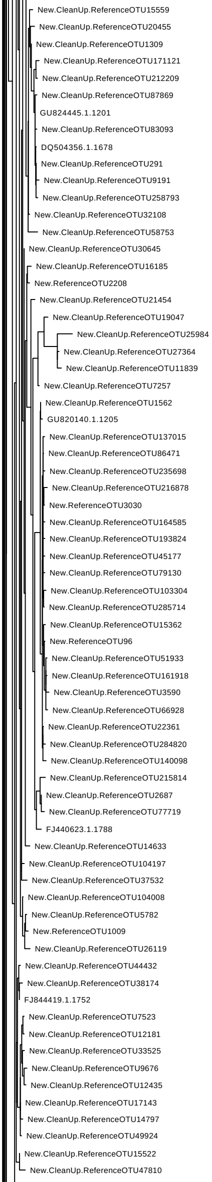
New.CleanUp.ReferenceOTU15559
New.CleanUp.ReferenceOTU20455
New.CleanUp.ReferenceOTU1309
New.CleanUp.ReferenceOTU171121
New.CleanUp.ReferenceOTU212209
New.CleanUp.ReferenceOTU87869
GU824445.1.1201
New.CleanUp.ReferenceOTU83093
DQ504356.1.1678
New.CleanUp.ReferenceOTU291
New.CleanUp.ReferenceOTU9191
New.CleanUp.ReferenceOTU258793
New.CleanUp.ReferenceOTU32108
New.CleanUp.ReferenceOTU58753
New.CleanUp.ReferenceOTU30645
New.CleanUp.ReferenceOTU16185
New.ReferenceOTU2208
New.CleanUp.ReferenceOTU21454
New.CleanUp.ReferenceOTU19047
New.CleanUp.ReferenceOTU25984
New.CleanUp.ReferenceOTU27364
New.CleanUp.ReferenceOTU11839
New.CleanUp.ReferenceOTU7257
New.CleanUp.ReferenceOTU1562
GU820140.1.1205
New.CleanUp.ReferenceOTU137015
New.CleanUp.ReferenceOTU86471
New.CleanUp.ReferenceOTU235698
New.CleanUp.ReferenceOTU216878
New.ReferenceOTU3030
New.CleanUp.ReferenceOTU164585
New.CleanUp.ReferenceOTU193824
New.CleanUp.ReferenceOTU45177
New.CleanUp.ReferenceOTU79130
New.CleanUp.ReferenceOTU103304
New.CleanUp.ReferenceOTU285714
New.CleanUp.ReferenceOTU15362
New.ReferenceOTU96
New.CleanUp.ReferenceOTU51933
New.CleanUp.ReferenceOTU161918
New.CleanUp.ReferenceOTU3590
New.CleanUp.ReferenceOTU66928
New.CleanUp.ReferenceOTU22361
New.CleanUp.ReferenceOTU284820
New.CleanUp.ReferenceOTU140098
New.CleanUp.ReferenceOTU215814
New.CleanUp.ReferenceOTU2687
New.CleanUp.ReferenceOTU77719
FJ440623.1.1788
New.CleanUp.ReferenceOTU14633
New.CleanUp.ReferenceOTU104197
New.CleanUp.ReferenceOTU37532
New.CleanUp.ReferenceOTU104008
New.CleanUp.ReferenceOTU5782
New.ReferenceOTU1009
New.CleanUp.ReferenceOTU26119
New.CleanUp.ReferenceOTU44432
New.CleanUp.ReferenceOTU38174
FJ844419.1.1752
New.CleanUp.ReferenceOTU7523
New.CleanUp.ReferenceOTU12181
New.CleanUp.ReferenceOTU33525
New.CleanUp.ReferenceOTU9676
New.CleanUp.ReferenceOTU12435
New.CleanUp.ReferenceOTU17143
New.CleanUp.ReferenceOTU14797
New.CleanUp.ReferenceOTU49924
New.CleanUp.ReferenceOTU15522
New.CleanUp.ReferenceOTU47810

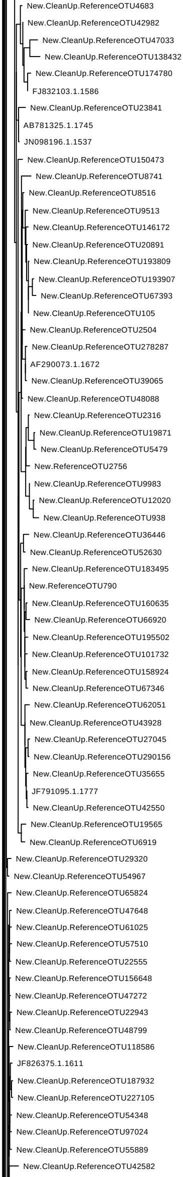
New.CleanUp.ReferenceOTU4683
New.CleanUp.ReferenceOTU42982
New.CleanUp.ReferenceOTU47033
New.CleanUp.ReferenceOTU138432
New.CleanUp.ReferenceOTU174780
FJ832103.1.1586
New.CleanUp.ReferenceOTU23841
AB781325.1.1745
JN098196.1.1537
New.CleanUp.ReferenceOTU150473
New.CleanUp.ReferenceOTU8741
New.CleanUp.ReferenceOTU8516
New.CleanUp.ReferenceOTU9513
New.CleanUp.ReferenceOTU146172
New.CleanUp.ReferenceOTU20891
New.CleanUp.ReferenceOTU193809
New.CleanUp.ReferenceOTU193907
New.CleanUp.ReferenceOTU67393
New.CleanUp.ReferenceOTU105
New.CleanUp.ReferenceOTU2504
New.CleanUp.ReferenceOTU278287
AF290073.1.1672
New.CleanUp.ReferenceOTU39065
New.CleanUp.ReferenceOTU48088
New.CleanUp.ReferenceOTU2316
New.CleanUp.ReferenceOTU19871
New.CleanUp.ReferenceOTU5479
New.ReferenceOTU2756
New.CleanUp.ReferenceOTU9983
New.CleanUp.ReferenceOTU12020
New.CleanUp.ReferenceOTU938
New.CleanUp.ReferenceOTU36446
New.CleanUp.ReferenceOTU52630
New.CleanUp.ReferenceOTU183495
New.ReferenceOTU790
New.CleanUp.ReferenceOTU160635
New.CleanUp.ReferenceOTU66920
New.CleanUp.ReferenceOTU195502
New.CleanUp.ReferenceOTU101732
New.CleanUp.ReferenceOTU158924
New.CleanUp.ReferenceOTU67346
New.CleanUp.ReferenceOTU62051
New.CleanUp.ReferenceOTU43928
New.CleanUp.ReferenceOTU27045
New.CleanUp.ReferenceOTU290156
New.CleanUp.ReferenceOTU35655
JF791095.1.1777
New.CleanUp.ReferenceOTU42550
New.CleanUp.ReferenceOTU19565
New.CleanUp.ReferenceOTU6919
New.CleanUp.ReferenceOTU29320
New.CleanUp.ReferenceOTU54967
New.CleanUp.ReferenceOTU65824
New.CleanUp.ReferenceOTU47648
New.CleanUp.ReferenceOTU61025
New.CleanUp.ReferenceOTU57510
New.CleanUp.ReferenceOTU22555
New.CleanUp.ReferenceOTU156648
New.CleanUp.ReferenceOTU47272
New.CleanUp.ReferenceOTU22943
New.CleanUp.ReferenceOTU48799
New.CleanUp.ReferenceOTU118586
JF826375.1.1611
New.CleanUp.ReferenceOTU187932
New.CleanUp.ReferenceOTU227105
New.CleanUp.ReferenceOTU54348
New.CleanUp.ReferenceOTU97024
New.CleanUp.ReferenceOTU55889
New.CleanUp.ReferenceOTU42582

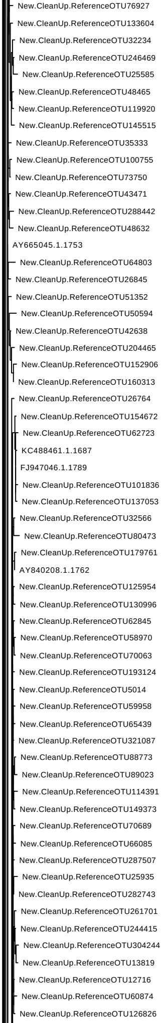
New.CleanUp.ReferenceOTU76927
New.CleanUp.ReferenceOTU133604
New.CleanUp.ReferenceOTU32234
New.CleanUp.ReferenceOTU246469
New.CleanUp.ReferenceOTU25585
New.CleanUp.ReferenceOTU48465
New.CleanUp.ReferenceOTU119920
New.CleanUp.ReferenceOTU145515
New.CleanUp.ReferenceOTU35333
New.CleanUp.ReferenceOTU100755
New.CleanUp.ReferenceOTU73750
New.CleanUp.ReferenceOTU43471
New.CleanUp.ReferenceOTU288442
New.CleanUp.ReferenceOTU48632
AY665045.1.1753
New.CleanUp.ReferenceOTU64803
New.CleanUp.ReferenceOTU26845
New.CleanUp.ReferenceOTU51352
New.CleanUp.ReferenceOTU50594
New.CleanUp.ReferenceOTU42638
New.CleanUp.ReferenceOTU204465
New.CleanUp.ReferenceOTU152906
New.CleanUp.ReferenceOTU160313
New.CleanUp.ReferenceOTU26764
New.CleanUp.ReferenceOTU154672
New.CleanUp.ReferenceOTU62723
KC488461.1.1687
FJ947046.1.1789
New.CleanUp.ReferenceOTU101836
New.CleanUp.ReferenceOTU137053
New.CleanUp.ReferenceOTU32566
New.CleanUp.ReferenceOTU80473
New.CleanUp.ReferenceOTU179761
AY840208.1.1762
New.CleanUp.ReferenceOTU125954
New.CleanUp.ReferenceOTU130996
New.CleanUp.ReferenceOTU62845
New.CleanUp.ReferenceOTU58970
New.CleanUp.ReferenceOTU70063
New.CleanUp.ReferenceOTU193124
New.CleanUp.ReferenceOTU5014
New.CleanUp.ReferenceOTU59958
New.CleanUp.ReferenceOTU65439
New.CleanUp.ReferenceOTU321087
New.CleanUp.ReferenceOTU88773
New.CleanUp.ReferenceOTU89023
New.CleanUp.ReferenceOTU114391
New.CleanUp.ReferenceOTU149373
New.CleanUp.ReferenceOTU70689
New.CleanUp.ReferenceOTU66085
New.CleanUp.ReferenceOTU287507
New.CleanUp.ReferenceOTU25935
New.CleanUp.ReferenceOTU282743
New.CleanUp.ReferenceOTU261701
New.CleanUp.ReferenceOTU244415
New.CleanUp.ReferenceOTU304244
New.CleanUp.ReferenceOTU13819
New.CleanUp.ReferenceOTU12716
New.CleanUp.ReferenceOTU60874
New.CleanUp.ReferenceOTU126826

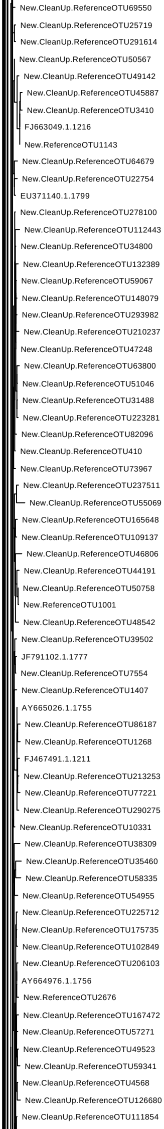
New.CleanUp.ReferenceOTU69550
New.CleanUp.ReferenceOTU25719
New.CleanUp.ReferenceOTU291614
New.CleanUp.ReferenceOTU50567
New.CleanUp.ReferenceOTU49142
New.CleanUp.ReferenceOTU45887
New.CleanUp.ReferenceOTU3410
FJ663049.1.1216
New.ReferenceOTU1143
New.CleanUp.ReferenceOTU64679
New.CleanUp.ReferenceOTU22754
EU371140.1.1799
New.CleanUp.ReferenceOTU278100
New.CleanUp.ReferenceOTU112443
New.CleanUp.ReferenceOTU34800
New.CleanUp.ReferenceOTU132389
New.CleanUp.ReferenceOTU59067
New.CleanUp.ReferenceOTU148079
New.CleanUp.ReferenceOTU293982
New.CleanUp.ReferenceOTU210237
New.CleanUp.ReferenceOTU47248
New.CleanUp.ReferenceOTU63800
New.CleanUp.ReferenceOTU51046
New.CleanUp.ReferenceOTU31488
New.CleanUp.ReferenceOTU223281
New.CleanUp.ReferenceOTU82096
New.CleanUp.ReferenceOTU410
New.CleanUp.ReferenceOTU73967
New.CleanUp.ReferenceOTU237511
New.CleanUp.ReferenceOTU55069
New.CleanUp.ReferenceOTU165648
New.CleanUp.ReferenceOTU109137
New.CleanUp.ReferenceOTU46806
New.CleanUp.ReferenceOTU44191
New.CleanUp.ReferenceOTU50758
New.ReferenceOTU1001
New.CleanUp.ReferenceOTU48542
New.CleanUp.ReferenceOTU39502
JF791102.1.1777
New.CleanUp.ReferenceOTU7554
New.CleanUp.ReferenceOTU1407
AY665026.1.1755
New.CleanUp.ReferenceOTU86187
New.CleanUp.ReferenceOTU1268
FJ467491.1.1211
New.CleanUp.ReferenceOTU213253
New.CleanUp.ReferenceOTU77221
New.CleanUp.ReferenceOTU290275
New.CleanUp.ReferenceOTU10331
New.CleanUp.ReferenceOTU38309
New.CleanUp.ReferenceOTU35460
New.CleanUp.ReferenceOTU58335
New.CleanUp.ReferenceOTU54955
New.CleanUp.ReferenceOTU225712
New.CleanUp.ReferenceOTU175735
New.CleanUp.ReferenceOTU102849
New.CleanUp.ReferenceOTU206103
AY664976.1.1756
New.ReferenceOTU2676
New.CleanUp.ReferenceOTU167472
New.CleanUp.ReferenceOTU57271
New.CleanUp.ReferenceOTU49523
New.CleanUp.ReferenceOTU59341
New.CleanUp.ReferenceOTU4568
New.CleanUp.ReferenceOTU126680
New.CleanUp.ReferenceOTU111854

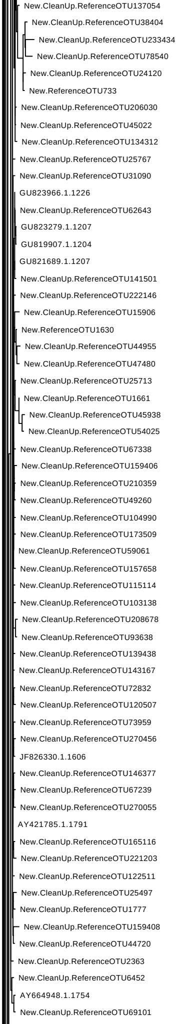
New.CleanUp.ReferenceOTU137054
New.CleanUp.ReferenceOTU38404
New.CleanUp.ReferenceOTU233434
New.CleanUp.ReferenceOTU78540
New.CleanUp.ReferenceOTU24120
New.ReferenceOTU733
New.CleanUp.ReferenceOTU206030
New.CleanUp.ReferenceOTU45022
New.CleanUp.ReferenceOTU134312
New.CleanUp.ReferenceOTU25767
New.CleanUp.ReferenceOTU31090
GU823966.1.1226
New.CleanUp.ReferenceOTU62643
GU823279.1.1207
GU819907.1.1204
GU821689.1.1207
New.CleanUp.ReferenceOTU141501
New.CleanUp.ReferenceOTU222146
New.CleanUp.ReferenceOTU15906
New.ReferenceOTU1630
New.CleanUp.ReferenceOTU44955
New.CleanUp.ReferenceOTU47480
New.CleanUp.ReferenceOTU25713
New.CleanUp.ReferenceOTU1661
New.CleanUp.ReferenceOTU45938
New.CleanUp.ReferenceOTU54025
New.CleanUp.ReferenceOTU67338
New.CleanUp.ReferenceOTU159406
New.CleanUp.ReferenceOTU210359
New.CleanUp.ReferenceOTU49260
New.CleanUp.ReferenceOTU104990
New.CleanUp.ReferenceOTU173509
New.CleanUp.ReferenceOTU59061
New.CleanUp.ReferenceOTU157658
New.CleanUp.ReferenceOTU115114
New.CleanUp.ReferenceOTU103138
New.CleanUp.ReferenceOTU208678
New.CleanUp.ReferenceOTU93638
New.CleanUp.ReferenceOTU139438
New.CleanUp.ReferenceOTU143167
New.CleanUp.ReferenceOTU72832
New.CleanUp.ReferenceOTU120507
New.CleanUp.ReferenceOTU73959
New.CleanUp.ReferenceOTU270456
JF826330.1.1606
New.CleanUp.ReferenceOTU146377
New.CleanUp.ReferenceOTU67239
New.CleanUp.ReferenceOTU270055
AY421785.1.1791
New.CleanUp.ReferenceOTU165116
New.CleanUp.ReferenceOTU221203
New.CleanUp.ReferenceOTU122511
New.CleanUp.ReferenceOTU25497
New.CleanUp.ReferenceOTU1777
New.CleanUp.ReferenceOTU159408
New.CleanUp.ReferenceOTU44720
New.CleanUp.ReferenceOTU2363
New.CleanUp.ReferenceOTU6452
AY664948.1.1754
New.CleanUp.ReferenceOTU69101

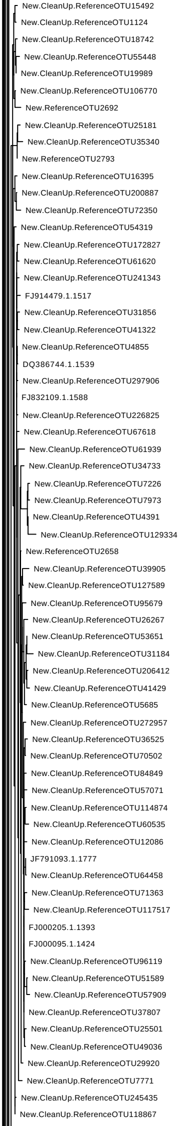
New.CleanUp.ReferenceOTU15492
New.CleanUp.ReferenceOTU1124
New.CleanUp.ReferenceOTU18742
New.CleanUp.ReferenceOTU55448
New.CleanUp.ReferenceOTU19989
New.CleanUp.ReferenceOTU106770
New.ReferenceOTU2692
New.CleanUp.ReferenceOTU25181
New.CleanUp.ReferenceOTU35340
New.ReferenceOTU2793
New.CleanUp.ReferenceOTU16395
New.CleanUp.ReferenceOTU200887
New.CleanUp.ReferenceOTU72350
New.CleanUp.ReferenceOTU54319
New.CleanUp.ReferenceOTU172827
New.CleanUp.ReferenceOTU61620
New.CleanUp.ReferenceOTU241343
FJ914479.1.1517
New.CleanUp.ReferenceOTU31856
New.CleanUp.ReferenceOTU41322
New.CleanUp.ReferenceOTU4855
DQ386744.1.1539
New.CleanUp.ReferenceOTU297906
FJ832109.1.1588
New.CleanUp.ReferenceOTU226825
New.CleanUp.ReferenceOTU67618
New.CleanUp.ReferenceOTU61939
New.CleanUp.ReferenceOTU34733
New.CleanUp.ReferenceOTU7226
New.CleanUp.ReferenceOTU7973
New.CleanUp.ReferenceOTU4391
New.CleanUp.ReferenceOTU129334
New.ReferenceOTU2658
New.CleanUp.ReferenceOTU39905
New.CleanUp.ReferenceOTU127589
New.CleanUp.ReferenceOTU95679
New.CleanUp.ReferenceOTU26267
New.CleanUp.ReferenceOTU53651
New.CleanUp.ReferenceOTU31184
New.CleanUp.ReferenceOTU206412
New.CleanUp.ReferenceOTU41429
New.CleanUp.ReferenceOTU5685
New.CleanUp.ReferenceOTU272957
New.CleanUp.ReferenceOTU36525
New.CleanUp.ReferenceOTU70502
New.CleanUp.ReferenceOTU84849
New.CleanUp.ReferenceOTU57071
New.CleanUp.ReferenceOTU114874
New.CleanUp.ReferenceOTU60535
New.CleanUp.ReferenceOTU12086
JF791093.1.1777
New.CleanUp.ReferenceOTU64458
New.CleanUp.ReferenceOTU71363
New.CleanUp.ReferenceOTU117517
FJ000205.1.1393
FJ000095.1.1424
New.CleanUp.ReferenceOTU96119
New.CleanUp.ReferenceOTU51589
New.CleanUp.ReferenceOTU57909
New.CleanUp.ReferenceOTU37807
New.CleanUp.ReferenceOTU25501
New.CleanUp.ReferenceOTU49036
New.CleanUp.ReferenceOTU29920
New.CleanUp.ReferenceOTU7771
New.CleanUp.ReferenceOTU245435
New.CleanUp.ReferenceOTU118867

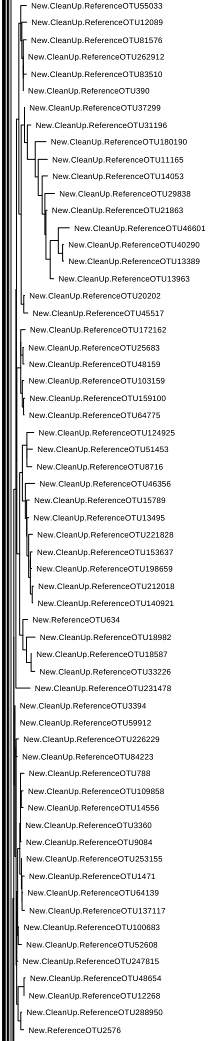
New.CleanUp.ReferenceOTU55033
New.CleanUp.ReferenceOTU12089
New.CleanUp.ReferenceOTU81576
New.CleanUp.ReferenceOTU262912
New.CleanUp.ReferenceOTU83510
New.CleanUp.ReferenceOTU390
New.CleanUp.ReferenceOTU37299
New.CleanUp.ReferenceOTU31196
New.CleanUp.ReferenceOTU180190
New.CleanUp.ReferenceOTU11165
New.CleanUp.ReferenceOTU14053
New.CleanUp.ReferenceOTU29838
New.CleanUp.ReferenceOTU21863
New.CleanUp.ReferenceOTU46601
New.CleanUp.ReferenceOTU40290
New.CleanUp.ReferenceOTU13389
New.CleanUp.ReferenceOTU13963
New.CleanUp.ReferenceOTU20202
New.CleanUp.ReferenceOTU45517
New.CleanUp.ReferenceOTU172162
New.CleanUp.ReferenceOTU25683
New.CleanUp.ReferenceOTU48159
New.CleanUp.ReferenceOTU103159
New.CleanUp.ReferenceOTU159100
New.CleanUp.ReferenceOTU64775
New.CleanUp.ReferenceOTU124925
New.CleanUp.ReferenceOTU51453
New.CleanUp.ReferenceOTU8716
New.CleanUp.ReferenceOTU46356
New.CleanUp.ReferenceOTU15789
New.CleanUp.ReferenceOTU13495
New.CleanUp.ReferenceOTU221828
New.CleanUp.ReferenceOTU153637
New.CleanUp.ReferenceOTU198659
New.CleanUp.ReferenceOTU212018
New.CleanUp.ReferenceOTU140921
New.ReferenceOTU634
New.CleanUp.ReferenceOTU18982
New.CleanUp.ReferenceOTU18587
New.CleanUp.ReferenceOTU33226
New.CleanUp.ReferenceOTU231478
New.CleanUp.ReferenceOTU3394
New.CleanUp.ReferenceOTU59912
New.CleanUp.ReferenceOTU226229
New.CleanUp.ReferenceOTU84223
New.CleanUp.ReferenceOTU788
New.CleanUp.ReferenceOTU109858
New.CleanUp.ReferenceOTU14556
New.CleanUp.ReferenceOTU3360
New.CleanUp.ReferenceOTU9084
New.CleanUp.ReferenceOTU253155
New.CleanUp.ReferenceOTU1471
New.CleanUp.ReferenceOTU64139
New.CleanUp.ReferenceOTU137117
New.CleanUp.ReferenceOTU100683
New.CleanUp.ReferenceOTU52608
New.CleanUp.ReferenceOTU247815
New.CleanUp.ReferenceOTU48654
New.CleanUp.ReferenceOTU12268
New.CleanUp.ReferenceOTU288950
New.ReferenceOTU2576

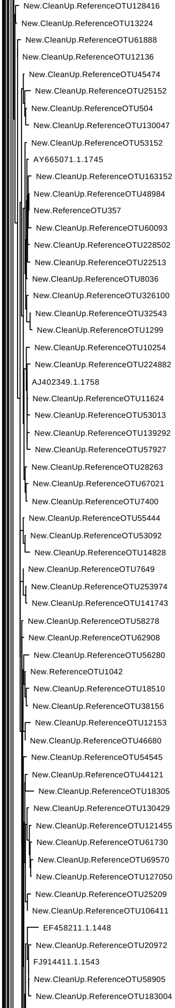
New.CleanUp.ReferenceOTU128416
New.CleanUp.ReferenceOTU13224
New.CleanUp.ReferenceOTU61888
New.CleanUp.ReferenceOTU12136
New.CleanUp.ReferenceOTU45474
New.CleanUp.ReferenceOTU25152
New.CleanUp.ReferenceOTU504
New.CleanUp.ReferenceOTU130047
New.CleanUp.ReferenceOTU53152
AY665071.1.1745
New.CleanUp.ReferenceOTU163152
New.CleanUp.ReferenceOTU48984
New.ReferenceOTU357
New.CleanUp.ReferenceOTU60093
New.CleanUp.ReferenceOTU228502
New.CleanUp.ReferenceOTU22513
New.CleanUp.ReferenceOTU8036
New.CleanUp.ReferenceOTU326100
New.CleanUp.ReferenceOTU32543
New.CleanUp.ReferenceOTU1299
New.CleanUp.ReferenceOTU10254
New.CleanUp.ReferenceOTU224882
AJ402349.1.1758
New.CleanUp.ReferenceOTU11624
New.CleanUp.ReferenceOTU53013
New.CleanUp.ReferenceOTU139292
New.CleanUp.ReferenceOTU57927
New.CleanUp.ReferenceOTU28263
New.CleanUp.ReferenceOTU67021
New.CleanUp.ReferenceOTU7400
New.CleanUp.ReferenceOTU55444
New.CleanUp.ReferenceOTU53092
New.CleanUp.ReferenceOTU14828
New.CleanUp.ReferenceOTU7649
New.CleanUp.ReferenceOTU253974
New.CleanUp.ReferenceOTU141743
New.CleanUp.ReferenceOTU58278
New.CleanUp.ReferenceOTU62908
New.CleanUp.ReferenceOTU56280
New.ReferenceOTU1042
New.CleanUp.ReferenceOTU18510
New.CleanUp.ReferenceOTU38156
New.CleanUp.ReferenceOTU12153
New.CleanUp.ReferenceOTU46680
New.CleanUp.ReferenceOTU54545
New.CleanUp.ReferenceOTU44121
New.CleanUp.ReferenceOTU18305
New.CleanUp.ReferenceOTU130429
New.CleanUp.ReferenceOTU121455
New.CleanUp.ReferenceOTU61730
New.CleanUp.ReferenceOTU69570
New.CleanUp.ReferenceOTU127050
New.CleanUp.ReferenceOTU25209
New.CleanUp.ReferenceOTU106411
EF458211.1.1448
New.CleanUp.ReferenceOTU20972
FJ914411.1.1543
New.CleanUp.ReferenceOTU58905
New.CleanUp.ReferenceOTU183004

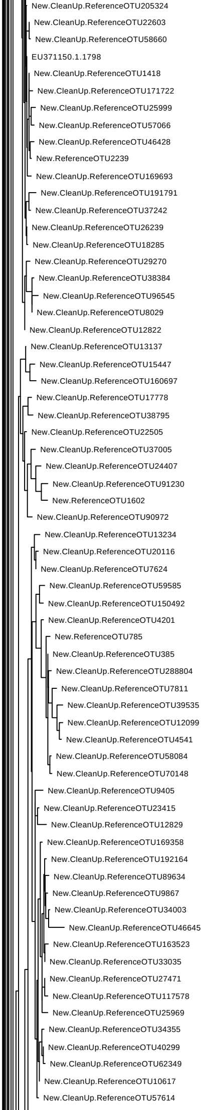
New.CleanUp.ReferenceOTU205324
New.CleanUp.ReferenceOTU22603
New.CleanUp.ReferenceOTU58660
EU371150.1.1798
New.CleanUp.ReferenceOTU1418
New.CleanUp.ReferenceOTU171722
New.CleanUp.ReferenceOTU25999
New.CleanUp.ReferenceOTU57066
New.CleanUp.ReferenceOTU46428
New.ReferenceOTU2239
New.CleanUp.ReferenceOTU169693
New.CleanUp.ReferenceOTU191791
New.CleanUp.ReferenceOTU37242
New.CleanUp.ReferenceOTU26239
New.CleanUp.ReferenceOTU18285
New.CleanUp.ReferenceOTU29270
New.CleanUp.ReferenceOTU38384
New.CleanUp.ReferenceOTU96545
New.CleanUp.ReferenceOTU8029
New.CleanUp.ReferenceOTU12822
New.CleanUp.ReferenceOTU13137
New.CleanUp.ReferenceOTU15447
New.CleanUp.ReferenceOTU160697
New.CleanUp.ReferenceOTU17778
New.CleanUp.ReferenceOTU38795
New.CleanUp.ReferenceOTU22505
New.CleanUp.ReferenceOTU37005
New.CleanUp.ReferenceOTU24407
New.CleanUp.ReferenceOTU91230
New.ReferenceOTU1602
New.CleanUp.ReferenceOTU90972
New.CleanUp.ReferenceOTU13234
New.CleanUp.ReferenceOTU20116
New.CleanUp.ReferenceOTU7624
New.CleanUp.ReferenceOTU59585
New.CleanUp.ReferenceOTU150492
New.CleanUp.ReferenceOTU4201
New.ReferenceOTU785
New.CleanUp.ReferenceOTU385
New.CleanUp.ReferenceOTU288804
New.CleanUp.ReferenceOTU7811
New.CleanUp.ReferenceOTU39535
New.CleanUp.ReferenceOTU12099
New.CleanUp.ReferenceOTU4541
New.CleanUp.ReferenceOTU58084
New.CleanUp.ReferenceOTU70148
New.CleanUp.ReferenceOTU9405
New.CleanUp.ReferenceOTU23415
New.CleanUp.ReferenceOTU12829
New.CleanUp.ReferenceOTU169358
New.CleanUp.ReferenceOTU192164
New.CleanUp.ReferenceOTU89634
New.CleanUp.ReferenceOTU9867
New.CleanUp.ReferenceOTU34003
New.CleanUp.ReferenceOTU46645
New.CleanUp.ReferenceOTU163523
New.CleanUp.ReferenceOTU33035
New.CleanUp.ReferenceOTU27471
New.CleanUp.ReferenceOTU117578
New.CleanUp.ReferenceOTU25969
New.CleanUp.ReferenceOTU34355
New.CleanUp.ReferenceOTU40299
New.CleanUp.ReferenceOTU62349
New.CleanUp.ReferenceOTU10617
New.CleanUp.ReferenceOTU57614

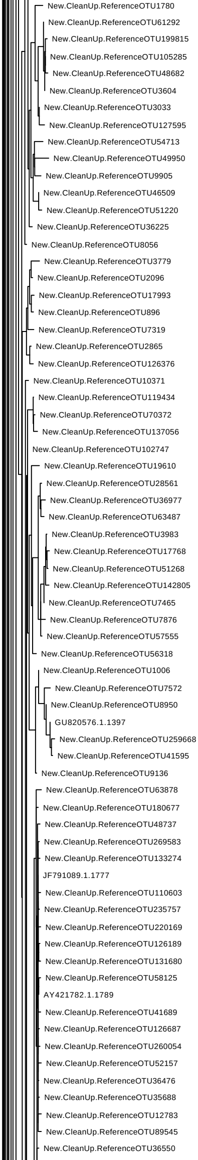
New.CleanUp.ReferenceOTU1780
New.CleanUp.ReferenceOTU61292
New.CleanUp.ReferenceOTU199815
New.CleanUp.ReferenceOTU105285
New.CleanUp.ReferenceOTU48682
New.CleanUp.ReferenceOTU3604
New.CleanUp.ReferenceOTU3033
New.CleanUp.ReferenceOTU127595
New.CleanUp.ReferenceOTU54713
New.CleanUp.ReferenceOTU49950
New.CleanUp.ReferenceOTU9905
New.CleanUp.ReferenceOTU46509
New.CleanUp.ReferenceOTU51220
New.CleanUp.ReferenceOTU36225
New.CleanUp.ReferenceOTU8056
New.CleanUp.ReferenceOTU3779
New.CleanUp.ReferenceOTU2096
New.CleanUp.ReferenceOTU17993
New.CleanUp.ReferenceOTU896
New.CleanUp.ReferenceOTU7319
New.CleanUp.ReferenceOTU2865
New.CleanUp.ReferenceOTU126376
New.CleanUp.ReferenceOTU10371
New.CleanUp.ReferenceOTU119434
New.CleanUp.ReferenceOTU70372
New.CleanUp.ReferenceOTU137056
New.CleanUp.ReferenceOTU102747
New.CleanUp.ReferenceOTU19610
New.CleanUp.ReferenceOTU28561
New.CleanUp.ReferenceOTU36977
New.CleanUp.ReferenceOTU63487
New.CleanUp.ReferenceOTU3983
New.CleanUp.ReferenceOTU17768
New.CleanUp.ReferenceOTU51268
New.CleanUp.ReferenceOTU142805
New.CleanUp.ReferenceOTU7465
New.CleanUp.ReferenceOTU7876
New.CleanUp.ReferenceOTU57555
New.CleanUp.ReferenceOTU56318
New.CleanUp.ReferenceOTU1006
New.CleanUp.ReferenceOTU7572
New.CleanUp.ReferenceOTU8950
GU820576.1.1397
New.CleanUp.ReferenceOTU259668
New.CleanUp.ReferenceOTU41595
New.CleanUp.ReferenceOTU9136
New.CleanUp.ReferenceOTU63878
New.CleanUp.ReferenceOTU180677
New.CleanUp.ReferenceOTU48737
New.CleanUp.ReferenceOTU269583
New.CleanUp.ReferenceOTU133274
JF791089.1.1777
New.CleanUp.ReferenceOTU110603
New.CleanUp.ReferenceOTU235757
New.CleanUp.ReferenceOTU220169
New.CleanUp.ReferenceOTU126189
New.CleanUp.ReferenceOTU131680
New.CleanUp.ReferenceOTU58125
AY421782.1.1789
New.CleanUp.ReferenceOTU41689
New.CleanUp.ReferenceOTU126687
New.CleanUp.ReferenceOTU260054
New.CleanUp.ReferenceOTU52157
New.CleanUp.ReferenceOTU36476
New.CleanUp.ReferenceOTU35688
New.CleanUp.ReferenceOTU12783
New.CleanUp.ReferenceOTU89545
New.CleanUp.ReferenceOTU36550

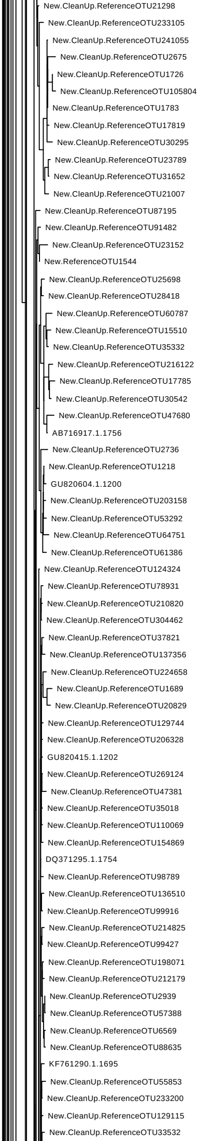
New.CleanUp.ReferenceOTU21298
New.CleanUp.ReferenceOTU233105
New.CleanUp.ReferenceOTU241055
New.CleanUp.ReferenceOTU2675
New.CleanUp.ReferenceOTU1726
New.CleanUp.ReferenceOTU105804
New.CleanUp.ReferenceOTU1783
New.CleanUp.ReferenceOTU17819
New.CleanUp.ReferenceOTU30295
New.CleanUp.ReferenceOTU23789
New.CleanUp.ReferenceOTU31652
New.CleanUp.ReferenceOTU21007
New.CleanUp.ReferenceOTU87195
New.CleanUp.ReferenceOTU91482
New.CleanUp.ReferenceOTU23152
New.ReferenceOTU1544
New.CleanUp.ReferenceOTU25698
New.CleanUp.ReferenceOTU28418
New.CleanUp.ReferenceOTU60787
New.CleanUp.ReferenceOTU15510
New.CleanUp.ReferenceOTU35332
New.CleanUp.ReferenceOTU216122
New.CleanUp.ReferenceOTU17785
New.CleanUp.ReferenceOTU30542
New.CleanUp.ReferenceOTU47680
AB716917.1.1756
New.CleanUp.ReferenceOTU2736
New.CleanUp.ReferenceOTU1218
GU820604.1.1200
New.CleanUp.ReferenceOTU203158
New.CleanUp.ReferenceOTU53292
New.CleanUp.ReferenceOTU64751
New.CleanUp.ReferenceOTU61386
New.CleanUp.ReferenceOTU124324
New.CleanUp.ReferenceOTU78931
New.CleanUp.ReferenceOTU210820
New.CleanUp.ReferenceOTU304462
New.CleanUp.ReferenceOTU37821
New.CleanUp.ReferenceOTU137356
New.CleanUp.ReferenceOTU224658
New.CleanUp.ReferenceOTU1689
New.CleanUp.ReferenceOTU20829
New.CleanUp.ReferenceOTU129744
New.CleanUp.ReferenceOTU206328
GU820415.1.1202
New.CleanUp.ReferenceOTU269124
New.CleanUp.ReferenceOTU47381
New.CleanUp.ReferenceOTU35018
New.CleanUp.ReferenceOTU110069
New.CleanUp.ReferenceOTU154869
DQ371295.1.1754
New.CleanUp.ReferenceOTU98789
New.CleanUp.ReferenceOTU136510
New.CleanUp.ReferenceOTU99916
New.CleanUp.ReferenceOTU214825
New.CleanUp.ReferenceOTU99427
New.CleanUp.ReferenceOTU198071
New.CleanUp.ReferenceOTU212179
New.CleanUp.ReferenceOTU2939
New.CleanUp.ReferenceOTU57388
New.CleanUp.ReferenceOTU6569
New.CleanUp.ReferenceOTU88635
KF761290.1.1695
New.CleanUp.ReferenceOTU55853
New.CleanUp.ReferenceOTU233200
New.CleanUp.ReferenceOTU129115
New.CleanUp.ReferenceOTU33532

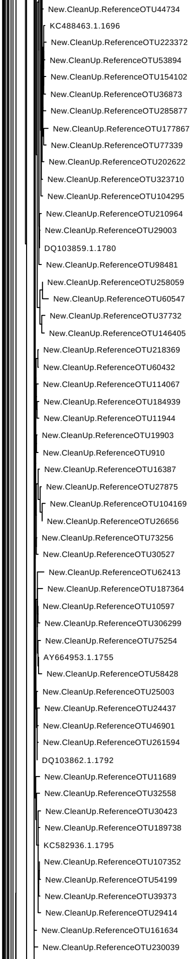
New.CleanUp.ReferenceOTU44734
KC488463.1.1696
New.CleanUp.ReferenceOTU223372
New.CleanUp.ReferenceOTU53894
New.CleanUp.ReferenceOTU154102
New.CleanUp.ReferenceOTU36873
New.CleanUp.ReferenceOTU285877
New.CleanUp.ReferenceOTU177867
New.CleanUp.ReferenceOTU77339
New.CleanUp.ReferenceOTU202622
New.CleanUp.ReferenceOTU323710
New.CleanUp.ReferenceOTU104295
New.CleanUp.ReferenceOTU210964
New.CleanUp.ReferenceOTU29003
DQ103859.1.1780
New.CleanUp.ReferenceOTU98481
New.CleanUp.ReferenceOTU258059
New.CleanUp.ReferenceOTU60547
New.CleanUp.ReferenceOTU37732
New.CleanUp.ReferenceOTU146405
New.CleanUp.ReferenceOTU218369
New.CleanUp.ReferenceOTU60432
New.CleanUp.ReferenceOTU114067
New.CleanUp.ReferenceOTU184939
New.CleanUp.ReferenceOTU11944
New.CleanUp.ReferenceOTU19903
New.CleanUp.ReferenceOTU910
New.CleanUp.ReferenceOTU16387
New.CleanUp.ReferenceOTU27875
New.CleanUp.ReferenceOTU104169
New.CleanUp.ReferenceOTU26656
New.CleanUp.ReferenceOTU73256
New.CleanUp.ReferenceOTU30527
New.CleanUp.ReferenceOTU62413
New.CleanUp.ReferenceOTU187364
New.CleanUp.ReferenceOTU10597
New.CleanUp.ReferenceOTU306299
New.CleanUp.ReferenceOTU75254
AY664953.1.1755
New.CleanUp.ReferenceOTU58428
New.CleanUp.ReferenceOTU25003
New.CleanUp.ReferenceOTU24437
New.CleanUp.ReferenceOTU46901
New.CleanUp.ReferenceOTU261594
DQ103862.1.1792
New.CleanUp.ReferenceOTU11689
New.CleanUp.ReferenceOTU32558
New.CleanUp.ReferenceOTU30423
New.CleanUp.ReferenceOTU189738
KC582936.1.1795
New.CleanUp.ReferenceOTU107352
New.CleanUp.ReferenceOTU54199
New.CleanUp.ReferenceOTU39373
New.CleanUp.ReferenceOTU29414
New.CleanUp.ReferenceOTU161634
New.CleanUp.ReferenceOTU230039

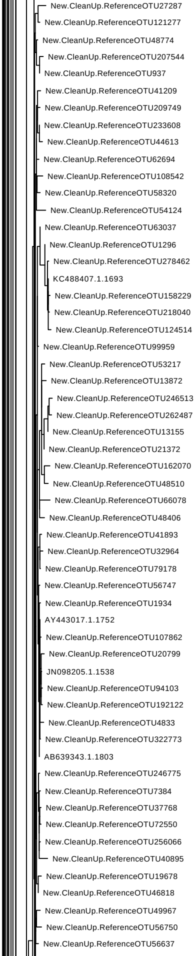
New.CleanUp.ReferenceOTU27287
New.CleanUp.ReferenceOTU121277
New.CleanUp.ReferenceOTU48774
New.CleanUp.ReferenceOTU207544
New.CleanUp.ReferenceOTU937
New.CleanUp.ReferenceOTU41209
New.CleanUp.ReferenceOTU209749
New.CleanUp.ReferenceOTU233608
New.CleanUp.ReferenceOTU44613
New.CleanUp.ReferenceOTU62694
New.CleanUp.ReferenceOTU108542
New.CleanUp.ReferenceOTU58320
New.CleanUp.ReferenceOTU54124
New.CleanUp.ReferenceOTU63037
New.CleanUp.ReferenceOTU1296
New.CleanUp.ReferenceOTU278462
KC488407.1.1693
New.CleanUp.ReferenceOTU158229
New.CleanUp.ReferenceOTU218040
New.CleanUp.ReferenceOTU124514
New.CleanUp.ReferenceOTU99959
New.CleanUp.ReferenceOTU53217
New.CleanUp.ReferenceOTU13872
New.CleanUp.ReferenceOTU246513
New.CleanUp.ReferenceOTU262487
New.CleanUp.ReferenceOTU13155
New.CleanUp.ReferenceOTU21372
New.CleanUp.ReferenceOTU162070
New.CleanUp.ReferenceOTU48510
New.CleanUp.ReferenceOTU66078
New.CleanUp.ReferenceOTU48406
New.CleanUp.ReferenceOTU41893
New.CleanUp.ReferenceOTU32964
New.CleanUp.ReferenceOTU79178
New.CleanUp.ReferenceOTU56747
New.CleanUp.ReferenceOTU1934
AY443017.1.1752
New.CleanUp.ReferenceOTU107862
New.CleanUp.ReferenceOTU20799
JN098205.1.1538
New.CleanUp.ReferenceOTU94103
New.CleanUp.ReferenceOTU192122
New.CleanUp.ReferenceOTU4833
New.CleanUp.ReferenceOTU322773
AB639343.1.1803
New.CleanUp.ReferenceOTU246775
New.CleanUp.ReferenceOTU7384
New.CleanUp.ReferenceOTU37768
New.CleanUp.ReferenceOTU72550
New.CleanUp.ReferenceOTU256066
New.CleanUp.ReferenceOTU40895
New.CleanUp.ReferenceOTU19678
New.CleanUp.ReferenceOTU46818
New.CleanUp.ReferenceOTU49967
New.CleanUp.ReferenceOTU56750
New.CleanUp.ReferenceOTU56637

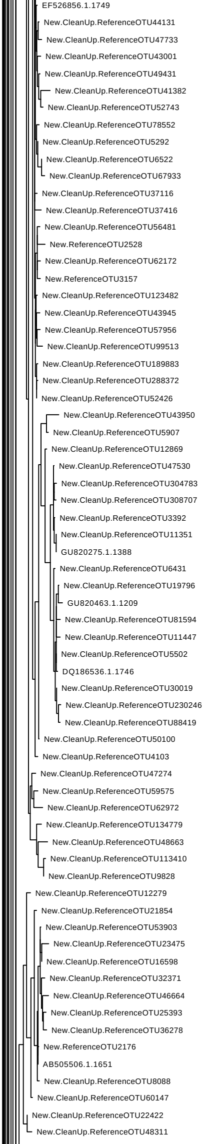
EF526856.1.1749
New.CleanUp.ReferenceOTU44131
New.CleanUp.ReferenceOTU47733
New.CleanUp.ReferenceOTU43001
New.CleanUp.ReferenceOTU49431
New.CleanUp.ReferenceOTU41382
New.CleanUp.ReferenceOTU52743
New.CleanUp.ReferenceOTU78552
New.CleanUp.ReferenceOTU5292
New.CleanUp.ReferenceOTU6522
New.CleanUp.ReferenceOTU67933
New.CleanUp.ReferenceOTU37116
New.CleanUp.ReferenceOTU37416
New.CleanUp.ReferenceOTU56481
New.ReferenceOTU2528
New.CleanUp.ReferenceOTU62172
New.ReferenceOTU3157
New.CleanUp.ReferenceOTU123482
New.CleanUp.ReferenceOTU43945
New.CleanUp.ReferenceOTU57956
New.CleanUp.ReferenceOTU99513
New.CleanUp.ReferenceOTU189883
New.CleanUp.ReferenceOTU288372
New.CleanUp.ReferenceOTU52426
New.CleanUp.ReferenceOTU43950
New.CleanUp.ReferenceOTU5907
New.CleanUp.ReferenceOTU12869
New.CleanUp.ReferenceOTU47530
New.CleanUp.ReferenceOTU304783
New.CleanUp.ReferenceOTU308707
New.CleanUp.ReferenceOTU3392
New.CleanUp.ReferenceOTU11351
GU820275.1.1388
New.CleanUp.ReferenceOTU6431
New.CleanUp.ReferenceOTU19796
GU820463.1.1209
New.CleanUp.ReferenceOTU81594
New.CleanUp.ReferenceOTU11447
New.CleanUp.ReferenceOTU5502
DQ186536.1.1746
New.CleanUp.ReferenceOTU30019
New.CleanUp.ReferenceOTU230246
New.CleanUp.ReferenceOTU88419
New.CleanUp.ReferenceOTU50100
New.CleanUp.ReferenceOTU4103
New.CleanUp.ReferenceOTU47274
New.CleanUp.ReferenceOTU59575
New.CleanUp.ReferenceOTU62972
New.CleanUp.ReferenceOTU134779
New.CleanUp.ReferenceOTU48663
New.CleanUp.ReferenceOTU113410
New.CleanUp.ReferenceOTU9828
New.CleanUp.ReferenceOTU12279
New.CleanUp.ReferenceOTU21854
New.CleanUp.ReferenceOTU53903
New.CleanUp.ReferenceOTU23475
New.CleanUp.ReferenceOTU16598
New.CleanUp.ReferenceOTU32371
New.CleanUp.ReferenceOTU46664
New.CleanUp.ReferenceOTU25393
New.CleanUp.ReferenceOTU36278
New.ReferenceOTU2176
AB505506.1.1651
New.CleanUp.ReferenceOTU8088
New.CleanUp.ReferenceOTU60147
New.CleanUp.ReferenceOTU22422
New.CleanUp.ReferenceOTU48311

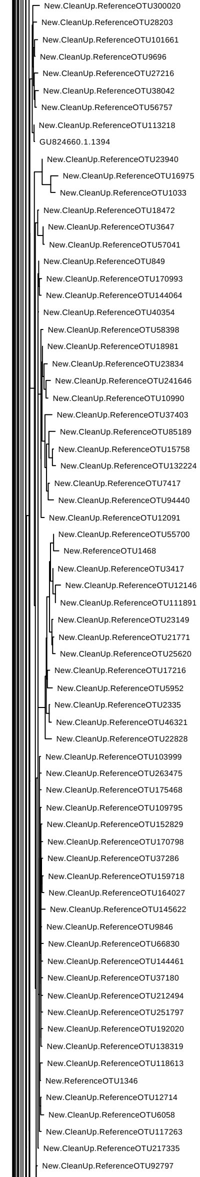
New.CleanUp.ReferenceOTU300020
New.CleanUp.ReferenceOTU28203
New.CleanUp.ReferenceOTU101661
New.CleanUp.ReferenceOTU9696
New.CleanUp.ReferenceOTU27216
New.CleanUp.ReferenceOTU38042
New.CleanUp.ReferenceOTU56757
New.CleanUp.ReferenceOTU113218
GU824660.1.1394
New.CleanUp.ReferenceOTU23940
New.CleanUp.ReferenceOTU16975
New.CleanUp.ReferenceOTU1033
New.CleanUp.ReferenceOTU18472
New.CleanUp.ReferenceOTU3647
New.CleanUp.ReferenceOTU57041
New.CleanUp.ReferenceOTU849
New.CleanUp.ReferenceOTU170993
New.CleanUp.ReferenceOTU144064
New.CleanUp.ReferenceOTU40354
New.CleanUp.ReferenceOTU58398
New.CleanUp.ReferenceOTU18981
New.CleanUp.ReferenceOTU23834
New.CleanUp.ReferenceOTU241646
New.CleanUp.ReferenceOTU10990
New.CleanUp.ReferenceOTU37403
New.CleanUp.ReferenceOTU85189
New.CleanUp.ReferenceOTU15758
New.CleanUp.ReferenceOTU132224
New.CleanUp.ReferenceOTU7417
New.CleanUp.ReferenceOTU94440
New.CleanUp.ReferenceOTU12091
New.CleanUp.ReferenceOTU55700
New.ReferenceOTU1468
New.CleanUp.ReferenceOTU3417
New.CleanUp.ReferenceOTU12146
New.CleanUp.ReferenceOTU111891
New.CleanUp.ReferenceOTU23149
New.CleanUp.ReferenceOTU21771
New.CleanUp.ReferenceOTU25620
New.CleanUp.ReferenceOTU17216
New.CleanUp.ReferenceOTU5952
New.CleanUp.ReferenceOTU2335
New.CleanUp.ReferenceOTU46321
New.CleanUp.ReferenceOTU22828
New.CleanUp.ReferenceOTU103999
New.CleanUp.ReferenceOTU263475
New.CleanUp.ReferenceOTU175468
New.CleanUp.ReferenceOTU109795
New.CleanUp.ReferenceOTU152829
New.CleanUp.ReferenceOTU170798
New.CleanUp.ReferenceOTU37286
New.CleanUp.ReferenceOTU159718
New.CleanUp.ReferenceOTU164027
New.CleanUp.ReferenceOTU145622
New.CleanUp.ReferenceOTU9846
New.CleanUp.ReferenceOTU66830
New.CleanUp.ReferenceOTU144461
New.CleanUp.ReferenceOTU37180
New.CleanUp.ReferenceOTU212494
New.CleanUp.ReferenceOTU251797
New.CleanUp.ReferenceOTU192020
New.CleanUp.ReferenceOTU138319
New.CleanUp.ReferenceOTU118613
New.ReferenceOTU1346
New.CleanUp.ReferenceOTU12714
New.CleanUp.ReferenceOTU6058
New.CleanUp.ReferenceOTU117263
New.CleanUp.ReferenceOTU217335
New.CleanUp.ReferenceOTU92797

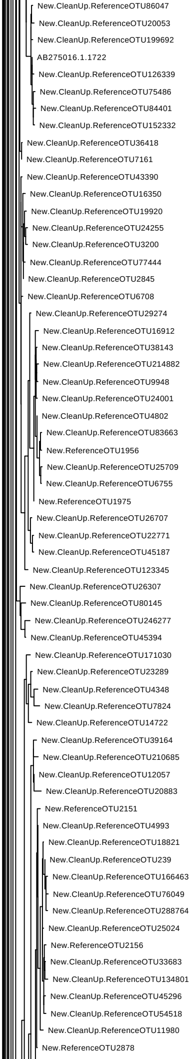
New.CleanUp.ReferenceOTU86047
New.CleanUp.ReferenceOTU20053
New.CleanUp.ReferenceOTU199692
AB275016.1.1722
New.CleanUp.ReferenceOTU126339
New.CleanUp.ReferenceOTU75486
New.CleanUp.ReferenceOTU84401
New.CleanUp.ReferenceOTU152332
New.CleanUp.ReferenceOTU36418
New.CleanUp.ReferenceOTU7161
New.CleanUp.ReferenceOTU43390
New.CleanUp.ReferenceOTU16350
New.CleanUp.ReferenceOTU19920
New.CleanUp.ReferenceOTU24255
New.CleanUp.ReferenceOTU3200
New.CleanUp.ReferenceOTU77444
New.CleanUp.ReferenceOTU2845
New.CleanUp.ReferenceOTU6708
New.CleanUp.ReferenceOTU29274
New.CleanUp.ReferenceOTU16912
New.CleanUp.ReferenceOTU38143
New.CleanUp.ReferenceOTU214882
New.CleanUp.ReferenceOTU9948
New.CleanUp.ReferenceOTU24001
New.CleanUp.ReferenceOTU4802
New.CleanUp.ReferenceOTU83663
New.ReferenceOTU1956
New.CleanUp.ReferenceOTU25709
New.CleanUp.ReferenceOTU6755
New.ReferenceOTU1975
New.CleanUp.ReferenceOTU26707
New.CleanUp.ReferenceOTU22771
New.CleanUp.ReferenceOTU45187
New.CleanUp.ReferenceOTU123345
New.CleanUp.ReferenceOTU26307
New.CleanUp.ReferenceOTU80145
New.CleanUp.ReferenceOTU246277
New.CleanUp.ReferenceOTU45394
New.CleanUp.ReferenceOTU171030
New.CleanUp.ReferenceOTU23289
New.CleanUp.ReferenceOTU4348
New.CleanUp.ReferenceOTU7824
New.CleanUp.ReferenceOTU14722
New.CleanUp.ReferenceOTU39164
New.CleanUp.ReferenceOTU210685
New.CleanUp.ReferenceOTU12057
New.CleanUp.ReferenceOTU20883
New.ReferenceOTU2151
New.CleanUp.ReferenceOTU4993
New.CleanUp.ReferenceOTU18821
New.CleanUp.ReferenceOTU239
New.CleanUp.ReferenceOTU166463
New.CleanUp.ReferenceOTU76049
New.CleanUp.ReferenceOTU288764
New.CleanUp.ReferenceOTU25024
New.ReferenceOTU2156
New.CleanUp.ReferenceOTU33683
New.CleanUp.ReferenceOTU134801
New.CleanUp.ReferenceOTU45296
New.CleanUp.ReferenceOTU54518
New.CleanUp.ReferenceOTU11980
New.ReferenceOTU2878

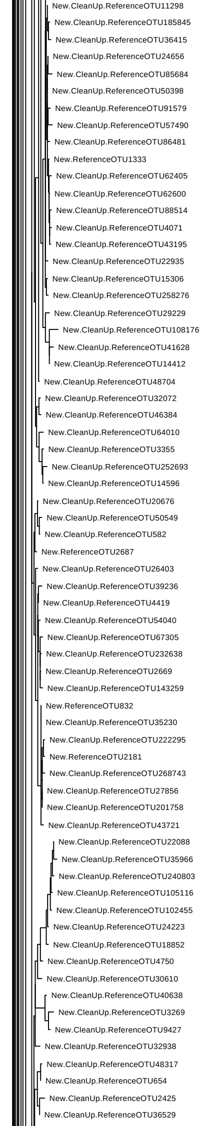
New.CleanUp.ReferenceOTU11298
New.CleanUp.ReferenceOTU185845
New.CleanUp.ReferenceOTU36415
New.CleanUp.ReferenceOTU24656
New.CleanUp.ReferenceOTU85684
New.CleanUp.ReferenceOTU50398
New.CleanUp.ReferenceOTU91579
New.CleanUp.ReferenceOTU57490
New.CleanUp.ReferenceOTU86481
New.ReferenceOTU1333
New.CleanUp.ReferenceOTU62405
New.CleanUp.ReferenceOTU62600
New.CleanUp.ReferenceOTU88514
New.CleanUp.ReferenceOTU4071
New.CleanUp.ReferenceOTU43195
New.CleanUp.ReferenceOTU22935
New.CleanUp.ReferenceOTU15306
New.CleanUp.ReferenceOTU258276
New.CleanUp.ReferenceOTU29229
New.CleanUp.ReferenceOTU108176
New.CleanUp.ReferenceOTU41628
New.CleanUp.ReferenceOTU14412
New.CleanUp.ReferenceOTU48704
New.CleanUp.ReferenceOTU32072
New.CleanUp.ReferenceOTU46384
New.CleanUp.ReferenceOTU64010
New.CleanUp.ReferenceOTU3355
New.CleanUp.ReferenceOTU252693
New.CleanUp.ReferenceOTU14596
New.CleanUp.ReferenceOTU20676
New.CleanUp.ReferenceOTU50549
New.CleanUp.ReferenceOTU582
New.ReferenceOTU2687
New.CleanUp.ReferenceOTU26403
New.CleanUp.ReferenceOTU39236
New.CleanUp.ReferenceOTU4419
New.CleanUp.ReferenceOTU54040
New.CleanUp.ReferenceOTU67305
New.CleanUp.ReferenceOTU232638
New.CleanUp.ReferenceOTU2669
New.CleanUp.ReferenceOTU143259
New.ReferenceOTU832
New.CleanUp.ReferenceOTU35230
New.CleanUp.ReferenceOTU222295
New.ReferenceOTU2181
New.CleanUp.ReferenceOTU268743
New.CleanUp.ReferenceOTU27856
New.CleanUp.ReferenceOTU201758
New.CleanUp.ReferenceOTU43721
New.CleanUp.ReferenceOTU22088
New.CleanUp.ReferenceOTU35966
New.CleanUp.ReferenceOTU240803
New.CleanUp.ReferenceOTU105116
New.CleanUp.ReferenceOTU102455
New.CleanUp.ReferenceOTU24223
New.CleanUp.ReferenceOTU18852
New.CleanUp.ReferenceOTU4750
New.CleanUp.ReferenceOTU30610
New.CleanUp.ReferenceOTU40638
New.CleanUp.ReferenceOTU3269
New.CleanUp.ReferenceOTU9427
New.CleanUp.ReferenceOTU32938
New.CleanUp.ReferenceOTU48317
New.CleanUp.ReferenceOTU654
New.CleanUp.ReferenceOTU2425
New.CleanUp.ReferenceOTU36529

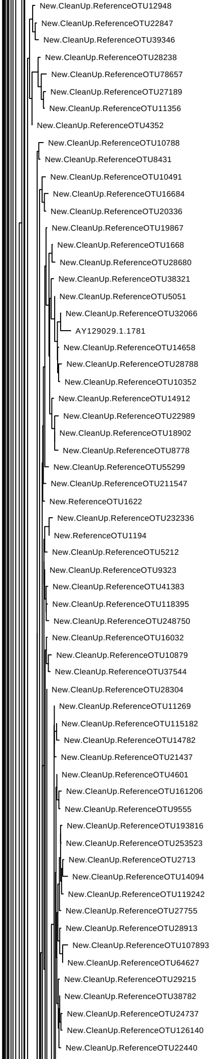
New.CleanUp.ReferenceOTU12948
New.CleanUp.ReferenceOTU22847
New.CleanUp.ReferenceOTU39346
New.CleanUp.ReferenceOTU28238
New.CleanUp.ReferenceOTU78657
New.CleanUp.ReferenceOTU27189
New.CleanUp.ReferenceOTU11356
New.CleanUp.ReferenceOTU4352
New.CleanUp.ReferenceOTU10788
New.CleanUp.ReferenceOTU8431
New.CleanUp.ReferenceOTU10491
New.CleanUp.ReferenceOTU16684
New.CleanUp.ReferenceOTU20336
New.CleanUp.ReferenceOTU19867
New.CleanUp.ReferenceOTU1668
New.CleanUp.ReferenceOTU28680
New.CleanUp.ReferenceOTU38321
New.CleanUp.ReferenceOTU5051
New.CleanUp.ReferenceOTU32066
AY129029.1.1781
New.CleanUp.ReferenceOTU14658
New.CleanUp.ReferenceOTU28788
New.CleanUp.ReferenceOTU10352
New.CleanUp.ReferenceOTU14912
New.CleanUp.ReferenceOTU22989
New.CleanUp.ReferenceOTU18902
New.CleanUp.ReferenceOTU8778
New.CleanUp.ReferenceOTU55299
New.CleanUp.ReferenceOTU211547
New.ReferenceOTU1622
New.CleanUp.ReferenceOTU232336
New.ReferenceOTU1194
New.CleanUp.ReferenceOTU5212
New.CleanUp.ReferenceOTU9323
New.CleanUp.ReferenceOTU41383
New.CleanUp.ReferenceOTU118395
New.CleanUp.ReferenceOTU248750
New.CleanUp.ReferenceOTU16032
New.CleanUp.ReferenceOTU10879
New.CleanUp.ReferenceOTU37544
New.CleanUp.ReferenceOTU28304
New.CleanUp.ReferenceOTU11269
New.CleanUp.ReferenceOTU115182
New.CleanUp.ReferenceOTU14782
New.CleanUp.ReferenceOTU21437
New.CleanUp.ReferenceOTU4601
New.CleanUp.ReferenceOTU161206
New.CleanUp.ReferenceOTU9555
New.CleanUp.ReferenceOTU193816
New.CleanUp.ReferenceOTU253523
New.CleanUp.ReferenceOTU2713
New.CleanUp.ReferenceOTU14094
New.CleanUp.ReferenceOTU119242
New.CleanUp.ReferenceOTU27755
New.CleanUp.ReferenceOTU28913
New.CleanUp.ReferenceOTU107893
New.CleanUp.ReferenceOTU64627
New.CleanUp.ReferenceOTU29215
New.CleanUp.ReferenceOTU38782
New.CleanUp.ReferenceOTU24737
New.CleanUp.ReferenceOTU126140
New.CleanUp.ReferenceOTU22440

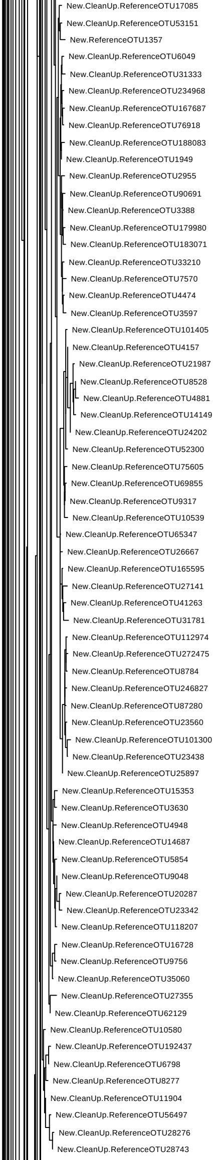
New.CleanUp.ReferenceOTU17085
New.CleanUp.ReferenceOTU53151
New.ReferenceOTU1357
New.CleanUp.ReferenceOTU6049
New.CleanUp.ReferenceOTU31333
New.CleanUp.ReferenceOTU234968
New.CleanUp.ReferenceOTU167687
New.CleanUp.ReferenceOTU76918
New.CleanUp.ReferenceOTU188083
New.CleanUp.ReferenceOTU1949
New.CleanUp.ReferenceOTU2955
New.CleanUp.ReferenceOTU90691
New.CleanUp.ReferenceOTU3388
New.CleanUp.ReferenceOTU179980
New.CleanUp.ReferenceOTU183071
New.CleanUp.ReferenceOTU33210
New.CleanUp.ReferenceOTU7570
New.CleanUp.ReferenceOTU4474
New.CleanUp.ReferenceOTU3597
New.CleanUp.ReferenceOTU101405
New.CleanUp.ReferenceOTU4157
New.CleanUp.ReferenceOTU21987
New.CleanUp.ReferenceOTU8528
New.CleanUp.ReferenceOTU4881
New.CleanUp.ReferenceOTU14149
New.CleanUp.ReferenceOTU24202
New.CleanUp.ReferenceOTU52300
New.CleanUp.ReferenceOTU75605
New.CleanUp.ReferenceOTU69855
New.CleanUp.ReferenceOTU9317
New.CleanUp.ReferenceOTU10539
New.CleanUp.ReferenceOTU65347
New.CleanUp.ReferenceOTU26667
New.CleanUp.ReferenceOTU165595
New.CleanUp.ReferenceOTU27141
New.CleanUp.ReferenceOTU41263
New.CleanUp.ReferenceOTU31781
New.CleanUp.ReferenceOTU112974
New.CleanUp.ReferenceOTU272475
New.CleanUp.ReferenceOTU8784
New.CleanUp.ReferenceOTU246827
New.CleanUp.ReferenceOTU87280
New.CleanUp.ReferenceOTU23560
New.CleanUp.ReferenceOTU101300
New.CleanUp.ReferenceOTU23438
New.CleanUp.ReferenceOTU25897
New.CleanUp.ReferenceOTU15353
New.CleanUp.ReferenceOTU3630
New.CleanUp.ReferenceOTU4948
New.CleanUp.ReferenceOTU14687
New.CleanUp.ReferenceOTU5854
New.CleanUp.ReferenceOTU9048
New.CleanUp.ReferenceOTU20287
New.CleanUp.ReferenceOTU23342
New.CleanUp.ReferenceOTU118207
New.CleanUp.ReferenceOTU16728
New.CleanUp.ReferenceOTU9756
New.CleanUp.ReferenceOTU35060
New.CleanUp.ReferenceOTU27355
New.CleanUp.ReferenceOTU62129
New.CleanUp.ReferenceOTU10580
New.CleanUp.ReferenceOTU192437
New.CleanUp.ReferenceOTU6798
New.CleanUp.ReferenceOTU8277
New.CleanUp.ReferenceOTU11904
New.CleanUp.ReferenceOTU56497
New.CleanUp.ReferenceOTU28276
New.CleanUp.ReferenceOTU28743

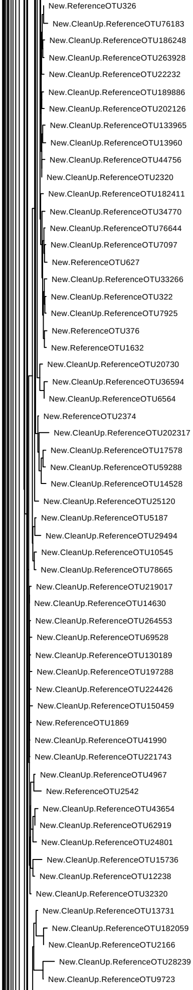
New.ReferenceOTU326
New.CleanUp.ReferenceOTU76183
New.CleanUp.ReferenceOTU186248
New.CleanUp.ReferenceOTU263928
New.CleanUp.ReferenceOTU22232
New.CleanUp.ReferenceOTU189886
New.CleanUp.ReferenceOTU202126
New.CleanUp.ReferenceOTU133965
New.CleanUp.ReferenceOTU13960
New.CleanUp.ReferenceOTU44756
New.CleanUp.ReferenceOTU2320
New.CleanUp.ReferenceOTU182411
New.CleanUp.ReferenceOTU34770
New.CleanUp.ReferenceOTU76644
New.CleanUp.ReferenceOTU7097
New.ReferenceOTU627
New.CleanUp.ReferenceOTU33266
New.CleanUp.ReferenceOTU322
New.CleanUp.ReferenceOTU7925
New.ReferenceOTU376
New.ReferenceOTU1632
New.CleanUp.ReferenceOTU20730
New.CleanUp.ReferenceOTU36594
New.CleanUp.ReferenceOTU6564
New.ReferenceOTU2374
New.CleanUp.ReferenceOTU202317
New.CleanUp.ReferenceOTU17578
New.CleanUp.ReferenceOTU59288
New.CleanUp.ReferenceOTU14528
New.CleanUp.ReferenceOTU25120
New.CleanUp.ReferenceOTU5187
New.CleanUp.ReferenceOTU29494
New.CleanUp.ReferenceOTU10545
New.CleanUp.ReferenceOTU78665
New.CleanUp.ReferenceOTU219017
New.CleanUp.ReferenceOTU14630
New.CleanUp.ReferenceOTU264553
New.CleanUp.ReferenceOTU69528
New.CleanUp.ReferenceOTU130189
New.CleanUp.ReferenceOTU197288
New.CleanUp.ReferenceOTU224426
New.CleanUp.ReferenceOTU150459
New.ReferenceOTU1869
New.CleanUp.ReferenceOTU41990
New.CleanUp.ReferenceOTU221743
New.CleanUp.ReferenceOTU4967
New.ReferenceOTU2542
New.CleanUp.ReferenceOTU43654
New.CleanUp.ReferenceOTU62919
New.CleanUp.ReferenceOTU24801
New.CleanUp.ReferenceOTU15736
New.CleanUp.ReferenceOTU12238
New.CleanUp.ReferenceOTU32320
New.CleanUp.ReferenceOTU13731
New.CleanUp.ReferenceOTU182059
New.CleanUp.ReferenceOTU2166
New.CleanUp.ReferenceOTU28239
New.CleanUp.ReferenceOTU9723

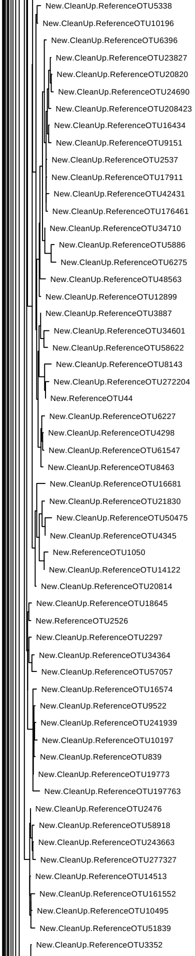
New.CleanUp.ReferenceOTU5338
New.CleanUp.ReferenceOTU10196
New.CleanUp.ReferenceOTU6396
New.CleanUp.ReferenceOTU23827
New.CleanUp.ReferenceOTU20820
New.CleanUp.ReferenceOTU24690
New.CleanUp.ReferenceOTU208423
New.CleanUp.ReferenceOTU16434
New.CleanUp.ReferenceOTU9151
New.CleanUp.ReferenceOTU2537
New.CleanUp.ReferenceOTU17911
New.CleanUp.ReferenceOTU42431
New.CleanUp.ReferenceOTU176461
New.CleanUp.ReferenceOTU34710
New.CleanUp.ReferenceOTU5886
New.CleanUp.ReferenceOTU6275
New.CleanUp.ReferenceOTU48563
New.CleanUp.ReferenceOTU12899
New.CleanUp.ReferenceOTU3887
New.CleanUp.ReferenceOTU34601
New.CleanUp.ReferenceOTU58622
New.CleanUp.ReferenceOTU8143
New.CleanUp.ReferenceOTU272204
New.ReferenceOTU44
New.CleanUp.ReferenceOTU6227
New.CleanUp.ReferenceOTU4298
New.CleanUp.ReferenceOTU61547
New.CleanUp.ReferenceOTU8463
New.CleanUp.ReferenceOTU16681
New.CleanUp.ReferenceOTU21830
New.CleanUp.ReferenceOTU50475
New.CleanUp.ReferenceOTU4345
New.ReferenceOTU1050
New.CleanUp.ReferenceOTU14122
New.CleanUp.ReferenceOTU20814
New.CleanUp.ReferenceOTU18645
New.ReferenceOTU2526
New.CleanUp.ReferenceOTU2297
New.CleanUp.ReferenceOTU34364
New.CleanUp.ReferenceOTU57057
New.CleanUp.ReferenceOTU16574
New.CleanUp.ReferenceOTU9522
New.CleanUp.ReferenceOTU241939
New.CleanUp.ReferenceOTU10197
New.CleanUp.ReferenceOTU839
New.CleanUp.ReferenceOTU19773
New.CleanUp.ReferenceOTU197763
New.CleanUp.ReferenceOTU2476
New.CleanUp.ReferenceOTU58918
New.CleanUp.ReferenceOTU243663
New.CleanUp.ReferenceOTU277327
New.CleanUp.ReferenceOTU14513
New.CleanUp.ReferenceOTU161552
New.CleanUp.ReferenceOTU10495
New.CleanUp.ReferenceOTU51839
New.CleanUp.ReferenceOTU3352

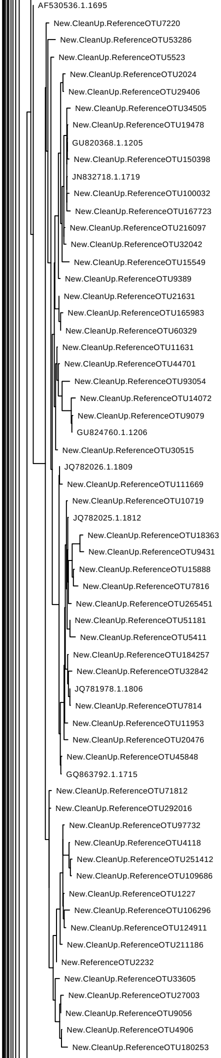
AF530536.1.1695
New.CleanUp.ReferenceOTU7220
New.CleanUp.ReferenceOTU53286
New.CleanUp.ReferenceOTU5523
New.CleanUp.ReferenceOTU2024
New.CleanUp.ReferenceOTU29406
New.CleanUp.ReferenceOTU34505
New.CleanUp.ReferenceOTU19478
GU820368.1.1205
New.CleanUp.ReferenceOTU150398
JN832718.1.1719
New.CleanUp.ReferenceOTU100032
New.CleanUp.ReferenceOTU167723
New.CleanUp.ReferenceOTU216097
New.CleanUp.ReferenceOTU32042
New.CleanUp.ReferenceOTU15549
New.CleanUp.ReferenceOTU9389
New.CleanUp.ReferenceOTU21631
New.CleanUp.ReferenceOTU165983
New.CleanUp.ReferenceOTU60329
New.CleanUp.ReferenceOTU11631
New.CleanUp.ReferenceOTU44701
New.CleanUp.ReferenceOTU93054
New.CleanUp.ReferenceOTU14072
New.CleanUp.ReferenceOTU9079
GU824760.1.1206
New.CleanUp.ReferenceOTU30515
JQ782026.1.1809
New.CleanUp.ReferenceOTU111669
New.CleanUp.ReferenceOTU10719
JQ782025.1.1812
New.CleanUp.ReferenceOTU18363
New.CleanUp.ReferenceOTU9431
New.CleanUp.ReferenceOTU15888
New.CleanUp.ReferenceOTU7816
New.CleanUp.ReferenceOTU265451
New.CleanUp.ReferenceOTU51181
New.CleanUp.ReferenceOTU5411
New.CleanUp.ReferenceOTU184257
New.CleanUp.ReferenceOTU32842
JQ781978.1.1806
New.CleanUp.ReferenceOTU7814
New.CleanUp.ReferenceOTU11953
New.CleanUp.ReferenceOTU20476
New.CleanUp.ReferenceOTU45848
GQ863792.1.1715
New.CleanUp.ReferenceOTU71812
New.CleanUp.ReferenceOTU292016
New.CleanUp.ReferenceOTU97732
New.CleanUp.ReferenceOTU4118
New.CleanUp.ReferenceOTU251412
New.CleanUp.ReferenceOTU109686
New.CleanUp.ReferenceOTU1227
New.CleanUp.ReferenceOTU106296
New.CleanUp.ReferenceOTU124911
New.CleanUp.ReferenceOTU211186
New.ReferenceOTU2232
New.CleanUp.ReferenceOTU33605
New.CleanUp.ReferenceOTU27003
New.CleanUp.ReferenceOTU9056
New.CleanUp.ReferenceOTU4906
New.CleanUp.ReferenceOTU180253

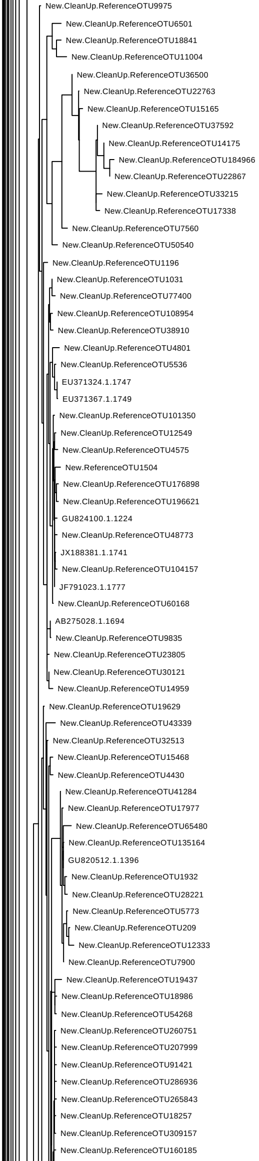
New.CleanUp.ReferenceOTU9975
New.CleanUp.ReferenceOTU6501
New.CleanUp.ReferenceOTU18841
New.CleanUp.ReferenceOTU11004
New.CleanUp.ReferenceOTU36500
New.CleanUp.ReferenceOTU22763
New.CleanUp.ReferenceOTU15165
New.CleanUp.ReferenceOTU37592
New.CleanUp.ReferenceOTU14175
New.CleanUp.ReferenceOTU184966
New.CleanUp.ReferenceOTU22867
New.CleanUp.ReferenceOTU33215
New.CleanUp.ReferenceOTU17338
New.CleanUp.ReferenceOTU7560
New.CleanUp.ReferenceOTU50540
New.CleanUp.ReferenceOTU1196
New.CleanUp.ReferenceOTU1031
New.CleanUp.ReferenceOTU77400
New.CleanUp.ReferenceOTU108954
New.CleanUp.ReferenceOTU38910
New.CleanUp.ReferenceOTU4801
New.CleanUp.ReferenceOTU5536
EU371324.1.1747
EU371367.1.1749
New.CleanUp.ReferenceOTU101350
New.CleanUp.ReferenceOTU12549
New.CleanUp.ReferenceOTU4575
New.ReferenceOTU1504
New.CleanUp.ReferenceOTU176898
New.CleanUp.ReferenceOTU196621
GU824100.1.1224
New.CleanUp.ReferenceOTU48773
JX188381.1.1741
New.CleanUp.ReferenceOTU104157
JF791023.1.1777
New.CleanUp.ReferenceOTU60168
AB275028.1.1694
New.CleanUp.ReferenceOTU9835
New.CleanUp.ReferenceOTU23805
New.CleanUp.ReferenceOTU30121
New.CleanUp.ReferenceOTU14959
New.CleanUp.ReferenceOTU19629
New.CleanUp.ReferenceOTU43339
New.CleanUp.ReferenceOTU32513
New.CleanUp.ReferenceOTU15468
New.CleanUp.ReferenceOTU4430
New.CleanUp.ReferenceOTU41284
New.CleanUp.ReferenceOTU17977
New.CleanUp.ReferenceOTU65480
New.CleanUp.ReferenceOTU135164
GU820512.1.1396
New.CleanUp.ReferenceOTU1932
New.CleanUp.ReferenceOTU28221
New.CleanUp.ReferenceOTU5773
New.CleanUp.ReferenceOTU209
New.CleanUp.ReferenceOTU12333
New.CleanUp.ReferenceOTU7900
New.CleanUp.ReferenceOTU19437
New.CleanUp.ReferenceOTU18986
New.CleanUp.ReferenceOTU54268
New.CleanUp.ReferenceOTU260751
New.CleanUp.ReferenceOTU207999
New.CleanUp.ReferenceOTU91421
New.CleanUp.ReferenceOTU286936
New.CleanUp.ReferenceOTU265843
New.CleanUp.ReferenceOTU18257
New.CleanUp.ReferenceOTU309157
New.CleanUp.ReferenceOTU160185

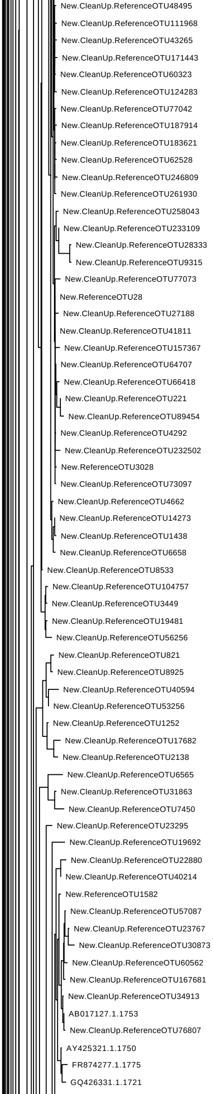
New.CleanUp.ReferenceOTU48495
New.CleanUp.ReferenceOTU111968
New.CleanUp.ReferenceOTU43265
New.CleanUp.ReferenceOTU171443
New.CleanUp.ReferenceOTU60323
New.CleanUp.ReferenceOTU124283
New.CleanUp.ReferenceOTU77042
New.CleanUp.ReferenceOTU187914
New.CleanUp.ReferenceOTU183621
New.CleanUp.ReferenceOTU62528
New.CleanUp.ReferenceOTU246809
New.CleanUp.ReferenceOTU261930
New.CleanUp.ReferenceOTU258043
New.CleanUp.ReferenceOTU233109
New.CleanUp.ReferenceOTU28333
New.CleanUp.ReferenceOTU9315
New.CleanUp.ReferenceOTU77073
New.ReferenceOTU28
New.CleanUp.ReferenceOTU27188
New.CleanUp.ReferenceOTU41811
New.CleanUp.ReferenceOTU157367
New.CleanUp.ReferenceOTU64707
New.CleanUp.ReferenceOTU66418
New.CleanUp.ReferenceOTU221
New.CleanUp.ReferenceOTU89454
New.CleanUp.ReferenceOTU4292
New.CleanUp.ReferenceOTU232502
New.ReferenceOTU3028
New.CleanUp.ReferenceOTU73097
New.CleanUp.ReferenceOTU4662
New.CleanUp.ReferenceOTU14273
New.CleanUp.ReferenceOTU1438
New.CleanUp.ReferenceOTU6658
New.CleanUp.ReferenceOTU8533
New.CleanUp.ReferenceOTU104757
New.CleanUp.ReferenceOTU3449
New.CleanUp.ReferenceOTU19481
New.CleanUp.ReferenceOTU56256
New.CleanUp.ReferenceOTU821
New.CleanUp.ReferenceOTU8925
New.CleanUp.ReferenceOTU40594
New.CleanUp.ReferenceOTU53256
New.CleanUp.ReferenceOTU1252
New.CleanUp.ReferenceOTU17682
New.CleanUp.ReferenceOTU2138
New.CleanUp.ReferenceOTU6565
New.CleanUp.ReferenceOTU31863
New.CleanUp.ReferenceOTU7450
New.CleanUp.ReferenceOTU23295
New.CleanUp.ReferenceOTU19692
New.CleanUp.ReferenceOTU22880
New.CleanUp.ReferenceOTU40214
New.ReferenceOTU1582
New.CleanUp.ReferenceOTU57087
New.CleanUp.ReferenceOTU23767
New.CleanUp.ReferenceOTU30873
New.CleanUp.ReferenceOTU60562
New.CleanUp.ReferenceOTU167681
New.CleanUp.ReferenceOTU34913
AB017127.1.1753
New.CleanUp.ReferenceOTU76807
AY425321.1.1750
FR874277.1.1775
GQ426331.1.1721

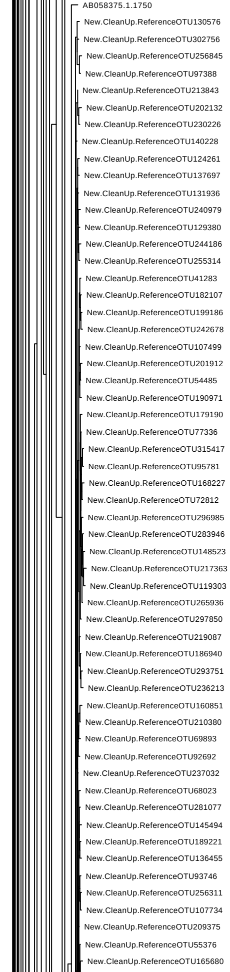
AB058375.1.1750
New.CleanUp.ReferenceOTU130576
New.CleanUp.ReferenceOTU302756
New.CleanUp.ReferenceOTU256845
New.CleanUp.ReferenceOTU97388
New.CleanUp.ReferenceOTU213843
New.CleanUp.ReferenceOTU202132
New.CleanUp.ReferenceOTU230226
New.CleanUp.ReferenceOTU140228
New.CleanUp.ReferenceOTU124261
New.CleanUp.ReferenceOTU137697
New.CleanUp.ReferenceOTU131936
New.CleanUp.ReferenceOTU240979
New.CleanUp.ReferenceOTU129380
New.CleanUp.ReferenceOTU244186
New.CleanUp.ReferenceOTU255314
New.CleanUp.ReferenceOTU41283
New.CleanUp.ReferenceOTU182107
New.CleanUp.ReferenceOTU199186
New.CleanUp.ReferenceOTU242678
New.CleanUp.ReferenceOTU107499
New.CleanUp.ReferenceOTU201912
New.CleanUp.ReferenceOTU54485
New.CleanUp.ReferenceOTU190971
New.CleanUp.ReferenceOTU179190
New.CleanUp.ReferenceOTU77336
New.CleanUp.ReferenceOTU315417
New.CleanUp.ReferenceOTU95781
New.CleanUp.ReferenceOTU168227
New.CleanUp.ReferenceOTU72812
New.CleanUp.ReferenceOTU296985
New.CleanUp.ReferenceOTU283946
New.CleanUp.ReferenceOTU148523
New.CleanUp.ReferenceOTU217363
New.CleanUp.ReferenceOTU119303
New.CleanUp.ReferenceOTU265936
New.CleanUp.ReferenceOTU297850
New.CleanUp.ReferenceOTU219087
New.CleanUp.ReferenceOTU186940
New.CleanUp.ReferenceOTU293751
New.CleanUp.ReferenceOTU236213
New.CleanUp.ReferenceOTU160851
New.CleanUp.ReferenceOTU210380
New.CleanUp.ReferenceOTU69893
New.CleanUp.ReferenceOTU92692
New.CleanUp.ReferenceOTU237032
New.CleanUp.ReferenceOTU68023
New.CleanUp.ReferenceOTU281077
New.CleanUp.ReferenceOTU145494
New.CleanUp.ReferenceOTU189221
New.CleanUp.ReferenceOTU136455
New.CleanUp.ReferenceOTU93746
New.CleanUp.ReferenceOTU256311
New.CleanUp.ReferenceOTU107734
New.CleanUp.ReferenceOTU209375
New.CleanUp.ReferenceOTU55376
New.CleanUp.ReferenceOTU165680

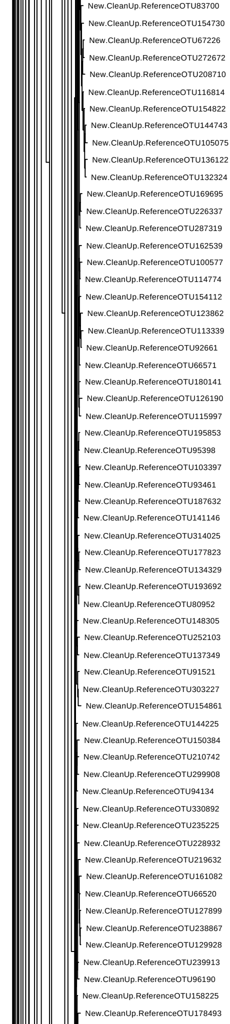
New.CleanUp.ReferenceOTU83700
New.CleanUp.ReferenceOTU154730
New.CleanUp.ReferenceOTU67226
New.CleanUp.ReferenceOTU272672
New.CleanUp.ReferenceOTU208710
New.CleanUp.ReferenceOTU116814
New.CleanUp.ReferenceOTU154822
New.CleanUp.ReferenceOTU144743
New.CleanUp.ReferenceOTU105075
New.CleanUp.ReferenceOTU136122
New.CleanUp.ReferenceOTU132324
New.CleanUp.ReferenceOTU169695
New.CleanUp.ReferenceOTU226337
New.CleanUp.ReferenceOTU287319
New.CleanUp.ReferenceOTU162539
New.CleanUp.ReferenceOTU100577
New.CleanUp.ReferenceOTU114774
New.CleanUp.ReferenceOTU154112
New.CleanUp.ReferenceOTU123862
New.CleanUp.ReferenceOTU113339
New.CleanUp.ReferenceOTU92661
New.CleanUp.ReferenceOTU66571
New.CleanUp.ReferenceOTU180141
New.CleanUp.ReferenceOTU126190
New.CleanUp.ReferenceOTU115997
New.CleanUp.ReferenceOTU195853
New.CleanUp.ReferenceOTU95398
New.CleanUp.ReferenceOTU103397
New.CleanUp.ReferenceOTU93461
New.CleanUp.ReferenceOTU187632
New.CleanUp.ReferenceOTU141146
New.CleanUp.ReferenceOTU314025
New.CleanUp.ReferenceOTU177823
New.CleanUp.ReferenceOTU134329
New.CleanUp.ReferenceOTU193692
New.CleanUp.ReferenceOTU80952
New.CleanUp.ReferenceOTU148305
New.CleanUp.ReferenceOTU252103
New.CleanUp.ReferenceOTU137349
New.CleanUp.ReferenceOTU91521
New.CleanUp.ReferenceOTU303227
New.CleanUp.ReferenceOTU154861
New.CleanUp.ReferenceOTU144225
New.CleanUp.ReferenceOTU150384
New.CleanUp.ReferenceOTU210742
New.CleanUp.ReferenceOTU299908
New.CleanUp.ReferenceOTU94134
New.CleanUp.ReferenceOTU330892
New.CleanUp.ReferenceOTU235225
New.CleanUp.ReferenceOTU228932
New.CleanUp.ReferenceOTU219632
New.CleanUp.ReferenceOTU161082
New.CleanUp.ReferenceOTU66520
New.CleanUp.ReferenceOTU127899
New.CleanUp.ReferenceOTU238867
New.CleanUp.ReferenceOTU129928
New.CleanUp.ReferenceOTU239913
New.CleanUp.ReferenceOTU96190
New.CleanUp.ReferenceOTU158225
New.CleanUp.ReferenceOTU178493

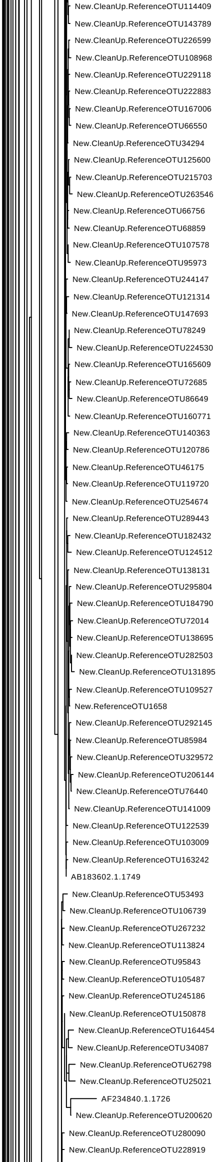
New.CleanUp.ReferenceOTU114409
New.CleanUp.ReferenceOTU143789
New.CleanUp.ReferenceOTU226599
New.CleanUp.ReferenceOTU108968
New.CleanUp.ReferenceOTU229118
New.CleanUp.ReferenceOTU222883
New.CleanUp.ReferenceOTU167006
New.CleanUp.ReferenceOTU66550
New.CleanUp.ReferenceOTU34294
New.CleanUp.ReferenceOTU125600
New.CleanUp.ReferenceOTU215703
New.CleanUp.ReferenceOTU263546
New.CleanUp.ReferenceOTU66756
New.CleanUp.ReferenceOTU68859
New.CleanUp.ReferenceOTU107578
New.CleanUp.ReferenceOTU95973
New.CleanUp.ReferenceOTU244147
New.CleanUp.ReferenceOTU121314
New.CleanUp.ReferenceOTU147693
New.CleanUp.ReferenceOTU78249
New.CleanUp.ReferenceOTU224530
New.CleanUp.ReferenceOTU165609
New.CleanUp.ReferenceOTU72685
New.CleanUp.ReferenceOTU86649
New.CleanUp.ReferenceOTU160771
New.CleanUp.ReferenceOTU140363
New.CleanUp.ReferenceOTU120786
New.CleanUp.ReferenceOTU46175
New.CleanUp.ReferenceOTU119720
New.CleanUp.ReferenceOTU254674
New.CleanUp.ReferenceOTU289443
New.CleanUp.ReferenceOTU182432
New.CleanUp.ReferenceOTU124512
New.CleanUp.ReferenceOTU138131
New.CleanUp.ReferenceOTU295804
New.CleanUp.ReferenceOTU184790
New.CleanUp.ReferenceOTU72014
New.CleanUp.ReferenceOTU138695
New.CleanUp.ReferenceOTU282503
New.CleanUp.ReferenceOTU131895
New.CleanUp.ReferenceOTU109527
New.ReferenceOTU1658
New.CleanUp.ReferenceOTU292145
New.CleanUp.ReferenceOTU85984
New.CleanUp.ReferenceOTU329572
New.CleanUp.ReferenceOTU206144
New.CleanUp.ReferenceOTU76440
New.CleanUp.ReferenceOTU141009
New.CleanUp.ReferenceOTU122539
New.CleanUp.ReferenceOTU103009
New.CleanUp.ReferenceOTU163242
AB183602.1.1749
New.CleanUp.ReferenceOTU53493
New.CleanUp.ReferenceOTU106739
New.CleanUp.ReferenceOTU267232
New.CleanUp.ReferenceOTU113824
New.CleanUp.ReferenceOTU95843
New.CleanUp.ReferenceOTU105487
New.CleanUp.ReferenceOTU245186
New.CleanUp.ReferenceOTU150878
New.CleanUp.ReferenceOTU164454
New.CleanUp.ReferenceOTU34087
New.CleanUp.ReferenceOTU62798
New.CleanUp.ReferenceOTU25021
AF234840.1.1726
New.CleanUp.ReferenceOTU200620
New.CleanUp.ReferenceOTU280090
New.CleanUp.ReferenceOTU228919

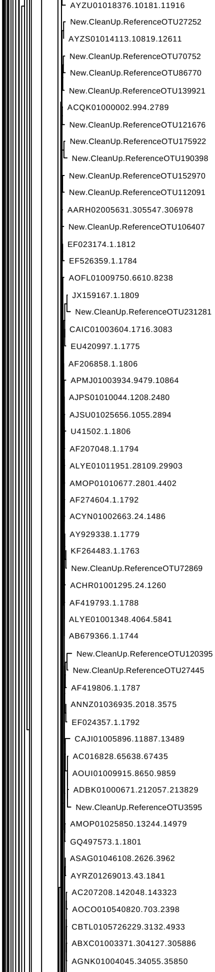
AYZU01018376.10181.11916
New.CleanUp.ReferenceOTU27252
AYZS01014113.10819.12611
New.CleanUp.ReferenceOTU70752
New.CleanUp.ReferenceOTU86770
New.CleanUp.ReferenceOTU139921
ACQK01000002.994.2789
New.CleanUp.ReferenceOTU121676
New.CleanUp.ReferenceOTU175922
New.CleanUp.ReferenceOTU190398
New.CleanUp.ReferenceOTU152970
New.CleanUp.ReferenceOTU112091
AARH02005631.305547.306978
New.CleanUp.ReferenceOTU106407
EF023174.1.1812
EF526359.1.1784
AOFL01009750.6610.8238
JX159167.1.1809
New.CleanUp.ReferenceOTU231281
CAIC01003604.1716.3083
EU420997.1.1775
AF206858.1.1806
APMJ01003934.9479.10864
AJPS01010044.1208.2480
AJSU01025656.1055.2894
U41502.1.1806
AF207048.1.1794
ALYE01011951.28109.29903
AMOP01010677.2801.4402
AF274604.1.1792
ACYN01002663.24.1486
AY929338.1.1779
KF264483.1.1763
New.CleanUp.ReferenceOTU72869
ACHR01001295.24.1260
AF419793.1.1788
ALYE01001348.4064.5841
AB679366.1.1744
New.CleanUp.ReferenceOTU120395
New.CleanUp.ReferenceOTU27445
AF419806.1.1787
ANNZ01036935.2018.3575
EF024357.1.1792
CAJI01005896.11887.13489
AC016828.65638.67435
AOUI01009915.8650.9859
ADBK01000671.212057.213829
New.CleanUp.ReferenceOTU3595
AMOP01025850.13244.14979
GQ497573.1.1801
ASAG01046108.2626.3962
AYRZ01269013.43.1841
AC207208.142048.143323
AOCO010540820.703.2398
CBTL0105726229.3132.4933
ABXC01003371.304127.305886
AGNK01004045.34055.35850

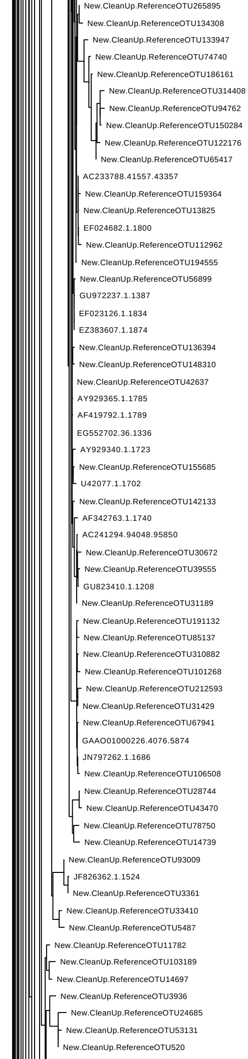
New.CleanUp.ReferenceOTU265895
New.CleanUp.ReferenceOTU134308
New.CleanUp.ReferenceOTU133947
New.CleanUp.ReferenceOTU74740
New.CleanUp.ReferenceOTU186161
New.CleanUp.ReferenceOTU314408
New.CleanUp.ReferenceOTU94762
New.CleanUp.ReferenceOTU150284
New.CleanUp.ReferenceOTU122176
New.CleanUp.ReferenceOTU65417
AC233788.41557.43357
New.CleanUp.ReferenceOTU159364
New.CleanUp.ReferenceOTU13825
EF024682.1.1800
New.CleanUp.ReferenceOTU112962
New.CleanUp.ReferenceOTU194555
New.CleanUp.ReferenceOTU56899
GU972237.1.1387
EF023126.1.1834
EZ383607.1.1874
New.CleanUp.ReferenceOTU136394
New.CleanUp.ReferenceOTU148310
New.CleanUp.ReferenceOTU42637
AY929365.1.1785
AF419792.1.1789
EG552702.36.1336
AY929340.1.1723
New.CleanUp.ReferenceOTU155685
U42077.1.1702
New.CleanUp.ReferenceOTU142133
AF342763.1.1740
AC241294.94048.95850
New.CleanUp.ReferenceOTU30672
New.CleanUp.ReferenceOTU39555
GU823410.1.1208
New.CleanUp.ReferenceOTU31189
New.CleanUp.ReferenceOTU191132
New.CleanUp.ReferenceOTU85137
New.CleanUp.ReferenceOTU310882
New.CleanUp.ReferenceOTU101268
New.CleanUp.ReferenceOTU212593
New.CleanUp.ReferenceOTU31429
New.CleanUp.ReferenceOTU67941
GAAO01000226.4076.5874
JN797262.1.1686
New.CleanUp.ReferenceOTU106508
New.CleanUp.ReferenceOTU28744
New.CleanUp.ReferenceOTU43470
New.CleanUp.ReferenceOTU78750
New.CleanUp.ReferenceOTU14739
New.CleanUp.ReferenceOTU93009
JF826362.1.1524
New.CleanUp.ReferenceOTU3361
New.CleanUp.ReferenceOTU33410
New.CleanUp.ReferenceOTU5487
New.CleanUp.ReferenceOTU11782
New.CleanUp.ReferenceOTU103189
New.CleanUp.ReferenceOTU14697
New.CleanUp.ReferenceOTU3936
New.CleanUp.ReferenceOTU24685
New.CleanUp.ReferenceOTU53131
New.CleanUp.ReferenceOTU520

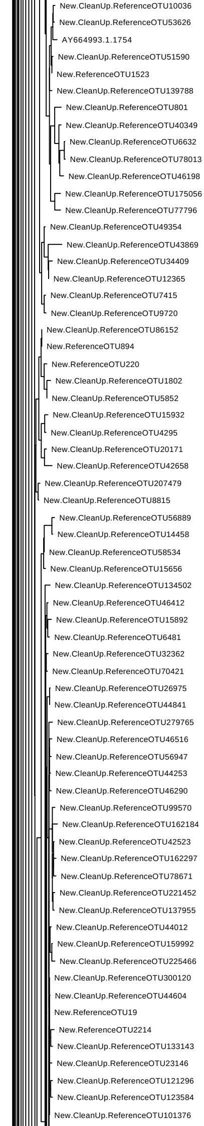
New.CleanUp.ReferenceOTU10036
New.CleanUp.ReferenceOTU53626
AY664993.1.1754
New.CleanUp.ReferenceOTU51590
New.ReferenceOTU1523
New.CleanUp.ReferenceOTU139788
New.CleanUp.ReferenceOTU801
New.CleanUp.ReferenceOTU40349
New.CleanUp.ReferenceOTU6632
New.CleanUp.ReferenceOTU78013
New.CleanUp.ReferenceOTU46198
New.CleanUp.ReferenceOTU175056
New.CleanUp.ReferenceOTU77796
New.CleanUp.ReferenceOTU49354
New.CleanUp.ReferenceOTU43869
New.CleanUp.ReferenceOTU34409
New.CleanUp.ReferenceOTU12365
New.CleanUp.ReferenceOTU7415
New.CleanUp.ReferenceOTU9720
New.CleanUp.ReferenceOTU86152
New.ReferenceOTU894
New.ReferenceOTU220
New.CleanUp.ReferenceOTU1802
New.CleanUp.ReferenceOTU5852
New.CleanUp.ReferenceOTU15932
New.CleanUp.ReferenceOTU4295
New.CleanUp.ReferenceOTU20171
New.CleanUp.ReferenceOTU42658
New.CleanUp.ReferenceOTU207479
New.CleanUp.ReferenceOTU8815
New.CleanUp.ReferenceOTU56889
New.CleanUp.ReferenceOTU14458
New.CleanUp.ReferenceOTU58534
New.CleanUp.ReferenceOTU15656
New.CleanUp.ReferenceOTU134502
New.CleanUp.ReferenceOTU46412
New.CleanUp.ReferenceOTU15892
New.CleanUp.ReferenceOTU6481
New.CleanUp.ReferenceOTU32362
New.CleanUp.ReferenceOTU70421
New.CleanUp.ReferenceOTU26975
New.CleanUp.ReferenceOTU44841
New.CleanUp.ReferenceOTU279765
New.CleanUp.ReferenceOTU46516
New.CleanUp.ReferenceOTU56947
New.CleanUp.ReferenceOTU44253
New.CleanUp.ReferenceOTU46290
New.CleanUp.ReferenceOTU99570
New.CleanUp.ReferenceOTU162184
New.CleanUp.ReferenceOTU42523
New.CleanUp.ReferenceOTU162297
New.CleanUp.ReferenceOTU78671
New.CleanUp.ReferenceOTU221452
New.CleanUp.ReferenceOTU137955
New.CleanUp.ReferenceOTU44012
New.CleanUp.ReferenceOTU159992
New.CleanUp.ReferenceOTU225466
New.CleanUp.ReferenceOTU300120
New.CleanUp.ReferenceOTU44604
New.ReferenceOTU19
New.ReferenceOTU2214
New.CleanUp.ReferenceOTU133143
New.CleanUp.ReferenceOTU23146
New.CleanUp.ReferenceOTU121296
New.CleanUp.ReferenceOTU123584
New.CleanUp.ReferenceOTU101376

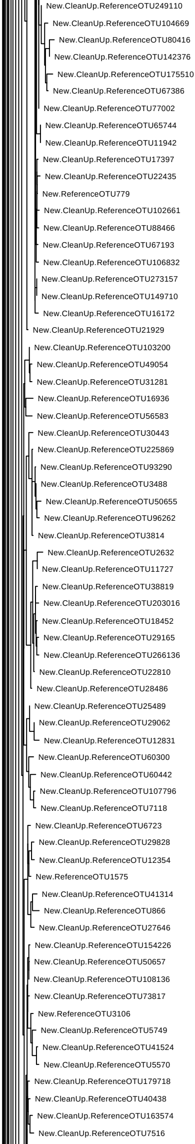
New.CleanUp.ReferenceOTU249110
New.CleanUp.ReferenceOTU104669
New.CleanUp.ReferenceOTU80416
New.CleanUp.ReferenceOTU142376
New.CleanUp.ReferenceOTU175510
New.CleanUp.ReferenceOTU67386
New.CleanUp.ReferenceOTU77002
New.CleanUp.ReferenceOTU65744
New.CleanUp.ReferenceOTU11942
New.CleanUp.ReferenceOTU17397
New.CleanUp.ReferenceOTU22435
New.ReferenceOTU779
New.CleanUp.ReferenceOTU102661
New.CleanUp.ReferenceOTU88466
New.CleanUp.ReferenceOTU67193
New.CleanUp.ReferenceOTU106832
New.CleanUp.ReferenceOTU273157
New.CleanUp.ReferenceOTU149710
New.CleanUp.ReferenceOTU16172
New.CleanUp.ReferenceOTU21929
New.CleanUp.ReferenceOTU103200
New.CleanUp.ReferenceOTU49054
New.CleanUp.ReferenceOTU31281
New.CleanUp.ReferenceOTU16936
New.CleanUp.ReferenceOTU56583
New.CleanUp.ReferenceOTU30443
New.CleanUp.ReferenceOTU225869
New.CleanUp.ReferenceOTU93290
New.CleanUp.ReferenceOTU3488
New.CleanUp.ReferenceOTU50655
New.CleanUp.ReferenceOTU96262
New.CleanUp.ReferenceOTU3814
New.CleanUp.ReferenceOTU2632
New.CleanUp.ReferenceOTU11727
New.CleanUp.ReferenceOTU38819
New.CleanUp.ReferenceOTU203016
New.CleanUp.ReferenceOTU18452
New.CleanUp.ReferenceOTU29165
New.CleanUp.ReferenceOTU266136
New.CleanUp.ReferenceOTU22810
New.CleanUp.ReferenceOTU28486
New.CleanUp.ReferenceOTU25489
New.CleanUp.ReferenceOTU29062
New.CleanUp.ReferenceOTU12831
New.CleanUp.ReferenceOTU60300
New.CleanUp.ReferenceOTU60442
New.CleanUp.ReferenceOTU107796
New.CleanUp.ReferenceOTU7118
New.CleanUp.ReferenceOTU6723
New.CleanUp.ReferenceOTU29828
New.CleanUp.ReferenceOTU12354
New.ReferenceOTU1575
New.CleanUp.ReferenceOTU41314
New.CleanUp.ReferenceOTU866
New.CleanUp.ReferenceOTU27646
New.CleanUp.ReferenceOTU154226
New.CleanUp.ReferenceOTU50657
New.CleanUp.ReferenceOTU108136
New.CleanUp.ReferenceOTU73817
New.ReferenceOTU3106
New.CleanUp.ReferenceOTU5749
New.CleanUp.ReferenceOTU41524
New.CleanUp.ReferenceOTU5570
New.CleanUp.ReferenceOTU179718
New.CleanUp.ReferenceOTU40438
New.CleanUp.ReferenceOTU163574
New.CleanUp.ReferenceOTU7516

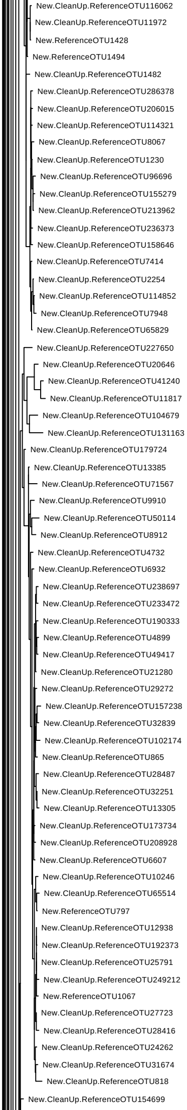
New.CleanUp.ReferenceOTU116062
New.CleanUp.ReferenceOTU11972
New.ReferenceOTU1428
New.ReferenceOTU1494
New.CleanUp.ReferenceOTU1482
New.CleanUp.ReferenceOTU286378
New.CleanUp.ReferenceOTU206015
New.CleanUp.ReferenceOTU114321
New.CleanUp.ReferenceOTU8067
New.CleanUp.ReferenceOTU1230
New.CleanUp.ReferenceOTU96696
New.CleanUp.ReferenceOTU155279
New.CleanUp.ReferenceOTU213962
New.CleanUp.ReferenceOTU236373
New.CleanUp.ReferenceOTU158646
New.CleanUp.ReferenceOTU7414
New.CleanUp.ReferenceOTU2254
New.CleanUp.ReferenceOTU114852
New.CleanUp.ReferenceOTU7948
New.CleanUp.ReferenceOTU65829
New.CleanUp.ReferenceOTU227650
New.CleanUp.ReferenceOTU20646
New.CleanUp.ReferenceOTU41240
New.CleanUp.ReferenceOTU11817
New.CleanUp.ReferenceOTU104679
New.CleanUp.ReferenceOTU131163
New.CleanUp.ReferenceOTU179724
New.CleanUp.ReferenceOTU13385
New.CleanUp.ReferenceOTU71567
New.CleanUp.ReferenceOTU9910
New.CleanUp.ReferenceOTU50114
New.CleanUp.ReferenceOTU8912
New.CleanUp.ReferenceOTU4732
New.CleanUp.ReferenceOTU6932
New.CleanUp.ReferenceOTU238697
New.CleanUp.ReferenceOTU233472
New.CleanUp.ReferenceOTU190333
New.CleanUp.ReferenceOTU4899
New.CleanUp.ReferenceOTU49417
New.CleanUp.ReferenceOTU21280
New.CleanUp.ReferenceOTU29272
New.CleanUp.ReferenceOTU157238
New.CleanUp.ReferenceOTU32839
New.CleanUp.ReferenceOTU102174
New.CleanUp.ReferenceOTU865
New.CleanUp.ReferenceOTU28487
New.CleanUp.ReferenceOTU32251
New.CleanUp.ReferenceOTU13305
New.CleanUp.ReferenceOTU173734
New.CleanUp.ReferenceOTU208928
New.CleanUp.ReferenceOTU6607
New.CleanUp.ReferenceOTU10246
New.CleanUp.ReferenceOTU65514
New.ReferenceOTU797
New.CleanUp.ReferenceOTU12938
New.CleanUp.ReferenceOTU192373
New.CleanUp.ReferenceOTU25791
New.CleanUp.ReferenceOTU249212
New.ReferenceOTU1067
New.CleanUp.ReferenceOTU27723
New.CleanUp.ReferenceOTU28416
New.CleanUp.ReferenceOTU24262
New.CleanUp.ReferenceOTU31674
New.CleanUp.ReferenceOTU818
New.CleanUp.ReferenceOTU154699

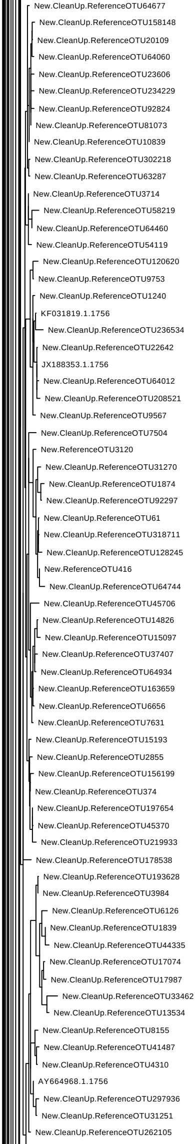

New.CleanUp.ReferenceOTU64677
New.CleanUp.ReferenceOTU158148
New.CleanUp.ReferenceOTU20109
New.CleanUp.ReferenceOTU64060
New.CleanUp.ReferenceOTU23606
New.CleanUp.ReferenceOTU234229
New.CleanUp.ReferenceOTU92824
New.CleanUp.ReferenceOTU81073
New.CleanUp.ReferenceOTU10839
New.CleanUp.ReferenceOTU302218
New.CleanUp.ReferenceOTU63287
New.CleanUp.ReferenceOTU3714
New.CleanUp.ReferenceOTU58219
New.CleanUp.ReferenceOTU64460
New.CleanUp.ReferenceOTU54119
New.CleanUp.ReferenceOTU120620
New.CleanUp.ReferenceOTU9753
New.CleanUp.ReferenceOTU1240
KF031819.1.1756
New.CleanUp.ReferenceOTU236534
New.CleanUp.ReferenceOTU22642
JX188353.1.1756
New.CleanUp.ReferenceOTU64012
New.CleanUp.ReferenceOTU208521
New.CleanUp.ReferenceOTU9567
New.CleanUp.ReferenceOTU7504
New.ReferenceOTU3120
New.CleanUp.ReferenceOTU31270
New.CleanUp.ReferenceOTU1874
New.CleanUp.ReferenceOTU92297
New.CleanUp.ReferenceOTU61
New.CleanUp.ReferenceOTU318711
New.CleanUp.ReferenceOTU128245
New.ReferenceOTU416
New.CleanUp.ReferenceOTU64744
New.CleanUp.ReferenceOTU45706
New.CleanUp.ReferenceOTU14826
New.CleanUp.ReferenceOTU15097
New.CleanUp.ReferenceOTU37407
New.CleanUp.ReferenceOTU64934
New.CleanUp.ReferenceOTU163659
New.CleanUp.ReferenceOTU6656
New.CleanUp.ReferenceOTU7631
New.CleanUp.ReferenceOTU15193
New.CleanUp.ReferenceOTU2855
New.CleanUp.ReferenceOTU156199
New.CleanUp.ReferenceOTU374
New.CleanUp.ReferenceOTU197654
New.CleanUp.ReferenceOTU45370
New.CleanUp.ReferenceOTU219933
New.CleanUp.ReferenceOTU178538
New.CleanUp.ReferenceOTU193628
New.CleanUp.ReferenceOTU3984
New.CleanUp.ReferenceOTU6126
New.CleanUp.ReferenceOTU1839
New.CleanUp.ReferenceOTU44335
New.CleanUp.ReferenceOTU17074
New.CleanUp.ReferenceOTU17987
New.CleanUp.ReferenceOTU33462
New.CleanUp.ReferenceOTU13534
New.CleanUp.ReferenceOTU8155
New.CleanUp.ReferenceOTU41487
New.CleanUp.ReferenceOTU4310
AY664968.1.1756
New.CleanUp.ReferenceOTU297936
New.CleanUp.ReferenceOTU31251
New.CleanUp.ReferenceOTU262105

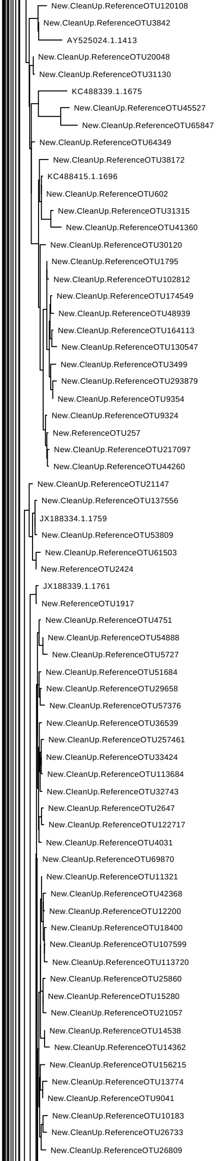
New.CleanUp.ReferenceOTU120108
New.CleanUp.ReferenceOTU3842
AY525024.1.1413
New.CleanUp.ReferenceOTU20048
New.CleanUp.ReferenceOTU31130
KC488339.1.1675
New.CleanUp.ReferenceOTU45527
New.CleanUp.ReferenceOTU65847
New.CleanUp.ReferenceOTU64349
New.CleanUp.ReferenceOTU38172
KC488415.1.1696
New.CleanUp.ReferenceOTU602
New.CleanUp.ReferenceOTU31315
New.CleanUp.ReferenceOTU41360
New.CleanUp.ReferenceOTU30120
New.CleanUp.ReferenceOTU1795
New.CleanUp.ReferenceOTU102812
New.CleanUp.ReferenceOTU174549
New.CleanUp.ReferenceOTU48939
New.CleanUp.ReferenceOTU164113
New.CleanUp.ReferenceOTU130547
New.CleanUp.ReferenceOTU3499
New.CleanUp.ReferenceOTU293879
New.CleanUp.ReferenceOTU9354
New.CleanUp.ReferenceOTU9324
New.ReferenceOTU257
New.CleanUp.ReferenceOTU217097
New.CleanUp.ReferenceOTU44260
New.CleanUp.ReferenceOTU21147
New.CleanUp.ReferenceOTU137556
JX188334.1.1759
New.CleanUp.ReferenceOTU53809
New.CleanUp.ReferenceOTU61503
New.ReferenceOTU2424
JX188339.1.1761
New.ReferenceOTU1917
New.CleanUp.ReferenceOTU4751
New.CleanUp.ReferenceOTU54888
New.CleanUp.ReferenceOTU5727
New.CleanUp.ReferenceOTU51684
New.CleanUp.ReferenceOTU29658
New.CleanUp.ReferenceOTU57376
New.CleanUp.ReferenceOTU36539
New.CleanUp.ReferenceOTU257461
New.CleanUp.ReferenceOTU33424
New.CleanUp.ReferenceOTU113684
New.CleanUp.ReferenceOTU32743
New.CleanUp.ReferenceOTU2647
New.CleanUp.ReferenceOTU122717
New.CleanUp.ReferenceOTU4031
New.CleanUp.ReferenceOTU69870
New.CleanUp.ReferenceOTU11321
New.CleanUp.ReferenceOTU42368
New.CleanUp.ReferenceOTU12200
New.CleanUp.ReferenceOTU18400
New.CleanUp.ReferenceOTU107599
New.CleanUp.ReferenceOTU113720
New.CleanUp.ReferenceOTU25860
New.CleanUp.ReferenceOTU15280
New.CleanUp.ReferenceOTU21057
New.CleanUp.ReferenceOTU14538
New.CleanUp.ReferenceOTU14362
New.CleanUp.ReferenceOTU156215
New.CleanUp.ReferenceOTU13774
New.CleanUp.ReferenceOTU9041
New.CleanUp.ReferenceOTU10183
New.CleanUp.ReferenceOTU26733
New.CleanUp.ReferenceOTU26809

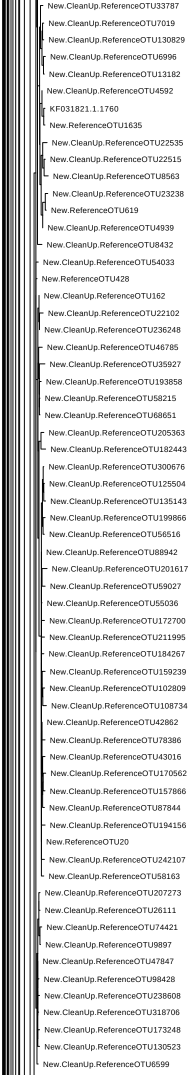
New.CleanUp.ReferenceOTU33787
New.CleanUp.ReferenceOTU7019
New.CleanUp.ReferenceOTU130829
New.CleanUp.ReferenceOTU6996
New.CleanUp.ReferenceOTU13182
New.CleanUp.ReferenceOTU4592
KF031821.1.1760
New.ReferenceOTU1635
New.CleanUp.ReferenceOTU22535
New.CleanUp.ReferenceOTU22515
New.CleanUp.ReferenceOTU8563
New.CleanUp.ReferenceOTU23238
New.ReferenceOTU619
New.CleanUp.ReferenceOTU4939
New.CleanUp.ReferenceOTU8432
New.CleanUp.ReferenceOTU54033
New.ReferenceOTU428
New.CleanUp.ReferenceOTU162
New.CleanUp.ReferenceOTU22102
New.CleanUp.ReferenceOTU236248
New.CleanUp.ReferenceOTU46785
New.CleanUp.ReferenceOTU35927
New.CleanUp.ReferenceOTU193858
New.CleanUp.ReferenceOTU58215
New.CleanUp.ReferenceOTU68651
New.CleanUp.ReferenceOTU205363
New.CleanUp.ReferenceOTU182443
New.CleanUp.ReferenceOTU300676
New.CleanUp.ReferenceOTU125504
New.CleanUp.ReferenceOTU135143
New.CleanUp.ReferenceOTU199866
New.CleanUp.ReferenceOTU56516
New.CleanUp.ReferenceOTU88942
New.CleanUp.ReferenceOTU201617
New.CleanUp.ReferenceOTU59027
New.CleanUp.ReferenceOTU55036
New.CleanUp.ReferenceOTU172700
New.CleanUp.ReferenceOTU211995
New.CleanUp.ReferenceOTU184267
New.CleanUp.ReferenceOTU159239
New.CleanUp.ReferenceOTU102809
New.CleanUp.ReferenceOTU108734
New.CleanUp.ReferenceOTU42862
New.CleanUp.ReferenceOTU78386
New.CleanUp.ReferenceOTU43016
New.CleanUp.ReferenceOTU170562
New.CleanUp.ReferenceOTU157866
New.CleanUp.ReferenceOTU87844
New.CleanUp.ReferenceOTU194156
New.ReferenceOTU20
New.CleanUp.ReferenceOTU242107
New.CleanUp.ReferenceOTU58163
New.CleanUp.ReferenceOTU207273
New.CleanUp.ReferenceOTU26111
New.CleanUp.ReferenceOTU74421
New.CleanUp.ReferenceOTU9897
New.CleanUp.ReferenceOTU47847
New.CleanUp.ReferenceOTU98428
New.CleanUp.ReferenceOTU238608
New.CleanUp.ReferenceOTU318706
New.CleanUp.ReferenceOTU173248
New.CleanUp.ReferenceOTU130523
New.CleanUp.ReferenceOTU6599

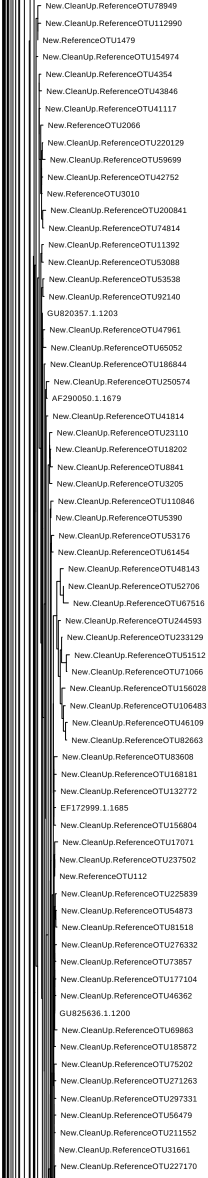
New.CleanUp.ReferenceOTU78949
New.CleanUp.ReferenceOTU112990
New.ReferenceOTU1479
New.CleanUp.ReferenceOTU154974
New.CleanUp.ReferenceOTU4354
New.CleanUp.ReferenceOTU43846
New.CleanUp.ReferenceOTU41117
New.ReferenceOTU2066
New.CleanUp.ReferenceOTU220129
New.CleanUp.ReferenceOTU59699
New.CleanUp.ReferenceOTU42752
New.ReferenceOTU3010
New.CleanUp.ReferenceOTU200841
New.CleanUp.ReferenceOTU74814
New.CleanUp.ReferenceOTU11392
New.CleanUp.ReferenceOTU53088
New.CleanUp.ReferenceOTU53538
New.CleanUp.ReferenceOTU92140
GU820357.1.1203
New.CleanUp.ReferenceOTU47961
New.CleanUp.ReferenceOTU65052
New.CleanUp.ReferenceOTU186844
New.CleanUp.ReferenceOTU250574
AF290050.1.1679
New.CleanUp.ReferenceOTU41814
New.CleanUp.ReferenceOTU23110
New.CleanUp.ReferenceOTU18202
New.CleanUp.ReferenceOTU8841
New.CleanUp.ReferenceOTU3205
New.CleanUp.ReferenceOTU110846
New.CleanUp.ReferenceOTU5390
New.CleanUp.ReferenceOTU53176
New.CleanUp.ReferenceOTU61454
New.CleanUp.ReferenceOTU48143
New.CleanUp.ReferenceOTU52706
New.CleanUp.ReferenceOTU67516
New.CleanUp.ReferenceOTU244593
New.CleanUp.ReferenceOTU233129
New.CleanUp.ReferenceOTU51512
New.CleanUp.ReferenceOTU71066
New.CleanUp.ReferenceOTU156028
New.CleanUp.ReferenceOTU106483
New.CleanUp.ReferenceOTU46109
New.CleanUp.ReferenceOTU82663
New.CleanUp.ReferenceOTU83608
New.CleanUp.ReferenceOTU168181
New.CleanUp.ReferenceOTU132772
EF172999.1.1685
New.CleanUp.ReferenceOTU156804
New.CleanUp.ReferenceOTU17071
New.CleanUp.ReferenceOTU237502
New.ReferenceOTU112
New.CleanUp.ReferenceOTU225839
New.CleanUp.ReferenceOTU54873
New.CleanUp.ReferenceOTU81518
New.CleanUp.ReferenceOTU276332
New.CleanUp.ReferenceOTU73857
New.CleanUp.ReferenceOTU177104
New.CleanUp.ReferenceOTU46362
GU825636.1.1200
New.CleanUp.ReferenceOTU69863
New.CleanUp.ReferenceOTU185872
New.CleanUp.ReferenceOTU75202
New.CleanUp.ReferenceOTU271263
New.CleanUp.ReferenceOTU297331
New.CleanUp.ReferenceOTU56479
New.CleanUp.ReferenceOTU211552
New.CleanUp.ReferenceOTU31661
New.CleanUp.ReferenceOTU227170

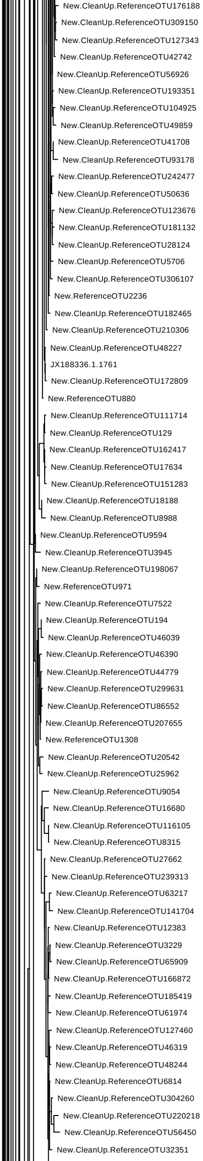
New.CleanUp.ReferenceOTU176188
New.CleanUp.ReferenceOTU309150
New.CleanUp.ReferenceOTU127343
New.CleanUp.ReferenceOTU42742
New.CleanUp.ReferenceOTU56926
New.CleanUp.ReferenceOTU193351
New.CleanUp.ReferenceOTU104925
New.CleanUp.ReferenceOTU49859
New.CleanUp.ReferenceOTU41708
New.CleanUp.ReferenceOTU93178
New.CleanUp.ReferenceOTU242477
New.CleanUp.ReferenceOTU50636
New.CleanUp.ReferenceOTU123676
New.CleanUp.ReferenceOTU181132
New.CleanUp.ReferenceOTU28124
New.CleanUp.ReferenceOTU5706
New.CleanUp.ReferenceOTU306107
New.ReferenceOTU2236
New.CleanUp.ReferenceOTU182465
New.CleanUp.ReferenceOTU210306
New.CleanUp.ReferenceOTU48227
JX188336.1.1761
New.CleanUp.ReferenceOTU172809
New.ReferenceOTU880
New.CleanUp.ReferenceOTU111714
New.CleanUp.ReferenceOTU129
New.CleanUp.ReferenceOTU162417
New.CleanUp.ReferenceOTU17634
New.CleanUp.ReferenceOTU151283
New.CleanUp.ReferenceOTU18188
New.CleanUp.ReferenceOTU8988
New.CleanUp.ReferenceOTU9594
New.CleanUp.ReferenceOTU3945
New.CleanUp.ReferenceOTU198067
New.ReferenceOTU971
New.CleanUp.ReferenceOTU7522
New.CleanUp.ReferenceOTU194
New.CleanUp.ReferenceOTU46039
New.CleanUp.ReferenceOTU46390
New.CleanUp.ReferenceOTU44779
New.CleanUp.ReferenceOTU299631
New.CleanUp.ReferenceOTU86552
New.CleanUp.ReferenceOTU207655
New.ReferenceOTU1308
New.CleanUp.ReferenceOTU20542
New.CleanUp.ReferenceOTU25962
New.CleanUp.ReferenceOTU9054
New.CleanUp.ReferenceOTU16680
New.CleanUp.ReferenceOTU116105
New.CleanUp.ReferenceOTU8315
New.CleanUp.ReferenceOTU27662
New.CleanUp.ReferenceOTU239313
New.CleanUp.ReferenceOTU63217
New.CleanUp.ReferenceOTU141704
New.CleanUp.ReferenceOTU12383
New.CleanUp.ReferenceOTU3229
New.CleanUp.ReferenceOTU65909
New.CleanUp.ReferenceOTU166872
New.CleanUp.ReferenceOTU185419
New.CleanUp.ReferenceOTU61974
New.CleanUp.ReferenceOTU127460
New.CleanUp.ReferenceOTU46319
New.CleanUp.ReferenceOTU48244
New.CleanUp.ReferenceOTU6814
New.CleanUp.ReferenceOTU304260
New.CleanUp.ReferenceOTU220218
New.CleanUp.ReferenceOTU56450
New.CleanUp.ReferenceOTU32351

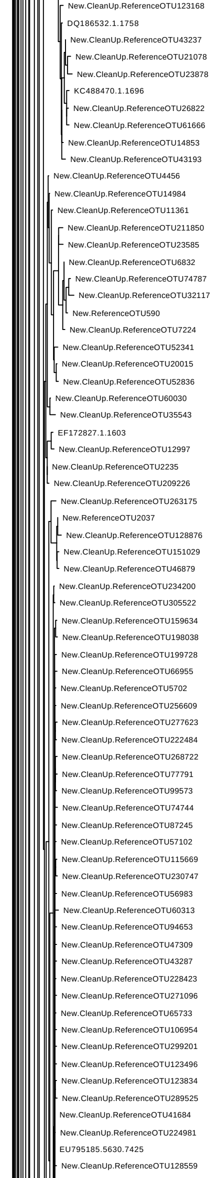
New.CleanUp.ReferenceOTU123168
DQ186532.1.1758
New.CleanUp.ReferenceOTU43237
New.CleanUp.ReferenceOTU21078
New.CleanUp.ReferenceOTU23878
KC488470.1.1696
New.CleanUp.ReferenceOTU26822
New.CleanUp.ReferenceOTU61666
New.CleanUp.ReferenceOTU14853
New.CleanUp.ReferenceOTU43193
New.CleanUp.ReferenceOTU4456
New.CleanUp.ReferenceOTU14984
New.CleanUp.ReferenceOTU11361
New.CleanUp.ReferenceOTU211850
New.CleanUp.ReferenceOTU23585
New.CleanUp.ReferenceOTU6832
New.CleanUp.ReferenceOTU74787
New.CleanUp.ReferenceOTU32117
New.ReferenceOTU590
New.CleanUp.ReferenceOTU7224
New.CleanUp.ReferenceOTU52341
New.CleanUp.ReferenceOTU20015
New.CleanUp.ReferenceOTU52836
New.CleanUp.ReferenceOTU60030
New.CleanUp.ReferenceOTU35543
EF172827.1.1603
New.CleanUp.ReferenceOTU12997
New.CleanUp.ReferenceOTU2235
New.CleanUp.ReferenceOTU209226
New.CleanUp.ReferenceOTU263175
New.ReferenceOTU2037
New.CleanUp.ReferenceOTU128876
New.CleanUp.ReferenceOTU151029
New.CleanUp.ReferenceOTU46879
New.CleanUp.ReferenceOTU234200
New.CleanUp.ReferenceOTU305522
New.CleanUp.ReferenceOTU159634
New.CleanUp.ReferenceOTU198038
New.CleanUp.ReferenceOTU199728
New.CleanUp.ReferenceOTU66955
New.CleanUp.ReferenceOTU5702
New.CleanUp.ReferenceOTU256609
New.CleanUp.ReferenceOTU277623
New.CleanUp.ReferenceOTU222484
New.CleanUp.ReferenceOTU268722
New.CleanUp.ReferenceOTU77791
New.CleanUp.ReferenceOTU99573
New.CleanUp.ReferenceOTU74744
New.CleanUp.ReferenceOTU87245
New.CleanUp.ReferenceOTU57102
New.CleanUp.ReferenceOTU115669
New.CleanUp.ReferenceOTU230747
New.CleanUp.ReferenceOTU56983
New.CleanUp.ReferenceOTU60313
New.CleanUp.ReferenceOTU94653
New.CleanUp.ReferenceOTU47309
New.CleanUp.ReferenceOTU43287
New.CleanUp.ReferenceOTU228423
New.CleanUp.ReferenceOTU271096
New.CleanUp.ReferenceOTU65733
New.CleanUp.ReferenceOTU106954
New.CleanUp.ReferenceOTU299201
New.CleanUp.ReferenceOTU123496
New.CleanUp.ReferenceOTU123834
New.CleanUp.ReferenceOTU289525
New.CleanUp.ReferenceOTU41684
New.CleanUp.ReferenceOTU224981
EU795185.5630.7425
New.CleanUp.ReferenceOTU128559

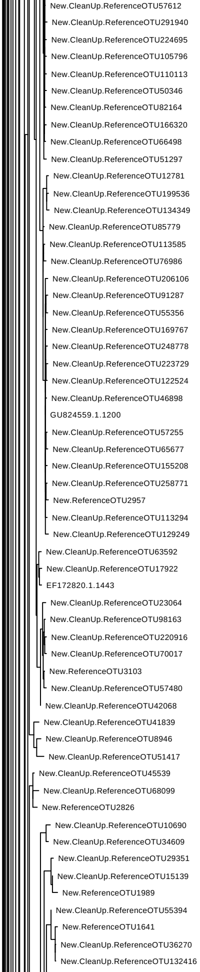
New.CleanUp.ReferenceOTU57612
New.CleanUp.ReferenceOTU291940
New.CleanUp.ReferenceOTU224695
New.CleanUp.ReferenceOTU105796
New.CleanUp.ReferenceOTU110113
New.CleanUp.ReferenceOTU50346
New.CleanUp.ReferenceOTU82164
New.CleanUp.ReferenceOTU166320
New.CleanUp.ReferenceOTU66498
New.CleanUp.ReferenceOTU51297
New.CleanUp.ReferenceOTU12781
New.CleanUp.ReferenceOTU199536
New.CleanUp.ReferenceOTU134349
New.CleanUp.ReferenceOTU85779
New.CleanUp.ReferenceOTU113585
New.CleanUp.ReferenceOTU76986
New.CleanUp.ReferenceOTU206106
New.CleanUp.ReferenceOTU91287
New.CleanUp.ReferenceOTU55356
New.CleanUp.ReferenceOTU169767
New.CleanUp.ReferenceOTU248778
New.CleanUp.ReferenceOTU223729
New.CleanUp.ReferenceOTU122524
New.CleanUp.ReferenceOTU46898
GU824559.1.1200
New.CleanUp.ReferenceOTU57255
New.CleanUp.ReferenceOTU65677
New.CleanUp.ReferenceOTU155208
New.CleanUp.ReferenceOTU258771
New.ReferenceOTU2957
New.CleanUp.ReferenceOTU113294
New.CleanUp.ReferenceOTU129249
New.CleanUp.ReferenceOTU63592
New.CleanUp.ReferenceOTU17922
EF172820.1.1443
New.CleanUp.ReferenceOTU23064
New.CleanUp.ReferenceOTU98163
New.CleanUp.ReferenceOTU220916
New.CleanUp.ReferenceOTU70017
New.ReferenceOTU3103
New.CleanUp.ReferenceOTU57480
New.CleanUp.ReferenceOTU42068
New.CleanUp.ReferenceOTU41839
New.CleanUp.ReferenceOTU8946
New.CleanUp.ReferenceOTU51417
New.CleanUp.ReferenceOTU45539
New.CleanUp.ReferenceOTU68099
New.ReferenceOTU2826
New.CleanUp.ReferenceOTU10690
New.CleanUp.ReferenceOTU34609
New.CleanUp.ReferenceOTU29351
New.CleanUp.ReferenceOTU15139
New.ReferenceOTU1989
New.CleanUp.ReferenceOTU55394
New.ReferenceOTU1641
New.CleanUp.ReferenceOTU36270
New.CleanUp.ReferenceOTU132416

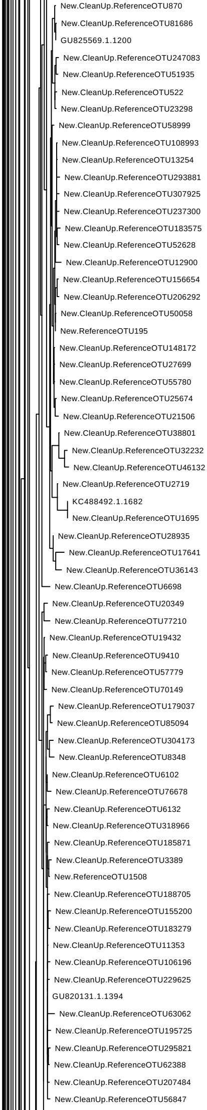
New.CleanUp.ReferenceOTU870
New.CleanUp.ReferenceOTU81686
GU825569.1.1200
New.CleanUp.ReferenceOTU247083
New.CleanUp.ReferenceOTU51935
New.CleanUp.ReferenceOTU522
New.CleanUp.ReferenceOTU23298
New.CleanUp.ReferenceOTU58999
New.CleanUp.ReferenceOTU108993
New.CleanUp.ReferenceOTU13254
New.CleanUp.ReferenceOTU293881
New.CleanUp.ReferenceOTU307925
New.CleanUp.ReferenceOTU237300
New.CleanUp.ReferenceOTU183575
New.CleanUp.ReferenceOTU52628
New.CleanUp.ReferenceOTU12900
New.CleanUp.ReferenceOTU156654
New.CleanUp.ReferenceOTU206292
New.CleanUp.ReferenceOTU50058
New.ReferenceOTU195
New.CleanUp.ReferenceOTU148172
New.CleanUp.ReferenceOTU27699
New.CleanUp.ReferenceOTU55780
New.CleanUp.ReferenceOTU25674
New.CleanUp.ReferenceOTU21506
New.CleanUp.ReferenceOTU38801
New.CleanUp.ReferenceOTU32232
New.CleanUp.ReferenceOTU46132
New.CleanUp.ReferenceOTU2719
KC488492.1.1682
New.CleanUp.ReferenceOTU1695
New.CleanUp.ReferenceOTU28935
New.CleanUp.ReferenceOTU17641
New.CleanUp.ReferenceOTU36143
New.CleanUp.ReferenceOTU6698
New.CleanUp.ReferenceOTU20349
New.CleanUp.ReferenceOTU77210
New.CleanUp.ReferenceOTU19432
New.CleanUp.ReferenceOTU9410
New.CleanUp.ReferenceOTU57779
New.CleanUp.ReferenceOTU70149
New.CleanUp.ReferenceOTU179037
New.CleanUp.ReferenceOTU85094
New.CleanUp.ReferenceOTU304173
New.CleanUp.ReferenceOTU8348
New.CleanUp.ReferenceOTU6102
New.CleanUp.ReferenceOTU76678
New.CleanUp.ReferenceOTU6132
New.CleanUp.ReferenceOTU318966
New.CleanUp.ReferenceOTU185871
New.CleanUp.ReferenceOTU3389
New.ReferenceOTU1508
New.CleanUp.ReferenceOTU188705
New.CleanUp.ReferenceOTU155200
New.CleanUp.ReferenceOTU183279
New.CleanUp.ReferenceOTU11353
New.CleanUp.ReferenceOTU106196
New.CleanUp.ReferenceOTU229625
GU820131.1.1394
New.CleanUp.ReferenceOTU63062
New.CleanUp.ReferenceOTU195725
New.CleanUp.ReferenceOTU295821
New.CleanUp.ReferenceOTU62388
New.CleanUp.ReferenceOTU207484
New.CleanUp.ReferenceOTU56847

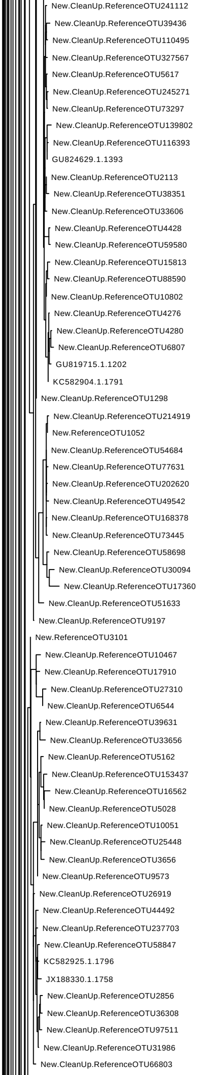
New.CleanUp.ReferenceOTU241112
New.CleanUp.ReferenceOTU39436
New.CleanUp.ReferenceOTU110495
New.CleanUp.ReferenceOTU327567
New.CleanUp.ReferenceOTU5617
New.CleanUp.ReferenceOTU245271
New.CleanUp.ReferenceOTU73297
New.CleanUp.ReferenceOTU139802
New.CleanUp.ReferenceOTU116393
GU824629.1.1393
New.CleanUp.ReferenceOTU2113
New.CleanUp.ReferenceOTU38351
New.CleanUp.ReferenceOTU33606
New.CleanUp.ReferenceOTU4428
New.CleanUp.ReferenceOTU59580
New.CleanUp.ReferenceOTU15813
New.CleanUp.ReferenceOTU88590
New.CleanUp.ReferenceOTU10802
New.CleanUp.ReferenceOTU4276
New.CleanUp.ReferenceOTU4280
New.CleanUp.ReferenceOTU6807
GU819715.1.1202
KC582904.1.1791
New.CleanUp.ReferenceOTU1298
New.CleanUp.ReferenceOTU214919
New.ReferenceOTU1052
New.CleanUp.ReferenceOTU54684
New.CleanUp.ReferenceOTU77631
New.CleanUp.ReferenceOTU202620
New.CleanUp.ReferenceOTU49542
New.CleanUp.ReferenceOTU168378
New.CleanUp.ReferenceOTU73445
New.CleanUp.ReferenceOTU58698
New.CleanUp.ReferenceOTU30094
New.CleanUp.ReferenceOTU17360
New.CleanUp.ReferenceOTU51633
New.CleanUp.ReferenceOTU9197
New.ReferenceOTU3101
New.CleanUp.ReferenceOTU10467
New.CleanUp.ReferenceOTU17910
New.CleanUp.ReferenceOTU27310
New.CleanUp.ReferenceOTU6544
New.CleanUp.ReferenceOTU39631
New.CleanUp.ReferenceOTU33656
New.CleanUp.ReferenceOTU5162
New.CleanUp.ReferenceOTU153437
New.CleanUp.ReferenceOTU16562
New.CleanUp.ReferenceOTU5028
New.CleanUp.ReferenceOTU10051
New.CleanUp.ReferenceOTU25448
New.CleanUp.ReferenceOTU3656
New.CleanUp.ReferenceOTU9573
New.CleanUp.ReferenceOTU26919
New.CleanUp.ReferenceOTU44492
New.CleanUp.ReferenceOTU237703
New.CleanUp.ReferenceOTU58847
KC582925.1.1796
JX188330.1.1758
New.CleanUp.ReferenceOTU2856
New.CleanUp.ReferenceOTU36308
New.CleanUp.ReferenceOTU97511
New.CleanUp.ReferenceOTU31986
New.CleanUp.ReferenceOTU66803

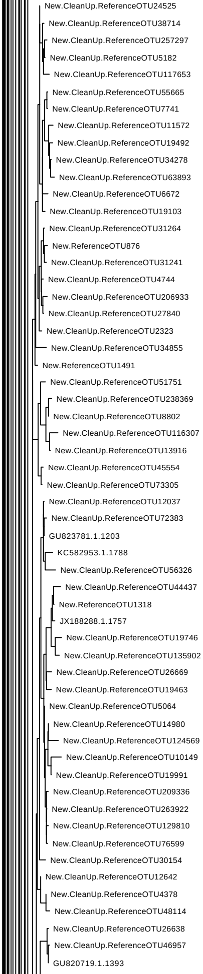
New.CleanUp.ReferenceOTU24525
New.CleanUp.ReferenceOTU38714
New.CleanUp.ReferenceOTU257297
New.CleanUp.ReferenceOTU5182
New.CleanUp.ReferenceOTU117653
New.CleanUp.ReferenceOTU55665
New.CleanUp.ReferenceOTU7741
New.CleanUp.ReferenceOTU11572
New.CleanUp.ReferenceOTU19492
New.CleanUp.ReferenceOTU34278
New.CleanUp.ReferenceOTU63893
New.CleanUp.ReferenceOTU6672
New.CleanUp.ReferenceOTU19103
New.CleanUp.ReferenceOTU31264
New.ReferenceOTU876
New.CleanUp.ReferenceOTU31241
New.CleanUp.ReferenceOTU4744
New.CleanUp.ReferenceOTU206933
New.CleanUp.ReferenceOTU27840
New.CleanUp.ReferenceOTU2323
New.CleanUp.ReferenceOTU34855
New.ReferenceOTU1491
New.CleanUp.ReferenceOTU51751
New.CleanUp.ReferenceOTU238369
New.CleanUp.ReferenceOTU8802
New.CleanUp.ReferenceOTU116307
New.CleanUp.ReferenceOTU13916
New.CleanUp.ReferenceOTU45554
New.CleanUp.ReferenceOTU73305
New.CleanUp.ReferenceOTU12037
New.CleanUp.ReferenceOTU72383
GU823781.1.1203
KC582953.1.1788
New.CleanUp.ReferenceOTU56326
New.CleanUp.ReferenceOTU44437
New.ReferenceOTU1318
JX188288.1.1757
New.CleanUp.ReferenceOTU19746
New.CleanUp.ReferenceOTU135902
New.CleanUp.ReferenceOTU26669
New.CleanUp.ReferenceOTU19463
New.CleanUp.ReferenceOTU5064
New.CleanUp.ReferenceOTU14980
New.CleanUp.ReferenceOTU124569
New.CleanUp.ReferenceOTU10149
New.CleanUp.ReferenceOTU19991
New.CleanUp.ReferenceOTU209336
New.CleanUp.ReferenceOTU263922
New.CleanUp.ReferenceOTU129810
New.CleanUp.ReferenceOTU76599
New.CleanUp.ReferenceOTU30154
New.CleanUp.ReferenceOTU12642
New.CleanUp.ReferenceOTU4378
New.CleanUp.ReferenceOTU48114
New.CleanUp.ReferenceOTU26638
New.CleanUp.ReferenceOTU46957
GU820719.1.1393

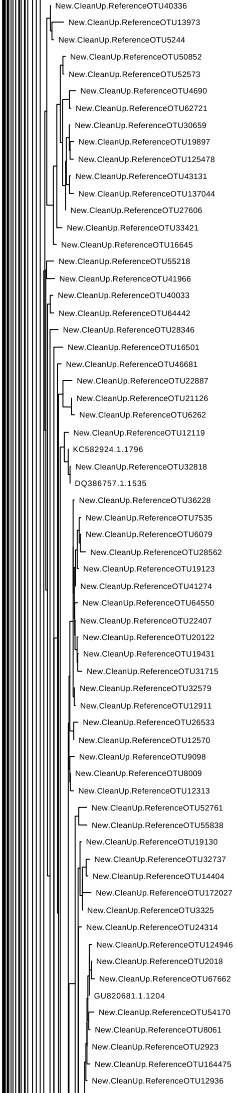
New.CleanUp.ReferenceOTU40336
New.CleanUp.ReferenceOTU13973
New.CleanUp.ReferenceOTU5244
New.CleanUp.ReferenceOTU50852
New.CleanUp.ReferenceOTU52573
New.CleanUp.ReferenceOTU4690
New.CleanUp.ReferenceOTU62721
New.CleanUp.ReferenceOTU30659
New.CleanUp.ReferenceOTU19897
New.CleanUp.ReferenceOTU125478
New.CleanUp.ReferenceOTU43131
New.CleanUp.ReferenceOTU137044
New.CleanUp.ReferenceOTU27606
New.CleanUp.ReferenceOTU33421
New.CleanUp.ReferenceOTU16645
New.CleanUp.ReferenceOTU55218
New.CleanUp.ReferenceOTU41966
New.CleanUp.ReferenceOTU40033
New.CleanUp.ReferenceOTU64442
New.CleanUp.ReferenceOTU28346
New.CleanUp.ReferenceOTU16501
New.CleanUp.ReferenceOTU46681
New.CleanUp.ReferenceOTU22887
New.CleanUp.ReferenceOTU21126
New.CleanUp.ReferenceOTU6262
New.CleanUp.ReferenceOTU12119
KC582924.1.1796
New.CleanUp.ReferenceOTU32818
DQ386757.1.1535
New.CleanUp.ReferenceOTU36228
New.CleanUp.ReferenceOTU7535
New.CleanUp.ReferenceOTU6079
New.CleanUp.ReferenceOTU28562
New.CleanUp.ReferenceOTU19123
New.CleanUp.ReferenceOTU41274
New.CleanUp.ReferenceOTU64550
New.CleanUp.ReferenceOTU22407
New.CleanUp.ReferenceOTU20122
New.CleanUp.ReferenceOTU19431
New.CleanUp.ReferenceOTU31715
New.CleanUp.ReferenceOTU32579
New.CleanUp.ReferenceOTU12911
New.CleanUp.ReferenceOTU26533
New.CleanUp.ReferenceOTU12570
New.CleanUp.ReferenceOTU9098
New.CleanUp.ReferenceOTU8009
New.CleanUp.ReferenceOTU12313
New.CleanUp.ReferenceOTU52761
New.CleanUp.ReferenceOTU55838
New.CleanUp.ReferenceOTU19130
New.CleanUp.ReferenceOTU32737
New.CleanUp.ReferenceOTU14404
New.CleanUp.ReferenceOTU172027
New.CleanUp.ReferenceOTU3325
New.CleanUp.ReferenceOTU24314
New.CleanUp.ReferenceOTU124946
New.CleanUp.ReferenceOTU2018
New.CleanUp.ReferenceOTU67662
GU820681.1.1204
New.CleanUp.ReferenceOTU54170
New.CleanUp.ReferenceOTU8061
New.CleanUp.ReferenceOTU2923
New.CleanUp.ReferenceOTU164475
New.CleanUp.ReferenceOTU12936

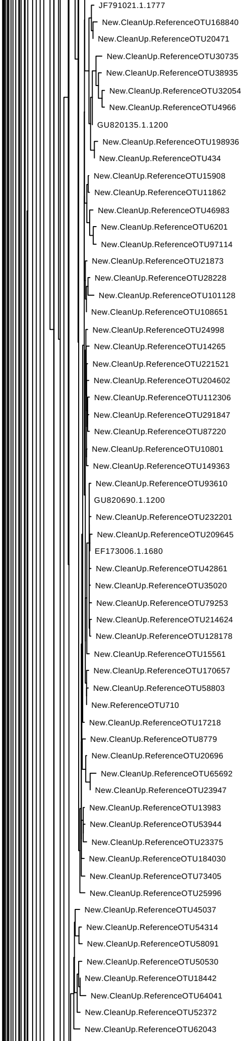
JF791021.1.1777
New.CleanUp.ReferenceOTU168840
New.CleanUp.ReferenceOTU20471
New.CleanUp.ReferenceOTU30735
New.CleanUp.ReferenceOTU38935
New.CleanUp.ReferenceOTU32054
New.CleanUp.ReferenceOTU4966
GU820135.1.1200
New.CleanUp.ReferenceOTU198936
New.CleanUp.ReferenceOTU434
New.CleanUp.ReferenceOTU15908
New.CleanUp.ReferenceOTU11862
New.CleanUp.ReferenceOTU46983
New.CleanUp.ReferenceOTU6201
New.CleanUp.ReferenceOTU97114
New.CleanUp.ReferenceOTU21873
New.CleanUp.ReferenceOTU28228
New.CleanUp.ReferenceOTU101128
New.CleanUp.ReferenceOTU108651
New.CleanUp.ReferenceOTU24998
New.CleanUp.ReferenceOTU14265
New.CleanUp.ReferenceOTU221521
New.CleanUp.ReferenceOTU204602
New.CleanUp.ReferenceOTU112306
New.CleanUp.ReferenceOTU291847
New.CleanUp.ReferenceOTU87220
New.CleanUp.ReferenceOTU10801
New.CleanUp.ReferenceOTU149363
New.CleanUp.ReferenceOTU93610
GU820690.1.1200
New.CleanUp.ReferenceOTU232201
New.CleanUp.ReferenceOTU209645
EF173006.1.1680
New.CleanUp.ReferenceOTU42861
New.CleanUp.ReferenceOTU35020
New.CleanUp.ReferenceOTU79253
New.CleanUp.ReferenceOTU214624
New.CleanUp.ReferenceOTU128178
New.CleanUp.ReferenceOTU15561
New.CleanUp.ReferenceOTU170657
New.CleanUp.ReferenceOTU58803
New.ReferenceOTU710
New.CleanUp.ReferenceOTU17218
New.CleanUp.ReferenceOTU8779
New.CleanUp.ReferenceOTU20696
New.CleanUp.ReferenceOTU65692
New.CleanUp.ReferenceOTU23947
New.CleanUp.ReferenceOTU13983
New.CleanUp.ReferenceOTU53944
New.CleanUp.ReferenceOTU23375
New.CleanUp.ReferenceOTU184030
New.CleanUp.ReferenceOTU73405
New.CleanUp.ReferenceOTU25996
New.CleanUp.ReferenceOTU45037
New.CleanUp.ReferenceOTU54314
New.CleanUp.ReferenceOTU58091
New.CleanUp.ReferenceOTU50530
New.CleanUp.ReferenceOTU18442
New.CleanUp.ReferenceOTU64041
New.CleanUp.ReferenceOTU52372
New.CleanUp.ReferenceOTU62043

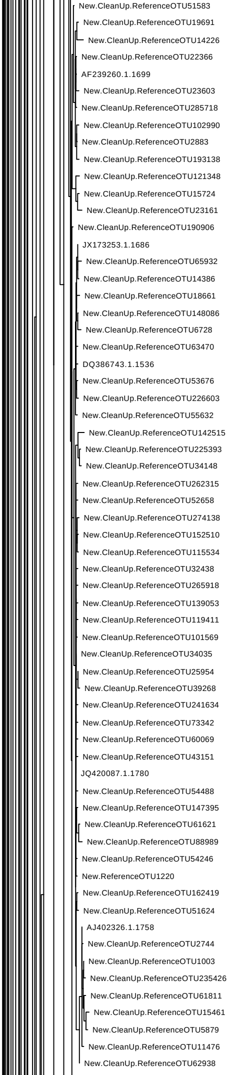

New.CleanUp.ReferenceOTU51583
New.CleanUp.ReferenceOTU19691
New.CleanUp.ReferenceOTU14226
New.CleanUp.ReferenceOTU22366
AF239260.1.1699
New.CleanUp.ReferenceOTU23603
New.CleanUp.ReferenceOTU285718
New.CleanUp.ReferenceOTU102990
New.CleanUp.ReferenceOTU2883
New.CleanUp.ReferenceOTU193138
New.CleanUp.ReferenceOTU121348
New.CleanUp.ReferenceOTU15724
New.CleanUp.ReferenceOTU23161
New.CleanUp.ReferenceOTU190906
JX173253.1.1686
New.CleanUp.ReferenceOTU65932
New.CleanUp.ReferenceOTU14386
New.CleanUp.ReferenceOTU18661
New.CleanUp.ReferenceOTU148086
New.CleanUp.ReferenceOTU6728
New.CleanUp.ReferenceOTU63470
DQ386743.1.1536
New.CleanUp.ReferenceOTU53676
New.CleanUp.ReferenceOTU226603
New.CleanUp.ReferenceOTU55632
New.CleanUp.ReferenceOTU142515
New.CleanUp.ReferenceOTU225393
New.CleanUp.ReferenceOTU34148
New.CleanUp.ReferenceOTU262315
New.CleanUp.ReferenceOTU52658
New.CleanUp.ReferenceOTU274138
New.CleanUp.ReferenceOTU152510
New.CleanUp.ReferenceOTU115534
New.CleanUp.ReferenceOTU32438
New.CleanUp.ReferenceOTU265918
New.CleanUp.ReferenceOTU139053
New.CleanUp.ReferenceOTU119411
New.CleanUp.ReferenceOTU101569
New.CleanUp.ReferenceOTU34035
New.CleanUp.ReferenceOTU25954
New.CleanUp.ReferenceOTU39268
New.CleanUp.ReferenceOTU241634
New.CleanUp.ReferenceOTU73342
New.CleanUp.ReferenceOTU60069
New.CleanUp.ReferenceOTU43151
JQ420087.1.1780
New.CleanUp.ReferenceOTU54488
New.CleanUp.ReferenceOTU147395
New.CleanUp.ReferenceOTU61621
New.CleanUp.ReferenceOTU88989
New.CleanUp.ReferenceOTU54246
New.ReferenceOTU1220
New.CleanUp.ReferenceOTU162419
New.CleanUp.ReferenceOTU51624
AJ402326.1.1758
New.CleanUp.ReferenceOTU2744
New.CleanUp.ReferenceOTU1003
New.CleanUp.ReferenceOTU235426
New.CleanUp.ReferenceOTU61811
New.CleanUp.ReferenceOTU15461
New.CleanUp.ReferenceOTU5879
New.CleanUp.ReferenceOTU11476
New.CleanUp.ReferenceOTU62938

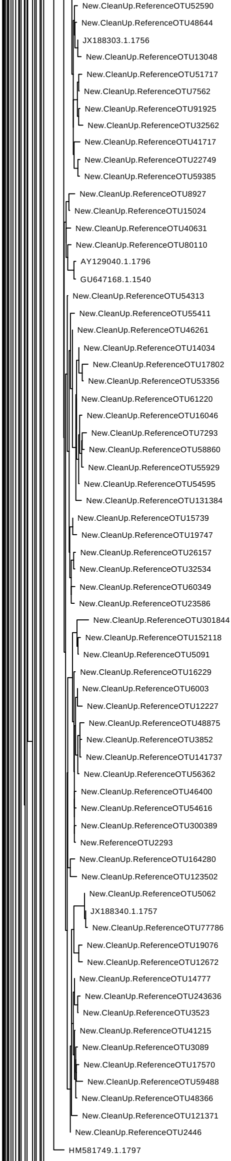
New.CleanUp.ReferenceOTU52590
New.CleanUp.ReferenceOTU48644
JX188303.1.1756
New.CleanUp.ReferenceOTU13048
New.CleanUp.ReferenceOTU51717
New.CleanUp.ReferenceOTU7562
New.CleanUp.ReferenceOTU91925
New.CleanUp.ReferenceOTU32562
New.CleanUp.ReferenceOTU41717
New.CleanUp.ReferenceOTU22749
New.CleanUp.ReferenceOTU59385
New.CleanUp.ReferenceOTU8927
New.CleanUp.ReferenceOTU15024
New.CleanUp.ReferenceOTU40631
New.CleanUp.ReferenceOTU80110
AY129040.1.1796
GU647168.1.1540
New.CleanUp.ReferenceOTU54313
New.CleanUp.ReferenceOTU55411
New.CleanUp.ReferenceOTU46261
New.CleanUp.ReferenceOTU14034
New.CleanUp.ReferenceOTU17802
New.CleanUp.ReferenceOTU53356
New.CleanUp.ReferenceOTU61220
New.CleanUp.ReferenceOTU16046
New.CleanUp.ReferenceOTU7293
New.CleanUp.ReferenceOTU58860
New.CleanUp.ReferenceOTU55929
New.CleanUp.ReferenceOTU54595
New.CleanUp.ReferenceOTU131384
New.CleanUp.ReferenceOTU15739
New.CleanUp.ReferenceOTU19747
New.CleanUp.ReferenceOTU26157
New.CleanUp.ReferenceOTU32534
New.CleanUp.ReferenceOTU60349
New.CleanUp.ReferenceOTU23586
New.CleanUp.ReferenceOTU301844
New.CleanUp.ReferenceOTU152118
New.CleanUp.ReferenceOTU5091
New.CleanUp.ReferenceOTU16229
New.CleanUp.ReferenceOTU6003
New.CleanUp.ReferenceOTU12227
New.CleanUp.ReferenceOTU48875
New.CleanUp.ReferenceOTU3852
New.CleanUp.ReferenceOTU141737
New.CleanUp.ReferenceOTU56362
New.CleanUp.ReferenceOTU46400
New.CleanUp.ReferenceOTU54616
New.CleanUp.ReferenceOTU300389
New.ReferenceOTU2293
New.CleanUp.ReferenceOTU164280
New.CleanUp.ReferenceOTU123502
New.CleanUp.ReferenceOTU5062
JX188340.1.1757
New.CleanUp.ReferenceOTU77786
New.CleanUp.ReferenceOTU19076
New.CleanUp.ReferenceOTU12672
New.CleanUp.ReferenceOTU14777
New.CleanUp.ReferenceOTU243636
New.CleanUp.ReferenceOTU3523
New.CleanUp.ReferenceOTU41215
New.CleanUp.ReferenceOTU3089
New.CleanUp.ReferenceOTU17570
New.CleanUp.ReferenceOTU59488
New.CleanUp.ReferenceOTU48366
New.CleanUp.ReferenceOTU121371
New.CleanUp.ReferenceOTU2446
HM581749.1.1797

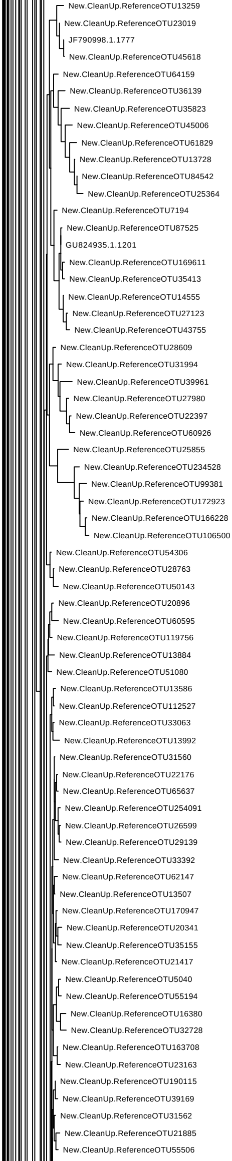
New.CleanUp.ReferenceOTU13259
New.CleanUp.ReferenceOTU23019
JF790998.1.1777
New.CleanUp.ReferenceOTU45618
New.CleanUp.ReferenceOTU64159
New.CleanUp.ReferenceOTU36139
New.CleanUp.ReferenceOTU35823
New.CleanUp.ReferenceOTU45006
New.CleanUp.ReferenceOTU61829
New.CleanUp.ReferenceOTU13728
New.CleanUp.ReferenceOTU84542
New.CleanUp.ReferenceOTU25364
New.CleanUp.ReferenceOTU7194
New.CleanUp.ReferenceOTU87525
GU824935.1.1201
New.CleanUp.ReferenceOTU169611
New.CleanUp.ReferenceOTU35413
New.CleanUp.ReferenceOTU14555
New.CleanUp.ReferenceOTU27123
New.CleanUp.ReferenceOTU43755
New.CleanUp.ReferenceOTU28609
New.CleanUp.ReferenceOTU31994
New.CleanUp.ReferenceOTU39961
New.CleanUp.ReferenceOTU27980
New.CleanUp.ReferenceOTU22397
New.CleanUp.ReferenceOTU60926
New.CleanUp.ReferenceOTU25855
New.CleanUp.ReferenceOTU234528
New.CleanUp.ReferenceOTU99381
New.CleanUp.ReferenceOTU172923
New.CleanUp.ReferenceOTU166228
New.CleanUp.ReferenceOTU106500
New.CleanUp.ReferenceOTU54306
New.CleanUp.ReferenceOTU28763
New.CleanUp.ReferenceOTU50143
New.CleanUp.ReferenceOTU20896
New.CleanUp.ReferenceOTU60595
New.CleanUp.ReferenceOTU119756
New.CleanUp.ReferenceOTU13884
New.CleanUp.ReferenceOTU51080
New.CleanUp.ReferenceOTU13586
New.CleanUp.ReferenceOTU112527
New.CleanUp.ReferenceOTU33063
New.CleanUp.ReferenceOTU13992
New.CleanUp.ReferenceOTU31560
New.CleanUp.ReferenceOTU22176
New.CleanUp.ReferenceOTU65637
New.CleanUp.ReferenceOTU254091
New.CleanUp.ReferenceOTU26599
New.CleanUp.ReferenceOTU29139
New.CleanUp.ReferenceOTU33392
New.CleanUp.ReferenceOTU62147
New.CleanUp.ReferenceOTU13507
New.CleanUp.ReferenceOTU170947
New.CleanUp.ReferenceOTU20341
New.CleanUp.ReferenceOTU35155
New.CleanUp.ReferenceOTU21417
New.CleanUp.ReferenceOTU5040
New.CleanUp.ReferenceOTU55194
New.CleanUp.ReferenceOTU16380
New.CleanUp.ReferenceOTU32728
New.CleanUp.ReferenceOTU163708
New.CleanUp.ReferenceOTU23163
New.CleanUp.ReferenceOTU190115
New.CleanUp.ReferenceOTU39169
New.CleanUp.ReferenceOTU31562
New.CleanUp.ReferenceOTU21885
New.CleanUp.ReferenceOTU55506

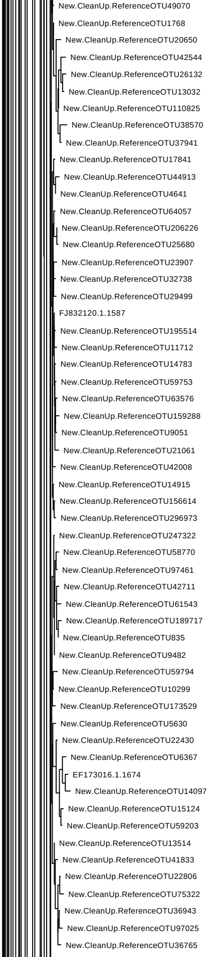
New.CleanUp.ReferenceOTU49070
New.CleanUp.ReferenceOTU1768
New.CleanUp.ReferenceOTU20650
New.CleanUp.ReferenceOTU42544
New.CleanUp.ReferenceOTU26132
New.CleanUp.ReferenceOTU13032
New.CleanUp.ReferenceOTU110825
New.CleanUp.ReferenceOTU38570
New.CleanUp.ReferenceOTU37941
New.CleanUp.ReferenceOTU17841
New.CleanUp.ReferenceOTU44913
New.CleanUp.ReferenceOTU4641
New.CleanUp.ReferenceOTU64057
New.CleanUp.ReferenceOTU206226
New.CleanUp.ReferenceOTU25680
New.CleanUp.ReferenceOTU23907
New.CleanUp.ReferenceOTU32738
New.CleanUp.ReferenceOTU29499
FJ832120.1.1587
New.CleanUp.ReferenceOTU195514
New.CleanUp.ReferenceOTU11712
New.CleanUp.ReferenceOTU14783
New.CleanUp.ReferenceOTU59753
New.CleanUp.ReferenceOTU63576
New.CleanUp.ReferenceOTU159288
New.CleanUp.ReferenceOTU9051
New.CleanUp.ReferenceOTU21061
New.CleanUp.ReferenceOTU42008
New.CleanUp.ReferenceOTU14915
New.CleanUp.ReferenceOTU156614
New.CleanUp.ReferenceOTU296973
New.CleanUp.ReferenceOTU247322
New.CleanUp.ReferenceOTU58770
New.CleanUp.ReferenceOTU97461
New.CleanUp.ReferenceOTU42711
New.CleanUp.ReferenceOTU61543
New.CleanUp.ReferenceOTU189717
New.CleanUp.ReferenceOTU835
New.CleanUp.ReferenceOTU9482
New.CleanUp.ReferenceOTU59794
New.CleanUp.ReferenceOTU10299
New.CleanUp.ReferenceOTU173529
New.CleanUp.ReferenceOTU5630
New.CleanUp.ReferenceOTU22430
New.CleanUp.ReferenceOTU6367
EF173016.1.1674
New.CleanUp.ReferenceOTU14097
New.CleanUp.ReferenceOTU15124
New.CleanUp.ReferenceOTU59203
New.CleanUp.ReferenceOTU13514
New.CleanUp.ReferenceOTU41833
New.CleanUp.ReferenceOTU22806
New.CleanUp.ReferenceOTU75322
New.CleanUp.ReferenceOTU36943
New.CleanUp.ReferenceOTU97025
New.CleanUp.ReferenceOTU36765

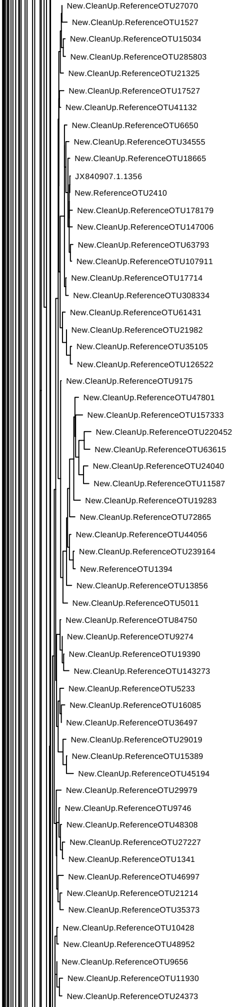
New.CleanUp.ReferenceOTU27070
New.CleanUp.ReferenceOTU1527
New.CleanUp.ReferenceOTU15034
New.CleanUp.ReferenceOTU285803
New.CleanUp.ReferenceOTU21325
New.CleanUp.ReferenceOTU17527
New.CleanUp.ReferenceOTU41132
New.CleanUp.ReferenceOTU6650
New.CleanUp.ReferenceOTU34555
New.CleanUp.ReferenceOTU18665
JX840907.1.1356
New.ReferenceOTU2410
New.CleanUp.ReferenceOTU178179
New.CleanUp.ReferenceOTU147006
New.CleanUp.ReferenceOTU63793
New.CleanUp.ReferenceOTU107911
New.CleanUp.ReferenceOTU17714
New.CleanUp.ReferenceOTU308334
New.CleanUp.ReferenceOTU61431
New.CleanUp.ReferenceOTU21982
New.CleanUp.ReferenceOTU35105
New.CleanUp.ReferenceOTU126522
New.CleanUp.ReferenceOTU9175
New.CleanUp.ReferenceOTU47801
New.CleanUp.ReferenceOTU157333
New.CleanUp.ReferenceOTU220452
New.CleanUp.ReferenceOTU63615
New.CleanUp.ReferenceOTU24040
New.CleanUp.ReferenceOTU11587
New.CleanUp.ReferenceOTU19283
New.CleanUp.ReferenceOTU72865
New.CleanUp.ReferenceOTU44056
New.CleanUp.ReferenceOTU239164
New.ReferenceOTU1394
New.CleanUp.ReferenceOTU13856
New.CleanUp.ReferenceOTU5011
New.CleanUp.ReferenceOTU84750
New.CleanUp.ReferenceOTU9274
New.CleanUp.ReferenceOTU19390
New.CleanUp.ReferenceOTU143273
New.CleanUp.ReferenceOTU5233
New.CleanUp.ReferenceOTU16085
New.CleanUp.ReferenceOTU36497
New.CleanUp.ReferenceOTU29019
New.CleanUp.ReferenceOTU15389
New.CleanUp.ReferenceOTU45194
New.CleanUp.ReferenceOTU29979
New.CleanUp.ReferenceOTU9746
New.CleanUp.ReferenceOTU48308
New.CleanUp.ReferenceOTU27227
New.CleanUp.ReferenceOTU1341
New.CleanUp.ReferenceOTU46997
New.CleanUp.ReferenceOTU21214
New.CleanUp.ReferenceOTU35373
New.CleanUp.ReferenceOTU10428
New.CleanUp.ReferenceOTU48952
New.CleanUp.ReferenceOTU9656
New.CleanUp.ReferenceOTU11930
New.CleanUp.ReferenceOTU24373

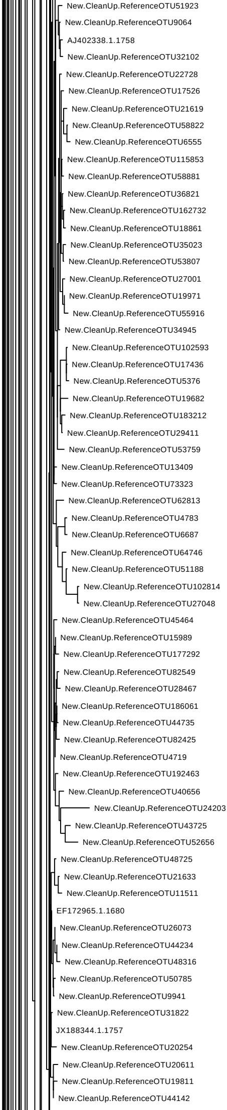
New.CleanUp.ReferenceOTU51923
New.CleanUp.ReferenceOTU9064
AJ402338.1.1758
New.CleanUp.ReferenceOTU32102
New.CleanUp.ReferenceOTU22728
New.CleanUp.ReferenceOTU17526
New.CleanUp.ReferenceOTU21619
New.CleanUp.ReferenceOTU58822
New.CleanUp.ReferenceOTU6555
New.CleanUp.ReferenceOTU115853
New.CleanUp.ReferenceOTU58881
New.CleanUp.ReferenceOTU36821
New.CleanUp.ReferenceOTU162732
New.CleanUp.ReferenceOTU18861
New.CleanUp.ReferenceOTU35023
New.CleanUp.ReferenceOTU53807
New.CleanUp.ReferenceOTU27001
New.CleanUp.ReferenceOTU19971
New.CleanUp.ReferenceOTU55916
New.CleanUp.ReferenceOTU34945
New.CleanUp.ReferenceOTU102593
New.CleanUp.ReferenceOTU17436
New.CleanUp.ReferenceOTU5376
New.CleanUp.ReferenceOTU19682
New.CleanUp.ReferenceOTU183212
New.CleanUp.ReferenceOTU29411
New.CleanUp.ReferenceOTU53759
New.CleanUp.ReferenceOTU13409
New.CleanUp.ReferenceOTU73323
New.CleanUp.ReferenceOTU62813
New.CleanUp.ReferenceOTU4783
New.CleanUp.ReferenceOTU6687
New.CleanUp.ReferenceOTU64746
New.CleanUp.ReferenceOTU51188
New.CleanUp.ReferenceOTU102814
New.CleanUp.ReferenceOTU27048
New.CleanUp.ReferenceOTU45464
New.CleanUp.ReferenceOTU15989
New.CleanUp.ReferenceOTU177292
New.CleanUp.ReferenceOTU82549
New.CleanUp.ReferenceOTU28467
New.CleanUp.ReferenceOTU186061
New.CleanUp.ReferenceOTU44735
New.CleanUp.ReferenceOTU82425
New.CleanUp.ReferenceOTU4719
New.CleanUp.ReferenceOTU192463
New.CleanUp.ReferenceOTU40656
New.CleanUp.ReferenceOTU24203
New.CleanUp.ReferenceOTU43725
New.CleanUp.ReferenceOTU52656
New.CleanUp.ReferenceOTU48725
New.CleanUp.ReferenceOTU21633
New.CleanUp.ReferenceOTU11511
EF172965.1.1680
New.CleanUp.ReferenceOTU26073
New.CleanUp.ReferenceOTU44234
New.CleanUp.ReferenceOTU48316
New.CleanUp.ReferenceOTU50785
New.CleanUp.ReferenceOTU9941
New.CleanUp.ReferenceOTU31822
JX188344.1.1757
New.CleanUp.ReferenceOTU20254
New.CleanUp.ReferenceOTU20611
New.CleanUp.ReferenceOTU19811
New.CleanUp.ReferenceOTU44142

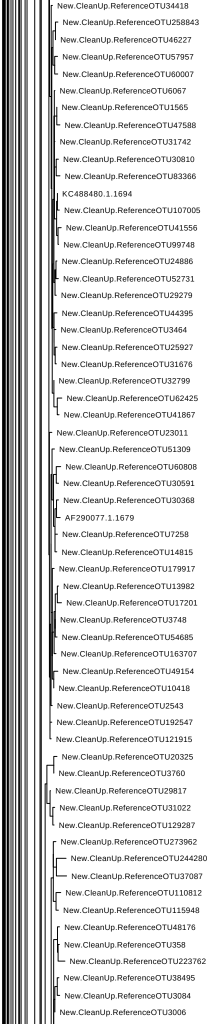
New.CleanUp.ReferenceOTU34418
New.CleanUp.ReferenceOTU258843
New.CleanUp.ReferenceOTU46227
New.CleanUp.ReferenceOTU57957
New.CleanUp.ReferenceOTU60007
New.CleanUp.ReferenceOTU6067
New.CleanUp.ReferenceOTU1565
New.CleanUp.ReferenceOTU47588
New.CleanUp.ReferenceOTU31742
New.CleanUp.ReferenceOTU30810
New.CleanUp.ReferenceOTU83366
KC488480.1.1694
New.CleanUp.ReferenceOTU107005
New.CleanUp.ReferenceOTU41556
New.CleanUp.ReferenceOTU99748
New.CleanUp.ReferenceOTU24886
New.CleanUp.ReferenceOTU52731
New.CleanUp.ReferenceOTU29279
New.CleanUp.ReferenceOTU44395
New.CleanUp.ReferenceOTU3464
New.CleanUp.ReferenceOTU25927
New.CleanUp.ReferenceOTU31676
New.CleanUp.ReferenceOTU32799
New.CleanUp.ReferenceOTU62425
New.CleanUp.ReferenceOTU41867
New.CleanUp.ReferenceOTU23011
New.CleanUp.ReferenceOTU51309
New.CleanUp.ReferenceOTU60808
New.CleanUp.ReferenceOTU30591
New.CleanUp.ReferenceOTU30368
AF290077.1.1679
New.CleanUp.ReferenceOTU7258
New.CleanUp.ReferenceOTU14815
New.CleanUp.ReferenceOTU179917
New.CleanUp.ReferenceOTU13982
New.CleanUp.ReferenceOTU17201
New.CleanUp.ReferenceOTU3748
New.CleanUp.ReferenceOTU54685
New.CleanUp.ReferenceOTU163707
New.CleanUp.ReferenceOTU49154
New.CleanUp.ReferenceOTU10418
New.CleanUp.ReferenceOTU2543
New.CleanUp.ReferenceOTU192547
New.CleanUp.ReferenceOTU121915
New.CleanUp.ReferenceOTU20325
New.CleanUp.ReferenceOTU3760
New.CleanUp.ReferenceOTU29817
New.CleanUp.ReferenceOTU31022
New.CleanUp.ReferenceOTU129287
New.CleanUp.ReferenceOTU273962
New.CleanUp.ReferenceOTU244280
New.CleanUp.ReferenceOTU37087
New.CleanUp.ReferenceOTU110812
New.CleanUp.ReferenceOTU115948
New.CleanUp.ReferenceOTU48176
New.CleanUp.ReferenceOTU358
New.CleanUp.ReferenceOTU223762
New.CleanUp.ReferenceOTU38495
New.CleanUp.ReferenceOTU3084
New.CleanUp.ReferenceOTU3006

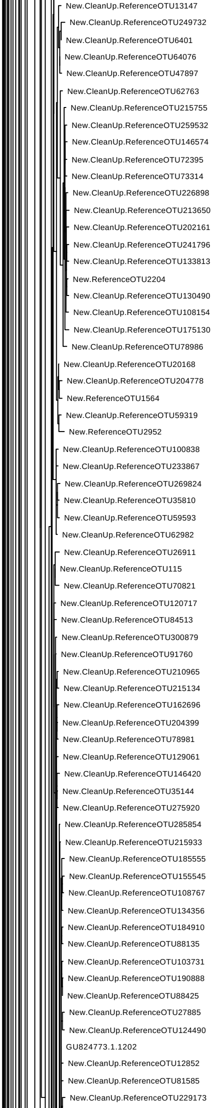
New.CleanUp.ReferenceOTU13147
New.CleanUp.ReferenceOTU249732
New.CleanUp.ReferenceOTU6401
New.CleanUp.ReferenceOTU64076
New.CleanUp.ReferenceOTU47897
New.CleanUp.ReferenceOTU62763
New.CleanUp.ReferenceOTU215755
New.CleanUp.ReferenceOTU259532
New.CleanUp.ReferenceOTU146574
New.CleanUp.ReferenceOTU72395
New.CleanUp.ReferenceOTU73314
New.CleanUp.ReferenceOTU226898
New.CleanUp.ReferenceOTU213650
New.CleanUp.ReferenceOTU202161
New.CleanUp.ReferenceOTU241796
New.CleanUp.ReferenceOTU133813
New.ReferenceOTU2204
New.CleanUp.ReferenceOTU130490
New.CleanUp.ReferenceOTU108154
New.CleanUp.ReferenceOTU175130
New.CleanUp.ReferenceOTU78986
New.CleanUp.ReferenceOTU20168
New.CleanUp.ReferenceOTU204778
New.ReferenceOTU1564
New.CleanUp.ReferenceOTU59319
New.ReferenceOTU2952
New.CleanUp.ReferenceOTU100838
New.CleanUp.ReferenceOTU233867
New.CleanUp.ReferenceOTU269824
New.CleanUp.ReferenceOTU35810
New.CleanUp.ReferenceOTU59593
New.CleanUp.ReferenceOTU62982
New.CleanUp.ReferenceOTU26911
New.CleanUp.ReferenceOTU115
New.CleanUp.ReferenceOTU70821
New.CleanUp.ReferenceOTU120717
New.CleanUp.ReferenceOTU84513
New.CleanUp.ReferenceOTU300879
New.CleanUp.ReferenceOTU91760
New.CleanUp.ReferenceOTU210965
New.CleanUp.ReferenceOTU215134
New.CleanUp.ReferenceOTU162696
New.CleanUp.ReferenceOTU204399
New.CleanUp.ReferenceOTU78981
New.CleanUp.ReferenceOTU129061
New.CleanUp.ReferenceOTU146420
New.CleanUp.ReferenceOTU35144
New.CleanUp.ReferenceOTU275920
New.CleanUp.ReferenceOTU285854
New.CleanUp.ReferenceOTU215933
New.CleanUp.ReferenceOTU185555
New.CleanUp.ReferenceOTU155545
New.CleanUp.ReferenceOTU108767
New.CleanUp.ReferenceOTU134356
New.CleanUp.ReferenceOTU184910
New.CleanUp.ReferenceOTU88135
New.CleanUp.ReferenceOTU103731
New.CleanUp.ReferenceOTU190888
New.CleanUp.ReferenceOTU88425
New.CleanUp.ReferenceOTU27885
New.CleanUp.ReferenceOTU124490
GU824773.1.1202
New.CleanUp.ReferenceOTU12852
New.CleanUp.ReferenceOTU81585
New.CleanUp.ReferenceOTU229173

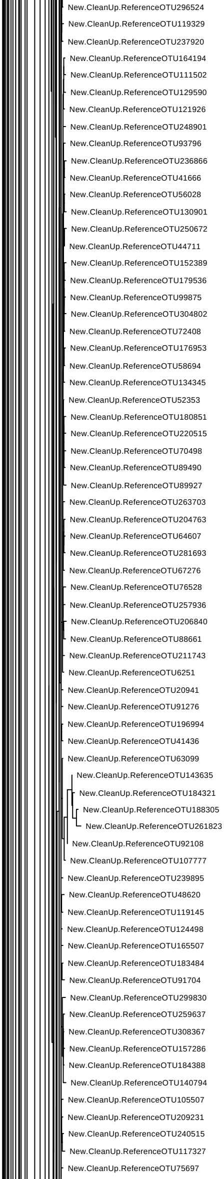
New.CleanUp.ReferenceOTU296524
New.CleanUp.ReferenceOTU119329
New.CleanUp.ReferenceOTU237920
New.CleanUp.ReferenceOTU164194
New.CleanUp.ReferenceOTU111502
New.CleanUp.ReferenceOTU129590
New.CleanUp.ReferenceOTU121926
New.CleanUp.ReferenceOTU248901
New.CleanUp.ReferenceOTU93796
New.CleanUp.ReferenceOTU236866
New.CleanUp.ReferenceOTU41666
New.CleanUp.ReferenceOTU56028
New.CleanUp.ReferenceOTU130901
New.CleanUp.ReferenceOTU250672
New.CleanUp.ReferenceOTU44711
New.CleanUp.ReferenceOTU152389
New.CleanUp.ReferenceOTU179536
New.CleanUp.ReferenceOTU99875
New.CleanUp.ReferenceOTU304802
New.CleanUp.ReferenceOTU72408
New.CleanUp.ReferenceOTU176953
New.CleanUp.ReferenceOTU58694
New.CleanUp.ReferenceOTU134345
New.CleanUp.ReferenceOTU52353
New.CleanUp.ReferenceOTU180851
New.CleanUp.ReferenceOTU220515
New.CleanUp.ReferenceOTU70498
New.CleanUp.ReferenceOTU89490
New.CleanUp.ReferenceOTU89927
New.CleanUp.ReferenceOTU263703
New.CleanUp.ReferenceOTU204763
New.CleanUp.ReferenceOTU64607
New.CleanUp.ReferenceOTU281693
New.CleanUp.ReferenceOTU67276
New.CleanUp.ReferenceOTU76528
New.CleanUp.ReferenceOTU257936
New.CleanUp.ReferenceOTU206840
New.CleanUp.ReferenceOTU88661
New.CleanUp.ReferenceOTU211743
New.CleanUp.ReferenceOTU6251
New.CleanUp.ReferenceOTU20941
New.CleanUp.ReferenceOTU91276
New.CleanUp.ReferenceOTU196994
New.CleanUp.ReferenceOTU41436
New.CleanUp.ReferenceOTU63099
New.CleanUp.ReferenceOTU143635
New.CleanUp.ReferenceOTU184321
New.CleanUp.ReferenceOTU188305
New.CleanUp.ReferenceOTU261823
New.CleanUp.ReferenceOTU92108
New.CleanUp.ReferenceOTU107777
New.CleanUp.ReferenceOTU239895
New.CleanUp.ReferenceOTU48620
New.CleanUp.ReferenceOTU119145
New.CleanUp.ReferenceOTU124498
New.CleanUp.ReferenceOTU165507
New.CleanUp.ReferenceOTU183484
New.CleanUp.ReferenceOTU91704
New.CleanUp.ReferenceOTU299830
New.CleanUp.ReferenceOTU259637
New.CleanUp.ReferenceOTU308367
New.CleanUp.ReferenceOTU157286
New.CleanUp.ReferenceOTU184388
New.CleanUp.ReferenceOTU140794
New.CleanUp.ReferenceOTU105507
New.CleanUp.ReferenceOTU209231
New.CleanUp.ReferenceOTU240515
New.CleanUp.ReferenceOTU117327
New.CleanUp.ReferenceOTU75697

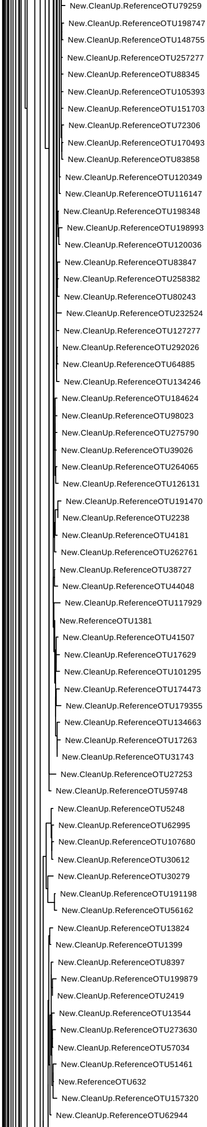
New.CleanUp.ReferenceOTU79259
New.CleanUp.ReferenceOTU198747
New.CleanUp.ReferenceOTU148755
New.CleanUp.ReferenceOTU257277
New.CleanUp.ReferenceOTU88345
New.CleanUp.ReferenceOTU105393
New.CleanUp.ReferenceOTU151703
New.CleanUp.ReferenceOTU72306
New.CleanUp.ReferenceOTU170493
New.CleanUp.ReferenceOTU83858
New.CleanUp.ReferenceOTU120349
New.CleanUp.ReferenceOTU116147
New.CleanUp.ReferenceOTU198348
New.CleanUp.ReferenceOTU198993
New.CleanUp.ReferenceOTU120036
New.CleanUp.ReferenceOTU83847
New.CleanUp.ReferenceOTU258382
New.CleanUp.ReferenceOTU80243
New.CleanUp.ReferenceOTU232524
New.CleanUp.ReferenceOTU127277
New.CleanUp.ReferenceOTU292026
New.CleanUp.ReferenceOTU64885
New.CleanUp.ReferenceOTU134246
New.CleanUp.ReferenceOTU184624
New.CleanUp.ReferenceOTU98023
New.CleanUp.ReferenceOTU275790
New.CleanUp.ReferenceOTU39026
New.CleanUp.ReferenceOTU264065
New.CleanUp.ReferenceOTU126131
New.CleanUp.ReferenceOTU191470
New.CleanUp.ReferenceOTU2238
New.CleanUp.ReferenceOTU4181
New.CleanUp.ReferenceOTU262761
New.CleanUp.ReferenceOTU38727
New.CleanUp.ReferenceOTU44048
New.CleanUp.ReferenceOTU117929
New.ReferenceOTU1381
New.CleanUp.ReferenceOTU41507
New.CleanUp.ReferenceOTU17629
New.CleanUp.ReferenceOTU101295
New.CleanUp.ReferenceOTU174473
New.CleanUp.ReferenceOTU179355
New.CleanUp.ReferenceOTU134663
New.CleanUp.ReferenceOTU17263
New.CleanUp.ReferenceOTU31743
New.CleanUp.ReferenceOTU27253
New.CleanUp.ReferenceOTU59748
New.CleanUp.ReferenceOTU5248
New.CleanUp.ReferenceOTU62995
New.CleanUp.ReferenceOTU107680
New.CleanUp.ReferenceOTU30612
New.CleanUp.ReferenceOTU30279
New.CleanUp.ReferenceOTU191198
New.CleanUp.ReferenceOTU56162
New.CleanUp.ReferenceOTU13824
New.CleanUp.ReferenceOTU1399
New.CleanUp.ReferenceOTU8397
New.CleanUp.ReferenceOTU199879
New.CleanUp.ReferenceOTU2419
New.CleanUp.ReferenceOTU13544
New.CleanUp.ReferenceOTU273630
New.CleanUp.ReferenceOTU57034
New.CleanUp.ReferenceOTU51461
New.ReferenceOTU632
New.CleanUp.ReferenceOTU157320
New.CleanUp.ReferenceOTU62944

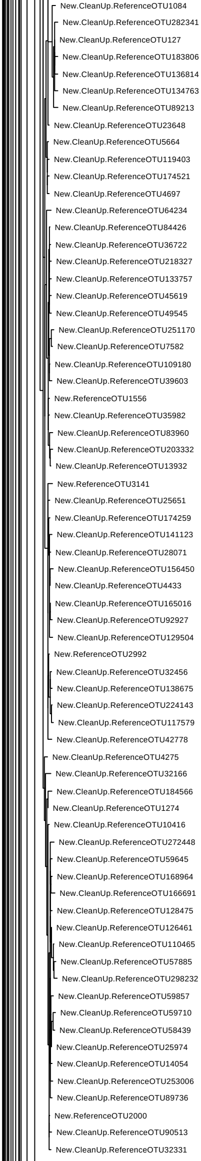
New.CleanUp.ReferenceOTU1084
New.CleanUp.ReferenceOTU282341
New.CleanUp.ReferenceOTU127
New.CleanUp.ReferenceOTU183806
New.CleanUp.ReferenceOTU136814
New.CleanUp.ReferenceOTU134763
New.CleanUp.ReferenceOTU89213
New.CleanUp.ReferenceOTU23648
New.CleanUp.ReferenceOTU5664
New.CleanUp.ReferenceOTU119403
New.CleanUp.ReferenceOTU174521
New.CleanUp.ReferenceOTU4697
New.CleanUp.ReferenceOTU64234
New.CleanUp.ReferenceOTU84426
New.CleanUp.ReferenceOTU36722
New.CleanUp.ReferenceOTU218327
New.CleanUp.ReferenceOTU133757
New.CleanUp.ReferenceOTU45619
New.CleanUp.ReferenceOTU49545
New.CleanUp.ReferenceOTU251170
New.CleanUp.ReferenceOTU7582
New.CleanUp.ReferenceOTU109180
New.CleanUp.ReferenceOTU39603
New.ReferenceOTU1556
New.CleanUp.ReferenceOTU35982
New.CleanUp.ReferenceOTU83960
New.CleanUp.ReferenceOTU203332
New.CleanUp.ReferenceOTU13932
New.ReferenceOTU3141
New.CleanUp.ReferenceOTU25651
New.CleanUp.ReferenceOTU174259
New.CleanUp.ReferenceOTU141123
New.CleanUp.ReferenceOTU28071
New.CleanUp.ReferenceOTU156450
New.CleanUp.ReferenceOTU4433
New.CleanUp.ReferenceOTU165016
New.CleanUp.ReferenceOTU92927
New.CleanUp.ReferenceOTU129504
New.ReferenceOTU2992
New.CleanUp.ReferenceOTU32456
New.CleanUp.ReferenceOTU138675
New.CleanUp.ReferenceOTU224143
New.CleanUp.ReferenceOTU117579
New.CleanUp.ReferenceOTU42778
New.CleanUp.ReferenceOTU4275
New.CleanUp.ReferenceOTU32166
New.CleanUp.ReferenceOTU184566
New.CleanUp.ReferenceOTU1274
New.CleanUp.ReferenceOTU10416
New.CleanUp.ReferenceOTU272448
New.CleanUp.ReferenceOTU59645
New.CleanUp.ReferenceOTU168964
New.CleanUp.ReferenceOTU166691
New.CleanUp.ReferenceOTU128475
New.CleanUp.ReferenceOTU126461
New.CleanUp.ReferenceOTU110465
New.CleanUp.ReferenceOTU57885
New.CleanUp.ReferenceOTU298232
New.CleanUp.ReferenceOTU59857
New.CleanUp.ReferenceOTU59710
New.CleanUp.ReferenceOTU58439
New.CleanUp.ReferenceOTU25974
New.CleanUp.ReferenceOTU14054
New.CleanUp.ReferenceOTU253006
New.CleanUp.ReferenceOTU89736
New.ReferenceOTU2000
New.CleanUp.ReferenceOTU90513
New.CleanUp.ReferenceOTU32331

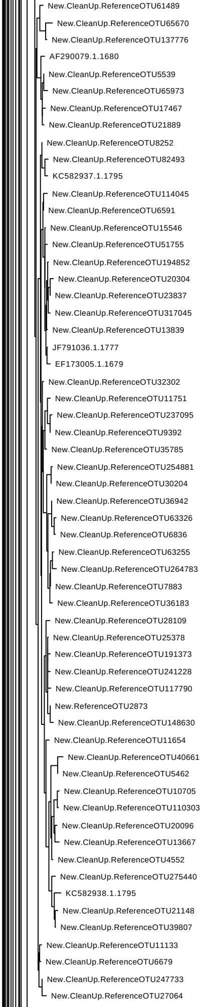
New.CleanUp.ReferenceOTU61489
New.CleanUp.ReferenceOTU65670
New.CleanUp.ReferenceOTU137776
AF290079.1.1680
New.CleanUp.ReferenceOTU5539
New.CleanUp.ReferenceOTU65973
New.CleanUp.ReferenceOTU17467
New.CleanUp.ReferenceOTU21889
New.CleanUp.ReferenceOTU8252
New.CleanUp.ReferenceOTU82493
KC582937.1.1795
New.CleanUp.ReferenceOTU114045
New.CleanUp.ReferenceOTU6591
New.CleanUp.ReferenceOTU15546
New.CleanUp.ReferenceOTU51755
New.CleanUp.ReferenceOTU194852
New.CleanUp.ReferenceOTU20304
New.CleanUp.ReferenceOTU23837
New.CleanUp.ReferenceOTU317045
New.CleanUp.ReferenceOTU13839
JF791036.1.1777
EF173005.1.1679
New.CleanUp.ReferenceOTU32302
New.CleanUp.ReferenceOTU11751
New.CleanUp.ReferenceOTU237095
New.CleanUp.ReferenceOTU9392
New.CleanUp.ReferenceOTU35785
New.CleanUp.ReferenceOTU254881
New.CleanUp.ReferenceOTU30204
New.CleanUp.ReferenceOTU36942
New.CleanUp.ReferenceOTU63326
New.CleanUp.ReferenceOTU6836
New.CleanUp.ReferenceOTU63255
New.CleanUp.ReferenceOTU264783
New.CleanUp.ReferenceOTU7883
New.CleanUp.ReferenceOTU36183
New.CleanUp.ReferenceOTU28109
New.CleanUp.ReferenceOTU25378
New.CleanUp.ReferenceOTU191373
New.CleanUp.ReferenceOTU241228
New.CleanUp.ReferenceOTU117790
New.ReferenceOTU2873
New.CleanUp.ReferenceOTU148630
New.CleanUp.ReferenceOTU11654
New.CleanUp.ReferenceOTU40661
New.CleanUp.ReferenceOTU5462
New.CleanUp.ReferenceOTU10705
New.CleanUp.ReferenceOTU110303
New.CleanUp.ReferenceOTU20096
New.CleanUp.ReferenceOTU13667
New.CleanUp.ReferenceOTU4552
New.CleanUp.ReferenceOTU275440
KC582938.1.1795
New.CleanUp.ReferenceOTU21148
New.CleanUp.ReferenceOTU39807
New.CleanUp.ReferenceOTU11133
New.CleanUp.ReferenceOTU6679
New.CleanUp.ReferenceOTU247733
New.CleanUp.ReferenceOTU27064

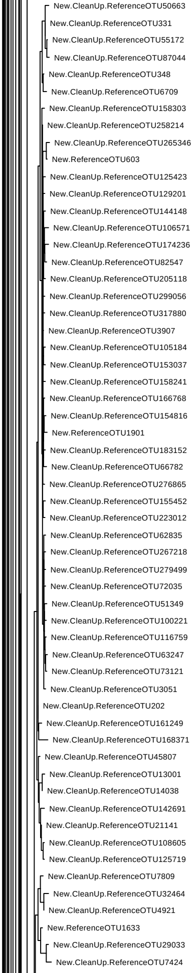
New.CleanUp.ReferenceOTU50663
New.CleanUp.ReferenceOTU331
New.CleanUp.ReferenceOTU55172
New.CleanUp.ReferenceOTU87044
New.CleanUp.ReferenceOTU348
New.CleanUp.ReferenceOTU6709
New.CleanUp.ReferenceOTU158303
New.CleanUp.ReferenceOTU258214
New.CleanUp.ReferenceOTU265346
New.ReferenceOTU603
New.CleanUp.ReferenceOTU125423
New.CleanUp.ReferenceOTU129201
New.CleanUp.ReferenceOTU144148
New.CleanUp.ReferenceOTU106571
New.CleanUp.ReferenceOTU174236
New.CleanUp.ReferenceOTU82547
New.CleanUp.ReferenceOTU205118
New.CleanUp.ReferenceOTU299056
New.CleanUp.ReferenceOTU317880
New.CleanUp.ReferenceOTU3907
New.CleanUp.ReferenceOTU105184
New.CleanUp.ReferenceOTU153037
New.CleanUp.ReferenceOTU158241
New.CleanUp.ReferenceOTU166768
New.CleanUp.ReferenceOTU154816
New.ReferenceOTU1901
New.CleanUp.ReferenceOTU183152
New.CleanUp.ReferenceOTU66782
New.CleanUp.ReferenceOTU276865
New.CleanUp.ReferenceOTU155452
New.CleanUp.ReferenceOTU223012
New.CleanUp.ReferenceOTU62835
New.CleanUp.ReferenceOTU267218
New.CleanUp.ReferenceOTU279499
New.CleanUp.ReferenceOTU72035
New.CleanUp.ReferenceOTU51349
New.CleanUp.ReferenceOTU100221
New.CleanUp.ReferenceOTU116759
New.CleanUp.ReferenceOTU63247
New.CleanUp.ReferenceOTU73121
New.CleanUp.ReferenceOTU3051
New.CleanUp.ReferenceOTU202
New.CleanUp.ReferenceOTU161249
New.CleanUp.ReferenceOTU168371
New.CleanUp.ReferenceOTU45807
New.CleanUp.ReferenceOTU13001
New.CleanUp.ReferenceOTU14038
New.CleanUp.ReferenceOTU142691
New.CleanUp.ReferenceOTU21141
New.CleanUp.ReferenceOTU108605
New.CleanUp.ReferenceOTU125719
New.CleanUp.ReferenceOTU7809
New.CleanUp.ReferenceOTU32464
New.CleanUp.ReferenceOTU4921
New.ReferenceOTU1633
New.CleanUp.ReferenceOTU29033
New.CleanUp.ReferenceOTU7424

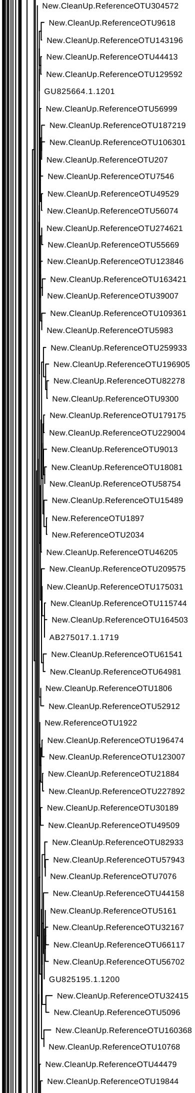
New.CleanUp.ReferenceOTU304572
New.CleanUp.ReferenceOTU9618
New.CleanUp.ReferenceOTU143196
New.CleanUp.ReferenceOTU44413
New.CleanUp.ReferenceOTU129592
GU825664.1.1201
New.CleanUp.ReferenceOTU56999
New.CleanUp.ReferenceOTU187219
New.CleanUp.ReferenceOTU106301
New.CleanUp.ReferenceOTU207
New.CleanUp.ReferenceOTU7546
New.CleanUp.ReferenceOTU49529
New.CleanUp.ReferenceOTU56074
New.CleanUp.ReferenceOTU274621
New.CleanUp.ReferenceOTU55669
New.CleanUp.ReferenceOTU123846
New.CleanUp.ReferenceOTU163421
New.CleanUp.ReferenceOTU39007
New.CleanUp.ReferenceOTU109361
New.CleanUp.ReferenceOTU5983
New.CleanUp.ReferenceOTU259933
New.CleanUp.ReferenceOTU196905
New.CleanUp.ReferenceOTU82278
New.CleanUp.ReferenceOTU9300
New.CleanUp.ReferenceOTU179175
New.CleanUp.ReferenceOTU229004
New.CleanUp.ReferenceOTU9013
New.CleanUp.ReferenceOTU18081
New.CleanUp.ReferenceOTU58754
New.CleanUp.ReferenceOTU15489
New.ReferenceOTU1897
New.ReferenceOTU2034
New.CleanUp.ReferenceOTU46205
New.CleanUp.ReferenceOTU209575
New.CleanUp.ReferenceOTU175031
New.CleanUp.ReferenceOTU115744
New.CleanUp.ReferenceOTU164503
AB275017.1.1719
New.CleanUp.ReferenceOTU61541
New.CleanUp.ReferenceOTU64981
New.CleanUp.ReferenceOTU1806
New.CleanUp.ReferenceOTU52912
New.ReferenceOTU1922
New.CleanUp.ReferenceOTU196474
New.CleanUp.ReferenceOTU123007
New.CleanUp.ReferenceOTU21884
New.CleanUp.ReferenceOTU227892
New.CleanUp.ReferenceOTU30189
New.CleanUp.ReferenceOTU49509
New.CleanUp.ReferenceOTU82933
New.CleanUp.ReferenceOTU57943
New.CleanUp.ReferenceOTU7076
New.CleanUp.ReferenceOTU44158
New.CleanUp.ReferenceOTU5161
New.CleanUp.ReferenceOTU32167
New.CleanUp.ReferenceOTU66117
New.CleanUp.ReferenceOTU56702
GU825195.1.1200
New.CleanUp.ReferenceOTU32415
New.CleanUp.ReferenceOTU5096
New.CleanUp.ReferenceOTU160368
New.CleanUp.ReferenceOTU10768
New.CleanUp.ReferenceOTU44479
New.CleanUp.ReferenceOTU19844

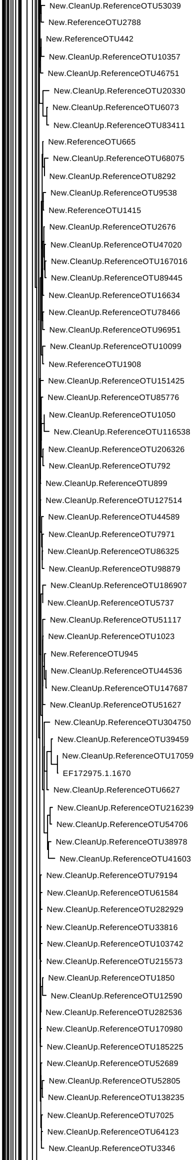
New.CleanUp.ReferenceOTU53039
New.ReferenceOTU2788
New.ReferenceOTU442
New.CleanUp.ReferenceOTU10357
New.CleanUp.ReferenceOTU46751
New.CleanUp.ReferenceOTU20330
New.CleanUp.ReferenceOTU6073
New.CleanUp.ReferenceOTU83411
New.ReferenceOTU665
New.CleanUp.ReferenceOTU68075
New.CleanUp.ReferenceOTU8292
New.CleanUp.ReferenceOTU9538
New.ReferenceOTU1415
New.CleanUp.ReferenceOTU2676
New.CleanUp.ReferenceOTU47020
New.CleanUp.ReferenceOTU167016
New.CleanUp.ReferenceOTU89445
New.CleanUp.ReferenceOTU16634
New.CleanUp.ReferenceOTU78466
New.CleanUp.ReferenceOTU96951
New.CleanUp.ReferenceOTU10099
New.ReferenceOTU1908
New.CleanUp.ReferenceOTU151425
New.CleanUp.ReferenceOTU85776
New.CleanUp.ReferenceOTU1050
New.CleanUp.ReferenceOTU116538
New.CleanUp.ReferenceOTU206326
New.CleanUp.ReferenceOTU792
New.CleanUp.ReferenceOTU899
New.CleanUp.ReferenceOTU127514
New.CleanUp.ReferenceOTU44589
New.CleanUp.ReferenceOTU7971
New.CleanUp.ReferenceOTU86325
New.CleanUp.ReferenceOTU98879
New.CleanUp.ReferenceOTU186907
New.CleanUp.ReferenceOTU5737
New.CleanUp.ReferenceOTU51117
New.CleanUp.ReferenceOTU1023
New.ReferenceOTU945
New.CleanUp.ReferenceOTU44536
New.CleanUp.ReferenceOTU147687
New.CleanUp.ReferenceOTU51627
New.CleanUp.ReferenceOTU304750
New.CleanUp.ReferenceOTU39459
New.CleanUp.ReferenceOTU17059
EF172975.1.1670
New.CleanUp.ReferenceOTU6627
New.CleanUp.ReferenceOTU216239
New.CleanUp.ReferenceOTU54706
New.CleanUp.ReferenceOTU38978
New.CleanUp.ReferenceOTU41603
New.CleanUp.ReferenceOTU79194
New.CleanUp.ReferenceOTU61584
New.CleanUp.ReferenceOTU282929
New.CleanUp.ReferenceOTU33816
New.CleanUp.ReferenceOTU103742
New.CleanUp.ReferenceOTU215573
New.CleanUp.ReferenceOTU1850
New.CleanUp.ReferenceOTU12590
New.CleanUp.ReferenceOTU282536
New.CleanUp.ReferenceOTU170980
New.CleanUp.ReferenceOTU185225
New.CleanUp.ReferenceOTU52689
New.CleanUp.ReferenceOTU52805
New.CleanUp.ReferenceOTU138235
New.CleanUp.ReferenceOTU7025
New.CleanUp.ReferenceOTU64123
New.CleanUp.ReferenceOTU3346

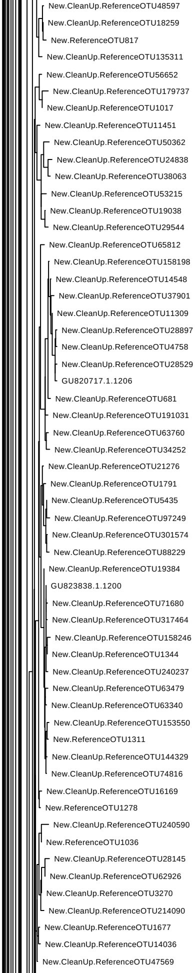
New.CleanUp.ReferenceOTU48597
New.CleanUp.ReferenceOTU18259
New.ReferenceOTU817
New.CleanUp.ReferenceOTU135311
New.CleanUp.ReferenceOTU56652
New.CleanUp.ReferenceOTU179737
New.CleanUp.ReferenceOTU1017
New.CleanUp.ReferenceOTU11451
New.CleanUp.ReferenceOTU50362
New.CleanUp.ReferenceOTU24838
New.CleanUp.ReferenceOTU38063
New.CleanUp.ReferenceOTU53215
New.CleanUp.ReferenceOTU19038
New.CleanUp.ReferenceOTU29544
New.CleanUp.ReferenceOTU65812
New.CleanUp.ReferenceOTU158198
New.CleanUp.ReferenceOTU14548
New.CleanUp.ReferenceOTU37901
New.CleanUp.ReferenceOTU11309
New.CleanUp.ReferenceOTU28897
New.CleanUp.ReferenceOTU4758
New.CleanUp.ReferenceOTU28529
GU820717.1.1206
New.CleanUp.ReferenceOTU681
New.CleanUp.ReferenceOTU191031
New.CleanUp.ReferenceOTU63760
New.CleanUp.ReferenceOTU34252
New.CleanUp.ReferenceOTU21276
New.CleanUp.ReferenceOTU1791
New.CleanUp.ReferenceOTU5435
New.CleanUp.ReferenceOTU97249
New.CleanUp.ReferenceOTU301574
New.CleanUp.ReferenceOTU88229
New.CleanUp.ReferenceOTU19384
GU823838.1.1200
New.CleanUp.ReferenceOTU71680
New.CleanUp.ReferenceOTU317464
New.CleanUp.ReferenceOTU158246
New.CleanUp.ReferenceOTU1344
New.CleanUp.ReferenceOTU240237
New.CleanUp.ReferenceOTU63479
New.CleanUp.ReferenceOTU63340
New.CleanUp.ReferenceOTU153550
New.ReferenceOTU1311
New.CleanUp.ReferenceOTU144329
New.CleanUp.ReferenceOTU74816
New.CleanUp.ReferenceOTU16169
New.ReferenceOTU1278
New.CleanUp.ReferenceOTU240590
New.ReferenceOTU1036
New.CleanUp.ReferenceOTU28145
New.CleanUp.ReferenceOTU62926
New.CleanUp.ReferenceOTU3270
New.CleanUp.ReferenceOTU214090
New.CleanUp.ReferenceOTU1677
New.CleanUp.ReferenceOTU14036
New.CleanUp.ReferenceOTU47569

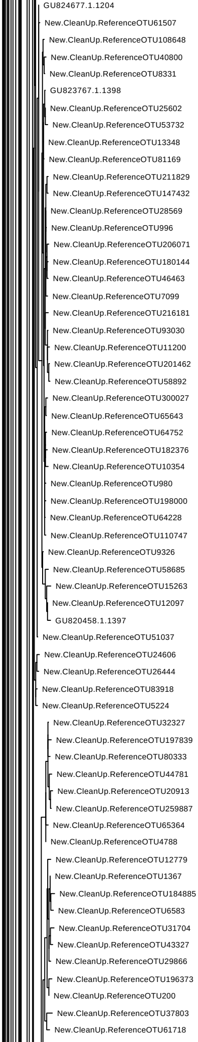
GU824677.1.1204
New.CleanUp.ReferenceOTU61507
New.CleanUp.ReferenceOTU108648
New.CleanUp.ReferenceOTU40800
New.CleanUp.ReferenceOTU8331
GU823767.1.1398
New.CleanUp.ReferenceOTU25602
New.CleanUp.ReferenceOTU53732
New.CleanUp.ReferenceOTU13348
New.CleanUp.ReferenceOTU81169
New.CleanUp.ReferenceOTU211829
New.CleanUp.ReferenceOTU147432
New.CleanUp.ReferenceOTU28569
New.CleanUp.ReferenceOTU996
New.CleanUp.ReferenceOTU206071
New.CleanUp.ReferenceOTU180144
New.CleanUp.ReferenceOTU46463
New.CleanUp.ReferenceOTU7099
New.CleanUp.ReferenceOTU216181
New.CleanUp.ReferenceOTU93030
New.CleanUp.ReferenceOTU11200
New.CleanUp.ReferenceOTU201462
New.CleanUp.ReferenceOTU58892
New.CleanUp.ReferenceOTU300027
New.CleanUp.ReferenceOTU65643
New.CleanUp.ReferenceOTU64752
New.CleanUp.ReferenceOTU182376
New.CleanUp.ReferenceOTU10354
New.CleanUp.ReferenceOTU980
New.CleanUp.ReferenceOTU198000
New.CleanUp.ReferenceOTU64228
New.CleanUp.ReferenceOTU110747
New.CleanUp.ReferenceOTU9326
New.CleanUp.ReferenceOTU58685
New.CleanUp.ReferenceOTU15263
New.CleanUp.ReferenceOTU12097
GU820458.1.1397
New.CleanUp.ReferenceOTU51037
New.CleanUp.ReferenceOTU24606
New.CleanUp.ReferenceOTU26444
New.CleanUp.ReferenceOTU83918
New.CleanUp.ReferenceOTU5224
New.CleanUp.ReferenceOTU32327
New.CleanUp.ReferenceOTU197839
New.CleanUp.ReferenceOTU80333
New.CleanUp.ReferenceOTU44781
New.CleanUp.ReferenceOTU20913
New.CleanUp.ReferenceOTU259887
New.CleanUp.ReferenceOTU65364
New.CleanUp.ReferenceOTU4788
New.CleanUp.ReferenceOTU12779
New.CleanUp.ReferenceOTU1367
New.CleanUp.ReferenceOTU184885
New.CleanUp.ReferenceOTU6583
New.CleanUp.ReferenceOTU31704
New.CleanUp.ReferenceOTU43327
New.CleanUp.ReferenceOTU29866
New.CleanUp.ReferenceOTU196373
New.CleanUp.ReferenceOTU200
New.CleanUp.ReferenceOTU37803
New.CleanUp.ReferenceOTU61718

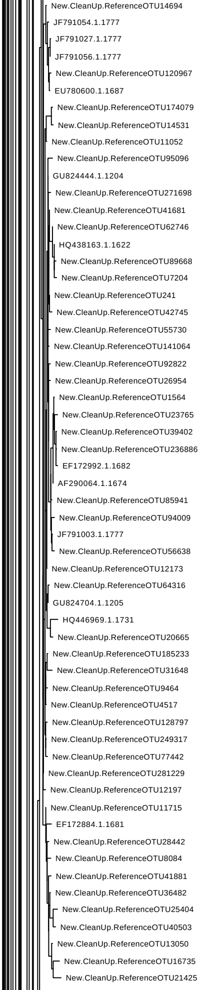
New.CleanUp.ReferenceOTU14694
JF791054.1.1777
JF791027.1.1777
JF791056.1.1777
New.CleanUp.ReferenceOTU120967
EU780600.1.1687
New.CleanUp.ReferenceOTU174079
New.CleanUp.ReferenceOTU14531
New.CleanUp.ReferenceOTU11052
New.CleanUp.ReferenceOTU95096
GU824444.1.1204
New.CleanUp.ReferenceOTU271698
New.CleanUp.ReferenceOTU41681
New.CleanUp.ReferenceOTU62746
HQ438163.1.1622
New.CleanUp.ReferenceOTU89668
New.CleanUp.ReferenceOTU7204
New.CleanUp.ReferenceOTU241
New.CleanUp.ReferenceOTU42745
New.CleanUp.ReferenceOTU55730
New.CleanUp.ReferenceOTU141064
New.CleanUp.ReferenceOTU92822
New.CleanUp.ReferenceOTU26954
New.CleanUp.ReferenceOTU1564
New.CleanUp.ReferenceOTU23765
New.CleanUp.ReferenceOTU39402
New.CleanUp.ReferenceOTU236886
EF172992.1.1682
AF290064.1.1674
New.CleanUp.ReferenceOTU85941
New.CleanUp.ReferenceOTU94009
JF791003.1.1777
New.CleanUp.ReferenceOTU56638
New.CleanUp.ReferenceOTU12173
New.CleanUp.ReferenceOTU64316
GU824704.1.1205
HQ446969.1.1731
New.CleanUp.ReferenceOTU20665
New.CleanUp.ReferenceOTU185233
New.CleanUp.ReferenceOTU31648
New.CleanUp.ReferenceOTU9464
New.CleanUp.ReferenceOTU4517
New.CleanUp.ReferenceOTU128797
New.CleanUp.ReferenceOTU249317
New.CleanUp.ReferenceOTU77442
New.CleanUp.ReferenceOTU281229
New.CleanUp.ReferenceOTU12197
New.CleanUp.ReferenceOTU11715
EF172884.1.1681
New.CleanUp.ReferenceOTU28442
New.CleanUp.ReferenceOTU8084
New.CleanUp.ReferenceOTU41881
New.CleanUp.ReferenceOTU36482
New.CleanUp.ReferenceOTU25404
New.CleanUp.ReferenceOTU40503
New.CleanUp.ReferenceOTU13050
New.CleanUp.ReferenceOTU16735
New.CleanUp.ReferenceOTU21425

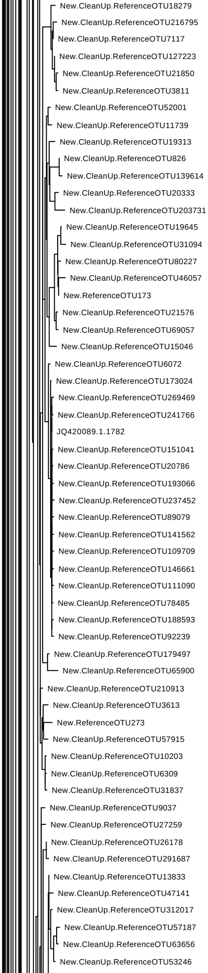
New.CleanUp.ReferenceOTU18279
New.CleanUp.ReferenceOTU216795
New.CleanUp.ReferenceOTU7117
New.CleanUp.ReferenceOTU127223
New.CleanUp.ReferenceOTU21850
New.CleanUp.ReferenceOTU3811
New.CleanUp.ReferenceOTU52001
New.CleanUp.ReferenceOTU11739
New.CleanUp.ReferenceOTU19313
New.CleanUp.ReferenceOTU826
New.CleanUp.ReferenceOTU139614
New.CleanUp.ReferenceOTU20333
New.CleanUp.ReferenceOTU203731
New.CleanUp.ReferenceOTU19645
New.CleanUp.ReferenceOTU31094
New.CleanUp.ReferenceOTU80227
New.CleanUp.ReferenceOTU46057
New.ReferenceOTU173
New.CleanUp.ReferenceOTU21576
New.CleanUp.ReferenceOTU69057
New.CleanUp.ReferenceOTU15046
New.CleanUp.ReferenceOTU6072
New.CleanUp.ReferenceOTU173024
New.CleanUp.ReferenceOTU269469
New.CleanUp.ReferenceOTU241766
JQ420089.1.1782
New.CleanUp.ReferenceOTU151041
New.CleanUp.ReferenceOTU20786
New.CleanUp.ReferenceOTU193066
New.CleanUp.ReferenceOTU237452
New.CleanUp.ReferenceOTU89079
New.CleanUp.ReferenceOTU141562
New.CleanUp.ReferenceOTU109709
New.CleanUp.ReferenceOTU146661
New.CleanUp.ReferenceOTU111090
New.CleanUp.ReferenceOTU78485
New.CleanUp.ReferenceOTU188593
New.CleanUp.ReferenceOTU92239
New.CleanUp.ReferenceOTU179497
New.CleanUp.ReferenceOTU65900
New.CleanUp.ReferenceOTU210913
New.CleanUp.ReferenceOTU3613
New.ReferenceOTU273
New.CleanUp.ReferenceOTU57915
New.CleanUp.ReferenceOTU10203
New.CleanUp.ReferenceOTU6309
New.CleanUp.ReferenceOTU31837
New.CleanUp.ReferenceOTU9037
New.CleanUp.ReferenceOTU27259
New.CleanUp.ReferenceOTU26178
New.CleanUp.ReferenceOTU291687
New.CleanUp.ReferenceOTU13833
New.CleanUp.ReferenceOTU47141
New.CleanUp.ReferenceOTU312017
New.CleanUp.ReferenceOTU57187
New.CleanUp.ReferenceOTU63656
New.CleanUp.ReferenceOTU53246

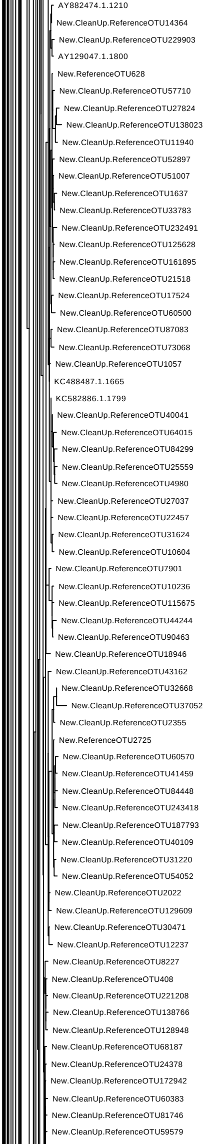

AY882474.1.1210
New.CleanUp.ReferenceOTU14364
New.CleanUp.ReferenceOTU229903
AY129047.1.1800
New.ReferenceOTU628
New.CleanUp.ReferenceOTU57710
New.CleanUp.ReferenceOTU27824
New.CleanUp.ReferenceOTU138023
New.CleanUp.ReferenceOTU11940
New.CleanUp.ReferenceOTU52897
New.CleanUp.ReferenceOTU51007
New.CleanUp.ReferenceOTU1637
New.CleanUp.ReferenceOTU33783
New.CleanUp.ReferenceOTU232491
New.CleanUp.ReferenceOTU125628
New.CleanUp.ReferenceOTU161895
New.CleanUp.ReferenceOTU21518
New.CleanUp.ReferenceOTU17524
New.CleanUp.ReferenceOTU60500
New.CleanUp.ReferenceOTU87083
New.CleanUp.ReferenceOTU73068
New.CleanUp.ReferenceOTU1057
KC488487.1.1665
KC582886.1.1799
New.CleanUp.ReferenceOTU40041
New.CleanUp.ReferenceOTU64015
New.CleanUp.ReferenceOTU84299
New.CleanUp.ReferenceOTU25559
New.CleanUp.ReferenceOTU4980
New.CleanUp.ReferenceOTU27037
New.CleanUp.ReferenceOTU22457
New.CleanUp.ReferenceOTU31624
New.CleanUp.ReferenceOTU10604
New.CleanUp.ReferenceOTU7901
New.CleanUp.ReferenceOTU10236
New.CleanUp.ReferenceOTU115675
New.CleanUp.ReferenceOTU44244
New.CleanUp.ReferenceOTU90463
New.CleanUp.ReferenceOTU18946
New.CleanUp.ReferenceOTU43162
New.CleanUp.ReferenceOTU32668
New.CleanUp.ReferenceOTU37052
New.CleanUp.ReferenceOTU2355
New.ReferenceOTU2725
New.CleanUp.ReferenceOTU60570
New.CleanUp.ReferenceOTU41459
New.CleanUp.ReferenceOTU84448
New.CleanUp.ReferenceOTU243418
New.CleanUp.ReferenceOTU187793
New.CleanUp.ReferenceOTU40109
New.CleanUp.ReferenceOTU31220
New.CleanUp.ReferenceOTU54052
New.CleanUp.ReferenceOTU2022
New.CleanUp.ReferenceOTU129609
New.CleanUp.ReferenceOTU30471
New.CleanUp.ReferenceOTU12237
New.CleanUp.ReferenceOTU8227
New.CleanUp.ReferenceOTU408
New.CleanUp.ReferenceOTU221208
New.CleanUp.ReferenceOTU138766
New.CleanUp.ReferenceOTU128948
New.CleanUp.ReferenceOTU68187
New.CleanUp.ReferenceOTU24378
New.CleanUp.ReferenceOTU172942
New.CleanUp.ReferenceOTU60383
New.CleanUp.ReferenceOTU81746
New.CleanUp.ReferenceOTU59579

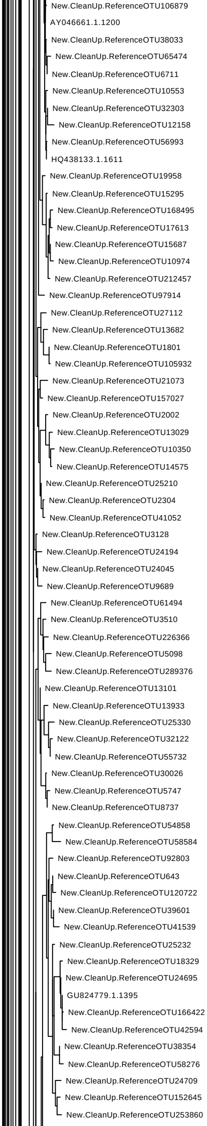
New.CleanUp.ReferenceOTU106879
AY046661.1.1200
New.CleanUp.ReferenceOTU38033
New.CleanUp.ReferenceOTU65474
New.CleanUp.ReferenceOTU6711
New.CleanUp.ReferenceOTU10553
New.CleanUp.ReferenceOTU32303
New.CleanUp.ReferenceOTU12158
New.CleanUp.ReferenceOTU56993
HQ438133.1.1611
New.CleanUp.ReferenceOTU19958
New.CleanUp.ReferenceOTU15295
New.CleanUp.ReferenceOTU168495
New.CleanUp.ReferenceOTU17613
New.CleanUp.ReferenceOTU15687
New.CleanUp.ReferenceOTU10974
New.CleanUp.ReferenceOTU212457
New.CleanUp.ReferenceOTU97914
New.CleanUp.ReferenceOTU27112
New.CleanUp.ReferenceOTU13682
New.CleanUp.ReferenceOTU1801
New.CleanUp.ReferenceOTU105932
New.CleanUp.ReferenceOTU21073
New.CleanUp.ReferenceOTU157027
New.CleanUp.ReferenceOTU2002
New.CleanUp.ReferenceOTU13029
New.CleanUp.ReferenceOTU10350
New.CleanUp.ReferenceOTU14575
New.CleanUp.ReferenceOTU25210
New.CleanUp.ReferenceOTU2304
New.CleanUp.ReferenceOTU41052
New.CleanUp.ReferenceOTU3128
New.CleanUp.ReferenceOTU24194
New.CleanUp.ReferenceOTU24045
New.CleanUp.ReferenceOTU9689
New.CleanUp.ReferenceOTU61494
New.CleanUp.ReferenceOTU3510
New.CleanUp.ReferenceOTU226366
New.CleanUp.ReferenceOTU5098
New.CleanUp.ReferenceOTU289376
New.CleanUp.ReferenceOTU13101
New.CleanUp.ReferenceOTU13933
New.CleanUp.ReferenceOTU25330
New.CleanUp.ReferenceOTU32122
New.CleanUp.ReferenceOTU55732
New.CleanUp.ReferenceOTU30026
New.CleanUp.ReferenceOTU5747
New.CleanUp.ReferenceOTU8737
New.CleanUp.ReferenceOTU54858
New.CleanUp.ReferenceOTU58584
New.CleanUp.ReferenceOTU92803
New.CleanUp.ReferenceOTU643
New.CleanUp.ReferenceOTU120722
New.CleanUp.ReferenceOTU39601
New.CleanUp.ReferenceOTU41539
New.CleanUp.ReferenceOTU25232
New.CleanUp.ReferenceOTU18329
New.CleanUp.ReferenceOTU24695
GU824779.1.1395
New.CleanUp.ReferenceOTU166422
New.CleanUp.ReferenceOTU42594
New.CleanUp.ReferenceOTU38354
New.CleanUp.ReferenceOTU58276
New.CleanUp.ReferenceOTU24709
New.CleanUp.ReferenceOTU152645
New.CleanUp.ReferenceOTU253860

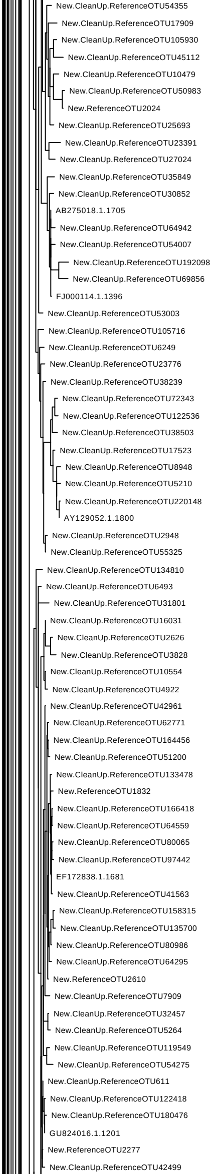
New.CleanUp.ReferenceOTU54355
New.CleanUp.ReferenceOTU17909
New.CleanUp.ReferenceOTU105930
New.CleanUp.ReferenceOTU45112
New.CleanUp.ReferenceOTU10479
New.CleanUp.ReferenceOTU50983
New.ReferenceOTU2024
New.CleanUp.ReferenceOTU25693
New.CleanUp.ReferenceOTU23391
New.CleanUp.ReferenceOTU27024
New.CleanUp.ReferenceOTU35849
New.CleanUp.ReferenceOTU30852
AB275018.1.1705
New.CleanUp.ReferenceOTU64942
New.CleanUp.ReferenceOTU54007
New.CleanUp.ReferenceOTU192098
New.CleanUp.ReferenceOTU69856
FJ000114.1.1396
New.CleanUp.ReferenceOTU53003
New.CleanUp.ReferenceOTU105716
New.CleanUp.ReferenceOTU6249
New.CleanUp.ReferenceOTU23776
New.CleanUp.ReferenceOTU38239
New.CleanUp.ReferenceOTU72343
New.CleanUp.ReferenceOTU122536
New.CleanUp.ReferenceOTU38503
New.CleanUp.ReferenceOTU17523
New.CleanUp.ReferenceOTU8948
New.CleanUp.ReferenceOTU5210
New.CleanUp.ReferenceOTU220148
AY129052.1.1800
New.CleanUp.ReferenceOTU2948
New.CleanUp.ReferenceOTU55325
New.CleanUp.ReferenceOTU134810
New.CleanUp.ReferenceOTU6493
New.CleanUp.ReferenceOTU31801
New.CleanUp.ReferenceOTU16031
New.CleanUp.ReferenceOTU2626
New.CleanUp.ReferenceOTU3828
New.CleanUp.ReferenceOTU10554
New.CleanUp.ReferenceOTU4922
New.CleanUp.ReferenceOTU42961
New.CleanUp.ReferenceOTU62771
New.CleanUp.ReferenceOTU164456
New.CleanUp.ReferenceOTU51200
New.CleanUp.ReferenceOTU133478
New.ReferenceOTU1832
New.CleanUp.ReferenceOTU166418
New.CleanUp.ReferenceOTU64559
New.CleanUp.ReferenceOTU80065
New.CleanUp.ReferenceOTU97442
EF172838.1.1681
New.CleanUp.ReferenceOTU41563
New.CleanUp.ReferenceOTU158315
New.CleanUp.ReferenceOTU135700
New.CleanUp.ReferenceOTU80986
New.CleanUp.ReferenceOTU64295
New.ReferenceOTU2610
New.CleanUp.ReferenceOTU7909
New.CleanUp.ReferenceOTU32457
New.CleanUp.ReferenceOTU5264
New.CleanUp.ReferenceOTU119549
New.CleanUp.ReferenceOTU54275
New.CleanUp.ReferenceOTU611
New.CleanUp.ReferenceOTU122418
New.CleanUp.ReferenceOTU180476
GU824016.1.1201
New.ReferenceOTU2277
New.CleanUp.ReferenceOTU42499

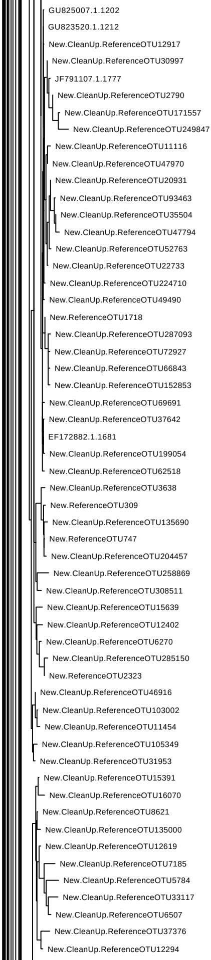
GU825007.1.1202
GU823520.1.1212
New.CleanUp.ReferenceOTU12917
New.CleanUp.ReferenceOTU30997
JF791107.1.1777
New.CleanUp.ReferenceOTU2790
New.CleanUp.ReferenceOTU171557
New.CleanUp.ReferenceOTU249847
New.CleanUp.ReferenceOTU11116
New.CleanUp.ReferenceOTU47970
New.CleanUp.ReferenceOTU20931
New.CleanUp.ReferenceOTU93463
New.CleanUp.ReferenceOTU35504
New.CleanUp.ReferenceOTU47794
New.CleanUp.ReferenceOTU52763
New.CleanUp.ReferenceOTU22733
New.CleanUp.ReferenceOTU224710
New.CleanUp.ReferenceOTU49490
New.ReferenceOTU1718
New.CleanUp.ReferenceOTU287093
New.CleanUp.ReferenceOTU72927
New.CleanUp.ReferenceOTU66843
New.CleanUp.ReferenceOTU152853
New.CleanUp.ReferenceOTU69691
New.CleanUp.ReferenceOTU37642
EF172882.1.1681
New.CleanUp.ReferenceOTU199054
New.CleanUp.ReferenceOTU62518
New.CleanUp.ReferenceOTU3638
New.ReferenceOTU309
New.CleanUp.ReferenceOTU135690
New.ReferenceOTU747
New.CleanUp.ReferenceOTU204457
New.CleanUp.ReferenceOTU258869
New.CleanUp.ReferenceOTU308511
New.CleanUp.ReferenceOTU15639
New.CleanUp.ReferenceOTU12402
New.CleanUp.ReferenceOTU6270
New.CleanUp.ReferenceOTU285150
New.ReferenceOTU2323
New.CleanUp.ReferenceOTU46916
New.CleanUp.ReferenceOTU103002
New.CleanUp.ReferenceOTU11454
New.CleanUp.ReferenceOTU105349
New.CleanUp.ReferenceOTU31953
New.CleanUp.ReferenceOTU15391
New.CleanUp.ReferenceOTU16070
New.CleanUp.ReferenceOTU8621
New.CleanUp.ReferenceOTU135000
New.CleanUp.ReferenceOTU12619
New.CleanUp.ReferenceOTU7185
New.CleanUp.ReferenceOTU5784
New.CleanUp.ReferenceOTU33117
New.CleanUp.ReferenceOTU6507
New.CleanUp.ReferenceOTU37376
New.CleanUp.ReferenceOTU12294

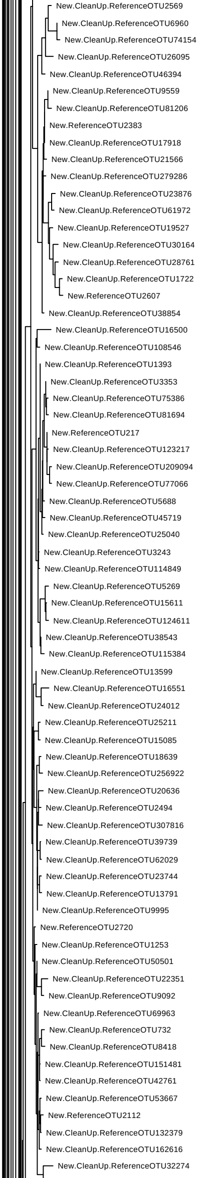
New.CleanUp.ReferenceOTU2569
New.CleanUp.ReferenceOTU6960
New.CleanUp.ReferenceOTU74154
New.CleanUp.ReferenceOTU26095
New.CleanUp.ReferenceOTU46394
New.CleanUp.ReferenceOTU9559
New.CleanUp.ReferenceOTU81206
New.ReferenceOTU2383
New.CleanUp.ReferenceOTU17918
New.CleanUp.ReferenceOTU21566
New.CleanUp.ReferenceOTU279286
New.CleanUp.ReferenceOTU23876
New.CleanUp.ReferenceOTU61972
New.CleanUp.ReferenceOTU19527
New.CleanUp.ReferenceOTU30164
New.CleanUp.ReferenceOTU28761
New.CleanUp.ReferenceOTU1722
New.ReferenceOTU2607
New.CleanUp.ReferenceOTU38854
New.CleanUp.ReferenceOTU16500
New.CleanUp.ReferenceOTU108546
New.CleanUp.ReferenceOTU1393
New.CleanUp.ReferenceOTU3353
New.CleanUp.ReferenceOTU75386
New.CleanUp.ReferenceOTU81694
New.ReferenceOTU217
New.CleanUp.ReferenceOTU123217
New.CleanUp.ReferenceOTU209094
New.CleanUp.ReferenceOTU77066
New.CleanUp.ReferenceOTU5688
New.CleanUp.ReferenceOTU45719
New.CleanUp.ReferenceOTU25040
New.CleanUp.ReferenceOTU3243
New.CleanUp.ReferenceOTU114849
New.CleanUp.ReferenceOTU5269
New.CleanUp.ReferenceOTU15611
New.CleanUp.ReferenceOTU124611
New.CleanUp.ReferenceOTU38543
New.CleanUp.ReferenceOTU115384
New.CleanUp.ReferenceOTU13599
New.CleanUp.ReferenceOTU16551
New.CleanUp.ReferenceOTU24012
New.CleanUp.ReferenceOTU25211
New.CleanUp.ReferenceOTU15085
New.CleanUp.ReferenceOTU18639
New.CleanUp.ReferenceOTU256922
New.CleanUp.ReferenceOTU20636
New.CleanUp.ReferenceOTU2494
New.CleanUp.ReferenceOTU307816
New.CleanUp.ReferenceOTU39739
New.CleanUp.ReferenceOTU62029
New.CleanUp.ReferenceOTU23744
New.CleanUp.ReferenceOTU13791
New.CleanUp.ReferenceOTU9995
New.ReferenceOTU2720
New.CleanUp.ReferenceOTU1253
New.CleanUp.ReferenceOTU50501
New.CleanUp.ReferenceOTU22351
New.CleanUp.ReferenceOTU9092
New.CleanUp.ReferenceOTU69963
New.CleanUp.ReferenceOTU732
New.CleanUp.ReferenceOTU8418
New.CleanUp.ReferenceOTU151481
New.CleanUp.ReferenceOTU42761
New.CleanUp.ReferenceOTU53667
New.ReferenceOTU2112
New.CleanUp.ReferenceOTU132379
New.CleanUp.ReferenceOTU162616
New.CleanUp.ReferenceOTU32274

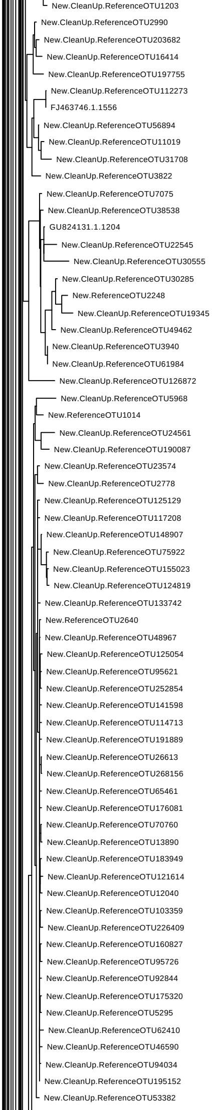
New.CleanUp.ReferenceOTU1203
New.CleanUp.ReferenceOTU2990
New.CleanUp.ReferenceOTU203682
New.CleanUp.ReferenceOTU16414
New.CleanUp.ReferenceOTU197755
New.CleanUp.ReferenceOTU112273
FJ463746.1.1556
New.CleanUp.ReferenceOTU56894
New.CleanUp.ReferenceOTU11019
New.CleanUp.ReferenceOTU31708
New.CleanUp.ReferenceOTU3822
New.CleanUp.ReferenceOTU7075
New.CleanUp.ReferenceOTU38538
GU824131.1.1204
New.CleanUp.ReferenceOTU22545
New.CleanUp.ReferenceOTU30555
New.CleanUp.ReferenceOTU30285
New.ReferenceOTU2248
New.CleanUp.ReferenceOTU19345
New.CleanUp.ReferenceOTU49462
New.CleanUp.ReferenceOTU3940
New.CleanUp.ReferenceOTU61984
New.CleanUp.ReferenceOTU126872
New.CleanUp.ReferenceOTU5968
New.ReferenceOTU1014
New.CleanUp.ReferenceOTU24561
New.CleanUp.ReferenceOTU190087
New.CleanUp.ReferenceOTU23574
New.CleanUp.ReferenceOTU2778
New.CleanUp.ReferenceOTU125129
New.CleanUp.ReferenceOTU117208
New.CleanUp.ReferenceOTU148907
New.CleanUp.ReferenceOTU75922
New.CleanUp.ReferenceOTU155023
New.CleanUp.ReferenceOTU124819
New.CleanUp.ReferenceOTU133742
New.ReferenceOTU2640
New.CleanUp.ReferenceOTU48967
New.CleanUp.ReferenceOTU125054
New.CleanUp.ReferenceOTU95621
New.CleanUp.ReferenceOTU252854
New.CleanUp.ReferenceOTU141598
New.CleanUp.ReferenceOTU114713
New.CleanUp.ReferenceOTU191889
New.CleanUp.ReferenceOTU26613
New.CleanUp.ReferenceOTU268156
New.CleanUp.ReferenceOTU65461
New.CleanUp.ReferenceOTU176081
New.CleanUp.ReferenceOTU70760
New.CleanUp.ReferenceOTU13890
New.CleanUp.ReferenceOTU183949
New.CleanUp.ReferenceOTU121614
New.CleanUp.ReferenceOTU12040
New.CleanUp.ReferenceOTU103359
New.CleanUp.ReferenceOTU226409
New.CleanUp.ReferenceOTU160827
New.CleanUp.ReferenceOTU95726
New.CleanUp.ReferenceOTU92844
New.CleanUp.ReferenceOTU175320
New.CleanUp.ReferenceOTU5295
New.CleanUp.ReferenceOTU62410
New.CleanUp.ReferenceOTU46590
New.CleanUp.ReferenceOTU94034
New.CleanUp.ReferenceOTU195152
New.CleanUp.ReferenceOTU53382

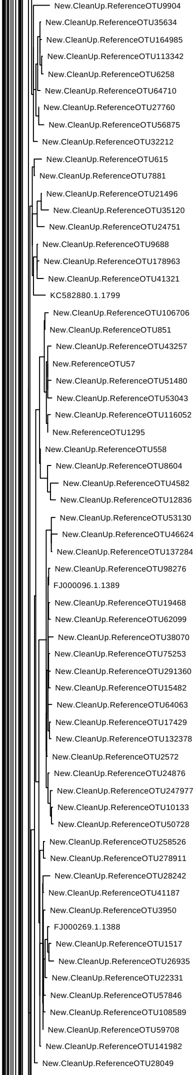
New.CleanUp.ReferenceOTU9904
New.CleanUp.ReferenceOTU35634
New.CleanUp.ReferenceOTU164985
New.CleanUp.ReferenceOTU113342
New.CleanUp.ReferenceOTU6258
New.CleanUp.ReferenceOTU64710
New.CleanUp.ReferenceOTU27760
New.CleanUp.ReferenceOTU56875
New.CleanUp.ReferenceOTU32212
New.CleanUp.ReferenceOTU615
New.CleanUp.ReferenceOTU7881
New.CleanUp.ReferenceOTU21496
New.CleanUp.ReferenceOTU35120
New.CleanUp.ReferenceOTU24751
New.CleanUp.ReferenceOTU9688
New.CleanUp.ReferenceOTU178963
New.CleanUp.ReferenceOTU41321
KC582880.1.1799
New.CleanUp.ReferenceOTU106706
New.CleanUp.ReferenceOTU851
New.CleanUp.ReferenceOTU43257
New.ReferenceOTU57
New.CleanUp.ReferenceOTU51480
New.CleanUp.ReferenceOTU53043
New.CleanUp.ReferenceOTU116052
New.ReferenceOTU1295
New.CleanUp.ReferenceOTU558
New.CleanUp.ReferenceOTU8604
New.CleanUp.ReferenceOTU4582
New.CleanUp.ReferenceOTU12836
New.CleanUp.ReferenceOTU53130
New.CleanUp.ReferenceOTU46624
New.CleanUp.ReferenceOTU137284
New.CleanUp.ReferenceOTU98276
FJ000096.1.1389
New.CleanUp.ReferenceOTU19468
New.CleanUp.ReferenceOTU62099
New.CleanUp.ReferenceOTU38070
New.CleanUp.ReferenceOTU75253
New.CleanUp.ReferenceOTU291360
New.CleanUp.ReferenceOTU15482
New.CleanUp.ReferenceOTU64063
New.CleanUp.ReferenceOTU17429
New.CleanUp.ReferenceOTU132378
New.CleanUp.ReferenceOTU2572
New.CleanUp.ReferenceOTU24876
New.CleanUp.ReferenceOTU247977
New.CleanUp.ReferenceOTU10133
New.CleanUp.ReferenceOTU50728
New.CleanUp.ReferenceOTU258526
New.CleanUp.ReferenceOTU278911
New.CleanUp.ReferenceOTU28242
New.CleanUp.ReferenceOTU41187
New.CleanUp.ReferenceOTU3950
FJ000269.1.1388
New.CleanUp.ReferenceOTU1517
New.CleanUp.ReferenceOTU26935
New.CleanUp.ReferenceOTU22331
New.CleanUp.ReferenceOTU57846
New.CleanUp.ReferenceOTU108589
New.CleanUp.ReferenceOTU59708
New.CleanUp.ReferenceOTU141982
New.CleanUp.ReferenceOTU28049

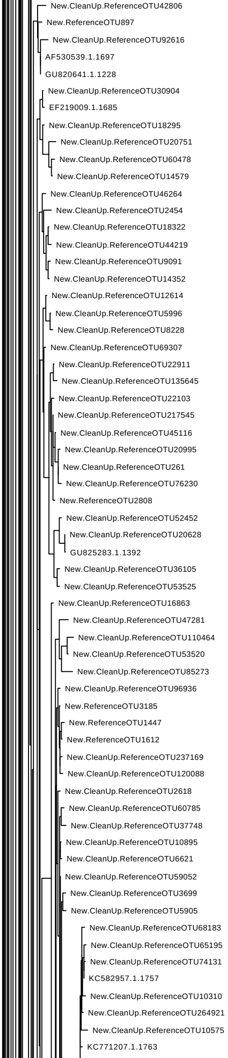
New.CleanUp.ReferenceOTU42806
New.ReferenceOTU897
New.CleanUp.ReferenceOTU92616
AF530539.1.1697
GU820641.1.1228
New.CleanUp.ReferenceOTU30904
EF219009.1.1685
New.CleanUp.ReferenceOTU18295
New.CleanUp.ReferenceOTU20751
New.CleanUp.ReferenceOTU60478
New.CleanUp.ReferenceOTU14579
New.CleanUp.ReferenceOTU46264
New.CleanUp.ReferenceOTU2454
New.CleanUp.ReferenceOTU18322
New.CleanUp.ReferenceOTU44219
New.CleanUp.ReferenceOTU9091
New.CleanUp.ReferenceOTU14352
New.CleanUp.ReferenceOTU12614
New.CleanUp.ReferenceOTU5996
New.CleanUp.ReferenceOTU8228
New.CleanUp.ReferenceOTU69307
New.CleanUp.ReferenceOTU22911
New.CleanUp.ReferenceOTU135645
New.CleanUp.ReferenceOTU22103
New.CleanUp.ReferenceOTU217545
New.CleanUp.ReferenceOTU45116
New.CleanUp.ReferenceOTU20995
New.CleanUp.ReferenceOTU261
New.CleanUp.ReferenceOTU76230
New.ReferenceOTU2808
New.CleanUp.ReferenceOTU52452
New.CleanUp.ReferenceOTU20628
GU825283.1.1392
New.CleanUp.ReferenceOTU36105
New.CleanUp.ReferenceOTU53525
New.CleanUp.ReferenceOTU16863
New.CleanUp.ReferenceOTU47281
New.CleanUp.ReferenceOTU110464
New.CleanUp.ReferenceOTU53520
New.CleanUp.ReferenceOTU85273
New.CleanUp.ReferenceOTU96936
New.ReferenceOTU3185
New.ReferenceOTU1447
New.ReferenceOTU1612
New.CleanUp.ReferenceOTU237169
New.CleanUp.ReferenceOTU120088
New.CleanUp.ReferenceOTU2618
New.CleanUp.ReferenceOTU60785
New.CleanUp.ReferenceOTU37748
New.CleanUp.ReferenceOTU10895
New.CleanUp.ReferenceOTU6621
New.CleanUp.ReferenceOTU59052
New.CleanUp.ReferenceOTU3699
New.CleanUp.ReferenceOTU5905
New.CleanUp.ReferenceOTU68183
New.CleanUp.ReferenceOTU65195
New.CleanUp.ReferenceOTU74131
KC582957.1.1757
New.CleanUp.ReferenceOTU10310
New.CleanUp.ReferenceOTU264921
New.CleanUp.ReferenceOTU10575
KC771207.1.1763

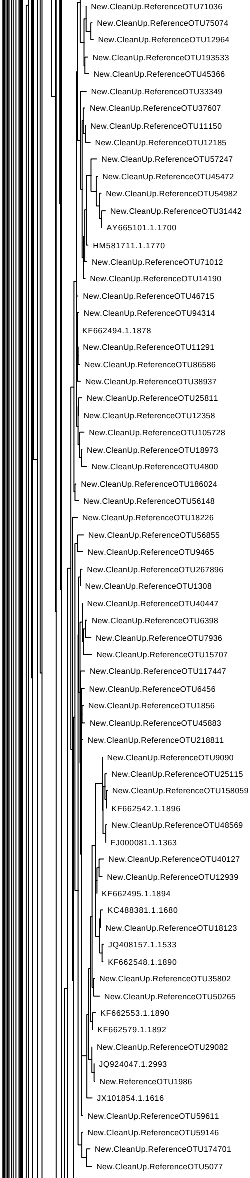
New.CleanUp.ReferenceOTU71036
New.CleanUp.ReferenceOTU75074
New.CleanUp.ReferenceOTU12964
New.CleanUp.ReferenceOTU193533
New.CleanUp.ReferenceOTU45366
New.CleanUp.ReferenceOTU33349
New.CleanUp.ReferenceOTU37607
New.CleanUp.ReferenceOTU11150
New.CleanUp.ReferenceOTU12185
New.CleanUp.ReferenceOTU57247
New.CleanUp.ReferenceOTU45472
New.CleanUp.ReferenceOTU54982
New.CleanUp.ReferenceOTU31442
AY665101.1.1700
HM581711.1.1770
New.CleanUp.ReferenceOTU71012
New.CleanUp.ReferenceOTU14190
New.CleanUp.ReferenceOTU46715
New.CleanUp.ReferenceOTU94314
KF662494.1.1878
New.CleanUp.ReferenceOTU11291
New.CleanUp.ReferenceOTU86586
New.CleanUp.ReferenceOTU38937
New.CleanUp.ReferenceOTU25811
New.CleanUp.ReferenceOTU12358
New.CleanUp.ReferenceOTU105728
New.CleanUp.ReferenceOTU18973
New.CleanUp.ReferenceOTU4800
New.CleanUp.ReferenceOTU186024
New.CleanUp.ReferenceOTU56148
New.CleanUp.ReferenceOTU18226
New.CleanUp.ReferenceOTU56855
New.CleanUp.ReferenceOTU9465
New.CleanUp.ReferenceOTU267896
New.CleanUp.ReferenceOTU1308
New.CleanUp.ReferenceOTU40447
New.CleanUp.ReferenceOTU6398
New.CleanUp.ReferenceOTU7936
New.CleanUp.ReferenceOTU15707
New.CleanUp.ReferenceOTU117447
New.CleanUp.ReferenceOTU6456
New.CleanUp.ReferenceOTU1856
New.CleanUp.ReferenceOTU45883
New.CleanUp.ReferenceOTU218811
New.CleanUp.ReferenceOTU9090
New.CleanUp.ReferenceOTU25115
New.CleanUp.ReferenceOTU158059
KF662542.1.1896
New.CleanUp.ReferenceOTU48569
FJ000081.1.1363
New.CleanUp.ReferenceOTU40127
New.CleanUp.ReferenceOTU12939
KF662495.1.1894
KC488381.1.1680
New.CleanUp.ReferenceOTU18123
JQ408157.1.1533
KF662548.1.1890
New.CleanUp.ReferenceOTU35802
New.CleanUp.ReferenceOTU50265
KF662553.1.1890
KF662579.1.1892
New.CleanUp.ReferenceOTU29082
JQ924047.1.2993
New.ReferenceOTU1986
JX101854.1.1616
New.CleanUp.ReferenceOTU59611
New.CleanUp.ReferenceOTU59146
New.CleanUp.ReferenceOTU174701
New.CleanUp.ReferenceOTU5077

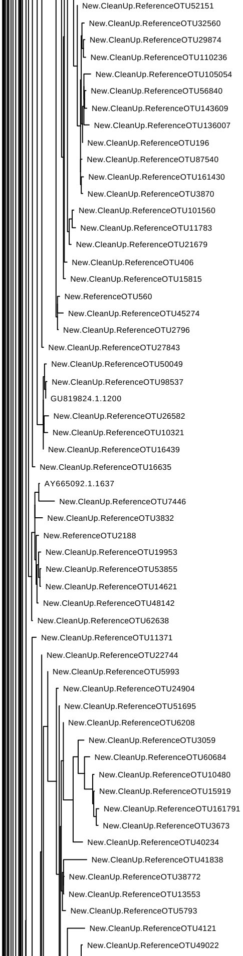
New.CleanUp.ReferenceOTU52151
New.CleanUp.ReferenceOTU32560
New.CleanUp.ReferenceOTU29874
New.CleanUp.ReferenceOTU110236
New.CleanUp.ReferenceOTU105054
New.CleanUp.ReferenceOTU56840
New.CleanUp.ReferenceOTU143609
New.CleanUp.ReferenceOTU136007
New.CleanUp.ReferenceOTU196
New.CleanUp.ReferenceOTU87540
New.CleanUp.ReferenceOTU161430
New.CleanUp.ReferenceOTU3870
New.CleanUp.ReferenceOTU101560
New.CleanUp.ReferenceOTU11783
New.CleanUp.ReferenceOTU21679
New.CleanUp.ReferenceOTU406
New.CleanUp.ReferenceOTU15815
New.ReferenceOTU560
New.CleanUp.ReferenceOTU45274
New.CleanUp.ReferenceOTU2796
New.CleanUp.ReferenceOTU27843
New.CleanUp.ReferenceOTU50049
New.CleanUp.ReferenceOTU98537
GU819824.1.1200
New.CleanUp.ReferenceOTU26582
New.CleanUp.ReferenceOTU10321
New.CleanUp.ReferenceOTU16439
New.CleanUp.ReferenceOTU16635
AY665092.1.1637
New.CleanUp.ReferenceOTU7446
New.CleanUp.ReferenceOTU3832
New.ReferenceOTU2188
New.CleanUp.ReferenceOTU19953
New.CleanUp.ReferenceOTU53855
New.CleanUp.ReferenceOTU14621
New.CleanUp.ReferenceOTU48142
New.CleanUp.ReferenceOTU62638
New.CleanUp.ReferenceOTU11371
New.CleanUp.ReferenceOTU22744
New.CleanUp.ReferenceOTU5993
New.CleanUp.ReferenceOTU24904
New.CleanUp.ReferenceOTU51695
New.CleanUp.ReferenceOTU6208
New.CleanUp.ReferenceOTU3059
New.CleanUp.ReferenceOTU60684
New.CleanUp.ReferenceOTU10480
New.CleanUp.ReferenceOTU15919
New.CleanUp.ReferenceOTU161791
New.CleanUp.ReferenceOTU3673
New.CleanUp.ReferenceOTU40234
New.CleanUp.ReferenceOTU41838
New.CleanUp.ReferenceOTU38772
New.CleanUp.ReferenceOTU13553
New.CleanUp.ReferenceOTU5793
New.CleanUp.ReferenceOTU4121
New.CleanUp.ReferenceOTU49022

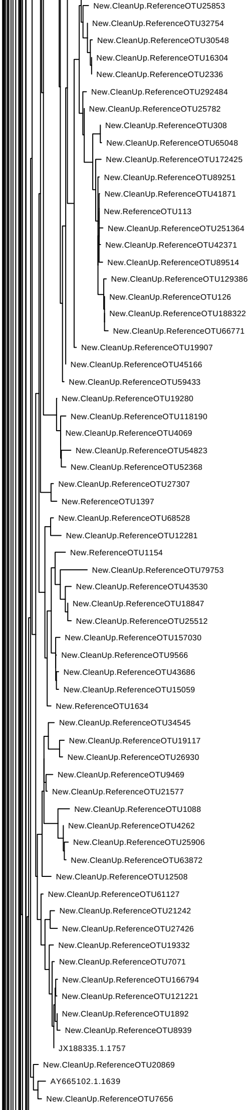
New.CleanUp.ReferenceOTU25853
New.CleanUp.ReferenceOTU32754
New.CleanUp.ReferenceOTU30548
New.CleanUp.ReferenceOTU16304
New.CleanUp.ReferenceOTU2336
New.CleanUp.ReferenceOTU292484
New.CleanUp.ReferenceOTU25782
New.CleanUp.ReferenceOTU308
New.CleanUp.ReferenceOTU65048
New.CleanUp.ReferenceOTU172425
New.CleanUp.ReferenceOTU89251
New.CleanUp.ReferenceOTU41871
New.ReferenceOTU113
New.CleanUp.ReferenceOTU251364
New.CleanUp.ReferenceOTU42371
New.CleanUp.ReferenceOTU89514
New.CleanUp.ReferenceOTU129386
New.CleanUp.ReferenceOTU126
New.CleanUp.ReferenceOTU188322
New.CleanUp.ReferenceOTU66771
New.CleanUp.ReferenceOTU19907
New.CleanUp.ReferenceOTU45166
New.CleanUp.ReferenceOTU59433
New.CleanUp.ReferenceOTU19280
New.CleanUp.ReferenceOTU118190
New.CleanUp.ReferenceOTU4069
New.CleanUp.ReferenceOTU54823
New.CleanUp.ReferenceOTU52368
New.CleanUp.ReferenceOTU27307
New.ReferenceOTU1397
New.CleanUp.ReferenceOTU68528
New.CleanUp.ReferenceOTU12281
New.ReferenceOTU1154
New.CleanUp.ReferenceOTU79753
New.CleanUp.ReferenceOTU43530
New.CleanUp.ReferenceOTU18847
New.CleanUp.ReferenceOTU25512
New.CleanUp.ReferenceOTU157030
New.CleanUp.ReferenceOTU9566
New.CleanUp.ReferenceOTU43686
New.CleanUp.ReferenceOTU15059
New.ReferenceOTU1634
New.CleanUp.ReferenceOTU34545
New.CleanUp.ReferenceOTU19117
New.CleanUp.ReferenceOTU26930
New.CleanUp.ReferenceOTU9469
New.CleanUp.ReferenceOTU21577
New.CleanUp.ReferenceOTU1088
New.CleanUp.ReferenceOTU4262
New.CleanUp.ReferenceOTU25906
New.CleanUp.ReferenceOTU63872
New.CleanUp.ReferenceOTU12508
New.CleanUp.ReferenceOTU61127
New.CleanUp.ReferenceOTU21242
New.CleanUp.ReferenceOTU27426
New.CleanUp.ReferenceOTU19332
New.CleanUp.ReferenceOTU7071
New.CleanUp.ReferenceOTU166794
New.CleanUp.ReferenceOTU121221
New.CleanUp.ReferenceOTU1892
New.CleanUp.ReferenceOTU8939
JX188335.1.1757
New.CleanUp.ReferenceOTU20869
AY665102.1.1639
New.CleanUp.ReferenceOTU7656

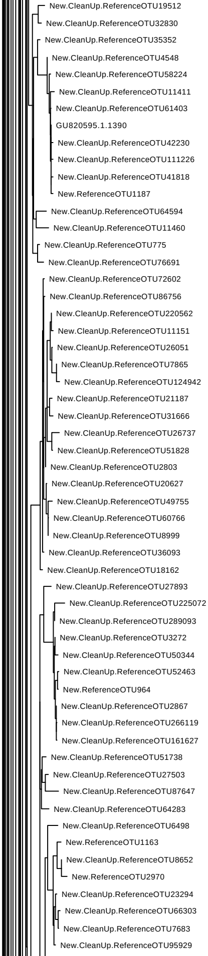
New.CleanUp.ReferenceOTU19512
New.CleanUp.ReferenceOTU32830
New.CleanUp.ReferenceOTU35352
New.CleanUp.ReferenceOTU4548
New.CleanUp.ReferenceOTU58224
New.CleanUp.ReferenceOTU11411
New.CleanUp.ReferenceOTU61403
GU820595.1.1390
New.CleanUp.ReferenceOTU42230
New.CleanUp.ReferenceOTU111226
New.CleanUp.ReferenceOTU41818
New.ReferenceOTU1187
New.CleanUp.ReferenceOTU64594
New.CleanUp.ReferenceOTU11460
New.CleanUp.ReferenceOTU775
New.CleanUp.ReferenceOTU76691
New.CleanUp.ReferenceOTU72602
New.CleanUp.ReferenceOTU86756
New.CleanUp.ReferenceOTU220562
New.CleanUp.ReferenceOTU11151
New.CleanUp.ReferenceOTU26051
New.CleanUp.ReferenceOTU7865
New.CleanUp.ReferenceOTU124942
New.CleanUp.ReferenceOTU21187
New.CleanUp.ReferenceOTU31666
New.CleanUp.ReferenceOTU26737
New.CleanUp.ReferenceOTU51828
New.CleanUp.ReferenceOTU2803
New.CleanUp.ReferenceOTU20627
New.CleanUp.ReferenceOTU49755
New.CleanUp.ReferenceOTU60766
New.CleanUp.ReferenceOTU8999
New.CleanUp.ReferenceOTU36093
New.CleanUp.ReferenceOTU18162
New.CleanUp.ReferenceOTU27893
New.CleanUp.ReferenceOTU225072
New.CleanUp.ReferenceOTU289093
New.CleanUp.ReferenceOTU3272
New.CleanUp.ReferenceOTU50344
New.CleanUp.ReferenceOTU52463
New.ReferenceOTU964
New.CleanUp.ReferenceOTU2867
New.CleanUp.ReferenceOTU266119
New.CleanUp.ReferenceOTU161627
New.CleanUp.ReferenceOTU51738
New.CleanUp.ReferenceOTU27503
New.CleanUp.ReferenceOTU87647
New.CleanUp.ReferenceOTU64283
New.CleanUp.ReferenceOTU6498
New.ReferenceOTU1163
New.CleanUp.ReferenceOTU8652
New.ReferenceOTU2970
New.CleanUp.ReferenceOTU23294
New.CleanUp.ReferenceOTU66303
New.CleanUp.ReferenceOTU7683
New.CleanUp.ReferenceOTU95929

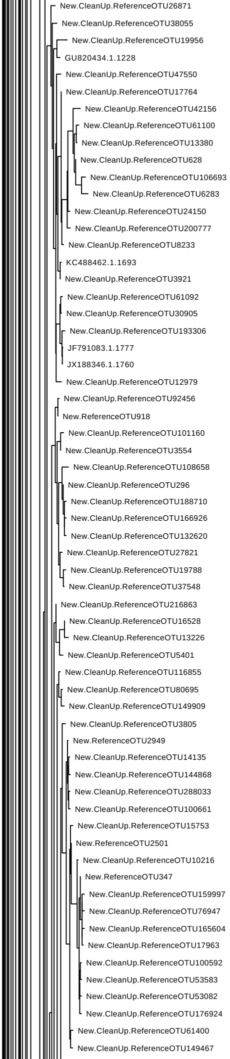
New.CleanUp.ReferenceOTU26871
New.CleanUp.ReferenceOTU38055
New.CleanUp.ReferenceOTU19956
GU820434.1.1228
New.CleanUp.ReferenceOTU47550
New.CleanUp.ReferenceOTU17764
New.CleanUp.ReferenceOTU42156
New.CleanUp.ReferenceOTU61100
New.CleanUp.ReferenceOTU13380
New.CleanUp.ReferenceOTU628
New.CleanUp.ReferenceOTU106693
New.CleanUp.ReferenceOTU6283
New.CleanUp.ReferenceOTU24150
New.CleanUp.ReferenceOTU200777
New.CleanUp.ReferenceOTU8233
KC488462.1.1693
New.CleanUp.ReferenceOTU3921
New.CleanUp.ReferenceOTU61092
New.CleanUp.ReferenceOTU30905
New.CleanUp.ReferenceOTU193306
JF791083.1.1777
JX188346.1.1760
New.CleanUp.ReferenceOTU12979
New.CleanUp.ReferenceOTU92456
New.ReferenceOTU918
New.CleanUp.ReferenceOTU101160
New.CleanUp.ReferenceOTU3554
New.CleanUp.ReferenceOTU108658
New.CleanUp.ReferenceOTU296
New.CleanUp.ReferenceOTU188710
New.CleanUp.ReferenceOTU166926
New.CleanUp.ReferenceOTU132620
New.CleanUp.ReferenceOTU27821
New.CleanUp.ReferenceOTU19788
New.CleanUp.ReferenceOTU37548
New.CleanUp.ReferenceOTU216863
New.CleanUp.ReferenceOTU16528
New.CleanUp.ReferenceOTU13226
New.CleanUp.ReferenceOTU5401
New.CleanUp.ReferenceOTU116855
New.CleanUp.ReferenceOTU80695
New.CleanUp.ReferenceOTU149909
New.CleanUp.ReferenceOTU3805
New.ReferenceOTU2949
New.CleanUp.ReferenceOTU14135
New.CleanUp.ReferenceOTU144868
New.CleanUp.ReferenceOTU288033
New.CleanUp.ReferenceOTU100661
New.CleanUp.ReferenceOTU15753
New.ReferenceOTU2501
New.CleanUp.ReferenceOTU10216
New.ReferenceOTU347
New.CleanUp.ReferenceOTU159997
New.CleanUp.ReferenceOTU76947
New.CleanUp.ReferenceOTU165604
New.CleanUp.ReferenceOTU17963
New.CleanUp.ReferenceOTU100592
New.CleanUp.ReferenceOTU53583
New.CleanUp.ReferenceOTU53082
New.CleanUp.ReferenceOTU176924
New.CleanUp.ReferenceOTU61400
New.CleanUp.ReferenceOTU149467

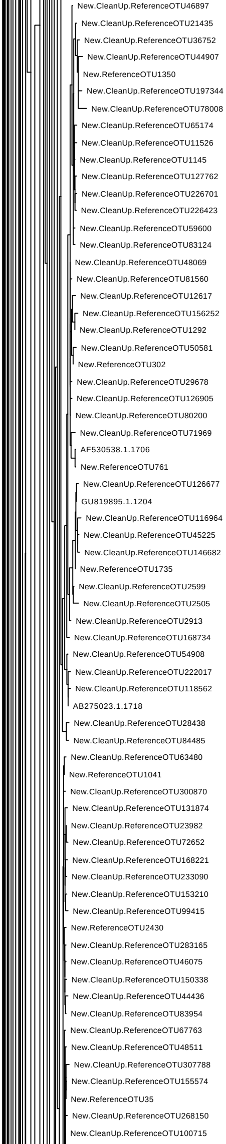
New.CleanUp.ReferenceOTU46897
New.CleanUp.ReferenceOTU21435
New.CleanUp.ReferenceOTU36752
New.CleanUp.ReferenceOTU44907
New.ReferenceOTU1350
New.CleanUp.ReferenceOTU197344
New.CleanUp.ReferenceOTU78008
New.CleanUp.ReferenceOTU65174
New.CleanUp.ReferenceOTU11526
New.CleanUp.ReferenceOTU1145
New.CleanUp.ReferenceOTU127762
New.CleanUp.ReferenceOTU226701
New.CleanUp.ReferenceOTU226423
New.CleanUp.ReferenceOTU59600
New.CleanUp.ReferenceOTU83124
New.CleanUp.ReferenceOTU48069
New.CleanUp.ReferenceOTU81560
New.CleanUp.ReferenceOTU12617
New.CleanUp.ReferenceOTU156252
New.CleanUp.ReferenceOTU1292
New.CleanUp.ReferenceOTU50581
New.ReferenceOTU302
New.CleanUp.ReferenceOTU29678
New.CleanUp.ReferenceOTU126905
New.CleanUp.ReferenceOTU80200
New.CleanUp.ReferenceOTU71969
AF530538.1.1706
New.ReferenceOTU761
New.CleanUp.ReferenceOTU126677
GU819895.1.1204
New.CleanUp.ReferenceOTU116964
New.CleanUp.ReferenceOTU45225
New.CleanUp.ReferenceOTU146682
New.ReferenceOTU1735
New.CleanUp.ReferenceOTU2599
New.CleanUp.ReferenceOTU2505
New.CleanUp.ReferenceOTU2913
New.CleanUp.ReferenceOTU168734
New.CleanUp.ReferenceOTU54908
New.CleanUp.ReferenceOTU222017
New.CleanUp.ReferenceOTU118562
AB275023.1.1718
New.CleanUp.ReferenceOTU28438
New.CleanUp.ReferenceOTU84485
New.CleanUp.ReferenceOTU63480
New.ReferenceOTU1041
New.CleanUp.ReferenceOTU300870
New.CleanUp.ReferenceOTU131874
New.CleanUp.ReferenceOTU23982
New.CleanUp.ReferenceOTU72652
New.CleanUp.ReferenceOTU168221
New.CleanUp.ReferenceOTU233090
New.CleanUp.ReferenceOTU153210
New.CleanUp.ReferenceOTU99415
New.ReferenceOTU2430
New.CleanUp.ReferenceOTU283165
New.CleanUp.ReferenceOTU46075
New.CleanUp.ReferenceOTU150338
New.CleanUp.ReferenceOTU44436
New.CleanUp.ReferenceOTU83954
New.CleanUp.ReferenceOTU67763
New.CleanUp.ReferenceOTU48511
New.CleanUp.ReferenceOTU307788
New.CleanUp.ReferenceOTU155574
New.ReferenceOTU35
New.CleanUp.ReferenceOTU268150
New.CleanUp.ReferenceOTU100715

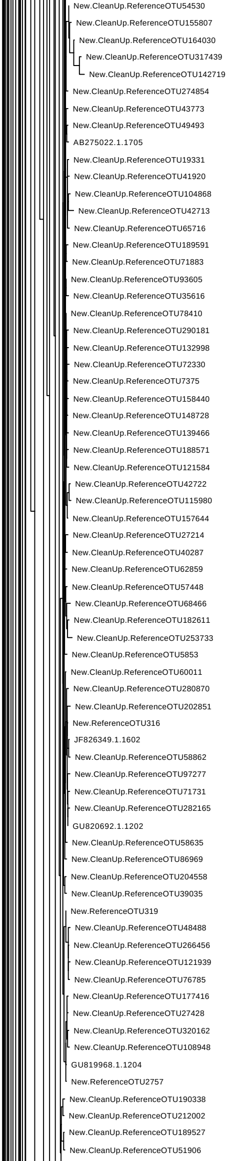
New.CleanUp.ReferenceOTU54530
New.CleanUp.ReferenceOTU155807
New.CleanUp.ReferenceOTU164030
New.CleanUp.ReferenceOTU317439
New.CleanUp.ReferenceOTU142719
New.CleanUp.ReferenceOTU274854
New.CleanUp.ReferenceOTU43773
New.CleanUp.ReferenceOTU49493
AB275022.1.1705
New.CleanUp.ReferenceOTU19331
New.CleanUp.ReferenceOTU41920
New.CleanUp.ReferenceOTU104868
New.CleanUp.ReferenceOTU42713
New.CleanUp.ReferenceOTU65716
New.CleanUp.ReferenceOTU189591
New.CleanUp.ReferenceOTU71883
New.CleanUp.ReferenceOTU93605
New.CleanUp.ReferenceOTU35616
New.CleanUp.ReferenceOTU78410
New.CleanUp.ReferenceOTU290181
New.CleanUp.ReferenceOTU132998
New.CleanUp.ReferenceOTU72330
New.CleanUp.ReferenceOTU7375
New.CleanUp.ReferenceOTU158440
New.CleanUp.ReferenceOTU148728
New.CleanUp.ReferenceOTU139466
New.CleanUp.ReferenceOTU188571
New.CleanUp.ReferenceOTU121584
New.CleanUp.ReferenceOTU42722
New.CleanUp.ReferenceOTU115980
New.CleanUp.ReferenceOTU157644
New.CleanUp.ReferenceOTU27214
New.CleanUp.ReferenceOTU40287
New.CleanUp.ReferenceOTU62859
New.CleanUp.ReferenceOTU57448
New.CleanUp.ReferenceOTU68466
New.CleanUp.ReferenceOTU182611
New.CleanUp.ReferenceOTU253733
New.CleanUp.ReferenceOTU5853
New.CleanUp.ReferenceOTU60011
New.CleanUp.ReferenceOTU280870
New.CleanUp.ReferenceOTU202851
New.ReferenceOTU316
JF826349.1.1602
New.CleanUp.ReferenceOTU58862
New.CleanUp.ReferenceOTU97277
New.CleanUp.ReferenceOTU71731
New.CleanUp.ReferenceOTU282165
GU820692.1.1202
New.CleanUp.ReferenceOTU58635
New.CleanUp.ReferenceOTU86969
New.CleanUp.ReferenceOTU204558
New.CleanUp.ReferenceOTU39035
New.ReferenceOTU319
New.CleanUp.ReferenceOTU48488
New.CleanUp.ReferenceOTU266456
New.CleanUp.ReferenceOTU121939
New.CleanUp.ReferenceOTU76785
New.CleanUp.ReferenceOTU177416
New.CleanUp.ReferenceOTU27428
New.CleanUp.ReferenceOTU320162
New.CleanUp.ReferenceOTU108948
GU819968.1.1204
New.ReferenceOTU2757
New.CleanUp.ReferenceOTU190338
New.CleanUp.ReferenceOTU212002
New.CleanUp.ReferenceOTU189527
New.CleanUp.ReferenceOTU51906

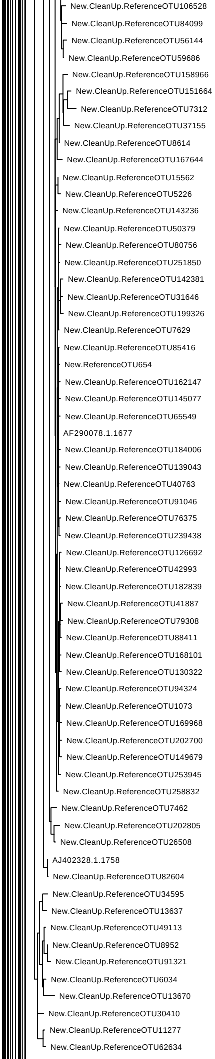
New.CleanUp.ReferenceOTU106528
New.CleanUp.ReferenceOTU84099
New.CleanUp.ReferenceOTU56144
New.CleanUp.ReferenceOTU59686
New.CleanUp.ReferenceOTU158966
New.CleanUp.ReferenceOTU151664
New.CleanUp.ReferenceOTU7312
New.CleanUp.ReferenceOTU37155
New.CleanUp.ReferenceOTU8614
New.CleanUp.ReferenceOTU167644
New.CleanUp.ReferenceOTU15562
New.CleanUp.ReferenceOTU5226
New.CleanUp.ReferenceOTU143236
New.CleanUp.ReferenceOTU50379
New.CleanUp.ReferenceOTU80756
New.CleanUp.ReferenceOTU251850
New.CleanUp.ReferenceOTU142381
New.CleanUp.ReferenceOTU31646
New.CleanUp.ReferenceOTU199326
New.CleanUp.ReferenceOTU7629
New.CleanUp.ReferenceOTU85416
New.ReferenceOTU654
New.CleanUp.ReferenceOTU162147
New.CleanUp.ReferenceOTU145077
New.CleanUp.ReferenceOTU65549
AF290078.1.1677
New.CleanUp.ReferenceOTU184006
New.CleanUp.ReferenceOTU139043
New.CleanUp.ReferenceOTU40763
New.CleanUp.ReferenceOTU91046
New.CleanUp.ReferenceOTU76375
New.CleanUp.ReferenceOTU239438
New.CleanUp.ReferenceOTU126692
New.CleanUp.ReferenceOTU42993
New.CleanUp.ReferenceOTU182839
New.CleanUp.ReferenceOTU41887
New.CleanUp.ReferenceOTU79308
New.CleanUp.ReferenceOTU88411
New.CleanUp.ReferenceOTU168101
New.CleanUp.ReferenceOTU130322
New.CleanUp.ReferenceOTU94324
New.CleanUp.ReferenceOTU1073
New.CleanUp.ReferenceOTU169968
New.CleanUp.ReferenceOTU202700
New.CleanUp.ReferenceOTU149679
New.CleanUp.ReferenceOTU253945
New.CleanUp.ReferenceOTU258832
New.CleanUp.ReferenceOTU7462
New.CleanUp.ReferenceOTU202805
New.CleanUp.ReferenceOTU26508
AJ402328.1.1758
New.CleanUp.ReferenceOTU82604
New.CleanUp.ReferenceOTU34595
New.CleanUp.ReferenceOTU13637
New.CleanUp.ReferenceOTU49113
New.CleanUp.ReferenceOTU8952
New.CleanUp.ReferenceOTU91321
New.CleanUp.ReferenceOTU6034
New.CleanUp.ReferenceOTU13670
New.CleanUp.ReferenceOTU30410
New.CleanUp.ReferenceOTU11277
New.CleanUp.ReferenceOTU62634

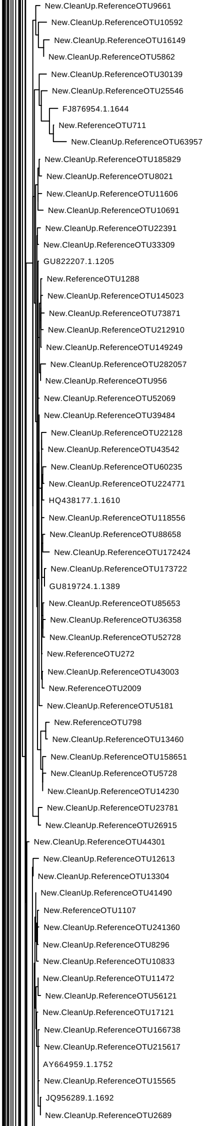
New.CleanUp.ReferenceOTU9661
New.CleanUp.ReferenceOTU10592
New.CleanUp.ReferenceOTU16149
New.CleanUp.ReferenceOTU5862
New.CleanUp.ReferenceOTU30139
New.CleanUp.ReferenceOTU25546
FJ876954.1.1644
New.ReferenceOTU711
New.CleanUp.ReferenceOTU63957
New.CleanUp.ReferenceOTU185829
New.CleanUp.ReferenceOTU8021
New.CleanUp.ReferenceOTU11606
New.CleanUp.ReferenceOTU10691
New.CleanUp.ReferenceOTU22391
New.CleanUp.ReferenceOTU33309
GU822207.1.1205
New.ReferenceOTU1288
New.CleanUp.ReferenceOTU145023
New.CleanUp.ReferenceOTU73871
New.CleanUp.ReferenceOTU212910
New.CleanUp.ReferenceOTU149249
New.CleanUp.ReferenceOTU282057
New.CleanUp.ReferenceOTU956
New.CleanUp.ReferenceOTU52069
New.CleanUp.ReferenceOTU39484
New.CleanUp.ReferenceOTU22128
New.CleanUp.ReferenceOTU43542
New.CleanUp.ReferenceOTU60235
New.CleanUp.ReferenceOTU224771
HQ438177.1.1610
New.CleanUp.ReferenceOTU118556
New.CleanUp.ReferenceOTU88658
New.CleanUp.ReferenceOTU172424
New.CleanUp.ReferenceOTU173722
GU819724.1.1389
New.CleanUp.ReferenceOTU85653
New.CleanUp.ReferenceOTU36358
New.CleanUp.ReferenceOTU52728
New.ReferenceOTU272
New.CleanUp.ReferenceOTU43003
New.ReferenceOTU2009
New.CleanUp.ReferenceOTU5181
New.ReferenceOTU798
New.CleanUp.ReferenceOTU13460
New.CleanUp.ReferenceOTU158651
New.CleanUp.ReferenceOTU5728
New.CleanUp.ReferenceOTU14230
New.CleanUp.ReferenceOTU23781
New.CleanUp.ReferenceOTU26915
New.CleanUp.ReferenceOTU44301
New.CleanUp.ReferenceOTU12613
New.CleanUp.ReferenceOTU13304
New.CleanUp.ReferenceOTU41490
New.ReferenceOTU1107
New.CleanUp.ReferenceOTU241360
New.CleanUp.ReferenceOTU8296
New.CleanUp.ReferenceOTU10833
New.CleanUp.ReferenceOTU11472
New.CleanUp.ReferenceOTU56121
New.CleanUp.ReferenceOTU17121
New.CleanUp.ReferenceOTU166738
New.CleanUp.ReferenceOTU215617
AY664959.1.1752
New.CleanUp.ReferenceOTU15565
JQ956289.1.1692
New.CleanUp.ReferenceOTU2689

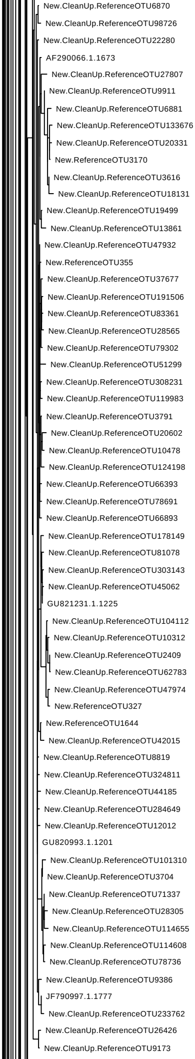
New.CleanUp.ReferenceOTU6870
New.CleanUp.ReferenceOTU98726
New.CleanUp.ReferenceOTU22280
AF290066.1.1673
New.CleanUp.ReferenceOTU27807
New.CleanUp.ReferenceOTU9911
New.CleanUp.ReferenceOTU6881
New.CleanUp.ReferenceOTU133676
New.CleanUp.ReferenceOTU20331
New.ReferenceOTU3170
New.CleanUp.ReferenceOTU3616
New.CleanUp.ReferenceOTU18131
New.CleanUp.ReferenceOTU19499
New.CleanUp.ReferenceOTU13861
New.CleanUp.ReferenceOTU47932
New.ReferenceOTU355
New.CleanUp.ReferenceOTU37677
New.CleanUp.ReferenceOTU191506
New.CleanUp.ReferenceOTU83361
New.CleanUp.ReferenceOTU28565
New.CleanUp.ReferenceOTU79302
New.CleanUp.ReferenceOTU51299
New.CleanUp.ReferenceOTU308231
New.CleanUp.ReferenceOTU119983
New.CleanUp.ReferenceOTU3791
New.CleanUp.ReferenceOTU20602
New.CleanUp.ReferenceOTU10478
New.CleanUp.ReferenceOTU124198
New.CleanUp.ReferenceOTU66393
New.CleanUp.ReferenceOTU78691
New.CleanUp.ReferenceOTU66893
New.CleanUp.ReferenceOTU178149
New.CleanUp.ReferenceOTU81078
New.CleanUp.ReferenceOTU303143
New.CleanUp.ReferenceOTU45062
GU821231.1.1225
New.CleanUp.ReferenceOTU104112
New.CleanUp.ReferenceOTU10312
New.CleanUp.ReferenceOTU2409
New.CleanUp.ReferenceOTU62783
New.CleanUp.ReferenceOTU47974
New.ReferenceOTU327
New.ReferenceOTU1644
New.CleanUp.ReferenceOTU42015
New.CleanUp.ReferenceOTU8819
New.CleanUp.ReferenceOTU324811
New.CleanUp.ReferenceOTU44185
New.CleanUp.ReferenceOTU284649
New.CleanUp.ReferenceOTU12012
GU820993.1.1201
New.CleanUp.ReferenceOTU101310
New.CleanUp.ReferenceOTU3704
New.CleanUp.ReferenceOTU71337
New.CleanUp.ReferenceOTU28305
New.CleanUp.ReferenceOTU114655
New.CleanUp.ReferenceOTU114608
New.CleanUp.ReferenceOTU78736
New.CleanUp.ReferenceOTU9386
JF790997.1.1777
New.CleanUp.ReferenceOTU233762
New.CleanUp.ReferenceOTU26426
New.CleanUp.ReferenceOTU9173

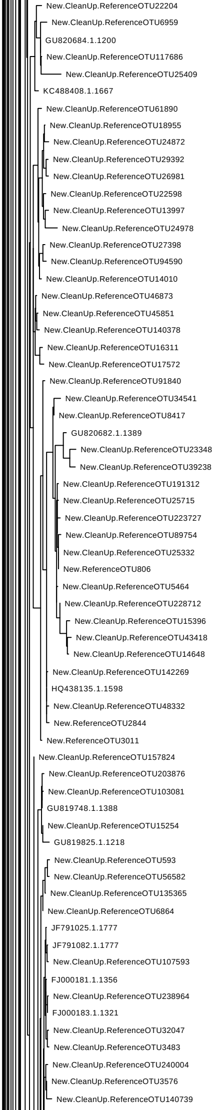
New.CleanUp.ReferenceOTU22204
New.CleanUp.ReferenceOTU6959
GU820684.1.1200
New.CleanUp.ReferenceOTU117686
New.CleanUp.ReferenceOTU25409
KC488408.1.1667
New.CleanUp.ReferenceOTU61890
New.CleanUp.ReferenceOTU18955
New.CleanUp.ReferenceOTU24872
New.CleanUp.ReferenceOTU29392
New.CleanUp.ReferenceOTU26981
New.CleanUp.ReferenceOTU22598
New.CleanUp.ReferenceOTU13997
New.CleanUp.ReferenceOTU24978
New.CleanUp.ReferenceOTU27398
New.CleanUp.ReferenceOTU94590
New.CleanUp.ReferenceOTU14010
New.CleanUp.ReferenceOTU46873
New.CleanUp.ReferenceOTU45851
New.CleanUp.ReferenceOTU140378
New.CleanUp.ReferenceOTU16311
New.CleanUp.ReferenceOTU17572
New.CleanUp.ReferenceOTU91840
New.CleanUp.ReferenceOTU34541
New.CleanUp.ReferenceOTU8417
GU820682.1.1389
New.CleanUp.ReferenceOTU23348
New.CleanUp.ReferenceOTU39238
New.CleanUp.ReferenceOTU191312
New.CleanUp.ReferenceOTU25715
New.CleanUp.ReferenceOTU223727
New.CleanUp.ReferenceOTU89754
New.CleanUp.ReferenceOTU25332
New.ReferenceOTU806
New.CleanUp.ReferenceOTU5464
New.CleanUp.ReferenceOTU228712
New.CleanUp.ReferenceOTU15396
New.CleanUp.ReferenceOTU43418
New.CleanUp.ReferenceOTU14648
New.CleanUp.ReferenceOTU142269
HQ438135.1.1598
New.CleanUp.ReferenceOTU48332
New.ReferenceOTU2844
New.ReferenceOTU3011
New.CleanUp.ReferenceOTU157824
New.CleanUp.ReferenceOTU203876
New.CleanUp.ReferenceOTU103081
GU819748.1.1388
New.CleanUp.ReferenceOTU15254
GU819825.1.1218
New.CleanUp.ReferenceOTU593
New.CleanUp.ReferenceOTU56582
New.CleanUp.ReferenceOTU135365
New.CleanUp.ReferenceOTU6864
JF791025.1.1777
JF791082.1.1777
New.CleanUp.ReferenceOTU107593
FJ000181.1.1356
New.CleanUp.ReferenceOTU238964
FJ000183.1.1321
New.CleanUp.ReferenceOTU32047
New.CleanUp.ReferenceOTU3483
New.CleanUp.ReferenceOTU240004
New.CleanUp.ReferenceOTU3576
New.CleanUp.ReferenceOTU140739

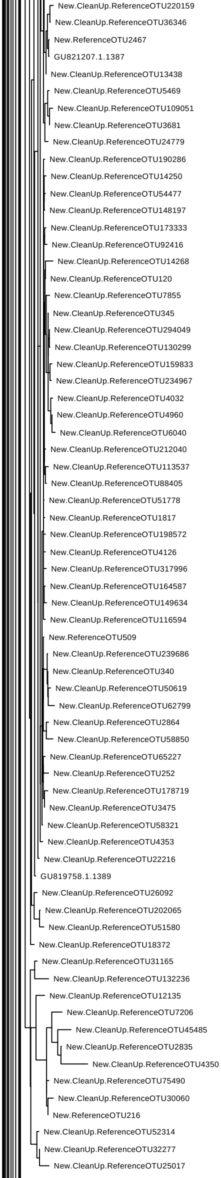
New.CleanUp.ReferenceOTU220159
New.CleanUp.ReferenceOTU36346
New.ReferenceOTU2467
GU821207.1.1387
New.CleanUp.ReferenceOTU13438
New.CleanUp.ReferenceOTU5469
New.CleanUp.ReferenceOTU109051
New.CleanUp.ReferenceOTU3681
New.CleanUp.ReferenceOTU24779
New.CleanUp.ReferenceOTU190286
New.CleanUp.ReferenceOTU14250
New.CleanUp.ReferenceOTU54477
New.CleanUp.ReferenceOTU148197
New.CleanUp.ReferenceOTU173333
New.CleanUp.ReferenceOTU92416
New.CleanUp.ReferenceOTU14268
New.CleanUp.ReferenceOTU120
New.CleanUp.ReferenceOTU7855
New.CleanUp.ReferenceOTU345
New.CleanUp.ReferenceOTU294049
New.CleanUp.ReferenceOTU130299
New.CleanUp.ReferenceOTU159833
New.CleanUp.ReferenceOTU234967
New.CleanUp.ReferenceOTU4032
New.CleanUp.ReferenceOTU4960
New.CleanUp.ReferenceOTU6040
New.CleanUp.ReferenceOTU212040
New.CleanUp.ReferenceOTU113537
New.CleanUp.ReferenceOTU88405
New.CleanUp.ReferenceOTU51778
New.CleanUp.ReferenceOTU1817
New.CleanUp.ReferenceOTU198572
New.CleanUp.ReferenceOTU4126
New.CleanUp.ReferenceOTU317996
New.CleanUp.ReferenceOTU164587
New.CleanUp.ReferenceOTU149634
New.CleanUp.ReferenceOTU116594
New.ReferenceOTU509
New.CleanUp.ReferenceOTU239686
New.CleanUp.ReferenceOTU340
New.CleanUp.ReferenceOTU50619
New.CleanUp.ReferenceOTU62799
New.CleanUp.ReferenceOTU2864
New.CleanUp.ReferenceOTU58850
New.CleanUp.ReferenceOTU65227
New.CleanUp.ReferenceOTU252
New.CleanUp.ReferenceOTU178719
New.CleanUp.ReferenceOTU3475
New.CleanUp.ReferenceOTU58321
New.CleanUp.ReferenceOTU4353
New.CleanUp.ReferenceOTU22216
GU819758.1.1389
New.CleanUp.ReferenceOTU26092
New.CleanUp.ReferenceOTU202065
New.CleanUp.ReferenceOTU51580
New.CleanUp.ReferenceOTU18372
New.CleanUp.ReferenceOTU31165
New.CleanUp.ReferenceOTU132236
New.CleanUp.ReferenceOTU12135
New.CleanUp.ReferenceOTU7206
New.CleanUp.ReferenceOTU45485
New.CleanUp.ReferenceOTU2835
New.CleanUp.ReferenceOTU4350
New.CleanUp.ReferenceOTU75490
New.CleanUp.ReferenceOTU30060
New.ReferenceOTU216
New.CleanUp.ReferenceOTU52314
New.CleanUp.ReferenceOTU32277
New.CleanUp.ReferenceOTU25017

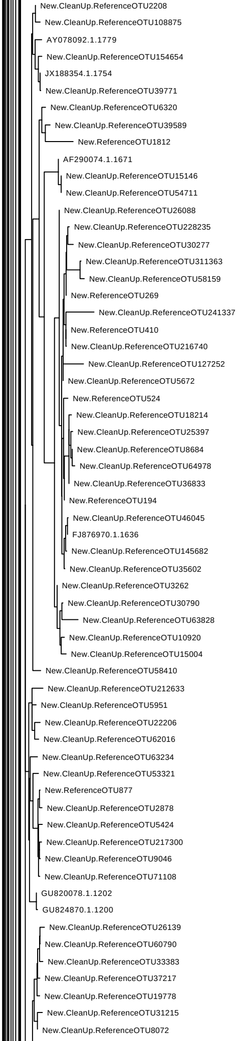
New.CleanUp.ReferenceOTU2208
New.CleanUp.ReferenceOTU108875
AY078092.1.1779
New.CleanUp.ReferenceOTU154654
JX188354.1.1754
New.CleanUp.ReferenceOTU39771
New.CleanUp.ReferenceOTU6320
New.CleanUp.ReferenceOTU39589
New.ReferenceOTU1812
AF290074.1.1671
New.CleanUp.ReferenceOTU15146
New.CleanUp.ReferenceOTU54711
New.CleanUp.ReferenceOTU26088
New.CleanUp.ReferenceOTU228235
New.CleanUp.ReferenceOTU30277
New.CleanUp.ReferenceOTU311363
New.CleanUp.ReferenceOTU58159
New.ReferenceOTU269
New.CleanUp.ReferenceOTU241337
New.ReferenceOTU410
New.CleanUp.ReferenceOTU216740
New.CleanUp.ReferenceOTU127252
New.CleanUp.ReferenceOTU5672
New.ReferenceOTU524
New.CleanUp.ReferenceOTU18214
New.CleanUp.ReferenceOTU25397
New.CleanUp.ReferenceOTU8684
New.CleanUp.ReferenceOTU64978
New.CleanUp.ReferenceOTU36833
New.ReferenceOTU194
New.CleanUp.ReferenceOTU46045
FJ876970.1.1636
New.CleanUp.ReferenceOTU145682
New.CleanUp.ReferenceOTU35602
New.CleanUp.ReferenceOTU3262
New.CleanUp.ReferenceOTU30790
New.CleanUp.ReferenceOTU63828
New.CleanUp.ReferenceOTU10920
New.CleanUp.ReferenceOTU15004
New.CleanUp.ReferenceOTU58410
New.CleanUp.ReferenceOTU212633
New.CleanUp.ReferenceOTU5951
New.CleanUp.ReferenceOTU22206
New.CleanUp.ReferenceOTU62016
New.CleanUp.ReferenceOTU63234
New.CleanUp.ReferenceOTU53321
New.ReferenceOTU877
New.CleanUp.ReferenceOTU2878
New.CleanUp.ReferenceOTU5424
New.CleanUp.ReferenceOTU217300
New.CleanUp.ReferenceOTU9046
New.CleanUp.ReferenceOTU71108
GU820078.1.1202
GU824870.1.1200
New.CleanUp.ReferenceOTU26139
New.CleanUp.ReferenceOTU60790
New.CleanUp.ReferenceOTU33383
New.CleanUp.ReferenceOTU37217
New.CleanUp.ReferenceOTU19778
New.CleanUp.ReferenceOTU31215
New.CleanUp.ReferenceOTU8072

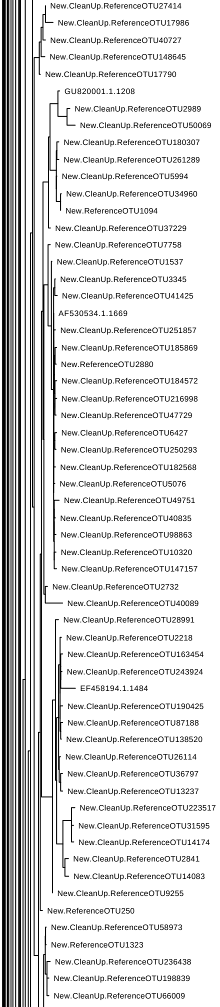
New.CleanUp.ReferenceOTU27414
New.CleanUp.ReferenceOTU17986
New.CleanUp.ReferenceOTU40727
New.CleanUp.ReferenceOTU148645
New.CleanUp.ReferenceOTU17790
GU820001.1.1208
New.CleanUp.ReferenceOTU2989
New.CleanUp.ReferenceOTU50069
New.CleanUp.ReferenceOTU180307
New.CleanUp.ReferenceOTU261289
New.CleanUp.ReferenceOTU5994
New.CleanUp.ReferenceOTU34960
New.ReferenceOTU1094
New.CleanUp.ReferenceOTU37229
New.CleanUp.ReferenceOTU7758
New.CleanUp.ReferenceOTU1537
New.CleanUp.ReferenceOTU3345
New.CleanUp.ReferenceOTU41425
AF530534.1.1669
New.CleanUp.ReferenceOTU251857
New.CleanUp.ReferenceOTU185869
New.ReferenceOTU2880
New.CleanUp.ReferenceOTU184572
New.CleanUp.ReferenceOTU216998
New.CleanUp.ReferenceOTU47729
New.CleanUp.ReferenceOTU6427
New.CleanUp.ReferenceOTU250293
New.CleanUp.ReferenceOTU182568
New.CleanUp.ReferenceOTU5076
New.CleanUp.ReferenceOTU49751
New.CleanUp.ReferenceOTU40835
New.CleanUp.ReferenceOTU98863
New.CleanUp.ReferenceOTU10320
New.CleanUp.ReferenceOTU147157
New.CleanUp.ReferenceOTU2732
New.CleanUp.ReferenceOTU40089
New.CleanUp.ReferenceOTU28991
New.CleanUp.ReferenceOTU2218
New.CleanUp.ReferenceOTU163454
New.CleanUp.ReferenceOTU243924
EF458194.1.1484
New.CleanUp.ReferenceOTU190425
New.CleanUp.ReferenceOTU87188
New.CleanUp.ReferenceOTU138520
New.CleanUp.ReferenceOTU26114
New.CleanUp.ReferenceOTU36797
New.CleanUp.ReferenceOTU13237
New.CleanUp.ReferenceOTU223517
New.CleanUp.ReferenceOTU31595
New.CleanUp.ReferenceOTU14174
New.CleanUp.ReferenceOTU2841
New.CleanUp.ReferenceOTU14083
New.CleanUp.ReferenceOTU9255
New.ReferenceOTU250
New.CleanUp.ReferenceOTU58973
New.ReferenceOTU1323
New.CleanUp.ReferenceOTU236438
New.CleanUp.ReferenceOTU198839
New.CleanUp.ReferenceOTU66009

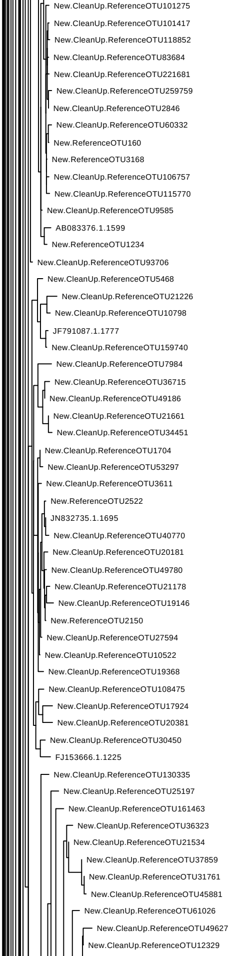
New.CleanUp.ReferenceOTU101275
New.CleanUp.ReferenceOTU101417
New.CleanUp.ReferenceOTU118852
New.CleanUp.ReferenceOTU83684
New.CleanUp.ReferenceOTU221681
New.CleanUp.ReferenceOTU259759
New.CleanUp.ReferenceOTU2846
New.CleanUp.ReferenceOTU60332
New.ReferenceOTU160
New.ReferenceOTU3168
New.CleanUp.ReferenceOTU106757
New.CleanUp.ReferenceOTU115770
New.CleanUp.ReferenceOTU9585
AB083376.1.1599
New.ReferenceOTU1234
New.CleanUp.ReferenceOTU93706
New.CleanUp.ReferenceOTU5468
New.CleanUp.ReferenceOTU21226
New.CleanUp.ReferenceOTU10798
JF791087.1.1777
New.CleanUp.ReferenceOTU159740
New.CleanUp.ReferenceOTU7984
New.CleanUp.ReferenceOTU36715
New.CleanUp.ReferenceOTU49186
New.CleanUp.ReferenceOTU21661
New.CleanUp.ReferenceOTU34451
New.CleanUp.ReferenceOTU1704
New.CleanUp.ReferenceOTU53297
New.CleanUp.ReferenceOTU3611
New.ReferenceOTU2522
JN832735.1.1695
New.CleanUp.ReferenceOTU40770
New.CleanUp.ReferenceOTU20181
New.CleanUp.ReferenceOTU49780
New.CleanUp.ReferenceOTU21178
New.CleanUp.ReferenceOTU19146
New.ReferenceOTU2150
New.CleanUp.ReferenceOTU27594
New.CleanUp.ReferenceOTU10522
New.CleanUp.ReferenceOTU19368
New.CleanUp.ReferenceOTU108475
New.CleanUp.ReferenceOTU17924
New.CleanUp.ReferenceOTU20381
New.CleanUp.ReferenceOTU30450
FJ153666.1.1225
New.CleanUp.ReferenceOTU130335
New.CleanUp.ReferenceOTU25197
New.CleanUp.ReferenceOTU161463
New.CleanUp.ReferenceOTU36323
New.CleanUp.ReferenceOTU21534
New.CleanUp.ReferenceOTU37859
New.CleanUp.ReferenceOTU31761
New.CleanUp.ReferenceOTU45881
New.CleanUp.ReferenceOTU61026
New.CleanUp.ReferenceOTU49627
New.CleanUp.ReferenceOTU12329

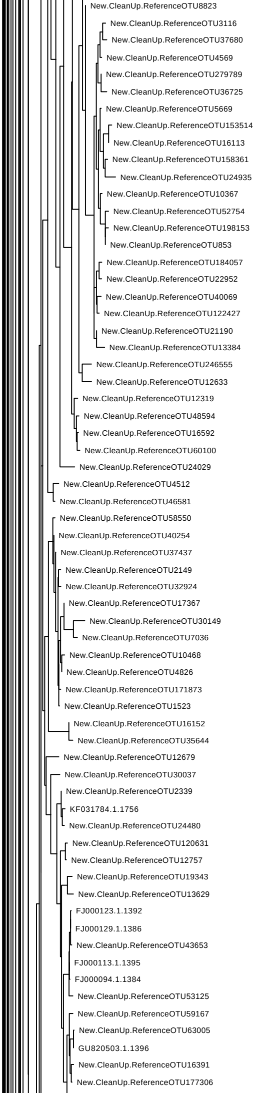
New.CleanUp.ReferenceOTU8823
New.CleanUp.ReferenceOTU3116
New.CleanUp.ReferenceOTU37680
New.CleanUp.ReferenceOTU4569
New.CleanUp.ReferenceOTU279789
New.CleanUp.ReferenceOTU36725
New.CleanUp.ReferenceOTU5669
New.CleanUp.ReferenceOTU153514
New.CleanUp.ReferenceOTU16113
New.CleanUp.ReferenceOTU158361
New.CleanUp.ReferenceOTU24935
New.CleanUp.ReferenceOTU10367
New.CleanUp.ReferenceOTU52754
New.CleanUp.ReferenceOTU198153
New.CleanUp.ReferenceOTU853
New.CleanUp.ReferenceOTU184057
New.CleanUp.ReferenceOTU22952
New.CleanUp.ReferenceOTU40069
New.CleanUp.ReferenceOTU122427
New.CleanUp.ReferenceOTU21190
New.CleanUp.ReferenceOTU13384
New.CleanUp.ReferenceOTU246555
New.CleanUp.ReferenceOTU12633
New.CleanUp.ReferenceOTU12319
New.CleanUp.ReferenceOTU48594
New.CleanUp.ReferenceOTU16592
New.CleanUp.ReferenceOTU60100
New.CleanUp.ReferenceOTU24029
New.CleanUp.ReferenceOTU4512
New.CleanUp.ReferenceOTU46581
New.CleanUp.ReferenceOTU58550
New.CleanUp.ReferenceOTU40254
New.CleanUp.ReferenceOTU37437
New.CleanUp.ReferenceOTU2149
New.CleanUp.ReferenceOTU32924
New.CleanUp.ReferenceOTU17367
New.CleanUp.ReferenceOTU30149
New.CleanUp.ReferenceOTU7036
New.CleanUp.ReferenceOTU10468
New.CleanUp.ReferenceOTU4826
New.CleanUp.ReferenceOTU171873
New.CleanUp.ReferenceOTU1523
New.CleanUp.ReferenceOTU16152
New.CleanUp.ReferenceOTU35644
New.CleanUp.ReferenceOTU12679
New.CleanUp.ReferenceOTU30037
New.CleanUp.ReferenceOTU2339
KF031784.1.1756
New.CleanUp.ReferenceOTU24480
New.CleanUp.ReferenceOTU120631
New.CleanUp.ReferenceOTU12757
New.CleanUp.ReferenceOTU19343
New.CleanUp.ReferenceOTU13629
FJ000123.1.1392
FJ000129.1.1386
New.CleanUp.ReferenceOTU43653
FJ000113.1.1395
FJ000094.1.1384
New.CleanUp.ReferenceOTU53125
New.CleanUp.ReferenceOTU59167
New.CleanUp.ReferenceOTU63005
GU820503.1.1396
New.CleanUp.ReferenceOTU16391
New.CleanUp.ReferenceOTU177306

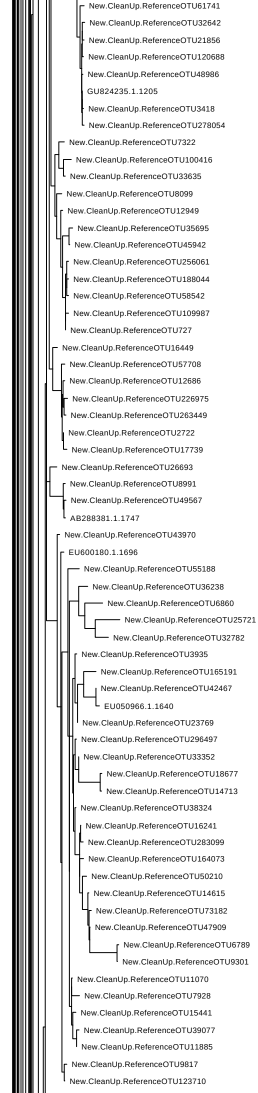
New.CleanUp.ReferenceOTU61741
New.CleanUp.ReferenceOTU32642
New.CleanUp.ReferenceOTU21856
New.CleanUp.ReferenceOTU120688
New.CleanUp.ReferenceOTU48986
GU824235.1.1205
New.CleanUp.ReferenceOTU3418
New.CleanUp.ReferenceOTU278054
New.CleanUp.ReferenceOTU7322
New.CleanUp.ReferenceOTU100416
New.CleanUp.ReferenceOTU33635
New.CleanUp.ReferenceOTU8099
New.CleanUp.ReferenceOTU12949
New.CleanUp.ReferenceOTU35695
New.CleanUp.ReferenceOTU45942
New.CleanUp.ReferenceOTU256061
New.CleanUp.ReferenceOTU188044
New.CleanUp.ReferenceOTU58542
New.CleanUp.ReferenceOTU109987
New.CleanUp.ReferenceOTU727
New.CleanUp.ReferenceOTU16449
New.CleanUp.ReferenceOTU57708
New.CleanUp.ReferenceOTU12686
New.CleanUp.ReferenceOTU226975
New.CleanUp.ReferenceOTU263449
New.CleanUp.ReferenceOTU2722
New.CleanUp.ReferenceOTU17739
New.CleanUp.ReferenceOTU26693
New.CleanUp.ReferenceOTU8991
New.CleanUp.ReferenceOTU49567
AB288381.1.1747
New.CleanUp.ReferenceOTU43970
EU600180.1.1696
New.CleanUp.ReferenceOTU55188
New.CleanUp.ReferenceOTU36238
New.CleanUp.ReferenceOTU6860
New.CleanUp.ReferenceOTU25721
New.CleanUp.ReferenceOTU32782
New.CleanUp.ReferenceOTU3935
New.CleanUp.ReferenceOTU165191
New.CleanUp.ReferenceOTU42467
EU050966.1.1640
New.CleanUp.ReferenceOTU23769
New.CleanUp.ReferenceOTU296497
New.CleanUp.ReferenceOTU33352
New.CleanUp.ReferenceOTU18677
New.CleanUp.ReferenceOTU14713
New.CleanUp.ReferenceOTU38324
New.CleanUp.ReferenceOTU16241
New.CleanUp.ReferenceOTU283099
New.CleanUp.ReferenceOTU164073
New.CleanUp.ReferenceOTU50210
New.CleanUp.ReferenceOTU14615
New.CleanUp.ReferenceOTU73182
New.CleanUp.ReferenceOTU47909
New.CleanUp.ReferenceOTU6789
New.CleanUp.ReferenceOTU9301
New.CleanUp.ReferenceOTU11070
New.CleanUp.ReferenceOTU7928
New.CleanUp.ReferenceOTU15441
New.CleanUp.ReferenceOTU39077
New.CleanUp.ReferenceOTU11885
New.CleanUp.ReferenceOTU9817
New.CleanUp.ReferenceOTU123710

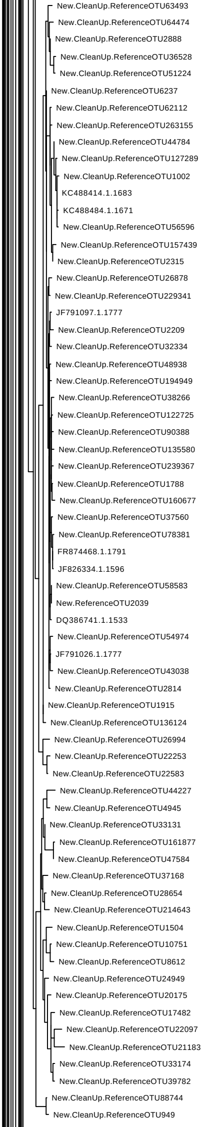
New.CleanUp.ReferenceOTU63493
New.CleanUp.ReferenceOTU64474
New.CleanUp.ReferenceOTU2888
New.CleanUp.ReferenceOTU36528
New.CleanUp.ReferenceOTU51224
New.CleanUp.ReferenceOTU6237
New.CleanUp.ReferenceOTU62112
New.CleanUp.ReferenceOTU263155
New.CleanUp.ReferenceOTU44784
New.CleanUp.ReferenceOTU127289
New.CleanUp.ReferenceOTU1002
KC488414.1.1683
KC488484.1.1671
New.CleanUp.ReferenceOTU56596
New.CleanUp.ReferenceOTU157439
New.CleanUp.ReferenceOTU2315
New.CleanUp.ReferenceOTU26878
New.CleanUp.ReferenceOTU229341
JF791097.1.1777
New.CleanUp.ReferenceOTU2209
New.CleanUp.ReferenceOTU32334
New.CleanUp.ReferenceOTU48938
New.CleanUp.ReferenceOTU194949
New.CleanUp.ReferenceOTU38266
New.CleanUp.ReferenceOTU122725
New.CleanUp.ReferenceOTU90388
New.CleanUp.ReferenceOTU135580
New.CleanUp.ReferenceOTU239367
New.CleanUp.ReferenceOTU1788
New.CleanUp.ReferenceOTU160677
New.CleanUp.ReferenceOTU37560
New.CleanUp.ReferenceOTU78381
FR874468.1.1791
JF826334.1.1596
New.CleanUp.ReferenceOTU58583
New.ReferenceOTU2039
DQ386741.1.1533
New.CleanUp.ReferenceOTU54974
JF791026.1.1777
New.CleanUp.ReferenceOTU43038
New.CleanUp.ReferenceOTU2814
New.CleanUp.ReferenceOTU1915
New.CleanUp.ReferenceOTU136124
New.CleanUp.ReferenceOTU26994
New.CleanUp.ReferenceOTU22253
New.CleanUp.ReferenceOTU22583
New.CleanUp.ReferenceOTU44227
New.CleanUp.ReferenceOTU4945
New.CleanUp.ReferenceOTU33131
New.CleanUp.ReferenceOTU161877
New.CleanUp.ReferenceOTU47584
New.CleanUp.ReferenceOTU37168
New.CleanUp.ReferenceOTU28654
New.CleanUp.ReferenceOTU214643
New.CleanUp.ReferenceOTU1504
New.CleanUp.ReferenceOTU10751
New.CleanUp.ReferenceOTU8612
New.CleanUp.ReferenceOTU24949
New.CleanUp.ReferenceOTU20175
New.CleanUp.ReferenceOTU17482
New.CleanUp.ReferenceOTU22097
New.CleanUp.ReferenceOTU21183
New.CleanUp.ReferenceOTU33174
New.CleanUp.ReferenceOTU39782
New.CleanUp.ReferenceOTU88744
New.CleanUp.ReferenceOTU949

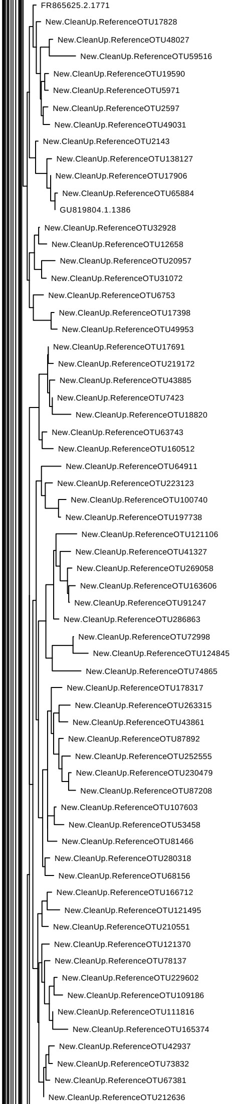
FR865625.2.1771
New.CleanUp.ReferenceOTU17828
New.CleanUp.ReferenceOTU48027
New.CleanUp.ReferenceOTU59516
New.CleanUp.ReferenceOTU19590
New.CleanUp.ReferenceOTU5971
New.CleanUp.ReferenceOTU2597
New.CleanUp.ReferenceOTU49031
New.CleanUp.ReferenceOTU2143
New.CleanUp.ReferenceOTU138127
New.CleanUp.ReferenceOTU17906
New.CleanUp.ReferenceOTU65884
GU819804.1.1386
New.CleanUp.ReferenceOTU32928
New.CleanUp.ReferenceOTU12658
New.CleanUp.ReferenceOTU20957
New.CleanUp.ReferenceOTU31072
New.CleanUp.ReferenceOTU6753
New.CleanUp.ReferenceOTU17398
New.CleanUp.ReferenceOTU49953
New.CleanUp.ReferenceOTU17691
New.CleanUp.ReferenceOTU219172
New.CleanUp.ReferenceOTU43885
New.CleanUp.ReferenceOTU7423
New.CleanUp.ReferenceOTU18820
New.CleanUp.ReferenceOTU63743
New.CleanUp.ReferenceOTU160512
New.CleanUp.ReferenceOTU64911
New.CleanUp.ReferenceOTU223123
New.CleanUp.ReferenceOTU100740
New.CleanUp.ReferenceOTU197738
New.CleanUp.ReferenceOTU121106
New.CleanUp.ReferenceOTU41327
New.CleanUp.ReferenceOTU269058
New.CleanUp.ReferenceOTU163606
New.CleanUp.ReferenceOTU91247
New.CleanUp.ReferenceOTU286863
New.CleanUp.ReferenceOTU72998
New.CleanUp.ReferenceOTU124845
New.CleanUp.ReferenceOTU74865
New.CleanUp.ReferenceOTU178317
New.CleanUp.ReferenceOTU263315
New.CleanUp.ReferenceOTU43861
New.CleanUp.ReferenceOTU87892
New.CleanUp.ReferenceOTU252555
New.CleanUp.ReferenceOTU230479
New.CleanUp.ReferenceOTU87208
New.CleanUp.ReferenceOTU107603
New.CleanUp.ReferenceOTU53458
New.CleanUp.ReferenceOTU81466
New.CleanUp.ReferenceOTU280318
New.CleanUp.ReferenceOTU68156
New.CleanUp.ReferenceOTU166712
New.CleanUp.ReferenceOTU121495
New.CleanUp.ReferenceOTU210551
New.CleanUp.ReferenceOTU121370
New.CleanUp.ReferenceOTU78137
New.CleanUp.ReferenceOTU229602
New.CleanUp.ReferenceOTU109186
New.CleanUp.ReferenceOTU111816
New.CleanUp.ReferenceOTU165374
New.CleanUp.ReferenceOTU42937
New.CleanUp.ReferenceOTU73832
New.CleanUp.ReferenceOTU67381
New.CleanUp.ReferenceOTU212636

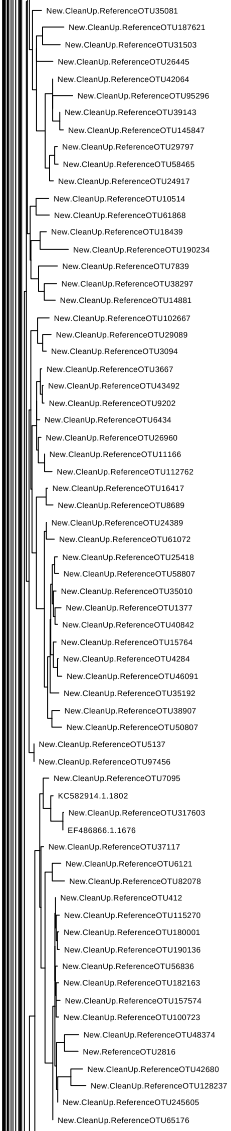

New.CleanUp.ReferenceOTU35081
New.CleanUp.ReferenceOTU187621
New.CleanUp.ReferenceOTU31503
New.CleanUp.ReferenceOTU26445
New.CleanUp.ReferenceOTU42064
New.CleanUp.ReferenceOTU95296
New.CleanUp.ReferenceOTU39143
New.CleanUp.ReferenceOTU145847
New.CleanUp.ReferenceOTU29797
New.CleanUp.ReferenceOTU58465
New.CleanUp.ReferenceOTU24917
New.CleanUp.ReferenceOTU10514
New.CleanUp.ReferenceOTU61868
New.CleanUp.ReferenceOTU18439
New.CleanUp.ReferenceOTU190234
New.CleanUp.ReferenceOTU7839
New.CleanUp.ReferenceOTU38297
New.CleanUp.ReferenceOTU14881
New.CleanUp.ReferenceOTU102667
New.CleanUp.ReferenceOTU29089
New.CleanUp.ReferenceOTU3094
New.CleanUp.ReferenceOTU3667
New.CleanUp.ReferenceOTU43492
New.CleanUp.ReferenceOTU9202
New.CleanUp.ReferenceOTU6434
New.CleanUp.ReferenceOTU26960
New.CleanUp.ReferenceOTU11166
New.CleanUp.ReferenceOTU112762
New.CleanUp.ReferenceOTU16417
New.CleanUp.ReferenceOTU8689
New.CleanUp.ReferenceOTU24389
New.CleanUp.ReferenceOTU61072
New.CleanUp.ReferenceOTU25418
New.CleanUp.ReferenceOTU58807
New.CleanUp.ReferenceOTU35010
New.CleanUp.ReferenceOTU1377
New.CleanUp.ReferenceOTU40842
New.CleanUp.ReferenceOTU15764
New.CleanUp.ReferenceOTU4284
New.CleanUp.ReferenceOTU46091
New.CleanUp.ReferenceOTU35192
New.CleanUp.ReferenceOTU38907
New.CleanUp.ReferenceOTU50807
New.CleanUp.ReferenceOTU5137
New.CleanUp.ReferenceOTU97456
New.CleanUp.ReferenceOTU7095
KC582914.1.1802
New.CleanUp.ReferenceOTU317603
EF486866.1.1676
New.CleanUp.ReferenceOTU37117
New.CleanUp.ReferenceOTU6121
New.CleanUp.ReferenceOTU82078
New.CleanUp.ReferenceOTU412
New.CleanUp.ReferenceOTU115270
New.CleanUp.ReferenceOTU180001
New.CleanUp.ReferenceOTU190136
New.CleanUp.ReferenceOTU56836
New.CleanUp.ReferenceOTU182163
New.CleanUp.ReferenceOTU157574
New.CleanUp.ReferenceOTU100723
New.CleanUp.ReferenceOTU48374
New.ReferenceOTU2816
New.CleanUp.ReferenceOTU42680
New.CleanUp.ReferenceOTU128237
New.CleanUp.ReferenceOTU245605
New.CleanUp.ReferenceOTU65176

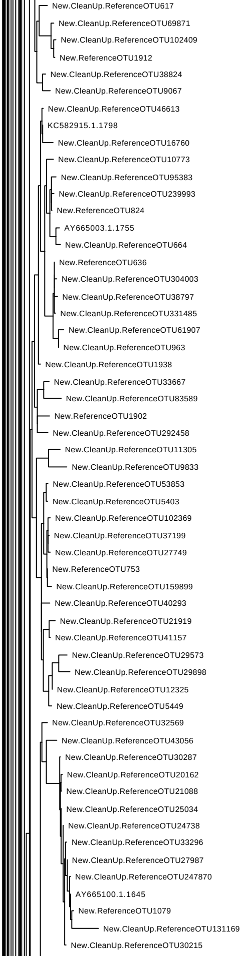
New.CleanUp.ReferenceOTU617
New.CleanUp.ReferenceOTU69871
New.CleanUp.ReferenceOTU102409
New.ReferenceOTU1912
New.CleanUp.ReferenceOTU38824
New.CleanUp.ReferenceOTU9067
New.CleanUp.ReferenceOTU46613
KC582915.1.1798
New.CleanUp.ReferenceOTU16760
New.CleanUp.ReferenceOTU10773
New.CleanUp.ReferenceOTU95383
New.CleanUp.ReferenceOTU239993
New.ReferenceOTU824
AY665003.1.1755
New.CleanUp.ReferenceOTU664
New.ReferenceOTU636
New.CleanUp.ReferenceOTU304003
New.CleanUp.ReferenceOTU38797
New.CleanUp.ReferenceOTU331485
New.CleanUp.ReferenceOTU61907
New.CleanUp.ReferenceOTU963
New.CleanUp.ReferenceOTU1938
New.CleanUp.ReferenceOTU33667
New.CleanUp.ReferenceOTU83589
New.ReferenceOTU1902
New.CleanUp.ReferenceOTU292458
New.CleanUp.ReferenceOTU11305
New.CleanUp.ReferenceOTU9833
New.CleanUp.ReferenceOTU53853
New.CleanUp.ReferenceOTU5403
New.CleanUp.ReferenceOTU102369
New.CleanUp.ReferenceOTU37199
New.CleanUp.ReferenceOTU27749
New.ReferenceOTU753
New.CleanUp.ReferenceOTU159899
New.CleanUp.ReferenceOTU40293
New.CleanUp.ReferenceOTU21919
New.CleanUp.ReferenceOTU41157
New.CleanUp.ReferenceOTU29573
New.CleanUp.ReferenceOTU29898
New.CleanUp.ReferenceOTU12325
New.CleanUp.ReferenceOTU5449
New.CleanUp.ReferenceOTU32569
New.CleanUp.ReferenceOTU43056
New.CleanUp.ReferenceOTU30287
New.CleanUp.ReferenceOTU20162
New.CleanUp.ReferenceOTU21088
New.CleanUp.ReferenceOTU25034
New.CleanUp.ReferenceOTU24738
New.CleanUp.ReferenceOTU33296
New.CleanUp.ReferenceOTU27987
New.CleanUp.ReferenceOTU247870
AY665100.1.1645
New.ReferenceOTU1079
New.CleanUp.ReferenceOTU131169
New.CleanUp.ReferenceOTU30215

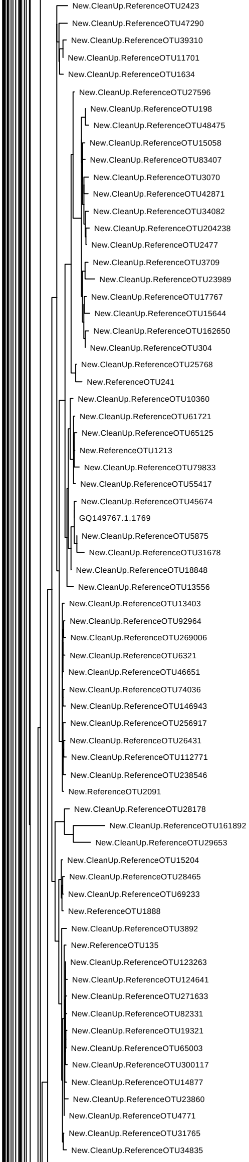
New.CleanUp.ReferenceOTU2423
New.CleanUp.ReferenceOTU47290
New.CleanUp.ReferenceOTU39310
New.CleanUp.ReferenceOTU11701
New.CleanUp.ReferenceOTU1634
New.CleanUp.ReferenceOTU27596
New.CleanUp.ReferenceOTU198
New.CleanUp.ReferenceOTU48475
New.CleanUp.ReferenceOTU15058
New.CleanUp.ReferenceOTU83407
New.CleanUp.ReferenceOTU3070
New.CleanUp.ReferenceOTU42871
New.CleanUp.ReferenceOTU34082
New.CleanUp.ReferenceOTU204238
New.CleanUp.ReferenceOTU2477
New.CleanUp.ReferenceOTU3709
New.CleanUp.ReferenceOTU23989
New.CleanUp.ReferenceOTU17767
New.CleanUp.ReferenceOTU15644
New.CleanUp.ReferenceOTU162650
New.CleanUp.ReferenceOTU304
New.CleanUp.ReferenceOTU25768
New.ReferenceOTU241
New.CleanUp.ReferenceOTU10360
New.CleanUp.ReferenceOTU61721
New.CleanUp.ReferenceOTU65125
New.ReferenceOTU1213
New.CleanUp.ReferenceOTU79833
New.CleanUp.ReferenceOTU55417
New.CleanUp.ReferenceOTU45674
GQ149767.1.1769
New.CleanUp.ReferenceOTU5875
New.CleanUp.ReferenceOTU31678
New.CleanUp.ReferenceOTU18848
New.CleanUp.ReferenceOTU13556
New.CleanUp.ReferenceOTU13403
New.CleanUp.ReferenceOTU92964
New.CleanUp.ReferenceOTU269006
New.CleanUp.ReferenceOTU6321
New.CleanUp.ReferenceOTU46651
New.CleanUp.ReferenceOTU74036
New.CleanUp.ReferenceOTU146943
New.CleanUp.ReferenceOTU256917
New.CleanUp.ReferenceOTU26431
New.CleanUp.ReferenceOTU112771
New.CleanUp.ReferenceOTU238546
New.ReferenceOTU2091
New.CleanUp.ReferenceOTU28178
New.CleanUp.ReferenceOTU161892
New.CleanUp.ReferenceOTU29653
New.CleanUp.ReferenceOTU15204
New.CleanUp.ReferenceOTU28465
New.CleanUp.ReferenceOTU69233
New.ReferenceOTU1888
New.CleanUp.ReferenceOTU3892
New.ReferenceOTU135
New.CleanUp.ReferenceOTU123263
New.CleanUp.ReferenceOTU124641
New.CleanUp.ReferenceOTU271633
New.CleanUp.ReferenceOTU82331
New.CleanUp.ReferenceOTU19321
New.CleanUp.ReferenceOTU65003
New.CleanUp.ReferenceOTU300117
New.CleanUp.ReferenceOTU14877
New.CleanUp.ReferenceOTU23860
New.CleanUp.ReferenceOTU4771
New.CleanUp.ReferenceOTU31765
New.CleanUp.ReferenceOTU34835

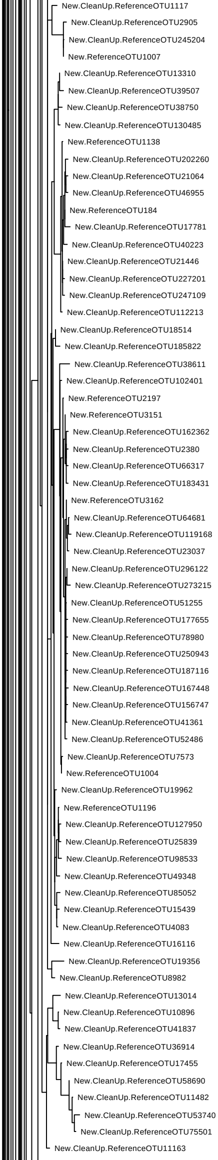
New.CleanUp.ReferenceOTU1117
New.CleanUp.ReferenceOTU2905
New.CleanUp.ReferenceOTU245204
New.ReferenceOTU1007
New.CleanUp.ReferenceOTU13310
New.CleanUp.ReferenceOTU39507
New.CleanUp.ReferenceOTU38750
New.CleanUp.ReferenceOTU130485
New.ReferenceOTU1138
New.CleanUp.ReferenceOTU202260
New.CleanUp.ReferenceOTU21064
New.CleanUp.ReferenceOTU46955
New.ReferenceOTU184
New.CleanUp.ReferenceOTU17781
New.CleanUp.ReferenceOTU40223
New.CleanUp.ReferenceOTU21446
New.CleanUp.ReferenceOTU227201
New.CleanUp.ReferenceOTU247109
New.CleanUp.ReferenceOTU112213
New.CleanUp.ReferenceOTU18514
New.CleanUp.ReferenceOTU185822
New.CleanUp.ReferenceOTU38611
New.CleanUp.ReferenceOTU102401
New.ReferenceOTU2197
New.ReferenceOTU3151
New.CleanUp.ReferenceOTU162362
New.CleanUp.ReferenceOTU2380
New.CleanUp.ReferenceOTU66317
New.CleanUp.ReferenceOTU183431
New.ReferenceOTU3162
New.CleanUp.ReferenceOTU64681
New.CleanUp.ReferenceOTU119168
New.CleanUp.ReferenceOTU23037
New.CleanUp.ReferenceOTU296122
New.CleanUp.ReferenceOTU273215
New.CleanUp.ReferenceOTU51255
New.CleanUp.ReferenceOTU177655
New.CleanUp.ReferenceOTU78980
New.CleanUp.ReferenceOTU250943
New.CleanUp.ReferenceOTU187116
New.CleanUp.ReferenceOTU167448
New.CleanUp.ReferenceOTU156747
New.CleanUp.ReferenceOTU41361
New.CleanUp.ReferenceOTU52486
New.CleanUp.ReferenceOTU7573
New.ReferenceOTU1004
New.CleanUp.ReferenceOTU19962
New.ReferenceOTU1196
New.CleanUp.ReferenceOTU127950
New.CleanUp.ReferenceOTU25839
New.CleanUp.ReferenceOTU98533
New.CleanUp.ReferenceOTU49348
New.CleanUp.ReferenceOTU85052
New.CleanUp.ReferenceOTU15439
New.CleanUp.ReferenceOTU4083
New.CleanUp.ReferenceOTU16116
New.CleanUp.ReferenceOTU19356
New.CleanUp.ReferenceOTU8982
New.CleanUp.ReferenceOTU13014
New.CleanUp.ReferenceOTU10896
New.CleanUp.ReferenceOTU41837
New.CleanUp.ReferenceOTU36914
New.CleanUp.ReferenceOTU17455
New.CleanUp.ReferenceOTU58690
New.CleanUp.ReferenceOTU11482
New.CleanUp.ReferenceOTU53740
New.CleanUp.ReferenceOTU75501
New.CleanUp.ReferenceOTU11163

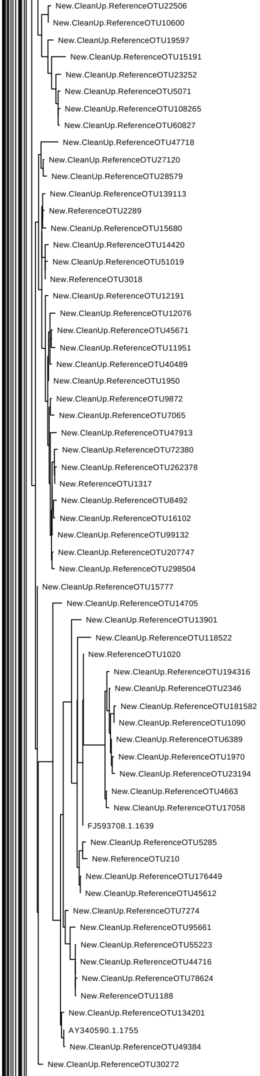
New.CleanUp.ReferenceOTU22506
New.CleanUp.ReferenceOTU10600
New.CleanUp.ReferenceOTU19597
New.CleanUp.ReferenceOTU15191
New.CleanUp.ReferenceOTU23252
New.CleanUp.ReferenceOTU5071
New.CleanUp.ReferenceOTU108265
New.CleanUp.ReferenceOTU60827
New.CleanUp.ReferenceOTU47718
New.CleanUp.ReferenceOTU27120
New.CleanUp.ReferenceOTU28579
New.CleanUp.ReferenceOTU139113
New.ReferenceOTU2289
New.CleanUp.ReferenceOTU15680
New.CleanUp.ReferenceOTU14420
New.CleanUp.ReferenceOTU51019
New.ReferenceOTU3018
New.CleanUp.ReferenceOTU12191
New.CleanUp.ReferenceOTU12076
New.CleanUp.ReferenceOTU45671
New.CleanUp.ReferenceOTU11951
New.CleanUp.ReferenceOTU40489
New.CleanUp.ReferenceOTU1950
New.CleanUp.ReferenceOTU9872
New.CleanUp.ReferenceOTU7065
New.CleanUp.ReferenceOTU47913
New.CleanUp.ReferenceOTU72380
New.CleanUp.ReferenceOTU262378
New.ReferenceOTU1317
New.CleanUp.ReferenceOTU8492
New.CleanUp.ReferenceOTU16102
New.CleanUp.ReferenceOTU99132
New.CleanUp.ReferenceOTU207747
New.CleanUp.ReferenceOTU298504
New.CleanUp.ReferenceOTU15777
New.CleanUp.ReferenceOTU14705
New.CleanUp.ReferenceOTU13901
New.CleanUp.ReferenceOTU118522
New.ReferenceOTU1020
New.CleanUp.ReferenceOTU194316
New.CleanUp.ReferenceOTU2346
New.CleanUp.ReferenceOTU181582
New.CleanUp.ReferenceOTU1090
New.CleanUp.ReferenceOTU6389
New.CleanUp.ReferenceOTU1970
New.CleanUp.ReferenceOTU23194
New.CleanUp.ReferenceOTU4663
New.CleanUp.ReferenceOTU17058
FJ593708.1.1639
New.CleanUp.ReferenceOTU5285
New.ReferenceOTU210
New.CleanUp.ReferenceOTU176449
New.CleanUp.ReferenceOTU45612
New.CleanUp.ReferenceOTU7274
New.CleanUp.ReferenceOTU95661
New.CleanUp.ReferenceOTU55223
New.CleanUp.ReferenceOTU44716
New.CleanUp.ReferenceOTU78624
New.ReferenceOTU1188
New.CleanUp.ReferenceOTU134201
AY340590.1.1755
New.CleanUp.ReferenceOTU49384
New.CleanUp.ReferenceOTU30272

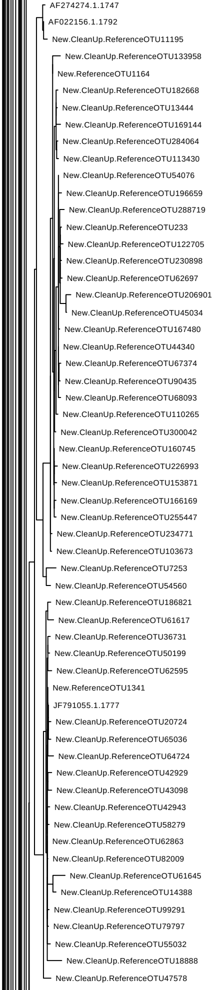
AF274274.1.1747
AF022156.1.1792
New.CleanUp.ReferenceOTU11195
New.CleanUp.ReferenceOTU133958
New.ReferenceOTU1164
New.CleanUp.ReferenceOTU182668
New.CleanUp.ReferenceOTU13444
New.CleanUp.ReferenceOTU169144
New.CleanUp.ReferenceOTU284064
New.CleanUp.ReferenceOTU113430
New.CleanUp.ReferenceOTU54076
New.CleanUp.ReferenceOTU196659
New.CleanUp.ReferenceOTU288719
New.CleanUp.ReferenceOTU233
New.CleanUp.ReferenceOTU122705
New.CleanUp.ReferenceOTU230898
New.CleanUp.ReferenceOTU62697
New.CleanUp.ReferenceOTU206901
New.CleanUp.ReferenceOTU45034
New.CleanUp.ReferenceOTU167480
New.CleanUp.ReferenceOTU44340
New.CleanUp.ReferenceOTU67374
New.CleanUp.ReferenceOTU90435
New.CleanUp.ReferenceOTU68093
New.CleanUp.ReferenceOTU110265
New.CleanUp.ReferenceOTU300042
New.CleanUp.ReferenceOTU160745
New.CleanUp.ReferenceOTU226993
New.CleanUp.ReferenceOTU153871
New.CleanUp.ReferenceOTU166169
New.CleanUp.ReferenceOTU255447
New.CleanUp.ReferenceOTU234771
New.CleanUp.ReferenceOTU103673
New.CleanUp.ReferenceOTU7253
New.CleanUp.ReferenceOTU54560
New.CleanUp.ReferenceOTU186821
New.CleanUp.ReferenceOTU61617
New.CleanUp.ReferenceOTU36731
New.CleanUp.ReferenceOTU50199
New.CleanUp.ReferenceOTU62595
New.ReferenceOTU1341
JF791055.1.1777
New.CleanUp.ReferenceOTU20724
New.CleanUp.ReferenceOTU65036
New.CleanUp.ReferenceOTU64724
New.CleanUp.ReferenceOTU42929
New.CleanUp.ReferenceOTU43098
New.CleanUp.ReferenceOTU42943
New.CleanUp.ReferenceOTU58279
New.CleanUp.ReferenceOTU62863
New.CleanUp.ReferenceOTU82009
New.CleanUp.ReferenceOTU61645
New.CleanUp.ReferenceOTU14388
New.CleanUp.ReferenceOTU99291
New.CleanUp.ReferenceOTU79797
New.CleanUp.ReferenceOTU55032
New.CleanUp.ReferenceOTU18888
New.CleanUp.ReferenceOTU47578

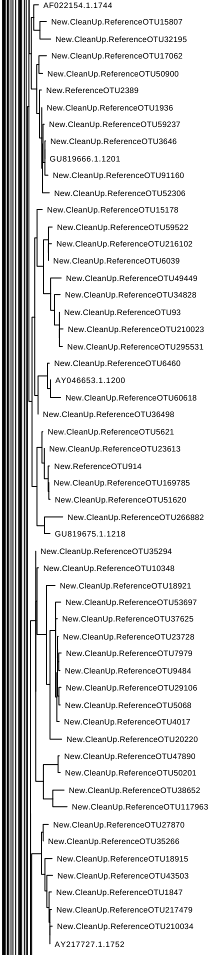

AF022154.1.1744
New.CleanUp.ReferenceOTU15807
New.CleanUp.ReferenceOTU32195
New.CleanUp.ReferenceOTU17062
New.CleanUp.ReferenceOTU50900
New.ReferenceOTU2389
New.CleanUp.ReferenceOTU1936
New.CleanUp.ReferenceOTU59237
New.CleanUp.ReferenceOTU3646
GU819666.1.1201
New.CleanUp.ReferenceOTU91160
New.CleanUp.ReferenceOTU52306
New.CleanUp.ReferenceOTU15178
New.CleanUp.ReferenceOTU59522
New.CleanUp.ReferenceOTU216102
New.CleanUp.ReferenceOTU6039
New.CleanUp.ReferenceOTU49449
New.CleanUp.ReferenceOTU34828
New.CleanUp.ReferenceOTU93
New.CleanUp.ReferenceOTU210023
New.CleanUp.ReferenceOTU295531
New.CleanUp.ReferenceOTU6460
AY046653.1.1200
New.CleanUp.ReferenceOTU60618
New.CleanUp.ReferenceOTU36498
New.CleanUp.ReferenceOTU5621
New.CleanUp.ReferenceOTU23613
New.ReferenceOTU914
New.CleanUp.ReferenceOTU169785
New.CleanUp.ReferenceOTU51620
New.CleanUp.ReferenceOTU266882
GU819675.1.1218
New.CleanUp.ReferenceOTU35294
New.CleanUp.ReferenceOTU10348
New.CleanUp.ReferenceOTU18921
New.CleanUp.ReferenceOTU53697
New.CleanUp.ReferenceOTU37625
New.CleanUp.ReferenceOTU23728
New.CleanUp.ReferenceOTU7979
New.CleanUp.ReferenceOTU9484
New.CleanUp.ReferenceOTU29106
New.CleanUp.ReferenceOTU5068
New.CleanUp.ReferenceOTU4017
New.CleanUp.ReferenceOTU20220
New.CleanUp.ReferenceOTU47890
New.CleanUp.ReferenceOTU50201
New.CleanUp.ReferenceOTU38652
New.CleanUp.ReferenceOTU117963
New.CleanUp.ReferenceOTU27870
New.CleanUp.ReferenceOTU35266
New.CleanUp.ReferenceOTU18915
New.CleanUp.ReferenceOTU43503
New.CleanUp.ReferenceOTU1847
New.CleanUp.ReferenceOTU217479
New.CleanUp.ReferenceOTU210034
AY217727.1.1752

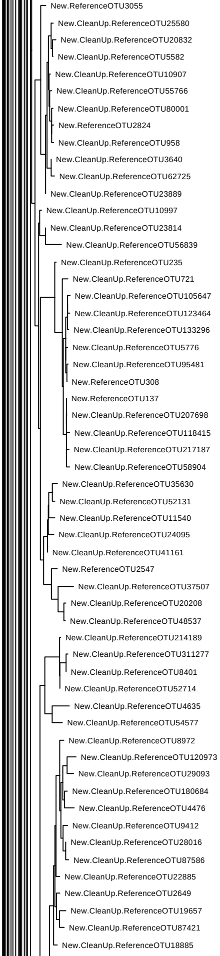
New.ReferenceOTU3055
New.CleanUp.ReferenceOTU25580
New.CleanUp.ReferenceOTU20832
New.CleanUp.ReferenceOTU5582
New.CleanUp.ReferenceOTU10907
New.CleanUp.ReferenceOTU55766
New.CleanUp.ReferenceOTU80001
New.ReferenceOTU2824
New.CleanUp.ReferenceOTU958
New.CleanUp.ReferenceOTU3640
New.CleanUp.ReferenceOTU62725
New.CleanUp.ReferenceOTU23889
New.CleanUp.ReferenceOTU10997
New.CleanUp.ReferenceOTU23814
New.CleanUp.ReferenceOTU56839
New.CleanUp.ReferenceOTU235
New.CleanUp.ReferenceOTU721
New.CleanUp.ReferenceOTU105647
New.CleanUp.ReferenceOTU123464
New.CleanUp.ReferenceOTU133296
New.CleanUp.ReferenceOTU5776
New.CleanUp.ReferenceOTU95481
New.ReferenceOTU308
New.ReferenceOTU137
New.CleanUp.ReferenceOTU207698
New.CleanUp.ReferenceOTU118415
New.CleanUp.ReferenceOTU217187
New.CleanUp.ReferenceOTU58904
New.CleanUp.ReferenceOTU35630
New.CleanUp.ReferenceOTU52131
New.CleanUp.ReferenceOTU11540
New.CleanUp.ReferenceOTU24095
New.CleanUp.ReferenceOTU41161
New.ReferenceOTU2547
New.CleanUp.ReferenceOTU37507
New.CleanUp.ReferenceOTU20208
New.CleanUp.ReferenceOTU48537
New.CleanUp.ReferenceOTU214189
New.CleanUp.ReferenceOTU311277
New.CleanUp.ReferenceOTU8401
New.CleanUp.ReferenceOTU52714
New.CleanUp.ReferenceOTU4635
New.CleanUp.ReferenceOTU54577
New.CleanUp.ReferenceOTU8972
New.CleanUp.ReferenceOTU120973
New.CleanUp.ReferenceOTU29093
New.CleanUp.ReferenceOTU180684
New.CleanUp.ReferenceOTU4476
New.CleanUp.ReferenceOTU9412
New.CleanUp.ReferenceOTU28016
New.CleanUp.ReferenceOTU87586
New.CleanUp.ReferenceOTU22885
New.CleanUp.ReferenceOTU2649
New.CleanUp.ReferenceOTU19657
New.CleanUp.ReferenceOTU87421
New.CleanUp.ReferenceOTU18885

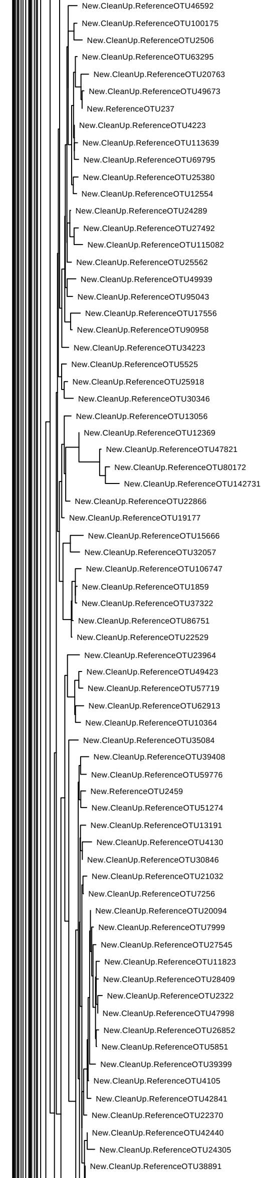
New.CleanUp.ReferenceOTU46592
New.CleanUp.ReferenceOTU100175
New.CleanUp.ReferenceOTU2506
New.CleanUp.ReferenceOTU63295
New.CleanUp.ReferenceOTU20763
New.CleanUp.ReferenceOTU49673
New.ReferenceOTU237
New.CleanUp.ReferenceOTU4223
New.CleanUp.ReferenceOTU113639
New.CleanUp.ReferenceOTU69795
New.CleanUp.ReferenceOTU25380
New.CleanUp.ReferenceOTU12554
New.CleanUp.ReferenceOTU24289
New.CleanUp.ReferenceOTU27492
New.CleanUp.ReferenceOTU115082
New.CleanUp.ReferenceOTU25562
New.CleanUp.ReferenceOTU49939
New.CleanUp.ReferenceOTU95043
New.CleanUp.ReferenceOTU17556
New.CleanUp.ReferenceOTU90958
New.CleanUp.ReferenceOTU34223
New.CleanUp.ReferenceOTU5525
New.CleanUp.ReferenceOTU25918
New.CleanUp.ReferenceOTU30346
New.CleanUp.ReferenceOTU13056
New.CleanUp.ReferenceOTU12369
New.CleanUp.ReferenceOTU47821
New.CleanUp.ReferenceOTU80172
New.CleanUp.ReferenceOTU142731
New.CleanUp.ReferenceOTU22866
New.CleanUp.ReferenceOTU19177
New.CleanUp.ReferenceOTU15666
New.CleanUp.ReferenceOTU32057
New.CleanUp.ReferenceOTU106747
New.CleanUp.ReferenceOTU1859
New.CleanUp.ReferenceOTU37322
New.CleanUp.ReferenceOTU86751
New.CleanUp.ReferenceOTU22529
New.CleanUp.ReferenceOTU23964
New.CleanUp.ReferenceOTU49423
New.CleanUp.ReferenceOTU57719
New.CleanUp.ReferenceOTU62913
New.CleanUp.ReferenceOTU10364
New.CleanUp.ReferenceOTU35084
New.CleanUp.ReferenceOTU39408
New.CleanUp.ReferenceOTU59776
New.ReferenceOTU2459
New.CleanUp.ReferenceOTU51274
New.CleanUp.ReferenceOTU13191
New.CleanUp.ReferenceOTU4130
New.CleanUp.ReferenceOTU30846
New.CleanUp.ReferenceOTU21032
New.CleanUp.ReferenceOTU7256
New.CleanUp.ReferenceOTU20094
New.CleanUp.ReferenceOTU7999
New.CleanUp.ReferenceOTU27545
New.CleanUp.ReferenceOTU11823
New.CleanUp.ReferenceOTU28409
New.CleanUp.ReferenceOTU2322
New.CleanUp.ReferenceOTU47998
New.CleanUp.ReferenceOTU26852
New.CleanUp.ReferenceOTU5851
New.CleanUp.ReferenceOTU39399
New.CleanUp.ReferenceOTU4105
New.CleanUp.ReferenceOTU42841
New.CleanUp.ReferenceOTU22370
New.CleanUp.ReferenceOTU42440
New.CleanUp.ReferenceOTU24305
New.CleanUp.ReferenceOTU38891

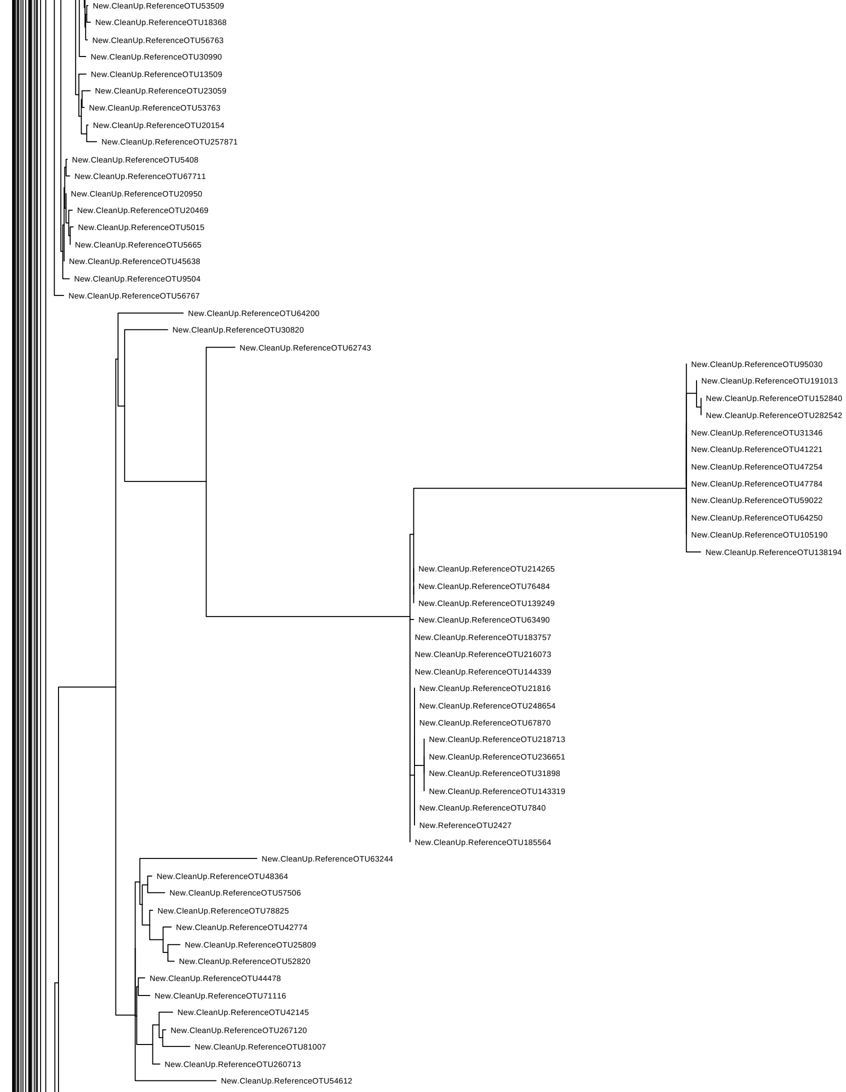
New.CleanUp.ReferenceOTU53509
New.CleanUp.ReferenceOTU18368
New.CleanUp.ReferenceOTU56763
New.CleanUp.ReferenceOTU30990
New.CleanUp.ReferenceOTU13509
New.CleanUp.ReferenceOTU23059
New.CleanUp.ReferenceOTU53763
New.CleanUp.ReferenceOTU20154
New.CleanUp.ReferenceOTU257871
New.CleanUp.ReferenceOTU5408
New.CleanUp.ReferenceOTU67711
New.CleanUp.ReferenceOTU20950
New.CleanUp.ReferenceOTU20469
New.CleanUp.ReferenceOTU5015
New.CleanUp.ReferenceOTU5665
New.CleanUp.ReferenceOTU45638
New.CleanUp.ReferenceOTU9504
New.CleanUp.ReferenceOTU56767
New.CleanUp.ReferenceOTU64200
New.CleanUp.ReferenceOTU30820
New.CleanUp.ReferenceOTU62743
New.CleanUp.ReferenceOTU95030
New.CleanUp.ReferenceOTU191013
New.CleanUp.ReferenceOTU152840
New.CleanUp.ReferenceOTU282542
New.CleanUp.ReferenceOTU31346
New.CleanUp.ReferenceOTU41221
New.CleanUp.ReferenceOTU47254
New.CleanUp.ReferenceOTU47784
New.CleanUp.ReferenceOTU59022
New.CleanUp.ReferenceOTU64250
New.CleanUp.ReferenceOTU105190
New.CleanUp.ReferenceOTU138194
New.CleanUp.ReferenceOTU214265
New.CleanUp.ReferenceOTU76484
New.CleanUp.ReferenceOTU139249
New.CleanUp.ReferenceOTU63490
New.CleanUp.ReferenceOTU183757
New.CleanUp.ReferenceOTU216073
New.CleanUp.ReferenceOTU144339
New.CleanUp.ReferenceOTU21816
New.CleanUp.ReferenceOTU248654
New.CleanUp.ReferenceOTU67870
New.CleanUp.ReferenceOTU218713
New.CleanUp.ReferenceOTU236651
New.CleanUp.ReferenceOTU31898
New.CleanUp.ReferenceOTU143319
New.CleanUp.ReferenceOTU7840
New.ReferenceOTU2427
New.CleanUp.ReferenceOTU185564
New.CleanUp.ReferenceOTU63244
New.CleanUp.ReferenceOTU48364
New.CleanUp.ReferenceOTU57506
New.CleanUp.ReferenceOTU78825
New.CleanUp.ReferenceOTU42774
New.CleanUp.ReferenceOTU25809
New.CleanUp.ReferenceOTU52820
New.CleanUp.ReferenceOTU44478
New.CleanUp.ReferenceOTU71116
New.CleanUp.ReferenceOTU42145
New.CleanUp.ReferenceOTU267120
New.CleanUp.ReferenceOTU81007
New.CleanUp.ReferenceOTU260713
New.CleanUp.ReferenceOTU54612

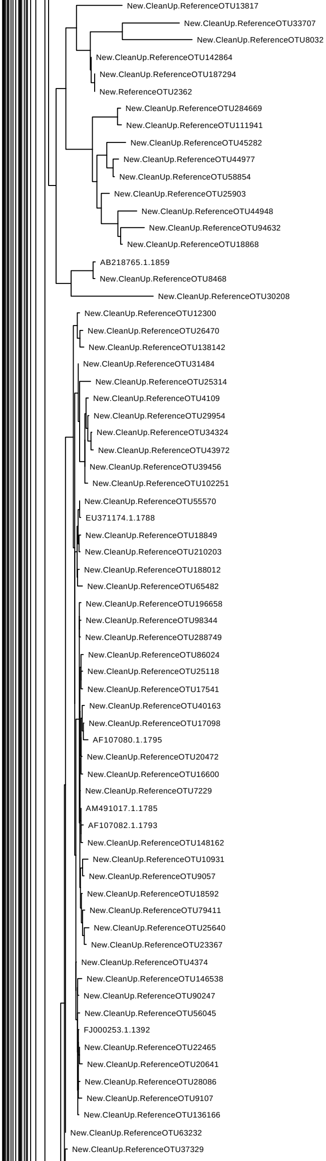
New.CleanUp.ReferenceOTU13817
New.CleanUp.ReferenceOTU33707
New.CleanUp.ReferenceOTU8032
New.CleanUp.ReferenceOTU142864
New.CleanUp.ReferenceOTU187294
New.ReferenceOTU2362
New.CleanUp.ReferenceOTU284669
New.CleanUp.ReferenceOTU111941
New.CleanUp.ReferenceOTU45282
New.CleanUp.ReferenceOTU44977
New.CleanUp.ReferenceOTU58854
New.CleanUp.ReferenceOTU25903
New.CleanUp.ReferenceOTU44948
New.CleanUp.ReferenceOTU94632
New.CleanUp.ReferenceOTU18868
AB218765.1.1859
New.CleanUp.ReferenceOTU8468
New.CleanUp.ReferenceOTU30208
New.CleanUp.ReferenceOTU12300
New.CleanUp.ReferenceOTU26470
New.CleanUp.ReferenceOTU138142
New.CleanUp.ReferenceOTU31484
New.CleanUp.ReferenceOTU25314
New.CleanUp.ReferenceOTU4109
New.CleanUp.ReferenceOTU29954
New.CleanUp.ReferenceOTU34324
New.CleanUp.ReferenceOTU43972
New.CleanUp.ReferenceOTU39456
New.CleanUp.ReferenceOTU102251
New.CleanUp.ReferenceOTU55570
EU371174.1.1788
New.CleanUp.ReferenceOTU18849
New.CleanUp.ReferenceOTU210203
New.CleanUp.ReferenceOTU188012
New.CleanUp.ReferenceOTU65482
New.CleanUp.ReferenceOTU196658
New.CleanUp.ReferenceOTU98344
New.CleanUp.ReferenceOTU288749
New.CleanUp.ReferenceOTU86024
New.CleanUp.ReferenceOTU25118
New.CleanUp.ReferenceOTU17541
New.CleanUp.ReferenceOTU40163
New.CleanUp.ReferenceOTU17098
AF107080.1.1795
New.CleanUp.ReferenceOTU20472
New.CleanUp.ReferenceOTU16600
New.CleanUp.ReferenceOTU7229
AM491017.1.1785
AF107082.1.1793
New.CleanUp.ReferenceOTU148162
New.CleanUp.ReferenceOTU10931
New.CleanUp.ReferenceOTU9057
New.CleanUp.ReferenceOTU18592
New.CleanUp.ReferenceOTU79411
New.CleanUp.ReferenceOTU25640
New.CleanUp.ReferenceOTU23367
New.CleanUp.ReferenceOTU4374
New.CleanUp.ReferenceOTU146538
New.CleanUp.ReferenceOTU90247
New.CleanUp.ReferenceOTU56045
FJ000253.1.1392
New.CleanUp.ReferenceOTU22465
New.CleanUp.ReferenceOTU20641
New.CleanUp.ReferenceOTU28086
New.CleanUp.ReferenceOTU9107
New.CleanUp.ReferenceOTU136166
New.CleanUp.ReferenceOTU63232
New.CleanUp.ReferenceOTU37329

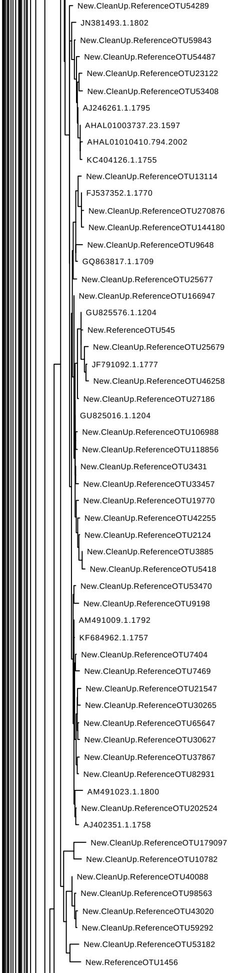
New.CleanUp.ReferenceOTU54289
JN381493.1.1802
New.CleanUp.ReferenceOTU59843
New.CleanUp.ReferenceOTU54487
New.CleanUp.ReferenceOTU23122
New.CleanUp.ReferenceOTU53408
AJ246261.1.1795
AHAL01003737.23.1597
AHAL01010410.794.2002
KC404126.1.1755
New.CleanUp.ReferenceOTU13114
FJ537352.1.1770
New.CleanUp.ReferenceOTU270876
New.CleanUp.ReferenceOTU144180
New.CleanUp.ReferenceOTU9648
GQ863817.1.1709
New.CleanUp.ReferenceOTU25677
New.CleanUp.ReferenceOTU166947
GU825576.1.1204
New.ReferenceOTU545
New.CleanUp.ReferenceOTU25679
JF791092.1.1777
New.CleanUp.ReferenceOTU46258
New.CleanUp.ReferenceOTU27186
GU825016.1.1204
New.CleanUp.ReferenceOTU106988
New.CleanUp.ReferenceOTU118856
New.CleanUp.ReferenceOTU3431
New.CleanUp.ReferenceOTU33457
New.CleanUp.ReferenceOTU19770
New.CleanUp.ReferenceOTU42255
New.CleanUp.ReferenceOTU2124
New.CleanUp.ReferenceOTU3885
New.CleanUp.ReferenceOTU5418
New.CleanUp.ReferenceOTU53470
New.CleanUp.ReferenceOTU9198
AM491009.1.1792
KF684962.1.1757
New.CleanUp.ReferenceOTU7404
New.CleanUp.ReferenceOTU7469
New.CleanUp.ReferenceOTU21547
New.CleanUp.ReferenceOTU30265
New.CleanUp.ReferenceOTU65647
New.CleanUp.ReferenceOTU30627
New.CleanUp.ReferenceOTU37867
New.CleanUp.ReferenceOTU82931
AM491023.1.1800
New.CleanUp.ReferenceOTU202524
AJ402351.1.1758
New.CleanUp.ReferenceOTU179097
New.CleanUp.ReferenceOTU10782
New.CleanUp.ReferenceOTU40088
New.CleanUp.ReferenceOTU98563
New.CleanUp.ReferenceOTU43020
New.CleanUp.ReferenceOTU59292
New.CleanUp.ReferenceOTU53182
New.ReferenceOTU1456

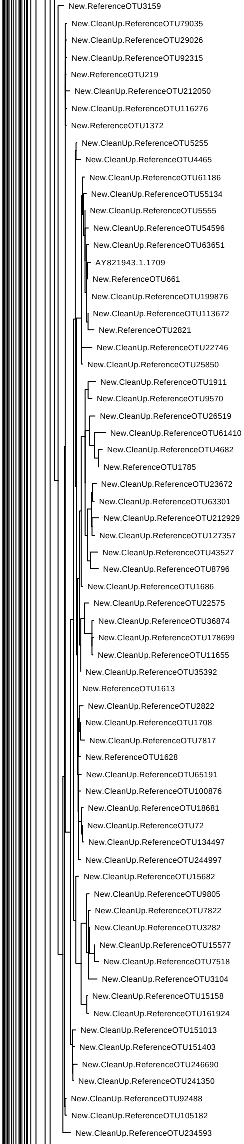
New.ReferenceOTU3159
New.CleanUp.ReferenceOTU79035
New.CleanUp.ReferenceOTU29026
New.CleanUp.ReferenceOTU92315
New.ReferenceOTU219
New.CleanUp.ReferenceOTU212050
New.CleanUp.ReferenceOTU116276
New.ReferenceOTU1372
New.CleanUp.ReferenceOTU5255
New.CleanUp.ReferenceOTU4465
New.CleanUp.ReferenceOTU61186
New.CleanUp.ReferenceOTU55134
New.CleanUp.ReferenceOTU5555
New.CleanUp.ReferenceOTU54596
New.CleanUp.ReferenceOTU63651
AY821943.1.1709
New.ReferenceOTU661
New.CleanUp.ReferenceOTU199876
New.CleanUp.ReferenceOTU113672
New.ReferenceOTU2821
New.CleanUp.ReferenceOTU22746
New.CleanUp.ReferenceOTU25850
New.CleanUp.ReferenceOTU1911
New.CleanUp.ReferenceOTU9570
New.CleanUp.ReferenceOTU26519
New.CleanUp.ReferenceOTU61410
New.CleanUp.ReferenceOTU4682
New.ReferenceOTU1785
New.CleanUp.ReferenceOTU23672
New.CleanUp.ReferenceOTU63301
New.CleanUp.ReferenceOTU212929
New.CleanUp.ReferenceOTU127357
New.CleanUp.ReferenceOTU43527
New.CleanUp.ReferenceOTU8796
New.CleanUp.ReferenceOTU1686
New.CleanUp.ReferenceOTU22575
New.CleanUp.ReferenceOTU36874
New.CleanUp.ReferenceOTU178699
New.CleanUp.ReferenceOTU11655
New.CleanUp.ReferenceOTU35392
New.ReferenceOTU1613
New.CleanUp.ReferenceOTU2822
New.CleanUp.ReferenceOTU1708
New.CleanUp.ReferenceOTU7817
New.ReferenceOTU1628
New.CleanUp.ReferenceOTU65191
New.CleanUp.ReferenceOTU100876
New.CleanUp.ReferenceOTU18681
New.CleanUp.ReferenceOTU72
New.CleanUp.ReferenceOTU134497
New.CleanUp.ReferenceOTU244997
New.CleanUp.ReferenceOTU15682
New.CleanUp.ReferenceOTU9805
New.CleanUp.ReferenceOTU7822
New.CleanUp.ReferenceOTU3282
New.CleanUp.ReferenceOTU15577
New.CleanUp.ReferenceOTU7518
New.CleanUp.ReferenceOTU3104
New.CleanUp.ReferenceOTU15158
New.CleanUp.ReferenceOTU161924
New.CleanUp.ReferenceOTU151013
New.CleanUp.ReferenceOTU151403
New.CleanUp.ReferenceOTU246690
New.CleanUp.ReferenceOTU241350
New.CleanUp.ReferenceOTU92488
New.CleanUp.ReferenceOTU105182
New.CleanUp.ReferenceOTU234593

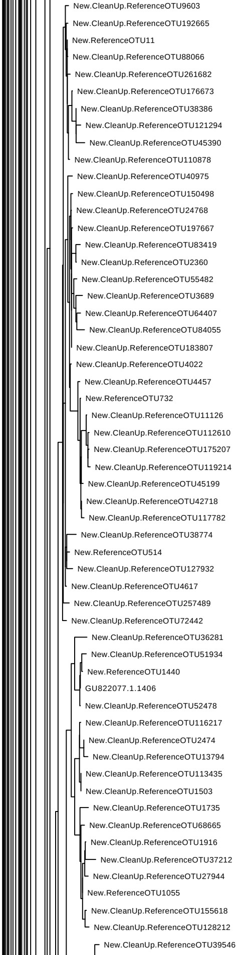
New.CleanUp.ReferenceOTU9603
New.CleanUp.ReferenceOTU192665
New.ReferenceOTU11
New.CleanUp.ReferenceOTU88066
New.CleanUp.ReferenceOTU261682
New.CleanUp.ReferenceOTU176673
New.CleanUp.ReferenceOTU38386
New.CleanUp.ReferenceOTU121294
New.CleanUp.ReferenceOTU45390
New.CleanUp.ReferenceOTU110878
New.CleanUp.ReferenceOTU40975
New.CleanUp.ReferenceOTU150498
New.CleanUp.ReferenceOTU24768
New.CleanUp.ReferenceOTU197667
New.CleanUp.ReferenceOTU83419
New.CleanUp.ReferenceOTU2360
New.CleanUp.ReferenceOTU55482
New.CleanUp.ReferenceOTU3689
New.CleanUp.ReferenceOTU64407
New.CleanUp.ReferenceOTU84055
New.CleanUp.ReferenceOTU183807
New.CleanUp.ReferenceOTU4022
New.CleanUp.ReferenceOTU4457
New.ReferenceOTU732
New.CleanUp.ReferenceOTU11126
New.CleanUp.ReferenceOTU112610
New.CleanUp.ReferenceOTU175207
New.CleanUp.ReferenceOTU119214
New.CleanUp.ReferenceOTU45199
New.CleanUp.ReferenceOTU42718
New.CleanUp.ReferenceOTU117782
New.CleanUp.ReferenceOTU38774
New.ReferenceOTU514
New.CleanUp.ReferenceOTU127932
New.CleanUp.ReferenceOTU4617
New.CleanUp.ReferenceOTU257489
New.CleanUp.ReferenceOTU72442
New.CleanUp.ReferenceOTU36281
New.CleanUp.ReferenceOTU51934
New.ReferenceOTU1440
GU822077.1.1406
New.CleanUp.ReferenceOTU52478
New.CleanUp.ReferenceOTU116217
New.CleanUp.ReferenceOTU2474
New.CleanUp.ReferenceOTU13794
New.CleanUp.ReferenceOTU113435
New.CleanUp.ReferenceOTU1503
New.CleanUp.ReferenceOTU1735
New.CleanUp.ReferenceOTU68665
New.CleanUp.ReferenceOTU1916
New.CleanUp.ReferenceOTU37212
New.CleanUp.ReferenceOTU27944
New.ReferenceOTU1055
New.CleanUp.ReferenceOTU155618
New.CleanUp.ReferenceOTU128212
New.CleanUp.ReferenceOTU39546

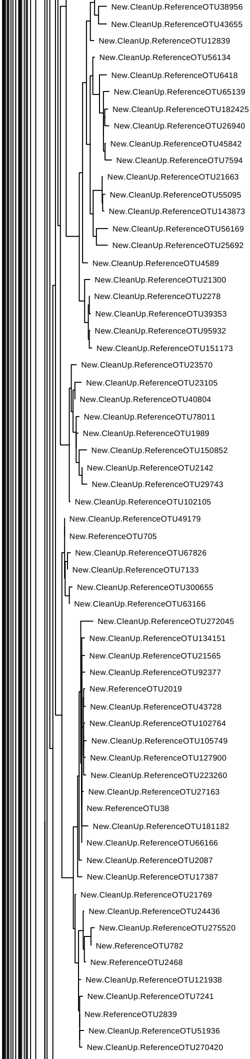
New.CleanUp.ReferenceOTU38956
New.CleanUp.ReferenceOTU43655
New.CleanUp.ReferenceOTU12839
New.CleanUp.ReferenceOTU56134
New.CleanUp.ReferenceOTU6418
New.CleanUp.ReferenceOTU65139
New.CleanUp.ReferenceOTU182425
New.CleanUp.ReferenceOTU26940
New.CleanUp.ReferenceOTU45842
New.CleanUp.ReferenceOTU7594
New.CleanUp.ReferenceOTU21663
New.CleanUp.ReferenceOTU55095
New.CleanUp.ReferenceOTU143873
New.CleanUp.ReferenceOTU56169
New.CleanUp.ReferenceOTU25692
New.CleanUp.ReferenceOTU4589
New.CleanUp.ReferenceOTU21300
New.CleanUp.ReferenceOTU2278
New.CleanUp.ReferenceOTU39353
New.CleanUp.ReferenceOTU95932
New.CleanUp.ReferenceOTU151173
New.CleanUp.ReferenceOTU23570
New.CleanUp.ReferenceOTU23105
New.CleanUp.ReferenceOTU40804
New.CleanUp.ReferenceOTU78011
New.CleanUp.ReferenceOTU1989
New.CleanUp.ReferenceOTU150852
New.CleanUp.ReferenceOTU2142
New.CleanUp.ReferenceOTU29743
New.CleanUp.ReferenceOTU102105
New.CleanUp.ReferenceOTU49179
New.ReferenceOTU705
New.CleanUp.ReferenceOTU67826
New.CleanUp.ReferenceOTU7133
New.CleanUp.ReferenceOTU300655
New.CleanUp.ReferenceOTU63166
New.CleanUp.ReferenceOTU272045
New.CleanUp.ReferenceOTU134151
New.CleanUp.ReferenceOTU21565
New.CleanUp.ReferenceOTU92377
New.ReferenceOTU2019
New.CleanUp.ReferenceOTU43728
New.CleanUp.ReferenceOTU102764
New.CleanUp.ReferenceOTU105749
New.CleanUp.ReferenceOTU127900
New.CleanUp.ReferenceOTU223260
New.CleanUp.ReferenceOTU27163
New.ReferenceOTU38
New.CleanUp.ReferenceOTU181182
New.CleanUp.ReferenceOTU66166
New.CleanUp.ReferenceOTU2087
New.CleanUp.ReferenceOTU17387
New.CleanUp.ReferenceOTU21769
New.CleanUp.ReferenceOTU24436
New.CleanUp.ReferenceOTU275520
New.ReferenceOTU782
New.ReferenceOTU2468
New.CleanUp.ReferenceOTU121938
New.CleanUp.ReferenceOTU7241
New.ReferenceOTU2839
New.CleanUp.ReferenceOTU51936
New.CleanUp.ReferenceOTU270420

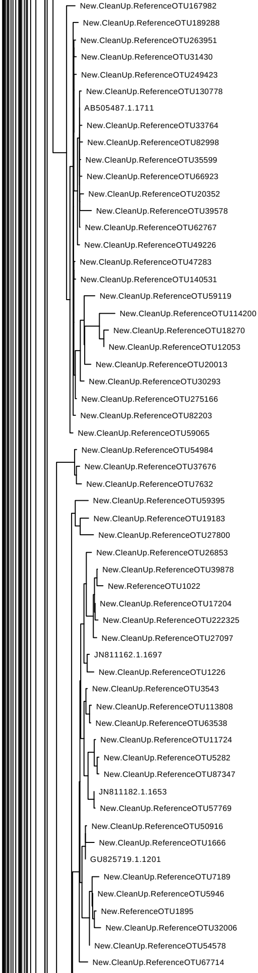
New.CleanUp.ReferenceOTU167982
New.CleanUp.ReferenceOTU189288
New.CleanUp.ReferenceOTU263951
New.CleanUp.ReferenceOTU31430
New.CleanUp.ReferenceOTU249423
New.CleanUp.ReferenceOTU130778
AB505487.1.1711
New.CleanUp.ReferenceOTU33764
New.CleanUp.ReferenceOTU82998
New.CleanUp.ReferenceOTU35599
New.CleanUp.ReferenceOTU66923
New.CleanUp.ReferenceOTU20352
New.CleanUp.ReferenceOTU39578
New.CleanUp.ReferenceOTU62767
New.CleanUp.ReferenceOTU49226
New.CleanUp.ReferenceOTU47283
New.CleanUp.ReferenceOTU140531
New.CleanUp.ReferenceOTU59119
New.CleanUp.ReferenceOTU114200
New.CleanUp.ReferenceOTU18270
New.CleanUp.ReferenceOTU12053
New.CleanUp.ReferenceOTU20013
New.CleanUp.ReferenceOTU30293
New.CleanUp.ReferenceOTU275166
New.CleanUp.ReferenceOTU82203
New.CleanUp.ReferenceOTU59065
New.CleanUp.ReferenceOTU54984
New.CleanUp.ReferenceOTU37676
New.CleanUp.ReferenceOTU7632
New.CleanUp.ReferenceOTU59395
New.CleanUp.ReferenceOTU19183
New.CleanUp.ReferenceOTU27800
New.CleanUp.ReferenceOTU26853
New.CleanUp.ReferenceOTU39878
New.ReferenceOTU1022
New.CleanUp.ReferenceOTU17204
New.CleanUp.ReferenceOTU222325
New.CleanUp.ReferenceOTU27097
JN811162.1.1697
New.CleanUp.ReferenceOTU1226
New.CleanUp.ReferenceOTU3543
New.CleanUp.ReferenceOTU113808
New.CleanUp.ReferenceOTU63538
New.CleanUp.ReferenceOTU11724
New.CleanUp.ReferenceOTU5282
New.CleanUp.ReferenceOTU87347
JN811182.1.1653
New.CleanUp.ReferenceOTU57769
New.CleanUp.ReferenceOTU50916
New.CleanUp.ReferenceOTU1666
GU825719.1.1201
New.CleanUp.ReferenceOTU7189
New.CleanUp.ReferenceOTU5946
New.ReferenceOTU1895
New.CleanUp.ReferenceOTU32006
New.CleanUp.ReferenceOTU54578
New.CleanUp.ReferenceOTU67714

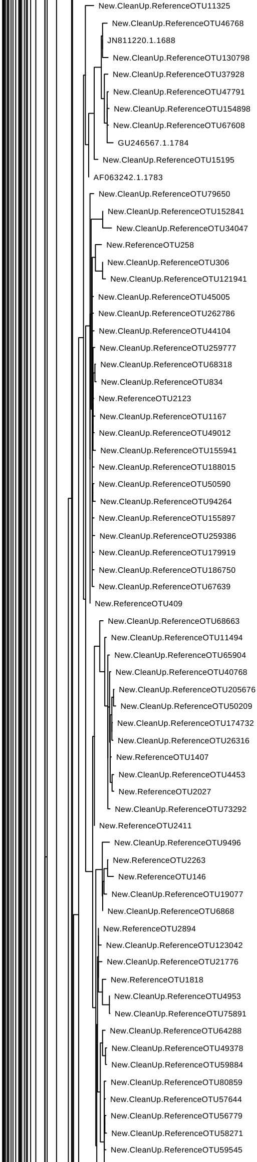
New.CleanUp.ReferenceOTU11325
New.CleanUp.ReferenceOTU46768
JN811220.1.1688
New.CleanUp.ReferenceOTU130798
New.CleanUp.ReferenceOTU37928
New.CleanUp.ReferenceOTU47791
New.CleanUp.ReferenceOTU154898
New.CleanUp.ReferenceOTU67608
GU246567.1.1784
New.CleanUp.ReferenceOTU15195
AF063242.1.1783
New.CleanUp.ReferenceOTU79650
New.CleanUp.ReferenceOTU152841
New.CleanUp.ReferenceOTU34047
New.ReferenceOTU258
New.CleanUp.ReferenceOTU306
New.CleanUp.ReferenceOTU121941
New.CleanUp.ReferenceOTU45005
New.CleanUp.ReferenceOTU262786
New.CleanUp.ReferenceOTU44104
New.CleanUp.ReferenceOTU259777
New.CleanUp.ReferenceOTU68318
New.CleanUp.ReferenceOTU834
New.ReferenceOTU2123
New.CleanUp.ReferenceOTU1167
New.CleanUp.ReferenceOTU49012
New.CleanUp.ReferenceOTU155941
New.CleanUp.ReferenceOTU188015
New.CleanUp.ReferenceOTU50590
New.CleanUp.ReferenceOTU94264
New.CleanUp.ReferenceOTU155897
New.CleanUp.ReferenceOTU259386
New.CleanUp.ReferenceOTU179919
New.CleanUp.ReferenceOTU186750
New.CleanUp.ReferenceOTU67639
New.ReferenceOTU409
New.CleanUp.ReferenceOTU68663
New.CleanUp.ReferenceOTU11494
New.CleanUp.ReferenceOTU65904
New.CleanUp.ReferenceOTU40768
New.CleanUp.ReferenceOTU205676
New.CleanUp.ReferenceOTU50209
New.CleanUp.ReferenceOTU174732
New.CleanUp.ReferenceOTU26316
New.ReferenceOTU1407
New.CleanUp.ReferenceOTU4453
New.ReferenceOTU2027
New.CleanUp.ReferenceOTU73292
New.ReferenceOTU2411
New.CleanUp.ReferenceOTU9496
New.ReferenceOTU2263
New.ReferenceOTU146
New.CleanUp.ReferenceOTU19077
New.CleanUp.ReferenceOTU6868
New.ReferenceOTU2894
New.CleanUp.ReferenceOTU123042
New.CleanUp.ReferenceOTU21776
New.ReferenceOTU1818
New.CleanUp.ReferenceOTU4953
New.CleanUp.ReferenceOTU75891
New.CleanUp.ReferenceOTU64288
New.CleanUp.ReferenceOTU49378
New.CleanUp.ReferenceOTU59884
New.CleanUp.ReferenceOTU80859
New.CleanUp.ReferenceOTU57644
New.CleanUp.ReferenceOTU56779
New.CleanUp.ReferenceOTU58271
New.CleanUp.ReferenceOTU59545

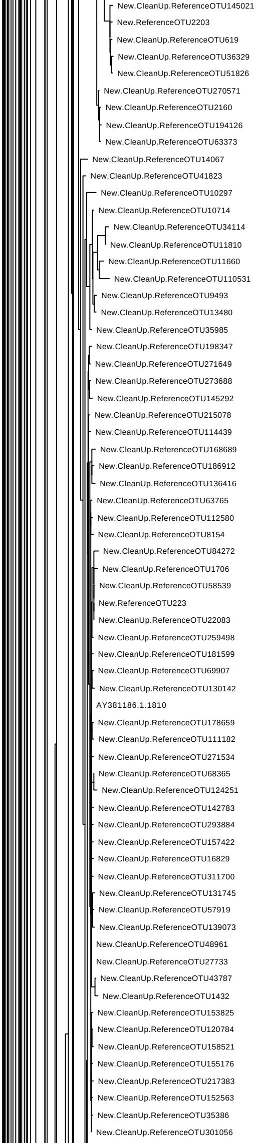
New.CleanUp.ReferenceOTU145021
New.ReferenceOTU2203
New.CleanUp.ReferenceOTU619
New.CleanUp.ReferenceOTU36329
New.CleanUp.ReferenceOTU51826
New.CleanUp.ReferenceOTU270571
New.CleanUp.ReferenceOTU2160
New.CleanUp.ReferenceOTU194126
New.CleanUp.ReferenceOTU63373
New.CleanUp.ReferenceOTU14067
New.CleanUp.ReferenceOTU41823
New.CleanUp.ReferenceOTU10297
New.CleanUp.ReferenceOTU10714
New.CleanUp.ReferenceOTU34114
New.CleanUp.ReferenceOTU11810
New.CleanUp.ReferenceOTU11660
New.CleanUp.ReferenceOTU110531
New.CleanUp.ReferenceOTU9493
New.CleanUp.ReferenceOTU13480
New.CleanUp.ReferenceOTU35985
New.CleanUp.ReferenceOTU198347
New.CleanUp.ReferenceOTU271649
New.CleanUp.ReferenceOTU273688
New.CleanUp.ReferenceOTU145292
New.CleanUp.ReferenceOTU215078
New.CleanUp.ReferenceOTU114439
New.CleanUp.ReferenceOTU168689
New.CleanUp.ReferenceOTU186912
New.CleanUp.ReferenceOTU136416
New.CleanUp.ReferenceOTU63765
New.CleanUp.ReferenceOTU112580
New.CleanUp.ReferenceOTU8154
New.CleanUp.ReferenceOTU84272
New.CleanUp.ReferenceOTU1706
New.CleanUp.ReferenceOTU58539
New.ReferenceOTU223
New.CleanUp.ReferenceOTU22083
New.CleanUp.ReferenceOTU259498
New.CleanUp.ReferenceOTU181599
New.CleanUp.ReferenceOTU69907
New.CleanUp.ReferenceOTU130142
AY381186.1.1810
New.CleanUp.ReferenceOTU178659
New.CleanUp.ReferenceOTU111182
New.CleanUp.ReferenceOTU271534
New.CleanUp.ReferenceOTU68365
New.CleanUp.ReferenceOTU124251
New.CleanUp.ReferenceOTU142783
New.CleanUp.ReferenceOTU293884
New.CleanUp.ReferenceOTU157422
New.CleanUp.ReferenceOTU16829
New.CleanUp.ReferenceOTU311700
New.CleanUp.ReferenceOTU131745
New.CleanUp.ReferenceOTU57919
New.CleanUp.ReferenceOTU139073
New.CleanUp.ReferenceOTU48961
New.CleanUp.ReferenceOTU27733
New.CleanUp.ReferenceOTU43787
New.CleanUp.ReferenceOTU1432
New.CleanUp.ReferenceOTU153825
New.CleanUp.ReferenceOTU120784
New.CleanUp.ReferenceOTU158521
New.CleanUp.ReferenceOTU155176
New.CleanUp.ReferenceOTU217383
New.CleanUp.ReferenceOTU152563
New.CleanUp.ReferenceOTU35386
New.CleanUp.ReferenceOTU301056

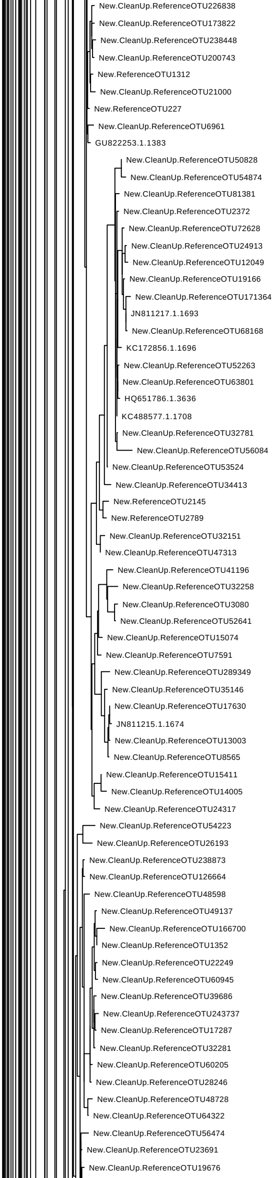
New.CleanUp.ReferenceOTU226838
New.CleanUp.ReferenceOTU173822
New.CleanUp.ReferenceOTU238448
New.CleanUp.ReferenceOTU200743
New.ReferenceOTU1312
New.CleanUp.ReferenceOTU21000
New.ReferenceOTU227
New.CleanUp.ReferenceOTU6961
GU822253.1.1383
New.CleanUp.ReferenceOTU50828
New.CleanUp.ReferenceOTU54874
New.CleanUp.ReferenceOTU81381
New.CleanUp.ReferenceOTU2372
New.CleanUp.ReferenceOTU72628
New.CleanUp.ReferenceOTU24913
New.CleanUp.ReferenceOTU12049
New.CleanUp.ReferenceOTU19166
New.CleanUp.ReferenceOTU171364
JN811217.1.1693
New.CleanUp.ReferenceOTU68168
KC172856.1.1696
New.CleanUp.ReferenceOTU52263
New.CleanUp.ReferenceOTU63801
HQ651786.1.3636
KC488577.1.1708
New.CleanUp.ReferenceOTU32781
New.CleanUp.ReferenceOTU56084
New.CleanUp.ReferenceOTU53524
New.CleanUp.ReferenceOTU34413
New.ReferenceOTU2145
New.ReferenceOTU2789
New.CleanUp.ReferenceOTU32151
New.CleanUp.ReferenceOTU47313
New.CleanUp.ReferenceOTU41196
New.CleanUp.ReferenceOTU32258
New.CleanUp.ReferenceOTU3080
New.CleanUp.ReferenceOTU52641
New.CleanUp.ReferenceOTU15074
New.CleanUp.ReferenceOTU7591
New.CleanUp.ReferenceOTU289349
New.CleanUp.ReferenceOTU35146
New.CleanUp.ReferenceOTU17630
JN811215.1.1674
New.CleanUp.ReferenceOTU13003
New.CleanUp.ReferenceOTU8565
New.CleanUp.ReferenceOTU15411
New.CleanUp.ReferenceOTU14005
New.CleanUp.ReferenceOTU24317
New.CleanUp.ReferenceOTU54223
New.CleanUp.ReferenceOTU26193
New.CleanUp.ReferenceOTU238873
New.CleanUp.ReferenceOTU126664
New.CleanUp.ReferenceOTU48598
New.CleanUp.ReferenceOTU49137
New.CleanUp.ReferenceOTU166700
New.CleanUp.ReferenceOTU1352
New.CleanUp.ReferenceOTU22249
New.CleanUp.ReferenceOTU60945
New.CleanUp.ReferenceOTU39686
New.CleanUp.ReferenceOTU243737
New.CleanUp.ReferenceOTU17287
New.CleanUp.ReferenceOTU32281
New.CleanUp.ReferenceOTU60205
New.CleanUp.ReferenceOTU28246
New.CleanUp.ReferenceOTU48728
New.CleanUp.ReferenceOTU64322
New.CleanUp.ReferenceOTU56474
New.CleanUp.ReferenceOTU23691
New.CleanUp.ReferenceOTU19676

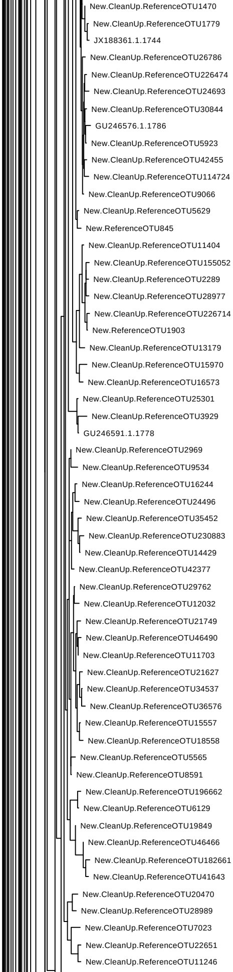
New.CleanUp.ReferenceOTU1470
New.CleanUp.ReferenceOTU1779
JX188361.1.1744
New.CleanUp.ReferenceOTU26786
New.CleanUp.ReferenceOTU226474
New.CleanUp.ReferenceOTU24693
New.CleanUp.ReferenceOTU30844
GU246576.1.1786
New.CleanUp.ReferenceOTU5923
New.CleanUp.ReferenceOTU42455
New.CleanUp.ReferenceOTU114724
New.CleanUp.ReferenceOTU9066
New.CleanUp.ReferenceOTU5629
New.ReferenceOTU845
New.CleanUp.ReferenceOTU11404
New.CleanUp.ReferenceOTU155052
New.CleanUp.ReferenceOTU2289
New.CleanUp.ReferenceOTU28977
New.CleanUp.ReferenceOTU226714
New.ReferenceOTU1903
New.CleanUp.ReferenceOTU13179
New.CleanUp.ReferenceOTU15970
New.CleanUp.ReferenceOTU16573
New.CleanUp.ReferenceOTU25301
New.CleanUp.ReferenceOTU3929
GU246591.1.1778
New.CleanUp.ReferenceOTU2969
New.CleanUp.ReferenceOTU9534
New.CleanUp.ReferenceOTU16244
New.CleanUp.ReferenceOTU24496
New.CleanUp.ReferenceOTU35452
New.CleanUp.ReferenceOTU230883
New.CleanUp.ReferenceOTU14429
New.CleanUp.ReferenceOTU42377
New.CleanUp.ReferenceOTU29762
New.CleanUp.ReferenceOTU12032
New.CleanUp.ReferenceOTU21749
New.CleanUp.ReferenceOTU46490
New.CleanUp.ReferenceOTU11703
New.CleanUp.ReferenceOTU21627
New.CleanUp.ReferenceOTU34537
New.CleanUp.ReferenceOTU36576
New.CleanUp.ReferenceOTU15557
New.CleanUp.ReferenceOTU18558
New.CleanUp.ReferenceOTU5565
New.CleanUp.ReferenceOTU8591
New.CleanUp.ReferenceOTU196662
New.CleanUp.ReferenceOTU6129
New.CleanUp.ReferenceOTU19849
New.CleanUp.ReferenceOTU46466
New.CleanUp.ReferenceOTU182661
New.CleanUp.ReferenceOTU41643
New.CleanUp.ReferenceOTU20470
New.CleanUp.ReferenceOTU28989
New.CleanUp.ReferenceOTU7023
New.CleanUp.ReferenceOTU22651
New.CleanUp.ReferenceOTU11246

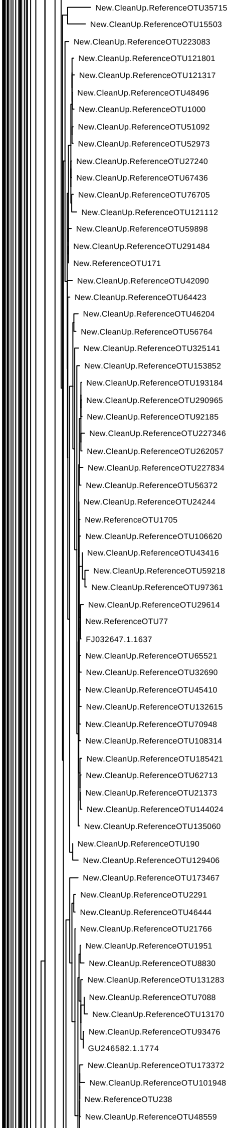
New.CleanUp.ReferenceOTU35715
New.CleanUp.ReferenceOTU15503
New.CleanUp.ReferenceOTU223083
New.CleanUp.ReferenceOTU121801
New.CleanUp.ReferenceOTU121317
New.CleanUp.ReferenceOTU48496
New.CleanUp.ReferenceOTU1000
New.CleanUp.ReferenceOTU51092
New.CleanUp.ReferenceOTU52973
New.CleanUp.ReferenceOTU27240
New.CleanUp.ReferenceOTU67436
New.CleanUp.ReferenceOTU76705
New.CleanUp.ReferenceOTU121112
New.CleanUp.ReferenceOTU59898
New.CleanUp.ReferenceOTU291484
New.ReferenceOTU171
New.CleanUp.ReferenceOTU42090
New.CleanUp.ReferenceOTU64423
New.CleanUp.ReferenceOTU46204
New.CleanUp.ReferenceOTU56764
New.CleanUp.ReferenceOTU325141
New.CleanUp.ReferenceOTU153852
New.CleanUp.ReferenceOTU193184
New.CleanUp.ReferenceOTU290965
New.CleanUp.ReferenceOTU92185
New.CleanUp.ReferenceOTU227346
New.CleanUp.ReferenceOTU262057
New.CleanUp.ReferenceOTU227834
New.CleanUp.ReferenceOTU56372
New.CleanUp.ReferenceOTU24244
New.ReferenceOTU1705
New.CleanUp.ReferenceOTU106620
New.CleanUp.ReferenceOTU43416
New.CleanUp.ReferenceOTU59218
New.CleanUp.ReferenceOTU97361
New.CleanUp.ReferenceOTU29614
New.ReferenceOTU77
FJ032647.1.1637
New.CleanUp.ReferenceOTU65521
New.CleanUp.ReferenceOTU32690
New.CleanUp.ReferenceOTU45410
New.CleanUp.ReferenceOTU132615
New.CleanUp.ReferenceOTU70948
New.CleanUp.ReferenceOTU108314
New.CleanUp.ReferenceOTU185421
New.CleanUp.ReferenceOTU62713
New.CleanUp.ReferenceOTU21373
New.CleanUp.ReferenceOTU144024
New.CleanUp.ReferenceOTU135060
New.CleanUp.ReferenceOTU190
New.CleanUp.ReferenceOTU129406
New.CleanUp.ReferenceOTU173467
New.CleanUp.ReferenceOTU2291
New.CleanUp.ReferenceOTU46444
New.CleanUp.ReferenceOTU21766
New.CleanUp.ReferenceOTU1951
New.CleanUp.ReferenceOTU8830
New.CleanUp.ReferenceOTU131283
New.CleanUp.ReferenceOTU7088
New.CleanUp.ReferenceOTU13170
New.CleanUp.ReferenceOTU93476
GU246582.1.1774
New.CleanUp.ReferenceOTU173372
New.CleanUp.ReferenceOTU101948
New.ReferenceOTU238
New.CleanUp.ReferenceOTU48559

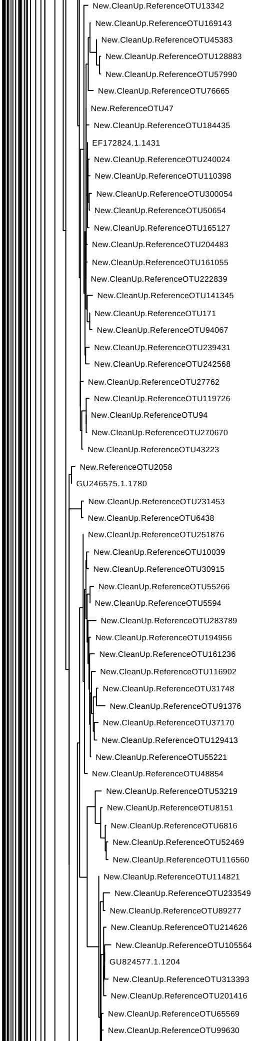
New.CleanUp.ReferenceOTU13342
New.CleanUp.ReferenceOTU169143
New.CleanUp.ReferenceOTU45383
New.CleanUp.ReferenceOTU128883
New.CleanUp.ReferenceOTU57990
New.CleanUp.ReferenceOTU76665
New.ReferenceOTU47
New.CleanUp.ReferenceOTU184435
EF172824.1.1431
New.CleanUp.ReferenceOTU240024
New.CleanUp.ReferenceOTU110398
New.CleanUp.ReferenceOTU300054
New.CleanUp.ReferenceOTU50654
New.CleanUp.ReferenceOTU165127
New.CleanUp.ReferenceOTU204483
New.CleanUp.ReferenceOTU161055
New.CleanUp.ReferenceOTU222839
New.CleanUp.ReferenceOTU141345
New.CleanUp.ReferenceOTU171
New.CleanUp.ReferenceOTU94067
New.CleanUp.ReferenceOTU239431
New.CleanUp.ReferenceOTU242568
New.CleanUp.ReferenceOTU27762
New.CleanUp.ReferenceOTU119726
New.CleanUp.ReferenceOTU94
New.CleanUp.ReferenceOTU270670
New.CleanUp.ReferenceOTU43223
New.ReferenceOTU2058
GU246575.1.1780
New.CleanUp.ReferenceOTU231453
New.CleanUp.ReferenceOTU6438
New.CleanUp.ReferenceOTU251876
New.CleanUp.ReferenceOTU10039
New.CleanUp.ReferenceOTU30915
New.CleanUp.ReferenceOTU55266
New.CleanUp.ReferenceOTU5594
New.CleanUp.ReferenceOTU283789
New.CleanUp.ReferenceOTU194956
New.CleanUp.ReferenceOTU161236
New.CleanUp.ReferenceOTU116902
New.CleanUp.ReferenceOTU31748
New.CleanUp.ReferenceOTU91376
New.CleanUp.ReferenceOTU37170
New.CleanUp.ReferenceOTU129413
New.CleanUp.ReferenceOTU55221
New.CleanUp.ReferenceOTU48854
New.CleanUp.ReferenceOTU53219
New.CleanUp.ReferenceOTU8151
New.CleanUp.ReferenceOTU6816
New.CleanUp.ReferenceOTU52469
New.CleanUp.ReferenceOTU116560
New.CleanUp.ReferenceOTU114821
New.CleanUp.ReferenceOTU233549
New.CleanUp.ReferenceOTU89277
New.CleanUp.ReferenceOTU214626
New.CleanUp.ReferenceOTU105564
GU824577.1.1204
New.CleanUp.ReferenceOTU313393
New.CleanUp.ReferenceOTU201416
New.CleanUp.ReferenceOTU65569
New.CleanUp.ReferenceOTU99630

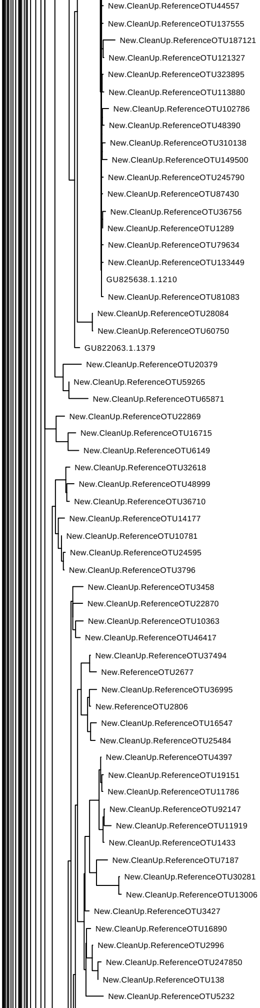
New.CleanUp.ReferenceOTU44557
New.CleanUp.ReferenceOTU137555
New.CleanUp.ReferenceOTU187121
New.CleanUp.ReferenceOTU121327
New.CleanUp.ReferenceOTU323895
New.CleanUp.ReferenceOTU113880
New.CleanUp.ReferenceOTU102786
New.CleanUp.ReferenceOTU48390
New.CleanUp.ReferenceOTU310138
New.CleanUp.ReferenceOTU149500
New.CleanUp.ReferenceOTU245790
New.CleanUp.ReferenceOTU87430
New.CleanUp.ReferenceOTU36756
New.CleanUp.ReferenceOTU1289
New.CleanUp.ReferenceOTU79634
New.CleanUp.ReferenceOTU133449
GU825638.1.1210
New.CleanUp.ReferenceOTU81083
New.CleanUp.ReferenceOTU28084
New.CleanUp.ReferenceOTU60750
GU822063.1.1379
New.CleanUp.ReferenceOTU20379
New.CleanUp.ReferenceOTU59265
New.CleanUp.ReferenceOTU65871
New.CleanUp.ReferenceOTU22869
New.CleanUp.ReferenceOTU16715
New.CleanUp.ReferenceOTU6149
New.CleanUp.ReferenceOTU32618
New.CleanUp.ReferenceOTU48999
New.CleanUp.ReferenceOTU36710
New.CleanUp.ReferenceOTU14177
New.CleanUp.ReferenceOTU10781
New.CleanUp.ReferenceOTU24595
New.CleanUp.ReferenceOTU3796
New.CleanUp.ReferenceOTU3458
New.CleanUp.ReferenceOTU22870
New.CleanUp.ReferenceOTU10363
New.CleanUp.ReferenceOTU46417
New.CleanUp.ReferenceOTU37494
New.ReferenceOTU2677
New.CleanUp.ReferenceOTU36995
New.ReferenceOTU2806
New.CleanUp.ReferenceOTU16547
New.CleanUp.ReferenceOTU25484
New.CleanUp.ReferenceOTU4397
New.CleanUp.ReferenceOTU19151
New.CleanUp.ReferenceOTU11786
New.CleanUp.ReferenceOTU92147
New.CleanUp.ReferenceOTU11919
New.CleanUp.ReferenceOTU1433
New.CleanUp.ReferenceOTU7187
New.CleanUp.ReferenceOTU30281
New.CleanUp.ReferenceOTU13006
New.CleanUp.ReferenceOTU3427
New.CleanUp.ReferenceOTU16890
New.CleanUp.ReferenceOTU2996
New.CleanUp.ReferenceOTU247850
New.CleanUp.ReferenceOTU138
New.CleanUp.ReferenceOTU5232

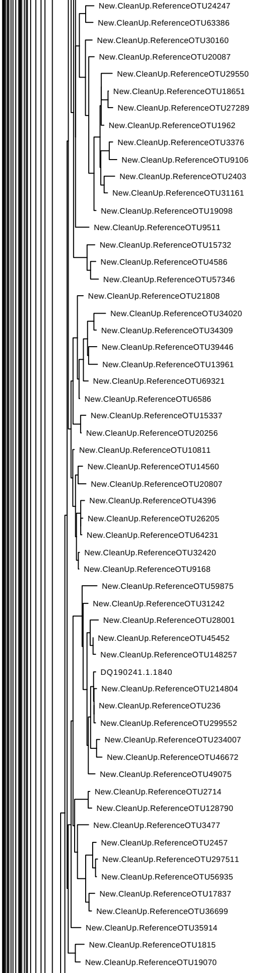
New.CleanUp.ReferenceOTU24247
New.CleanUp.ReferenceOTU63386
New.CleanUp.ReferenceOTU30160
New.CleanUp.ReferenceOTU20087
New.CleanUp.ReferenceOTU29550
New.CleanUp.ReferenceOTU18651
New.CleanUp.ReferenceOTU27289
New.CleanUp.ReferenceOTU1962
New.CleanUp.ReferenceOTU3376
New.CleanUp.ReferenceOTU9106
New.CleanUp.ReferenceOTU2403
New.CleanUp.ReferenceOTU31161
New.CleanUp.ReferenceOTU19098
New.CleanUp.ReferenceOTU9511
New.CleanUp.ReferenceOTU15732
New.CleanUp.ReferenceOTU4586
New.CleanUp.ReferenceOTU57346
New.CleanUp.ReferenceOTU21808
New.CleanUp.ReferenceOTU34020
New.CleanUp.ReferenceOTU34309
New.CleanUp.ReferenceOTU39446
New.CleanUp.ReferenceOTU13961
New.CleanUp.ReferenceOTU69321
New.CleanUp.ReferenceOTU6586
New.CleanUp.ReferenceOTU15337
New.CleanUp.ReferenceOTU20256
New.CleanUp.ReferenceOTU10811
New.CleanUp.ReferenceOTU14560
New.CleanUp.ReferenceOTU20807
New.CleanUp.ReferenceOTU4396
New.CleanUp.ReferenceOTU26205
New.CleanUp.ReferenceOTU64231
New.CleanUp.ReferenceOTU32420
New.CleanUp.ReferenceOTU9168
New.CleanUp.ReferenceOTU59875
New.CleanUp.ReferenceOTU31242
New.CleanUp.ReferenceOTU28001
New.CleanUp.ReferenceOTU45452
New.CleanUp.ReferenceOTU148257
DQ190241.1.1840
New.CleanUp.ReferenceOTU214804
New.CleanUp.ReferenceOTU236
New.CleanUp.ReferenceOTU299552
New.CleanUp.ReferenceOTU234007
New.CleanUp.ReferenceOTU46672
New.CleanUp.ReferenceOTU49075
New.CleanUp.ReferenceOTU2714
New.CleanUp.ReferenceOTU128790
New.CleanUp.ReferenceOTU3477
New.CleanUp.ReferenceOTU2457
New.CleanUp.ReferenceOTU297511
New.CleanUp.ReferenceOTU56935
New.CleanUp.ReferenceOTU17837
New.CleanUp.ReferenceOTU36699
New.CleanUp.ReferenceOTU35914
New.CleanUp.ReferenceOTU1815
New.CleanUp.ReferenceOTU19070

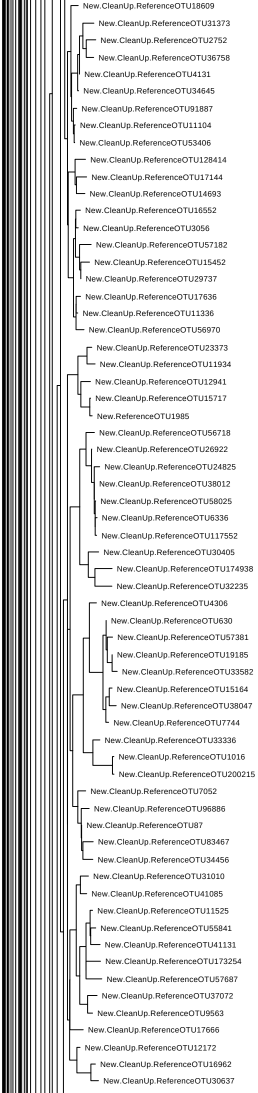
New.CleanUp.ReferenceOTU18609
New.CleanUp.ReferenceOTU31373
New.CleanUp.ReferenceOTU2752
New.CleanUp.ReferenceOTU36758
New.CleanUp.ReferenceOTU4131
New.CleanUp.ReferenceOTU34645
New.CleanUp.ReferenceOTU91887
New.CleanUp.ReferenceOTU11104
New.CleanUp.ReferenceOTU53406
New.CleanUp.ReferenceOTU128414
New.CleanUp.ReferenceOTU17144
New.CleanUp.ReferenceOTU14693
New.CleanUp.ReferenceOTU16552
New.CleanUp.ReferenceOTU3056
New.CleanUp.ReferenceOTU57182
New.CleanUp.ReferenceOTU15452
New.CleanUp.ReferenceOTU29737
New.CleanUp.ReferenceOTU17636
New.CleanUp.ReferenceOTU11336
New.CleanUp.ReferenceOTU56970
New.CleanUp.ReferenceOTU23373
New.CleanUp.ReferenceOTU11934
New.CleanUp.ReferenceOTU12941
New.CleanUp.ReferenceOTU15717
New.ReferenceOTU1985
New.CleanUp.ReferenceOTU56718
New.CleanUp.ReferenceOTU26922
New.CleanUp.ReferenceOTU24825
New.CleanUp.ReferenceOTU38012
New.CleanUp.ReferenceOTU58025
New.CleanUp.ReferenceOTU6336
New.CleanUp.ReferenceOTU117552
New.CleanUp.ReferenceOTU30405
New.CleanUp.ReferenceOTU174938
New.CleanUp.ReferenceOTU32235
New.CleanUp.ReferenceOTU4306
New.CleanUp.ReferenceOTU630
New.CleanUp.ReferenceOTU57381
New.CleanUp.ReferenceOTU19185
New.CleanUp.ReferenceOTU33582
New.CleanUp.ReferenceOTU15164
New.CleanUp.ReferenceOTU38047
New.CleanUp.ReferenceOTU7744
New.CleanUp.ReferenceOTU33336
New.CleanUp.ReferenceOTU1016
New.CleanUp.ReferenceOTU200215
New.CleanUp.ReferenceOTU7052
New.CleanUp.ReferenceOTU96886
New.CleanUp.ReferenceOTU87
New.CleanUp.ReferenceOTU83467
New.CleanUp.ReferenceOTU34456
New.CleanUp.ReferenceOTU31010
New.CleanUp.ReferenceOTU41085
New.CleanUp.ReferenceOTU11525
New.CleanUp.ReferenceOTU55841
New.CleanUp.ReferenceOTU41131
New.CleanUp.ReferenceOTU173254
New.CleanUp.ReferenceOTU57687
New.CleanUp.ReferenceOTU37072
New.CleanUp.ReferenceOTU9563
New.CleanUp.ReferenceOTU17666
New.CleanUp.ReferenceOTU12172
New.CleanUp.ReferenceOTU16962
New.CleanUp.ReferenceOTU30637

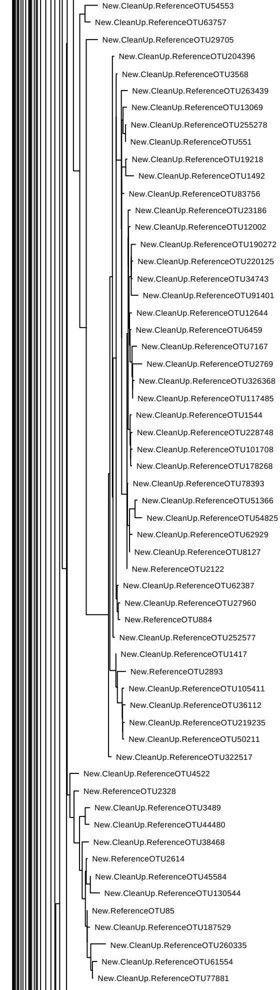
New.CleanUp.ReferenceOTU54553
New.CleanUp.ReferenceOTU63757
New.CleanUp.ReferenceOTU29705
New.CleanUp.ReferenceOTU204396
New.CleanUp.ReferenceOTU3568
New.CleanUp.ReferenceOTU263439
New.CleanUp.ReferenceOTU13069
New.CleanUp.ReferenceOTU255278
New.CleanUp.ReferenceOTU551
New.CleanUp.ReferenceOTU19218
New.CleanUp.ReferenceOTU1492
New.CleanUp.ReferenceOTU83756
New.CleanUp.ReferenceOTU23186
New.CleanUp.ReferenceOTU12002
New.CleanUp.ReferenceOTU190272
New.CleanUp.ReferenceOTU220125
New.CleanUp.ReferenceOTU34743
New.CleanUp.ReferenceOTU91401
New.CleanUp.ReferenceOTU12644
New.CleanUp.ReferenceOTU6459
New.CleanUp.ReferenceOTU7167
New.CleanUp.ReferenceOTU2769
New.CleanUp.ReferenceOTU326368
New.CleanUp.ReferenceOTU117485
New.CleanUp.ReferenceOTU1544
New.CleanUp.ReferenceOTU228748
New.CleanUp.ReferenceOTU101708
New.CleanUp.ReferenceOTU178268
New.CleanUp.ReferenceOTU78393
New.CleanUp.ReferenceOTU51366
New.CleanUp.ReferenceOTU54825
New.CleanUp.ReferenceOTU62929
New.CleanUp.ReferenceOTU8127
New.ReferenceOTU2122
New.CleanUp.ReferenceOTU62387
New.CleanUp.ReferenceOTU27960
New.ReferenceOTU884
New.CleanUp.ReferenceOTU252577
New.CleanUp.ReferenceOTU1417
New.ReferenceOTU2893
New.CleanUp.ReferenceOTU105411
New.CleanUp.ReferenceOTU36112
New.CleanUp.ReferenceOTU219235
New.CleanUp.ReferenceOTU50211
New.CleanUp.ReferenceOTU322517
New.CleanUp.ReferenceOTU4522
New.ReferenceOTU2328
New.CleanUp.ReferenceOTU3489
New.CleanUp.ReferenceOTU44480
New.CleanUp.ReferenceOTU38468
New.ReferenceOTU2614
New.CleanUp.ReferenceOTU45584
New.CleanUp.ReferenceOTU130544
New.ReferenceOTU85
New.CleanUp.ReferenceOTU187529
New.CleanUp.ReferenceOTU260335
New.CleanUp.ReferenceOTU61554
New.CleanUp.ReferenceOTU77881

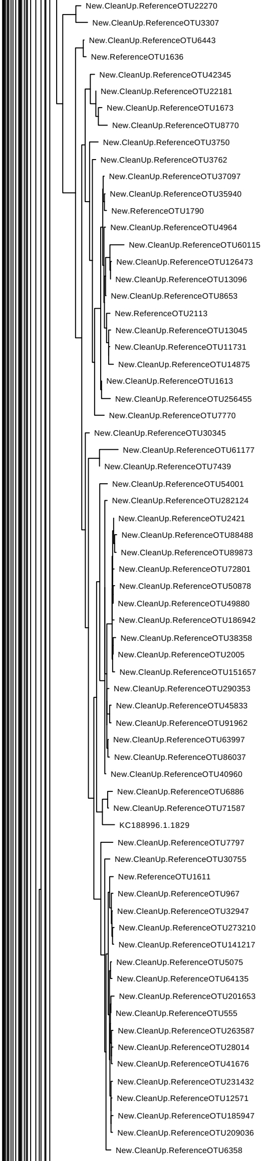

New.CleanUp.ReferenceOTU22270
New.CleanUp.ReferenceOTU3307
New.CleanUp.ReferenceOTU6443
New.ReferenceOTU1636
New.CleanUp.ReferenceOTU42345
New.CleanUp.ReferenceOTU22181
New.CleanUp.ReferenceOTU1673
New.CleanUp.ReferenceOTU8770
New.CleanUp.ReferenceOTU3750
New.CleanUp.ReferenceOTU3762
New.CleanUp.ReferenceOTU37097
New.CleanUp.ReferenceOTU35940
New.ReferenceOTU1790
New.CleanUp.ReferenceOTU4964
New.CleanUp.ReferenceOTU60115
New.CleanUp.ReferenceOTU126473
New.CleanUp.ReferenceOTU13096
New.CleanUp.ReferenceOTU8653
New.ReferenceOTU2113
New.CleanUp.ReferenceOTU13045
New.CleanUp.ReferenceOTU11731
New.CleanUp.ReferenceOTU14875
New.CleanUp.ReferenceOTU1613
New.CleanUp.ReferenceOTU256455
New.CleanUp.ReferenceOTU7770
New.CleanUp.ReferenceOTU30345
New.CleanUp.ReferenceOTU61177
New.CleanUp.ReferenceOTU7439
New.CleanUp.ReferenceOTU54001
New.CleanUp.ReferenceOTU282124
New.CleanUp.ReferenceOTU2421
New.CleanUp.ReferenceOTU88488
New.CleanUp.ReferenceOTU89873
New.CleanUp.ReferenceOTU72801
New.CleanUp.ReferenceOTU50878
New.CleanUp.ReferenceOTU49880
New.CleanUp.ReferenceOTU186942
New.CleanUp.ReferenceOTU38358
New.CleanUp.ReferenceOTU2005
New.CleanUp.ReferenceOTU151657
New.CleanUp.ReferenceOTU290353
New.CleanUp.ReferenceOTU45833
New.CleanUp.ReferenceOTU91962
New.CleanUp.ReferenceOTU63997
New.CleanUp.ReferenceOTU86037
New.CleanUp.ReferenceOTU40960
New.CleanUp.ReferenceOTU6886
New.CleanUp.ReferenceOTU71587
KC188996.1.1829
New.CleanUp.ReferenceOTU7797
New.CleanUp.ReferenceOTU30755
New.ReferenceOTU1611
New.CleanUp.ReferenceOTU967
New.CleanUp.ReferenceOTU32947
New.CleanUp.ReferenceOTU273210
New.CleanUp.ReferenceOTU141217
New.CleanUp.ReferenceOTU5075
New.CleanUp.ReferenceOTU64135
New.CleanUp.ReferenceOTU201653
New.CleanUp.ReferenceOTU555
New.CleanUp.ReferenceOTU263587
New.CleanUp.ReferenceOTU28014
New.CleanUp.ReferenceOTU41676
New.CleanUp.ReferenceOTU231432
New.CleanUp.ReferenceOTU12571
New.CleanUp.ReferenceOTU185947
New.CleanUp.ReferenceOTU209036
New.CleanUp.ReferenceOTU6358

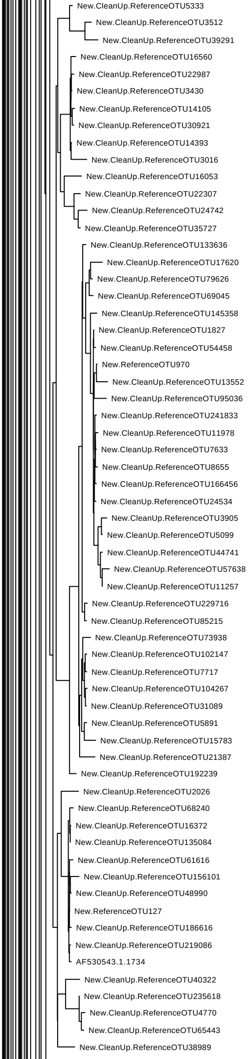
New.CleanUp.ReferenceOTU5333
New.CleanUp.ReferenceOTU3512
New.CleanUp.ReferenceOTU39291
New.CleanUp.ReferenceOTU16560
New.CleanUp.ReferenceOTU22987
New.CleanUp.ReferenceOTU3430
New.CleanUp.ReferenceOTU14105
New.CleanUp.ReferenceOTU30921
New.CleanUp.ReferenceOTU14393
New.CleanUp.ReferenceOTU3016
New.CleanUp.ReferenceOTU16053
New.CleanUp.ReferenceOTU22307
New.CleanUp.ReferenceOTU24742
New.CleanUp.ReferenceOTU35727
New.CleanUp.ReferenceOTU133636
New.CleanUp.ReferenceOTU17620
New.CleanUp.ReferenceOTU79626
New.CleanUp.ReferenceOTU69045
New.CleanUp.ReferenceOTU145358
New.CleanUp.ReferenceOTU1827
New.CleanUp.ReferenceOTU54458
New.ReferenceOTU970
New.CleanUp.ReferenceOTU13552
New.CleanUp.ReferenceOTU95036
New.CleanUp.ReferenceOTU241833
New.CleanUp.ReferenceOTU11978
New.CleanUp.ReferenceOTU7633
New.CleanUp.ReferenceOTU8655
New.CleanUp.ReferenceOTU166456
New.CleanUp.ReferenceOTU24534
New.CleanUp.ReferenceOTU3905
New.CleanUp.ReferenceOTU5099
New.CleanUp.ReferenceOTU44741
New.CleanUp.ReferenceOTU57638
New.CleanUp.ReferenceOTU11257
New.CleanUp.ReferenceOTU229716
New.CleanUp.ReferenceOTU85215
New.CleanUp.ReferenceOTU73938
New.CleanUp.ReferenceOTU102147
New.CleanUp.ReferenceOTU7717
New.CleanUp.ReferenceOTU104267
New.CleanUp.ReferenceOTU31089
New.CleanUp.ReferenceOTU5891
New.CleanUp.ReferenceOTU15783
New.CleanUp.ReferenceOTU21387
New.CleanUp.ReferenceOTU192239
New.CleanUp.ReferenceOTU2026
New.CleanUp.ReferenceOTU68240
New.CleanUp.ReferenceOTU16372
New.CleanUp.ReferenceOTU135084
New.CleanUp.ReferenceOTU61616
New.CleanUp.ReferenceOTU156101
New.CleanUp.ReferenceOTU48990
New.ReferenceOTU127
New.CleanUp.ReferenceOTU186616
New.CleanUp.ReferenceOTU219086
AF530543.1.1734
New.CleanUp.ReferenceOTU40322
New.CleanUp.ReferenceOTU235618
New.CleanUp.ReferenceOTU4770
New.CleanUp.ReferenceOTU65443
New.CleanUp.ReferenceOTU38989

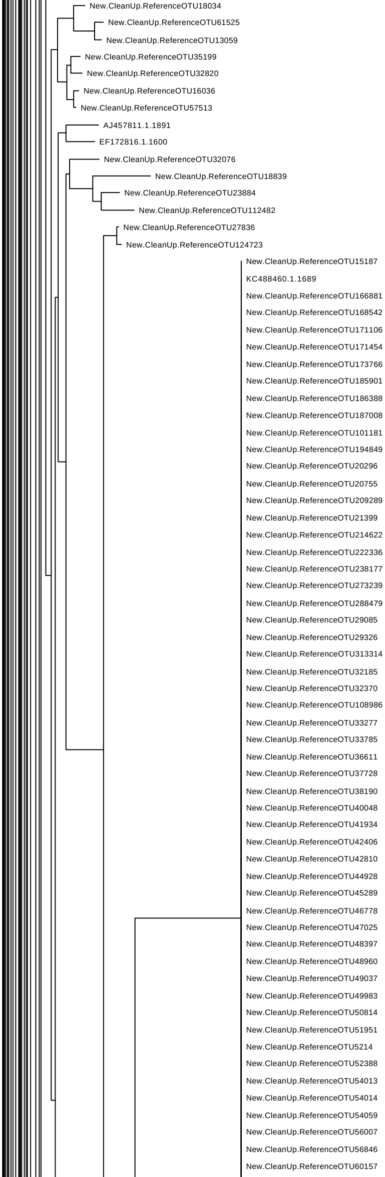
New.CleanUp.ReferenceOTU18034
New.CleanUp.ReferenceOTU61525
New.CleanUp.ReferenceOTU13059
New.CleanUp.ReferenceOTU35199
New.CleanUp.ReferenceOTU32820
New.CleanUp.ReferenceOTU16036
New.CleanUp.ReferenceOTU57513
AJ457811.1.1891
EF172816.1.1600
New.CleanUp.ReferenceOTU32076
New.CleanUp.ReferenceOTU18839
New.CleanUp.ReferenceOTU23884
New.CleanUp.ReferenceOTU112482
New.CleanUp.ReferenceOTU27836
New.CleanUp.ReferenceOTU124723
New.CleanUp.ReferenceOTU15187
KC488460.1.1689
New.CleanUp.ReferenceOTU166881
New.CleanUp.ReferenceOTU168542
New.CleanUp.ReferenceOTU171106
New.CleanUp.ReferenceOTU171454
New.CleanUp.ReferenceOTU173766
New.CleanUp.ReferenceOTU185901
New.CleanUp.ReferenceOTU186388
New.CleanUp.ReferenceOTU187008
New.CleanUp.ReferenceOTU101181
New.CleanUp.ReferenceOTU194849
New.CleanUp.ReferenceOTU20296
New.CleanUp.ReferenceOTU20755
New.CleanUp.ReferenceOTU209289
New.CleanUp.ReferenceOTU21399
New.CleanUp.ReferenceOTU214622
New.CleanUp.ReferenceOTU222336
New.CleanUp.ReferenceOTU238177
New.CleanUp.ReferenceOTU273239
New.CleanUp.ReferenceOTU288479
New.CleanUp.ReferenceOTU29085
New.CleanUp.ReferenceOTU29326
New.CleanUp.ReferenceOTU313314
New.CleanUp.ReferenceOTU32185
New.CleanUp.ReferenceOTU32370
New.CleanUp.ReferenceOTU108986
New.CleanUp.ReferenceOTU33277
New.CleanUp.ReferenceOTU33785
New.CleanUp.ReferenceOTU36611
New.CleanUp.ReferenceOTU37728
New.CleanUp.ReferenceOTU38190
New.CleanUp.ReferenceOTU40048
New.CleanUp.ReferenceOTU41934
New.CleanUp.ReferenceOTU42406
New.CleanUp.ReferenceOTU42810
New.CleanUp.ReferenceOTU44928
New.CleanUp.ReferenceOTU45289
New.CleanUp.ReferenceOTU46778
New.CleanUp.ReferenceOTU47025
New.CleanUp.ReferenceOTU48397
New.CleanUp.ReferenceOTU48960
New.CleanUp.ReferenceOTU49037
New.CleanUp.ReferenceOTU49983
New.CleanUp.ReferenceOTU50814
New.CleanUp.ReferenceOTU51951
New.CleanUp.ReferenceOTU5214
New.CleanUp.ReferenceOTU52388
New.CleanUp.ReferenceOTU54013
New.CleanUp.ReferenceOTU54014
New.CleanUp.ReferenceOTU54059
New.CleanUp.ReferenceOTU56007
New.CleanUp.ReferenceOTU56846
New.CleanUp.ReferenceOTU60157

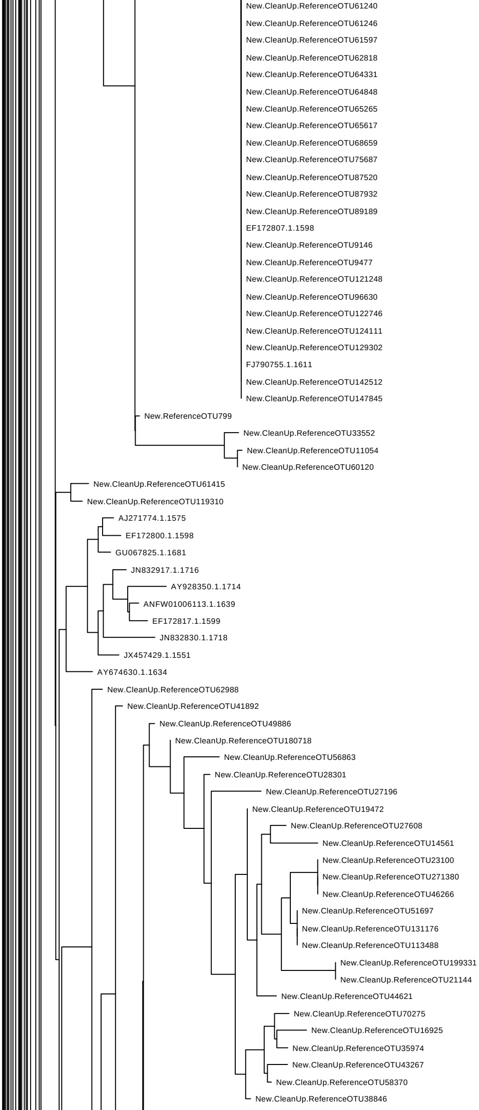
New.CleanUp.ReferenceOTU61240
New.CleanUp.ReferenceOTU61246
New.CleanUp.ReferenceOTU61597
New.CleanUp.ReferenceOTU62818
New.CleanUp.ReferenceOTU64331
New.CleanUp.ReferenceOTU64848
New.CleanUp.ReferenceOTU65265
New.CleanUp.ReferenceOTU65617
New.CleanUp.ReferenceOTU68659
New.CleanUp.ReferenceOTU75687
New.CleanUp.ReferenceOTU87520
New.CleanUp.ReferenceOTU87932
New.CleanUp.ReferenceOTU89189
EF172807.1.1598
New.CleanUp.ReferenceOTU9146
New.CleanUp.ReferenceOTU9477
New.CleanUp.ReferenceOTU121248
New.CleanUp.ReferenceOTU96630
New.CleanUp.ReferenceOTU122746
New.CleanUp.ReferenceOTU124111
New.CleanUp.ReferenceOTU129302
FJ790755.1.1611
New.CleanUp.ReferenceOTU142512
New.CleanUp.ReferenceOTU147845
New.ReferenceOTU799
New.CleanUp.ReferenceOTU33552
New.CleanUp.ReferenceOTU11054
New.CleanUp.ReferenceOTU60120
New.CleanUp.ReferenceOTU61415
New.CleanUp.ReferenceOTU119310
AJ271774.1.1575
EF172800.1.1598
GU067825.1.1681
JN832917.1.1716
AY928350.1.1714
ANFW01006113.1.1639
EF172817.1.1599
JN832830.1.1718
JX457429.1.1551
AY674630.1.1634
New.CleanUp.ReferenceOTU62988
New.CleanUp.ReferenceOTU41892
New.CleanUp.ReferenceOTU49886
New.CleanUp.ReferenceOTU180718
New.CleanUp.ReferenceOTU56863
New.CleanUp.ReferenceOTU28301
New.CleanUp.ReferenceOTU27196
New.CleanUp.ReferenceOTU19472
New.CleanUp.ReferenceOTU27608
New.CleanUp.ReferenceOTU14561
New.CleanUp.ReferenceOTU23100
New.CleanUp.ReferenceOTU271380
New.CleanUp.ReferenceOTU46266
New.CleanUp.ReferenceOTU51697
New.CleanUp.ReferenceOTU131176
New.CleanUp.ReferenceOTU113488
New.CleanUp.ReferenceOTU199331
New.CleanUp.ReferenceOTU21144
New.CleanUp.ReferenceOTU44621
New.CleanUp.ReferenceOTU70275
New.CleanUp.ReferenceOTU16925
New.CleanUp.ReferenceOTU35974
New.CleanUp.ReferenceOTU43267
New.CleanUp.ReferenceOTU58370
New.CleanUp.ReferenceOTU38846

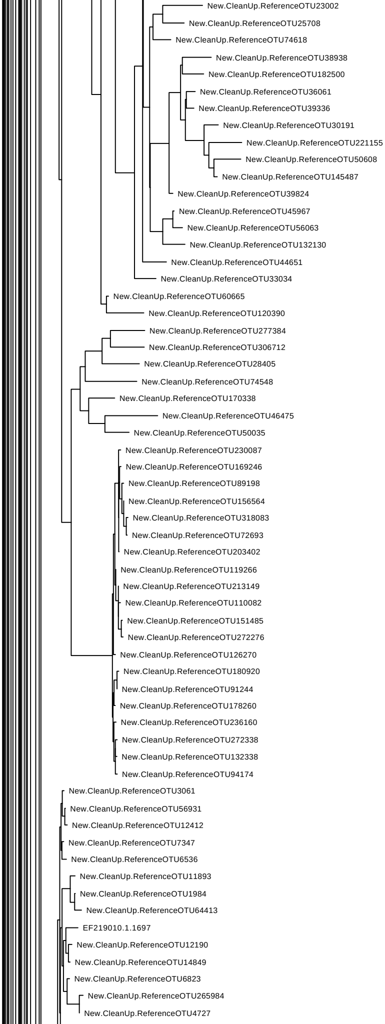
New.CleanUp.ReferenceOTU23002
New.CleanUp.ReferenceOTU25708
New.CleanUp.ReferenceOTU74618
New.CleanUp.ReferenceOTU38938
New.CleanUp.ReferenceOTU182500
New.CleanUp.ReferenceOTU36061
New.CleanUp.ReferenceOTU39336
New.CleanUp.ReferenceOTU30191
New.CleanUp.ReferenceOTU221155
New.CleanUp.ReferenceOTU50608
New.CleanUp.ReferenceOTU145487
New.CleanUp.ReferenceOTU39824
New.CleanUp.ReferenceOTU45967
New.CleanUp.ReferenceOTU56063
New.CleanUp.ReferenceOTU132130
New.CleanUp.ReferenceOTU44651
New.CleanUp.ReferenceOTU33034
New.CleanUp.ReferenceOTU60665
New.CleanUp.ReferenceOTU120390
New.CleanUp.ReferenceOTU277384
New.CleanUp.ReferenceOTU306712
New.CleanUp.ReferenceOTU28405
New.CleanUp.ReferenceOTU74548
New.CleanUp.ReferenceOTU170338
New.CleanUp.ReferenceOTU46475
New.CleanUp.ReferenceOTU50035
New.CleanUp.ReferenceOTU230087
New.CleanUp.ReferenceOTU169246
New.CleanUp.ReferenceOTU89198
New.CleanUp.ReferenceOTU156564
New.CleanUp.ReferenceOTU318083
New.CleanUp.ReferenceOTU72693
New.CleanUp.ReferenceOTU203402
New.CleanUp.ReferenceOTU119266
New.CleanUp.ReferenceOTU213149
New.CleanUp.ReferenceOTU110082
New.CleanUp.ReferenceOTU151485
New.CleanUp.ReferenceOTU272276
New.CleanUp.ReferenceOTU126270
New.CleanUp.ReferenceOTU180920
New.CleanUp.ReferenceOTU91244
New.CleanUp.ReferenceOTU178260
New.CleanUp.ReferenceOTU236160
New.CleanUp.ReferenceOTU272338
New.CleanUp.ReferenceOTU132338
New.CleanUp.ReferenceOTU94174
New.CleanUp.ReferenceOTU3061
New.CleanUp.ReferenceOTU56931
New.CleanUp.ReferenceOTU12412
New.CleanUp.ReferenceOTU7347
New.CleanUp.ReferenceOTU6536
New.CleanUp.ReferenceOTU11893
New.CleanUp.ReferenceOTU1984
New.CleanUp.ReferenceOTU64413
EF219010.1.1697
New.CleanUp.ReferenceOTU12190
New.CleanUp.ReferenceOTU14849
New.CleanUp.ReferenceOTU6823
New.CleanUp.ReferenceOTU265984
New.CleanUp.ReferenceOTU4727

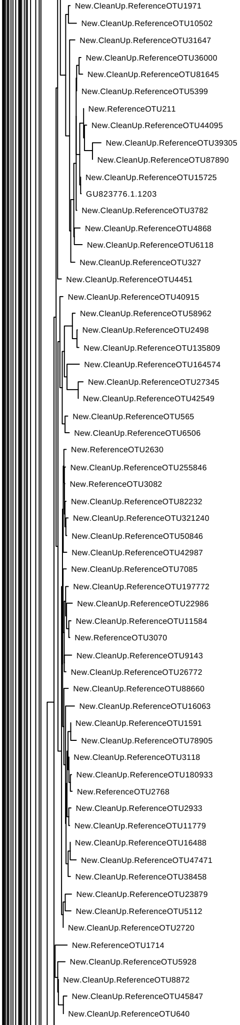
New.CleanUp.ReferenceOTU1971
New.CleanUp.ReferenceOTU10502
New.CleanUp.ReferenceOTU31647
New.CleanUp.ReferenceOTU36000
New.CleanUp.ReferenceOTU81645
New.CleanUp.ReferenceOTU5399
New.ReferenceOTU211
New.CleanUp.ReferenceOTU44095
New.CleanUp.ReferenceOTU39305
New.CleanUp.ReferenceOTU87890
New.CleanUp.ReferenceOTU15725
GU823776.1.1203
New.CleanUp.ReferenceOTU3782
New.CleanUp.ReferenceOTU4868
New.CleanUp.ReferenceOTU6118
New.CleanUp.ReferenceOTU327
New.CleanUp.ReferenceOTU4451
New.CleanUp.ReferenceOTU40915
New.CleanUp.ReferenceOTU58962
New.CleanUp.ReferenceOTU2498
New.CleanUp.ReferenceOTU135809
New.CleanUp.ReferenceOTU164574
New.CleanUp.ReferenceOTU27345
New.CleanUp.ReferenceOTU42549
New.CleanUp.ReferenceOTU565
New.CleanUp.ReferenceOTU6506
New.ReferenceOTU2630
New.CleanUp.ReferenceOTU255846
New.ReferenceOTU3082
New.CleanUp.ReferenceOTU82232
New.CleanUp.ReferenceOTU321240
New.CleanUp.ReferenceOTU50846
New.CleanUp.ReferenceOTU42987
New.CleanUp.ReferenceOTU7085
New.CleanUp.ReferenceOTU197772
New.CleanUp.ReferenceOTU22986
New.CleanUp.ReferenceOTU11584
New.ReferenceOTU3070
New.CleanUp.ReferenceOTU9143
New.CleanUp.ReferenceOTU26772
New.CleanUp.ReferenceOTU88660
New.CleanUp.ReferenceOTU16063
New.CleanUp.ReferenceOTU1591
New.CleanUp.ReferenceOTU78905
New.CleanUp.ReferenceOTU3118
New.CleanUp.ReferenceOTU180933
New.ReferenceOTU2768
New.CleanUp.ReferenceOTU2933
New.CleanUp.ReferenceOTU11779
New.CleanUp.ReferenceOTU16488
New.CleanUp.ReferenceOTU47471
New.CleanUp.ReferenceOTU38458
New.CleanUp.ReferenceOTU23879
New.CleanUp.ReferenceOTU5112
New.CleanUp.ReferenceOTU2720
New.ReferenceOTU1714
New.CleanUp.ReferenceOTU5928
New.CleanUp.ReferenceOTU8872
New.CleanUp.ReferenceOTU45847
New.CleanUp.ReferenceOTU640

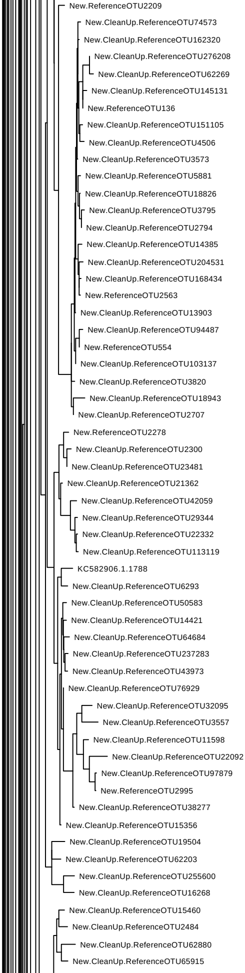
New.ReferenceOTU2209
New.CleanUp.ReferenceOTU74573
New.CleanUp.ReferenceOTU162320
New.CleanUp.ReferenceOTU276208
New.CleanUp.ReferenceOTU62269
New.CleanUp.ReferenceOTU145131
New.ReferenceOTU136
New.CleanUp.ReferenceOTU151105
New.CleanUp.ReferenceOTU4506
New.CleanUp.ReferenceOTU3573
New.CleanUp.ReferenceOTU5881
New.CleanUp.ReferenceOTU18826
New.CleanUp.ReferenceOTU3795
New.CleanUp.ReferenceOTU2794
New.CleanUp.ReferenceOTU14385
New.CleanUp.ReferenceOTU204531
New.CleanUp.ReferenceOTU168434
New.ReferenceOTU2563
New.CleanUp.ReferenceOTU13903
New.CleanUp.ReferenceOTU94487
New.ReferenceOTU554
New.CleanUp.ReferenceOTU103137
New.CleanUp.ReferenceOTU3820
New.CleanUp.ReferenceOTU18943
New.CleanUp.ReferenceOTU2707
New.ReferenceOTU2278
New.CleanUp.ReferenceOTU2300
New.CleanUp.ReferenceOTU23481
New.CleanUp.ReferenceOTU21362
New.CleanUp.ReferenceOTU42059
New.CleanUp.ReferenceOTU29344
New.CleanUp.ReferenceOTU22332
New.CleanUp.ReferenceOTU113119
KC582906.1.1788
New.CleanUp.ReferenceOTU6293
New.CleanUp.ReferenceOTU50583
New.CleanUp.ReferenceOTU14421
New.CleanUp.ReferenceOTU64684
New.CleanUp.ReferenceOTU237283
New.CleanUp.ReferenceOTU43973
New.CleanUp.ReferenceOTU76929
New.CleanUp.ReferenceOTU32095
New.CleanUp.ReferenceOTU3557
New.CleanUp.ReferenceOTU11598
New.CleanUp.ReferenceOTU22092
New.CleanUp.ReferenceOTU97879
New.ReferenceOTU2995
New.CleanUp.ReferenceOTU38277
New.CleanUp.ReferenceOTU15356
New.CleanUp.ReferenceOTU19504
New.CleanUp.ReferenceOTU62203
New.CleanUp.ReferenceOTU255600
New.CleanUp.ReferenceOTU16268
New.CleanUp.ReferenceOTU15460
New.CleanUp.ReferenceOTU2484
New.CleanUp.ReferenceOTU62880
New.CleanUp.ReferenceOTU65915

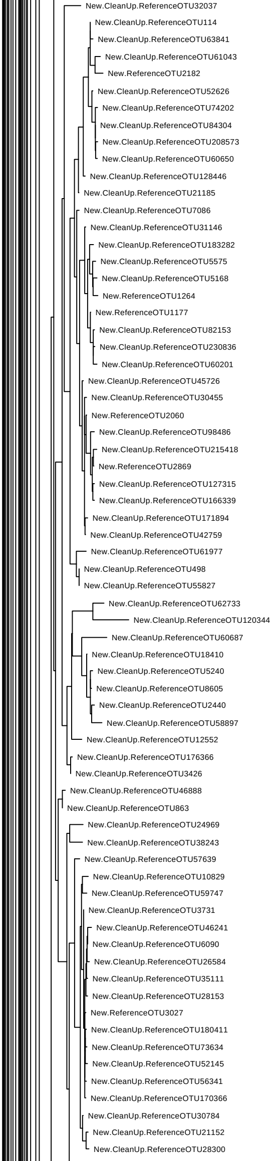
New.CleanUp.ReferenceOTU32037
New.CleanUp.ReferenceOTU114
New.CleanUp.ReferenceOTU63841
New.CleanUp.ReferenceOTU61043
New.ReferenceOTU2182
New.CleanUp.ReferenceOTU52626
New.CleanUp.ReferenceOTU74202
New.CleanUp.ReferenceOTU84304
New.CleanUp.ReferenceOTU208573
New.CleanUp.ReferenceOTU60650
New.CleanUp.ReferenceOTU128446
New.CleanUp.ReferenceOTU21185
New.CleanUp.ReferenceOTU7086
New.CleanUp.ReferenceOTU31146
New.CleanUp.ReferenceOTU183282
New.CleanUp.ReferenceOTU5575
New.CleanUp.ReferenceOTU5168
New.ReferenceOTU1264
New.ReferenceOTU1177
New.CleanUp.ReferenceOTU82153
New.CleanUp.ReferenceOTU230836
New.CleanUp.ReferenceOTU60201
New.CleanUp.ReferenceOTU45726
New.CleanUp.ReferenceOTU30455
New.ReferenceOTU2060
New.CleanUp.ReferenceOTU98486
New.CleanUp.ReferenceOTU215418
New.ReferenceOTU2869
New.CleanUp.ReferenceOTU127315
New.CleanUp.ReferenceOTU166339
New.CleanUp.ReferenceOTU171894
New.CleanUp.ReferenceOTU42759
New.CleanUp.ReferenceOTU61977
New.CleanUp.ReferenceOTU498
New.CleanUp.ReferenceOTU55827
New.CleanUp.ReferenceOTU62733
New.CleanUp.ReferenceOTU120344
New.CleanUp.ReferenceOTU60687
New.CleanUp.ReferenceOTU18410
New.CleanUp.ReferenceOTU5240
New.CleanUp.ReferenceOTU8605
New.CleanUp.ReferenceOTU2440
New.CleanUp.ReferenceOTU58897
New.CleanUp.ReferenceOTU12552
New.CleanUp.ReferenceOTU176366
New.CleanUp.ReferenceOTU3426
New.CleanUp.ReferenceOTU46888
New.CleanUp.ReferenceOTU863
New.CleanUp.ReferenceOTU24969
New.CleanUp.ReferenceOTU38243
New.CleanUp.ReferenceOTU57639
New.CleanUp.ReferenceOTU10829
New.CleanUp.ReferenceOTU59747
New.CleanUp.ReferenceOTU3731
New.CleanUp.ReferenceOTU46241
New.CleanUp.ReferenceOTU6090
New.CleanUp.ReferenceOTU26584
New.CleanUp.ReferenceOTU35111
New.CleanUp.ReferenceOTU28153
New.ReferenceOTU3027
New.CleanUp.ReferenceOTU180411
New.CleanUp.ReferenceOTU73634
New.CleanUp.ReferenceOTU52145
New.CleanUp.ReferenceOTU56341
New.CleanUp.ReferenceOTU170366
New.CleanUp.ReferenceOTU30784
New.CleanUp.ReferenceOTU21152
New.CleanUp.ReferenceOTU28300

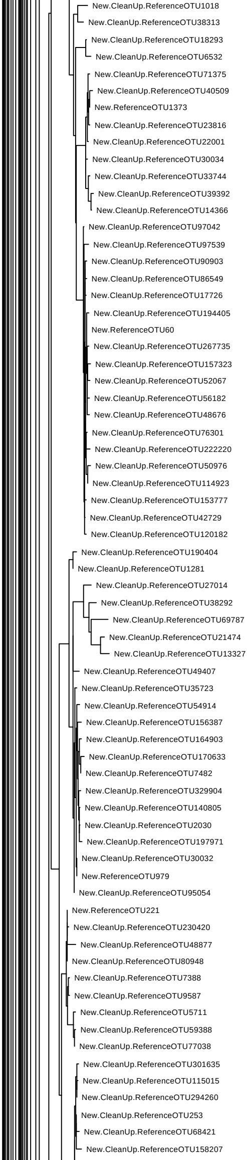
New.CleanUp.ReferenceOTU1018
New.CleanUp.ReferenceOTU38313
New.CleanUp.ReferenceOTU18293
New.CleanUp.ReferenceOTU6532
New.CleanUp.ReferenceOTU71375
New.CleanUp.ReferenceOTU40509
New.ReferenceOTU1373
New.CleanUp.ReferenceOTU23816
New.CleanUp.ReferenceOTU22001
New.CleanUp.ReferenceOTU30034
New.CleanUp.ReferenceOTU33744
New.CleanUp.ReferenceOTU39392
New.CleanUp.ReferenceOTU14366
New.CleanUp.ReferenceOTU97042
New.CleanUp.ReferenceOTU97539
New.CleanUp.ReferenceOTU90903
New.CleanUp.ReferenceOTU86549
New.CleanUp.ReferenceOTU17726
New.CleanUp.ReferenceOTU194405
New.ReferenceOTU60
New.CleanUp.ReferenceOTU267735
New.CleanUp.ReferenceOTU157323
New.CleanUp.ReferenceOTU52067
New.CleanUp.ReferenceOTU56182
New.CleanUp.ReferenceOTU48676
New.CleanUp.ReferenceOTU76301
New.CleanUp.ReferenceOTU222220
New.CleanUp.ReferenceOTU50976
New.CleanUp.ReferenceOTU114923
New.CleanUp.ReferenceOTU153777
New.CleanUp.ReferenceOTU42729
New.CleanUp.ReferenceOTU120182
New.CleanUp.ReferenceOTU190404
New.CleanUp.ReferenceOTU1281
New.CleanUp.ReferenceOTU27014
New.CleanUp.ReferenceOTU38292
New.CleanUp.ReferenceOTU69787
New.CleanUp.ReferenceOTU21474
New.CleanUp.ReferenceOTU13327
New.CleanUp.ReferenceOTU49407
New.CleanUp.ReferenceOTU35723
New.CleanUp.ReferenceOTU54914
New.CleanUp.ReferenceOTU156387
New.CleanUp.ReferenceOTU164903
New.CleanUp.ReferenceOTU170633
New.CleanUp.ReferenceOTU7482
New.CleanUp.ReferenceOTU329904
New.CleanUp.ReferenceOTU140805
New.CleanUp.ReferenceOTU2030
New.CleanUp.ReferenceOTU197971
New.CleanUp.ReferenceOTU30032
New.ReferenceOTU979
New.CleanUp.ReferenceOTU95054
New.ReferenceOTU221
New.CleanUp.ReferenceOTU230420
New.CleanUp.ReferenceOTU48877
New.CleanUp.ReferenceOTU80948
New.CleanUp.ReferenceOTU7388
New.CleanUp.ReferenceOTU9587
New.CleanUp.ReferenceOTU5711
New.CleanUp.ReferenceOTU59388
New.CleanUp.ReferenceOTU77038
New.CleanUp.ReferenceOTU301635
New.CleanUp.ReferenceOTU115015
New.CleanUp.ReferenceOTU294260
New.CleanUp.ReferenceOTU253
New.CleanUp.ReferenceOTU68421
New.CleanUp.ReferenceOTU158207

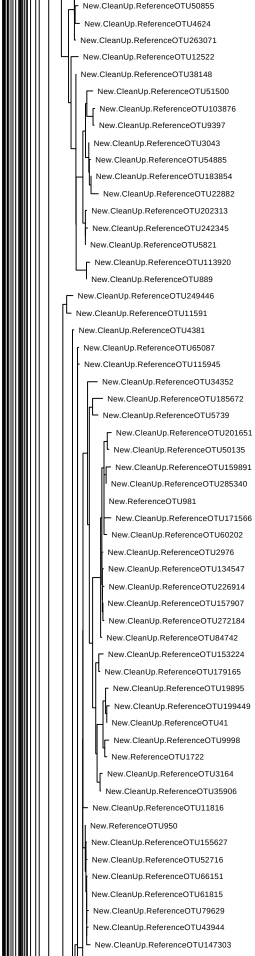
New.CleanUp.ReferenceOTU50855
New.CleanUp.ReferenceOTU4624
New.CleanUp.ReferenceOTU263071
New.CleanUp.ReferenceOTU12522
New.CleanUp.ReferenceOTU38148
New.CleanUp.ReferenceOTU51500
New.CleanUp.ReferenceOTU103876
New.CleanUp.ReferenceOTU9397
New.CleanUp.ReferenceOTU3043
New.CleanUp.ReferenceOTU54885
New.CleanUp.ReferenceOTU183854
New.CleanUp.ReferenceOTU22882
New.CleanUp.ReferenceOTU202313
New.CleanUp.ReferenceOTU242345
New.CleanUp.ReferenceOTU5821
New.CleanUp.ReferenceOTU113920
New.CleanUp.ReferenceOTU889
New.CleanUp.ReferenceOTU249446
New.CleanUp.ReferenceOTU11591
New.CleanUp.ReferenceOTU4381
New.CleanUp.ReferenceOTU65087
New.CleanUp.ReferenceOTU115945
New.CleanUp.ReferenceOTU34352
New.CleanUp.ReferenceOTU185672
New.CleanUp.ReferenceOTU5739
New.CleanUp.ReferenceOTU201651
New.CleanUp.ReferenceOTU50135
New.CleanUp.ReferenceOTU159891
New.CleanUp.ReferenceOTU285340
New.ReferenceOTU981
New.CleanUp.ReferenceOTU171566
New.CleanUp.ReferenceOTU60202
New.CleanUp.ReferenceOTU2976
New.CleanUp.ReferenceOTU134547
New.CleanUp.ReferenceOTU226914
New.CleanUp.ReferenceOTU157907
New.CleanUp.ReferenceOTU272184
New.CleanUp.ReferenceOTU84742
New.CleanUp.ReferenceOTU153224
New.CleanUp.ReferenceOTU179165
New.CleanUp.ReferenceOTU19895
New.CleanUp.ReferenceOTU199449
New.CleanUp.ReferenceOTU41
New.CleanUp.ReferenceOTU9998
New.ReferenceOTU1722
New.CleanUp.ReferenceOTU3164
New.CleanUp.ReferenceOTU35906
New.CleanUp.ReferenceOTU11816
New.ReferenceOTU950
New.CleanUp.ReferenceOTU155627
New.CleanUp.ReferenceOTU52716
New.CleanUp.ReferenceOTU66151
New.CleanUp.ReferenceOTU61815
New.CleanUp.ReferenceOTU79629
New.CleanUp.ReferenceOTU43944
New.CleanUp.ReferenceOTU147303

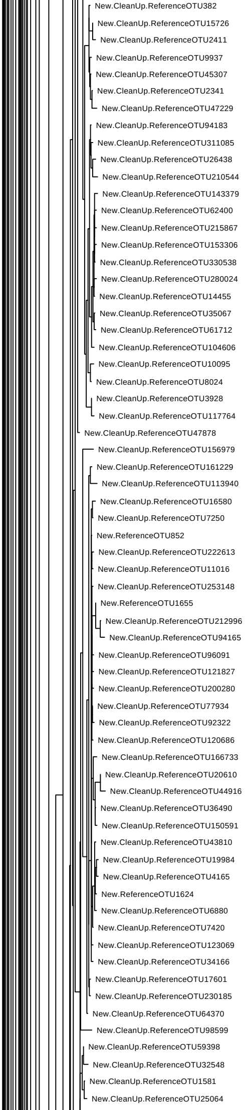
New.CleanUp.ReferenceOTU382
New.CleanUp.ReferenceOTU15726
New.CleanUp.ReferenceOTU2411
New.CleanUp.ReferenceOTU9937
New.CleanUp.ReferenceOTU45307
New.CleanUp.ReferenceOTU2341
New.CleanUp.ReferenceOTU47229
New.CleanUp.ReferenceOTU94183
New.CleanUp.ReferenceOTU311085
New.CleanUp.ReferenceOTU26438
New.CleanUp.ReferenceOTU210544
New.CleanUp.ReferenceOTU143379
New.CleanUp.ReferenceOTU62400
New.CleanUp.ReferenceOTU215867
New.CleanUp.ReferenceOTU153306
New.CleanUp.ReferenceOTU330538
New.CleanUp.ReferenceOTU280024
New.CleanUp.ReferenceOTU14455
New.CleanUp.ReferenceOTU35067
New.CleanUp.ReferenceOTU61712
New.CleanUp.ReferenceOTU104606
New.CleanUp.ReferenceOTU10095
New.CleanUp.ReferenceOTU8024
New.CleanUp.ReferenceOTU3928
New.CleanUp.ReferenceOTU117764
New.CleanUp.ReferenceOTU47878
New.CleanUp.ReferenceOTU156979
New.CleanUp.ReferenceOTU161229
New.CleanUp.ReferenceOTU113940
New.CleanUp.ReferenceOTU16580
New.CleanUp.ReferenceOTU7250
New.ReferenceOTU852
New.CleanUp.ReferenceOTU222613
New.CleanUp.ReferenceOTU11016
New.CleanUp.ReferenceOTU253148
New.ReferenceOTU1655
New.CleanUp.ReferenceOTU212996
New.CleanUp.ReferenceOTU94165
New.CleanUp.ReferenceOTU96091
New.CleanUp.ReferenceOTU121827
New.CleanUp.ReferenceOTU200280
New.CleanUp.ReferenceOTU77934
New.CleanUp.ReferenceOTU92322
New.CleanUp.ReferenceOTU120686
New.CleanUp.ReferenceOTU166733
New.CleanUp.ReferenceOTU20610
New.CleanUp.ReferenceOTU44916
New.CleanUp.ReferenceOTU36490
New.CleanUp.ReferenceOTU150591
New.CleanUp.ReferenceOTU43810
New.CleanUp.ReferenceOTU19984
New.CleanUp.ReferenceOTU4165
New.ReferenceOTU1624
New.CleanUp.ReferenceOTU6880
New.CleanUp.ReferenceOTU7420
New.CleanUp.ReferenceOTU123069
New.CleanUp.ReferenceOTU34166
New.CleanUp.ReferenceOTU17601
New.CleanUp.ReferenceOTU230185
New.CleanUp.ReferenceOTU64370
New.CleanUp.ReferenceOTU98599
New.CleanUp.ReferenceOTU59398
New.CleanUp.ReferenceOTU32548
New.CleanUp.ReferenceOTU1581
New.CleanUp.ReferenceOTU25064

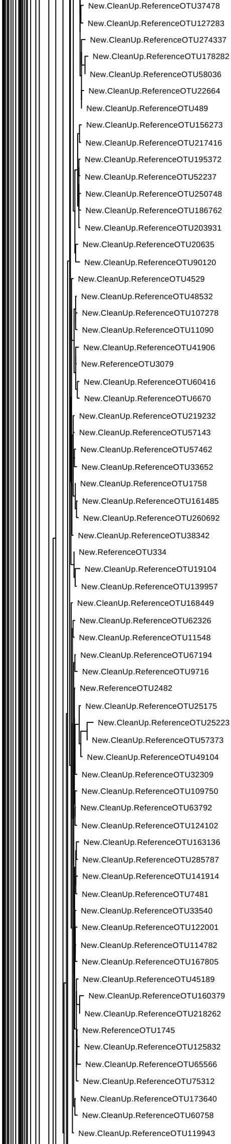
New.CleanUp.ReferenceOTU37478
New.CleanUp.ReferenceOTU127283
New.CleanUp.ReferenceOTU274337
New.CleanUp.ReferenceOTU178282
New.CleanUp.ReferenceOTU58036
New.CleanUp.ReferenceOTU22664
New.CleanUp.ReferenceOTU489
New.CleanUp.ReferenceOTU156273
New.CleanUp.ReferenceOTU217416
New.CleanUp.ReferenceOTU195372
New.CleanUp.ReferenceOTU52237
New.CleanUp.ReferenceOTU250748
New.CleanUp.ReferenceOTU186762
New.CleanUp.ReferenceOTU203931
New.CleanUp.ReferenceOTU20635
New.CleanUp.ReferenceOTU90120
New.CleanUp.ReferenceOTU4529
New.CleanUp.ReferenceOTU48532
New.CleanUp.ReferenceOTU107278
New.CleanUp.ReferenceOTU11090
New.CleanUp.ReferenceOTU41906
New.ReferenceOTU3079
New.CleanUp.ReferenceOTU60416
New.CleanUp.ReferenceOTU6670
New.CleanUp.ReferenceOTU219232
New.CleanUp.ReferenceOTU57143
New.CleanUp.ReferenceOTU57462
New.CleanUp.ReferenceOTU33652
New.CleanUp.ReferenceOTU1758
New.CleanUp.ReferenceOTU161485
New.CleanUp.ReferenceOTU260692
New.CleanUp.ReferenceOTU38342
New.ReferenceOTU334
New.CleanUp.ReferenceOTU19104
New.CleanUp.ReferenceOTU139957
New.CleanUp.ReferenceOTU168449
New.CleanUp.ReferenceOTU62326
New.CleanUp.ReferenceOTU11548
New.CleanUp.ReferenceOTU67194
New.CleanUp.ReferenceOTU9716
New.ReferenceOTU2482
New.CleanUp.ReferenceOTU25175
New.CleanUp.ReferenceOTU25223
New.CleanUp.ReferenceOTU57373
New.CleanUp.ReferenceOTU49104
New.CleanUp.ReferenceOTU32309
New.CleanUp.ReferenceOTU109750
New.CleanUp.ReferenceOTU63792
New.CleanUp.ReferenceOTU124102
New.CleanUp.ReferenceOTU163136
New.CleanUp.ReferenceOTU285787
New.CleanUp.ReferenceOTU141914
New.CleanUp.ReferenceOTU7481
New.CleanUp.ReferenceOTU33540
New.CleanUp.ReferenceOTU122001
New.CleanUp.ReferenceOTU114782
New.CleanUp.ReferenceOTU167805
New.CleanUp.ReferenceOTU45189
New.CleanUp.ReferenceOTU160379
New.CleanUp.ReferenceOTU218262
New.ReferenceOTU1745
New.CleanUp.ReferenceOTU125832
New.CleanUp.ReferenceOTU65566
New.CleanUp.ReferenceOTU75312
New.CleanUp.ReferenceOTU173640
New.CleanUp.ReferenceOTU60758
New.CleanUp.ReferenceOTU119943

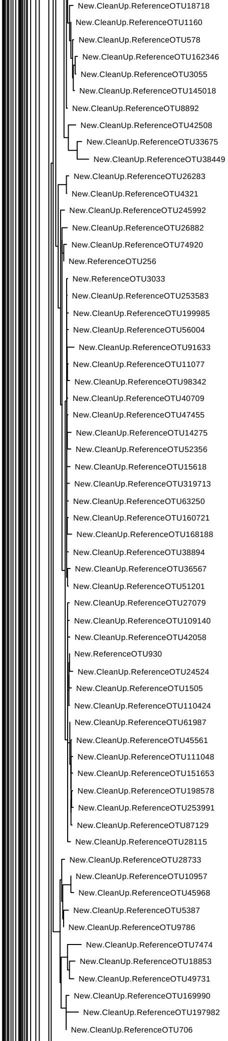
New.CleanUp.ReferenceOTU18718
New.CleanUp.ReferenceOTU1160
New.CleanUp.ReferenceOTU578
New.CleanUp.ReferenceOTU162346
New.CleanUp.ReferenceOTU3055
New.CleanUp.ReferenceOTU145018
New.CleanUp.ReferenceOTU8892
New.CleanUp.ReferenceOTU42508
New.CleanUp.ReferenceOTU33675
New.CleanUp.ReferenceOTU38449
New.CleanUp.ReferenceOTU26283
New.CleanUp.ReferenceOTU4321
New.CleanUp.ReferenceOTU245992
New.CleanUp.ReferenceOTU26882
New.CleanUp.ReferenceOTU74920
New.ReferenceOTU256
New.ReferenceOTU3033
New.CleanUp.ReferenceOTU253583
New.CleanUp.ReferenceOTU199985
New.CleanUp.ReferenceOTU56004
New.CleanUp.ReferenceOTU91633
New.CleanUp.ReferenceOTU11077
New.CleanUp.ReferenceOTU98342
New.CleanUp.ReferenceOTU40709
New.CleanUp.ReferenceOTU47455
New.CleanUp.ReferenceOTU14275
New.CleanUp.ReferenceOTU52356
New.CleanUp.ReferenceOTU15618
New.CleanUp.ReferenceOTU319713
New.CleanUp.ReferenceOTU63250
New.CleanUp.ReferenceOTU160721
New.CleanUp.ReferenceOTU168188
New.CleanUp.ReferenceOTU38894
New.CleanUp.ReferenceOTU36567
New.CleanUp.ReferenceOTU51201
New.CleanUp.ReferenceOTU27079
New.CleanUp.ReferenceOTU109140
New.CleanUp.ReferenceOTU42058
New.ReferenceOTU930
New.CleanUp.ReferenceOTU24524
New.CleanUp.ReferenceOTU1505
New.CleanUp.ReferenceOTU110424
New.CleanUp.ReferenceOTU61987
New.CleanUp.ReferenceOTU45561
New.CleanUp.ReferenceOTU111048
New.CleanUp.ReferenceOTU151653
New.CleanUp.ReferenceOTU198578
New.CleanUp.ReferenceOTU253991
New.CleanUp.ReferenceOTU87129
New.CleanUp.ReferenceOTU28115
New.CleanUp.ReferenceOTU28733
New.CleanUp.ReferenceOTU10957
New.CleanUp.ReferenceOTU45968
New.CleanUp.ReferenceOTU5387
New.CleanUp.ReferenceOTU9786
New.CleanUp.ReferenceOTU7474
New.CleanUp.ReferenceOTU18853
New.CleanUp.ReferenceOTU49731
New.CleanUp.ReferenceOTU169990
New.CleanUp.ReferenceOTU197982
New.CleanUp.ReferenceOTU706

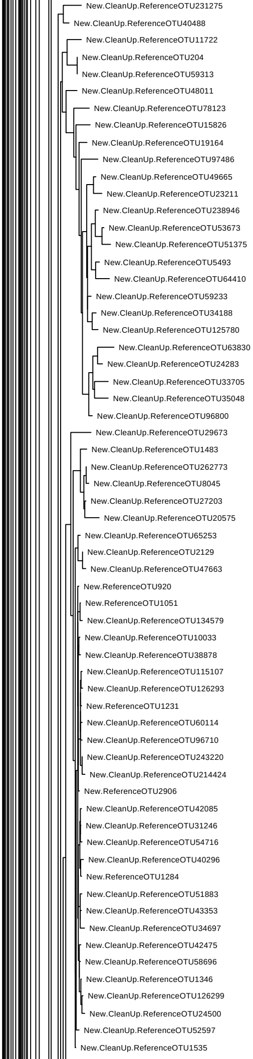
New.CleanUp.ReferenceOTU231275
New.CleanUp.ReferenceOTU40488
New.CleanUp.ReferenceOTU11722
New.CleanUp.ReferenceOTU204
New.CleanUp.ReferenceOTU59313
New.CleanUp.ReferenceOTU48011
New.CleanUp.ReferenceOTU78123
New.CleanUp.ReferenceOTU15826
New.CleanUp.ReferenceOTU19164
New.CleanUp.ReferenceOTU97486
New.CleanUp.ReferenceOTU49665
New.CleanUp.ReferenceOTU23211
New.CleanUp.ReferenceOTU238946
New.CleanUp.ReferenceOTU53673
New.CleanUp.ReferenceOTU51375
New.CleanUp.ReferenceOTU5493
New.CleanUp.ReferenceOTU64410
New.CleanUp.ReferenceOTU59233
New.CleanUp.ReferenceOTU34188
New.CleanUp.ReferenceOTU125780
New.CleanUp.ReferenceOTU63830
New.CleanUp.ReferenceOTU24283
New.CleanUp.ReferenceOTU33705
New.CleanUp.ReferenceOTU35048
New.CleanUp.ReferenceOTU96800
New.CleanUp.ReferenceOTU29673
New.CleanUp.ReferenceOTU1483
New.CleanUp.ReferenceOTU262773
New.CleanUp.ReferenceOTU8045
New.CleanUp.ReferenceOTU27203
New.CleanUp.ReferenceOTU20575
New.CleanUp.ReferenceOTU65253
New.CleanUp.ReferenceOTU2129
New.CleanUp.ReferenceOTU47663
New.ReferenceOTU920
New.ReferenceOTU1051
New.CleanUp.ReferenceOTU134579
New.CleanUp.ReferenceOTU10033
New.CleanUp.ReferenceOTU38878
New.CleanUp.ReferenceOTU115107
New.CleanUp.ReferenceOTU126293
New.ReferenceOTU1231
New.CleanUp.ReferenceOTU60114
New.CleanUp.ReferenceOTU96710
New.CleanUp.ReferenceOTU243220
New.CleanUp.ReferenceOTU214424
New.ReferenceOTU2906
New.CleanUp.ReferenceOTU42085
New.CleanUp.ReferenceOTU31246
New.CleanUp.ReferenceOTU54716
New.CleanUp.ReferenceOTU40296
New.ReferenceOTU1284
New.CleanUp.ReferenceOTU51883
New.CleanUp.ReferenceOTU43353
New.CleanUp.ReferenceOTU34697
New.CleanUp.ReferenceOTU42475
New.CleanUp.ReferenceOTU58696
New.CleanUp.ReferenceOTU1346
New.CleanUp.ReferenceOTU126299
New.CleanUp.ReferenceOTU24500
New.CleanUp.ReferenceOTU52597
New.CleanUp.ReferenceOTU1535

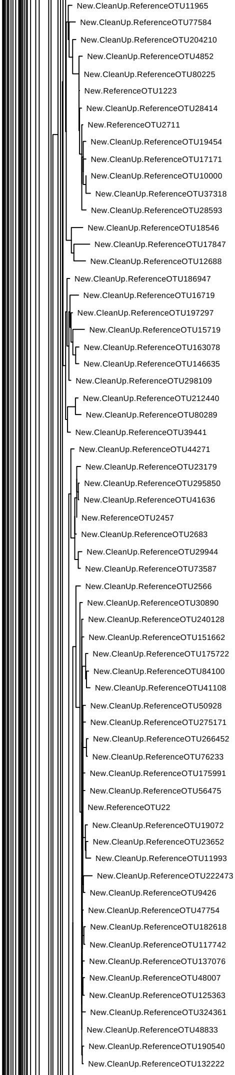

New.CleanUp.ReferenceOTU11965
New.CleanUp.ReferenceOTU77584
New.CleanUp.ReferenceOTU204210
New.CleanUp.ReferenceOTU4852
New.CleanUp.ReferenceOTU80225
New.ReferenceOTU1223
New.CleanUp.ReferenceOTU28414
New.ReferenceOTU2711
New.CleanUp.ReferenceOTU19454
New.CleanUp.ReferenceOTU17171
New.CleanUp.ReferenceOTU10000
New.CleanUp.ReferenceOTU37318
New.CleanUp.ReferenceOTU28593
New.CleanUp.ReferenceOTU18546
New.CleanUp.ReferenceOTU17847
New.CleanUp.ReferenceOTU12688
New.CleanUp.ReferenceOTU186947
New.CleanUp.ReferenceOTU16719
New.CleanUp.ReferenceOTU197297
New.CleanUp.ReferenceOTU15719
New.CleanUp.ReferenceOTU163078
New.CleanUp.ReferenceOTU146635
New.CleanUp.ReferenceOTU298109
New.CleanUp.ReferenceOTU212440
New.CleanUp.ReferenceOTU80289
New.CleanUp.ReferenceOTU39441
New.CleanUp.ReferenceOTU44271
New.CleanUp.ReferenceOTU23179
New.CleanUp.ReferenceOTU295850
New.CleanUp.ReferenceOTU41636
New.ReferenceOTU2457
New.CleanUp.ReferenceOTU2683
New.CleanUp.ReferenceOTU29944
New.CleanUp.ReferenceOTU73587
New.CleanUp.ReferenceOTU2566
New.CleanUp.ReferenceOTU30890
New.CleanUp.ReferenceOTU240128
New.CleanUp.ReferenceOTU151662
New.CleanUp.ReferenceOTU175722
New.CleanUp.ReferenceOTU84100
New.CleanUp.ReferenceOTU41108
New.CleanUp.ReferenceOTU50928
New.CleanUp.ReferenceOTU275171
New.CleanUp.ReferenceOTU266452
New.CleanUp.ReferenceOTU76233
New.CleanUp.ReferenceOTU175991
New.CleanUp.ReferenceOTU56475
New.ReferenceOTU22
New.CleanUp.ReferenceOTU19072
New.CleanUp.ReferenceOTU23652
New.CleanUp.ReferenceOTU11993
New.CleanUp.ReferenceOTU222473
New.CleanUp.ReferenceOTU9426
New.CleanUp.ReferenceOTU47754
New.CleanUp.ReferenceOTU182618
New.CleanUp.ReferenceOTU117742
New.CleanUp.ReferenceOTU137076
New.CleanUp.ReferenceOTU48007
New.CleanUp.ReferenceOTU125363
New.CleanUp.ReferenceOTU324361
New.CleanUp.ReferenceOTU48833
New.CleanUp.ReferenceOTU190540
New.CleanUp.ReferenceOTU132222

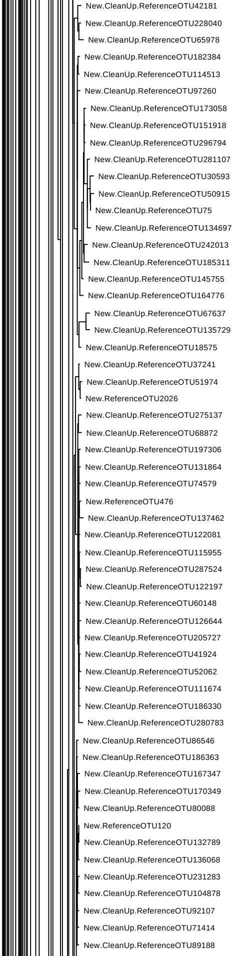
New.CleanUp.ReferenceOTU42181
New.CleanUp.ReferenceOTU228040
New.CleanUp.ReferenceOTU65978
New.CleanUp.ReferenceOTU182384
New.CleanUp.ReferenceOTU114513
New.CleanUp.ReferenceOTU97260
New.CleanUp.ReferenceOTU173058
New.CleanUp.ReferenceOTU151918
New.CleanUp.ReferenceOTU296794
New.CleanUp.ReferenceOTU281107
New.CleanUp.ReferenceOTU30593
New.CleanUp.ReferenceOTU50915
New.CleanUp.ReferenceOTU75
New.CleanUp.ReferenceOTU134697
New.CleanUp.ReferenceOTU242013
New.CleanUp.ReferenceOTU185311
New.CleanUp.ReferenceOTU145755
New.CleanUp.ReferenceOTU164776
New.CleanUp.ReferenceOTU67637
New.CleanUp.ReferenceOTU135729
New.CleanUp.ReferenceOTU18575
New.CleanUp.ReferenceOTU37241
New.CleanUp.ReferenceOTU51974
New.ReferenceOTU2026
New.CleanUp.ReferenceOTU275137
New.CleanUp.ReferenceOTU68872
New.CleanUp.ReferenceOTU197306
New.CleanUp.ReferenceOTU131864
New.CleanUp.ReferenceOTU74579
New.ReferenceOTU476
New.CleanUp.ReferenceOTU137462
New.CleanUp.ReferenceOTU122081
New.CleanUp.ReferenceOTU115955
New.CleanUp.ReferenceOTU287524
New.CleanUp.ReferenceOTU122197
New.CleanUp.ReferenceOTU60148
New.CleanUp.ReferenceOTU126644
New.CleanUp.ReferenceOTU205727
New.CleanUp.ReferenceOTU41924
New.CleanUp.ReferenceOTU52062
New.CleanUp.ReferenceOTU111674
New.CleanUp.ReferenceOTU186330
New.CleanUp.ReferenceOTU280783
New.CleanUp.ReferenceOTU86546
New.CleanUp.ReferenceOTU186363
New.CleanUp.ReferenceOTU167347
New.CleanUp.ReferenceOTU170349
New.CleanUp.ReferenceOTU80088
New.ReferenceOTU120
New.CleanUp.ReferenceOTU132789
New.CleanUp.ReferenceOTU136068
New.CleanUp.ReferenceOTU231283
New.CleanUp.ReferenceOTU104878
New.CleanUp.ReferenceOTU92107
New.CleanUp.ReferenceOTU71414
New.CleanUp.ReferenceOTU89188

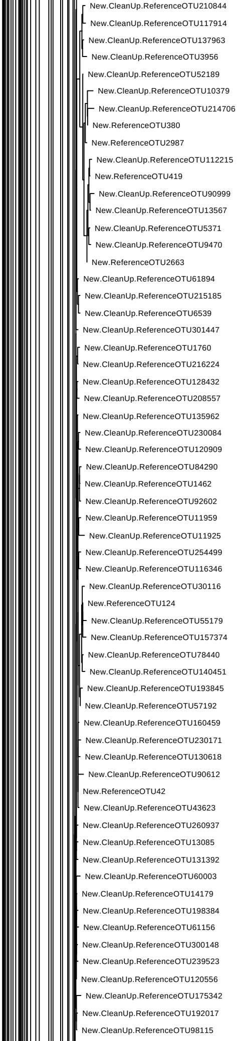
New.CleanUp.ReferenceOTU210844
New.CleanUp.ReferenceOTU117914
New.CleanUp.ReferenceOTU137963
New.CleanUp.ReferenceOTU3956
New.CleanUp.ReferenceOTU52189
New.CleanUp.ReferenceOTU10379
New.CleanUp.ReferenceOTU214706
New.ReferenceOTU380
New.ReferenceOTU2987
New.CleanUp.ReferenceOTU112215
New.ReferenceOTU419
New.CleanUp.ReferenceOTU90999
New.CleanUp.ReferenceOTU13567
New.CleanUp.ReferenceOTU5371
New.CleanUp.ReferenceOTU9470
New.ReferenceOTU2663
New.CleanUp.ReferenceOTU61894
New.CleanUp.ReferenceOTU215185
New.CleanUp.ReferenceOTU6539
New.CleanUp.ReferenceOTU301447
New.CleanUp.ReferenceOTU1760
New.CleanUp.ReferenceOTU216224
New.CleanUp.ReferenceOTU128432
New.CleanUp.ReferenceOTU208557
New.CleanUp.ReferenceOTU135962
New.CleanUp.ReferenceOTU230084
New.CleanUp.ReferenceOTU120909
New.CleanUp.ReferenceOTU84290
New.CleanUp.ReferenceOTU1462
New.CleanUp.ReferenceOTU92602
New.CleanUp.ReferenceOTU11959
New.CleanUp.ReferenceOTU11925
New.CleanUp.ReferenceOTU254499
New.CleanUp.ReferenceOTU116346
New.CleanUp.ReferenceOTU30116
New.ReferenceOTU124
New.CleanUp.ReferenceOTU55179
New.CleanUp.ReferenceOTU157374
New.CleanUp.ReferenceOTU78440
New.CleanUp.ReferenceOTU140451
New.CleanUp.ReferenceOTU193845
New.CleanUp.ReferenceOTU57192
New.CleanUp.ReferenceOTU160459
New.CleanUp.ReferenceOTU230171
New.CleanUp.ReferenceOTU130618
New.CleanUp.ReferenceOTU90612
New.ReferenceOTU42
New.CleanUp.ReferenceOTU43623
New.CleanUp.ReferenceOTU260937
New.CleanUp.ReferenceOTU13085
New.CleanUp.ReferenceOTU131392
New.CleanUp.ReferenceOTU60003
New.CleanUp.ReferenceOTU14179
New.CleanUp.ReferenceOTU198384
New.CleanUp.ReferenceOTU61156
New.CleanUp.ReferenceOTU300148
New.CleanUp.ReferenceOTU239523
New.CleanUp.ReferenceOTU120556
New.CleanUp.ReferenceOTU175342
New.CleanUp.ReferenceOTU192017
New.CleanUp.ReferenceOTU98115

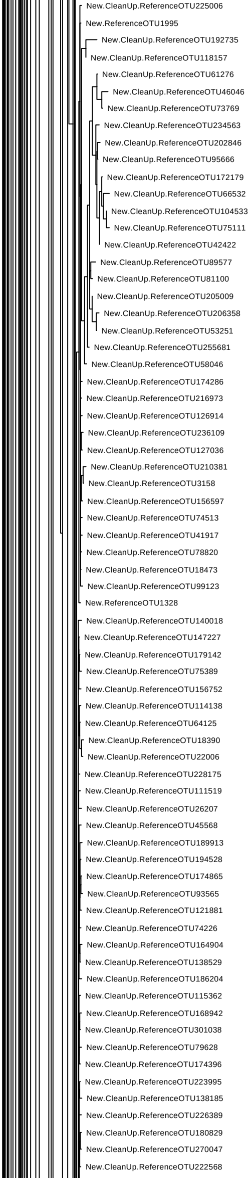
New.CleanUp.ReferenceOTU225006
New.ReferenceOTU1995
New.CleanUp.ReferenceOTU192735
New.CleanUp.ReferenceOTU118157
New.CleanUp.ReferenceOTU61276
New.CleanUp.ReferenceOTU46046
New.CleanUp.ReferenceOTU73769
New.CleanUp.ReferenceOTU234563
New.CleanUp.ReferenceOTU202846
New.CleanUp.ReferenceOTU95666
New.CleanUp.ReferenceOTU172179
New.CleanUp.ReferenceOTU66532
New.CleanUp.ReferenceOTU104533
New.CleanUp.ReferenceOTU75111
New.CleanUp.ReferenceOTU42422
New.CleanUp.ReferenceOTU89577
New.CleanUp.ReferenceOTU81100
New.CleanUp.ReferenceOTU205009
New.CleanUp.ReferenceOTU206358
New.CleanUp.ReferenceOTU53251
New.CleanUp.ReferenceOTU255681
New.CleanUp.ReferenceOTU58046
New.CleanUp.ReferenceOTU174286
New.CleanUp.ReferenceOTU216973
New.CleanUp.ReferenceOTU126914
New.CleanUp.ReferenceOTU236109
New.CleanUp.ReferenceOTU127036
New.CleanUp.ReferenceOTU210381
New.CleanUp.ReferenceOTU3158
New.CleanUp.ReferenceOTU156597
New.CleanUp.ReferenceOTU74513
New.CleanUp.ReferenceOTU41917
New.CleanUp.ReferenceOTU78820
New.CleanUp.ReferenceOTU18473
New.CleanUp.ReferenceOTU99123
New.ReferenceOTU1328
New.CleanUp.ReferenceOTU140018
New.CleanUp.ReferenceOTU147227
New.CleanUp.ReferenceOTU179142
New.CleanUp.ReferenceOTU75389
New.CleanUp.ReferenceOTU156752
New.CleanUp.ReferenceOTU114138
New.CleanUp.ReferenceOTU64125
New.CleanUp.ReferenceOTU18390
New.CleanUp.ReferenceOTU22006
New.CleanUp.ReferenceOTU228175
New.CleanUp.ReferenceOTU111519
New.CleanUp.ReferenceOTU26207
New.CleanUp.ReferenceOTU45568
New.CleanUp.ReferenceOTU189913
New.CleanUp.ReferenceOTU194528
New.CleanUp.ReferenceOTU174865
New.CleanUp.ReferenceOTU93565
New.CleanUp.ReferenceOTU121881
New.CleanUp.ReferenceOTU74226
New.CleanUp.ReferenceOTU164904
New.CleanUp.ReferenceOTU138529
New.CleanUp.ReferenceOTU186204
New.CleanUp.ReferenceOTU115362
New.CleanUp.ReferenceOTU168942
New.CleanUp.ReferenceOTU301038
New.CleanUp.ReferenceOTU79628
New.CleanUp.ReferenceOTU174396
New.CleanUp.ReferenceOTU223995
New.CleanUp.ReferenceOTU138185
New.CleanUp.ReferenceOTU226389
New.CleanUp.ReferenceOTU180829
New.CleanUp.ReferenceOTU270047
New.CleanUp.ReferenceOTU222568

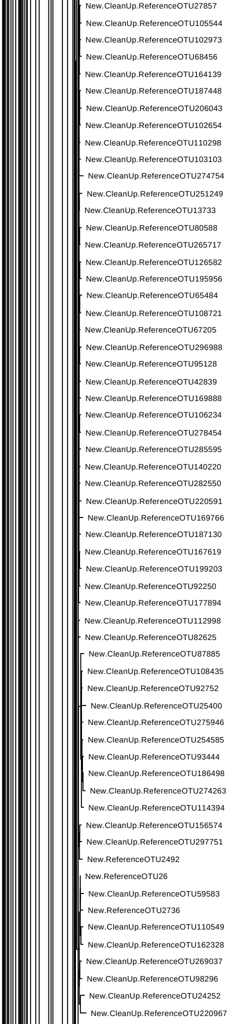
New.CleanUp.ReferenceOTU27857
New.CleanUp.ReferenceOTU105544
New.CleanUp.ReferenceOTU102973
New.CleanUp.ReferenceOTU68456
New.CleanUp.ReferenceOTU164139
New.CleanUp.ReferenceOTU187448
New.CleanUp.ReferenceOTU206043
New.CleanUp.ReferenceOTU102654
New.CleanUp.ReferenceOTU110298
New.CleanUp.ReferenceOTU103103
New.CleanUp.ReferenceOTU274754
New.CleanUp.ReferenceOTU251249
New.CleanUp.ReferenceOTU13733
New.CleanUp.ReferenceOTU80588
New.CleanUp.ReferenceOTU265717
New.CleanUp.ReferenceOTU126582
New.CleanUp.ReferenceOTU195956
New.CleanUp.ReferenceOTU65484
New.CleanUp.ReferenceOTU108721
New.CleanUp.ReferenceOTU67205
New.CleanUp.ReferenceOTU296988
New.CleanUp.ReferenceOTU95128
New.CleanUp.ReferenceOTU42839
New.CleanUp.ReferenceOTU169888
New.CleanUp.ReferenceOTU106234
New.CleanUp.ReferenceOTU278454
New.CleanUp.ReferenceOTU285595
New.CleanUp.ReferenceOTU140220
New.CleanUp.ReferenceOTU282550
New.CleanUp.ReferenceOTU220591
New.CleanUp.ReferenceOTU169766
New.CleanUp.ReferenceOTU187130
New.CleanUp.ReferenceOTU167619
New.CleanUp.ReferenceOTU199203
New.CleanUp.ReferenceOTU92250
New.CleanUp.ReferenceOTU177894
New.CleanUp.ReferenceOTU112998
New.CleanUp.ReferenceOTU82625
New.CleanUp.ReferenceOTU87885
New.CleanUp.ReferenceOTU108435
New.CleanUp.ReferenceOTU92752
New.CleanUp.ReferenceOTU25400
New.CleanUp.ReferenceOTU275946
New.CleanUp.ReferenceOTU254585
New.CleanUp.ReferenceOTU93444
New.CleanUp.ReferenceOTU186498
New.CleanUp.ReferenceOTU274263
New.CleanUp.ReferenceOTU114394
New.CleanUp.ReferenceOTU156574
New.CleanUp.ReferenceOTU297751
New.ReferenceOTU2492
New.ReferenceOTU26
New.CleanUp.ReferenceOTU59583
New.ReferenceOTU2736
New.CleanUp.ReferenceOTU110549
New.CleanUp.ReferenceOTU162328
New.CleanUp.ReferenceOTU269037
New.CleanUp.ReferenceOTU98296
New.CleanUp.ReferenceOTU24252
New.CleanUp.ReferenceOTU220967

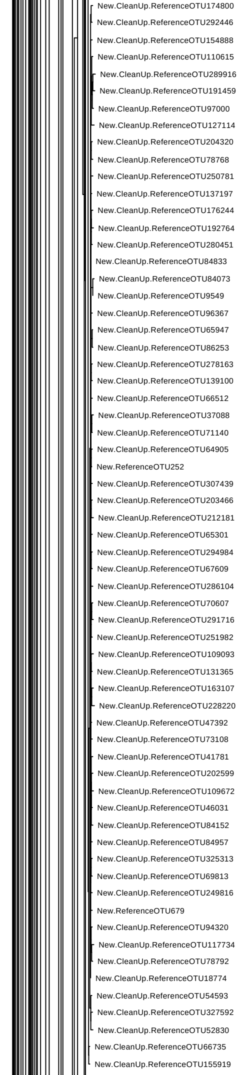
New.CleanUp.ReferenceOTU174800
New.CleanUp.ReferenceOTU292446
New.CleanUp.ReferenceOTU154888
New.CleanUp.ReferenceOTU110615
New.CleanUp.ReferenceOTU289916
New.CleanUp.ReferenceOTU191459
New.CleanUp.ReferenceOTU97000
New.CleanUp.ReferenceOTU127114
New.CleanUp.ReferenceOTU204320
New.CleanUp.ReferenceOTU78768
New.CleanUp.ReferenceOTU250781
New.CleanUp.ReferenceOTU137197
New.CleanUp.ReferenceOTU176244
New.CleanUp.ReferenceOTU192764
New.CleanUp.ReferenceOTU280451
New.CleanUp.ReferenceOTU84833
New.CleanUp.ReferenceOTU84073
New.CleanUp.ReferenceOTU9549
New.CleanUp.ReferenceOTU96367
New.CleanUp.ReferenceOTU65947
New.CleanUp.ReferenceOTU86253
New.CleanUp.ReferenceOTU278163
New.CleanUp.ReferenceOTU139100
New.CleanUp.ReferenceOTU66512
New.CleanUp.ReferenceOTU37088
New.CleanUp.ReferenceOTU71140
New.CleanUp.ReferenceOTU64905
New.ReferenceOTU252
New.CleanUp.ReferenceOTU307439
New.CleanUp.ReferenceOTU203466
New.CleanUp.ReferenceOTU212181
New.CleanUp.ReferenceOTU65301
New.CleanUp.ReferenceOTU294984
New.CleanUp.ReferenceOTU67609
New.CleanUp.ReferenceOTU286104
New.CleanUp.ReferenceOTU70607
New.CleanUp.ReferenceOTU291716
New.CleanUp.ReferenceOTU251982
New.CleanUp.ReferenceOTU109093
New.CleanUp.ReferenceOTU131365
New.CleanUp.ReferenceOTU163107
New.CleanUp.ReferenceOTU228220
New.CleanUp.ReferenceOTU47392
New.CleanUp.ReferenceOTU73108
New.CleanUp.ReferenceOTU41781
New.CleanUp.ReferenceOTU202599
New.CleanUp.ReferenceOTU109672
New.CleanUp.ReferenceOTU46031
New.CleanUp.ReferenceOTU84152
New.CleanUp.ReferenceOTU84957
New.CleanUp.ReferenceOTU325313
New.CleanUp.ReferenceOTU69813
New.CleanUp.ReferenceOTU249816
New.ReferenceOTU679
New.CleanUp.ReferenceOTU94320
New.CleanUp.ReferenceOTU117734
New.CleanUp.ReferenceOTU78792
New.CleanUp.ReferenceOTU18774
New.CleanUp.ReferenceOTU54593
New.CleanUp.ReferenceOTU327592
New.CleanUp.ReferenceOTU52830
New.CleanUp.ReferenceOTU66735
New.CleanUp.ReferenceOTU155919

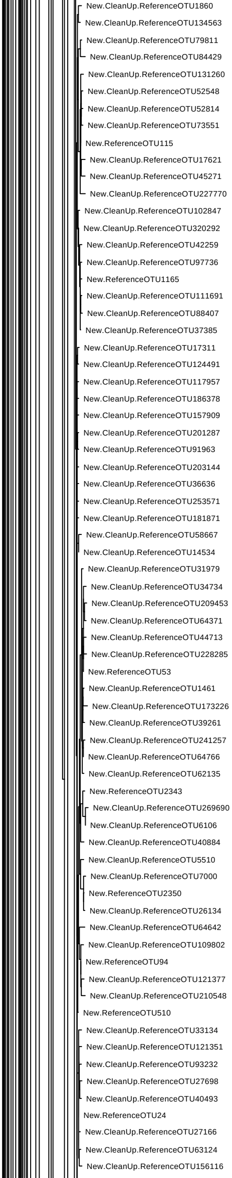
New.CleanUp.ReferenceOTU1860
New.CleanUp.ReferenceOTU134563
New.CleanUp.ReferenceOTU79811
New.CleanUp.ReferenceOTU84429
New.CleanUp.ReferenceOTU131260
New.CleanUp.ReferenceOTU52548
New.CleanUp.ReferenceOTU52814
New.CleanUp.ReferenceOTU73551
New.ReferenceOTU115
New.CleanUp.ReferenceOTU17621
New.CleanUp.ReferenceOTU45271
New.CleanUp.ReferenceOTU227770
New.CleanUp.ReferenceOTU102847
New.CleanUp.ReferenceOTU320292
New.CleanUp.ReferenceOTU42259
New.CleanUp.ReferenceOTU97736
New.ReferenceOTU1165
New.CleanUp.ReferenceOTU111691
New.CleanUp.ReferenceOTU88407
New.CleanUp.ReferenceOTU37385
New.CleanUp.ReferenceOTU17311
New.CleanUp.ReferenceOTU124491
New.CleanUp.ReferenceOTU117957
New.CleanUp.ReferenceOTU186378
New.CleanUp.ReferenceOTU157909
New.CleanUp.ReferenceOTU201287
New.CleanUp.ReferenceOTU91963
New.CleanUp.ReferenceOTU203144
New.CleanUp.ReferenceOTU36636
New.CleanUp.ReferenceOTU253571
New.CleanUp.ReferenceOTU181871
New.CleanUp.ReferenceOTU58667
New.CleanUp.ReferenceOTU14534
New.CleanUp.ReferenceOTU31979
New.CleanUp.ReferenceOTU34734
New.CleanUp.ReferenceOTU209453
New.CleanUp.ReferenceOTU64371
New.CleanUp.ReferenceOTU44713
New.CleanUp.ReferenceOTU228285
New.ReferenceOTU53
New.CleanUp.ReferenceOTU1461
New.CleanUp.ReferenceOTU173226
New.CleanUp.ReferenceOTU39261
New.CleanUp.ReferenceOTU241257
New.CleanUp.ReferenceOTU64766
New.CleanUp.ReferenceOTU62135
New.ReferenceOTU2343
New.CleanUp.ReferenceOTU269690
New.CleanUp.ReferenceOTU6106
New.CleanUp.ReferenceOTU40884
New.CleanUp.ReferenceOTU5510
New.CleanUp.ReferenceOTU7000
New.ReferenceOTU2350
New.CleanUp.ReferenceOTU26134
New.CleanUp.ReferenceOTU64642
New.CleanUp.ReferenceOTU109802
New.ReferenceOTU94
New.CleanUp.ReferenceOTU121377
New.CleanUp.ReferenceOTU210548
New.ReferenceOTU510
New.CleanUp.ReferenceOTU33134
New.CleanUp.ReferenceOTU121351
New.CleanUp.ReferenceOTU93232
New.CleanUp.ReferenceOTU27698
New.CleanUp.ReferenceOTU40493
New.ReferenceOTU24
New.CleanUp.ReferenceOTU27166
New.CleanUp.ReferenceOTU63124
New.CleanUp.ReferenceOTU156116

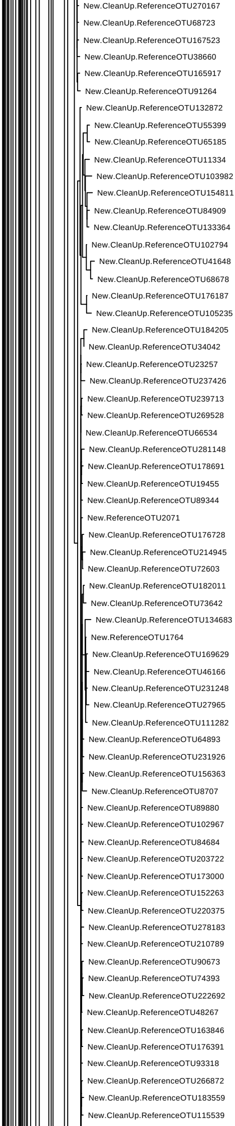

New.CleanUp.ReferenceOTU270167
New.CleanUp.ReferenceOTU68723
New.CleanUp.ReferenceOTU167523
New.CleanUp.ReferenceOTU38660
New.CleanUp.ReferenceOTU165917
New.CleanUp.ReferenceOTU91264
New.CleanUp.ReferenceOTU132872
New.CleanUp.ReferenceOTU55399
New.CleanUp.ReferenceOTU65185
New.CleanUp.ReferenceOTU11334
New.CleanUp.ReferenceOTU103982
New.CleanUp.ReferenceOTU154811
New.CleanUp.ReferenceOTU84909
New.CleanUp.ReferenceOTU133364
New.CleanUp.ReferenceOTU102794
New.CleanUp.ReferenceOTU41648
New.CleanUp.ReferenceOTU68678
New.CleanUp.ReferenceOTU176187
New.CleanUp.ReferenceOTU105235
New.CleanUp.ReferenceOTU184205
New.CleanUp.ReferenceOTU34042
New.CleanUp.ReferenceOTU23257
New.CleanUp.ReferenceOTU237426
New.CleanUp.ReferenceOTU239713
New.CleanUp.ReferenceOTU269528
New.CleanUp.ReferenceOTU66534
New.CleanUp.ReferenceOTU281148
New.CleanUp.ReferenceOTU178691
New.CleanUp.ReferenceOTU19455
New.CleanUp.ReferenceOTU89344
New.ReferenceOTU2071
New.CleanUp.ReferenceOTU176728
New.CleanUp.ReferenceOTU214945
New.CleanUp.ReferenceOTU72603
New.CleanUp.ReferenceOTU182011
New.CleanUp.ReferenceOTU73642
New.CleanUp.ReferenceOTU134683
New.ReferenceOTU1764
New.CleanUp.ReferenceOTU169629
New.CleanUp.ReferenceOTU46166
New.CleanUp.ReferenceOTU231248
New.CleanUp.ReferenceOTU27965
New.CleanUp.ReferenceOTU111282
New.CleanUp.ReferenceOTU64893
New.CleanUp.ReferenceOTU231926
New.CleanUp.ReferenceOTU156363
New.CleanUp.ReferenceOTU8707
New.CleanUp.ReferenceOTU89880
New.CleanUp.ReferenceOTU102967
New.CleanUp.ReferenceOTU84684
New.CleanUp.ReferenceOTU203722
New.CleanUp.ReferenceOTU173000
New.CleanUp.ReferenceOTU152263
New.CleanUp.ReferenceOTU220375
New.CleanUp.ReferenceOTU278183
New.CleanUp.ReferenceOTU210789
New.CleanUp.ReferenceOTU90673
New.CleanUp.ReferenceOTU74393
New.CleanUp.ReferenceOTU222692
New.CleanUp.ReferenceOTU48267
New.CleanUp.ReferenceOTU163846
New.CleanUp.ReferenceOTU176391
New.CleanUp.ReferenceOTU93318
New.CleanUp.ReferenceOTU266872
New.CleanUp.ReferenceOTU183559
New.CleanUp.ReferenceOTU115539

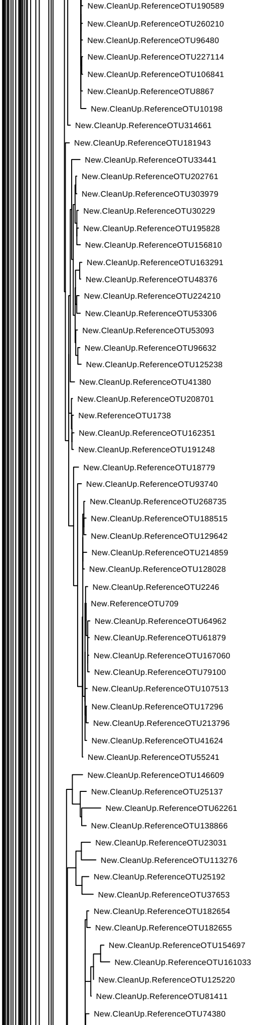
New.CleanUp.ReferenceOTU190589
New.CleanUp.ReferenceOTU260210
New.CleanUp.ReferenceOTU96480
New.CleanUp.ReferenceOTU227114
New.CleanUp.ReferenceOTU106841
New.CleanUp.ReferenceOTU8867
New.CleanUp.ReferenceOTU10198
New.CleanUp.ReferenceOTU314661
New.CleanUp.ReferenceOTU181943
New.CleanUp.ReferenceOTU33441
New.CleanUp.ReferenceOTU202761
New.CleanUp.ReferenceOTU303979
New.CleanUp.ReferenceOTU30229
New.CleanUp.ReferenceOTU195828
New.CleanUp.ReferenceOTU156810
New.CleanUp.ReferenceOTU163291
New.CleanUp.ReferenceOTU48376
New.CleanUp.ReferenceOTU224210
New.CleanUp.ReferenceOTU53306
New.CleanUp.ReferenceOTU53093
New.CleanUp.ReferenceOTU96632
New.CleanUp.ReferenceOTU125238
New.CleanUp.ReferenceOTU41380
New.CleanUp.ReferenceOTU208701
New.ReferenceOTU1738
New.CleanUp.ReferenceOTU162351
New.CleanUp.ReferenceOTU191248
New.CleanUp.ReferenceOTU18779
New.CleanUp.ReferenceOTU93740
New.CleanUp.ReferenceOTU268735
New.CleanUp.ReferenceOTU188515
New.CleanUp.ReferenceOTU129642
New.CleanUp.ReferenceOTU214859
New.CleanUp.ReferenceOTU128028
New.CleanUp.ReferenceOTU2246
New.ReferenceOTU709
New.CleanUp.ReferenceOTU64962
New.CleanUp.ReferenceOTU61879
New.CleanUp.ReferenceOTU167060
New.CleanUp.ReferenceOTU79100
New.CleanUp.ReferenceOTU107513
New.CleanUp.ReferenceOTU17296
New.CleanUp.ReferenceOTU213796
New.CleanUp.ReferenceOTU41624
New.CleanUp.ReferenceOTU55241
New.CleanUp.ReferenceOTU146609
New.CleanUp.ReferenceOTU25137
New.CleanUp.ReferenceOTU62261
New.CleanUp.ReferenceOTU138866
New.CleanUp.ReferenceOTU23031
New.CleanUp.ReferenceOTU113276
New.CleanUp.ReferenceOTU25192
New.CleanUp.ReferenceOTU37653
New.CleanUp.ReferenceOTU182654
New.CleanUp.ReferenceOTU182655
New.CleanUp.ReferenceOTU154697
New.CleanUp.ReferenceOTU161033
New.CleanUp.ReferenceOTU125220
New.CleanUp.ReferenceOTU81411
New.CleanUp.ReferenceOTU74380

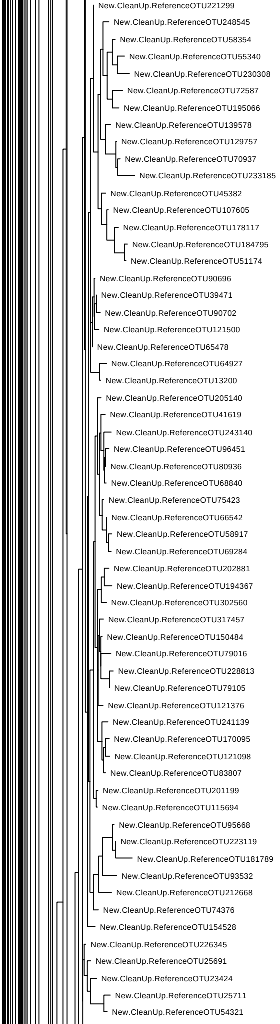
New.CleanUp.ReferenceOTU221299
New.CleanUp.ReferenceOTU248545
New.CleanUp.ReferenceOTU58354
New.CleanUp.ReferenceOTU55340
New.CleanUp.ReferenceOTU230308
New.CleanUp.ReferenceOTU72587
New.CleanUp.ReferenceOTU195066
New.CleanUp.ReferenceOTU139578
New.CleanUp.ReferenceOTU129757
New.CleanUp.ReferenceOTU70937
New.CleanUp.ReferenceOTU233185
New.CleanUp.ReferenceOTU45382
New.CleanUp.ReferenceOTU107605
New.CleanUp.ReferenceOTU178117
New.CleanUp.ReferenceOTU184795
New.CleanUp.ReferenceOTU51174
New.CleanUp.ReferenceOTU90696
New.CleanUp.ReferenceOTU39471
New.CleanUp.ReferenceOTU90702
New.CleanUp.ReferenceOTU121500
New.CleanUp.ReferenceOTU65478
New.CleanUp.ReferenceOTU64927
New.CleanUp.ReferenceOTU13200
New.CleanUp.ReferenceOTU205140
New.CleanUp.ReferenceOTU41619
New.CleanUp.ReferenceOTU243140
New.CleanUp.ReferenceOTU96451
New.CleanUp.ReferenceOTU80936
New.CleanUp.ReferenceOTU68840
New.CleanUp.ReferenceOTU75423
New.CleanUp.ReferenceOTU66542
New.CleanUp.ReferenceOTU58917
New.CleanUp.ReferenceOTU69284
New.CleanUp.ReferenceOTU202881
New.CleanUp.ReferenceOTU194367
New.CleanUp.ReferenceOTU302560
New.CleanUp.ReferenceOTU317457
New.CleanUp.ReferenceOTU150484
New.CleanUp.ReferenceOTU79016
New.CleanUp.ReferenceOTU228813
New.CleanUp.ReferenceOTU79105
New.CleanUp.ReferenceOTU121376
New.CleanUp.ReferenceOTU241139
New.CleanUp.ReferenceOTU170095
New.CleanUp.ReferenceOTU121098
New.CleanUp.ReferenceOTU83807
New.CleanUp.ReferenceOTU201199
New.CleanUp.ReferenceOTU115694
New.CleanUp.ReferenceOTU95668
New.CleanUp.ReferenceOTU223119
New.CleanUp.ReferenceOTU181789
New.CleanUp.ReferenceOTU93532
New.CleanUp.ReferenceOTU212668
New.CleanUp.ReferenceOTU74376
New.CleanUp.ReferenceOTU154528
New.CleanUp.ReferenceOTU226345
New.CleanUp.ReferenceOTU25691
New.CleanUp.ReferenceOTU23424
New.CleanUp.ReferenceOTU25711
New.CleanUp.ReferenceOTU54321

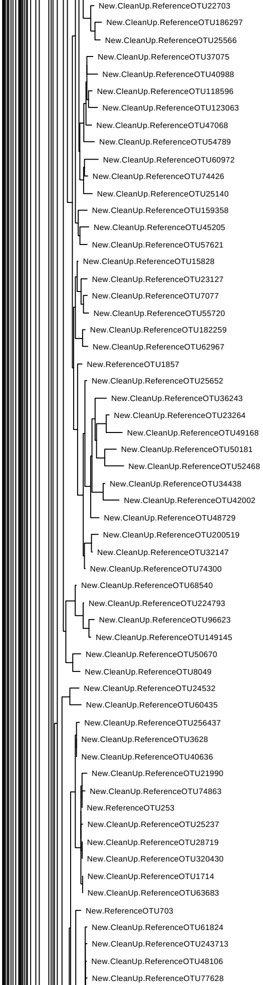
New.CleanUp.ReferenceOTU22703
New.CleanUp.ReferenceOTU186297
New.CleanUp.ReferenceOTU25566
New.CleanUp.ReferenceOTU37075
New.CleanUp.ReferenceOTU40988
New.CleanUp.ReferenceOTU118596
New.CleanUp.ReferenceOTU123063
New.CleanUp.ReferenceOTU47068
New.CleanUp.ReferenceOTU54789
New.CleanUp.ReferenceOTU60972
New.CleanUp.ReferenceOTU74426
New.CleanUp.ReferenceOTU25140
New.CleanUp.ReferenceOTU159358
New.CleanUp.ReferenceOTU45205
New.CleanUp.ReferenceOTU57621
New.CleanUp.ReferenceOTU15828
New.CleanUp.ReferenceOTU23127
New.CleanUp.ReferenceOTU7077
New.CleanUp.ReferenceOTU55720
New.CleanUp.ReferenceOTU182259
New.CleanUp.ReferenceOTU62967
New.ReferenceOTU1857
New.CleanUp.ReferenceOTU25652
New.CleanUp.ReferenceOTU36243
New.CleanUp.ReferenceOTU23264
New.CleanUp.ReferenceOTU49168
New.CleanUp.ReferenceOTU50181
New.CleanUp.ReferenceOTU52468
New.CleanUp.ReferenceOTU34438
New.CleanUp.ReferenceOTU42002
New.CleanUp.ReferenceOTU48729
New.CleanUp.ReferenceOTU200519
New.CleanUp.ReferenceOTU32147
New.CleanUp.ReferenceOTU74300
New.CleanUp.ReferenceOTU68540
New.CleanUp.ReferenceOTU224793
New.CleanUp.ReferenceOTU96623
New.CleanUp.ReferenceOTU149145
New.CleanUp.ReferenceOTU50670
New.CleanUp.ReferenceOTU8049
New.CleanUp.ReferenceOTU24532
New.CleanUp.ReferenceOTU60435
New.CleanUp.ReferenceOTU256437
New.CleanUp.ReferenceOTU3628
New.CleanUp.ReferenceOTU40636
New.CleanUp.ReferenceOTU21990
New.CleanUp.ReferenceOTU74863
New.ReferenceOTU253
New.CleanUp.ReferenceOTU25237
New.CleanUp.ReferenceOTU28719
New.CleanUp.ReferenceOTU320430
New.CleanUp.ReferenceOTU1714
New.CleanUp.ReferenceOTU63683
New.ReferenceOTU703
New.CleanUp.ReferenceOTU61824
New.CleanUp.ReferenceOTU243713
New.CleanUp.ReferenceOTU48106
New.CleanUp.ReferenceOTU77628

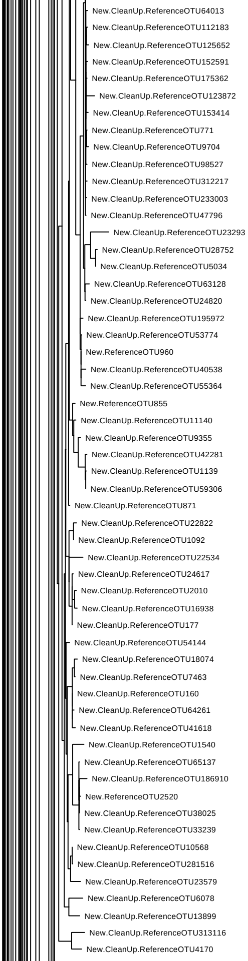
New.CleanUp.ReferenceOTU64013
New.CleanUp.ReferenceOTU112183
New.CleanUp.ReferenceOTU125652
New.CleanUp.ReferenceOTU152591
New.CleanUp.ReferenceOTU175362
New.CleanUp.ReferenceOTU123872
New.CleanUp.ReferenceOTU153414
New.CleanUp.ReferenceOTU771
New.CleanUp.ReferenceOTU9704
New.CleanUp.ReferenceOTU98527
New.CleanUp.ReferenceOTU312217
New.CleanUp.ReferenceOTU233003
New.CleanUp.ReferenceOTU47796
New.CleanUp.ReferenceOTU23293
New.CleanUp.ReferenceOTU28752
New.CleanUp.ReferenceOTU5034
New.CleanUp.ReferenceOTU63128
New.CleanUp.ReferenceOTU24820
New.CleanUp.ReferenceOTU195972
New.CleanUp.ReferenceOTU53774
New.ReferenceOTU960
New.CleanUp.ReferenceOTU40538
New.CleanUp.ReferenceOTU55364
New.ReferenceOTU855
New.CleanUp.ReferenceOTU11140
New.CleanUp.ReferenceOTU9355
New.CleanUp.ReferenceOTU42281
New.CleanUp.ReferenceOTU1139
New.CleanUp.ReferenceOTU59306
New.CleanUp.ReferenceOTU871
New.CleanUp.ReferenceOTU22822
New.CleanUp.ReferenceOTU1092
New.CleanUp.ReferenceOTU22534
New.CleanUp.ReferenceOTU24617
New.CleanUp.ReferenceOTU2010
New.CleanUp.ReferenceOTU16938
New.CleanUp.ReferenceOTU177
New.CleanUp.ReferenceOTU54144
New.CleanUp.ReferenceOTU18074
New.CleanUp.ReferenceOTU7463
New.CleanUp.ReferenceOTU160
New.CleanUp.ReferenceOTU64261
New.CleanUp.ReferenceOTU41618
New.CleanUp.ReferenceOTU1540
New.CleanUp.ReferenceOTU65137
New.CleanUp.ReferenceOTU186910
New.ReferenceOTU2520
New.CleanUp.ReferenceOTU38025
New.CleanUp.ReferenceOTU33239
New.CleanUp.ReferenceOTU10568
New.CleanUp.ReferenceOTU281516
New.CleanUp.ReferenceOTU23579
New.CleanUp.ReferenceOTU6078
New.CleanUp.ReferenceOTU13899
New.CleanUp.ReferenceOTU313116
New.CleanUp.ReferenceOTU4170

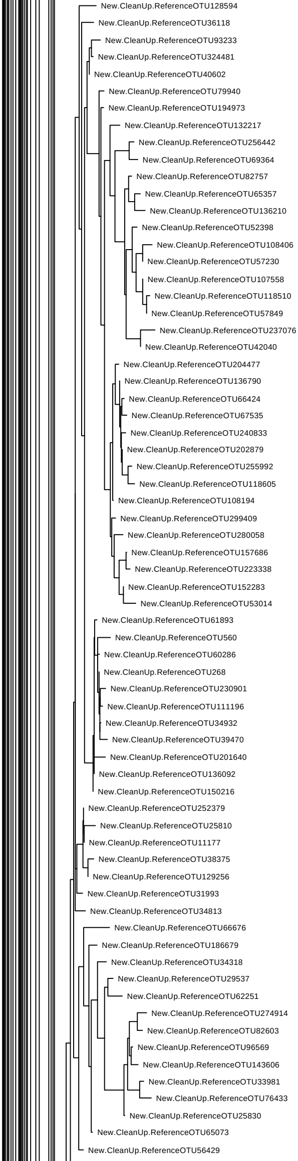
New.CleanUp.ReferenceOTU128594
New.CleanUp.ReferenceOTU36118
New.CleanUp.ReferenceOTU93233
New.CleanUp.ReferenceOTU324481
New.CleanUp.ReferenceOTU40602
New.CleanUp.ReferenceOTU79940
New.CleanUp.ReferenceOTU194973
New.CleanUp.ReferenceOTU132217
New.CleanUp.ReferenceOTU256442
New.CleanUp.ReferenceOTU69364
New.CleanUp.ReferenceOTU82757
New.CleanUp.ReferenceOTU65357
New.CleanUp.ReferenceOTU136210
New.CleanUp.ReferenceOTU52398
New.CleanUp.ReferenceOTU108406
New.CleanUp.ReferenceOTU57230
New.CleanUp.ReferenceOTU107558
New.CleanUp.ReferenceOTU118510
New.CleanUp.ReferenceOTU57849
New.CleanUp.ReferenceOTU237076
New.CleanUp.ReferenceOTU42040
New.CleanUp.ReferenceOTU204477
New.CleanUp.ReferenceOTU136790
New.CleanUp.ReferenceOTU66424
New.CleanUp.ReferenceOTU67535
New.CleanUp.ReferenceOTU240833
New.CleanUp.ReferenceOTU202879
New.CleanUp.ReferenceOTU255992
New.CleanUp.ReferenceOTU118605
New.CleanUp.ReferenceOTU108194
New.CleanUp.ReferenceOTU299409
New.CleanUp.ReferenceOTU280058
New.CleanUp.ReferenceOTU157686
New.CleanUp.ReferenceOTU223338
New.CleanUp.ReferenceOTU152283
New.CleanUp.ReferenceOTU53014
New.CleanUp.ReferenceOTU61893
New.CleanUp.ReferenceOTU560
New.CleanUp.ReferenceOTU60286
New.CleanUp.ReferenceOTU268
New.CleanUp.ReferenceOTU230901
New.CleanUp.ReferenceOTU111196
New.CleanUp.ReferenceOTU34932
New.CleanUp.ReferenceOTU39470
New.CleanUp.ReferenceOTU201640
New.CleanUp.ReferenceOTU136092
New.CleanUp.ReferenceOTU150216
New.CleanUp.ReferenceOTU252379
New.CleanUp.ReferenceOTU25810
New.CleanUp.ReferenceOTU11177
New.CleanUp.ReferenceOTU38375
New.CleanUp.ReferenceOTU129256
New.CleanUp.ReferenceOTU31993
New.CleanUp.ReferenceOTU34813
New.CleanUp.ReferenceOTU66676
New.CleanUp.ReferenceOTU186679
New.CleanUp.ReferenceOTU34318
New.CleanUp.ReferenceOTU29537
New.CleanUp.ReferenceOTU62251
New.CleanUp.ReferenceOTU274914
New.CleanUp.ReferenceOTU82603
New.CleanUp.ReferenceOTU96569
New.CleanUp.ReferenceOTU143606
New.CleanUp.ReferenceOTU33981
New.CleanUp.ReferenceOTU76433
New.CleanUp.ReferenceOTU25830
New.CleanUp.ReferenceOTU65073
New.CleanUp.ReferenceOTU56429

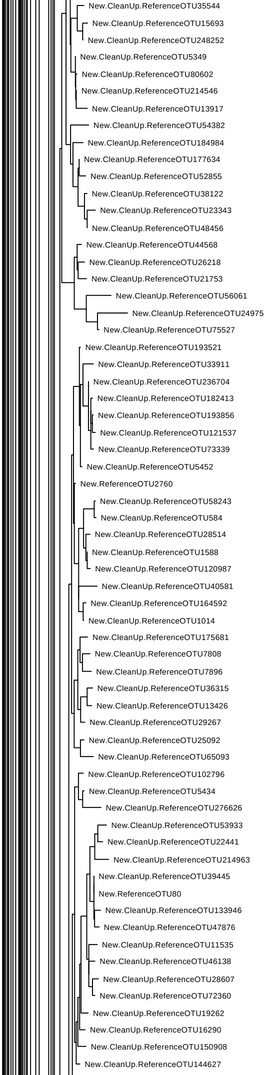
New.CleanUp.ReferenceOTU35544
New.CleanUp.ReferenceOTU15693
New.CleanUp.ReferenceOTU248252
New.CleanUp.ReferenceOTU5349
New.CleanUp.ReferenceOTU80602
New.CleanUp.ReferenceOTU214546
New.CleanUp.ReferenceOTU13917
New.CleanUp.ReferenceOTU54382
New.CleanUp.ReferenceOTU184984
New.CleanUp.ReferenceOTU177634
New.CleanUp.ReferenceOTU52855
New.CleanUp.ReferenceOTU38122
New.CleanUp.ReferenceOTU23343
New.CleanUp.ReferenceOTU48456
New.CleanUp.ReferenceOTU44568
New.CleanUp.ReferenceOTU26218
New.CleanUp.ReferenceOTU21753
New.CleanUp.ReferenceOTU56061
New.CleanUp.ReferenceOTU24975
New.CleanUp.ReferenceOTU75527
New.CleanUp.ReferenceOTU193521
New.CleanUp.ReferenceOTU33911
New.CleanUp.ReferenceOTU236704
New.CleanUp.ReferenceOTU182413
New.CleanUp.ReferenceOTU193856
New.CleanUp.ReferenceOTU121537
New.CleanUp.ReferenceOTU73339
New.CleanUp.ReferenceOTU5452
New.ReferenceOTU2760
New.CleanUp.ReferenceOTU58243
New.CleanUp.ReferenceOTU584
New.CleanUp.ReferenceOTU28514
New.CleanUp.ReferenceOTU1588
New.CleanUp.ReferenceOTU120987
New.CleanUp.ReferenceOTU40581
New.CleanUp.ReferenceOTU164592
New.CleanUp.ReferenceOTU1014
New.CleanUp.ReferenceOTU175681
New.CleanUp.ReferenceOTU7808
New.CleanUp.ReferenceOTU7896
New.CleanUp.ReferenceOTU36315
New.CleanUp.ReferenceOTU13426
New.CleanUp.ReferenceOTU29267
New.CleanUp.ReferenceOTU25092
New.CleanUp.ReferenceOTU65093
New.CleanUp.ReferenceOTU102796
New.CleanUp.ReferenceOTU5434
New.CleanUp.ReferenceOTU276626
New.CleanUp.ReferenceOTU53933
New.CleanUp.ReferenceOTU22441
New.CleanUp.ReferenceOTU214963
New.CleanUp.ReferenceOTU39445
New.ReferenceOTU80
New.CleanUp.ReferenceOTU133946
New.CleanUp.ReferenceOTU47876
New.CleanUp.ReferenceOTU11535
New.CleanUp.ReferenceOTU46138
New.CleanUp.ReferenceOTU28607
New.CleanUp.ReferenceOTU72360
New.CleanUp.ReferenceOTU19262
New.CleanUp.ReferenceOTU16290
New.CleanUp.ReferenceOTU150908
New.CleanUp.ReferenceOTU144627

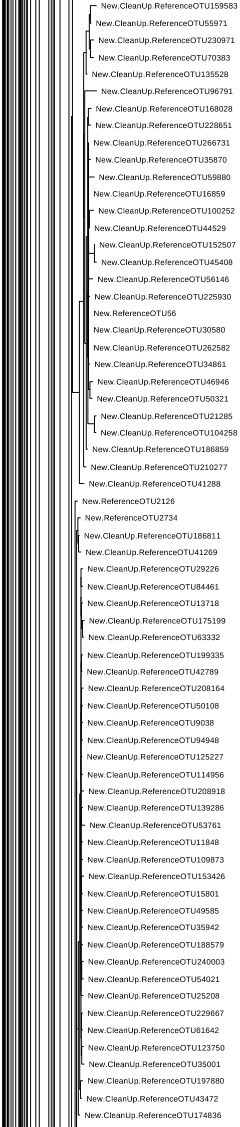
New.CleanUp.ReferenceOTU159583
New.CleanUp.ReferenceOTU55971
New.CleanUp.ReferenceOTU230971
New.CleanUp.ReferenceOTU70383
New.CleanUp.ReferenceOTU135528
New.CleanUp.ReferenceOTU96791
New.CleanUp.ReferenceOTU168028
New.CleanUp.ReferenceOTU228651
New.CleanUp.ReferenceOTU266731
New.CleanUp.ReferenceOTU35870
New.CleanUp.ReferenceOTU59880
New.CleanUp.ReferenceOTU16859
New.CleanUp.ReferenceOTU100252
New.CleanUp.ReferenceOTU44529
New.CleanUp.ReferenceOTU152507
New.CleanUp.ReferenceOTU45408
New.CleanUp.ReferenceOTU56146
New.CleanUp.ReferenceOTU225930
New.ReferenceOTU56
New.CleanUp.ReferenceOTU30580
New.CleanUp.ReferenceOTU262582
New.CleanUp.ReferenceOTU34861
New.CleanUp.ReferenceOTU46946
New.CleanUp.ReferenceOTU50321
New.CleanUp.ReferenceOTU21285
New.CleanUp.ReferenceOTU104258
New.CleanUp.ReferenceOTU186859
New.CleanUp.ReferenceOTU210277
New.CleanUp.ReferenceOTU41288
New.ReferenceOTU2126
New.ReferenceOTU2734
New.CleanUp.ReferenceOTU186811
New.CleanUp.ReferenceOTU41269
New.CleanUp.ReferenceOTU29226
New.CleanUp.ReferenceOTU84461
New.CleanUp.ReferenceOTU13718
New.CleanUp.ReferenceOTU175199
New.CleanUp.ReferenceOTU63332
New.CleanUp.ReferenceOTU199335
New.CleanUp.ReferenceOTU42789
New.CleanUp.ReferenceOTU208164
New.CleanUp.ReferenceOTU50108
New.CleanUp.ReferenceOTU9038
New.CleanUp.ReferenceOTU94948
New.CleanUp.ReferenceOTU125227
New.CleanUp.ReferenceOTU114956
New.CleanUp.ReferenceOTU208918
New.CleanUp.ReferenceOTU139286
New.CleanUp.ReferenceOTU53761
New.CleanUp.ReferenceOTU11848
New.CleanUp.ReferenceOTU109873
New.CleanUp.ReferenceOTU153426
New.CleanUp.ReferenceOTU15801
New.CleanUp.ReferenceOTU49585
New.CleanUp.ReferenceOTU35942
New.CleanUp.ReferenceOTU188579
New.CleanUp.ReferenceOTU240003
New.CleanUp.ReferenceOTU54021
New.CleanUp.ReferenceOTU25208
New.CleanUp.ReferenceOTU229667
New.CleanUp.ReferenceOTU61642
New.CleanUp.ReferenceOTU123750
New.CleanUp.ReferenceOTU35001
New.CleanUp.ReferenceOTU197880
New.CleanUp.ReferenceOTU43472
New.CleanUp.ReferenceOTU174836

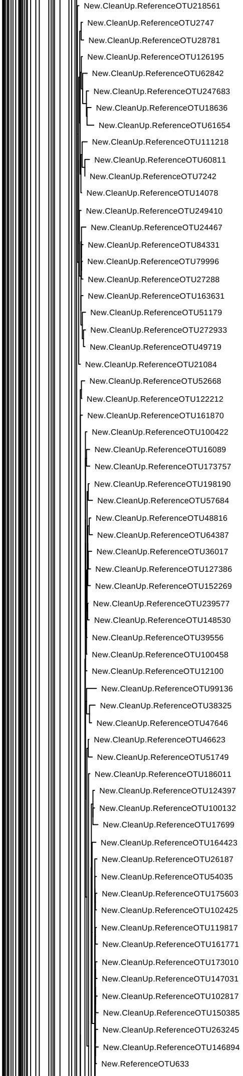
New.CleanUp.ReferenceOTU218561
New.CleanUp.ReferenceOTU2747
New.CleanUp.ReferenceOTU28781
New.CleanUp.ReferenceOTU126195
New.CleanUp.ReferenceOTU62842
New.CleanUp.ReferenceOTU247683
New.CleanUp.ReferenceOTU18636
New.CleanUp.ReferenceOTU61654
New.CleanUp.ReferenceOTU111218
New.CleanUp.ReferenceOTU60811
New.CleanUp.ReferenceOTU7242
New.CleanUp.ReferenceOTU14078
New.CleanUp.ReferenceOTU249410
New.CleanUp.ReferenceOTU24467
New.CleanUp.ReferenceOTU84331
New.CleanUp.ReferenceOTU79996
New.CleanUp.ReferenceOTU27288
New.CleanUp.ReferenceOTU163631
New.CleanUp.ReferenceOTU51179
New.CleanUp.ReferenceOTU272933
New.CleanUp.ReferenceOTU49719
New.CleanUp.ReferenceOTU21084
New.CleanUp.ReferenceOTU52668
New.CleanUp.ReferenceOTU122212
New.CleanUp.ReferenceOTU161870
New.CleanUp.ReferenceOTU100422
New.CleanUp.ReferenceOTU16089
New.CleanUp.ReferenceOTU173757
New.CleanUp.ReferenceOTU198190
New.CleanUp.ReferenceOTU57684
New.CleanUp.ReferenceOTU48816
New.CleanUp.ReferenceOTU64387
New.CleanUp.ReferenceOTU36017
New.CleanUp.ReferenceOTU127386
New.CleanUp.ReferenceOTU152269
New.CleanUp.ReferenceOTU239577
New.CleanUp.ReferenceOTU148530
New.CleanUp.ReferenceOTU39556
New.CleanUp.ReferenceOTU100458
New.CleanUp.ReferenceOTU12100
New.CleanUp.ReferenceOTU99136
New.CleanUp.ReferenceOTU38325
New.CleanUp.ReferenceOTU47646
New.CleanUp.ReferenceOTU46623
New.CleanUp.ReferenceOTU51749
New.CleanUp.ReferenceOTU186011
New.CleanUp.ReferenceOTU124397
New.CleanUp.ReferenceOTU100132
New.CleanUp.ReferenceOTU17699
New.CleanUp.ReferenceOTU164423
New.CleanUp.ReferenceOTU26187
New.CleanUp.ReferenceOTU54035
New.CleanUp.ReferenceOTU175603
New.CleanUp.ReferenceOTU102425
New.CleanUp.ReferenceOTU119817
New.CleanUp.ReferenceOTU161771
New.CleanUp.ReferenceOTU173010
New.CleanUp.ReferenceOTU147031
New.CleanUp.ReferenceOTU102817
New.CleanUp.ReferenceOTU150385
New.CleanUp.ReferenceOTU263245
New.CleanUp.ReferenceOTU146894
New.ReferenceOTU633

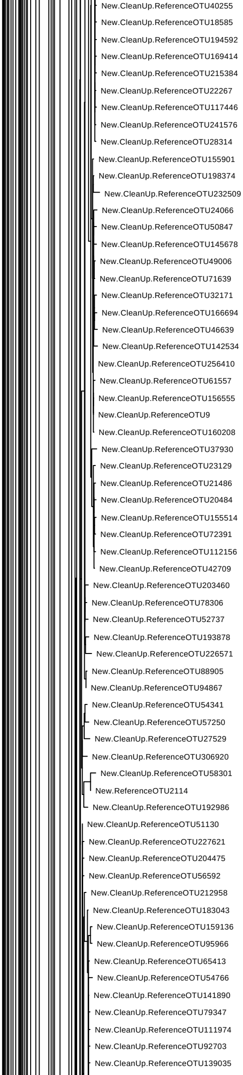
New.CleanUp.ReferenceOTU40255
New.CleanUp.ReferenceOTU18585
New.CleanUp.ReferenceOTU194592
New.CleanUp.ReferenceOTU169414
New.CleanUp.ReferenceOTU215384
New.CleanUp.ReferenceOTU22267
New.CleanUp.ReferenceOTU117446
New.CleanUp.ReferenceOTU241576
New.CleanUp.ReferenceOTU28314
New.CleanUp.ReferenceOTU155901
New.CleanUp.ReferenceOTU198374
New.CleanUp.ReferenceOTU232509
New.CleanUp.ReferenceOTU24066
New.CleanUp.ReferenceOTU50847
New.CleanUp.ReferenceOTU145678
New.CleanUp.ReferenceOTU49006
New.CleanUp.ReferenceOTU71639
New.CleanUp.ReferenceOTU32171
New.CleanUp.ReferenceOTU166694
New.CleanUp.ReferenceOTU46639
New.CleanUp.ReferenceOTU142534
New.CleanUp.ReferenceOTU256410
New.CleanUp.ReferenceOTU61557
New.CleanUp.ReferenceOTU156555
New.CleanUp.ReferenceOTU9
New.CleanUp.ReferenceOTU160208
New.CleanUp.ReferenceOTU37930
New.CleanUp.ReferenceOTU23129
New.CleanUp.ReferenceOTU21486
New.CleanUp.ReferenceOTU20484
New.CleanUp.ReferenceOTU155514
New.CleanUp.ReferenceOTU72391
New.CleanUp.ReferenceOTU112156
New.CleanUp.ReferenceOTU42709
New.CleanUp.ReferenceOTU203460
New.CleanUp.ReferenceOTU78306
New.CleanUp.ReferenceOTU52737
New.CleanUp.ReferenceOTU193878
New.CleanUp.ReferenceOTU226571
New.CleanUp.ReferenceOTU88905
New.CleanUp.ReferenceOTU94867
New.CleanUp.ReferenceOTU54341
New.CleanUp.ReferenceOTU57250
New.CleanUp.ReferenceOTU27529
New.CleanUp.ReferenceOTU306920
New.CleanUp.ReferenceOTU58301
New.ReferenceOTU2114
New.CleanUp.ReferenceOTU192986
New.CleanUp.ReferenceOTU51130
New.CleanUp.ReferenceOTU227621
New.CleanUp.ReferenceOTU204475
New.CleanUp.ReferenceOTU56592
New.CleanUp.ReferenceOTU212958
New.CleanUp.ReferenceOTU183043
New.CleanUp.ReferenceOTU159136
New.CleanUp.ReferenceOTU95966
New.CleanUp.ReferenceOTU65413
New.CleanUp.ReferenceOTU54766
New.CleanUp.ReferenceOTU141890
New.CleanUp.ReferenceOTU79347
New.CleanUp.ReferenceOTU111974
New.CleanUp.ReferenceOTU92703
New.CleanUp.ReferenceOTU139035

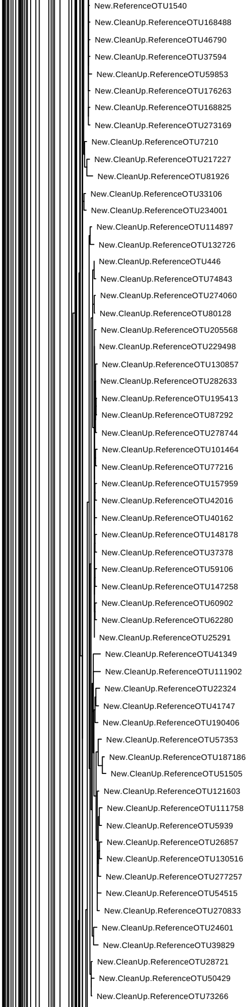
New.ReferenceOTU1540
New.CleanUp.ReferenceOTU168488
New.CleanUp.ReferenceOTU46790
New.CleanUp.ReferenceOTU37594
New.CleanUp.ReferenceOTU59853
New.CleanUp.ReferenceOTU176263
New.CleanUp.ReferenceOTU168825
New.CleanUp.ReferenceOTU273169
New.CleanUp.ReferenceOTU7210
New.CleanUp.ReferenceOTU217227
New.CleanUp.ReferenceOTU81926
New.CleanUp.ReferenceOTU33106
New.CleanUp.ReferenceOTU234001
New.CleanUp.ReferenceOTU114897
New.CleanUp.ReferenceOTU132726
New.CleanUp.ReferenceOTU446
New.CleanUp.ReferenceOTU74843
New.CleanUp.ReferenceOTU274060
New.CleanUp.ReferenceOTU80128
New.CleanUp.ReferenceOTU205568
New.CleanUp.ReferenceOTU229498
New.CleanUp.ReferenceOTU130857
New.CleanUp.ReferenceOTU282633
New.CleanUp.ReferenceOTU195413
New.CleanUp.ReferenceOTU87292
New.CleanUp.ReferenceOTU278744
New.CleanUp.ReferenceOTU101464
New.CleanUp.ReferenceOTU77216
New.CleanUp.ReferenceOTU157959
New.CleanUp.ReferenceOTU42016
New.CleanUp.ReferenceOTU40162
New.CleanUp.ReferenceOTU148178
New.CleanUp.ReferenceOTU37378
New.CleanUp.ReferenceOTU59106
New.CleanUp.ReferenceOTU147258
New.CleanUp.ReferenceOTU60902
New.CleanUp.ReferenceOTU62280
New.CleanUp.ReferenceOTU25291
New.CleanUp.ReferenceOTU41349
New.CleanUp.ReferenceOTU111902
New.CleanUp.ReferenceOTU22324
New.CleanUp.ReferenceOTU41747
New.CleanUp.ReferenceOTU190406
New.CleanUp.ReferenceOTU57353
New.CleanUp.ReferenceOTU187186
New.CleanUp.ReferenceOTU51505
New.CleanUp.ReferenceOTU121603
New.CleanUp.ReferenceOTU111758
New.CleanUp.ReferenceOTU5939
New.CleanUp.ReferenceOTU26857
New.CleanUp.ReferenceOTU130516
New.CleanUp.ReferenceOTU277257
New.CleanUp.ReferenceOTU54515
New.CleanUp.ReferenceOTU270833
New.CleanUp.ReferenceOTU24601
New.CleanUp.ReferenceOTU39829
New.CleanUp.ReferenceOTU28721
New.CleanUp.ReferenceOTU50429
New.CleanUp.ReferenceOTU73266

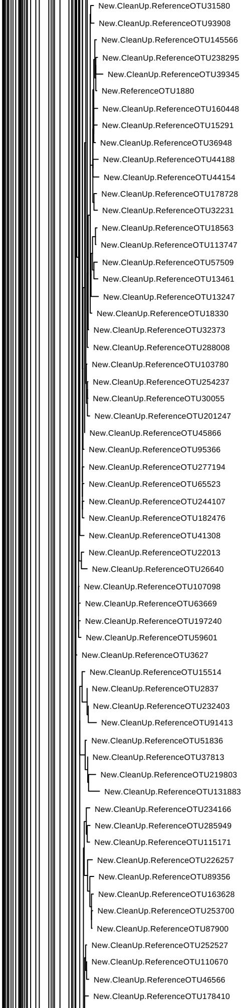
New.CleanUp.ReferenceOTU31580
New.CleanUp.ReferenceOTU93908
New.CleanUp.ReferenceOTU145566
New.CleanUp.ReferenceOTU238295
New.CleanUp.ReferenceOTU39345
New.ReferenceOTU1880
New.CleanUp.ReferenceOTU160448
New.CleanUp.ReferenceOTU15291
New.CleanUp.ReferenceOTU36948
New.CleanUp.ReferenceOTU44188
New.CleanUp.ReferenceOTU44154
New.CleanUp.ReferenceOTU178728
New.CleanUp.ReferenceOTU32231
New.CleanUp.ReferenceOTU18563
New.CleanUp.ReferenceOTU113747
New.CleanUp.ReferenceOTU57509
New.CleanUp.ReferenceOTU13461
New.CleanUp.ReferenceOTU13247
New.CleanUp.ReferenceOTU18330
New.CleanUp.ReferenceOTU32373
New.CleanUp.ReferenceOTU288008
New.CleanUp.ReferenceOTU103780
New.CleanUp.ReferenceOTU254237
New.CleanUp.ReferenceOTU30055
New.CleanUp.ReferenceOTU201247
New.CleanUp.ReferenceOTU45866
New.CleanUp.ReferenceOTU95366
New.CleanUp.ReferenceOTU277194
New.CleanUp.ReferenceOTU65523
New.CleanUp.ReferenceOTU244107
New.CleanUp.ReferenceOTU182476
New.CleanUp.ReferenceOTU41308
New.CleanUp.ReferenceOTU22013
New.CleanUp.ReferenceOTU26640
New.CleanUp.ReferenceOTU107098
New.CleanUp.ReferenceOTU63669
New.CleanUp.ReferenceOTU197240
New.CleanUp.ReferenceOTU59601
New.CleanUp.ReferenceOTU3627
New.CleanUp.ReferenceOTU15514
New.CleanUp.ReferenceOTU2837
New.CleanUp.ReferenceOTU232403
New.CleanUp.ReferenceOTU91413
New.CleanUp.ReferenceOTU51836
New.CleanUp.ReferenceOTU37813
New.CleanUp.ReferenceOTU219803
New.CleanUp.ReferenceOTU131883
New.CleanUp.ReferenceOTU234166
New.CleanUp.ReferenceOTU285949
New.CleanUp.ReferenceOTU115171
New.CleanUp.ReferenceOTU226257
New.CleanUp.ReferenceOTU89356
New.CleanUp.ReferenceOTU163628
New.CleanUp.ReferenceOTU253700
New.CleanUp.ReferenceOTU87900
New.CleanUp.ReferenceOTU252527
New.CleanUp.ReferenceOTU110670
New.CleanUp.ReferenceOTU46566
New.CleanUp.ReferenceOTU178410

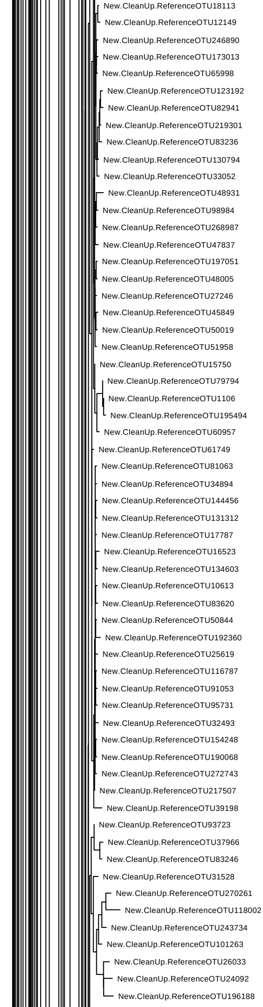
New.CleanUp.ReferenceOTU18113
New.CleanUp.ReferenceOTU12149
New.CleanUp.ReferenceOTU246890
New.CleanUp.ReferenceOTU173013
New.CleanUp.ReferenceOTU65998
New.CleanUp.ReferenceOTU123192
New.CleanUp.ReferenceOTU82941
New.CleanUp.ReferenceOTU219301
New.CleanUp.ReferenceOTU83236
New.CleanUp.ReferenceOTU130794
New.CleanUp.ReferenceOTU33052
New.CleanUp.ReferenceOTU48931
New.CleanUp.ReferenceOTU98984
New.CleanUp.ReferenceOTU268987
New.CleanUp.ReferenceOTU47837
New.CleanUp.ReferenceOTU197051
New.CleanUp.ReferenceOTU48005
New.CleanUp.ReferenceOTU27246
New.CleanUp.ReferenceOTU45849
New.CleanUp.ReferenceOTU50019
New.CleanUp.ReferenceOTU51958
New.CleanUp.ReferenceOTU15750
New.CleanUp.ReferenceOTU79794
New.CleanUp.ReferenceOTU1106
New.CleanUp.ReferenceOTU195494
New.CleanUp.ReferenceOTU60957
New.CleanUp.ReferenceOTU61749
New.CleanUp.ReferenceOTU81063
New.CleanUp.ReferenceOTU34894
New.CleanUp.ReferenceOTU144456
New.CleanUp.ReferenceOTU131312
New.CleanUp.ReferenceOTU17787
New.CleanUp.ReferenceOTU16523
New.CleanUp.ReferenceOTU134603
New.CleanUp.ReferenceOTU10613
New.CleanUp.ReferenceOTU83620
New.CleanUp.ReferenceOTU50844
New.CleanUp.ReferenceOTU192360
New.CleanUp.ReferenceOTU25619
New.CleanUp.ReferenceOTU116787
New.CleanUp.ReferenceOTU91053
New.CleanUp.ReferenceOTU95731
New.CleanUp.ReferenceOTU32493
New.CleanUp.ReferenceOTU154248
New.CleanUp.ReferenceOTU190068
New.CleanUp.ReferenceOTU272743
New.CleanUp.ReferenceOTU217507
New.CleanUp.ReferenceOTU39198
New.CleanUp.ReferenceOTU93723
New.CleanUp.ReferenceOTU37966
New.CleanUp.ReferenceOTU83246
New.CleanUp.ReferenceOTU31528
New.CleanUp.ReferenceOTU270261
New.CleanUp.ReferenceOTU118002
New.CleanUp.ReferenceOTU243734
New.CleanUp.ReferenceOTU101263
New.CleanUp.ReferenceOTU26033
New.CleanUp.ReferenceOTU24092
New.CleanUp.ReferenceOTU196188

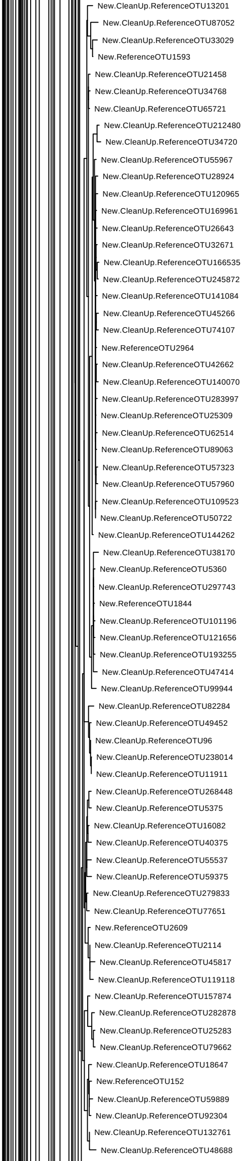
New.CleanUp.ReferenceOTU13201
New.CleanUp.ReferenceOTU87052
New.CleanUp.ReferenceOTU33029
New.ReferenceOTU1593
New.CleanUp.ReferenceOTU21458
New.CleanUp.ReferenceOTU34768
New.CleanUp.ReferenceOTU65721
New.CleanUp.ReferenceOTU212480
New.CleanUp.ReferenceOTU34720
New.CleanUp.ReferenceOTU55967
New.CleanUp.ReferenceOTU28924
New.CleanUp.ReferenceOTU120965
New.CleanUp.ReferenceOTU169961
New.CleanUp.ReferenceOTU26643
New.CleanUp.ReferenceOTU32671
New.CleanUp.ReferenceOTU166535
New.CleanUp.ReferenceOTU245872
New.CleanUp.ReferenceOTU141084
New.CleanUp.ReferenceOTU45266
New.CleanUp.ReferenceOTU74107
New.ReferenceOTU2964
New.CleanUp.ReferenceOTU42662
New.CleanUp.ReferenceOTU140070
New.CleanUp.ReferenceOTU283997
New.CleanUp.ReferenceOTU25309
New.CleanUp.ReferenceOTU62514
New.CleanUp.ReferenceOTU89063
New.CleanUp.ReferenceOTU57323
New.CleanUp.ReferenceOTU57960
New.CleanUp.ReferenceOTU109523
New.CleanUp.ReferenceOTU50722
New.CleanUp.ReferenceOTU144262
New.CleanUp.ReferenceOTU38170
New.CleanUp.ReferenceOTU5360
New.CleanUp.ReferenceOTU297743
New.ReferenceOTU1844
New.CleanUp.ReferenceOTU101196
New.CleanUp.ReferenceOTU121656
New.CleanUp.ReferenceOTU193255
New.CleanUp.ReferenceOTU47414
New.CleanUp.ReferenceOTU99944
New.CleanUp.ReferenceOTU82284
New.CleanUp.ReferenceOTU49452
New.CleanUp.ReferenceOTU96
New.CleanUp.ReferenceOTU238014
New.CleanUp.ReferenceOTU11911
New.CleanUp.ReferenceOTU268448
New.CleanUp.ReferenceOTU5375
New.CleanUp.ReferenceOTU16082
New.CleanUp.ReferenceOTU40375
New.CleanUp.ReferenceOTU55537
New.CleanUp.ReferenceOTU59375
New.CleanUp.ReferenceOTU279833
New.CleanUp.ReferenceOTU77651
New.ReferenceOTU2609
New.CleanUp.ReferenceOTU2114
New.CleanUp.ReferenceOTU45817
New.CleanUp.ReferenceOTU119118
New.CleanUp.ReferenceOTU157874
New.CleanUp.ReferenceOTU282878
New.CleanUp.ReferenceOTU25283
New.CleanUp.ReferenceOTU79662
New.CleanUp.ReferenceOTU18647
New.ReferenceOTU152
New.CleanUp.ReferenceOTU59889
New.CleanUp.ReferenceOTU92304
New.CleanUp.ReferenceOTU132761
New.CleanUp.ReferenceOTU48688

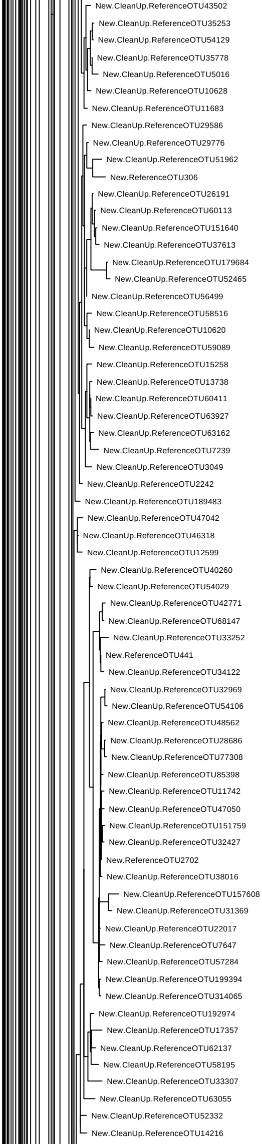
New.CleanUp.ReferenceOTU43502
New.CleanUp.ReferenceOTU35253
New.CleanUp.ReferenceOTU54129
New.CleanUp.ReferenceOTU35778
New.CleanUp.ReferenceOTU5016
New.CleanUp.ReferenceOTU10628
New.CleanUp.ReferenceOTU11683
New.CleanUp.ReferenceOTU29586
New.CleanUp.ReferenceOTU29776
New.CleanUp.ReferenceOTU51962
New.ReferenceOTU306
New.CleanUp.ReferenceOTU26191
New.CleanUp.ReferenceOTU60113
New.CleanUp.ReferenceOTU151640
New.CleanUp.ReferenceOTU37613
New.CleanUp.ReferenceOTU179684
New.CleanUp.ReferenceOTU52465
New.CleanUp.ReferenceOTU56499
New.CleanUp.ReferenceOTU58516
New.CleanUp.ReferenceOTU10620
New.CleanUp.ReferenceOTU59089
New.CleanUp.ReferenceOTU15258
New.CleanUp.ReferenceOTU13738
New.CleanUp.ReferenceOTU60411
New.CleanUp.ReferenceOTU63927
New.CleanUp.ReferenceOTU63162
New.CleanUp.ReferenceOTU7239
New.CleanUp.ReferenceOTU3049
New.CleanUp.ReferenceOTU2242
New.CleanUp.ReferenceOTU189483
New.CleanUp.ReferenceOTU47042
New.CleanUp.ReferenceOTU46318
New.CleanUp.ReferenceOTU12599
New.CleanUp.ReferenceOTU40260
New.CleanUp.ReferenceOTU54029
New.CleanUp.ReferenceOTU42771
New.CleanUp.ReferenceOTU68147
New.CleanUp.ReferenceOTU33252
New.ReferenceOTU441
New.CleanUp.ReferenceOTU34122
New.CleanUp.ReferenceOTU32969
New.CleanUp.ReferenceOTU54106
New.CleanUp.ReferenceOTU48562
New.CleanUp.ReferenceOTU28686
New.CleanUp.ReferenceOTU77308
New.CleanUp.ReferenceOTU85398
New.CleanUp.ReferenceOTU11742
New.CleanUp.ReferenceOTU47050
New.CleanUp.ReferenceOTU151759
New.CleanUp.ReferenceOTU32427
New.ReferenceOTU2702
New.CleanUp.ReferenceOTU38016
New.CleanUp.ReferenceOTU157608
New.CleanUp.ReferenceOTU31369
New.CleanUp.ReferenceOTU22017
New.CleanUp.ReferenceOTU7647
New.CleanUp.ReferenceOTU57284
New.CleanUp.ReferenceOTU199394
New.CleanUp.ReferenceOTU314065
New.CleanUp.ReferenceOTU192974
New.CleanUp.ReferenceOTU17357
New.CleanUp.ReferenceOTU62137
New.CleanUp.ReferenceOTU58195
New.CleanUp.ReferenceOTU33307
New.CleanUp.ReferenceOTU63055
New.CleanUp.ReferenceOTU52332
New.CleanUp.ReferenceOTU14216

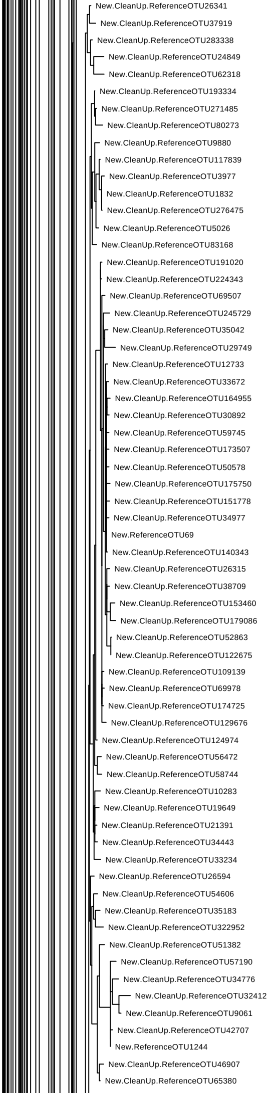
New.CleanUp.ReferenceOTU26341
New.CleanUp.ReferenceOTU37919
New.CleanUp.ReferenceOTU283338
New.CleanUp.ReferenceOTU24849
New.CleanUp.ReferenceOTU62318
New.CleanUp.ReferenceOTU193334
New.CleanUp.ReferenceOTU271485
New.CleanUp.ReferenceOTU80273
New.CleanUp.ReferenceOTU9880
New.CleanUp.ReferenceOTU117839
New.CleanUp.ReferenceOTU3977
New.CleanUp.ReferenceOTU1832
New.CleanUp.ReferenceOTU276475
New.CleanUp.ReferenceOTU5026
New.CleanUp.ReferenceOTU83168
New.CleanUp.ReferenceOTU191020
New.CleanUp.ReferenceOTU224343
New.CleanUp.ReferenceOTU69507
New.CleanUp.ReferenceOTU245729
New.CleanUp.ReferenceOTU35042
New.CleanUp.ReferenceOTU29749
New.CleanUp.ReferenceOTU12733
New.CleanUp.ReferenceOTU33672
New.CleanUp.ReferenceOTU164955
New.CleanUp.ReferenceOTU30892
New.CleanUp.ReferenceOTU59745
New.CleanUp.ReferenceOTU173507
New.CleanUp.ReferenceOTU50578
New.CleanUp.ReferenceOTU175750
New.CleanUp.ReferenceOTU151778
New.CleanUp.ReferenceOTU34977
New.ReferenceOTU69
New.CleanUp.ReferenceOTU140343
New.CleanUp.ReferenceOTU26315
New.CleanUp.ReferenceOTU38709
New.CleanUp.ReferenceOTU153460
New.CleanUp.ReferenceOTU179086
New.CleanUp.ReferenceOTU52863
New.CleanUp.ReferenceOTU122675
New.CleanUp.ReferenceOTU109139
New.CleanUp.ReferenceOTU69978
New.CleanUp.ReferenceOTU174725
New.CleanUp.ReferenceOTU129676
New.CleanUp.ReferenceOTU124974
New.CleanUp.ReferenceOTU56472
New.CleanUp.ReferenceOTU58744
New.CleanUp.ReferenceOTU10283
New.CleanUp.ReferenceOTU19649
New.CleanUp.ReferenceOTU21391
New.CleanUp.ReferenceOTU34443
New.CleanUp.ReferenceOTU33234
New.CleanUp.ReferenceOTU26594
New.CleanUp.ReferenceOTU54606
New.CleanUp.ReferenceOTU35183
New.CleanUp.ReferenceOTU322952
New.CleanUp.ReferenceOTU51382
New.CleanUp.ReferenceOTU57190
New.CleanUp.ReferenceOTU34776
New.CleanUp.ReferenceOTU32412
New.CleanUp.ReferenceOTU9061
New.CleanUp.ReferenceOTU42707
New.ReferenceOTU1244
New.CleanUp.ReferenceOTU46907
New.CleanUp.ReferenceOTU65380

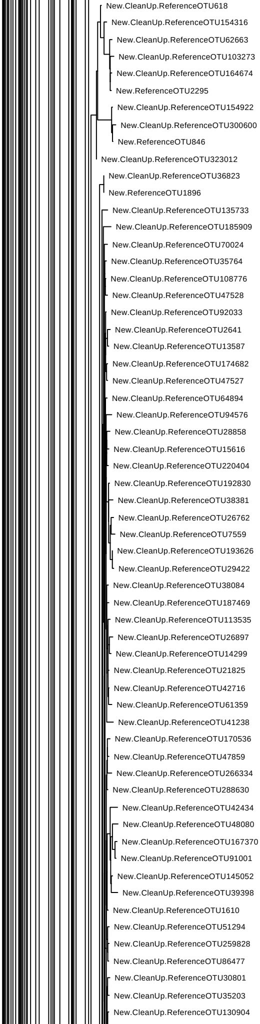

New.CleanUp.ReferenceOTU618
New.CleanUp.ReferenceOTU154316
New.CleanUp.ReferenceOTU62663
New.CleanUp.ReferenceOTU103273
New.CleanUp.ReferenceOTU164674
New.ReferenceOTU2295
New.CleanUp.ReferenceOTU154922
New.CleanUp.ReferenceOTU300600
New.ReferenceOTU846
New.CleanUp.ReferenceOTU323012
New.CleanUp.ReferenceOTU36823
New.ReferenceOTU1896
New.CleanUp.ReferenceOTU135733
New.CleanUp.ReferenceOTU185909
New.CleanUp.ReferenceOTU70024
New.CleanUp.ReferenceOTU35764
New.CleanUp.ReferenceOTU108776
New.CleanUp.ReferenceOTU47528
New.CleanUp.ReferenceOTU92033
New.CleanUp.ReferenceOTU2641
New.CleanUp.ReferenceOTU13587
New.CleanUp.ReferenceOTU174682
New.CleanUp.ReferenceOTU47527
New.CleanUp.ReferenceOTU64894
New.CleanUp.ReferenceOTU94576
New.CleanUp.ReferenceOTU28858
New.CleanUp.ReferenceOTU15616
New.CleanUp.ReferenceOTU220404
New.CleanUp.ReferenceOTU192830
New.CleanUp.ReferenceOTU38381
New.CleanUp.ReferenceOTU26762
New.CleanUp.ReferenceOTU7559
New.CleanUp.ReferenceOTU193626
New.CleanUp.ReferenceOTU29422
New.CleanUp.ReferenceOTU38084
New.CleanUp.ReferenceOTU187469
New.CleanUp.ReferenceOTU113535
New.CleanUp.ReferenceOTU26897
New.CleanUp.ReferenceOTU14299
New.CleanUp.ReferenceOTU21825
New.CleanUp.ReferenceOTU42716
New.CleanUp.ReferenceOTU61359
New.CleanUp.ReferenceOTU41238
New.CleanUp.ReferenceOTU170536
New.CleanUp.ReferenceOTU47859
New.CleanUp.ReferenceOTU266334
New.CleanUp.ReferenceOTU288630
New.CleanUp.ReferenceOTU42434
New.CleanUp.ReferenceOTU48080
New.CleanUp.ReferenceOTU167370
New.CleanUp.ReferenceOTU91001
New.CleanUp.ReferenceOTU145052
New.CleanUp.ReferenceOTU39398
New.CleanUp.ReferenceOTU1610
New.CleanUp.ReferenceOTU51294
New.CleanUp.ReferenceOTU259828
New.CleanUp.ReferenceOTU86477
New.CleanUp.ReferenceOTU30801
New.CleanUp.ReferenceOTU35203
New.CleanUp.ReferenceOTU130904

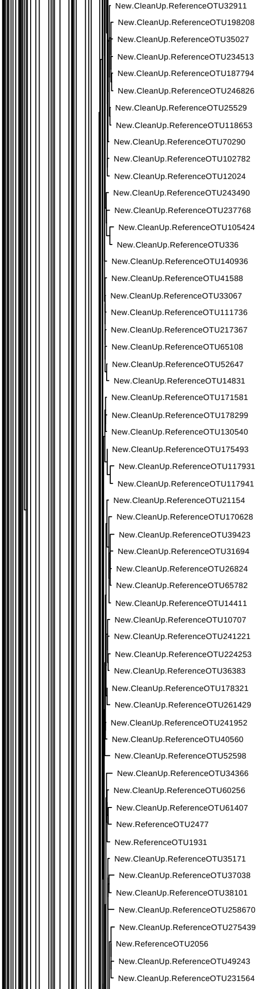
New.CleanUp.ReferenceOTU32911
New.CleanUp.ReferenceOTU198208
New.CleanUp.ReferenceOTU35027
New.CleanUp.ReferenceOTU234513
New.CleanUp.ReferenceOTU187794
New.CleanUp.ReferenceOTU246826
New.CleanUp.ReferenceOTU25529
New.CleanUp.ReferenceOTU118653
New.CleanUp.ReferenceOTU70290
New.CleanUp.ReferenceOTU102782
New.CleanUp.ReferenceOTU12024
New.CleanUp.ReferenceOTU243490
New.CleanUp.ReferenceOTU237768
New.CleanUp.ReferenceOTU105424
New.CleanUp.ReferenceOTU336
New.CleanUp.ReferenceOTU140936
New.CleanUp.ReferenceOTU41588
New.CleanUp.ReferenceOTU33067
New.CleanUp.ReferenceOTU111736
New.CleanUp.ReferenceOTU217367
New.CleanUp.ReferenceOTU65108
New.CleanUp.ReferenceOTU52647
New.CleanUp.ReferenceOTU14831
New.CleanUp.ReferenceOTU171581
New.CleanUp.ReferenceOTU178299
New.CleanUp.ReferenceOTU130540
New.CleanUp.ReferenceOTU175493
New.CleanUp.ReferenceOTU117931
New.CleanUp.ReferenceOTU117941
New.CleanUp.ReferenceOTU21154
New.CleanUp.ReferenceOTU170628
New.CleanUp.ReferenceOTU39423
New.CleanUp.ReferenceOTU31694
New.CleanUp.ReferenceOTU26824
New.CleanUp.ReferenceOTU65782
New.CleanUp.ReferenceOTU14411
New.CleanUp.ReferenceOTU10707
New.CleanUp.ReferenceOTU241221
New.CleanUp.ReferenceOTU224253
New.CleanUp.ReferenceOTU36383
New.CleanUp.ReferenceOTU178321
New.CleanUp.ReferenceOTU261429
New.CleanUp.ReferenceOTU241952
New.CleanUp.ReferenceOTU40560
New.CleanUp.ReferenceOTU52598
New.CleanUp.ReferenceOTU34366
New.CleanUp.ReferenceOTU60256
New.CleanUp.ReferenceOTU61407
New.ReferenceOTU2477
New.ReferenceOTU1931
New.CleanUp.ReferenceOTU35171
New.CleanUp.ReferenceOTU37038
New.CleanUp.ReferenceOTU38101
New.CleanUp.ReferenceOTU258670
New.CleanUp.ReferenceOTU275439
New.ReferenceOTU2056
New.CleanUp.ReferenceOTU49243
New.CleanUp.ReferenceOTU231564

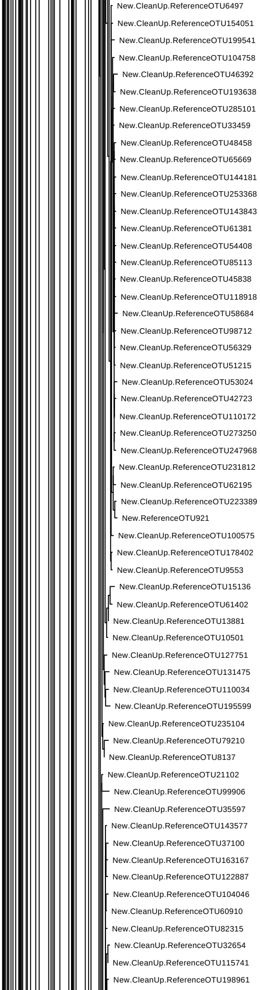
New.CleanUp.ReferenceOTU6497
New.CleanUp.ReferenceOTU154051
New.CleanUp.ReferenceOTU199541
New.CleanUp.ReferenceOTU104758
New.CleanUp.ReferenceOTU46392
New.CleanUp.ReferenceOTU193638
New.CleanUp.ReferenceOTU285101
New.CleanUp.ReferenceOTU33459
New.CleanUp.ReferenceOTU48458
New.CleanUp.ReferenceOTU65669
New.CleanUp.ReferenceOTU144181
New.CleanUp.ReferenceOTU253368
New.CleanUp.ReferenceOTU143843
New.CleanUp.ReferenceOTU61381
New.CleanUp.ReferenceOTU54408
New.CleanUp.ReferenceOTU85113
New.CleanUp.ReferenceOTU45838
New.CleanUp.ReferenceOTU118918
New.CleanUp.ReferenceOTU58684
New.CleanUp.ReferenceOTU98712
New.CleanUp.ReferenceOTU56329
New.CleanUp.ReferenceOTU51215
New.CleanUp.ReferenceOTU53024
New.CleanUp.ReferenceOTU42723
New.CleanUp.ReferenceOTU110172
New.CleanUp.ReferenceOTU273250
New.CleanUp.ReferenceOTU247968
New.CleanUp.ReferenceOTU231812
New.CleanUp.ReferenceOTU62195
New.CleanUp.ReferenceOTU223389
New.ReferenceOTU921
New.CleanUp.ReferenceOTU100575
New.CleanUp.ReferenceOTU178402
New.CleanUp.ReferenceOTU9553
New.CleanUp.ReferenceOTU15136
New.CleanUp.ReferenceOTU61402
New.CleanUp.ReferenceOTU13881
New.CleanUp.ReferenceOTU10501
New.CleanUp.ReferenceOTU127751
New.CleanUp.ReferenceOTU131475
New.CleanUp.ReferenceOTU110034
New.CleanUp.ReferenceOTU195599
New.CleanUp.ReferenceOTU235104
New.CleanUp.ReferenceOTU79210
New.CleanUp.ReferenceOTU8137
New.CleanUp.ReferenceOTU21102
New.CleanUp.ReferenceOTU99906
New.CleanUp.ReferenceOTU35597
New.CleanUp.ReferenceOTU143577
New.CleanUp.ReferenceOTU37100
New.CleanUp.ReferenceOTU163167
New.CleanUp.ReferenceOTU122887
New.CleanUp.ReferenceOTU104046
New.CleanUp.ReferenceOTU60910
New.CleanUp.ReferenceOTU82315
New.CleanUp.ReferenceOTU32654
New.CleanUp.ReferenceOTU115741
New.CleanUp.ReferenceOTU198961

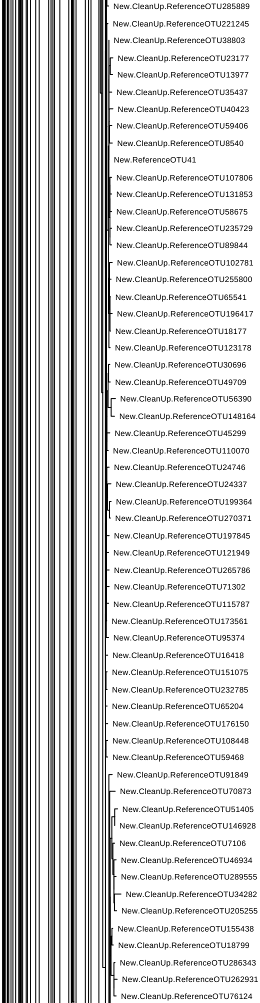
New.CleanUp.ReferenceOTU285889
New.CleanUp.ReferenceOTU221245
New.CleanUp.ReferenceOTU38803
New.CleanUp.ReferenceOTU23177
New.CleanUp.ReferenceOTU13977
New.CleanUp.ReferenceOTU35437
New.CleanUp.ReferenceOTU40423
New.CleanUp.ReferenceOTU59406
New.CleanUp.ReferenceOTU8540
New.ReferenceOTU41
New.CleanUp.ReferenceOTU107806
New.CleanUp.ReferenceOTU131853
New.CleanUp.ReferenceOTU58675
New.CleanUp.ReferenceOTU235729
New.CleanUp.ReferenceOTU89844
New.CleanUp.ReferenceOTU102781
New.CleanUp.ReferenceOTU255800
New.CleanUp.ReferenceOTU65541
New.CleanUp.ReferenceOTU196417
New.CleanUp.ReferenceOTU18177
New.CleanUp.ReferenceOTU123178
New.CleanUp.ReferenceOTU30696
New.CleanUp.ReferenceOTU49709
New.CleanUp.ReferenceOTU56390
New.CleanUp.ReferenceOTU148164
New.CleanUp.ReferenceOTU45299
New.CleanUp.ReferenceOTU110070
New.CleanUp.ReferenceOTU24746
New.CleanUp.ReferenceOTU24337
New.CleanUp.ReferenceOTU199364
New.CleanUp.ReferenceOTU270371
New.CleanUp.ReferenceOTU197845
New.CleanUp.ReferenceOTU121949
New.CleanUp.ReferenceOTU265786
New.CleanUp.ReferenceOTU71302
New.CleanUp.ReferenceOTU115787
New.CleanUp.ReferenceOTU173561
New.CleanUp.ReferenceOTU95374
New.CleanUp.ReferenceOTU16418
New.CleanUp.ReferenceOTU151075
New.CleanUp.ReferenceOTU232785
New.CleanUp.ReferenceOTU65204
New.CleanUp.ReferenceOTU176150
New.CleanUp.ReferenceOTU108448
New.CleanUp.ReferenceOTU59468
New.CleanUp.ReferenceOTU91849
New.CleanUp.ReferenceOTU70873
New.CleanUp.ReferenceOTU51405
New.CleanUp.ReferenceOTU146928
New.CleanUp.ReferenceOTU7106
New.CleanUp.ReferenceOTU46934
New.CleanUp.ReferenceOTU289555
New.CleanUp.ReferenceOTU34282
New.CleanUp.ReferenceOTU205255
New.CleanUp.ReferenceOTU155438
New.CleanUp.ReferenceOTU18799
New.CleanUp.ReferenceOTU286343
New.CleanUp.ReferenceOTU262931
New.CleanUp.ReferenceOTU76124

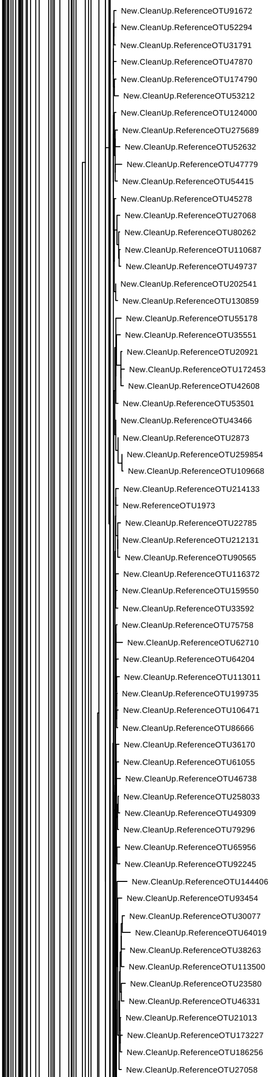
New.CleanUp.ReferenceOTU91672
New.CleanUp.ReferenceOTU52294
New.CleanUp.ReferenceOTU31791
New.CleanUp.ReferenceOTU47870
New.CleanUp.ReferenceOTU174790
New.CleanUp.ReferenceOTU53212
New.CleanUp.ReferenceOTU124000
New.CleanUp.ReferenceOTU275689
New.CleanUp.ReferenceOTU52632
New.CleanUp.ReferenceOTU47779
New.CleanUp.ReferenceOTU54415
New.CleanUp.ReferenceOTU45278
New.CleanUp.ReferenceOTU27068
New.CleanUp.ReferenceOTU80262
New.CleanUp.ReferenceOTU110687
New.CleanUp.ReferenceOTU49737
New.CleanUp.ReferenceOTU202541
New.CleanUp.ReferenceOTU130859
New.CleanUp.ReferenceOTU55178
New.CleanUp.ReferenceOTU35551
New.CleanUp.ReferenceOTU20921
New.CleanUp.ReferenceOTU172453
New.CleanUp.ReferenceOTU42608
New.CleanUp.ReferenceOTU53501
New.CleanUp.ReferenceOTU43466
New.CleanUp.ReferenceOTU2873
New.CleanUp.ReferenceOTU259854
New.CleanUp.ReferenceOTU109668
New.CleanUp.ReferenceOTU214133
New.ReferenceOTU1973
New.CleanUp.ReferenceOTU22785
New.CleanUp.ReferenceOTU212131
New.CleanUp.ReferenceOTU90565
New.CleanUp.ReferenceOTU116372
New.CleanUp.ReferenceOTU159550
New.CleanUp.ReferenceOTU33592
New.CleanUp.ReferenceOTU75758
New.CleanUp.ReferenceOTU62710
New.CleanUp.ReferenceOTU64204
New.CleanUp.ReferenceOTU113011
New.CleanUp.ReferenceOTU199735
New.CleanUp.ReferenceOTU106471
New.CleanUp.ReferenceOTU86666
New.CleanUp.ReferenceOTU36170
New.CleanUp.ReferenceOTU61055
New.CleanUp.ReferenceOTU46738
New.CleanUp.ReferenceOTU258033
New.CleanUp.ReferenceOTU49309
New.CleanUp.ReferenceOTU79296
New.CleanUp.ReferenceOTU65956
New.CleanUp.ReferenceOTU92245
New.CleanUp.ReferenceOTU144406
New.CleanUp.ReferenceOTU93454
New.CleanUp.ReferenceOTU30077
New.CleanUp.ReferenceOTU64019
New.CleanUp.ReferenceOTU38263
New.CleanUp.ReferenceOTU113500
New.CleanUp.ReferenceOTU23580
New.CleanUp.ReferenceOTU46331
New.CleanUp.ReferenceOTU21013
New.CleanUp.ReferenceOTU173227
New.CleanUp.ReferenceOTU186256
New.CleanUp.ReferenceOTU27058

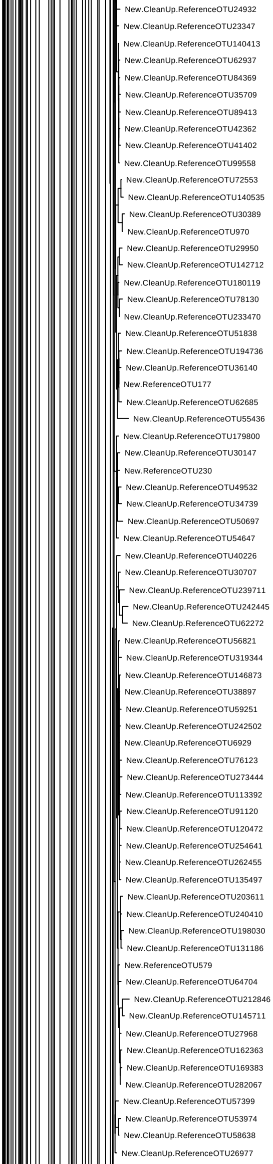
New.CleanUp.ReferenceOTU24932
New.CleanUp.ReferenceOTU23347
New.CleanUp.ReferenceOTU140413
New.CleanUp.ReferenceOTU62937
New.CleanUp.ReferenceOTU84369
New.CleanUp.ReferenceOTU35709
New.CleanUp.ReferenceOTU89413
New.CleanUp.ReferenceOTU42362
New.CleanUp.ReferenceOTU41402
New.CleanUp.ReferenceOTU99558
New.CleanUp.ReferenceOTU72553
New.CleanUp.ReferenceOTU140535
New.CleanUp.ReferenceOTU30389
New.CleanUp.ReferenceOTU970
New.CleanUp.ReferenceOTU29950
New.CleanUp.ReferenceOTU142712
New.CleanUp.ReferenceOTU180119
New.CleanUp.ReferenceOTU78130
New.CleanUp.ReferenceOTU233470
New.CleanUp.ReferenceOTU51838
New.CleanUp.ReferenceOTU194736
New.CleanUp.ReferenceOTU36140
New.ReferenceOTU177
New.CleanUp.ReferenceOTU62685
New.CleanUp.ReferenceOTU55436
New.CleanUp.ReferenceOTU179800
New.CleanUp.ReferenceOTU30147
New.ReferenceOTU230
New.CleanUp.ReferenceOTU49532
New.CleanUp.ReferenceOTU34739
New.CleanUp.ReferenceOTU50697
New.CleanUp.ReferenceOTU54647
New.CleanUp.ReferenceOTU40226
New.CleanUp.ReferenceOTU30707
New.CleanUp.ReferenceOTU239711
New.CleanUp.ReferenceOTU242445
New.CleanUp.ReferenceOTU62272
New.CleanUp.ReferenceOTU56821
New.CleanUp.ReferenceOTU319344
New.CleanUp.ReferenceOTU146873
New.CleanUp.ReferenceOTU38897
New.CleanUp.ReferenceOTU59251
New.CleanUp.ReferenceOTU242502
New.CleanUp.ReferenceOTU6929
New.CleanUp.ReferenceOTU76123
New.CleanUp.ReferenceOTU273444
New.CleanUp.ReferenceOTU113392
New.CleanUp.ReferenceOTU91120
New.CleanUp.ReferenceOTU120472
New.CleanUp.ReferenceOTU254641
New.CleanUp.ReferenceOTU262455
New.CleanUp.ReferenceOTU135497
New.CleanUp.ReferenceOTU203611
New.CleanUp.ReferenceOTU240410
New.CleanUp.ReferenceOTU198030
New.CleanUp.ReferenceOTU131186
New.ReferenceOTU579
New.CleanUp.ReferenceOTU64704
New.CleanUp.ReferenceOTU212846
New.CleanUp.ReferenceOTU145711
New.CleanUp.ReferenceOTU27968
New.CleanUp.ReferenceOTU162363
New.CleanUp.ReferenceOTU169383
New.CleanUp.ReferenceOTU282067
New.CleanUp.ReferenceOTU57399
New.CleanUp.ReferenceOTU53974
New.CleanUp.ReferenceOTU58638
New.CleanUp.ReferenceOTU26977

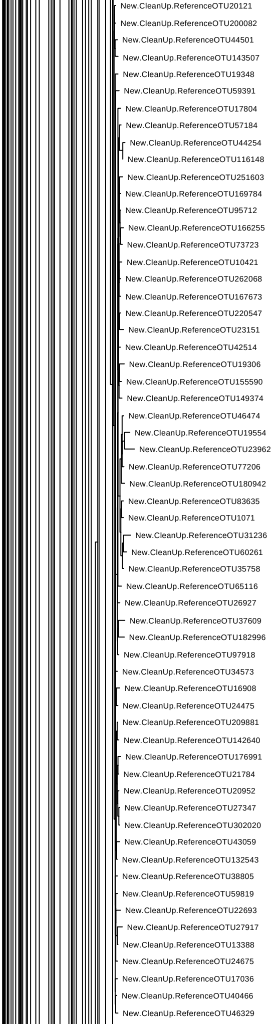
New.CleanUp.ReferenceOTU20121
New.CleanUp.ReferenceOTU200082
New.CleanUp.ReferenceOTU44501
New.CleanUp.ReferenceOTU143507
New.CleanUp.ReferenceOTU19348
New.CleanUp.ReferenceOTU59391
New.CleanUp.ReferenceOTU17804
New.CleanUp.ReferenceOTU57184
New.CleanUp.ReferenceOTU44254
New.CleanUp.ReferenceOTU116148
New.CleanUp.ReferenceOTU251603
New.CleanUp.ReferenceOTU169784
New.CleanUp.ReferenceOTU95712
New.CleanUp.ReferenceOTU166255
New.CleanUp.ReferenceOTU73723
New.CleanUp.ReferenceOTU10421
New.CleanUp.ReferenceOTU262068
New.CleanUp.ReferenceOTU167673
New.CleanUp.ReferenceOTU220547
New.CleanUp.ReferenceOTU23151
New.CleanUp.ReferenceOTU42514
New.CleanUp.ReferenceOTU19306
New.CleanUp.ReferenceOTU155590
New.CleanUp.ReferenceOTU149374
New.CleanUp.ReferenceOTU46474
New.CleanUp.ReferenceOTU19554
New.CleanUp.ReferenceOTU23962
New.CleanUp.ReferenceOTU77206
New.CleanUp.ReferenceOTU180942
New.CleanUp.ReferenceOTU83635
New.CleanUp.ReferenceOTU1071
New.CleanUp.ReferenceOTU31236
New.CleanUp.ReferenceOTU60261
New.CleanUp.ReferenceOTU35758
New.CleanUp.ReferenceOTU65116
New.CleanUp.ReferenceOTU26927
New.CleanUp.ReferenceOTU37609
New.CleanUp.ReferenceOTU182996
New.CleanUp.ReferenceOTU97918
New.CleanUp.ReferenceOTU34573
New.CleanUp.ReferenceOTU16908
New.CleanUp.ReferenceOTU24475
New.CleanUp.ReferenceOTU209881
New.CleanUp.ReferenceOTU142640
New.CleanUp.ReferenceOTU176991
New.CleanUp.ReferenceOTU21784
New.CleanUp.ReferenceOTU20952
New.CleanUp.ReferenceOTU27347
New.CleanUp.ReferenceOTU302020
New.CleanUp.ReferenceOTU43059
New.CleanUp.ReferenceOTU132543
New.CleanUp.ReferenceOTU38805
New.CleanUp.ReferenceOTU59819
New.CleanUp.ReferenceOTU22693
New.CleanUp.ReferenceOTU27917
New.CleanUp.ReferenceOTU13388
New.CleanUp.ReferenceOTU24675
New.CleanUp.ReferenceOTU17036
New.CleanUp.ReferenceOTU40466
New.CleanUp.ReferenceOTU46329

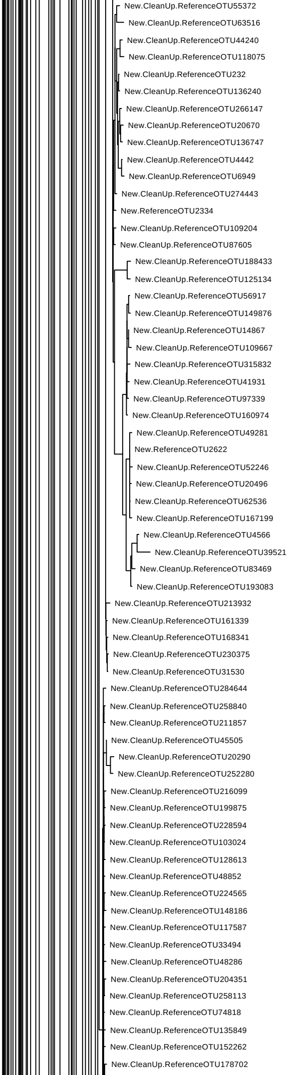
New.CleanUp.ReferenceOTU55372
New.CleanUp.ReferenceOTU63516
New.CleanUp.ReferenceOTU44240
New.CleanUp.ReferenceOTU118075
New.CleanUp.ReferenceOTU232
New.CleanUp.ReferenceOTU136240
New.CleanUp.ReferenceOTU266147
New.CleanUp.ReferenceOTU20670
New.CleanUp.ReferenceOTU136747
New.CleanUp.ReferenceOTU4442
New.CleanUp.ReferenceOTU6949
New.CleanUp.ReferenceOTU274443
New.ReferenceOTU2334
New.CleanUp.ReferenceOTU109204
New.CleanUp.ReferenceOTU87605
New.CleanUp.ReferenceOTU188433
New.CleanUp.ReferenceOTU125134
New.CleanUp.ReferenceOTU56917
New.CleanUp.ReferenceOTU149876
New.CleanUp.ReferenceOTU14867
New.CleanUp.ReferenceOTU109667
New.CleanUp.ReferenceOTU315832
New.CleanUp.ReferenceOTU41931
New.CleanUp.ReferenceOTU97339
New.CleanUp.ReferenceOTU160974
New.CleanUp.ReferenceOTU49281
New.ReferenceOTU2622
New.CleanUp.ReferenceOTU52246
New.CleanUp.ReferenceOTU20496
New.CleanUp.ReferenceOTU62536
New.CleanUp.ReferenceOTU167199
New.CleanUp.ReferenceOTU4566
New.CleanUp.ReferenceOTU39521
New.CleanUp.ReferenceOTU83469
New.CleanUp.ReferenceOTU193083
New.CleanUp.ReferenceOTU213932
New.CleanUp.ReferenceOTU161339
New.CleanUp.ReferenceOTU168341
New.CleanUp.ReferenceOTU230375
New.CleanUp.ReferenceOTU31530
New.CleanUp.ReferenceOTU284644
New.CleanUp.ReferenceOTU258840
New.CleanUp.ReferenceOTU211857
New.CleanUp.ReferenceOTU45505
New.CleanUp.ReferenceOTU20290
New.CleanUp.ReferenceOTU252280
New.CleanUp.ReferenceOTU216099
New.CleanUp.ReferenceOTU199875
New.CleanUp.ReferenceOTU228594
New.CleanUp.ReferenceOTU103024
New.CleanUp.ReferenceOTU128613
New.CleanUp.ReferenceOTU48852
New.CleanUp.ReferenceOTU224565
New.CleanUp.ReferenceOTU148186
New.CleanUp.ReferenceOTU117587
New.CleanUp.ReferenceOTU33494
New.CleanUp.ReferenceOTU48286
New.CleanUp.ReferenceOTU204351
New.CleanUp.ReferenceOTU258113
New.CleanUp.ReferenceOTU74818
New.CleanUp.ReferenceOTU135849
New.CleanUp.ReferenceOTU152262
New.CleanUp.ReferenceOTU178702

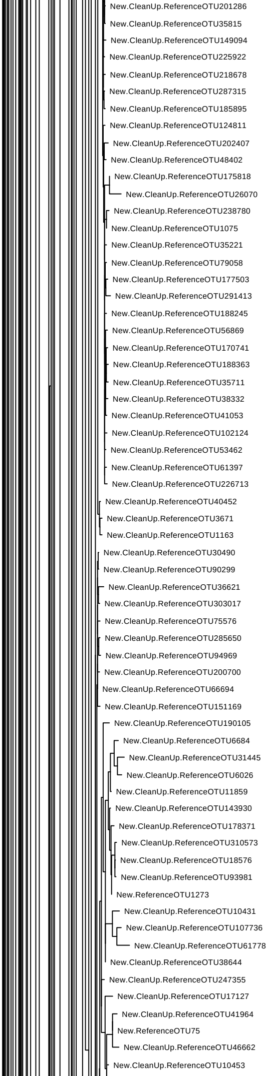
New.CleanUp.ReferenceOTU201286
New.CleanUp.ReferenceOTU35815
New.CleanUp.ReferenceOTU149094
New.CleanUp.ReferenceOTU225922
New.CleanUp.ReferenceOTU218678
New.CleanUp.ReferenceOTU287315
New.CleanUp.ReferenceOTU185895
New.CleanUp.ReferenceOTU124811
New.CleanUp.ReferenceOTU202407
New.CleanUp.ReferenceOTU48402
New.CleanUp.ReferenceOTU175818
New.CleanUp.ReferenceOTU26070
New.CleanUp.ReferenceOTU238780
New.CleanUp.ReferenceOTU1075
New.CleanUp.ReferenceOTU35221
New.CleanUp.ReferenceOTU79058
New.CleanUp.ReferenceOTU177503
New.CleanUp.ReferenceOTU291413
New.CleanUp.ReferenceOTU188245
New.CleanUp.ReferenceOTU56869
New.CleanUp.ReferenceOTU170741
New.CleanUp.ReferenceOTU188363
New.CleanUp.ReferenceOTU35711
New.CleanUp.ReferenceOTU38332
New.CleanUp.ReferenceOTU41053
New.CleanUp.ReferenceOTU102124
New.CleanUp.ReferenceOTU53462
New.CleanUp.ReferenceOTU61397
New.CleanUp.ReferenceOTU226713
New.CleanUp.ReferenceOTU40452
New.CleanUp.ReferenceOTU3671
New.CleanUp.ReferenceOTU1163
New.CleanUp.ReferenceOTU30490
New.CleanUp.ReferenceOTU90299
New.CleanUp.ReferenceOTU36621
New.CleanUp.ReferenceOTU303017
New.CleanUp.ReferenceOTU75576
New.CleanUp.ReferenceOTU285650
New.CleanUp.ReferenceOTU94969
New.CleanUp.ReferenceOTU200700
New.CleanUp.ReferenceOTU66694
New.CleanUp.ReferenceOTU151169
New.CleanUp.ReferenceOTU190105
New.CleanUp.ReferenceOTU6684
New.CleanUp.ReferenceOTU31445
New.CleanUp.ReferenceOTU6026
New.CleanUp.ReferenceOTU11859
New.CleanUp.ReferenceOTU143930
New.CleanUp.ReferenceOTU178371
New.CleanUp.ReferenceOTU310573
New.CleanUp.ReferenceOTU18576
New.CleanUp.ReferenceOTU93981
New.ReferenceOTU1273
New.CleanUp.ReferenceOTU10431
New.CleanUp.ReferenceOTU107736
New.CleanUp.ReferenceOTU61778
New.CleanUp.ReferenceOTU38644
New.CleanUp.ReferenceOTU247355
New.CleanUp.ReferenceOTU17127
New.CleanUp.ReferenceOTU41964
New.ReferenceOTU75
New.CleanUp.ReferenceOTU46662
New.CleanUp.ReferenceOTU10453

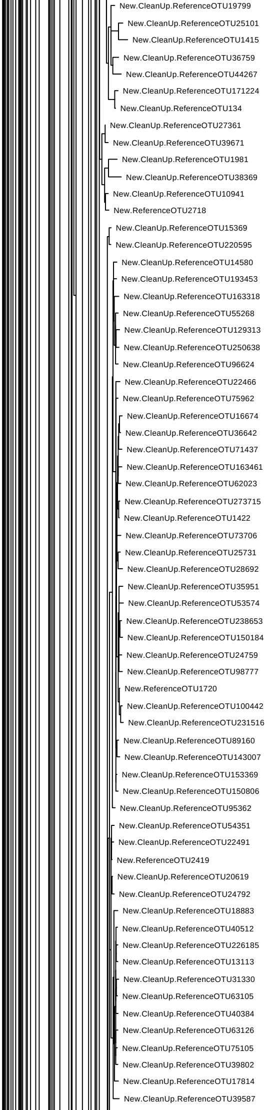
New.CleanUp.ReferenceOTU19799
New.CleanUp.ReferenceOTU25101
New.CleanUp.ReferenceOTU1415
New.CleanUp.ReferenceOTU36759
New.CleanUp.ReferenceOTU44267
New.CleanUp.ReferenceOTU171224
New.CleanUp.ReferenceOTU134
New.CleanUp.ReferenceOTU27361
New.CleanUp.ReferenceOTU39671
New.CleanUp.ReferenceOTU1981
New.CleanUp.ReferenceOTU38369
New.CleanUp.ReferenceOTU10941
New.ReferenceOTU2718
New.CleanUp.ReferenceOTU15369
New.CleanUp.ReferenceOTU220595
New.CleanUp.ReferenceOTU14580
New.CleanUp.ReferenceOTU193453
New.CleanUp.ReferenceOTU163318
New.CleanUp.ReferenceOTU55268
New.CleanUp.ReferenceOTU129313
New.CleanUp.ReferenceOTU250638
New.CleanUp.ReferenceOTU96624
New.CleanUp.ReferenceOTU22466
New.CleanUp.ReferenceOTU75962
New.CleanUp.ReferenceOTU16674
New.CleanUp.ReferenceOTU36642
New.CleanUp.ReferenceOTU71437
New.CleanUp.ReferenceOTU163461
New.CleanUp.ReferenceOTU62023
New.CleanUp.ReferenceOTU273715
New.CleanUp.ReferenceOTU1422
New.CleanUp.ReferenceOTU73706
New.CleanUp.ReferenceOTU25731
New.CleanUp.ReferenceOTU28692
New.CleanUp.ReferenceOTU35951
New.CleanUp.ReferenceOTU53574
New.CleanUp.ReferenceOTU238653
New.CleanUp.ReferenceOTU150184
New.CleanUp.ReferenceOTU24759
New.CleanUp.ReferenceOTU98777
New.ReferenceOTU1720
New.CleanUp.ReferenceOTU100442
New.CleanUp.ReferenceOTU231516
New.CleanUp.ReferenceOTU89160
New.CleanUp.ReferenceOTU143007
New.CleanUp.ReferenceOTU153369
New.CleanUp.ReferenceOTU150806
New.CleanUp.ReferenceOTU95362
New.CleanUp.ReferenceOTU54351
New.CleanUp.ReferenceOTU22491
New.ReferenceOTU2419
New.CleanUp.ReferenceOTU20619
New.CleanUp.ReferenceOTU24792
New.CleanUp.ReferenceOTU18883
New.CleanUp.ReferenceOTU40512
New.CleanUp.ReferenceOTU226185
New.CleanUp.ReferenceOTU13113
New.CleanUp.ReferenceOTU31330
New.CleanUp.ReferenceOTU63105
New.CleanUp.ReferenceOTU40384
New.CleanUp.ReferenceOTU63126
New.CleanUp.ReferenceOTU75105
New.CleanUp.ReferenceOTU39802
New.CleanUp.ReferenceOTU17814
New.CleanUp.ReferenceOTU39587

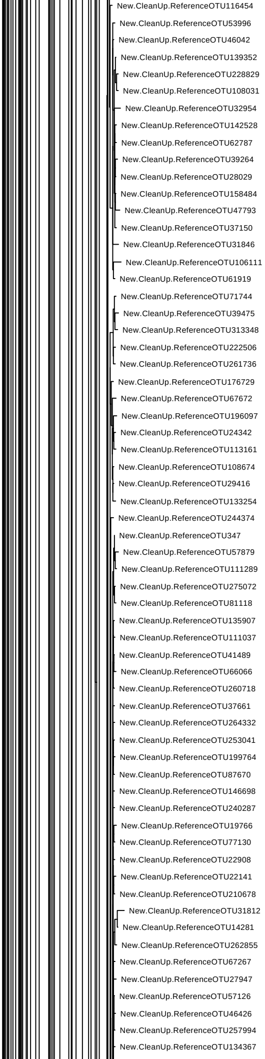
New.CleanUp.ReferenceOTU116454
New.CleanUp.ReferenceOTU53996
New.CleanUp.ReferenceOTU46042
New.CleanUp.ReferenceOTU139352
New.CleanUp.ReferenceOTU228829
New.CleanUp.ReferenceOTU108031
New.CleanUp.ReferenceOTU32954
New.CleanUp.ReferenceOTU142528
New.CleanUp.ReferenceOTU62787
New.CleanUp.ReferenceOTU39264
New.CleanUp.ReferenceOTU28029
New.CleanUp.ReferenceOTU158484
New.CleanUp.ReferenceOTU47793
New.CleanUp.ReferenceOTU37150
New.CleanUp.ReferenceOTU31846
New.CleanUp.ReferenceOTU106111
New.CleanUp.ReferenceOTU61919
New.CleanUp.ReferenceOTU71744
New.CleanUp.ReferenceOTU39475
New.CleanUp.ReferenceOTU313348
New.CleanUp.ReferenceOTU222506
New.CleanUp.ReferenceOTU261736
New.CleanUp.ReferenceOTU176729
New.CleanUp.ReferenceOTU67672
New.CleanUp.ReferenceOTU196097
New.CleanUp.ReferenceOTU24342
New.CleanUp.ReferenceOTU113161
New.CleanUp.ReferenceOTU108674
New.CleanUp.ReferenceOTU29416
New.CleanUp.ReferenceOTU133254
New.CleanUp.ReferenceOTU244374
New.CleanUp.ReferenceOTU347
New.CleanUp.ReferenceOTU57879
New.CleanUp.ReferenceOTU111289
New.CleanUp.ReferenceOTU275072
New.CleanUp.ReferenceOTU81118
New.CleanUp.ReferenceOTU135907
New.CleanUp.ReferenceOTU111037
New.CleanUp.ReferenceOTU41489
New.CleanUp.ReferenceOTU66066
New.CleanUp.ReferenceOTU260718
New.CleanUp.ReferenceOTU37661
New.CleanUp.ReferenceOTU264332
New.CleanUp.ReferenceOTU253041
New.CleanUp.ReferenceOTU199764
New.CleanUp.ReferenceOTU87670
New.CleanUp.ReferenceOTU146698
New.CleanUp.ReferenceOTU240287
New.CleanUp.ReferenceOTU19766
New.CleanUp.ReferenceOTU77130
New.CleanUp.ReferenceOTU22908
New.CleanUp.ReferenceOTU22141
New.CleanUp.ReferenceOTU210678
New.CleanUp.ReferenceOTU31812
New.CleanUp.ReferenceOTU14281
New.CleanUp.ReferenceOTU262855
New.CleanUp.ReferenceOTU67267
New.CleanUp.ReferenceOTU27947
New.CleanUp.ReferenceOTU57126
New.CleanUp.ReferenceOTU46426
New.CleanUp.ReferenceOTU257994
New.CleanUp.ReferenceOTU134367

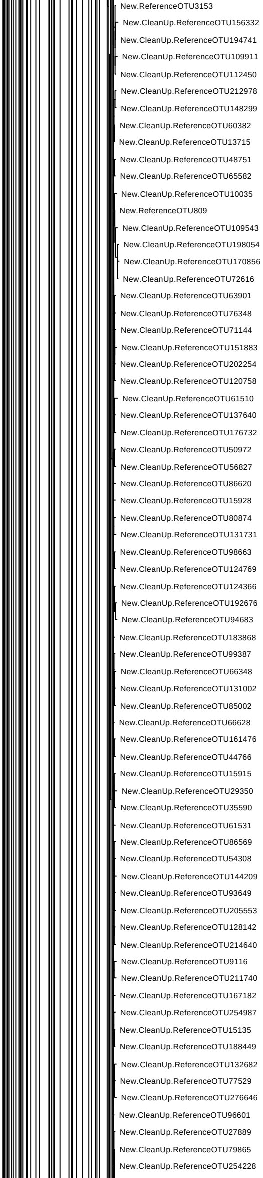
New.ReferenceOTU3153
New.CleanUp.ReferenceOTU156332
New.CleanUp.ReferenceOTU194741
New.CleanUp.ReferenceOTU109911
New.CleanUp.ReferenceOTU112450
New.CleanUp.ReferenceOTU212978
New.CleanUp.ReferenceOTU148299
New.CleanUp.ReferenceOTU60382
New.CleanUp.ReferenceOTU13715
New.CleanUp.ReferenceOTU48751
New.CleanUp.ReferenceOTU65582
New.CleanUp.ReferenceOTU10035
New.ReferenceOTU809
New.CleanUp.ReferenceOTU109543
New.CleanUp.ReferenceOTU198054
New.CleanUp.ReferenceOTU170856
New.CleanUp.ReferenceOTU72616
New.CleanUp.ReferenceOTU63901
New.CleanUp.ReferenceOTU76348
New.CleanUp.ReferenceOTU71144
New.CleanUp.ReferenceOTU151883
New.CleanUp.ReferenceOTU202254
New.CleanUp.ReferenceOTU120758
New.CleanUp.ReferenceOTU61510
New.CleanUp.ReferenceOTU137640
New.CleanUp.ReferenceOTU176732
New.CleanUp.ReferenceOTU50972
New.CleanUp.ReferenceOTU56827
New.CleanUp.ReferenceOTU86620
New.CleanUp.ReferenceOTU15928
New.CleanUp.ReferenceOTU80874
New.CleanUp.ReferenceOTU131731
New.CleanUp.ReferenceOTU98663
New.CleanUp.ReferenceOTU124769
New.CleanUp.ReferenceOTU124366
New.CleanUp.ReferenceOTU192676
New.CleanUp.ReferenceOTU94683
New.CleanUp.ReferenceOTU183868
New.CleanUp.ReferenceOTU99387
New.CleanUp.ReferenceOTU66348
New.CleanUp.ReferenceOTU131002
New.CleanUp.ReferenceOTU85002
New.CleanUp.ReferenceOTU66628
New.CleanUp.ReferenceOTU161476
New.CleanUp.ReferenceOTU44766
New.CleanUp.ReferenceOTU15915
New.CleanUp.ReferenceOTU29350
New.CleanUp.ReferenceOTU35590
New.CleanUp.ReferenceOTU61531
New.CleanUp.ReferenceOTU86569
New.CleanUp.ReferenceOTU54308
New.CleanUp.ReferenceOTU144209
New.CleanUp.ReferenceOTU93649
New.CleanUp.ReferenceOTU205553
New.CleanUp.ReferenceOTU128142
New.CleanUp.ReferenceOTU214640
New.CleanUp.ReferenceOTU9116
New.CleanUp.ReferenceOTU211740
New.CleanUp.ReferenceOTU167182
New.CleanUp.ReferenceOTU254987
New.CleanUp.ReferenceOTU15135
New.CleanUp.ReferenceOTU188449
New.CleanUp.ReferenceOTU132682
New.CleanUp.ReferenceOTU77529
New.CleanUp.ReferenceOTU276646
New.CleanUp.ReferenceOTU96601
New.CleanUp.ReferenceOTU27889
New.CleanUp.ReferenceOTU79865
New.CleanUp.ReferenceOTU254228

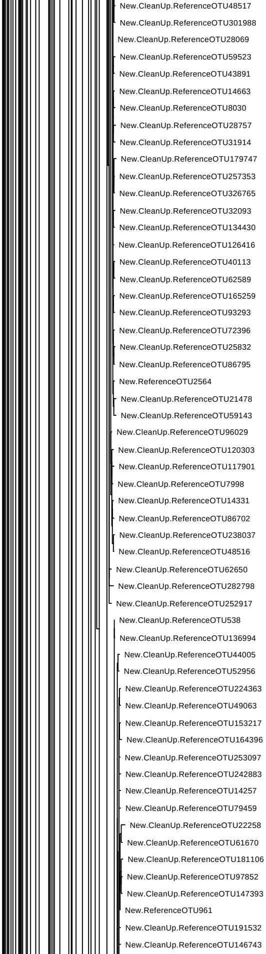
New.CleanUp.ReferenceOTU48517
New.CleanUp.ReferenceOTU301988
New.CleanUp.ReferenceOTU28069
New.CleanUp.ReferenceOTU59523
New.CleanUp.ReferenceOTU43891
New.CleanUp.ReferenceOTU14663
New.CleanUp.ReferenceOTU8030
New.CleanUp.ReferenceOTU28757
New.CleanUp.ReferenceOTU31914
New.CleanUp.ReferenceOTU179747
New.CleanUp.ReferenceOTU257353
New.CleanUp.ReferenceOTU326765
New.CleanUp.ReferenceOTU32093
New.CleanUp.ReferenceOTU134430
New.CleanUp.ReferenceOTU126416
New.CleanUp.ReferenceOTU40113
New.CleanUp.ReferenceOTU62589
New.CleanUp.ReferenceOTU165259
New.CleanUp.ReferenceOTU93293
New.CleanUp.ReferenceOTU72396
New.CleanUp.ReferenceOTU25832
New.CleanUp.ReferenceOTU86795
New.ReferenceOTU2564
New.CleanUp.ReferenceOTU21478
New.CleanUp.ReferenceOTU59143
New.CleanUp.ReferenceOTU96029
New.CleanUp.ReferenceOTU120303
New.CleanUp.ReferenceOTU117901
New.CleanUp.ReferenceOTU7998
New.CleanUp.ReferenceOTU14331
New.CleanUp.ReferenceOTU86702
New.CleanUp.ReferenceOTU238037
New.CleanUp.ReferenceOTU48516
New.CleanUp.ReferenceOTU62650
New.CleanUp.ReferenceOTU282798
New.CleanUp.ReferenceOTU252917
New.CleanUp.ReferenceOTU538
New.CleanUp.ReferenceOTU136994
New.CleanUp.ReferenceOTU44005
New.CleanUp.ReferenceOTU52956
New.CleanUp.ReferenceOTU224363
New.CleanUp.ReferenceOTU49063
New.CleanUp.ReferenceOTU153217
New.CleanUp.ReferenceOTU164396
New.CleanUp.ReferenceOTU253097
New.CleanUp.ReferenceOTU242883
New.CleanUp.ReferenceOTU14257
New.CleanUp.ReferenceOTU79459
New.CleanUp.ReferenceOTU22258
New.CleanUp.ReferenceOTU61670
New.CleanUp.ReferenceOTU181106
New.CleanUp.ReferenceOTU97852
New.CleanUp.ReferenceOTU147393
New.ReferenceOTU961
New.CleanUp.ReferenceOTU191532
New.CleanUp.ReferenceOTU146743

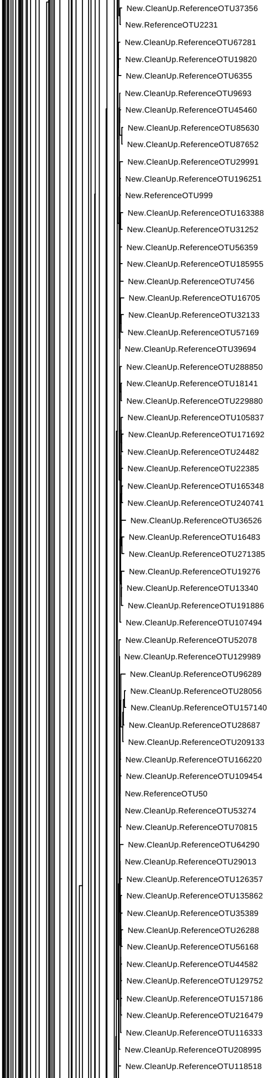
New.CleanUp.ReferenceOTU37356
New.ReferenceOTU2231
New.CleanUp.ReferenceOTU67281
New.CleanUp.ReferenceOTU19820
New.CleanUp.ReferenceOTU6355
New.CleanUp.ReferenceOTU9693
New.CleanUp.ReferenceOTU45460
New.CleanUp.ReferenceOTU85630
New.CleanUp.ReferenceOTU87652
New.CleanUp.ReferenceOTU29991
New.CleanUp.ReferenceOTU196251
New.ReferenceOTU999
New.CleanUp.ReferenceOTU163388
New.CleanUp.ReferenceOTU31252
New.CleanUp.ReferenceOTU56359
New.CleanUp.ReferenceOTU185955
New.CleanUp.ReferenceOTU7456
New.CleanUp.ReferenceOTU16705
New.CleanUp.ReferenceOTU32133
New.CleanUp.ReferenceOTU57169
New.CleanUp.ReferenceOTU39694
New.CleanUp.ReferenceOTU288850
New.CleanUp.ReferenceOTU18141
New.CleanUp.ReferenceOTU229880
New.CleanUp.ReferenceOTU105837
New.CleanUp.ReferenceOTU171692
New.CleanUp.ReferenceOTU24482
New.CleanUp.ReferenceOTU22385
New.CleanUp.ReferenceOTU165348
New.CleanUp.ReferenceOTU240741
New.CleanUp.ReferenceOTU36526
New.CleanUp.ReferenceOTU16483
New.CleanUp.ReferenceOTU271385
New.CleanUp.ReferenceOTU19276
New.CleanUp.ReferenceOTU13340
New.CleanUp.ReferenceOTU191886
New.CleanUp.ReferenceOTU107494
New.CleanUp.ReferenceOTU52078
New.CleanUp.ReferenceOTU129989
New.CleanUp.ReferenceOTU96289
New.CleanUp.ReferenceOTU28056
New.CleanUp.ReferenceOTU157140
New.CleanUp.ReferenceOTU28687
New.CleanUp.ReferenceOTU209133
New.CleanUp.ReferenceOTU166220
New.CleanUp.ReferenceOTU109454
New.ReferenceOTU50
New.CleanUp.ReferenceOTU53274
New.CleanUp.ReferenceOTU70815
New.CleanUp.ReferenceOTU64290
New.CleanUp.ReferenceOTU29013
New.CleanUp.ReferenceOTU126357
New.CleanUp.ReferenceOTU135862
New.CleanUp.ReferenceOTU35389
New.CleanUp.ReferenceOTU26288
New.CleanUp.ReferenceOTU56168
New.CleanUp.ReferenceOTU44582
New.CleanUp.ReferenceOTU129752
New.CleanUp.ReferenceOTU157186
New.CleanUp.ReferenceOTU216479
New.CleanUp.ReferenceOTU116333
New.CleanUp.ReferenceOTU208995
New.CleanUp.ReferenceOTU118518

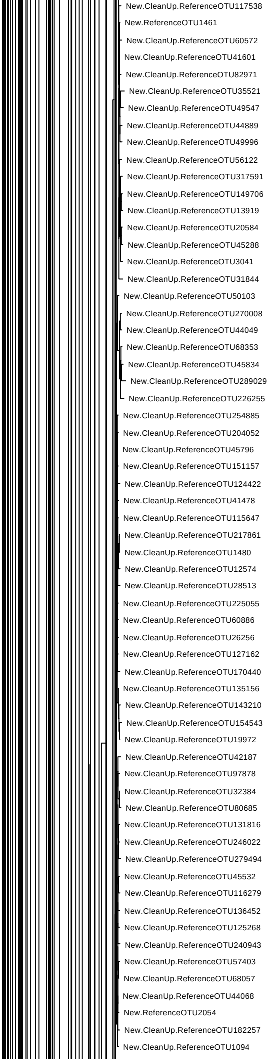
New.CleanUp.ReferenceOTU117538
New.ReferenceOTU1461
New.CleanUp.ReferenceOTU60572
New.CleanUp.ReferenceOTU41601
New.CleanUp.ReferenceOTU82971
New.CleanUp.ReferenceOTU35521
New.CleanUp.ReferenceOTU49547
New.CleanUp.ReferenceOTU44889
New.CleanUp.ReferenceOTU49996
New.CleanUp.ReferenceOTU56122
New.CleanUp.ReferenceOTU317591
New.CleanUp.ReferenceOTU149706
New.CleanUp.ReferenceOTU13919
New.CleanUp.ReferenceOTU20584
New.CleanUp.ReferenceOTU45288
New.CleanUp.ReferenceOTU3041
New.CleanUp.ReferenceOTU31844
New.CleanUp.ReferenceOTU50103
New.CleanUp.ReferenceOTU270008
New.CleanUp.ReferenceOTU44049
New.CleanUp.ReferenceOTU68353
New.CleanUp.ReferenceOTU45834
New.CleanUp.ReferenceOTU289029
New.CleanUp.ReferenceOTU226255
New.CleanUp.ReferenceOTU254885
New.CleanUp.ReferenceOTU204052
New.CleanUp.ReferenceOTU45796
New.CleanUp.ReferenceOTU151157
New.CleanUp.ReferenceOTU124422
New.CleanUp.ReferenceOTU41478
New.CleanUp.ReferenceOTU115647
New.CleanUp.ReferenceOTU217861
New.CleanUp.ReferenceOTU1480
New.CleanUp.ReferenceOTU12574
New.CleanUp.ReferenceOTU28513
New.CleanUp.ReferenceOTU225055
New.CleanUp.ReferenceOTU60886
New.CleanUp.ReferenceOTU26256
New.CleanUp.ReferenceOTU127162
New.CleanUp.ReferenceOTU170440
New.CleanUp.ReferenceOTU135156
New.CleanUp.ReferenceOTU143210
New.CleanUp.ReferenceOTU154543
New.CleanUp.ReferenceOTU19972
New.CleanUp.ReferenceOTU42187
New.CleanUp.ReferenceOTU97878
New.CleanUp.ReferenceOTU32384
New.CleanUp.ReferenceOTU80685
New.CleanUp.ReferenceOTU131816
New.CleanUp.ReferenceOTU246022
New.CleanUp.ReferenceOTU279494
New.CleanUp.ReferenceOTU45532
New.CleanUp.ReferenceOTU116279
New.CleanUp.ReferenceOTU136452
New.CleanUp.ReferenceOTU125268
New.CleanUp.ReferenceOTU240943
New.CleanUp.ReferenceOTU57403
New.CleanUp.ReferenceOTU68057
New.CleanUp.ReferenceOTU44068
New.ReferenceOTU2054
New.CleanUp.ReferenceOTU182257
New.CleanUp.ReferenceOTU1094

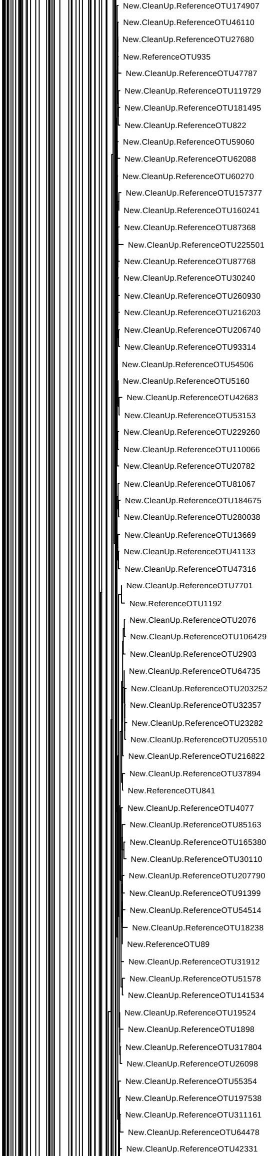
New.CleanUp.ReferenceOTU174907
New.CleanUp.ReferenceOTU46110
New.CleanUp.ReferenceOTU27680
New.ReferenceOTU935
New.CleanUp.ReferenceOTU47787
New.CleanUp.ReferenceOTU119729
New.CleanUp.ReferenceOTU181495
New.CleanUp.ReferenceOTU822
New.CleanUp.ReferenceOTU59060
New.CleanUp.ReferenceOTU62088
New.CleanUp.ReferenceOTU60270
New.CleanUp.ReferenceOTU157377
New.CleanUp.ReferenceOTU160241
New.CleanUp.ReferenceOTU87368
New.CleanUp.ReferenceOTU225501
New.CleanUp.ReferenceOTU87768
New.CleanUp.ReferenceOTU30240
New.CleanUp.ReferenceOTU260930
New.CleanUp.ReferenceOTU216203
New.CleanUp.ReferenceOTU206740
New.CleanUp.ReferenceOTU93314
New.CleanUp.ReferenceOTU54506
New.CleanUp.ReferenceOTU5160
New.CleanUp.ReferenceOTU42683
New.CleanUp.ReferenceOTU53153
New.CleanUp.ReferenceOTU229260
New.CleanUp.ReferenceOTU110066
New.CleanUp.ReferenceOTU20782
New.CleanUp.ReferenceOTU81067
New.CleanUp.ReferenceOTU184675
New.CleanUp.ReferenceOTU280038
New.CleanUp.ReferenceOTU13669
New.CleanUp.ReferenceOTU41133
New.CleanUp.ReferenceOTU47316
New.CleanUp.ReferenceOTU7701
New.ReferenceOTU1192
New.CleanUp.ReferenceOTU2076
New.CleanUp.ReferenceOTU106429
New.CleanUp.ReferenceOTU2903
New.CleanUp.ReferenceOTU64735
New.CleanUp.ReferenceOTU203252
New.CleanUp.ReferenceOTU32357
New.CleanUp.ReferenceOTU23282
New.CleanUp.ReferenceOTU205510
New.CleanUp.ReferenceOTU216822
New.CleanUp.ReferenceOTU37894
New.ReferenceOTU841
New.CleanUp.ReferenceOTU4077
New.CleanUp.ReferenceOTU85163
New.CleanUp.ReferenceOTU165380
New.CleanUp.ReferenceOTU30110
New.CleanUp.ReferenceOTU207790
New.CleanUp.ReferenceOTU91399
New.CleanUp.ReferenceOTU54514
New.CleanUp.ReferenceOTU18238
New.ReferenceOTU89
New.CleanUp.ReferenceOTU31912
New.CleanUp.ReferenceOTU51578
New.CleanUp.ReferenceOTU141534
New.CleanUp.ReferenceOTU19524
New.CleanUp.ReferenceOTU1898
New.CleanUp.ReferenceOTU317804
New.CleanUp.ReferenceOTU26098
New.CleanUp.ReferenceOTU55354
New.CleanUp.ReferenceOTU197538
New.CleanUp.ReferenceOTU311161
New.CleanUp.ReferenceOTU64478
New.CleanUp.ReferenceOTU42331

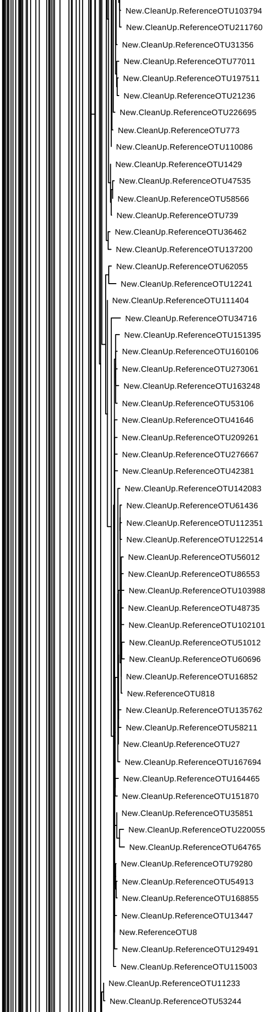
New.CleanUp.ReferenceOTU103794
New.CleanUp.ReferenceOTU211760
New.CleanUp.ReferenceOTU31356
New.CleanUp.ReferenceOTU77011
New.CleanUp.ReferenceOTU197511
New.CleanUp.ReferenceOTU21236
New.CleanUp.ReferenceOTU226695
New.CleanUp.ReferenceOTU773
New.CleanUp.ReferenceOTU110086
New.CleanUp.ReferenceOTU1429
New.CleanUp.ReferenceOTU47535
New.CleanUp.ReferenceOTU58566
New.CleanUp.ReferenceOTU739
New.CleanUp.ReferenceOTU36462
New.CleanUp.ReferenceOTU137200
New.CleanUp.ReferenceOTU62055
New.CleanUp.ReferenceOTU12241
New.CleanUp.ReferenceOTU111404
New.CleanUp.ReferenceOTU34716
New.CleanUp.ReferenceOTU151395
New.CleanUp.ReferenceOTU160106
New.CleanUp.ReferenceOTU273061
New.CleanUp.ReferenceOTU163248
New.CleanUp.ReferenceOTU53106
New.CleanUp.ReferenceOTU41646
New.CleanUp.ReferenceOTU209261
New.CleanUp.ReferenceOTU276667
New.CleanUp.ReferenceOTU42381
New.CleanUp.ReferenceOTU142083
New.CleanUp.ReferenceOTU61436
New.CleanUp.ReferenceOTU112351
New.CleanUp.ReferenceOTU122514
New.CleanUp.ReferenceOTU56012
New.CleanUp.ReferenceOTU86553
New.CleanUp.ReferenceOTU103988
New.CleanUp.ReferenceOTU48735
New.CleanUp.ReferenceOTU102101
New.CleanUp.ReferenceOTU51012
New.CleanUp.ReferenceOTU60696
New.CleanUp.ReferenceOTU16852
New.ReferenceOTU818
New.CleanUp.ReferenceOTU135762
New.CleanUp.ReferenceOTU58211
New.CleanUp.ReferenceOTU27
New.CleanUp.ReferenceOTU167694
New.CleanUp.ReferenceOTU164465
New.CleanUp.ReferenceOTU151870
New.CleanUp.ReferenceOTU35851
New.CleanUp.ReferenceOTU220055
New.CleanUp.ReferenceOTU64765
New.CleanUp.ReferenceOTU79280
New.CleanUp.ReferenceOTU54913
New.CleanUp.ReferenceOTU168855
New.CleanUp.ReferenceOTU13447
New.ReferenceOTU8
New.CleanUp.ReferenceOTU129491
New.CleanUp.ReferenceOTU115003
New.CleanUp.ReferenceOTU11233
New.CleanUp.ReferenceOTU53244

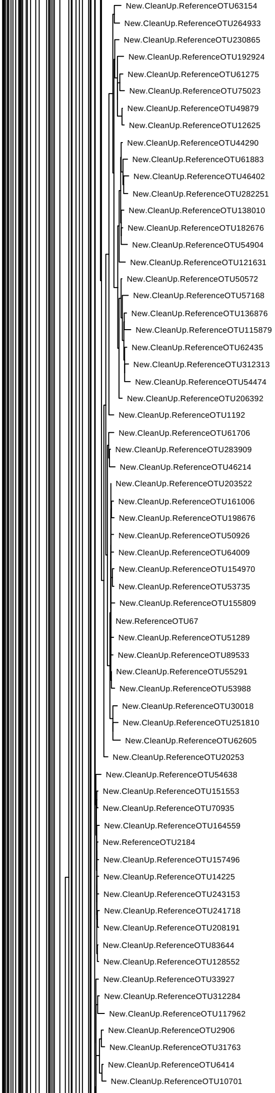
New.CleanUp.ReferenceOTU63154
New.CleanUp.ReferenceOTU264933
New.CleanUp.ReferenceOTU230865
New.CleanUp.ReferenceOTU192924
New.CleanUp.ReferenceOTU61275
New.CleanUp.ReferenceOTU75023
New.CleanUp.ReferenceOTU49879
New.CleanUp.ReferenceOTU12625
New.CleanUp.ReferenceOTU44290
New.CleanUp.ReferenceOTU61883
New.CleanUp.ReferenceOTU46402
New.CleanUp.ReferenceOTU282251
New.CleanUp.ReferenceOTU138010
New.CleanUp.ReferenceOTU182676
New.CleanUp.ReferenceOTU54904
New.CleanUp.ReferenceOTU121631
New.CleanUp.ReferenceOTU50572
New.CleanUp.ReferenceOTU57168
New.CleanUp.ReferenceOTU136876
New.CleanUp.ReferenceOTU115879
New.CleanUp.ReferenceOTU62435
New.CleanUp.ReferenceOTU312313
New.CleanUp.ReferenceOTU54474
New.CleanUp.ReferenceOTU206392
New.CleanUp.ReferenceOTU1192
New.CleanUp.ReferenceOTU61706
New.CleanUp.ReferenceOTU283909
New.CleanUp.ReferenceOTU46214
New.CleanUp.ReferenceOTU203522
New.CleanUp.ReferenceOTU161006
New.CleanUp.ReferenceOTU198676
New.CleanUp.ReferenceOTU50926
New.CleanUp.ReferenceOTU64009
New.CleanUp.ReferenceOTU154970
New.CleanUp.ReferenceOTU53735
New.CleanUp.ReferenceOTU155809
New.ReferenceOTU67
New.CleanUp.ReferenceOTU51289
New.CleanUp.ReferenceOTU89533
New.CleanUp.ReferenceOTU55291
New.CleanUp.ReferenceOTU53988
New.CleanUp.ReferenceOTU30018
New.CleanUp.ReferenceOTU251810
New.CleanUp.ReferenceOTU62605
New.CleanUp.ReferenceOTU20253
New.CleanUp.ReferenceOTU54638
New.CleanUp.ReferenceOTU151553
New.CleanUp.ReferenceOTU70935
New.CleanUp.ReferenceOTU164559
New.ReferenceOTU2184
New.CleanUp.ReferenceOTU157496
New.CleanUp.ReferenceOTU14225
New.CleanUp.ReferenceOTU243153
New.CleanUp.ReferenceOTU241718
New.CleanUp.ReferenceOTU208191
New.CleanUp.ReferenceOTU83644
New.CleanUp.ReferenceOTU128552
New.CleanUp.ReferenceOTU33927
New.CleanUp.ReferenceOTU312284
New.CleanUp.ReferenceOTU117962
New.CleanUp.ReferenceOTU2906
New.CleanUp.ReferenceOTU31763
New.CleanUp.ReferenceOTU6414
New.CleanUp.ReferenceOTU10701

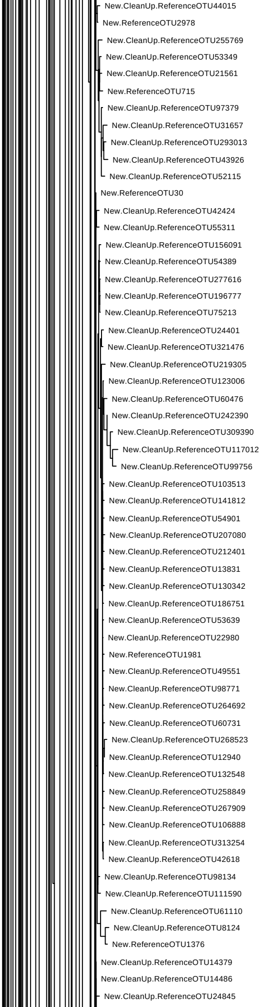
New.CleanUp.ReferenceOTU44015
New.ReferenceOTU2978
New.CleanUp.ReferenceOTU255769
New.CleanUp.ReferenceOTU53349
New.CleanUp.ReferenceOTU21561
New.ReferenceOTU715
New.CleanUp.ReferenceOTU97379
New.CleanUp.ReferenceOTU31657
New.CleanUp.ReferenceOTU293013
New.CleanUp.ReferenceOTU43926
New.CleanUp.ReferenceOTU52115
New.ReferenceOTU30
New.CleanUp.ReferenceOTU42424
New.CleanUp.ReferenceOTU55311
New.CleanUp.ReferenceOTU156091
New.CleanUp.ReferenceOTU54389
New.CleanUp.ReferenceOTU277616
New.CleanUp.ReferenceOTU196777
New.CleanUp.ReferenceOTU75213
New.CleanUp.ReferenceOTU24401
New.CleanUp.ReferenceOTU321476
New.CleanUp.ReferenceOTU219305
New.CleanUp.ReferenceOTU123006
New.CleanUp.ReferenceOTU60476
New.CleanUp.ReferenceOTU242390
New.CleanUp.ReferenceOTU309390
New.CleanUp.ReferenceOTU117012
New.CleanUp.ReferenceOTU99756
New.CleanUp.ReferenceOTU103513
New.CleanUp.ReferenceOTU141812
New.CleanUp.ReferenceOTU54901
New.CleanUp.ReferenceOTU207080
New.CleanUp.ReferenceOTU212401
New.CleanUp.ReferenceOTU13831
New.CleanUp.ReferenceOTU130342
New.CleanUp.ReferenceOTU186751
New.CleanUp.ReferenceOTU53639
New.CleanUp.ReferenceOTU22980
New.ReferenceOTU1981
New.CleanUp.ReferenceOTU49551
New.CleanUp.ReferenceOTU98771
New.CleanUp.ReferenceOTU264692
New.CleanUp.ReferenceOTU60731
New.CleanUp.ReferenceOTU268523
New.CleanUp.ReferenceOTU12940
New.CleanUp.ReferenceOTU132548
New.CleanUp.ReferenceOTU258849
New.CleanUp.ReferenceOTU267909
New.CleanUp.ReferenceOTU106888
New.CleanUp.ReferenceOTU313254
New.CleanUp.ReferenceOTU42618
New.CleanUp.ReferenceOTU98134
New.CleanUp.ReferenceOTU111590
New.CleanUp.ReferenceOTU61110
New.CleanUp.ReferenceOTU8124
New.ReferenceOTU1376
New.CleanUp.ReferenceOTU14379
New.CleanUp.ReferenceOTU14486
New.CleanUp.ReferenceOTU24845

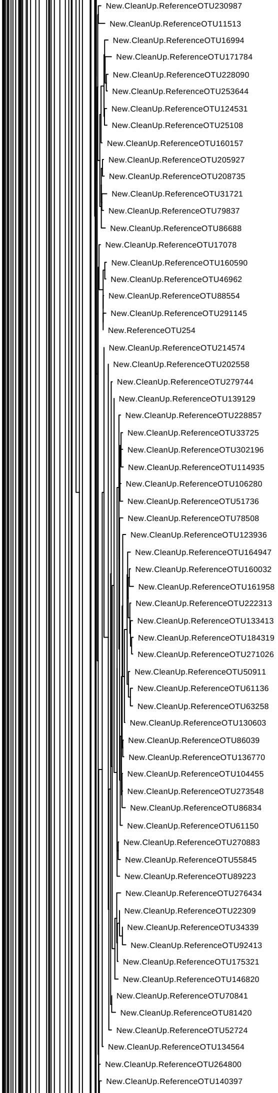
New.CleanUp.ReferenceOTU230987
New.CleanUp.ReferenceOTU11513
New.CleanUp.ReferenceOTU16994
New.CleanUp.ReferenceOTU171784
New.CleanUp.ReferenceOTU228090
New.CleanUp.ReferenceOTU253644
New.CleanUp.ReferenceOTU124531
New.CleanUp.ReferenceOTU25108
New.CleanUp.ReferenceOTU160157
New.CleanUp.ReferenceOTU205927
New.CleanUp.ReferenceOTU208735
New.CleanUp.ReferenceOTU31721
New.CleanUp.ReferenceOTU79837
New.CleanUp.ReferenceOTU86688
New.CleanUp.ReferenceOTU17078
New.CleanUp.ReferenceOTU160590
New.CleanUp.ReferenceOTU46962
New.CleanUp.ReferenceOTU88554
New.CleanUp.ReferenceOTU291145
New.ReferenceOTU254
New.CleanUp.ReferenceOTU214574
New.CleanUp.ReferenceOTU202558
New.CleanUp.ReferenceOTU279744
New.CleanUp.ReferenceOTU139129
New.CleanUp.ReferenceOTU228857
New.CleanUp.ReferenceOTU33725
New.CleanUp.ReferenceOTU302196
New.CleanUp.ReferenceOTU114935
New.CleanUp.ReferenceOTU106280
New.CleanUp.ReferenceOTU51736
New.CleanUp.ReferenceOTU78508
New.CleanUp.ReferenceOTU123936
New.CleanUp.ReferenceOTU164947
New.CleanUp.ReferenceOTU160032
New.CleanUp.ReferenceOTU161958
New.CleanUp.ReferenceOTU222313
New.CleanUp.ReferenceOTU133413
New.CleanUp.ReferenceOTU184319
New.CleanUp.ReferenceOTU271026
New.CleanUp.ReferenceOTU50911
New.CleanUp.ReferenceOTU61136
New.CleanUp.ReferenceOTU63258
New.CleanUp.ReferenceOTU130603
New.CleanUp.ReferenceOTU86039
New.CleanUp.ReferenceOTU136770
New.CleanUp.ReferenceOTU104455
New.CleanUp.ReferenceOTU273548
New.CleanUp.ReferenceOTU86834
New.CleanUp.ReferenceOTU61150
New.CleanUp.ReferenceOTU270883
New.CleanUp.ReferenceOTU55845
New.CleanUp.ReferenceOTU89223
New.CleanUp.ReferenceOTU276434
New.CleanUp.ReferenceOTU22309
New.CleanUp.ReferenceOTU34339
New.CleanUp.ReferenceOTU92413
New.CleanUp.ReferenceOTU175321
New.CleanUp.ReferenceOTU146820
New.CleanUp.ReferenceOTU70841
New.CleanUp.ReferenceOTU81420
New.CleanUp.ReferenceOTU52724
New.CleanUp.ReferenceOTU134564
New.CleanUp.ReferenceOTU264800
New.CleanUp.ReferenceOTU140397

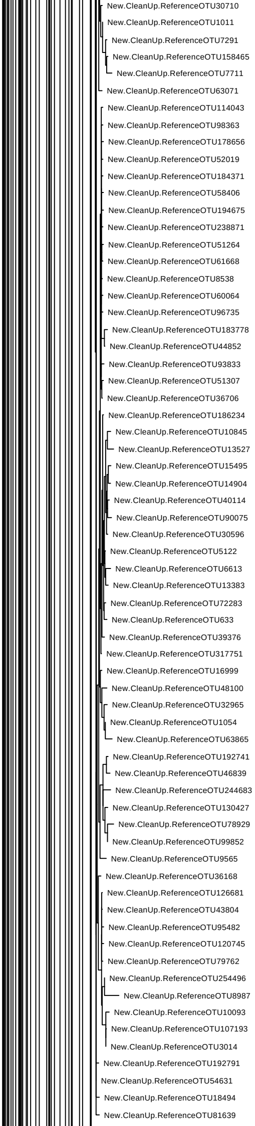
New.CleanUp.ReferenceOTU30710
New.CleanUp.ReferenceOTU1011
New.CleanUp.ReferenceOTU7291
New.CleanUp.ReferenceOTU158465
New.CleanUp.ReferenceOTU7711
New.CleanUp.ReferenceOTU63071
New.CleanUp.ReferenceOTU114043
New.CleanUp.ReferenceOTU98363
New.CleanUp.ReferenceOTU178656
New.CleanUp.ReferenceOTU52019
New.CleanUp.ReferenceOTU184371
New.CleanUp.ReferenceOTU58406
New.CleanUp.ReferenceOTU194675
New.CleanUp.ReferenceOTU238871
New.CleanUp.ReferenceOTU51264
New.CleanUp.ReferenceOTU61668
New.CleanUp.ReferenceOTU8538
New.CleanUp.ReferenceOTU60064
New.CleanUp.ReferenceOTU96735
New.CleanUp.ReferenceOTU183778
New.CleanUp.ReferenceOTU44852
New.CleanUp.ReferenceOTU93833
New.CleanUp.ReferenceOTU51307
New.CleanUp.ReferenceOTU36706
New.CleanUp.ReferenceOTU186234
New.CleanUp.ReferenceOTU10845
New.CleanUp.ReferenceOTU13527
New.CleanUp.ReferenceOTU15495
New.CleanUp.ReferenceOTU14904
New.CleanUp.ReferenceOTU40114
New.CleanUp.ReferenceOTU90075
New.CleanUp.ReferenceOTU30596
New.CleanUp.ReferenceOTU5122
New.CleanUp.ReferenceOTU6613
New.CleanUp.ReferenceOTU13383
New.CleanUp.ReferenceOTU72283
New.CleanUp.ReferenceOTU633
New.CleanUp.ReferenceOTU39376
New.CleanUp.ReferenceOTU317751
New.CleanUp.ReferenceOTU16999
New.CleanUp.ReferenceOTU48100
New.CleanUp.ReferenceOTU32965
New.CleanUp.ReferenceOTU1054
New.CleanUp.ReferenceOTU63865
New.CleanUp.ReferenceOTU192741
New.CleanUp.ReferenceOTU46839
New.CleanUp.ReferenceOTU244683
New.CleanUp.ReferenceOTU130427
New.CleanUp.ReferenceOTU78929
New.CleanUp.ReferenceOTU99852
New.CleanUp.ReferenceOTU9565
New.CleanUp.ReferenceOTU36168
New.CleanUp.ReferenceOTU126681
New.CleanUp.ReferenceOTU43804
New.CleanUp.ReferenceOTU95482
New.CleanUp.ReferenceOTU120745
New.CleanUp.ReferenceOTU79762
New.CleanUp.ReferenceOTU254496
New.CleanUp.ReferenceOTU8987
New.CleanUp.ReferenceOTU10093
New.CleanUp.ReferenceOTU107193
New.CleanUp.ReferenceOTU3014
New.CleanUp.ReferenceOTU192791
New.CleanUp.ReferenceOTU54631
New.CleanUp.ReferenceOTU18494
New.CleanUp.ReferenceOTU81639

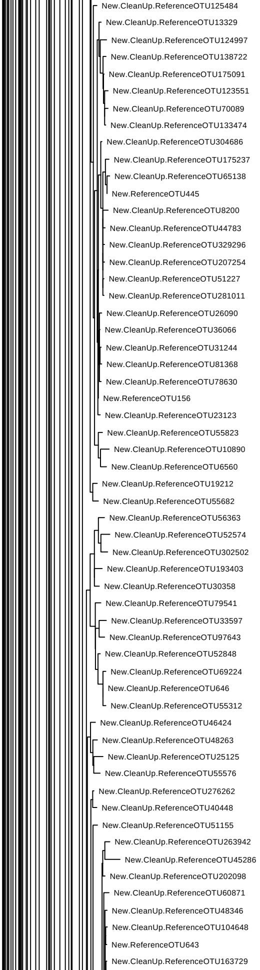
New.CleanUp.ReferenceOTU125484
New.CleanUp.ReferenceOTU13329
New.CleanUp.ReferenceOTU124997
New.CleanUp.ReferenceOTU138722
New.CleanUp.ReferenceOTU175091
New.CleanUp.ReferenceOTU123551
New.CleanUp.ReferenceOTU70089
New.CleanUp.ReferenceOTU133474
New.CleanUp.ReferenceOTU304686
New.CleanUp.ReferenceOTU175237
New.CleanUp.ReferenceOTU65138
New.ReferenceOTU445
New.CleanUp.ReferenceOTU8200
New.CleanUp.ReferenceOTU44783
New.CleanUp.ReferenceOTU329296
New.CleanUp.ReferenceOTU207254
New.CleanUp.ReferenceOTU51227
New.CleanUp.ReferenceOTU281011
New.CleanUp.ReferenceOTU26090
New.CleanUp.ReferenceOTU36066
New.CleanUp.ReferenceOTU31244
New.CleanUp.ReferenceOTU81368
New.CleanUp.ReferenceOTU78630
New.ReferenceOTU156
New.CleanUp.ReferenceOTU23123
New.CleanUp.ReferenceOTU55823
New.CleanUp.ReferenceOTU10890
New.CleanUp.ReferenceOTU6560
New.CleanUp.ReferenceOTU19212
New.CleanUp.ReferenceOTU55682
New.CleanUp.ReferenceOTU56363
New.CleanUp.ReferenceOTU52574
New.CleanUp.ReferenceOTU302502
New.CleanUp.ReferenceOTU193403
New.CleanUp.ReferenceOTU30358
New.CleanUp.ReferenceOTU79541
New.CleanUp.ReferenceOTU33597
New.CleanUp.ReferenceOTU97643
New.CleanUp.ReferenceOTU52848
New.CleanUp.ReferenceOTU69224
New.CleanUp.ReferenceOTU646
New.CleanUp.ReferenceOTU55312
New.CleanUp.ReferenceOTU46424
New.CleanUp.ReferenceOTU48263
New.CleanUp.ReferenceOTU25125
New.CleanUp.ReferenceOTU55576
New.CleanUp.ReferenceOTU276262
New.CleanUp.ReferenceOTU40448
New.CleanUp.ReferenceOTU51155
New.CleanUp.ReferenceOTU263942
New.CleanUp.ReferenceOTU45286
New.CleanUp.ReferenceOTU202098
New.CleanUp.ReferenceOTU60871
New.CleanUp.ReferenceOTU48346
New.CleanUp.ReferenceOTU104648
New.ReferenceOTU643
New.CleanUp.ReferenceOTU163729

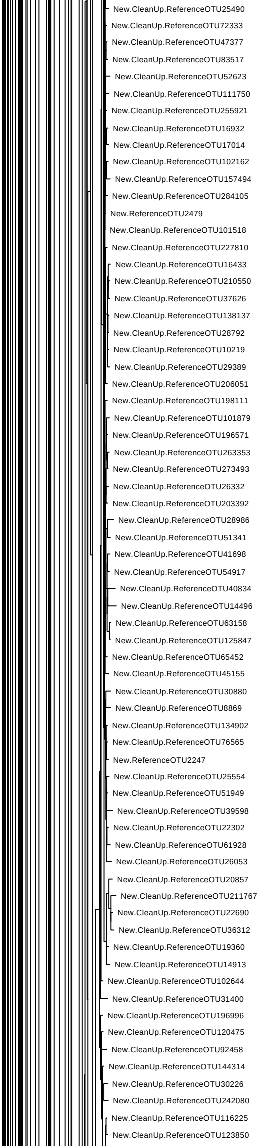
New.CleanUp.ReferenceOTU25490
New.CleanUp.ReferenceOTU72333
New.CleanUp.ReferenceOTU47377
New.CleanUp.ReferenceOTU83517
New.CleanUp.ReferenceOTU52623
New.CleanUp.ReferenceOTU111750
New.CleanUp.ReferenceOTU255921
New.CleanUp.ReferenceOTU16932
New.CleanUp.ReferenceOTU17014
New.CleanUp.ReferenceOTU102162
New.CleanUp.ReferenceOTU157494
New.CleanUp.ReferenceOTU284105
New.ReferenceOTU2479
New.CleanUp.ReferenceOTU101518
New.CleanUp.ReferenceOTU227810
New.CleanUp.ReferenceOTU16433
New.CleanUp.ReferenceOTU210550
New.CleanUp.ReferenceOTU37626
New.CleanUp.ReferenceOTU138137
New.CleanUp.ReferenceOTU28792
New.CleanUp.ReferenceOTU10219
New.CleanUp.ReferenceOTU29389
New.CleanUp.ReferenceOTU206051
New.CleanUp.ReferenceOTU198111
New.CleanUp.ReferenceOTU101879
New.CleanUp.ReferenceOTU196571
New.CleanUp.ReferenceOTU263353
New.CleanUp.ReferenceOTU273493
New.CleanUp.ReferenceOTU26332
New.CleanUp.ReferenceOTU203392
New.CleanUp.ReferenceOTU28986
New.CleanUp.ReferenceOTU51341
New.CleanUp.ReferenceOTU41698
New.CleanUp.ReferenceOTU54917
New.CleanUp.ReferenceOTU40834
New.CleanUp.ReferenceOTU14496
New.CleanUp.ReferenceOTU63158
New.CleanUp.ReferenceOTU125847
New.CleanUp.ReferenceOTU65452
New.CleanUp.ReferenceOTU45155
New.CleanUp.ReferenceOTU30880
New.CleanUp.ReferenceOTU8869
New.CleanUp.ReferenceOTU134902
New.CleanUp.ReferenceOTU76565
New.ReferenceOTU2247
New.CleanUp.ReferenceOTU25554
New.CleanUp.ReferenceOTU51949
New.CleanUp.ReferenceOTU39598
New.CleanUp.ReferenceOTU22302
New.CleanUp.ReferenceOTU61928
New.CleanUp.ReferenceOTU26053
New.CleanUp.ReferenceOTU20857
New.CleanUp.ReferenceOTU211767
New.CleanUp.ReferenceOTU22690
New.CleanUp.ReferenceOTU36312
New.CleanUp.ReferenceOTU19360
New.CleanUp.ReferenceOTU14913
New.CleanUp.ReferenceOTU102644
New.CleanUp.ReferenceOTU31400
New.CleanUp.ReferenceOTU196996
New.CleanUp.ReferenceOTU120475
New.CleanUp.ReferenceOTU92458
New.CleanUp.ReferenceOTU144314
New.CleanUp.ReferenceOTU30226
New.CleanUp.ReferenceOTU242080
New.CleanUp.ReferenceOTU116225
New.CleanUp.ReferenceOTU123850

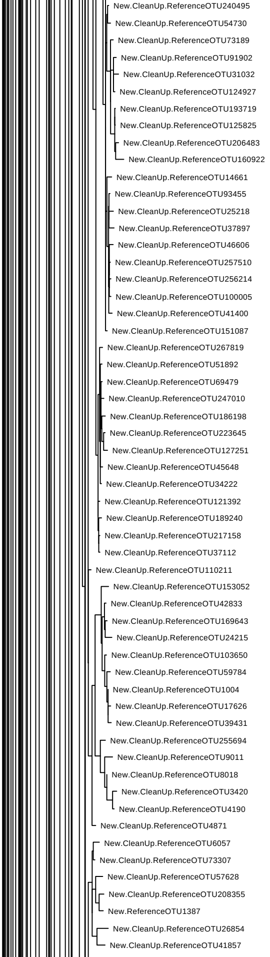
New.CleanUp.ReferenceOTU240495
New.CleanUp.ReferenceOTU54730
New.CleanUp.ReferenceOTU73189
New.CleanUp.ReferenceOTU91902
New.CleanUp.ReferenceOTU31032
New.CleanUp.ReferenceOTU124927
New.CleanUp.ReferenceOTU193719
New.CleanUp.ReferenceOTU125825
New.CleanUp.ReferenceOTU206483
New.CleanUp.ReferenceOTU160922
New.CleanUp.ReferenceOTU14661
New.CleanUp.ReferenceOTU93455
New.CleanUp.ReferenceOTU25218
New.CleanUp.ReferenceOTU37897
New.CleanUp.ReferenceOTU46606
New.CleanUp.ReferenceOTU257510
New.CleanUp.ReferenceOTU256214
New.CleanUp.ReferenceOTU100005
New.CleanUp.ReferenceOTU41400
New.CleanUp.ReferenceOTU151087
New.CleanUp.ReferenceOTU267819
New.CleanUp.ReferenceOTU51892
New.CleanUp.ReferenceOTU69479
New.CleanUp.ReferenceOTU247010
New.CleanUp.ReferenceOTU186198
New.CleanUp.ReferenceOTU223645
New.CleanUp.ReferenceOTU127251
New.CleanUp.ReferenceOTU45648
New.CleanUp.ReferenceOTU34222
New.CleanUp.ReferenceOTU121392
New.CleanUp.ReferenceOTU189240
New.CleanUp.ReferenceOTU217158
New.CleanUp.ReferenceOTU37112
New.CleanUp.ReferenceOTU110211
New.CleanUp.ReferenceOTU153052
New.CleanUp.ReferenceOTU42833
New.CleanUp.ReferenceOTU169643
New.CleanUp.ReferenceOTU24215
New.CleanUp.ReferenceOTU103650
New.CleanUp.ReferenceOTU59784
New.CleanUp.ReferenceOTU1004
New.CleanUp.ReferenceOTU17626
New.CleanUp.ReferenceOTU39431
New.CleanUp.ReferenceOTU255694
New.CleanUp.ReferenceOTU9011
New.CleanUp.ReferenceOTU8018
New.CleanUp.ReferenceOTU3420
New.CleanUp.ReferenceOTU4190
New.CleanUp.ReferenceOTU4871
New.CleanUp.ReferenceOTU6057
New.CleanUp.ReferenceOTU73307
New.CleanUp.ReferenceOTU57628
New.CleanUp.ReferenceOTU208355
New.ReferenceOTU1387
New.CleanUp.ReferenceOTU26854
New.CleanUp.ReferenceOTU41857

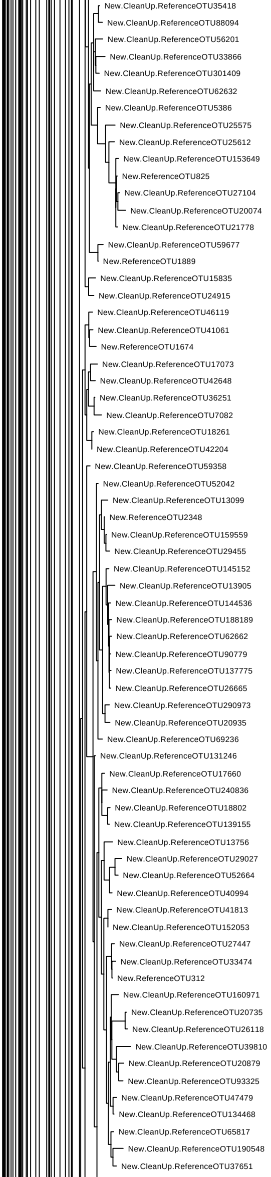
New.CleanUp.ReferenceOTU35418
New.CleanUp.ReferenceOTU88094
New.CleanUp.ReferenceOTU56201
New.CleanUp.ReferenceOTU33866
New.CleanUp.ReferenceOTU301409
New.CleanUp.ReferenceOTU62632
New.CleanUp.ReferenceOTU5386
New.CleanUp.ReferenceOTU25575
New.CleanUp.ReferenceOTU25612
New.CleanUp.ReferenceOTU153649
New.ReferenceOTU825
New.CleanUp.ReferenceOTU27104
New.CleanUp.ReferenceOTU20074
New.CleanUp.ReferenceOTU21778
New.CleanUp.ReferenceOTU59677
New.ReferenceOTU1889
New.CleanUp.ReferenceOTU15835
New.CleanUp.ReferenceOTU24915
New.CleanUp.ReferenceOTU46119
New.CleanUp.ReferenceOTU41061
New.ReferenceOTU1674
New.CleanUp.ReferenceOTU17073
New.CleanUp.ReferenceOTU42648
New.CleanUp.ReferenceOTU36251
New.CleanUp.ReferenceOTU7082
New.CleanUp.ReferenceOTU18261
New.CleanUp.ReferenceOTU42204
New.CleanUp.ReferenceOTU59358
New.CleanUp.ReferenceOTU52042
New.CleanUp.ReferenceOTU13099
New.ReferenceOTU2348
New.CleanUp.ReferenceOTU159559
New.CleanUp.ReferenceOTU29455
New.CleanUp.ReferenceOTU145152
New.CleanUp.ReferenceOTU13905
New.CleanUp.ReferenceOTU144536
New.CleanUp.ReferenceOTU188189
New.CleanUp.ReferenceOTU62662
New.CleanUp.ReferenceOTU90779
New.CleanUp.ReferenceOTU137775
New.CleanUp.ReferenceOTU26665
New.CleanUp.ReferenceOTU290973
New.CleanUp.ReferenceOTU20935
New.CleanUp.ReferenceOTU69236
New.CleanUp.ReferenceOTU131246
New.CleanUp.ReferenceOTU17660
New.CleanUp.ReferenceOTU240836
New.CleanUp.ReferenceOTU18802
New.CleanUp.ReferenceOTU139155
New.CleanUp.ReferenceOTU13756
New.CleanUp.ReferenceOTU29027
New.CleanUp.ReferenceOTU52664
New.CleanUp.ReferenceOTU40994
New.CleanUp.ReferenceOTU41813
New.CleanUp.ReferenceOTU152053
New.CleanUp.ReferenceOTU27447
New.CleanUp.ReferenceOTU33474
New.ReferenceOTU312
New.CleanUp.ReferenceOTU160971
New.CleanUp.ReferenceOTU20735
New.CleanUp.ReferenceOTU26118
New.CleanUp.ReferenceOTU39810
New.CleanUp.ReferenceOTU20879
New.CleanUp.ReferenceOTU93325
New.CleanUp.ReferenceOTU47479
New.CleanUp.ReferenceOTU134468
New.CleanUp.ReferenceOTU65817
New.CleanUp.ReferenceOTU190548
New.CleanUp.ReferenceOTU37651

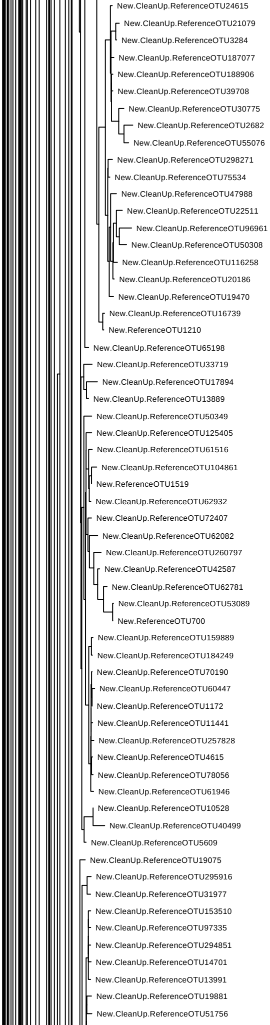
New.CleanUp.ReferenceOTU24615
New.CleanUp.ReferenceOTU21079
New.CleanUp.ReferenceOTU3284
New.CleanUp.ReferenceOTU187077
New.CleanUp.ReferenceOTU188906
New.CleanUp.ReferenceOTU39708
New.CleanUp.ReferenceOTU30775
New.CleanUp.ReferenceOTU2682
New.CleanUp.ReferenceOTU55076
New.CleanUp.ReferenceOTU298271
New.CleanUp.ReferenceOTU75534
New.CleanUp.ReferenceOTU47988
New.CleanUp.ReferenceOTU22511
New.CleanUp.ReferenceOTU96961
New.CleanUp.ReferenceOTU50308
New.CleanUp.ReferenceOTU116258
New.CleanUp.ReferenceOTU20186
New.CleanUp.ReferenceOTU19470
New.CleanUp.ReferenceOTU16739
New.ReferenceOTU1210
New.CleanUp.ReferenceOTU65198
New.CleanUp.ReferenceOTU33719
New.CleanUp.ReferenceOTU17894
New.CleanUp.ReferenceOTU13889
New.CleanUp.ReferenceOTU50349
New.CleanUp.ReferenceOTU125405
New.CleanUp.ReferenceOTU61516
New.CleanUp.ReferenceOTU104861
New.ReferenceOTU1519
New.CleanUp.ReferenceOTU62932
New.CleanUp.ReferenceOTU72407
New.CleanUp.ReferenceOTU62082
New.CleanUp.ReferenceOTU260797
New.CleanUp.ReferenceOTU42587
New.CleanUp.ReferenceOTU62781
New.CleanUp.ReferenceOTU53089
New.ReferenceOTU700
New.CleanUp.ReferenceOTU159889
New.CleanUp.ReferenceOTU184249
New.CleanUp.ReferenceOTU70190
New.CleanUp.ReferenceOTU60447
New.CleanUp.ReferenceOTU1172
New.CleanUp.ReferenceOTU11441
New.CleanUp.ReferenceOTU257828
New.CleanUp.ReferenceOTU4615
New.CleanUp.ReferenceOTU78056
New.CleanUp.ReferenceOTU61946
New.CleanUp.ReferenceOTU10528
New.CleanUp.ReferenceOTU40499
New.CleanUp.ReferenceOTU5609
New.CleanUp.ReferenceOTU19075
New.CleanUp.ReferenceOTU295916
New.CleanUp.ReferenceOTU31977
New.CleanUp.ReferenceOTU153510
New.CleanUp.ReferenceOTU97335
New.CleanUp.ReferenceOTU294851
New.CleanUp.ReferenceOTU14701
New.CleanUp.ReferenceOTU13991
New.CleanUp.ReferenceOTU19881
New.CleanUp.ReferenceOTU51756

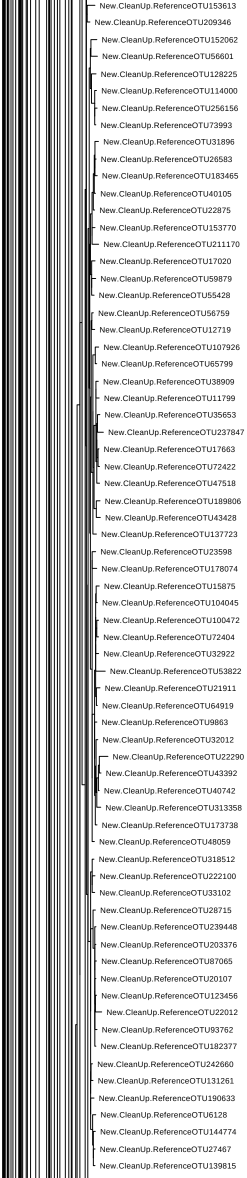
New.CleanUp.ReferenceOTU153613
New.CleanUp.ReferenceOTU209346
New.CleanUp.ReferenceOTU152062
New.CleanUp.ReferenceOTU56601
New.CleanUp.ReferenceOTU128225
New.CleanUp.ReferenceOTU114000
New.CleanUp.ReferenceOTU256156
New.CleanUp.ReferenceOTU73993
New.CleanUp.ReferenceOTU31896
New.CleanUp.ReferenceOTU26583
New.CleanUp.ReferenceOTU183465
New.CleanUp.ReferenceOTU40105
New.CleanUp.ReferenceOTU22875
New.CleanUp.ReferenceOTU153770
New.CleanUp.ReferenceOTU211170
New.CleanUp.ReferenceOTU17020
New.CleanUp.ReferenceOTU59879
New.CleanUp.ReferenceOTU55428
New.CleanUp.ReferenceOTU56759
New.CleanUp.ReferenceOTU12719
New.CleanUp.ReferenceOTU107926
New.CleanUp.ReferenceOTU65799
New.CleanUp.ReferenceOTU38909
New.CleanUp.ReferenceOTU11799
New.CleanUp.ReferenceOTU35653
New.CleanUp.ReferenceOTU237847
New.CleanUp.ReferenceOTU17663
New.CleanUp.ReferenceOTU72422
New.CleanUp.ReferenceOTU47518
New.CleanUp.ReferenceOTU189806
New.CleanUp.ReferenceOTU43428
New.CleanUp.ReferenceOTU137723
New.CleanUp.ReferenceOTU23598
New.CleanUp.ReferenceOTU178074
New.CleanUp.ReferenceOTU15875
New.CleanUp.ReferenceOTU104045
New.CleanUp.ReferenceOTU100472
New.CleanUp.ReferenceOTU72404
New.CleanUp.ReferenceOTU32922
New.CleanUp.ReferenceOTU53822
New.CleanUp.ReferenceOTU21911
New.CleanUp.ReferenceOTU64919
New.CleanUp.ReferenceOTU9863
New.CleanUp.ReferenceOTU32012
New.CleanUp.ReferenceOTU22290
New.CleanUp.ReferenceOTU43392
New.CleanUp.ReferenceOTU40742
New.CleanUp.ReferenceOTU313358
New.CleanUp.ReferenceOTU173738
New.CleanUp.ReferenceOTU48059
New.CleanUp.ReferenceOTU318512
New.CleanUp.ReferenceOTU222100
New.CleanUp.ReferenceOTU33102
New.CleanUp.ReferenceOTU28715
New.CleanUp.ReferenceOTU239448
New.CleanUp.ReferenceOTU203376
New.CleanUp.ReferenceOTU87065
New.CleanUp.ReferenceOTU20107
New.CleanUp.ReferenceOTU123456
New.CleanUp.ReferenceOTU22012
New.CleanUp.ReferenceOTU93762
New.CleanUp.ReferenceOTU182377
New.CleanUp.ReferenceOTU242660
New.CleanUp.ReferenceOTU131261
New.CleanUp.ReferenceOTU190633
New.CleanUp.ReferenceOTU6128
New.CleanUp.ReferenceOTU144774
New.CleanUp.ReferenceOTU27467
New.CleanUp.ReferenceOTU139815

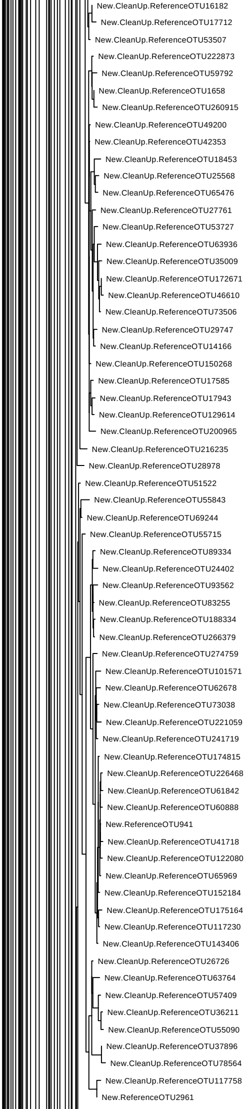
New.CleanUp.ReferenceOTU16182
New.CleanUp.ReferenceOTU17712
New.CleanUp.ReferenceOTU53507
New.CleanUp.ReferenceOTU222873
New.CleanUp.ReferenceOTU59792
New.CleanUp.ReferenceOTU1658
New.CleanUp.ReferenceOTU260915
New.CleanUp.ReferenceOTU49200
New.CleanUp.ReferenceOTU42353
New.CleanUp.ReferenceOTU18453
New.CleanUp.ReferenceOTU25568
New.CleanUp.ReferenceOTU65476
New.CleanUp.ReferenceOTU27761
New.CleanUp.ReferenceOTU53727
New.CleanUp.ReferenceOTU63936
New.CleanUp.ReferenceOTU35009
New.CleanUp.ReferenceOTU172671
New.CleanUp.ReferenceOTU46610
New.CleanUp.ReferenceOTU73506
New.CleanUp.ReferenceOTU29747
New.CleanUp.ReferenceOTU14166
New.CleanUp.ReferenceOTU150268
New.CleanUp.ReferenceOTU17585
New.CleanUp.ReferenceOTU17943
New.CleanUp.ReferenceOTU129614
New.CleanUp.ReferenceOTU200965
New.CleanUp.ReferenceOTU216235
New.CleanUp.ReferenceOTU28978
New.CleanUp.ReferenceOTU51522
New.CleanUp.ReferenceOTU55843
New.CleanUp.ReferenceOTU69244
New.CleanUp.ReferenceOTU55715
New.CleanUp.ReferenceOTU89334
New.CleanUp.ReferenceOTU24402
New.CleanUp.ReferenceOTU93562
New.CleanUp.ReferenceOTU83255
New.CleanUp.ReferenceOTU188334
New.CleanUp.ReferenceOTU266379
New.CleanUp.ReferenceOTU274759
New.CleanUp.ReferenceOTU101571
New.CleanUp.ReferenceOTU62678
New.CleanUp.ReferenceOTU73038
New.CleanUp.ReferenceOTU221059
New.CleanUp.ReferenceOTU241719
New.CleanUp.ReferenceOTU174815
New.CleanUp.ReferenceOTU226468
New.CleanUp.ReferenceOTU61842
New.CleanUp.ReferenceOTU60888
New.ReferenceOTU941
New.CleanUp.ReferenceOTU41718
New.CleanUp.ReferenceOTU122080
New.CleanUp.ReferenceOTU65969
New.CleanUp.ReferenceOTU152184
New.CleanUp.ReferenceOTU175164
New.CleanUp.ReferenceOTU117230
New.CleanUp.ReferenceOTU143406
New.CleanUp.ReferenceOTU26726
New.CleanUp.ReferenceOTU63764
New.CleanUp.ReferenceOTU57409
New.CleanUp.ReferenceOTU36211
New.CleanUp.ReferenceOTU55090
New.CleanUp.ReferenceOTU37896
New.CleanUp.ReferenceOTU78564
New.CleanUp.ReferenceOTU117758
New.ReferenceOTU2961

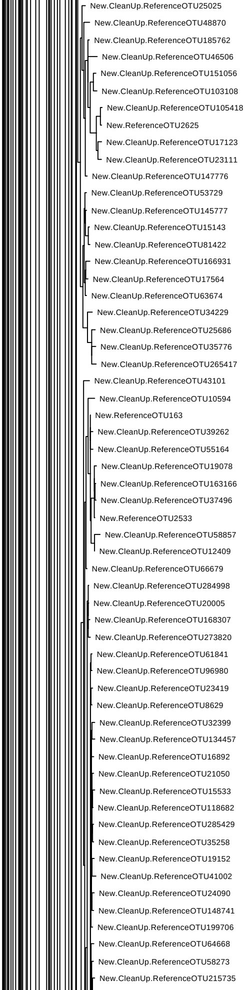
New.CleanUp.ReferenceOTU25025
New.CleanUp.ReferenceOTU48870
New.CleanUp.ReferenceOTU185762
New.CleanUp.ReferenceOTU46506
New.CleanUp.ReferenceOTU151056
New.CleanUp.ReferenceOTU103108
New.CleanUp.ReferenceOTU105418
New.ReferenceOTU2625
New.CleanUp.ReferenceOTU17123
New.CleanUp.ReferenceOTU23111
New.CleanUp.ReferenceOTU147776
New.CleanUp.ReferenceOTU53729
New.CleanUp.ReferenceOTU145777
New.CleanUp.ReferenceOTU15143
New.CleanUp.ReferenceOTU81422
New.CleanUp.ReferenceOTU166931
New.CleanUp.ReferenceOTU17564
New.CleanUp.ReferenceOTU63674
New.CleanUp.ReferenceOTU34229
New.CleanUp.ReferenceOTU25686
New.CleanUp.ReferenceOTU35776
New.CleanUp.ReferenceOTU265417
New.CleanUp.ReferenceOTU43101
New.CleanUp.ReferenceOTU10594
New.ReferenceOTU163
New.CleanUp.ReferenceOTU39262
New.CleanUp.ReferenceOTU55164
New.CleanUp.ReferenceOTU19078
New.CleanUp.ReferenceOTU163166
New.CleanUp.ReferenceOTU37496
New.ReferenceOTU2533
New.CleanUp.ReferenceOTU58857
New.CleanUp.ReferenceOTU12409
New.CleanUp.ReferenceOTU66679
New.CleanUp.ReferenceOTU284998
New.CleanUp.ReferenceOTU20005
New.CleanUp.ReferenceOTU168307
New.CleanUp.ReferenceOTU273820
New.CleanUp.ReferenceOTU61841
New.CleanUp.ReferenceOTU96980
New.CleanUp.ReferenceOTU23419
New.CleanUp.ReferenceOTU8629
New.CleanUp.ReferenceOTU32399
New.CleanUp.ReferenceOTU134457
New.CleanUp.ReferenceOTU16892
New.CleanUp.ReferenceOTU21050
New.CleanUp.ReferenceOTU15533
New.CleanUp.ReferenceOTU118682
New.CleanUp.ReferenceOTU285429
New.CleanUp.ReferenceOTU35258
New.CleanUp.ReferenceOTU19152
New.CleanUp.ReferenceOTU41002
New.CleanUp.ReferenceOTU24090
New.CleanUp.ReferenceOTU148741
New.CleanUp.ReferenceOTU199706
New.CleanUp.ReferenceOTU64668
New.CleanUp.ReferenceOTU58273
New.CleanUp.ReferenceOTU215735

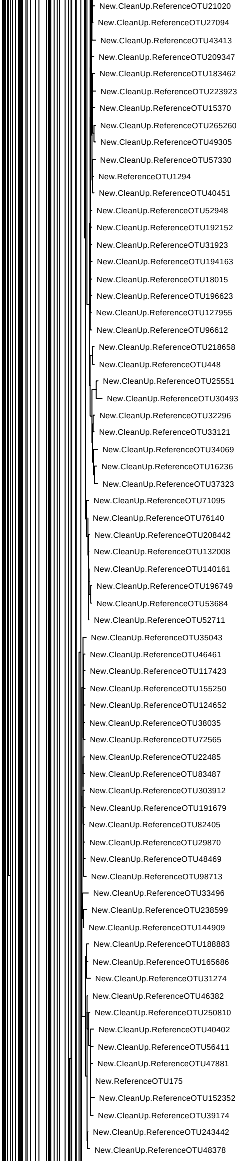
New.CleanUp.ReferenceOTU21020
New.CleanUp.ReferenceOTU27094
New.CleanUp.ReferenceOTU43413
New.CleanUp.ReferenceOTU209347
New.CleanUp.ReferenceOTU183462
New.CleanUp.ReferenceOTU223923
New.CleanUp.ReferenceOTU15370
New.CleanUp.ReferenceOTU265260
New.CleanUp.ReferenceOTU49305
New.CleanUp.ReferenceOTU57330
New.ReferenceOTU1294
New.CleanUp.ReferenceOTU40451
New.CleanUp.ReferenceOTU52948
New.CleanUp.ReferenceOTU192152
New.CleanUp.ReferenceOTU31923
New.CleanUp.ReferenceOTU194163
New.CleanUp.ReferenceOTU18015
New.CleanUp.ReferenceOTU196623
New.CleanUp.ReferenceOTU127955
New.CleanUp.ReferenceOTU96612
New.CleanUp.ReferenceOTU218658
New.CleanUp.ReferenceOTU448
New.CleanUp.ReferenceOTU25551
New.CleanUp.ReferenceOTU30493
New.CleanUp.ReferenceOTU32296
New.CleanUp.ReferenceOTU33121
New.CleanUp.ReferenceOTU34069
New.CleanUp.ReferenceOTU16236
New.CleanUp.ReferenceOTU37323
New.CleanUp.ReferenceOTU71095
New.CleanUp.ReferenceOTU76140
New.CleanUp.ReferenceOTU208442
New.CleanUp.ReferenceOTU132008
New.CleanUp.ReferenceOTU140161
New.CleanUp.ReferenceOTU196749
New.CleanUp.ReferenceOTU53684
New.CleanUp.ReferenceOTU52711
New.CleanUp.ReferenceOTU35043
New.CleanUp.ReferenceOTU46461
New.CleanUp.ReferenceOTU117423
New.CleanUp.ReferenceOTU155250
New.CleanUp.ReferenceOTU124652
New.CleanUp.ReferenceOTU38035
New.CleanUp.ReferenceOTU72565
New.CleanUp.ReferenceOTU22485
New.CleanUp.ReferenceOTU83487
New.CleanUp.ReferenceOTU303912
New.CleanUp.ReferenceOTU191679
New.CleanUp.ReferenceOTU82405
New.CleanUp.ReferenceOTU29870
New.CleanUp.ReferenceOTU48469
New.CleanUp.ReferenceOTU98713
New.CleanUp.ReferenceOTU33496
New.CleanUp.ReferenceOTU238599
New.CleanUp.ReferenceOTU144909
New.CleanUp.ReferenceOTU188883
New.CleanUp.ReferenceOTU165686
New.CleanUp.ReferenceOTU31274
New.CleanUp.ReferenceOTU46382
New.CleanUp.ReferenceOTU250810
New.CleanUp.ReferenceOTU40402
New.CleanUp.ReferenceOTU56411
New.CleanUp.ReferenceOTU47881
New.ReferenceOTU175
New.CleanUp.ReferenceOTU152352
New.CleanUp.ReferenceOTU39174
New.CleanUp.ReferenceOTU243442
New.CleanUp.ReferenceOTU48378

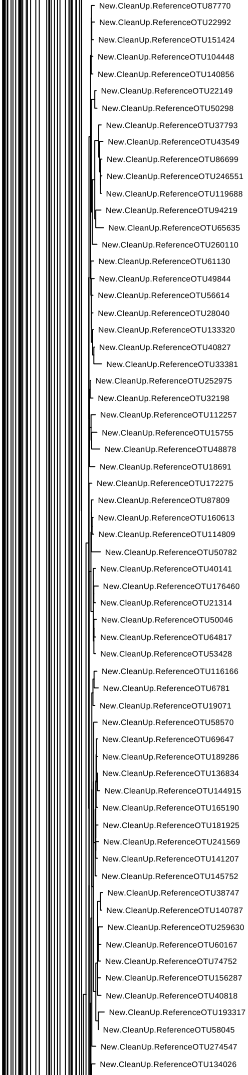
New.CleanUp.ReferenceOTU87770
New.CleanUp.ReferenceOTU22992
New.CleanUp.ReferenceOTU151424
New.CleanUp.ReferenceOTU104448
New.CleanUp.ReferenceOTU140856
New.CleanUp.ReferenceOTU22149
New.CleanUp.ReferenceOTU50298
New.CleanUp.ReferenceOTU37793
New.CleanUp.ReferenceOTU43549
New.CleanUp.ReferenceOTU86699
New.CleanUp.ReferenceOTU246551
New.CleanUp.ReferenceOTU119688
New.CleanUp.ReferenceOTU94219
New.CleanUp.ReferenceOTU65635
New.CleanUp.ReferenceOTU260110
New.CleanUp.ReferenceOTU61130
New.CleanUp.ReferenceOTU49844
New.CleanUp.ReferenceOTU56614
New.CleanUp.ReferenceOTU28040
New.CleanUp.ReferenceOTU133320
New.CleanUp.ReferenceOTU40827
New.CleanUp.ReferenceOTU33381
New.CleanUp.ReferenceOTU252975
New.CleanUp.ReferenceOTU32198
New.CleanUp.ReferenceOTU112257
New.CleanUp.ReferenceOTU15755
New.CleanUp.ReferenceOTU48878
New.CleanUp.ReferenceOTU18691
New.CleanUp.ReferenceOTU172275
New.CleanUp.ReferenceOTU87809
New.CleanUp.ReferenceOTU160613
New.CleanUp.ReferenceOTU114809
New.CleanUp.ReferenceOTU50782
New.CleanUp.ReferenceOTU40141
New.CleanUp.ReferenceOTU176460
New.CleanUp.ReferenceOTU21314
New.CleanUp.ReferenceOTU50046
New.CleanUp.ReferenceOTU64817
New.CleanUp.ReferenceOTU53428
New.CleanUp.ReferenceOTU116166
New.CleanUp.ReferenceOTU6781
New.CleanUp.ReferenceOTU19071
New.CleanUp.ReferenceOTU58570
New.CleanUp.ReferenceOTU69647
New.CleanUp.ReferenceOTU189286
New.CleanUp.ReferenceOTU136834
New.CleanUp.ReferenceOTU144915
New.CleanUp.ReferenceOTU165190
New.CleanUp.ReferenceOTU181925
New.CleanUp.ReferenceOTU241569
New.CleanUp.ReferenceOTU141207
New.CleanUp.ReferenceOTU145752
New.CleanUp.ReferenceOTU38747
New.CleanUp.ReferenceOTU140787
New.CleanUp.ReferenceOTU259630
New.CleanUp.ReferenceOTU60167
New.CleanUp.ReferenceOTU74752
New.CleanUp.ReferenceOTU156287
New.CleanUp.ReferenceOTU40818
New.CleanUp.ReferenceOTU193317
New.CleanUp.ReferenceOTU58045
New.CleanUp.ReferenceOTU274547
New.CleanUp.ReferenceOTU134026

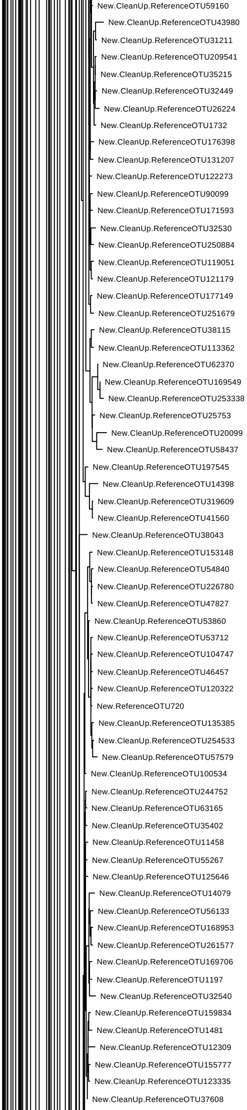
New.CleanUp.ReferenceOTU59160
New.CleanUp.ReferenceOTU43980
New.CleanUp.ReferenceOTU31211
New.CleanUp.ReferenceOTU209541
New.CleanUp.ReferenceOTU35215
New.CleanUp.ReferenceOTU32449
New.CleanUp.ReferenceOTU26224
New.CleanUp.ReferenceOTU1732
New.CleanUp.ReferenceOTU176398
New.CleanUp.ReferenceOTU131207
New.CleanUp.ReferenceOTU122273
New.CleanUp.ReferenceOTU90099
New.CleanUp.ReferenceOTU171593
New.CleanUp.ReferenceOTU32530
New.CleanUp.ReferenceOTU250884
New.CleanUp.ReferenceOTU119051
New.CleanUp.ReferenceOTU121179
New.CleanUp.ReferenceOTU177149
New.CleanUp.ReferenceOTU251679
New.CleanUp.ReferenceOTU38115
New.CleanUp.ReferenceOTU113362
New.CleanUp.ReferenceOTU62370
New.CleanUp.ReferenceOTU169549
New.CleanUp.ReferenceOTU253338
New.CleanUp.ReferenceOTU25753
New.CleanUp.ReferenceOTU20099
New.CleanUp.ReferenceOTU58437
New.CleanUp.ReferenceOTU197545
New.CleanUp.ReferenceOTU14398
New.CleanUp.ReferenceOTU319609
New.CleanUp.ReferenceOTU41560
New.CleanUp.ReferenceOTU38043
New.CleanUp.ReferenceOTU153148
New.CleanUp.ReferenceOTU54840
New.CleanUp.ReferenceOTU226780
New.CleanUp.ReferenceOTU47827
New.CleanUp.ReferenceOTU53860
New.CleanUp.ReferenceOTU53712
New.CleanUp.ReferenceOTU104747
New.CleanUp.ReferenceOTU46457
New.CleanUp.ReferenceOTU120322
New.ReferenceOTU720
New.CleanUp.ReferenceOTU135385
New.CleanUp.ReferenceOTU254533
New.CleanUp.ReferenceOTU57579
New.CleanUp.ReferenceOTU100534
New.CleanUp.ReferenceOTU244752
New.CleanUp.ReferenceOTU63165
New.CleanUp.ReferenceOTU35402
New.CleanUp.ReferenceOTU11458
New.CleanUp.ReferenceOTU55267
New.CleanUp.ReferenceOTU125646
New.CleanUp.ReferenceOTU14079
New.CleanUp.ReferenceOTU56133
New.CleanUp.ReferenceOTU168953
New.CleanUp.ReferenceOTU261577
New.CleanUp.ReferenceOTU169706
New.CleanUp.ReferenceOTU1197
New.CleanUp.ReferenceOTU32540
New.CleanUp.ReferenceOTU159834
New.CleanUp.ReferenceOTU1481
New.CleanUp.ReferenceOTU12309
New.CleanUp.ReferenceOTU155777
New.CleanUp.ReferenceOTU123335
New.CleanUp.ReferenceOTU37608

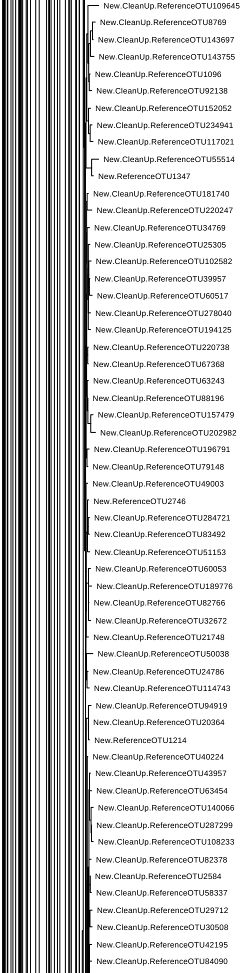
New.CleanUp.ReferenceOTU109645
New.CleanUp.ReferenceOTU8769
New.CleanUp.ReferenceOTU143697
New.CleanUp.ReferenceOTU143755
New.CleanUp.ReferenceOTU1096
New.CleanUp.ReferenceOTU92138
New.CleanUp.ReferenceOTU152052
New.CleanUp.ReferenceOTU234941
New.CleanUp.ReferenceOTU117021
New.CleanUp.ReferenceOTU55514
New.ReferenceOTU1347
New.CleanUp.ReferenceOTU181740
New.CleanUp.ReferenceOTU220247
New.CleanUp.ReferenceOTU34769
New.CleanUp.ReferenceOTU25305
New.CleanUp.ReferenceOTU102582
New.CleanUp.ReferenceOTU39957
New.CleanUp.ReferenceOTU60517
New.CleanUp.ReferenceOTU278040
New.CleanUp.ReferenceOTU194125
New.CleanUp.ReferenceOTU220738
New.CleanUp.ReferenceOTU67368
New.CleanUp.ReferenceOTU63243
New.CleanUp.ReferenceOTU88196
New.CleanUp.ReferenceOTU157479
New.CleanUp.ReferenceOTU202982
New.CleanUp.ReferenceOTU196791
New.CleanUp.ReferenceOTU79148
New.CleanUp.ReferenceOTU49003
New.ReferenceOTU2746
New.CleanUp.ReferenceOTU284721
New.CleanUp.ReferenceOTU83492
New.CleanUp.ReferenceOTU51153
New.CleanUp.ReferenceOTU60053
New.CleanUp.ReferenceOTU189776
New.CleanUp.ReferenceOTU82766
New.CleanUp.ReferenceOTU32672
New.CleanUp.ReferenceOTU21748
New.CleanUp.ReferenceOTU50038
New.CleanUp.ReferenceOTU24786
New.CleanUp.ReferenceOTU114743
New.CleanUp.ReferenceOTU94919
New.CleanUp.ReferenceOTU20364
New.ReferenceOTU1214
New.CleanUp.ReferenceOTU40224
New.CleanUp.ReferenceOTU43957
New.CleanUp.ReferenceOTU63454
New.CleanUp.ReferenceOTU140066
New.CleanUp.ReferenceOTU287299
New.CleanUp.ReferenceOTU108233
New.CleanUp.ReferenceOTU82378
New.CleanUp.ReferenceOTU2584
New.CleanUp.ReferenceOTU58337
New.CleanUp.ReferenceOTU29712
New.CleanUp.ReferenceOTU30508
New.CleanUp.ReferenceOTU42195
New.CleanUp.ReferenceOTU84090

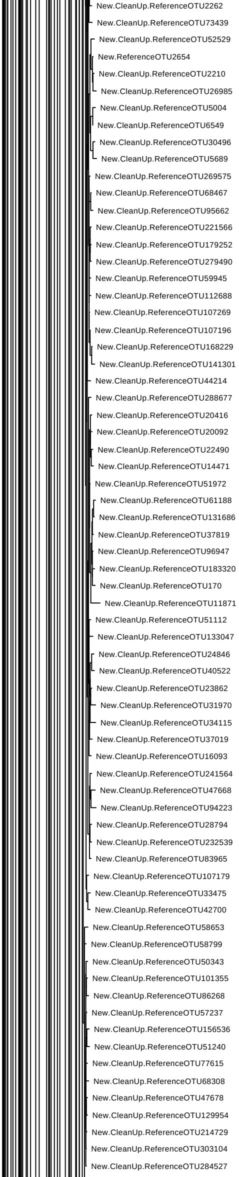
New.CleanUp.ReferenceOTU2262
New.CleanUp.ReferenceOTU73439
New.CleanUp.ReferenceOTU52529
New.ReferenceOTU2654
New.CleanUp.ReferenceOTU2210
New.CleanUp.ReferenceOTU26985
New.CleanUp.ReferenceOTU5004
New.CleanUp.ReferenceOTU6549
New.CleanUp.ReferenceOTU30496
New.CleanUp.ReferenceOTU5689
New.CleanUp.ReferenceOTU269575
New.CleanUp.ReferenceOTU68467
New.CleanUp.ReferenceOTU95662
New.CleanUp.ReferenceOTU221566
New.CleanUp.ReferenceOTU179252
New.CleanUp.ReferenceOTU279490
New.CleanUp.ReferenceOTU59945
New.CleanUp.ReferenceOTU112688
New.CleanUp.ReferenceOTU107269
New.CleanUp.ReferenceOTU107196
New.CleanUp.ReferenceOTU168229
New.CleanUp.ReferenceOTU141301
New.CleanUp.ReferenceOTU44214
New.CleanUp.ReferenceOTU288677
New.CleanUp.ReferenceOTU20416
New.CleanUp.ReferenceOTU20092
New.CleanUp.ReferenceOTU22490
New.CleanUp.ReferenceOTU14471
New.CleanUp.ReferenceOTU51972
New.CleanUp.ReferenceOTU61188
New.CleanUp.ReferenceOTU131686
New.CleanUp.ReferenceOTU37819
New.CleanUp.ReferenceOTU96947
New.CleanUp.ReferenceOTU183320
New.CleanUp.ReferenceOTU170
New.CleanUp.ReferenceOTU11871
New.CleanUp.ReferenceOTU51112
New.CleanUp.ReferenceOTU133047
New.CleanUp.ReferenceOTU24846
New.CleanUp.ReferenceOTU40522
New.CleanUp.ReferenceOTU23862
New.CleanUp.ReferenceOTU31970
New.CleanUp.ReferenceOTU34115
New.CleanUp.ReferenceOTU37019
New.CleanUp.ReferenceOTU16093
New.CleanUp.ReferenceOTU241564
New.CleanUp.ReferenceOTU47668
New.CleanUp.ReferenceOTU94223
New.CleanUp.ReferenceOTU28794
New.CleanUp.ReferenceOTU232539
New.CleanUp.ReferenceOTU83965
New.CleanUp.ReferenceOTU107179
New.CleanUp.ReferenceOTU33475
New.CleanUp.ReferenceOTU42700
New.CleanUp.ReferenceOTU58653
New.CleanUp.ReferenceOTU58799
New.CleanUp.ReferenceOTU50343
New.CleanUp.ReferenceOTU101355
New.CleanUp.ReferenceOTU86268
New.CleanUp.ReferenceOTU57237
New.CleanUp.ReferenceOTU156536
New.CleanUp.ReferenceOTU51240
New.CleanUp.ReferenceOTU77615
New.CleanUp.ReferenceOTU68308
New.CleanUp.ReferenceOTU47678
New.CleanUp.ReferenceOTU129954
New.CleanUp.ReferenceOTU214729
New.CleanUp.ReferenceOTU303104
New.CleanUp.ReferenceOTU284527

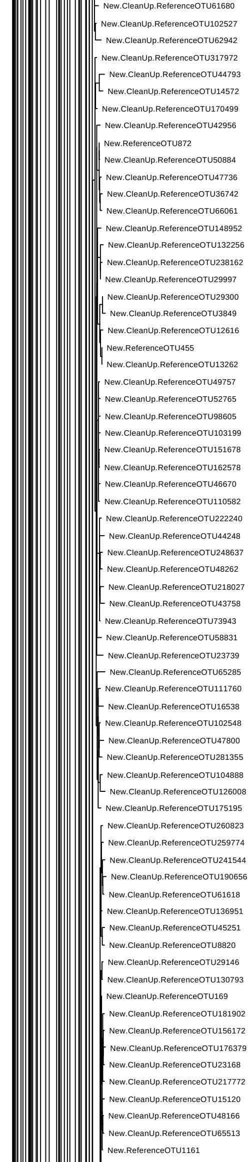
New.CleanUp.ReferenceOTU61680
New.CleanUp.ReferenceOTU102527
New.CleanUp.ReferenceOTU62942
New.CleanUp.ReferenceOTU317972
New.CleanUp.ReferenceOTU44793
New.CleanUp.ReferenceOTU14572
New.CleanUp.ReferenceOTU170499
New.CleanUp.ReferenceOTU42956
New.ReferenceOTU872
New.CleanUp.ReferenceOTU50884
New.CleanUp.ReferenceOTU47736
New.CleanUp.ReferenceOTU36742
New.CleanUp.ReferenceOTU66061
New.CleanUp.ReferenceOTU148952
New.CleanUp.ReferenceOTU132256
New.CleanUp.ReferenceOTU238162
New.CleanUp.ReferenceOTU29997
New.CleanUp.ReferenceOTU29300
New.CleanUp.ReferenceOTU3849
New.CleanUp.ReferenceOTU12616
New.ReferenceOTU455
New.CleanUp.ReferenceOTU13262
New.CleanUp.ReferenceOTU49757
New.CleanUp.ReferenceOTU52765
New.CleanUp.ReferenceOTU98605
New.CleanUp.ReferenceOTU103199
New.CleanUp.ReferenceOTU151678
New.CleanUp.ReferenceOTU162578
New.CleanUp.ReferenceOTU46670
New.CleanUp.ReferenceOTU110582
New.CleanUp.ReferenceOTU222240
New.CleanUp.ReferenceOTU44248
New.CleanUp.ReferenceOTU248637
New.CleanUp.ReferenceOTU48262
New.CleanUp.ReferenceOTU218027
New.CleanUp.ReferenceOTU43758
New.CleanUp.ReferenceOTU73943
New.CleanUp.ReferenceOTU58831
New.CleanUp.ReferenceOTU23739
New.CleanUp.ReferenceOTU65285
New.CleanUp.ReferenceOTU111760
New.CleanUp.ReferenceOTU16538
New.CleanUp.ReferenceOTU102548
New.CleanUp.ReferenceOTU47800
New.CleanUp.ReferenceOTU281355
New.CleanUp.ReferenceOTU104888
New.CleanUp.ReferenceOTU126008
New.CleanUp.ReferenceOTU175195
New.CleanUp.ReferenceOTU260823
New.CleanUp.ReferenceOTU259774
New.CleanUp.ReferenceOTU241544
New.CleanUp.ReferenceOTU190656
New.CleanUp.ReferenceOTU61618
New.CleanUp.ReferenceOTU136951
New.CleanUp.ReferenceOTU45251
New.CleanUp.ReferenceOTU8820
New.CleanUp.ReferenceOTU29146
New.CleanUp.ReferenceOTU130793
New.CleanUp.ReferenceOTU169
New.CleanUp.ReferenceOTU181902
New.CleanUp.ReferenceOTU156172
New.CleanUp.ReferenceOTU176379
New.CleanUp.ReferenceOTU23168
New.CleanUp.ReferenceOTU217772
New.CleanUp.ReferenceOTU15120
New.CleanUp.ReferenceOTU48166
New.CleanUp.ReferenceOTU65513
New.ReferenceOTU1161

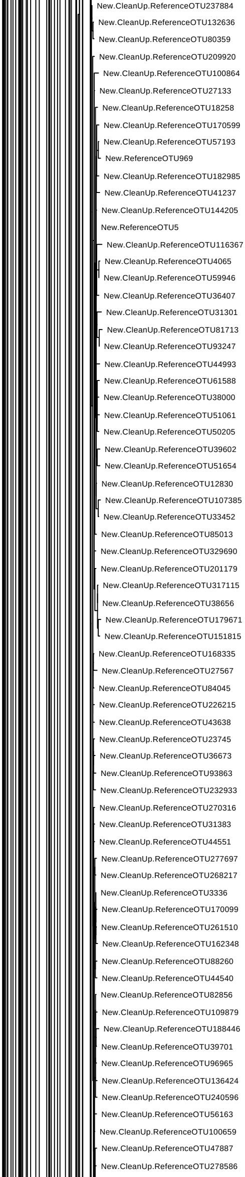
New.CleanUp.ReferenceOTU237884
New.CleanUp.ReferenceOTU132636
New.CleanUp.ReferenceOTU80359
New.CleanUp.ReferenceOTU209920
New.CleanUp.ReferenceOTU100864
New.CleanUp.ReferenceOTU27133
New.CleanUp.ReferenceOTU18258
New.CleanUp.ReferenceOTU170599
New.CleanUp.ReferenceOTU57193
New.ReferenceOTU969
New.CleanUp.ReferenceOTU182985
New.CleanUp.ReferenceOTU41237
New.CleanUp.ReferenceOTU144205
New.ReferenceOTU5
New.CleanUp.ReferenceOTU116367
New.CleanUp.ReferenceOTU4065
New.CleanUp.ReferenceOTU59946
New.CleanUp.ReferenceOTU36407
New.CleanUp.ReferenceOTU31301
New.CleanUp.ReferenceOTU81713
New.CleanUp.ReferenceOTU93247
New.CleanUp.ReferenceOTU44993
New.CleanUp.ReferenceOTU61588
New.CleanUp.ReferenceOTU38000
New.CleanUp.ReferenceOTU51061
New.CleanUp.ReferenceOTU50205
New.CleanUp.ReferenceOTU39602
New.CleanUp.ReferenceOTU51654
New.CleanUp.ReferenceOTU12830
New.CleanUp.ReferenceOTU107385
New.CleanUp.ReferenceOTU33452
New.CleanUp.ReferenceOTU85013
New.CleanUp.ReferenceOTU329690
New.CleanUp.ReferenceOTU201179
New.CleanUp.ReferenceOTU317115
New.CleanUp.ReferenceOTU38656
New.CleanUp.ReferenceOTU179671
New.CleanUp.ReferenceOTU151815
New.CleanUp.ReferenceOTU168335
New.CleanUp.ReferenceOTU27567
New.CleanUp.ReferenceOTU84045
New.CleanUp.ReferenceOTU226215
New.CleanUp.ReferenceOTU43638
New.CleanUp.ReferenceOTU23745
New.CleanUp.ReferenceOTU36673
New.CleanUp.ReferenceOTU93863
New.CleanUp.ReferenceOTU232933
New.CleanUp.ReferenceOTU270316
New.CleanUp.ReferenceOTU31383
New.CleanUp.ReferenceOTU44551
New.CleanUp.ReferenceOTU277697
New.CleanUp.ReferenceOTU268217
New.CleanUp.ReferenceOTU3336
New.CleanUp.ReferenceOTU170099
New.CleanUp.ReferenceOTU261510
New.CleanUp.ReferenceOTU162348
New.CleanUp.ReferenceOTU88260
New.CleanUp.ReferenceOTU44540
New.CleanUp.ReferenceOTU82856
New.CleanUp.ReferenceOTU109879
New.CleanUp.ReferenceOTU188446
New.CleanUp.ReferenceOTU39701
New.CleanUp.ReferenceOTU96965
New.CleanUp.ReferenceOTU136424
New.CleanUp.ReferenceOTU240596
New.CleanUp.ReferenceOTU56163
New.CleanUp.ReferenceOTU100659
New.CleanUp.ReferenceOTU47887
New.CleanUp.ReferenceOTU278586

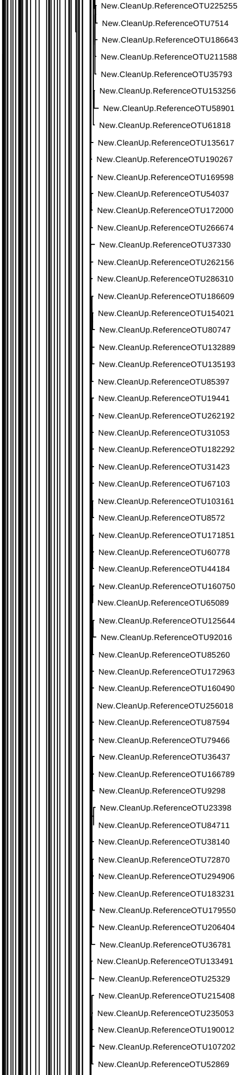
New.CleanUp.ReferenceOTU225255
New.CleanUp.ReferenceOTU7514
New.CleanUp.ReferenceOTU186643
New.CleanUp.ReferenceOTU211588
New.CleanUp.ReferenceOTU35793
New.CleanUp.ReferenceOTU153256
New.CleanUp.ReferenceOTU58901
New.CleanUp.ReferenceOTU61818
New.CleanUp.ReferenceOTU135617
New.CleanUp.ReferenceOTU190267
New.CleanUp.ReferenceOTU169598
New.CleanUp.ReferenceOTU54037
New.CleanUp.ReferenceOTU172000
New.CleanUp.ReferenceOTU266674
New.CleanUp.ReferenceOTU37330
New.CleanUp.ReferenceOTU262156
New.CleanUp.ReferenceOTU286310
New.CleanUp.ReferenceOTU186609
New.CleanUp.ReferenceOTU154021
New.CleanUp.ReferenceOTU80747
New.CleanUp.ReferenceOTU132889
New.CleanUp.ReferenceOTU135193
New.CleanUp.ReferenceOTU85397
New.CleanUp.ReferenceOTU19441
New.CleanUp.ReferenceOTU262192
New.CleanUp.ReferenceOTU31053
New.CleanUp.ReferenceOTU182292
New.CleanUp.ReferenceOTU31423
New.CleanUp.ReferenceOTU67103
New.CleanUp.ReferenceOTU103161
New.CleanUp.ReferenceOTU8572
New.CleanUp.ReferenceOTU171851
New.CleanUp.ReferenceOTU60778
New.CleanUp.ReferenceOTU44184
New.CleanUp.ReferenceOTU160750
New.CleanUp.ReferenceOTU65089
New.CleanUp.ReferenceOTU125644
New.CleanUp.ReferenceOTU92016
New.CleanUp.ReferenceOTU85260
New.CleanUp.ReferenceOTU172963
New.CleanUp.ReferenceOTU160490
New.CleanUp.ReferenceOTU256018
New.CleanUp.ReferenceOTU87594
New.CleanUp.ReferenceOTU79466
New.CleanUp.ReferenceOTU36437
New.CleanUp.ReferenceOTU166789
New.CleanUp.ReferenceOTU9298
New.CleanUp.ReferenceOTU23398
New.CleanUp.ReferenceOTU84711
New.CleanUp.ReferenceOTU38140
New.CleanUp.ReferenceOTU72870
New.CleanUp.ReferenceOTU294906
New.CleanUp.ReferenceOTU183231
New.CleanUp.ReferenceOTU179550
New.CleanUp.ReferenceOTU206404
New.CleanUp.ReferenceOTU36781
New.CleanUp.ReferenceOTU133491
New.CleanUp.ReferenceOTU25329
New.CleanUp.ReferenceOTU215408
New.CleanUp.ReferenceOTU235053
New.CleanUp.ReferenceOTU190012
New.CleanUp.ReferenceOTU107202
New.CleanUp.ReferenceOTU52869

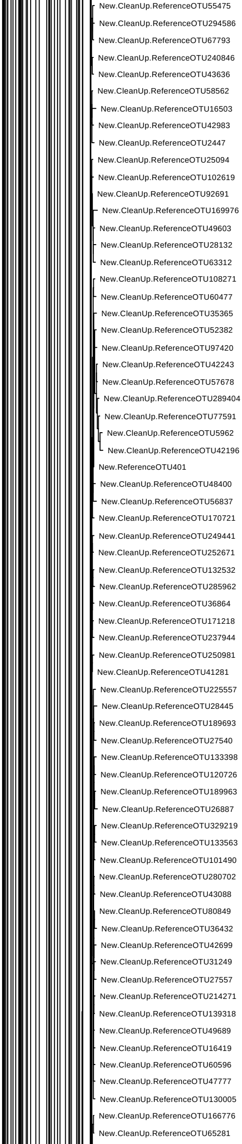
New.CleanUp.ReferenceOTU55475
New.CleanUp.ReferenceOTU294586
New.CleanUp.ReferenceOTU67793
New.CleanUp.ReferenceOTU240846
New.CleanUp.ReferenceOTU43636
New.CleanUp.ReferenceOTU58562
New.CleanUp.ReferenceOTU16503
New.CleanUp.ReferenceOTU42983
New.CleanUp.ReferenceOTU2447
New.CleanUp.ReferenceOTU25094
New.CleanUp.ReferenceOTU102619
New.CleanUp.ReferenceOTU92691
New.CleanUp.ReferenceOTU169976
New.CleanUp.ReferenceOTU49603
New.CleanUp.ReferenceOTU28132
New.CleanUp.ReferenceOTU63312
New.CleanUp.ReferenceOTU108271
New.CleanUp.ReferenceOTU60477
New.CleanUp.ReferenceOTU35365
New.CleanUp.ReferenceOTU52382
New.CleanUp.ReferenceOTU97420
New.CleanUp.ReferenceOTU42243
New.CleanUp.ReferenceOTU57678
New.CleanUp.ReferenceOTU289404
New.CleanUp.ReferenceOTU77591
New.CleanUp.ReferenceOTU5962
New.CleanUp.ReferenceOTU42196
New.ReferenceOTU401
New.CleanUp.ReferenceOTU48400
New.CleanUp.ReferenceOTU56837
New.CleanUp.ReferenceOTU170721
New.CleanUp.ReferenceOTU249441
New.CleanUp.ReferenceOTU252671
New.CleanUp.ReferenceOTU132532
New.CleanUp.ReferenceOTU285962
New.CleanUp.ReferenceOTU36864
New.CleanUp.ReferenceOTU171218
New.CleanUp.ReferenceOTU237944
New.CleanUp.ReferenceOTU250981
New.CleanUp.ReferenceOTU41281
New.CleanUp.ReferenceOTU225557
New.CleanUp.ReferenceOTU28445
New.CleanUp.ReferenceOTU189693
New.CleanUp.ReferenceOTU27540
New.CleanUp.ReferenceOTU133398
New.CleanUp.ReferenceOTU120726
New.CleanUp.ReferenceOTU189963
New.CleanUp.ReferenceOTU26887
New.CleanUp.ReferenceOTU329219
New.CleanUp.ReferenceOTU133563
New.CleanUp.ReferenceOTU101490
New.CleanUp.ReferenceOTU280702
New.CleanUp.ReferenceOTU43088
New.CleanUp.ReferenceOTU80849
New.CleanUp.ReferenceOTU36432
New.CleanUp.ReferenceOTU42699
New.CleanUp.ReferenceOTU31249
New.CleanUp.ReferenceOTU27557
New.CleanUp.ReferenceOTU214271
New.CleanUp.ReferenceOTU139318
New.CleanUp.ReferenceOTU49689
New.CleanUp.ReferenceOTU16419
New.CleanUp.ReferenceOTU60596
New.CleanUp.ReferenceOTU47777
New.CleanUp.ReferenceOTU130005
New.CleanUp.ReferenceOTU166776
New.CleanUp.ReferenceOTU65281

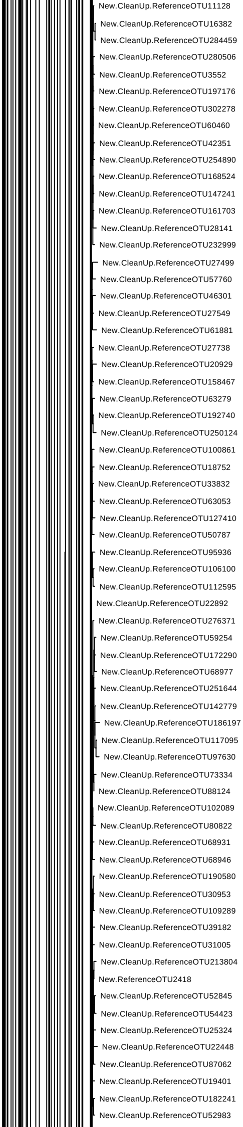
New.CleanUp.ReferenceOTU11128
New.CleanUp.ReferenceOTU16382
New.CleanUp.ReferenceOTU284459
New.CleanUp.ReferenceOTU280506
New.CleanUp.ReferenceOTU3552
New.CleanUp.ReferenceOTU197176
New.CleanUp.ReferenceOTU302278
New.CleanUp.ReferenceOTU60460
New.CleanUp.ReferenceOTU42351
New.CleanUp.ReferenceOTU254890
New.CleanUp.ReferenceOTU168524
New.CleanUp.ReferenceOTU147241
New.CleanUp.ReferenceOTU161703
New.CleanUp.ReferenceOTU28141
New.CleanUp.ReferenceOTU232999
New.CleanUp.ReferenceOTU27499
New.CleanUp.ReferenceOTU57760
New.CleanUp.ReferenceOTU46301
New.CleanUp.ReferenceOTU27549
New.CleanUp.ReferenceOTU61881
New.CleanUp.ReferenceOTU27738
New.CleanUp.ReferenceOTU20929
New.CleanUp.ReferenceOTU158467
New.CleanUp.ReferenceOTU63279
New.CleanUp.ReferenceOTU192740
New.CleanUp.ReferenceOTU250124
New.CleanUp.ReferenceOTU100861
New.CleanUp.ReferenceOTU18752
New.CleanUp.ReferenceOTU33832
New.CleanUp.ReferenceOTU63053
New.CleanUp.ReferenceOTU127410
New.CleanUp.ReferenceOTU50787
New.CleanUp.ReferenceOTU95936
New.CleanUp.ReferenceOTU106100
New.CleanUp.ReferenceOTU112595
New.CleanUp.ReferenceOTU22892
New.CleanUp.ReferenceOTU276371
New.CleanUp.ReferenceOTU59254
New.CleanUp.ReferenceOTU172290
New.CleanUp.ReferenceOTU68977
New.CleanUp.ReferenceOTU251644
New.CleanUp.ReferenceOTU142779
New.CleanUp.ReferenceOTU186197
New.CleanUp.ReferenceOTU117095
New.CleanUp.ReferenceOTU97630
New.CleanUp.ReferenceOTU73334
New.CleanUp.ReferenceOTU88124
New.CleanUp.ReferenceOTU102089
New.CleanUp.ReferenceOTU80822
New.CleanUp.ReferenceOTU68931
New.CleanUp.ReferenceOTU68946
New.CleanUp.ReferenceOTU190580
New.CleanUp.ReferenceOTU30953
New.CleanUp.ReferenceOTU109289
New.CleanUp.ReferenceOTU39182
New.CleanUp.ReferenceOTU31005
New.CleanUp.ReferenceOTU213804
New.ReferenceOTU2418
New.CleanUp.ReferenceOTU52845
New.CleanUp.ReferenceOTU54423
New.CleanUp.ReferenceOTU25324
New.CleanUp.ReferenceOTU22448
New.CleanUp.ReferenceOTU87062
New.CleanUp.ReferenceOTU19401
New.CleanUp.ReferenceOTU182241
New.CleanUp.ReferenceOTU52983

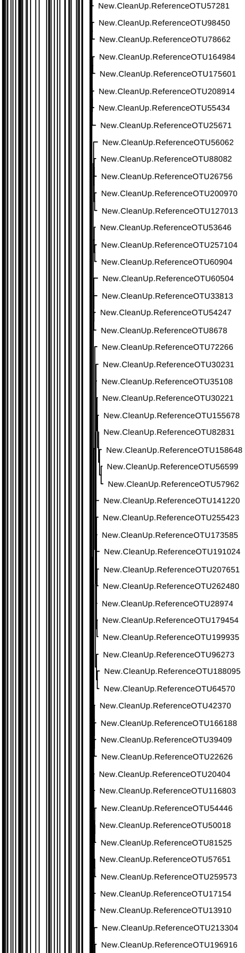
New.CleanUp.ReferenceOTU57281
New.CleanUp.ReferenceOTU98450
New.CleanUp.ReferenceOTU78662
New.CleanUp.ReferenceOTU164984
New.CleanUp.ReferenceOTU175601
New.CleanUp.ReferenceOTU208914
New.CleanUp.ReferenceOTU55434
New.CleanUp.ReferenceOTU25671
New.CleanUp.ReferenceOTU56062
New.CleanUp.ReferenceOTU88082
New.CleanUp.ReferenceOTU26756
New.CleanUp.ReferenceOTU200970
New.CleanUp.ReferenceOTU127013
New.CleanUp.ReferenceOTU53646
New.CleanUp.ReferenceOTU257104
New.CleanUp.ReferenceOTU60904
New.CleanUp.ReferenceOTU60504
New.CleanUp.ReferenceOTU33813
New.CleanUp.ReferenceOTU54247
New.CleanUp.ReferenceOTU8678
New.CleanUp.ReferenceOTU72266
New.CleanUp.ReferenceOTU30231
New.CleanUp.ReferenceOTU35108
New.CleanUp.ReferenceOTU30221
New.CleanUp.ReferenceOTU155678
New.CleanUp.ReferenceOTU82831
New.CleanUp.ReferenceOTU158648
New.CleanUp.ReferenceOTU56599
New.CleanUp.ReferenceOTU57962
New.CleanUp.ReferenceOTU141220
New.CleanUp.ReferenceOTU255423
New.CleanUp.ReferenceOTU173585
New.CleanUp.ReferenceOTU191024
New.CleanUp.ReferenceOTU207651
New.CleanUp.ReferenceOTU262480
New.CleanUp.ReferenceOTU28974
New.CleanUp.ReferenceOTU179454
New.CleanUp.ReferenceOTU199935
New.CleanUp.ReferenceOTU96273
New.CleanUp.ReferenceOTU188095
New.CleanUp.ReferenceOTU64570
New.CleanUp.ReferenceOTU42370
New.CleanUp.ReferenceOTU166188
New.CleanUp.ReferenceOTU39409
New.CleanUp.ReferenceOTU22626
New.CleanUp.ReferenceOTU20404
New.CleanUp.ReferenceOTU116803
New.CleanUp.ReferenceOTU54446
New.CleanUp.ReferenceOTU50018
New.CleanUp.ReferenceOTU81525
New.CleanUp.ReferenceOTU57651
New.CleanUp.ReferenceOTU259573
New.CleanUp.ReferenceOTU17154
New.CleanUp.ReferenceOTU13910
New.CleanUp.ReferenceOTU213304
New.CleanUp.ReferenceOTU196916

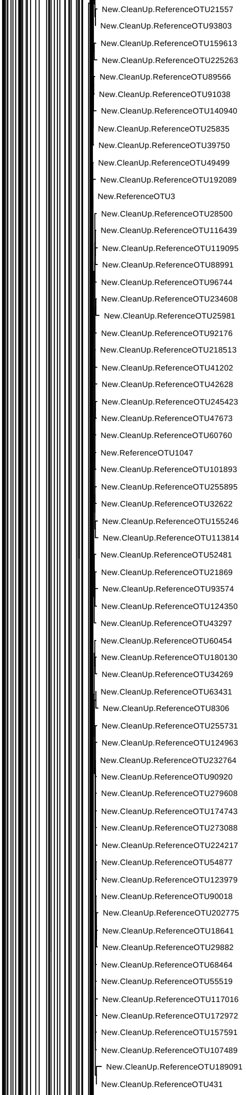
New.CleanUp.ReferenceOTU21557
New.CleanUp.ReferenceOTU93803
New.CleanUp.ReferenceOTU159613
New.CleanUp.ReferenceOTU225263
New.CleanUp.ReferenceOTU89566
New.CleanUp.ReferenceOTU91038
New.CleanUp.ReferenceOTU140940
New.CleanUp.ReferenceOTU25835
New.CleanUp.ReferenceOTU39750
New.CleanUp.ReferenceOTU49499
New.CleanUp.ReferenceOTU192089
New.ReferenceOTU3
New.CleanUp.ReferenceOTU28500
New.CleanUp.ReferenceOTU116439
New.CleanUp.ReferenceOTU119095
New.CleanUp.ReferenceOTU88991
New.CleanUp.ReferenceOTU96744
New.CleanUp.ReferenceOTU234608
New.CleanUp.ReferenceOTU25981
New.CleanUp.ReferenceOTU92176
New.CleanUp.ReferenceOTU218513
New.CleanUp.ReferenceOTU41202
New.CleanUp.ReferenceOTU42628
New.CleanUp.ReferenceOTU245423
New.CleanUp.ReferenceOTU47673
New.CleanUp.ReferenceOTU60760
New.ReferenceOTU1047
New.CleanUp.ReferenceOTU101893
New.CleanUp.ReferenceOTU255895
New.CleanUp.ReferenceOTU32622
New.CleanUp.ReferenceOTU155246
New.CleanUp.ReferenceOTU113814
New.CleanUp.ReferenceOTU52481
New.CleanUp.ReferenceOTU21869
New.CleanUp.ReferenceOTU93574
New.CleanUp.ReferenceOTU124350
New.CleanUp.ReferenceOTU43297
New.CleanUp.ReferenceOTU60454
New.CleanUp.ReferenceOTU180130
New.CleanUp.ReferenceOTU34269
New.CleanUp.ReferenceOTU63431
New.CleanUp.ReferenceOTU8306
New.CleanUp.ReferenceOTU255731
New.CleanUp.ReferenceOTU124963
New.CleanUp.ReferenceOTU232764
New.CleanUp.ReferenceOTU90920
New.CleanUp.ReferenceOTU279608
New.CleanUp.ReferenceOTU174743
New.CleanUp.ReferenceOTU273088
New.CleanUp.ReferenceOTU224217
New.CleanUp.ReferenceOTU54877
New.CleanUp.ReferenceOTU123979
New.CleanUp.ReferenceOTU90018
New.CleanUp.ReferenceOTU202775
New.CleanUp.ReferenceOTU18641
New.CleanUp.ReferenceOTU29882
New.CleanUp.ReferenceOTU68464
New.CleanUp.ReferenceOTU55519
New.CleanUp.ReferenceOTU117016
New.CleanUp.ReferenceOTU172972
New.CleanUp.ReferenceOTU157591
New.CleanUp.ReferenceOTU107489
New.CleanUp.ReferenceOTU189091
New.CleanUp.ReferenceOTU431

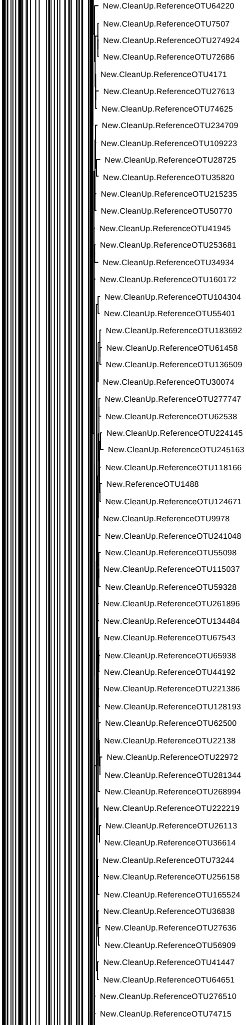
New.CleanUp.ReferenceOTU64220
New.CleanUp.ReferenceOTU7507
New.CleanUp.ReferenceOTU274924
New.CleanUp.ReferenceOTU72686
New.CleanUp.ReferenceOTU4171
New.CleanUp.ReferenceOTU27613
New.CleanUp.ReferenceOTU74625
New.CleanUp.ReferenceOTU234709
New.CleanUp.ReferenceOTU109223
New.CleanUp.ReferenceOTU28725
New.CleanUp.ReferenceOTU35820
New.CleanUp.ReferenceOTU215235
New.CleanUp.ReferenceOTU50770
New.CleanUp.ReferenceOTU41945
New.CleanUp.ReferenceOTU253681
New.CleanUp.ReferenceOTU34934
New.CleanUp.ReferenceOTU160172
New.CleanUp.ReferenceOTU104304
New.CleanUp.ReferenceOTU55401
New.CleanUp.ReferenceOTU183692
New.CleanUp.ReferenceOTU61458
New.CleanUp.ReferenceOTU136509
New.CleanUp.ReferenceOTU30074
New.CleanUp.ReferenceOTU277747
New.CleanUp.ReferenceOTU62538
New.CleanUp.ReferenceOTU224145
New.CleanUp.ReferenceOTU245163
New.CleanUp.ReferenceOTU118166
New.ReferenceOTU1488
New.CleanUp.ReferenceOTU124671
New.CleanUp.ReferenceOTU9978
New.CleanUp.ReferenceOTU241048
New.CleanUp.ReferenceOTU55098
New.CleanUp.ReferenceOTU115037
New.CleanUp.ReferenceOTU59328
New.CleanUp.ReferenceOTU261896
New.CleanUp.ReferenceOTU134484
New.CleanUp.ReferenceOTU67543
New.CleanUp.ReferenceOTU65938
New.CleanUp.ReferenceOTU44192
New.CleanUp.ReferenceOTU221386
New.CleanUp.ReferenceOTU128193
New.CleanUp.ReferenceOTU62500
New.CleanUp.ReferenceOTU22138
New.CleanUp.ReferenceOTU22972
New.CleanUp.ReferenceOTU281344
New.CleanUp.ReferenceOTU268994
New.CleanUp.ReferenceOTU222219
New.CleanUp.ReferenceOTU26113
New.CleanUp.ReferenceOTU36614
New.CleanUp.ReferenceOTU73244
New.CleanUp.ReferenceOTU256158
New.CleanUp.ReferenceOTU165524
New.CleanUp.ReferenceOTU36838
New.CleanUp.ReferenceOTU27636
New.CleanUp.ReferenceOTU56909
New.CleanUp.ReferenceOTU41447
New.CleanUp.ReferenceOTU64651
New.CleanUp.ReferenceOTU276510
New.CleanUp.ReferenceOTU74715

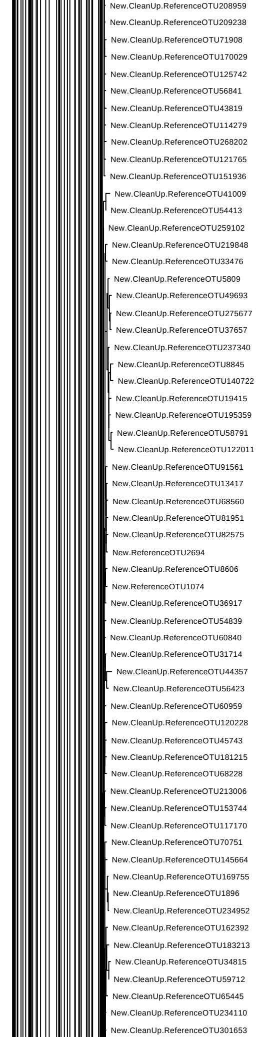
New.CleanUp.ReferenceOTU208959
New.CleanUp.ReferenceOTU209238
New.CleanUp.ReferenceOTU71908
New.CleanUp.ReferenceOTU170029
New.CleanUp.ReferenceOTU125742
New.CleanUp.ReferenceOTU56841
New.CleanUp.ReferenceOTU43819
New.CleanUp.ReferenceOTU114279
New.CleanUp.ReferenceOTU268202
New.CleanUp.ReferenceOTU121765
New.CleanUp.ReferenceOTU151936
New.CleanUp.ReferenceOTU41009
New.CleanUp.ReferenceOTU54413
New.CleanUp.ReferenceOTU259102
New.CleanUp.ReferenceOTU219848
New.CleanUp.ReferenceOTU33476
New.CleanUp.ReferenceOTU5809
New.CleanUp.ReferenceOTU49693
New.CleanUp.ReferenceOTU275677
New.CleanUp.ReferenceOTU37657
New.CleanUp.ReferenceOTU237340
New.CleanUp.ReferenceOTU8845
New.CleanUp.ReferenceOTU140722
New.CleanUp.ReferenceOTU19415
New.CleanUp.ReferenceOTU195359
New.CleanUp.ReferenceOTU58791
New.CleanUp.ReferenceOTU122011
New.CleanUp.ReferenceOTU91561
New.CleanUp.ReferenceOTU13417
New.CleanUp.ReferenceOTU68560
New.CleanUp.ReferenceOTU81951
New.CleanUp.ReferenceOTU82575
New.ReferenceOTU2694
New.CleanUp.ReferenceOTU8606
New.ReferenceOTU1074
New.CleanUp.ReferenceOTU36917
New.CleanUp.ReferenceOTU54839
New.CleanUp.ReferenceOTU60840
New.CleanUp.ReferenceOTU31714
New.CleanUp.ReferenceOTU44357
New.CleanUp.ReferenceOTU56423
New.CleanUp.ReferenceOTU60959
New.CleanUp.ReferenceOTU120228
New.CleanUp.ReferenceOTU45743
New.CleanUp.ReferenceOTU181215
New.CleanUp.ReferenceOTU68228
New.CleanUp.ReferenceOTU213006
New.CleanUp.ReferenceOTU153744
New.CleanUp.ReferenceOTU117170
New.CleanUp.ReferenceOTU70751
New.CleanUp.ReferenceOTU145664
New.CleanUp.ReferenceOTU169755
New.CleanUp.ReferenceOTU1896
New.CleanUp.ReferenceOTU234952
New.CleanUp.ReferenceOTU162392
New.CleanUp.ReferenceOTU183213
New.CleanUp.ReferenceOTU34815
New.CleanUp.ReferenceOTU59712
New.CleanUp.ReferenceOTU65445
New.CleanUp.ReferenceOTU234110
New.CleanUp.ReferenceOTU301653

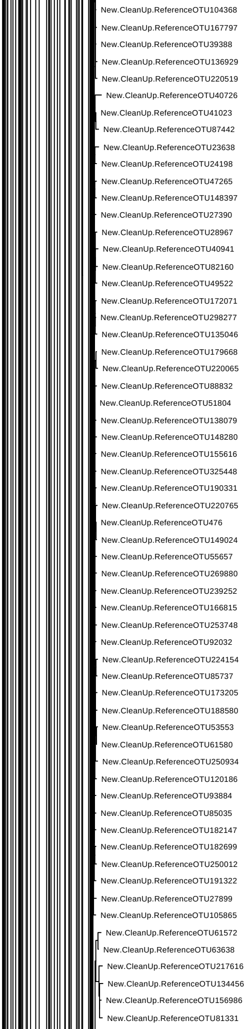
New.CleanUp.ReferenceOTU104368
New.CleanUp.ReferenceOTU167797
New.CleanUp.ReferenceOTU39388
New.CleanUp.ReferenceOTU136929
New.CleanUp.ReferenceOTU220519
New.CleanUp.ReferenceOTU40726
New.CleanUp.ReferenceOTU41023
New.CleanUp.ReferenceOTU87442
New.CleanUp.ReferenceOTU23638
New.CleanUp.ReferenceOTU24198
New.CleanUp.ReferenceOTU47265
New.CleanUp.ReferenceOTU148397
New.CleanUp.ReferenceOTU27390
New.CleanUp.ReferenceOTU28967
New.CleanUp.ReferenceOTU40941
New.CleanUp.ReferenceOTU82160
New.CleanUp.ReferenceOTU49522
New.CleanUp.ReferenceOTU172071
New.CleanUp.ReferenceOTU298277
New.CleanUp.ReferenceOTU135046
New.CleanUp.ReferenceOTU179668
New.CleanUp.ReferenceOTU220065
New.CleanUp.ReferenceOTU88832
New.CleanUp.ReferenceOTU51804
New.CleanUp.ReferenceOTU138079
New.CleanUp.ReferenceOTU148280
New.CleanUp.ReferenceOTU155616
New.CleanUp.ReferenceOTU325448
New.CleanUp.ReferenceOTU190331
New.CleanUp.ReferenceOTU220765
New.CleanUp.ReferenceOTU476
New.CleanUp.ReferenceOTU149024
New.CleanUp.ReferenceOTU55657
New.CleanUp.ReferenceOTU269880
New.CleanUp.ReferenceOTU239252
New.CleanUp.ReferenceOTU166815
New.CleanUp.ReferenceOTU253748
New.CleanUp.ReferenceOTU92032
New.CleanUp.ReferenceOTU224154
New.CleanUp.ReferenceOTU85737
New.CleanUp.ReferenceOTU173205
New.CleanUp.ReferenceOTU188580
New.CleanUp.ReferenceOTU53553
New.CleanUp.ReferenceOTU61580
New.CleanUp.ReferenceOTU250934
New.CleanUp.ReferenceOTU120186
New.CleanUp.ReferenceOTU93884
New.CleanUp.ReferenceOTU85035
New.CleanUp.ReferenceOTU182147
New.CleanUp.ReferenceOTU182699
New.CleanUp.ReferenceOTU250012
New.CleanUp.ReferenceOTU191322
New.CleanUp.ReferenceOTU27899
New.CleanUp.ReferenceOTU105865
New.CleanUp.ReferenceOTU61572
New.CleanUp.ReferenceOTU63638
New.CleanUp.ReferenceOTU217616
New.CleanUp.ReferenceOTU134456
New.CleanUp.ReferenceOTU156986
New.CleanUp.ReferenceOTU81331

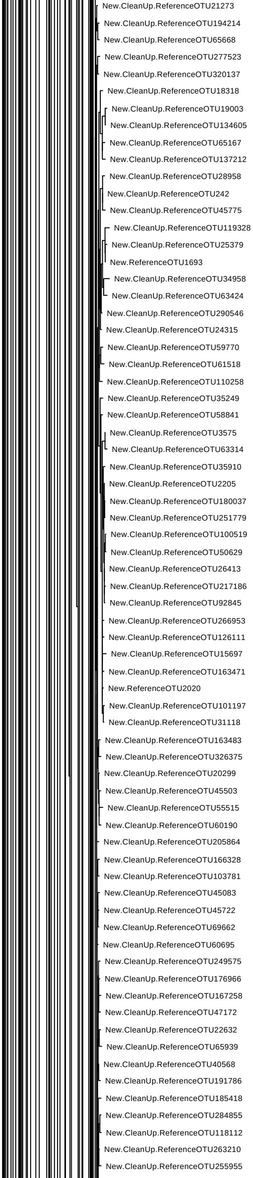
New.CleanUp.ReferenceOTU21273
New.CleanUp.ReferenceOTU194214
New.CleanUp.ReferenceOTU65668
New.CleanUp.ReferenceOTU277523
New.CleanUp.ReferenceOTU320137
New.CleanUp.ReferenceOTU18318
New.CleanUp.ReferenceOTU19003
New.CleanUp.ReferenceOTU134605
New.CleanUp.ReferenceOTU65167
New.CleanUp.ReferenceOTU137212
New.CleanUp.ReferenceOTU28958
New.CleanUp.ReferenceOTU242
New.CleanUp.ReferenceOTU45775
New.CleanUp.ReferenceOTU119328
New.CleanUp.ReferenceOTU25379
New.ReferenceOTU1693
New.CleanUp.ReferenceOTU34958
New.CleanUp.ReferenceOTU63424
New.CleanUp.ReferenceOTU290546
New.CleanUp.ReferenceOTU24315
New.CleanUp.ReferenceOTU59770
New.CleanUp.ReferenceOTU61518
New.CleanUp.ReferenceOTU110258
New.CleanUp.ReferenceOTU35249
New.CleanUp.ReferenceOTU58841
New.CleanUp.ReferenceOTU3575
New.CleanUp.ReferenceOTU63314
New.CleanUp.ReferenceOTU35910
New.CleanUp.ReferenceOTU2205
New.CleanUp.ReferenceOTU180037
New.CleanUp.ReferenceOTU251779
New.CleanUp.ReferenceOTU100519
New.CleanUp.ReferenceOTU50629
New.CleanUp.ReferenceOTU26413
New.CleanUp.ReferenceOTU217186
New.CleanUp.ReferenceOTU92845
New.CleanUp.ReferenceOTU266953
New.CleanUp.ReferenceOTU126111
New.CleanUp.ReferenceOTU15697
New.CleanUp.ReferenceOTU163471
New.ReferenceOTU2020
New.CleanUp.ReferenceOTU101197
New.CleanUp.ReferenceOTU31118
New.CleanUp.ReferenceOTU163483
New.CleanUp.ReferenceOTU326375
New.CleanUp.ReferenceOTU20299
New.CleanUp.ReferenceOTU45503
New.CleanUp.ReferenceOTU55515
New.CleanUp.ReferenceOTU60190
New.CleanUp.ReferenceOTU205864
New.CleanUp.ReferenceOTU166328
New.CleanUp.ReferenceOTU103781
New.CleanUp.ReferenceOTU45083
New.CleanUp.ReferenceOTU45722
New.CleanUp.ReferenceOTU69662
New.CleanUp.ReferenceOTU60695
New.CleanUp.ReferenceOTU249575
New.CleanUp.ReferenceOTU176966
New.CleanUp.ReferenceOTU167258
New.CleanUp.ReferenceOTU47172
New.CleanUp.ReferenceOTU22632
New.CleanUp.ReferenceOTU65939
New.CleanUp.ReferenceOTU40568
New.CleanUp.ReferenceOTU191786
New.CleanUp.ReferenceOTU185418
New.CleanUp.ReferenceOTU284855
New.CleanUp.ReferenceOTU118112
New.CleanUp.ReferenceOTU263210
New.CleanUp.ReferenceOTU255955

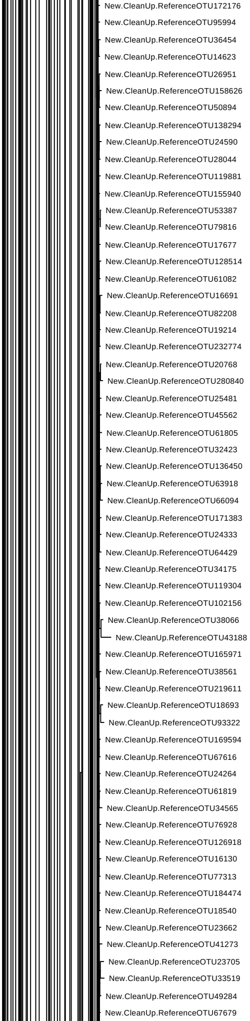
New.CleanUp.ReferenceOTU172176
New.CleanUp.ReferenceOTU95994
New.CleanUp.ReferenceOTU36454
New.CleanUp.ReferenceOTU14623
New.CleanUp.ReferenceOTU26951
New.CleanUp.ReferenceOTU158626
New.CleanUp.ReferenceOTU50894
New.CleanUp.ReferenceOTU138294
New.CleanUp.ReferenceOTU24590
New.CleanUp.ReferenceOTU28044
New.CleanUp.ReferenceOTU119881
New.CleanUp.ReferenceOTU155940
New.CleanUp.ReferenceOTU53387
New.CleanUp.ReferenceOTU79816
New.CleanUp.ReferenceOTU17677
New.CleanUp.ReferenceOTU128514
New.CleanUp.ReferenceOTU61082
New.CleanUp.ReferenceOTU16691
New.CleanUp.ReferenceOTU82208
New.CleanUp.ReferenceOTU19214
New.CleanUp.ReferenceOTU232774
New.CleanUp.ReferenceOTU20768
New.CleanUp.ReferenceOTU280840
New.CleanUp.ReferenceOTU25481
New.CleanUp.ReferenceOTU45562
New.CleanUp.ReferenceOTU61805
New.CleanUp.ReferenceOTU32423
New.CleanUp.ReferenceOTU136450
New.CleanUp.ReferenceOTU63918
New.CleanUp.ReferenceOTU66094
New.CleanUp.ReferenceOTU171383
New.CleanUp.ReferenceOTU24333
New.CleanUp.ReferenceOTU64429
New.CleanUp.ReferenceOTU34175
New.CleanUp.ReferenceOTU119304
New.CleanUp.ReferenceOTU102156
New.CleanUp.ReferenceOTU38066
New.CleanUp.ReferenceOTU43188
New.CleanUp.ReferenceOTU165971
New.CleanUp.ReferenceOTU38561
New.CleanUp.ReferenceOTU219611
New.CleanUp.ReferenceOTU18693
New.CleanUp.ReferenceOTU93322
New.CleanUp.ReferenceOTU169594
New.CleanUp.ReferenceOTU67616
New.CleanUp.ReferenceOTU24264
New.CleanUp.ReferenceOTU61819
New.CleanUp.ReferenceOTU34565
New.CleanUp.ReferenceOTU76928
New.CleanUp.ReferenceOTU126918
New.CleanUp.ReferenceOTU16130
New.CleanUp.ReferenceOTU77313
New.CleanUp.ReferenceOTU184474
New.CleanUp.ReferenceOTU18540
New.CleanUp.ReferenceOTU23662
New.CleanUp.ReferenceOTU41273
New.CleanUp.ReferenceOTU23705
New.CleanUp.ReferenceOTU33519
New.CleanUp.ReferenceOTU49284
New.CleanUp.ReferenceOTU67679

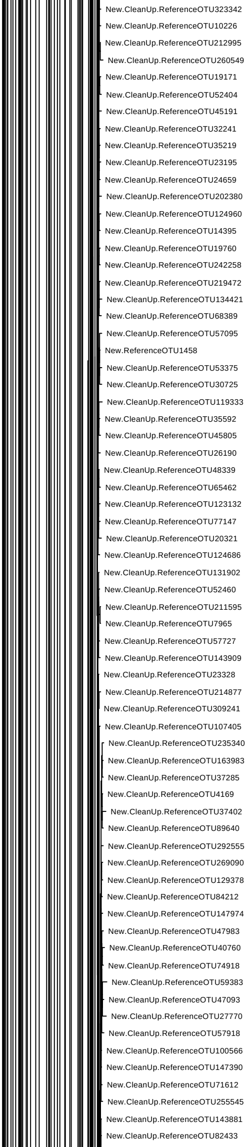
New.CleanUp.ReferenceOTU323342
New.CleanUp.ReferenceOTU10226
New.CleanUp.ReferenceOTU212995
New.CleanUp.ReferenceOTU260549
New.CleanUp.ReferenceOTU19171
New.CleanUp.ReferenceOTU52404
New.CleanUp.ReferenceOTU45191
New.CleanUp.ReferenceOTU32241
New.CleanUp.ReferenceOTU35219
New.CleanUp.ReferenceOTU23195
New.CleanUp.ReferenceOTU24659
New.CleanUp.ReferenceOTU202380
New.CleanUp.ReferenceOTU124960
New.CleanUp.ReferenceOTU14395
New.CleanUp.ReferenceOTU19760
New.CleanUp.ReferenceOTU242258
New.CleanUp.ReferenceOTU219472
New.CleanUp.ReferenceOTU134421
New.CleanUp.ReferenceOTU68389
New.CleanUp.ReferenceOTU57095
New.ReferenceOTU1458
New.CleanUp.ReferenceOTU53375
New.CleanUp.ReferenceOTU30725
New.CleanUp.ReferenceOTU119333
New.CleanUp.ReferenceOTU35592
New.CleanUp.ReferenceOTU45805
New.CleanUp.ReferenceOTU26190
New.CleanUp.ReferenceOTU48339
New.CleanUp.ReferenceOTU65462
New.CleanUp.ReferenceOTU123132
New.CleanUp.ReferenceOTU77147
New.CleanUp.ReferenceOTU20321
New.CleanUp.ReferenceOTU124686
New.CleanUp.ReferenceOTU131902
New.CleanUp.ReferenceOTU52460
New.CleanUp.ReferenceOTU211595
New.CleanUp.ReferenceOTU7965
New.CleanUp.ReferenceOTU57727
New.CleanUp.ReferenceOTU143909
New.CleanUp.ReferenceOTU23328
New.CleanUp.ReferenceOTU214877
New.CleanUp.ReferenceOTU309241
New.CleanUp.ReferenceOTU107405
New.CleanUp.ReferenceOTU235340
New.CleanUp.ReferenceOTU163983
New.CleanUp.ReferenceOTU37285
New.CleanUp.ReferenceOTU4169
New.CleanUp.ReferenceOTU37402
New.CleanUp.ReferenceOTU89640
New.CleanUp.ReferenceOTU292555
New.CleanUp.ReferenceOTU269090
New.CleanUp.ReferenceOTU129378
New.CleanUp.ReferenceOTU84212
New.CleanUp.ReferenceOTU147974
New.CleanUp.ReferenceOTU47983
New.CleanUp.ReferenceOTU40760
New.CleanUp.ReferenceOTU74918
New.CleanUp.ReferenceOTU59383
New.CleanUp.ReferenceOTU47093
New.CleanUp.ReferenceOTU27770
New.CleanUp.ReferenceOTU57918
New.CleanUp.ReferenceOTU100566
New.CleanUp.ReferenceOTU147390
New.CleanUp.ReferenceOTU71612
New.CleanUp.ReferenceOTU255545
New.CleanUp.ReferenceOTU143881
New.CleanUp.ReferenceOTU82433

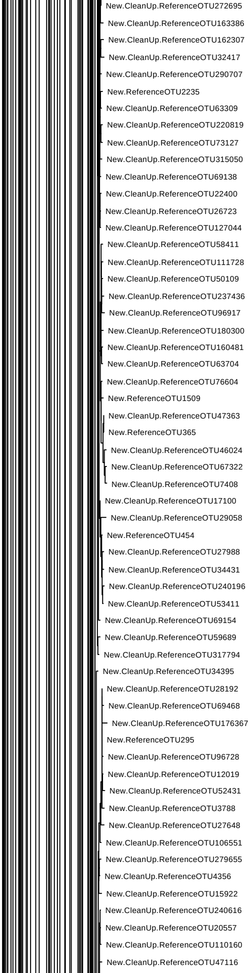
New.CleanUp.ReferenceOTU272695
New.CleanUp.ReferenceOTU163386
New.CleanUp.ReferenceOTU162307
New.CleanUp.ReferenceOTU32417
New.CleanUp.ReferenceOTU290707
New.ReferenceOTU2235
New.CleanUp.ReferenceOTU63309
New.CleanUp.ReferenceOTU220819
New.CleanUp.ReferenceOTU73127
New.CleanUp.ReferenceOTU315050
New.CleanUp.ReferenceOTU69138
New.CleanUp.ReferenceOTU22400
New.CleanUp.ReferenceOTU26723
New.CleanUp.ReferenceOTU127044
New.CleanUp.ReferenceOTU58411
New.CleanUp.ReferenceOTU111728
New.CleanUp.ReferenceOTU50109
New.CleanUp.ReferenceOTU237436
New.CleanUp.ReferenceOTU96917
New.CleanUp.ReferenceOTU180300
New.CleanUp.ReferenceOTU160481
New.CleanUp.ReferenceOTU63704
New.CleanUp.ReferenceOTU76604
New.ReferenceOTU1509
New.CleanUp.ReferenceOTU47363
New.ReferenceOTU365
New.CleanUp.ReferenceOTU46024
New.CleanUp.ReferenceOTU67322
New.CleanUp.ReferenceOTU7408
New.CleanUp.ReferenceOTU17100
New.CleanUp.ReferenceOTU29058
New.ReferenceOTU454
New.CleanUp.ReferenceOTU27988
New.CleanUp.ReferenceOTU34431
New.CleanUp.ReferenceOTU240196
New.CleanUp.ReferenceOTU53411
New.CleanUp.ReferenceOTU69154
New.CleanUp.ReferenceOTU59689
New.CleanUp.ReferenceOTU317794
New.CleanUp.ReferenceOTU34395
New.CleanUp.ReferenceOTU28192
New.CleanUp.ReferenceOTU69468
New.CleanUp.ReferenceOTU176367
New.ReferenceOTU295
New.CleanUp.ReferenceOTU96728
New.CleanUp.ReferenceOTU12019
New.CleanUp.ReferenceOTU52431
New.CleanUp.ReferenceOTU3788
New.CleanUp.ReferenceOTU27648
New.CleanUp.ReferenceOTU106551
New.CleanUp.ReferenceOTU279655
New.CleanUp.ReferenceOTU4356
New.CleanUp.ReferenceOTU15922
New.CleanUp.ReferenceOTU240616
New.CleanUp.ReferenceOTU20557
New.CleanUp.ReferenceOTU110160
New.CleanUp.ReferenceOTU47116

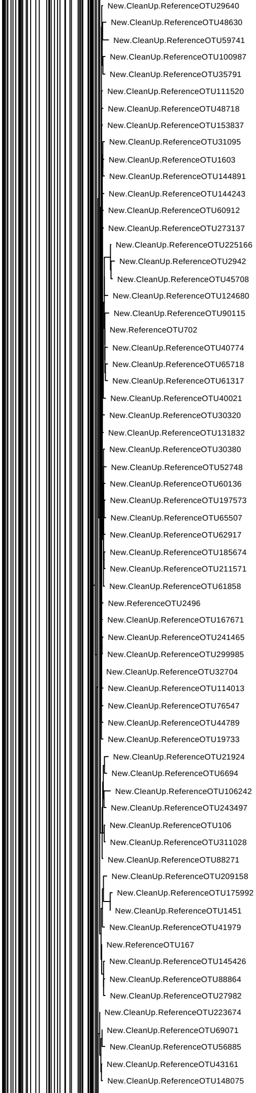
New.CleanUp.ReferenceOTU29640
New.CleanUp.ReferenceOTU48630
New.CleanUp.ReferenceOTU59741
New.CleanUp.ReferenceOTU100987
New.CleanUp.ReferenceOTU35791
New.CleanUp.ReferenceOTU111520
New.CleanUp.ReferenceOTU48718
New.CleanUp.ReferenceOTU153837
New.CleanUp.ReferenceOTU31095
New.CleanUp.ReferenceOTU1603
New.CleanUp.ReferenceOTU144891
New.CleanUp.ReferenceOTU144243
New.CleanUp.ReferenceOTU60912
New.CleanUp.ReferenceOTU273137
New.CleanUp.ReferenceOTU225166
New.CleanUp.ReferenceOTU2942
New.CleanUp.ReferenceOTU45708
New.CleanUp.ReferenceOTU124680
New.CleanUp.ReferenceOTU90115
New.ReferenceOTU702
New.CleanUp.ReferenceOTU40774
New.CleanUp.ReferenceOTU65718
New.CleanUp.ReferenceOTU61317
New.CleanUp.ReferenceOTU40021
New.CleanUp.ReferenceOTU30320
New.CleanUp.ReferenceOTU131832
New.CleanUp.ReferenceOTU30380
New.CleanUp.ReferenceOTU52748
New.CleanUp.ReferenceOTU60136
New.CleanUp.ReferenceOTU197573
New.CleanUp.ReferenceOTU65507
New.CleanUp.ReferenceOTU62917
New.CleanUp.ReferenceOTU185674
New.CleanUp.ReferenceOTU211571
New.CleanUp.ReferenceOTU61858
New.ReferenceOTU2496
New.CleanUp.ReferenceOTU167671
New.CleanUp.ReferenceOTU241465
New.CleanUp.ReferenceOTU299985
New.CleanUp.ReferenceOTU32704
New.CleanUp.ReferenceOTU114013
New.CleanUp.ReferenceOTU76547
New.CleanUp.ReferenceOTU44789
New.CleanUp.ReferenceOTU19733
New.CleanUp.ReferenceOTU21924
New.CleanUp.ReferenceOTU6694
New.CleanUp.ReferenceOTU106242
New.CleanUp.ReferenceOTU243497
New.CleanUp.ReferenceOTU106
New.CleanUp.ReferenceOTU311028
New.CleanUp.ReferenceOTU88271
New.CleanUp.ReferenceOTU209158
New.CleanUp.ReferenceOTU175992
New.CleanUp.ReferenceOTU1451
New.CleanUp.ReferenceOTU41979
New.ReferenceOTU167
New.CleanUp.ReferenceOTU145426
New.CleanUp.ReferenceOTU88864
New.CleanUp.ReferenceOTU27982
New.CleanUp.ReferenceOTU223674
New.CleanUp.ReferenceOTU69071
New.CleanUp.ReferenceOTU56885
New.CleanUp.ReferenceOTU43161
New.CleanUp.ReferenceOTU148075

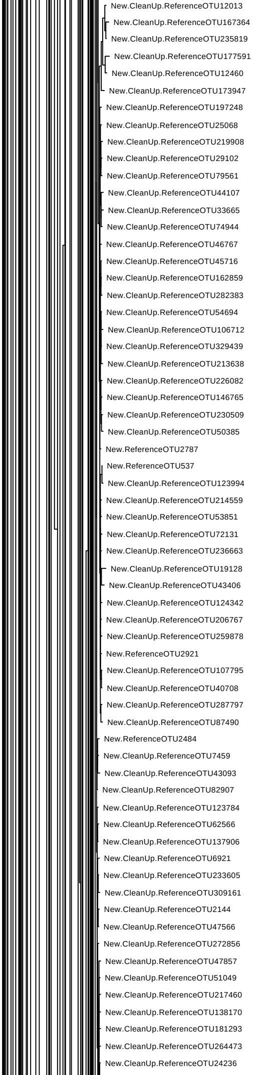
New.CleanUp.ReferenceOTU12013
New.CleanUp.ReferenceOTU167364
New.CleanUp.ReferenceOTU235819
New.CleanUp.ReferenceOTU177591
New.CleanUp.ReferenceOTU12460
New.CleanUp.ReferenceOTU173947
New.CleanUp.ReferenceOTU197248
New.CleanUp.ReferenceOTU25068
New.CleanUp.ReferenceOTU219908
New.CleanUp.ReferenceOTU29102
New.CleanUp.ReferenceOTU79561
New.CleanUp.ReferenceOTU44107
New.CleanUp.ReferenceOTU33665
New.CleanUp.ReferenceOTU74944
New.CleanUp.ReferenceOTU46767
New.CleanUp.ReferenceOTU45716
New.CleanUp.ReferenceOTU162859
New.CleanUp.ReferenceOTU282383
New.CleanUp.ReferenceOTU54694
New.CleanUp.ReferenceOTU106712
New.CleanUp.ReferenceOTU329439
New.CleanUp.ReferenceOTU213638
New.CleanUp.ReferenceOTU226082
New.CleanUp.ReferenceOTU146765
New.CleanUp.ReferenceOTU230509
New.CleanUp.ReferenceOTU50385
New.ReferenceOTU2787
New.ReferenceOTU537
New.CleanUp.ReferenceOTU123994
New.CleanUp.ReferenceOTU214559
New.CleanUp.ReferenceOTU53851
New.CleanUp.ReferenceOTU72131
New.CleanUp.ReferenceOTU236663
New.CleanUp.ReferenceOTU19128
New.CleanUp.ReferenceOTU43406
New.CleanUp.ReferenceOTU124342
New.CleanUp.ReferenceOTU206767
New.CleanUp.ReferenceOTU259878
New.ReferenceOTU2921
New.CleanUp.ReferenceOTU107795
New.CleanUp.ReferenceOTU40708
New.CleanUp.ReferenceOTU287797
New.CleanUp.ReferenceOTU87490
New.ReferenceOTU2484
New.CleanUp.ReferenceOTU7459
New.CleanUp.ReferenceOTU43093
New.CleanUp.ReferenceOTU82907
New.CleanUp.ReferenceOTU123784
New.CleanUp.ReferenceOTU62566
New.CleanUp.ReferenceOTU137906
New.CleanUp.ReferenceOTU6921
New.CleanUp.ReferenceOTU233605
New.CleanUp.ReferenceOTU309161
New.CleanUp.ReferenceOTU2144
New.CleanUp.ReferenceOTU47566
New.CleanUp.ReferenceOTU272856
New.CleanUp.ReferenceOTU47857
New.CleanUp.ReferenceOTU51049
New.CleanUp.ReferenceOTU217460
New.CleanUp.ReferenceOTU138170
New.CleanUp.ReferenceOTU181293
New.CleanUp.ReferenceOTU264473
New.CleanUp.ReferenceOTU24236

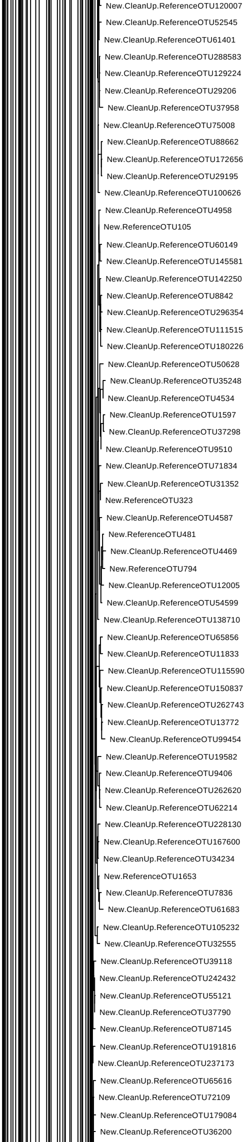
New.CleanUp.ReferenceOTU120007
New.CleanUp.ReferenceOTU52545
New.CleanUp.ReferenceOTU61401
New.CleanUp.ReferenceOTU288583
New.CleanUp.ReferenceOTU129224
New.CleanUp.ReferenceOTU29206
New.CleanUp.ReferenceOTU37958
New.CleanUp.ReferenceOTU75008
New.CleanUp.ReferenceOTU88662
New.CleanUp.ReferenceOTU172656
New.CleanUp.ReferenceOTU29195
New.CleanUp.ReferenceOTU100626
New.CleanUp.ReferenceOTU4958
New.ReferenceOTU105
New.CleanUp.ReferenceOTU60149
New.CleanUp.ReferenceOTU145581
New.CleanUp.ReferenceOTU142250
New.CleanUp.ReferenceOTU8842
New.CleanUp.ReferenceOTU296354
New.CleanUp.ReferenceOTU111515
New.CleanUp.ReferenceOTU180226
New.CleanUp.ReferenceOTU50628
New.CleanUp.ReferenceOTU35248
New.CleanUp.ReferenceOTU4534
New.CleanUp.ReferenceOTU1597
New.CleanUp.ReferenceOTU37298
New.CleanUp.ReferenceOTU9510
New.CleanUp.ReferenceOTU71834
New.CleanUp.ReferenceOTU31352
New.ReferenceOTU323
New.CleanUp.ReferenceOTU4587
New.ReferenceOTU481
New.CleanUp.ReferenceOTU4469
New.ReferenceOTU794
New.CleanUp.ReferenceOTU12005
New.CleanUp.ReferenceOTU54599
New.CleanUp.ReferenceOTU138710
New.CleanUp.ReferenceOTU65856
New.CleanUp.ReferenceOTU11833
New.CleanUp.ReferenceOTU115590
New.CleanUp.ReferenceOTU150837
New.CleanUp.ReferenceOTU262743
New.CleanUp.ReferenceOTU13772
New.CleanUp.ReferenceOTU99454
New.CleanUp.ReferenceOTU19582
New.CleanUp.ReferenceOTU9406
New.CleanUp.ReferenceOTU262620
New.CleanUp.ReferenceOTU62214
New.CleanUp.ReferenceOTU228130
New.CleanUp.ReferenceOTU167600
New.CleanUp.ReferenceOTU34234
New.ReferenceOTU1653
New.CleanUp.ReferenceOTU7836
New.CleanUp.ReferenceOTU61683
New.CleanUp.ReferenceOTU105232
New.CleanUp.ReferenceOTU32555
New.CleanUp.ReferenceOTU39118
New.CleanUp.ReferenceOTU242432
New.CleanUp.ReferenceOTU55121
New.CleanUp.ReferenceOTU37790
New.CleanUp.ReferenceOTU87145
New.CleanUp.ReferenceOTU191816
New.CleanUp.ReferenceOTU237173
New.CleanUp.ReferenceOTU65616
New.CleanUp.ReferenceOTU72109
New.CleanUp.ReferenceOTU179084
New.CleanUp.ReferenceOTU36200

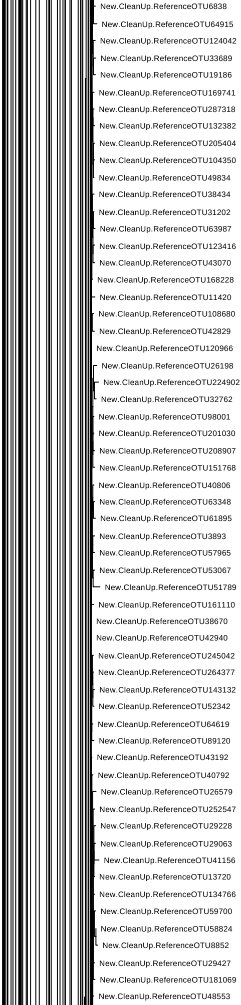
New.CleanUp.ReferenceOTU6838
New.CleanUp.ReferenceOTU64915
New.CleanUp.ReferenceOTU124042
New.CleanUp.ReferenceOTU33689
New.CleanUp.ReferenceOTU19186
New.CleanUp.ReferenceOTU169741
New.CleanUp.ReferenceOTU287318
New.CleanUp.ReferenceOTU132382
New.CleanUp.ReferenceOTU205404
New.CleanUp.ReferenceOTU104350
New.CleanUp.ReferenceOTU49834
New.CleanUp.ReferenceOTU38434
New.CleanUp.ReferenceOTU31202
New.CleanUp.ReferenceOTU63987
New.CleanUp.ReferenceOTU123416
New.CleanUp.ReferenceOTU43070
New.CleanUp.ReferenceOTU168228
New.CleanUp.ReferenceOTU11420
New.CleanUp.ReferenceOTU108680
New.CleanUp.ReferenceOTU42829
New.CleanUp.ReferenceOTU120966
New.CleanUp.ReferenceOTU26198
New.CleanUp.ReferenceOTU224902
New.CleanUp.ReferenceOTU32762
New.CleanUp.ReferenceOTU98001
New.CleanUp.ReferenceOTU201030
New.CleanUp.ReferenceOTU208907
New.CleanUp.ReferenceOTU151768
New.CleanUp.ReferenceOTU40806
New.CleanUp.ReferenceOTU63348
New.CleanUp.ReferenceOTU61895
New.CleanUp.ReferenceOTU3893
New.CleanUp.ReferenceOTU57965
New.CleanUp.ReferenceOTU53067
New.CleanUp.ReferenceOTU51789
New.CleanUp.ReferenceOTU161110
New.CleanUp.ReferenceOTU38670
New.CleanUp.ReferenceOTU42940
New.CleanUp.ReferenceOTU245042
New.CleanUp.ReferenceOTU264377
New.CleanUp.ReferenceOTU143132
New.CleanUp.ReferenceOTU52342
New.CleanUp.ReferenceOTU64619
New.CleanUp.ReferenceOTU89120
New.CleanUp.ReferenceOTU43192
New.CleanUp.ReferenceOTU40792
New.CleanUp.ReferenceOTU26579
New.CleanUp.ReferenceOTU252547
New.CleanUp.ReferenceOTU29228
New.CleanUp.ReferenceOTU29063
New.CleanUp.ReferenceOTU41156
New.CleanUp.ReferenceOTU13720
New.CleanUp.ReferenceOTU134766
New.CleanUp.ReferenceOTU59700
New.CleanUp.ReferenceOTU58824
New.CleanUp.ReferenceOTU8852
New.CleanUp.ReferenceOTU29427
New.CleanUp.ReferenceOTU181069
New.CleanUp.ReferenceOTU48553

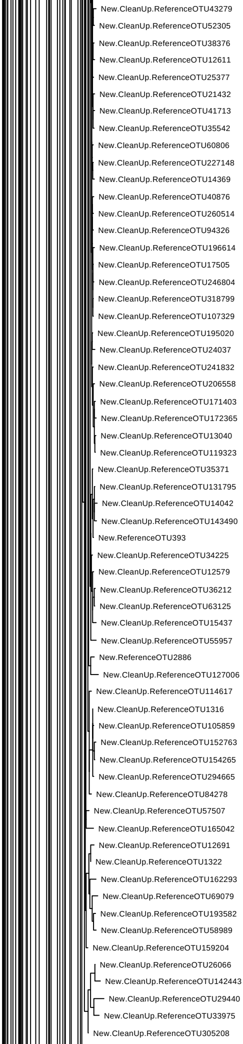
New.CleanUp.ReferenceOTU43279
New.CleanUp.ReferenceOTU52305
New.CleanUp.ReferenceOTU38376
New.CleanUp.ReferenceOTU12611
New.CleanUp.ReferenceOTU25377
New.CleanUp.ReferenceOTU21432
New.CleanUp.ReferenceOTU41713
New.CleanUp.ReferenceOTU35542
New.CleanUp.ReferenceOTU60806
New.CleanUp.ReferenceOTU227148
New.CleanUp.ReferenceOTU14369
New.CleanUp.ReferenceOTU40876
New.CleanUp.ReferenceOTU260514
New.CleanUp.ReferenceOTU94326
New.CleanUp.ReferenceOTU196614
New.CleanUp.ReferenceOTU17505
New.CleanUp.ReferenceOTU246804
New.CleanUp.ReferenceOTU318799
New.CleanUp.ReferenceOTU107329
New.CleanUp.ReferenceOTU195020
New.CleanUp.ReferenceOTU24037
New.CleanUp.ReferenceOTU241832
New.CleanUp.ReferenceOTU206558
New.CleanUp.ReferenceOTU171403
New.CleanUp.ReferenceOTU172365
New.CleanUp.ReferenceOTU13040
New.CleanUp.ReferenceOTU119323
New.CleanUp.ReferenceOTU35371
New.CleanUp.ReferenceOTU131795
New.CleanUp.ReferenceOTU14042
New.CleanUp.ReferenceOTU143490
New.ReferenceOTU393
New.CleanUp.ReferenceOTU34225
New.CleanUp.ReferenceOTU12579
New.CleanUp.ReferenceOTU36212
New.CleanUp.ReferenceOTU63125
New.CleanUp.ReferenceOTU15437
New.CleanUp.ReferenceOTU55957
New.ReferenceOTU2886
New.CleanUp.ReferenceOTU127006
New.CleanUp.ReferenceOTU114617
New.CleanUp.ReferenceOTU1316
New.CleanUp.ReferenceOTU105859
New.CleanUp.ReferenceOTU152763
New.CleanUp.ReferenceOTU154265
New.CleanUp.ReferenceOTU294665
New.CleanUp.ReferenceOTU84278
New.CleanUp.ReferenceOTU57507
New.CleanUp.ReferenceOTU165042
New.CleanUp.ReferenceOTU12691
New.CleanUp.ReferenceOTU1322
New.CleanUp.ReferenceOTU162293
New.CleanUp.ReferenceOTU69079
New.CleanUp.ReferenceOTU193582
New.CleanUp.ReferenceOTU58989
New.CleanUp.ReferenceOTU159204
New.CleanUp.ReferenceOTU26066
New.CleanUp.ReferenceOTU142443
New.CleanUp.ReferenceOTU29440
New.CleanUp.ReferenceOTU33975
New.CleanUp.ReferenceOTU305208

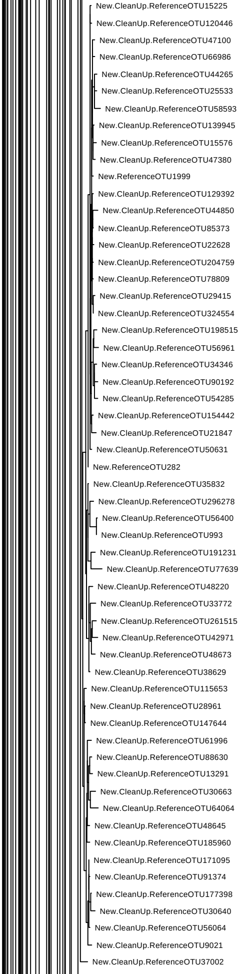
New.CleanUp.ReferenceOTU15225
New.CleanUp.ReferenceOTU120446
New.CleanUp.ReferenceOTU47100
New.CleanUp.ReferenceOTU66986
New.CleanUp.ReferenceOTU44265
New.CleanUp.ReferenceOTU25533
New.CleanUp.ReferenceOTU58593
New.CleanUp.ReferenceOTU139945
New.CleanUp.ReferenceOTU15576
New.CleanUp.ReferenceOTU47380
New.ReferenceOTU1999
New.CleanUp.ReferenceOTU129392
New.CleanUp.ReferenceOTU44850
New.CleanUp.ReferenceOTU85373
New.CleanUp.ReferenceOTU22628
New.CleanUp.ReferenceOTU204759
New.CleanUp.ReferenceOTU78809
New.CleanUp.ReferenceOTU29415
New.CleanUp.ReferenceOTU324554
New.CleanUp.ReferenceOTU198515
New.CleanUp.ReferenceOTU56961
New.CleanUp.ReferenceOTU34346
New.CleanUp.ReferenceOTU90192
New.CleanUp.ReferenceOTU54285
New.CleanUp.ReferenceOTU154442
New.CleanUp.ReferenceOTU21847
New.CleanUp.ReferenceOTU50631
New.ReferenceOTU282
New.CleanUp.ReferenceOTU35832
New.CleanUp.ReferenceOTU296278
New.CleanUp.ReferenceOTU56400
New.CleanUp.ReferenceOTU993
New.CleanUp.ReferenceOTU191231
New.CleanUp.ReferenceOTU77639
New.CleanUp.ReferenceOTU48220
New.CleanUp.ReferenceOTU33772
New.CleanUp.ReferenceOTU261515
New.CleanUp.ReferenceOTU42971
New.CleanUp.ReferenceOTU48673
New.CleanUp.ReferenceOTU38629
New.CleanUp.ReferenceOTU115653
New.CleanUp.ReferenceOTU28961
New.CleanUp.ReferenceOTU147644
New.CleanUp.ReferenceOTU61996
New.CleanUp.ReferenceOTU88630
New.CleanUp.ReferenceOTU13291
New.CleanUp.ReferenceOTU30663
New.CleanUp.ReferenceOTU64064
New.CleanUp.ReferenceOTU48645
New.CleanUp.ReferenceOTU185960
New.CleanUp.ReferenceOTU171095
New.CleanUp.ReferenceOTU91374
New.CleanUp.ReferenceOTU177398
New.CleanUp.ReferenceOTU30640
New.CleanUp.ReferenceOTU56064
New.CleanUp.ReferenceOTU9021
New.CleanUp.ReferenceOTU37002

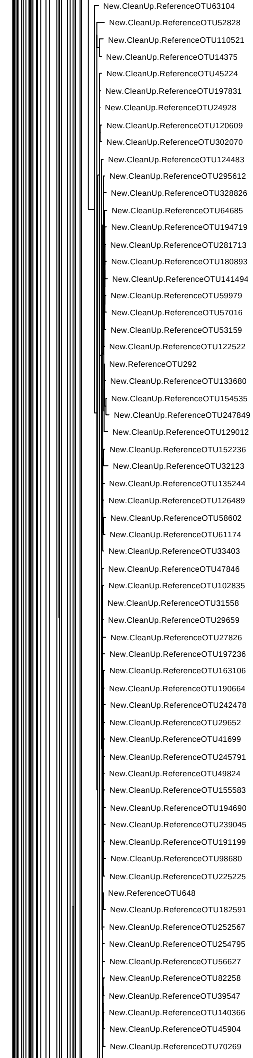

New.CleanUp.ReferenceOTU63104
New.CleanUp.ReferenceOTU52828
New.CleanUp.ReferenceOTU110521
New.CleanUp.ReferenceOTU14375
New.CleanUp.ReferenceOTU45224
New.CleanUp.ReferenceOTU197831
New.CleanUp.ReferenceOTU24928
New.CleanUp.ReferenceOTU120609
New.CleanUp.ReferenceOTU302070
New.CleanUp.ReferenceOTU124483
New.CleanUp.ReferenceOTU295612
New.CleanUp.ReferenceOTU328826
New.CleanUp.ReferenceOTU64685
New.CleanUp.ReferenceOTU194719
New.CleanUp.ReferenceOTU281713
New.CleanUp.ReferenceOTU180893
New.CleanUp.ReferenceOTU141494
New.CleanUp.ReferenceOTU59979
New.CleanUp.ReferenceOTU57016
New.CleanUp.ReferenceOTU53159
New.CleanUp.ReferenceOTU122522
New.ReferenceOTU292
New.CleanUp.ReferenceOTU133680
New.CleanUp.ReferenceOTU154535
New.CleanUp.ReferenceOTU247849
New.CleanUp.ReferenceOTU129012
New.CleanUp.ReferenceOTU152236
New.CleanUp.ReferenceOTU32123
New.CleanUp.ReferenceOTU135244
New.CleanUp.ReferenceOTU126489
New.CleanUp.ReferenceOTU58602
New.CleanUp.ReferenceOTU61174
New.CleanUp.ReferenceOTU33403
New.CleanUp.ReferenceOTU47846
New.CleanUp.ReferenceOTU102835
New.CleanUp.ReferenceOTU31558
New.CleanUp.ReferenceOTU29659
New.CleanUp.ReferenceOTU27826
New.CleanUp.ReferenceOTU197236
New.CleanUp.ReferenceOTU163106
New.CleanUp.ReferenceOTU190664
New.CleanUp.ReferenceOTU242478
New.CleanUp.ReferenceOTU29652
New.CleanUp.ReferenceOTU41699
New.CleanUp.ReferenceOTU245791
New.CleanUp.ReferenceOTU49824
New.CleanUp.ReferenceOTU155583
New.CleanUp.ReferenceOTU194690
New.CleanUp.ReferenceOTU239045
New.CleanUp.ReferenceOTU191199
New.CleanUp.ReferenceOTU98680
New.CleanUp.ReferenceOTU225225
New.ReferenceOTU648
New.CleanUp.ReferenceOTU182591
New.CleanUp.ReferenceOTU252567
New.CleanUp.ReferenceOTU254795
New.CleanUp.ReferenceOTU56627
New.CleanUp.ReferenceOTU82258
New.CleanUp.ReferenceOTU39547
New.CleanUp.ReferenceOTU140366
New.CleanUp.ReferenceOTU45904
New.CleanUp.ReferenceOTU70269

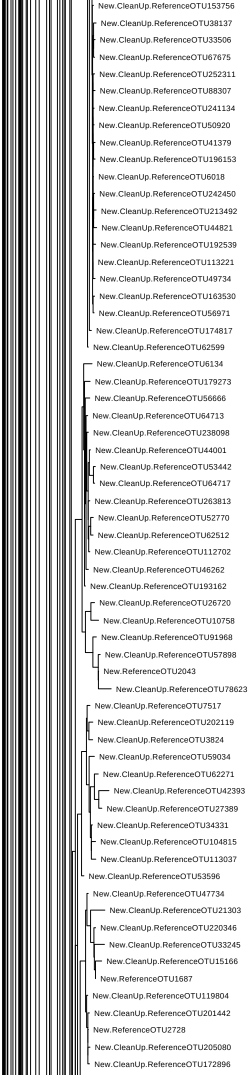
New.CleanUp.ReferenceOTU153756
New.CleanUp.ReferenceOTU38137
New.CleanUp.ReferenceOTU33506
New.CleanUp.ReferenceOTU67675
New.CleanUp.ReferenceOTU252311
New.CleanUp.ReferenceOTU88307
New.CleanUp.ReferenceOTU241134
New.CleanUp.ReferenceOTU50920
New.CleanUp.ReferenceOTU41379
New.CleanUp.ReferenceOTU196153
New.CleanUp.ReferenceOTU6018
New.CleanUp.ReferenceOTU242450
New.CleanUp.ReferenceOTU213492
New.CleanUp.ReferenceOTU44821
New.CleanUp.ReferenceOTU192539
New.CleanUp.ReferenceOTU113221
New.CleanUp.ReferenceOTU49734
New.CleanUp.ReferenceOTU163530
New.CleanUp.ReferenceOTU56971
New.CleanUp.ReferenceOTU174817
New.CleanUp.ReferenceOTU62599
New.CleanUp.ReferenceOTU6134
New.CleanUp.ReferenceOTU179273
New.CleanUp.ReferenceOTU56666
New.CleanUp.ReferenceOTU64713
New.CleanUp.ReferenceOTU238098
New.CleanUp.ReferenceOTU44001
New.CleanUp.ReferenceOTU53442
New.CleanUp.ReferenceOTU64717
New.CleanUp.ReferenceOTU263813
New.CleanUp.ReferenceOTU52770
New.CleanUp.ReferenceOTU62512
New.CleanUp.ReferenceOTU112702
New.CleanUp.ReferenceOTU46262
New.CleanUp.ReferenceOTU193162
New.CleanUp.ReferenceOTU26720
New.CleanUp.ReferenceOTU10758
New.CleanUp.ReferenceOTU91968
New.CleanUp.ReferenceOTU57898
New.ReferenceOTU2043
New.CleanUp.ReferenceOTU78623
New.CleanUp.ReferenceOTU7517
New.CleanUp.ReferenceOTU202119
New.CleanUp.ReferenceOTU3824
New.CleanUp.ReferenceOTU59034
New.CleanUp.ReferenceOTU62271
New.CleanUp.ReferenceOTU42393
New.CleanUp.ReferenceOTU27389
New.CleanUp.ReferenceOTU34331
New.CleanUp.ReferenceOTU104815
New.CleanUp.ReferenceOTU113037
New.CleanUp.ReferenceOTU53596
New.CleanUp.ReferenceOTU47734
New.CleanUp.ReferenceOTU21303
New.CleanUp.ReferenceOTU220346
New.CleanUp.ReferenceOTU33245
New.CleanUp.ReferenceOTU15166
New.ReferenceOTU1687
New.CleanUp.ReferenceOTU119804
New.CleanUp.ReferenceOTU201442
New.ReferenceOTU2728
New.CleanUp.ReferenceOTU205080
New.CleanUp.ReferenceOTU172896

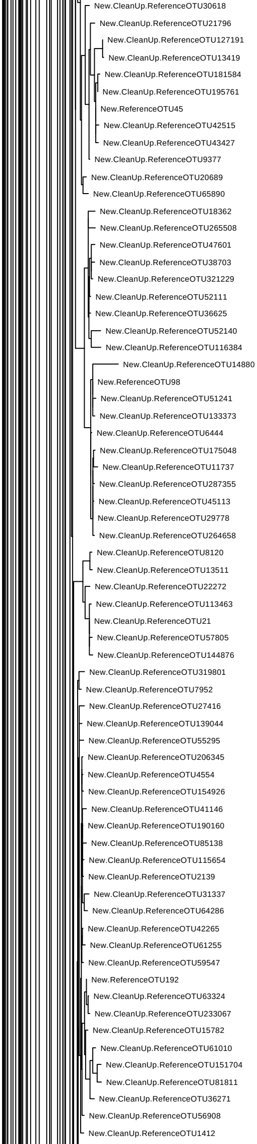
New.CleanUp.ReferenceOTU30618
New.CleanUp.ReferenceOTU21796
New.CleanUp.ReferenceOTU127191
New.CleanUp.ReferenceOTU13419
New.CleanUp.ReferenceOTU181584
New.CleanUp.ReferenceOTU195761
New.ReferenceOTU45
New.CleanUp.ReferenceOTU42515
New.CleanUp.ReferenceOTU43427
New.CleanUp.ReferenceOTU9377
New.CleanUp.ReferenceOTU20689
New.CleanUp.ReferenceOTU65890
New.CleanUp.ReferenceOTU18362
New.CleanUp.ReferenceOTU265508
New.CleanUp.ReferenceOTU47601
New.CleanUp.ReferenceOTU38703
New.CleanUp.ReferenceOTU321229
New.CleanUp.ReferenceOTU52111
New.CleanUp.ReferenceOTU36625
New.CleanUp.ReferenceOTU52140
New.CleanUp.ReferenceOTU116384
New.CleanUp.ReferenceOTU14880
New.ReferenceOTU98
New.CleanUp.ReferenceOTU51241
New.CleanUp.ReferenceOTU133373
New.CleanUp.ReferenceOTU6444
New.CleanUp.ReferenceOTU175048
New.CleanUp.ReferenceOTU11737
New.CleanUp.ReferenceOTU287355
New.CleanUp.ReferenceOTU45113
New.CleanUp.ReferenceOTU29778
New.CleanUp.ReferenceOTU264658
New.CleanUp.ReferenceOTU8120
New.CleanUp.ReferenceOTU13511
New.CleanUp.ReferenceOTU22272
New.CleanUp.ReferenceOTU113463
New.CleanUp.ReferenceOTU21
New.CleanUp.ReferenceOTU57805
New.CleanUp.ReferenceOTU144876
New.CleanUp.ReferenceOTU319801
New.CleanUp.ReferenceOTU7952
New.CleanUp.ReferenceOTU27416
New.CleanUp.ReferenceOTU139044
New.CleanUp.ReferenceOTU55295
New.CleanUp.ReferenceOTU206345
New.CleanUp.ReferenceOTU4554
New.CleanUp.ReferenceOTU154926
New.CleanUp.ReferenceOTU41146
New.CleanUp.ReferenceOTU190160
New.CleanUp.ReferenceOTU85138
New.CleanUp.ReferenceOTU115654
New.CleanUp.ReferenceOTU2139
New.CleanUp.ReferenceOTU31337
New.CleanUp.ReferenceOTU64286
New.CleanUp.ReferenceOTU42265
New.CleanUp.ReferenceOTU61255
New.CleanUp.ReferenceOTU59547
New.ReferenceOTU192
New.CleanUp.ReferenceOTU63324
New.CleanUp.ReferenceOTU233067
New.CleanUp.ReferenceOTU15782
New.CleanUp.ReferenceOTU61010
New.CleanUp.ReferenceOTU151704
New.CleanUp.ReferenceOTU81811
New.CleanUp.ReferenceOTU36271
New.CleanUp.ReferenceOTU56908
New.CleanUp.ReferenceOTU1412

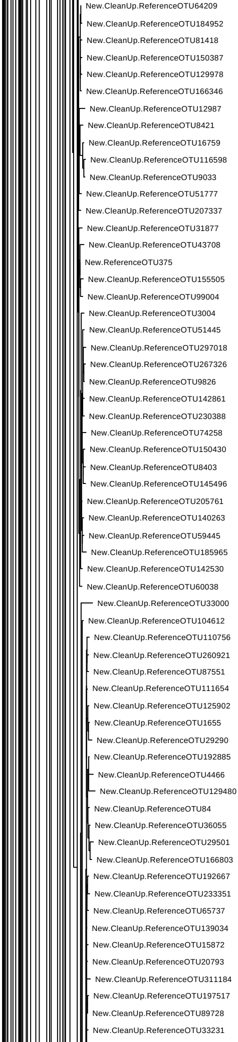
New.CleanUp.ReferenceOTU64209
New.CleanUp.ReferenceOTU184952
New.CleanUp.ReferenceOTU81418
New.CleanUp.ReferenceOTU150387
New.CleanUp.ReferenceOTU129978
New.CleanUp.ReferenceOTU166346
New.CleanUp.ReferenceOTU12987
New.CleanUp.ReferenceOTU8421
New.CleanUp.ReferenceOTU16759
New.CleanUp.ReferenceOTU116598
New.CleanUp.ReferenceOTU9033
New.CleanUp.ReferenceOTU51777
New.CleanUp.ReferenceOTU207337
New.CleanUp.ReferenceOTU31877
New.CleanUp.ReferenceOTU43708
New.ReferenceOTU375
New.CleanUp.ReferenceOTU155505
New.CleanUp.ReferenceOTU99004
New.CleanUp.ReferenceOTU3004
New.CleanUp.ReferenceOTU51445
New.CleanUp.ReferenceOTU297018
New.CleanUp.ReferenceOTU267326
New.CleanUp.ReferenceOTU9826
New.CleanUp.ReferenceOTU142861
New.CleanUp.ReferenceOTU230388
New.CleanUp.ReferenceOTU74258
New.CleanUp.ReferenceOTU150430
New.CleanUp.ReferenceOTU8403
New.CleanUp.ReferenceOTU145496
New.CleanUp.ReferenceOTU205761
New.CleanUp.ReferenceOTU140263
New.CleanUp.ReferenceOTU59445
New.CleanUp.ReferenceOTU185965
New.CleanUp.ReferenceOTU142530
New.CleanUp.ReferenceOTU60038
New.CleanUp.ReferenceOTU33000
New.CleanUp.ReferenceOTU104612
New.CleanUp.ReferenceOTU110756
New.CleanUp.ReferenceOTU260921
New.CleanUp.ReferenceOTU87551
New.CleanUp.ReferenceOTU111654
New.CleanUp.ReferenceOTU125902
New.CleanUp.ReferenceOTU1655
New.CleanUp.ReferenceOTU29290
New.CleanUp.ReferenceOTU192885
New.CleanUp.ReferenceOTU4466
New.CleanUp.ReferenceOTU129480
New.CleanUp.ReferenceOTU84
New.CleanUp.ReferenceOTU36055
New.CleanUp.ReferenceOTU29501
New.CleanUp.ReferenceOTU166803
New.CleanUp.ReferenceOTU192667
New.CleanUp.ReferenceOTU233351
New.CleanUp.ReferenceOTU65737
New.CleanUp.ReferenceOTU139034
New.CleanUp.ReferenceOTU15872
New.CleanUp.ReferenceOTU20793
New.CleanUp.ReferenceOTU311184
New.CleanUp.ReferenceOTU197517
New.CleanUp.ReferenceOTU89728
New.CleanUp.ReferenceOTU33231

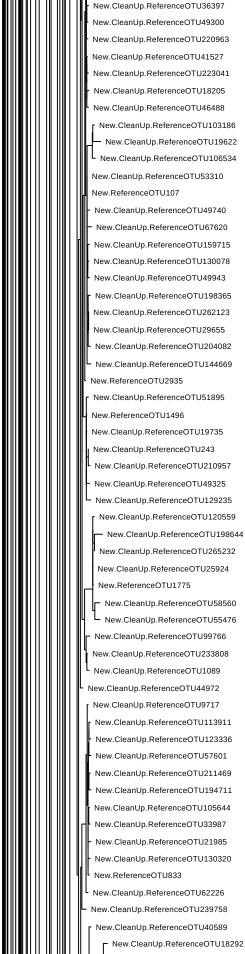
New.CleanUp.ReferenceOTU36397
New.CleanUp.ReferenceOTU49300
New.CleanUp.ReferenceOTU220963
New.CleanUp.ReferenceOTU41527
New.CleanUp.ReferenceOTU223041
New.CleanUp.ReferenceOTU18205
New.CleanUp.ReferenceOTU46488
New.CleanUp.ReferenceOTU103186
New.CleanUp.ReferenceOTU19622
New.CleanUp.ReferenceOTU106534
New.CleanUp.ReferenceOTU53310
New.ReferenceOTU107
New.CleanUp.ReferenceOTU49740
New.CleanUp.ReferenceOTU67620
New.CleanUp.ReferenceOTU159715
New.CleanUp.ReferenceOTU130078
New.CleanUp.ReferenceOTU49943
New.CleanUp.ReferenceOTU198365
New.CleanUp.ReferenceOTU262123
New.CleanUp.ReferenceOTU29655
New.CleanUp.ReferenceOTU204082
New.CleanUp.ReferenceOTU144669
New.ReferenceOTU2935
New.CleanUp.ReferenceOTU51895
New.ReferenceOTU1496
New.CleanUp.ReferenceOTU19735
New.CleanUp.ReferenceOTU243
New.CleanUp.ReferenceOTU210957
New.CleanUp.ReferenceOTU49325
New.CleanUp.ReferenceOTU129235
New.CleanUp.ReferenceOTU120559
New.CleanUp.ReferenceOTU198644
New.CleanUp.ReferenceOTU265232
New.CleanUp.ReferenceOTU25924
New.ReferenceOTU1775
New.CleanUp.ReferenceOTU58560
New.CleanUp.ReferenceOTU55476
New.CleanUp.ReferenceOTU99766
New.CleanUp.ReferenceOTU233808
New.CleanUp.ReferenceOTU1089
New.CleanUp.ReferenceOTU44972
New.CleanUp.ReferenceOTU9717
New.CleanUp.ReferenceOTU113911
New.CleanUp.ReferenceOTU123336
New.CleanUp.ReferenceOTU57601
New.CleanUp.ReferenceOTU211469
New.CleanUp.ReferenceOTU194711
New.CleanUp.ReferenceOTU105644
New.CleanUp.ReferenceOTU33987
New.CleanUp.ReferenceOTU21985
New.CleanUp.ReferenceOTU130320
New.ReferenceOTU833
New.CleanUp.ReferenceOTU62226
New.CleanUp.ReferenceOTU239758
New.CleanUp.ReferenceOTU40589
New.CleanUp.ReferenceOTU18292

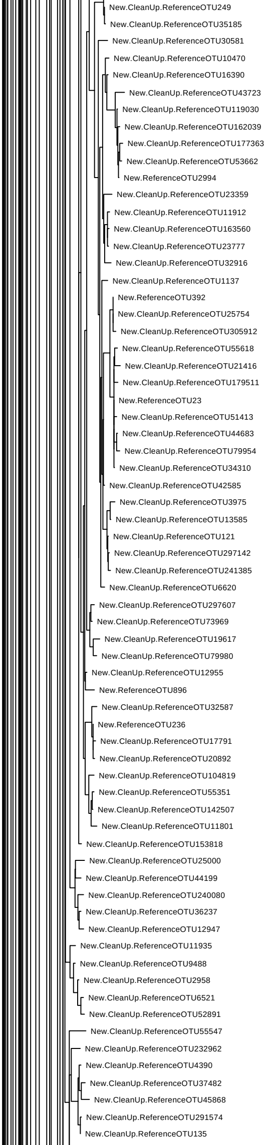
New.CleanUp.ReferenceOTU249
New.CleanUp.ReferenceOTU35185
New.CleanUp.ReferenceOTU30581
New.CleanUp.ReferenceOTU10470
New.CleanUp.ReferenceOTU16390
New.CleanUp.ReferenceOTU43723
New.CleanUp.ReferenceOTU119030
New.CleanUp.ReferenceOTU162039
New.CleanUp.ReferenceOTU177363
New.CleanUp.ReferenceOTU53662
New.ReferenceOTU2994
New.CleanUp.ReferenceOTU23359
New.CleanUp.ReferenceOTU11912
New.CleanUp.ReferenceOTU163560
New.CleanUp.ReferenceOTU23777
New.CleanUp.ReferenceOTU32916
New.CleanUp.ReferenceOTU1137
New.ReferenceOTU392
New.CleanUp.ReferenceOTU25754
New.CleanUp.ReferenceOTU305912
New.CleanUp.ReferenceOTU55618
New.CleanUp.ReferenceOTU21416
New.CleanUp.ReferenceOTU179511
New.ReferenceOTU23
New.CleanUp.ReferenceOTU51413
New.CleanUp.ReferenceOTU44683
New.CleanUp.ReferenceOTU79954
New.CleanUp.ReferenceOTU34310
New.CleanUp.ReferenceOTU42585
New.CleanUp.ReferenceOTU3975
New.CleanUp.ReferenceOTU13585
New.CleanUp.ReferenceOTU121
New.CleanUp.ReferenceOTU297142
New.CleanUp.ReferenceOTU241385
New.CleanUp.ReferenceOTU6620
New.CleanUp.ReferenceOTU297607
New.CleanUp.ReferenceOTU73969
New.CleanUp.ReferenceOTU19617
New.CleanUp.ReferenceOTU79980
New.CleanUp.ReferenceOTU12955
New.ReferenceOTU896
New.CleanUp.ReferenceOTU32587
New.ReferenceOTU236
New.CleanUp.ReferenceOTU17791
New.CleanUp.ReferenceOTU20892
New.CleanUp.ReferenceOTU104819
New.CleanUp.ReferenceOTU55351
New.CleanUp.ReferenceOTU142507
New.CleanUp.ReferenceOTU11801
New.CleanUp.ReferenceOTU153818
New.CleanUp.ReferenceOTU25000
New.CleanUp.ReferenceOTU44199
New.CleanUp.ReferenceOTU240080
New.CleanUp.ReferenceOTU36237
New.CleanUp.ReferenceOTU12947
New.CleanUp.ReferenceOTU11935
New.CleanUp.ReferenceOTU9488
New.CleanUp.ReferenceOTU2958
New.CleanUp.ReferenceOTU6521
New.CleanUp.ReferenceOTU52891
New.CleanUp.ReferenceOTU55547
New.CleanUp.ReferenceOTU232962
New.CleanUp.ReferenceOTU4390
New.CleanUp.ReferenceOTU37482
New.CleanUp.ReferenceOTU45868
New.CleanUp.ReferenceOTU291574
New.CleanUp.ReferenceOTU135

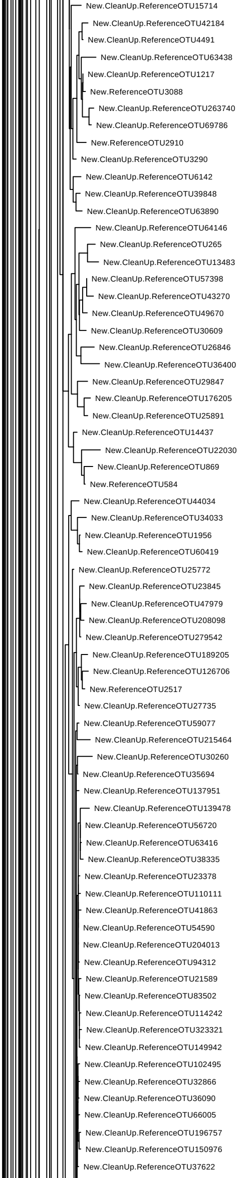

New.CleanUp.ReferenceOTU15714
New.CleanUp.ReferenceOTU42184
New.CleanUp.ReferenceOTU4491
New.CleanUp.ReferenceOTU63438
New.CleanUp.ReferenceOTU1217
New.ReferenceOTU3088
New.CleanUp.ReferenceOTU263740
New.CleanUp.ReferenceOTU69786
New.ReferenceOTU2910
New.CleanUp.ReferenceOTU3290
New.CleanUp.ReferenceOTU6142
New.CleanUp.ReferenceOTU39848
New.CleanUp.ReferenceOTU63890
New.CleanUp.ReferenceOTU64146
New.CleanUp.ReferenceOTU265
New.CleanUp.ReferenceOTU13483
New.CleanUp.ReferenceOTU57398
New.CleanUp.ReferenceOTU43270
New.CleanUp.ReferenceOTU49670
New.CleanUp.ReferenceOTU30609
New.CleanUp.ReferenceOTU26846
New.CleanUp.ReferenceOTU36400
New.CleanUp.ReferenceOTU29847
New.CleanUp.ReferenceOTU176205
New.CleanUp.ReferenceOTU25891
New.CleanUp.ReferenceOTU14437
New.CleanUp.ReferenceOTU22030
New.CleanUp.ReferenceOTU869
New.ReferenceOTU584
New.CleanUp.ReferenceOTU44034
New.CleanUp.ReferenceOTU34033
New.CleanUp.ReferenceOTU1956
New.CleanUp.ReferenceOTU60419
New.CleanUp.ReferenceOTU25772
New.CleanUp.ReferenceOTU23845
New.CleanUp.ReferenceOTU47979
New.CleanUp.ReferenceOTU208098
New.CleanUp.ReferenceOTU279542
New.CleanUp.ReferenceOTU189205
New.CleanUp.ReferenceOTU126706
New.ReferenceOTU2517
New.CleanUp.ReferenceOTU27735
New.CleanUp.ReferenceOTU59077
New.CleanUp.ReferenceOTU215464
New.CleanUp.ReferenceOTU30260
New.CleanUp.ReferenceOTU35694
New.CleanUp.ReferenceOTU137951
New.CleanUp.ReferenceOTU139478
New.CleanUp.ReferenceOTU56720
New.CleanUp.ReferenceOTU63416
New.CleanUp.ReferenceOTU38335
New.CleanUp.ReferenceOTU23378
New.CleanUp.ReferenceOTU110111
New.CleanUp.ReferenceOTU41863
New.CleanUp.ReferenceOTU54590
New.CleanUp.ReferenceOTU204013
New.CleanUp.ReferenceOTU94312
New.CleanUp.ReferenceOTU21589
New.CleanUp.ReferenceOTU83502
New.CleanUp.ReferenceOTU114242
New.CleanUp.ReferenceOTU323321
New.CleanUp.ReferenceOTU149942
New.CleanUp.ReferenceOTU102495
New.CleanUp.ReferenceOTU32866
New.CleanUp.ReferenceOTU36090
New.CleanUp.ReferenceOTU66005
New.CleanUp.ReferenceOTU196757
New.CleanUp.ReferenceOTU150976
New.CleanUp.ReferenceOTU37622

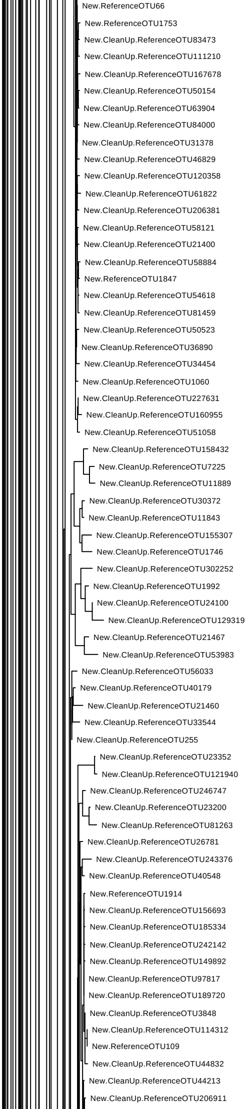
New.ReferenceOTU66
New.ReferenceOTU1753
New.CleanUp.ReferenceOTU83473
New.CleanUp.ReferenceOTU111210
New.CleanUp.ReferenceOTU167678
New.CleanUp.ReferenceOTU50154
New.CleanUp.ReferenceOTU63904
New.CleanUp.ReferenceOTU84000
New.CleanUp.ReferenceOTU31378
New.CleanUp.ReferenceOTU46829
New.CleanUp.ReferenceOTU120358
New.CleanUp.ReferenceOTU61822
New.CleanUp.ReferenceOTU206381
New.CleanUp.ReferenceOTU58121
New.CleanUp.ReferenceOTU21400
New.CleanUp.ReferenceOTU58884
New.ReferenceOTU1847
New.CleanUp.ReferenceOTU54618
New.CleanUp.ReferenceOTU81459
New.CleanUp.ReferenceOTU50523
New.CleanUp.ReferenceOTU36890
New.CleanUp.ReferenceOTU34454
New.CleanUp.ReferenceOTU1060
New.CleanUp.ReferenceOTU227631
New.CleanUp.ReferenceOTU160955
New.CleanUp.ReferenceOTU51058
New.CleanUp.ReferenceOTU158432
New.CleanUp.ReferenceOTU7225
New.CleanUp.ReferenceOTU11889
New.CleanUp.ReferenceOTU30372
New.CleanUp.ReferenceOTU11843
New.CleanUp.ReferenceOTU155307
New.CleanUp.ReferenceOTU1746
New.CleanUp.ReferenceOTU302252
New.CleanUp.ReferenceOTU1992
New.CleanUp.ReferenceOTU24100
New.CleanUp.ReferenceOTU129319
New.CleanUp.ReferenceOTU21467
New.CleanUp.ReferenceOTU53983
New.CleanUp.ReferenceOTU56033
New.CleanUp.ReferenceOTU40179
New.CleanUp.ReferenceOTU21460
New.CleanUp.ReferenceOTU33544
New.CleanUp.ReferenceOTU255
New.CleanUp.ReferenceOTU23352
New.CleanUp.ReferenceOTU121940
New.CleanUp.ReferenceOTU246747
New.CleanUp.ReferenceOTU23200
New.CleanUp.ReferenceOTU81263
New.CleanUp.ReferenceOTU26781
New.CleanUp.ReferenceOTU243376
New.CleanUp.ReferenceOTU40548
New.ReferenceOTU1914
New.CleanUp.ReferenceOTU156693
New.CleanUp.ReferenceOTU185334
New.CleanUp.ReferenceOTU242142
New.CleanUp.ReferenceOTU149892
New.CleanUp.ReferenceOTU97817
New.CleanUp.ReferenceOTU189720
New.CleanUp.ReferenceOTU3848
New.CleanUp.ReferenceOTU114312
New.ReferenceOTU109
New.CleanUp.ReferenceOTU44832
New.CleanUp.ReferenceOTU44213
New.CleanUp.ReferenceOTU206911

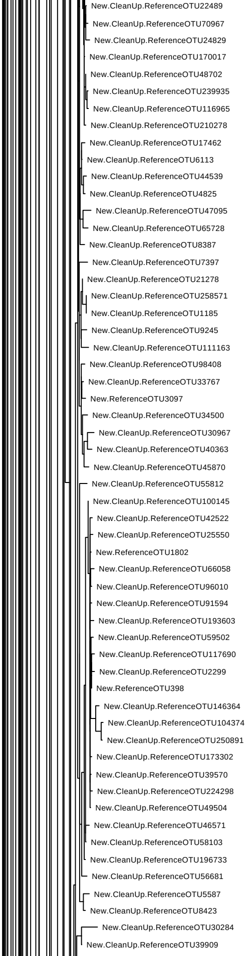

New.CleanUp.ReferenceOTU22489
New.CleanUp.ReferenceOTU70967
New.CleanUp.ReferenceOTU24829
New.CleanUp.ReferenceOTU170017
New.CleanUp.ReferenceOTU48702
New.CleanUp.ReferenceOTU239935
New.CleanUp.ReferenceOTU116965
New.CleanUp.ReferenceOTU210278
New.CleanUp.ReferenceOTU17462
New.CleanUp.ReferenceOTU6113
New.CleanUp.ReferenceOTU44539
New.CleanUp.ReferenceOTU4825
New.CleanUp.ReferenceOTU47095
New.CleanUp.ReferenceOTU65728
New.CleanUp.ReferenceOTU8387
New.CleanUp.ReferenceOTU7397
New.CleanUp.ReferenceOTU21278
New.CleanUp.ReferenceOTU258571
New.CleanUp.ReferenceOTU1185
New.CleanUp.ReferenceOTU9245
New.CleanUp.ReferenceOTU111163
New.CleanUp.ReferenceOTU98408
New.CleanUp.ReferenceOTU33767
New.ReferenceOTU3097
New.CleanUp.ReferenceOTU34500
New.CleanUp.ReferenceOTU30967
New.CleanUp.ReferenceOTU40363
New.CleanUp.ReferenceOTU45870
New.CleanUp.ReferenceOTU55812
New.CleanUp.ReferenceOTU100145
New.CleanUp.ReferenceOTU42522
New.CleanUp.ReferenceOTU25550
New.ReferenceOTU1802
New.CleanUp.ReferenceOTU66058
New.CleanUp.ReferenceOTU96010
New.CleanUp.ReferenceOTU91594
New.CleanUp.ReferenceOTU193603
New.CleanUp.ReferenceOTU59502
New.CleanUp.ReferenceOTU117690
New.CleanUp.ReferenceOTU2299
New.ReferenceOTU398
New.CleanUp.ReferenceOTU146364
New.CleanUp.ReferenceOTU104374
New.CleanUp.ReferenceOTU250891
New.CleanUp.ReferenceOTU173302
New.CleanUp.ReferenceOTU39570
New.CleanUp.ReferenceOTU224298
New.CleanUp.ReferenceOTU49504
New.CleanUp.ReferenceOTU46571
New.CleanUp.ReferenceOTU58103
New.CleanUp.ReferenceOTU196733
New.CleanUp.ReferenceOTU56681
New.CleanUp.ReferenceOTU5587
New.CleanUp.ReferenceOTU8423
New.CleanUp.ReferenceOTU30284
New.CleanUp.ReferenceOTU39909

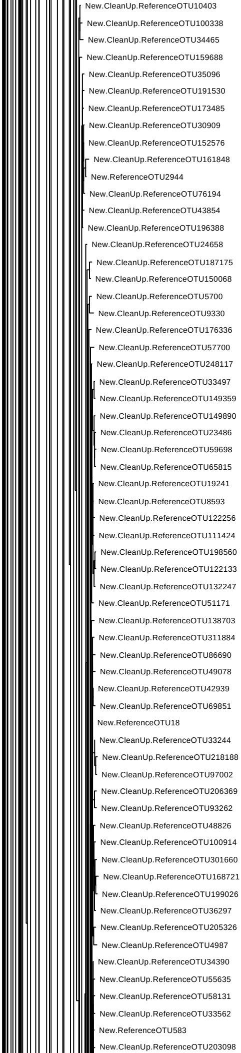
New.CleanUp.ReferenceOTU10403
New.CleanUp.ReferenceOTU100338
New.CleanUp.ReferenceOTU34465
New.CleanUp.ReferenceOTU159688
New.CleanUp.ReferenceOTU35096
New.CleanUp.ReferenceOTU191530
New.CleanUp.ReferenceOTU173485
New.CleanUp.ReferenceOTU30909
New.CleanUp.ReferenceOTU152576
New.CleanUp.ReferenceOTU161848
New.ReferenceOTU2944
New.CleanUp.ReferenceOTU76194
New.CleanUp.ReferenceOTU43854
New.CleanUp.ReferenceOTU196388
New.CleanUp.ReferenceOTU24658
New.CleanUp.ReferenceOTU187175
New.CleanUp.ReferenceOTU150068
New.CleanUp.ReferenceOTU5700
New.CleanUp.ReferenceOTU9330
New.CleanUp.ReferenceOTU176336
New.CleanUp.ReferenceOTU57700
New.CleanUp.ReferenceOTU248117
New.CleanUp.ReferenceOTU33497
New.CleanUp.ReferenceOTU149359
New.CleanUp.ReferenceOTU149890
New.CleanUp.ReferenceOTU23486
New.CleanUp.ReferenceOTU59698
New.CleanUp.ReferenceOTU65815
New.CleanUp.ReferenceOTU19241
New.CleanUp.ReferenceOTU8593
New.CleanUp.ReferenceOTU122256
New.CleanUp.ReferenceOTU111424
New.CleanUp.ReferenceOTU198560
New.CleanUp.ReferenceOTU122133
New.CleanUp.ReferenceOTU132247
New.CleanUp.ReferenceOTU51171
New.CleanUp.ReferenceOTU138703
New.CleanUp.ReferenceOTU311884
New.CleanUp.ReferenceOTU86690
New.CleanUp.ReferenceOTU49078
New.CleanUp.ReferenceOTU42939
New.CleanUp.ReferenceOTU69851
New.ReferenceOTU18
New.CleanUp.ReferenceOTU33244
New.CleanUp.ReferenceOTU218188
New.CleanUp.ReferenceOTU97002
New.CleanUp.ReferenceOTU206369
New.CleanUp.ReferenceOTU93262
New.CleanUp.ReferenceOTU48826
New.CleanUp.ReferenceOTU100914
New.CleanUp.ReferenceOTU301660
New.CleanUp.ReferenceOTU168721
New.CleanUp.ReferenceOTU199026
New.CleanUp.ReferenceOTU36297
New.CleanUp.ReferenceOTU205326
New.CleanUp.ReferenceOTU4987
New.CleanUp.ReferenceOTU34390
New.CleanUp.ReferenceOTU55635
New.CleanUp.ReferenceOTU58131
New.CleanUp.ReferenceOTU33562
New.ReferenceOTU583
New.CleanUp.ReferenceOTU203098

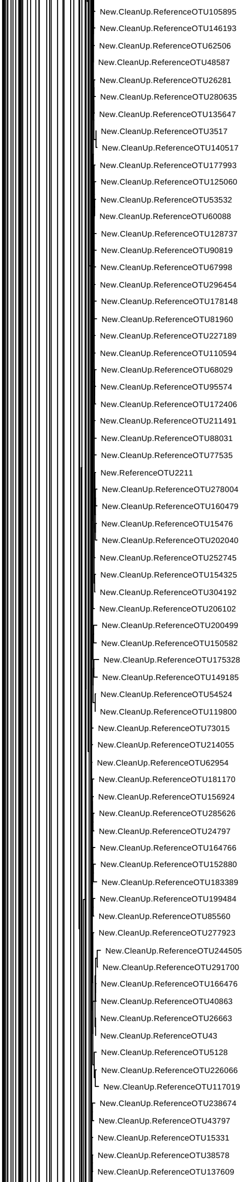
New.CleanUp.ReferenceOTU105895
New.CleanUp.ReferenceOTU146193
New.CleanUp.ReferenceOTU62506
New.CleanUp.ReferenceOTU48587
New.CleanUp.ReferenceOTU26281
New.CleanUp.ReferenceOTU280635
New.CleanUp.ReferenceOTU135647
New.CleanUp.ReferenceOTU3517
New.CleanUp.ReferenceOTU140517
New.CleanUp.ReferenceOTU177993
New.CleanUp.ReferenceOTU125060
New.CleanUp.ReferenceOTU53532
New.CleanUp.ReferenceOTU60088
New.CleanUp.ReferenceOTU128737
New.CleanUp.ReferenceOTU90819
New.CleanUp.ReferenceOTU67998
New.CleanUp.ReferenceOTU296454
New.CleanUp.ReferenceOTU178148
New.CleanUp.ReferenceOTU81960
New.CleanUp.ReferenceOTU227189
New.CleanUp.ReferenceOTU110594
New.CleanUp.ReferenceOTU68029
New.CleanUp.ReferenceOTU95574
New.CleanUp.ReferenceOTU172406
New.CleanUp.ReferenceOTU211491
New.CleanUp.ReferenceOTU88031
New.CleanUp.ReferenceOTU77535
New.ReferenceOTU2211
New.CleanUp.ReferenceOTU278004
New.CleanUp.ReferenceOTU160479
New.CleanUp.ReferenceOTU15476
New.CleanUp.ReferenceOTU202040
New.CleanUp.ReferenceOTU252745
New.CleanUp.ReferenceOTU154325
New.CleanUp.ReferenceOTU304192
New.CleanUp.ReferenceOTU206102
New.CleanUp.ReferenceOTU200499
New.CleanUp.ReferenceOTU150582
New.CleanUp.ReferenceOTU175328
New.CleanUp.ReferenceOTU149185
New.CleanUp.ReferenceOTU54524
New.CleanUp.ReferenceOTU119800
New.CleanUp.ReferenceOTU73015
New.CleanUp.ReferenceOTU214055
New.CleanUp.ReferenceOTU62954
New.CleanUp.ReferenceOTU181170
New.CleanUp.ReferenceOTU156924
New.CleanUp.ReferenceOTU285626
New.CleanUp.ReferenceOTU24797
New.CleanUp.ReferenceOTU164766
New.CleanUp.ReferenceOTU152880
New.CleanUp.ReferenceOTU183389
New.CleanUp.ReferenceOTU199484
New.CleanUp.ReferenceOTU85560
New.CleanUp.ReferenceOTU277923
New.CleanUp.ReferenceOTU244505
New.CleanUp.ReferenceOTU291700
New.CleanUp.ReferenceOTU166476
New.CleanUp.ReferenceOTU40863
New.CleanUp.ReferenceOTU26663
New.CleanUp.ReferenceOTU43
New.CleanUp.ReferenceOTU5128
New.CleanUp.ReferenceOTU226066
New.CleanUp.ReferenceOTU117019
New.CleanUp.ReferenceOTU238674
New.CleanUp.ReferenceOTU43797
New.CleanUp.ReferenceOTU15331
New.CleanUp.ReferenceOTU38578
New.CleanUp.ReferenceOTU137609

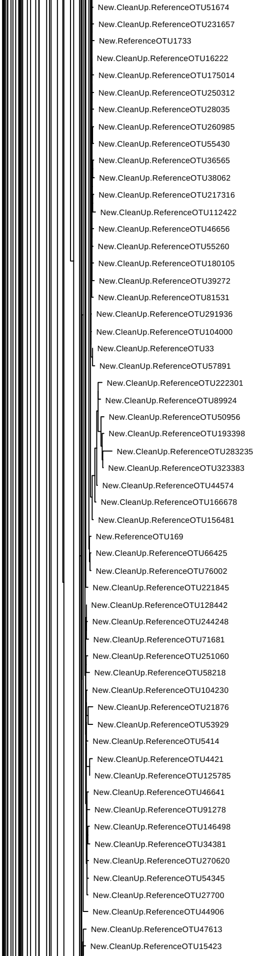
New.CleanUp.ReferenceOTU51674
New.CleanUp.ReferenceOTU231657
New.ReferenceOTU1733
New.CleanUp.ReferenceOTU16222
New.CleanUp.ReferenceOTU175014
New.CleanUp.ReferenceOTU250312
New.CleanUp.ReferenceOTU28035
New.CleanUp.ReferenceOTU260985
New.CleanUp.ReferenceOTU55430
New.CleanUp.ReferenceOTU36565
New.CleanUp.ReferenceOTU38062
New.CleanUp.ReferenceOTU217316
New.CleanUp.ReferenceOTU112422
New.CleanUp.ReferenceOTU46656
New.CleanUp.ReferenceOTU55260
New.CleanUp.ReferenceOTU180105
New.CleanUp.ReferenceOTU39272
New.CleanUp.ReferenceOTU81531
New.CleanUp.ReferenceOTU291936
New.CleanUp.ReferenceOTU104000
New.CleanUp.ReferenceOTU33
New.CleanUp.ReferenceOTU57891
New.CleanUp.ReferenceOTU222301
New.CleanUp.ReferenceOTU89924
New.CleanUp.ReferenceOTU50956
New.CleanUp.ReferenceOTU193398
New.CleanUp.ReferenceOTU283235
New.CleanUp.ReferenceOTU323383
New.CleanUp.ReferenceOTU44574
New.CleanUp.ReferenceOTU166678
New.CleanUp.ReferenceOTU156481
New.ReferenceOTU169
New.CleanUp.ReferenceOTU66425
New.CleanUp.ReferenceOTU76002
New.CleanUp.ReferenceOTU221845
New.CleanUp.ReferenceOTU128442
New.CleanUp.ReferenceOTU244248
New.CleanUp.ReferenceOTU71681
New.CleanUp.ReferenceOTU251060
New.CleanUp.ReferenceOTU58218
New.CleanUp.ReferenceOTU104230
New.CleanUp.ReferenceOTU21876
New.CleanUp.ReferenceOTU53929
New.CleanUp.ReferenceOTU5414
New.CleanUp.ReferenceOTU4421
New.CleanUp.ReferenceOTU125785
New.CleanUp.ReferenceOTU46641
New.CleanUp.ReferenceOTU91278
New.CleanUp.ReferenceOTU146498
New.CleanUp.ReferenceOTU34381
New.CleanUp.ReferenceOTU270620
New.CleanUp.ReferenceOTU54345
New.CleanUp.ReferenceOTU27700
New.CleanUp.ReferenceOTU44906
New.CleanUp.ReferenceOTU47613
New.CleanUp.ReferenceOTU15423

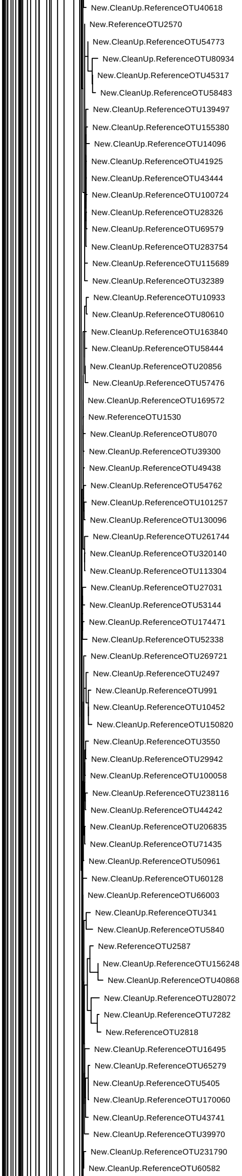
New.CleanUp.ReferenceOTU40618
New.ReferenceOTU2570
New.CleanUp.ReferenceOTU54773
New.CleanUp.ReferenceOTU80934
New.CleanUp.ReferenceOTU45317
New.CleanUp.ReferenceOTU58483
New.CleanUp.ReferenceOTU139497
New.CleanUp.ReferenceOTU155380
New.CleanUp.ReferenceOTU14096
New.CleanUp.ReferenceOTU41925
New.CleanUp.ReferenceOTU43444
New.CleanUp.ReferenceOTU100724
New.CleanUp.ReferenceOTU28326
New.CleanUp.ReferenceOTU69579
New.CleanUp.ReferenceOTU283754
New.CleanUp.ReferenceOTU115689
New.CleanUp.ReferenceOTU32389
New.CleanUp.ReferenceOTU10933
New.CleanUp.ReferenceOTU80610
New.CleanUp.ReferenceOTU163840
New.CleanUp.ReferenceOTU58444
New.CleanUp.ReferenceOTU20856
New.CleanUp.ReferenceOTU57476
New.CleanUp.ReferenceOTU169572
New.ReferenceOTU1530
New.CleanUp.ReferenceOTU8070
New.CleanUp.ReferenceOTU39300
New.CleanUp.ReferenceOTU49438
New.CleanUp.ReferenceOTU54762
New.CleanUp.ReferenceOTU101257
New.CleanUp.ReferenceOTU130096
New.CleanUp.ReferenceOTU261744
New.CleanUp.ReferenceOTU320140
New.CleanUp.ReferenceOTU113304
New.CleanUp.ReferenceOTU27031
New.CleanUp.ReferenceOTU53144
New.CleanUp.ReferenceOTU174471
New.CleanUp.ReferenceOTU52338
New.CleanUp.ReferenceOTU269721
New.CleanUp.ReferenceOTU2497
New.CleanUp.ReferenceOTU991
New.CleanUp.ReferenceOTU10452
New.CleanUp.ReferenceOTU150820
New.CleanUp.ReferenceOTU3550
New.CleanUp.ReferenceOTU29942
New.CleanUp.ReferenceOTU100058
New.CleanUp.ReferenceOTU238116
New.CleanUp.ReferenceOTU44242
New.CleanUp.ReferenceOTU206835
New.CleanUp.ReferenceOTU71435
New.CleanUp.ReferenceOTU50961
New.CleanUp.ReferenceOTU60128
New.CleanUp.ReferenceOTU66003
New.CleanUp.ReferenceOTU341
New.CleanUp.ReferenceOTU5840
New.ReferenceOTU2587
New.CleanUp.ReferenceOTU156248
New.CleanUp.ReferenceOTU40868
New.CleanUp.ReferenceOTU28072
New.CleanUp.ReferenceOTU7282
New.ReferenceOTU2818
New.CleanUp.ReferenceOTU16495
New.CleanUp.ReferenceOTU65279
New.CleanUp.ReferenceOTU5405
New.CleanUp.ReferenceOTU170060
New.CleanUp.ReferenceOTU43741
New.CleanUp.ReferenceOTU39970
New.CleanUp.ReferenceOTU231790
New.CleanUp.ReferenceOTU60582

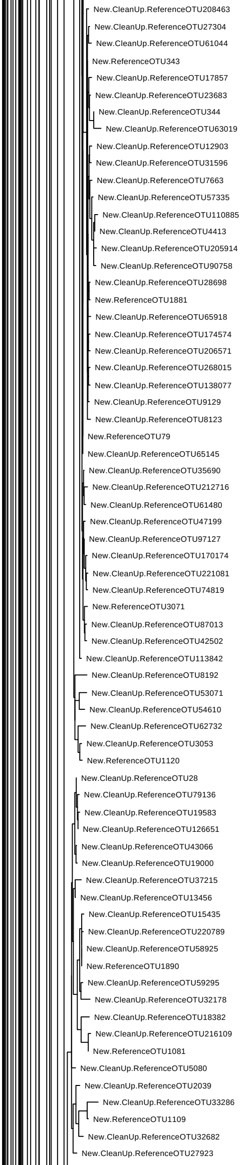
New.CleanUp.ReferenceOTU208463
New.CleanUp.ReferenceOTU27304
New.CleanUp.ReferenceOTU61044
New.ReferenceOTU343
New.CleanUp.ReferenceOTU17857
New.CleanUp.ReferenceOTU23683
New.CleanUp.ReferenceOTU344
New.CleanUp.ReferenceOTU63019
New.CleanUp.ReferenceOTU12903
New.CleanUp.ReferenceOTU31596
New.CleanUp.ReferenceOTU7663
New.CleanUp.ReferenceOTU57335
New.CleanUp.ReferenceOTU110885
New.CleanUp.ReferenceOTU4413
New.CleanUp.ReferenceOTU205914
New.CleanUp.ReferenceOTU90758
New.CleanUp.ReferenceOTU28698
New.ReferenceOTU1881
New.CleanUp.ReferenceOTU65918
New.CleanUp.ReferenceOTU174574
New.CleanUp.ReferenceOTU206571
New.CleanUp.ReferenceOTU268015
New.CleanUp.ReferenceOTU138077
New.CleanUp.ReferenceOTU9129
New.CleanUp.ReferenceOTU8123
New.ReferenceOTU79
New.CleanUp.ReferenceOTU65145
New.CleanUp.ReferenceOTU35690
New.CleanUp.ReferenceOTU212716
New.CleanUp.ReferenceOTU61480
New.CleanUp.ReferenceOTU47199
New.CleanUp.ReferenceOTU97127
New.CleanUp.ReferenceOTU170174
New.CleanUp.ReferenceOTU221081
New.CleanUp.ReferenceOTU74819
New.ReferenceOTU3071
New.CleanUp.ReferenceOTU87013
New.CleanUp.ReferenceOTU42502
New.CleanUp.ReferenceOTU113842
New.CleanUp.ReferenceOTU8192
New.CleanUp.ReferenceOTU53071
New.CleanUp.ReferenceOTU54610
New.CleanUp.ReferenceOTU62732
New.CleanUp.ReferenceOTU3053
New.ReferenceOTU1120
New.CleanUp.ReferenceOTU28
New.CleanUp.ReferenceOTU79136
New.CleanUp.ReferenceOTU19583
New.CleanUp.ReferenceOTU126651
New.CleanUp.ReferenceOTU43066
New.CleanUp.ReferenceOTU19000
New.CleanUp.ReferenceOTU37215
New.CleanUp.ReferenceOTU13456
New.CleanUp.ReferenceOTU15435
New.CleanUp.ReferenceOTU220789
New.CleanUp.ReferenceOTU58925
New.ReferenceOTU1890
New.CleanUp.ReferenceOTU59295
New.CleanUp.ReferenceOTU32178
New.CleanUp.ReferenceOTU18382
New.CleanUp.ReferenceOTU216109
New.ReferenceOTU1081
New.CleanUp.ReferenceOTU5080
New.CleanUp.ReferenceOTU2039
New.CleanUp.ReferenceOTU33286
New.ReferenceOTU1109
New.CleanUp.ReferenceOTU32682
New.CleanUp.ReferenceOTU27923

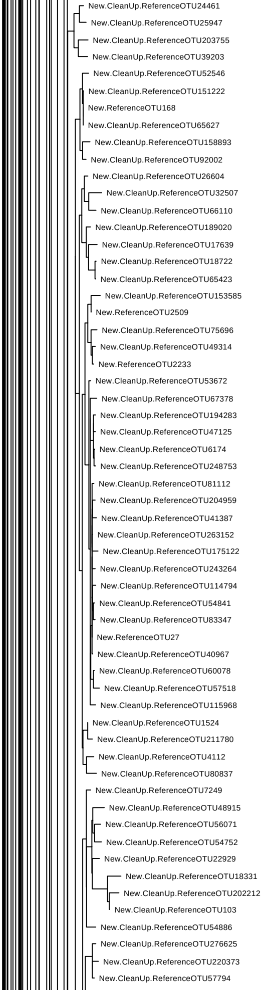
New.CleanUp.ReferenceOTU24461
New.CleanUp.ReferenceOTU25947
New.CleanUp.ReferenceOTU203755
New.CleanUp.ReferenceOTU39203
New.CleanUp.ReferenceOTU52546
New.CleanUp.ReferenceOTU151222
New.ReferenceOTU168
New.CleanUp.ReferenceOTU65627
New.CleanUp.ReferenceOTU158893
New.CleanUp.ReferenceOTU92002
New.CleanUp.ReferenceOTU26604
New.CleanUp.ReferenceOTU32507
New.CleanUp.ReferenceOTU66110
New.CleanUp.ReferenceOTU189020
New.CleanUp.ReferenceOTU17639
New.CleanUp.ReferenceOTU18722
New.CleanUp.ReferenceOTU65423
New.CleanUp.ReferenceOTU153585
New.ReferenceOTU2509
New.CleanUp.ReferenceOTU75696
New.CleanUp.ReferenceOTU49314
New.ReferenceOTU2233
New.CleanUp.ReferenceOTU53672
New.CleanUp.ReferenceOTU67378
New.CleanUp.ReferenceOTU194283
New.CleanUp.ReferenceOTU47125
New.CleanUp.ReferenceOTU6174
New.CleanUp.ReferenceOTU248753
New.CleanUp.ReferenceOTU81112
New.CleanUp.ReferenceOTU204959
New.CleanUp.ReferenceOTU41387
New.CleanUp.ReferenceOTU263152
New.CleanUp.ReferenceOTU175122
New.CleanUp.ReferenceOTU243264
New.CleanUp.ReferenceOTU114794
New.CleanUp.ReferenceOTU54841
New.CleanUp.ReferenceOTU83347
New.ReferenceOTU27
New.CleanUp.ReferenceOTU40967
New.CleanUp.ReferenceOTU60078
New.CleanUp.ReferenceOTU57518
New.CleanUp.ReferenceOTU115968
New.CleanUp.ReferenceOTU1524
New.CleanUp.ReferenceOTU211780
New.CleanUp.ReferenceOTU4112
New.CleanUp.ReferenceOTU80837
New.CleanUp.ReferenceOTU7249
New.CleanUp.ReferenceOTU48915
New.CleanUp.ReferenceOTU56071
New.CleanUp.ReferenceOTU54752
New.CleanUp.ReferenceOTU22929
New.CleanUp.ReferenceOTU18331
New.CleanUp.ReferenceOTU202212
New.CleanUp.ReferenceOTU103
New.CleanUp.ReferenceOTU54886
New.CleanUp.ReferenceOTU276625
New.CleanUp.ReferenceOTU220373
New.CleanUp.ReferenceOTU57794

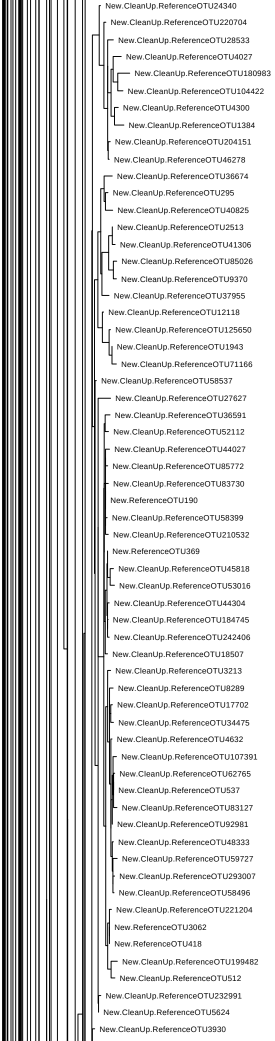
New.CleanUp.ReferenceOTU24340
New.CleanUp.ReferenceOTU220704
New.CleanUp.ReferenceOTU28533
New.CleanUp.ReferenceOTU4027
New.CleanUp.ReferenceOTU180983
New.CleanUp.ReferenceOTU104422
New.CleanUp.ReferenceOTU4300
New.CleanUp.ReferenceOTU1384
New.CleanUp.ReferenceOTU204151
New.CleanUp.ReferenceOTU46278
New.CleanUp.ReferenceOTU36674
New.CleanUp.ReferenceOTU295
New.CleanUp.ReferenceOTU40825
New.CleanUp.ReferenceOTU2513
New.CleanUp.ReferenceOTU41306
New.CleanUp.ReferenceOTU85026
New.CleanUp.ReferenceOTU9370
New.CleanUp.ReferenceOTU37955
New.CleanUp.ReferenceOTU12118
New.CleanUp.ReferenceOTU125650
New.CleanUp.ReferenceOTU1943
New.CleanUp.ReferenceOTU71166
New.CleanUp.ReferenceOTU58537
New.CleanUp.ReferenceOTU27627
New.CleanUp.ReferenceOTU36591
New.CleanUp.ReferenceOTU52112
New.CleanUp.ReferenceOTU44027
New.CleanUp.ReferenceOTU85772
New.CleanUp.ReferenceOTU83730
New.ReferenceOTU190
New.CleanUp.ReferenceOTU58399
New.CleanUp.ReferenceOTU210532
New.ReferenceOTU369
New.CleanUp.ReferenceOTU45818
New.CleanUp.ReferenceOTU53016
New.CleanUp.ReferenceOTU44304
New.CleanUp.ReferenceOTU184745
New.CleanUp.ReferenceOTU242406
New.CleanUp.ReferenceOTU18507
New.CleanUp.ReferenceOTU3213
New.CleanUp.ReferenceOTU8289
New.CleanUp.ReferenceOTU17702
New.CleanUp.ReferenceOTU34475
New.CleanUp.ReferenceOTU4632
New.CleanUp.ReferenceOTU107391
New.CleanUp.ReferenceOTU62765
New.CleanUp.ReferenceOTU537
New.CleanUp.ReferenceOTU83127
New.CleanUp.ReferenceOTU92981
New.CleanUp.ReferenceOTU48333
New.CleanUp.ReferenceOTU59727
New.CleanUp.ReferenceOTU293007
New.CleanUp.ReferenceOTU58496
New.CleanUp.ReferenceOTU221204
New.ReferenceOTU3062
New.ReferenceOTU418
New.CleanUp.ReferenceOTU199482
New.CleanUp.ReferenceOTU512
New.CleanUp.ReferenceOTU232991
New.CleanUp.ReferenceOTU5624
New.CleanUp.ReferenceOTU3930

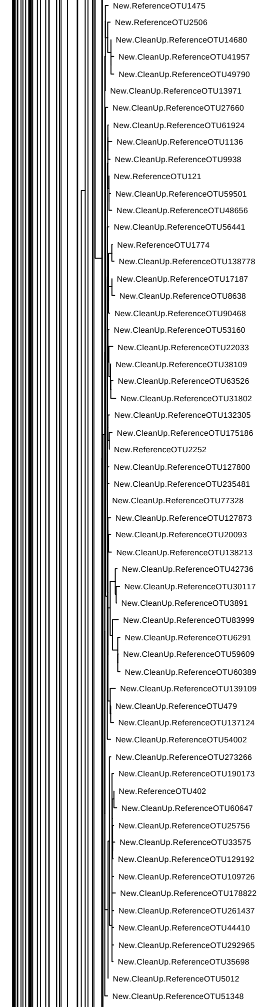
New.ReferenceOTU1475
New.ReferenceOTU2506
New.CleanUp.ReferenceOTU14680
New.CleanUp.ReferenceOTU41957
New.CleanUp.ReferenceOTU49790
New.CleanUp.ReferenceOTU13971
New.CleanUp.ReferenceOTU27660
New.CleanUp.ReferenceOTU61924
New.CleanUp.ReferenceOTU1136
New.CleanUp.ReferenceOTU9938
New.ReferenceOTU121
New.CleanUp.ReferenceOTU59501
New.CleanUp.ReferenceOTU48656
New.CleanUp.ReferenceOTU56441
New.ReferenceOTU1774
New.CleanUp.ReferenceOTU138778
New.CleanUp.ReferenceOTU17187
New.CleanUp.ReferenceOTU8638
New.CleanUp.ReferenceOTU90468
New.CleanUp.ReferenceOTU53160
New.CleanUp.ReferenceOTU22033
New.CleanUp.ReferenceOTU38109
New.CleanUp.ReferenceOTU63526
New.CleanUp.ReferenceOTU31802
New.CleanUp.ReferenceOTU132305
New.CleanUp.ReferenceOTU175186
New.ReferenceOTU2252
New.CleanUp.ReferenceOTU127800
New.CleanUp.ReferenceOTU235481
New.CleanUp.ReferenceOTU77328
New.CleanUp.ReferenceOTU127873
New.CleanUp.ReferenceOTU20093
New.CleanUp.ReferenceOTU138213
New.CleanUp.ReferenceOTU42736
New.CleanUp.ReferenceOTU30117
New.CleanUp.ReferenceOTU3891
New.CleanUp.ReferenceOTU83999
New.CleanUp.ReferenceOTU6291
New.CleanUp.ReferenceOTU59609
New.CleanUp.ReferenceOTU60389
New.CleanUp.ReferenceOTU139109
New.CleanUp.ReferenceOTU479
New.CleanUp.ReferenceOTU137124
New.CleanUp.ReferenceOTU54002
New.CleanUp.ReferenceOTU273266
New.CleanUp.ReferenceOTU190173
New.ReferenceOTU402
New.CleanUp.ReferenceOTU60647
New.CleanUp.ReferenceOTU25756
New.CleanUp.ReferenceOTU33575
New.CleanUp.ReferenceOTU129192
New.CleanUp.ReferenceOTU109726
New.CleanUp.ReferenceOTU178822
New.CleanUp.ReferenceOTU261437
New.CleanUp.ReferenceOTU44410
New.CleanUp.ReferenceOTU292965
New.CleanUp.ReferenceOTU35698
New.CleanUp.ReferenceOTU5012
New.CleanUp.ReferenceOTU51348

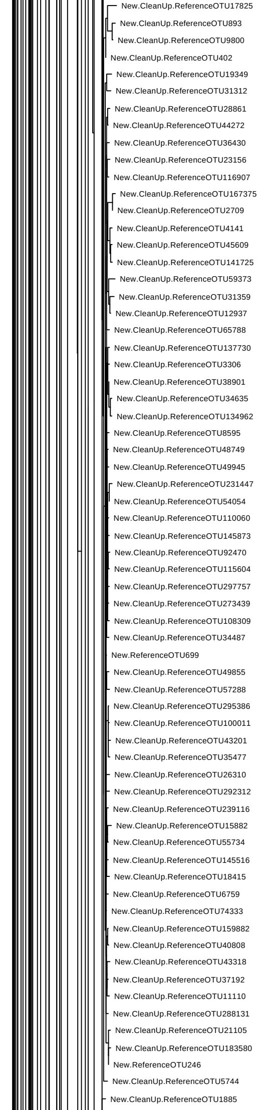
New.CleanUp.ReferenceOTU17825
New.CleanUp.ReferenceOTU893
New.CleanUp.ReferenceOTU9800
New.CleanUp.ReferenceOTU402
New.CleanUp.ReferenceOTU19349
New.CleanUp.ReferenceOTU31312
New.CleanUp.ReferenceOTU28861
New.CleanUp.ReferenceOTU44272
New.CleanUp.ReferenceOTU36430
New.CleanUp.ReferenceOTU23156
New.CleanUp.ReferenceOTU116907
New.CleanUp.ReferenceOTU167375
New.CleanUp.ReferenceOTU2709
New.CleanUp.ReferenceOTU4141
New.CleanUp.ReferenceOTU45609
New.CleanUp.ReferenceOTU141725
New.CleanUp.ReferenceOTU59373
New.CleanUp.ReferenceOTU31359
New.CleanUp.ReferenceOTU12937
New.CleanUp.ReferenceOTU65788
New.CleanUp.ReferenceOTU137730
New.CleanUp.ReferenceOTU3306
New.CleanUp.ReferenceOTU38901
New.CleanUp.ReferenceOTU34635
New.CleanUp.ReferenceOTU134962
New.CleanUp.ReferenceOTU8595
New.CleanUp.ReferenceOTU48749
New.CleanUp.ReferenceOTU49945
New.CleanUp.ReferenceOTU231447
New.CleanUp.ReferenceOTU54054
New.CleanUp.ReferenceOTU110060
New.CleanUp.ReferenceOTU145873
New.CleanUp.ReferenceOTU92470
New.CleanUp.ReferenceOTU115604
New.CleanUp.ReferenceOTU297757
New.CleanUp.ReferenceOTU273439
New.CleanUp.ReferenceOTU108309
New.CleanUp.ReferenceOTU34487
New.ReferenceOTU699
New.CleanUp.ReferenceOTU49855
New.CleanUp.ReferenceOTU57288
New.CleanUp.ReferenceOTU295386
New.CleanUp.ReferenceOTU100011
New.CleanUp.ReferenceOTU43201
New.CleanUp.ReferenceOTU35477
New.CleanUp.ReferenceOTU26310
New.CleanUp.ReferenceOTU292312
New.CleanUp.ReferenceOTU239116
New.CleanUp.ReferenceOTU15882
New.CleanUp.ReferenceOTU55734
New.CleanUp.ReferenceOTU145516
New.CleanUp.ReferenceOTU18415
New.CleanUp.ReferenceOTU6759
New.CleanUp.ReferenceOTU74333
New.CleanUp.ReferenceOTU159882
New.CleanUp.ReferenceOTU40808
New.CleanUp.ReferenceOTU43318
New.CleanUp.ReferenceOTU37192
New.CleanUp.ReferenceOTU11110
New.CleanUp.ReferenceOTU288131
New.CleanUp.ReferenceOTU21105
New.CleanUp.ReferenceOTU183580
New.ReferenceOTU246
New.CleanUp.ReferenceOTU5744
New.CleanUp.ReferenceOTU1885

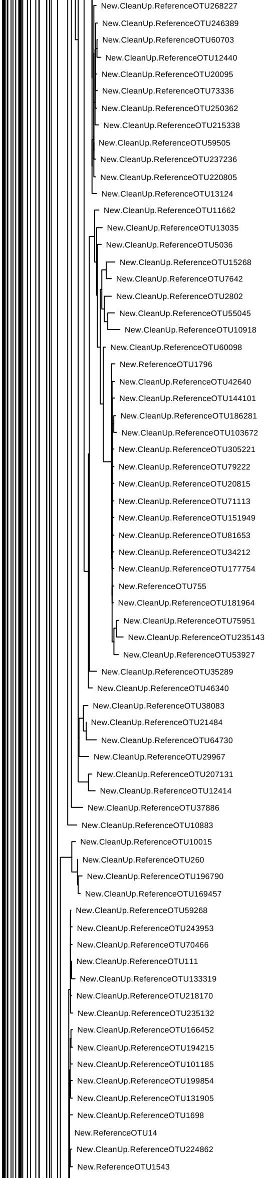
New.CleanUp.ReferenceOTU268227
New.CleanUp.ReferenceOTU246389
New.CleanUp.ReferenceOTU60703
New.CleanUp.ReferenceOTU12440
New.CleanUp.ReferenceOTU20095
New.CleanUp.ReferenceOTU73336
New.CleanUp.ReferenceOTU250362
New.CleanUp.ReferenceOTU215338
New.CleanUp.ReferenceOTU59505
New.CleanUp.ReferenceOTU237236
New.CleanUp.ReferenceOTU220805
New.CleanUp.ReferenceOTU13124
New.CleanUp.ReferenceOTU11662
New.CleanUp.ReferenceOTU13035
New.CleanUp.ReferenceOTU5036
New.CleanUp.ReferenceOTU15268
New.CleanUp.ReferenceOTU7642
New.CleanUp.ReferenceOTU2802
New.CleanUp.ReferenceOTU55045
New.CleanUp.ReferenceOTU10918
New.CleanUp.ReferenceOTU60098
New.ReferenceOTU1796
New.CleanUp.ReferenceOTU42640
New.CleanUp.ReferenceOTU144101
New.CleanUp.ReferenceOTU186281
New.CleanUp.ReferenceOTU103672
New.CleanUp.ReferenceOTU305221
New.CleanUp.ReferenceOTU79222
New.CleanUp.ReferenceOTU20815
New.CleanUp.ReferenceOTU71113
New.CleanUp.ReferenceOTU151949
New.CleanUp.ReferenceOTU81653
New.CleanUp.ReferenceOTU34212
New.CleanUp.ReferenceOTU177754
New.ReferenceOTU755
New.CleanUp.ReferenceOTU181964
New.CleanUp.ReferenceOTU75951
New.CleanUp.ReferenceOTU235143
New.CleanUp.ReferenceOTU53927
New.CleanUp.ReferenceOTU35289
New.CleanUp.ReferenceOTU46340
New.CleanUp.ReferenceOTU38083
New.CleanUp.ReferenceOTU21484
New.CleanUp.ReferenceOTU64730
New.CleanUp.ReferenceOTU29967
New.CleanUp.ReferenceOTU207131
New.CleanUp.ReferenceOTU12414
New.CleanUp.ReferenceOTU37886
New.CleanUp.ReferenceOTU10883
New.CleanUp.ReferenceOTU10015
New.CleanUp.ReferenceOTU260
New.CleanUp.ReferenceOTU196790
New.CleanUp.ReferenceOTU169457
New.CleanUp.ReferenceOTU59268
New.CleanUp.ReferenceOTU243953
New.CleanUp.ReferenceOTU70466
New.CleanUp.ReferenceOTU111
New.CleanUp.ReferenceOTU133319
New.CleanUp.ReferenceOTU218170
New.CleanUp.ReferenceOTU235132
New.CleanUp.ReferenceOTU166452
New.CleanUp.ReferenceOTU194215
New.CleanUp.ReferenceOTU101185
New.CleanUp.ReferenceOTU199854
New.CleanUp.ReferenceOTU131905
New.CleanUp.ReferenceOTU1698
New.ReferenceOTU14
New.CleanUp.ReferenceOTU224862
New.ReferenceOTU1543

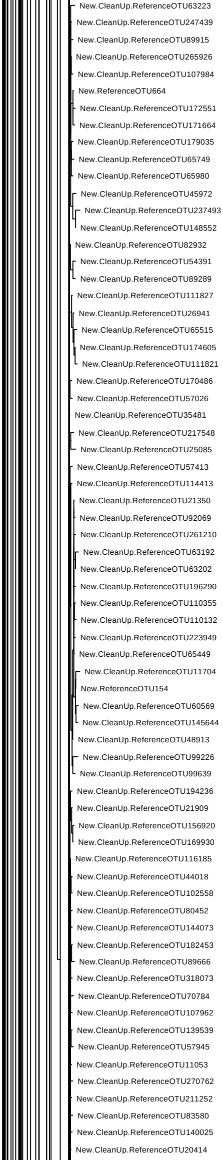
New.CleanUp.ReferenceOTU63223
New.CleanUp.ReferenceOTU247439
New.CleanUp.ReferenceOTU89915
New.CleanUp.ReferenceOTU265926
New.CleanUp.ReferenceOTU107984
New.ReferenceOTU664
New.CleanUp.ReferenceOTU172551
New.CleanUp.ReferenceOTU171664
New.CleanUp.ReferenceOTU179035
New.CleanUp.ReferenceOTU65749
New.CleanUp.ReferenceOTU65980
New.CleanUp.ReferenceOTU45972
New.CleanUp.ReferenceOTU237493
New.CleanUp.ReferenceOTU148552
New.CleanUp.ReferenceOTU82932
New.CleanUp.ReferenceOTU54391
New.CleanUp.ReferenceOTU89289
New.CleanUp.ReferenceOTU111827
New.CleanUp.ReferenceOTU26941
New.CleanUp.ReferenceOTU65515
New.CleanUp.ReferenceOTU174605
New.CleanUp.ReferenceOTU111821
New.CleanUp.ReferenceOTU170486
New.CleanUp.ReferenceOTU57026
New.CleanUp.ReferenceOTU35481
New.CleanUp.ReferenceOTU217548
New.CleanUp.ReferenceOTU25085
New.CleanUp.ReferenceOTU57413
New.CleanUp.ReferenceOTU114413
New.CleanUp.ReferenceOTU21350
New.CleanUp.ReferenceOTU92069
New.CleanUp.ReferenceOTU261210
New.CleanUp.ReferenceOTU63192
New.CleanUp.ReferenceOTU63202
New.CleanUp.ReferenceOTU196290
New.CleanUp.ReferenceOTU110355
New.CleanUp.ReferenceOTU110132
New.CleanUp.ReferenceOTU223949
New.CleanUp.ReferenceOTU65449
New.CleanUp.ReferenceOTU11704
New.ReferenceOTU154
New.CleanUp.ReferenceOTU60569
New.CleanUp.ReferenceOTU145644
New.CleanUp.ReferenceOTU48913
New.CleanUp.ReferenceOTU99226
New.CleanUp.ReferenceOTU99639
New.CleanUp.ReferenceOTU194236
New.CleanUp.ReferenceOTU21909
New.CleanUp.ReferenceOTU156920
New.CleanUp.ReferenceOTU169930
New.CleanUp.ReferenceOTU116185
New.CleanUp.ReferenceOTU44018
New.CleanUp.ReferenceOTU102558
New.CleanUp.ReferenceOTU80452
New.CleanUp.ReferenceOTU144073
New.CleanUp.ReferenceOTU182453
New.CleanUp.ReferenceOTU89666
New.CleanUp.ReferenceOTU318073
New.CleanUp.ReferenceOTU70784
New.CleanUp.ReferenceOTU107962
New.CleanUp.ReferenceOTU139539
New.CleanUp.ReferenceOTU57945
New.CleanUp.ReferenceOTU11053
New.CleanUp.ReferenceOTU270762
New.CleanUp.ReferenceOTU211252
New.CleanUp.ReferenceOTU83580
New.CleanUp.ReferenceOTU140025
New.CleanUp.ReferenceOTU20414

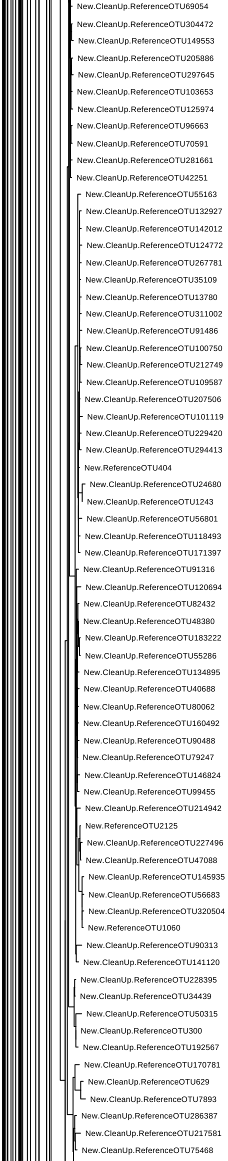
New.CleanUp.ReferenceOTU69054
New.CleanUp.ReferenceOTU304472
New.CleanUp.ReferenceOTU149553
New.CleanUp.ReferenceOTU205886
New.CleanUp.ReferenceOTU297645
New.CleanUp.ReferenceOTU103653
New.CleanUp.ReferenceOTU125974
New.CleanUp.ReferenceOTU96663
New.CleanUp.ReferenceOTU70591
New.CleanUp.ReferenceOTU281661
New.CleanUp.ReferenceOTU42251
New.CleanUp.ReferenceOTU55163
New.CleanUp.ReferenceOTU132927
New.CleanUp.ReferenceOTU142012
New.CleanUp.ReferenceOTU124772
New.CleanUp.ReferenceOTU267781
New.CleanUp.ReferenceOTU35109
New.CleanUp.ReferenceOTU13780
New.CleanUp.ReferenceOTU311002
New.CleanUp.ReferenceOTU91486
New.CleanUp.ReferenceOTU100750
New.CleanUp.ReferenceOTU212749
New.CleanUp.ReferenceOTU109587
New.CleanUp.ReferenceOTU207506
New.CleanUp.ReferenceOTU101119
New.CleanUp.ReferenceOTU229420
New.CleanUp.ReferenceOTU294413
New.ReferenceOTU404
New.CleanUp.ReferenceOTU24680
New.CleanUp.ReferenceOTU1243
New.CleanUp.ReferenceOTU56801
New.CleanUp.ReferenceOTU118493
New.CleanUp.ReferenceOTU171397
New.CleanUp.ReferenceOTU91316
New.CleanUp.ReferenceOTU120694
New.CleanUp.ReferenceOTU82432
New.CleanUp.ReferenceOTU48380
New.CleanUp.ReferenceOTU183222
New.CleanUp.ReferenceOTU55286
New.CleanUp.ReferenceOTU134895
New.CleanUp.ReferenceOTU40688
New.CleanUp.ReferenceOTU80062
New.CleanUp.ReferenceOTU160492
New.CleanUp.ReferenceOTU90488
New.CleanUp.ReferenceOTU79247
New.CleanUp.ReferenceOTU146824
New.CleanUp.ReferenceOTU99455
New.CleanUp.ReferenceOTU214942
New.ReferenceOTU2125
New.CleanUp.ReferenceOTU227496
New.CleanUp.ReferenceOTU47088
New.CleanUp.ReferenceOTU145935
New.CleanUp.ReferenceOTU56683
New.CleanUp.ReferenceOTU320504
New.ReferenceOTU1060
New.CleanUp.ReferenceOTU90313
New.CleanUp.ReferenceOTU141120
New.CleanUp.ReferenceOTU228395
New.CleanUp.ReferenceOTU34439
New.CleanUp.ReferenceOTU50315
New.CleanUp.ReferenceOTU300
New.CleanUp.ReferenceOTU192567
New.CleanUp.ReferenceOTU170781
New.CleanUp.ReferenceOTU629
New.CleanUp.ReferenceOTU7893
New.CleanUp.ReferenceOTU286387
New.CleanUp.ReferenceOTU217581
New.CleanUp.ReferenceOTU75468

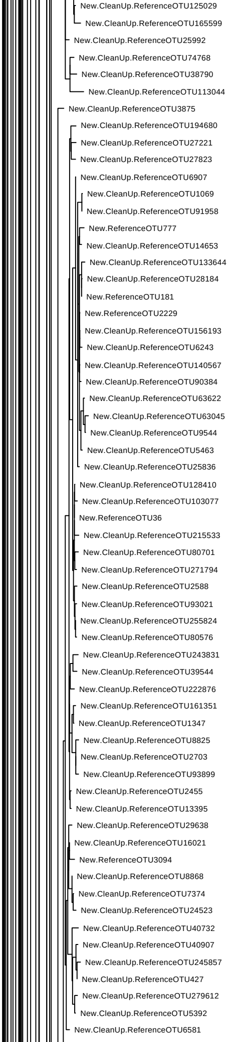
New.CleanUp.ReferenceOTU125029
New.CleanUp.ReferenceOTU165599
New.CleanUp.ReferenceOTU25992
New.CleanUp.ReferenceOTU74768
New.CleanUp.ReferenceOTU38790
New.CleanUp.ReferenceOTU113044
New.CleanUp.ReferenceOTU3875
New.CleanUp.ReferenceOTU194680
New.CleanUp.ReferenceOTU27221
New.CleanUp.ReferenceOTU27823
New.CleanUp.ReferenceOTU6907
New.CleanUp.ReferenceOTU1069
New.CleanUp.ReferenceOTU91958
New.ReferenceOTU777
New.CleanUp.ReferenceOTU14653
New.CleanUp.ReferenceOTU133644
New.CleanUp.ReferenceOTU28184
New.ReferenceOTU181
New.ReferenceOTU2229
New.CleanUp.ReferenceOTU156193
New.CleanUp.ReferenceOTU6243
New.CleanUp.ReferenceOTU140567
New.CleanUp.ReferenceOTU90384
New.CleanUp.ReferenceOTU63622
New.CleanUp.ReferenceOTU63045
New.CleanUp.ReferenceOTU9544
New.CleanUp.ReferenceOTU5463
New.CleanUp.ReferenceOTU25836
New.CleanUp.ReferenceOTU128410
New.CleanUp.ReferenceOTU103077
New.ReferenceOTU36
New.CleanUp.ReferenceOTU215533
New.CleanUp.ReferenceOTU80701
New.CleanUp.ReferenceOTU271794
New.CleanUp.ReferenceOTU2588
New.CleanUp.ReferenceOTU93021
New.CleanUp.ReferenceOTU255824
New.CleanUp.ReferenceOTU80576
New.CleanUp.ReferenceOTU243831
New.CleanUp.ReferenceOTU39544
New.CleanUp.ReferenceOTU222876
New.CleanUp.ReferenceOTU161351
New.CleanUp.ReferenceOTU1347
New.CleanUp.ReferenceOTU8825
New.CleanUp.ReferenceOTU2703
New.CleanUp.ReferenceOTU93899
New.CleanUp.ReferenceOTU2455
New.CleanUp.ReferenceOTU13395
New.CleanUp.ReferenceOTU29638
New.CleanUp.ReferenceOTU16021
New.ReferenceOTU3094
New.CleanUp.ReferenceOTU8868
New.CleanUp.ReferenceOTU7374
New.CleanUp.ReferenceOTU24523
New.CleanUp.ReferenceOTU40732
New.CleanUp.ReferenceOTU40907
New.CleanUp.ReferenceOTU245857
New.CleanUp.ReferenceOTU427
New.CleanUp.ReferenceOTU279612
New.CleanUp.ReferenceOTU5392
New.CleanUp.ReferenceOTU6581

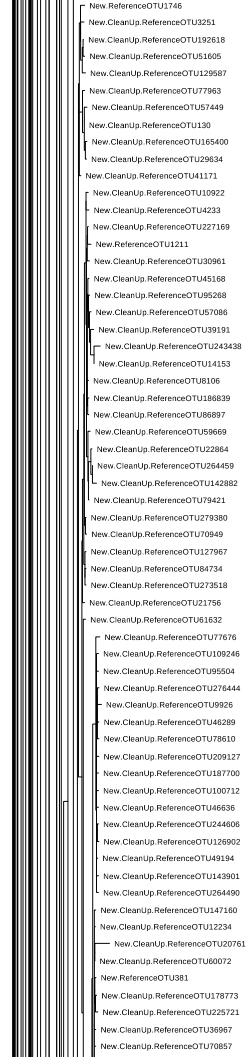
New.ReferenceOTU1746
New.CleanUp.ReferenceOTU3251
New.CleanUp.ReferenceOTU192618
New.CleanUp.ReferenceOTU51605
New.CleanUp.ReferenceOTU129587
New.CleanUp.ReferenceOTU77963
New.CleanUp.ReferenceOTU57449
New.CleanUp.ReferenceOTU130
New.CleanUp.ReferenceOTU165400
New.CleanUp.ReferenceOTU29634
New.CleanUp.ReferenceOTU41171
New.CleanUp.ReferenceOTU10922
New.CleanUp.ReferenceOTU4233
New.CleanUp.ReferenceOTU227169
New.ReferenceOTU1211
New.CleanUp.ReferenceOTU30961
New.CleanUp.ReferenceOTU45168
New.CleanUp.ReferenceOTU95268
New.CleanUp.ReferenceOTU57086
New.CleanUp.ReferenceOTU39191
New.CleanUp.ReferenceOTU243438
New.CleanUp.ReferenceOTU14153
New.CleanUp.ReferenceOTU8106
New.CleanUp.ReferenceOTU186839
New.CleanUp.ReferenceOTU86897
New.CleanUp.ReferenceOTU59669
New.CleanUp.ReferenceOTU22864
New.CleanUp.ReferenceOTU264459
New.CleanUp.ReferenceOTU142882
New.CleanUp.ReferenceOTU79421
New.CleanUp.ReferenceOTU279380
New.CleanUp.ReferenceOTU70949
New.CleanUp.ReferenceOTU127967
New.CleanUp.ReferenceOTU84734
New.CleanUp.ReferenceOTU273518
New.CleanUp.ReferenceOTU21756
New.CleanUp.ReferenceOTU61632
New.CleanUp.ReferenceOTU77676
New.CleanUp.ReferenceOTU109246
New.CleanUp.ReferenceOTU95504
New.CleanUp.ReferenceOTU276444
New.CleanUp.ReferenceOTU9926
New.CleanUp.ReferenceOTU46289
New.CleanUp.ReferenceOTU78610
New.CleanUp.ReferenceOTU209127
New.CleanUp.ReferenceOTU187700
New.CleanUp.ReferenceOTU100712
New.CleanUp.ReferenceOTU46636
New.CleanUp.ReferenceOTU244606
New.CleanUp.ReferenceOTU126902
New.CleanUp.ReferenceOTU49194
New.CleanUp.ReferenceOTU143901
New.CleanUp.ReferenceOTU264490
New.CleanUp.ReferenceOTU147160
New.CleanUp.ReferenceOTU12234
New.CleanUp.ReferenceOTU20761
New.CleanUp.ReferenceOTU60072
New.ReferenceOTU381
New.CleanUp.ReferenceOTU178773
New.CleanUp.ReferenceOTU225721
New.CleanUp.ReferenceOTU36967
New.CleanUp.ReferenceOTU70857

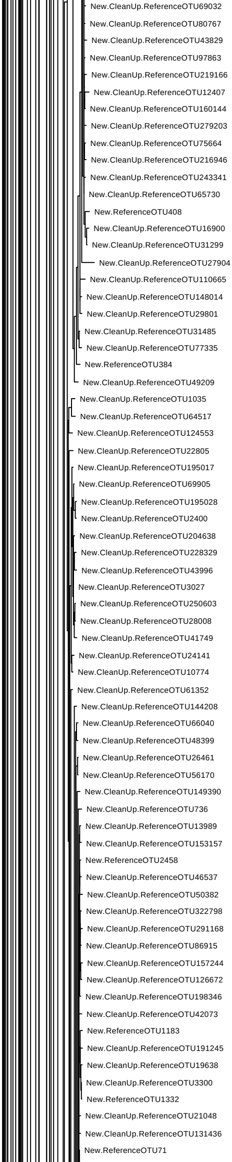
New.CleanUp.ReferenceOTU69032
New.CleanUp.ReferenceOTU80767
New.CleanUp.ReferenceOTU43829
New.CleanUp.ReferenceOTU97863
New.CleanUp.ReferenceOTU219166
New.CleanUp.ReferenceOTU12407
New.CleanUp.ReferenceOTU160144
New.CleanUp.ReferenceOTU279203
New.CleanUp.ReferenceOTU75664
New.CleanUp.ReferenceOTU216946
New.CleanUp.ReferenceOTU243341
New.CleanUp.ReferenceOTU65730
New.ReferenceOTU408
New.CleanUp.ReferenceOTU16900
New.CleanUp.ReferenceOTU31299
New.CleanUp.ReferenceOTU27904
New.CleanUp.ReferenceOTU110665
New.CleanUp.ReferenceOTU148014
New.CleanUp.ReferenceOTU29801
New.CleanUp.ReferenceOTU31485
New.CleanUp.ReferenceOTU77335
New.ReferenceOTU384
New.CleanUp.ReferenceOTU49209
New.CleanUp.ReferenceOTU1035
New.CleanUp.ReferenceOTU64517
New.CleanUp.ReferenceOTU124553
New.CleanUp.ReferenceOTU22805
New.CleanUp.ReferenceOTU195017
New.CleanUp.ReferenceOTU69905
New.CleanUp.ReferenceOTU195028
New.CleanUp.ReferenceOTU2400
New.CleanUp.ReferenceOTU204638
New.CleanUp.ReferenceOTU228329
New.CleanUp.ReferenceOTU43996
New.CleanUp.ReferenceOTU3027
New.CleanUp.ReferenceOTU250603
New.CleanUp.ReferenceOTU28008
New.CleanUp.ReferenceOTU41749
New.CleanUp.ReferenceOTU24141
New.CleanUp.ReferenceOTU10774
New.CleanUp.ReferenceOTU61352
New.CleanUp.ReferenceOTU144208
New.CleanUp.ReferenceOTU66040
New.CleanUp.ReferenceOTU48399
New.CleanUp.ReferenceOTU26461
New.CleanUp.ReferenceOTU56170
New.CleanUp.ReferenceOTU149390
New.CleanUp.ReferenceOTU736
New.CleanUp.ReferenceOTU13989
New.CleanUp.ReferenceOTU153157
New.ReferenceOTU2458
New.CleanUp.ReferenceOTU46537
New.CleanUp.ReferenceOTU50382
New.CleanUp.ReferenceOTU322798
New.CleanUp.ReferenceOTU291168
New.CleanUp.ReferenceOTU86915
New.CleanUp.ReferenceOTU157244
New.CleanUp.ReferenceOTU126672
New.CleanUp.ReferenceOTU198346
New.CleanUp.ReferenceOTU42073
New.ReferenceOTU1183
New.CleanUp.ReferenceOTU191245
New.CleanUp.ReferenceOTU19638
New.CleanUp.ReferenceOTU3300
New.ReferenceOTU1332
New.CleanUp.ReferenceOTU21048
New.CleanUp.ReferenceOTU131436
New.ReferenceOTU71

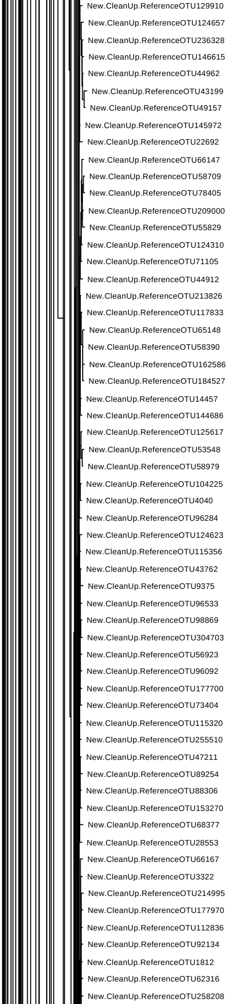
New.CleanUp.ReferenceOTU129910
New.CleanUp.ReferenceOTU124657
New.CleanUp.ReferenceOTU236328
New.CleanUp.ReferenceOTU146615
New.CleanUp.ReferenceOTU44962
New.CleanUp.ReferenceOTU43199
New.CleanUp.ReferenceOTU49157
New.CleanUp.ReferenceOTU145972
New.CleanUp.ReferenceOTU22692
New.CleanUp.ReferenceOTU66147
New.CleanUp.ReferenceOTU58709
New.CleanUp.ReferenceOTU78405
New.CleanUp.ReferenceOTU209000
New.CleanUp.ReferenceOTU55829
New.CleanUp.ReferenceOTU124310
New.CleanUp.ReferenceOTU71105
New.CleanUp.ReferenceOTU44912
New.CleanUp.ReferenceOTU213826
New.CleanUp.ReferenceOTU117833
New.CleanUp.ReferenceOTU65148
New.CleanUp.ReferenceOTU58390
New.CleanUp.ReferenceOTU162586
New.CleanUp.ReferenceOTU184527
New.CleanUp.ReferenceOTU14457
New.CleanUp.ReferenceOTU144686
New.CleanUp.ReferenceOTU125617
New.CleanUp.ReferenceOTU53548
New.CleanUp.ReferenceOTU58979
New.CleanUp.ReferenceOTU104225
New.CleanUp.ReferenceOTU4040
New.CleanUp.ReferenceOTU96284
New.CleanUp.ReferenceOTU124623
New.CleanUp.ReferenceOTU115356
New.CleanUp.ReferenceOTU43762
New.CleanUp.ReferenceOTU9375
New.CleanUp.ReferenceOTU96533
New.CleanUp.ReferenceOTU98869
New.CleanUp.ReferenceOTU304703
New.CleanUp.ReferenceOTU56923
New.CleanUp.ReferenceOTU96092
New.CleanUp.ReferenceOTU177700
New.CleanUp.ReferenceOTU73404
New.CleanUp.ReferenceOTU115320
New.CleanUp.ReferenceOTU255510
New.CleanUp.ReferenceOTU47211
New.CleanUp.ReferenceOTU89254
New.CleanUp.ReferenceOTU88306
New.CleanUp.ReferenceOTU153270
New.CleanUp.ReferenceOTU68377
New.CleanUp.ReferenceOTU28553
New.CleanUp.ReferenceOTU66167
New.CleanUp.ReferenceOTU3322
New.CleanUp.ReferenceOTU214995
New.CleanUp.ReferenceOTU177970
New.CleanUp.ReferenceOTU112836
New.CleanUp.ReferenceOTU92134
New.CleanUp.ReferenceOTU1812
New.CleanUp.ReferenceOTU62316
New.CleanUp.ReferenceOTU258208

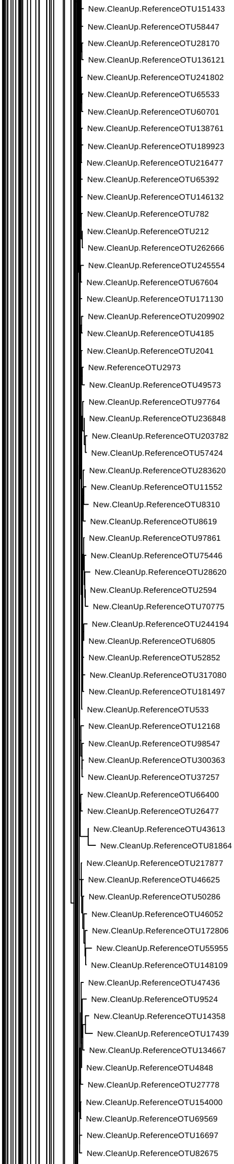
New.CleanUp.ReferenceOTU151433
New.CleanUp.ReferenceOTU58447
New.CleanUp.ReferenceOTU28170
New.CleanUp.ReferenceOTU136121
New.CleanUp.ReferenceOTU241802
New.CleanUp.ReferenceOTU65533
New.CleanUp.ReferenceOTU60701
New.CleanUp.ReferenceOTU138761
New.CleanUp.ReferenceOTU189923
New.CleanUp.ReferenceOTU216477
New.CleanUp.ReferenceOTU65392
New.CleanUp.ReferenceOTU146132
New.CleanUp.ReferenceOTU782
New.CleanUp.ReferenceOTU212
New.CleanUp.ReferenceOTU262666
New.CleanUp.ReferenceOTU245554
New.CleanUp.ReferenceOTU67604
New.CleanUp.ReferenceOTU171130
New.CleanUp.ReferenceOTU209902
New.CleanUp.ReferenceOTU4185
New.CleanUp.ReferenceOTU2041
New.ReferenceOTU2973
New.CleanUp.ReferenceOTU49573
New.CleanUp.ReferenceOTU97764
New.CleanUp.ReferenceOTU236848
New.CleanUp.ReferenceOTU203782
New.CleanUp.ReferenceOTU57424
New.CleanUp.ReferenceOTU283620
New.CleanUp.ReferenceOTU11552
New.CleanUp.ReferenceOTU8310
New.CleanUp.ReferenceOTU8619
New.CleanUp.ReferenceOTU97861
New.CleanUp.ReferenceOTU75446
New.CleanUp.ReferenceOTU28620
New.CleanUp.ReferenceOTU2594
New.CleanUp.ReferenceOTU70775
New.CleanUp.ReferenceOTU244194
New.CleanUp.ReferenceOTU6805
New.CleanUp.ReferenceOTU52852
New.CleanUp.ReferenceOTU317080
New.CleanUp.ReferenceOTU181497
New.CleanUp.ReferenceOTU533
New.CleanUp.ReferenceOTU12168
New.CleanUp.ReferenceOTU98547
New.CleanUp.ReferenceOTU300363
New.CleanUp.ReferenceOTU37257
New.CleanUp.ReferenceOTU66400
New.CleanUp.ReferenceOTU26477
New.CleanUp.ReferenceOTU43613
New.CleanUp.ReferenceOTU81864
New.CleanUp.ReferenceOTU217877
New.CleanUp.ReferenceOTU46625
New.CleanUp.ReferenceOTU50286
New.CleanUp.ReferenceOTU46052
New.CleanUp.ReferenceOTU172806
New.CleanUp.ReferenceOTU55955
New.CleanUp.ReferenceOTU148109
New.CleanUp.ReferenceOTU47436
New.CleanUp.ReferenceOTU9524
New.CleanUp.ReferenceOTU14358
New.CleanUp.ReferenceOTU17439
New.CleanUp.ReferenceOTU134667
New.CleanUp.ReferenceOTU4848
New.CleanUp.ReferenceOTU27778
New.CleanUp.ReferenceOTU154000
New.CleanUp.ReferenceOTU69569
New.CleanUp.ReferenceOTU16697
New.CleanUp.ReferenceOTU82675

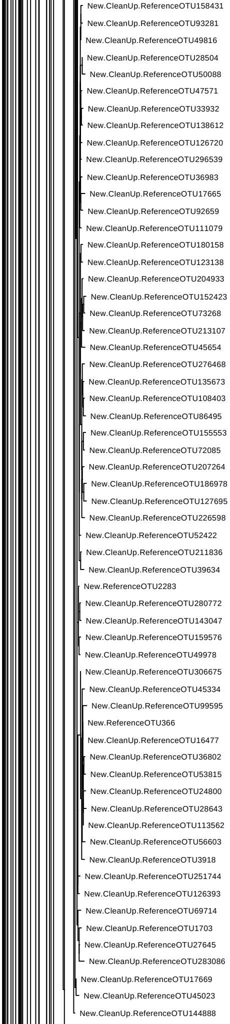
New.CleanUp.ReferenceOTU158431
New.CleanUp.ReferenceOTU93281
New.CleanUp.ReferenceOTU49816
New.CleanUp.ReferenceOTU28504
New.CleanUp.ReferenceOTU50088
New.CleanUp.ReferenceOTU47571
New.CleanUp.ReferenceOTU33932
New.CleanUp.ReferenceOTU138612
New.CleanUp.ReferenceOTU126720
New.CleanUp.ReferenceOTU296539
New.CleanUp.ReferenceOTU36983
New.CleanUp.ReferenceOTU17665
New.CleanUp.ReferenceOTU92659
New.CleanUp.ReferenceOTU111079
New.CleanUp.ReferenceOTU180158
New.CleanUp.ReferenceOTU123138
New.CleanUp.ReferenceOTU204933
New.CleanUp.ReferenceOTU152423
New.CleanUp.ReferenceOTU73268
New.CleanUp.ReferenceOTU213107
New.CleanUp.ReferenceOTU45654
New.CleanUp.ReferenceOTU276468
New.CleanUp.ReferenceOTU135673
New.CleanUp.ReferenceOTU108403
New.CleanUp.ReferenceOTU86495
New.CleanUp.ReferenceOTU155553
New.CleanUp.ReferenceOTU72085
New.CleanUp.ReferenceOTU207264
New.CleanUp.ReferenceOTU186978
New.CleanUp.ReferenceOTU127695
New.CleanUp.ReferenceOTU226598
New.CleanUp.ReferenceOTU52422
New.CleanUp.ReferenceOTU211836
New.CleanUp.ReferenceOTU39634
New.ReferenceOTU2283
New.CleanUp.ReferenceOTU280772
New.CleanUp.ReferenceOTU143047
New.CleanUp.ReferenceOTU159576
New.CleanUp.ReferenceOTU49978
New.CleanUp.ReferenceOTU306675
New.CleanUp.ReferenceOTU45334
New.CleanUp.ReferenceOTU99595
New.ReferenceOTU366
New.CleanUp.ReferenceOTU16477
New.CleanUp.ReferenceOTU36802
New.CleanUp.ReferenceOTU53815
New.CleanUp.ReferenceOTU24800
New.CleanUp.ReferenceOTU28643
New.CleanUp.ReferenceOTU113562
New.CleanUp.ReferenceOTU56603
New.CleanUp.ReferenceOTU3918
New.CleanUp.ReferenceOTU251744
New.CleanUp.ReferenceOTU126393
New.CleanUp.ReferenceOTU69714
New.CleanUp.ReferenceOTU1703
New.CleanUp.ReferenceOTU27645
New.CleanUp.ReferenceOTU283086
New.CleanUp.ReferenceOTU17669
New.CleanUp.ReferenceOTU45023
New.CleanUp.ReferenceOTU144888

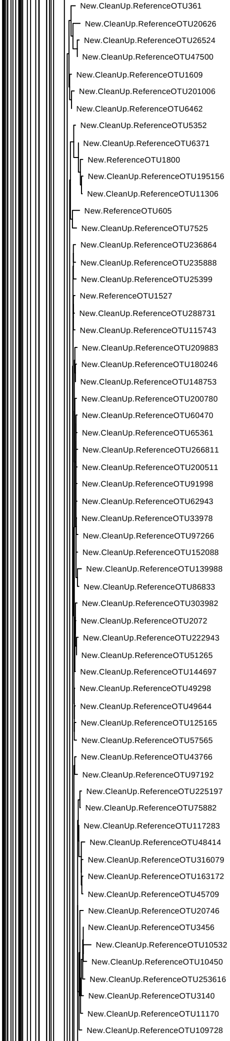
New.CleanUp.ReferenceOTU361
New.CleanUp.ReferenceOTU20626
New.CleanUp.ReferenceOTU26524
New.CleanUp.ReferenceOTU47500
New.CleanUp.ReferenceOTU1609
New.CleanUp.ReferenceOTU201006
New.CleanUp.ReferenceOTU6462
New.CleanUp.ReferenceOTU5352
New.CleanUp.ReferenceOTU6371
New.ReferenceOTU1800
New.CleanUp.ReferenceOTU195156
New.CleanUp.ReferenceOTU11306
New.ReferenceOTU605
New.CleanUp.ReferenceOTU7525
New.CleanUp.ReferenceOTU236864
New.CleanUp.ReferenceOTU235888
New.CleanUp.ReferenceOTU25399
New.ReferenceOTU1527
New.CleanUp.ReferenceOTU288731
New.CleanUp.ReferenceOTU115743
New.CleanUp.ReferenceOTU209883
New.CleanUp.ReferenceOTU180246
New.CleanUp.ReferenceOTU148753
New.CleanUp.ReferenceOTU200780
New.CleanUp.ReferenceOTU60470
New.CleanUp.ReferenceOTU65361
New.CleanUp.ReferenceOTU266811
New.CleanUp.ReferenceOTU200511
New.CleanUp.ReferenceOTU91998
New.CleanUp.ReferenceOTU62943
New.CleanUp.ReferenceOTU33978
New.CleanUp.ReferenceOTU97266
New.CleanUp.ReferenceOTU152088
New.CleanUp.ReferenceOTU139988
New.CleanUp.ReferenceOTU86833
New.CleanUp.ReferenceOTU303982
New.CleanUp.ReferenceOTU2072
New.CleanUp.ReferenceOTU222943
New.CleanUp.ReferenceOTU51265
New.CleanUp.ReferenceOTU144697
New.CleanUp.ReferenceOTU49298
New.CleanUp.ReferenceOTU49644
New.CleanUp.ReferenceOTU125165
New.CleanUp.ReferenceOTU57565
New.CleanUp.ReferenceOTU43766
New.CleanUp.ReferenceOTU97192
New.CleanUp.ReferenceOTU225197
New.CleanUp.ReferenceOTU75882
New.CleanUp.ReferenceOTU117283
New.CleanUp.ReferenceOTU48414
New.CleanUp.ReferenceOTU316079
New.CleanUp.ReferenceOTU163172
New.CleanUp.ReferenceOTU45709
New.CleanUp.ReferenceOTU20746
New.CleanUp.ReferenceOTU3456
New.CleanUp.ReferenceOTU10532
New.CleanUp.ReferenceOTU10450
New.CleanUp.ReferenceOTU253616
New.CleanUp.ReferenceOTU3140
New.CleanUp.ReferenceOTU11170
New.CleanUp.ReferenceOTU109728

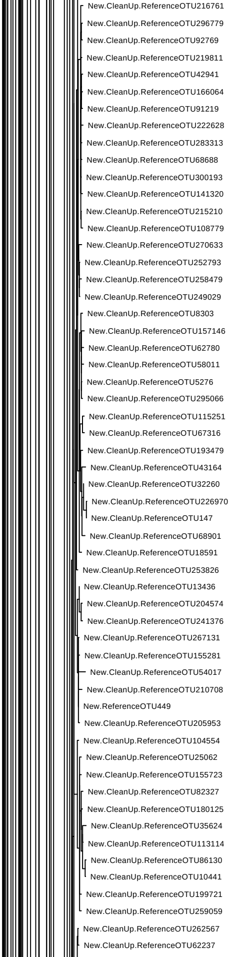
New.CleanUp.ReferenceOTU216761
New.CleanUp.ReferenceOTU296779
New.CleanUp.ReferenceOTU92769
New.CleanUp.ReferenceOTU219811
New.CleanUp.ReferenceOTU42941
New.CleanUp.ReferenceOTU166064
New.CleanUp.ReferenceOTU91219
New.CleanUp.ReferenceOTU222628
New.CleanUp.ReferenceOTU283313
New.CleanUp.ReferenceOTU68688
New.CleanUp.ReferenceOTU300193
New.CleanUp.ReferenceOTU141320
New.CleanUp.ReferenceOTU215210
New.CleanUp.ReferenceOTU108779
New.CleanUp.ReferenceOTU270633
New.CleanUp.ReferenceOTU252793
New.CleanUp.ReferenceOTU258479
New.CleanUp.ReferenceOTU249029
New.CleanUp.ReferenceOTU8303
New.CleanUp.ReferenceOTU157146
New.CleanUp.ReferenceOTU62780
New.CleanUp.ReferenceOTU58011
New.CleanUp.ReferenceOTU5276
New.CleanUp.ReferenceOTU295066
New.CleanUp.ReferenceOTU115251
New.CleanUp.ReferenceOTU67316
New.CleanUp.ReferenceOTU193479
New.CleanUp.ReferenceOTU43164
New.CleanUp.ReferenceOTU32260
New.CleanUp.ReferenceOTU226970
New.CleanUp.ReferenceOTU147
New.CleanUp.ReferenceOTU68901
New.CleanUp.ReferenceOTU18591
New.CleanUp.ReferenceOTU253826
New.CleanUp.ReferenceOTU13436
New.CleanUp.ReferenceOTU204574
New.CleanUp.ReferenceOTU241376
New.CleanUp.ReferenceOTU267131
New.CleanUp.ReferenceOTU155281
New.CleanUp.ReferenceOTU54017
New.CleanUp.ReferenceOTU210708
New.ReferenceOTU449
New.CleanUp.ReferenceOTU205953
New.CleanUp.ReferenceOTU104554
New.CleanUp.ReferenceOTU25062
New.CleanUp.ReferenceOTU155723
New.CleanUp.ReferenceOTU82327
New.CleanUp.ReferenceOTU180125
New.CleanUp.ReferenceOTU35624
New.CleanUp.ReferenceOTU113114
New.CleanUp.ReferenceOTU86130
New.CleanUp.ReferenceOTU10441
New.CleanUp.ReferenceOTU199721
New.CleanUp.ReferenceOTU259059
New.CleanUp.ReferenceOTU262567
New.CleanUp.ReferenceOTU62237

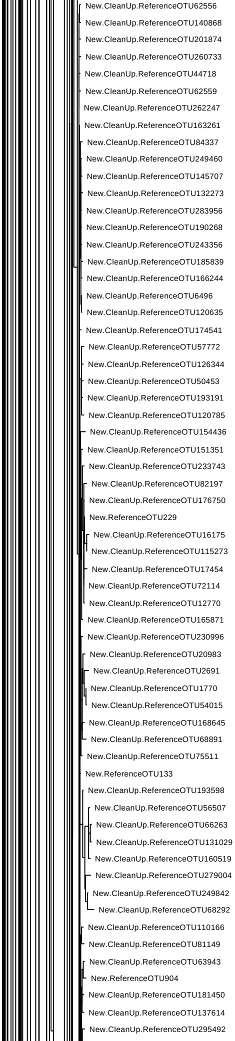
New.CleanUp.ReferenceOTU62556
New.CleanUp.ReferenceOTU140868
New.CleanUp.ReferenceOTU201874
New.CleanUp.ReferenceOTU260733
New.CleanUp.ReferenceOTU44718
New.CleanUp.ReferenceOTU62559
New.CleanUp.ReferenceOTU262247
New.CleanUp.ReferenceOTU163261
New.CleanUp.ReferenceOTU84337
New.CleanUp.ReferenceOTU249460
New.CleanUp.ReferenceOTU145707
New.CleanUp.ReferenceOTU132273
New.CleanUp.ReferenceOTU283956
New.CleanUp.ReferenceOTU190268
New.CleanUp.ReferenceOTU243356
New.CleanUp.ReferenceOTU185839
New.CleanUp.ReferenceOTU166244
New.CleanUp.ReferenceOTU6496
New.CleanUp.ReferenceOTU120635
New.CleanUp.ReferenceOTU174541
New.CleanUp.ReferenceOTU57772
New.CleanUp.ReferenceOTU126344
New.CleanUp.ReferenceOTU50453
New.CleanUp.ReferenceOTU193191
New.CleanUp.ReferenceOTU120785
New.CleanUp.ReferenceOTU154436
New.CleanUp.ReferenceOTU151351
New.CleanUp.ReferenceOTU233743
New.CleanUp.ReferenceOTU82197
New.CleanUp.ReferenceOTU176750
New.ReferenceOTU229
New.CleanUp.ReferenceOTU16175
New.CleanUp.ReferenceOTU115273
New.CleanUp.ReferenceOTU17454
New.CleanUp.ReferenceOTU72114
New.CleanUp.ReferenceOTU12770
New.CleanUp.ReferenceOTU165871
New.CleanUp.ReferenceOTU230996
New.CleanUp.ReferenceOTU20983
New.CleanUp.ReferenceOTU2691
New.CleanUp.ReferenceOTU1770
New.CleanUp.ReferenceOTU54015
New.CleanUp.ReferenceOTU168645
New.CleanUp.ReferenceOTU68891
New.CleanUp.ReferenceOTU75511
New.ReferenceOTU133
New.CleanUp.ReferenceOTU193598
New.CleanUp.ReferenceOTU56507
New.CleanUp.ReferenceOTU66263
New.CleanUp.ReferenceOTU131029
New.CleanUp.ReferenceOTU160519
New.CleanUp.ReferenceOTU279004
New.CleanUp.ReferenceOTU249842
New.CleanUp.ReferenceOTU68292
New.CleanUp.ReferenceOTU110166
New.CleanUp.ReferenceOTU81149
New.CleanUp.ReferenceOTU63943
New.ReferenceOTU904
New.CleanUp.ReferenceOTU181450
New.CleanUp.ReferenceOTU137614
New.CleanUp.ReferenceOTU295492

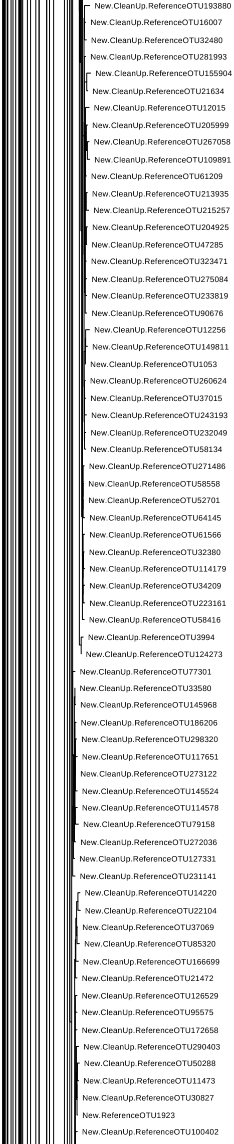
New.CleanUp.ReferenceOTU193880
New.CleanUp.ReferenceOTU16007
New.CleanUp.ReferenceOTU32480
New.CleanUp.ReferenceOTU281993
New.CleanUp.ReferenceOTU155904
New.CleanUp.ReferenceOTU21634
New.CleanUp.ReferenceOTU12015
New.CleanUp.ReferenceOTU205999
New.CleanUp.ReferenceOTU267058
New.CleanUp.ReferenceOTU109891
New.CleanUp.ReferenceOTU61209
New.CleanUp.ReferenceOTU213935
New.CleanUp.ReferenceOTU215257
New.CleanUp.ReferenceOTU204925
New.CleanUp.ReferenceOTU47285
New.CleanUp.ReferenceOTU323471
New.CleanUp.ReferenceOTU275084
New.CleanUp.ReferenceOTU233819
New.CleanUp.ReferenceOTU90676
New.CleanUp.ReferenceOTU12256
New.CleanUp.ReferenceOTU149811
New.CleanUp.ReferenceOTU1053
New.CleanUp.ReferenceOTU260624
New.CleanUp.ReferenceOTU37015
New.CleanUp.ReferenceOTU243193
New.CleanUp.ReferenceOTU232049
New.CleanUp.ReferenceOTU58134
New.CleanUp.ReferenceOTU271486
New.CleanUp.ReferenceOTU58558
New.CleanUp.ReferenceOTU52701
New.CleanUp.ReferenceOTU64145
New.CleanUp.ReferenceOTU61566
New.CleanUp.ReferenceOTU32380
New.CleanUp.ReferenceOTU114179
New.CleanUp.ReferenceOTU34209
New.CleanUp.ReferenceOTU223161
New.CleanUp.ReferenceOTU58416
New.CleanUp.ReferenceOTU3994
New.CleanUp.ReferenceOTU124273
New.CleanUp.ReferenceOTU77301
New.CleanUp.ReferenceOTU33580
New.CleanUp.ReferenceOTU145968
New.CleanUp.ReferenceOTU186206
New.CleanUp.ReferenceOTU298320
New.CleanUp.ReferenceOTU117651
New.CleanUp.ReferenceOTU273122
New.CleanUp.ReferenceOTU145524
New.CleanUp.ReferenceOTU114578
New.CleanUp.ReferenceOTU79158
New.CleanUp.ReferenceOTU272036
New.CleanUp.ReferenceOTU127331
New.CleanUp.ReferenceOTU231141
New.CleanUp.ReferenceOTU14220
New.CleanUp.ReferenceOTU22104
New.CleanUp.ReferenceOTU37069
New.CleanUp.ReferenceOTU85320
New.CleanUp.ReferenceOTU166699
New.CleanUp.ReferenceOTU21472
New.CleanUp.ReferenceOTU126529
New.CleanUp.ReferenceOTU95575
New.CleanUp.ReferenceOTU172658
New.CleanUp.ReferenceOTU290403
New.CleanUp.ReferenceOTU50288
New.CleanUp.ReferenceOTU11473
New.CleanUp.ReferenceOTU30827
New.ReferenceOTU1923
New.CleanUp.ReferenceOTU100402

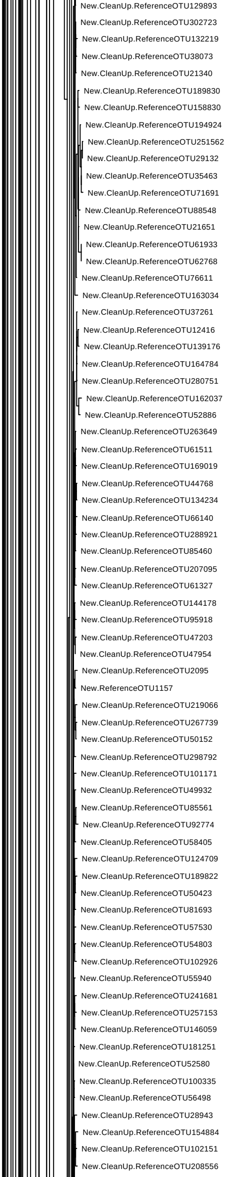
New.CleanUp.ReferenceOTU129893
New.CleanUp.ReferenceOTU302723
New.CleanUp.ReferenceOTU132219
New.CleanUp.ReferenceOTU38073
New.CleanUp.ReferenceOTU21340
New.CleanUp.ReferenceOTU189830
New.CleanUp.ReferenceOTU158830
New.CleanUp.ReferenceOTU194924
New.CleanUp.ReferenceOTU251562
New.CleanUp.ReferenceOTU29132
New.CleanUp.ReferenceOTU35463
New.CleanUp.ReferenceOTU71691
New.CleanUp.ReferenceOTU88548
New.CleanUp.ReferenceOTU21651
New.CleanUp.ReferenceOTU61933
New.CleanUp.ReferenceOTU62768
New.CleanUp.ReferenceOTU76611
New.CleanUp.ReferenceOTU163034
New.CleanUp.ReferenceOTU37261
New.CleanUp.ReferenceOTU12416
New.CleanUp.ReferenceOTU139176
New.CleanUp.ReferenceOTU164784
New.CleanUp.ReferenceOTU280751
New.CleanUp.ReferenceOTU162037
New.CleanUp.ReferenceOTU52886
New.CleanUp.ReferenceOTU263649
New.CleanUp.ReferenceOTU61511
New.CleanUp.ReferenceOTU169019
New.CleanUp.ReferenceOTU44768
New.CleanUp.ReferenceOTU134234
New.CleanUp.ReferenceOTU66140
New.CleanUp.ReferenceOTU288921
New.CleanUp.ReferenceOTU85460
New.CleanUp.ReferenceOTU207095
New.CleanUp.ReferenceOTU61327
New.CleanUp.ReferenceOTU144178
New.CleanUp.ReferenceOTU95918
New.CleanUp.ReferenceOTU47203
New.CleanUp.ReferenceOTU47954
New.CleanUp.ReferenceOTU2095
New.ReferenceOTU1157
New.CleanUp.ReferenceOTU219066
New.CleanUp.ReferenceOTU267739
New.CleanUp.ReferenceOTU50152
New.CleanUp.ReferenceOTU298792
New.CleanUp.ReferenceOTU101171
New.CleanUp.ReferenceOTU49932
New.CleanUp.ReferenceOTU85561
New.CleanUp.ReferenceOTU92774
New.CleanUp.ReferenceOTU58405
New.CleanUp.ReferenceOTU124709
New.CleanUp.ReferenceOTU189822
New.CleanUp.ReferenceOTU50423
New.CleanUp.ReferenceOTU81693
New.CleanUp.ReferenceOTU57530
New.CleanUp.ReferenceOTU54803
New.CleanUp.ReferenceOTU102926
New.CleanUp.ReferenceOTU55940
New.CleanUp.ReferenceOTU241681
New.CleanUp.ReferenceOTU257153
New.CleanUp.ReferenceOTU146059
New.CleanUp.ReferenceOTU181251
New.CleanUp.ReferenceOTU52580
New.CleanUp.ReferenceOTU100335
New.CleanUp.ReferenceOTU56498
New.CleanUp.ReferenceOTU28943
New.CleanUp.ReferenceOTU154884
New.CleanUp.ReferenceOTU102151
New.CleanUp.ReferenceOTU208556

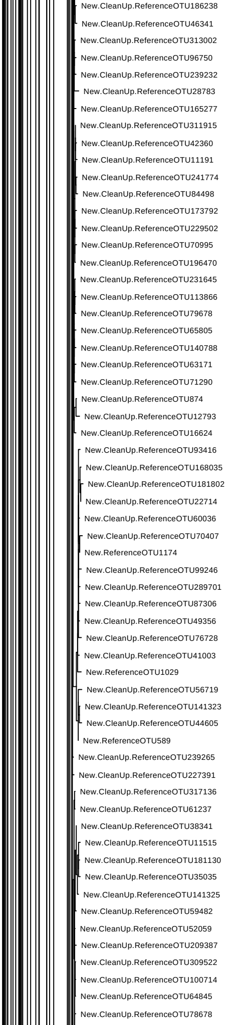
New.CleanUp.ReferenceOTU186238
New.CleanUp.ReferenceOTU46341
New.CleanUp.ReferenceOTU313002
New.CleanUp.ReferenceOTU96750
New.CleanUp.ReferenceOTU239232
New.CleanUp.ReferenceOTU28783
New.CleanUp.ReferenceOTU165277
New.CleanUp.ReferenceOTU311915
New.CleanUp.ReferenceOTU42360
New.CleanUp.ReferenceOTU11191
New.CleanUp.ReferenceOTU241774
New.CleanUp.ReferenceOTU84498
New.CleanUp.ReferenceOTU173792
New.CleanUp.ReferenceOTU229502
New.CleanUp.ReferenceOTU70995
New.CleanUp.ReferenceOTU196470
New.CleanUp.ReferenceOTU231645
New.CleanUp.ReferenceOTU113866
New.CleanUp.ReferenceOTU79678
New.CleanUp.ReferenceOTU65805
New.CleanUp.ReferenceOTU140788
New.CleanUp.ReferenceOTU63171
New.CleanUp.ReferenceOTU71290
New.CleanUp.ReferenceOTU874
New.CleanUp.ReferenceOTU12793
New.CleanUp.ReferenceOTU16624
New.CleanUp.ReferenceOTU93416
New.CleanUp.ReferenceOTU168035
New.CleanUp.ReferenceOTU181802
New.CleanUp.ReferenceOTU22714
New.CleanUp.ReferenceOTU60036
New.CleanUp.ReferenceOTU70407
New.ReferenceOTU1174
New.CleanUp.ReferenceOTU99246
New.CleanUp.ReferenceOTU289701
New.CleanUp.ReferenceOTU87306
New.CleanUp.ReferenceOTU49356
New.CleanUp.ReferenceOTU76728
New.CleanUp.ReferenceOTU41003
New.ReferenceOTU1029
New.CleanUp.ReferenceOTU56719
New.CleanUp.ReferenceOTU141323
New.CleanUp.ReferenceOTU44605
New.ReferenceOTU589
New.CleanUp.ReferenceOTU239265
New.CleanUp.ReferenceOTU227391
New.CleanUp.ReferenceOTU317136
New.CleanUp.ReferenceOTU61237
New.CleanUp.ReferenceOTU38341
New.CleanUp.ReferenceOTU11515
New.CleanUp.ReferenceOTU181130
New.CleanUp.ReferenceOTU35035
New.CleanUp.ReferenceOTU141325
New.CleanUp.ReferenceOTU59482
New.CleanUp.ReferenceOTU52059
New.CleanUp.ReferenceOTU209387
New.CleanUp.ReferenceOTU309522
New.CleanUp.ReferenceOTU100714
New.CleanUp.ReferenceOTU64845
New.CleanUp.ReferenceOTU78678

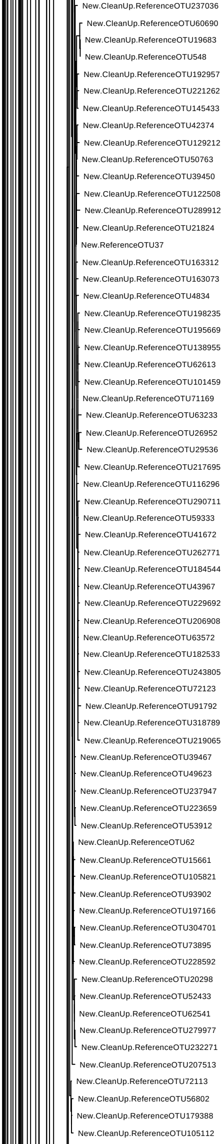
New.CleanUp.ReferenceOTU237036
New.CleanUp.ReferenceOTU60690
New.CleanUp.ReferenceOTU19683
New.CleanUp.ReferenceOTU548
New.CleanUp.ReferenceOTU192957
New.CleanUp.ReferenceOTU221262
New.CleanUp.ReferenceOTU145433
New.CleanUp.ReferenceOTU42374
New.CleanUp.ReferenceOTU129212
New.CleanUp.ReferenceOTU50763
New.CleanUp.ReferenceOTU39450
New.CleanUp.ReferenceOTU122508
New.CleanUp.ReferenceOTU289912
New.CleanUp.ReferenceOTU21824
New.ReferenceOTU37
New.CleanUp.ReferenceOTU163312
New.CleanUp.ReferenceOTU163073
New.CleanUp.ReferenceOTU4834
New.CleanUp.ReferenceOTU198235
New.CleanUp.ReferenceOTU195669
New.CleanUp.ReferenceOTU138955
New.CleanUp.ReferenceOTU62613
New.CleanUp.ReferenceOTU101459
New.CleanUp.ReferenceOTU71169
New.CleanUp.ReferenceOTU63233
New.CleanUp.ReferenceOTU26952
New.CleanUp.ReferenceOTU29536
New.CleanUp.ReferenceOTU217695
New.CleanUp.ReferenceOTU116296
New.CleanUp.ReferenceOTU290711
New.CleanUp.ReferenceOTU59333
New.CleanUp.ReferenceOTU41672
New.CleanUp.ReferenceOTU262771
New.CleanUp.ReferenceOTU184544
New.CleanUp.ReferenceOTU43967
New.CleanUp.ReferenceOTU229692
New.CleanUp.ReferenceOTU206908
New.CleanUp.ReferenceOTU63572
New.CleanUp.ReferenceOTU182533
New.CleanUp.ReferenceOTU243805
New.CleanUp.ReferenceOTU72123
New.CleanUp.ReferenceOTU91792
New.CleanUp.ReferenceOTU318789
New.CleanUp.ReferenceOTU219065
New.CleanUp.ReferenceOTU39467
New.CleanUp.ReferenceOTU49623
New.CleanUp.ReferenceOTU237947
New.CleanUp.ReferenceOTU223659
New.CleanUp.ReferenceOTU53912
New.CleanUp.ReferenceOTU62
New.CleanUp.ReferenceOTU15661
New.CleanUp.ReferenceOTU105821
New.CleanUp.ReferenceOTU93902
New.CleanUp.ReferenceOTU197166
New.CleanUp.ReferenceOTU304701
New.CleanUp.ReferenceOTU73895
New.CleanUp.ReferenceOTU228592
New.CleanUp.ReferenceOTU20298
New.CleanUp.ReferenceOTU52433
New.CleanUp.ReferenceOTU62541
New.CleanUp.ReferenceOTU279977
New.CleanUp.ReferenceOTU232271
New.CleanUp.ReferenceOTU207513
New.CleanUp.ReferenceOTU72113
New.CleanUp.ReferenceOTU56802
New.CleanUp.ReferenceOTU179388
New.CleanUp.ReferenceOTU105112

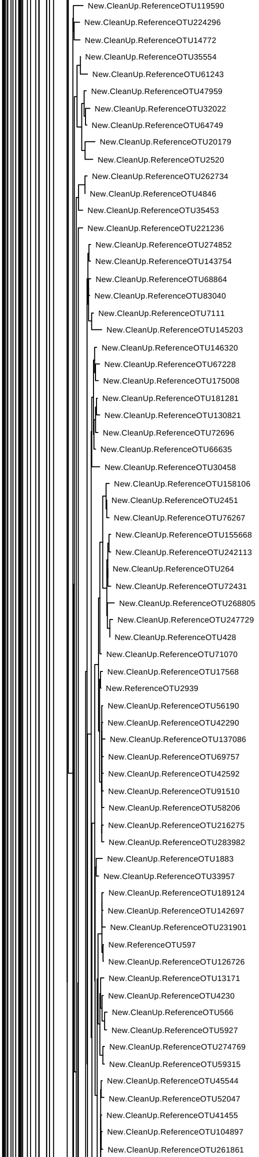
New.CleanUp.ReferenceOTU119590
New.CleanUp.ReferenceOTU224296
New.CleanUp.ReferenceOTU14772
New.CleanUp.ReferenceOTU35554
New.CleanUp.ReferenceOTU61243
New.CleanUp.ReferenceOTU47959
New.CleanUp.ReferenceOTU32022
New.CleanUp.ReferenceOTU64749
New.CleanUp.ReferenceOTU20179
New.CleanUp.ReferenceOTU2520
New.CleanUp.ReferenceOTU262734
New.CleanUp.ReferenceOTU4846
New.CleanUp.ReferenceOTU35453
New.CleanUp.ReferenceOTU221236
New.CleanUp.ReferenceOTU274852
New.CleanUp.ReferenceOTU143754
New.CleanUp.ReferenceOTU68864
New.CleanUp.ReferenceOTU83040
New.CleanUp.ReferenceOTU7111
New.CleanUp.ReferenceOTU145203
New.CleanUp.ReferenceOTU146320
New.CleanUp.ReferenceOTU67228
New.CleanUp.ReferenceOTU175008
New.CleanUp.ReferenceOTU181281
New.CleanUp.ReferenceOTU130821
New.CleanUp.ReferenceOTU72696
New.CleanUp.ReferenceOTU66635
New.CleanUp.ReferenceOTU30458
New.CleanUp.ReferenceOTU158106
New.CleanUp.ReferenceOTU2451
New.CleanUp.ReferenceOTU76267
New.CleanUp.ReferenceOTU155668
New.CleanUp.ReferenceOTU242113
New.CleanUp.ReferenceOTU264
New.CleanUp.ReferenceOTU72431
New.CleanUp.ReferenceOTU268805
New.CleanUp.ReferenceOTU247729
New.CleanUp.ReferenceOTU428
New.CleanUp.ReferenceOTU71070
New.CleanUp.ReferenceOTU17568
New.ReferenceOTU2939
New.CleanUp.ReferenceOTU56190
New.CleanUp.ReferenceOTU42290
New.CleanUp.ReferenceOTU137086
New.CleanUp.ReferenceOTU69757
New.CleanUp.ReferenceOTU42592
New.CleanUp.ReferenceOTU91510
New.CleanUp.ReferenceOTU58206
New.CleanUp.ReferenceOTU216275
New.CleanUp.ReferenceOTU283982
New.CleanUp.ReferenceOTU1883
New.CleanUp.ReferenceOTU33957
New.CleanUp.ReferenceOTU189124
New.CleanUp.ReferenceOTU142697
New.CleanUp.ReferenceOTU231901
New.ReferenceOTU597
New.CleanUp.ReferenceOTU126726
New.CleanUp.ReferenceOTU13171
New.CleanUp.ReferenceOTU4230
New.CleanUp.ReferenceOTU566
New.CleanUp.ReferenceOTU5927
New.CleanUp.ReferenceOTU274769
New.CleanUp.ReferenceOTU59315
New.CleanUp.ReferenceOTU45544
New.CleanUp.ReferenceOTU52047
New.CleanUp.ReferenceOTU41455
New.CleanUp.ReferenceOTU104897
New.CleanUp.ReferenceOTU261861

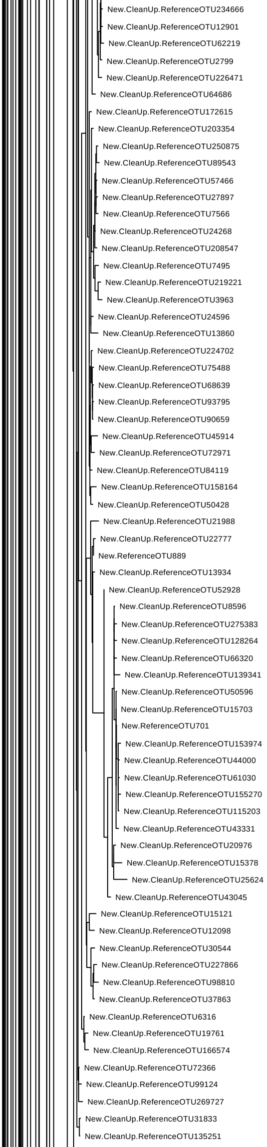

New.CleanUp.ReferenceOTU234666
New.CleanUp.ReferenceOTU12901
New.CleanUp.ReferenceOTU62219
New.CleanUp.ReferenceOTU2799
New.CleanUp.ReferenceOTU226471
New.CleanUp.ReferenceOTU64686
New.CleanUp.ReferenceOTU172615
New.CleanUp.ReferenceOTU203354
New.CleanUp.ReferenceOTU250875
New.CleanUp.ReferenceOTU89543
New.CleanUp.ReferenceOTU57466
New.CleanUp.ReferenceOTU27897
New.CleanUp.ReferenceOTU7566
New.CleanUp.ReferenceOTU24268
New.CleanUp.ReferenceOTU208547
New.CleanUp.ReferenceOTU7495
New.CleanUp.ReferenceOTU219221
New.CleanUp.ReferenceOTU3963
New.CleanUp.ReferenceOTU24596
New.CleanUp.ReferenceOTU13860
New.CleanUp.ReferenceOTU224702
New.CleanUp.ReferenceOTU75488
New.CleanUp.ReferenceOTU68639
New.CleanUp.ReferenceOTU93795
New.CleanUp.ReferenceOTU90659
New.CleanUp.ReferenceOTU45914
New.CleanUp.ReferenceOTU72971
New.CleanUp.ReferenceOTU84119
New.CleanUp.ReferenceOTU158164
New.CleanUp.ReferenceOTU50428
New.CleanUp.ReferenceOTU21988
New.CleanUp.ReferenceOTU22777
New.ReferenceOTU889
New.CleanUp.ReferenceOTU13934
New.CleanUp.ReferenceOTU52928
New.CleanUp.ReferenceOTU8596
New.CleanUp.ReferenceOTU275383
New.CleanUp.ReferenceOTU128264
New.CleanUp.ReferenceOTU66320
New.CleanUp.ReferenceOTU139341
New.CleanUp.ReferenceOTU50596
New.CleanUp.ReferenceOTU15703
New.ReferenceOTU701
New.CleanUp.ReferenceOTU153974
New.CleanUp.ReferenceOTU44000
New.CleanUp.ReferenceOTU61030
New.CleanUp.ReferenceOTU155270
New.CleanUp.ReferenceOTU115203
New.CleanUp.ReferenceOTU43331
New.CleanUp.ReferenceOTU20976
New.CleanUp.ReferenceOTU15378
New.CleanUp.ReferenceOTU25624
New.CleanUp.ReferenceOTU43045
New.CleanUp.ReferenceOTU15121
New.CleanUp.ReferenceOTU12098
New.CleanUp.ReferenceOTU30544
New.CleanUp.ReferenceOTU227866
New.CleanUp.ReferenceOTU98810
New.CleanUp.ReferenceOTU37863
New.CleanUp.ReferenceOTU6316
New.CleanUp.ReferenceOTU19761
New.CleanUp.ReferenceOTU166574
New.CleanUp.ReferenceOTU72366
New.CleanUp.ReferenceOTU99124
New.CleanUp.ReferenceOTU269727
New.CleanUp.ReferenceOTU31833
New.CleanUp.ReferenceOTU135251

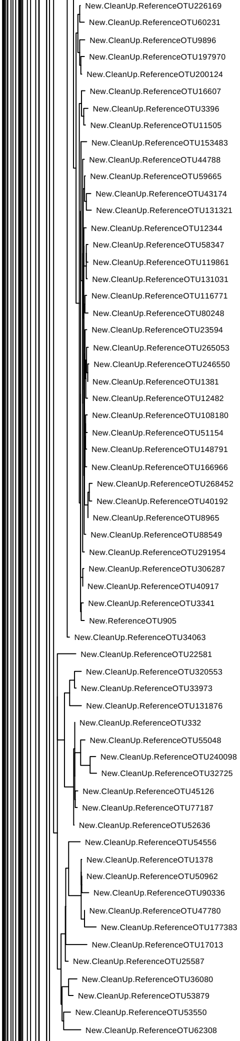
New.CleanUp.ReferenceOTU226169
New.CleanUp.ReferenceOTU60231
New.CleanUp.ReferenceOTU9896
New.CleanUp.ReferenceOTU197970
New.CleanUp.ReferenceOTU200124
New.CleanUp.ReferenceOTU16607
New.CleanUp.ReferenceOTU3396
New.CleanUp.ReferenceOTU11505
New.CleanUp.ReferenceOTU153483
New.CleanUp.ReferenceOTU44788
New.CleanUp.ReferenceOTU59665
New.CleanUp.ReferenceOTU43174
New.CleanUp.ReferenceOTU131321
New.CleanUp.ReferenceOTU12344
New.CleanUp.ReferenceOTU58347
New.CleanUp.ReferenceOTU119861
New.CleanUp.ReferenceOTU131031
New.CleanUp.ReferenceOTU116771
New.CleanUp.ReferenceOTU80248
New.CleanUp.ReferenceOTU23594
New.CleanUp.ReferenceOTU265053
New.CleanUp.ReferenceOTU246550
New.CleanUp.ReferenceOTU1381
New.CleanUp.ReferenceOTU12482
New.CleanUp.ReferenceOTU108180
New.CleanUp.ReferenceOTU51154
New.CleanUp.ReferenceOTU148791
New.CleanUp.ReferenceOTU166966
New.CleanUp.ReferenceOTU268452
New.CleanUp.ReferenceOTU40192
New.CleanUp.ReferenceOTU8965
New.CleanUp.ReferenceOTU88549
New.CleanUp.ReferenceOTU291954
New.CleanUp.ReferenceOTU306287
New.CleanUp.ReferenceOTU40917
New.CleanUp.ReferenceOTU3341
New.ReferenceOTU905
New.CleanUp.ReferenceOTU34063
New.CleanUp.ReferenceOTU22581
New.CleanUp.ReferenceOTU320553
New.CleanUp.ReferenceOTU33973
New.CleanUp.ReferenceOTU131876
New.CleanUp.ReferenceOTU332
New.CleanUp.ReferenceOTU55048
New.CleanUp.ReferenceOTU240098
New.CleanUp.ReferenceOTU32725
New.CleanUp.ReferenceOTU45126
New.CleanUp.ReferenceOTU77187
New.CleanUp.ReferenceOTU52636
New.CleanUp.ReferenceOTU54556
New.CleanUp.ReferenceOTU1378
New.CleanUp.ReferenceOTU50962
New.CleanUp.ReferenceOTU90336
New.CleanUp.ReferenceOTU47780
New.CleanUp.ReferenceOTU177383
New.CleanUp.ReferenceOTU17013
New.CleanUp.ReferenceOTU25587
New.CleanUp.ReferenceOTU36080
New.CleanUp.ReferenceOTU53879
New.CleanUp.ReferenceOTU53550
New.CleanUp.ReferenceOTU62308

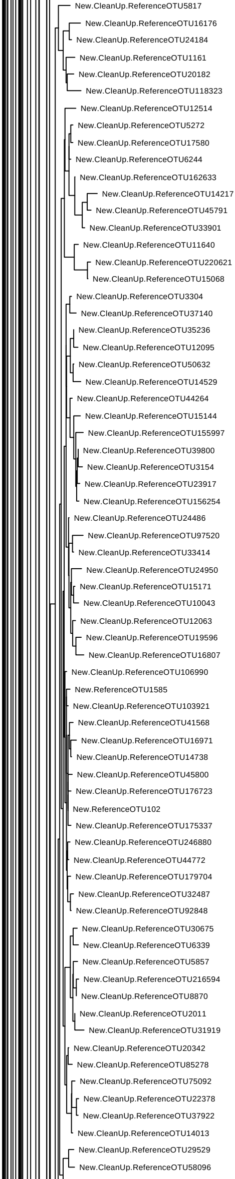
New.CleanUp.ReferenceOTU5817
New.CleanUp.ReferenceOTU16176
New.CleanUp.ReferenceOTU24184
New.CleanUp.ReferenceOTU1161
New.CleanUp.ReferenceOTU20182
New.CleanUp.ReferenceOTU118323
New.CleanUp.ReferenceOTU12514
New.CleanUp.ReferenceOTU5272
New.CleanUp.ReferenceOTU17580
New.CleanUp.ReferenceOTU6244
New.CleanUp.ReferenceOTU162633
New.CleanUp.ReferenceOTU14217
New.CleanUp.ReferenceOTU45791
New.CleanUp.ReferenceOTU33901
New.CleanUp.ReferenceOTU11640
New.CleanUp.ReferenceOTU220621
New.CleanUp.ReferenceOTU15068
New.CleanUp.ReferenceOTU3304
New.CleanUp.ReferenceOTU37140
New.CleanUp.ReferenceOTU35236
New.CleanUp.ReferenceOTU12095
New.CleanUp.ReferenceOTU50632
New.CleanUp.ReferenceOTU14529
New.CleanUp.ReferenceOTU44264
New.CleanUp.ReferenceOTU15144
New.CleanUp.ReferenceOTU155997
New.CleanUp.ReferenceOTU39800
New.CleanUp.ReferenceOTU3154
New.CleanUp.ReferenceOTU23917
New.CleanUp.ReferenceOTU156254
New.CleanUp.ReferenceOTU24486
New.CleanUp.ReferenceOTU97520
New.CleanUp.ReferenceOTU33414
New.CleanUp.ReferenceOTU24950
New.CleanUp.ReferenceOTU15171
New.CleanUp.ReferenceOTU10043
New.CleanUp.ReferenceOTU12063
New.CleanUp.ReferenceOTU19596
New.CleanUp.ReferenceOTU16807
New.CleanUp.ReferenceOTU106990
New.ReferenceOTU1585
New.CleanUp.ReferenceOTU103921
New.CleanUp.ReferenceOTU41568
New.CleanUp.ReferenceOTU16971
New.CleanUp.ReferenceOTU14738
New.CleanUp.ReferenceOTU45800
New.CleanUp.ReferenceOTU176723
New.ReferenceOTU102
New.CleanUp.ReferenceOTU175337
New.CleanUp.ReferenceOTU246880
New.CleanUp.ReferenceOTU44772
New.CleanUp.ReferenceOTU179704
New.CleanUp.ReferenceOTU32487
New.CleanUp.ReferenceOTU92848
New.CleanUp.ReferenceOTU30675
New.CleanUp.ReferenceOTU6339
New.CleanUp.ReferenceOTU5857
New.CleanUp.ReferenceOTU216594
New.CleanUp.ReferenceOTU8870
New.CleanUp.ReferenceOTU2011
New.CleanUp.ReferenceOTU31919
New.CleanUp.ReferenceOTU20342
New.CleanUp.ReferenceOTU85278
New.CleanUp.ReferenceOTU75092
New.CleanUp.ReferenceOTU22378
New.CleanUp.ReferenceOTU37922
New.CleanUp.ReferenceOTU14013
New.CleanUp.ReferenceOTU29529
New.CleanUp.ReferenceOTU58096

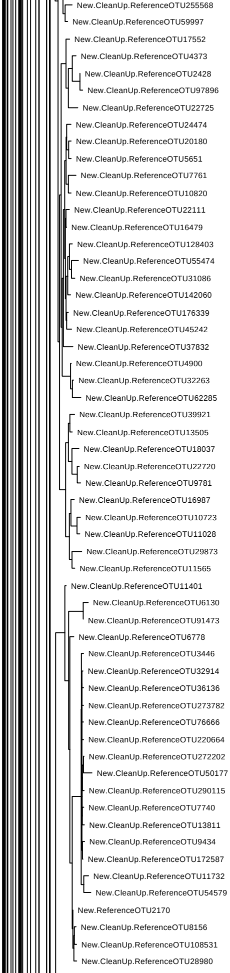

New.CleanUp.ReferenceOTU255568
New.CleanUp.ReferenceOTU59997
New.CleanUp.ReferenceOTU17552
New.CleanUp.ReferenceOTU4373
New.CleanUp.ReferenceOTU2428
New.CleanUp.ReferenceOTU97896
New.CleanUp.ReferenceOTU22725
New.CleanUp.ReferenceOTU24474
New.CleanUp.ReferenceOTU20180
New.CleanUp.ReferenceOTU5651
New.CleanUp.ReferenceOTU7761
New.CleanUp.ReferenceOTU10820
New.CleanUp.ReferenceOTU22111
New.CleanUp.ReferenceOTU16479
New.CleanUp.ReferenceOTU128403
New.CleanUp.ReferenceOTU55474
New.CleanUp.ReferenceOTU31086
New.CleanUp.ReferenceOTU142060
New.CleanUp.ReferenceOTU176339
New.CleanUp.ReferenceOTU45242
New.CleanUp.ReferenceOTU37832
New.CleanUp.ReferenceOTU4900
New.CleanUp.ReferenceOTU32263
New.CleanUp.ReferenceOTU62285
New.CleanUp.ReferenceOTU39921
New.CleanUp.ReferenceOTU13505
New.CleanUp.ReferenceOTU18037
New.CleanUp.ReferenceOTU22720
New.CleanUp.ReferenceOTU9781
New.CleanUp.ReferenceOTU16987
New.CleanUp.ReferenceOTU10723
New.CleanUp.ReferenceOTU11028
New.CleanUp.ReferenceOTU29873
New.CleanUp.ReferenceOTU11565
New.CleanUp.ReferenceOTU11401
New.CleanUp.ReferenceOTU6130
New.CleanUp.ReferenceOTU91473
New.CleanUp.ReferenceOTU6778
New.CleanUp.ReferenceOTU3446
New.CleanUp.ReferenceOTU32914
New.CleanUp.ReferenceOTU36136
New.CleanUp.ReferenceOTU273782
New.CleanUp.ReferenceOTU76666
New.CleanUp.ReferenceOTU220664
New.CleanUp.ReferenceOTU272202
New.CleanUp.ReferenceOTU50177
New.CleanUp.ReferenceOTU290115
New.CleanUp.ReferenceOTU7740
New.CleanUp.ReferenceOTU13811
New.CleanUp.ReferenceOTU9434
New.CleanUp.ReferenceOTU172587
New.CleanUp.ReferenceOTU11732
New.CleanUp.ReferenceOTU54579
New.ReferenceOTU2170
New.CleanUp.ReferenceOTU8156
New.CleanUp.ReferenceOTU108531
New.CleanUp.ReferenceOTU28980

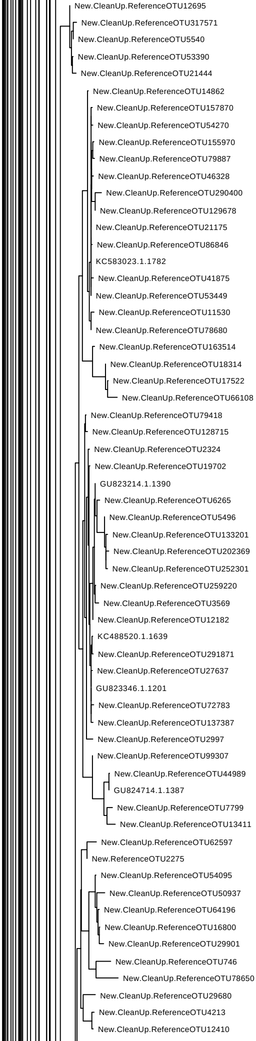
New.CleanUp.ReferenceOTU12695
New.CleanUp.ReferenceOTU317571
New.CleanUp.ReferenceOTU5540
New.CleanUp.ReferenceOTU53390
New.CleanUp.ReferenceOTU21444
New.CleanUp.ReferenceOTU14862
New.CleanUp.ReferenceOTU157870
New.CleanUp.ReferenceOTU54270
New.CleanUp.ReferenceOTU155970
New.CleanUp.ReferenceOTU79887
New.CleanUp.ReferenceOTU46328
New.CleanUp.ReferenceOTU290400
New.CleanUp.ReferenceOTU129678
New.CleanUp.ReferenceOTU21175
New.CleanUp.ReferenceOTU86846
KC583023.1.1782
New.CleanUp.ReferenceOTU41875
New.CleanUp.ReferenceOTU53449
New.CleanUp.ReferenceOTU11530
New.CleanUp.ReferenceOTU78680
New.CleanUp.ReferenceOTU163514
New.CleanUp.ReferenceOTU18314
New.CleanUp.ReferenceOTU17522
New.CleanUp.ReferenceOTU66108
New.CleanUp.ReferenceOTU79418
New.CleanUp.ReferenceOTU128715
New.CleanUp.ReferenceOTU2324
New.CleanUp.ReferenceOTU19702
GU823214.1.1390
New.CleanUp.ReferenceOTU6265
New.CleanUp.ReferenceOTU5496
New.CleanUp.ReferenceOTU133201
New.CleanUp.ReferenceOTU202369
New.CleanUp.ReferenceOTU252301
New.CleanUp.ReferenceOTU259220
New.CleanUp.ReferenceOTU3569
New.CleanUp.ReferenceOTU12182
KC488520.1.1639
New.CleanUp.ReferenceOTU291871
New.CleanUp.ReferenceOTU27637
GU823346.1.1201
New.CleanUp.ReferenceOTU72783
New.CleanUp.ReferenceOTU137387
New.CleanUp.ReferenceOTU2997
New.CleanUp.ReferenceOTU99307
New.CleanUp.ReferenceOTU44989
GU824714.1.1387
New.CleanUp.ReferenceOTU7799
New.CleanUp.ReferenceOTU13411
New.CleanUp.ReferenceOTU62597
New.ReferenceOTU2275
New.CleanUp.ReferenceOTU54095
New.CleanUp.ReferenceOTU50937
New.CleanUp.ReferenceOTU64196
New.CleanUp.ReferenceOTU16800
New.CleanUp.ReferenceOTU29901
New.CleanUp.ReferenceOTU746
New.CleanUp.ReferenceOTU78650
New.CleanUp.ReferenceOTU29680
New.CleanUp.ReferenceOTU4213
New.CleanUp.ReferenceOTU12410

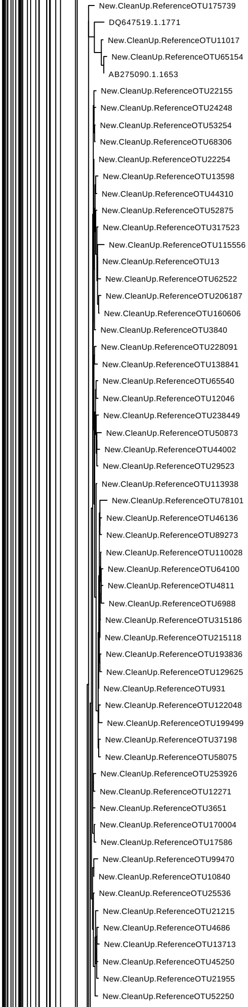
New.CleanUp.ReferenceOTU175739
DQ647519.1.1771
New.CleanUp.ReferenceOTU11017
New.CleanUp.ReferenceOTU65154
AB275090.1.1653
New.CleanUp.ReferenceOTU22155
New.CleanUp.ReferenceOTU24248
New.CleanUp.ReferenceOTU53254
New.CleanUp.ReferenceOTU68306
New.CleanUp.ReferenceOTU22254
New.CleanUp.ReferenceOTU13598
New.CleanUp.ReferenceOTU44310
New.CleanUp.ReferenceOTU52875
New.CleanUp.ReferenceOTU317523
New.CleanUp.ReferenceOTU115556
New.CleanUp.ReferenceOTU13
New.CleanUp.ReferenceOTU62522
New.CleanUp.ReferenceOTU206187
New.CleanUp.ReferenceOTU160606
New.CleanUp.ReferenceOTU3840
New.CleanUp.ReferenceOTU228091
New.CleanUp.ReferenceOTU138841
New.CleanUp.ReferenceOTU65540
New.CleanUp.ReferenceOTU12046
New.CleanUp.ReferenceOTU238449
New.CleanUp.ReferenceOTU50873
New.CleanUp.ReferenceOTU44002
New.CleanUp.ReferenceOTU29523
New.CleanUp.ReferenceOTU113938
New.CleanUp.ReferenceOTU78101
New.CleanUp.ReferenceOTU46136
New.CleanUp.ReferenceOTU89273
New.CleanUp.ReferenceOTU110028
New.CleanUp.ReferenceOTU64100
New.CleanUp.ReferenceOTU4811
New.CleanUp.ReferenceOTU6988
New.CleanUp.ReferenceOTU315186
New.CleanUp.ReferenceOTU215118
New.CleanUp.ReferenceOTU193836
New.CleanUp.ReferenceOTU129625
New.CleanUp.ReferenceOTU931
New.CleanUp.ReferenceOTU122048
New.CleanUp.ReferenceOTU199499
New.CleanUp.ReferenceOTU37198
New.CleanUp.ReferenceOTU58075
New.CleanUp.ReferenceOTU253926
New.CleanUp.ReferenceOTU12271
New.CleanUp.ReferenceOTU3651
New.CleanUp.ReferenceOTU170004
New.CleanUp.ReferenceOTU17586
New.CleanUp.ReferenceOTU99470
New.CleanUp.ReferenceOTU10840
New.CleanUp.ReferenceOTU25536
New.CleanUp.ReferenceOTU21215
New.CleanUp.ReferenceOTU4686
New.CleanUp.ReferenceOTU13713
New.CleanUp.ReferenceOTU45250
New.CleanUp.ReferenceOTU21955
New.CleanUp.ReferenceOTU52250

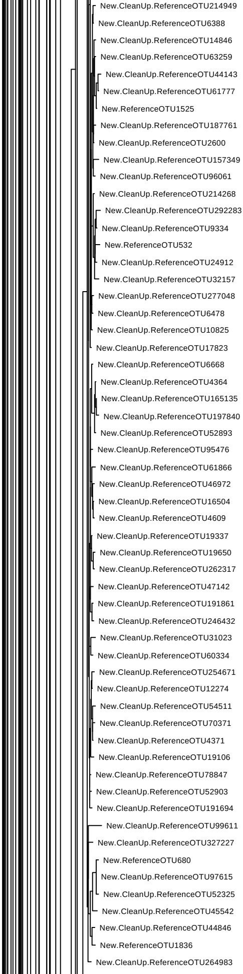
New.CleanUp.ReferenceOTU214949
New.CleanUp.ReferenceOTU6388
New.CleanUp.ReferenceOTU14846
New.CleanUp.ReferenceOTU63259
New.CleanUp.ReferenceOTU44143
New.CleanUp.ReferenceOTU61777
New.ReferenceOTU1525
New.CleanUp.ReferenceOTU187761
New.CleanUp.ReferenceOTU2600
New.CleanUp.ReferenceOTU157349
New.CleanUp.ReferenceOTU96061
New.CleanUp.ReferenceOTU214268
New.CleanUp.ReferenceOTU292283
New.CleanUp.ReferenceOTU9334
New.ReferenceOTU532
New.CleanUp.ReferenceOTU24912
New.CleanUp.ReferenceOTU32157
New.CleanUp.ReferenceOTU277048
New.CleanUp.ReferenceOTU6478
New.CleanUp.ReferenceOTU10825
New.CleanUp.ReferenceOTU17823
New.CleanUp.ReferenceOTU6668
New.CleanUp.ReferenceOTU4364
New.CleanUp.ReferenceOTU165135
New.CleanUp.ReferenceOTU197840
New.CleanUp.ReferenceOTU52893
New.CleanUp.ReferenceOTU95476
New.CleanUp.ReferenceOTU61866
New.CleanUp.ReferenceOTU46972
New.CleanUp.ReferenceOTU16504
New.CleanUp.ReferenceOTU4609
New.CleanUp.ReferenceOTU19337
New.CleanUp.ReferenceOTU19650
New.CleanUp.ReferenceOTU262317
New.CleanUp.ReferenceOTU47142
New.CleanUp.ReferenceOTU191861
New.CleanUp.ReferenceOTU246432
New.CleanUp.ReferenceOTU31023
New.CleanUp.ReferenceOTU60334
New.CleanUp.ReferenceOTU254671
New.CleanUp.ReferenceOTU12274
New.CleanUp.ReferenceOTU54511
New.CleanUp.ReferenceOTU70371
New.CleanUp.ReferenceOTU4371
New.CleanUp.ReferenceOTU19106
New.CleanUp.ReferenceOTU78847
New.CleanUp.ReferenceOTU52903
New.CleanUp.ReferenceOTU191694
New.CleanUp.ReferenceOTU99611
New.CleanUp.ReferenceOTU327227
New.ReferenceOTU680
New.CleanUp.ReferenceOTU97615
New.CleanUp.ReferenceOTU52325
New.CleanUp.ReferenceOTU45542
New.CleanUp.ReferenceOTU44846
New.ReferenceOTU1836
New.CleanUp.ReferenceOTU264983

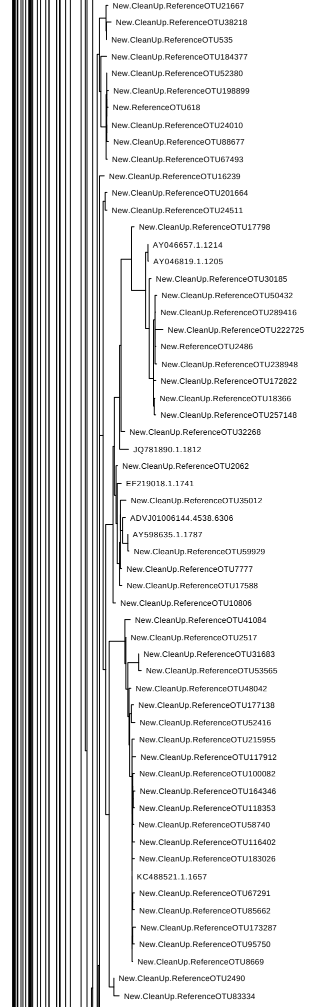
New.CleanUp.ReferenceOTU21667
New.CleanUp.ReferenceOTU38218
New.CleanUp.ReferenceOTU535
New.CleanUp.ReferenceOTU184377
New.CleanUp.ReferenceOTU52380
New.CleanUp.ReferenceOTU198899
New.ReferenceOTU618
New.CleanUp.ReferenceOTU24010
New.CleanUp.ReferenceOTU88677
New.CleanUp.ReferenceOTU67493
New.CleanUp.ReferenceOTU16239
New.CleanUp.ReferenceOTU201664
New.CleanUp.ReferenceOTU24511
New.CleanUp.ReferenceOTU17798
AY046657.1.1214
AY046819.1.1205
New.CleanUp.ReferenceOTU30185
New.CleanUp.ReferenceOTU50432
New.CleanUp.ReferenceOTU289416
New.CleanUp.ReferenceOTU222725
New.ReferenceOTU2486
New.CleanUp.ReferenceOTU238948
New.CleanUp.ReferenceOTU172822
New.CleanUp.ReferenceOTU18366
New.CleanUp.ReferenceOTU257148
New.CleanUp.ReferenceOTU32268
JQ781890.1.1812
New.CleanUp.ReferenceOTU2062
EF219018.1.1741
New.CleanUp.ReferenceOTU35012
ADVJ01006144.4538.6306
AY598635.1.1787
New.CleanUp.ReferenceOTU59929
New.CleanUp.ReferenceOTU7777
New.CleanUp.ReferenceOTU17588
New.CleanUp.ReferenceOTU10806
New.CleanUp.ReferenceOTU41084
New.CleanUp.ReferenceOTU2517
New.CleanUp.ReferenceOTU31683
New.CleanUp.ReferenceOTU53565
New.CleanUp.ReferenceOTU48042
New.CleanUp.ReferenceOTU177138
New.CleanUp.ReferenceOTU52416
New.CleanUp.ReferenceOTU215955
New.CleanUp.ReferenceOTU117912
New.CleanUp.ReferenceOTU100082
New.CleanUp.ReferenceOTU164346
New.CleanUp.ReferenceOTU118353
New.CleanUp.ReferenceOTU58740
New.CleanUp.ReferenceOTU116402
New.CleanUp.ReferenceOTU183026
KC488521.1.1657
New.CleanUp.ReferenceOTU67291
New.CleanUp.ReferenceOTU85662
New.CleanUp.ReferenceOTU173287
New.CleanUp.ReferenceOTU95750
New.CleanUp.ReferenceOTU8669
New.CleanUp.ReferenceOTU2490
New.CleanUp.ReferenceOTU83334

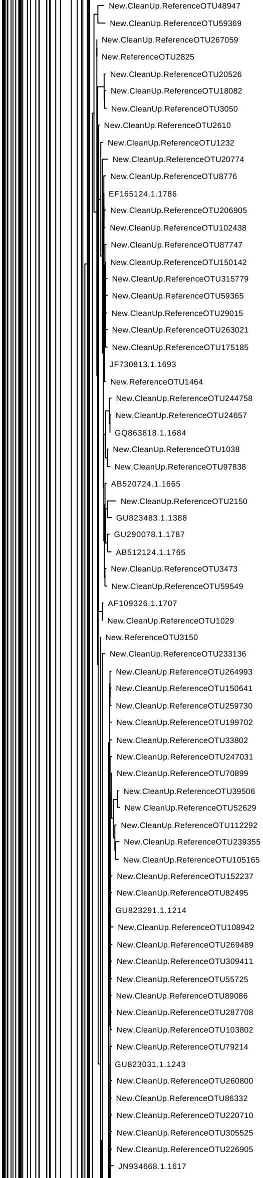
New.CleanUp.ReferenceOTU48947
New.CleanUp.ReferenceOTU59369
New.CleanUp.ReferenceOTU267059
New.ReferenceOTU2825
New.CleanUp.ReferenceOTU20526
New.CleanUp.ReferenceOTU18082
New.CleanUp.ReferenceOTU3050
New.CleanUp.ReferenceOTU2610
New.CleanUp.ReferenceOTU1232
New.CleanUp.ReferenceOTU20774
New.CleanUp.ReferenceOTU8776
EF165124.1.1786
New.CleanUp.ReferenceOTU206905
New.CleanUp.ReferenceOTU102438
New.CleanUp.ReferenceOTU87747
New.CleanUp.ReferenceOTU150142
New.CleanUp.ReferenceOTU315779
New.CleanUp.ReferenceOTU59365
New.CleanUp.ReferenceOTU29015
New.CleanUp.ReferenceOTU263021
New.CleanUp.ReferenceOTU175185
JF730813.1.1693
New.ReferenceOTU1464
New.CleanUp.ReferenceOTU244758
New.CleanUp.ReferenceOTU24657
GQ863818.1.1684
New.CleanUp.ReferenceOTU1038
New.CleanUp.ReferenceOTU97838
AB520724.1.1665
New.CleanUp.ReferenceOTU2150
GU823483.1.1388
GU290078.1.1787
AB512124.1.1765
New.CleanUp.ReferenceOTU3473
New.CleanUp.ReferenceOTU59549
AF109326.1.1707
New.CleanUp.ReferenceOTU1029
New.ReferenceOTU3150
New.CleanUp.ReferenceOTU233136
New.CleanUp.ReferenceOTU264993
New.CleanUp.ReferenceOTU150641
New.CleanUp.ReferenceOTU259730
New.CleanUp.ReferenceOTU199702
New.CleanUp.ReferenceOTU33802
New.CleanUp.ReferenceOTU247031
New.CleanUp.ReferenceOTU70899
New.CleanUp.ReferenceOTU39506
New.CleanUp.ReferenceOTU52629
New.CleanUp.ReferenceOTU112292
New.CleanUp.ReferenceOTU239355
New.CleanUp.ReferenceOTU105165
New.CleanUp.ReferenceOTU152237
New.CleanUp.ReferenceOTU82495
GU823291.1.1214
New.CleanUp.ReferenceOTU108942
New.CleanUp.ReferenceOTU269489
New.CleanUp.ReferenceOTU309411
New.CleanUp.ReferenceOTU55725
New.CleanUp.ReferenceOTU89086
New.CleanUp.ReferenceOTU287708
New.CleanUp.ReferenceOTU103802
New.CleanUp.ReferenceOTU79214
GU823031.1.1243
New.CleanUp.ReferenceOTU260800
New.CleanUp.ReferenceOTU86332
New.CleanUp.ReferenceOTU220710
New.CleanUp.ReferenceOTU305525
New.CleanUp.ReferenceOTU226905
JN934668.1.1617

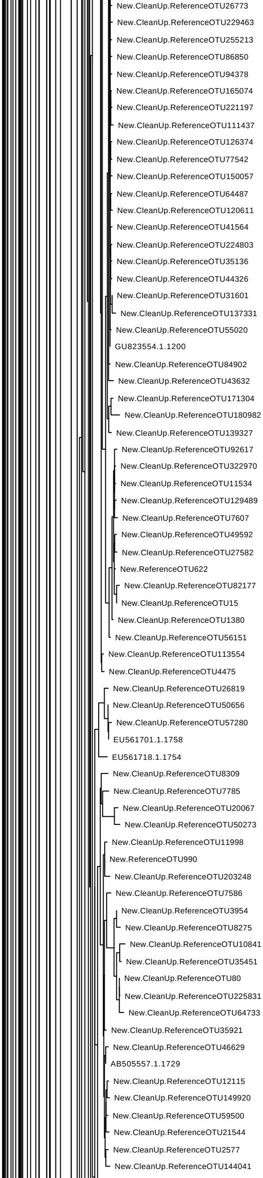
New.CleanUp.ReferenceOTU26773
New.CleanUp.ReferenceOTU229463
New.CleanUp.ReferenceOTU255213
New.CleanUp.ReferenceOTU86850
New.CleanUp.ReferenceOTU94378
New.CleanUp.ReferenceOTU165074
New.CleanUp.ReferenceOTU221197
New.CleanUp.ReferenceOTU111437
New.CleanUp.ReferenceOTU126374
New.CleanUp.ReferenceOTU77542
New.CleanUp.ReferenceOTU150057
New.CleanUp.ReferenceOTU64487
New.CleanUp.ReferenceOTU120611
New.CleanUp.ReferenceOTU41564
New.CleanUp.ReferenceOTU224803
New.CleanUp.ReferenceOTU35136
New.CleanUp.ReferenceOTU44326
New.CleanUp.ReferenceOTU31601
New.CleanUp.ReferenceOTU137331
New.CleanUp.ReferenceOTU55020
GU823554.1.1200
New.CleanUp.ReferenceOTU84902
New.CleanUp.ReferenceOTU43632
New.CleanUp.ReferenceOTU171304
New.CleanUp.ReferenceOTU180982
New.CleanUp.ReferenceOTU139327
New.CleanUp.ReferenceOTU92617
New.CleanUp.ReferenceOTU322970
New.CleanUp.ReferenceOTU11534
New.CleanUp.ReferenceOTU129489
New.CleanUp.ReferenceOTU7607
New.CleanUp.ReferenceOTU49592
New.CleanUp.ReferenceOTU27582
New.ReferenceOTU622
New.CleanUp.ReferenceOTU82177
New.CleanUp.ReferenceOTU15
New.CleanUp.ReferenceOTU1380
New.CleanUp.ReferenceOTU56151
New.CleanUp.ReferenceOTU113554
New.CleanUp.ReferenceOTU4475
New.CleanUp.ReferenceOTU26819
New.CleanUp.ReferenceOTU50656
New.CleanUp.ReferenceOTU57280
EU561701.1.1758
EU561718.1.1754
New.CleanUp.ReferenceOTU8309
New.CleanUp.ReferenceOTU7785
New.CleanUp.ReferenceOTU20067
New.CleanUp.ReferenceOTU50273
New.CleanUp.ReferenceOTU11998
New.ReferenceOTU990
New.CleanUp.ReferenceOTU203248
New.CleanUp.ReferenceOTU7586
New.CleanUp.ReferenceOTU3954
New.CleanUp.ReferenceOTU8275
New.CleanUp.ReferenceOTU10841
New.CleanUp.ReferenceOTU35451
New.CleanUp.ReferenceOTU80
New.CleanUp.ReferenceOTU225831
New.CleanUp.ReferenceOTU64733
New.CleanUp.ReferenceOTU35921
New.CleanUp.ReferenceOTU46629
AB505557.1.1729
New.CleanUp.ReferenceOTU12115
New.CleanUp.ReferenceOTU149920
New.CleanUp.ReferenceOTU59500
New.CleanUp.ReferenceOTU21544
New.CleanUp.ReferenceOTU2577
New.CleanUp.ReferenceOTU144041

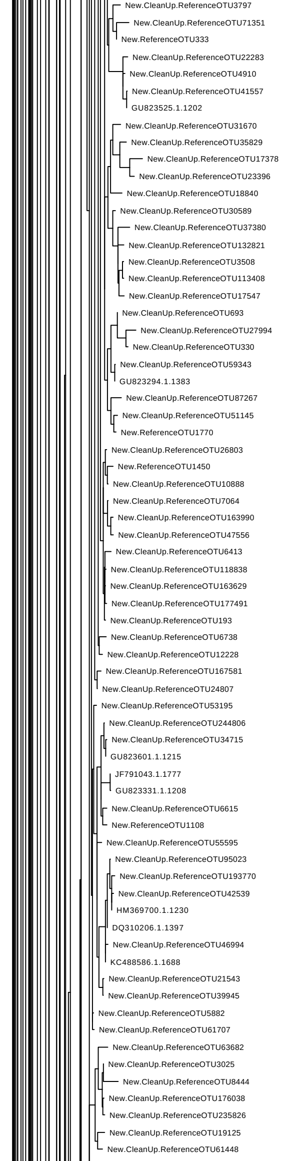
New.CleanUp.ReferenceOTU3797
New.CleanUp.ReferenceOTU71351
New.ReferenceOTU333
New.CleanUp.ReferenceOTU22283
New.CleanUp.ReferenceOTU4910
New.CleanUp.ReferenceOTU41557
GU823525.1.1202
New.CleanUp.ReferenceOTU31670
New.CleanUp.ReferenceOTU35829
New.CleanUp.ReferenceOTU17378
New.CleanUp.ReferenceOTU23396
New.CleanUp.ReferenceOTU18840
New.CleanUp.ReferenceOTU30589
New.CleanUp.ReferenceOTU37380
New.CleanUp.ReferenceOTU132821
New.CleanUp.ReferenceOTU3508
New.CleanUp.ReferenceOTU113408
New.CleanUp.ReferenceOTU17547
New.CleanUp.ReferenceOTU693
New.CleanUp.ReferenceOTU27994
New.CleanUp.ReferenceOTU330
New.CleanUp.ReferenceOTU59343
GU823294.1.1383
New.CleanUp.ReferenceOTU87267
New.CleanUp.ReferenceOTU51145
New.ReferenceOTU1770
New.CleanUp.ReferenceOTU26803
New.ReferenceOTU1450
New.CleanUp.ReferenceOTU10888
New.CleanUp.ReferenceOTU7064
New.CleanUp.ReferenceOTU163990
New.CleanUp.ReferenceOTU47556
New.CleanUp.ReferenceOTU6413
New.CleanUp.ReferenceOTU118838
New.CleanUp.ReferenceOTU163629
New.CleanUp.ReferenceOTU177491
New.CleanUp.ReferenceOTU193
New.CleanUp.ReferenceOTU6738
New.CleanUp.ReferenceOTU12228
New.CleanUp.ReferenceOTU167581
New.CleanUp.ReferenceOTU24807
New.CleanUp.ReferenceOTU53195
New.CleanUp.ReferenceOTU244806
New.CleanUp.ReferenceOTU34715
GU823601.1.1215
JF791043.1.1777
GU823331.1.1208
New.CleanUp.ReferenceOTU6615
New.ReferenceOTU1108
New.CleanUp.ReferenceOTU55595
New.CleanUp.ReferenceOTU95023
New.CleanUp.ReferenceOTU193770
New.CleanUp.ReferenceOTU42539
HM369700.1.1230
DQ310206.1.1397
New.CleanUp.ReferenceOTU46994
KC488586.1.1688
New.CleanUp.ReferenceOTU21543
New.CleanUp.ReferenceOTU39945
New.CleanUp.ReferenceOTU5882
New.CleanUp.ReferenceOTU61707
New.CleanUp.ReferenceOTU63682
New.CleanUp.ReferenceOTU3025
New.CleanUp.ReferenceOTU8444
New.CleanUp.ReferenceOTU176038
New.CleanUp.ReferenceOTU235826
New.CleanUp.ReferenceOTU19125
New.CleanUp.ReferenceOTU61448

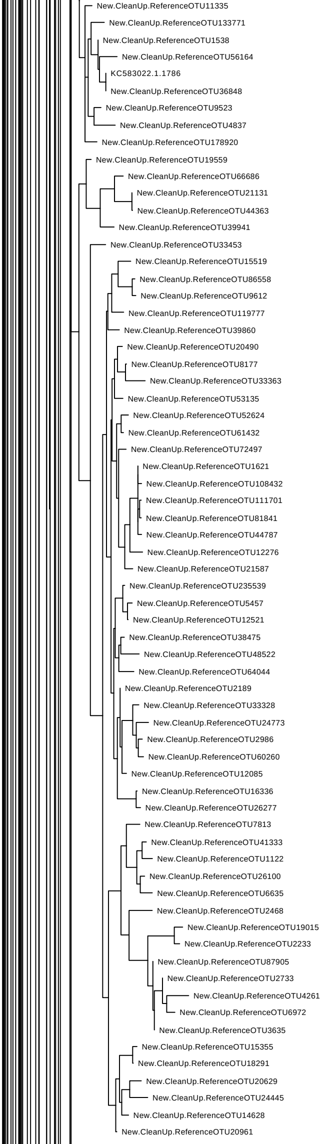
New.CleanUp.ReferenceOTU11335
New.CleanUp.ReferenceOTU133771
New.CleanUp.ReferenceOTU1538
New.CleanUp.ReferenceOTU56164
KC583022.1.1786
New.CleanUp.ReferenceOTU36848
New.CleanUp.ReferenceOTU9523
New.CleanUp.ReferenceOTU4837
New.CleanUp.ReferenceOTU178920
New.CleanUp.ReferenceOTU19559
New.CleanUp.ReferenceOTU66686
New.CleanUp.ReferenceOTU21131
New.CleanUp.ReferenceOTU44363
New.CleanUp.ReferenceOTU39941
New.CleanUp.ReferenceOTU33453
New.CleanUp.ReferenceOTU15519
New.CleanUp.ReferenceOTU86558
New.CleanUp.ReferenceOTU9612
New.CleanUp.ReferenceOTU119777
New.CleanUp.ReferenceOTU39860
New.CleanUp.ReferenceOTU20490
New.CleanUp.ReferenceOTU8177
New.CleanUp.ReferenceOTU33363
New.CleanUp.ReferenceOTU53135
New.CleanUp.ReferenceOTU52624
New.CleanUp.ReferenceOTU61432
New.CleanUp.ReferenceOTU72497
New.CleanUp.ReferenceOTU1621
New.CleanUp.ReferenceOTU108432
New.CleanUp.ReferenceOTU111701
New.CleanUp.ReferenceOTU81841
New.CleanUp.ReferenceOTU44787
New.CleanUp.ReferenceOTU12276
New.CleanUp.ReferenceOTU21587
New.CleanUp.ReferenceOTU235539
New.CleanUp.ReferenceOTU5457
New.CleanUp.ReferenceOTU12521
New.CleanUp.ReferenceOTU38475
New.CleanUp.ReferenceOTU48522
New.CleanUp.ReferenceOTU64044
New.CleanUp.ReferenceOTU2189
New.CleanUp.ReferenceOTU33328
New.CleanUp.ReferenceOTU24773
New.CleanUp.ReferenceOTU2986
New.CleanUp.ReferenceOTU60260
New.CleanUp.ReferenceOTU12085
New.CleanUp.ReferenceOTU16336
New.CleanUp.ReferenceOTU26277
New.CleanUp.ReferenceOTU7813
New.CleanUp.ReferenceOTU41333
New.CleanUp.ReferenceOTU1122
New.CleanUp.ReferenceOTU26100
New.CleanUp.ReferenceOTU6635
New.CleanUp.ReferenceOTU2468
New.CleanUp.ReferenceOTU19015
New.CleanUp.ReferenceOTU2233
New.CleanUp.ReferenceOTU87905
New.CleanUp.ReferenceOTU2733
New.CleanUp.ReferenceOTU4261
New.CleanUp.ReferenceOTU6972
New.CleanUp.ReferenceOTU3635
New.CleanUp.ReferenceOTU15355
New.CleanUp.ReferenceOTU18291
New.CleanUp.ReferenceOTU20629
New.CleanUp.ReferenceOTU24445
New.CleanUp.ReferenceOTU14628
New.CleanUp.ReferenceOTU20961

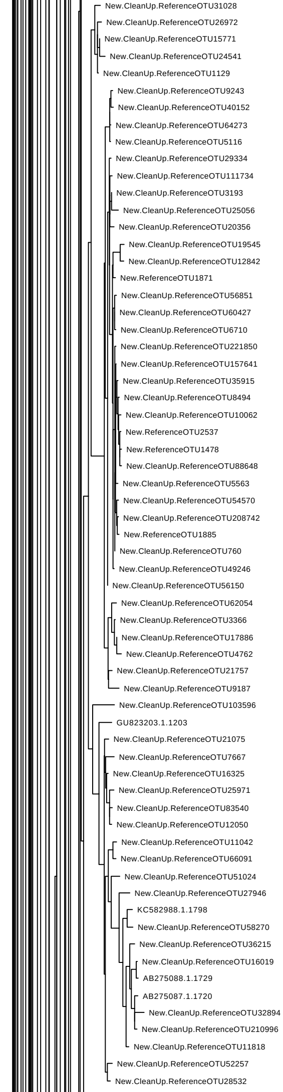
New.CleanUp.ReferenceOTU31028
New.CleanUp.ReferenceOTU26972
New.CleanUp.ReferenceOTU15771
New.CleanUp.ReferenceOTU24541
New.CleanUp.ReferenceOTU1129
New.CleanUp.ReferenceOTU9243
New.CleanUp.ReferenceOTU40152
New.CleanUp.ReferenceOTU64273
New.CleanUp.ReferenceOTU5116
New.CleanUp.ReferenceOTU29334
New.CleanUp.ReferenceOTU111734
New.CleanUp.ReferenceOTU3193
New.CleanUp.ReferenceOTU25056
New.CleanUp.ReferenceOTU20356
New.CleanUp.ReferenceOTU19545
New.CleanUp.ReferenceOTU12842
New.ReferenceOTU1871
New.CleanUp.ReferenceOTU56851
New.CleanUp.ReferenceOTU60427
New.CleanUp.ReferenceOTU6710
New.CleanUp.ReferenceOTU221850
New.CleanUp.ReferenceOTU157641
New.CleanUp.ReferenceOTU35915
New.CleanUp.ReferenceOTU8494
New.CleanUp.ReferenceOTU10062
New.ReferenceOTU2537
New.ReferenceOTU1478
New.CleanUp.ReferenceOTU88648
New.CleanUp.ReferenceOTU5563
New.CleanUp.ReferenceOTU54570
New.CleanUp.ReferenceOTU208742
New.ReferenceOTU1885
New.CleanUp.ReferenceOTU760
New.CleanUp.ReferenceOTU49246
New.CleanUp.ReferenceOTU56150
New.CleanUp.ReferenceOTU62054
New.CleanUp.ReferenceOTU3366
New.CleanUp.ReferenceOTU17886
New.CleanUp.ReferenceOTU4762
New.CleanUp.ReferenceOTU21757
New.CleanUp.ReferenceOTU9187
New.CleanUp.ReferenceOTU103596
GU823203.1.1203
New.CleanUp.ReferenceOTU21075
New.CleanUp.ReferenceOTU7667
New.CleanUp.ReferenceOTU16325
New.CleanUp.ReferenceOTU25971
New.CleanUp.ReferenceOTU83540
New.CleanUp.ReferenceOTU12050
New.CleanUp.ReferenceOTU11042
New.CleanUp.ReferenceOTU66091
New.CleanUp.ReferenceOTU51024
New.CleanUp.ReferenceOTU27946
KC582988.1.1798
New.CleanUp.ReferenceOTU58270
New.CleanUp.ReferenceOTU36215
New.CleanUp.ReferenceOTU16019
AB275088.1.1729
AB275087.1.1720
New.CleanUp.ReferenceOTU32894
New.CleanUp.ReferenceOTU210996
New.CleanUp.ReferenceOTU11818
New.CleanUp.ReferenceOTU52257
New.CleanUp.ReferenceOTU28532

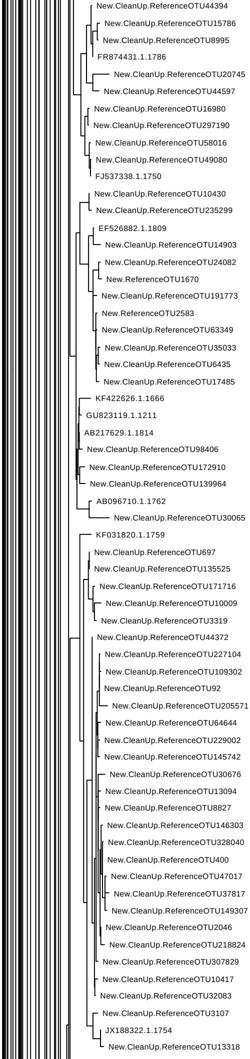
New.CleanUp.ReferenceOTU44394
New.CleanUp.ReferenceOTU15786
New.CleanUp.ReferenceOTU8995
FR874431.1.1786
New.CleanUp.ReferenceOTU20745
New.CleanUp.ReferenceOTU44597
New.CleanUp.ReferenceOTU16980
New.CleanUp.ReferenceOTU297190
New.CleanUp.ReferenceOTU58016
New.CleanUp.ReferenceOTU49080
FJ537338.1.1750
New.CleanUp.ReferenceOTU10430
New.CleanUp.ReferenceOTU235299
EF526882.1.1809
New.CleanUp.ReferenceOTU14903
New.CleanUp.ReferenceOTU24082
New.ReferenceOTU1670
New.CleanUp.ReferenceOTU191773
New.ReferenceOTU2583
New.CleanUp.ReferenceOTU63349
New.CleanUp.ReferenceOTU35033
New.CleanUp.ReferenceOTU6435
New.CleanUp.ReferenceOTU17485
KF422626.1.1666
GU823119.1.1211
AB217629.1.1814
New.CleanUp.ReferenceOTU98406
New.CleanUp.ReferenceOTU172910
New.CleanUp.ReferenceOTU139964
AB096710.1.1762
New.CleanUp.ReferenceOTU30065
KF031820.1.1759
New.CleanUp.ReferenceOTU697
New.CleanUp.ReferenceOTU135525
New.CleanUp.ReferenceOTU171716
New.CleanUp.ReferenceOTU10009
New.CleanUp.ReferenceOTU3319
New.CleanUp.ReferenceOTU44372
New.CleanUp.ReferenceOTU227104
New.CleanUp.ReferenceOTU109302
New.CleanUp.ReferenceOTU92
New.CleanUp.ReferenceOTU205571
New.CleanUp.ReferenceOTU64644
New.CleanUp.ReferenceOTU229002
New.CleanUp.ReferenceOTU145742
New.CleanUp.ReferenceOTU30676
New.CleanUp.ReferenceOTU13094
New.CleanUp.ReferenceOTU8827
New.CleanUp.ReferenceOTU146303
New.CleanUp.ReferenceOTU328040
New.CleanUp.ReferenceOTU400
New.CleanUp.ReferenceOTU47017
New.CleanUp.ReferenceOTU37817
New.CleanUp.ReferenceOTU149307
New.CleanUp.ReferenceOTU2046
New.CleanUp.ReferenceOTU218824
New.CleanUp.ReferenceOTU307829
New.CleanUp.ReferenceOTU10417
New.CleanUp.ReferenceOTU32083
New.CleanUp.ReferenceOTU3107
JX188322.1.1754
New.CleanUp.ReferenceOTU13318

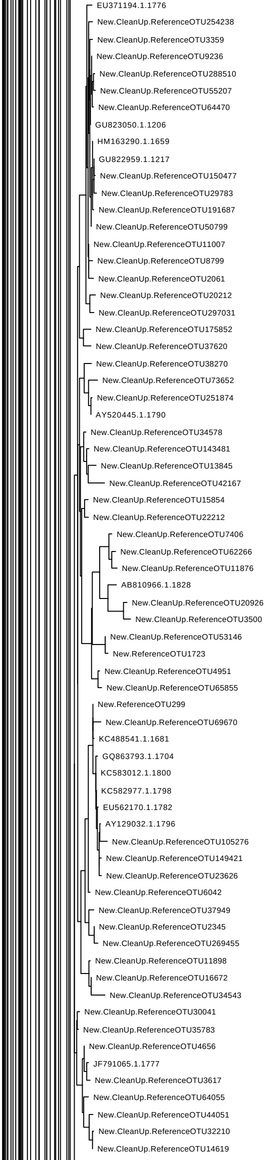
EU371194.1.1776
New.CleanUp.ReferenceOTU254238
New.CleanUp.ReferenceOTU3359
New.CleanUp.ReferenceOTU9236
New.CleanUp.ReferenceOTU288510
New.CleanUp.ReferenceOTU55207
New.CleanUp.ReferenceOTU64470
GU823050.1.1206
HM163290.1.1659
GU822959.1.1217
New.CleanUp.ReferenceOTU150477
New.CleanUp.ReferenceOTU29783
New.CleanUp.ReferenceOTU191687
New.CleanUp.ReferenceOTU50799
New.CleanUp.ReferenceOTU11007
New.CleanUp.ReferenceOTU8799
New.CleanUp.ReferenceOTU2061
New.CleanUp.ReferenceOTU20212
New.CleanUp.ReferenceOTU297031
New.CleanUp.ReferenceOTU175852
New.CleanUp.ReferenceOTU37620
New.CleanUp.ReferenceOTU38270
New.CleanUp.ReferenceOTU73652
New.CleanUp.ReferenceOTU251874
AY520445.1.1790
New.CleanUp.ReferenceOTU34578
New.CleanUp.ReferenceOTU143481
New.CleanUp.ReferenceOTU13845
New.CleanUp.ReferenceOTU42167
New.CleanUp.ReferenceOTU15854
New.CleanUp.ReferenceOTU22212
New.CleanUp.ReferenceOTU7406
New.CleanUp.ReferenceOTU62266
New.CleanUp.ReferenceOTU11876
AB810966.1.1828
New.CleanUp.ReferenceOTU20926
New.CleanUp.ReferenceOTU3500
New.CleanUp.ReferenceOTU53146
New.ReferenceOTU1723
New.CleanUp.ReferenceOTU4951
New.CleanUp.ReferenceOTU65855
New.ReferenceOTU299
New.CleanUp.ReferenceOTU69670
KC488541.1.1681
GQ863793.1.1704
KC583012.1.1800
KC582977.1.1798
EU562170.1.1782
AY129032.1.1796
New.CleanUp.ReferenceOTU105276
New.CleanUp.ReferenceOTU149421
New.CleanUp.ReferenceOTU23626
New.CleanUp.ReferenceOTU6042
New.CleanUp.ReferenceOTU37949
New.CleanUp.ReferenceOTU2345
New.CleanUp.ReferenceOTU269455
New.CleanUp.ReferenceOTU11898
New.CleanUp.ReferenceOTU16672
New.CleanUp.ReferenceOTU34543
New.CleanUp.ReferenceOTU30041
New.CleanUp.ReferenceOTU35783
New.CleanUp.ReferenceOTU4656
JF791065.1.1777
New.CleanUp.ReferenceOTU3617
New.CleanUp.ReferenceOTU64055
New.CleanUp.ReferenceOTU44051
New.CleanUp.ReferenceOTU32210
New.CleanUp.ReferenceOTU14619

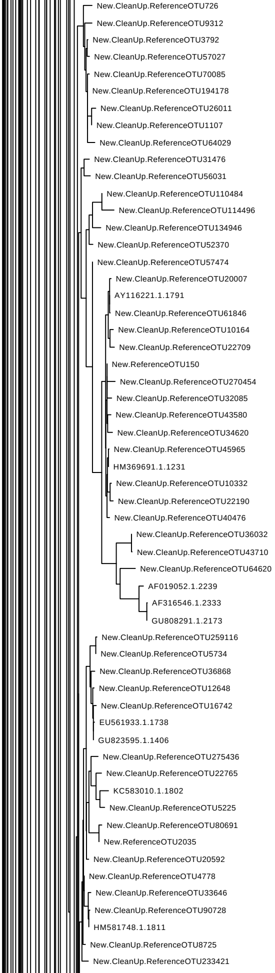
New.CleanUp.ReferenceOTU726
New.CleanUp.ReferenceOTU9312
New.CleanUp.ReferenceOTU3792
New.CleanUp.ReferenceOTU57027
New.CleanUp.ReferenceOTU70085
New.CleanUp.ReferenceOTU194178
New.CleanUp.ReferenceOTU26011
New.CleanUp.ReferenceOTU1107
New.CleanUp.ReferenceOTU64029
New.CleanUp.ReferenceOTU31476
New.CleanUp.ReferenceOTU56031
New.CleanUp.ReferenceOTU110484
New.CleanUp.ReferenceOTU114496
New.CleanUp.ReferenceOTU134946
New.CleanUp.ReferenceOTU52370
New.CleanUp.ReferenceOTU57474
New.CleanUp.ReferenceOTU20007
AY116221.1.1791
New.CleanUp.ReferenceOTU61846
New.CleanUp.ReferenceOTU10164
New.CleanUp.ReferenceOTU22709
New.ReferenceOTU150
New.CleanUp.ReferenceOTU270454
New.CleanUp.ReferenceOTU32085
New.CleanUp.ReferenceOTU43580
New.CleanUp.ReferenceOTU34620
New.CleanUp.ReferenceOTU45965
HM369691.1.1231
New.CleanUp.ReferenceOTU10332
New.CleanUp.ReferenceOTU22190
New.CleanUp.ReferenceOTU40476
New.CleanUp.ReferenceOTU36032
New.CleanUp.ReferenceOTU43710
New.CleanUp.ReferenceOTU64620
AF019052.1.2239
AF316546.1.2333
GU808291.1.2173
New.CleanUp.ReferenceOTU259116
New.CleanUp.ReferenceOTU5734
New.CleanUp.ReferenceOTU36868
New.CleanUp.ReferenceOTU12648
New.CleanUp.ReferenceOTU16742
EU561933.1.1738
GU823595.1.1406
New.CleanUp.ReferenceOTU275436
New.CleanUp.ReferenceOTU22765
KC583010.1.1802
New.CleanUp.ReferenceOTU5225
New.CleanUp.ReferenceOTU80691
New.ReferenceOTU2035
New.CleanUp.ReferenceOTU20592
New.CleanUp.ReferenceOTU4778
New.CleanUp.ReferenceOTU33646
New.CleanUp.ReferenceOTU90728
HM581748.1.1811
New.CleanUp.ReferenceOTU8725
New.CleanUp.ReferenceOTU233421

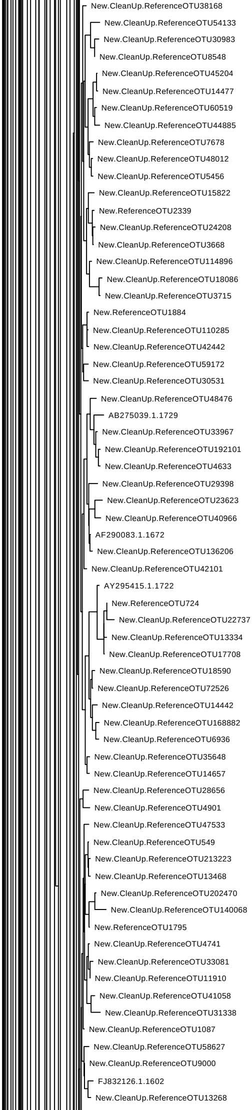
New.CleanUp.ReferenceOTU38168
New.CleanUp.ReferenceOTU54133
New.CleanUp.ReferenceOTU30983
New.CleanUp.ReferenceOTU8548
New.CleanUp.ReferenceOTU45204
New.CleanUp.ReferenceOTU14477
New.CleanUp.ReferenceOTU60519
New.CleanUp.ReferenceOTU44885
New.CleanUp.ReferenceOTU7678
New.CleanUp.ReferenceOTU48012
New.CleanUp.ReferenceOTU5456
New.CleanUp.ReferenceOTU15822
New.ReferenceOTU2339
New.CleanUp.ReferenceOTU24208
New.CleanUp.ReferenceOTU3668
New.CleanUp.ReferenceOTU114896
New.CleanUp.ReferenceOTU18086
New.CleanUp.ReferenceOTU3715
New.ReferenceOTU1884
New.CleanUp.ReferenceOTU110285
New.CleanUp.ReferenceOTU42442
New.CleanUp.ReferenceOTU59172
New.CleanUp.ReferenceOTU30531
New.CleanUp.ReferenceOTU48476
AB275039.1.1729
New.CleanUp.ReferenceOTU33967
New.CleanUp.ReferenceOTU192101
New.CleanUp.ReferenceOTU4633
New.CleanUp.ReferenceOTU29398
New.CleanUp.ReferenceOTU23623
New.CleanUp.ReferenceOTU40966
AF290083.1.1672
New.CleanUp.ReferenceOTU136206
New.CleanUp.ReferenceOTU42101
AY295415.1.1722
New.ReferenceOTU724
New.CleanUp.ReferenceOTU22737
New.CleanUp.ReferenceOTU13334
New.CleanUp.ReferenceOTU17708
New.CleanUp.ReferenceOTU18590
New.CleanUp.ReferenceOTU72526
New.CleanUp.ReferenceOTU14442
New.CleanUp.ReferenceOTU168882
New.CleanUp.ReferenceOTU6936
New.CleanUp.ReferenceOTU35648
New.CleanUp.ReferenceOTU14657
New.CleanUp.ReferenceOTU28656
New.CleanUp.ReferenceOTU4901
New.CleanUp.ReferenceOTU47533
New.CleanUp.ReferenceOTU549
New.CleanUp.ReferenceOTU213223
New.CleanUp.ReferenceOTU13468
New.CleanUp.ReferenceOTU202470
New.CleanUp.ReferenceOTU140068
New.ReferenceOTU1795
New.CleanUp.ReferenceOTU4741
New.CleanUp.ReferenceOTU33081
New.CleanUp.ReferenceOTU11910
New.CleanUp.ReferenceOTU41058
New.CleanUp.ReferenceOTU31338
New.CleanUp.ReferenceOTU1087
New.CleanUp.ReferenceOTU58627
New.CleanUp.ReferenceOTU9000
FJ832126.1.1602
New.CleanUp.ReferenceOTU13268

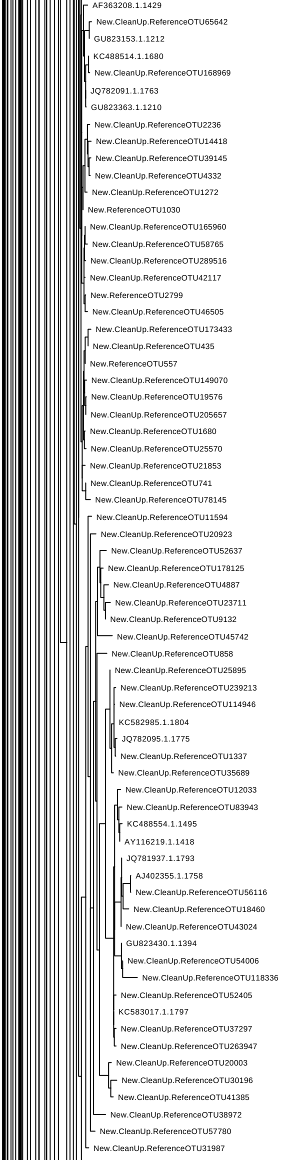
AF363208.1.1429
New.CleanUp.ReferenceOTU65642
GU823153.1.1212
KC488514.1.1680
New.CleanUp.ReferenceOTU168969
JQ782091.1.1763
GU823363.1.1210
New.CleanUp.ReferenceOTU2236
New.CleanUp.ReferenceOTU14418
New.CleanUp.ReferenceOTU39145
New.CleanUp.ReferenceOTU4332
New.CleanUp.ReferenceOTU1272
New.ReferenceOTU1030
New.CleanUp.ReferenceOTU165960
New.CleanUp.ReferenceOTU58765
New.CleanUp.ReferenceOTU289516
New.CleanUp.ReferenceOTU42117
New.ReferenceOTU2799
New.CleanUp.ReferenceOTU46505
New.CleanUp.ReferenceOTU173433
New.CleanUp.ReferenceOTU435
New.ReferenceOTU557
New.CleanUp.ReferenceOTU149070
New.CleanUp.ReferenceOTU19576
New.CleanUp.ReferenceOTU205657
New.CleanUp.ReferenceOTU1680
New.CleanUp.ReferenceOTU25570
New.CleanUp.ReferenceOTU21853
New.CleanUp.ReferenceOTU741
New.CleanUp.ReferenceOTU78145
New.CleanUp.ReferenceOTU11594
New.CleanUp.ReferenceOTU20923
New.CleanUp.ReferenceOTU52637
New.CleanUp.ReferenceOTU178125
New.CleanUp.ReferenceOTU4887
New.CleanUp.ReferenceOTU23711
New.CleanUp.ReferenceOTU9132
New.CleanUp.ReferenceOTU45742
New.CleanUp.ReferenceOTU858
New.CleanUp.ReferenceOTU25895
New.CleanUp.ReferenceOTU239213
New.CleanUp.ReferenceOTU114946
KC582985.1.1804
JQ782095.1.1775
New.CleanUp.ReferenceOTU1337
New.CleanUp.ReferenceOTU35689
New.CleanUp.ReferenceOTU12033
New.CleanUp.ReferenceOTU83943
KC488554.1.1495
AY116219.1.1418
JQ781937.1.1793
AJ402355.1.1758
New.CleanUp.ReferenceOTU56116
New.CleanUp.ReferenceOTU18460
New.CleanUp.ReferenceOTU43024
GU823430.1.1394
New.CleanUp.ReferenceOTU54006
New.CleanUp.ReferenceOTU118336
New.CleanUp.ReferenceOTU52405
KC583017.1.1797
New.CleanUp.ReferenceOTU37297
New.CleanUp.ReferenceOTU263947
New.CleanUp.ReferenceOTU20003
New.CleanUp.ReferenceOTU30196
New.CleanUp.ReferenceOTU41385
New.CleanUp.ReferenceOTU38972
New.CleanUp.ReferenceOTU57780
New.CleanUp.ReferenceOTU31987

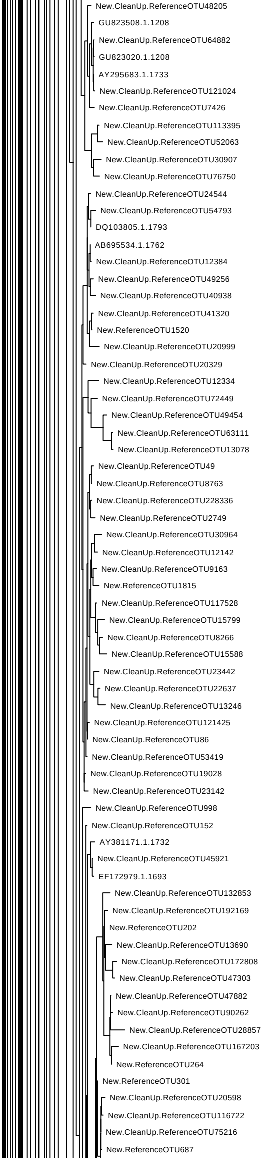
New.CleanUp.ReferenceOTU48205
GU823508.1.1208
New.CleanUp.ReferenceOTU64882
GU823020.1.1208
AY295683.1.1733
New.CleanUp.ReferenceOTU121024
New.CleanUp.ReferenceOTU7426
New.CleanUp.ReferenceOTU113395
New.CleanUp.ReferenceOTU52063
New.CleanUp.ReferenceOTU30907
New.CleanUp.ReferenceOTU76750
New.CleanUp.ReferenceOTU24544
New.CleanUp.ReferenceOTU54793
DQ103805.1.1793
AB695534.1.1762
New.CleanUp.ReferenceOTU12384
New.CleanUp.ReferenceOTU49256
New.CleanUp.ReferenceOTU40938
New.CleanUp.ReferenceOTU41320
New.ReferenceOTU1520
New.CleanUp.ReferenceOTU20999
New.CleanUp.ReferenceOTU20329
New.CleanUp.ReferenceOTU12334
New.CleanUp.ReferenceOTU72449
New.CleanUp.ReferenceOTU49454
New.CleanUp.ReferenceOTU63111
New.CleanUp.ReferenceOTU13078
New.CleanUp.ReferenceOTU49
New.CleanUp.ReferenceOTU8763
New.CleanUp.ReferenceOTU228336
New.CleanUp.ReferenceOTU2749
New.CleanUp.ReferenceOTU30964
New.CleanUp.ReferenceOTU12142
New.CleanUp.ReferenceOTU9163
New.ReferenceOTU1815
New.CleanUp.ReferenceOTU117528
New.CleanUp.ReferenceOTU15799
New.CleanUp.ReferenceOTU8266
New.CleanUp.ReferenceOTU15588
New.CleanUp.ReferenceOTU23442
New.CleanUp.ReferenceOTU22637
New.CleanUp.ReferenceOTU13246
New.CleanUp.ReferenceOTU121425
New.CleanUp.ReferenceOTU86
New.CleanUp.ReferenceOTU53419
New.CleanUp.ReferenceOTU19028
New.CleanUp.ReferenceOTU23142
New.CleanUp.ReferenceOTU998
New.CleanUp.ReferenceOTU152
AY381171.1.1732
New.CleanUp.ReferenceOTU45921
EF172979.1.1693
New.CleanUp.ReferenceOTU132853
New.CleanUp.ReferenceOTU192169
New.ReferenceOTU202
New.CleanUp.ReferenceOTU13690
New.CleanUp.ReferenceOTU172808
New.CleanUp.ReferenceOTU47303
New.CleanUp.ReferenceOTU47882
New.CleanUp.ReferenceOTU90262
New.CleanUp.ReferenceOTU28857
New.CleanUp.ReferenceOTU167203
New.ReferenceOTU264
New.ReferenceOTU301
New.CleanUp.ReferenceOTU20598
New.CleanUp.ReferenceOTU116722
New.CleanUp.ReferenceOTU75216
New.ReferenceOTU687

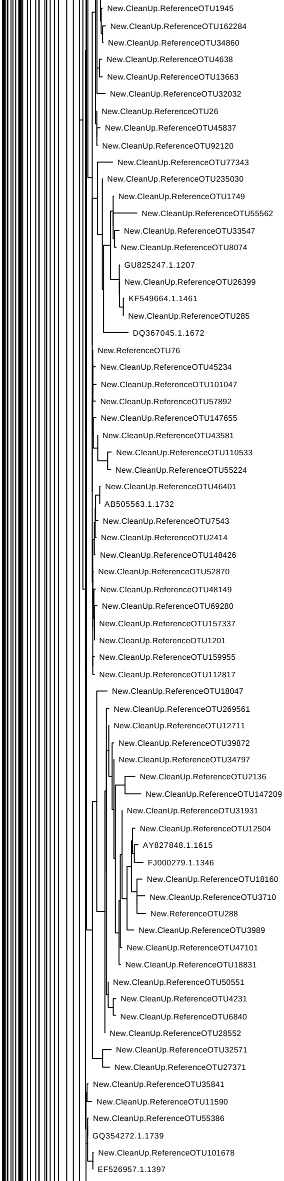
New.CleanUp.ReferenceOTU1945
New.CleanUp.ReferenceOTU162284
New.CleanUp.ReferenceOTU34860
New.CleanUp.ReferenceOTU4638
New.CleanUp.ReferenceOTU13663
New.CleanUp.ReferenceOTU32032
New.CleanUp.ReferenceOTU26
New.CleanUp.ReferenceOTU45837
New.CleanUp.ReferenceOTU92120
New.CleanUp.ReferenceOTU77343
New.CleanUp.ReferenceOTU235030
New.CleanUp.ReferenceOTU1749
New.CleanUp.ReferenceOTU55562
New.CleanUp.ReferenceOTU33547
New.CleanUp.ReferenceOTU8074
GU825247.1.1207
New.CleanUp.ReferenceOTU26399
KF549664.1.1461
New.CleanUp.ReferenceOTU285
DQ367045.1.1672
New.ReferenceOTU76
New.CleanUp.ReferenceOTU45234
New.CleanUp.ReferenceOTU101047
New.CleanUp.ReferenceOTU57892
New.CleanUp.ReferenceOTU147655
New.CleanUp.ReferenceOTU43581
New.CleanUp.ReferenceOTU110533
New.CleanUp.ReferenceOTU55224
New.CleanUp.ReferenceOTU46401
AB505563.1.1732
New.CleanUp.ReferenceOTU7543
New.CleanUp.ReferenceOTU2414
New.CleanUp.ReferenceOTU148426
New.CleanUp.ReferenceOTU52870
New.CleanUp.ReferenceOTU48149
New.CleanUp.ReferenceOTU69280
New.CleanUp.ReferenceOTU157337
New.CleanUp.ReferenceOTU1201
New.CleanUp.ReferenceOTU159955
New.CleanUp.ReferenceOTU112817
New.CleanUp.ReferenceOTU18047
New.CleanUp.ReferenceOTU269561
New.CleanUp.ReferenceOTU12711
New.CleanUp.ReferenceOTU39872
New.CleanUp.ReferenceOTU34797
New.CleanUp.ReferenceOTU2136
New.CleanUp.ReferenceOTU147209
New.CleanUp.ReferenceOTU31931
New.CleanUp.ReferenceOTU12504
AY827848.1.1615
FJ000279.1.1346
New.CleanUp.ReferenceOTU18160
New.CleanUp.ReferenceOTU3710
New.ReferenceOTU288
New.CleanUp.ReferenceOTU3989
New.CleanUp.ReferenceOTU47101
New.CleanUp.ReferenceOTU18831
New.CleanUp.ReferenceOTU50551
New.CleanUp.ReferenceOTU4231
New.CleanUp.ReferenceOTU6840
New.CleanUp.ReferenceOTU28552
New.CleanUp.ReferenceOTU32571
New.CleanUp.ReferenceOTU27371
New.CleanUp.ReferenceOTU35841
New.CleanUp.ReferenceOTU11590
New.CleanUp.ReferenceOTU55386
GQ354272.1.1739
New.CleanUp.ReferenceOTU101678
EF526957.1.1397

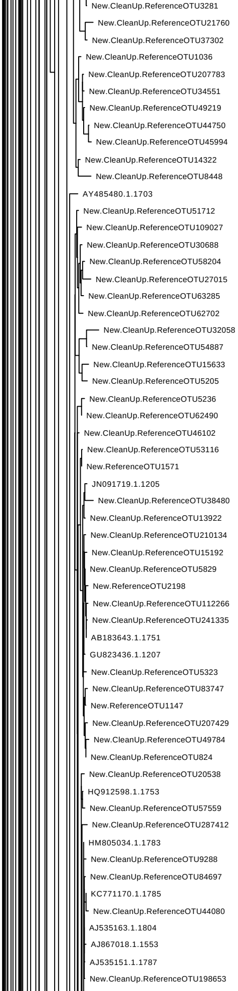
New.CleanUp.ReferenceOTU3281
New.CleanUp.ReferenceOTU21760
New.CleanUp.ReferenceOTU37302
New.CleanUp.ReferenceOTU1036
New.CleanUp.ReferenceOTU207783
New.CleanUp.ReferenceOTU34551
New.CleanUp.ReferenceOTU49219
New.CleanUp.ReferenceOTU44750
New.CleanUp.ReferenceOTU45994
New.CleanUp.ReferenceOTU14322
New.CleanUp.ReferenceOTU8448
AY485480.1.1703
New.CleanUp.ReferenceOTU51712
New.CleanUp.ReferenceOTU109027
New.CleanUp.ReferenceOTU30688
New.CleanUp.ReferenceOTU58204
New.CleanUp.ReferenceOTU27015
New.CleanUp.ReferenceOTU63285
New.CleanUp.ReferenceOTU62702
New.CleanUp.ReferenceOTU32058
New.CleanUp.ReferenceOTU54887
New.CleanUp.ReferenceOTU15633
New.CleanUp.ReferenceOTU5205
New.CleanUp.ReferenceOTU5236
New.CleanUp.ReferenceOTU62490
New.CleanUp.ReferenceOTU46102
New.CleanUp.ReferenceOTU53116
New.ReferenceOTU1571
JN091719.1.1205
New.CleanUp.ReferenceOTU38480
New.CleanUp.ReferenceOTU13922
New.CleanUp.ReferenceOTU210134
New.CleanUp.ReferenceOTU15192
New.CleanUp.ReferenceOTU5829
New.ReferenceOTU2198
New.CleanUp.ReferenceOTU112266
New.CleanUp.ReferenceOTU241335
AB183643.1.1751
GU823436.1.1207
New.CleanUp.ReferenceOTU5323
New.CleanUp.ReferenceOTU83747
New.ReferenceOTU1147
New.CleanUp.ReferenceOTU207429
New.CleanUp.ReferenceOTU49784
New.CleanUp.ReferenceOTU824
New.CleanUp.ReferenceOTU20538
HQ912598.1.1753
New.CleanUp.ReferenceOTU57559
New.CleanUp.ReferenceOTU287412
HM805034.1.1783
New.CleanUp.ReferenceOTU9288
New.CleanUp.ReferenceOTU84697
KC771170.1.1785
New.CleanUp.ReferenceOTU44080
AJ535163.1.1804
AJ867018.1.1553
AJ535151.1.1787
New.CleanUp.ReferenceOTU198653

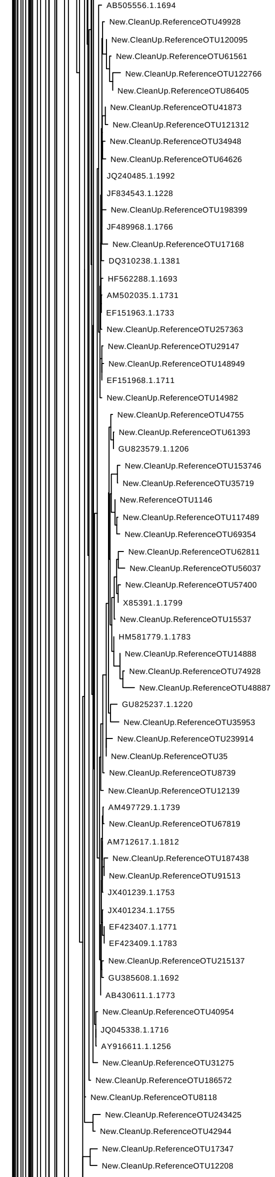
AB505556.1.1694
New.CleanUp.ReferenceOTU49928
New.CleanUp.ReferenceOTU120095
New.CleanUp.ReferenceOTU61561
New.CleanUp.ReferenceOTU122766
New.CleanUp.ReferenceOTU86405
New.CleanUp.ReferenceOTU41873
New.CleanUp.ReferenceOTU121312
New.CleanUp.ReferenceOTU34948
New.CleanUp.ReferenceOTU64626
JQ240485.1.1992
JF834543.1.1228
New.CleanUp.ReferenceOTU198399
JF489968.1.1766
New.CleanUp.ReferenceOTU17168
DQ310238.1.1381
HF562288.1.1693
AM502035.1.1731
EF151963.1.1733
New.CleanUp.ReferenceOTU257363
New.CleanUp.ReferenceOTU29147
New.CleanUp.ReferenceOTU148949
EF151968.1.1711
New.CleanUp.ReferenceOTU14982
New.CleanUp.ReferenceOTU4755
New.CleanUp.ReferenceOTU61393
GU823579.1.1206
New.CleanUp.ReferenceOTU153746
New.CleanUp.ReferenceOTU35719
New.ReferenceOTU1146
New.CleanUp.ReferenceOTU117489
New.CleanUp.ReferenceOTU69354
New.CleanUp.ReferenceOTU62811
New.CleanUp.ReferenceOTU56037
New.CleanUp.ReferenceOTU57400
X85391.1.1799
New.CleanUp.ReferenceOTU15537
HM581779.1.1783
New.CleanUp.ReferenceOTU14888
New.CleanUp.ReferenceOTU74928
New.CleanUp.ReferenceOTU48887
GU825237.1.1220
New.CleanUp.ReferenceOTU35953
New.CleanUp.ReferenceOTU239914
New.CleanUp.ReferenceOTU35
New.CleanUp.ReferenceOTU8739
New.CleanUp.ReferenceOTU12139
AM497729.1.1739
New.CleanUp.ReferenceOTU67819
AM712617.1.1812
New.CleanUp.ReferenceOTU187438
New.CleanUp.ReferenceOTU91513
JX401239.1.1753
JX401234.1.1755
EF423407.1.1771
EF423409.1.1783
New.CleanUp.ReferenceOTU215137
GU385608.1.1692
AB430611.1.1773
New.CleanUp.ReferenceOTU40954
JQ045338.1.1716
AY916611.1.1256
New.CleanUp.ReferenceOTU31275
New.CleanUp.ReferenceOTU186572
New.CleanUp.ReferenceOTU8118
New.CleanUp.ReferenceOTU243425
New.CleanUp.ReferenceOTU42944
New.CleanUp.ReferenceOTU17347
New.CleanUp.ReferenceOTU12208

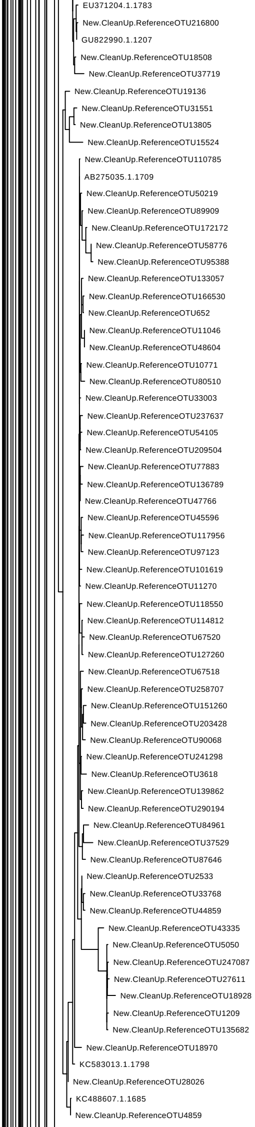
EU371204.1.1783
New.CleanUp.ReferenceOTU216800
GU822990.1.1207
New.CleanUp.ReferenceOTU18508
New.CleanUp.ReferenceOTU37719
New.CleanUp.ReferenceOTU19136
New.CleanUp.ReferenceOTU31551
New.CleanUp.ReferenceOTU13805
New.CleanUp.ReferenceOTU15524
New.CleanUp.ReferenceOTU110785
AB275035.1.1709
New.CleanUp.ReferenceOTU50219
New.CleanUp.ReferenceOTU89909
New.CleanUp.ReferenceOTU172172
New.CleanUp.ReferenceOTU58776
New.CleanUp.ReferenceOTU95388
New.CleanUp.ReferenceOTU133057
New.CleanUp.ReferenceOTU166530
New.CleanUp.ReferenceOTU652
New.CleanUp.ReferenceOTU11046
New.CleanUp.ReferenceOTU48604
New.CleanUp.ReferenceOTU10771
New.CleanUp.ReferenceOTU80510
New.CleanUp.ReferenceOTU33003
New.CleanUp.ReferenceOTU237637
New.CleanUp.ReferenceOTU54105
New.CleanUp.ReferenceOTU209504
New.CleanUp.ReferenceOTU77883
New.CleanUp.ReferenceOTU136789
New.CleanUp.ReferenceOTU47766
New.CleanUp.ReferenceOTU45596
New.CleanUp.ReferenceOTU117956
New.CleanUp.ReferenceOTU97123
New.CleanUp.ReferenceOTU101619
New.CleanUp.ReferenceOTU11270
New.CleanUp.ReferenceOTU118550
New.CleanUp.ReferenceOTU114812
New.CleanUp.ReferenceOTU67520
New.CleanUp.ReferenceOTU127260
New.CleanUp.ReferenceOTU67518
New.CleanUp.ReferenceOTU258707
New.CleanUp.ReferenceOTU151260
New.CleanUp.ReferenceOTU203428
New.CleanUp.ReferenceOTU90068
New.CleanUp.ReferenceOTU241298
New.CleanUp.ReferenceOTU3618
New.CleanUp.ReferenceOTU139862
New.CleanUp.ReferenceOTU290194
New.CleanUp.ReferenceOTU84961
New.CleanUp.ReferenceOTU37529
New.CleanUp.ReferenceOTU87646
New.CleanUp.ReferenceOTU2533
New.CleanUp.ReferenceOTU33768
New.CleanUp.ReferenceOTU44859
New.CleanUp.ReferenceOTU43335
New.CleanUp.ReferenceOTU5050
New.CleanUp.ReferenceOTU247087
New.CleanUp.ReferenceOTU27611
New.CleanUp.ReferenceOTU18928
New.CleanUp.ReferenceOTU1209
New.CleanUp.ReferenceOTU135682
New.CleanUp.ReferenceOTU18970
KC583013.1.1798
New.CleanUp.ReferenceOTU28026
KC488607.1.1685
New.CleanUp.ReferenceOTU4859

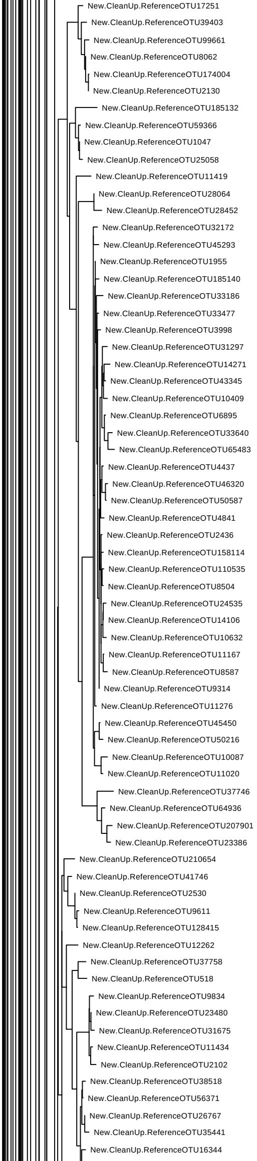
New.CleanUp.ReferenceOTU17251
New.CleanUp.ReferenceOTU39403
New.CleanUp.ReferenceOTU99661
New.CleanUp.ReferenceOTU8062
New.CleanUp.ReferenceOTU174004
New.CleanUp.ReferenceOTU2130
New.CleanUp.ReferenceOTU185132
New.CleanUp.ReferenceOTU59366
New.CleanUp.ReferenceOTU1047
New.CleanUp.ReferenceOTU25058
New.CleanUp.ReferenceOTU11419
New.CleanUp.ReferenceOTU28064
New.CleanUp.ReferenceOTU28452
New.CleanUp.ReferenceOTU32172
New.CleanUp.ReferenceOTU45293
New.CleanUp.ReferenceOTU1955
New.CleanUp.ReferenceOTU185140
New.CleanUp.ReferenceOTU33186
New.CleanUp.ReferenceOTU33477
New.CleanUp.ReferenceOTU3998
New.CleanUp.ReferenceOTU31297
New.CleanUp.ReferenceOTU14271
New.CleanUp.ReferenceOTU43345
New.CleanUp.ReferenceOTU10409
New.CleanUp.ReferenceOTU6895
New.CleanUp.ReferenceOTU33640
New.CleanUp.ReferenceOTU65483
New.CleanUp.ReferenceOTU4437
New.CleanUp.ReferenceOTU46320
New.CleanUp.ReferenceOTU50587
New.CleanUp.ReferenceOTU4841
New.CleanUp.ReferenceOTU2436
New.CleanUp.ReferenceOTU158114
New.CleanUp.ReferenceOTU110535
New.CleanUp.ReferenceOTU8504
New.CleanUp.ReferenceOTU24535
New.CleanUp.ReferenceOTU14106
New.CleanUp.ReferenceOTU10632
New.CleanUp.ReferenceOTU11167
New.CleanUp.ReferenceOTU8587
New.CleanUp.ReferenceOTU9314
New.CleanUp.ReferenceOTU11276
New.CleanUp.ReferenceOTU45450
New.CleanUp.ReferenceOTU50216
New.CleanUp.ReferenceOTU10087
New.CleanUp.ReferenceOTU11020
New.CleanUp.ReferenceOTU37746
New.CleanUp.ReferenceOTU64936
New.CleanUp.ReferenceOTU207901
New.CleanUp.ReferenceOTU23386
New.CleanUp.ReferenceOTU210654
New.CleanUp.ReferenceOTU41746
New.CleanUp.ReferenceOTU2530
New.CleanUp.ReferenceOTU9611
New.CleanUp.ReferenceOTU128415
New.CleanUp.ReferenceOTU12262
New.CleanUp.ReferenceOTU37758
New.CleanUp.ReferenceOTU518
New.CleanUp.ReferenceOTU9834
New.CleanUp.ReferenceOTU23480
New.CleanUp.ReferenceOTU31675
New.CleanUp.ReferenceOTU11434
New.CleanUp.ReferenceOTU2102
New.CleanUp.ReferenceOTU38518
New.CleanUp.ReferenceOTU56371
New.CleanUp.ReferenceOTU26767
New.CleanUp.ReferenceOTU35441
New.CleanUp.ReferenceOTU16344

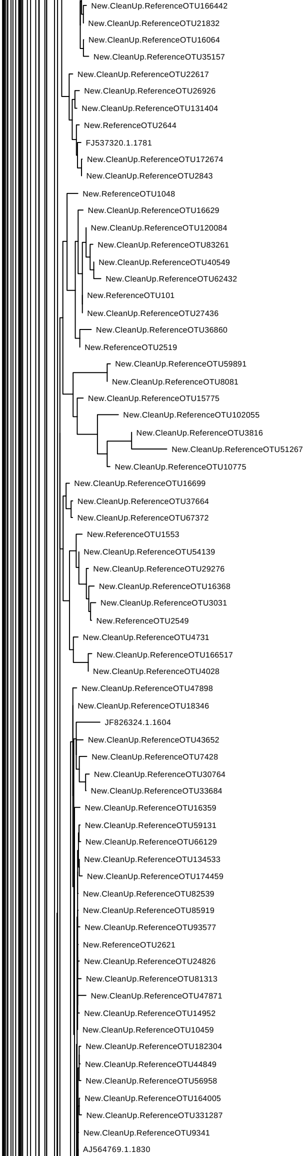
New.CleanUp.ReferenceOTU166442
New.CleanUp.ReferenceOTU21832
New.CleanUp.ReferenceOTU16064
New.CleanUp.ReferenceOTU35157
New.CleanUp.ReferenceOTU22617
New.CleanUp.ReferenceOTU26926
New.CleanUp.ReferenceOTU131404
New.ReferenceOTU2644
FJ537320.1.1781
New.CleanUp.ReferenceOTU172674
New.CleanUp.ReferenceOTU2843
New.ReferenceOTU1048
New.CleanUp.ReferenceOTU16629
New.CleanUp.ReferenceOTU120084
New.CleanUp.ReferenceOTU83261
New.CleanUp.ReferenceOTU40549
New.CleanUp.ReferenceOTU62432
New.ReferenceOTU101
New.CleanUp.ReferenceOTU27436
New.CleanUp.ReferenceOTU36860
New.ReferenceOTU2519
New.CleanUp.ReferenceOTU59891
New.CleanUp.ReferenceOTU8081
New.CleanUp.ReferenceOTU15775
New.CleanUp.ReferenceOTU102055
New.CleanUp.ReferenceOTU3816
New.CleanUp.ReferenceOTU51267
New.CleanUp.ReferenceOTU10775
New.CleanUp.ReferenceOTU16699
New.CleanUp.ReferenceOTU37664
New.CleanUp.ReferenceOTU67372
New.ReferenceOTU1553
New.CleanUp.ReferenceOTU54139
New.CleanUp.ReferenceOTU29276
New.CleanUp.ReferenceOTU16368
New.CleanUp.ReferenceOTU3031
New.ReferenceOTU2549
New.CleanUp.ReferenceOTU4731
New.CleanUp.ReferenceOTU166517
New.CleanUp.ReferenceOTU4028
New.CleanUp.ReferenceOTU47898
New.CleanUp.ReferenceOTU18346
JF826324.1.1604
New.CleanUp.ReferenceOTU43652
New.CleanUp.ReferenceOTU7428
New.CleanUp.ReferenceOTU30764
New.CleanUp.ReferenceOTU33684
New.CleanUp.ReferenceOTU16359
New.CleanUp.ReferenceOTU59131
New.CleanUp.ReferenceOTU66129
New.CleanUp.ReferenceOTU134533
New.CleanUp.ReferenceOTU174459
New.CleanUp.ReferenceOTU82539
New.CleanUp.ReferenceOTU85919
New.CleanUp.ReferenceOTU93577
New.ReferenceOTU2621
New.CleanUp.ReferenceOTU24826
New.CleanUp.ReferenceOTU81313
New.CleanUp.ReferenceOTU47871
New.CleanUp.ReferenceOTU14952
New.CleanUp.ReferenceOTU10459
New.CleanUp.ReferenceOTU182304
New.CleanUp.ReferenceOTU44849
New.CleanUp.ReferenceOTU56958
New.CleanUp.ReferenceOTU164005
New.CleanUp.ReferenceOTU331287
New.CleanUp.ReferenceOTU9341
AJ564769.1.1830

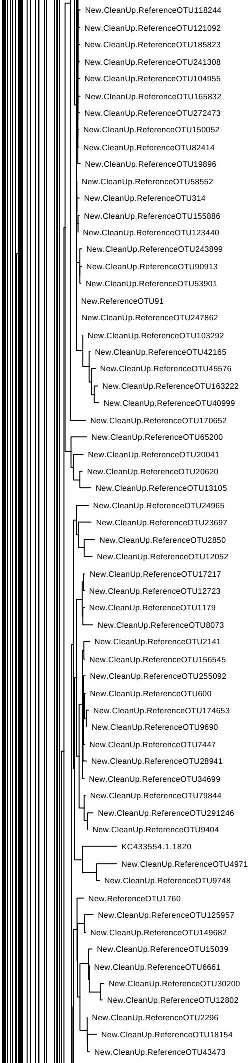
New.CleanUp.ReferenceOTU118244
New.CleanUp.ReferenceOTU121092
New.CleanUp.ReferenceOTU185823
New.CleanUp.ReferenceOTU241308
New.CleanUp.ReferenceOTU104955
New.CleanUp.ReferenceOTU165832
New.CleanUp.ReferenceOTU272473
New.CleanUp.ReferenceOTU150052
New.CleanUp.ReferenceOTU82414
New.CleanUp.ReferenceOTU19896
New.CleanUp.ReferenceOTU58552
New.CleanUp.ReferenceOTU314
New.CleanUp.ReferenceOTU155886
New.CleanUp.ReferenceOTU123440
New.CleanUp.ReferenceOTU243899
New.CleanUp.ReferenceOTU90913
New.CleanUp.ReferenceOTU53901
New.ReferenceOTU91
New.CleanUp.ReferenceOTU247862
New.CleanUp.ReferenceOTU103292
New.CleanUp.ReferenceOTU42165
New.CleanUp.ReferenceOTU45576
New.CleanUp.ReferenceOTU163222
New.CleanUp.ReferenceOTU40999
New.CleanUp.ReferenceOTU170652
New.CleanUp.ReferenceOTU65200
New.CleanUp.ReferenceOTU20041
New.CleanUp.ReferenceOTU20620
New.CleanUp.ReferenceOTU13105
New.CleanUp.ReferenceOTU24965
New.CleanUp.ReferenceOTU23697
New.CleanUp.ReferenceOTU2850
New.CleanUp.ReferenceOTU12052
New.CleanUp.ReferenceOTU17217
New.CleanUp.ReferenceOTU12723
New.CleanUp.ReferenceOTU1179
New.CleanUp.ReferenceOTU8073
New.CleanUp.ReferenceOTU2141
New.CleanUp.ReferenceOTU156545
New.CleanUp.ReferenceOTU255092
New.CleanUp.ReferenceOTU600
New.CleanUp.ReferenceOTU174653
New.CleanUp.ReferenceOTU9690
New.CleanUp.ReferenceOTU7447
New.CleanUp.ReferenceOTU28941
New.CleanUp.ReferenceOTU34699
New.CleanUp.ReferenceOTU79844
New.CleanUp.ReferenceOTU291246
New.CleanUp.ReferenceOTU9404
KC433554.1.1820
New.CleanUp.ReferenceOTU4971
New.CleanUp.ReferenceOTU9748
New.ReferenceOTU1760
New.CleanUp.ReferenceOTU125957
New.CleanUp.ReferenceOTU149682
New.CleanUp.ReferenceOTU15039
New.CleanUp.ReferenceOTU6661
New.CleanUp.ReferenceOTU30200
New.CleanUp.ReferenceOTU12802
New.CleanUp.ReferenceOTU2296
New.CleanUp.ReferenceOTU18154
New.CleanUp.ReferenceOTU43473

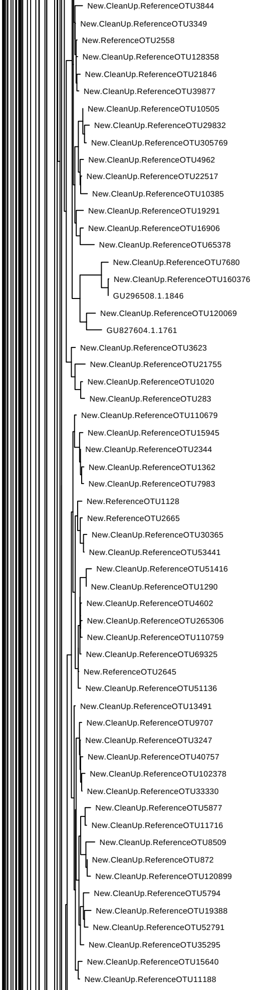
New.CleanUp.ReferenceOTU3844
New.CleanUp.ReferenceOTU3349
New.ReferenceOTU2558
New.CleanUp.ReferenceOTU128358
New.CleanUp.ReferenceOTU21846
New.CleanUp.ReferenceOTU39877
New.CleanUp.ReferenceOTU10505
New.CleanUp.ReferenceOTU29832
New.CleanUp.ReferenceOTU305769
New.CleanUp.ReferenceOTU4962
New.CleanUp.ReferenceOTU22517
New.CleanUp.ReferenceOTU10385
New.CleanUp.ReferenceOTU19291
New.CleanUp.ReferenceOTU16906
New.CleanUp.ReferenceOTU65378
New.CleanUp.ReferenceOTU7680
New.CleanUp.ReferenceOTU160376
GU296508.1.1846
New.CleanUp.ReferenceOTU120069
GU827604.1.1761
New.CleanUp.ReferenceOTU3623
New.CleanUp.ReferenceOTU21755
New.CleanUp.ReferenceOTU1020
New.CleanUp.ReferenceOTU283
New.CleanUp.ReferenceOTU110679
New.CleanUp.ReferenceOTU15945
New.CleanUp.ReferenceOTU2344
New.CleanUp.ReferenceOTU1362
New.CleanUp.ReferenceOTU7983
New.ReferenceOTU1128
New.ReferenceOTU2665
New.CleanUp.ReferenceOTU30365
New.CleanUp.ReferenceOTU53441
New.CleanUp.ReferenceOTU51416
New.CleanUp.ReferenceOTU1290
New.CleanUp.ReferenceOTU4602
New.CleanUp.ReferenceOTU265306
New.CleanUp.ReferenceOTU110759
New.CleanUp.ReferenceOTU69325
New.ReferenceOTU2645
New.CleanUp.ReferenceOTU51136
New.CleanUp.ReferenceOTU13491
New.CleanUp.ReferenceOTU9707
New.CleanUp.ReferenceOTU3247
New.CleanUp.ReferenceOTU40757
New.CleanUp.ReferenceOTU102378
New.CleanUp.ReferenceOTU33330
New.CleanUp.ReferenceOTU5877
New.CleanUp.ReferenceOTU11716
New.CleanUp.ReferenceOTU8509
New.CleanUp.ReferenceOTU872
New.CleanUp.ReferenceOTU120899
New.CleanUp.ReferenceOTU5794
New.CleanUp.ReferenceOTU19388
New.CleanUp.ReferenceOTU52791
New.CleanUp.ReferenceOTU35295
New.CleanUp.ReferenceOTU15640
New.CleanUp.ReferenceOTU11188

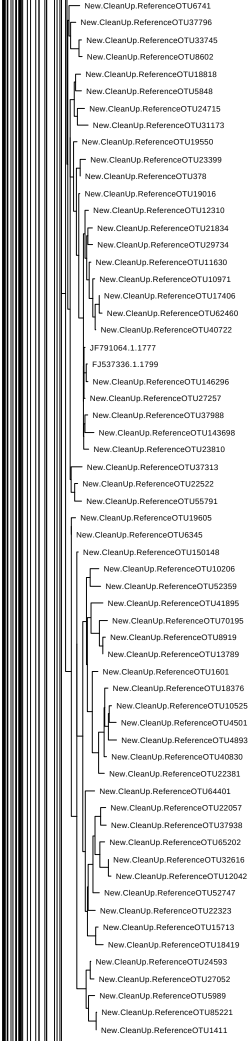
New.CleanUp.ReferenceOTU6741
New.CleanUp.ReferenceOTU37796
New.CleanUp.ReferenceOTU33745
New.CleanUp.ReferenceOTU8602
New.CleanUp.ReferenceOTU18818
New.CleanUp.ReferenceOTU5848
New.CleanUp.ReferenceOTU24715
New.CleanUp.ReferenceOTU31173
New.CleanUp.ReferenceOTU19550
New.CleanUp.ReferenceOTU23399
New.CleanUp.ReferenceOTU378
New.CleanUp.ReferenceOTU19016
New.CleanUp.ReferenceOTU12310
New.CleanUp.ReferenceOTU21834
New.CleanUp.ReferenceOTU29734
New.CleanUp.ReferenceOTU11630
New.CleanUp.ReferenceOTU10971
New.CleanUp.ReferenceOTU17406
New.CleanUp.ReferenceOTU62460
New.CleanUp.ReferenceOTU40722
JF791064.1.1777
FJ537336.1.1799
New.CleanUp.ReferenceOTU146296
New.CleanUp.ReferenceOTU27257
New.CleanUp.ReferenceOTU37988
New.CleanUp.ReferenceOTU143698
New.CleanUp.ReferenceOTU23810
New.CleanUp.ReferenceOTU37313
New.CleanUp.ReferenceOTU22522
New.CleanUp.ReferenceOTU55791
New.CleanUp.ReferenceOTU19605
New.CleanUp.ReferenceOTU6345
New.CleanUp.ReferenceOTU150148
New.CleanUp.ReferenceOTU10206
New.CleanUp.ReferenceOTU52359
New.CleanUp.ReferenceOTU41895
New.CleanUp.ReferenceOTU70195
New.CleanUp.ReferenceOTU8919
New.CleanUp.ReferenceOTU13789
New.CleanUp.ReferenceOTU1601
New.CleanUp.ReferenceOTU18376
New.CleanUp.ReferenceOTU10525
New.CleanUp.ReferenceOTU4501
New.CleanUp.ReferenceOTU4893
New.CleanUp.ReferenceOTU40830
New.CleanUp.ReferenceOTU22381
New.CleanUp.ReferenceOTU64401
New.CleanUp.ReferenceOTU22057
New.CleanUp.ReferenceOTU37938
New.CleanUp.ReferenceOTU65202
New.CleanUp.ReferenceOTU32616
New.CleanUp.ReferenceOTU12042
New.CleanUp.ReferenceOTU52747
New.CleanUp.ReferenceOTU22323
New.CleanUp.ReferenceOTU15713
New.CleanUp.ReferenceOTU18419
New.CleanUp.ReferenceOTU24593
New.CleanUp.ReferenceOTU27052
New.CleanUp.ReferenceOTU5989
New.CleanUp.ReferenceOTU85221
New.CleanUp.ReferenceOTU1411

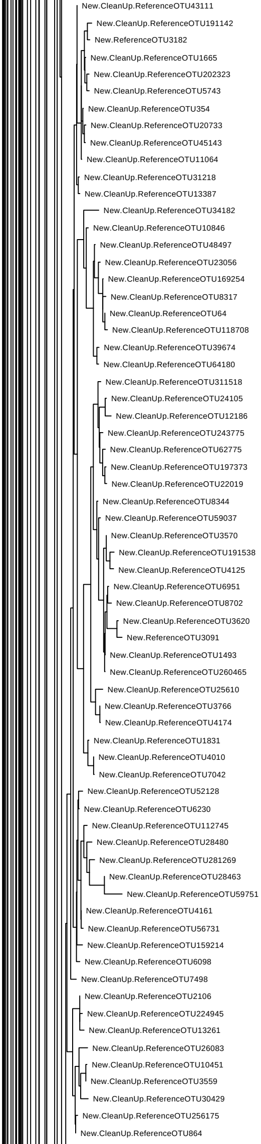
New.CleanUp.ReferenceOTU43111
New.CleanUp.ReferenceOTU191142
New.ReferenceOTU3182
New.CleanUp.ReferenceOTU1665
New.CleanUp.ReferenceOTU202323
New.CleanUp.ReferenceOTU5743
New.CleanUp.ReferenceOTU354
New.CleanUp.ReferenceOTU20733
New.CleanUp.ReferenceOTU45143
New.CleanUp.ReferenceOTU11064
New.CleanUp.ReferenceOTU31218
New.CleanUp.ReferenceOTU13387
New.CleanUp.ReferenceOTU34182
New.CleanUp.ReferenceOTU10846
New.CleanUp.ReferenceOTU48497
New.CleanUp.ReferenceOTU23056
New.CleanUp.ReferenceOTU169254
New.CleanUp.ReferenceOTU8317
New.CleanUp.ReferenceOTU64
New.CleanUp.ReferenceOTU118708
New.CleanUp.ReferenceOTU39674
New.CleanUp.ReferenceOTU64180
New.CleanUp.ReferenceOTU311518
New.CleanUp.ReferenceOTU24105
New.CleanUp.ReferenceOTU12186
New.CleanUp.ReferenceOTU243775
New.CleanUp.ReferenceOTU62775
New.CleanUp.ReferenceOTU197373
New.CleanUp.ReferenceOTU22019
New.CleanUp.ReferenceOTU8344
New.CleanUp.ReferenceOTU59037
New.CleanUp.ReferenceOTU3570
New.CleanUp.ReferenceOTU191538
New.CleanUp.ReferenceOTU4125
New.CleanUp.ReferenceOTU6951
New.CleanUp.ReferenceOTU8702
New.CleanUp.ReferenceOTU3620
New.ReferenceOTU3091
New.CleanUp.ReferenceOTU1493
New.CleanUp.ReferenceOTU260465
New.CleanUp.ReferenceOTU25610
New.CleanUp.ReferenceOTU3766
New.CleanUp.ReferenceOTU4174
New.CleanUp.ReferenceOTU1831
New.CleanUp.ReferenceOTU4010
New.CleanUp.ReferenceOTU7042
New.CleanUp.ReferenceOTU52128
New.CleanUp.ReferenceOTU6230
New.CleanUp.ReferenceOTU112745
New.CleanUp.ReferenceOTU28480
New.CleanUp.ReferenceOTU281269
New.CleanUp.ReferenceOTU28463
New.CleanUp.ReferenceOTU59751
New.CleanUp.ReferenceOTU4161
New.CleanUp.ReferenceOTU56731
New.CleanUp.ReferenceOTU159214
New.CleanUp.ReferenceOTU6098
New.CleanUp.ReferenceOTU7498
New.CleanUp.ReferenceOTU2106
New.CleanUp.ReferenceOTU224945
New.CleanUp.ReferenceOTU13261
New.CleanUp.ReferenceOTU26083
New.CleanUp.ReferenceOTU10451
New.CleanUp.ReferenceOTU3559
New.CleanUp.ReferenceOTU30429
New.CleanUp.ReferenceOTU256175
New.CleanUp.ReferenceOTU864

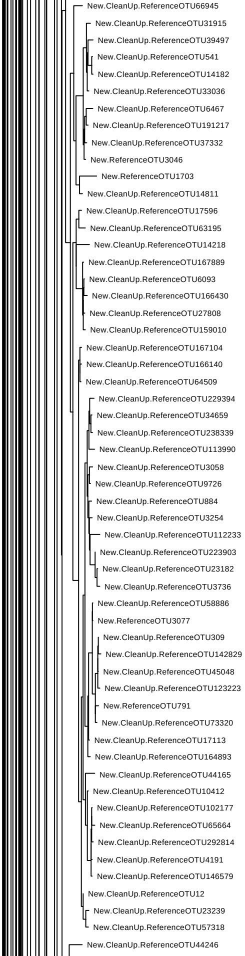
New.CleanUp.ReferenceOTU66945
New.CleanUp.ReferenceOTU31915
New.CleanUp.ReferenceOTU39497
New.CleanUp.ReferenceOTU541
New.CleanUp.ReferenceOTU14182
New.CleanUp.ReferenceOTU33036
New.CleanUp.ReferenceOTU6467
New.CleanUp.ReferenceOTU191217
New.CleanUp.ReferenceOTU37332
New.ReferenceOTU3046
New.ReferenceOTU1703
New.CleanUp.ReferenceOTU14811
New.CleanUp.ReferenceOTU17596
New.CleanUp.ReferenceOTU63195
New.CleanUp.ReferenceOTU14218
New.CleanUp.ReferenceOTU167889
New.CleanUp.ReferenceOTU6093
New.CleanUp.ReferenceOTU166430
New.CleanUp.ReferenceOTU27808
New.CleanUp.ReferenceOTU159010
New.CleanUp.ReferenceOTU167104
New.CleanUp.ReferenceOTU166140
New.CleanUp.ReferenceOTU64509
New.CleanUp.ReferenceOTU229394
New.CleanUp.ReferenceOTU34659
New.CleanUp.ReferenceOTU238339
New.CleanUp.ReferenceOTU113990
New.CleanUp.ReferenceOTU3058
New.CleanUp.ReferenceOTU9726
New.CleanUp.ReferenceOTU884
New.CleanUp.ReferenceOTU3254
New.CleanUp.ReferenceOTU112233
New.CleanUp.ReferenceOTU223903
New.CleanUp.ReferenceOTU23182
New.CleanUp.ReferenceOTU3736
New.CleanUp.ReferenceOTU58886
New.ReferenceOTU3077
New.CleanUp.ReferenceOTU309
New.CleanUp.ReferenceOTU142829
New.CleanUp.ReferenceOTU45048
New.CleanUp.ReferenceOTU123223
New.ReferenceOTU791
New.CleanUp.ReferenceOTU73320
New.CleanUp.ReferenceOTU17113
New.CleanUp.ReferenceOTU164893
New.CleanUp.ReferenceOTU44165
New.CleanUp.ReferenceOTU10412
New.CleanUp.ReferenceOTU102177
New.CleanUp.ReferenceOTU65664
New.CleanUp.ReferenceOTU292814
New.CleanUp.ReferenceOTU4191
New.CleanUp.ReferenceOTU146579
New.CleanUp.ReferenceOTU12
New.CleanUp.ReferenceOTU23239
New.CleanUp.ReferenceOTU57318
New.CleanUp.ReferenceOTU44246

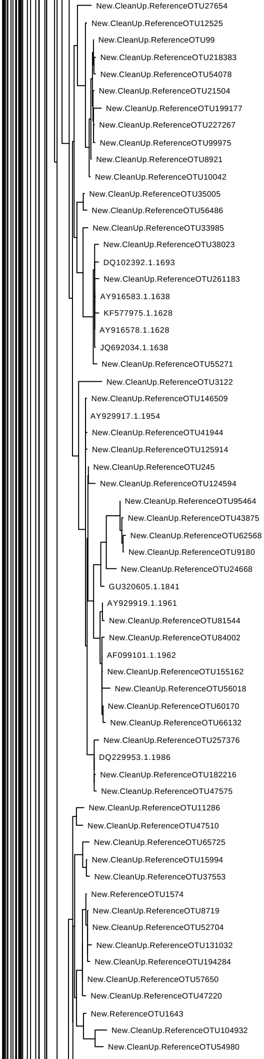
New.CleanUp.ReferenceOTU27654
New.CleanUp.ReferenceOTU12525
New.CleanUp.ReferenceOTU99
New.CleanUp.ReferenceOTU218383
New.CleanUp.ReferenceOTU54078
New.CleanUp.ReferenceOTU21504
New.CleanUp.ReferenceOTU199177
New.CleanUp.ReferenceOTU227267
New.CleanUp.ReferenceOTU99975
New.CleanUp.ReferenceOTU8921
New.CleanUp.ReferenceOTU10042
New.CleanUp.ReferenceOTU35005
New.CleanUp.ReferenceOTU56486
New.CleanUp.ReferenceOTU33985
New.CleanUp.ReferenceOTU38023
DQ102392.1.1693
New.CleanUp.ReferenceOTU261183
AY916583.1.1638
KF577975.1.1628
AY916578.1.1628
JQ692034.1.1638
New.CleanUp.ReferenceOTU55271
New.CleanUp.ReferenceOTU3122
New.CleanUp.ReferenceOTU146509
AY929917.1.1954
New.CleanUp.ReferenceOTU41944
New.CleanUp.ReferenceOTU125914
New.CleanUp.ReferenceOTU245
New.CleanUp.ReferenceOTU124594
New.CleanUp.ReferenceOTU95464
New.CleanUp.ReferenceOTU43875
New.CleanUp.ReferenceOTU62568
New.CleanUp.ReferenceOTU9180
New.CleanUp.ReferenceOTU24668
GU320605.1.1841
AY929919.1.1961
New.CleanUp.ReferenceOTU81544
New.CleanUp.ReferenceOTU84002
AF099101.1.1962
New.CleanUp.ReferenceOTU155162
New.CleanUp.ReferenceOTU56018
New.CleanUp.ReferenceOTU60170
New.CleanUp.ReferenceOTU66132
New.CleanUp.ReferenceOTU257376
DQ229953.1.1986
New.CleanUp.ReferenceOTU182216
New.CleanUp.ReferenceOTU47575
New.CleanUp.ReferenceOTU11286
New.CleanUp.ReferenceOTU47510
New.CleanUp.ReferenceOTU65725
New.CleanUp.ReferenceOTU15994
New.CleanUp.ReferenceOTU37553
New.ReferenceOTU1574
New.CleanUp.ReferenceOTU8719
New.CleanUp.ReferenceOTU52704
New.CleanUp.ReferenceOTU131032
New.CleanUp.ReferenceOTU194284
New.CleanUp.ReferenceOTU57650
New.CleanUp.ReferenceOTU47220
New.ReferenceOTU1643
New.CleanUp.ReferenceOTU104932
New.CleanUp.ReferenceOTU54980

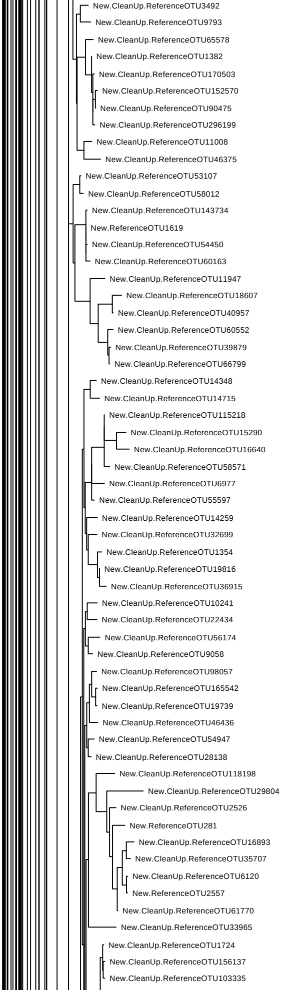
New.CleanUp.ReferenceOTU3492
New.CleanUp.ReferenceOTU9793
New.CleanUp.ReferenceOTU65578
New.CleanUp.ReferenceOTU1382
New.CleanUp.ReferenceOTU170503
New.CleanUp.ReferenceOTU152570
New.CleanUp.ReferenceOTU90475
New.CleanUp.ReferenceOTU296199
New.CleanUp.ReferenceOTU11008
New.CleanUp.ReferenceOTU46375
New.CleanUp.ReferenceOTU53107
New.CleanUp.ReferenceOTU58012
New.CleanUp.ReferenceOTU143734
New.ReferenceOTU1619
New.CleanUp.ReferenceOTU54450
New.CleanUp.ReferenceOTU60163
New.CleanUp.ReferenceOTU11947
New.CleanUp.ReferenceOTU18607
New.CleanUp.ReferenceOTU40957
New.CleanUp.ReferenceOTU60552
New.CleanUp.ReferenceOTU39879
New.CleanUp.ReferenceOTU66799
New.CleanUp.ReferenceOTU14348
New.CleanUp.ReferenceOTU14715
New.CleanUp.ReferenceOTU115218
New.CleanUp.ReferenceOTU15290
New.CleanUp.ReferenceOTU16640
New.CleanUp.ReferenceOTU58571
New.CleanUp.ReferenceOTU6977
New.CleanUp.ReferenceOTU55597
New.CleanUp.ReferenceOTU14259
New.CleanUp.ReferenceOTU32699
New.CleanUp.ReferenceOTU1354
New.CleanUp.ReferenceOTU19816
New.CleanUp.ReferenceOTU36915
New.CleanUp.ReferenceOTU10241
New.CleanUp.ReferenceOTU22434
New.CleanUp.ReferenceOTU56174
New.CleanUp.ReferenceOTU9058
New.CleanUp.ReferenceOTU98057
New.CleanUp.ReferenceOTU165542
New.CleanUp.ReferenceOTU19739
New.CleanUp.ReferenceOTU46436
New.CleanUp.ReferenceOTU54947
New.CleanUp.ReferenceOTU28138
New.CleanUp.ReferenceOTU118198
New.CleanUp.ReferenceOTU29804
New.CleanUp.ReferenceOTU2526
New.ReferenceOTU281
New.CleanUp.ReferenceOTU16893
New.CleanUp.ReferenceOTU35707
New.CleanUp.ReferenceOTU6120
New.ReferenceOTU2557
New.CleanUp.ReferenceOTU61770
New.CleanUp.ReferenceOTU33965
New.CleanUp.ReferenceOTU1724
New.CleanUp.ReferenceOTU156137
New.CleanUp.ReferenceOTU103335

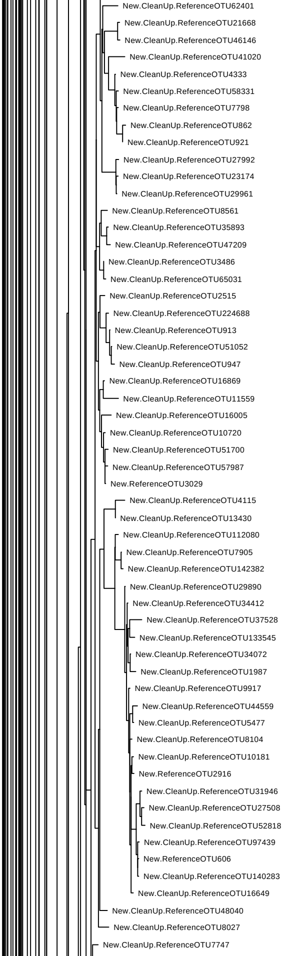
New.CleanUp.ReferenceOTU62401
New.CleanUp.ReferenceOTU21668
New.CleanUp.ReferenceOTU46146
New.CleanUp.ReferenceOTU41020
New.CleanUp.ReferenceOTU4333
New.CleanUp.ReferenceOTU58331
New.CleanUp.ReferenceOTU7798
New.CleanUp.ReferenceOTU862
New.CleanUp.ReferenceOTU921
New.CleanUp.ReferenceOTU27992
New.CleanUp.ReferenceOTU23174
New.CleanUp.ReferenceOTU29961
New.CleanUp.ReferenceOTU8561
New.CleanUp.ReferenceOTU35893
New.CleanUp.ReferenceOTU47209
New.CleanUp.ReferenceOTU3486
New.CleanUp.ReferenceOTU65031
New.CleanUp.ReferenceOTU2515
New.CleanUp.ReferenceOTU224688
New.CleanUp.ReferenceOTU913
New.CleanUp.ReferenceOTU51052
New.CleanUp.ReferenceOTU947
New.CleanUp.ReferenceOTU16869
New.CleanUp.ReferenceOTU11559
New.CleanUp.ReferenceOTU16005
New.CleanUp.ReferenceOTU10720
New.CleanUp.ReferenceOTU51700
New.CleanUp.ReferenceOTU57987
New.ReferenceOTU3029
New.CleanUp.ReferenceOTU4115
New.CleanUp.ReferenceOTU13430
New.CleanUp.ReferenceOTU112080
New.CleanUp.ReferenceOTU7905
New.CleanUp.ReferenceOTU142382
New.CleanUp.ReferenceOTU29890
New.CleanUp.ReferenceOTU34412
New.CleanUp.ReferenceOTU37528
New.CleanUp.ReferenceOTU133545
New.CleanUp.ReferenceOTU34072
New.CleanUp.ReferenceOTU1987
New.CleanUp.ReferenceOTU9917
New.CleanUp.ReferenceOTU44559
New.CleanUp.ReferenceOTU5477
New.CleanUp.ReferenceOTU8104
New.CleanUp.ReferenceOTU10181
New.ReferenceOTU2916
New.CleanUp.ReferenceOTU31946
New.CleanUp.ReferenceOTU27508
New.CleanUp.ReferenceOTU52818
New.CleanUp.ReferenceOTU97439
New.ReferenceOTU606
New.CleanUp.ReferenceOTU140283
New.CleanUp.ReferenceOTU16649
New.CleanUp.ReferenceOTU48040
New.CleanUp.ReferenceOTU8027
New.CleanUp.ReferenceOTU7747

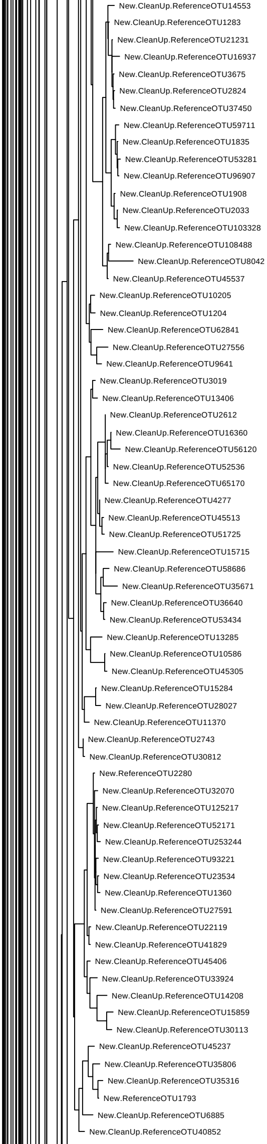

New.CleanUp.ReferenceOTU14553
New.CleanUp.ReferenceOTU1283
New.CleanUp.ReferenceOTU21231
New.CleanUp.ReferenceOTU16937
New.CleanUp.ReferenceOTU3675
New.CleanUp.ReferenceOTU2824
New.CleanUp.ReferenceOTU37450
New.CleanUp.ReferenceOTU59711
New.CleanUp.ReferenceOTU1835
New.CleanUp.ReferenceOTU53281
New.CleanUp.ReferenceOTU96907
New.CleanUp.ReferenceOTU1908
New.CleanUp.ReferenceOTU2033
New.CleanUp.ReferenceOTU103328
New.CleanUp.ReferenceOTU108488
New.CleanUp.ReferenceOTU8042
New.CleanUp.ReferenceOTU45537
New.CleanUp.ReferenceOTU10205
New.CleanUp.ReferenceOTU1204
New.CleanUp.ReferenceOTU62841
New.CleanUp.ReferenceOTU27556
New.CleanUp.ReferenceOTU9641
New.CleanUp.ReferenceOTU3019
New.CleanUp.ReferenceOTU13406
New.CleanUp.ReferenceOTU2612
New.CleanUp.ReferenceOTU16360
New.CleanUp.ReferenceOTU56120
New.CleanUp.ReferenceOTU52536
New.CleanUp.ReferenceOTU65170
New.CleanUp.ReferenceOTU4277
New.CleanUp.ReferenceOTU45513
New.CleanUp.ReferenceOTU51725
New.CleanUp.ReferenceOTU15715
New.CleanUp.ReferenceOTU58686
New.CleanUp.ReferenceOTU35671
New.CleanUp.ReferenceOTU36640
New.CleanUp.ReferenceOTU53434
New.CleanUp.ReferenceOTU13285
New.CleanUp.ReferenceOTU10586
New.CleanUp.ReferenceOTU45305
New.CleanUp.ReferenceOTU15284
New.CleanUp.ReferenceOTU28027
New.CleanUp.ReferenceOTU11370
New.CleanUp.ReferenceOTU2743
New.CleanUp.ReferenceOTU30812
New.ReferenceOTU2280
New.CleanUp.ReferenceOTU32070
New.CleanUp.ReferenceOTU125217
New.CleanUp.ReferenceOTU52171
New.CleanUp.ReferenceOTU253244
New.CleanUp.ReferenceOTU93221
New.CleanUp.ReferenceOTU23534
New.CleanUp.ReferenceOTU1360
New.CleanUp.ReferenceOTU27591
New.CleanUp.ReferenceOTU22119
New.CleanUp.ReferenceOTU41829
New.CleanUp.ReferenceOTU45406
New.CleanUp.ReferenceOTU33924
New.CleanUp.ReferenceOTU14208
New.CleanUp.ReferenceOTU15859
New.CleanUp.ReferenceOTU30113
New.CleanUp.ReferenceOTU45237
New.CleanUp.ReferenceOTU35806
New.CleanUp.ReferenceOTU35316
New.ReferenceOTU1793
New.CleanUp.ReferenceOTU6885
New.CleanUp.ReferenceOTU40852

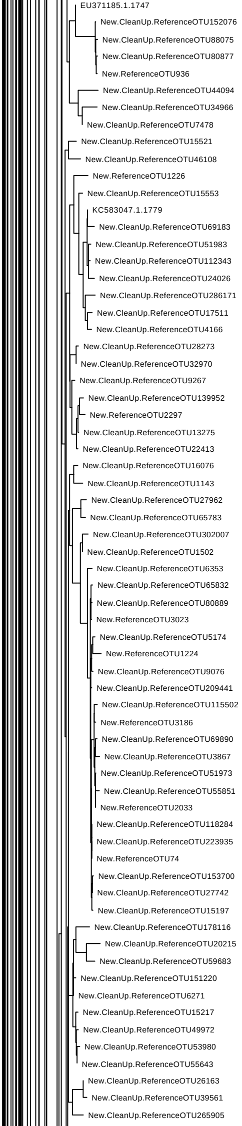
EU371185.1.1747
New.CleanUp.ReferenceOTU152076
New.CleanUp.ReferenceOTU88075
New.CleanUp.ReferenceOTU80877
New.ReferenceOTU936
New.CleanUp.ReferenceOTU44094
New.CleanUp.ReferenceOTU34966
New.CleanUp.ReferenceOTU7478
New.CleanUp.ReferenceOTU15521
New.CleanUp.ReferenceOTU46108
New.ReferenceOTU1226
New.CleanUp.ReferenceOTU15553
KC583047.1.1779
New.CleanUp.ReferenceOTU69183
New.CleanUp.ReferenceOTU51983
New.CleanUp.ReferenceOTU112343
New.CleanUp.ReferenceOTU24026
New.CleanUp.ReferenceOTU286171
New.CleanUp.ReferenceOTU17511
New.CleanUp.ReferenceOTU4166
New.CleanUp.ReferenceOTU28273
New.CleanUp.ReferenceOTU32970
New.CleanUp.ReferenceOTU9267
New.CleanUp.ReferenceOTU139952
New.ReferenceOTU2297
New.CleanUp.ReferenceOTU13275
New.CleanUp.ReferenceOTU22413
New.CleanUp.ReferenceOTU16076
New.CleanUp.ReferenceOTU1143
New.CleanUp.ReferenceOTU27962
New.CleanUp.ReferenceOTU65783
New.CleanUp.ReferenceOTU302007
New.CleanUp.ReferenceOTU1502
New.CleanUp.ReferenceOTU6353
New.CleanUp.ReferenceOTU65832
New.CleanUp.ReferenceOTU80889
New.ReferenceOTU3023
New.CleanUp.ReferenceOTU5174
New.ReferenceOTU1224
New.CleanUp.ReferenceOTU9076
New.CleanUp.ReferenceOTU209441
New.CleanUp.ReferenceOTU115502
New.ReferenceOTU3186
New.CleanUp.ReferenceOTU69890
New.CleanUp.ReferenceOTU3867
New.CleanUp.ReferenceOTU51973
New.CleanUp.ReferenceOTU55851
New.ReferenceOTU2033
New.CleanUp.ReferenceOTU118284
New.CleanUp.ReferenceOTU223935
New.ReferenceOTU74
New.CleanUp.ReferenceOTU153700
New.CleanUp.ReferenceOTU27742
New.CleanUp.ReferenceOTU15197
New.CleanUp.ReferenceOTU178116
New.CleanUp.ReferenceOTU20215
New.CleanUp.ReferenceOTU59683
New.CleanUp.ReferenceOTU151220
New.CleanUp.ReferenceOTU6271
New.CleanUp.ReferenceOTU15217
New.CleanUp.ReferenceOTU49972
New.CleanUp.ReferenceOTU53980
New.CleanUp.ReferenceOTU55643
New.CleanUp.ReferenceOTU26163
New.CleanUp.ReferenceOTU39561
New.CleanUp.ReferenceOTU265905

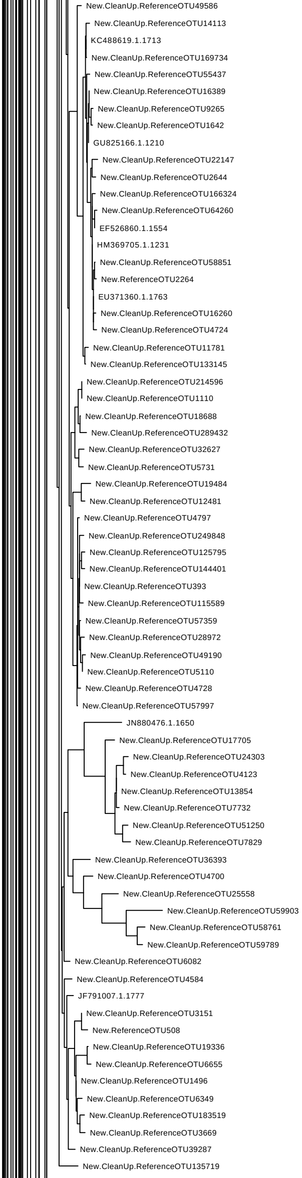
New.CleanUp.ReferenceOTU49586
New.CleanUp.ReferenceOTU14113
KC488619.1.1713
New.CleanUp.ReferenceOTU169734
New.CleanUp.ReferenceOTU55437
New.CleanUp.ReferenceOTU16389
New.CleanUp.ReferenceOTU9265
New.CleanUp.ReferenceOTU1642
GU825166.1.1210
New.CleanUp.ReferenceOTU22147
New.CleanUp.ReferenceOTU2644
New.CleanUp.ReferenceOTU166324
New.CleanUp.ReferenceOTU64260
EF526860.1.1554
HM369705.1.1231
New.CleanUp.ReferenceOTU58851
New.ReferenceOTU2264
EU371360.1.1763
New.CleanUp.ReferenceOTU16260
New.CleanUp.ReferenceOTU4724
New.CleanUp.ReferenceOTU11781
New.CleanUp.ReferenceOTU133145
New.CleanUp.ReferenceOTU214596
New.CleanUp.ReferenceOTU1110
New.CleanUp.ReferenceOTU18688
New.CleanUp.ReferenceOTU289432
New.CleanUp.ReferenceOTU32627
New.CleanUp.ReferenceOTU5731
New.CleanUp.ReferenceOTU19484
New.CleanUp.ReferenceOTU12481
New.CleanUp.ReferenceOTU4797
New.CleanUp.ReferenceOTU249848
New.CleanUp.ReferenceOTU125795
New.CleanUp.ReferenceOTU144401
New.CleanUp.ReferenceOTU393
New.CleanUp.ReferenceOTU115589
New.CleanUp.ReferenceOTU57359
New.CleanUp.ReferenceOTU28972
New.CleanUp.ReferenceOTU49190
New.CleanUp.ReferenceOTU5110
New.CleanUp.ReferenceOTU4728
New.CleanUp.ReferenceOTU57997
JN880476.1.1650
New.CleanUp.ReferenceOTU17705
New.CleanUp.ReferenceOTU24303
New.CleanUp.ReferenceOTU4123
New.CleanUp.ReferenceOTU13854
New.CleanUp.ReferenceOTU7732
New.CleanUp.ReferenceOTU51250
New.CleanUp.ReferenceOTU7829
New.CleanUp.ReferenceOTU36393
New.CleanUp.ReferenceOTU4700
New.CleanUp.ReferenceOTU25558
New.CleanUp.ReferenceOTU59903
New.CleanUp.ReferenceOTU58761
New.CleanUp.ReferenceOTU59789
New.CleanUp.ReferenceOTU6082
New.CleanUp.ReferenceOTU4584
JF791007.1.1777
New.CleanUp.ReferenceOTU3151
New.ReferenceOTU508
New.CleanUp.ReferenceOTU19336
New.CleanUp.ReferenceOTU6655
New.CleanUp.ReferenceOTU1496
New.CleanUp.ReferenceOTU6349
New.CleanUp.ReferenceOTU183519
New.CleanUp.ReferenceOTU3669
New.CleanUp.ReferenceOTU39287
New.CleanUp.ReferenceOTU135719

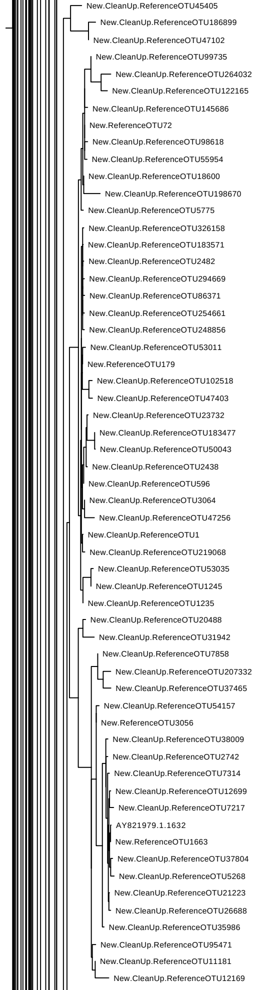
New.CleanUp.ReferenceOTU45405
New.CleanUp.ReferenceOTU186899
New.CleanUp.ReferenceOTU47102
New.CleanUp.ReferenceOTU99735
New.CleanUp.ReferenceOTU264032
New.CleanUp.ReferenceOTU122165
New.CleanUp.ReferenceOTU145686
New.ReferenceOTU72
New.CleanUp.ReferenceOTU98618
New.CleanUp.ReferenceOTU55954
New.CleanUp.ReferenceOTU18600
New.CleanUp.ReferenceOTU198670
New.CleanUp.ReferenceOTU5775
New.CleanUp.ReferenceOTU326158
New.CleanUp.ReferenceOTU183571
New.CleanUp.ReferenceOTU2482
New.CleanUp.ReferenceOTU294669
New.CleanUp.ReferenceOTU86371
New.CleanUp.ReferenceOTU254661
New.CleanUp.ReferenceOTU248856
New.CleanUp.ReferenceOTU53011
New.ReferenceOTU179
New.CleanUp.ReferenceOTU102518
New.CleanUp.ReferenceOTU47403
New.CleanUp.ReferenceOTU23732
New.CleanUp.ReferenceOTU183477
New.CleanUp.ReferenceOTU50043
New.CleanUp.ReferenceOTU2438
New.CleanUp.ReferenceOTU596
New.CleanUp.ReferenceOTU3064
New.CleanUp.ReferenceOTU47256
New.CleanUp.ReferenceOTU1
New.CleanUp.ReferenceOTU219068
New.CleanUp.ReferenceOTU53035
New.CleanUp.ReferenceOTU1245
New.CleanUp.ReferenceOTU1235
New.CleanUp.ReferenceOTU20488
New.CleanUp.ReferenceOTU31942
New.CleanUp.ReferenceOTU7858
New.CleanUp.ReferenceOTU207332
New.CleanUp.ReferenceOTU37465
New.CleanUp.ReferenceOTU54157
New.ReferenceOTU3056
New.CleanUp.ReferenceOTU38009
New.CleanUp.ReferenceOTU2742
New.CleanUp.ReferenceOTU7314
New.CleanUp.ReferenceOTU12699
New.CleanUp.ReferenceOTU7217
AY821979.1.1632
New.ReferenceOTU1663
New.CleanUp.ReferenceOTU37804
New.CleanUp.ReferenceOTU5268
New.CleanUp.ReferenceOTU21223
New.CleanUp.ReferenceOTU26688
New.CleanUp.ReferenceOTU35986
New.CleanUp.ReferenceOTU95471
New.CleanUp.ReferenceOTU11181
New.CleanUp.ReferenceOTU12169

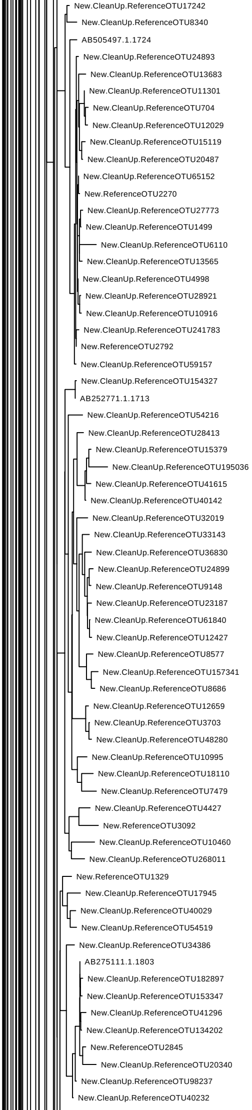
New.CleanUp.ReferenceOTU17242
New.CleanUp.ReferenceOTU8340
AB505497.1.1724
New.CleanUp.ReferenceOTU24893
New.CleanUp.ReferenceOTU13683
New.CleanUp.ReferenceOTU11301
New.CleanUp.ReferenceOTU704
New.CleanUp.ReferenceOTU12029
New.CleanUp.ReferenceOTU15119
New.CleanUp.ReferenceOTU20487
New.CleanUp.ReferenceOTU65152
New.ReferenceOTU2270
New.CleanUp.ReferenceOTU27773
New.CleanUp.ReferenceOTU1499
New.CleanUp.ReferenceOTU6110
New.CleanUp.ReferenceOTU13565
New.CleanUp.ReferenceOTU4998
New.CleanUp.ReferenceOTU28921
New.CleanUp.ReferenceOTU10916
New.CleanUp.ReferenceOTU241783
New.ReferenceOTU2792
New.CleanUp.ReferenceOTU59157
New.CleanUp.ReferenceOTU154327
AB252771.1.1713
New.CleanUp.ReferenceOTU54216
New.CleanUp.ReferenceOTU28413
New.CleanUp.ReferenceOTU15379
New.CleanUp.ReferenceOTU195036
New.CleanUp.ReferenceOTU41615
New.CleanUp.ReferenceOTU40142
New.CleanUp.ReferenceOTU32019
New.CleanUp.ReferenceOTU33143
New.CleanUp.ReferenceOTU36830
New.CleanUp.ReferenceOTU24899
New.CleanUp.ReferenceOTU9148
New.CleanUp.ReferenceOTU23187
New.CleanUp.ReferenceOTU61840
New.CleanUp.ReferenceOTU12427
New.CleanUp.ReferenceOTU8577
New.CleanUp.ReferenceOTU157341
New.CleanUp.ReferenceOTU8686
New.CleanUp.ReferenceOTU12659
New.CleanUp.ReferenceOTU3703
New.CleanUp.ReferenceOTU48280
New.CleanUp.ReferenceOTU10995
New.CleanUp.ReferenceOTU18110
New.CleanUp.ReferenceOTU7479
New.CleanUp.ReferenceOTU4427
New.ReferenceOTU3092
New.CleanUp.ReferenceOTU10460
New.CleanUp.ReferenceOTU268011
New.ReferenceOTU1329
New.CleanUp.ReferenceOTU17945
New.CleanUp.ReferenceOTU40029
New.CleanUp.ReferenceOTU54519
New.CleanUp.ReferenceOTU34386
AB275111.1.1803
New.CleanUp.ReferenceOTU182897
New.CleanUp.ReferenceOTU153347
New.CleanUp.ReferenceOTU41296
New.CleanUp.ReferenceOTU134202
New.ReferenceOTU2845
New.CleanUp.ReferenceOTU20340
New.CleanUp.ReferenceOTU98237
New.CleanUp.ReferenceOTU40232

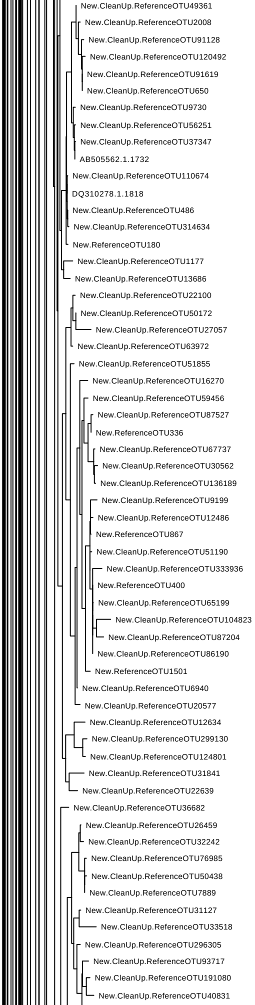
New.CleanUp.ReferenceOTU49361
New.CleanUp.ReferenceOTU2008
New.CleanUp.ReferenceOTU91128
New.CleanUp.ReferenceOTU120492
New.CleanUp.ReferenceOTU91619
New.CleanUp.ReferenceOTU650
New.CleanUp.ReferenceOTU9730
New.CleanUp.ReferenceOTU56251
New.CleanUp.ReferenceOTU37347
AB505562.1.1732
New.CleanUp.ReferenceOTU110674
DQ310278.1.1818
New.CleanUp.ReferenceOTU486
New.CleanUp.ReferenceOTU314634
New.ReferenceOTU180
New.CleanUp.ReferenceOTU1177
New.CleanUp.ReferenceOTU13686
New.CleanUp.ReferenceOTU22100
New.CleanUp.ReferenceOTU50172
New.CleanUp.ReferenceOTU27057
New.CleanUp.ReferenceOTU63972
New.CleanUp.ReferenceOTU51855
New.CleanUp.ReferenceOTU16270
New.CleanUp.ReferenceOTU59456
New.CleanUp.ReferenceOTU87527
New.ReferenceOTU336
New.CleanUp.ReferenceOTU67737
New.CleanUp.ReferenceOTU30562
New.CleanUp.ReferenceOTU136189
New.CleanUp.ReferenceOTU9199
New.CleanUp.ReferenceOTU12486
New.ReferenceOTU867
New.CleanUp.ReferenceOTU51190
New.CleanUp.ReferenceOTU333936
New.ReferenceOTU400
New.CleanUp.ReferenceOTU65199
New.CleanUp.ReferenceOTU104823
New.CleanUp.ReferenceOTU87204
New.CleanUp.ReferenceOTU86190
New.ReferenceOTU1501
New.CleanUp.ReferenceOTU6940
New.CleanUp.ReferenceOTU20577
New.CleanUp.ReferenceOTU12634
New.CleanUp.ReferenceOTU299130
New.CleanUp.ReferenceOTU124801
New.CleanUp.ReferenceOTU31841
New.CleanUp.ReferenceOTU22639
New.CleanUp.ReferenceOTU36682
New.CleanUp.ReferenceOTU26459
New.CleanUp.ReferenceOTU32242
New.CleanUp.ReferenceOTU76985
New.CleanUp.ReferenceOTU50438
New.CleanUp.ReferenceOTU7889
New.CleanUp.ReferenceOTU31127
New.CleanUp.ReferenceOTU33518
New.CleanUp.ReferenceOTU296305
New.CleanUp.ReferenceOTU93717
New.CleanUp.ReferenceOTU191080
New.CleanUp.ReferenceOTU40831

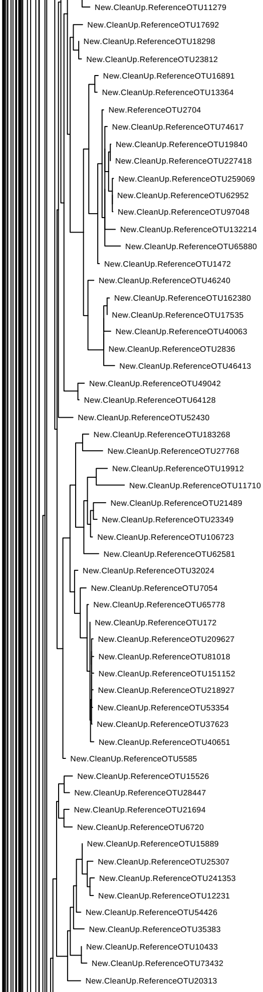
New.CleanUp.ReferenceOTU11279
New.CleanUp.ReferenceOTU17692
New.CleanUp.ReferenceOTU18298
New.CleanUp.ReferenceOTU23812
New.CleanUp.ReferenceOTU16891
New.CleanUp.ReferenceOTU13364
New.ReferenceOTU2704
New.CleanUp.ReferenceOTU74617
New.CleanUp.ReferenceOTU19840
New.CleanUp.ReferenceOTU227418
New.CleanUp.ReferenceOTU259069
New.CleanUp.ReferenceOTU62952
New.CleanUp.ReferenceOTU97048
New.CleanUp.ReferenceOTU132214
New.CleanUp.ReferenceOTU65880
New.CleanUp.ReferenceOTU1472
New.CleanUp.ReferenceOTU46240
New.CleanUp.ReferenceOTU162380
New.CleanUp.ReferenceOTU17535
New.CleanUp.ReferenceOTU40063
New.CleanUp.ReferenceOTU2836
New.CleanUp.ReferenceOTU46413
New.CleanUp.ReferenceOTU49042
New.CleanUp.ReferenceOTU64128
New.CleanUp.ReferenceOTU52430
New.CleanUp.ReferenceOTU183268
New.CleanUp.ReferenceOTU27768
New.CleanUp.ReferenceOTU19912
New.CleanUp.ReferenceOTU11710
New.CleanUp.ReferenceOTU21489
New.CleanUp.ReferenceOTU23349
New.CleanUp.ReferenceOTU106723
New.CleanUp.ReferenceOTU62581
New.CleanUp.ReferenceOTU32024
New.CleanUp.ReferenceOTU7054
New.CleanUp.ReferenceOTU65778
New.CleanUp.ReferenceOTU172
New.CleanUp.ReferenceOTU209627
New.CleanUp.ReferenceOTU81018
New.CleanUp.ReferenceOTU151152
New.CleanUp.ReferenceOTU218927
New.CleanUp.ReferenceOTU53354
New.CleanUp.ReferenceOTU37623
New.CleanUp.ReferenceOTU40651
New.CleanUp.ReferenceOTU5585
New.CleanUp.ReferenceOTU15526
New.CleanUp.ReferenceOTU28447
New.CleanUp.ReferenceOTU21694
New.CleanUp.ReferenceOTU6720
New.CleanUp.ReferenceOTU15889
New.CleanUp.ReferenceOTU25307
New.CleanUp.ReferenceOTU241353
New.CleanUp.ReferenceOTU12231
New.CleanUp.ReferenceOTU54426
New.CleanUp.ReferenceOTU35383
New.CleanUp.ReferenceOTU10433
New.CleanUp.ReferenceOTU73432
New.CleanUp.ReferenceOTU20313

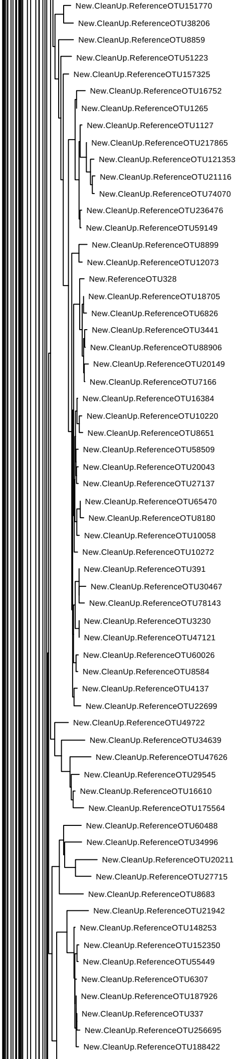
New.CleanUp.ReferenceOTU151770
New.CleanUp.ReferenceOTU38206
New.CleanUp.ReferenceOTU8859
New.CleanUp.ReferenceOTU51223
New.CleanUp.ReferenceOTU157325
New.CleanUp.ReferenceOTU16752
New.CleanUp.ReferenceOTU1265
New.CleanUp.ReferenceOTU1127
New.CleanUp.ReferenceOTU217865
New.CleanUp.ReferenceOTU121353
New.CleanUp.ReferenceOTU21116
New.CleanUp.ReferenceOTU74070
New.CleanUp.ReferenceOTU236476
New.CleanUp.ReferenceOTU59149
New.CleanUp.ReferenceOTU8899
New.CleanUp.ReferenceOTU12073
New.ReferenceOTU328
New.CleanUp.ReferenceOTU18705
New.CleanUp.ReferenceOTU6826
New.CleanUp.ReferenceOTU3441
New.CleanUp.ReferenceOTU88906
New.CleanUp.ReferenceOTU20149
New.CleanUp.ReferenceOTU7166
New.CleanUp.ReferenceOTU16384
New.CleanUp.ReferenceOTU10220
New.CleanUp.ReferenceOTU8651
New.CleanUp.ReferenceOTU58509
New.CleanUp.ReferenceOTU20043
New.CleanUp.ReferenceOTU27137
New.CleanUp.ReferenceOTU65470
New.CleanUp.ReferenceOTU8180
New.CleanUp.ReferenceOTU10058
New.CleanUp.ReferenceOTU10272
New.CleanUp.ReferenceOTU391
New.CleanUp.ReferenceOTU30467
New.CleanUp.ReferenceOTU78143
New.CleanUp.ReferenceOTU3230
New.CleanUp.ReferenceOTU47121
New.CleanUp.ReferenceOTU60026
New.CleanUp.ReferenceOTU8584
New.CleanUp.ReferenceOTU4137
New.CleanUp.ReferenceOTU22699
New.CleanUp.ReferenceOTU49722
New.CleanUp.ReferenceOTU34639
New.CleanUp.ReferenceOTU47626
New.CleanUp.ReferenceOTU29545
New.CleanUp.ReferenceOTU16610
New.CleanUp.ReferenceOTU175564
New.CleanUp.ReferenceOTU60488
New.CleanUp.ReferenceOTU34996
New.CleanUp.ReferenceOTU20211
New.CleanUp.ReferenceOTU27715
New.CleanUp.ReferenceOTU8683
New.CleanUp.ReferenceOTU21942
New.CleanUp.ReferenceOTU148253
New.CleanUp.ReferenceOTU152350
New.CleanUp.ReferenceOTU55449
New.CleanUp.ReferenceOTU6307
New.CleanUp.ReferenceOTU187926
New.CleanUp.ReferenceOTU337
New.CleanUp.ReferenceOTU256695
New.CleanUp.ReferenceOTU188422

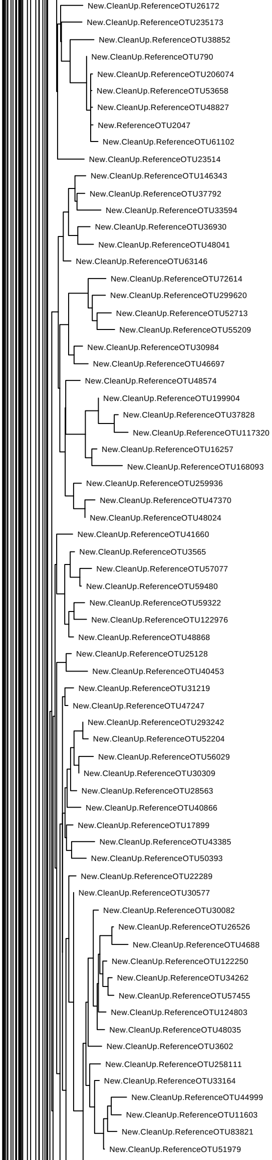
New.CleanUp.ReferenceOTU26172
New.CleanUp.ReferenceOTU235173
New.CleanUp.ReferenceOTU38852
New.CleanUp.ReferenceOTU790
New.CleanUp.ReferenceOTU206074
New.CleanUp.ReferenceOTU53658
New.CleanUp.ReferenceOTU48827
New.ReferenceOTU2047
New.CleanUp.ReferenceOTU61102
New.CleanUp.ReferenceOTU23514
New.CleanUp.ReferenceOTU146343
New.CleanUp.ReferenceOTU37792
New.CleanUp.ReferenceOTU33594
New.CleanUp.ReferenceOTU36930
New.CleanUp.ReferenceOTU48041
New.CleanUp.ReferenceOTU63146
New.CleanUp.ReferenceOTU72614
New.CleanUp.ReferenceOTU299620
New.CleanUp.ReferenceOTU52713
New.CleanUp.ReferenceOTU55209
New.CleanUp.ReferenceOTU30984
New.CleanUp.ReferenceOTU46697
New.CleanUp.ReferenceOTU48574
New.CleanUp.ReferenceOTU199904
New.CleanUp.ReferenceOTU37828
New.CleanUp.ReferenceOTU117320
New.CleanUp.ReferenceOTU16257
New.CleanUp.ReferenceOTU168093
New.CleanUp.ReferenceOTU259936
New.CleanUp.ReferenceOTU47370
New.CleanUp.ReferenceOTU48024
New.CleanUp.ReferenceOTU41660
New.CleanUp.ReferenceOTU3565
New.CleanUp.ReferenceOTU57077
New.CleanUp.ReferenceOTU59480
New.CleanUp.ReferenceOTU59322
New.CleanUp.ReferenceOTU122976
New.CleanUp.ReferenceOTU48868
New.CleanUp.ReferenceOTU25128
New.CleanUp.ReferenceOTU40453
New.CleanUp.ReferenceOTU31219
New.CleanUp.ReferenceOTU47247
New.CleanUp.ReferenceOTU293242
New.CleanUp.ReferenceOTU52204
New.CleanUp.ReferenceOTU56029
New.CleanUp.ReferenceOTU30309
New.CleanUp.ReferenceOTU28563
New.CleanUp.ReferenceOTU40866
New.CleanUp.ReferenceOTU17899
New.CleanUp.ReferenceOTU43385
New.CleanUp.ReferenceOTU50393
New.CleanUp.ReferenceOTU22289
New.CleanUp.ReferenceOTU30577
New.CleanUp.ReferenceOTU30082
New.CleanUp.ReferenceOTU26526
New.CleanUp.ReferenceOTU4688
New.CleanUp.ReferenceOTU122250
New.CleanUp.ReferenceOTU34262
New.CleanUp.ReferenceOTU57455
New.CleanUp.ReferenceOTU124803
New.CleanUp.ReferenceOTU48035
New.CleanUp.ReferenceOTU3602
New.CleanUp.ReferenceOTU258111
New.CleanUp.ReferenceOTU33164
New.CleanUp.ReferenceOTU44999
New.CleanUp.ReferenceOTU11603
New.CleanUp.ReferenceOTU83821
New.CleanUp.ReferenceOTU51979

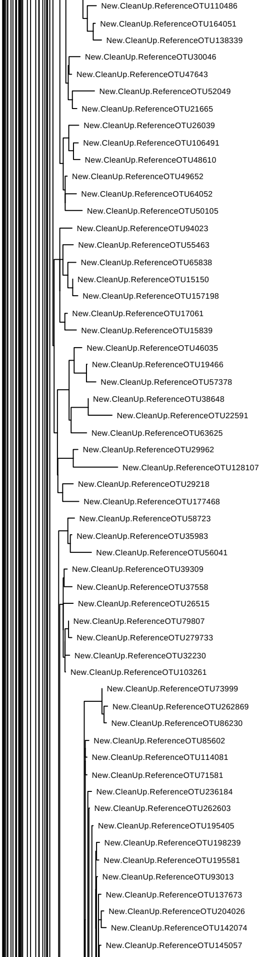
New.CleanUp.ReferenceOTU110486
New.CleanUp.ReferenceOTU164051
New.CleanUp.ReferenceOTU138339
New.CleanUp.ReferenceOTU30046
New.CleanUp.ReferenceOTU47643
New.CleanUp.ReferenceOTU52049
New.CleanUp.ReferenceOTU21665
New.CleanUp.ReferenceOTU26039
New.CleanUp.ReferenceOTU106491
New.CleanUp.ReferenceOTU48610
New.CleanUp.ReferenceOTU49652
New.CleanUp.ReferenceOTU64052
New.CleanUp.ReferenceOTU50105
New.CleanUp.ReferenceOTU94023
New.CleanUp.ReferenceOTU55463
New.CleanUp.ReferenceOTU65838
New.CleanUp.ReferenceOTU15150
New.CleanUp.ReferenceOTU157198
New.CleanUp.ReferenceOTU17061
New.CleanUp.ReferenceOTU15839
New.CleanUp.ReferenceOTU46035
New.CleanUp.ReferenceOTU19466
New.CleanUp.ReferenceOTU57378
New.CleanUp.ReferenceOTU38648
New.CleanUp.ReferenceOTU22591
New.CleanUp.ReferenceOTU63625
New.CleanUp.ReferenceOTU29962
New.CleanUp.ReferenceOTU128107
New.CleanUp.ReferenceOTU29218
New.CleanUp.ReferenceOTU177468
New.CleanUp.ReferenceOTU58723
New.CleanUp.ReferenceOTU35983
New.CleanUp.ReferenceOTU56041
New.CleanUp.ReferenceOTU39309
New.CleanUp.ReferenceOTU37558
New.CleanUp.ReferenceOTU26515
New.CleanUp.ReferenceOTU79807
New.CleanUp.ReferenceOTU279733
New.CleanUp.ReferenceOTU32230
New.CleanUp.ReferenceOTU103261
New.CleanUp.ReferenceOTU73999
New.CleanUp.ReferenceOTU262869
New.CleanUp.ReferenceOTU86230
New.CleanUp.ReferenceOTU85602
New.CleanUp.ReferenceOTU114081
New.CleanUp.ReferenceOTU71581
New.CleanUp.ReferenceOTU236184
New.CleanUp.ReferenceOTU262603
New.CleanUp.ReferenceOTU195405
New.CleanUp.ReferenceOTU198239
New.CleanUp.ReferenceOTU195581
New.CleanUp.ReferenceOTU93013
New.CleanUp.ReferenceOTU137673
New.CleanUp.ReferenceOTU204026
New.CleanUp.ReferenceOTU142074
New.CleanUp.ReferenceOTU145057

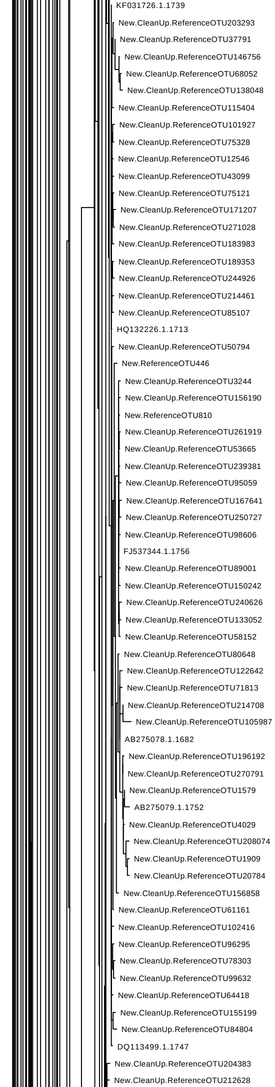
KF031726.1.1739
New.CleanUp.ReferenceOTU203293
New.CleanUp.ReferenceOTU37791
New.CleanUp.ReferenceOTU146756
New.CleanUp.ReferenceOTU68052
New.CleanUp.ReferenceOTU138048
New.CleanUp.ReferenceOTU115404
New.CleanUp.ReferenceOTU101927
New.CleanUp.ReferenceOTU75328
New.CleanUp.ReferenceOTU12546
New.CleanUp.ReferenceOTU43099
New.CleanUp.ReferenceOTU75121
New.CleanUp.ReferenceOTU171207
New.CleanUp.ReferenceOTU271028
New.CleanUp.ReferenceOTU183983
New.CleanUp.ReferenceOTU189353
New.CleanUp.ReferenceOTU244926
New.CleanUp.ReferenceOTU214461
New.CleanUp.ReferenceOTU85107
HQ132226.1.1713
New.CleanUp.ReferenceOTU50794
New.ReferenceOTU446
New.CleanUp.ReferenceOTU3244
New.CleanUp.ReferenceOTU156190
New.ReferenceOTU810
New.CleanUp.ReferenceOTU261919
New.CleanUp.ReferenceOTU53665
New.CleanUp.ReferenceOTU239381
New.CleanUp.ReferenceOTU95059
New.CleanUp.ReferenceOTU167641
New.CleanUp.ReferenceOTU250727
New.CleanUp.ReferenceOTU98606
FJ537344.1.1756
New.CleanUp.ReferenceOTU89001
New.CleanUp.ReferenceOTU150242
New.CleanUp.ReferenceOTU240626
New.CleanUp.ReferenceOTU133052
New.CleanUp.ReferenceOTU58152
New.CleanUp.ReferenceOTU80648
New.CleanUp.ReferenceOTU122642
New.CleanUp.ReferenceOTU71813
New.CleanUp.ReferenceOTU214708
New.CleanUp.ReferenceOTU105987
AB275078.1.1682
New.CleanUp.ReferenceOTU196192
New.CleanUp.ReferenceOTU270791
New.CleanUp.ReferenceOTU1579
AB275079.1.1752
New.CleanUp.ReferenceOTU4029
New.CleanUp.ReferenceOTU208074
New.CleanUp.ReferenceOTU1909
New.CleanUp.ReferenceOTU20784
New.CleanUp.ReferenceOTU156858
New.CleanUp.ReferenceOTU61161
New.CleanUp.ReferenceOTU102416
New.CleanUp.ReferenceOTU96295
New.CleanUp.ReferenceOTU78303
New.CleanUp.ReferenceOTU99632
New.CleanUp.ReferenceOTU64418
New.CleanUp.ReferenceOTU155199
New.CleanUp.ReferenceOTU84804
DQ113499.1.1747
New.CleanUp.ReferenceOTU204383
New.CleanUp.ReferenceOTU212628

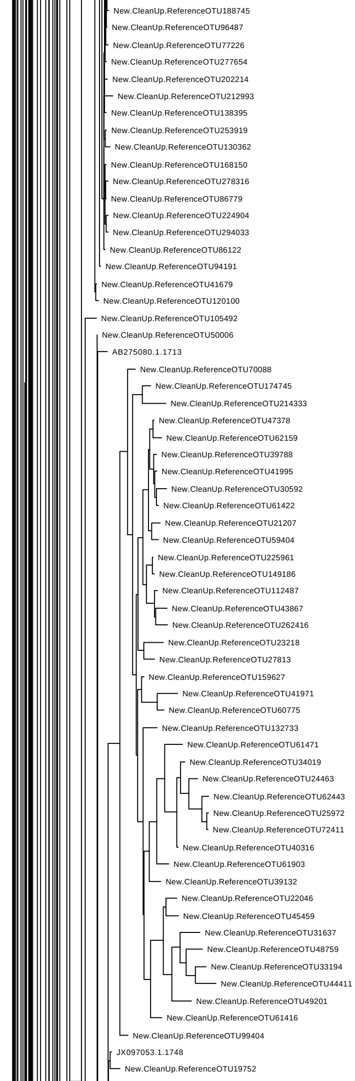
New.CleanUp.ReferenceOTU188745
New.CleanUp.ReferenceOTU96487
New.CleanUp.ReferenceOTU77226
New.CleanUp.ReferenceOTU277654
New.CleanUp.ReferenceOTU202214
New.CleanUp.ReferenceOTU212993
New.CleanUp.ReferenceOTU138395
New.CleanUp.ReferenceOTU253919
New.CleanUp.ReferenceOTU130362
New.CleanUp.ReferenceOTU168150
New.CleanUp.ReferenceOTU278316
New.CleanUp.ReferenceOTU86779
New.CleanUp.ReferenceOTU224904
New.CleanUp.ReferenceOTU294033
New.CleanUp.ReferenceOTU86122
New.CleanUp.ReferenceOTU94191
New.CleanUp.ReferenceOTU41679
New.CleanUp.ReferenceOTU120100
New.CleanUp.ReferenceOTU105492
New.CleanUp.ReferenceOTU50006
AB275080.1.1713
New.CleanUp.ReferenceOTU70088
New.CleanUp.ReferenceOTU174745
New.CleanUp.ReferenceOTU214333
New.CleanUp.ReferenceOTU47378
New.CleanUp.ReferenceOTU62159
New.CleanUp.ReferenceOTU39788
New.CleanUp.ReferenceOTU41995
New.CleanUp.ReferenceOTU30592
New.CleanUp.ReferenceOTU61422
New.CleanUp.ReferenceOTU21207
New.CleanUp.ReferenceOTU59404
New.CleanUp.ReferenceOTU225961
New.CleanUp.ReferenceOTU149186
New.CleanUp.ReferenceOTU112487
New.CleanUp.ReferenceOTU43867
New.CleanUp.ReferenceOTU262416
New.CleanUp.ReferenceOTU23218
New.CleanUp.ReferenceOTU27813
New.CleanUp.ReferenceOTU159627
New.CleanUp.ReferenceOTU41971
New.CleanUp.ReferenceOTU60775
New.CleanUp.ReferenceOTU132733
New.CleanUp.ReferenceOTU61471
New.CleanUp.ReferenceOTU34019
New.CleanUp.ReferenceOTU24463
New.CleanUp.ReferenceOTU62443
New.CleanUp.ReferenceOTU25972
New.CleanUp.ReferenceOTU72411
New.CleanUp.ReferenceOTU40316
New.CleanUp.ReferenceOTU61903
New.CleanUp.ReferenceOTU39132
New.CleanUp.ReferenceOTU22046
New.CleanUp.ReferenceOTU45459
New.CleanUp.ReferenceOTU31637
New.CleanUp.ReferenceOTU48759
New.CleanUp.ReferenceOTU33194
New.CleanUp.ReferenceOTU44411
New.CleanUp.ReferenceOTU49201
New.CleanUp.ReferenceOTU61416
New.CleanUp.ReferenceOTU99404
JX097053.1.1748
New.CleanUp.ReferenceOTU19752

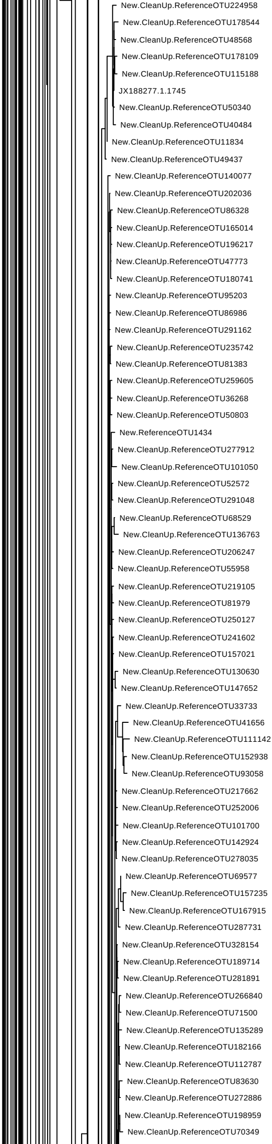
New.CleanUp.ReferenceOTU224958
New.CleanUp.ReferenceOTU178544
New.CleanUp.ReferenceOTU48568
New.CleanUp.ReferenceOTU178109
New.CleanUp.ReferenceOTU115188
JX188277.1.1745
New.CleanUp.ReferenceOTU50340
New.CleanUp.ReferenceOTU40484
New.CleanUp.ReferenceOTU11834
New.CleanUp.ReferenceOTU49437
New.CleanUp.ReferenceOTU140077
New.CleanUp.ReferenceOTU202036
New.CleanUp.ReferenceOTU86328
New.CleanUp.ReferenceOTU165014
New.CleanUp.ReferenceOTU196217
New.CleanUp.ReferenceOTU47773
New.CleanUp.ReferenceOTU180741
New.CleanUp.ReferenceOTU95203
New.CleanUp.ReferenceOTU86986
New.CleanUp.ReferenceOTU291162
New.CleanUp.ReferenceOTU235742
New.CleanUp.ReferenceOTU81383
New.CleanUp.ReferenceOTU259605
New.CleanUp.ReferenceOTU36268
New.CleanUp.ReferenceOTU50803
New.ReferenceOTU1434
New.CleanUp.ReferenceOTU277912
New.CleanUp.ReferenceOTU101050
New.CleanUp.ReferenceOTU52572
New.CleanUp.ReferenceOTU291048
New.CleanUp.ReferenceOTU68529
New.CleanUp.ReferenceOTU136763
New.CleanUp.ReferenceOTU206247
New.CleanUp.ReferenceOTU55958
New.CleanUp.ReferenceOTU219105
New.CleanUp.ReferenceOTU81979
New.CleanUp.ReferenceOTU250127
New.CleanUp.ReferenceOTU241602
New.CleanUp.ReferenceOTU157021
New.CleanUp.ReferenceOTU130630
New.CleanUp.ReferenceOTU147652
New.CleanUp.ReferenceOTU33733
New.CleanUp.ReferenceOTU41656
New.CleanUp.ReferenceOTU111142
New.CleanUp.ReferenceOTU152938
New.CleanUp.ReferenceOTU93058
New.CleanUp.ReferenceOTU217662
New.CleanUp.ReferenceOTU252006
New.CleanUp.ReferenceOTU101700
New.CleanUp.ReferenceOTU142924
New.CleanUp.ReferenceOTU278035
New.CleanUp.ReferenceOTU69577
New.CleanUp.ReferenceOTU157235
New.CleanUp.ReferenceOTU167915
New.CleanUp.ReferenceOTU287731
New.CleanUp.ReferenceOTU328154
New.CleanUp.ReferenceOTU189714
New.CleanUp.ReferenceOTU281891
New.CleanUp.ReferenceOTU266840
New.CleanUp.ReferenceOTU71500
New.CleanUp.ReferenceOTU135289
New.CleanUp.ReferenceOTU182166
New.CleanUp.ReferenceOTU112787
New.CleanUp.ReferenceOTU83630
New.CleanUp.ReferenceOTU272886
New.CleanUp.ReferenceOTU198959
New.CleanUp.ReferenceOTU70349

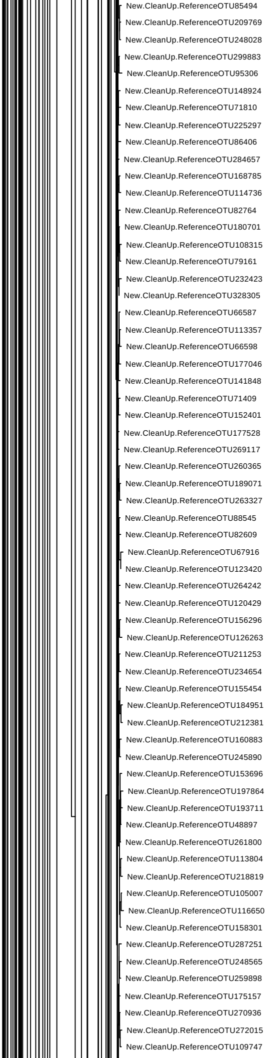
New.CleanUp.ReferenceOTU85494
New.CleanUp.ReferenceOTU209769
New.CleanUp.ReferenceOTU248028
New.CleanUp.ReferenceOTU299883
New.CleanUp.ReferenceOTU95306
New.CleanUp.ReferenceOTU148924
New.CleanUp.ReferenceOTU71810
New.CleanUp.ReferenceOTU225297
New.CleanUp.ReferenceOTU86406
New.CleanUp.ReferenceOTU284657
New.CleanUp.ReferenceOTU168785
New.CleanUp.ReferenceOTU114736
New.CleanUp.ReferenceOTU82764
New.CleanUp.ReferenceOTU180701
New.CleanUp.ReferenceOTU108315
New.CleanUp.ReferenceOTU79161
New.CleanUp.ReferenceOTU232423
New.CleanUp.ReferenceOTU328305
New.CleanUp.ReferenceOTU66587
New.CleanUp.ReferenceOTU113357
New.CleanUp.ReferenceOTU66598
New.CleanUp.ReferenceOTU177046
New.CleanUp.ReferenceOTU141848
New.CleanUp.ReferenceOTU71409
New.CleanUp.ReferenceOTU152401
New.CleanUp.ReferenceOTU177528
New.CleanUp.ReferenceOTU269117
New.CleanUp.ReferenceOTU260365
New.CleanUp.ReferenceOTU189071
New.CleanUp.ReferenceOTU263327
New.CleanUp.ReferenceOTU88545
New.CleanUp.ReferenceOTU82609
New.CleanUp.ReferenceOTU67916
New.CleanUp.ReferenceOTU123420
New.CleanUp.ReferenceOTU264242
New.CleanUp.ReferenceOTU120429
New.CleanUp.ReferenceOTU156296
New.CleanUp.ReferenceOTU126263
New.CleanUp.ReferenceOTU211253
New.CleanUp.ReferenceOTU234654
New.CleanUp.ReferenceOTU155454
New.CleanUp.ReferenceOTU184951
New.CleanUp.ReferenceOTU212381
New.CleanUp.ReferenceOTU160883
New.CleanUp.ReferenceOTU245890
New.CleanUp.ReferenceOTU153696
New.CleanUp.ReferenceOTU197864
New.CleanUp.ReferenceOTU193711
New.CleanUp.ReferenceOTU48897
New.CleanUp.ReferenceOTU261800
New.CleanUp.ReferenceOTU113804
New.CleanUp.ReferenceOTU218819
New.CleanUp.ReferenceOTU105007
New.CleanUp.ReferenceOTU116650
New.CleanUp.ReferenceOTU158301
New.CleanUp.ReferenceOTU287251
New.CleanUp.ReferenceOTU248565
New.CleanUp.ReferenceOTU259898
New.CleanUp.ReferenceOTU175157
New.CleanUp.ReferenceOTU270936
New.CleanUp.ReferenceOTU272015
New.CleanUp.ReferenceOTU109747

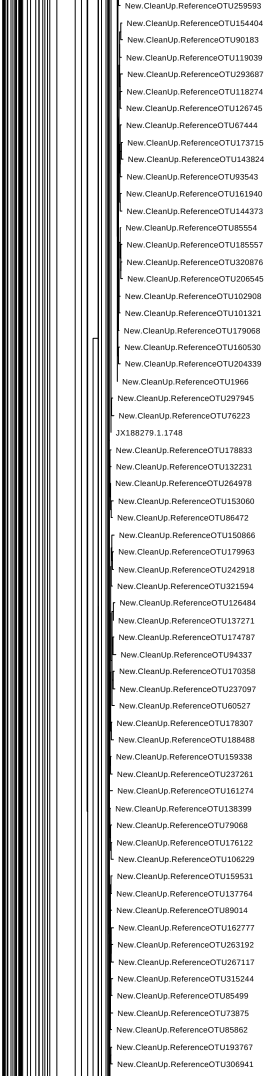
New.CleanUp.ReferenceOTU259593
New.CleanUp.ReferenceOTU154404
New.CleanUp.ReferenceOTU90183
New.CleanUp.ReferenceOTU119039
New.CleanUp.ReferenceOTU293687
New.CleanUp.ReferenceOTU118274
New.CleanUp.ReferenceOTU126745
New.CleanUp.ReferenceOTU67444
New.CleanUp.ReferenceOTU173715
New.CleanUp.ReferenceOTU143824
New.CleanUp.ReferenceOTU93543
New.CleanUp.ReferenceOTU161940
New.CleanUp.ReferenceOTU144373
New.CleanUp.ReferenceOTU85554
New.CleanUp.ReferenceOTU185557
New.CleanUp.ReferenceOTU320876
New.CleanUp.ReferenceOTU206545
New.CleanUp.ReferenceOTU102908
New.CleanUp.ReferenceOTU101321
New.CleanUp.ReferenceOTU179068
New.CleanUp.ReferenceOTU160530
New.CleanUp.ReferenceOTU204339
New.CleanUp.ReferenceOTU1966
New.CleanUp.ReferenceOTU297945
New.CleanUp.ReferenceOTU76223
JX188279.1.1748
New.CleanUp.ReferenceOTU178833
New.CleanUp.ReferenceOTU132231
New.CleanUp.ReferenceOTU264978
New.CleanUp.ReferenceOTU153060
New.CleanUp.ReferenceOTU86472
New.CleanUp.ReferenceOTU150866
New.CleanUp.ReferenceOTU179963
New.CleanUp.ReferenceOTU242918
New.CleanUp.ReferenceOTU321594
New.CleanUp.ReferenceOTU126484
New.CleanUp.ReferenceOTU137271
New.CleanUp.ReferenceOTU174787
New.CleanUp.ReferenceOTU94337
New.CleanUp.ReferenceOTU170358
New.CleanUp.ReferenceOTU237097
New.CleanUp.ReferenceOTU60527
New.CleanUp.ReferenceOTU178307
New.CleanUp.ReferenceOTU188488
New.CleanUp.ReferenceOTU159338
New.CleanUp.ReferenceOTU237261
New.CleanUp.ReferenceOTU161274
New.CleanUp.ReferenceOTU138399
New.CleanUp.ReferenceOTU79068
New.CleanUp.ReferenceOTU176122
New.CleanUp.ReferenceOTU106229
New.CleanUp.ReferenceOTU159531
New.CleanUp.ReferenceOTU137764
New.CleanUp.ReferenceOTU89014
New.CleanUp.ReferenceOTU162777
New.CleanUp.ReferenceOTU263192
New.CleanUp.ReferenceOTU267117
New.CleanUp.ReferenceOTU315244
New.CleanUp.ReferenceOTU85499
New.CleanUp.ReferenceOTU73875
New.CleanUp.ReferenceOTU85862
New.CleanUp.ReferenceOTU193767
New.CleanUp.ReferenceOTU306941

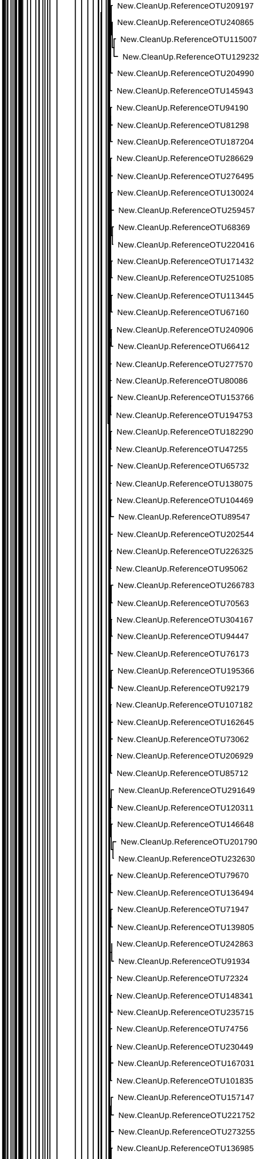
New.CleanUp.ReferenceOTU209197
New.CleanUp.ReferenceOTU240865
New.CleanUp.ReferenceOTU115007
New.CleanUp.ReferenceOTU129232
New.CleanUp.ReferenceOTU204990
New.CleanUp.ReferenceOTU145943
New.CleanUp.ReferenceOTU94190
New.CleanUp.ReferenceOTU81298
New.CleanUp.ReferenceOTU187204
New.CleanUp.ReferenceOTU286629
New.CleanUp.ReferenceOTU276495
New.CleanUp.ReferenceOTU130024
New.CleanUp.ReferenceOTU259457
New.CleanUp.ReferenceOTU68369
New.CleanUp.ReferenceOTU220416
New.CleanUp.ReferenceOTU171432
New.CleanUp.ReferenceOTU251085
New.CleanUp.ReferenceOTU113445
New.CleanUp.ReferenceOTU67160
New.CleanUp.ReferenceOTU240906
New.CleanUp.ReferenceOTU66412
New.CleanUp.ReferenceOTU277570
New.CleanUp.ReferenceOTU80086
New.CleanUp.ReferenceOTU153766
New.CleanUp.ReferenceOTU194753
New.CleanUp.ReferenceOTU182290
New.CleanUp.ReferenceOTU47255
New.CleanUp.ReferenceOTU65732
New.CleanUp.ReferenceOTU138075
New.CleanUp.ReferenceOTU104469
New.CleanUp.ReferenceOTU89547
New.CleanUp.ReferenceOTU202544
New.CleanUp.ReferenceOTU226325
New.CleanUp.ReferenceOTU95062
New.CleanUp.ReferenceOTU266783
New.CleanUp.ReferenceOTU70563
New.CleanUp.ReferenceOTU304167
New.CleanUp.ReferenceOTU94447
New.CleanUp.ReferenceOTU76173
New.CleanUp.ReferenceOTU195366
New.CleanUp.ReferenceOTU92179
New.CleanUp.ReferenceOTU107182
New.CleanUp.ReferenceOTU162645
New.CleanUp.ReferenceOTU73062
New.CleanUp.ReferenceOTU206929
New.CleanUp.ReferenceOTU85712
New.CleanUp.ReferenceOTU291649
New.CleanUp.ReferenceOTU120311
New.CleanUp.ReferenceOTU146648
New.CleanUp.ReferenceOTU201790
New.CleanUp.ReferenceOTU232630
New.CleanUp.ReferenceOTU79670
New.CleanUp.ReferenceOTU136494
New.CleanUp.ReferenceOTU71947
New.CleanUp.ReferenceOTU139805
New.CleanUp.ReferenceOTU242863
New.CleanUp.ReferenceOTU91934
New.CleanUp.ReferenceOTU72324
New.CleanUp.ReferenceOTU148341
New.CleanUp.ReferenceOTU235715
New.CleanUp.ReferenceOTU74756
New.CleanUp.ReferenceOTU230449
New.CleanUp.ReferenceOTU167031
New.CleanUp.ReferenceOTU101835
New.CleanUp.ReferenceOTU157147
New.CleanUp.ReferenceOTU221752
New.CleanUp.ReferenceOTU273255
New.CleanUp.ReferenceOTU136985

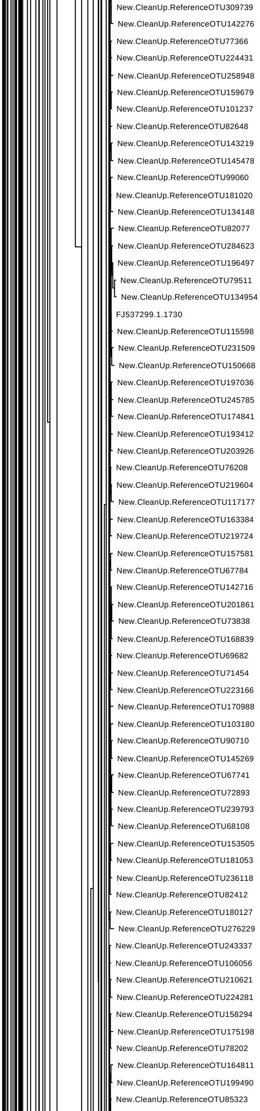
New.CleanUp.ReferenceOTU309739
New.CleanUp.ReferenceOTU142276
New.CleanUp.ReferenceOTU77366
New.CleanUp.ReferenceOTU224431
New.CleanUp.ReferenceOTU258948
New.CleanUp.ReferenceOTU159679
New.CleanUp.ReferenceOTU101237
New.CleanUp.ReferenceOTU82648
New.CleanUp.ReferenceOTU143219
New.CleanUp.ReferenceOTU145478
New.CleanUp.ReferenceOTU99060
New.CleanUp.ReferenceOTU181020
New.CleanUp.ReferenceOTU134148
New.CleanUp.ReferenceOTU82077
New.CleanUp.ReferenceOTU284623
New.CleanUp.ReferenceOTU196497
New.CleanUp.ReferenceOTU79511
New.CleanUp.ReferenceOTU134954
FJ537299.1.1730
New.CleanUp.ReferenceOTU115598
New.CleanUp.ReferenceOTU231509
New.CleanUp.ReferenceOTU150668
New.CleanUp.ReferenceOTU197036
New.CleanUp.ReferenceOTU245785
New.CleanUp.ReferenceOTU174841
New.CleanUp.ReferenceOTU193412
New.CleanUp.ReferenceOTU203926
New.CleanUp.ReferenceOTU76208
New.CleanUp.ReferenceOTU219604
New.CleanUp.ReferenceOTU117177
New.CleanUp.ReferenceOTU163384
New.CleanUp.ReferenceOTU219724
New.CleanUp.ReferenceOTU157581
New.CleanUp.ReferenceOTU67784
New.CleanUp.ReferenceOTU142716
New.CleanUp.ReferenceOTU201861
New.CleanUp.ReferenceOTU73838
New.CleanUp.ReferenceOTU168839
New.CleanUp.ReferenceOTU69682
New.CleanUp.ReferenceOTU71454
New.CleanUp.ReferenceOTU223166
New.CleanUp.ReferenceOTU170988
New.CleanUp.ReferenceOTU103180
New.CleanUp.ReferenceOTU90710
New.CleanUp.ReferenceOTU145269
New.CleanUp.ReferenceOTU67741
New.CleanUp.ReferenceOTU72893
New.CleanUp.ReferenceOTU239793
New.CleanUp.ReferenceOTU68108
New.CleanUp.ReferenceOTU153505
New.CleanUp.ReferenceOTU181053
New.CleanUp.ReferenceOTU236118
New.CleanUp.ReferenceOTU82412
New.CleanUp.ReferenceOTU180127
New.CleanUp.ReferenceOTU276229
New.CleanUp.ReferenceOTU243337
New.CleanUp.ReferenceOTU106056
New.CleanUp.ReferenceOTU210621
New.CleanUp.ReferenceOTU224281
New.CleanUp.ReferenceOTU158294
New.CleanUp.ReferenceOTU175198
New.CleanUp.ReferenceOTU78202
New.CleanUp.ReferenceOTU164811
New.CleanUp.ReferenceOTU199490
New.CleanUp.ReferenceOTU85323

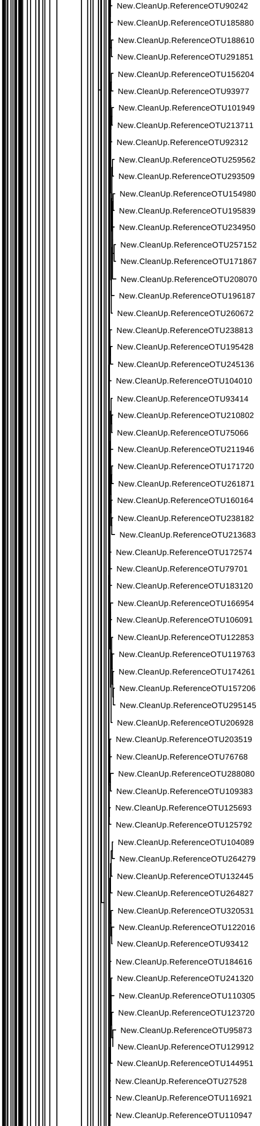
New.CleanUp.ReferenceOTU90242
New.CleanUp.ReferenceOTU185880
New.CleanUp.ReferenceOTU188610
New.CleanUp.ReferenceOTU291851
New.CleanUp.ReferenceOTU156204
New.CleanUp.ReferenceOTU93977
New.CleanUp.ReferenceOTU101949
New.CleanUp.ReferenceOTU213711
New.CleanUp.ReferenceOTU92312
New.CleanUp.ReferenceOTU259562
New.CleanUp.ReferenceOTU293509
New.CleanUp.ReferenceOTU154980
New.CleanUp.ReferenceOTU195839
New.CleanUp.ReferenceOTU234950
New.CleanUp.ReferenceOTU257152
New.CleanUp.ReferenceOTU171867
New.CleanUp.ReferenceOTU208070
New.CleanUp.ReferenceOTU196187
New.CleanUp.ReferenceOTU260672
New.CleanUp.ReferenceOTU238813
New.CleanUp.ReferenceOTU195428
New.CleanUp.ReferenceOTU245136
New.CleanUp.ReferenceOTU104010
New.CleanUp.ReferenceOTU93414
New.CleanUp.ReferenceOTU210802
New.CleanUp.ReferenceOTU75066
New.CleanUp.ReferenceOTU211946
New.CleanUp.ReferenceOTU171720
New.CleanUp.ReferenceOTU261871
New.CleanUp.ReferenceOTU160164
New.CleanUp.ReferenceOTU238182
New.CleanUp.ReferenceOTU213683
New.CleanUp.ReferenceOTU172574
New.CleanUp.ReferenceOTU79701
New.CleanUp.ReferenceOTU183120
New.CleanUp.ReferenceOTU166954
New.CleanUp.ReferenceOTU106091
New.CleanUp.ReferenceOTU122853
New.CleanUp.ReferenceOTU119763
New.CleanUp.ReferenceOTU174261
New.CleanUp.ReferenceOTU157206
New.CleanUp.ReferenceOTU295145
New.CleanUp.ReferenceOTU206928
New.CleanUp.ReferenceOTU203519
New.CleanUp.ReferenceOTU76768
New.CleanUp.ReferenceOTU288080
New.CleanUp.ReferenceOTU109383
New.CleanUp.ReferenceOTU125693
New.CleanUp.ReferenceOTU125792
New.CleanUp.ReferenceOTU104089
New.CleanUp.ReferenceOTU264279
New.CleanUp.ReferenceOTU132445
New.CleanUp.ReferenceOTU264827
New.CleanUp.ReferenceOTU320531
New.CleanUp.ReferenceOTU122016
New.CleanUp.ReferenceOTU93412
New.CleanUp.ReferenceOTU184616
New.CleanUp.ReferenceOTU241320
New.CleanUp.ReferenceOTU110305
New.CleanUp.ReferenceOTU123720
New.CleanUp.ReferenceOTU95873
New.CleanUp.ReferenceOTU129912
New.CleanUp.ReferenceOTU144951
New.CleanUp.ReferenceOTU27528
New.CleanUp.ReferenceOTU116921
New.CleanUp.ReferenceOTU110947

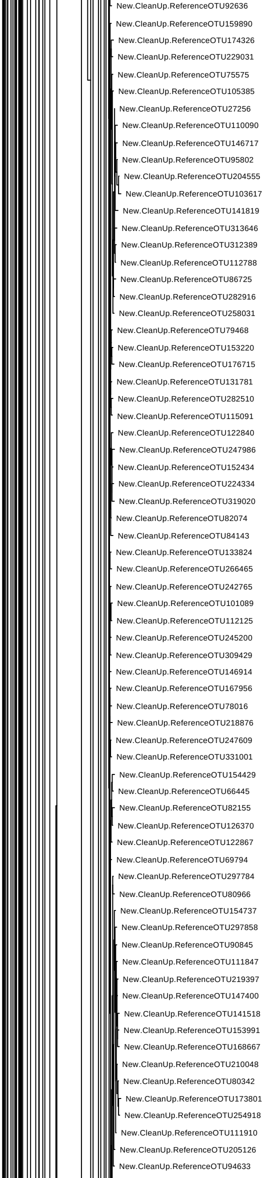

New.CleanUp.ReferenceOTU92636
New.CleanUp.ReferenceOTU159890
New.CleanUp.ReferenceOTU174326
New.CleanUp.ReferenceOTU229031
New.CleanUp.ReferenceOTU75575
New.CleanUp.ReferenceOTU105385
New.CleanUp.ReferenceOTU27256
New.CleanUp.ReferenceOTU110090
New.CleanUp.ReferenceOTU146717
New.CleanUp.ReferenceOTU95802
New.CleanUp.ReferenceOTU204555
New.CleanUp.ReferenceOTU103617
New.CleanUp.ReferenceOTU141819
New.CleanUp.ReferenceOTU313646
New.CleanUp.ReferenceOTU312389
New.CleanUp.ReferenceOTU112788
New.CleanUp.ReferenceOTU86725
New.CleanUp.ReferenceOTU282916
New.CleanUp.ReferenceOTU258031
New.CleanUp.ReferenceOTU79468
New.CleanUp.ReferenceOTU153220
New.CleanUp.ReferenceOTU176715
New.CleanUp.ReferenceOTU131781
New.CleanUp.ReferenceOTU282510
New.CleanUp.ReferenceOTU115091
New.CleanUp.ReferenceOTU122840
New.CleanUp.ReferenceOTU247986
New.CleanUp.ReferenceOTU152434
New.CleanUp.ReferenceOTU224334
New.CleanUp.ReferenceOTU319020
New.CleanUp.ReferenceOTU82074
New.CleanUp.ReferenceOTU84143
New.CleanUp.ReferenceOTU133824
New.CleanUp.ReferenceOTU266465
New.CleanUp.ReferenceOTU242765
New.CleanUp.ReferenceOTU101089
New.CleanUp.ReferenceOTU112125
New.CleanUp.ReferenceOTU245200
New.CleanUp.ReferenceOTU309429
New.CleanUp.ReferenceOTU146914
New.CleanUp.ReferenceOTU167956
New.CleanUp.ReferenceOTU78016
New.CleanUp.ReferenceOTU218876
New.CleanUp.ReferenceOTU247609
New.CleanUp.ReferenceOTU331001
New.CleanUp.ReferenceOTU154429
New.CleanUp.ReferenceOTU66445
New.CleanUp.ReferenceOTU82155
New.CleanUp.ReferenceOTU126370
New.CleanUp.ReferenceOTU122867
New.CleanUp.ReferenceOTU69794
New.CleanUp.ReferenceOTU297784
New.CleanUp.ReferenceOTU80966
New.CleanUp.ReferenceOTU154737
New.CleanUp.ReferenceOTU297858
New.CleanUp.ReferenceOTU90845
New.CleanUp.ReferenceOTU111847
New.CleanUp.ReferenceOTU219397
New.CleanUp.ReferenceOTU147400
New.CleanUp.ReferenceOTU141518
New.CleanUp.ReferenceOTU153991
New.CleanUp.ReferenceOTU168667
New.CleanUp.ReferenceOTU210048
New.CleanUp.ReferenceOTU80342
New.CleanUp.ReferenceOTU173801
New.CleanUp.ReferenceOTU254918
New.CleanUp.ReferenceOTU111910
New.CleanUp.ReferenceOTU205126
New.CleanUp.ReferenceOTU94633

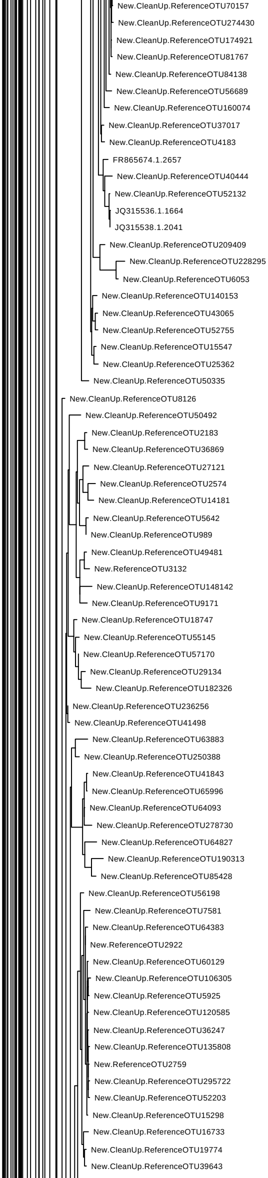
New.CleanUp.ReferenceOTU70157
New.CleanUp.ReferenceOTU274430
New.CleanUp.ReferenceOTU174921
New.CleanUp.ReferenceOTU81767
New.CleanUp.ReferenceOTU84138
New.CleanUp.ReferenceOTU56689
New.CleanUp.ReferenceOTU160074
New.CleanUp.ReferenceOTU37017
New.CleanUp.ReferenceOTU4183
FR865674.1.2657
New.CleanUp.ReferenceOTU40444
New.CleanUp.ReferenceOTU52132
JQ315536.1.1664
JQ315538.1.2041
New.CleanUp.ReferenceOTU209409
New.CleanUp.ReferenceOTU228295
New.CleanUp.ReferenceOTU6053
New.CleanUp.ReferenceOTU140153
New.CleanUp.ReferenceOTU43065
New.CleanUp.ReferenceOTU52755
New.CleanUp.ReferenceOTU15547
New.CleanUp.ReferenceOTU25362
New.CleanUp.ReferenceOTU50335
New.CleanUp.ReferenceOTU8126
New.CleanUp.ReferenceOTU50492
New.CleanUp.ReferenceOTU2183
New.CleanUp.ReferenceOTU36869
New.CleanUp.ReferenceOTU27121
New.CleanUp.ReferenceOTU2574
New.CleanUp.ReferenceOTU14181
New.CleanUp.ReferenceOTU5642
New.CleanUp.ReferenceOTU989
New.CleanUp.ReferenceOTU49481
New.ReferenceOTU3132
New.CleanUp.ReferenceOTU148142
New.CleanUp.ReferenceOTU9171
New.CleanUp.ReferenceOTU18747
New.CleanUp.ReferenceOTU55145
New.CleanUp.ReferenceOTU57170
New.CleanUp.ReferenceOTU29134
New.CleanUp.ReferenceOTU182326
New.CleanUp.ReferenceOTU236256
New.CleanUp.ReferenceOTU41498
New.CleanUp.ReferenceOTU63883
New.CleanUp.ReferenceOTU250388
New.CleanUp.ReferenceOTU41843
New.CleanUp.ReferenceOTU65996
New.CleanUp.ReferenceOTU64093
New.CleanUp.ReferenceOTU278730
New.CleanUp.ReferenceOTU64827
New.CleanUp.ReferenceOTU190313
New.CleanUp.ReferenceOTU85428
New.CleanUp.ReferenceOTU56198
New.CleanUp.ReferenceOTU7581
New.CleanUp.ReferenceOTU64383
New.ReferenceOTU2922
New.CleanUp.ReferenceOTU60129
New.CleanUp.ReferenceOTU106305
New.CleanUp.ReferenceOTU5925
New.CleanUp.ReferenceOTU120585
New.CleanUp.ReferenceOTU36247
New.CleanUp.ReferenceOTU135808
New.ReferenceOTU2759
New.CleanUp.ReferenceOTU295722
New.CleanUp.ReferenceOTU52203
New.CleanUp.ReferenceOTU15298
New.CleanUp.ReferenceOTU16733
New.CleanUp.ReferenceOTU19774
New.CleanUp.ReferenceOTU39643

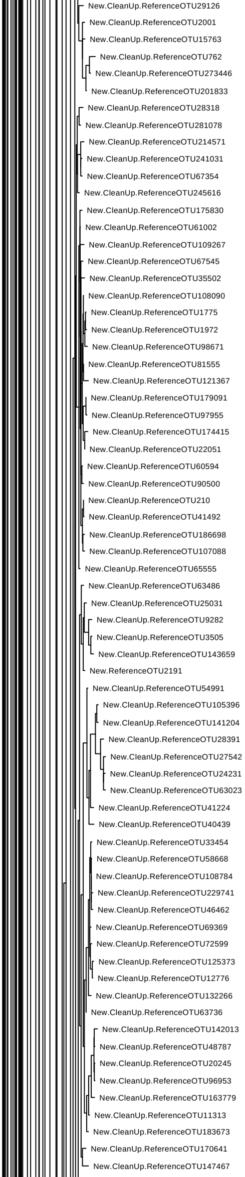
New.CleanUp.ReferenceOTU29126
New.CleanUp.ReferenceOTU2001
New.CleanUp.ReferenceOTU15763
New.CleanUp.ReferenceOTU762
New.CleanUp.ReferenceOTU273446
New.CleanUp.ReferenceOTU201833
New.CleanUp.ReferenceOTU28318
New.CleanUp.ReferenceOTU281078
New.CleanUp.ReferenceOTU214571
New.CleanUp.ReferenceOTU241031
New.CleanUp.ReferenceOTU67354
New.CleanUp.ReferenceOTU245616
New.CleanUp.ReferenceOTU175830
New.CleanUp.ReferenceOTU61002
New.CleanUp.ReferenceOTU109267
New.CleanUp.ReferenceOTU67545
New.CleanUp.ReferenceOTU35502
New.CleanUp.ReferenceOTU108090
New.CleanUp.ReferenceOTU1775
New.CleanUp.ReferenceOTU1972
New.CleanUp.ReferenceOTU98671
New.CleanUp.ReferenceOTU81555
New.CleanUp.ReferenceOTU121367
New.CleanUp.ReferenceOTU179091
New.CleanUp.ReferenceOTU97955
New.CleanUp.ReferenceOTU174415
New.CleanUp.ReferenceOTU22051
New.CleanUp.ReferenceOTU60594
New.CleanUp.ReferenceOTU90500
New.CleanUp.ReferenceOTU210
New.CleanUp.ReferenceOTU41492
New.CleanUp.ReferenceOTU186698
New.CleanUp.ReferenceOTU107088
New.CleanUp.ReferenceOTU65555
New.CleanUp.ReferenceOTU63486
New.CleanUp.ReferenceOTU25031
New.CleanUp.ReferenceOTU9282
New.CleanUp.ReferenceOTU3505
New.CleanUp.ReferenceOTU143659
New.ReferenceOTU2191
New.CleanUp.ReferenceOTU54991
New.CleanUp.ReferenceOTU105396
New.CleanUp.ReferenceOTU141204
New.CleanUp.ReferenceOTU28391
New.CleanUp.ReferenceOTU27542
New.CleanUp.ReferenceOTU24231
New.CleanUp.ReferenceOTU63023
New.CleanUp.ReferenceOTU41224
New.CleanUp.ReferenceOTU40439
New.CleanUp.ReferenceOTU33454
New.CleanUp.ReferenceOTU58668
New.CleanUp.ReferenceOTU108784
New.CleanUp.ReferenceOTU229741
New.CleanUp.ReferenceOTU46462
New.CleanUp.ReferenceOTU69369
New.CleanUp.ReferenceOTU72599
New.CleanUp.ReferenceOTU125373
New.CleanUp.ReferenceOTU12776
New.CleanUp.ReferenceOTU132266
New.CleanUp.ReferenceOTU63736
New.CleanUp.ReferenceOTU142013
New.CleanUp.ReferenceOTU48787
New.CleanUp.ReferenceOTU20245
New.CleanUp.ReferenceOTU96953
New.CleanUp.ReferenceOTU163779
New.CleanUp.ReferenceOTU11313
New.CleanUp.ReferenceOTU183673
New.CleanUp.ReferenceOTU170641
New.CleanUp.ReferenceOTU147467

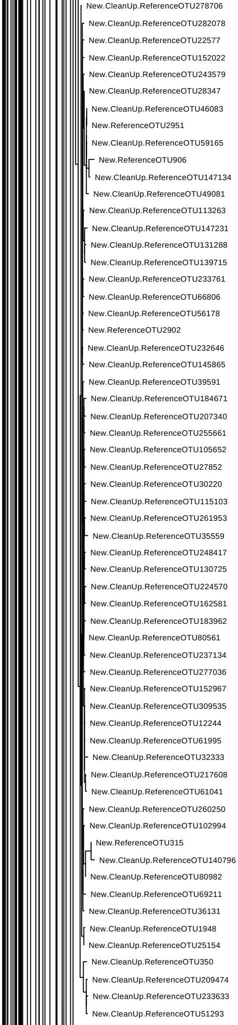
New.CleanUp.ReferenceOTU278706
New.CleanUp.ReferenceOTU282078
New.CleanUp.ReferenceOTU22577
New.CleanUp.ReferenceOTU152022
New.CleanUp.ReferenceOTU243579
New.CleanUp.ReferenceOTU28347
New.CleanUp.ReferenceOTU46083
New.ReferenceOTU2951
New.CleanUp.ReferenceOTU59165
New.ReferenceOTU906
New.CleanUp.ReferenceOTU147134
New.CleanUp.ReferenceOTU49081
New.CleanUp.ReferenceOTU113263
New.CleanUp.ReferenceOTU147231
New.CleanUp.ReferenceOTU131288
New.CleanUp.ReferenceOTU139715
New.CleanUp.ReferenceOTU233761
New.CleanUp.ReferenceOTU66806
New.CleanUp.ReferenceOTU56178
New.ReferenceOTU2902
New.CleanUp.ReferenceOTU232646
New.CleanUp.ReferenceOTU145865
New.CleanUp.ReferenceOTU39591
New.CleanUp.ReferenceOTU184671
New.CleanUp.ReferenceOTU207340
New.CleanUp.ReferenceOTU255661
New.CleanUp.ReferenceOTU105652
New.CleanUp.ReferenceOTU27852
New.CleanUp.ReferenceOTU30220
New.CleanUp.ReferenceOTU115103
New.CleanUp.ReferenceOTU261953
New.CleanUp.ReferenceOTU35559
New.CleanUp.ReferenceOTU248417
New.CleanUp.ReferenceOTU130725
New.CleanUp.ReferenceOTU224570
New.CleanUp.ReferenceOTU162581
New.CleanUp.ReferenceOTU183962
New.CleanUp.ReferenceOTU80561
New.CleanUp.ReferenceOTU237134
New.CleanUp.ReferenceOTU277036
New.CleanUp.ReferenceOTU152967
New.CleanUp.ReferenceOTU309535
New.CleanUp.ReferenceOTU12244
New.CleanUp.ReferenceOTU61995
New.CleanUp.ReferenceOTU32333
New.CleanUp.ReferenceOTU217608
New.CleanUp.ReferenceOTU61041
New.CleanUp.ReferenceOTU260250
New.CleanUp.ReferenceOTU102994
New.ReferenceOTU315
New.CleanUp.ReferenceOTU140796
New.CleanUp.ReferenceOTU80982
New.CleanUp.ReferenceOTU69211
New.CleanUp.ReferenceOTU36131
New.CleanUp.ReferenceOTU1948
New.CleanUp.ReferenceOTU25154
New.CleanUp.ReferenceOTU350
New.CleanUp.ReferenceOTU209474
New.CleanUp.ReferenceOTU233633
New.CleanUp.ReferenceOTU51293

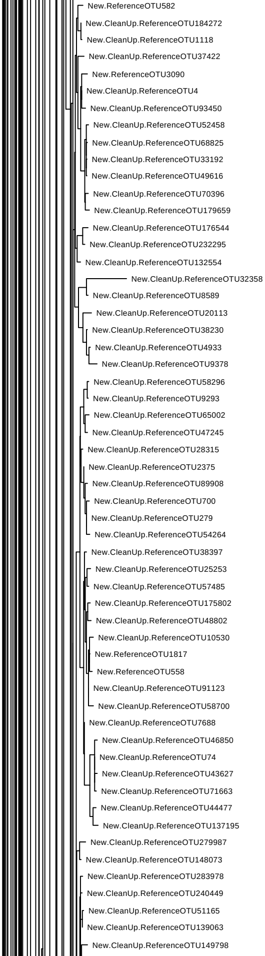
New.ReferenceOTU582
New.CleanUp.ReferenceOTU184272
New.CleanUp.ReferenceOTU1118
New.CleanUp.ReferenceOTU37422
New.ReferenceOTU3090
New.CleanUp.ReferenceOTU4
New.CleanUp.ReferenceOTU93450
New.CleanUp.ReferenceOTU52458
New.CleanUp.ReferenceOTU68825
New.CleanUp.ReferenceOTU33192
New.CleanUp.ReferenceOTU49616
New.CleanUp.ReferenceOTU70396
New.CleanUp.ReferenceOTU179659
New.CleanUp.ReferenceOTU176544
New.CleanUp.ReferenceOTU232295
New.CleanUp.ReferenceOTU132554
New.CleanUp.ReferenceOTU32358
New.CleanUp.ReferenceOTU8589
New.CleanUp.ReferenceOTU20113
New.CleanUp.ReferenceOTU38230
New.CleanUp.ReferenceOTU4933
New.CleanUp.ReferenceOTU9378
New.CleanUp.ReferenceOTU58296
New.CleanUp.ReferenceOTU9293
New.CleanUp.ReferenceOTU65002
New.CleanUp.ReferenceOTU47245
New.CleanUp.ReferenceOTU28315
New.CleanUp.ReferenceOTU2375
New.CleanUp.ReferenceOTU89908
New.CleanUp.ReferenceOTU700
New.CleanUp.ReferenceOTU279
New.CleanUp.ReferenceOTU54264
New.CleanUp.ReferenceOTU38397
New.CleanUp.ReferenceOTU25253
New.CleanUp.ReferenceOTU57485
New.CleanUp.ReferenceOTU175802
New.CleanUp.ReferenceOTU48802
New.CleanUp.ReferenceOTU10530
New.ReferenceOTU1817
New.ReferenceOTU558
New.CleanUp.ReferenceOTU91123
New.CleanUp.ReferenceOTU58700
New.CleanUp.ReferenceOTU7688
New.CleanUp.ReferenceOTU46850
New.CleanUp.ReferenceOTU74
New.CleanUp.ReferenceOTU43627
New.CleanUp.ReferenceOTU71663
New.CleanUp.ReferenceOTU44477
New.CleanUp.ReferenceOTU137195
New.CleanUp.ReferenceOTU279987
New.CleanUp.ReferenceOTU148073
New.CleanUp.ReferenceOTU283978
New.CleanUp.ReferenceOTU240449
New.CleanUp.ReferenceOTU51165
New.CleanUp.ReferenceOTU139063
New.CleanUp.ReferenceOTU149798

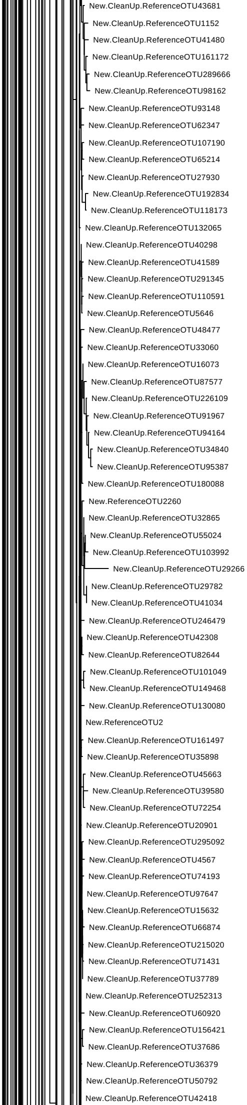
New.CleanUp.ReferenceOTU43681
New.CleanUp.ReferenceOTU1152
New.CleanUp.ReferenceOTU41480
New.CleanUp.ReferenceOTU161172
New.CleanUp.ReferenceOTU289666
New.CleanUp.ReferenceOTU98162
New.CleanUp.ReferenceOTU93148
New.CleanUp.ReferenceOTU62347
New.CleanUp.ReferenceOTU107190
New.CleanUp.ReferenceOTU65214
New.CleanUp.ReferenceOTU27930
New.CleanUp.ReferenceOTU192834
New.CleanUp.ReferenceOTU118173
New.CleanUp.ReferenceOTU132065
New.CleanUp.ReferenceOTU40298
New.CleanUp.ReferenceOTU41589
New.CleanUp.ReferenceOTU291345
New.CleanUp.ReferenceOTU110591
New.CleanUp.ReferenceOTU5646
New.CleanUp.ReferenceOTU48477
New.CleanUp.ReferenceOTU33060
New.CleanUp.ReferenceOTU16073
New.CleanUp.ReferenceOTU87577
New.CleanUp.ReferenceOTU226109
New.CleanUp.ReferenceOTU91967
New.CleanUp.ReferenceOTU94164
New.CleanUp.ReferenceOTU34840
New.CleanUp.ReferenceOTU95387
New.CleanUp.ReferenceOTU180088
New.ReferenceOTU2260
New.CleanUp.ReferenceOTU32865
New.CleanUp.ReferenceOTU55024
New.CleanUp.ReferenceOTU103992
New.CleanUp.ReferenceOTU29266
New.CleanUp.ReferenceOTU29782
New.CleanUp.ReferenceOTU41034
New.CleanUp.ReferenceOTU246479
New.CleanUp.ReferenceOTU42308
New.CleanUp.ReferenceOTU82644
New.CleanUp.ReferenceOTU101049
New.CleanUp.ReferenceOTU149468
New.CleanUp.ReferenceOTU130080
New.ReferenceOTU2
New.CleanUp.ReferenceOTU161497
New.CleanUp.ReferenceOTU35898
New.CleanUp.ReferenceOTU45663
New.CleanUp.ReferenceOTU39580
New.CleanUp.ReferenceOTU72254
New.CleanUp.ReferenceOTU20901
New.CleanUp.ReferenceOTU295092
New.CleanUp.ReferenceOTU4567
New.CleanUp.ReferenceOTU74193
New.CleanUp.ReferenceOTU97647
New.CleanUp.ReferenceOTU15632
New.CleanUp.ReferenceOTU66874
New.CleanUp.ReferenceOTU215020
New.CleanUp.ReferenceOTU71431
New.CleanUp.ReferenceOTU37789
New.CleanUp.ReferenceOTU252313
New.CleanUp.ReferenceOTU60920
New.CleanUp.ReferenceOTU156421
New.CleanUp.ReferenceOTU37686
New.CleanUp.ReferenceOTU36379
New.CleanUp.ReferenceOTU50792
New.CleanUp.ReferenceOTU42418

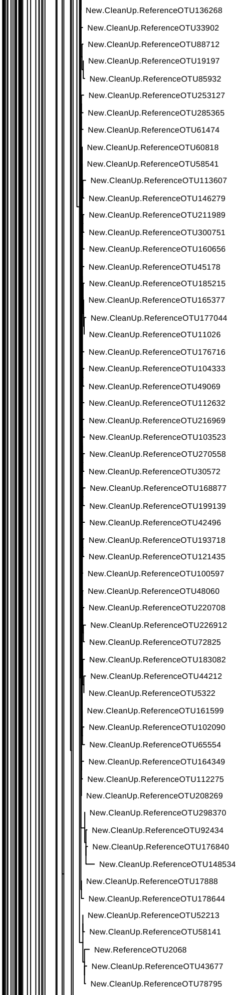
New.CleanUp.ReferenceOTU136268
New.CleanUp.ReferenceOTU33902
New.CleanUp.ReferenceOTU88712
New.CleanUp.ReferenceOTU19197
New.CleanUp.ReferenceOTU85932
New.CleanUp.ReferenceOTU253127
New.CleanUp.ReferenceOTU285365
New.CleanUp.ReferenceOTU61474
New.CleanUp.ReferenceOTU60818
New.CleanUp.ReferenceOTU58541
New.CleanUp.ReferenceOTU113607
New.CleanUp.ReferenceOTU146279
New.CleanUp.ReferenceOTU211989
New.CleanUp.ReferenceOTU300751
New.CleanUp.ReferenceOTU160656
New.CleanUp.ReferenceOTU45178
New.CleanUp.ReferenceOTU185215
New.CleanUp.ReferenceOTU165377
New.CleanUp.ReferenceOTU177044
New.CleanUp.ReferenceOTU11026
New.CleanUp.ReferenceOTU176716
New.CleanUp.ReferenceOTU104333
New.CleanUp.ReferenceOTU49069
New.CleanUp.ReferenceOTU112632
New.CleanUp.ReferenceOTU216969
New.CleanUp.ReferenceOTU103523
New.CleanUp.ReferenceOTU270558
New.CleanUp.ReferenceOTU30572
New.CleanUp.ReferenceOTU168877
New.CleanUp.ReferenceOTU199139
New.CleanUp.ReferenceOTU42496
New.CleanUp.ReferenceOTU193718
New.CleanUp.ReferenceOTU121435
New.CleanUp.ReferenceOTU100597
New.CleanUp.ReferenceOTU48060
New.CleanUp.ReferenceOTU220708
New.CleanUp.ReferenceOTU226912
New.CleanUp.ReferenceOTU72825
New.CleanUp.ReferenceOTU183082
New.CleanUp.ReferenceOTU44212
New.CleanUp.ReferenceOTU5322
New.CleanUp.ReferenceOTU161599
New.CleanUp.ReferenceOTU102090
New.CleanUp.ReferenceOTU65554
New.CleanUp.ReferenceOTU164349
New.CleanUp.ReferenceOTU112275
New.CleanUp.ReferenceOTU208269
New.CleanUp.ReferenceOTU298370
New.CleanUp.ReferenceOTU92434
New.CleanUp.ReferenceOTU176840
New.CleanUp.ReferenceOTU148534
New.CleanUp.ReferenceOTU17888
New.CleanUp.ReferenceOTU178644
New.CleanUp.ReferenceOTU52213
New.CleanUp.ReferenceOTU58141
New.ReferenceOTU2068
New.CleanUp.ReferenceOTU43677
New.CleanUp.ReferenceOTU78795

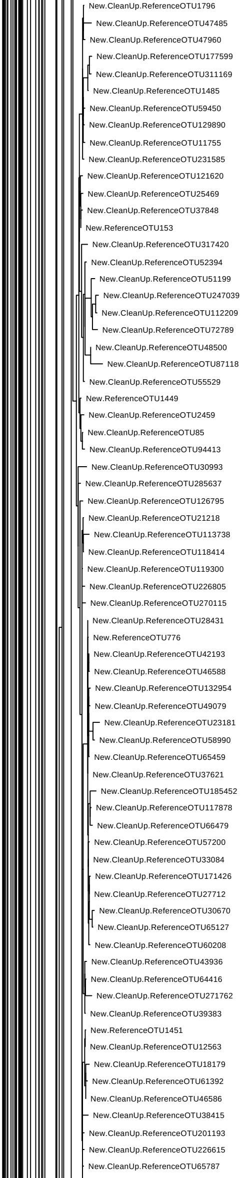
New.CleanUp.ReferenceOTU1796
New.CleanUp.ReferenceOTU47485
New.CleanUp.ReferenceOTU47960
New.CleanUp.ReferenceOTU177599
New.CleanUp.ReferenceOTU311169
New.CleanUp.ReferenceOTU1485
New.CleanUp.ReferenceOTU59450
New.CleanUp.ReferenceOTU129890
New.CleanUp.ReferenceOTU11755
New.CleanUp.ReferenceOTU231585
New.CleanUp.ReferenceOTU121620
New.CleanUp.ReferenceOTU25469
New.CleanUp.ReferenceOTU37848
New.ReferenceOTU153
New.CleanUp.ReferenceOTU317420
New.CleanUp.ReferenceOTU52394
New.CleanUp.ReferenceOTU51199
New.CleanUp.ReferenceOTU247039
New.CleanUp.ReferenceOTU112209
New.CleanUp.ReferenceOTU72789
New.CleanUp.ReferenceOTU48500
New.CleanUp.ReferenceOTU87118
New.CleanUp.ReferenceOTU55529
New.ReferenceOTU1449
New.CleanUp.ReferenceOTU2459
New.CleanUp.ReferenceOTU85
New.CleanUp.ReferenceOTU94413
New.CleanUp.ReferenceOTU30993
New.CleanUp.ReferenceOTU285637
New.CleanUp.ReferenceOTU126795
New.CleanUp.ReferenceOTU21218
New.CleanUp.ReferenceOTU113738
New.CleanUp.ReferenceOTU118414
New.CleanUp.ReferenceOTU119300
New.CleanUp.ReferenceOTU226805
New.CleanUp.ReferenceOTU270115
New.CleanUp.ReferenceOTU28431
New.ReferenceOTU776
New.CleanUp.ReferenceOTU42193
New.CleanUp.ReferenceOTU46588
New.CleanUp.ReferenceOTU132954
New.CleanUp.ReferenceOTU49079
New.CleanUp.ReferenceOTU23181
New.CleanUp.ReferenceOTU58990
New.CleanUp.ReferenceOTU65459
New.CleanUp.ReferenceOTU37621
New.CleanUp.ReferenceOTU185452
New.CleanUp.ReferenceOTU117878
New.CleanUp.ReferenceOTU66479
New.CleanUp.ReferenceOTU57200
New.CleanUp.ReferenceOTU33084
New.CleanUp.ReferenceOTU171426
New.CleanUp.ReferenceOTU27712
New.CleanUp.ReferenceOTU30670
New.CleanUp.ReferenceOTU65127
New.CleanUp.ReferenceOTU60208
New.CleanUp.ReferenceOTU43936
New.CleanUp.ReferenceOTU64416
New.CleanUp.ReferenceOTU271762
New.CleanUp.ReferenceOTU39383
New.ReferenceOTU1451
New.CleanUp.ReferenceOTU12563
New.CleanUp.ReferenceOTU18179
New.CleanUp.ReferenceOTU61392
New.CleanUp.ReferenceOTU46586
New.CleanUp.ReferenceOTU38415
New.CleanUp.ReferenceOTU201193
New.CleanUp.ReferenceOTU226615
New.CleanUp.ReferenceOTU65787

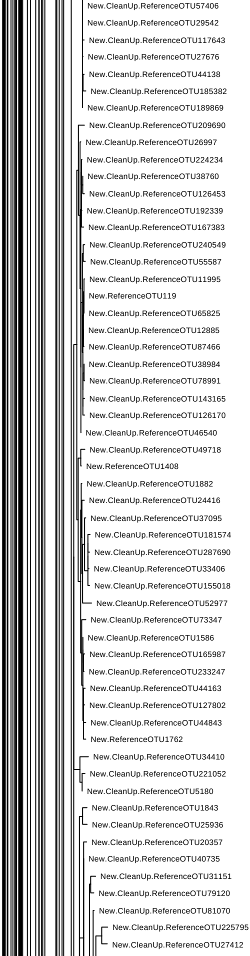
New.CleanUp.ReferenceOTU57406
New.CleanUp.ReferenceOTU29542
New.CleanUp.ReferenceOTU117643
New.CleanUp.ReferenceOTU27676
New.CleanUp.ReferenceOTU44138
New.CleanUp.ReferenceOTU185382
New.CleanUp.ReferenceOTU189869
New.CleanUp.ReferenceOTU209690
New.CleanUp.ReferenceOTU26997
New.CleanUp.ReferenceOTU224234
New.CleanUp.ReferenceOTU38760
New.CleanUp.ReferenceOTU126453
New.CleanUp.ReferenceOTU192339
New.CleanUp.ReferenceOTU167383
New.CleanUp.ReferenceOTU240549
New.CleanUp.ReferenceOTU55587
New.CleanUp.ReferenceOTU11995
New.ReferenceOTU119
New.CleanUp.ReferenceOTU65825
New.CleanUp.ReferenceOTU12885
New.CleanUp.ReferenceOTU87466
New.CleanUp.ReferenceOTU38984
New.CleanUp.ReferenceOTU78991
New.CleanUp.ReferenceOTU143165
New.CleanUp.ReferenceOTU126170
New.CleanUp.ReferenceOTU46540
New.CleanUp.ReferenceOTU49718
New.ReferenceOTU1408
New.CleanUp.ReferenceOTU1882
New.CleanUp.ReferenceOTU24416
New.CleanUp.ReferenceOTU37095
New.CleanUp.ReferenceOTU181574
New.CleanUp.ReferenceOTU287690
New.CleanUp.ReferenceOTU33406
New.CleanUp.ReferenceOTU155018
New.CleanUp.ReferenceOTU52977
New.CleanUp.ReferenceOTU73347
New.CleanUp.ReferenceOTU1586
New.CleanUp.ReferenceOTU165987
New.CleanUp.ReferenceOTU233247
New.CleanUp.ReferenceOTU44163
New.CleanUp.ReferenceOTU127802
New.CleanUp.ReferenceOTU44843
New.ReferenceOTU1762
New.CleanUp.ReferenceOTU34410
New.CleanUp.ReferenceOTU221052
New.CleanUp.ReferenceOTU5180
New.CleanUp.ReferenceOTU1843
New.CleanUp.ReferenceOTU25936
New.CleanUp.ReferenceOTU20357
New.CleanUp.ReferenceOTU40735
New.CleanUp.ReferenceOTU31151
New.CleanUp.ReferenceOTU79120
New.CleanUp.ReferenceOTU81070
New.CleanUp.ReferenceOTU225795
New.CleanUp.ReferenceOTU27412

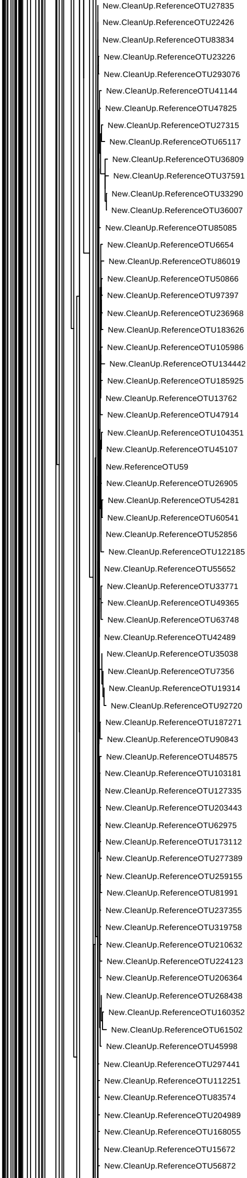
New.CleanUp.ReferenceOTU27835
New.CleanUp.ReferenceOTU22426
New.CleanUp.ReferenceOTU83834
New.CleanUp.ReferenceOTU23226
New.CleanUp.ReferenceOTU293076
New.CleanUp.ReferenceOTU41144
New.CleanUp.ReferenceOTU47825
New.CleanUp.ReferenceOTU27315
New.CleanUp.ReferenceOTU65117
New.CleanUp.ReferenceOTU36809
New.CleanUp.ReferenceOTU37591
New.CleanUp.ReferenceOTU33290
New.CleanUp.ReferenceOTU36007
New.CleanUp.ReferenceOTU85085
New.CleanUp.ReferenceOTU6654
New.CleanUp.ReferenceOTU86019
New.CleanUp.ReferenceOTU50866
New.CleanUp.ReferenceOTU97397
New.CleanUp.ReferenceOTU236968
New.CleanUp.ReferenceOTU183626
New.CleanUp.ReferenceOTU105986
New.CleanUp.ReferenceOTU134442
New.CleanUp.ReferenceOTU185925
New.CleanUp.ReferenceOTU13762
New.CleanUp.ReferenceOTU47914
New.CleanUp.ReferenceOTU104351
New.CleanUp.ReferenceOTU45107
New.ReferenceOTU59
New.CleanUp.ReferenceOTU26905
New.CleanUp.ReferenceOTU54281
New.CleanUp.ReferenceOTU60541
New.CleanUp.ReferenceOTU52856
New.CleanUp.ReferenceOTU122185
New.CleanUp.ReferenceOTU55652
New.CleanUp.ReferenceOTU33771
New.CleanUp.ReferenceOTU49365
New.CleanUp.ReferenceOTU63748
New.CleanUp.ReferenceOTU42489
New.CleanUp.ReferenceOTU35038
New.CleanUp.ReferenceOTU7356
New.CleanUp.ReferenceOTU19314
New.CleanUp.ReferenceOTU92720
New.CleanUp.ReferenceOTU187271
New.CleanUp.ReferenceOTU90843
New.CleanUp.ReferenceOTU48575
New.CleanUp.ReferenceOTU103181
New.CleanUp.ReferenceOTU127335
New.CleanUp.ReferenceOTU203443
New.CleanUp.ReferenceOTU62975
New.CleanUp.ReferenceOTU173112
New.CleanUp.ReferenceOTU277389
New.CleanUp.ReferenceOTU259155
New.CleanUp.ReferenceOTU81991
New.CleanUp.ReferenceOTU237355
New.CleanUp.ReferenceOTU319758
New.CleanUp.ReferenceOTU210632
New.CleanUp.ReferenceOTU224123
New.CleanUp.ReferenceOTU206364
New.CleanUp.ReferenceOTU268438
New.CleanUp.ReferenceOTU160352
New.CleanUp.ReferenceOTU61502
New.CleanUp.ReferenceOTU45998
New.CleanUp.ReferenceOTU297441
New.CleanUp.ReferenceOTU112251
New.CleanUp.ReferenceOTU83574
New.CleanUp.ReferenceOTU204989
New.CleanUp.ReferenceOTU168055
New.CleanUp.ReferenceOTU15672
New.CleanUp.ReferenceOTU56872

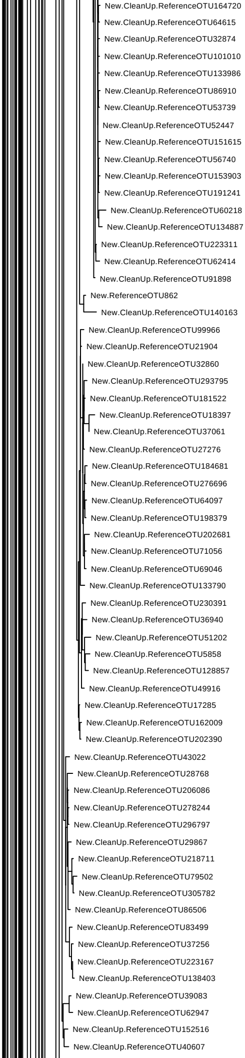
New.CleanUp.ReferenceOTU164720
New.CleanUp.ReferenceOTU64615
New.CleanUp.ReferenceOTU32874
New.CleanUp.ReferenceOTU101010
New.CleanUp.ReferenceOTU133986
New.CleanUp.ReferenceOTU86910
New.CleanUp.ReferenceOTU53739
New.CleanUp.ReferenceOTU52447
New.CleanUp.ReferenceOTU151615
New.CleanUp.ReferenceOTU56740
New.CleanUp.ReferenceOTU153903
New.CleanUp.ReferenceOTU191241
New.CleanUp.ReferenceOTU60218
New.CleanUp.ReferenceOTU134887
New.CleanUp.ReferenceOTU223311
New.CleanUp.ReferenceOTU62414
New.CleanUp.ReferenceOTU91898
New.ReferenceOTU862
New.CleanUp.ReferenceOTU140163
New.CleanUp.ReferenceOTU99966
New.CleanUp.ReferenceOTU21904
New.CleanUp.ReferenceOTU32860
New.CleanUp.ReferenceOTU293795
New.CleanUp.ReferenceOTU181522
New.CleanUp.ReferenceOTU18397
New.CleanUp.ReferenceOTU37061
New.CleanUp.ReferenceOTU27276
New.CleanUp.ReferenceOTU184681
New.CleanUp.ReferenceOTU276696
New.CleanUp.ReferenceOTU64097
New.CleanUp.ReferenceOTU198379
New.CleanUp.ReferenceOTU202681
New.CleanUp.ReferenceOTU71056
New.CleanUp.ReferenceOTU69046
New.CleanUp.ReferenceOTU133790
New.CleanUp.ReferenceOTU230391
New.CleanUp.ReferenceOTU36940
New.CleanUp.ReferenceOTU51202
New.CleanUp.ReferenceOTU5858
New.CleanUp.ReferenceOTU128857
New.CleanUp.ReferenceOTU49916
New.CleanUp.ReferenceOTU17285
New.CleanUp.ReferenceOTU162009
New.CleanUp.ReferenceOTU202390
New.CleanUp.ReferenceOTU43022
New.CleanUp.ReferenceOTU28768
New.CleanUp.ReferenceOTU206086
New.CleanUp.ReferenceOTU278244
New.CleanUp.ReferenceOTU296797
New.CleanUp.ReferenceOTU29867
New.CleanUp.ReferenceOTU218711
New.CleanUp.ReferenceOTU79502
New.CleanUp.ReferenceOTU305782
New.CleanUp.ReferenceOTU86506
New.CleanUp.ReferenceOTU83499
New.CleanUp.ReferenceOTU37256
New.CleanUp.ReferenceOTU223167
New.CleanUp.ReferenceOTU138403
New.CleanUp.ReferenceOTU39083
New.CleanUp.ReferenceOTU62947
New.CleanUp.ReferenceOTU152516
New.CleanUp.ReferenceOTU40607

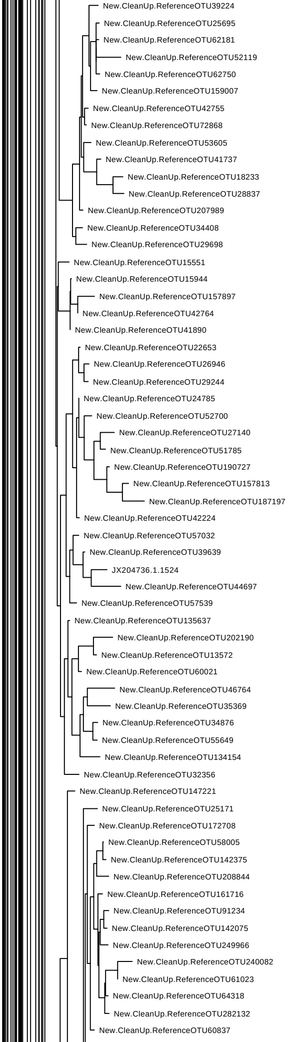
New.CleanUp.ReferenceOTU39224
New.CleanUp.ReferenceOTU25695
New.CleanUp.ReferenceOTU62181
New.CleanUp.ReferenceOTU52119
New.CleanUp.ReferenceOTU62750
New.CleanUp.ReferenceOTU159007
New.CleanUp.ReferenceOTU42755
New.CleanUp.ReferenceOTU72868
New.CleanUp.ReferenceOTU53605
New.CleanUp.ReferenceOTU41737
New.CleanUp.ReferenceOTU18233
New.CleanUp.ReferenceOTU28837
New.CleanUp.ReferenceOTU207989
New.CleanUp.ReferenceOTU34408
New.CleanUp.ReferenceOTU29698
New.CleanUp.ReferenceOTU15551
New.CleanUp.ReferenceOTU15944
New.CleanUp.ReferenceOTU157897
New.CleanUp.ReferenceOTU42764
New.CleanUp.ReferenceOTU41890
New.CleanUp.ReferenceOTU22653
New.CleanUp.ReferenceOTU26946
New.CleanUp.ReferenceOTU29244
New.CleanUp.ReferenceOTU24785
New.CleanUp.ReferenceOTU52700
New.CleanUp.ReferenceOTU27140
New.CleanUp.ReferenceOTU51785
New.CleanUp.ReferenceOTU190727
New.CleanUp.ReferenceOTU157813
New.CleanUp.ReferenceOTU187197
New.CleanUp.ReferenceOTU42224
New.CleanUp.ReferenceOTU57032
New.CleanUp.ReferenceOTU39639
JX204736.1.1524
New.CleanUp.ReferenceOTU44697
New.CleanUp.ReferenceOTU57539
New.CleanUp.ReferenceOTU135637
New.CleanUp.ReferenceOTU202190
New.CleanUp.ReferenceOTU13572
New.CleanUp.ReferenceOTU60021
New.CleanUp.ReferenceOTU46764
New.CleanUp.ReferenceOTU35369
New.CleanUp.ReferenceOTU34876
New.CleanUp.ReferenceOTU55649
New.CleanUp.ReferenceOTU134154
New.CleanUp.ReferenceOTU32356
New.CleanUp.ReferenceOTU147221
New.CleanUp.ReferenceOTU25171
New.CleanUp.ReferenceOTU172708
New.CleanUp.ReferenceOTU58005
New.CleanUp.ReferenceOTU142375
New.CleanUp.ReferenceOTU208844
New.CleanUp.ReferenceOTU161716
New.CleanUp.ReferenceOTU91234
New.CleanUp.ReferenceOTU142075
New.CleanUp.ReferenceOTU249966
New.CleanUp.ReferenceOTU240082
New.CleanUp.ReferenceOTU61023
New.CleanUp.ReferenceOTU64318
New.CleanUp.ReferenceOTU282132
New.CleanUp.ReferenceOTU60837

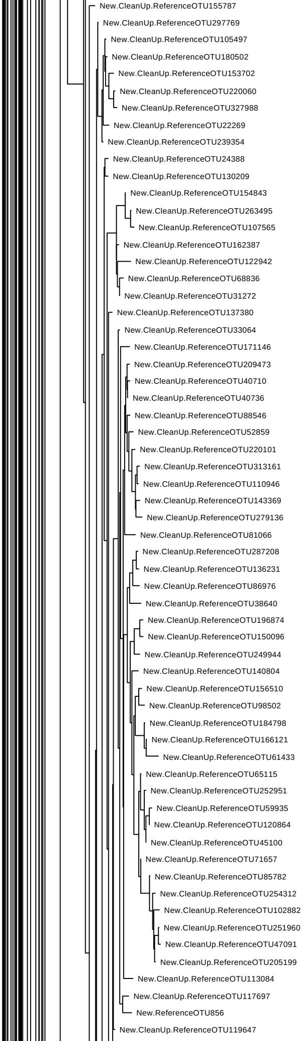
New.CleanUp.ReferenceOTU155787
New.CleanUp.ReferenceOTU297769
New.CleanUp.ReferenceOTU105497
New.CleanUp.ReferenceOTU180502
New.CleanUp.ReferenceOTU153702
New.CleanUp.ReferenceOTU220060
New.CleanUp.ReferenceOTU327988
New.CleanUp.ReferenceOTU22269
New.CleanUp.ReferenceOTU239354
New.CleanUp.ReferenceOTU24388
New.CleanUp.ReferenceOTU130209
New.CleanUp.ReferenceOTU154843
New.CleanUp.ReferenceOTU263495
New.CleanUp.ReferenceOTU107565
New.CleanUp.ReferenceOTU162387
New.CleanUp.ReferenceOTU122942
New.CleanUp.ReferenceOTU68836
New.CleanUp.ReferenceOTU31272
New.CleanUp.ReferenceOTU137380
New.CleanUp.ReferenceOTU33064
New.CleanUp.ReferenceOTU171146
New.CleanUp.ReferenceOTU209473
New.CleanUp.ReferenceOTU40710
New.CleanUp.ReferenceOTU40736
New.CleanUp.ReferenceOTU88546
New.CleanUp.ReferenceOTU52859
New.CleanUp.ReferenceOTU220101
New.CleanUp.ReferenceOTU313161
New.CleanUp.ReferenceOTU110946
New.CleanUp.ReferenceOTU143369
New.CleanUp.ReferenceOTU279136
New.CleanUp.ReferenceOTU81066
New.CleanUp.ReferenceOTU287208
New.CleanUp.ReferenceOTU136231
New.CleanUp.ReferenceOTU86976
New.CleanUp.ReferenceOTU38640
New.CleanUp.ReferenceOTU196874
New.CleanUp.ReferenceOTU150096
New.CleanUp.ReferenceOTU249944
New.CleanUp.ReferenceOTU140804
New.CleanUp.ReferenceOTU156510
New.CleanUp.ReferenceOTU98502
New.CleanUp.ReferenceOTU184798
New.CleanUp.ReferenceOTU166121
New.CleanUp.ReferenceOTU61433
New.CleanUp.ReferenceOTU65115
New.CleanUp.ReferenceOTU252951
New.CleanUp.ReferenceOTU59935
New.CleanUp.ReferenceOTU120864
New.CleanUp.ReferenceOTU45100
New.CleanUp.ReferenceOTU71657
New.CleanUp.ReferenceOTU85782
New.CleanUp.ReferenceOTU254312
New.CleanUp.ReferenceOTU102882
New.CleanUp.ReferenceOTU251960
New.CleanUp.ReferenceOTU47091
New.CleanUp.ReferenceOTU205199
New.CleanUp.ReferenceOTU113084
New.CleanUp.ReferenceOTU117697
New.ReferenceOTU856
New.CleanUp.ReferenceOTU119647

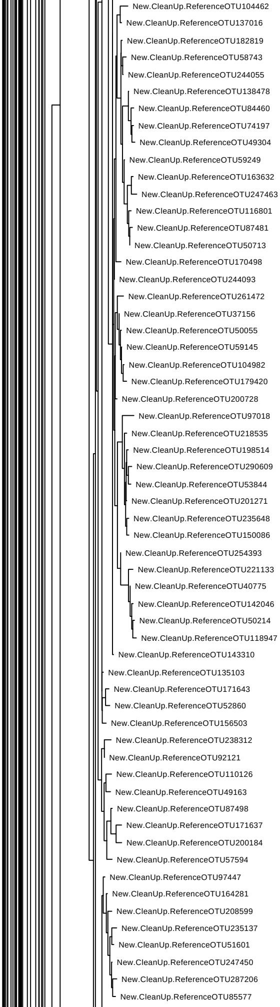
New.CleanUp.ReferenceOTU104462
New.CleanUp.ReferenceOTU137016
New.CleanUp.ReferenceOTU182819
New.CleanUp.ReferenceOTU58743
New.CleanUp.ReferenceOTU244055
New.CleanUp.ReferenceOTU138478
New.CleanUp.ReferenceOTU84460
New.CleanUp.ReferenceOTU74197
New.CleanUp.ReferenceOTU49304
New.CleanUp.ReferenceOTU59249
New.CleanUp.ReferenceOTU163632
New.CleanUp.ReferenceOTU247463
New.CleanUp.ReferenceOTU116801
New.CleanUp.ReferenceOTU87481
New.CleanUp.ReferenceOTU50713
New.CleanUp.ReferenceOTU170498
New.CleanUp.ReferenceOTU244093
New.CleanUp.ReferenceOTU261472
New.CleanUp.ReferenceOTU37156
New.CleanUp.ReferenceOTU50055
New.CleanUp.ReferenceOTU59145
New.CleanUp.ReferenceOTU104982
New.CleanUp.ReferenceOTU179420
New.CleanUp.ReferenceOTU200728
New.CleanUp.ReferenceOTU97018
New.CleanUp.ReferenceOTU218535
New.CleanUp.ReferenceOTU198514
New.CleanUp.ReferenceOTU290609
New.CleanUp.ReferenceOTU53844
New.CleanUp.ReferenceOTU201271
New.CleanUp.ReferenceOTU235648
New.CleanUp.ReferenceOTU150086
New.CleanUp.ReferenceOTU254393
New.CleanUp.ReferenceOTU221133
New.CleanUp.ReferenceOTU40775
New.CleanUp.ReferenceOTU142046
New.CleanUp.ReferenceOTU50214
New.CleanUp.ReferenceOTU118947
New.CleanUp.ReferenceOTU143310
New.CleanUp.ReferenceOTU135103
New.CleanUp.ReferenceOTU171643
New.CleanUp.ReferenceOTU52860
New.CleanUp.ReferenceOTU156503
New.CleanUp.ReferenceOTU238312
New.CleanUp.ReferenceOTU92121
New.CleanUp.ReferenceOTU110126
New.CleanUp.ReferenceOTU49163
New.CleanUp.ReferenceOTU87498
New.CleanUp.ReferenceOTU171637
New.CleanUp.ReferenceOTU200184
New.CleanUp.ReferenceOTU57594
New.CleanUp.ReferenceOTU97447
New.CleanUp.ReferenceOTU164281
New.CleanUp.ReferenceOTU208599
New.CleanUp.ReferenceOTU235137
New.CleanUp.ReferenceOTU51601
New.CleanUp.ReferenceOTU247450
New.CleanUp.ReferenceOTU287206
New.CleanUp.ReferenceOTU85577

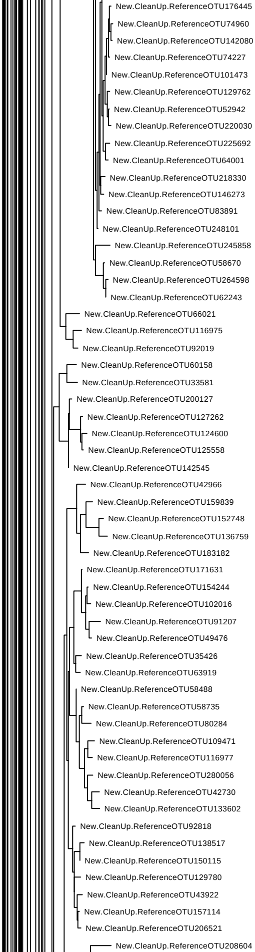
New.CleanUp.ReferenceOTU176445
New.CleanUp.ReferenceOTU74960
New.CleanUp.ReferenceOTU142080
New.CleanUp.ReferenceOTU74227
New.CleanUp.ReferenceOTU101473
New.CleanUp.ReferenceOTU129762
New.CleanUp.ReferenceOTU52942
New.CleanUp.ReferenceOTU220030
New.CleanUp.ReferenceOTU225692
New.CleanUp.ReferenceOTU64001
New.CleanUp.ReferenceOTU218330
New.CleanUp.ReferenceOTU146273
New.CleanUp.ReferenceOTU83891
New.CleanUp.ReferenceOTU248101
New.CleanUp.ReferenceOTU245858
New.CleanUp.ReferenceOTU58670
New.CleanUp.ReferenceOTU264598
New.CleanUp.ReferenceOTU62243
New.CleanUp.ReferenceOTU66021
New.CleanUp.ReferenceOTU116975
New.CleanUp.ReferenceOTU92019
New.CleanUp.ReferenceOTU60158
New.CleanUp.ReferenceOTU33581
New.CleanUp.ReferenceOTU200127
New.CleanUp.ReferenceOTU127262
New.CleanUp.ReferenceOTU124600
New.CleanUp.ReferenceOTU125558
New.CleanUp.ReferenceOTU142545
New.CleanUp.ReferenceOTU42966
New.CleanUp.ReferenceOTU159839
New.CleanUp.ReferenceOTU152748
New.CleanUp.ReferenceOTU136759
New.CleanUp.ReferenceOTU183182
New.CleanUp.ReferenceOTU171631
New.CleanUp.ReferenceOTU154244
New.CleanUp.ReferenceOTU102016
New.CleanUp.ReferenceOTU91207
New.CleanUp.ReferenceOTU49476
New.CleanUp.ReferenceOTU35426
New.CleanUp.ReferenceOTU63919
New.CleanUp.ReferenceOTU58488
New.CleanUp.ReferenceOTU58735
New.CleanUp.ReferenceOTU80284
New.CleanUp.ReferenceOTU109471
New.CleanUp.ReferenceOTU116977
New.CleanUp.ReferenceOTU280056
New.CleanUp.ReferenceOTU42730
New.CleanUp.ReferenceOTU133602
New.CleanUp.ReferenceOTU92818
New.CleanUp.ReferenceOTU138517
New.CleanUp.ReferenceOTU150115
New.CleanUp.ReferenceOTU129780
New.CleanUp.ReferenceOTU43922
New.CleanUp.ReferenceOTU157114
New.CleanUp.ReferenceOTU206521
New.CleanUp.ReferenceOTU208604

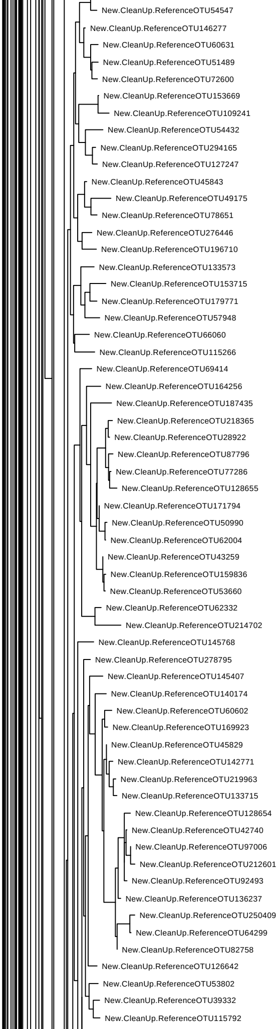
New.CleanUp.ReferenceOTU54547
New.CleanUp.ReferenceOTU146277
New.CleanUp.ReferenceOTU60631
New.CleanUp.ReferenceOTU51489
New.CleanUp.ReferenceOTU72600
New.CleanUp.ReferenceOTU153669
New.CleanUp.ReferenceOTU109241
New.CleanUp.ReferenceOTU54432
New.CleanUp.ReferenceOTU294165
New.CleanUp.ReferenceOTU127247
New.CleanUp.ReferenceOTU45843
New.CleanUp.ReferenceOTU49175
New.CleanUp.ReferenceOTU78651
New.CleanUp.ReferenceOTU276446
New.CleanUp.ReferenceOTU196710
New.CleanUp.ReferenceOTU133573
New.CleanUp.ReferenceOTU153715
New.CleanUp.ReferenceOTU179771
New.CleanUp.ReferenceOTU57948
New.CleanUp.ReferenceOTU66060
New.CleanUp.ReferenceOTU115266
New.CleanUp.ReferenceOTU69414
New.CleanUp.ReferenceOTU164256
New.CleanUp.ReferenceOTU187435
New.CleanUp.ReferenceOTU218365
New.CleanUp.ReferenceOTU28922
New.CleanUp.ReferenceOTU87796
New.CleanUp.ReferenceOTU77286
New.CleanUp.ReferenceOTU128655
New.CleanUp.ReferenceOTU171794
New.CleanUp.ReferenceOTU50990
New.CleanUp.ReferenceOTU62004
New.CleanUp.ReferenceOTU43259
New.CleanUp.ReferenceOTU159836
New.CleanUp.ReferenceOTU53660
New.CleanUp.ReferenceOTU62332
New.CleanUp.ReferenceOTU214702
New.CleanUp.ReferenceOTU145768
New.CleanUp.ReferenceOTU278795
New.CleanUp.ReferenceOTU145407
New.CleanUp.ReferenceOTU140174
New.CleanUp.ReferenceOTU60602
New.CleanUp.ReferenceOTU169923
New.CleanUp.ReferenceOTU45829
New.CleanUp.ReferenceOTU142771
New.CleanUp.ReferenceOTU219963
New.CleanUp.ReferenceOTU133715
New.CleanUp.ReferenceOTU128654
New.CleanUp.ReferenceOTU42740
New.CleanUp.ReferenceOTU97006
New.CleanUp.ReferenceOTU212601
New.CleanUp.ReferenceOTU92493
New.CleanUp.ReferenceOTU136237
New.CleanUp.ReferenceOTU250409
New.CleanUp.ReferenceOTU64299
New.CleanUp.ReferenceOTU82758
New.CleanUp.ReferenceOTU126642
New.CleanUp.ReferenceOTU53802
New.CleanUp.ReferenceOTU39332
New.CleanUp.ReferenceOTU115792

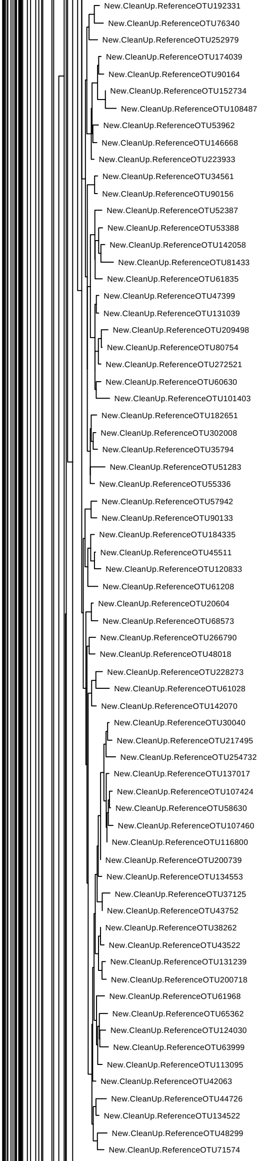
New.CleanUp.ReferenceOTU192331
New.CleanUp.ReferenceOTU76340
New.CleanUp.ReferenceOTU252979
New.CleanUp.ReferenceOTU174039
New.CleanUp.ReferenceOTU90164
New.CleanUp.ReferenceOTU152734
New.CleanUp.ReferenceOTU108487
New.CleanUp.ReferenceOTU53962
New.CleanUp.ReferenceOTU146668
New.CleanUp.ReferenceOTU223933
New.CleanUp.ReferenceOTU34561
New.CleanUp.ReferenceOTU90156
New.CleanUp.ReferenceOTU52387
New.CleanUp.ReferenceOTU53388
New.CleanUp.ReferenceOTU142058
New.CleanUp.ReferenceOTU81433
New.CleanUp.ReferenceOTU61835
New.CleanUp.ReferenceOTU47399
New.CleanUp.ReferenceOTU131039
New.CleanUp.ReferenceOTU209498
New.CleanUp.ReferenceOTU80754
New.CleanUp.ReferenceOTU272521
New.CleanUp.ReferenceOTU60630
New.CleanUp.ReferenceOTU101403
New.CleanUp.ReferenceOTU182651
New.CleanUp.ReferenceOTU302008
New.CleanUp.ReferenceOTU35794
New.CleanUp.ReferenceOTU51283
New.CleanUp.ReferenceOTU55336
New.CleanUp.ReferenceOTU57942
New.CleanUp.ReferenceOTU90133
New.CleanUp.ReferenceOTU184335
New.CleanUp.ReferenceOTU45511
New.CleanUp.ReferenceOTU120833
New.CleanUp.ReferenceOTU61208
New.CleanUp.ReferenceOTU20604
New.CleanUp.ReferenceOTU68573
New.CleanUp.ReferenceOTU266790
New.CleanUp.ReferenceOTU48018
New.CleanUp.ReferenceOTU228273
New.CleanUp.ReferenceOTU61028
New.CleanUp.ReferenceOTU142070
New.CleanUp.ReferenceOTU30040
New.CleanUp.ReferenceOTU217495
New.CleanUp.ReferenceOTU254732
New.CleanUp.ReferenceOTU137017
New.CleanUp.ReferenceOTU107424
New.CleanUp.ReferenceOTU58630
New.CleanUp.ReferenceOTU107460
New.CleanUp.ReferenceOTU116800
New.CleanUp.ReferenceOTU200739
New.CleanUp.ReferenceOTU134553
New.CleanUp.ReferenceOTU37125
New.CleanUp.ReferenceOTU43752
New.CleanUp.ReferenceOTU38262
New.CleanUp.ReferenceOTU43522
New.CleanUp.ReferenceOTU131239
New.CleanUp.ReferenceOTU200718
New.CleanUp.ReferenceOTU61968
New.CleanUp.ReferenceOTU65362
New.CleanUp.ReferenceOTU124030
New.CleanUp.ReferenceOTU63999
New.CleanUp.ReferenceOTU113095
New.CleanUp.ReferenceOTU42063
New.CleanUp.ReferenceOTU44726
New.CleanUp.ReferenceOTU134522
New.CleanUp.ReferenceOTU48299
New.CleanUp.ReferenceOTU71574

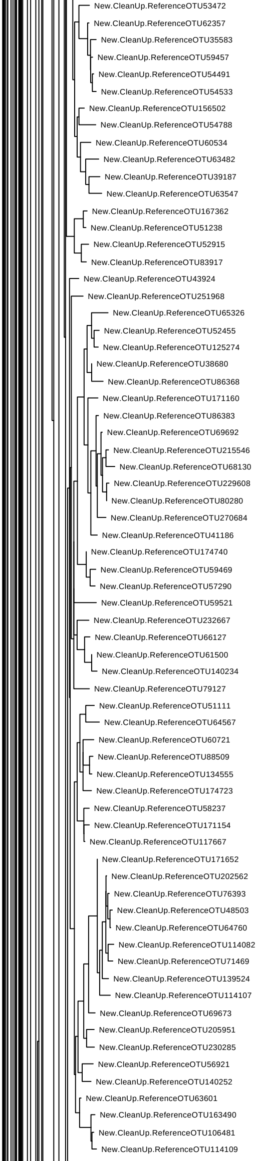
New.CleanUp.ReferenceOTU53472
New.CleanUp.ReferenceOTU62357
New.CleanUp.ReferenceOTU35583
New.CleanUp.ReferenceOTU59457
New.CleanUp.ReferenceOTU54491
New.CleanUp.ReferenceOTU54533
New.CleanUp.ReferenceOTU156502
New.CleanUp.ReferenceOTU54788
New.CleanUp.ReferenceOTU60534
New.CleanUp.ReferenceOTU63482
New.CleanUp.ReferenceOTU39187
New.CleanUp.ReferenceOTU63547
New.CleanUp.ReferenceOTU167362
New.CleanUp.ReferenceOTU51238
New.CleanUp.ReferenceOTU52915
New.CleanUp.ReferenceOTU83917
New.CleanUp.ReferenceOTU43924
New.CleanUp.ReferenceOTU251968
New.CleanUp.ReferenceOTU65326
New.CleanUp.ReferenceOTU52455
New.CleanUp.ReferenceOTU125274
New.CleanUp.ReferenceOTU38680
New.CleanUp.ReferenceOTU86368
New.CleanUp.ReferenceOTU171160
New.CleanUp.ReferenceOTU86383
New.CleanUp.ReferenceOTU69692
New.CleanUp.ReferenceOTU215546
New.CleanUp.ReferenceOTU68130
New.CleanUp.ReferenceOTU229608
New.CleanUp.ReferenceOTU80280
New.CleanUp.ReferenceOTU270684
New.CleanUp.ReferenceOTU41186
New.CleanUp.ReferenceOTU174740
New.CleanUp.ReferenceOTU59469
New.CleanUp.ReferenceOTU57290
New.CleanUp.ReferenceOTU59521
New.CleanUp.ReferenceOTU232667
New.CleanUp.ReferenceOTU66127
New.CleanUp.ReferenceOTU61500
New.CleanUp.ReferenceOTU140234
New.CleanUp.ReferenceOTU79127
New.CleanUp.ReferenceOTU51111
New.CleanUp.ReferenceOTU64567
New.CleanUp.ReferenceOTU60721
New.CleanUp.ReferenceOTU88509
New.CleanUp.ReferenceOTU134555
New.CleanUp.ReferenceOTU174723
New.CleanUp.ReferenceOTU58237
New.CleanUp.ReferenceOTU171154
New.CleanUp.ReferenceOTU117667
New.CleanUp.ReferenceOTU171652
New.CleanUp.ReferenceOTU202562
New.CleanUp.ReferenceOTU76393
New.CleanUp.ReferenceOTU48503
New.CleanUp.ReferenceOTU64760
New.CleanUp.ReferenceOTU114082
New.CleanUp.ReferenceOTU71469
New.CleanUp.ReferenceOTU139524
New.CleanUp.ReferenceOTU114107
New.CleanUp.ReferenceOTU69673
New.CleanUp.ReferenceOTU205951
New.CleanUp.ReferenceOTU230285
New.CleanUp.ReferenceOTU56921
New.CleanUp.ReferenceOTU140252
New.CleanUp.ReferenceOTU63601
New.CleanUp.ReferenceOTU163490
New.CleanUp.ReferenceOTU106481
New.CleanUp.ReferenceOTU114109

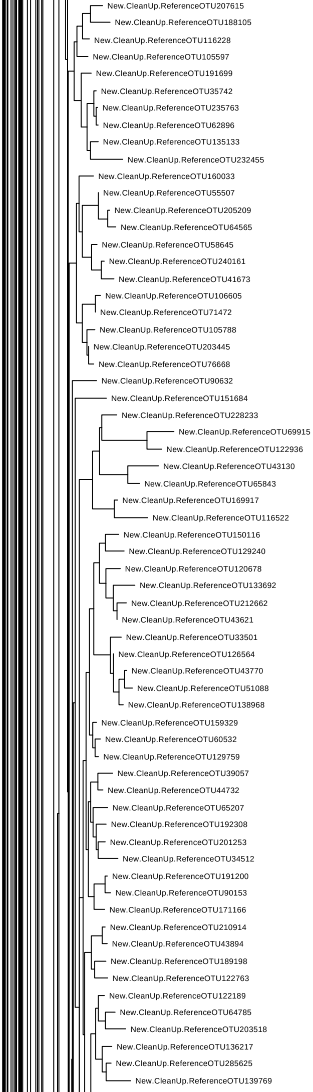
New.CleanUp.ReferenceOTU207615
New.CleanUp.ReferenceOTU188105
New.CleanUp.ReferenceOTU116228
New.CleanUp.ReferenceOTU105597
New.CleanUp.ReferenceOTU191699
New.CleanUp.ReferenceOTU35742
New.CleanUp.ReferenceOTU235763
New.CleanUp.ReferenceOTU62896
New.CleanUp.ReferenceOTU135133
New.CleanUp.ReferenceOTU232455
New.CleanUp.ReferenceOTU160033
New.CleanUp.ReferenceOTU55507
New.CleanUp.ReferenceOTU205209
New.CleanUp.ReferenceOTU64565
New.CleanUp.ReferenceOTU58645
New.CleanUp.ReferenceOTU240161
New.CleanUp.ReferenceOTU41673
New.CleanUp.ReferenceOTU106605
New.CleanUp.ReferenceOTU71472
New.CleanUp.ReferenceOTU105788
New.CleanUp.ReferenceOTU203445
New.CleanUp.ReferenceOTU76668
New.CleanUp.ReferenceOTU90632
New.CleanUp.ReferenceOTU151684
New.CleanUp.ReferenceOTU228233
New.CleanUp.ReferenceOTU69915
New.CleanUp.ReferenceOTU122936
New.CleanUp.ReferenceOTU43130
New.CleanUp.ReferenceOTU65843
New.CleanUp.ReferenceOTU169917
New.CleanUp.ReferenceOTU116522
New.CleanUp.ReferenceOTU150116
New.CleanUp.ReferenceOTU129240
New.CleanUp.ReferenceOTU120678
New.CleanUp.ReferenceOTU133692
New.CleanUp.ReferenceOTU212662
New.CleanUp.ReferenceOTU43621
New.CleanUp.ReferenceOTU33501
New.CleanUp.ReferenceOTU126564
New.CleanUp.ReferenceOTU43770
New.CleanUp.ReferenceOTU51088
New.CleanUp.ReferenceOTU138968
New.CleanUp.ReferenceOTU159329
New.CleanUp.ReferenceOTU60532
New.CleanUp.ReferenceOTU129759
New.CleanUp.ReferenceOTU39057
New.CleanUp.ReferenceOTU44732
New.CleanUp.ReferenceOTU65207
New.CleanUp.ReferenceOTU192308
New.CleanUp.ReferenceOTU201253
New.CleanUp.ReferenceOTU34512
New.CleanUp.ReferenceOTU191200
New.CleanUp.ReferenceOTU90153
New.CleanUp.ReferenceOTU171166
New.CleanUp.ReferenceOTU210914
New.CleanUp.ReferenceOTU43894
New.CleanUp.ReferenceOTU189198
New.CleanUp.ReferenceOTU122763
New.CleanUp.ReferenceOTU122189
New.CleanUp.ReferenceOTU64785
New.CleanUp.ReferenceOTU203518
New.CleanUp.ReferenceOTU136217
New.CleanUp.ReferenceOTU285625
New.CleanUp.ReferenceOTU139769

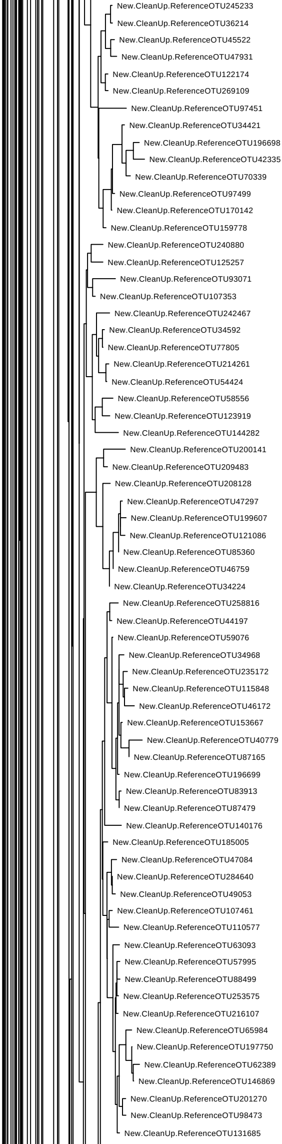
New.CleanUp.ReferenceOTU245233
New.CleanUp.ReferenceOTU36214
New.CleanUp.ReferenceOTU45522
New.CleanUp.ReferenceOTU47931
New.CleanUp.ReferenceOTU122174
New.CleanUp.ReferenceOTU269109
New.CleanUp.ReferenceOTU97451
New.CleanUp.ReferenceOTU34421
New.CleanUp.ReferenceOTU196698
New.CleanUp.ReferenceOTU42335
New.CleanUp.ReferenceOTU70339
New.CleanUp.ReferenceOTU97499
New.CleanUp.ReferenceOTU170142
New.CleanUp.ReferenceOTU159778
New.CleanUp.ReferenceOTU240880
New.CleanUp.ReferenceOTU125257
New.CleanUp.ReferenceOTU93071
New.CleanUp.ReferenceOTU107353
New.CleanUp.ReferenceOTU242467
New.CleanUp.ReferenceOTU34592
New.CleanUp.ReferenceOTU77805
New.CleanUp.ReferenceOTU214261
New.CleanUp.ReferenceOTU54424
New.CleanUp.ReferenceOTU58556
New.CleanUp.ReferenceOTU123919
New.CleanUp.ReferenceOTU144282
New.CleanUp.ReferenceOTU200141
New.CleanUp.ReferenceOTU209483
New.CleanUp.ReferenceOTU208128
New.CleanUp.ReferenceOTU47297
New.CleanUp.ReferenceOTU199607
New.CleanUp.ReferenceOTU121086
New.CleanUp.ReferenceOTU85360
New.CleanUp.ReferenceOTU46759
New.CleanUp.ReferenceOTU34224
New.CleanUp.ReferenceOTU258816
New.CleanUp.ReferenceOTU44197
New.CleanUp.ReferenceOTU59076
New.CleanUp.ReferenceOTU34968
New.CleanUp.ReferenceOTU235172
New.CleanUp.ReferenceOTU115848
New.CleanUp.ReferenceOTU46172
New.CleanUp.ReferenceOTU153667
New.CleanUp.ReferenceOTU40779
New.CleanUp.ReferenceOTU87165
New.CleanUp.ReferenceOTU196699
New.CleanUp.ReferenceOTU83913
New.CleanUp.ReferenceOTU87479
New.CleanUp.ReferenceOTU140176
New.CleanUp.ReferenceOTU185005
New.CleanUp.ReferenceOTU47084
New.CleanUp.ReferenceOTU284640
New.CleanUp.ReferenceOTU49053
New.CleanUp.ReferenceOTU107461
New.CleanUp.ReferenceOTU110577
New.CleanUp.ReferenceOTU63093
New.CleanUp.ReferenceOTU57995
New.CleanUp.ReferenceOTU88499
New.CleanUp.ReferenceOTU253575
New.CleanUp.ReferenceOTU216107
New.CleanUp.ReferenceOTU65984
New.CleanUp.ReferenceOTU197750
New.CleanUp.ReferenceOTU62389
New.CleanUp.ReferenceOTU146869
New.CleanUp.ReferenceOTU201270
New.CleanUp.ReferenceOTU98473
New.CleanUp.ReferenceOTU131685

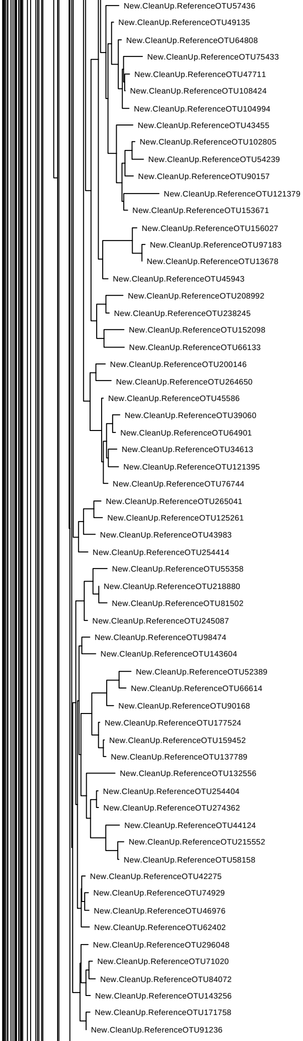
New.CleanUp.ReferenceOTU57436
New.CleanUp.ReferenceOTU49135
New.CleanUp.ReferenceOTU64808
New.CleanUp.ReferenceOTU75433
New.CleanUp.ReferenceOTU47711
New.CleanUp.ReferenceOTU108424
New.CleanUp.ReferenceOTU104994
New.CleanUp.ReferenceOTU43455
New.CleanUp.ReferenceOTU102805
New.CleanUp.ReferenceOTU54239
New.CleanUp.ReferenceOTU90157
New.CleanUp.ReferenceOTU121379
New.CleanUp.ReferenceOTU153671
New.CleanUp.ReferenceOTU156027
New.CleanUp.ReferenceOTU97183
New.CleanUp.ReferenceOTU13678
New.CleanUp.ReferenceOTU45943
New.CleanUp.ReferenceOTU208992
New.CleanUp.ReferenceOTU238245
New.CleanUp.ReferenceOTU152098
New.CleanUp.ReferenceOTU66133
New.CleanUp.ReferenceOTU200146
New.CleanUp.ReferenceOTU264650
New.CleanUp.ReferenceOTU45586
New.CleanUp.ReferenceOTU39060
New.CleanUp.ReferenceOTU64901
New.CleanUp.ReferenceOTU34613
New.CleanUp.ReferenceOTU121395
New.CleanUp.ReferenceOTU76744
New.CleanUp.ReferenceOTU265041
New.CleanUp.ReferenceOTU125261
New.CleanUp.ReferenceOTU43983
New.CleanUp.ReferenceOTU254414
New.CleanUp.ReferenceOTU55358
New.CleanUp.ReferenceOTU218880
New.CleanUp.ReferenceOTU81502
New.CleanUp.ReferenceOTU245087
New.CleanUp.ReferenceOTU98474
New.CleanUp.ReferenceOTU143604
New.CleanUp.ReferenceOTU52389
New.CleanUp.ReferenceOTU66614
New.CleanUp.ReferenceOTU90168
New.CleanUp.ReferenceOTU177524
New.CleanUp.ReferenceOTU159452
New.CleanUp.ReferenceOTU137789
New.CleanUp.ReferenceOTU132556
New.CleanUp.ReferenceOTU254404
New.CleanUp.ReferenceOTU274362
New.CleanUp.ReferenceOTU44124
New.CleanUp.ReferenceOTU215552
New.CleanUp.ReferenceOTU58158
New.CleanUp.ReferenceOTU42275
New.CleanUp.ReferenceOTU74929
New.CleanUp.ReferenceOTU46976
New.CleanUp.ReferenceOTU62402
New.CleanUp.ReferenceOTU296048
New.CleanUp.ReferenceOTU71020
New.CleanUp.ReferenceOTU84072
New.CleanUp.ReferenceOTU143256
New.CleanUp.ReferenceOTU171758
New.CleanUp.ReferenceOTU91236

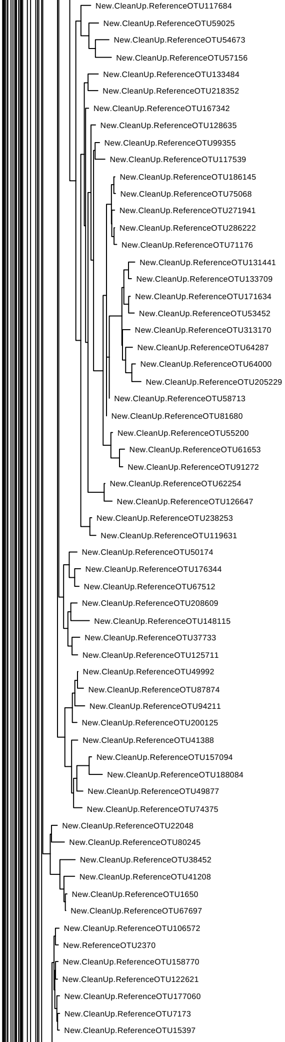
New.CleanUp.ReferenceOTU117684
New.CleanUp.ReferenceOTU59025
New.CleanUp.ReferenceOTU54673
New.CleanUp.ReferenceOTU57156
New.CleanUp.ReferenceOTU133484
New.CleanUp.ReferenceOTU218352
New.CleanUp.ReferenceOTU167342
New.CleanUp.ReferenceOTU128635
New.CleanUp.ReferenceOTU99355
New.CleanUp.ReferenceOTU117539
New.CleanUp.ReferenceOTU186145
New.CleanUp.ReferenceOTU75068
New.CleanUp.ReferenceOTU271941
New.CleanUp.ReferenceOTU286222
New.CleanUp.ReferenceOTU71176
New.CleanUp.ReferenceOTU131441
New.CleanUp.ReferenceOTU133709
New.CleanUp.ReferenceOTU171634
New.CleanUp.ReferenceOTU53452
New.CleanUp.ReferenceOTU313170
New.CleanUp.ReferenceOTU64287
New.CleanUp.ReferenceOTU64000
New.CleanUp.ReferenceOTU205229
New.CleanUp.ReferenceOTU58713
New.CleanUp.ReferenceOTU81680
New.CleanUp.ReferenceOTU55200
New.CleanUp.ReferenceOTU61653
New.CleanUp.ReferenceOTU91272
New.CleanUp.ReferenceOTU62254
New.CleanUp.ReferenceOTU126647
New.CleanUp.ReferenceOTU238253
New.CleanUp.ReferenceOTU119631
New.CleanUp.ReferenceOTU50174
New.CleanUp.ReferenceOTU176344
New.CleanUp.ReferenceOTU67512
New.CleanUp.ReferenceOTU208609
New.CleanUp.ReferenceOTU148115
New.CleanUp.ReferenceOTU37733
New.CleanUp.ReferenceOTU125711
New.CleanUp.ReferenceOTU49992
New.CleanUp.ReferenceOTU87874
New.CleanUp.ReferenceOTU94211
New.CleanUp.ReferenceOTU200125
New.CleanUp.ReferenceOTU41388
New.CleanUp.ReferenceOTU157094
New.CleanUp.ReferenceOTU188084
New.CleanUp.ReferenceOTU49877
New.CleanUp.ReferenceOTU74375
New.CleanUp.ReferenceOTU22048
New.CleanUp.ReferenceOTU80245
New.CleanUp.ReferenceOTU38452
New.CleanUp.ReferenceOTU41208
New.CleanUp.ReferenceOTU1650
New.CleanUp.ReferenceOTU67697
New.CleanUp.ReferenceOTU106572
New.ReferenceOTU2370
New.CleanUp.ReferenceOTU158770
New.CleanUp.ReferenceOTU122621
New.CleanUp.ReferenceOTU177060
New.CleanUp.ReferenceOTU7173
New.CleanUp.ReferenceOTU15397

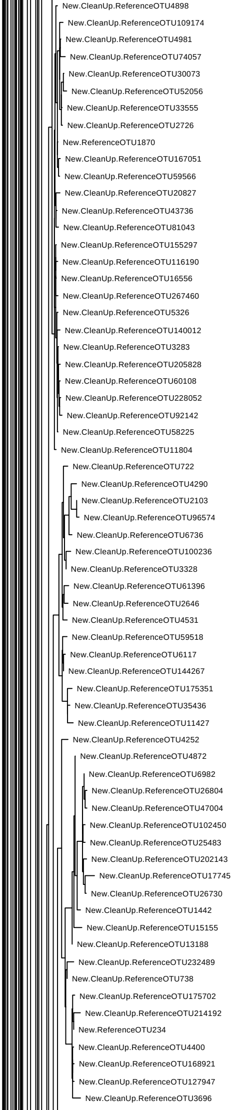

New.CleanUp.ReferenceOTU4898
New.CleanUp.ReferenceOTU109174
New.CleanUp.ReferenceOTU4981
New.CleanUp.ReferenceOTU74057
New.CleanUp.ReferenceOTU30073
New.CleanUp.ReferenceOTU52056
New.CleanUp.ReferenceOTU33555
New.CleanUp.ReferenceOTU2726
New.ReferenceOTU1870
New.CleanUp.ReferenceOTU167051
New.CleanUp.ReferenceOTU59566
New.CleanUp.ReferenceOTU20827
New.CleanUp.ReferenceOTU43736
New.CleanUp.ReferenceOTU81043
New.CleanUp.ReferenceOTU155297
New.CleanUp.ReferenceOTU116190
New.CleanUp.ReferenceOTU16556
New.CleanUp.ReferenceOTU267460
New.CleanUp.ReferenceOTU5326
New.CleanUp.ReferenceOTU140012
New.CleanUp.ReferenceOTU3283
New.CleanUp.ReferenceOTU205828
New.CleanUp.ReferenceOTU60108
New.CleanUp.ReferenceOTU228052
New.CleanUp.ReferenceOTU92142
New.CleanUp.ReferenceOTU58225
New.CleanUp.ReferenceOTU11804
New.CleanUp.ReferenceOTU722
New.CleanUp.ReferenceOTU4290
New.CleanUp.ReferenceOTU2103
New.CleanUp.ReferenceOTU96574
New.CleanUp.ReferenceOTU6736
New.CleanUp.ReferenceOTU100236
New.CleanUp.ReferenceOTU3328
New.CleanUp.ReferenceOTU61396
New.CleanUp.ReferenceOTU2646
New.CleanUp.ReferenceOTU4531
New.CleanUp.ReferenceOTU59518
New.CleanUp.ReferenceOTU6117
New.CleanUp.ReferenceOTU144267
New.CleanUp.ReferenceOTU175351
New.CleanUp.ReferenceOTU35436
New.CleanUp.ReferenceOTU11427
New.CleanUp.ReferenceOTU4252
New.CleanUp.ReferenceOTU4872
New.CleanUp.ReferenceOTU6982
New.CleanUp.ReferenceOTU26804
New.CleanUp.ReferenceOTU47004
New.CleanUp.ReferenceOTU102450
New.CleanUp.ReferenceOTU25483
New.CleanUp.ReferenceOTU202143
New.CleanUp.ReferenceOTU17745
New.CleanUp.ReferenceOTU26730
New.CleanUp.ReferenceOTU1442
New.CleanUp.ReferenceOTU15155
New.CleanUp.ReferenceOTU13188
New.CleanUp.ReferenceOTU232489
New.CleanUp.ReferenceOTU738
New.CleanUp.ReferenceOTU175702
New.CleanUp.ReferenceOTU214192
New.ReferenceOTU234
New.CleanUp.ReferenceOTU4400
New.CleanUp.ReferenceOTU168921
New.CleanUp.ReferenceOTU127947
New.CleanUp.ReferenceOTU3696

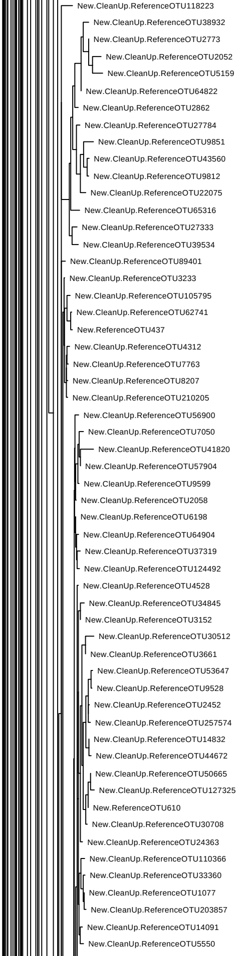
New.CleanUp.ReferenceOTU118223
New.CleanUp.ReferenceOTU38932
New.CleanUp.ReferenceOTU2773
New.CleanUp.ReferenceOTU2052
New.CleanUp.ReferenceOTU5159
New.CleanUp.ReferenceOTU64822
New.CleanUp.ReferenceOTU2862
New.CleanUp.ReferenceOTU27784
New.CleanUp.ReferenceOTU9851
New.CleanUp.ReferenceOTU43560
New.CleanUp.ReferenceOTU9812
New.CleanUp.ReferenceOTU22075
New.CleanUp.ReferenceOTU65316
New.CleanUp.ReferenceOTU27333
New.CleanUp.ReferenceOTU39534
New.CleanUp.ReferenceOTU89401
New.CleanUp.ReferenceOTU3233
New.CleanUp.ReferenceOTU105795
New.CleanUp.ReferenceOTU62741
New.ReferenceOTU437
New.CleanUp.ReferenceOTU4312
New.CleanUp.ReferenceOTU7763
New.CleanUp.ReferenceOTU8207
New.CleanUp.ReferenceOTU210205
New.CleanUp.ReferenceOTU56900
New.CleanUp.ReferenceOTU7050
New.CleanUp.ReferenceOTU41820
New.CleanUp.ReferenceOTU57904
New.CleanUp.ReferenceOTU9599
New.CleanUp.ReferenceOTU2058
New.CleanUp.ReferenceOTU6198
New.CleanUp.ReferenceOTU64904
New.CleanUp.ReferenceOTU37319
New.CleanUp.ReferenceOTU124492
New.CleanUp.ReferenceOTU4528
New.CleanUp.ReferenceOTU34845
New.CleanUp.ReferenceOTU3152
New.CleanUp.ReferenceOTU30512
New.CleanUp.ReferenceOTU3661
New.CleanUp.ReferenceOTU53647
New.CleanUp.ReferenceOTU9528
New.CleanUp.ReferenceOTU2452
New.CleanUp.ReferenceOTU257574
New.CleanUp.ReferenceOTU14832
New.CleanUp.ReferenceOTU44672
New.CleanUp.ReferenceOTU50665
New.CleanUp.ReferenceOTU127325
New.ReferenceOTU610
New.CleanUp.ReferenceOTU30708
New.CleanUp.ReferenceOTU24363
New.CleanUp.ReferenceOTU110366
New.CleanUp.ReferenceOTU33360
New.CleanUp.ReferenceOTU1077
New.CleanUp.ReferenceOTU203857
New.CleanUp.ReferenceOTU14091
New.CleanUp.ReferenceOTU5550

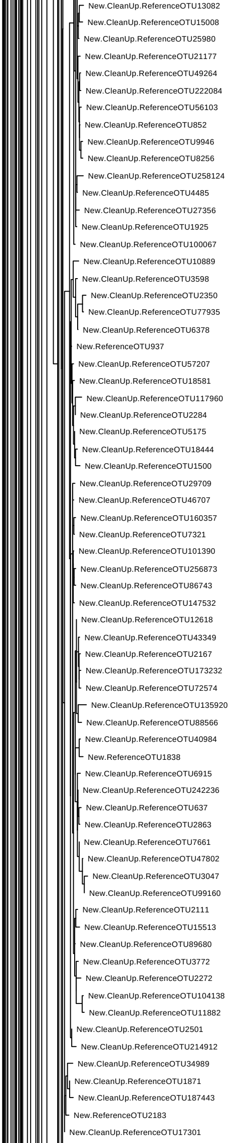
New.CleanUp.ReferenceOTU13082
New.CleanUp.ReferenceOTU15008
New.CleanUp.ReferenceOTU25980
New.CleanUp.ReferenceOTU21177
New.CleanUp.ReferenceOTU49264
New.CleanUp.ReferenceOTU222084
New.CleanUp.ReferenceOTU56103
New.CleanUp.ReferenceOTU852
New.CleanUp.ReferenceOTU9946
New.CleanUp.ReferenceOTU8256
New.CleanUp.ReferenceOTU258124
New.CleanUp.ReferenceOTU4485
New.CleanUp.ReferenceOTU27356
New.CleanUp.ReferenceOTU1925
New.CleanUp.ReferenceOTU100067
New.CleanUp.ReferenceOTU10889
New.CleanUp.ReferenceOTU3598
New.CleanUp.ReferenceOTU2350
New.CleanUp.ReferenceOTU77935
New.CleanUp.ReferenceOTU6378
New.ReferenceOTU937
New.CleanUp.ReferenceOTU57207
New.CleanUp.ReferenceOTU18581
New.CleanUp.ReferenceOTU117960
New.CleanUp.ReferenceOTU2284
New.CleanUp.ReferenceOTU5175
New.CleanUp.ReferenceOTU18444
New.CleanUp.ReferenceOTU1500
New.CleanUp.ReferenceOTU29709
New.CleanUp.ReferenceOTU46707
New.CleanUp.ReferenceOTU160357
New.CleanUp.ReferenceOTU7321
New.CleanUp.ReferenceOTU101390
New.CleanUp.ReferenceOTU256873
New.CleanUp.ReferenceOTU86743
New.CleanUp.ReferenceOTU147532
New.CleanUp.ReferenceOTU12618
New.CleanUp.ReferenceOTU43349
New.CleanUp.ReferenceOTU2167
New.CleanUp.ReferenceOTU173232
New.CleanUp.ReferenceOTU72574
New.CleanUp.ReferenceOTU135920
New.CleanUp.ReferenceOTU88566
New.CleanUp.ReferenceOTU40984
New.ReferenceOTU1838
New.CleanUp.ReferenceOTU6915
New.CleanUp.ReferenceOTU242236
New.CleanUp.ReferenceOTU637
New.CleanUp.ReferenceOTU2863
New.CleanUp.ReferenceOTU7661
New.CleanUp.ReferenceOTU47802
New.CleanUp.ReferenceOTU3047
New.CleanUp.ReferenceOTU99160
New.CleanUp.ReferenceOTU2111
New.CleanUp.ReferenceOTU15513
New.CleanUp.ReferenceOTU89680
New.CleanUp.ReferenceOTU3772
New.CleanUp.ReferenceOTU2272
New.CleanUp.ReferenceOTU104138
New.CleanUp.ReferenceOTU11882
New.CleanUp.ReferenceOTU2501
New.CleanUp.ReferenceOTU214912
New.CleanUp.ReferenceOTU34989
New.CleanUp.ReferenceOTU1871
New.CleanUp.ReferenceOTU187443
New.ReferenceOTU2183
New.CleanUp.ReferenceOTU17301

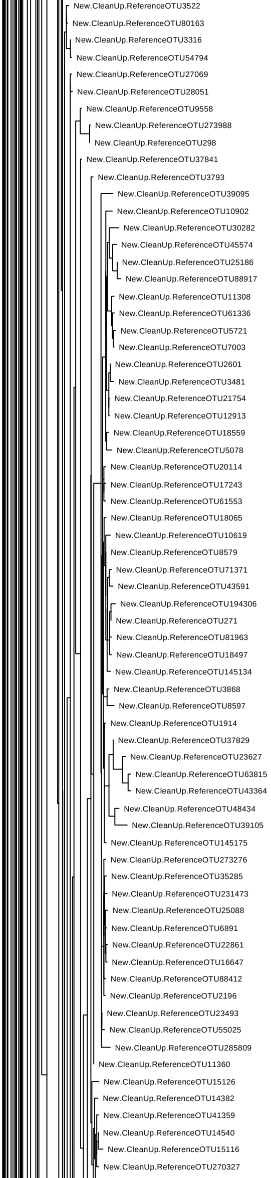
New.CleanUp.ReferenceOTU3522
New.CleanUp.ReferenceOTU80163
New.CleanUp.ReferenceOTU3316
New.CleanUp.ReferenceOTU54794
New.CleanUp.ReferenceOTU27069
New.CleanUp.ReferenceOTU28051
New.CleanUp.ReferenceOTU9558
New.CleanUp.ReferenceOTU273988
New.CleanUp.ReferenceOTU298
New.CleanUp.ReferenceOTU37841
New.CleanUp.ReferenceOTU3793
New.CleanUp.ReferenceOTU39095
New.CleanUp.ReferenceOTU10902
New.CleanUp.ReferenceOTU30282
New.CleanUp.ReferenceOTU45574
New.CleanUp.ReferenceOTU25186
New.CleanUp.ReferenceOTU88917
New.CleanUp.ReferenceOTU11308
New.CleanUp.ReferenceOTU61336
New.CleanUp.ReferenceOTU5721
New.CleanUp.ReferenceOTU7003
New.CleanUp.ReferenceOTU2601
New.CleanUp.ReferenceOTU3481
New.CleanUp.ReferenceOTU21754
New.CleanUp.ReferenceOTU12913
New.CleanUp.ReferenceOTU18559
New.CleanUp.ReferenceOTU5078
New.CleanUp.ReferenceOTU20114
New.CleanUp.ReferenceOTU17243
New.CleanUp.ReferenceOTU61553
New.CleanUp.ReferenceOTU18065
New.CleanUp.ReferenceOTU10619
New.CleanUp.ReferenceOTU8579
New.CleanUp.ReferenceOTU71371
New.CleanUp.ReferenceOTU43591
New.CleanUp.ReferenceOTU194306
New.CleanUp.ReferenceOTU271
New.CleanUp.ReferenceOTU81963
New.CleanUp.ReferenceOTU18497
New.CleanUp.ReferenceOTU145134
New.CleanUp.ReferenceOTU3868
New.CleanUp.ReferenceOTU8597
New.CleanUp.ReferenceOTU1914
New.CleanUp.ReferenceOTU37829
New.CleanUp.ReferenceOTU23627
New.CleanUp.ReferenceOTU63815
New.CleanUp.ReferenceOTU43364
New.CleanUp.ReferenceOTU48434
New.CleanUp.ReferenceOTU39105
New.CleanUp.ReferenceOTU145175
New.CleanUp.ReferenceOTU273276
New.CleanUp.ReferenceOTU35285
New.CleanUp.ReferenceOTU231473
New.CleanUp.ReferenceOTU25088
New.CleanUp.ReferenceOTU6891
New.CleanUp.ReferenceOTU22861
New.CleanUp.ReferenceOTU16647
New.CleanUp.ReferenceOTU88412
New.CleanUp.ReferenceOTU2196
New.CleanUp.ReferenceOTU23493
New.CleanUp.ReferenceOTU55025
New.CleanUp.ReferenceOTU285809
New.CleanUp.ReferenceOTU11360
New.CleanUp.ReferenceOTU15126
New.CleanUp.ReferenceOTU14382
New.CleanUp.ReferenceOTU41359
New.CleanUp.ReferenceOTU14540
New.CleanUp.ReferenceOTU15116
New.CleanUp.ReferenceOTU270327

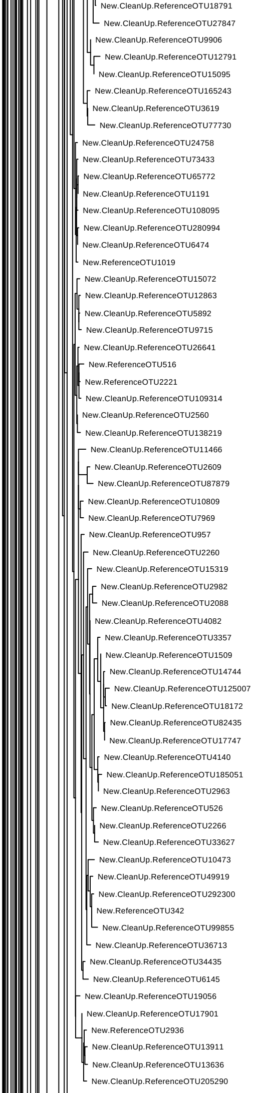
New.CleanUp.ReferenceOTU18791
New.CleanUp.ReferenceOTU27847
New.CleanUp.ReferenceOTU9906
New.CleanUp.ReferenceOTU12791
New.CleanUp.ReferenceOTU15095
New.CleanUp.ReferenceOTU165243
New.CleanUp.ReferenceOTU3619
New.CleanUp.ReferenceOTU77730
New.CleanUp.ReferenceOTU24758
New.CleanUp.ReferenceOTU73433
New.CleanUp.ReferenceOTU65772
New.CleanUp.ReferenceOTU1191
New.CleanUp.ReferenceOTU108095
New.CleanUp.ReferenceOTU280994
New.CleanUp.ReferenceOTU6474
New.ReferenceOTU1019
New.CleanUp.ReferenceOTU15072
New.CleanUp.ReferenceOTU12863
New.CleanUp.ReferenceOTU5892
New.CleanUp.ReferenceOTU9715
New.CleanUp.ReferenceOTU26641
New.ReferenceOTU516
New.ReferenceOTU2221
New.CleanUp.ReferenceOTU109314
New.CleanUp.ReferenceOTU2560
New.CleanUp.ReferenceOTU138219
New.CleanUp.ReferenceOTU11466
New.CleanUp.ReferenceOTU2609
New.CleanUp.ReferenceOTU87879
New.CleanUp.ReferenceOTU10809
New.CleanUp.ReferenceOTU7969
New.CleanUp.ReferenceOTU957
New.CleanUp.ReferenceOTU2260
New.CleanUp.ReferenceOTU15319
New.CleanUp.ReferenceOTU2982
New.CleanUp.ReferenceOTU2088
New.CleanUp.ReferenceOTU4082
New.CleanUp.ReferenceOTU3357
New.CleanUp.ReferenceOTU1509
New.CleanUp.ReferenceOTU14744
New.CleanUp.ReferenceOTU125007
New.CleanUp.ReferenceOTU18172
New.CleanUp.ReferenceOTU82435
New.CleanUp.ReferenceOTU17747
New.CleanUp.ReferenceOTU4140
New.CleanUp.ReferenceOTU185051
New.CleanUp.ReferenceOTU2963
New.CleanUp.ReferenceOTU526
New.CleanUp.ReferenceOTU2266
New.CleanUp.ReferenceOTU33627
New.CleanUp.ReferenceOTU10473
New.CleanUp.ReferenceOTU49919
New.CleanUp.ReferenceOTU292300
New.ReferenceOTU342
New.CleanUp.ReferenceOTU99855
New.CleanUp.ReferenceOTU36713
New.CleanUp.ReferenceOTU34435
New.CleanUp.ReferenceOTU6145
New.CleanUp.ReferenceOTU19056
New.CleanUp.ReferenceOTU17901
New.ReferenceOTU2936
New.CleanUp.ReferenceOTU13911
New.CleanUp.ReferenceOTU13636
New.CleanUp.ReferenceOTU205290

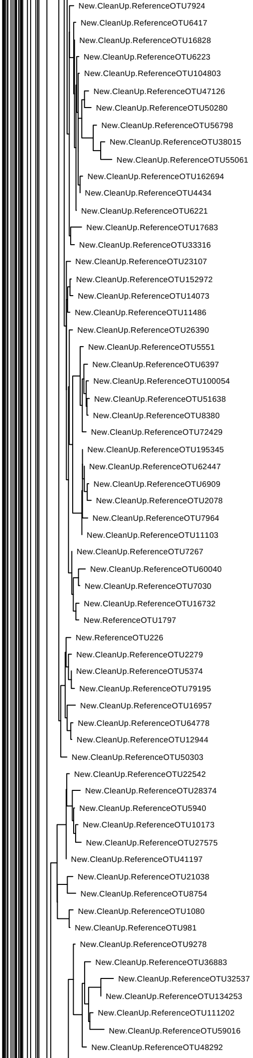
New.CleanUp.ReferenceOTU7924
New.CleanUp.ReferenceOTU6417
New.CleanUp.ReferenceOTU16828
New.CleanUp.ReferenceOTU6223
New.CleanUp.ReferenceOTU104803
New.CleanUp.ReferenceOTU47126
New.CleanUp.ReferenceOTU50280
New.CleanUp.ReferenceOTU56798
New.CleanUp.ReferenceOTU38015
New.CleanUp.ReferenceOTU55061
New.CleanUp.ReferenceOTU162694
New.CleanUp.ReferenceOTU4434
New.CleanUp.ReferenceOTU6221
New.CleanUp.ReferenceOTU17683
New.CleanUp.ReferenceOTU33316
New.CleanUp.ReferenceOTU23107
New.CleanUp.ReferenceOTU152972
New.CleanUp.ReferenceOTU14073
New.CleanUp.ReferenceOTU11486
New.CleanUp.ReferenceOTU26390
New.CleanUp.ReferenceOTU5551
New.CleanUp.ReferenceOTU6397
New.CleanUp.ReferenceOTU100054
New.CleanUp.ReferenceOTU51638
New.CleanUp.ReferenceOTU8380
New.CleanUp.ReferenceOTU72429
New.CleanUp.ReferenceOTU195345
New.CleanUp.ReferenceOTU62447
New.CleanUp.ReferenceOTU6909
New.CleanUp.ReferenceOTU2078
New.CleanUp.ReferenceOTU7964
New.CleanUp.ReferenceOTU11103
New.CleanUp.ReferenceOTU7267
New.CleanUp.ReferenceOTU60040
New.CleanUp.ReferenceOTU7030
New.CleanUp.ReferenceOTU16732
New.ReferenceOTU1797
New.ReferenceOTU226
New.CleanUp.ReferenceOTU2279
New.CleanUp.ReferenceOTU5374
New.CleanUp.ReferenceOTU79195
New.CleanUp.ReferenceOTU16957
New.CleanUp.ReferenceOTU64778
New.CleanUp.ReferenceOTU12944
New.CleanUp.ReferenceOTU50303
New.CleanUp.ReferenceOTU22542
New.CleanUp.ReferenceOTU28374
New.CleanUp.ReferenceOTU5940
New.CleanUp.ReferenceOTU10173
New.CleanUp.ReferenceOTU27575
New.CleanUp.ReferenceOTU41197
New.CleanUp.ReferenceOTU21038
New.CleanUp.ReferenceOTU8754
New.CleanUp.ReferenceOTU1080
New.CleanUp.ReferenceOTU981
New.CleanUp.ReferenceOTU9278
New.CleanUp.ReferenceOTU36883
New.CleanUp.ReferenceOTU32537
New.CleanUp.ReferenceOTU134253
New.CleanUp.ReferenceOTU111202
New.CleanUp.ReferenceOTU59016
New.CleanUp.ReferenceOTU48292

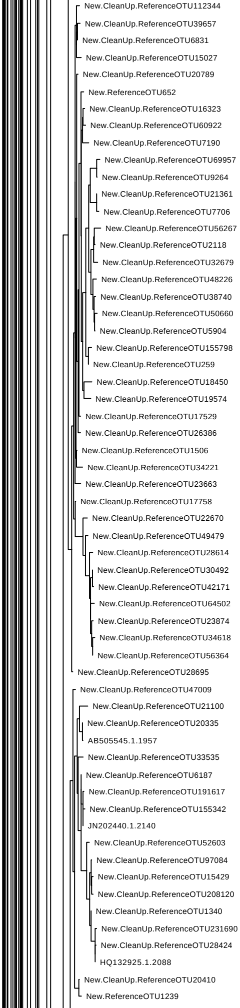
New.CleanUp.ReferenceOTU112344
New.CleanUp.ReferenceOTU39657
New.CleanUp.ReferenceOTU6831
New.CleanUp.ReferenceOTU15027
New.CleanUp.ReferenceOTU20789
New.ReferenceOTU652
New.CleanUp.ReferenceOTU16323
New.CleanUp.ReferenceOTU60922
New.CleanUp.ReferenceOTU7190
New.CleanUp.ReferenceOTU69957
New.CleanUp.ReferenceOTU9264
New.CleanUp.ReferenceOTU21361
New.CleanUp.ReferenceOTU7706
New.CleanUp.ReferenceOTU56267
New.CleanUp.ReferenceOTU2118
New.CleanUp.ReferenceOTU32679
New.CleanUp.ReferenceOTU48226
New.CleanUp.ReferenceOTU38740
New.CleanUp.ReferenceOTU50660
New.CleanUp.ReferenceOTU5904
New.CleanUp.ReferenceOTU155798
New.CleanUp.ReferenceOTU259
New.CleanUp.ReferenceOTU18450
New.CleanUp.ReferenceOTU19574
New.CleanUp.ReferenceOTU17529
New.CleanUp.ReferenceOTU26386
New.CleanUp.ReferenceOTU1506
New.CleanUp.ReferenceOTU34221
New.CleanUp.ReferenceOTU23663
New.CleanUp.ReferenceOTU17758
New.CleanUp.ReferenceOTU22670
New.CleanUp.ReferenceOTU49479
New.CleanUp.ReferenceOTU28614
New.CleanUp.ReferenceOTU30492
New.CleanUp.ReferenceOTU42171
New.CleanUp.ReferenceOTU64502
New.CleanUp.ReferenceOTU23874
New.CleanUp.ReferenceOTU34618
New.CleanUp.ReferenceOTU56364
New.CleanUp.ReferenceOTU28695
New.CleanUp.ReferenceOTU47009
New.CleanUp.ReferenceOTU21100
New.CleanUp.ReferenceOTU20335
AB505545.1.1957
New.CleanUp.ReferenceOTU33535
New.CleanUp.ReferenceOTU6187
New.CleanUp.ReferenceOTU191617
New.CleanUp.ReferenceOTU155342
JN202440.1.2140
New.CleanUp.ReferenceOTU52603
New.CleanUp.ReferenceOTU97084
New.CleanUp.ReferenceOTU15429
New.CleanUp.ReferenceOTU208120
New.CleanUp.ReferenceOTU1340
New.CleanUp.ReferenceOTU231690
New.CleanUp.ReferenceOTU28424
HQ132925.1.2088
New.CleanUp.ReferenceOTU20410
New.ReferenceOTU1239

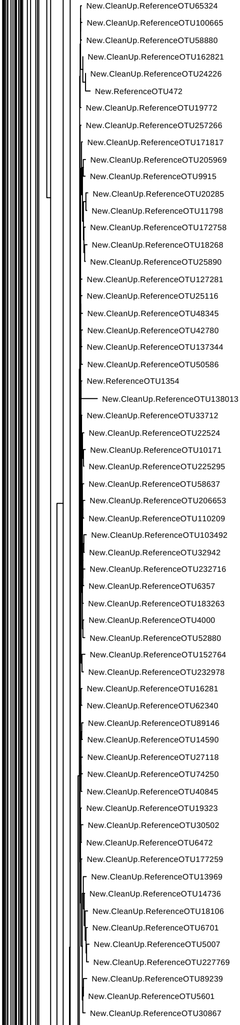
New.CleanUp.ReferenceOTU65324
New.CleanUp.ReferenceOTU100665
New.CleanUp.ReferenceOTU58880
New.CleanUp.ReferenceOTU162821
New.CleanUp.ReferenceOTU24226
New.ReferenceOTU472
New.CleanUp.ReferenceOTU19772
New.CleanUp.ReferenceOTU257266
New.CleanUp.ReferenceOTU171817
New.CleanUp.ReferenceOTU205969
New.CleanUp.ReferenceOTU9915
New.CleanUp.ReferenceOTU20285
New.CleanUp.ReferenceOTU11798
New.CleanUp.ReferenceOTU172758
New.CleanUp.ReferenceOTU18268
New.CleanUp.ReferenceOTU25890
New.CleanUp.ReferenceOTU127281
New.CleanUp.ReferenceOTU25116
New.CleanUp.ReferenceOTU48345
New.CleanUp.ReferenceOTU42780
New.CleanUp.ReferenceOTU137344
New.CleanUp.ReferenceOTU50586
New.ReferenceOTU1354
New.CleanUp.ReferenceOTU138013
New.CleanUp.ReferenceOTU33712
New.CleanUp.ReferenceOTU22524
New.CleanUp.ReferenceOTU10171
New.CleanUp.ReferenceOTU225295
New.CleanUp.ReferenceOTU58637
New.CleanUp.ReferenceOTU206653
New.CleanUp.ReferenceOTU110209
New.CleanUp.ReferenceOTU103492
New.CleanUp.ReferenceOTU32942
New.CleanUp.ReferenceOTU232716
New.CleanUp.ReferenceOTU6357
New.CleanUp.ReferenceOTU183263
New.CleanUp.ReferenceOTU4000
New.CleanUp.ReferenceOTU52880
New.CleanUp.ReferenceOTU152764
New.CleanUp.ReferenceOTU232978
New.CleanUp.ReferenceOTU16281
New.CleanUp.ReferenceOTU62340
New.CleanUp.ReferenceOTU89146
New.CleanUp.ReferenceOTU14590
New.CleanUp.ReferenceOTU27118
New.CleanUp.ReferenceOTU74250
New.CleanUp.ReferenceOTU40845
New.CleanUp.ReferenceOTU19323
New.CleanUp.ReferenceOTU30502
New.CleanUp.ReferenceOTU6472
New.CleanUp.ReferenceOTU177259
New.CleanUp.ReferenceOTU13969
New.CleanUp.ReferenceOTU14736
New.CleanUp.ReferenceOTU18106
New.CleanUp.ReferenceOTU6701
New.CleanUp.ReferenceOTU5007
New.CleanUp.ReferenceOTU227769
New.CleanUp.ReferenceOTU89239
New.CleanUp.ReferenceOTU5601
New.CleanUp.ReferenceOTU30867

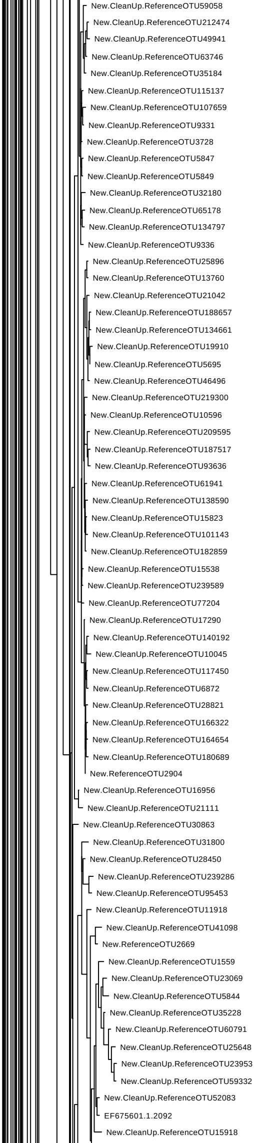
New.CleanUp.ReferenceOTU59058
New.CleanUp.ReferenceOTU212474
New.CleanUp.ReferenceOTU49941
New.CleanUp.ReferenceOTU63746
New.CleanUp.ReferenceOTU35184
New.CleanUp.ReferenceOTU115137
New.CleanUp.ReferenceOTU107659
New.CleanUp.ReferenceOTU9331
New.CleanUp.ReferenceOTU3728
New.CleanUp.ReferenceOTU5847
New.CleanUp.ReferenceOTU5849
New.CleanUp.ReferenceOTU32180
New.CleanUp.ReferenceOTU65178
New.CleanUp.ReferenceOTU134797
New.CleanUp.ReferenceOTU9336
New.CleanUp.ReferenceOTU25896
New.CleanUp.ReferenceOTU13760
New.CleanUp.ReferenceOTU21042
New.CleanUp.ReferenceOTU188657
New.CleanUp.ReferenceOTU134661
New.CleanUp.ReferenceOTU19910
New.CleanUp.ReferenceOTU5695
New.CleanUp.ReferenceOTU46496
New.CleanUp.ReferenceOTU219300
New.CleanUp.ReferenceOTU10596
New.CleanUp.ReferenceOTU209595
New.CleanUp.ReferenceOTU187517
New.CleanUp.ReferenceOTU93636
New.CleanUp.ReferenceOTU61941
New.CleanUp.ReferenceOTU138590
New.CleanUp.ReferenceOTU15823
New.CleanUp.ReferenceOTU101143
New.CleanUp.ReferenceOTU182859
New.CleanUp.ReferenceOTU15538
New.CleanUp.ReferenceOTU239589
New.CleanUp.ReferenceOTU77204
New.CleanUp.ReferenceOTU17290
New.CleanUp.ReferenceOTU140192
New.CleanUp.ReferenceOTU10045
New.CleanUp.ReferenceOTU117450
New.CleanUp.ReferenceOTU6872
New.CleanUp.ReferenceOTU28821
New.CleanUp.ReferenceOTU166322
New.CleanUp.ReferenceOTU164654
New.CleanUp.ReferenceOTU180689
New.ReferenceOTU2904
New.CleanUp.ReferenceOTU16956
New.CleanUp.ReferenceOTU21111
New.CleanUp.ReferenceOTU30863
New.CleanUp.ReferenceOTU31800
New.CleanUp.ReferenceOTU28450
New.CleanUp.ReferenceOTU239286
New.CleanUp.ReferenceOTU95453
New.CleanUp.ReferenceOTU11918
New.CleanUp.ReferenceOTU41098
New.ReferenceOTU2669
New.CleanUp.ReferenceOTU1559
New.CleanUp.ReferenceOTU23069
New.CleanUp.ReferenceOTU5844
New.CleanUp.ReferenceOTU35228
New.CleanUp.ReferenceOTU60791
New.CleanUp.ReferenceOTU25648
New.CleanUp.ReferenceOTU23953
New.CleanUp.ReferenceOTU59332
New.CleanUp.ReferenceOTU52083
EF675601.1.2092
New.CleanUp.ReferenceOTU15918

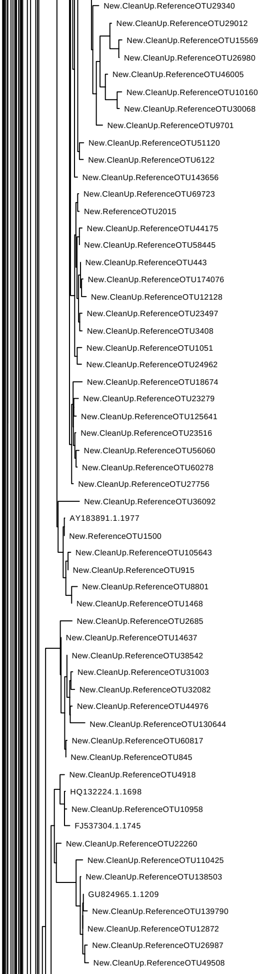
New.CleanUp.ReferenceOTU29340
New.CleanUp.ReferenceOTU29012
New.CleanUp.ReferenceOTU15569
New.CleanUp.ReferenceOTU26980
New.CleanUp.ReferenceOTU46005
New.CleanUp.ReferenceOTU10160
New.CleanUp.ReferenceOTU30068
New.CleanUp.ReferenceOTU9701
New.CleanUp.ReferenceOTU51120
New.CleanUp.ReferenceOTU6122
New.CleanUp.ReferenceOTU143656
New.CleanUp.ReferenceOTU69723
New.ReferenceOTU2015
New.CleanUp.ReferenceOTU44175
New.CleanUp.ReferenceOTU58445
New.CleanUp.ReferenceOTU443
New.CleanUp.ReferenceOTU174076
New.CleanUp.ReferenceOTU12128
New.CleanUp.ReferenceOTU23497
New.CleanUp.ReferenceOTU3408
New.CleanUp.ReferenceOTU1051
New.CleanUp.ReferenceOTU24962
New.CleanUp.ReferenceOTU18674
New.CleanUp.ReferenceOTU23279
New.CleanUp.ReferenceOTU125641
New.CleanUp.ReferenceOTU23516
New.CleanUp.ReferenceOTU56060
New.CleanUp.ReferenceOTU60278
New.CleanUp.ReferenceOTU27756
New.CleanUp.ReferenceOTU36092
AY183891.1.1977
New.ReferenceOTU1500
New.CleanUp.ReferenceOTU105643
New.CleanUp.ReferenceOTU915
New.CleanUp.ReferenceOTU8801
New.CleanUp.ReferenceOTU1468
New.CleanUp.ReferenceOTU2685
New.CleanUp.ReferenceOTU14637
New.CleanUp.ReferenceOTU38542
New.CleanUp.ReferenceOTU31003
New.CleanUp.ReferenceOTU32082
New.CleanUp.ReferenceOTU44976
New.CleanUp.ReferenceOTU130644
New.CleanUp.ReferenceOTU60817
New.CleanUp.ReferenceOTU845
New.CleanUp.ReferenceOTU4918
HQ132224.1.1698
New.CleanUp.ReferenceOTU10958
FJ537304.1.1745
New.CleanUp.ReferenceOTU22260
New.CleanUp.ReferenceOTU110425
New.CleanUp.ReferenceOTU138503
GU824965.1.1209
New.CleanUp.ReferenceOTU139790
New.CleanUp.ReferenceOTU12872
New.CleanUp.ReferenceOTU26987
New.CleanUp.ReferenceOTU49508

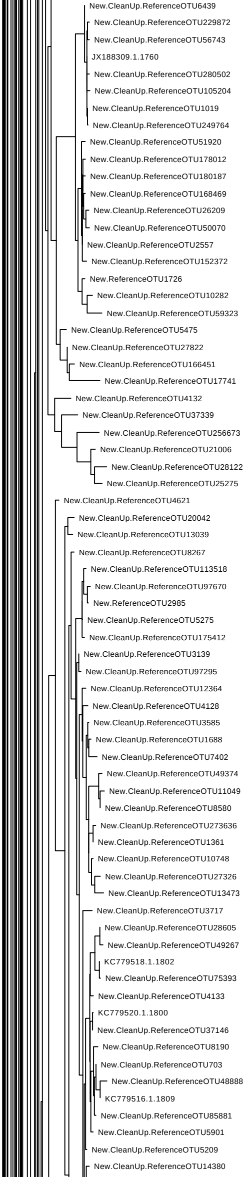
New.CleanUp.ReferenceOTU6439
New.CleanUp.ReferenceOTU229872
New.CleanUp.ReferenceOTU56743
JX188309.1.1760
New.CleanUp.ReferenceOTU280502
New.CleanUp.ReferenceOTU105204
New.CleanUp.ReferenceOTU1019
New.CleanUp.ReferenceOTU249764
New.CleanUp.ReferenceOTU51920
New.CleanUp.ReferenceOTU178012
New.CleanUp.ReferenceOTU180187
New.CleanUp.ReferenceOTU168469
New.CleanUp.ReferenceOTU26209
New.CleanUp.ReferenceOTU50070
New.CleanUp.ReferenceOTU2557
New.CleanUp.ReferenceOTU152372
New.ReferenceOTU1726
New.CleanUp.ReferenceOTU10282
New.CleanUp.ReferenceOTU59323
New.CleanUp.ReferenceOTU5475
New.CleanUp.ReferenceOTU27822
New.CleanUp.ReferenceOTU166451
New.CleanUp.ReferenceOTU17741
New.CleanUp.ReferenceOTU4132
New.CleanUp.ReferenceOTU37339
New.CleanUp.ReferenceOTU256673
New.CleanUp.ReferenceOTU21006
New.CleanUp.ReferenceOTU28122
New.CleanUp.ReferenceOTU25275
New.CleanUp.ReferenceOTU4621
New.CleanUp.ReferenceOTU20042
New.CleanUp.ReferenceOTU13039
New.CleanUp.ReferenceOTU8267
New.CleanUp.ReferenceOTU113518
New.CleanUp.ReferenceOTU97670
New.ReferenceOTU2985
New.CleanUp.ReferenceOTU5275
New.CleanUp.ReferenceOTU175412
New.CleanUp.ReferenceOTU3139
New.CleanUp.ReferenceOTU97295
New.CleanUp.ReferenceOTU12364
New.CleanUp.ReferenceOTU4128
New.CleanUp.ReferenceOTU3585
New.CleanUp.ReferenceOTU1688
New.CleanUp.ReferenceOTU7402
New.CleanUp.ReferenceOTU49374
New.CleanUp.ReferenceOTU11049
New.CleanUp.ReferenceOTU8580
New.CleanUp.ReferenceOTU273636
New.CleanUp.ReferenceOTU1361
New.CleanUp.ReferenceOTU10748
New.CleanUp.ReferenceOTU27326
New.CleanUp.ReferenceOTU13473
New.CleanUp.ReferenceOTU3717
New.CleanUp.ReferenceOTU28605
New.CleanUp.ReferenceOTU49267
KC779518.1.1802
New.CleanUp.ReferenceOTU75393
New.CleanUp.ReferenceOTU4133
KC779520.1.1800
New.CleanUp.ReferenceOTU37146
New.CleanUp.ReferenceOTU8190
New.CleanUp.ReferenceOTU703
New.CleanUp.ReferenceOTU48888
KC779516.1.1809
New.CleanUp.ReferenceOTU85881
New.CleanUp.ReferenceOTU5901
New.CleanUp.ReferenceOTU5209
New.CleanUp.ReferenceOTU14380

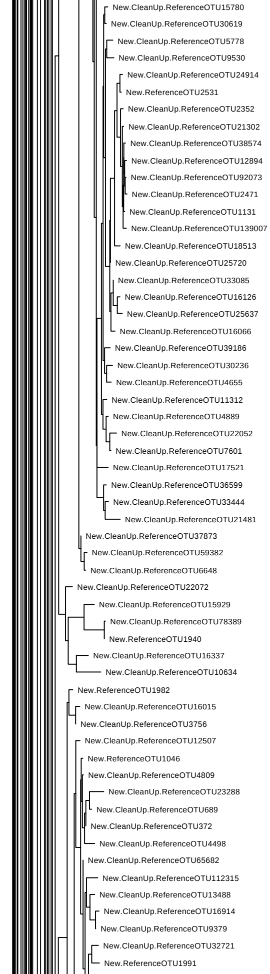
New.CleanUp.ReferenceOTU15780
New.CleanUp.ReferenceOTU30619
New.CleanUp.ReferenceOTU5778
New.CleanUp.ReferenceOTU9530
New.CleanUp.ReferenceOTU24914
New.ReferenceOTU2531
New.CleanUp.ReferenceOTU2352
New.CleanUp.ReferenceOTU21302
New.CleanUp.ReferenceOTU38574
New.CleanUp.ReferenceOTU12894
New.CleanUp.ReferenceOTU92073
New.CleanUp.ReferenceOTU2471
New.CleanUp.ReferenceOTU1131
New.CleanUp.ReferenceOTU139007
New.CleanUp.ReferenceOTU18513
New.CleanUp.ReferenceOTU25720
New.CleanUp.ReferenceOTU33085
New.CleanUp.ReferenceOTU16126
New.CleanUp.ReferenceOTU25637
New.CleanUp.ReferenceOTU16066
New.CleanUp.ReferenceOTU39186
New.CleanUp.ReferenceOTU30236
New.CleanUp.ReferenceOTU4655
New.CleanUp.ReferenceOTU11312
New.CleanUp.ReferenceOTU4889
New.CleanUp.ReferenceOTU22052
New.CleanUp.ReferenceOTU7601
New.CleanUp.ReferenceOTU17521
New.CleanUp.ReferenceOTU36599
New.CleanUp.ReferenceOTU33444
New.CleanUp.ReferenceOTU21481
New.CleanUp.ReferenceOTU37873
New.CleanUp.ReferenceOTU59382
New.CleanUp.ReferenceOTU6648
New.CleanUp.ReferenceOTU22072
New.CleanUp.ReferenceOTU15929
New.CleanUp.ReferenceOTU78389
New.ReferenceOTU1940
New.CleanUp.ReferenceOTU16337
New.CleanUp.ReferenceOTU10634
New.ReferenceOTU1982
New.CleanUp.ReferenceOTU16015
New.CleanUp.ReferenceOTU3756
New.CleanUp.ReferenceOTU12507
New.ReferenceOTU1046
New.CleanUp.ReferenceOTU4809
New.CleanUp.ReferenceOTU23288
New.CleanUp.ReferenceOTU689
New.CleanUp.ReferenceOTU372
New.CleanUp.ReferenceOTU4498
New.CleanUp.ReferenceOTU65682
New.CleanUp.ReferenceOTU112315
New.CleanUp.ReferenceOTU13488
New.CleanUp.ReferenceOTU16914
New.CleanUp.ReferenceOTU9379
New.CleanUp.ReferenceOTU32721
New.ReferenceOTU1991

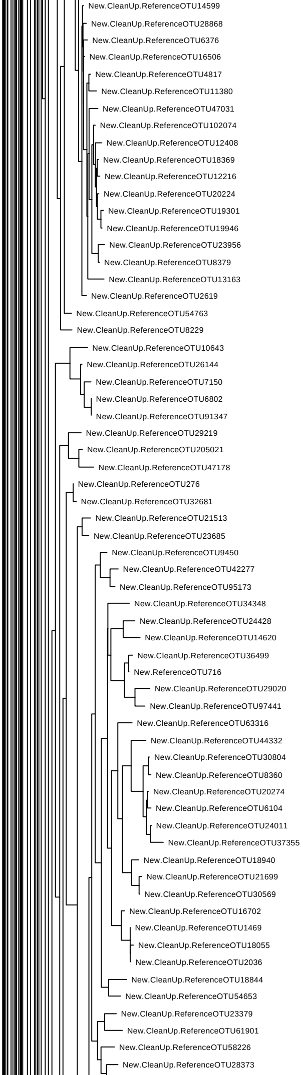
New.CleanUp.ReferenceOTU14599
New.CleanUp.ReferenceOTU28868
New.CleanUp.ReferenceOTU6376
New.CleanUp.ReferenceOTU16506
New.CleanUp.ReferenceOTU4817
New.CleanUp.ReferenceOTU11380
New.CleanUp.ReferenceOTU47031
New.CleanUp.ReferenceOTU102074
New.CleanUp.ReferenceOTU12408
New.CleanUp.ReferenceOTU18369
New.CleanUp.ReferenceOTU12216
New.CleanUp.ReferenceOTU20224
New.CleanUp.ReferenceOTU19301
New.CleanUp.ReferenceOTU19946
New.CleanUp.ReferenceOTU23956
New.CleanUp.ReferenceOTU8379
New.CleanUp.ReferenceOTU13163
New.CleanUp.ReferenceOTU2619
New.CleanUp.ReferenceOTU54763
New.CleanUp.ReferenceOTU8229
New.CleanUp.ReferenceOTU10643
New.CleanUp.ReferenceOTU26144
New.CleanUp.ReferenceOTU7150
New.CleanUp.ReferenceOTU6802
New.CleanUp.ReferenceOTU91347
New.CleanUp.ReferenceOTU29219
New.CleanUp.ReferenceOTU205021
New.CleanUp.ReferenceOTU47178
New.CleanUp.ReferenceOTU276
New.CleanUp.ReferenceOTU32681
New.CleanUp.ReferenceOTU21513
New.CleanUp.ReferenceOTU23685
New.CleanUp.ReferenceOTU9450
New.CleanUp.ReferenceOTU42277
New.CleanUp.ReferenceOTU95173
New.CleanUp.ReferenceOTU34348
New.CleanUp.ReferenceOTU24428
New.CleanUp.ReferenceOTU14620
New.CleanUp.ReferenceOTU36499
New.ReferenceOTU716
New.CleanUp.ReferenceOTU29020
New.CleanUp.ReferenceOTU97441
New.CleanUp.ReferenceOTU63316
New.CleanUp.ReferenceOTU44332
New.CleanUp.ReferenceOTU30804
New.CleanUp.ReferenceOTU8360
New.CleanUp.ReferenceOTU20274
New.CleanUp.ReferenceOTU6104
New.CleanUp.ReferenceOTU24011
New.CleanUp.ReferenceOTU37355
New.CleanUp.ReferenceOTU18940
New.CleanUp.ReferenceOTU21699
New.CleanUp.ReferenceOTU30569
New.CleanUp.ReferenceOTU16702
New.CleanUp.ReferenceOTU1469
New.CleanUp.ReferenceOTU18055
New.CleanUp.ReferenceOTU2036
New.CleanUp.ReferenceOTU18844
New.CleanUp.ReferenceOTU54653
New.CleanUp.ReferenceOTU23379
New.CleanUp.ReferenceOTU61901
New.CleanUp.ReferenceOTU58226
New.CleanUp.ReferenceOTU28373

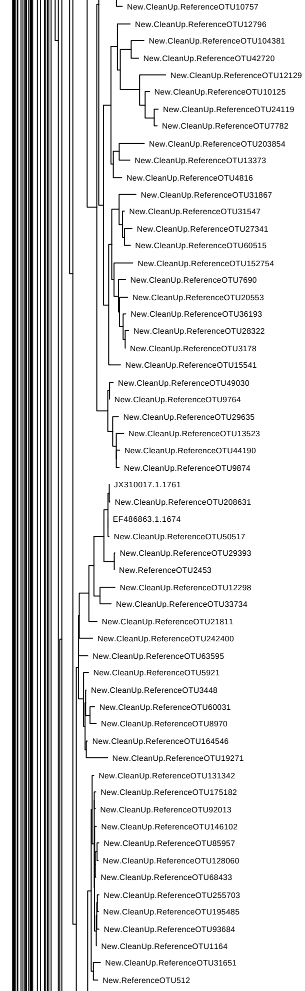

New.CleanUp.ReferenceOTU10757
New.CleanUp.ReferenceOTU12796
New.CleanUp.ReferenceOTU104381
New.CleanUp.ReferenceOTU42720
New.CleanUp.ReferenceOTU12129
New.CleanUp.ReferenceOTU10125
New.CleanUp.ReferenceOTU24119
New.CleanUp.ReferenceOTU7782
New.CleanUp.ReferenceOTU203854
New.CleanUp.ReferenceOTU13373
New.CleanUp.ReferenceOTU4816
New.CleanUp.ReferenceOTU31867
New.CleanUp.ReferenceOTU31547
New.CleanUp.ReferenceOTU27341
New.CleanUp.ReferenceOTU60515
New.CleanUp.ReferenceOTU152754
New.CleanUp.ReferenceOTU7690
New.CleanUp.ReferenceOTU20553
New.CleanUp.ReferenceOTU36193
New.CleanUp.ReferenceOTU28322
New.CleanUp.ReferenceOTU3178
New.CleanUp.ReferenceOTU15541
New.CleanUp.ReferenceOTU49030
New.CleanUp.ReferenceOTU9764
New.CleanUp.ReferenceOTU29635
New.CleanUp.ReferenceOTU13523
New.CleanUp.ReferenceOTU44190
New.CleanUp.ReferenceOTU9874
JX310017.1.1761
New.CleanUp.ReferenceOTU208631
EF486863.1.1674
New.CleanUp.ReferenceOTU50517
New.CleanUp.ReferenceOTU29393
New.ReferenceOTU2453
New.CleanUp.ReferenceOTU12298
New.CleanUp.ReferenceOTU33734
New.CleanUp.ReferenceOTU21811
New.CleanUp.ReferenceOTU242400
New.CleanUp.ReferenceOTU63595
New.CleanUp.ReferenceOTU5921
New.CleanUp.ReferenceOTU3448
New.CleanUp.ReferenceOTU60031
New.CleanUp.ReferenceOTU8970
New.CleanUp.ReferenceOTU164546
New.CleanUp.ReferenceOTU19271
New.CleanUp.ReferenceOTU131342
New.CleanUp.ReferenceOTU175182
New.CleanUp.ReferenceOTU92013
New.CleanUp.ReferenceOTU146102
New.CleanUp.ReferenceOTU85957
New.CleanUp.ReferenceOTU128060
New.CleanUp.ReferenceOTU68433
New.CleanUp.ReferenceOTU255703
New.CleanUp.ReferenceOTU195485
New.CleanUp.ReferenceOTU93684
New.CleanUp.ReferenceOTU1164
New.CleanUp.ReferenceOTU31651
New.ReferenceOTU512

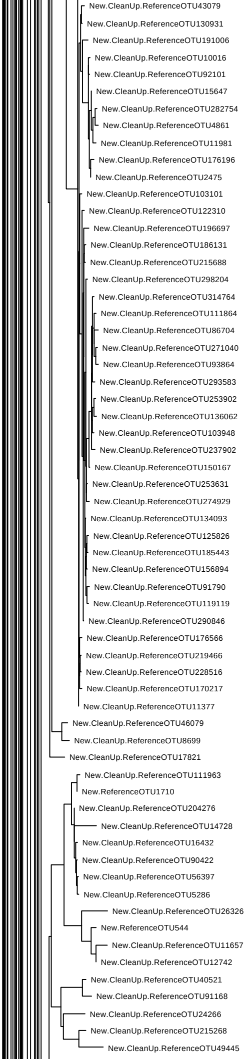
New.CleanUp.ReferenceOTU43079
New.CleanUp.ReferenceOTU130931
New.CleanUp.ReferenceOTU191006
New.CleanUp.ReferenceOTU10016
New.CleanUp.ReferenceOTU92101
New.CleanUp.ReferenceOTU15647
New.CleanUp.ReferenceOTU282754
New.CleanUp.ReferenceOTU4861
New.CleanUp.ReferenceOTU11981
New.CleanUp.ReferenceOTU176196
New.CleanUp.ReferenceOTU2475
New.CleanUp.ReferenceOTU103101
New.CleanUp.ReferenceOTU122310
New.CleanUp.ReferenceOTU196697
New.CleanUp.ReferenceOTU186131
New.CleanUp.ReferenceOTU215688
New.CleanUp.ReferenceOTU298204
New.CleanUp.ReferenceOTU314764
New.CleanUp.ReferenceOTU111864
New.CleanUp.ReferenceOTU86704
New.CleanUp.ReferenceOTU271040
New.CleanUp.ReferenceOTU93864
New.CleanUp.ReferenceOTU293583
New.CleanUp.ReferenceOTU253902
New.CleanUp.ReferenceOTU136062
New.CleanUp.ReferenceOTU103948
New.CleanUp.ReferenceOTU237902
New.CleanUp.ReferenceOTU150167
New.CleanUp.ReferenceOTU253631
New.CleanUp.ReferenceOTU274929
New.CleanUp.ReferenceOTU134093
New.CleanUp.ReferenceOTU125826
New.CleanUp.ReferenceOTU185443
New.CleanUp.ReferenceOTU156894
New.CleanUp.ReferenceOTU91790
New.CleanUp.ReferenceOTU119119
New.CleanUp.ReferenceOTU290846
New.CleanUp.ReferenceOTU176566
New.CleanUp.ReferenceOTU219466
New.CleanUp.ReferenceOTU228516
New.CleanUp.ReferenceOTU170217
New.CleanUp.ReferenceOTU11377
New.CleanUp.ReferenceOTU46079
New.CleanUp.ReferenceOTU8699
New.CleanUp.ReferenceOTU17821
New.CleanUp.ReferenceOTU111963
New.ReferenceOTU1710
New.CleanUp.ReferenceOTU204276
New.CleanUp.ReferenceOTU14728
New.CleanUp.ReferenceOTU16432
New.CleanUp.ReferenceOTU90422
New.CleanUp.ReferenceOTU56397
New.CleanUp.ReferenceOTU5286
New.CleanUp.ReferenceOTU26326
New.ReferenceOTU544
New.CleanUp.ReferenceOTU11657
New.CleanUp.ReferenceOTU12742
New.CleanUp.ReferenceOTU40521
New.CleanUp.ReferenceOTU91168
New.CleanUp.ReferenceOTU24266
New.CleanUp.ReferenceOTU215268
New.CleanUp.ReferenceOTU49445

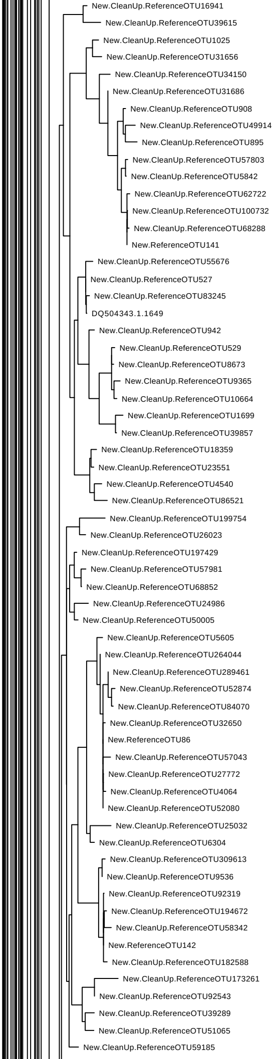
New.CleanUp.ReferenceOTU16941
New.CleanUp.ReferenceOTU39615
New.CleanUp.ReferenceOTU1025
New.CleanUp.ReferenceOTU31656
New.CleanUp.ReferenceOTU34150
New.CleanUp.ReferenceOTU31686
New.CleanUp.ReferenceOTU908
New.CleanUp.ReferenceOTU49914
New.CleanUp.ReferenceOTU895
New.CleanUp.ReferenceOTU57803
New.CleanUp.ReferenceOTU5842
New.CleanUp.ReferenceOTU62722
New.CleanUp.ReferenceOTU100732
New.CleanUp.ReferenceOTU68288
New.ReferenceOTU141
New.CleanUp.ReferenceOTU55676
New.CleanUp.ReferenceOTU527
New.CleanUp.ReferenceOTU83245
DQ504343.1.1649
New.CleanUp.ReferenceOTU942
New.CleanUp.ReferenceOTU529
New.CleanUp.ReferenceOTU8673
New.CleanUp.ReferenceOTU9365
New.CleanUp.ReferenceOTU10664
New.CleanUp.ReferenceOTU1699
New.CleanUp.ReferenceOTU39857
New.CleanUp.ReferenceOTU18359
New.CleanUp.ReferenceOTU23551
New.CleanUp.ReferenceOTU4540
New.CleanUp.ReferenceOTU86521
New.CleanUp.ReferenceOTU199754
New.CleanUp.ReferenceOTU26023
New.CleanUp.ReferenceOTU197429
New.CleanUp.ReferenceOTU57981
New.CleanUp.ReferenceOTU68852
New.CleanUp.ReferenceOTU24986
New.CleanUp.ReferenceOTU50005
New.CleanUp.ReferenceOTU5605
New.CleanUp.ReferenceOTU264044
New.CleanUp.ReferenceOTU289461
New.CleanUp.ReferenceOTU52874
New.CleanUp.ReferenceOTU84070
New.CleanUp.ReferenceOTU32650
New.ReferenceOTU86
New.CleanUp.ReferenceOTU57043
New.CleanUp.ReferenceOTU27772
New.CleanUp.ReferenceOTU4064
New.CleanUp.ReferenceOTU52080
New.CleanUp.ReferenceOTU25032
New.CleanUp.ReferenceOTU6304
New.CleanUp.ReferenceOTU309613
New.CleanUp.ReferenceOTU9536
New.CleanUp.ReferenceOTU92319
New.CleanUp.ReferenceOTU194672
New.CleanUp.ReferenceOTU58342
New.ReferenceOTU142
New.CleanUp.ReferenceOTU182588
New.CleanUp.ReferenceOTU173261
New.CleanUp.ReferenceOTU92543
New.CleanUp.ReferenceOTU39289
New.CleanUp.ReferenceOTU51065
New.CleanUp.ReferenceOTU59185

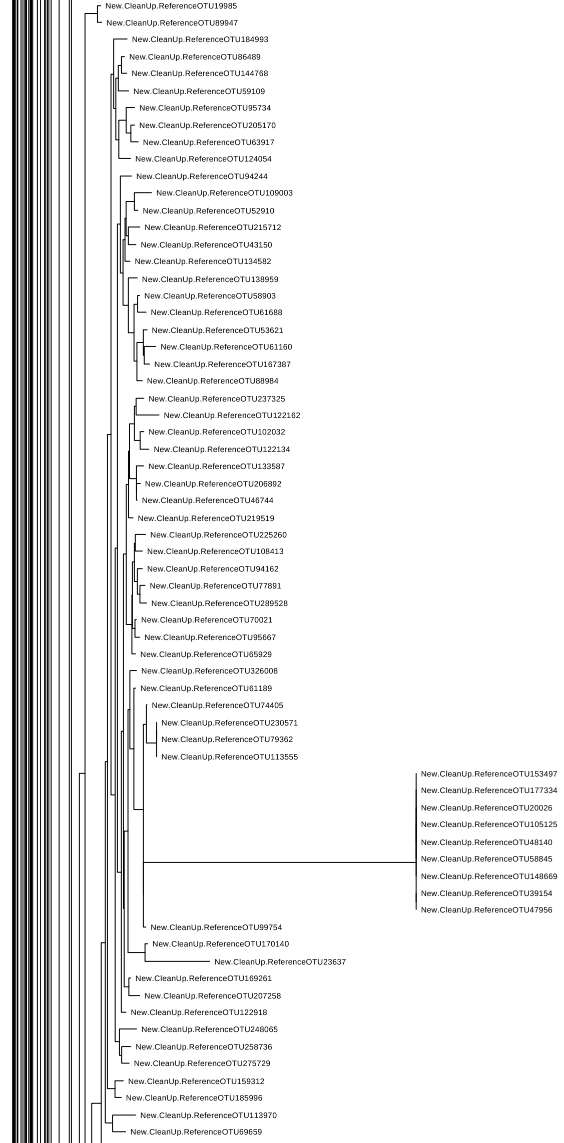
New.CleanUp.ReferenceOTU19985
New.CleanUp.ReferenceOTU89947
New.CleanUp.ReferenceOTU184993
New.CleanUp.ReferenceOTU86489
New.CleanUp.ReferenceOTU144768
New.CleanUp.ReferenceOTU59109
New.CleanUp.ReferenceOTU95734
New.CleanUp.ReferenceOTU205170
New.CleanUp.ReferenceOTU63917
New.CleanUp.ReferenceOTU124054
New.CleanUp.ReferenceOTU94244
New.CleanUp.ReferenceOTU109003
New.CleanUp.ReferenceOTU52910
New.CleanUp.ReferenceOTU215712
New.CleanUp.ReferenceOTU43150
New.CleanUp.ReferenceOTU134582
New.CleanUp.ReferenceOTU138959
New.CleanUp.ReferenceOTU58903
New.CleanUp.ReferenceOTU61688
New.CleanUp.ReferenceOTU53621
New.CleanUp.ReferenceOTU61160
New.CleanUp.ReferenceOTU167387
New.CleanUp.ReferenceOTU88984
New.CleanUp.ReferenceOTU237325
New.CleanUp.ReferenceOTU122162
New.CleanUp.ReferenceOTU102032
New.CleanUp.ReferenceOTU122134
New.CleanUp.ReferenceOTU133587
New.CleanUp.ReferenceOTU206892
New.CleanUp.ReferenceOTU46744
New.CleanUp.ReferenceOTU219519
New.CleanUp.ReferenceOTU225260
New.CleanUp.ReferenceOTU108413
New.CleanUp.ReferenceOTU94162
New.CleanUp.ReferenceOTU77891
New.CleanUp.ReferenceOTU289528
New.CleanUp.ReferenceOTU70021
New.CleanUp.ReferenceOTU95667
New.CleanUp.ReferenceOTU65929
New.CleanUp.ReferenceOTU326008
New.CleanUp.ReferenceOTU61189
New.CleanUp.ReferenceOTU74405
New.CleanUp.ReferenceOTU230571
New.CleanUp.ReferenceOTU79362
New.CleanUp.ReferenceOTU113555
New.CleanUp.ReferenceOTU153497
New.CleanUp.ReferenceOTU177334
New.CleanUp.ReferenceOTU20026
New.CleanUp.ReferenceOTU105125
New.CleanUp.ReferenceOTU48140
New.CleanUp.ReferenceOTU58845
New.CleanUp.ReferenceOTU148669
New.CleanUp.ReferenceOTU39154
New.CleanUp.ReferenceOTU47956
New.CleanUp.ReferenceOTU99754
New.CleanUp.ReferenceOTU170140
New.CleanUp.ReferenceOTU23637
New.CleanUp.ReferenceOTU169261
New.CleanUp.ReferenceOTU207258
New.CleanUp.ReferenceOTU122918
New.CleanUp.ReferenceOTU248065
New.CleanUp.ReferenceOTU258736
New.CleanUp.ReferenceOTU275729
New.CleanUp.ReferenceOTU159312
New.CleanUp.ReferenceOTU185996
New.CleanUp.ReferenceOTU113970
New.CleanUp.ReferenceOTU69659

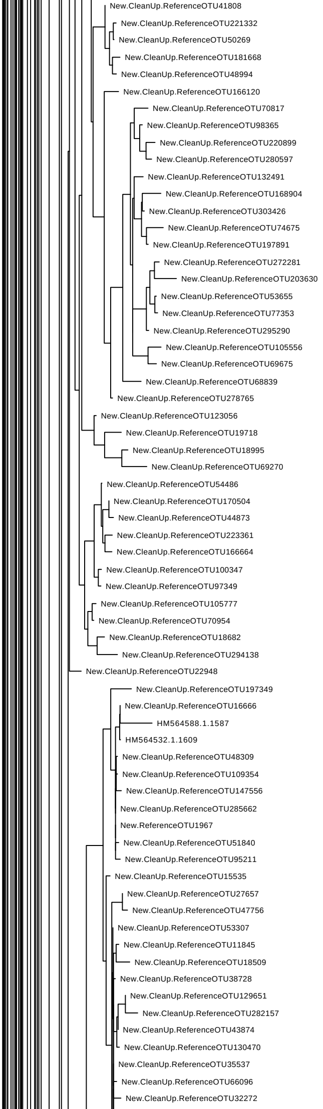
New.CleanUp.ReferenceOTU41808
New.CleanUp.ReferenceOTU221332
New.CleanUp.ReferenceOTU50269
New.CleanUp.ReferenceOTU181668
New.CleanUp.ReferenceOTU48994
New.CleanUp.ReferenceOTU166120
New.CleanUp.ReferenceOTU70817
New.CleanUp.ReferenceOTU98365
New.CleanUp.ReferenceOTU220899
New.CleanUp.ReferenceOTU280597
New.CleanUp.ReferenceOTU132491
New.CleanUp.ReferenceOTU168904
New.CleanUp.ReferenceOTU303426
New.CleanUp.ReferenceOTU74675
New.CleanUp.ReferenceOTU197891
New.CleanUp.ReferenceOTU272281
New.CleanUp.ReferenceOTU203630
New.CleanUp.ReferenceOTU53655
New.CleanUp.ReferenceOTU77353
New.CleanUp.ReferenceOTU295290
New.CleanUp.ReferenceOTU105556
New.CleanUp.ReferenceOTU69675
New.CleanUp.ReferenceOTU68839
New.CleanUp.ReferenceOTU278765
New.CleanUp.ReferenceOTU123056
New.CleanUp.ReferenceOTU19718
New.CleanUp.ReferenceOTU18995
New.CleanUp.ReferenceOTU69270
New.CleanUp.ReferenceOTU54486
New.CleanUp.ReferenceOTU170504
New.CleanUp.ReferenceOTU44873
New.CleanUp.ReferenceOTU223361
New.CleanUp.ReferenceOTU166664
New.CleanUp.ReferenceOTU100347
New.CleanUp.ReferenceOTU97349
New.CleanUp.ReferenceOTU105777
New.CleanUp.ReferenceOTU70954
New.CleanUp.ReferenceOTU18682
New.CleanUp.ReferenceOTU294138
New.CleanUp.ReferenceOTU22948
New.CleanUp.ReferenceOTU197349
New.CleanUp.ReferenceOTU16666
HM564588.1.1587
HM564532.1.1609
New.CleanUp.ReferenceOTU48309
New.CleanUp.ReferenceOTU109354
New.CleanUp.ReferenceOTU147556
New.CleanUp.ReferenceOTU285662
New.ReferenceOTU1967
New.CleanUp.ReferenceOTU51840
New.CleanUp.ReferenceOTU95211
New.CleanUp.ReferenceOTU15535
New.CleanUp.ReferenceOTU27657
New.CleanUp.ReferenceOTU47756
New.CleanUp.ReferenceOTU53307
New.CleanUp.ReferenceOTU11845
New.CleanUp.ReferenceOTU18509
New.CleanUp.ReferenceOTU38728
New.CleanUp.ReferenceOTU129651
New.CleanUp.ReferenceOTU282157
New.CleanUp.ReferenceOTU43874
New.CleanUp.ReferenceOTU130470
New.CleanUp.ReferenceOTU35537
New.CleanUp.ReferenceOTU66096
New.CleanUp.ReferenceOTU32272

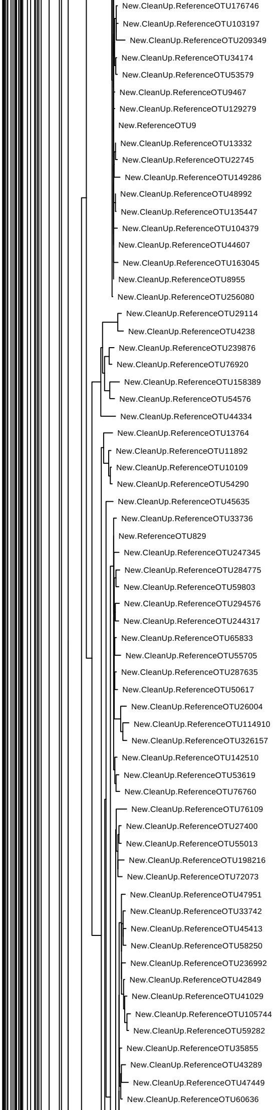
New.CleanUp.ReferenceOTU176746
New.CleanUp.ReferenceOTU103197
New.CleanUp.ReferenceOTU209349
New.CleanUp.ReferenceOTU34174
New.CleanUp.ReferenceOTU53579
New.CleanUp.ReferenceOTU9467
New.CleanUp.ReferenceOTU129279
New.ReferenceOTU9
New.CleanUp.ReferenceOTU13332
New.CleanUp.ReferenceOTU22745
New.CleanUp.ReferenceOTU149286
New.CleanUp.ReferenceOTU48992
New.CleanUp.ReferenceOTU135447
New.CleanUp.ReferenceOTU104379
New.CleanUp.ReferenceOTU44607
New.CleanUp.ReferenceOTU163045
New.CleanUp.ReferenceOTU8955
New.CleanUp.ReferenceOTU256080
New.CleanUp.ReferenceOTU29114
New.CleanUp.ReferenceOTU4238
New.CleanUp.ReferenceOTU239876
New.CleanUp.ReferenceOTU76920
New.CleanUp.ReferenceOTU158389
New.CleanUp.ReferenceOTU54576
New.CleanUp.ReferenceOTU44334
New.CleanUp.ReferenceOTU13764
New.CleanUp.ReferenceOTU11892
New.CleanUp.ReferenceOTU10109
New.CleanUp.ReferenceOTU54290
New.CleanUp.ReferenceOTU45635
New.CleanUp.ReferenceOTU33736
New.ReferenceOTU829
New.CleanUp.ReferenceOTU247345
New.CleanUp.ReferenceOTU284775
New.CleanUp.ReferenceOTU59803
New.CleanUp.ReferenceOTU294576
New.CleanUp.ReferenceOTU244317
New.CleanUp.ReferenceOTU65833
New.CleanUp.ReferenceOTU55705
New.CleanUp.ReferenceOTU287635
New.CleanUp.ReferenceOTU50617
New.CleanUp.ReferenceOTU26004
New.CleanUp.ReferenceOTU114910
New.CleanUp.ReferenceOTU326157
New.CleanUp.ReferenceOTU142510
New.CleanUp.ReferenceOTU53619
New.CleanUp.ReferenceOTU76760
New.CleanUp.ReferenceOTU76109
New.CleanUp.ReferenceOTU27400
New.CleanUp.ReferenceOTU55013
New.CleanUp.ReferenceOTU198216
New.CleanUp.ReferenceOTU72073
New.CleanUp.ReferenceOTU47951
New.CleanUp.ReferenceOTU33742
New.CleanUp.ReferenceOTU45413
New.CleanUp.ReferenceOTU58250
New.CleanUp.ReferenceOTU236992
New.CleanUp.ReferenceOTU42849
New.CleanUp.ReferenceOTU41029
New.CleanUp.ReferenceOTU105744
New.CleanUp.ReferenceOTU59282
New.CleanUp.ReferenceOTU35855
New.CleanUp.ReferenceOTU43289
New.CleanUp.ReferenceOTU47449
New.CleanUp.ReferenceOTU60636

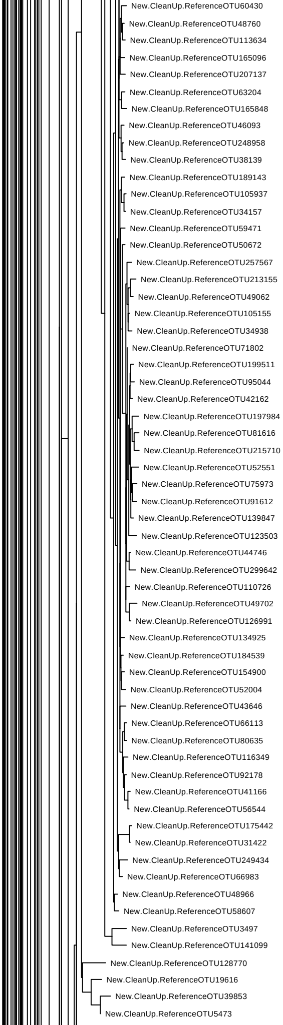
New.CleanUp.ReferenceOTU60430
New.CleanUp.ReferenceOTU48760
New.CleanUp.ReferenceOTU113634
New.CleanUp.ReferenceOTU165096
New.CleanUp.ReferenceOTU207137
New.CleanUp.ReferenceOTU63204
New.CleanUp.ReferenceOTU165848
New.CleanUp.ReferenceOTU46093
New.CleanUp.ReferenceOTU248958
New.CleanUp.ReferenceOTU38139
New.CleanUp.ReferenceOTU189143
New.CleanUp.ReferenceOTU105937
New.CleanUp.ReferenceOTU34157
New.CleanUp.ReferenceOTU59471
New.CleanUp.ReferenceOTU50672
New.CleanUp.ReferenceOTU257567
New.CleanUp.ReferenceOTU213155
New.CleanUp.ReferenceOTU49062
New.CleanUp.ReferenceOTU105155
New.CleanUp.ReferenceOTU34938
New.CleanUp.ReferenceOTU71802
New.CleanUp.ReferenceOTU199511
New.CleanUp.ReferenceOTU95044
New.CleanUp.ReferenceOTU42162
New.CleanUp.ReferenceOTU197984
New.CleanUp.ReferenceOTU81616
New.CleanUp.ReferenceOTU215710
New.CleanUp.ReferenceOTU52551
New.CleanUp.ReferenceOTU75973
New.CleanUp.ReferenceOTU91612
New.CleanUp.ReferenceOTU139847
New.CleanUp.ReferenceOTU123503
New.CleanUp.ReferenceOTU44746
New.CleanUp.ReferenceOTU299642
New.CleanUp.ReferenceOTU110726
New.CleanUp.ReferenceOTU49702
New.CleanUp.ReferenceOTU126991
New.CleanUp.ReferenceOTU134925
New.CleanUp.ReferenceOTU184539
New.CleanUp.ReferenceOTU154900
New.CleanUp.ReferenceOTU52004
New.CleanUp.ReferenceOTU43646
New.CleanUp.ReferenceOTU66113
New.CleanUp.ReferenceOTU80635
New.CleanUp.ReferenceOTU116349
New.CleanUp.ReferenceOTU92178
New.CleanUp.ReferenceOTU41166
New.CleanUp.ReferenceOTU56544
New.CleanUp.ReferenceOTU175442
New.CleanUp.ReferenceOTU31422
New.CleanUp.ReferenceOTU249434
New.CleanUp.ReferenceOTU66983
New.CleanUp.ReferenceOTU48966
New.CleanUp.ReferenceOTU58607
New.CleanUp.ReferenceOTU3497
New.CleanUp.ReferenceOTU141099
New.CleanUp.ReferenceOTU128770
New.CleanUp.ReferenceOTU19616
New.CleanUp.ReferenceOTU39853
New.CleanUp.ReferenceOTU5473

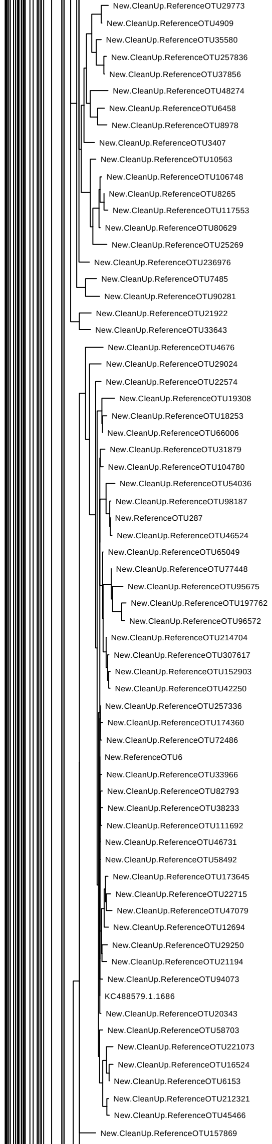
New.CleanUp.ReferenceOTU29773
New.CleanUp.ReferenceOTU4909
New.CleanUp.ReferenceOTU35580
New.CleanUp.ReferenceOTU257836
New.CleanUp.ReferenceOTU37856
New.CleanUp.ReferenceOTU48274
New.CleanUp.ReferenceOTU6458
New.CleanUp.ReferenceOTU8978
New.CleanUp.ReferenceOTU3407
New.CleanUp.ReferenceOTU10563
New.CleanUp.ReferenceOTU106748
New.CleanUp.ReferenceOTU8265
New.CleanUp.ReferenceOTU117553
New.CleanUp.ReferenceOTU80629
New.CleanUp.ReferenceOTU25269
New.CleanUp.ReferenceOTU236976
New.CleanUp.ReferenceOTU7485
New.CleanUp.ReferenceOTU90281
New.CleanUp.ReferenceOTU21922
New.CleanUp.ReferenceOTU33643
New.CleanUp.ReferenceOTU4676
New.CleanUp.ReferenceOTU29024
New.CleanUp.ReferenceOTU22574
New.CleanUp.ReferenceOTU19308
New.CleanUp.ReferenceOTU18253
New.CleanUp.ReferenceOTU66006
New.CleanUp.ReferenceOTU31879
New.CleanUp.ReferenceOTU104780
New.CleanUp.ReferenceOTU54036
New.CleanUp.ReferenceOTU98187
New.ReferenceOTU287
New.CleanUp.ReferenceOTU46524
New.CleanUp.ReferenceOTU65049
New.CleanUp.ReferenceOTU77448
New.CleanUp.ReferenceOTU95675
New.CleanUp.ReferenceOTU197762
New.CleanUp.ReferenceOTU96572
New.CleanUp.ReferenceOTU214704
New.CleanUp.ReferenceOTU307617
New.CleanUp.ReferenceOTU152903
New.CleanUp.ReferenceOTU42250
New.CleanUp.ReferenceOTU257336
New.CleanUp.ReferenceOTU174360
New.CleanUp.ReferenceOTU72486
New.ReferenceOTU6
New.CleanUp.ReferenceOTU33966
New.CleanUp.ReferenceOTU82793
New.CleanUp.ReferenceOTU38233
New.CleanUp.ReferenceOTU111692
New.CleanUp.ReferenceOTU46731
New.CleanUp.ReferenceOTU58492
New.CleanUp.ReferenceOTU173645
New.CleanUp.ReferenceOTU22715
New.CleanUp.ReferenceOTU47079
New.CleanUp.ReferenceOTU12694
New.CleanUp.ReferenceOTU29250
New.CleanUp.ReferenceOTU21194
New.CleanUp.ReferenceOTU94073
KC488579.1.1686
New.CleanUp.ReferenceOTU20343
New.CleanUp.ReferenceOTU58703
New.CleanUp.ReferenceOTU221073
New.CleanUp.ReferenceOTU16524
New.CleanUp.ReferenceOTU6153
New.CleanUp.ReferenceOTU212321
New.CleanUp.ReferenceOTU45466
New.CleanUp.ReferenceOTU157869

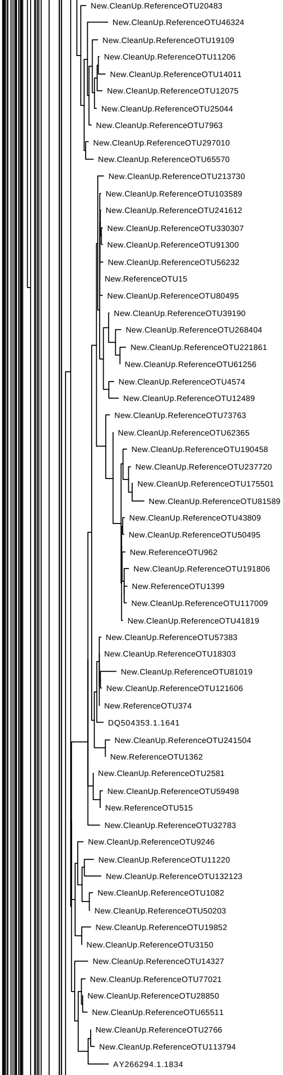
New.CleanUp.ReferenceOTU20483
New.CleanUp.ReferenceOTU46324
New.CleanUp.ReferenceOTU19109
New.CleanUp.ReferenceOTU11206
New.CleanUp.ReferenceOTU14011
New.CleanUp.ReferenceOTU12075
New.CleanUp.ReferenceOTU25044
New.CleanUp.ReferenceOTU7963
New.CleanUp.ReferenceOTU297010
New.CleanUp.ReferenceOTU65570
New.CleanUp.ReferenceOTU213730
New.CleanUp.ReferenceOTU103589
New.CleanUp.ReferenceOTU241612
New.CleanUp.ReferenceOTU330307
New.CleanUp.ReferenceOTU91300
New.CleanUp.ReferenceOTU56232
New.ReferenceOTU15
New.CleanUp.ReferenceOTU80495
New.CleanUp.ReferenceOTU39190
New.CleanUp.ReferenceOTU268404
New.CleanUp.ReferenceOTU221861
New.CleanUp.ReferenceOTU61256
New.CleanUp.ReferenceOTU4574
New.CleanUp.ReferenceOTU12489
New.CleanUp.ReferenceOTU73763
New.CleanUp.ReferenceOTU62365
New.CleanUp.ReferenceOTU190458
New.CleanUp.ReferenceOTU237720
New.CleanUp.ReferenceOTU175501
New.CleanUp.ReferenceOTU81589
New.CleanUp.ReferenceOTU43809
New.CleanUp.ReferenceOTU50495
New.ReferenceOTU962
New.CleanUp.ReferenceOTU191806
New.ReferenceOTU1399
New.CleanUp.ReferenceOTU117009
New.CleanUp.ReferenceOTU41819
New.CleanUp.ReferenceOTU57383
New.CleanUp.ReferenceOTU18303
New.CleanUp.ReferenceOTU81019
New.CleanUp.ReferenceOTU121606
New.ReferenceOTU374
DQ504353.1.1641
New.CleanUp.ReferenceOTU241504
New.ReferenceOTU1362
New.CleanUp.ReferenceOTU2581
New.CleanUp.ReferenceOTU59498
New.ReferenceOTU515
New.CleanUp.ReferenceOTU32783
New.CleanUp.ReferenceOTU9246
New.CleanUp.ReferenceOTU11220
New.CleanUp.ReferenceOTU132123
New.CleanUp.ReferenceOTU1082
New.CleanUp.ReferenceOTU50203
New.CleanUp.ReferenceOTU19852
New.CleanUp.ReferenceOTU3150
New.CleanUp.ReferenceOTU14327
New.CleanUp.ReferenceOTU77021
New.CleanUp.ReferenceOTU28850
New.CleanUp.ReferenceOTU65511
New.CleanUp.ReferenceOTU2766
New.CleanUp.ReferenceOTU113794
AY266294.1.1834

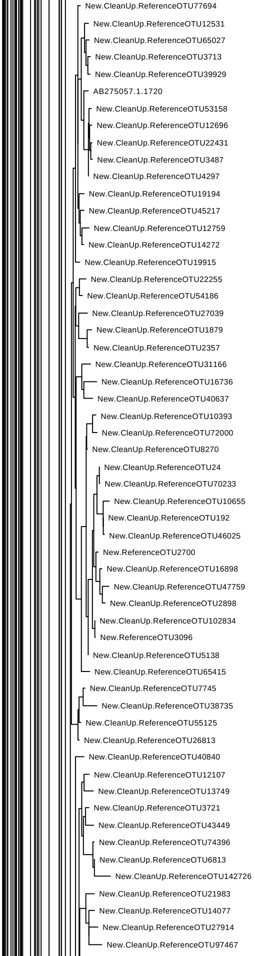
New.CleanUp.ReferenceOTU77694
New.CleanUp.ReferenceOTU12531
New.CleanUp.ReferenceOTU65027
New.CleanUp.ReferenceOTU3713
New.CleanUp.ReferenceOTU39929
AB275057.1.1720
New.CleanUp.ReferenceOTU53158
New.CleanUp.ReferenceOTU12696
New.CleanUp.ReferenceOTU22431
New.CleanUp.ReferenceOTU3487
New.CleanUp.ReferenceOTU4297
New.CleanUp.ReferenceOTU19194
New.CleanUp.ReferenceOTU45217
New.CleanUp.ReferenceOTU12759
New.CleanUp.ReferenceOTU14272
New.CleanUp.ReferenceOTU19915
New.CleanUp.ReferenceOTU22255
New.CleanUp.ReferenceOTU54186
New.CleanUp.ReferenceOTU27039
New.CleanUp.ReferenceOTU1879
New.CleanUp.ReferenceOTU2357
New.CleanUp.ReferenceOTU31166
New.CleanUp.ReferenceOTU16736
New.CleanUp.ReferenceOTU40637
New.CleanUp.ReferenceOTU10393
New.CleanUp.ReferenceOTU72000
New.CleanUp.ReferenceOTU8270
New.CleanUp.ReferenceOTU24
New.CleanUp.ReferenceOTU70233
New.CleanUp.ReferenceOTU10655
New.CleanUp.ReferenceOTU192
New.CleanUp.ReferenceOTU46025
New.ReferenceOTU2700
New.CleanUp.ReferenceOTU16898
New.CleanUp.ReferenceOTU47759
New.CleanUp.ReferenceOTU2898
New.CleanUp.ReferenceOTU102834
New.ReferenceOTU3096
New.CleanUp.ReferenceOTU5138
New.CleanUp.ReferenceOTU65415
New.CleanUp.ReferenceOTU7745
New.CleanUp.ReferenceOTU38735
New.CleanUp.ReferenceOTU55125
New.CleanUp.ReferenceOTU26813
New.CleanUp.ReferenceOTU40840
New.CleanUp.ReferenceOTU12107
New.CleanUp.ReferenceOTU13749
New.CleanUp.ReferenceOTU3721
New.CleanUp.ReferenceOTU43449
New.CleanUp.ReferenceOTU74396
New.CleanUp.ReferenceOTU6813
New.CleanUp.ReferenceOTU142726
New.CleanUp.ReferenceOTU21983
New.CleanUp.ReferenceOTU14077
New.CleanUp.ReferenceOTU27914
New.CleanUp.ReferenceOTU97467

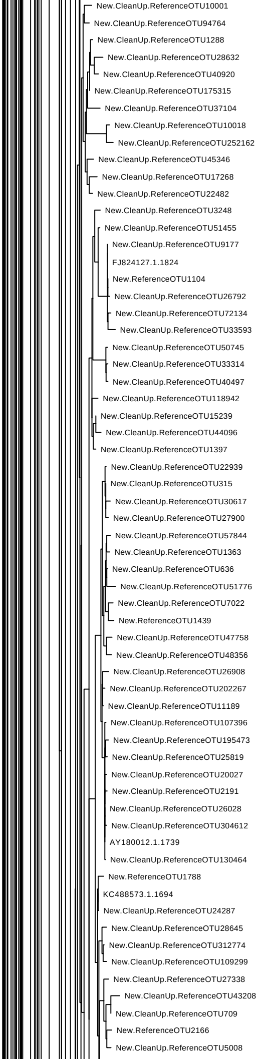
New.CleanUp.ReferenceOTU10001
New.CleanUp.ReferenceOTU94764
New.CleanUp.ReferenceOTU1288
New.CleanUp.ReferenceOTU28632
New.CleanUp.ReferenceOTU40920
New.CleanUp.ReferenceOTU175315
New.CleanUp.ReferenceOTU37104
New.CleanUp.ReferenceOTU10018
New.CleanUp.ReferenceOTU252162
New.CleanUp.ReferenceOTU45346
New.CleanUp.ReferenceOTU17268
New.CleanUp.ReferenceOTU22482
New.CleanUp.ReferenceOTU3248
New.CleanUp.ReferenceOTU51455
New.CleanUp.ReferenceOTU9177
FJ824127.1.1824
New.ReferenceOTU1104
New.CleanUp.ReferenceOTU26792
New.CleanUp.ReferenceOTU72134
New.CleanUp.ReferenceOTU33593
New.CleanUp.ReferenceOTU50745
New.CleanUp.ReferenceOTU33314
New.CleanUp.ReferenceOTU40497
New.CleanUp.ReferenceOTU118942
New.CleanUp.ReferenceOTU15239
New.CleanUp.ReferenceOTU44096
New.CleanUp.ReferenceOTU1397
New.CleanUp.ReferenceOTU22939
New.CleanUp.ReferenceOTU315
New.CleanUp.ReferenceOTU30617
New.CleanUp.ReferenceOTU27900
New.CleanUp.ReferenceOTU57844
New.CleanUp.ReferenceOTU1363
New.CleanUp.ReferenceOTU636
New.CleanUp.ReferenceOTU51776
New.CleanUp.ReferenceOTU7022
New.ReferenceOTU1439
New.CleanUp.ReferenceOTU47758
New.CleanUp.ReferenceOTU48356
New.CleanUp.ReferenceOTU26908
New.CleanUp.ReferenceOTU202267
New.CleanUp.ReferenceOTU11189
New.CleanUp.ReferenceOTU107396
New.CleanUp.ReferenceOTU195473
New.CleanUp.ReferenceOTU25819
New.CleanUp.ReferenceOTU20027
New.CleanUp.ReferenceOTU2191
New.CleanUp.ReferenceOTU26028
New.CleanUp.ReferenceOTU304612
AY180012.1.1739
New.CleanUp.ReferenceOTU130464
New.ReferenceOTU1788
KC488573.1.1694
New.CleanUp.ReferenceOTU24287
New.CleanUp.ReferenceOTU28645
New.CleanUp.ReferenceOTU312774
New.CleanUp.ReferenceOTU109299
New.CleanUp.ReferenceOTU27338
New.CleanUp.ReferenceOTU43208
New.CleanUp.ReferenceOTU709
New.ReferenceOTU2166
New.CleanUp.ReferenceOTU5008

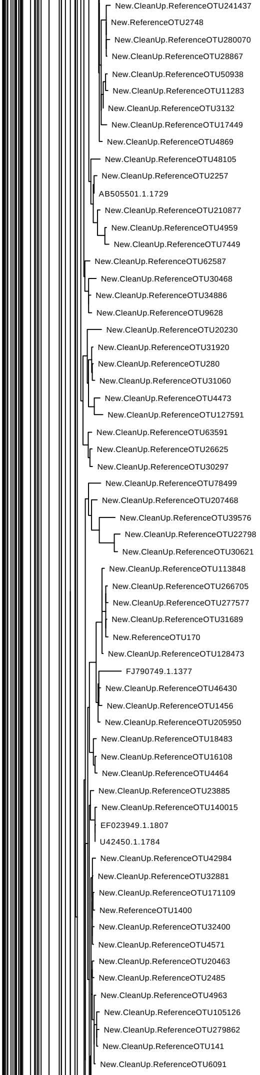

New.CleanUp.ReferenceOTU241437
New.ReferenceOTU2748
New.CleanUp.ReferenceOTU280070
New.CleanUp.ReferenceOTU28867
New.CleanUp.ReferenceOTU50938
New.CleanUp.ReferenceOTU11283
New.CleanUp.ReferenceOTU3132
New.CleanUp.ReferenceOTU17449
New.CleanUp.ReferenceOTU4869
New.CleanUp.ReferenceOTU48105
New.CleanUp.ReferenceOTU2257
AB505501.1.1729
New.CleanUp.ReferenceOTU210877
New.CleanUp.ReferenceOTU4959
New.CleanUp.ReferenceOTU7449
New.CleanUp.ReferenceOTU62587
New.CleanUp.ReferenceOTU30468
New.CleanUp.ReferenceOTU34886
New.CleanUp.ReferenceOTU9628
New.CleanUp.ReferenceOTU20230
New.CleanUp.ReferenceOTU31920
New.CleanUp.ReferenceOTU280
New.CleanUp.ReferenceOTU31060
New.CleanUp.ReferenceOTU4473
New.CleanUp.ReferenceOTU127591
New.CleanUp.ReferenceOTU63591
New.CleanUp.ReferenceOTU26625
New.CleanUp.ReferenceOTU30297
New.CleanUp.ReferenceOTU78499
New.CleanUp.ReferenceOTU207468
New.CleanUp.ReferenceOTU39576
New.CleanUp.ReferenceOTU22798
New.CleanUp.ReferenceOTU30621
New.CleanUp.ReferenceOTU113848
New.CleanUp.ReferenceOTU266705
New.CleanUp.ReferenceOTU277577
New.CleanUp.ReferenceOTU31689
New.ReferenceOTU170
New.CleanUp.ReferenceOTU128473
FJ790749.1.1377
New.CleanUp.ReferenceOTU46430
New.CleanUp.ReferenceOTU1456
New.CleanUp.ReferenceOTU205950
New.CleanUp.ReferenceOTU18483
New.CleanUp.ReferenceOTU16108
New.CleanUp.ReferenceOTU4464
New.CleanUp.ReferenceOTU23885
New.CleanUp.ReferenceOTU140015
EF023949.1.1807
U42450.1.1784
New.CleanUp.ReferenceOTU42984
New.CleanUp.ReferenceOTU32881
New.CleanUp.ReferenceOTU171109
New.ReferenceOTU1400
New.CleanUp.ReferenceOTU32400
New.CleanUp.ReferenceOTU4571
New.CleanUp.ReferenceOTU20463
New.CleanUp.ReferenceOTU2485
New.CleanUp.ReferenceOTU4963
New.CleanUp.ReferenceOTU105126
New.CleanUp.ReferenceOTU279862
New.CleanUp.ReferenceOTU141
New.CleanUp.ReferenceOTU6091

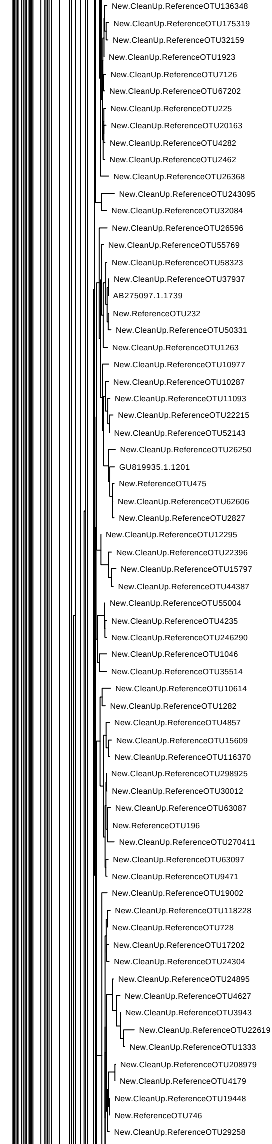
New.CleanUp.ReferenceOTU136348
New.CleanUp.ReferenceOTU175319
New.CleanUp.ReferenceOTU32159
New.CleanUp.ReferenceOTU1923
New.CleanUp.ReferenceOTU7126
New.CleanUp.ReferenceOTU67202
New.CleanUp.ReferenceOTU225
New.CleanUp.ReferenceOTU20163
New.CleanUp.ReferenceOTU4282
New.CleanUp.ReferenceOTU2462
New.CleanUp.ReferenceOTU26368
New.CleanUp.ReferenceOTU243095
New.CleanUp.ReferenceOTU32084
New.CleanUp.ReferenceOTU26596
New.CleanUp.ReferenceOTU55769
New.CleanUp.ReferenceOTU58323
New.CleanUp.ReferenceOTU37937
AB275097.1.1739
New.ReferenceOTU232
New.CleanUp.ReferenceOTU50331
New.CleanUp.ReferenceOTU1263
New.CleanUp.ReferenceOTU10977
New.CleanUp.ReferenceOTU10287
New.CleanUp.ReferenceOTU11093
New.CleanUp.ReferenceOTU22215
New.CleanUp.ReferenceOTU52143
New.CleanUp.ReferenceOTU26250
GU819935.1.1201
New.ReferenceOTU475
New.CleanUp.ReferenceOTU62606
New.CleanUp.ReferenceOTU2827
New.CleanUp.ReferenceOTU12295
New.CleanUp.ReferenceOTU22396
New.CleanUp.ReferenceOTU15797
New.CleanUp.ReferenceOTU44387
New.CleanUp.ReferenceOTU55004
New.CleanUp.ReferenceOTU4235
New.CleanUp.ReferenceOTU246290
New.CleanUp.ReferenceOTU1046
New.CleanUp.ReferenceOTU35514
New.CleanUp.ReferenceOTU10614
New.CleanUp.ReferenceOTU1282
New.CleanUp.ReferenceOTU4857
New.CleanUp.ReferenceOTU15609
New.CleanUp.ReferenceOTU116370
New.CleanUp.ReferenceOTU298925
New.CleanUp.ReferenceOTU30012
New.CleanUp.ReferenceOTU63087
New.ReferenceOTU196
New.CleanUp.ReferenceOTU270411
New.CleanUp.ReferenceOTU63097
New.CleanUp.ReferenceOTU9471
New.CleanUp.ReferenceOTU19002
New.CleanUp.ReferenceOTU118228
New.CleanUp.ReferenceOTU728
New.CleanUp.ReferenceOTU17202
New.CleanUp.ReferenceOTU24304
New.CleanUp.ReferenceOTU24895
New.CleanUp.ReferenceOTU4627
New.CleanUp.ReferenceOTU3943
New.CleanUp.ReferenceOTU22619
New.CleanUp.ReferenceOTU1333
New.CleanUp.ReferenceOTU208979
New.CleanUp.ReferenceOTU4179
New.CleanUp.ReferenceOTU19448
New.ReferenceOTU746
New.CleanUp.ReferenceOTU29258

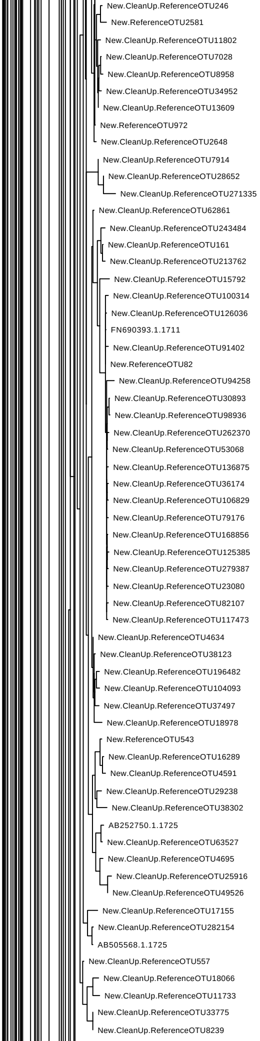

New.CleanUp.ReferenceOTU246
New.ReferenceOTU2581
New.CleanUp.ReferenceOTU11802
New.CleanUp.ReferenceOTU7028
New.CleanUp.ReferenceOTU8958
New.CleanUp.ReferenceOTU34952
New.CleanUp.ReferenceOTU13609
New.ReferenceOTU972
New.CleanUp.ReferenceOTU2648
New.CleanUp.ReferenceOTU7914
New.CleanUp.ReferenceOTU28652
New.CleanUp.ReferenceOTU271335
New.CleanUp.ReferenceOTU62861
New.CleanUp.ReferenceOTU243484
New.CleanUp.ReferenceOTU161
New.CleanUp.ReferenceOTU213762
New.CleanUp.ReferenceOTU15792
New.CleanUp.ReferenceOTU100314
New.CleanUp.ReferenceOTU126036
FN690393.1.1711
New.CleanUp.ReferenceOTU91402
New.ReferenceOTU82
New.CleanUp.ReferenceOTU94258
New.CleanUp.ReferenceOTU30893
New.CleanUp.ReferenceOTU98936
New.CleanUp.ReferenceOTU262370
New.CleanUp.ReferenceOTU53068
New.CleanUp.ReferenceOTU136875
New.CleanUp.ReferenceOTU36174
New.CleanUp.ReferenceOTU106829
New.CleanUp.ReferenceOTU79176
New.CleanUp.ReferenceOTU168856
New.CleanUp.ReferenceOTU125385
New.CleanUp.ReferenceOTU279387
New.CleanUp.ReferenceOTU23080
New.CleanUp.ReferenceOTU82107
New.CleanUp.ReferenceOTU117473
New.CleanUp.ReferenceOTU4634
New.CleanUp.ReferenceOTU38123
New.CleanUp.ReferenceOTU196482
New.CleanUp.ReferenceOTU104093
New.CleanUp.ReferenceOTU37497
New.CleanUp.ReferenceOTU18978
New.ReferenceOTU543
New.CleanUp.ReferenceOTU16289
New.CleanUp.ReferenceOTU4591
New.CleanUp.ReferenceOTU29238
New.CleanUp.ReferenceOTU38302
AB252750.1.1725
New.CleanUp.ReferenceOTU63527
New.CleanUp.ReferenceOTU4695
New.CleanUp.ReferenceOTU25916
New.CleanUp.ReferenceOTU49526
New.CleanUp.ReferenceOTU17155
New.CleanUp.ReferenceOTU282154
AB505568.1.1725
New.CleanUp.ReferenceOTU557
New.CleanUp.ReferenceOTU18066
New.CleanUp.ReferenceOTU11733
New.CleanUp.ReferenceOTU33775
New.CleanUp.ReferenceOTU8239

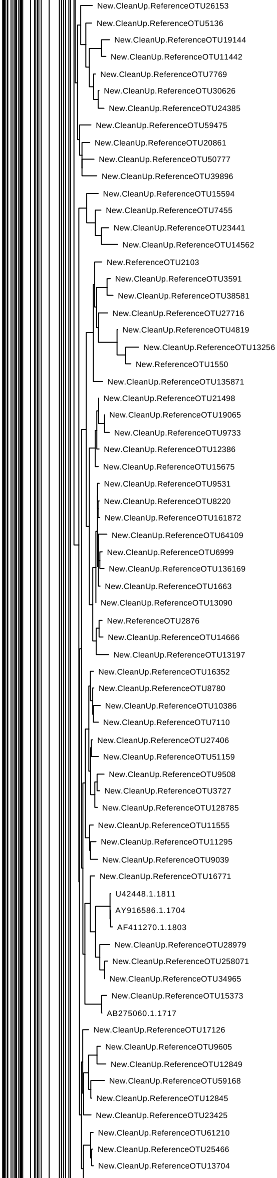
New.CleanUp.ReferenceOTU26153
New.CleanUp.ReferenceOTU5136
New.CleanUp.ReferenceOTU19144
New.CleanUp.ReferenceOTU11442
New.CleanUp.ReferenceOTU7769
New.CleanUp.ReferenceOTU30626
New.CleanUp.ReferenceOTU24385
New.CleanUp.ReferenceOTU59475
New.CleanUp.ReferenceOTU20861
New.CleanUp.ReferenceOTU50777
New.CleanUp.ReferenceOTU39896
New.CleanUp.ReferenceOTU15594
New.CleanUp.ReferenceOTU7455
New.CleanUp.ReferenceOTU23441
New.CleanUp.ReferenceOTU14562
New.ReferenceOTU2103
New.CleanUp.ReferenceOTU3591
New.CleanUp.ReferenceOTU38581
New.CleanUp.ReferenceOTU27716
New.CleanUp.ReferenceOTU4819
New.CleanUp.ReferenceOTU13256
New.ReferenceOTU1550
New.CleanUp.ReferenceOTU135871
New.CleanUp.ReferenceOTU21498
New.CleanUp.ReferenceOTU19065
New.CleanUp.ReferenceOTU9733
New.CleanUp.ReferenceOTU12386
New.CleanUp.ReferenceOTU15675
New.CleanUp.ReferenceOTU9531
New.CleanUp.ReferenceOTU8220
New.CleanUp.ReferenceOTU161872
New.CleanUp.ReferenceOTU64109
New.CleanUp.ReferenceOTU6999
New.CleanUp.ReferenceOTU136169
New.CleanUp.ReferenceOTU1663
New.CleanUp.ReferenceOTU13090
New.ReferenceOTU2876
New.CleanUp.ReferenceOTU14666
New.CleanUp.ReferenceOTU13197
New.CleanUp.ReferenceOTU16352
New.CleanUp.ReferenceOTU8780
New.CleanUp.ReferenceOTU10386
New.CleanUp.ReferenceOTU7110
New.CleanUp.ReferenceOTU27406
New.CleanUp.ReferenceOTU51159
New.CleanUp.ReferenceOTU9508
New.CleanUp.ReferenceOTU3727
New.CleanUp.ReferenceOTU128785
New.CleanUp.ReferenceOTU11555
New.CleanUp.ReferenceOTU11295
New.CleanUp.ReferenceOTU9039
New.CleanUp.ReferenceOTU16771
U42448.1.1811
AY916586.1.1704
AF411270.1.1803
New.CleanUp.ReferenceOTU28979
New.CleanUp.ReferenceOTU258071
New.CleanUp.ReferenceOTU34965
New.CleanUp.ReferenceOTU15373
AB275060.1.1717
New.CleanUp.ReferenceOTU17126
New.CleanUp.ReferenceOTU9605
New.CleanUp.ReferenceOTU12849
New.CleanUp.ReferenceOTU59168
New.CleanUp.ReferenceOTU12845
New.CleanUp.ReferenceOTU23425
New.CleanUp.ReferenceOTU61210
New.CleanUp.ReferenceOTU25466
New.CleanUp.ReferenceOTU13704

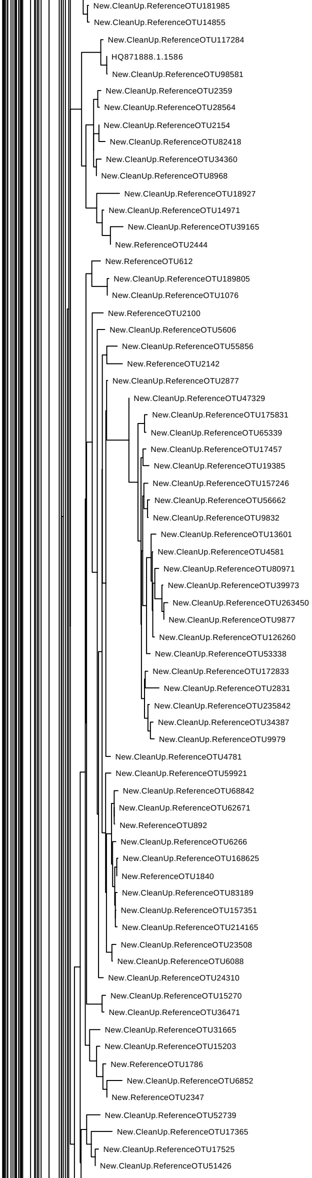
New.CleanUp.ReferenceOTU181985
New.CleanUp.ReferenceOTU14855
New.CleanUp.ReferenceOTU117284
HQ871888.1.1586
New.CleanUp.ReferenceOTU98581
New.CleanUp.ReferenceOTU2359
New.CleanUp.ReferenceOTU28564
New.CleanUp.ReferenceOTU2154
New.CleanUp.ReferenceOTU82418
New.CleanUp.ReferenceOTU34360
New.CleanUp.ReferenceOTU8968
New.CleanUp.ReferenceOTU18927
New.CleanUp.ReferenceOTU14971
New.CleanUp.ReferenceOTU39165
New.ReferenceOTU2444
New.ReferenceOTU612
New.CleanUp.ReferenceOTU189805
New.CleanUp.ReferenceOTU1076
New.ReferenceOTU2100
New.CleanUp.ReferenceOTU5606
New.CleanUp.ReferenceOTU55856
New.ReferenceOTU2142
New.CleanUp.ReferenceOTU2877
New.CleanUp.ReferenceOTU47329
New.CleanUp.ReferenceOTU175831
New.CleanUp.ReferenceOTU65339
New.CleanUp.ReferenceOTU17457
New.CleanUp.ReferenceOTU19385
New.CleanUp.ReferenceOTU157246
New.CleanUp.ReferenceOTU56662
New.CleanUp.ReferenceOTU9832
New.CleanUp.ReferenceOTU13601
New.CleanUp.ReferenceOTU4581
New.CleanUp.ReferenceOTU80971
New.CleanUp.ReferenceOTU39973
New.CleanUp.ReferenceOTU263450
New.CleanUp.ReferenceOTU9877
New.CleanUp.ReferenceOTU126260
New.CleanUp.ReferenceOTU53338
New.CleanUp.ReferenceOTU172833
New.CleanUp.ReferenceOTU2831
New.CleanUp.ReferenceOTU235842
New.CleanUp.ReferenceOTU34387
New.CleanUp.ReferenceOTU9979
New.CleanUp.ReferenceOTU4781
New.CleanUp.ReferenceOTU59921
New.CleanUp.ReferenceOTU68842
New.CleanUp.ReferenceOTU62671
New.ReferenceOTU892
New.CleanUp.ReferenceOTU6266
New.CleanUp.ReferenceOTU168625
New.ReferenceOTU1840
New.CleanUp.ReferenceOTU83189
New.CleanUp.ReferenceOTU157351
New.CleanUp.ReferenceOTU214165
New.CleanUp.ReferenceOTU23508
New.CleanUp.ReferenceOTU6088
New.CleanUp.ReferenceOTU24310
New.CleanUp.ReferenceOTU15270
New.CleanUp.ReferenceOTU36471
New.CleanUp.ReferenceOTU31665
New.CleanUp.ReferenceOTU15203
New.ReferenceOTU1786
New.CleanUp.ReferenceOTU6852
New.ReferenceOTU2347
New.CleanUp.ReferenceOTU52739
New.CleanUp.ReferenceOTU17365
New.CleanUp.ReferenceOTU17525
New.CleanUp.ReferenceOTU51426

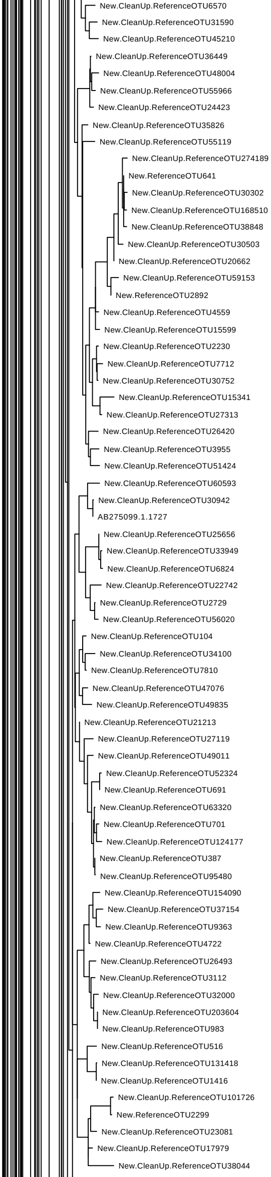
New.CleanUp.ReferenceOTU6570
New.CleanUp.ReferenceOTU31590
New.CleanUp.ReferenceOTU45210
New.CleanUp.ReferenceOTU36449
New.CleanUp.ReferenceOTU48004
New.CleanUp.ReferenceOTU55966
New.CleanUp.ReferenceOTU24423
New.CleanUp.ReferenceOTU35826
New.CleanUp.ReferenceOTU55119
New.CleanUp.ReferenceOTU274189
New.ReferenceOTU641
New.CleanUp.ReferenceOTU30302
New.CleanUp.ReferenceOTU168510
New.CleanUp.ReferenceOTU38848
New.CleanUp.ReferenceOTU30503
New.CleanUp.ReferenceOTU20662
New.CleanUp.ReferenceOTU59153
New.ReferenceOTU2892
New.CleanUp.ReferenceOTU4559
New.CleanUp.ReferenceOTU15599
New.CleanUp.ReferenceOTU2230
New.CleanUp.ReferenceOTU7712
New.CleanUp.ReferenceOTU30752
New.CleanUp.ReferenceOTU15341
New.CleanUp.ReferenceOTU27313
New.CleanUp.ReferenceOTU26420
New.CleanUp.ReferenceOTU3955
New.CleanUp.ReferenceOTU51424
New.CleanUp.ReferenceOTU60593
New.CleanUp.ReferenceOTU30942
AB275099.1.1727
New.CleanUp.ReferenceOTU25656
New.CleanUp.ReferenceOTU33949
New.CleanUp.ReferenceOTU6824
New.CleanUp.ReferenceOTU22742
New.CleanUp.ReferenceOTU2729
New.CleanUp.ReferenceOTU56020
New.CleanUp.ReferenceOTU104
New.CleanUp.ReferenceOTU34100
New.CleanUp.ReferenceOTU7810
New.CleanUp.ReferenceOTU47076
New.CleanUp.ReferenceOTU49835
New.CleanUp.ReferenceOTU21213
New.CleanUp.ReferenceOTU27119
New.CleanUp.ReferenceOTU49011
New.CleanUp.ReferenceOTU52324
New.CleanUp.ReferenceOTU691
New.CleanUp.ReferenceOTU63320
New.CleanUp.ReferenceOTU701
New.CleanUp.ReferenceOTU124177
New.CleanUp.ReferenceOTU387
New.CleanUp.ReferenceOTU95480
New.CleanUp.ReferenceOTU154090
New.CleanUp.ReferenceOTU37154
New.CleanUp.ReferenceOTU9363
New.CleanUp.ReferenceOTU4722
New.CleanUp.ReferenceOTU26493
New.CleanUp.ReferenceOTU3112
New.CleanUp.ReferenceOTU32000
New.CleanUp.ReferenceOTU203604
New.CleanUp.ReferenceOTU983
New.CleanUp.ReferenceOTU516
New.CleanUp.ReferenceOTU131418
New.CleanUp.ReferenceOTU1416
New.CleanUp.ReferenceOTU101726
New.ReferenceOTU2299
New.CleanUp.ReferenceOTU23081
New.CleanUp.ReferenceOTU17979
New.CleanUp.ReferenceOTU38044

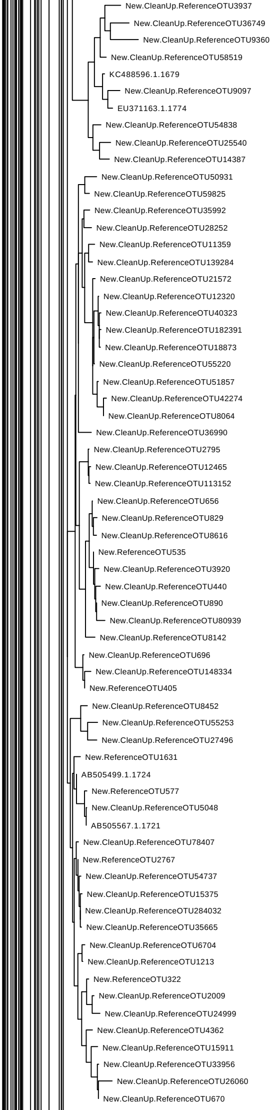
New.CleanUp.ReferenceOTU3937
New.CleanUp.ReferenceOTU36749
New.CleanUp.ReferenceOTU9360
New.CleanUp.ReferenceOTU58519
KC488596.1.1679
New.CleanUp.ReferenceOTU9097
EU371163.1.1774
New.CleanUp.ReferenceOTU54838
New.CleanUp.ReferenceOTU25540
New.CleanUp.ReferenceOTU14387
New.CleanUp.ReferenceOTU50931
New.CleanUp.ReferenceOTU59825
New.CleanUp.ReferenceOTU35992
New.CleanUp.ReferenceOTU28252
New.CleanUp.ReferenceOTU11359
New.CleanUp.ReferenceOTU139284
New.CleanUp.ReferenceOTU21572
New.CleanUp.ReferenceOTU12320
New.CleanUp.ReferenceOTU40323
New.CleanUp.ReferenceOTU182391
New.CleanUp.ReferenceOTU18873
New.CleanUp.ReferenceOTU55220
New.CleanUp.ReferenceOTU51857
New.CleanUp.ReferenceOTU42274
New.CleanUp.ReferenceOTU8064
New.CleanUp.ReferenceOTU36990
New.CleanUp.ReferenceOTU2795
New.CleanUp.ReferenceOTU12465
New.CleanUp.ReferenceOTU113152
New.CleanUp.ReferenceOTU656
New.CleanUp.ReferenceOTU829
New.CleanUp.ReferenceOTU8616
New.ReferenceOTU535
New.CleanUp.ReferenceOTU3920
New.CleanUp.ReferenceOTU440
New.CleanUp.ReferenceOTU890
New.CleanUp.ReferenceOTU80939
New.CleanUp.ReferenceOTU8142
New.CleanUp.ReferenceOTU696
New.CleanUp.ReferenceOTU148334
New.ReferenceOTU405
New.CleanUp.ReferenceOTU8452
New.CleanUp.ReferenceOTU55253
New.CleanUp.ReferenceOTU27496
New.ReferenceOTU1631
AB505499.1.1724
New.ReferenceOTU577
New.CleanUp.ReferenceOTU5048
AB505567.1.1721
New.CleanUp.ReferenceOTU78407
New.ReferenceOTU2767
New.CleanUp.ReferenceOTU54737
New.CleanUp.ReferenceOTU15375
New.CleanUp.ReferenceOTU284032
New.CleanUp.ReferenceOTU35665
New.CleanUp.ReferenceOTU6704
New.CleanUp.ReferenceOTU1213
New.ReferenceOTU322
New.CleanUp.ReferenceOTU2009
New.CleanUp.ReferenceOTU24999
New.CleanUp.ReferenceOTU4362
New.CleanUp.ReferenceOTU15911
New.CleanUp.ReferenceOTU33956
New.CleanUp.ReferenceOTU26060
New.CleanUp.ReferenceOTU670

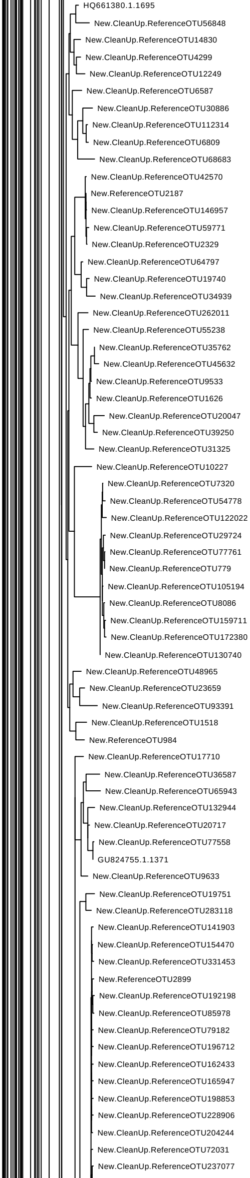
HQ661380.1.1695
New.CleanUp.ReferenceOTU56848
New.CleanUp.ReferenceOTU14830
New.CleanUp.ReferenceOTU4299
New.CleanUp.ReferenceOTU12249
New.CleanUp.ReferenceOTU6587
New.CleanUp.ReferenceOTU30886
New.CleanUp.ReferenceOTU112314
New.CleanUp.ReferenceOTU6809
New.CleanUp.ReferenceOTU68683
New.CleanUp.ReferenceOTU42570
New.ReferenceOTU2187
New.CleanUp.ReferenceOTU146957
New.CleanUp.ReferenceOTU59771
New.CleanUp.ReferenceOTU2329
New.CleanUp.ReferenceOTU64797
New.CleanUp.ReferenceOTU19740
New.CleanUp.ReferenceOTU34939
New.CleanUp.ReferenceOTU262011
New.CleanUp.ReferenceOTU55238
New.CleanUp.ReferenceOTU35762
New.CleanUp.ReferenceOTU45632
New.CleanUp.ReferenceOTU9533
New.CleanUp.ReferenceOTU1626
New.CleanUp.ReferenceOTU20047
New.CleanUp.ReferenceOTU39250
New.CleanUp.ReferenceOTU31325
New.CleanUp.ReferenceOTU10227
New.CleanUp.ReferenceOTU7320
New.CleanUp.ReferenceOTU54778
New.CleanUp.ReferenceOTU122022
New.CleanUp.ReferenceOTU29724
New.CleanUp.ReferenceOTU77761
New.CleanUp.ReferenceOTU779
New.CleanUp.ReferenceOTU105194
New.CleanUp.ReferenceOTU8086
New.CleanUp.ReferenceOTU159711
New.CleanUp.ReferenceOTU172380
New.CleanUp.ReferenceOTU130740
New.CleanUp.ReferenceOTU48965
New.CleanUp.ReferenceOTU23659
New.CleanUp.ReferenceOTU93391
New.CleanUp.ReferenceOTU1518
New.ReferenceOTU984
New.CleanUp.ReferenceOTU17710
New.CleanUp.ReferenceOTU36587
New.CleanUp.ReferenceOTU65943
New.CleanUp.ReferenceOTU132944
New.CleanUp.ReferenceOTU20717
New.CleanUp.ReferenceOTU77558
GU824755.1.1371
New.CleanUp.ReferenceOTU9633
New.CleanUp.ReferenceOTU19751
New.CleanUp.ReferenceOTU283118
New.CleanUp.ReferenceOTU141903
New.CleanUp.ReferenceOTU154470
New.CleanUp.ReferenceOTU331453
New.ReferenceOTU2899
New.CleanUp.ReferenceOTU192198
New.CleanUp.ReferenceOTU85978
New.CleanUp.ReferenceOTU79182
New.CleanUp.ReferenceOTU196712
New.CleanUp.ReferenceOTU162433
New.CleanUp.ReferenceOTU165947
New.CleanUp.ReferenceOTU198853
New.CleanUp.ReferenceOTU228906
New.CleanUp.ReferenceOTU204244
New.CleanUp.ReferenceOTU72031
New.CleanUp.ReferenceOTU237077

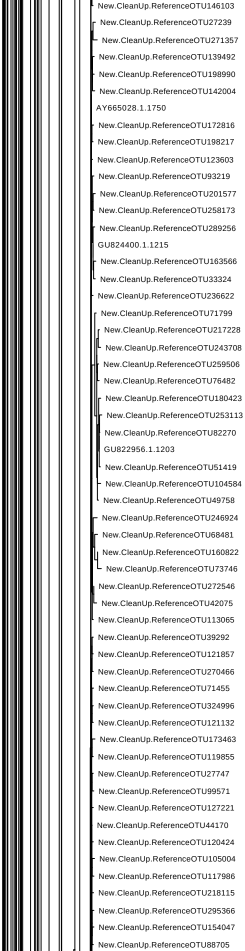
New.CleanUp.ReferenceOTU146103
New.CleanUp.ReferenceOTU27239
New.CleanUp.ReferenceOTU271357
New.CleanUp.ReferenceOTU139492
New.CleanUp.ReferenceOTU198990
New.CleanUp.ReferenceOTU142004
AY665028.1.1750
New.CleanUp.ReferenceOTU172816
New.CleanUp.ReferenceOTU198217
New.CleanUp.ReferenceOTU123603
New.CleanUp.ReferenceOTU93219
New.CleanUp.ReferenceOTU201577
New.CleanUp.ReferenceOTU258173
New.CleanUp.ReferenceOTU289256
GU824400.1.1215
New.CleanUp.ReferenceOTU163566
New.CleanUp.ReferenceOTU33324
New.CleanUp.ReferenceOTU236622
New.CleanUp.ReferenceOTU71799
New.CleanUp.ReferenceOTU217228
New.CleanUp.ReferenceOTU243708
New.CleanUp.ReferenceOTU259506
New.CleanUp.ReferenceOTU76482
New.CleanUp.ReferenceOTU180423
New.CleanUp.ReferenceOTU253113
New.CleanUp.ReferenceOTU82270
GU822956.1.1203
New.CleanUp.ReferenceOTU51419
New.CleanUp.ReferenceOTU104584
New.CleanUp.ReferenceOTU49758
New.CleanUp.ReferenceOTU246924
New.CleanUp.ReferenceOTU68481
New.CleanUp.ReferenceOTU160822
New.CleanUp.ReferenceOTU73746
New.CleanUp.ReferenceOTU272546
New.CleanUp.ReferenceOTU42075
New.CleanUp.ReferenceOTU113065
New.CleanUp.ReferenceOTU39292
New.CleanUp.ReferenceOTU121857
New.CleanUp.ReferenceOTU270466
New.CleanUp.ReferenceOTU71455
New.CleanUp.ReferenceOTU324996
New.CleanUp.ReferenceOTU121132
New.CleanUp.ReferenceOTU173463
New.CleanUp.ReferenceOTU119855
New.CleanUp.ReferenceOTU27747
New.CleanUp.ReferenceOTU99571
New.CleanUp.ReferenceOTU127221
New.CleanUp.ReferenceOTU44170
New.CleanUp.ReferenceOTU120424
New.CleanUp.ReferenceOTU105004
New.CleanUp.ReferenceOTU117986
New.CleanUp.ReferenceOTU218115
New.CleanUp.ReferenceOTU295366
New.CleanUp.ReferenceOTU154047
New.CleanUp.ReferenceOTU88705

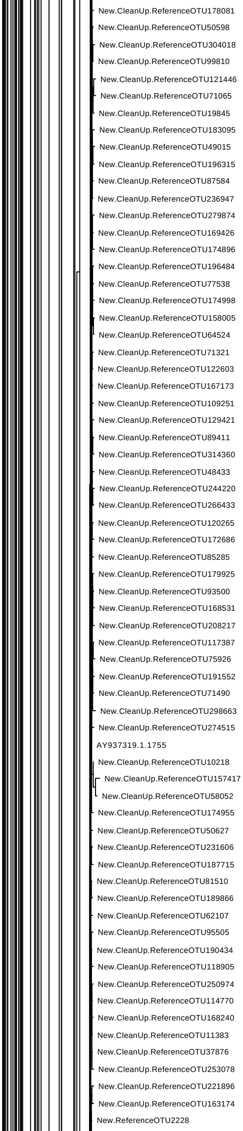
New.CleanUp.ReferenceOTU178081
New.CleanUp.ReferenceOTU50598
New.CleanUp.ReferenceOTU304018
New.CleanUp.ReferenceOTU99810
New.CleanUp.ReferenceOTU121446
New.CleanUp.ReferenceOTU71065
New.CleanUp.ReferenceOTU19845
New.CleanUp.ReferenceOTU183095
New.CleanUp.ReferenceOTU49015
New.CleanUp.ReferenceOTU196315
New.CleanUp.ReferenceOTU87584
New.CleanUp.ReferenceOTU236947
New.CleanUp.ReferenceOTU279874
New.CleanUp.ReferenceOTU169426
New.CleanUp.ReferenceOTU174896
New.CleanUp.ReferenceOTU196484
New.CleanUp.ReferenceOTU77538
New.CleanUp.ReferenceOTU174998
New.CleanUp.ReferenceOTU158005
New.CleanUp.ReferenceOTU64524
New.CleanUp.ReferenceOTU71321
New.CleanUp.ReferenceOTU122603
New.CleanUp.ReferenceOTU167173
New.CleanUp.ReferenceOTU109251
New.CleanUp.ReferenceOTU129421
New.CleanUp.ReferenceOTU89411
New.CleanUp.ReferenceOTU314360
New.CleanUp.ReferenceOTU48433
New.CleanUp.ReferenceOTU244220
New.CleanUp.ReferenceOTU266433
New.CleanUp.ReferenceOTU120265
New.CleanUp.ReferenceOTU172686
New.CleanUp.ReferenceOTU85285
New.CleanUp.ReferenceOTU179925
New.CleanUp.ReferenceOTU93500
New.CleanUp.ReferenceOTU168531
New.CleanUp.ReferenceOTU208217
New.CleanUp.ReferenceOTU117387
New.CleanUp.ReferenceOTU75926
New.CleanUp.ReferenceOTU191552
New.CleanUp.ReferenceOTU71490
New.CleanUp.ReferenceOTU298663
New.CleanUp.ReferenceOTU274515
AY937319.1.1755
New.CleanUp.ReferenceOTU10218
New.CleanUp.ReferenceOTU157417
New.CleanUp.ReferenceOTU58052
New.CleanUp.ReferenceOTU174955
New.CleanUp.ReferenceOTU50627
New.CleanUp.ReferenceOTU231606
New.CleanUp.ReferenceOTU187715
New.CleanUp.ReferenceOTU81510
New.CleanUp.ReferenceOTU189866
New.CleanUp.ReferenceOTU62107
New.CleanUp.ReferenceOTU95505
New.CleanUp.ReferenceOTU190434
New.CleanUp.ReferenceOTU118905
New.CleanUp.ReferenceOTU250974
New.CleanUp.ReferenceOTU114770
New.CleanUp.ReferenceOTU168240
New.CleanUp.ReferenceOTU11383
New.CleanUp.ReferenceOTU37876
New.CleanUp.ReferenceOTU253078
New.CleanUp.ReferenceOTU221896
New.CleanUp.ReferenceOTU163174
New.ReferenceOTU2228

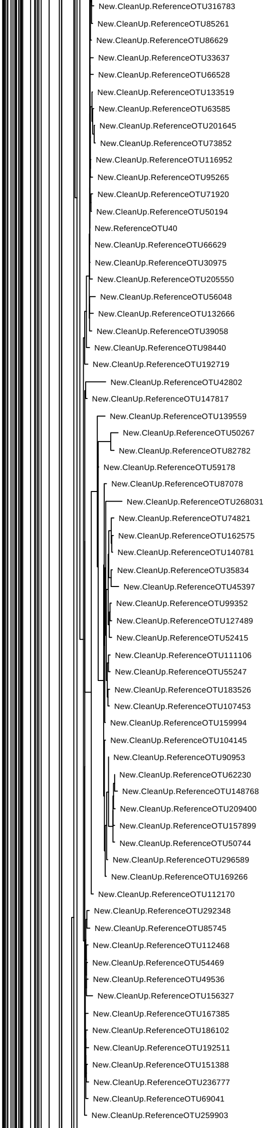
New.CleanUp.ReferenceOTU316783
New.CleanUp.ReferenceOTU85261
New.CleanUp.ReferenceOTU86629
New.CleanUp.ReferenceOTU33637
New.CleanUp.ReferenceOTU66528
New.CleanUp.ReferenceOTU133519
New.CleanUp.ReferenceOTU63585
New.CleanUp.ReferenceOTU201645
New.CleanUp.ReferenceOTU73852
New.CleanUp.ReferenceOTU116952
New.CleanUp.ReferenceOTU95265
New.CleanUp.ReferenceOTU71920
New.CleanUp.ReferenceOTU50194
New.ReferenceOTU40
New.CleanUp.ReferenceOTU66629
New.CleanUp.ReferenceOTU30975
New.CleanUp.ReferenceOTU205550
New.CleanUp.ReferenceOTU56048
New.CleanUp.ReferenceOTU132666
New.CleanUp.ReferenceOTU39058
New.CleanUp.ReferenceOTU98440
New.CleanUp.ReferenceOTU192719
New.CleanUp.ReferenceOTU42802
New.CleanUp.ReferenceOTU147817
New.CleanUp.ReferenceOTU139559
New.CleanUp.ReferenceOTU50267
New.CleanUp.ReferenceOTU82782
New.CleanUp.ReferenceOTU59178
New.CleanUp.ReferenceOTU87078
New.CleanUp.ReferenceOTU268031
New.CleanUp.ReferenceOTU74821
New.CleanUp.ReferenceOTU162575
New.CleanUp.ReferenceOTU140781
New.CleanUp.ReferenceOTU35834
New.CleanUp.ReferenceOTU45397
New.CleanUp.ReferenceOTU99352
New.CleanUp.ReferenceOTU127489
New.CleanUp.ReferenceOTU52415
New.CleanUp.ReferenceOTU111106
New.CleanUp.ReferenceOTU55247
New.CleanUp.ReferenceOTU183526
New.CleanUp.ReferenceOTU107453
New.CleanUp.ReferenceOTU159994
New.CleanUp.ReferenceOTU104145
New.CleanUp.ReferenceOTU90953
New.CleanUp.ReferenceOTU62230
New.CleanUp.ReferenceOTU148768
New.CleanUp.ReferenceOTU209400
New.CleanUp.ReferenceOTU157899
New.CleanUp.ReferenceOTU50744
New.CleanUp.ReferenceOTU296589
New.CleanUp.ReferenceOTU169266
New.CleanUp.ReferenceOTU112170
New.CleanUp.ReferenceOTU292348
New.CleanUp.ReferenceOTU85745
New.CleanUp.ReferenceOTU112468
New.CleanUp.ReferenceOTU54469
New.CleanUp.ReferenceOTU49536
New.CleanUp.ReferenceOTU156327
New.CleanUp.ReferenceOTU167385
New.CleanUp.ReferenceOTU186102
New.CleanUp.ReferenceOTU192511
New.CleanUp.ReferenceOTU151388
New.CleanUp.ReferenceOTU236777
New.CleanUp.ReferenceOTU69041
New.CleanUp.ReferenceOTU259903

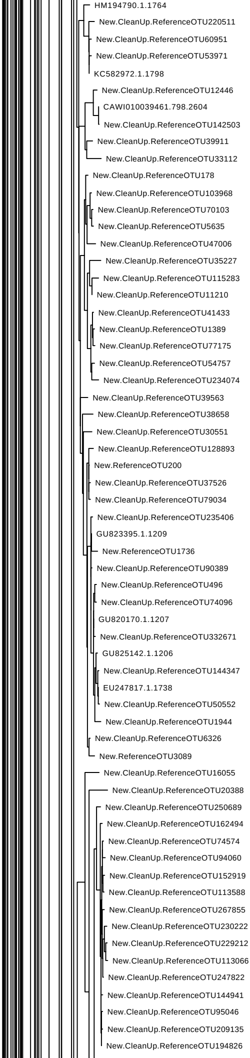
HM194790.1.1764
New.CleanUp.ReferenceOTU220511
New.CleanUp.ReferenceOTU60951
New.CleanUp.ReferenceOTU53971
KC582972.1.1798
New.CleanUp.ReferenceOTU12446
CAWI010039461.798.2604
New.CleanUp.ReferenceOTU142503
New.CleanUp.ReferenceOTU39911
New.CleanUp.ReferenceOTU33112
New.CleanUp.ReferenceOTU178
New.CleanUp.ReferenceOTU103968
New.CleanUp.ReferenceOTU70103
New.CleanUp.ReferenceOTU5635
New.CleanUp.ReferenceOTU47006
New.CleanUp.ReferenceOTU35227
New.CleanUp.ReferenceOTU115283
New.CleanUp.ReferenceOTU11210
New.CleanUp.ReferenceOTU41433
New.CleanUp.ReferenceOTU1389
New.CleanUp.ReferenceOTU77175
New.CleanUp.ReferenceOTU54757
New.CleanUp.ReferenceOTU234074
New.CleanUp.ReferenceOTU39563
New.CleanUp.ReferenceOTU38658
New.CleanUp.ReferenceOTU30551
New.CleanUp.ReferenceOTU128893
New.ReferenceOTU200
New.CleanUp.ReferenceOTU37526
New.CleanUp.ReferenceOTU79034
New.CleanUp.ReferenceOTU235406
GU823395.1.1209
New.ReferenceOTU1736
New.CleanUp.ReferenceOTU90389
New.CleanUp.ReferenceOTU496
New.CleanUp.ReferenceOTU74096
GU820170.1.1207
New.CleanUp.ReferenceOTU332671
GU825142.1.1206
New.CleanUp.ReferenceOTU144347
EU247817.1.1738
New.CleanUp.ReferenceOTU50552
New.CleanUp.ReferenceOTU1944
New.CleanUp.ReferenceOTU6326
New.ReferenceOTU3089
New.CleanUp.ReferenceOTU16055
New.CleanUp.ReferenceOTU20388
New.CleanUp.ReferenceOTU250689
New.CleanUp.ReferenceOTU162494
New.CleanUp.ReferenceOTU74574
New.CleanUp.ReferenceOTU94060
New.CleanUp.ReferenceOTU152919
New.CleanUp.ReferenceOTU113588
New.CleanUp.ReferenceOTU267855
New.CleanUp.ReferenceOTU230222
New.CleanUp.ReferenceOTU229212
New.CleanUp.ReferenceOTU113066
New.CleanUp.ReferenceOTU247822
New.CleanUp.ReferenceOTU144941
New.CleanUp.ReferenceOTU95046
New.CleanUp.ReferenceOTU209135
New.CleanUp.ReferenceOTU194826

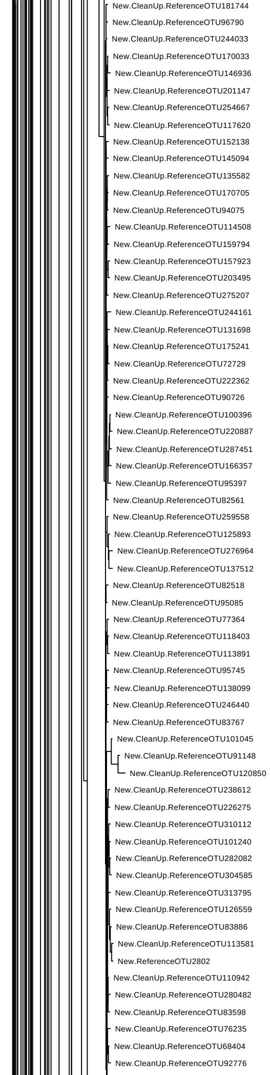
New.CleanUp.ReferenceOTU181744
New.CleanUp.ReferenceOTU96790
New.CleanUp.ReferenceOTU244033
New.CleanUp.ReferenceOTU170033
New.CleanUp.ReferenceOTU146936
New.CleanUp.ReferenceOTU201147
New.CleanUp.ReferenceOTU254667
New.CleanUp.ReferenceOTU117620
New.CleanUp.ReferenceOTU152138
New.CleanUp.ReferenceOTU145094
New.CleanUp.ReferenceOTU135582
New.CleanUp.ReferenceOTU170705
New.CleanUp.ReferenceOTU94075
New.CleanUp.ReferenceOTU114508
New.CleanUp.ReferenceOTU159794
New.CleanUp.ReferenceOTU157923
New.CleanUp.ReferenceOTU203495
New.CleanUp.ReferenceOTU275207
New.CleanUp.ReferenceOTU244161
New.CleanUp.ReferenceOTU131698
New.CleanUp.ReferenceOTU175241
New.CleanUp.ReferenceOTU72729
New.CleanUp.ReferenceOTU222362
New.CleanUp.ReferenceOTU90726
New.CleanUp.ReferenceOTU100396
New.CleanUp.ReferenceOTU220887
New.CleanUp.ReferenceOTU287451
New.CleanUp.ReferenceOTU166357
New.CleanUp.ReferenceOTU95397
New.CleanUp.ReferenceOTU82561
New.CleanUp.ReferenceOTU259558
New.CleanUp.ReferenceOTU125893
New.CleanUp.ReferenceOTU276964
New.CleanUp.ReferenceOTU137512
New.CleanUp.ReferenceOTU82518
New.CleanUp.ReferenceOTU95085
New.CleanUp.ReferenceOTU77364
New.CleanUp.ReferenceOTU118403
New.CleanUp.ReferenceOTU113891
New.CleanUp.ReferenceOTU95745
New.CleanUp.ReferenceOTU138099
New.CleanUp.ReferenceOTU246440
New.CleanUp.ReferenceOTU83767
New.CleanUp.ReferenceOTU101045
New.CleanUp.ReferenceOTU91148
New.CleanUp.ReferenceOTU120850
New.CleanUp.ReferenceOTU238612
New.CleanUp.ReferenceOTU226275
New.CleanUp.ReferenceOTU310112
New.CleanUp.ReferenceOTU101240
New.CleanUp.ReferenceOTU282082
New.CleanUp.ReferenceOTU304585
New.CleanUp.ReferenceOTU313795
New.CleanUp.ReferenceOTU126559
New.CleanUp.ReferenceOTU83886
New.CleanUp.ReferenceOTU113581
New.ReferenceOTU2802
New.CleanUp.ReferenceOTU110942
New.CleanUp.ReferenceOTU280482
New.CleanUp.ReferenceOTU83598
New.CleanUp.ReferenceOTU76235
New.CleanUp.ReferenceOTU68404
New.CleanUp.ReferenceOTU92776

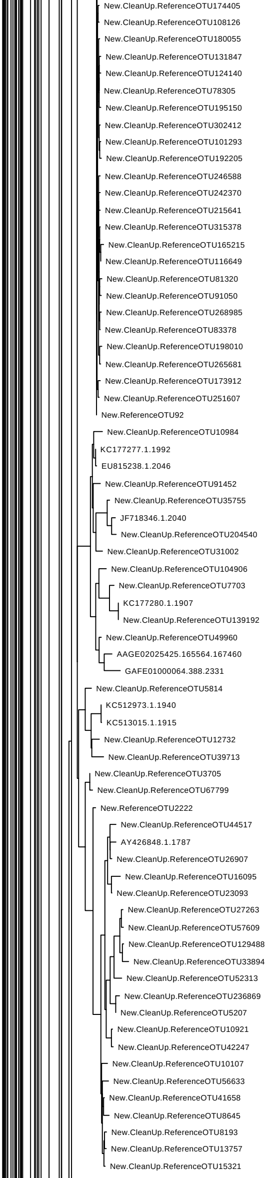
New.CleanUp.ReferenceOTU174405
New.CleanUp.ReferenceOTU108126
New.CleanUp.ReferenceOTU180055
New.CleanUp.ReferenceOTU131847
New.CleanUp.ReferenceOTU124140
New.CleanUp.ReferenceOTU78305
New.CleanUp.ReferenceOTU195150
New.CleanUp.ReferenceOTU302412
New.CleanUp.ReferenceOTU101293
New.CleanUp.ReferenceOTU192205
New.CleanUp.ReferenceOTU246588
New.CleanUp.ReferenceOTU242370
New.CleanUp.ReferenceOTU215641
New.CleanUp.ReferenceOTU315378
New.CleanUp.ReferenceOTU165215
New.CleanUp.ReferenceOTU116649
New.CleanUp.ReferenceOTU81320
New.CleanUp.ReferenceOTU91050
New.CleanUp.ReferenceOTU268985
New.CleanUp.ReferenceOTU83378
New.CleanUp.ReferenceOTU198010
New.CleanUp.ReferenceOTU265681
New.CleanUp.ReferenceOTU173912
New.CleanUp.ReferenceOTU251607
New.ReferenceOTU92
New.CleanUp.ReferenceOTU10984
KC177277.1.1992
EU815238.1.2046
New.CleanUp.ReferenceOTU91452
New.CleanUp.ReferenceOTU35755
JF718346.1.2040
New.CleanUp.ReferenceOTU204540
New.CleanUp.ReferenceOTU31002
New.CleanUp.ReferenceOTU104906
New.CleanUp.ReferenceOTU7703
KC177280.1.1907
New.CleanUp.ReferenceOTU139192
New.CleanUp.ReferenceOTU49960
AAGE02025425.165564.167460
GAFE01000064.388.2331
New.CleanUp.ReferenceOTU5814
KC512973.1.1940
KC513015.1.1915
New.CleanUp.ReferenceOTU12732
New.CleanUp.ReferenceOTU39713
New.CleanUp.ReferenceOTU3705
New.CleanUp.ReferenceOTU67799
New.ReferenceOTU2222
New.CleanUp.ReferenceOTU44517
AY426848.1.1787
New.CleanUp.ReferenceOTU26907
New.CleanUp.ReferenceOTU16095
New.CleanUp.ReferenceOTU23093
New.CleanUp.ReferenceOTU27263
New.CleanUp.ReferenceOTU57609
New.CleanUp.ReferenceOTU129488
New.CleanUp.ReferenceOTU33894
New.CleanUp.ReferenceOTU52313
New.CleanUp.ReferenceOTU236869
New.CleanUp.ReferenceOTU5207
New.CleanUp.ReferenceOTU10921
New.CleanUp.ReferenceOTU42247
New.CleanUp.ReferenceOTU10107
New.CleanUp.ReferenceOTU56633
New.CleanUp.ReferenceOTU41658
New.CleanUp.ReferenceOTU8645
New.CleanUp.ReferenceOTU8193
New.CleanUp.ReferenceOTU13757
New.CleanUp.ReferenceOTU15321

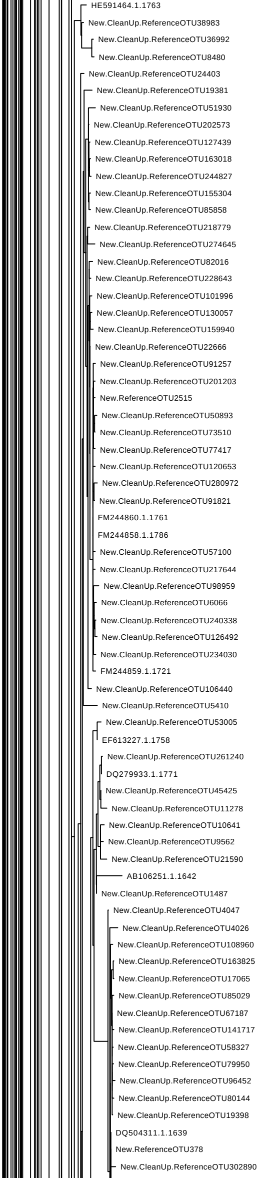

HE591464.1.1763
New.CleanUp.ReferenceOTU38983
New.CleanUp.ReferenceOTU36992
New.CleanUp.ReferenceOTU8480
New.CleanUp.ReferenceOTU24403
New.CleanUp.ReferenceOTU19381
New.CleanUp.ReferenceOTU51930
New.CleanUp.ReferenceOTU202573
New.CleanUp.ReferenceOTU127439
New.CleanUp.ReferenceOTU163018
New.CleanUp.ReferenceOTU244827
New.CleanUp.ReferenceOTU155304
New.CleanUp.ReferenceOTU85858
New.CleanUp.ReferenceOTU218779
New.CleanUp.ReferenceOTU274645
New.CleanUp.ReferenceOTU82016
New.CleanUp.ReferenceOTU228643
New.CleanUp.ReferenceOTU101996
New.CleanUp.ReferenceOTU130057
New.CleanUp.ReferenceOTU159940
New.CleanUp.ReferenceOTU22666
New.CleanUp.ReferenceOTU91257
New.CleanUp.ReferenceOTU201203
New.ReferenceOTU2515
New.CleanUp.ReferenceOTU50893
New.CleanUp.ReferenceOTU73510
New.CleanUp.ReferenceOTU77417
New.CleanUp.ReferenceOTU120653
New.CleanUp.ReferenceOTU280972
New.CleanUp.ReferenceOTU91821
FM244860.1.1761
FM244858.1.1786
New.CleanUp.ReferenceOTU57100
New.CleanUp.ReferenceOTU217644
New.CleanUp.ReferenceOTU98959
New.CleanUp.ReferenceOTU6066
New.CleanUp.ReferenceOTU240338
New.CleanUp.ReferenceOTU126492
New.CleanUp.ReferenceOTU234030
FM244859.1.1721
New.CleanUp.ReferenceOTU106440
New.CleanUp.ReferenceOTU5410
New.CleanUp.ReferenceOTU53005
EF613227.1.1758
New.CleanUp.ReferenceOTU261240
DQ279933.1.1771
New.CleanUp.ReferenceOTU45425
New.CleanUp.ReferenceOTU11278
New.CleanUp.ReferenceOTU10641
New.CleanUp.ReferenceOTU9562
New.CleanUp.ReferenceOTU21590
AB106251.1.1642
New.CleanUp.ReferenceOTU1487
New.CleanUp.ReferenceOTU4047
New.CleanUp.ReferenceOTU4026
New.CleanUp.ReferenceOTU108960
New.CleanUp.ReferenceOTU163825
New.CleanUp.ReferenceOTU17065
New.CleanUp.ReferenceOTU85029
New.CleanUp.ReferenceOTU67187
New.CleanUp.ReferenceOTU141717
New.CleanUp.ReferenceOTU58327
New.CleanUp.ReferenceOTU79950
New.CleanUp.ReferenceOTU96452
New.CleanUp.ReferenceOTU80144
New.CleanUp.ReferenceOTU19398
DQ504311.1.1639
New.ReferenceOTU378
New.CleanUp.ReferenceOTU302890

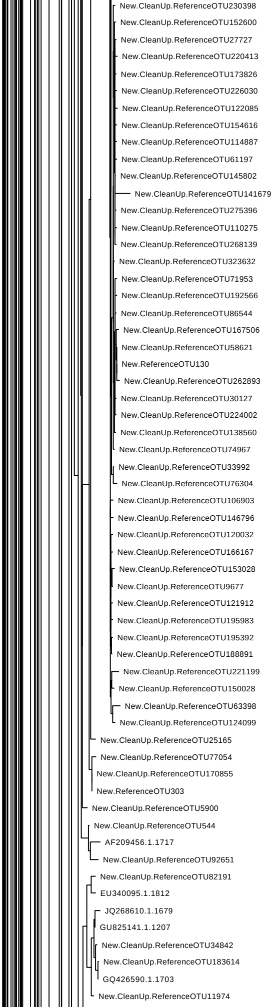
New.CleanUp.ReferenceOTU230398
New.CleanUp.ReferenceOTU152600
New.CleanUp.ReferenceOTU27727
New.CleanUp.ReferenceOTU220413
New.CleanUp.ReferenceOTU173826
New.CleanUp.ReferenceOTU226030
New.CleanUp.ReferenceOTU122085
New.CleanUp.ReferenceOTU154616
New.CleanUp.ReferenceOTU114887
New.CleanUp.ReferenceOTU61197
New.CleanUp.ReferenceOTU145802
New.CleanUp.ReferenceOTU141679
New.CleanUp.ReferenceOTU275396
New.CleanUp.ReferenceOTU110275
New.CleanUp.ReferenceOTU268139
New.CleanUp.ReferenceOTU323632
New.CleanUp.ReferenceOTU71953
New.CleanUp.ReferenceOTU192566
New.CleanUp.ReferenceOTU86544
New.CleanUp.ReferenceOTU167506
New.CleanUp.ReferenceOTU58621
New.ReferenceOTU130
New.CleanUp.ReferenceOTU262893
New.CleanUp.ReferenceOTU30127
New.CleanUp.ReferenceOTU224002
New.CleanUp.ReferenceOTU138560
New.CleanUp.ReferenceOTU74967
New.CleanUp.ReferenceOTU33992
New.CleanUp.ReferenceOTU76304
New.CleanUp.ReferenceOTU106903
New.CleanUp.ReferenceOTU146796
New.CleanUp.ReferenceOTU120032
New.CleanUp.ReferenceOTU166167
New.CleanUp.ReferenceOTU153028
New.CleanUp.ReferenceOTU9677
New.CleanUp.ReferenceOTU121912
New.CleanUp.ReferenceOTU195983
New.CleanUp.ReferenceOTU195392
New.CleanUp.ReferenceOTU188891
New.CleanUp.ReferenceOTU221199
New.CleanUp.ReferenceOTU150028
New.CleanUp.ReferenceOTU63398
New.CleanUp.ReferenceOTU124099
New.CleanUp.ReferenceOTU25165
New.CleanUp.ReferenceOTU77054
New.CleanUp.ReferenceOTU170855
New.ReferenceOTU303
New.CleanUp.ReferenceOTU5900
New.CleanUp.ReferenceOTU544
AF209456.1.1717
New.CleanUp.ReferenceOTU92651
New.CleanUp.ReferenceOTU82191
EU340095.1.1812
JQ268610.1.1679
GU825141.1.1207
New.CleanUp.ReferenceOTU34842
New.CleanUp.ReferenceOTU183614
GQ426590.1.1703
New.CleanUp.ReferenceOTU11974

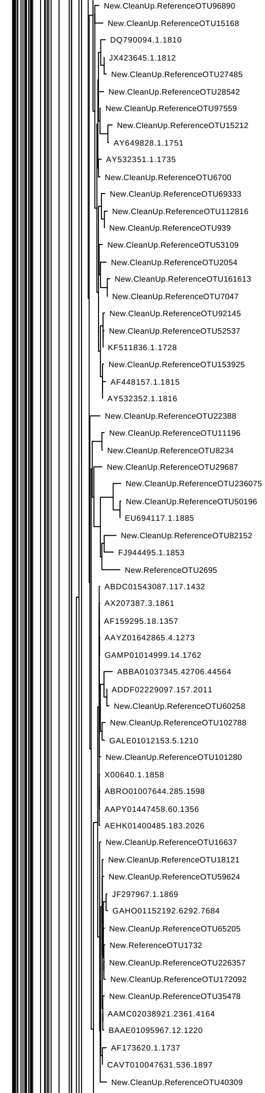
New.CleanUp.ReferenceOTU96890
New.CleanUp.ReferenceOTU15168
DQ790094.1.1810
JX423645.1.1812
New.CleanUp.ReferenceOTU27485
New.CleanUp.ReferenceOTU28542
New.CleanUp.ReferenceOTU97559
New.CleanUp.ReferenceOTU15212
AY649828.1.1751
AY532351.1.1735
New.CleanUp.ReferenceOTU6700
New.CleanUp.ReferenceOTU69333
New.CleanUp.ReferenceOTU112816
New.CleanUp.ReferenceOTU939
New.CleanUp.ReferenceOTU53109
New.CleanUp.ReferenceOTU2054
New.CleanUp.ReferenceOTU161613
New.CleanUp.ReferenceOTU7047
New.CleanUp.ReferenceOTU92145
New.CleanUp.ReferenceOTU52537
KF511836.1.1728
New.CleanUp.ReferenceOTU153925
AF448157.1.1815
AY532352.1.1816
New.CleanUp.ReferenceOTU22388
New.CleanUp.ReferenceOTU11196
New.CleanUp.ReferenceOTU8234
New.CleanUp.ReferenceOTU29687
New.CleanUp.ReferenceOTU236075
New.CleanUp.ReferenceOTU50196
EU694117.1.1885
New.CleanUp.ReferenceOTU82152
FJ944495.1.1853
New.ReferenceOTU2695
ABDC01543087.117.1432
AX207387.3.1861
AF159295.18.1357
AAYZ01642865.4.1273
GAMP01014999.14.1762
ABBA01037345.42706.44564
ADDF02229097.157.2011
New.CleanUp.ReferenceOTU60258
New.CleanUp.ReferenceOTU102788
GALE01012153.5.1210
New.CleanUp.ReferenceOTU101280
X00640.1.1858
ABRO01007644.285.1598
AAPY01447458.60.1356
AEHK01400485.183.2026
New.CleanUp.ReferenceOTU16637
New.CleanUp.ReferenceOTU18121
New.CleanUp.ReferenceOTU59624
JF297967.1.1869
GAHO01152192.6292.7684
New.CleanUp.ReferenceOTU65205
New.ReferenceOTU1732
New.CleanUp.ReferenceOTU226357
New.CleanUp.ReferenceOTU172092
New.CleanUp.ReferenceOTU35478
AAMC02038921.2361.4164
BAAE01095967.12.1220
AF173620.1.1737
CAVT010047631.536.1897
New.CleanUp.ReferenceOTU40309

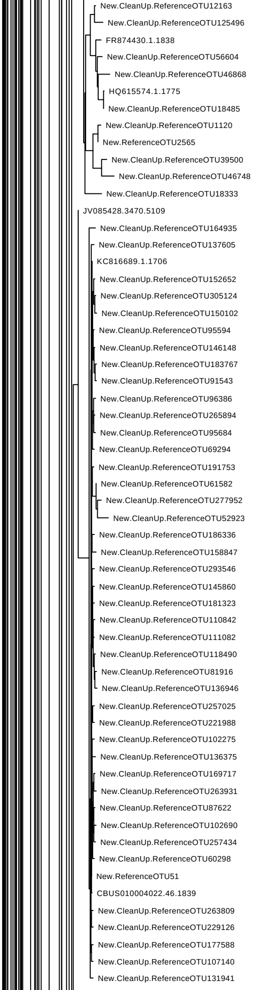

New.CleanUp.ReferenceOTU12163
New.CleanUp.ReferenceOTU125496
FR874430.1.1838
New.CleanUp.ReferenceOTU56604
New.CleanUp.ReferenceOTU46868
HQ615574.1.1775
New.CleanUp.ReferenceOTU18485
New.CleanUp.ReferenceOTU1120
New.ReferenceOTU2565
New.CleanUp.ReferenceOTU39500
New.CleanUp.ReferenceOTU46748
New.CleanUp.ReferenceOTU18333
JV085428.3470.5109
New.CleanUp.ReferenceOTU164935
New.CleanUp.ReferenceOTU137605
KC816689.1.1706
New.CleanUp.ReferenceOTU152652
New.CleanUp.ReferenceOTU305124
New.CleanUp.ReferenceOTU150102
New.CleanUp.ReferenceOTU95594
New.CleanUp.ReferenceOTU146148
New.CleanUp.ReferenceOTU183767
New.CleanUp.ReferenceOTU91543
New.CleanUp.ReferenceOTU96386
New.CleanUp.ReferenceOTU265894
New.CleanUp.ReferenceOTU95684
New.CleanUp.ReferenceOTU69294
New.CleanUp.ReferenceOTU191753
New.CleanUp.ReferenceOTU61582
New.CleanUp.ReferenceOTU277952
New.CleanUp.ReferenceOTU52923
New.CleanUp.ReferenceOTU186336
New.CleanUp.ReferenceOTU158847
New.CleanUp.ReferenceOTU293546
New.CleanUp.ReferenceOTU145860
New.CleanUp.ReferenceOTU181323
New.CleanUp.ReferenceOTU110842
New.CleanUp.ReferenceOTU111082
New.CleanUp.ReferenceOTU118490
New.CleanUp.ReferenceOTU81916
New.CleanUp.ReferenceOTU136946
New.CleanUp.ReferenceOTU257025
New.CleanUp.ReferenceOTU221988
New.CleanUp.ReferenceOTU102275
New.CleanUp.ReferenceOTU136375
New.CleanUp.ReferenceOTU169717
New.CleanUp.ReferenceOTU263931
New.CleanUp.ReferenceOTU87622
New.CleanUp.ReferenceOTU102690
New.CleanUp.ReferenceOTU257434
New.CleanUp.ReferenceOTU60298
New.ReferenceOTU51
CBUS010004022.46.1839
New.CleanUp.ReferenceOTU263809
New.CleanUp.ReferenceOTU229126
New.CleanUp.ReferenceOTU177588
New.CleanUp.ReferenceOTU107140
New.CleanUp.ReferenceOTU131941

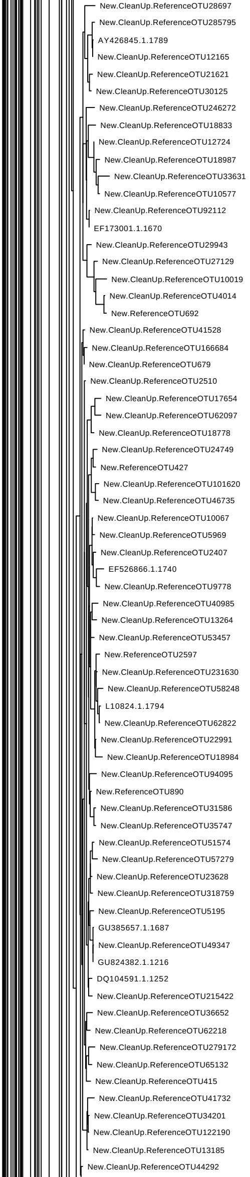
New.CleanUp.ReferenceOTU28697
New.CleanUp.ReferenceOTU285795
AY426845.1.1789
New.CleanUp.ReferenceOTU12165
New.CleanUp.ReferenceOTU21621
New.CleanUp.ReferenceOTU30125
New.CleanUp.ReferenceOTU246272
New.CleanUp.ReferenceOTU18833
New.CleanUp.ReferenceOTU12724
New.CleanUp.ReferenceOTU18987
New.CleanUp.ReferenceOTU33631
New.CleanUp.ReferenceOTU10577
New.CleanUp.ReferenceOTU92112
EF173001.1.1670
New.CleanUp.ReferenceOTU29943
New.CleanUp.ReferenceOTU27129
New.CleanUp.ReferenceOTU10019
New.CleanUp.ReferenceOTU4014
New.ReferenceOTU692
New.CleanUp.ReferenceOTU41528
New.CleanUp.ReferenceOTU166684
New.CleanUp.ReferenceOTU679
New.CleanUp.ReferenceOTU2510
New.CleanUp.ReferenceOTU17654
New.CleanUp.ReferenceOTU62097
New.CleanUp.ReferenceOTU18778
New.CleanUp.ReferenceOTU24749
New.ReferenceOTU427
New.CleanUp.ReferenceOTU101620
New.CleanUp.ReferenceOTU46735
New.CleanUp.ReferenceOTU10067
New.CleanUp.ReferenceOTU5969
New.CleanUp.ReferenceOTU2407
EF526866.1.1740
New.CleanUp.ReferenceOTU9778
New.CleanUp.ReferenceOTU40985
New.CleanUp.ReferenceOTU13264
New.CleanUp.ReferenceOTU53457
New.ReferenceOTU2597
New.CleanUp.ReferenceOTU231630
New.CleanUp.ReferenceOTU58248
L10824.1.1794
New.CleanUp.ReferenceOTU62822
New.CleanUp.ReferenceOTU22991
New.CleanUp.ReferenceOTU18984
New.CleanUp.ReferenceOTU94095
New.ReferenceOTU890
New.CleanUp.ReferenceOTU31586
New.CleanUp.ReferenceOTU35747
New.CleanUp.ReferenceOTU51574
New.CleanUp.ReferenceOTU57279
New.CleanUp.ReferenceOTU23628
New.CleanUp.ReferenceOTU318759
New.CleanUp.ReferenceOTU5195
GU385657.1.1687
New.CleanUp.ReferenceOTU49347
GU824382.1.1216
DQ104591.1.1252
New.CleanUp.ReferenceOTU215422
New.CleanUp.ReferenceOTU36652
New.CleanUp.ReferenceOTU62218
New.CleanUp.ReferenceOTU279172
New.CleanUp.ReferenceOTU65132
New.CleanUp.ReferenceOTU415
New.CleanUp.ReferenceOTU41732
New.CleanUp.ReferenceOTU34201
New.CleanUp.ReferenceOTU122190
New.CleanUp.ReferenceOTU13185
New.CleanUp.ReferenceOTU44292

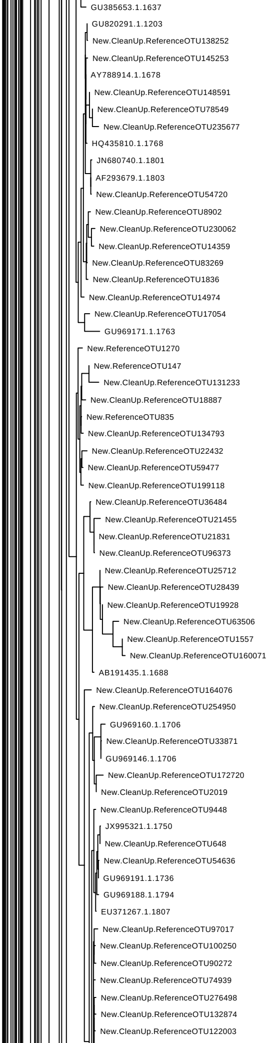
GU385653.1.1637
GU820291.1.1203
New.CleanUp.ReferenceOTU138252
New.CleanUp.ReferenceOTU145253
AY788914.1.1678
New.CleanUp.ReferenceOTU148591
New.CleanUp.ReferenceOTU78549
New.CleanUp.ReferenceOTU235677
HQ435810.1.1768
JN680740.1.1801
AF293679.1.1803
New.CleanUp.ReferenceOTU54720
New.CleanUp.ReferenceOTU8902
New.CleanUp.ReferenceOTU230062
New.CleanUp.ReferenceOTU14359
New.CleanUp.ReferenceOTU83269
New.CleanUp.ReferenceOTU1836
New.CleanUp.ReferenceOTU14974
New.CleanUp.ReferenceOTU17054
GU969171.1.1763
New.ReferenceOTU1270
New.ReferenceOTU147
New.CleanUp.ReferenceOTU131233
New.CleanUp.ReferenceOTU18887
New.ReferenceOTU835
New.CleanUp.ReferenceOTU134793
New.CleanUp.ReferenceOTU22432
New.CleanUp.ReferenceOTU59477
New.CleanUp.ReferenceOTU199118
New.CleanUp.ReferenceOTU36484
New.CleanUp.ReferenceOTU21455
New.CleanUp.ReferenceOTU21831
New.CleanUp.ReferenceOTU96373
New.CleanUp.ReferenceOTU25712
New.CleanUp.ReferenceOTU28439
New.CleanUp.ReferenceOTU19928
New.CleanUp.ReferenceOTU63506
New.CleanUp.ReferenceOTU1557
New.CleanUp.ReferenceOTU160071
AB191435.1.1688
New.CleanUp.ReferenceOTU164076
New.CleanUp.ReferenceOTU254950
GU969160.1.1706
New.CleanUp.ReferenceOTU33871
GU969146.1.1706
New.CleanUp.ReferenceOTU172720
New.CleanUp.ReferenceOTU2019
New.CleanUp.ReferenceOTU9448
JX995321.1.1750
New.CleanUp.ReferenceOTU648
New.CleanUp.ReferenceOTU54636
GU969191.1.1736
GU969188.1.1794
EU371267.1.1807
New.CleanUp.ReferenceOTU97017
New.CleanUp.ReferenceOTU100250
New.CleanUp.ReferenceOTU90272
New.CleanUp.ReferenceOTU74939
New.CleanUp.ReferenceOTU276498
New.CleanUp.ReferenceOTU132874
New.CleanUp.ReferenceOTU122003

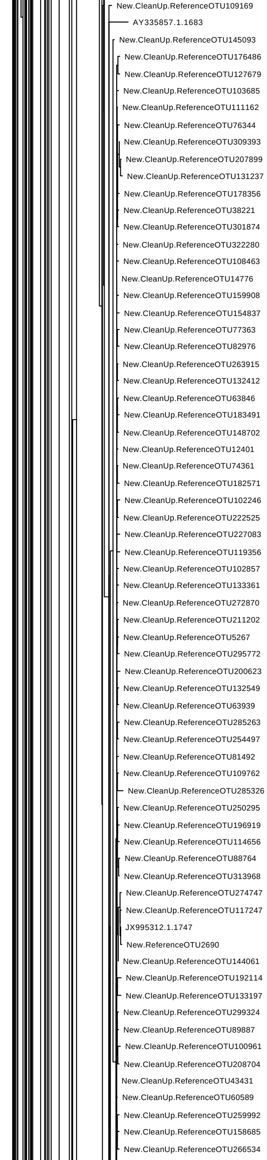

New.CleanUp.ReferenceOTU109169
AY335857.1.1683
New.CleanUp.ReferenceOTU145093
New.CleanUp.ReferenceOTU176486
New.CleanUp.ReferenceOTU127679
New.CleanUp.ReferenceOTU103685
New.CleanUp.ReferenceOTU111162
New.CleanUp.ReferenceOTU76344
New.CleanUp.ReferenceOTU309393
New.CleanUp.ReferenceOTU207899
New.CleanUp.ReferenceOTU131237
New.CleanUp.ReferenceOTU178356
New.CleanUp.ReferenceOTU38221
New.CleanUp.ReferenceOTU301874
New.CleanUp.ReferenceOTU322280
New.CleanUp.ReferenceOTU108463
New.CleanUp.ReferenceOTU14776
New.CleanUp.ReferenceOTU159908
New.CleanUp.ReferenceOTU154837
New.CleanUp.ReferenceOTU77363
New.CleanUp.ReferenceOTU82976
New.CleanUp.ReferenceOTU263915
New.CleanUp.ReferenceOTU132412
New.CleanUp.ReferenceOTU63846
New.CleanUp.ReferenceOTU183491
New.CleanUp.ReferenceOTU148702
New.CleanUp.ReferenceOTU12401
New.CleanUp.ReferenceOTU74361
New.CleanUp.ReferenceOTU182571
New.CleanUp.ReferenceOTU102246
New.CleanUp.ReferenceOTU222525
New.CleanUp.ReferenceOTU227083
New.CleanUp.ReferenceOTU119356
New.CleanUp.ReferenceOTU102857
New.CleanUp.ReferenceOTU133361
New.CleanUp.ReferenceOTU272870
New.CleanUp.ReferenceOTU211202
New.CleanUp.ReferenceOTU5267
New.CleanUp.ReferenceOTU295772
New.CleanUp.ReferenceOTU200623
New.CleanUp.ReferenceOTU132549
New.CleanUp.ReferenceOTU63939
New.CleanUp.ReferenceOTU285263
New.CleanUp.ReferenceOTU254497
New.CleanUp.ReferenceOTU81492
New.CleanUp.ReferenceOTU109762
New.CleanUp.ReferenceOTU285326
New.CleanUp.ReferenceOTU250295
New.CleanUp.ReferenceOTU196919
New.CleanUp.ReferenceOTU114656
New.CleanUp.ReferenceOTU88764
New.CleanUp.ReferenceOTU313968
New.CleanUp.ReferenceOTU274747
New.CleanUp.ReferenceOTU117247
JX995312.1.1747
New.ReferenceOTU2690
New.CleanUp.ReferenceOTU144061
New.CleanUp.ReferenceOTU192114
New.CleanUp.ReferenceOTU133197
New.CleanUp.ReferenceOTU299324
New.CleanUp.ReferenceOTU89887
New.CleanUp.ReferenceOTU100961
New.CleanUp.ReferenceOTU208704
New.CleanUp.ReferenceOTU43431
New.CleanUp.ReferenceOTU60589
New.CleanUp.ReferenceOTU259992
New.CleanUp.ReferenceOTU158685
New.CleanUp.ReferenceOTU266534

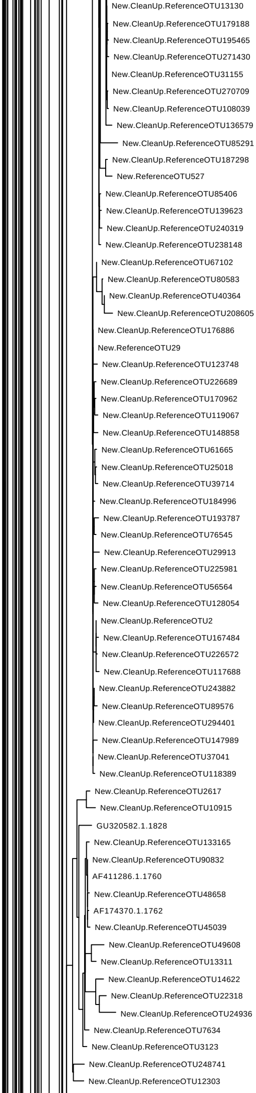
New.CleanUp.ReferenceOTU13130
New.CleanUp.ReferenceOTU179188
New.CleanUp.ReferenceOTU195465
New.CleanUp.ReferenceOTU271430
New.CleanUp.ReferenceOTU31155
New.CleanUp.ReferenceOTU270709
New.CleanUp.ReferenceOTU108039
New.CleanUp.ReferenceOTU136579
New.CleanUp.ReferenceOTU85291
New.CleanUp.ReferenceOTU187298
New.ReferenceOTU527
New.CleanUp.ReferenceOTU85406
New.CleanUp.ReferenceOTU139623
New.CleanUp.ReferenceOTU240319
New.CleanUp.ReferenceOTU238148
New.CleanUp.ReferenceOTU67102
New.CleanUp.ReferenceOTU80583
New.CleanUp.ReferenceOTU40364
New.CleanUp.ReferenceOTU208605
New.CleanUp.ReferenceOTU176886
New.ReferenceOTU29
New.CleanUp.ReferenceOTU123748
New.CleanUp.ReferenceOTU226689
New.CleanUp.ReferenceOTU170962
New.CleanUp.ReferenceOTU119067
New.CleanUp.ReferenceOTU148858
New.CleanUp.ReferenceOTU61665
New.CleanUp.ReferenceOTU25018
New.CleanUp.ReferenceOTU39714
New.CleanUp.ReferenceOTU184996
New.CleanUp.ReferenceOTU193787
New.CleanUp.ReferenceOTU76545
New.CleanUp.ReferenceOTU29913
New.CleanUp.ReferenceOTU225981
New.CleanUp.ReferenceOTU56564
New.CleanUp.ReferenceOTU128054
New.CleanUp.ReferenceOTU2
New.CleanUp.ReferenceOTU167484
New.CleanUp.ReferenceOTU226572
New.CleanUp.ReferenceOTU117688
New.CleanUp.ReferenceOTU243882
New.CleanUp.ReferenceOTU89576
New.CleanUp.ReferenceOTU294401
New.CleanUp.ReferenceOTU147989
New.CleanUp.ReferenceOTU37041
New.CleanUp.ReferenceOTU118389
New.CleanUp.ReferenceOTU2617
New.CleanUp.ReferenceOTU10915
GU320582.1.1828
New.CleanUp.ReferenceOTU133165
New.CleanUp.ReferenceOTU90832
AF411286.1.1760
New.CleanUp.ReferenceOTU48658
AF174370.1.1762
New.CleanUp.ReferenceOTU45039
New.CleanUp.ReferenceOTU49608
New.CleanUp.ReferenceOTU13311
New.CleanUp.ReferenceOTU14622
New.CleanUp.ReferenceOTU22318
New.CleanUp.ReferenceOTU24936
New.CleanUp.ReferenceOTU7634
New.CleanUp.ReferenceOTU3123
New.CleanUp.ReferenceOTU248741
New.CleanUp.ReferenceOTU12303

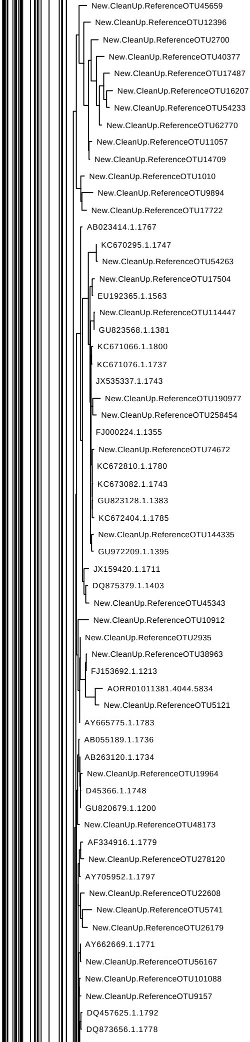

New.CleanUp.ReferenceOTU45659
New.CleanUp.ReferenceOTU12396
New.CleanUp.ReferenceOTU2700
New.CleanUp.ReferenceOTU40377
New.CleanUp.ReferenceOTU17487
New.CleanUp.ReferenceOTU16207
New.CleanUp.ReferenceOTU54233
New.CleanUp.ReferenceOTU62770
New.CleanUp.ReferenceOTU11057
New.CleanUp.ReferenceOTU14709
New.CleanUp.ReferenceOTU1010
New.CleanUp.ReferenceOTU9894
New.CleanUp.ReferenceOTU17722
AB023414.1.1767
KC670295.1.1747
New.CleanUp.ReferenceOTU54263
New.CleanUp.ReferenceOTU17504
EU192365.1.1563
New.CleanUp.ReferenceOTU114447
GU823568.1.1381
KC671066.1.1800
KC671076.1.1737
JX535337.1.1743
New.CleanUp.ReferenceOTU190977
New.CleanUp.ReferenceOTU258454
FJ000224.1.1355
New.CleanUp.ReferenceOTU74672
KC672810.1.1780
KC673082.1.1743
GU823128.1.1383
KC672404.1.1785
New.CleanUp.ReferenceOTU144335
GU972209.1.1395
JX159420.1.1711
DQ875379.1.1403
New.CleanUp.ReferenceOTU45343
New.CleanUp.ReferenceOTU10912
New.CleanUp.ReferenceOTU2935
New.CleanUp.ReferenceOTU38963
FJ153692.1.1213
AORR01011381.4044.5834
New.CleanUp.ReferenceOTU5121
AY665775.1.1783
AB055189.1.1736
AB263120.1.1734
New.CleanUp.ReferenceOTU19964
D45366.1.1748
GU820679.1.1200
New.CleanUp.ReferenceOTU48173
AF334916.1.1779
New.CleanUp.ReferenceOTU278120
AY705952.1.1797
New.CleanUp.ReferenceOTU22608
New.CleanUp.ReferenceOTU5741
New.CleanUp.ReferenceOTU26179
AY662669.1.1771
New.CleanUp.ReferenceOTU56167
New.CleanUp.ReferenceOTU101088
New.CleanUp.ReferenceOTU9157
DQ457625.1.1792
DQ873656.1.1778

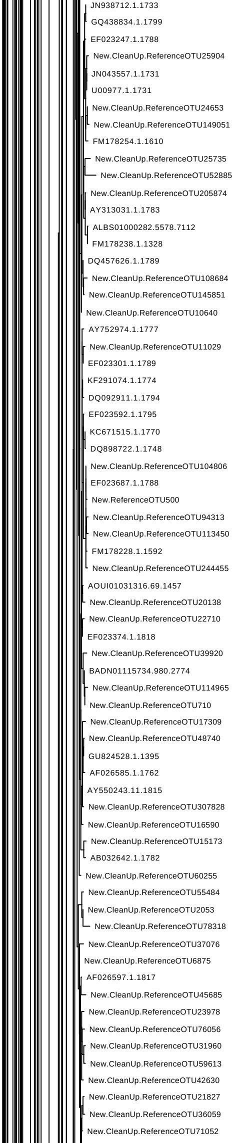
JN938712.1.1733
GQ438834.1.1799
EF023247.1.1788
New.CleanUp.ReferenceOTU25904
JN043557.1.1731
U00977.1.1731
New.CleanUp.ReferenceOTU24653
New.CleanUp.ReferenceOTU149051
FM178254.1.1610
New.CleanUp.ReferenceOTU25735
New.CleanUp.ReferenceOTU52885
New.CleanUp.ReferenceOTU205874
AY313031.1.1783
ALBS01000282.5578.7112
FM178238.1.1328
DQ457626.1.1789
New.CleanUp.ReferenceOTU108684
New.CleanUp.ReferenceOTU145851
New.CleanUp.ReferenceOTU10640
AY752974.1.1777
New.CleanUp.ReferenceOTU11029
EF023301.1.1789
KF291074.1.1774
DQ092911.1.1794
EF023592.1.1795
KC671515.1.1770
DQ898722.1.1748
New.CleanUp.ReferenceOTU104806
EF023687.1.1788
New.ReferenceOTU500
New.CleanUp.ReferenceOTU94313
New.CleanUp.ReferenceOTU113450
FM178228.1.1592
New.CleanUp.ReferenceOTU244455
AOUI01031316.69.1457
New.CleanUp.ReferenceOTU20138
New.CleanUp.ReferenceOTU22710
EF023374.1.1818
New.CleanUp.ReferenceOTU39920
BADN01115734.980.2774
New.CleanUp.ReferenceOTU114965
New.CleanUp.ReferenceOTU710
New.CleanUp.ReferenceOTU17309
New.CleanUp.ReferenceOTU48740
GU824528.1.1395
AF026585.1.1762
AY550243.11.1815
New.CleanUp.ReferenceOTU307828
New.CleanUp.ReferenceOTU16590
New.CleanUp.ReferenceOTU15173
AB032642.1.1782
New.CleanUp.ReferenceOTU60255
New.CleanUp.ReferenceOTU55484
New.CleanUp.ReferenceOTU2053
New.CleanUp.ReferenceOTU78318
New.CleanUp.ReferenceOTU37076
New.CleanUp.ReferenceOTU6875
AF026597.1.1817
New.CleanUp.ReferenceOTU45685
New.CleanUp.ReferenceOTU23978
New.CleanUp.ReferenceOTU76056
New.CleanUp.ReferenceOTU31960
New.CleanUp.ReferenceOTU59613
New.CleanUp.ReferenceOTU42630
New.CleanUp.ReferenceOTU21827
New.CleanUp.ReferenceOTU36059
New.CleanUp.ReferenceOTU71052

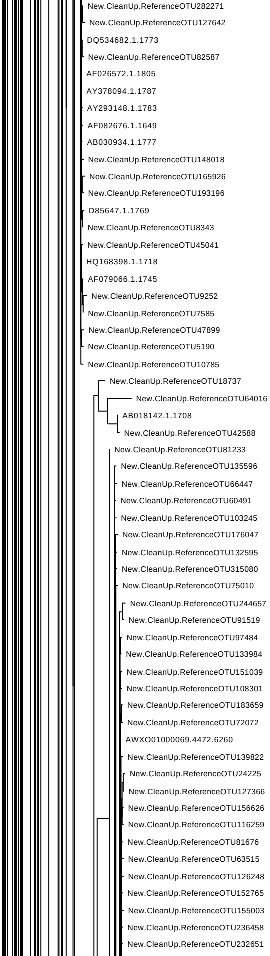

New.CleanUp.ReferenceOTU282271
New.CleanUp.ReferenceOTU127642
DQ534682.1.1773
New.CleanUp.ReferenceOTU82587
AF026572.1.1805
AY378094.1.1787
AY293148.1.1783
AF082676.1.1649
AB030934.1.1777
New.CleanUp.ReferenceOTU148018
New.CleanUp.ReferenceOTU165926
New.CleanUp.ReferenceOTU193196
D85647.1.1769
New.CleanUp.ReferenceOTU8343
New.CleanUp.ReferenceOTU45041
HQ168398.1.1718
AF079066.1.1745
New.CleanUp.ReferenceOTU9252
New.CleanUp.ReferenceOTU7585
New.CleanUp.ReferenceOTU47899
New.CleanUp.ReferenceOTU5190
New.CleanUp.ReferenceOTU10785
New.CleanUp.ReferenceOTU18737
New.CleanUp.ReferenceOTU64016
AB018142.1.1708
New.CleanUp.ReferenceOTU42588
New.CleanUp.ReferenceOTU81233
New.CleanUp.ReferenceOTU135596
New.CleanUp.ReferenceOTU66447
New.CleanUp.ReferenceOTU60491
New.CleanUp.ReferenceOTU103245
New.CleanUp.ReferenceOTU176047
New.CleanUp.ReferenceOTU132595
New.CleanUp.ReferenceOTU315080
New.CleanUp.ReferenceOTU75010
New.CleanUp.ReferenceOTU244657
New.CleanUp.ReferenceOTU91519
New.CleanUp.ReferenceOTU97484
New.CleanUp.ReferenceOTU133984
New.CleanUp.ReferenceOTU151039
New.CleanUp.ReferenceOTU108301
New.CleanUp.ReferenceOTU183659
New.CleanUp.ReferenceOTU72072
AWXO01000069.4472.6260
New.CleanUp.ReferenceOTU139822
New.CleanUp.ReferenceOTU24225
New.CleanUp.ReferenceOTU127366
New.CleanUp.ReferenceOTU156626
New.CleanUp.ReferenceOTU116259
New.CleanUp.ReferenceOTU81676
New.CleanUp.ReferenceOTU63515
New.CleanUp.ReferenceOTU126248
New.CleanUp.ReferenceOTU152765
New.CleanUp.ReferenceOTU155003
New.CleanUp.ReferenceOTU236458
New.CleanUp.ReferenceOTU232651

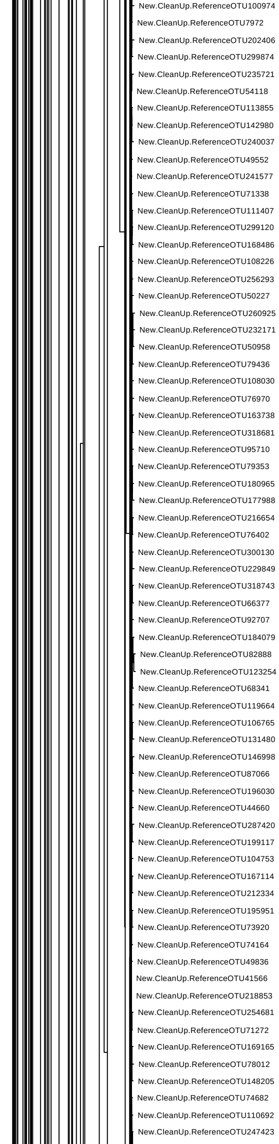
New.CleanUp.ReferenceOTU100974
New.CleanUp.ReferenceOTU7972
New.CleanUp.ReferenceOTU202406
New.CleanUp.ReferenceOTU299874
New.CleanUp.ReferenceOTU235721
New.CleanUp.ReferenceOTU54118
New.CleanUp.ReferenceOTU113855
New.CleanUp.ReferenceOTU142980
New.CleanUp.ReferenceOTU240037
New.CleanUp.ReferenceOTU49552
New.CleanUp.ReferenceOTU241577
New.CleanUp.ReferenceOTU71338
New.CleanUp.ReferenceOTU111407
New.CleanUp.ReferenceOTU299120
New.CleanUp.ReferenceOTU168486
New.CleanUp.ReferenceOTU108226
New.CleanUp.ReferenceOTU256293
New.CleanUp.ReferenceOTU50227
New.CleanUp.ReferenceOTU260925
New.CleanUp.ReferenceOTU232171
New.CleanUp.ReferenceOTU50958
New.CleanUp.ReferenceOTU79436
New.CleanUp.ReferenceOTU108030
New.CleanUp.ReferenceOTU76970
New.CleanUp.ReferenceOTU163738
New.CleanUp.ReferenceOTU318681
New.CleanUp.ReferenceOTU95710
New.CleanUp.ReferenceOTU79353
New.CleanUp.ReferenceOTU180965
New.CleanUp.ReferenceOTU177988
New.CleanUp.ReferenceOTU216654
New.CleanUp.ReferenceOTU76402
New.CleanUp.ReferenceOTU300130
New.CleanUp.ReferenceOTU229849
New.CleanUp.ReferenceOTU318743
New.CleanUp.ReferenceOTU66377
New.CleanUp.ReferenceOTU92707
New.CleanUp.ReferenceOTU184079
New.CleanUp.ReferenceOTU82888
New.CleanUp.ReferenceOTU123254
New.CleanUp.ReferenceOTU68341
New.CleanUp.ReferenceOTU119664
New.CleanUp.ReferenceOTU106765
New.CleanUp.ReferenceOTU131480
New.CleanUp.ReferenceOTU146998
New.CleanUp.ReferenceOTU87066
New.CleanUp.ReferenceOTU196030
New.CleanUp.ReferenceOTU44660
New.CleanUp.ReferenceOTU287420
New.CleanUp.ReferenceOTU199117
New.CleanUp.ReferenceOTU104753
New.CleanUp.ReferenceOTU167114
New.CleanUp.ReferenceOTU212334
New.CleanUp.ReferenceOTU195951
New.CleanUp.ReferenceOTU73920
New.CleanUp.ReferenceOTU74164
New.CleanUp.ReferenceOTU49836
New.CleanUp.ReferenceOTU41566
New.CleanUp.ReferenceOTU218853
New.CleanUp.ReferenceOTU254681
New.CleanUp.ReferenceOTU71272
New.CleanUp.ReferenceOTU169165
New.CleanUp.ReferenceOTU78012
New.CleanUp.ReferenceOTU148205
New.CleanUp.ReferenceOTU74682
New.CleanUp.ReferenceOTU110692
New.CleanUp.ReferenceOTU247423

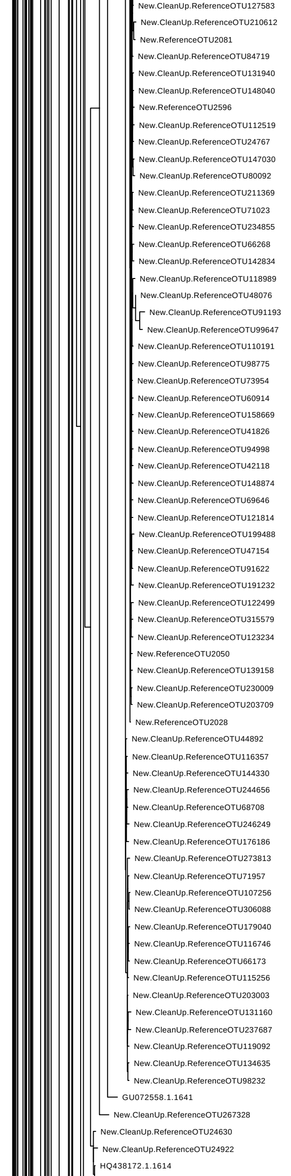
New.CleanUp.ReferenceOTU127583
New.CleanUp.ReferenceOTU210612
New.ReferenceOTU2081
New.CleanUp.ReferenceOTU84719
New.CleanUp.ReferenceOTU131940
New.CleanUp.ReferenceOTU148040
New.ReferenceOTU2596
New.CleanUp.ReferenceOTU112519
New.CleanUp.ReferenceOTU24767
New.CleanUp.ReferenceOTU147030
New.CleanUp.ReferenceOTU80092
New.CleanUp.ReferenceOTU211369
New.CleanUp.ReferenceOTU71023
New.CleanUp.ReferenceOTU234855
New.CleanUp.ReferenceOTU66268
New.CleanUp.ReferenceOTU142834
New.CleanUp.ReferenceOTU118989
New.CleanUp.ReferenceOTU48076
New.CleanUp.ReferenceOTU91193
New.CleanUp.ReferenceOTU99647
New.CleanUp.ReferenceOTU110191
New.CleanUp.ReferenceOTU98775
New.CleanUp.ReferenceOTU73954
New.CleanUp.ReferenceOTU60914
New.CleanUp.ReferenceOTU158669
New.CleanUp.ReferenceOTU41826
New.CleanUp.ReferenceOTU94998
New.CleanUp.ReferenceOTU42118
New.CleanUp.ReferenceOTU148874
New.CleanUp.ReferenceOTU69646
New.CleanUp.ReferenceOTU121814
New.CleanUp.ReferenceOTU199488
New.CleanUp.ReferenceOTU47154
New.CleanUp.ReferenceOTU91622
New.CleanUp.ReferenceOTU191232
New.CleanUp.ReferenceOTU122499
New.CleanUp.ReferenceOTU315579
New.CleanUp.ReferenceOTU123234
New.ReferenceOTU2050
New.CleanUp.ReferenceOTU139158
New.CleanUp.ReferenceOTU230009
New.CleanUp.ReferenceOTU203709
New.ReferenceOTU2028
New.CleanUp.ReferenceOTU44892
New.CleanUp.ReferenceOTU116357
New.CleanUp.ReferenceOTU144330
New.CleanUp.ReferenceOTU244656
New.CleanUp.ReferenceOTU68708
New.CleanUp.ReferenceOTU246249
New.CleanUp.ReferenceOTU176186
New.CleanUp.ReferenceOTU273813
New.CleanUp.ReferenceOTU71957
New.CleanUp.ReferenceOTU107256
New.CleanUp.ReferenceOTU306088
New.CleanUp.ReferenceOTU179040
New.CleanUp.ReferenceOTU116746
New.CleanUp.ReferenceOTU66173
New.CleanUp.ReferenceOTU115256
New.CleanUp.ReferenceOTU203003
New.CleanUp.ReferenceOTU131160
New.CleanUp.ReferenceOTU237687
New.CleanUp.ReferenceOTU119092
New.CleanUp.ReferenceOTU134635
New.CleanUp.ReferenceOTU98232
GU072558.1.1641
New.CleanUp.ReferenceOTU267328
New.CleanUp.ReferenceOTU24630
New.CleanUp.ReferenceOTU24922
HQ438172.1.1614

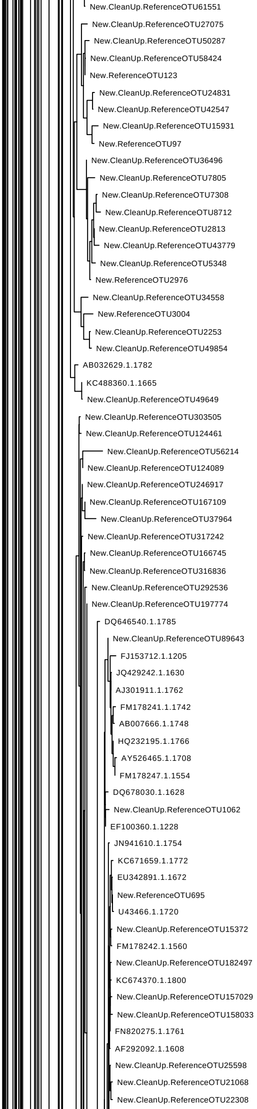
New.CleanUp.ReferenceOTU61551
New.CleanUp.ReferenceOTU27075
New.CleanUp.ReferenceOTU50287
New.CleanUp.ReferenceOTU58424
New.ReferenceOTU123
New.CleanUp.ReferenceOTU24831
New.CleanUp.ReferenceOTU42547
New.CleanUp.ReferenceOTU15931
New.ReferenceOTU97
New.CleanUp.ReferenceOTU36496
New.CleanUp.ReferenceOTU7805
New.CleanUp.ReferenceOTU7308
New.CleanUp.ReferenceOTU8712
New.CleanUp.ReferenceOTU2813
New.CleanUp.ReferenceOTU43779
New.CleanUp.ReferenceOTU5348
New.ReferenceOTU2976
New.CleanUp.ReferenceOTU34558
New.ReferenceOTU3004
New.CleanUp.ReferenceOTU2253
New.CleanUp.ReferenceOTU49854
AB032629.1.1782
KC488360.1.1665
New.CleanUp.ReferenceOTU49649
New.CleanUp.ReferenceOTU303505
New.CleanUp.ReferenceOTU124461
New.CleanUp.ReferenceOTU56214
New.CleanUp.ReferenceOTU124089
New.CleanUp.ReferenceOTU246917
New.CleanUp.ReferenceOTU167109
New.CleanUp.ReferenceOTU37964
New.CleanUp.ReferenceOTU317242
New.CleanUp.ReferenceOTU166745
New.CleanUp.ReferenceOTU316836
New.CleanUp.ReferenceOTU292536
New.CleanUp.ReferenceOTU197774
DQ646540.1.1785
New.CleanUp.ReferenceOTU89643
FJ153712.1.1205
JQ429242.1.1630
AJ301911.1.1762
FM178241.1.1742
AB007666.1.1748
HQ232195.1.1766
AY526465.1.1708
FM178247.1.1554
DQ678030.1.1628
New.CleanUp.ReferenceOTU1062
EF100360.1.1228
JN941610.1.1754
KC671659.1.1772
EU342891.1.1672
New.ReferenceOTU695
U43466.1.1720
New.CleanUp.ReferenceOTU15372
FM178242.1.1560
New.CleanUp.ReferenceOTU182497
KC674370.1.1800
New.CleanUp.ReferenceOTU157029
New.CleanUp.ReferenceOTU158033
FN820275.1.1761
AF292092.1.1608
New.CleanUp.ReferenceOTU25598
New.CleanUp.ReferenceOTU21068
New.CleanUp.ReferenceOTU22308

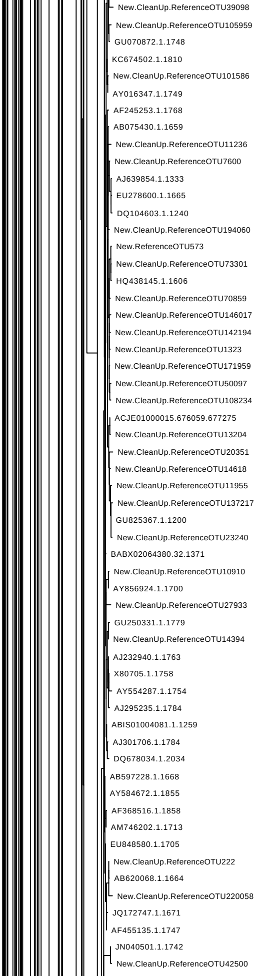
New.CleanUp.ReferenceOTU39098
New.CleanUp.ReferenceOTU105959
GU070872.1.1748
KC674502.1.1810
New.CleanUp.ReferenceOTU101586
AY016347.1.1749
AF245253.1.1768
AB075430.1.1659
New.CleanUp.ReferenceOTU11236
New.CleanUp.ReferenceOTU7600
AJ639854.1.1333
EU278600.1.1665
DQ104603.1.1240
New.CleanUp.ReferenceOTU194060
New.ReferenceOTU573
New.CleanUp.ReferenceOTU73301
HQ438145.1.1606
New.CleanUp.ReferenceOTU70859
New.CleanUp.ReferenceOTU146017
New.CleanUp.ReferenceOTU142194
New.CleanUp.ReferenceOTU1323
New.CleanUp.ReferenceOTU171959
New.CleanUp.ReferenceOTU50097
New.CleanUp.ReferenceOTU108234
ACJE01000015.676059.677275
New.CleanUp.ReferenceOTU13204
New.CleanUp.ReferenceOTU20351
New.CleanUp.ReferenceOTU14618
New.CleanUp.ReferenceOTU11955
New.CleanUp.ReferenceOTU137217
GU825367.1.1200
New.CleanUp.ReferenceOTU23240
BABX02064380.32.1371
New.CleanUp.ReferenceOTU10910
AY856924.1.1700
New.CleanUp.ReferenceOTU27933
GU250331.1.1779
New.CleanUp.ReferenceOTU14394
AJ232940.1.1763
X80705.1.1758
AY554287.1.1754
AJ295235.1.1784
ABIS01004081.1.1259
AJ301706.1.1784
DQ678034.1.2034
AB597228.1.1668
AY584672.1.1855
AF368516.1.1858
AM746202.1.1713
EU848580.1.1705
New.CleanUp.ReferenceOTU222
AB620068.1.1664
New.CleanUp.ReferenceOTU220058
JQ172747.1.1671
AF455135.1.1747
JN040501.1.1742
New.CleanUp.ReferenceOTU42500

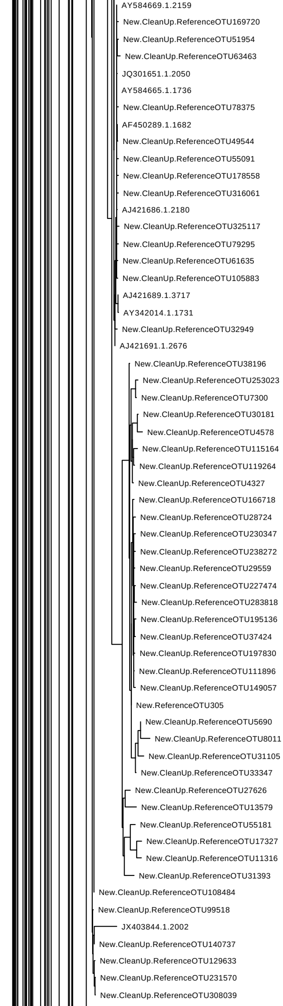
AY584669.1.2159
New.CleanUp.ReferenceOTU169720
New.CleanUp.ReferenceOTU51954
New.CleanUp.ReferenceOTU63463
JQ301651.1.2050
AY584665.1.1736
New.CleanUp.ReferenceOTU78375
AF450289.1.1682
New.CleanUp.ReferenceOTU49544
New.CleanUp.ReferenceOTU55091
New.CleanUp.ReferenceOTU178558
New.CleanUp.ReferenceOTU316061
AJ421686.1.2180
New.CleanUp.ReferenceOTU325117
New.CleanUp.ReferenceOTU79295
New.CleanUp.ReferenceOTU61635
New.CleanUp.ReferenceOTU105883
AJ421689.1.3717
AY342014.1.1731
New.CleanUp.ReferenceOTU32949
AJ421691.1.2676
New.CleanUp.ReferenceOTU38196
New.CleanUp.ReferenceOTU253023
New.CleanUp.ReferenceOTU7300
New.CleanUp.ReferenceOTU30181
New.CleanUp.ReferenceOTU4578
New.CleanUp.ReferenceOTU115164
New.CleanUp.ReferenceOTU119264
New.CleanUp.ReferenceOTU4327
New.CleanUp.ReferenceOTU166718
New.CleanUp.ReferenceOTU28724
New.CleanUp.ReferenceOTU230347
New.CleanUp.ReferenceOTU238272
New.CleanUp.ReferenceOTU29559
New.CleanUp.ReferenceOTU227474
New.CleanUp.ReferenceOTU283818
New.CleanUp.ReferenceOTU195136
New.CleanUp.ReferenceOTU37424
New.CleanUp.ReferenceOTU197830
New.CleanUp.ReferenceOTU111896
New.CleanUp.ReferenceOTU149057
New.ReferenceOTU305
New.CleanUp.ReferenceOTU5690
New.CleanUp.ReferenceOTU8011
New.CleanUp.ReferenceOTU31105
New.CleanUp.ReferenceOTU33347
New.CleanUp.ReferenceOTU27626
New.CleanUp.ReferenceOTU13579
New.CleanUp.ReferenceOTU55181
New.CleanUp.ReferenceOTU17327
New.CleanUp.ReferenceOTU11316
New.CleanUp.ReferenceOTU31393
New.CleanUp.ReferenceOTU108484
New.CleanUp.ReferenceOTU99518
JX403844.1.2002
New.CleanUp.ReferenceOTU140737
New.CleanUp.ReferenceOTU129633
New.CleanUp.ReferenceOTU231570
New.CleanUp.ReferenceOTU308039

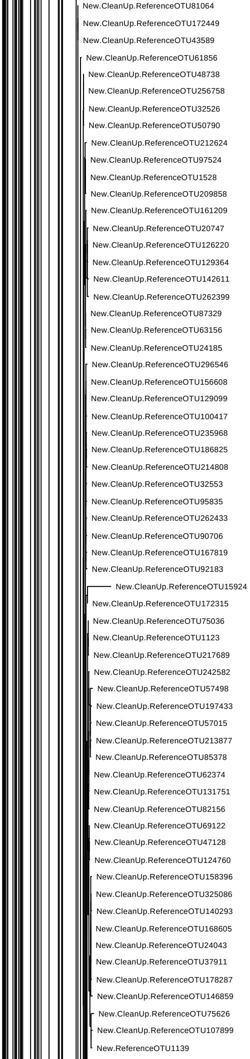
New.CleanUp.ReferenceOTU81064
New.CleanUp.ReferenceOTU172449
New.CleanUp.ReferenceOTU43589
New.CleanUp.ReferenceOTU61856
New.CleanUp.ReferenceOTU48738
New.CleanUp.ReferenceOTU256758
New.CleanUp.ReferenceOTU32526
New.CleanUp.ReferenceOTU50790
New.CleanUp.ReferenceOTU212624
New.CleanUp.ReferenceOTU97524
New.CleanUp.ReferenceOTU1528
New.CleanUp.ReferenceOTU209858
New.CleanUp.ReferenceOTU161209
New.CleanUp.ReferenceOTU20747
New.CleanUp.ReferenceOTU126220
New.CleanUp.ReferenceOTU129364
New.CleanUp.ReferenceOTU142611
New.CleanUp.ReferenceOTU262399
New.CleanUp.ReferenceOTU87329
New.CleanUp.ReferenceOTU63156
New.CleanUp.ReferenceOTU24185
New.CleanUp.ReferenceOTU296546
New.CleanUp.ReferenceOTU156608
New.CleanUp.ReferenceOTU129099
New.CleanUp.ReferenceOTU100417
New.CleanUp.ReferenceOTU235968
New.CleanUp.ReferenceOTU186825
New.CleanUp.ReferenceOTU214808
New.CleanUp.ReferenceOTU32553
New.CleanUp.ReferenceOTU95835
New.CleanUp.ReferenceOTU262433
New.CleanUp.ReferenceOTU90706
New.CleanUp.ReferenceOTU167819
New.CleanUp.ReferenceOTU92183
New.CleanUp.ReferenceOTU15924
New.CleanUp.ReferenceOTU172315
New.CleanUp.ReferenceOTU75036
New.CleanUp.ReferenceOTU1123
New.CleanUp.ReferenceOTU217689
New.CleanUp.ReferenceOTU242582
New.CleanUp.ReferenceOTU57498
New.CleanUp.ReferenceOTU197433
New.CleanUp.ReferenceOTU57015
New.CleanUp.ReferenceOTU213877
New.CleanUp.ReferenceOTU85378
New.CleanUp.ReferenceOTU62374
New.CleanUp.ReferenceOTU131751
New.CleanUp.ReferenceOTU82156
New.CleanUp.ReferenceOTU69122
New.CleanUp.ReferenceOTU47128
New.CleanUp.ReferenceOTU124760
New.CleanUp.ReferenceOTU158396
New.CleanUp.ReferenceOTU325086
New.CleanUp.ReferenceOTU140293
New.CleanUp.ReferenceOTU168605
New.CleanUp.ReferenceOTU24043
New.CleanUp.ReferenceOTU37911
New.CleanUp.ReferenceOTU178287
New.CleanUp.ReferenceOTU146859
New.CleanUp.ReferenceOTU75626
New.CleanUp.ReferenceOTU107899
New.ReferenceOTU1139

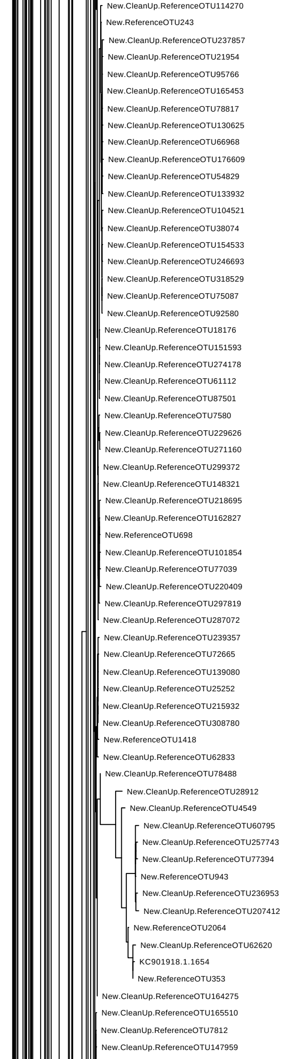
New.CleanUp.ReferenceOTU114270
New.ReferenceOTU243
New.CleanUp.ReferenceOTU237857
New.CleanUp.ReferenceOTU21954
New.CleanUp.ReferenceOTU95766
New.CleanUp.ReferenceOTU165453
New.CleanUp.ReferenceOTU78817
New.CleanUp.ReferenceOTU130625
New.CleanUp.ReferenceOTU66968
New.CleanUp.ReferenceOTU176609
New.CleanUp.ReferenceOTU54829
New.CleanUp.ReferenceOTU133932
New.CleanUp.ReferenceOTU104521
New.CleanUp.ReferenceOTU38074
New.CleanUp.ReferenceOTU154533
New.CleanUp.ReferenceOTU246693
New.CleanUp.ReferenceOTU318529
New.CleanUp.ReferenceOTU75087
New.CleanUp.ReferenceOTU92580
New.CleanUp.ReferenceOTU18176
New.CleanUp.ReferenceOTU151593
New.CleanUp.ReferenceOTU274178
New.CleanUp.ReferenceOTU61112
New.CleanUp.ReferenceOTU87501
New.CleanUp.ReferenceOTU7580
New.CleanUp.ReferenceOTU229626
New.CleanUp.ReferenceOTU271160
New.CleanUp.ReferenceOTU299372
New.CleanUp.ReferenceOTU148321
New.CleanUp.ReferenceOTU218695
New.CleanUp.ReferenceOTU162827
New.ReferenceOTU698
New.CleanUp.ReferenceOTU101854
New.CleanUp.ReferenceOTU77039
New.CleanUp.ReferenceOTU220409
New.CleanUp.ReferenceOTU297819
New.CleanUp.ReferenceOTU287072
New.CleanUp.ReferenceOTU239357
New.CleanUp.ReferenceOTU72665
New.CleanUp.ReferenceOTU139080
New.CleanUp.ReferenceOTU25252
New.CleanUp.ReferenceOTU215932
New.CleanUp.ReferenceOTU308780
New.ReferenceOTU1418
New.CleanUp.ReferenceOTU62833
New.CleanUp.ReferenceOTU78488
New.CleanUp.ReferenceOTU28912
New.CleanUp.ReferenceOTU4549
New.CleanUp.ReferenceOTU60795
New.CleanUp.ReferenceOTU257743
New.CleanUp.ReferenceOTU77394
New.ReferenceOTU943
New.CleanUp.ReferenceOTU236953
New.CleanUp.ReferenceOTU207412
New.ReferenceOTU2064
New.CleanUp.ReferenceOTU62620
KC901918.1.1654
New.ReferenceOTU353
New.CleanUp.ReferenceOTU164275
New.CleanUp.ReferenceOTU165510
New.CleanUp.ReferenceOTU7812
New.CleanUp.ReferenceOTU147959

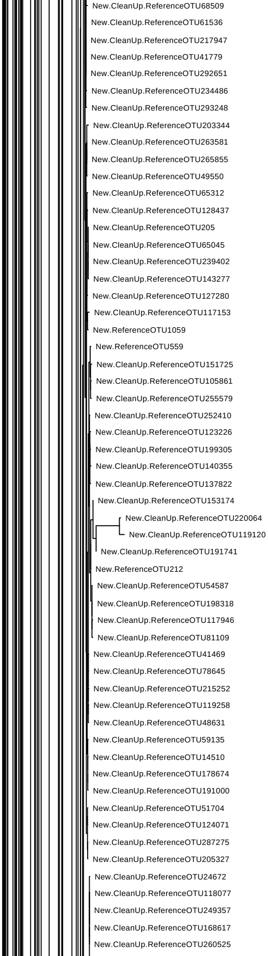
New.CleanUp.ReferenceOTU68509
New.CleanUp.ReferenceOTU61536
New.CleanUp.ReferenceOTU217947
New.CleanUp.ReferenceOTU41779
New.CleanUp.ReferenceOTU292651
New.CleanUp.ReferenceOTU234486
New.CleanUp.ReferenceOTU293248
New.CleanUp.ReferenceOTU203344
New.CleanUp.ReferenceOTU263581
New.CleanUp.ReferenceOTU265855
New.CleanUp.ReferenceOTU49550
New.CleanUp.ReferenceOTU65312
New.CleanUp.ReferenceOTU128437
New.CleanUp.ReferenceOTU205
New.CleanUp.ReferenceOTU65045
New.CleanUp.ReferenceOTU239402
New.CleanUp.ReferenceOTU143277
New.CleanUp.ReferenceOTU127280
New.CleanUp.ReferenceOTU117153
New.ReferenceOTU1059
New.ReferenceOTU559
New.CleanUp.ReferenceOTU151725
New.CleanUp.ReferenceOTU105861
New.CleanUp.ReferenceOTU255579
New.CleanUp.ReferenceOTU252410
New.CleanUp.ReferenceOTU123226
New.CleanUp.ReferenceOTU199305
New.CleanUp.ReferenceOTU140355
New.CleanUp.ReferenceOTU137822
New.CleanUp.ReferenceOTU153174
New.CleanUp.ReferenceOTU220064
New.CleanUp.ReferenceOTU119120
New.CleanUp.ReferenceOTU191741
New.ReferenceOTU212
New.CleanUp.ReferenceOTU54587
New.CleanUp.ReferenceOTU198318
New.CleanUp.ReferenceOTU117946
New.CleanUp.ReferenceOTU81109
New.CleanUp.ReferenceOTU41469
New.CleanUp.ReferenceOTU78645
New.CleanUp.ReferenceOTU215252
New.CleanUp.ReferenceOTU119258
New.CleanUp.ReferenceOTU48631
New.CleanUp.ReferenceOTU59135
New.CleanUp.ReferenceOTU14510
New.CleanUp.ReferenceOTU178674
New.CleanUp.ReferenceOTU191000
New.CleanUp.ReferenceOTU51704
New.CleanUp.ReferenceOTU124071
New.CleanUp.ReferenceOTU287275
New.CleanUp.ReferenceOTU205327
New.CleanUp.ReferenceOTU24672
New.CleanUp.ReferenceOTU118077
New.CleanUp.ReferenceOTU249357
New.CleanUp.ReferenceOTU168617
New.CleanUp.ReferenceOTU260525

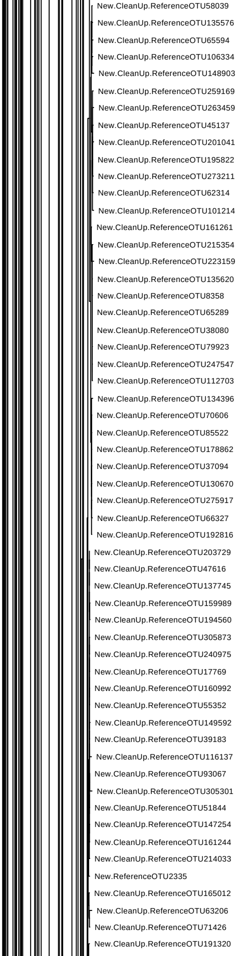
New.CleanUp.ReferenceOTU58039
New.CleanUp.ReferenceOTU135576
New.CleanUp.ReferenceOTU65594
New.CleanUp.ReferenceOTU106334
New.CleanUp.ReferenceOTU148903
New.CleanUp.ReferenceOTU259169
New.CleanUp.ReferenceOTU263459
New.CleanUp.ReferenceOTU45137
New.CleanUp.ReferenceOTU201041
New.CleanUp.ReferenceOTU195822
New.CleanUp.ReferenceOTU273211
New.CleanUp.ReferenceOTU62314
New.CleanUp.ReferenceOTU101214
New.CleanUp.ReferenceOTU161261
New.CleanUp.ReferenceOTU215354
New.CleanUp.ReferenceOTU223159
New.CleanUp.ReferenceOTU135620
New.CleanUp.ReferenceOTU8358
New.CleanUp.ReferenceOTU65289
New.CleanUp.ReferenceOTU38080
New.CleanUp.ReferenceOTU79923
New.CleanUp.ReferenceOTU247547
New.CleanUp.ReferenceOTU112703
New.CleanUp.ReferenceOTU134396
New.CleanUp.ReferenceOTU70606
New.CleanUp.ReferenceOTU85522
New.CleanUp.ReferenceOTU178862
New.CleanUp.ReferenceOTU37094
New.CleanUp.ReferenceOTU130670
New.CleanUp.ReferenceOTU275917
New.CleanUp.ReferenceOTU66327
New.CleanUp.ReferenceOTU192816
New.CleanUp.ReferenceOTU203729
New.CleanUp.ReferenceOTU47616
New.CleanUp.ReferenceOTU137745
New.CleanUp.ReferenceOTU159989
New.CleanUp.ReferenceOTU194560
New.CleanUp.ReferenceOTU305873
New.CleanUp.ReferenceOTU240975
New.CleanUp.ReferenceOTU17769
New.CleanUp.ReferenceOTU160992
New.CleanUp.ReferenceOTU55352
New.CleanUp.ReferenceOTU149592
New.CleanUp.ReferenceOTU39183
New.CleanUp.ReferenceOTU116137
New.CleanUp.ReferenceOTU93067
New.CleanUp.ReferenceOTU305301
New.CleanUp.ReferenceOTU51844
New.CleanUp.ReferenceOTU147254
New.CleanUp.ReferenceOTU161244
New.CleanUp.ReferenceOTU214033
New.ReferenceOTU2335
New.CleanUp.ReferenceOTU165012
New.CleanUp.ReferenceOTU63206
New.CleanUp.ReferenceOTU71426
New.CleanUp.ReferenceOTU191320

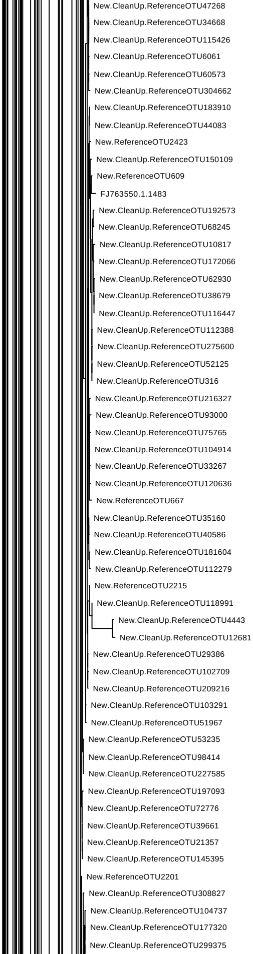
New.CleanUp.ReferenceOTU47268
New.CleanUp.ReferenceOTU34668
New.CleanUp.ReferenceOTU115426
New.CleanUp.ReferenceOTU6061
New.CleanUp.ReferenceOTU60573
New.CleanUp.ReferenceOTU304662
New.CleanUp.ReferenceOTU183910
New.CleanUp.ReferenceOTU44083
New.ReferenceOTU2423
New.CleanUp.ReferenceOTU150109
New.ReferenceOTU609
FJ763550.1.1483
New.CleanUp.ReferenceOTU192573
New.CleanUp.ReferenceOTU68245
New.CleanUp.ReferenceOTU10817
New.CleanUp.ReferenceOTU172066
New.CleanUp.ReferenceOTU62930
New.CleanUp.ReferenceOTU38679
New.CleanUp.ReferenceOTU116447
New.CleanUp.ReferenceOTU112388
New.CleanUp.ReferenceOTU275600
New.CleanUp.ReferenceOTU52125
New.CleanUp.ReferenceOTU316
New.CleanUp.ReferenceOTU216327
New.CleanUp.ReferenceOTU93000
New.CleanUp.ReferenceOTU75765
New.CleanUp.ReferenceOTU104914
New.CleanUp.ReferenceOTU33267
New.CleanUp.ReferenceOTU120636
New.ReferenceOTU667
New.CleanUp.ReferenceOTU35160
New.CleanUp.ReferenceOTU40586
New.CleanUp.ReferenceOTU181604
New.CleanUp.ReferenceOTU112279
New.ReferenceOTU2215
New.CleanUp.ReferenceOTU118991
New.CleanUp.ReferenceOTU4443
New.CleanUp.ReferenceOTU12681
New.CleanUp.ReferenceOTU29386
New.CleanUp.ReferenceOTU102709
New.CleanUp.ReferenceOTU209216
New.CleanUp.ReferenceOTU103291
New.CleanUp.ReferenceOTU51967
New.CleanUp.ReferenceOTU53235
New.CleanUp.ReferenceOTU98414
New.CleanUp.ReferenceOTU227585
New.CleanUp.ReferenceOTU197093
New.CleanUp.ReferenceOTU72776
New.CleanUp.ReferenceOTU39661
New.CleanUp.ReferenceOTU21357
New.CleanUp.ReferenceOTU145395
New.ReferenceOTU2201
New.CleanUp.ReferenceOTU308827
New.CleanUp.ReferenceOTU104737
New.CleanUp.ReferenceOTU177320
New.CleanUp.ReferenceOTU299375

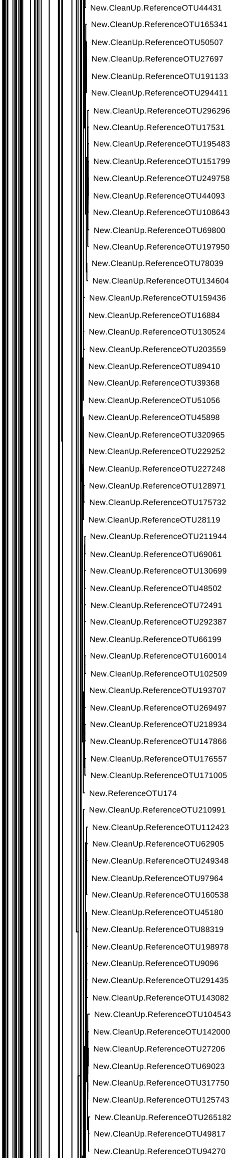
New.CleanUp.ReferenceOTU44431
New.CleanUp.ReferenceOTU165341
New.CleanUp.ReferenceOTU50507
New.CleanUp.ReferenceOTU27697
New.CleanUp.ReferenceOTU191133
New.CleanUp.ReferenceOTU294411
New.CleanUp.ReferenceOTU296296
New.CleanUp.ReferenceOTU17531
New.CleanUp.ReferenceOTU195483
New.CleanUp.ReferenceOTU151799
New.CleanUp.ReferenceOTU249758
New.CleanUp.ReferenceOTU44093
New.CleanUp.ReferenceOTU108643
New.CleanUp.ReferenceOTU69800
New.CleanUp.ReferenceOTU197950
New.CleanUp.ReferenceOTU78039
New.CleanUp.ReferenceOTU134604
New.CleanUp.ReferenceOTU159436
New.CleanUp.ReferenceOTU16884
New.CleanUp.ReferenceOTU130524
New.CleanUp.ReferenceOTU203559
New.CleanUp.ReferenceOTU89410
New.CleanUp.ReferenceOTU39368
New.CleanUp.ReferenceOTU51056
New.CleanUp.ReferenceOTU45898
New.CleanUp.ReferenceOTU320965
New.CleanUp.ReferenceOTU229252
New.CleanUp.ReferenceOTU227248
New.CleanUp.ReferenceOTU128971
New.CleanUp.ReferenceOTU175732
New.CleanUp.ReferenceOTU28119
New.CleanUp.ReferenceOTU211944
New.CleanUp.ReferenceOTU69061
New.CleanUp.ReferenceOTU130699
New.CleanUp.ReferenceOTU48502
New.CleanUp.ReferenceOTU72491
New.CleanUp.ReferenceOTU292387
New.CleanUp.ReferenceOTU66199
New.CleanUp.ReferenceOTU160014
New.CleanUp.ReferenceOTU102509
New.CleanUp.ReferenceOTU193707
New.CleanUp.ReferenceOTU269497
New.CleanUp.ReferenceOTU218934
New.CleanUp.ReferenceOTU147866
New.CleanUp.ReferenceOTU176557
New.CleanUp.ReferenceOTU171005
New.ReferenceOTU174
New.CleanUp.ReferenceOTU210991
New.CleanUp.ReferenceOTU112423
New.CleanUp.ReferenceOTU62905
New.CleanUp.ReferenceOTU249348
New.CleanUp.ReferenceOTU97964
New.CleanUp.ReferenceOTU160538
New.CleanUp.ReferenceOTU45180
New.CleanUp.ReferenceOTU88319
New.CleanUp.ReferenceOTU198978
New.CleanUp.ReferenceOTU9096
New.CleanUp.ReferenceOTU291435
New.CleanUp.ReferenceOTU143082
New.CleanUp.ReferenceOTU104543
New.CleanUp.ReferenceOTU142000
New.CleanUp.ReferenceOTU27206
New.CleanUp.ReferenceOTU69023
New.CleanUp.ReferenceOTU317750
New.CleanUp.ReferenceOTU125743
New.CleanUp.ReferenceOTU265182
New.CleanUp.ReferenceOTU49817
New.CleanUp.ReferenceOTU94270

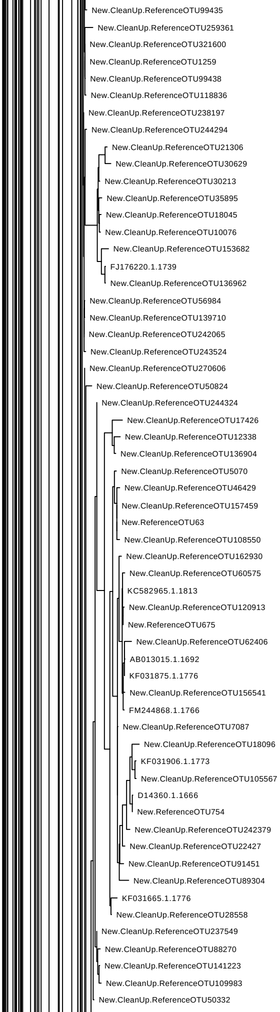
New.CleanUp.ReferenceOTU99435
New.CleanUp.ReferenceOTU259361
New.CleanUp.ReferenceOTU321600
New.CleanUp.ReferenceOTU1259
New.CleanUp.ReferenceOTU99438
New.CleanUp.ReferenceOTU118836
New.CleanUp.ReferenceOTU238197
New.CleanUp.ReferenceOTU244294
New.CleanUp.ReferenceOTU21306
New.CleanUp.ReferenceOTU30629
New.CleanUp.ReferenceOTU30213
New.CleanUp.ReferenceOTU35895
New.CleanUp.ReferenceOTU18045
New.CleanUp.ReferenceOTU10076
New.CleanUp.ReferenceOTU153682
FJ176220.1.1739
New.CleanUp.ReferenceOTU136962
New.CleanUp.ReferenceOTU56984
New.CleanUp.ReferenceOTU139710
New.CleanUp.ReferenceOTU242065
New.CleanUp.ReferenceOTU243524
New.CleanUp.ReferenceOTU270606
New.CleanUp.ReferenceOTU50824
New.CleanUp.ReferenceOTU244324
New.CleanUp.ReferenceOTU17426
New.CleanUp.ReferenceOTU12338
New.CleanUp.ReferenceOTU136904
New.CleanUp.ReferenceOTU5070
New.CleanUp.ReferenceOTU46429
New.CleanUp.ReferenceOTU157459
New.ReferenceOTU63
New.CleanUp.ReferenceOTU108550
New.CleanUp.ReferenceOTU162930
New.CleanUp.ReferenceOTU60575
KC582965.1.1813
New.CleanUp.ReferenceOTU120913
New.ReferenceOTU675
New.CleanUp.ReferenceOTU62406
AB013015.1.1692
KF031875.1.1776
New.CleanUp.ReferenceOTU156541
FM244868.1.1766
New.CleanUp.ReferenceOTU7087
New.CleanUp.ReferenceOTU18096
KF031906.1.1773
New.CleanUp.ReferenceOTU105567
D14360.1.1666
New.ReferenceOTU754
New.CleanUp.ReferenceOTU242379
New.CleanUp.ReferenceOTU22427
New.CleanUp.ReferenceOTU91451
New.CleanUp.ReferenceOTU89304
KF031665.1.1776
New.CleanUp.ReferenceOTU28558
New.CleanUp.ReferenceOTU237549
New.CleanUp.ReferenceOTU88270
New.CleanUp.ReferenceOTU141223
New.CleanUp.ReferenceOTU109983
New.CleanUp.ReferenceOTU50332

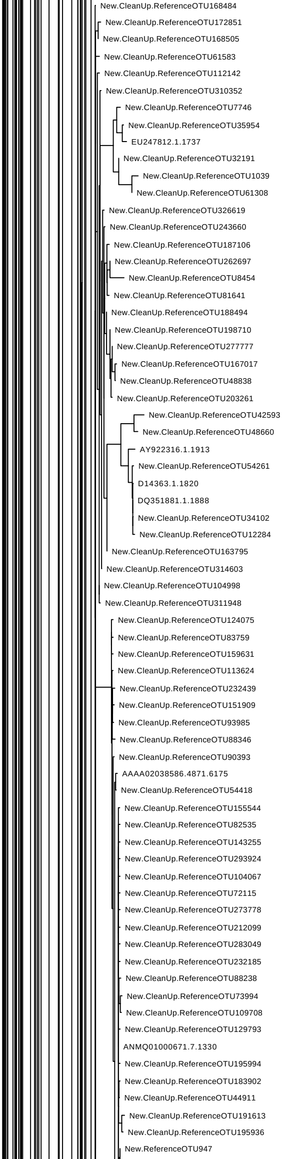
New.CleanUp.ReferenceOTU168484
New.CleanUp.ReferenceOTU172851
New.CleanUp.ReferenceOTU168505
New.CleanUp.ReferenceOTU61583
New.CleanUp.ReferenceOTU112142
New.CleanUp.ReferenceOTU310352
New.CleanUp.ReferenceOTU7746
New.CleanUp.ReferenceOTU35954
EU247812.1.1737
New.CleanUp.ReferenceOTU32191
New.CleanUp.ReferenceOTU1039
New.CleanUp.ReferenceOTU61308
New.CleanUp.ReferenceOTU326619
New.CleanUp.ReferenceOTU243660
New.CleanUp.ReferenceOTU187106
New.CleanUp.ReferenceOTU262697
New.CleanUp.ReferenceOTU8454
New.CleanUp.ReferenceOTU81641
New.CleanUp.ReferenceOTU188494
New.CleanUp.ReferenceOTU198710
New.CleanUp.ReferenceOTU277777
New.CleanUp.ReferenceOTU167017
New.CleanUp.ReferenceOTU48838
New.CleanUp.ReferenceOTU203261
New.CleanUp.ReferenceOTU42593
New.CleanUp.ReferenceOTU48660
AY922316.1.1913
New.CleanUp.ReferenceOTU54261
D14363.1.1820
DQ351881.1.1888
New.CleanUp.ReferenceOTU34102
New.CleanUp.ReferenceOTU12284
New.CleanUp.ReferenceOTU163795
New.CleanUp.ReferenceOTU314603
New.CleanUp.ReferenceOTU104998
New.CleanUp.ReferenceOTU311948
New.CleanUp.ReferenceOTU124075
New.CleanUp.ReferenceOTU83759
New.CleanUp.ReferenceOTU159631
New.CleanUp.ReferenceOTU113624
New.CleanUp.ReferenceOTU232439
New.CleanUp.ReferenceOTU151909
New.CleanUp.ReferenceOTU93985
New.CleanUp.ReferenceOTU88346
New.CleanUp.ReferenceOTU90393
AAAA02038586.4871.6175
New.CleanUp.ReferenceOTU54418
New.CleanUp.ReferenceOTU155544
New.CleanUp.ReferenceOTU82535
New.CleanUp.ReferenceOTU143255
New.CleanUp.ReferenceOTU293924
New.CleanUp.ReferenceOTU104067
New.CleanUp.ReferenceOTU72115
New.CleanUp.ReferenceOTU273778
New.CleanUp.ReferenceOTU212099
New.CleanUp.ReferenceOTU283049
New.CleanUp.ReferenceOTU232185
New.CleanUp.ReferenceOTU88238
New.CleanUp.ReferenceOTU73994
New.CleanUp.ReferenceOTU109708
New.CleanUp.ReferenceOTU129793
ANMQ01000671.7.1330
New.CleanUp.ReferenceOTU195994
New.CleanUp.ReferenceOTU183902
New.CleanUp.ReferenceOTU44911
New.CleanUp.ReferenceOTU191613
New.CleanUp.ReferenceOTU195936
New.ReferenceOTU947

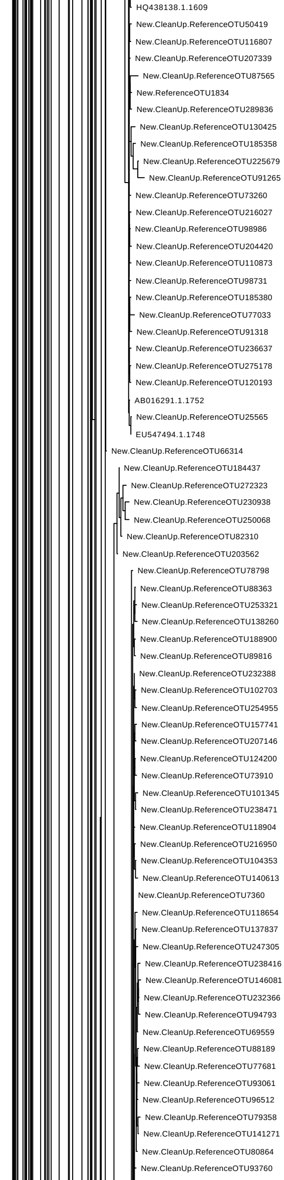
HQ438138.1.1609
New.CleanUp.ReferenceOTU50419
New.CleanUp.ReferenceOTU116807
New.CleanUp.ReferenceOTU207339
New.CleanUp.ReferenceOTU87565
New.ReferenceOTU1834
New.CleanUp.ReferenceOTU289836
New.CleanUp.ReferenceOTU130425
New.CleanUp.ReferenceOTU185358
New.CleanUp.ReferenceOTU225679
New.CleanUp.ReferenceOTU91265
New.CleanUp.ReferenceOTU73260
New.CleanUp.ReferenceOTU216027
New.CleanUp.ReferenceOTU98986
New.CleanUp.ReferenceOTU204420
New.CleanUp.ReferenceOTU110873
New.CleanUp.ReferenceOTU98731
New.CleanUp.ReferenceOTU185380
New.CleanUp.ReferenceOTU77033
New.CleanUp.ReferenceOTU91318
New.CleanUp.ReferenceOTU236637
New.CleanUp.ReferenceOTU275178
New.CleanUp.ReferenceOTU120193
AB016291.1.1752
New.CleanUp.ReferenceOTU25565
EU547494.1.1748
New.CleanUp.ReferenceOTU66314
New.CleanUp.ReferenceOTU184437
New.CleanUp.ReferenceOTU272323
New.CleanUp.ReferenceOTU230938
New.CleanUp.ReferenceOTU250068
New.CleanUp.ReferenceOTU82310
New.CleanUp.ReferenceOTU203562
New.CleanUp.ReferenceOTU78798
New.CleanUp.ReferenceOTU88363
New.CleanUp.ReferenceOTU253321
New.CleanUp.ReferenceOTU138260
New.CleanUp.ReferenceOTU188900
New.CleanUp.ReferenceOTU89816
New.CleanUp.ReferenceOTU232388
New.CleanUp.ReferenceOTU102703
New.CleanUp.ReferenceOTU254955
New.CleanUp.ReferenceOTU157741
New.CleanUp.ReferenceOTU207146
New.CleanUp.ReferenceOTU124200
New.CleanUp.ReferenceOTU73910
New.CleanUp.ReferenceOTU101345
New.CleanUp.ReferenceOTU238471
New.CleanUp.ReferenceOTU118904
New.CleanUp.ReferenceOTU216950
New.CleanUp.ReferenceOTU104353
New.CleanUp.ReferenceOTU140613
New.CleanUp.ReferenceOTU7360
New.CleanUp.ReferenceOTU118654
New.CleanUp.ReferenceOTU137837
New.CleanUp.ReferenceOTU247305
New.CleanUp.ReferenceOTU238416
New.CleanUp.ReferenceOTU146081
New.CleanUp.ReferenceOTU232366
New.CleanUp.ReferenceOTU94793
New.CleanUp.ReferenceOTU69559
New.CleanUp.ReferenceOTU88189
New.CleanUp.ReferenceOTU77681
New.CleanUp.ReferenceOTU93061
New.CleanUp.ReferenceOTU96512
New.CleanUp.ReferenceOTU79358
New.CleanUp.ReferenceOTU141271
New.CleanUp.ReferenceOTU80864
New.CleanUp.ReferenceOTU93760

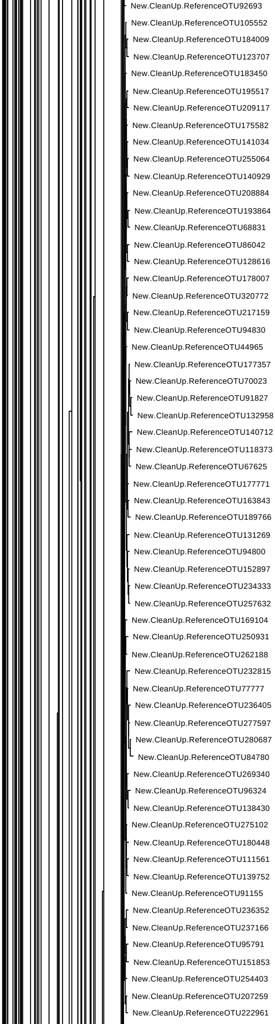
New.CleanUp.ReferenceOTU92693
New.CleanUp.ReferenceOTU105552
New.CleanUp.ReferenceOTU184009
New.CleanUp.ReferenceOTU123707
New.CleanUp.ReferenceOTU183450
New.CleanUp.ReferenceOTU195517
New.CleanUp.ReferenceOTU209117
New.CleanUp.ReferenceOTU175582
New.CleanUp.ReferenceOTU141034
New.CleanUp.ReferenceOTU255064
New.CleanUp.ReferenceOTU140929
New.CleanUp.ReferenceOTU208884
New.CleanUp.ReferenceOTU193864
New.CleanUp.ReferenceOTU68831
New.CleanUp.ReferenceOTU86042
New.CleanUp.ReferenceOTU128616
New.CleanUp.ReferenceOTU178007
New.CleanUp.ReferenceOTU320772
New.CleanUp.ReferenceOTU217159
New.CleanUp.ReferenceOTU94830
New.CleanUp.ReferenceOTU44965
New.CleanUp.ReferenceOTU177357
New.CleanUp.ReferenceOTU70023
New.CleanUp.ReferenceOTU91827
New.CleanUp.ReferenceOTU132958
New.CleanUp.ReferenceOTU140712
New.CleanUp.ReferenceOTU118373
New.CleanUp.ReferenceOTU67625
New.CleanUp.ReferenceOTU177771
New.CleanUp.ReferenceOTU163843
New.CleanUp.ReferenceOTU189766
New.CleanUp.ReferenceOTU131269
New.CleanUp.ReferenceOTU94800
New.CleanUp.ReferenceOTU152897
New.CleanUp.ReferenceOTU234333
New.CleanUp.ReferenceOTU257632
New.CleanUp.ReferenceOTU169104
New.CleanUp.ReferenceOTU250931
New.CleanUp.ReferenceOTU262188
New.CleanUp.ReferenceOTU232815
New.CleanUp.ReferenceOTU77777
New.CleanUp.ReferenceOTU236405
New.CleanUp.ReferenceOTU277597
New.CleanUp.ReferenceOTU280687
New.CleanUp.ReferenceOTU84780
New.CleanUp.ReferenceOTU269340
New.CleanUp.ReferenceOTU96324
New.CleanUp.ReferenceOTU138430
New.CleanUp.ReferenceOTU275102
New.CleanUp.ReferenceOTU180448
New.CleanUp.ReferenceOTU111561
New.CleanUp.ReferenceOTU139752
New.CleanUp.ReferenceOTU91155
New.CleanUp.ReferenceOTU236352
New.CleanUp.ReferenceOTU237166
New.CleanUp.ReferenceOTU95791
New.CleanUp.ReferenceOTU151853
New.CleanUp.ReferenceOTU254403
New.CleanUp.ReferenceOTU207259
New.CleanUp.ReferenceOTU222961

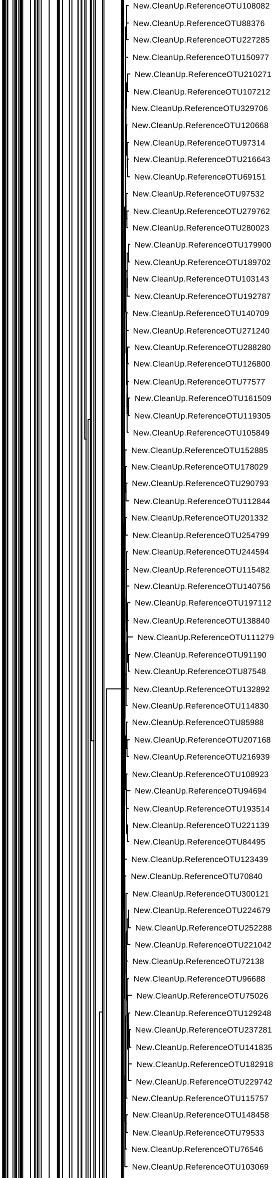
New.CleanUp.ReferenceOTU108082
New.CleanUp.ReferenceOTU88376
New.CleanUp.ReferenceOTU227285
New.CleanUp.ReferenceOTU150977
New.CleanUp.ReferenceOTU210271
New.CleanUp.ReferenceOTU107212
New.CleanUp.ReferenceOTU329706
New.CleanUp.ReferenceOTU120668
New.CleanUp.ReferenceOTU97314
New.CleanUp.ReferenceOTU216643
New.CleanUp.ReferenceOTU69151
New.CleanUp.ReferenceOTU97532
New.CleanUp.ReferenceOTU279762
New.CleanUp.ReferenceOTU280023
New.CleanUp.ReferenceOTU179900
New.CleanUp.ReferenceOTU189702
New.CleanUp.ReferenceOTU103143
New.CleanUp.ReferenceOTU192787
New.CleanUp.ReferenceOTU140709
New.CleanUp.ReferenceOTU271240
New.CleanUp.ReferenceOTU288280
New.CleanUp.ReferenceOTU126800
New.CleanUp.ReferenceOTU77577
New.CleanUp.ReferenceOTU161509
New.CleanUp.ReferenceOTU119305
New.CleanUp.ReferenceOTU105849
New.CleanUp.ReferenceOTU152885
New.CleanUp.ReferenceOTU178029
New.CleanUp.ReferenceOTU290793
New.CleanUp.ReferenceOTU112844
New.CleanUp.ReferenceOTU201332
New.CleanUp.ReferenceOTU254799
New.CleanUp.ReferenceOTU244594
New.CleanUp.ReferenceOTU115482
New.CleanUp.ReferenceOTU140756
New.CleanUp.ReferenceOTU197112
New.CleanUp.ReferenceOTU138840
New.CleanUp.ReferenceOTU111279
New.CleanUp.ReferenceOTU91190
New.CleanUp.ReferenceOTU87548
New.CleanUp.ReferenceOTU132892
New.CleanUp.ReferenceOTU114830
New.CleanUp.ReferenceOTU85988
New.CleanUp.ReferenceOTU207168
New.CleanUp.ReferenceOTU216939
New.CleanUp.ReferenceOTU108923
New.CleanUp.ReferenceOTU94694
New.CleanUp.ReferenceOTU193514
New.CleanUp.ReferenceOTU221139
New.CleanUp.ReferenceOTU84495
New.CleanUp.ReferenceOTU123439
New.CleanUp.ReferenceOTU70840
New.CleanUp.ReferenceOTU300121
New.CleanUp.ReferenceOTU224679
New.CleanUp.ReferenceOTU252288
New.CleanUp.ReferenceOTU221042
New.CleanUp.ReferenceOTU72138
New.CleanUp.ReferenceOTU96688
New.CleanUp.ReferenceOTU75026
New.CleanUp.ReferenceOTU129248
New.CleanUp.ReferenceOTU237281
New.CleanUp.ReferenceOTU141835
New.CleanUp.ReferenceOTU182918
New.CleanUp.ReferenceOTU229742
New.CleanUp.ReferenceOTU115757
New.CleanUp.ReferenceOTU148458
New.CleanUp.ReferenceOTU79533
New.CleanUp.ReferenceOTU76546
New.CleanUp.ReferenceOTU103069

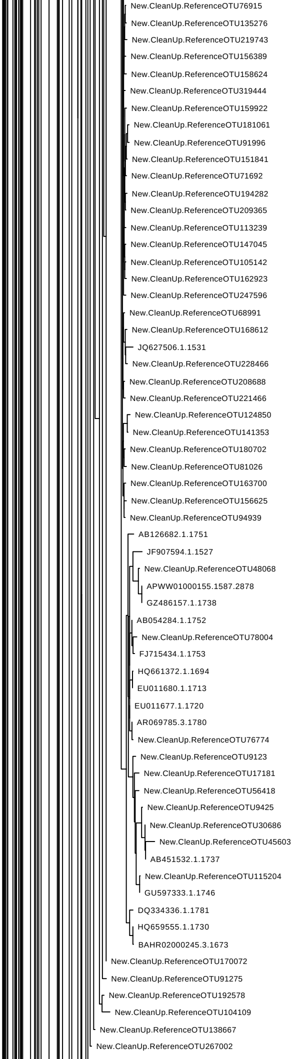
New.CleanUp.ReferenceOTU76915
New.CleanUp.ReferenceOTU135276
New.CleanUp.ReferenceOTU219743
New.CleanUp.ReferenceOTU156389
New.CleanUp.ReferenceOTU158624
New.CleanUp.ReferenceOTU319444
New.CleanUp.ReferenceOTU159922
New.CleanUp.ReferenceOTU181061
New.CleanUp.ReferenceOTU91996
New.CleanUp.ReferenceOTU151841
New.CleanUp.ReferenceOTU71692
New.CleanUp.ReferenceOTU194282
New.CleanUp.ReferenceOTU209365
New.CleanUp.ReferenceOTU113239
New.CleanUp.ReferenceOTU147045
New.CleanUp.ReferenceOTU105142
New.CleanUp.ReferenceOTU162923
New.CleanUp.ReferenceOTU247596
New.CleanUp.ReferenceOTU68991
New.CleanUp.ReferenceOTU168612
JQ627506.1.1531
New.CleanUp.ReferenceOTU228466
New.CleanUp.ReferenceOTU208688
New.CleanUp.ReferenceOTU221466
New.CleanUp.ReferenceOTU124850
New.CleanUp.ReferenceOTU141353
New.CleanUp.ReferenceOTU180702
New.CleanUp.ReferenceOTU81026
New.CleanUp.ReferenceOTU163700
New.CleanUp.ReferenceOTU156625
New.CleanUp.ReferenceOTU94939
AB126682.1.1751
JF907594.1.1527
New.CleanUp.ReferenceOTU48068
APWW01000155.1587.2878
GZ486157.1.1738
AB054284.1.1752
New.CleanUp.ReferenceOTU78004
FJ715434.1.1753
HQ661372.1.1694
EU011680.1.1713
EU011677.1.1720
AR069785.3.1780
New.CleanUp.ReferenceOTU76774
New.CleanUp.ReferenceOTU9123
New.CleanUp.ReferenceOTU17181
New.CleanUp.ReferenceOTU56418
New.CleanUp.ReferenceOTU9425
New.CleanUp.ReferenceOTU30686
New.CleanUp.ReferenceOTU45603
AB451532.1.1737
New.CleanUp.ReferenceOTU115204
GU597333.1.1746
DQ334336.1.1781
HQ659555.1.1730
BAHR02000245.3.1673
New.CleanUp.ReferenceOTU170072
New.CleanUp.ReferenceOTU91275
New.CleanUp.ReferenceOTU192578
New.CleanUp.ReferenceOTU104109
New.CleanUp.ReferenceOTU138667
New.CleanUp.ReferenceOTU267002

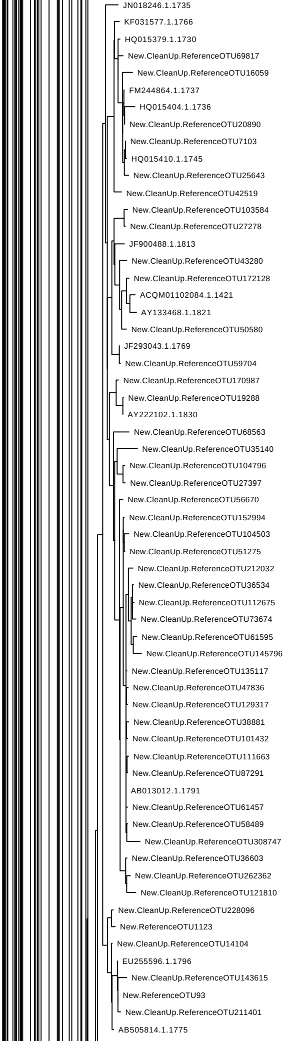
JN018246.1.1735
KF031577.1.1766
HQ015379.1.1730
New.CleanUp.ReferenceOTU69817
New.CleanUp.ReferenceOTU16059
FM244864.1.1737
HQ015404.1.1736
New.CleanUp.ReferenceOTU20890
New.CleanUp.ReferenceOTU7103
HQ015410.1.1745
New.CleanUp.ReferenceOTU25643
New.CleanUp.ReferenceOTU42519
New.CleanUp.ReferenceOTU103584
New.CleanUp.ReferenceOTU27278
JF900488.1.1813
New.CleanUp.ReferenceOTU43280
New.CleanUp.ReferenceOTU172128
ACQM01102084.1.1421
AY133468.1.1821
New.CleanUp.ReferenceOTU50580
JF293043.1.1769
New.CleanUp.ReferenceOTU59704
New.CleanUp.ReferenceOTU170987
New.CleanUp.ReferenceOTU19288
AY222102.1.1830
New.CleanUp.ReferenceOTU68563
New.CleanUp.ReferenceOTU35140
New.CleanUp.ReferenceOTU104796
New.CleanUp.ReferenceOTU27397
New.CleanUp.ReferenceOTU56670
New.CleanUp.ReferenceOTU152994
New.CleanUp.ReferenceOTU104503
New.CleanUp.ReferenceOTU51275
New.CleanUp.ReferenceOTU212032
New.CleanUp.ReferenceOTU36534
New.CleanUp.ReferenceOTU112675
New.CleanUp.ReferenceOTU73674
New.CleanUp.ReferenceOTU61595
New.CleanUp.ReferenceOTU145796
New.CleanUp.ReferenceOTU135117
New.CleanUp.ReferenceOTU47836
New.CleanUp.ReferenceOTU129317
New.CleanUp.ReferenceOTU38881
New.CleanUp.ReferenceOTU101432
New.CleanUp.ReferenceOTU111663
New.CleanUp.ReferenceOTU87291
AB013012.1.1791
New.CleanUp.ReferenceOTU61457
New.CleanUp.ReferenceOTU58489
New.CleanUp.ReferenceOTU308747
New.CleanUp.ReferenceOTU36603
New.CleanUp.ReferenceOTU262362
New.CleanUp.ReferenceOTU121810
New.CleanUp.ReferenceOTU228096
New.ReferenceOTU1123
New.CleanUp.ReferenceOTU14104
EU255596.1.1796
New.CleanUp.ReferenceOTU143615
New.ReferenceOTU93
New.CleanUp.ReferenceOTU211401
AB505814.1.1775

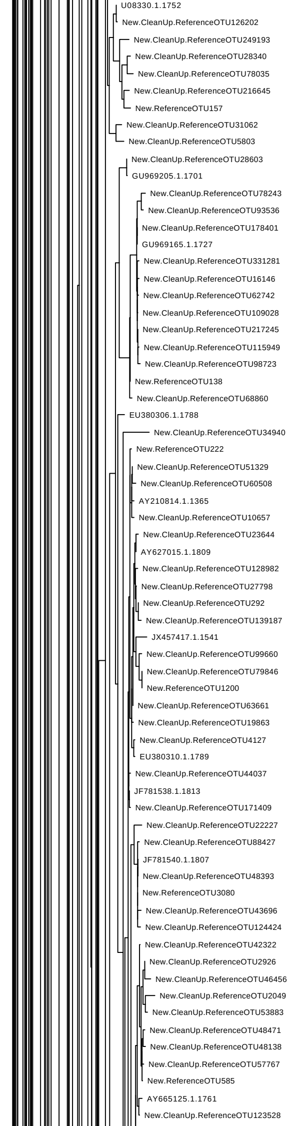
U08330.1.1752
New.CleanUp.ReferenceOTU126202
New.CleanUp.ReferenceOTU249193
New.CleanUp.ReferenceOTU28340
New.CleanUp.ReferenceOTU78035
New.CleanUp.ReferenceOTU216645
New.ReferenceOTU157
New.CleanUp.ReferenceOTU31062
New.CleanUp.ReferenceOTU5803
New.CleanUp.ReferenceOTU28603
GU969205.1.1701
New.CleanUp.ReferenceOTU78243
New.CleanUp.ReferenceOTU93536
New.CleanUp.ReferenceOTU178401
GU969165.1.1727
New.CleanUp.ReferenceOTU331281
New.CleanUp.ReferenceOTU16146
New.CleanUp.ReferenceOTU62742
New.CleanUp.ReferenceOTU109028
New.CleanUp.ReferenceOTU217245
New.CleanUp.ReferenceOTU115949
New.CleanUp.ReferenceOTU98723
New.ReferenceOTU138
New.CleanUp.ReferenceOTU68860
EU380306.1.1788
New.CleanUp.ReferenceOTU34940
New.ReferenceOTU222
New.CleanUp.ReferenceOTU51329
New.CleanUp.ReferenceOTU60508
AY210814.1.1365
New.CleanUp.ReferenceOTU10657
New.CleanUp.ReferenceOTU23644
AY627015.1.1809
New.CleanUp.ReferenceOTU128982
New.CleanUp.ReferenceOTU27798
New.CleanUp.ReferenceOTU292
New.CleanUp.ReferenceOTU139187
JX457417.1.1541
New.CleanUp.ReferenceOTU99660
New.CleanUp.ReferenceOTU79846
New.ReferenceOTU1200
New.CleanUp.ReferenceOTU63661
New.CleanUp.ReferenceOTU19863
New.CleanUp.ReferenceOTU4127
EU380310.1.1789
New.CleanUp.ReferenceOTU44037
JF781538.1.1813
New.CleanUp.ReferenceOTU171409
New.CleanUp.ReferenceOTU22227
New.CleanUp.ReferenceOTU88427
JF781540.1.1807
New.CleanUp.ReferenceOTU48393
New.ReferenceOTU3080
New.CleanUp.ReferenceOTU43696
New.CleanUp.ReferenceOTU124424
New.CleanUp.ReferenceOTU42322
New.CleanUp.ReferenceOTU2926
New.CleanUp.ReferenceOTU46456
New.CleanUp.ReferenceOTU2049
New.CleanUp.ReferenceOTU53883
New.CleanUp.ReferenceOTU48471
New.CleanUp.ReferenceOTU48138
New.CleanUp.ReferenceOTU57767
New.ReferenceOTU585
AY665125.1.1761
New.CleanUp.ReferenceOTU123528

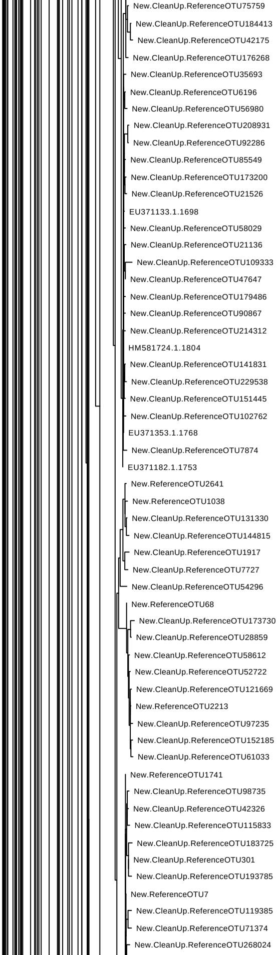
New.CleanUp.ReferenceOTU75759
New.CleanUp.ReferenceOTU184413
New.CleanUp.ReferenceOTU42175
New.CleanUp.ReferenceOTU176268
New.CleanUp.ReferenceOTU35693
New.CleanUp.ReferenceOTU6196
New.CleanUp.ReferenceOTU56980
New.CleanUp.ReferenceOTU208931
New.CleanUp.ReferenceOTU92286
New.CleanUp.ReferenceOTU85549
New.CleanUp.ReferenceOTU173200
New.CleanUp.ReferenceOTU21526
EU371133.1.1698
New.CleanUp.ReferenceOTU58029
New.CleanUp.ReferenceOTU21136
New.CleanUp.ReferenceOTU109333
New.CleanUp.ReferenceOTU47647
New.CleanUp.ReferenceOTU179486
New.CleanUp.ReferenceOTU90867
New.CleanUp.ReferenceOTU214312
HM581724.1.1804
New.CleanUp.ReferenceOTU141831
New.CleanUp.ReferenceOTU229538
New.CleanUp.ReferenceOTU151445
New.CleanUp.ReferenceOTU102762
EU371353.1.1768
New.CleanUp.ReferenceOTU7874
EU371182.1.1753
New.ReferenceOTU2641
New.ReferenceOTU1038
New.CleanUp.ReferenceOTU131330
New.CleanUp.ReferenceOTU144815
New.CleanUp.ReferenceOTU1917
New.CleanUp.ReferenceOTU7727
New.CleanUp.ReferenceOTU54296
New.ReferenceOTU68
New.CleanUp.ReferenceOTU173730
New.CleanUp.ReferenceOTU28859
New.CleanUp.ReferenceOTU58612
New.CleanUp.ReferenceOTU52722
New.CleanUp.ReferenceOTU121669
New.ReferenceOTU2213
New.CleanUp.ReferenceOTU97235
New.CleanUp.ReferenceOTU152185
New.CleanUp.ReferenceOTU61033
New.ReferenceOTU1741
New.CleanUp.ReferenceOTU98735
New.CleanUp.ReferenceOTU42326
New.CleanUp.ReferenceOTU115833
New.CleanUp.ReferenceOTU183725
New.CleanUp.ReferenceOTU301
New.CleanUp.ReferenceOTU193785
New.ReferenceOTU7
New.CleanUp.ReferenceOTU119385
New.CleanUp.ReferenceOTU71374
New.CleanUp.ReferenceOTU268024

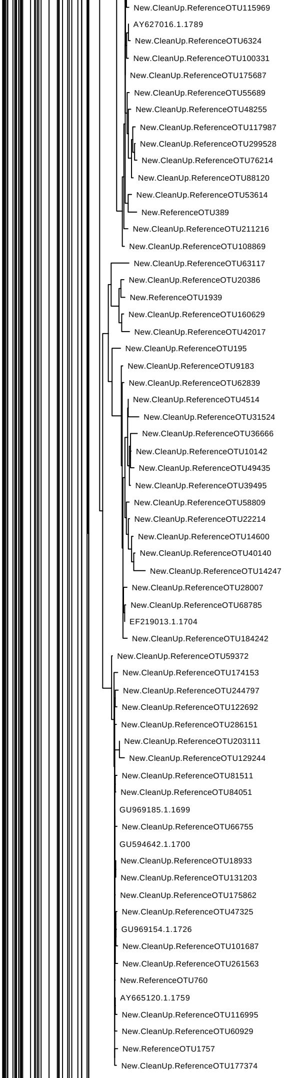
New.CleanUp.ReferenceOTU115969
AY627016.1.1789
New.CleanUp.ReferenceOTU6324
New.CleanUp.ReferenceOTU100331
New.CleanUp.ReferenceOTU175687
New.CleanUp.ReferenceOTU55689
New.CleanUp.ReferenceOTU48255
New.CleanUp.ReferenceOTU117987
New.CleanUp.ReferenceOTU299528
New.CleanUp.ReferenceOTU76214
New.CleanUp.ReferenceOTU88120
New.CleanUp.ReferenceOTU53614
New.ReferenceOTU389
New.CleanUp.ReferenceOTU211216
New.CleanUp.ReferenceOTU108869
New.CleanUp.ReferenceOTU63117
New.CleanUp.ReferenceOTU20386
New.ReferenceOTU1939
New.CleanUp.ReferenceOTU160629
New.CleanUp.ReferenceOTU42017
New.CleanUp.ReferenceOTU195
New.CleanUp.ReferenceOTU9183
New.CleanUp.ReferenceOTU62839
New.CleanUp.ReferenceOTU4514
New.CleanUp.ReferenceOTU31524
New.CleanUp.ReferenceOTU36666
New.CleanUp.ReferenceOTU10142
New.CleanUp.ReferenceOTU49435
New.CleanUp.ReferenceOTU39495
New.CleanUp.ReferenceOTU58809
New.CleanUp.ReferenceOTU22214
New.CleanUp.ReferenceOTU14600
New.CleanUp.ReferenceOTU40140
New.CleanUp.ReferenceOTU14247
New.CleanUp.ReferenceOTU28007
New.CleanUp.ReferenceOTU68785
EF219013.1.1704
New.CleanUp.ReferenceOTU184242
New.CleanUp.ReferenceOTU59372
New.CleanUp.ReferenceOTU174153
New.CleanUp.ReferenceOTU244797
New.CleanUp.ReferenceOTU122692
New.CleanUp.ReferenceOTU286151
New.CleanUp.ReferenceOTU203111
New.CleanUp.ReferenceOTU129244
New.CleanUp.ReferenceOTU81511
New.CleanUp.ReferenceOTU84051
GU969185.1.1699
New.CleanUp.ReferenceOTU66755
GU594642.1.1700
New.CleanUp.ReferenceOTU18933
New.CleanUp.ReferenceOTU131203
New.CleanUp.ReferenceOTU175862
New.CleanUp.ReferenceOTU47325
GU969154.1.1726
New.CleanUp.ReferenceOTU101687
New.CleanUp.ReferenceOTU261563
New.ReferenceOTU760
AY665120.1.1759
New.CleanUp.ReferenceOTU116995
New.CleanUp.ReferenceOTU60929
New.ReferenceOTU1757
New.CleanUp.ReferenceOTU177374

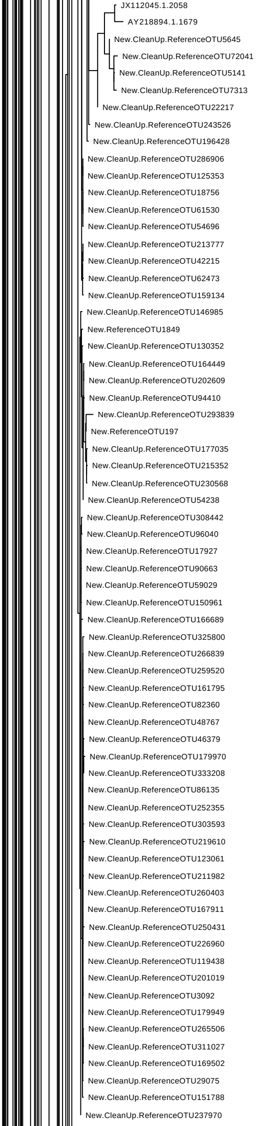
JX112045.1.2058
AY218894.1.1679
New.CleanUp.ReferenceOTU5645
New.CleanUp.ReferenceOTU72041
New.CleanUp.ReferenceOTU5141
New.CleanUp.ReferenceOTU7313
New.CleanUp.ReferenceOTU22217
New.CleanUp.ReferenceOTU243526
New.CleanUp.ReferenceOTU196428
New.CleanUp.ReferenceOTU286906
New.CleanUp.ReferenceOTU125353
New.CleanUp.ReferenceOTU18756
New.CleanUp.ReferenceOTU61530
New.CleanUp.ReferenceOTU54696
New.CleanUp.ReferenceOTU213777
New.CleanUp.ReferenceOTU42215
New.CleanUp.ReferenceOTU62473
New.CleanUp.ReferenceOTU159134
New.CleanUp.ReferenceOTU146985
New.ReferenceOTU1849
New.CleanUp.ReferenceOTU130352
New.CleanUp.ReferenceOTU164449
New.CleanUp.ReferenceOTU202609
New.CleanUp.ReferenceOTU94410
New.CleanUp.ReferenceOTU293839
New.ReferenceOTU197
New.CleanUp.ReferenceOTU177035
New.CleanUp.ReferenceOTU215352
New.CleanUp.ReferenceOTU230568
New.CleanUp.ReferenceOTU54238
New.CleanUp.ReferenceOTU308442
New.CleanUp.ReferenceOTU96040
New.CleanUp.ReferenceOTU17927
New.CleanUp.ReferenceOTU90663
New.CleanUp.ReferenceOTU59029
New.CleanUp.ReferenceOTU150961
New.CleanUp.ReferenceOTU166689
New.CleanUp.ReferenceOTU325800
New.CleanUp.ReferenceOTU266839
New.CleanUp.ReferenceOTU259520
New.CleanUp.ReferenceOTU161795
New.CleanUp.ReferenceOTU82360
New.CleanUp.ReferenceOTU48767
New.CleanUp.ReferenceOTU46379
New.CleanUp.ReferenceOTU179970
New.CleanUp.ReferenceOTU333208
New.CleanUp.ReferenceOTU86135
New.CleanUp.ReferenceOTU252355
New.CleanUp.ReferenceOTU303593
New.CleanUp.ReferenceOTU219610
New.CleanUp.ReferenceOTU123061
New.CleanUp.ReferenceOTU211982
New.CleanUp.ReferenceOTU260403
New.CleanUp.ReferenceOTU167911
New.CleanUp.ReferenceOTU250431
New.CleanUp.ReferenceOTU226960
New.CleanUp.ReferenceOTU119438
New.CleanUp.ReferenceOTU201019
New.CleanUp.ReferenceOTU3092
New.CleanUp.ReferenceOTU179949
New.CleanUp.ReferenceOTU265506
New.CleanUp.ReferenceOTU311027
New.CleanUp.ReferenceOTU169502
New.CleanUp.ReferenceOTU29075
New.CleanUp.ReferenceOTU151788
New.CleanUp.ReferenceOTU237970

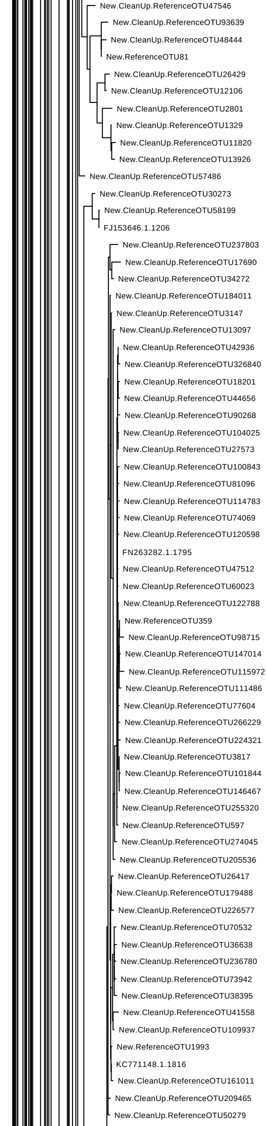
New.CleanUp.ReferenceOTU47546
New.CleanUp.ReferenceOTU93639
New.CleanUp.ReferenceOTU48444
New.ReferenceOTU81
New.CleanUp.ReferenceOTU26429
New.CleanUp.ReferenceOTU12106
New.CleanUp.ReferenceOTU2801
New.CleanUp.ReferenceOTU1329
New.CleanUp.ReferenceOTU11820
New.CleanUp.ReferenceOTU13926
New.CleanUp.ReferenceOTU57486
New.CleanUp.ReferenceOTU30273
New.CleanUp.ReferenceOTU58199
FJ153646.1.1206
New.CleanUp.ReferenceOTU237803
New.CleanUp.ReferenceOTU17690
New.CleanUp.ReferenceOTU34272
New.CleanUp.ReferenceOTU184011
New.CleanUp.ReferenceOTU3147
New.CleanUp.ReferenceOTU13097
New.CleanUp.ReferenceOTU42936
New.CleanUp.ReferenceOTU326840
New.CleanUp.ReferenceOTU18201
New.CleanUp.ReferenceOTU44656
New.CleanUp.ReferenceOTU90268
New.CleanUp.ReferenceOTU104025
New.CleanUp.ReferenceOTU27573
New.CleanUp.ReferenceOTU100843
New.CleanUp.ReferenceOTU81096
New.CleanUp.ReferenceOTU114783
New.CleanUp.ReferenceOTU74069
New.CleanUp.ReferenceOTU120598
FN263282.1.1795
New.CleanUp.ReferenceOTU47512
New.CleanUp.ReferenceOTU60023
New.CleanUp.ReferenceOTU122788
New.ReferenceOTU359
New.CleanUp.ReferenceOTU98715
New.CleanUp.ReferenceOTU147014
New.CleanUp.ReferenceOTU115972
New.CleanUp.ReferenceOTU111486
New.CleanUp.ReferenceOTU77604
New.CleanUp.ReferenceOTU266229
New.CleanUp.ReferenceOTU224321
New.CleanUp.ReferenceOTU3817
New.CleanUp.ReferenceOTU101844
New.CleanUp.ReferenceOTU146467
New.CleanUp.ReferenceOTU255320
New.CleanUp.ReferenceOTU597
New.CleanUp.ReferenceOTU274045
New.CleanUp.ReferenceOTU205536
New.CleanUp.ReferenceOTU26417
New.CleanUp.ReferenceOTU179488
New.CleanUp.ReferenceOTU226577
New.CleanUp.ReferenceOTU70532
New.CleanUp.ReferenceOTU36638
New.CleanUp.ReferenceOTU236780
New.CleanUp.ReferenceOTU73942
New.CleanUp.ReferenceOTU38395
New.CleanUp.ReferenceOTU41558
New.CleanUp.ReferenceOTU109937
New.ReferenceOTU1993
KC771148.1.1816
New.CleanUp.ReferenceOTU161011
New.CleanUp.ReferenceOTU209465
New.CleanUp.ReferenceOTU50279

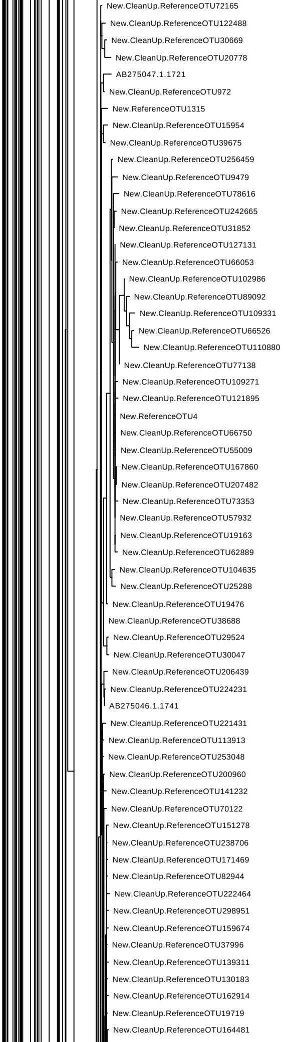
New.CleanUp.ReferenceOTU72165
New.CleanUp.ReferenceOTU122488
New.CleanUp.ReferenceOTU30669
New.CleanUp.ReferenceOTU20778
AB275047.1.1721
New.CleanUp.ReferenceOTU972
New.ReferenceOTU1315
New.CleanUp.ReferenceOTU15954
New.CleanUp.ReferenceOTU39675
New.CleanUp.ReferenceOTU256459
New.CleanUp.ReferenceOTU9479
New.CleanUp.ReferenceOTU78616
New.CleanUp.ReferenceOTU242665
New.CleanUp.ReferenceOTU31852
New.CleanUp.ReferenceOTU127131
New.CleanUp.ReferenceOTU66053
New.CleanUp.ReferenceOTU102986
New.CleanUp.ReferenceOTU89092
New.CleanUp.ReferenceOTU109331
New.CleanUp.ReferenceOTU66526
New.CleanUp.ReferenceOTU110880
New.CleanUp.ReferenceOTU77138
New.CleanUp.ReferenceOTU109271
New.CleanUp.ReferenceOTU121895
New.ReferenceOTU4
New.CleanUp.ReferenceOTU66750
New.CleanUp.ReferenceOTU55009
New.CleanUp.ReferenceOTU167860
New.CleanUp.ReferenceOTU207482
New.CleanUp.ReferenceOTU73353
New.CleanUp.ReferenceOTU57932
New.CleanUp.ReferenceOTU19163
New.CleanUp.ReferenceOTU62889
New.CleanUp.ReferenceOTU104635
New.CleanUp.ReferenceOTU25288
New.CleanUp.ReferenceOTU19476
New.CleanUp.ReferenceOTU38688
New.CleanUp.ReferenceOTU29524
New.CleanUp.ReferenceOTU30047
New.CleanUp.ReferenceOTU206439
New.CleanUp.ReferenceOTU224231
AB275046.1.1741
New.CleanUp.ReferenceOTU221431
New.CleanUp.ReferenceOTU113913
New.CleanUp.ReferenceOTU253048
New.CleanUp.ReferenceOTU200960
New.CleanUp.ReferenceOTU141232
New.CleanUp.ReferenceOTU70122
New.CleanUp.ReferenceOTU151278
New.CleanUp.ReferenceOTU238706
New.CleanUp.ReferenceOTU171469
New.CleanUp.ReferenceOTU82944
New.CleanUp.ReferenceOTU222464
New.CleanUp.ReferenceOTU298951
New.CleanUp.ReferenceOTU159674
New.CleanUp.ReferenceOTU37996
New.CleanUp.ReferenceOTU139311
New.CleanUp.ReferenceOTU130183
New.CleanUp.ReferenceOTU162914
New.CleanUp.ReferenceOTU19719
New.CleanUp.ReferenceOTU164481

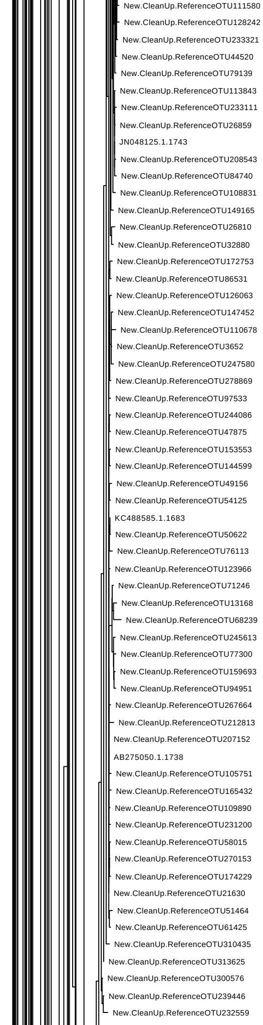
New.CleanUp.ReferenceOTU111580
New.CleanUp.ReferenceOTU128242
New.CleanUp.ReferenceOTU233321
New.CleanUp.ReferenceOTU44520
New.CleanUp.ReferenceOTU79139
New.CleanUp.ReferenceOTU113843
New.CleanUp.ReferenceOTU233111
New.CleanUp.ReferenceOTU26859
JN048125.1.1743
New.CleanUp.ReferenceOTU208543
New.CleanUp.ReferenceOTU84740
New.CleanUp.ReferenceOTU108831
New.CleanUp.ReferenceOTU149165
New.CleanUp.ReferenceOTU26810
New.CleanUp.ReferenceOTU32880
New.CleanUp.ReferenceOTU172753
New.CleanUp.ReferenceOTU86531
New.CleanUp.ReferenceOTU126063
New.CleanUp.ReferenceOTU147452
New.CleanUp.ReferenceOTU110678
New.CleanUp.ReferenceOTU3652
New.CleanUp.ReferenceOTU247580
New.CleanUp.ReferenceOTU278869
New.CleanUp.ReferenceOTU97533
New.CleanUp.ReferenceOTU244086
New.CleanUp.ReferenceOTU47875
New.CleanUp.ReferenceOTU153553
New.CleanUp.ReferenceOTU144599
New.CleanUp.ReferenceOTU49156
New.CleanUp.ReferenceOTU54125
KC488585.1.1683
New.CleanUp.ReferenceOTU50622
New.CleanUp.ReferenceOTU76113
New.CleanUp.ReferenceOTU123966
New.CleanUp.ReferenceOTU71246
New.CleanUp.ReferenceOTU13168
New.CleanUp.ReferenceOTU68239
New.CleanUp.ReferenceOTU245613
New.CleanUp.ReferenceOTU77300
New.CleanUp.ReferenceOTU159693
New.CleanUp.ReferenceOTU94951
New.CleanUp.ReferenceOTU267664
New.CleanUp.ReferenceOTU212813
New.CleanUp.ReferenceOTU207152
AB275050.1.1738
New.CleanUp.ReferenceOTU105751
New.CleanUp.ReferenceOTU165432
New.CleanUp.ReferenceOTU109890
New.CleanUp.ReferenceOTU231200
New.CleanUp.ReferenceOTU58015
New.CleanUp.ReferenceOTU270153
New.CleanUp.ReferenceOTU174229
New.CleanUp.ReferenceOTU21630
New.CleanUp.ReferenceOTU51464
New.CleanUp.ReferenceOTU61425
New.CleanUp.ReferenceOTU310435
New.CleanUp.ReferenceOTU313625
New.CleanUp.ReferenceOTU300576
New.CleanUp.ReferenceOTU239446
New.CleanUp.ReferenceOTU232559

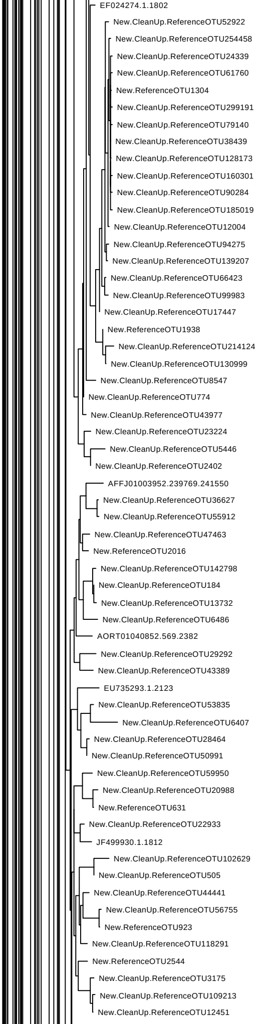
EF024274.1.1802
New.CleanUp.ReferenceOTU52922
New.CleanUp.ReferenceOTU254458
New.CleanUp.ReferenceOTU24339
New.CleanUp.ReferenceOTU61760
New.ReferenceOTU1304
New.CleanUp.ReferenceOTU299191
New.CleanUp.ReferenceOTU79140
New.CleanUp.ReferenceOTU38439
New.CleanUp.ReferenceOTU128173
New.CleanUp.ReferenceOTU160301
New.CleanUp.ReferenceOTU90284
New.CleanUp.ReferenceOTU185019
New.CleanUp.ReferenceOTU12004
New.CleanUp.ReferenceOTU94275
New.CleanUp.ReferenceOTU139207
New.CleanUp.ReferenceOTU66423
New.CleanUp.ReferenceOTU99983
New.CleanUp.ReferenceOTU17447
New.ReferenceOTU1938
New.CleanUp.ReferenceOTU214124
New.CleanUp.ReferenceOTU130999
New.CleanUp.ReferenceOTU8547
New.CleanUp.ReferenceOTU774
New.CleanUp.ReferenceOTU43977
New.CleanUp.ReferenceOTU23224
New.CleanUp.ReferenceOTU5446
New.CleanUp.ReferenceOTU2402
AFFJ01003952.239769.241550
New.CleanUp.ReferenceOTU36627
New.CleanUp.ReferenceOTU55912
New.CleanUp.ReferenceOTU47463
New.ReferenceOTU2016
New.CleanUp.ReferenceOTU142798
New.CleanUp.ReferenceOTU184
New.CleanUp.ReferenceOTU13732
New.CleanUp.ReferenceOTU6486
AORT01040852.569.2382
New.CleanUp.ReferenceOTU29292
New.CleanUp.ReferenceOTU43389
EU735293.1.2123
New.CleanUp.ReferenceOTU53835
New.CleanUp.ReferenceOTU6407
New.CleanUp.ReferenceOTU28464
New.CleanUp.ReferenceOTU50991
New.CleanUp.ReferenceOTU59950
New.CleanUp.ReferenceOTU20988
New.ReferenceOTU631
New.CleanUp.ReferenceOTU22933
JF499930.1.1812
New.CleanUp.ReferenceOTU102629
New.CleanUp.ReferenceOTU505
New.CleanUp.ReferenceOTU44441
New.CleanUp.ReferenceOTU56755
New.ReferenceOTU923
New.CleanUp.ReferenceOTU118291
New.ReferenceOTU2544
New.CleanUp.ReferenceOTU3175
New.CleanUp.ReferenceOTU109213
New.CleanUp.ReferenceOTU12451

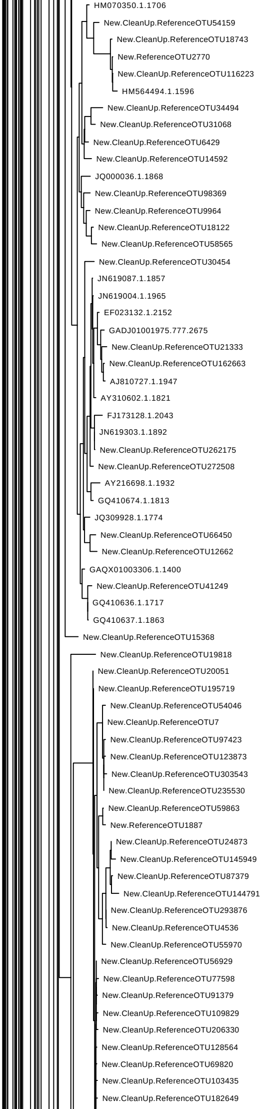
HM070350.1.1706
New.CleanUp.ReferenceOTU54159
New.CleanUp.ReferenceOTU18743
New.ReferenceOTU2770
New.CleanUp.ReferenceOTU116223
HM564494.1.1596
New.CleanUp.ReferenceOTU34494
New.CleanUp.ReferenceOTU31068
New.CleanUp.ReferenceOTU6429
New.CleanUp.ReferenceOTU14592
JQ000036.1.1868
New.CleanUp.ReferenceOTU98369
New.CleanUp.ReferenceOTU9964
New.CleanUp.ReferenceOTU18122
New.CleanUp.ReferenceOTU58565
New.CleanUp.ReferenceOTU30454
JN619087.1.1857
JN619004.1.1965
EF023132.1.2152
GADJ01001975.777.2675
New.CleanUp.ReferenceOTU21333
New.CleanUp.ReferenceOTU162663
AJ810727.1.1947
AY310602.1.1821
FJ173128.1.2043
JN619303.1.1892
New.CleanUp.ReferenceOTU262175
New.CleanUp.ReferenceOTU272508
AY216698.1.1932
GQ410674.1.1813
JQ309928.1.1774
New.CleanUp.ReferenceOTU66450
New.CleanUp.ReferenceOTU12662
GAQX01003306.1.1400
New.CleanUp.ReferenceOTU41249
GQ410636.1.1717
GQ410637.1.1863
New.CleanUp.ReferenceOTU15368
New.CleanUp.ReferenceOTU19818
New.CleanUp.ReferenceOTU20051
New.CleanUp.ReferenceOTU195719
New.CleanUp.ReferenceOTU54046
New.CleanUp.ReferenceOTU7
New.CleanUp.ReferenceOTU97423
New.CleanUp.ReferenceOTU123873
New.CleanUp.ReferenceOTU303543
New.CleanUp.ReferenceOTU235530
New.CleanUp.ReferenceOTU59863
New.ReferenceOTU1887
New.CleanUp.ReferenceOTU24873
New.CleanUp.ReferenceOTU145949
New.CleanUp.ReferenceOTU87379
New.CleanUp.ReferenceOTU144791
New.CleanUp.ReferenceOTU293876
New.CleanUp.ReferenceOTU4536
New.CleanUp.ReferenceOTU55970
New.CleanUp.ReferenceOTU56929
New.CleanUp.ReferenceOTU77598
New.CleanUp.ReferenceOTU91379
New.CleanUp.ReferenceOTU109829
New.CleanUp.ReferenceOTU206330
New.CleanUp.ReferenceOTU128564
New.CleanUp.ReferenceOTU69820
New.CleanUp.ReferenceOTU103435
New.CleanUp.ReferenceOTU182649

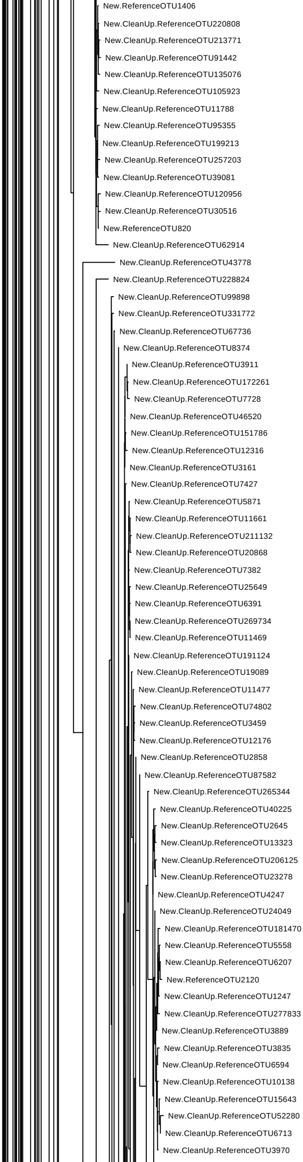
New.ReferenceOTU1406
New.CleanUp.ReferenceOTU220808
New.CleanUp.ReferenceOTU213771
New.CleanUp.ReferenceOTU91442
New.CleanUp.ReferenceOTU135076
New.CleanUp.ReferenceOTU105923
New.CleanUp.ReferenceOTU11788
New.CleanUp.ReferenceOTU95355
New.CleanUp.ReferenceOTU199213
New.CleanUp.ReferenceOTU257203
New.CleanUp.ReferenceOTU39081
New.CleanUp.ReferenceOTU120956
New.CleanUp.ReferenceOTU30516
New.ReferenceOTU820
New.CleanUp.ReferenceOTU62914
New.CleanUp.ReferenceOTU43778
New.CleanUp.ReferenceOTU228824
New.CleanUp.ReferenceOTU99898
New.CleanUp.ReferenceOTU331772
New.CleanUp.ReferenceOTU67736
New.CleanUp.ReferenceOTU8374
New.CleanUp.ReferenceOTU3911
New.CleanUp.ReferenceOTU172261
New.CleanUp.ReferenceOTU7728
New.CleanUp.ReferenceOTU46520
New.CleanUp.ReferenceOTU151786
New.CleanUp.ReferenceOTU12316
New.CleanUp.ReferenceOTU3161
New.CleanUp.ReferenceOTU7427
New.CleanUp.ReferenceOTU5871
New.CleanUp.ReferenceOTU11661
New.CleanUp.ReferenceOTU211132
New.CleanUp.ReferenceOTU20868
New.CleanUp.ReferenceOTU7382
New.CleanUp.ReferenceOTU25649
New.CleanUp.ReferenceOTU6391
New.CleanUp.ReferenceOTU269734
New.CleanUp.ReferenceOTU11469
New.CleanUp.ReferenceOTU191124
New.CleanUp.ReferenceOTU19089
New.CleanUp.ReferenceOTU11477
New.CleanUp.ReferenceOTU74802
New.CleanUp.ReferenceOTU3459
New.CleanUp.ReferenceOTU12176
New.CleanUp.ReferenceOTU2858
New.CleanUp.ReferenceOTU87582
New.CleanUp.ReferenceOTU265344
New.CleanUp.ReferenceOTU40225
New.CleanUp.ReferenceOTU2645
New.CleanUp.ReferenceOTU13323
New.CleanUp.ReferenceOTU206125
New.CleanUp.ReferenceOTU23278
New.CleanUp.ReferenceOTU4247
New.CleanUp.ReferenceOTU24049
New.CleanUp.ReferenceOTU181470
New.CleanUp.ReferenceOTU5558
New.CleanUp.ReferenceOTU6207
New.ReferenceOTU2120
New.CleanUp.ReferenceOTU1247
New.CleanUp.ReferenceOTU277833
New.CleanUp.ReferenceOTU3889
New.CleanUp.ReferenceOTU3835
New.CleanUp.ReferenceOTU6594
New.CleanUp.ReferenceOTU10138
New.CleanUp.ReferenceOTU15643
New.CleanUp.ReferenceOTU52280
New.CleanUp.ReferenceOTU6713
New.CleanUp.ReferenceOTU3970

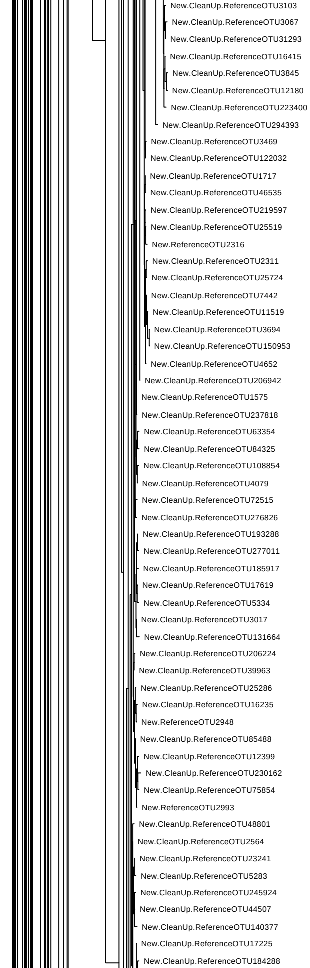

New.CleanUp.ReferenceOTU3103
New.CleanUp.ReferenceOTU3067
New.CleanUp.ReferenceOTU31293
New.CleanUp.ReferenceOTU16415
New.CleanUp.ReferenceOTU3845
New.CleanUp.ReferenceOTU12180
New.CleanUp.ReferenceOTU223400
New.CleanUp.ReferenceOTU294393
New.CleanUp.ReferenceOTU3469
New.CleanUp.ReferenceOTU122032
New.CleanUp.ReferenceOTU1717
New.CleanUp.ReferenceOTU46535
New.CleanUp.ReferenceOTU219597
New.CleanUp.ReferenceOTU25519
New.ReferenceOTU2316
New.CleanUp.ReferenceOTU2311
New.CleanUp.ReferenceOTU25724
New.CleanUp.ReferenceOTU7442
New.CleanUp.ReferenceOTU11519
New.CleanUp.ReferenceOTU3694
New.CleanUp.ReferenceOTU150953
New.CleanUp.ReferenceOTU4652
New.CleanUp.ReferenceOTU206942
New.CleanUp.ReferenceOTU1575
New.CleanUp.ReferenceOTU237818
New.CleanUp.ReferenceOTU63354
New.CleanUp.ReferenceOTU84325
New.CleanUp.ReferenceOTU108854
New.CleanUp.ReferenceOTU4079
New.CleanUp.ReferenceOTU72515
New.CleanUp.ReferenceOTU276826
New.CleanUp.ReferenceOTU193288
New.CleanUp.ReferenceOTU277011
New.CleanUp.ReferenceOTU185917
New.CleanUp.ReferenceOTU17619
New.CleanUp.ReferenceOTU5334
New.CleanUp.ReferenceOTU3017
New.CleanUp.ReferenceOTU131664
New.CleanUp.ReferenceOTU206224
New.CleanUp.ReferenceOTU39963
New.CleanUp.ReferenceOTU25286
New.CleanUp.ReferenceOTU16235
New.ReferenceOTU2948
New.CleanUp.ReferenceOTU85488
New.CleanUp.ReferenceOTU12399
New.CleanUp.ReferenceOTU230162
New.CleanUp.ReferenceOTU75854
New.ReferenceOTU2993
New.CleanUp.ReferenceOTU48801
New.CleanUp.ReferenceOTU2564
New.CleanUp.ReferenceOTU23241
New.CleanUp.ReferenceOTU5283
New.CleanUp.ReferenceOTU245924
New.CleanUp.ReferenceOTU44507
New.CleanUp.ReferenceOTU140377
New.CleanUp.ReferenceOTU17225
New.CleanUp.ReferenceOTU184288

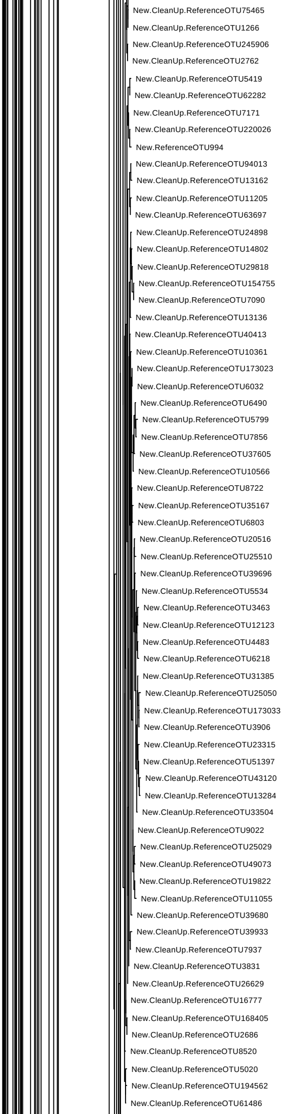

New.CleanUp.ReferenceOTU75465
New.CleanUp.ReferenceOTU1266
New.CleanUp.ReferenceOTU245906
New.CleanUp.ReferenceOTU2762
New.CleanUp.ReferenceOTU5419
New.CleanUp.ReferenceOTU62282
New.CleanUp.ReferenceOTU7171
New.CleanUp.ReferenceOTU220026
New.ReferenceOTU994
New.CleanUp.ReferenceOTU94013
New.CleanUp.ReferenceOTU13162
New.CleanUp.ReferenceOTU11205
New.CleanUp.ReferenceOTU63697
New.CleanUp.ReferenceOTU24898
New.CleanUp.ReferenceOTU14802
New.CleanUp.ReferenceOTU29818
New.CleanUp.ReferenceOTU154755
New.CleanUp.ReferenceOTU7090
New.CleanUp.ReferenceOTU13136
New.CleanUp.ReferenceOTU40413
New.CleanUp.ReferenceOTU10361
New.CleanUp.ReferenceOTU173023
New.CleanUp.ReferenceOTU6032
New.CleanUp.ReferenceOTU6490
New.CleanUp.ReferenceOTU5799
New.CleanUp.ReferenceOTU7856
New.CleanUp.ReferenceOTU37605
New.CleanUp.ReferenceOTU10566
New.CleanUp.ReferenceOTU8722
New.CleanUp.ReferenceOTU35167
New.CleanUp.ReferenceOTU6803
New.CleanUp.ReferenceOTU20516
New.CleanUp.ReferenceOTU25510
New.CleanUp.ReferenceOTU39696
New.CleanUp.ReferenceOTU5534
New.CleanUp.ReferenceOTU3463
New.CleanUp.ReferenceOTU12123
New.CleanUp.ReferenceOTU4483
New.CleanUp.ReferenceOTU6218
New.CleanUp.ReferenceOTU31385
New.CleanUp.ReferenceOTU25050
New.CleanUp.ReferenceOTU173033
New.CleanUp.ReferenceOTU3906
New.CleanUp.ReferenceOTU23315
New.CleanUp.ReferenceOTU51397
New.CleanUp.ReferenceOTU43120
New.CleanUp.ReferenceOTU13284
New.CleanUp.ReferenceOTU33504
New.CleanUp.ReferenceOTU9022
New.CleanUp.ReferenceOTU25029
New.CleanUp.ReferenceOTU49073
New.CleanUp.ReferenceOTU19822
New.CleanUp.ReferenceOTU11055
New.CleanUp.ReferenceOTU39680
New.CleanUp.ReferenceOTU39933
New.CleanUp.ReferenceOTU7937
New.CleanUp.ReferenceOTU3831
New.CleanUp.ReferenceOTU26629
New.CleanUp.ReferenceOTU16777
New.CleanUp.ReferenceOTU168405
New.CleanUp.ReferenceOTU2686
New.CleanUp.ReferenceOTU8520
New.CleanUp.ReferenceOTU5020
New.CleanUp.ReferenceOTU194562
New.CleanUp.ReferenceOTU61486

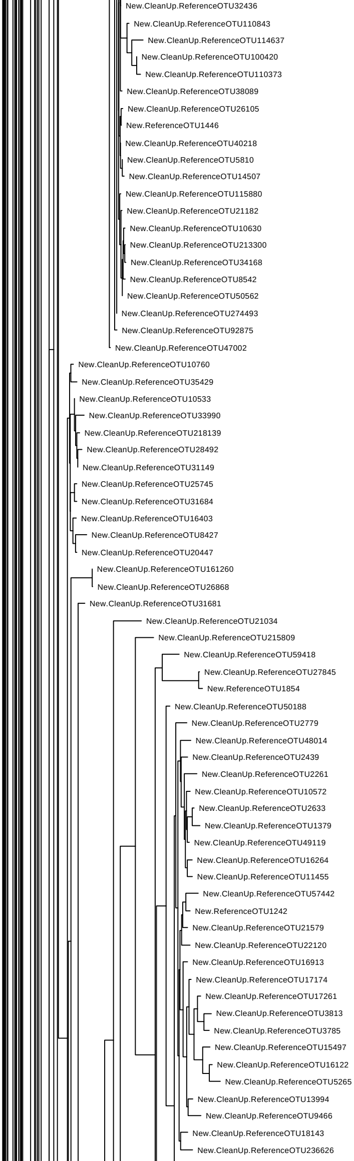
New.CleanUp.ReferenceOTU32436
New.CleanUp.ReferenceOTU110843
New.CleanUp.ReferenceOTU114637
New.CleanUp.ReferenceOTU100420
New.CleanUp.ReferenceOTU110373
New.CleanUp.ReferenceOTU38089
New.CleanUp.ReferenceOTU26105
New.ReferenceOTU1446
New.CleanUp.ReferenceOTU40218
New.CleanUp.ReferenceOTU5810
New.CleanUp.ReferenceOTU14507
New.CleanUp.ReferenceOTU115880
New.CleanUp.ReferenceOTU21182
New.CleanUp.ReferenceOTU10630
New.CleanUp.ReferenceOTU213300
New.CleanUp.ReferenceOTU34168
New.CleanUp.ReferenceOTU8542
New.CleanUp.ReferenceOTU50562
New.CleanUp.ReferenceOTU274493
New.CleanUp.ReferenceOTU92875
New.CleanUp.ReferenceOTU47002
New.CleanUp.ReferenceOTU10760
New.CleanUp.ReferenceOTU35429
New.CleanUp.ReferenceOTU10533
New.CleanUp.ReferenceOTU33990
New.CleanUp.ReferenceOTU218139
New.CleanUp.ReferenceOTU28492
New.CleanUp.ReferenceOTU31149
New.CleanUp.ReferenceOTU25745
New.CleanUp.ReferenceOTU31684
New.CleanUp.ReferenceOTU16403
New.CleanUp.ReferenceOTU8427
New.CleanUp.ReferenceOTU20447
New.CleanUp.ReferenceOTU161260
New.CleanUp.ReferenceOTU26868
New.CleanUp.ReferenceOTU31681
New.CleanUp.ReferenceOTU21034
New.CleanUp.ReferenceOTU215809
New.CleanUp.ReferenceOTU59418
New.CleanUp.ReferenceOTU27845
New.ReferenceOTU1854
New.CleanUp.ReferenceOTU50188
New.CleanUp.ReferenceOTU2779
New.CleanUp.ReferenceOTU48014
New.CleanUp.ReferenceOTU2439
New.CleanUp.ReferenceOTU2261
New.CleanUp.ReferenceOTU10572
New.CleanUp.ReferenceOTU2633
New.CleanUp.ReferenceOTU1379
New.CleanUp.ReferenceOTU49119
New.CleanUp.ReferenceOTU16264
New.CleanUp.ReferenceOTU11455
New.CleanUp.ReferenceOTU57442
New.ReferenceOTU1242
New.CleanUp.ReferenceOTU21579
New.CleanUp.ReferenceOTU22120
New.CleanUp.ReferenceOTU16913
New.CleanUp.ReferenceOTU17174
New.CleanUp.ReferenceOTU17261
New.CleanUp.ReferenceOTU3813
New.CleanUp.ReferenceOTU3785
New.CleanUp.ReferenceOTU15497
New.CleanUp.ReferenceOTU16122
New.CleanUp.ReferenceOTU5265
New.CleanUp.ReferenceOTU13994
New.CleanUp.ReferenceOTU9466
New.CleanUp.ReferenceOTU18143
New.CleanUp.ReferenceOTU236626

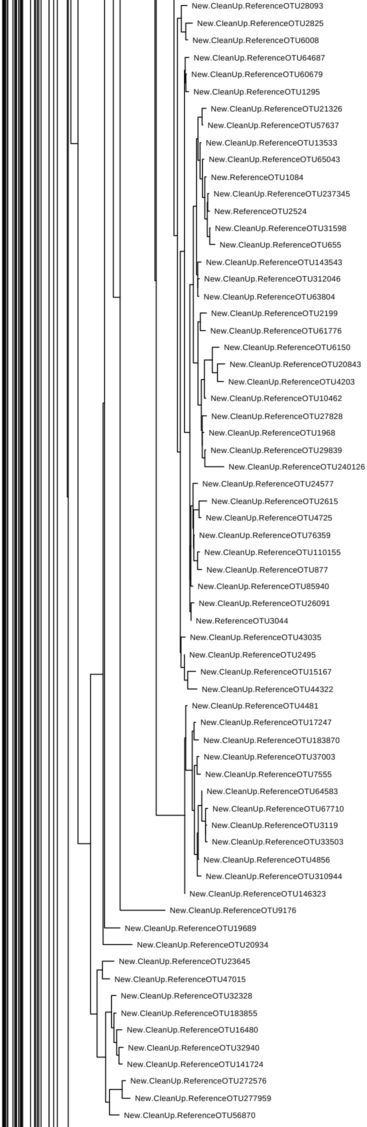
New.CleanUp.ReferenceOTU28093
New.CleanUp.ReferenceOTU2825
New.CleanUp.ReferenceOTU6008
New.CleanUp.ReferenceOTU64687
New.CleanUp.ReferenceOTU60679
New.CleanUp.ReferenceOTU1295
New.CleanUp.ReferenceOTU21326
New.CleanUp.ReferenceOTU57637
New.CleanUp.ReferenceOTU13533
New.CleanUp.ReferenceOTU65043
New.ReferenceOTU1084
New.CleanUp.ReferenceOTU237345
New.ReferenceOTU2524
New.CleanUp.ReferenceOTU31598
New.CleanUp.ReferenceOTU655
New.CleanUp.ReferenceOTU143543
New.CleanUp.ReferenceOTU312046
New.CleanUp.ReferenceOTU63804
New.CleanUp.ReferenceOTU2199
New.CleanUp.ReferenceOTU61776
New.CleanUp.ReferenceOTU6150
New.CleanUp.ReferenceOTU20843
New.CleanUp.ReferenceOTU4203
New.CleanUp.ReferenceOTU10462
New.CleanUp.ReferenceOTU27828
New.CleanUp.ReferenceOTU1968
New.CleanUp.ReferenceOTU29839
New.CleanUp.ReferenceOTU240126
New.CleanUp.ReferenceOTU24577
New.CleanUp.ReferenceOTU2615
New.CleanUp.ReferenceOTU4725
New.CleanUp.ReferenceOTU76359
New.CleanUp.ReferenceOTU110155
New.CleanUp.ReferenceOTU877
New.CleanUp.ReferenceOTU85940
New.CleanUp.ReferenceOTU26091
New.ReferenceOTU3044
New.CleanUp.ReferenceOTU43035
New.CleanUp.ReferenceOTU2495
New.CleanUp.ReferenceOTU15167
New.CleanUp.ReferenceOTU44322
New.CleanUp.ReferenceOTU4481
New.CleanUp.ReferenceOTU17247
New.CleanUp.ReferenceOTU183870
New.CleanUp.ReferenceOTU37003
New.CleanUp.ReferenceOTU7555
New.CleanUp.ReferenceOTU64583
New.CleanUp.ReferenceOTU67710
New.CleanUp.ReferenceOTU3119
New.CleanUp.ReferenceOTU33503
New.CleanUp.ReferenceOTU4856
New.CleanUp.ReferenceOTU310944
New.CleanUp.ReferenceOTU146323
New.CleanUp.ReferenceOTU9176
New.CleanUp.ReferenceOTU19689
New.CleanUp.ReferenceOTU20934
New.CleanUp.ReferenceOTU23645
New.CleanUp.ReferenceOTU47015
New.CleanUp.ReferenceOTU32328
New.CleanUp.ReferenceOTU183855
New.CleanUp.ReferenceOTU16480
New.CleanUp.ReferenceOTU32940
New.CleanUp.ReferenceOTU141724
New.CleanUp.ReferenceOTU272576
New.CleanUp.ReferenceOTU277959
New.CleanUp.ReferenceOTU56870

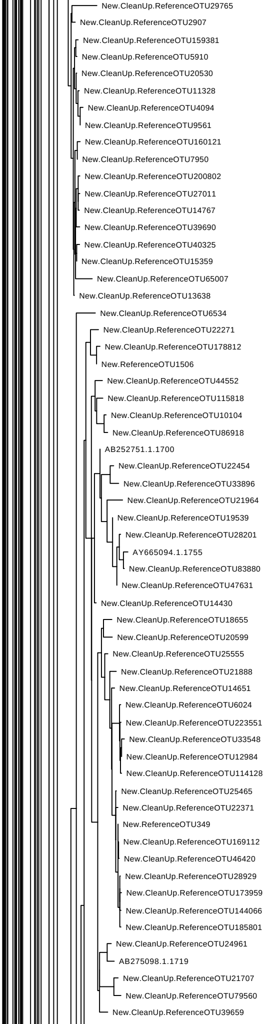
New.CleanUp.ReferenceOTU29765
New.CleanUp.ReferenceOTU2907
New.CleanUp.ReferenceOTU159381
New.CleanUp.ReferenceOTU5910
New.CleanUp.ReferenceOTU20530
New.CleanUp.ReferenceOTU11328
New.CleanUp.ReferenceOTU4094
New.CleanUp.ReferenceOTU9561
New.CleanUp.ReferenceOTU160121
New.CleanUp.ReferenceOTU7950
New.CleanUp.ReferenceOTU200802
New.CleanUp.ReferenceOTU27011
New.CleanUp.ReferenceOTU14767
New.CleanUp.ReferenceOTU39690
New.CleanUp.ReferenceOTU40325
New.CleanUp.ReferenceOTU15359
New.CleanUp.ReferenceOTU65007
New.CleanUp.ReferenceOTU13638
New.CleanUp.ReferenceOTU6534
New.CleanUp.ReferenceOTU22271
New.CleanUp.ReferenceOTU178812
New.ReferenceOTU1506
New.CleanUp.ReferenceOTU44552
New.CleanUp.ReferenceOTU115818
New.CleanUp.ReferenceOTU10104
New.CleanUp.ReferenceOTU86918
AB252751.1.1700
New.CleanUp.ReferenceOTU22454
New.CleanUp.ReferenceOTU33896
New.CleanUp.ReferenceOTU21964
New.CleanUp.ReferenceOTU19539
New.CleanUp.ReferenceOTU28201
AY665094.1.1755
New.CleanUp.ReferenceOTU83880
New.CleanUp.ReferenceOTU47631
New.CleanUp.ReferenceOTU14430
New.CleanUp.ReferenceOTU18655
New.CleanUp.ReferenceOTU20599
New.CleanUp.ReferenceOTU25555
New.CleanUp.ReferenceOTU21888
New.CleanUp.ReferenceOTU14651
New.CleanUp.ReferenceOTU6024
New.CleanUp.ReferenceOTU223551
New.CleanUp.ReferenceOTU33548
New.CleanUp.ReferenceOTU12984
New.CleanUp.ReferenceOTU114128
New.CleanUp.ReferenceOTU25465
New.CleanUp.ReferenceOTU22371
New.ReferenceOTU349
New.CleanUp.ReferenceOTU169112
New.CleanUp.ReferenceOTU46420
New.CleanUp.ReferenceOTU28929
New.CleanUp.ReferenceOTU173959
New.CleanUp.ReferenceOTU144066
New.CleanUp.ReferenceOTU185801
New.CleanUp.ReferenceOTU24961
AB275098.1.1719
New.CleanUp.ReferenceOTU21707
New.CleanUp.ReferenceOTU79560
New.CleanUp.ReferenceOTU39659

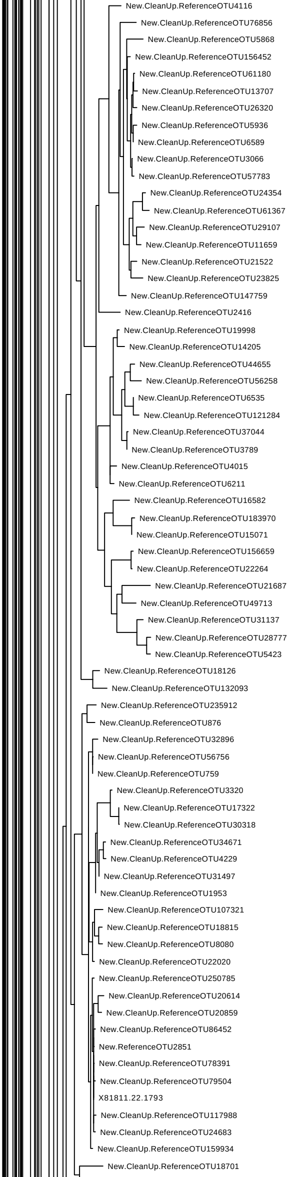
New.CleanUp.ReferenceOTU4116
New.CleanUp.ReferenceOTU76856
New.CleanUp.ReferenceOTU5868
New.CleanUp.ReferenceOTU156452
New.CleanUp.ReferenceOTU61180
New.CleanUp.ReferenceOTU13707
New.CleanUp.ReferenceOTU26320
New.CleanUp.ReferenceOTU5936
New.CleanUp.ReferenceOTU6589
New.CleanUp.ReferenceOTU3066
New.CleanUp.ReferenceOTU57783
New.CleanUp.ReferenceOTU24354
New.CleanUp.ReferenceOTU61367
New.CleanUp.ReferenceOTU29107
New.CleanUp.ReferenceOTU11659
New.CleanUp.ReferenceOTU21522
New.CleanUp.ReferenceOTU23825
New.CleanUp.ReferenceOTU147759
New.CleanUp.ReferenceOTU2416
New.CleanUp.ReferenceOTU19998
New.CleanUp.ReferenceOTU14205
New.CleanUp.ReferenceOTU44655
New.CleanUp.ReferenceOTU56258
New.CleanUp.ReferenceOTU6535
New.CleanUp.ReferenceOTU121284
New.CleanUp.ReferenceOTU37044
New.CleanUp.ReferenceOTU3789
New.CleanUp.ReferenceOTU4015
New.CleanUp.ReferenceOTU6211
New.CleanUp.ReferenceOTU16582
New.CleanUp.ReferenceOTU183970
New.CleanUp.ReferenceOTU15071
New.CleanUp.ReferenceOTU156659
New.CleanUp.ReferenceOTU22264
New.CleanUp.ReferenceOTU21687
New.CleanUp.ReferenceOTU49713
New.CleanUp.ReferenceOTU31137
New.CleanUp.ReferenceOTU28777
New.CleanUp.ReferenceOTU5423
New.CleanUp.ReferenceOTU18126
New.CleanUp.ReferenceOTU132093
New.CleanUp.ReferenceOTU235912
New.CleanUp.ReferenceOTU876
New.CleanUp.ReferenceOTU32896
New.CleanUp.ReferenceOTU56756
New.CleanUp.ReferenceOTU759
New.CleanUp.ReferenceOTU3320
New.CleanUp.ReferenceOTU17322
New.CleanUp.ReferenceOTU30318
New.CleanUp.ReferenceOTU34671
New.CleanUp.ReferenceOTU4229
New.CleanUp.ReferenceOTU31497
New.CleanUp.ReferenceOTU1953
New.CleanUp.ReferenceOTU107321
New.CleanUp.ReferenceOTU18815
New.CleanUp.ReferenceOTU8080
New.CleanUp.ReferenceOTU22020
New.CleanUp.ReferenceOTU250785
New.CleanUp.ReferenceOTU20614
New.CleanUp.ReferenceOTU20859
New.CleanUp.ReferenceOTU86452
New.ReferenceOTU2851
New.CleanUp.ReferenceOTU78391
New.CleanUp.ReferenceOTU79504
X81811.22.1793
New.CleanUp.ReferenceOTU117988
New.CleanUp.ReferenceOTU24683
New.CleanUp.ReferenceOTU159934
New.CleanUp.ReferenceOTU18701

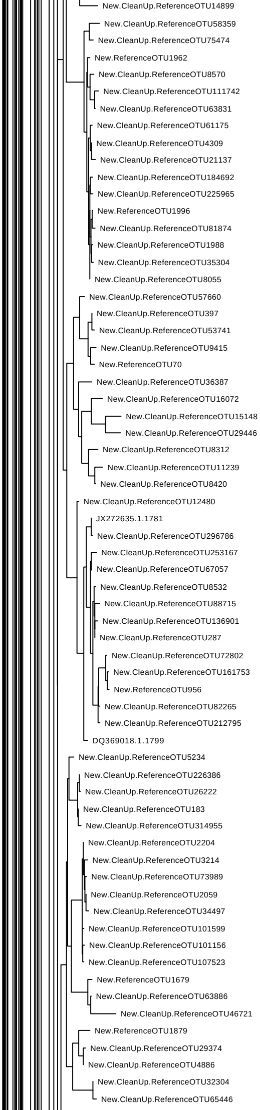
New.CleanUp.ReferenceOTU14899
New.CleanUp.ReferenceOTU58359
New.CleanUp.ReferenceOTU75474
New.ReferenceOTU1962
New.CleanUp.ReferenceOTU8570
New.CleanUp.ReferenceOTU111742
New.CleanUp.ReferenceOTU63831
New.CleanUp.ReferenceOTU61175
New.CleanUp.ReferenceOTU4309
New.CleanUp.ReferenceOTU21137
New.CleanUp.ReferenceOTU184692
New.CleanUp.ReferenceOTU225965
New.ReferenceOTU1996
New.CleanUp.ReferenceOTU81874
New.CleanUp.ReferenceOTU1988
New.CleanUp.ReferenceOTU35304
New.CleanUp.ReferenceOTU8055
New.CleanUp.ReferenceOTU57660
New.CleanUp.ReferenceOTU397
New.CleanUp.ReferenceOTU53741
New.CleanUp.ReferenceOTU9415
New.ReferenceOTU70
New.CleanUp.ReferenceOTU36387
New.CleanUp.ReferenceOTU16072
New.CleanUp.ReferenceOTU15148
New.CleanUp.ReferenceOTU29446
New.CleanUp.ReferenceOTU8312
New.CleanUp.ReferenceOTU11239
New.CleanUp.ReferenceOTU8420
New.CleanUp.ReferenceOTU12480
JX272635.1.1781
New.CleanUp.ReferenceOTU296786
New.CleanUp.ReferenceOTU253167
New.CleanUp.ReferenceOTU67057
New.CleanUp.ReferenceOTU8532
New.CleanUp.ReferenceOTU88715
New.CleanUp.ReferenceOTU136901
New.CleanUp.ReferenceOTU287
New.CleanUp.ReferenceOTU72802
New.CleanUp.ReferenceOTU161753
New.ReferenceOTU956
New.CleanUp.ReferenceOTU82265
New.CleanUp.ReferenceOTU212795
DQ369018.1.1799
New.CleanUp.ReferenceOTU5234
New.CleanUp.ReferenceOTU226386
New.CleanUp.ReferenceOTU26222
New.CleanUp.ReferenceOTU183
New.CleanUp.ReferenceOTU314955
New.CleanUp.ReferenceOTU2204
New.CleanUp.ReferenceOTU3214
New.CleanUp.ReferenceOTU73989
New.CleanUp.ReferenceOTU2059
New.CleanUp.ReferenceOTU34497
New.CleanUp.ReferenceOTU101599
New.CleanUp.ReferenceOTU101156
New.CleanUp.ReferenceOTU107523
New.ReferenceOTU1679
New.CleanUp.ReferenceOTU63886
New.CleanUp.ReferenceOTU46721
New.ReferenceOTU1879
New.CleanUp.ReferenceOTU29374
New.CleanUp.ReferenceOTU4886
New.CleanUp.ReferenceOTU32304
New.CleanUp.ReferenceOTU65446

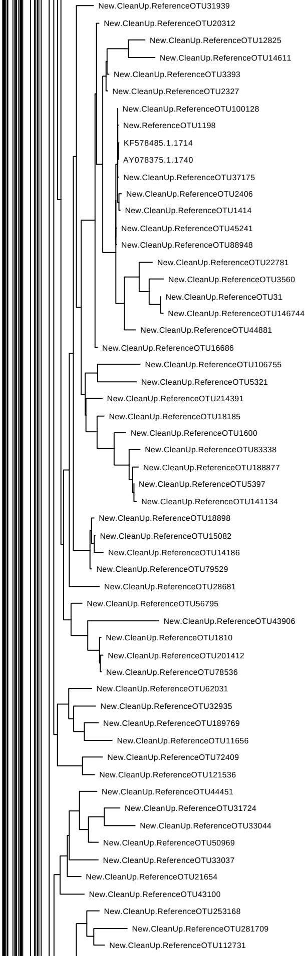
New.CleanUp.ReferenceOTU31939
New.CleanUp.ReferenceOTU20312
New.CleanUp.ReferenceOTU12825
New.CleanUp.ReferenceOTU14611
New.CleanUp.ReferenceOTU3393
New.CleanUp.ReferenceOTU2327
New.CleanUp.ReferenceOTU100128
New.ReferenceOTU1198
KF578485.1.1714
AY078375.1.1740
New.CleanUp.ReferenceOTU37175
New.CleanUp.ReferenceOTU2406
New.CleanUp.ReferenceOTU1414
New.CleanUp.ReferenceOTU45241
New.CleanUp.ReferenceOTU88948
New.CleanUp.ReferenceOTU22781
New.CleanUp.ReferenceOTU3560
New.CleanUp.ReferenceOTU31
New.CleanUp.ReferenceOTU146744
New.CleanUp.ReferenceOTU44881
New.CleanUp.ReferenceOTU16686
New.CleanUp.ReferenceOTU106755
New.CleanUp.ReferenceOTU5321
New.CleanUp.ReferenceOTU214391
New.CleanUp.ReferenceOTU18185
New.CleanUp.ReferenceOTU1600
New.CleanUp.ReferenceOTU83338
New.CleanUp.ReferenceOTU188877
New.CleanUp.ReferenceOTU5397
New.CleanUp.ReferenceOTU141134
New.CleanUp.ReferenceOTU18898
New.CleanUp.ReferenceOTU15082
New.CleanUp.ReferenceOTU14186
New.CleanUp.ReferenceOTU79529
New.CleanUp.ReferenceOTU28681
New.CleanUp.ReferenceOTU56795
New.CleanUp.ReferenceOTU43906
New.CleanUp.ReferenceOTU1810
New.CleanUp.ReferenceOTU201412
New.CleanUp.ReferenceOTU78536
New.CleanUp.ReferenceOTU62031
New.CleanUp.ReferenceOTU32935
New.CleanUp.ReferenceOTU189769
New.CleanUp.ReferenceOTU11656
New.CleanUp.ReferenceOTU72409
New.CleanUp.ReferenceOTU121536
New.CleanUp.ReferenceOTU44451
New.CleanUp.ReferenceOTU31724
New.CleanUp.ReferenceOTU33044
New.CleanUp.ReferenceOTU50969
New.CleanUp.ReferenceOTU33037
New.CleanUp.ReferenceOTU21654
New.CleanUp.ReferenceOTU43100
New.CleanUp.ReferenceOTU253168
New.CleanUp.ReferenceOTU281709
New.CleanUp.ReferenceOTU112731

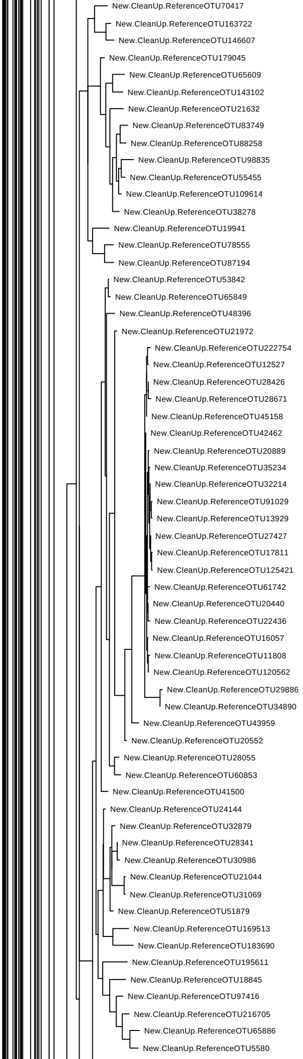
New.CleanUp.ReferenceOTU70417
New.CleanUp.ReferenceOTU163722
New.CleanUp.ReferenceOTU146607
New.CleanUp.ReferenceOTU179045
New.CleanUp.ReferenceOTU65609
New.CleanUp.ReferenceOTU143102
New.CleanUp.ReferenceOTU21632
New.CleanUp.ReferenceOTU83749
New.CleanUp.ReferenceOTU88258
New.CleanUp.ReferenceOTU98835
New.CleanUp.ReferenceOTU55455
New.CleanUp.ReferenceOTU109614
New.CleanUp.ReferenceOTU38278
New.CleanUp.ReferenceOTU19941
New.CleanUp.ReferenceOTU78555
New.CleanUp.ReferenceOTU87194
New.CleanUp.ReferenceOTU53842
New.CleanUp.ReferenceOTU65849
New.CleanUp.ReferenceOTU48396
New.CleanUp.ReferenceOTU21972
New.CleanUp.ReferenceOTU222754
New.CleanUp.ReferenceOTU12527
New.CleanUp.ReferenceOTU28426
New.CleanUp.ReferenceOTU28671
New.CleanUp.ReferenceOTU45158
New.CleanUp.ReferenceOTU42462
New.CleanUp.ReferenceOTU20889
New.CleanUp.ReferenceOTU35234
New.CleanUp.ReferenceOTU32214
New.CleanUp.ReferenceOTU91029
New.CleanUp.ReferenceOTU13929
New.CleanUp.ReferenceOTU27427
New.CleanUp.ReferenceOTU17811
New.CleanUp.ReferenceOTU125421
New.CleanUp.ReferenceOTU61742
New.CleanUp.ReferenceOTU20440
New.CleanUp.ReferenceOTU22436
New.CleanUp.ReferenceOTU16057
New.CleanUp.ReferenceOTU11808
New.CleanUp.ReferenceOTU120562
New.CleanUp.ReferenceOTU29886
New.CleanUp.ReferenceOTU34890
New.CleanUp.ReferenceOTU43959
New.CleanUp.ReferenceOTU20552
New.CleanUp.ReferenceOTU28055
New.CleanUp.ReferenceOTU60853
New.CleanUp.ReferenceOTU41500
New.CleanUp.ReferenceOTU24144
New.CleanUp.ReferenceOTU32879
New.CleanUp.ReferenceOTU28341
New.CleanUp.ReferenceOTU30986
New.CleanUp.ReferenceOTU21044
New.CleanUp.ReferenceOTU31069
New.CleanUp.ReferenceOTU51879
New.CleanUp.ReferenceOTU169513
New.CleanUp.ReferenceOTU183690
New.CleanUp.ReferenceOTU195611
New.CleanUp.ReferenceOTU18845
New.CleanUp.ReferenceOTU97416
New.CleanUp.ReferenceOTU216705
New.CleanUp.ReferenceOTU65886
New.CleanUp.ReferenceOTU5580

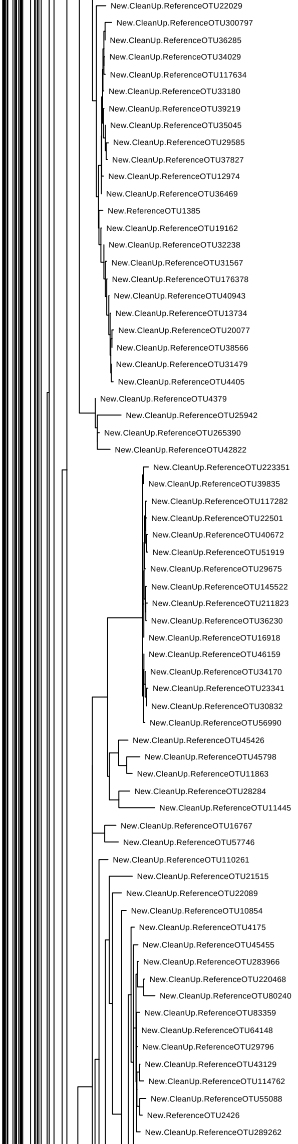
New.CleanUp.ReferenceOTU22029
New.CleanUp.ReferenceOTU300797
New.CleanUp.ReferenceOTU36285
New.CleanUp.ReferenceOTU34029
New.CleanUp.ReferenceOTU117634
New.CleanUp.ReferenceOTU33180
New.CleanUp.ReferenceOTU39219
New.CleanUp.ReferenceOTU35045
New.CleanUp.ReferenceOTU29585
New.CleanUp.ReferenceOTU37827
New.CleanUp.ReferenceOTU12974
New.CleanUp.ReferenceOTU36469
New.ReferenceOTU1385
New.CleanUp.ReferenceOTU19162
New.CleanUp.ReferenceOTU32238
New.CleanUp.ReferenceOTU31567
New.CleanUp.ReferenceOTU176378
New.CleanUp.ReferenceOTU40943
New.CleanUp.ReferenceOTU13734
New.CleanUp.ReferenceOTU20077
New.CleanUp.ReferenceOTU38566
New.CleanUp.ReferenceOTU31479
New.CleanUp.ReferenceOTU4405
New.CleanUp.ReferenceOTU4379
New.CleanUp.ReferenceOTU25942
New.CleanUp.ReferenceOTU265390
New.CleanUp.ReferenceOTU42822
New.CleanUp.ReferenceOTU223351
New.CleanUp.ReferenceOTU39835
New.CleanUp.ReferenceOTU117282
New.CleanUp.ReferenceOTU22501
New.CleanUp.ReferenceOTU40672
New.CleanUp.ReferenceOTU51919
New.CleanUp.ReferenceOTU29675
New.CleanUp.ReferenceOTU145522
New.CleanUp.ReferenceOTU211823
New.CleanUp.ReferenceOTU36230
New.CleanUp.ReferenceOTU16918
New.CleanUp.ReferenceOTU46159
New.CleanUp.ReferenceOTU34170
New.CleanUp.ReferenceOTU23341
New.CleanUp.ReferenceOTU30832
New.CleanUp.ReferenceOTU56990
New.CleanUp.ReferenceOTU45426
New.CleanUp.ReferenceOTU45798
New.CleanUp.ReferenceOTU11863
New.CleanUp.ReferenceOTU28284
New.CleanUp.ReferenceOTU11445
New.CleanUp.ReferenceOTU16767
New.CleanUp.ReferenceOTU57746
New.CleanUp.ReferenceOTU110261
New.CleanUp.ReferenceOTU21515
New.CleanUp.ReferenceOTU22089
New.CleanUp.ReferenceOTU10854
New.CleanUp.ReferenceOTU4175
New.CleanUp.ReferenceOTU45455
New.CleanUp.ReferenceOTU283966
New.CleanUp.ReferenceOTU220468
New.CleanUp.ReferenceOTU80240
New.CleanUp.ReferenceOTU83359
New.CleanUp.ReferenceOTU64148
New.CleanUp.ReferenceOTU29796
New.CleanUp.ReferenceOTU43129
New.CleanUp.ReferenceOTU114762
New.CleanUp.ReferenceOTU55088
New.ReferenceOTU2426
New.CleanUp.ReferenceOTU289262

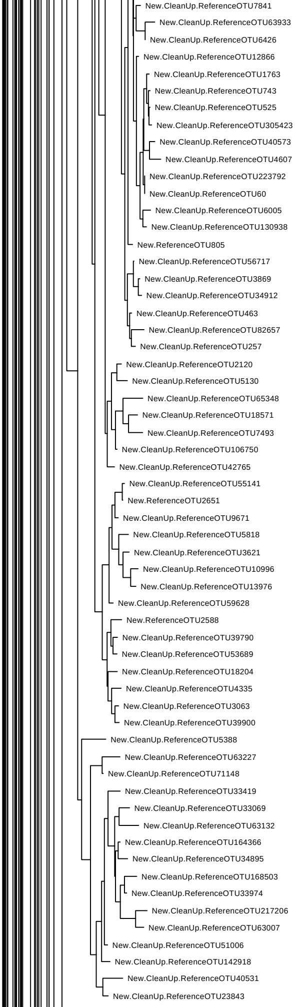
New.CleanUp.ReferenceOTU7841
New.CleanUp.ReferenceOTU63933
New.CleanUp.ReferenceOTU6426
New.CleanUp.ReferenceOTU12866
New.CleanUp.ReferenceOTU1763
New.CleanUp.ReferenceOTU743
New.CleanUp.ReferenceOTU525
New.CleanUp.ReferenceOTU305423
New.CleanUp.ReferenceOTU40573
New.CleanUp.ReferenceOTU4607
New.CleanUp.ReferenceOTU223792
New.CleanUp.ReferenceOTU60
New.CleanUp.ReferenceOTU6005
New.CleanUp.ReferenceOTU130938
New.ReferenceOTU805
New.CleanUp.ReferenceOTU56717
New.CleanUp.ReferenceOTU3869
New.CleanUp.ReferenceOTU34912
New.CleanUp.ReferenceOTU463
New.CleanUp.ReferenceOTU82657
New.CleanUp.ReferenceOTU257
New.CleanUp.ReferenceOTU2120
New.CleanUp.ReferenceOTU5130
New.CleanUp.ReferenceOTU65348
New.CleanUp.ReferenceOTU18571
New.CleanUp.ReferenceOTU7493
New.CleanUp.ReferenceOTU106750
New.CleanUp.ReferenceOTU42765
New.CleanUp.ReferenceOTU55141
New.ReferenceOTU2651
New.CleanUp.ReferenceOTU9671
New.CleanUp.ReferenceOTU5818
New.CleanUp.ReferenceOTU3621
New.CleanUp.ReferenceOTU10996
New.CleanUp.ReferenceOTU13976
New.CleanUp.ReferenceOTU59628
New.ReferenceOTU2588
New.CleanUp.ReferenceOTU39790
New.CleanUp.ReferenceOTU53689
New.CleanUp.ReferenceOTU18204
New.CleanUp.ReferenceOTU4335
New.CleanUp.ReferenceOTU3063
New.CleanUp.ReferenceOTU39900
New.CleanUp.ReferenceOTU5388
New.CleanUp.ReferenceOTU63227
New.CleanUp.ReferenceOTU71148
New.CleanUp.ReferenceOTU33419
New.CleanUp.ReferenceOTU33069
New.CleanUp.ReferenceOTU63132
New.CleanUp.ReferenceOTU164366
New.CleanUp.ReferenceOTU34895
New.CleanUp.ReferenceOTU168503
New.CleanUp.ReferenceOTU33974
New.CleanUp.ReferenceOTU217206
New.CleanUp.ReferenceOTU63007
New.CleanUp.ReferenceOTU51006
New.CleanUp.ReferenceOTU142918
New.CleanUp.ReferenceOTU40531
New.CleanUp.ReferenceOTU23843

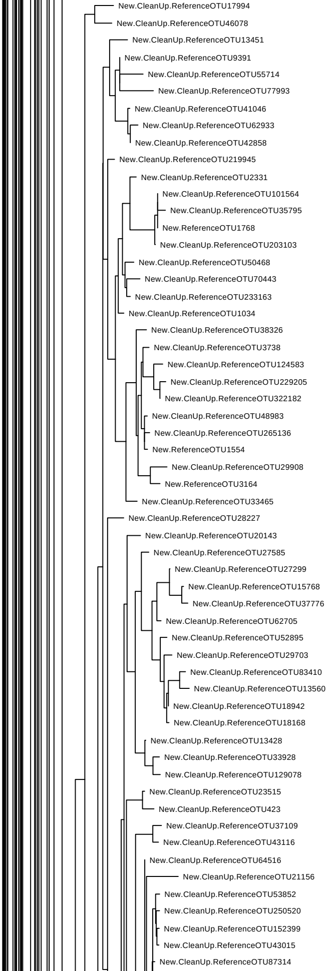
New.CleanUp.ReferenceOTU17994
New.CleanUp.ReferenceOTU46078
New.CleanUp.ReferenceOTU13451
New.CleanUp.ReferenceOTU9391
New.CleanUp.ReferenceOTU55714
New.CleanUp.ReferenceOTU77993
New.CleanUp.ReferenceOTU41046
New.CleanUp.ReferenceOTU62933
New.CleanUp.ReferenceOTU42858
New.CleanUp.ReferenceOTU219945
New.CleanUp.ReferenceOTU2331
New.CleanUp.ReferenceOTU101564
New.CleanUp.ReferenceOTU35795
New.ReferenceOTU1768
New.CleanUp.ReferenceOTU203103
New.CleanUp.ReferenceOTU50468
New.CleanUp.ReferenceOTU70443
New.CleanUp.ReferenceOTU233163
New.CleanUp.ReferenceOTU1034
New.CleanUp.ReferenceOTU38326
New.CleanUp.ReferenceOTU3738
New.CleanUp.ReferenceOTU124583
New.CleanUp.ReferenceOTU229205
New.CleanUp.ReferenceOTU322182
New.CleanUp.ReferenceOTU48983
New.CleanUp.ReferenceOTU265136
New.ReferenceOTU1554
New.CleanUp.ReferenceOTU29908
New.ReferenceOTU3164
New.CleanUp.ReferenceOTU33465
New.CleanUp.ReferenceOTU28227
New.CleanUp.ReferenceOTU20143
New.CleanUp.ReferenceOTU27585
New.CleanUp.ReferenceOTU27299
New.CleanUp.ReferenceOTU15768
New.CleanUp.ReferenceOTU37776
New.CleanUp.ReferenceOTU62705
New.CleanUp.ReferenceOTU52895
New.CleanUp.ReferenceOTU29703
New.CleanUp.ReferenceOTU83410
New.CleanUp.ReferenceOTU13560
New.CleanUp.ReferenceOTU18942
New.CleanUp.ReferenceOTU18168
New.CleanUp.ReferenceOTU13428
New.CleanUp.ReferenceOTU33928
New.CleanUp.ReferenceOTU129078
New.CleanUp.ReferenceOTU23515
New.CleanUp.ReferenceOTU423
New.CleanUp.ReferenceOTU37109
New.CleanUp.ReferenceOTU43116
New.CleanUp.ReferenceOTU64516
New.CleanUp.ReferenceOTU21156
New.CleanUp.ReferenceOTU53852
New.CleanUp.ReferenceOTU250520
New.CleanUp.ReferenceOTU152399
New.CleanUp.ReferenceOTU43015
New.CleanUp.ReferenceOTU87314

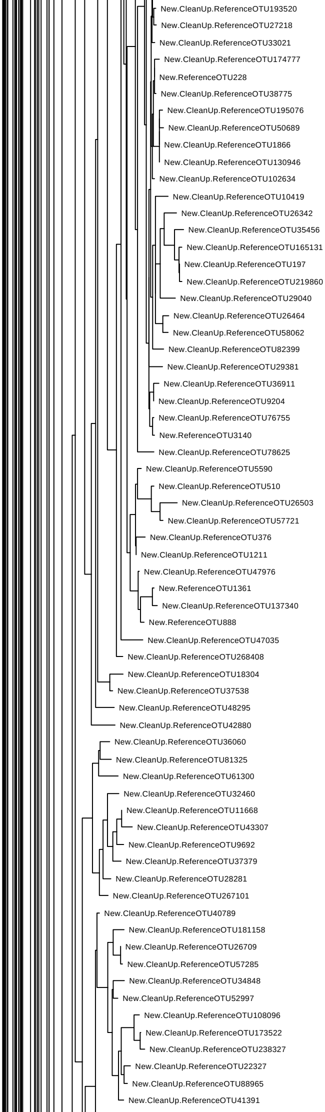
New.CleanUp.ReferenceOTU193520
New.CleanUp.ReferenceOTU27218
New.CleanUp.ReferenceOTU33021
New.CleanUp.ReferenceOTU174777
New.ReferenceOTU228
New.CleanUp.ReferenceOTU38775
New.CleanUp.ReferenceOTU195076
New.CleanUp.ReferenceOTU50689
New.CleanUp.ReferenceOTU1866
New.CleanUp.ReferenceOTU130946
New.CleanUp.ReferenceOTU102634
New.CleanUp.ReferenceOTU10419
New.CleanUp.ReferenceOTU26342
New.CleanUp.ReferenceOTU35456
New.CleanUp.ReferenceOTU165131
New.CleanUp.ReferenceOTU197
New.CleanUp.ReferenceOTU219860
New.CleanUp.ReferenceOTU29040
New.CleanUp.ReferenceOTU26464
New.CleanUp.ReferenceOTU58062
New.CleanUp.ReferenceOTU82399
New.CleanUp.ReferenceOTU29381
New.CleanUp.ReferenceOTU36911
New.CleanUp.ReferenceOTU9204
New.CleanUp.ReferenceOTU76755
New.ReferenceOTU3140
New.CleanUp.ReferenceOTU78625
New.CleanUp.ReferenceOTU5590
New.CleanUp.ReferenceOTU510
New.CleanUp.ReferenceOTU26503
New.CleanUp.ReferenceOTU57721
New.CleanUp.ReferenceOTU376
New.CleanUp.ReferenceOTU1211
New.CleanUp.ReferenceOTU47976
New.ReferenceOTU1361
New.CleanUp.ReferenceOTU137340
New.ReferenceOTU888
New.CleanUp.ReferenceOTU47035
New.CleanUp.ReferenceOTU268408
New.CleanUp.ReferenceOTU18304
New.CleanUp.ReferenceOTU37538
New.CleanUp.ReferenceOTU48295
New.CleanUp.ReferenceOTU42880
New.CleanUp.ReferenceOTU36060
New.CleanUp.ReferenceOTU81325
New.CleanUp.ReferenceOTU61300
New.CleanUp.ReferenceOTU32460
New.CleanUp.ReferenceOTU11668
New.CleanUp.ReferenceOTU43307
New.CleanUp.ReferenceOTU9692
New.CleanUp.ReferenceOTU37379
New.CleanUp.ReferenceOTU28281
New.CleanUp.ReferenceOTU267101
New.CleanUp.ReferenceOTU40789
New.CleanUp.ReferenceOTU181158
New.CleanUp.ReferenceOTU26709
New.CleanUp.ReferenceOTU57285
New.CleanUp.ReferenceOTU34848
New.CleanUp.ReferenceOTU52997
New.CleanUp.ReferenceOTU108096
New.CleanUp.ReferenceOTU173522
New.CleanUp.ReferenceOTU238327
New.CleanUp.ReferenceOTU22327
New.CleanUp.ReferenceOTU88965
New.CleanUp.ReferenceOTU41391

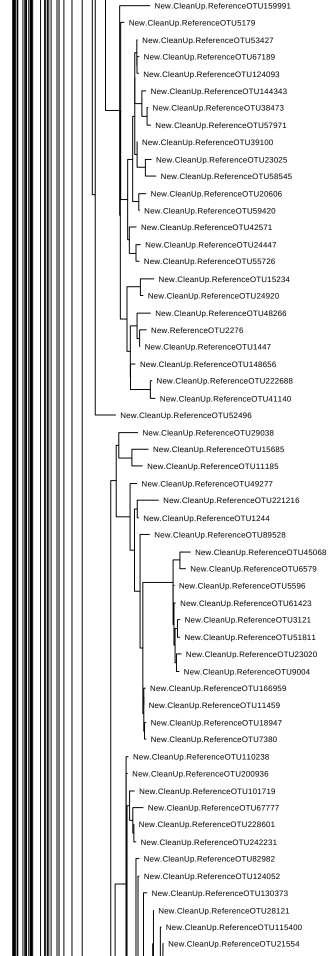
New.CleanUp.ReferenceOTU159991
New.CleanUp.ReferenceOTU5179
New.CleanUp.ReferenceOTU53427
New.CleanUp.ReferenceOTU67189
New.CleanUp.ReferenceOTU124093
New.CleanUp.ReferenceOTU144343
New.CleanUp.ReferenceOTU38473
New.CleanUp.ReferenceOTU57971
New.CleanUp.ReferenceOTU39100
New.CleanUp.ReferenceOTU23025
New.CleanUp.ReferenceOTU58545
New.CleanUp.ReferenceOTU20606
New.CleanUp.ReferenceOTU59420
New.CleanUp.ReferenceOTU42571
New.CleanUp.ReferenceOTU24447
New.CleanUp.ReferenceOTU55726
New.CleanUp.ReferenceOTU15234
New.CleanUp.ReferenceOTU24920
New.CleanUp.ReferenceOTU48266
New.ReferenceOTU2276
New.CleanUp.ReferenceOTU1447
New.CleanUp.ReferenceOTU148656
New.CleanUp.ReferenceOTU222688
New.CleanUp.ReferenceOTU41140
New.CleanUp.ReferenceOTU52496
New.CleanUp.ReferenceOTU29038
New.CleanUp.ReferenceOTU15685
New.CleanUp.ReferenceOTU11185
New.CleanUp.ReferenceOTU49277
New.CleanUp.ReferenceOTU221216
New.CleanUp.ReferenceOTU1244
New.CleanUp.ReferenceOTU89528
New.CleanUp.ReferenceOTU45068
New.CleanUp.ReferenceOTU6579
New.CleanUp.ReferenceOTU5596
New.CleanUp.ReferenceOTU61423
New.CleanUp.ReferenceOTU3121
New.CleanUp.ReferenceOTU51811
New.CleanUp.ReferenceOTU23020
New.CleanUp.ReferenceOTU9004
New.CleanUp.ReferenceOTU166959
New.CleanUp.ReferenceOTU11459
New.CleanUp.ReferenceOTU18947
New.CleanUp.ReferenceOTU7380
New.CleanUp.ReferenceOTU110238
New.CleanUp.ReferenceOTU200936
New.CleanUp.ReferenceOTU101719
New.CleanUp.ReferenceOTU67777
New.CleanUp.ReferenceOTU228601
New.CleanUp.ReferenceOTU242231
New.CleanUp.ReferenceOTU82982
New.CleanUp.ReferenceOTU124052
New.CleanUp.ReferenceOTU130373
New.CleanUp.ReferenceOTU28121
New.CleanUp.ReferenceOTU115400
New.CleanUp.ReferenceOTU21554

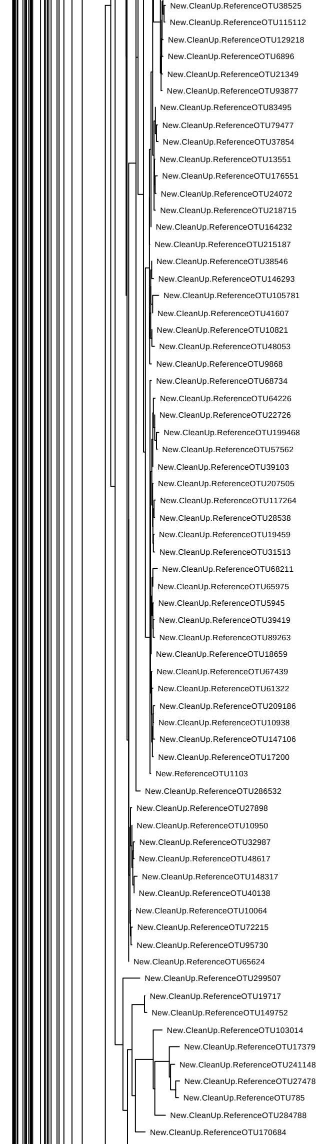
New.CleanUp.ReferenceOTU38525
New.CleanUp.ReferenceOTU115112
New.CleanUp.ReferenceOTU129218
New.CleanUp.ReferenceOTU6896
New.CleanUp.ReferenceOTU21349
New.CleanUp.ReferenceOTU93877
New.CleanUp.ReferenceOTU83495
New.CleanUp.ReferenceOTU79477
New.CleanUp.ReferenceOTU37854
New.CleanUp.ReferenceOTU13551
New.CleanUp.ReferenceOTU176551
New.CleanUp.ReferenceOTU24072
New.CleanUp.ReferenceOTU218715
New.CleanUp.ReferenceOTU164232
New.CleanUp.ReferenceOTU215187
New.CleanUp.ReferenceOTU38546
New.CleanUp.ReferenceOTU146293
New.CleanUp.ReferenceOTU105781
New.CleanUp.ReferenceOTU41607
New.CleanUp.ReferenceOTU10821
New.CleanUp.ReferenceOTU48053
New.CleanUp.ReferenceOTU9868
New.CleanUp.ReferenceOTU68734
New.CleanUp.ReferenceOTU64226
New.CleanUp.ReferenceOTU22726
New.CleanUp.ReferenceOTU199468
New.CleanUp.ReferenceOTU57562
New.CleanUp.ReferenceOTU39103
New.CleanUp.ReferenceOTU207505
New.CleanUp.ReferenceOTU117264
New.CleanUp.ReferenceOTU28538
New.CleanUp.ReferenceOTU19459
New.CleanUp.ReferenceOTU31513
New.CleanUp.ReferenceOTU68211
New.CleanUp.ReferenceOTU65975
New.CleanUp.ReferenceOTU5945
New.CleanUp.ReferenceOTU39419
New.CleanUp.ReferenceOTU89263
New.CleanUp.ReferenceOTU18659
New.CleanUp.ReferenceOTU67439
New.CleanUp.ReferenceOTU61322
New.CleanUp.ReferenceOTU209186
New.CleanUp.ReferenceOTU10938
New.CleanUp.ReferenceOTU147106
New.CleanUp.ReferenceOTU17200
New.ReferenceOTU1103
New.CleanUp.ReferenceOTU286532
New.CleanUp.ReferenceOTU27898
New.CleanUp.ReferenceOTU10950
New.CleanUp.ReferenceOTU32987
New.CleanUp.ReferenceOTU48617
New.CleanUp.ReferenceOTU148317
New.CleanUp.ReferenceOTU40138
New.CleanUp.ReferenceOTU10064
New.CleanUp.ReferenceOTU72215
New.CleanUp.ReferenceOTU95730
New.CleanUp.ReferenceOTU65624
New.CleanUp.ReferenceOTU299507
New.CleanUp.ReferenceOTU19717
New.CleanUp.ReferenceOTU149752
New.CleanUp.ReferenceOTU103014
New.CleanUp.ReferenceOTU17379
New.CleanUp.ReferenceOTU241148
New.CleanUp.ReferenceOTU27478
New.CleanUp.ReferenceOTU785
New.CleanUp.ReferenceOTU284788
New.CleanUp.ReferenceOTU170684

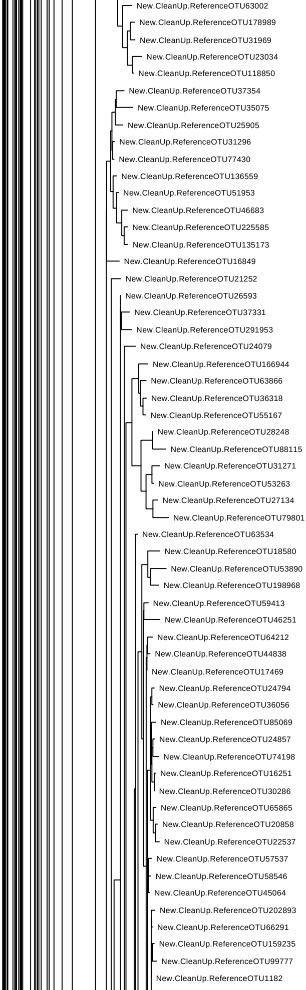
New.CleanUp.ReferenceOTU63002
New.CleanUp.ReferenceOTU178989
New.CleanUp.ReferenceOTU31969
New.CleanUp.ReferenceOTU23034
New.CleanUp.ReferenceOTU118850
New.CleanUp.ReferenceOTU37354
New.CleanUp.ReferenceOTU35075
New.CleanUp.ReferenceOTU25905
New.CleanUp.ReferenceOTU31296
New.CleanUp.ReferenceOTU77430
New.CleanUp.ReferenceOTU136559
New.CleanUp.ReferenceOTU51953
New.CleanUp.ReferenceOTU46683
New.CleanUp.ReferenceOTU225585
New.CleanUp.ReferenceOTU135173
New.CleanUp.ReferenceOTU16849
New.CleanUp.ReferenceOTU21252
New.CleanUp.ReferenceOTU26593
New.CleanUp.ReferenceOTU37331
New.CleanUp.ReferenceOTU291953
New.CleanUp.ReferenceOTU24079
New.CleanUp.ReferenceOTU166944
New.CleanUp.ReferenceOTU63866
New.CleanUp.ReferenceOTU36318
New.CleanUp.ReferenceOTU55167
New.CleanUp.ReferenceOTU28248
New.CleanUp.ReferenceOTU88115
New.CleanUp.ReferenceOTU31271
New.CleanUp.ReferenceOTU53263
New.CleanUp.ReferenceOTU27134
New.CleanUp.ReferenceOTU79801
New.CleanUp.ReferenceOTU63534
New.CleanUp.ReferenceOTU18580
New.CleanUp.ReferenceOTU53890
New.CleanUp.ReferenceOTU198968
New.CleanUp.ReferenceOTU59413
New.CleanUp.ReferenceOTU46251
New.CleanUp.ReferenceOTU64212
New.CleanUp.ReferenceOTU44838
New.CleanUp.ReferenceOTU17469
New.CleanUp.ReferenceOTU24794
New.CleanUp.ReferenceOTU36056
New.CleanUp.ReferenceOTU85069
New.CleanUp.ReferenceOTU24857
New.CleanUp.ReferenceOTU74198
New.CleanUp.ReferenceOTU16251
New.CleanUp.ReferenceOTU30286
New.CleanUp.ReferenceOTU65865
New.CleanUp.ReferenceOTU20858
New.CleanUp.ReferenceOTU22537
New.CleanUp.ReferenceOTU57537
New.CleanUp.ReferenceOTU58546
New.CleanUp.ReferenceOTU45064
New.CleanUp.ReferenceOTU202893
New.CleanUp.ReferenceOTU66291
New.CleanUp.ReferenceOTU159235
New.CleanUp.ReferenceOTU99777
New.CleanUp.ReferenceOTU1182

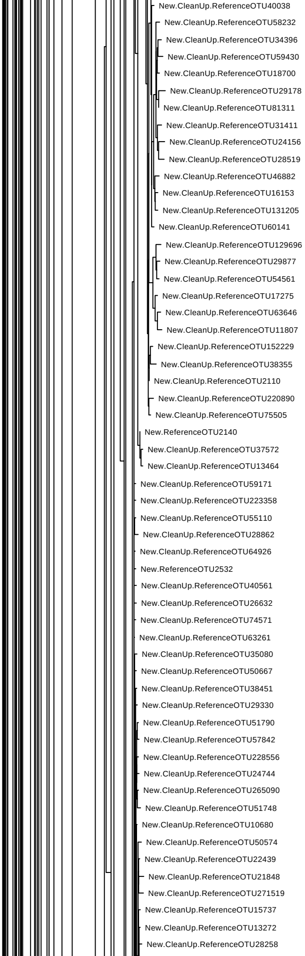
New.CleanUp.ReferenceOTU40038
New.CleanUp.ReferenceOTU58232
New.CleanUp.ReferenceOTU34396
New.CleanUp.ReferenceOTU59430
New.CleanUp.ReferenceOTU18700
New.CleanUp.ReferenceOTU29178
New.CleanUp.ReferenceOTU81311
New.CleanUp.ReferenceOTU31411
New.CleanUp.ReferenceOTU24156
New.CleanUp.ReferenceOTU28519
New.CleanUp.ReferenceOTU46882
New.CleanUp.ReferenceOTU16153
New.CleanUp.ReferenceOTU131205
New.CleanUp.ReferenceOTU60141
New.CleanUp.ReferenceOTU129696
New.CleanUp.ReferenceOTU29877
New.CleanUp.ReferenceOTU54561
New.CleanUp.ReferenceOTU17275
New.CleanUp.ReferenceOTU63646
New.CleanUp.ReferenceOTU11807
New.CleanUp.ReferenceOTU152229
New.CleanUp.ReferenceOTU38355
New.CleanUp.ReferenceOTU2110
New.CleanUp.ReferenceOTU220890
New.CleanUp.ReferenceOTU75505
New.ReferenceOTU2140
New.CleanUp.ReferenceOTU37572
New.CleanUp.ReferenceOTU13464
New.CleanUp.ReferenceOTU59171
New.CleanUp.ReferenceOTU223358
New.CleanUp.ReferenceOTU55110
New.CleanUp.ReferenceOTU28862
New.CleanUp.ReferenceOTU64926
New.ReferenceOTU2532
New.CleanUp.ReferenceOTU40561
New.CleanUp.ReferenceOTU26632
New.CleanUp.ReferenceOTU74571
New.CleanUp.ReferenceOTU63261
New.CleanUp.ReferenceOTU35080
New.CleanUp.ReferenceOTU50667
New.CleanUp.ReferenceOTU38451
New.CleanUp.ReferenceOTU29330
New.CleanUp.ReferenceOTU51790
New.CleanUp.ReferenceOTU57842
New.CleanUp.ReferenceOTU228556
New.CleanUp.ReferenceOTU24744
New.CleanUp.ReferenceOTU265090
New.CleanUp.ReferenceOTU51748
New.CleanUp.ReferenceOTU10680
New.CleanUp.ReferenceOTU50574
New.CleanUp.ReferenceOTU22439
New.CleanUp.ReferenceOTU21848
New.CleanUp.ReferenceOTU271519
New.CleanUp.ReferenceOTU15737
New.CleanUp.ReferenceOTU13272
New.CleanUp.ReferenceOTU28258

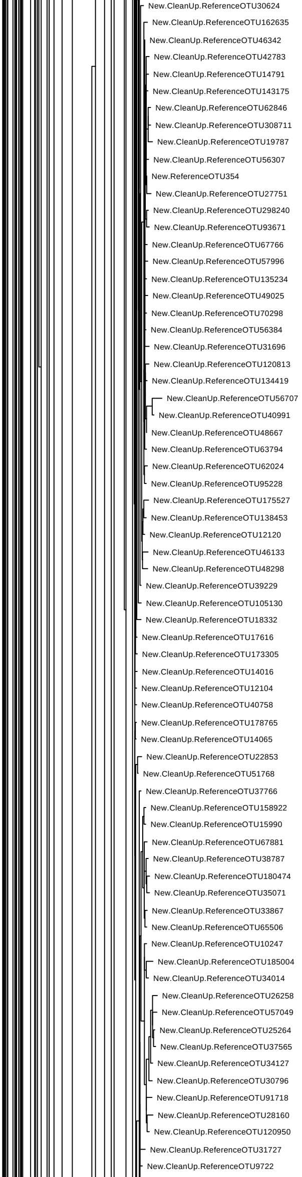
New.CleanUp.ReferenceOTU30624
New.CleanUp.ReferenceOTU162635
New.CleanUp.ReferenceOTU46342
New.CleanUp.ReferenceOTU42783
New.CleanUp.ReferenceOTU14791
New.CleanUp.ReferenceOTU143175
New.CleanUp.ReferenceOTU62846
New.CleanUp.ReferenceOTU308711
New.CleanUp.ReferenceOTU19787
New.CleanUp.ReferenceOTU56307
New.ReferenceOTU354
New.CleanUp.ReferenceOTU27751
New.CleanUp.ReferenceOTU298240
New.CleanUp.ReferenceOTU93671
New.CleanUp.ReferenceOTU67766
New.CleanUp.ReferenceOTU57996
New.CleanUp.ReferenceOTU135234
New.CleanUp.ReferenceOTU49025
New.CleanUp.ReferenceOTU70298
New.CleanUp.ReferenceOTU56384
New.CleanUp.ReferenceOTU31696
New.CleanUp.ReferenceOTU120813
New.CleanUp.ReferenceOTU134419
New.CleanUp.ReferenceOTU56707
New.CleanUp.ReferenceOTU40991
New.CleanUp.ReferenceOTU48667
New.CleanUp.ReferenceOTU63794
New.CleanUp.ReferenceOTU62024
New.CleanUp.ReferenceOTU95228
New.CleanUp.ReferenceOTU175527
New.CleanUp.ReferenceOTU138453
New.CleanUp.ReferenceOTU12120
New.CleanUp.ReferenceOTU46133
New.CleanUp.ReferenceOTU48298
New.CleanUp.ReferenceOTU39229
New.CleanUp.ReferenceOTU105130
New.CleanUp.ReferenceOTU18332
New.CleanUp.ReferenceOTU17616
New.CleanUp.ReferenceOTU173305
New.CleanUp.ReferenceOTU14016
New.CleanUp.ReferenceOTU12104
New.CleanUp.ReferenceOTU40758
New.CleanUp.ReferenceOTU178765
New.CleanUp.ReferenceOTU14065
New.CleanUp.ReferenceOTU22853
New.CleanUp.ReferenceOTU51768
New.CleanUp.ReferenceOTU37766
New.CleanUp.ReferenceOTU158922
New.CleanUp.ReferenceOTU15990
New.CleanUp.ReferenceOTU67881
New.CleanUp.ReferenceOTU38787
New.CleanUp.ReferenceOTU180474
New.CleanUp.ReferenceOTU35071
New.CleanUp.ReferenceOTU33867
New.CleanUp.ReferenceOTU65506
New.CleanUp.ReferenceOTU10247
New.CleanUp.ReferenceOTU185004
New.CleanUp.ReferenceOTU34014
New.CleanUp.ReferenceOTU26258
New.CleanUp.ReferenceOTU57049
New.CleanUp.ReferenceOTU25264
New.CleanUp.ReferenceOTU37565
New.CleanUp.ReferenceOTU34127
New.CleanUp.ReferenceOTU30796
New.CleanUp.ReferenceOTU91718
New.CleanUp.ReferenceOTU28160
New.CleanUp.ReferenceOTU120950
New.CleanUp.ReferenceOTU31727
New.CleanUp.ReferenceOTU9722

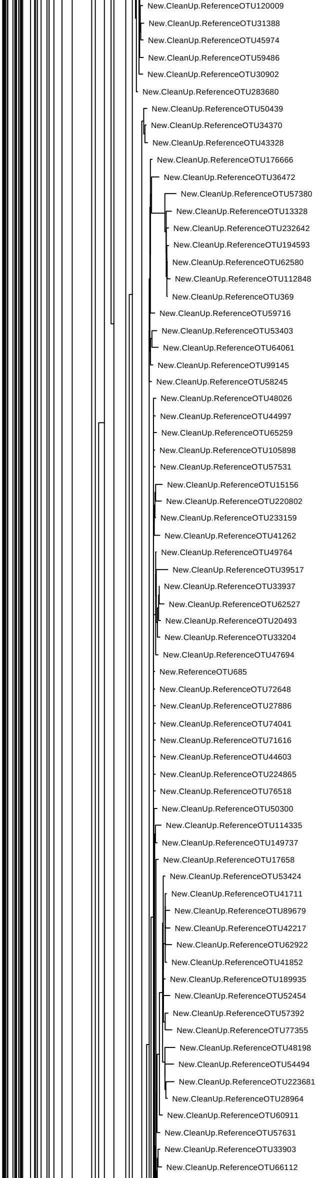
New.CleanUp.ReferenceOTU120009
New.CleanUp.ReferenceOTU31388
New.CleanUp.ReferenceOTU45974
New.CleanUp.ReferenceOTU59486
New.CleanUp.ReferenceOTU30902
New.CleanUp.ReferenceOTU283680
New.CleanUp.ReferenceOTU50439
New.CleanUp.ReferenceOTU34370
New.CleanUp.ReferenceOTU43328
New.CleanUp.ReferenceOTU176666
New.CleanUp.ReferenceOTU36472
New.CleanUp.ReferenceOTU57380
New.CleanUp.ReferenceOTU13328
New.CleanUp.ReferenceOTU232642
New.CleanUp.ReferenceOTU194593
New.CleanUp.ReferenceOTU62580
New.CleanUp.ReferenceOTU112848
New.CleanUp.ReferenceOTU369
New.CleanUp.ReferenceOTU59716
New.CleanUp.ReferenceOTU53403
New.CleanUp.ReferenceOTU64061
New.CleanUp.ReferenceOTU99145
New.CleanUp.ReferenceOTU58245
New.CleanUp.ReferenceOTU48026
New.CleanUp.ReferenceOTU44997
New.CleanUp.ReferenceOTU65259
New.CleanUp.ReferenceOTU105898
New.CleanUp.ReferenceOTU57531
New.CleanUp.ReferenceOTU15156
New.CleanUp.ReferenceOTU220802
New.CleanUp.ReferenceOTU233159
New.CleanUp.ReferenceOTU41262
New.CleanUp.ReferenceOTU49764
New.CleanUp.ReferenceOTU39517
New.CleanUp.ReferenceOTU33937
New.CleanUp.ReferenceOTU62527
New.CleanUp.ReferenceOTU20493
New.CleanUp.ReferenceOTU33204
New.CleanUp.ReferenceOTU47694
New.ReferenceOTU685
New.CleanUp.ReferenceOTU72648
New.CleanUp.ReferenceOTU27886
New.CleanUp.ReferenceOTU74041
New.CleanUp.ReferenceOTU71616
New.CleanUp.ReferenceOTU44603
New.CleanUp.ReferenceOTU224865
New.CleanUp.ReferenceOTU76518
New.CleanUp.ReferenceOTU50300
New.CleanUp.ReferenceOTU114335
New.CleanUp.ReferenceOTU149737
New.CleanUp.ReferenceOTU17658
New.CleanUp.ReferenceOTU53424
New.CleanUp.ReferenceOTU41711
New.CleanUp.ReferenceOTU89679
New.CleanUp.ReferenceOTU42217
New.CleanUp.ReferenceOTU62922
New.CleanUp.ReferenceOTU41852
New.CleanUp.ReferenceOTU189935
New.CleanUp.ReferenceOTU52454
New.CleanUp.ReferenceOTU57392
New.CleanUp.ReferenceOTU77355
New.CleanUp.ReferenceOTU48198
New.CleanUp.ReferenceOTU54494
New.CleanUp.ReferenceOTU223681
New.CleanUp.ReferenceOTU28964
New.CleanUp.ReferenceOTU60911
New.CleanUp.ReferenceOTU57631
New.CleanUp.ReferenceOTU33903
New.CleanUp.ReferenceOTU66112

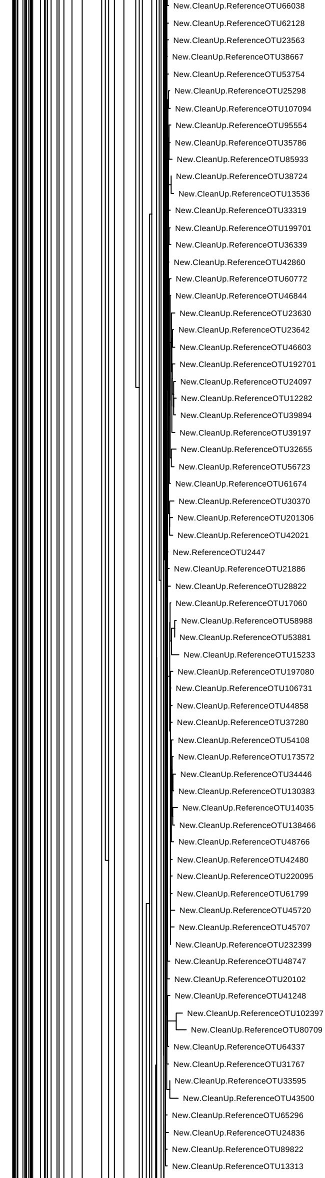

New.CleanUp.ReferenceOTU66038
New.CleanUp.ReferenceOTU62128
New.CleanUp.ReferenceOTU23563
New.CleanUp.ReferenceOTU38667
New.CleanUp.ReferenceOTU53754
New.CleanUp.ReferenceOTU25298
New.CleanUp.ReferenceOTU107094
New.CleanUp.ReferenceOTU95554
New.CleanUp.ReferenceOTU35786
New.CleanUp.ReferenceOTU85933
New.CleanUp.ReferenceOTU38724
New.CleanUp.ReferenceOTU13536
New.CleanUp.ReferenceOTU33319
New.CleanUp.ReferenceOTU199701
New.CleanUp.ReferenceOTU36339
New.CleanUp.ReferenceOTU42860
New.CleanUp.ReferenceOTU60772
New.CleanUp.ReferenceOTU46844
New.CleanUp.ReferenceOTU23630
New.CleanUp.ReferenceOTU23642
New.CleanUp.ReferenceOTU46603
New.CleanUp.ReferenceOTU192701
New.CleanUp.ReferenceOTU24097
New.CleanUp.ReferenceOTU12282
New.CleanUp.ReferenceOTU39894
New.CleanUp.ReferenceOTU39197
New.CleanUp.ReferenceOTU32655
New.CleanUp.ReferenceOTU56723
New.CleanUp.ReferenceOTU61674
New.CleanUp.ReferenceOTU30370
New.CleanUp.ReferenceOTU201306
New.CleanUp.ReferenceOTU42021
New.ReferenceOTU2447
New.CleanUp.ReferenceOTU21886
New.CleanUp.ReferenceOTU28822
New.CleanUp.ReferenceOTU17060
New.CleanUp.ReferenceOTU58988
New.CleanUp.ReferenceOTU53881
New.CleanUp.ReferenceOTU15233
New.CleanUp.ReferenceOTU197080
New.CleanUp.ReferenceOTU106731
New.CleanUp.ReferenceOTU44858
New.CleanUp.ReferenceOTU37280
New.CleanUp.ReferenceOTU54108
New.CleanUp.ReferenceOTU173572
New.CleanUp.ReferenceOTU34446
New.CleanUp.ReferenceOTU130383
New.CleanUp.ReferenceOTU14035
New.CleanUp.ReferenceOTU138466
New.CleanUp.ReferenceOTU48766
New.CleanUp.ReferenceOTU42480
New.CleanUp.ReferenceOTU220095
New.CleanUp.ReferenceOTU61799
New.CleanUp.ReferenceOTU45720
New.CleanUp.ReferenceOTU45707
New.CleanUp.ReferenceOTU232399
New.CleanUp.ReferenceOTU48747
New.CleanUp.ReferenceOTU20102
New.CleanUp.ReferenceOTU41248
New.CleanUp.ReferenceOTU102397
New.CleanUp.ReferenceOTU80709
New.CleanUp.ReferenceOTU64337
New.CleanUp.ReferenceOTU31767
New.CleanUp.ReferenceOTU33595
New.CleanUp.ReferenceOTU43500
New.CleanUp.ReferenceOTU65296
New.CleanUp.ReferenceOTU24836
New.CleanUp.ReferenceOTU89822
New.CleanUp.ReferenceOTU13313

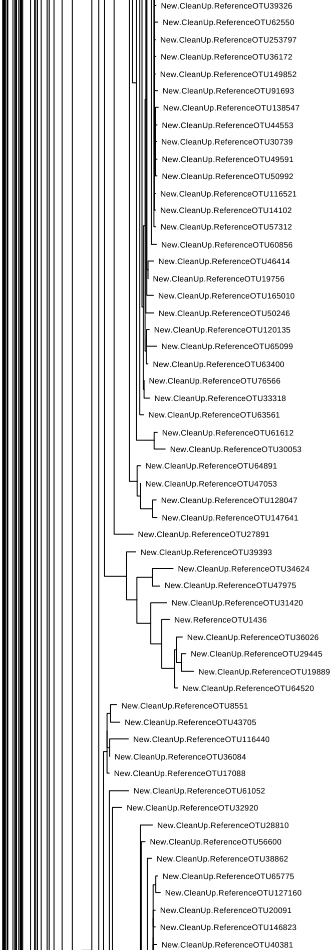
New.CleanUp.ReferenceOTU39326
New.CleanUp.ReferenceOTU62550
New.CleanUp.ReferenceOTU253797
New.CleanUp.ReferenceOTU36172
New.CleanUp.ReferenceOTU149852
New.CleanUp.ReferenceOTU91693
New.CleanUp.ReferenceOTU138547
New.CleanUp.ReferenceOTU44553
New.CleanUp.ReferenceOTU30739
New.CleanUp.ReferenceOTU49591
New.CleanUp.ReferenceOTU50992
New.CleanUp.ReferenceOTU116521
New.CleanUp.ReferenceOTU14102
New.CleanUp.ReferenceOTU57312
New.CleanUp.ReferenceOTU60856
New.CleanUp.ReferenceOTU46414
New.CleanUp.ReferenceOTU19756
New.CleanUp.ReferenceOTU165010
New.CleanUp.ReferenceOTU50246
New.CleanUp.ReferenceOTU120135
New.CleanUp.ReferenceOTU65099
New.CleanUp.ReferenceOTU63400
New.CleanUp.ReferenceOTU76566
New.CleanUp.ReferenceOTU33318
New.CleanUp.ReferenceOTU63561
New.CleanUp.ReferenceOTU61612
New.CleanUp.ReferenceOTU30053
New.CleanUp.ReferenceOTU64891
New.CleanUp.ReferenceOTU47053
New.CleanUp.ReferenceOTU128047
New.CleanUp.ReferenceOTU147641
New.CleanUp.ReferenceOTU27891
New.CleanUp.ReferenceOTU39393
New.CleanUp.ReferenceOTU34624
New.CleanUp.ReferenceOTU47975
New.CleanUp.ReferenceOTU31420
New.ReferenceOTU1436
New.CleanUp.ReferenceOTU36026
New.CleanUp.ReferenceOTU29445
New.CleanUp.ReferenceOTU19889
New.CleanUp.ReferenceOTU64520
New.CleanUp.ReferenceOTU8551
New.CleanUp.ReferenceOTU43705
New.CleanUp.ReferenceOTU116440
New.CleanUp.ReferenceOTU36084
New.CleanUp.ReferenceOTU17088
New.CleanUp.ReferenceOTU61052
New.CleanUp.ReferenceOTU32920
New.CleanUp.ReferenceOTU28810
New.CleanUp.ReferenceOTU56600
New.CleanUp.ReferenceOTU38862
New.CleanUp.ReferenceOTU65775
New.CleanUp.ReferenceOTU127160
New.CleanUp.ReferenceOTU20091
New.CleanUp.ReferenceOTU146823
New.CleanUp.ReferenceOTU40381

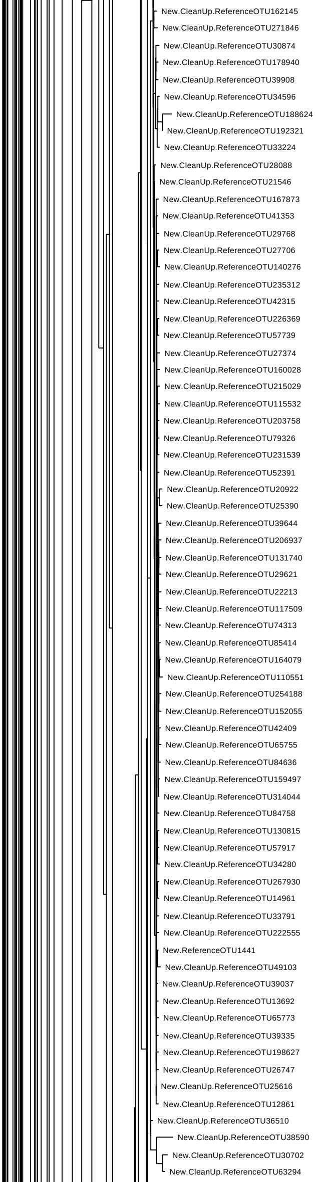
New.CleanUp.ReferenceOTU162145
New.CleanUp.ReferenceOTU271846
New.CleanUp.ReferenceOTU30874
New.CleanUp.ReferenceOTU178940
New.CleanUp.ReferenceOTU39908
New.CleanUp.ReferenceOTU34596
New.CleanUp.ReferenceOTU188624
New.CleanUp.ReferenceOTU192321
New.CleanUp.ReferenceOTU33224
New.CleanUp.ReferenceOTU28088
New.CleanUp.ReferenceOTU21546
New.CleanUp.ReferenceOTU167873
New.CleanUp.ReferenceOTU41353
New.CleanUp.ReferenceOTU29768
New.CleanUp.ReferenceOTU27706
New.CleanUp.ReferenceOTU140276
New.CleanUp.ReferenceOTU235312
New.CleanUp.ReferenceOTU42315
New.CleanUp.ReferenceOTU226369
New.CleanUp.ReferenceOTU57739
New.CleanUp.ReferenceOTU27374
New.CleanUp.ReferenceOTU160028
New.CleanUp.ReferenceOTU215029
New.CleanUp.ReferenceOTU115532
New.CleanUp.ReferenceOTU203758
New.CleanUp.ReferenceOTU79326
New.CleanUp.ReferenceOTU231539
New.CleanUp.ReferenceOTU52391
New.CleanUp.ReferenceOTU20922
New.CleanUp.ReferenceOTU25390
New.CleanUp.ReferenceOTU39644
New.CleanUp.ReferenceOTU206937
New.CleanUp.ReferenceOTU131740
New.CleanUp.ReferenceOTU29621
New.CleanUp.ReferenceOTU22213
New.CleanUp.ReferenceOTU117509
New.CleanUp.ReferenceOTU74313
New.CleanUp.ReferenceOTU85414
New.CleanUp.ReferenceOTU164079
New.CleanUp.ReferenceOTU110551
New.CleanUp.ReferenceOTU254188
New.CleanUp.ReferenceOTU152055
New.CleanUp.ReferenceOTU42409
New.CleanUp.ReferenceOTU65755
New.CleanUp.ReferenceOTU84636
New.CleanUp.ReferenceOTU159497
New.CleanUp.ReferenceOTU314044
New.CleanUp.ReferenceOTU84758
New.CleanUp.ReferenceOTU130815
New.CleanUp.ReferenceOTU57917
New.CleanUp.ReferenceOTU34280
New.CleanUp.ReferenceOTU267930
New.CleanUp.ReferenceOTU14961
New.CleanUp.ReferenceOTU33791
New.CleanUp.ReferenceOTU222555
New.ReferenceOTU1441
New.CleanUp.ReferenceOTU49103
New.CleanUp.ReferenceOTU39037
New.CleanUp.ReferenceOTU13692
New.CleanUp.ReferenceOTU65773
New.CleanUp.ReferenceOTU39335
New.CleanUp.ReferenceOTU198627
New.CleanUp.ReferenceOTU26747
New.CleanUp.ReferenceOTU25616
New.CleanUp.ReferenceOTU12861
New.CleanUp.ReferenceOTU36510
New.CleanUp.ReferenceOTU38590
New.CleanUp.ReferenceOTU30702
New.CleanUp.ReferenceOTU63294

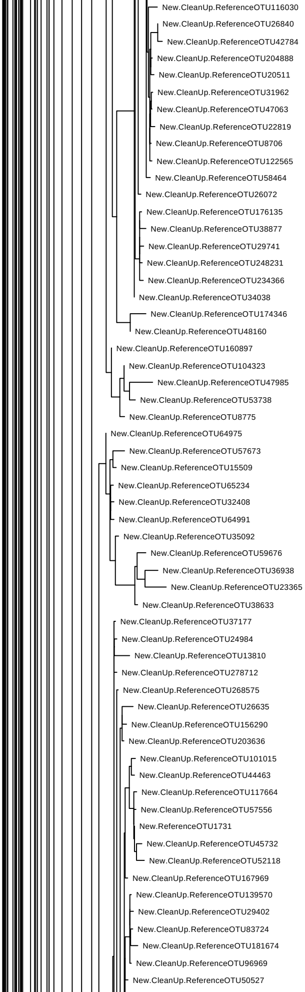
New.CleanUp.ReferenceOTU116030
New.CleanUp.ReferenceOTU26840
New.CleanUp.ReferenceOTU42784
New.CleanUp.ReferenceOTU204888
New.CleanUp.ReferenceOTU20511
New.CleanUp.ReferenceOTU31962
New.CleanUp.ReferenceOTU47063
New.CleanUp.ReferenceOTU22819
New.CleanUp.ReferenceOTU8706
New.CleanUp.ReferenceOTU122565
New.CleanUp.ReferenceOTU58464
New.CleanUp.ReferenceOTU26072
New.CleanUp.ReferenceOTU176135
New.CleanUp.ReferenceOTU38877
New.CleanUp.ReferenceOTU29741
New.CleanUp.ReferenceOTU248231
New.CleanUp.ReferenceOTU234366
New.CleanUp.ReferenceOTU34038
New.CleanUp.ReferenceOTU174346
New.CleanUp.ReferenceOTU48160
New.CleanUp.ReferenceOTU160897
New.CleanUp.ReferenceOTU104323
New.CleanUp.ReferenceOTU47985
New.CleanUp.ReferenceOTU53738
New.CleanUp.ReferenceOTU8775
New.CleanUp.ReferenceOTU64975
New.CleanUp.ReferenceOTU57673
New.CleanUp.ReferenceOTU15509
New.CleanUp.ReferenceOTU65234
New.CleanUp.ReferenceOTU32408
New.CleanUp.ReferenceOTU64991
New.CleanUp.ReferenceOTU35092
New.CleanUp.ReferenceOTU59676
New.CleanUp.ReferenceOTU36938
New.CleanUp.ReferenceOTU23365
New.CleanUp.ReferenceOTU38633
New.CleanUp.ReferenceOTU37177
New.CleanUp.ReferenceOTU24984
New.CleanUp.ReferenceOTU13810
New.CleanUp.ReferenceOTU278712
New.CleanUp.ReferenceOTU268575
New.CleanUp.ReferenceOTU26635
New.CleanUp.ReferenceOTU156290
New.CleanUp.ReferenceOTU203636
New.CleanUp.ReferenceOTU101015
New.CleanUp.ReferenceOTU44463
New.CleanUp.ReferenceOTU117664
New.CleanUp.ReferenceOTU57556
New.ReferenceOTU1731
New.CleanUp.ReferenceOTU45732
New.CleanUp.ReferenceOTU52118
New.CleanUp.ReferenceOTU167969
New.CleanUp.ReferenceOTU139570
New.CleanUp.ReferenceOTU29402
New.CleanUp.ReferenceOTU83724
New.CleanUp.ReferenceOTU181674
New.CleanUp.ReferenceOTU96969
New.CleanUp.ReferenceOTU50527

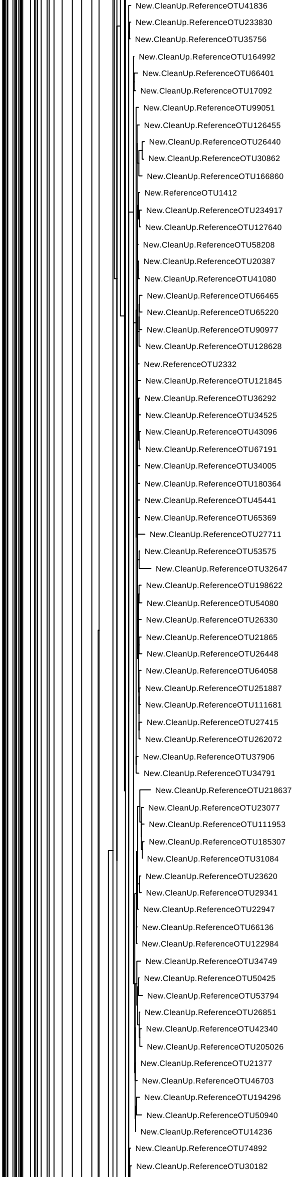
New.CleanUp.ReferenceOTU41836
New.CleanUp.ReferenceOTU233830
New.CleanUp.ReferenceOTU35756
New.CleanUp.ReferenceOTU164992
New.CleanUp.ReferenceOTU66401
New.CleanUp.ReferenceOTU17092
New.CleanUp.ReferenceOTU99051
New.CleanUp.ReferenceOTU126455
New.CleanUp.ReferenceOTU26440
New.CleanUp.ReferenceOTU30862
New.CleanUp.ReferenceOTU166860
New.ReferenceOTU1412
New.CleanUp.ReferenceOTU234917
New.CleanUp.ReferenceOTU127640
New.CleanUp.ReferenceOTU58208
New.CleanUp.ReferenceOTU20387
New.CleanUp.ReferenceOTU41080
New.CleanUp.ReferenceOTU66465
New.CleanUp.ReferenceOTU65220
New.CleanUp.ReferenceOTU90977
New.CleanUp.ReferenceOTU128628
New.ReferenceOTU2332
New.CleanUp.ReferenceOTU121845
New.CleanUp.ReferenceOTU36292
New.CleanUp.ReferenceOTU34525
New.CleanUp.ReferenceOTU43096
New.CleanUp.ReferenceOTU67191
New.CleanUp.ReferenceOTU34005
New.CleanUp.ReferenceOTU180364
New.CleanUp.ReferenceOTU45441
New.CleanUp.ReferenceOTU65369
New.CleanUp.ReferenceOTU27711
New.CleanUp.ReferenceOTU53575
New.CleanUp.ReferenceOTU32647
New.CleanUp.ReferenceOTU198622
New.CleanUp.ReferenceOTU54080
New.CleanUp.ReferenceOTU26330
New.CleanUp.ReferenceOTU21865
New.CleanUp.ReferenceOTU26448
New.CleanUp.ReferenceOTU64058
New.CleanUp.ReferenceOTU251887
New.CleanUp.ReferenceOTU111681
New.CleanUp.ReferenceOTU27415
New.CleanUp.ReferenceOTU262072
New.CleanUp.ReferenceOTU37906
New.CleanUp.ReferenceOTU34791
New.CleanUp.ReferenceOTU218637
New.CleanUp.ReferenceOTU23077
New.CleanUp.ReferenceOTU111953
New.CleanUp.ReferenceOTU185307
New.CleanUp.ReferenceOTU31084
New.CleanUp.ReferenceOTU23620
New.CleanUp.ReferenceOTU29341
New.CleanUp.ReferenceOTU22947
New.CleanUp.ReferenceOTU66136
New.CleanUp.ReferenceOTU122984
New.CleanUp.ReferenceOTU34749
New.CleanUp.ReferenceOTU50425
New.CleanUp.ReferenceOTU53794
New.CleanUp.ReferenceOTU26851
New.CleanUp.ReferenceOTU42340
New.CleanUp.ReferenceOTU205026
New.CleanUp.ReferenceOTU21377
New.CleanUp.ReferenceOTU46703
New.CleanUp.ReferenceOTU194296
New.CleanUp.ReferenceOTU50940
New.CleanUp.ReferenceOTU14236
New.CleanUp.ReferenceOTU74892
New.CleanUp.ReferenceOTU30182

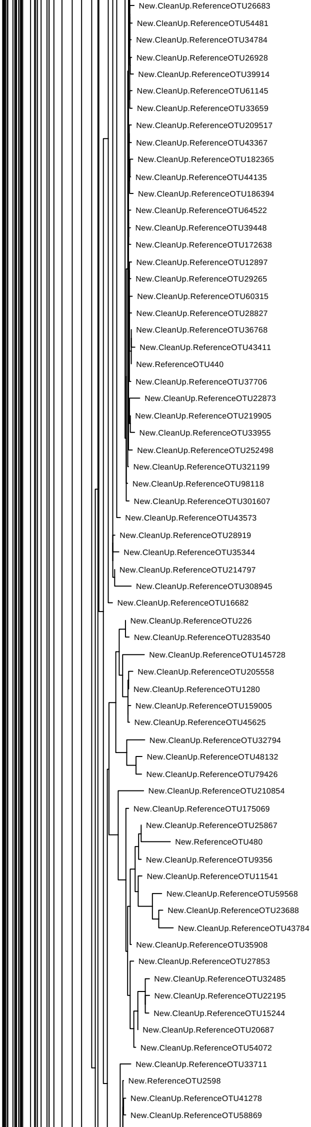
New.CleanUp.ReferenceOTU26683
New.CleanUp.ReferenceOTU54481
New.CleanUp.ReferenceOTU34784
New.CleanUp.ReferenceOTU26928
New.CleanUp.ReferenceOTU39914
New.CleanUp.ReferenceOTU61145
New.CleanUp.ReferenceOTU33659
New.CleanUp.ReferenceOTU209517
New.CleanUp.ReferenceOTU43367
New.CleanUp.ReferenceOTU182365
New.CleanUp.ReferenceOTU44135
New.CleanUp.ReferenceOTU186394
New.CleanUp.ReferenceOTU64522
New.CleanUp.ReferenceOTU39448
New.CleanUp.ReferenceOTU172638
New.CleanUp.ReferenceOTU12897
New.CleanUp.ReferenceOTU29265
New.CleanUp.ReferenceOTU60315
New.CleanUp.ReferenceOTU28827
New.CleanUp.ReferenceOTU36768
New.CleanUp.ReferenceOTU43411
New.ReferenceOTU440
New.CleanUp.ReferenceOTU37706
New.CleanUp.ReferenceOTU22873
New.CleanUp.ReferenceOTU219905
New.CleanUp.ReferenceOTU33955
New.CleanUp.ReferenceOTU252498
New.CleanUp.ReferenceOTU321199
New.CleanUp.ReferenceOTU98118
New.CleanUp.ReferenceOTU301607
New.CleanUp.ReferenceOTU43573
New.CleanUp.ReferenceOTU28919
New.CleanUp.ReferenceOTU35344
New.CleanUp.ReferenceOTU214797
New.CleanUp.ReferenceOTU308945
New.CleanUp.ReferenceOTU16682
New.CleanUp.ReferenceOTU226
New.CleanUp.ReferenceOTU283540
New.CleanUp.ReferenceOTU145728
New.CleanUp.ReferenceOTU205558
New.CleanUp.ReferenceOTU1280
New.CleanUp.ReferenceOTU159005
New.CleanUp.ReferenceOTU45625
New.CleanUp.ReferenceOTU32794
New.CleanUp.ReferenceOTU48132
New.CleanUp.ReferenceOTU79426
New.CleanUp.ReferenceOTU210854
New.CleanUp.ReferenceOTU175069
New.CleanUp.ReferenceOTU25867
New.ReferenceOTU480
New.CleanUp.ReferenceOTU9356
New.CleanUp.ReferenceOTU11541
New.CleanUp.ReferenceOTU59568
New.CleanUp.ReferenceOTU23688
New.CleanUp.ReferenceOTU43784
New.CleanUp.ReferenceOTU35908
New.CleanUp.ReferenceOTU27853
New.CleanUp.ReferenceOTU32485
New.CleanUp.ReferenceOTU22195
New.CleanUp.ReferenceOTU15244
New.CleanUp.ReferenceOTU20687
New.CleanUp.ReferenceOTU54072
New.CleanUp.ReferenceOTU33711
New.ReferenceOTU2598
New.CleanUp.ReferenceOTU41278
New.CleanUp.ReferenceOTU58869

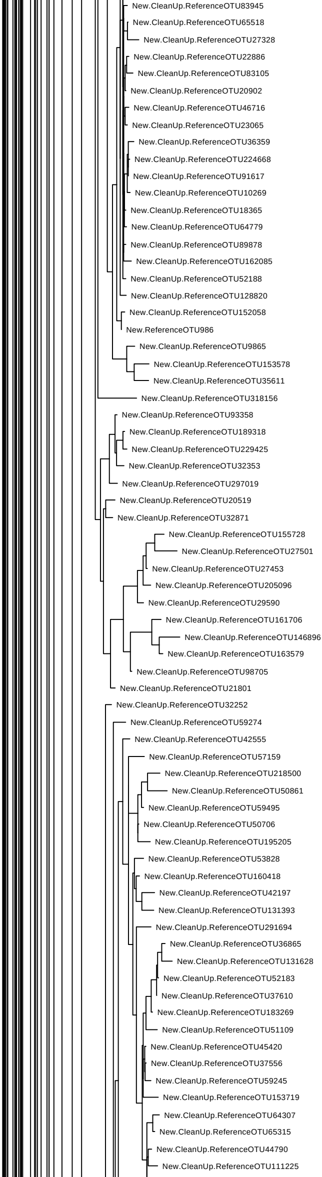
New.CleanUp.ReferenceOTU83945
New.CleanUp.ReferenceOTU65518
New.CleanUp.ReferenceOTU27328
New.CleanUp.ReferenceOTU22886
New.CleanUp.ReferenceOTU83105
New.CleanUp.ReferenceOTU20902
New.CleanUp.ReferenceOTU46716
New.CleanUp.ReferenceOTU23065
New.CleanUp.ReferenceOTU36359
New.CleanUp.ReferenceOTU224668
New.CleanUp.ReferenceOTU91617
New.CleanUp.ReferenceOTU10269
New.CleanUp.ReferenceOTU18365
New.CleanUp.ReferenceOTU64779
New.CleanUp.ReferenceOTU89878
New.CleanUp.ReferenceOTU162085
New.CleanUp.ReferenceOTU52188
New.CleanUp.ReferenceOTU128820
New.CleanUp.ReferenceOTU152058
New.ReferenceOTU986
New.CleanUp.ReferenceOTU9865
New.CleanUp.ReferenceOTU153578
New.CleanUp.ReferenceOTU35611
New.CleanUp.ReferenceOTU318156
New.CleanUp.ReferenceOTU93358
New.CleanUp.ReferenceOTU189318
New.CleanUp.ReferenceOTU229425
New.CleanUp.ReferenceOTU32353
New.CleanUp.ReferenceOTU297019
New.CleanUp.ReferenceOTU20519
New.CleanUp.ReferenceOTU32871
New.CleanUp.ReferenceOTU155728
New.CleanUp.ReferenceOTU27501
New.CleanUp.ReferenceOTU27453
New.CleanUp.ReferenceOTU205096
New.CleanUp.ReferenceOTU29590
New.CleanUp.ReferenceOTU161706
New.CleanUp.ReferenceOTU146896
New.CleanUp.ReferenceOTU163579
New.CleanUp.ReferenceOTU98705
New.CleanUp.ReferenceOTU21801
New.CleanUp.ReferenceOTU32252
New.CleanUp.ReferenceOTU59274
New.CleanUp.ReferenceOTU42555
New.CleanUp.ReferenceOTU57159
New.CleanUp.ReferenceOTU218500
New.CleanUp.ReferenceOTU50861
New.CleanUp.ReferenceOTU59495
New.CleanUp.ReferenceOTU50706
New.CleanUp.ReferenceOTU195205
New.CleanUp.ReferenceOTU53828
New.CleanUp.ReferenceOTU160418
New.CleanUp.ReferenceOTU42197
New.CleanUp.ReferenceOTU131393
New.CleanUp.ReferenceOTU291694
New.CleanUp.ReferenceOTU36865
New.CleanUp.ReferenceOTU131628
New.CleanUp.ReferenceOTU52183
New.CleanUp.ReferenceOTU37610
New.CleanUp.ReferenceOTU183269
New.CleanUp.ReferenceOTU51109
New.CleanUp.ReferenceOTU45420
New.CleanUp.ReferenceOTU37556
New.CleanUp.ReferenceOTU59245
New.CleanUp.ReferenceOTU153719
New.CleanUp.ReferenceOTU64307
New.CleanUp.ReferenceOTU65315
New.CleanUp.ReferenceOTU44790
New.CleanUp.ReferenceOTU111225

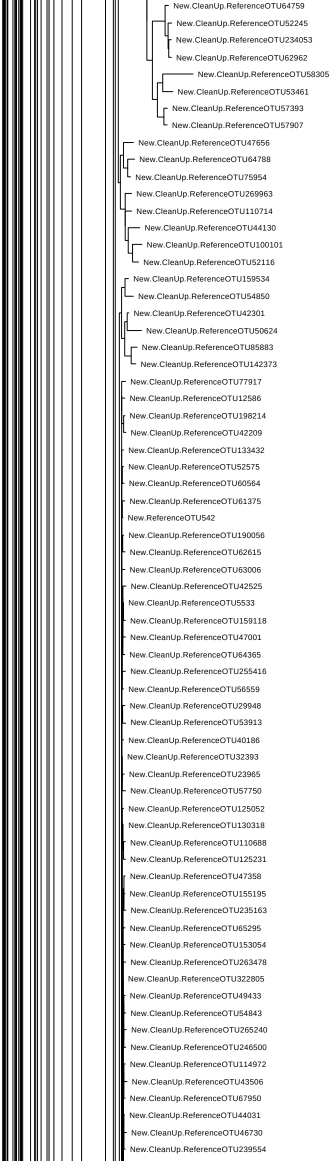
New.CleanUp.ReferenceOTU64759
New.CleanUp.ReferenceOTU52245
New.CleanUp.ReferenceOTU234053
New.CleanUp.ReferenceOTU62962
New.CleanUp.ReferenceOTU58305
New.CleanUp.ReferenceOTU53461
New.CleanUp.ReferenceOTU57393
New.CleanUp.ReferenceOTU57907
New.CleanUp.ReferenceOTU47656
New.CleanUp.ReferenceOTU64788
New.CleanUp.ReferenceOTU75954
New.CleanUp.ReferenceOTU269963
New.CleanUp.ReferenceOTU110714
New.CleanUp.ReferenceOTU44130
New.CleanUp.ReferenceOTU100101
New.CleanUp.ReferenceOTU52116
New.CleanUp.ReferenceOTU159534
New.CleanUp.ReferenceOTU54850
New.CleanUp.ReferenceOTU42301
New.CleanUp.ReferenceOTU50624
New.CleanUp.ReferenceOTU85883
New.CleanUp.ReferenceOTU142373
New.CleanUp.ReferenceOTU77917
New.CleanUp.ReferenceOTU12586
New.CleanUp.ReferenceOTU198214
New.CleanUp.ReferenceOTU42209
New.CleanUp.ReferenceOTU133432
New.CleanUp.ReferenceOTU52575
New.CleanUp.ReferenceOTU60564
New.CleanUp.ReferenceOTU61375
New.ReferenceOTU542
New.CleanUp.ReferenceOTU190056
New.CleanUp.ReferenceOTU62615
New.CleanUp.ReferenceOTU63006
New.CleanUp.ReferenceOTU42525
New.CleanUp.ReferenceOTU5533
New.CleanUp.ReferenceOTU159118
New.CleanUp.ReferenceOTU47001
New.CleanUp.ReferenceOTU64365
New.CleanUp.ReferenceOTU255416
New.CleanUp.ReferenceOTU56559
New.CleanUp.ReferenceOTU29948
New.CleanUp.ReferenceOTU53913
New.CleanUp.ReferenceOTU40186
New.CleanUp.ReferenceOTU32393
New.CleanUp.ReferenceOTU23965
New.CleanUp.ReferenceOTU57750
New.CleanUp.ReferenceOTU125052
New.CleanUp.ReferenceOTU130318
New.CleanUp.ReferenceOTU110688
New.CleanUp.ReferenceOTU125231
New.CleanUp.ReferenceOTU47358
New.CleanUp.ReferenceOTU155195
New.CleanUp.ReferenceOTU235163
New.CleanUp.ReferenceOTU65295
New.CleanUp.ReferenceOTU153054
New.CleanUp.ReferenceOTU263478
New.CleanUp.ReferenceOTU322805
New.CleanUp.ReferenceOTU49433
New.CleanUp.ReferenceOTU54843
New.CleanUp.ReferenceOTU265240
New.CleanUp.ReferenceOTU246500
New.CleanUp.ReferenceOTU114972
New.CleanUp.ReferenceOTU43506
New.CleanUp.ReferenceOTU67950
New.CleanUp.ReferenceOTU44031
New.CleanUp.ReferenceOTU46730
New.CleanUp.ReferenceOTU239554

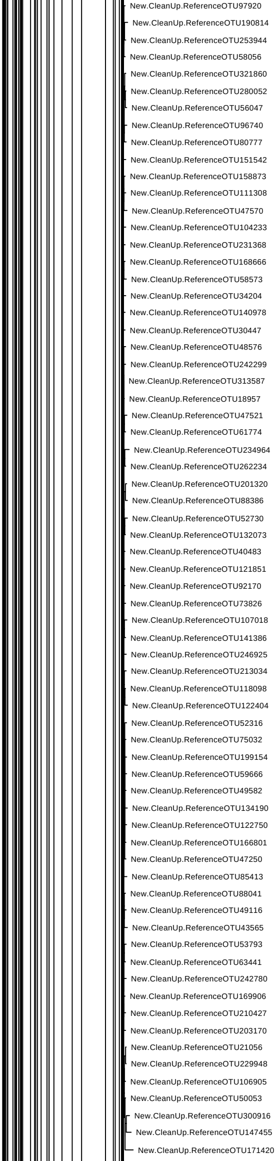
New.CleanUp.ReferenceOTU97920
New.CleanUp.ReferenceOTU190814
New.CleanUp.ReferenceOTU253944
New.CleanUp.ReferenceOTU58056
New.CleanUp.ReferenceOTU321860
New.CleanUp.ReferenceOTU280052
New.CleanUp.ReferenceOTU56047
New.CleanUp.ReferenceOTU96740
New.CleanUp.ReferenceOTU80777
New.CleanUp.ReferenceOTU151542
New.CleanUp.ReferenceOTU158873
New.CleanUp.ReferenceOTU111308
New.CleanUp.ReferenceOTU47570
New.CleanUp.ReferenceOTU104233
New.CleanUp.ReferenceOTU231368
New.CleanUp.ReferenceOTU168666
New.CleanUp.ReferenceOTU58573
New.CleanUp.ReferenceOTU34204
New.CleanUp.ReferenceOTU140978
New.CleanUp.ReferenceOTU30447
New.CleanUp.ReferenceOTU48576
New.CleanUp.ReferenceOTU242299
New.CleanUp.ReferenceOTU313587
New.CleanUp.ReferenceOTU18957
New.CleanUp.ReferenceOTU47521
New.CleanUp.ReferenceOTU61774
New.CleanUp.ReferenceOTU234964
New.CleanUp.ReferenceOTU262234
New.CleanUp.ReferenceOTU201320
New.CleanUp.ReferenceOTU88386
New.CleanUp.ReferenceOTU52730
New.CleanUp.ReferenceOTU132073
New.CleanUp.ReferenceOTU40483
New.CleanUp.ReferenceOTU121851
New.CleanUp.ReferenceOTU92170
New.CleanUp.ReferenceOTU73826
New.CleanUp.ReferenceOTU107018
New.CleanUp.ReferenceOTU141386
New.CleanUp.ReferenceOTU246925
New.CleanUp.ReferenceOTU213034
New.CleanUp.ReferenceOTU118098
New.CleanUp.ReferenceOTU122404
New.CleanUp.ReferenceOTU52316
New.CleanUp.ReferenceOTU75032
New.CleanUp.ReferenceOTU199154
New.CleanUp.ReferenceOTU59666
New.CleanUp.ReferenceOTU49582
New.CleanUp.ReferenceOTU134190
New.CleanUp.ReferenceOTU122750
New.CleanUp.ReferenceOTU166801
New.CleanUp.ReferenceOTU47250
New.CleanUp.ReferenceOTU85413
New.CleanUp.ReferenceOTU88041
New.CleanUp.ReferenceOTU49116
New.CleanUp.ReferenceOTU43565
New.CleanUp.ReferenceOTU53793
New.CleanUp.ReferenceOTU63441
New.CleanUp.ReferenceOTU242780
New.CleanUp.ReferenceOTU169906
New.CleanUp.ReferenceOTU210427
New.CleanUp.ReferenceOTU203170
New.CleanUp.ReferenceOTU21056
New.CleanUp.ReferenceOTU229948
New.CleanUp.ReferenceOTU106905
New.CleanUp.ReferenceOTU50053
New.CleanUp.ReferenceOTU300916
New.CleanUp.ReferenceOTU147455
New.CleanUp.ReferenceOTU171420

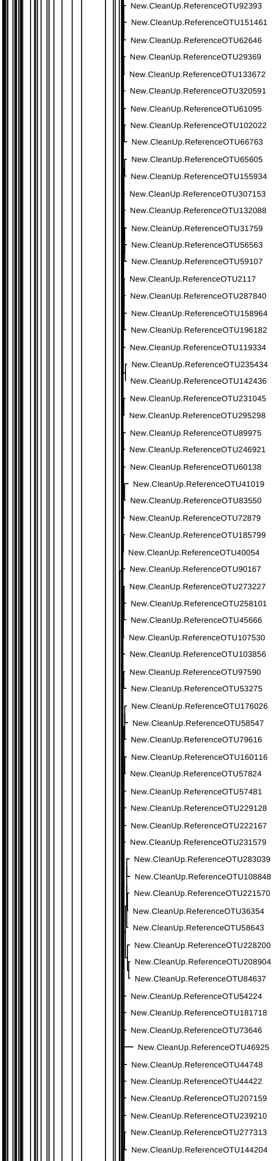
New.CleanUp.ReferenceOTU92393
New.CleanUp.ReferenceOTU151461
New.CleanUp.ReferenceOTU62646
New.CleanUp.ReferenceOTU29369
New.CleanUp.ReferenceOTU133672
New.CleanUp.ReferenceOTU320591
New.CleanUp.ReferenceOTU61095
New.CleanUp.ReferenceOTU102022
New.CleanUp.ReferenceOTU66763
New.CleanUp.ReferenceOTU65605
New.CleanUp.ReferenceOTU155934
New.CleanUp.ReferenceOTU307153
New.CleanUp.ReferenceOTU132088
New.CleanUp.ReferenceOTU31759
New.CleanUp.ReferenceOTU56563
New.CleanUp.ReferenceOTU59107
New.CleanUp.ReferenceOTU2117
New.CleanUp.ReferenceOTU287840
New.CleanUp.ReferenceOTU158964
New.CleanUp.ReferenceOTU196182
New.CleanUp.ReferenceOTU119334
New.CleanUp.ReferenceOTU235434
New.CleanUp.ReferenceOTU142436
New.CleanUp.ReferenceOTU231045
New.CleanUp.ReferenceOTU295298
New.CleanUp.ReferenceOTU89975
New.CleanUp.ReferenceOTU246921
New.CleanUp.ReferenceOTU60138
New.CleanUp.ReferenceOTU41019
New.CleanUp.ReferenceOTU83550
New.CleanUp.ReferenceOTU72879
New.CleanUp.ReferenceOTU185799
New.CleanUp.ReferenceOTU40054
New.CleanUp.ReferenceOTU90167
New.CleanUp.ReferenceOTU273227
New.CleanUp.ReferenceOTU258101
New.CleanUp.ReferenceOTU45666
New.CleanUp.ReferenceOTU107530
New.CleanUp.ReferenceOTU103856
New.CleanUp.ReferenceOTU97590
New.CleanUp.ReferenceOTU53275
New.CleanUp.ReferenceOTU176026
New.CleanUp.ReferenceOTU58547
New.CleanUp.ReferenceOTU79616
New.CleanUp.ReferenceOTU160116
New.CleanUp.ReferenceOTU57824
New.CleanUp.ReferenceOTU57481
New.CleanUp.ReferenceOTU229128
New.CleanUp.ReferenceOTU222167
New.CleanUp.ReferenceOTU231579
New.CleanUp.ReferenceOTU283039
New.CleanUp.ReferenceOTU108848
New.CleanUp.ReferenceOTU221570
New.CleanUp.ReferenceOTU36354
New.CleanUp.ReferenceOTU58643
New.CleanUp.ReferenceOTU228200
New.CleanUp.ReferenceOTU208904
New.CleanUp.ReferenceOTU84637
New.CleanUp.ReferenceOTU54224
New.CleanUp.ReferenceOTU181718
New.CleanUp.ReferenceOTU73646
New.CleanUp.ReferenceOTU46925
New.CleanUp.ReferenceOTU44748
New.CleanUp.ReferenceOTU44422
New.CleanUp.ReferenceOTU207159
New.CleanUp.ReferenceOTU239210
New.CleanUp.ReferenceOTU277313
New.CleanUp.ReferenceOTU144204

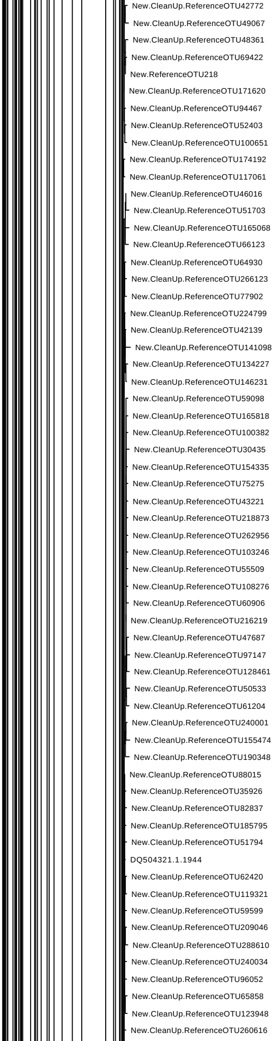
New.CleanUp.ReferenceOTU42772
New.CleanUp.ReferenceOTU49067
New.CleanUp.ReferenceOTU48361
New.CleanUp.ReferenceOTU69422
New.ReferenceOTU218
New.CleanUp.ReferenceOTU171620
New.CleanUp.ReferenceOTU94467
New.CleanUp.ReferenceOTU52403
New.CleanUp.ReferenceOTU100651
New.CleanUp.ReferenceOTU174192
New.CleanUp.ReferenceOTU117061
New.CleanUp.ReferenceOTU46016
New.CleanUp.ReferenceOTU51703
New.CleanUp.ReferenceOTU165068
New.CleanUp.ReferenceOTU66123
New.CleanUp.ReferenceOTU64930
New.CleanUp.ReferenceOTU266123
New.CleanUp.ReferenceOTU77902
New.CleanUp.ReferenceOTU224799
New.CleanUp.ReferenceOTU42139
New.CleanUp.ReferenceOTU141098
New.CleanUp.ReferenceOTU134227
New.CleanUp.ReferenceOTU146231
New.CleanUp.ReferenceOTU59098
New.CleanUp.ReferenceOTU165818
New.CleanUp.ReferenceOTU100382
New.CleanUp.ReferenceOTU30435
New.CleanUp.ReferenceOTU154335
New.CleanUp.ReferenceOTU75275
New.CleanUp.ReferenceOTU43221
New.CleanUp.ReferenceOTU218873
New.CleanUp.ReferenceOTU262956
New.CleanUp.ReferenceOTU103246
New.CleanUp.ReferenceOTU55509
New.CleanUp.ReferenceOTU108276
New.CleanUp.ReferenceOTU60906
New.CleanUp.ReferenceOTU216219
New.CleanUp.ReferenceOTU47687
New.CleanUp.ReferenceOTU97147
New.CleanUp.ReferenceOTU128461
New.CleanUp.ReferenceOTU50533
New.CleanUp.ReferenceOTU61204
New.CleanUp.ReferenceOTU240001
New.CleanUp.ReferenceOTU155474
New.CleanUp.ReferenceOTU190348
New.CleanUp.ReferenceOTU88015
New.CleanUp.ReferenceOTU35926
New.CleanUp.ReferenceOTU82837
New.CleanUp.ReferenceOTU185795
New.CleanUp.ReferenceOTU51794
DQ504321.1.1944
New.CleanUp.ReferenceOTU62420
New.CleanUp.ReferenceOTU119321
New.CleanUp.ReferenceOTU59599
New.CleanUp.ReferenceOTU209046
New.CleanUp.ReferenceOTU288610
New.CleanUp.ReferenceOTU240034
New.CleanUp.ReferenceOTU96052
New.CleanUp.ReferenceOTU65858
New.CleanUp.ReferenceOTU123948
New.CleanUp.ReferenceOTU260616

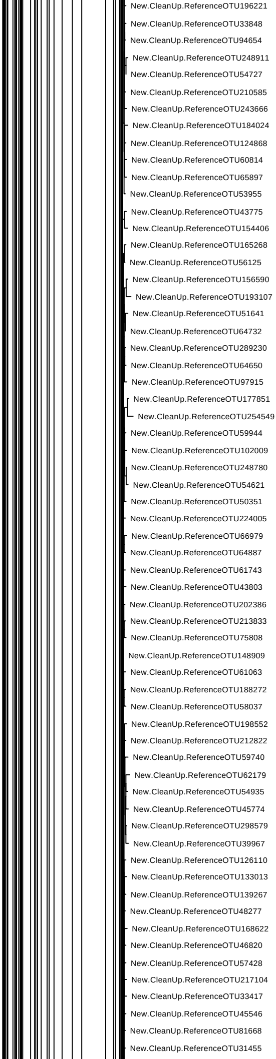
New.CleanUp.ReferenceOTU196221
New.CleanUp.ReferenceOTU33848
New.CleanUp.ReferenceOTU94654
New.CleanUp.ReferenceOTU248911
New.CleanUp.ReferenceOTU54727
New.CleanUp.ReferenceOTU210585
New.CleanUp.ReferenceOTU243666
New.CleanUp.ReferenceOTU184024
New.CleanUp.ReferenceOTU124868
New.CleanUp.ReferenceOTU60814
New.CleanUp.ReferenceOTU65897
New.CleanUp.ReferenceOTU53955
New.CleanUp.ReferenceOTU43775
New.CleanUp.ReferenceOTU154406
New.CleanUp.ReferenceOTU165268
New.CleanUp.ReferenceOTU56125
New.CleanUp.ReferenceOTU156590
New.CleanUp.ReferenceOTU193107
New.CleanUp.ReferenceOTU51641
New.CleanUp.ReferenceOTU64732
New.CleanUp.ReferenceOTU289230
New.CleanUp.ReferenceOTU64650
New.CleanUp.ReferenceOTU97915
New.CleanUp.ReferenceOTU177851
New.CleanUp.ReferenceOTU254549
New.CleanUp.ReferenceOTU59944
New.CleanUp.ReferenceOTU102009
New.CleanUp.ReferenceOTU248780
New.CleanUp.ReferenceOTU54621
New.CleanUp.ReferenceOTU50351
New.CleanUp.ReferenceOTU224005
New.CleanUp.ReferenceOTU66979
New.CleanUp.ReferenceOTU64887
New.CleanUp.ReferenceOTU61743
New.CleanUp.ReferenceOTU43803
New.CleanUp.ReferenceOTU202386
New.CleanUp.ReferenceOTU213833
New.CleanUp.ReferenceOTU75808
New.CleanUp.ReferenceOTU148909
New.CleanUp.ReferenceOTU61063
New.CleanUp.ReferenceOTU188272
New.CleanUp.ReferenceOTU58037
New.CleanUp.ReferenceOTU198552
New.CleanUp.ReferenceOTU212822
New.CleanUp.ReferenceOTU59740
New.CleanUp.ReferenceOTU62179
New.CleanUp.ReferenceOTU54935
New.CleanUp.ReferenceOTU45774
New.CleanUp.ReferenceOTU298579
New.CleanUp.ReferenceOTU39967
New.CleanUp.ReferenceOTU126110
New.CleanUp.ReferenceOTU133013
New.CleanUp.ReferenceOTU139267
New.CleanUp.ReferenceOTU48277
New.CleanUp.ReferenceOTU168622
New.CleanUp.ReferenceOTU46820
New.CleanUp.ReferenceOTU57428
New.CleanUp.ReferenceOTU217104
New.CleanUp.ReferenceOTU33417
New.CleanUp.ReferenceOTU45546
New.CleanUp.ReferenceOTU81668
New.CleanUp.ReferenceOTU31455

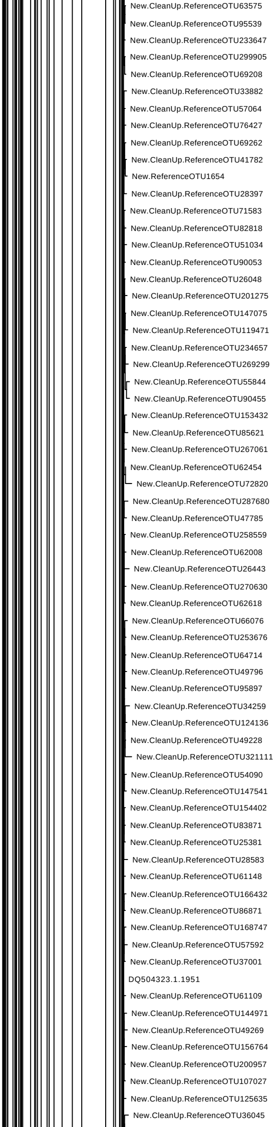
New.CleanUp.ReferenceOTU63575
New.CleanUp.ReferenceOTU95539
New.CleanUp.ReferenceOTU233647
New.CleanUp.ReferenceOTU299905
New.CleanUp.ReferenceOTU69208
New.CleanUp.ReferenceOTU33882
New.CleanUp.ReferenceOTU57064
New.CleanUp.ReferenceOTU76427
New.CleanUp.ReferenceOTU69262
New.CleanUp.ReferenceOTU41782
New.ReferenceOTU1654
New.CleanUp.ReferenceOTU28397
New.CleanUp.ReferenceOTU71583
New.CleanUp.ReferenceOTU82818
New.CleanUp.ReferenceOTU51034
New.CleanUp.ReferenceOTU90053
New.CleanUp.ReferenceOTU26048
New.CleanUp.ReferenceOTU201275
New.CleanUp.ReferenceOTU147075
New.CleanUp.ReferenceOTU119471
New.CleanUp.ReferenceOTU234657
New.CleanUp.ReferenceOTU269299
New.CleanUp.ReferenceOTU55844
New.CleanUp.ReferenceOTU90455
New.CleanUp.ReferenceOTU153432
New.CleanUp.ReferenceOTU85621
New.CleanUp.ReferenceOTU267061
New.CleanUp.ReferenceOTU62454
New.CleanUp.ReferenceOTU72820
New.CleanUp.ReferenceOTU287680
New.CleanUp.ReferenceOTU47785
New.CleanUp.ReferenceOTU258559
New.CleanUp.ReferenceOTU62008
New.CleanUp.ReferenceOTU26443
New.CleanUp.ReferenceOTU270630
New.CleanUp.ReferenceOTU62618
New.CleanUp.ReferenceOTU66076
New.CleanUp.ReferenceOTU253676
New.CleanUp.ReferenceOTU64714
New.CleanUp.ReferenceOTU49796
New.CleanUp.ReferenceOTU95897
New.CleanUp.ReferenceOTU34259
New.CleanUp.ReferenceOTU124136
New.CleanUp.ReferenceOTU49228
New.CleanUp.ReferenceOTU321111
New.CleanUp.ReferenceOTU54090
New.CleanUp.ReferenceOTU147541
New.CleanUp.ReferenceOTU154402
New.CleanUp.ReferenceOTU83871
New.CleanUp.ReferenceOTU25381
New.CleanUp.ReferenceOTU28583
New.CleanUp.ReferenceOTU61148
New.CleanUp.ReferenceOTU166432
New.CleanUp.ReferenceOTU86871
New.CleanUp.ReferenceOTU168747
New.CleanUp.ReferenceOTU57592
New.CleanUp.ReferenceOTU37001
DQ504323.1.1951
New.CleanUp.ReferenceOTU61109
New.CleanUp.ReferenceOTU144971
New.CleanUp.ReferenceOTU49269
New.CleanUp.ReferenceOTU156764
New.CleanUp.ReferenceOTU200957
New.CleanUp.ReferenceOTU107027
New.CleanUp.ReferenceOTU125635
New.CleanUp.ReferenceOTU36045

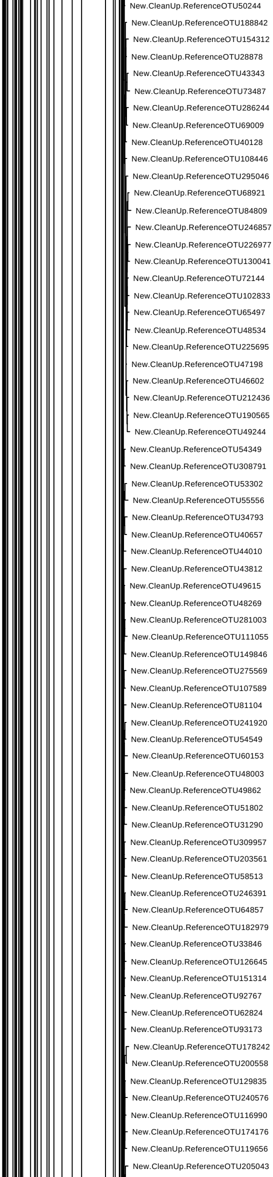
New.CleanUp.ReferenceOTU50244
New.CleanUp.ReferenceOTU188842
New.CleanUp.ReferenceOTU154312
New.CleanUp.ReferenceOTU28878
New.CleanUp.ReferenceOTU43343
New.CleanUp.ReferenceOTU73487
New.CleanUp.ReferenceOTU286244
New.CleanUp.ReferenceOTU69009
New.CleanUp.ReferenceOTU40128
New.CleanUp.ReferenceOTU108446
New.CleanUp.ReferenceOTU295046
New.CleanUp.ReferenceOTU68921
New.CleanUp.ReferenceOTU84809
New.CleanUp.ReferenceOTU246857
New.CleanUp.ReferenceOTU226977
New.CleanUp.ReferenceOTU130041
New.CleanUp.ReferenceOTU72144
New.CleanUp.ReferenceOTU102833
New.CleanUp.ReferenceOTU65497
New.CleanUp.ReferenceOTU48534
New.CleanUp.ReferenceOTU225695
New.CleanUp.ReferenceOTU47198
New.CleanUp.ReferenceOTU46602
New.CleanUp.ReferenceOTU212436
New.CleanUp.ReferenceOTU190565
New.CleanUp.ReferenceOTU49244
New.CleanUp.ReferenceOTU54349
New.CleanUp.ReferenceOTU308791
New.CleanUp.ReferenceOTU53302
New.CleanUp.ReferenceOTU55556
New.CleanUp.ReferenceOTU34793
New.CleanUp.ReferenceOTU40657
New.CleanUp.ReferenceOTU44010
New.CleanUp.ReferenceOTU43812
New.CleanUp.ReferenceOTU49615
New.CleanUp.ReferenceOTU48269
New.CleanUp.ReferenceOTU281003
New.CleanUp.ReferenceOTU111055
New.CleanUp.ReferenceOTU149846
New.CleanUp.ReferenceOTU275569
New.CleanUp.ReferenceOTU107589
New.CleanUp.ReferenceOTU81104
New.CleanUp.ReferenceOTU241920
New.CleanUp.ReferenceOTU54549
New.CleanUp.ReferenceOTU60153
New.CleanUp.ReferenceOTU48003
New.CleanUp.ReferenceOTU49862
New.CleanUp.ReferenceOTU51802
New.CleanUp.ReferenceOTU31290
New.CleanUp.ReferenceOTU309957
New.CleanUp.ReferenceOTU203561
New.CleanUp.ReferenceOTU58513
New.CleanUp.ReferenceOTU246391
New.CleanUp.ReferenceOTU64857
New.CleanUp.ReferenceOTU182979
New.CleanUp.ReferenceOTU33846
New.CleanUp.ReferenceOTU126645
New.CleanUp.ReferenceOTU151314
New.CleanUp.ReferenceOTU92767
New.CleanUp.ReferenceOTU62824
New.CleanUp.ReferenceOTU93173
New.CleanUp.ReferenceOTU178242
New.CleanUp.ReferenceOTU200558
New.CleanUp.ReferenceOTU129835
New.CleanUp.ReferenceOTU240576
New.CleanUp.ReferenceOTU116990
New.CleanUp.ReferenceOTU174176
New.CleanUp.ReferenceOTU119656
New.CleanUp.ReferenceOTU205043

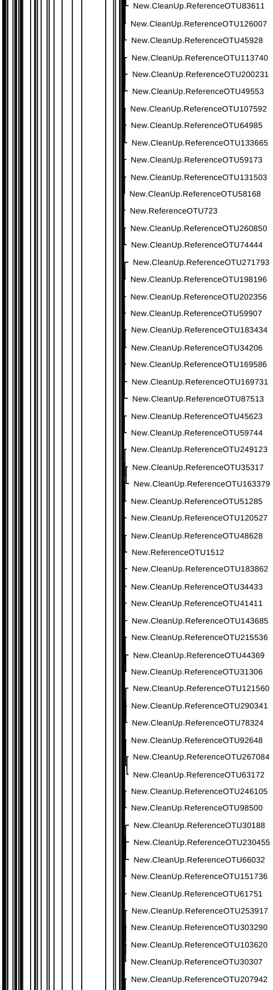
New.CleanUp.ReferenceOTU83611
New.CleanUp.ReferenceOTU126007
New.CleanUp.ReferenceOTU45928
New.CleanUp.ReferenceOTU113740
New.CleanUp.ReferenceOTU200231
New.CleanUp.ReferenceOTU49553
New.CleanUp.ReferenceOTU107592
New.CleanUp.ReferenceOTU64985
New.CleanUp.ReferenceOTU133665
New.CleanUp.ReferenceOTU59173
New.CleanUp.ReferenceOTU131503
New.CleanUp.ReferenceOTU58168
New.ReferenceOTU723
New.CleanUp.ReferenceOTU260850
New.CleanUp.ReferenceOTU74444
New.CleanUp.ReferenceOTU271793
New.CleanUp.ReferenceOTU198196
New.CleanUp.ReferenceOTU202356
New.CleanUp.ReferenceOTU59907
New.CleanUp.ReferenceOTU183434
New.CleanUp.ReferenceOTU34206
New.CleanUp.ReferenceOTU169586
New.CleanUp.ReferenceOTU169731
New.CleanUp.ReferenceOTU87513
New.CleanUp.ReferenceOTU45623
New.CleanUp.ReferenceOTU59744
New.CleanUp.ReferenceOTU249123
New.CleanUp.ReferenceOTU35317
New.CleanUp.ReferenceOTU163379
New.CleanUp.ReferenceOTU51285
New.CleanUp.ReferenceOTU120527
New.CleanUp.ReferenceOTU48628
New.ReferenceOTU1512
New.CleanUp.ReferenceOTU183862
New.CleanUp.ReferenceOTU34433
New.CleanUp.ReferenceOTU41411
New.CleanUp.ReferenceOTU143685
New.CleanUp.ReferenceOTU215536
New.CleanUp.ReferenceOTU44369
New.CleanUp.ReferenceOTU31306
New.CleanUp.ReferenceOTU121560
New.CleanUp.ReferenceOTU290341
New.CleanUp.ReferenceOTU78324
New.CleanUp.ReferenceOTU92648
New.CleanUp.ReferenceOTU267084
New.CleanUp.ReferenceOTU63172
New.CleanUp.ReferenceOTU246105
New.CleanUp.ReferenceOTU98500
New.CleanUp.ReferenceOTU30188
New.CleanUp.ReferenceOTU230455
New.CleanUp.ReferenceOTU66032
New.CleanUp.ReferenceOTU151736
New.CleanUp.ReferenceOTU61751
New.CleanUp.ReferenceOTU253917
New.CleanUp.ReferenceOTU303290
New.CleanUp.ReferenceOTU103620
New.CleanUp.ReferenceOTU30307
New.CleanUp.ReferenceOTU207942

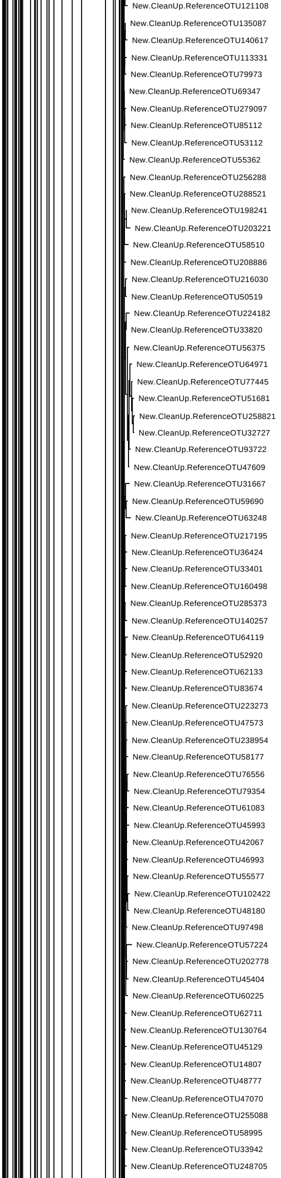
New.CleanUp.ReferenceOTU121108
New.CleanUp.ReferenceOTU135087
New.CleanUp.ReferenceOTU140617
New.CleanUp.ReferenceOTU113331
New.CleanUp.ReferenceOTU79973
New.CleanUp.ReferenceOTU69347
New.CleanUp.ReferenceOTU279097
New.CleanUp.ReferenceOTU85112
New.CleanUp.ReferenceOTU53112
New.CleanUp.ReferenceOTU55362
New.CleanUp.ReferenceOTU256288
New.CleanUp.ReferenceOTU288521
New.CleanUp.ReferenceOTU198241
New.CleanUp.ReferenceOTU203221
New.CleanUp.ReferenceOTU58510
New.CleanUp.ReferenceOTU208886
New.CleanUp.ReferenceOTU216030
New.CleanUp.ReferenceOTU50519
New.CleanUp.ReferenceOTU224182
New.CleanUp.ReferenceOTU33820
New.CleanUp.ReferenceOTU56375
New.CleanUp.ReferenceOTU64971
New.CleanUp.ReferenceOTU77445
New.CleanUp.ReferenceOTU51681
New.CleanUp.ReferenceOTU258821
New.CleanUp.ReferenceOTU32727
New.CleanUp.ReferenceOTU93722
New.CleanUp.ReferenceOTU47609
New.CleanUp.ReferenceOTU31667
New.CleanUp.ReferenceOTU59690
New.CleanUp.ReferenceOTU63248
New.CleanUp.ReferenceOTU217195
New.CleanUp.ReferenceOTU36424
New.CleanUp.ReferenceOTU33401
New.CleanUp.ReferenceOTU160498
New.CleanUp.ReferenceOTU285373
New.CleanUp.ReferenceOTU140257
New.CleanUp.ReferenceOTU64119
New.CleanUp.ReferenceOTU52920
New.CleanUp.ReferenceOTU62133
New.CleanUp.ReferenceOTU83674
New.CleanUp.ReferenceOTU223273
New.CleanUp.ReferenceOTU47573
New.CleanUp.ReferenceOTU238954
New.CleanUp.ReferenceOTU58177
New.CleanUp.ReferenceOTU76556
New.CleanUp.ReferenceOTU79354
New.CleanUp.ReferenceOTU61083
New.CleanUp.ReferenceOTU45993
New.CleanUp.ReferenceOTU42067
New.CleanUp.ReferenceOTU46993
New.CleanUp.ReferenceOTU55577
New.CleanUp.ReferenceOTU102422
New.CleanUp.ReferenceOTU48180
New.CleanUp.ReferenceOTU97498
New.CleanUp.ReferenceOTU57224
New.CleanUp.ReferenceOTU202778
New.CleanUp.ReferenceOTU45404
New.CleanUp.ReferenceOTU60225
New.CleanUp.ReferenceOTU62711
New.CleanUp.ReferenceOTU130764
New.CleanUp.ReferenceOTU45129
New.CleanUp.ReferenceOTU14807
New.CleanUp.ReferenceOTU48777
New.CleanUp.ReferenceOTU47070
New.CleanUp.ReferenceOTU255088
New.CleanUp.ReferenceOTU58995
New.CleanUp.ReferenceOTU33942
New.CleanUp.ReferenceOTU248705

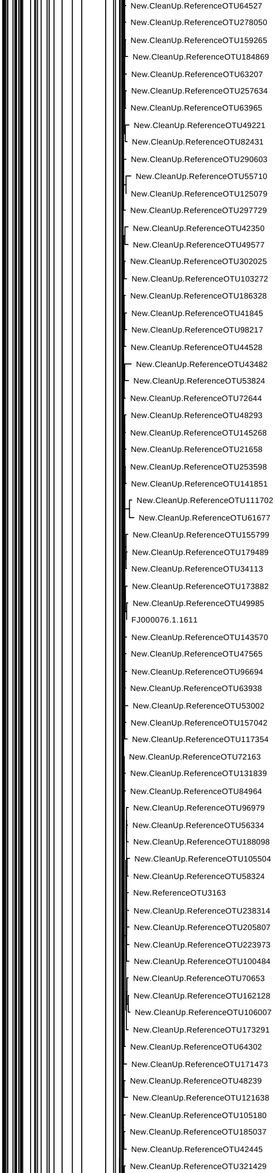
New.CleanUp.ReferenceOTU64527
New.CleanUp.ReferenceOTU278050
New.CleanUp.ReferenceOTU159265
New.CleanUp.ReferenceOTU184869
New.CleanUp.ReferenceOTU63207
New.CleanUp.ReferenceOTU257634
New.CleanUp.ReferenceOTU63965
New.CleanUp.ReferenceOTU49221
New.CleanUp.ReferenceOTU82431
New.CleanUp.ReferenceOTU290603
New.CleanUp.ReferenceOTU55710
New.CleanUp.ReferenceOTU125079
New.CleanUp.ReferenceOTU297729
New.CleanUp.ReferenceOTU42350
New.CleanUp.ReferenceOTU49577
New.CleanUp.ReferenceOTU302025
New.CleanUp.ReferenceOTU103272
New.CleanUp.ReferenceOTU186328
New.CleanUp.ReferenceOTU41845
New.CleanUp.ReferenceOTU98217
New.CleanUp.ReferenceOTU44528
New.CleanUp.ReferenceOTU43482
New.CleanUp.ReferenceOTU53824
New.CleanUp.ReferenceOTU72644
New.CleanUp.ReferenceOTU48293
New.CleanUp.ReferenceOTU145268
New.CleanUp.ReferenceOTU21658
New.CleanUp.ReferenceOTU253598
New.CleanUp.ReferenceOTU141851
New.CleanUp.ReferenceOTU111702
New.CleanUp.ReferenceOTU61677
New.CleanUp.ReferenceOTU155799
New.CleanUp.ReferenceOTU179489
New.CleanUp.ReferenceOTU34113
New.CleanUp.ReferenceOTU173882
New.CleanUp.ReferenceOTU49985
FJ000076.1.1611
New.CleanUp.ReferenceOTU143570
New.CleanUp.ReferenceOTU47565
New.CleanUp.ReferenceOTU96694
New.CleanUp.ReferenceOTU63938
New.CleanUp.ReferenceOTU53002
New.CleanUp.ReferenceOTU157042
New.CleanUp.ReferenceOTU117354
New.CleanUp.ReferenceOTU72163
New.CleanUp.ReferenceOTU131839
New.CleanUp.ReferenceOTU84964
New.CleanUp.ReferenceOTU96979
New.CleanUp.ReferenceOTU56334
New.CleanUp.ReferenceOTU188098
New.CleanUp.ReferenceOTU105504
New.CleanUp.ReferenceOTU58324
New.ReferenceOTU3163
New.CleanUp.ReferenceOTU238314
New.CleanUp.ReferenceOTU205807
New.CleanUp.ReferenceOTU223973
New.CleanUp.ReferenceOTU100484
New.CleanUp.ReferenceOTU70653
New.CleanUp.ReferenceOTU162128
New.CleanUp.ReferenceOTU106007
New.CleanUp.ReferenceOTU173291
New.CleanUp.ReferenceOTU64302
New.CleanUp.ReferenceOTU171473
New.CleanUp.ReferenceOTU48239
New.CleanUp.ReferenceOTU121638
New.CleanUp.ReferenceOTU105180
New.CleanUp.ReferenceOTU185037
New.CleanUp.ReferenceOTU42445
New.CleanUp.ReferenceOTU321429

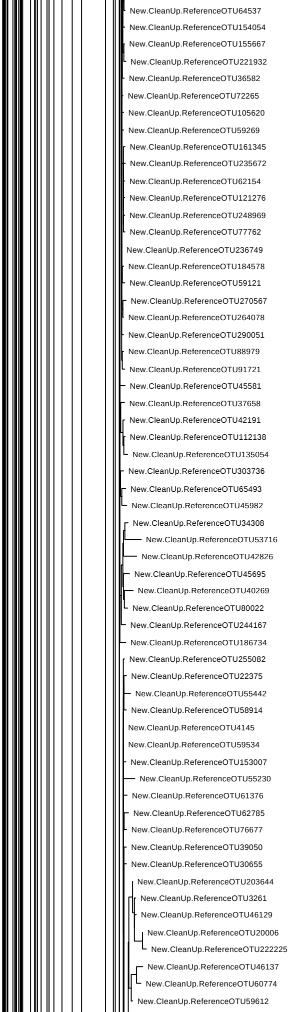
New.CleanUp.ReferenceOTU64537
New.CleanUp.ReferenceOTU154054
New.CleanUp.ReferenceOTU155667
New.CleanUp.ReferenceOTU221932
New.CleanUp.ReferenceOTU36582
New.CleanUp.ReferenceOTU72265
New.CleanUp.ReferenceOTU105620
New.CleanUp.ReferenceOTU59269
New.CleanUp.ReferenceOTU161345
New.CleanUp.ReferenceOTU235672
New.CleanUp.ReferenceOTU62154
New.CleanUp.ReferenceOTU121276
New.CleanUp.ReferenceOTU248969
New.CleanUp.ReferenceOTU77762
New.CleanUp.ReferenceOTU236749
New.CleanUp.ReferenceOTU184578
New.CleanUp.ReferenceOTU59121
New.CleanUp.ReferenceOTU270567
New.CleanUp.ReferenceOTU264078
New.CleanUp.ReferenceOTU290051
New.CleanUp.ReferenceOTU88979
New.CleanUp.ReferenceOTU91721
New.CleanUp.ReferenceOTU45581
New.CleanUp.ReferenceOTU37658
New.CleanUp.ReferenceOTU42191
New.CleanUp.ReferenceOTU112138
New.CleanUp.ReferenceOTU135054
New.CleanUp.ReferenceOTU303736
New.CleanUp.ReferenceOTU65493
New.CleanUp.ReferenceOTU45982
New.CleanUp.ReferenceOTU34308
New.CleanUp.ReferenceOTU53716
New.CleanUp.ReferenceOTU42826
New.CleanUp.ReferenceOTU45695
New.CleanUp.ReferenceOTU40269
New.CleanUp.ReferenceOTU80022
New.CleanUp.ReferenceOTU244167
New.CleanUp.ReferenceOTU186734
New.CleanUp.ReferenceOTU255082
New.CleanUp.ReferenceOTU22375
New.CleanUp.ReferenceOTU55442
New.CleanUp.ReferenceOTU58914
New.CleanUp.ReferenceOTU4145
New.CleanUp.ReferenceOTU59534
New.CleanUp.ReferenceOTU153007
New.CleanUp.ReferenceOTU55230
New.CleanUp.ReferenceOTU61376
New.CleanUp.ReferenceOTU62785
New.CleanUp.ReferenceOTU76677
New.CleanUp.ReferenceOTU39050
New.CleanUp.ReferenceOTU30655
New.CleanUp.ReferenceOTU203644
New.CleanUp.ReferenceOTU3261
New.CleanUp.ReferenceOTU46129
New.CleanUp.ReferenceOTU20006
New.CleanUp.ReferenceOTU222225
New.CleanUp.ReferenceOTU46137
New.CleanUp.ReferenceOTU60774
New.CleanUp.ReferenceOTU59612

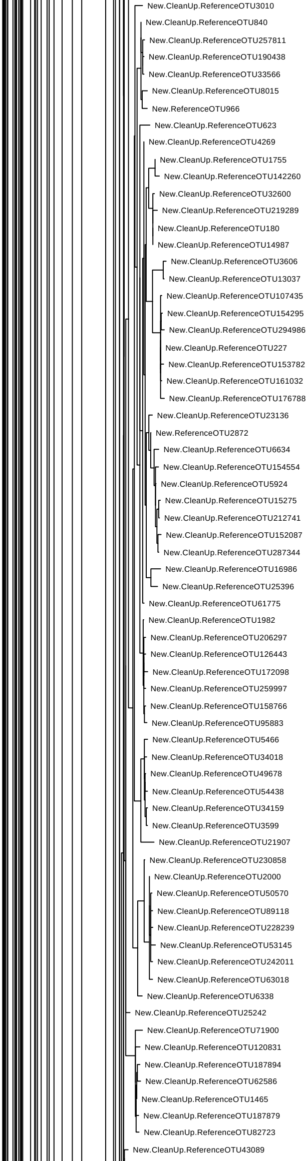
New.CleanUp.ReferenceOTU3010
New.CleanUp.ReferenceOTU840
New.CleanUp.ReferenceOTU257811
New.CleanUp.ReferenceOTU190438
New.CleanUp.ReferenceOTU33566
New.CleanUp.ReferenceOTU8015
New.ReferenceOTU966
New.CleanUp.ReferenceOTU623
New.CleanUp.ReferenceOTU4269
New.CleanUp.ReferenceOTU1755
New.CleanUp.ReferenceOTU142260
New.CleanUp.ReferenceOTU32600
New.CleanUp.ReferenceOTU219289
New.CleanUp.ReferenceOTU180
New.CleanUp.ReferenceOTU14987
New.CleanUp.ReferenceOTU3606
New.CleanUp.ReferenceOTU13037
New.CleanUp.ReferenceOTU107435
New.CleanUp.ReferenceOTU154295
New.CleanUp.ReferenceOTU294986
New.CleanUp.ReferenceOTU227
New.CleanUp.ReferenceOTU153782
New.CleanUp.ReferenceOTU161032
New.CleanUp.ReferenceOTU176788
New.CleanUp.ReferenceOTU23136
New.ReferenceOTU2872
New.CleanUp.ReferenceOTU6634
New.CleanUp.ReferenceOTU154554
New.CleanUp.ReferenceOTU5924
New.CleanUp.ReferenceOTU15275
New.CleanUp.ReferenceOTU212741
New.CleanUp.ReferenceOTU152087
New.CleanUp.ReferenceOTU287344
New.CleanUp.ReferenceOTU16986
New.CleanUp.ReferenceOTU25396
New.CleanUp.ReferenceOTU61775
New.CleanUp.ReferenceOTU1982
New.CleanUp.ReferenceOTU206297
New.CleanUp.ReferenceOTU126443
New.CleanUp.ReferenceOTU172098
New.CleanUp.ReferenceOTU259997
New.CleanUp.ReferenceOTU158766
New.CleanUp.ReferenceOTU95883
New.CleanUp.ReferenceOTU5466
New.CleanUp.ReferenceOTU34018
New.CleanUp.ReferenceOTU49678
New.CleanUp.ReferenceOTU54438
New.CleanUp.ReferenceOTU34159
New.CleanUp.ReferenceOTU3599
New.CleanUp.ReferenceOTU21907
New.CleanUp.ReferenceOTU230858
New.CleanUp.ReferenceOTU2000
New.CleanUp.ReferenceOTU50570
New.CleanUp.ReferenceOTU89118
New.CleanUp.ReferenceOTU228239
New.CleanUp.ReferenceOTU53145
New.CleanUp.ReferenceOTU242011
New.CleanUp.ReferenceOTU63018
New.CleanUp.ReferenceOTU6338
New.CleanUp.ReferenceOTU25242
New.CleanUp.ReferenceOTU71900
New.CleanUp.ReferenceOTU120831
New.CleanUp.ReferenceOTU187894
New.CleanUp.ReferenceOTU62586
New.CleanUp.ReferenceOTU1465
New.CleanUp.ReferenceOTU187879
New.CleanUp.ReferenceOTU82723
New.CleanUp.ReferenceOTU43089

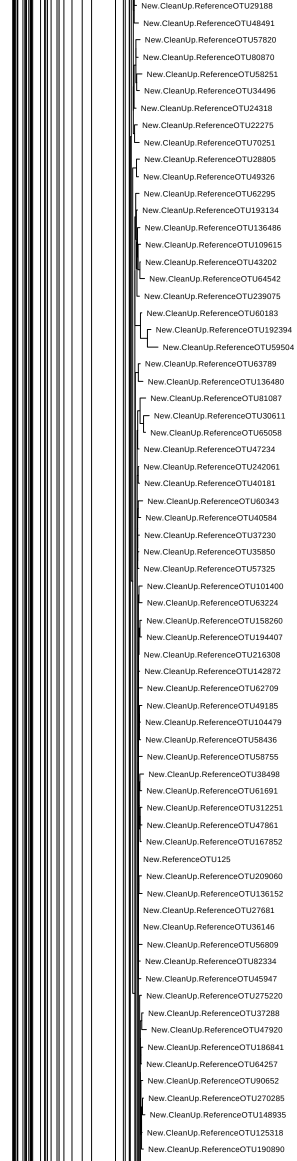
New.CleanUp.ReferenceOTU29188
New.CleanUp.ReferenceOTU48491
New.CleanUp.ReferenceOTU57820
New.CleanUp.ReferenceOTU80870
New.CleanUp.ReferenceOTU58251
New.CleanUp.ReferenceOTU34496
New.CleanUp.ReferenceOTU24318
New.CleanUp.ReferenceOTU22275
New.CleanUp.ReferenceOTU70251
New.CleanUp.ReferenceOTU28805
New.CleanUp.ReferenceOTU49326
New.CleanUp.ReferenceOTU62295
New.CleanUp.ReferenceOTU193134
New.CleanUp.ReferenceOTU136486
New.CleanUp.ReferenceOTU109615
New.CleanUp.ReferenceOTU43202
New.CleanUp.ReferenceOTU64542
New.CleanUp.ReferenceOTU239075
New.CleanUp.ReferenceOTU60183
New.CleanUp.ReferenceOTU192394
New.CleanUp.ReferenceOTU59504
New.CleanUp.ReferenceOTU63789
New.CleanUp.ReferenceOTU136480
New.CleanUp.ReferenceOTU81087
New.CleanUp.ReferenceOTU30611
New.CleanUp.ReferenceOTU65058
New.CleanUp.ReferenceOTU47234
New.CleanUp.ReferenceOTU242061
New.CleanUp.ReferenceOTU40181
New.CleanUp.ReferenceOTU60343
New.CleanUp.ReferenceOTU40584
New.CleanUp.ReferenceOTU37230
New.CleanUp.ReferenceOTU35850
New.CleanUp.ReferenceOTU57325
New.CleanUp.ReferenceOTU101400
New.CleanUp.ReferenceOTU63224
New.CleanUp.ReferenceOTU158260
New.CleanUp.ReferenceOTU194407
New.CleanUp.ReferenceOTU216308
New.CleanUp.ReferenceOTU142872
New.CleanUp.ReferenceOTU62709
New.CleanUp.ReferenceOTU49185
New.CleanUp.ReferenceOTU104479
New.CleanUp.ReferenceOTU58436
New.CleanUp.ReferenceOTU58755
New.CleanUp.ReferenceOTU38498
New.CleanUp.ReferenceOTU61691
New.CleanUp.ReferenceOTU312251
New.CleanUp.ReferenceOTU47861
New.CleanUp.ReferenceOTU167852
New.ReferenceOTU125
New.CleanUp.ReferenceOTU209060
New.CleanUp.ReferenceOTU136152
New.CleanUp.ReferenceOTU27681
New.CleanUp.ReferenceOTU36146
New.CleanUp.ReferenceOTU56809
New.CleanUp.ReferenceOTU82334
New.CleanUp.ReferenceOTU45947
New.CleanUp.ReferenceOTU275220
New.CleanUp.ReferenceOTU37288
New.CleanUp.ReferenceOTU47920
New.CleanUp.ReferenceOTU186841
New.CleanUp.ReferenceOTU64257
New.CleanUp.ReferenceOTU90652
New.CleanUp.ReferenceOTU270285
New.CleanUp.ReferenceOTU148935
New.CleanUp.ReferenceOTU125318
New.CleanUp.ReferenceOTU190890

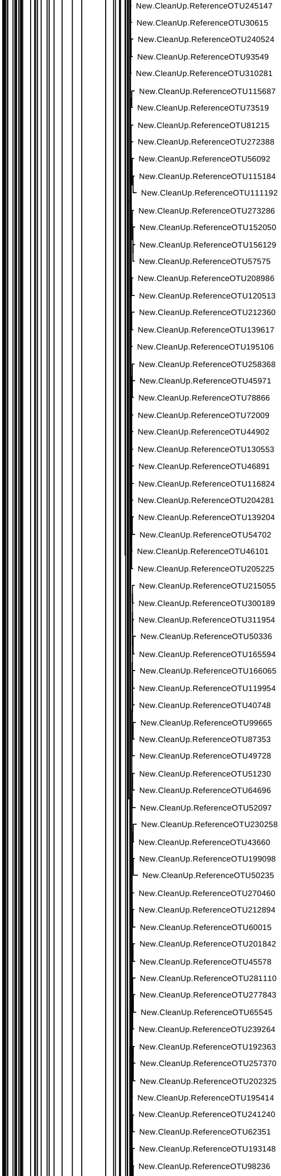
New.CleanUp.ReferenceOTU245147
New.CleanUp.ReferenceOTU30615
New.CleanUp.ReferenceOTU240524
New.CleanUp.ReferenceOTU93549
New.CleanUp.ReferenceOTU310281
New.CleanUp.ReferenceOTU115687
New.CleanUp.ReferenceOTU73519
New.CleanUp.ReferenceOTU81215
New.CleanUp.ReferenceOTU272388
New.CleanUp.ReferenceOTU56092
New.CleanUp.ReferenceOTU115184
New.CleanUp.ReferenceOTU111192
New.CleanUp.ReferenceOTU273286
New.CleanUp.ReferenceOTU152050
New.CleanUp.ReferenceOTU156129
New.CleanUp.ReferenceOTU57575
New.CleanUp.ReferenceOTU208986
New.CleanUp.ReferenceOTU120513
New.CleanUp.ReferenceOTU212360
New.CleanUp.ReferenceOTU139617
New.CleanUp.ReferenceOTU195106
New.CleanUp.ReferenceOTU258368
New.CleanUp.ReferenceOTU45971
New.CleanUp.ReferenceOTU78866
New.CleanUp.ReferenceOTU72009
New.CleanUp.ReferenceOTU44902
New.CleanUp.ReferenceOTU130553
New.CleanUp.ReferenceOTU46891
New.CleanUp.ReferenceOTU116824
New.CleanUp.ReferenceOTU204281
New.CleanUp.ReferenceOTU139204
New.CleanUp.ReferenceOTU54702
New.CleanUp.ReferenceOTU46101
New.CleanUp.ReferenceOTU205225
New.CleanUp.ReferenceOTU215055
New.CleanUp.ReferenceOTU300189
New.CleanUp.ReferenceOTU311954
New.CleanUp.ReferenceOTU50336
New.CleanUp.ReferenceOTU165594
New.CleanUp.ReferenceOTU166065
New.CleanUp.ReferenceOTU119954
New.CleanUp.ReferenceOTU40748
New.CleanUp.ReferenceOTU99665
New.CleanUp.ReferenceOTU87353
New.CleanUp.ReferenceOTU49728
New.CleanUp.ReferenceOTU51230
New.CleanUp.ReferenceOTU64696
New.CleanUp.ReferenceOTU52097
New.CleanUp.ReferenceOTU230258
New.CleanUp.ReferenceOTU43660
New.CleanUp.ReferenceOTU199098
New.CleanUp.ReferenceOTU50235
New.CleanUp.ReferenceOTU270460
New.CleanUp.ReferenceOTU212894
New.CleanUp.ReferenceOTU60015
New.CleanUp.ReferenceOTU201842
New.CleanUp.ReferenceOTU45578
New.CleanUp.ReferenceOTU281110
New.CleanUp.ReferenceOTU277843
New.CleanUp.ReferenceOTU65545
New.CleanUp.ReferenceOTU239264
New.CleanUp.ReferenceOTU192363
New.CleanUp.ReferenceOTU257370
New.CleanUp.ReferenceOTU202325
New.CleanUp.ReferenceOTU195414
New.CleanUp.ReferenceOTU241240
New.CleanUp.ReferenceOTU62351
New.CleanUp.ReferenceOTU193148
New.CleanUp.ReferenceOTU98236

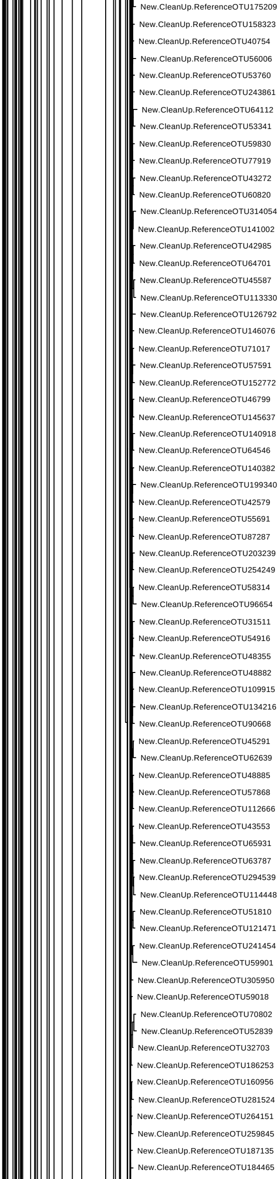
New.CleanUp.ReferenceOTU175209
New.CleanUp.ReferenceOTU158323
New.CleanUp.ReferenceOTU40754
New.CleanUp.ReferenceOTU56006
New.CleanUp.ReferenceOTU53760
New.CleanUp.ReferenceOTU243861
New.CleanUp.ReferenceOTU64112
New.CleanUp.ReferenceOTU53341
New.CleanUp.ReferenceOTU59830
New.CleanUp.ReferenceOTU77919
New.CleanUp.ReferenceOTU43272
New.CleanUp.ReferenceOTU60820
New.CleanUp.ReferenceOTU314054
New.CleanUp.ReferenceOTU141002
New.CleanUp.ReferenceOTU42985
New.CleanUp.ReferenceOTU64701
New.CleanUp.ReferenceOTU45587
New.CleanUp.ReferenceOTU113330
New.CleanUp.ReferenceOTU126792
New.CleanUp.ReferenceOTU146076
New.CleanUp.ReferenceOTU71017
New.CleanUp.ReferenceOTU57591
New.CleanUp.ReferenceOTU152772
New.CleanUp.ReferenceOTU46799
New.CleanUp.ReferenceOTU145637
New.CleanUp.ReferenceOTU140918
New.CleanUp.ReferenceOTU64546
New.CleanUp.ReferenceOTU140382
New.CleanUp.ReferenceOTU199340
New.CleanUp.ReferenceOTU42579
New.CleanUp.ReferenceOTU55691
New.CleanUp.ReferenceOTU87287
New.CleanUp.ReferenceOTU203239
New.CleanUp.ReferenceOTU254249
New.CleanUp.ReferenceOTU58314
New.CleanUp.ReferenceOTU96654
New.CleanUp.ReferenceOTU31511
New.CleanUp.ReferenceOTU54916
New.CleanUp.ReferenceOTU48355
New.CleanUp.ReferenceOTU48882
New.CleanUp.ReferenceOTU109915
New.CleanUp.ReferenceOTU134216
New.CleanUp.ReferenceOTU90668
New.CleanUp.ReferenceOTU45291
New.CleanUp.ReferenceOTU62639
New.CleanUp.ReferenceOTU48885
New.CleanUp.ReferenceOTU57868
New.CleanUp.ReferenceOTU112666
New.CleanUp.ReferenceOTU43553
New.CleanUp.ReferenceOTU65931
New.CleanUp.ReferenceOTU63787
New.CleanUp.ReferenceOTU294539
New.CleanUp.ReferenceOTU114448
New.CleanUp.ReferenceOTU51810
New.CleanUp.ReferenceOTU121471
New.CleanUp.ReferenceOTU241454
New.CleanUp.ReferenceOTU59901
New.CleanUp.ReferenceOTU305950
New.CleanUp.ReferenceOTU59018
New.CleanUp.ReferenceOTU70802
New.CleanUp.ReferenceOTU52839
New.CleanUp.ReferenceOTU32703
New.CleanUp.ReferenceOTU186253
New.CleanUp.ReferenceOTU160956
New.CleanUp.ReferenceOTU281524
New.CleanUp.ReferenceOTU264151
New.CleanUp.ReferenceOTU259845
New.CleanUp.ReferenceOTU187135
New.CleanUp.ReferenceOTU184465

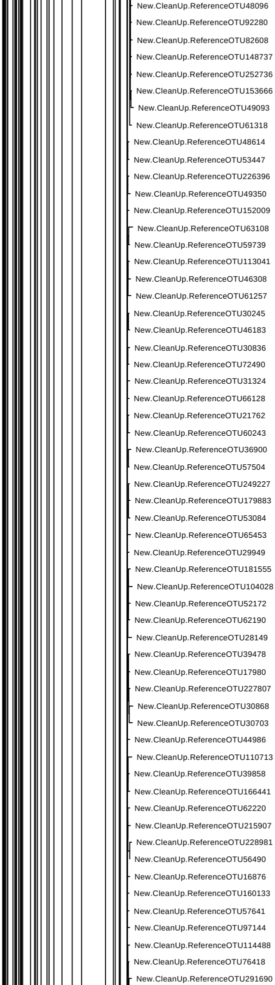
New.CleanUp.ReferenceOTU48096
New.CleanUp.ReferenceOTU92280
New.CleanUp.ReferenceOTU82608
New.CleanUp.ReferenceOTU148737
New.CleanUp.ReferenceOTU252736
New.CleanUp.ReferenceOTU153666
New.CleanUp.ReferenceOTU49093
New.CleanUp.ReferenceOTU61318
New.CleanUp.ReferenceOTU48614
New.CleanUp.ReferenceOTU53447
New.CleanUp.ReferenceOTU226396
New.CleanUp.ReferenceOTU49350
New.CleanUp.ReferenceOTU152009
New.CleanUp.ReferenceOTU63108
New.CleanUp.ReferenceOTU59739
New.CleanUp.ReferenceOTU113041
New.CleanUp.ReferenceOTU46308
New.CleanUp.ReferenceOTU61257
New.CleanUp.ReferenceOTU30245
New.CleanUp.ReferenceOTU46183
New.CleanUp.ReferenceOTU30836
New.CleanUp.ReferenceOTU72490
New.CleanUp.ReferenceOTU31324
New.CleanUp.ReferenceOTU66128
New.CleanUp.ReferenceOTU21762
New.CleanUp.ReferenceOTU60243
New.CleanUp.ReferenceOTU36900
New.CleanUp.ReferenceOTU57504
New.CleanUp.ReferenceOTU249227
New.CleanUp.ReferenceOTU179883
New.CleanUp.ReferenceOTU53084
New.CleanUp.ReferenceOTU65453
New.CleanUp.ReferenceOTU29949
New.CleanUp.ReferenceOTU181555
New.CleanUp.ReferenceOTU104028
New.CleanUp.ReferenceOTU52172
New.CleanUp.ReferenceOTU62190
New.CleanUp.ReferenceOTU28149
New.CleanUp.ReferenceOTU39478
New.CleanUp.ReferenceOTU17980
New.CleanUp.ReferenceOTU227807
New.CleanUp.ReferenceOTU30868
New.CleanUp.ReferenceOTU30703
New.CleanUp.ReferenceOTU44986
New.CleanUp.ReferenceOTU110713
New.CleanUp.ReferenceOTU39858
New.CleanUp.ReferenceOTU166441
New.CleanUp.ReferenceOTU62220
New.CleanUp.ReferenceOTU215907
New.CleanUp.ReferenceOTU228981
New.CleanUp.ReferenceOTU56490
New.CleanUp.ReferenceOTU16876
New.CleanUp.ReferenceOTU160133
New.CleanUp.ReferenceOTU57641
New.CleanUp.ReferenceOTU97144
New.CleanUp.ReferenceOTU114488
New.CleanUp.ReferenceOTU76418
New.CleanUp.ReferenceOTU291690

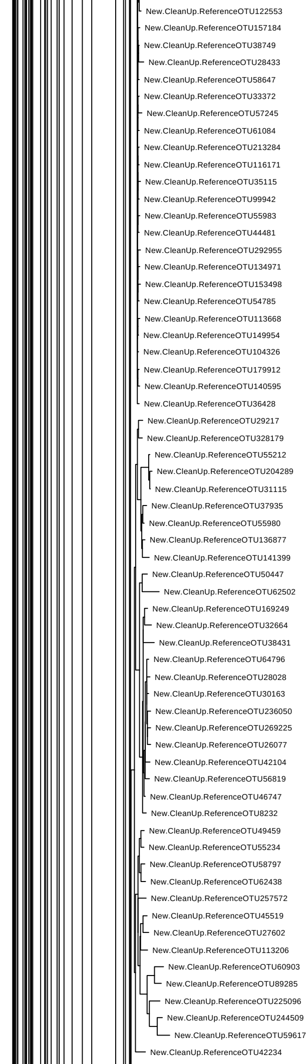
New.CleanUp.ReferenceOTU122553
New.CleanUp.ReferenceOTU157184
New.CleanUp.ReferenceOTU38749
New.CleanUp.ReferenceOTU28433
New.CleanUp.ReferenceOTU58647
New.CleanUp.ReferenceOTU33372
New.CleanUp.ReferenceOTU57245
New.CleanUp.ReferenceOTU61084
New.CleanUp.ReferenceOTU213284
New.CleanUp.ReferenceOTU116171
New.CleanUp.ReferenceOTU35115
New.CleanUp.ReferenceOTU99942
New.CleanUp.ReferenceOTU55983
New.CleanUp.ReferenceOTU44481
New.CleanUp.ReferenceOTU292955
New.CleanUp.ReferenceOTU134971
New.CleanUp.ReferenceOTU153498
New.CleanUp.ReferenceOTU54785
New.CleanUp.ReferenceOTU113668
New.CleanUp.ReferenceOTU149954
New.CleanUp.ReferenceOTU104326
New.CleanUp.ReferenceOTU179912
New.CleanUp.ReferenceOTU140595
New.CleanUp.ReferenceOTU36428
New.CleanUp.ReferenceOTU29217
New.CleanUp.ReferenceOTU328179
New.CleanUp.ReferenceOTU55212
New.CleanUp.ReferenceOTU204289
New.CleanUp.ReferenceOTU31115
New.CleanUp.ReferenceOTU37935
New.CleanUp.ReferenceOTU55980
New.CleanUp.ReferenceOTU136877
New.CleanUp.ReferenceOTU141399
New.CleanUp.ReferenceOTU50447
New.CleanUp.ReferenceOTU62502
New.CleanUp.ReferenceOTU169249
New.CleanUp.ReferenceOTU32664
New.CleanUp.ReferenceOTU38431
New.CleanUp.ReferenceOTU64796
New.CleanUp.ReferenceOTU28028
New.CleanUp.ReferenceOTU30163
New.CleanUp.ReferenceOTU236050
New.CleanUp.ReferenceOTU269225
New.CleanUp.ReferenceOTU26077
New.CleanUp.ReferenceOTU42104
New.CleanUp.ReferenceOTU56819
New.CleanUp.ReferenceOTU46747
New.CleanUp.ReferenceOTU8232
New.CleanUp.ReferenceOTU49459
New.CleanUp.ReferenceOTU55234
New.CleanUp.ReferenceOTU58797
New.CleanUp.ReferenceOTU62438
New.CleanUp.ReferenceOTU257572
New.CleanUp.ReferenceOTU45519
New.CleanUp.ReferenceOTU27602
New.CleanUp.ReferenceOTU113206
New.CleanUp.ReferenceOTU60903
New.CleanUp.ReferenceOTU89285
New.CleanUp.ReferenceOTU225096
New.CleanUp.ReferenceOTU244509
New.CleanUp.ReferenceOTU59617
New.CleanUp.ReferenceOTU42234

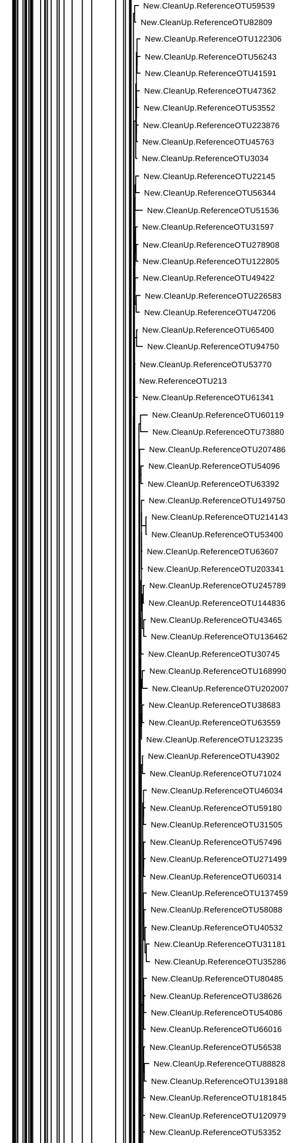
New.CleanUp.ReferenceOTU59539
New.CleanUp.ReferenceOTU82809
New.CleanUp.ReferenceOTU122306
New.CleanUp.ReferenceOTU56243
New.CleanUp.ReferenceOTU41591
New.CleanUp.ReferenceOTU47362
New.CleanUp.ReferenceOTU53552
New.CleanUp.ReferenceOTU223876
New.CleanUp.ReferenceOTU45763
New.CleanUp.ReferenceOTU3034
New.CleanUp.ReferenceOTU22145
New.CleanUp.ReferenceOTU56344
New.CleanUp.ReferenceOTU51536
New.CleanUp.ReferenceOTU31597
New.CleanUp.ReferenceOTU278908
New.CleanUp.ReferenceOTU122805
New.CleanUp.ReferenceOTU49422
New.CleanUp.ReferenceOTU226583
New.CleanUp.ReferenceOTU47206
New.CleanUp.ReferenceOTU65400
New.CleanUp.ReferenceOTU94750
New.CleanUp.ReferenceOTU53770
New.ReferenceOTU213
New.CleanUp.ReferenceOTU61341
New.CleanUp.ReferenceOTU60119
New.CleanUp.ReferenceOTU73880
New.CleanUp.ReferenceOTU207486
New.CleanUp.ReferenceOTU54096
New.CleanUp.ReferenceOTU63392
New.CleanUp.ReferenceOTU149750
New.CleanUp.ReferenceOTU214143
New.CleanUp.ReferenceOTU53400
New.CleanUp.ReferenceOTU63607
New.CleanUp.ReferenceOTU203341
New.CleanUp.ReferenceOTU245789
New.CleanUp.ReferenceOTU144836
New.CleanUp.ReferenceOTU43465
New.CleanUp.ReferenceOTU136462
New.CleanUp.ReferenceOTU30745
New.CleanUp.ReferenceOTU168990
New.CleanUp.ReferenceOTU202007
New.CleanUp.ReferenceOTU38683
New.CleanUp.ReferenceOTU63559
New.CleanUp.ReferenceOTU123235
New.CleanUp.ReferenceOTU43902
New.CleanUp.ReferenceOTU71024
New.CleanUp.ReferenceOTU46034
New.CleanUp.ReferenceOTU59180
New.CleanUp.ReferenceOTU31505
New.CleanUp.ReferenceOTU57496
New.CleanUp.ReferenceOTU271499
New.CleanUp.ReferenceOTU60314
New.CleanUp.ReferenceOTU137459
New.CleanUp.ReferenceOTU58088
New.CleanUp.ReferenceOTU40532
New.CleanUp.ReferenceOTU31181
New.CleanUp.ReferenceOTU35286
New.CleanUp.ReferenceOTU80485
New.CleanUp.ReferenceOTU38626
New.CleanUp.ReferenceOTU54086
New.CleanUp.ReferenceOTU66016
New.CleanUp.ReferenceOTU56538
New.CleanUp.ReferenceOTU88828
New.CleanUp.ReferenceOTU139188
New.CleanUp.ReferenceOTU181845
New.CleanUp.ReferenceOTU120979
New.CleanUp.ReferenceOTU53352

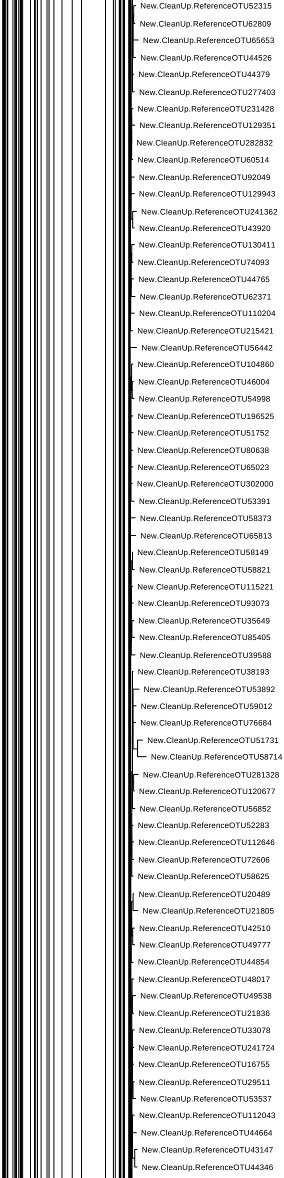

New.CleanUp.ReferenceOTU52315
New.CleanUp.ReferenceOTU62809
New.CleanUp.ReferenceOTU65653
New.CleanUp.ReferenceOTU44526
New.CleanUp.ReferenceOTU44379
New.CleanUp.ReferenceOTU277403
New.CleanUp.ReferenceOTU231428
New.CleanUp.ReferenceOTU129351
New.CleanUp.ReferenceOTU282832
New.CleanUp.ReferenceOTU60514
New.CleanUp.ReferenceOTU92049
New.CleanUp.ReferenceOTU129943
New.CleanUp.ReferenceOTU241362
New.CleanUp.ReferenceOTU43920
New.CleanUp.ReferenceOTU130411
New.CleanUp.ReferenceOTU74093
New.CleanUp.ReferenceOTU44765
New.CleanUp.ReferenceOTU62371
New.CleanUp.ReferenceOTU110204
New.CleanUp.ReferenceOTU215421
New.CleanUp.ReferenceOTU56442
New.CleanUp.ReferenceOTU104860
New.CleanUp.ReferenceOTU46004
New.CleanUp.ReferenceOTU54998
New.CleanUp.ReferenceOTU196525
New.CleanUp.ReferenceOTU51752
New.CleanUp.ReferenceOTU80638
New.CleanUp.ReferenceOTU65023
New.CleanUp.ReferenceOTU302000
New.CleanUp.ReferenceOTU53391
New.CleanUp.ReferenceOTU58373
New.CleanUp.ReferenceOTU65813
New.CleanUp.ReferenceOTU58149
New.CleanUp.ReferenceOTU58821
New.CleanUp.ReferenceOTU115221
New.CleanUp.ReferenceOTU93073
New.CleanUp.ReferenceOTU35649
New.CleanUp.ReferenceOTU85405
New.CleanUp.ReferenceOTU39588
New.CleanUp.ReferenceOTU38193
New.CleanUp.ReferenceOTU53892
New.CleanUp.ReferenceOTU59012
New.CleanUp.ReferenceOTU76684
New.CleanUp.ReferenceOTU51731
New.CleanUp.ReferenceOTU58714
New.CleanUp.ReferenceOTU281328
New.CleanUp.ReferenceOTU120677
New.CleanUp.ReferenceOTU56852
New.CleanUp.ReferenceOTU52283
New.CleanUp.ReferenceOTU112646
New.CleanUp.ReferenceOTU72606
New.CleanUp.ReferenceOTU58625
New.CleanUp.ReferenceOTU20489
New.CleanUp.ReferenceOTU21805
New.CleanUp.ReferenceOTU42510
New.CleanUp.ReferenceOTU49777
New.CleanUp.ReferenceOTU44854
New.CleanUp.ReferenceOTU48017
New.CleanUp.ReferenceOTU49538
New.CleanUp.ReferenceOTU21836
New.CleanUp.ReferenceOTU33078
New.CleanUp.ReferenceOTU241724
New.CleanUp.ReferenceOTU16755
New.CleanUp.ReferenceOTU29511
New.CleanUp.ReferenceOTU53537
New.CleanUp.ReferenceOTU112043
New.CleanUp.ReferenceOTU44664
New.CleanUp.ReferenceOTU43147
New.CleanUp.ReferenceOTU44346

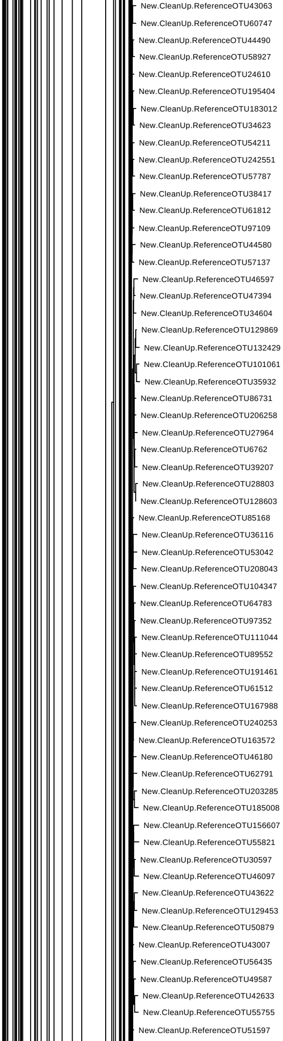
New.CleanUp.ReferenceOTU43063
New.CleanUp.ReferenceOTU60747
New.CleanUp.ReferenceOTU44490
New.CleanUp.ReferenceOTU58927
New.CleanUp.ReferenceOTU24610
New.CleanUp.ReferenceOTU195404
New.CleanUp.ReferenceOTU183012
New.CleanUp.ReferenceOTU34623
New.CleanUp.ReferenceOTU54211
New.CleanUp.ReferenceOTU242551
New.CleanUp.ReferenceOTU57787
New.CleanUp.ReferenceOTU38417
New.CleanUp.ReferenceOTU61812
New.CleanUp.ReferenceOTU97109
New.CleanUp.ReferenceOTU44580
New.CleanUp.ReferenceOTU57137
New.CleanUp.ReferenceOTU46597
New.CleanUp.ReferenceOTU47394
New.CleanUp.ReferenceOTU34604
New.CleanUp.ReferenceOTU129869
New.CleanUp.ReferenceOTU132429
New.CleanUp.ReferenceOTU101061
New.CleanUp.ReferenceOTU35932
New.CleanUp.ReferenceOTU86731
New.CleanUp.ReferenceOTU206258
New.CleanUp.ReferenceOTU27964
New.CleanUp.ReferenceOTU6762
New.CleanUp.ReferenceOTU39207
New.CleanUp.ReferenceOTU28803
New.CleanUp.ReferenceOTU128603
New.CleanUp.ReferenceOTU85168
New.CleanUp.ReferenceOTU36116
New.CleanUp.ReferenceOTU53042
New.CleanUp.ReferenceOTU208043
New.CleanUp.ReferenceOTU104347
New.CleanUp.ReferenceOTU64783
New.CleanUp.ReferenceOTU97352
New.CleanUp.ReferenceOTU111044
New.CleanUp.ReferenceOTU89552
New.CleanUp.ReferenceOTU191461
New.CleanUp.ReferenceOTU61512
New.CleanUp.ReferenceOTU167988
New.CleanUp.ReferenceOTU240253
New.CleanUp.ReferenceOTU163572
New.CleanUp.ReferenceOTU46180
New.CleanUp.ReferenceOTU62791
New.CleanUp.ReferenceOTU203285
New.CleanUp.ReferenceOTU185008
New.CleanUp.ReferenceOTU156607
New.CleanUp.ReferenceOTU55821
New.CleanUp.ReferenceOTU30597
New.CleanUp.ReferenceOTU46097
New.CleanUp.ReferenceOTU43622
New.CleanUp.ReferenceOTU129453
New.CleanUp.ReferenceOTU50879
New.CleanUp.ReferenceOTU43007
New.CleanUp.ReferenceOTU56435
New.CleanUp.ReferenceOTU49587
New.CleanUp.ReferenceOTU42633
New.CleanUp.ReferenceOTU55755
New.CleanUp.ReferenceOTU51597

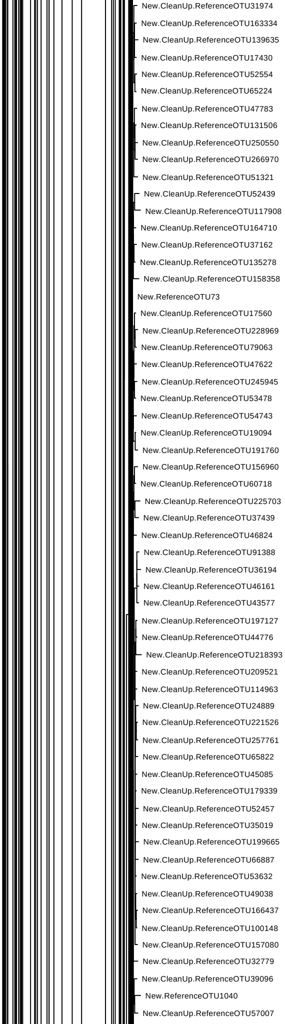
New.CleanUp.ReferenceOTU31974
New.CleanUp.ReferenceOTU163334
New.CleanUp.ReferenceOTU139635
New.CleanUp.ReferenceOTU17430
New.CleanUp.ReferenceOTU52554
New.CleanUp.ReferenceOTU65224
New.CleanUp.ReferenceOTU47783
New.CleanUp.ReferenceOTU131506
New.CleanUp.ReferenceOTU250550
New.CleanUp.ReferenceOTU266970
New.CleanUp.ReferenceOTU51321
New.CleanUp.ReferenceOTU52439
New.CleanUp.ReferenceOTU117908
New.CleanUp.ReferenceOTU164710
New.CleanUp.ReferenceOTU37162
New.CleanUp.ReferenceOTU135278
New.CleanUp.ReferenceOTU158358
New.ReferenceOTU73
New.CleanUp.ReferenceOTU17560
New.CleanUp.ReferenceOTU228969
New.CleanUp.ReferenceOTU79063
New.CleanUp.ReferenceOTU47622
New.CleanUp.ReferenceOTU245945
New.CleanUp.ReferenceOTU53478
New.CleanUp.ReferenceOTU54743
New.CleanUp.ReferenceOTU19094
New.CleanUp.ReferenceOTU191760
New.CleanUp.ReferenceOTU156960
New.CleanUp.ReferenceOTU60718
New.CleanUp.ReferenceOTU225703
New.CleanUp.ReferenceOTU37439
New.CleanUp.ReferenceOTU46824
New.CleanUp.ReferenceOTU91388
New.CleanUp.ReferenceOTU36194
New.CleanUp.ReferenceOTU46161
New.CleanUp.ReferenceOTU43577
New.CleanUp.ReferenceOTU197127
New.CleanUp.ReferenceOTU44776
New.CleanUp.ReferenceOTU218393
New.CleanUp.ReferenceOTU209521
New.CleanUp.ReferenceOTU114963
New.CleanUp.ReferenceOTU24889
New.CleanUp.ReferenceOTU221526
New.CleanUp.ReferenceOTU257761
New.CleanUp.ReferenceOTU65822
New.CleanUp.ReferenceOTU45085
New.CleanUp.ReferenceOTU179339
New.CleanUp.ReferenceOTU52457
New.CleanUp.ReferenceOTU35019
New.CleanUp.ReferenceOTU199665
New.CleanUp.ReferenceOTU66887
New.CleanUp.ReferenceOTU53632
New.CleanUp.ReferenceOTU49038
New.CleanUp.ReferenceOTU166437
New.CleanUp.ReferenceOTU100148
New.CleanUp.ReferenceOTU157080
New.CleanUp.ReferenceOTU32779
New.CleanUp.ReferenceOTU39096
New.ReferenceOTU1040
New.CleanUp.ReferenceOTU57007

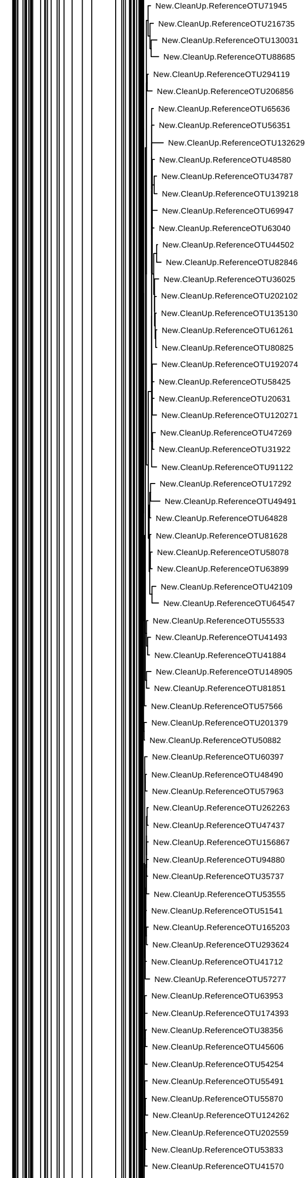
New.CleanUp.ReferenceOTU71945
New.CleanUp.ReferenceOTU216735
New.CleanUp.ReferenceOTU130031
New.CleanUp.ReferenceOTU88685
New.CleanUp.ReferenceOTU294119
New.CleanUp.ReferenceOTU206856
New.CleanUp.ReferenceOTU65636
New.CleanUp.ReferenceOTU56351
New.CleanUp.ReferenceOTU132629
New.CleanUp.ReferenceOTU48580
New.CleanUp.ReferenceOTU34787
New.CleanUp.ReferenceOTU139218
New.CleanUp.ReferenceOTU69947
New.CleanUp.ReferenceOTU63040
New.CleanUp.ReferenceOTU44502
New.CleanUp.ReferenceOTU82846
New.CleanUp.ReferenceOTU36025
New.CleanUp.ReferenceOTU202102
New.CleanUp.ReferenceOTU135130
New.CleanUp.ReferenceOTU61261
New.CleanUp.ReferenceOTU80825
New.CleanUp.ReferenceOTU192074
New.CleanUp.ReferenceOTU58425
New.CleanUp.ReferenceOTU20631
New.CleanUp.ReferenceOTU120271
New.CleanUp.ReferenceOTU47269
New.CleanUp.ReferenceOTU31922
New.CleanUp.ReferenceOTU91122
New.CleanUp.ReferenceOTU17292
New.CleanUp.ReferenceOTU49491
New.CleanUp.ReferenceOTU64828
New.CleanUp.ReferenceOTU81628
New.CleanUp.ReferenceOTU58078
New.CleanUp.ReferenceOTU63899
New.CleanUp.ReferenceOTU42109
New.CleanUp.ReferenceOTU64547
New.CleanUp.ReferenceOTU55533
New.CleanUp.ReferenceOTU41493
New.CleanUp.ReferenceOTU41884
New.CleanUp.ReferenceOTU148905
New.CleanUp.ReferenceOTU81851
New.CleanUp.ReferenceOTU57566
New.CleanUp.ReferenceOTU201379
New.CleanUp.ReferenceOTU50882
New.CleanUp.ReferenceOTU60397
New.CleanUp.ReferenceOTU48490
New.CleanUp.ReferenceOTU57963
New.CleanUp.ReferenceOTU262263
New.CleanUp.ReferenceOTU47437
New.CleanUp.ReferenceOTU156867
New.CleanUp.ReferenceOTU94880
New.CleanUp.ReferenceOTU35737
New.CleanUp.ReferenceOTU53555
New.CleanUp.ReferenceOTU51541
New.CleanUp.ReferenceOTU165203
New.CleanUp.ReferenceOTU293624
New.CleanUp.ReferenceOTU41712
New.CleanUp.ReferenceOTU57277
New.CleanUp.ReferenceOTU63953
New.CleanUp.ReferenceOTU174393
New.CleanUp.ReferenceOTU38356
New.CleanUp.ReferenceOTU45606
New.CleanUp.ReferenceOTU54254
New.CleanUp.ReferenceOTU55491
New.CleanUp.ReferenceOTU55870
New.CleanUp.ReferenceOTU124262
New.CleanUp.ReferenceOTU202559
New.CleanUp.ReferenceOTU53833
New.CleanUp.ReferenceOTU41570

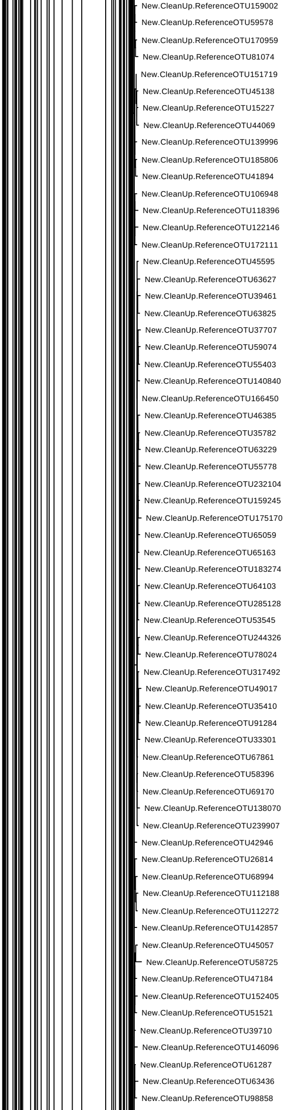
New.CleanUp.ReferenceOTU159002
New.CleanUp.ReferenceOTU59578
New.CleanUp.ReferenceOTU170959
New.CleanUp.ReferenceOTU81074
New.CleanUp.ReferenceOTU151719
New.CleanUp.ReferenceOTU45138
New.CleanUp.ReferenceOTU15227
New.CleanUp.ReferenceOTU44069
New.CleanUp.ReferenceOTU139996
New.CleanUp.ReferenceOTU185806
New.CleanUp.ReferenceOTU41894
New.CleanUp.ReferenceOTU106948
New.CleanUp.ReferenceOTU118396
New.CleanUp.ReferenceOTU122146
New.CleanUp.ReferenceOTU172111
New.CleanUp.ReferenceOTU45595
New.CleanUp.ReferenceOTU63627
New.CleanUp.ReferenceOTU39461
New.CleanUp.ReferenceOTU63825
New.CleanUp.ReferenceOTU37707
New.CleanUp.ReferenceOTU59074
New.CleanUp.ReferenceOTU55403
New.CleanUp.ReferenceOTU140840
New.CleanUp.ReferenceOTU166450
New.CleanUp.ReferenceOTU46385
New.CleanUp.ReferenceOTU35782
New.CleanUp.ReferenceOTU63229
New.CleanUp.ReferenceOTU55778
New.CleanUp.ReferenceOTU232104
New.CleanUp.ReferenceOTU159245
New.CleanUp.ReferenceOTU175170
New.CleanUp.ReferenceOTU65059
New.CleanUp.ReferenceOTU65163
New.CleanUp.ReferenceOTU183274
New.CleanUp.ReferenceOTU64103
New.CleanUp.ReferenceOTU285128
New.CleanUp.ReferenceOTU53545
New.CleanUp.ReferenceOTU244326
New.CleanUp.ReferenceOTU78024
New.CleanUp.ReferenceOTU317492
New.CleanUp.ReferenceOTU49017
New.CleanUp.ReferenceOTU35410
New.CleanUp.ReferenceOTU91284
New.CleanUp.ReferenceOTU33301
New.CleanUp.ReferenceOTU67861
New.CleanUp.ReferenceOTU58396
New.CleanUp.ReferenceOTU69170
New.CleanUp.ReferenceOTU138070
New.CleanUp.ReferenceOTU239907
New.CleanUp.ReferenceOTU42946
New.CleanUp.ReferenceOTU26814
New.CleanUp.ReferenceOTU68994
New.CleanUp.ReferenceOTU112188
New.CleanUp.ReferenceOTU112272
New.CleanUp.ReferenceOTU142857
New.CleanUp.ReferenceOTU45057
New.CleanUp.ReferenceOTU58725
New.CleanUp.ReferenceOTU47184
New.CleanUp.ReferenceOTU152405
New.CleanUp.ReferenceOTU51521
New.CleanUp.ReferenceOTU39710
New.CleanUp.ReferenceOTU146096
New.CleanUp.ReferenceOTU61287
New.CleanUp.ReferenceOTU63436
New.CleanUp.ReferenceOTU98858

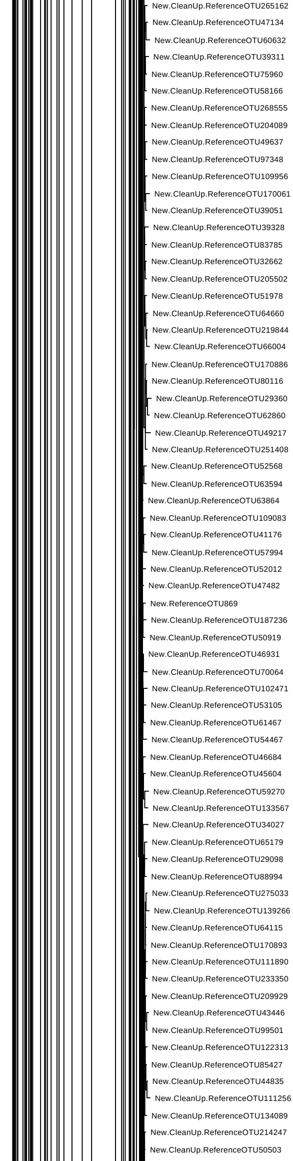
New.CleanUp.ReferenceOTU265162
New.CleanUp.ReferenceOTU47134
New.CleanUp.ReferenceOTU60632
New.CleanUp.ReferenceOTU39311
New.CleanUp.ReferenceOTU75960
New.CleanUp.ReferenceOTU58166
New.CleanUp.ReferenceOTU268555
New.CleanUp.ReferenceOTU204089
New.CleanUp.ReferenceOTU49637
New.CleanUp.ReferenceOTU97348
New.CleanUp.ReferenceOTU109956
New.CleanUp.ReferenceOTU170061
New.CleanUp.ReferenceOTU39051
New.CleanUp.ReferenceOTU39328
New.CleanUp.ReferenceOTU83785
New.CleanUp.ReferenceOTU32662
New.CleanUp.ReferenceOTU205502
New.CleanUp.ReferenceOTU51978
New.CleanUp.ReferenceOTU64660
New.CleanUp.ReferenceOTU219844
New.CleanUp.ReferenceOTU66004
New.CleanUp.ReferenceOTU170886
New.CleanUp.ReferenceOTU80116
New.CleanUp.ReferenceOTU29360
New.CleanUp.ReferenceOTU62860
New.CleanUp.ReferenceOTU49217
New.CleanUp.ReferenceOTU251408
New.CleanUp.ReferenceOTU52568
New.CleanUp.ReferenceOTU63594
New.CleanUp.ReferenceOTU63864
New.CleanUp.ReferenceOTU109083
New.CleanUp.ReferenceOTU41176
New.CleanUp.ReferenceOTU57994
New.CleanUp.ReferenceOTU52012
New.CleanUp.ReferenceOTU47482
New.ReferenceOTU869
New.CleanUp.ReferenceOTU187236
New.CleanUp.ReferenceOTU50919
New.CleanUp.ReferenceOTU46931
New.CleanUp.ReferenceOTU70064
New.CleanUp.ReferenceOTU102471
New.CleanUp.ReferenceOTU53105
New.CleanUp.ReferenceOTU61467
New.CleanUp.ReferenceOTU54467
New.CleanUp.ReferenceOTU46684
New.CleanUp.ReferenceOTU45604
New.CleanUp.ReferenceOTU59270
New.CleanUp.ReferenceOTU133567
New.CleanUp.ReferenceOTU34027
New.CleanUp.ReferenceOTU65179
New.CleanUp.ReferenceOTU29098
New.CleanUp.ReferenceOTU88994
New.CleanUp.ReferenceOTU275033
New.CleanUp.ReferenceOTU139266
New.CleanUp.ReferenceOTU64115
New.CleanUp.ReferenceOTU170893
New.CleanUp.ReferenceOTU111890
New.CleanUp.ReferenceOTU233350
New.CleanUp.ReferenceOTU209929
New.CleanUp.ReferenceOTU43446
New.CleanUp.ReferenceOTU99501
New.CleanUp.ReferenceOTU122313
New.CleanUp.ReferenceOTU85427
New.CleanUp.ReferenceOTU44835
New.CleanUp.ReferenceOTU111256
New.CleanUp.ReferenceOTU134089
New.CleanUp.ReferenceOTU214247
New.CleanUp.ReferenceOTU50503

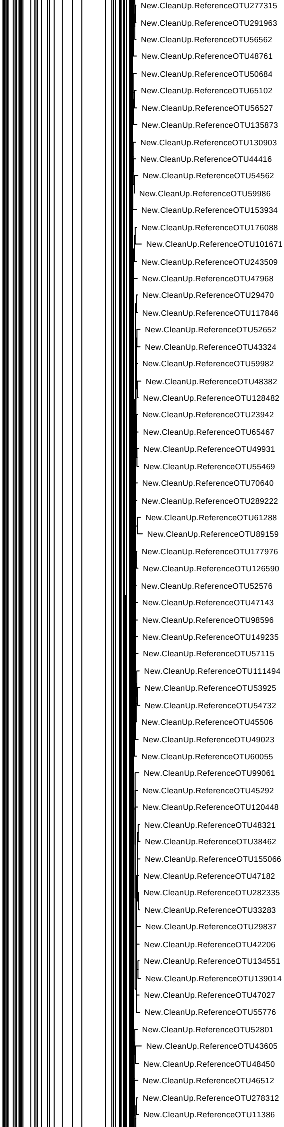
New.CleanUp.ReferenceOTU277315
New.CleanUp.ReferenceOTU291963
New.CleanUp.ReferenceOTU56562
New.CleanUp.ReferenceOTU48761
New.CleanUp.ReferenceOTU50684
New.CleanUp.ReferenceOTU65102
New.CleanUp.ReferenceOTU56527
New.CleanUp.ReferenceOTU135873
New.CleanUp.ReferenceOTU130903
New.CleanUp.ReferenceOTU44416
New.CleanUp.ReferenceOTU54562
New.CleanUp.ReferenceOTU59986
New.CleanUp.ReferenceOTU153934
New.CleanUp.ReferenceOTU176088
New.CleanUp.ReferenceOTU101671
New.CleanUp.ReferenceOTU243509
New.CleanUp.ReferenceOTU47968
New.CleanUp.ReferenceOTU29470
New.CleanUp.ReferenceOTU117846
New.CleanUp.ReferenceOTU52652
New.CleanUp.ReferenceOTU43324
New.CleanUp.ReferenceOTU59982
New.CleanUp.ReferenceOTU48382
New.CleanUp.ReferenceOTU128482
New.CleanUp.ReferenceOTU23942
New.CleanUp.ReferenceOTU65467
New.CleanUp.ReferenceOTU49931
New.CleanUp.ReferenceOTU55469
New.CleanUp.ReferenceOTU70640
New.CleanUp.ReferenceOTU289222
New.CleanUp.ReferenceOTU61288
New.CleanUp.ReferenceOTU89159
New.CleanUp.ReferenceOTU177976
New.CleanUp.ReferenceOTU126590
New.CleanUp.ReferenceOTU52576
New.CleanUp.ReferenceOTU47143
New.CleanUp.ReferenceOTU98596
New.CleanUp.ReferenceOTU149235
New.CleanUp.ReferenceOTU57115
New.CleanUp.ReferenceOTU111494
New.CleanUp.ReferenceOTU53925
New.CleanUp.ReferenceOTU54732
New.CleanUp.ReferenceOTU45506
New.CleanUp.ReferenceOTU49023
New.CleanUp.ReferenceOTU60055
New.CleanUp.ReferenceOTU99061
New.CleanUp.ReferenceOTU45292
New.CleanUp.ReferenceOTU120448
New.CleanUp.ReferenceOTU48321
New.CleanUp.ReferenceOTU38462
New.CleanUp.ReferenceOTU155066
New.CleanUp.ReferenceOTU47182
New.CleanUp.ReferenceOTU282335
New.CleanUp.ReferenceOTU33283
New.CleanUp.ReferenceOTU29837
New.CleanUp.ReferenceOTU42206
New.CleanUp.ReferenceOTU134551
New.CleanUp.ReferenceOTU139014
New.CleanUp.ReferenceOTU47027
New.CleanUp.ReferenceOTU55776
New.CleanUp.ReferenceOTU52801
New.CleanUp.ReferenceOTU43605
New.CleanUp.ReferenceOTU48450
New.CleanUp.ReferenceOTU46512
New.CleanUp.ReferenceOTU278312
New.CleanUp.ReferenceOTU11386

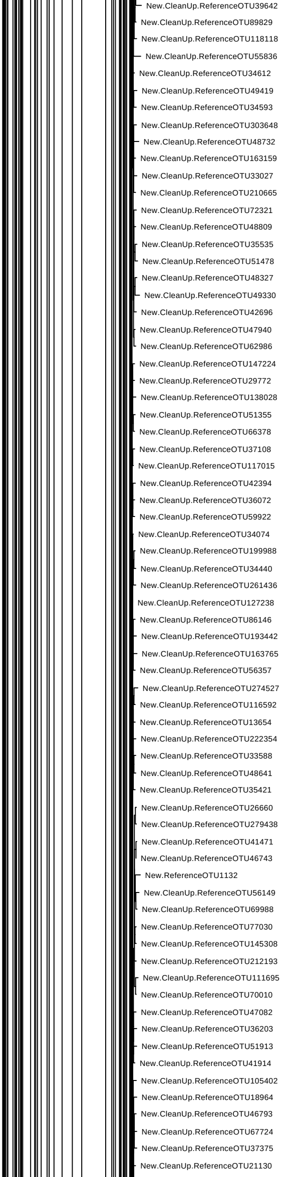
New.CleanUp.ReferenceOTU39642
New.CleanUp.ReferenceOTU89829
New.CleanUp.ReferenceOTU118118
New.CleanUp.ReferenceOTU55836
New.CleanUp.ReferenceOTU34612
New.CleanUp.ReferenceOTU49419
New.CleanUp.ReferenceOTU34593
New.CleanUp.ReferenceOTU303648
New.CleanUp.ReferenceOTU48732
New.CleanUp.ReferenceOTU163159
New.CleanUp.ReferenceOTU33027
New.CleanUp.ReferenceOTU210665
New.CleanUp.ReferenceOTU72321
New.CleanUp.ReferenceOTU48809
New.CleanUp.ReferenceOTU35535
New.CleanUp.ReferenceOTU51478
New.CleanUp.ReferenceOTU48327
New.CleanUp.ReferenceOTU49330
New.CleanUp.ReferenceOTU42696
New.CleanUp.ReferenceOTU47940
New.CleanUp.ReferenceOTU62986
New.CleanUp.ReferenceOTU147224
New.CleanUp.ReferenceOTU29772
New.CleanUp.ReferenceOTU138028
New.CleanUp.ReferenceOTU51355
New.CleanUp.ReferenceOTU66378
New.CleanUp.ReferenceOTU37108
New.CleanUp.ReferenceOTU117015
New.CleanUp.ReferenceOTU42394
New.CleanUp.ReferenceOTU36072
New.CleanUp.ReferenceOTU59922
New.CleanUp.ReferenceOTU34074
New.CleanUp.ReferenceOTU199988
New.CleanUp.ReferenceOTU34440
New.CleanUp.ReferenceOTU261436
New.CleanUp.ReferenceOTU127238
New.CleanUp.ReferenceOTU86146
New.CleanUp.ReferenceOTU193442
New.CleanUp.ReferenceOTU163765
New.CleanUp.ReferenceOTU56357
New.CleanUp.ReferenceOTU274527
New.CleanUp.ReferenceOTU116592
New.CleanUp.ReferenceOTU13654
New.CleanUp.ReferenceOTU222354
New.CleanUp.ReferenceOTU33588
New.CleanUp.ReferenceOTU48641
New.CleanUp.ReferenceOTU35421
New.CleanUp.ReferenceOTU26660
New.CleanUp.ReferenceOTU279438
New.CleanUp.ReferenceOTU41471
New.CleanUp.ReferenceOTU46743
New.ReferenceOTU1132
New.CleanUp.ReferenceOTU56149
New.CleanUp.ReferenceOTU69988
New.CleanUp.ReferenceOTU77030
New.CleanUp.ReferenceOTU145308
New.CleanUp.ReferenceOTU212193
New.CleanUp.ReferenceOTU111695
New.CleanUp.ReferenceOTU70010
New.CleanUp.ReferenceOTU47082
New.CleanUp.ReferenceOTU36203
New.CleanUp.ReferenceOTU51913
New.CleanUp.ReferenceOTU41914
New.CleanUp.ReferenceOTU105402
New.CleanUp.ReferenceOTU18964
New.CleanUp.ReferenceOTU46793
New.CleanUp.ReferenceOTU67724
New.CleanUp.ReferenceOTU37375
New.CleanUp.ReferenceOTU21130

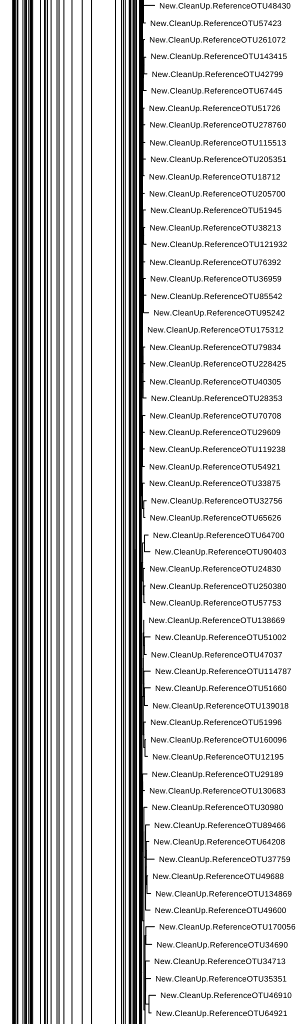
New.CleanUp.ReferenceOTU48430
New.CleanUp.ReferenceOTU57423
New.CleanUp.ReferenceOTU261072
New.CleanUp.ReferenceOTU143415
New.CleanUp.ReferenceOTU42799
New.CleanUp.ReferenceOTU67445
New.CleanUp.ReferenceOTU51726
New.CleanUp.ReferenceOTU278760
New.CleanUp.ReferenceOTU115513
New.CleanUp.ReferenceOTU205351
New.CleanUp.ReferenceOTU18712
New.CleanUp.ReferenceOTU205700
New.CleanUp.ReferenceOTU51945
New.CleanUp.ReferenceOTU38213
New.CleanUp.ReferenceOTU121932
New.CleanUp.ReferenceOTU76392
New.CleanUp.ReferenceOTU36959
New.CleanUp.ReferenceOTU85542
New.CleanUp.ReferenceOTU95242
New.CleanUp.ReferenceOTU175312
New.CleanUp.ReferenceOTU79834
New.CleanUp.ReferenceOTU228425
New.CleanUp.ReferenceOTU40305
New.CleanUp.ReferenceOTU28353
New.CleanUp.ReferenceOTU70708
New.CleanUp.ReferenceOTU29609
New.CleanUp.ReferenceOTU119238
New.CleanUp.ReferenceOTU54921
New.CleanUp.ReferenceOTU33875
New.CleanUp.ReferenceOTU32756
New.CleanUp.ReferenceOTU65626
New.CleanUp.ReferenceOTU64700
New.CleanUp.ReferenceOTU90403
New.CleanUp.ReferenceOTU24830
New.CleanUp.ReferenceOTU250380
New.CleanUp.ReferenceOTU57753
New.CleanUp.ReferenceOTU138669
New.CleanUp.ReferenceOTU51002
New.CleanUp.ReferenceOTU47037
New.CleanUp.ReferenceOTU114787
New.CleanUp.ReferenceOTU51660
New.CleanUp.ReferenceOTU139018
New.CleanUp.ReferenceOTU51996
New.CleanUp.ReferenceOTU160096
New.CleanUp.ReferenceOTU12195
New.CleanUp.ReferenceOTU29189
New.CleanUp.ReferenceOTU130683
New.CleanUp.ReferenceOTU30980
New.CleanUp.ReferenceOTU89466
New.CleanUp.ReferenceOTU64208
New.CleanUp.ReferenceOTU37759
New.CleanUp.ReferenceOTU49688
New.CleanUp.ReferenceOTU134869
New.CleanUp.ReferenceOTU49600
New.CleanUp.ReferenceOTU170056
New.CleanUp.ReferenceOTU34690
New.CleanUp.ReferenceOTU34713
New.CleanUp.ReferenceOTU35351
New.CleanUp.ReferenceOTU46910
New.CleanUp.ReferenceOTU64921

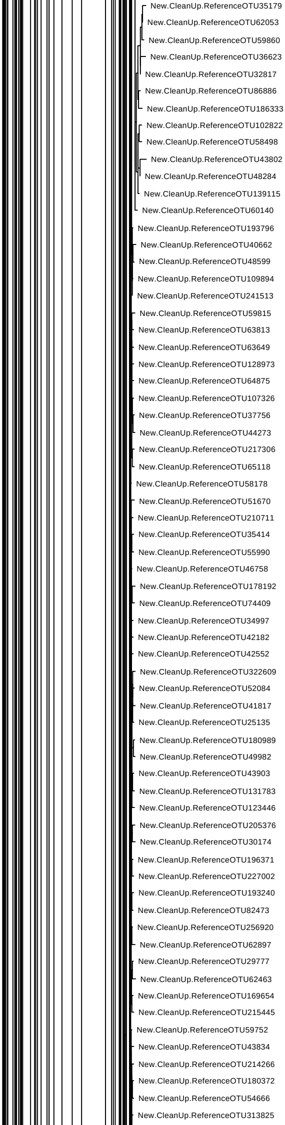
New.CleanUp.ReferenceOTU35179
New.CleanUp.ReferenceOTU62053
New.CleanUp.ReferenceOTU59860
New.CleanUp.ReferenceOTU36623
New.CleanUp.ReferenceOTU32817
New.CleanUp.ReferenceOTU86886
New.CleanUp.ReferenceOTU186333
New.CleanUp.ReferenceOTU102822
New.CleanUp.ReferenceOTU58498
New.CleanUp.ReferenceOTU43802
New.CleanUp.ReferenceOTU48284
New.CleanUp.ReferenceOTU139115
New.CleanUp.ReferenceOTU60140
New.CleanUp.ReferenceOTU193796
New.CleanUp.ReferenceOTU40662
New.CleanUp.ReferenceOTU48599
New.CleanUp.ReferenceOTU109894
New.CleanUp.ReferenceOTU241513
New.CleanUp.ReferenceOTU59815
New.CleanUp.ReferenceOTU63813
New.CleanUp.ReferenceOTU63649
New.CleanUp.ReferenceOTU128973
New.CleanUp.ReferenceOTU64875
New.CleanUp.ReferenceOTU107326
New.CleanUp.ReferenceOTU37756
New.CleanUp.ReferenceOTU44273
New.CleanUp.ReferenceOTU217306
New.CleanUp.ReferenceOTU65118
New.CleanUp.ReferenceOTU58178
New.CleanUp.ReferenceOTU51670
New.CleanUp.ReferenceOTU210711
New.CleanUp.ReferenceOTU35414
New.CleanUp.ReferenceOTU55990
New.CleanUp.ReferenceOTU46758
New.CleanUp.ReferenceOTU178192
New.CleanUp.ReferenceOTU74409
New.CleanUp.ReferenceOTU34997
New.CleanUp.ReferenceOTU42182
New.CleanUp.ReferenceOTU42552
New.CleanUp.ReferenceOTU322609
New.CleanUp.ReferenceOTU52084
New.CleanUp.ReferenceOTU41817
New.CleanUp.ReferenceOTU25135
New.CleanUp.ReferenceOTU180989
New.CleanUp.ReferenceOTU49982
New.CleanUp.ReferenceOTU43903
New.CleanUp.ReferenceOTU131783
New.CleanUp.ReferenceOTU123446
New.CleanUp.ReferenceOTU205376
New.CleanUp.ReferenceOTU30174
New.CleanUp.ReferenceOTU196371
New.CleanUp.ReferenceOTU227002
New.CleanUp.ReferenceOTU193240
New.CleanUp.ReferenceOTU82473
New.CleanUp.ReferenceOTU256920
New.CleanUp.ReferenceOTU62897
New.CleanUp.ReferenceOTU29777
New.CleanUp.ReferenceOTU62463
New.CleanUp.ReferenceOTU169654
New.CleanUp.ReferenceOTU215445
New.CleanUp.ReferenceOTU59752
New.CleanUp.ReferenceOTU43834
New.CleanUp.ReferenceOTU214266
New.CleanUp.ReferenceOTU180372
New.CleanUp.ReferenceOTU54666
New.CleanUp.ReferenceOTU313825

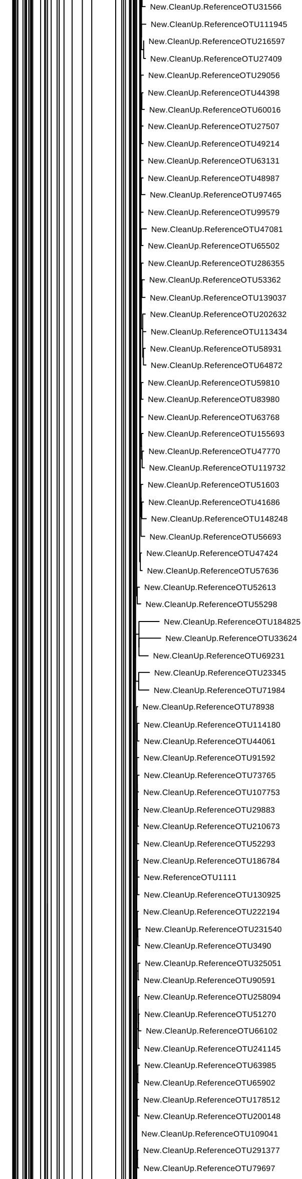
New.CleanUp.ReferenceOTU31566
New.CleanUp.ReferenceOTU111945
New.CleanUp.ReferenceOTU216597
New.CleanUp.ReferenceOTU27409
New.CleanUp.ReferenceOTU29056
New.CleanUp.ReferenceOTU44398
New.CleanUp.ReferenceOTU60016
New.CleanUp.ReferenceOTU27507
New.CleanUp.ReferenceOTU49214
New.CleanUp.ReferenceOTU63131
New.CleanUp.ReferenceOTU48987
New.CleanUp.ReferenceOTU97465
New.CleanUp.ReferenceOTU99579
New.CleanUp.ReferenceOTU47081
New.CleanUp.ReferenceOTU65502
New.CleanUp.ReferenceOTU286355
New.CleanUp.ReferenceOTU53362
New.CleanUp.ReferenceOTU139037
New.CleanUp.ReferenceOTU202632
New.CleanUp.ReferenceOTU113434
New.CleanUp.ReferenceOTU58931
New.CleanUp.ReferenceOTU64872
New.CleanUp.ReferenceOTU59810
New.CleanUp.ReferenceOTU83980
New.CleanUp.ReferenceOTU63768
New.CleanUp.ReferenceOTU155693
New.CleanUp.ReferenceOTU47770
New.CleanUp.ReferenceOTU119732
New.CleanUp.ReferenceOTU51603
New.CleanUp.ReferenceOTU41686
New.CleanUp.ReferenceOTU148248
New.CleanUp.ReferenceOTU56693
New.CleanUp.ReferenceOTU47424
New.CleanUp.ReferenceOTU57636
New.CleanUp.ReferenceOTU52613
New.CleanUp.ReferenceOTU55298
New.CleanUp.ReferenceOTU184825
New.CleanUp.ReferenceOTU33624
New.CleanUp.ReferenceOTU69231
New.CleanUp.ReferenceOTU23345
New.CleanUp.ReferenceOTU71984
New.CleanUp.ReferenceOTU78938
New.CleanUp.ReferenceOTU114180
New.CleanUp.ReferenceOTU44061
New.CleanUp.ReferenceOTU91592
New.CleanUp.ReferenceOTU73765
New.CleanUp.ReferenceOTU107753
New.CleanUp.ReferenceOTU29883
New.CleanUp.ReferenceOTU210673
New.CleanUp.ReferenceOTU52293
New.CleanUp.ReferenceOTU186784
New.ReferenceOTU1111
New.CleanUp.ReferenceOTU130925
New.CleanUp.ReferenceOTU222194
New.CleanUp.ReferenceOTU231540
New.CleanUp.ReferenceOTU3490
New.CleanUp.ReferenceOTU325051
New.CleanUp.ReferenceOTU90591
New.CleanUp.ReferenceOTU258094
New.CleanUp.ReferenceOTU51270
New.CleanUp.ReferenceOTU66102
New.CleanUp.ReferenceOTU241145
New.CleanUp.ReferenceOTU63985
New.CleanUp.ReferenceOTU65902
New.CleanUp.ReferenceOTU178512
New.CleanUp.ReferenceOTU200148
New.CleanUp.ReferenceOTU109041
New.CleanUp.ReferenceOTU291377
New.CleanUp.ReferenceOTU79697

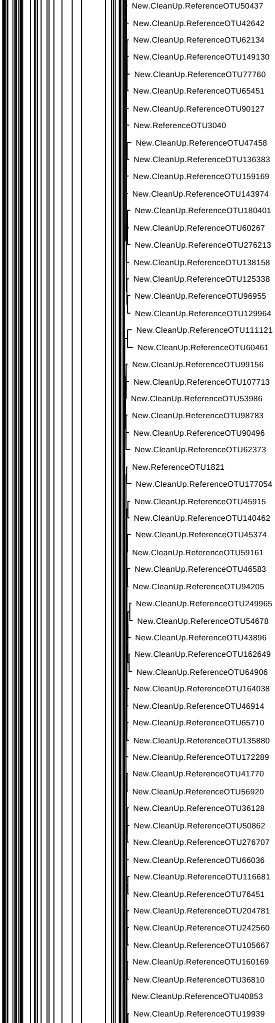
New.CleanUp.ReferenceOTU50437
New.CleanUp.ReferenceOTU42642
New.CleanUp.ReferenceOTU62134
New.CleanUp.ReferenceOTU149130
New.CleanUp.ReferenceOTU77760
New.CleanUp.ReferenceOTU65451
New.CleanUp.ReferenceOTU90127
New.ReferenceOTU3040
New.CleanUp.ReferenceOTU47458
New.CleanUp.ReferenceOTU136383
New.CleanUp.ReferenceOTU159169
New.CleanUp.ReferenceOTU143974
New.CleanUp.ReferenceOTU180401
New.CleanUp.ReferenceOTU60267
New.CleanUp.ReferenceOTU276213
New.CleanUp.ReferenceOTU138158
New.CleanUp.ReferenceOTU125338
New.CleanUp.ReferenceOTU96955
New.CleanUp.ReferenceOTU129964
New.CleanUp.ReferenceOTU111121
New.CleanUp.ReferenceOTU60461
New.CleanUp.ReferenceOTU99156
New.CleanUp.ReferenceOTU107713
New.CleanUp.ReferenceOTU53986
New.CleanUp.ReferenceOTU98783
New.CleanUp.ReferenceOTU90496
New.CleanUp.ReferenceOTU62373
New.ReferenceOTU1821
New.CleanUp.ReferenceOTU177054
New.CleanUp.ReferenceOTU45915
New.CleanUp.ReferenceOTU140462
New.CleanUp.ReferenceOTU45374
New.CleanUp.ReferenceOTU59161
New.CleanUp.ReferenceOTU46583
New.CleanUp.ReferenceOTU94205
New.CleanUp.ReferenceOTU249965
New.CleanUp.ReferenceOTU54678
New.CleanUp.ReferenceOTU43896
New.CleanUp.ReferenceOTU162649
New.CleanUp.ReferenceOTU64906
New.CleanUp.ReferenceOTU164038
New.CleanUp.ReferenceOTU46914
New.CleanUp.ReferenceOTU65710
New.CleanUp.ReferenceOTU135880
New.CleanUp.ReferenceOTU172289
New.CleanUp.ReferenceOTU41770
New.CleanUp.ReferenceOTU56920
New.CleanUp.ReferenceOTU36128
New.CleanUp.ReferenceOTU50862
New.CleanUp.ReferenceOTU276707
New.CleanUp.ReferenceOTU66036
New.CleanUp.ReferenceOTU116681
New.CleanUp.ReferenceOTU76451
New.CleanUp.ReferenceOTU204781
New.CleanUp.ReferenceOTU242560
New.CleanUp.ReferenceOTU105667
New.CleanUp.ReferenceOTU160169
New.CleanUp.ReferenceOTU36810
New.CleanUp.ReferenceOTU40853
New.CleanUp.ReferenceOTU19939

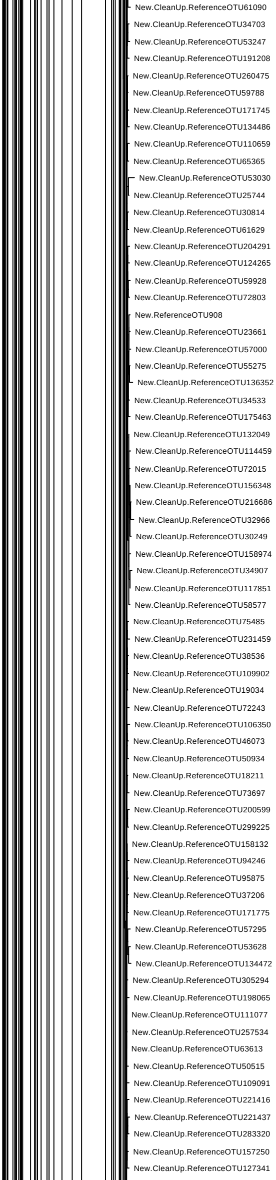
New.CleanUp.ReferenceOTU61090
New.CleanUp.ReferenceOTU34703
New.CleanUp.ReferenceOTU53247
New.CleanUp.ReferenceOTU191208
New.CleanUp.ReferenceOTU260475
New.CleanUp.ReferenceOTU59788
New.CleanUp.ReferenceOTU171745
New.CleanUp.ReferenceOTU134486
New.CleanUp.ReferenceOTU110659
New.CleanUp.ReferenceOTU65365
New.CleanUp.ReferenceOTU53030
New.CleanUp.ReferenceOTU25744
New.CleanUp.ReferenceOTU30814
New.CleanUp.ReferenceOTU61629
New.CleanUp.ReferenceOTU204291
New.CleanUp.ReferenceOTU124265
New.CleanUp.ReferenceOTU59928
New.CleanUp.ReferenceOTU72803
New.ReferenceOTU908
New.CleanUp.ReferenceOTU23661
New.CleanUp.ReferenceOTU57000
New.CleanUp.ReferenceOTU55275
New.CleanUp.ReferenceOTU136352
New.CleanUp.ReferenceOTU34533
New.CleanUp.ReferenceOTU175463
New.CleanUp.ReferenceOTU132049
New.CleanUp.ReferenceOTU114459
New.CleanUp.ReferenceOTU72015
New.CleanUp.ReferenceOTU156348
New.CleanUp.ReferenceOTU216686
New.CleanUp.ReferenceOTU32966
New.CleanUp.ReferenceOTU30249
New.CleanUp.ReferenceOTU158974
New.CleanUp.ReferenceOTU34907
New.CleanUp.ReferenceOTU117851
New.CleanUp.ReferenceOTU58577
New.CleanUp.ReferenceOTU75485
New.CleanUp.ReferenceOTU231459
New.CleanUp.ReferenceOTU38536
New.CleanUp.ReferenceOTU109902
New.CleanUp.ReferenceOTU19034
New.CleanUp.ReferenceOTU72243
New.CleanUp.ReferenceOTU106350
New.CleanUp.ReferenceOTU46073
New.CleanUp.ReferenceOTU50934
New.CleanUp.ReferenceOTU18211
New.CleanUp.ReferenceOTU73697
New.CleanUp.ReferenceOTU200599
New.CleanUp.ReferenceOTU299225
New.CleanUp.ReferenceOTU158132
New.CleanUp.ReferenceOTU94246
New.CleanUp.ReferenceOTU95875
New.CleanUp.ReferenceOTU37206
New.CleanUp.ReferenceOTU171775
New.CleanUp.ReferenceOTU57295
New.CleanUp.ReferenceOTU53628
New.CleanUp.ReferenceOTU134472
New.CleanUp.ReferenceOTU305294
New.CleanUp.ReferenceOTU198065
New.CleanUp.ReferenceOTU111077
New.CleanUp.ReferenceOTU257534
New.CleanUp.ReferenceOTU63613
New.CleanUp.ReferenceOTU50515
New.CleanUp.ReferenceOTU109091
New.CleanUp.ReferenceOTU221416
New.CleanUp.ReferenceOTU221437
New.CleanUp.ReferenceOTU283320
New.CleanUp.ReferenceOTU157250
New.CleanUp.ReferenceOTU127341

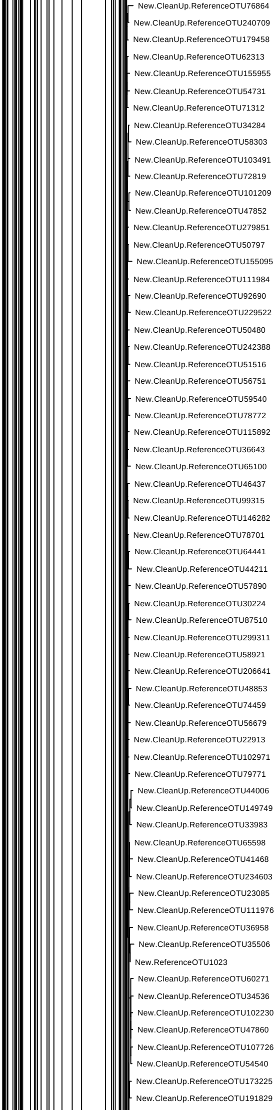
New.CleanUp.ReferenceOTU76864
New.CleanUp.ReferenceOTU240709
New.CleanUp.ReferenceOTU179458
New.CleanUp.ReferenceOTU62313
New.CleanUp.ReferenceOTU155955
New.CleanUp.ReferenceOTU54731
New.CleanUp.ReferenceOTU71312
New.CleanUp.ReferenceOTU34284
New.CleanUp.ReferenceOTU58303
New.CleanUp.ReferenceOTU103491
New.CleanUp.ReferenceOTU72819
New.CleanUp.ReferenceOTU101209
New.CleanUp.ReferenceOTU47852
New.CleanUp.ReferenceOTU279851
New.CleanUp.ReferenceOTU50797
New.CleanUp.ReferenceOTU155095
New.CleanUp.ReferenceOTU111984
New.CleanUp.ReferenceOTU92690
New.CleanUp.ReferenceOTU229522
New.CleanUp.ReferenceOTU50480
New.CleanUp.ReferenceOTU242388
New.CleanUp.ReferenceOTU51516
New.CleanUp.ReferenceOTU56751
New.CleanUp.ReferenceOTU59540
New.CleanUp.ReferenceOTU78772
New.CleanUp.ReferenceOTU115892
New.CleanUp.ReferenceOTU36643
New.CleanUp.ReferenceOTU65100
New.CleanUp.ReferenceOTU46437
New.CleanUp.ReferenceOTU99315
New.CleanUp.ReferenceOTU146282
New.CleanUp.ReferenceOTU78701
New.CleanUp.ReferenceOTU64441
New.CleanUp.ReferenceOTU44211
New.CleanUp.ReferenceOTU57890
New.CleanUp.ReferenceOTU30224
New.CleanUp.ReferenceOTU87510
New.CleanUp.ReferenceOTU299311
New.CleanUp.ReferenceOTU58921
New.CleanUp.ReferenceOTU206641
New.CleanUp.ReferenceOTU48853
New.CleanUp.ReferenceOTU74459
New.CleanUp.ReferenceOTU56679
New.CleanUp.ReferenceOTU22913
New.CleanUp.ReferenceOTU102971
New.CleanUp.ReferenceOTU79771
New.CleanUp.ReferenceOTU44006
New.CleanUp.ReferenceOTU149749
New.CleanUp.ReferenceOTU33983
New.CleanUp.ReferenceOTU65598
New.CleanUp.ReferenceOTU41468
New.CleanUp.ReferenceOTU234603
New.CleanUp.ReferenceOTU23085
New.CleanUp.ReferenceOTU111976
New.CleanUp.ReferenceOTU36958
New.CleanUp.ReferenceOTU35506
New.ReferenceOTU1023
New.CleanUp.ReferenceOTU60271
New.CleanUp.ReferenceOTU34536
New.CleanUp.ReferenceOTU102230
New.CleanUp.ReferenceOTU47860
New.CleanUp.ReferenceOTU107726
New.CleanUp.ReferenceOTU54540
New.CleanUp.ReferenceOTU173225
New.CleanUp.ReferenceOTU191829

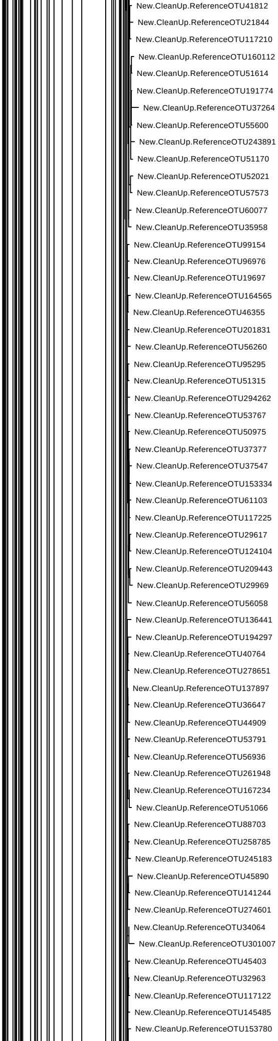
New.CleanUp.ReferenceOTU41812
New.CleanUp.ReferenceOTU21844
New.CleanUp.ReferenceOTU117210
New.CleanUp.ReferenceOTU160112
New.CleanUp.ReferenceOTU51614
New.CleanUp.ReferenceOTU191774
New.CleanUp.ReferenceOTU37264
New.CleanUp.ReferenceOTU55600
New.CleanUp.ReferenceOTU243891
New.CleanUp.ReferenceOTU51170
New.CleanUp.ReferenceOTU52021
New.CleanUp.ReferenceOTU57573
New.CleanUp.ReferenceOTU60077
New.CleanUp.ReferenceOTU35958
New.CleanUp.ReferenceOTU99154
New.CleanUp.ReferenceOTU96976
New.CleanUp.ReferenceOTU19697
New.CleanUp.ReferenceOTU164565
New.CleanUp.ReferenceOTU46355
New.CleanUp.ReferenceOTU201831
New.CleanUp.ReferenceOTU56260
New.CleanUp.ReferenceOTU95295
New.CleanUp.ReferenceOTU51315
New.CleanUp.ReferenceOTU294262
New.CleanUp.ReferenceOTU53767
New.CleanUp.ReferenceOTU50975
New.CleanUp.ReferenceOTU37377
New.CleanUp.ReferenceOTU37547
New.CleanUp.ReferenceOTU153334
New.CleanUp.ReferenceOTU61103
New.CleanUp.ReferenceOTU117225
New.CleanUp.ReferenceOTU29617
New.CleanUp.ReferenceOTU124104
New.CleanUp.ReferenceOTU209443
New.CleanUp.ReferenceOTU29969
New.CleanUp.ReferenceOTU56058
New.CleanUp.ReferenceOTU136441
New.CleanUp.ReferenceOTU194297
New.CleanUp.ReferenceOTU40764
New.CleanUp.ReferenceOTU278651
New.CleanUp.ReferenceOTU137897
New.CleanUp.ReferenceOTU36647
New.CleanUp.ReferenceOTU44909
New.CleanUp.ReferenceOTU53791
New.CleanUp.ReferenceOTU56936
New.CleanUp.ReferenceOTU261948
New.CleanUp.ReferenceOTU167234
New.CleanUp.ReferenceOTU51066
New.CleanUp.ReferenceOTU88703
New.CleanUp.ReferenceOTU258785
New.CleanUp.ReferenceOTU245183
New.CleanUp.ReferenceOTU45890
New.CleanUp.ReferenceOTU141244
New.CleanUp.ReferenceOTU274601
New.CleanUp.ReferenceOTU34064
New.CleanUp.ReferenceOTU301007
New.CleanUp.ReferenceOTU45403
New.CleanUp.ReferenceOTU32963
New.CleanUp.ReferenceOTU117122
New.CleanUp.ReferenceOTU145485
New.CleanUp.ReferenceOTU153780

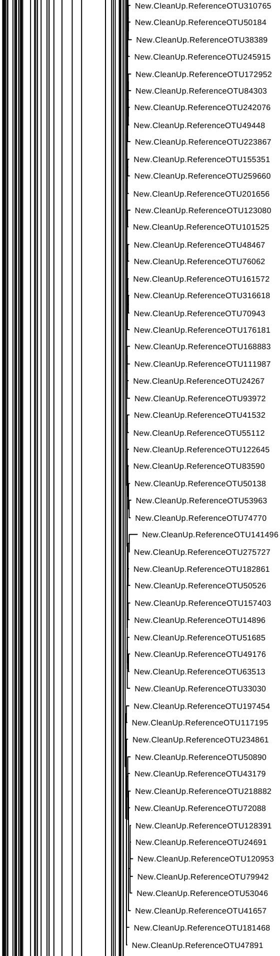
New.CleanUp.ReferenceOTU310765
New.CleanUp.ReferenceOTU50184
New.CleanUp.ReferenceOTU38389
New.CleanUp.ReferenceOTU245915
New.CleanUp.ReferenceOTU172952
New.CleanUp.ReferenceOTU84303
New.CleanUp.ReferenceOTU242076
New.CleanUp.ReferenceOTU49448
New.CleanUp.ReferenceOTU223867
New.CleanUp.ReferenceOTU155351
New.CleanUp.ReferenceOTU259660
New.CleanUp.ReferenceOTU201656
New.CleanUp.ReferenceOTU123080
New.CleanUp.ReferenceOTU101525
New.CleanUp.ReferenceOTU48467
New.CleanUp.ReferenceOTU76062
New.CleanUp.ReferenceOTU161572
New.CleanUp.ReferenceOTU316618
New.CleanUp.ReferenceOTU70943
New.CleanUp.ReferenceOTU176181
New.CleanUp.ReferenceOTU168883
New.CleanUp.ReferenceOTU111987
New.CleanUp.ReferenceOTU24267
New.CleanUp.ReferenceOTU93972
New.CleanUp.ReferenceOTU41532
New.CleanUp.ReferenceOTU55112
New.CleanUp.ReferenceOTU122645
New.CleanUp.ReferenceOTU83590
New.CleanUp.ReferenceOTU50138
New.CleanUp.ReferenceOTU53963
New.CleanUp.ReferenceOTU74770
New.CleanUp.ReferenceOTU141496
New.CleanUp.ReferenceOTU275727
New.CleanUp.ReferenceOTU182861
New.CleanUp.ReferenceOTU50526
New.CleanUp.ReferenceOTU157403
New.CleanUp.ReferenceOTU14896
New.CleanUp.ReferenceOTU51685
New.CleanUp.ReferenceOTU49176
New.CleanUp.ReferenceOTU63513
New.CleanUp.ReferenceOTU33030
New.CleanUp.ReferenceOTU197454
New.CleanUp.ReferenceOTU117195
New.CleanUp.ReferenceOTU234861
New.CleanUp.ReferenceOTU50890
New.CleanUp.ReferenceOTU43179
New.CleanUp.ReferenceOTU218882
New.CleanUp.ReferenceOTU72088
New.CleanUp.ReferenceOTU128391
New.CleanUp.ReferenceOTU24691
New.CleanUp.ReferenceOTU120953
New.CleanUp.ReferenceOTU79942
New.CleanUp.ReferenceOTU53046
New.CleanUp.ReferenceOTU41657
New.CleanUp.ReferenceOTU181468
New.CleanUp.ReferenceOTU47891

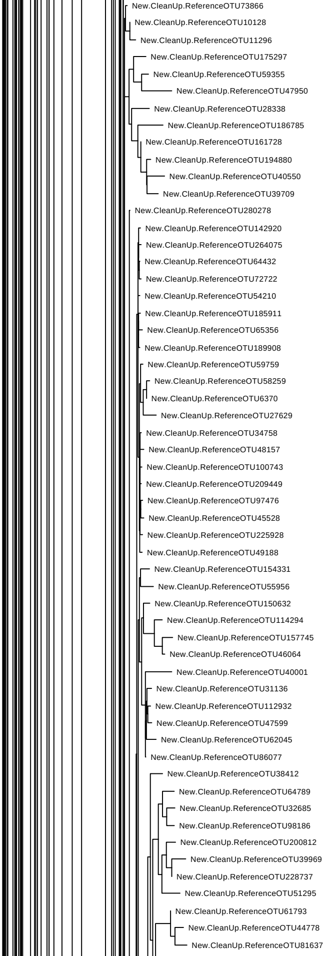
New.CleanUp.ReferenceOTU73866
New.CleanUp.ReferenceOTU10128
New.CleanUp.ReferenceOTU11296
New.CleanUp.ReferenceOTU175297
New.CleanUp.ReferenceOTU59355
New.CleanUp.ReferenceOTU47950
New.CleanUp.ReferenceOTU28338
New.CleanUp.ReferenceOTU186785
New.CleanUp.ReferenceOTU161728
New.CleanUp.ReferenceOTU194880
New.CleanUp.ReferenceOTU40550
New.CleanUp.ReferenceOTU39709
New.CleanUp.ReferenceOTU280278
New.CleanUp.ReferenceOTU142920
New.CleanUp.ReferenceOTU264075
New.CleanUp.ReferenceOTU64432
New.CleanUp.ReferenceOTU72722
New.CleanUp.ReferenceOTU54210
New.CleanUp.ReferenceOTU185911
New.CleanUp.ReferenceOTU65356
New.CleanUp.ReferenceOTU189908
New.CleanUp.ReferenceOTU59759
New.CleanUp.ReferenceOTU58259
New.CleanUp.ReferenceOTU6370
New.CleanUp.ReferenceOTU27629
New.CleanUp.ReferenceOTU34758
New.CleanUp.ReferenceOTU48157
New.CleanUp.ReferenceOTU100743
New.CleanUp.ReferenceOTU209449
New.CleanUp.ReferenceOTU97476
New.CleanUp.ReferenceOTU45528
New.CleanUp.ReferenceOTU225928
New.CleanUp.ReferenceOTU49188
New.CleanUp.ReferenceOTU154331
New.CleanUp.ReferenceOTU55956
New.CleanUp.ReferenceOTU150632
New.CleanUp.ReferenceOTU114294
New.CleanUp.ReferenceOTU157745
New.CleanUp.ReferenceOTU46064
New.CleanUp.ReferenceOTU40001
New.CleanUp.ReferenceOTU31136
New.CleanUp.ReferenceOTU112932
New.CleanUp.ReferenceOTU47599
New.CleanUp.ReferenceOTU62045
New.CleanUp.ReferenceOTU86077
New.CleanUp.ReferenceOTU38412
New.CleanUp.ReferenceOTU64789
New.CleanUp.ReferenceOTU32685
New.CleanUp.ReferenceOTU98186
New.CleanUp.ReferenceOTU200812
New.CleanUp.ReferenceOTU39969
New.CleanUp.ReferenceOTU228737
New.CleanUp.ReferenceOTU51295
New.CleanUp.ReferenceOTU61793
New.CleanUp.ReferenceOTU44778
New.CleanUp.ReferenceOTU81637

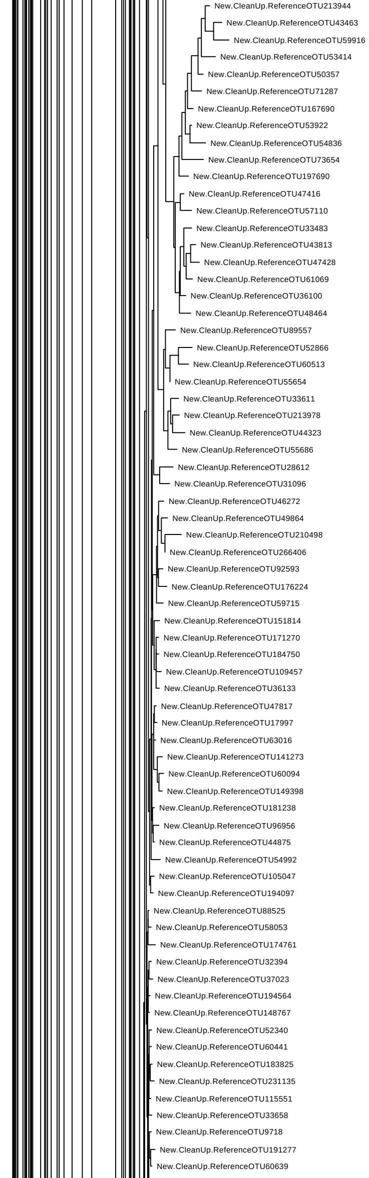
New.CleanUp.ReferenceOTU213944
New.CleanUp.ReferenceOTU43463
New.CleanUp.ReferenceOTU59916
New.CleanUp.ReferenceOTU53414
New.CleanUp.ReferenceOTU50357
New.CleanUp.ReferenceOTU71287
New.CleanUp.ReferenceOTU167690
New.CleanUp.ReferenceOTU53922
New.CleanUp.ReferenceOTU54836
New.CleanUp.ReferenceOTU73654
New.CleanUp.ReferenceOTU197690
New.CleanUp.ReferenceOTU47416
New.CleanUp.ReferenceOTU57110
New.CleanUp.ReferenceOTU33483
New.CleanUp.ReferenceOTU43813
New.CleanUp.ReferenceOTU47428
New.CleanUp.ReferenceOTU61069
New.CleanUp.ReferenceOTU36100
New.CleanUp.ReferenceOTU48464
New.CleanUp.ReferenceOTU89557
New.CleanUp.ReferenceOTU52866
New.CleanUp.ReferenceOTU60513
New.CleanUp.ReferenceOTU55654
New.CleanUp.ReferenceOTU33611
New.CleanUp.ReferenceOTU213978
New.CleanUp.ReferenceOTU44323
New.CleanUp.ReferenceOTU55686
New.CleanUp.ReferenceOTU28612
New.CleanUp.ReferenceOTU31096
New.CleanUp.ReferenceOTU46272
New.CleanUp.ReferenceOTU49864
New.CleanUp.ReferenceOTU210498
New.CleanUp.ReferenceOTU266406
New.CleanUp.ReferenceOTU92593
New.CleanUp.ReferenceOTU176224
New.CleanUp.ReferenceOTU59715
New.CleanUp.ReferenceOTU151814
New.CleanUp.ReferenceOTU171270
New.CleanUp.ReferenceOTU184750
New.CleanUp.ReferenceOTU109457
New.CleanUp.ReferenceOTU36133
New.CleanUp.ReferenceOTU47817
New.CleanUp.ReferenceOTU17997
New.CleanUp.ReferenceOTU63016
New.CleanUp.ReferenceOTU141273
New.CleanUp.ReferenceOTU60094
New.CleanUp.ReferenceOTU149398
New.CleanUp.ReferenceOTU181238
New.CleanUp.ReferenceOTU96956
New.CleanUp.ReferenceOTU44875
New.CleanUp.ReferenceOTU54992
New.CleanUp.ReferenceOTU105047
New.CleanUp.ReferenceOTU194097
New.CleanUp.ReferenceOTU88525
New.CleanUp.ReferenceOTU58053
New.CleanUp.ReferenceOTU174761
New.CleanUp.ReferenceOTU32394
New.CleanUp.ReferenceOTU37023
New.CleanUp.ReferenceOTU194564
New.CleanUp.ReferenceOTU148767
New.CleanUp.ReferenceOTU52340
New.CleanUp.ReferenceOTU60441
New.CleanUp.ReferenceOTU183825
New.CleanUp.ReferenceOTU231135
New.CleanUp.ReferenceOTU115551
New.CleanUp.ReferenceOTU33658
New.CleanUp.ReferenceOTU9718
New.CleanUp.ReferenceOTU191277
New.CleanUp.ReferenceOTU60639

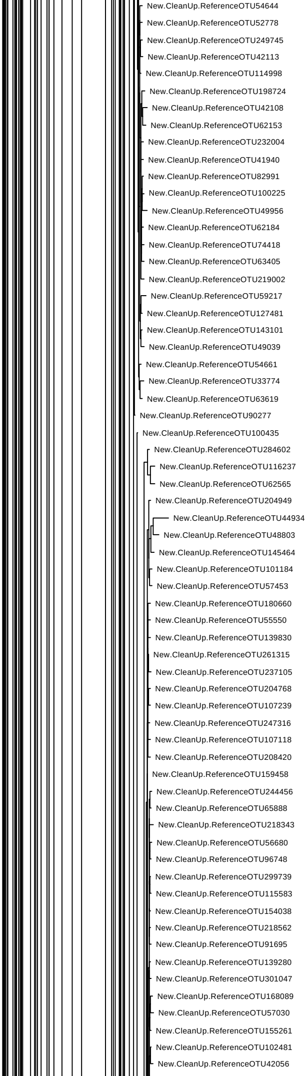
New.CleanUp.ReferenceOTU54644
New.CleanUp.ReferenceOTU52778
New.CleanUp.ReferenceOTU249745
New.CleanUp.ReferenceOTU42113
New.CleanUp.ReferenceOTU114998
New.CleanUp.ReferenceOTU198724
New.CleanUp.ReferenceOTU42108
New.CleanUp.ReferenceOTU62153
New.CleanUp.ReferenceOTU232004
New.CleanUp.ReferenceOTU41940
New.CleanUp.ReferenceOTU82991
New.CleanUp.ReferenceOTU100225
New.CleanUp.ReferenceOTU49956
New.CleanUp.ReferenceOTU62184
New.CleanUp.ReferenceOTU74418
New.CleanUp.ReferenceOTU63405
New.CleanUp.ReferenceOTU219002
New.CleanUp.ReferenceOTU59217
New.CleanUp.ReferenceOTU127481
New.CleanUp.ReferenceOTU143101
New.CleanUp.ReferenceOTU49039
New.CleanUp.ReferenceOTU54661
New.CleanUp.ReferenceOTU33774
New.CleanUp.ReferenceOTU63619
New.CleanUp.ReferenceOTU90277
New.CleanUp.ReferenceOTU100435
New.CleanUp.ReferenceOTU284602
New.CleanUp.ReferenceOTU116237
New.CleanUp.ReferenceOTU62565
New.CleanUp.ReferenceOTU204949
New.CleanUp.ReferenceOTU44934
New.CleanUp.ReferenceOTU48803
New.CleanUp.ReferenceOTU145464
New.CleanUp.ReferenceOTU101184
New.CleanUp.ReferenceOTU57453
New.CleanUp.ReferenceOTU180660
New.CleanUp.ReferenceOTU55550
New.CleanUp.ReferenceOTU139830
New.CleanUp.ReferenceOTU261315
New.CleanUp.ReferenceOTU237105
New.CleanUp.ReferenceOTU204768
New.CleanUp.ReferenceOTU107239
New.CleanUp.ReferenceOTU247316
New.CleanUp.ReferenceOTU107118
New.CleanUp.ReferenceOTU208420
New.CleanUp.ReferenceOTU159458
New.CleanUp.ReferenceOTU244456
New.CleanUp.ReferenceOTU65888
New.CleanUp.ReferenceOTU218343
New.CleanUp.ReferenceOTU56680
New.CleanUp.ReferenceOTU96748
New.CleanUp.ReferenceOTU299739
New.CleanUp.ReferenceOTU115583
New.CleanUp.ReferenceOTU154038
New.CleanUp.ReferenceOTU218562
New.CleanUp.ReferenceOTU91695
New.CleanUp.ReferenceOTU139280
New.CleanUp.ReferenceOTU301047
New.CleanUp.ReferenceOTU168089
New.CleanUp.ReferenceOTU57030
New.CleanUp.ReferenceOTU155261
New.CleanUp.ReferenceOTU102481
New.CleanUp.ReferenceOTU42056

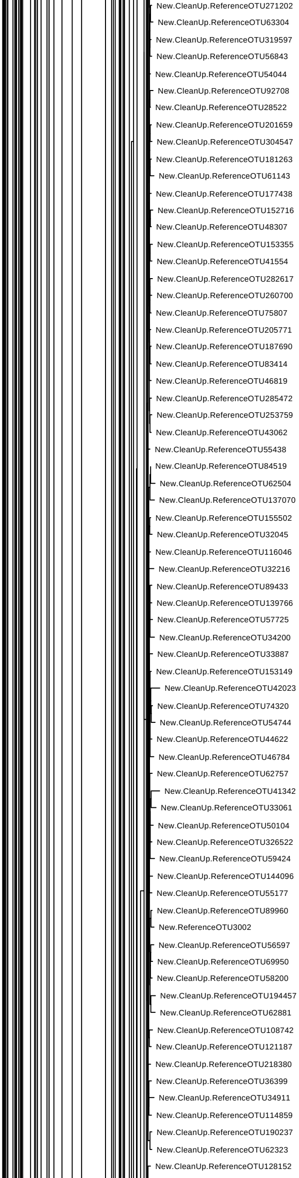
New.CleanUp.ReferenceOTU271202
New.CleanUp.ReferenceOTU63304
New.CleanUp.ReferenceOTU319597
New.CleanUp.ReferenceOTU56843
New.CleanUp.ReferenceOTU54044
New.CleanUp.ReferenceOTU92708
New.CleanUp.ReferenceOTU28522
New.CleanUp.ReferenceOTU201659
New.CleanUp.ReferenceOTU304547
New.CleanUp.ReferenceOTU181263
New.CleanUp.ReferenceOTU61143
New.CleanUp.ReferenceOTU177438
New.CleanUp.ReferenceOTU152716
New.CleanUp.ReferenceOTU48307
New.CleanUp.ReferenceOTU153355
New.CleanUp.ReferenceOTU41554
New.CleanUp.ReferenceOTU282617
New.CleanUp.ReferenceOTU260700
New.CleanUp.ReferenceOTU75807
New.CleanUp.ReferenceOTU205771
New.CleanUp.ReferenceOTU187690
New.CleanUp.ReferenceOTU83414
New.CleanUp.ReferenceOTU46819
New.CleanUp.ReferenceOTU285472
New.CleanUp.ReferenceOTU253759
New.CleanUp.ReferenceOTU43062
New.CleanUp.ReferenceOTU55438
New.CleanUp.ReferenceOTU84519
New.CleanUp.ReferenceOTU62504
New.CleanUp.ReferenceOTU137070
New.CleanUp.ReferenceOTU155502
New.CleanUp.ReferenceOTU32045
New.CleanUp.ReferenceOTU116046
New.CleanUp.ReferenceOTU32216
New.CleanUp.ReferenceOTU89433
New.CleanUp.ReferenceOTU139766
New.CleanUp.ReferenceOTU57725
New.CleanUp.ReferenceOTU34200
New.CleanUp.ReferenceOTU33887
New.CleanUp.ReferenceOTU153149
New.CleanUp.ReferenceOTU42023
New.CleanUp.ReferenceOTU74320
New.CleanUp.ReferenceOTU54744
New.CleanUp.ReferenceOTU44622
New.CleanUp.ReferenceOTU46784
New.CleanUp.ReferenceOTU62757
New.CleanUp.ReferenceOTU41342
New.CleanUp.ReferenceOTU33061
New.CleanUp.ReferenceOTU50104
New.CleanUp.ReferenceOTU326522
New.CleanUp.ReferenceOTU59424
New.CleanUp.ReferenceOTU144096
New.CleanUp.ReferenceOTU55177
New.CleanUp.ReferenceOTU89960
New.ReferenceOTU3002
New.CleanUp.ReferenceOTU56597
New.CleanUp.ReferenceOTU69950
New.CleanUp.ReferenceOTU58200
New.CleanUp.ReferenceOTU194457
New.CleanUp.ReferenceOTU62881
New.CleanUp.ReferenceOTU108742
New.CleanUp.ReferenceOTU121187
New.CleanUp.ReferenceOTU218380
New.CleanUp.ReferenceOTU36399
New.CleanUp.ReferenceOTU34911
New.CleanUp.ReferenceOTU114859
New.CleanUp.ReferenceOTU190237
New.CleanUp.ReferenceOTU62323
New.CleanUp.ReferenceOTU128152

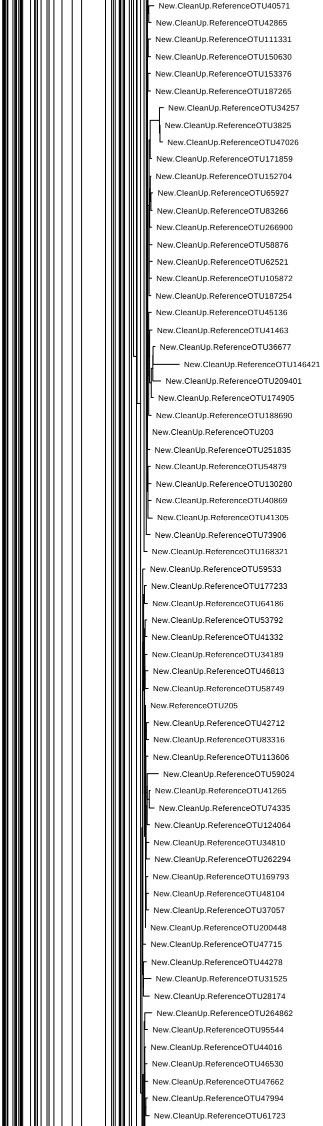
New.CleanUp.ReferenceOTU40571
New.CleanUp.ReferenceOTU42865
New.CleanUp.ReferenceOTU111331
New.CleanUp.ReferenceOTU150630
New.CleanUp.ReferenceOTU153376
New.CleanUp.ReferenceOTU187265
New.CleanUp.ReferenceOTU34257
New.CleanUp.ReferenceOTU3825
New.CleanUp.ReferenceOTU47026
New.CleanUp.ReferenceOTU171859
New.CleanUp.ReferenceOTU152704
New.CleanUp.ReferenceOTU65927
New.CleanUp.ReferenceOTU83266
New.CleanUp.ReferenceOTU266900
New.CleanUp.ReferenceOTU58876
New.CleanUp.ReferenceOTU62521
New.CleanUp.ReferenceOTU105872
New.CleanUp.ReferenceOTU187254
New.CleanUp.ReferenceOTU45136
New.CleanUp.ReferenceOTU41463
New.CleanUp.ReferenceOTU36677
New.CleanUp.ReferenceOTU146421
New.CleanUp.ReferenceOTU209401
New.CleanUp.ReferenceOTU174905
New.CleanUp.ReferenceOTU188690
New.CleanUp.ReferenceOTU203
New.CleanUp.ReferenceOTU251835
New.CleanUp.ReferenceOTU54879
New.CleanUp.ReferenceOTU130280
New.CleanUp.ReferenceOTU40869
New.CleanUp.ReferenceOTU41305
New.CleanUp.ReferenceOTU73906
New.CleanUp.ReferenceOTU168321
New.CleanUp.ReferenceOTU59533
New.CleanUp.ReferenceOTU177233
New.CleanUp.ReferenceOTU64186
New.CleanUp.ReferenceOTU53792
New.CleanUp.ReferenceOTU41332
New.CleanUp.ReferenceOTU34189
New.CleanUp.ReferenceOTU46813
New.CleanUp.ReferenceOTU58749
New.ReferenceOTU205
New.CleanUp.ReferenceOTU42712
New.CleanUp.ReferenceOTU83316
New.CleanUp.ReferenceOTU113606
New.CleanUp.ReferenceOTU59024
New.CleanUp.ReferenceOTU41265
New.CleanUp.ReferenceOTU74335
New.CleanUp.ReferenceOTU124064
New.CleanUp.ReferenceOTU34810
New.CleanUp.ReferenceOTU262294
New.CleanUp.ReferenceOTU169793
New.CleanUp.ReferenceOTU48104
New.CleanUp.ReferenceOTU37057
New.CleanUp.ReferenceOTU200448
New.CleanUp.ReferenceOTU47715
New.CleanUp.ReferenceOTU44278
New.CleanUp.ReferenceOTU31525
New.CleanUp.ReferenceOTU28174
New.CleanUp.ReferenceOTU264862
New.CleanUp.ReferenceOTU95544
New.CleanUp.ReferenceOTU44016
New.CleanUp.ReferenceOTU46530
New.CleanUp.ReferenceOTU47662
New.CleanUp.ReferenceOTU47994
New.CleanUp.ReferenceOTU61723

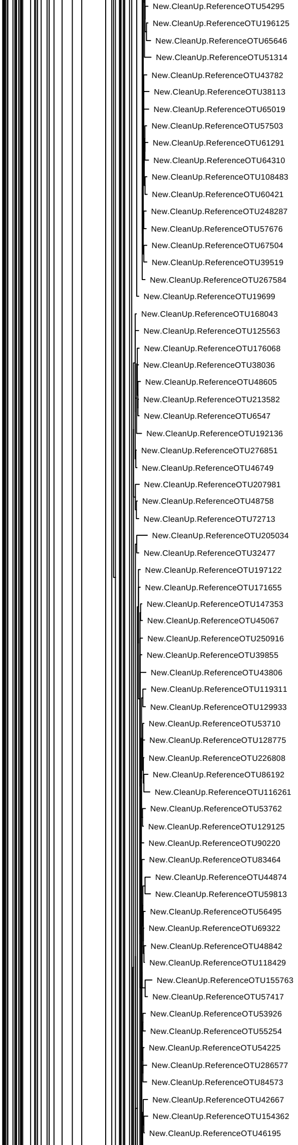
New.CleanUp.ReferenceOTU54295
New.CleanUp.ReferenceOTU196125
New.CleanUp.ReferenceOTU65646
New.CleanUp.ReferenceOTU51314
New.CleanUp.ReferenceOTU43782
New.CleanUp.ReferenceOTU38113
New.CleanUp.ReferenceOTU65019
New.CleanUp.ReferenceOTU57503
New.CleanUp.ReferenceOTU61291
New.CleanUp.ReferenceOTU64310
New.CleanUp.ReferenceOTU108483
New.CleanUp.ReferenceOTU60421
New.CleanUp.ReferenceOTU248287
New.CleanUp.ReferenceOTU57676
New.CleanUp.ReferenceOTU67504
New.CleanUp.ReferenceOTU39519
New.CleanUp.ReferenceOTU267584
New.CleanUp.ReferenceOTU19699
New.CleanUp.ReferenceOTU168043
New.CleanUp.ReferenceOTU125563
New.CleanUp.ReferenceOTU176068
New.CleanUp.ReferenceOTU38036
New.CleanUp.ReferenceOTU48605
New.CleanUp.ReferenceOTU213582
New.CleanUp.ReferenceOTU6547
New.CleanUp.ReferenceOTU192136
New.CleanUp.ReferenceOTU276851
New.CleanUp.ReferenceOTU46749
New.CleanUp.ReferenceOTU207981
New.CleanUp.ReferenceOTU48758
New.CleanUp.ReferenceOTU72713
New.CleanUp.ReferenceOTU205034
New.CleanUp.ReferenceOTU32477
New.CleanUp.ReferenceOTU197122
New.CleanUp.ReferenceOTU171655
New.CleanUp.ReferenceOTU147353
New.CleanUp.ReferenceOTU45067
New.CleanUp.ReferenceOTU250916
New.CleanUp.ReferenceOTU39855
New.CleanUp.ReferenceOTU43806
New.CleanUp.ReferenceOTU119311
New.CleanUp.ReferenceOTU129933
New.CleanUp.ReferenceOTU53710
New.CleanUp.ReferenceOTU128775
New.CleanUp.ReferenceOTU226808
New.CleanUp.ReferenceOTU86192
New.CleanUp.ReferenceOTU116261
New.CleanUp.ReferenceOTU53762
New.CleanUp.ReferenceOTU129125
New.CleanUp.ReferenceOTU90220
New.CleanUp.ReferenceOTU83464
New.CleanUp.ReferenceOTU44874
New.CleanUp.ReferenceOTU59813
New.CleanUp.ReferenceOTU56495
New.CleanUp.ReferenceOTU69322
New.CleanUp.ReferenceOTU48842
New.CleanUp.ReferenceOTU118429
New.CleanUp.ReferenceOTU155763
New.CleanUp.ReferenceOTU57417
New.CleanUp.ReferenceOTU53926
New.CleanUp.ReferenceOTU55254
New.CleanUp.ReferenceOTU54225
New.CleanUp.ReferenceOTU286577
New.CleanUp.ReferenceOTU84573
New.CleanUp.ReferenceOTU42667
New.CleanUp.ReferenceOTU154362
New.CleanUp.ReferenceOTU46195

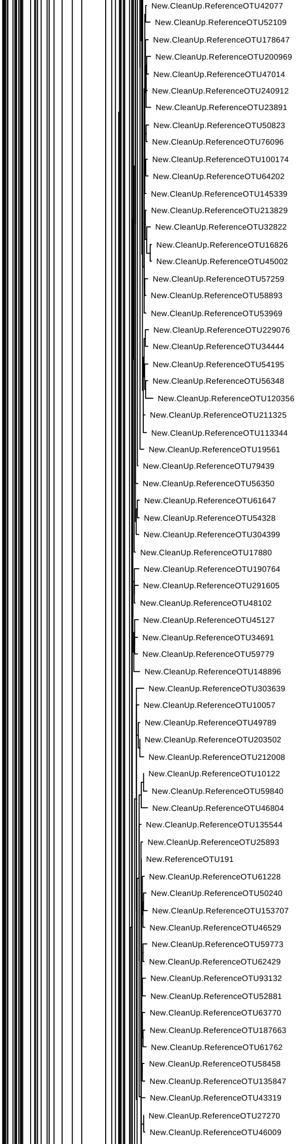
New.CleanUp.ReferenceOTU42077
New.CleanUp.ReferenceOTU52109
New.CleanUp.ReferenceOTU178647
New.CleanUp.ReferenceOTU200969
New.CleanUp.ReferenceOTU47014
New.CleanUp.ReferenceOTU240912
New.CleanUp.ReferenceOTU23891
New.CleanUp.ReferenceOTU50823
New.CleanUp.ReferenceOTU76096
New.CleanUp.ReferenceOTU100174
New.CleanUp.ReferenceOTU64202
New.CleanUp.ReferenceOTU145339
New.CleanUp.ReferenceOTU213829
New.CleanUp.ReferenceOTU32822
New.CleanUp.ReferenceOTU16826
New.CleanUp.ReferenceOTU45002
New.CleanUp.ReferenceOTU57259
New.CleanUp.ReferenceOTU58893
New.CleanUp.ReferenceOTU53969
New.CleanUp.ReferenceOTU229076
New.CleanUp.ReferenceOTU34444
New.CleanUp.ReferenceOTU54195
New.CleanUp.ReferenceOTU56348
New.CleanUp.ReferenceOTU120356
New.CleanUp.ReferenceOTU211325
New.CleanUp.ReferenceOTU113344
New.CleanUp.ReferenceOTU19561
New.CleanUp.ReferenceOTU79439
New.CleanUp.ReferenceOTU56350
New.CleanUp.ReferenceOTU61647
New.CleanUp.ReferenceOTU54328
New.CleanUp.ReferenceOTU304399
New.CleanUp.ReferenceOTU17880
New.CleanUp.ReferenceOTU190764
New.CleanUp.ReferenceOTU291605
New.CleanUp.ReferenceOTU48102
New.CleanUp.ReferenceOTU45127
New.CleanUp.ReferenceOTU34691
New.CleanUp.ReferenceOTU59779
New.CleanUp.ReferenceOTU148896
New.CleanUp.ReferenceOTU303639
New.CleanUp.ReferenceOTU10057
New.CleanUp.ReferenceOTU49789
New.CleanUp.ReferenceOTU203502
New.CleanUp.ReferenceOTU212008
New.CleanUp.ReferenceOTU10122
New.CleanUp.ReferenceOTU59840
New.CleanUp.ReferenceOTU46804
New.CleanUp.ReferenceOTU135544
New.CleanUp.ReferenceOTU25893
New.ReferenceOTU191
New.CleanUp.ReferenceOTU61228
New.CleanUp.ReferenceOTU50240
New.CleanUp.ReferenceOTU153707
New.CleanUp.ReferenceOTU46529
New.CleanUp.ReferenceOTU59773
New.CleanUp.ReferenceOTU62429
New.CleanUp.ReferenceOTU93132
New.CleanUp.ReferenceOTU52881
New.CleanUp.ReferenceOTU63770
New.CleanUp.ReferenceOTU187663
New.CleanUp.ReferenceOTU61762
New.CleanUp.ReferenceOTU58458
New.CleanUp.ReferenceOTU135847
New.CleanUp.ReferenceOTU43319
New.CleanUp.ReferenceOTU27270
New.CleanUp.ReferenceOTU46009

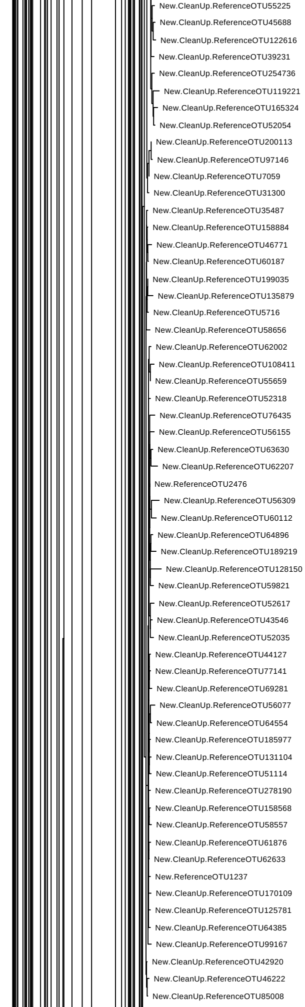
New.CleanUp.ReferenceOTU55225
New.CleanUp.ReferenceOTU45688
New.CleanUp.ReferenceOTU122616
New.CleanUp.ReferenceOTU39231
New.CleanUp.ReferenceOTU254736
New.CleanUp.ReferenceOTU119221
New.CleanUp.ReferenceOTU165324
New.CleanUp.ReferenceOTU52054
New.CleanUp.ReferenceOTU200113
New.CleanUp.ReferenceOTU97146
New.CleanUp.ReferenceOTU7059
New.CleanUp.ReferenceOTU31300
New.CleanUp.ReferenceOTU35487
New.CleanUp.ReferenceOTU158884
New.CleanUp.ReferenceOTU46771
New.CleanUp.ReferenceOTU60187
New.CleanUp.ReferenceOTU199035
New.CleanUp.ReferenceOTU135879
New.CleanUp.ReferenceOTU5716
New.CleanUp.ReferenceOTU58656
New.CleanUp.ReferenceOTU62002
New.CleanUp.ReferenceOTU108411
New.CleanUp.ReferenceOTU55659
New.CleanUp.ReferenceOTU52318
New.CleanUp.ReferenceOTU76435
New.CleanUp.ReferenceOTU56155
New.CleanUp.ReferenceOTU63630
New.CleanUp.ReferenceOTU62207
New.ReferenceOTU2476
New.CleanUp.ReferenceOTU56309
New.CleanUp.ReferenceOTU60112
New.CleanUp.ReferenceOTU64896
New.CleanUp.ReferenceOTU189219
New.CleanUp.ReferenceOTU128150
New.CleanUp.ReferenceOTU59821
New.CleanUp.ReferenceOTU52617
New.CleanUp.ReferenceOTU43546
New.CleanUp.ReferenceOTU52035
New.CleanUp.ReferenceOTU44127
New.CleanUp.ReferenceOTU77141
New.CleanUp.ReferenceOTU69281
New.CleanUp.ReferenceOTU56077
New.CleanUp.ReferenceOTU64554
New.CleanUp.ReferenceOTU185977
New.CleanUp.ReferenceOTU131104
New.CleanUp.ReferenceOTU51114
New.CleanUp.ReferenceOTU278190
New.CleanUp.ReferenceOTU158568
New.CleanUp.ReferenceOTU58557
New.CleanUp.ReferenceOTU61876
New.CleanUp.ReferenceOTU62633
New.ReferenceOTU1237
New.CleanUp.ReferenceOTU170109
New.CleanUp.ReferenceOTU125781
New.CleanUp.ReferenceOTU64385
New.CleanUp.ReferenceOTU99167
New.CleanUp.ReferenceOTU42920
New.CleanUp.ReferenceOTU46222
New.CleanUp.ReferenceOTU85008

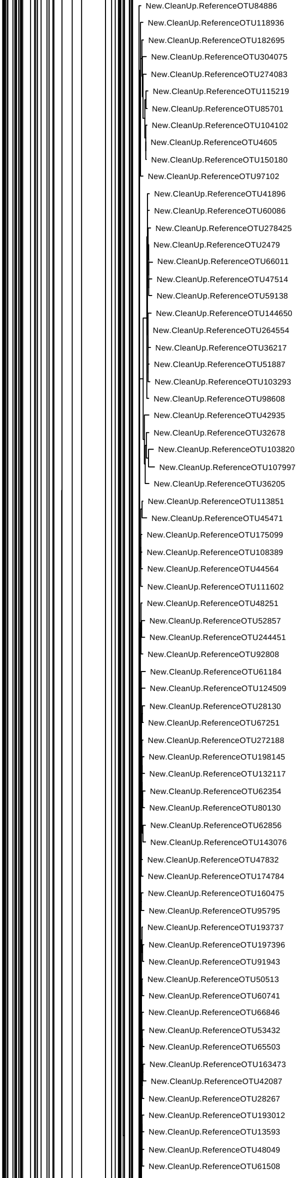
New.CleanUp.ReferenceOTU84886
New.CleanUp.ReferenceOTU118936
New.CleanUp.ReferenceOTU182695
New.CleanUp.ReferenceOTU304075
New.CleanUp.ReferenceOTU274083
New.CleanUp.ReferenceOTU115219
New.CleanUp.ReferenceOTU85701
New.CleanUp.ReferenceOTU104102
New.CleanUp.ReferenceOTU4605
New.CleanUp.ReferenceOTU150180
New.CleanUp.ReferenceOTU97102
New.CleanUp.ReferenceOTU41896
New.CleanUp.ReferenceOTU60086
New.CleanUp.ReferenceOTU278425
New.CleanUp.ReferenceOTU2479
New.CleanUp.ReferenceOTU66011
New.CleanUp.ReferenceOTU47514
New.CleanUp.ReferenceOTU59138
New.CleanUp.ReferenceOTU144650
New.CleanUp.ReferenceOTU264554
New.CleanUp.ReferenceOTU36217
New.CleanUp.ReferenceOTU51887
New.CleanUp.ReferenceOTU103293
New.CleanUp.ReferenceOTU98608
New.CleanUp.ReferenceOTU42935
New.CleanUp.ReferenceOTU32678
New.CleanUp.ReferenceOTU103820
New.CleanUp.ReferenceOTU107997
New.CleanUp.ReferenceOTU36205
New.CleanUp.ReferenceOTU113851
New.CleanUp.ReferenceOTU45471
New.CleanUp.ReferenceOTU175099
New.CleanUp.ReferenceOTU108389
New.CleanUp.ReferenceOTU44564
New.CleanUp.ReferenceOTU111602
New.CleanUp.ReferenceOTU48251
New.CleanUp.ReferenceOTU52857
New.CleanUp.ReferenceOTU244451
New.CleanUp.ReferenceOTU92808
New.CleanUp.ReferenceOTU61184
New.CleanUp.ReferenceOTU124509
New.CleanUp.ReferenceOTU28130
New.CleanUp.ReferenceOTU67251
New.CleanUp.ReferenceOTU272188
New.CleanUp.ReferenceOTU198145
New.CleanUp.ReferenceOTU132117
New.CleanUp.ReferenceOTU62354
New.CleanUp.ReferenceOTU80130
New.CleanUp.ReferenceOTU62856
New.CleanUp.ReferenceOTU143076
New.CleanUp.ReferenceOTU47832
New.CleanUp.ReferenceOTU174784
New.CleanUp.ReferenceOTU160475
New.CleanUp.ReferenceOTU95795
New.CleanUp.ReferenceOTU193737
New.CleanUp.ReferenceOTU197396
New.CleanUp.ReferenceOTU91943
New.CleanUp.ReferenceOTU50513
New.CleanUp.ReferenceOTU60741
New.CleanUp.ReferenceOTU66846
New.CleanUp.ReferenceOTU53432
New.CleanUp.ReferenceOTU65503
New.CleanUp.ReferenceOTU163473
New.CleanUp.ReferenceOTU42087
New.CleanUp.ReferenceOTU28267
New.CleanUp.ReferenceOTU193012
New.CleanUp.ReferenceOTU13593
New.CleanUp.ReferenceOTU48049
New.CleanUp.ReferenceOTU61508

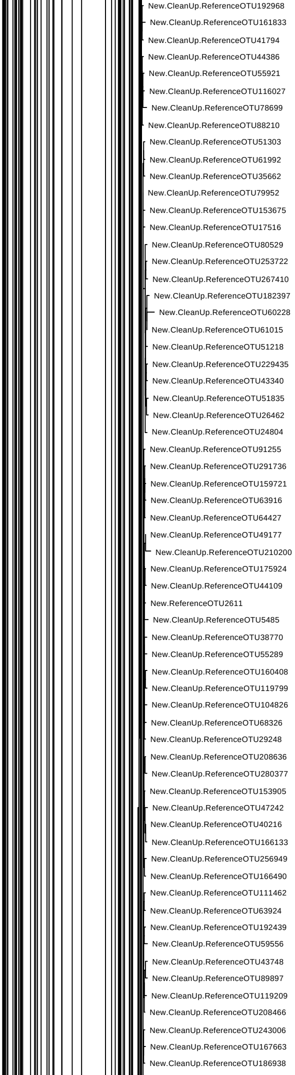
New.CleanUp.ReferenceOTU192968
New.CleanUp.ReferenceOTU161833
New.CleanUp.ReferenceOTU41794
New.CleanUp.ReferenceOTU44386
New.CleanUp.ReferenceOTU55921
New.CleanUp.ReferenceOTU116027
New.CleanUp.ReferenceOTU78699
New.CleanUp.ReferenceOTU88210
New.CleanUp.ReferenceOTU51303
New.CleanUp.ReferenceOTU61992
New.CleanUp.ReferenceOTU35662
New.CleanUp.ReferenceOTU79952
New.CleanUp.ReferenceOTU153675
New.CleanUp.ReferenceOTU17516
New.CleanUp.ReferenceOTU80529
New.CleanUp.ReferenceOTU253722
New.CleanUp.ReferenceOTU267410
New.CleanUp.ReferenceOTU182397
New.CleanUp.ReferenceOTU60228
New.CleanUp.ReferenceOTU61015
New.CleanUp.ReferenceOTU51218
New.CleanUp.ReferenceOTU229435
New.CleanUp.ReferenceOTU43340
New.CleanUp.ReferenceOTU51835
New.CleanUp.ReferenceOTU26462
New.CleanUp.ReferenceOTU24804
New.CleanUp.ReferenceOTU91255
New.CleanUp.ReferenceOTU291736
New.CleanUp.ReferenceOTU159721
New.CleanUp.ReferenceOTU63916
New.CleanUp.ReferenceOTU64427
New.CleanUp.ReferenceOTU49177
New.CleanUp.ReferenceOTU210200
New.CleanUp.ReferenceOTU175924
New.CleanUp.ReferenceOTU44109
New.ReferenceOTU2611
New.CleanUp.ReferenceOTU5485
New.CleanUp.ReferenceOTU38770
New.CleanUp.ReferenceOTU55289
New.CleanUp.ReferenceOTU160408
New.CleanUp.ReferenceOTU119799
New.CleanUp.ReferenceOTU104826
New.CleanUp.ReferenceOTU68326
New.CleanUp.ReferenceOTU29248
New.CleanUp.ReferenceOTU208636
New.CleanUp.ReferenceOTU280377
New.CleanUp.ReferenceOTU153905
New.CleanUp.ReferenceOTU47242
New.CleanUp.ReferenceOTU40216
New.CleanUp.ReferenceOTU166133
New.CleanUp.ReferenceOTU256949
New.CleanUp.ReferenceOTU166490
New.CleanUp.ReferenceOTU111462
New.CleanUp.ReferenceOTU63924
New.CleanUp.ReferenceOTU192439
New.CleanUp.ReferenceOTU59556
New.CleanUp.ReferenceOTU43748
New.CleanUp.ReferenceOTU89897
New.CleanUp.ReferenceOTU119209
New.CleanUp.ReferenceOTU208466
New.CleanUp.ReferenceOTU243006
New.CleanUp.ReferenceOTU167663
New.CleanUp.ReferenceOTU186938

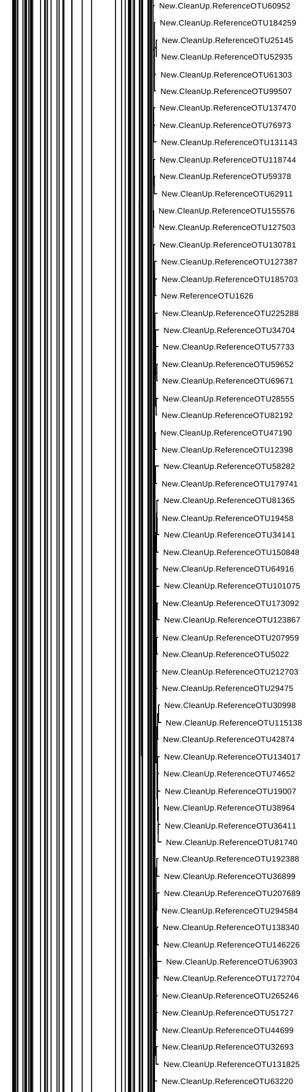

New.CleanUp.ReferenceOTU60952
New.CleanUp.ReferenceOTU184259
New.CleanUp.ReferenceOTU25145
New.CleanUp.ReferenceOTU52935
New.CleanUp.ReferenceOTU61303
New.CleanUp.ReferenceOTU99507
New.CleanUp.ReferenceOTU137470
New.CleanUp.ReferenceOTU76973
New.CleanUp.ReferenceOTU131143
New.CleanUp.ReferenceOTU118744
New.CleanUp.ReferenceOTU59378
New.CleanUp.ReferenceOTU62911
New.CleanUp.ReferenceOTU155576
New.CleanUp.ReferenceOTU127503
New.CleanUp.ReferenceOTU130781
New.CleanUp.ReferenceOTU127387
New.CleanUp.ReferenceOTU185703
New.ReferenceOTU1626
New.CleanUp.ReferenceOTU225288
New.CleanUp.ReferenceOTU34704
New.CleanUp.ReferenceOTU57733
New.CleanUp.ReferenceOTU59652
New.CleanUp.ReferenceOTU69671
New.CleanUp.ReferenceOTU28555
New.CleanUp.ReferenceOTU82192
New.CleanUp.ReferenceOTU47190
New.CleanUp.ReferenceOTU12398
New.CleanUp.ReferenceOTU58282
New.CleanUp.ReferenceOTU179741
New.CleanUp.ReferenceOTU81365
New.CleanUp.ReferenceOTU19458
New.CleanUp.ReferenceOTU34141
New.CleanUp.ReferenceOTU150848
New.CleanUp.ReferenceOTU64916
New.CleanUp.ReferenceOTU101075
New.CleanUp.ReferenceOTU173092
New.CleanUp.ReferenceOTU123867
New.CleanUp.ReferenceOTU207959
New.CleanUp.ReferenceOTU5022
New.CleanUp.ReferenceOTU212703
New.CleanUp.ReferenceOTU29475
New.CleanUp.ReferenceOTU30998
New.CleanUp.ReferenceOTU115138
New.CleanUp.ReferenceOTU42874
New.CleanUp.ReferenceOTU134017
New.CleanUp.ReferenceOTU74652
New.CleanUp.ReferenceOTU19007
New.CleanUp.ReferenceOTU38964
New.CleanUp.ReferenceOTU36411
New.CleanUp.ReferenceOTU81740
New.CleanUp.ReferenceOTU192388
New.CleanUp.ReferenceOTU36899
New.CleanUp.ReferenceOTU207689
New.CleanUp.ReferenceOTU294584
New.CleanUp.ReferenceOTU138340
New.CleanUp.ReferenceOTU146226
New.CleanUp.ReferenceOTU63903
New.CleanUp.ReferenceOTU172704
New.CleanUp.ReferenceOTU265246
New.CleanUp.ReferenceOTU51727
New.CleanUp.ReferenceOTU44699
New.CleanUp.ReferenceOTU32693
New.CleanUp.ReferenceOTU131825
New.CleanUp.ReferenceOTU63220

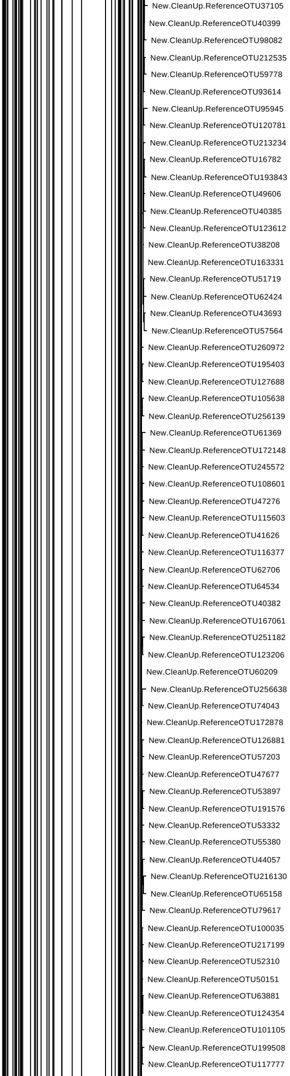
New.CleanUp.ReferenceOTU37105
New.CleanUp.ReferenceOTU40399
New.CleanUp.ReferenceOTU98082
New.CleanUp.ReferenceOTU212535
New.CleanUp.ReferenceOTU59778
New.CleanUp.ReferenceOTU93614
New.CleanUp.ReferenceOTU95945
New.CleanUp.ReferenceOTU120781
New.CleanUp.ReferenceOTU213234
New.CleanUp.ReferenceOTU16782
New.CleanUp.ReferenceOTU193843
New.CleanUp.ReferenceOTU49606
New.CleanUp.ReferenceOTU40385
New.CleanUp.ReferenceOTU123612
New.CleanUp.ReferenceOTU38208
New.CleanUp.ReferenceOTU163331
New.CleanUp.ReferenceOTU51719
New.CleanUp.ReferenceOTU62424
New.CleanUp.ReferenceOTU43693
New.CleanUp.ReferenceOTU57564
New.CleanUp.ReferenceOTU260972
New.CleanUp.ReferenceOTU195403
New.CleanUp.ReferenceOTU127688
New.CleanUp.ReferenceOTU105638
New.CleanUp.ReferenceOTU256139
New.CleanUp.ReferenceOTU61369
New.CleanUp.ReferenceOTU172148
New.CleanUp.ReferenceOTU245572
New.CleanUp.ReferenceOTU108601
New.CleanUp.ReferenceOTU47276
New.CleanUp.ReferenceOTU115603
New.CleanUp.ReferenceOTU41626
New.CleanUp.ReferenceOTU116377
New.CleanUp.ReferenceOTU62706
New.CleanUp.ReferenceOTU64534
New.CleanUp.ReferenceOTU40382
New.CleanUp.ReferenceOTU167061
New.CleanUp.ReferenceOTU251182
New.CleanUp.ReferenceOTU123206
New.CleanUp.ReferenceOTU60209
New.CleanUp.ReferenceOTU256638
New.CleanUp.ReferenceOTU74043
New.CleanUp.ReferenceOTU172878
New.CleanUp.ReferenceOTU126881
New.CleanUp.ReferenceOTU57203
New.CleanUp.ReferenceOTU47677
New.CleanUp.ReferenceOTU53897
New.CleanUp.ReferenceOTU191576
New.CleanUp.ReferenceOTU53332
New.CleanUp.ReferenceOTU55380
New.CleanUp.ReferenceOTU44057
New.CleanUp.ReferenceOTU216130
New.CleanUp.ReferenceOTU65158
New.CleanUp.ReferenceOTU79617
New.CleanUp.ReferenceOTU100035
New.CleanUp.ReferenceOTU217199
New.CleanUp.ReferenceOTU52310
New.CleanUp.ReferenceOTU50151
New.CleanUp.ReferenceOTU63881
New.CleanUp.ReferenceOTU124354
New.CleanUp.ReferenceOTU101105
New.CleanUp.ReferenceOTU199508
New.CleanUp.ReferenceOTU117777

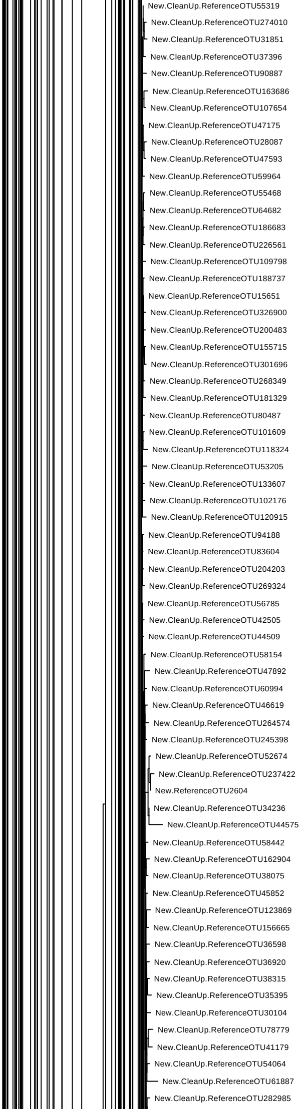
New.CleanUp.ReferenceOTU55319
New.CleanUp.ReferenceOTU274010
New.CleanUp.ReferenceOTU31851
New.CleanUp.ReferenceOTU37396
New.CleanUp.ReferenceOTU90887
New.CleanUp.ReferenceOTU163686
New.CleanUp.ReferenceOTU107654
New.CleanUp.ReferenceOTU47175
New.CleanUp.ReferenceOTU28087
New.CleanUp.ReferenceOTU47593
New.CleanUp.ReferenceOTU59964
New.CleanUp.ReferenceOTU55468
New.CleanUp.ReferenceOTU64682
New.CleanUp.ReferenceOTU186683
New.CleanUp.ReferenceOTU226561
New.CleanUp.ReferenceOTU109798
New.CleanUp.ReferenceOTU188737
New.CleanUp.ReferenceOTU15651
New.CleanUp.ReferenceOTU326900
New.CleanUp.ReferenceOTU200483
New.CleanUp.ReferenceOTU155715
New.CleanUp.ReferenceOTU301696
New.CleanUp.ReferenceOTU268349
New.CleanUp.ReferenceOTU181329
New.CleanUp.ReferenceOTU80487
New.CleanUp.ReferenceOTU101609
New.CleanUp.ReferenceOTU118324
New.CleanUp.ReferenceOTU53205
New.CleanUp.ReferenceOTU133607
New.CleanUp.ReferenceOTU102176
New.CleanUp.ReferenceOTU120915
New.CleanUp.ReferenceOTU94188
New.CleanUp.ReferenceOTU83604
New.CleanUp.ReferenceOTU204203
New.CleanUp.ReferenceOTU269324
New.CleanUp.ReferenceOTU56785
New.CleanUp.ReferenceOTU42505
New.CleanUp.ReferenceOTU44509
New.CleanUp.ReferenceOTU58154
New.CleanUp.ReferenceOTU47892
New.CleanUp.ReferenceOTU60994
New.CleanUp.ReferenceOTU46619
New.CleanUp.ReferenceOTU264574
New.CleanUp.ReferenceOTU245398
New.CleanUp.ReferenceOTU52674
New.CleanUp.ReferenceOTU237422
New.ReferenceOTU2604
New.CleanUp.ReferenceOTU34236
New.CleanUp.ReferenceOTU44575
New.CleanUp.ReferenceOTU58442
New.CleanUp.ReferenceOTU162904
New.CleanUp.ReferenceOTU38075
New.CleanUp.ReferenceOTU45852
New.CleanUp.ReferenceOTU123869
New.CleanUp.ReferenceOTU156665
New.CleanUp.ReferenceOTU36598
New.CleanUp.ReferenceOTU36920
New.CleanUp.ReferenceOTU38315
New.CleanUp.ReferenceOTU35395
New.CleanUp.ReferenceOTU30104
New.CleanUp.ReferenceOTU78779
New.CleanUp.ReferenceOTU41179
New.CleanUp.ReferenceOTU54064
New.CleanUp.ReferenceOTU61887
New.CleanUp.ReferenceOTU282985

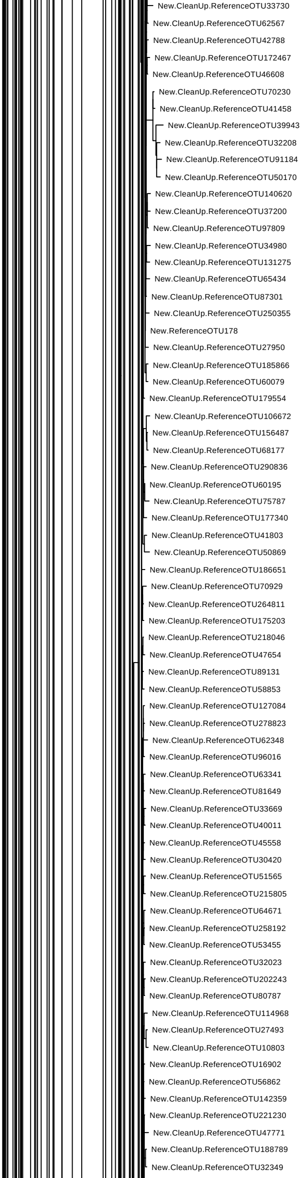
New.CleanUp.ReferenceOTU33730
New.CleanUp.ReferenceOTU62567
New.CleanUp.ReferenceOTU42788
New.CleanUp.ReferenceOTU172467
New.CleanUp.ReferenceOTU46608
New.CleanUp.ReferenceOTU70230
New.CleanUp.ReferenceOTU41458
New.CleanUp.ReferenceOTU39943
New.CleanUp.ReferenceOTU32208
New.CleanUp.ReferenceOTU91184
New.CleanUp.ReferenceOTU50170
New.CleanUp.ReferenceOTU140620
New.CleanUp.ReferenceOTU37200
New.CleanUp.ReferenceOTU97809
New.CleanUp.ReferenceOTU34980
New.CleanUp.ReferenceOTU131275
New.CleanUp.ReferenceOTU65434
New.CleanUp.ReferenceOTU87301
New.CleanUp.ReferenceOTU250355
New.ReferenceOTU178
New.CleanUp.ReferenceOTU27950
New.CleanUp.ReferenceOTU185866
New.CleanUp.ReferenceOTU60079
New.CleanUp.ReferenceOTU179554
New.CleanUp.ReferenceOTU106672
New.CleanUp.ReferenceOTU156487
New.CleanUp.ReferenceOTU68177
New.CleanUp.ReferenceOTU290836
New.CleanUp.ReferenceOTU60195
New.CleanUp.ReferenceOTU75787
New.CleanUp.ReferenceOTU177340
New.CleanUp.ReferenceOTU41803
New.CleanUp.ReferenceOTU50869
New.CleanUp.ReferenceOTU186651
New.CleanUp.ReferenceOTU70929
New.CleanUp.ReferenceOTU264811
New.CleanUp.ReferenceOTU175203
New.CleanUp.ReferenceOTU218046
New.CleanUp.ReferenceOTU47654
New.CleanUp.ReferenceOTU89131
New.CleanUp.ReferenceOTU58853
New.CleanUp.ReferenceOTU127084
New.CleanUp.ReferenceOTU278823
New.CleanUp.ReferenceOTU62348
New.CleanUp.ReferenceOTU96016
New.CleanUp.ReferenceOTU63341
New.CleanUp.ReferenceOTU81649
New.CleanUp.ReferenceOTU33669
New.CleanUp.ReferenceOTU40011
New.CleanUp.ReferenceOTU45558
New.CleanUp.ReferenceOTU30420
New.CleanUp.ReferenceOTU51565
New.CleanUp.ReferenceOTU215805
New.CleanUp.ReferenceOTU64671
New.CleanUp.ReferenceOTU258192
New.CleanUp.ReferenceOTU53455
New.CleanUp.ReferenceOTU32023
New.CleanUp.ReferenceOTU202243
New.CleanUp.ReferenceOTU80787
New.CleanUp.ReferenceOTU114968
New.CleanUp.ReferenceOTU27493
New.CleanUp.ReferenceOTU10803
New.CleanUp.ReferenceOTU16902
New.CleanUp.ReferenceOTU56862
New.CleanUp.ReferenceOTU142359
New.CleanUp.ReferenceOTU221230
New.CleanUp.ReferenceOTU47771
New.CleanUp.ReferenceOTU188789
New.CleanUp.ReferenceOTU32349

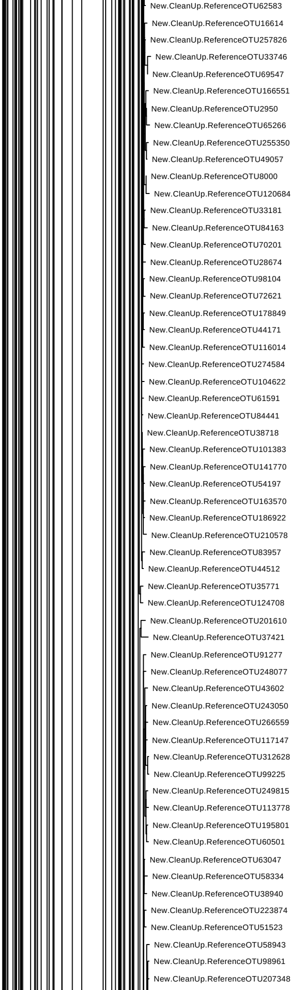
New.CleanUp.ReferenceOTU62583
New.CleanUp.ReferenceOTU16614
New.CleanUp.ReferenceOTU257826
New.CleanUp.ReferenceOTU33746
New.CleanUp.ReferenceOTU69547
New.CleanUp.ReferenceOTU166551
New.CleanUp.ReferenceOTU2950
New.CleanUp.ReferenceOTU65266
New.CleanUp.ReferenceOTU255350
New.CleanUp.ReferenceOTU49057
New.CleanUp.ReferenceOTU8000
New.CleanUp.ReferenceOTU120684
New.CleanUp.ReferenceOTU33181
New.CleanUp.ReferenceOTU84163
New.CleanUp.ReferenceOTU70201
New.CleanUp.ReferenceOTU28674
New.CleanUp.ReferenceOTU98104
New.CleanUp.ReferenceOTU72621
New.CleanUp.ReferenceOTU178849
New.CleanUp.ReferenceOTU44171
New.CleanUp.ReferenceOTU116014
New.CleanUp.ReferenceOTU274584
New.CleanUp.ReferenceOTU104622
New.CleanUp.ReferenceOTU61591
New.CleanUp.ReferenceOTU84441
New.CleanUp.ReferenceOTU38718
New.CleanUp.ReferenceOTU101383
New.CleanUp.ReferenceOTU141770
New.CleanUp.ReferenceOTU54197
New.CleanUp.ReferenceOTU163570
New.CleanUp.ReferenceOTU186922
New.CleanUp.ReferenceOTU210578
New.CleanUp.ReferenceOTU83957
New.CleanUp.ReferenceOTU44512
New.CleanUp.ReferenceOTU35771
New.CleanUp.ReferenceOTU124708
New.CleanUp.ReferenceOTU201610
New.CleanUp.ReferenceOTU37421
New.CleanUp.ReferenceOTU91277
New.CleanUp.ReferenceOTU248077
New.CleanUp.ReferenceOTU43602
New.CleanUp.ReferenceOTU243050
New.CleanUp.ReferenceOTU266559
New.CleanUp.ReferenceOTU117147
New.CleanUp.ReferenceOTU312628
New.CleanUp.ReferenceOTU99225
New.CleanUp.ReferenceOTU249815
New.CleanUp.ReferenceOTU113778
New.CleanUp.ReferenceOTU195801
New.CleanUp.ReferenceOTU60501
New.CleanUp.ReferenceOTU63047
New.CleanUp.ReferenceOTU58334
New.CleanUp.ReferenceOTU38940
New.CleanUp.ReferenceOTU223874
New.CleanUp.ReferenceOTU51523
New.CleanUp.ReferenceOTU58943
New.CleanUp.ReferenceOTU98961
New.CleanUp.ReferenceOTU207348

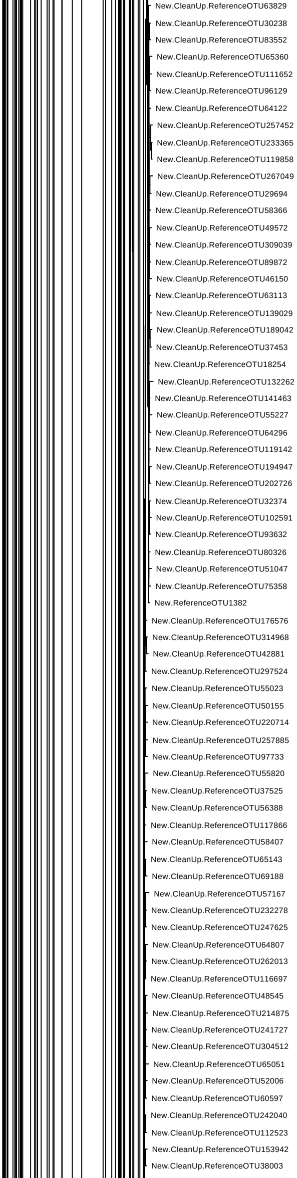
New.CleanUp.ReferenceOTU63829
New.CleanUp.ReferenceOTU30238
New.CleanUp.ReferenceOTU83552
New.CleanUp.ReferenceOTU65360
New.CleanUp.ReferenceOTU111652
New.CleanUp.ReferenceOTU96129
New.CleanUp.ReferenceOTU64122
New.CleanUp.ReferenceOTU257452
New.CleanUp.ReferenceOTU233365
New.CleanUp.ReferenceOTU119858
New.CleanUp.ReferenceOTU267049
New.CleanUp.ReferenceOTU29694
New.CleanUp.ReferenceOTU58366
New.CleanUp.ReferenceOTU49572
New.CleanUp.ReferenceOTU309039
New.CleanUp.ReferenceOTU89872
New.CleanUp.ReferenceOTU46150
New.CleanUp.ReferenceOTU63113
New.CleanUp.ReferenceOTU139029
New.CleanUp.ReferenceOTU189042
New.CleanUp.ReferenceOTU37453
New.CleanUp.ReferenceOTU18254
New.CleanUp.ReferenceOTU132262
New.CleanUp.ReferenceOTU141463
New.CleanUp.ReferenceOTU55227
New.CleanUp.ReferenceOTU64296
New.CleanUp.ReferenceOTU119142
New.CleanUp.ReferenceOTU194947
New.CleanUp.ReferenceOTU202726
New.CleanUp.ReferenceOTU32374
New.CleanUp.ReferenceOTU102591
New.CleanUp.ReferenceOTU93632
New.CleanUp.ReferenceOTU80326
New.CleanUp.ReferenceOTU51047
New.CleanUp.ReferenceOTU75358
New.ReferenceOTU1382
New.CleanUp.ReferenceOTU176576
New.CleanUp.ReferenceOTU314968
New.CleanUp.ReferenceOTU42881
New.CleanUp.ReferenceOTU297524
New.CleanUp.ReferenceOTU55023
New.CleanUp.ReferenceOTU50155
New.CleanUp.ReferenceOTU220714
New.CleanUp.ReferenceOTU257885
New.CleanUp.ReferenceOTU97733
New.CleanUp.ReferenceOTU55820
New.CleanUp.ReferenceOTU37525
New.CleanUp.ReferenceOTU56388
New.CleanUp.ReferenceOTU117866
New.CleanUp.ReferenceOTU58407
New.CleanUp.ReferenceOTU65143
New.CleanUp.ReferenceOTU69188
New.CleanUp.ReferenceOTU57167
New.CleanUp.ReferenceOTU232278
New.CleanUp.ReferenceOTU247625
New.CleanUp.ReferenceOTU64807
New.CleanUp.ReferenceOTU262013
New.CleanUp.ReferenceOTU116697
New.CleanUp.ReferenceOTU48545
New.CleanUp.ReferenceOTU214875
New.CleanUp.ReferenceOTU241727
New.CleanUp.ReferenceOTU304512
New.CleanUp.ReferenceOTU65051
New.CleanUp.ReferenceOTU52006
New.CleanUp.ReferenceOTU60597
New.CleanUp.ReferenceOTU242040
New.CleanUp.ReferenceOTU112523
New.CleanUp.ReferenceOTU153942
New.CleanUp.ReferenceOTU38003

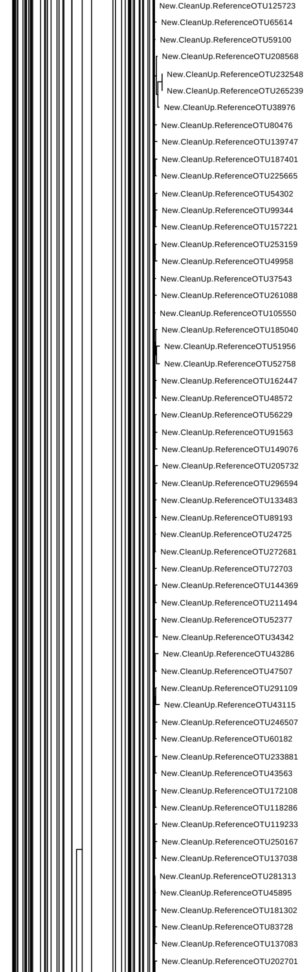
New.CleanUp.ReferenceOTU125723
New.CleanUp.ReferenceOTU65614
New.CleanUp.ReferenceOTU59100
New.CleanUp.ReferenceOTU208568
New.CleanUp.ReferenceOTU232548
New.CleanUp.ReferenceOTU265239
New.CleanUp.ReferenceOTU38976
New.CleanUp.ReferenceOTU80476
New.CleanUp.ReferenceOTU139747
New.CleanUp.ReferenceOTU187401
New.CleanUp.ReferenceOTU225665
New.CleanUp.ReferenceOTU54302
New.CleanUp.ReferenceOTU99344
New.CleanUp.ReferenceOTU157221
New.CleanUp.ReferenceOTU253159
New.CleanUp.ReferenceOTU49958
New.CleanUp.ReferenceOTU37543
New.CleanUp.ReferenceOTU261088
New.CleanUp.ReferenceOTU105550
New.CleanUp.ReferenceOTU185040
New.CleanUp.ReferenceOTU51956
New.CleanUp.ReferenceOTU52758
New.CleanUp.ReferenceOTU162447
New.CleanUp.ReferenceOTU48572
New.CleanUp.ReferenceOTU56229
New.CleanUp.ReferenceOTU91563
New.CleanUp.ReferenceOTU149076
New.CleanUp.ReferenceOTU205732
New.CleanUp.ReferenceOTU296594
New.CleanUp.ReferenceOTU133483
New.CleanUp.ReferenceOTU89193
New.CleanUp.ReferenceOTU24725
New.CleanUp.ReferenceOTU272681
New.CleanUp.ReferenceOTU72703
New.CleanUp.ReferenceOTU144369
New.CleanUp.ReferenceOTU211494
New.CleanUp.ReferenceOTU52377
New.CleanUp.ReferenceOTU34342
New.CleanUp.ReferenceOTU43286
New.CleanUp.ReferenceOTU47507
New.CleanUp.ReferenceOTU291109
New.CleanUp.ReferenceOTU43115
New.CleanUp.ReferenceOTU246507
New.CleanUp.ReferenceOTU60182
New.CleanUp.ReferenceOTU233881
New.CleanUp.ReferenceOTU43563
New.CleanUp.ReferenceOTU172108
New.CleanUp.ReferenceOTU118286
New.CleanUp.ReferenceOTU119233
New.CleanUp.ReferenceOTU250167
New.CleanUp.ReferenceOTU137038
New.CleanUp.ReferenceOTU281313
New.CleanUp.ReferenceOTU45895
New.CleanUp.ReferenceOTU181302
New.CleanUp.ReferenceOTU83728
New.CleanUp.ReferenceOTU137083
New.CleanUp.ReferenceOTU202701

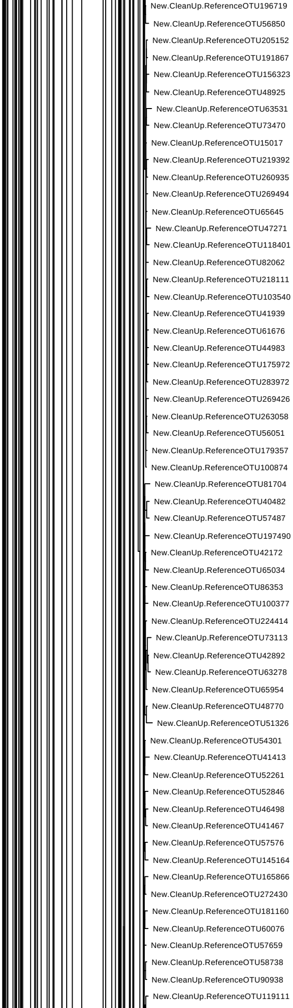
New.CleanUp.ReferenceOTU196719
New.CleanUp.ReferenceOTU56850
New.CleanUp.ReferenceOTU205152
New.CleanUp.ReferenceOTU191867
New.CleanUp.ReferenceOTU156323
New.CleanUp.ReferenceOTU48925
New.CleanUp.ReferenceOTU63531
New.CleanUp.ReferenceOTU73470
New.CleanUp.ReferenceOTU15017
New.CleanUp.ReferenceOTU219392
New.CleanUp.ReferenceOTU260935
New.CleanUp.ReferenceOTU269494
New.CleanUp.ReferenceOTU65645
New.CleanUp.ReferenceOTU47271
New.CleanUp.ReferenceOTU118401
New.CleanUp.ReferenceOTU82062
New.CleanUp.ReferenceOTU218111
New.CleanUp.ReferenceOTU103540
New.CleanUp.ReferenceOTU41939
New.CleanUp.ReferenceOTU61676
New.CleanUp.ReferenceOTU44983
New.CleanUp.ReferenceOTU175972
New.CleanUp.ReferenceOTU283972
New.CleanUp.ReferenceOTU269426
New.CleanUp.ReferenceOTU263058
New.CleanUp.ReferenceOTU56051
New.CleanUp.ReferenceOTU179357
New.CleanUp.ReferenceOTU100874
New.CleanUp.ReferenceOTU81704
New.CleanUp.ReferenceOTU40482
New.CleanUp.ReferenceOTU57487
New.CleanUp.ReferenceOTU197490
New.CleanUp.ReferenceOTU42172
New.CleanUp.ReferenceOTU65034
New.CleanUp.ReferenceOTU86353
New.CleanUp.ReferenceOTU100377
New.CleanUp.ReferenceOTU224414
New.CleanUp.ReferenceOTU73113
New.CleanUp.ReferenceOTU42892
New.CleanUp.ReferenceOTU63278
New.CleanUp.ReferenceOTU65954
New.CleanUp.ReferenceOTU48770
New.CleanUp.ReferenceOTU51326
New.CleanUp.ReferenceOTU54301
New.CleanUp.ReferenceOTU41413
New.CleanUp.ReferenceOTU52261
New.CleanUp.ReferenceOTU52846
New.CleanUp.ReferenceOTU46498
New.CleanUp.ReferenceOTU41467
New.CleanUp.ReferenceOTU57576
New.CleanUp.ReferenceOTU145164
New.CleanUp.ReferenceOTU165866
New.CleanUp.ReferenceOTU272430
New.CleanUp.ReferenceOTU181160
New.CleanUp.ReferenceOTU60076
New.CleanUp.ReferenceOTU57659
New.CleanUp.ReferenceOTU58738
New.CleanUp.ReferenceOTU90938
New.CleanUp.ReferenceOTU119111

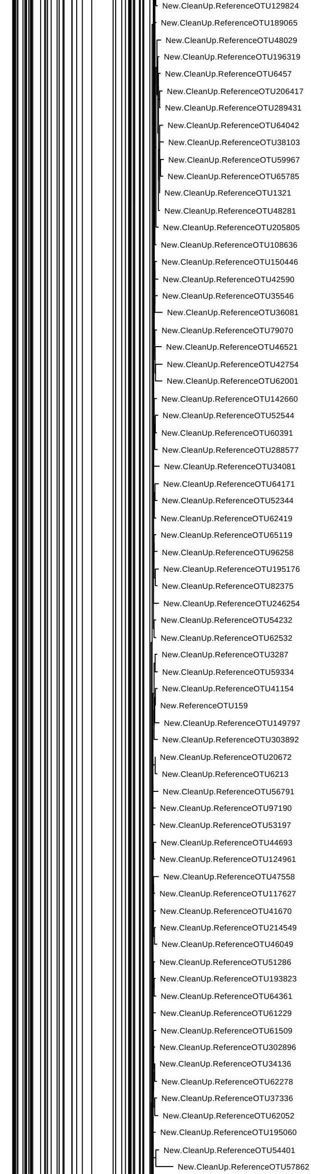
New.CleanUp.ReferenceOTU129824
New.CleanUp.ReferenceOTU189065
New.CleanUp.ReferenceOTU48029
New.CleanUp.ReferenceOTU196319
New.CleanUp.ReferenceOTU6457
New.CleanUp.ReferenceOTU206417
New.CleanUp.ReferenceOTU289431
New.CleanUp.ReferenceOTU64042
New.CleanUp.ReferenceOTU38103
New.CleanUp.ReferenceOTU59967
New.CleanUp.ReferenceOTU65785
New.CleanUp.ReferenceOTU1321
New.CleanUp.ReferenceOTU48281
New.CleanUp.ReferenceOTU205805
New.CleanUp.ReferenceOTU108636
New.CleanUp.ReferenceOTU150446
New.CleanUp.ReferenceOTU42590
New.CleanUp.ReferenceOTU35546
New.CleanUp.ReferenceOTU36081
New.CleanUp.ReferenceOTU79070
New.CleanUp.ReferenceOTU46521
New.CleanUp.ReferenceOTU42754
New.CleanUp.ReferenceOTU62001
New.CleanUp.ReferenceOTU142660
New.CleanUp.ReferenceOTU52544
New.CleanUp.ReferenceOTU60391
New.CleanUp.ReferenceOTU288577
New.CleanUp.ReferenceOTU34081
New.CleanUp.ReferenceOTU64171
New.CleanUp.ReferenceOTU52344
New.CleanUp.ReferenceOTU62419
New.CleanUp.ReferenceOTU65119
New.CleanUp.ReferenceOTU96258
New.CleanUp.ReferenceOTU195176
New.CleanUp.ReferenceOTU82375
New.CleanUp.ReferenceOTU246254
New.CleanUp.ReferenceOTU54232
New.CleanUp.ReferenceOTU62532
New.CleanUp.ReferenceOTU3287
New.CleanUp.ReferenceOTU59334
New.CleanUp.ReferenceOTU41154
New.ReferenceOTU159
New.CleanUp.ReferenceOTU149797
New.CleanUp.ReferenceOTU303892
New.CleanUp.ReferenceOTU20672
New.CleanUp.ReferenceOTU6213
New.CleanUp.ReferenceOTU56791
New.CleanUp.ReferenceOTU97190
New.CleanUp.ReferenceOTU53197
New.CleanUp.ReferenceOTU44693
New.CleanUp.ReferenceOTU124961
New.CleanUp.ReferenceOTU47558
New.CleanUp.ReferenceOTU117627
New.CleanUp.ReferenceOTU41670
New.CleanUp.ReferenceOTU214549
New.CleanUp.ReferenceOTU46049
New.CleanUp.ReferenceOTU51286
New.CleanUp.ReferenceOTU193823
New.CleanUp.ReferenceOTU64361
New.CleanUp.ReferenceOTU61229
New.CleanUp.ReferenceOTU61509
New.CleanUp.ReferenceOTU302896
New.CleanUp.ReferenceOTU34136
New.CleanUp.ReferenceOTU62278
New.CleanUp.ReferenceOTU37336
New.CleanUp.ReferenceOTU62052
New.CleanUp.ReferenceOTU195060
New.CleanUp.ReferenceOTU54401
New.CleanUp.ReferenceOTU57862

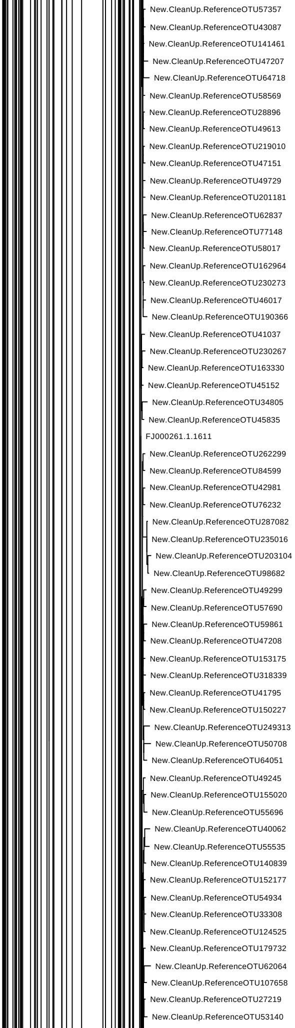
New.CleanUp.ReferenceOTU57357
New.CleanUp.ReferenceOTU43087
New.CleanUp.ReferenceOTU141461
New.CleanUp.ReferenceOTU47207
New.CleanUp.ReferenceOTU64718
New.CleanUp.ReferenceOTU58569
New.CleanUp.ReferenceOTU28896
New.CleanUp.ReferenceOTU49613
New.CleanUp.ReferenceOTU219010
New.CleanUp.ReferenceOTU47151
New.CleanUp.ReferenceOTU49729
New.CleanUp.ReferenceOTU201181
New.CleanUp.ReferenceOTU62837
New.CleanUp.ReferenceOTU77148
New.CleanUp.ReferenceOTU58017
New.CleanUp.ReferenceOTU162964
New.CleanUp.ReferenceOTU230273
New.CleanUp.ReferenceOTU46017
New.CleanUp.ReferenceOTU190366
New.CleanUp.ReferenceOTU41037
New.CleanUp.ReferenceOTU230267
New.CleanUp.ReferenceOTU163330
New.CleanUp.ReferenceOTU45152
New.CleanUp.ReferenceOTU34805
New.CleanUp.ReferenceOTU45835
FJ000261.1.1611
New.CleanUp.ReferenceOTU262299
New.CleanUp.ReferenceOTU84599
New.CleanUp.ReferenceOTU42981
New.CleanUp.ReferenceOTU76232
New.CleanUp.ReferenceOTU287082
New.CleanUp.ReferenceOTU235016
New.CleanUp.ReferenceOTU203104
New.CleanUp.ReferenceOTU98682
New.CleanUp.ReferenceOTU49299
New.CleanUp.ReferenceOTU57690
New.CleanUp.ReferenceOTU59861
New.CleanUp.ReferenceOTU47208
New.CleanUp.ReferenceOTU153175
New.CleanUp.ReferenceOTU318339
New.CleanUp.ReferenceOTU41795
New.CleanUp.ReferenceOTU150227
New.CleanUp.ReferenceOTU249313
New.CleanUp.ReferenceOTU50708
New.CleanUp.ReferenceOTU64051
New.CleanUp.ReferenceOTU49245
New.CleanUp.ReferenceOTU155020
New.CleanUp.ReferenceOTU55696
New.CleanUp.ReferenceOTU40062
New.CleanUp.ReferenceOTU55535
New.CleanUp.ReferenceOTU140839
New.CleanUp.ReferenceOTU152177
New.CleanUp.ReferenceOTU54934
New.CleanUp.ReferenceOTU33308
New.CleanUp.ReferenceOTU124525
New.CleanUp.ReferenceOTU179732
New.CleanUp.ReferenceOTU62064
New.CleanUp.ReferenceOTU107658
New.CleanUp.ReferenceOTU27219
New.CleanUp.ReferenceOTU53140

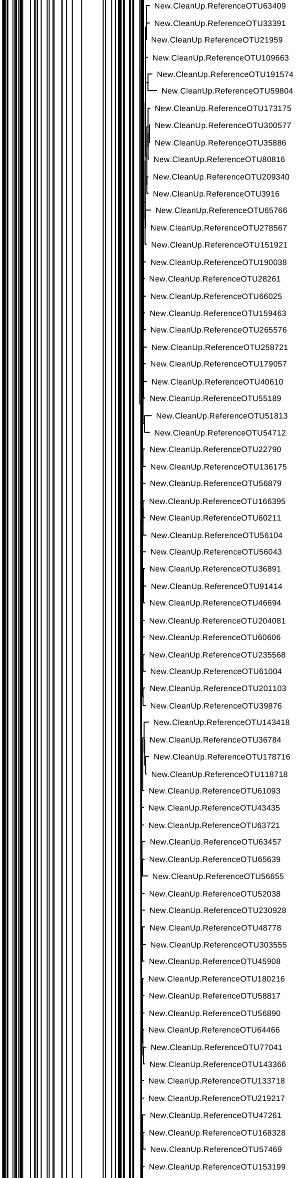
New.CleanUp.ReferenceOTU63409
New.CleanUp.ReferenceOTU33391
New.CleanUp.ReferenceOTU21959
New.CleanUp.ReferenceOTU109663
New.CleanUp.ReferenceOTU191574
New.CleanUp.ReferenceOTU59804
New.CleanUp.ReferenceOTU173175
New.CleanUp.ReferenceOTU300577
New.CleanUp.ReferenceOTU35886
New.CleanUp.ReferenceOTU80816
New.CleanUp.ReferenceOTU209340
New.CleanUp.ReferenceOTU3916
New.CleanUp.ReferenceOTU65766
New.CleanUp.ReferenceOTU278567
New.CleanUp.ReferenceOTU151921
New.CleanUp.ReferenceOTU190038
New.CleanUp.ReferenceOTU28261
New.CleanUp.ReferenceOTU66025
New.CleanUp.ReferenceOTU159463
New.CleanUp.ReferenceOTU265576
New.CleanUp.ReferenceOTU258721
New.CleanUp.ReferenceOTU179057
New.CleanUp.ReferenceOTU40610
New.CleanUp.ReferenceOTU55189
New.CleanUp.ReferenceOTU51813
New.CleanUp.ReferenceOTU54712
New.CleanUp.ReferenceOTU22790
New.CleanUp.ReferenceOTU136175
New.CleanUp.ReferenceOTU56879
New.CleanUp.ReferenceOTU166395
New.CleanUp.ReferenceOTU60211
New.CleanUp.ReferenceOTU56104
New.CleanUp.ReferenceOTU56043
New.CleanUp.ReferenceOTU36891
New.CleanUp.ReferenceOTU91414
New.CleanUp.ReferenceOTU46694
New.CleanUp.ReferenceOTU204081
New.CleanUp.ReferenceOTU60606
New.CleanUp.ReferenceOTU235568
New.CleanUp.ReferenceOTU61004
New.CleanUp.ReferenceOTU201103
New.CleanUp.ReferenceOTU39876
New.CleanUp.ReferenceOTU143418
New.CleanUp.ReferenceOTU36784
New.CleanUp.ReferenceOTU178716
New.CleanUp.ReferenceOTU118718
New.CleanUp.ReferenceOTU61093
New.CleanUp.ReferenceOTU43435
New.CleanUp.ReferenceOTU63721
New.CleanUp.ReferenceOTU63457
New.CleanUp.ReferenceOTU65639
New.CleanUp.ReferenceOTU56655
New.CleanUp.ReferenceOTU52038
New.CleanUp.ReferenceOTU230928
New.CleanUp.ReferenceOTU48778
New.CleanUp.ReferenceOTU303555
New.CleanUp.ReferenceOTU45908
New.CleanUp.ReferenceOTU180216
New.CleanUp.ReferenceOTU58817
New.CleanUp.ReferenceOTU56890
New.CleanUp.ReferenceOTU64466
New.CleanUp.ReferenceOTU77041
New.CleanUp.ReferenceOTU143366
New.CleanUp.ReferenceOTU133718
New.CleanUp.ReferenceOTU219217
New.CleanUp.ReferenceOTU47261
New.CleanUp.ReferenceOTU168328
New.CleanUp.ReferenceOTU57469
New.CleanUp.ReferenceOTU153199

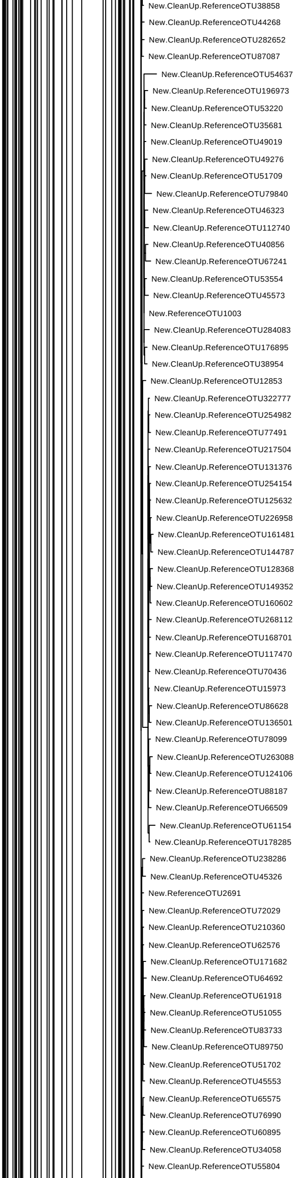
New.CleanUp.ReferenceOTU38858
New.CleanUp.ReferenceOTU44268
New.CleanUp.ReferenceOTU282652
New.CleanUp.ReferenceOTU87087
New.CleanUp.ReferenceOTU54637
New.CleanUp.ReferenceOTU196973
New.CleanUp.ReferenceOTU53220
New.CleanUp.ReferenceOTU35681
New.CleanUp.ReferenceOTU49019
New.CleanUp.ReferenceOTU49276
New.CleanUp.ReferenceOTU51709
New.CleanUp.ReferenceOTU79840
New.CleanUp.ReferenceOTU46323
New.CleanUp.ReferenceOTU112740
New.CleanUp.ReferenceOTU40856
New.CleanUp.ReferenceOTU67241
New.CleanUp.ReferenceOTU53554
New.CleanUp.ReferenceOTU45573
New.ReferenceOTU1003
New.CleanUp.ReferenceOTU284083
New.CleanUp.ReferenceOTU176895
New.CleanUp.ReferenceOTU38954
New.CleanUp.ReferenceOTU12853
New.CleanUp.ReferenceOTU322777
New.CleanUp.ReferenceOTU254982
New.CleanUp.ReferenceOTU77491
New.CleanUp.ReferenceOTU217504
New.CleanUp.ReferenceOTU131376
New.CleanUp.ReferenceOTU254154
New.CleanUp.ReferenceOTU125632
New.CleanUp.ReferenceOTU226958
New.CleanUp.ReferenceOTU161481
New.CleanUp.ReferenceOTU144787
New.CleanUp.ReferenceOTU128368
New.CleanUp.ReferenceOTU149352
New.CleanUp.ReferenceOTU160602
New.CleanUp.ReferenceOTU268112
New.CleanUp.ReferenceOTU168701
New.CleanUp.ReferenceOTU117470
New.CleanUp.ReferenceOTU70436
New.CleanUp.ReferenceOTU15973
New.CleanUp.ReferenceOTU86628
New.CleanUp.ReferenceOTU136501
New.CleanUp.ReferenceOTU78099
New.CleanUp.ReferenceOTU263088
New.CleanUp.ReferenceOTU124106
New.CleanUp.ReferenceOTU88187
New.CleanUp.ReferenceOTU66509
New.CleanUp.ReferenceOTU61154
New.CleanUp.ReferenceOTU178285
New.CleanUp.ReferenceOTU238286
New.CleanUp.ReferenceOTU45326
New.ReferenceOTU2691
New.CleanUp.ReferenceOTU72029
New.CleanUp.ReferenceOTU210360
New.CleanUp.ReferenceOTU62576
New.CleanUp.ReferenceOTU171682
New.CleanUp.ReferenceOTU64692
New.CleanUp.ReferenceOTU61918
New.CleanUp.ReferenceOTU51055
New.CleanUp.ReferenceOTU83733
New.CleanUp.ReferenceOTU89750
New.CleanUp.ReferenceOTU51702
New.CleanUp.ReferenceOTU45553
New.CleanUp.ReferenceOTU65575
New.CleanUp.ReferenceOTU76990
New.CleanUp.ReferenceOTU60895
New.CleanUp.ReferenceOTU34058
New.CleanUp.ReferenceOTU55804

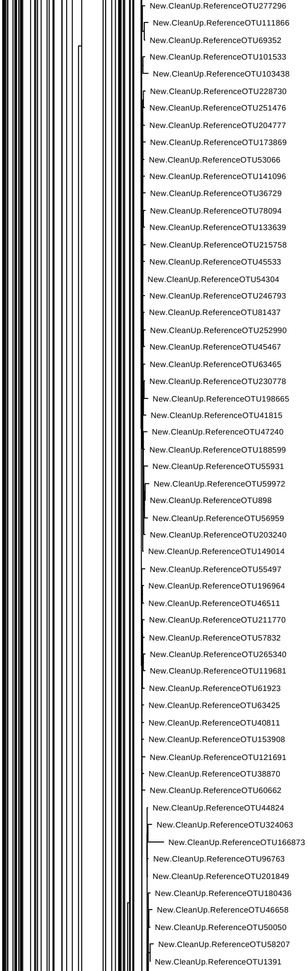
New.CleanUp.ReferenceOTU277296
New.CleanUp.ReferenceOTU111866
New.CleanUp.ReferenceOTU69352
New.CleanUp.ReferenceOTU101533
New.CleanUp.ReferenceOTU103438
New.CleanUp.ReferenceOTU228730
New.CleanUp.ReferenceOTU251476
New.CleanUp.ReferenceOTU204777
New.CleanUp.ReferenceOTU173869
New.CleanUp.ReferenceOTU53066
New.CleanUp.ReferenceOTU141096
New.CleanUp.ReferenceOTU36729
New.CleanUp.ReferenceOTU78094
New.CleanUp.ReferenceOTU133639
New.CleanUp.ReferenceOTU215758
New.CleanUp.ReferenceOTU45533
New.CleanUp.ReferenceOTU54304
New.CleanUp.ReferenceOTU246793
New.CleanUp.ReferenceOTU81437
New.CleanUp.ReferenceOTU252990
New.CleanUp.ReferenceOTU45467
New.CleanUp.ReferenceOTU63465
New.CleanUp.ReferenceOTU230778
New.CleanUp.ReferenceOTU198665
New.CleanUp.ReferenceOTU41815
New.CleanUp.ReferenceOTU47240
New.CleanUp.ReferenceOTU188599
New.CleanUp.ReferenceOTU55931
New.CleanUp.ReferenceOTU59972
New.CleanUp.ReferenceOTU898
New.CleanUp.ReferenceOTU56959
New.CleanUp.ReferenceOTU203240
New.CleanUp.ReferenceOTU149014
New.CleanUp.ReferenceOTU55497
New.CleanUp.ReferenceOTU196964
New.CleanUp.ReferenceOTU46511
New.CleanUp.ReferenceOTU211770
New.CleanUp.ReferenceOTU57832
New.CleanUp.ReferenceOTU265340
New.CleanUp.ReferenceOTU119681
New.CleanUp.ReferenceOTU61923
New.CleanUp.ReferenceOTU63425
New.CleanUp.ReferenceOTU40811
New.CleanUp.ReferenceOTU153908
New.CleanUp.ReferenceOTU121691
New.CleanUp.ReferenceOTU38870
New.CleanUp.ReferenceOTU60662
New.CleanUp.ReferenceOTU44824
New.CleanUp.ReferenceOTU324063
New.CleanUp.ReferenceOTU166873
New.CleanUp.ReferenceOTU96763
New.CleanUp.ReferenceOTU201849
New.CleanUp.ReferenceOTU180436
New.CleanUp.ReferenceOTU46658
New.CleanUp.ReferenceOTU50050
New.CleanUp.ReferenceOTU58207
New.CleanUp.ReferenceOTU1391

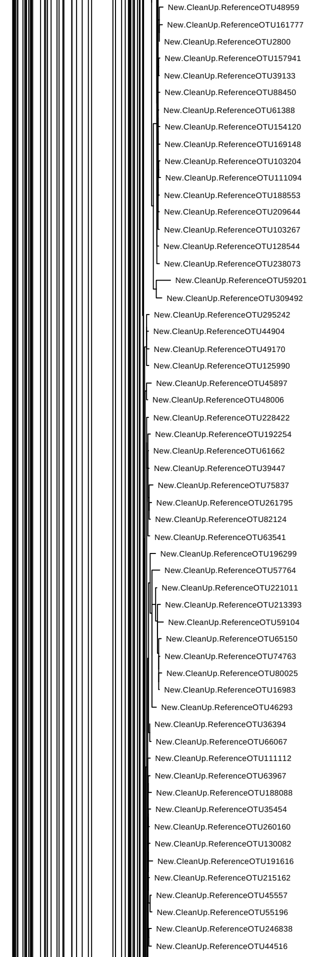
New.CleanUp.ReferenceOTU48959
New.CleanUp.ReferenceOTU161777
New.CleanUp.ReferenceOTU2800
New.CleanUp.ReferenceOTU157941
New.CleanUp.ReferenceOTU39133
New.CleanUp.ReferenceOTU88450
New.CleanUp.ReferenceOTU61388
New.CleanUp.ReferenceOTU154120
New.CleanUp.ReferenceOTU169148
New.CleanUp.ReferenceOTU103204
New.CleanUp.ReferenceOTU111094
New.CleanUp.ReferenceOTU188553
New.CleanUp.ReferenceOTU209644
New.CleanUp.ReferenceOTU103267
New.CleanUp.ReferenceOTU128544
New.CleanUp.ReferenceOTU238073
New.CleanUp.ReferenceOTU59201
New.CleanUp.ReferenceOTU309492
New.CleanUp.ReferenceOTU295242
New.CleanUp.ReferenceOTU44904
New.CleanUp.ReferenceOTU49170
New.CleanUp.ReferenceOTU125990
New.CleanUp.ReferenceOTU45897
New.CleanUp.ReferenceOTU48006
New.CleanUp.ReferenceOTU228422
New.CleanUp.ReferenceOTU192254
New.CleanUp.ReferenceOTU61662
New.CleanUp.ReferenceOTU39447
New.CleanUp.ReferenceOTU75837
New.CleanUp.ReferenceOTU261795
New.CleanUp.ReferenceOTU82124
New.CleanUp.ReferenceOTU63541
New.CleanUp.ReferenceOTU196299
New.CleanUp.ReferenceOTU57764
New.CleanUp.ReferenceOTU221011
New.CleanUp.ReferenceOTU213393
New.CleanUp.ReferenceOTU59104
New.CleanUp.ReferenceOTU65150
New.CleanUp.ReferenceOTU74763
New.CleanUp.ReferenceOTU80025
New.CleanUp.ReferenceOTU16983
New.CleanUp.ReferenceOTU46293
New.CleanUp.ReferenceOTU36394
New.CleanUp.ReferenceOTU66067
New.CleanUp.ReferenceOTU111112
New.CleanUp.ReferenceOTU63967
New.CleanUp.ReferenceOTU188088
New.CleanUp.ReferenceOTU35454
New.CleanUp.ReferenceOTU260160
New.CleanUp.ReferenceOTU130082
New.CleanUp.ReferenceOTU191616
New.CleanUp.ReferenceOTU215162
New.CleanUp.ReferenceOTU45557
New.CleanUp.ReferenceOTU55196
New.CleanUp.ReferenceOTU246838
New.CleanUp.ReferenceOTU44516

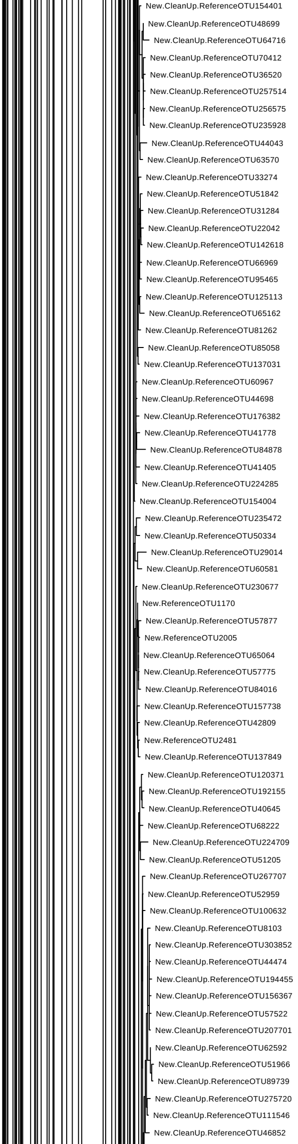

New.CleanUp.ReferenceOTU154401
New.CleanUp.ReferenceOTU48699
New.CleanUp.ReferenceOTU64716
New.CleanUp.ReferenceOTU70412
New.CleanUp.ReferenceOTU36520
New.CleanUp.ReferenceOTU257514
New.CleanUp.ReferenceOTU256575
New.CleanUp.ReferenceOTU235928
New.CleanUp.ReferenceOTU44043
New.CleanUp.ReferenceOTU63570
New.CleanUp.ReferenceOTU33274
New.CleanUp.ReferenceOTU51842
New.CleanUp.ReferenceOTU31284
New.CleanUp.ReferenceOTU22042
New.CleanUp.ReferenceOTU142618
New.CleanUp.ReferenceOTU66969
New.CleanUp.ReferenceOTU95465
New.CleanUp.ReferenceOTU125113
New.CleanUp.ReferenceOTU65162
New.CleanUp.ReferenceOTU81262
New.CleanUp.ReferenceOTU85058
New.CleanUp.ReferenceOTU137031
New.CleanUp.ReferenceOTU60967
New.CleanUp.ReferenceOTU44698
New.CleanUp.ReferenceOTU176382
New.CleanUp.ReferenceOTU41778
New.CleanUp.ReferenceOTU84878
New.CleanUp.ReferenceOTU41405
New.CleanUp.ReferenceOTU224285
New.CleanUp.ReferenceOTU154004
New.CleanUp.ReferenceOTU235472
New.CleanUp.ReferenceOTU50334
New.CleanUp.ReferenceOTU29014
New.CleanUp.ReferenceOTU60581
New.CleanUp.ReferenceOTU230677
New.ReferenceOTU1170
New.CleanUp.ReferenceOTU57877
New.ReferenceOTU2005
New.CleanUp.ReferenceOTU65064
New.CleanUp.ReferenceOTU57775
New.CleanUp.ReferenceOTU84016
New.CleanUp.ReferenceOTU157738
New.CleanUp.ReferenceOTU42809
New.ReferenceOTU2481
New.CleanUp.ReferenceOTU137849
New.CleanUp.ReferenceOTU120371
New.CleanUp.ReferenceOTU192155
New.CleanUp.ReferenceOTU40645
New.CleanUp.ReferenceOTU68222
New.CleanUp.ReferenceOTU224709
New.CleanUp.ReferenceOTU51205
New.CleanUp.ReferenceOTU267707
New.CleanUp.ReferenceOTU52959
New.CleanUp.ReferenceOTU100632
New.CleanUp.ReferenceOTU8103
New.CleanUp.ReferenceOTU303852
New.CleanUp.ReferenceOTU44474
New.CleanUp.ReferenceOTU194455
New.CleanUp.ReferenceOTU156367
New.CleanUp.ReferenceOTU57522
New.CleanUp.ReferenceOTU207701
New.CleanUp.ReferenceOTU62592
New.CleanUp.ReferenceOTU51966
New.CleanUp.ReferenceOTU89739
New.CleanUp.ReferenceOTU275720
New.CleanUp.ReferenceOTU111546
New.CleanUp.ReferenceOTU46852

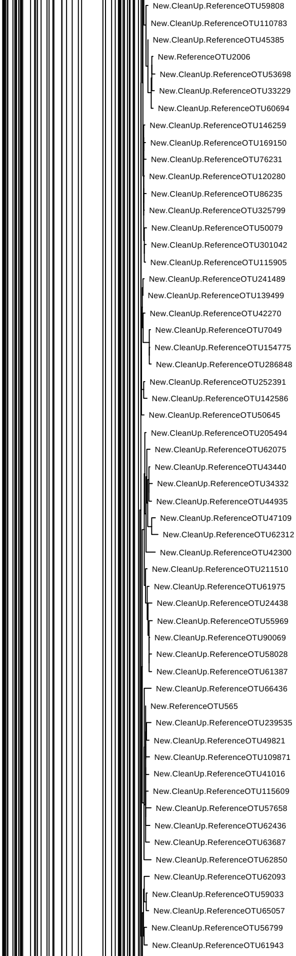
New.CleanUp.ReferenceOTU59808
New.CleanUp.ReferenceOTU110783
New.CleanUp.ReferenceOTU45385
New.ReferenceOTU2006
New.CleanUp.ReferenceOTU53698
New.CleanUp.ReferenceOTU33229
New.CleanUp.ReferenceOTU60694
New.CleanUp.ReferenceOTU146259
New.CleanUp.ReferenceOTU169150
New.CleanUp.ReferenceOTU76231
New.CleanUp.ReferenceOTU120280
New.CleanUp.ReferenceOTU86235
New.CleanUp.ReferenceOTU325799
New.CleanUp.ReferenceOTU50079
New.CleanUp.ReferenceOTU301042
New.CleanUp.ReferenceOTU115905
New.CleanUp.ReferenceOTU241489
New.CleanUp.ReferenceOTU139499
New.CleanUp.ReferenceOTU42270
New.CleanUp.ReferenceOTU7049
New.CleanUp.ReferenceOTU154775
New.CleanUp.ReferenceOTU286848
New.CleanUp.ReferenceOTU252391
New.CleanUp.ReferenceOTU142586
New.CleanUp.ReferenceOTU50645
New.CleanUp.ReferenceOTU205494
New.CleanUp.ReferenceOTU62075
New.CleanUp.ReferenceOTU43440
New.CleanUp.ReferenceOTU34332
New.CleanUp.ReferenceOTU44935
New.CleanUp.ReferenceOTU47109
New.CleanUp.ReferenceOTU62312
New.CleanUp.ReferenceOTU42300
New.CleanUp.ReferenceOTU211510
New.CleanUp.ReferenceOTU61975
New.CleanUp.ReferenceOTU24438
New.CleanUp.ReferenceOTU55969
New.CleanUp.ReferenceOTU90069
New.CleanUp.ReferenceOTU58028
New.CleanUp.ReferenceOTU61387
New.CleanUp.ReferenceOTU66436
New.ReferenceOTU565
New.CleanUp.ReferenceOTU239535
New.CleanUp.ReferenceOTU49821
New.CleanUp.ReferenceOTU109871
New.CleanUp.ReferenceOTU41016
New.CleanUp.ReferenceOTU115609
New.CleanUp.ReferenceOTU57658
New.CleanUp.ReferenceOTU62436
New.CleanUp.ReferenceOTU63687
New.CleanUp.ReferenceOTU62850
New.CleanUp.ReferenceOTU62093
New.CleanUp.ReferenceOTU59033
New.CleanUp.ReferenceOTU65057
New.CleanUp.ReferenceOTU56799
New.CleanUp.ReferenceOTU61943

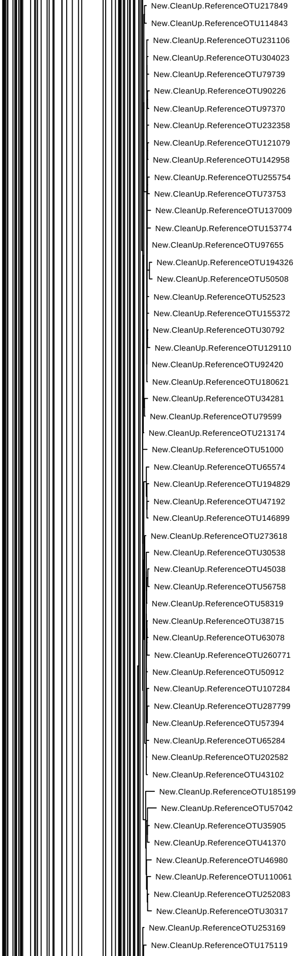
New.CleanUp.ReferenceOTU217849
New.CleanUp.ReferenceOTU114843
New.CleanUp.ReferenceOTU231106
New.CleanUp.ReferenceOTU304023
New.CleanUp.ReferenceOTU79739
New.CleanUp.ReferenceOTU90226
New.CleanUp.ReferenceOTU97370
New.CleanUp.ReferenceOTU232358
New.CleanUp.ReferenceOTU121079
New.CleanUp.ReferenceOTU142958
New.CleanUp.ReferenceOTU255754
New.CleanUp.ReferenceOTU73753
New.CleanUp.ReferenceOTU137009
New.CleanUp.ReferenceOTU153774
New.CleanUp.ReferenceOTU97655
New.CleanUp.ReferenceOTU194326
New.CleanUp.ReferenceOTU50508
New.CleanUp.ReferenceOTU52523
New.CleanUp.ReferenceOTU155372
New.CleanUp.ReferenceOTU30792
New.CleanUp.ReferenceOTU129110
New.CleanUp.ReferenceOTU92420
New.CleanUp.ReferenceOTU180621
New.CleanUp.ReferenceOTU34281
New.CleanUp.ReferenceOTU79599
New.CleanUp.ReferenceOTU213174
New.CleanUp.ReferenceOTU51000
New.CleanUp.ReferenceOTU65574
New.CleanUp.ReferenceOTU194829
New.CleanUp.ReferenceOTU47192
New.CleanUp.ReferenceOTU146899
New.CleanUp.ReferenceOTU273618
New.CleanUp.ReferenceOTU30538
New.CleanUp.ReferenceOTU45038
New.CleanUp.ReferenceOTU56758
New.CleanUp.ReferenceOTU58319
New.CleanUp.ReferenceOTU38715
New.CleanUp.ReferenceOTU63078
New.CleanUp.ReferenceOTU260771
New.CleanUp.ReferenceOTU50912
New.CleanUp.ReferenceOTU107284
New.CleanUp.ReferenceOTU287799
New.CleanUp.ReferenceOTU57394
New.CleanUp.ReferenceOTU65284
New.CleanUp.ReferenceOTU202582
New.CleanUp.ReferenceOTU43102
New.CleanUp.ReferenceOTU185199
New.CleanUp.ReferenceOTU57042
New.CleanUp.ReferenceOTU35905
New.CleanUp.ReferenceOTU41370
New.CleanUp.ReferenceOTU46980
New.CleanUp.ReferenceOTU110061
New.CleanUp.ReferenceOTU252083
New.CleanUp.ReferenceOTU30317
New.CleanUp.ReferenceOTU253169
New.CleanUp.ReferenceOTU175119

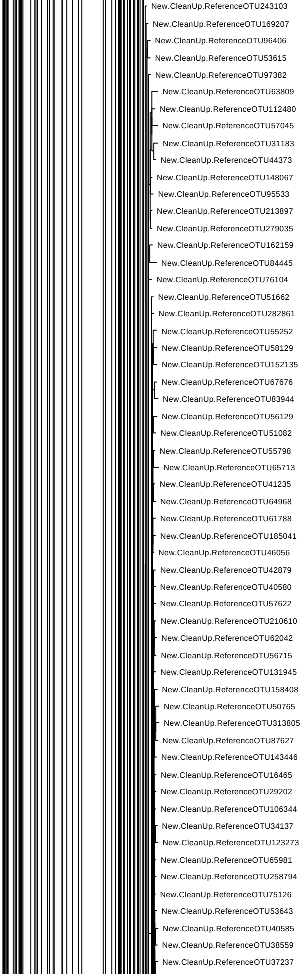
New.CleanUp.ReferenceOTU243103
New.CleanUp.ReferenceOTU169207
New.CleanUp.ReferenceOTU96406
New.CleanUp.ReferenceOTU53615
New.CleanUp.ReferenceOTU97382
New.CleanUp.ReferenceOTU63809
New.CleanUp.ReferenceOTU112480
New.CleanUp.ReferenceOTU57045
New.CleanUp.ReferenceOTU31183
New.CleanUp.ReferenceOTU44373
New.CleanUp.ReferenceOTU148067
New.CleanUp.ReferenceOTU95533
New.CleanUp.ReferenceOTU213897
New.CleanUp.ReferenceOTU279035
New.CleanUp.ReferenceOTU162159
New.CleanUp.ReferenceOTU84445
New.CleanUp.ReferenceOTU76104
New.CleanUp.ReferenceOTU51662
New.CleanUp.ReferenceOTU282861
New.CleanUp.ReferenceOTU55252
New.CleanUp.ReferenceOTU58129
New.CleanUp.ReferenceOTU152135
New.CleanUp.ReferenceOTU67676
New.CleanUp.ReferenceOTU83944
New.CleanUp.ReferenceOTU56129
New.CleanUp.ReferenceOTU51082
New.CleanUp.ReferenceOTU55798
New.CleanUp.ReferenceOTU65713
New.CleanUp.ReferenceOTU41235
New.CleanUp.ReferenceOTU64968
New.CleanUp.ReferenceOTU61788
New.CleanUp.ReferenceOTU185041
New.CleanUp.ReferenceOTU46056
New.CleanUp.ReferenceOTU42879
New.CleanUp.ReferenceOTU40580
New.CleanUp.ReferenceOTU57622
New.CleanUp.ReferenceOTU210610
New.CleanUp.ReferenceOTU62042
New.CleanUp.ReferenceOTU56715
New.CleanUp.ReferenceOTU131945
New.CleanUp.ReferenceOTU158408
New.CleanUp.ReferenceOTU50765
New.CleanUp.ReferenceOTU313805
New.CleanUp.ReferenceOTU87627
New.CleanUp.ReferenceOTU143446
New.CleanUp.ReferenceOTU16465
New.CleanUp.ReferenceOTU29202
New.CleanUp.ReferenceOTU106344
New.CleanUp.ReferenceOTU34137
New.CleanUp.ReferenceOTU123273
New.CleanUp.ReferenceOTU65981
New.CleanUp.ReferenceOTU258794
New.CleanUp.ReferenceOTU75126
New.CleanUp.ReferenceOTU53643
New.CleanUp.ReferenceOTU40585
New.CleanUp.ReferenceOTU38559
New.CleanUp.ReferenceOTU37237

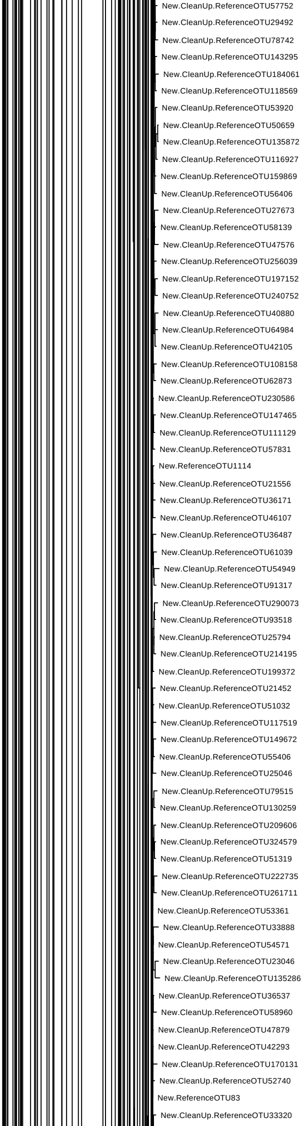
New.CleanUp.ReferenceOTU57752
New.CleanUp.ReferenceOTU29492
New.CleanUp.ReferenceOTU78742
New.CleanUp.ReferenceOTU143295
New.CleanUp.ReferenceOTU184061
New.CleanUp.ReferenceOTU118569
New.CleanUp.ReferenceOTU53920
New.CleanUp.ReferenceOTU50659
New.CleanUp.ReferenceOTU135872
New.CleanUp.ReferenceOTU116927
New.CleanUp.ReferenceOTU159869
New.CleanUp.ReferenceOTU56406
New.CleanUp.ReferenceOTU27673
New.CleanUp.ReferenceOTU58139
New.CleanUp.ReferenceOTU47576
New.CleanUp.ReferenceOTU256039
New.CleanUp.ReferenceOTU197152
New.CleanUp.ReferenceOTU240752
New.CleanUp.ReferenceOTU40880
New.CleanUp.ReferenceOTU64984
New.CleanUp.ReferenceOTU42105
New.CleanUp.ReferenceOTU108158
New.CleanUp.ReferenceOTU62873
New.CleanUp.ReferenceOTU230586
New.CleanUp.ReferenceOTU147465
New.CleanUp.ReferenceOTU111129
New.CleanUp.ReferenceOTU57831
New.ReferenceOTU1114
New.CleanUp.ReferenceOTU21556
New.CleanUp.ReferenceOTU36171
New.CleanUp.ReferenceOTU46107
New.CleanUp.ReferenceOTU36487
New.CleanUp.ReferenceOTU61039
New.CleanUp.ReferenceOTU54949
New.CleanUp.ReferenceOTU91317
New.CleanUp.ReferenceOTU290073
New.CleanUp.ReferenceOTU93518
New.CleanUp.ReferenceOTU25794
New.CleanUp.ReferenceOTU214195
New.CleanUp.ReferenceOTU199372
New.CleanUp.ReferenceOTU21452
New.CleanUp.ReferenceOTU51032
New.CleanUp.ReferenceOTU117519
New.CleanUp.ReferenceOTU149672
New.CleanUp.ReferenceOTU55406
New.CleanUp.ReferenceOTU25046
New.CleanUp.ReferenceOTU79515
New.CleanUp.ReferenceOTU130259
New.CleanUp.ReferenceOTU209606
New.CleanUp.ReferenceOTU324579
New.CleanUp.ReferenceOTU51319
New.CleanUp.ReferenceOTU222735
New.CleanUp.ReferenceOTU261711
New.CleanUp.ReferenceOTU53361
New.CleanUp.ReferenceOTU33888
New.CleanUp.ReferenceOTU54571
New.CleanUp.ReferenceOTU23046
New.CleanUp.ReferenceOTU135286
New.CleanUp.ReferenceOTU36537
New.CleanUp.ReferenceOTU58960
New.CleanUp.ReferenceOTU47879
New.CleanUp.ReferenceOTU42293
New.CleanUp.ReferenceOTU170131
New.CleanUp.ReferenceOTU52740
New.ReferenceOTU83
New.CleanUp.ReferenceOTU33320

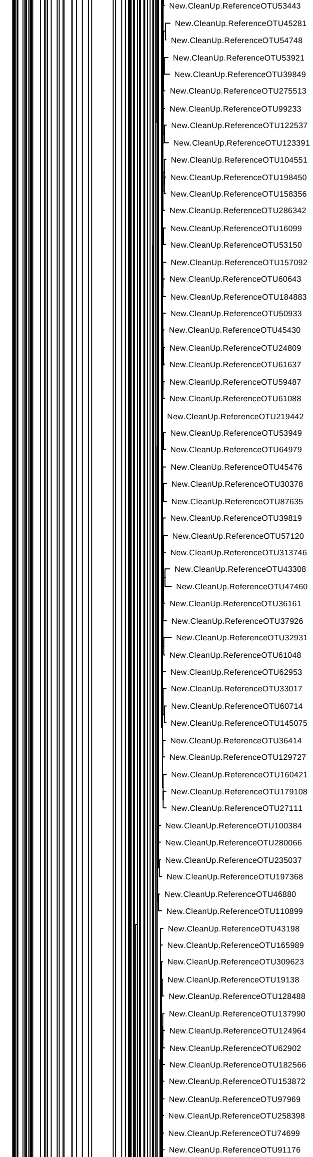
New.CleanUp.ReferenceOTU53443
New.CleanUp.ReferenceOTU45281
New.CleanUp.ReferenceOTU54748
New.CleanUp.ReferenceOTU53921
New.CleanUp.ReferenceOTU39849
New.CleanUp.ReferenceOTU275513
New.CleanUp.ReferenceOTU99233
New.CleanUp.ReferenceOTU122537
New.CleanUp.ReferenceOTU123391
New.CleanUp.ReferenceOTU104551
New.CleanUp.ReferenceOTU198450
New.CleanUp.ReferenceOTU158356
New.CleanUp.ReferenceOTU286342
New.CleanUp.ReferenceOTU16099
New.CleanUp.ReferenceOTU53150
New.CleanUp.ReferenceOTU157092
New.CleanUp.ReferenceOTU60643
New.CleanUp.ReferenceOTU184883
New.CleanUp.ReferenceOTU50933
New.CleanUp.ReferenceOTU45430
New.CleanUp.ReferenceOTU24809
New.CleanUp.ReferenceOTU61637
New.CleanUp.ReferenceOTU59487
New.CleanUp.ReferenceOTU61088
New.CleanUp.ReferenceOTU219442
New.CleanUp.ReferenceOTU53949
New.CleanUp.ReferenceOTU64979
New.CleanUp.ReferenceOTU45476
New.CleanUp.ReferenceOTU30378
New.CleanUp.ReferenceOTU87635
New.CleanUp.ReferenceOTU39819
New.CleanUp.ReferenceOTU57120
New.CleanUp.ReferenceOTU313746
New.CleanUp.ReferenceOTU43308
New.CleanUp.ReferenceOTU47460
New.CleanUp.ReferenceOTU36161
New.CleanUp.ReferenceOTU37926
New.CleanUp.ReferenceOTU32931
New.CleanUp.ReferenceOTU61048
New.CleanUp.ReferenceOTU62953
New.CleanUp.ReferenceOTU33017
New.CleanUp.ReferenceOTU60714
New.CleanUp.ReferenceOTU145075
New.CleanUp.ReferenceOTU36414
New.CleanUp.ReferenceOTU129727
New.CleanUp.ReferenceOTU160421
New.CleanUp.ReferenceOTU179108
New.CleanUp.ReferenceOTU27111
New.CleanUp.ReferenceOTU100384
New.CleanUp.ReferenceOTU280066
New.CleanUp.ReferenceOTU235037
New.CleanUp.ReferenceOTU197368
New.CleanUp.ReferenceOTU46880
New.CleanUp.ReferenceOTU110899
New.CleanUp.ReferenceOTU43198
New.CleanUp.ReferenceOTU165989
New.CleanUp.ReferenceOTU309623
New.CleanUp.ReferenceOTU19138
New.CleanUp.ReferenceOTU128488
New.CleanUp.ReferenceOTU137990
New.CleanUp.ReferenceOTU124964
New.CleanUp.ReferenceOTU62902
New.CleanUp.ReferenceOTU182566
New.CleanUp.ReferenceOTU153872
New.CleanUp.ReferenceOTU97969
New.CleanUp.ReferenceOTU258398
New.CleanUp.ReferenceOTU74699
New.CleanUp.ReferenceOTU91176

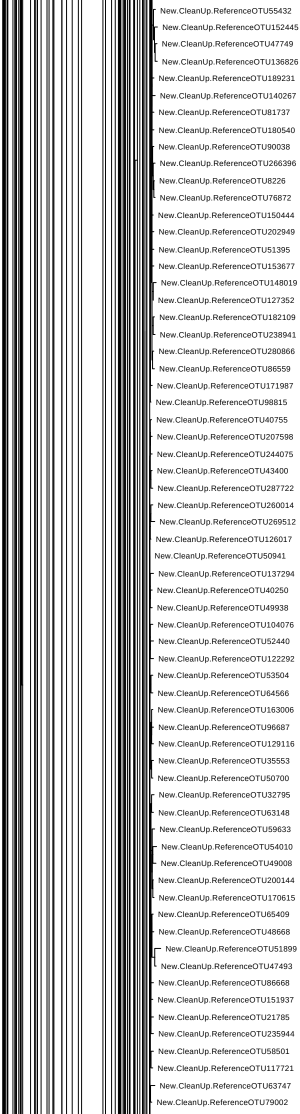

New.CleanUp.ReferenceOTU55432
New.CleanUp.ReferenceOTU152445
New.CleanUp.ReferenceOTU47749
New.CleanUp.ReferenceOTU136826
New.CleanUp.ReferenceOTU189231
New.CleanUp.ReferenceOTU140267
New.CleanUp.ReferenceOTU81737
New.CleanUp.ReferenceOTU180540
New.CleanUp.ReferenceOTU90038
New.CleanUp.ReferenceOTU266396
New.CleanUp.ReferenceOTU8226
New.CleanUp.ReferenceOTU76872
New.CleanUp.ReferenceOTU150444
New.CleanUp.ReferenceOTU202949
New.CleanUp.ReferenceOTU51395
New.CleanUp.ReferenceOTU153677
New.CleanUp.ReferenceOTU148019
New.CleanUp.ReferenceOTU127352
New.CleanUp.ReferenceOTU182109
New.CleanUp.ReferenceOTU238941
New.CleanUp.ReferenceOTU280866
New.CleanUp.ReferenceOTU86559
New.CleanUp.ReferenceOTU171987
New.CleanUp.ReferenceOTU98815
New.CleanUp.ReferenceOTU40755
New.CleanUp.ReferenceOTU207598
New.CleanUp.ReferenceOTU244075
New.CleanUp.ReferenceOTU43400
New.CleanUp.ReferenceOTU287722
New.CleanUp.ReferenceOTU260014
New.CleanUp.ReferenceOTU269512
New.CleanUp.ReferenceOTU126017
New.CleanUp.ReferenceOTU50941
New.CleanUp.ReferenceOTU137294
New.CleanUp.ReferenceOTU40250
New.CleanUp.ReferenceOTU49938
New.CleanUp.ReferenceOTU104076
New.CleanUp.ReferenceOTU52440
New.CleanUp.ReferenceOTU122292
New.CleanUp.ReferenceOTU53504
New.CleanUp.ReferenceOTU64566
New.CleanUp.ReferenceOTU163006
New.CleanUp.ReferenceOTU96687
New.CleanUp.ReferenceOTU129116
New.CleanUp.ReferenceOTU35553
New.CleanUp.ReferenceOTU50700
New.CleanUp.ReferenceOTU32795
New.CleanUp.ReferenceOTU63148
New.CleanUp.ReferenceOTU59633
New.CleanUp.ReferenceOTU54010
New.CleanUp.ReferenceOTU49008
New.CleanUp.ReferenceOTU200144
New.CleanUp.ReferenceOTU170615
New.CleanUp.ReferenceOTU65409
New.CleanUp.ReferenceOTU48668
New.CleanUp.ReferenceOTU51899
New.CleanUp.ReferenceOTU47493
New.CleanUp.ReferenceOTU86668
New.CleanUp.ReferenceOTU151937
New.CleanUp.ReferenceOTU21785
New.CleanUp.ReferenceOTU235944
New.CleanUp.ReferenceOTU58501
New.CleanUp.ReferenceOTU117721
New.CleanUp.ReferenceOTU63747
New.CleanUp.ReferenceOTU79002

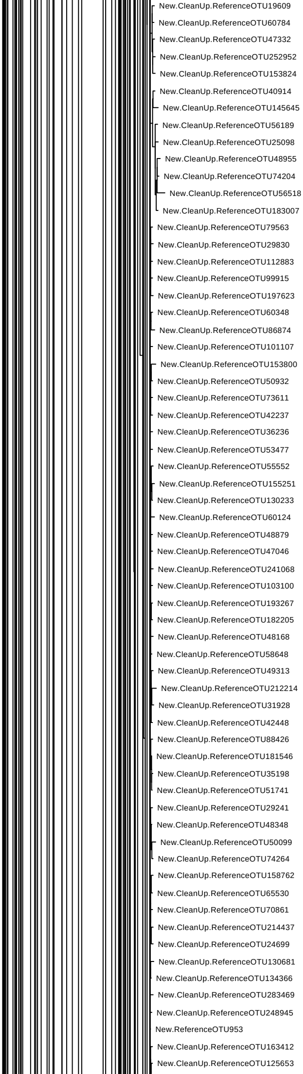
New.CleanUp.ReferenceOTU19609
New.CleanUp.ReferenceOTU60784
New.CleanUp.ReferenceOTU47332
New.CleanUp.ReferenceOTU252952
New.CleanUp.ReferenceOTU153824
New.CleanUp.ReferenceOTU40914
New.CleanUp.ReferenceOTU145645
New.CleanUp.ReferenceOTU56189
New.CleanUp.ReferenceOTU25098
New.CleanUp.ReferenceOTU48955
New.CleanUp.ReferenceOTU74204
New.CleanUp.ReferenceOTU56518
New.CleanUp.ReferenceOTU183007
New.CleanUp.ReferenceOTU79563
New.CleanUp.ReferenceOTU29830
New.CleanUp.ReferenceOTU112883
New.CleanUp.ReferenceOTU99915
New.CleanUp.ReferenceOTU197623
New.CleanUp.ReferenceOTU60348
New.CleanUp.ReferenceOTU86874
New.CleanUp.ReferenceOTU101107
New.CleanUp.ReferenceOTU153800
New.CleanUp.ReferenceOTU50932
New.CleanUp.ReferenceOTU73611
New.CleanUp.ReferenceOTU42237
New.CleanUp.ReferenceOTU36236
New.CleanUp.ReferenceOTU53477
New.CleanUp.ReferenceOTU55552
New.CleanUp.ReferenceOTU155251
New.CleanUp.ReferenceOTU130233
New.CleanUp.ReferenceOTU60124
New.CleanUp.ReferenceOTU48879
New.CleanUp.ReferenceOTU47046
New.CleanUp.ReferenceOTU241068
New.CleanUp.ReferenceOTU103100
New.CleanUp.ReferenceOTU193267
New.CleanUp.ReferenceOTU182205
New.CleanUp.ReferenceOTU48168
New.CleanUp.ReferenceOTU58648
New.CleanUp.ReferenceOTU49313
New.CleanUp.ReferenceOTU212214
New.CleanUp.ReferenceOTU31928
New.CleanUp.ReferenceOTU42448
New.CleanUp.ReferenceOTU88426
New.CleanUp.ReferenceOTU181546
New.CleanUp.ReferenceOTU35198
New.CleanUp.ReferenceOTU51741
New.CleanUp.ReferenceOTU29241
New.CleanUp.ReferenceOTU48348
New.CleanUp.ReferenceOTU50099
New.CleanUp.ReferenceOTU74264
New.CleanUp.ReferenceOTU158762
New.CleanUp.ReferenceOTU65530
New.CleanUp.ReferenceOTU70861
New.CleanUp.ReferenceOTU214437
New.CleanUp.ReferenceOTU24699
New.CleanUp.ReferenceOTU130681
New.CleanUp.ReferenceOTU134366
New.CleanUp.ReferenceOTU283469
New.CleanUp.ReferenceOTU248945
New.ReferenceOTU953
New.CleanUp.ReferenceOTU163412
New.CleanUp.ReferenceOTU125653

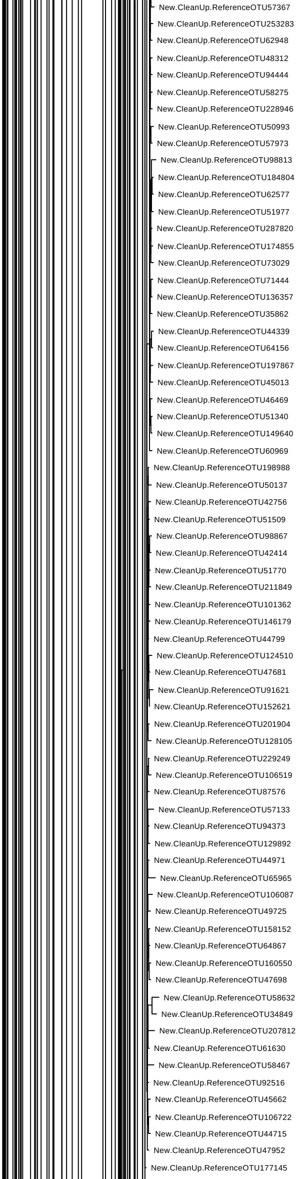
New.CleanUp.ReferenceOTU57367
New.CleanUp.ReferenceOTU253283
New.CleanUp.ReferenceOTU62948
New.CleanUp.ReferenceOTU48312
New.CleanUp.ReferenceOTU94444
New.CleanUp.ReferenceOTU58275
New.CleanUp.ReferenceOTU228946
New.CleanUp.ReferenceOTU50993
New.CleanUp.ReferenceOTU57973
New.CleanUp.ReferenceOTU98813
New.CleanUp.ReferenceOTU184804
New.CleanUp.ReferenceOTU62577
New.CleanUp.ReferenceOTU51977
New.CleanUp.ReferenceOTU287820
New.CleanUp.ReferenceOTU174855
New.CleanUp.ReferenceOTU73029
New.CleanUp.ReferenceOTU71444
New.CleanUp.ReferenceOTU136357
New.CleanUp.ReferenceOTU35862
New.CleanUp.ReferenceOTU44339
New.CleanUp.ReferenceOTU64156
New.CleanUp.ReferenceOTU197867
New.CleanUp.ReferenceOTU45013
New.CleanUp.ReferenceOTU46469
New.CleanUp.ReferenceOTU51340
New.CleanUp.ReferenceOTU149640
New.CleanUp.ReferenceOTU60969
New.CleanUp.ReferenceOTU198988
New.CleanUp.ReferenceOTU50137
New.CleanUp.ReferenceOTU42756
New.CleanUp.ReferenceOTU51509
New.CleanUp.ReferenceOTU98867
New.CleanUp.ReferenceOTU42414
New.CleanUp.ReferenceOTU51770
New.CleanUp.ReferenceOTU211849
New.CleanUp.ReferenceOTU101362
New.CleanUp.ReferenceOTU146179
New.CleanUp.ReferenceOTU44799
New.CleanUp.ReferenceOTU124510
New.CleanUp.ReferenceOTU47681
New.CleanUp.ReferenceOTU91621
New.CleanUp.ReferenceOTU152621
New.CleanUp.ReferenceOTU201904
New.CleanUp.ReferenceOTU128105
New.CleanUp.ReferenceOTU229249
New.CleanUp.ReferenceOTU106519
New.CleanUp.ReferenceOTU87576
New.CleanUp.ReferenceOTU57133
New.CleanUp.ReferenceOTU94373
New.CleanUp.ReferenceOTU129892
New.CleanUp.ReferenceOTU44971
New.CleanUp.ReferenceOTU65965
New.CleanUp.ReferenceOTU106087
New.CleanUp.ReferenceOTU49725
New.CleanUp.ReferenceOTU158152
New.CleanUp.ReferenceOTU64867
New.CleanUp.ReferenceOTU160550
New.CleanUp.ReferenceOTU47698
New.CleanUp.ReferenceOTU58632
New.CleanUp.ReferenceOTU34849
New.CleanUp.ReferenceOTU207812
New.CleanUp.ReferenceOTU61630
New.CleanUp.ReferenceOTU58467
New.CleanUp.ReferenceOTU92516
New.CleanUp.ReferenceOTU45662
New.CleanUp.ReferenceOTU106722
New.CleanUp.ReferenceOTU44715
New.CleanUp.ReferenceOTU47952
New.CleanUp.ReferenceOTU177145

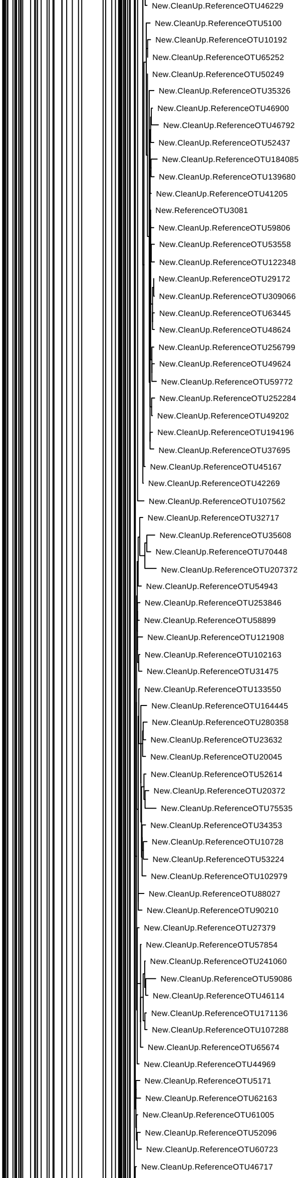
New.CleanUp.ReferenceOTU46229
New.CleanUp.ReferenceOTU5100
New.CleanUp.ReferenceOTU10192
New.CleanUp.ReferenceOTU65252
New.CleanUp.ReferenceOTU50249
New.CleanUp.ReferenceOTU35326
New.CleanUp.ReferenceOTU46900
New.CleanUp.ReferenceOTU46792
New.CleanUp.ReferenceOTU52437
New.CleanUp.ReferenceOTU184085
New.CleanUp.ReferenceOTU139680
New.CleanUp.ReferenceOTU41205
New.ReferenceOTU3081
New.CleanUp.ReferenceOTU59806
New.CleanUp.ReferenceOTU53558
New.CleanUp.ReferenceOTU122348
New.CleanUp.ReferenceOTU29172
New.CleanUp.ReferenceOTU309066
New.CleanUp.ReferenceOTU63445
New.CleanUp.ReferenceOTU48624
New.CleanUp.ReferenceOTU256799
New.CleanUp.ReferenceOTU49624
New.CleanUp.ReferenceOTU59772
New.CleanUp.ReferenceOTU252284
New.CleanUp.ReferenceOTU49202
New.CleanUp.ReferenceOTU194196
New.CleanUp.ReferenceOTU37695
New.CleanUp.ReferenceOTU45167
New.CleanUp.ReferenceOTU42269
New.CleanUp.ReferenceOTU107562
New.CleanUp.ReferenceOTU32717
New.CleanUp.ReferenceOTU35608
New.CleanUp.ReferenceOTU70448
New.CleanUp.ReferenceOTU207372
New.CleanUp.ReferenceOTU54943
New.CleanUp.ReferenceOTU253846
New.CleanUp.ReferenceOTU58899
New.CleanUp.ReferenceOTU121908
New.CleanUp.ReferenceOTU102163
New.CleanUp.ReferenceOTU31475
New.CleanUp.ReferenceOTU133550
New.CleanUp.ReferenceOTU164445
New.CleanUp.ReferenceOTU280358
New.CleanUp.ReferenceOTU23632
New.CleanUp.ReferenceOTU20045
New.CleanUp.ReferenceOTU52614
New.CleanUp.ReferenceOTU20372
New.CleanUp.ReferenceOTU75535
New.CleanUp.ReferenceOTU34353
New.CleanUp.ReferenceOTU10728
New.CleanUp.ReferenceOTU53224
New.CleanUp.ReferenceOTU102979
New.CleanUp.ReferenceOTU88027
New.CleanUp.ReferenceOTU90210
New.CleanUp.ReferenceOTU27379
New.CleanUp.ReferenceOTU57854
New.CleanUp.ReferenceOTU241060
New.CleanUp.ReferenceOTU59086
New.CleanUp.ReferenceOTU46114
New.CleanUp.ReferenceOTU171136
New.CleanUp.ReferenceOTU107288
New.CleanUp.ReferenceOTU65674
New.CleanUp.ReferenceOTU44969
New.CleanUp.ReferenceOTU5171
New.CleanUp.ReferenceOTU62163
New.CleanUp.ReferenceOTU61005
New.CleanUp.ReferenceOTU52096
New.CleanUp.ReferenceOTU60723
New.CleanUp.ReferenceOTU46717

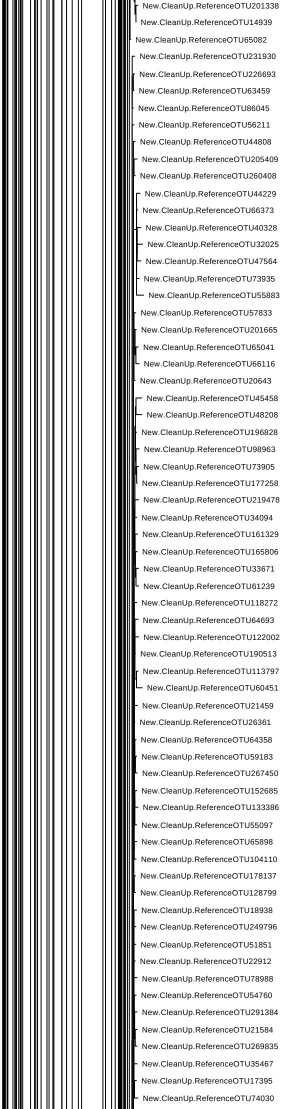
New.CleanUp.ReferenceOTU201338
New.CleanUp.ReferenceOTU14939
New.CleanUp.ReferenceOTU65082
New.CleanUp.ReferenceOTU231930
New.CleanUp.ReferenceOTU226693
New.CleanUp.ReferenceOTU63459
New.CleanUp.ReferenceOTU86045
New.CleanUp.ReferenceOTU56211
New.CleanUp.ReferenceOTU44808
New.CleanUp.ReferenceOTU205409
New.CleanUp.ReferenceOTU260408
New.CleanUp.ReferenceOTU44229
New.CleanUp.ReferenceOTU66373
New.CleanUp.ReferenceOTU40328
New.CleanUp.ReferenceOTU32025
New.CleanUp.ReferenceOTU47564
New.CleanUp.ReferenceOTU73935
New.CleanUp.ReferenceOTU55883
New.CleanUp.ReferenceOTU57833
New.CleanUp.ReferenceOTU201665
New.CleanUp.ReferenceOTU65041
New.CleanUp.ReferenceOTU66116
New.CleanUp.ReferenceOTU20643
New.CleanUp.ReferenceOTU45458
New.CleanUp.ReferenceOTU48208
New.CleanUp.ReferenceOTU196828
New.CleanUp.ReferenceOTU98963
New.CleanUp.ReferenceOTU73905
New.CleanUp.ReferenceOTU177258
New.CleanUp.ReferenceOTU219478
New.CleanUp.ReferenceOTU34094
New.CleanUp.ReferenceOTU161329
New.CleanUp.ReferenceOTU165806
New.CleanUp.ReferenceOTU33671
New.CleanUp.ReferenceOTU61239
New.CleanUp.ReferenceOTU118272
New.CleanUp.ReferenceOTU64693
New.CleanUp.ReferenceOTU122002
New.CleanUp.ReferenceOTU190513
New.CleanUp.ReferenceOTU113797
New.CleanUp.ReferenceOTU60451
New.CleanUp.ReferenceOTU21459
New.CleanUp.ReferenceOTU26361
New.CleanUp.ReferenceOTU64358
New.CleanUp.ReferenceOTU59183
New.CleanUp.ReferenceOTU267450
New.CleanUp.ReferenceOTU152685
New.CleanUp.ReferenceOTU133386
New.CleanUp.ReferenceOTU55097
New.CleanUp.ReferenceOTU65898
New.CleanUp.ReferenceOTU104110
New.CleanUp.ReferenceOTU178137
New.CleanUp.ReferenceOTU128799
New.CleanUp.ReferenceOTU18938
New.CleanUp.ReferenceOTU249796
New.CleanUp.ReferenceOTU51851
New.CleanUp.ReferenceOTU22912
New.CleanUp.ReferenceOTU78988
New.CleanUp.ReferenceOTU54760
New.CleanUp.ReferenceOTU291384
New.CleanUp.ReferenceOTU21584
New.CleanUp.ReferenceOTU269835
New.CleanUp.ReferenceOTU35467
New.CleanUp.ReferenceOTU17395
New.CleanUp.ReferenceOTU74030

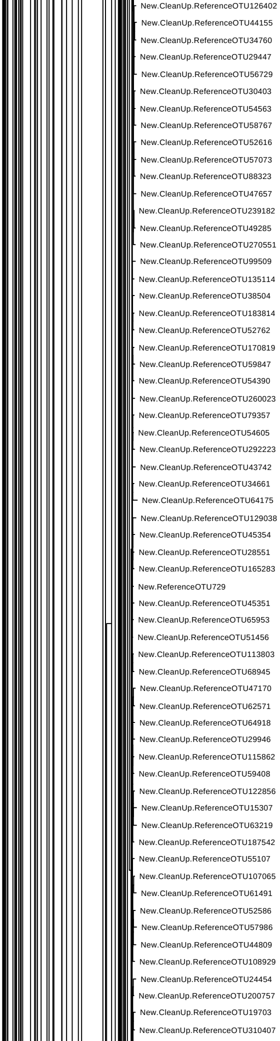
New.CleanUp.ReferenceOTU126402
New.CleanUp.ReferenceOTU44155
New.CleanUp.ReferenceOTU34760
New.CleanUp.ReferenceOTU29447
New.CleanUp.ReferenceOTU56729
New.CleanUp.ReferenceOTU30403
New.CleanUp.ReferenceOTU54563
New.CleanUp.ReferenceOTU58767
New.CleanUp.ReferenceOTU52616
New.CleanUp.ReferenceOTU57073
New.CleanUp.ReferenceOTU88323
New.CleanUp.ReferenceOTU47657
New.CleanUp.ReferenceOTU239182
New.CleanUp.ReferenceOTU49285
New.CleanUp.ReferenceOTU270551
New.CleanUp.ReferenceOTU99509
New.CleanUp.ReferenceOTU135114
New.CleanUp.ReferenceOTU38504
New.CleanUp.ReferenceOTU183814
New.CleanUp.ReferenceOTU52762
New.CleanUp.ReferenceOTU170819
New.CleanUp.ReferenceOTU59847
New.CleanUp.ReferenceOTU54390
New.CleanUp.ReferenceOTU260023
New.CleanUp.ReferenceOTU79357
New.CleanUp.ReferenceOTU54605
New.CleanUp.ReferenceOTU292223
New.CleanUp.ReferenceOTU43742
New.CleanUp.ReferenceOTU34661
New.CleanUp.ReferenceOTU64175
New.CleanUp.ReferenceOTU129038
New.CleanUp.ReferenceOTU45354
New.CleanUp.ReferenceOTU28551
New.CleanUp.ReferenceOTU165283
New.ReferenceOTU729
New.CleanUp.ReferenceOTU45351
New.CleanUp.ReferenceOTU65953
New.CleanUp.ReferenceOTU51456
New.CleanUp.ReferenceOTU113803
New.CleanUp.ReferenceOTU68945
New.CleanUp.ReferenceOTU47170
New.CleanUp.ReferenceOTU62571
New.CleanUp.ReferenceOTU64918
New.CleanUp.ReferenceOTU29946
New.CleanUp.ReferenceOTU115862
New.CleanUp.ReferenceOTU59408
New.CleanUp.ReferenceOTU122856
New.CleanUp.ReferenceOTU15307
New.CleanUp.ReferenceOTU63219
New.CleanUp.ReferenceOTU187542
New.CleanUp.ReferenceOTU55107
New.CleanUp.ReferenceOTU107065
New.CleanUp.ReferenceOTU61491
New.CleanUp.ReferenceOTU52586
New.CleanUp.ReferenceOTU57986
New.CleanUp.ReferenceOTU44809
New.CleanUp.ReferenceOTU108929
New.CleanUp.ReferenceOTU24454
New.CleanUp.ReferenceOTU200757
New.CleanUp.ReferenceOTU19703
New.CleanUp.ReferenceOTU310407

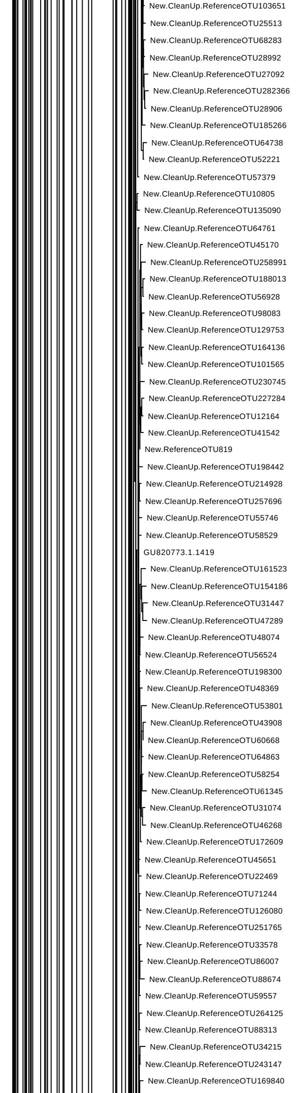
New.CleanUp.ReferenceOTU103651
New.CleanUp.ReferenceOTU25513
New.CleanUp.ReferenceOTU68283
New.CleanUp.ReferenceOTU28992
New.CleanUp.ReferenceOTU27092
New.CleanUp.ReferenceOTU282366
New.CleanUp.ReferenceOTU28906
New.CleanUp.ReferenceOTU185266
New.CleanUp.ReferenceOTU64738
New.CleanUp.ReferenceOTU52221
New.CleanUp.ReferenceOTU57379
New.CleanUp.ReferenceOTU10805
New.CleanUp.ReferenceOTU135090
New.CleanUp.ReferenceOTU64761
New.CleanUp.ReferenceOTU45170
New.CleanUp.ReferenceOTU258991
New.CleanUp.ReferenceOTU188013
New.CleanUp.ReferenceOTU56928
New.CleanUp.ReferenceOTU98083
New.CleanUp.ReferenceOTU129753
New.CleanUp.ReferenceOTU164136
New.CleanUp.ReferenceOTU101565
New.CleanUp.ReferenceOTU230745
New.CleanUp.ReferenceOTU227284
New.CleanUp.ReferenceOTU12164
New.CleanUp.ReferenceOTU41542
New.ReferenceOTU819
New.CleanUp.ReferenceOTU198442
New.CleanUp.ReferenceOTU214928
New.CleanUp.ReferenceOTU257696
New.CleanUp.ReferenceOTU55746
New.CleanUp.ReferenceOTU58529
GU820773.1.1419
New.CleanUp.ReferenceOTU161523
New.CleanUp.ReferenceOTU154186
New.CleanUp.ReferenceOTU31447
New.CleanUp.ReferenceOTU47289
New.CleanUp.ReferenceOTU48074
New.CleanUp.ReferenceOTU56524
New.CleanUp.ReferenceOTU198300
New.CleanUp.ReferenceOTU48369
New.CleanUp.ReferenceOTU53801
New.CleanUp.ReferenceOTU43908
New.CleanUp.ReferenceOTU60668
New.CleanUp.ReferenceOTU64863
New.CleanUp.ReferenceOTU58254
New.CleanUp.ReferenceOTU61345
New.CleanUp.ReferenceOTU31074
New.CleanUp.ReferenceOTU46268
New.CleanUp.ReferenceOTU172609
New.CleanUp.ReferenceOTU45651
New.CleanUp.ReferenceOTU22469
New.CleanUp.ReferenceOTU71244
New.CleanUp.ReferenceOTU126080
New.CleanUp.ReferenceOTU251765
New.CleanUp.ReferenceOTU33578
New.CleanUp.ReferenceOTU86007
New.CleanUp.ReferenceOTU88674
New.CleanUp.ReferenceOTU59557
New.CleanUp.ReferenceOTU264125
New.CleanUp.ReferenceOTU88313
New.CleanUp.ReferenceOTU34215
New.CleanUp.ReferenceOTU243147
New.CleanUp.ReferenceOTU169840

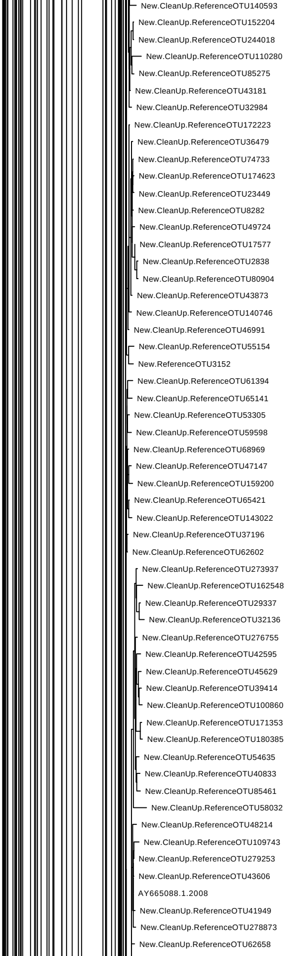
New.CleanUp.ReferenceOTU140593
New.CleanUp.ReferenceOTU152204
New.CleanUp.ReferenceOTU244018
New.CleanUp.ReferenceOTU110280
New.CleanUp.ReferenceOTU85275
New.CleanUp.ReferenceOTU43181
New.CleanUp.ReferenceOTU32984
New.CleanUp.ReferenceOTU172223
New.CleanUp.ReferenceOTU36479
New.CleanUp.ReferenceOTU74733
New.CleanUp.ReferenceOTU174623
New.CleanUp.ReferenceOTU23449
New.CleanUp.ReferenceOTU8282
New.CleanUp.ReferenceOTU49724
New.CleanUp.ReferenceOTU17577
New.CleanUp.ReferenceOTU2838
New.CleanUp.ReferenceOTU80904
New.CleanUp.ReferenceOTU43873
New.CleanUp.ReferenceOTU140746
New.CleanUp.ReferenceOTU46991
New.CleanUp.ReferenceOTU55154
New.ReferenceOTU3152
New.CleanUp.ReferenceOTU61394
New.CleanUp.ReferenceOTU65141
New.CleanUp.ReferenceOTU53305
New.CleanUp.ReferenceOTU59598
New.CleanUp.ReferenceOTU68969
New.CleanUp.ReferenceOTU47147
New.CleanUp.ReferenceOTU159200
New.CleanUp.ReferenceOTU65421
New.CleanUp.ReferenceOTU143022
New.CleanUp.ReferenceOTU37196
New.CleanUp.ReferenceOTU62602
New.CleanUp.ReferenceOTU273937
New.CleanUp.ReferenceOTU162548
New.CleanUp.ReferenceOTU29337
New.CleanUp.ReferenceOTU32136
New.CleanUp.ReferenceOTU276755
New.CleanUp.ReferenceOTU42595
New.CleanUp.ReferenceOTU45629
New.CleanUp.ReferenceOTU39414
New.CleanUp.ReferenceOTU100860
New.CleanUp.ReferenceOTU171353
New.CleanUp.ReferenceOTU180385
New.CleanUp.ReferenceOTU54635
New.CleanUp.ReferenceOTU40833
New.CleanUp.ReferenceOTU85461
New.CleanUp.ReferenceOTU58032
New.CleanUp.ReferenceOTU48214
New.CleanUp.ReferenceOTU109743
New.CleanUp.ReferenceOTU279253
New.CleanUp.ReferenceOTU43606
AY665088.1.2008
New.CleanUp.ReferenceOTU41949
New.CleanUp.ReferenceOTU278873
New.CleanUp.ReferenceOTU62658

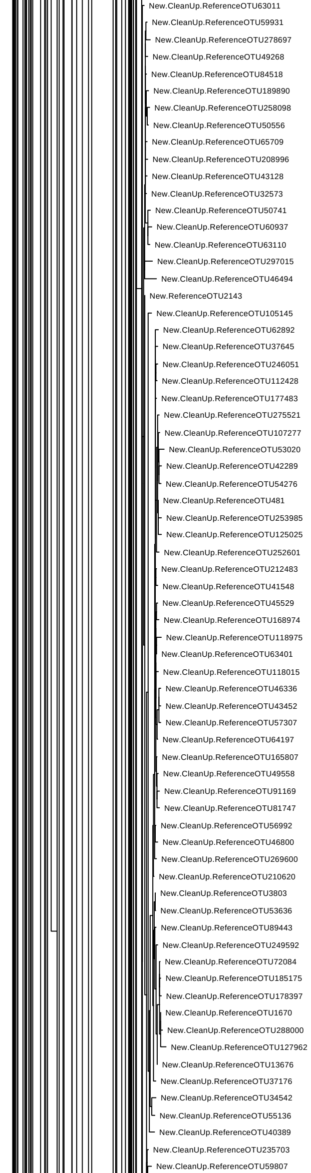
New.CleanUp.ReferenceOTU63011
New.CleanUp.ReferenceOTU59931
New.CleanUp.ReferenceOTU278697
New.CleanUp.ReferenceOTU49268
New.CleanUp.ReferenceOTU84518
New.CleanUp.ReferenceOTU189890
New.CleanUp.ReferenceOTU258098
New.CleanUp.ReferenceOTU50556
New.CleanUp.ReferenceOTU65709
New.CleanUp.ReferenceOTU208996
New.CleanUp.ReferenceOTU43128
New.CleanUp.ReferenceOTU32573
New.CleanUp.ReferenceOTU50741
New.CleanUp.ReferenceOTU60937
New.CleanUp.ReferenceOTU63110
New.CleanUp.ReferenceOTU297015
New.CleanUp.ReferenceOTU46494
New.ReferenceOTU2143
New.CleanUp.ReferenceOTU105145
New.CleanUp.ReferenceOTU62892
New.CleanUp.ReferenceOTU37645
New.CleanUp.ReferenceOTU246051
New.CleanUp.ReferenceOTU112428
New.CleanUp.ReferenceOTU177483
New.CleanUp.ReferenceOTU275521
New.CleanUp.ReferenceOTU107277
New.CleanUp.ReferenceOTU53020
New.CleanUp.ReferenceOTU42289
New.CleanUp.ReferenceOTU54276
New.CleanUp.ReferenceOTU481
New.CleanUp.ReferenceOTU253985
New.CleanUp.ReferenceOTU125025
New.CleanUp.ReferenceOTU252601
New.CleanUp.ReferenceOTU212483
New.CleanUp.ReferenceOTU41548
New.CleanUp.ReferenceOTU45529
New.CleanUp.ReferenceOTU168974
New.CleanUp.ReferenceOTU118975
New.CleanUp.ReferenceOTU63401
New.CleanUp.ReferenceOTU118015
New.CleanUp.ReferenceOTU46336
New.CleanUp.ReferenceOTU43452
New.CleanUp.ReferenceOTU57307
New.CleanUp.ReferenceOTU64197
New.CleanUp.ReferenceOTU165807
New.CleanUp.ReferenceOTU49558
New.CleanUp.ReferenceOTU91169
New.CleanUp.ReferenceOTU81747
New.CleanUp.ReferenceOTU56992
New.CleanUp.ReferenceOTU46800
New.CleanUp.ReferenceOTU269600
New.CleanUp.ReferenceOTU210620
New.CleanUp.ReferenceOTU3803
New.CleanUp.ReferenceOTU53636
New.CleanUp.ReferenceOTU89443
New.CleanUp.ReferenceOTU249592
New.CleanUp.ReferenceOTU72084
New.CleanUp.ReferenceOTU185175
New.CleanUp.ReferenceOTU178397
New.CleanUp.ReferenceOTU1670
New.CleanUp.ReferenceOTU288000
New.CleanUp.ReferenceOTU127962
New.CleanUp.ReferenceOTU13676
New.CleanUp.ReferenceOTU37176
New.CleanUp.ReferenceOTU34542
New.CleanUp.ReferenceOTU55136
New.CleanUp.ReferenceOTU40389
New.CleanUp.ReferenceOTU235703
New.CleanUp.ReferenceOTU59807

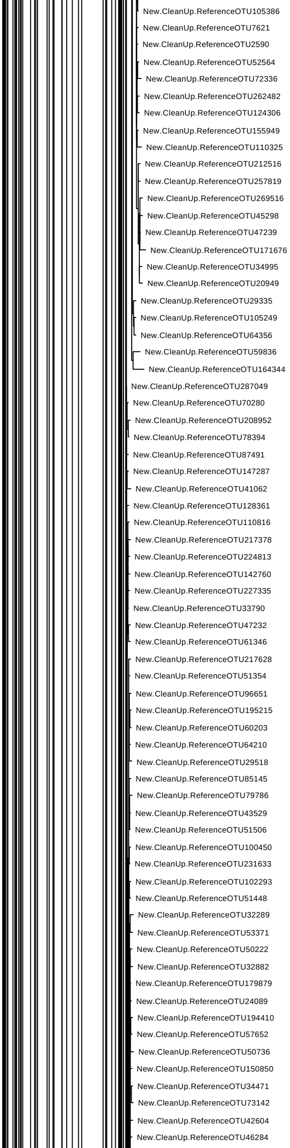
New.CleanUp.ReferenceOTU105386
New.CleanUp.ReferenceOTU7621
New.CleanUp.ReferenceOTU2590
New.CleanUp.ReferenceOTU52564
New.CleanUp.ReferenceOTU72336
New.CleanUp.ReferenceOTU262482
New.CleanUp.ReferenceOTU124306
New.CleanUp.ReferenceOTU155949
New.CleanUp.ReferenceOTU110325
New.CleanUp.ReferenceOTU212516
New.CleanUp.ReferenceOTU257819
New.CleanUp.ReferenceOTU269516
New.CleanUp.ReferenceOTU45298
New.CleanUp.ReferenceOTU47239
New.CleanUp.ReferenceOTU171676
New.CleanUp.ReferenceOTU34995
New.CleanUp.ReferenceOTU20949
New.CleanUp.ReferenceOTU29335
New.CleanUp.ReferenceOTU105249
New.CleanUp.ReferenceOTU64356
New.CleanUp.ReferenceOTU59836
New.CleanUp.ReferenceOTU164344
New.CleanUp.ReferenceOTU287049
New.CleanUp.ReferenceOTU70280
New.CleanUp.ReferenceOTU208952
New.CleanUp.ReferenceOTU78394
New.CleanUp.ReferenceOTU87491
New.CleanUp.ReferenceOTU147287
New.CleanUp.ReferenceOTU41062
New.CleanUp.ReferenceOTU128361
New.CleanUp.ReferenceOTU110816
New.CleanUp.ReferenceOTU217378
New.CleanUp.ReferenceOTU224813
New.CleanUp.ReferenceOTU142760
New.CleanUp.ReferenceOTU227335
New.CleanUp.ReferenceOTU33790
New.CleanUp.ReferenceOTU47232
New.CleanUp.ReferenceOTU61346
New.CleanUp.ReferenceOTU217628
New.CleanUp.ReferenceOTU51354
New.CleanUp.ReferenceOTU96651
New.CleanUp.ReferenceOTU195215
New.CleanUp.ReferenceOTU60203
New.CleanUp.ReferenceOTU64210
New.CleanUp.ReferenceOTU29518
New.CleanUp.ReferenceOTU85145
New.CleanUp.ReferenceOTU79786
New.CleanUp.ReferenceOTU43529
New.CleanUp.ReferenceOTU51506
New.CleanUp.ReferenceOTU100450
New.CleanUp.ReferenceOTU231633
New.CleanUp.ReferenceOTU102293
New.CleanUp.ReferenceOTU51448
New.CleanUp.ReferenceOTU32289
New.CleanUp.ReferenceOTU53371
New.CleanUp.ReferenceOTU50222
New.CleanUp.ReferenceOTU32882
New.CleanUp.ReferenceOTU179879
New.CleanUp.ReferenceOTU24089
New.CleanUp.ReferenceOTU194410
New.CleanUp.ReferenceOTU57652
New.CleanUp.ReferenceOTU50736
New.CleanUp.ReferenceOTU150850
New.CleanUp.ReferenceOTU34471
New.CleanUp.ReferenceOTU73142
New.CleanUp.ReferenceOTU42604
New.CleanUp.ReferenceOTU46284

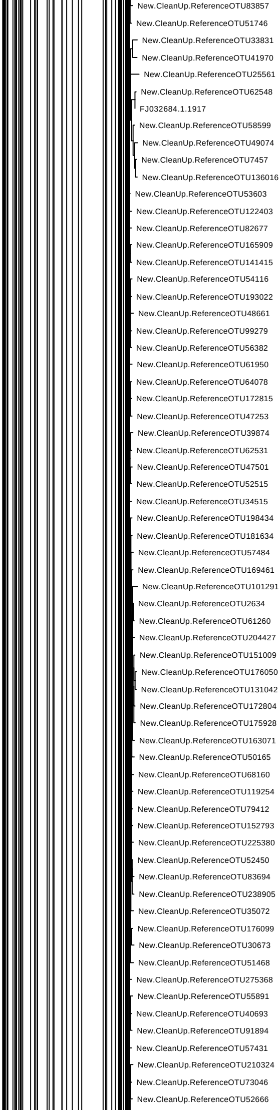
New.CleanUp.ReferenceOTU83857
New.CleanUp.ReferenceOTU51746
New.CleanUp.ReferenceOTU33831
New.CleanUp.ReferenceOTU41970
New.CleanUp.ReferenceOTU25561
New.CleanUp.ReferenceOTU62548
FJ032684.1.1917
New.CleanUp.ReferenceOTU58599
New.CleanUp.ReferenceOTU49074
New.CleanUp.ReferenceOTU7457
New.CleanUp.ReferenceOTU136016
New.CleanUp.ReferenceOTU53603
New.CleanUp.ReferenceOTU122403
New.CleanUp.ReferenceOTU82677
New.CleanUp.ReferenceOTU165909
New.CleanUp.ReferenceOTU141415
New.CleanUp.ReferenceOTU54116
New.CleanUp.ReferenceOTU193022
New.CleanUp.ReferenceOTU48661
New.CleanUp.ReferenceOTU99279
New.CleanUp.ReferenceOTU56382
New.CleanUp.ReferenceOTU61950
New.CleanUp.ReferenceOTU64078
New.CleanUp.ReferenceOTU172815
New.CleanUp.ReferenceOTU47253
New.CleanUp.ReferenceOTU39874
New.CleanUp.ReferenceOTU62531
New.CleanUp.ReferenceOTU47501
New.CleanUp.ReferenceOTU52515
New.CleanUp.ReferenceOTU34515
New.CleanUp.ReferenceOTU198434
New.CleanUp.ReferenceOTU181634
New.CleanUp.ReferenceOTU57484
New.CleanUp.ReferenceOTU169461
New.CleanUp.ReferenceOTU101291
New.CleanUp.ReferenceOTU2634
New.CleanUp.ReferenceOTU61260
New.CleanUp.ReferenceOTU204427
New.CleanUp.ReferenceOTU151009
New.CleanUp.ReferenceOTU176050
New.CleanUp.ReferenceOTU131042
New.CleanUp.ReferenceOTU172804
New.CleanUp.ReferenceOTU175928
New.CleanUp.ReferenceOTU163071
New.CleanUp.ReferenceOTU50165
New.CleanUp.ReferenceOTU68160
New.CleanUp.ReferenceOTU119254
New.CleanUp.ReferenceOTU79412
New.CleanUp.ReferenceOTU152793
New.CleanUp.ReferenceOTU225380
New.CleanUp.ReferenceOTU52450
New.CleanUp.ReferenceOTU83694
New.CleanUp.ReferenceOTU238905
New.CleanUp.ReferenceOTU35072
New.CleanUp.ReferenceOTU176099
New.CleanUp.ReferenceOTU30673
New.CleanUp.ReferenceOTU51468
New.CleanUp.ReferenceOTU275368
New.CleanUp.ReferenceOTU55891
New.CleanUp.ReferenceOTU40693
New.CleanUp.ReferenceOTU91894
New.CleanUp.ReferenceOTU57431
New.CleanUp.ReferenceOTU210324
New.CleanUp.ReferenceOTU73046
New.CleanUp.ReferenceOTU52666

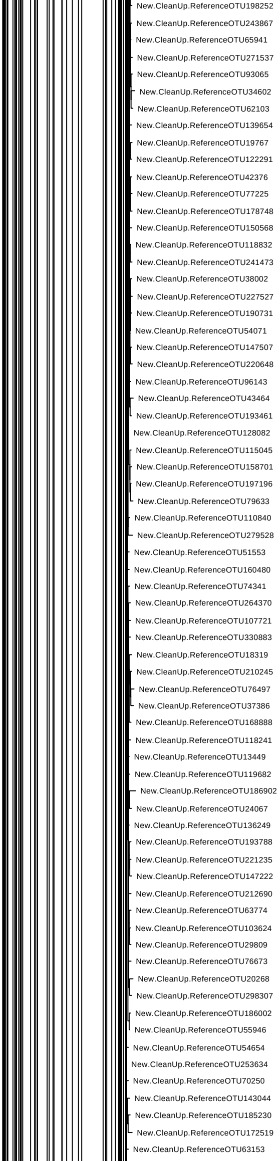
New.CleanUp.ReferenceOTU198252
New.CleanUp.ReferenceOTU243867
New.CleanUp.ReferenceOTU65941
New.CleanUp.ReferenceOTU271537
New.CleanUp.ReferenceOTU93065
New.CleanUp.ReferenceOTU34602
New.CleanUp.ReferenceOTU62103
New.CleanUp.ReferenceOTU139654
New.CleanUp.ReferenceOTU19767
New.CleanUp.ReferenceOTU122291
New.CleanUp.ReferenceOTU42376
New.CleanUp.ReferenceOTU77225
New.CleanUp.ReferenceOTU178748
New.CleanUp.ReferenceOTU150568
New.CleanUp.ReferenceOTU118832
New.CleanUp.ReferenceOTU241473
New.CleanUp.ReferenceOTU38002
New.CleanUp.ReferenceOTU227527
New.CleanUp.ReferenceOTU190731
New.CleanUp.ReferenceOTU54071
New.CleanUp.ReferenceOTU147507
New.CleanUp.ReferenceOTU220648
New.CleanUp.ReferenceOTU96143
New.CleanUp.ReferenceOTU43464
New.CleanUp.ReferenceOTU193461
New.CleanUp.ReferenceOTU128082
New.CleanUp.ReferenceOTU115045
New.CleanUp.ReferenceOTU158701
New.CleanUp.ReferenceOTU197196
New.CleanUp.ReferenceOTU79633
New.CleanUp.ReferenceOTU110840
New.CleanUp.ReferenceOTU279528
New.CleanUp.ReferenceOTU51553
New.CleanUp.ReferenceOTU160480
New.CleanUp.ReferenceOTU74341
New.CleanUp.ReferenceOTU264370
New.CleanUp.ReferenceOTU107721
New.CleanUp.ReferenceOTU330883
New.CleanUp.ReferenceOTU18319
New.CleanUp.ReferenceOTU210245
New.CleanUp.ReferenceOTU76497
New.CleanUp.ReferenceOTU37386
New.CleanUp.ReferenceOTU168888
New.CleanUp.ReferenceOTU118241
New.CleanUp.ReferenceOTU13449
New.CleanUp.ReferenceOTU119682
New.CleanUp.ReferenceOTU186902
New.CleanUp.ReferenceOTU24067
New.CleanUp.ReferenceOTU136249
New.CleanUp.ReferenceOTU193788
New.CleanUp.ReferenceOTU221235
New.CleanUp.ReferenceOTU147222
New.CleanUp.ReferenceOTU212690
New.CleanUp.ReferenceOTU63774
New.CleanUp.ReferenceOTU103624
New.CleanUp.ReferenceOTU29809
New.CleanUp.ReferenceOTU76673
New.CleanUp.ReferenceOTU20268
New.CleanUp.ReferenceOTU298307
New.CleanUp.ReferenceOTU186002
New.CleanUp.ReferenceOTU55946
New.CleanUp.ReferenceOTU54654
New.CleanUp.ReferenceOTU253634
New.CleanUp.ReferenceOTU70250
New.CleanUp.ReferenceOTU143044
New.CleanUp.ReferenceOTU185230
New.CleanUp.ReferenceOTU172519
New.CleanUp.ReferenceOTU63153

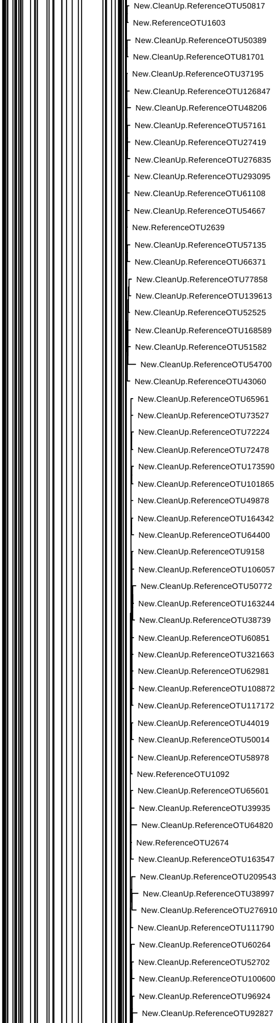
New.CleanUp.ReferenceOTU50817
New.ReferenceOTU1603
New.CleanUp.ReferenceOTU50389
New.CleanUp.ReferenceOTU81701
New.CleanUp.ReferenceOTU37195
New.CleanUp.ReferenceOTU126847
New.CleanUp.ReferenceOTU48206
New.CleanUp.ReferenceOTU57161
New.CleanUp.ReferenceOTU27419
New.CleanUp.ReferenceOTU276835
New.CleanUp.ReferenceOTU293095
New.CleanUp.ReferenceOTU61108
New.CleanUp.ReferenceOTU54667
New.ReferenceOTU2639
New.CleanUp.ReferenceOTU57135
New.CleanUp.ReferenceOTU66371
New.CleanUp.ReferenceOTU77858
New.CleanUp.ReferenceOTU139613
New.CleanUp.ReferenceOTU52525
New.CleanUp.ReferenceOTU168589
New.CleanUp.ReferenceOTU51582
New.CleanUp.ReferenceOTU54700
New.CleanUp.ReferenceOTU43060
New.CleanUp.ReferenceOTU65961
New.CleanUp.ReferenceOTU73527
New.CleanUp.ReferenceOTU72224
New.CleanUp.ReferenceOTU72478
New.CleanUp.ReferenceOTU173590
New.CleanUp.ReferenceOTU101865
New.CleanUp.ReferenceOTU49878
New.CleanUp.ReferenceOTU164342
New.CleanUp.ReferenceOTU64400
New.CleanUp.ReferenceOTU9158
New.CleanUp.ReferenceOTU106057
New.CleanUp.ReferenceOTU50772
New.CleanUp.ReferenceOTU163244
New.CleanUp.ReferenceOTU38739
New.CleanUp.ReferenceOTU60851
New.CleanUp.ReferenceOTU321663
New.CleanUp.ReferenceOTU62981
New.CleanUp.ReferenceOTU108872
New.CleanUp.ReferenceOTU117172
New.CleanUp.ReferenceOTU44019
New.CleanUp.ReferenceOTU50014
New.CleanUp.ReferenceOTU58978
New.ReferenceOTU1092
New.CleanUp.ReferenceOTU65601
New.CleanUp.ReferenceOTU39935
New.CleanUp.ReferenceOTU64820
New.ReferenceOTU2674
New.CleanUp.ReferenceOTU163547
New.CleanUp.ReferenceOTU209543
New.CleanUp.ReferenceOTU38997
New.CleanUp.ReferenceOTU276910
New.CleanUp.ReferenceOTU111790
New.CleanUp.ReferenceOTU60264
New.CleanUp.ReferenceOTU52702
New.CleanUp.ReferenceOTU100600
New.CleanUp.ReferenceOTU96924
New.CleanUp.ReferenceOTU92827

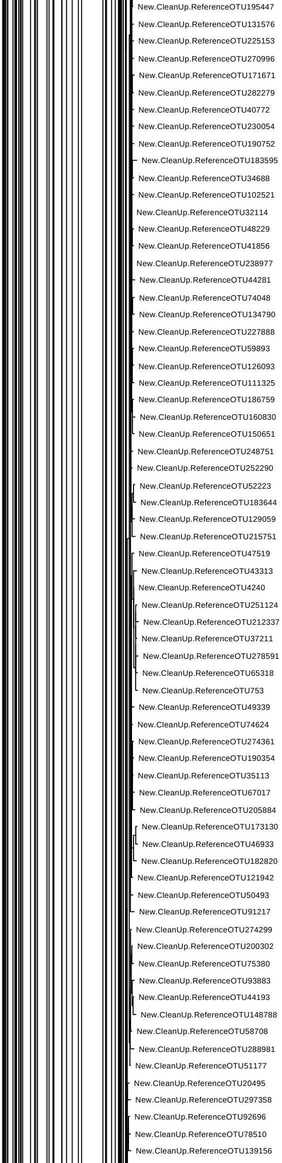
New.CleanUp.ReferenceOTU195447
New.CleanUp.ReferenceOTU131576
New.CleanUp.ReferenceOTU225153
New.CleanUp.ReferenceOTU270996
New.CleanUp.ReferenceOTU171671
New.CleanUp.ReferenceOTU282279
New.CleanUp.ReferenceOTU40772
New.CleanUp.ReferenceOTU230054
New.CleanUp.ReferenceOTU190752
New.CleanUp.ReferenceOTU183595
New.CleanUp.ReferenceOTU34688
New.CleanUp.ReferenceOTU102521
New.CleanUp.ReferenceOTU32114
New.CleanUp.ReferenceOTU48229
New.CleanUp.ReferenceOTU41856
New.CleanUp.ReferenceOTU238977
New.CleanUp.ReferenceOTU44281
New.CleanUp.ReferenceOTU74048
New.CleanUp.ReferenceOTU134790
New.CleanUp.ReferenceOTU227888
New.CleanUp.ReferenceOTU59893
New.CleanUp.ReferenceOTU126093
New.CleanUp.ReferenceOTU111325
New.CleanUp.ReferenceOTU186759
New.CleanUp.ReferenceOTU160830
New.CleanUp.ReferenceOTU150651
New.CleanUp.ReferenceOTU248751
New.CleanUp.ReferenceOTU252290
New.CleanUp.ReferenceOTU52223
New.CleanUp.ReferenceOTU183644
New.CleanUp.ReferenceOTU129059
New.CleanUp.ReferenceOTU215751
New.CleanUp.ReferenceOTU47519
New.CleanUp.ReferenceOTU43313
New.CleanUp.ReferenceOTU4240
New.CleanUp.ReferenceOTU251124
New.CleanUp.ReferenceOTU212337
New.CleanUp.ReferenceOTU37211
New.CleanUp.ReferenceOTU278591
New.CleanUp.ReferenceOTU65318
New.CleanUp.ReferenceOTU753
New.CleanUp.ReferenceOTU49339
New.CleanUp.ReferenceOTU74624
New.CleanUp.ReferenceOTU274361
New.CleanUp.ReferenceOTU190354
New.CleanUp.ReferenceOTU35113
New.CleanUp.ReferenceOTU67017
New.CleanUp.ReferenceOTU205884
New.CleanUp.ReferenceOTU173130
New.CleanUp.ReferenceOTU46933
New.CleanUp.ReferenceOTU182820
New.CleanUp.ReferenceOTU121942
New.CleanUp.ReferenceOTU50493
New.CleanUp.ReferenceOTU91217
New.CleanUp.ReferenceOTU274299
New.CleanUp.ReferenceOTU200302
New.CleanUp.ReferenceOTU75380
New.CleanUp.ReferenceOTU93883
New.CleanUp.ReferenceOTU44193
New.CleanUp.ReferenceOTU148788
New.CleanUp.ReferenceOTU58708
New.CleanUp.ReferenceOTU288981
New.CleanUp.ReferenceOTU51177
New.CleanUp.ReferenceOTU20495
New.CleanUp.ReferenceOTU297358
New.CleanUp.ReferenceOTU92696
New.CleanUp.ReferenceOTU78510
New.CleanUp.ReferenceOTU139156

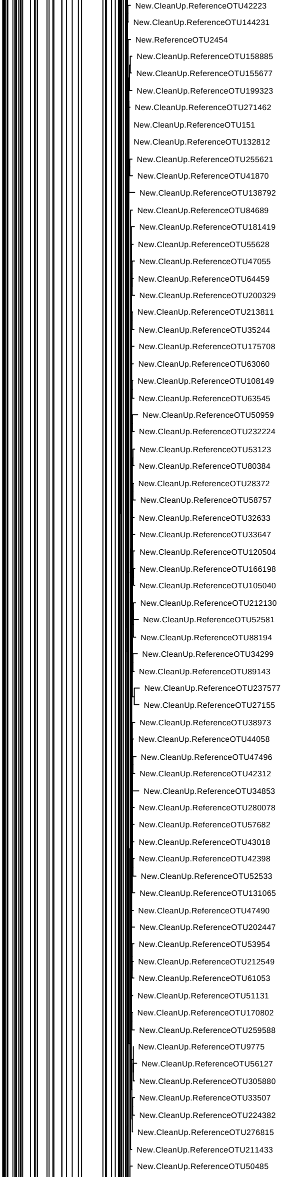
New.CleanUp.ReferenceOTU42223
New.CleanUp.ReferenceOTU144231
New.ReferenceOTU2454
New.CleanUp.ReferenceOTU158885
New.CleanUp.ReferenceOTU155677
New.CleanUp.ReferenceOTU199323
New.CleanUp.ReferenceOTU271462
New.CleanUp.ReferenceOTU151
New.CleanUp.ReferenceOTU132812
New.CleanUp.ReferenceOTU255621
New.CleanUp.ReferenceOTU41870
New.CleanUp.ReferenceOTU138792
New.CleanUp.ReferenceOTU84689
New.CleanUp.ReferenceOTU181419
New.CleanUp.ReferenceOTU55628
New.CleanUp.ReferenceOTU47055
New.CleanUp.ReferenceOTU64459
New.CleanUp.ReferenceOTU200329
New.CleanUp.ReferenceOTU213811
New.CleanUp.ReferenceOTU35244
New.CleanUp.ReferenceOTU175708
New.CleanUp.ReferenceOTU63060
New.CleanUp.ReferenceOTU108149
New.CleanUp.ReferenceOTU63545
New.CleanUp.ReferenceOTU50959
New.CleanUp.ReferenceOTU232224
New.CleanUp.ReferenceOTU53123
New.CleanUp.ReferenceOTU80384
New.CleanUp.ReferenceOTU28372
New.CleanUp.ReferenceOTU58757
New.CleanUp.ReferenceOTU32633
New.CleanUp.ReferenceOTU33647
New.CleanUp.ReferenceOTU120504
New.CleanUp.ReferenceOTU166198
New.CleanUp.ReferenceOTU105040
New.CleanUp.ReferenceOTU212130
New.CleanUp.ReferenceOTU52581
New.CleanUp.ReferenceOTU88194
New.CleanUp.ReferenceOTU34299
New.CleanUp.ReferenceOTU89143
New.CleanUp.ReferenceOTU237577
New.CleanUp.ReferenceOTU27155
New.CleanUp.ReferenceOTU38973
New.CleanUp.ReferenceOTU44058
New.CleanUp.ReferenceOTU47496
New.CleanUp.ReferenceOTU42312
New.CleanUp.ReferenceOTU34853
New.CleanUp.ReferenceOTU280078
New.CleanUp.ReferenceOTU57682
New.CleanUp.ReferenceOTU43018
New.CleanUp.ReferenceOTU42398
New.CleanUp.ReferenceOTU52533
New.CleanUp.ReferenceOTU131065
New.CleanUp.ReferenceOTU47490
New.CleanUp.ReferenceOTU202447
New.CleanUp.ReferenceOTU53954
New.CleanUp.ReferenceOTU212549
New.CleanUp.ReferenceOTU61053
New.CleanUp.ReferenceOTU51131
New.CleanUp.ReferenceOTU170802
New.CleanUp.ReferenceOTU259588
New.CleanUp.ReferenceOTU9775
New.CleanUp.ReferenceOTU56127
New.CleanUp.ReferenceOTU305880
New.CleanUp.ReferenceOTU33507
New.CleanUp.ReferenceOTU224382
New.CleanUp.ReferenceOTU276815
New.CleanUp.ReferenceOTU211433
New.CleanUp.ReferenceOTU50485

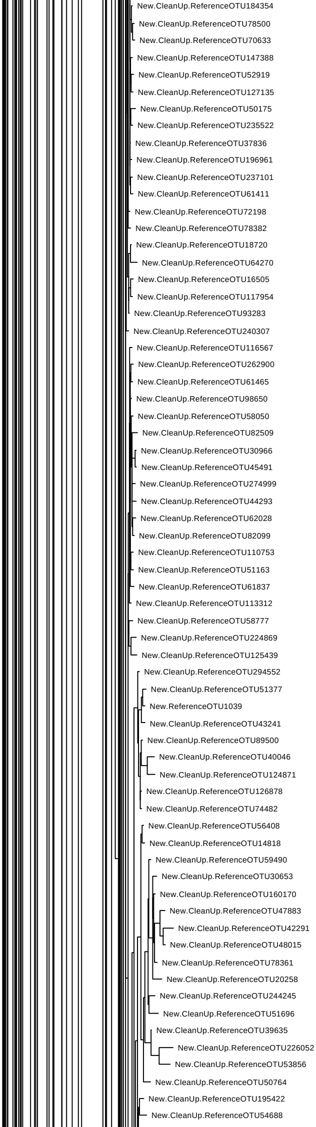
New.CleanUp.ReferenceOTU184354
New.CleanUp.ReferenceOTU78500
New.CleanUp.ReferenceOTU70633
New.CleanUp.ReferenceOTU147388
New.CleanUp.ReferenceOTU52919
New.CleanUp.ReferenceOTU127135
New.CleanUp.ReferenceOTU50175
New.CleanUp.ReferenceOTU235522
New.CleanUp.ReferenceOTU37836
New.CleanUp.ReferenceOTU196961
New.CleanUp.ReferenceOTU237101
New.CleanUp.ReferenceOTU61411
New.CleanUp.ReferenceOTU72198
New.CleanUp.ReferenceOTU78382
New.CleanUp.ReferenceOTU18720
New.CleanUp.ReferenceOTU64270
New.CleanUp.ReferenceOTU16505
New.CleanUp.ReferenceOTU117954
New.CleanUp.ReferenceOTU93283
New.CleanUp.ReferenceOTU240307
New.CleanUp.ReferenceOTU116567
New.CleanUp.ReferenceOTU262900
New.CleanUp.ReferenceOTU61465
New.CleanUp.ReferenceOTU98650
New.CleanUp.ReferenceOTU58050
New.CleanUp.ReferenceOTU82509
New.CleanUp.ReferenceOTU30966
New.CleanUp.ReferenceOTU45491
New.CleanUp.ReferenceOTU274999
New.CleanUp.ReferenceOTU44293
New.CleanUp.ReferenceOTU62028
New.CleanUp.ReferenceOTU82099
New.CleanUp.ReferenceOTU110753
New.CleanUp.ReferenceOTU51163
New.CleanUp.ReferenceOTU61837
New.CleanUp.ReferenceOTU113312
New.CleanUp.ReferenceOTU58777
New.CleanUp.ReferenceOTU224869
New.CleanUp.ReferenceOTU125439
New.CleanUp.ReferenceOTU294552
New.CleanUp.ReferenceOTU51377
New.ReferenceOTU1039
New.CleanUp.ReferenceOTU43241
New.CleanUp.ReferenceOTU89500
New.CleanUp.ReferenceOTU40046
New.CleanUp.ReferenceOTU124871
New.CleanUp.ReferenceOTU126878
New.CleanUp.ReferenceOTU74482
New.CleanUp.ReferenceOTU56408
New.CleanUp.ReferenceOTU14818
New.CleanUp.ReferenceOTU59490
New.CleanUp.ReferenceOTU30653
New.CleanUp.ReferenceOTU160170
New.CleanUp.ReferenceOTU47883
New.CleanUp.ReferenceOTU42291
New.CleanUp.ReferenceOTU48015
New.CleanUp.ReferenceOTU78361
New.CleanUp.ReferenceOTU20258
New.CleanUp.ReferenceOTU244245
New.CleanUp.ReferenceOTU51696
New.CleanUp.ReferenceOTU39635
New.CleanUp.ReferenceOTU226052
New.CleanUp.ReferenceOTU53856
New.CleanUp.ReferenceOTU50764
New.CleanUp.ReferenceOTU195422
New.CleanUp.ReferenceOTU54688

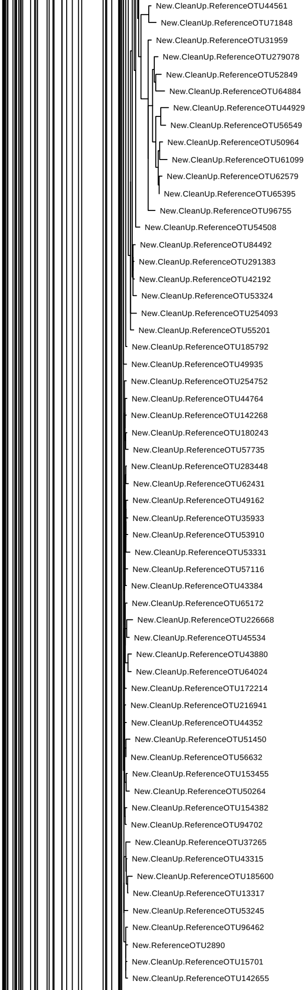
New.CleanUp.ReferenceOTU44561
New.CleanUp.ReferenceOTU71848
New.CleanUp.ReferenceOTU31959
New.CleanUp.ReferenceOTU279078
New.CleanUp.ReferenceOTU52849
New.CleanUp.ReferenceOTU64884
New.CleanUp.ReferenceOTU44929
New.CleanUp.ReferenceOTU56549
New.CleanUp.ReferenceOTU50964
New.CleanUp.ReferenceOTU61099
New.CleanUp.ReferenceOTU62579
New.CleanUp.ReferenceOTU65395
New.CleanUp.ReferenceOTU96755
New.CleanUp.ReferenceOTU54508
New.CleanUp.ReferenceOTU84492
New.CleanUp.ReferenceOTU291383
New.CleanUp.ReferenceOTU42192
New.CleanUp.ReferenceOTU53324
New.CleanUp.ReferenceOTU254093
New.CleanUp.ReferenceOTU55201
New.CleanUp.ReferenceOTU185792
New.CleanUp.ReferenceOTU49935
New.CleanUp.ReferenceOTU254752
New.CleanUp.ReferenceOTU44764
New.CleanUp.ReferenceOTU142268
New.CleanUp.ReferenceOTU180243
New.CleanUp.ReferenceOTU57735
New.CleanUp.ReferenceOTU283448
New.CleanUp.ReferenceOTU62431
New.CleanUp.ReferenceOTU49162
New.CleanUp.ReferenceOTU35933
New.CleanUp.ReferenceOTU53910
New.CleanUp.ReferenceOTU53331
New.CleanUp.ReferenceOTU57116
New.CleanUp.ReferenceOTU43384
New.CleanUp.ReferenceOTU65172
New.CleanUp.ReferenceOTU226668
New.CleanUp.ReferenceOTU45534
New.CleanUp.ReferenceOTU43880
New.CleanUp.ReferenceOTU64024
New.CleanUp.ReferenceOTU172214
New.CleanUp.ReferenceOTU216941
New.CleanUp.ReferenceOTU44352
New.CleanUp.ReferenceOTU51450
New.CleanUp.ReferenceOTU56632
New.CleanUp.ReferenceOTU153455
New.CleanUp.ReferenceOTU50264
New.CleanUp.ReferenceOTU154382
New.CleanUp.ReferenceOTU94702
New.CleanUp.ReferenceOTU37265
New.CleanUp.ReferenceOTU43315
New.CleanUp.ReferenceOTU185600
New.CleanUp.ReferenceOTU13317
New.CleanUp.ReferenceOTU53245
New.CleanUp.ReferenceOTU96462
New.ReferenceOTU2890
New.CleanUp.ReferenceOTU15701
New.CleanUp.ReferenceOTU142655

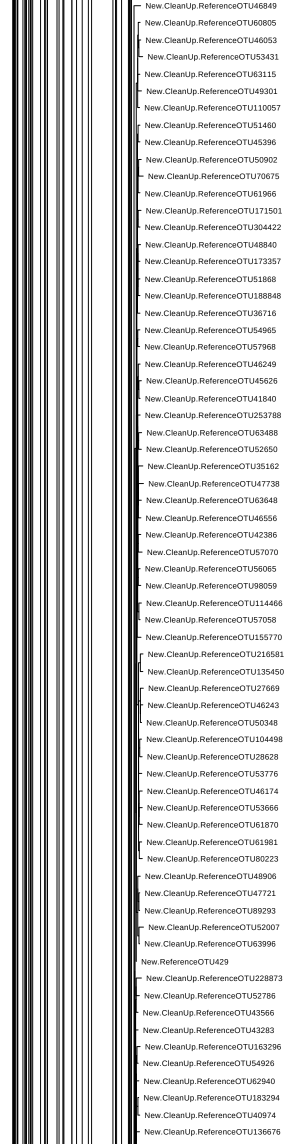
New.CleanUp.ReferenceOTU46849
New.CleanUp.ReferenceOTU60805
New.CleanUp.ReferenceOTU46053
New.CleanUp.ReferenceOTU53431
New.CleanUp.ReferenceOTU63115
New.CleanUp.ReferenceOTU49301
New.CleanUp.ReferenceOTU110057
New.CleanUp.ReferenceOTU51460
New.CleanUp.ReferenceOTU45396
New.CleanUp.ReferenceOTU50902
New.CleanUp.ReferenceOTU70675
New.CleanUp.ReferenceOTU61966
New.CleanUp.ReferenceOTU171501
New.CleanUp.ReferenceOTU304422
New.CleanUp.ReferenceOTU48840
New.CleanUp.ReferenceOTU173357
New.CleanUp.ReferenceOTU51868
New.CleanUp.ReferenceOTU188848
New.CleanUp.ReferenceOTU36716
New.CleanUp.ReferenceOTU54965
New.CleanUp.ReferenceOTU57968
New.CleanUp.ReferenceOTU46249
New.CleanUp.ReferenceOTU45626
New.CleanUp.ReferenceOTU41840
New.CleanUp.ReferenceOTU253788
New.CleanUp.ReferenceOTU63488
New.CleanUp.ReferenceOTU52650
New.CleanUp.ReferenceOTU35162
New.CleanUp.ReferenceOTU47738
New.CleanUp.ReferenceOTU63648
New.CleanUp.ReferenceOTU46556
New.CleanUp.ReferenceOTU42386
New.CleanUp.ReferenceOTU57070
New.CleanUp.ReferenceOTU56065
New.CleanUp.ReferenceOTU98059
New.CleanUp.ReferenceOTU114466
New.CleanUp.ReferenceOTU57058
New.CleanUp.ReferenceOTU155770
New.CleanUp.ReferenceOTU216581
New.CleanUp.ReferenceOTU135450
New.CleanUp.ReferenceOTU27669
New.CleanUp.ReferenceOTU46243
New.CleanUp.ReferenceOTU50348
New.CleanUp.ReferenceOTU104498
New.CleanUp.ReferenceOTU28628
New.CleanUp.ReferenceOTU53776
New.CleanUp.ReferenceOTU46174
New.CleanUp.ReferenceOTU53666
New.CleanUp.ReferenceOTU61870
New.CleanUp.ReferenceOTU61981
New.CleanUp.ReferenceOTU80223
New.CleanUp.ReferenceOTU48906
New.CleanUp.ReferenceOTU47721
New.CleanUp.ReferenceOTU89293
New.CleanUp.ReferenceOTU52007
New.CleanUp.ReferenceOTU63996
New.ReferenceOTU429
New.CleanUp.ReferenceOTU228873
New.CleanUp.ReferenceOTU52786
New.CleanUp.ReferenceOTU43566
New.CleanUp.ReferenceOTU43283
New.CleanUp.ReferenceOTU163296
New.CleanUp.ReferenceOTU54926
New.CleanUp.ReferenceOTU62940
New.CleanUp.ReferenceOTU183294
New.CleanUp.ReferenceOTU40974
New.CleanUp.ReferenceOTU136676

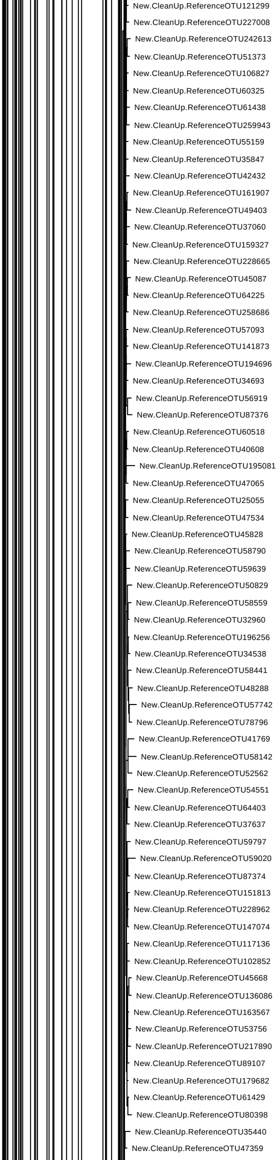
New.CleanUp.ReferenceOTU121299
New.CleanUp.ReferenceOTU227008
New.CleanUp.ReferenceOTU242613
New.CleanUp.ReferenceOTU51373
New.CleanUp.ReferenceOTU106827
New.CleanUp.ReferenceOTU60325
New.CleanUp.ReferenceOTU61438
New.CleanUp.ReferenceOTU259943
New.CleanUp.ReferenceOTU55159
New.CleanUp.ReferenceOTU35847
New.CleanUp.ReferenceOTU42432
New.CleanUp.ReferenceOTU161907
New.CleanUp.ReferenceOTU49403
New.CleanUp.ReferenceOTU37060
New.CleanUp.ReferenceOTU159327
New.CleanUp.ReferenceOTU228665
New.CleanUp.ReferenceOTU45087
New.CleanUp.ReferenceOTU64225
New.CleanUp.ReferenceOTU258686
New.CleanUp.ReferenceOTU57093
New.CleanUp.ReferenceOTU141873
New.CleanUp.ReferenceOTU194696
New.CleanUp.ReferenceOTU34693
New.CleanUp.ReferenceOTU56919
New.CleanUp.ReferenceOTU87376
New.CleanUp.ReferenceOTU60518
New.CleanUp.ReferenceOTU40608
New.CleanUp.ReferenceOTU195081
New.CleanUp.ReferenceOTU47065
New.CleanUp.ReferenceOTU25055
New.CleanUp.ReferenceOTU47534
New.CleanUp.ReferenceOTU45828
New.CleanUp.ReferenceOTU58790
New.CleanUp.ReferenceOTU59639
New.CleanUp.ReferenceOTU50829
New.CleanUp.ReferenceOTU58559
New.CleanUp.ReferenceOTU32960
New.CleanUp.ReferenceOTU196256
New.CleanUp.ReferenceOTU34538
New.CleanUp.ReferenceOTU58441
New.CleanUp.ReferenceOTU48288
New.CleanUp.ReferenceOTU57742
New.CleanUp.ReferenceOTU78796
New.CleanUp.ReferenceOTU41769
New.CleanUp.ReferenceOTU58142
New.CleanUp.ReferenceOTU52562
New.CleanUp.ReferenceOTU54551
New.CleanUp.ReferenceOTU64403
New.CleanUp.ReferenceOTU37637
New.CleanUp.ReferenceOTU59797
New.CleanUp.ReferenceOTU59020
New.CleanUp.ReferenceOTU87374
New.CleanUp.ReferenceOTU151813
New.CleanUp.ReferenceOTU228962
New.CleanUp.ReferenceOTU147074
New.CleanUp.ReferenceOTU117136
New.CleanUp.ReferenceOTU102852
New.CleanUp.ReferenceOTU45668
New.CleanUp.ReferenceOTU136086
New.CleanUp.ReferenceOTU163567
New.CleanUp.ReferenceOTU53756
New.CleanUp.ReferenceOTU217890
New.CleanUp.ReferenceOTU89107
New.CleanUp.ReferenceOTU179682
New.CleanUp.ReferenceOTU61429
New.CleanUp.ReferenceOTU80398
New.CleanUp.ReferenceOTU35440
New.CleanUp.ReferenceOTU47359

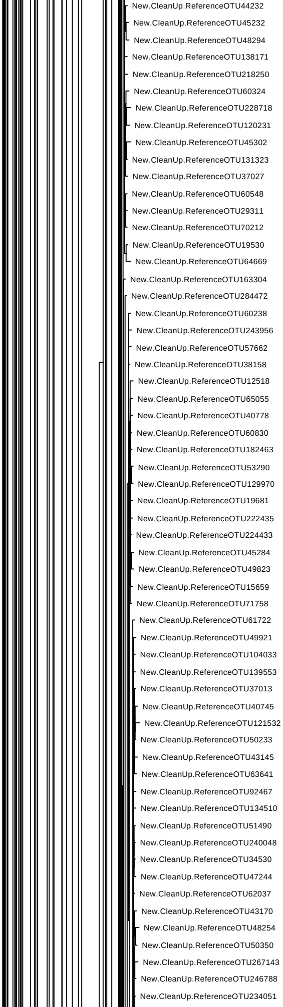
New.CleanUp.ReferenceOTU44232
New.CleanUp.ReferenceOTU45232
New.CleanUp.ReferenceOTU48294
New.CleanUp.ReferenceOTU138171
New.CleanUp.ReferenceOTU218250
New.CleanUp.ReferenceOTU60324
New.CleanUp.ReferenceOTU228718
New.CleanUp.ReferenceOTU120231
New.CleanUp.ReferenceOTU45302
New.CleanUp.ReferenceOTU131323
New.CleanUp.ReferenceOTU37027
New.CleanUp.ReferenceOTU60548
New.CleanUp.ReferenceOTU29311
New.CleanUp.ReferenceOTU70212
New.CleanUp.ReferenceOTU19530
New.CleanUp.ReferenceOTU64669
New.CleanUp.ReferenceOTU163304
New.CleanUp.ReferenceOTU284472
New.CleanUp.ReferenceOTU60238
New.CleanUp.ReferenceOTU243956
New.CleanUp.ReferenceOTU57662
New.CleanUp.ReferenceOTU38158
New.CleanUp.ReferenceOTU12518
New.CleanUp.ReferenceOTU65055
New.CleanUp.ReferenceOTU40778
New.CleanUp.ReferenceOTU60830
New.CleanUp.ReferenceOTU182463
New.CleanUp.ReferenceOTU53290
New.CleanUp.ReferenceOTU129970
New.CleanUp.ReferenceOTU19681
New.CleanUp.ReferenceOTU222435
New.CleanUp.ReferenceOTU224433
New.CleanUp.ReferenceOTU45284
New.CleanUp.ReferenceOTU49823
New.CleanUp.ReferenceOTU15659
New.CleanUp.ReferenceOTU71758
New.CleanUp.ReferenceOTU61722
New.CleanUp.ReferenceOTU49921
New.CleanUp.ReferenceOTU104033
New.CleanUp.ReferenceOTU139553
New.CleanUp.ReferenceOTU37013
New.CleanUp.ReferenceOTU40745
New.CleanUp.ReferenceOTU121532
New.CleanUp.ReferenceOTU50233
New.CleanUp.ReferenceOTU43145
New.CleanUp.ReferenceOTU63641
New.CleanUp.ReferenceOTU92467
New.CleanUp.ReferenceOTU134510
New.CleanUp.ReferenceOTU51490
New.CleanUp.ReferenceOTU240048
New.CleanUp.ReferenceOTU34530
New.CleanUp.ReferenceOTU47244
New.CleanUp.ReferenceOTU62037
New.CleanUp.ReferenceOTU43170
New.CleanUp.ReferenceOTU48254
New.CleanUp.ReferenceOTU50350
New.CleanUp.ReferenceOTU267143
New.CleanUp.ReferenceOTU246788
New.CleanUp.ReferenceOTU234051

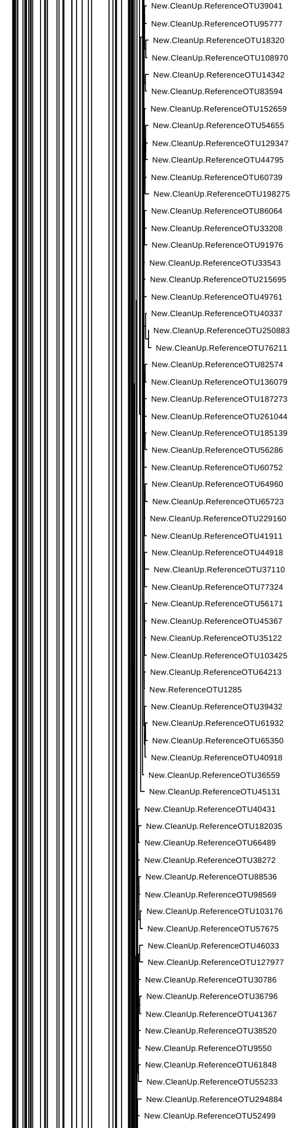
New.CleanUp.ReferenceOTU39041
New.CleanUp.ReferenceOTU95777
New.CleanUp.ReferenceOTU18320
New.CleanUp.ReferenceOTU108970
New.CleanUp.ReferenceOTU14342
New.CleanUp.ReferenceOTU83594
New.CleanUp.ReferenceOTU152659
New.CleanUp.ReferenceOTU54655
New.CleanUp.ReferenceOTU129347
New.CleanUp.ReferenceOTU44795
New.CleanUp.ReferenceOTU60739
New.CleanUp.ReferenceOTU198275
New.CleanUp.ReferenceOTU86064
New.CleanUp.ReferenceOTU33208
New.CleanUp.ReferenceOTU91976
New.CleanUp.ReferenceOTU33543
New.CleanUp.ReferenceOTU215695
New.CleanUp.ReferenceOTU49761
New.CleanUp.ReferenceOTU40337
New.CleanUp.ReferenceOTU250883
New.CleanUp.ReferenceOTU76211
New.CleanUp.ReferenceOTU82574
New.CleanUp.ReferenceOTU136079
New.CleanUp.ReferenceOTU187273
New.CleanUp.ReferenceOTU261044
New.CleanUp.ReferenceOTU185139
New.CleanUp.ReferenceOTU56286
New.CleanUp.ReferenceOTU60752
New.CleanUp.ReferenceOTU64960
New.CleanUp.ReferenceOTU65723
New.CleanUp.ReferenceOTU229160
New.CleanUp.ReferenceOTU41911
New.CleanUp.ReferenceOTU44918
New.CleanUp.ReferenceOTU37110
New.CleanUp.ReferenceOTU77324
New.CleanUp.ReferenceOTU56171
New.CleanUp.ReferenceOTU45367
New.CleanUp.ReferenceOTU35122
New.CleanUp.ReferenceOTU103425
New.CleanUp.ReferenceOTU64213
New.ReferenceOTU1285
New.CleanUp.ReferenceOTU39432
New.CleanUp.ReferenceOTU61932
New.CleanUp.ReferenceOTU65350
New.CleanUp.ReferenceOTU40918
New.CleanUp.ReferenceOTU36559
New.CleanUp.ReferenceOTU45131
New.CleanUp.ReferenceOTU40431
New.CleanUp.ReferenceOTU182035
New.CleanUp.ReferenceOTU66489
New.CleanUp.ReferenceOTU38272
New.CleanUp.ReferenceOTU88536
New.CleanUp.ReferenceOTU98569
New.CleanUp.ReferenceOTU103176
New.CleanUp.ReferenceOTU57675
New.CleanUp.ReferenceOTU46033
New.CleanUp.ReferenceOTU127977
New.CleanUp.ReferenceOTU30786
New.CleanUp.ReferenceOTU36796
New.CleanUp.ReferenceOTU41367
New.CleanUp.ReferenceOTU38520
New.CleanUp.ReferenceOTU9550
New.CleanUp.ReferenceOTU61848
New.CleanUp.ReferenceOTU55233
New.CleanUp.ReferenceOTU294884
New.CleanUp.ReferenceOTU52499

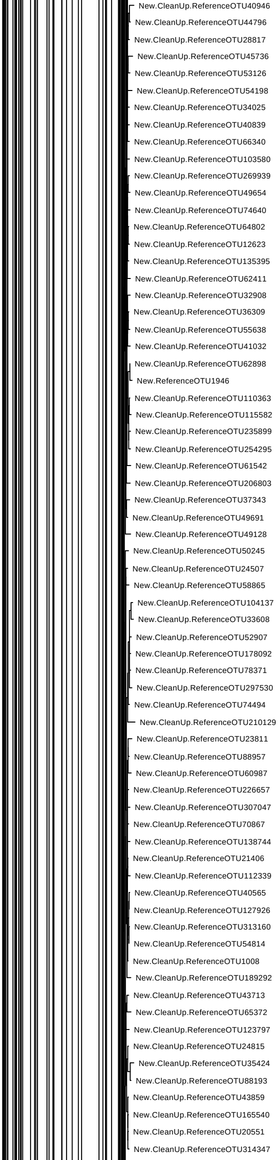
New.CleanUp.ReferenceOTU40946
New.CleanUp.ReferenceOTU44796
New.CleanUp.ReferenceOTU28817
New.CleanUp.ReferenceOTU45736
New.CleanUp.ReferenceOTU53126
New.CleanUp.ReferenceOTU54198
New.CleanUp.ReferenceOTU34025
New.CleanUp.ReferenceOTU40839
New.CleanUp.ReferenceOTU66340
New.CleanUp.ReferenceOTU103580
New.CleanUp.ReferenceOTU269939
New.CleanUp.ReferenceOTU49654
New.CleanUp.ReferenceOTU74640
New.CleanUp.ReferenceOTU64802
New.CleanUp.ReferenceOTU12623
New.CleanUp.ReferenceOTU135395
New.CleanUp.ReferenceOTU62411
New.CleanUp.ReferenceOTU32908
New.CleanUp.ReferenceOTU36309
New.CleanUp.ReferenceOTU55638
New.CleanUp.ReferenceOTU41032
New.CleanUp.ReferenceOTU62898
New.ReferenceOTU1946
New.CleanUp.ReferenceOTU110363
New.CleanUp.ReferenceOTU115582
New.CleanUp.ReferenceOTU235899
New.CleanUp.ReferenceOTU254295
New.CleanUp.ReferenceOTU61542
New.CleanUp.ReferenceOTU206803
New.CleanUp.ReferenceOTU37343
New.CleanUp.ReferenceOTU49691
New.CleanUp.ReferenceOTU49128
New.CleanUp.ReferenceOTU50245
New.CleanUp.ReferenceOTU24507
New.CleanUp.ReferenceOTU58865
New.CleanUp.ReferenceOTU104137
New.CleanUp.ReferenceOTU33608
New.CleanUp.ReferenceOTU52907
New.CleanUp.ReferenceOTU178092
New.CleanUp.ReferenceOTU78371
New.CleanUp.ReferenceOTU297530
New.CleanUp.ReferenceOTU74494
New.CleanUp.ReferenceOTU210129
New.CleanUp.ReferenceOTU23811
New.CleanUp.ReferenceOTU88957
New.CleanUp.ReferenceOTU60987
New.CleanUp.ReferenceOTU226657
New.CleanUp.ReferenceOTU307047
New.CleanUp.ReferenceOTU70867
New.CleanUp.ReferenceOTU138744
New.CleanUp.ReferenceOTU21406
New.CleanUp.ReferenceOTU112339
New.CleanUp.ReferenceOTU40565
New.CleanUp.ReferenceOTU127926
New.CleanUp.ReferenceOTU313160
New.CleanUp.ReferenceOTU54814
New.CleanUp.ReferenceOTU1008
New.CleanUp.ReferenceOTU189292
New.CleanUp.ReferenceOTU43713
New.CleanUp.ReferenceOTU65372
New.CleanUp.ReferenceOTU123797
New.CleanUp.ReferenceOTU24815
New.CleanUp.ReferenceOTU35424
New.CleanUp.ReferenceOTU88193
New.CleanUp.ReferenceOTU43859
New.CleanUp.ReferenceOTU165540
New.CleanUp.ReferenceOTU20551
New.CleanUp.ReferenceOTU314347

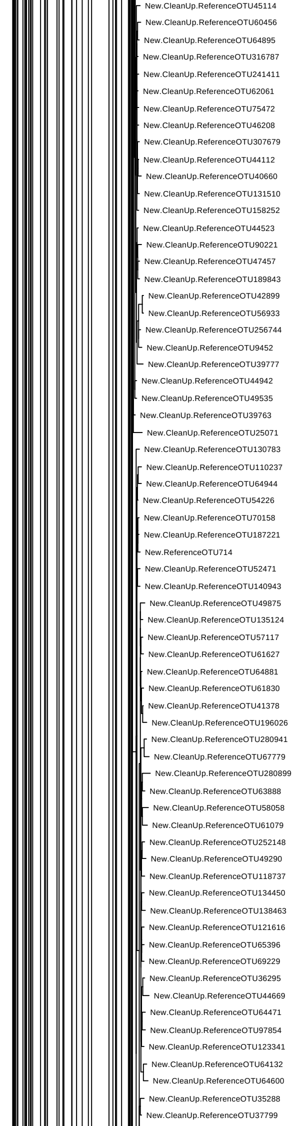
New.CleanUp.ReferenceOTU45114
New.CleanUp.ReferenceOTU60456
New.CleanUp.ReferenceOTU64895
New.CleanUp.ReferenceOTU316787
New.CleanUp.ReferenceOTU241411
New.CleanUp.ReferenceOTU62061
New.CleanUp.ReferenceOTU75472
New.CleanUp.ReferenceOTU46208
New.CleanUp.ReferenceOTU307679
New.CleanUp.ReferenceOTU44112
New.CleanUp.ReferenceOTU40660
New.CleanUp.ReferenceOTU131510
New.CleanUp.ReferenceOTU158252
New.CleanUp.ReferenceOTU44523
New.CleanUp.ReferenceOTU90221
New.CleanUp.ReferenceOTU47457
New.CleanUp.ReferenceOTU189843
New.CleanUp.ReferenceOTU42899
New.CleanUp.ReferenceOTU56933
New.CleanUp.ReferenceOTU256744
New.CleanUp.ReferenceOTU9452
New.CleanUp.ReferenceOTU39777
New.CleanUp.ReferenceOTU44942
New.CleanUp.ReferenceOTU49535
New.CleanUp.ReferenceOTU39763
New.CleanUp.ReferenceOTU25071
New.CleanUp.ReferenceOTU130783
New.CleanUp.ReferenceOTU110237
New.CleanUp.ReferenceOTU64944
New.CleanUp.ReferenceOTU54226
New.CleanUp.ReferenceOTU70158
New.CleanUp.ReferenceOTU187221
New.ReferenceOTU714
New.CleanUp.ReferenceOTU52471
New.CleanUp.ReferenceOTU140943
New.CleanUp.ReferenceOTU49875
New.CleanUp.ReferenceOTU135124
New.CleanUp.ReferenceOTU57117
New.CleanUp.ReferenceOTU61627
New.CleanUp.ReferenceOTU64881
New.CleanUp.ReferenceOTU61830
New.CleanUp.ReferenceOTU41378
New.CleanUp.ReferenceOTU196026
New.CleanUp.ReferenceOTU280941
New.CleanUp.ReferenceOTU67779
New.CleanUp.ReferenceOTU280899
New.CleanUp.ReferenceOTU63888
New.CleanUp.ReferenceOTU58058
New.CleanUp.ReferenceOTU61079
New.CleanUp.ReferenceOTU252148
New.CleanUp.ReferenceOTU49290
New.CleanUp.ReferenceOTU118737
New.CleanUp.ReferenceOTU134450
New.CleanUp.ReferenceOTU138463
New.CleanUp.ReferenceOTU121616
New.CleanUp.ReferenceOTU65396
New.CleanUp.ReferenceOTU69229
New.CleanUp.ReferenceOTU36295
New.CleanUp.ReferenceOTU44669
New.CleanUp.ReferenceOTU64471
New.CleanUp.ReferenceOTU97854
New.CleanUp.ReferenceOTU123341
New.CleanUp.ReferenceOTU64132
New.CleanUp.ReferenceOTU64600
New.CleanUp.ReferenceOTU35288
New.CleanUp.ReferenceOTU37799

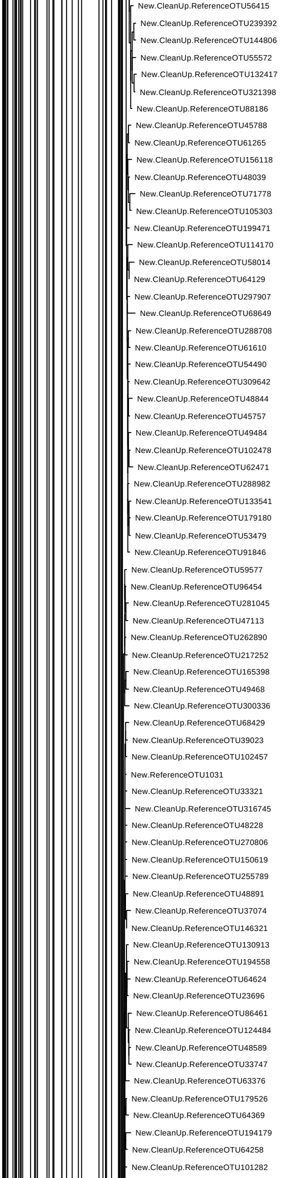
New.CleanUp.ReferenceOTU56415
New.CleanUp.ReferenceOTU239392
New.CleanUp.ReferenceOTU144806
New.CleanUp.ReferenceOTU55572
New.CleanUp.ReferenceOTU132417
New.CleanUp.ReferenceOTU321398
New.CleanUp.ReferenceOTU88186
New.CleanUp.ReferenceOTU45788
New.CleanUp.ReferenceOTU61265
New.CleanUp.ReferenceOTU156118
New.CleanUp.ReferenceOTU48039
New.CleanUp.ReferenceOTU71778
New.CleanUp.ReferenceOTU105303
New.CleanUp.ReferenceOTU199471
New.CleanUp.ReferenceOTU114170
New.CleanUp.ReferenceOTU58014
New.CleanUp.ReferenceOTU64129
New.CleanUp.ReferenceOTU297907
New.CleanUp.ReferenceOTU68649
New.CleanUp.ReferenceOTU288708
New.CleanUp.ReferenceOTU61610
New.CleanUp.ReferenceOTU54490
New.CleanUp.ReferenceOTU309642
New.CleanUp.ReferenceOTU48844
New.CleanUp.ReferenceOTU45757
New.CleanUp.ReferenceOTU49484
New.CleanUp.ReferenceOTU102478
New.CleanUp.ReferenceOTU62471
New.CleanUp.ReferenceOTU288982
New.CleanUp.ReferenceOTU133541
New.CleanUp.ReferenceOTU179180
New.CleanUp.ReferenceOTU53479
New.CleanUp.ReferenceOTU91846
New.CleanUp.ReferenceOTU59577
New.CleanUp.ReferenceOTU96454
New.CleanUp.ReferenceOTU281045
New.CleanUp.ReferenceOTU47113
New.CleanUp.ReferenceOTU262890
New.CleanUp.ReferenceOTU217252
New.CleanUp.ReferenceOTU165398
New.CleanUp.ReferenceOTU49468
New.CleanUp.ReferenceOTU300336
New.CleanUp.ReferenceOTU68429
New.CleanUp.ReferenceOTU39023
New.CleanUp.ReferenceOTU102457
New.ReferenceOTU1031
New.CleanUp.ReferenceOTU33321
New.CleanUp.ReferenceOTU316745
New.CleanUp.ReferenceOTU48228
New.CleanUp.ReferenceOTU270806
New.CleanUp.ReferenceOTU150619
New.CleanUp.ReferenceOTU255789
New.CleanUp.ReferenceOTU48891
New.CleanUp.ReferenceOTU37074
New.CleanUp.ReferenceOTU146321
New.CleanUp.ReferenceOTU130913
New.CleanUp.ReferenceOTU194558
New.CleanUp.ReferenceOTU64624
New.CleanUp.ReferenceOTU23696
New.CleanUp.ReferenceOTU86461
New.CleanUp.ReferenceOTU124484
New.CleanUp.ReferenceOTU48589
New.CleanUp.ReferenceOTU33747
New.CleanUp.ReferenceOTU63376
New.CleanUp.ReferenceOTU179526
New.CleanUp.ReferenceOTU64369
New.CleanUp.ReferenceOTU194179
New.CleanUp.ReferenceOTU64258
New.CleanUp.ReferenceOTU101282

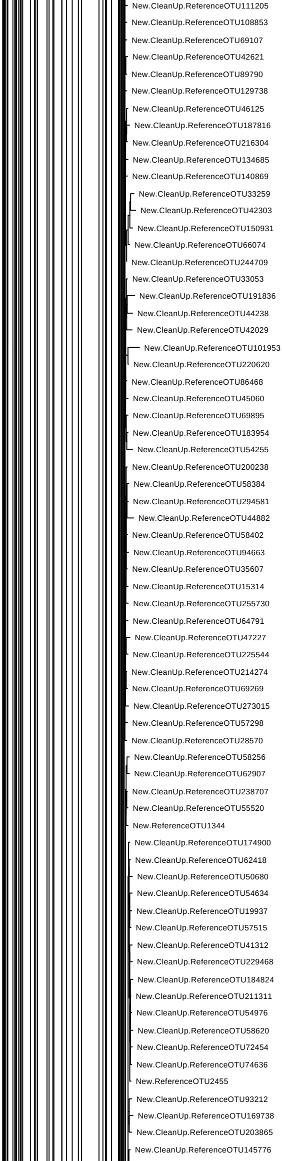
New.CleanUp.ReferenceOTU111205
New.CleanUp.ReferenceOTU108853
New.CleanUp.ReferenceOTU69107
New.CleanUp.ReferenceOTU42621
New.CleanUp.ReferenceOTU89790
New.CleanUp.ReferenceOTU129738
New.CleanUp.ReferenceOTU46125
New.CleanUp.ReferenceOTU187816
New.CleanUp.ReferenceOTU216304
New.CleanUp.ReferenceOTU134685
New.CleanUp.ReferenceOTU140869
New.CleanUp.ReferenceOTU33259
New.CleanUp.ReferenceOTU42303
New.CleanUp.ReferenceOTU150931
New.CleanUp.ReferenceOTU66074
New.CleanUp.ReferenceOTU244709
New.CleanUp.ReferenceOTU33053
New.CleanUp.ReferenceOTU191836
New.CleanUp.ReferenceOTU44238
New.CleanUp.ReferenceOTU42029
New.CleanUp.ReferenceOTU101953
New.CleanUp.ReferenceOTU220620
New.CleanUp.ReferenceOTU86468
New.CleanUp.ReferenceOTU45060
New.CleanUp.ReferenceOTU69895
New.CleanUp.ReferenceOTU183954
New.CleanUp.ReferenceOTU54255
New.CleanUp.ReferenceOTU200238
New.CleanUp.ReferenceOTU58384
New.CleanUp.ReferenceOTU294581
New.CleanUp.ReferenceOTU44882
New.CleanUp.ReferenceOTU58402
New.CleanUp.ReferenceOTU94663
New.CleanUp.ReferenceOTU35607
New.CleanUp.ReferenceOTU15314
New.CleanUp.ReferenceOTU255730
New.CleanUp.ReferenceOTU64791
New.CleanUp.ReferenceOTU47227
New.CleanUp.ReferenceOTU225544
New.CleanUp.ReferenceOTU214274
New.CleanUp.ReferenceOTU69269
New.CleanUp.ReferenceOTU273015
New.CleanUp.ReferenceOTU57298
New.CleanUp.ReferenceOTU28570
New.CleanUp.ReferenceOTU58256
New.CleanUp.ReferenceOTU62907
New.CleanUp.ReferenceOTU238707
New.CleanUp.ReferenceOTU55520
New.ReferenceOTU1344
New.CleanUp.ReferenceOTU174900
New.CleanUp.ReferenceOTU62418
New.CleanUp.ReferenceOTU50680
New.CleanUp.ReferenceOTU54634
New.CleanUp.ReferenceOTU19937
New.CleanUp.ReferenceOTU57515
New.CleanUp.ReferenceOTU41312
New.CleanUp.ReferenceOTU229468
New.CleanUp.ReferenceOTU184824
New.CleanUp.ReferenceOTU211311
New.CleanUp.ReferenceOTU54976
New.CleanUp.ReferenceOTU58620
New.CleanUp.ReferenceOTU72454
New.CleanUp.ReferenceOTU74636
New.ReferenceOTU2455
New.CleanUp.ReferenceOTU93212
New.CleanUp.ReferenceOTU169738
New.CleanUp.ReferenceOTU203865
New.CleanUp.ReferenceOTU145776

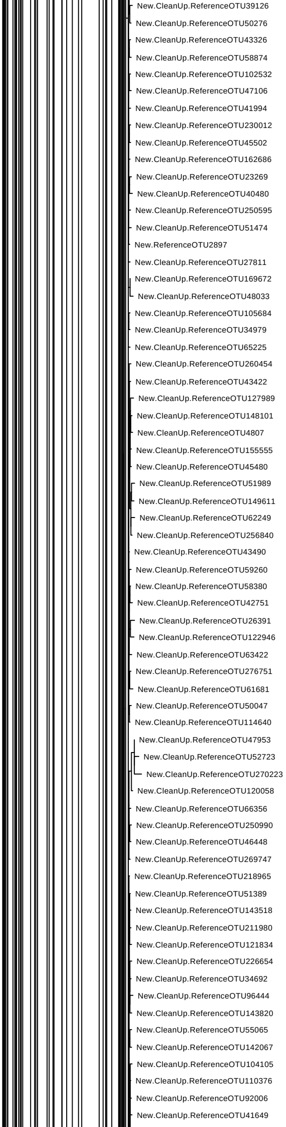
New.CleanUp.ReferenceOTU39126
New.CleanUp.ReferenceOTU50276
New.CleanUp.ReferenceOTU43326
New.CleanUp.ReferenceOTU58874
New.CleanUp.ReferenceOTU102532
New.CleanUp.ReferenceOTU47106
New.CleanUp.ReferenceOTU41994
New.CleanUp.ReferenceOTU230012
New.CleanUp.ReferenceOTU45502
New.CleanUp.ReferenceOTU162686
New.CleanUp.ReferenceOTU23269
New.CleanUp.ReferenceOTU40480
New.CleanUp.ReferenceOTU250595
New.CleanUp.ReferenceOTU51474
New.ReferenceOTU2897
New.CleanUp.ReferenceOTU27811
New.CleanUp.ReferenceOTU169672
New.CleanUp.ReferenceOTU48033
New.CleanUp.ReferenceOTU105684
New.CleanUp.ReferenceOTU34979
New.CleanUp.ReferenceOTU65225
New.CleanUp.ReferenceOTU260454
New.CleanUp.ReferenceOTU43422
New.CleanUp.ReferenceOTU127989
New.CleanUp.ReferenceOTU148101
New.CleanUp.ReferenceOTU4807
New.CleanUp.ReferenceOTU155555
New.CleanUp.ReferenceOTU45480
New.CleanUp.ReferenceOTU51989
New.CleanUp.ReferenceOTU149611
New.CleanUp.ReferenceOTU62249
New.CleanUp.ReferenceOTU256840
New.CleanUp.ReferenceOTU43490
New.CleanUp.ReferenceOTU59260
New.CleanUp.ReferenceOTU58380
New.CleanUp.ReferenceOTU42751
New.CleanUp.ReferenceOTU26391
New.CleanUp.ReferenceOTU122946
New.CleanUp.ReferenceOTU63422
New.CleanUp.ReferenceOTU276751
New.CleanUp.ReferenceOTU61681
New.CleanUp.ReferenceOTU50047
New.CleanUp.ReferenceOTU114640
New.CleanUp.ReferenceOTU47953
New.CleanUp.ReferenceOTU52723
New.CleanUp.ReferenceOTU270223
New.CleanUp.ReferenceOTU120058
New.CleanUp.ReferenceOTU66356
New.CleanUp.ReferenceOTU250990
New.CleanUp.ReferenceOTU46448
New.CleanUp.ReferenceOTU269747
New.CleanUp.ReferenceOTU218965
New.CleanUp.ReferenceOTU51389
New.CleanUp.ReferenceOTU143518
New.CleanUp.ReferenceOTU211980
New.CleanUp.ReferenceOTU121834
New.CleanUp.ReferenceOTU226654
New.CleanUp.ReferenceOTU34692
New.CleanUp.ReferenceOTU96444
New.CleanUp.ReferenceOTU143820
New.CleanUp.ReferenceOTU55065
New.CleanUp.ReferenceOTU142067
New.CleanUp.ReferenceOTU104105
New.CleanUp.ReferenceOTU110376
New.CleanUp.ReferenceOTU92006
New.CleanUp.ReferenceOTU41649

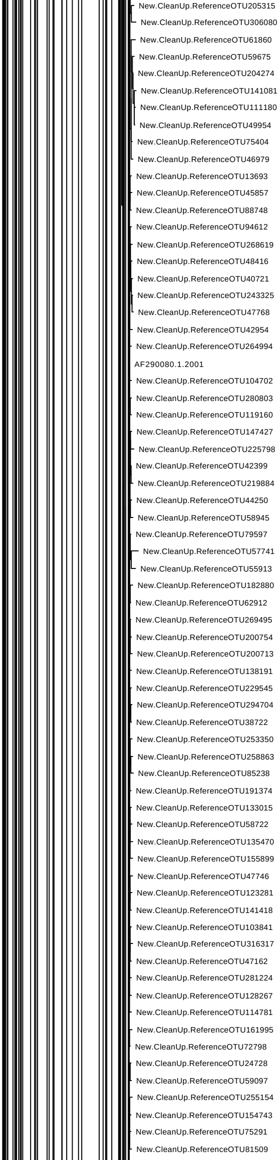
New.CleanUp.ReferenceOTU205315
New.CleanUp.ReferenceOTU306080
New.CleanUp.ReferenceOTU61860
New.CleanUp.ReferenceOTU59675
New.CleanUp.ReferenceOTU204274
New.CleanUp.ReferenceOTU141081
New.CleanUp.ReferenceOTU111180
New.CleanUp.ReferenceOTU49954
New.CleanUp.ReferenceOTU75404
New.CleanUp.ReferenceOTU46979
New.CleanUp.ReferenceOTU13693
New.CleanUp.ReferenceOTU45857
New.CleanUp.ReferenceOTU88748
New.CleanUp.ReferenceOTU94612
New.CleanUp.ReferenceOTU268619
New.CleanUp.ReferenceOTU48416
New.CleanUp.ReferenceOTU40721
New.CleanUp.ReferenceOTU243325
New.CleanUp.ReferenceOTU47768
New.CleanUp.ReferenceOTU42954
New.CleanUp.ReferenceOTU264994
AF290080.1.2001
New.CleanUp.ReferenceOTU104702
New.CleanUp.ReferenceOTU280803
New.CleanUp.ReferenceOTU119160
New.CleanUp.ReferenceOTU147427
New.CleanUp.ReferenceOTU225798
New.CleanUp.ReferenceOTU42399
New.CleanUp.ReferenceOTU219884
New.CleanUp.ReferenceOTU44250
New.CleanUp.ReferenceOTU58945
New.CleanUp.ReferenceOTU79597
New.CleanUp.ReferenceOTU57741
New.CleanUp.ReferenceOTU55913
New.CleanUp.ReferenceOTU182880
New.CleanUp.ReferenceOTU62912
New.CleanUp.ReferenceOTU269495
New.CleanUp.ReferenceOTU200754
New.CleanUp.ReferenceOTU200713
New.CleanUp.ReferenceOTU138191
New.CleanUp.ReferenceOTU229545
New.CleanUp.ReferenceOTU294704
New.CleanUp.ReferenceOTU38722
New.CleanUp.ReferenceOTU253350
New.CleanUp.ReferenceOTU258863
New.CleanUp.ReferenceOTU85238
New.CleanUp.ReferenceOTU191374
New.CleanUp.ReferenceOTU133015
New.CleanUp.ReferenceOTU58722
New.CleanUp.ReferenceOTU135470
New.CleanUp.ReferenceOTU155899
New.CleanUp.ReferenceOTU47746
New.CleanUp.ReferenceOTU123281
New.CleanUp.ReferenceOTU141418
New.CleanUp.ReferenceOTU103841
New.CleanUp.ReferenceOTU316317
New.CleanUp.ReferenceOTU47162
New.CleanUp.ReferenceOTU281224
New.CleanUp.ReferenceOTU128267
New.CleanUp.ReferenceOTU114781
New.CleanUp.ReferenceOTU161995
New.CleanUp.ReferenceOTU72798
New.CleanUp.ReferenceOTU24728
New.CleanUp.ReferenceOTU59097
New.CleanUp.ReferenceOTU255154
New.CleanUp.ReferenceOTU154743
New.CleanUp.ReferenceOTU75291
New.CleanUp.ReferenceOTU81509

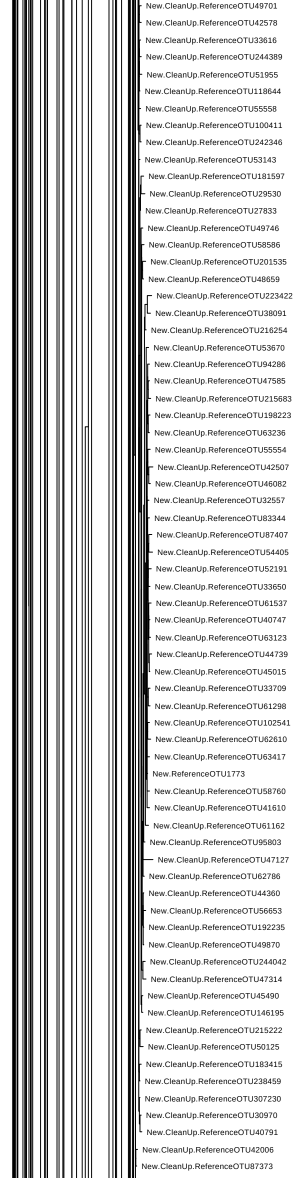
New.CleanUp.ReferenceOTU49701
New.CleanUp.ReferenceOTU42578
New.CleanUp.ReferenceOTU33616
New.CleanUp.ReferenceOTU244389
New.CleanUp.ReferenceOTU51955
New.CleanUp.ReferenceOTU118644
New.CleanUp.ReferenceOTU55558
New.CleanUp.ReferenceOTU100411
New.CleanUp.ReferenceOTU242346
New.CleanUp.ReferenceOTU53143
New.CleanUp.ReferenceOTU181597
New.CleanUp.ReferenceOTU29530
New.CleanUp.ReferenceOTU27833
New.CleanUp.ReferenceOTU49746
New.CleanUp.ReferenceOTU58586
New.CleanUp.ReferenceOTU201535
New.CleanUp.ReferenceOTU48659
New.CleanUp.ReferenceOTU223422
New.CleanUp.ReferenceOTU38091
New.CleanUp.ReferenceOTU216254
New.CleanUp.ReferenceOTU53670
New.CleanUp.ReferenceOTU94286
New.CleanUp.ReferenceOTU47585
New.CleanUp.ReferenceOTU215683
New.CleanUp.ReferenceOTU198223
New.CleanUp.ReferenceOTU63236
New.CleanUp.ReferenceOTU55554
New.CleanUp.ReferenceOTU42507
New.CleanUp.ReferenceOTU46082
New.CleanUp.ReferenceOTU32557
New.CleanUp.ReferenceOTU83344
New.CleanUp.ReferenceOTU87407
New.CleanUp.ReferenceOTU54405
New.CleanUp.ReferenceOTU52191
New.CleanUp.ReferenceOTU33650
New.CleanUp.ReferenceOTU61537
New.CleanUp.ReferenceOTU40747
New.CleanUp.ReferenceOTU63123
New.CleanUp.ReferenceOTU44739
New.CleanUp.ReferenceOTU45015
New.CleanUp.ReferenceOTU33709
New.CleanUp.ReferenceOTU61298
New.CleanUp.ReferenceOTU102541
New.CleanUp.ReferenceOTU62610
New.CleanUp.ReferenceOTU63417
New.ReferenceOTU1773
New.CleanUp.ReferenceOTU58760
New.CleanUp.ReferenceOTU41610
New.CleanUp.ReferenceOTU61162
New.CleanUp.ReferenceOTU95803
New.CleanUp.ReferenceOTU47127
New.CleanUp.ReferenceOTU62786
New.CleanUp.ReferenceOTU44360
New.CleanUp.ReferenceOTU56653
New.CleanUp.ReferenceOTU192235
New.CleanUp.ReferenceOTU49870
New.CleanUp.ReferenceOTU244042
New.CleanUp.ReferenceOTU47314
New.CleanUp.ReferenceOTU45490
New.CleanUp.ReferenceOTU146195
New.CleanUp.ReferenceOTU215222
New.CleanUp.ReferenceOTU50125
New.CleanUp.ReferenceOTU183415
New.CleanUp.ReferenceOTU238459
New.CleanUp.ReferenceOTU307230
New.CleanUp.ReferenceOTU30970
New.CleanUp.ReferenceOTU40791
New.CleanUp.ReferenceOTU42006
New.CleanUp.ReferenceOTU87373

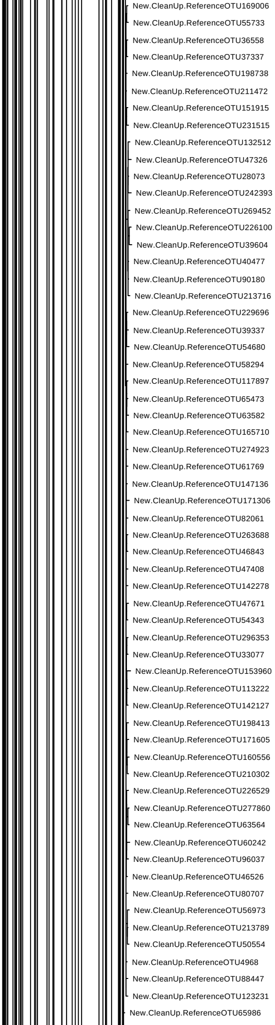
New.CleanUp.ReferenceOTU169006
New.CleanUp.ReferenceOTU55733
New.CleanUp.ReferenceOTU36558
New.CleanUp.ReferenceOTU37337
New.CleanUp.ReferenceOTU198738
New.CleanUp.ReferenceOTU211472
New.CleanUp.ReferenceOTU151915
New.CleanUp.ReferenceOTU231515
New.CleanUp.ReferenceOTU132512
New.CleanUp.ReferenceOTU47326
New.CleanUp.ReferenceOTU28073
New.CleanUp.ReferenceOTU242393
New.CleanUp.ReferenceOTU269452
New.CleanUp.ReferenceOTU226100
New.CleanUp.ReferenceOTU39604
New.CleanUp.ReferenceOTU40477
New.CleanUp.ReferenceOTU90180
New.CleanUp.ReferenceOTU213716
New.CleanUp.ReferenceOTU229696
New.CleanUp.ReferenceOTU39337
New.CleanUp.ReferenceOTU54680
New.CleanUp.ReferenceOTU58294
New.CleanUp.ReferenceOTU117897
New.CleanUp.ReferenceOTU65473
New.CleanUp.ReferenceOTU63582
New.CleanUp.ReferenceOTU165710
New.CleanUp.ReferenceOTU274923
New.CleanUp.ReferenceOTU61769
New.CleanUp.ReferenceOTU147136
New.CleanUp.ReferenceOTU171306
New.CleanUp.ReferenceOTU82061
New.CleanUp.ReferenceOTU263688
New.CleanUp.ReferenceOTU46843
New.CleanUp.ReferenceOTU47408
New.CleanUp.ReferenceOTU142278
New.CleanUp.ReferenceOTU47671
New.CleanUp.ReferenceOTU54343
New.CleanUp.ReferenceOTU296353
New.CleanUp.ReferenceOTU33077
New.CleanUp.ReferenceOTU153960
New.CleanUp.ReferenceOTU113222
New.CleanUp.ReferenceOTU142127
New.CleanUp.ReferenceOTU198413
New.CleanUp.ReferenceOTU171605
New.CleanUp.ReferenceOTU160556
New.CleanUp.ReferenceOTU210302
New.CleanUp.ReferenceOTU226529
New.CleanUp.ReferenceOTU277860
New.CleanUp.ReferenceOTU63564
New.CleanUp.ReferenceOTU60242
New.CleanUp.ReferenceOTU96037
New.CleanUp.ReferenceOTU46526
New.CleanUp.ReferenceOTU80707
New.CleanUp.ReferenceOTU56973
New.CleanUp.ReferenceOTU213789
New.CleanUp.ReferenceOTU50554
New.CleanUp.ReferenceOTU4968
New.CleanUp.ReferenceOTU88447
New.CleanUp.ReferenceOTU123231
New.CleanUp.ReferenceOTU65986

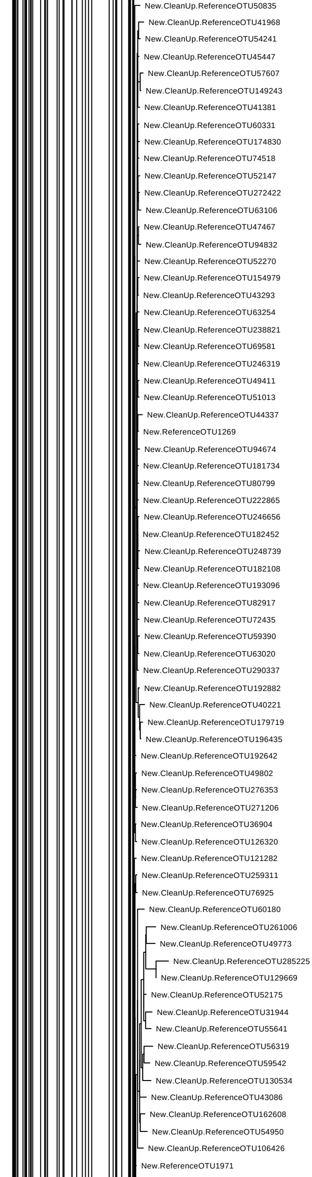
New.CleanUp.ReferenceOTU50835
New.CleanUp.ReferenceOTU41968
New.CleanUp.ReferenceOTU54241
New.CleanUp.ReferenceOTU45447
New.CleanUp.ReferenceOTU57607
New.CleanUp.ReferenceOTU149243
New.CleanUp.ReferenceOTU41381
New.CleanUp.ReferenceOTU60331
New.CleanUp.ReferenceOTU174830
New.CleanUp.ReferenceOTU74518
New.CleanUp.ReferenceOTU52147
New.CleanUp.ReferenceOTU272422
New.CleanUp.ReferenceOTU63106
New.CleanUp.ReferenceOTU47467
New.CleanUp.ReferenceOTU94832
New.CleanUp.ReferenceOTU52270
New.CleanUp.ReferenceOTU154979
New.CleanUp.ReferenceOTU43293
New.CleanUp.ReferenceOTU63254
New.CleanUp.ReferenceOTU238821
New.CleanUp.ReferenceOTU69581
New.CleanUp.ReferenceOTU246319
New.CleanUp.ReferenceOTU49411
New.CleanUp.ReferenceOTU51013
New.CleanUp.ReferenceOTU44337
New.ReferenceOTU1269
New.CleanUp.ReferenceOTU94674
New.CleanUp.ReferenceOTU181734
New.CleanUp.ReferenceOTU80799
New.CleanUp.ReferenceOTU222865
New.CleanUp.ReferenceOTU246656
New.CleanUp.ReferenceOTU182452
New.CleanUp.ReferenceOTU248739
New.CleanUp.ReferenceOTU182108
New.CleanUp.ReferenceOTU193096
New.CleanUp.ReferenceOTU82917
New.CleanUp.ReferenceOTU72435
New.CleanUp.ReferenceOTU59390
New.CleanUp.ReferenceOTU63020
New.CleanUp.ReferenceOTU290337
New.CleanUp.ReferenceOTU192882
New.CleanUp.ReferenceOTU40221
New.CleanUp.ReferenceOTU179719
New.CleanUp.ReferenceOTU196435
New.CleanUp.ReferenceOTU192642
New.CleanUp.ReferenceOTU49802
New.CleanUp.ReferenceOTU276353
New.CleanUp.ReferenceOTU271206
New.CleanUp.ReferenceOTU36904
New.CleanUp.ReferenceOTU126320
New.CleanUp.ReferenceOTU121282
New.CleanUp.ReferenceOTU259311
New.CleanUp.ReferenceOTU76925
New.CleanUp.ReferenceOTU60180
New.CleanUp.ReferenceOTU261006
New.CleanUp.ReferenceOTU49773
New.CleanUp.ReferenceOTU285225
New.CleanUp.ReferenceOTU129669
New.CleanUp.ReferenceOTU52175
New.CleanUp.ReferenceOTU31944
New.CleanUp.ReferenceOTU55641
New.CleanUp.ReferenceOTU56319
New.CleanUp.ReferenceOTU59542
New.CleanUp.ReferenceOTU130534
New.CleanUp.ReferenceOTU43086
New.CleanUp.ReferenceOTU162608
New.CleanUp.ReferenceOTU54950
New.CleanUp.ReferenceOTU106426
New.ReferenceOTU1971

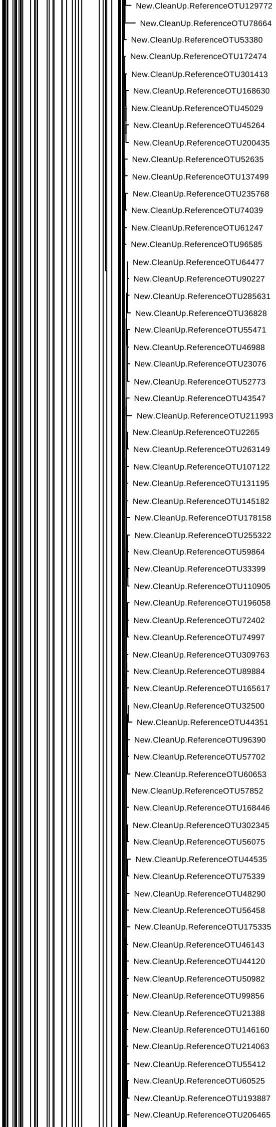
New.CleanUp.ReferenceOTU129772
New.CleanUp.ReferenceOTU78664
New.CleanUp.ReferenceOTU53380
New.CleanUp.ReferenceOTU172474
New.CleanUp.ReferenceOTU301413
New.CleanUp.ReferenceOTU168630
New.CleanUp.ReferenceOTU45029
New.CleanUp.ReferenceOTU45264
New.CleanUp.ReferenceOTU200435
New.CleanUp.ReferenceOTU52635
New.CleanUp.ReferenceOTU137499
New.CleanUp.ReferenceOTU235768
New.CleanUp.ReferenceOTU74039
New.CleanUp.ReferenceOTU61247
New.CleanUp.ReferenceOTU96585
New.CleanUp.ReferenceOTU64477
New.CleanUp.ReferenceOTU90227
New.CleanUp.ReferenceOTU285631
New.CleanUp.ReferenceOTU36828
New.CleanUp.ReferenceOTU55471
New.CleanUp.ReferenceOTU46988
New.CleanUp.ReferenceOTU23076
New.CleanUp.ReferenceOTU52773
New.CleanUp.ReferenceOTU43547
New.CleanUp.ReferenceOTU211993
New.CleanUp.ReferenceOTU2265
New.CleanUp.ReferenceOTU263149
New.CleanUp.ReferenceOTU107122
New.CleanUp.ReferenceOTU131195
New.CleanUp.ReferenceOTU145182
New.CleanUp.ReferenceOTU178158
New.CleanUp.ReferenceOTU255322
New.CleanUp.ReferenceOTU59864
New.CleanUp.ReferenceOTU33399
New.CleanUp.ReferenceOTU110905
New.CleanUp.ReferenceOTU196058
New.CleanUp.ReferenceOTU72402
New.CleanUp.ReferenceOTU74997
New.CleanUp.ReferenceOTU309763
New.CleanUp.ReferenceOTU89884
New.CleanUp.ReferenceOTU165617
New.CleanUp.ReferenceOTU32500
New.CleanUp.ReferenceOTU44351
New.CleanUp.ReferenceOTU96390
New.CleanUp.ReferenceOTU57702
New.CleanUp.ReferenceOTU60653
New.CleanUp.ReferenceOTU57852
New.CleanUp.ReferenceOTU168446
New.CleanUp.ReferenceOTU302345
New.CleanUp.ReferenceOTU56075
New.CleanUp.ReferenceOTU44535
New.CleanUp.ReferenceOTU75339
New.CleanUp.ReferenceOTU48290
New.CleanUp.ReferenceOTU56458
New.CleanUp.ReferenceOTU175335
New.CleanUp.ReferenceOTU46143
New.CleanUp.ReferenceOTU44120
New.CleanUp.ReferenceOTU50982
New.CleanUp.ReferenceOTU99856
New.CleanUp.ReferenceOTU21388
New.CleanUp.ReferenceOTU146160
New.CleanUp.ReferenceOTU214063
New.CleanUp.ReferenceOTU55412
New.CleanUp.ReferenceOTU60525
New.CleanUp.ReferenceOTU193887
New.CleanUp.ReferenceOTU206465

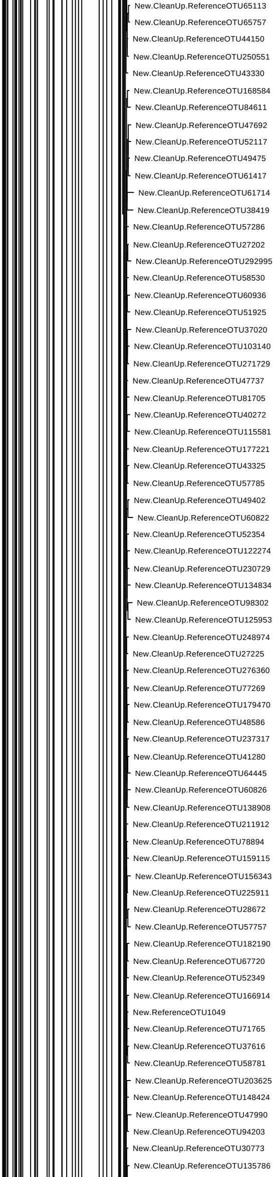
New.CleanUp.ReferenceOTU65113
New.CleanUp.ReferenceOTU65757
New.CleanUp.ReferenceOTU44150
New.CleanUp.ReferenceOTU250551
New.CleanUp.ReferenceOTU43330
New.CleanUp.ReferenceOTU168584
New.CleanUp.ReferenceOTU84611
New.CleanUp.ReferenceOTU47692
New.CleanUp.ReferenceOTU52117
New.CleanUp.ReferenceOTU49475
New.CleanUp.ReferenceOTU61417
New.CleanUp.ReferenceOTU61714
New.CleanUp.ReferenceOTU38419
New.CleanUp.ReferenceOTU57286
New.CleanUp.ReferenceOTU27202
New.CleanUp.ReferenceOTU292995
New.CleanUp.ReferenceOTU58530
New.CleanUp.ReferenceOTU60936
New.CleanUp.ReferenceOTU51925
New.CleanUp.ReferenceOTU37020
New.CleanUp.ReferenceOTU103140
New.CleanUp.ReferenceOTU271729
New.CleanUp.ReferenceOTU47737
New.CleanUp.ReferenceOTU81705
New.CleanUp.ReferenceOTU40272
New.CleanUp.ReferenceOTU115581
New.CleanUp.ReferenceOTU177221
New.CleanUp.ReferenceOTU43325
New.CleanUp.ReferenceOTU57785
New.CleanUp.ReferenceOTU49402
New.CleanUp.ReferenceOTU60822
New.CleanUp.ReferenceOTU52354
New.CleanUp.ReferenceOTU122274
New.CleanUp.ReferenceOTU230729
New.CleanUp.ReferenceOTU134834
New.CleanUp.ReferenceOTU98302
New.CleanUp.ReferenceOTU125953
New.CleanUp.ReferenceOTU248974
New.CleanUp.ReferenceOTU27225
New.CleanUp.ReferenceOTU276360
New.CleanUp.ReferenceOTU77269
New.CleanUp.ReferenceOTU179470
New.CleanUp.ReferenceOTU48586
New.CleanUp.ReferenceOTU237317
New.CleanUp.ReferenceOTU41280
New.CleanUp.ReferenceOTU64445
New.CleanUp.ReferenceOTU60826
New.CleanUp.ReferenceOTU138908
New.CleanUp.ReferenceOTU211912
New.CleanUp.ReferenceOTU78894
New.CleanUp.ReferenceOTU159115
New.CleanUp.ReferenceOTU156343
New.CleanUp.ReferenceOTU225911
New.CleanUp.ReferenceOTU28672
New.CleanUp.ReferenceOTU57757
New.CleanUp.ReferenceOTU182190
New.CleanUp.ReferenceOTU67720
New.CleanUp.ReferenceOTU52349
New.CleanUp.ReferenceOTU166914
New.ReferenceOTU1049
New.CleanUp.ReferenceOTU71765
New.CleanUp.ReferenceOTU37616
New.CleanUp.ReferenceOTU58781
New.CleanUp.ReferenceOTU203625
New.CleanUp.ReferenceOTU148424
New.CleanUp.ReferenceOTU47990
New.CleanUp.ReferenceOTU94203
New.CleanUp.ReferenceOTU30773
New.CleanUp.ReferenceOTU135786

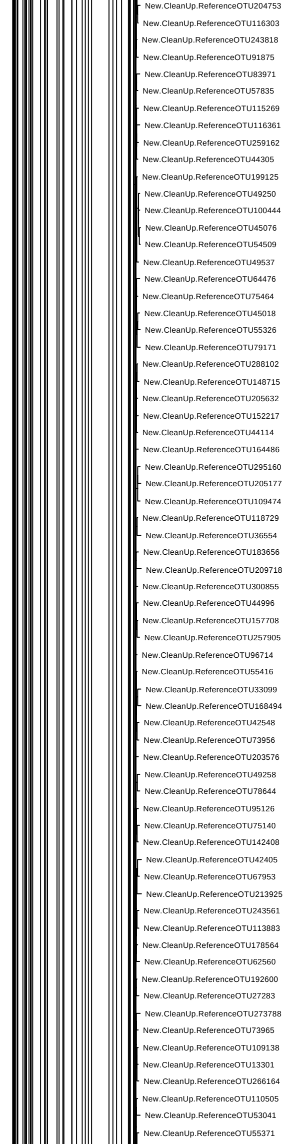
New.CleanUp.ReferenceOTU204753
New.CleanUp.ReferenceOTU116303
New.CleanUp.ReferenceOTU243818
New.CleanUp.ReferenceOTU91875
New.CleanUp.ReferenceOTU83971
New.CleanUp.ReferenceOTU57835
New.CleanUp.ReferenceOTU115269
New.CleanUp.ReferenceOTU116361
New.CleanUp.ReferenceOTU259162
New.CleanUp.ReferenceOTU44305
New.CleanUp.ReferenceOTU199125
New.CleanUp.ReferenceOTU49250
New.CleanUp.ReferenceOTU100444
New.CleanUp.ReferenceOTU45076
New.CleanUp.ReferenceOTU54509
New.CleanUp.ReferenceOTU49537
New.CleanUp.ReferenceOTU64476
New.CleanUp.ReferenceOTU75464
New.CleanUp.ReferenceOTU45018
New.CleanUp.ReferenceOTU55326
New.CleanUp.ReferenceOTU79171
New.CleanUp.ReferenceOTU288102
New.CleanUp.ReferenceOTU148715
New.CleanUp.ReferenceOTU205632
New.CleanUp.ReferenceOTU152217
New.CleanUp.ReferenceOTU44114
New.CleanUp.ReferenceOTU164486
New.CleanUp.ReferenceOTU295160
New.CleanUp.ReferenceOTU205177
New.CleanUp.ReferenceOTU109474
New.CleanUp.ReferenceOTU118729
New.CleanUp.ReferenceOTU36554
New.CleanUp.ReferenceOTU183656
New.CleanUp.ReferenceOTU209718
New.CleanUp.ReferenceOTU300855
New.CleanUp.ReferenceOTU44996
New.CleanUp.ReferenceOTU157708
New.CleanUp.ReferenceOTU257905
New.CleanUp.ReferenceOTU96714
New.CleanUp.ReferenceOTU55416
New.CleanUp.ReferenceOTU33099
New.CleanUp.ReferenceOTU168494
New.CleanUp.ReferenceOTU42548
New.CleanUp.ReferenceOTU73956
New.CleanUp.ReferenceOTU203576
New.CleanUp.ReferenceOTU49258
New.CleanUp.ReferenceOTU78644
New.CleanUp.ReferenceOTU95126
New.CleanUp.ReferenceOTU75140
New.CleanUp.ReferenceOTU142408
New.CleanUp.ReferenceOTU42405
New.CleanUp.ReferenceOTU67953
New.CleanUp.ReferenceOTU213925
New.CleanUp.ReferenceOTU243561
New.CleanUp.ReferenceOTU113883
New.CleanUp.ReferenceOTU178564
New.CleanUp.ReferenceOTU62560
New.CleanUp.ReferenceOTU192600
New.CleanUp.ReferenceOTU27283
New.CleanUp.ReferenceOTU273788
New.CleanUp.ReferenceOTU73965
New.CleanUp.ReferenceOTU109138
New.CleanUp.ReferenceOTU13301
New.CleanUp.ReferenceOTU266164
New.CleanUp.ReferenceOTU110505
New.CleanUp.ReferenceOTU53041
New.CleanUp.ReferenceOTU55371

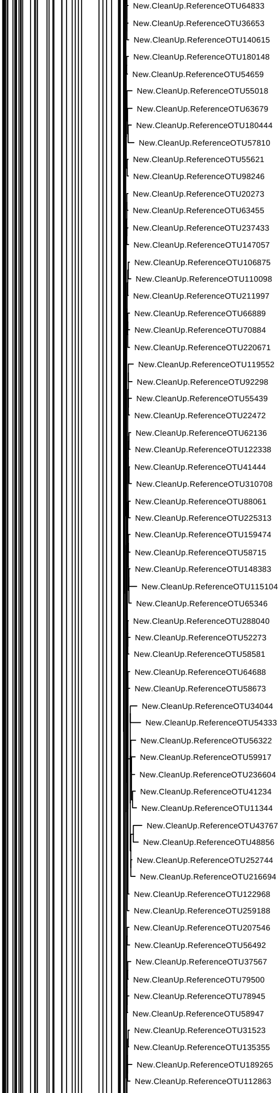
New.CleanUp.ReferenceOTU64833
New.CleanUp.ReferenceOTU36653
New.CleanUp.ReferenceOTU140615
New.CleanUp.ReferenceOTU180148
New.CleanUp.ReferenceOTU54659
New.CleanUp.ReferenceOTU55018
New.CleanUp.ReferenceOTU63679
New.CleanUp.ReferenceOTU180444
New.CleanUp.ReferenceOTU57810
New.CleanUp.ReferenceOTU55621
New.CleanUp.ReferenceOTU98246
New.CleanUp.ReferenceOTU20273
New.CleanUp.ReferenceOTU63455
New.CleanUp.ReferenceOTU237433
New.CleanUp.ReferenceOTU147057
New.CleanUp.ReferenceOTU106875
New.CleanUp.ReferenceOTU110098
New.CleanUp.ReferenceOTU211997
New.CleanUp.ReferenceOTU66889
New.CleanUp.ReferenceOTU70884
New.CleanUp.ReferenceOTU220671
New.CleanUp.ReferenceOTU119552
New.CleanUp.ReferenceOTU92298
New.CleanUp.ReferenceOTU55439
New.CleanUp.ReferenceOTU22472
New.CleanUp.ReferenceOTU62136
New.CleanUp.ReferenceOTU122338
New.CleanUp.ReferenceOTU41444
New.CleanUp.ReferenceOTU310708
New.CleanUp.ReferenceOTU88061
New.CleanUp.ReferenceOTU225313
New.CleanUp.ReferenceOTU159474
New.CleanUp.ReferenceOTU58715
New.CleanUp.ReferenceOTU148383
New.CleanUp.ReferenceOTU115104
New.CleanUp.ReferenceOTU65346
New.CleanUp.ReferenceOTU288040
New.CleanUp.ReferenceOTU52273
New.CleanUp.ReferenceOTU58581
New.CleanUp.ReferenceOTU64688
New.CleanUp.ReferenceOTU58673
New.CleanUp.ReferenceOTU34044
New.CleanUp.ReferenceOTU54333
New.CleanUp.ReferenceOTU56322
New.CleanUp.ReferenceOTU59917
New.CleanUp.ReferenceOTU236604
New.CleanUp.ReferenceOTU41234
New.CleanUp.ReferenceOTU11344
New.CleanUp.ReferenceOTU43767
New.CleanUp.ReferenceOTU48856
New.CleanUp.ReferenceOTU252744
New.CleanUp.ReferenceOTU216694
New.CleanUp.ReferenceOTU122968
New.CleanUp.ReferenceOTU259188
New.CleanUp.ReferenceOTU207546
New.CleanUp.ReferenceOTU56492
New.CleanUp.ReferenceOTU37567
New.CleanUp.ReferenceOTU79500
New.CleanUp.ReferenceOTU78945
New.CleanUp.ReferenceOTU58947
New.CleanUp.ReferenceOTU31523
New.CleanUp.ReferenceOTU135355
New.CleanUp.ReferenceOTU189265
New.CleanUp.ReferenceOTU112863

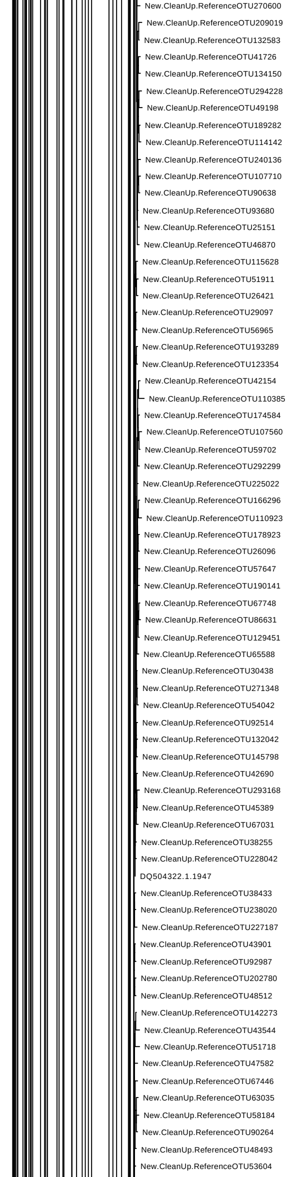
New.CleanUp.ReferenceOTU270600
New.CleanUp.ReferenceOTU209019
New.CleanUp.ReferenceOTU132583
New.CleanUp.ReferenceOTU41726
New.CleanUp.ReferenceOTU134150
New.CleanUp.ReferenceOTU294228
New.CleanUp.ReferenceOTU49198
New.CleanUp.ReferenceOTU189282
New.CleanUp.ReferenceOTU114142
New.CleanUp.ReferenceOTU240136
New.CleanUp.ReferenceOTU107710
New.CleanUp.ReferenceOTU90638
New.CleanUp.ReferenceOTU93680
New.CleanUp.ReferenceOTU25151
New.CleanUp.ReferenceOTU46870
New.CleanUp.ReferenceOTU115628
New.CleanUp.ReferenceOTU51911
New.CleanUp.ReferenceOTU26421
New.CleanUp.ReferenceOTU29097
New.CleanUp.ReferenceOTU56965
New.CleanUp.ReferenceOTU193289
New.CleanUp.ReferenceOTU123354
New.CleanUp.ReferenceOTU42154
New.CleanUp.ReferenceOTU110385
New.CleanUp.ReferenceOTU174584
New.CleanUp.ReferenceOTU107560
New.CleanUp.ReferenceOTU59702
New.CleanUp.ReferenceOTU292299
New.CleanUp.ReferenceOTU225022
New.CleanUp.ReferenceOTU166296
New.CleanUp.ReferenceOTU110923
New.CleanUp.ReferenceOTU178923
New.CleanUp.ReferenceOTU26096
New.CleanUp.ReferenceOTU57647
New.CleanUp.ReferenceOTU190141
New.CleanUp.ReferenceOTU67748
New.CleanUp.ReferenceOTU86631
New.CleanUp.ReferenceOTU129451
New.CleanUp.ReferenceOTU65588
New.CleanUp.ReferenceOTU30438
New.CleanUp.ReferenceOTU271348
New.CleanUp.ReferenceOTU54042
New.CleanUp.ReferenceOTU92514
New.CleanUp.ReferenceOTU132042
New.CleanUp.ReferenceOTU145798
New.CleanUp.ReferenceOTU42690
New.CleanUp.ReferenceOTU293168
New.CleanUp.ReferenceOTU45389
New.CleanUp.ReferenceOTU67031
New.CleanUp.ReferenceOTU38255
New.CleanUp.ReferenceOTU228042
DQ504322.1.1947
New.CleanUp.ReferenceOTU38433
New.CleanUp.ReferenceOTU238020
New.CleanUp.ReferenceOTU227187
New.CleanUp.ReferenceOTU43901
New.CleanUp.ReferenceOTU92987
New.CleanUp.ReferenceOTU202780
New.CleanUp.ReferenceOTU48512
New.CleanUp.ReferenceOTU142273
New.CleanUp.ReferenceOTU43544
New.CleanUp.ReferenceOTU51718
New.CleanUp.ReferenceOTU47582
New.CleanUp.ReferenceOTU67446
New.CleanUp.ReferenceOTU63035
New.CleanUp.ReferenceOTU58184
New.CleanUp.ReferenceOTU90264
New.CleanUp.ReferenceOTU48493
New.CleanUp.ReferenceOTU53604

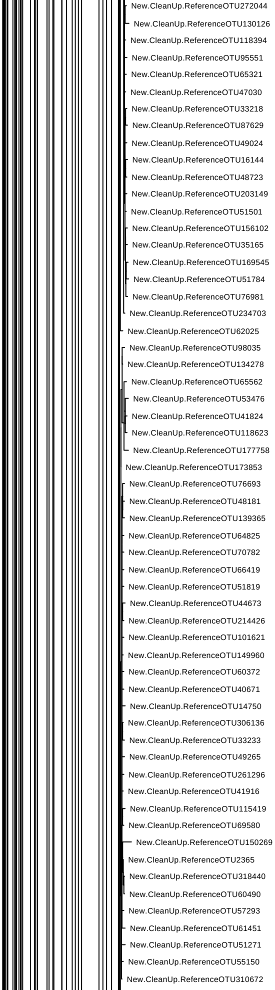
New.CleanUp.ReferenceOTU272044
New.CleanUp.ReferenceOTU130126
New.CleanUp.ReferenceOTU118394
New.CleanUp.ReferenceOTU95551
New.CleanUp.ReferenceOTU65321
New.CleanUp.ReferenceOTU47030
New.CleanUp.ReferenceOTU33218
New.CleanUp.ReferenceOTU87629
New.CleanUp.ReferenceOTU49024
New.CleanUp.ReferenceOTU16144
New.CleanUp.ReferenceOTU48723
New.CleanUp.ReferenceOTU203149
New.CleanUp.ReferenceOTU51501
New.CleanUp.ReferenceOTU156102
New.CleanUp.ReferenceOTU35165
New.CleanUp.ReferenceOTU169545
New.CleanUp.ReferenceOTU51784
New.CleanUp.ReferenceOTU76981
New.CleanUp.ReferenceOTU234703
New.CleanUp.ReferenceOTU62025
New.CleanUp.ReferenceOTU98035
New.CleanUp.ReferenceOTU134278
New.CleanUp.ReferenceOTU65562
New.CleanUp.ReferenceOTU53476
New.CleanUp.ReferenceOTU41824
New.CleanUp.ReferenceOTU118623
New.CleanUp.ReferenceOTU177758
New.CleanUp.ReferenceOTU173853
New.CleanUp.ReferenceOTU76693
New.CleanUp.ReferenceOTU48181
New.CleanUp.ReferenceOTU139365
New.CleanUp.ReferenceOTU64825
New.CleanUp.ReferenceOTU70782
New.CleanUp.ReferenceOTU66419
New.CleanUp.ReferenceOTU51819
New.CleanUp.ReferenceOTU44673
New.CleanUp.ReferenceOTU214426
New.CleanUp.ReferenceOTU101621
New.CleanUp.ReferenceOTU149960
New.CleanUp.ReferenceOTU60372
New.CleanUp.ReferenceOTU40671
New.CleanUp.ReferenceOTU14750
New.CleanUp.ReferenceOTU306136
New.CleanUp.ReferenceOTU33233
New.CleanUp.ReferenceOTU49265
New.CleanUp.ReferenceOTU261296
New.CleanUp.ReferenceOTU41916
New.CleanUp.ReferenceOTU115419
New.CleanUp.ReferenceOTU69580
New.CleanUp.ReferenceOTU150269
New.CleanUp.ReferenceOTU2365
New.CleanUp.ReferenceOTU318440
New.CleanUp.ReferenceOTU60490
New.CleanUp.ReferenceOTU57293
New.CleanUp.ReferenceOTU61451
New.CleanUp.ReferenceOTU51271
New.CleanUp.ReferenceOTU55150
New.CleanUp.ReferenceOTU310672

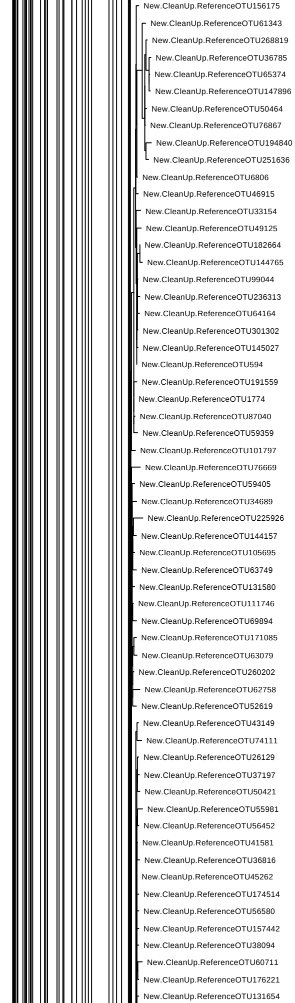
New.CleanUp.ReferenceOTU156175
New.CleanUp.ReferenceOTU61343
New.CleanUp.ReferenceOTU268819
New.CleanUp.ReferenceOTU36785
New.CleanUp.ReferenceOTU65374
New.CleanUp.ReferenceOTU147896
New.CleanUp.ReferenceOTU50464
New.CleanUp.ReferenceOTU76867
New.CleanUp.ReferenceOTU194840
New.CleanUp.ReferenceOTU251636
New.CleanUp.ReferenceOTU6806
New.CleanUp.ReferenceOTU46915
New.CleanUp.ReferenceOTU33154
New.CleanUp.ReferenceOTU49125
New.CleanUp.ReferenceOTU182664
New.CleanUp.ReferenceOTU144765
New.CleanUp.ReferenceOTU99044
New.CleanUp.ReferenceOTU236313
New.CleanUp.ReferenceOTU64164
New.CleanUp.ReferenceOTU301302
New.CleanUp.ReferenceOTU145027
New.CleanUp.ReferenceOTU594
New.CleanUp.ReferenceOTU191559
New.CleanUp.ReferenceOTU1774
New.CleanUp.ReferenceOTU87040
New.CleanUp.ReferenceOTU59359
New.CleanUp.ReferenceOTU101797
New.CleanUp.ReferenceOTU76669
New.CleanUp.ReferenceOTU59405
New.CleanUp.ReferenceOTU34689
New.CleanUp.ReferenceOTU225926
New.CleanUp.ReferenceOTU144157
New.CleanUp.ReferenceOTU105695
New.CleanUp.ReferenceOTU63749
New.CleanUp.ReferenceOTU131580
New.CleanUp.ReferenceOTU111746
New.CleanUp.ReferenceOTU69894
New.CleanUp.ReferenceOTU171085
New.CleanUp.ReferenceOTU63079
New.CleanUp.ReferenceOTU260202
New.CleanUp.ReferenceOTU62758
New.CleanUp.ReferenceOTU52619
New.CleanUp.ReferenceOTU43149
New.CleanUp.ReferenceOTU74111
New.CleanUp.ReferenceOTU26129
New.CleanUp.ReferenceOTU37197
New.CleanUp.ReferenceOTU50421
New.CleanUp.ReferenceOTU55981
New.CleanUp.ReferenceOTU56452
New.CleanUp.ReferenceOTU41581
New.CleanUp.ReferenceOTU36816
New.CleanUp.ReferenceOTU45262
New.CleanUp.ReferenceOTU174514
New.CleanUp.ReferenceOTU56580
New.CleanUp.ReferenceOTU157442
New.CleanUp.ReferenceOTU38094
New.CleanUp.ReferenceOTU60711
New.CleanUp.ReferenceOTU176221
New.CleanUp.ReferenceOTU131654

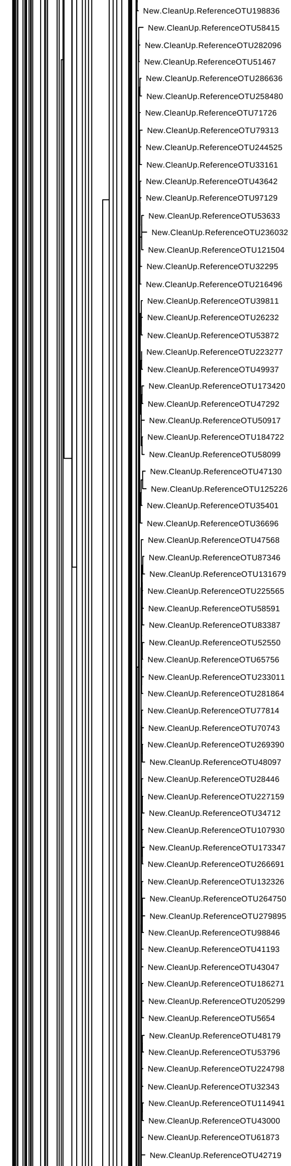
New.CleanUp.ReferenceOTU198836
New.CleanUp.ReferenceOTU58415
New.CleanUp.ReferenceOTU282096
New.CleanUp.ReferenceOTU51467
New.CleanUp.ReferenceOTU286636
New.CleanUp.ReferenceOTU258480
New.CleanUp.ReferenceOTU71726
New.CleanUp.ReferenceOTU79313
New.CleanUp.ReferenceOTU244525
New.CleanUp.ReferenceOTU33161
New.CleanUp.ReferenceOTU43642
New.CleanUp.ReferenceOTU97129
New.CleanUp.ReferenceOTU53633
New.CleanUp.ReferenceOTU236032
New.CleanUp.ReferenceOTU121504
New.CleanUp.ReferenceOTU32295
New.CleanUp.ReferenceOTU216496
New.CleanUp.ReferenceOTU39811
New.CleanUp.ReferenceOTU26232
New.CleanUp.ReferenceOTU53872
New.CleanUp.ReferenceOTU223277
New.CleanUp.ReferenceOTU49937
New.CleanUp.ReferenceOTU173420
New.CleanUp.ReferenceOTU47292
New.CleanUp.ReferenceOTU50917
New.CleanUp.ReferenceOTU184722
New.CleanUp.ReferenceOTU58099
New.CleanUp.ReferenceOTU47130
New.CleanUp.ReferenceOTU125226
New.CleanUp.ReferenceOTU35401
New.CleanUp.ReferenceOTU36696
New.CleanUp.ReferenceOTU47568
New.CleanUp.ReferenceOTU87346
New.CleanUp.ReferenceOTU131679
New.CleanUp.ReferenceOTU225565
New.CleanUp.ReferenceOTU58591
New.CleanUp.ReferenceOTU83387
New.CleanUp.ReferenceOTU52550
New.CleanUp.ReferenceOTU65756
New.CleanUp.ReferenceOTU233011
New.CleanUp.ReferenceOTU281864
New.CleanUp.ReferenceOTU77814
New.CleanUp.ReferenceOTU70743
New.CleanUp.ReferenceOTU269390
New.CleanUp.ReferenceOTU48097
New.CleanUp.ReferenceOTU28446
New.CleanUp.ReferenceOTU227159
New.CleanUp.ReferenceOTU34712
New.CleanUp.ReferenceOTU107930
New.CleanUp.ReferenceOTU173347
New.CleanUp.ReferenceOTU266691
New.CleanUp.ReferenceOTU132326
New.CleanUp.ReferenceOTU264750
New.CleanUp.ReferenceOTU279895
New.CleanUp.ReferenceOTU98846
New.CleanUp.ReferenceOTU41193
New.CleanUp.ReferenceOTU43047
New.CleanUp.ReferenceOTU186271
New.CleanUp.ReferenceOTU205299
New.CleanUp.ReferenceOTU5654
New.CleanUp.ReferenceOTU48179
New.CleanUp.ReferenceOTU53796
New.CleanUp.ReferenceOTU224798
New.CleanUp.ReferenceOTU32343
New.CleanUp.ReferenceOTU114941
New.CleanUp.ReferenceOTU43000
New.CleanUp.ReferenceOTU61873
New.CleanUp.ReferenceOTU42719

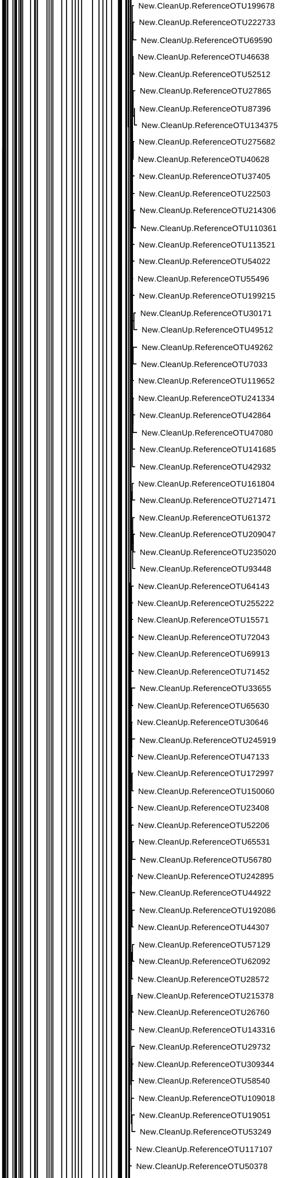
New.CleanUp.ReferenceOTU199678
New.CleanUp.ReferenceOTU222733
New.CleanUp.ReferenceOTU69590
New.CleanUp.ReferenceOTU46638
New.CleanUp.ReferenceOTU52512
New.CleanUp.ReferenceOTU27865
New.CleanUp.ReferenceOTU87396
New.CleanUp.ReferenceOTU134375
New.CleanUp.ReferenceOTU275682
New.CleanUp.ReferenceOTU40628
New.CleanUp.ReferenceOTU37405
New.CleanUp.ReferenceOTU22503
New.CleanUp.ReferenceOTU214306
New.CleanUp.ReferenceOTU110361
New.CleanUp.ReferenceOTU113521
New.CleanUp.ReferenceOTU54022
New.CleanUp.ReferenceOTU55496
New.CleanUp.ReferenceOTU199215
New.CleanUp.ReferenceOTU30171
New.CleanUp.ReferenceOTU49512
New.CleanUp.ReferenceOTU49262
New.CleanUp.ReferenceOTU7033
New.CleanUp.ReferenceOTU119652
New.CleanUp.ReferenceOTU241334
New.CleanUp.ReferenceOTU42864
New.CleanUp.ReferenceOTU47080
New.CleanUp.ReferenceOTU141685
New.CleanUp.ReferenceOTU42932
New.CleanUp.ReferenceOTU161804
New.CleanUp.ReferenceOTU271471
New.CleanUp.ReferenceOTU61372
New.CleanUp.ReferenceOTU209047
New.CleanUp.ReferenceOTU235020
New.CleanUp.ReferenceOTU93448
New.CleanUp.ReferenceOTU64143
New.CleanUp.ReferenceOTU255222
New.CleanUp.ReferenceOTU15571
New.CleanUp.ReferenceOTU72043
New.CleanUp.ReferenceOTU69913
New.CleanUp.ReferenceOTU71452
New.CleanUp.ReferenceOTU33655
New.CleanUp.ReferenceOTU65630
New.CleanUp.ReferenceOTU30646
New.CleanUp.ReferenceOTU245919
New.CleanUp.ReferenceOTU47133
New.CleanUp.ReferenceOTU172997
New.CleanUp.ReferenceOTU150060
New.CleanUp.ReferenceOTU23408
New.CleanUp.ReferenceOTU52206
New.CleanUp.ReferenceOTU65531
New.CleanUp.ReferenceOTU56780
New.CleanUp.ReferenceOTU242895
New.CleanUp.ReferenceOTU44922
New.CleanUp.ReferenceOTU192086
New.CleanUp.ReferenceOTU44307
New.CleanUp.ReferenceOTU57129
New.CleanUp.ReferenceOTU62092
New.CleanUp.ReferenceOTU28572
New.CleanUp.ReferenceOTU215378
New.CleanUp.ReferenceOTU26760
New.CleanUp.ReferenceOTU143316
New.CleanUp.ReferenceOTU29732
New.CleanUp.ReferenceOTU309344
New.CleanUp.ReferenceOTU58540
New.CleanUp.ReferenceOTU109018
New.CleanUp.ReferenceOTU19051
New.CleanUp.ReferenceOTU53249
New.CleanUp.ReferenceOTU117107
New.CleanUp.ReferenceOTU50378

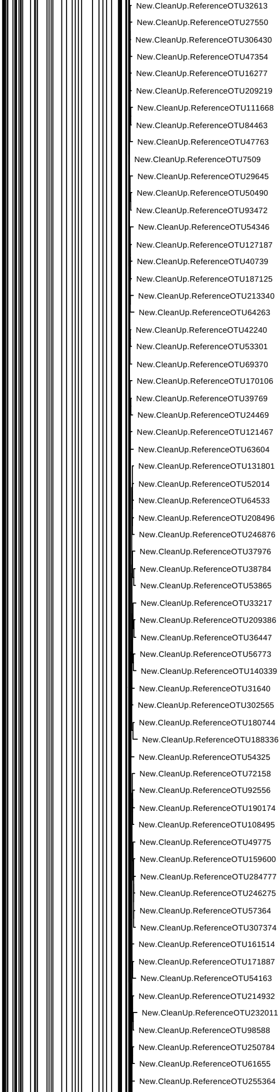
New.CleanUp.ReferenceOTU32613
New.CleanUp.ReferenceOTU27550
New.CleanUp.ReferenceOTU306430
New.CleanUp.ReferenceOTU47354
New.CleanUp.ReferenceOTU16277
New.CleanUp.ReferenceOTU209219
New.CleanUp.ReferenceOTU111668
New.CleanUp.ReferenceOTU84463
New.CleanUp.ReferenceOTU47763
New.CleanUp.ReferenceOTU7509
New.CleanUp.ReferenceOTU29645
New.CleanUp.ReferenceOTU50490
New.CleanUp.ReferenceOTU93472
New.CleanUp.ReferenceOTU54346
New.CleanUp.ReferenceOTU127187
New.CleanUp.ReferenceOTU40739
New.CleanUp.ReferenceOTU187125
New.CleanUp.ReferenceOTU213340
New.CleanUp.ReferenceOTU64263
New.CleanUp.ReferenceOTU42240
New.CleanUp.ReferenceOTU53301
New.CleanUp.ReferenceOTU69370
New.CleanUp.ReferenceOTU170106
New.CleanUp.ReferenceOTU39769
New.CleanUp.ReferenceOTU24469
New.CleanUp.ReferenceOTU121467
New.CleanUp.ReferenceOTU63604
New.CleanUp.ReferenceOTU131801
New.CleanUp.ReferenceOTU52014
New.CleanUp.ReferenceOTU64533
New.CleanUp.ReferenceOTU208496
New.CleanUp.ReferenceOTU246876
New.CleanUp.ReferenceOTU37976
New.CleanUp.ReferenceOTU38784
New.CleanUp.ReferenceOTU53865
New.CleanUp.ReferenceOTU33217
New.CleanUp.ReferenceOTU209386
New.CleanUp.ReferenceOTU36447
New.CleanUp.ReferenceOTU56773
New.CleanUp.ReferenceOTU140339
New.CleanUp.ReferenceOTU31640
New.CleanUp.ReferenceOTU302565
New.CleanUp.ReferenceOTU180744
New.CleanUp.ReferenceOTU188336
New.CleanUp.ReferenceOTU54325
New.CleanUp.ReferenceOTU72158
New.CleanUp.ReferenceOTU92556
New.CleanUp.ReferenceOTU190174
New.CleanUp.ReferenceOTU108495
New.CleanUp.ReferenceOTU49775
New.CleanUp.ReferenceOTU159600
New.CleanUp.ReferenceOTU284777
New.CleanUp.ReferenceOTU246275
New.CleanUp.ReferenceOTU57364
New.CleanUp.ReferenceOTU307374
New.CleanUp.ReferenceOTU161514
New.CleanUp.ReferenceOTU171887
New.CleanUp.ReferenceOTU54163
New.CleanUp.ReferenceOTU214932
New.CleanUp.ReferenceOTU232011
New.CleanUp.ReferenceOTU98588
New.CleanUp.ReferenceOTU250784
New.CleanUp.ReferenceOTU61655
New.CleanUp.ReferenceOTU255364

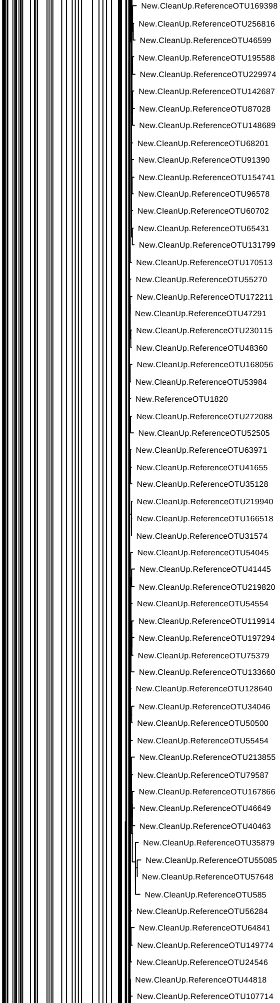
New.CleanUp.ReferenceOTU169398
New.CleanUp.ReferenceOTU256816
New.CleanUp.ReferenceOTU46599
New.CleanUp.ReferenceOTU195588
New.CleanUp.ReferenceOTU229974
New.CleanUp.ReferenceOTU142687
New.CleanUp.ReferenceOTU87028
New.CleanUp.ReferenceOTU148689
New.CleanUp.ReferenceOTU68201
New.CleanUp.ReferenceOTU91390
New.CleanUp.ReferenceOTU154741
New.CleanUp.ReferenceOTU96578
New.CleanUp.ReferenceOTU60702
New.CleanUp.ReferenceOTU65431
New.CleanUp.ReferenceOTU131799
New.CleanUp.ReferenceOTU170513
New.CleanUp.ReferenceOTU55270
New.CleanUp.ReferenceOTU172211
New.CleanUp.ReferenceOTU47291
New.CleanUp.ReferenceOTU230115
New.CleanUp.ReferenceOTU48360
New.CleanUp.ReferenceOTU168056
New.CleanUp.ReferenceOTU53984
New.ReferenceOTU1820
New.CleanUp.ReferenceOTU272088
New.CleanUp.ReferenceOTU52505
New.CleanUp.ReferenceOTU63971
New.CleanUp.ReferenceOTU41655
New.CleanUp.ReferenceOTU35128
New.CleanUp.ReferenceOTU219940
New.CleanUp.ReferenceOTU166518
New.CleanUp.ReferenceOTU31574
New.CleanUp.ReferenceOTU54045
New.CleanUp.ReferenceOTU41445
New.CleanUp.ReferenceOTU219820
New.CleanUp.ReferenceOTU54554
New.CleanUp.ReferenceOTU119914
New.CleanUp.ReferenceOTU197294
New.CleanUp.ReferenceOTU75379
New.CleanUp.ReferenceOTU133660
New.CleanUp.ReferenceOTU128640
New.CleanUp.ReferenceOTU34046
New.CleanUp.ReferenceOTU50500
New.CleanUp.ReferenceOTU55454
New.CleanUp.ReferenceOTU213855
New.CleanUp.ReferenceOTU79587
New.CleanUp.ReferenceOTU167866
New.CleanUp.ReferenceOTU46649
New.CleanUp.ReferenceOTU40463
New.CleanUp.ReferenceOTU35879
New.CleanUp.ReferenceOTU55085
New.CleanUp.ReferenceOTU57648
New.CleanUp.ReferenceOTU585
New.CleanUp.ReferenceOTU56284
New.CleanUp.ReferenceOTU64841
New.CleanUp.ReferenceOTU149774
New.CleanUp.ReferenceOTU24546
New.CleanUp.ReferenceOTU44818
New.CleanUp.ReferenceOTU107714

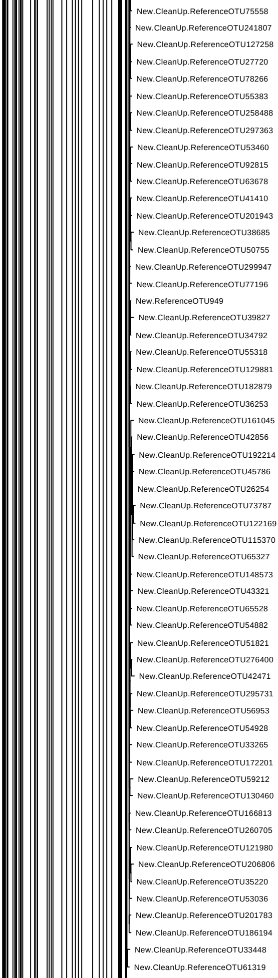
New.CleanUp.ReferenceOTU75558
New.CleanUp.ReferenceOTU241807
New.CleanUp.ReferenceOTU127258
New.CleanUp.ReferenceOTU27720
New.CleanUp.ReferenceOTU78266
New.CleanUp.ReferenceOTU55383
New.CleanUp.ReferenceOTU258488
New.CleanUp.ReferenceOTU297363
New.CleanUp.ReferenceOTU53460
New.CleanUp.ReferenceOTU92815
New.CleanUp.ReferenceOTU63678
New.CleanUp.ReferenceOTU41410
New.CleanUp.ReferenceOTU201943
New.CleanUp.ReferenceOTU38685
New.CleanUp.ReferenceOTU50755
New.CleanUp.ReferenceOTU299947
New.CleanUp.ReferenceOTU77196
New.ReferenceOTU949
New.CleanUp.ReferenceOTU39827
New.CleanUp.ReferenceOTU34792
New.CleanUp.ReferenceOTU55318
New.CleanUp.ReferenceOTU129881
New.CleanUp.ReferenceOTU182879
New.CleanUp.ReferenceOTU36253
New.CleanUp.ReferenceOTU161045
New.CleanUp.ReferenceOTU42856
New.CleanUp.ReferenceOTU192214
New.CleanUp.ReferenceOTU45786
New.CleanUp.ReferenceOTU26254
New.CleanUp.ReferenceOTU73787
New.CleanUp.ReferenceOTU122169
New.CleanUp.ReferenceOTU115370
New.CleanUp.ReferenceOTU65327
New.CleanUp.ReferenceOTU148573
New.CleanUp.ReferenceOTU43321
New.CleanUp.ReferenceOTU65528
New.CleanUp.ReferenceOTU54882
New.CleanUp.ReferenceOTU51821
New.CleanUp.ReferenceOTU276400
New.CleanUp.ReferenceOTU42471
New.CleanUp.ReferenceOTU295731
New.CleanUp.ReferenceOTU56953
New.CleanUp.ReferenceOTU54928
New.CleanUp.ReferenceOTU33265
New.CleanUp.ReferenceOTU172201
New.CleanUp.ReferenceOTU59212
New.CleanUp.ReferenceOTU130460
New.CleanUp.ReferenceOTU166813
New.CleanUp.ReferenceOTU260705
New.CleanUp.ReferenceOTU121980
New.CleanUp.ReferenceOTU206806
New.CleanUp.ReferenceOTU35220
New.CleanUp.ReferenceOTU53036
New.CleanUp.ReferenceOTU201783
New.CleanUp.ReferenceOTU186194
New.CleanUp.ReferenceOTU33448
New.CleanUp.ReferenceOTU61319

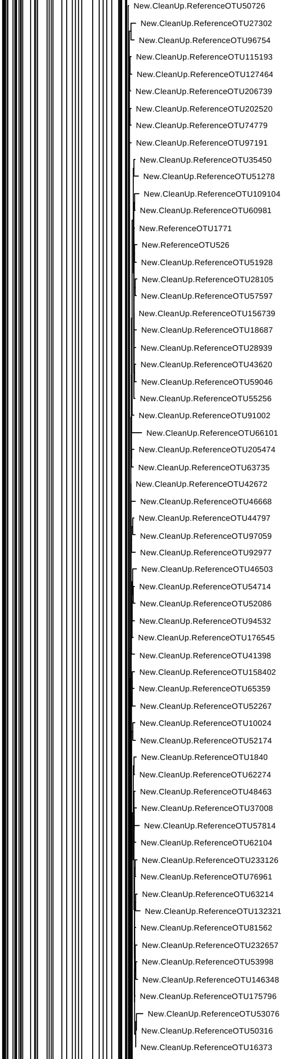
New.CleanUp.ReferenceOTU50726
New.CleanUp.ReferenceOTU27302
New.CleanUp.ReferenceOTU96754
New.CleanUp.ReferenceOTU115193
New.CleanUp.ReferenceOTU127464
New.CleanUp.ReferenceOTU206739
New.CleanUp.ReferenceOTU202520
New.CleanUp.ReferenceOTU74779
New.CleanUp.ReferenceOTU97191
New.CleanUp.ReferenceOTU35450
New.CleanUp.ReferenceOTU51278
New.CleanUp.ReferenceOTU109104
New.CleanUp.ReferenceOTU60981
New.ReferenceOTU1771
New.ReferenceOTU526
New.CleanUp.ReferenceOTU51928
New.CleanUp.ReferenceOTU28105
New.CleanUp.ReferenceOTU57597
New.CleanUp.ReferenceOTU156739
New.CleanUp.ReferenceOTU18687
New.CleanUp.ReferenceOTU28939
New.CleanUp.ReferenceOTU43620
New.CleanUp.ReferenceOTU59046
New.CleanUp.ReferenceOTU55256
New.CleanUp.ReferenceOTU91002
New.CleanUp.ReferenceOTU66101
New.CleanUp.ReferenceOTU205474
New.CleanUp.ReferenceOTU63735
New.CleanUp.ReferenceOTU42672
New.CleanUp.ReferenceOTU46668
New.CleanUp.ReferenceOTU44797
New.CleanUp.ReferenceOTU97059
New.CleanUp.ReferenceOTU92977
New.CleanUp.ReferenceOTU46503
New.CleanUp.ReferenceOTU54714
New.CleanUp.ReferenceOTU52086
New.CleanUp.ReferenceOTU94532
New.CleanUp.ReferenceOTU176545
New.CleanUp.ReferenceOTU41398
New.CleanUp.ReferenceOTU158402
New.CleanUp.ReferenceOTU65359
New.CleanUp.ReferenceOTU52267
New.CleanUp.ReferenceOTU10024
New.CleanUp.ReferenceOTU52174
New.CleanUp.ReferenceOTU1840
New.CleanUp.ReferenceOTU62274
New.CleanUp.ReferenceOTU48463
New.CleanUp.ReferenceOTU37008
New.CleanUp.ReferenceOTU57814
New.CleanUp.ReferenceOTU62104
New.CleanUp.ReferenceOTU233126
New.CleanUp.ReferenceOTU76961
New.CleanUp.ReferenceOTU63214
New.CleanUp.ReferenceOTU132321
New.CleanUp.ReferenceOTU81562
New.CleanUp.ReferenceOTU232657
New.CleanUp.ReferenceOTU53998
New.CleanUp.ReferenceOTU146348
New.CleanUp.ReferenceOTU175796
New.CleanUp.ReferenceOTU53076
New.CleanUp.ReferenceOTU50316
New.CleanUp.ReferenceOTU16373

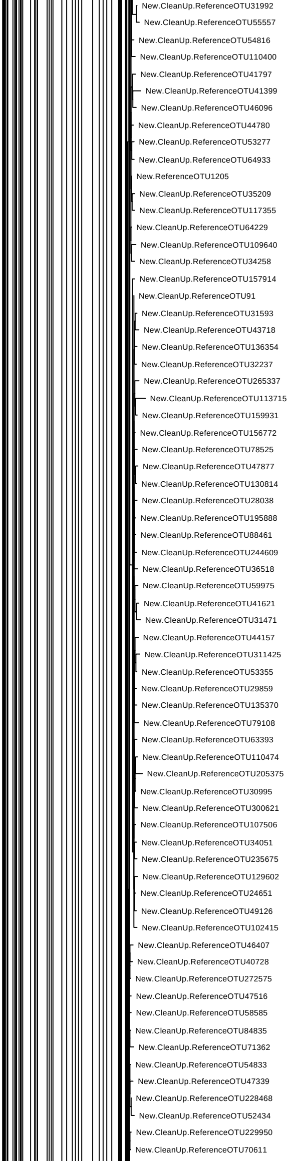
New.CleanUp.ReferenceOTU31992
New.CleanUp.ReferenceOTU55557
New.CleanUp.ReferenceOTU54816
New.CleanUp.ReferenceOTU110400
New.CleanUp.ReferenceOTU41797
New.CleanUp.ReferenceOTU41399
New.CleanUp.ReferenceOTU46096
New.CleanUp.ReferenceOTU44780
New.CleanUp.ReferenceOTU53277
New.CleanUp.ReferenceOTU64933
New.ReferenceOTU1205
New.CleanUp.ReferenceOTU35209
New.CleanUp.ReferenceOTU117355
New.CleanUp.ReferenceOTU64229
New.CleanUp.ReferenceOTU109640
New.CleanUp.ReferenceOTU34258
New.CleanUp.ReferenceOTU157914
New.CleanUp.ReferenceOTU91
New.CleanUp.ReferenceOTU31593
New.CleanUp.ReferenceOTU43718
New.CleanUp.ReferenceOTU136354
New.CleanUp.ReferenceOTU32237
New.CleanUp.ReferenceOTU265337
New.CleanUp.ReferenceOTU113715
New.CleanUp.ReferenceOTU159931
New.CleanUp.ReferenceOTU156772
New.CleanUp.ReferenceOTU78525
New.CleanUp.ReferenceOTU47877
New.CleanUp.ReferenceOTU130814
New.CleanUp.ReferenceOTU28038
New.CleanUp.ReferenceOTU195888
New.CleanUp.ReferenceOTU88461
New.CleanUp.ReferenceOTU244609
New.CleanUp.ReferenceOTU36518
New.CleanUp.ReferenceOTU59975
New.CleanUp.ReferenceOTU41621
New.CleanUp.ReferenceOTU31471
New.CleanUp.ReferenceOTU44157
New.CleanUp.ReferenceOTU311425
New.CleanUp.ReferenceOTU53355
New.CleanUp.ReferenceOTU29859
New.CleanUp.ReferenceOTU135370
New.CleanUp.ReferenceOTU79108
New.CleanUp.ReferenceOTU63393
New.CleanUp.ReferenceOTU110474
New.CleanUp.ReferenceOTU205375
New.CleanUp.ReferenceOTU30995
New.CleanUp.ReferenceOTU300621
New.CleanUp.ReferenceOTU107506
New.CleanUp.ReferenceOTU34051
New.CleanUp.ReferenceOTU235675
New.CleanUp.ReferenceOTU129602
New.CleanUp.ReferenceOTU24651
New.CleanUp.ReferenceOTU49126
New.CleanUp.ReferenceOTU102415
New.CleanUp.ReferenceOTU46407
New.CleanUp.ReferenceOTU40728
New.CleanUp.ReferenceOTU272575
New.CleanUp.ReferenceOTU47516
New.CleanUp.ReferenceOTU58585
New.CleanUp.ReferenceOTU84835
New.CleanUp.ReferenceOTU71362
New.CleanUp.ReferenceOTU54833
New.CleanUp.ReferenceOTU47339
New.CleanUp.ReferenceOTU228468
New.CleanUp.ReferenceOTU52434
New.CleanUp.ReferenceOTU229950
New.CleanUp.ReferenceOTU70611

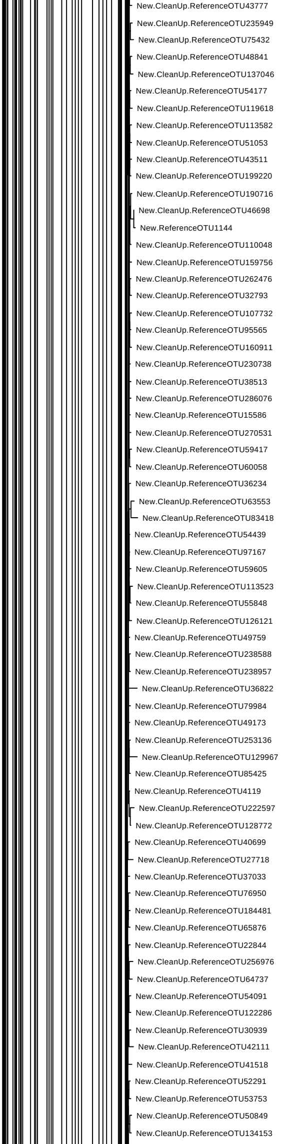
New.CleanUp.ReferenceOTU43777
New.CleanUp.ReferenceOTU235949
New.CleanUp.ReferenceOTU75432
New.CleanUp.ReferenceOTU48841
New.CleanUp.ReferenceOTU137046
New.CleanUp.ReferenceOTU54177
New.CleanUp.ReferenceOTU119618
New.CleanUp.ReferenceOTU113582
New.CleanUp.ReferenceOTU51053
New.CleanUp.ReferenceOTU43511
New.CleanUp.ReferenceOTU199220
New.CleanUp.ReferenceOTU190716
New.CleanUp.ReferenceOTU46698
New.ReferenceOTU1144
New.CleanUp.ReferenceOTU110048
New.CleanUp.ReferenceOTU159756
New.CleanUp.ReferenceOTU262476
New.CleanUp.ReferenceOTU32793
New.CleanUp.ReferenceOTU107732
New.CleanUp.ReferenceOTU95565
New.CleanUp.ReferenceOTU160911
New.CleanUp.ReferenceOTU230738
New.CleanUp.ReferenceOTU38513
New.CleanUp.ReferenceOTU286076
New.CleanUp.ReferenceOTU15586
New.CleanUp.ReferenceOTU270531
New.CleanUp.ReferenceOTU59417
New.CleanUp.ReferenceOTU60058
New.CleanUp.ReferenceOTU36234
New.CleanUp.ReferenceOTU63553
New.CleanUp.ReferenceOTU83418
New.CleanUp.ReferenceOTU54439
New.CleanUp.ReferenceOTU97167
New.CleanUp.ReferenceOTU59605
New.CleanUp.ReferenceOTU113523
New.CleanUp.ReferenceOTU55848
New.CleanUp.ReferenceOTU126121
New.CleanUp.ReferenceOTU49759
New.CleanUp.ReferenceOTU238588
New.CleanUp.ReferenceOTU238957
New.CleanUp.ReferenceOTU36822
New.CleanUp.ReferenceOTU79984
New.CleanUp.ReferenceOTU49173
New.CleanUp.ReferenceOTU253136
New.CleanUp.ReferenceOTU129967
New.CleanUp.ReferenceOTU85425
New.CleanUp.ReferenceOTU4119
New.CleanUp.ReferenceOTU222597
New.CleanUp.ReferenceOTU128772
New.CleanUp.ReferenceOTU40699
New.CleanUp.ReferenceOTU27718
New.CleanUp.ReferenceOTU37033
New.CleanUp.ReferenceOTU76950
New.CleanUp.ReferenceOTU184481
New.CleanUp.ReferenceOTU65876
New.CleanUp.ReferenceOTU22844
New.CleanUp.ReferenceOTU256976
New.CleanUp.ReferenceOTU64737
New.CleanUp.ReferenceOTU54091
New.CleanUp.ReferenceOTU122286
New.CleanUp.ReferenceOTU30939
New.CleanUp.ReferenceOTU42111
New.CleanUp.ReferenceOTU41518
New.CleanUp.ReferenceOTU52291
New.CleanUp.ReferenceOTU53753
New.CleanUp.ReferenceOTU50849
New.CleanUp.ReferenceOTU134153

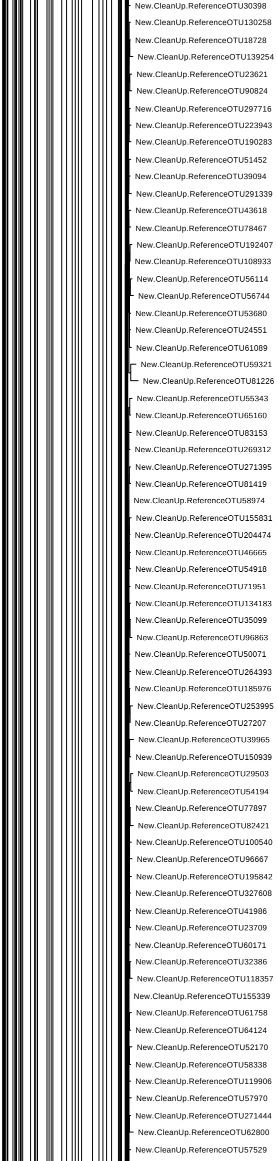
New.CleanUp.ReferenceOTU30398
New.CleanUp.ReferenceOTU130258
New.CleanUp.ReferenceOTU18728
New.CleanUp.ReferenceOTU139254
New.CleanUp.ReferenceOTU23621
New.CleanUp.ReferenceOTU90824
New.CleanUp.ReferenceOTU297716
New.CleanUp.ReferenceOTU223943
New.CleanUp.ReferenceOTU190283
New.CleanUp.ReferenceOTU51452
New.CleanUp.ReferenceOTU39094
New.CleanUp.ReferenceOTU291339
New.CleanUp.ReferenceOTU43618
New.CleanUp.ReferenceOTU78467
New.CleanUp.ReferenceOTU192407
New.CleanUp.ReferenceOTU108933
New.CleanUp.ReferenceOTU56114
New.CleanUp.ReferenceOTU56744
New.CleanUp.ReferenceOTU53680
New.CleanUp.ReferenceOTU24551
New.CleanUp.ReferenceOTU61089
New.CleanUp.ReferenceOTU59321
New.CleanUp.ReferenceOTU81226
New.CleanUp.ReferenceOTU55343
New.CleanUp.ReferenceOTU65160
New.CleanUp.ReferenceOTU83153
New.CleanUp.ReferenceOTU269312
New.CleanUp.ReferenceOTU271395
New.CleanUp.ReferenceOTU81419
New.CleanUp.ReferenceOTU58974
New.CleanUp.ReferenceOTU155831
New.CleanUp.ReferenceOTU204474
New.CleanUp.ReferenceOTU46665
New.CleanUp.ReferenceOTU54918
New.CleanUp.ReferenceOTU71951
New.CleanUp.ReferenceOTU134183
New.CleanUp.ReferenceOTU35099
New.CleanUp.ReferenceOTU96863
New.CleanUp.ReferenceOTU50071
New.CleanUp.ReferenceOTU264393
New.CleanUp.ReferenceOTU185976
New.CleanUp.ReferenceOTU253995
New.CleanUp.ReferenceOTU27207
New.CleanUp.ReferenceOTU39965
New.CleanUp.ReferenceOTU150939
New.CleanUp.ReferenceOTU29503
New.CleanUp.ReferenceOTU54194
New.CleanUp.ReferenceOTU77897
New.CleanUp.ReferenceOTU82421
New.CleanUp.ReferenceOTU100540
New.CleanUp.ReferenceOTU96667
New.CleanUp.ReferenceOTU195842
New.CleanUp.ReferenceOTU327608
New.CleanUp.ReferenceOTU41986
New.CleanUp.ReferenceOTU23709
New.CleanUp.ReferenceOTU60171
New.CleanUp.ReferenceOTU32386
New.CleanUp.ReferenceOTU118357
New.CleanUp.ReferenceOTU155339
New.CleanUp.ReferenceOTU61758
New.CleanUp.ReferenceOTU64124
New.CleanUp.ReferenceOTU52170
New.CleanUp.ReferenceOTU58338
New.CleanUp.ReferenceOTU119906
New.CleanUp.ReferenceOTU57970
New.CleanUp.ReferenceOTU271444
New.CleanUp.ReferenceOTU62800
New.CleanUp.ReferenceOTU57529

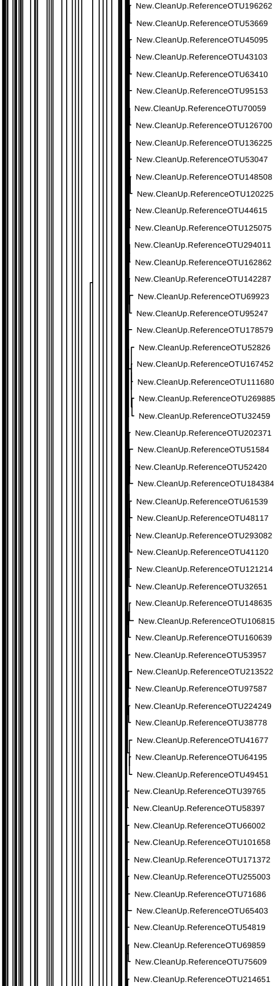
New.CleanUp.ReferenceOTU196262
New.CleanUp.ReferenceOTU53669
New.CleanUp.ReferenceOTU45095
New.CleanUp.ReferenceOTU43103
New.CleanUp.ReferenceOTU63410
New.CleanUp.ReferenceOTU95153
New.CleanUp.ReferenceOTU70059
New.CleanUp.ReferenceOTU126700
New.CleanUp.ReferenceOTU136225
New.CleanUp.ReferenceOTU53047
New.CleanUp.ReferenceOTU148508
New.CleanUp.ReferenceOTU120225
New.CleanUp.ReferenceOTU44615
New.CleanUp.ReferenceOTU125075
New.CleanUp.ReferenceOTU294011
New.CleanUp.ReferenceOTU162862
New.CleanUp.ReferenceOTU142287
New.CleanUp.ReferenceOTU69923
New.CleanUp.ReferenceOTU95247
New.CleanUp.ReferenceOTU178579
New.CleanUp.ReferenceOTU52826
New.CleanUp.ReferenceOTU167452
New.CleanUp.ReferenceOTU111680
New.CleanUp.ReferenceOTU269885
New.CleanUp.ReferenceOTU32459
New.CleanUp.ReferenceOTU202371
New.CleanUp.ReferenceOTU51584
New.CleanUp.ReferenceOTU52420
New.CleanUp.ReferenceOTU184384
New.CleanUp.ReferenceOTU61539
New.CleanUp.ReferenceOTU48117
New.CleanUp.ReferenceOTU293082
New.CleanUp.ReferenceOTU41120
New.CleanUp.ReferenceOTU121214
New.CleanUp.ReferenceOTU32651
New.CleanUp.ReferenceOTU148635
New.CleanUp.ReferenceOTU106815
New.CleanUp.ReferenceOTU160639
New.CleanUp.ReferenceOTU53957
New.CleanUp.ReferenceOTU213522
New.CleanUp.ReferenceOTU97587
New.CleanUp.ReferenceOTU224249
New.CleanUp.ReferenceOTU38778
New.CleanUp.ReferenceOTU41677
New.CleanUp.ReferenceOTU64195
New.CleanUp.ReferenceOTU49451
New.CleanUp.ReferenceOTU39765
New.CleanUp.ReferenceOTU58397
New.CleanUp.ReferenceOTU66002
New.CleanUp.ReferenceOTU101658
New.CleanUp.ReferenceOTU171372
New.CleanUp.ReferenceOTU255003
New.CleanUp.ReferenceOTU71686
New.CleanUp.ReferenceOTU65403
New.CleanUp.ReferenceOTU54819
New.CleanUp.ReferenceOTU69859
New.CleanUp.ReferenceOTU75609
New.CleanUp.ReferenceOTU214651

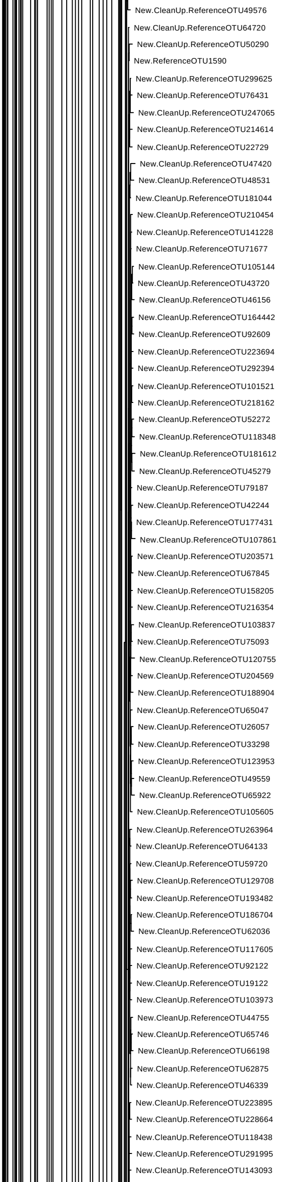

New.CleanUp.ReferenceOTU49576
New.CleanUp.ReferenceOTU64720
New.CleanUp.ReferenceOTU50290
New.ReferenceOTU1590
New.CleanUp.ReferenceOTU299625
New.CleanUp.ReferenceOTU76431
New.CleanUp.ReferenceOTU247065
New.CleanUp.ReferenceOTU214614
New.CleanUp.ReferenceOTU22729
New.CleanUp.ReferenceOTU47420
New.CleanUp.ReferenceOTU48531
New.CleanUp.ReferenceOTU181044
New.CleanUp.ReferenceOTU210454
New.CleanUp.ReferenceOTU141228
New.CleanUp.ReferenceOTU71677
New.CleanUp.ReferenceOTU105144
New.CleanUp.ReferenceOTU43720
New.CleanUp.ReferenceOTU46156
New.CleanUp.ReferenceOTU164442
New.CleanUp.ReferenceOTU92609
New.CleanUp.ReferenceOTU223694
New.CleanUp.ReferenceOTU292394
New.CleanUp.ReferenceOTU101521
New.CleanUp.ReferenceOTU218162
New.CleanUp.ReferenceOTU52272
New.CleanUp.ReferenceOTU118348
New.CleanUp.ReferenceOTU181612
New.CleanUp.ReferenceOTU45279
New.CleanUp.ReferenceOTU79187
New.CleanUp.ReferenceOTU42244
New.CleanUp.ReferenceOTU177431
New.CleanUp.ReferenceOTU107861
New.CleanUp.ReferenceOTU203571
New.CleanUp.ReferenceOTU67845
New.CleanUp.ReferenceOTU158205
New.CleanUp.ReferenceOTU216354
New.CleanUp.ReferenceOTU103837
New.CleanUp.ReferenceOTU75093
New.CleanUp.ReferenceOTU120755
New.CleanUp.ReferenceOTU204569
New.CleanUp.ReferenceOTU188904
New.CleanUp.ReferenceOTU65047
New.CleanUp.ReferenceOTU26057
New.CleanUp.ReferenceOTU33298
New.CleanUp.ReferenceOTU123953
New.CleanUp.ReferenceOTU49559
New.CleanUp.ReferenceOTU65922
New.CleanUp.ReferenceOTU105605
New.CleanUp.ReferenceOTU263964
New.CleanUp.ReferenceOTU64133
New.CleanUp.ReferenceOTU59720
New.CleanUp.ReferenceOTU129708
New.CleanUp.ReferenceOTU193482
New.CleanUp.ReferenceOTU186704
New.CleanUp.ReferenceOTU62036
New.CleanUp.ReferenceOTU117605
New.CleanUp.ReferenceOTU92122
New.CleanUp.ReferenceOTU19122
New.CleanUp.ReferenceOTU103973
New.CleanUp.ReferenceOTU44755
New.CleanUp.ReferenceOTU65746
New.CleanUp.ReferenceOTU66198
New.CleanUp.ReferenceOTU62875
New.CleanUp.ReferenceOTU46339
New.CleanUp.ReferenceOTU223895
New.CleanUp.ReferenceOTU228664
New.CleanUp.ReferenceOTU118438
New.CleanUp.ReferenceOTU291995
New.CleanUp.ReferenceOTU143093

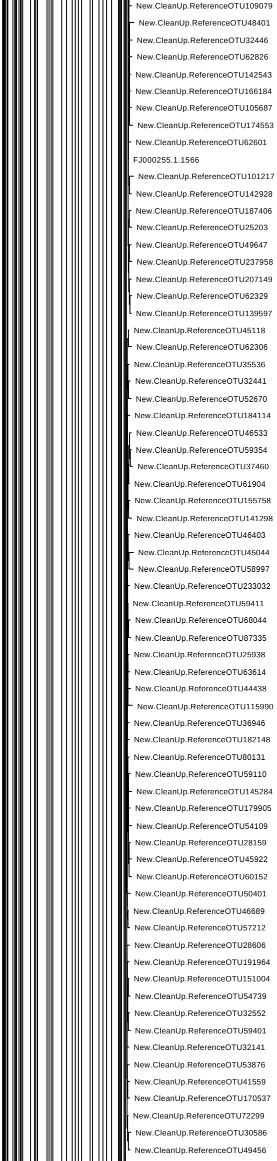

New.CleanUp.ReferenceOTU109079
New.CleanUp.ReferenceOTU48401
New.CleanUp.ReferenceOTU32446
New.CleanUp.ReferenceOTU62826
New.CleanUp.ReferenceOTU142543
New.CleanUp.ReferenceOTU166184
New.CleanUp.ReferenceOTU105687
New.CleanUp.ReferenceOTU174553
New.CleanUp.ReferenceOTU62601
FJ000255.1.1566
New.CleanUp.ReferenceOTU101217
New.CleanUp.ReferenceOTU142928
New.CleanUp.ReferenceOTU187406
New.CleanUp.ReferenceOTU25203
New.CleanUp.ReferenceOTU49647
New.CleanUp.ReferenceOTU237958
New.CleanUp.ReferenceOTU207149
New.CleanUp.ReferenceOTU62329
New.CleanUp.ReferenceOTU139597
New.CleanUp.ReferenceOTU45118
New.CleanUp.ReferenceOTU62306
New.CleanUp.ReferenceOTU35536
New.CleanUp.ReferenceOTU32441
New.CleanUp.ReferenceOTU52670
New.CleanUp.ReferenceOTU184114
New.CleanUp.ReferenceOTU46533
New.CleanUp.ReferenceOTU59354
New.CleanUp.ReferenceOTU37460
New.CleanUp.ReferenceOTU61904
New.CleanUp.ReferenceOTU155758
New.CleanUp.ReferenceOTU141298
New.CleanUp.ReferenceOTU46403
New.CleanUp.ReferenceOTU45044
New.CleanUp.ReferenceOTU58997
New.CleanUp.ReferenceOTU233032
New.CleanUp.ReferenceOTU59411
New.CleanUp.ReferenceOTU68044
New.CleanUp.ReferenceOTU87335
New.CleanUp.ReferenceOTU25938
New.CleanUp.ReferenceOTU63614
New.CleanUp.ReferenceOTU44438
New.CleanUp.ReferenceOTU115990
New.CleanUp.ReferenceOTU36946
New.CleanUp.ReferenceOTU182148
New.CleanUp.ReferenceOTU80131
New.CleanUp.ReferenceOTU59110
New.CleanUp.ReferenceOTU145284
New.CleanUp.ReferenceOTU179905
New.CleanUp.ReferenceOTU54109
New.CleanUp.ReferenceOTU28159
New.CleanUp.ReferenceOTU45922
New.CleanUp.ReferenceOTU60152
New.CleanUp.ReferenceOTU50401
New.CleanUp.ReferenceOTU46689
New.CleanUp.ReferenceOTU57212
New.CleanUp.ReferenceOTU28606
New.CleanUp.ReferenceOTU191964
New.CleanUp.ReferenceOTU151004
New.CleanUp.ReferenceOTU54739
New.CleanUp.ReferenceOTU32552
New.CleanUp.ReferenceOTU59401
New.CleanUp.ReferenceOTU32141
New.CleanUp.ReferenceOTU53876
New.CleanUp.ReferenceOTU41559
New.CleanUp.ReferenceOTU170537
New.CleanUp.ReferenceOTU72299
New.CleanUp.ReferenceOTU30586
New.CleanUp.ReferenceOTU49456

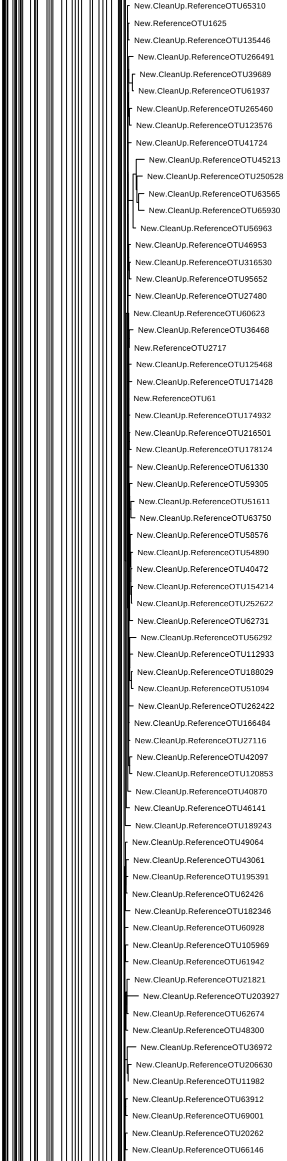
New.CleanUp.ReferenceOTU65310
New.ReferenceOTU1625
New.CleanUp.ReferenceOTU135446
New.CleanUp.ReferenceOTU266491
New.CleanUp.ReferenceOTU39689
New.CleanUp.ReferenceOTU61937
New.CleanUp.ReferenceOTU265460
New.CleanUp.ReferenceOTU123576
New.CleanUp.ReferenceOTU41724
New.CleanUp.ReferenceOTU45213
New.CleanUp.ReferenceOTU250528
New.CleanUp.ReferenceOTU63565
New.CleanUp.ReferenceOTU65930
New.CleanUp.ReferenceOTU56963
New.CleanUp.ReferenceOTU46953
New.CleanUp.ReferenceOTU316530
New.CleanUp.ReferenceOTU95652
New.CleanUp.ReferenceOTU27480
New.CleanUp.ReferenceOTU60623
New.CleanUp.ReferenceOTU36468
New.ReferenceOTU2717
New.CleanUp.ReferenceOTU125468
New.CleanUp.ReferenceOTU171428
New.ReferenceOTU61
New.CleanUp.ReferenceOTU174932
New.CleanUp.ReferenceOTU216501
New.CleanUp.ReferenceOTU178124
New.CleanUp.ReferenceOTU61330
New.CleanUp.ReferenceOTU59305
New.CleanUp.ReferenceOTU51611
New.CleanUp.ReferenceOTU63750
New.CleanUp.ReferenceOTU58576
New.CleanUp.ReferenceOTU54890
New.CleanUp.ReferenceOTU40472
New.CleanUp.ReferenceOTU154214
New.CleanUp.ReferenceOTU252622
New.CleanUp.ReferenceOTU62731
New.CleanUp.ReferenceOTU56292
New.CleanUp.ReferenceOTU112933
New.CleanUp.ReferenceOTU188029
New.CleanUp.ReferenceOTU51094
New.CleanUp.ReferenceOTU262422
New.CleanUp.ReferenceOTU166484
New.CleanUp.ReferenceOTU27116
New.CleanUp.ReferenceOTU42097
New.CleanUp.ReferenceOTU120853
New.CleanUp.ReferenceOTU40870
New.CleanUp.ReferenceOTU46141
New.CleanUp.ReferenceOTU189243
New.CleanUp.ReferenceOTU49064
New.CleanUp.ReferenceOTU43061
New.CleanUp.ReferenceOTU195391
New.CleanUp.ReferenceOTU62426
New.CleanUp.ReferenceOTU182346
New.CleanUp.ReferenceOTU60928
New.CleanUp.ReferenceOTU105969
New.CleanUp.ReferenceOTU61942
New.CleanUp.ReferenceOTU21821
New.CleanUp.ReferenceOTU203927
New.CleanUp.ReferenceOTU62674
New.CleanUp.ReferenceOTU48300
New.CleanUp.ReferenceOTU36972
New.CleanUp.ReferenceOTU206630
New.CleanUp.ReferenceOTU11982
New.CleanUp.ReferenceOTU63912
New.CleanUp.ReferenceOTU69001
New.CleanUp.ReferenceOTU20262
New.CleanUp.ReferenceOTU66146

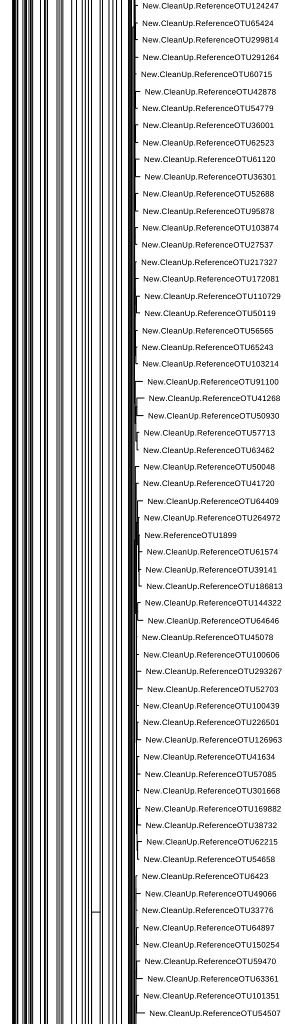
New.CleanUp.ReferenceOTU124247
New.CleanUp.ReferenceOTU65424
New.CleanUp.ReferenceOTU299814
New.CleanUp.ReferenceOTU291264
New.CleanUp.ReferenceOTU60715
New.CleanUp.ReferenceOTU42878
New.CleanUp.ReferenceOTU54779
New.CleanUp.ReferenceOTU36001
New.CleanUp.ReferenceOTU62523
New.CleanUp.ReferenceOTU61120
New.CleanUp.ReferenceOTU36301
New.CleanUp.ReferenceOTU52688
New.CleanUp.ReferenceOTU95878
New.CleanUp.ReferenceOTU103874
New.CleanUp.ReferenceOTU27537
New.CleanUp.ReferenceOTU217327
New.CleanUp.ReferenceOTU172081
New.CleanUp.ReferenceOTU110729
New.CleanUp.ReferenceOTU50119
New.CleanUp.ReferenceOTU56565
New.CleanUp.ReferenceOTU65243
New.CleanUp.ReferenceOTU103214
New.CleanUp.ReferenceOTU91100
New.CleanUp.ReferenceOTU41268
New.CleanUp.ReferenceOTU50930
New.CleanUp.ReferenceOTU57713
New.CleanUp.ReferenceOTU63462
New.CleanUp.ReferenceOTU50048
New.CleanUp.ReferenceOTU41720
New.CleanUp.ReferenceOTU64409
New.CleanUp.ReferenceOTU264972
New.ReferenceOTU1899
New.CleanUp.ReferenceOTU61574
New.CleanUp.ReferenceOTU39141
New.CleanUp.ReferenceOTU186813
New.CleanUp.ReferenceOTU144322
New.CleanUp.ReferenceOTU64646
New.CleanUp.ReferenceOTU45078
New.CleanUp.ReferenceOTU100606
New.CleanUp.ReferenceOTU293267
New.CleanUp.ReferenceOTU52703
New.CleanUp.ReferenceOTU100439
New.CleanUp.ReferenceOTU226501
New.CleanUp.ReferenceOTU126963
New.CleanUp.ReferenceOTU41634
New.CleanUp.ReferenceOTU57085
New.CleanUp.ReferenceOTU301668
New.CleanUp.ReferenceOTU169882
New.CleanUp.ReferenceOTU38732
New.CleanUp.ReferenceOTU62215
New.CleanUp.ReferenceOTU54658
New.CleanUp.ReferenceOTU6423
New.CleanUp.ReferenceOTU49066
New.CleanUp.ReferenceOTU33776
New.CleanUp.ReferenceOTU64897
New.CleanUp.ReferenceOTU150254
New.CleanUp.ReferenceOTU59470
New.CleanUp.ReferenceOTU63361
New.CleanUp.ReferenceOTU101351
New.CleanUp.ReferenceOTU54507

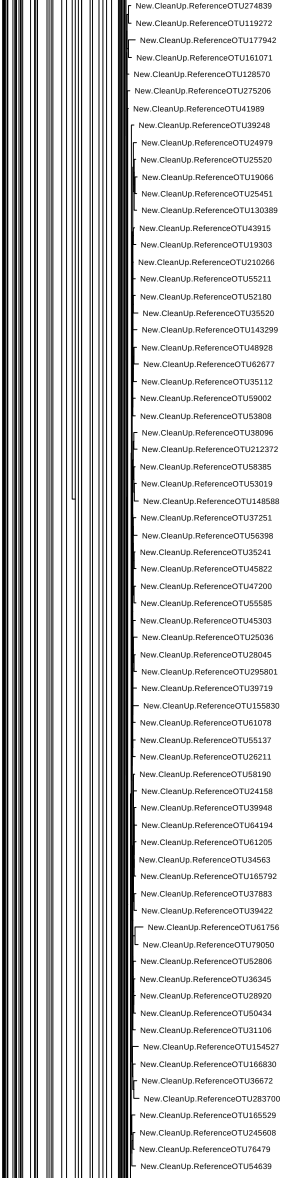
New.CleanUp.ReferenceOTU274839
New.CleanUp.ReferenceOTU119272
New.CleanUp.ReferenceOTU177942
New.CleanUp.ReferenceOTU161071
New.CleanUp.ReferenceOTU128570
New.CleanUp.ReferenceOTU275206
New.CleanUp.ReferenceOTU41989
New.CleanUp.ReferenceOTU39248
New.CleanUp.ReferenceOTU24979
New.CleanUp.ReferenceOTU25520
New.CleanUp.ReferenceOTU19066
New.CleanUp.ReferenceOTU25451
New.CleanUp.ReferenceOTU130389
New.CleanUp.ReferenceOTU43915
New.CleanUp.ReferenceOTU19303
New.CleanUp.ReferenceOTU210266
New.CleanUp.ReferenceOTU55211
New.CleanUp.ReferenceOTU52180
New.CleanUp.ReferenceOTU35520
New.CleanUp.ReferenceOTU143299
New.CleanUp.ReferenceOTU48928
New.CleanUp.ReferenceOTU62677
New.CleanUp.ReferenceOTU35112
New.CleanUp.ReferenceOTU59002
New.CleanUp.ReferenceOTU53808
New.CleanUp.ReferenceOTU38096
New.CleanUp.ReferenceOTU212372
New.CleanUp.ReferenceOTU58385
New.CleanUp.ReferenceOTU53019
New.CleanUp.ReferenceOTU148588
New.CleanUp.ReferenceOTU37251
New.CleanUp.ReferenceOTU56398
New.CleanUp.ReferenceOTU35241
New.CleanUp.ReferenceOTU45822
New.CleanUp.ReferenceOTU47200
New.CleanUp.ReferenceOTU55585
New.CleanUp.ReferenceOTU45303
New.CleanUp.ReferenceOTU25036
New.CleanUp.ReferenceOTU28045
New.CleanUp.ReferenceOTU295801
New.CleanUp.ReferenceOTU39719
New.CleanUp.ReferenceOTU155830
New.CleanUp.ReferenceOTU61078
New.CleanUp.ReferenceOTU55137
New.CleanUp.ReferenceOTU26211
New.CleanUp.ReferenceOTU58190
New.CleanUp.ReferenceOTU24158
New.CleanUp.ReferenceOTU39948
New.CleanUp.ReferenceOTU64194
New.CleanUp.ReferenceOTU61205
New.CleanUp.ReferenceOTU34563
New.CleanUp.ReferenceOTU165792
New.CleanUp.ReferenceOTU37883
New.CleanUp.ReferenceOTU39422
New.CleanUp.ReferenceOTU61756
New.CleanUp.ReferenceOTU79050
New.CleanUp.ReferenceOTU52806
New.CleanUp.ReferenceOTU36345
New.CleanUp.ReferenceOTU28920
New.CleanUp.ReferenceOTU50434
New.CleanUp.ReferenceOTU31106
New.CleanUp.ReferenceOTU154527
New.CleanUp.ReferenceOTU166830
New.CleanUp.ReferenceOTU36672
New.CleanUp.ReferenceOTU283700
New.CleanUp.ReferenceOTU165529
New.CleanUp.ReferenceOTU245608
New.CleanUp.ReferenceOTU76479
New.CleanUp.ReferenceOTU54639

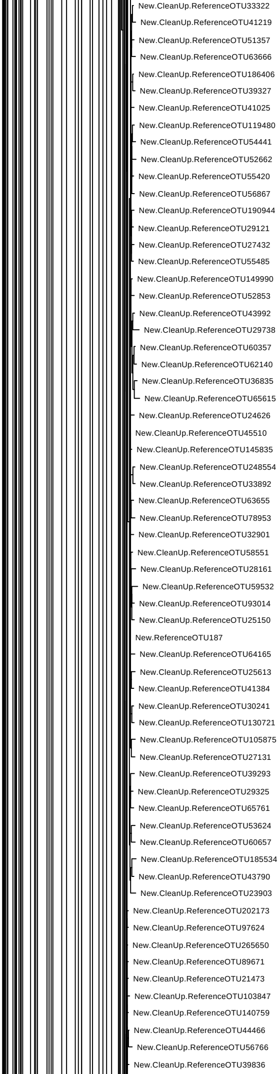
New.CleanUp.ReferenceOTU33322
New.CleanUp.ReferenceOTU41219
New.CleanUp.ReferenceOTU51357
New.CleanUp.ReferenceOTU63666
New.CleanUp.ReferenceOTU186406
New.CleanUp.ReferenceOTU39327
New.CleanUp.ReferenceOTU41025
New.CleanUp.ReferenceOTU119480
New.CleanUp.ReferenceOTU54441
New.CleanUp.ReferenceOTU52662
New.CleanUp.ReferenceOTU55420
New.CleanUp.ReferenceOTU56867
New.CleanUp.ReferenceOTU190944
New.CleanUp.ReferenceOTU29121
New.CleanUp.ReferenceOTU27432
New.CleanUp.ReferenceOTU55485
New.CleanUp.ReferenceOTU149990
New.CleanUp.ReferenceOTU52853
New.CleanUp.ReferenceOTU43992
New.CleanUp.ReferenceOTU29738
New.CleanUp.ReferenceOTU60357
New.CleanUp.ReferenceOTU62140
New.CleanUp.ReferenceOTU36835
New.CleanUp.ReferenceOTU65615
New.CleanUp.ReferenceOTU24626
New.CleanUp.ReferenceOTU45510
New.CleanUp.ReferenceOTU145835
New.CleanUp.ReferenceOTU248554
New.CleanUp.ReferenceOTU33892
New.CleanUp.ReferenceOTU63655
New.CleanUp.ReferenceOTU78953
New.CleanUp.ReferenceOTU32901
New.CleanUp.ReferenceOTU58551
New.CleanUp.ReferenceOTU28161
New.CleanUp.ReferenceOTU59532
New.CleanUp.ReferenceOTU93014
New.CleanUp.ReferenceOTU25150
New.ReferenceOTU187
New.CleanUp.ReferenceOTU64165
New.CleanUp.ReferenceOTU25613
New.CleanUp.ReferenceOTU41384
New.CleanUp.ReferenceOTU30241
New.CleanUp.ReferenceOTU130721
New.CleanUp.ReferenceOTU105875
New.CleanUp.ReferenceOTU27131
New.CleanUp.ReferenceOTU39293
New.CleanUp.ReferenceOTU29325
New.CleanUp.ReferenceOTU65761
New.CleanUp.ReferenceOTU53624
New.CleanUp.ReferenceOTU60657
New.CleanUp.ReferenceOTU185534
New.CleanUp.ReferenceOTU43790
New.CleanUp.ReferenceOTU23903
New.CleanUp.ReferenceOTU202173
New.CleanUp.ReferenceOTU97624
New.CleanUp.ReferenceOTU265650
New.CleanUp.ReferenceOTU89671
New.CleanUp.ReferenceOTU21473
New.CleanUp.ReferenceOTU103847
New.CleanUp.ReferenceOTU140759
New.CleanUp.ReferenceOTU44466
New.CleanUp.ReferenceOTU56766
New.CleanUp.ReferenceOTU39836

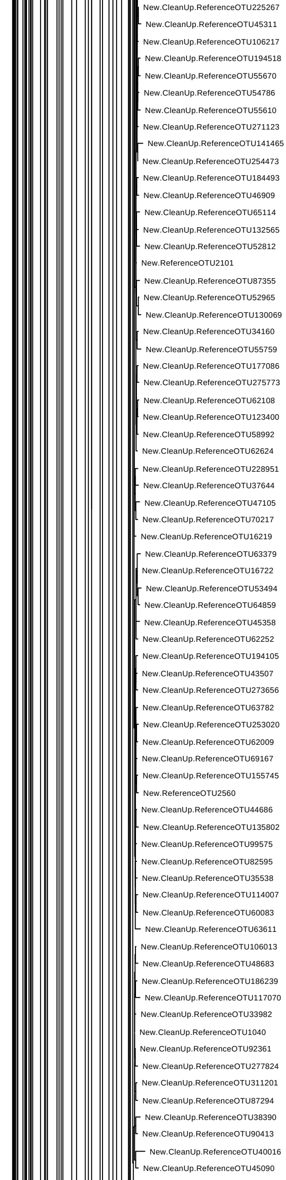
New.CleanUp.ReferenceOTU225267
New.CleanUp.ReferenceOTU45311
New.CleanUp.ReferenceOTU106217
New.CleanUp.ReferenceOTU194518
New.CleanUp.ReferenceOTU55670
New.CleanUp.ReferenceOTU54786
New.CleanUp.ReferenceOTU55610
New.CleanUp.ReferenceOTU271123
New.CleanUp.ReferenceOTU141465
New.CleanUp.ReferenceOTU254473
New.CleanUp.ReferenceOTU184493
New.CleanUp.ReferenceOTU46909
New.CleanUp.ReferenceOTU65114
New.CleanUp.ReferenceOTU132565
New.CleanUp.ReferenceOTU52812
New.ReferenceOTU2101
New.CleanUp.ReferenceOTU87355
New.CleanUp.ReferenceOTU52965
New.CleanUp.ReferenceOTU130069
New.CleanUp.ReferenceOTU34160
New.CleanUp.ReferenceOTU55759
New.CleanUp.ReferenceOTU177086
New.CleanUp.ReferenceOTU275773
New.CleanUp.ReferenceOTU62108
New.CleanUp.ReferenceOTU123400
New.CleanUp.ReferenceOTU58992
New.CleanUp.ReferenceOTU62624
New.CleanUp.ReferenceOTU228951
New.CleanUp.ReferenceOTU37644
New.CleanUp.ReferenceOTU47105
New.CleanUp.ReferenceOTU70217
New.CleanUp.ReferenceOTU16219
New.CleanUp.ReferenceOTU63379
New.CleanUp.ReferenceOTU16722
New.CleanUp.ReferenceOTU53494
New.CleanUp.ReferenceOTU64859
New.CleanUp.ReferenceOTU45358
New.CleanUp.ReferenceOTU62252
New.CleanUp.ReferenceOTU194105
New.CleanUp.ReferenceOTU43507
New.CleanUp.ReferenceOTU273656
New.CleanUp.ReferenceOTU63782
New.CleanUp.ReferenceOTU253020
New.CleanUp.ReferenceOTU62009
New.CleanUp.ReferenceOTU69167
New.CleanUp.ReferenceOTU155745
New.ReferenceOTU2560
New.CleanUp.ReferenceOTU44686
New.CleanUp.ReferenceOTU135802
New.CleanUp.ReferenceOTU99575
New.CleanUp.ReferenceOTU82595
New.CleanUp.ReferenceOTU35538
New.CleanUp.ReferenceOTU114007
New.CleanUp.ReferenceOTU60083
New.CleanUp.ReferenceOTU63611
New.CleanUp.ReferenceOTU106013
New.CleanUp.ReferenceOTU48683
New.CleanUp.ReferenceOTU186239
New.CleanUp.ReferenceOTU117070
New.CleanUp.ReferenceOTU33982
New.CleanUp.ReferenceOTU1040
New.CleanUp.ReferenceOTU92361
New.CleanUp.ReferenceOTU277824
New.CleanUp.ReferenceOTU311201
New.CleanUp.ReferenceOTU87294
New.CleanUp.ReferenceOTU38390
New.CleanUp.ReferenceOTU90413
New.CleanUp.ReferenceOTU40016
New.CleanUp.ReferenceOTU45090

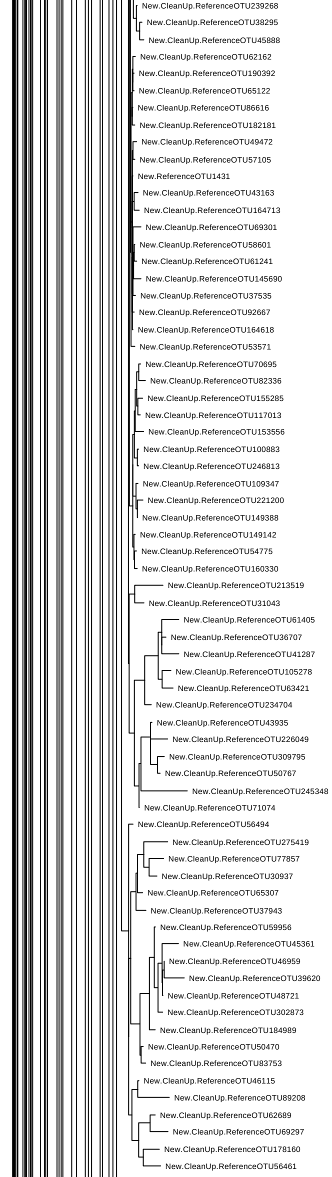
New.CleanUp.ReferenceOTU239268
New.CleanUp.ReferenceOTU38295
New.CleanUp.ReferenceOTU45888
New.CleanUp.ReferenceOTU62162
New.CleanUp.ReferenceOTU190392
New.CleanUp.ReferenceOTU65122
New.CleanUp.ReferenceOTU86616
New.CleanUp.ReferenceOTU182181
New.CleanUp.ReferenceOTU49472
New.CleanUp.ReferenceOTU57105
New.ReferenceOTU1431
New.CleanUp.ReferenceOTU43163
New.CleanUp.ReferenceOTU164713
New.CleanUp.ReferenceOTU69301
New.CleanUp.ReferenceOTU58601
New.CleanUp.ReferenceOTU61241
New.CleanUp.ReferenceOTU145690
New.CleanUp.ReferenceOTU37535
New.CleanUp.ReferenceOTU92667
New.CleanUp.ReferenceOTU164618
New.CleanUp.ReferenceOTU53571
New.CleanUp.ReferenceOTU70695
New.CleanUp.ReferenceOTU82336
New.CleanUp.ReferenceOTU155285
New.CleanUp.ReferenceOTU117013
New.CleanUp.ReferenceOTU153556
New.CleanUp.ReferenceOTU100883
New.CleanUp.ReferenceOTU246813
New.CleanUp.ReferenceOTU109347
New.CleanUp.ReferenceOTU221200
New.CleanUp.ReferenceOTU149388
New.CleanUp.ReferenceOTU149142
New.CleanUp.ReferenceOTU54775
New.CleanUp.ReferenceOTU160330
New.CleanUp.ReferenceOTU213519
New.CleanUp.ReferenceOTU31043
New.CleanUp.ReferenceOTU61405
New.CleanUp.ReferenceOTU36707
New.CleanUp.ReferenceOTU41287
New.CleanUp.ReferenceOTU105278
New.CleanUp.ReferenceOTU63421
New.CleanUp.ReferenceOTU234704
New.CleanUp.ReferenceOTU43935
New.CleanUp.ReferenceOTU226049
New.CleanUp.ReferenceOTU309795
New.CleanUp.ReferenceOTU50767
New.CleanUp.ReferenceOTU245348
New.CleanUp.ReferenceOTU71074
New.CleanUp.ReferenceOTU56494
New.CleanUp.ReferenceOTU275419
New.CleanUp.ReferenceOTU77857
New.CleanUp.ReferenceOTU30937
New.CleanUp.ReferenceOTU65307
New.CleanUp.ReferenceOTU37943
New.CleanUp.ReferenceOTU59956
New.CleanUp.ReferenceOTU45361
New.CleanUp.ReferenceOTU46959
New.CleanUp.ReferenceOTU39620
New.CleanUp.ReferenceOTU48721
New.CleanUp.ReferenceOTU302873
New.CleanUp.ReferenceOTU184989
New.CleanUp.ReferenceOTU50470
New.CleanUp.ReferenceOTU83753
New.CleanUp.ReferenceOTU46115
New.CleanUp.ReferenceOTU89208
New.CleanUp.ReferenceOTU62689
New.CleanUp.ReferenceOTU69297
New.CleanUp.ReferenceOTU178160
New.CleanUp.ReferenceOTU56461

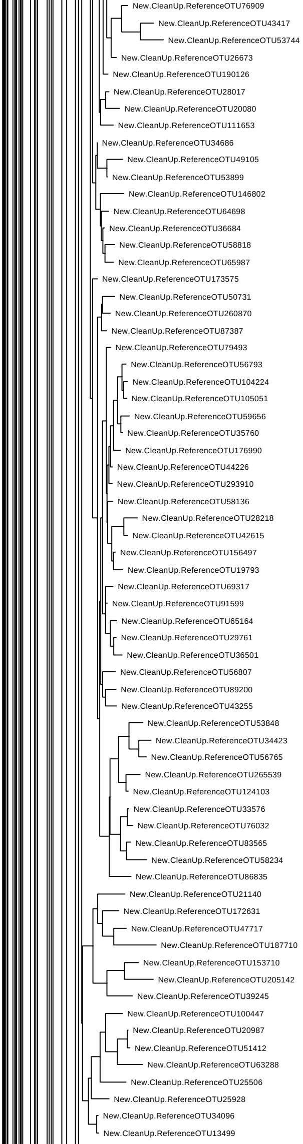
New.CleanUp.ReferenceOTU76909
New.CleanUp.ReferenceOTU43417
New.CleanUp.ReferenceOTU53744
New.CleanUp.ReferenceOTU26673
New.CleanUp.ReferenceOTU190126
New.CleanUp.ReferenceOTU28017
New.CleanUp.ReferenceOTU20080
New.CleanUp.ReferenceOTU111653
New.CleanUp.ReferenceOTU34686
New.CleanUp.ReferenceOTU49105
New.CleanUp.ReferenceOTU53899
New.CleanUp.ReferenceOTU146802
New.CleanUp.ReferenceOTU64698
New.CleanUp.ReferenceOTU36684
New.CleanUp.ReferenceOTU58818
New.CleanUp.ReferenceOTU65987
New.CleanUp.ReferenceOTU173575
New.CleanUp.ReferenceOTU50731
New.CleanUp.ReferenceOTU260870
New.CleanUp.ReferenceOTU87387
New.CleanUp.ReferenceOTU79493
New.CleanUp.ReferenceOTU56793
New.CleanUp.ReferenceOTU104224
New.CleanUp.ReferenceOTU105051
New.CleanUp.ReferenceOTU59656
New.CleanUp.ReferenceOTU35760
New.CleanUp.ReferenceOTU176990
New.CleanUp.ReferenceOTU44226
New.CleanUp.ReferenceOTU293910
New.CleanUp.ReferenceOTU58136
New.CleanUp.ReferenceOTU28218
New.CleanUp.ReferenceOTU42615
New.CleanUp.ReferenceOTU156497
New.CleanUp.ReferenceOTU19793
New.CleanUp.ReferenceOTU69317
New.CleanUp.ReferenceOTU91599
New.CleanUp.ReferenceOTU65164
New.CleanUp.ReferenceOTU29761
New.CleanUp.ReferenceOTU36501
New.CleanUp.ReferenceOTU56807
New.CleanUp.ReferenceOTU89200
New.CleanUp.ReferenceOTU43255
New.CleanUp.ReferenceOTU53848
New.CleanUp.ReferenceOTU34423
New.CleanUp.ReferenceOTU56765
New.CleanUp.ReferenceOTU265539
New.CleanUp.ReferenceOTU124103
New.CleanUp.ReferenceOTU33576
New.CleanUp.ReferenceOTU76032
New.CleanUp.ReferenceOTU83565
New.CleanUp.ReferenceOTU58234
New.CleanUp.ReferenceOTU86835
New.CleanUp.ReferenceOTU21140
New.CleanUp.ReferenceOTU172631
New.CleanUp.ReferenceOTU47717
New.CleanUp.ReferenceOTU187710
New.CleanUp.ReferenceOTU153710
New.CleanUp.ReferenceOTU205142
New.CleanUp.ReferenceOTU39245
New.CleanUp.ReferenceOTU100447
New.CleanUp.ReferenceOTU20987
New.CleanUp.ReferenceOTU51412
New.CleanUp.ReferenceOTU63288
New.CleanUp.ReferenceOTU25506
New.CleanUp.ReferenceOTU25928
New.CleanUp.ReferenceOTU34096
New.CleanUp.ReferenceOTU13499

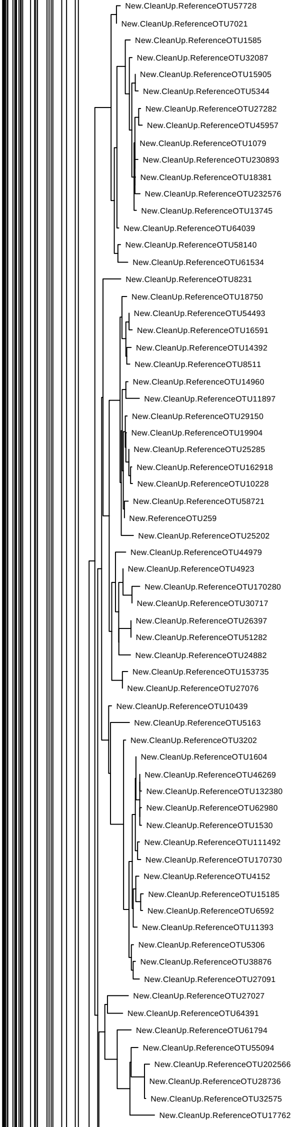
New.CleanUp.ReferenceOTU57728
New.CleanUp.ReferenceOTU7021
New.CleanUp.ReferenceOTU1585
New.CleanUp.ReferenceOTU32087
New.CleanUp.ReferenceOTU15905
New.CleanUp.ReferenceOTU5344
New.CleanUp.ReferenceOTU27282
New.CleanUp.ReferenceOTU45957
New.CleanUp.ReferenceOTU1079
New.CleanUp.ReferenceOTU230893
New.CleanUp.ReferenceOTU18381
New.CleanUp.ReferenceOTU232576
New.CleanUp.ReferenceOTU13745
New.CleanUp.ReferenceOTU64039
New.CleanUp.ReferenceOTU58140
New.CleanUp.ReferenceOTU61534
New.CleanUp.ReferenceOTU8231
New.CleanUp.ReferenceOTU18750
New.CleanUp.ReferenceOTU54493
New.CleanUp.ReferenceOTU16591
New.CleanUp.ReferenceOTU14392
New.CleanUp.ReferenceOTU8511
New.CleanUp.ReferenceOTU14960
New.CleanUp.ReferenceOTU11897
New.CleanUp.ReferenceOTU29150
New.CleanUp.ReferenceOTU19904
New.CleanUp.ReferenceOTU25285
New.CleanUp.ReferenceOTU162918
New.CleanUp.ReferenceOTU10228
New.CleanUp.ReferenceOTU58721
New.ReferenceOTU259
New.CleanUp.ReferenceOTU25202
New.CleanUp.ReferenceOTU44979
New.CleanUp.ReferenceOTU4923
New.CleanUp.ReferenceOTU170280
New.CleanUp.ReferenceOTU30717
New.CleanUp.ReferenceOTU26397
New.CleanUp.ReferenceOTU51282
New.CleanUp.ReferenceOTU24882
New.CleanUp.ReferenceOTU153735
New.CleanUp.ReferenceOTU27076
New.CleanUp.ReferenceOTU10439
New.CleanUp.ReferenceOTU5163
New.CleanUp.ReferenceOTU3202
New.CleanUp.ReferenceOTU1604
New.CleanUp.ReferenceOTU46269
New.CleanUp.ReferenceOTU132380
New.CleanUp.ReferenceOTU62980
New.CleanUp.ReferenceOTU1530
New.CleanUp.ReferenceOTU111492
New.CleanUp.ReferenceOTU170730
New.CleanUp.ReferenceOTU4152
New.CleanUp.ReferenceOTU15185
New.CleanUp.ReferenceOTU6592
New.CleanUp.ReferenceOTU11393
New.CleanUp.ReferenceOTU5306
New.CleanUp.ReferenceOTU38876
New.CleanUp.ReferenceOTU27091
New.CleanUp.ReferenceOTU27027
New.CleanUp.ReferenceOTU64391
New.CleanUp.ReferenceOTU61794
New.CleanUp.ReferenceOTU55094
New.CleanUp.ReferenceOTU202566
New.CleanUp.ReferenceOTU28736
New.CleanUp.ReferenceOTU32575
New.CleanUp.ReferenceOTU17762

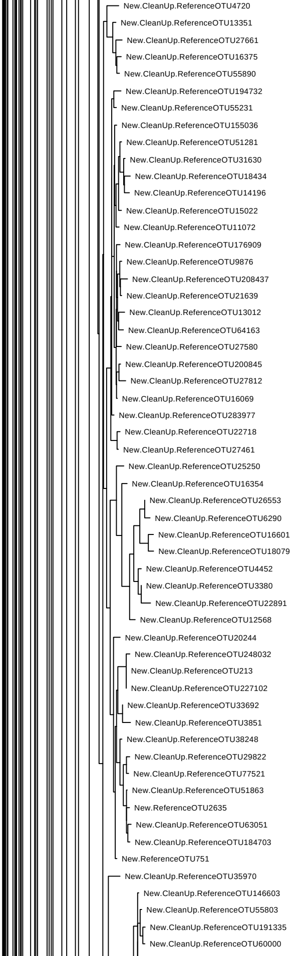
New.CleanUp.ReferenceOTU4720
New.CleanUp.ReferenceOTU13351
New.CleanUp.ReferenceOTU27661
New.CleanUp.ReferenceOTU16375
New.CleanUp.ReferenceOTU55890
New.CleanUp.ReferenceOTU194732
New.CleanUp.ReferenceOTU55231
New.CleanUp.ReferenceOTU155036
New.CleanUp.ReferenceOTU51281
New.CleanUp.ReferenceOTU31630
New.CleanUp.ReferenceOTU18434
New.CleanUp.ReferenceOTU14196
New.CleanUp.ReferenceOTU15022
New.CleanUp.ReferenceOTU11072
New.CleanUp.ReferenceOTU176909
New.CleanUp.ReferenceOTU9876
New.CleanUp.ReferenceOTU208437
New.CleanUp.ReferenceOTU21639
New.CleanUp.ReferenceOTU13012
New.CleanUp.ReferenceOTU64163
New.CleanUp.ReferenceOTU27580
New.CleanUp.ReferenceOTU200845
New.CleanUp.ReferenceOTU27812
New.CleanUp.ReferenceOTU16069
New.CleanUp.ReferenceOTU283977
New.CleanUp.ReferenceOTU22718
New.CleanUp.ReferenceOTU27461
New.CleanUp.ReferenceOTU25250
New.CleanUp.ReferenceOTU16354
New.CleanUp.ReferenceOTU26553
New.CleanUp.ReferenceOTU6290
New.CleanUp.ReferenceOTU16601
New.CleanUp.ReferenceOTU18079
New.CleanUp.ReferenceOTU4452
New.CleanUp.ReferenceOTU3380
New.CleanUp.ReferenceOTU22891
New.CleanUp.ReferenceOTU12568
New.CleanUp.ReferenceOTU20244
New.CleanUp.ReferenceOTU248032
New.CleanUp.ReferenceOTU213
New.CleanUp.ReferenceOTU227102
New.CleanUp.ReferenceOTU33692
New.CleanUp.ReferenceOTU3851
New.CleanUp.ReferenceOTU38248
New.CleanUp.ReferenceOTU29822
New.CleanUp.ReferenceOTU77521
New.CleanUp.ReferenceOTU51863
New.ReferenceOTU2635
New.CleanUp.ReferenceOTU63051
New.CleanUp.ReferenceOTU184703
New.ReferenceOTU751
New.CleanUp.ReferenceOTU35970
New.CleanUp.ReferenceOTU146603
New.CleanUp.ReferenceOTU55803
New.CleanUp.ReferenceOTU191335
New.CleanUp.ReferenceOTU60000

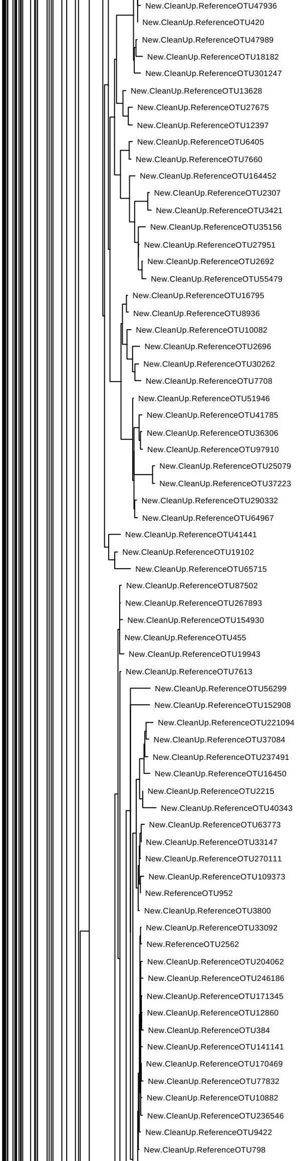
New.CleanUp.ReferenceOTU47936
New.CleanUp.ReferenceOTU420
New.CleanUp.ReferenceOTU47989
New.CleanUp.ReferenceOTU18182
New.CleanUp.ReferenceOTU301247
New.CleanUp.ReferenceOTU13628
New.CleanUp.ReferenceOTU27675
New.CleanUp.ReferenceOTU12397
New.CleanUp.ReferenceOTU6405
New.CleanUp.ReferenceOTU7660
New.CleanUp.ReferenceOTU164452
New.CleanUp.ReferenceOTU2307
New.CleanUp.ReferenceOTU3421
New.CleanUp.ReferenceOTU35156
New.CleanUp.ReferenceOTU27951
New.CleanUp.ReferenceOTU2692
New.CleanUp.ReferenceOTU55479
New.CleanUp.ReferenceOTU16795
New.CleanUp.ReferenceOTU8936
New.CleanUp.ReferenceOTU10082
New.CleanUp.ReferenceOTU2696
New.CleanUp.ReferenceOTU30262
New.CleanUp.ReferenceOTU7708
New.CleanUp.ReferenceOTU51946
New.CleanUp.ReferenceOTU41785
New.CleanUp.ReferenceOTU36306
New.CleanUp.ReferenceOTU97910
New.CleanUp.ReferenceOTU25079
New.CleanUp.ReferenceOTU37223
New.CleanUp.ReferenceOTU290332
New.CleanUp.ReferenceOTU64967
New.CleanUp.ReferenceOTU41441
New.CleanUp.ReferenceOTU19102
New.CleanUp.ReferenceOTU65715
New.CleanUp.ReferenceOTU87502
New.CleanUp.ReferenceOTU267893
New.CleanUp.ReferenceOTU154930
New.CleanUp.ReferenceOTU455
New.CleanUp.ReferenceOTU19943
New.CleanUp.ReferenceOTU7613
New.CleanUp.ReferenceOTU56299
New.CleanUp.ReferenceOTU152908
New.CleanUp.ReferenceOTU221094
New.CleanUp.ReferenceOTU37084
New.CleanUp.ReferenceOTU237491
New.CleanUp.ReferenceOTU16450
New.CleanUp.ReferenceOTU2215
New.CleanUp.ReferenceOTU40343
New.CleanUp.ReferenceOTU63773
New.CleanUp.ReferenceOTU33147
New.CleanUp.ReferenceOTU270111
New.CleanUp.ReferenceOTU109373
New.ReferenceOTU952
New.CleanUp.ReferenceOTU3800
New.CleanUp.ReferenceOTU33092
New.ReferenceOTU2562
New.CleanUp.ReferenceOTU204062
New.CleanUp.ReferenceOTU246186
New.CleanUp.ReferenceOTU171345
New.CleanUp.ReferenceOTU12860
New.CleanUp.ReferenceOTU384
New.CleanUp.ReferenceOTU141141
New.CleanUp.ReferenceOTU170469
New.CleanUp.ReferenceOTU77832
New.CleanUp.ReferenceOTU10882
New.CleanUp.ReferenceOTU236546
New.CleanUp.ReferenceOTU9422
New.CleanUp.ReferenceOTU798

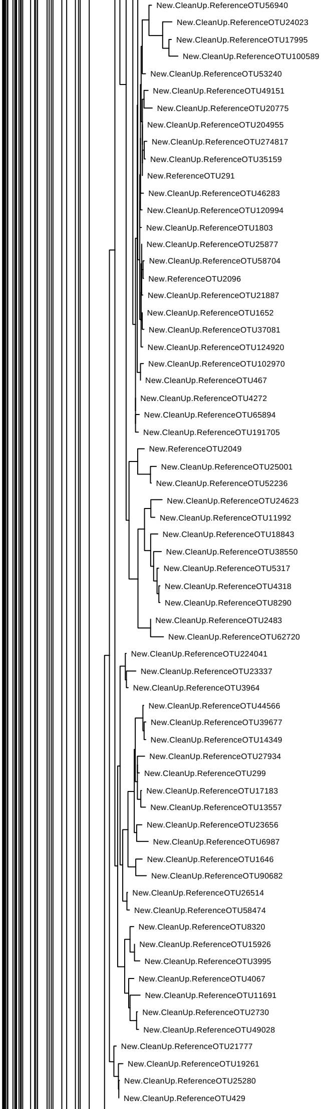
New.CleanUp.ReferenceOTU56940
New.CleanUp.ReferenceOTU24023
New.CleanUp.ReferenceOTU17995
New.CleanUp.ReferenceOTU100589
New.CleanUp.ReferenceOTU53240
New.CleanUp.ReferenceOTU49151
New.CleanUp.ReferenceOTU20775
New.CleanUp.ReferenceOTU204955
New.CleanUp.ReferenceOTU274817
New.CleanUp.ReferenceOTU35159
New.ReferenceOTU291
New.CleanUp.ReferenceOTU46283
New.CleanUp.ReferenceOTU120994
New.CleanUp.ReferenceOTU1803
New.CleanUp.ReferenceOTU25877
New.CleanUp.ReferenceOTU58704
New.ReferenceOTU2096
New.CleanUp.ReferenceOTU21887
New.CleanUp.ReferenceOTU1652
New.CleanUp.ReferenceOTU37081
New.CleanUp.ReferenceOTU124920
New.CleanUp.ReferenceOTU102970
New.CleanUp.ReferenceOTU467
New.CleanUp.ReferenceOTU4272
New.CleanUp.ReferenceOTU65894
New.CleanUp.ReferenceOTU191705
New.ReferenceOTU2049
New.CleanUp.ReferenceOTU25001
New.CleanUp.ReferenceOTU52236
New.CleanUp.ReferenceOTU24623
New.CleanUp.ReferenceOTU11992
New.CleanUp.ReferenceOTU18843
New.CleanUp.ReferenceOTU38550
New.CleanUp.ReferenceOTU5317
New.CleanUp.ReferenceOTU4318
New.CleanUp.ReferenceOTU8290
New.CleanUp.ReferenceOTU2483
New.CleanUp.ReferenceOTU62720
New.CleanUp.ReferenceOTU224041
New.CleanUp.ReferenceOTU23337
New.CleanUp.ReferenceOTU3964
New.CleanUp.ReferenceOTU44566
New.CleanUp.ReferenceOTU39677
New.CleanUp.ReferenceOTU14349
New.CleanUp.ReferenceOTU27934
New.CleanUp.ReferenceOTU299
New.CleanUp.ReferenceOTU17183
New.CleanUp.ReferenceOTU13557
New.CleanUp.ReferenceOTU23656
New.CleanUp.ReferenceOTU6987
New.CleanUp.ReferenceOTU1646
New.CleanUp.ReferenceOTU90682
New.CleanUp.ReferenceOTU26514
New.CleanUp.ReferenceOTU58474
New.CleanUp.ReferenceOTU8320
New.CleanUp.ReferenceOTU15926
New.CleanUp.ReferenceOTU3995
New.CleanUp.ReferenceOTU4067
New.CleanUp.ReferenceOTU11691
New.CleanUp.ReferenceOTU2730
New.CleanUp.ReferenceOTU49028
New.CleanUp.ReferenceOTU21777
New.CleanUp.ReferenceOTU19261
New.CleanUp.ReferenceOTU25280
New.CleanUp.ReferenceOTU429

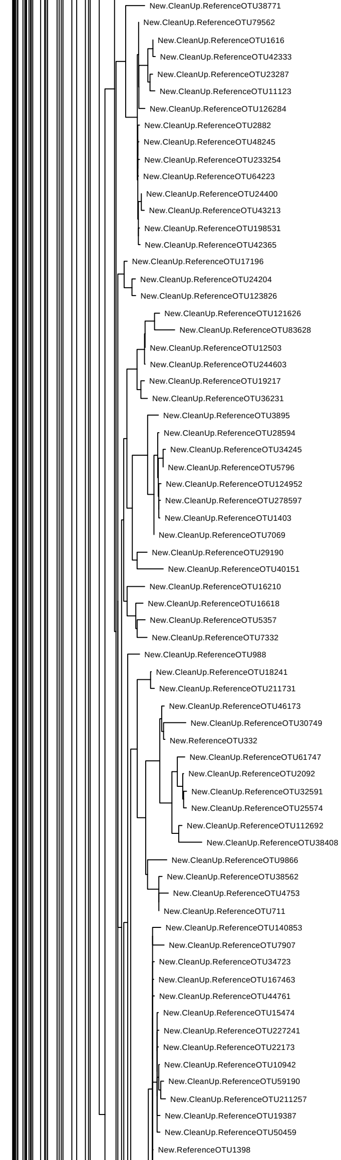
New.CleanUp.ReferenceOTU38771
New.CleanUp.ReferenceOTU79562
New.CleanUp.ReferenceOTU1616
New.CleanUp.ReferenceOTU42333
New.CleanUp.ReferenceOTU23287
New.CleanUp.ReferenceOTU11123
New.CleanUp.ReferenceOTU126284
New.CleanUp.ReferenceOTU2882
New.CleanUp.ReferenceOTU48245
New.CleanUp.ReferenceOTU233254
New.CleanUp.ReferenceOTU64223
New.CleanUp.ReferenceOTU24400
New.CleanUp.ReferenceOTU43213
New.CleanUp.ReferenceOTU198531
New.CleanUp.ReferenceOTU42365
New.CleanUp.ReferenceOTU17196
New.CleanUp.ReferenceOTU24204
New.CleanUp.ReferenceOTU123826
New.CleanUp.ReferenceOTU121626
New.CleanUp.ReferenceOTU83628
New.CleanUp.ReferenceOTU12503
New.CleanUp.ReferenceOTU244603
New.CleanUp.ReferenceOTU19217
New.CleanUp.ReferenceOTU36231
New.CleanUp.ReferenceOTU3895
New.CleanUp.ReferenceOTU28594
New.CleanUp.ReferenceOTU34245
New.CleanUp.ReferenceOTU5796
New.CleanUp.ReferenceOTU124952
New.CleanUp.ReferenceOTU278597
New.CleanUp.ReferenceOTU1403
New.CleanUp.ReferenceOTU7069
New.CleanUp.ReferenceOTU29190
New.CleanUp.ReferenceOTU40151
New.CleanUp.ReferenceOTU16210
New.CleanUp.ReferenceOTU16618
New.CleanUp.ReferenceOTU5357
New.CleanUp.ReferenceOTU7332
New.CleanUp.ReferenceOTU988
New.CleanUp.ReferenceOTU18241
New.CleanUp.ReferenceOTU211731
New.CleanUp.ReferenceOTU46173
New.CleanUp.ReferenceOTU30749
New.ReferenceOTU332
New.CleanUp.ReferenceOTU61747
New.CleanUp.ReferenceOTU2092
New.CleanUp.ReferenceOTU32591
New.CleanUp.ReferenceOTU25574
New.CleanUp.ReferenceOTU112692
New.CleanUp.ReferenceOTU38408
New.CleanUp.ReferenceOTU9866
New.CleanUp.ReferenceOTU38562
New.CleanUp.ReferenceOTU4753
New.CleanUp.ReferenceOTU711
New.CleanUp.ReferenceOTU140853
New.CleanUp.ReferenceOTU7907
New.CleanUp.ReferenceOTU34723
New.CleanUp.ReferenceOTU167463
New.CleanUp.ReferenceOTU44761
New.CleanUp.ReferenceOTU15474
New.CleanUp.ReferenceOTU227241
New.CleanUp.ReferenceOTU22173
New.CleanUp.ReferenceOTU10942
New.CleanUp.ReferenceOTU59190
New.CleanUp.ReferenceOTU211257
New.CleanUp.ReferenceOTU19387
New.CleanUp.ReferenceOTU50459
New.ReferenceOTU1398

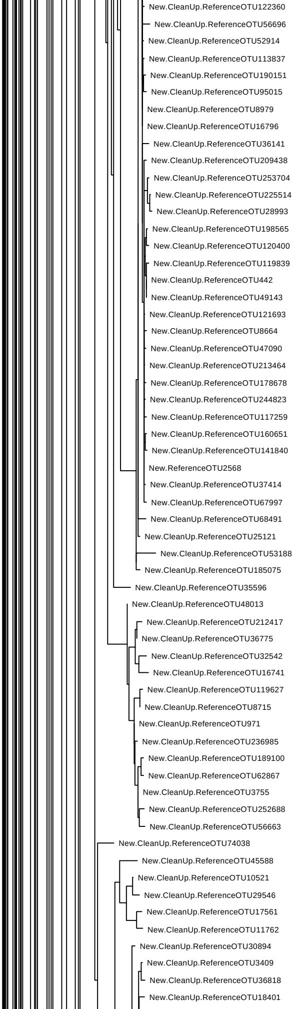
New.CleanUp.ReferenceOTU122360
New.CleanUp.ReferenceOTU56696
New.CleanUp.ReferenceOTU52914
New.CleanUp.ReferenceOTU113837
New.CleanUp.ReferenceOTU190151
New.CleanUp.ReferenceOTU95015
New.CleanUp.ReferenceOTU8979
New.CleanUp.ReferenceOTU16796
New.CleanUp.ReferenceOTU36141
New.CleanUp.ReferenceOTU209438
New.CleanUp.ReferenceOTU253704
New.CleanUp.ReferenceOTU225514
New.CleanUp.ReferenceOTU28993
New.CleanUp.ReferenceOTU198565
New.CleanUp.ReferenceOTU120400
New.CleanUp.ReferenceOTU119839
New.CleanUp.ReferenceOTU442
New.CleanUp.ReferenceOTU49143
New.CleanUp.ReferenceOTU121693
New.CleanUp.ReferenceOTU8664
New.CleanUp.ReferenceOTU47090
New.CleanUp.ReferenceOTU213464
New.CleanUp.ReferenceOTU178678
New.CleanUp.ReferenceOTU244823
New.CleanUp.ReferenceOTU117259
New.CleanUp.ReferenceOTU160651
New.CleanUp.ReferenceOTU141840
New.ReferenceOTU2568
New.CleanUp.ReferenceOTU37414
New.CleanUp.ReferenceOTU67997
New.CleanUp.ReferenceOTU68491
New.CleanUp.ReferenceOTU25121
New.CleanUp.ReferenceOTU53188
New.CleanUp.ReferenceOTU185075
New.CleanUp.ReferenceOTU35596
New.CleanUp.ReferenceOTU48013
New.CleanUp.ReferenceOTU212417
New.CleanUp.ReferenceOTU36775
New.CleanUp.ReferenceOTU32542
New.CleanUp.ReferenceOTU16741
New.CleanUp.ReferenceOTU119627
New.CleanUp.ReferenceOTU8715
New.CleanUp.ReferenceOTU971
New.CleanUp.ReferenceOTU236985
New.CleanUp.ReferenceOTU189100
New.CleanUp.ReferenceOTU62867
New.CleanUp.ReferenceOTU3755
New.CleanUp.ReferenceOTU252688
New.CleanUp.ReferenceOTU56663
New.CleanUp.ReferenceOTU74038
New.CleanUp.ReferenceOTU45588
New.CleanUp.ReferenceOTU10521
New.CleanUp.ReferenceOTU29546
New.CleanUp.ReferenceOTU17561
New.CleanUp.ReferenceOTU11762
New.CleanUp.ReferenceOTU30894
New.CleanUp.ReferenceOTU3409
New.CleanUp.ReferenceOTU36818
New.CleanUp.ReferenceOTU18401

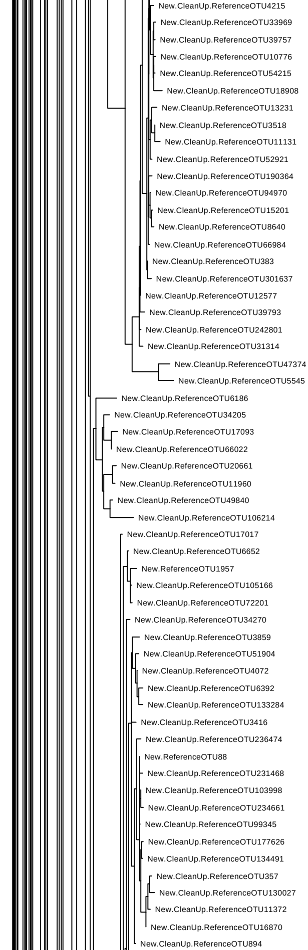
New.CleanUp.ReferenceOTU4215
New.CleanUp.ReferenceOTU33969
New.CleanUp.ReferenceOTU39757
New.CleanUp.ReferenceOTU10776
New.CleanUp.ReferenceOTU54215
New.CleanUp.ReferenceOTU18908
New.CleanUp.ReferenceOTU13231
New.CleanUp.ReferenceOTU3518
New.CleanUp.ReferenceOTU11131
New.CleanUp.ReferenceOTU52921
New.CleanUp.ReferenceOTU190364
New.CleanUp.ReferenceOTU94970
New.CleanUp.ReferenceOTU15201
New.CleanUp.ReferenceOTU8640
New.CleanUp.ReferenceOTU66984
New.CleanUp.ReferenceOTU383
New.CleanUp.ReferenceOTU301637
New.CleanUp.ReferenceOTU12577
New.CleanUp.ReferenceOTU39793
New.CleanUp.ReferenceOTU242801
New.CleanUp.ReferenceOTU31314
New.CleanUp.ReferenceOTU47374
New.CleanUp.ReferenceOTU5545
New.CleanUp.ReferenceOTU6186
New.CleanUp.ReferenceOTU34205
New.CleanUp.ReferenceOTU17093
New.CleanUp.ReferenceOTU66022
New.CleanUp.ReferenceOTU20661
New.CleanUp.ReferenceOTU11960
New.CleanUp.ReferenceOTU49840
New.CleanUp.ReferenceOTU106214
New.CleanUp.ReferenceOTU17017
New.CleanUp.ReferenceOTU6652
New.ReferenceOTU1957
New.CleanUp.ReferenceOTU105166
New.CleanUp.ReferenceOTU72201
New.CleanUp.ReferenceOTU34270
New.CleanUp.ReferenceOTU3859
New.CleanUp.ReferenceOTU51904
New.CleanUp.ReferenceOTU4072
New.CleanUp.ReferenceOTU6392
New.CleanUp.ReferenceOTU133284
New.CleanUp.ReferenceOTU3416
New.CleanUp.ReferenceOTU236474
New.ReferenceOTU88
New.CleanUp.ReferenceOTU231468
New.CleanUp.ReferenceOTU103998
New.CleanUp.ReferenceOTU234661
New.CleanUp.ReferenceOTU99345
New.CleanUp.ReferenceOTU177626
New.CleanUp.ReferenceOTU134491
New.CleanUp.ReferenceOTU357
New.CleanUp.ReferenceOTU130027
New.CleanUp.ReferenceOTU11372
New.CleanUp.ReferenceOTU16870
New.CleanUp.ReferenceOTU894

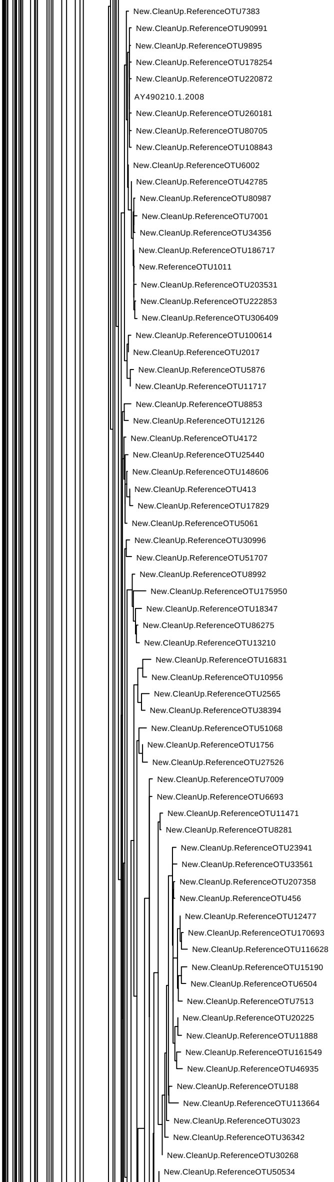
New.CleanUp.ReferenceOTU7383
New.CleanUp.ReferenceOTU90991
New.CleanUp.ReferenceOTU9895
New.CleanUp.ReferenceOTU178254
New.CleanUp.ReferenceOTU220872
AY490210.1.2008
New.CleanUp.ReferenceOTU260181
New.CleanUp.ReferenceOTU80705
New.CleanUp.ReferenceOTU108843
New.CleanUp.ReferenceOTU6002
New.CleanUp.ReferenceOTU42785
New.CleanUp.ReferenceOTU80987
New.CleanUp.ReferenceOTU7001
New.CleanUp.ReferenceOTU34356
New.CleanUp.ReferenceOTU186717
New.ReferenceOTU1011
New.CleanUp.ReferenceOTU203531
New.CleanUp.ReferenceOTU222853
New.CleanUp.ReferenceOTU306409
New.CleanUp.ReferenceOTU100614
New.CleanUp.ReferenceOTU2017
New.CleanUp.ReferenceOTU5876
New.CleanUp.ReferenceOTU11717
New.CleanUp.ReferenceOTU8853
New.CleanUp.ReferenceOTU12126
New.CleanUp.ReferenceOTU4172
New.CleanUp.ReferenceOTU25440
New.CleanUp.ReferenceOTU148606
New.CleanUp.ReferenceOTU413
New.CleanUp.ReferenceOTU17829
New.CleanUp.ReferenceOTU5061
New.CleanUp.ReferenceOTU30996
New.CleanUp.ReferenceOTU51707
New.CleanUp.ReferenceOTU8992
New.CleanUp.ReferenceOTU175950
New.CleanUp.ReferenceOTU18347
New.CleanUp.ReferenceOTU86275
New.CleanUp.ReferenceOTU13210
New.CleanUp.ReferenceOTU16831
New.CleanUp.ReferenceOTU10956
New.CleanUp.ReferenceOTU2565
New.CleanUp.ReferenceOTU38394
New.CleanUp.ReferenceOTU51068
New.CleanUp.ReferenceOTU1756
New.CleanUp.ReferenceOTU27526
New.CleanUp.ReferenceOTU7009
New.CleanUp.ReferenceOTU6693
New.CleanUp.ReferenceOTU11471
New.CleanUp.ReferenceOTU8281
New.CleanUp.ReferenceOTU23941
New.CleanUp.ReferenceOTU33561
New.CleanUp.ReferenceOTU207358
New.CleanUp.ReferenceOTU456
New.CleanUp.ReferenceOTU12477
New.CleanUp.ReferenceOTU170693
New.CleanUp.ReferenceOTU116628
New.CleanUp.ReferenceOTU15190
New.CleanUp.ReferenceOTU6504
New.CleanUp.ReferenceOTU7513
New.CleanUp.ReferenceOTU20225
New.CleanUp.ReferenceOTU11888
New.CleanUp.ReferenceOTU161549
New.CleanUp.ReferenceOTU46935
New.CleanUp.ReferenceOTU188
New.CleanUp.ReferenceOTU113664
New.CleanUp.ReferenceOTU3023
New.CleanUp.ReferenceOTU36342
New.CleanUp.ReferenceOTU30268
New.CleanUp.ReferenceOTU50534

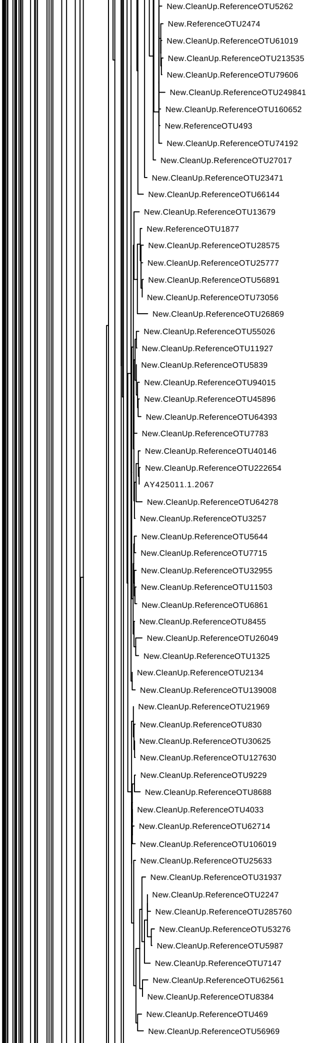
New.CleanUp.ReferenceOTU5262
New.ReferenceOTU2474
New.CleanUp.ReferenceOTU61019
New.CleanUp.ReferenceOTU213535
New.CleanUp.ReferenceOTU79606
New.CleanUp.ReferenceOTU249841
New.CleanUp.ReferenceOTU160652
New.ReferenceOTU493
New.CleanUp.ReferenceOTU74192
New.CleanUp.ReferenceOTU27017
New.CleanUp.ReferenceOTU23471
New.CleanUp.ReferenceOTU66144
New.CleanUp.ReferenceOTU13679
New.ReferenceOTU1877
New.CleanUp.ReferenceOTU28575
New.CleanUp.ReferenceOTU25777
New.CleanUp.ReferenceOTU56891
New.CleanUp.ReferenceOTU73056
New.CleanUp.ReferenceOTU26869
New.CleanUp.ReferenceOTU55026
New.CleanUp.ReferenceOTU11927
New.CleanUp.ReferenceOTU5839
New.CleanUp.ReferenceOTU94015
New.CleanUp.ReferenceOTU45896
New.CleanUp.ReferenceOTU64393
New.CleanUp.ReferenceOTU7783
New.CleanUp.ReferenceOTU40146
New.CleanUp.ReferenceOTU222654
AY425011.1.2067
New.CleanUp.ReferenceOTU64278
New.CleanUp.ReferenceOTU3257
New.CleanUp.ReferenceOTU5644
New.CleanUp.ReferenceOTU7715
New.CleanUp.ReferenceOTU32955
New.CleanUp.ReferenceOTU11503
New.CleanUp.ReferenceOTU6861
New.CleanUp.ReferenceOTU8455
New.CleanUp.ReferenceOTU26049
New.CleanUp.ReferenceOTU1325
New.CleanUp.ReferenceOTU2134
New.CleanUp.ReferenceOTU139008
New.CleanUp.ReferenceOTU21969
New.CleanUp.ReferenceOTU830
New.CleanUp.ReferenceOTU30625
New.CleanUp.ReferenceOTU127630
New.CleanUp.ReferenceOTU9229
New.CleanUp.ReferenceOTU8688
New.CleanUp.ReferenceOTU4033
New.CleanUp.ReferenceOTU62714
New.CleanUp.ReferenceOTU106019
New.CleanUp.ReferenceOTU25633
New.CleanUp.ReferenceOTU31937
New.CleanUp.ReferenceOTU2247
New.CleanUp.ReferenceOTU285760
New.CleanUp.ReferenceOTU53276
New.CleanUp.ReferenceOTU5987
New.CleanUp.ReferenceOTU7147
New.CleanUp.ReferenceOTU62561
New.CleanUp.ReferenceOTU8384
New.CleanUp.ReferenceOTU469
New.CleanUp.ReferenceOTU56969

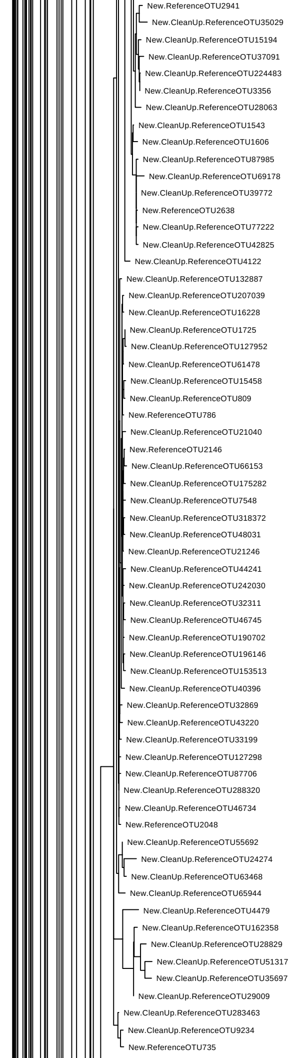
New.ReferenceOTU2941
New.CleanUp.ReferenceOTU35029
New.CleanUp.ReferenceOTU15194
New.CleanUp.ReferenceOTU37091
New.CleanUp.ReferenceOTU224483
New.CleanUp.ReferenceOTU3356
New.CleanUp.ReferenceOTU28063
New.CleanUp.ReferenceOTU1543
New.CleanUp.ReferenceOTU1606
New.CleanUp.ReferenceOTU87985
New.CleanUp.ReferenceOTU69178
New.CleanUp.ReferenceOTU39772
New.ReferenceOTU2638
New.CleanUp.ReferenceOTU77222
New.CleanUp.ReferenceOTU42825
New.CleanUp.ReferenceOTU4122
New.CleanUp.ReferenceOTU132887
New.CleanUp.ReferenceOTU207039
New.CleanUp.ReferenceOTU16228
New.CleanUp.ReferenceOTU1725
New.CleanUp.ReferenceOTU127952
New.CleanUp.ReferenceOTU61478
New.CleanUp.ReferenceOTU15458
New.CleanUp.ReferenceOTU809
New.ReferenceOTU786
New.CleanUp.ReferenceOTU21040
New.ReferenceOTU2146
New.CleanUp.ReferenceOTU66153
New.CleanUp.ReferenceOTU175282
New.CleanUp.ReferenceOTU7548
New.CleanUp.ReferenceOTU318372
New.CleanUp.ReferenceOTU48031
New.CleanUp.ReferenceOTU21246
New.CleanUp.ReferenceOTU44241
New.CleanUp.ReferenceOTU242030
New.CleanUp.ReferenceOTU32311
New.CleanUp.ReferenceOTU46745
New.CleanUp.ReferenceOTU190702
New.CleanUp.ReferenceOTU196146
New.CleanUp.ReferenceOTU153513
New.CleanUp.ReferenceOTU40396
New.CleanUp.ReferenceOTU32869
New.CleanUp.ReferenceOTU43220
New.CleanUp.ReferenceOTU33199
New.CleanUp.ReferenceOTU127298
New.CleanUp.ReferenceOTU87706
New.CleanUp.ReferenceOTU288320
New.CleanUp.ReferenceOTU46734
New.ReferenceOTU2048
New.CleanUp.ReferenceOTU55692
New.CleanUp.ReferenceOTU24274
New.CleanUp.ReferenceOTU63468
New.CleanUp.ReferenceOTU65944
New.CleanUp.ReferenceOTU4479
New.CleanUp.ReferenceOTU162358
New.CleanUp.ReferenceOTU28829
New.CleanUp.ReferenceOTU51317
New.CleanUp.ReferenceOTU35697
New.CleanUp.ReferenceOTU29009
New.CleanUp.ReferenceOTU283463
New.CleanUp.ReferenceOTU9234
New.ReferenceOTU735

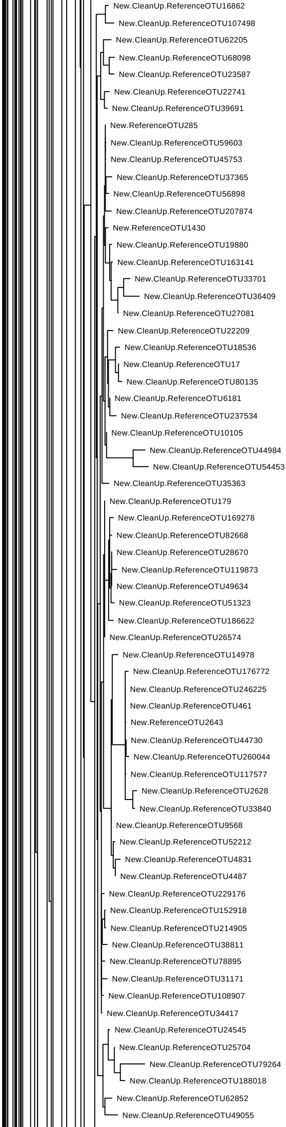
New.CleanUp.ReferenceOTU16862
New.CleanUp.ReferenceOTU107498
New.CleanUp.ReferenceOTU62205
New.CleanUp.ReferenceOTU68098
New.CleanUp.ReferenceOTU23587
New.CleanUp.ReferenceOTU22741
New.CleanUp.ReferenceOTU39691
New.ReferenceOTU285
New.CleanUp.ReferenceOTU59603
New.CleanUp.ReferenceOTU45753
New.CleanUp.ReferenceOTU37365
New.CleanUp.ReferenceOTU56898
New.CleanUp.ReferenceOTU207874
New.ReferenceOTU1430
New.CleanUp.ReferenceOTU19880
New.CleanUp.ReferenceOTU163141
New.CleanUp.ReferenceOTU33701
New.CleanUp.ReferenceOTU36409
New.CleanUp.ReferenceOTU27081
New.CleanUp.ReferenceOTU22209
New.CleanUp.ReferenceOTU18536
New.CleanUp.ReferenceOTU17
New.CleanUp.ReferenceOTU80135
New.CleanUp.ReferenceOTU6181
New.CleanUp.ReferenceOTU237534
New.CleanUp.ReferenceOTU10105
New.CleanUp.ReferenceOTU44984
New.CleanUp.ReferenceOTU54453
New.CleanUp.ReferenceOTU35363
New.CleanUp.ReferenceOTU179
New.CleanUp.ReferenceOTU169278
New.CleanUp.ReferenceOTU82668
New.CleanUp.ReferenceOTU28670
New.CleanUp.ReferenceOTU119873
New.CleanUp.ReferenceOTU49634
New.CleanUp.ReferenceOTU51323
New.CleanUp.ReferenceOTU186622
New.CleanUp.ReferenceOTU26574
New.CleanUp.ReferenceOTU14978
New.CleanUp.ReferenceOTU176772
New.CleanUp.ReferenceOTU246225
New.CleanUp.ReferenceOTU461
New.ReferenceOTU2643
New.CleanUp.ReferenceOTU44730
New.CleanUp.ReferenceOTU260044
New.CleanUp.ReferenceOTU117577
New.CleanUp.ReferenceOTU2628
New.CleanUp.ReferenceOTU33840
New.CleanUp.ReferenceOTU9568
New.CleanUp.ReferenceOTU52212
New.CleanUp.ReferenceOTU4831
New.CleanUp.ReferenceOTU4487
New.CleanUp.ReferenceOTU229176
New.CleanUp.ReferenceOTU152918
New.CleanUp.ReferenceOTU214905
New.CleanUp.ReferenceOTU38811
New.CleanUp.ReferenceOTU78895
New.CleanUp.ReferenceOTU31171
New.CleanUp.ReferenceOTU108907
New.CleanUp.ReferenceOTU34417
New.CleanUp.ReferenceOTU24545
New.CleanUp.ReferenceOTU25704
New.CleanUp.ReferenceOTU79264
New.CleanUp.ReferenceOTU188018
New.CleanUp.ReferenceOTU62852
New.CleanUp.ReferenceOTU49055

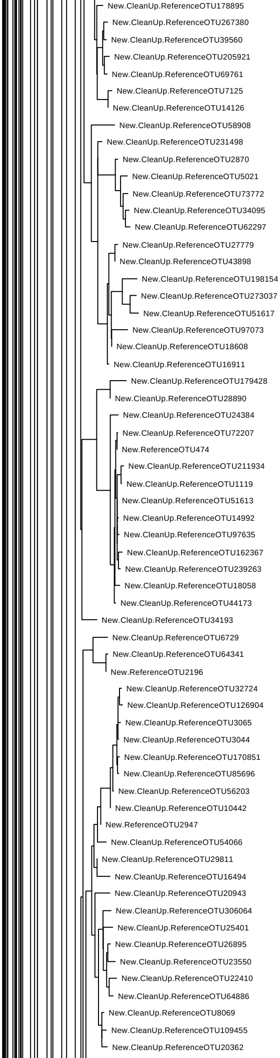
New.CleanUp.ReferenceOTU178895
New.CleanUp.ReferenceOTU267380
New.CleanUp.ReferenceOTU39560
New.CleanUp.ReferenceOTU205921
New.CleanUp.ReferenceOTU69761
New.CleanUp.ReferenceOTU7125
New.CleanUp.ReferenceOTU14126
New.CleanUp.ReferenceOTU58908
New.CleanUp.ReferenceOTU231498
New.CleanUp.ReferenceOTU2870
New.CleanUp.ReferenceOTU5021
New.CleanUp.ReferenceOTU73772
New.CleanUp.ReferenceOTU34095
New.CleanUp.ReferenceOTU62297
New.CleanUp.ReferenceOTU27779
New.CleanUp.ReferenceOTU43898
New.CleanUp.ReferenceOTU198154
New.CleanUp.ReferenceOTU273037
New.CleanUp.ReferenceOTU51617
New.CleanUp.ReferenceOTU97073
New.CleanUp.ReferenceOTU18608
New.CleanUp.ReferenceOTU16911
New.CleanUp.ReferenceOTU179428
New.CleanUp.ReferenceOTU28890
New.CleanUp.ReferenceOTU24384
New.CleanUp.ReferenceOTU72207
New.ReferenceOTU474
New.CleanUp.ReferenceOTU211934
New.CleanUp.ReferenceOTU1119
New.CleanUp.ReferenceOTU51613
New.CleanUp.ReferenceOTU14992
New.CleanUp.ReferenceOTU97635
New.CleanUp.ReferenceOTU162367
New.CleanUp.ReferenceOTU239263
New.CleanUp.ReferenceOTU18058
New.CleanUp.ReferenceOTU44173
New.CleanUp.ReferenceOTU34193
New.CleanUp.ReferenceOTU6729
New.CleanUp.ReferenceOTU64341
New.ReferenceOTU2196
New.CleanUp.ReferenceOTU32724
New.CleanUp.ReferenceOTU126904
New.CleanUp.ReferenceOTU3065
New.CleanUp.ReferenceOTU3044
New.CleanUp.ReferenceOTU170851
New.CleanUp.ReferenceOTU85696
New.CleanUp.ReferenceOTU56203
New.CleanUp.ReferenceOTU10442
New.ReferenceOTU2947
New.CleanUp.ReferenceOTU54066
New.CleanUp.ReferenceOTU29811
New.CleanUp.ReferenceOTU16494
New.CleanUp.ReferenceOTU20943
New.CleanUp.ReferenceOTU306064
New.CleanUp.ReferenceOTU25401
New.CleanUp.ReferenceOTU26895
New.CleanUp.ReferenceOTU23550
New.CleanUp.ReferenceOTU22410
New.CleanUp.ReferenceOTU64886
New.CleanUp.ReferenceOTU8069
New.CleanUp.ReferenceOTU109455
New.CleanUp.ReferenceOTU20362

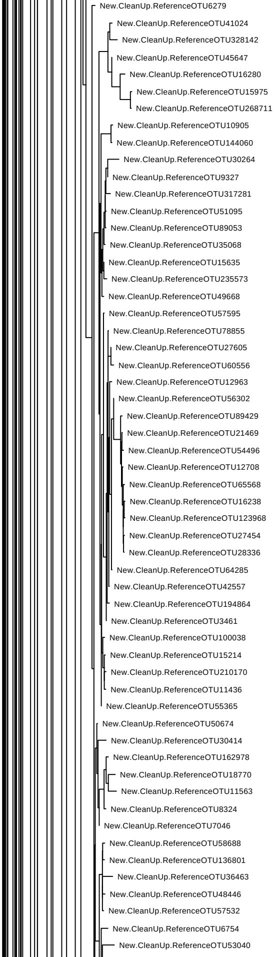
New.CleanUp.ReferenceOTU6279
New.CleanUp.ReferenceOTU41024
New.CleanUp.ReferenceOTU328142
New.CleanUp.ReferenceOTU45647
New.CleanUp.ReferenceOTU16280
New.CleanUp.ReferenceOTU15975
New.CleanUp.ReferenceOTU268711
New.CleanUp.ReferenceOTU10905
New.CleanUp.ReferenceOTU144060
New.CleanUp.ReferenceOTU30264
New.CleanUp.ReferenceOTU9327
New.CleanUp.ReferenceOTU317281
New.CleanUp.ReferenceOTU51095
New.CleanUp.ReferenceOTU89053
New.CleanUp.ReferenceOTU35068
New.CleanUp.ReferenceOTU15635
New.CleanUp.ReferenceOTU235573
New.CleanUp.ReferenceOTU49668
New.CleanUp.ReferenceOTU57595
New.CleanUp.ReferenceOTU78855
New.CleanUp.ReferenceOTU27605
New.CleanUp.ReferenceOTU60556
New.CleanUp.ReferenceOTU12963
New.CleanUp.ReferenceOTU56302
New.CleanUp.ReferenceOTU89429
New.CleanUp.ReferenceOTU21469
New.CleanUp.ReferenceOTU54496
New.CleanUp.ReferenceOTU12708
New.CleanUp.ReferenceOTU65568
New.CleanUp.ReferenceOTU16238
New.CleanUp.ReferenceOTU123968
New.CleanUp.ReferenceOTU27454
New.CleanUp.ReferenceOTU28336
New.CleanUp.ReferenceOTU64285
New.CleanUp.ReferenceOTU42557
New.CleanUp.ReferenceOTU194864
New.CleanUp.ReferenceOTU3461
New.CleanUp.ReferenceOTU100038
New.CleanUp.ReferenceOTU15214
New.CleanUp.ReferenceOTU210170
New.CleanUp.ReferenceOTU11436
New.CleanUp.ReferenceOTU55365
New.CleanUp.ReferenceOTU50674
New.CleanUp.ReferenceOTU30414
New.CleanUp.ReferenceOTU162978
New.CleanUp.ReferenceOTU18770
New.CleanUp.ReferenceOTU11563
New.CleanUp.ReferenceOTU8324
New.CleanUp.ReferenceOTU7046
New.CleanUp.ReferenceOTU58688
New.CleanUp.ReferenceOTU136801
New.CleanUp.ReferenceOTU36463
New.CleanUp.ReferenceOTU48446
New.CleanUp.ReferenceOTU57532
New.CleanUp.ReferenceOTU6754
New.CleanUp.ReferenceOTU53040

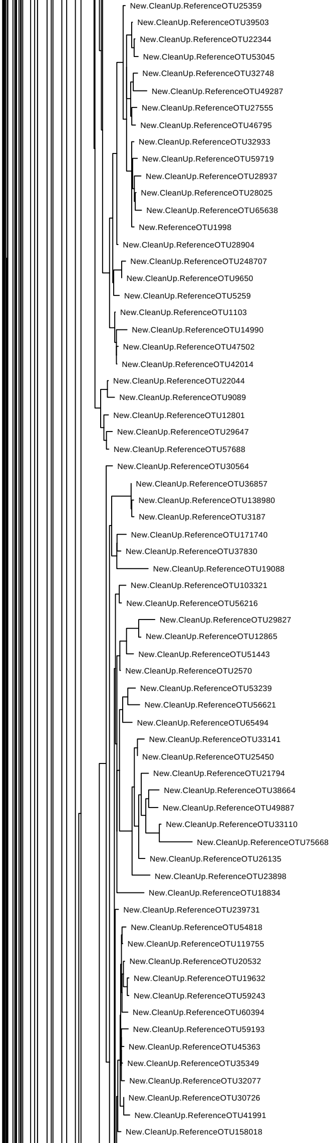
New.CleanUp.ReferenceOTU25359
New.CleanUp.ReferenceOTU39503
New.CleanUp.ReferenceOTU22344
New.CleanUp.ReferenceOTU53045
New.CleanUp.ReferenceOTU32748
New.CleanUp.ReferenceOTU49287
New.CleanUp.ReferenceOTU27555
New.CleanUp.ReferenceOTU46795
New.CleanUp.ReferenceOTU32933
New.CleanUp.ReferenceOTU59719
New.CleanUp.ReferenceOTU28937
New.CleanUp.ReferenceOTU28025
New.CleanUp.ReferenceOTU65638
New.ReferenceOTU1998
New.CleanUp.ReferenceOTU28904
New.CleanUp.ReferenceOTU248707
New.CleanUp.ReferenceOTU9650
New.CleanUp.ReferenceOTU5259
New.CleanUp.ReferenceOTU1103
New.CleanUp.ReferenceOTU14990
New.CleanUp.ReferenceOTU47502
New.CleanUp.ReferenceOTU42014
New.CleanUp.ReferenceOTU22044
New.CleanUp.ReferenceOTU9089
New.CleanUp.ReferenceOTU12801
New.CleanUp.ReferenceOTU29647
New.CleanUp.ReferenceOTU57688
New.CleanUp.ReferenceOTU30564
New.CleanUp.ReferenceOTU36857
New.CleanUp.ReferenceOTU138980
New.CleanUp.ReferenceOTU3187
New.CleanUp.ReferenceOTU171740
New.CleanUp.ReferenceOTU37830
New.CleanUp.ReferenceOTU19088
New.CleanUp.ReferenceOTU103321
New.CleanUp.ReferenceOTU56216
New.CleanUp.ReferenceOTU29827
New.CleanUp.ReferenceOTU12865
New.CleanUp.ReferenceOTU51443
New.CleanUp.ReferenceOTU2570
New.CleanUp.ReferenceOTU53239
New.CleanUp.ReferenceOTU56621
New.CleanUp.ReferenceOTU65494
New.CleanUp.ReferenceOTU33141
New.CleanUp.ReferenceOTU25450
New.CleanUp.ReferenceOTU21794
New.CleanUp.ReferenceOTU38664
New.CleanUp.ReferenceOTU49887
New.CleanUp.ReferenceOTU33110
New.CleanUp.ReferenceOTU75668
New.CleanUp.ReferenceOTU26135
New.CleanUp.ReferenceOTU23898
New.CleanUp.ReferenceOTU18834
New.CleanUp.ReferenceOTU239731
New.CleanUp.ReferenceOTU54818
New.CleanUp.ReferenceOTU119755
New.CleanUp.ReferenceOTU20532
New.CleanUp.ReferenceOTU19632
New.CleanUp.ReferenceOTU59243
New.CleanUp.ReferenceOTU60394
New.CleanUp.ReferenceOTU59193
New.CleanUp.ReferenceOTU45363
New.CleanUp.ReferenceOTU35349
New.CleanUp.ReferenceOTU32077
New.CleanUp.ReferenceOTU30726
New.CleanUp.ReferenceOTU41991
New.CleanUp.ReferenceOTU158018

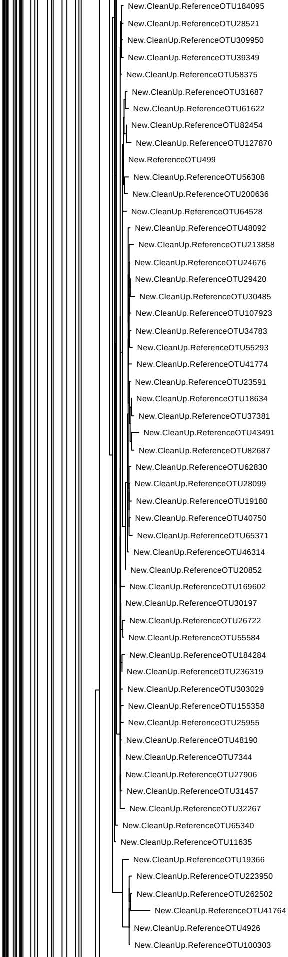
New.CleanUp.ReferenceOTU184095
New.CleanUp.ReferenceOTU28521
New.CleanUp.ReferenceOTU309950
New.CleanUp.ReferenceOTU39349
New.CleanUp.ReferenceOTU58375
New.CleanUp.ReferenceOTU31687
New.CleanUp.ReferenceOTU61622
New.CleanUp.ReferenceOTU82454
New.CleanUp.ReferenceOTU127870
New.ReferenceOTU499
New.CleanUp.ReferenceOTU56308
New.CleanUp.ReferenceOTU200636
New.CleanUp.ReferenceOTU64528
New.CleanUp.ReferenceOTU48092
New.CleanUp.ReferenceOTU213858
New.CleanUp.ReferenceOTU24676
New.CleanUp.ReferenceOTU29420
New.CleanUp.ReferenceOTU30485
New.CleanUp.ReferenceOTU107923
New.CleanUp.ReferenceOTU34783
New.CleanUp.ReferenceOTU55293
New.CleanUp.ReferenceOTU41774
New.CleanUp.ReferenceOTU23591
New.CleanUp.ReferenceOTU18634
New.CleanUp.ReferenceOTU37381
New.CleanUp.ReferenceOTU43491
New.CleanUp.ReferenceOTU82687
New.CleanUp.ReferenceOTU62830
New.CleanUp.ReferenceOTU28099
New.CleanUp.ReferenceOTU19180
New.CleanUp.ReferenceOTU40750
New.CleanUp.ReferenceOTU65371
New.CleanUp.ReferenceOTU46314
New.CleanUp.ReferenceOTU20852
New.CleanUp.ReferenceOTU169602
New.CleanUp.ReferenceOTU30197
New.CleanUp.ReferenceOTU26722
New.CleanUp.ReferenceOTU55584
New.CleanUp.ReferenceOTU184284
New.CleanUp.ReferenceOTU236319
New.CleanUp.ReferenceOTU303029
New.CleanUp.ReferenceOTU155358
New.CleanUp.ReferenceOTU25955
New.CleanUp.ReferenceOTU48190
New.CleanUp.ReferenceOTU7344
New.CleanUp.ReferenceOTU27906
New.CleanUp.ReferenceOTU31457
New.CleanUp.ReferenceOTU32267
New.CleanUp.ReferenceOTU65340
New.CleanUp.ReferenceOTU11635
New.CleanUp.ReferenceOTU19366
New.CleanUp.ReferenceOTU223950
New.CleanUp.ReferenceOTU262502
New.CleanUp.ReferenceOTU41764
New.CleanUp.ReferenceOTU4926
New.CleanUp.ReferenceOTU100303

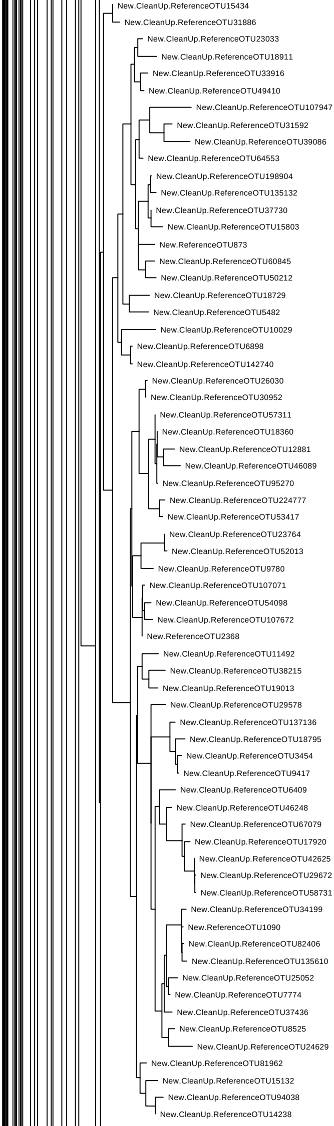
New.CleanUp.ReferenceOTU15434
New.CleanUp.ReferenceOTU31886
New.CleanUp.ReferenceOTU23033
New.CleanUp.ReferenceOTU18911
New.CleanUp.ReferenceOTU33916
New.CleanUp.ReferenceOTU49410
New.CleanUp.ReferenceOTU107947
New.CleanUp.ReferenceOTU31592
New.CleanUp.ReferenceOTU39086
New.CleanUp.ReferenceOTU64553
New.CleanUp.ReferenceOTU198904
New.CleanUp.ReferenceOTU135132
New.CleanUp.ReferenceOTU37730
New.CleanUp.ReferenceOTU15803
New.ReferenceOTU873
New.CleanUp.ReferenceOTU60845
New.CleanUp.ReferenceOTU50212
New.CleanUp.ReferenceOTU18729
New.CleanUp.ReferenceOTU5482
New.CleanUp.ReferenceOTU10029
New.CleanUp.ReferenceOTU6898
New.CleanUp.ReferenceOTU142740
New.CleanUp.ReferenceOTU26030
New.CleanUp.ReferenceOTU30952
New.CleanUp.ReferenceOTU57311
New.CleanUp.ReferenceOTU18360
New.CleanUp.ReferenceOTU12881
New.CleanUp.ReferenceOTU46089
New.CleanUp.ReferenceOTU95270
New.CleanUp.ReferenceOTU224777
New.CleanUp.ReferenceOTU53417
New.CleanUp.ReferenceOTU23764
New.CleanUp.ReferenceOTU52013
New.CleanUp.ReferenceOTU9780
New.CleanUp.ReferenceOTU107071
New.CleanUp.ReferenceOTU54098
New.CleanUp.ReferenceOTU107672
New.ReferenceOTU2368
New.CleanUp.ReferenceOTU11492
New.CleanUp.ReferenceOTU38215
New.CleanUp.ReferenceOTU19013
New.CleanUp.ReferenceOTU29578
New.CleanUp.ReferenceOTU137136
New.CleanUp.ReferenceOTU18795
New.CleanUp.ReferenceOTU3454
New.CleanUp.ReferenceOTU9417
New.CleanUp.ReferenceOTU6409
New.CleanUp.ReferenceOTU46248
New.CleanUp.ReferenceOTU67079
New.CleanUp.ReferenceOTU17920
New.CleanUp.ReferenceOTU42625
New.CleanUp.ReferenceOTU29672
New.CleanUp.ReferenceOTU58731
New.CleanUp.ReferenceOTU34199
New.ReferenceOTU1090
New.CleanUp.ReferenceOTU82406
New.CleanUp.ReferenceOTU135610
New.CleanUp.ReferenceOTU25052
New.CleanUp.ReferenceOTU7774
New.CleanUp.ReferenceOTU37436
New.CleanUp.ReferenceOTU8525
New.CleanUp.ReferenceOTU24629
New.CleanUp.ReferenceOTU81962
New.CleanUp.ReferenceOTU15132
New.CleanUp.ReferenceOTU94038
New.CleanUp.ReferenceOTU14238

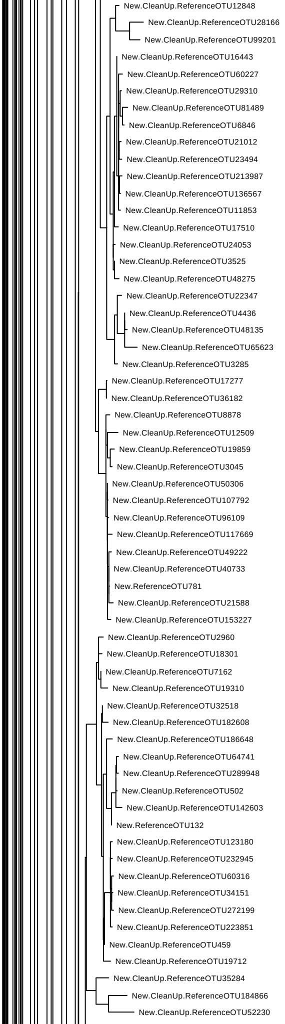
New.CleanUp.ReferenceOTU12848
New.CleanUp.ReferenceOTU28166
New.CleanUp.ReferenceOTU99201
New.CleanUp.ReferenceOTU16443
New.CleanUp.ReferenceOTU60227
New.CleanUp.ReferenceOTU29310
New.CleanUp.ReferenceOTU81489
New.CleanUp.ReferenceOTU6846
New.CleanUp.ReferenceOTU21012
New.CleanUp.ReferenceOTU23494
New.CleanUp.ReferenceOTU213987
New.CleanUp.ReferenceOTU136567
New.CleanUp.ReferenceOTU11853
New.CleanUp.ReferenceOTU17510
New.CleanUp.ReferenceOTU24053
New.CleanUp.ReferenceOTU3525
New.CleanUp.ReferenceOTU48275
New.CleanUp.ReferenceOTU22347
New.CleanUp.ReferenceOTU4436
New.CleanUp.ReferenceOTU48135
New.CleanUp.ReferenceOTU65623
New.CleanUp.ReferenceOTU3285
New.CleanUp.ReferenceOTU17277
New.CleanUp.ReferenceOTU36182
New.CleanUp.ReferenceOTU8878
New.CleanUp.ReferenceOTU12509
New.CleanUp.ReferenceOTU19859
New.CleanUp.ReferenceOTU3045
New.CleanUp.ReferenceOTU50306
New.CleanUp.ReferenceOTU107792
New.CleanUp.ReferenceOTU96109
New.CleanUp.ReferenceOTU117669
New.CleanUp.ReferenceOTU49222
New.CleanUp.ReferenceOTU40733
New.ReferenceOTU781
New.CleanUp.ReferenceOTU21588
New.CleanUp.ReferenceOTU153227
New.CleanUp.ReferenceOTU2960
New.CleanUp.ReferenceOTU18301
New.CleanUp.ReferenceOTU7162
New.CleanUp.ReferenceOTU19310
New.CleanUp.ReferenceOTU32518
New.CleanUp.ReferenceOTU182608
New.CleanUp.ReferenceOTU186648
New.CleanUp.ReferenceOTU64741
New.CleanUp.ReferenceOTU289948
New.CleanUp.ReferenceOTU502
New.CleanUp.ReferenceOTU142603
New.ReferenceOTU132
New.CleanUp.ReferenceOTU123180
New.CleanUp.ReferenceOTU232945
New.CleanUp.ReferenceOTU60316
New.CleanUp.ReferenceOTU34151
New.CleanUp.ReferenceOTU272199
New.CleanUp.ReferenceOTU223851
New.CleanUp.ReferenceOTU459
New.CleanUp.ReferenceOTU19712
New.CleanUp.ReferenceOTU35284
New.CleanUp.ReferenceOTU184866
New.CleanUp.ReferenceOTU52230

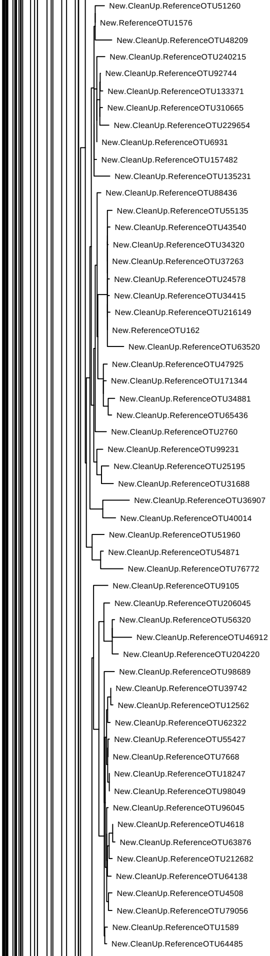
New.CleanUp.ReferenceOTU51260
New.ReferenceOTU1576
New.CleanUp.ReferenceOTU48209
New.CleanUp.ReferenceOTU240215
New.CleanUp.ReferenceOTU92744
New.CleanUp.ReferenceOTU133371
New.CleanUp.ReferenceOTU310665
New.CleanUp.ReferenceOTU229654
New.CleanUp.ReferenceOTU6931
New.CleanUp.ReferenceOTU157482
New.CleanUp.ReferenceOTU135231
New.CleanUp.ReferenceOTU88436
New.CleanUp.ReferenceOTU55135
New.CleanUp.ReferenceOTU43540
New.CleanUp.ReferenceOTU34320
New.CleanUp.ReferenceOTU37263
New.CleanUp.ReferenceOTU24578
New.CleanUp.ReferenceOTU34415
New.CleanUp.ReferenceOTU216149
New.ReferenceOTU162
New.CleanUp.ReferenceOTU63520
New.CleanUp.ReferenceOTU47925
New.CleanUp.ReferenceOTU171344
New.CleanUp.ReferenceOTU34881
New.CleanUp.ReferenceOTU65436
New.CleanUp.ReferenceOTU2760
New.CleanUp.ReferenceOTU99231
New.CleanUp.ReferenceOTU25195
New.CleanUp.ReferenceOTU31688
New.CleanUp.ReferenceOTU36907
New.CleanUp.ReferenceOTU40014
New.CleanUp.ReferenceOTU51960
New.CleanUp.ReferenceOTU54871
New.CleanUp.ReferenceOTU76772
New.CleanUp.ReferenceOTU9105
New.CleanUp.ReferenceOTU206045
New.CleanUp.ReferenceOTU56320
New.CleanUp.ReferenceOTU46912
New.CleanUp.ReferenceOTU204220
New.CleanUp.ReferenceOTU98689
New.CleanUp.ReferenceOTU39742
New.CleanUp.ReferenceOTU12562
New.CleanUp.ReferenceOTU62322
New.CleanUp.ReferenceOTU55427
New.CleanUp.ReferenceOTU7668
New.CleanUp.ReferenceOTU18247
New.CleanUp.ReferenceOTU98049
New.CleanUp.ReferenceOTU96045
New.CleanUp.ReferenceOTU4618
New.CleanUp.ReferenceOTU63876
New.CleanUp.ReferenceOTU212682
New.CleanUp.ReferenceOTU64138
New.CleanUp.ReferenceOTU4508
New.CleanUp.ReferenceOTU79056
New.CleanUp.ReferenceOTU1589
New.CleanUp.ReferenceOTU64485

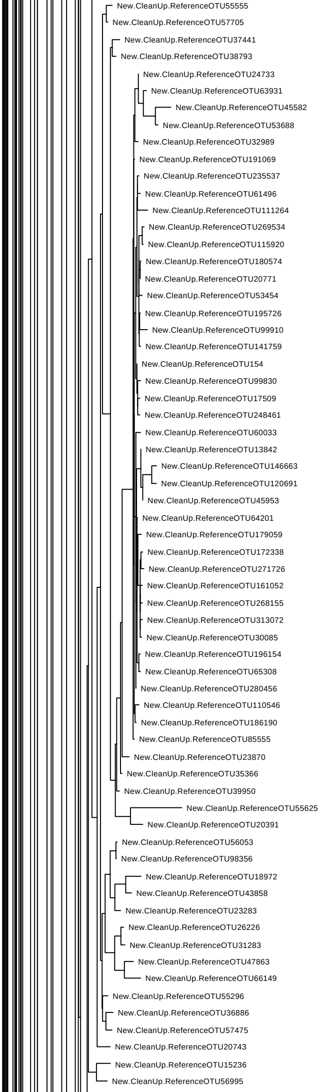
New.CleanUp.ReferenceOTU55555
New.CleanUp.ReferenceOTU57705
New.CleanUp.ReferenceOTU37441
New.CleanUp.ReferenceOTU38793
New.CleanUp.ReferenceOTU24733
New.CleanUp.ReferenceOTU63931
New.CleanUp.ReferenceOTU45582
New.CleanUp.ReferenceOTU53688
New.CleanUp.ReferenceOTU32989
New.CleanUp.ReferenceOTU191069
New.CleanUp.ReferenceOTU235537
New.CleanUp.ReferenceOTU61496
New.CleanUp.ReferenceOTU111264
New.CleanUp.ReferenceOTU269534
New.CleanUp.ReferenceOTU115920
New.CleanUp.ReferenceOTU180574
New.CleanUp.ReferenceOTU20771
New.CleanUp.ReferenceOTU53454
New.CleanUp.ReferenceOTU195726
New.CleanUp.ReferenceOTU99910
New.CleanUp.ReferenceOTU141759
New.CleanUp.ReferenceOTU154
New.CleanUp.ReferenceOTU99830
New.CleanUp.ReferenceOTU17509
New.CleanUp.ReferenceOTU248461
New.CleanUp.ReferenceOTU60033
New.CleanUp.ReferenceOTU13842
New.CleanUp.ReferenceOTU146663
New.CleanUp.ReferenceOTU120691
New.CleanUp.ReferenceOTU45953
New.CleanUp.ReferenceOTU64201
New.CleanUp.ReferenceOTU179059
New.CleanUp.ReferenceOTU172338
New.CleanUp.ReferenceOTU271726
New.CleanUp.ReferenceOTU161052
New.CleanUp.ReferenceOTU268155
New.CleanUp.ReferenceOTU313072
New.CleanUp.ReferenceOTU30085
New.CleanUp.ReferenceOTU196154
New.CleanUp.ReferenceOTU65308
New.CleanUp.ReferenceOTU280456
New.CleanUp.ReferenceOTU110546
New.CleanUp.ReferenceOTU186190
New.CleanUp.ReferenceOTU85555
New.CleanUp.ReferenceOTU23870
New.CleanUp.ReferenceOTU35366
New.CleanUp.ReferenceOTU39950
New.CleanUp.ReferenceOTU55625
New.CleanUp.ReferenceOTU20391
New.CleanUp.ReferenceOTU56053
New.CleanUp.ReferenceOTU98356
New.CleanUp.ReferenceOTU18972
New.CleanUp.ReferenceOTU43858
New.CleanUp.ReferenceOTU23283
New.CleanUp.ReferenceOTU26226
New.CleanUp.ReferenceOTU31283
New.CleanUp.ReferenceOTU47863
New.CleanUp.ReferenceOTU66149
New.CleanUp.ReferenceOTU55296
New.CleanUp.ReferenceOTU36886
New.CleanUp.ReferenceOTU57475
New.CleanUp.ReferenceOTU20743
New.CleanUp.ReferenceOTU15236
New.CleanUp.ReferenceOTU56995

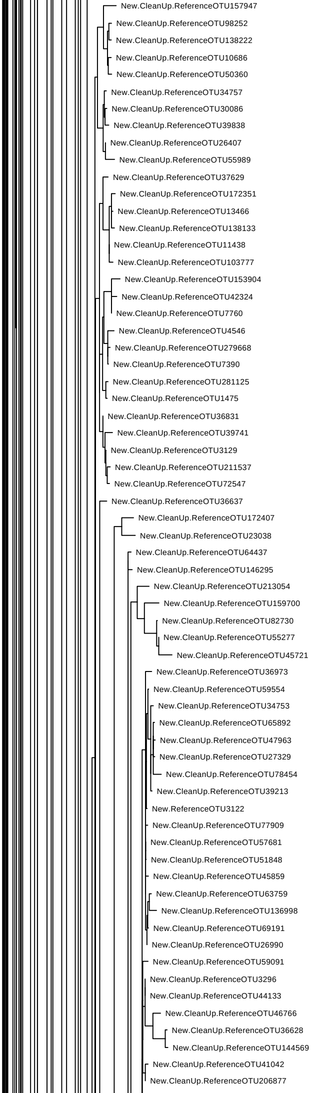
New.CleanUp.ReferenceOTU157947
New.CleanUp.ReferenceOTU98252
New.CleanUp.ReferenceOTU138222
New.CleanUp.ReferenceOTU10686
New.CleanUp.ReferenceOTU50360
New.CleanUp.ReferenceOTU34757
New.CleanUp.ReferenceOTU30086
New.CleanUp.ReferenceOTU39838
New.CleanUp.ReferenceOTU26407
New.CleanUp.ReferenceOTU55989
New.CleanUp.ReferenceOTU37629
New.CleanUp.ReferenceOTU172351
New.CleanUp.ReferenceOTU13466
New.CleanUp.ReferenceOTU138133
New.CleanUp.ReferenceOTU11438
New.CleanUp.ReferenceOTU103777
New.CleanUp.ReferenceOTU153904
New.CleanUp.ReferenceOTU42324
New.CleanUp.ReferenceOTU7760
New.CleanUp.ReferenceOTU4546
New.CleanUp.ReferenceOTU279668
New.CleanUp.ReferenceOTU7390
New.CleanUp.ReferenceOTU281125
New.CleanUp.ReferenceOTU1475
New.CleanUp.ReferenceOTU36831
New.CleanUp.ReferenceOTU39741
New.CleanUp.ReferenceOTU3129
New.CleanUp.ReferenceOTU211537
New.CleanUp.ReferenceOTU72547
New.CleanUp.ReferenceOTU36637
New.CleanUp.ReferenceOTU172407
New.CleanUp.ReferenceOTU23038
New.CleanUp.ReferenceOTU64437
New.CleanUp.ReferenceOTU146295
New.CleanUp.ReferenceOTU213054
New.CleanUp.ReferenceOTU159700
New.CleanUp.ReferenceOTU82730
New.CleanUp.ReferenceOTU55277
New.CleanUp.ReferenceOTU45721
New.CleanUp.ReferenceOTU36973
New.CleanUp.ReferenceOTU59554
New.CleanUp.ReferenceOTU34753
New.CleanUp.ReferenceOTU65892
New.CleanUp.ReferenceOTU47963
New.CleanUp.ReferenceOTU27329
New.CleanUp.ReferenceOTU78454
New.CleanUp.ReferenceOTU39213
New.ReferenceOTU3122
New.CleanUp.ReferenceOTU77909
New.CleanUp.ReferenceOTU57681
New.CleanUp.ReferenceOTU51848
New.CleanUp.ReferenceOTU45859
New.CleanUp.ReferenceOTU63759
New.CleanUp.ReferenceOTU136998
New.CleanUp.ReferenceOTU69191
New.CleanUp.ReferenceOTU26990
New.CleanUp.ReferenceOTU59091
New.CleanUp.ReferenceOTU3296
New.CleanUp.ReferenceOTU44133
New.CleanUp.ReferenceOTU46766
New.CleanUp.ReferenceOTU36628
New.CleanUp.ReferenceOTU144569
New.CleanUp.ReferenceOTU41042
New.CleanUp.ReferenceOTU206877

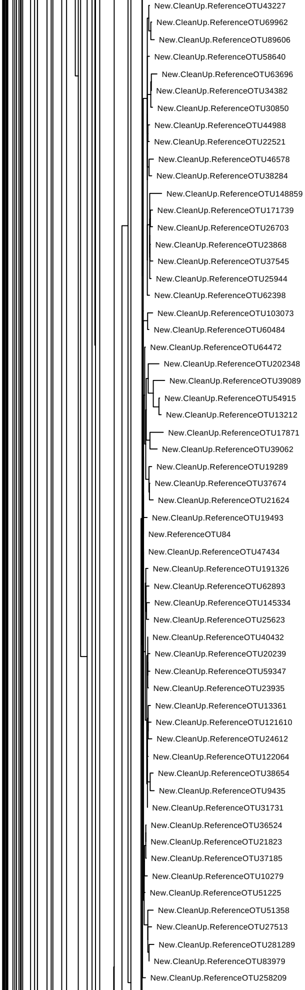
New.CleanUp.ReferenceOTU43227
New.CleanUp.ReferenceOTU69962
New.CleanUp.ReferenceOTU89606
New.CleanUp.ReferenceOTU58640
New.CleanUp.ReferenceOTU63696
New.CleanUp.ReferenceOTU34382
New.CleanUp.ReferenceOTU30850
New.CleanUp.ReferenceOTU44988
New.CleanUp.ReferenceOTU22521
New.CleanUp.ReferenceOTU46578
New.CleanUp.ReferenceOTU38284
New.CleanUp.ReferenceOTU148859
New.CleanUp.ReferenceOTU171739
New.CleanUp.ReferenceOTU26703
New.CleanUp.ReferenceOTU23868
New.CleanUp.ReferenceOTU37545
New.CleanUp.ReferenceOTU25944
New.CleanUp.ReferenceOTU62398
New.CleanUp.ReferenceOTU103073
New.CleanUp.ReferenceOTU60484
New.CleanUp.ReferenceOTU64472
New.CleanUp.ReferenceOTU202348
New.CleanUp.ReferenceOTU39089
New.CleanUp.ReferenceOTU54915
New.CleanUp.ReferenceOTU13212
New.CleanUp.ReferenceOTU17871
New.CleanUp.ReferenceOTU39062
New.CleanUp.ReferenceOTU19289
New.CleanUp.ReferenceOTU37674
New.CleanUp.ReferenceOTU21624
New.CleanUp.ReferenceOTU19493
New.ReferenceOTU84
New.CleanUp.ReferenceOTU47434
New.CleanUp.ReferenceOTU191326
New.CleanUp.ReferenceOTU62893
New.CleanUp.ReferenceOTU145334
New.CleanUp.ReferenceOTU25623
New.CleanUp.ReferenceOTU40432
New.CleanUp.ReferenceOTU20239
New.CleanUp.ReferenceOTU59347
New.CleanUp.ReferenceOTU23935
New.CleanUp.ReferenceOTU13361
New.CleanUp.ReferenceOTU121610
New.CleanUp.ReferenceOTU24612
New.CleanUp.ReferenceOTU122064
New.CleanUp.ReferenceOTU38654
New.CleanUp.ReferenceOTU9435
New.CleanUp.ReferenceOTU31731
New.CleanUp.ReferenceOTU36524
New.CleanUp.ReferenceOTU21823
New.CleanUp.ReferenceOTU37185
New.CleanUp.ReferenceOTU10279
New.CleanUp.ReferenceOTU51225
New.CleanUp.ReferenceOTU51358
New.CleanUp.ReferenceOTU27513
New.CleanUp.ReferenceOTU281289
New.CleanUp.ReferenceOTU83979
New.CleanUp.ReferenceOTU258209

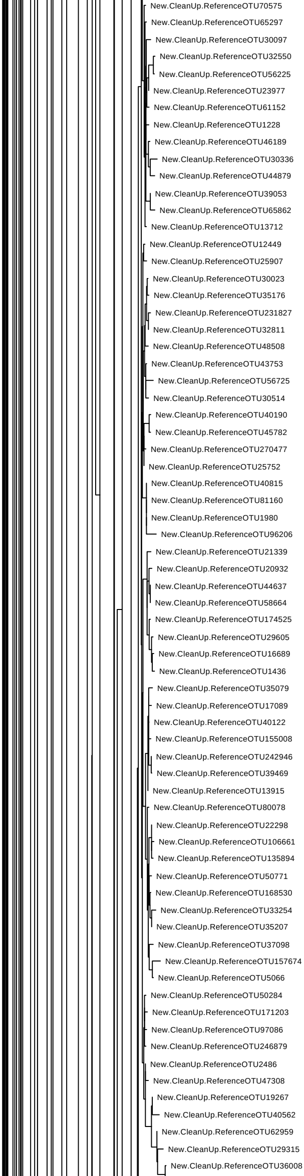
New.CleanUp.ReferenceOTU70575
New.CleanUp.ReferenceOTU65297
New.CleanUp.ReferenceOTU30097
New.CleanUp.ReferenceOTU32550
New.CleanUp.ReferenceOTU56225
New.CleanUp.ReferenceOTU23977
New.CleanUp.ReferenceOTU61152
New.CleanUp.ReferenceOTU1228
New.CleanUp.ReferenceOTU46189
New.CleanUp.ReferenceOTU30336
New.CleanUp.ReferenceOTU44879
New.CleanUp.ReferenceOTU39053
New.CleanUp.ReferenceOTU65862
New.CleanUp.ReferenceOTU13712
New.CleanUp.ReferenceOTU12449
New.CleanUp.ReferenceOTU25907
New.CleanUp.ReferenceOTU30023
New.CleanUp.ReferenceOTU35176
New.CleanUp.ReferenceOTU231827
New.CleanUp.ReferenceOTU32811
New.CleanUp.ReferenceOTU48508
New.CleanUp.ReferenceOTU43753
New.CleanUp.ReferenceOTU56725
New.CleanUp.ReferenceOTU30514
New.CleanUp.ReferenceOTU40190
New.CleanUp.ReferenceOTU45782
New.CleanUp.ReferenceOTU270477
New.CleanUp.ReferenceOTU25752
New.CleanUp.ReferenceOTU40815
New.CleanUp.ReferenceOTU81160
New.CleanUp.ReferenceOTU1980
New.CleanUp.ReferenceOTU96206
New.CleanUp.ReferenceOTU21339
New.CleanUp.ReferenceOTU20932
New.CleanUp.ReferenceOTU44637
New.CleanUp.ReferenceOTU58664
New.CleanUp.ReferenceOTU174525
New.CleanUp.ReferenceOTU29605
New.CleanUp.ReferenceOTU16689
New.CleanUp.ReferenceOTU1436
New.CleanUp.ReferenceOTU35079
New.CleanUp.ReferenceOTU17089
New.CleanUp.ReferenceOTU40122
New.CleanUp.ReferenceOTU155008
New.CleanUp.ReferenceOTU242946
New.CleanUp.ReferenceOTU39469
New.CleanUp.ReferenceOTU13915
New.CleanUp.ReferenceOTU80078
New.CleanUp.ReferenceOTU22298
New.CleanUp.ReferenceOTU106661
New.CleanUp.ReferenceOTU135894
New.CleanUp.ReferenceOTU50771
New.CleanUp.ReferenceOTU168530
New.CleanUp.ReferenceOTU33254
New.CleanUp.ReferenceOTU35207
New.CleanUp.ReferenceOTU37098
New.CleanUp.ReferenceOTU157674
New.CleanUp.ReferenceOTU5066
New.CleanUp.ReferenceOTU50284
New.CleanUp.ReferenceOTU171203
New.CleanUp.ReferenceOTU97086
New.CleanUp.ReferenceOTU246879
New.CleanUp.ReferenceOTU2486
New.CleanUp.ReferenceOTU47308
New.CleanUp.ReferenceOTU19267
New.CleanUp.ReferenceOTU40562
New.CleanUp.ReferenceOTU62959
New.CleanUp.ReferenceOTU29315
New.CleanUp.ReferenceOTU36008

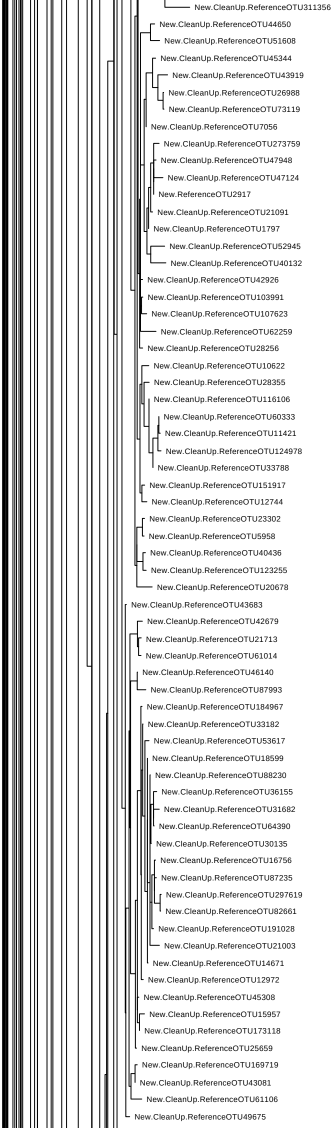
New.CleanUp.ReferenceOTU311356
New.CleanUp.ReferenceOTU44650
New.CleanUp.ReferenceOTU51608
New.CleanUp.ReferenceOTU45344
New.CleanUp.ReferenceOTU43919
New.CleanUp.ReferenceOTU26988
New.CleanUp.ReferenceOTU73119
New.CleanUp.ReferenceOTU7056
New.CleanUp.ReferenceOTU273759
New.CleanUp.ReferenceOTU47948
New.CleanUp.ReferenceOTU47124
New.ReferenceOTU2917
New.CleanUp.ReferenceOTU21091
New.CleanUp.ReferenceOTU1797
New.CleanUp.ReferenceOTU52945
New.CleanUp.ReferenceOTU40132
New.CleanUp.ReferenceOTU42926
New.CleanUp.ReferenceOTU103991
New.CleanUp.ReferenceOTU107623
New.CleanUp.ReferenceOTU62259
New.CleanUp.ReferenceOTU28256
New.CleanUp.ReferenceOTU10622
New.CleanUp.ReferenceOTU28355
New.CleanUp.ReferenceOTU116106
New.CleanUp.ReferenceOTU60333
New.CleanUp.ReferenceOTU11421
New.CleanUp.ReferenceOTU124978
New.CleanUp.ReferenceOTU33788
New.CleanUp.ReferenceOTU151917
New.CleanUp.ReferenceOTU12744
New.CleanUp.ReferenceOTU23302
New.CleanUp.ReferenceOTU5958
New.CleanUp.ReferenceOTU40436
New.CleanUp.ReferenceOTU123255
New.CleanUp.ReferenceOTU20678
New.CleanUp.ReferenceOTU43683
New.CleanUp.ReferenceOTU42679
New.CleanUp.ReferenceOTU21713
New.CleanUp.ReferenceOTU61014
New.CleanUp.ReferenceOTU46140
New.CleanUp.ReferenceOTU87993
New.CleanUp.ReferenceOTU184967
New.CleanUp.ReferenceOTU33182
New.CleanUp.ReferenceOTU53617
New.CleanUp.ReferenceOTU18599
New.CleanUp.ReferenceOTU88230
New.CleanUp.ReferenceOTU36155
New.CleanUp.ReferenceOTU31682
New.CleanUp.ReferenceOTU64390
New.CleanUp.ReferenceOTU30135
New.CleanUp.ReferenceOTU16756
New.CleanUp.ReferenceOTU87235
New.CleanUp.ReferenceOTU297619
New.CleanUp.ReferenceOTU82661
New.CleanUp.ReferenceOTU191028
New.CleanUp.ReferenceOTU21003
New.CleanUp.ReferenceOTU14671
New.CleanUp.ReferenceOTU12972
New.CleanUp.ReferenceOTU45308
New.CleanUp.ReferenceOTU15957
New.CleanUp.ReferenceOTU173118
New.CleanUp.ReferenceOTU25659
New.CleanUp.ReferenceOTU169719
New.CleanUp.ReferenceOTU43081
New.CleanUp.ReferenceOTU61106
New.CleanUp.ReferenceOTU49675

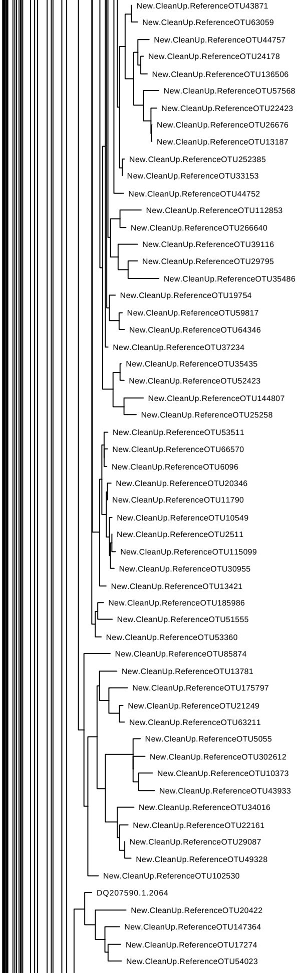
New.CleanUp.ReferenceOTU43871
New.CleanUp.ReferenceOTU63059
New.CleanUp.ReferenceOTU44757
New.CleanUp.ReferenceOTU24178
New.CleanUp.ReferenceOTU136506
New.CleanUp.ReferenceOTU57568
New.CleanUp.ReferenceOTU22423
New.CleanUp.ReferenceOTU26676
New.CleanUp.ReferenceOTU13187
New.CleanUp.ReferenceOTU252385
New.CleanUp.ReferenceOTU33153
New.CleanUp.ReferenceOTU44752
New.CleanUp.ReferenceOTU112853
New.CleanUp.ReferenceOTU266640
New.CleanUp.ReferenceOTU39116
New.CleanUp.ReferenceOTU29795
New.CleanUp.ReferenceOTU35486
New.CleanUp.ReferenceOTU19754
New.CleanUp.ReferenceOTU59817
New.CleanUp.ReferenceOTU64346
New.CleanUp.ReferenceOTU37234
New.CleanUp.ReferenceOTU35435
New.CleanUp.ReferenceOTU52423
New.CleanUp.ReferenceOTU144807
New.CleanUp.ReferenceOTU25258
New.CleanUp.ReferenceOTU53511
New.CleanUp.ReferenceOTU66570
New.CleanUp.ReferenceOTU6096
New.CleanUp.ReferenceOTU20346
New.CleanUp.ReferenceOTU11790
New.CleanUp.ReferenceOTU10549
New.CleanUp.ReferenceOTU2511
New.CleanUp.ReferenceOTU115099
New.CleanUp.ReferenceOTU30955
New.CleanUp.ReferenceOTU13421
New.CleanUp.ReferenceOTU185986
New.CleanUp.ReferenceOTU51555
New.CleanUp.ReferenceOTU53360
New.CleanUp.ReferenceOTU85874
New.CleanUp.ReferenceOTU13781
New.CleanUp.ReferenceOTU175797
New.CleanUp.ReferenceOTU21249
New.CleanUp.ReferenceOTU63211
New.CleanUp.ReferenceOTU5055
New.CleanUp.ReferenceOTU302612
New.CleanUp.ReferenceOTU10373
New.CleanUp.ReferenceOTU43933
New.CleanUp.ReferenceOTU34016
New.CleanUp.ReferenceOTU22161
New.CleanUp.ReferenceOTU29087
New.CleanUp.ReferenceOTU49328
New.CleanUp.ReferenceOTU102530
DQ207590.1.2064
New.CleanUp.ReferenceOTU20422
New.CleanUp.ReferenceOTU147364
New.CleanUp.ReferenceOTU17274
New.CleanUp.ReferenceOTU54023

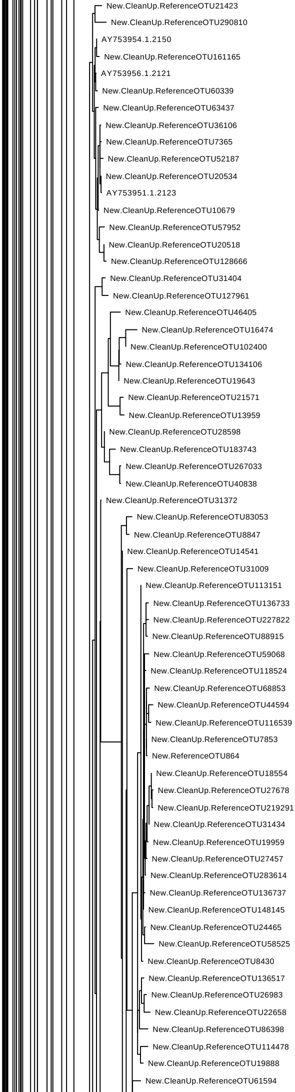
New.CleanUp.ReferenceOTU21423
New.CleanUp.ReferenceOTU290810
AY753954.1.2150
New.CleanUp.ReferenceOTU161165
AY753956.1.2121
New.CleanUp.ReferenceOTU60339
New.CleanUp.ReferenceOTU63437
New.CleanUp.ReferenceOTU36106
New.CleanUp.ReferenceOTU7365
New.CleanUp.ReferenceOTU52187
New.CleanUp.ReferenceOTU20534
AY753951.1.2123
New.CleanUp.ReferenceOTU10679
New.CleanUp.ReferenceOTU57952
New.CleanUp.ReferenceOTU20518
New.CleanUp.ReferenceOTU128666
New.CleanUp.ReferenceOTU31404
New.CleanUp.ReferenceOTU127961
New.CleanUp.ReferenceOTU46405
New.CleanUp.ReferenceOTU16474
New.CleanUp.ReferenceOTU102400
New.CleanUp.ReferenceOTU134106
New.CleanUp.ReferenceOTU19643
New.CleanUp.ReferenceOTU21571
New.CleanUp.ReferenceOTU13959
New.CleanUp.ReferenceOTU28598
New.CleanUp.ReferenceOTU183743
New.CleanUp.ReferenceOTU267033
New.CleanUp.ReferenceOTU40838
New.CleanUp.ReferenceOTU31372
New.CleanUp.ReferenceOTU83053
New.CleanUp.ReferenceOTU8847
New.CleanUp.ReferenceOTU14541
New.CleanUp.ReferenceOTU31009
New.CleanUp.ReferenceOTU113151
New.CleanUp.ReferenceOTU136733
New.CleanUp.ReferenceOTU227822
New.CleanUp.ReferenceOTU88915
New.CleanUp.ReferenceOTU59068
New.CleanUp.ReferenceOTU118524
New.CleanUp.ReferenceOTU68853
New.CleanUp.ReferenceOTU44594
New.CleanUp.ReferenceOTU116539
New.CleanUp.ReferenceOTU7853
New.ReferenceOTU864
New.CleanUp.ReferenceOTU18554
New.CleanUp.ReferenceOTU27678
New.CleanUp.ReferenceOTU219291
New.CleanUp.ReferenceOTU31434
New.CleanUp.ReferenceOTU19959
New.CleanUp.ReferenceOTU27457
New.CleanUp.ReferenceOTU283614
New.CleanUp.ReferenceOTU136737
New.CleanUp.ReferenceOTU148145
New.CleanUp.ReferenceOTU24465
New.CleanUp.ReferenceOTU58525
New.CleanUp.ReferenceOTU8430
New.CleanUp.ReferenceOTU136517
New.CleanUp.ReferenceOTU26983
New.CleanUp.ReferenceOTU22658
New.CleanUp.ReferenceOTU86398
New.CleanUp.ReferenceOTU114478
New.CleanUp.ReferenceOTU19888
New.CleanUp.ReferenceOTU61594

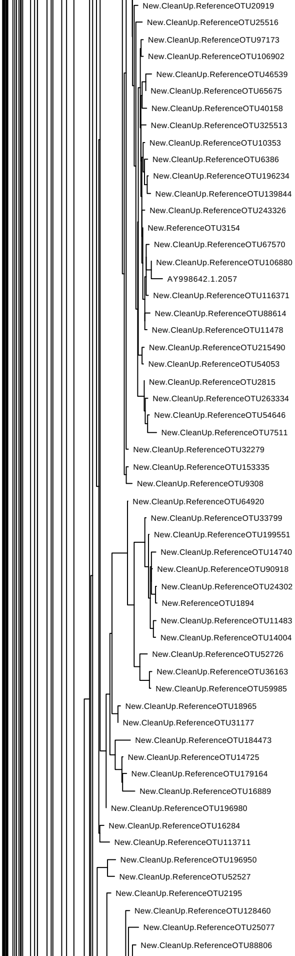
New.CleanUp.ReferenceOTU20919
New.CleanUp.ReferenceOTU25516
New.CleanUp.ReferenceOTU97173
New.CleanUp.ReferenceOTU106902
New.CleanUp.ReferenceOTU46539
New.CleanUp.ReferenceOTU65675
New.CleanUp.ReferenceOTU40158
New.CleanUp.ReferenceOTU325513
New.CleanUp.ReferenceOTU10353
New.CleanUp.ReferenceOTU6386
New.CleanUp.ReferenceOTU196234
New.CleanUp.ReferenceOTU139844
New.CleanUp.ReferenceOTU243326
New.ReferenceOTU3154
New.CleanUp.ReferenceOTU67570
New.CleanUp.ReferenceOTU106880
AY998642.1.2057
New.CleanUp.ReferenceOTU116371
New.CleanUp.ReferenceOTU88614
New.CleanUp.ReferenceOTU11478
New.CleanUp.ReferenceOTU215490
New.CleanUp.ReferenceOTU54053
New.CleanUp.ReferenceOTU2815
New.CleanUp.ReferenceOTU263334
New.CleanUp.ReferenceOTU54646
New.CleanUp.ReferenceOTU7511
New.CleanUp.ReferenceOTU32279
New.CleanUp.ReferenceOTU153335
New.CleanUp.ReferenceOTU9308
New.CleanUp.ReferenceOTU64920
New.CleanUp.ReferenceOTU33799
New.CleanUp.ReferenceOTU199551
New.CleanUp.ReferenceOTU14740
New.CleanUp.ReferenceOTU90918
New.CleanUp.ReferenceOTU24302
New.ReferenceOTU1894
New.CleanUp.ReferenceOTU11483
New.CleanUp.ReferenceOTU14004
New.CleanUp.ReferenceOTU52726
New.CleanUp.ReferenceOTU36163
New.CleanUp.ReferenceOTU59985
New.CleanUp.ReferenceOTU18965
New.CleanUp.ReferenceOTU31177
New.CleanUp.ReferenceOTU184473
New.CleanUp.ReferenceOTU14725
New.CleanUp.ReferenceOTU179164
New.CleanUp.ReferenceOTU16889
New.CleanUp.ReferenceOTU196980
New.CleanUp.ReferenceOTU16284
New.CleanUp.ReferenceOTU113711
New.CleanUp.ReferenceOTU196950
New.CleanUp.ReferenceOTU52527
New.CleanUp.ReferenceOTU2195
New.CleanUp.ReferenceOTU128460
New.CleanUp.ReferenceOTU25077
New.CleanUp.ReferenceOTU88806

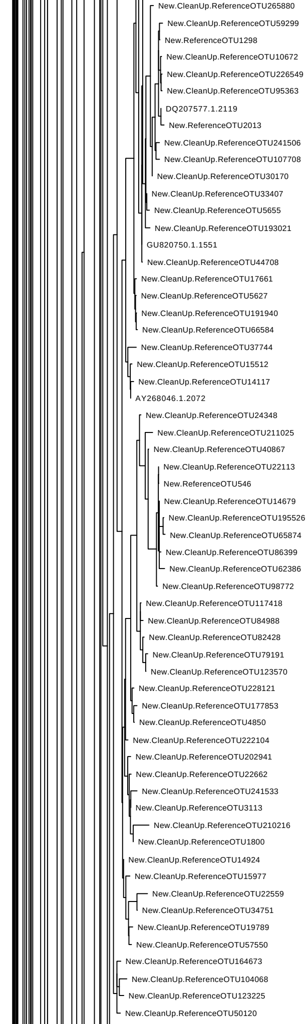
New.CleanUp.ReferenceOTU265880
New.CleanUp.ReferenceOTU59299
New.ReferenceOTU1298
New.CleanUp.ReferenceOTU10672
New.CleanUp.ReferenceOTU226549
New.CleanUp.ReferenceOTU95363
DQ207577.1.2119
New.ReferenceOTU2013
New.CleanUp.ReferenceOTU241506
New.CleanUp.ReferenceOTU107708
New.CleanUp.ReferenceOTU30170
New.CleanUp.ReferenceOTU33407
New.CleanUp.ReferenceOTU5655
New.CleanUp.ReferenceOTU193021
GU820750.1.1551
New.CleanUp.ReferenceOTU44708
New.CleanUp.ReferenceOTU17661
New.CleanUp.ReferenceOTU5627
New.CleanUp.ReferenceOTU191940
New.CleanUp.ReferenceOTU66584
New.CleanUp.ReferenceOTU37744
New.CleanUp.ReferenceOTU15512
New.CleanUp.ReferenceOTU14117
AY268046.1.2072
New.CleanUp.ReferenceOTU24348
New.CleanUp.ReferenceOTU211025
New.CleanUp.ReferenceOTU40867
New.CleanUp.ReferenceOTU22113
New.ReferenceOTU546
New.CleanUp.ReferenceOTU14679
New.CleanUp.ReferenceOTU195526
New.CleanUp.ReferenceOTU65874
New.CleanUp.ReferenceOTU86399
New.CleanUp.ReferenceOTU62386
New.CleanUp.ReferenceOTU98772
New.CleanUp.ReferenceOTU117418
New.CleanUp.ReferenceOTU84988
New.CleanUp.ReferenceOTU82428
New.CleanUp.ReferenceOTU79191
New.CleanUp.ReferenceOTU123570
New.CleanUp.ReferenceOTU228121
New.CleanUp.ReferenceOTU177853
New.CleanUp.ReferenceOTU4850
New.CleanUp.ReferenceOTU222104
New.CleanUp.ReferenceOTU202941
New.CleanUp.ReferenceOTU22662
New.CleanUp.ReferenceOTU241533
New.CleanUp.ReferenceOTU3113
New.CleanUp.ReferenceOTU210216
New.CleanUp.ReferenceOTU1800
New.CleanUp.ReferenceOTU14924
New.CleanUp.ReferenceOTU15977
New.CleanUp.ReferenceOTU22559
New.CleanUp.ReferenceOTU34751
New.CleanUp.ReferenceOTU19789
New.CleanUp.ReferenceOTU57550
New.CleanUp.ReferenceOTU164673
New.CleanUp.ReferenceOTU104068
New.CleanUp.ReferenceOTU123225
New.CleanUp.ReferenceOTU50120

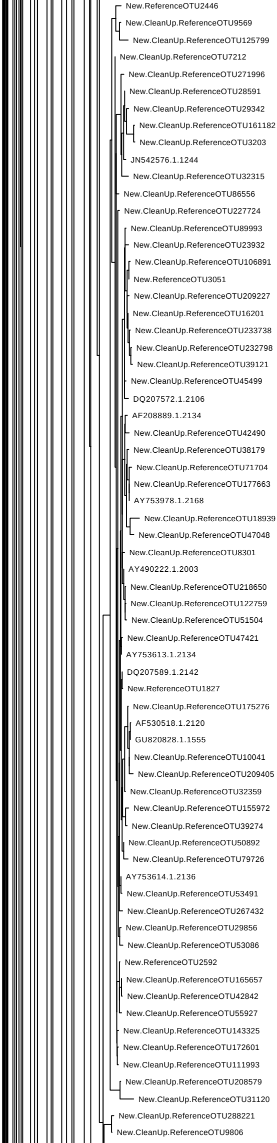
New.ReferenceOTU2446
New.CleanUp.ReferenceOTU9569
New.CleanUp.ReferenceOTU125799
New.CleanUp.ReferenceOTU7212
New.CleanUp.ReferenceOTU271996
New.CleanUp.ReferenceOTU28591
New.CleanUp.ReferenceOTU29342
New.CleanUp.ReferenceOTU161182
New.CleanUp.ReferenceOTU3203
JN542576.1.1244
New.CleanUp.ReferenceOTU32315
New.CleanUp.ReferenceOTU86556
New.CleanUp.ReferenceOTU227724
New.CleanUp.ReferenceOTU89993
New.CleanUp.ReferenceOTU23932
New.CleanUp.ReferenceOTU106891
New.ReferenceOTU3051
New.CleanUp.ReferenceOTU209227
New.CleanUp.ReferenceOTU16201
New.CleanUp.ReferenceOTU233738
New.CleanUp.ReferenceOTU232798
New.CleanUp.ReferenceOTU39121
New.CleanUp.ReferenceOTU45499
DQ207572.1.2106
AF208889.1.2134
New.CleanUp.ReferenceOTU42490
New.CleanUp.ReferenceOTU38179
New.CleanUp.ReferenceOTU71704
New.CleanUp.ReferenceOTU177663
AY753978.1.2168
New.CleanUp.ReferenceOTU18939
New.CleanUp.ReferenceOTU47048
New.CleanUp.ReferenceOTU8301
AY490222.1.2003
New.CleanUp.ReferenceOTU218650
New.CleanUp.ReferenceOTU122759
New.CleanUp.ReferenceOTU51504
New.CleanUp.ReferenceOTU47421
AY753613.1.2134
DQ207589.1.2142
New.ReferenceOTU1827
New.CleanUp.ReferenceOTU175276
AF530518.1.2120
GU820828.1.1555
New.CleanUp.ReferenceOTU10041
New.CleanUp.ReferenceOTU209405
New.CleanUp.ReferenceOTU32359
New.CleanUp.ReferenceOTU155972
New.CleanUp.ReferenceOTU39274
New.CleanUp.ReferenceOTU50892
New.CleanUp.ReferenceOTU79726
AY753614.1.2136
New.CleanUp.ReferenceOTU53491
New.CleanUp.ReferenceOTU267432
New.CleanUp.ReferenceOTU29856
New.CleanUp.ReferenceOTU53086
New.ReferenceOTU2592
New.CleanUp.ReferenceOTU165657
New.CleanUp.ReferenceOTU42842
New.CleanUp.ReferenceOTU55927
New.CleanUp.ReferenceOTU143325
New.CleanUp.ReferenceOTU172601
New.CleanUp.ReferenceOTU111993
New.CleanUp.ReferenceOTU208579
New.CleanUp.ReferenceOTU31120
New.CleanUp.ReferenceOTU288221
New.CleanUp.ReferenceOTU9806

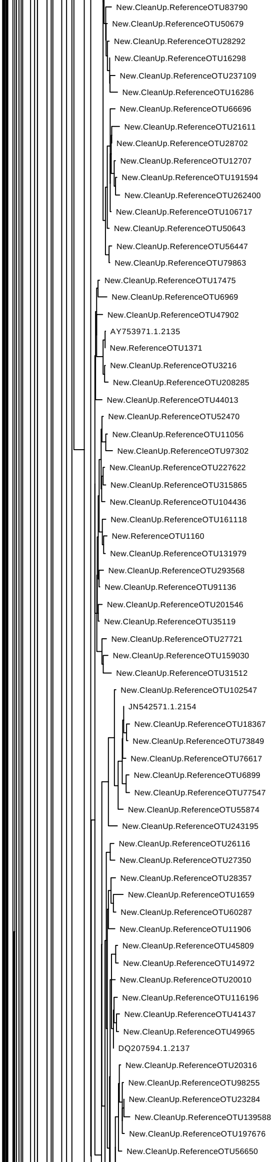
New.CleanUp.ReferenceOTU83790
New.CleanUp.ReferenceOTU50679
New.CleanUp.ReferenceOTU28292
New.CleanUp.ReferenceOTU16298
New.CleanUp.ReferenceOTU237109
New.CleanUp.ReferenceOTU16286
New.CleanUp.ReferenceOTU66696
New.CleanUp.ReferenceOTU21611
New.CleanUp.ReferenceOTU28702
New.CleanUp.ReferenceOTU12707
New.CleanUp.ReferenceOTU191594
New.CleanUp.ReferenceOTU262400
New.CleanUp.ReferenceOTU106717
New.CleanUp.ReferenceOTU50643
New.CleanUp.ReferenceOTU56447
New.CleanUp.ReferenceOTU79863
New.CleanUp.ReferenceOTU17475
New.CleanUp.ReferenceOTU6969
New.CleanUp.ReferenceOTU47902
AY753971.1.2135
New.ReferenceOTU1371
New.CleanUp.ReferenceOTU3216
New.CleanUp.ReferenceOTU208285
New.CleanUp.ReferenceOTU44013
New.CleanUp.ReferenceOTU52470
New.CleanUp.ReferenceOTU11056
New.CleanUp.ReferenceOTU97302
New.CleanUp.ReferenceOTU227622
New.CleanUp.ReferenceOTU315865
New.CleanUp.ReferenceOTU104436
New.CleanUp.ReferenceOTU161118
New.ReferenceOTU1160
New.CleanUp.ReferenceOTU131979
New.CleanUp.ReferenceOTU293568
New.CleanUp.ReferenceOTU91136
New.CleanUp.ReferenceOTU201546
New.CleanUp.ReferenceOTU35119
New.CleanUp.ReferenceOTU27721
New.CleanUp.ReferenceOTU159030
New.CleanUp.ReferenceOTU31512
New.CleanUp.ReferenceOTU102547
JN542571.1.2154
New.CleanUp.ReferenceOTU18367
New.CleanUp.ReferenceOTU73849
New.CleanUp.ReferenceOTU76617
New.CleanUp.ReferenceOTU6899
New.CleanUp.ReferenceOTU77547
New.CleanUp.ReferenceOTU55874
New.CleanUp.ReferenceOTU243195
New.CleanUp.ReferenceOTU26116
New.CleanUp.ReferenceOTU27350
New.CleanUp.ReferenceOTU28357
New.CleanUp.ReferenceOTU1659
New.CleanUp.ReferenceOTU60287
New.CleanUp.ReferenceOTU11906
New.CleanUp.ReferenceOTU45809
New.CleanUp.ReferenceOTU14972
New.CleanUp.ReferenceOTU20010
New.CleanUp.ReferenceOTU116196
New.CleanUp.ReferenceOTU41437
New.CleanUp.ReferenceOTU49965
DQ207594.1.2137
New.CleanUp.ReferenceOTU20316
New.CleanUp.ReferenceOTU98255
New.CleanUp.ReferenceOTU23284
New.CleanUp.ReferenceOTU139588
New.CleanUp.ReferenceOTU197676
New.CleanUp.ReferenceOTU56650

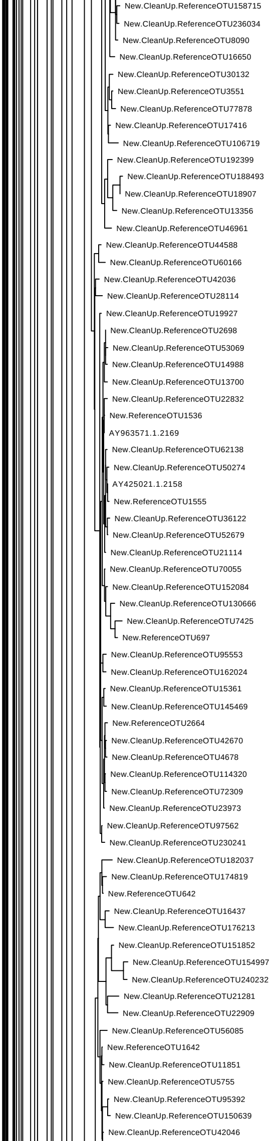
New.CleanUp.ReferenceOTU158715
New.CleanUp.ReferenceOTU236034
New.CleanUp.ReferenceOTU8090
New.CleanUp.ReferenceOTU16650
New.CleanUp.ReferenceOTU30132
New.CleanUp.ReferenceOTU3551
New.CleanUp.ReferenceOTU77878
New.CleanUp.ReferenceOTU17416
New.CleanUp.ReferenceOTU106719
New.CleanUp.ReferenceOTU192399
New.CleanUp.ReferenceOTU188493
New.CleanUp.ReferenceOTU18907
New.CleanUp.ReferenceOTU13356
New.CleanUp.ReferenceOTU46961
New.CleanUp.ReferenceOTU44588
New.CleanUp.ReferenceOTU60166
New.CleanUp.ReferenceOTU42036
New.CleanUp.ReferenceOTU28114
New.CleanUp.ReferenceOTU19927
New.CleanUp.ReferenceOTU2698
New.CleanUp.ReferenceOTU53069
New.CleanUp.ReferenceOTU14988
New.CleanUp.ReferenceOTU13700
New.CleanUp.ReferenceOTU22832
New.ReferenceOTU1536
AY963571.1.2169
New.CleanUp.ReferenceOTU62138
New.CleanUp.ReferenceOTU50274
AY425021.1.2158
New.ReferenceOTU1555
New.CleanUp.ReferenceOTU36122
New.CleanUp.ReferenceOTU52679
New.CleanUp.ReferenceOTU21114
New.CleanUp.ReferenceOTU70055
New.CleanUp.ReferenceOTU152084
New.CleanUp.ReferenceOTU130666
New.CleanUp.ReferenceOTU7425
New.ReferenceOTU697
New.CleanUp.ReferenceOTU95553
New.CleanUp.ReferenceOTU162024
New.CleanUp.ReferenceOTU15361
New.CleanUp.ReferenceOTU145469
New.ReferenceOTU2664
New.CleanUp.ReferenceOTU42670
New.CleanUp.ReferenceOTU4678
New.CleanUp.ReferenceOTU114320
New.CleanUp.ReferenceOTU72309
New.CleanUp.ReferenceOTU23973
New.CleanUp.ReferenceOTU97562
New.CleanUp.ReferenceOTU230241
New.CleanUp.ReferenceOTU182037
New.CleanUp.ReferenceOTU174819
New.ReferenceOTU642
New.CleanUp.ReferenceOTU16437
New.CleanUp.ReferenceOTU176213
New.CleanUp.ReferenceOTU151852
New.CleanUp.ReferenceOTU154997
New.CleanUp.ReferenceOTU240232
New.CleanUp.ReferenceOTU21281
New.CleanUp.ReferenceOTU22909
New.CleanUp.ReferenceOTU56085
New.ReferenceOTU1642
New.CleanUp.ReferenceOTU11851
New.CleanUp.ReferenceOTU5755
New.CleanUp.ReferenceOTU95392
New.CleanUp.ReferenceOTU150639
New.CleanUp.ReferenceOTU42046

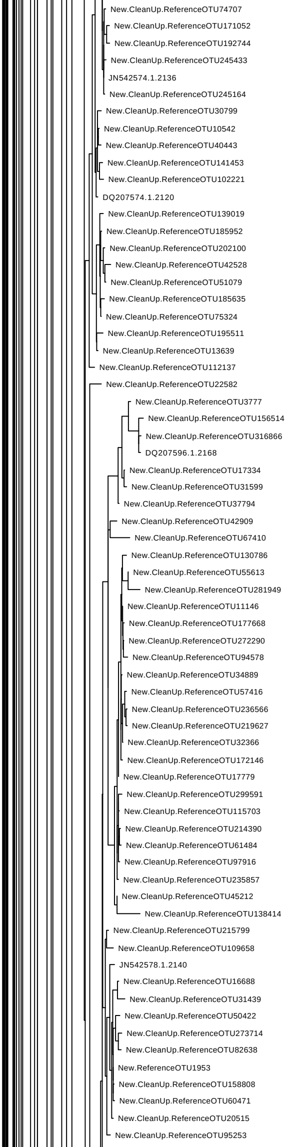
New.CleanUp.ReferenceOTU74707
New.CleanUp.ReferenceOTU171052
New.CleanUp.ReferenceOTU192744
New.CleanUp.ReferenceOTU245433
JN542574.1.2136
New.CleanUp.ReferenceOTU245164
New.CleanUp.ReferenceOTU30799
New.CleanUp.ReferenceOTU10542
New.CleanUp.ReferenceOTU40443
New.CleanUp.ReferenceOTU141453
New.CleanUp.ReferenceOTU102221
DQ207574.1.2120
New.CleanUp.ReferenceOTU139019
New.CleanUp.ReferenceOTU185952
New.CleanUp.ReferenceOTU202100
New.CleanUp.ReferenceOTU42528
New.CleanUp.ReferenceOTU51079
New.CleanUp.ReferenceOTU185635
New.CleanUp.ReferenceOTU75324
New.CleanUp.ReferenceOTU195511
New.CleanUp.ReferenceOTU13639
New.CleanUp.ReferenceOTU112137
New.CleanUp.ReferenceOTU22582
New.CleanUp.ReferenceOTU3777
New.CleanUp.ReferenceOTU156514
New.CleanUp.ReferenceOTU316866
DQ207596.1.2168
New.CleanUp.ReferenceOTU17334
New.CleanUp.ReferenceOTU31599
New.CleanUp.ReferenceOTU37794
New.CleanUp.ReferenceOTU42909
New.CleanUp.ReferenceOTU67410
New.CleanUp.ReferenceOTU130786
New.CleanUp.ReferenceOTU55613
New.CleanUp.ReferenceOTU281949
New.CleanUp.ReferenceOTU11146
New.CleanUp.ReferenceOTU177668
New.CleanUp.ReferenceOTU272290
New.CleanUp.ReferenceOTU94578
New.CleanUp.ReferenceOTU34889
New.CleanUp.ReferenceOTU57416
New.CleanUp.ReferenceOTU236566
New.CleanUp.ReferenceOTU219627
New.CleanUp.ReferenceOTU32366
New.CleanUp.ReferenceOTU172146
New.CleanUp.ReferenceOTU17779
New.CleanUp.ReferenceOTU299591
New.CleanUp.ReferenceOTU115703
New.CleanUp.ReferenceOTU214390
New.CleanUp.ReferenceOTU61484
New.CleanUp.ReferenceOTU97916
New.CleanUp.ReferenceOTU235857
New.CleanUp.ReferenceOTU45212
New.CleanUp.ReferenceOTU138414
New.CleanUp.ReferenceOTU215799
New.CleanUp.ReferenceOTU109658
JN542578.1.2140
New.CleanUp.ReferenceOTU16688
New.CleanUp.ReferenceOTU31439
New.CleanUp.ReferenceOTU50422
New.CleanUp.ReferenceOTU273714
New.CleanUp.ReferenceOTU82638
New.ReferenceOTU1953
New.CleanUp.ReferenceOTU158808
New.CleanUp.ReferenceOTU60471
New.CleanUp.ReferenceOTU20515
New.CleanUp.ReferenceOTU95253

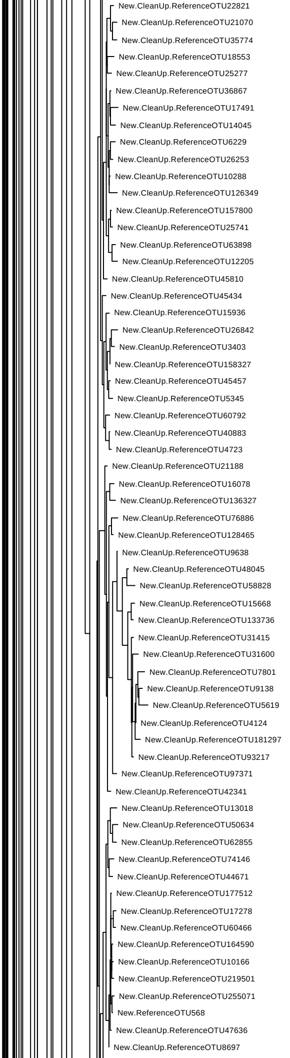
New.CleanUp.ReferenceOTU22821
New.CleanUp.ReferenceOTU21070
New.CleanUp.ReferenceOTU35774
New.CleanUp.ReferenceOTU18553
New.CleanUp.ReferenceOTU25277
New.CleanUp.ReferenceOTU36867
New.CleanUp.ReferenceOTU17491
New.CleanUp.ReferenceOTU14045
New.CleanUp.ReferenceOTU6229
New.CleanUp.ReferenceOTU26253
New.CleanUp.ReferenceOTU10288
New.CleanUp.ReferenceOTU126349
New.CleanUp.ReferenceOTU157800
New.CleanUp.ReferenceOTU25741
New.CleanUp.ReferenceOTU63898
New.CleanUp.ReferenceOTU12205
New.CleanUp.ReferenceOTU45810
New.CleanUp.ReferenceOTU45434
New.CleanUp.ReferenceOTU15936
New.CleanUp.ReferenceOTU26842
New.CleanUp.ReferenceOTU3403
New.CleanUp.ReferenceOTU158327
New.CleanUp.ReferenceOTU45457
New.CleanUp.ReferenceOTU5345
New.CleanUp.ReferenceOTU60792
New.CleanUp.ReferenceOTU40883
New.CleanUp.ReferenceOTU4723
New.CleanUp.ReferenceOTU21188
New.CleanUp.ReferenceOTU16078
New.CleanUp.ReferenceOTU136327
New.CleanUp.ReferenceOTU76886
New.CleanUp.ReferenceOTU128465
New.CleanUp.ReferenceOTU9638
New.CleanUp.ReferenceOTU48045
New.CleanUp.ReferenceOTU58828
New.CleanUp.ReferenceOTU15668
New.CleanUp.ReferenceOTU133736
New.CleanUp.ReferenceOTU31415
New.CleanUp.ReferenceOTU31600
New.CleanUp.ReferenceOTU7801
New.CleanUp.ReferenceOTU9138
New.CleanUp.ReferenceOTU5619
New.CleanUp.ReferenceOTU4124
New.CleanUp.ReferenceOTU181297
New.CleanUp.ReferenceOTU93217
New.CleanUp.ReferenceOTU97371
New.CleanUp.ReferenceOTU42341
New.CleanUp.ReferenceOTU13018
New.CleanUp.ReferenceOTU50634
New.CleanUp.ReferenceOTU62855
New.CleanUp.ReferenceOTU74146
New.CleanUp.ReferenceOTU44671
New.CleanUp.ReferenceOTU177512
New.CleanUp.ReferenceOTU17278
New.CleanUp.ReferenceOTU60466
New.CleanUp.ReferenceOTU164590
New.CleanUp.ReferenceOTU10166
New.CleanUp.ReferenceOTU219501
New.CleanUp.ReferenceOTU255071
New.ReferenceOTU568
New.CleanUp.ReferenceOTU47636
New.CleanUp.ReferenceOTU8697

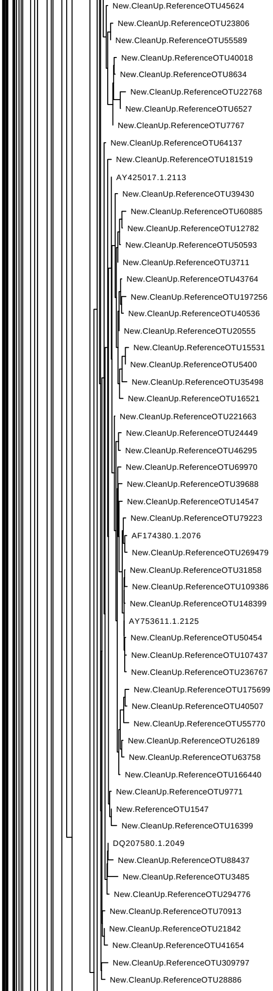
New.CleanUp.ReferenceOTU45624
New.CleanUp.ReferenceOTU23806
New.CleanUp.ReferenceOTU55589
New.CleanUp.ReferenceOTU40018
New.CleanUp.ReferenceOTU8634
New.CleanUp.ReferenceOTU22768
New.CleanUp.ReferenceOTU6527
New.CleanUp.ReferenceOTU7767
New.CleanUp.ReferenceOTU64137
New.CleanUp.ReferenceOTU181519
AY425017.1.2113
New.CleanUp.ReferenceOTU39430
New.CleanUp.ReferenceOTU60885
New.CleanUp.ReferenceOTU12782
New.CleanUp.ReferenceOTU50593
New.CleanUp.ReferenceOTU3711
New.CleanUp.ReferenceOTU43764
New.CleanUp.ReferenceOTU197256
New.CleanUp.ReferenceOTU40536
New.CleanUp.ReferenceOTU20555
New.CleanUp.ReferenceOTU15531
New.CleanUp.ReferenceOTU5400
New.CleanUp.ReferenceOTU35498
New.CleanUp.ReferenceOTU16521
New.CleanUp.ReferenceOTU221663
New.CleanUp.ReferenceOTU24449
New.CleanUp.ReferenceOTU46295
New.CleanUp.ReferenceOTU69970
New.CleanUp.ReferenceOTU39688
New.CleanUp.ReferenceOTU14547
New.CleanUp.ReferenceOTU79223
AF174380.1.2076
New.CleanUp.ReferenceOTU269479
New.CleanUp.ReferenceOTU31858
New.CleanUp.ReferenceOTU109386
New.CleanUp.ReferenceOTU148399
AY753611.1.2125
New.CleanUp.ReferenceOTU50454
New.CleanUp.ReferenceOTU107437
New.CleanUp.ReferenceOTU236767
New.CleanUp.ReferenceOTU175699
New.CleanUp.ReferenceOTU40507
New.CleanUp.ReferenceOTU55770
New.CleanUp.ReferenceOTU26189
New.CleanUp.ReferenceOTU63758
New.CleanUp.ReferenceOTU166440
New.CleanUp.ReferenceOTU9771
New.ReferenceOTU1547
New.CleanUp.ReferenceOTU16399
DQ207580.1.2049
New.CleanUp.ReferenceOTU88437
New.CleanUp.ReferenceOTU3485
New.CleanUp.ReferenceOTU294776
New.CleanUp.ReferenceOTU70913
New.CleanUp.ReferenceOTU21842
New.CleanUp.ReferenceOTU41654
New.CleanUp.ReferenceOTU309797
New.CleanUp.ReferenceOTU28886

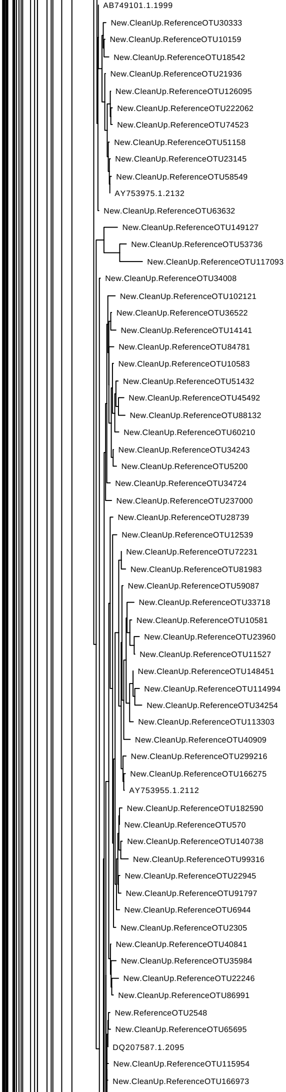
AB749101.1.1999
New.CleanUp.ReferenceOTU30333
New.CleanUp.ReferenceOTU10159
New.CleanUp.ReferenceOTU18542
New.CleanUp.ReferenceOTU21936
New.CleanUp.ReferenceOTU126095
New.CleanUp.ReferenceOTU222062
New.CleanUp.ReferenceOTU74523
New.CleanUp.ReferenceOTU51158
New.CleanUp.ReferenceOTU23145
New.CleanUp.ReferenceOTU58549
AY753975.1.2132
New.CleanUp.ReferenceOTU63632
New.CleanUp.ReferenceOTU149127
New.CleanUp.ReferenceOTU53736
New.CleanUp.ReferenceOTU117093
New.CleanUp.ReferenceOTU34008
New.CleanUp.ReferenceOTU102121
New.CleanUp.ReferenceOTU36522
New.CleanUp.ReferenceOTU14141
New.CleanUp.ReferenceOTU84781
New.CleanUp.ReferenceOTU10583
New.CleanUp.ReferenceOTU51432
New.CleanUp.ReferenceOTU45492
New.CleanUp.ReferenceOTU88132
New.CleanUp.ReferenceOTU60210
New.CleanUp.ReferenceOTU34243
New.CleanUp.ReferenceOTU5200
New.CleanUp.ReferenceOTU34724
New.CleanUp.ReferenceOTU237000
New.CleanUp.ReferenceOTU28739
New.CleanUp.ReferenceOTU12539
New.CleanUp.ReferenceOTU72231
New.CleanUp.ReferenceOTU81983
New.CleanUp.ReferenceOTU59087
New.CleanUp.ReferenceOTU33718
New.CleanUp.ReferenceOTU10581
New.CleanUp.ReferenceOTU23960
New.CleanUp.ReferenceOTU11527
New.CleanUp.ReferenceOTU148451
New.CleanUp.ReferenceOTU114994
New.CleanUp.ReferenceOTU34254
New.CleanUp.ReferenceOTU113303
New.CleanUp.ReferenceOTU40909
New.CleanUp.ReferenceOTU299216
New.CleanUp.ReferenceOTU166275
AY753955.1.2112
New.CleanUp.ReferenceOTU182590
New.CleanUp.ReferenceOTU570
New.CleanUp.ReferenceOTU140738
New.CleanUp.ReferenceOTU99316
New.CleanUp.ReferenceOTU22945
New.CleanUp.ReferenceOTU91797
New.CleanUp.ReferenceOTU6944
New.CleanUp.ReferenceOTU2305
New.CleanUp.ReferenceOTU40841
New.CleanUp.ReferenceOTU35984
New.CleanUp.ReferenceOTU22246
New.CleanUp.ReferenceOTU86991
New.ReferenceOTU2548
New.CleanUp.ReferenceOTU65695
DQ207587.1.2095
New.CleanUp.ReferenceOTU115954
New.CleanUp.ReferenceOTU166973

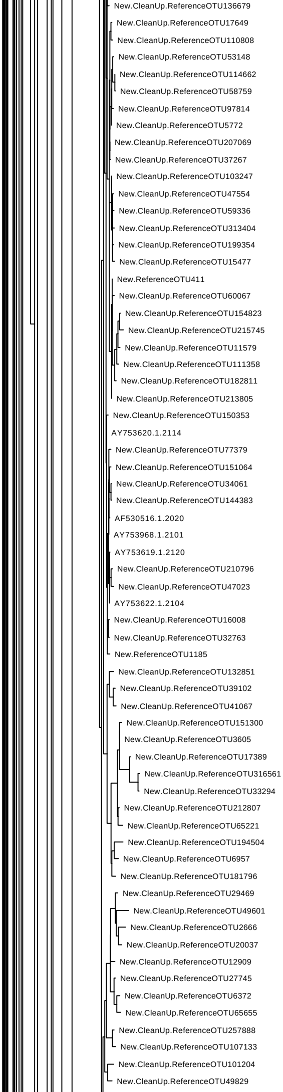
New.CleanUp.ReferenceOTU136679
New.CleanUp.ReferenceOTU17649
New.CleanUp.ReferenceOTU110808
New.CleanUp.ReferenceOTU53148
New.CleanUp.ReferenceOTU114662
New.CleanUp.ReferenceOTU58759
New.CleanUp.ReferenceOTU97814
New.CleanUp.ReferenceOTU5772
New.CleanUp.ReferenceOTU207069
New.CleanUp.ReferenceOTU37267
New.CleanUp.ReferenceOTU103247
New.CleanUp.ReferenceOTU47554
New.CleanUp.ReferenceOTU59336
New.CleanUp.ReferenceOTU313404
New.CleanUp.ReferenceOTU199354
New.CleanUp.ReferenceOTU15477
New.ReferenceOTU411
New.CleanUp.ReferenceOTU60067
New.CleanUp.ReferenceOTU154823
New.CleanUp.ReferenceOTU215745
New.CleanUp.ReferenceOTU11579
New.CleanUp.ReferenceOTU111358
New.CleanUp.ReferenceOTU182811
New.CleanUp.ReferenceOTU213805
New.CleanUp.ReferenceOTU150353
AY753620.1.2114
New.CleanUp.ReferenceOTU77379
New.CleanUp.ReferenceOTU151064
New.CleanUp.ReferenceOTU34061
New.CleanUp.ReferenceOTU144383
AF530516.1.2020
AY753968.1.2101
AY753619.1.2120
New.CleanUp.ReferenceOTU210796
New.CleanUp.ReferenceOTU47023
AY753622.1.2104
New.CleanUp.ReferenceOTU16008
New.CleanUp.ReferenceOTU32763
New.ReferenceOTU1185
New.CleanUp.ReferenceOTU132851
New.CleanUp.ReferenceOTU39102
New.CleanUp.ReferenceOTU41067
New.CleanUp.ReferenceOTU151300
New.CleanUp.ReferenceOTU3605
New.CleanUp.ReferenceOTU17389
New.CleanUp.ReferenceOTU316561
New.CleanUp.ReferenceOTU33294
New.CleanUp.ReferenceOTU212807
New.CleanUp.ReferenceOTU65221
New.CleanUp.ReferenceOTU194504
New.CleanUp.ReferenceOTU6957
New.CleanUp.ReferenceOTU181796
New.CleanUp.ReferenceOTU29469
New.CleanUp.ReferenceOTU49601
New.CleanUp.ReferenceOTU2666
New.CleanUp.ReferenceOTU20037
New.CleanUp.ReferenceOTU12909
New.CleanUp.ReferenceOTU27745
New.CleanUp.ReferenceOTU6372
New.CleanUp.ReferenceOTU65655
New.CleanUp.ReferenceOTU257888
New.CleanUp.ReferenceOTU107133
New.CleanUp.ReferenceOTU101204
New.CleanUp.ReferenceOTU49829

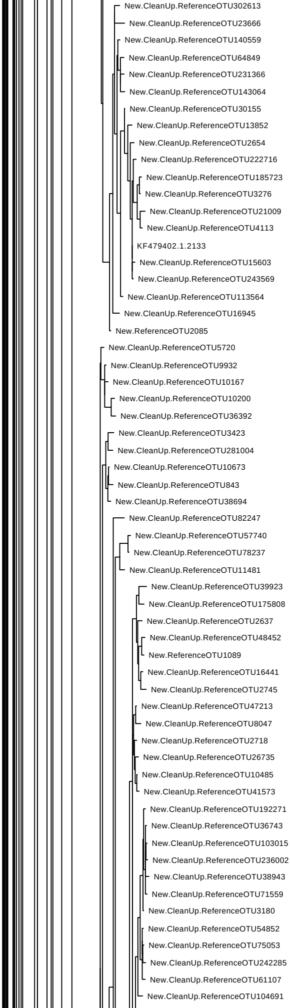
New.CleanUp.ReferenceOTU302613
New.CleanUp.ReferenceOTU23666
New.CleanUp.ReferenceOTU140559
New.CleanUp.ReferenceOTU64849
New.CleanUp.ReferenceOTU231366
New.CleanUp.ReferenceOTU143064
New.CleanUp.ReferenceOTU30155
New.CleanUp.ReferenceOTU13852
New.CleanUp.ReferenceOTU2654
New.CleanUp.ReferenceOTU222716
New.CleanUp.ReferenceOTU185723
New.CleanUp.ReferenceOTU3276
New.CleanUp.ReferenceOTU21009
New.CleanUp.ReferenceOTU4113
KF479402.1.2133
New.CleanUp.ReferenceOTU15603
New.CleanUp.ReferenceOTU243569
New.CleanUp.ReferenceOTU113564
New.CleanUp.ReferenceOTU16945
New.ReferenceOTU2085
New.CleanUp.ReferenceOTU5720
New.CleanUp.ReferenceOTU9932
New.CleanUp.ReferenceOTU10167
New.CleanUp.ReferenceOTU10200
New.CleanUp.ReferenceOTU36392
New.CleanUp.ReferenceOTU3423
New.CleanUp.ReferenceOTU281004
New.CleanUp.ReferenceOTU10673
New.CleanUp.ReferenceOTU843
New.CleanUp.ReferenceOTU38694
New.CleanUp.ReferenceOTU82247
New.CleanUp.ReferenceOTU57740
New.CleanUp.ReferenceOTU78237
New.CleanUp.ReferenceOTU11481
New.CleanUp.ReferenceOTU39923
New.CleanUp.ReferenceOTU175808
New.CleanUp.ReferenceOTU2637
New.CleanUp.ReferenceOTU48452
New.ReferenceOTU1089
New.CleanUp.ReferenceOTU16441
New.CleanUp.ReferenceOTU2745
New.CleanUp.ReferenceOTU47213
New.CleanUp.ReferenceOTU8047
New.CleanUp.ReferenceOTU2718
New.CleanUp.ReferenceOTU26735
New.CleanUp.ReferenceOTU10485
New.CleanUp.ReferenceOTU41573
New.CleanUp.ReferenceOTU192271
New.CleanUp.ReferenceOTU36743
New.CleanUp.ReferenceOTU103015
New.CleanUp.ReferenceOTU236002
New.CleanUp.ReferenceOTU38943
New.CleanUp.ReferenceOTU71559
New.CleanUp.ReferenceOTU3180
New.CleanUp.ReferenceOTU54852
New.CleanUp.ReferenceOTU75053
New.CleanUp.ReferenceOTU242285
New.CleanUp.ReferenceOTU61107
New.CleanUp.ReferenceOTU104691

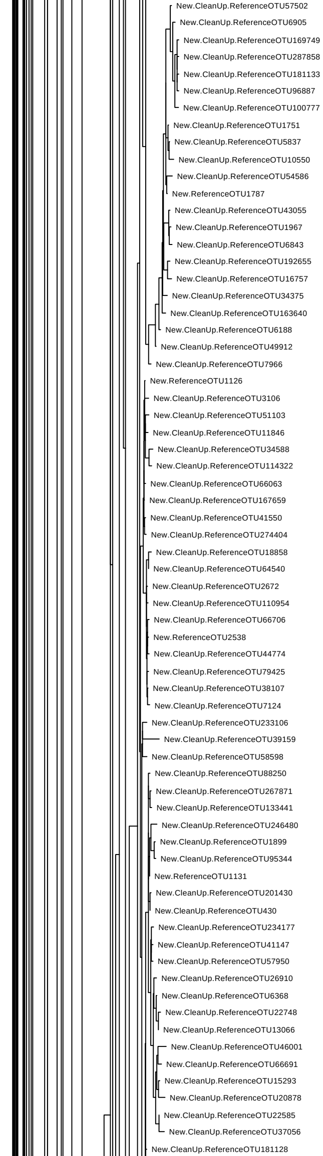
New.CleanUp.ReferenceOTU57502
New.CleanUp.ReferenceOTU6905
New.CleanUp.ReferenceOTU169749
New.CleanUp.ReferenceOTU287858
New.CleanUp.ReferenceOTU181133
New.CleanUp.ReferenceOTU96887
New.CleanUp.ReferenceOTU100777
New.CleanUp.ReferenceOTU1751
New.CleanUp.ReferenceOTU5837
New.CleanUp.ReferenceOTU10550
New.CleanUp.ReferenceOTU54586
New.ReferenceOTU1787
New.CleanUp.ReferenceOTU43055
New.CleanUp.ReferenceOTU1967
New.CleanUp.ReferenceOTU6843
New.CleanUp.ReferenceOTU192655
New.CleanUp.ReferenceOTU16757
New.CleanUp.ReferenceOTU34375
New.CleanUp.ReferenceOTU163640
New.CleanUp.ReferenceOTU6188
New.CleanUp.ReferenceOTU49912
New.CleanUp.ReferenceOTU7966
New.ReferenceOTU1126
New.CleanUp.ReferenceOTU3106
New.CleanUp.ReferenceOTU51103
New.CleanUp.ReferenceOTU11846
New.CleanUp.ReferenceOTU34588
New.CleanUp.ReferenceOTU114322
New.CleanUp.ReferenceOTU66063
New.CleanUp.ReferenceOTU167659
New.CleanUp.ReferenceOTU41550
New.CleanUp.ReferenceOTU274404
New.CleanUp.ReferenceOTU18858
New.CleanUp.ReferenceOTU64540
New.CleanUp.ReferenceOTU2672
New.CleanUp.ReferenceOTU110954
New.CleanUp.ReferenceOTU66706
New.ReferenceOTU2538
New.CleanUp.ReferenceOTU44774
New.CleanUp.ReferenceOTU79425
New.CleanUp.ReferenceOTU38107
New.CleanUp.ReferenceOTU7124
New.CleanUp.ReferenceOTU233106
New.CleanUp.ReferenceOTU39159
New.CleanUp.ReferenceOTU58598
New.CleanUp.ReferenceOTU88250
New.CleanUp.ReferenceOTU267871
New.CleanUp.ReferenceOTU133441
New.CleanUp.ReferenceOTU246480
New.CleanUp.ReferenceOTU1899
New.CleanUp.ReferenceOTU95344
New.ReferenceOTU1131
New.CleanUp.ReferenceOTU201430
New.CleanUp.ReferenceOTU430
New.CleanUp.ReferenceOTU234177
New.CleanUp.ReferenceOTU41147
New.CleanUp.ReferenceOTU57950
New.CleanUp.ReferenceOTU26910
New.CleanUp.ReferenceOTU6368
New.CleanUp.ReferenceOTU22748
New.CleanUp.ReferenceOTU13066
New.CleanUp.ReferenceOTU46001
New.CleanUp.ReferenceOTU66691
New.CleanUp.ReferenceOTU15293
New.CleanUp.ReferenceOTU20878
New.CleanUp.ReferenceOTU22585
New.CleanUp.ReferenceOTU37056
New.CleanUp.ReferenceOTU181128

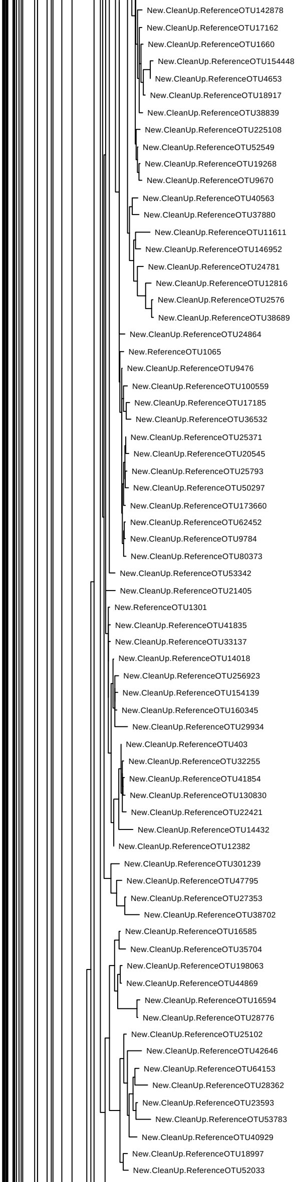

New.CleanUp.ReferenceOTU142878
New.CleanUp.ReferenceOTU17162
New.CleanUp.ReferenceOTU1660
New.CleanUp.ReferenceOTU154448
New.CleanUp.ReferenceOTU4653
New.CleanUp.ReferenceOTU18917
New.CleanUp.ReferenceOTU38839
New.CleanUp.ReferenceOTU225108
New.CleanUp.ReferenceOTU52549
New.CleanUp.ReferenceOTU19268
New.CleanUp.ReferenceOTU9670
New.CleanUp.ReferenceOTU40563
New.CleanUp.ReferenceOTU37880
New.CleanUp.ReferenceOTU11611
New.CleanUp.ReferenceOTU146952
New.CleanUp.ReferenceOTU24781
New.CleanUp.ReferenceOTU12816
New.CleanUp.ReferenceOTU2576
New.CleanUp.ReferenceOTU38689
New.CleanUp.ReferenceOTU24864
New.ReferenceOTU1065
New.CleanUp.ReferenceOTU9476
New.CleanUp.ReferenceOTU100559
New.CleanUp.ReferenceOTU17185
New.CleanUp.ReferenceOTU36532
New.CleanUp.ReferenceOTU25371
New.CleanUp.ReferenceOTU20545
New.CleanUp.ReferenceOTU25793
New.CleanUp.ReferenceOTU50297
New.CleanUp.ReferenceOTU173660
New.CleanUp.ReferenceOTU62452
New.CleanUp.ReferenceOTU9784
New.CleanUp.ReferenceOTU80373
New.CleanUp.ReferenceOTU53342
New.CleanUp.ReferenceOTU21405
New.ReferenceOTU1301
New.CleanUp.ReferenceOTU41835
New.CleanUp.ReferenceOTU33137
New.CleanUp.ReferenceOTU14018
New.CleanUp.ReferenceOTU256923
New.CleanUp.ReferenceOTU154139
New.CleanUp.ReferenceOTU160345
New.CleanUp.ReferenceOTU29934
New.CleanUp.ReferenceOTU403
New.CleanUp.ReferenceOTU32255
New.CleanUp.ReferenceOTU41854
New.CleanUp.ReferenceOTU130830
New.CleanUp.ReferenceOTU22421
New.CleanUp.ReferenceOTU14432
New.CleanUp.ReferenceOTU12382
New.CleanUp.ReferenceOTU301239
New.CleanUp.ReferenceOTU47795
New.CleanUp.ReferenceOTU27353
New.CleanUp.ReferenceOTU38702
New.CleanUp.ReferenceOTU16585
New.CleanUp.ReferenceOTU35704
New.CleanUp.ReferenceOTU198063
New.CleanUp.ReferenceOTU44869
New.CleanUp.ReferenceOTU16594
New.CleanUp.ReferenceOTU28776
New.CleanUp.ReferenceOTU25102
New.CleanUp.ReferenceOTU42646
New.CleanUp.ReferenceOTU64153
New.CleanUp.ReferenceOTU28362
New.CleanUp.ReferenceOTU23593
New.CleanUp.ReferenceOTU53783
New.CleanUp.ReferenceOTU40929
New.CleanUp.ReferenceOTU18997
New.CleanUp.ReferenceOTU52033

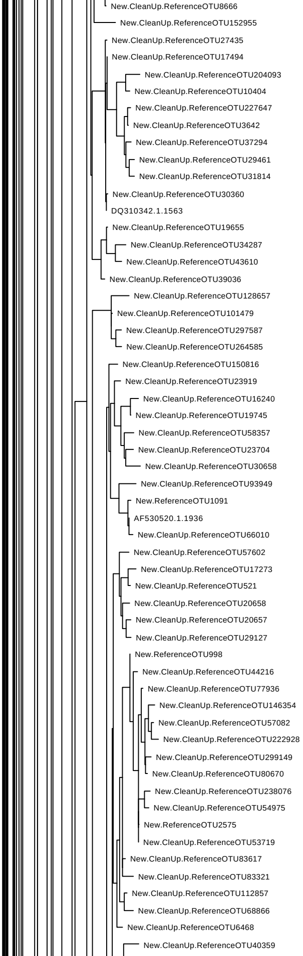
New.CleanUp.ReferenceOTU8666
New.CleanUp.ReferenceOTU152955
New.CleanUp.ReferenceOTU27435
New.CleanUp.ReferenceOTU17494
New.CleanUp.ReferenceOTU204093
New.CleanUp.ReferenceOTU10404
New.CleanUp.ReferenceOTU227647
New.CleanUp.ReferenceOTU3642
New.CleanUp.ReferenceOTU37294
New.CleanUp.ReferenceOTU29461
New.CleanUp.ReferenceOTU31814
New.CleanUp.ReferenceOTU30360
DQ310342.1.1563
New.CleanUp.ReferenceOTU19655
New.CleanUp.ReferenceOTU34287
New.CleanUp.ReferenceOTU43610
New.CleanUp.ReferenceOTU39036
New.CleanUp.ReferenceOTU128657
New.CleanUp.ReferenceOTU101479
New.CleanUp.ReferenceOTU297587
New.CleanUp.ReferenceOTU264585
New.CleanUp.ReferenceOTU150816
New.CleanUp.ReferenceOTU23919
New.CleanUp.ReferenceOTU16240
New.CleanUp.ReferenceOTU19745
New.CleanUp.ReferenceOTU58357
New.CleanUp.ReferenceOTU23704
New.CleanUp.ReferenceOTU30658
New.CleanUp.ReferenceOTU93949
New.ReferenceOTU1091
AF530520.1.1936
New.CleanUp.ReferenceOTU66010
New.CleanUp.ReferenceOTU57602
New.CleanUp.ReferenceOTU17273
New.CleanUp.ReferenceOTU521
New.CleanUp.ReferenceOTU20658
New.CleanUp.ReferenceOTU20657
New.CleanUp.ReferenceOTU29127
New.ReferenceOTU998
New.CleanUp.ReferenceOTU44216
New.CleanUp.ReferenceOTU77936
New.CleanUp.ReferenceOTU146354
New.CleanUp.ReferenceOTU57082
New.CleanUp.ReferenceOTU222928
New.CleanUp.ReferenceOTU299149
New.CleanUp.ReferenceOTU80670
New.CleanUp.ReferenceOTU238076
New.CleanUp.ReferenceOTU54975
New.ReferenceOTU2575
New.CleanUp.ReferenceOTU53719
New.CleanUp.ReferenceOTU83617
New.CleanUp.ReferenceOTU83321
New.CleanUp.ReferenceOTU112857
New.CleanUp.ReferenceOTU68866
New.CleanUp.ReferenceOTU6468
New.CleanUp.ReferenceOTU40359

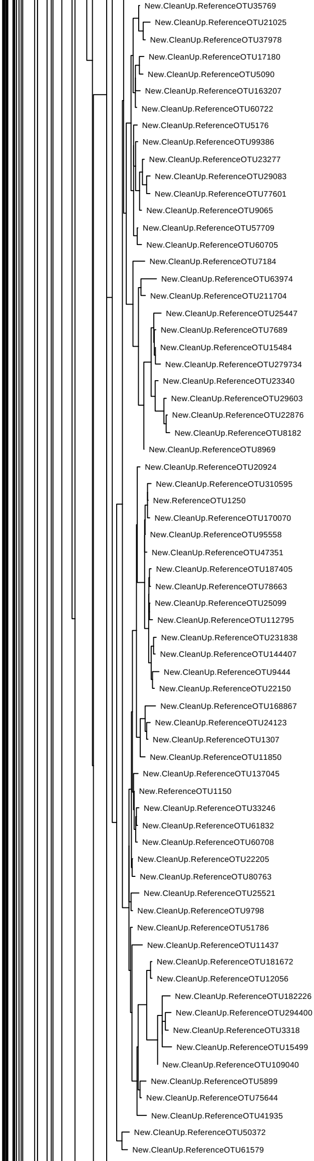
New.CleanUp.ReferenceOTU35769
New.CleanUp.ReferenceOTU21025
New.CleanUp.ReferenceOTU37978
New.CleanUp.ReferenceOTU17180
New.CleanUp.ReferenceOTU5090
New.CleanUp.ReferenceOTU163207
New.CleanUp.ReferenceOTU60722
New.CleanUp.ReferenceOTU5176
New.CleanUp.ReferenceOTU99386
New.CleanUp.ReferenceOTU23277
New.CleanUp.ReferenceOTU29083
New.CleanUp.ReferenceOTU77601
New.CleanUp.ReferenceOTU9065
New.CleanUp.ReferenceOTU57709
New.CleanUp.ReferenceOTU60705
New.CleanUp.ReferenceOTU7184
New.CleanUp.ReferenceOTU63974
New.CleanUp.ReferenceOTU211704
New.CleanUp.ReferenceOTU25447
New.CleanUp.ReferenceOTU7689
New.CleanUp.ReferenceOTU15484
New.CleanUp.ReferenceOTU279734
New.CleanUp.ReferenceOTU23340
New.CleanUp.ReferenceOTU29603
New.CleanUp.ReferenceOTU22876
New.CleanUp.ReferenceOTU8182
New.CleanUp.ReferenceOTU8969
New.CleanUp.ReferenceOTU20924
New.CleanUp.ReferenceOTU310595
New.ReferenceOTU1250
New.CleanUp.ReferenceOTU170070
New.CleanUp.ReferenceOTU95558
New.CleanUp.ReferenceOTU47351
New.CleanUp.ReferenceOTU187405
New.CleanUp.ReferenceOTU78663
New.CleanUp.ReferenceOTU25099
New.CleanUp.ReferenceOTU112795
New.CleanUp.ReferenceOTU231838
New.CleanUp.ReferenceOTU144407
New.CleanUp.ReferenceOTU9444
New.CleanUp.ReferenceOTU22150
New.CleanUp.ReferenceOTU168867
New.CleanUp.ReferenceOTU24123
New.CleanUp.ReferenceOTU1307
New.CleanUp.ReferenceOTU11850
New.CleanUp.ReferenceOTU137045
New.ReferenceOTU1150
New.CleanUp.ReferenceOTU33246
New.CleanUp.ReferenceOTU61832
New.CleanUp.ReferenceOTU60708
New.CleanUp.ReferenceOTU22205
New.CleanUp.ReferenceOTU80763
New.CleanUp.ReferenceOTU25521
New.CleanUp.ReferenceOTU9798
New.CleanUp.ReferenceOTU51786
New.CleanUp.ReferenceOTU11437
New.CleanUp.ReferenceOTU181672
New.CleanUp.ReferenceOTU12056
New.CleanUp.ReferenceOTU182226
New.CleanUp.ReferenceOTU294400
New.CleanUp.ReferenceOTU3318
New.CleanUp.ReferenceOTU15499
New.CleanUp.ReferenceOTU109040
New.CleanUp.ReferenceOTU5899
New.CleanUp.ReferenceOTU75644
New.CleanUp.ReferenceOTU41935
New.CleanUp.ReferenceOTU50372
New.CleanUp.ReferenceOTU61579

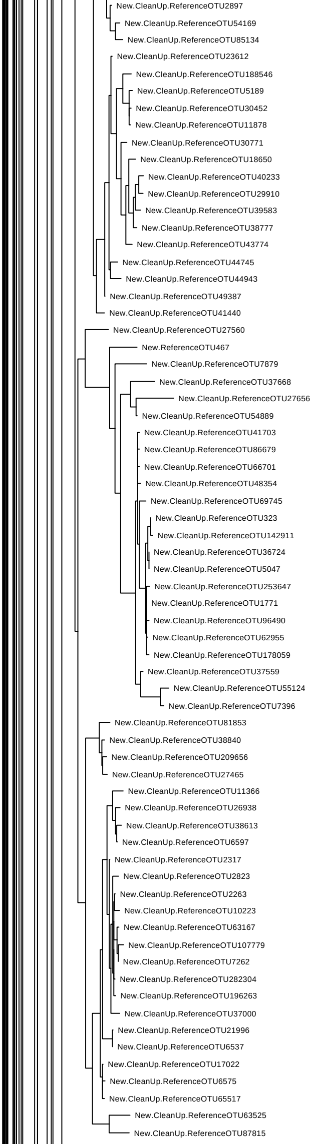
New.CleanUp.ReferenceOTU2897
New.CleanUp.ReferenceOTU54169
New.CleanUp.ReferenceOTU85134
New.CleanUp.ReferenceOTU23612
New.CleanUp.ReferenceOTU188546
New.CleanUp.ReferenceOTU5189
New.CleanUp.ReferenceOTU30452
New.CleanUp.ReferenceOTU11878
New.CleanUp.ReferenceOTU30771
New.CleanUp.ReferenceOTU18650
New.CleanUp.ReferenceOTU40233
New.CleanUp.ReferenceOTU29910
New.CleanUp.ReferenceOTU39583
New.CleanUp.ReferenceOTU38777
New.CleanUp.ReferenceOTU43774
New.CleanUp.ReferenceOTU44745
New.CleanUp.ReferenceOTU44943
New.CleanUp.ReferenceOTU49387
New.CleanUp.ReferenceOTU41440
New.CleanUp.ReferenceOTU27560
New.ReferenceOTU467
New.CleanUp.ReferenceOTU7879
New.CleanUp.ReferenceOTU37668
New.CleanUp.ReferenceOTU27656
New.CleanUp.ReferenceOTU54889
New.CleanUp.ReferenceOTU41703
New.CleanUp.ReferenceOTU86679
New.CleanUp.ReferenceOTU66701
New.CleanUp.ReferenceOTU48354
New.CleanUp.ReferenceOTU69745
New.CleanUp.ReferenceOTU323
New.CleanUp.ReferenceOTU142911
New.CleanUp.ReferenceOTU36724
New.CleanUp.ReferenceOTU5047
New.CleanUp.ReferenceOTU253647
New.CleanUp.ReferenceOTU1771
New.CleanUp.ReferenceOTU96490
New.CleanUp.ReferenceOTU62955
New.CleanUp.ReferenceOTU178059
New.CleanUp.ReferenceOTU37559
New.CleanUp.ReferenceOTU55124
New.CleanUp.ReferenceOTU7396
New.CleanUp.ReferenceOTU81853
New.CleanUp.ReferenceOTU38840
New.CleanUp.ReferenceOTU209656
New.CleanUp.ReferenceOTU27465
New.CleanUp.ReferenceOTU11366
New.CleanUp.ReferenceOTU26938
New.CleanUp.ReferenceOTU38613
New.CleanUp.ReferenceOTU6597
New.CleanUp.ReferenceOTU2317
New.CleanUp.ReferenceOTU2823
New.CleanUp.ReferenceOTU2263
New.CleanUp.ReferenceOTU10223
New.CleanUp.ReferenceOTU63167
New.CleanUp.ReferenceOTU107779
New.CleanUp.ReferenceOTU7262
New.CleanUp.ReferenceOTU282304
New.CleanUp.ReferenceOTU196263
New.CleanUp.ReferenceOTU37000
New.CleanUp.ReferenceOTU21996
New.CleanUp.ReferenceOTU6537
New.CleanUp.ReferenceOTU17022
New.CleanUp.ReferenceOTU6575
New.CleanUp.ReferenceOTU65517
New.CleanUp.ReferenceOTU63525
New.CleanUp.ReferenceOTU87815

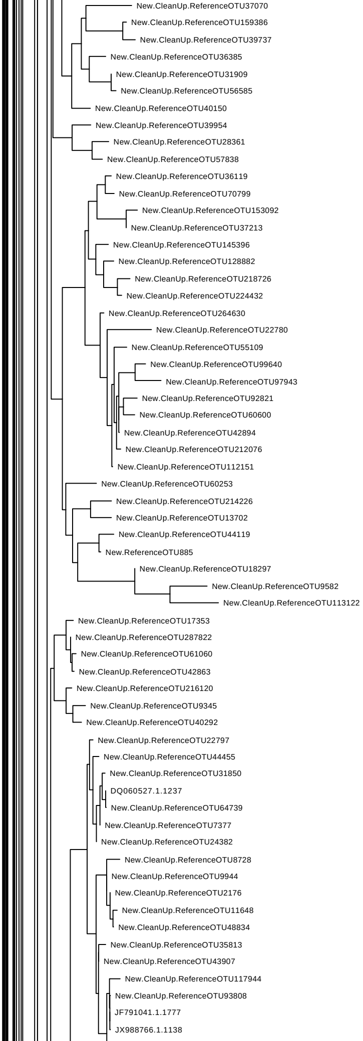
New.CleanUp.ReferenceOTU37070
New.CleanUp.ReferenceOTU159386
New.CleanUp.ReferenceOTU39737
New.CleanUp.ReferenceOTU36385
New.CleanUp.ReferenceOTU31909
New.CleanUp.ReferenceOTU56585
New.CleanUp.ReferenceOTU40150
New.CleanUp.ReferenceOTU39954
New.CleanUp.ReferenceOTU28361
New.CleanUp.ReferenceOTU57838
New.CleanUp.ReferenceOTU36119
New.CleanUp.ReferenceOTU70799
New.CleanUp.ReferenceOTU153092
New.CleanUp.ReferenceOTU37213
New.CleanUp.ReferenceOTU145396
New.CleanUp.ReferenceOTU128882
New.CleanUp.ReferenceOTU218726
New.CleanUp.ReferenceOTU224432
New.CleanUp.ReferenceOTU264630
New.CleanUp.ReferenceOTU22780
New.CleanUp.ReferenceOTU55109
New.CleanUp.ReferenceOTU99640
New.CleanUp.ReferenceOTU97943
New.CleanUp.ReferenceOTU92821
New.CleanUp.ReferenceOTU60600
New.CleanUp.ReferenceOTU42894
New.CleanUp.ReferenceOTU212076
New.CleanUp.ReferenceOTU112151
New.CleanUp.ReferenceOTU60253
New.CleanUp.ReferenceOTU214226
New.CleanUp.ReferenceOTU13702
New.CleanUp.ReferenceOTU44119
New.ReferenceOTU885
New.CleanUp.ReferenceOTU18297
New.CleanUp.ReferenceOTU9582
New.CleanUp.ReferenceOTU113122
New.CleanUp.ReferenceOTU17353
New.CleanUp.ReferenceOTU287822
New.CleanUp.ReferenceOTU61060
New.CleanUp.ReferenceOTU42863
New.CleanUp.ReferenceOTU216120
New.CleanUp.ReferenceOTU9345
New.CleanUp.ReferenceOTU40292
New.CleanUp.ReferenceOTU22797
New.CleanUp.ReferenceOTU44455
New.CleanUp.ReferenceOTU31850
DQ060527.1.1237
New.CleanUp.ReferenceOTU64739
New.CleanUp.ReferenceOTU7377
New.CleanUp.ReferenceOTU24382
New.CleanUp.ReferenceOTU8728
New.CleanUp.ReferenceOTU9944
New.CleanUp.ReferenceOTU2176
New.CleanUp.ReferenceOTU11648
New.CleanUp.ReferenceOTU48834
New.CleanUp.ReferenceOTU35813
New.CleanUp.ReferenceOTU43907
New.CleanUp.ReferenceOTU117944
New.CleanUp.ReferenceOTU93808
JF791041.1.1777
JX988766.1.1138

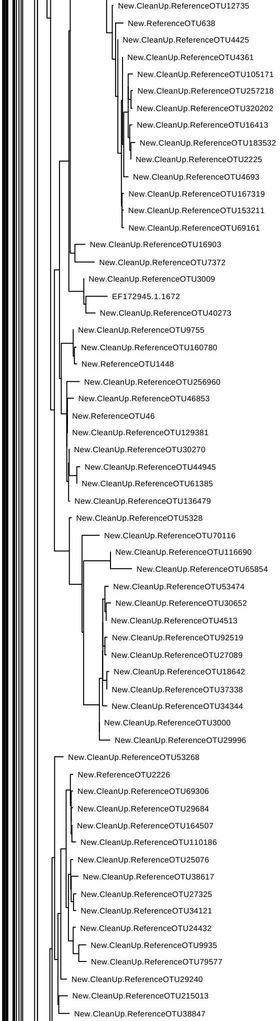
New.CleanUp.ReferenceOTU12735
New.ReferenceOTU638
New.CleanUp.ReferenceOTU4425
New.CleanUp.ReferenceOTU4361
New.CleanUp.ReferenceOTU105171
New.CleanUp.ReferenceOTU257218
New.CleanUp.ReferenceOTU320202
New.CleanUp.ReferenceOTU16413
New.CleanUp.ReferenceOTU183532
New.CleanUp.ReferenceOTU2225
New.CleanUp.ReferenceOTU4693
New.CleanUp.ReferenceOTU167319
New.CleanUp.ReferenceOTU153211
New.CleanUp.ReferenceOTU69161
New.CleanUp.ReferenceOTU16903
New.CleanUp.ReferenceOTU7372
New.CleanUp.ReferenceOTU3009
EF172945.1.1672
New.CleanUp.ReferenceOTU40273
New.CleanUp.ReferenceOTU9755
New.CleanUp.ReferenceOTU160780
New.ReferenceOTU1448
New.CleanUp.ReferenceOTU256960
New.CleanUp.ReferenceOTU46853
New.ReferenceOTU46
New.CleanUp.ReferenceOTU129381
New.CleanUp.ReferenceOTU30270
New.CleanUp.ReferenceOTU44945
New.CleanUp.ReferenceOTU61385
New.CleanUp.ReferenceOTU136479
New.CleanUp.ReferenceOTU5328
New.CleanUp.ReferenceOTU70116
New.CleanUp.ReferenceOTU116690
New.CleanUp.ReferenceOTU65854
New.CleanUp.ReferenceOTU53474
New.CleanUp.ReferenceOTU30652
New.CleanUp.ReferenceOTU4513
New.CleanUp.ReferenceOTU92519
New.CleanUp.ReferenceOTU27089
New.CleanUp.ReferenceOTU18642
New.CleanUp.ReferenceOTU37338
New.CleanUp.ReferenceOTU34344
New.CleanUp.ReferenceOTU3000
New.CleanUp.ReferenceOTU29996
New.CleanUp.ReferenceOTU53268
New.ReferenceOTU2226
New.CleanUp.ReferenceOTU69306
New.CleanUp.ReferenceOTU29684
New.CleanUp.ReferenceOTU164507
New.CleanUp.ReferenceOTU110186
New.CleanUp.ReferenceOTU25076
New.CleanUp.ReferenceOTU38617
New.CleanUp.ReferenceOTU27325
New.CleanUp.ReferenceOTU34121
New.CleanUp.ReferenceOTU24432
New.CleanUp.ReferenceOTU9935
New.CleanUp.ReferenceOTU79577
New.CleanUp.ReferenceOTU29240
New.CleanUp.ReferenceOTU215013
New.CleanUp.ReferenceOTU38847

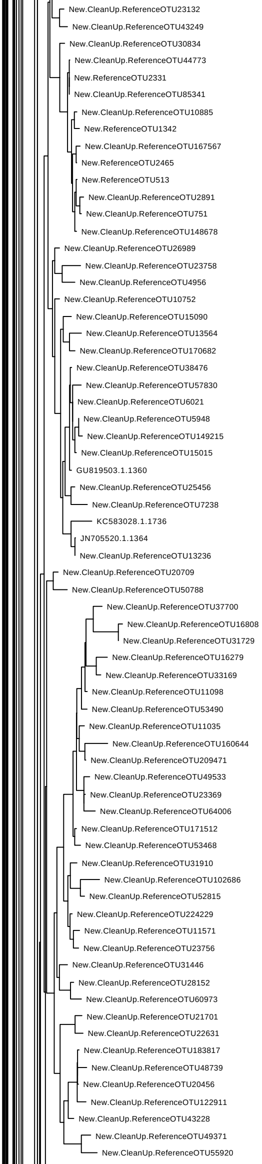
New.CleanUp.ReferenceOTU23132
New.CleanUp.ReferenceOTU43249
New.CleanUp.ReferenceOTU30834
New.CleanUp.ReferenceOTU44773
New.ReferenceOTU2331
New.CleanUp.ReferenceOTU85341
New.CleanUp.ReferenceOTU10885
New.ReferenceOTU1342
New.CleanUp.ReferenceOTU167567
New.ReferenceOTU2465
New.ReferenceOTU513
New.CleanUp.ReferenceOTU2891
New.CleanUp.ReferenceOTU751
New.CleanUp.ReferenceOTU148678
New.CleanUp.ReferenceOTU26989
New.CleanUp.ReferenceOTU23758
New.CleanUp.ReferenceOTU4956
New.CleanUp.ReferenceOTU10752
New.CleanUp.ReferenceOTU15090
New.CleanUp.ReferenceOTU13564
New.CleanUp.ReferenceOTU170682
New.CleanUp.ReferenceOTU38476
New.CleanUp.ReferenceOTU57830
New.CleanUp.ReferenceOTU6021
New.CleanUp.ReferenceOTU5948
New.CleanUp.ReferenceOTU149215
New.CleanUp.ReferenceOTU15015
GU819503.1.1360
New.CleanUp.ReferenceOTU25456
New.CleanUp.ReferenceOTU7238
KC583028.1.1736
JN705520.1.1364
New.CleanUp.ReferenceOTU13236
New.CleanUp.ReferenceOTU20709
New.CleanUp.ReferenceOTU50788
New.CleanUp.ReferenceOTU37700
New.CleanUp.ReferenceOTU16808
New.CleanUp.ReferenceOTU31729
New.CleanUp.ReferenceOTU16279
New.CleanUp.ReferenceOTU33169
New.CleanUp.ReferenceOTU11098
New.CleanUp.ReferenceOTU53490
New.CleanUp.ReferenceOTU11035
New.CleanUp.ReferenceOTU160644
New.CleanUp.ReferenceOTU209471
New.CleanUp.ReferenceOTU49533
New.CleanUp.ReferenceOTU23369
New.CleanUp.ReferenceOTU64006
New.CleanUp.ReferenceOTU171512
New.CleanUp.ReferenceOTU53468
New.CleanUp.ReferenceOTU31910
New.CleanUp.ReferenceOTU102686
New.CleanUp.ReferenceOTU52815
New.CleanUp.ReferenceOTU224229
New.CleanUp.ReferenceOTU11571
New.CleanUp.ReferenceOTU23756
New.CleanUp.ReferenceOTU31446
New.CleanUp.ReferenceOTU28152
New.CleanUp.ReferenceOTU60973
New.CleanUp.ReferenceOTU21701
New.CleanUp.ReferenceOTU22631
New.CleanUp.ReferenceOTU183817
New.CleanUp.ReferenceOTU48739
New.CleanUp.ReferenceOTU20456
New.CleanUp.ReferenceOTU122911
New.CleanUp.ReferenceOTU43228
New.CleanUp.ReferenceOTU49371
New.CleanUp.ReferenceOTU55920

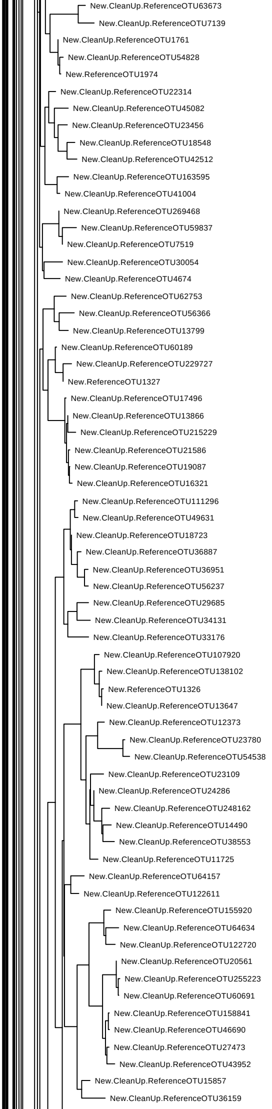
New.CleanUp.ReferenceOTU63673
New.CleanUp.ReferenceOTU7139
New.CleanUp.ReferenceOTU1761
New.CleanUp.ReferenceOTU54828
New.ReferenceOTU1974
New.CleanUp.ReferenceOTU22314
New.CleanUp.ReferenceOTU45082
New.CleanUp.ReferenceOTU23456
New.CleanUp.ReferenceOTU18548
New.CleanUp.ReferenceOTU42512
New.CleanUp.ReferenceOTU163595
New.CleanUp.ReferenceOTU41004
New.CleanUp.ReferenceOTU269468
New.CleanUp.ReferenceOTU59837
New.CleanUp.ReferenceOTU7519
New.CleanUp.ReferenceOTU30054
New.CleanUp.ReferenceOTU4674
New.CleanUp.ReferenceOTU62753
New.CleanUp.ReferenceOTU56366
New.CleanUp.ReferenceOTU13799
New.CleanUp.ReferenceOTU60189
New.CleanUp.ReferenceOTU229727
New.ReferenceOTU1327
New.CleanUp.ReferenceOTU17496
New.CleanUp.ReferenceOTU13866
New.CleanUp.ReferenceOTU215229
New.CleanUp.ReferenceOTU21586
New.CleanUp.ReferenceOTU19087
New.CleanUp.ReferenceOTU16321
New.CleanUp.ReferenceOTU111296
New.CleanUp.ReferenceOTU49631
New.CleanUp.ReferenceOTU18723
New.CleanUp.ReferenceOTU36887
New.CleanUp.ReferenceOTU36951
New.CleanUp.ReferenceOTU56237
New.CleanUp.ReferenceOTU29685
New.CleanUp.ReferenceOTU34131
New.CleanUp.ReferenceOTU33176
New.CleanUp.ReferenceOTU107920
New.CleanUp.ReferenceOTU138102
New.ReferenceOTU1326
New.CleanUp.ReferenceOTU13647
New.CleanUp.ReferenceOTU12373
New.CleanUp.ReferenceOTU23780
New.CleanUp.ReferenceOTU54538
New.CleanUp.ReferenceOTU23109
New.CleanUp.ReferenceOTU24286
New.CleanUp.ReferenceOTU248162
New.CleanUp.ReferenceOTU14490
New.CleanUp.ReferenceOTU38553
New.CleanUp.ReferenceOTU11725
New.CleanUp.ReferenceOTU64157
New.CleanUp.ReferenceOTU122611
New.CleanUp.ReferenceOTU155920
New.CleanUp.ReferenceOTU64634
New.CleanUp.ReferenceOTU122720
New.CleanUp.ReferenceOTU20561
New.CleanUp.ReferenceOTU255223
New.CleanUp.ReferenceOTU60691
New.CleanUp.ReferenceOTU158841
New.CleanUp.ReferenceOTU46690
New.CleanUp.ReferenceOTU27473
New.CleanUp.ReferenceOTU43952
New.CleanUp.ReferenceOTU15857
New.CleanUp.ReferenceOTU36159

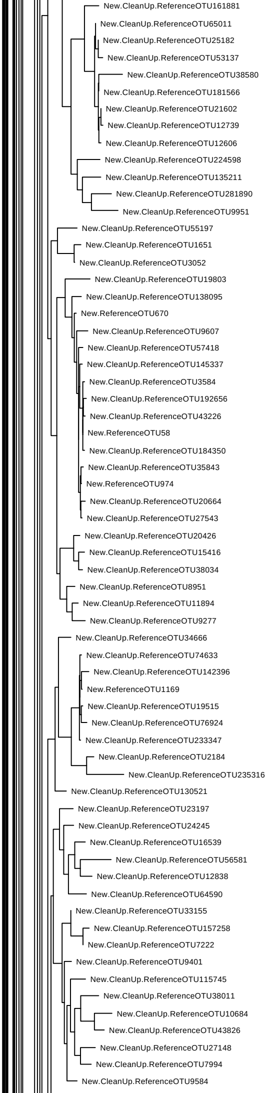
New.CleanUp.ReferenceOTU161881
New.CleanUp.ReferenceOTU65011
New.CleanUp.ReferenceOTU25182
New.CleanUp.ReferenceOTU53137
New.CleanUp.ReferenceOTU38580
New.CleanUp.ReferenceOTU181566
New.CleanUp.ReferenceOTU21602
New.CleanUp.ReferenceOTU12739
New.CleanUp.ReferenceOTU12606
New.CleanUp.ReferenceOTU224598
New.CleanUp.ReferenceOTU135211
New.CleanUp.ReferenceOTU281890
New.CleanUp.ReferenceOTU9951
New.CleanUp.ReferenceOTU55197
New.CleanUp.ReferenceOTU1651
New.CleanUp.ReferenceOTU3052
New.CleanUp.ReferenceOTU19803
New.CleanUp.ReferenceOTU138095
New.ReferenceOTU670
New.CleanUp.ReferenceOTU9607
New.CleanUp.ReferenceOTU57418
New.CleanUp.ReferenceOTU145337
New.CleanUp.ReferenceOTU3584
New.CleanUp.ReferenceOTU192656
New.CleanUp.ReferenceOTU43226
New.ReferenceOTU58
New.CleanUp.ReferenceOTU184350
New.CleanUp.ReferenceOTU35843
New.ReferenceOTU974
New.CleanUp.ReferenceOTU20664
New.CleanUp.ReferenceOTU27543
New.CleanUp.ReferenceOTU20426
New.CleanUp.ReferenceOTU15416
New.CleanUp.ReferenceOTU38034
New.CleanUp.ReferenceOTU8951
New.CleanUp.ReferenceOTU11894
New.CleanUp.ReferenceOTU9277
New.CleanUp.ReferenceOTU34666
New.CleanUp.ReferenceOTU74633
New.CleanUp.ReferenceOTU142396
New.ReferenceOTU1169
New.CleanUp.ReferenceOTU19515
New.CleanUp.ReferenceOTU76924
New.CleanUp.ReferenceOTU233347
New.CleanUp.ReferenceOTU2184
New.CleanUp.ReferenceOTU235316
New.CleanUp.ReferenceOTU130521
New.CleanUp.ReferenceOTU23197
New.CleanUp.ReferenceOTU24245
New.CleanUp.ReferenceOTU16539
New.CleanUp.ReferenceOTU56581
New.CleanUp.ReferenceOTU12838
New.CleanUp.ReferenceOTU64590
New.CleanUp.ReferenceOTU33155
New.CleanUp.ReferenceOTU157258
New.CleanUp.ReferenceOTU7222
New.CleanUp.ReferenceOTU9401
New.CleanUp.ReferenceOTU115745
New.CleanUp.ReferenceOTU38011
New.CleanUp.ReferenceOTU10684
New.CleanUp.ReferenceOTU43826
New.CleanUp.ReferenceOTU27148
New.CleanUp.ReferenceOTU7994
New.CleanUp.ReferenceOTU9584

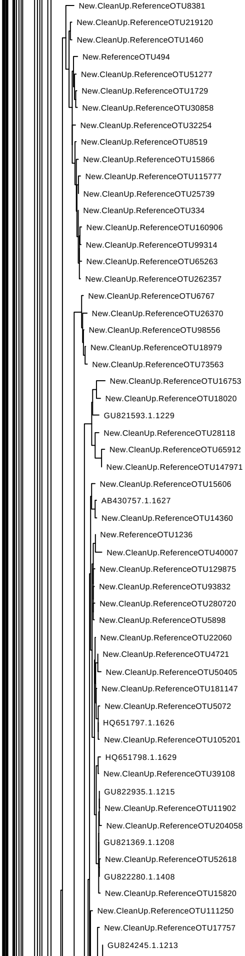
New.CleanUp.ReferenceOTU8381
New.CleanUp.ReferenceOTU219120
New.CleanUp.ReferenceOTU1460
New.ReferenceOTU494
New.CleanUp.ReferenceOTU51277
New.CleanUp.ReferenceOTU1729
New.CleanUp.ReferenceOTU30858
New.CleanUp.ReferenceOTU32254
New.CleanUp.ReferenceOTU8519
New.CleanUp.ReferenceOTU15866
New.CleanUp.ReferenceOTU115777
New.CleanUp.ReferenceOTU25739
New.CleanUp.ReferenceOTU334
New.CleanUp.ReferenceOTU160906
New.CleanUp.ReferenceOTU99314
New.CleanUp.ReferenceOTU65263
New.CleanUp.ReferenceOTU262357
New.CleanUp.ReferenceOTU6767
New.CleanUp.ReferenceOTU26370
New.CleanUp.ReferenceOTU98556
New.CleanUp.ReferenceOTU18979
New.CleanUp.ReferenceOTU73563
New.CleanUp.ReferenceOTU16753
New.CleanUp.ReferenceOTU18020
GU821593.1.1229
New.CleanUp.ReferenceOTU28118
New.CleanUp.ReferenceOTU65912
New.CleanUp.ReferenceOTU147971
New.CleanUp.ReferenceOTU15606
AB430757.1.1627
New.CleanUp.ReferenceOTU14360
New.ReferenceOTU1236
New.CleanUp.ReferenceOTU40007
New.CleanUp.ReferenceOTU129875
New.CleanUp.ReferenceOTU93832
New.CleanUp.ReferenceOTU280720
New.CleanUp.ReferenceOTU5898
New.CleanUp.ReferenceOTU22060
New.CleanUp.ReferenceOTU4721
New.CleanUp.ReferenceOTU50405
New.CleanUp.ReferenceOTU181147
New.CleanUp.ReferenceOTU5072
HQ651797.1.1626
New.CleanUp.ReferenceOTU105201
HQ651798.1.1629
New.CleanUp.ReferenceOTU39108
GU822935.1.1215
New.CleanUp.ReferenceOTU11902
New.CleanUp.ReferenceOTU204058
GU821369.1.1208
New.CleanUp.ReferenceOTU52618
GU822280.1.1408
New.CleanUp.ReferenceOTU15820
New.CleanUp.ReferenceOTU111250
New.CleanUp.ReferenceOTU17757
GU824245.1.1213

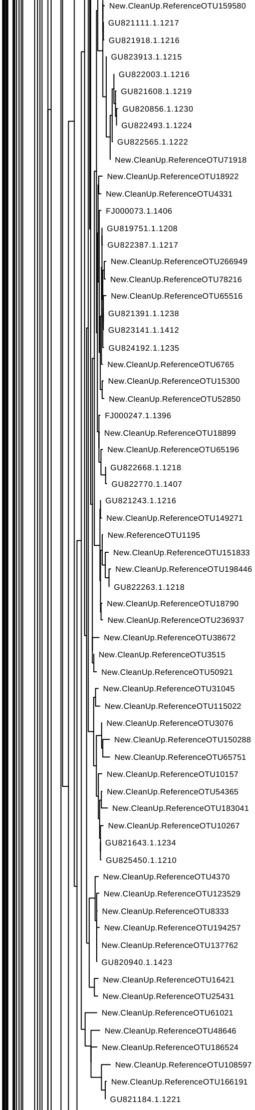
New.CleanUp.ReferenceOTU159580
GU821111.1.1217
GU821918.1.1216
GU823913.1.1215
GU822003.1.1216
GU821608.1.1219
GU820856.1.1230
GU822493.1.1224
GU822565.1.1222
New.CleanUp.ReferenceOTU71918
New.CleanUp.ReferenceOTU18922
New.CleanUp.ReferenceOTU4331
FJ000073.1.1406
GU819751.1.1208
GU822387.1.1217
New.CleanUp.ReferenceOTU266949
New.CleanUp.ReferenceOTU78216
New.CleanUp.ReferenceOTU65516
GU821391.1.1238
GU823141.1.1412
GU824192.1.1235
New.CleanUp.ReferenceOTU6765
New.CleanUp.ReferenceOTU15300
New.CleanUp.ReferenceOTU52850
FJ000247.1.1396
New.CleanUp.ReferenceOTU18899
New.CleanUp.ReferenceOTU65196
GU822668.1.1218
GU822770.1.1407
GU821243.1.1216
New.CleanUp.ReferenceOTU149271
New.ReferenceOTU1195
New.CleanUp.ReferenceOTU151833
New.CleanUp.ReferenceOTU198446
GU822263.1.1218
New.CleanUp.ReferenceOTU18790
New.CleanUp.ReferenceOTU236937
New.CleanUp.ReferenceOTU38672
New.CleanUp.ReferenceOTU3515
New.CleanUp.ReferenceOTU50921
New.CleanUp.ReferenceOTU31045
New.CleanUp.ReferenceOTU115022
New.CleanUp.ReferenceOTU3076
New.CleanUp.ReferenceOTU150288
New.CleanUp.ReferenceOTU65751
New.CleanUp.ReferenceOTU10157
New.CleanUp.ReferenceOTU54365
New.CleanUp.ReferenceOTU183041
New.CleanUp.ReferenceOTU10267
GU821643.1.1234
GU825450.1.1210
New.CleanUp.ReferenceOTU4370
New.CleanUp.ReferenceOTU123529
New.CleanUp.ReferenceOTU8333
New.CleanUp.ReferenceOTU194257
New.CleanUp.ReferenceOTU137762
GU820940.1.1423
New.CleanUp.ReferenceOTU16421
New.CleanUp.ReferenceOTU25431
New.CleanUp.ReferenceOTU61021
New.CleanUp.ReferenceOTU48646
New.CleanUp.ReferenceOTU186524
New.CleanUp.ReferenceOTU108597
New.CleanUp.ReferenceOTU166191
GU821184.1.1221

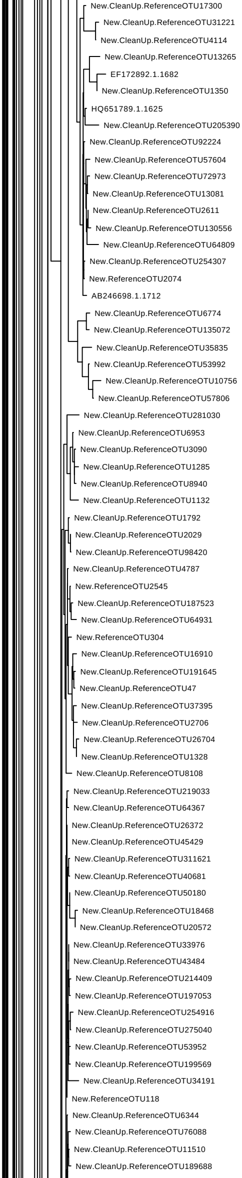
New.CleanUp.ReferenceOTU17300
New.CleanUp.ReferenceOTU31221
New.CleanUp.ReferenceOTU4114
New.CleanUp.ReferenceOTU13265
EF172892.1.1682
New.CleanUp.ReferenceOTU1350
HQ651789.1.1625
New.CleanUp.ReferenceOTU205390
New.CleanUp.ReferenceOTU92224
New.CleanUp.ReferenceOTU57604
New.CleanUp.ReferenceOTU72973
New.CleanUp.ReferenceOTU13081
New.CleanUp.ReferenceOTU2611
New.CleanUp.ReferenceOTU130556
New.CleanUp.ReferenceOTU64809
New.CleanUp.ReferenceOTU254307
New.ReferenceOTU2074
AB246698.1.1712
New.CleanUp.ReferenceOTU6774
New.CleanUp.ReferenceOTU135072
New.CleanUp.ReferenceOTU35835
New.CleanUp.ReferenceOTU53992
New.CleanUp.ReferenceOTU10756
New.CleanUp.ReferenceOTU57806
New.CleanUp.ReferenceOTU281030
New.CleanUp.ReferenceOTU6953
New.CleanUp.ReferenceOTU3090
New.CleanUp.ReferenceOTU1285
New.CleanUp.ReferenceOTU8940
New.CleanUp.ReferenceOTU1132
New.CleanUp.ReferenceOTU1792
New.CleanUp.ReferenceOTU2029
New.CleanUp.ReferenceOTU98420
New.CleanUp.ReferenceOTU4787
New.ReferenceOTU2545
New.CleanUp.ReferenceOTU187523
New.CleanUp.ReferenceOTU64931
New.ReferenceOTU304
New.CleanUp.ReferenceOTU16910
New.CleanUp.ReferenceOTU191645
New.CleanUp.ReferenceOTU47
New.CleanUp.ReferenceOTU37395
New.CleanUp.ReferenceOTU2706
New.CleanUp.ReferenceOTU26704
New.CleanUp.ReferenceOTU1328
New.CleanUp.ReferenceOTU8108
New.CleanUp.ReferenceOTU219033
New.CleanUp.ReferenceOTU64367
New.CleanUp.ReferenceOTU26372
New.CleanUp.ReferenceOTU45429
New.CleanUp.ReferenceOTU311621
New.CleanUp.ReferenceOTU40681
New.CleanUp.ReferenceOTU50180
New.CleanUp.ReferenceOTU18468
New.CleanUp.ReferenceOTU20572
New.CleanUp.ReferenceOTU33976
New.CleanUp.ReferenceOTU43484
New.CleanUp.ReferenceOTU214409
New.CleanUp.ReferenceOTU197053
New.CleanUp.ReferenceOTU254916
New.CleanUp.ReferenceOTU275040
New.CleanUp.ReferenceOTU53952
New.CleanUp.ReferenceOTU199569
New.CleanUp.ReferenceOTU34191
New.ReferenceOTU118
New.CleanUp.ReferenceOTU6344
New.CleanUp.ReferenceOTU76088
New.CleanUp.ReferenceOTU11510
New.CleanUp.ReferenceOTU189688

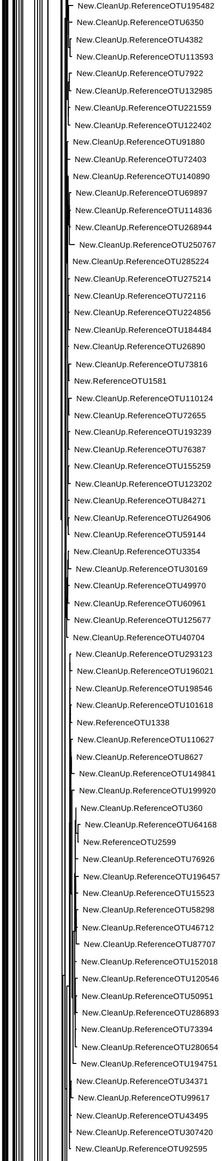
New.CleanUp.ReferenceOTU195482
New.CleanUp.ReferenceOTU6350
New.CleanUp.ReferenceOTU4382
New.CleanUp.ReferenceOTU113593
New.CleanUp.ReferenceOTU7922
New.CleanUp.ReferenceOTU132985
New.CleanUp.ReferenceOTU221559
New.CleanUp.ReferenceOTU122402
New.CleanUp.ReferenceOTU91880
New.CleanUp.ReferenceOTU72403
New.CleanUp.ReferenceOTU140890
New.CleanUp.ReferenceOTU69897
New.CleanUp.ReferenceOTU114836
New.CleanUp.ReferenceOTU268944
New.CleanUp.ReferenceOTU250767
New.CleanUp.ReferenceOTU285224
New.CleanUp.ReferenceOTU275214
New.CleanUp.ReferenceOTU72116
New.CleanUp.ReferenceOTU224856
New.CleanUp.ReferenceOTU184484
New.CleanUp.ReferenceOTU26890
New.CleanUp.ReferenceOTU73816
New.ReferenceOTU1581
New.CleanUp.ReferenceOTU110124
New.CleanUp.ReferenceOTU72655
New.CleanUp.ReferenceOTU193239
New.CleanUp.ReferenceOTU76387
New.CleanUp.ReferenceOTU155259
New.CleanUp.ReferenceOTU123202
New.CleanUp.ReferenceOTU84271
New.CleanUp.ReferenceOTU264906
New.CleanUp.ReferenceOTU59144
New.CleanUp.ReferenceOTU3354
New.CleanUp.ReferenceOTU30169
New.CleanUp.ReferenceOTU49970
New.CleanUp.ReferenceOTU60961
New.CleanUp.ReferenceOTU125677
New.CleanUp.ReferenceOTU40704
New.CleanUp.ReferenceOTU293123
New.CleanUp.ReferenceOTU196021
New.CleanUp.ReferenceOTU198546
New.CleanUp.ReferenceOTU101618
New.ReferenceOTU1338
New.CleanUp.ReferenceOTU110627
New.CleanUp.ReferenceOTU8627
New.CleanUp.ReferenceOTU149841
New.CleanUp.ReferenceOTU199920
New.CleanUp.ReferenceOTU360
New.CleanUp.ReferenceOTU64168
New.ReferenceOTU2599
New.CleanUp.ReferenceOTU76926
New.CleanUp.ReferenceOTU196457
New.CleanUp.ReferenceOTU15523
New.CleanUp.ReferenceOTU58298
New.CleanUp.ReferenceOTU46712
New.CleanUp.ReferenceOTU87707
New.CleanUp.ReferenceOTU152018
New.CleanUp.ReferenceOTU120546
New.CleanUp.ReferenceOTU50951
New.CleanUp.ReferenceOTU286893
New.CleanUp.ReferenceOTU73394
New.CleanUp.ReferenceOTU280654
New.CleanUp.ReferenceOTU194751
New.CleanUp.ReferenceOTU34371
New.CleanUp.ReferenceOTU99617
New.CleanUp.ReferenceOTU43495
New.CleanUp.ReferenceOTU307420
New.CleanUp.ReferenceOTU92595

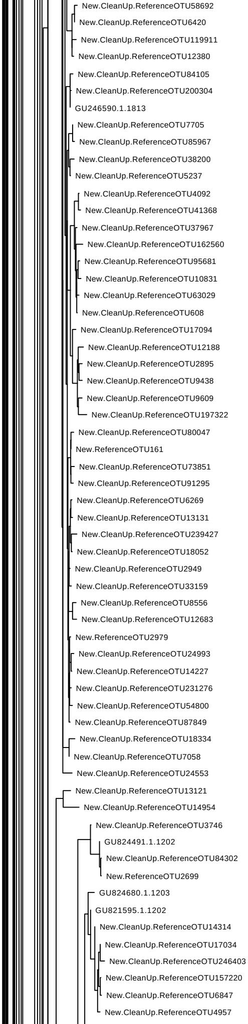
New.CleanUp.ReferenceOTU58692
New.CleanUp.ReferenceOTU6420
New.CleanUp.ReferenceOTU119911
New.CleanUp.ReferenceOTU12380
New.CleanUp.ReferenceOTU84105
New.CleanUp.ReferenceOTU200304
GU246590.1.1813
New.CleanUp.ReferenceOTU7705
New.CleanUp.ReferenceOTU85967
New.CleanUp.ReferenceOTU38200
New.CleanUp.ReferenceOTU5237
New.CleanUp.ReferenceOTU4092
New.CleanUp.ReferenceOTU41368
New.CleanUp.ReferenceOTU37967
New.CleanUp.ReferenceOTU162560
New.CleanUp.ReferenceOTU95681
New.CleanUp.ReferenceOTU10831
New.CleanUp.ReferenceOTU63029
New.CleanUp.ReferenceOTU608
New.CleanUp.ReferenceOTU17094
New.CleanUp.ReferenceOTU12188
New.CleanUp.ReferenceOTU2895
New.CleanUp.ReferenceOTU9438
New.CleanUp.ReferenceOTU9609
New.CleanUp.ReferenceOTU197322
New.CleanUp.ReferenceOTU80047
New.ReferenceOTU161
New.CleanUp.ReferenceOTU73851
New.CleanUp.ReferenceOTU91295
New.CleanUp.ReferenceOTU6269
New.CleanUp.ReferenceOTU13131
New.CleanUp.ReferenceOTU239427
New.CleanUp.ReferenceOTU18052
New.CleanUp.ReferenceOTU2949
New.CleanUp.ReferenceOTU33159
New.CleanUp.ReferenceOTU8556
New.CleanUp.ReferenceOTU12683
New.ReferenceOTU2979
New.CleanUp.ReferenceOTU24993
New.CleanUp.ReferenceOTU14227
New.CleanUp.ReferenceOTU231276
New.CleanUp.ReferenceOTU54800
New.CleanUp.ReferenceOTU87849
New.CleanUp.ReferenceOTU18334
New.CleanUp.ReferenceOTU7058
New.CleanUp.ReferenceOTU24553
New.CleanUp.ReferenceOTU13121
New.CleanUp.ReferenceOTU14954
New.CleanUp.ReferenceOTU3746
GU824491.1.1202
New.CleanUp.ReferenceOTU84302
New.ReferenceOTU2699
GU824680.1.1203
GU821595.1.1202
New.CleanUp.ReferenceOTU14314
New.CleanUp.ReferenceOTU17034
New.CleanUp.ReferenceOTU246403
New.CleanUp.ReferenceOTU157220
New.CleanUp.ReferenceOTU6847
New.CleanUp.ReferenceOTU4957

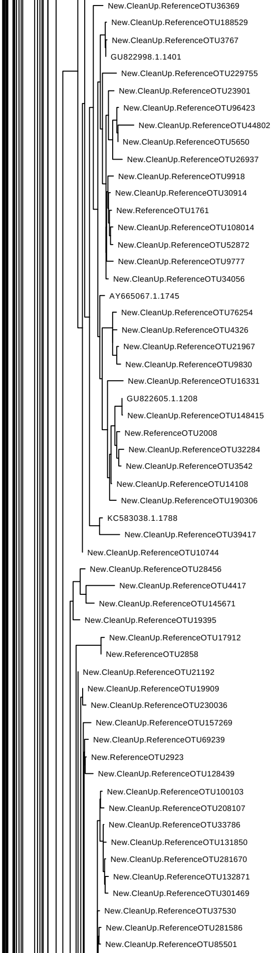
New.CleanUp.ReferenceOTU36369
New.CleanUp.ReferenceOTU188529
New.CleanUp.ReferenceOTU3767
GU822998.1.1401
New.CleanUp.ReferenceOTU229755
New.CleanUp.ReferenceOTU23901
New.CleanUp.ReferenceOTU96423
New.CleanUp.ReferenceOTU44802
New.CleanUp.ReferenceOTU5650
New.CleanUp.ReferenceOTU26937
New.CleanUp.ReferenceOTU9918
New.CleanUp.ReferenceOTU30914
New.ReferenceOTU1761
New.CleanUp.ReferenceOTU108014
New.CleanUp.ReferenceOTU52872
New.CleanUp.ReferenceOTU9777
New.CleanUp.ReferenceOTU34056
AY665067.1.1745
New.CleanUp.ReferenceOTU76254
New.CleanUp.ReferenceOTU4326
New.CleanUp.ReferenceOTU21967
New.CleanUp.ReferenceOTU9830
New.CleanUp.ReferenceOTU16331
GU822605.1.1208
New.CleanUp.ReferenceOTU148415
New.ReferenceOTU2008
New.CleanUp.ReferenceOTU32284
New.CleanUp.ReferenceOTU3542
New.CleanUp.ReferenceOTU14108
New.CleanUp.ReferenceOTU190306
KC583038.1.1788
New.CleanUp.ReferenceOTU39417
New.CleanUp.ReferenceOTU10744
New.CleanUp.ReferenceOTU28456
New.CleanUp.ReferenceOTU4417
New.CleanUp.ReferenceOTU145671
New.CleanUp.ReferenceOTU19395
New.CleanUp.ReferenceOTU17912
New.ReferenceOTU2858
New.CleanUp.ReferenceOTU21192
New.CleanUp.ReferenceOTU19909
New.CleanUp.ReferenceOTU230036
New.CleanUp.ReferenceOTU157269
New.CleanUp.ReferenceOTU69239
New.ReferenceOTU2923
New.CleanUp.ReferenceOTU128439
New.CleanUp.ReferenceOTU100103
New.CleanUp.ReferenceOTU208107
New.CleanUp.ReferenceOTU33786
New.CleanUp.ReferenceOTU131850
New.CleanUp.ReferenceOTU281670
New.CleanUp.ReferenceOTU132871
New.CleanUp.ReferenceOTU301469
New.CleanUp.ReferenceOTU37530
New.CleanUp.ReferenceOTU281586
New.CleanUp.ReferenceOTU85501

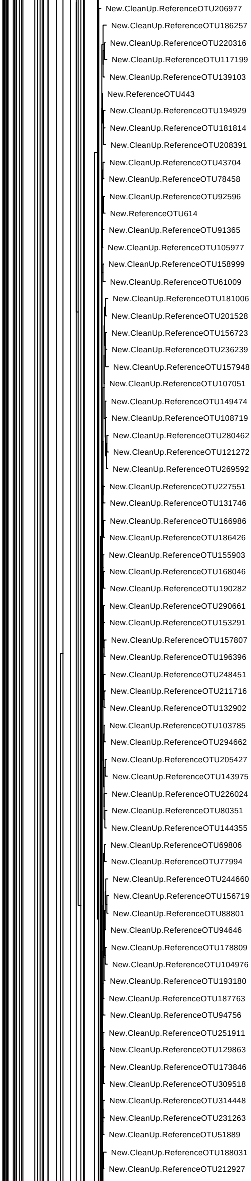
New.CleanUp.ReferenceOTU206977
New.CleanUp.ReferenceOTU186257
New.CleanUp.ReferenceOTU220316
New.CleanUp.ReferenceOTU117199
New.CleanUp.ReferenceOTU139103
New.ReferenceOTU443
New.CleanUp.ReferenceOTU194929
New.CleanUp.ReferenceOTU181814
New.CleanUp.ReferenceOTU208391
New.CleanUp.ReferenceOTU43704
New.CleanUp.ReferenceOTU78458
New.CleanUp.ReferenceOTU92596
New.ReferenceOTU614
New.CleanUp.ReferenceOTU91365
New.CleanUp.ReferenceOTU105977
New.CleanUp.ReferenceOTU158999
New.CleanUp.ReferenceOTU61009
New.CleanUp.ReferenceOTU181006
New.CleanUp.ReferenceOTU201528
New.CleanUp.ReferenceOTU156723
New.CleanUp.ReferenceOTU236239
New.CleanUp.ReferenceOTU157948
New.CleanUp.ReferenceOTU107051
New.CleanUp.ReferenceOTU149474
New.CleanUp.ReferenceOTU108719
New.CleanUp.ReferenceOTU280462
New.CleanUp.ReferenceOTU121272
New.CleanUp.ReferenceOTU269592
New.CleanUp.ReferenceOTU227551
New.CleanUp.ReferenceOTU131746
New.CleanUp.ReferenceOTU166986
New.CleanUp.ReferenceOTU186426
New.CleanUp.ReferenceOTU155903
New.CleanUp.ReferenceOTU168046
New.CleanUp.ReferenceOTU190282
New.CleanUp.ReferenceOTU290661
New.CleanUp.ReferenceOTU153291
New.CleanUp.ReferenceOTU157807
New.CleanUp.ReferenceOTU196396
New.CleanUp.ReferenceOTU248451
New.CleanUp.ReferenceOTU211716
New.CleanUp.ReferenceOTU132902
New.CleanUp.ReferenceOTU103785
New.CleanUp.ReferenceOTU294662
New.CleanUp.ReferenceOTU205427
New.CleanUp.ReferenceOTU143975
New.CleanUp.ReferenceOTU226024
New.CleanUp.ReferenceOTU80351
New.CleanUp.ReferenceOTU144355
New.CleanUp.ReferenceOTU69806
New.CleanUp.ReferenceOTU77994
New.CleanUp.ReferenceOTU244660
New.CleanUp.ReferenceOTU156719
New.CleanUp.ReferenceOTU88801
New.CleanUp.ReferenceOTU94646
New.CleanUp.ReferenceOTU178809
New.CleanUp.ReferenceOTU104976
New.CleanUp.ReferenceOTU193180
New.CleanUp.ReferenceOTU187763
New.CleanUp.ReferenceOTU94756
New.CleanUp.ReferenceOTU251911
New.CleanUp.ReferenceOTU129863
New.CleanUp.ReferenceOTU173846
New.CleanUp.ReferenceOTU309518
New.CleanUp.ReferenceOTU314448
New.CleanUp.ReferenceOTU231263
New.CleanUp.ReferenceOTU51889
New.CleanUp.ReferenceOTU188031
New.CleanUp.ReferenceOTU212927

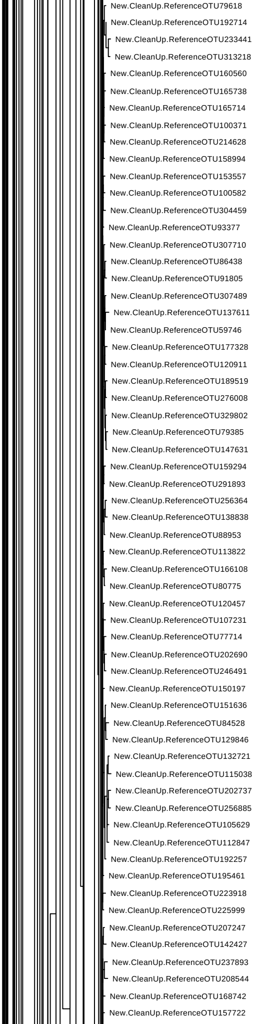
New.CleanUp.ReferenceOTU79618
New.CleanUp.ReferenceOTU192714
New.CleanUp.ReferenceOTU233441
New.CleanUp.ReferenceOTU313218
New.CleanUp.ReferenceOTU160560
New.CleanUp.ReferenceOTU165738
New.CleanUp.ReferenceOTU165714
New.CleanUp.ReferenceOTU100371
New.CleanUp.ReferenceOTU214628
New.CleanUp.ReferenceOTU158994
New.CleanUp.ReferenceOTU153557
New.CleanUp.ReferenceOTU100582
New.CleanUp.ReferenceOTU304459
New.CleanUp.ReferenceOTU93377
New.CleanUp.ReferenceOTU307710
New.CleanUp.ReferenceOTU86438
New.CleanUp.ReferenceOTU91805
New.CleanUp.ReferenceOTU307489
New.CleanUp.ReferenceOTU137611
New.CleanUp.ReferenceOTU59746
New.CleanUp.ReferenceOTU177328
New.CleanUp.ReferenceOTU120911
New.CleanUp.ReferenceOTU189519
New.CleanUp.ReferenceOTU276008
New.CleanUp.ReferenceOTU329802
New.CleanUp.ReferenceOTU79385
New.CleanUp.ReferenceOTU147631
New.CleanUp.ReferenceOTU159294
New.CleanUp.ReferenceOTU291893
New.CleanUp.ReferenceOTU256364
New.CleanUp.ReferenceOTU138838
New.CleanUp.ReferenceOTU88953
New.CleanUp.ReferenceOTU113822
New.CleanUp.ReferenceOTU166108
New.CleanUp.ReferenceOTU80775
New.CleanUp.ReferenceOTU120457
New.CleanUp.ReferenceOTU107231
New.CleanUp.ReferenceOTU77714
New.CleanUp.ReferenceOTU202690
New.CleanUp.ReferenceOTU246491
New.CleanUp.ReferenceOTU150197
New.CleanUp.ReferenceOTU151636
New.CleanUp.ReferenceOTU84528
New.CleanUp.ReferenceOTU129846
New.CleanUp.ReferenceOTU132721
New.CleanUp.ReferenceOTU115038
New.CleanUp.ReferenceOTU202737
New.CleanUp.ReferenceOTU256885
New.CleanUp.ReferenceOTU105629
New.CleanUp.ReferenceOTU112847
New.CleanUp.ReferenceOTU192257
New.CleanUp.ReferenceOTU195461
New.CleanUp.ReferenceOTU223918
New.CleanUp.ReferenceOTU225999
New.CleanUp.ReferenceOTU207247
New.CleanUp.ReferenceOTU142427
New.CleanUp.ReferenceOTU237893
New.CleanUp.ReferenceOTU208544
New.CleanUp.ReferenceOTU168742
New.CleanUp.ReferenceOTU157722

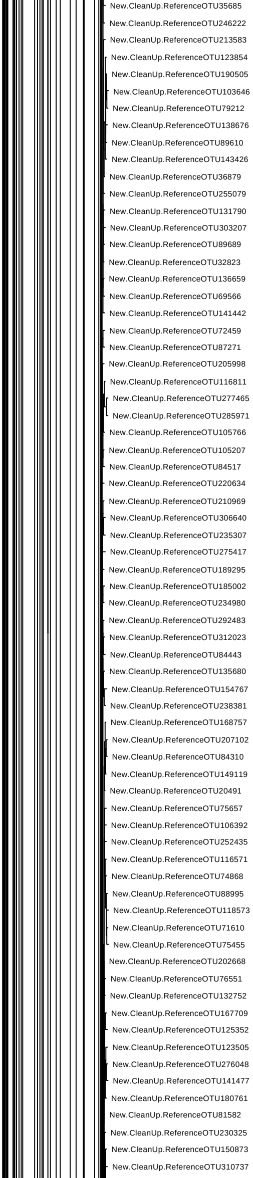
New.CleanUp.ReferenceOTU35685
New.CleanUp.ReferenceOTU246222
New.CleanUp.ReferenceOTU213583
New.CleanUp.ReferenceOTU123854
New.CleanUp.ReferenceOTU190505
New.CleanUp.ReferenceOTU103646
New.CleanUp.ReferenceOTU79212
New.CleanUp.ReferenceOTU138676
New.CleanUp.ReferenceOTU89610
New.CleanUp.ReferenceOTU143426
New.CleanUp.ReferenceOTU36879
New.CleanUp.ReferenceOTU255079
New.CleanUp.ReferenceOTU131790
New.CleanUp.ReferenceOTU303207
New.CleanUp.ReferenceOTU89689
New.CleanUp.ReferenceOTU32823
New.CleanUp.ReferenceOTU136659
New.CleanUp.ReferenceOTU69566
New.CleanUp.ReferenceOTU141442
New.CleanUp.ReferenceOTU72459
New.CleanUp.ReferenceOTU87271
New.CleanUp.ReferenceOTU205998
New.CleanUp.ReferenceOTU116811
New.CleanUp.ReferenceOTU277465
New.CleanUp.ReferenceOTU285971
New.CleanUp.ReferenceOTU105766
New.CleanUp.ReferenceOTU105207
New.CleanUp.ReferenceOTU84517
New.CleanUp.ReferenceOTU220634
New.CleanUp.ReferenceOTU210969
New.CleanUp.ReferenceOTU306640
New.CleanUp.ReferenceOTU235307
New.CleanUp.ReferenceOTU275417
New.CleanUp.ReferenceOTU189295
New.CleanUp.ReferenceOTU185002
New.CleanUp.ReferenceOTU234980
New.CleanUp.ReferenceOTU292483
New.CleanUp.ReferenceOTU312023
New.CleanUp.ReferenceOTU84443
New.CleanUp.ReferenceOTU135680
New.CleanUp.ReferenceOTU154767
New.CleanUp.ReferenceOTU238381
New.CleanUp.ReferenceOTU168757
New.CleanUp.ReferenceOTU207102
New.CleanUp.ReferenceOTU84310
New.CleanUp.ReferenceOTU149119
New.CleanUp.ReferenceOTU20491
New.CleanUp.ReferenceOTU75657
New.CleanUp.ReferenceOTU106392
New.CleanUp.ReferenceOTU252435
New.CleanUp.ReferenceOTU116571
New.CleanUp.ReferenceOTU74868
New.CleanUp.ReferenceOTU88995
New.CleanUp.ReferenceOTU118573
New.CleanUp.ReferenceOTU71610
New.CleanUp.ReferenceOTU75455
New.CleanUp.ReferenceOTU202668
New.CleanUp.ReferenceOTU76551
New.CleanUp.ReferenceOTU132752
New.CleanUp.ReferenceOTU167709
New.CleanUp.ReferenceOTU125352
New.CleanUp.ReferenceOTU123505
New.CleanUp.ReferenceOTU276048
New.CleanUp.ReferenceOTU141477
New.CleanUp.ReferenceOTU180761
New.CleanUp.ReferenceOTU81582
New.CleanUp.ReferenceOTU230325
New.CleanUp.ReferenceOTU150873
New.CleanUp.ReferenceOTU310737

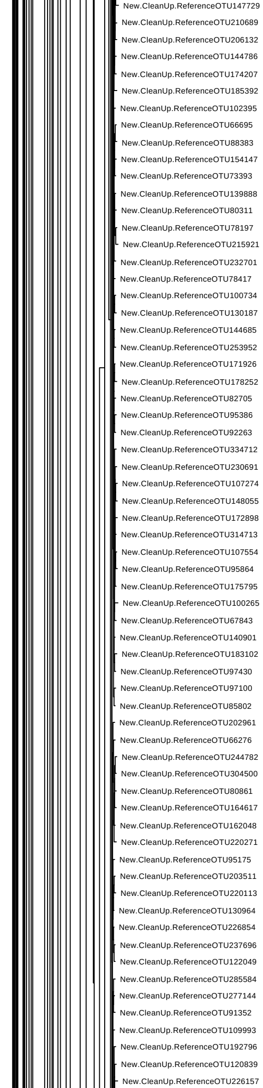
New.CleanUp.ReferenceOTU147729
New.CleanUp.ReferenceOTU210689
New.CleanUp.ReferenceOTU206132
New.CleanUp.ReferenceOTU144786
New.CleanUp.ReferenceOTU174207
New.CleanUp.ReferenceOTU185392
New.CleanUp.ReferenceOTU102395
New.CleanUp.ReferenceOTU66695
New.CleanUp.ReferenceOTU88383
New.CleanUp.ReferenceOTU154147
New.CleanUp.ReferenceOTU73393
New.CleanUp.ReferenceOTU139888
New.CleanUp.ReferenceOTU80311
New.CleanUp.ReferenceOTU78197
New.CleanUp.ReferenceOTU215921
New.CleanUp.ReferenceOTU232701
New.CleanUp.ReferenceOTU78417
New.CleanUp.ReferenceOTU100734
New.CleanUp.ReferenceOTU130187
New.CleanUp.ReferenceOTU144685
New.CleanUp.ReferenceOTU253952
New.CleanUp.ReferenceOTU171926
New.CleanUp.ReferenceOTU178252
New.CleanUp.ReferenceOTU82705
New.CleanUp.ReferenceOTU95386
New.CleanUp.ReferenceOTU92263
New.CleanUp.ReferenceOTU334712
New.CleanUp.ReferenceOTU230691
New.CleanUp.ReferenceOTU107274
New.CleanUp.ReferenceOTU148055
New.CleanUp.ReferenceOTU172898
New.CleanUp.ReferenceOTU314713
New.CleanUp.ReferenceOTU107554
New.CleanUp.ReferenceOTU95864
New.CleanUp.ReferenceOTU175795
New.CleanUp.ReferenceOTU100265
New.CleanUp.ReferenceOTU67843
New.CleanUp.ReferenceOTU140901
New.CleanUp.ReferenceOTU183102
New.CleanUp.ReferenceOTU97430
New.CleanUp.ReferenceOTU97100
New.CleanUp.ReferenceOTU85802
New.CleanUp.ReferenceOTU202961
New.CleanUp.ReferenceOTU66276
New.CleanUp.ReferenceOTU244782
New.CleanUp.ReferenceOTU304500
New.CleanUp.ReferenceOTU80861
New.CleanUp.ReferenceOTU164617
New.CleanUp.ReferenceOTU162048
New.CleanUp.ReferenceOTU220271
New.CleanUp.ReferenceOTU95175
New.CleanUp.ReferenceOTU203511
New.CleanUp.ReferenceOTU220113
New.CleanUp.ReferenceOTU130964
New.CleanUp.ReferenceOTU226854
New.CleanUp.ReferenceOTU237696
New.CleanUp.ReferenceOTU122049
New.CleanUp.ReferenceOTU285584
New.CleanUp.ReferenceOTU277144
New.CleanUp.ReferenceOTU91352
New.CleanUp.ReferenceOTU109993
New.CleanUp.ReferenceOTU192796
New.CleanUp.ReferenceOTU120839
New.CleanUp.ReferenceOTU226157

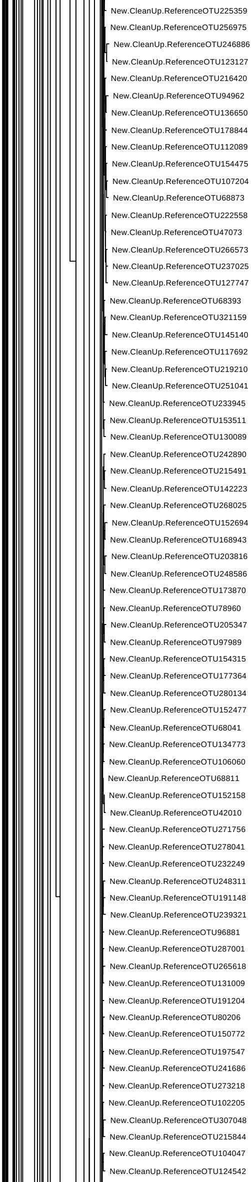
New.CleanUp.ReferenceOTU225359
New.CleanUp.ReferenceOTU256975
New.CleanUp.ReferenceOTU246886
New.CleanUp.ReferenceOTU123127
New.CleanUp.ReferenceOTU216420
New.CleanUp.ReferenceOTU94962
New.CleanUp.ReferenceOTU136650
New.CleanUp.ReferenceOTU178844
New.CleanUp.ReferenceOTU112089
New.CleanUp.ReferenceOTU154475
New.CleanUp.ReferenceOTU107204
New.CleanUp.ReferenceOTU68873
New.CleanUp.ReferenceOTU222558
New.CleanUp.ReferenceOTU47073
New.CleanUp.ReferenceOTU266573
New.CleanUp.ReferenceOTU237025
New.CleanUp.ReferenceOTU127747
New.CleanUp.ReferenceOTU68393
New.CleanUp.ReferenceOTU321159
New.CleanUp.ReferenceOTU145140
New.CleanUp.ReferenceOTU117692
New.CleanUp.ReferenceOTU219210
New.CleanUp.ReferenceOTU251041
New.CleanUp.ReferenceOTU233945
New.CleanUp.ReferenceOTU153511
New.CleanUp.ReferenceOTU130089
New.CleanUp.ReferenceOTU242890
New.CleanUp.ReferenceOTU215491
New.CleanUp.ReferenceOTU142223
New.CleanUp.ReferenceOTU268025
New.CleanUp.ReferenceOTU152694
New.CleanUp.ReferenceOTU168943
New.CleanUp.ReferenceOTU203816
New.CleanUp.ReferenceOTU248586
New.CleanUp.ReferenceOTU173870
New.CleanUp.ReferenceOTU78960
New.CleanUp.ReferenceOTU205347
New.CleanUp.ReferenceOTU97989
New.CleanUp.ReferenceOTU154315
New.CleanUp.ReferenceOTU177364
New.CleanUp.ReferenceOTU280134
New.CleanUp.ReferenceOTU152477
New.CleanUp.ReferenceOTU68041
New.CleanUp.ReferenceOTU134773
New.CleanUp.ReferenceOTU106060
New.CleanUp.ReferenceOTU68811
New.CleanUp.ReferenceOTU152158
New.CleanUp.ReferenceOTU42010
New.CleanUp.ReferenceOTU271756
New.CleanUp.ReferenceOTU278041
New.CleanUp.ReferenceOTU232249
New.CleanUp.ReferenceOTU248311
New.CleanUp.ReferenceOTU191148
New.CleanUp.ReferenceOTU239321
New.CleanUp.ReferenceOTU96881
New.CleanUp.ReferenceOTU287001
New.CleanUp.ReferenceOTU265618
New.CleanUp.ReferenceOTU131009
New.CleanUp.ReferenceOTU191204
New.CleanUp.ReferenceOTU80206
New.CleanUp.ReferenceOTU150772
New.CleanUp.ReferenceOTU197547
New.CleanUp.ReferenceOTU241686
New.CleanUp.ReferenceOTU273218
New.CleanUp.ReferenceOTU102205
New.CleanUp.ReferenceOTU307048
New.CleanUp.ReferenceOTU215844
New.CleanUp.ReferenceOTU104047
New.CleanUp.ReferenceOTU124542

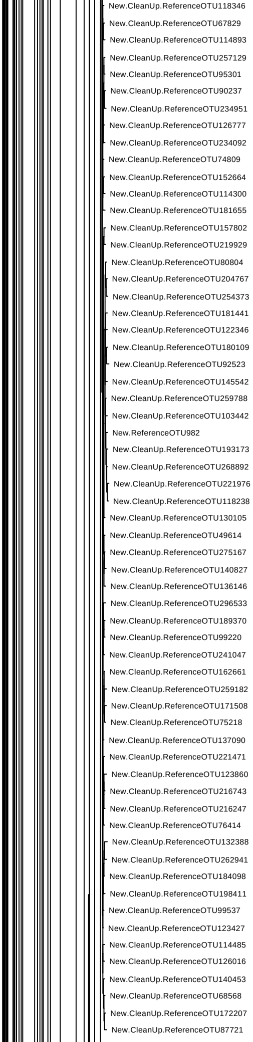
New.CleanUp.ReferenceOTU118346
New.CleanUp.ReferenceOTU67829
New.CleanUp.ReferenceOTU114893
New.CleanUp.ReferenceOTU257129
New.CleanUp.ReferenceOTU95301
New.CleanUp.ReferenceOTU90237
New.CleanUp.ReferenceOTU234951
New.CleanUp.ReferenceOTU126777
New.CleanUp.ReferenceOTU234092
New.CleanUp.ReferenceOTU74809
New.CleanUp.ReferenceOTU152664
New.CleanUp.ReferenceOTU114300
New.CleanUp.ReferenceOTU181655
New.CleanUp.ReferenceOTU157802
New.CleanUp.ReferenceOTU219929
New.CleanUp.ReferenceOTU80804
New.CleanUp.ReferenceOTU204767
New.CleanUp.ReferenceOTU254373
New.CleanUp.ReferenceOTU181441
New.CleanUp.ReferenceOTU122346
New.CleanUp.ReferenceOTU180109
New.CleanUp.ReferenceOTU92523
New.CleanUp.ReferenceOTU145542
New.CleanUp.ReferenceOTU259788
New.CleanUp.ReferenceOTU103442
New.ReferenceOTU982
New.CleanUp.ReferenceOTU193173
New.CleanUp.ReferenceOTU268892
New.CleanUp.ReferenceOTU221976
New.CleanUp.ReferenceOTU118238
New.CleanUp.ReferenceOTU130105
New.CleanUp.ReferenceOTU49614
New.CleanUp.ReferenceOTU275167
New.CleanUp.ReferenceOTU140827
New.CleanUp.ReferenceOTU136146
New.CleanUp.ReferenceOTU296533
New.CleanUp.ReferenceOTU189370
New.CleanUp.ReferenceOTU99220
New.CleanUp.ReferenceOTU241047
New.CleanUp.ReferenceOTU162661
New.CleanUp.ReferenceOTU259182
New.CleanUp.ReferenceOTU171508
New.CleanUp.ReferenceOTU75218
New.CleanUp.ReferenceOTU137090
New.CleanUp.ReferenceOTU221471
New.CleanUp.ReferenceOTU123860
New.CleanUp.ReferenceOTU216743
New.CleanUp.ReferenceOTU216247
New.CleanUp.ReferenceOTU76414
New.CleanUp.ReferenceOTU132388
New.CleanUp.ReferenceOTU262941
New.CleanUp.ReferenceOTU184098
New.CleanUp.ReferenceOTU198411
New.CleanUp.ReferenceOTU99537
New.CleanUp.ReferenceOTU123427
New.CleanUp.ReferenceOTU114485
New.CleanUp.ReferenceOTU126016
New.CleanUp.ReferenceOTU140453
New.CleanUp.ReferenceOTU68568
New.CleanUp.ReferenceOTU172207
New.CleanUp.ReferenceOTU87721

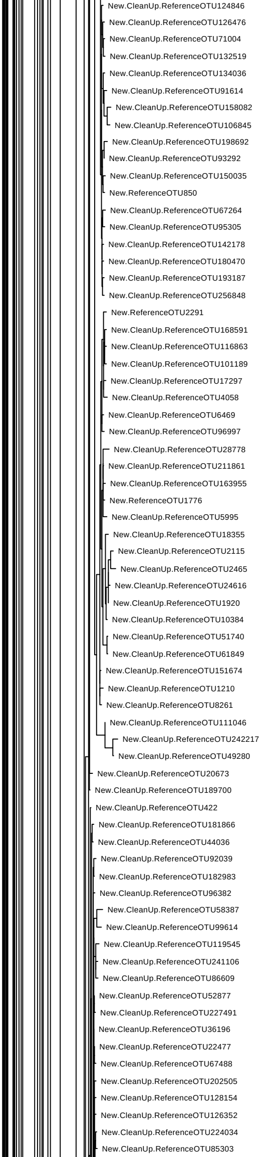
New.CleanUp.ReferenceOTU124846
New.CleanUp.ReferenceOTU126476
New.CleanUp.ReferenceOTU71004
New.CleanUp.ReferenceOTU132519
New.CleanUp.ReferenceOTU134036
New.CleanUp.ReferenceOTU91614
New.CleanUp.ReferenceOTU158082
New.CleanUp.ReferenceOTU106845
New.CleanUp.ReferenceOTU198692
New.CleanUp.ReferenceOTU93292
New.CleanUp.ReferenceOTU150035
New.ReferenceOTU850
New.CleanUp.ReferenceOTU67264
New.CleanUp.ReferenceOTU95305
New.CleanUp.ReferenceOTU142178
New.CleanUp.ReferenceOTU180470
New.CleanUp.ReferenceOTU193187
New.CleanUp.ReferenceOTU256848
New.ReferenceOTU2291
New.CleanUp.ReferenceOTU168591
New.CleanUp.ReferenceOTU116863
New.CleanUp.ReferenceOTU101189
New.CleanUp.ReferenceOTU17297
New.CleanUp.ReferenceOTU4058
New.CleanUp.ReferenceOTU6469
New.CleanUp.ReferenceOTU96997
New.CleanUp.ReferenceOTU28778
New.CleanUp.ReferenceOTU211861
New.CleanUp.ReferenceOTU163955
New.ReferenceOTU1776
New.CleanUp.ReferenceOTU5995
New.CleanUp.ReferenceOTU18355
New.CleanUp.ReferenceOTU2115
New.CleanUp.ReferenceOTU2465
New.CleanUp.ReferenceOTU24616
New.CleanUp.ReferenceOTU1920
New.CleanUp.ReferenceOTU10384
New.CleanUp.ReferenceOTU51740
New.CleanUp.ReferenceOTU61849
New.CleanUp.ReferenceOTU151674
New.CleanUp.ReferenceOTU1210
New.CleanUp.ReferenceOTU8261
New.CleanUp.ReferenceOTU111046
New.CleanUp.ReferenceOTU242217
New.CleanUp.ReferenceOTU49280
New.CleanUp.ReferenceOTU20673
New.CleanUp.ReferenceOTU189700
New.CleanUp.ReferenceOTU422
New.CleanUp.ReferenceOTU181866
New.CleanUp.ReferenceOTU44036
New.CleanUp.ReferenceOTU92039
New.CleanUp.ReferenceOTU182983
New.CleanUp.ReferenceOTU96382
New.CleanUp.ReferenceOTU58387
New.CleanUp.ReferenceOTU99614
New.CleanUp.ReferenceOTU119545
New.CleanUp.ReferenceOTU241106
New.CleanUp.ReferenceOTU86609
New.CleanUp.ReferenceOTU52877
New.CleanUp.ReferenceOTU227491
New.CleanUp.ReferenceOTU36196
New.CleanUp.ReferenceOTU22477
New.CleanUp.ReferenceOTU67488
New.CleanUp.ReferenceOTU202505
New.CleanUp.ReferenceOTU128154
New.CleanUp.ReferenceOTU126352
New.CleanUp.ReferenceOTU224034
New.CleanUp.ReferenceOTU85303

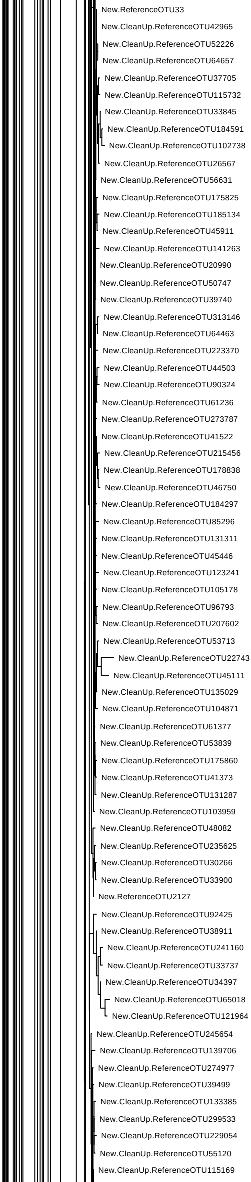
New.ReferenceOTU33
New.CleanUp.ReferenceOTU42965
New.CleanUp.ReferenceOTU52226
New.CleanUp.ReferenceOTU64657
New.CleanUp.ReferenceOTU37705
New.CleanUp.ReferenceOTU115732
New.CleanUp.ReferenceOTU33845
New.CleanUp.ReferenceOTU184591
New.CleanUp.ReferenceOTU102738
New.CleanUp.ReferenceOTU26567
New.CleanUp.ReferenceOTU56631
New.CleanUp.ReferenceOTU175825
New.CleanUp.ReferenceOTU185134
New.CleanUp.ReferenceOTU45911
New.CleanUp.ReferenceOTU141263
New.CleanUp.ReferenceOTU20990
New.CleanUp.ReferenceOTU50747
New.CleanUp.ReferenceOTU39740
New.CleanUp.ReferenceOTU313146
New.CleanUp.ReferenceOTU64463
New.CleanUp.ReferenceOTU223370
New.CleanUp.ReferenceOTU44503
New.CleanUp.ReferenceOTU90324
New.CleanUp.ReferenceOTU61236
New.CleanUp.ReferenceOTU273787
New.CleanUp.ReferenceOTU41522
New.CleanUp.ReferenceOTU215456
New.CleanUp.ReferenceOTU178838
New.CleanUp.ReferenceOTU46750
New.CleanUp.ReferenceOTU184297
New.CleanUp.ReferenceOTU85296
New.CleanUp.ReferenceOTU131311
New.CleanUp.ReferenceOTU45446
New.CleanUp.ReferenceOTU123241
New.CleanUp.ReferenceOTU105178
New.CleanUp.ReferenceOTU96793
New.CleanUp.ReferenceOTU207602
New.CleanUp.ReferenceOTU53713
New.CleanUp.ReferenceOTU22743
New.CleanUp.ReferenceOTU45111
New.CleanUp.ReferenceOTU135029
New.CleanUp.ReferenceOTU104871
New.CleanUp.ReferenceOTU61377
New.CleanUp.ReferenceOTU53839
New.CleanUp.ReferenceOTU175860
New.CleanUp.ReferenceOTU41373
New.CleanUp.ReferenceOTU131287
New.CleanUp.ReferenceOTU103959
New.CleanUp.ReferenceOTU48082
New.CleanUp.ReferenceOTU235625
New.CleanUp.ReferenceOTU30266
New.CleanUp.ReferenceOTU33900
New.ReferenceOTU2127
New.CleanUp.ReferenceOTU92425
New.CleanUp.ReferenceOTU38911
New.CleanUp.ReferenceOTU241160
New.CleanUp.ReferenceOTU33737
New.CleanUp.ReferenceOTU34397
New.CleanUp.ReferenceOTU65018
New.CleanUp.ReferenceOTU121964
New.CleanUp.ReferenceOTU245654
New.CleanUp.ReferenceOTU139706
New.CleanUp.ReferenceOTU274977
New.CleanUp.ReferenceOTU39499
New.CleanUp.ReferenceOTU133385
New.CleanUp.ReferenceOTU299533
New.CleanUp.ReferenceOTU229054
New.CleanUp.ReferenceOTU55120
New.CleanUp.ReferenceOTU115169

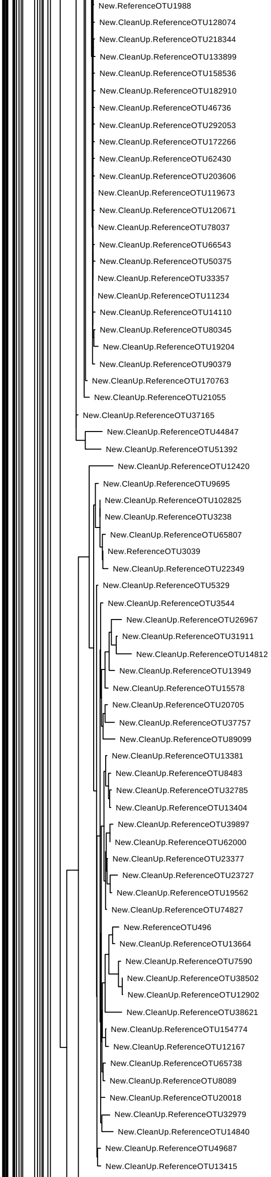
New.ReferenceOTU1988
New.CleanUp.ReferenceOTU128074
New.CleanUp.ReferenceOTU218344
New.CleanUp.ReferenceOTU133899
New.CleanUp.ReferenceOTU158536
New.CleanUp.ReferenceOTU182910
New.CleanUp.ReferenceOTU46736
New.CleanUp.ReferenceOTU292053
New.CleanUp.ReferenceOTU172266
New.CleanUp.ReferenceOTU62430
New.CleanUp.ReferenceOTU203606
New.CleanUp.ReferenceOTU119673
New.CleanUp.ReferenceOTU120671
New.CleanUp.ReferenceOTU78037
New.CleanUp.ReferenceOTU66543
New.CleanUp.ReferenceOTU50375
New.CleanUp.ReferenceOTU33357
New.CleanUp.ReferenceOTU11234
New.CleanUp.ReferenceOTU14110
New.CleanUp.ReferenceOTU80345
New.CleanUp.ReferenceOTU19204
New.CleanUp.ReferenceOTU90379
New.CleanUp.ReferenceOTU170763
New.CleanUp.ReferenceOTU21055
New.CleanUp.ReferenceOTU37165
New.CleanUp.ReferenceOTU44847
New.CleanUp.ReferenceOTU51392
New.CleanUp.ReferenceOTU12420
New.CleanUp.ReferenceOTU9695
New.CleanUp.ReferenceOTU102825
New.CleanUp.ReferenceOTU3238
New.CleanUp.ReferenceOTU65807
New.ReferenceOTU3039
New.CleanUp.ReferenceOTU22349
New.CleanUp.ReferenceOTU5329
New.CleanUp.ReferenceOTU3544
New.CleanUp.ReferenceOTU26967
New.CleanUp.ReferenceOTU31911
New.CleanUp.ReferenceOTU14812
New.CleanUp.ReferenceOTU13949
New.CleanUp.ReferenceOTU15578
New.CleanUp.ReferenceOTU20705
New.CleanUp.ReferenceOTU37757
New.CleanUp.ReferenceOTU89099
New.CleanUp.ReferenceOTU13381
New.CleanUp.ReferenceOTU8483
New.CleanUp.ReferenceOTU32785
New.CleanUp.ReferenceOTU13404
New.CleanUp.ReferenceOTU39897
New.CleanUp.ReferenceOTU62000
New.CleanUp.ReferenceOTU23377
New.CleanUp.ReferenceOTU23727
New.CleanUp.ReferenceOTU19562
New.CleanUp.ReferenceOTU74827
New.ReferenceOTU496
New.CleanUp.ReferenceOTU13664
New.CleanUp.ReferenceOTU7590
New.CleanUp.ReferenceOTU38502
New.CleanUp.ReferenceOTU12902
New.CleanUp.ReferenceOTU38621
New.CleanUp.ReferenceOTU154774
New.CleanUp.ReferenceOTU12167
New.CleanUp.ReferenceOTU65738
New.CleanUp.ReferenceOTU8089
New.CleanUp.ReferenceOTU20018
New.CleanUp.ReferenceOTU32979
New.CleanUp.ReferenceOTU14840
New.CleanUp.ReferenceOTU49687
New.CleanUp.ReferenceOTU13415

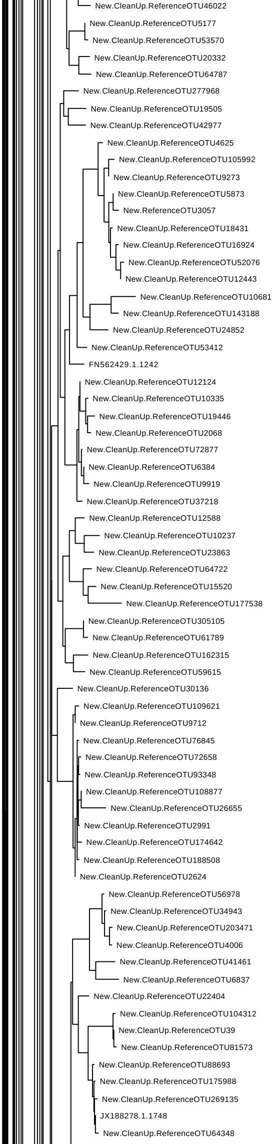
New.CleanUp.ReferenceOTU46022
New.CleanUp.ReferenceOTU5177
New.CleanUp.ReferenceOTU53570
New.CleanUp.ReferenceOTU20332
New.CleanUp.ReferenceOTU64787
New.CleanUp.ReferenceOTU277968
New.CleanUp.ReferenceOTU19505
New.CleanUp.ReferenceOTU42977
New.CleanUp.ReferenceOTU4625
New.CleanUp.ReferenceOTU105992
New.CleanUp.ReferenceOTU9273
New.CleanUp.ReferenceOTU5873
New.ReferenceOTU3057
New.CleanUp.ReferenceOTU18431
New.CleanUp.ReferenceOTU16924
New.CleanUp.ReferenceOTU52076
New.CleanUp.ReferenceOTU12443
New.CleanUp.ReferenceOTU10681
New.CleanUp.ReferenceOTU143188
New.CleanUp.ReferenceOTU24852
New.CleanUp.ReferenceOTU53412
FN562429.1.1242
New.CleanUp.ReferenceOTU12124
New.CleanUp.ReferenceOTU10335
New.CleanUp.ReferenceOTU19446
New.CleanUp.ReferenceOTU2068
New.CleanUp.ReferenceOTU72877
New.CleanUp.ReferenceOTU6384
New.CleanUp.ReferenceOTU9919
New.CleanUp.ReferenceOTU37218
New.CleanUp.ReferenceOTU12588
New.CleanUp.ReferenceOTU10237
New.CleanUp.ReferenceOTU23863
New.CleanUp.ReferenceOTU64722
New.CleanUp.ReferenceOTU15520
New.CleanUp.ReferenceOTU177538
New.CleanUp.ReferenceOTU305105
New.CleanUp.ReferenceOTU61789
New.CleanUp.ReferenceOTU162315
New.CleanUp.ReferenceOTU59615
New.CleanUp.ReferenceOTU30136
New.CleanUp.ReferenceOTU109621
New.CleanUp.ReferenceOTU9712
New.CleanUp.ReferenceOTU76845
New.CleanUp.ReferenceOTU72658
New.CleanUp.ReferenceOTU93348
New.CleanUp.ReferenceOTU108877
New.CleanUp.ReferenceOTU26655
New.CleanUp.ReferenceOTU2991
New.CleanUp.ReferenceOTU174642
New.CleanUp.ReferenceOTU188508
New.CleanUp.ReferenceOTU2624
New.CleanUp.ReferenceOTU56978
New.CleanUp.ReferenceOTU34943
New.CleanUp.ReferenceOTU203471
New.CleanUp.ReferenceOTU4006
New.CleanUp.ReferenceOTU41461
New.CleanUp.ReferenceOTU6837
New.CleanUp.ReferenceOTU22404
New.CleanUp.ReferenceOTU104312
New.CleanUp.ReferenceOTU39
New.CleanUp.ReferenceOTU81573
New.CleanUp.ReferenceOTU88693
New.CleanUp.ReferenceOTU175988
New.CleanUp.ReferenceOTU269135
JX188278.1.1748
New.CleanUp.ReferenceOTU64348

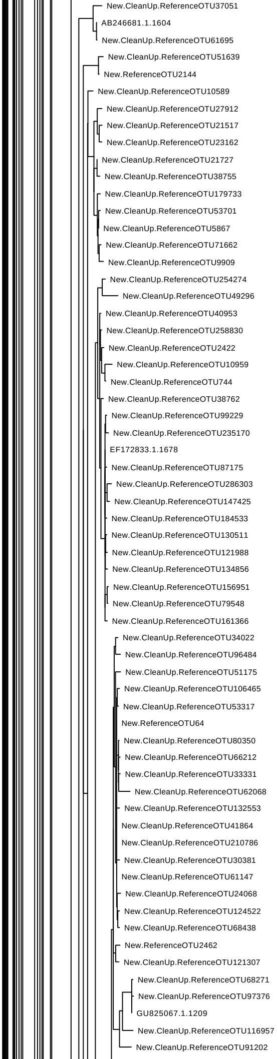
New.CleanUp.ReferenceOTU37051
AB246681.1.1604
New.CleanUp.ReferenceOTU61695
New.CleanUp.ReferenceOTU51639
New.ReferenceOTU2144
New.CleanUp.ReferenceOTU10589
New.CleanUp.ReferenceOTU27912
New.CleanUp.ReferenceOTU21517
New.CleanUp.ReferenceOTU23162
New.CleanUp.ReferenceOTU21727
New.CleanUp.ReferenceOTU38755
New.CleanUp.ReferenceOTU179733
New.CleanUp.ReferenceOTU53701
New.CleanUp.ReferenceOTU5867
New.CleanUp.ReferenceOTU71662
New.CleanUp.ReferenceOTU9909
New.CleanUp.ReferenceOTU254274
New.CleanUp.ReferenceOTU49296
New.CleanUp.ReferenceOTU40953
New.CleanUp.ReferenceOTU258830
New.CleanUp.ReferenceOTU2422
New.CleanUp.ReferenceOTU10959
New.CleanUp.ReferenceOTU744
New.CleanUp.ReferenceOTU38762
New.CleanUp.ReferenceOTU99229
New.CleanUp.ReferenceOTU235170
EF172833.1.1678
New.CleanUp.ReferenceOTU87175
New.CleanUp.ReferenceOTU286303
New.CleanUp.ReferenceOTU147425
New.CleanUp.ReferenceOTU184533
New.CleanUp.ReferenceOTU130511
New.CleanUp.ReferenceOTU121988
New.CleanUp.ReferenceOTU134856
New.CleanUp.ReferenceOTU156951
New.CleanUp.ReferenceOTU79548
New.CleanUp.ReferenceOTU161366
New.CleanUp.ReferenceOTU34022
New.CleanUp.ReferenceOTU96484
New.CleanUp.ReferenceOTU51175
New.CleanUp.ReferenceOTU106465
New.CleanUp.ReferenceOTU53317
New.ReferenceOTU64
New.CleanUp.ReferenceOTU80350
New.CleanUp.ReferenceOTU66212
New.CleanUp.ReferenceOTU33331
New.CleanUp.ReferenceOTU62068
New.CleanUp.ReferenceOTU132553
New.CleanUp.ReferenceOTU41864
New.CleanUp.ReferenceOTU210786
New.CleanUp.ReferenceOTU30381
New.CleanUp.ReferenceOTU61147
New.CleanUp.ReferenceOTU24068
New.CleanUp.ReferenceOTU124522
New.CleanUp.ReferenceOTU68438
New.ReferenceOTU2462
New.CleanUp.ReferenceOTU121307
New.CleanUp.ReferenceOTU68271
New.CleanUp.ReferenceOTU97376
GU825067.1.1209
New.CleanUp.ReferenceOTU116957
New.CleanUp.ReferenceOTU91202

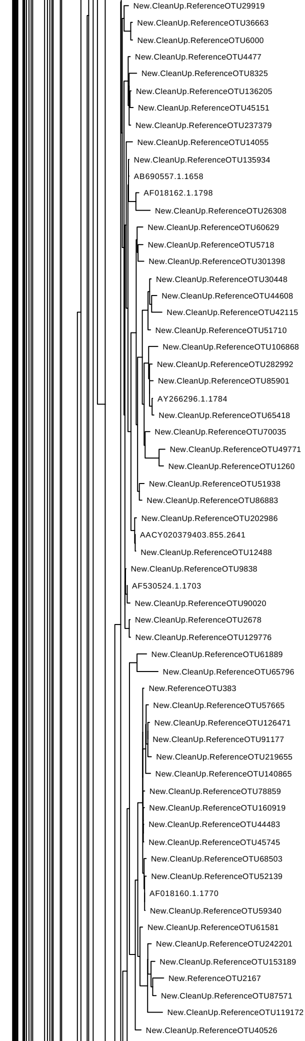
New.CleanUp.ReferenceOTU29919
New.CleanUp.ReferenceOTU36663
New.CleanUp.ReferenceOTU6000
New.CleanUp.ReferenceOTU4477
New.CleanUp.ReferenceOTU8325
New.CleanUp.ReferenceOTU136205
New.CleanUp.ReferenceOTU45151
New.CleanUp.ReferenceOTU237379
New.CleanUp.ReferenceOTU14055
New.CleanUp.ReferenceOTU135934
AB690557.1.1658
AF018162.1.1798
New.CleanUp.ReferenceOTU26308
New.CleanUp.ReferenceOTU60629
New.CleanUp.ReferenceOTU5718
New.CleanUp.ReferenceOTU301398
New.CleanUp.ReferenceOTU30448
New.CleanUp.ReferenceOTU44608
New.CleanUp.ReferenceOTU42115
New.CleanUp.ReferenceOTU51710
New.CleanUp.ReferenceOTU106868
New.CleanUp.ReferenceOTU282992
New.CleanUp.ReferenceOTU85901
AY266296.1.1784
New.CleanUp.ReferenceOTU65418
New.CleanUp.ReferenceOTU70035
New.CleanUp.ReferenceOTU49771
New.CleanUp.ReferenceOTU1260
New.CleanUp.ReferenceOTU51938
New.CleanUp.ReferenceOTU86883
New.CleanUp.ReferenceOTU202986
AACY020379403.855.2641
New.CleanUp.ReferenceOTU12488
New.CleanUp.ReferenceOTU9838
AF530524.1.1703
New.CleanUp.ReferenceOTU90020
New.CleanUp.ReferenceOTU2678
New.CleanUp.ReferenceOTU129776
New.CleanUp.ReferenceOTU61889
New.CleanUp.ReferenceOTU65796
New.ReferenceOTU383
New.CleanUp.ReferenceOTU57665
New.CleanUp.ReferenceOTU126471
New.CleanUp.ReferenceOTU91177
New.CleanUp.ReferenceOTU219655
New.CleanUp.ReferenceOTU140865
New.CleanUp.ReferenceOTU78859
New.CleanUp.ReferenceOTU160919
New.CleanUp.ReferenceOTU44483
New.CleanUp.ReferenceOTU45745
New.CleanUp.ReferenceOTU68503
New.CleanUp.ReferenceOTU52139
AF018160.1.1770
New.CleanUp.ReferenceOTU59340
New.CleanUp.ReferenceOTU61581
New.CleanUp.ReferenceOTU242201
New.CleanUp.ReferenceOTU153189
New.ReferenceOTU2167
New.CleanUp.ReferenceOTU87571
New.CleanUp.ReferenceOTU119172
New.CleanUp.ReferenceOTU40526

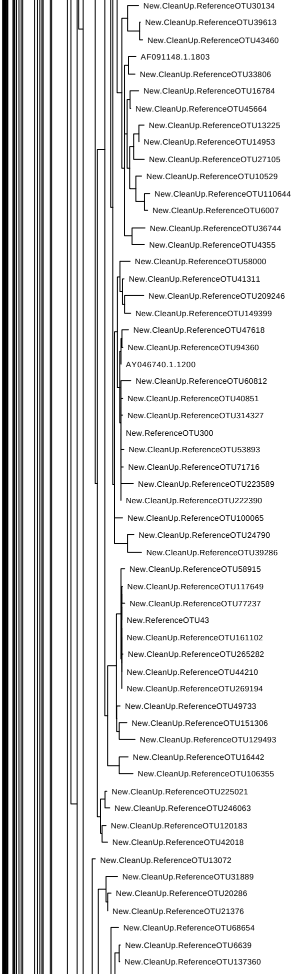
New.CleanUp.ReferenceOTU30134
New.CleanUp.ReferenceOTU39613
New.CleanUp.ReferenceOTU43460
AF091148.1.1803
New.CleanUp.ReferenceOTU33806
New.CleanUp.ReferenceOTU16784
New.CleanUp.ReferenceOTU45664
New.CleanUp.ReferenceOTU13225
New.CleanUp.ReferenceOTU14953
New.CleanUp.ReferenceOTU27105
New.CleanUp.ReferenceOTU10529
New.CleanUp.ReferenceOTU110644
New.CleanUp.ReferenceOTU6007
New.CleanUp.ReferenceOTU36744
New.CleanUp.ReferenceOTU4355
New.CleanUp.ReferenceOTU58000
New.CleanUp.ReferenceOTU41311
New.CleanUp.ReferenceOTU209246
New.CleanUp.ReferenceOTU149399
New.CleanUp.ReferenceOTU47618
New.CleanUp.ReferenceOTU94360
AY046740.1.1200
New.CleanUp.ReferenceOTU60812
New.CleanUp.ReferenceOTU40851
New.CleanUp.ReferenceOTU314327
New.ReferenceOTU300
New.CleanUp.ReferenceOTU53893
New.CleanUp.ReferenceOTU71716
New.CleanUp.ReferenceOTU223589
New.CleanUp.ReferenceOTU222390
New.CleanUp.ReferenceOTU100065
New.CleanUp.ReferenceOTU24790
New.CleanUp.ReferenceOTU39286
New.CleanUp.ReferenceOTU58915
New.CleanUp.ReferenceOTU117649
New.CleanUp.ReferenceOTU77237
New.ReferenceOTU43
New.CleanUp.ReferenceOTU161102
New.CleanUp.ReferenceOTU265282
New.CleanUp.ReferenceOTU44210
New.CleanUp.ReferenceOTU269194
New.CleanUp.ReferenceOTU49733
New.CleanUp.ReferenceOTU151306
New.CleanUp.ReferenceOTU129493
New.CleanUp.ReferenceOTU16442
New.CleanUp.ReferenceOTU106355
New.CleanUp.ReferenceOTU225021
New.CleanUp.ReferenceOTU246063
New.CleanUp.ReferenceOTU120183
New.CleanUp.ReferenceOTU42018
New.CleanUp.ReferenceOTU13072
New.CleanUp.ReferenceOTU31889
New.CleanUp.ReferenceOTU20286
New.CleanUp.ReferenceOTU21376
New.CleanUp.ReferenceOTU68654
New.CleanUp.ReferenceOTU6639
New.CleanUp.ReferenceOTU137360

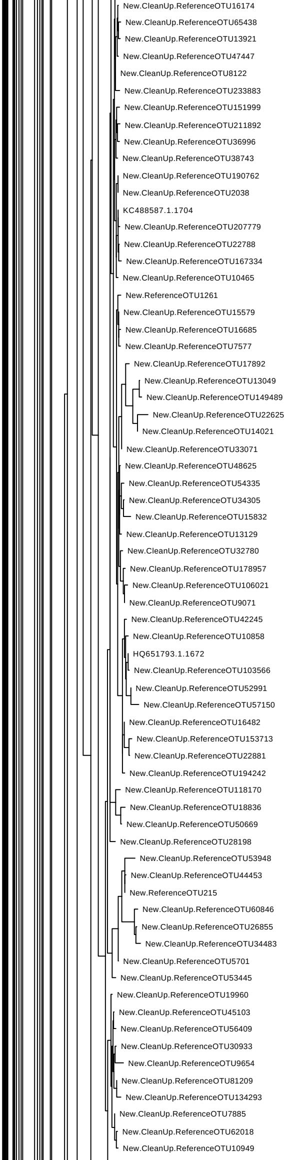
New.CleanUp.ReferenceOTU16174
New.CleanUp.ReferenceOTU65438
New.CleanUp.ReferenceOTU13921
New.CleanUp.ReferenceOTU47447
New.CleanUp.ReferenceOTU8122
New.CleanUp.ReferenceOTU233883
New.CleanUp.ReferenceOTU151999
New.CleanUp.ReferenceOTU211892
New.CleanUp.ReferenceOTU36996
New.CleanUp.ReferenceOTU38743
New.CleanUp.ReferenceOTU190762
New.CleanUp.ReferenceOTU2038
KC488587.1.1704
New.CleanUp.ReferenceOTU207779
New.CleanUp.ReferenceOTU22788
New.CleanUp.ReferenceOTU167334
New.CleanUp.ReferenceOTU10465
New.ReferenceOTU1261
New.CleanUp.ReferenceOTU15579
New.CleanUp.ReferenceOTU16685
New.CleanUp.ReferenceOTU7577
New.CleanUp.ReferenceOTU17892
New.CleanUp.ReferenceOTU13049
New.CleanUp.ReferenceOTU149489
New.CleanUp.ReferenceOTU22625
New.CleanUp.ReferenceOTU14021
New.CleanUp.ReferenceOTU33071
New.CleanUp.ReferenceOTU48625
New.CleanUp.ReferenceOTU54335
New.CleanUp.ReferenceOTU34305
New.CleanUp.ReferenceOTU15832
New.CleanUp.ReferenceOTU13129
New.CleanUp.ReferenceOTU32780
New.CleanUp.ReferenceOTU178957
New.CleanUp.ReferenceOTU106021
New.CleanUp.ReferenceOTU9071
New.CleanUp.ReferenceOTU42245
New.CleanUp.ReferenceOTU10858
HQ651793.1.1672
New.CleanUp.ReferenceOTU103566
New.CleanUp.ReferenceOTU52991
New.CleanUp.ReferenceOTU57150
New.CleanUp.ReferenceOTU16482
New.CleanUp.ReferenceOTU153713
New.CleanUp.ReferenceOTU22881
New.CleanUp.ReferenceOTU194242
New.CleanUp.ReferenceOTU118170
New.CleanUp.ReferenceOTU18836
New.CleanUp.ReferenceOTU50669
New.CleanUp.ReferenceOTU28198
New.CleanUp.ReferenceOTU53948
New.CleanUp.ReferenceOTU44453
New.ReferenceOTU215
New.CleanUp.ReferenceOTU60846
New.CleanUp.ReferenceOTU26855
New.CleanUp.ReferenceOTU34483
New.CleanUp.ReferenceOTU5701
New.CleanUp.ReferenceOTU53445
New.CleanUp.ReferenceOTU19960
New.CleanUp.ReferenceOTU45103
New.CleanUp.ReferenceOTU56409
New.CleanUp.ReferenceOTU30933
New.CleanUp.ReferenceOTU9654
New.CleanUp.ReferenceOTU81209
New.CleanUp.ReferenceOTU134293
New.CleanUp.ReferenceOTU7885
New.CleanUp.ReferenceOTU62018
New.CleanUp.ReferenceOTU10949

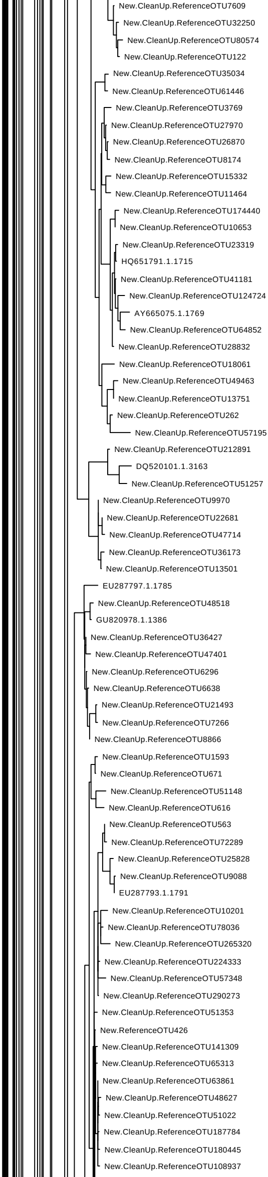
New.CleanUp.ReferenceOTU7609
New.CleanUp.ReferenceOTU32250
New.CleanUp.ReferenceOTU80574
New.CleanUp.ReferenceOTU122
New.CleanUp.ReferenceOTU35034
New.CleanUp.ReferenceOTU61446
New.CleanUp.ReferenceOTU3769
New.CleanUp.ReferenceOTU27970
New.CleanUp.ReferenceOTU26870
New.CleanUp.ReferenceOTU8174
New.CleanUp.ReferenceOTU15332
New.CleanUp.ReferenceOTU11464
New.CleanUp.ReferenceOTU174440
New.CleanUp.ReferenceOTU10653
New.CleanUp.ReferenceOTU23319
HQ651791.1.1715
New.CleanUp.ReferenceOTU41181
New.CleanUp.ReferenceOTU124724
AY665075.1.1769
New.CleanUp.ReferenceOTU64852
New.CleanUp.ReferenceOTU28832
New.CleanUp.ReferenceOTU18061
New.CleanUp.ReferenceOTU49463
New.CleanUp.ReferenceOTU13751
New.CleanUp.ReferenceOTU262
New.CleanUp.ReferenceOTU57195
New.CleanUp.ReferenceOTU212891
DQ520101.1.3163
New.CleanUp.ReferenceOTU51257
New.CleanUp.ReferenceOTU9970
New.CleanUp.ReferenceOTU22681
New.CleanUp.ReferenceOTU47714
New.CleanUp.ReferenceOTU36173
New.CleanUp.ReferenceOTU13501
EU287797.1.1785
New.CleanUp.ReferenceOTU48518
GU820978.1.1386
New.CleanUp.ReferenceOTU36427
New.CleanUp.ReferenceOTU47401
New.CleanUp.ReferenceOTU6296
New.CleanUp.ReferenceOTU6638
New.CleanUp.ReferenceOTU21493
New.CleanUp.ReferenceOTU7266
New.CleanUp.ReferenceOTU8866
New.CleanUp.ReferenceOTU1593
New.CleanUp.ReferenceOTU671
New.CleanUp.ReferenceOTU51148
New.CleanUp.ReferenceOTU616
New.CleanUp.ReferenceOTU563
New.CleanUp.ReferenceOTU72289
New.CleanUp.ReferenceOTU25828
New.CleanUp.ReferenceOTU9088
EU287793.1.1791
New.CleanUp.ReferenceOTU10201
New.CleanUp.ReferenceOTU78036
New.CleanUp.ReferenceOTU265320
New.CleanUp.ReferenceOTU224333
New.CleanUp.ReferenceOTU57348
New.CleanUp.ReferenceOTU290273
New.CleanUp.ReferenceOTU51353
New.ReferenceOTU426
New.CleanUp.ReferenceOTU141309
New.CleanUp.ReferenceOTU65313
New.CleanUp.ReferenceOTU63861
New.CleanUp.ReferenceOTU48627
New.CleanUp.ReferenceOTU51022
New.CleanUp.ReferenceOTU187784
New.CleanUp.ReferenceOTU180445
New.CleanUp.ReferenceOTU108937

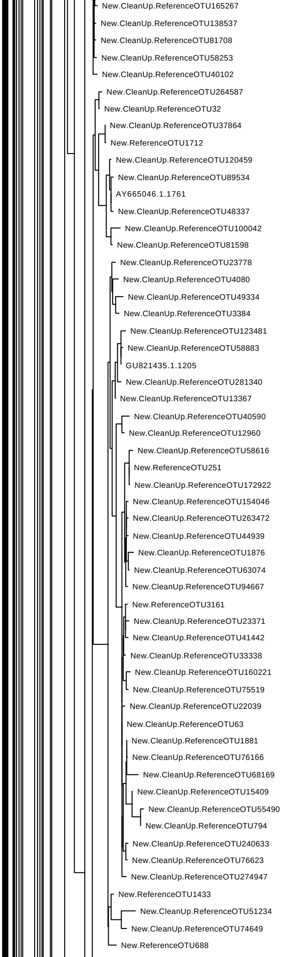
New.CleanUp.ReferenceOTU165267
New.CleanUp.ReferenceOTU138537
New.CleanUp.ReferenceOTU81708
New.CleanUp.ReferenceOTU58253
New.CleanUp.ReferenceOTU40102
New.CleanUp.ReferenceOTU264587
New.CleanUp.ReferenceOTU32
New.CleanUp.ReferenceOTU37864
New.ReferenceOTU1712
New.CleanUp.ReferenceOTU120459
New.CleanUp.ReferenceOTU89534
AY665046.1.1761
New.CleanUp.ReferenceOTU48337
New.CleanUp.ReferenceOTU100042
New.CleanUp.ReferenceOTU81598
New.CleanUp.ReferenceOTU23778
New.CleanUp.ReferenceOTU4080
New.CleanUp.ReferenceOTU49334
New.CleanUp.ReferenceOTU3384
New.CleanUp.ReferenceOTU123481
New.CleanUp.ReferenceOTU58883
GU821435.1.1205
New.CleanUp.ReferenceOTU281340
New.CleanUp.ReferenceOTU13367
New.CleanUp.ReferenceOTU40590
New.CleanUp.ReferenceOTU12960
New.CleanUp.ReferenceOTU58616
New.ReferenceOTU251
New.CleanUp.ReferenceOTU172922
New.CleanUp.ReferenceOTU154046
New.CleanUp.ReferenceOTU263472
New.CleanUp.ReferenceOTU44939
New.CleanUp.ReferenceOTU1876
New.CleanUp.ReferenceOTU63074
New.CleanUp.ReferenceOTU94667
New.ReferenceOTU3161
New.CleanUp.ReferenceOTU23371
New.CleanUp.ReferenceOTU41442
New.CleanUp.ReferenceOTU33338
New.CleanUp.ReferenceOTU160221
New.CleanUp.ReferenceOTU75519
New.CleanUp.ReferenceOTU22039
New.CleanUp.ReferenceOTU63
New.CleanUp.ReferenceOTU1881
New.CleanUp.ReferenceOTU76166
New.CleanUp.ReferenceOTU68169
New.CleanUp.ReferenceOTU15409
New.CleanUp.ReferenceOTU55490
New.CleanUp.ReferenceOTU794
New.CleanUp.ReferenceOTU240633
New.CleanUp.ReferenceOTU76623
New.CleanUp.ReferenceOTU274947
New.ReferenceOTU1433
New.CleanUp.ReferenceOTU51234
New.CleanUp.ReferenceOTU74649
New.ReferenceOTU688

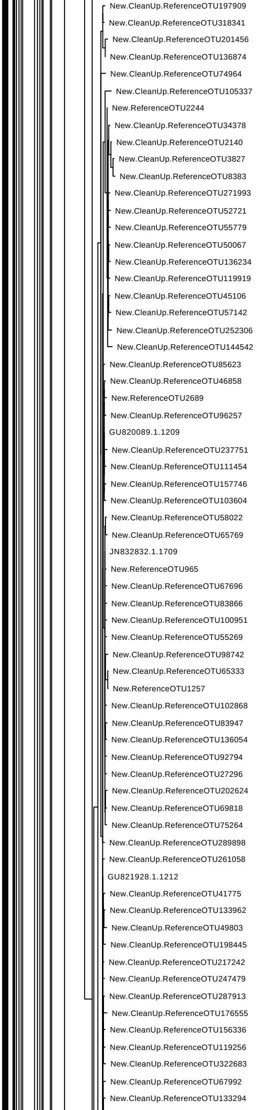

New.CleanUp.ReferenceOTU197909
New.CleanUp.ReferenceOTU318341
New.CleanUp.ReferenceOTU201456
New.CleanUp.ReferenceOTU136874
New.CleanUp.ReferenceOTU74964
New.CleanUp.ReferenceOTU105337
New.ReferenceOTU2244
New.CleanUp.ReferenceOTU34378
New.CleanUp.ReferenceOTU2140
New.CleanUp.ReferenceOTU3827
New.CleanUp.ReferenceOTU8383
New.CleanUp.ReferenceOTU271993
New.CleanUp.ReferenceOTU52721
New.CleanUp.ReferenceOTU55779
New.CleanUp.ReferenceOTU50067
New.CleanUp.ReferenceOTU136234
New.CleanUp.ReferenceOTU119919
New.CleanUp.ReferenceOTU45106
New.CleanUp.ReferenceOTU57142
New.CleanUp.ReferenceOTU252306
New.CleanUp.ReferenceOTU144542
New.CleanUp.ReferenceOTU85623
New.CleanUp.ReferenceOTU46858
New.ReferenceOTU2689
New.CleanUp.ReferenceOTU96257
GU820089.1.1209
New.CleanUp.ReferenceOTU237751
New.CleanUp.ReferenceOTU111454
New.CleanUp.ReferenceOTU157746
New.CleanUp.ReferenceOTU103604
New.CleanUp.ReferenceOTU58022
New.CleanUp.ReferenceOTU65769
JN832832.1.1709
New.ReferenceOTU965
New.CleanUp.ReferenceOTU67696
New.CleanUp.ReferenceOTU83866
New.CleanUp.ReferenceOTU100951
New.CleanUp.ReferenceOTU55269
New.CleanUp.ReferenceOTU98742
New.CleanUp.ReferenceOTU65333
New.ReferenceOTU1257
New.CleanUp.ReferenceOTU102868
New.CleanUp.ReferenceOTU83947
New.CleanUp.ReferenceOTU136054
New.CleanUp.ReferenceOTU92794
New.CleanUp.ReferenceOTU27296
New.CleanUp.ReferenceOTU202624
New.CleanUp.ReferenceOTU69818
New.CleanUp.ReferenceOTU75264
New.CleanUp.ReferenceOTU289898
New.CleanUp.ReferenceOTU261058
GU821928.1.1212
New.CleanUp.ReferenceOTU41775
New.CleanUp.ReferenceOTU133962
New.CleanUp.ReferenceOTU49803
New.CleanUp.ReferenceOTU198445
New.CleanUp.ReferenceOTU217242
New.CleanUp.ReferenceOTU247479
New.CleanUp.ReferenceOTU287913
New.CleanUp.ReferenceOTU176555
New.CleanUp.ReferenceOTU156336
New.CleanUp.ReferenceOTU119256
New.CleanUp.ReferenceOTU322683
New.CleanUp.ReferenceOTU67992
New.CleanUp.ReferenceOTU133294

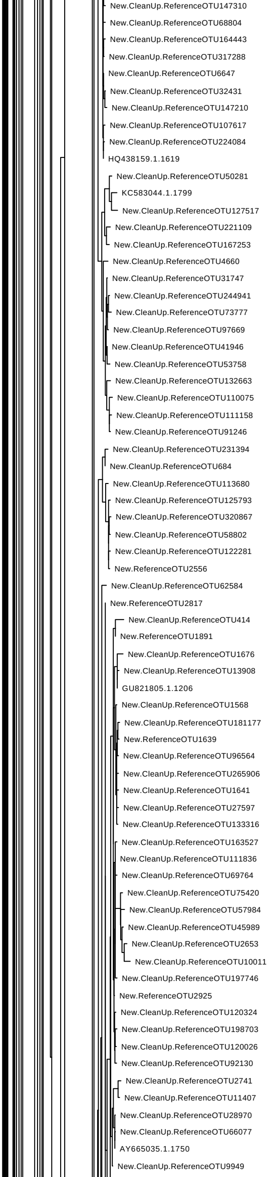
New.CleanUp.ReferenceOTU147310
New.CleanUp.ReferenceOTU68804
New.CleanUp.ReferenceOTU164443
New.CleanUp.ReferenceOTU317288
New.CleanUp.ReferenceOTU6647
New.CleanUp.ReferenceOTU32431
New.CleanUp.ReferenceOTU147210
New.CleanUp.ReferenceOTU107617
New.CleanUp.ReferenceOTU224084
HQ438159.1.1619
New.CleanUp.ReferenceOTU50281
KC583044.1.1799
New.CleanUp.ReferenceOTU127517
New.CleanUp.ReferenceOTU221109
New.CleanUp.ReferenceOTU167253
New.CleanUp.ReferenceOTU4660
New.CleanUp.ReferenceOTU31747
New.CleanUp.ReferenceOTU244941
New.CleanUp.ReferenceOTU73777
New.CleanUp.ReferenceOTU97669
New.CleanUp.ReferenceOTU41946
New.CleanUp.ReferenceOTU53758
New.CleanUp.ReferenceOTU132663
New.CleanUp.ReferenceOTU110075
New.CleanUp.ReferenceOTU111158
New.CleanUp.ReferenceOTU91246
New.CleanUp.ReferenceOTU231394
New.CleanUp.ReferenceOTU684
New.CleanUp.ReferenceOTU113680
New.CleanUp.ReferenceOTU125793
New.CleanUp.ReferenceOTU320867
New.CleanUp.ReferenceOTU58802
New.CleanUp.ReferenceOTU122281
New.ReferenceOTU2556
New.CleanUp.ReferenceOTU62584
New.ReferenceOTU2817
New.CleanUp.ReferenceOTU414
New.ReferenceOTU1891
New.CleanUp.ReferenceOTU1676
New.CleanUp.ReferenceOTU13908
GU821805.1.1206
New.CleanUp.ReferenceOTU1568
New.CleanUp.ReferenceOTU181177
New.ReferenceOTU1639
New.CleanUp.ReferenceOTU96564
New.CleanUp.ReferenceOTU265906
New.CleanUp.ReferenceOTU1641
New.CleanUp.ReferenceOTU27597
New.CleanUp.ReferenceOTU133316
New.CleanUp.ReferenceOTU163527
New.CleanUp.ReferenceOTU111836
New.CleanUp.ReferenceOTU69764
New.CleanUp.ReferenceOTU75420
New.CleanUp.ReferenceOTU57984
New.CleanUp.ReferenceOTU45989
New.CleanUp.ReferenceOTU2653
New.CleanUp.ReferenceOTU10011
New.CleanUp.ReferenceOTU197746
New.ReferenceOTU2925
New.CleanUp.ReferenceOTU120324
New.CleanUp.ReferenceOTU198703
New.CleanUp.ReferenceOTU120026
New.CleanUp.ReferenceOTU92130
New.CleanUp.ReferenceOTU2741
New.CleanUp.ReferenceOTU11407
New.CleanUp.ReferenceOTU28970
New.CleanUp.ReferenceOTU66077
AY665035.1.1750
New.CleanUp.ReferenceOTU9949

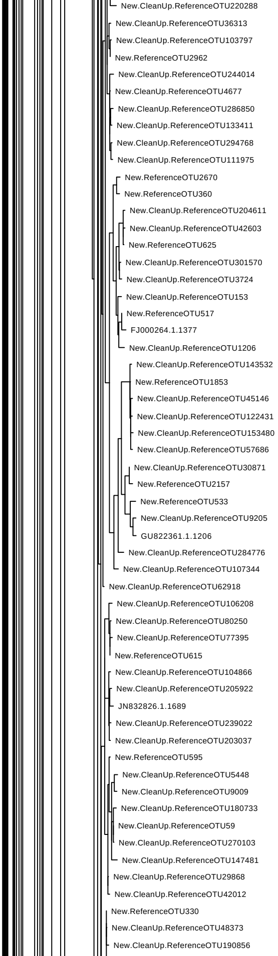
New.CleanUp.ReferenceOTU220288
New.CleanUp.ReferenceOTU36313
New.CleanUp.ReferenceOTU103797
New.ReferenceOTU2962
New.CleanUp.ReferenceOTU244014
New.CleanUp.ReferenceOTU4677
New.CleanUp.ReferenceOTU286850
New.CleanUp.ReferenceOTU133411
New.CleanUp.ReferenceOTU294768
New.CleanUp.ReferenceOTU111975
New.ReferenceOTU2670
New.ReferenceOTU360
New.CleanUp.ReferenceOTU204611
New.CleanUp.ReferenceOTU42603
New.ReferenceOTU625
New.CleanUp.ReferenceOTU301570
New.CleanUp.ReferenceOTU3724
New.CleanUp.ReferenceOTU153
New.ReferenceOTU517
FJ000264.1.1377
New.CleanUp.ReferenceOTU1206
New.CleanUp.ReferenceOTU143532
New.ReferenceOTU1853
New.CleanUp.ReferenceOTU45146
New.CleanUp.ReferenceOTU122431
New.CleanUp.ReferenceOTU153480
New.CleanUp.ReferenceOTU57686
New.CleanUp.ReferenceOTU30871
New.ReferenceOTU2157
New.ReferenceOTU533
New.CleanUp.ReferenceOTU9205
GU822361.1.1206
New.CleanUp.ReferenceOTU284776
New.CleanUp.ReferenceOTU107344
New.CleanUp.ReferenceOTU62918
New.CleanUp.ReferenceOTU106208
New.CleanUp.ReferenceOTU80250
New.CleanUp.ReferenceOTU77395
New.ReferenceOTU615
New.CleanUp.ReferenceOTU104866
New.CleanUp.ReferenceOTU205922
JN832826.1.1689
New.CleanUp.ReferenceOTU239022
New.CleanUp.ReferenceOTU203037
New.ReferenceOTU595
New.CleanUp.ReferenceOTU5448
New.CleanUp.ReferenceOTU9009
New.CleanUp.ReferenceOTU180733
New.CleanUp.ReferenceOTU59
New.CleanUp.ReferenceOTU270103
New.CleanUp.ReferenceOTU147481
New.CleanUp.ReferenceOTU29868
New.CleanUp.ReferenceOTU42012
New.ReferenceOTU330
New.CleanUp.ReferenceOTU48373
New.CleanUp.ReferenceOTU190856

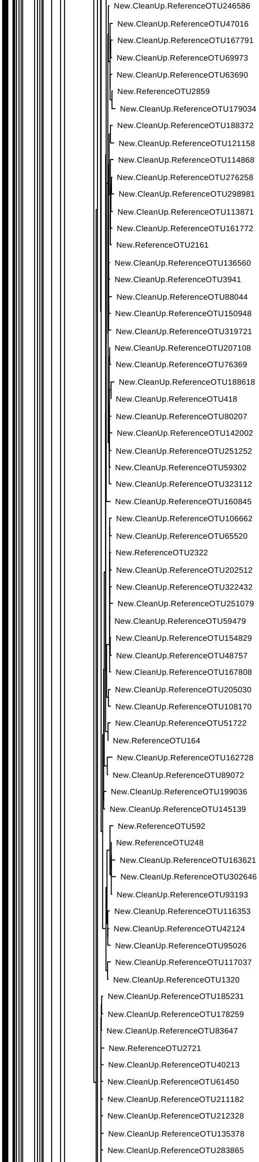
New.CleanUp.ReferenceOTU246586
New.CleanUp.ReferenceOTU47016
New.CleanUp.ReferenceOTU167791
New.CleanUp.ReferenceOTU69973
New.CleanUp.ReferenceOTU63690
New.ReferenceOTU2859
New.CleanUp.ReferenceOTU179034
New.CleanUp.ReferenceOTU188372
New.CleanUp.ReferenceOTU121158
New.CleanUp.ReferenceOTU114868
New.CleanUp.ReferenceOTU276258
New.CleanUp.ReferenceOTU298981
New.CleanUp.ReferenceOTU113871
New.CleanUp.ReferenceOTU161772
New.ReferenceOTU2161
New.CleanUp.ReferenceOTU136560
New.CleanUp.ReferenceOTU3941
New.CleanUp.ReferenceOTU88044
New.CleanUp.ReferenceOTU150948
New.CleanUp.ReferenceOTU319721
New.CleanUp.ReferenceOTU207108
New.CleanUp.ReferenceOTU76369
New.CleanUp.ReferenceOTU188618
New.CleanUp.ReferenceOTU418
New.CleanUp.ReferenceOTU80207
New.CleanUp.ReferenceOTU142002
New.CleanUp.ReferenceOTU251252
New.CleanUp.ReferenceOTU59302
New.CleanUp.ReferenceOTU323112
New.CleanUp.ReferenceOTU160845
New.CleanUp.ReferenceOTU106662
New.CleanUp.ReferenceOTU65520
New.ReferenceOTU2322
New.CleanUp.ReferenceOTU202512
New.CleanUp.ReferenceOTU322432
New.CleanUp.ReferenceOTU251079
New.CleanUp.ReferenceOTU59479
New.CleanUp.ReferenceOTU154829
New.CleanUp.ReferenceOTU48757
New.CleanUp.ReferenceOTU167808
New.CleanUp.ReferenceOTU205030
New.CleanUp.ReferenceOTU108170
New.CleanUp.ReferenceOTU51722
New.ReferenceOTU164
New.CleanUp.ReferenceOTU162728
New.CleanUp.ReferenceOTU89072
New.CleanUp.ReferenceOTU199036
New.CleanUp.ReferenceOTU145139
New.ReferenceOTU592
New.ReferenceOTU248
New.CleanUp.ReferenceOTU163621
New.CleanUp.ReferenceOTU302646
New.CleanUp.ReferenceOTU93193
New.CleanUp.ReferenceOTU116353
New.CleanUp.ReferenceOTU42124
New.CleanUp.ReferenceOTU95026
New.CleanUp.ReferenceOTU117037
New.CleanUp.ReferenceOTU1320
New.CleanUp.ReferenceOTU185231
New.CleanUp.ReferenceOTU178259
New.CleanUp.ReferenceOTU83647
New.ReferenceOTU2721
New.CleanUp.ReferenceOTU40213
New.CleanUp.ReferenceOTU61450
New.CleanUp.ReferenceOTU211182
New.CleanUp.ReferenceOTU212328
New.CleanUp.ReferenceOTU135378
New.CleanUp.ReferenceOTU283865

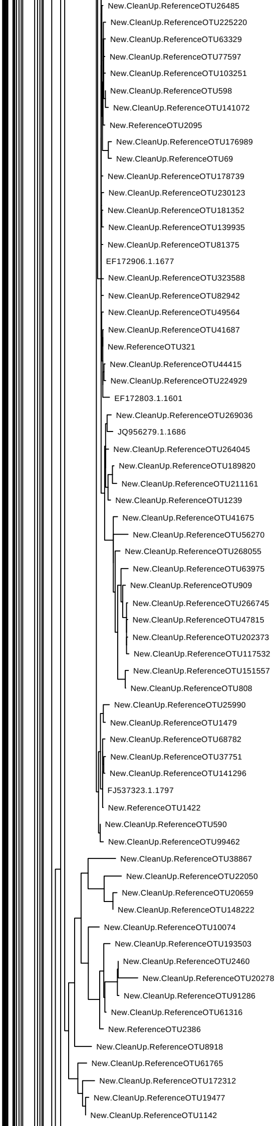
New.CleanUp.ReferenceOTU26485
New.CleanUp.ReferenceOTU225220
New.CleanUp.ReferenceOTU63329
New.CleanUp.ReferenceOTU77597
New.CleanUp.ReferenceOTU103251
New.CleanUp.ReferenceOTU598
New.CleanUp.ReferenceOTU141072
New.ReferenceOTU2095
New.CleanUp.ReferenceOTU176989
New.CleanUp.ReferenceOTU69
New.CleanUp.ReferenceOTU178739
New.CleanUp.ReferenceOTU230123
New.CleanUp.ReferenceOTU181352
New.CleanUp.ReferenceOTU139935
New.CleanUp.ReferenceOTU81375
EF172906.1.1677
New.CleanUp.ReferenceOTU323588
New.CleanUp.ReferenceOTU82942
New.CleanUp.ReferenceOTU49564
New.CleanUp.ReferenceOTU41687
New.ReferenceOTU321
New.CleanUp.ReferenceOTU44415
New.CleanUp.ReferenceOTU224929
EF172803.1.1601
New.CleanUp.ReferenceOTU269036
JQ956279.1.1686
New.CleanUp.ReferenceOTU264045
New.CleanUp.ReferenceOTU189820
New.CleanUp.ReferenceOTU211161
New.CleanUp.ReferenceOTU1239
New.CleanUp.ReferenceOTU41675
New.CleanUp.ReferenceOTU56270
New.CleanUp.ReferenceOTU268055
New.CleanUp.ReferenceOTU63975
New.CleanUp.ReferenceOTU909
New.CleanUp.ReferenceOTU266745
New.CleanUp.ReferenceOTU47815
New.CleanUp.ReferenceOTU202373
New.CleanUp.ReferenceOTU117532
New.CleanUp.ReferenceOTU151557
New.CleanUp.ReferenceOTU808
New.CleanUp.ReferenceOTU25990
New.CleanUp.ReferenceOTU1479
New.CleanUp.ReferenceOTU68782
New.CleanUp.ReferenceOTU37751
New.CleanUp.ReferenceOTU141296
FJ537323.1.1797
New.ReferenceOTU1422
New.CleanUp.ReferenceOTU590
New.CleanUp.ReferenceOTU99462
New.CleanUp.ReferenceOTU38867
New.CleanUp.ReferenceOTU22050
New.CleanUp.ReferenceOTU20659
New.CleanUp.ReferenceOTU148222
New.CleanUp.ReferenceOTU10074
New.CleanUp.ReferenceOTU193503
New.CleanUp.ReferenceOTU2460
New.CleanUp.ReferenceOTU20278
New.CleanUp.ReferenceOTU91286
New.CleanUp.ReferenceOTU61316
New.ReferenceOTU2386
New.CleanUp.ReferenceOTU8918
New.CleanUp.ReferenceOTU61765
New.CleanUp.ReferenceOTU172312
New.CleanUp.ReferenceOTU19477
New.CleanUp.ReferenceOTU1142

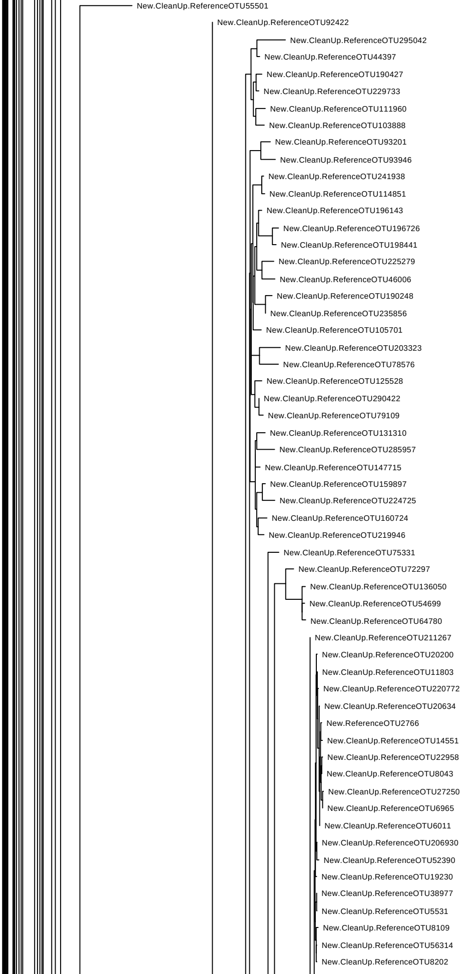
New.CleanUp.ReferenceOTU55501
New.CleanUp.ReferenceOTU92422
New.CleanUp.ReferenceOTU295042
New.CleanUp.ReferenceOTU44397
New.CleanUp.ReferenceOTU190427
New.CleanUp.ReferenceOTU229733
New.CleanUp.ReferenceOTU111960
New.CleanUp.ReferenceOTU103888
New.CleanUp.ReferenceOTU93201
New.CleanUp.ReferenceOTU93946
New.CleanUp.ReferenceOTU241938
New.CleanUp.ReferenceOTU114851
New.CleanUp.ReferenceOTU196143
New.CleanUp.ReferenceOTU196726
New.CleanUp.ReferenceOTU198441
New.CleanUp.ReferenceOTU225279
New.CleanUp.ReferenceOTU46006
New.CleanUp.ReferenceOTU190248
New.CleanUp.ReferenceOTU235856
New.CleanUp.ReferenceOTU105701
New.CleanUp.ReferenceOTU203323
New.CleanUp.ReferenceOTU78576
New.CleanUp.ReferenceOTU125528
New.CleanUp.ReferenceOTU290422
New.CleanUp.ReferenceOTU79109
New.CleanUp.ReferenceOTU131310
New.CleanUp.ReferenceOTU285957
New.CleanUp.ReferenceOTU147715
New.CleanUp.ReferenceOTU159897
New.CleanUp.ReferenceOTU224725
New.CleanUp.ReferenceOTU160724
New.CleanUp.ReferenceOTU219946
New.CleanUp.ReferenceOTU75331
New.CleanUp.ReferenceOTU72297
New.CleanUp.ReferenceOTU136050
New.CleanUp.ReferenceOTU54699
New.CleanUp.ReferenceOTU64780
New.CleanUp.ReferenceOTU211267
New.CleanUp.ReferenceOTU20200
New.CleanUp.ReferenceOTU11803
New.CleanUp.ReferenceOTU220772
New.CleanUp.ReferenceOTU20634
New.ReferenceOTU2766
New.CleanUp.ReferenceOTU14551
New.CleanUp.ReferenceOTU22958
New.CleanUp.ReferenceOTU8043
New.CleanUp.ReferenceOTU27250
New.CleanUp.ReferenceOTU6965
New.CleanUp.ReferenceOTU6011
New.CleanUp.ReferenceOTU206930
New.CleanUp.ReferenceOTU52390
New.CleanUp.ReferenceOTU19230
New.CleanUp.ReferenceOTU38977
New.CleanUp.ReferenceOTU5531
New.CleanUp.ReferenceOTU8109
New.CleanUp.ReferenceOTU56314
New.CleanUp.ReferenceOTU8202

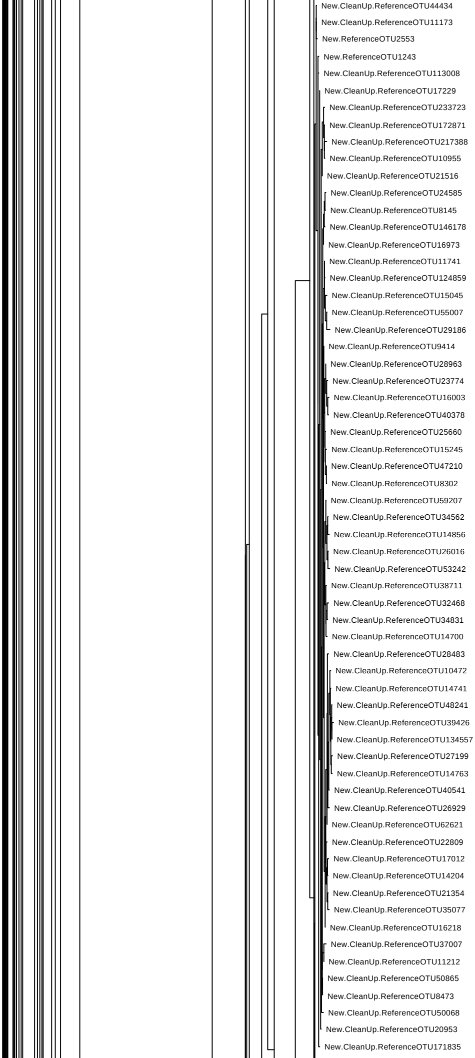
New.CleanUp.ReferenceOTU44434
New.CleanUp.ReferenceOTU11173
New.ReferenceOTU2553
New.ReferenceOTU1243
New.CleanUp.ReferenceOTU113008
New.CleanUp.ReferenceOTU17229
New.CleanUp.ReferenceOTU233723
New.CleanUp.ReferenceOTU172871
New.CleanUp.ReferenceOTU217388
New.CleanUp.ReferenceOTU10955
New.CleanUp.ReferenceOTU21516
New.CleanUp.ReferenceOTU24585
New.CleanUp.ReferenceOTU8145
New.CleanUp.ReferenceOTU146178
New.CleanUp.ReferenceOTU16973
New.CleanUp.ReferenceOTU11741
New.CleanUp.ReferenceOTU124859
New.CleanUp.ReferenceOTU15045
New.CleanUp.ReferenceOTU55007
New.CleanUp.ReferenceOTU29186
New.CleanUp.ReferenceOTU9414
New.CleanUp.ReferenceOTU28963
New.CleanUp.ReferenceOTU23774
New.CleanUp.ReferenceOTU16003
New.CleanUp.ReferenceOTU40378
New.CleanUp.ReferenceOTU25660
New.CleanUp.ReferenceOTU15245
New.CleanUp.ReferenceOTU47210
New.CleanUp.ReferenceOTU8302
New.CleanUp.ReferenceOTU59207
New.CleanUp.ReferenceOTU34562
New.CleanUp.ReferenceOTU14856
New.CleanUp.ReferenceOTU26016
New.CleanUp.ReferenceOTU53242
New.CleanUp.ReferenceOTU38711
New.CleanUp.ReferenceOTU32468
New.CleanUp.ReferenceOTU34831
New.CleanUp.ReferenceOTU14700
New.CleanUp.ReferenceOTU28483
New.CleanUp.ReferenceOTU10472
New.CleanUp.ReferenceOTU14741
New.CleanUp.ReferenceOTU48241
New.CleanUp.ReferenceOTU39426
New.CleanUp.ReferenceOTU134557
New.CleanUp.ReferenceOTU27199
New.CleanUp.ReferenceOTU14763
New.CleanUp.ReferenceOTU40541
New.CleanUp.ReferenceOTU26929
New.CleanUp.ReferenceOTU62621
New.CleanUp.ReferenceOTU22809
New.CleanUp.ReferenceOTU17012
New.CleanUp.ReferenceOTU14204
New.CleanUp.ReferenceOTU21354
New.CleanUp.ReferenceOTU35077
New.CleanUp.ReferenceOTU16218
New.CleanUp.ReferenceOTU37007
New.CleanUp.ReferenceOTU11212
New.CleanUp.ReferenceOTU50865
New.CleanUp.ReferenceOTU8473
New.CleanUp.ReferenceOTU50068
New.CleanUp.ReferenceOTU20953
New.CleanUp.ReferenceOTU171835

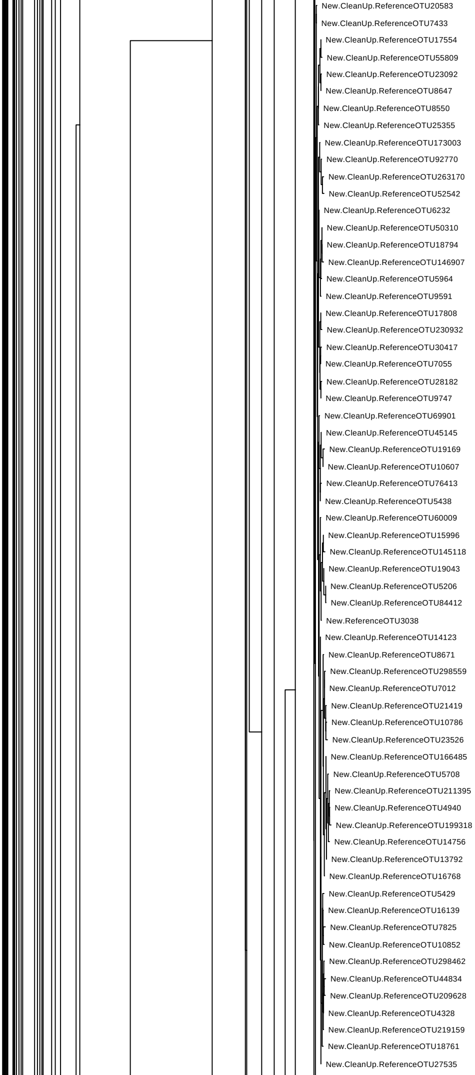
New.CleanUp.ReferenceOTU20583
New.CleanUp.ReferenceOTU7433
New.CleanUp.ReferenceOTU17554
New.CleanUp.ReferenceOTU55809
New.CleanUp.ReferenceOTU23092
New.CleanUp.ReferenceOTU8647
New.CleanUp.ReferenceOTU8550
New.CleanUp.ReferenceOTU25355
New.CleanUp.ReferenceOTU173003
New.CleanUp.ReferenceOTU92770
New.CleanUp.ReferenceOTU263170
New.CleanUp.ReferenceOTU52542
New.CleanUp.ReferenceOTU6232
New.CleanUp.ReferenceOTU50310
New.CleanUp.ReferenceOTU18794
New.CleanUp.ReferenceOTU146907
New.CleanUp.ReferenceOTU5964
New.CleanUp.ReferenceOTU9591
New.CleanUp.ReferenceOTU17808
New.CleanUp.ReferenceOTU230932
New.CleanUp.ReferenceOTU30417
New.CleanUp.ReferenceOTU7055
New.CleanUp.ReferenceOTU28182
New.CleanUp.ReferenceOTU9747
New.CleanUp.ReferenceOTU69901
New.CleanUp.ReferenceOTU45145
New.CleanUp.ReferenceOTU19169
New.CleanUp.ReferenceOTU10607
New.CleanUp.ReferenceOTU76413
New.CleanUp.ReferenceOTU5438
New.CleanUp.ReferenceOTU60009
New.CleanUp.ReferenceOTU15996
New.CleanUp.ReferenceOTU145118
New.CleanUp.ReferenceOTU19043
New.CleanUp.ReferenceOTU5206
New.CleanUp.ReferenceOTU84412
New.ReferenceOTU3038
New.CleanUp.ReferenceOTU14123
New.CleanUp.ReferenceOTU8671
New.CleanUp.ReferenceOTU298559
New.CleanUp.ReferenceOTU7012
New.CleanUp.ReferenceOTU21419
New.CleanUp.ReferenceOTU10786
New.CleanUp.ReferenceOTU23526
New.CleanUp.ReferenceOTU166485
New.CleanUp.ReferenceOTU5708
New.CleanUp.ReferenceOTU211395
New.CleanUp.ReferenceOTU4940
New.CleanUp.ReferenceOTU199318
New.CleanUp.ReferenceOTU14756
New.CleanUp.ReferenceOTU13792
New.CleanUp.ReferenceOTU16768
New.CleanUp.ReferenceOTU5429
New.CleanUp.ReferenceOTU16139
New.CleanUp.ReferenceOTU7825
New.CleanUp.ReferenceOTU10852
New.CleanUp.ReferenceOTU298462
New.CleanUp.ReferenceOTU44834
New.CleanUp.ReferenceOTU209628
New.CleanUp.ReferenceOTU4328
New.CleanUp.ReferenceOTU219159
New.CleanUp.ReferenceOTU18761
New.CleanUp.ReferenceOTU27535

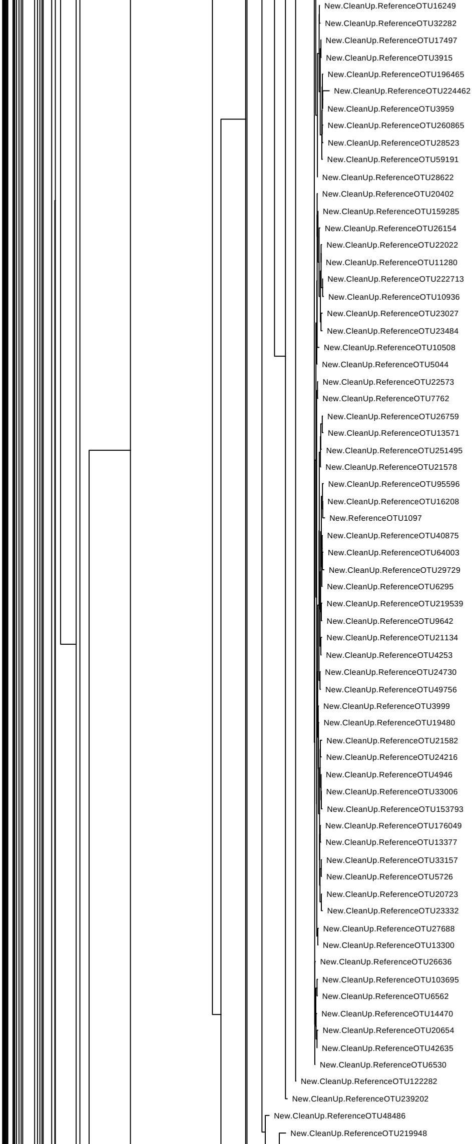
New.CleanUp.ReferenceOTU16249
New.CleanUp.ReferenceOTU32282
New.CleanUp.ReferenceOTU17497
New.CleanUp.ReferenceOTU3915
New.CleanUp.ReferenceOTU196465
New.CleanUp.ReferenceOTU224462
New.CleanUp.ReferenceOTU3959
New.CleanUp.ReferenceOTU260865
New.CleanUp.ReferenceOTU28523
New.CleanUp.ReferenceOTU59191
New.CleanUp.ReferenceOTU28622
New.CleanUp.ReferenceOTU20402
New.CleanUp.ReferenceOTU159285
New.CleanUp.ReferenceOTU26154
New.CleanUp.ReferenceOTU22022
New.CleanUp.ReferenceOTU11280
New.CleanUp.ReferenceOTU222713
New.CleanUp.ReferenceOTU10936
New.CleanUp.ReferenceOTU23027
New.CleanUp.ReferenceOTU23484
New.CleanUp.ReferenceOTU10508
New.CleanUp.ReferenceOTU5044
New.CleanUp.ReferenceOTU22573
New.CleanUp.ReferenceOTU7762
New.CleanUp.ReferenceOTU26759
New.CleanUp.ReferenceOTU13571
New.CleanUp.ReferenceOTU251495
New.CleanUp.ReferenceOTU21578
New.CleanUp.ReferenceOTU95596
New.CleanUp.ReferenceOTU16208
New.ReferenceOTU1097
New.CleanUp.ReferenceOTU40875
New.CleanUp.ReferenceOTU64003
New.CleanUp.ReferenceOTU29729
New.CleanUp.ReferenceOTU6295
New.CleanUp.ReferenceOTU219539
New.CleanUp.ReferenceOTU9642
New.CleanUp.ReferenceOTU21134
New.CleanUp.ReferenceOTU4253
New.CleanUp.ReferenceOTU24730
New.CleanUp.ReferenceOTU49756
New.CleanUp.ReferenceOTU3999
New.CleanUp.ReferenceOTU19480
New.CleanUp.ReferenceOTU21582
New.CleanUp.ReferenceOTU24216
New.CleanUp.ReferenceOTU4946
New.CleanUp.ReferenceOTU33006
New.CleanUp.ReferenceOTU153793
New.CleanUp.ReferenceOTU176049
New.CleanUp.ReferenceOTU13377
New.CleanUp.ReferenceOTU33157
New.CleanUp.ReferenceOTU5726
New.CleanUp.ReferenceOTU20723
New.CleanUp.ReferenceOTU23332
New.CleanUp.ReferenceOTU27688
New.CleanUp.ReferenceOTU13300
New.CleanUp.ReferenceOTU26636
New.CleanUp.ReferenceOTU103695
New.CleanUp.ReferenceOTU6562
New.CleanUp.ReferenceOTU14470
New.CleanUp.ReferenceOTU20654
New.CleanUp.ReferenceOTU42635
New.CleanUp.ReferenceOTU6530
New.CleanUp.ReferenceOTU122282
New.CleanUp.ReferenceOTU239202
New.CleanUp.ReferenceOTU48486
New.CleanUp.ReferenceOTU219948

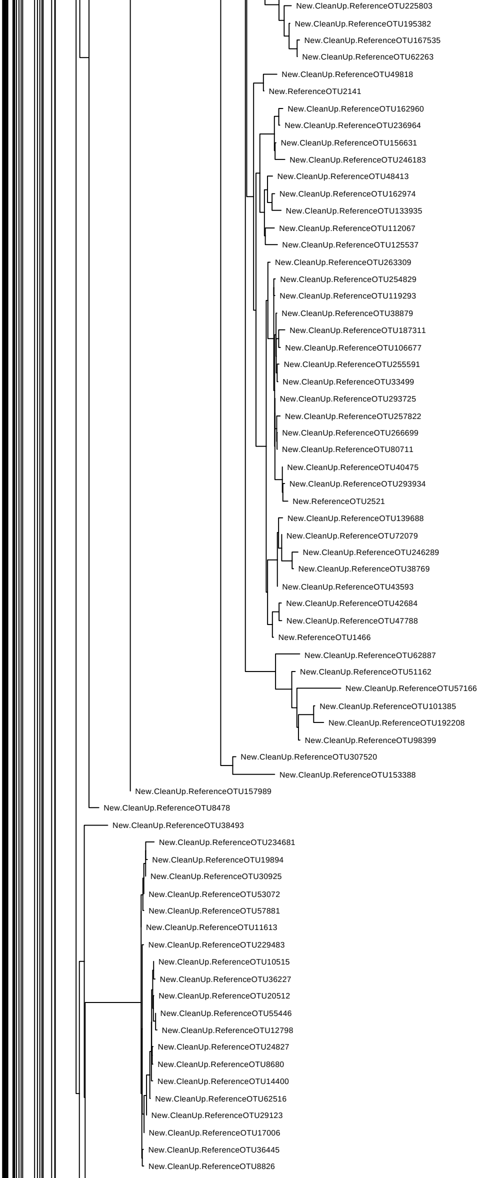
New.CleanUp.ReferenceOTU225803
New.CleanUp.ReferenceOTU195382
New.CleanUp.ReferenceOTU167535
New.CleanUp.ReferenceOTU62263
New.CleanUp.ReferenceOTU49818
New.ReferenceOTU2141
New.CleanUp.ReferenceOTU162960
New.CleanUp.ReferenceOTU236964
New.CleanUp.ReferenceOTU156631
New.CleanUp.ReferenceOTU246183
New.CleanUp.ReferenceOTU48413
New.CleanUp.ReferenceOTU162974
New.CleanUp.ReferenceOTU133935
New.CleanUp.ReferenceOTU112067
New.CleanUp.ReferenceOTU125537
New.CleanUp.ReferenceOTU263309
New.CleanUp.ReferenceOTU254829
New.CleanUp.ReferenceOTU119293
New.CleanUp.ReferenceOTU38879
New.CleanUp.ReferenceOTU187311
New.CleanUp.ReferenceOTU106677
New.CleanUp.ReferenceOTU255591
New.CleanUp.ReferenceOTU33499
New.CleanUp.ReferenceOTU293725
New.CleanUp.ReferenceOTU257822
New.CleanUp.ReferenceOTU266699
New.CleanUp.ReferenceOTU80711
New.CleanUp.ReferenceOTU40475
New.CleanUp.ReferenceOTU293934
New.ReferenceOTU2521
New.CleanUp.ReferenceOTU139688
New.CleanUp.ReferenceOTU72079
New.CleanUp.ReferenceOTU246289
New.CleanUp.ReferenceOTU38769
New.CleanUp.ReferenceOTU43593
New.CleanUp.ReferenceOTU42684
New.CleanUp.ReferenceOTU47788
New.ReferenceOTU1466
New.CleanUp.ReferenceOTU62887
New.CleanUp.ReferenceOTU51162
New.CleanUp.ReferenceOTU57166
New.CleanUp.ReferenceOTU101385
New.CleanUp.ReferenceOTU192208
New.CleanUp.ReferenceOTU98399
New.CleanUp.ReferenceOTU307520
New.CleanUp.ReferenceOTU153388
New.CleanUp.ReferenceOTU157989
New.CleanUp.ReferenceOTU8478
New.CleanUp.ReferenceOTU38493
New.CleanUp.ReferenceOTU234681
New.CleanUp.ReferenceOTU19894
New.CleanUp.ReferenceOTU30925
New.CleanUp.ReferenceOTU53072
New.CleanUp.ReferenceOTU57881
New.CleanUp.ReferenceOTU11613
New.CleanUp.ReferenceOTU229483
New.CleanUp.ReferenceOTU10515
New.CleanUp.ReferenceOTU36227
New.CleanUp.ReferenceOTU20512
New.CleanUp.ReferenceOTU55446
New.CleanUp.ReferenceOTU12798
New.CleanUp.ReferenceOTU24827
New.CleanUp.ReferenceOTU8680
New.CleanUp.ReferenceOTU14400
New.CleanUp.ReferenceOTU62516
New.CleanUp.ReferenceOTU29123
New.CleanUp.ReferenceOTU17006
New.CleanUp.ReferenceOTU36445
New.CleanUp.ReferenceOTU8826

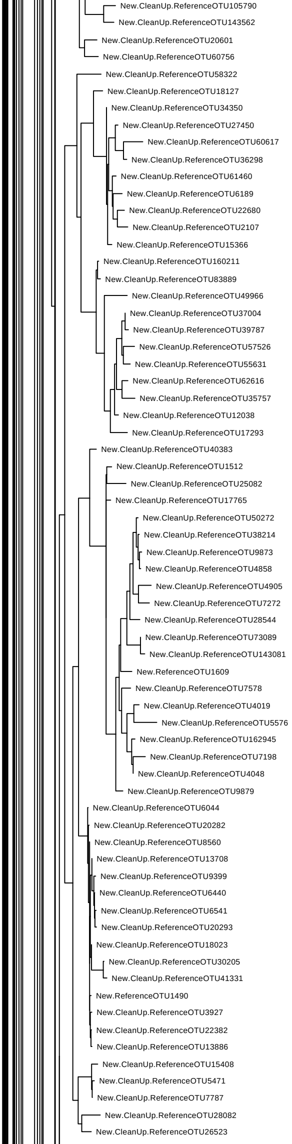
New.CleanUp.ReferenceOTU105790
New.CleanUp.ReferenceOTU143562
New.CleanUp.ReferenceOTU20601
New.CleanUp.ReferenceOTU60756
New.CleanUp.ReferenceOTU58322
New.CleanUp.ReferenceOTU18127
New.CleanUp.ReferenceOTU34350
New.CleanUp.ReferenceOTU27450
New.CleanUp.ReferenceOTU60617
New.CleanUp.ReferenceOTU36298
New.CleanUp.ReferenceOTU61460
New.CleanUp.ReferenceOTU6189
New.CleanUp.ReferenceOTU22680
New.CleanUp.ReferenceOTU2107
New.CleanUp.ReferenceOTU15366
New.CleanUp.ReferenceOTU160211
New.CleanUp.ReferenceOTU83889
New.CleanUp.ReferenceOTU49966
New.CleanUp.ReferenceOTU37004
New.CleanUp.ReferenceOTU39787
New.CleanUp.ReferenceOTU57526
New.CleanUp.ReferenceOTU55631
New.CleanUp.ReferenceOTU62616
New.CleanUp.ReferenceOTU35757
New.CleanUp.ReferenceOTU12038
New.CleanUp.ReferenceOTU17293
New.CleanUp.ReferenceOTU40383
New.CleanUp.ReferenceOTU1512
New.CleanUp.ReferenceOTU25082
New.CleanUp.ReferenceOTU17765
New.CleanUp.ReferenceOTU50272
New.CleanUp.ReferenceOTU38214
New.CleanUp.ReferenceOTU9873
New.CleanUp.ReferenceOTU4858
New.CleanUp.ReferenceOTU4905
New.CleanUp.ReferenceOTU7272
New.CleanUp.ReferenceOTU28544
New.CleanUp.ReferenceOTU73089
New.CleanUp.ReferenceOTU143081
New.ReferenceOTU1609
New.CleanUp.ReferenceOTU7578
New.CleanUp.ReferenceOTU4019
New.CleanUp.ReferenceOTU5576
New.CleanUp.ReferenceOTU162945
New.CleanUp.ReferenceOTU7198
New.CleanUp.ReferenceOTU4048
New.CleanUp.ReferenceOTU9879
New.CleanUp.ReferenceOTU6044
New.CleanUp.ReferenceOTU20282
New.CleanUp.ReferenceOTU8560
New.CleanUp.ReferenceOTU13708
New.CleanUp.ReferenceOTU9399
New.CleanUp.ReferenceOTU6440
New.CleanUp.ReferenceOTU6541
New.CleanUp.ReferenceOTU20293
New.CleanUp.ReferenceOTU18023
New.CleanUp.ReferenceOTU30205
New.CleanUp.ReferenceOTU41331
New.ReferenceOTU1490
New.CleanUp.ReferenceOTU3927
New.CleanUp.ReferenceOTU22382
New.CleanUp.ReferenceOTU13886
New.CleanUp.ReferenceOTU15408
New.CleanUp.ReferenceOTU5471
New.CleanUp.ReferenceOTU7787
New.CleanUp.ReferenceOTU28082
New.CleanUp.ReferenceOTU26523

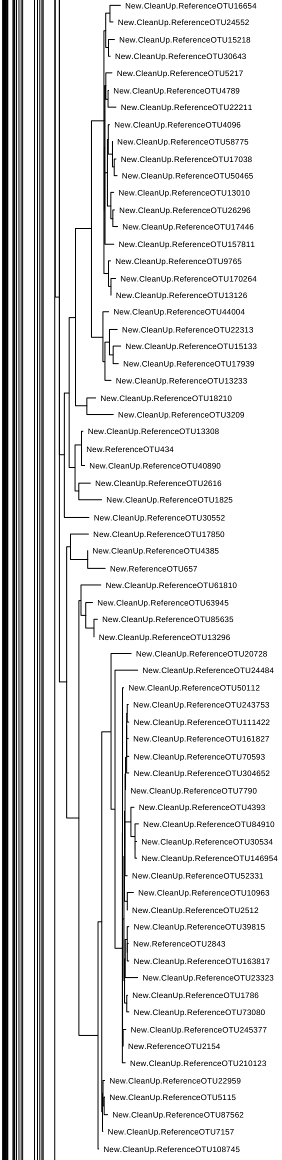

New.CleanUp.ReferenceOTU16654
New.CleanUp.ReferenceOTU24552
New.CleanUp.ReferenceOTU15218
New.CleanUp.ReferenceOTU30643
New.CleanUp.ReferenceOTU5217
New.CleanUp.ReferenceOTU4789
New.CleanUp.ReferenceOTU22211
New.CleanUp.ReferenceOTU4096
New.CleanUp.ReferenceOTU58775
New.CleanUp.ReferenceOTU17038
New.CleanUp.ReferenceOTU50465
New.CleanUp.ReferenceOTU13010
New.CleanUp.ReferenceOTU26296
New.CleanUp.ReferenceOTU17446
New.CleanUp.ReferenceOTU157811
New.CleanUp.ReferenceOTU9765
New.CleanUp.ReferenceOTU170264
New.CleanUp.ReferenceOTU13126
New.CleanUp.ReferenceOTU44004
New.CleanUp.ReferenceOTU22313
New.CleanUp.ReferenceOTU15133
New.CleanUp.ReferenceOTU17939
New.CleanUp.ReferenceOTU13233
New.CleanUp.ReferenceOTU18210
New.CleanUp.ReferenceOTU3209
New.CleanUp.ReferenceOTU13308
New.ReferenceOTU434
New.CleanUp.ReferenceOTU40890
New.CleanUp.ReferenceOTU2616
New.CleanUp.ReferenceOTU1825
New.CleanUp.ReferenceOTU30552
New.CleanUp.ReferenceOTU17850
New.CleanUp.ReferenceOTU4385
New.ReferenceOTU657
New.CleanUp.ReferenceOTU61810
New.CleanUp.ReferenceOTU63945
New.CleanUp.ReferenceOTU85635
New.CleanUp.ReferenceOTU13296
New.CleanUp.ReferenceOTU20728
New.CleanUp.ReferenceOTU24484
New.CleanUp.ReferenceOTU50112
New.CleanUp.ReferenceOTU243753
New.CleanUp.ReferenceOTU111422
New.CleanUp.ReferenceOTU161827
New.CleanUp.ReferenceOTU70593
New.CleanUp.ReferenceOTU304652
New.CleanUp.ReferenceOTU7790
New.CleanUp.ReferenceOTU4393
New.CleanUp.ReferenceOTU84910
New.CleanUp.ReferenceOTU30534
New.CleanUp.ReferenceOTU146954
New.CleanUp.ReferenceOTU52331
New.CleanUp.ReferenceOTU10963
New.CleanUp.ReferenceOTU2512
New.CleanUp.ReferenceOTU39815
New.ReferenceOTU2843
New.CleanUp.ReferenceOTU163817
New.CleanUp.ReferenceOTU23323
New.CleanUp.ReferenceOTU1786
New.CleanUp.ReferenceOTU73080
New.CleanUp.ReferenceOTU245377
New.ReferenceOTU2154
New.CleanUp.ReferenceOTU210123
New.CleanUp.ReferenceOTU22959
New.CleanUp.ReferenceOTU5115
New.CleanUp.ReferenceOTU87562
New.CleanUp.ReferenceOTU7157
New.CleanUp.ReferenceOTU108745

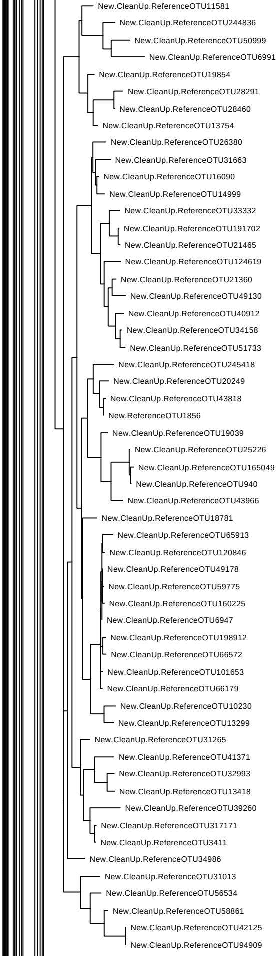
New.CleanUp.ReferenceOTU11581
New.CleanUp.ReferenceOTU244836
New.CleanUp.ReferenceOTU50999
New.CleanUp.ReferenceOTU6991
New.CleanUp.ReferenceOTU19854
New.CleanUp.ReferenceOTU28291
New.CleanUp.ReferenceOTU28460
New.CleanUp.ReferenceOTU13754
New.CleanUp.ReferenceOTU26380
New.CleanUp.ReferenceOTU31663
New.CleanUp.ReferenceOTU16090
New.CleanUp.ReferenceOTU14999
New.CleanUp.ReferenceOTU33332
New.CleanUp.ReferenceOTU191702
New.CleanUp.ReferenceOTU21465
New.CleanUp.ReferenceOTU124619
New.CleanUp.ReferenceOTU21360
New.CleanUp.ReferenceOTU49130
New.CleanUp.ReferenceOTU40912
New.CleanUp.ReferenceOTU34158
New.CleanUp.ReferenceOTU51733
New.CleanUp.ReferenceOTU245418
New.CleanUp.ReferenceOTU20249
New.CleanUp.ReferenceOTU43818
New.ReferenceOTU1856
New.CleanUp.ReferenceOTU19039
New.CleanUp.ReferenceOTU25226
New.CleanUp.ReferenceOTU165049
New.CleanUp.ReferenceOTU940
New.CleanUp.ReferenceOTU43966
New.CleanUp.ReferenceOTU18781
New.CleanUp.ReferenceOTU65913
New.CleanUp.ReferenceOTU120846
New.CleanUp.ReferenceOTU49178
New.CleanUp.ReferenceOTU59775
New.CleanUp.ReferenceOTU160225
New.CleanUp.ReferenceOTU6947
New.CleanUp.ReferenceOTU198912
New.CleanUp.ReferenceOTU66572
New.CleanUp.ReferenceOTU101653
New.CleanUp.ReferenceOTU66179
New.CleanUp.ReferenceOTU10230
New.CleanUp.ReferenceOTU13299
New.CleanUp.ReferenceOTU31265
New.CleanUp.ReferenceOTU41371
New.CleanUp.ReferenceOTU32993
New.CleanUp.ReferenceOTU13418
New.CleanUp.ReferenceOTU39260
New.CleanUp.ReferenceOTU317171
New.CleanUp.ReferenceOTU3411
New.CleanUp.ReferenceOTU34986
New.CleanUp.ReferenceOTU31013
New.CleanUp.ReferenceOTU56534
New.CleanUp.ReferenceOTU58861
New.CleanUp.ReferenceOTU42125
New.CleanUp.ReferenceOTU94909

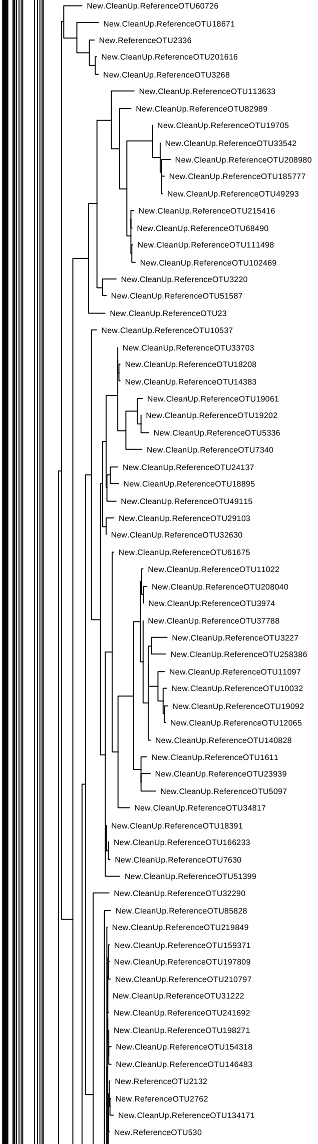
New.CleanUp.ReferenceOTU60726
New.CleanUp.ReferenceOTU18671
New.ReferenceOTU2336
New.CleanUp.ReferenceOTU201616
New.CleanUp.ReferenceOTU3268
New.CleanUp.ReferenceOTU113633
New.CleanUp.ReferenceOTU82989
New.CleanUp.ReferenceOTU19705
New.CleanUp.ReferenceOTU33542
New.CleanUp.ReferenceOTU208980
New.CleanUp.ReferenceOTU185777
New.CleanUp.ReferenceOTU49293
New.CleanUp.ReferenceOTU215416
New.CleanUp.ReferenceOTU68490
New.CleanUp.ReferenceOTU111498
New.CleanUp.ReferenceOTU102469
New.CleanUp.ReferenceOTU3220
New.CleanUp.ReferenceOTU51587
New.CleanUp.ReferenceOTU23
New.CleanUp.ReferenceOTU10537
New.CleanUp.ReferenceOTU33703
New.CleanUp.ReferenceOTU18208
New.CleanUp.ReferenceOTU14383
New.CleanUp.ReferenceOTU19061
New.CleanUp.ReferenceOTU19202
New.CleanUp.ReferenceOTU5336
New.CleanUp.ReferenceOTU7340
New.CleanUp.ReferenceOTU24137
New.CleanUp.ReferenceOTU18895
New.CleanUp.ReferenceOTU49115
New.CleanUp.ReferenceOTU29103
New.CleanUp.ReferenceOTU32630
New.CleanUp.ReferenceOTU61675
New.CleanUp.ReferenceOTU11022
New.CleanUp.ReferenceOTU208040
New.CleanUp.ReferenceOTU3974
New.CleanUp.ReferenceOTU37788
New.CleanUp.ReferenceOTU3227
New.CleanUp.ReferenceOTU258386
New.CleanUp.ReferenceOTU11097
New.CleanUp.ReferenceOTU10032
New.CleanUp.ReferenceOTU19092
New.CleanUp.ReferenceOTU12065
New.CleanUp.ReferenceOTU140828
New.CleanUp.ReferenceOTU1611
New.CleanUp.ReferenceOTU23939
New.CleanUp.ReferenceOTU5097
New.CleanUp.ReferenceOTU34817
New.CleanUp.ReferenceOTU18391
New.CleanUp.ReferenceOTU166233
New.CleanUp.ReferenceOTU7630
New.CleanUp.ReferenceOTU51399
New.CleanUp.ReferenceOTU32290
New.CleanUp.ReferenceOTU85828
New.CleanUp.ReferenceOTU219849
New.CleanUp.ReferenceOTU159371
New.CleanUp.ReferenceOTU197809
New.CleanUp.ReferenceOTU210797
New.CleanUp.ReferenceOTU31222
New.CleanUp.ReferenceOTU241692
New.CleanUp.ReferenceOTU198271
New.CleanUp.ReferenceOTU154318
New.CleanUp.ReferenceOTU146483
New.ReferenceOTU2132
New.ReferenceOTU2762
New.CleanUp.ReferenceOTU134171
New.ReferenceOTU530

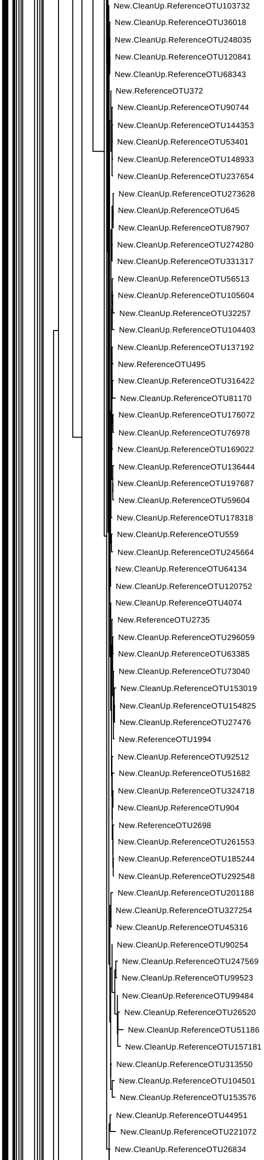
New.CleanUp.ReferenceOTU103732
New.CleanUp.ReferenceOTU36018
New.CleanUp.ReferenceOTU248035
New.CleanUp.ReferenceOTU120841
New.CleanUp.ReferenceOTU68343
New.ReferenceOTU372
New.CleanUp.ReferenceOTU90744
New.CleanUp.ReferenceOTU144353
New.CleanUp.ReferenceOTU53401
New.CleanUp.ReferenceOTU148933
New.CleanUp.ReferenceOTU237654
New.CleanUp.ReferenceOTU273628
New.CleanUp.ReferenceOTU645
New.CleanUp.ReferenceOTU87907
New.CleanUp.ReferenceOTU274280
New.CleanUp.ReferenceOTU331317
New.CleanUp.ReferenceOTU56513
New.CleanUp.ReferenceOTU105604
New.CleanUp.ReferenceOTU32257
New.CleanUp.ReferenceOTU104403
New.CleanUp.ReferenceOTU137192
New.ReferenceOTU495
New.CleanUp.ReferenceOTU316422
New.CleanUp.ReferenceOTU81170
New.CleanUp.ReferenceOTU176072
New.CleanUp.ReferenceOTU76978
New.CleanUp.ReferenceOTU169022
New.CleanUp.ReferenceOTU136444
New.CleanUp.ReferenceOTU197687
New.CleanUp.ReferenceOTU59604
New.CleanUp.ReferenceOTU178318
New.CleanUp.ReferenceOTU559
New.CleanUp.ReferenceOTU245664
New.CleanUp.ReferenceOTU64134
New.CleanUp.ReferenceOTU120752
New.CleanUp.ReferenceOTU4074
New.ReferenceOTU2735
New.CleanUp.ReferenceOTU296059
New.CleanUp.ReferenceOTU63385
New.CleanUp.ReferenceOTU73040
New.CleanUp.ReferenceOTU153019
New.CleanUp.ReferenceOTU154825
New.CleanUp.ReferenceOTU27476
New.ReferenceOTU1994
New.CleanUp.ReferenceOTU92512
New.CleanUp.ReferenceOTU51682
New.CleanUp.ReferenceOTU324718
New.CleanUp.ReferenceOTU904
New.ReferenceOTU2698
New.CleanUp.ReferenceOTU261553
New.CleanUp.ReferenceOTU185244
New.CleanUp.ReferenceOTU292548
New.CleanUp.ReferenceOTU201188
New.CleanUp.ReferenceOTU327254
New.CleanUp.ReferenceOTU45316
New.CleanUp.ReferenceOTU90254
New.CleanUp.ReferenceOTU247569
New.CleanUp.ReferenceOTU99523
New.CleanUp.ReferenceOTU99484
New.CleanUp.ReferenceOTU26520
New.CleanUp.ReferenceOTU51186
New.CleanUp.ReferenceOTU157181
New.CleanUp.ReferenceOTU313550
New.CleanUp.ReferenceOTU104501
New.CleanUp.ReferenceOTU153576
New.CleanUp.ReferenceOTU44951
New.CleanUp.ReferenceOTU221072
New.CleanUp.ReferenceOTU26834

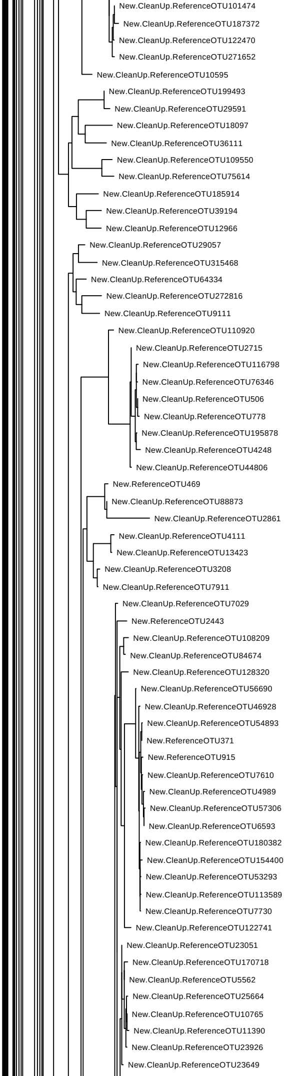
New.CleanUp.ReferenceOTU101474
New.CleanUp.ReferenceOTU187372
New.CleanUp.ReferenceOTU122470
New.CleanUp.ReferenceOTU271652
New.CleanUp.ReferenceOTU10595
New.CleanUp.ReferenceOTU199493
New.CleanUp.ReferenceOTU29591
New.CleanUp.ReferenceOTU18097
New.CleanUp.ReferenceOTU36111
New.CleanUp.ReferenceOTU109550
New.CleanUp.ReferenceOTU75614
New.CleanUp.ReferenceOTU185914
New.CleanUp.ReferenceOTU39194
New.CleanUp.ReferenceOTU12966
New.CleanUp.ReferenceOTU29057
New.CleanUp.ReferenceOTU315468
New.CleanUp.ReferenceOTU64334
New.CleanUp.ReferenceOTU272816
New.CleanUp.ReferenceOTU9111
New.CleanUp.ReferenceOTU110920
New.CleanUp.ReferenceOTU2715
New.CleanUp.ReferenceOTU116798
New.CleanUp.ReferenceOTU76346
New.CleanUp.ReferenceOTU506
New.CleanUp.ReferenceOTU778
New.CleanUp.ReferenceOTU195878
New.CleanUp.ReferenceOTU4248
New.CleanUp.ReferenceOTU44806
New.ReferenceOTU469
New.CleanUp.ReferenceOTU88873
New.CleanUp.ReferenceOTU2861
New.CleanUp.ReferenceOTU4111
New.CleanUp.ReferenceOTU13423
New.CleanUp.ReferenceOTU3208
New.CleanUp.ReferenceOTU7911
New.CleanUp.ReferenceOTU7029
New.ReferenceOTU2443
New.CleanUp.ReferenceOTU108209
New.CleanUp.ReferenceOTU84674
New.CleanUp.ReferenceOTU128320
New.CleanUp.ReferenceOTU56690
New.CleanUp.ReferenceOTU46928
New.CleanUp.ReferenceOTU54893
New.ReferenceOTU371
New.ReferenceOTU915
New.CleanUp.ReferenceOTU7610
New.CleanUp.ReferenceOTU4989
New.CleanUp.ReferenceOTU57306
New.CleanUp.ReferenceOTU6593
New.CleanUp.ReferenceOTU180382
New.CleanUp.ReferenceOTU154400
New.CleanUp.ReferenceOTU53293
New.CleanUp.ReferenceOTU113589
New.CleanUp.ReferenceOTU7730
New.CleanUp.ReferenceOTU122741
New.CleanUp.ReferenceOTU23051
New.CleanUp.ReferenceOTU170718
New.CleanUp.ReferenceOTU5562
New.CleanUp.ReferenceOTU25664
New.CleanUp.ReferenceOTU10765
New.CleanUp.ReferenceOTU11390
New.CleanUp.ReferenceOTU23926
New.CleanUp.ReferenceOTU23649

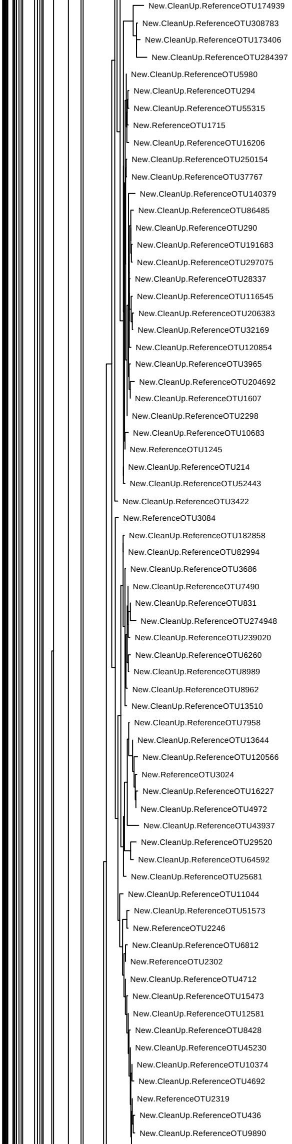
New.CleanUp.ReferenceOTU174939
New.CleanUp.ReferenceOTU308783
New.CleanUp.ReferenceOTU173406
New.CleanUp.ReferenceOTU284397
New.CleanUp.ReferenceOTU5980
New.CleanUp.ReferenceOTU294
New.CleanUp.ReferenceOTU55315
New.ReferenceOTU1715
New.CleanUp.ReferenceOTU16206
New.CleanUp.ReferenceOTU250154
New.CleanUp.ReferenceOTU37767
New.CleanUp.ReferenceOTU140379
New.CleanUp.ReferenceOTU86485
New.CleanUp.ReferenceOTU290
New.CleanUp.ReferenceOTU191683
New.CleanUp.ReferenceOTU297075
New.CleanUp.ReferenceOTU28337
New.CleanUp.ReferenceOTU116545
New.CleanUp.ReferenceOTU206383
New.CleanUp.ReferenceOTU32169
New.CleanUp.ReferenceOTU120854
New.CleanUp.ReferenceOTU3965
New.CleanUp.ReferenceOTU204692
New.CleanUp.ReferenceOTU1607
New.CleanUp.ReferenceOTU2298
New.CleanUp.ReferenceOTU10683
New.ReferenceOTU1245
New.CleanUp.ReferenceOTU214
New.CleanUp.ReferenceOTU52443
New.CleanUp.ReferenceOTU3422
New.ReferenceOTU3084
New.CleanUp.ReferenceOTU182858
New.CleanUp.ReferenceOTU82994
New.CleanUp.ReferenceOTU3686
New.CleanUp.ReferenceOTU7490
New.CleanUp.ReferenceOTU831
New.CleanUp.ReferenceOTU274948
New.CleanUp.ReferenceOTU239020
New.CleanUp.ReferenceOTU6260
New.CleanUp.ReferenceOTU8989
New.CleanUp.ReferenceOTU8962
New.CleanUp.ReferenceOTU13510
New.CleanUp.ReferenceOTU7958
New.CleanUp.ReferenceOTU13644
New.CleanUp.ReferenceOTU120566
New.ReferenceOTU3024
New.CleanUp.ReferenceOTU16227
New.CleanUp.ReferenceOTU4972
New.CleanUp.ReferenceOTU43937
New.CleanUp.ReferenceOTU29520
New.CleanUp.ReferenceOTU64592
New.CleanUp.ReferenceOTU25681
New.CleanUp.ReferenceOTU11044
New.CleanUp.ReferenceOTU51573
New.ReferenceOTU2246
New.CleanUp.ReferenceOTU6812
New.ReferenceOTU2302
New.CleanUp.ReferenceOTU4712
New.CleanUp.ReferenceOTU15473
New.CleanUp.ReferenceOTU12581
New.CleanUp.ReferenceOTU8428
New.CleanUp.ReferenceOTU45230
New.CleanUp.ReferenceOTU10374
New.CleanUp.ReferenceOTU4692
New.ReferenceOTU2319
New.CleanUp.ReferenceOTU436
New.CleanUp.ReferenceOTU9890

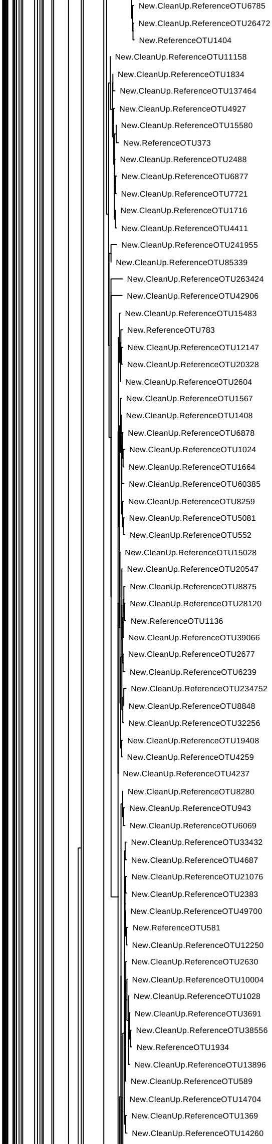
New.CleanUp.ReferenceOTU6785
New.CleanUp.ReferenceOTU26472
New.ReferenceOTU1404
New.CleanUp.ReferenceOTU11158
New.CleanUp.ReferenceOTU1834
New.CleanUp.ReferenceOTU137464
New.CleanUp.ReferenceOTU4927
New.CleanUp.ReferenceOTU15580
New.ReferenceOTU373
New.CleanUp.ReferenceOTU2488
New.CleanUp.ReferenceOTU6877
New.CleanUp.ReferenceOTU7721
New.CleanUp.ReferenceOTU1716
New.CleanUp.ReferenceOTU4411
New.CleanUp.ReferenceOTU241955
New.CleanUp.ReferenceOTU85339
New.CleanUp.ReferenceOTU263424
New.CleanUp.ReferenceOTU42906
New.CleanUp.ReferenceOTU15483
New.ReferenceOTU783
New.CleanUp.ReferenceOTU12147
New.CleanUp.ReferenceOTU20328
New.CleanUp.ReferenceOTU2604
New.CleanUp.ReferenceOTU1567
New.CleanUp.ReferenceOTU1408
New.CleanUp.ReferenceOTU6878
New.CleanUp.ReferenceOTU1024
New.CleanUp.ReferenceOTU1664
New.CleanUp.ReferenceOTU60385
New.CleanUp.ReferenceOTU8259
New.CleanUp.ReferenceOTU5081
New.CleanUp.ReferenceOTU552
New.CleanUp.ReferenceOTU15028
New.CleanUp.ReferenceOTU20547
New.CleanUp.ReferenceOTU8875
New.CleanUp.ReferenceOTU28120
New.ReferenceOTU1136
New.CleanUp.ReferenceOTU39066
New.CleanUp.ReferenceOTU2677
New.CleanUp.ReferenceOTU6239
New.CleanUp.ReferenceOTU234752
New.CleanUp.ReferenceOTU8848
New.CleanUp.ReferenceOTU32256
New.CleanUp.ReferenceOTU19408
New.CleanUp.ReferenceOTU4259
New.CleanUp.ReferenceOTU4237
New.CleanUp.ReferenceOTU8280
New.CleanUp.ReferenceOTU943
New.CleanUp.ReferenceOTU6069
New.CleanUp.ReferenceOTU33432
New.CleanUp.ReferenceOTU4687
New.CleanUp.ReferenceOTU21076
New.CleanUp.ReferenceOTU2383
New.CleanUp.ReferenceOTU49700
New.ReferenceOTU581
New.CleanUp.ReferenceOTU12250
New.CleanUp.ReferenceOTU2630
New.CleanUp.ReferenceOTU10004
New.CleanUp.ReferenceOTU1028
New.CleanUp.ReferenceOTU3691
New.CleanUp.ReferenceOTU38556
New.ReferenceOTU1934
New.CleanUp.ReferenceOTU13896
New.CleanUp.ReferenceOTU589
New.CleanUp.ReferenceOTU14704
New.CleanUp.ReferenceOTU1369
New.CleanUp.ReferenceOTU14260

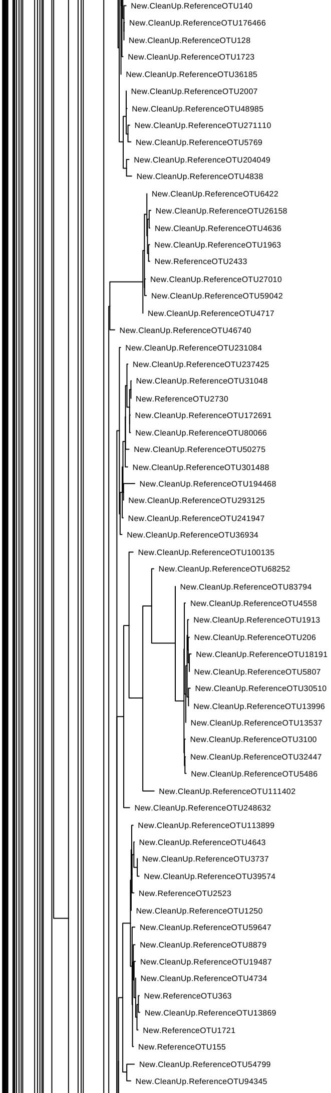
New.CleanUp.ReferenceOTU140
New.CleanUp.ReferenceOTU176466
New.CleanUp.ReferenceOTU128
New.CleanUp.ReferenceOTU1723
New.CleanUp.ReferenceOTU36185
New.CleanUp.ReferenceOTU2007
New.CleanUp.ReferenceOTU48985
New.CleanUp.ReferenceOTU271110
New.CleanUp.ReferenceOTU5769
New.CleanUp.ReferenceOTU204049
New.CleanUp.ReferenceOTU4838
New.CleanUp.ReferenceOTU6422
New.CleanUp.ReferenceOTU26158
New.CleanUp.ReferenceOTU4636
New.CleanUp.ReferenceOTU1963
New.ReferenceOTU2433
New.CleanUp.ReferenceOTU27010
New.CleanUp.ReferenceOTU59042
New.CleanUp.ReferenceOTU4717
New.CleanUp.ReferenceOTU46740
New.CleanUp.ReferenceOTU231084
New.CleanUp.ReferenceOTU237425
New.CleanUp.ReferenceOTU31048
New.ReferenceOTU2730
New.CleanUp.ReferenceOTU172691
New.CleanUp.ReferenceOTU80066
New.CleanUp.ReferenceOTU50275
New.CleanUp.ReferenceOTU301488
New.CleanUp.ReferenceOTU194468
New.CleanUp.ReferenceOTU293125
New.CleanUp.ReferenceOTU241947
New.CleanUp.ReferenceOTU36934
New.CleanUp.ReferenceOTU100135
New.CleanUp.ReferenceOTU68252
New.CleanUp.ReferenceOTU83794
New.CleanUp.ReferenceOTU4558
New.CleanUp.ReferenceOTU1913
New.CleanUp.ReferenceOTU206
New.CleanUp.ReferenceOTU18191
New.CleanUp.ReferenceOTU5807
New.CleanUp.ReferenceOTU30510
New.CleanUp.ReferenceOTU13996
New.CleanUp.ReferenceOTU13537
New.CleanUp.ReferenceOTU3100
New.CleanUp.ReferenceOTU32447
New.CleanUp.ReferenceOTU5486
New.CleanUp.ReferenceOTU111402
New.CleanUp.ReferenceOTU248632
New.CleanUp.ReferenceOTU113899
New.CleanUp.ReferenceOTU4643
New.CleanUp.ReferenceOTU3737
New.CleanUp.ReferenceOTU39574
New.ReferenceOTU2523
New.CleanUp.ReferenceOTU1250
New.CleanUp.ReferenceOTU59647
New.CleanUp.ReferenceOTU8879
New.CleanUp.ReferenceOTU19487
New.CleanUp.ReferenceOTU4734
New.ReferenceOTU363
New.CleanUp.ReferenceOTU13869
New.ReferenceOTU1721
New.ReferenceOTU155
New.CleanUp.ReferenceOTU54799
New.CleanUp.ReferenceOTU94345

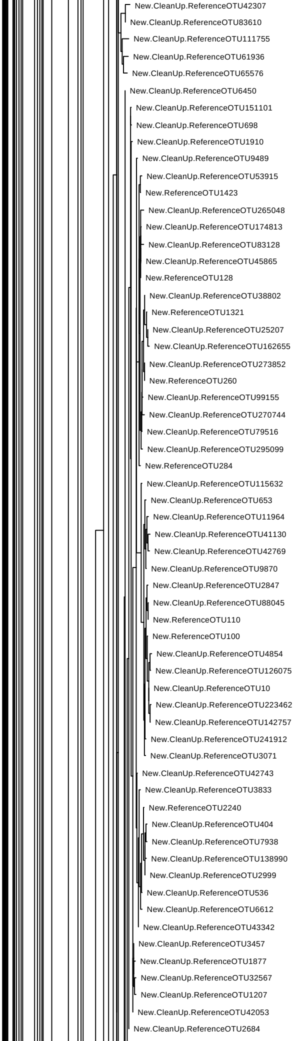
New.CleanUp.ReferenceOTU42307
New.CleanUp.ReferenceOTU83610
New.CleanUp.ReferenceOTU111755
New.CleanUp.ReferenceOTU61936
New.CleanUp.ReferenceOTU65576
New.CleanUp.ReferenceOTU6450
New.CleanUp.ReferenceOTU151101
New.CleanUp.ReferenceOTU698
New.CleanUp.ReferenceOTU1910
New.CleanUp.ReferenceOTU9489
New.CleanUp.ReferenceOTU53915
New.ReferenceOTU1423
New.CleanUp.ReferenceOTU265048
New.CleanUp.ReferenceOTU174813
New.CleanUp.ReferenceOTU83128
New.CleanUp.ReferenceOTU45865
New.ReferenceOTU128
New.CleanUp.ReferenceOTU38802
New.ReferenceOTU1321
New.CleanUp.ReferenceOTU25207
New.CleanUp.ReferenceOTU162655
New.CleanUp.ReferenceOTU273852
New.ReferenceOTU260
New.CleanUp.ReferenceOTU99155
New.CleanUp.ReferenceOTU270744
New.CleanUp.ReferenceOTU79516
New.CleanUp.ReferenceOTU295099
New.ReferenceOTU284
New.CleanUp.ReferenceOTU115632
New.CleanUp.ReferenceOTU653
New.CleanUp.ReferenceOTU11964
New.CleanUp.ReferenceOTU41130
New.CleanUp.ReferenceOTU42769
New.CleanUp.ReferenceOTU9870
New.CleanUp.ReferenceOTU2847
New.CleanUp.ReferenceOTU88045
New.ReferenceOTU110
New.ReferenceOTU100
New.CleanUp.ReferenceOTU4854
New.CleanUp.ReferenceOTU126075
New.CleanUp.ReferenceOTU10
New.CleanUp.ReferenceOTU223462
New.CleanUp.ReferenceOTU142757
New.CleanUp.ReferenceOTU241912
New.CleanUp.ReferenceOTU3071
New.CleanUp.ReferenceOTU42743
New.CleanUp.ReferenceOTU3833
New.ReferenceOTU2240
New.CleanUp.ReferenceOTU404
New.CleanUp.ReferenceOTU7938
New.CleanUp.ReferenceOTU138990
New.CleanUp.ReferenceOTU2999
New.CleanUp.ReferenceOTU536
New.CleanUp.ReferenceOTU6612
New.CleanUp.ReferenceOTU43342
New.CleanUp.ReferenceOTU3457
New.CleanUp.ReferenceOTU1877
New.CleanUp.ReferenceOTU32567
New.CleanUp.ReferenceOTU1207
New.CleanUp.ReferenceOTU42053
New.CleanUp.ReferenceOTU2684

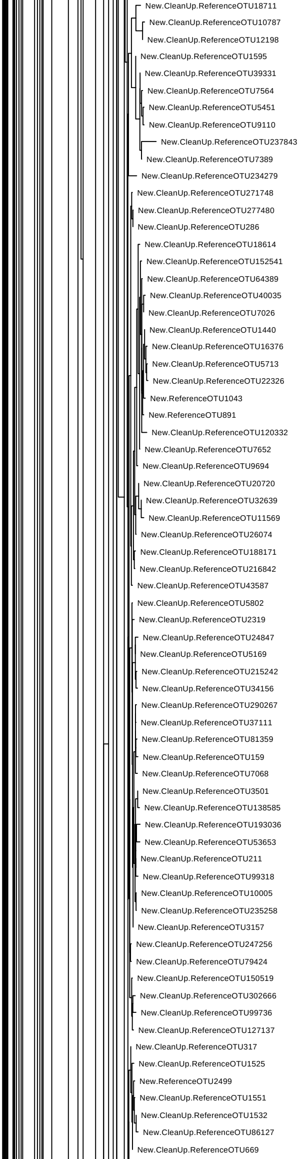

New.CleanUp.ReferenceOTU18711
New.CleanUp.ReferenceOTU10787
New.CleanUp.ReferenceOTU12198
New.CleanUp.ReferenceOTU1595
New.CleanUp.ReferenceOTU39331
New.CleanUp.ReferenceOTU7564
New.CleanUp.ReferenceOTU5451
New.CleanUp.ReferenceOTU9110
New.CleanUp.ReferenceOTU237843
New.CleanUp.ReferenceOTU7389
New.CleanUp.ReferenceOTU234279
New.CleanUp.ReferenceOTU271748
New.CleanUp.ReferenceOTU277480
New.CleanUp.ReferenceOTU286
New.CleanUp.ReferenceOTU18614
New.CleanUp.ReferenceOTU152541
New.CleanUp.ReferenceOTU64389
New.CleanUp.ReferenceOTU40035
New.CleanUp.ReferenceOTU7026
New.CleanUp.ReferenceOTU1440
New.CleanUp.ReferenceOTU16376
New.CleanUp.ReferenceOTU5713
New.CleanUp.ReferenceOTU22326
New.ReferenceOTU1043
New.ReferenceOTU891
New.CleanUp.ReferenceOTU120332
New.CleanUp.ReferenceOTU7652
New.CleanUp.ReferenceOTU9694
New.CleanUp.ReferenceOTU20720
New.CleanUp.ReferenceOTU32639
New.CleanUp.ReferenceOTU11569
New.CleanUp.ReferenceOTU26074
New.CleanUp.ReferenceOTU188171
New.CleanUp.ReferenceOTU216842
New.CleanUp.ReferenceOTU43587
New.CleanUp.ReferenceOTU5802
New.CleanUp.ReferenceOTU2319
New.CleanUp.ReferenceOTU24847
New.CleanUp.ReferenceOTU5169
New.CleanUp.ReferenceOTU215242
New.CleanUp.ReferenceOTU34156
New.CleanUp.ReferenceOTU290267
New.CleanUp.ReferenceOTU37111
New.CleanUp.ReferenceOTU81359
New.CleanUp.ReferenceOTU159
New.CleanUp.ReferenceOTU7068
New.CleanUp.ReferenceOTU3501
New.CleanUp.ReferenceOTU138585
New.CleanUp.ReferenceOTU193036
New.CleanUp.ReferenceOTU53653
New.CleanUp.ReferenceOTU211
New.CleanUp.ReferenceOTU99318
New.CleanUp.ReferenceOTU10005
New.CleanUp.ReferenceOTU235258
New.CleanUp.ReferenceOTU3157
New.CleanUp.ReferenceOTU247256
New.CleanUp.ReferenceOTU79424
New.CleanUp.ReferenceOTU150519
New.CleanUp.ReferenceOTU302666
New.CleanUp.ReferenceOTU99736
New.CleanUp.ReferenceOTU127137
New.CleanUp.ReferenceOTU317
New.CleanUp.ReferenceOTU1525
New.ReferenceOTU2499
New.CleanUp.ReferenceOTU1551
New.CleanUp.ReferenceOTU1532
New.CleanUp.ReferenceOTU86127
New.CleanUp.ReferenceOTU669

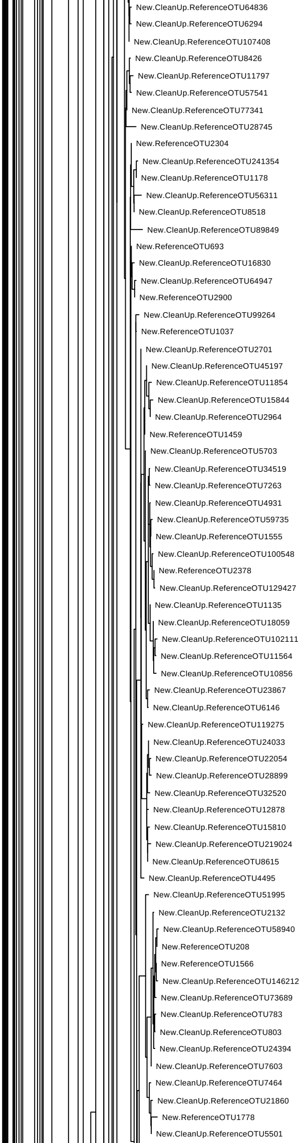
New.CleanUp.ReferenceOTU64836
New.CleanUp.ReferenceOTU6294
New.CleanUp.ReferenceOTU107408
New.CleanUp.ReferenceOTU8426
New.CleanUp.ReferenceOTU11797
New.CleanUp.ReferenceOTU57541
New.CleanUp.ReferenceOTU77341
New.CleanUp.ReferenceOTU28745
New.ReferenceOTU2304
New.CleanUp.ReferenceOTU241354
New.CleanUp.ReferenceOTU1178
New.CleanUp.ReferenceOTU56311
New.CleanUp.ReferenceOTU8518
New.CleanUp.ReferenceOTU89849
New.ReferenceOTU693
New.CleanUp.ReferenceOTU16830
New.CleanUp.ReferenceOTU64947
New.ReferenceOTU2900
New.CleanUp.ReferenceOTU99264
New.ReferenceOTU1037
New.CleanUp.ReferenceOTU2701
New.CleanUp.ReferenceOTU45197
New.CleanUp.ReferenceOTU11854
New.CleanUp.ReferenceOTU15844
New.CleanUp.ReferenceOTU2964
New.ReferenceOTU1459
New.CleanUp.ReferenceOTU5703
New.CleanUp.ReferenceOTU34519
New.CleanUp.ReferenceOTU7263
New.CleanUp.ReferenceOTU4931
New.CleanUp.ReferenceOTU59735
New.CleanUp.ReferenceOTU1555
New.CleanUp.ReferenceOTU100548
New.ReferenceOTU2378
New.CleanUp.ReferenceOTU129427
New.CleanUp.ReferenceOTU1135
New.CleanUp.ReferenceOTU18059
New.CleanUp.ReferenceOTU102111
New.CleanUp.ReferenceOTU11564
New.CleanUp.ReferenceOTU10856
New.CleanUp.ReferenceOTU23867
New.CleanUp.ReferenceOTU6146
New.CleanUp.ReferenceOTU119275
New.CleanUp.ReferenceOTU24033
New.CleanUp.ReferenceOTU22054
New.CleanUp.ReferenceOTU28899
New.CleanUp.ReferenceOTU32520
New.CleanUp.ReferenceOTU12878
New.CleanUp.ReferenceOTU15810
New.CleanUp.ReferenceOTU219024
New.CleanUp.ReferenceOTU8615
New.CleanUp.ReferenceOTU4495
New.CleanUp.ReferenceOTU51995
New.CleanUp.ReferenceOTU2132
New.CleanUp.ReferenceOTU58940
New.ReferenceOTU208
New.ReferenceOTU1566
New.CleanUp.ReferenceOTU146212
New.CleanUp.ReferenceOTU73689
New.CleanUp.ReferenceOTU783
New.CleanUp.ReferenceOTU803
New.CleanUp.ReferenceOTU24394
New.CleanUp.ReferenceOTU7603
New.CleanUp.ReferenceOTU7464
New.CleanUp.ReferenceOTU21860
New.ReferenceOTU1778
New.CleanUp.ReferenceOTU5501

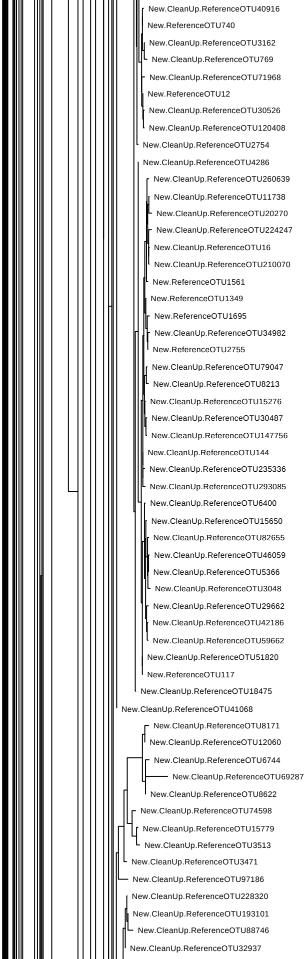
New.CleanUp.ReferenceOTU40916
New.ReferenceOTU740
New.CleanUp.ReferenceOTU3162
New.CleanUp.ReferenceOTU769
New.CleanUp.ReferenceOTU71968
New.ReferenceOTU12
New.CleanUp.ReferenceOTU30526
New.CleanUp.ReferenceOTU120408
New.CleanUp.ReferenceOTU2754
New.CleanUp.ReferenceOTU4286
New.CleanUp.ReferenceOTU260639
New.CleanUp.ReferenceOTU11738
New.CleanUp.ReferenceOTU20270
New.CleanUp.ReferenceOTU224247
New.CleanUp.ReferenceOTU16
New.CleanUp.ReferenceOTU210070
New.ReferenceOTU1561
New.ReferenceOTU1349
New.ReferenceOTU1695
New.CleanUp.ReferenceOTU34982
New.ReferenceOTU2755
New.CleanUp.ReferenceOTU79047
New.CleanUp.ReferenceOTU8213
New.CleanUp.ReferenceOTU15276
New.CleanUp.ReferenceOTU30487
New.CleanUp.ReferenceOTU147756
New.CleanUp.ReferenceOTU144
New.CleanUp.ReferenceOTU235336
New.CleanUp.ReferenceOTU293085
New.CleanUp.ReferenceOTU6400
New.CleanUp.ReferenceOTU15650
New.CleanUp.ReferenceOTU82655
New.CleanUp.ReferenceOTU46059
New.CleanUp.ReferenceOTU5366
New.CleanUp.ReferenceOTU3048
New.CleanUp.ReferenceOTU29662
New.CleanUp.ReferenceOTU42186
New.CleanUp.ReferenceOTU59662
New.CleanUp.ReferenceOTU51820
New.ReferenceOTU117
New.CleanUp.ReferenceOTU18475
New.CleanUp.ReferenceOTU41068
New.CleanUp.ReferenceOTU8171
New.CleanUp.ReferenceOTU12060
New.CleanUp.ReferenceOTU6744
New.CleanUp.ReferenceOTU69287
New.CleanUp.ReferenceOTU8622
New.CleanUp.ReferenceOTU74598
New.CleanUp.ReferenceOTU15779
New.CleanUp.ReferenceOTU3513
New.CleanUp.ReferenceOTU3471
New.CleanUp.ReferenceOTU97186
New.CleanUp.ReferenceOTU228320
New.CleanUp.ReferenceOTU193101
New.CleanUp.ReferenceOTU88746
New.CleanUp.ReferenceOTU32937

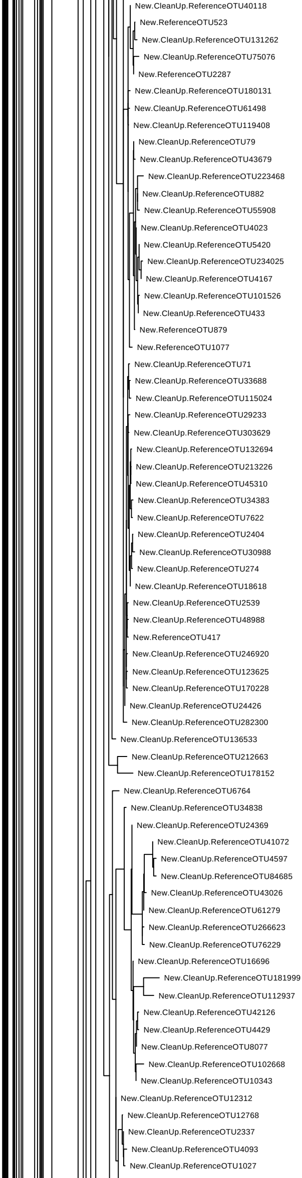
New.CleanUp.ReferenceOTU40118
New.ReferenceOTU523
New.CleanUp.ReferenceOTU131262
New.CleanUp.ReferenceOTU75076
New.ReferenceOTU2287
New.CleanUp.ReferenceOTU180131
New.CleanUp.ReferenceOTU61498
New.CleanUp.ReferenceOTU119408
New.CleanUp.ReferenceOTU79
New.CleanUp.ReferenceOTU43679
New.CleanUp.ReferenceOTU223468
New.CleanUp.ReferenceOTU882
New.CleanUp.ReferenceOTU55908
New.CleanUp.ReferenceOTU4023
New.CleanUp.ReferenceOTU5420
New.CleanUp.ReferenceOTU234025
New.CleanUp.ReferenceOTU4167
New.CleanUp.ReferenceOTU101526
New.CleanUp.ReferenceOTU433
New.ReferenceOTU879
New.ReferenceOTU1077
New.CleanUp.ReferenceOTU71
New.CleanUp.ReferenceOTU33688
New.CleanUp.ReferenceOTU115024
New.CleanUp.ReferenceOTU29233
New.CleanUp.ReferenceOTU303629
New.CleanUp.ReferenceOTU132694
New.CleanUp.ReferenceOTU213226
New.CleanUp.ReferenceOTU45310
New.CleanUp.ReferenceOTU34383
New.CleanUp.ReferenceOTU7622
New.CleanUp.ReferenceOTU2404
New.CleanUp.ReferenceOTU30988
New.CleanUp.ReferenceOTU274
New.CleanUp.ReferenceOTU18618
New.CleanUp.ReferenceOTU2539
New.CleanUp.ReferenceOTU48988
New.ReferenceOTU417
New.CleanUp.ReferenceOTU246920
New.CleanUp.ReferenceOTU123625
New.CleanUp.ReferenceOTU170228
New.CleanUp.ReferenceOTU24426
New.CleanUp.ReferenceOTU282300
New.CleanUp.ReferenceOTU136533
New.CleanUp.ReferenceOTU212663
New.CleanUp.ReferenceOTU178152
New.CleanUp.ReferenceOTU6764
New.CleanUp.ReferenceOTU34838
New.CleanUp.ReferenceOTU24369
New.CleanUp.ReferenceOTU41072
New.CleanUp.ReferenceOTU4597
New.CleanUp.ReferenceOTU84685
New.CleanUp.ReferenceOTU43026
New.CleanUp.ReferenceOTU61279
New.CleanUp.ReferenceOTU266623
New.CleanUp.ReferenceOTU76229
New.CleanUp.ReferenceOTU16696
New.CleanUp.ReferenceOTU181999
New.CleanUp.ReferenceOTU112937
New.CleanUp.ReferenceOTU42126
New.CleanUp.ReferenceOTU4429
New.CleanUp.ReferenceOTU8077
New.CleanUp.ReferenceOTU102668
New.CleanUp.ReferenceOTU10343
New.CleanUp.ReferenceOTU12312
New.CleanUp.ReferenceOTU12768
New.CleanUp.ReferenceOTU2337
New.CleanUp.ReferenceOTU4093
New.CleanUp.ReferenceOTU1027

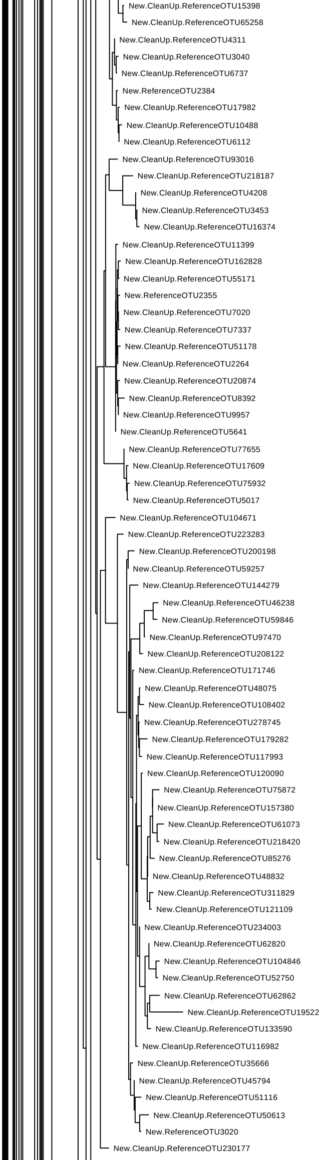
New.CleanUp.ReferenceOTU15398
New.CleanUp.ReferenceOTU65258
New.CleanUp.ReferenceOTU4311
New.CleanUp.ReferenceOTU3040
New.CleanUp.ReferenceOTU6737
New.ReferenceOTU2384
New.CleanUp.ReferenceOTU17982
New.CleanUp.ReferenceOTU10488
New.CleanUp.ReferenceOTU6112
New.CleanUp.ReferenceOTU93016
New.CleanUp.ReferenceOTU218187
New.CleanUp.ReferenceOTU4208
New.CleanUp.ReferenceOTU3453
New.CleanUp.ReferenceOTU16374
New.CleanUp.ReferenceOTU11399
New.CleanUp.ReferenceOTU162828
New.CleanUp.ReferenceOTU55171
New.ReferenceOTU2355
New.CleanUp.ReferenceOTU7020
New.CleanUp.ReferenceOTU7337
New.CleanUp.ReferenceOTU51178
New.CleanUp.ReferenceOTU2264
New.CleanUp.ReferenceOTU20874
New.CleanUp.ReferenceOTU8392
New.CleanUp.ReferenceOTU9957
New.CleanUp.ReferenceOTU5641
New.CleanUp.ReferenceOTU77655
New.CleanUp.ReferenceOTU17609
New.CleanUp.ReferenceOTU75932
New.CleanUp.ReferenceOTU5017
New.CleanUp.ReferenceOTU104671
New.CleanUp.ReferenceOTU223283
New.CleanUp.ReferenceOTU200198
New.CleanUp.ReferenceOTU59257
New.CleanUp.ReferenceOTU144279
New.CleanUp.ReferenceOTU46238
New.CleanUp.ReferenceOTU59846
New.CleanUp.ReferenceOTU97470
New.CleanUp.ReferenceOTU208122
New.CleanUp.ReferenceOTU171746
New.CleanUp.ReferenceOTU48075
New.CleanUp.ReferenceOTU108402
New.CleanUp.ReferenceOTU278745
New.CleanUp.ReferenceOTU179282
New.CleanUp.ReferenceOTU117993
New.CleanUp.ReferenceOTU120090
New.CleanUp.ReferenceOTU75872
New.CleanUp.ReferenceOTU157380
New.CleanUp.ReferenceOTU61073
New.CleanUp.ReferenceOTU218420
New.CleanUp.ReferenceOTU85276
New.CleanUp.ReferenceOTU48832
New.CleanUp.ReferenceOTU311829
New.CleanUp.ReferenceOTU121109
New.CleanUp.ReferenceOTU234003
New.CleanUp.ReferenceOTU62820
New.CleanUp.ReferenceOTU104846
New.CleanUp.ReferenceOTU52750
New.CleanUp.ReferenceOTU62862
New.CleanUp.ReferenceOTU19522
New.CleanUp.ReferenceOTU133590
New.CleanUp.ReferenceOTU116982
New.CleanUp.ReferenceOTU35666
New.CleanUp.ReferenceOTU45794
New.CleanUp.ReferenceOTU51116
New.CleanUp.ReferenceOTU50613
New.ReferenceOTU3020
New.CleanUp.ReferenceOTU230177

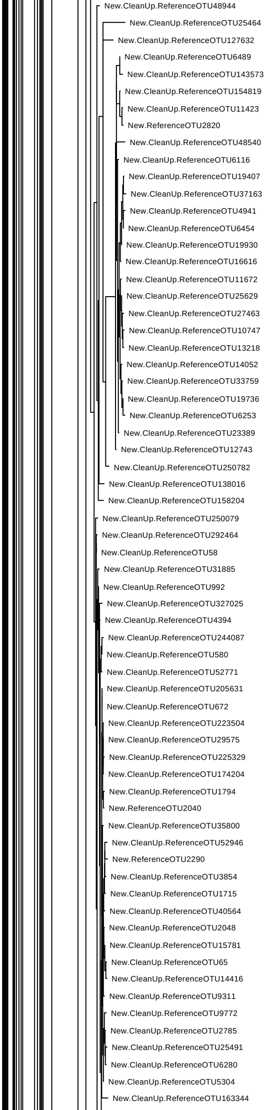
New.CleanUp.ReferenceOTU48944
New.CleanUp.ReferenceOTU25464
New.CleanUp.ReferenceOTU127632
New.CleanUp.ReferenceOTU6489
New.CleanUp.ReferenceOTU143573
New.CleanUp.ReferenceOTU154819
New.CleanUp.ReferenceOTU11423
New.ReferenceOTU2820
New.CleanUp.ReferenceOTU48540
New.CleanUp.ReferenceOTU6116
New.CleanUp.ReferenceOTU19407
New.CleanUp.ReferenceOTU37163
New.CleanUp.ReferenceOTU4941
New.CleanUp.ReferenceOTU6454
New.CleanUp.ReferenceOTU19930
New.CleanUp.ReferenceOTU16616
New.CleanUp.ReferenceOTU11672
New.CleanUp.ReferenceOTU25629
New.CleanUp.ReferenceOTU27463
New.CleanUp.ReferenceOTU10747
New.CleanUp.ReferenceOTU13218
New.CleanUp.ReferenceOTU14052
New.CleanUp.ReferenceOTU33759
New.CleanUp.ReferenceOTU19736
New.CleanUp.ReferenceOTU6253
New.CleanUp.ReferenceOTU23389
New.CleanUp.ReferenceOTU12743
New.CleanUp.ReferenceOTU250782
New.CleanUp.ReferenceOTU138016
New.CleanUp.ReferenceOTU158204
New.CleanUp.ReferenceOTU250079
New.CleanUp.ReferenceOTU292464
New.CleanUp.ReferenceOTU58
New.CleanUp.ReferenceOTU31885
New.CleanUp.ReferenceOTU992
New.CleanUp.ReferenceOTU327025
New.CleanUp.ReferenceOTU4394
New.CleanUp.ReferenceOTU244087
New.CleanUp.ReferenceOTU580
New.CleanUp.ReferenceOTU52771
New.CleanUp.ReferenceOTU205631
New.CleanUp.ReferenceOTU672
New.CleanUp.ReferenceOTU223504
New.CleanUp.ReferenceOTU29575
New.CleanUp.ReferenceOTU225329
New.CleanUp.ReferenceOTU174204
New.CleanUp.ReferenceOTU1794
New.ReferenceOTU2040
New.CleanUp.ReferenceOTU35800
New.CleanUp.ReferenceOTU52946
New.ReferenceOTU2290
New.CleanUp.ReferenceOTU3854
New.CleanUp.ReferenceOTU1715
New.CleanUp.ReferenceOTU40564
New.CleanUp.ReferenceOTU2048
New.CleanUp.ReferenceOTU15781
New.CleanUp.ReferenceOTU65
New.CleanUp.ReferenceOTU14416
New.CleanUp.ReferenceOTU9311
New.CleanUp.ReferenceOTU9772
New.CleanUp.ReferenceOTU2785
New.CleanUp.ReferenceOTU25491
New.CleanUp.ReferenceOTU6280
New.CleanUp.ReferenceOTU5304
New.CleanUp.ReferenceOTU163344

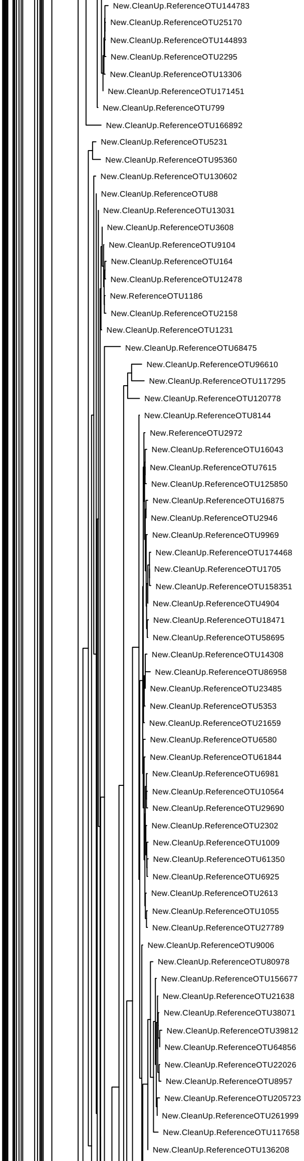
New.CleanUp.ReferenceOTU144783
New.CleanUp.ReferenceOTU25170
New.CleanUp.ReferenceOTU144893
New.CleanUp.ReferenceOTU2295
New.CleanUp.ReferenceOTU13306
New.CleanUp.ReferenceOTU171451
New.CleanUp.ReferenceOTU799
New.CleanUp.ReferenceOTU166892
New.CleanUp.ReferenceOTU5231
New.CleanUp.ReferenceOTU95360
New.CleanUp.ReferenceOTU130602
New.CleanUp.ReferenceOTU88
New.CleanUp.ReferenceOTU13031
New.CleanUp.ReferenceOTU3608
New.CleanUp.ReferenceOTU9104
New.CleanUp.ReferenceOTU164
New.CleanUp.ReferenceOTU12478
New.ReferenceOTU1186
New.CleanUp.ReferenceOTU2158
New.CleanUp.ReferenceOTU1231
New.CleanUp.ReferenceOTU68475
New.CleanUp.ReferenceOTU96610
New.CleanUp.ReferenceOTU117295
New.CleanUp.ReferenceOTU120778
New.CleanUp.ReferenceOTU8144
New.ReferenceOTU2972
New.CleanUp.ReferenceOTU16043
New.CleanUp.ReferenceOTU7615
New.CleanUp.ReferenceOTU125850
New.CleanUp.ReferenceOTU16875
New.CleanUp.ReferenceOTU2946
New.CleanUp.ReferenceOTU9969
New.CleanUp.ReferenceOTU174468
New.CleanUp.ReferenceOTU1705
New.CleanUp.ReferenceOTU158351
New.CleanUp.ReferenceOTU4904
New.CleanUp.ReferenceOTU18471
New.CleanUp.ReferenceOTU58695
New.CleanUp.ReferenceOTU14308
New.CleanUp.ReferenceOTU86958
New.CleanUp.ReferenceOTU23485
New.CleanUp.ReferenceOTU5353
New.CleanUp.ReferenceOTU21659
New.CleanUp.ReferenceOTU6580
New.CleanUp.ReferenceOTU61844
New.CleanUp.ReferenceOTU6981
New.CleanUp.ReferenceOTU10564
New.CleanUp.ReferenceOTU29690
New.CleanUp.ReferenceOTU2302
New.CleanUp.ReferenceOTU1009
New.CleanUp.ReferenceOTU61350
New.CleanUp.ReferenceOTU6925
New.CleanUp.ReferenceOTU2613
New.CleanUp.ReferenceOTU1055
New.CleanUp.ReferenceOTU27789
New.CleanUp.ReferenceOTU9006
New.CleanUp.ReferenceOTU80978
New.CleanUp.ReferenceOTU156677
New.CleanUp.ReferenceOTU21638
New.CleanUp.ReferenceOTU38071
New.CleanUp.ReferenceOTU39812
New.CleanUp.ReferenceOTU64856
New.CleanUp.ReferenceOTU22026
New.CleanUp.ReferenceOTU8957
New.CleanUp.ReferenceOTU205723
New.CleanUp.ReferenceOTU261999
New.CleanUp.ReferenceOTU117658
New.CleanUp.ReferenceOTU136208

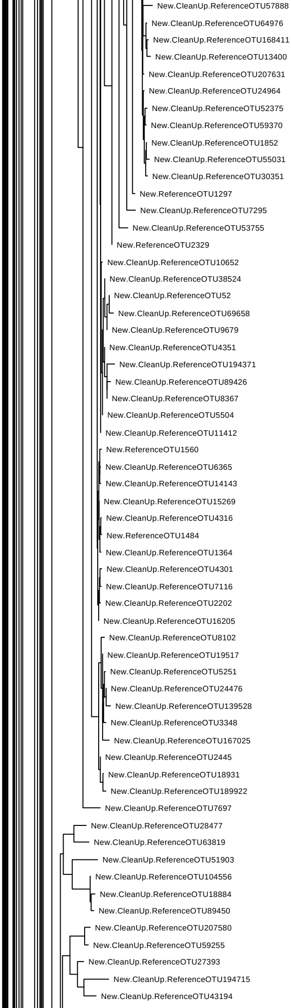
New.CleanUp.ReferenceOTU57888
New.CleanUp.ReferenceOTU64976
New.CleanUp.ReferenceOTU168411
New.CleanUp.ReferenceOTU13400
New.CleanUp.ReferenceOTU207631
New.CleanUp.ReferenceOTU24964
New.CleanUp.ReferenceOTU52375
New.CleanUp.ReferenceOTU59370
New.CleanUp.ReferenceOTU1852
New.CleanUp.ReferenceOTU55031
New.CleanUp.ReferenceOTU30351
New.ReferenceOTU1297
New.CleanUp.ReferenceOTU7295
New.CleanUp.ReferenceOTU53755
New.ReferenceOTU2329
New.CleanUp.ReferenceOTU10652
New.CleanUp.ReferenceOTU38524
New.CleanUp.ReferenceOTU52
New.CleanUp.ReferenceOTU69658
New.CleanUp.ReferenceOTU9679
New.CleanUp.ReferenceOTU4351
New.CleanUp.ReferenceOTU194371
New.CleanUp.ReferenceOTU89426
New.CleanUp.ReferenceOTU8367
New.CleanUp.ReferenceOTU5504
New.CleanUp.ReferenceOTU11412
New.ReferenceOTU1560
New.CleanUp.ReferenceOTU6365
New.CleanUp.ReferenceOTU14143
New.CleanUp.ReferenceOTU15269
New.CleanUp.ReferenceOTU4316
New.ReferenceOTU1484
New.CleanUp.ReferenceOTU1364
New.CleanUp.ReferenceOTU4301
New.CleanUp.ReferenceOTU7116
New.CleanUp.ReferenceOTU2202
New.CleanUp.ReferenceOTU16205
New.CleanUp.ReferenceOTU8102
New.CleanUp.ReferenceOTU19517
New.CleanUp.ReferenceOTU5251
New.CleanUp.ReferenceOTU24476
New.CleanUp.ReferenceOTU139528
New.CleanUp.ReferenceOTU3348
New.CleanUp.ReferenceOTU167025
New.CleanUp.ReferenceOTU2445
New.CleanUp.ReferenceOTU18931
New.CleanUp.ReferenceOTU189922
New.CleanUp.ReferenceOTU7697
New.CleanUp.ReferenceOTU28477
New.CleanUp.ReferenceOTU63819
New.CleanUp.ReferenceOTU51903
New.CleanUp.ReferenceOTU104556
New.CleanUp.ReferenceOTU18884
New.CleanUp.ReferenceOTU89450
New.CleanUp.ReferenceOTU207580
New.CleanUp.ReferenceOTU59255
New.CleanUp.ReferenceOTU27393
New.CleanUp.ReferenceOTU194715
New.CleanUp.ReferenceOTU43194

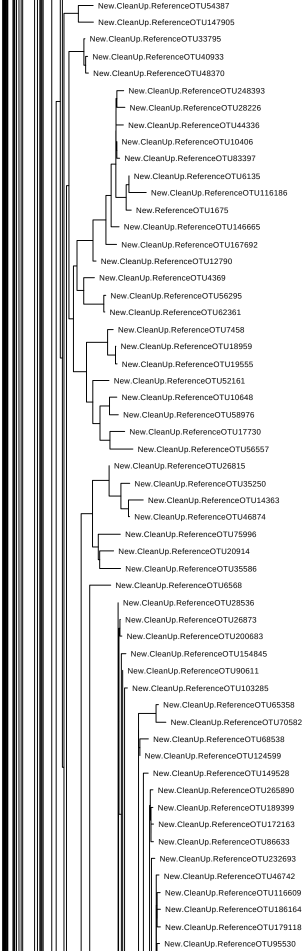
New.CleanUp.ReferenceOTU54387
New.CleanUp.ReferenceOTU147905
New.CleanUp.ReferenceOTU33795
New.CleanUp.ReferenceOTU40933
New.CleanUp.ReferenceOTU48370
New.CleanUp.ReferenceOTU248393
New.CleanUp.ReferenceOTU28226
New.CleanUp.ReferenceOTU44336
New.CleanUp.ReferenceOTU10406
New.CleanUp.ReferenceOTU83397
New.CleanUp.ReferenceOTU6135
New.CleanUp.ReferenceOTU116186
New.ReferenceOTU1675
New.CleanUp.ReferenceOTU146665
New.CleanUp.ReferenceOTU167692
New.CleanUp.ReferenceOTU12790
New.CleanUp.ReferenceOTU4369
New.CleanUp.ReferenceOTU56295
New.CleanUp.ReferenceOTU62361
New.CleanUp.ReferenceOTU7458
New.CleanUp.ReferenceOTU18959
New.CleanUp.ReferenceOTU19555
New.CleanUp.ReferenceOTU52161
New.CleanUp.ReferenceOTU10648
New.CleanUp.ReferenceOTU58976
New.CleanUp.ReferenceOTU17730
New.CleanUp.ReferenceOTU56557
New.CleanUp.ReferenceOTU26815
New.CleanUp.ReferenceOTU35250
New.CleanUp.ReferenceOTU14363
New.CleanUp.ReferenceOTU46874
New.CleanUp.ReferenceOTU75996
New.CleanUp.ReferenceOTU20914
New.CleanUp.ReferenceOTU35586
New.CleanUp.ReferenceOTU6568
New.CleanUp.ReferenceOTU28536
New.CleanUp.ReferenceOTU26873
New.CleanUp.ReferenceOTU200683
New.CleanUp.ReferenceOTU154845
New.CleanUp.ReferenceOTU90611
New.CleanUp.ReferenceOTU103285
New.CleanUp.ReferenceOTU65358
New.CleanUp.ReferenceOTU70582
New.CleanUp.ReferenceOTU68538
New.CleanUp.ReferenceOTU124599
New.CleanUp.ReferenceOTU149528
New.CleanUp.ReferenceOTU265890
New.CleanUp.ReferenceOTU189399
New.CleanUp.ReferenceOTU172163
New.CleanUp.ReferenceOTU86633
New.CleanUp.ReferenceOTU232693
New.CleanUp.ReferenceOTU46742
New.CleanUp.ReferenceOTU116609
New.CleanUp.ReferenceOTU186164
New.CleanUp.ReferenceOTU179118
New.CleanUp.ReferenceOTU95530

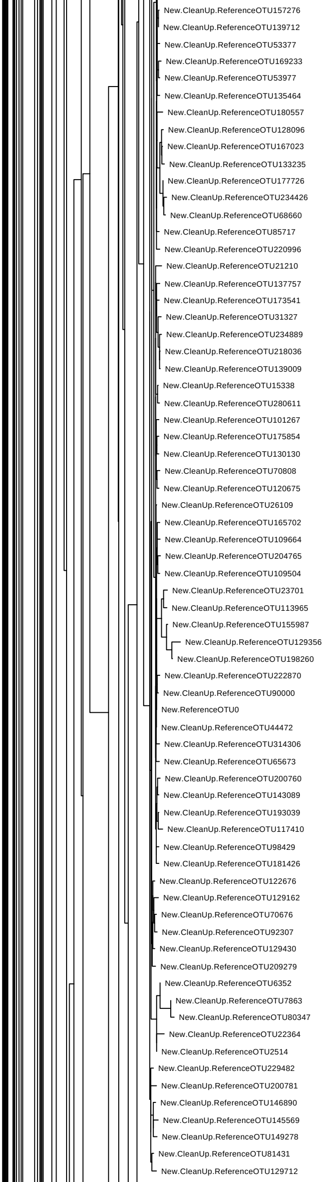
New.CleanUp.ReferenceOTU157276
New.CleanUp.ReferenceOTU139712
New.CleanUp.ReferenceOTU53377
New.CleanUp.ReferenceOTU169233
New.CleanUp.ReferenceOTU53977
New.CleanUp.ReferenceOTU135464
New.CleanUp.ReferenceOTU180557
New.CleanUp.ReferenceOTU128096
New.CleanUp.ReferenceOTU167023
New.CleanUp.ReferenceOTU133235
New.CleanUp.ReferenceOTU177726
New.CleanUp.ReferenceOTU234426
New.CleanUp.ReferenceOTU68660
New.CleanUp.ReferenceOTU85717
New.CleanUp.ReferenceOTU220996
New.CleanUp.ReferenceOTU21210
New.CleanUp.ReferenceOTU137757
New.CleanUp.ReferenceOTU173541
New.CleanUp.ReferenceOTU31327
New.CleanUp.ReferenceOTU234889
New.CleanUp.ReferenceOTU218036
New.CleanUp.ReferenceOTU139009
New.CleanUp.ReferenceOTU15338
New.CleanUp.ReferenceOTU280611
New.CleanUp.ReferenceOTU101267
New.CleanUp.ReferenceOTU175854
New.CleanUp.ReferenceOTU130130
New.CleanUp.ReferenceOTU70808
New.CleanUp.ReferenceOTU120675
New.CleanUp.ReferenceOTU26109
New.CleanUp.ReferenceOTU165702
New.CleanUp.ReferenceOTU109664
New.CleanUp.ReferenceOTU204765
New.CleanUp.ReferenceOTU109504
New.CleanUp.ReferenceOTU23701
New.CleanUp.ReferenceOTU113965
New.CleanUp.ReferenceOTU155987
New.CleanUp.ReferenceOTU129356
New.CleanUp.ReferenceOTU198260
New.CleanUp.ReferenceOTU222870
New.CleanUp.ReferenceOTU90000
New.ReferenceOTU0
New.CleanUp.ReferenceOTU44472
New.CleanUp.ReferenceOTU314306
New.CleanUp.ReferenceOTU65673
New.CleanUp.ReferenceOTU200760
New.CleanUp.ReferenceOTU143089
New.CleanUp.ReferenceOTU193039
New.CleanUp.ReferenceOTU117410
New.CleanUp.ReferenceOTU98429
New.CleanUp.ReferenceOTU181426
New.CleanUp.ReferenceOTU122676
New.CleanUp.ReferenceOTU129162
New.CleanUp.ReferenceOTU70676
New.CleanUp.ReferenceOTU92307
New.CleanUp.ReferenceOTU129430
New.CleanUp.ReferenceOTU209279
New.CleanUp.ReferenceOTU6352
New.CleanUp.ReferenceOTU7863
New.CleanUp.ReferenceOTU80347
New.CleanUp.ReferenceOTU22364
New.CleanUp.ReferenceOTU2514
New.CleanUp.ReferenceOTU229482
New.CleanUp.ReferenceOTU200781
New.CleanUp.ReferenceOTU146890
New.CleanUp.ReferenceOTU145569
New.CleanUp.ReferenceOTU149278
New.CleanUp.ReferenceOTU81431
New.CleanUp.ReferenceOTU129712

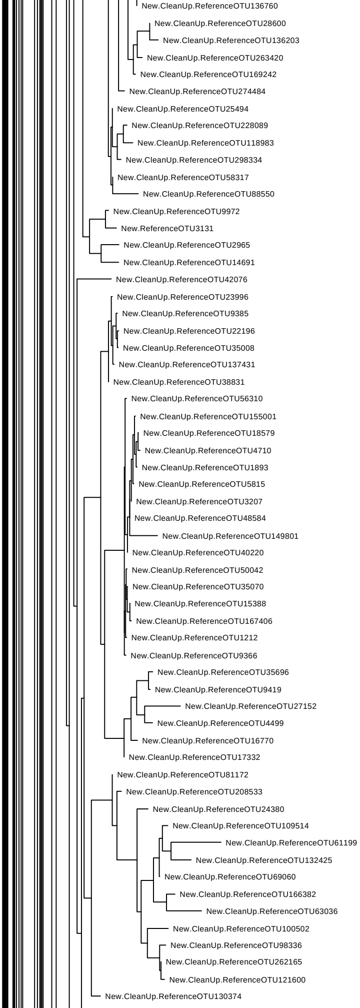
New.CleanUp.ReferenceOTU136760
New.CleanUp.ReferenceOTU28600
New.CleanUp.ReferenceOTU136203
New.CleanUp.ReferenceOTU263420
New.CleanUp.ReferenceOTU169242
New.CleanUp.ReferenceOTU274484
New.CleanUp.ReferenceOTU25494
New.CleanUp.ReferenceOTU228089
New.CleanUp.ReferenceOTU118983
New.CleanUp.ReferenceOTU298334
New.CleanUp.ReferenceOTU58317
New.CleanUp.ReferenceOTU88550
New.CleanUp.ReferenceOTU9972
New.ReferenceOTU3131
New.CleanUp.ReferenceOTU2965
New.CleanUp.ReferenceOTU14691
New.CleanUp.ReferenceOTU42076
New.CleanUp.ReferenceOTU23996
New.CleanUp.ReferenceOTU9385
New.CleanUp.ReferenceOTU22196
New.CleanUp.ReferenceOTU35008
New.CleanUp.ReferenceOTU137431
New.CleanUp.ReferenceOTU38831
New.CleanUp.ReferenceOTU56310
New.CleanUp.ReferenceOTU155001
New.CleanUp.ReferenceOTU18579
New.CleanUp.ReferenceOTU4710
New.CleanUp.ReferenceOTU1893
New.CleanUp.ReferenceOTU5815
New.CleanUp.ReferenceOTU3207
New.CleanUp.ReferenceOTU48584
New.CleanUp.ReferenceOTU149801
New.CleanUp.ReferenceOTU40220
New.CleanUp.ReferenceOTU50042
New.CleanUp.ReferenceOTU35070
New.CleanUp.ReferenceOTU15388
New.CleanUp.ReferenceOTU167406
New.CleanUp.ReferenceOTU1212
New.CleanUp.ReferenceOTU9366
New.CleanUp.ReferenceOTU35696
New.CleanUp.ReferenceOTU9419
New.CleanUp.ReferenceOTU27152
New.CleanUp.ReferenceOTU4499
New.CleanUp.ReferenceOTU16770
New.CleanUp.ReferenceOTU17332
New.CleanUp.ReferenceOTU81172
New.CleanUp.ReferenceOTU208533
New.CleanUp.ReferenceOTU24380
New.CleanUp.ReferenceOTU109514
New.CleanUp.ReferenceOTU61199
New.CleanUp.ReferenceOTU132425
New.CleanUp.ReferenceOTU69060
New.CleanUp.ReferenceOTU166382
New.CleanUp.ReferenceOTU63036
New.CleanUp.ReferenceOTU100502
New.CleanUp.ReferenceOTU98336
New.CleanUp.ReferenceOTU262165
New.CleanUp.ReferenceOTU121600
New.CleanUp.ReferenceOTU130374

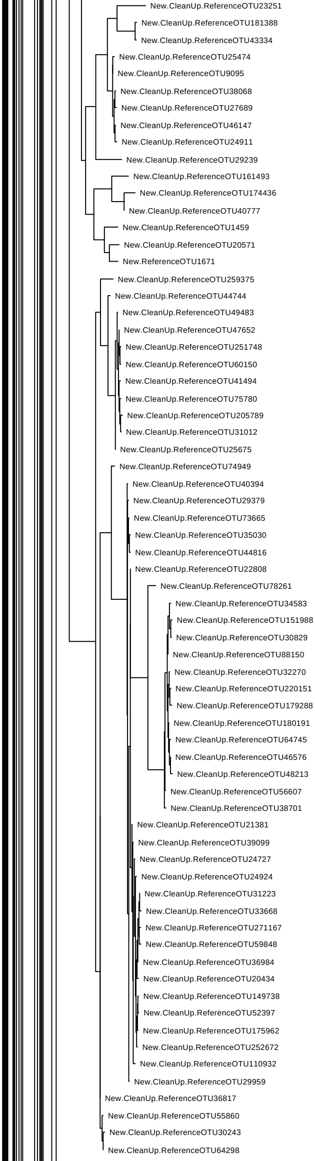
New.CleanUp.ReferenceOTU23251
New.CleanUp.ReferenceOTU181388
New.CleanUp.ReferenceOTU43334
New.CleanUp.ReferenceOTU25474
New.CleanUp.ReferenceOTU9095
New.CleanUp.ReferenceOTU38068
New.CleanUp.ReferenceOTU27689
New.CleanUp.ReferenceOTU46147
New.CleanUp.ReferenceOTU24911
New.CleanUp.ReferenceOTU29239
New.CleanUp.ReferenceOTU161493
New.CleanUp.ReferenceOTU174436
New.CleanUp.ReferenceOTU40777
New.CleanUp.ReferenceOTU1459
New.CleanUp.ReferenceOTU20571
New.ReferenceOTU1671
New.CleanUp.ReferenceOTU259375
New.CleanUp.ReferenceOTU44744
New.CleanUp.ReferenceOTU49483
New.CleanUp.ReferenceOTU47652
New.CleanUp.ReferenceOTU251748
New.CleanUp.ReferenceOTU60150
New.CleanUp.ReferenceOTU41494
New.CleanUp.ReferenceOTU75780
New.CleanUp.ReferenceOTU205789
New.CleanUp.ReferenceOTU31012
New.CleanUp.ReferenceOTU25675
New.CleanUp.ReferenceOTU74949
New.CleanUp.ReferenceOTU40394
New.CleanUp.ReferenceOTU29379
New.CleanUp.ReferenceOTU73665
New.CleanUp.ReferenceOTU35030
New.CleanUp.ReferenceOTU44816
New.CleanUp.ReferenceOTU22808
New.CleanUp.ReferenceOTU78261
New.CleanUp.ReferenceOTU34583
New.CleanUp.ReferenceOTU151988
New.CleanUp.ReferenceOTU30829
New.CleanUp.ReferenceOTU88150
New.CleanUp.ReferenceOTU32270
New.CleanUp.ReferenceOTU220151
New.CleanUp.ReferenceOTU179288
New.CleanUp.ReferenceOTU180191
New.CleanUp.ReferenceOTU64745
New.CleanUp.ReferenceOTU46576
New.CleanUp.ReferenceOTU48213
New.CleanUp.ReferenceOTU56607
New.CleanUp.ReferenceOTU38701
New.CleanUp.ReferenceOTU21381
New.CleanUp.ReferenceOTU39099
New.CleanUp.ReferenceOTU24727
New.CleanUp.ReferenceOTU24924
New.CleanUp.ReferenceOTU31223
New.CleanUp.ReferenceOTU33668
New.CleanUp.ReferenceOTU271167
New.CleanUp.ReferenceOTU59848
New.CleanUp.ReferenceOTU36984
New.CleanUp.ReferenceOTU20434
New.CleanUp.ReferenceOTU149738
New.CleanUp.ReferenceOTU52397
New.CleanUp.ReferenceOTU175962
New.CleanUp.ReferenceOTU252672
New.CleanUp.ReferenceOTU110932
New.CleanUp.ReferenceOTU29959
New.CleanUp.ReferenceOTU36817
New.CleanUp.ReferenceOTU55860
New.CleanUp.ReferenceOTU30243
New.CleanUp.ReferenceOTU64298

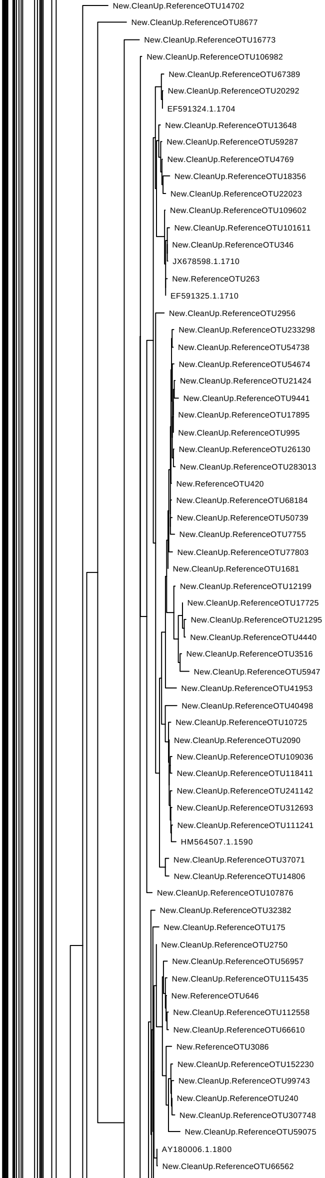
New.CleanUp.ReferenceOTU14702
New.CleanUp.ReferenceOTU8677
New.CleanUp.ReferenceOTU16773
New.CleanUp.ReferenceOTU106982
New.CleanUp.ReferenceOTU67389
New.CleanUp.ReferenceOTU20292
EF591324.1.1704
New.CleanUp.ReferenceOTU13648
New.CleanUp.ReferenceOTU59287
New.CleanUp.ReferenceOTU4769
New.CleanUp.ReferenceOTU18356
New.CleanUp.ReferenceOTU22023
New.CleanUp.ReferenceOTU109602
New.CleanUp.ReferenceOTU101611
New.CleanUp.ReferenceOTU346
JX678598.1.1710
New.ReferenceOTU263
EF591325.1.1710
New.CleanUp.ReferenceOTU2956
New.CleanUp.ReferenceOTU233298
New.CleanUp.ReferenceOTU54738
New.CleanUp.ReferenceOTU54674
New.CleanUp.ReferenceOTU21424
New.CleanUp.ReferenceOTU9441
New.CleanUp.ReferenceOTU17895
New.CleanUp.ReferenceOTU995
New.CleanUp.ReferenceOTU26130
New.CleanUp.ReferenceOTU283013
New.ReferenceOTU420
New.CleanUp.ReferenceOTU68184
New.CleanUp.ReferenceOTU50739
New.CleanUp.ReferenceOTU7755
New.CleanUp.ReferenceOTU77803
New.CleanUp.ReferenceOTU1681
New.CleanUp.ReferenceOTU12199
New.CleanUp.ReferenceOTU17725
New.CleanUp.ReferenceOTU21295
New.CleanUp.ReferenceOTU4440
New.CleanUp.ReferenceOTU3516
New.CleanUp.ReferenceOTU5947
New.CleanUp.ReferenceOTU41953
New.CleanUp.ReferenceOTU40498
New.CleanUp.ReferenceOTU10725
New.CleanUp.ReferenceOTU2090
New.CleanUp.ReferenceOTU109036
New.CleanUp.ReferenceOTU118411
New.CleanUp.ReferenceOTU241142
New.CleanUp.ReferenceOTU312693
New.CleanUp.ReferenceOTU111241
HM564507.1.1590
New.CleanUp.ReferenceOTU37071
New.CleanUp.ReferenceOTU14806
New.CleanUp.ReferenceOTU107876
New.CleanUp.ReferenceOTU32382
New.CleanUp.ReferenceOTU175
New.CleanUp.ReferenceOTU2750
New.CleanUp.ReferenceOTU56957
New.CleanUp.ReferenceOTU115435
New.ReferenceOTU646
New.CleanUp.ReferenceOTU112558
New.CleanUp.ReferenceOTU66610
New.ReferenceOTU3086
New.CleanUp.ReferenceOTU152230
New.CleanUp.ReferenceOTU99743
New.CleanUp.ReferenceOTU240
New.CleanUp.ReferenceOTU307748
New.CleanUp.ReferenceOTU59075
AY180006.1.1800
New.CleanUp.ReferenceOTU66562

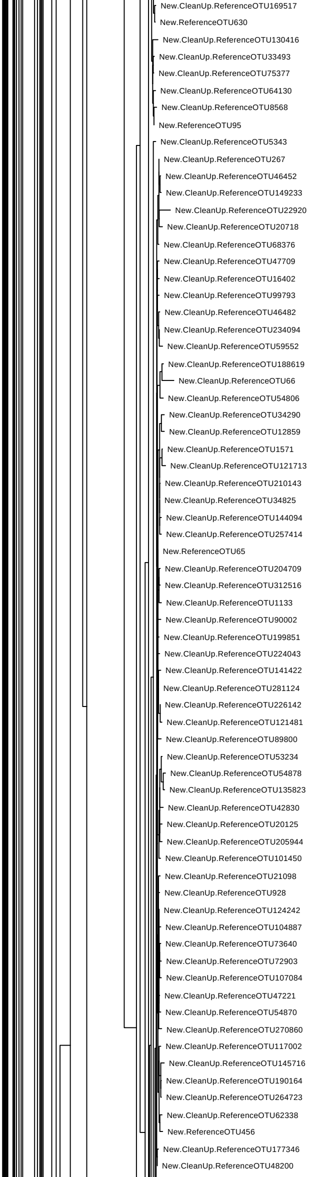
New.CleanUp.ReferenceOTU169517
New.ReferenceOTU630
New.CleanUp.ReferenceOTU130416
New.CleanUp.ReferenceOTU33493
New.CleanUp.ReferenceOTU75377
New.CleanUp.ReferenceOTU64130
New.CleanUp.ReferenceOTU8568
New.ReferenceOTU95
New.CleanUp.ReferenceOTU5343
New.CleanUp.ReferenceOTU267
New.CleanUp.ReferenceOTU46452
New.CleanUp.ReferenceOTU149233
New.CleanUp.ReferenceOTU22920
New.CleanUp.ReferenceOTU20718
New.CleanUp.ReferenceOTU68376
New.CleanUp.ReferenceOTU47709
New.CleanUp.ReferenceOTU16402
New.CleanUp.ReferenceOTU99793
New.CleanUp.ReferenceOTU46482
New.CleanUp.ReferenceOTU234094
New.CleanUp.ReferenceOTU59552
New.CleanUp.ReferenceOTU188619
New.CleanUp.ReferenceOTU66
New.CleanUp.ReferenceOTU54806
New.CleanUp.ReferenceOTU34290
New.CleanUp.ReferenceOTU12859
New.CleanUp.ReferenceOTU1571
New.CleanUp.ReferenceOTU121713
New.CleanUp.ReferenceOTU210143
New.CleanUp.ReferenceOTU34825
New.CleanUp.ReferenceOTU144094
New.CleanUp.ReferenceOTU257414
New.ReferenceOTU65
New.CleanUp.ReferenceOTU204709
New.CleanUp.ReferenceOTU312516
New.CleanUp.ReferenceOTU1133
New.CleanUp.ReferenceOTU90002
New.CleanUp.ReferenceOTU199851
New.CleanUp.ReferenceOTU224043
New.CleanUp.ReferenceOTU141422
New.CleanUp.ReferenceOTU281124
New.CleanUp.ReferenceOTU226142
New.CleanUp.ReferenceOTU121481
New.CleanUp.ReferenceOTU89800
New.CleanUp.ReferenceOTU53234
New.CleanUp.ReferenceOTU54878
New.CleanUp.ReferenceOTU135823
New.CleanUp.ReferenceOTU42830
New.CleanUp.ReferenceOTU20125
New.CleanUp.ReferenceOTU205944
New.CleanUp.ReferenceOTU101450
New.CleanUp.ReferenceOTU21098
New.CleanUp.ReferenceOTU928
New.CleanUp.ReferenceOTU124242
New.CleanUp.ReferenceOTU104887
New.CleanUp.ReferenceOTU73640
New.CleanUp.ReferenceOTU72903
New.CleanUp.ReferenceOTU107084
New.CleanUp.ReferenceOTU47221
New.CleanUp.ReferenceOTU54870
New.CleanUp.ReferenceOTU270860
New.CleanUp.ReferenceOTU117002
New.CleanUp.ReferenceOTU145716
New.CleanUp.ReferenceOTU190164
New.CleanUp.ReferenceOTU264723
New.CleanUp.ReferenceOTU62338
New.ReferenceOTU456
New.CleanUp.ReferenceOTU177346
New.CleanUp.ReferenceOTU48200

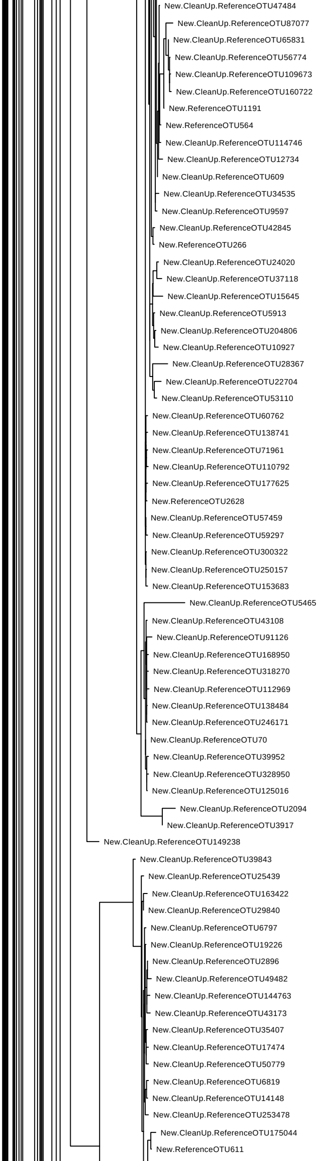
New.CleanUp.ReferenceOTU47484
New.CleanUp.ReferenceOTU87077
New.CleanUp.ReferenceOTU65831
New.CleanUp.ReferenceOTU56774
New.CleanUp.ReferenceOTU109673
New.CleanUp.ReferenceOTU160722
New.ReferenceOTU1191
New.ReferenceOTU564
New.CleanUp.ReferenceOTU114746
New.CleanUp.ReferenceOTU12734
New.CleanUp.ReferenceOTU609
New.CleanUp.ReferenceOTU34535
New.CleanUp.ReferenceOTU9597
New.CleanUp.ReferenceOTU42845
New.ReferenceOTU266
New.CleanUp.ReferenceOTU24020
New.CleanUp.ReferenceOTU37118
New.CleanUp.ReferenceOTU15645
New.CleanUp.ReferenceOTU5913
New.CleanUp.ReferenceOTU204806
New.CleanUp.ReferenceOTU10927
New.CleanUp.ReferenceOTU28367
New.CleanUp.ReferenceOTU22704
New.CleanUp.ReferenceOTU53110
New.CleanUp.ReferenceOTU60762
New.CleanUp.ReferenceOTU138741
New.CleanUp.ReferenceOTU71961
New.CleanUp.ReferenceOTU110792
New.CleanUp.ReferenceOTU177625
New.ReferenceOTU2628
New.CleanUp.ReferenceOTU57459
New.CleanUp.ReferenceOTU59297
New.CleanUp.ReferenceOTU300322
New.CleanUp.ReferenceOTU250157
New.CleanUp.ReferenceOTU153683
New.CleanUp.ReferenceOTU5465
New.CleanUp.ReferenceOTU43108
New.CleanUp.ReferenceOTU91126
New.CleanUp.ReferenceOTU168950
New.CleanUp.ReferenceOTU318270
New.CleanUp.ReferenceOTU112969
New.CleanUp.ReferenceOTU138484
New.CleanUp.ReferenceOTU246171
New.CleanUp.ReferenceOTU70
New.CleanUp.ReferenceOTU39952
New.CleanUp.ReferenceOTU328950
New.CleanUp.ReferenceOTU125016
New.CleanUp.ReferenceOTU2094
New.CleanUp.ReferenceOTU3917
New.CleanUp.ReferenceOTU149238
New.CleanUp.ReferenceOTU39843
New.CleanUp.ReferenceOTU25439
New.CleanUp.ReferenceOTU163422
New.CleanUp.ReferenceOTU29840
New.CleanUp.ReferenceOTU6797
New.CleanUp.ReferenceOTU19226
New.CleanUp.ReferenceOTU2896
New.CleanUp.ReferenceOTU49482
New.CleanUp.ReferenceOTU144763
New.CleanUp.ReferenceOTU43173
New.CleanUp.ReferenceOTU35407
New.CleanUp.ReferenceOTU17474
New.CleanUp.ReferenceOTU50779
New.CleanUp.ReferenceOTU6819
New.CleanUp.ReferenceOTU14148
New.CleanUp.ReferenceOTU253478
New.CleanUp.ReferenceOTU175044
New.ReferenceOTU611

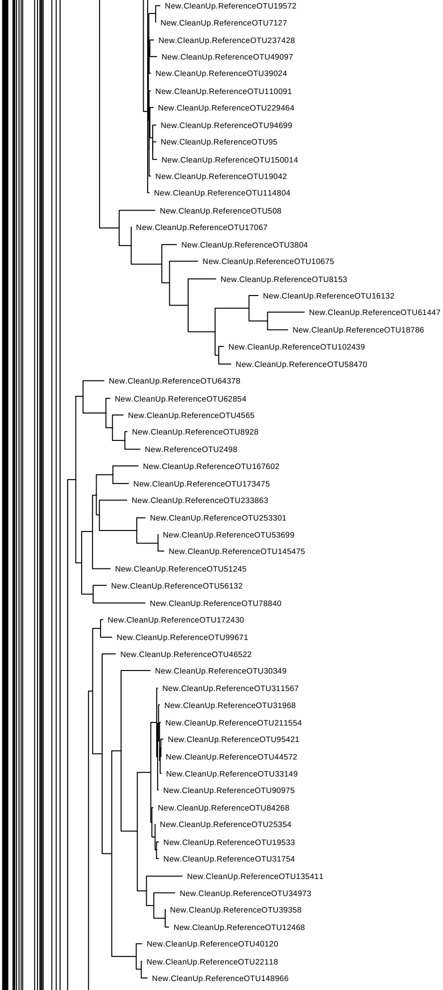
New.CleanUp.ReferenceOTU19572
New.CleanUp.ReferenceOTU7127
New.CleanUp.ReferenceOTU237428
New.CleanUp.ReferenceOTU49097
New.CleanUp.ReferenceOTU39024
New.CleanUp.ReferenceOTU110091
New.CleanUp.ReferenceOTU229464
New.CleanUp.ReferenceOTU94699
New.CleanUp.ReferenceOTU95
New.CleanUp.ReferenceOTU150014
New.CleanUp.ReferenceOTU19042
New.CleanUp.ReferenceOTU114804
New.CleanUp.ReferenceOTU508
New.CleanUp.ReferenceOTU17067
New.CleanUp.ReferenceOTU3804
New.CleanUp.ReferenceOTU10675
New.CleanUp.ReferenceOTU8153
New.CleanUp.ReferenceOTU16132
New.CleanUp.ReferenceOTU61447
New.CleanUp.ReferenceOTU18786
New.CleanUp.ReferenceOTU102439
New.CleanUp.ReferenceOTU58470
New.CleanUp.ReferenceOTU64378
New.CleanUp.ReferenceOTU62854
New.CleanUp.ReferenceOTU4565
New.CleanUp.ReferenceOTU8928
New.ReferenceOTU2498
New.CleanUp.ReferenceOTU167602
New.CleanUp.ReferenceOTU173475
New.CleanUp.ReferenceOTU233863
New.CleanUp.ReferenceOTU253301
New.CleanUp.ReferenceOTU53699
New.CleanUp.ReferenceOTU145475
New.CleanUp.ReferenceOTU51245
New.CleanUp.ReferenceOTU56132
New.CleanUp.ReferenceOTU78840
New.CleanUp.ReferenceOTU172430
New.CleanUp.ReferenceOTU99671
New.CleanUp.ReferenceOTU46522
New.CleanUp.ReferenceOTU30349
New.CleanUp.ReferenceOTU311567
New.CleanUp.ReferenceOTU31968
New.CleanUp.ReferenceOTU211554
New.CleanUp.ReferenceOTU95421
New.CleanUp.ReferenceOTU44572
New.CleanUp.ReferenceOTU33149
New.CleanUp.ReferenceOTU90975
New.CleanUp.ReferenceOTU84268
New.CleanUp.ReferenceOTU25354
New.CleanUp.ReferenceOTU19533
New.CleanUp.ReferenceOTU31754
New.CleanUp.ReferenceOTU135411
New.CleanUp.ReferenceOTU34973
New.CleanUp.ReferenceOTU39358
New.CleanUp.ReferenceOTU12468
New.CleanUp.ReferenceOTU40120
New.CleanUp.ReferenceOTU22118
New.CleanUp.ReferenceOTU148966

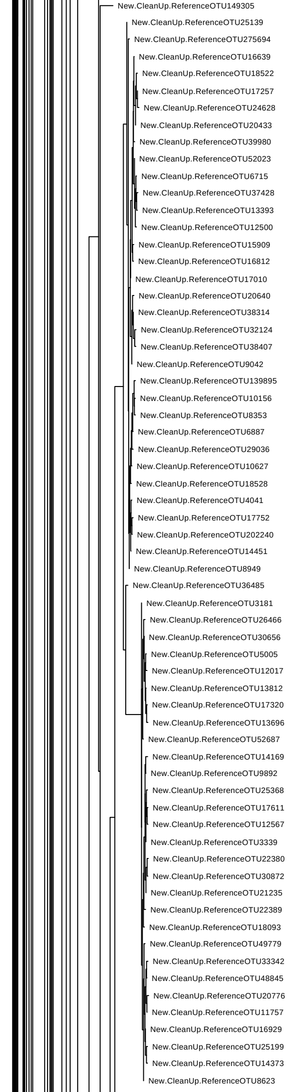

New.CleanUp.ReferenceOTU149305
New.CleanUp.ReferenceOTU25139
New.CleanUp.ReferenceOTU275694
New.CleanUp.ReferenceOTU16639
New.CleanUp.ReferenceOTU18522
New.CleanUp.ReferenceOTU17257
New.CleanUp.ReferenceOTU24628
New.CleanUp.ReferenceOTU20433
New.CleanUp.ReferenceOTU39980
New.CleanUp.ReferenceOTU52023
New.CleanUp.ReferenceOTU6715
New.CleanUp.ReferenceOTU37428
New.CleanUp.ReferenceOTU13393
New.CleanUp.ReferenceOTU12500
New.CleanUp.ReferenceOTU15909
New.CleanUp.ReferenceOTU16812
New.CleanUp.ReferenceOTU17010
New.CleanUp.ReferenceOTU20640
New.CleanUp.ReferenceOTU38314
New.CleanUp.ReferenceOTU32124
New.CleanUp.ReferenceOTU38407
New.CleanUp.ReferenceOTU9042
New.CleanUp.ReferenceOTU139895
New.CleanUp.ReferenceOTU10156
New.CleanUp.ReferenceOTU8353
New.CleanUp.ReferenceOTU6887
New.CleanUp.ReferenceOTU29036
New.CleanUp.ReferenceOTU10627
New.CleanUp.ReferenceOTU18528
New.CleanUp.ReferenceOTU4041
New.CleanUp.ReferenceOTU17752
New.CleanUp.ReferenceOTU202240
New.CleanUp.ReferenceOTU14451
New.CleanUp.ReferenceOTU8949
New.CleanUp.ReferenceOTU36485
New.CleanUp.ReferenceOTU3181
New.CleanUp.ReferenceOTU26466
New.CleanUp.ReferenceOTU30656
New.CleanUp.ReferenceOTU5005
New.CleanUp.ReferenceOTU12017
New.CleanUp.ReferenceOTU13812
New.CleanUp.ReferenceOTU17320
New.CleanUp.ReferenceOTU13696
New.CleanUp.ReferenceOTU52687
New.CleanUp.ReferenceOTU14169
New.CleanUp.ReferenceOTU9892
New.CleanUp.ReferenceOTU25368
New.CleanUp.ReferenceOTU17611
New.CleanUp.ReferenceOTU12567
New.CleanUp.ReferenceOTU3339
New.CleanUp.ReferenceOTU22380
New.CleanUp.ReferenceOTU30872
New.CleanUp.ReferenceOTU21235
New.CleanUp.ReferenceOTU22389
New.CleanUp.ReferenceOTU18093
New.CleanUp.ReferenceOTU49779
New.CleanUp.ReferenceOTU33342
New.CleanUp.ReferenceOTU48845
New.CleanUp.ReferenceOTU20776
New.CleanUp.ReferenceOTU11757
New.CleanUp.ReferenceOTU16929
New.CleanUp.ReferenceOTU25199
New.CleanUp.ReferenceOTU14373
New.CleanUp.ReferenceOTU8623

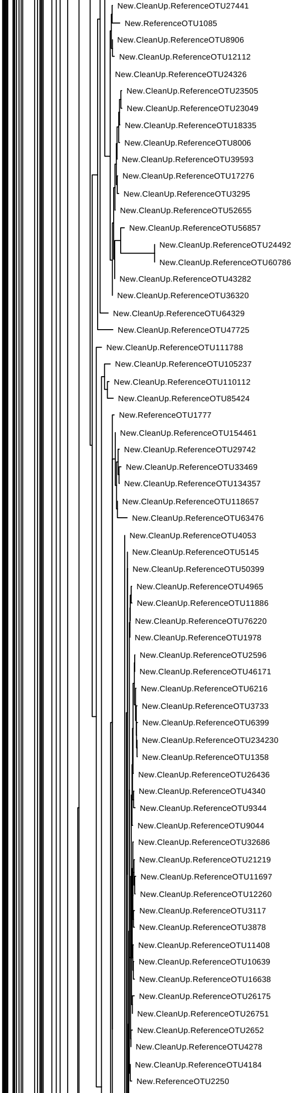
New.CleanUp.ReferenceOTU27441
New.ReferenceOTU1085
New.CleanUp.ReferenceOTU8906
New.CleanUp.ReferenceOTU12112
New.CleanUp.ReferenceOTU24326
New.CleanUp.ReferenceOTU23505
New.CleanUp.ReferenceOTU23049
New.CleanUp.ReferenceOTU18335
New.CleanUp.ReferenceOTU8006
New.CleanUp.ReferenceOTU39593
New.CleanUp.ReferenceOTU17276
New.CleanUp.ReferenceOTU3295
New.CleanUp.ReferenceOTU52655
New.CleanUp.ReferenceOTU56857
New.CleanUp.ReferenceOTU24492
New.CleanUp.ReferenceOTU60786
New.CleanUp.ReferenceOTU43282
New.CleanUp.ReferenceOTU36320
New.CleanUp.ReferenceOTU64329
New.CleanUp.ReferenceOTU47725
New.CleanUp.ReferenceOTU111788
New.CleanUp.ReferenceOTU105237
New.CleanUp.ReferenceOTU110112
New.CleanUp.ReferenceOTU85424
New.ReferenceOTU1777
New.CleanUp.ReferenceOTU154461
New.CleanUp.ReferenceOTU29742
New.CleanUp.ReferenceOTU33469
New.CleanUp.ReferenceOTU134357
New.CleanUp.ReferenceOTU118657
New.CleanUp.ReferenceOTU63476
New.CleanUp.ReferenceOTU4053
New.CleanUp.ReferenceOTU5145
New.CleanUp.ReferenceOTU50399
New.CleanUp.ReferenceOTU4965
New.CleanUp.ReferenceOTU11886
New.CleanUp.ReferenceOTU76220
New.CleanUp.ReferenceOTU1978
New.CleanUp.ReferenceOTU2596
New.CleanUp.ReferenceOTU46171
New.CleanUp.ReferenceOTU6216
New.CleanUp.ReferenceOTU3733
New.CleanUp.ReferenceOTU6399
New.CleanUp.ReferenceOTU234230
New.CleanUp.ReferenceOTU1358
New.CleanUp.ReferenceOTU26436
New.CleanUp.ReferenceOTU4340
New.CleanUp.ReferenceOTU9344
New.CleanUp.ReferenceOTU9044
New.CleanUp.ReferenceOTU32686
New.CleanUp.ReferenceOTU21219
New.CleanUp.ReferenceOTU11697
New.CleanUp.ReferenceOTU12260
New.CleanUp.ReferenceOTU3117
New.CleanUp.ReferenceOTU3878
New.CleanUp.ReferenceOTU11408
New.CleanUp.ReferenceOTU10639
New.CleanUp.ReferenceOTU16638
New.CleanUp.ReferenceOTU26175
New.CleanUp.ReferenceOTU26751
New.CleanUp.ReferenceOTU2652
New.CleanUp.ReferenceOTU4278
New.CleanUp.ReferenceOTU4184
New.ReferenceOTU2250

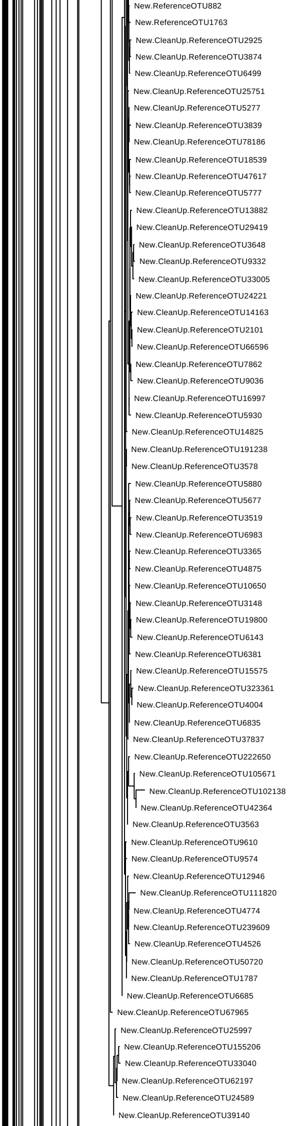

New.ReferenceOTU882
New.ReferenceOTU1763
New.CleanUp.ReferenceOTU2925
New.CleanUp.ReferenceOTU3874
New.CleanUp.ReferenceOTU6499
New.CleanUp.ReferenceOTU25751
New.CleanUp.ReferenceOTU5277
New.CleanUp.ReferenceOTU3839
New.CleanUp.ReferenceOTU78186
New.CleanUp.ReferenceOTU18539
New.CleanUp.ReferenceOTU47617
New.CleanUp.ReferenceOTU5777
New.CleanUp.ReferenceOTU13882
New.CleanUp.ReferenceOTU29419
New.CleanUp.ReferenceOTU3648
New.CleanUp.ReferenceOTU9332
New.CleanUp.ReferenceOTU33005
New.CleanUp.ReferenceOTU24221
New.CleanUp.ReferenceOTU14163
New.CleanUp.ReferenceOTU2101
New.CleanUp.ReferenceOTU66596
New.CleanUp.ReferenceOTU7862
New.CleanUp.ReferenceOTU9036
New.CleanUp.ReferenceOTU16997
New.CleanUp.ReferenceOTU5930
New.CleanUp.ReferenceOTU14825
New.CleanUp.ReferenceOTU191238
New.CleanUp.ReferenceOTU3578
New.CleanUp.ReferenceOTU5880
New.CleanUp.ReferenceOTU5677
New.CleanUp.ReferenceOTU3519
New.CleanUp.ReferenceOTU6983
New.CleanUp.ReferenceOTU3365
New.CleanUp.ReferenceOTU4875
New.CleanUp.ReferenceOTU10650
New.CleanUp.ReferenceOTU3148
New.CleanUp.ReferenceOTU19800
New.CleanUp.ReferenceOTU6143
New.CleanUp.ReferenceOTU6381
New.CleanUp.ReferenceOTU15575
New.CleanUp.ReferenceOTU323361
New.CleanUp.ReferenceOTU4004
New.CleanUp.ReferenceOTU6835
New.CleanUp.ReferenceOTU37837
New.CleanUp.ReferenceOTU222650
New.CleanUp.ReferenceOTU105671
New.CleanUp.ReferenceOTU102138
New.CleanUp.ReferenceOTU42364
New.CleanUp.ReferenceOTU3563
New.CleanUp.ReferenceOTU9610
New.CleanUp.ReferenceOTU9574
New.CleanUp.ReferenceOTU12946
New.CleanUp.ReferenceOTU111820
New.CleanUp.ReferenceOTU4774
New.CleanUp.ReferenceOTU239609
New.CleanUp.ReferenceOTU4526
New.CleanUp.ReferenceOTU50720
New.CleanUp.ReferenceOTU1787
New.CleanUp.ReferenceOTU6685
New.CleanUp.ReferenceOTU67965
New.CleanUp.ReferenceOTU25997
New.CleanUp.ReferenceOTU155206
New.CleanUp.ReferenceOTU33040
New.CleanUp.ReferenceOTU62197
New.CleanUp.ReferenceOTU24589
New.CleanUp.ReferenceOTU39140

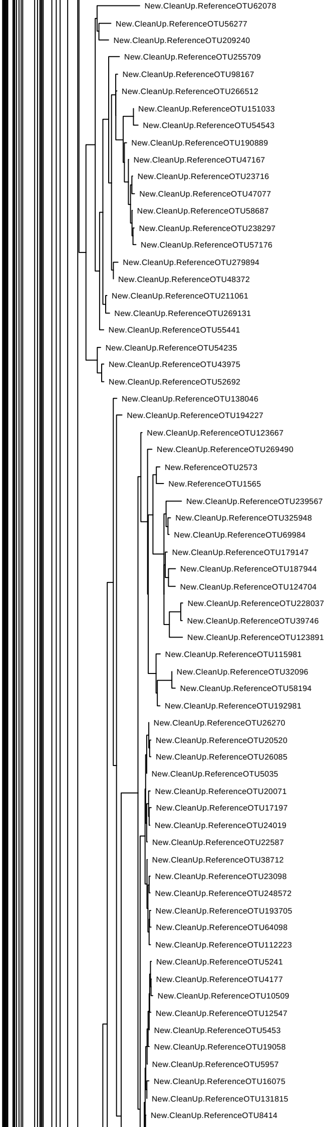
New.CleanUp.ReferenceOTU62078
New.CleanUp.ReferenceOTU56277
New.CleanUp.ReferenceOTU209240
New.CleanUp.ReferenceOTU255709
New.CleanUp.ReferenceOTU98167
New.CleanUp.ReferenceOTU266512
New.CleanUp.ReferenceOTU151033
New.CleanUp.ReferenceOTU54543
New.CleanUp.ReferenceOTU190889
New.CleanUp.ReferenceOTU47167
New.CleanUp.ReferenceOTU23716
New.CleanUp.ReferenceOTU47077
New.CleanUp.ReferenceOTU58687
New.CleanUp.ReferenceOTU238297
New.CleanUp.ReferenceOTU57176
New.CleanUp.ReferenceOTU279894
New.CleanUp.ReferenceOTU48372
New.CleanUp.ReferenceOTU211061
New.CleanUp.ReferenceOTU269131
New.CleanUp.ReferenceOTU55441
New.CleanUp.ReferenceOTU54235
New.CleanUp.ReferenceOTU43975
New.CleanUp.ReferenceOTU52692
New.CleanUp.ReferenceOTU138046
New.CleanUp.ReferenceOTU194227
New.CleanUp.ReferenceOTU123667
New.CleanUp.ReferenceOTU269490
New.ReferenceOTU2573
New.ReferenceOTU1565
New.CleanUp.ReferenceOTU239567
New.CleanUp.ReferenceOTU325948
New.CleanUp.ReferenceOTU69984
New.CleanUp.ReferenceOTU179147
New.CleanUp.ReferenceOTU187944
New.CleanUp.ReferenceOTU124704
New.CleanUp.ReferenceOTU228037
New.CleanUp.ReferenceOTU39746
New.CleanUp.ReferenceOTU123891
New.CleanUp.ReferenceOTU115981
New.CleanUp.ReferenceOTU32096
New.CleanUp.ReferenceOTU58194
New.CleanUp.ReferenceOTU192981
New.CleanUp.ReferenceOTU26270
New.CleanUp.ReferenceOTU20520
New.CleanUp.ReferenceOTU26085
New.CleanUp.ReferenceOTU5035
New.CleanUp.ReferenceOTU20071
New.CleanUp.ReferenceOTU17197
New.CleanUp.ReferenceOTU24019
New.CleanUp.ReferenceOTU22587
New.CleanUp.ReferenceOTU38712
New.CleanUp.ReferenceOTU23098
New.CleanUp.ReferenceOTU248572
New.CleanUp.ReferenceOTU193705
New.CleanUp.ReferenceOTU64098
New.CleanUp.ReferenceOTU112223
New.CleanUp.ReferenceOTU5241
New.CleanUp.ReferenceOTU4177
New.CleanUp.ReferenceOTU10509
New.CleanUp.ReferenceOTU12547
New.CleanUp.ReferenceOTU5453
New.CleanUp.ReferenceOTU19058
New.CleanUp.ReferenceOTU5957
New.CleanUp.ReferenceOTU16075
New.CleanUp.ReferenceOTU131815
New.CleanUp.ReferenceOTU8414

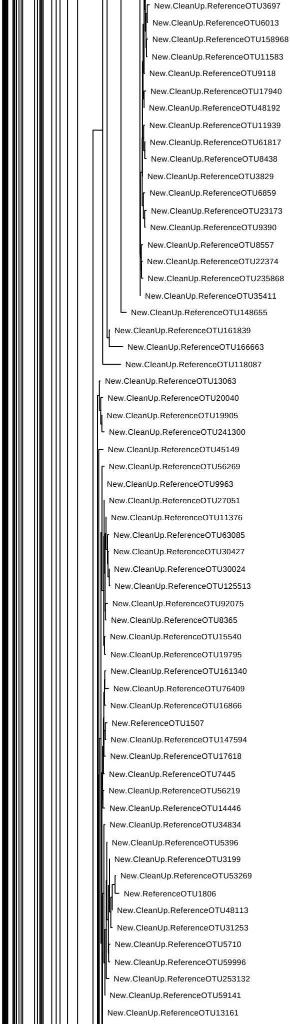
New.CleanUp.ReferenceOTU3697
New.CleanUp.ReferenceOTU6013
New.CleanUp.ReferenceOTU158968
New.CleanUp.ReferenceOTU11583
New.CleanUp.ReferenceOTU9118
New.CleanUp.ReferenceOTU17940
New.CleanUp.ReferenceOTU48192
New.CleanUp.ReferenceOTU11939
New.CleanUp.ReferenceOTU61817
New.CleanUp.ReferenceOTU8438
New.CleanUp.ReferenceOTU3829
New.CleanUp.ReferenceOTU6859
New.CleanUp.ReferenceOTU23173
New.CleanUp.ReferenceOTU9390
New.CleanUp.ReferenceOTU8557
New.CleanUp.ReferenceOTU22374
New.CleanUp.ReferenceOTU235868
New.CleanUp.ReferenceOTU35411
New.CleanUp.ReferenceOTU148655
New.CleanUp.ReferenceOTU161839
New.CleanUp.ReferenceOTU166663
New.CleanUp.ReferenceOTU118087
New.CleanUp.ReferenceOTU13063
New.CleanUp.ReferenceOTU20040
New.CleanUp.ReferenceOTU19905
New.CleanUp.ReferenceOTU241300
New.CleanUp.ReferenceOTU45149
New.CleanUp.ReferenceOTU56269
New.CleanUp.ReferenceOTU9963
New.CleanUp.ReferenceOTU27051
New.CleanUp.ReferenceOTU11376
New.CleanUp.ReferenceOTU63085
New.CleanUp.ReferenceOTU30427
New.CleanUp.ReferenceOTU30024
New.CleanUp.ReferenceOTU125513
New.CleanUp.ReferenceOTU92075
New.CleanUp.ReferenceOTU8365
New.CleanUp.ReferenceOTU15540
New.CleanUp.ReferenceOTU19795
New.CleanUp.ReferenceOTU161340
New.CleanUp.ReferenceOTU76409
New.CleanUp.ReferenceOTU16866
New.ReferenceOTU1507
New.CleanUp.ReferenceOTU147594
New.CleanUp.ReferenceOTU17618
New.CleanUp.ReferenceOTU7445
New.CleanUp.ReferenceOTU56219
New.CleanUp.ReferenceOTU14446
New.CleanUp.ReferenceOTU34834
New.CleanUp.ReferenceOTU5396
New.CleanUp.ReferenceOTU3199
New.CleanUp.ReferenceOTU53269
New.ReferenceOTU1806
New.CleanUp.ReferenceOTU48113
New.CleanUp.ReferenceOTU31253
New.CleanUp.ReferenceOTU5710
New.CleanUp.ReferenceOTU59996
New.CleanUp.ReferenceOTU253132
New.CleanUp.ReferenceOTU59141
New.CleanUp.ReferenceOTU13161

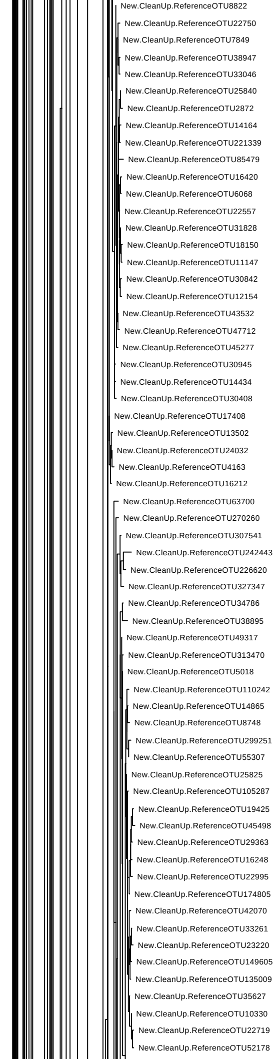
New.CleanUp.ReferenceOTU8822
New.CleanUp.ReferenceOTU22750
New.CleanUp.ReferenceOTU7849
New.CleanUp.ReferenceOTU38947
New.CleanUp.ReferenceOTU33046
New.CleanUp.ReferenceOTU25840
New.CleanUp.ReferenceOTU2872
New.CleanUp.ReferenceOTU14164
New.CleanUp.ReferenceOTU221339
New.CleanUp.ReferenceOTU85479
New.CleanUp.ReferenceOTU16420
New.CleanUp.ReferenceOTU6068
New.CleanUp.ReferenceOTU22557
New.CleanUp.ReferenceOTU31828
New.CleanUp.ReferenceOTU18150
New.CleanUp.ReferenceOTU11147
New.CleanUp.ReferenceOTU30842
New.CleanUp.ReferenceOTU12154
New.CleanUp.ReferenceOTU43532
New.CleanUp.ReferenceOTU47712
New.CleanUp.ReferenceOTU45277
New.CleanUp.ReferenceOTU30945
New.CleanUp.ReferenceOTU14434
New.CleanUp.ReferenceOTU30408
New.CleanUp.ReferenceOTU17408
New.CleanUp.ReferenceOTU13502
New.CleanUp.ReferenceOTU24032
New.CleanUp.ReferenceOTU4163
New.CleanUp.ReferenceOTU16212
New.CleanUp.ReferenceOTU63700
New.CleanUp.ReferenceOTU270260
New.CleanUp.ReferenceOTU307541
New.CleanUp.ReferenceOTU242443
New.CleanUp.ReferenceOTU226620
New.CleanUp.ReferenceOTU327347
New.CleanUp.ReferenceOTU34786
New.CleanUp.ReferenceOTU38895
New.CleanUp.ReferenceOTU49317
New.CleanUp.ReferenceOTU313470
New.CleanUp.ReferenceOTU5018
New.CleanUp.ReferenceOTU110242
New.CleanUp.ReferenceOTU14865
New.CleanUp.ReferenceOTU8748
New.CleanUp.ReferenceOTU299251
New.CleanUp.ReferenceOTU55307
New.CleanUp.ReferenceOTU25825
New.CleanUp.ReferenceOTU105287
New.CleanUp.ReferenceOTU19425
New.CleanUp.ReferenceOTU45498
New.CleanUp.ReferenceOTU29363
New.CleanUp.ReferenceOTU16248
New.CleanUp.ReferenceOTU22995
New.CleanUp.ReferenceOTU174805
New.CleanUp.ReferenceOTU42070
New.CleanUp.ReferenceOTU33261
New.CleanUp.ReferenceOTU23220
New.CleanUp.ReferenceOTU149605
New.CleanUp.ReferenceOTU135009
New.CleanUp.ReferenceOTU35627
New.CleanUp.ReferenceOTU10330
New.CleanUp.ReferenceOTU22719
New.CleanUp.ReferenceOTU52178

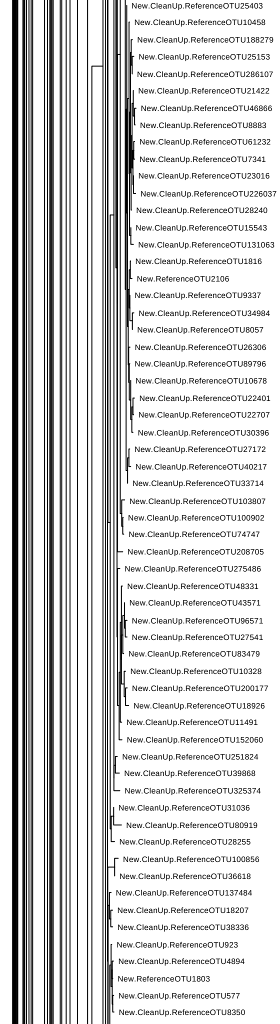
New.CleanUp.ReferenceOTU25403
New.CleanUp.ReferenceOTU10458
New.CleanUp.ReferenceOTU188279
New.CleanUp.ReferenceOTU25153
New.CleanUp.ReferenceOTU286107
New.CleanUp.ReferenceOTU21422
New.CleanUp.ReferenceOTU46866
New.CleanUp.ReferenceOTU8883
New.CleanUp.ReferenceOTU61232
New.CleanUp.ReferenceOTU7341
New.CleanUp.ReferenceOTU23016
New.CleanUp.ReferenceOTU226037
New.CleanUp.ReferenceOTU28240
New.CleanUp.ReferenceOTU15543
New.CleanUp.ReferenceOTU131063
New.CleanUp.ReferenceOTU1816
New.ReferenceOTU2106
New.CleanUp.ReferenceOTU9337
New.CleanUp.ReferenceOTU34984
New.CleanUp.ReferenceOTU8057
New.CleanUp.ReferenceOTU26306
New.CleanUp.ReferenceOTU89796
New.CleanUp.ReferenceOTU10678
New.CleanUp.ReferenceOTU22401
New.CleanUp.ReferenceOTU22707
New.CleanUp.ReferenceOTU30396
New.CleanUp.ReferenceOTU27172
New.CleanUp.ReferenceOTU40217
New.CleanUp.ReferenceOTU33714
New.CleanUp.ReferenceOTU103807
New.CleanUp.ReferenceOTU100902
New.CleanUp.ReferenceOTU74747
New.CleanUp.ReferenceOTU208705
New.CleanUp.ReferenceOTU275486
New.CleanUp.ReferenceOTU48331
New.CleanUp.ReferenceOTU43571
New.CleanUp.ReferenceOTU96571
New.CleanUp.ReferenceOTU27541
New.CleanUp.ReferenceOTU83479
New.CleanUp.ReferenceOTU10328
New.CleanUp.ReferenceOTU200177
New.CleanUp.ReferenceOTU18926
New.CleanUp.ReferenceOTU11491
New.CleanUp.ReferenceOTU152060
New.CleanUp.ReferenceOTU251824
New.CleanUp.ReferenceOTU39868
New.CleanUp.ReferenceOTU325374
New.CleanUp.ReferenceOTU31036
New.CleanUp.ReferenceOTU80919
New.CleanUp.ReferenceOTU28255
New.CleanUp.ReferenceOTU100856
New.CleanUp.ReferenceOTU36618
New.CleanUp.ReferenceOTU137484
New.CleanUp.ReferenceOTU18207
New.CleanUp.ReferenceOTU38336
New.CleanUp.ReferenceOTU923
New.CleanUp.ReferenceOTU4894
New.ReferenceOTU1803
New.CleanUp.ReferenceOTU577
New.CleanUp.ReferenceOTU8350

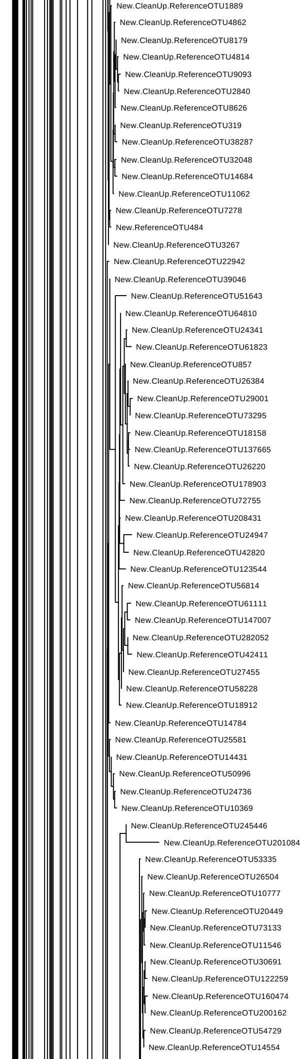
New.CleanUp.ReferenceOTU1889
New.CleanUp.ReferenceOTU4862
New.CleanUp.ReferenceOTU8179
New.CleanUp.ReferenceOTU4814
New.CleanUp.ReferenceOTU9093
New.CleanUp.ReferenceOTU2840
New.CleanUp.ReferenceOTU8626
New.CleanUp.ReferenceOTU319
New.CleanUp.ReferenceOTU38287
New.CleanUp.ReferenceOTU32048
New.CleanUp.ReferenceOTU14684
New.CleanUp.ReferenceOTU11062
New.CleanUp.ReferenceOTU7278
New.ReferenceOTU484
New.CleanUp.ReferenceOTU3267
New.CleanUp.ReferenceOTU22942
New.CleanUp.ReferenceOTU39046
New.CleanUp.ReferenceOTU51643
New.CleanUp.ReferenceOTU64810
New.CleanUp.ReferenceOTU24341
New.CleanUp.ReferenceOTU61823
New.CleanUp.ReferenceOTU857
New.CleanUp.ReferenceOTU26384
New.CleanUp.ReferenceOTU29001
New.CleanUp.ReferenceOTU73295
New.CleanUp.ReferenceOTU18158
New.CleanUp.ReferenceOTU137665
New.CleanUp.ReferenceOTU26220
New.CleanUp.ReferenceOTU178903
New.CleanUp.ReferenceOTU72755
New.CleanUp.ReferenceOTU208431
New.CleanUp.ReferenceOTU24947
New.CleanUp.ReferenceOTU42820
New.CleanUp.ReferenceOTU123544
New.CleanUp.ReferenceOTU56814
New.CleanUp.ReferenceOTU61111
New.CleanUp.ReferenceOTU147007
New.CleanUp.ReferenceOTU282052
New.CleanUp.ReferenceOTU42411
New.CleanUp.ReferenceOTU27455
New.CleanUp.ReferenceOTU58228
New.CleanUp.ReferenceOTU18912
New.CleanUp.ReferenceOTU14784
New.CleanUp.ReferenceOTU25581
New.CleanUp.ReferenceOTU14431
New.CleanUp.ReferenceOTU50996
New.CleanUp.ReferenceOTU24736
New.CleanUp.ReferenceOTU10369
New.CleanUp.ReferenceOTU245446
New.CleanUp.ReferenceOTU201084
New.CleanUp.ReferenceOTU53335
New.CleanUp.ReferenceOTU26504
New.CleanUp.ReferenceOTU10777
New.CleanUp.ReferenceOTU20449
New.CleanUp.ReferenceOTU73133
New.CleanUp.ReferenceOTU11546
New.CleanUp.ReferenceOTU30691
New.CleanUp.ReferenceOTU122259
New.CleanUp.ReferenceOTU160474
New.CleanUp.ReferenceOTU200162
New.CleanUp.ReferenceOTU54729
New.CleanUp.ReferenceOTU14554

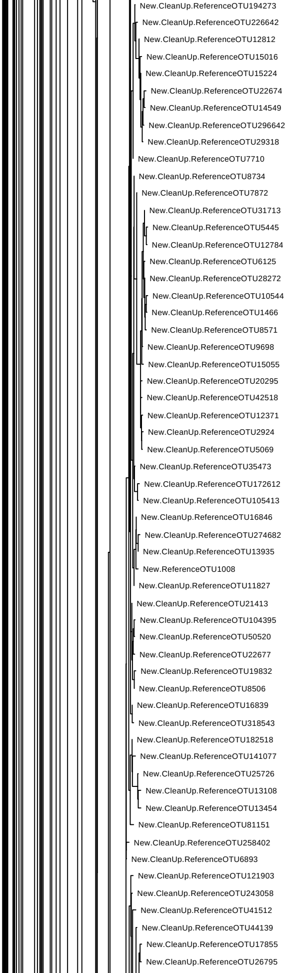
New.CleanUp.ReferenceOTU194273
New.CleanUp.ReferenceOTU226642
New.CleanUp.ReferenceOTU12812
New.CleanUp.ReferenceOTU15016
New.CleanUp.ReferenceOTU15224
New.CleanUp.ReferenceOTU22674
New.CleanUp.ReferenceOTU14549
New.CleanUp.ReferenceOTU296642
New.CleanUp.ReferenceOTU29318
New.CleanUp.ReferenceOTU7710
New.CleanUp.ReferenceOTU8734
New.CleanUp.ReferenceOTU7872
New.CleanUp.ReferenceOTU31713
New.CleanUp.ReferenceOTU5445
New.CleanUp.ReferenceOTU12784
New.CleanUp.ReferenceOTU6125
New.CleanUp.ReferenceOTU28272
New.CleanUp.ReferenceOTU10544
New.CleanUp.ReferenceOTU1466
New.CleanUp.ReferenceOTU8571
New.CleanUp.ReferenceOTU9698
New.CleanUp.ReferenceOTU15055
New.CleanUp.ReferenceOTU20295
New.CleanUp.ReferenceOTU42518
New.CleanUp.ReferenceOTU12371
New.CleanUp.ReferenceOTU2924
New.CleanUp.ReferenceOTU5069
New.CleanUp.ReferenceOTU35473
New.CleanUp.ReferenceOTU172612
New.CleanUp.ReferenceOTU105413
New.CleanUp.ReferenceOTU16846
New.CleanUp.ReferenceOTU274682
New.CleanUp.ReferenceOTU13935
New.ReferenceOTU1008
New.CleanUp.ReferenceOTU11827
New.CleanUp.ReferenceOTU21413
New.CleanUp.ReferenceOTU104395
New.CleanUp.ReferenceOTU50520
New.CleanUp.ReferenceOTU22677
New.CleanUp.ReferenceOTU19832
New.CleanUp.ReferenceOTU8506
New.CleanUp.ReferenceOTU16839
New.CleanUp.ReferenceOTU318543
New.CleanUp.ReferenceOTU182518
New.CleanUp.ReferenceOTU141077
New.CleanUp.ReferenceOTU25726
New.CleanUp.ReferenceOTU13108
New.CleanUp.ReferenceOTU13454
New.CleanUp.ReferenceOTU81151
New.CleanUp.ReferenceOTU258402
New.CleanUp.ReferenceOTU6893
New.CleanUp.ReferenceOTU121903
New.CleanUp.ReferenceOTU243058
New.CleanUp.ReferenceOTU41512
New.CleanUp.ReferenceOTU44139
New.CleanUp.ReferenceOTU17855
New.CleanUp.ReferenceOTU26795

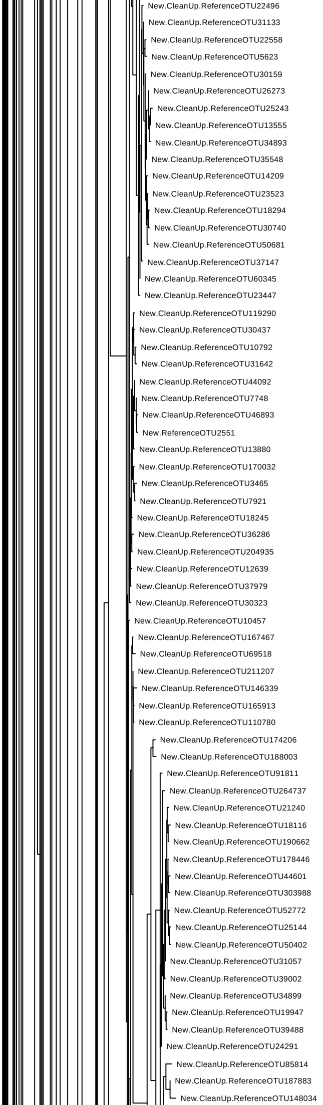

New.CleanUp.ReferenceOTU22496
New.CleanUp.ReferenceOTU31133
New.CleanUp.ReferenceOTU22558
New.CleanUp.ReferenceOTU5623
New.CleanUp.ReferenceOTU30159
New.CleanUp.ReferenceOTU26273
New.CleanUp.ReferenceOTU25243
New.CleanUp.ReferenceOTU13555
New.CleanUp.ReferenceOTU34893
New.CleanUp.ReferenceOTU35548
New.CleanUp.ReferenceOTU14209
New.CleanUp.ReferenceOTU23523
New.CleanUp.ReferenceOTU18294
New.CleanUp.ReferenceOTU30740
New.CleanUp.ReferenceOTU50681
New.CleanUp.ReferenceOTU37147
New.CleanUp.ReferenceOTU60345
New.CleanUp.ReferenceOTU23447
New.CleanUp.ReferenceOTU119290
New.CleanUp.ReferenceOTU30437
New.CleanUp.ReferenceOTU10792
New.CleanUp.ReferenceOTU31642
New.CleanUp.ReferenceOTU44092
New.CleanUp.ReferenceOTU7748
New.CleanUp.ReferenceOTU46893
New.ReferenceOTU2551
New.CleanUp.ReferenceOTU13880
New.CleanUp.ReferenceOTU170032
New.CleanUp.ReferenceOTU3465
New.CleanUp.ReferenceOTU7921
New.CleanUp.ReferenceOTU18245
New.CleanUp.ReferenceOTU36286
New.CleanUp.ReferenceOTU204935
New.CleanUp.ReferenceOTU12639
New.CleanUp.ReferenceOTU37979
New.CleanUp.ReferenceOTU30323
New.CleanUp.ReferenceOTU10457
New.CleanUp.ReferenceOTU167467
New.CleanUp.ReferenceOTU69518
New.CleanUp.ReferenceOTU211207
New.CleanUp.ReferenceOTU146339
New.CleanUp.ReferenceOTU165913
New.CleanUp.ReferenceOTU110780
New.CleanUp.ReferenceOTU174206
New.CleanUp.ReferenceOTU188003
New.CleanUp.ReferenceOTU91811
New.CleanUp.ReferenceOTU264737
New.CleanUp.ReferenceOTU21240
New.CleanUp.ReferenceOTU18116
New.CleanUp.ReferenceOTU190662
New.CleanUp.ReferenceOTU178446
New.CleanUp.ReferenceOTU44601
New.CleanUp.ReferenceOTU303988
New.CleanUp.ReferenceOTU52772
New.CleanUp.ReferenceOTU25144
New.CleanUp.ReferenceOTU50402
New.CleanUp.ReferenceOTU31057
New.CleanUp.ReferenceOTU39002
New.CleanUp.ReferenceOTU34899
New.CleanUp.ReferenceOTU19947
New.CleanUp.ReferenceOTU39488
New.CleanUp.ReferenceOTU24291
New.CleanUp.ReferenceOTU85814
New.CleanUp.ReferenceOTU187883
New.CleanUp.ReferenceOTU148034

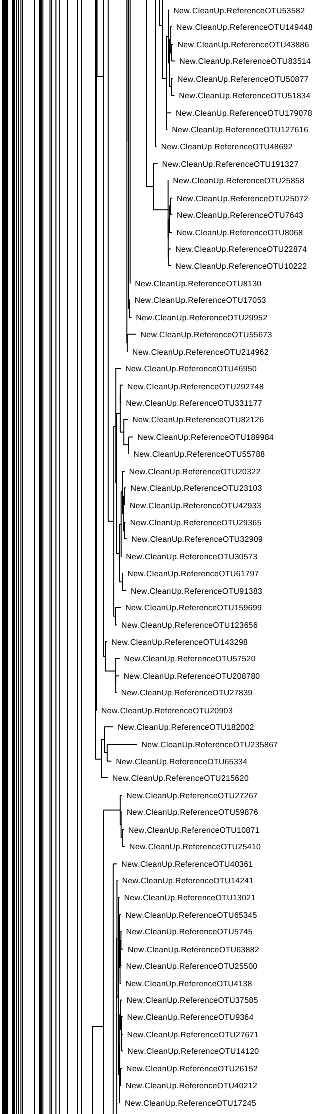
New.CleanUp.ReferenceOTU53582
New.CleanUp.ReferenceOTU149448
New.CleanUp.ReferenceOTU43886
New.CleanUp.ReferenceOTU83514
New.CleanUp.ReferenceOTU50877
New.CleanUp.ReferenceOTU51834
New.CleanUp.ReferenceOTU179078
New.CleanUp.ReferenceOTU127616
New.CleanUp.ReferenceOTU48692
New.CleanUp.ReferenceOTU191327
New.CleanUp.ReferenceOTU25858
New.CleanUp.ReferenceOTU25072
New.CleanUp.ReferenceOTU7643
New.CleanUp.ReferenceOTU8068
New.CleanUp.ReferenceOTU22874
New.CleanUp.ReferenceOTU10222
New.CleanUp.ReferenceOTU8130
New.CleanUp.ReferenceOTU17053
New.CleanUp.ReferenceOTU29952
New.CleanUp.ReferenceOTU55673
New.CleanUp.ReferenceOTU214962
New.CleanUp.ReferenceOTU46950
New.CleanUp.ReferenceOTU292748
New.CleanUp.ReferenceOTU331177
New.CleanUp.ReferenceOTU82126
New.CleanUp.ReferenceOTU189984
New.CleanUp.ReferenceOTU55788
New.CleanUp.ReferenceOTU20322
New.CleanUp.ReferenceOTU23103
New.CleanUp.ReferenceOTU42933
New.CleanUp.ReferenceOTU29365
New.CleanUp.ReferenceOTU32909
New.CleanUp.ReferenceOTU30573
New.CleanUp.ReferenceOTU61797
New.CleanUp.ReferenceOTU91383
New.CleanUp.ReferenceOTU159699
New.CleanUp.ReferenceOTU123656
New.CleanUp.ReferenceOTU143298
New.CleanUp.ReferenceOTU57520
New.CleanUp.ReferenceOTU208780
New.CleanUp.ReferenceOTU27839
New.CleanUp.ReferenceOTU20903
New.CleanUp.ReferenceOTU182002
New.CleanUp.ReferenceOTU235867
New.CleanUp.ReferenceOTU65334
New.CleanUp.ReferenceOTU215620
New.CleanUp.ReferenceOTU27267
New.CleanUp.ReferenceOTU59876
New.CleanUp.ReferenceOTU10871
New.CleanUp.ReferenceOTU25410
New.CleanUp.ReferenceOTU40361
New.CleanUp.ReferenceOTU14241
New.CleanUp.ReferenceOTU13021
New.CleanUp.ReferenceOTU65345
New.CleanUp.ReferenceOTU5745
New.CleanUp.ReferenceOTU63882
New.CleanUp.ReferenceOTU25500
New.CleanUp.ReferenceOTU4138
New.CleanUp.ReferenceOTU37585
New.CleanUp.ReferenceOTU9364
New.CleanUp.ReferenceOTU27671
New.CleanUp.ReferenceOTU14120
New.CleanUp.ReferenceOTU26152
New.CleanUp.ReferenceOTU40212
New.CleanUp.ReferenceOTU17245

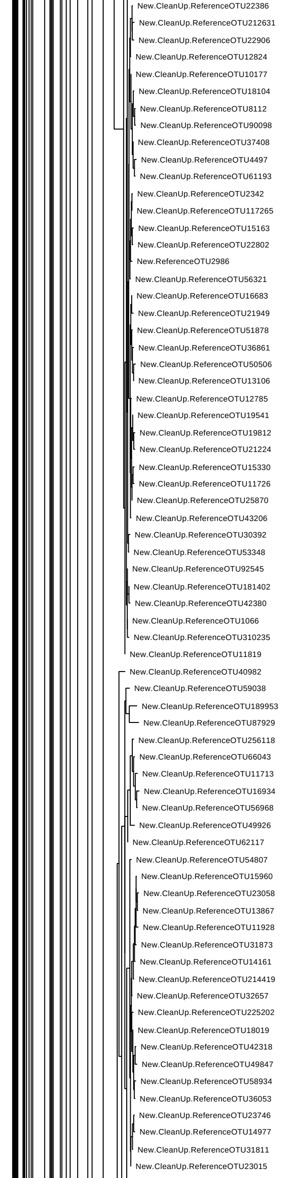
New.CleanUp.ReferenceOTU22386
New.CleanUp.ReferenceOTU212631
New.CleanUp.ReferenceOTU22906
New.CleanUp.ReferenceOTU12824
New.CleanUp.ReferenceOTU10177
New.CleanUp.ReferenceOTU18104
New.CleanUp.ReferenceOTU8112
New.CleanUp.ReferenceOTU90098
New.CleanUp.ReferenceOTU37408
New.CleanUp.ReferenceOTU4497
New.CleanUp.ReferenceOTU61193
New.CleanUp.ReferenceOTU2342
New.CleanUp.ReferenceOTU117265
New.CleanUp.ReferenceOTU15163
New.CleanUp.ReferenceOTU22802
New.ReferenceOTU2986
New.CleanUp.ReferenceOTU56321
New.CleanUp.ReferenceOTU16683
New.CleanUp.ReferenceOTU21949
New.CleanUp.ReferenceOTU51878
New.CleanUp.ReferenceOTU36861
New.CleanUp.ReferenceOTU50506
New.CleanUp.ReferenceOTU13106
New.CleanUp.ReferenceOTU12785
New.CleanUp.ReferenceOTU19541
New.CleanUp.ReferenceOTU19812
New.CleanUp.ReferenceOTU21224
New.CleanUp.ReferenceOTU15330
New.CleanUp.ReferenceOTU11726
New.CleanUp.ReferenceOTU25870
New.CleanUp.ReferenceOTU43206
New.CleanUp.ReferenceOTU30392
New.CleanUp.ReferenceOTU53348
New.CleanUp.ReferenceOTU92545
New.CleanUp.ReferenceOTU181402
New.CleanUp.ReferenceOTU42380
New.CleanUp.ReferenceOTU1066
New.CleanUp.ReferenceOTU310235
New.CleanUp.ReferenceOTU11819
New.CleanUp.ReferenceOTU40982
New.CleanUp.ReferenceOTU59038
New.CleanUp.ReferenceOTU189953
New.CleanUp.ReferenceOTU87929
New.CleanUp.ReferenceOTU256118
New.CleanUp.ReferenceOTU66043
New.CleanUp.ReferenceOTU11713
New.CleanUp.ReferenceOTU16934
New.CleanUp.ReferenceOTU56968
New.CleanUp.ReferenceOTU49926
New.CleanUp.ReferenceOTU62117
New.CleanUp.ReferenceOTU54807
New.CleanUp.ReferenceOTU15960
New.CleanUp.ReferenceOTU23058
New.CleanUp.ReferenceOTU13867
New.CleanUp.ReferenceOTU11928
New.CleanUp.ReferenceOTU31873
New.CleanUp.ReferenceOTU14161
New.CleanUp.ReferenceOTU214419
New.CleanUp.ReferenceOTU32657
New.CleanUp.ReferenceOTU225202
New.CleanUp.ReferenceOTU18019
New.CleanUp.ReferenceOTU42318
New.CleanUp.ReferenceOTU49847
New.CleanUp.ReferenceOTU58934
New.CleanUp.ReferenceOTU36053
New.CleanUp.ReferenceOTU23746
New.CleanUp.ReferenceOTU14977
New.CleanUp.ReferenceOTU31811
New.CleanUp.ReferenceOTU23015

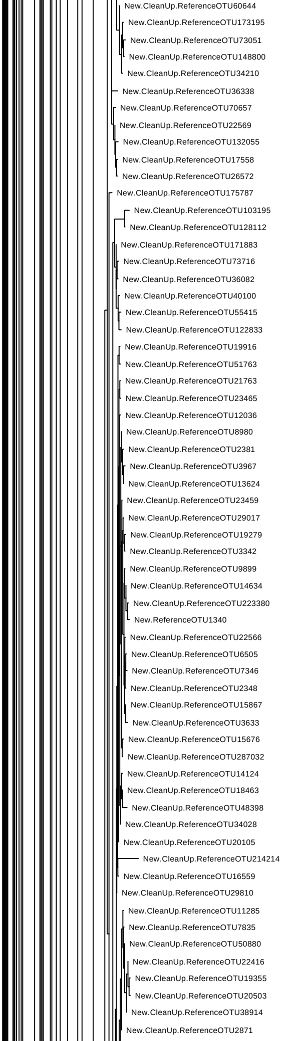
New.CleanUp.ReferenceOTU60644
New.CleanUp.ReferenceOTU173195
New.CleanUp.ReferenceOTU73051
New.CleanUp.ReferenceOTU148800
New.CleanUp.ReferenceOTU34210
New.CleanUp.ReferenceOTU36338
New.CleanUp.ReferenceOTU70657
New.CleanUp.ReferenceOTU22569
New.CleanUp.ReferenceOTU132055
New.CleanUp.ReferenceOTU17558
New.CleanUp.ReferenceOTU26572
New.CleanUp.ReferenceOTU175787
New.CleanUp.ReferenceOTU103195
New.CleanUp.ReferenceOTU128112
New.CleanUp.ReferenceOTU171883
New.CleanUp.ReferenceOTU73716
New.CleanUp.ReferenceOTU36082
New.CleanUp.ReferenceOTU40100
New.CleanUp.ReferenceOTU55415
New.CleanUp.ReferenceOTU122833
New.CleanUp.ReferenceOTU19916
New.CleanUp.ReferenceOTU51763
New.CleanUp.ReferenceOTU21763
New.CleanUp.ReferenceOTU23465
New.CleanUp.ReferenceOTU12036
New.CleanUp.ReferenceOTU8980
New.CleanUp.ReferenceOTU2381
New.CleanUp.ReferenceOTU3967
New.CleanUp.ReferenceOTU13624
New.CleanUp.ReferenceOTU23459
New.CleanUp.ReferenceOTU29017
New.CleanUp.ReferenceOTU19279
New.CleanUp.ReferenceOTU3342
New.CleanUp.ReferenceOTU9899
New.CleanUp.ReferenceOTU14634
New.CleanUp.ReferenceOTU223380
New.ReferenceOTU1340
New.CleanUp.ReferenceOTU22566
New.CleanUp.ReferenceOTU6505
New.CleanUp.ReferenceOTU7346
New.CleanUp.ReferenceOTU2348
New.CleanUp.ReferenceOTU15867
New.CleanUp.ReferenceOTU3633
New.CleanUp.ReferenceOTU15676
New.CleanUp.ReferenceOTU287032
New.CleanUp.ReferenceOTU14124
New.CleanUp.ReferenceOTU18463
New.CleanUp.ReferenceOTU48398
New.CleanUp.ReferenceOTU34028
New.CleanUp.ReferenceOTU20105
New.CleanUp.ReferenceOTU214214
New.CleanUp.ReferenceOTU16559
New.CleanUp.ReferenceOTU29810
New.CleanUp.ReferenceOTU11285
New.CleanUp.ReferenceOTU7835
New.CleanUp.ReferenceOTU50880
New.CleanUp.ReferenceOTU22416
New.CleanUp.ReferenceOTU19355
New.CleanUp.ReferenceOTU20503
New.CleanUp.ReferenceOTU38914
New.CleanUp.ReferenceOTU2871

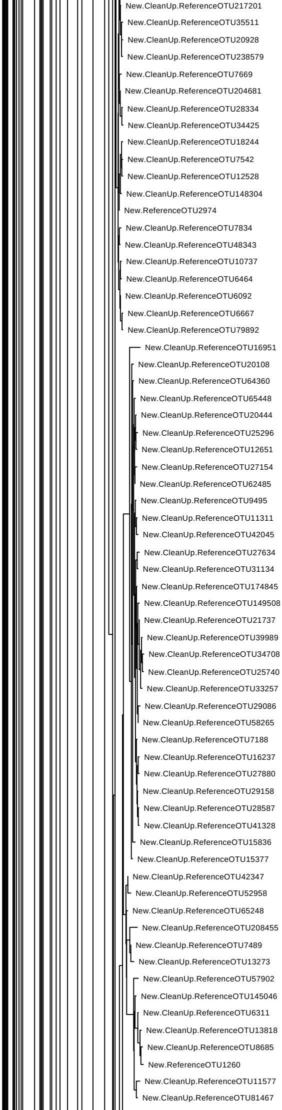
New.CleanUp.ReferenceOTU217201
New.CleanUp.ReferenceOTU35511
New.CleanUp.ReferenceOTU20928
New.CleanUp.ReferenceOTU238579
New.CleanUp.ReferenceOTU7669
New.CleanUp.ReferenceOTU204681
New.CleanUp.ReferenceOTU28334
New.CleanUp.ReferenceOTU34425
New.CleanUp.ReferenceOTU18244
New.CleanUp.ReferenceOTU7542
New.CleanUp.ReferenceOTU12528
New.CleanUp.ReferenceOTU148304
New.ReferenceOTU2974
New.CleanUp.ReferenceOTU7834
New.CleanUp.ReferenceOTU48343
New.CleanUp.ReferenceOTU10737
New.CleanUp.ReferenceOTU6464
New.CleanUp.ReferenceOTU6092
New.CleanUp.ReferenceOTU6667
New.CleanUp.ReferenceOTU79892
New.CleanUp.ReferenceOTU16951
New.CleanUp.ReferenceOTU20108
New.CleanUp.ReferenceOTU64360
New.CleanUp.ReferenceOTU65448
New.CleanUp.ReferenceOTU20444
New.CleanUp.ReferenceOTU25296
New.CleanUp.ReferenceOTU12651
New.CleanUp.ReferenceOTU27154
New.CleanUp.ReferenceOTU62485
New.CleanUp.ReferenceOTU9495
New.CleanUp.ReferenceOTU11311
New.CleanUp.ReferenceOTU42045
New.CleanUp.ReferenceOTU27634
New.CleanUp.ReferenceOTU31134
New.CleanUp.ReferenceOTU174845
New.CleanUp.ReferenceOTU149508
New.CleanUp.ReferenceOTU21737
New.CleanUp.ReferenceOTU39989
New.CleanUp.ReferenceOTU34708
New.CleanUp.ReferenceOTU25740
New.CleanUp.ReferenceOTU33257
New.CleanUp.ReferenceOTU29086
New.CleanUp.ReferenceOTU58265
New.CleanUp.ReferenceOTU7188
New.CleanUp.ReferenceOTU16237
New.CleanUp.ReferenceOTU27880
New.CleanUp.ReferenceOTU29158
New.CleanUp.ReferenceOTU28587
New.CleanUp.ReferenceOTU41328
New.CleanUp.ReferenceOTU15836
New.CleanUp.ReferenceOTU15377
New.CleanUp.ReferenceOTU42347
New.CleanUp.ReferenceOTU52958
New.CleanUp.ReferenceOTU65248
New.CleanUp.ReferenceOTU208455
New.CleanUp.ReferenceOTU7489
New.CleanUp.ReferenceOTU13273
New.CleanUp.ReferenceOTU57902
New.CleanUp.ReferenceOTU145046
New.CleanUp.ReferenceOTU6311
New.CleanUp.ReferenceOTU13818
New.CleanUp.ReferenceOTU8685
New.ReferenceOTU1260
New.CleanUp.ReferenceOTU11577
New.CleanUp.ReferenceOTU81467

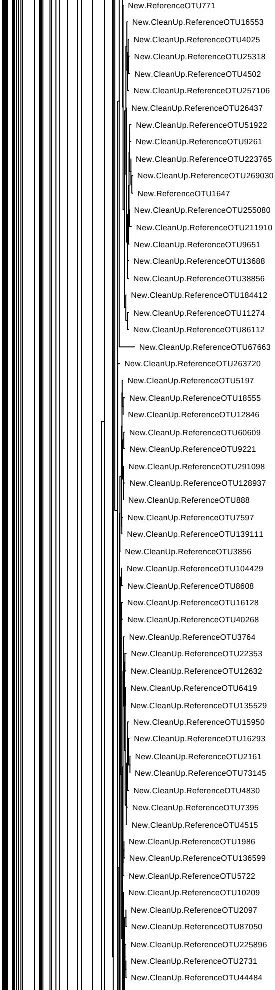
New.ReferenceOTU771
New.CleanUp.ReferenceOTU16553
New.CleanUp.ReferenceOTU4025
New.CleanUp.ReferenceOTU25318
New.CleanUp.ReferenceOTU4502
New.CleanUp.ReferenceOTU257106
New.CleanUp.ReferenceOTU26437
New.CleanUp.ReferenceOTU51922
New.CleanUp.ReferenceOTU9261
New.CleanUp.ReferenceOTU223765
New.CleanUp.ReferenceOTU269030
New.ReferenceOTU1647
New.CleanUp.ReferenceOTU255080
New.CleanUp.ReferenceOTU211910
New.CleanUp.ReferenceOTU9651
New.CleanUp.ReferenceOTU13688
New.CleanUp.ReferenceOTU38856
New.CleanUp.ReferenceOTU184412
New.CleanUp.ReferenceOTU11274
New.CleanUp.ReferenceOTU86112
New.CleanUp.ReferenceOTU67663
New.CleanUp.ReferenceOTU263720
New.CleanUp.ReferenceOTU5197
New.CleanUp.ReferenceOTU18555
New.CleanUp.ReferenceOTU12846
New.CleanUp.ReferenceOTU60609
New.CleanUp.ReferenceOTU9221
New.CleanUp.ReferenceOTU291098
New.CleanUp.ReferenceOTU128937
New.CleanUp.ReferenceOTU888
New.CleanUp.ReferenceOTU7597
New.CleanUp.ReferenceOTU139111
New.CleanUp.ReferenceOTU3856
New.CleanUp.ReferenceOTU104429
New.CleanUp.ReferenceOTU8608
New.CleanUp.ReferenceOTU16128
New.CleanUp.ReferenceOTU40268
New.CleanUp.ReferenceOTU3764
New.CleanUp.ReferenceOTU22353
New.CleanUp.ReferenceOTU12632
New.CleanUp.ReferenceOTU6419
New.CleanUp.ReferenceOTU135529
New.CleanUp.ReferenceOTU15950
New.CleanUp.ReferenceOTU16293
New.CleanUp.ReferenceOTU2161
New.CleanUp.ReferenceOTU73145
New.CleanUp.ReferenceOTU4830
New.CleanUp.ReferenceOTU7395
New.CleanUp.ReferenceOTU4515
New.CleanUp.ReferenceOTU1986
New.CleanUp.ReferenceOTU136599
New.CleanUp.ReferenceOTU5722
New.CleanUp.ReferenceOTU10209
New.CleanUp.ReferenceOTU2097
New.CleanUp.ReferenceOTU87050
New.CleanUp.ReferenceOTU225896
New.CleanUp.ReferenceOTU2731
New.CleanUp.ReferenceOTU44484

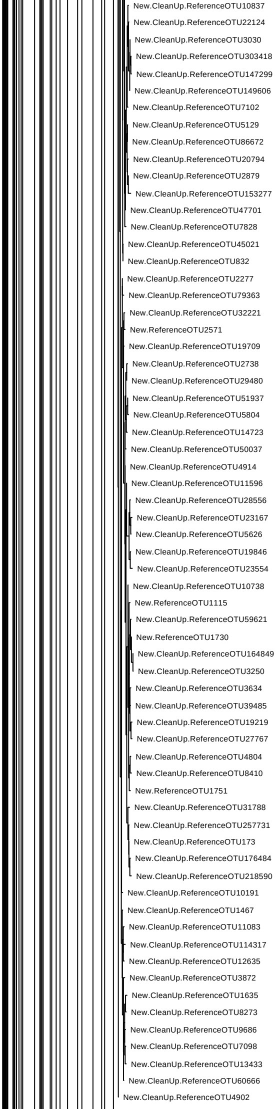
New.CleanUp.ReferenceOTU10837
New.CleanUp.ReferenceOTU22124
New.CleanUp.ReferenceOTU3030
New.CleanUp.ReferenceOTU303418
New.CleanUp.ReferenceOTU147299
New.CleanUp.ReferenceOTU149606
New.CleanUp.ReferenceOTU7102
New.CleanUp.ReferenceOTU5129
New.CleanUp.ReferenceOTU86672
New.CleanUp.ReferenceOTU20794
New.CleanUp.ReferenceOTU2879
New.CleanUp.ReferenceOTU153277
New.CleanUp.ReferenceOTU47701
New.CleanUp.ReferenceOTU7828
New.CleanUp.ReferenceOTU45021
New.CleanUp.ReferenceOTU832
New.CleanUp.ReferenceOTU2277
New.CleanUp.ReferenceOTU79363
New.CleanUp.ReferenceOTU32221
New.ReferenceOTU2571
New.CleanUp.ReferenceOTU19709
New.CleanUp.ReferenceOTU2738
New.CleanUp.ReferenceOTU29480
New.CleanUp.ReferenceOTU51937
New.CleanUp.ReferenceOTU5804
New.CleanUp.ReferenceOTU14723
New.CleanUp.ReferenceOTU50037
New.CleanUp.ReferenceOTU4914
New.CleanUp.ReferenceOTU11596
New.CleanUp.ReferenceOTU28556
New.CleanUp.ReferenceOTU23167
New.CleanUp.ReferenceOTU5626
New.CleanUp.ReferenceOTU19846
New.CleanUp.ReferenceOTU23554
New.CleanUp.ReferenceOTU10738
New.ReferenceOTU1115
New.CleanUp.ReferenceOTU59621
New.ReferenceOTU1730
New.CleanUp.ReferenceOTU164849
New.CleanUp.ReferenceOTU3250
New.CleanUp.ReferenceOTU3634
New.CleanUp.ReferenceOTU39485
New.CleanUp.ReferenceOTU19219
New.CleanUp.ReferenceOTU27767
New.CleanUp.ReferenceOTU4804
New.CleanUp.ReferenceOTU8410
New.ReferenceOTU1751
New.CleanUp.ReferenceOTU31788
New.CleanUp.ReferenceOTU257731
New.CleanUp.ReferenceOTU173
New.CleanUp.ReferenceOTU176484
New.CleanUp.ReferenceOTU218590
New.CleanUp.ReferenceOTU10191
New.CleanUp.ReferenceOTU1467
New.CleanUp.ReferenceOTU11083
New.CleanUp.ReferenceOTU114317
New.CleanUp.ReferenceOTU12635
New.CleanUp.ReferenceOTU3872
New.CleanUp.ReferenceOTU1635
New.CleanUp.ReferenceOTU8273
New.CleanUp.ReferenceOTU9686
New.CleanUp.ReferenceOTU7098
New.CleanUp.ReferenceOTU13433
New.CleanUp.ReferenceOTU60666
New.CleanUp.ReferenceOTU4902

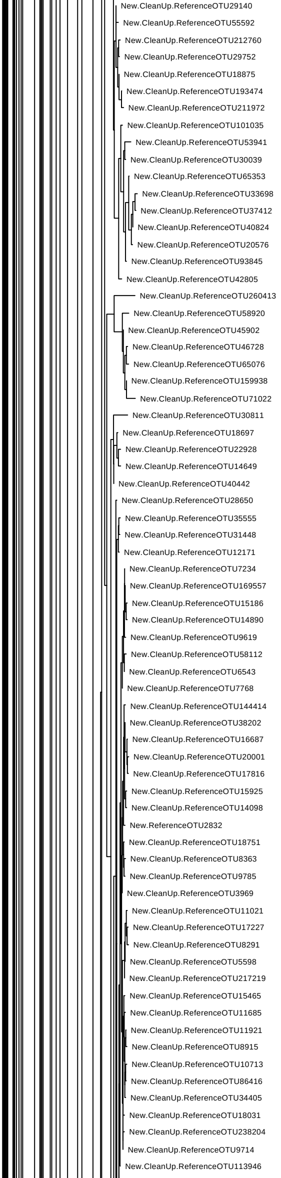

New.CleanUp.ReferenceOTU29140
New.CleanUp.ReferenceOTU55592
New.CleanUp.ReferenceOTU212760
New.CleanUp.ReferenceOTU29752
New.CleanUp.ReferenceOTU18875
New.CleanUp.ReferenceOTU193474
New.CleanUp.ReferenceOTU211972
New.CleanUp.ReferenceOTU101035
New.CleanUp.ReferenceOTU53941
New.CleanUp.ReferenceOTU30039
New.CleanUp.ReferenceOTU65353
New.CleanUp.ReferenceOTU33698
New.CleanUp.ReferenceOTU37412
New.CleanUp.ReferenceOTU40824
New.CleanUp.ReferenceOTU20576
New.CleanUp.ReferenceOTU93845
New.CleanUp.ReferenceOTU42805
New.CleanUp.ReferenceOTU260413
New.CleanUp.ReferenceOTU58920
New.CleanUp.ReferenceOTU45902
New.CleanUp.ReferenceOTU46728
New.CleanUp.ReferenceOTU65076
New.CleanUp.ReferenceOTU159938
New.CleanUp.ReferenceOTU71022
New.CleanUp.ReferenceOTU30811
New.CleanUp.ReferenceOTU18697
New.CleanUp.ReferenceOTU22928
New.CleanUp.ReferenceOTU14649
New.CleanUp.ReferenceOTU40442
New.CleanUp.ReferenceOTU28650
New.CleanUp.ReferenceOTU35555
New.CleanUp.ReferenceOTU31448
New.CleanUp.ReferenceOTU12171
New.CleanUp.ReferenceOTU7234
New.CleanUp.ReferenceOTU169557
New.CleanUp.ReferenceOTU15186
New.CleanUp.ReferenceOTU14890
New.CleanUp.ReferenceOTU9619
New.CleanUp.ReferenceOTU58112
New.CleanUp.ReferenceOTU6543
New.CleanUp.ReferenceOTU7768
New.CleanUp.ReferenceOTU144414
New.CleanUp.ReferenceOTU38202
New.CleanUp.ReferenceOTU16687
New.CleanUp.ReferenceOTU20001
New.CleanUp.ReferenceOTU17816
New.CleanUp.ReferenceOTU15925
New.CleanUp.ReferenceOTU14098
New.ReferenceOTU2832
New.CleanUp.ReferenceOTU18751
New.CleanUp.ReferenceOTU8363
New.CleanUp.ReferenceOTU9785
New.CleanUp.ReferenceOTU3969
New.CleanUp.ReferenceOTU11021
New.CleanUp.ReferenceOTU17227
New.CleanUp.ReferenceOTU8291
New.CleanUp.ReferenceOTU5598
New.CleanUp.ReferenceOTU217219
New.CleanUp.ReferenceOTU15465
New.CleanUp.ReferenceOTU11685
New.CleanUp.ReferenceOTU11921
New.CleanUp.ReferenceOTU8915
New.CleanUp.ReferenceOTU10713
New.CleanUp.ReferenceOTU86416
New.CleanUp.ReferenceOTU34405
New.CleanUp.ReferenceOTU18031
New.CleanUp.ReferenceOTU238204
New.CleanUp.ReferenceOTU9714
New.CleanUp.ReferenceOTU113946

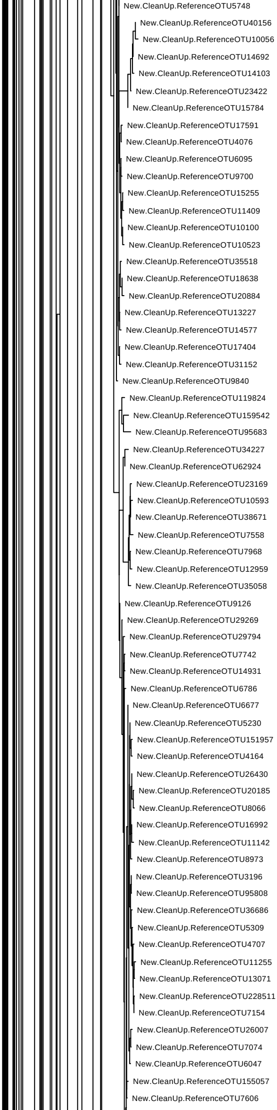
New.CleanUp.ReferenceOTU5748
New.CleanUp.ReferenceOTU40156
New.CleanUp.ReferenceOTU10056
New.CleanUp.ReferenceOTU14692
New.CleanUp.ReferenceOTU14103
New.CleanUp.ReferenceOTU23422
New.CleanUp.ReferenceOTU15784
New.CleanUp.ReferenceOTU17591
New.CleanUp.ReferenceOTU4076
New.CleanUp.ReferenceOTU6095
New.CleanUp.ReferenceOTU9700
New.CleanUp.ReferenceOTU15255
New.CleanUp.ReferenceOTU11409
New.CleanUp.ReferenceOTU10100
New.CleanUp.ReferenceOTU10523
New.CleanUp.ReferenceOTU35518
New.CleanUp.ReferenceOTU18638
New.CleanUp.ReferenceOTU20884
New.CleanUp.ReferenceOTU13227
New.CleanUp.ReferenceOTU14577
New.CleanUp.ReferenceOTU17404
New.CleanUp.ReferenceOTU31152
New.CleanUp.ReferenceOTU9840
New.CleanUp.ReferenceOTU119824
New.CleanUp.ReferenceOTU159542
New.CleanUp.ReferenceOTU95683
New.CleanUp.ReferenceOTU34227
New.CleanUp.ReferenceOTU62924
New.CleanUp.ReferenceOTU23169
New.CleanUp.ReferenceOTU10593
New.CleanUp.ReferenceOTU38671
New.CleanUp.ReferenceOTU7558
New.CleanUp.ReferenceOTU7968
New.CleanUp.ReferenceOTU12959
New.CleanUp.ReferenceOTU35058
New.CleanUp.ReferenceOTU9126
New.CleanUp.ReferenceOTU29269
New.CleanUp.ReferenceOTU29794
New.CleanUp.ReferenceOTU7742
New.CleanUp.ReferenceOTU14931
New.CleanUp.ReferenceOTU6786
New.CleanUp.ReferenceOTU6677
New.CleanUp.ReferenceOTU5230
New.CleanUp.ReferenceOTU151957
New.CleanUp.ReferenceOTU4164
New.CleanUp.ReferenceOTU26430
New.CleanUp.ReferenceOTU20185
New.CleanUp.ReferenceOTU8066
New.CleanUp.ReferenceOTU16992
New.CleanUp.ReferenceOTU11142
New.CleanUp.ReferenceOTU8973
New.CleanUp.ReferenceOTU3196
New.CleanUp.ReferenceOTU95808
New.CleanUp.ReferenceOTU36686
New.CleanUp.ReferenceOTU5309
New.CleanUp.ReferenceOTU4707
New.CleanUp.ReferenceOTU11255
New.CleanUp.ReferenceOTU13071
New.CleanUp.ReferenceOTU228511
New.CleanUp.ReferenceOTU7154
New.CleanUp.ReferenceOTU26007
New.CleanUp.ReferenceOTU7074
New.CleanUp.ReferenceOTU6047
New.CleanUp.ReferenceOTU155057
New.CleanUp.ReferenceOTU7606

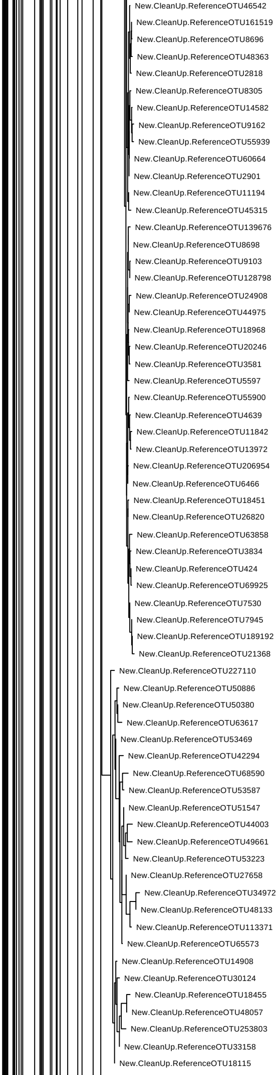
New.CleanUp.ReferenceOTU46542
New.CleanUp.ReferenceOTU161519
New.CleanUp.ReferenceOTU8696
New.CleanUp.ReferenceOTU48363
New.CleanUp.ReferenceOTU2818
New.CleanUp.ReferenceOTU8305
New.CleanUp.ReferenceOTU14582
New.CleanUp.ReferenceOTU9162
New.CleanUp.ReferenceOTU55939
New.CleanUp.ReferenceOTU60664
New.CleanUp.ReferenceOTU2901
New.CleanUp.ReferenceOTU11194
New.CleanUp.ReferenceOTU45315
New.CleanUp.ReferenceOTU139676
New.CleanUp.ReferenceOTU8698
New.CleanUp.ReferenceOTU9103
New.CleanUp.ReferenceOTU128798
New.CleanUp.ReferenceOTU24908
New.CleanUp.ReferenceOTU44975
New.CleanUp.ReferenceOTU18968
New.CleanUp.ReferenceOTU20246
New.CleanUp.ReferenceOTU3581
New.CleanUp.ReferenceOTU5597
New.CleanUp.ReferenceOTU55900
New.CleanUp.ReferenceOTU4639
New.CleanUp.ReferenceOTU11842
New.CleanUp.ReferenceOTU13972
New.CleanUp.ReferenceOTU206954
New.CleanUp.ReferenceOTU6466
New.CleanUp.ReferenceOTU18451
New.CleanUp.ReferenceOTU26820
New.CleanUp.ReferenceOTU63858
New.CleanUp.ReferenceOTU3834
New.CleanUp.ReferenceOTU424
New.CleanUp.ReferenceOTU69925
New.CleanUp.ReferenceOTU7530
New.CleanUp.ReferenceOTU7945
New.CleanUp.ReferenceOTU189192
New.CleanUp.ReferenceOTU21368
New.CleanUp.ReferenceOTU227110
New.CleanUp.ReferenceOTU50886
New.CleanUp.ReferenceOTU50380
New.CleanUp.ReferenceOTU63617
New.CleanUp.ReferenceOTU53469
New.CleanUp.ReferenceOTU42294
New.CleanUp.ReferenceOTU68590
New.CleanUp.ReferenceOTU53587
New.CleanUp.ReferenceOTU51547
New.CleanUp.ReferenceOTU44003
New.CleanUp.ReferenceOTU49661
New.CleanUp.ReferenceOTU53223
New.CleanUp.ReferenceOTU27658
New.CleanUp.ReferenceOTU34972
New.CleanUp.ReferenceOTU48133
New.CleanUp.ReferenceOTU113371
New.CleanUp.ReferenceOTU65573
New.CleanUp.ReferenceOTU14908
New.CleanUp.ReferenceOTU30124
New.CleanUp.ReferenceOTU18455
New.CleanUp.ReferenceOTU48057
New.CleanUp.ReferenceOTU253803
New.CleanUp.ReferenceOTU33158
New.CleanUp.ReferenceOTU18115

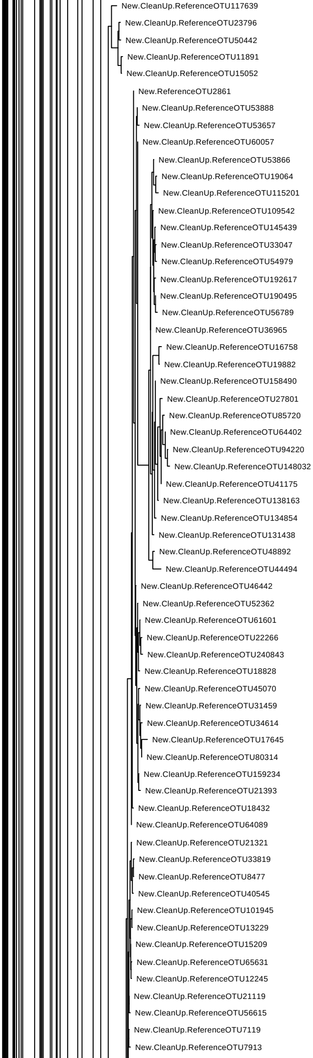
New.CleanUp.ReferenceOTU117639
New.CleanUp.ReferenceOTU23796
New.CleanUp.ReferenceOTU50442
New.CleanUp.ReferenceOTU11891
New.CleanUp.ReferenceOTU15052
New.ReferenceOTU2861
New.CleanUp.ReferenceOTU53888
New.CleanUp.ReferenceOTU53657
New.CleanUp.ReferenceOTU60057
New.CleanUp.ReferenceOTU53866
New.CleanUp.ReferenceOTU19064
New.CleanUp.ReferenceOTU115201
New.CleanUp.ReferenceOTU109542
New.CleanUp.ReferenceOTU145439
New.CleanUp.ReferenceOTU33047
New.CleanUp.ReferenceOTU54979
New.CleanUp.ReferenceOTU192617
New.CleanUp.ReferenceOTU190495
New.CleanUp.ReferenceOTU56789
New.CleanUp.ReferenceOTU36965
New.CleanUp.ReferenceOTU16758
New.CleanUp.ReferenceOTU19882
New.CleanUp.ReferenceOTU158490
New.CleanUp.ReferenceOTU27801
New.CleanUp.ReferenceOTU85720
New.CleanUp.ReferenceOTU64402
New.CleanUp.ReferenceOTU94220
New.CleanUp.ReferenceOTU148032
New.CleanUp.ReferenceOTU41175
New.CleanUp.ReferenceOTU138163
New.CleanUp.ReferenceOTU134854
New.CleanUp.ReferenceOTU131438
New.CleanUp.ReferenceOTU48892
New.CleanUp.ReferenceOTU44494
New.CleanUp.ReferenceOTU46442
New.CleanUp.ReferenceOTU52362
New.CleanUp.ReferenceOTU61601
New.CleanUp.ReferenceOTU22266
New.CleanUp.ReferenceOTU240843
New.CleanUp.ReferenceOTU18828
New.CleanUp.ReferenceOTU45070
New.CleanUp.ReferenceOTU31459
New.CleanUp.ReferenceOTU34614
New.CleanUp.ReferenceOTU17645
New.CleanUp.ReferenceOTU80314
New.CleanUp.ReferenceOTU159234
New.CleanUp.ReferenceOTU21393
New.CleanUp.ReferenceOTU18432
New.CleanUp.ReferenceOTU64089
New.CleanUp.ReferenceOTU21321
New.CleanUp.ReferenceOTU33819
New.CleanUp.ReferenceOTU8477
New.CleanUp.ReferenceOTU40545
New.CleanUp.ReferenceOTU101945
New.CleanUp.ReferenceOTU13229
New.CleanUp.ReferenceOTU15209
New.CleanUp.ReferenceOTU65631
New.CleanUp.ReferenceOTU12245
New.CleanUp.ReferenceOTU21119
New.CleanUp.ReferenceOTU56615
New.CleanUp.ReferenceOTU7119
New.CleanUp.ReferenceOTU7913

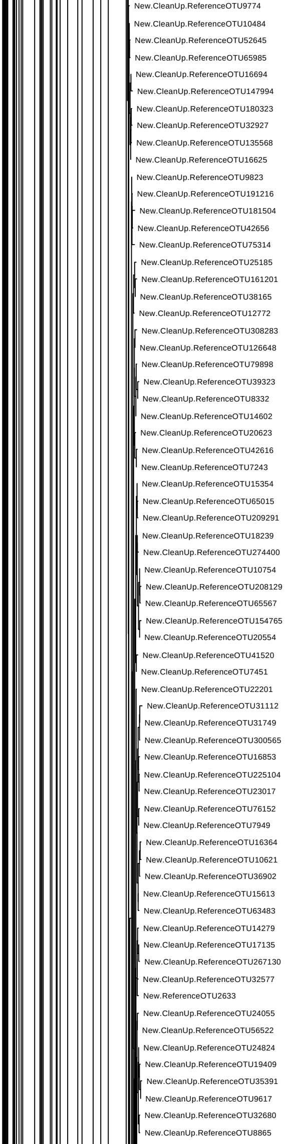
New.CleanUp.ReferenceOTU9774
New.CleanUp.ReferenceOTU10484
New.CleanUp.ReferenceOTU52645
New.CleanUp.ReferenceOTU65985
New.CleanUp.ReferenceOTU16694
New.CleanUp.ReferenceOTU147994
New.CleanUp.ReferenceOTU180323
New.CleanUp.ReferenceOTU32927
New.CleanUp.ReferenceOTU135568
New.CleanUp.ReferenceOTU16625
New.CleanUp.ReferenceOTU9823
New.CleanUp.ReferenceOTU191216
New.CleanUp.ReferenceOTU181504
New.CleanUp.ReferenceOTU42656
New.CleanUp.ReferenceOTU75314
New.CleanUp.ReferenceOTU25185
New.CleanUp.ReferenceOTU161201
New.CleanUp.ReferenceOTU38165
New.CleanUp.ReferenceOTU12772
New.CleanUp.ReferenceOTU308283
New.CleanUp.ReferenceOTU126648
New.CleanUp.ReferenceOTU79898
New.CleanUp.ReferenceOTU39323
New.CleanUp.ReferenceOTU8332
New.CleanUp.ReferenceOTU14602
New.CleanUp.ReferenceOTU20623
New.CleanUp.ReferenceOTU42616
New.CleanUp.ReferenceOTU7243
New.CleanUp.ReferenceOTU15354
New.CleanUp.ReferenceOTU65015
New.CleanUp.ReferenceOTU209291
New.CleanUp.ReferenceOTU18239
New.CleanUp.ReferenceOTU274400
New.CleanUp.ReferenceOTU10754
New.CleanUp.ReferenceOTU208129
New.CleanUp.ReferenceOTU65567
New.CleanUp.ReferenceOTU154765
New.CleanUp.ReferenceOTU20554
New.CleanUp.ReferenceOTU41520
New.CleanUp.ReferenceOTU7451
New.CleanUp.ReferenceOTU22201
New.CleanUp.ReferenceOTU31112
New.CleanUp.ReferenceOTU31749
New.CleanUp.ReferenceOTU300565
New.CleanUp.ReferenceOTU16853
New.CleanUp.ReferenceOTU225104
New.CleanUp.ReferenceOTU23017
New.CleanUp.ReferenceOTU76152
New.CleanUp.ReferenceOTU7949
New.CleanUp.ReferenceOTU16364
New.CleanUp.ReferenceOTU10621
New.CleanUp.ReferenceOTU36902
New.CleanUp.ReferenceOTU15613
New.CleanUp.ReferenceOTU63483
New.CleanUp.ReferenceOTU14279
New.CleanUp.ReferenceOTU17135
New.CleanUp.ReferenceOTU267130
New.CleanUp.ReferenceOTU32577
New.ReferenceOTU2633
New.CleanUp.ReferenceOTU24055
New.CleanUp.ReferenceOTU56522
New.CleanUp.ReferenceOTU24824
New.CleanUp.ReferenceOTU19409
New.CleanUp.ReferenceOTU35391
New.CleanUp.ReferenceOTU9617
New.CleanUp.ReferenceOTU32680
New.CleanUp.ReferenceOTU8865

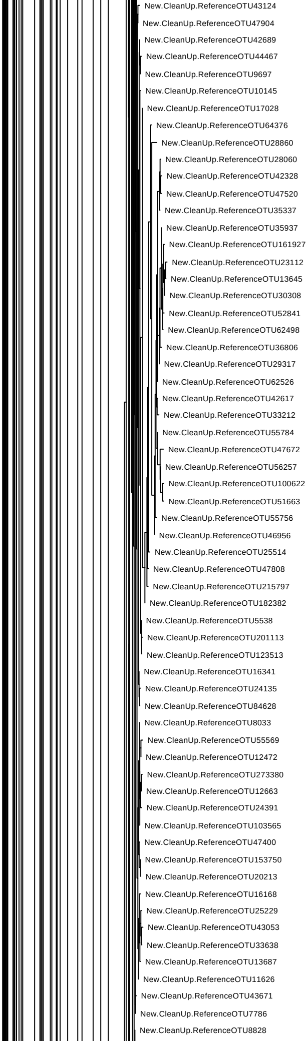
New.CleanUp.ReferenceOTU43124
New.CleanUp.ReferenceOTU47904
New.CleanUp.ReferenceOTU42689
New.CleanUp.ReferenceOTU44467
New.CleanUp.ReferenceOTU9697
New.CleanUp.ReferenceOTU10145
New.CleanUp.ReferenceOTU17028
New.CleanUp.ReferenceOTU64376
New.CleanUp.ReferenceOTU28860
New.CleanUp.ReferenceOTU28060
New.CleanUp.ReferenceOTU42328
New.CleanUp.ReferenceOTU47520
New.CleanUp.ReferenceOTU35337
New.CleanUp.ReferenceOTU35937
New.CleanUp.ReferenceOTU161927
New.CleanUp.ReferenceOTU23112
New.CleanUp.ReferenceOTU13645
New.CleanUp.ReferenceOTU30308
New.CleanUp.ReferenceOTU52841
New.CleanUp.ReferenceOTU62498
New.CleanUp.ReferenceOTU36806
New.CleanUp.ReferenceOTU29317
New.CleanUp.ReferenceOTU62526
New.CleanUp.ReferenceOTU42617
New.CleanUp.ReferenceOTU33212
New.CleanUp.ReferenceOTU55784
New.CleanUp.ReferenceOTU47672
New.CleanUp.ReferenceOTU56257
New.CleanUp.ReferenceOTU100622
New.CleanUp.ReferenceOTU51663
New.CleanUp.ReferenceOTU55756
New.CleanUp.ReferenceOTU46956
New.CleanUp.ReferenceOTU25514
New.CleanUp.ReferenceOTU47808
New.CleanUp.ReferenceOTU215797
New.CleanUp.ReferenceOTU182382
New.CleanUp.ReferenceOTU5538
New.CleanUp.ReferenceOTU201113
New.CleanUp.ReferenceOTU123513
New.CleanUp.ReferenceOTU16341
New.CleanUp.ReferenceOTU24135
New.CleanUp.ReferenceOTU84628
New.CleanUp.ReferenceOTU8033
New.CleanUp.ReferenceOTU55569
New.CleanUp.ReferenceOTU12472
New.CleanUp.ReferenceOTU273380
New.CleanUp.ReferenceOTU12663
New.CleanUp.ReferenceOTU24391
New.CleanUp.ReferenceOTU103565
New.CleanUp.ReferenceOTU47400
New.CleanUp.ReferenceOTU153750
New.CleanUp.ReferenceOTU20213
New.CleanUp.ReferenceOTU16168
New.CleanUp.ReferenceOTU25229
New.CleanUp.ReferenceOTU43053
New.CleanUp.ReferenceOTU33638
New.CleanUp.ReferenceOTU13687
New.CleanUp.ReferenceOTU11626
New.CleanUp.ReferenceOTU43671
New.CleanUp.ReferenceOTU7786
New.CleanUp.ReferenceOTU8828

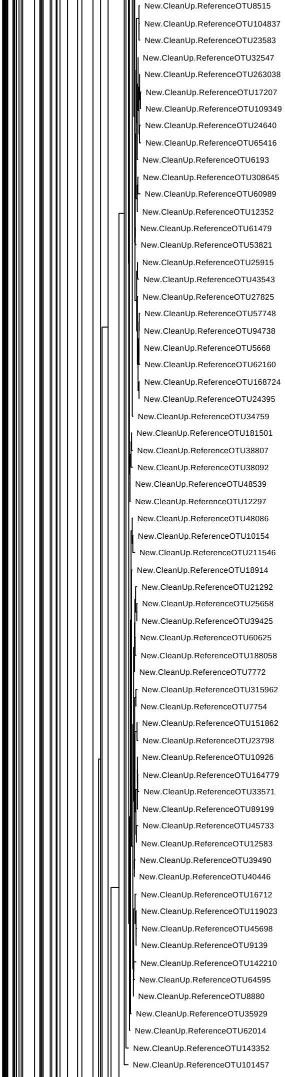
New.CleanUp.ReferenceOTU8515
New.CleanUp.ReferenceOTU104837
New.CleanUp.ReferenceOTU23583
New.CleanUp.ReferenceOTU32547
New.CleanUp.ReferenceOTU263038
New.CleanUp.ReferenceOTU17207
New.CleanUp.ReferenceOTU109349
New.CleanUp.ReferenceOTU24640
New.CleanUp.ReferenceOTU65416
New.CleanUp.ReferenceOTU6193
New.CleanUp.ReferenceOTU308645
New.CleanUp.ReferenceOTU60989
New.CleanUp.ReferenceOTU12352
New.CleanUp.ReferenceOTU61479
New.CleanUp.ReferenceOTU53821
New.CleanUp.ReferenceOTU25915
New.CleanUp.ReferenceOTU43543
New.CleanUp.ReferenceOTU27825
New.CleanUp.ReferenceOTU57748
New.CleanUp.ReferenceOTU94738
New.CleanUp.ReferenceOTU5668
New.CleanUp.ReferenceOTU62160
New.CleanUp.ReferenceOTU168724
New.CleanUp.ReferenceOTU24395
New.CleanUp.ReferenceOTU34759
New.CleanUp.ReferenceOTU181501
New.CleanUp.ReferenceOTU38807
New.CleanUp.ReferenceOTU38092
New.CleanUp.ReferenceOTU48539
New.CleanUp.ReferenceOTU12297
New.CleanUp.ReferenceOTU48086
New.CleanUp.ReferenceOTU10154
New.CleanUp.ReferenceOTU211546
New.CleanUp.ReferenceOTU18914
New.CleanUp.ReferenceOTU21292
New.CleanUp.ReferenceOTU25658
New.CleanUp.ReferenceOTU39425
New.CleanUp.ReferenceOTU60625
New.CleanUp.ReferenceOTU188058
New.CleanUp.ReferenceOTU7772
New.CleanUp.ReferenceOTU315962
New.CleanUp.ReferenceOTU7754
New.CleanUp.ReferenceOTU151862
New.CleanUp.ReferenceOTU23798
New.CleanUp.ReferenceOTU10926
New.CleanUp.ReferenceOTU164779
New.CleanUp.ReferenceOTU33571
New.CleanUp.ReferenceOTU89199
New.CleanUp.ReferenceOTU45733
New.CleanUp.ReferenceOTU12583
New.CleanUp.ReferenceOTU39490
New.CleanUp.ReferenceOTU40446
New.CleanUp.ReferenceOTU16712
New.CleanUp.ReferenceOTU119023
New.CleanUp.ReferenceOTU45698
New.CleanUp.ReferenceOTU9139
New.CleanUp.ReferenceOTU142210
New.CleanUp.ReferenceOTU64595
New.CleanUp.ReferenceOTU8880
New.CleanUp.ReferenceOTU35929
New.CleanUp.ReferenceOTU62014
New.CleanUp.ReferenceOTU143352
New.CleanUp.ReferenceOTU101457

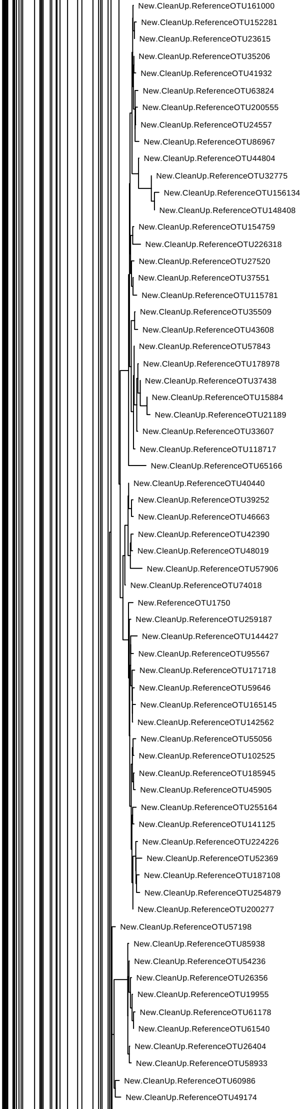
New.CleanUp.ReferenceOTU161000
New.CleanUp.ReferenceOTU152281
New.CleanUp.ReferenceOTU23615
New.CleanUp.ReferenceOTU35206
New.CleanUp.ReferenceOTU41932
New.CleanUp.ReferenceOTU63824
New.CleanUp.ReferenceOTU200555
New.CleanUp.ReferenceOTU24557
New.CleanUp.ReferenceOTU86967
New.CleanUp.ReferenceOTU44804
New.CleanUp.ReferenceOTU32775
New.CleanUp.ReferenceOTU156134
New.CleanUp.ReferenceOTU148408
New.CleanUp.ReferenceOTU154759
New.CleanUp.ReferenceOTU226318
New.CleanUp.ReferenceOTU27520
New.CleanUp.ReferenceOTU37551
New.CleanUp.ReferenceOTU115781
New.CleanUp.ReferenceOTU35509
New.CleanUp.ReferenceOTU43608
New.CleanUp.ReferenceOTU57843
New.CleanUp.ReferenceOTU178978
New.CleanUp.ReferenceOTU37438
New.CleanUp.ReferenceOTU15884
New.CleanUp.ReferenceOTU21189
New.CleanUp.ReferenceOTU33607
New.CleanUp.ReferenceOTU118717
New.CleanUp.ReferenceOTU65166
New.CleanUp.ReferenceOTU40440
New.CleanUp.ReferenceOTU39252
New.CleanUp.ReferenceOTU46663
New.CleanUp.ReferenceOTU42390
New.CleanUp.ReferenceOTU48019
New.CleanUp.ReferenceOTU57906
New.CleanUp.ReferenceOTU74018
New.ReferenceOTU1750
New.CleanUp.ReferenceOTU259187
New.CleanUp.ReferenceOTU144427
New.CleanUp.ReferenceOTU95567
New.CleanUp.ReferenceOTU171718
New.CleanUp.ReferenceOTU59646
New.CleanUp.ReferenceOTU165145
New.CleanUp.ReferenceOTU142562
New.CleanUp.ReferenceOTU55056
New.CleanUp.ReferenceOTU102525
New.CleanUp.ReferenceOTU185945
New.CleanUp.ReferenceOTU45905
New.CleanUp.ReferenceOTU255164
New.CleanUp.ReferenceOTU141125
New.CleanUp.ReferenceOTU224226
New.CleanUp.ReferenceOTU52369
New.CleanUp.ReferenceOTU187108
New.CleanUp.ReferenceOTU254879
New.CleanUp.ReferenceOTU200277
New.CleanUp.ReferenceOTU57198
New.CleanUp.ReferenceOTU85938
New.CleanUp.ReferenceOTU54236
New.CleanUp.ReferenceOTU26356
New.CleanUp.ReferenceOTU19955
New.CleanUp.ReferenceOTU61178
New.CleanUp.ReferenceOTU61540
New.CleanUp.ReferenceOTU26404
New.CleanUp.ReferenceOTU58933
New.CleanUp.ReferenceOTU60986
New.CleanUp.ReferenceOTU49174

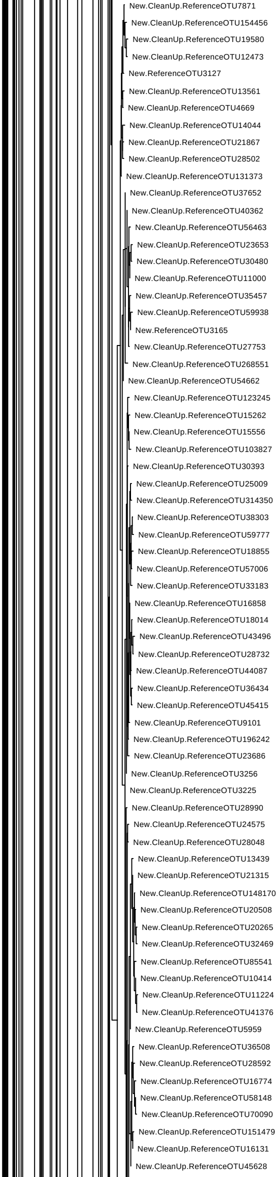
New.CleanUp.ReferenceOTU7871
New.CleanUp.ReferenceOTU154456
New.CleanUp.ReferenceOTU19580
New.CleanUp.ReferenceOTU12473
New.ReferenceOTU3127
New.CleanUp.ReferenceOTU13561
New.CleanUp.ReferenceOTU4669
New.CleanUp.ReferenceOTU14044
New.CleanUp.ReferenceOTU21867
New.CleanUp.ReferenceOTU28502
New.CleanUp.ReferenceOTU131373
New.CleanUp.ReferenceOTU37652
New.CleanUp.ReferenceOTU40362
New.CleanUp.ReferenceOTU56463
New.CleanUp.ReferenceOTU23653
New.CleanUp.ReferenceOTU30480
New.CleanUp.ReferenceOTU11000
New.CleanUp.ReferenceOTU35457
New.CleanUp.ReferenceOTU59938
New.ReferenceOTU3165
New.CleanUp.ReferenceOTU27753
New.CleanUp.ReferenceOTU268551
New.CleanUp.ReferenceOTU54662
New.CleanUp.ReferenceOTU123245
New.CleanUp.ReferenceOTU15262
New.CleanUp.ReferenceOTU15556
New.CleanUp.ReferenceOTU103827
New.CleanUp.ReferenceOTU30393
New.CleanUp.ReferenceOTU25009
New.CleanUp.ReferenceOTU314350
New.CleanUp.ReferenceOTU38303
New.CleanUp.ReferenceOTU59777
New.CleanUp.ReferenceOTU18855
New.CleanUp.ReferenceOTU57006
New.CleanUp.ReferenceOTU33183
New.CleanUp.ReferenceOTU16858
New.CleanUp.ReferenceOTU18014
New.CleanUp.ReferenceOTU43496
New.CleanUp.ReferenceOTU28732
New.CleanUp.ReferenceOTU44087
New.CleanUp.ReferenceOTU36434
New.CleanUp.ReferenceOTU45415
New.CleanUp.ReferenceOTU9101
New.CleanUp.ReferenceOTU196242
New.CleanUp.ReferenceOTU23686
New.CleanUp.ReferenceOTU3256
New.CleanUp.ReferenceOTU3225
New.CleanUp.ReferenceOTU28990
New.CleanUp.ReferenceOTU24575
New.CleanUp.ReferenceOTU28048
New.CleanUp.ReferenceOTU13439
New.CleanUp.ReferenceOTU21315
New.CleanUp.ReferenceOTU148170
New.CleanUp.ReferenceOTU20508
New.CleanUp.ReferenceOTU20265
New.CleanUp.ReferenceOTU32469
New.CleanUp.ReferenceOTU85541
New.CleanUp.ReferenceOTU10414
New.CleanUp.ReferenceOTU11224
New.CleanUp.ReferenceOTU41376
New.CleanUp.ReferenceOTU5959
New.CleanUp.ReferenceOTU36508
New.CleanUp.ReferenceOTU28592
New.CleanUp.ReferenceOTU16774
New.CleanUp.ReferenceOTU58148
New.CleanUp.ReferenceOTU70090
New.CleanUp.ReferenceOTU151479
New.CleanUp.ReferenceOTU16131
New.CleanUp.ReferenceOTU45628

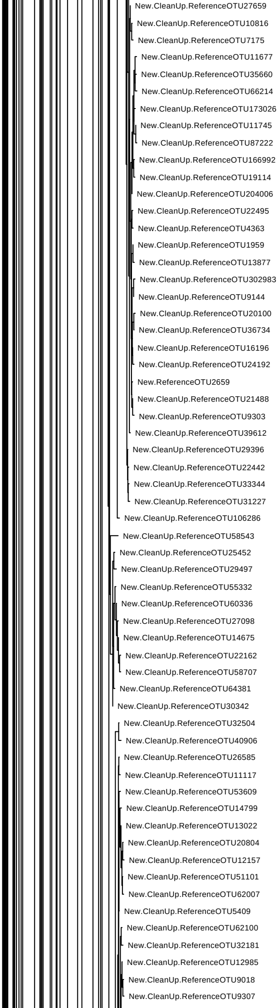
New.CleanUp.ReferenceOTU27659
New.CleanUp.ReferenceOTU10816
New.CleanUp.ReferenceOTU7175
New.CleanUp.ReferenceOTU11677
New.CleanUp.ReferenceOTU35660
New.CleanUp.ReferenceOTU66214
New.CleanUp.ReferenceOTU173026
New.CleanUp.ReferenceOTU11745
New.CleanUp.ReferenceOTU87222
New.CleanUp.ReferenceOTU166992
New.CleanUp.ReferenceOTU19114
New.CleanUp.ReferenceOTU204006
New.CleanUp.ReferenceOTU22495
New.CleanUp.ReferenceOTU4363
New.CleanUp.ReferenceOTU1959
New.CleanUp.ReferenceOTU13877
New.CleanUp.ReferenceOTU302983
New.CleanUp.ReferenceOTU9144
New.CleanUp.ReferenceOTU20100
New.CleanUp.ReferenceOTU36734
New.CleanUp.ReferenceOTU16196
New.CleanUp.ReferenceOTU24192
New.ReferenceOTU2659
New.CleanUp.ReferenceOTU21488
New.CleanUp.ReferenceOTU9303
New.CleanUp.ReferenceOTU39612
New.CleanUp.ReferenceOTU29396
New.CleanUp.ReferenceOTU22442
New.CleanUp.ReferenceOTU33344
New.CleanUp.ReferenceOTU31227
New.CleanUp.ReferenceOTU106286
New.CleanUp.ReferenceOTU58543
New.CleanUp.ReferenceOTU25452
New.CleanUp.ReferenceOTU29497
New.CleanUp.ReferenceOTU55332
New.CleanUp.ReferenceOTU60336
New.CleanUp.ReferenceOTU27098
New.CleanUp.ReferenceOTU14675
New.CleanUp.ReferenceOTU22162
New.CleanUp.ReferenceOTU58707
New.CleanUp.ReferenceOTU64381
New.CleanUp.ReferenceOTU30342
New.CleanUp.ReferenceOTU32504
New.CleanUp.ReferenceOTU40906
New.CleanUp.ReferenceOTU26585
New.CleanUp.ReferenceOTU11117
New.CleanUp.ReferenceOTU53609
New.CleanUp.ReferenceOTU14799
New.CleanUp.ReferenceOTU13022
New.CleanUp.ReferenceOTU20804
New.CleanUp.ReferenceOTU12157
New.CleanUp.ReferenceOTU51101
New.CleanUp.ReferenceOTU62007
New.CleanUp.ReferenceOTU5409
New.CleanUp.ReferenceOTU62100
New.CleanUp.ReferenceOTU32181
New.CleanUp.ReferenceOTU12985
New.CleanUp.ReferenceOTU9018
New.CleanUp.ReferenceOTU9307

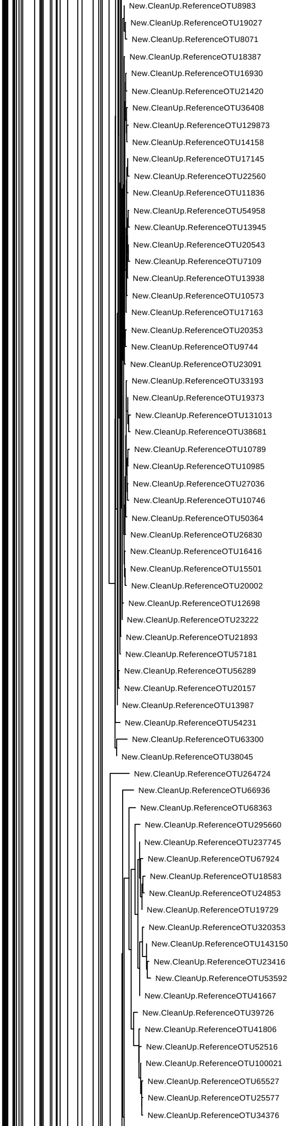

New.CleanUp.ReferenceOTU8983
New.CleanUp.ReferenceOTU19027
New.CleanUp.ReferenceOTU8071
New.CleanUp.ReferenceOTU18387
New.CleanUp.ReferenceOTU16930
New.CleanUp.ReferenceOTU21420
New.CleanUp.ReferenceOTU36408
New.CleanUp.ReferenceOTU129873
New.CleanUp.ReferenceOTU14158
New.CleanUp.ReferenceOTU17145
New.CleanUp.ReferenceOTU22560
New.CleanUp.ReferenceOTU11836
New.CleanUp.ReferenceOTU54958
New.CleanUp.ReferenceOTU13945
New.CleanUp.ReferenceOTU20543
New.CleanUp.ReferenceOTU7109
New.CleanUp.ReferenceOTU13938
New.CleanUp.ReferenceOTU10573
New.CleanUp.ReferenceOTU17163
New.CleanUp.ReferenceOTU20353
New.CleanUp.ReferenceOTU9744
New.CleanUp.ReferenceOTU23091
New.CleanUp.ReferenceOTU33193
New.CleanUp.ReferenceOTU19373
New.CleanUp.ReferenceOTU131013
New.CleanUp.ReferenceOTU38681
New.CleanUp.ReferenceOTU10789
New.CleanUp.ReferenceOTU10985
New.CleanUp.ReferenceOTU27036
New.CleanUp.ReferenceOTU10746
New.CleanUp.ReferenceOTU50364
New.CleanUp.ReferenceOTU26830
New.CleanUp.ReferenceOTU16416
New.CleanUp.ReferenceOTU15501
New.CleanUp.ReferenceOTU20002
New.CleanUp.ReferenceOTU12698
New.CleanUp.ReferenceOTU23222
New.CleanUp.ReferenceOTU21893
New.CleanUp.ReferenceOTU57181
New.CleanUp.ReferenceOTU56289
New.CleanUp.ReferenceOTU20157
New.CleanUp.ReferenceOTU13987
New.CleanUp.ReferenceOTU54231
New.CleanUp.ReferenceOTU63300
New.CleanUp.ReferenceOTU38045
New.CleanUp.ReferenceOTU264724
New.CleanUp.ReferenceOTU66936
New.CleanUp.ReferenceOTU68363
New.CleanUp.ReferenceOTU295660
New.CleanUp.ReferenceOTU237745
New.CleanUp.ReferenceOTU67924
New.CleanUp.ReferenceOTU18583
New.CleanUp.ReferenceOTU24853
New.CleanUp.ReferenceOTU19729
New.CleanUp.ReferenceOTU320353
New.CleanUp.ReferenceOTU143150
New.CleanUp.ReferenceOTU23416
New.CleanUp.ReferenceOTU53592
New.CleanUp.ReferenceOTU41667
New.CleanUp.ReferenceOTU39726
New.CleanUp.ReferenceOTU41806
New.CleanUp.ReferenceOTU52516
New.CleanUp.ReferenceOTU100021
New.CleanUp.ReferenceOTU65527
New.CleanUp.ReferenceOTU25577
New.CleanUp.ReferenceOTU34376

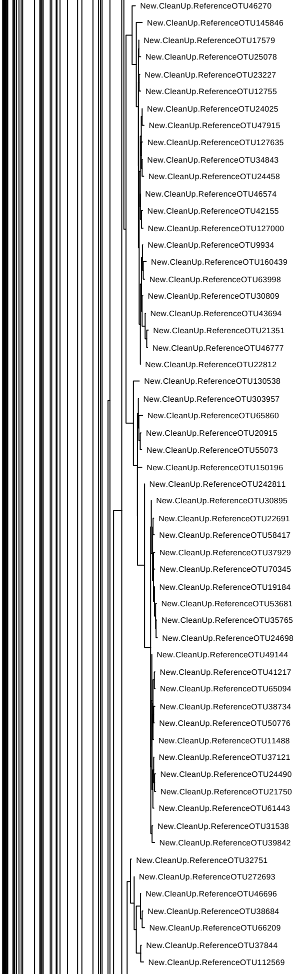
New.CleanUp.ReferenceOTU46270
New.CleanUp.ReferenceOTU145846
New.CleanUp.ReferenceOTU17579
New.CleanUp.ReferenceOTU25078
New.CleanUp.ReferenceOTU23227
New.CleanUp.ReferenceOTU12755
New.CleanUp.ReferenceOTU24025
New.CleanUp.ReferenceOTU47915
New.CleanUp.ReferenceOTU127635
New.CleanUp.ReferenceOTU34843
New.CleanUp.ReferenceOTU24458
New.CleanUp.ReferenceOTU46574
New.CleanUp.ReferenceOTU42155
New.CleanUp.ReferenceOTU127000
New.CleanUp.ReferenceOTU9934
New.CleanUp.ReferenceOTU160439
New.CleanUp.ReferenceOTU63998
New.CleanUp.ReferenceOTU30809
New.CleanUp.ReferenceOTU43694
New.CleanUp.ReferenceOTU21351
New.CleanUp.ReferenceOTU46777
New.CleanUp.ReferenceOTU22812
New.CleanUp.ReferenceOTU130538
New.CleanUp.ReferenceOTU303957
New.CleanUp.ReferenceOTU65860
New.CleanUp.ReferenceOTU20915
New.CleanUp.ReferenceOTU55073
New.CleanUp.ReferenceOTU150196
New.CleanUp.ReferenceOTU242811
New.CleanUp.ReferenceOTU30895
New.CleanUp.ReferenceOTU22691
New.CleanUp.ReferenceOTU58417
New.CleanUp.ReferenceOTU37929
New.CleanUp.ReferenceOTU70345
New.CleanUp.ReferenceOTU19184
New.CleanUp.ReferenceOTU53681
New.CleanUp.ReferenceOTU35765
New.CleanUp.ReferenceOTU24698
New.CleanUp.ReferenceOTU49144
New.CleanUp.ReferenceOTU41217
New.CleanUp.ReferenceOTU65094
New.CleanUp.ReferenceOTU38734
New.CleanUp.ReferenceOTU50776
New.CleanUp.ReferenceOTU11488
New.CleanUp.ReferenceOTU37121
New.CleanUp.ReferenceOTU24490
New.CleanUp.ReferenceOTU21750
New.CleanUp.ReferenceOTU61443
New.CleanUp.ReferenceOTU31538
New.CleanUp.ReferenceOTU39842
New.CleanUp.ReferenceOTU32751
New.CleanUp.ReferenceOTU272693
New.CleanUp.ReferenceOTU46696
New.CleanUp.ReferenceOTU38684
New.CleanUp.ReferenceOTU66209
New.CleanUp.ReferenceOTU37844
New.CleanUp.ReferenceOTU112569

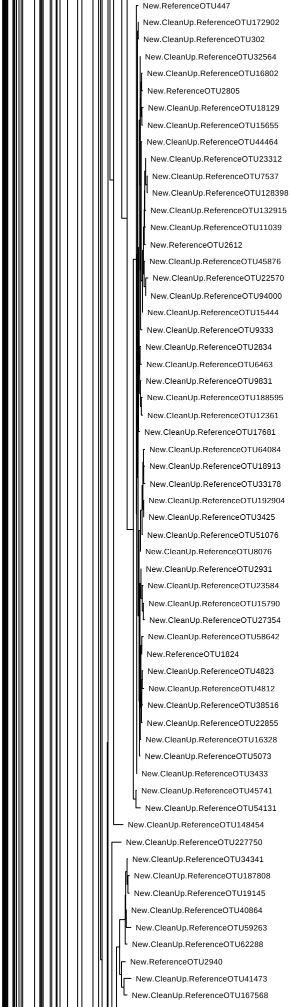
New.ReferenceOTU447
New.CleanUp.ReferenceOTU172902
New.CleanUp.ReferenceOTU302
New.CleanUp.ReferenceOTU32564
New.CleanUp.ReferenceOTU16802
New.ReferenceOTU2805
New.CleanUp.ReferenceOTU18129
New.CleanUp.ReferenceOTU15655
New.CleanUp.ReferenceOTU44464
New.CleanUp.ReferenceOTU23312
New.CleanUp.ReferenceOTU7537
New.CleanUp.ReferenceOTU128398
New.CleanUp.ReferenceOTU132915
New.CleanUp.ReferenceOTU11039
New.ReferenceOTU2612
New.CleanUp.ReferenceOTU45876
New.CleanUp.ReferenceOTU22570
New.CleanUp.ReferenceOTU94000
New.CleanUp.ReferenceOTU15444
New.CleanUp.ReferenceOTU9333
New.CleanUp.ReferenceOTU2834
New.CleanUp.ReferenceOTU6463
New.CleanUp.ReferenceOTU9831
New.CleanUp.ReferenceOTU188595
New.CleanUp.ReferenceOTU12361
New.CleanUp.ReferenceOTU17681
New.CleanUp.ReferenceOTU64084
New.CleanUp.ReferenceOTU18913
New.CleanUp.ReferenceOTU33178
New.CleanUp.ReferenceOTU192904
New.CleanUp.ReferenceOTU3425
New.CleanUp.ReferenceOTU51076
New.CleanUp.ReferenceOTU8076
New.CleanUp.ReferenceOTU2931
New.CleanUp.ReferenceOTU23584
New.CleanUp.ReferenceOTU15790
New.CleanUp.ReferenceOTU27354
New.CleanUp.ReferenceOTU58642
New.ReferenceOTU1824
New.CleanUp.ReferenceOTU4823
New.CleanUp.ReferenceOTU4812
New.CleanUp.ReferenceOTU38516
New.CleanUp.ReferenceOTU22855
New.CleanUp.ReferenceOTU16328
New.CleanUp.ReferenceOTU5073
New.CleanUp.ReferenceOTU3433
New.CleanUp.ReferenceOTU45741
New.CleanUp.ReferenceOTU54131
New.CleanUp.ReferenceOTU148454
New.CleanUp.ReferenceOTU227750
New.CleanUp.ReferenceOTU34341
New.CleanUp.ReferenceOTU187808
New.CleanUp.ReferenceOTU19145
New.CleanUp.ReferenceOTU40864
New.CleanUp.ReferenceOTU59263
New.CleanUp.ReferenceOTU62288
New.ReferenceOTU2940
New.CleanUp.ReferenceOTU41473
New.CleanUp.ReferenceOTU167568

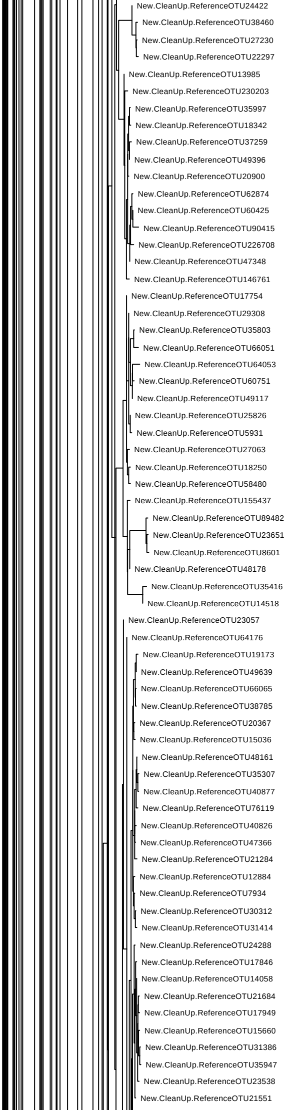
New.CleanUp.ReferenceOTU24422
New.CleanUp.ReferenceOTU38460
New.CleanUp.ReferenceOTU27230
New.CleanUp.ReferenceOTU22297
New.CleanUp.ReferenceOTU13985
New.CleanUp.ReferenceOTU230203
New.CleanUp.ReferenceOTU35997
New.CleanUp.ReferenceOTU18342
New.CleanUp.ReferenceOTU37259
New.CleanUp.ReferenceOTU49396
New.CleanUp.ReferenceOTU20900
New.CleanUp.ReferenceOTU62874
New.CleanUp.ReferenceOTU60425
New.CleanUp.ReferenceOTU90415
New.CleanUp.ReferenceOTU226708
New.CleanUp.ReferenceOTU47348
New.CleanUp.ReferenceOTU146761
New.CleanUp.ReferenceOTU17754
New.CleanUp.ReferenceOTU29308
New.CleanUp.ReferenceOTU35803
New.CleanUp.ReferenceOTU66051
New.CleanUp.ReferenceOTU64053
New.CleanUp.ReferenceOTU60751
New.CleanUp.ReferenceOTU49117
New.CleanUp.ReferenceOTU25826
New.CleanUp.ReferenceOTU5931
New.CleanUp.ReferenceOTU27063
New.CleanUp.ReferenceOTU18250
New.CleanUp.ReferenceOTU58480
New.CleanUp.ReferenceOTU155437
New.CleanUp.ReferenceOTU89482
New.CleanUp.ReferenceOTU23651
New.CleanUp.ReferenceOTU8601
New.CleanUp.ReferenceOTU48178
New.CleanUp.ReferenceOTU35416
New.CleanUp.ReferenceOTU14518
New.CleanUp.ReferenceOTU23057
New.CleanUp.ReferenceOTU64176
New.CleanUp.ReferenceOTU19173
New.CleanUp.ReferenceOTU49639
New.CleanUp.ReferenceOTU66065
New.CleanUp.ReferenceOTU38785
New.CleanUp.ReferenceOTU20367
New.CleanUp.ReferenceOTU15036
New.CleanUp.ReferenceOTU48161
New.CleanUp.ReferenceOTU35307
New.CleanUp.ReferenceOTU40877
New.CleanUp.ReferenceOTU76119
New.CleanUp.ReferenceOTU40826
New.CleanUp.ReferenceOTU47366
New.CleanUp.ReferenceOTU21284
New.CleanUp.ReferenceOTU12884
New.CleanUp.ReferenceOTU7934
New.CleanUp.ReferenceOTU30312
New.CleanUp.ReferenceOTU31414
New.CleanUp.ReferenceOTU24288
New.CleanUp.ReferenceOTU17846
New.CleanUp.ReferenceOTU14058
New.CleanUp.ReferenceOTU21684
New.CleanUp.ReferenceOTU17949
New.CleanUp.ReferenceOTU15660
New.CleanUp.ReferenceOTU31386
New.CleanUp.ReferenceOTU35947
New.CleanUp.ReferenceOTU23538
New.CleanUp.ReferenceOTU21551

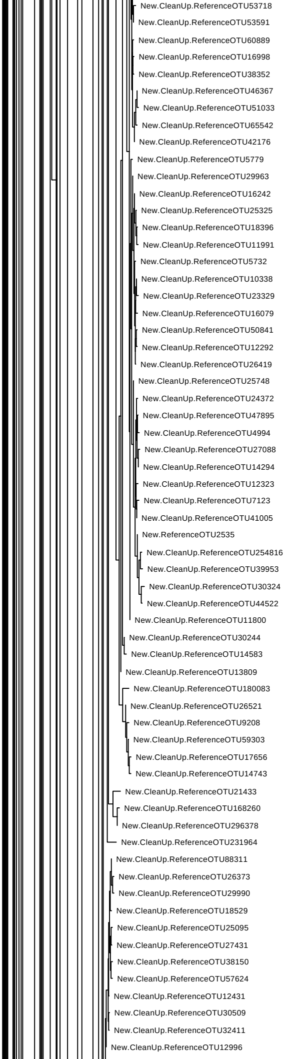
New.CleanUp.ReferenceOTU53718
New.CleanUp.ReferenceOTU53591
New.CleanUp.ReferenceOTU60889
New.CleanUp.ReferenceOTU16998
New.CleanUp.ReferenceOTU38352
New.CleanUp.ReferenceOTU46367
New.CleanUp.ReferenceOTU51033
New.CleanUp.ReferenceOTU65542
New.CleanUp.ReferenceOTU42176
New.CleanUp.ReferenceOTU5779
New.CleanUp.ReferenceOTU29963
New.CleanUp.ReferenceOTU16242
New.CleanUp.ReferenceOTU25325
New.CleanUp.ReferenceOTU18396
New.CleanUp.ReferenceOTU11991
New.CleanUp.ReferenceOTU5732
New.CleanUp.ReferenceOTU10338
New.CleanUp.ReferenceOTU23329
New.CleanUp.ReferenceOTU16079
New.CleanUp.ReferenceOTU50841
New.CleanUp.ReferenceOTU12292
New.CleanUp.ReferenceOTU26419
New.CleanUp.ReferenceOTU25748
New.CleanUp.ReferenceOTU24372
New.CleanUp.ReferenceOTU47895
New.CleanUp.ReferenceOTU4994
New.CleanUp.ReferenceOTU27088
New.CleanUp.ReferenceOTU14294
New.CleanUp.ReferenceOTU12323
New.CleanUp.ReferenceOTU7123
New.CleanUp.ReferenceOTU41005
New.ReferenceOTU2535
New.CleanUp.ReferenceOTU254816
New.CleanUp.ReferenceOTU39953
New.CleanUp.ReferenceOTU30324
New.CleanUp.ReferenceOTU44522
New.CleanUp.ReferenceOTU11800
New.CleanUp.ReferenceOTU30244
New.CleanUp.ReferenceOTU14583
New.CleanUp.ReferenceOTU13809
New.CleanUp.ReferenceOTU180083
New.CleanUp.ReferenceOTU26521
New.CleanUp.ReferenceOTU9208
New.CleanUp.ReferenceOTU59303
New.CleanUp.ReferenceOTU17656
New.CleanUp.ReferenceOTU14743
New.CleanUp.ReferenceOTU21433
New.CleanUp.ReferenceOTU168260
New.CleanUp.ReferenceOTU296378
New.CleanUp.ReferenceOTU231964
New.CleanUp.ReferenceOTU88311
New.CleanUp.ReferenceOTU26373
New.CleanUp.ReferenceOTU29990
New.CleanUp.ReferenceOTU18529
New.CleanUp.ReferenceOTU25095
New.CleanUp.ReferenceOTU27431
New.CleanUp.ReferenceOTU38150
New.CleanUp.ReferenceOTU57624
New.CleanUp.ReferenceOTU12431
New.CleanUp.ReferenceOTU30509
New.CleanUp.ReferenceOTU32411
New.CleanUp.ReferenceOTU12996

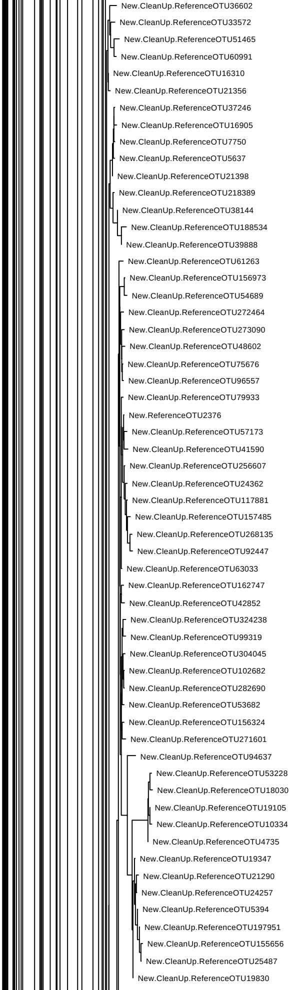
New.CleanUp.ReferenceOTU36602
New.CleanUp.ReferenceOTU33572
New.CleanUp.ReferenceOTU51465
New.CleanUp.ReferenceOTU60991
New.CleanUp.ReferenceOTU16310
New.CleanUp.ReferenceOTU21356
New.CleanUp.ReferenceOTU37246
New.CleanUp.ReferenceOTU16905
New.CleanUp.ReferenceOTU7750
New.CleanUp.ReferenceOTU5637
New.CleanUp.ReferenceOTU21398
New.CleanUp.ReferenceOTU218389
New.CleanUp.ReferenceOTU38144
New.CleanUp.ReferenceOTU188534
New.CleanUp.ReferenceOTU39888
New.CleanUp.ReferenceOTU61263
New.CleanUp.ReferenceOTU156973
New.CleanUp.ReferenceOTU54689
New.CleanUp.ReferenceOTU272464
New.CleanUp.ReferenceOTU273090
New.CleanUp.ReferenceOTU48602
New.CleanUp.ReferenceOTU75676
New.CleanUp.ReferenceOTU96557
New.CleanUp.ReferenceOTU79933
New.ReferenceOTU2376
New.CleanUp.ReferenceOTU57173
New.CleanUp.ReferenceOTU41590
New.CleanUp.ReferenceOTU256607
New.CleanUp.ReferenceOTU24362
New.CleanUp.ReferenceOTU117881
New.CleanUp.ReferenceOTU157485
New.CleanUp.ReferenceOTU268135
New.CleanUp.ReferenceOTU92447
New.CleanUp.ReferenceOTU63033
New.CleanUp.ReferenceOTU162747
New.CleanUp.ReferenceOTU42852
New.CleanUp.ReferenceOTU324238
New.CleanUp.ReferenceOTU99319
New.CleanUp.ReferenceOTU304045
New.CleanUp.ReferenceOTU102682
New.CleanUp.ReferenceOTU282690
New.CleanUp.ReferenceOTU53682
New.CleanUp.ReferenceOTU156324
New.CleanUp.ReferenceOTU271601
New.CleanUp.ReferenceOTU94637
New.CleanUp.ReferenceOTU53228
New.CleanUp.ReferenceOTU18030
New.CleanUp.ReferenceOTU19105
New.CleanUp.ReferenceOTU10334
New.CleanUp.ReferenceOTU4735
New.CleanUp.ReferenceOTU19347
New.CleanUp.ReferenceOTU21290
New.CleanUp.ReferenceOTU24257
New.CleanUp.ReferenceOTU5394
New.CleanUp.ReferenceOTU197951
New.CleanUp.ReferenceOTU155656
New.CleanUp.ReferenceOTU25487
New.CleanUp.ReferenceOTU19830

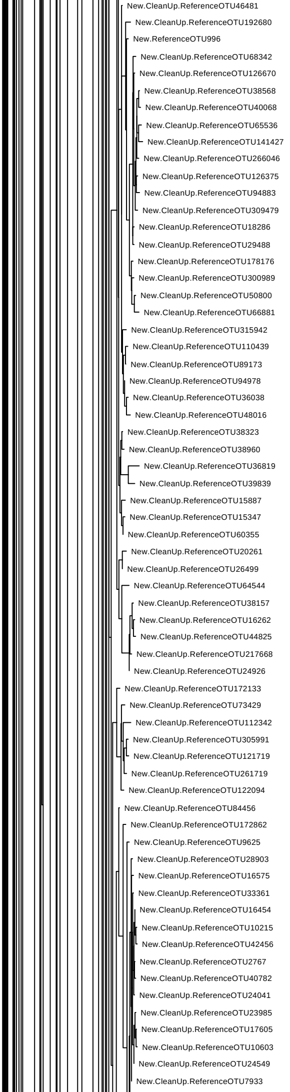
New.CleanUp.ReferenceOTU46481
New.CleanUp.ReferenceOTU192680
New.ReferenceOTU996
New.CleanUp.ReferenceOTU68342
New.CleanUp.ReferenceOTU126670
New.CleanUp.ReferenceOTU38568
New.CleanUp.ReferenceOTU40068
New.CleanUp.ReferenceOTU65536
New.CleanUp.ReferenceOTU141427
New.CleanUp.ReferenceOTU266046
New.CleanUp.ReferenceOTU126375
New.CleanUp.ReferenceOTU94883
New.CleanUp.ReferenceOTU309479
New.CleanUp.ReferenceOTU18286
New.CleanUp.ReferenceOTU29488
New.CleanUp.ReferenceOTU178176
New.CleanUp.ReferenceOTU300989
New.CleanUp.ReferenceOTU50800
New.CleanUp.ReferenceOTU66881
New.CleanUp.ReferenceOTU315942
New.CleanUp.ReferenceOTU110439
New.CleanUp.ReferenceOTU89173
New.CleanUp.ReferenceOTU94978
New.CleanUp.ReferenceOTU36038
New.CleanUp.ReferenceOTU48016
New.CleanUp.ReferenceOTU38323
New.CleanUp.ReferenceOTU38960
New.CleanUp.ReferenceOTU36819
New.CleanUp.ReferenceOTU39839
New.CleanUp.ReferenceOTU15887
New.CleanUp.ReferenceOTU15347
New.CleanUp.ReferenceOTU60355
New.CleanUp.ReferenceOTU20261
New.CleanUp.ReferenceOTU26499
New.CleanUp.ReferenceOTU64544
New.CleanUp.ReferenceOTU38157
New.CleanUp.ReferenceOTU16262
New.CleanUp.ReferenceOTU44825
New.CleanUp.ReferenceOTU217668
New.CleanUp.ReferenceOTU24926
New.CleanUp.ReferenceOTU172133
New.CleanUp.ReferenceOTU73429
New.CleanUp.ReferenceOTU112342
New.CleanUp.ReferenceOTU305991
New.CleanUp.ReferenceOTU121719
New.CleanUp.ReferenceOTU261719
New.CleanUp.ReferenceOTU122094
New.CleanUp.ReferenceOTU84456
New.CleanUp.ReferenceOTU172862
New.CleanUp.ReferenceOTU9625
New.CleanUp.ReferenceOTU28903
New.CleanUp.ReferenceOTU16575
New.CleanUp.ReferenceOTU33361
New.CleanUp.ReferenceOTU16454
New.CleanUp.ReferenceOTU10215
New.CleanUp.ReferenceOTU42456
New.CleanUp.ReferenceOTU2767
New.CleanUp.ReferenceOTU40782
New.CleanUp.ReferenceOTU24041
New.CleanUp.ReferenceOTU23985
New.CleanUp.ReferenceOTU17605
New.CleanUp.ReferenceOTU10603
New.CleanUp.ReferenceOTU24549
New.CleanUp.ReferenceOTU7933

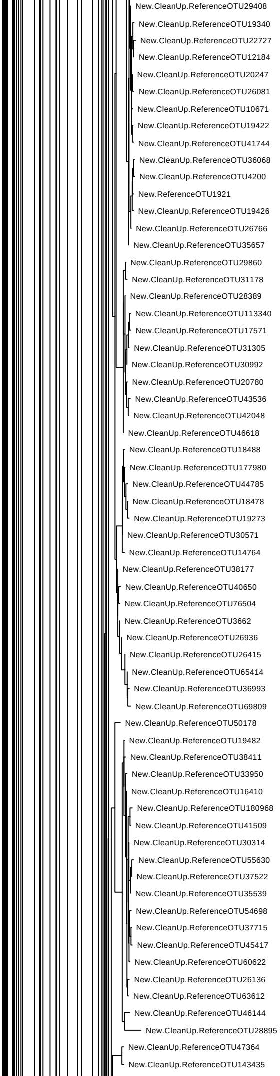
New.CleanUp.ReferenceOTU29408
New.CleanUp.ReferenceOTU19340
New.CleanUp.ReferenceOTU22727
New.CleanUp.ReferenceOTU12184
New.CleanUp.ReferenceOTU20247
New.CleanUp.ReferenceOTU26081
New.CleanUp.ReferenceOTU10671
New.CleanUp.ReferenceOTU19422
New.CleanUp.ReferenceOTU41744
New.CleanUp.ReferenceOTU36068
New.CleanUp.ReferenceOTU4200
New.ReferenceOTU1921
New.CleanUp.ReferenceOTU19426
New.CleanUp.ReferenceOTU26766
New.CleanUp.ReferenceOTU35657
New.CleanUp.ReferenceOTU29860
New.CleanUp.ReferenceOTU31178
New.CleanUp.ReferenceOTU28389
New.CleanUp.ReferenceOTU113340
New.CleanUp.ReferenceOTU17571
New.CleanUp.ReferenceOTU31305
New.CleanUp.ReferenceOTU30992
New.CleanUp.ReferenceOTU20780
New.CleanUp.ReferenceOTU43536
New.CleanUp.ReferenceOTU42048
New.CleanUp.ReferenceOTU46618
New.CleanUp.ReferenceOTU18488
New.CleanUp.ReferenceOTU177980
New.CleanUp.ReferenceOTU44785
New.CleanUp.ReferenceOTU18478
New.CleanUp.ReferenceOTU19273
New.CleanUp.ReferenceOTU30571
New.CleanUp.ReferenceOTU14764
New.CleanUp.ReferenceOTU38177
New.CleanUp.ReferenceOTU40650
New.CleanUp.ReferenceOTU76504
New.CleanUp.ReferenceOTU3662
New.CleanUp.ReferenceOTU26936
New.CleanUp.ReferenceOTU26415
New.CleanUp.ReferenceOTU65414
New.CleanUp.ReferenceOTU36993
New.CleanUp.ReferenceOTU69809
New.CleanUp.ReferenceOTU50178
New.CleanUp.ReferenceOTU19482
New.CleanUp.ReferenceOTU38411
New.CleanUp.ReferenceOTU33950
New.CleanUp.ReferenceOTU16410
New.CleanUp.ReferenceOTU180968
New.CleanUp.ReferenceOTU41509
New.CleanUp.ReferenceOTU30314
New.CleanUp.ReferenceOTU55630
New.CleanUp.ReferenceOTU37522
New.CleanUp.ReferenceOTU35539
New.CleanUp.ReferenceOTU54698
New.CleanUp.ReferenceOTU37715
New.CleanUp.ReferenceOTU45417
New.CleanUp.ReferenceOTU60622
New.CleanUp.ReferenceOTU26136
New.CleanUp.ReferenceOTU63612
New.CleanUp.ReferenceOTU46144
New.CleanUp.ReferenceOTU28895
New.CleanUp.ReferenceOTU47364
New.CleanUp.ReferenceOTU143435

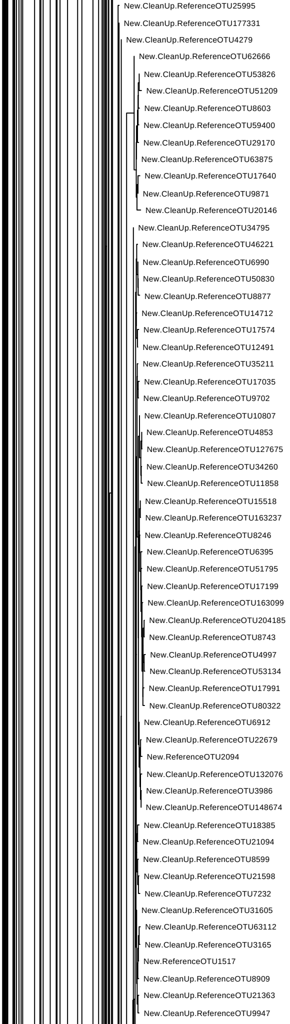
New.CleanUp.ReferenceOTU25995
New.CleanUp.ReferenceOTU177331
New.CleanUp.ReferenceOTU4279
New.CleanUp.ReferenceOTU62666
New.CleanUp.ReferenceOTU53826
New.CleanUp.ReferenceOTU51209
New.CleanUp.ReferenceOTU8603
New.CleanUp.ReferenceOTU59400
New.CleanUp.ReferenceOTU29170
New.CleanUp.ReferenceOTU63875
New.CleanUp.ReferenceOTU17640
New.CleanUp.ReferenceOTU9871
New.CleanUp.ReferenceOTU20146
New.CleanUp.ReferenceOTU34795
New.CleanUp.ReferenceOTU46221
New.CleanUp.ReferenceOTU6990
New.CleanUp.ReferenceOTU50830
New.CleanUp.ReferenceOTU8877
New.CleanUp.ReferenceOTU14712
New.CleanUp.ReferenceOTU17574
New.CleanUp.ReferenceOTU12491
New.CleanUp.ReferenceOTU35211
New.CleanUp.ReferenceOTU17035
New.CleanUp.ReferenceOTU9702
New.CleanUp.ReferenceOTU10807
New.CleanUp.ReferenceOTU4853
New.CleanUp.ReferenceOTU127675
New.CleanUp.ReferenceOTU34260
New.CleanUp.ReferenceOTU11858
New.CleanUp.ReferenceOTU15518
New.CleanUp.ReferenceOTU163237
New.CleanUp.ReferenceOTU8246
New.CleanUp.ReferenceOTU6395
New.CleanUp.ReferenceOTU51795
New.CleanUp.ReferenceOTU17199
New.CleanUp.ReferenceOTU163099
New.CleanUp.ReferenceOTU204185
New.CleanUp.ReferenceOTU8743
New.CleanUp.ReferenceOTU4997
New.CleanUp.ReferenceOTU53134
New.CleanUp.ReferenceOTU17991
New.CleanUp.ReferenceOTU80322
New.CleanUp.ReferenceOTU6912
New.CleanUp.ReferenceOTU22679
New.ReferenceOTU2094
New.CleanUp.ReferenceOTU132076
New.CleanUp.ReferenceOTU3986
New.CleanUp.ReferenceOTU148674
New.CleanUp.ReferenceOTU18385
New.CleanUp.ReferenceOTU21094
New.CleanUp.ReferenceOTU8599
New.CleanUp.ReferenceOTU21598
New.CleanUp.ReferenceOTU7232
New.CleanUp.ReferenceOTU31605
New.CleanUp.ReferenceOTU63112
New.CleanUp.ReferenceOTU3165
New.ReferenceOTU1517
New.CleanUp.ReferenceOTU8909
New.CleanUp.ReferenceOTU21363
New.CleanUp.ReferenceOTU9947

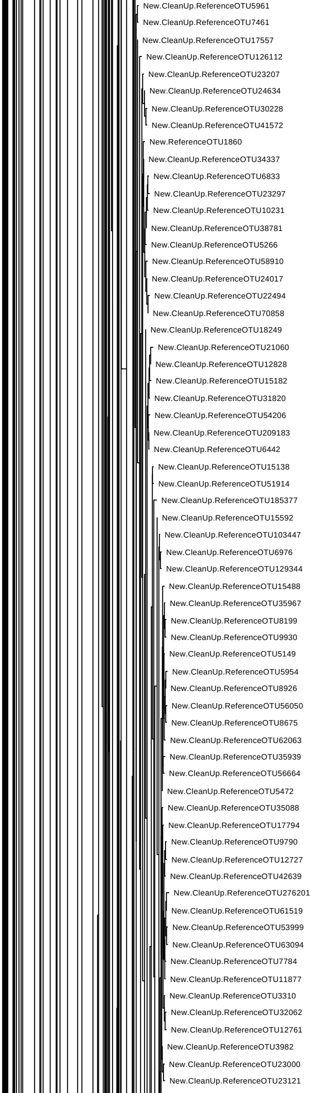
New.CleanUp.ReferenceOTU5961
New.CleanUp.ReferenceOTU7461
New.CleanUp.ReferenceOTU17557
New.CleanUp.ReferenceOTU126112
New.CleanUp.ReferenceOTU23207
New.CleanUp.ReferenceOTU24634
New.CleanUp.ReferenceOTU30228
New.CleanUp.ReferenceOTU41572
New.ReferenceOTU1860
New.CleanUp.ReferenceOTU34337
New.CleanUp.ReferenceOTU6833
New.CleanUp.ReferenceOTU23297
New.CleanUp.ReferenceOTU10231
New.CleanUp.ReferenceOTU38781
New.CleanUp.ReferenceOTU5266
New.CleanUp.ReferenceOTU58910
New.CleanUp.ReferenceOTU24017
New.CleanUp.ReferenceOTU22494
New.CleanUp.ReferenceOTU70858
New.CleanUp.ReferenceOTU18249
New.CleanUp.ReferenceOTU21060
New.CleanUp.ReferenceOTU12828
New.CleanUp.ReferenceOTU15182
New.CleanUp.ReferenceOTU31820
New.CleanUp.ReferenceOTU54206
New.CleanUp.ReferenceOTU209183
New.CleanUp.ReferenceOTU6442
New.CleanUp.ReferenceOTU15138
New.CleanUp.ReferenceOTU51914
New.CleanUp.ReferenceOTU185377
New.CleanUp.ReferenceOTU15592
New.CleanUp.ReferenceOTU103447
New.CleanUp.ReferenceOTU6976
New.CleanUp.ReferenceOTU129344
New.CleanUp.ReferenceOTU15488
New.CleanUp.ReferenceOTU35967
New.CleanUp.ReferenceOTU8199
New.CleanUp.ReferenceOTU9930
New.CleanUp.ReferenceOTU5149
New.CleanUp.ReferenceOTU5954
New.CleanUp.ReferenceOTU8926
New.CleanUp.ReferenceOTU56050
New.CleanUp.ReferenceOTU8675
New.CleanUp.ReferenceOTU62063
New.CleanUp.ReferenceOTU35939
New.CleanUp.ReferenceOTU56664
New.CleanUp.ReferenceOTU5472
New.CleanUp.ReferenceOTU35088
New.CleanUp.ReferenceOTU17794
New.CleanUp.ReferenceOTU9790
New.CleanUp.ReferenceOTU12727
New.CleanUp.ReferenceOTU42639
New.CleanUp.ReferenceOTU276201
New.CleanUp.ReferenceOTU61519
New.CleanUp.ReferenceOTU53999
New.CleanUp.ReferenceOTU63094
New.CleanUp.ReferenceOTU7784
New.CleanUp.ReferenceOTU11877
New.CleanUp.ReferenceOTU3310
New.CleanUp.ReferenceOTU32062
New.CleanUp.ReferenceOTU12761
New.CleanUp.ReferenceOTU3982
New.CleanUp.ReferenceOTU23000
New.CleanUp.ReferenceOTU23121

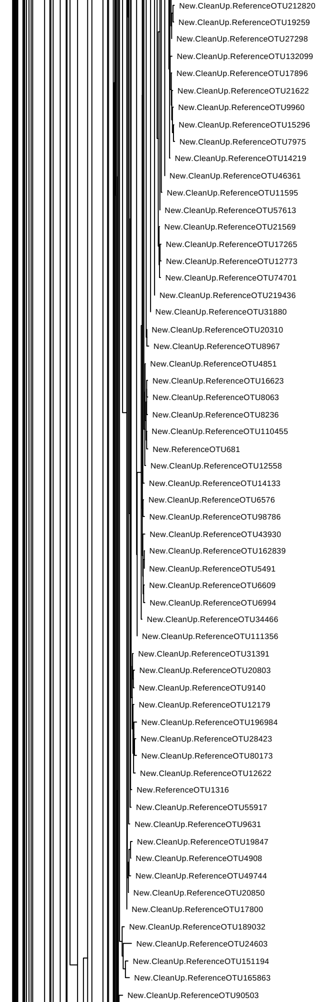
New.CleanUp.ReferenceOTU212820
New.CleanUp.ReferenceOTU19259
New.CleanUp.ReferenceOTU27298
New.CleanUp.ReferenceOTU132099
New.CleanUp.ReferenceOTU17896
New.CleanUp.ReferenceOTU21622
New.CleanUp.ReferenceOTU9960
New.CleanUp.ReferenceOTU15296
New.CleanUp.ReferenceOTU7975
New.CleanUp.ReferenceOTU14219
New.CleanUp.ReferenceOTU46361
New.CleanUp.ReferenceOTU11595
New.CleanUp.ReferenceOTU57613
New.CleanUp.ReferenceOTU21569
New.CleanUp.ReferenceOTU17265
New.CleanUp.ReferenceOTU12773
New.CleanUp.ReferenceOTU74701
New.CleanUp.ReferenceOTU219436
New.CleanUp.ReferenceOTU31880
New.CleanUp.ReferenceOTU20310
New.CleanUp.ReferenceOTU8967
New.CleanUp.ReferenceOTU4851
New.CleanUp.ReferenceOTU16623
New.CleanUp.ReferenceOTU8063
New.CleanUp.ReferenceOTU8236
New.CleanUp.ReferenceOTU110455
New.ReferenceOTU681
New.CleanUp.ReferenceOTU12558
New.CleanUp.ReferenceOTU14133
New.CleanUp.ReferenceOTU6576
New.CleanUp.ReferenceOTU98786
New.CleanUp.ReferenceOTU43930
New.CleanUp.ReferenceOTU162839
New.CleanUp.ReferenceOTU5491
New.CleanUp.ReferenceOTU6609
New.CleanUp.ReferenceOTU6994
New.CleanUp.ReferenceOTU34466
New.CleanUp.ReferenceOTU111356
New.CleanUp.ReferenceOTU31391
New.CleanUp.ReferenceOTU20803
New.CleanUp.ReferenceOTU9140
New.CleanUp.ReferenceOTU12179
New.CleanUp.ReferenceOTU196984
New.CleanUp.ReferenceOTU28423
New.CleanUp.ReferenceOTU80173
New.CleanUp.ReferenceOTU12622
New.ReferenceOTU1316
New.CleanUp.ReferenceOTU55917
New.CleanUp.ReferenceOTU9631
New.CleanUp.ReferenceOTU19847
New.CleanUp.ReferenceOTU4908
New.CleanUp.ReferenceOTU49744
New.CleanUp.ReferenceOTU20850
New.CleanUp.ReferenceOTU17800
New.CleanUp.ReferenceOTU189032
New.CleanUp.ReferenceOTU24603
New.CleanUp.ReferenceOTU151194
New.CleanUp.ReferenceOTU165863
New.CleanUp.ReferenceOTU90503

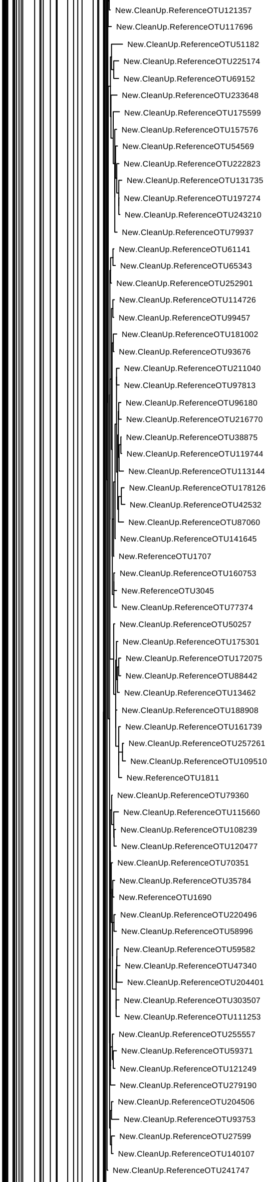

New.CleanUp.ReferenceOTU121357
New.CleanUp.ReferenceOTU117696
New.CleanUp.ReferenceOTU51182
New.CleanUp.ReferenceOTU225174
New.CleanUp.ReferenceOTU69152
New.CleanUp.ReferenceOTU233648
New.CleanUp.ReferenceOTU175599
New.CleanUp.ReferenceOTU157576
New.CleanUp.ReferenceOTU54569
New.CleanUp.ReferenceOTU222823
New.CleanUp.ReferenceOTU131735
New.CleanUp.ReferenceOTU197274
New.CleanUp.ReferenceOTU243210
New.CleanUp.ReferenceOTU79937
New.CleanUp.ReferenceOTU61141
New.CleanUp.ReferenceOTU65343
New.CleanUp.ReferenceOTU252901
New.CleanUp.ReferenceOTU114726
New.CleanUp.ReferenceOTU99457
New.CleanUp.ReferenceOTU181002
New.CleanUp.ReferenceOTU93676
New.CleanUp.ReferenceOTU211040
New.CleanUp.ReferenceOTU97813
New.CleanUp.ReferenceOTU96180
New.CleanUp.ReferenceOTU216770
New.CleanUp.ReferenceOTU38875
New.CleanUp.ReferenceOTU119744
New.CleanUp.ReferenceOTU113144
New.CleanUp.ReferenceOTU178126
New.CleanUp.ReferenceOTU42532
New.CleanUp.ReferenceOTU87060
New.CleanUp.ReferenceOTU141645
New.ReferenceOTU1707
New.CleanUp.ReferenceOTU160753
New.ReferenceOTU3045
New.CleanUp.ReferenceOTU77374
New.CleanUp.ReferenceOTU50257
New.CleanUp.ReferenceOTU175301
New.CleanUp.ReferenceOTU172075
New.CleanUp.ReferenceOTU88442
New.CleanUp.ReferenceOTU13462
New.CleanUp.ReferenceOTU188908
New.CleanUp.ReferenceOTU161739
New.CleanUp.ReferenceOTU257261
New.CleanUp.ReferenceOTU109510
New.ReferenceOTU1811
New.CleanUp.ReferenceOTU79360
New.CleanUp.ReferenceOTU115660
New.CleanUp.ReferenceOTU108239
New.CleanUp.ReferenceOTU120477
New.CleanUp.ReferenceOTU70351
New.CleanUp.ReferenceOTU35784
New.ReferenceOTU1690
New.CleanUp.ReferenceOTU220496
New.CleanUp.ReferenceOTU58996
New.CleanUp.ReferenceOTU59582
New.CleanUp.ReferenceOTU47340
New.CleanUp.ReferenceOTU204401
New.CleanUp.ReferenceOTU303507
New.CleanUp.ReferenceOTU111253
New.CleanUp.ReferenceOTU255557
New.CleanUp.ReferenceOTU59371
New.CleanUp.ReferenceOTU121249
New.CleanUp.ReferenceOTU279190
New.CleanUp.ReferenceOTU204506
New.CleanUp.ReferenceOTU93753
New.CleanUp.ReferenceOTU27599
New.CleanUp.ReferenceOTU140107
New.CleanUp.ReferenceOTU241747

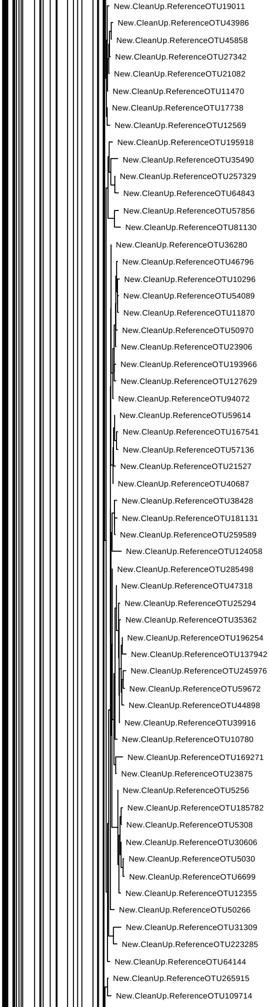
New.CleanUp.ReferenceOTU19011
New.CleanUp.ReferenceOTU43986
New.CleanUp.ReferenceOTU45858
New.CleanUp.ReferenceOTU27342
New.CleanUp.ReferenceOTU21082
New.CleanUp.ReferenceOTU11470
New.CleanUp.ReferenceOTU17738
New.CleanUp.ReferenceOTU12569
New.CleanUp.ReferenceOTU195918
New.CleanUp.ReferenceOTU35490
New.CleanUp.ReferenceOTU257329
New.CleanUp.ReferenceOTU64843
New.CleanUp.ReferenceOTU57856
New.CleanUp.ReferenceOTU81130
New.CleanUp.ReferenceOTU36280
New.CleanUp.ReferenceOTU46796
New.CleanUp.ReferenceOTU10296
New.CleanUp.ReferenceOTU54089
New.CleanUp.ReferenceOTU11870
New.CleanUp.ReferenceOTU50970
New.CleanUp.ReferenceOTU23906
New.CleanUp.ReferenceOTU193966
New.CleanUp.ReferenceOTU127629
New.CleanUp.ReferenceOTU94072
New.CleanUp.ReferenceOTU59614
New.CleanUp.ReferenceOTU167541
New.CleanUp.ReferenceOTU57136
New.CleanUp.ReferenceOTU21527
New.CleanUp.ReferenceOTU40687
New.CleanUp.ReferenceOTU38428
New.CleanUp.ReferenceOTU181131
New.CleanUp.ReferenceOTU259589
New.CleanUp.ReferenceOTU124058
New.CleanUp.ReferenceOTU285498
New.CleanUp.ReferenceOTU47318
New.CleanUp.ReferenceOTU25294
New.CleanUp.ReferenceOTU35362
New.CleanUp.ReferenceOTU196254
New.CleanUp.ReferenceOTU137942
New.CleanUp.ReferenceOTU245976
New.CleanUp.ReferenceOTU59672
New.CleanUp.ReferenceOTU44898
New.CleanUp.ReferenceOTU39916
New.CleanUp.ReferenceOTU10780
New.CleanUp.ReferenceOTU169271
New.CleanUp.ReferenceOTU23875
New.CleanUp.ReferenceOTU5256
New.CleanUp.ReferenceOTU185782
New.CleanUp.ReferenceOTU5308
New.CleanUp.ReferenceOTU30606
New.CleanUp.ReferenceOTU5030
New.CleanUp.ReferenceOTU6699
New.CleanUp.ReferenceOTU12355
New.CleanUp.ReferenceOTU50266
New.CleanUp.ReferenceOTU31309
New.CleanUp.ReferenceOTU223285
New.CleanUp.ReferenceOTU64144
New.CleanUp.ReferenceOTU265915
New.CleanUp.ReferenceOTU109714

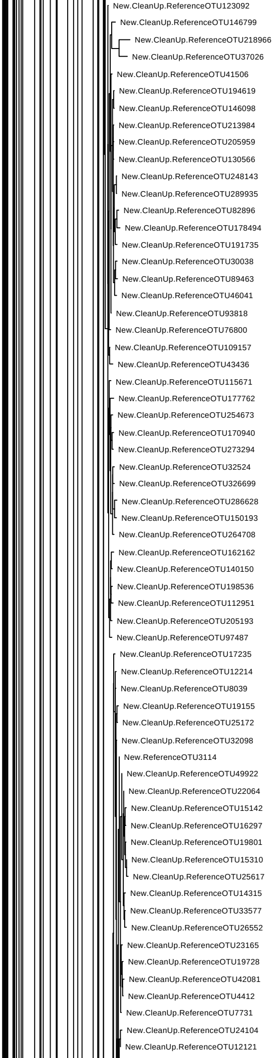
New.CleanUp.ReferenceOTU123092
New.CleanUp.ReferenceOTU146799
New.CleanUp.ReferenceOTU218966
New.CleanUp.ReferenceOTU37026
New.CleanUp.ReferenceOTU41506
New.CleanUp.ReferenceOTU194619
New.CleanUp.ReferenceOTU146098
New.CleanUp.ReferenceOTU213984
New.CleanUp.ReferenceOTU205959
New.CleanUp.ReferenceOTU130566
New.CleanUp.ReferenceOTU248143
New.CleanUp.ReferenceOTU289935
New.CleanUp.ReferenceOTU82896
New.CleanUp.ReferenceOTU178494
New.CleanUp.ReferenceOTU191735
New.CleanUp.ReferenceOTU30038
New.CleanUp.ReferenceOTU89463
New.CleanUp.ReferenceOTU46041
New.CleanUp.ReferenceOTU93818
New.CleanUp.ReferenceOTU76800
New.CleanUp.ReferenceOTU109157
New.CleanUp.ReferenceOTU43436
New.CleanUp.ReferenceOTU115671
New.CleanUp.ReferenceOTU177762
New.CleanUp.ReferenceOTU254673
New.CleanUp.ReferenceOTU170940
New.CleanUp.ReferenceOTU273294
New.CleanUp.ReferenceOTU32524
New.CleanUp.ReferenceOTU326699
New.CleanUp.ReferenceOTU286628
New.CleanUp.ReferenceOTU150193
New.CleanUp.ReferenceOTU264708
New.CleanUp.ReferenceOTU162162
New.CleanUp.ReferenceOTU140150
New.CleanUp.ReferenceOTU198536
New.CleanUp.ReferenceOTU112951
New.CleanUp.ReferenceOTU205193
New.CleanUp.ReferenceOTU97487
New.CleanUp.ReferenceOTU17235
New.CleanUp.ReferenceOTU12214
New.CleanUp.ReferenceOTU8039
New.CleanUp.ReferenceOTU19155
New.CleanUp.ReferenceOTU25172
New.CleanUp.ReferenceOTU32098
New.ReferenceOTU3114
New.CleanUp.ReferenceOTU49922
New.CleanUp.ReferenceOTU22064
New.CleanUp.ReferenceOTU15142
New.CleanUp.ReferenceOTU16297
New.CleanUp.ReferenceOTU19801
New.CleanUp.ReferenceOTU15310
New.CleanUp.ReferenceOTU25617
New.CleanUp.ReferenceOTU14315
New.CleanUp.ReferenceOTU33577
New.CleanUp.ReferenceOTU26552
New.CleanUp.ReferenceOTU23165
New.CleanUp.ReferenceOTU19728
New.CleanUp.ReferenceOTU42081
New.CleanUp.ReferenceOTU4412
New.CleanUp.ReferenceOTU7731
New.CleanUp.ReferenceOTU24104
New.CleanUp.ReferenceOTU12121

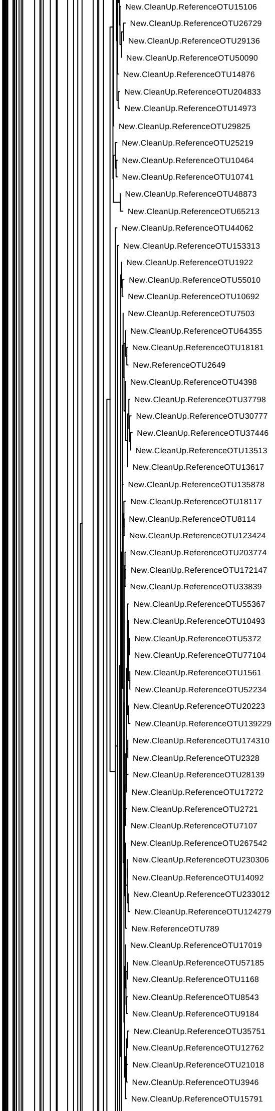
New.CleanUp.ReferenceOTU15106
New.CleanUp.ReferenceOTU26729
New.CleanUp.ReferenceOTU29136
New.CleanUp.ReferenceOTU50090
New.CleanUp.ReferenceOTU14876
New.CleanUp.ReferenceOTU204833
New.CleanUp.ReferenceOTU14973
New.CleanUp.ReferenceOTU29825
New.CleanUp.ReferenceOTU25219
New.CleanUp.ReferenceOTU10464
New.CleanUp.ReferenceOTU10741
New.CleanUp.ReferenceOTU48873
New.CleanUp.ReferenceOTU65213
New.CleanUp.ReferenceOTU44062
New.CleanUp.ReferenceOTU153313
New.CleanUp.ReferenceOTU1922
New.CleanUp.ReferenceOTU55010
New.CleanUp.ReferenceOTU10692
New.CleanUp.ReferenceOTU7503
New.CleanUp.ReferenceOTU64355
New.CleanUp.ReferenceOTU18181
New.ReferenceOTU2649
New.CleanUp.ReferenceOTU4398
New.CleanUp.ReferenceOTU37798
New.CleanUp.ReferenceOTU30777
New.CleanUp.ReferenceOTU37446
New.CleanUp.ReferenceOTU13513
New.CleanUp.ReferenceOTU13617
New.CleanUp.ReferenceOTU135878
New.CleanUp.ReferenceOTU18117
New.CleanUp.ReferenceOTU8114
New.CleanUp.ReferenceOTU123424
New.CleanUp.ReferenceOTU203774
New.CleanUp.ReferenceOTU172147
New.CleanUp.ReferenceOTU33839
New.CleanUp.ReferenceOTU55367
New.CleanUp.ReferenceOTU10493
New.CleanUp.ReferenceOTU5372
New.CleanUp.ReferenceOTU77104
New.CleanUp.ReferenceOTU1561
New.CleanUp.ReferenceOTU52234
New.CleanUp.ReferenceOTU20223
New.CleanUp.ReferenceOTU139229
New.CleanUp.ReferenceOTU174310
New.CleanUp.ReferenceOTU2328
New.CleanUp.ReferenceOTU28139
New.CleanUp.ReferenceOTU17272
New.CleanUp.ReferenceOTU2721
New.CleanUp.ReferenceOTU7107
New.CleanUp.ReferenceOTU267542
New.CleanUp.ReferenceOTU230306
New.CleanUp.ReferenceOTU14092
New.CleanUp.ReferenceOTU233012
New.CleanUp.ReferenceOTU124279
New.ReferenceOTU789
New.CleanUp.ReferenceOTU17019
New.CleanUp.ReferenceOTU57185
New.CleanUp.ReferenceOTU1168
New.CleanUp.ReferenceOTU8543
New.CleanUp.ReferenceOTU9184
New.CleanUp.ReferenceOTU35751
New.CleanUp.ReferenceOTU12762
New.CleanUp.ReferenceOTU21018
New.CleanUp.ReferenceOTU3946
New.CleanUp.ReferenceOTU15791

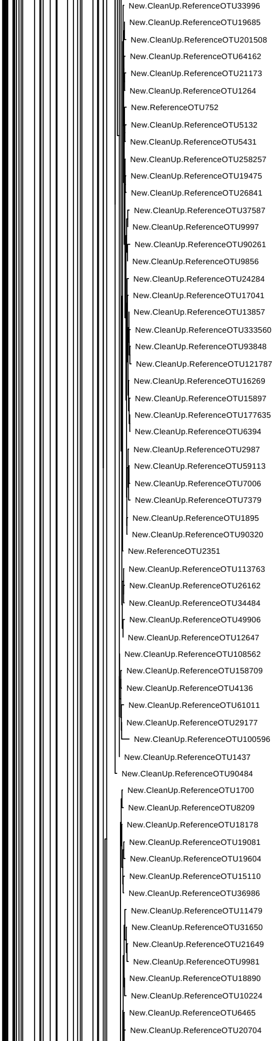
New.CleanUp.ReferenceOTU33996
New.CleanUp.ReferenceOTU19685
New.CleanUp.ReferenceOTU201508
New.CleanUp.ReferenceOTU64162
New.CleanUp.ReferenceOTU21173
New.CleanUp.ReferenceOTU1264
New.ReferenceOTU752
New.CleanUp.ReferenceOTU5132
New.CleanUp.ReferenceOTU5431
New.CleanUp.ReferenceOTU258257
New.CleanUp.ReferenceOTU19475
New.CleanUp.ReferenceOTU26841
New.CleanUp.ReferenceOTU37587
New.CleanUp.ReferenceOTU9997
New.CleanUp.ReferenceOTU90261
New.CleanUp.ReferenceOTU9856
New.CleanUp.ReferenceOTU24284
New.CleanUp.ReferenceOTU17041
New.CleanUp.ReferenceOTU13857
New.CleanUp.ReferenceOTU333560
New.CleanUp.ReferenceOTU93848
New.CleanUp.ReferenceOTU121787
New.CleanUp.ReferenceOTU16269
New.CleanUp.ReferenceOTU15897
New.CleanUp.ReferenceOTU177635
New.CleanUp.ReferenceOTU6394
New.CleanUp.ReferenceOTU2987
New.CleanUp.ReferenceOTU59113
New.CleanUp.ReferenceOTU7006
New.CleanUp.ReferenceOTU7379
New.CleanUp.ReferenceOTU1895
New.CleanUp.ReferenceOTU90320
New.ReferenceOTU2351
New.CleanUp.ReferenceOTU113763
New.CleanUp.ReferenceOTU26162
New.CleanUp.ReferenceOTU34484
New.CleanUp.ReferenceOTU49906
New.CleanUp.ReferenceOTU12647
New.CleanUp.ReferenceOTU108562
New.CleanUp.ReferenceOTU158709
New.CleanUp.ReferenceOTU4136
New.CleanUp.ReferenceOTU61011
New.CleanUp.ReferenceOTU29177
New.CleanUp.ReferenceOTU100596
New.CleanUp.ReferenceOTU1437
New.CleanUp.ReferenceOTU90484
New.CleanUp.ReferenceOTU1700
New.CleanUp.ReferenceOTU8209
New.CleanUp.ReferenceOTU18178
New.CleanUp.ReferenceOTU19081
New.CleanUp.ReferenceOTU19604
New.CleanUp.ReferenceOTU15110
New.CleanUp.ReferenceOTU36986
New.CleanUp.ReferenceOTU11479
New.CleanUp.ReferenceOTU31650
New.CleanUp.ReferenceOTU21649
New.CleanUp.ReferenceOTU9981
New.CleanUp.ReferenceOTU18890
New.CleanUp.ReferenceOTU10224
New.CleanUp.ReferenceOTU6465
New.CleanUp.ReferenceOTU20704

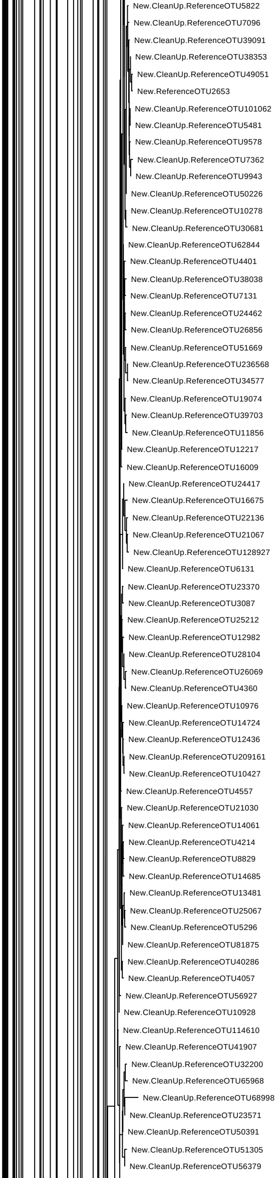
New.CleanUp.ReferenceOTU5822
New.CleanUp.ReferenceOTU7096
New.CleanUp.ReferenceOTU39091
New.CleanUp.ReferenceOTU38353
New.CleanUp.ReferenceOTU49051
New.ReferenceOTU2653
New.CleanUp.ReferenceOTU101062
New.CleanUp.ReferenceOTU5481
New.CleanUp.ReferenceOTU9578
New.CleanUp.ReferenceOTU7362
New.CleanUp.ReferenceOTU9943
New.CleanUp.ReferenceOTU50226
New.CleanUp.ReferenceOTU10278
New.CleanUp.ReferenceOTU30681
New.CleanUp.ReferenceOTU62844
New.CleanUp.ReferenceOTU4401
New.CleanUp.ReferenceOTU38038
New.CleanUp.ReferenceOTU7131
New.CleanUp.ReferenceOTU24462
New.CleanUp.ReferenceOTU26856
New.CleanUp.ReferenceOTU51669
New.CleanUp.ReferenceOTU236568
New.CleanUp.ReferenceOTU34577
New.CleanUp.ReferenceOTU19074
New.CleanUp.ReferenceOTU39703
New.CleanUp.ReferenceOTU11856
New.CleanUp.ReferenceOTU12217
New.CleanUp.ReferenceOTU16009
New.CleanUp.ReferenceOTU24417
New.CleanUp.ReferenceOTU16675
New.CleanUp.ReferenceOTU22136
New.CleanUp.ReferenceOTU21067
New.CleanUp.ReferenceOTU128927
New.CleanUp.ReferenceOTU6131
New.CleanUp.ReferenceOTU23370
New.CleanUp.ReferenceOTU3087
New.CleanUp.ReferenceOTU25212
New.CleanUp.ReferenceOTU12982
New.CleanUp.ReferenceOTU28104
New.CleanUp.ReferenceOTU26069
New.CleanUp.ReferenceOTU4360
New.CleanUp.ReferenceOTU10976
New.CleanUp.ReferenceOTU14724
New.CleanUp.ReferenceOTU12436
New.CleanUp.ReferenceOTU209161
New.CleanUp.ReferenceOTU10427
New.CleanUp.ReferenceOTU4557
New.CleanUp.ReferenceOTU21030
New.CleanUp.ReferenceOTU14061
New.CleanUp.ReferenceOTU4214
New.CleanUp.ReferenceOTU8829
New.CleanUp.ReferenceOTU14685
New.CleanUp.ReferenceOTU13481
New.CleanUp.ReferenceOTU25067
New.CleanUp.ReferenceOTU5296
New.CleanUp.ReferenceOTU81875
New.CleanUp.ReferenceOTU40286
New.CleanUp.ReferenceOTU4057
New.CleanUp.ReferenceOTU56927
New.CleanUp.ReferenceOTU10928
New.CleanUp.ReferenceOTU114610
New.CleanUp.ReferenceOTU41907
New.CleanUp.ReferenceOTU32200
New.CleanUp.ReferenceOTU65968
New.CleanUp.ReferenceOTU68998
New.CleanUp.ReferenceOTU23571
New.CleanUp.ReferenceOTU50391
New.CleanUp.ReferenceOTU51305
New.CleanUp.ReferenceOTU56379

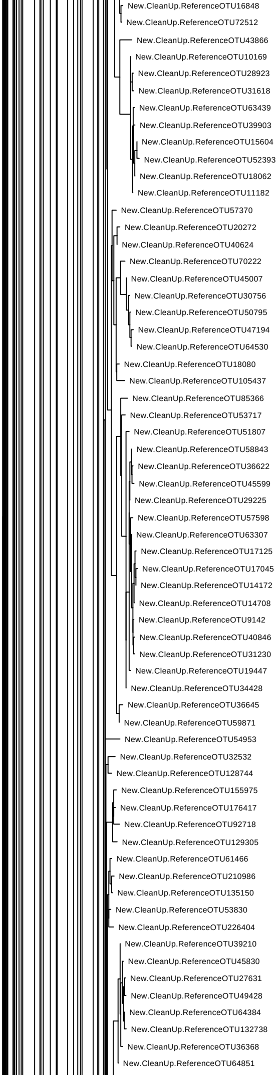
New.CleanUp.ReferenceOTU16848
New.CleanUp.ReferenceOTU72512
New.CleanUp.ReferenceOTU43866
New.CleanUp.ReferenceOTU10169
New.CleanUp.ReferenceOTU28923
New.CleanUp.ReferenceOTU31618
New.CleanUp.ReferenceOTU63439
New.CleanUp.ReferenceOTU39903
New.CleanUp.ReferenceOTU15604
New.CleanUp.ReferenceOTU52393
New.CleanUp.ReferenceOTU18062
New.CleanUp.ReferenceOTU11182
New.CleanUp.ReferenceOTU57370
New.CleanUp.ReferenceOTU20272
New.CleanUp.ReferenceOTU40624
New.CleanUp.ReferenceOTU70222
New.CleanUp.ReferenceOTU45007
New.CleanUp.ReferenceOTU30756
New.CleanUp.ReferenceOTU50795
New.CleanUp.ReferenceOTU47194
New.CleanUp.ReferenceOTU64530
New.CleanUp.ReferenceOTU18080
New.CleanUp.ReferenceOTU105437
New.CleanUp.ReferenceOTU85366
New.CleanUp.ReferenceOTU53717
New.CleanUp.ReferenceOTU51807
New.CleanUp.ReferenceOTU58843
New.CleanUp.ReferenceOTU36622
New.CleanUp.ReferenceOTU45599
New.CleanUp.ReferenceOTU29225
New.CleanUp.ReferenceOTU57598
New.CleanUp.ReferenceOTU63307
New.CleanUp.ReferenceOTU17125
New.CleanUp.ReferenceOTU17045
New.CleanUp.ReferenceOTU14172
New.CleanUp.ReferenceOTU14708
New.CleanUp.ReferenceOTU9142
New.CleanUp.ReferenceOTU40846
New.CleanUp.ReferenceOTU31230
New.CleanUp.ReferenceOTU19447
New.CleanUp.ReferenceOTU34428
New.CleanUp.ReferenceOTU36645
New.CleanUp.ReferenceOTU59871
New.CleanUp.ReferenceOTU54953
New.CleanUp.ReferenceOTU32532
New.CleanUp.ReferenceOTU128744
New.CleanUp.ReferenceOTU155975
New.CleanUp.ReferenceOTU176417
New.CleanUp.ReferenceOTU92718
New.CleanUp.ReferenceOTU129305
New.CleanUp.ReferenceOTU61466
New.CleanUp.ReferenceOTU210986
New.CleanUp.ReferenceOTU135150
New.CleanUp.ReferenceOTU53830
New.CleanUp.ReferenceOTU226404
New.CleanUp.ReferenceOTU39210
New.CleanUp.ReferenceOTU45830
New.CleanUp.ReferenceOTU27631
New.CleanUp.ReferenceOTU49428
New.CleanUp.ReferenceOTU64384
New.CleanUp.ReferenceOTU132738
New.CleanUp.ReferenceOTU36368
New.CleanUp.ReferenceOTU64851

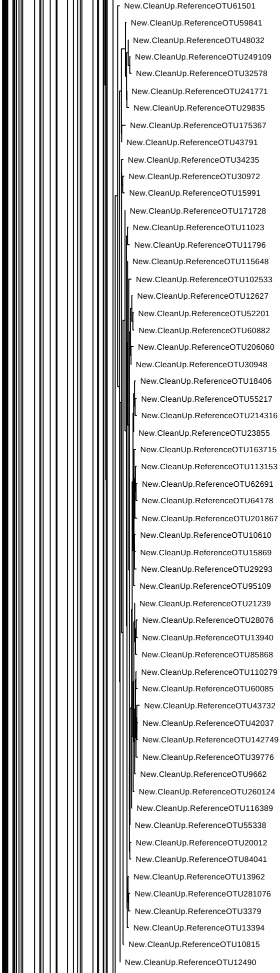
New.CleanUp.ReferenceOTU61501
New.CleanUp.ReferenceOTU59841
New.CleanUp.ReferenceOTU48032
New.CleanUp.ReferenceOTU249109
New.CleanUp.ReferenceOTU32578
New.CleanUp.ReferenceOTU241771
New.CleanUp.ReferenceOTU29835
New.CleanUp.ReferenceOTU175367
New.CleanUp.ReferenceOTU43791
New.CleanUp.ReferenceOTU34235
New.CleanUp.ReferenceOTU30972
New.CleanUp.ReferenceOTU15991
New.CleanUp.ReferenceOTU171728
New.CleanUp.ReferenceOTU11023
New.CleanUp.ReferenceOTU11796
New.CleanUp.ReferenceOTU115648
New.CleanUp.ReferenceOTU102533
New.CleanUp.ReferenceOTU12627
New.CleanUp.ReferenceOTU52201
New.CleanUp.ReferenceOTU60882
New.CleanUp.ReferenceOTU206060
New.CleanUp.ReferenceOTU30948
New.CleanUp.ReferenceOTU18406
New.CleanUp.ReferenceOTU55217
New.CleanUp.ReferenceOTU214316
New.CleanUp.ReferenceOTU23855
New.CleanUp.ReferenceOTU163715
New.CleanUp.ReferenceOTU113153
New.CleanUp.ReferenceOTU62691
New.CleanUp.ReferenceOTU64178
New.CleanUp.ReferenceOTU201867
New.CleanUp.ReferenceOTU10610
New.CleanUp.ReferenceOTU15869
New.CleanUp.ReferenceOTU29293
New.CleanUp.ReferenceOTU95109
New.CleanUp.ReferenceOTU21239
New.CleanUp.ReferenceOTU28076
New.CleanUp.ReferenceOTU13940
New.CleanUp.ReferenceOTU85868
New.CleanUp.ReferenceOTU110279
New.CleanUp.ReferenceOTU60085
New.CleanUp.ReferenceOTU43732
New.CleanUp.ReferenceOTU42037
New.CleanUp.ReferenceOTU142749
New.CleanUp.ReferenceOTU39776
New.CleanUp.ReferenceOTU9662
New.CleanUp.ReferenceOTU260124
New.CleanUp.ReferenceOTU116389
New.CleanUp.ReferenceOTU55338
New.CleanUp.ReferenceOTU20012
New.CleanUp.ReferenceOTU84041
New.CleanUp.ReferenceOTU13962
New.CleanUp.ReferenceOTU281076
New.CleanUp.ReferenceOTU3379
New.CleanUp.ReferenceOTU13394
New.CleanUp.ReferenceOTU10815
New.CleanUp.ReferenceOTU12490

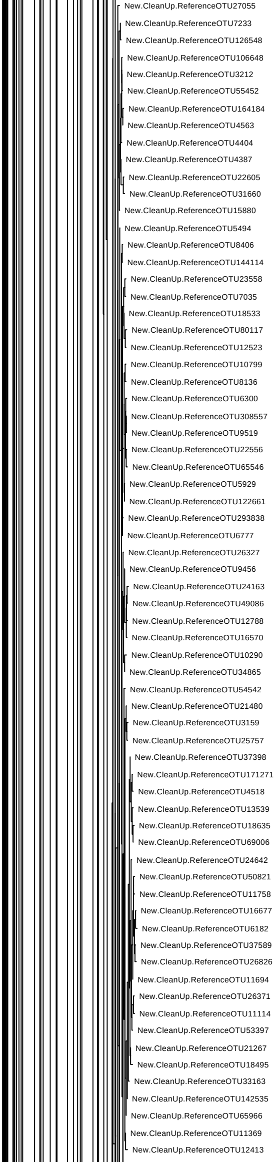
New.CleanUp.ReferenceOTU27055
New.CleanUp.ReferenceOTU7233
New.CleanUp.ReferenceOTU126548
New.CleanUp.ReferenceOTU106648
New.CleanUp.ReferenceOTU3212
New.CleanUp.ReferenceOTU55452
New.CleanUp.ReferenceOTU164184
New.CleanUp.ReferenceOTU4563
New.CleanUp.ReferenceOTU4404
New.CleanUp.ReferenceOTU4387
New.CleanUp.ReferenceOTU22605
New.CleanUp.ReferenceOTU31660
New.CleanUp.ReferenceOTU15880
New.CleanUp.ReferenceOTU5494
New.CleanUp.ReferenceOTU8406
New.CleanUp.ReferenceOTU144114
New.CleanUp.ReferenceOTU23558
New.CleanUp.ReferenceOTU7035
New.CleanUp.ReferenceOTU18533
New.CleanUp.ReferenceOTU80117
New.CleanUp.ReferenceOTU12523
New.CleanUp.ReferenceOTU10799
New.CleanUp.ReferenceOTU8136
New.CleanUp.ReferenceOTU6300
New.CleanUp.ReferenceOTU308557
New.CleanUp.ReferenceOTU9519
New.CleanUp.ReferenceOTU22556
New.CleanUp.ReferenceOTU65546
New.CleanUp.ReferenceOTU5929
New.CleanUp.ReferenceOTU122661
New.CleanUp.ReferenceOTU293838
New.CleanUp.ReferenceOTU6777
New.CleanUp.ReferenceOTU26327
New.CleanUp.ReferenceOTU9456
New.CleanUp.ReferenceOTU24163
New.CleanUp.ReferenceOTU49086
New.CleanUp.ReferenceOTU12788
New.CleanUp.ReferenceOTU16570
New.CleanUp.ReferenceOTU10290
New.CleanUp.ReferenceOTU34865
New.CleanUp.ReferenceOTU54542
New.CleanUp.ReferenceOTU21480
New.CleanUp.ReferenceOTU3159
New.CleanUp.ReferenceOTU25757
New.CleanUp.ReferenceOTU37398
New.CleanUp.ReferenceOTU171271
New.CleanUp.ReferenceOTU4518
New.CleanUp.ReferenceOTU13539
New.CleanUp.ReferenceOTU18635
New.CleanUp.ReferenceOTU69006
New.CleanUp.ReferenceOTU24642
New.CleanUp.ReferenceOTU50821
New.CleanUp.ReferenceOTU11758
New.CleanUp.ReferenceOTU16677
New.CleanUp.ReferenceOTU6182
New.CleanUp.ReferenceOTU37589
New.CleanUp.ReferenceOTU26826
New.CleanUp.ReferenceOTU11694
New.CleanUp.ReferenceOTU26371
New.CleanUp.ReferenceOTU11114
New.CleanUp.ReferenceOTU53397
New.CleanUp.ReferenceOTU21267
New.CleanUp.ReferenceOTU18495
New.CleanUp.ReferenceOTU33163
New.CleanUp.ReferenceOTU142535
New.CleanUp.ReferenceOTU65966
New.CleanUp.ReferenceOTU11369
New.CleanUp.ReferenceOTU12413

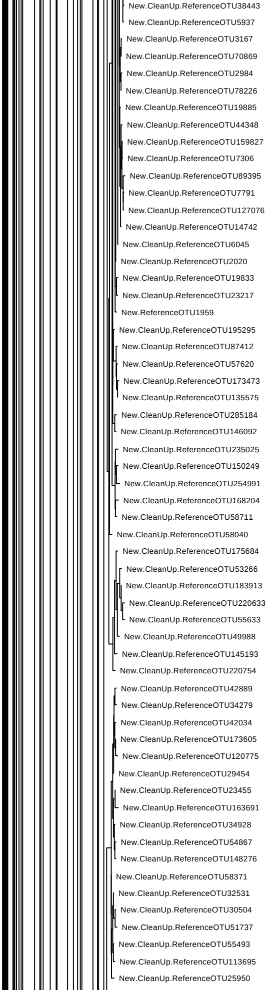
New.CleanUp.ReferenceOTU38443
New.CleanUp.ReferenceOTU5937
New.CleanUp.ReferenceOTU3167
New.CleanUp.ReferenceOTU70869
New.CleanUp.ReferenceOTU2984
New.CleanUp.ReferenceOTU78226
New.CleanUp.ReferenceOTU19885
New.CleanUp.ReferenceOTU44348
New.CleanUp.ReferenceOTU159827
New.CleanUp.ReferenceOTU7306
New.CleanUp.ReferenceOTU89395
New.CleanUp.ReferenceOTU7791
New.CleanUp.ReferenceOTU127076
New.CleanUp.ReferenceOTU14742
New.CleanUp.ReferenceOTU6045
New.CleanUp.ReferenceOTU2020
New.CleanUp.ReferenceOTU19833
New.CleanUp.ReferenceOTU23217
New.ReferenceOTU1959
New.CleanUp.ReferenceOTU195295
New.CleanUp.ReferenceOTU87412
New.CleanUp.ReferenceOTU57620
New.CleanUp.ReferenceOTU173473
New.CleanUp.ReferenceOTU135575
New.CleanUp.ReferenceOTU285184
New.CleanUp.ReferenceOTU146092
New.CleanUp.ReferenceOTU235025
New.CleanUp.ReferenceOTU150249
New.CleanUp.ReferenceOTU254991
New.CleanUp.ReferenceOTU168204
New.CleanUp.ReferenceOTU58711
New.CleanUp.ReferenceOTU58040
New.CleanUp.ReferenceOTU175684
New.CleanUp.ReferenceOTU53266
New.CleanUp.ReferenceOTU183913
New.CleanUp.ReferenceOTU220633
New.CleanUp.ReferenceOTU55633
New.CleanUp.ReferenceOTU49988
New.CleanUp.ReferenceOTU145193
New.CleanUp.ReferenceOTU220754
New.CleanUp.ReferenceOTU42889
New.CleanUp.ReferenceOTU34279
New.CleanUp.ReferenceOTU42034
New.CleanUp.ReferenceOTU173605
New.CleanUp.ReferenceOTU120775
New.CleanUp.ReferenceOTU29454
New.CleanUp.ReferenceOTU23455
New.CleanUp.ReferenceOTU163691
New.CleanUp.ReferenceOTU34928
New.CleanUp.ReferenceOTU54867
New.CleanUp.ReferenceOTU148276
New.CleanUp.ReferenceOTU58371
New.CleanUp.ReferenceOTU32531
New.CleanUp.ReferenceOTU30504
New.CleanUp.ReferenceOTU51737
New.CleanUp.ReferenceOTU55493
New.CleanUp.ReferenceOTU113695
New.CleanUp.ReferenceOTU25950

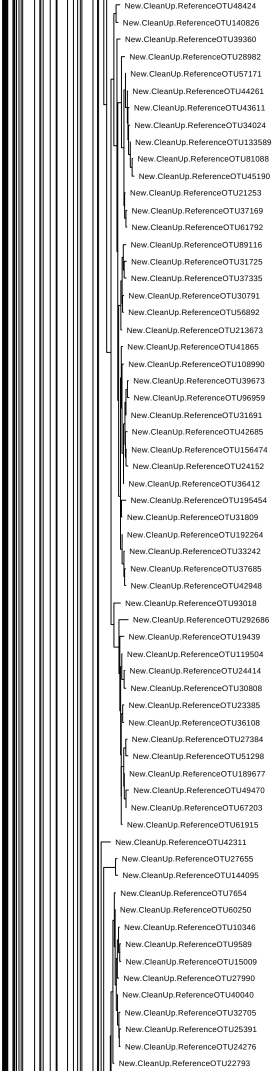
New.CleanUp.ReferenceOTU48424
New.CleanUp.ReferenceOTU140826
New.CleanUp.ReferenceOTU39360
New.CleanUp.ReferenceOTU28982
New.CleanUp.ReferenceOTU57171
New.CleanUp.ReferenceOTU44261
New.CleanUp.ReferenceOTU43611
New.CleanUp.ReferenceOTU34024
New.CleanUp.ReferenceOTU133589
New.CleanUp.ReferenceOTU81088
New.CleanUp.ReferenceOTU45190
New.CleanUp.ReferenceOTU21253
New.CleanUp.ReferenceOTU37169
New.CleanUp.ReferenceOTU61792
New.CleanUp.ReferenceOTU89116
New.CleanUp.ReferenceOTU31725
New.CleanUp.ReferenceOTU37335
New.CleanUp.ReferenceOTU30791
New.CleanUp.ReferenceOTU56892
New.CleanUp.ReferenceOTU213673
New.CleanUp.ReferenceOTU41865
New.CleanUp.ReferenceOTU108990
New.CleanUp.ReferenceOTU39673
New.CleanUp.ReferenceOTU96959
New.CleanUp.ReferenceOTU31691
New.CleanUp.ReferenceOTU42685
New.CleanUp.ReferenceOTU156474
New.CleanUp.ReferenceOTU24152
New.CleanUp.ReferenceOTU36412
New.CleanUp.ReferenceOTU195454
New.CleanUp.ReferenceOTU31809
New.CleanUp.ReferenceOTU192264
New.CleanUp.ReferenceOTU33242
New.CleanUp.ReferenceOTU37685
New.CleanUp.ReferenceOTU42948
New.CleanUp.ReferenceOTU93018
New.CleanUp.ReferenceOTU292686
New.CleanUp.ReferenceOTU19439
New.CleanUp.ReferenceOTU119504
New.CleanUp.ReferenceOTU24414
New.CleanUp.ReferenceOTU30808
New.CleanUp.ReferenceOTU23385
New.CleanUp.ReferenceOTU36108
New.CleanUp.ReferenceOTU27384
New.CleanUp.ReferenceOTU51298
New.CleanUp.ReferenceOTU189677
New.CleanUp.ReferenceOTU49470
New.CleanUp.ReferenceOTU67203
New.CleanUp.ReferenceOTU61915
New.CleanUp.ReferenceOTU42311
New.CleanUp.ReferenceOTU27655
New.CleanUp.ReferenceOTU144095
New.CleanUp.ReferenceOTU7654
New.CleanUp.ReferenceOTU60250
New.CleanUp.ReferenceOTU10346
New.CleanUp.ReferenceOTU9589
New.CleanUp.ReferenceOTU15009
New.CleanUp.ReferenceOTU27990
New.CleanUp.ReferenceOTU40040
New.CleanUp.ReferenceOTU32705
New.CleanUp.ReferenceOTU25391
New.CleanUp.ReferenceOTU24276
New.CleanUp.ReferenceOTU22793

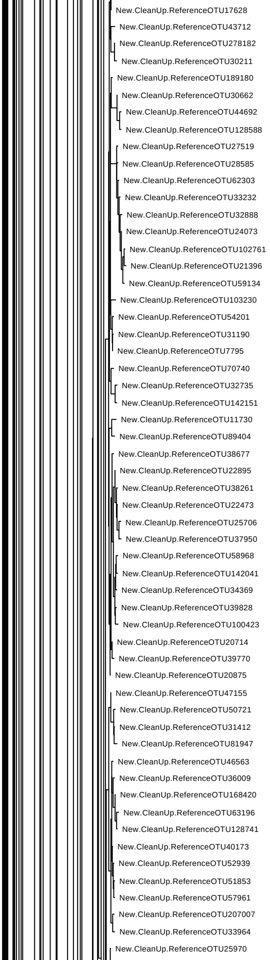
New.CleanUp.ReferenceOTU17628
New.CleanUp.ReferenceOTU43712
New.CleanUp.ReferenceOTU278182
New.CleanUp.ReferenceOTU30211
New.CleanUp.ReferenceOTU189180
New.CleanUp.ReferenceOTU30662
New.CleanUp.ReferenceOTU44692
New.CleanUp.ReferenceOTU128588
New.CleanUp.ReferenceOTU27519
New.CleanUp.ReferenceOTU28585
New.CleanUp.ReferenceOTU62303
New.CleanUp.ReferenceOTU33232
New.CleanUp.ReferenceOTU32888
New.CleanUp.ReferenceOTU24073
New.CleanUp.ReferenceOTU102761
New.CleanUp.ReferenceOTU21396
New.CleanUp.ReferenceOTU59134
New.CleanUp.ReferenceOTU103230
New.CleanUp.ReferenceOTU54201
New.CleanUp.ReferenceOTU31190
New.CleanUp.ReferenceOTU7795
New.CleanUp.ReferenceOTU70740
New.CleanUp.ReferenceOTU32735
New.CleanUp.ReferenceOTU142151
New.CleanUp.ReferenceOTU11730
New.CleanUp.ReferenceOTU89404
New.CleanUp.ReferenceOTU38677
New.CleanUp.ReferenceOTU22895
New.CleanUp.ReferenceOTU38261
New.CleanUp.ReferenceOTU22473
New.CleanUp.ReferenceOTU25706
New.CleanUp.ReferenceOTU37950
New.CleanUp.ReferenceOTU58968
New.CleanUp.ReferenceOTU142041
New.CleanUp.ReferenceOTU34369
New.CleanUp.ReferenceOTU39828
New.CleanUp.ReferenceOTU100423
New.CleanUp.ReferenceOTU20714
New.CleanUp.ReferenceOTU39770
New.CleanUp.ReferenceOTU20875
New.CleanUp.ReferenceOTU47155
New.CleanUp.ReferenceOTU50721
New.CleanUp.ReferenceOTU31412
New.CleanUp.ReferenceOTU81947
New.CleanUp.ReferenceOTU46563
New.CleanUp.ReferenceOTU36009
New.CleanUp.ReferenceOTU168420
New.CleanUp.ReferenceOTU63196
New.CleanUp.ReferenceOTU128741
New.CleanUp.ReferenceOTU40173
New.CleanUp.ReferenceOTU52939
New.CleanUp.ReferenceOTU51853
New.CleanUp.ReferenceOTU57961
New.CleanUp.ReferenceOTU207007
New.CleanUp.ReferenceOTU33964
New.CleanUp.ReferenceOTU25970

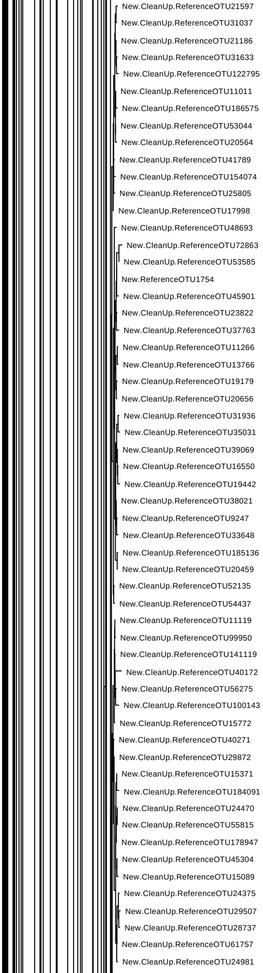
New.CleanUp.ReferenceOTU21597
New.CleanUp.ReferenceOTU31037
New.CleanUp.ReferenceOTU21186
New.CleanUp.ReferenceOTU31633
New.CleanUp.ReferenceOTU122795
New.CleanUp.ReferenceOTU11011
New.CleanUp.ReferenceOTU186575
New.CleanUp.ReferenceOTU53044
New.CleanUp.ReferenceOTU20564
New.CleanUp.ReferenceOTU41789
New.CleanUp.ReferenceOTU154074
New.CleanUp.ReferenceOTU25805
New.CleanUp.ReferenceOTU17998
New.CleanUp.ReferenceOTU48693
New.CleanUp.ReferenceOTU72863
New.CleanUp.ReferenceOTU53585
New.ReferenceOTU1754
New.CleanUp.ReferenceOTU45901
New.CleanUp.ReferenceOTU23822
New.CleanUp.ReferenceOTU37763
New.CleanUp.ReferenceOTU11266
New.CleanUp.ReferenceOTU13766
New.CleanUp.ReferenceOTU19179
New.CleanUp.ReferenceOTU20656
New.CleanUp.ReferenceOTU31936
New.CleanUp.ReferenceOTU35031
New.CleanUp.ReferenceOTU39069
New.CleanUp.ReferenceOTU16550
New.CleanUp.ReferenceOTU19442
New.CleanUp.ReferenceOTU38021
New.CleanUp.ReferenceOTU9247
New.CleanUp.ReferenceOTU33648
New.CleanUp.ReferenceOTU185136
New.CleanUp.ReferenceOTU20459
New.CleanUp.ReferenceOTU52135
New.CleanUp.ReferenceOTU54437
New.CleanUp.ReferenceOTU11119
New.CleanUp.ReferenceOTU99950
New.CleanUp.ReferenceOTU141119
New.CleanUp.ReferenceOTU40172
New.CleanUp.ReferenceOTU56275
New.CleanUp.ReferenceOTU100143
New.CleanUp.ReferenceOTU15772
New.CleanUp.ReferenceOTU40271
New.CleanUp.ReferenceOTU29872
New.CleanUp.ReferenceOTU15371
New.CleanUp.ReferenceOTU184091
New.CleanUp.ReferenceOTU24470
New.CleanUp.ReferenceOTU55815
New.CleanUp.ReferenceOTU178947
New.CleanUp.ReferenceOTU45304
New.CleanUp.ReferenceOTU15089
New.CleanUp.ReferenceOTU24375
New.CleanUp.ReferenceOTU29507
New.CleanUp.ReferenceOTU28737
New.CleanUp.ReferenceOTU61757
New.CleanUp.ReferenceOTU24981

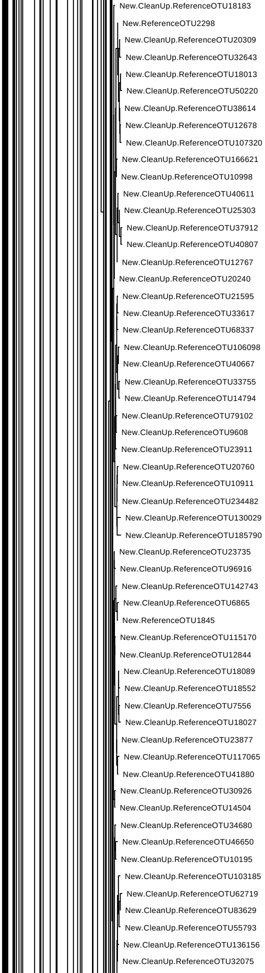
New.CleanUp.ReferenceOTU18183
New.ReferenceOTU2298
New.CleanUp.ReferenceOTU20309
New.CleanUp.ReferenceOTU32643
New.CleanUp.ReferenceOTU18013
New.CleanUp.ReferenceOTU50220
New.CleanUp.ReferenceOTU38614
New.CleanUp.ReferenceOTU12678
New.CleanUp.ReferenceOTU107320
New.CleanUp.ReferenceOTU166621
New.CleanUp.ReferenceOTU10998
New.CleanUp.ReferenceOTU40611
New.CleanUp.ReferenceOTU25303
New.CleanUp.ReferenceOTU37912
New.CleanUp.ReferenceOTU40807
New.CleanUp.ReferenceOTU12767
New.CleanUp.ReferenceOTU20240
New.CleanUp.ReferenceOTU21595
New.CleanUp.ReferenceOTU33617
New.CleanUp.ReferenceOTU68337
New.CleanUp.ReferenceOTU106098
New.CleanUp.ReferenceOTU40667
New.CleanUp.ReferenceOTU33755
New.CleanUp.ReferenceOTU14794
New.CleanUp.ReferenceOTU79102
New.CleanUp.ReferenceOTU9608
New.CleanUp.ReferenceOTU23911
New.CleanUp.ReferenceOTU20760
New.CleanUp.ReferenceOTU10911
New.CleanUp.ReferenceOTU234482
New.CleanUp.ReferenceOTU130029
New.CleanUp.ReferenceOTU185790
New.CleanUp.ReferenceOTU23735
New.CleanUp.ReferenceOTU96916
New.CleanUp.ReferenceOTU142743
New.CleanUp.ReferenceOTU6865
New.ReferenceOTU1845
New.CleanUp.ReferenceOTU115170
New.CleanUp.ReferenceOTU12844
New.CleanUp.ReferenceOTU18089
New.CleanUp.ReferenceOTU18552
New.CleanUp.ReferenceOTU7556
New.CleanUp.ReferenceOTU18027
New.CleanUp.ReferenceOTU23877
New.CleanUp.ReferenceOTU117065
New.CleanUp.ReferenceOTU41880
New.CleanUp.ReferenceOTU30926
New.CleanUp.ReferenceOTU14504
New.CleanUp.ReferenceOTU34680
New.CleanUp.ReferenceOTU46650
New.CleanUp.ReferenceOTU10195
New.CleanUp.ReferenceOTU103185
New.CleanUp.ReferenceOTU62719
New.CleanUp.ReferenceOTU83629
New.CleanUp.ReferenceOTU55793
New.CleanUp.ReferenceOTU136156
New.CleanUp.ReferenceOTU32075

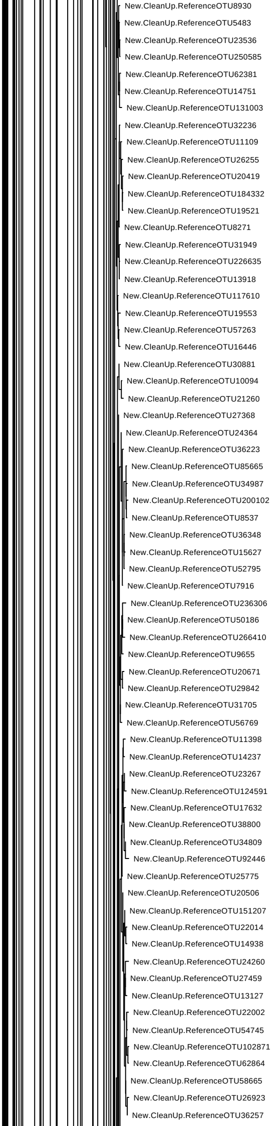
New.CleanUp.ReferenceOTU8930
New.CleanUp.ReferenceOTU5483
New.CleanUp.ReferenceOTU23536
New.CleanUp.ReferenceOTU250585
New.CleanUp.ReferenceOTU62381
New.CleanUp.ReferenceOTU14751
New.CleanUp.ReferenceOTU131003
New.CleanUp.ReferenceOTU32236
New.CleanUp.ReferenceOTU11109
New.CleanUp.ReferenceOTU26255
New.CleanUp.ReferenceOTU20419
New.CleanUp.ReferenceOTU184332
New.CleanUp.ReferenceOTU19521
New.CleanUp.ReferenceOTU8271
New.CleanUp.ReferenceOTU31949
New.CleanUp.ReferenceOTU226635
New.CleanUp.ReferenceOTU13918
New.CleanUp.ReferenceOTU117610
New.CleanUp.ReferenceOTU19553
New.CleanUp.ReferenceOTU57263
New.CleanUp.ReferenceOTU16446
New.CleanUp.ReferenceOTU30881
New.CleanUp.ReferenceOTU10094
New.CleanUp.ReferenceOTU21260
New.CleanUp.ReferenceOTU27368
New.CleanUp.ReferenceOTU24364
New.CleanUp.ReferenceOTU36223
New.CleanUp.ReferenceOTU85665
New.CleanUp.ReferenceOTU34987
New.CleanUp.ReferenceOTU200102
New.CleanUp.ReferenceOTU8537
New.CleanUp.ReferenceOTU36348
New.CleanUp.ReferenceOTU15627
New.CleanUp.ReferenceOTU52795
New.CleanUp.ReferenceOTU7916
New.CleanUp.ReferenceOTU236306
New.CleanUp.ReferenceOTU50186
New.CleanUp.ReferenceOTU266410
New.CleanUp.ReferenceOTU9655
New.CleanUp.ReferenceOTU20671
New.CleanUp.ReferenceOTU29842
New.CleanUp.ReferenceOTU31705
New.CleanUp.ReferenceOTU56769
New.CleanUp.ReferenceOTU11398
New.CleanUp.ReferenceOTU14237
New.CleanUp.ReferenceOTU23267
New.CleanUp.ReferenceOTU124591
New.CleanUp.ReferenceOTU17632
New.CleanUp.ReferenceOTU38800
New.CleanUp.ReferenceOTU34809
New.CleanUp.ReferenceOTU92446
New.CleanUp.ReferenceOTU25775
New.CleanUp.ReferenceOTU20506
New.CleanUp.ReferenceOTU151207
New.CleanUp.ReferenceOTU22014
New.CleanUp.ReferenceOTU14938
New.CleanUp.ReferenceOTU24260
New.CleanUp.ReferenceOTU27459
New.CleanUp.ReferenceOTU13127
New.CleanUp.ReferenceOTU22002
New.CleanUp.ReferenceOTU54745
New.CleanUp.ReferenceOTU102871
New.CleanUp.ReferenceOTU62864
New.CleanUp.ReferenceOTU58665
New.CleanUp.ReferenceOTU26923
New.CleanUp.ReferenceOTU36257

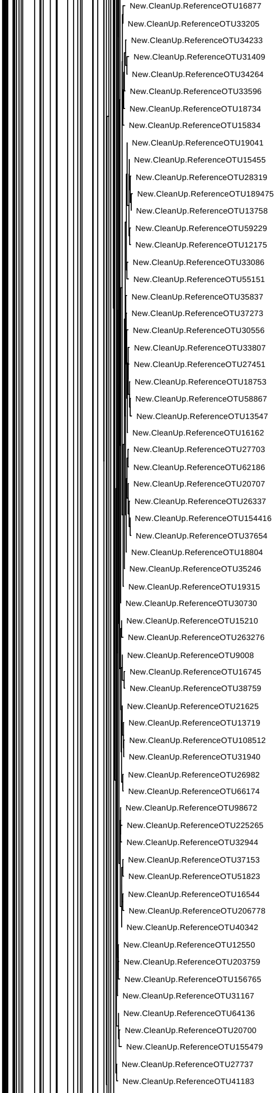
New.CleanUp.ReferenceOTU16877
New.CleanUp.ReferenceOTU33205
New.CleanUp.ReferenceOTU34233
New.CleanUp.ReferenceOTU31409
New.CleanUp.ReferenceOTU34264
New.CleanUp.ReferenceOTU33596
New.CleanUp.ReferenceOTU18734
New.CleanUp.ReferenceOTU15834
New.CleanUp.ReferenceOTU19041
New.CleanUp.ReferenceOTU15455
New.CleanUp.ReferenceOTU28319
New.CleanUp.ReferenceOTU189475
New.CleanUp.ReferenceOTU13758
New.CleanUp.ReferenceOTU59229
New.CleanUp.ReferenceOTU12175
New.CleanUp.ReferenceOTU33086
New.CleanUp.ReferenceOTU55151
New.CleanUp.ReferenceOTU35837
New.CleanUp.ReferenceOTU37273
New.CleanUp.ReferenceOTU30556
New.CleanUp.ReferenceOTU33807
New.CleanUp.ReferenceOTU27451
New.CleanUp.ReferenceOTU18753
New.CleanUp.ReferenceOTU58867
New.CleanUp.ReferenceOTU13547
New.CleanUp.ReferenceOTU16162
New.CleanUp.ReferenceOTU27703
New.CleanUp.ReferenceOTU62186
New.CleanUp.ReferenceOTU20707
New.CleanUp.ReferenceOTU26337
New.CleanUp.ReferenceOTU154416
New.CleanUp.ReferenceOTU37654
New.CleanUp.ReferenceOTU18804
New.CleanUp.ReferenceOTU35246
New.CleanUp.ReferenceOTU19315
New.CleanUp.ReferenceOTU30730
New.CleanUp.ReferenceOTU15210
New.CleanUp.ReferenceOTU263276
New.CleanUp.ReferenceOTU9008
New.CleanUp.ReferenceOTU16745
New.CleanUp.ReferenceOTU38759
New.CleanUp.ReferenceOTU21625
New.CleanUp.ReferenceOTU13719
New.CleanUp.ReferenceOTU108512
New.CleanUp.ReferenceOTU31940
New.CleanUp.ReferenceOTU26982
New.CleanUp.ReferenceOTU66174
New.CleanUp.ReferenceOTU98672
New.CleanUp.ReferenceOTU225265
New.CleanUp.ReferenceOTU32944
New.CleanUp.ReferenceOTU37153
New.CleanUp.ReferenceOTU51823
New.CleanUp.ReferenceOTU16544
New.CleanUp.ReferenceOTU206778
New.CleanUp.ReferenceOTU40342
New.CleanUp.ReferenceOTU12550
New.CleanUp.ReferenceOTU203759
New.CleanUp.ReferenceOTU156765
New.CleanUp.ReferenceOTU31167
New.CleanUp.ReferenceOTU64136
New.CleanUp.ReferenceOTU20700
New.CleanUp.ReferenceOTU155479
New.CleanUp.ReferenceOTU27737
New.CleanUp.ReferenceOTU41183

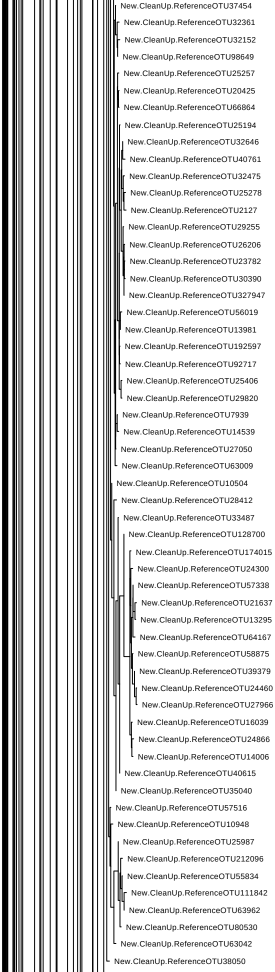
New.CleanUp.ReferenceOTU37454
New.CleanUp.ReferenceOTU32361
New.CleanUp.ReferenceOTU32152
New.CleanUp.ReferenceOTU98649
New.CleanUp.ReferenceOTU25257
New.CleanUp.ReferenceOTU20425
New.CleanUp.ReferenceOTU66864
New.CleanUp.ReferenceOTU25194
New.CleanUp.ReferenceOTU32646
New.CleanUp.ReferenceOTU40761
New.CleanUp.ReferenceOTU32475
New.CleanUp.ReferenceOTU25278
New.CleanUp.ReferenceOTU2127
New.CleanUp.ReferenceOTU29255
New.CleanUp.ReferenceOTU26206
New.CleanUp.ReferenceOTU23782
New.CleanUp.ReferenceOTU30390
New.CleanUp.ReferenceOTU327947
New.CleanUp.ReferenceOTU56019
New.CleanUp.ReferenceOTU13981
New.CleanUp.ReferenceOTU192597
New.CleanUp.ReferenceOTU92717
New.CleanUp.ReferenceOTU25406
New.CleanUp.ReferenceOTU29820
New.CleanUp.ReferenceOTU7939
New.CleanUp.ReferenceOTU14539
New.CleanUp.ReferenceOTU27050
New.CleanUp.ReferenceOTU63009
New.CleanUp.ReferenceOTU10504
New.CleanUp.ReferenceOTU28412
New.CleanUp.ReferenceOTU33487
New.CleanUp.ReferenceOTU128700
New.CleanUp.ReferenceOTU174015
New.CleanUp.ReferenceOTU24300
New.CleanUp.ReferenceOTU57338
New.CleanUp.ReferenceOTU21637
New.CleanUp.ReferenceOTU13295
New.CleanUp.ReferenceOTU64167
New.CleanUp.ReferenceOTU58875
New.CleanUp.ReferenceOTU39379
New.CleanUp.ReferenceOTU24460
New.CleanUp.ReferenceOTU27966
New.CleanUp.ReferenceOTU16039
New.CleanUp.ReferenceOTU24866
New.CleanUp.ReferenceOTU14006
New.CleanUp.ReferenceOTU40615
New.CleanUp.ReferenceOTU35040
New.CleanUp.ReferenceOTU57516
New.CleanUp.ReferenceOTU10948
New.CleanUp.ReferenceOTU25987
New.CleanUp.ReferenceOTU212096
New.CleanUp.ReferenceOTU55834
New.CleanUp.ReferenceOTU111842
New.CleanUp.ReferenceOTU63962
New.CleanUp.ReferenceOTU80530
New.CleanUp.ReferenceOTU63042
New.CleanUp.ReferenceOTU38050

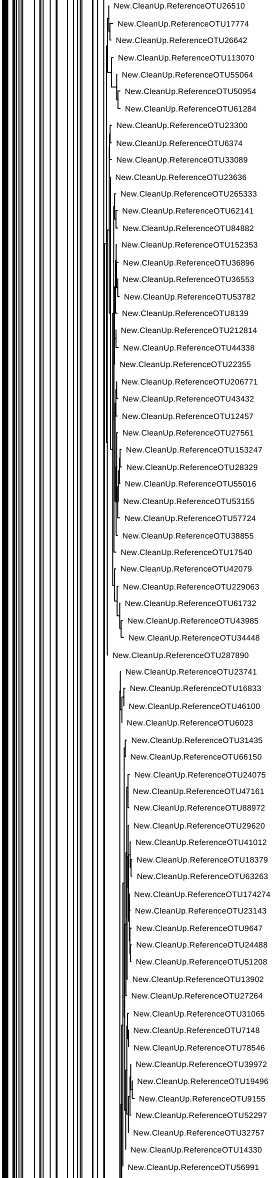
New.CleanUp.ReferenceOTU26510
New.CleanUp.ReferenceOTU17774
New.CleanUp.ReferenceOTU26642
New.CleanUp.ReferenceOTU113070
New.CleanUp.ReferenceOTU55064
New.CleanUp.ReferenceOTU50954
New.CleanUp.ReferenceOTU61284
New.CleanUp.ReferenceOTU23300
New.CleanUp.ReferenceOTU6374
New.CleanUp.ReferenceOTU33089
New.CleanUp.ReferenceOTU23636
New.CleanUp.ReferenceOTU265333
New.CleanUp.ReferenceOTU62141
New.CleanUp.ReferenceOTU84882
New.CleanUp.ReferenceOTU152353
New.CleanUp.ReferenceOTU36896
New.CleanUp.ReferenceOTU36553
New.CleanUp.ReferenceOTU53782
New.CleanUp.ReferenceOTU8139
New.CleanUp.ReferenceOTU212814
New.CleanUp.ReferenceOTU44338
New.CleanUp.ReferenceOTU22355
New.CleanUp.ReferenceOTU206771
New.CleanUp.ReferenceOTU43432
New.CleanUp.ReferenceOTU12457
New.CleanUp.ReferenceOTU27561
New.CleanUp.ReferenceOTU153247
New.CleanUp.ReferenceOTU28329
New.CleanUp.ReferenceOTU55016
New.CleanUp.ReferenceOTU53155
New.CleanUp.ReferenceOTU57724
New.CleanUp.ReferenceOTU38855
New.CleanUp.ReferenceOTU17540
New.CleanUp.ReferenceOTU42079
New.CleanUp.ReferenceOTU229063
New.CleanUp.ReferenceOTU61732
New.CleanUp.ReferenceOTU43985
New.CleanUp.ReferenceOTU34448
New.CleanUp.ReferenceOTU287890
New.CleanUp.ReferenceOTU23741
New.CleanUp.ReferenceOTU16833
New.CleanUp.ReferenceOTU46100
New.CleanUp.ReferenceOTU6023
New.CleanUp.ReferenceOTU31435
New.CleanUp.ReferenceOTU66150
New.CleanUp.ReferenceOTU24075
New.CleanUp.ReferenceOTU47161
New.CleanUp.ReferenceOTU88972
New.CleanUp.ReferenceOTU29620
New.CleanUp.ReferenceOTU41012
New.CleanUp.ReferenceOTU18379
New.CleanUp.ReferenceOTU63263
New.CleanUp.ReferenceOTU174274
New.CleanUp.ReferenceOTU23143
New.CleanUp.ReferenceOTU9647
New.CleanUp.ReferenceOTU24488
New.CleanUp.ReferenceOTU51208
New.CleanUp.ReferenceOTU13902
New.CleanUp.ReferenceOTU27264
New.CleanUp.ReferenceOTU31065
New.CleanUp.ReferenceOTU7148
New.CleanUp.ReferenceOTU78546
New.CleanUp.ReferenceOTU39972
New.CleanUp.ReferenceOTU19496
New.CleanUp.ReferenceOTU9155
New.CleanUp.ReferenceOTU52297
New.CleanUp.ReferenceOTU32757
New.CleanUp.ReferenceOTU14330
New.CleanUp.ReferenceOTU56991

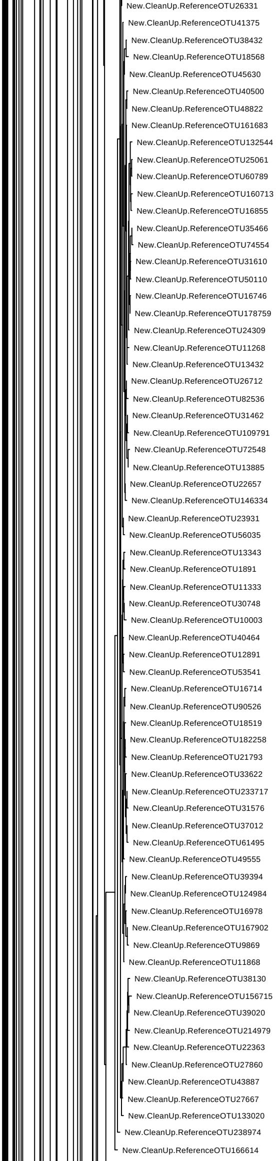
New.CleanUp.ReferenceOTU26331
New.CleanUp.ReferenceOTU41375
New.CleanUp.ReferenceOTU38432
New.CleanUp.ReferenceOTU18568
New.CleanUp.ReferenceOTU45630
New.CleanUp.ReferenceOTU40500
New.CleanUp.ReferenceOTU48822
New.CleanUp.ReferenceOTU161683
New.CleanUp.ReferenceOTU132544
New.CleanUp.ReferenceOTU25061
New.CleanUp.ReferenceOTU60789
New.CleanUp.ReferenceOTU160713
New.CleanUp.ReferenceOTU16855
New.CleanUp.ReferenceOTU35466
New.CleanUp.ReferenceOTU74554
New.CleanUp.ReferenceOTU31610
New.CleanUp.ReferenceOTU50110
New.CleanUp.ReferenceOTU16746
New.CleanUp.ReferenceOTU178759
New.CleanUp.ReferenceOTU24309
New.CleanUp.ReferenceOTU11268
New.CleanUp.ReferenceOTU13432
New.CleanUp.ReferenceOTU26712
New.CleanUp.ReferenceOTU82536
New.CleanUp.ReferenceOTU31462
New.CleanUp.ReferenceOTU109791
New.CleanUp.ReferenceOTU72548
New.CleanUp.ReferenceOTU13885
New.CleanUp.ReferenceOTU22657
New.CleanUp.ReferenceOTU146334
New.CleanUp.ReferenceOTU23931
New.CleanUp.ReferenceOTU56035
New.CleanUp.ReferenceOTU13343
New.CleanUp.ReferenceOTU1891
New.CleanUp.ReferenceOTU11333
New.CleanUp.ReferenceOTU30748
New.CleanUp.ReferenceOTU10003
New.CleanUp.ReferenceOTU40464
New.CleanUp.ReferenceOTU12891
New.CleanUp.ReferenceOTU53541
New.CleanUp.ReferenceOTU16714
New.CleanUp.ReferenceOTU90526
New.CleanUp.ReferenceOTU18519
New.CleanUp.ReferenceOTU182258
New.CleanUp.ReferenceOTU21793
New.CleanUp.ReferenceOTU33622
New.CleanUp.ReferenceOTU233717
New.CleanUp.ReferenceOTU31576
New.CleanUp.ReferenceOTU37012
New.CleanUp.ReferenceOTU61495
New.CleanUp.ReferenceOTU49555
New.CleanUp.ReferenceOTU39394
New.CleanUp.ReferenceOTU124984
New.CleanUp.ReferenceOTU16978
New.CleanUp.ReferenceOTU167902
New.CleanUp.ReferenceOTU9869
New.CleanUp.ReferenceOTU11868
New.CleanUp.ReferenceOTU38130
New.CleanUp.ReferenceOTU156715
New.CleanUp.ReferenceOTU39020
New.CleanUp.ReferenceOTU214979
New.CleanUp.ReferenceOTU22363
New.CleanUp.ReferenceOTU27860
New.CleanUp.ReferenceOTU43887
New.CleanUp.ReferenceOTU27667
New.CleanUp.ReferenceOTU133020
New.CleanUp.ReferenceOTU238974
New.CleanUp.ReferenceOTU166614

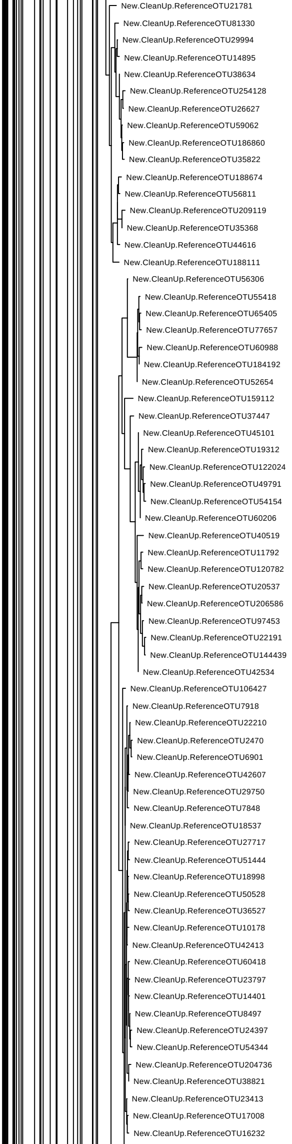
New.CleanUp.ReferenceOTU21781
New.CleanUp.ReferenceOTU81330
New.CleanUp.ReferenceOTU29994
New.CleanUp.ReferenceOTU14895
New.CleanUp.ReferenceOTU38634
New.CleanUp.ReferenceOTU254128
New.CleanUp.ReferenceOTU26627
New.CleanUp.ReferenceOTU59062
New.CleanUp.ReferenceOTU186860
New.CleanUp.ReferenceOTU35822
New.CleanUp.ReferenceOTU188674
New.CleanUp.ReferenceOTU56811
New.CleanUp.ReferenceOTU209119
New.CleanUp.ReferenceOTU35368
New.CleanUp.ReferenceOTU44616
New.CleanUp.ReferenceOTU188111
New.CleanUp.ReferenceOTU56306
New.CleanUp.ReferenceOTU55418
New.CleanUp.ReferenceOTU65405
New.CleanUp.ReferenceOTU77657
New.CleanUp.ReferenceOTU60988
New.CleanUp.ReferenceOTU184192
New.CleanUp.ReferenceOTU52654
New.CleanUp.ReferenceOTU159112
New.CleanUp.ReferenceOTU37447
New.CleanUp.ReferenceOTU45101
New.CleanUp.ReferenceOTU19312
New.CleanUp.ReferenceOTU122024
New.CleanUp.ReferenceOTU49791
New.CleanUp.ReferenceOTU54154
New.CleanUp.ReferenceOTU60206
New.CleanUp.ReferenceOTU40519
New.CleanUp.ReferenceOTU11792
New.CleanUp.ReferenceOTU120782
New.CleanUp.ReferenceOTU20537
New.CleanUp.ReferenceOTU206586
New.CleanUp.ReferenceOTU97453
New.CleanUp.ReferenceOTU22191
New.CleanUp.ReferenceOTU144439
New.CleanUp.ReferenceOTU42534
New.CleanUp.ReferenceOTU106427
New.CleanUp.ReferenceOTU7918
New.CleanUp.ReferenceOTU22210
New.CleanUp.ReferenceOTU2470
New.CleanUp.ReferenceOTU6901
New.CleanUp.ReferenceOTU42607
New.CleanUp.ReferenceOTU29750
New.CleanUp.ReferenceOTU7848
New.CleanUp.ReferenceOTU18537
New.CleanUp.ReferenceOTU27717
New.CleanUp.ReferenceOTU51444
New.CleanUp.ReferenceOTU18998
New.CleanUp.ReferenceOTU50528
New.CleanUp.ReferenceOTU36527
New.CleanUp.ReferenceOTU10178
New.CleanUp.ReferenceOTU42413
New.CleanUp.ReferenceOTU60418
New.CleanUp.ReferenceOTU23797
New.CleanUp.ReferenceOTU14401
New.CleanUp.ReferenceOTU8497
New.CleanUp.ReferenceOTU24397
New.CleanUp.ReferenceOTU54344
New.CleanUp.ReferenceOTU204736
New.CleanUp.ReferenceOTU38821
New.CleanUp.ReferenceOTU23413
New.CleanUp.ReferenceOTU17008
New.CleanUp.ReferenceOTU16232

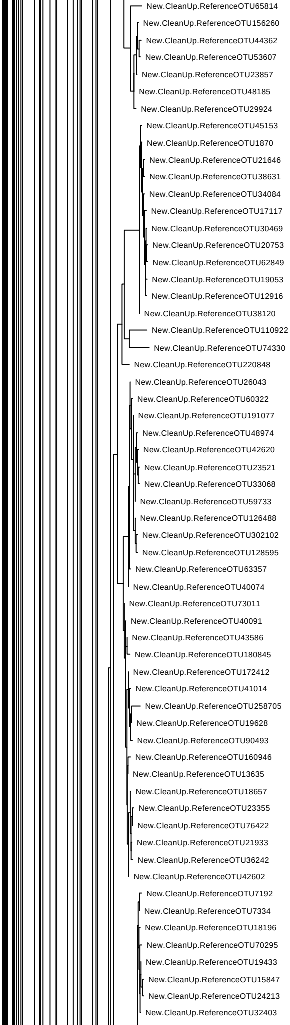
New.CleanUp.ReferenceOTU65814
New.CleanUp.ReferenceOTU156260
New.CleanUp.ReferenceOTU44362
New.CleanUp.ReferenceOTU53607
New.CleanUp.ReferenceOTU23857
New.CleanUp.ReferenceOTU48185
New.CleanUp.ReferenceOTU29924
New.CleanUp.ReferenceOTU45153
New.CleanUp.ReferenceOTU1870
New.CleanUp.ReferenceOTU21646
New.CleanUp.ReferenceOTU38631
New.CleanUp.ReferenceOTU34084
New.CleanUp.ReferenceOTU17117
New.CleanUp.ReferenceOTU30469
New.CleanUp.ReferenceOTU20753
New.CleanUp.ReferenceOTU62849
New.CleanUp.ReferenceOTU19053
New.CleanUp.ReferenceOTU12916
New.CleanUp.ReferenceOTU38120
New.CleanUp.ReferenceOTU110922
New.CleanUp.ReferenceOTU74330
New.CleanUp.ReferenceOTU220848
New.CleanUp.ReferenceOTU26043
New.CleanUp.ReferenceOTU60322
New.CleanUp.ReferenceOTU191077
New.CleanUp.ReferenceOTU48974
New.CleanUp.ReferenceOTU42620
New.CleanUp.ReferenceOTU23521
New.CleanUp.ReferenceOTU33068
New.CleanUp.ReferenceOTU59733
New.CleanUp.ReferenceOTU126488
New.CleanUp.ReferenceOTU302102
New.CleanUp.ReferenceOTU128595
New.CleanUp.ReferenceOTU63357
New.CleanUp.ReferenceOTU40074
New.CleanUp.ReferenceOTU73011
New.CleanUp.ReferenceOTU40091
New.CleanUp.ReferenceOTU43586
New.CleanUp.ReferenceOTU180845
New.CleanUp.ReferenceOTU172412
New.CleanUp.ReferenceOTU41014
New.CleanUp.ReferenceOTU258705
New.CleanUp.ReferenceOTU19628
New.CleanUp.ReferenceOTU90493
New.CleanUp.ReferenceOTU160946
New.CleanUp.ReferenceOTU13635
New.CleanUp.ReferenceOTU18657
New.CleanUp.ReferenceOTU23355
New.CleanUp.ReferenceOTU76422
New.CleanUp.ReferenceOTU21933
New.CleanUp.ReferenceOTU36242
New.CleanUp.ReferenceOTU42602
New.CleanUp.ReferenceOTU7192
New.CleanUp.ReferenceOTU7334
New.CleanUp.ReferenceOTU18196
New.CleanUp.ReferenceOTU70295
New.CleanUp.ReferenceOTU19433
New.CleanUp.ReferenceOTU15847
New.CleanUp.ReferenceOTU24213
New.CleanUp.ReferenceOTU32403

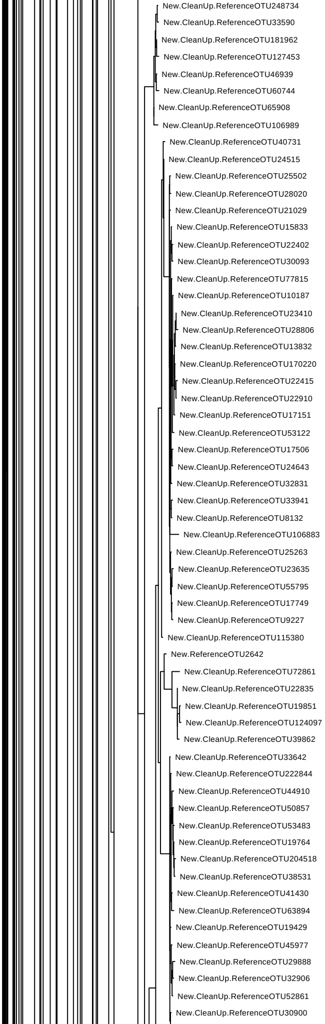

New.CleanUp.ReferenceOTU248734
New.CleanUp.ReferenceOTU33590
New.CleanUp.ReferenceOTU181962
New.CleanUp.ReferenceOTU127453
New.CleanUp.ReferenceOTU46939
New.CleanUp.ReferenceOTU60744
New.CleanUp.ReferenceOTU65908
New.CleanUp.ReferenceOTU106989
New.CleanUp.ReferenceOTU40731
New.CleanUp.ReferenceOTU24515
New.CleanUp.ReferenceOTU25502
New.CleanUp.ReferenceOTU28020
New.CleanUp.ReferenceOTU21029
New.CleanUp.ReferenceOTU15833
New.CleanUp.ReferenceOTU22402
New.CleanUp.ReferenceOTU30093
New.CleanUp.ReferenceOTU77815
New.CleanUp.ReferenceOTU10187
New.CleanUp.ReferenceOTU23410
New.CleanUp.ReferenceOTU28806
New.CleanUp.ReferenceOTU13832
New.CleanUp.ReferenceOTU170220
New.CleanUp.ReferenceOTU22415
New.CleanUp.ReferenceOTU22910
New.CleanUp.ReferenceOTU17151
New.CleanUp.ReferenceOTU53122
New.CleanUp.ReferenceOTU17506
New.CleanUp.ReferenceOTU24643
New.CleanUp.ReferenceOTU32831
New.CleanUp.ReferenceOTU33941
New.CleanUp.ReferenceOTU8132
New.CleanUp.ReferenceOTU106883
New.CleanUp.ReferenceOTU25263
New.CleanUp.ReferenceOTU23635
New.CleanUp.ReferenceOTU55795
New.CleanUp.ReferenceOTU17749
New.CleanUp.ReferenceOTU9227
New.CleanUp.ReferenceOTU115380
New.ReferenceOTU2642
New.CleanUp.ReferenceOTU72861
New.CleanUp.ReferenceOTU22835
New.CleanUp.ReferenceOTU19851
New.CleanUp.ReferenceOTU124097
New.CleanUp.ReferenceOTU39862
New.CleanUp.ReferenceOTU33642
New.CleanUp.ReferenceOTU222844
New.CleanUp.ReferenceOTU44910
New.CleanUp.ReferenceOTU50857
New.CleanUp.ReferenceOTU53483
New.CleanUp.ReferenceOTU19764
New.CleanUp.ReferenceOTU204518
New.CleanUp.ReferenceOTU38531
New.CleanUp.ReferenceOTU41430
New.CleanUp.ReferenceOTU63894
New.CleanUp.ReferenceOTU19429
New.CleanUp.ReferenceOTU45977
New.CleanUp.ReferenceOTU29888
New.CleanUp.ReferenceOTU32906
New.CleanUp.ReferenceOTU52861
New.CleanUp.ReferenceOTU30900

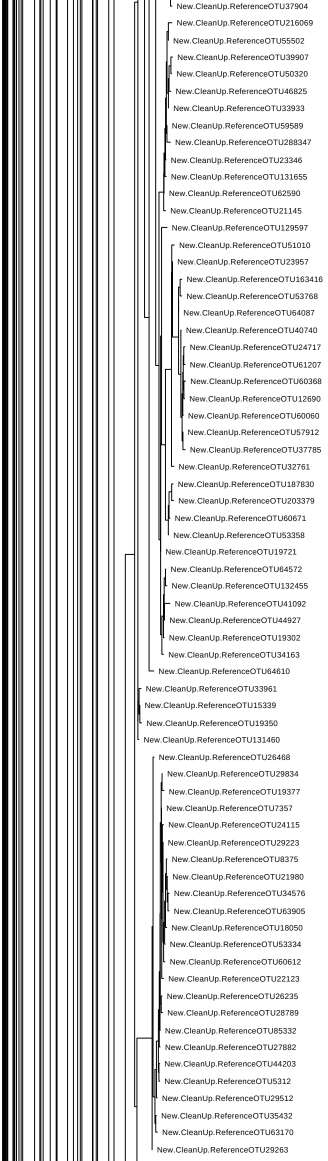

New.CleanUp.ReferenceOTU37904
New.CleanUp.ReferenceOTU216069
New.CleanUp.ReferenceOTU55502
New.CleanUp.ReferenceOTU39907
New.CleanUp.ReferenceOTU50320
New.CleanUp.ReferenceOTU46825
New.CleanUp.ReferenceOTU33933
New.CleanUp.ReferenceOTU59589
New.CleanUp.ReferenceOTU288347
New.CleanUp.ReferenceOTU23346
New.CleanUp.ReferenceOTU131655
New.CleanUp.ReferenceOTU62590
New.CleanUp.ReferenceOTU21145
New.CleanUp.ReferenceOTU129597
New.CleanUp.ReferenceOTU51010
New.CleanUp.ReferenceOTU23957
New.CleanUp.ReferenceOTU163416
New.CleanUp.ReferenceOTU53768
New.CleanUp.ReferenceOTU64087
New.CleanUp.ReferenceOTU40740
New.CleanUp.ReferenceOTU24717
New.CleanUp.ReferenceOTU61207
New.CleanUp.ReferenceOTU60368
New.CleanUp.ReferenceOTU12690
New.CleanUp.ReferenceOTU60060
New.CleanUp.ReferenceOTU57912
New.CleanUp.ReferenceOTU37785
New.CleanUp.ReferenceOTU32761
New.CleanUp.ReferenceOTU187830
New.CleanUp.ReferenceOTU203379
New.CleanUp.ReferenceOTU60671
New.CleanUp.ReferenceOTU53358
New.CleanUp.ReferenceOTU19721
New.CleanUp.ReferenceOTU64572
New.CleanUp.ReferenceOTU132455
New.CleanUp.ReferenceOTU41092
New.CleanUp.ReferenceOTU44927
New.CleanUp.ReferenceOTU19302
New.CleanUp.ReferenceOTU34163
New.CleanUp.ReferenceOTU64610
New.CleanUp.ReferenceOTU33961
New.CleanUp.ReferenceOTU15339
New.CleanUp.ReferenceOTU19350
New.CleanUp.ReferenceOTU131460
New.CleanUp.ReferenceOTU26468
New.CleanUp.ReferenceOTU29834
New.CleanUp.ReferenceOTU19377
New.CleanUp.ReferenceOTU7357
New.CleanUp.ReferenceOTU24115
New.CleanUp.ReferenceOTU29223
New.CleanUp.ReferenceOTU8375
New.CleanUp.ReferenceOTU21980
New.CleanUp.ReferenceOTU34576
New.CleanUp.ReferenceOTU63905
New.CleanUp.ReferenceOTU18050
New.CleanUp.ReferenceOTU53334
New.CleanUp.ReferenceOTU60612
New.CleanUp.ReferenceOTU22123
New.CleanUp.ReferenceOTU26235
New.CleanUp.ReferenceOTU28789
New.CleanUp.ReferenceOTU85332
New.CleanUp.ReferenceOTU27882
New.CleanUp.ReferenceOTU44203
New.CleanUp.ReferenceOTU5312
New.CleanUp.ReferenceOTU29512
New.CleanUp.ReferenceOTU35432
New.CleanUp.ReferenceOTU63170
New.CleanUp.ReferenceOTU29263

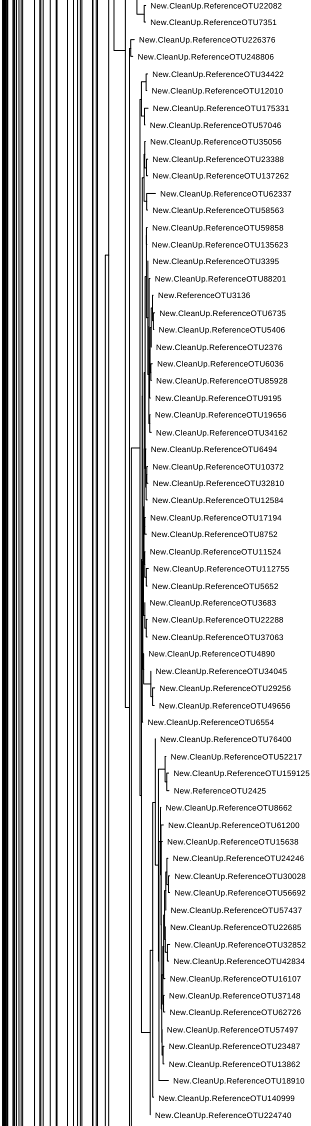
New.CleanUp.ReferenceOTU22082
New.CleanUp.ReferenceOTU7351
New.CleanUp.ReferenceOTU226376
New.CleanUp.ReferenceOTU248806
New.CleanUp.ReferenceOTU34422
New.CleanUp.ReferenceOTU12010
New.CleanUp.ReferenceOTU175331
New.CleanUp.ReferenceOTU57046
New.CleanUp.ReferenceOTU35056
New.CleanUp.ReferenceOTU23388
New.CleanUp.ReferenceOTU137262
New.CleanUp.ReferenceOTU62337
New.CleanUp.ReferenceOTU58563
New.CleanUp.ReferenceOTU59858
New.CleanUp.ReferenceOTU135623
New.CleanUp.ReferenceOTU3395
New.CleanUp.ReferenceOTU88201
New.ReferenceOTU3136
New.CleanUp.ReferenceOTU6735
New.CleanUp.ReferenceOTU5406
New.CleanUp.ReferenceOTU2376
New.CleanUp.ReferenceOTU6036
New.CleanUp.ReferenceOTU85928
New.CleanUp.ReferenceOTU9195
New.CleanUp.ReferenceOTU19656
New.CleanUp.ReferenceOTU34162
New.CleanUp.ReferenceOTU6494
New.CleanUp.ReferenceOTU10372
New.CleanUp.ReferenceOTU32810
New.CleanUp.ReferenceOTU12584
New.CleanUp.ReferenceOTU17194
New.CleanUp.ReferenceOTU8752
New.CleanUp.ReferenceOTU11524
New.CleanUp.ReferenceOTU112755
New.CleanUp.ReferenceOTU5652
New.CleanUp.ReferenceOTU3683
New.CleanUp.ReferenceOTU22288
New.CleanUp.ReferenceOTU37063
New.CleanUp.ReferenceOTU4890
New.CleanUp.ReferenceOTU34045
New.CleanUp.ReferenceOTU29256
New.CleanUp.ReferenceOTU49656
New.CleanUp.ReferenceOTU6554
New.CleanUp.ReferenceOTU76400
New.CleanUp.ReferenceOTU52217
New.CleanUp.ReferenceOTU159125
New.ReferenceOTU2425
New.CleanUp.ReferenceOTU8662
New.CleanUp.ReferenceOTU61200
New.CleanUp.ReferenceOTU15638
New.CleanUp.ReferenceOTU24246
New.CleanUp.ReferenceOTU30028
New.CleanUp.ReferenceOTU56692
New.CleanUp.ReferenceOTU57437
New.CleanUp.ReferenceOTU22685
New.CleanUp.ReferenceOTU32852
New.CleanUp.ReferenceOTU42834
New.CleanUp.ReferenceOTU16107
New.CleanUp.ReferenceOTU37148
New.CleanUp.ReferenceOTU62726
New.CleanUp.ReferenceOTU57497
New.CleanUp.ReferenceOTU23487
New.CleanUp.ReferenceOTU13862
New.CleanUp.ReferenceOTU18910
New.CleanUp.ReferenceOTU140999
New.CleanUp.ReferenceOTU224740

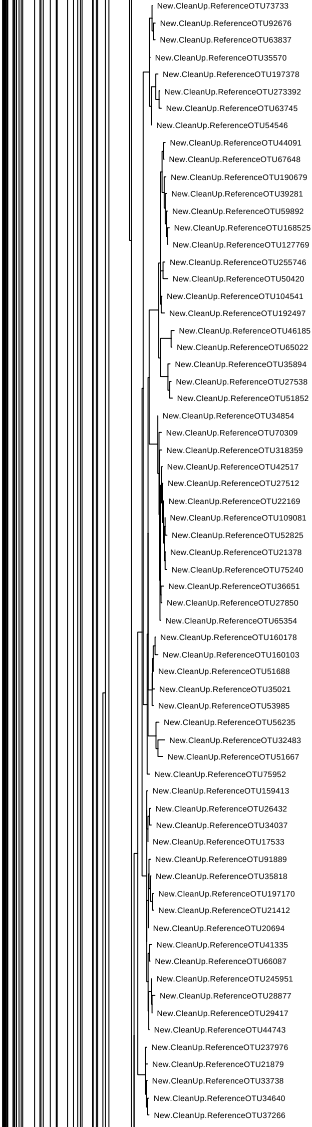
New.CleanUp.ReferenceOTU73733
New.CleanUp.ReferenceOTU92676
New.CleanUp.ReferenceOTU63837
New.CleanUp.ReferenceOTU35570
New.CleanUp.ReferenceOTU197378
New.CleanUp.ReferenceOTU273392
New.CleanUp.ReferenceOTU63745
New.CleanUp.ReferenceOTU54546
New.CleanUp.ReferenceOTU44091
New.CleanUp.ReferenceOTU67648
New.CleanUp.ReferenceOTU190679
New.CleanUp.ReferenceOTU39281
New.CleanUp.ReferenceOTU59892
New.CleanUp.ReferenceOTU168525
New.CleanUp.ReferenceOTU127769
New.CleanUp.ReferenceOTU255746
New.CleanUp.ReferenceOTU50420
New.CleanUp.ReferenceOTU104541
New.CleanUp.ReferenceOTU192497
New.CleanUp.ReferenceOTU46185
New.CleanUp.ReferenceOTU65022
New.CleanUp.ReferenceOTU35894
New.CleanUp.ReferenceOTU27538
New.CleanUp.ReferenceOTU51852
New.CleanUp.ReferenceOTU34854
New.CleanUp.ReferenceOTU70309
New.CleanUp.ReferenceOTU318359
New.CleanUp.ReferenceOTU42517
New.CleanUp.ReferenceOTU27512
New.CleanUp.ReferenceOTU22169
New.CleanUp.ReferenceOTU109081
New.CleanUp.ReferenceOTU52825
New.CleanUp.ReferenceOTU21378
New.CleanUp.ReferenceOTU75240
New.CleanUp.ReferenceOTU36651
New.CleanUp.ReferenceOTU27850
New.CleanUp.ReferenceOTU65354
New.CleanUp.ReferenceOTU160178
New.CleanUp.ReferenceOTU160103
New.CleanUp.ReferenceOTU51688
New.CleanUp.ReferenceOTU35021
New.CleanUp.ReferenceOTU53985
New.CleanUp.ReferenceOTU56235
New.CleanUp.ReferenceOTU32483
New.CleanUp.ReferenceOTU51667
New.CleanUp.ReferenceOTU75952
New.CleanUp.ReferenceOTU159413
New.CleanUp.ReferenceOTU26432
New.CleanUp.ReferenceOTU34037
New.CleanUp.ReferenceOTU17533
New.CleanUp.ReferenceOTU91889
New.CleanUp.ReferenceOTU35818
New.CleanUp.ReferenceOTU197170
New.CleanUp.ReferenceOTU21412
New.CleanUp.ReferenceOTU20694
New.CleanUp.ReferenceOTU41335
New.CleanUp.ReferenceOTU66087
New.CleanUp.ReferenceOTU245951
New.CleanUp.ReferenceOTU28877
New.CleanUp.ReferenceOTU29417
New.CleanUp.ReferenceOTU44743
New.CleanUp.ReferenceOTU237976
New.CleanUp.ReferenceOTU21879
New.CleanUp.ReferenceOTU33738
New.CleanUp.ReferenceOTU34640
New.CleanUp.ReferenceOTU37266

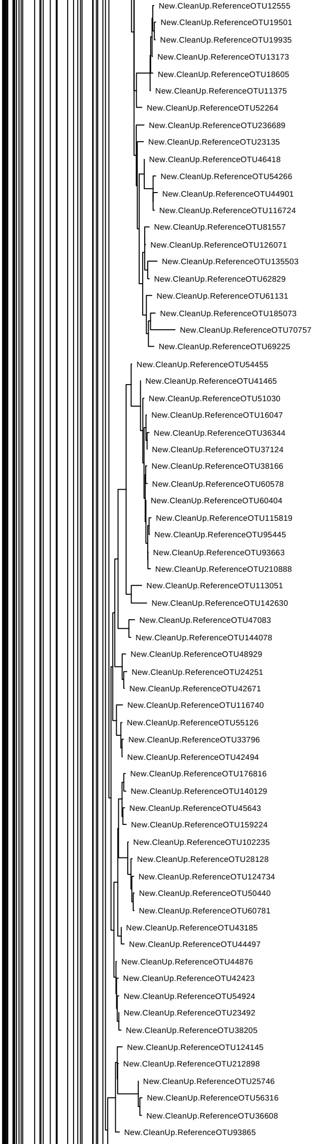
New.CleanUp.ReferenceOTU12555
New.CleanUp.ReferenceOTU19501
New.CleanUp.ReferenceOTU19935
New.CleanUp.ReferenceOTU13173
New.CleanUp.ReferenceOTU18605
New.CleanUp.ReferenceOTU11375
New.CleanUp.ReferenceOTU52264
New.CleanUp.ReferenceOTU236689
New.CleanUp.ReferenceOTU23135
New.CleanUp.ReferenceOTU46418
New.CleanUp.ReferenceOTU54266
New.CleanUp.ReferenceOTU44901
New.CleanUp.ReferenceOTU116724
New.CleanUp.ReferenceOTU81557
New.CleanUp.ReferenceOTU126071
New.CleanUp.ReferenceOTU135503
New.CleanUp.ReferenceOTU62829
New.CleanUp.ReferenceOTU61131
New.CleanUp.ReferenceOTU185073
New.CleanUp.ReferenceOTU70757
New.CleanUp.ReferenceOTU69225
New.CleanUp.ReferenceOTU54455
New.CleanUp.ReferenceOTU41465
New.CleanUp.ReferenceOTU51030
New.CleanUp.ReferenceOTU16047
New.CleanUp.ReferenceOTU36344
New.CleanUp.ReferenceOTU37124
New.CleanUp.ReferenceOTU38166
New.CleanUp.ReferenceOTU60578
New.CleanUp.ReferenceOTU60404
New.CleanUp.ReferenceOTU115819
New.CleanUp.ReferenceOTU95445
New.CleanUp.ReferenceOTU93663
New.CleanUp.ReferenceOTU210888
New.CleanUp.ReferenceOTU113051
New.CleanUp.ReferenceOTU142630
New.CleanUp.ReferenceOTU47083
New.CleanUp.ReferenceOTU144078
New.CleanUp.ReferenceOTU48929
New.CleanUp.ReferenceOTU24251
New.CleanUp.ReferenceOTU42671
New.CleanUp.ReferenceOTU116740
New.CleanUp.ReferenceOTU55126
New.CleanUp.ReferenceOTU33796
New.CleanUp.ReferenceOTU42494
New.CleanUp.ReferenceOTU176816
New.CleanUp.ReferenceOTU140129
New.CleanUp.ReferenceOTU45643
New.CleanUp.ReferenceOTU159224
New.CleanUp.ReferenceOTU102235
New.CleanUp.ReferenceOTU28128
New.CleanUp.ReferenceOTU124734
New.CleanUp.ReferenceOTU50440
New.CleanUp.ReferenceOTU60781
New.CleanUp.ReferenceOTU43185
New.CleanUp.ReferenceOTU44497
New.CleanUp.ReferenceOTU44876
New.CleanUp.ReferenceOTU42423
New.CleanUp.ReferenceOTU54924
New.CleanUp.ReferenceOTU23492
New.CleanUp.ReferenceOTU38205
New.CleanUp.ReferenceOTU124145
New.CleanUp.ReferenceOTU212898
New.CleanUp.ReferenceOTU25746
New.CleanUp.ReferenceOTU56316
New.CleanUp.ReferenceOTU36608
New.CleanUp.ReferenceOTU93865

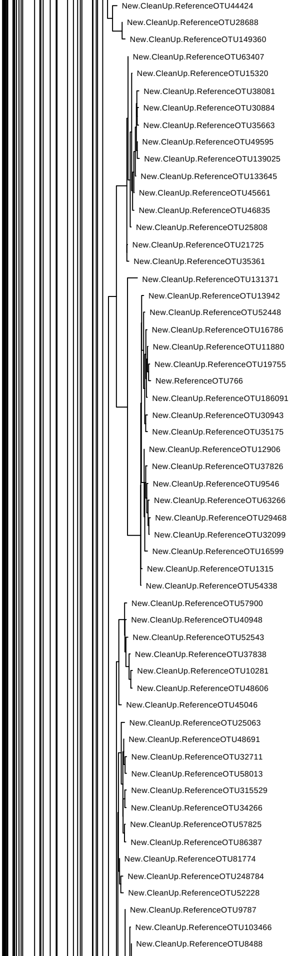
New.CleanUp.ReferenceOTU44424
New.CleanUp.ReferenceOTU28688
New.CleanUp.ReferenceOTU149360
New.CleanUp.ReferenceOTU63407
New.CleanUp.ReferenceOTU15320
New.CleanUp.ReferenceOTU38081
New.CleanUp.ReferenceOTU30884
New.CleanUp.ReferenceOTU35663
New.CleanUp.ReferenceOTU49595
New.CleanUp.ReferenceOTU139025
New.CleanUp.ReferenceOTU133645
New.CleanUp.ReferenceOTU45661
New.CleanUp.ReferenceOTU46835
New.CleanUp.ReferenceOTU25808
New.CleanUp.ReferenceOTU21725
New.CleanUp.ReferenceOTU35361
New.CleanUp.ReferenceOTU131371
New.CleanUp.ReferenceOTU13942
New.CleanUp.ReferenceOTU52448
New.CleanUp.ReferenceOTU16786
New.CleanUp.ReferenceOTU11880
New.CleanUp.ReferenceOTU19755
New.ReferenceOTU766
New.CleanUp.ReferenceOTU186091
New.CleanUp.ReferenceOTU30943
New.CleanUp.ReferenceOTU35175
New.CleanUp.ReferenceOTU12906
New.CleanUp.ReferenceOTU37826
New.CleanUp.ReferenceOTU9546
New.CleanUp.ReferenceOTU63266
New.CleanUp.ReferenceOTU29468
New.CleanUp.ReferenceOTU32099
New.CleanUp.ReferenceOTU16599
New.CleanUp.ReferenceOTU1315
New.CleanUp.ReferenceOTU54338
New.CleanUp.ReferenceOTU57900
New.CleanUp.ReferenceOTU40948
New.CleanUp.ReferenceOTU52543
New.CleanUp.ReferenceOTU37838
New.CleanUp.ReferenceOTU10281
New.CleanUp.ReferenceOTU48606
New.CleanUp.ReferenceOTU45046
New.CleanUp.ReferenceOTU25063
New.CleanUp.ReferenceOTU48691
New.CleanUp.ReferenceOTU32711
New.CleanUp.ReferenceOTU58013
New.CleanUp.ReferenceOTU315529
New.CleanUp.ReferenceOTU34266
New.CleanUp.ReferenceOTU57825
New.CleanUp.ReferenceOTU86387
New.CleanUp.ReferenceOTU81774
New.CleanUp.ReferenceOTU248784
New.CleanUp.ReferenceOTU52228
New.CleanUp.ReferenceOTU9787
New.CleanUp.ReferenceOTU103466
New.CleanUp.ReferenceOTU8488

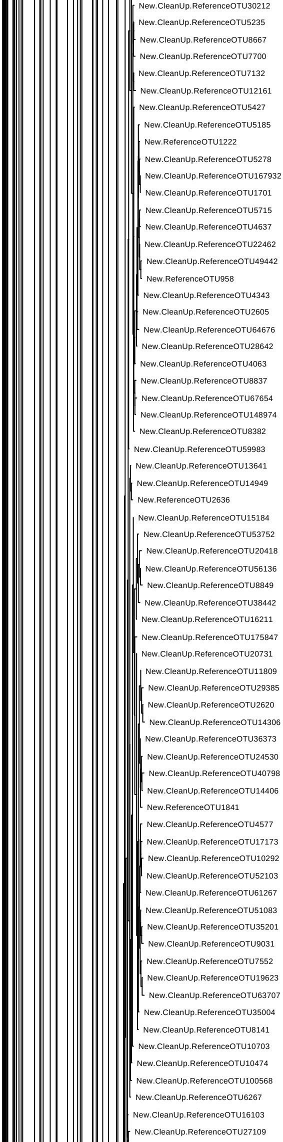
New.CleanUp.ReferenceOTU30212
New.CleanUp.ReferenceOTU5235
New.CleanUp.ReferenceOTU8667
New.CleanUp.ReferenceOTU7700
New.CleanUp.ReferenceOTU7132
New.CleanUp.ReferenceOTU12161
New.CleanUp.ReferenceOTU5427
New.CleanUp.ReferenceOTU5185
New.ReferenceOTU1222
New.CleanUp.ReferenceOTU5278
New.CleanUp.ReferenceOTU167932
New.CleanUp.ReferenceOTU1701
New.CleanUp.ReferenceOTU5715
New.CleanUp.ReferenceOTU4637
New.CleanUp.ReferenceOTU22462
New.CleanUp.ReferenceOTU49442
New.ReferenceOTU958
New.CleanUp.ReferenceOTU4343
New.CleanUp.ReferenceOTU2605
New.CleanUp.ReferenceOTU64676
New.CleanUp.ReferenceOTU28642
New.CleanUp.ReferenceOTU4063
New.CleanUp.ReferenceOTU8837
New.CleanUp.ReferenceOTU67654
New.CleanUp.ReferenceOTU148974
New.CleanUp.ReferenceOTU8382
New.CleanUp.ReferenceOTU59983
New.CleanUp.ReferenceOTU13641
New.CleanUp.ReferenceOTU14949
New.ReferenceOTU2636
New.CleanUp.ReferenceOTU15184
New.CleanUp.ReferenceOTU53752
New.CleanUp.ReferenceOTU20418
New.CleanUp.ReferenceOTU56136
New.CleanUp.ReferenceOTU8849
New.CleanUp.ReferenceOTU38442
New.CleanUp.ReferenceOTU16211
New.CleanUp.ReferenceOTU175847
New.CleanUp.ReferenceOTU20731
New.CleanUp.ReferenceOTU11809
New.CleanUp.ReferenceOTU29385
New.CleanUp.ReferenceOTU2620
New.CleanUp.ReferenceOTU14306
New.CleanUp.ReferenceOTU36373
New.CleanUp.ReferenceOTU24530
New.CleanUp.ReferenceOTU40798
New.CleanUp.ReferenceOTU14406
New.ReferenceOTU1841
New.CleanUp.ReferenceOTU4577
New.CleanUp.ReferenceOTU17173
New.CleanUp.ReferenceOTU10292
New.CleanUp.ReferenceOTU52103
New.CleanUp.ReferenceOTU61267
New.CleanUp.ReferenceOTU51083
New.CleanUp.ReferenceOTU35201
New.CleanUp.ReferenceOTU9031
New.CleanUp.ReferenceOTU7552
New.CleanUp.ReferenceOTU19623
New.CleanUp.ReferenceOTU63707
New.CleanUp.ReferenceOTU35004
New.CleanUp.ReferenceOTU8141
New.CleanUp.ReferenceOTU10703
New.CleanUp.ReferenceOTU10474
New.CleanUp.ReferenceOTU100568
New.CleanUp.ReferenceOTU6267
New.CleanUp.ReferenceOTU16103
New.CleanUp.ReferenceOTU27109

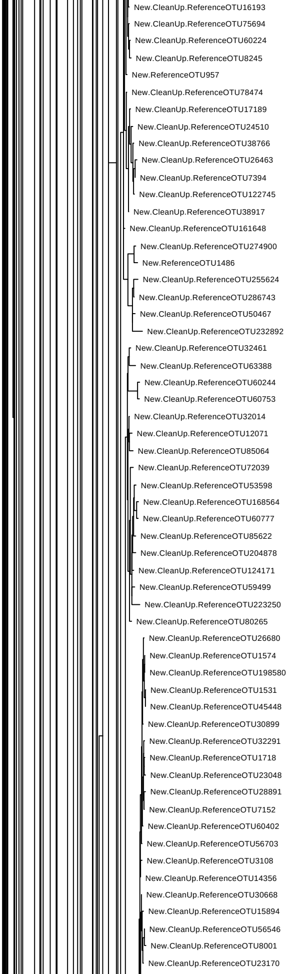
New.CleanUp.ReferenceOTU16193
New.CleanUp.ReferenceOTU75694
New.CleanUp.ReferenceOTU60224
New.CleanUp.ReferenceOTU8245
New.ReferenceOTU957
New.CleanUp.ReferenceOTU78474
New.CleanUp.ReferenceOTU17189
New.CleanUp.ReferenceOTU24510
New.CleanUp.ReferenceOTU38766
New.CleanUp.ReferenceOTU26463
New.CleanUp.ReferenceOTU7394
New.CleanUp.ReferenceOTU122745
New.CleanUp.ReferenceOTU38917
New.CleanUp.ReferenceOTU161648
New.CleanUp.ReferenceOTU274900
New.ReferenceOTU1486
New.CleanUp.ReferenceOTU255624
New.CleanUp.ReferenceOTU286743
New.CleanUp.ReferenceOTU50467
New.CleanUp.ReferenceOTU232892
New.CleanUp.ReferenceOTU32461
New.CleanUp.ReferenceOTU63388
New.CleanUp.ReferenceOTU60244
New.CleanUp.ReferenceOTU60753
New.CleanUp.ReferenceOTU32014
New.CleanUp.ReferenceOTU12071
New.CleanUp.ReferenceOTU85064
New.CleanUp.ReferenceOTU72039
New.CleanUp.ReferenceOTU53598
New.CleanUp.ReferenceOTU168564
New.CleanUp.ReferenceOTU60777
New.CleanUp.ReferenceOTU85622
New.CleanUp.ReferenceOTU204878
New.CleanUp.ReferenceOTU124171
New.CleanUp.ReferenceOTU59499
New.CleanUp.ReferenceOTU223250
New.CleanUp.ReferenceOTU80265
New.CleanUp.ReferenceOTU26680
New.CleanUp.ReferenceOTU1574
New.CleanUp.ReferenceOTU198580
New.CleanUp.ReferenceOTU1531
New.CleanUp.ReferenceOTU45448
New.CleanUp.ReferenceOTU30899
New.CleanUp.ReferenceOTU32291
New.CleanUp.ReferenceOTU1718
New.CleanUp.ReferenceOTU23048
New.CleanUp.ReferenceOTU28891
New.CleanUp.ReferenceOTU7152
New.CleanUp.ReferenceOTU60402
New.CleanUp.ReferenceOTU56703
New.CleanUp.ReferenceOTU3108
New.CleanUp.ReferenceOTU14356
New.CleanUp.ReferenceOTU30668
New.CleanUp.ReferenceOTU15894
New.CleanUp.ReferenceOTU56546
New.CleanUp.ReferenceOTU8001
New.CleanUp.ReferenceOTU23170

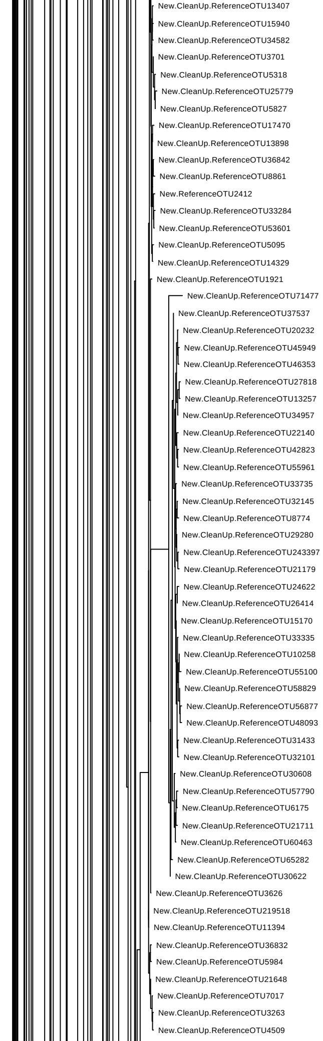
New.CleanUp.ReferenceOTU13407
New.CleanUp.ReferenceOTU15940
New.CleanUp.ReferenceOTU34582
New.CleanUp.ReferenceOTU3701
New.CleanUp.ReferenceOTU5318
New.CleanUp.ReferenceOTU25779
New.CleanUp.ReferenceOTU5827
New.CleanUp.ReferenceOTU17470
New.CleanUp.ReferenceOTU13898
New.CleanUp.ReferenceOTU36842
New.CleanUp.ReferenceOTU8861
New.ReferenceOTU2412
New.CleanUp.ReferenceOTU33284
New.CleanUp.ReferenceOTU53601
New.CleanUp.ReferenceOTU5095
New.CleanUp.ReferenceOTU14329
New.CleanUp.ReferenceOTU1921
New.CleanUp.ReferenceOTU71477
New.CleanUp.ReferenceOTU37537
New.CleanUp.ReferenceOTU20232
New.CleanUp.ReferenceOTU45949
New.CleanUp.ReferenceOTU46353
New.CleanUp.ReferenceOTU27818
New.CleanUp.ReferenceOTU13257
New.CleanUp.ReferenceOTU34957
New.CleanUp.ReferenceOTU22140
New.CleanUp.ReferenceOTU42823
New.CleanUp.ReferenceOTU55961
New.CleanUp.ReferenceOTU33735
New.CleanUp.ReferenceOTU32145
New.CleanUp.ReferenceOTU8774
New.CleanUp.ReferenceOTU29280
New.CleanUp.ReferenceOTU243397
New.CleanUp.ReferenceOTU21179
New.CleanUp.ReferenceOTU24622
New.CleanUp.ReferenceOTU26414
New.CleanUp.ReferenceOTU15170
New.CleanUp.ReferenceOTU33335
New.CleanUp.ReferenceOTU10258
New.CleanUp.ReferenceOTU55100
New.CleanUp.ReferenceOTU58829
New.CleanUp.ReferenceOTU56877
New.CleanUp.ReferenceOTU48093
New.CleanUp.ReferenceOTU31433
New.CleanUp.ReferenceOTU32101
New.CleanUp.ReferenceOTU30608
New.CleanUp.ReferenceOTU57790
New.CleanUp.ReferenceOTU6175
New.CleanUp.ReferenceOTU21711
New.CleanUp.ReferenceOTU60463
New.CleanUp.ReferenceOTU65282
New.CleanUp.ReferenceOTU30622
New.CleanUp.ReferenceOTU3626
New.CleanUp.ReferenceOTU219518
New.CleanUp.ReferenceOTU11394
New.CleanUp.ReferenceOTU36832
New.CleanUp.ReferenceOTU5984
New.CleanUp.ReferenceOTU21648
New.CleanUp.ReferenceOTU7017
New.CleanUp.ReferenceOTU3263
New.CleanUp.ReferenceOTU4509

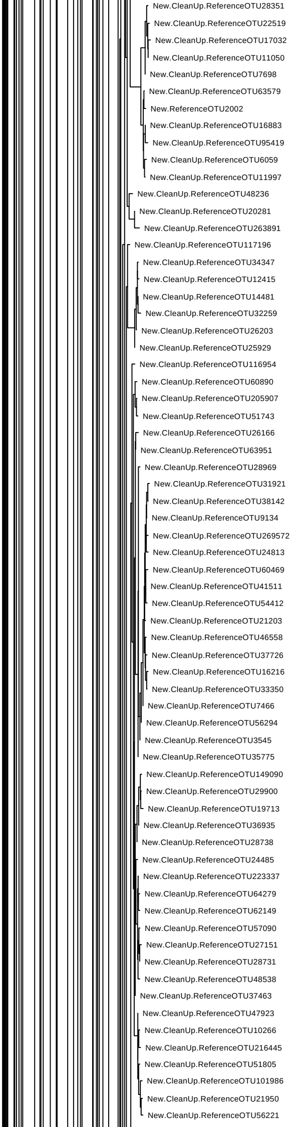
New.CleanUp.ReferenceOTU28351
New.CleanUp.ReferenceOTU22519
New.CleanUp.ReferenceOTU17032
New.CleanUp.ReferenceOTU11050
New.CleanUp.ReferenceOTU7698
New.CleanUp.ReferenceOTU63579
New.ReferenceOTU2002
New.CleanUp.ReferenceOTU16883
New.CleanUp.ReferenceOTU95419
New.CleanUp.ReferenceOTU6059
New.CleanUp.ReferenceOTU11997
New.CleanUp.ReferenceOTU48236
New.CleanUp.ReferenceOTU20281
New.CleanUp.ReferenceOTU263891
New.CleanUp.ReferenceOTU117196
New.CleanUp.ReferenceOTU34347
New.CleanUp.ReferenceOTU12415
New.CleanUp.ReferenceOTU14481
New.CleanUp.ReferenceOTU32259
New.CleanUp.ReferenceOTU26203
New.CleanUp.ReferenceOTU25929
New.CleanUp.ReferenceOTU116954
New.CleanUp.ReferenceOTU60890
New.CleanUp.ReferenceOTU205907
New.CleanUp.ReferenceOTU51743
New.CleanUp.ReferenceOTU26166
New.CleanUp.ReferenceOTU63951
New.CleanUp.ReferenceOTU28969
New.CleanUp.ReferenceOTU31921
New.CleanUp.ReferenceOTU38142
New.CleanUp.ReferenceOTU9134
New.CleanUp.ReferenceOTU269572
New.CleanUp.ReferenceOTU24813
New.CleanUp.ReferenceOTU60469
New.CleanUp.ReferenceOTU41511
New.CleanUp.ReferenceOTU54412
New.CleanUp.ReferenceOTU21203
New.CleanUp.ReferenceOTU46558
New.CleanUp.ReferenceOTU37726
New.CleanUp.ReferenceOTU16216
New.CleanUp.ReferenceOTU33350
New.CleanUp.ReferenceOTU7466
New.CleanUp.ReferenceOTU56294
New.CleanUp.ReferenceOTU3545
New.CleanUp.ReferenceOTU35775
New.CleanUp.ReferenceOTU149090
New.CleanUp.ReferenceOTU29900
New.CleanUp.ReferenceOTU19713
New.CleanUp.ReferenceOTU36935
New.CleanUp.ReferenceOTU28738
New.CleanUp.ReferenceOTU24485
New.CleanUp.ReferenceOTU223337
New.CleanUp.ReferenceOTU64279
New.CleanUp.ReferenceOTU62149
New.CleanUp.ReferenceOTU57090
New.CleanUp.ReferenceOTU27151
New.CleanUp.ReferenceOTU28731
New.CleanUp.ReferenceOTU48538
New.CleanUp.ReferenceOTU37463
New.CleanUp.ReferenceOTU47923
New.CleanUp.ReferenceOTU10266
New.CleanUp.ReferenceOTU216445
New.CleanUp.ReferenceOTU51805
New.CleanUp.ReferenceOTU101986
New.CleanUp.ReferenceOTU21950
New.CleanUp.ReferenceOTU56221

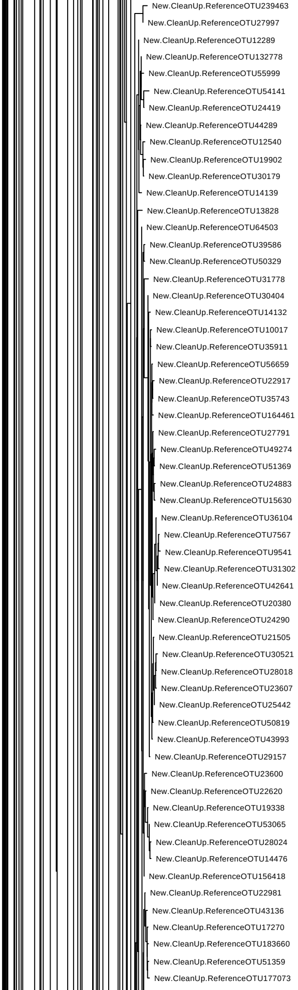
New.CleanUp.ReferenceOTU239463
New.CleanUp.ReferenceOTU27997
New.CleanUp.ReferenceOTU12289
New.CleanUp.ReferenceOTU132778
New.CleanUp.ReferenceOTU55999
New.CleanUp.ReferenceOTU54141
New.CleanUp.ReferenceOTU24419
New.CleanUp.ReferenceOTU44289
New.CleanUp.ReferenceOTU12540
New.CleanUp.ReferenceOTU19902
New.CleanUp.ReferenceOTU30179
New.CleanUp.ReferenceOTU14139
New.CleanUp.ReferenceOTU13828
New.CleanUp.ReferenceOTU64503
New.CleanUp.ReferenceOTU39586
New.CleanUp.ReferenceOTU50329
New.CleanUp.ReferenceOTU31778
New.CleanUp.ReferenceOTU30404
New.CleanUp.ReferenceOTU14132
New.CleanUp.ReferenceOTU10017
New.CleanUp.ReferenceOTU35911
New.CleanUp.ReferenceOTU56659
New.CleanUp.ReferenceOTU22917
New.CleanUp.ReferenceOTU35743
New.CleanUp.ReferenceOTU164461
New.CleanUp.ReferenceOTU27791
New.CleanUp.ReferenceOTU49274
New.CleanUp.ReferenceOTU51369
New.CleanUp.ReferenceOTU24883
New.CleanUp.ReferenceOTU15630
New.CleanUp.ReferenceOTU36104
New.CleanUp.ReferenceOTU7567
New.CleanUp.ReferenceOTU9541
New.CleanUp.ReferenceOTU31302
New.CleanUp.ReferenceOTU42641
New.CleanUp.ReferenceOTU20380
New.CleanUp.ReferenceOTU24290
New.CleanUp.ReferenceOTU21505
New.CleanUp.ReferenceOTU30521
New.CleanUp.ReferenceOTU28018
New.CleanUp.ReferenceOTU23607
New.CleanUp.ReferenceOTU25442
New.CleanUp.ReferenceOTU50819
New.CleanUp.ReferenceOTU43993
New.CleanUp.ReferenceOTU29157
New.CleanUp.ReferenceOTU23600
New.CleanUp.ReferenceOTU22620
New.CleanUp.ReferenceOTU19338
New.CleanUp.ReferenceOTU53065
New.CleanUp.ReferenceOTU28024
New.CleanUp.ReferenceOTU14476
New.CleanUp.ReferenceOTU156418
New.CleanUp.ReferenceOTU22981
New.CleanUp.ReferenceOTU43136
New.CleanUp.ReferenceOTU17270
New.CleanUp.ReferenceOTU183660
New.CleanUp.ReferenceOTU51359
New.CleanUp.ReferenceOTU177073

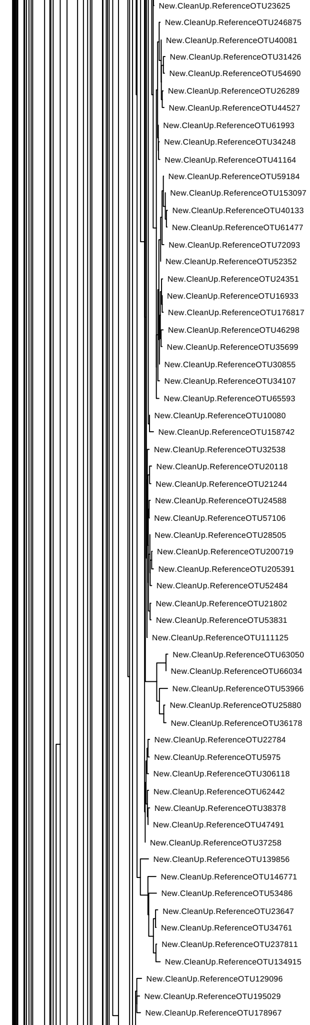
New.CleanUp.ReferenceOTU23625
New.CleanUp.ReferenceOTU246875
New.CleanUp.ReferenceOTU40081
New.CleanUp.ReferenceOTU31426
New.CleanUp.ReferenceOTU54690
New.CleanUp.ReferenceOTU26289
New.CleanUp.ReferenceOTU44527
New.CleanUp.ReferenceOTU61993
New.CleanUp.ReferenceOTU34248
New.CleanUp.ReferenceOTU41164
New.CleanUp.ReferenceOTU59184
New.CleanUp.ReferenceOTU153097
New.CleanUp.ReferenceOTU40133
New.CleanUp.ReferenceOTU61477
New.CleanUp.ReferenceOTU72093
New.CleanUp.ReferenceOTU52352
New.CleanUp.ReferenceOTU24351
New.CleanUp.ReferenceOTU16933
New.CleanUp.ReferenceOTU176817
New.CleanUp.ReferenceOTU46298
New.CleanUp.ReferenceOTU35699
New.CleanUp.ReferenceOTU30855
New.CleanUp.ReferenceOTU34107
New.CleanUp.ReferenceOTU65593
New.CleanUp.ReferenceOTU10080
New.CleanUp.ReferenceOTU158742
New.CleanUp.ReferenceOTU32538
New.CleanUp.ReferenceOTU20118
New.CleanUp.ReferenceOTU21244
New.CleanUp.ReferenceOTU24588
New.CleanUp.ReferenceOTU57106
New.CleanUp.ReferenceOTU28505
New.CleanUp.ReferenceOTU200719
New.CleanUp.ReferenceOTU205391
New.CleanUp.ReferenceOTU52484
New.CleanUp.ReferenceOTU21802
New.CleanUp.ReferenceOTU53831
New.CleanUp.ReferenceOTU111125
New.CleanUp.ReferenceOTU63050
New.CleanUp.ReferenceOTU66034
New.CleanUp.ReferenceOTU53966
New.CleanUp.ReferenceOTU25880
New.CleanUp.ReferenceOTU36178
New.CleanUp.ReferenceOTU22784
New.CleanUp.ReferenceOTU5975
New.CleanUp.ReferenceOTU306118
New.CleanUp.ReferenceOTU62442
New.CleanUp.ReferenceOTU38378
New.CleanUp.ReferenceOTU47491
New.CleanUp.ReferenceOTU37258
New.CleanUp.ReferenceOTU139856
New.CleanUp.ReferenceOTU146771
New.CleanUp.ReferenceOTU53486
New.CleanUp.ReferenceOTU23647
New.CleanUp.ReferenceOTU34761
New.CleanUp.ReferenceOTU237811
New.CleanUp.ReferenceOTU134915
New.CleanUp.ReferenceOTU129096
New.CleanUp.ReferenceOTU195029
New.CleanUp.ReferenceOTU178967

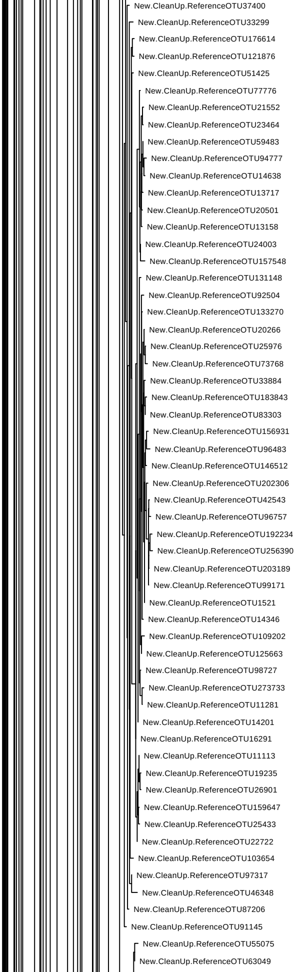
New.CleanUp.ReferenceOTU37400
New.CleanUp.ReferenceOTU33299
New.CleanUp.ReferenceOTU176614
New.CleanUp.ReferenceOTU121876
New.CleanUp.ReferenceOTU51425
New.CleanUp.ReferenceOTU77776
New.CleanUp.ReferenceOTU21552
New.CleanUp.ReferenceOTU23464
New.CleanUp.ReferenceOTU59483
New.CleanUp.ReferenceOTU94777
New.CleanUp.ReferenceOTU14638
New.CleanUp.ReferenceOTU13717
New.CleanUp.ReferenceOTU20501
New.CleanUp.ReferenceOTU13158
New.CleanUp.ReferenceOTU24003
New.CleanUp.ReferenceOTU157548
New.CleanUp.ReferenceOTU131148
New.CleanUp.ReferenceOTU92504
New.CleanUp.ReferenceOTU133270
New.CleanUp.ReferenceOTU20266
New.CleanUp.ReferenceOTU25976
New.CleanUp.ReferenceOTU73768
New.CleanUp.ReferenceOTU33884
New.CleanUp.ReferenceOTU183843
New.CleanUp.ReferenceOTU83303
New.CleanUp.ReferenceOTU156931
New.CleanUp.ReferenceOTU96483
New.CleanUp.ReferenceOTU146512
New.CleanUp.ReferenceOTU202306
New.CleanUp.ReferenceOTU42543
New.CleanUp.ReferenceOTU96757
New.CleanUp.ReferenceOTU192234
New.CleanUp.ReferenceOTU256390
New.CleanUp.ReferenceOTU203189
New.CleanUp.ReferenceOTU99171
New.CleanUp.ReferenceOTU1521
New.CleanUp.ReferenceOTU14346
New.CleanUp.ReferenceOTU109202
New.CleanUp.ReferenceOTU125663
New.CleanUp.ReferenceOTU98727
New.CleanUp.ReferenceOTU273733
New.CleanUp.ReferenceOTU11281
New.CleanUp.ReferenceOTU14201
New.CleanUp.ReferenceOTU16291
New.CleanUp.ReferenceOTU11113
New.CleanUp.ReferenceOTU19235
New.CleanUp.ReferenceOTU26901
New.CleanUp.ReferenceOTU159647
New.CleanUp.ReferenceOTU25433
New.CleanUp.ReferenceOTU22722
New.CleanUp.ReferenceOTU103654
New.CleanUp.ReferenceOTU97317
New.CleanUp.ReferenceOTU46348
New.CleanUp.ReferenceOTU87206
New.CleanUp.ReferenceOTU91145
New.CleanUp.ReferenceOTU55075
New.CleanUp.ReferenceOTU63049

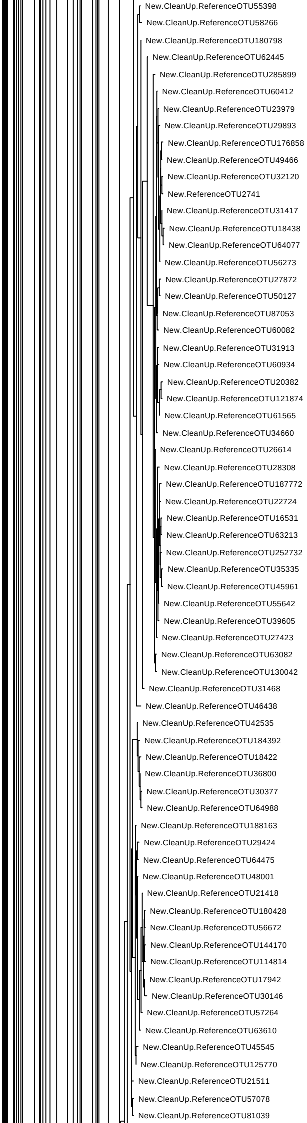
New.CleanUp.ReferenceOTU55398
New.CleanUp.ReferenceOTU58266
New.CleanUp.ReferenceOTU180798
New.CleanUp.ReferenceOTU62445
New.CleanUp.ReferenceOTU285899
New.CleanUp.ReferenceOTU60412
New.CleanUp.ReferenceOTU23979
New.CleanUp.ReferenceOTU29893
New.CleanUp.ReferenceOTU176858
New.CleanUp.ReferenceOTU49466
New.CleanUp.ReferenceOTU32120
New.ReferenceOTU2741
New.CleanUp.ReferenceOTU31417
New.CleanUp.ReferenceOTU18438
New.CleanUp.ReferenceOTU64077
New.CleanUp.ReferenceOTU56273
New.CleanUp.ReferenceOTU27872
New.CleanUp.ReferenceOTU50127
New.CleanUp.ReferenceOTU87053
New.CleanUp.ReferenceOTU60082
New.CleanUp.ReferenceOTU31913
New.CleanUp.ReferenceOTU60934
New.CleanUp.ReferenceOTU20382
New.CleanUp.ReferenceOTU121874
New.CleanUp.ReferenceOTU61565
New.CleanUp.ReferenceOTU34660
New.CleanUp.ReferenceOTU26614
New.CleanUp.ReferenceOTU28308
New.CleanUp.ReferenceOTU187772
New.CleanUp.ReferenceOTU22724
New.CleanUp.ReferenceOTU16531
New.CleanUp.ReferenceOTU63213
New.CleanUp.ReferenceOTU252732
New.CleanUp.ReferenceOTU35335
New.CleanUp.ReferenceOTU45961
New.CleanUp.ReferenceOTU55642
New.CleanUp.ReferenceOTU39605
New.CleanUp.ReferenceOTU27423
New.CleanUp.ReferenceOTU63082
New.CleanUp.ReferenceOTU130042
New.CleanUp.ReferenceOTU31468
New.CleanUp.ReferenceOTU46438
New.CleanUp.ReferenceOTU42535
New.CleanUp.ReferenceOTU184392
New.CleanUp.ReferenceOTU18422
New.CleanUp.ReferenceOTU36800
New.CleanUp.ReferenceOTU30377
New.CleanUp.ReferenceOTU64988
New.CleanUp.ReferenceOTU188163
New.CleanUp.ReferenceOTU29424
New.CleanUp.ReferenceOTU64475
New.CleanUp.ReferenceOTU48001
New.CleanUp.ReferenceOTU21418
New.CleanUp.ReferenceOTU180428
New.CleanUp.ReferenceOTU56672
New.CleanUp.ReferenceOTU144170
New.CleanUp.ReferenceOTU114814
New.CleanUp.ReferenceOTU17942
New.CleanUp.ReferenceOTU30146
New.CleanUp.ReferenceOTU57264
New.CleanUp.ReferenceOTU63610
New.CleanUp.ReferenceOTU45545
New.CleanUp.ReferenceOTU125770
New.CleanUp.ReferenceOTU21511
New.CleanUp.ReferenceOTU57078
New.CleanUp.ReferenceOTU81039

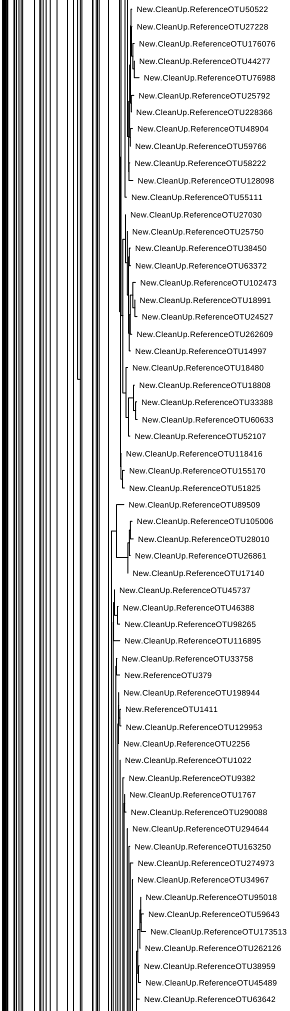
New.CleanUp.ReferenceOTU50522
New.CleanUp.ReferenceOTU27228
New.CleanUp.ReferenceOTU176076
New.CleanUp.ReferenceOTU44277
New.CleanUp.ReferenceOTU76988
New.CleanUp.ReferenceOTU25792
New.CleanUp.ReferenceOTU228366
New.CleanUp.ReferenceOTU48904
New.CleanUp.ReferenceOTU59766
New.CleanUp.ReferenceOTU58222
New.CleanUp.ReferenceOTU128098
New.CleanUp.ReferenceOTU55111
New.CleanUp.ReferenceOTU27030
New.CleanUp.ReferenceOTU25750
New.CleanUp.ReferenceOTU38450
New.CleanUp.ReferenceOTU63372
New.CleanUp.ReferenceOTU102473
New.CleanUp.ReferenceOTU18991
New.CleanUp.ReferenceOTU24527
New.CleanUp.ReferenceOTU262609
New.CleanUp.ReferenceOTU14997
New.CleanUp.ReferenceOTU18480
New.CleanUp.ReferenceOTU18808
New.CleanUp.ReferenceOTU33388
New.CleanUp.ReferenceOTU60633
New.CleanUp.ReferenceOTU52107
New.CleanUp.ReferenceOTU118416
New.CleanUp.ReferenceOTU155170
New.CleanUp.ReferenceOTU51825
New.CleanUp.ReferenceOTU89509
New.CleanUp.ReferenceOTU105006
New.CleanUp.ReferenceOTU28010
New.CleanUp.ReferenceOTU26861
New.CleanUp.ReferenceOTU17140
New.CleanUp.ReferenceOTU45737
New.CleanUp.ReferenceOTU46388
New.CleanUp.ReferenceOTU98265
New.CleanUp.ReferenceOTU116895
New.CleanUp.ReferenceOTU33758
New.ReferenceOTU379
New.CleanUp.ReferenceOTU198944
New.ReferenceOTU1411
New.CleanUp.ReferenceOTU129953
New.CleanUp.ReferenceOTU2256
New.CleanUp.ReferenceOTU1022
New.CleanUp.ReferenceOTU9382
New.CleanUp.ReferenceOTU1767
New.CleanUp.ReferenceOTU290088
New.CleanUp.ReferenceOTU294644
New.CleanUp.ReferenceOTU163250
New.CleanUp.ReferenceOTU274973
New.CleanUp.ReferenceOTU34967
New.CleanUp.ReferenceOTU95018
New.CleanUp.ReferenceOTU59643
New.CleanUp.ReferenceOTU173513
New.CleanUp.ReferenceOTU262126
New.CleanUp.ReferenceOTU38959
New.CleanUp.ReferenceOTU45489
New.CleanUp.ReferenceOTU63642

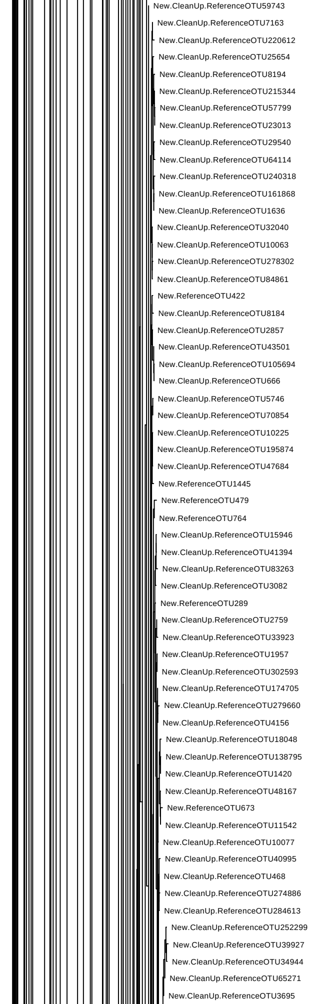
New.CleanUp.ReferenceOTU59743
New.CleanUp.ReferenceOTU7163
New.CleanUp.ReferenceOTU220612
New.CleanUp.ReferenceOTU25654
New.CleanUp.ReferenceOTU8194
New.CleanUp.ReferenceOTU215344
New.CleanUp.ReferenceOTU57799
New.CleanUp.ReferenceOTU23013
New.CleanUp.ReferenceOTU29540
New.CleanUp.ReferenceOTU64114
New.CleanUp.ReferenceOTU240318
New.CleanUp.ReferenceOTU161868
New.CleanUp.ReferenceOTU1636
New.CleanUp.ReferenceOTU32040
New.CleanUp.ReferenceOTU10063
New.CleanUp.ReferenceOTU278302
New.CleanUp.ReferenceOTU84861
New.ReferenceOTU422
New.CleanUp.ReferenceOTU8184
New.CleanUp.ReferenceOTU2857
New.CleanUp.ReferenceOTU43501
New.CleanUp.ReferenceOTU105694
New.CleanUp.ReferenceOTU666
New.CleanUp.ReferenceOTU5746
New.CleanUp.ReferenceOTU70854
New.CleanUp.ReferenceOTU10225
New.CleanUp.ReferenceOTU195874
New.CleanUp.ReferenceOTU47684
New.ReferenceOTU1445
New.ReferenceOTU479
New.ReferenceOTU764
New.CleanUp.ReferenceOTU15946
New.CleanUp.ReferenceOTU41394
New.CleanUp.ReferenceOTU83263
New.CleanUp.ReferenceOTU3082
New.ReferenceOTU289
New.CleanUp.ReferenceOTU2759
New.CleanUp.ReferenceOTU33923
New.CleanUp.ReferenceOTU1957
New.CleanUp.ReferenceOTU302593
New.CleanUp.ReferenceOTU174705
New.CleanUp.ReferenceOTU279660
New.CleanUp.ReferenceOTU4156
New.CleanUp.ReferenceOTU18048
New.CleanUp.ReferenceOTU138795
New.CleanUp.ReferenceOTU1420
New.CleanUp.ReferenceOTU48167
New.ReferenceOTU673
New.CleanUp.ReferenceOTU11542
New.CleanUp.ReferenceOTU10077
New.CleanUp.ReferenceOTU40995
New.CleanUp.ReferenceOTU468
New.CleanUp.ReferenceOTU274886
New.CleanUp.ReferenceOTU284613
New.CleanUp.ReferenceOTU252299
New.CleanUp.ReferenceOTU39927
New.CleanUp.ReferenceOTU34944
New.CleanUp.ReferenceOTU65271
New.CleanUp.ReferenceOTU3695

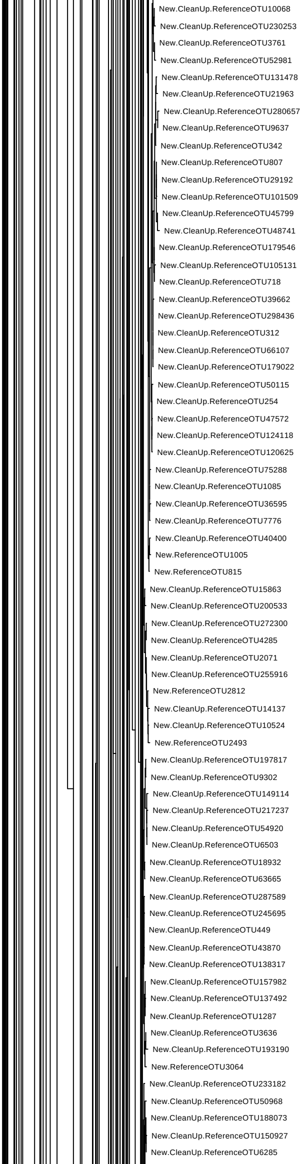
New.CleanUp.ReferenceOTU10068
New.CleanUp.ReferenceOTU230253
New.CleanUp.ReferenceOTU3761
New.CleanUp.ReferenceOTU52981
New.CleanUp.ReferenceOTU131478
New.CleanUp.ReferenceOTU21963
New.CleanUp.ReferenceOTU280657
New.CleanUp.ReferenceOTU9637
New.CleanUp.ReferenceOTU342
New.CleanUp.ReferenceOTU807
New.CleanUp.ReferenceOTU29192
New.CleanUp.ReferenceOTU101509
New.CleanUp.ReferenceOTU45799
New.CleanUp.ReferenceOTU48741
New.CleanUp.ReferenceOTU179546
New.CleanUp.ReferenceOTU105131
New.CleanUp.ReferenceOTU718
New.CleanUp.ReferenceOTU39662
New.CleanUp.ReferenceOTU298436
New.CleanUp.ReferenceOTU312
New.CleanUp.ReferenceOTU66107
New.CleanUp.ReferenceOTU179022
New.CleanUp.ReferenceOTU50115
New.CleanUp.ReferenceOTU254
New.CleanUp.ReferenceOTU47572
New.CleanUp.ReferenceOTU124118
New.CleanUp.ReferenceOTU120625
New.CleanUp.ReferenceOTU75288
New.CleanUp.ReferenceOTU1085
New.CleanUp.ReferenceOTU36595
New.CleanUp.ReferenceOTU7776
New.CleanUp.ReferenceOTU40400
New.ReferenceOTU1005
New.ReferenceOTU815
New.CleanUp.ReferenceOTU15863
New.CleanUp.ReferenceOTU200533
New.CleanUp.ReferenceOTU272300
New.CleanUp.ReferenceOTU4285
New.CleanUp.ReferenceOTU2071
New.CleanUp.ReferenceOTU255916
New.ReferenceOTU2812
New.CleanUp.ReferenceOTU14137
New.CleanUp.ReferenceOTU10524
New.ReferenceOTU2493
New.CleanUp.ReferenceOTU197817
New.CleanUp.ReferenceOTU9302
New.CleanUp.ReferenceOTU149114
New.CleanUp.ReferenceOTU217237
New.CleanUp.ReferenceOTU54920
New.CleanUp.ReferenceOTU6503
New.CleanUp.ReferenceOTU18932
New.CleanUp.ReferenceOTU63665
New.CleanUp.ReferenceOTU287589
New.CleanUp.ReferenceOTU245695
New.CleanUp.ReferenceOTU449
New.CleanUp.ReferenceOTU43870
New.CleanUp.ReferenceOTU138317
New.CleanUp.ReferenceOTU157982
New.CleanUp.ReferenceOTU137492
New.CleanUp.ReferenceOTU1287
New.CleanUp.ReferenceOTU3636
New.CleanUp.ReferenceOTU193190
New.ReferenceOTU3064
New.CleanUp.ReferenceOTU233182
New.CleanUp.ReferenceOTU50968
New.CleanUp.ReferenceOTU188073
New.CleanUp.ReferenceOTU150927
New.CleanUp.ReferenceOTU6285

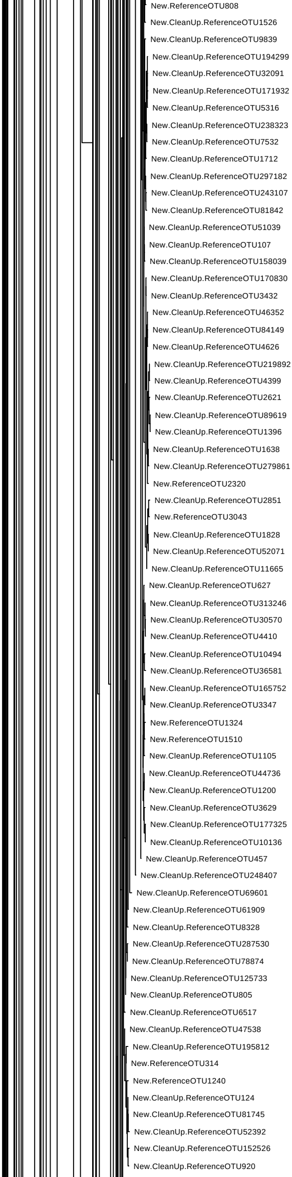
New.ReferenceOTU808
New.CleanUp.ReferenceOTU1526
New.CleanUp.ReferenceOTU9839
New.CleanUp.ReferenceOTU194299
New.CleanUp.ReferenceOTU32091
New.CleanUp.ReferenceOTU171932
New.CleanUp.ReferenceOTU5316
New.CleanUp.ReferenceOTU238323
New.CleanUp.ReferenceOTU7532
New.CleanUp.ReferenceOTU1712
New.CleanUp.ReferenceOTU297182
New.CleanUp.ReferenceOTU243107
New.CleanUp.ReferenceOTU81842
New.CleanUp.ReferenceOTU51039
New.CleanUp.ReferenceOTU107
New.CleanUp.ReferenceOTU158039
New.CleanUp.ReferenceOTU170830
New.CleanUp.ReferenceOTU3432
New.CleanUp.ReferenceOTU46352
New.CleanUp.ReferenceOTU84149
New.CleanUp.ReferenceOTU4626
New.CleanUp.ReferenceOTU219892
New.CleanUp.ReferenceOTU4399
New.CleanUp.ReferenceOTU2621
New.CleanUp.ReferenceOTU89619
New.CleanUp.ReferenceOTU1396
New.CleanUp.ReferenceOTU1638
New.CleanUp.ReferenceOTU279861
New.ReferenceOTU2320
New.CleanUp.ReferenceOTU2851
New.ReferenceOTU3043
New.CleanUp.ReferenceOTU1828
New.CleanUp.ReferenceOTU52071
New.CleanUp.ReferenceOTU11665
New.CleanUp.ReferenceOTU627
New.CleanUp.ReferenceOTU313246
New.CleanUp.ReferenceOTU30570
New.CleanUp.ReferenceOTU4410
New.CleanUp.ReferenceOTU10494
New.CleanUp.ReferenceOTU36581
New.CleanUp.ReferenceOTU165752
New.CleanUp.ReferenceOTU3347
New.ReferenceOTU1324
New.ReferenceOTU1510
New.CleanUp.ReferenceOTU1105
New.CleanUp.ReferenceOTU44736
New.CleanUp.ReferenceOTU1200
New.CleanUp.ReferenceOTU3629
New.CleanUp.ReferenceOTU177325
New.CleanUp.ReferenceOTU10136
New.CleanUp.ReferenceOTU457
New.CleanUp.ReferenceOTU248407
New.CleanUp.ReferenceOTU69601
New.CleanUp.ReferenceOTU61909
New.CleanUp.ReferenceOTU8328
New.CleanUp.ReferenceOTU287530
New.CleanUp.ReferenceOTU78874
New.CleanUp.ReferenceOTU125733
New.CleanUp.ReferenceOTU805
New.CleanUp.ReferenceOTU6517
New.CleanUp.ReferenceOTU47538
New.CleanUp.ReferenceOTU195812
New.ReferenceOTU314
New.ReferenceOTU1240
New.CleanUp.ReferenceOTU124
New.CleanUp.ReferenceOTU81745
New.CleanUp.ReferenceOTU52392
New.CleanUp.ReferenceOTU152526
New.CleanUp.ReferenceOTU920

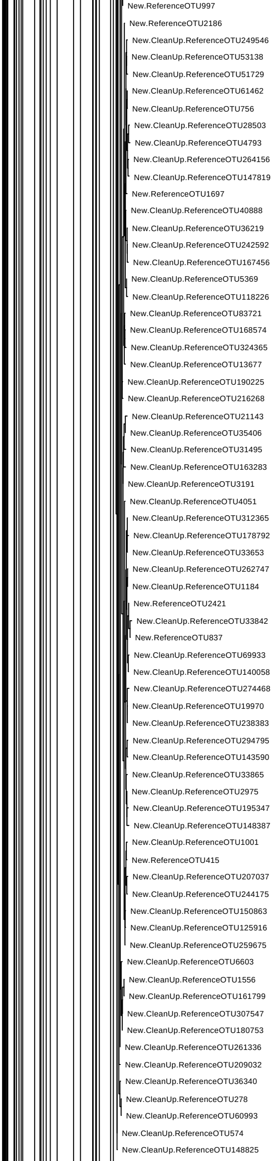
New.ReferenceOTU997
New.ReferenceOTU2186
New.CleanUp.ReferenceOTU249546
New.CleanUp.ReferenceOTU53138
New.CleanUp.ReferenceOTU51729
New.CleanUp.ReferenceOTU61462
New.CleanUp.ReferenceOTU756
New.CleanUp.ReferenceOTU28503
New.CleanUp.ReferenceOTU4793
New.CleanUp.ReferenceOTU264156
New.CleanUp.ReferenceOTU147819
New.ReferenceOTU1697
New.CleanUp.ReferenceOTU40888
New.CleanUp.ReferenceOTU36219
New.CleanUp.ReferenceOTU242592
New.CleanUp.ReferenceOTU167456
New.CleanUp.ReferenceOTU5369
New.CleanUp.ReferenceOTU118226
New.CleanUp.ReferenceOTU83721
New.CleanUp.ReferenceOTU168574
New.CleanUp.ReferenceOTU324365
New.CleanUp.ReferenceOTU13677
New.CleanUp.ReferenceOTU190225
New.CleanUp.ReferenceOTU216268
New.CleanUp.ReferenceOTU21143
New.CleanUp.ReferenceOTU35406
New.CleanUp.ReferenceOTU31495
New.CleanUp.ReferenceOTU163283
New.CleanUp.ReferenceOTU3191
New.CleanUp.ReferenceOTU4051
New.CleanUp.ReferenceOTU312365
New.CleanUp.ReferenceOTU178792
New.CleanUp.ReferenceOTU33653
New.CleanUp.ReferenceOTU262747
New.CleanUp.ReferenceOTU1184
New.ReferenceOTU2421
New.CleanUp.ReferenceOTU33842
New.ReferenceOTU837
New.CleanUp.ReferenceOTU69933
New.CleanUp.ReferenceOTU140058
New.CleanUp.ReferenceOTU274468
New.CleanUp.ReferenceOTU19970
New.CleanUp.ReferenceOTU238383
New.CleanUp.ReferenceOTU294795
New.CleanUp.ReferenceOTU143590
New.CleanUp.ReferenceOTU33865
New.CleanUp.ReferenceOTU2975
New.CleanUp.ReferenceOTU195347
New.CleanUp.ReferenceOTU148387
New.CleanUp.ReferenceOTU1001
New.ReferenceOTU415
New.CleanUp.ReferenceOTU207037
New.CleanUp.ReferenceOTU244175
New.CleanUp.ReferenceOTU150863
New.CleanUp.ReferenceOTU125916
New.CleanUp.ReferenceOTU259675
New.CleanUp.ReferenceOTU6603
New.CleanUp.ReferenceOTU1556
New.CleanUp.ReferenceOTU161799
New.CleanUp.ReferenceOTU307547
New.CleanUp.ReferenceOTU180753
New.CleanUp.ReferenceOTU261336
New.CleanUp.ReferenceOTU209032
New.CleanUp.ReferenceOTU36340
New.CleanUp.ReferenceOTU278
New.CleanUp.ReferenceOTU60993
New.CleanUp.ReferenceOTU574
New.CleanUp.ReferenceOTU148825

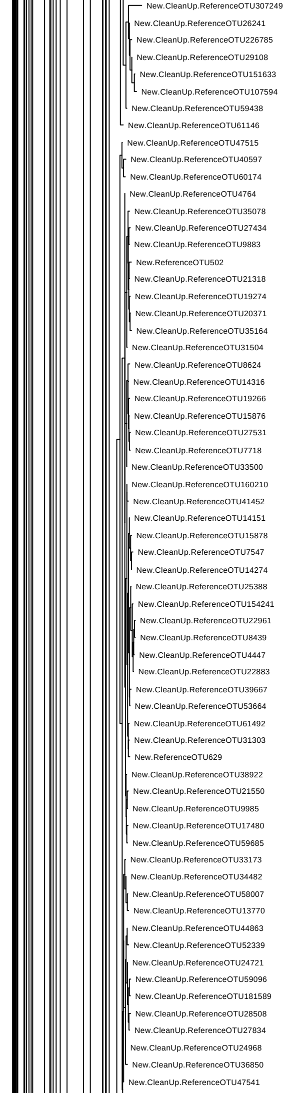
New.CleanUp.ReferenceOTU307249
New.CleanUp.ReferenceOTU26241
New.CleanUp.ReferenceOTU226785
New.CleanUp.ReferenceOTU29108
New.CleanUp.ReferenceOTU151633
New.CleanUp.ReferenceOTU107594
New.CleanUp.ReferenceOTU59438
New.CleanUp.ReferenceOTU61146
New.CleanUp.ReferenceOTU47515
New.CleanUp.ReferenceOTU40597
New.CleanUp.ReferenceOTU60174
New.CleanUp.ReferenceOTU4764
New.CleanUp.ReferenceOTU35078
New.CleanUp.ReferenceOTU27434
New.CleanUp.ReferenceOTU9883
New.ReferenceOTU502
New.CleanUp.ReferenceOTU21318
New.CleanUp.ReferenceOTU19274
New.CleanUp.ReferenceOTU20371
New.CleanUp.ReferenceOTU35164
New.CleanUp.ReferenceOTU31504
New.CleanUp.ReferenceOTU8624
New.CleanUp.ReferenceOTU14316
New.CleanUp.ReferenceOTU19266
New.CleanUp.ReferenceOTU15876
New.CleanUp.ReferenceOTU27531
New.CleanUp.ReferenceOTU7718
New.CleanUp.ReferenceOTU33500
New.CleanUp.ReferenceOTU160210
New.CleanUp.ReferenceOTU41452
New.CleanUp.ReferenceOTU14151
New.CleanUp.ReferenceOTU15878
New.CleanUp.ReferenceOTU7547
New.CleanUp.ReferenceOTU14274
New.CleanUp.ReferenceOTU25388
New.CleanUp.ReferenceOTU154241
New.CleanUp.ReferenceOTU22961
New.CleanUp.ReferenceOTU8439
New.CleanUp.ReferenceOTU4447
New.CleanUp.ReferenceOTU22883
New.CleanUp.ReferenceOTU39667
New.CleanUp.ReferenceOTU53664
New.CleanUp.ReferenceOTU61492
New.CleanUp.ReferenceOTU31303
New.ReferenceOTU629
New.CleanUp.ReferenceOTU38922
New.CleanUp.ReferenceOTU21550
New.CleanUp.ReferenceOTU9985
New.CleanUp.ReferenceOTU17480
New.CleanUp.ReferenceOTU59685
New.CleanUp.ReferenceOTU33173
New.CleanUp.ReferenceOTU34482
New.CleanUp.ReferenceOTU58007
New.CleanUp.ReferenceOTU13770
New.CleanUp.ReferenceOTU44863
New.CleanUp.ReferenceOTU52339
New.CleanUp.ReferenceOTU24721
New.CleanUp.ReferenceOTU59096
New.CleanUp.ReferenceOTU181589
New.CleanUp.ReferenceOTU28508
New.CleanUp.ReferenceOTU27834
New.CleanUp.ReferenceOTU24968
New.CleanUp.ReferenceOTU36850
New.CleanUp.ReferenceOTU47541

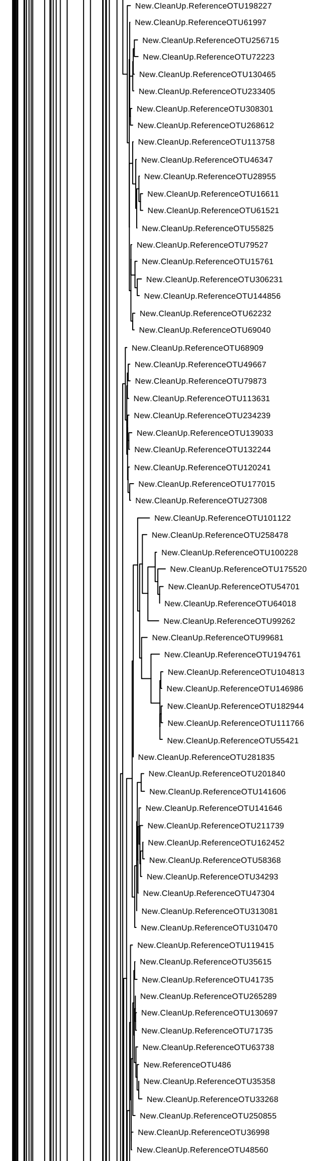
New.CleanUp.ReferenceOTU198227
New.CleanUp.ReferenceOTU61997
New.CleanUp.ReferenceOTU256715
New.CleanUp.ReferenceOTU72223
New.CleanUp.ReferenceOTU130465
New.CleanUp.ReferenceOTU233405
New.CleanUp.ReferenceOTU308301
New.CleanUp.ReferenceOTU268612
New.CleanUp.ReferenceOTU113758
New.CleanUp.ReferenceOTU46347
New.CleanUp.ReferenceOTU28955
New.CleanUp.ReferenceOTU16611
New.CleanUp.ReferenceOTU61521
New.CleanUp.ReferenceOTU55825
New.CleanUp.ReferenceOTU79527
New.CleanUp.ReferenceOTU15761
New.CleanUp.ReferenceOTU306231
New.CleanUp.ReferenceOTU144856
New.CleanUp.ReferenceOTU62232
New.CleanUp.ReferenceOTU69040
New.CleanUp.ReferenceOTU68909
New.CleanUp.ReferenceOTU49667
New.CleanUp.ReferenceOTU79873
New.CleanUp.ReferenceOTU113631
New.CleanUp.ReferenceOTU234239
New.CleanUp.ReferenceOTU139033
New.CleanUp.ReferenceOTU132244
New.CleanUp.ReferenceOTU120241
New.CleanUp.ReferenceOTU177015
New.CleanUp.ReferenceOTU27308
New.CleanUp.ReferenceOTU101122
New.CleanUp.ReferenceOTU258478
New.CleanUp.ReferenceOTU100228
New.CleanUp.ReferenceOTU175520
New.CleanUp.ReferenceOTU54701
New.CleanUp.ReferenceOTU64018
New.CleanUp.ReferenceOTU99262
New.CleanUp.ReferenceOTU99681
New.CleanUp.ReferenceOTU194761
New.CleanUp.ReferenceOTU104813
New.CleanUp.ReferenceOTU146986
New.CleanUp.ReferenceOTU182944
New.CleanUp.ReferenceOTU111766
New.CleanUp.ReferenceOTU55421
New.CleanUp.ReferenceOTU281835
New.CleanUp.ReferenceOTU201840
New.CleanUp.ReferenceOTU141606
New.CleanUp.ReferenceOTU141646
New.CleanUp.ReferenceOTU211739
New.CleanUp.ReferenceOTU162452
New.CleanUp.ReferenceOTU58368
New.CleanUp.ReferenceOTU34293
New.CleanUp.ReferenceOTU47304
New.CleanUp.ReferenceOTU313081
New.CleanUp.ReferenceOTU310470
New.CleanUp.ReferenceOTU119415
New.CleanUp.ReferenceOTU35615
New.CleanUp.ReferenceOTU41735
New.CleanUp.ReferenceOTU265289
New.CleanUp.ReferenceOTU130697
New.CleanUp.ReferenceOTU71735
New.CleanUp.ReferenceOTU63738
New.ReferenceOTU486
New.CleanUp.ReferenceOTU35358
New.CleanUp.ReferenceOTU33268
New.CleanUp.ReferenceOTU250855
New.CleanUp.ReferenceOTU36998
New.CleanUp.ReferenceOTU48560

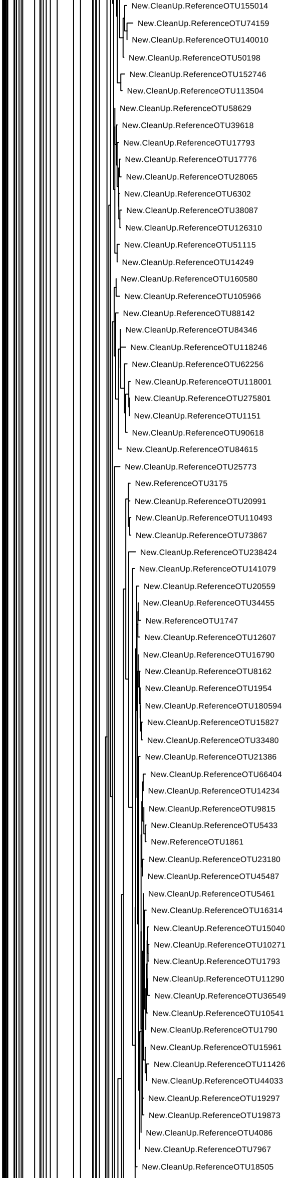
New.CleanUp.ReferenceOTU155014
New.CleanUp.ReferenceOTU74159
New.CleanUp.ReferenceOTU140010
New.CleanUp.ReferenceOTU50198
New.CleanUp.ReferenceOTU152746
New.CleanUp.ReferenceOTU113504
New.CleanUp.ReferenceOTU58629
New.CleanUp.ReferenceOTU39618
New.CleanUp.ReferenceOTU17793
New.CleanUp.ReferenceOTU17776
New.CleanUp.ReferenceOTU28065
New.CleanUp.ReferenceOTU6302
New.CleanUp.ReferenceOTU38087
New.CleanUp.ReferenceOTU126310
New.CleanUp.ReferenceOTU51115
New.CleanUp.ReferenceOTU14249
New.CleanUp.ReferenceOTU160580
New.CleanUp.ReferenceOTU105966
New.CleanUp.ReferenceOTU88142
New.CleanUp.ReferenceOTU84346
New.CleanUp.ReferenceOTU118246
New.CleanUp.ReferenceOTU62256
New.CleanUp.ReferenceOTU118001
New.CleanUp.ReferenceOTU275801
New.CleanUp.ReferenceOTU1151
New.CleanUp.ReferenceOTU90618
New.CleanUp.ReferenceOTU84615
New.CleanUp.ReferenceOTU25773
New.ReferenceOTU3175
New.CleanUp.ReferenceOTU20991
New.CleanUp.ReferenceOTU110493
New.CleanUp.ReferenceOTU73867
New.CleanUp.ReferenceOTU238424
New.CleanUp.ReferenceOTU141079
New.CleanUp.ReferenceOTU20559
New.CleanUp.ReferenceOTU34455
New.ReferenceOTU1747
New.CleanUp.ReferenceOTU12607
New.CleanUp.ReferenceOTU16790
New.CleanUp.ReferenceOTU8162
New.CleanUp.ReferenceOTU1954
New.CleanUp.ReferenceOTU180594
New.CleanUp.ReferenceOTU15827
New.CleanUp.ReferenceOTU33480
New.CleanUp.ReferenceOTU21386
New.CleanUp.ReferenceOTU66404
New.CleanUp.ReferenceOTU14234
New.CleanUp.ReferenceOTU9815
New.CleanUp.ReferenceOTU5433
New.ReferenceOTU1861
New.CleanUp.ReferenceOTU23180
New.CleanUp.ReferenceOTU45487
New.CleanUp.ReferenceOTU5461
New.CleanUp.ReferenceOTU16314
New.CleanUp.ReferenceOTU15040
New.CleanUp.ReferenceOTU10271
New.CleanUp.ReferenceOTU1793
New.CleanUp.ReferenceOTU11290
New.CleanUp.ReferenceOTU36549
New.CleanUp.ReferenceOTU10541
New.CleanUp.ReferenceOTU1790
New.CleanUp.ReferenceOTU15961
New.CleanUp.ReferenceOTU11426
New.CleanUp.ReferenceOTU44033
New.CleanUp.ReferenceOTU19297
New.CleanUp.ReferenceOTU19873
New.CleanUp.ReferenceOTU4086
New.CleanUp.ReferenceOTU7967
New.CleanUp.ReferenceOTU18505

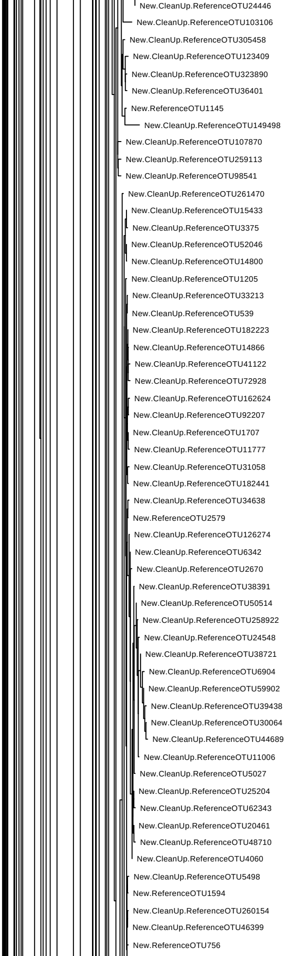
New.CleanUp.ReferenceOTU24446
New.CleanUp.ReferenceOTU103106
New.CleanUp.ReferenceOTU305458
New.CleanUp.ReferenceOTU123409
New.CleanUp.ReferenceOTU323890
New.CleanUp.ReferenceOTU36401
New.ReferenceOTU1145
New.CleanUp.ReferenceOTU149498
New.CleanUp.ReferenceOTU107870
New.CleanUp.ReferenceOTU259113
New.CleanUp.ReferenceOTU98541
New.CleanUp.ReferenceOTU261470
New.CleanUp.ReferenceOTU15433
New.CleanUp.ReferenceOTU3375
New.CleanUp.ReferenceOTU52046
New.CleanUp.ReferenceOTU14800
New.CleanUp.ReferenceOTU1205
New.CleanUp.ReferenceOTU33213
New.CleanUp.ReferenceOTU539
New.CleanUp.ReferenceOTU182223
New.CleanUp.ReferenceOTU14866
New.CleanUp.ReferenceOTU41122
New.CleanUp.ReferenceOTU72928
New.CleanUp.ReferenceOTU162624
New.CleanUp.ReferenceOTU92207
New.CleanUp.ReferenceOTU1707
New.CleanUp.ReferenceOTU11777
New.CleanUp.ReferenceOTU31058
New.CleanUp.ReferenceOTU182441
New.CleanUp.ReferenceOTU34638
New.ReferenceOTU2579
New.CleanUp.ReferenceOTU126274
New.CleanUp.ReferenceOTU6342
New.CleanUp.ReferenceOTU2670
New.CleanUp.ReferenceOTU38391
New.CleanUp.ReferenceOTU50514
New.CleanUp.ReferenceOTU258922
New.CleanUp.ReferenceOTU24548
New.CleanUp.ReferenceOTU38721
New.CleanUp.ReferenceOTU6904
New.CleanUp.ReferenceOTU59902
New.CleanUp.ReferenceOTU39438
New.CleanUp.ReferenceOTU30064
New.CleanUp.ReferenceOTU44689
New.CleanUp.ReferenceOTU11006
New.CleanUp.ReferenceOTU5027
New.CleanUp.ReferenceOTU25204
New.CleanUp.ReferenceOTU62343
New.CleanUp.ReferenceOTU20461
New.CleanUp.ReferenceOTU48710
New.CleanUp.ReferenceOTU4060
New.CleanUp.ReferenceOTU5498
New.ReferenceOTU1594
New.CleanUp.ReferenceOTU260154
New.CleanUp.ReferenceOTU46399
New.ReferenceOTU756

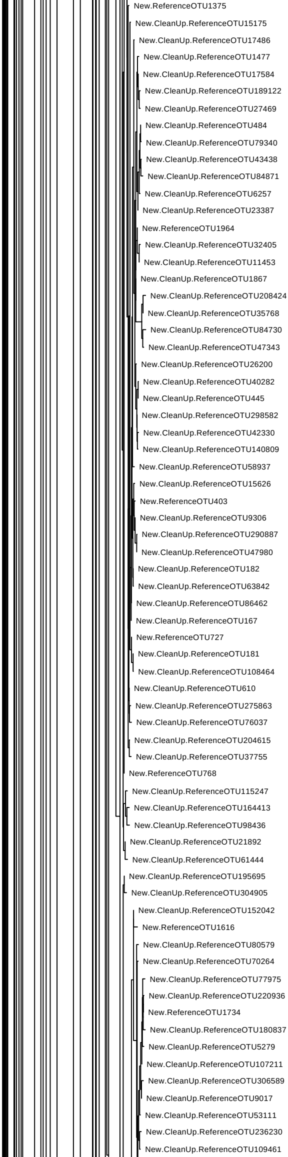
New.ReferenceOTU1375
New.CleanUp.ReferenceOTU15175
New.CleanUp.ReferenceOTU17486
New.CleanUp.ReferenceOTU1477
New.CleanUp.ReferenceOTU17584
New.CleanUp.ReferenceOTU189122
New.CleanUp.ReferenceOTU27469
New.CleanUp.ReferenceOTU484
New.CleanUp.ReferenceOTU79340
New.CleanUp.ReferenceOTU43438
New.CleanUp.ReferenceOTU84871
New.CleanUp.ReferenceOTU6257
New.CleanUp.ReferenceOTU23387
New.ReferenceOTU1964
New.CleanUp.ReferenceOTU32405
New.CleanUp.ReferenceOTU11453
New.CleanUp.ReferenceOTU1867
New.CleanUp.ReferenceOTU208424
New.CleanUp.ReferenceOTU35768
New.CleanUp.ReferenceOTU84730
New.CleanUp.ReferenceOTU47343
New.CleanUp.ReferenceOTU26200
New.CleanUp.ReferenceOTU40282
New.CleanUp.ReferenceOTU445
New.CleanUp.ReferenceOTU298582
New.CleanUp.ReferenceOTU42330
New.CleanUp.ReferenceOTU140809
New.CleanUp.ReferenceOTU58937
New.CleanUp.ReferenceOTU15626
New.ReferenceOTU403
New.CleanUp.ReferenceOTU9306
New.CleanUp.ReferenceOTU290887
New.CleanUp.ReferenceOTU47980
New.CleanUp.ReferenceOTU182
New.CleanUp.ReferenceOTU63842
New.CleanUp.ReferenceOTU86462
New.CleanUp.ReferenceOTU167
New.ReferenceOTU727
New.CleanUp.ReferenceOTU181
New.CleanUp.ReferenceOTU108464
New.CleanUp.ReferenceOTU610
New.CleanUp.ReferenceOTU275863
New.CleanUp.ReferenceOTU76037
New.CleanUp.ReferenceOTU204615
New.CleanUp.ReferenceOTU37755
New.ReferenceOTU768
New.CleanUp.ReferenceOTU115247
New.CleanUp.ReferenceOTU164413
New.CleanUp.ReferenceOTU98436
New.CleanUp.ReferenceOTU21892
New.CleanUp.ReferenceOTU61444
New.CleanUp.ReferenceOTU195695
New.CleanUp.ReferenceOTU304905
New.CleanUp.ReferenceOTU152042
New.ReferenceOTU1616
New.CleanUp.ReferenceOTU80579
New.CleanUp.ReferenceOTU70264
New.CleanUp.ReferenceOTU77975
New.CleanUp.ReferenceOTU220936
New.ReferenceOTU1734
New.CleanUp.ReferenceOTU180837
New.CleanUp.ReferenceOTU5279
New.CleanUp.ReferenceOTU107211
New.CleanUp.ReferenceOTU306589
New.CleanUp.ReferenceOTU9017
New.CleanUp.ReferenceOTU53111
New.CleanUp.ReferenceOTU236230
New.CleanUp.ReferenceOTU109461

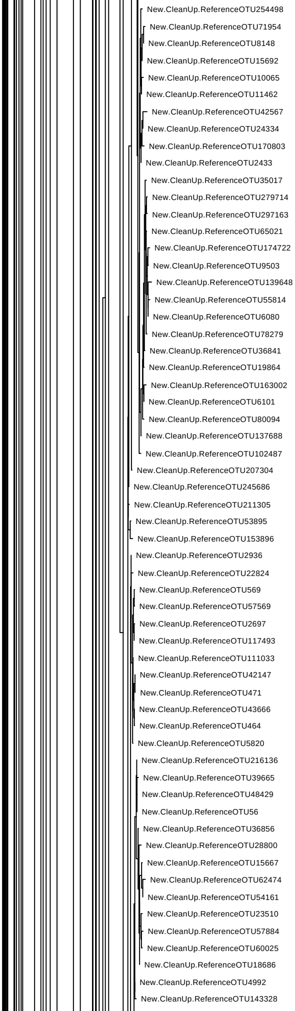
New.CleanUp.ReferenceOTU254498
New.CleanUp.ReferenceOTU71954
New.CleanUp.ReferenceOTU8148
New.CleanUp.ReferenceOTU15692
New.CleanUp.ReferenceOTU10065
New.CleanUp.ReferenceOTU11462
New.CleanUp.ReferenceOTU42567
New.CleanUp.ReferenceOTU24334
New.CleanUp.ReferenceOTU170803
New.CleanUp.ReferenceOTU2433
New.CleanUp.ReferenceOTU35017
New.CleanUp.ReferenceOTU279714
New.CleanUp.ReferenceOTU297163
New.CleanUp.ReferenceOTU65021
New.CleanUp.ReferenceOTU174722
New.CleanUp.ReferenceOTU9503
New.CleanUp.ReferenceOTU139648
New.CleanUp.ReferenceOTU55814
New.CleanUp.ReferenceOTU6080
New.CleanUp.ReferenceOTU78279
New.CleanUp.ReferenceOTU36841
New.CleanUp.ReferenceOTU19864
New.CleanUp.ReferenceOTU163002
New.CleanUp.ReferenceOTU6101
New.CleanUp.ReferenceOTU80094
New.CleanUp.ReferenceOTU137688
New.CleanUp.ReferenceOTU102487
New.CleanUp.ReferenceOTU207304
New.CleanUp.ReferenceOTU245686
New.CleanUp.ReferenceOTU211305
New.CleanUp.ReferenceOTU53895
New.CleanUp.ReferenceOTU153896
New.CleanUp.ReferenceOTU2936
New.CleanUp.ReferenceOTU22824
New.CleanUp.ReferenceOTU569
New.CleanUp.ReferenceOTU57569
New.CleanUp.ReferenceOTU2697
New.CleanUp.ReferenceOTU117493
New.CleanUp.ReferenceOTU111033
New.CleanUp.ReferenceOTU42147
New.CleanUp.ReferenceOTU471
New.CleanUp.ReferenceOTU43666
New.CleanUp.ReferenceOTU464
New.CleanUp.ReferenceOTU5820
New.CleanUp.ReferenceOTU216136
New.CleanUp.ReferenceOTU39665
New.CleanUp.ReferenceOTU48429
New.CleanUp.ReferenceOTU56
New.CleanUp.ReferenceOTU36856
New.CleanUp.ReferenceOTU28800
New.CleanUp.ReferenceOTU15667
New.CleanUp.ReferenceOTU62474
New.CleanUp.ReferenceOTU54161
New.CleanUp.ReferenceOTU23510
New.CleanUp.ReferenceOTU57884
New.CleanUp.ReferenceOTU60025
New.CleanUp.ReferenceOTU18686
New.CleanUp.ReferenceOTU4992
New.CleanUp.ReferenceOTU143328

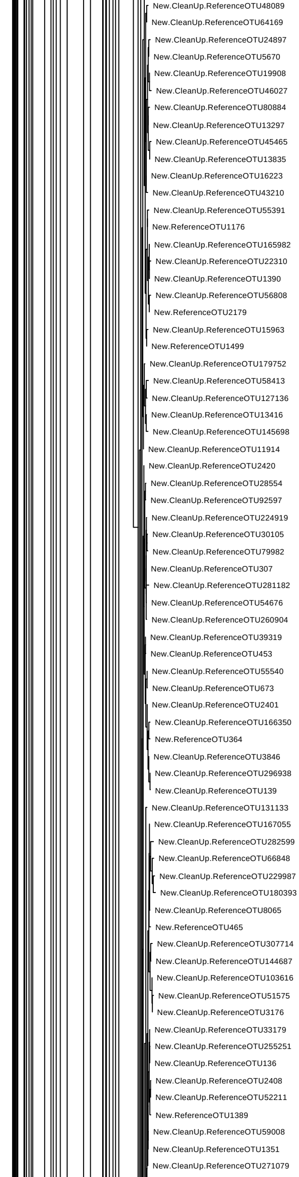
New.CleanUp.ReferenceOTU48089
New.CleanUp.ReferenceOTU64169
New.CleanUp.ReferenceOTU24897
New.CleanUp.ReferenceOTU5670
New.CleanUp.ReferenceOTU19908
New.CleanUp.ReferenceOTU46027
New.CleanUp.ReferenceOTU80884
New.CleanUp.ReferenceOTU13297
New.CleanUp.ReferenceOTU45465
New.CleanUp.ReferenceOTU13835
New.CleanUp.ReferenceOTU16223
New.CleanUp.ReferenceOTU43210
New.CleanUp.ReferenceOTU55391
New.ReferenceOTU1176
New.CleanUp.ReferenceOTU165982
New.CleanUp.ReferenceOTU22310
New.CleanUp.ReferenceOTU1390
New.CleanUp.ReferenceOTU56808
New.ReferenceOTU2179
New.CleanUp.ReferenceOTU15963
New.ReferenceOTU1499
New.CleanUp.ReferenceOTU179752
New.CleanUp.ReferenceOTU58413
New.CleanUp.ReferenceOTU127136
New.CleanUp.ReferenceOTU13416
New.CleanUp.ReferenceOTU145698
New.CleanUp.ReferenceOTU11914
New.CleanUp.ReferenceOTU2420
New.CleanUp.ReferenceOTU28554
New.CleanUp.ReferenceOTU92597
New.CleanUp.ReferenceOTU224919
New.CleanUp.ReferenceOTU30105
New.CleanUp.ReferenceOTU79982
New.CleanUp.ReferenceOTU307
New.CleanUp.ReferenceOTU281182
New.CleanUp.ReferenceOTU54676
New.CleanUp.ReferenceOTU260904
New.CleanUp.ReferenceOTU39319
New.CleanUp.ReferenceOTU453
New.CleanUp.ReferenceOTU55540
New.CleanUp.ReferenceOTU673
New.CleanUp.ReferenceOTU2401
New.CleanUp.ReferenceOTU166350
New.ReferenceOTU364
New.CleanUp.ReferenceOTU3846
New.CleanUp.ReferenceOTU296938
New.CleanUp.ReferenceOTU139
New.CleanUp.ReferenceOTU131133
New.CleanUp.ReferenceOTU167055
New.CleanUp.ReferenceOTU282599
New.CleanUp.ReferenceOTU66848
New.CleanUp.ReferenceOTU229987
New.CleanUp.ReferenceOTU180393
New.CleanUp.ReferenceOTU8065
New.ReferenceOTU465
New.CleanUp.ReferenceOTU307714
New.CleanUp.ReferenceOTU144687
New.CleanUp.ReferenceOTU103616
New.CleanUp.ReferenceOTU51575
New.CleanUp.ReferenceOTU3176
New.CleanUp.ReferenceOTU33179
New.CleanUp.ReferenceOTU255251
New.CleanUp.ReferenceOTU136
New.CleanUp.ReferenceOTU2408
New.CleanUp.ReferenceOTU52211
New.ReferenceOTU1389
New.CleanUp.ReferenceOTU59008
New.CleanUp.ReferenceOTU1351
New.CleanUp.ReferenceOTU271079

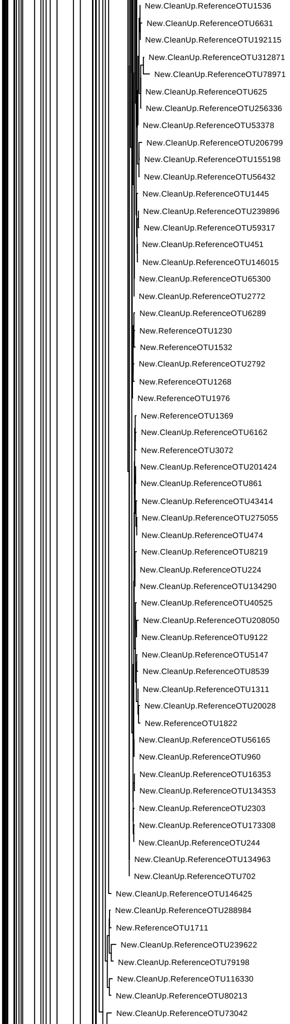
New.CleanUp.ReferenceOTU1536
New.CleanUp.ReferenceOTU6631
New.CleanUp.ReferenceOTU192115
New.CleanUp.ReferenceOTU312871
New.CleanUp.ReferenceOTU78971
New.CleanUp.ReferenceOTU625
New.CleanUp.ReferenceOTU256336
New.CleanUp.ReferenceOTU53378
New.CleanUp.ReferenceOTU206799
New.CleanUp.ReferenceOTU155198
New.CleanUp.ReferenceOTU56432
New.CleanUp.ReferenceOTU1445
New.CleanUp.ReferenceOTU239896
New.CleanUp.ReferenceOTU59317
New.CleanUp.ReferenceOTU451
New.CleanUp.ReferenceOTU146015
New.CleanUp.ReferenceOTU65300
New.CleanUp.ReferenceOTU2772
New.CleanUp.ReferenceOTU6289
New.ReferenceOTU1230
New.ReferenceOTU1532
New.CleanUp.ReferenceOTU2792
New.ReferenceOTU1268
New.ReferenceOTU1976
New.ReferenceOTU1369
New.CleanUp.ReferenceOTU6162
New.ReferenceOTU3072
New.CleanUp.ReferenceOTU201424
New.CleanUp.ReferenceOTU861
New.CleanUp.ReferenceOTU43414
New.CleanUp.ReferenceOTU275055
New.CleanUp.ReferenceOTU474
New.CleanUp.ReferenceOTU8219
New.CleanUp.ReferenceOTU224
New.CleanUp.ReferenceOTU134290
New.CleanUp.ReferenceOTU40525
New.CleanUp.ReferenceOTU208050
New.CleanUp.ReferenceOTU9122
New.CleanUp.ReferenceOTU5147
New.CleanUp.ReferenceOTU8539
New.CleanUp.ReferenceOTU1311
New.CleanUp.ReferenceOTU20028
New.ReferenceOTU1822
New.CleanUp.ReferenceOTU56165
New.CleanUp.ReferenceOTU960
New.CleanUp.ReferenceOTU16353
New.CleanUp.ReferenceOTU134353
New.CleanUp.ReferenceOTU2303
New.CleanUp.ReferenceOTU173308
New.CleanUp.ReferenceOTU244
New.CleanUp.ReferenceOTU134963
New.CleanUp.ReferenceOTU702
New.CleanUp.ReferenceOTU146425
New.CleanUp.ReferenceOTU288984
New.ReferenceOTU1711
New.CleanUp.ReferenceOTU239622
New.CleanUp.ReferenceOTU79198
New.CleanUp.ReferenceOTU116330
New.CleanUp.ReferenceOTU80213
New.CleanUp.ReferenceOTU73042

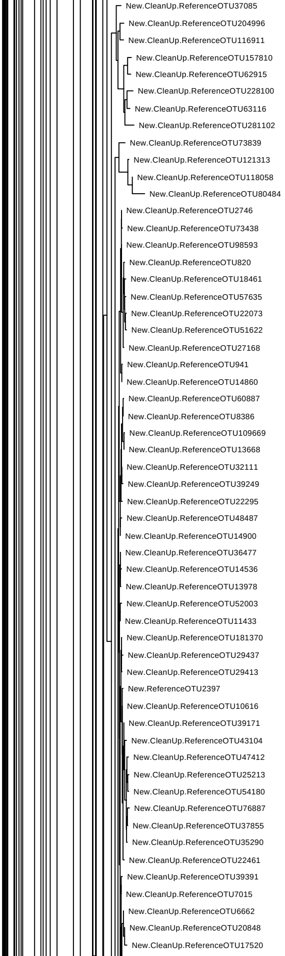
New.CleanUp.ReferenceOTU37085
New.CleanUp.ReferenceOTU204996
New.CleanUp.ReferenceOTU116911
New.CleanUp.ReferenceOTU157810
New.CleanUp.ReferenceOTU62915
New.CleanUp.ReferenceOTU228100
New.CleanUp.ReferenceOTU63116
New.CleanUp.ReferenceOTU281102
New.CleanUp.ReferenceOTU73839
New.CleanUp.ReferenceOTU121313
New.CleanUp.ReferenceOTU118058
New.CleanUp.ReferenceOTU80484
New.CleanUp.ReferenceOTU2746
New.CleanUp.ReferenceOTU73438
New.CleanUp.ReferenceOTU98593
New.CleanUp.ReferenceOTU820
New.CleanUp.ReferenceOTU18461
New.CleanUp.ReferenceOTU57635
New.CleanUp.ReferenceOTU22073
New.CleanUp.ReferenceOTU51622
New.CleanUp.ReferenceOTU27168
New.CleanUp.ReferenceOTU941
New.CleanUp.ReferenceOTU14860
New.CleanUp.ReferenceOTU60887
New.CleanUp.ReferenceOTU8386
New.CleanUp.ReferenceOTU109669
New.CleanUp.ReferenceOTU13668
New.CleanUp.ReferenceOTU32111
New.CleanUp.ReferenceOTU39249
New.CleanUp.ReferenceOTU22295
New.CleanUp.ReferenceOTU48487
New.CleanUp.ReferenceOTU14900
New.CleanUp.ReferenceOTU36477
New.CleanUp.ReferenceOTU14536
New.CleanUp.ReferenceOTU13978
New.CleanUp.ReferenceOTU52003
New.CleanUp.ReferenceOTU11433
New.CleanUp.ReferenceOTU181370
New.CleanUp.ReferenceOTU29437
New.CleanUp.ReferenceOTU29413
New.ReferenceOTU2397
New.CleanUp.ReferenceOTU10616
New.CleanUp.ReferenceOTU39171
New.CleanUp.ReferenceOTU43104
New.CleanUp.ReferenceOTU47412
New.CleanUp.ReferenceOTU25213
New.CleanUp.ReferenceOTU54180
New.CleanUp.ReferenceOTU76887
New.CleanUp.ReferenceOTU37855
New.CleanUp.ReferenceOTU35290
New.CleanUp.ReferenceOTU22461
New.CleanUp.ReferenceOTU39391
New.CleanUp.ReferenceOTU7015
New.CleanUp.ReferenceOTU6662
New.CleanUp.ReferenceOTU20848
New.CleanUp.ReferenceOTU17520

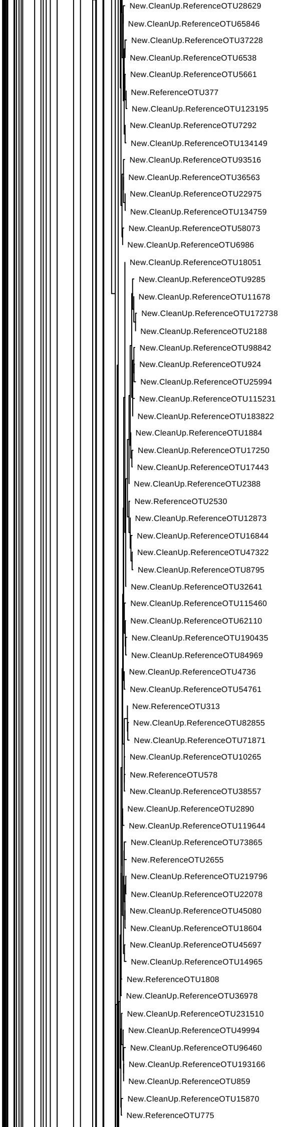
New.CleanUp.ReferenceOTU28629
New.CleanUp.ReferenceOTU65846
New.CleanUp.ReferenceOTU37228
New.CleanUp.ReferenceOTU6538
New.CleanUp.ReferenceOTU5661
New.ReferenceOTU377
New.CleanUp.ReferenceOTU123195
New.CleanUp.ReferenceOTU7292
New.CleanUp.ReferenceOTU134149
New.CleanUp.ReferenceOTU93516
New.CleanUp.ReferenceOTU36563
New.CleanUp.ReferenceOTU22975
New.CleanUp.ReferenceOTU134759
New.CleanUp.ReferenceOTU58073
New.CleanUp.ReferenceOTU6986
New.CleanUp.ReferenceOTU18051
New.CleanUp.ReferenceOTU9285
New.CleanUp.ReferenceOTU11678
New.CleanUp.ReferenceOTU172738
New.CleanUp.ReferenceOTU2188
New.CleanUp.ReferenceOTU98842
New.CleanUp.ReferenceOTU924
New.CleanUp.ReferenceOTU25994
New.CleanUp.ReferenceOTU115231
New.CleanUp.ReferenceOTU183822
New.CleanUp.ReferenceOTU1884
New.CleanUp.ReferenceOTU17250
New.CleanUp.ReferenceOTU17443
New.CleanUp.ReferenceOTU2388
New.ReferenceOTU2530
New.CleanUp.ReferenceOTU12873
New.CleanUp.ReferenceOTU16844
New.CleanUp.ReferenceOTU47322
New.CleanUp.ReferenceOTU8795
New.CleanUp.ReferenceOTU32641
New.CleanUp.ReferenceOTU115460
New.CleanUp.ReferenceOTU62110
New.CleanUp.ReferenceOTU190435
New.CleanUp.ReferenceOTU84969
New.CleanUp.ReferenceOTU4736
New.CleanUp.ReferenceOTU54761
New.ReferenceOTU313
New.CleanUp.ReferenceOTU82855
New.CleanUp.ReferenceOTU71871
New.CleanUp.ReferenceOTU10265
New.ReferenceOTU578
New.CleanUp.ReferenceOTU38557
New.CleanUp.ReferenceOTU2890
New.CleanUp.ReferenceOTU119644
New.CleanUp.ReferenceOTU73865
New.ReferenceOTU2655
New.CleanUp.ReferenceOTU219796
New.CleanUp.ReferenceOTU22078
New.CleanUp.ReferenceOTU45080
New.CleanUp.ReferenceOTU18604
New.CleanUp.ReferenceOTU45697
New.CleanUp.ReferenceOTU14965
New.ReferenceOTU1808
New.CleanUp.ReferenceOTU36978
New.CleanUp.ReferenceOTU231510
New.CleanUp.ReferenceOTU49994
New.CleanUp.ReferenceOTU96460
New.CleanUp.ReferenceOTU193166
New.CleanUp.ReferenceOTU859
New.CleanUp.ReferenceOTU15870
New.ReferenceOTU775

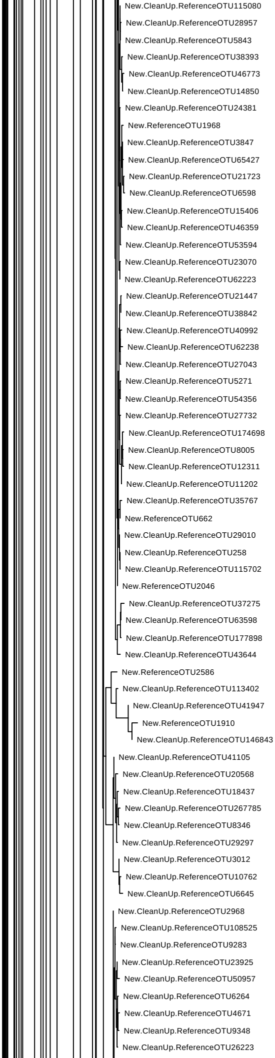
New.CleanUp.ReferenceOTU115080
New.CleanUp.ReferenceOTU28957
New.CleanUp.ReferenceOTU5843
New.CleanUp.ReferenceOTU38393
New.CleanUp.ReferenceOTU46773
New.CleanUp.ReferenceOTU14850
New.CleanUp.ReferenceOTU24381
New.ReferenceOTU1968
New.CleanUp.ReferenceOTU3847
New.CleanUp.ReferenceOTU65427
New.CleanUp.ReferenceOTU21723
New.CleanUp.ReferenceOTU6598
New.CleanUp.ReferenceOTU15406
New.CleanUp.ReferenceOTU46359
New.CleanUp.ReferenceOTU53594
New.CleanUp.ReferenceOTU23070
New.CleanUp.ReferenceOTU62223
New.CleanUp.ReferenceOTU21447
New.CleanUp.ReferenceOTU38842
New.CleanUp.ReferenceOTU40992
New.CleanUp.ReferenceOTU62238
New.CleanUp.ReferenceOTU27043
New.CleanUp.ReferenceOTU5271
New.CleanUp.ReferenceOTU54356
New.CleanUp.ReferenceOTU27732
New.CleanUp.ReferenceOTU174698
New.CleanUp.ReferenceOTU8005
New.CleanUp.ReferenceOTU12311
New.CleanUp.ReferenceOTU11202
New.CleanUp.ReferenceOTU35767
New.ReferenceOTU662
New.CleanUp.ReferenceOTU29010
New.CleanUp.ReferenceOTU258
New.CleanUp.ReferenceOTU115702
New.ReferenceOTU2046
New.CleanUp.ReferenceOTU37275
New.CleanUp.ReferenceOTU63598
New.CleanUp.ReferenceOTU177898
New.CleanUp.ReferenceOTU43644
New.ReferenceOTU2586
New.CleanUp.ReferenceOTU113402
New.CleanUp.ReferenceOTU41947
New.ReferenceOTU1910
New.CleanUp.ReferenceOTU146843
New.CleanUp.ReferenceOTU41105
New.CleanUp.ReferenceOTU20568
New.CleanUp.ReferenceOTU18437
New.CleanUp.ReferenceOTU267785
New.CleanUp.ReferenceOTU8346
New.CleanUp.ReferenceOTU29297
New.CleanUp.ReferenceOTU3012
New.CleanUp.ReferenceOTU10762
New.CleanUp.ReferenceOTU6645
New.CleanUp.ReferenceOTU2968
New.CleanUp.ReferenceOTU108525
New.CleanUp.ReferenceOTU9283
New.CleanUp.ReferenceOTU23925
New.CleanUp.ReferenceOTU50957
New.CleanUp.ReferenceOTU6264
New.CleanUp.ReferenceOTU4671
New.CleanUp.ReferenceOTU9348
New.CleanUp.ReferenceOTU26223

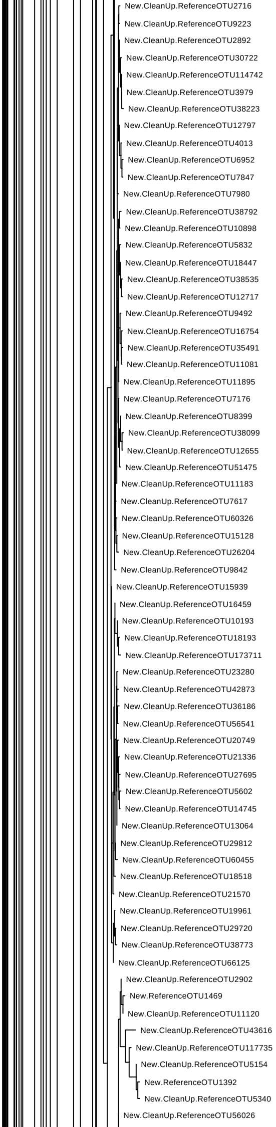
New.CleanUp.ReferenceOTU2716
New.CleanUp.ReferenceOTU9223
New.CleanUp.ReferenceOTU2892
New.CleanUp.ReferenceOTU30722
New.CleanUp.ReferenceOTU114742
New.CleanUp.ReferenceOTU3979
New.CleanUp.ReferenceOTU38223
New.CleanUp.ReferenceOTU12797
New.CleanUp.ReferenceOTU4013
New.CleanUp.ReferenceOTU6952
New.CleanUp.ReferenceOTU7847
New.CleanUp.ReferenceOTU7980
New.CleanUp.ReferenceOTU38792
New.CleanUp.ReferenceOTU10898
New.CleanUp.ReferenceOTU5832
New.CleanUp.ReferenceOTU18447
New.CleanUp.ReferenceOTU38535
New.CleanUp.ReferenceOTU12717
New.CleanUp.ReferenceOTU9492
New.CleanUp.ReferenceOTU16754
New.CleanUp.ReferenceOTU35491
New.CleanUp.ReferenceOTU11081
New.CleanUp.ReferenceOTU11895
New.CleanUp.ReferenceOTU7176
New.CleanUp.ReferenceOTU8399
New.CleanUp.ReferenceOTU38099
New.CleanUp.ReferenceOTU12655
New.CleanUp.ReferenceOTU51475
New.CleanUp.ReferenceOTU11183
New.CleanUp.ReferenceOTU7617
New.CleanUp.ReferenceOTU60326
New.CleanUp.ReferenceOTU15128
New.CleanUp.ReferenceOTU26204
New.CleanUp.ReferenceOTU9842
New.CleanUp.ReferenceOTU15939
New.CleanUp.ReferenceOTU16459
New.CleanUp.ReferenceOTU10193
New.CleanUp.ReferenceOTU18193
New.CleanUp.ReferenceOTU173711
New.CleanUp.ReferenceOTU23280
New.CleanUp.ReferenceOTU42873
New.CleanUp.ReferenceOTU36186
New.CleanUp.ReferenceOTU56541
New.CleanUp.ReferenceOTU20749
New.CleanUp.ReferenceOTU21336
New.CleanUp.ReferenceOTU27695
New.CleanUp.ReferenceOTU5602
New.CleanUp.ReferenceOTU14745
New.CleanUp.ReferenceOTU13064
New.CleanUp.ReferenceOTU29812
New.CleanUp.ReferenceOTU60455
New.CleanUp.ReferenceOTU18518
New.CleanUp.ReferenceOTU21570
New.CleanUp.ReferenceOTU19961
New.CleanUp.ReferenceOTU29720
New.CleanUp.ReferenceOTU38773
New.CleanUp.ReferenceOTU66125
New.CleanUp.ReferenceOTU2902
New.ReferenceOTU1469
New.CleanUp.ReferenceOTU11120
New.CleanUp.ReferenceOTU43616
New.CleanUp.ReferenceOTU117735
New.CleanUp.ReferenceOTU5154
New.ReferenceOTU1392
New.CleanUp.ReferenceOTU5340
New.CleanUp.ReferenceOTU56026

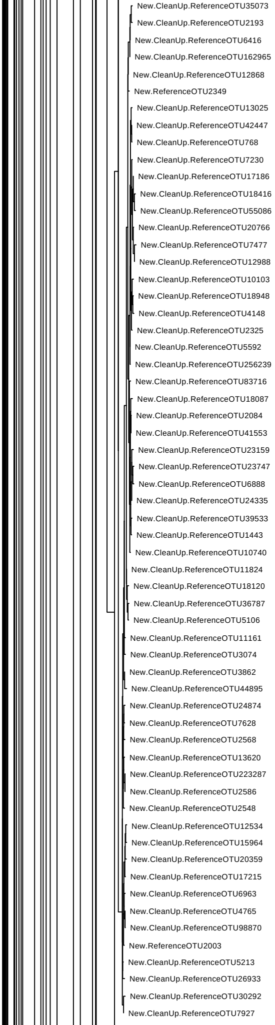
New.CleanUp.ReferenceOTU35073
New.CleanUp.ReferenceOTU2193
New.CleanUp.ReferenceOTU6416
New.CleanUp.ReferenceOTU162965
New.CleanUp.ReferenceOTU12868
New.ReferenceOTU2349
New.CleanUp.ReferenceOTU13025
New.CleanUp.ReferenceOTU42447
New.CleanUp.ReferenceOTU768
New.CleanUp.ReferenceOTU7230
New.CleanUp.ReferenceOTU17186
New.CleanUp.ReferenceOTU18416
New.CleanUp.ReferenceOTU55086
New.CleanUp.ReferenceOTU20766
New.CleanUp.ReferenceOTU7477
New.CleanUp.ReferenceOTU12988
New.CleanUp.ReferenceOTU10103
New.CleanUp.ReferenceOTU18948
New.CleanUp.ReferenceOTU4148
New.CleanUp.ReferenceOTU2325
New.CleanUp.ReferenceOTU5592
New.CleanUp.ReferenceOTU256239
New.CleanUp.ReferenceOTU83716
New.CleanUp.ReferenceOTU18087
New.CleanUp.ReferenceOTU2084
New.CleanUp.ReferenceOTU41553
New.CleanUp.ReferenceOTU23159
New.CleanUp.ReferenceOTU23747
New.CleanUp.ReferenceOTU6888
New.CleanUp.ReferenceOTU24335
New.CleanUp.ReferenceOTU39533
New.CleanUp.ReferenceOTU1443
New.CleanUp.ReferenceOTU10740
New.CleanUp.ReferenceOTU11824
New.CleanUp.ReferenceOTU18120
New.CleanUp.ReferenceOTU36787
New.CleanUp.ReferenceOTU5106
New.CleanUp.ReferenceOTU11161
New.CleanUp.ReferenceOTU3074
New.CleanUp.ReferenceOTU3862
New.CleanUp.ReferenceOTU44895
New.CleanUp.ReferenceOTU24874
New.CleanUp.ReferenceOTU7628
New.CleanUp.ReferenceOTU2568
New.CleanUp.ReferenceOTU13620
New.CleanUp.ReferenceOTU223287
New.CleanUp.ReferenceOTU2586
New.CleanUp.ReferenceOTU2548
New.CleanUp.ReferenceOTU12534
New.CleanUp.ReferenceOTU15964
New.CleanUp.ReferenceOTU20359
New.CleanUp.ReferenceOTU17215
New.CleanUp.ReferenceOTU6963
New.CleanUp.ReferenceOTU4765
New.CleanUp.ReferenceOTU98870
New.ReferenceOTU2003
New.CleanUp.ReferenceOTU5213
New.CleanUp.ReferenceOTU26933
New.CleanUp.ReferenceOTU30292
New.CleanUp.ReferenceOTU7927

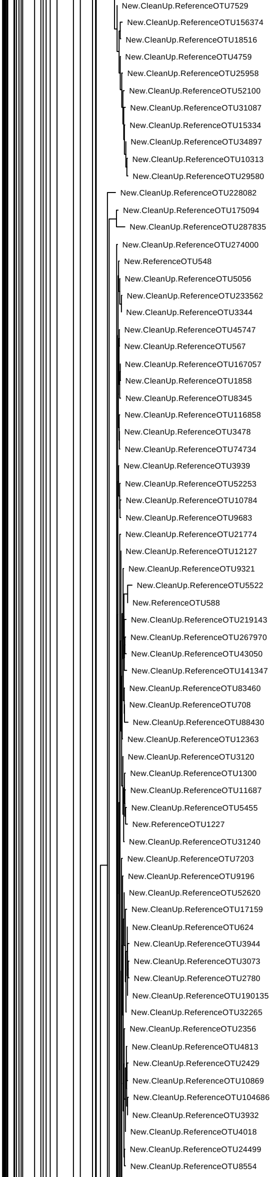
New.CleanUp.ReferenceOTU7529
New.CleanUp.ReferenceOTU156374
New.CleanUp.ReferenceOTU18516
New.CleanUp.ReferenceOTU4759
New.CleanUp.ReferenceOTU25958
New.CleanUp.ReferenceOTU52100
New.CleanUp.ReferenceOTU31087
New.CleanUp.ReferenceOTU15334
New.CleanUp.ReferenceOTU34897
New.CleanUp.ReferenceOTU10313
New.CleanUp.ReferenceOTU29580
New.CleanUp.ReferenceOTU228082
New.CleanUp.ReferenceOTU175094
New.CleanUp.ReferenceOTU287835
New.CleanUp.ReferenceOTU274000
New.ReferenceOTU548
New.CleanUp.ReferenceOTU5056
New.CleanUp.ReferenceOTU233562
New.CleanUp.ReferenceOTU3344
New.CleanUp.ReferenceOTU45747
New.CleanUp.ReferenceOTU567
New.CleanUp.ReferenceOTU167057
New.CleanUp.ReferenceOTU1858
New.CleanUp.ReferenceOTU8345
New.CleanUp.ReferenceOTU116858
New.CleanUp.ReferenceOTU3478
New.CleanUp.ReferenceOTU74734
New.CleanUp.ReferenceOTU3939
New.CleanUp.ReferenceOTU52253
New.CleanUp.ReferenceOTU10784
New.CleanUp.ReferenceOTU9683
New.CleanUp.ReferenceOTU21774
New.CleanUp.ReferenceOTU12127
New.CleanUp.ReferenceOTU9321
New.CleanUp.ReferenceOTU5522
New.ReferenceOTU588
New.CleanUp.ReferenceOTU219143
New.CleanUp.ReferenceOTU267970
New.CleanUp.ReferenceOTU43050
New.CleanUp.ReferenceOTU141347
New.CleanUp.ReferenceOTU83460
New.CleanUp.ReferenceOTU708
New.CleanUp.ReferenceOTU88430
New.CleanUp.ReferenceOTU12363
New.CleanUp.ReferenceOTU3120
New.CleanUp.ReferenceOTU1300
New.CleanUp.ReferenceOTU11687
New.CleanUp.ReferenceOTU5455
New.ReferenceOTU1227
New.CleanUp.ReferenceOTU31240
New.CleanUp.ReferenceOTU7203
New.CleanUp.ReferenceOTU9196
New.CleanUp.ReferenceOTU52620
New.CleanUp.ReferenceOTU17159
New.CleanUp.ReferenceOTU624
New.CleanUp.ReferenceOTU3944
New.CleanUp.ReferenceOTU3073
New.CleanUp.ReferenceOTU2780
New.CleanUp.ReferenceOTU190135
New.CleanUp.ReferenceOTU32265
New.CleanUp.ReferenceOTU2356
New.CleanUp.ReferenceOTU4813
New.CleanUp.ReferenceOTU2429
New.CleanUp.ReferenceOTU10869
New.CleanUp.ReferenceOTU104686
New.CleanUp.ReferenceOTU3932
New.CleanUp.ReferenceOTU4018
New.CleanUp.ReferenceOTU24499
New.CleanUp.ReferenceOTU8554

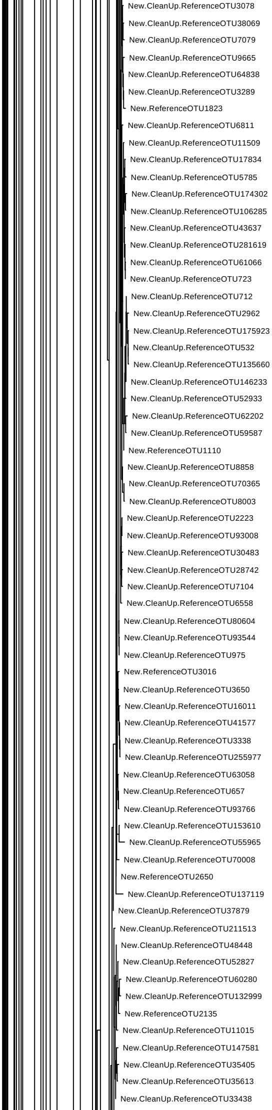
New.CleanUp.ReferenceOTU3078
New.CleanUp.ReferenceOTU38069
New.CleanUp.ReferenceOTU7079
New.CleanUp.ReferenceOTU9665
New.CleanUp.ReferenceOTU64838
New.CleanUp.ReferenceOTU3289
New.ReferenceOTU1823
New.CleanUp.ReferenceOTU6811
New.CleanUp.ReferenceOTU11509
New.CleanUp.ReferenceOTU17834
New.CleanUp.ReferenceOTU5785
New.CleanUp.ReferenceOTU174302
New.CleanUp.ReferenceOTU106285
New.CleanUp.ReferenceOTU43637
New.CleanUp.ReferenceOTU281619
New.CleanUp.ReferenceOTU61066
New.CleanUp.ReferenceOTU723
New.CleanUp.ReferenceOTU712
New.CleanUp.ReferenceOTU2962
New.CleanUp.ReferenceOTU175923
New.CleanUp.ReferenceOTU532
New.CleanUp.ReferenceOTU135660
New.CleanUp.ReferenceOTU146233
New.CleanUp.ReferenceOTU52933
New.CleanUp.ReferenceOTU62202
New.CleanUp.ReferenceOTU59587
New.ReferenceOTU1110
New.CleanUp.ReferenceOTU8858
New.CleanUp.ReferenceOTU70365
New.CleanUp.ReferenceOTU8003
New.CleanUp.ReferenceOTU2223
New.CleanUp.ReferenceOTU93008
New.CleanUp.ReferenceOTU30483
New.CleanUp.ReferenceOTU28742
New.CleanUp.ReferenceOTU7104
New.CleanUp.ReferenceOTU6558
New.CleanUp.ReferenceOTU80604
New.CleanUp.ReferenceOTU93544
New.CleanUp.ReferenceOTU975
New.ReferenceOTU3016
New.CleanUp.ReferenceOTU3650
New.CleanUp.ReferenceOTU16011
New.CleanUp.ReferenceOTU41577
New.CleanUp.ReferenceOTU3338
New.CleanUp.ReferenceOTU255977
New.CleanUp.ReferenceOTU63058
New.CleanUp.ReferenceOTU657
New.CleanUp.ReferenceOTU93766
New.CleanUp.ReferenceOTU153610
New.CleanUp.ReferenceOTU55965
New.CleanUp.ReferenceOTU70008
New.ReferenceOTU2650
New.CleanUp.ReferenceOTU137119
New.CleanUp.ReferenceOTU37879
New.CleanUp.ReferenceOTU211513
New.CleanUp.ReferenceOTU48448
New.CleanUp.ReferenceOTU52827
New.CleanUp.ReferenceOTU60280
New.CleanUp.ReferenceOTU132999
New.ReferenceOTU2135
New.CleanUp.ReferenceOTU11015
New.CleanUp.ReferenceOTU147581
New.CleanUp.ReferenceOTU35405
New.CleanUp.ReferenceOTU35613
New.CleanUp.ReferenceOTU33438

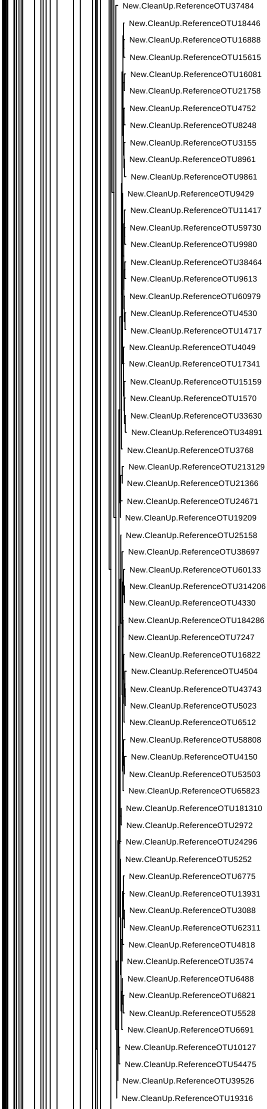
New.CleanUp.ReferenceOTU37484
New.CleanUp.ReferenceOTU18446
New.CleanUp.ReferenceOTU16888
New.CleanUp.ReferenceOTU15615
New.CleanUp.ReferenceOTU16081
New.CleanUp.ReferenceOTU21758
New.CleanUp.ReferenceOTU4752
New.CleanUp.ReferenceOTU8248
New.CleanUp.ReferenceOTU3155
New.CleanUp.ReferenceOTU8961
New.CleanUp.ReferenceOTU9861
New.CleanUp.ReferenceOTU9429
New.CleanUp.ReferenceOTU11417
New.CleanUp.ReferenceOTU59730
New.CleanUp.ReferenceOTU9980
New.CleanUp.ReferenceOTU38464
New.CleanUp.ReferenceOTU9613
New.CleanUp.ReferenceOTU60979
New.CleanUp.ReferenceOTU4530
New.CleanUp.ReferenceOTU14717
New.CleanUp.ReferenceOTU4049
New.CleanUp.ReferenceOTU17341
New.CleanUp.ReferenceOTU15159
New.CleanUp.ReferenceOTU1570
New.CleanUp.ReferenceOTU33630
New.CleanUp.ReferenceOTU34891
New.CleanUp.ReferenceOTU3768
New.CleanUp.ReferenceOTU213129
New.CleanUp.ReferenceOTU21366
New.CleanUp.ReferenceOTU24671
New.CleanUp.ReferenceOTU19209
New.CleanUp.ReferenceOTU25158
New.CleanUp.ReferenceOTU38697
New.CleanUp.ReferenceOTU60133
New.CleanUp.ReferenceOTU314206
New.CleanUp.ReferenceOTU4330
New.CleanUp.ReferenceOTU184286
New.CleanUp.ReferenceOTU7247
New.CleanUp.ReferenceOTU16822
New.CleanUp.ReferenceOTU4504
New.CleanUp.ReferenceOTU43743
New.CleanUp.ReferenceOTU5023
New.CleanUp.ReferenceOTU6512
New.CleanUp.ReferenceOTU58808
New.CleanUp.ReferenceOTU4150
New.CleanUp.ReferenceOTU53503
New.CleanUp.ReferenceOTU65823
New.CleanUp.ReferenceOTU181310
New.CleanUp.ReferenceOTU2972
New.CleanUp.ReferenceOTU24296
New.CleanUp.ReferenceOTU5252
New.CleanUp.ReferenceOTU6775
New.CleanUp.ReferenceOTU13931
New.CleanUp.ReferenceOTU3088
New.CleanUp.ReferenceOTU62311
New.CleanUp.ReferenceOTU4818
New.CleanUp.ReferenceOTU3574
New.CleanUp.ReferenceOTU6488
New.CleanUp.ReferenceOTU6821
New.CleanUp.ReferenceOTU5528
New.CleanUp.ReferenceOTU6691
New.CleanUp.ReferenceOTU10127
New.CleanUp.ReferenceOTU54475
New.CleanUp.ReferenceOTU39526
New.CleanUp.ReferenceOTU19316

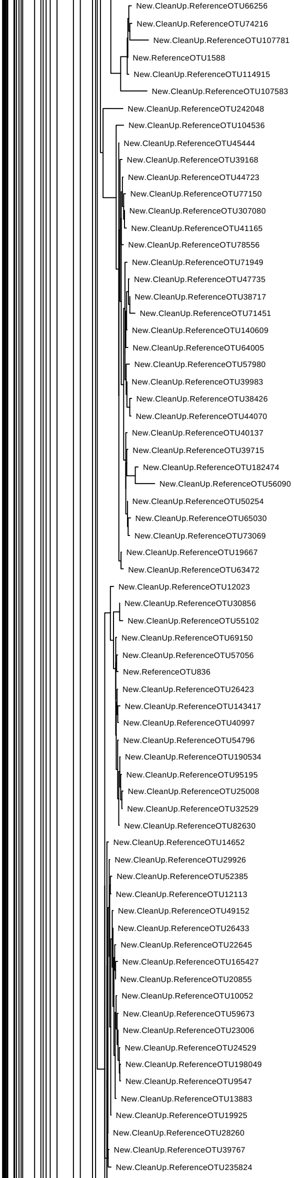
New.CleanUp.ReferenceOTU66256
New.CleanUp.ReferenceOTU74216
New.CleanUp.ReferenceOTU107781
New.ReferenceOTU1588
New.CleanUp.ReferenceOTU114915
New.CleanUp.ReferenceOTU107583
New.CleanUp.ReferenceOTU242048
New.CleanUp.ReferenceOTU104536
New.CleanUp.ReferenceOTU45444
New.CleanUp.ReferenceOTU39168
New.CleanUp.ReferenceOTU44723
New.CleanUp.ReferenceOTU77150
New.CleanUp.ReferenceOTU307080
New.CleanUp.ReferenceOTU41165
New.CleanUp.ReferenceOTU78556
New.CleanUp.ReferenceOTU71949
New.CleanUp.ReferenceOTU47735
New.CleanUp.ReferenceOTU38717
New.CleanUp.ReferenceOTU71451
New.CleanUp.ReferenceOTU140609
New.CleanUp.ReferenceOTU64005
New.CleanUp.ReferenceOTU57980
New.CleanUp.ReferenceOTU39983
New.CleanUp.ReferenceOTU38426
New.CleanUp.ReferenceOTU44070
New.CleanUp.ReferenceOTU40137
New.CleanUp.ReferenceOTU39715
New.CleanUp.ReferenceOTU182474
New.CleanUp.ReferenceOTU56090
New.CleanUp.ReferenceOTU50254
New.CleanUp.ReferenceOTU65030
New.CleanUp.ReferenceOTU73069
New.CleanUp.ReferenceOTU19667
New.CleanUp.ReferenceOTU63472
New.CleanUp.ReferenceOTU12023
New.CleanUp.ReferenceOTU30856
New.CleanUp.ReferenceOTU55102
New.CleanUp.ReferenceOTU69150
New.CleanUp.ReferenceOTU57056
New.ReferenceOTU836
New.CleanUp.ReferenceOTU26423
New.CleanUp.ReferenceOTU143417
New.CleanUp.ReferenceOTU40997
New.CleanUp.ReferenceOTU54796
New.CleanUp.ReferenceOTU190534
New.CleanUp.ReferenceOTU95195
New.CleanUp.ReferenceOTU25008
New.CleanUp.ReferenceOTU32529
New.CleanUp.ReferenceOTU82630
New.CleanUp.ReferenceOTU14652
New.CleanUp.ReferenceOTU29926
New.CleanUp.ReferenceOTU52385
New.CleanUp.ReferenceOTU12113
New.CleanUp.ReferenceOTU49152
New.CleanUp.ReferenceOTU26433
New.CleanUp.ReferenceOTU22645
New.CleanUp.ReferenceOTU165427
New.CleanUp.ReferenceOTU20855
New.CleanUp.ReferenceOTU10052
New.CleanUp.ReferenceOTU59673
New.CleanUp.ReferenceOTU23006
New.CleanUp.ReferenceOTU24529
New.CleanUp.ReferenceOTU198049
New.CleanUp.ReferenceOTU9547
New.CleanUp.ReferenceOTU13883
New.CleanUp.ReferenceOTU19925
New.CleanUp.ReferenceOTU28260
New.CleanUp.ReferenceOTU39767
New.CleanUp.ReferenceOTU235824

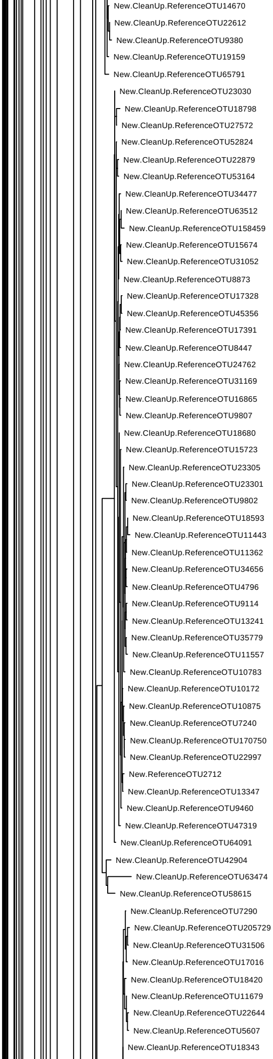
New.CleanUp.ReferenceOTU14670
New.CleanUp.ReferenceOTU22612
New.CleanUp.ReferenceOTU9380
New.CleanUp.ReferenceOTU19159
New.CleanUp.ReferenceOTU65791
New.CleanUp.ReferenceOTU23030
New.CleanUp.ReferenceOTU18798
New.CleanUp.ReferenceOTU27572
New.CleanUp.ReferenceOTU52824
New.CleanUp.ReferenceOTU22879
New.CleanUp.ReferenceOTU53164
New.CleanUp.ReferenceOTU34477
New.CleanUp.ReferenceOTU63512
New.CleanUp.ReferenceOTU158459
New.CleanUp.ReferenceOTU15674
New.CleanUp.ReferenceOTU31052
New.CleanUp.ReferenceOTU8873
New.CleanUp.ReferenceOTU17328
New.CleanUp.ReferenceOTU45356
New.CleanUp.ReferenceOTU17391
New.CleanUp.ReferenceOTU8447
New.CleanUp.ReferenceOTU24762
New.CleanUp.ReferenceOTU31169
New.CleanUp.ReferenceOTU16865
New.CleanUp.ReferenceOTU9807
New.CleanUp.ReferenceOTU18680
New.CleanUp.ReferenceOTU15723
New.CleanUp.ReferenceOTU23305
New.CleanUp.ReferenceOTU23301
New.CleanUp.ReferenceOTU9802
New.CleanUp.ReferenceOTU18593
New.CleanUp.ReferenceOTU11443
New.CleanUp.ReferenceOTU11362
New.CleanUp.ReferenceOTU34656
New.CleanUp.ReferenceOTU4796
New.CleanUp.ReferenceOTU9114
New.CleanUp.ReferenceOTU13241
New.CleanUp.ReferenceOTU35779
New.CleanUp.ReferenceOTU11557
New.CleanUp.ReferenceOTU10783
New.CleanUp.ReferenceOTU10172
New.CleanUp.ReferenceOTU10875
New.CleanUp.ReferenceOTU7240
New.CleanUp.ReferenceOTU170750
New.CleanUp.ReferenceOTU22997
New.ReferenceOTU2712
New.CleanUp.ReferenceOTU13347
New.CleanUp.ReferenceOTU9460
New.CleanUp.ReferenceOTU47319
New.CleanUp.ReferenceOTU64091
New.CleanUp.ReferenceOTU42904
New.CleanUp.ReferenceOTU63474
New.CleanUp.ReferenceOTU58615
New.CleanUp.ReferenceOTU7290
New.CleanUp.ReferenceOTU205729
New.CleanUp.ReferenceOTU31506
New.CleanUp.ReferenceOTU17016
New.CleanUp.ReferenceOTU18420
New.CleanUp.ReferenceOTU11679
New.CleanUp.ReferenceOTU22644
New.CleanUp.ReferenceOTU5607
New.CleanUp.ReferenceOTU18343

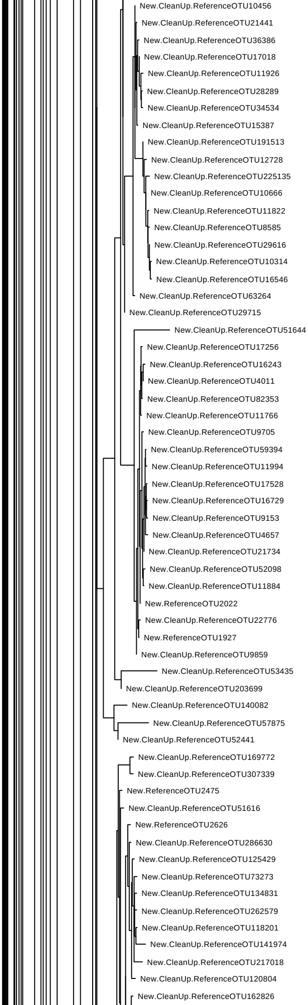
New.CleanUp.ReferenceOTU10456
New.CleanUp.ReferenceOTU21441
New.CleanUp.ReferenceOTU36386
New.CleanUp.ReferenceOTU17018
New.CleanUp.ReferenceOTU11926
New.CleanUp.ReferenceOTU28289
New.CleanUp.ReferenceOTU34534
New.CleanUp.ReferenceOTU15387
New.CleanUp.ReferenceOTU191513
New.CleanUp.ReferenceOTU12728
New.CleanUp.ReferenceOTU225135
New.CleanUp.ReferenceOTU10666
New.CleanUp.ReferenceOTU11822
New.CleanUp.ReferenceOTU8585
New.CleanUp.ReferenceOTU29616
New.CleanUp.ReferenceOTU10314
New.CleanUp.ReferenceOTU16546
New.CleanUp.ReferenceOTU63264
New.CleanUp.ReferenceOTU29715
New.CleanUp.ReferenceOTU51644
New.CleanUp.ReferenceOTU17256
New.CleanUp.ReferenceOTU16243
New.CleanUp.ReferenceOTU4011
New.CleanUp.ReferenceOTU82353
New.CleanUp.ReferenceOTU11766
New.CleanUp.ReferenceOTU9705
New.CleanUp.ReferenceOTU59394
New.CleanUp.ReferenceOTU11994
New.CleanUp.ReferenceOTU17528
New.CleanUp.ReferenceOTU16729
New.CleanUp.ReferenceOTU9153
New.CleanUp.ReferenceOTU4657
New.CleanUp.ReferenceOTU21734
New.CleanUp.ReferenceOTU52098
New.CleanUp.ReferenceOTU11884
New.ReferenceOTU2022
New.CleanUp.ReferenceOTU22776
New.ReferenceOTU1927
New.CleanUp.ReferenceOTU9859
New.CleanUp.ReferenceOTU53435
New.CleanUp.ReferenceOTU203699
New.CleanUp.ReferenceOTU140082
New.CleanUp.ReferenceOTU57875
New.CleanUp.ReferenceOTU52441
New.CleanUp.ReferenceOTU169772
New.CleanUp.ReferenceOTU307339
New.ReferenceOTU2475
New.CleanUp.ReferenceOTU51616
New.ReferenceOTU2626
New.CleanUp.ReferenceOTU286630
New.CleanUp.ReferenceOTU125429
New.CleanUp.ReferenceOTU73273
New.CleanUp.ReferenceOTU134831
New.CleanUp.ReferenceOTU262579
New.CleanUp.ReferenceOTU118201
New.CleanUp.ReferenceOTU141974
New.CleanUp.ReferenceOTU217018
New.CleanUp.ReferenceOTU120804
New.CleanUp.ReferenceOTU162826

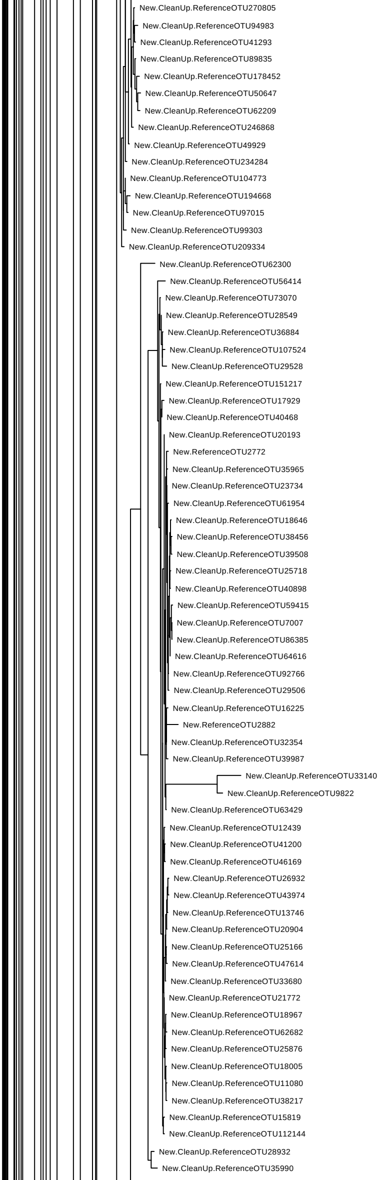
New.CleanUp.ReferenceOTU270805
New.CleanUp.ReferenceOTU94983
New.CleanUp.ReferenceOTU41293
New.CleanUp.ReferenceOTU89835
New.CleanUp.ReferenceOTU178452
New.CleanUp.ReferenceOTU50647
New.CleanUp.ReferenceOTU62209
New.CleanUp.ReferenceOTU246868
New.CleanUp.ReferenceOTU49929
New.CleanUp.ReferenceOTU234284
New.CleanUp.ReferenceOTU104773
New.CleanUp.ReferenceOTU194668
New.CleanUp.ReferenceOTU97015
New.CleanUp.ReferenceOTU99303
New.CleanUp.ReferenceOTU209334
New.CleanUp.ReferenceOTU62300
New.CleanUp.ReferenceOTU56414
New.CleanUp.ReferenceOTU73070
New.CleanUp.ReferenceOTU28549
New.CleanUp.ReferenceOTU36884
New.CleanUp.ReferenceOTU107524
New.CleanUp.ReferenceOTU29528
New.CleanUp.ReferenceOTU151217
New.CleanUp.ReferenceOTU17929
New.CleanUp.ReferenceOTU40468
New.CleanUp.ReferenceOTU20193
New.ReferenceOTU2772
New.CleanUp.ReferenceOTU35965
New.CleanUp.ReferenceOTU23734
New.CleanUp.ReferenceOTU61954
New.CleanUp.ReferenceOTU18646
New.CleanUp.ReferenceOTU38456
New.CleanUp.ReferenceOTU39508
New.CleanUp.ReferenceOTU25718
New.CleanUp.ReferenceOTU40898
New.CleanUp.ReferenceOTU59415
New.CleanUp.ReferenceOTU7007
New.CleanUp.ReferenceOTU86385
New.CleanUp.ReferenceOTU64616
New.CleanUp.ReferenceOTU92766
New.CleanUp.ReferenceOTU29506
New.CleanUp.ReferenceOTU16225
New.ReferenceOTU2882
New.CleanUp.ReferenceOTU32354
New.CleanUp.ReferenceOTU39987
New.CleanUp.ReferenceOTU33140
New.CleanUp.ReferenceOTU9822
New.CleanUp.ReferenceOTU63429
New.CleanUp.ReferenceOTU12439
New.CleanUp.ReferenceOTU41200
New.CleanUp.ReferenceOTU46169
New.CleanUp.ReferenceOTU26932
New.CleanUp.ReferenceOTU43974
New.CleanUp.ReferenceOTU13746
New.CleanUp.ReferenceOTU20904
New.CleanUp.ReferenceOTU25166
New.CleanUp.ReferenceOTU47614
New.CleanUp.ReferenceOTU33680
New.CleanUp.ReferenceOTU21772
New.CleanUp.ReferenceOTU18967
New.CleanUp.ReferenceOTU62682
New.CleanUp.ReferenceOTU25876
New.CleanUp.ReferenceOTU18005
New.CleanUp.ReferenceOTU11080
New.CleanUp.ReferenceOTU38217
New.CleanUp.ReferenceOTU15819
New.CleanUp.ReferenceOTU112144
New.CleanUp.ReferenceOTU28932
New.CleanUp.ReferenceOTU35990

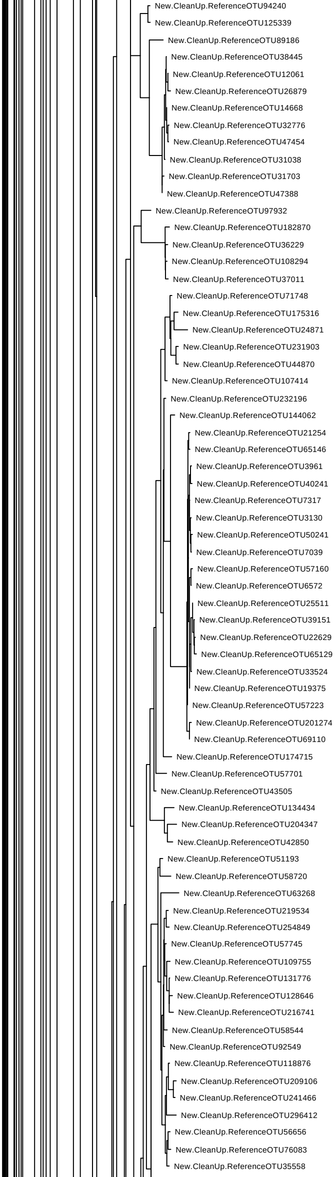
New.CleanUp.ReferenceOTU94240
New.CleanUp.ReferenceOTU125339
New.CleanUp.ReferenceOTU89186
New.CleanUp.ReferenceOTU38445
New.CleanUp.ReferenceOTU12061
New.CleanUp.ReferenceOTU26879
New.CleanUp.ReferenceOTU14668
New.CleanUp.ReferenceOTU32776
New.CleanUp.ReferenceOTU47454
New.CleanUp.ReferenceOTU31038
New.CleanUp.ReferenceOTU31703
New.CleanUp.ReferenceOTU47388
New.CleanUp.ReferenceOTU97932
New.CleanUp.ReferenceOTU182870
New.CleanUp.ReferenceOTU36229
New.CleanUp.ReferenceOTU108294
New.CleanUp.ReferenceOTU37011
New.CleanUp.ReferenceOTU71748
New.CleanUp.ReferenceOTU175316
New.CleanUp.ReferenceOTU24871
New.CleanUp.ReferenceOTU231903
New.CleanUp.ReferenceOTU44870
New.CleanUp.ReferenceOTU107414
New.CleanUp.ReferenceOTU232196
New.CleanUp.ReferenceOTU144062
New.CleanUp.ReferenceOTU21254
New.CleanUp.ReferenceOTU65146
New.CleanUp.ReferenceOTU3961
New.CleanUp.ReferenceOTU40241
New.CleanUp.ReferenceOTU7317
New.CleanUp.ReferenceOTU3130
New.CleanUp.ReferenceOTU50241
New.CleanUp.ReferenceOTU7039
New.CleanUp.ReferenceOTU57160
New.CleanUp.ReferenceOTU6572
New.CleanUp.ReferenceOTU25511
New.CleanUp.ReferenceOTU39151
New.CleanUp.ReferenceOTU22629
New.CleanUp.ReferenceOTU65129
New.CleanUp.ReferenceOTU33524
New.CleanUp.ReferenceOTU19375
New.CleanUp.ReferenceOTU57223
New.CleanUp.ReferenceOTU201274
New.CleanUp.ReferenceOTU69110
New.CleanUp.ReferenceOTU174715
New.CleanUp.ReferenceOTU57701
New.CleanUp.ReferenceOTU43505
New.CleanUp.ReferenceOTU134434
New.CleanUp.ReferenceOTU204347
New.CleanUp.ReferenceOTU42850
New.CleanUp.ReferenceOTU51193
New.CleanUp.ReferenceOTU58720
New.CleanUp.ReferenceOTU63268
New.CleanUp.ReferenceOTU219534
New.CleanUp.ReferenceOTU254849
New.CleanUp.ReferenceOTU57745
New.CleanUp.ReferenceOTU109755
New.CleanUp.ReferenceOTU131776
New.CleanUp.ReferenceOTU128646
New.CleanUp.ReferenceOTU216741
New.CleanUp.ReferenceOTU58544
New.CleanUp.ReferenceOTU92549
New.CleanUp.ReferenceOTU118876
New.CleanUp.ReferenceOTU209106
New.CleanUp.ReferenceOTU241466
New.CleanUp.ReferenceOTU296412
New.CleanUp.ReferenceOTU56656
New.CleanUp.ReferenceOTU76083
New.CleanUp.ReferenceOTU35558

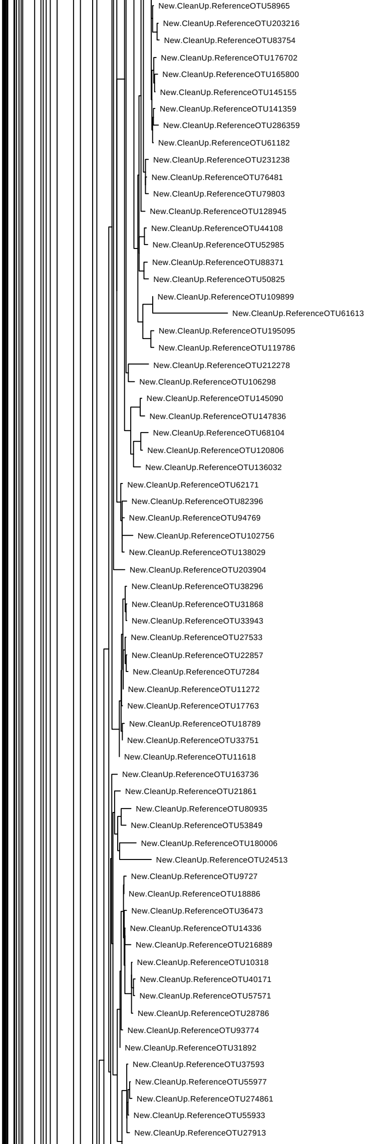
New.CleanUp.ReferenceOTU58965
New.CleanUp.ReferenceOTU203216
New.CleanUp.ReferenceOTU83754
New.CleanUp.ReferenceOTU176702
New.CleanUp.ReferenceOTU165800
New.CleanUp.ReferenceOTU145155
New.CleanUp.ReferenceOTU141359
New.CleanUp.ReferenceOTU286359
New.CleanUp.ReferenceOTU61182
New.CleanUp.ReferenceOTU231238
New.CleanUp.ReferenceOTU76481
New.CleanUp.ReferenceOTU79803
New.CleanUp.ReferenceOTU128945
New.CleanUp.ReferenceOTU44108
New.CleanUp.ReferenceOTU52985
New.CleanUp.ReferenceOTU88371
New.CleanUp.ReferenceOTU50825
New.CleanUp.ReferenceOTU109899
New.CleanUp.ReferenceOTU61613
New.CleanUp.ReferenceOTU195095
New.CleanUp.ReferenceOTU119786
New.CleanUp.ReferenceOTU212278
New.CleanUp.ReferenceOTU106298
New.CleanUp.ReferenceOTU145090
New.CleanUp.ReferenceOTU147836
New.CleanUp.ReferenceOTU68104
New.CleanUp.ReferenceOTU120806
New.CleanUp.ReferenceOTU136032
New.CleanUp.ReferenceOTU62171
New.CleanUp.ReferenceOTU82396
New.CleanUp.ReferenceOTU94769
New.CleanUp.ReferenceOTU102756
New.CleanUp.ReferenceOTU138029
New.CleanUp.ReferenceOTU203904
New.CleanUp.ReferenceOTU38296
New.CleanUp.ReferenceOTU31868
New.CleanUp.ReferenceOTU33943
New.CleanUp.ReferenceOTU27533
New.CleanUp.ReferenceOTU22857
New.CleanUp.ReferenceOTU7284
New.CleanUp.ReferenceOTU11272
New.CleanUp.ReferenceOTU17763
New.CleanUp.ReferenceOTU18789
New.CleanUp.ReferenceOTU33751
New.CleanUp.ReferenceOTU11618
New.CleanUp.ReferenceOTU163736
New.CleanUp.ReferenceOTU21861
New.CleanUp.ReferenceOTU80935
New.CleanUp.ReferenceOTU53849
New.CleanUp.ReferenceOTU180006
New.CleanUp.ReferenceOTU24513
New.CleanUp.ReferenceOTU9727
New.CleanUp.ReferenceOTU18886
New.CleanUp.ReferenceOTU36473
New.CleanUp.ReferenceOTU14336
New.CleanUp.ReferenceOTU216889
New.CleanUp.ReferenceOTU10318
New.CleanUp.ReferenceOTU40171
New.CleanUp.ReferenceOTU57571
New.CleanUp.ReferenceOTU28786
New.CleanUp.ReferenceOTU93774
New.CleanUp.ReferenceOTU31892
New.CleanUp.ReferenceOTU37593
New.CleanUp.ReferenceOTU55977
New.CleanUp.ReferenceOTU274861
New.CleanUp.ReferenceOTU55933
New.CleanUp.ReferenceOTU27913

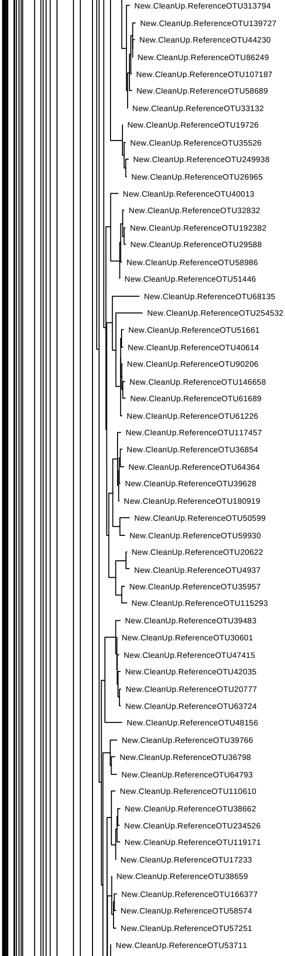
New.CleanUp.ReferenceOTU313794
New.CleanUp.ReferenceOTU139727
New.CleanUp.ReferenceOTU44230
New.CleanUp.ReferenceOTU86249
New.CleanUp.ReferenceOTU107187
New.CleanUp.ReferenceOTU58689
New.CleanUp.ReferenceOTU33132
New.CleanUp.ReferenceOTU19726
New.CleanUp.ReferenceOTU35526
New.CleanUp.ReferenceOTU249938
New.CleanUp.ReferenceOTU26965
New.CleanUp.ReferenceOTU40013
New.CleanUp.ReferenceOTU32832
New.CleanUp.ReferenceOTU192382
New.CleanUp.ReferenceOTU29588
New.CleanUp.ReferenceOTU58986
New.CleanUp.ReferenceOTU51446
New.CleanUp.ReferenceOTU68135
New.CleanUp.ReferenceOTU254532
New.CleanUp.ReferenceOTU51661
New.CleanUp.ReferenceOTU40614
New.CleanUp.ReferenceOTU90206
New.CleanUp.ReferenceOTU146658
New.CleanUp.ReferenceOTU61689
New.CleanUp.ReferenceOTU61226
New.CleanUp.ReferenceOTU117457
New.CleanUp.ReferenceOTU36854
New.CleanUp.ReferenceOTU64364
New.CleanUp.ReferenceOTU39628
New.CleanUp.ReferenceOTU180919
New.CleanUp.ReferenceOTU50599
New.CleanUp.ReferenceOTU59930
New.CleanUp.ReferenceOTU20622
New.CleanUp.ReferenceOTU4937
New.CleanUp.ReferenceOTU35957
New.CleanUp.ReferenceOTU115293
New.CleanUp.ReferenceOTU39483
New.CleanUp.ReferenceOTU30601
New.CleanUp.ReferenceOTU47415
New.CleanUp.ReferenceOTU42035
New.CleanUp.ReferenceOTU20777
New.CleanUp.ReferenceOTU63724
New.CleanUp.ReferenceOTU48156
New.CleanUp.ReferenceOTU39766
New.CleanUp.ReferenceOTU36798
New.CleanUp.ReferenceOTU64793
New.CleanUp.ReferenceOTU110610
New.CleanUp.ReferenceOTU38662
New.CleanUp.ReferenceOTU234526
New.CleanUp.ReferenceOTU119171
New.CleanUp.ReferenceOTU17233
New.CleanUp.ReferenceOTU38659
New.CleanUp.ReferenceOTU166377
New.CleanUp.ReferenceOTU58574
New.CleanUp.ReferenceOTU57251
New.CleanUp.ReferenceOTU53711

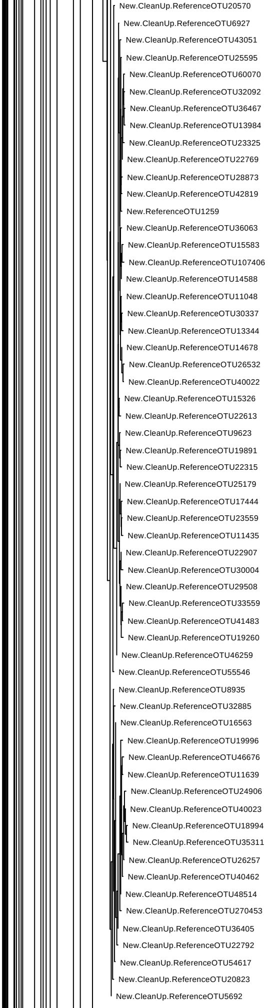
New.CleanUp.ReferenceOTU20570
New.CleanUp.ReferenceOTU6927
New.CleanUp.ReferenceOTU43051
New.CleanUp.ReferenceOTU25595
New.CleanUp.ReferenceOTU60070
New.CleanUp.ReferenceOTU32092
New.CleanUp.ReferenceOTU36467
New.CleanUp.ReferenceOTU13984
New.CleanUp.ReferenceOTU23325
New.CleanUp.ReferenceOTU22769
New.CleanUp.ReferenceOTU28873
New.CleanUp.ReferenceOTU42819
New.ReferenceOTU1259
New.CleanUp.ReferenceOTU36063
New.CleanUp.ReferenceOTU15583
New.CleanUp.ReferenceOTU107406
New.CleanUp.ReferenceOTU14588
New.CleanUp.ReferenceOTU11048
New.CleanUp.ReferenceOTU30337
New.CleanUp.ReferenceOTU13344
New.CleanUp.ReferenceOTU14678
New.CleanUp.ReferenceOTU26532
New.CleanUp.ReferenceOTU40022
New.CleanUp.ReferenceOTU15326
New.CleanUp.ReferenceOTU22613
New.CleanUp.ReferenceOTU9623
New.CleanUp.ReferenceOTU19891
New.CleanUp.ReferenceOTU22315
New.CleanUp.ReferenceOTU25179
New.CleanUp.ReferenceOTU17444
New.CleanUp.ReferenceOTU23559
New.CleanUp.ReferenceOTU11435
New.CleanUp.ReferenceOTU22907
New.CleanUp.ReferenceOTU30004
New.CleanUp.ReferenceOTU29508
New.CleanUp.ReferenceOTU33559
New.CleanUp.ReferenceOTU41483
New.CleanUp.ReferenceOTU19260
New.CleanUp.ReferenceOTU46259
New.CleanUp.ReferenceOTU55546
New.CleanUp.ReferenceOTU8935
New.CleanUp.ReferenceOTU32885
New.CleanUp.ReferenceOTU16563
New.CleanUp.ReferenceOTU19996
New.CleanUp.ReferenceOTU46676
New.CleanUp.ReferenceOTU11639
New.CleanUp.ReferenceOTU24906
New.CleanUp.ReferenceOTU40023
New.CleanUp.ReferenceOTU18994
New.CleanUp.ReferenceOTU35311
New.CleanUp.ReferenceOTU26257
New.CleanUp.ReferenceOTU40462
New.CleanUp.ReferenceOTU48514
New.CleanUp.ReferenceOTU270453
New.CleanUp.ReferenceOTU36405
New.CleanUp.ReferenceOTU22792
New.CleanUp.ReferenceOTU54617
New.CleanUp.ReferenceOTU20823
New.CleanUp.ReferenceOTU5692

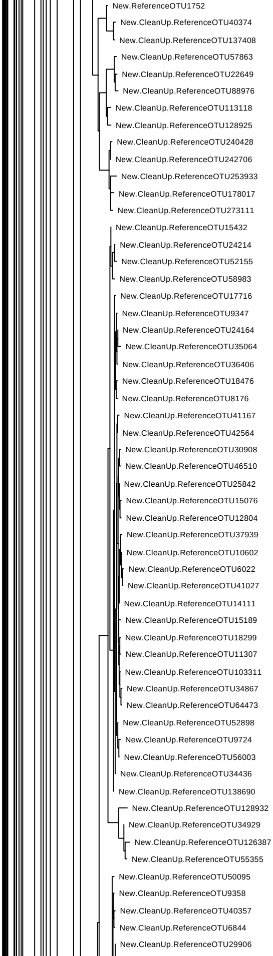
New.ReferenceOTU1752
New.CleanUp.ReferenceOTU40374
New.CleanUp.ReferenceOTU137408
New.CleanUp.ReferenceOTU57863
New.CleanUp.ReferenceOTU22649
New.CleanUp.ReferenceOTU88976
New.CleanUp.ReferenceOTU113118
New.CleanUp.ReferenceOTU128925
New.CleanUp.ReferenceOTU240428
New.CleanUp.ReferenceOTU242706
New.CleanUp.ReferenceOTU253933
New.CleanUp.ReferenceOTU178017
New.CleanUp.ReferenceOTU273111
New.CleanUp.ReferenceOTU15432
New.CleanUp.ReferenceOTU24214
New.CleanUp.ReferenceOTU52155
New.CleanUp.ReferenceOTU58983
New.CleanUp.ReferenceOTU17716
New.CleanUp.ReferenceOTU9347
New.CleanUp.ReferenceOTU24164
New.CleanUp.ReferenceOTU35064
New.CleanUp.ReferenceOTU36406
New.CleanUp.ReferenceOTU18476
New.CleanUp.ReferenceOTU8176
New.CleanUp.ReferenceOTU41167
New.CleanUp.ReferenceOTU42564
New.CleanUp.ReferenceOTU30908
New.CleanUp.ReferenceOTU46510
New.CleanUp.ReferenceOTU25842
New.CleanUp.ReferenceOTU15076
New.CleanUp.ReferenceOTU12804
New.CleanUp.ReferenceOTU37939
New.CleanUp.ReferenceOTU10602
New.CleanUp.ReferenceOTU6022
New.CleanUp.ReferenceOTU41027
New.CleanUp.ReferenceOTU14111
New.CleanUp.ReferenceOTU15189
New.CleanUp.ReferenceOTU18299
New.CleanUp.ReferenceOTU11307
New.CleanUp.ReferenceOTU103311
New.CleanUp.ReferenceOTU34867
New.CleanUp.ReferenceOTU64473
New.CleanUp.ReferenceOTU52898
New.CleanUp.ReferenceOTU9724
New.CleanUp.ReferenceOTU56003
New.CleanUp.ReferenceOTU34436
New.CleanUp.ReferenceOTU138690
New.CleanUp.ReferenceOTU128932
New.CleanUp.ReferenceOTU34929
New.CleanUp.ReferenceOTU126387
New.CleanUp.ReferenceOTU55355
New.CleanUp.ReferenceOTU50095
New.CleanUp.ReferenceOTU9358
New.CleanUp.ReferenceOTU40357
New.CleanUp.ReferenceOTU6844
New.CleanUp.ReferenceOTU29906

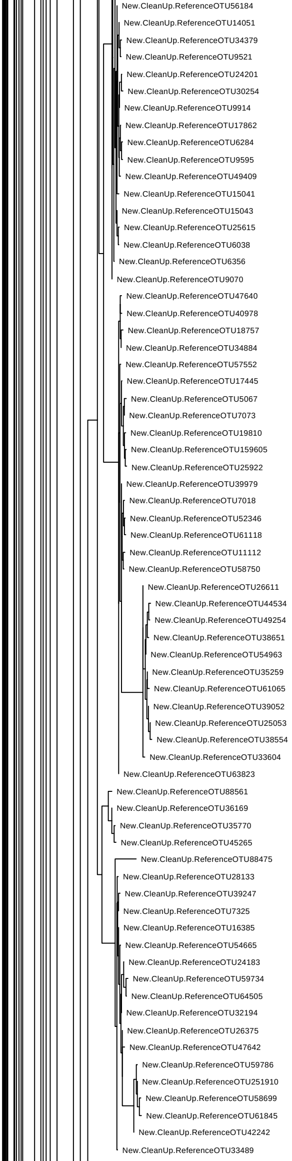
New.CleanUp.ReferenceOTU56184
New.CleanUp.ReferenceOTU14051
New.CleanUp.ReferenceOTU34379
New.CleanUp.ReferenceOTU9521
New.CleanUp.ReferenceOTU24201
New.CleanUp.ReferenceOTU30254
New.CleanUp.ReferenceOTU9914
New.CleanUp.ReferenceOTU17862
New.CleanUp.ReferenceOTU6284
New.CleanUp.ReferenceOTU9595
New.CleanUp.ReferenceOTU49409
New.CleanUp.ReferenceOTU15041
New.CleanUp.ReferenceOTU15043
New.CleanUp.ReferenceOTU25615
New.CleanUp.ReferenceOTU6038
New.CleanUp.ReferenceOTU6356
New.CleanUp.ReferenceOTU9070
New.CleanUp.ReferenceOTU47640
New.CleanUp.ReferenceOTU40978
New.CleanUp.ReferenceOTU18757
New.CleanUp.ReferenceOTU34884
New.CleanUp.ReferenceOTU57552
New.CleanUp.ReferenceOTU17445
New.CleanUp.ReferenceOTU5067
New.CleanUp.ReferenceOTU7073
New.CleanUp.ReferenceOTU19810
New.CleanUp.ReferenceOTU159605
New.CleanUp.ReferenceOTU25922
New.CleanUp.ReferenceOTU39979
New.CleanUp.ReferenceOTU7018
New.CleanUp.ReferenceOTU52346
New.CleanUp.ReferenceOTU61118
New.CleanUp.ReferenceOTU11112
New.CleanUp.ReferenceOTU58750
New.CleanUp.ReferenceOTU26611
New.CleanUp.ReferenceOTU44534
New.CleanUp.ReferenceOTU49254
New.CleanUp.ReferenceOTU38651
New.CleanUp.ReferenceOTU54963
New.CleanUp.ReferenceOTU35259
New.CleanUp.ReferenceOTU61065
New.CleanUp.ReferenceOTU39052
New.CleanUp.ReferenceOTU25053
New.CleanUp.ReferenceOTU38554
New.CleanUp.ReferenceOTU33604
New.CleanUp.ReferenceOTU63823
New.CleanUp.ReferenceOTU88561
New.CleanUp.ReferenceOTU36169
New.CleanUp.ReferenceOTU35770
New.CleanUp.ReferenceOTU45265
New.CleanUp.ReferenceOTU88475
New.CleanUp.ReferenceOTU28133
New.CleanUp.ReferenceOTU39247
New.CleanUp.ReferenceOTU7325
New.CleanUp.ReferenceOTU16385
New.CleanUp.ReferenceOTU54665
New.CleanUp.ReferenceOTU24183
New.CleanUp.ReferenceOTU59734
New.CleanUp.ReferenceOTU64505
New.CleanUp.ReferenceOTU32194
New.CleanUp.ReferenceOTU26375
New.CleanUp.ReferenceOTU47642
New.CleanUp.ReferenceOTU59786
New.CleanUp.ReferenceOTU251910
New.CleanUp.ReferenceOTU58699
New.CleanUp.ReferenceOTU61845
New.CleanUp.ReferenceOTU42242
New.CleanUp.ReferenceOTU33489

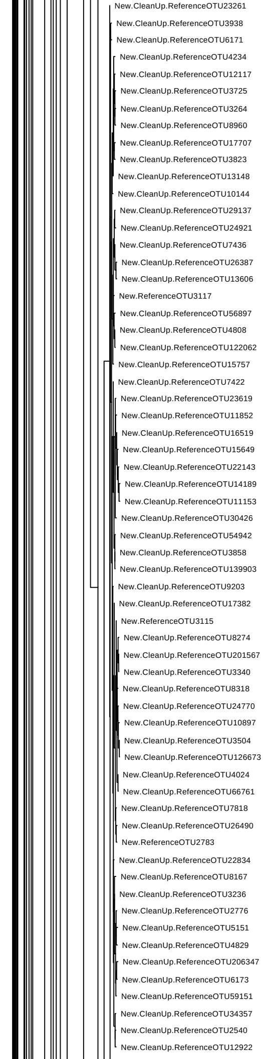
New.CleanUp.ReferenceOTU23261
New.CleanUp.ReferenceOTU3938
New.CleanUp.ReferenceOTU6171
New.CleanUp.ReferenceOTU4234
New.CleanUp.ReferenceOTU12117
New.CleanUp.ReferenceOTU3725
New.CleanUp.ReferenceOTU3264
New.CleanUp.ReferenceOTU8960
New.CleanUp.ReferenceOTU17707
New.CleanUp.ReferenceOTU3823
New.CleanUp.ReferenceOTU13148
New.CleanUp.ReferenceOTU10144
New.CleanUp.ReferenceOTU29137
New.CleanUp.ReferenceOTU24921
New.CleanUp.ReferenceOTU7436
New.CleanUp.ReferenceOTU26387
New.CleanUp.ReferenceOTU13606
New.ReferenceOTU3117
New.CleanUp.ReferenceOTU56897
New.CleanUp.ReferenceOTU4808
New.CleanUp.ReferenceOTU122062
New.CleanUp.ReferenceOTU15757
New.CleanUp.ReferenceOTU7422
New.CleanUp.ReferenceOTU23619
New.CleanUp.ReferenceOTU11852
New.CleanUp.ReferenceOTU16519
New.CleanUp.ReferenceOTU15649
New.CleanUp.ReferenceOTU22143
New.CleanUp.ReferenceOTU14189
New.CleanUp.ReferenceOTU11153
New.CleanUp.ReferenceOTU30426
New.CleanUp.ReferenceOTU54942
New.CleanUp.ReferenceOTU3858
New.CleanUp.ReferenceOTU139903
New.CleanUp.ReferenceOTU9203
New.CleanUp.ReferenceOTU17382
New.ReferenceOTU3115
New.CleanUp.ReferenceOTU8274
New.CleanUp.ReferenceOTU201567
New.CleanUp.ReferenceOTU3340
New.CleanUp.ReferenceOTU8318
New.CleanUp.ReferenceOTU24770
New.CleanUp.ReferenceOTU10897
New.CleanUp.ReferenceOTU3504
New.CleanUp.ReferenceOTU126673
New.CleanUp.ReferenceOTU4024
New.CleanUp.ReferenceOTU66761
New.CleanUp.ReferenceOTU7818
New.CleanUp.ReferenceOTU26490
New.ReferenceOTU2783
New.CleanUp.ReferenceOTU22834
New.CleanUp.ReferenceOTU8167
New.CleanUp.ReferenceOTU3236
New.CleanUp.ReferenceOTU2776
New.CleanUp.ReferenceOTU5151
New.CleanUp.ReferenceOTU4829
New.CleanUp.ReferenceOTU206347
New.CleanUp.ReferenceOTU6173
New.CleanUp.ReferenceOTU59151
New.CleanUp.ReferenceOTU34357
New.CleanUp.ReferenceOTU2540
New.CleanUp.ReferenceOTU12922

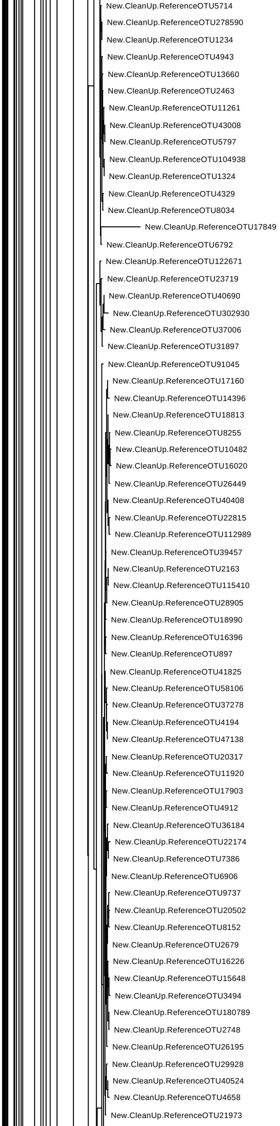
New.CleanUp.ReferenceOTU5714
New.CleanUp.ReferenceOTU278590
New.CleanUp.ReferenceOTU1234
New.CleanUp.ReferenceOTU4943
New.CleanUp.ReferenceOTU13660
New.CleanUp.ReferenceOTU2463
New.CleanUp.ReferenceOTU11261
New.CleanUp.ReferenceOTU43008
New.CleanUp.ReferenceOTU5797
New.CleanUp.ReferenceOTU104938
New.CleanUp.ReferenceOTU1324
New.CleanUp.ReferenceOTU4329
New.CleanUp.ReferenceOTU8034
New.CleanUp.ReferenceOTU17849
New.CleanUp.ReferenceOTU6792
New.CleanUp.ReferenceOTU122671
New.CleanUp.ReferenceOTU23719
New.CleanUp.ReferenceOTU40690
New.CleanUp.ReferenceOTU302930
New.CleanUp.ReferenceOTU37006
New.CleanUp.ReferenceOTU31897
New.CleanUp.ReferenceOTU91045
New.CleanUp.ReferenceOTU17160
New.CleanUp.ReferenceOTU14396
New.CleanUp.ReferenceOTU18813
New.CleanUp.ReferenceOTU8255
New.CleanUp.ReferenceOTU10482
New.CleanUp.ReferenceOTU16020
New.CleanUp.ReferenceOTU26449
New.CleanUp.ReferenceOTU40408
New.CleanUp.ReferenceOTU22815
New.CleanUp.ReferenceOTU112989
New.CleanUp.ReferenceOTU39457
New.CleanUp.ReferenceOTU2163
New.CleanUp.ReferenceOTU115410
New.CleanUp.ReferenceOTU28905
New.CleanUp.ReferenceOTU18990
New.CleanUp.ReferenceOTU16396
New.CleanUp.ReferenceOTU897
New.CleanUp.ReferenceOTU41825
New.CleanUp.ReferenceOTU58106
New.CleanUp.ReferenceOTU37278
New.CleanUp.ReferenceOTU4194
New.CleanUp.ReferenceOTU47138
New.CleanUp.ReferenceOTU20317
New.CleanUp.ReferenceOTU11920
New.CleanUp.ReferenceOTU17903
New.CleanUp.ReferenceOTU4912
New.CleanUp.ReferenceOTU36184
New.CleanUp.ReferenceOTU22174
New.CleanUp.ReferenceOTU7386
New.CleanUp.ReferenceOTU6906
New.CleanUp.ReferenceOTU9737
New.CleanUp.ReferenceOTU20502
New.CleanUp.ReferenceOTU8152
New.CleanUp.ReferenceOTU2679
New.CleanUp.ReferenceOTU16226
New.CleanUp.ReferenceOTU15648
New.CleanUp.ReferenceOTU3494
New.CleanUp.ReferenceOTU180789
New.CleanUp.ReferenceOTU2748
New.CleanUp.ReferenceOTU26195
New.CleanUp.ReferenceOTU29928
New.CleanUp.ReferenceOTU40524
New.CleanUp.ReferenceOTU4658
New.CleanUp.ReferenceOTU21973

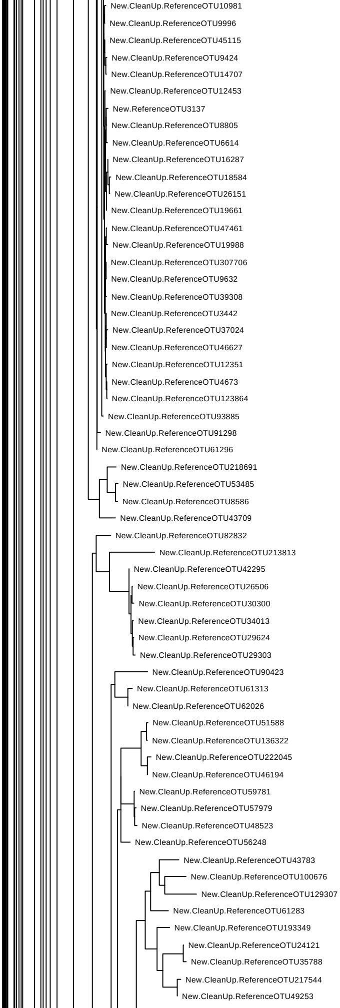
New.CleanUp.ReferenceOTU10981
New.CleanUp.ReferenceOTU9996
New.CleanUp.ReferenceOTU45115
New.CleanUp.ReferenceOTU9424
New.CleanUp.ReferenceOTU14707
New.CleanUp.ReferenceOTU12453
New.ReferenceOTU3137
New.CleanUp.ReferenceOTU8805
New.CleanUp.ReferenceOTU6614
New.CleanUp.ReferenceOTU16287
New.CleanUp.ReferenceOTU18584
New.CleanUp.ReferenceOTU26151
New.CleanUp.ReferenceOTU19661
New.CleanUp.ReferenceOTU47461
New.CleanUp.ReferenceOTU19988
New.CleanUp.ReferenceOTU307706
New.CleanUp.ReferenceOTU9632
New.CleanUp.ReferenceOTU39308
New.CleanUp.ReferenceOTU3442
New.CleanUp.ReferenceOTU37024
New.CleanUp.ReferenceOTU46627
New.CleanUp.ReferenceOTU12351
New.CleanUp.ReferenceOTU4673
New.CleanUp.ReferenceOTU123864
New.CleanUp.ReferenceOTU93885
New.CleanUp.ReferenceOTU91298
New.CleanUp.ReferenceOTU61296
New.CleanUp.ReferenceOTU218691
New.CleanUp.ReferenceOTU53485
New.CleanUp.ReferenceOTU8586
New.CleanUp.ReferenceOTU43709
New.CleanUp.ReferenceOTU82832
New.CleanUp.ReferenceOTU213813
New.CleanUp.ReferenceOTU42295
New.CleanUp.ReferenceOTU26506
New.CleanUp.ReferenceOTU30300
New.CleanUp.ReferenceOTU34013
New.CleanUp.ReferenceOTU29624
New.CleanUp.ReferenceOTU29303
New.CleanUp.ReferenceOTU90423
New.CleanUp.ReferenceOTU61313
New.CleanUp.ReferenceOTU62026
New.CleanUp.ReferenceOTU51588
New.CleanUp.ReferenceOTU136322
New.CleanUp.ReferenceOTU222045
New.CleanUp.ReferenceOTU46194
New.CleanUp.ReferenceOTU59781
New.CleanUp.ReferenceOTU57979
New.CleanUp.ReferenceOTU48523
New.CleanUp.ReferenceOTU56248
New.CleanUp.ReferenceOTU43783
New.CleanUp.ReferenceOTU100676
New.CleanUp.ReferenceOTU129307
New.CleanUp.ReferenceOTU61283
New.CleanUp.ReferenceOTU193349
New.CleanUp.ReferenceOTU24121
New.CleanUp.ReferenceOTU35788
New.CleanUp.ReferenceOTU217544
New.CleanUp.ReferenceOTU49253

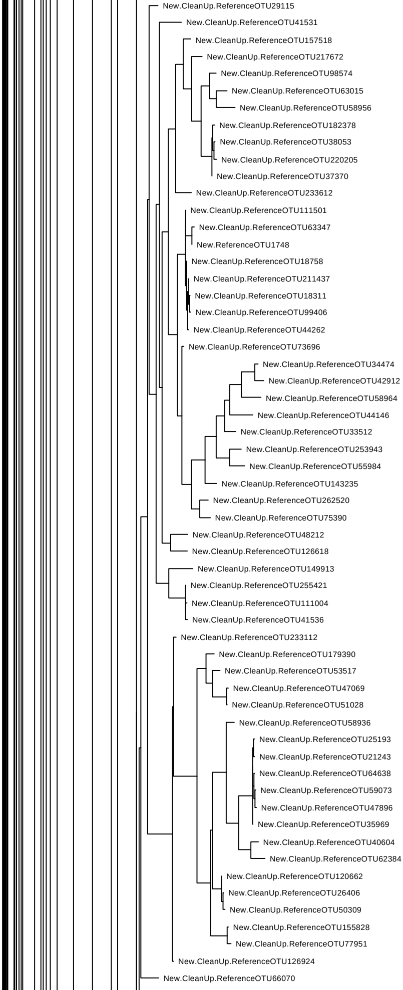
New.CleanUp.ReferenceOTU29115
New.CleanUp.ReferenceOTU41531
New.CleanUp.ReferenceOTU157518
New.CleanUp.ReferenceOTU217672
New.CleanUp.ReferenceOTU98574
New.CleanUp.ReferenceOTU63015
New.CleanUp.ReferenceOTU58956
New.CleanUp.ReferenceOTU182378
New.CleanUp.ReferenceOTU38053
New.CleanUp.ReferenceOTU220205
New.CleanUp.ReferenceOTU37370
New.CleanUp.ReferenceOTU233612
New.CleanUp.ReferenceOTU111501
New.CleanUp.ReferenceOTU63347
New.ReferenceOTU1748
New.CleanUp.ReferenceOTU18758
New.CleanUp.ReferenceOTU211437
New.CleanUp.ReferenceOTU18311
New.CleanUp.ReferenceOTU99406
New.CleanUp.ReferenceOTU44262
New.CleanUp.ReferenceOTU73696
New.CleanUp.ReferenceOTU34474
New.CleanUp.ReferenceOTU42912
New.CleanUp.ReferenceOTU58964
New.CleanUp.ReferenceOTU44146
New.CleanUp.ReferenceOTU33512
New.CleanUp.ReferenceOTU253943
New.CleanUp.ReferenceOTU55984
New.CleanUp.ReferenceOTU143235
New.CleanUp.ReferenceOTU262520
New.CleanUp.ReferenceOTU75390
New.CleanUp.ReferenceOTU48212
New.CleanUp.ReferenceOTU126618
New.CleanUp.ReferenceOTU149913
New.CleanUp.ReferenceOTU255421
New.CleanUp.ReferenceOTU111004
New.CleanUp.ReferenceOTU41536
New.CleanUp.ReferenceOTU233112
New.CleanUp.ReferenceOTU179390
New.CleanUp.ReferenceOTU53517
New.CleanUp.ReferenceOTU47069
New.CleanUp.ReferenceOTU51028
New.CleanUp.ReferenceOTU58936
New.CleanUp.ReferenceOTU25193
New.CleanUp.ReferenceOTU21243
New.CleanUp.ReferenceOTU64638
New.CleanUp.ReferenceOTU59073
New.CleanUp.ReferenceOTU47896
New.CleanUp.ReferenceOTU35969
New.CleanUp.ReferenceOTU40604
New.CleanUp.ReferenceOTU62384
New.CleanUp.ReferenceOTU120662
New.CleanUp.ReferenceOTU26406
New.CleanUp.ReferenceOTU50309
New.CleanUp.ReferenceOTU155828
New.CleanUp.ReferenceOTU77951
New.CleanUp.ReferenceOTU126924
New.CleanUp.ReferenceOTU66070

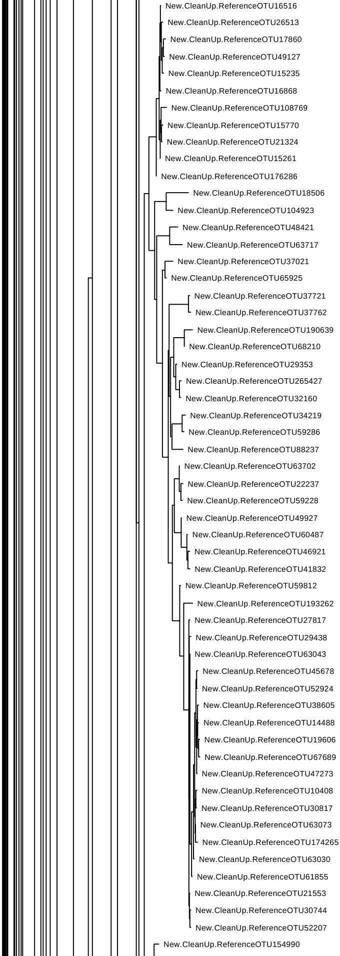
New.CleanUp.ReferenceOTU16516
New.CleanUp.ReferenceOTU26513
New.CleanUp.ReferenceOTU17860
New.CleanUp.ReferenceOTU49127
New.CleanUp.ReferenceOTU15235
New.CleanUp.ReferenceOTU16868
New.CleanUp.ReferenceOTU108769
New.CleanUp.ReferenceOTU15770
New.CleanUp.ReferenceOTU21324
New.CleanUp.ReferenceOTU15261
New.CleanUp.ReferenceOTU176286
New.CleanUp.ReferenceOTU18506
New.CleanUp.ReferenceOTU104923
New.CleanUp.ReferenceOTU48421
New.CleanUp.ReferenceOTU63717
New.CleanUp.ReferenceOTU37021
New.CleanUp.ReferenceOTU65925
New.CleanUp.ReferenceOTU37721
New.CleanUp.ReferenceOTU37762
New.CleanUp.ReferenceOTU190639
New.CleanUp.ReferenceOTU68210
New.CleanUp.ReferenceOTU29353
New.CleanUp.ReferenceOTU265427
New.CleanUp.ReferenceOTU32160
New.CleanUp.ReferenceOTU34219
New.CleanUp.ReferenceOTU59286
New.CleanUp.ReferenceOTU88237
New.CleanUp.ReferenceOTU63702
New.CleanUp.ReferenceOTU22237
New.CleanUp.ReferenceOTU59228
New.CleanUp.ReferenceOTU49927
New.CleanUp.ReferenceOTU60487
New.CleanUp.ReferenceOTU46921
New.CleanUp.ReferenceOTU41832
New.CleanUp.ReferenceOTU59812
New.CleanUp.ReferenceOTU193262
New.CleanUp.ReferenceOTU27817
New.CleanUp.ReferenceOTU29438
New.CleanUp.ReferenceOTU63043
New.CleanUp.ReferenceOTU45678
New.CleanUp.ReferenceOTU52924
New.CleanUp.ReferenceOTU38605
New.CleanUp.ReferenceOTU14488
New.CleanUp.ReferenceOTU19606
New.CleanUp.ReferenceOTU67689
New.CleanUp.ReferenceOTU47273
New.CleanUp.ReferenceOTU10408
New.CleanUp.ReferenceOTU30817
New.CleanUp.ReferenceOTU63073
New.CleanUp.ReferenceOTU174265
New.CleanUp.ReferenceOTU63030
New.CleanUp.ReferenceOTU61855
New.CleanUp.ReferenceOTU21553
New.CleanUp.ReferenceOTU30744
New.CleanUp.ReferenceOTU52207
New.CleanUp.ReferenceOTU154990

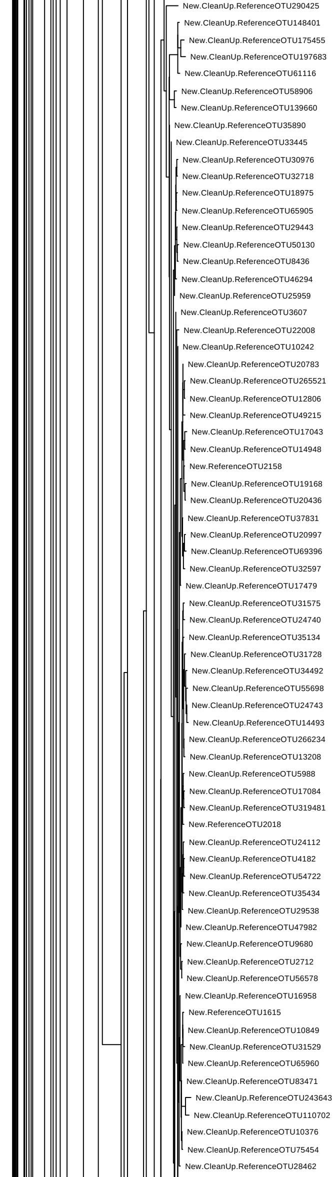
New.CleanUp.ReferenceOTU290425
New.CleanUp.ReferenceOTU148401
New.CleanUp.ReferenceOTU175455
New.CleanUp.ReferenceOTU197683
New.CleanUp.ReferenceOTU61116
New.CleanUp.ReferenceOTU58906
New.CleanUp.ReferenceOTU139660
New.CleanUp.ReferenceOTU35890
New.CleanUp.ReferenceOTU33445
New.CleanUp.ReferenceOTU30976
New.CleanUp.ReferenceOTU32718
New.CleanUp.ReferenceOTU18975
New.CleanUp.ReferenceOTU65905
New.CleanUp.ReferenceOTU29443
New.CleanUp.ReferenceOTU50130
New.CleanUp.ReferenceOTU8436
New.CleanUp.ReferenceOTU46294
New.CleanUp.ReferenceOTU25959
New.CleanUp.ReferenceOTU3607
New.CleanUp.ReferenceOTU22008
New.CleanUp.ReferenceOTU10242
New.CleanUp.ReferenceOTU20783
New.CleanUp.ReferenceOTU265521
New.CleanUp.ReferenceOTU12806
New.CleanUp.ReferenceOTU49215
New.CleanUp.ReferenceOTU17043
New.CleanUp.ReferenceOTU14948
New.ReferenceOTU2158
New.CleanUp.ReferenceOTU19168
New.CleanUp.ReferenceOTU20436
New.CleanUp.ReferenceOTU37831
New.CleanUp.ReferenceOTU20997
New.CleanUp.ReferenceOTU69396
New.CleanUp.ReferenceOTU32597
New.CleanUp.ReferenceOTU17479
New.CleanUp.ReferenceOTU31575
New.CleanUp.ReferenceOTU24740
New.CleanUp.ReferenceOTU35134
New.CleanUp.ReferenceOTU31728
New.CleanUp.ReferenceOTU34492
New.CleanUp.ReferenceOTU55698
New.CleanUp.ReferenceOTU24743
New.CleanUp.ReferenceOTU14493
New.CleanUp.ReferenceOTU266234
New.CleanUp.ReferenceOTU13208
New.CleanUp.ReferenceOTU5988
New.CleanUp.ReferenceOTU17084
New.CleanUp.ReferenceOTU319481
New.ReferenceOTU2018
New.CleanUp.ReferenceOTU24112
New.CleanUp.ReferenceOTU4182
New.CleanUp.ReferenceOTU54722
New.CleanUp.ReferenceOTU35434
New.CleanUp.ReferenceOTU29538
New.CleanUp.ReferenceOTU47982
New.CleanUp.ReferenceOTU9680
New.CleanUp.ReferenceOTU2712
New.CleanUp.ReferenceOTU56578
New.CleanUp.ReferenceOTU16958
New.ReferenceOTU1615
New.CleanUp.ReferenceOTU10849
New.CleanUp.ReferenceOTU31529
New.CleanUp.ReferenceOTU65960
New.CleanUp.ReferenceOTU83471
New.CleanUp.ReferenceOTU243643
New.CleanUp.ReferenceOTU110702
New.CleanUp.ReferenceOTU10376
New.CleanUp.ReferenceOTU75454
New.CleanUp.ReferenceOTU28462

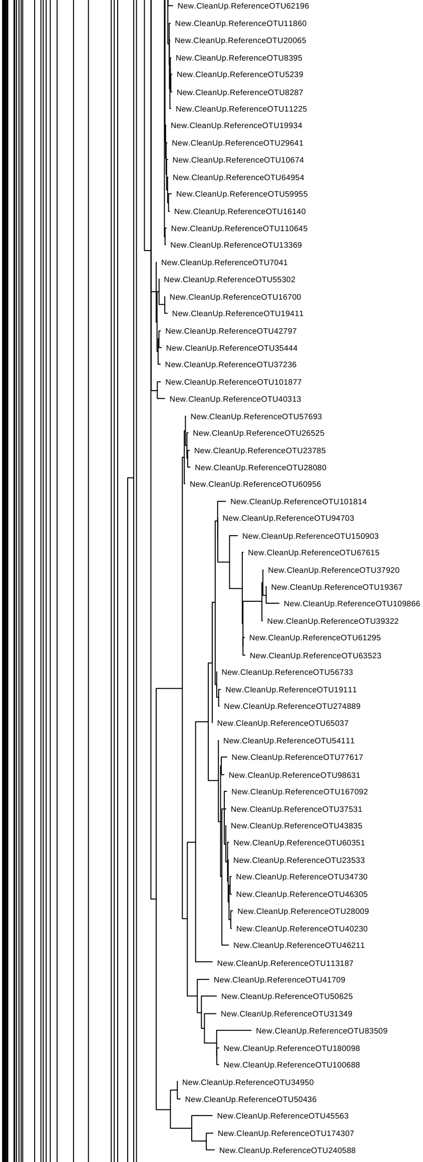
New.CleanUp.ReferenceOTU62196
New.CleanUp.ReferenceOTU11860
New.CleanUp.ReferenceOTU20065
New.CleanUp.ReferenceOTU8395
New.CleanUp.ReferenceOTU5239
New.CleanUp.ReferenceOTU8287
New.CleanUp.ReferenceOTU11225
New.CleanUp.ReferenceOTU19934
New.CleanUp.ReferenceOTU29641
New.CleanUp.ReferenceOTU10674
New.CleanUp.ReferenceOTU64954
New.CleanUp.ReferenceOTU59955
New.CleanUp.ReferenceOTU16140
New.CleanUp.ReferenceOTU110645
New.CleanUp.ReferenceOTU13369
New.CleanUp.ReferenceOTU7041
New.CleanUp.ReferenceOTU55302
New.CleanUp.ReferenceOTU16700
New.CleanUp.ReferenceOTU19411
New.CleanUp.ReferenceOTU42797
New.CleanUp.ReferenceOTU35444
New.CleanUp.ReferenceOTU37236
New.CleanUp.ReferenceOTU101877
New.CleanUp.ReferenceOTU40313
New.CleanUp.ReferenceOTU57693
New.CleanUp.ReferenceOTU26525
New.CleanUp.ReferenceOTU23785
New.CleanUp.ReferenceOTU28080
New.CleanUp.ReferenceOTU60956
New.CleanUp.ReferenceOTU101814
New.CleanUp.ReferenceOTU94703
New.CleanUp.ReferenceOTU150903
New.CleanUp.ReferenceOTU67615
New.CleanUp.ReferenceOTU37920
New.CleanUp.ReferenceOTU19367
New.CleanUp.ReferenceOTU109866
New.CleanUp.ReferenceOTU39322
New.CleanUp.ReferenceOTU61295
New.CleanUp.ReferenceOTU63523
New.CleanUp.ReferenceOTU56733
New.CleanUp.ReferenceOTU19111
New.CleanUp.ReferenceOTU274889
New.CleanUp.ReferenceOTU65037
New.CleanUp.ReferenceOTU54111
New.CleanUp.ReferenceOTU77617
New.CleanUp.ReferenceOTU98631
New.CleanUp.ReferenceOTU167092
New.CleanUp.ReferenceOTU37531
New.CleanUp.ReferenceOTU43835
New.CleanUp.ReferenceOTU60351
New.CleanUp.ReferenceOTU23533
New.CleanUp.ReferenceOTU34730
New.CleanUp.ReferenceOTU46305
New.CleanUp.ReferenceOTU28009
New.CleanUp.ReferenceOTU40230
New.CleanUp.ReferenceOTU46211
New.CleanUp.ReferenceOTU113187
New.CleanUp.ReferenceOTU41709
New.CleanUp.ReferenceOTU50625
New.CleanUp.ReferenceOTU31349
New.CleanUp.ReferenceOTU83509
New.CleanUp.ReferenceOTU180098
New.CleanUp.ReferenceOTU100688
New.CleanUp.ReferenceOTU34950
New.CleanUp.ReferenceOTU50436
New.CleanUp.ReferenceOTU45563
New.CleanUp.ReferenceOTU174307
New.CleanUp.ReferenceOTU240588

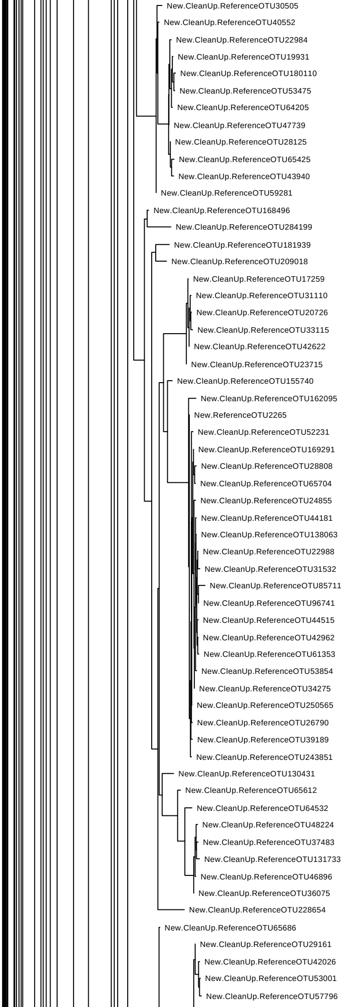
New.CleanUp.ReferenceOTU30505
New.CleanUp.ReferenceOTU40552
New.CleanUp.ReferenceOTU22984
New.CleanUp.ReferenceOTU19931
New.CleanUp.ReferenceOTU180110
New.CleanUp.ReferenceOTU53475
New.CleanUp.ReferenceOTU64205
New.CleanUp.ReferenceOTU47739
New.CleanUp.ReferenceOTU28125
New.CleanUp.ReferenceOTU65425
New.CleanUp.ReferenceOTU43940
New.CleanUp.ReferenceOTU59281
New.CleanUp.ReferenceOTU168496
New.CleanUp.ReferenceOTU284199
New.CleanUp.ReferenceOTU181939
New.CleanUp.ReferenceOTU209018
New.CleanUp.ReferenceOTU17259
New.CleanUp.ReferenceOTU31110
New.CleanUp.ReferenceOTU20726
New.CleanUp.ReferenceOTU33115
New.CleanUp.ReferenceOTU42622
New.CleanUp.ReferenceOTU23715
New.CleanUp.ReferenceOTU155740
New.CleanUp.ReferenceOTU162095
New.ReferenceOTU2265
New.CleanUp.ReferenceOTU52231
New.CleanUp.ReferenceOTU169291
New.CleanUp.ReferenceOTU28808
New.CleanUp.ReferenceOTU65704
New.CleanUp.ReferenceOTU24855
New.CleanUp.ReferenceOTU44181
New.CleanUp.ReferenceOTU138063
New.CleanUp.ReferenceOTU22988
New.CleanUp.ReferenceOTU31532
New.CleanUp.ReferenceOTU85711
New.CleanUp.ReferenceOTU96741
New.CleanUp.ReferenceOTU44515
New.CleanUp.ReferenceOTU42962
New.CleanUp.ReferenceOTU61353
New.CleanUp.ReferenceOTU53854
New.CleanUp.ReferenceOTU34275
New.CleanUp.ReferenceOTU250565
New.CleanUp.ReferenceOTU26790
New.CleanUp.ReferenceOTU39189
New.CleanUp.ReferenceOTU243851
New.CleanUp.ReferenceOTU130431
New.CleanUp.ReferenceOTU65612
New.CleanUp.ReferenceOTU64532
New.CleanUp.ReferenceOTU48224
New.CleanUp.ReferenceOTU37483
New.CleanUp.ReferenceOTU131733
New.CleanUp.ReferenceOTU46896
New.CleanUp.ReferenceOTU36075
New.CleanUp.ReferenceOTU228654
New.CleanUp.ReferenceOTU65686
New.CleanUp.ReferenceOTU29161
New.CleanUp.ReferenceOTU42026
New.CleanUp.ReferenceOTU53001
New.CleanUp.ReferenceOTU57796

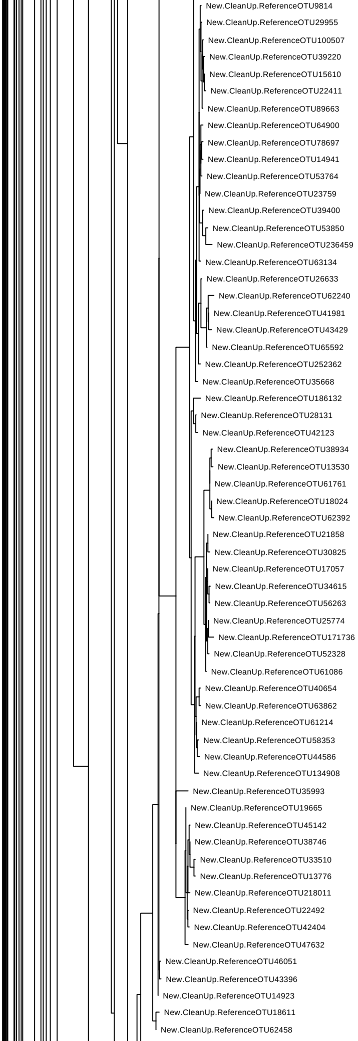
New.CleanUp.ReferenceOTU9814
New.CleanUp.ReferenceOTU29955
New.CleanUp.ReferenceOTU100507
New.CleanUp.ReferenceOTU39220
New.CleanUp.ReferenceOTU15610
New.CleanUp.ReferenceOTU22411
New.CleanUp.ReferenceOTU89663
New.CleanUp.ReferenceOTU64900
New.CleanUp.ReferenceOTU78697
New.CleanUp.ReferenceOTU14941
New.CleanUp.ReferenceOTU53764
New.CleanUp.ReferenceOTU23759
New.CleanUp.ReferenceOTU39400
New.CleanUp.ReferenceOTU53850
New.CleanUp.ReferenceOTU236459
New.CleanUp.ReferenceOTU63134
New.CleanUp.ReferenceOTU26633
New.CleanUp.ReferenceOTU62240
New.CleanUp.ReferenceOTU41981
New.CleanUp.ReferenceOTU43429
New.CleanUp.ReferenceOTU65592
New.CleanUp.ReferenceOTU252362
New.CleanUp.ReferenceOTU35668
New.CleanUp.ReferenceOTU186132
New.CleanUp.ReferenceOTU28131
New.CleanUp.ReferenceOTU42123
New.CleanUp.ReferenceOTU38934
New.CleanUp.ReferenceOTU13530
New.CleanUp.ReferenceOTU61761
New.CleanUp.ReferenceOTU18024
New.CleanUp.ReferenceOTU62392
New.CleanUp.ReferenceOTU21858
New.CleanUp.ReferenceOTU30825
New.CleanUp.ReferenceOTU17057
New.CleanUp.ReferenceOTU34615
New.CleanUp.ReferenceOTU56263
New.CleanUp.ReferenceOTU25774
New.CleanUp.ReferenceOTU171736
New.CleanUp.ReferenceOTU52328
New.CleanUp.ReferenceOTU61086
New.CleanUp.ReferenceOTU40654
New.CleanUp.ReferenceOTU63862
New.CleanUp.ReferenceOTU61214
New.CleanUp.ReferenceOTU58353
New.CleanUp.ReferenceOTU44586
New.CleanUp.ReferenceOTU134908
New.CleanUp.ReferenceOTU35993
New.CleanUp.ReferenceOTU19665
New.CleanUp.ReferenceOTU45142
New.CleanUp.ReferenceOTU38746
New.CleanUp.ReferenceOTU33510
New.CleanUp.ReferenceOTU13776
New.CleanUp.ReferenceOTU218011
New.CleanUp.ReferenceOTU22492
New.CleanUp.ReferenceOTU42404
New.CleanUp.ReferenceOTU47632
New.CleanUp.ReferenceOTU46051
New.CleanUp.ReferenceOTU43396
New.CleanUp.ReferenceOTU14923
New.CleanUp.ReferenceOTU18611
New.CleanUp.ReferenceOTU62458

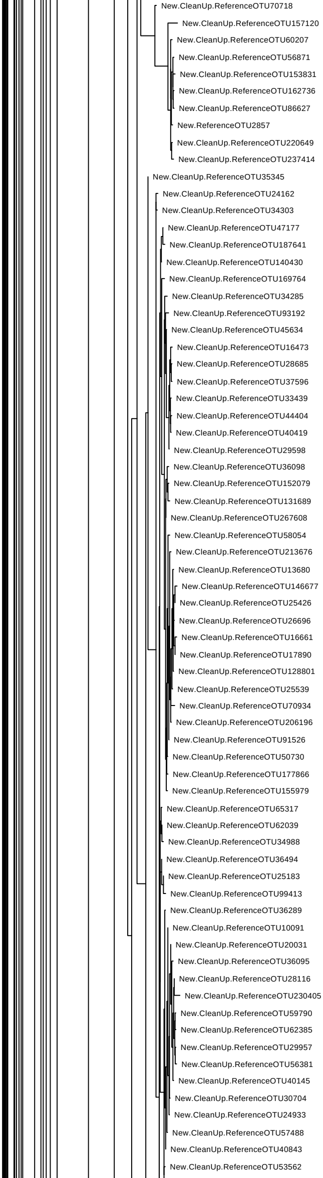
New.CleanUp.ReferenceOTU70718
New.CleanUp.ReferenceOTU157120
New.CleanUp.ReferenceOTU60207
New.CleanUp.ReferenceOTU56871
New.CleanUp.ReferenceOTU153831
New.CleanUp.ReferenceOTU162736
New.CleanUp.ReferenceOTU86627
New.ReferenceOTU2857
New.CleanUp.ReferenceOTU220649
New.CleanUp.ReferenceOTU237414
New.CleanUp.ReferenceOTU35345
New.CleanUp.ReferenceOTU24162
New.CleanUp.ReferenceOTU34303
New.CleanUp.ReferenceOTU47177
New.CleanUp.ReferenceOTU187641
New.CleanUp.ReferenceOTU140430
New.CleanUp.ReferenceOTU169764
New.CleanUp.ReferenceOTU34285
New.CleanUp.ReferenceOTU93192
New.CleanUp.ReferenceOTU45634
New.CleanUp.ReferenceOTU16473
New.CleanUp.ReferenceOTU28685
New.CleanUp.ReferenceOTU37596
New.CleanUp.ReferenceOTU33439
New.CleanUp.ReferenceOTU44404
New.CleanUp.ReferenceOTU40419
New.CleanUp.ReferenceOTU29598
New.CleanUp.ReferenceOTU36098
New.CleanUp.ReferenceOTU152079
New.CleanUp.ReferenceOTU131689
New.CleanUp.ReferenceOTU267608
New.CleanUp.ReferenceOTU58054
New.CleanUp.ReferenceOTU213676
New.CleanUp.ReferenceOTU13680
New.CleanUp.ReferenceOTU146677
New.CleanUp.ReferenceOTU25426
New.CleanUp.ReferenceOTU26696
New.CleanUp.ReferenceOTU16661
New.CleanUp.ReferenceOTU17890
New.CleanUp.ReferenceOTU128801
New.CleanUp.ReferenceOTU25539
New.CleanUp.ReferenceOTU70934
New.CleanUp.ReferenceOTU206196
New.CleanUp.ReferenceOTU91526
New.CleanUp.ReferenceOTU50730
New.CleanUp.ReferenceOTU177866
New.CleanUp.ReferenceOTU155979
New.CleanUp.ReferenceOTU65317
New.CleanUp.ReferenceOTU62039
New.CleanUp.ReferenceOTU34988
New.CleanUp.ReferenceOTU36494
New.CleanUp.ReferenceOTU25183
New.CleanUp.ReferenceOTU99413
New.CleanUp.ReferenceOTU36289
New.CleanUp.ReferenceOTU10091
New.CleanUp.ReferenceOTU20031
New.CleanUp.ReferenceOTU36095
New.CleanUp.ReferenceOTU28116
New.CleanUp.ReferenceOTU230405
New.CleanUp.ReferenceOTU59790
New.CleanUp.ReferenceOTU62385
New.CleanUp.ReferenceOTU29957
New.CleanUp.ReferenceOTU56381
New.CleanUp.ReferenceOTU40145
New.CleanUp.ReferenceOTU30704
New.CleanUp.ReferenceOTU24933
New.CleanUp.ReferenceOTU57488
New.CleanUp.ReferenceOTU40843
New.CleanUp.ReferenceOTU53562

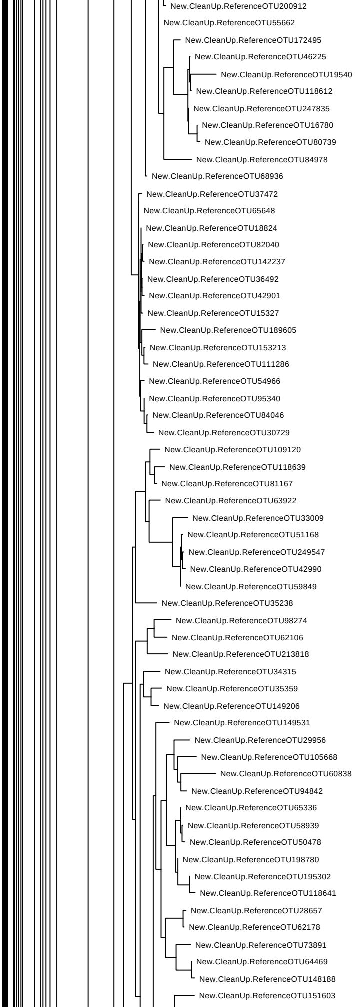
New.CleanUp.ReferenceOTU200912
New.CleanUp.ReferenceOTU55662
New.CleanUp.ReferenceOTU172495
New.CleanUp.ReferenceOTU46225
New.CleanUp.ReferenceOTU19540
New.CleanUp.ReferenceOTU118612
New.CleanUp.ReferenceOTU247835
New.CleanUp.ReferenceOTU16780
New.CleanUp.ReferenceOTU80739
New.CleanUp.ReferenceOTU84978
New.CleanUp.ReferenceOTU68936
New.CleanUp.ReferenceOTU37472
New.CleanUp.ReferenceOTU65648
New.CleanUp.ReferenceOTU18824
New.CleanUp.ReferenceOTU82040
New.CleanUp.ReferenceOTU142237
New.CleanUp.ReferenceOTU36492
New.CleanUp.ReferenceOTU42901
New.CleanUp.ReferenceOTU15327
New.CleanUp.ReferenceOTU189605
New.CleanUp.ReferenceOTU153213
New.CleanUp.ReferenceOTU111286
New.CleanUp.ReferenceOTU54966
New.CleanUp.ReferenceOTU95340
New.CleanUp.ReferenceOTU84046
New.CleanUp.ReferenceOTU30729
New.CleanUp.ReferenceOTU109120
New.CleanUp.ReferenceOTU118639
New.CleanUp.ReferenceOTU81167
New.CleanUp.ReferenceOTU63922
New.CleanUp.ReferenceOTU33009
New.CleanUp.ReferenceOTU51168
New.CleanUp.ReferenceOTU249547
New.CleanUp.ReferenceOTU42990
New.CleanUp.ReferenceOTU59849
New.CleanUp.ReferenceOTU35238
New.CleanUp.ReferenceOTU98274
New.CleanUp.ReferenceOTU62106
New.CleanUp.ReferenceOTU213818
New.CleanUp.ReferenceOTU34315
New.CleanUp.ReferenceOTU35359
New.CleanUp.ReferenceOTU149206
New.CleanUp.ReferenceOTU149531
New.CleanUp.ReferenceOTU29956
New.CleanUp.ReferenceOTU105668
New.CleanUp.ReferenceOTU60838
New.CleanUp.ReferenceOTU94842
New.CleanUp.ReferenceOTU65336
New.CleanUp.ReferenceOTU58939
New.CleanUp.ReferenceOTU50478
New.CleanUp.ReferenceOTU198780
New.CleanUp.ReferenceOTU195302
New.CleanUp.ReferenceOTU118641
New.CleanUp.ReferenceOTU28657
New.CleanUp.ReferenceOTU62178
New.CleanUp.ReferenceOTU73891
New.CleanUp.ReferenceOTU64469
New.CleanUp.ReferenceOTU148188
New.CleanUp.ReferenceOTU151603

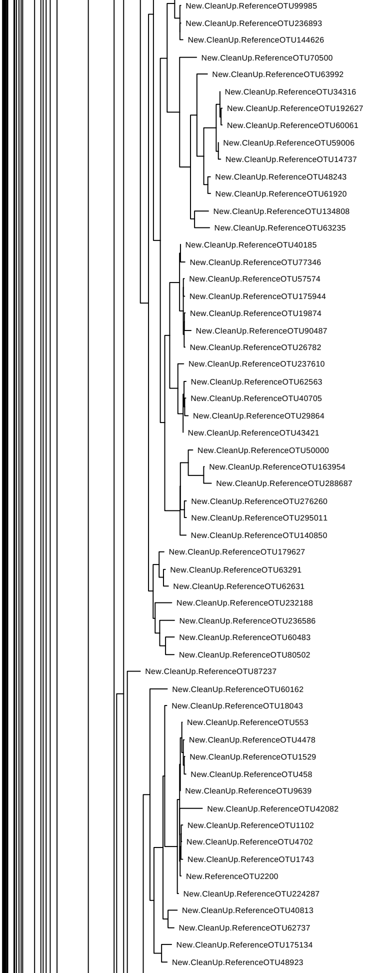
New.CleanUp.ReferenceOTU99985
New.CleanUp.ReferenceOTU236893
New.CleanUp.ReferenceOTU144626
New.CleanUp.ReferenceOTU70500
New.CleanUp.ReferenceOTU63992
New.CleanUp.ReferenceOTU34316
New.CleanUp.ReferenceOTU192627
New.CleanUp.ReferenceOTU60061
New.CleanUp.ReferenceOTU59006
New.CleanUp.ReferenceOTU14737
New.CleanUp.ReferenceOTU48243
New.CleanUp.ReferenceOTU61920
New.CleanUp.ReferenceOTU134808
New.CleanUp.ReferenceOTU63235
New.CleanUp.ReferenceOTU40185
New.CleanUp.ReferenceOTU77346
New.CleanUp.ReferenceOTU57574
New.CleanUp.ReferenceOTU175944
New.CleanUp.ReferenceOTU19874
New.CleanUp.ReferenceOTU90487
New.CleanUp.ReferenceOTU26782
New.CleanUp.ReferenceOTU237610
New.CleanUp.ReferenceOTU62563
New.CleanUp.ReferenceOTU40705
New.CleanUp.ReferenceOTU29864
New.CleanUp.ReferenceOTU43421
New.CleanUp.ReferenceOTU50000
New.CleanUp.ReferenceOTU163954
New.CleanUp.ReferenceOTU288687
New.CleanUp.ReferenceOTU276260
New.CleanUp.ReferenceOTU295011
New.CleanUp.ReferenceOTU140850
New.CleanUp.ReferenceOTU179627
New.CleanUp.ReferenceOTU63291
New.CleanUp.ReferenceOTU62631
New.CleanUp.ReferenceOTU232188
New.CleanUp.ReferenceOTU236586
New.CleanUp.ReferenceOTU60483
New.CleanUp.ReferenceOTU80502
New.CleanUp.ReferenceOTU87237
New.CleanUp.ReferenceOTU60162
New.CleanUp.ReferenceOTU18043
New.CleanUp.ReferenceOTU553
New.CleanUp.ReferenceOTU4478
New.CleanUp.ReferenceOTU1529
New.CleanUp.ReferenceOTU458
New.CleanUp.ReferenceOTU9639
New.CleanUp.ReferenceOTU42082
New.CleanUp.ReferenceOTU1102
New.CleanUp.ReferenceOTU4702
New.CleanUp.ReferenceOTU1743
New.ReferenceOTU2200
New.CleanUp.ReferenceOTU224287
New.CleanUp.ReferenceOTU40813
New.CleanUp.ReferenceOTU62737
New.CleanUp.ReferenceOTU175134
New.CleanUp.ReferenceOTU48923

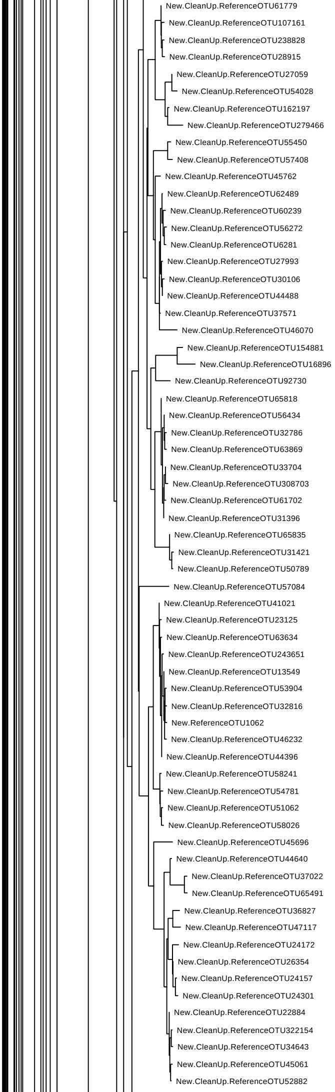

New.CleanUp.ReferenceOTU61779
New.CleanUp.ReferenceOTU107161
New.CleanUp.ReferenceOTU238828
New.CleanUp.ReferenceOTU28915
New.CleanUp.ReferenceOTU27059
New.CleanUp.ReferenceOTU54028
New.CleanUp.ReferenceOTU162197
New.CleanUp.ReferenceOTU279466
New.CleanUp.ReferenceOTU55450
New.CleanUp.ReferenceOTU57408
New.CleanUp.ReferenceOTU45762
New.CleanUp.ReferenceOTU62489
New.CleanUp.ReferenceOTU60239
New.CleanUp.ReferenceOTU56272
New.CleanUp.ReferenceOTU6281
New.CleanUp.ReferenceOTU27993
New.CleanUp.ReferenceOTU30106
New.CleanUp.ReferenceOTU44488
New.CleanUp.ReferenceOTU37571
New.CleanUp.ReferenceOTU46070
New.CleanUp.ReferenceOTU154881
New.CleanUp.ReferenceOTU16896
New.CleanUp.ReferenceOTU92730
New.CleanUp.ReferenceOTU65818
New.CleanUp.ReferenceOTU56434
New.CleanUp.ReferenceOTU32786
New.CleanUp.ReferenceOTU63869
New.CleanUp.ReferenceOTU33704
New.CleanUp.ReferenceOTU308703
New.CleanUp.ReferenceOTU61702
New.CleanUp.ReferenceOTU31396
New.CleanUp.ReferenceOTU65835
New.CleanUp.ReferenceOTU31421
New.CleanUp.ReferenceOTU50789
New.CleanUp.ReferenceOTU57084
New.CleanUp.ReferenceOTU41021
New.CleanUp.ReferenceOTU23125
New.CleanUp.ReferenceOTU63634
New.CleanUp.ReferenceOTU243651
New.CleanUp.ReferenceOTU13549
New.CleanUp.ReferenceOTU53904
New.CleanUp.ReferenceOTU32816
New.ReferenceOTU1062
New.CleanUp.ReferenceOTU46232
New.CleanUp.ReferenceOTU44396
New.CleanUp.ReferenceOTU58241
New.CleanUp.ReferenceOTU54781
New.CleanUp.ReferenceOTU51062
New.CleanUp.ReferenceOTU58026
New.CleanUp.ReferenceOTU45696
New.CleanUp.ReferenceOTU44640
New.CleanUp.ReferenceOTU37022
New.CleanUp.ReferenceOTU65491
New.CleanUp.ReferenceOTU36827
New.CleanUp.ReferenceOTU47117
New.CleanUp.ReferenceOTU24172
New.CleanUp.ReferenceOTU26354
New.CleanUp.ReferenceOTU24157
New.CleanUp.ReferenceOTU24301
New.CleanUp.ReferenceOTU22884
New.CleanUp.ReferenceOTU322154
New.CleanUp.ReferenceOTU34643
New.CleanUp.ReferenceOTU45061
New.CleanUp.ReferenceOTU52882

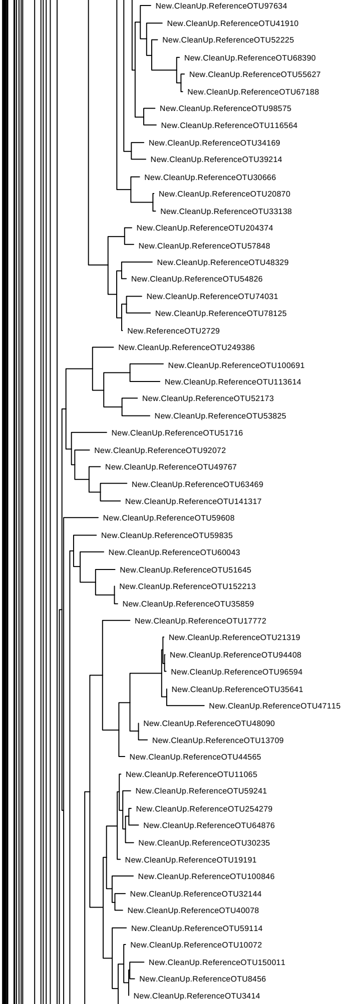
New.CleanUp.ReferenceOTU97634
New.CleanUp.ReferenceOTU41910
New.CleanUp.ReferenceOTU52225
New.CleanUp.ReferenceOTU68390
New.CleanUp.ReferenceOTU55627
New.CleanUp.ReferenceOTU67188
New.CleanUp.ReferenceOTU98575
New.CleanUp.ReferenceOTU116564
New.CleanUp.ReferenceOTU34169
New.CleanUp.ReferenceOTU39214
New.CleanUp.ReferenceOTU30666
New.CleanUp.ReferenceOTU20870
New.CleanUp.ReferenceOTU33138
New.CleanUp.ReferenceOTU204374
New.CleanUp.ReferenceOTU57848
New.CleanUp.ReferenceOTU48329
New.CleanUp.ReferenceOTU54826
New.CleanUp.ReferenceOTU74031
New.CleanUp.ReferenceOTU78125
New.ReferenceOTU2729
New.CleanUp.ReferenceOTU249386
New.CleanUp.ReferenceOTU100691
New.CleanUp.ReferenceOTU113614
New.CleanUp.ReferenceOTU52173
New.CleanUp.ReferenceOTU53825
New.CleanUp.ReferenceOTU51716
New.CleanUp.ReferenceOTU92072
New.CleanUp.ReferenceOTU49767
New.CleanUp.ReferenceOTU63469
New.CleanUp.ReferenceOTU141317
New.CleanUp.ReferenceOTU59608
New.CleanUp.ReferenceOTU59835
New.CleanUp.ReferenceOTU60043
New.CleanUp.ReferenceOTU51645
New.CleanUp.ReferenceOTU152213
New.CleanUp.ReferenceOTU35859
New.CleanUp.ReferenceOTU17772
New.CleanUp.ReferenceOTU21319
New.CleanUp.ReferenceOTU94408
New.CleanUp.ReferenceOTU96594
New.CleanUp.ReferenceOTU35641
New.CleanUp.ReferenceOTU47115
New.CleanUp.ReferenceOTU48090
New.CleanUp.ReferenceOTU13709
New.CleanUp.ReferenceOTU44565
New.CleanUp.ReferenceOTU11065
New.CleanUp.ReferenceOTU59241
New.CleanUp.ReferenceOTU254279
New.CleanUp.ReferenceOTU64876
New.CleanUp.ReferenceOTU30235
New.CleanUp.ReferenceOTU19191
New.CleanUp.ReferenceOTU100846
New.CleanUp.ReferenceOTU32144
New.CleanUp.ReferenceOTU40078
New.CleanUp.ReferenceOTU59114
New.CleanUp.ReferenceOTU10072
New.CleanUp.ReferenceOTU150011
New.CleanUp.ReferenceOTU8456
New.CleanUp.ReferenceOTU3414

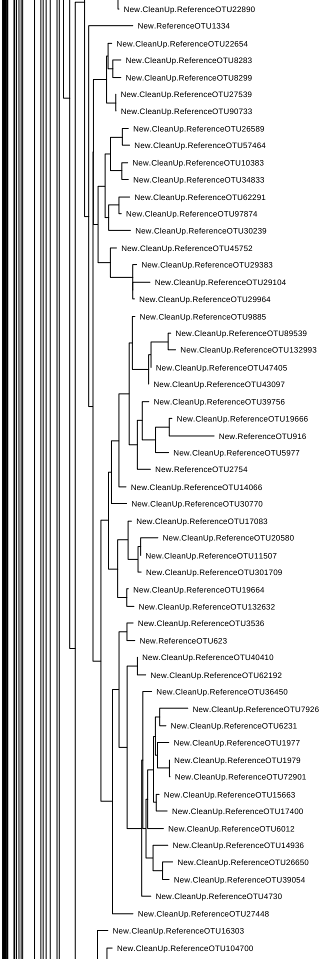
New.CleanUp.ReferenceOTU22890
New.ReferenceOTU1334
New.CleanUp.ReferenceOTU22654
New.CleanUp.ReferenceOTU8283
New.CleanUp.ReferenceOTU8299
New.CleanUp.ReferenceOTU27539
New.CleanUp.ReferenceOTU90733
New.CleanUp.ReferenceOTU26589
New.CleanUp.ReferenceOTU57464
New.CleanUp.ReferenceOTU10383
New.CleanUp.ReferenceOTU34833
New.CleanUp.ReferenceOTU62291
New.CleanUp.ReferenceOTU97874
New.CleanUp.ReferenceOTU30239
New.CleanUp.ReferenceOTU45752
New.CleanUp.ReferenceOTU29383
New.CleanUp.ReferenceOTU29104
New.CleanUp.ReferenceOTU29964
New.CleanUp.ReferenceOTU9885
New.CleanUp.ReferenceOTU89539
New.CleanUp.ReferenceOTU132993
New.CleanUp.ReferenceOTU47405
New.CleanUp.ReferenceOTU43097
New.CleanUp.ReferenceOTU39756
New.CleanUp.ReferenceOTU19666
New.ReferenceOTU916
New.CleanUp.ReferenceOTU5977
New.ReferenceOTU2754
New.CleanUp.ReferenceOTU14066
New.CleanUp.ReferenceOTU30770
New.CleanUp.ReferenceOTU17083
New.CleanUp.ReferenceOTU20580
New.CleanUp.ReferenceOTU11507
New.CleanUp.ReferenceOTU301709
New.CleanUp.ReferenceOTU19664
New.CleanUp.ReferenceOTU132632
New.CleanUp.ReferenceOTU3536
New.ReferenceOTU623
New.CleanUp.ReferenceOTU40410
New.CleanUp.ReferenceOTU62192
New.CleanUp.ReferenceOTU36450
New.CleanUp.ReferenceOTU7926
New.CleanUp.ReferenceOTU6231
New.CleanUp.ReferenceOTU1977
New.CleanUp.ReferenceOTU1979
New.CleanUp.ReferenceOTU72901
New.CleanUp.ReferenceOTU15663
New.CleanUp.ReferenceOTU17400
New.CleanUp.ReferenceOTU6012
New.CleanUp.ReferenceOTU14936
New.CleanUp.ReferenceOTU26650
New.CleanUp.ReferenceOTU39054
New.CleanUp.ReferenceOTU4730
New.CleanUp.ReferenceOTU27448
New.CleanUp.ReferenceOTU16303
New.CleanUp.ReferenceOTU104700

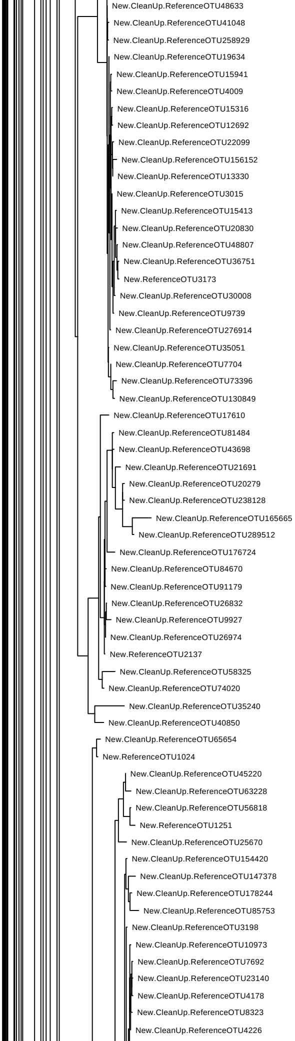
New.CleanUp.ReferenceOTU48633
New.CleanUp.ReferenceOTU41048
New.CleanUp.ReferenceOTU258929
New.CleanUp.ReferenceOTU19634
New.CleanUp.ReferenceOTU15941
New.CleanUp.ReferenceOTU4009
New.CleanUp.ReferenceOTU15316
New.CleanUp.ReferenceOTU12692
New.CleanUp.ReferenceOTU22099
New.CleanUp.ReferenceOTU156152
New.CleanUp.ReferenceOTU13330
New.CleanUp.ReferenceOTU3015
New.CleanUp.ReferenceOTU15413
New.CleanUp.ReferenceOTU20830
New.CleanUp.ReferenceOTU48807
New.CleanUp.ReferenceOTU36751
New.ReferenceOTU3173
New.CleanUp.ReferenceOTU30008
New.CleanUp.ReferenceOTU9739
New.CleanUp.ReferenceOTU276914
New.CleanUp.ReferenceOTU35051
New.CleanUp.ReferenceOTU7704
New.CleanUp.ReferenceOTU73396
New.CleanUp.ReferenceOTU130849
New.CleanUp.ReferenceOTU17610
New.CleanUp.ReferenceOTU81484
New.CleanUp.ReferenceOTU43698
New.CleanUp.ReferenceOTU21691
New.CleanUp.ReferenceOTU20279
New.CleanUp.ReferenceOTU238128
New.CleanUp.ReferenceOTU165665
New.CleanUp.ReferenceOTU289512
New.CleanUp.ReferenceOTU176724
New.CleanUp.ReferenceOTU84670
New.CleanUp.ReferenceOTU91179
New.CleanUp.ReferenceOTU26832
New.CleanUp.ReferenceOTU9927
New.CleanUp.ReferenceOTU26974
New.ReferenceOTU2137
New.CleanUp.ReferenceOTU58325
New.CleanUp.ReferenceOTU74020
New.CleanUp.ReferenceOTU35240
New.CleanUp.ReferenceOTU40850
New.CleanUp.ReferenceOTU65654
New.ReferenceOTU1024
New.CleanUp.ReferenceOTU45220
New.CleanUp.ReferenceOTU63228
New.CleanUp.ReferenceOTU56818
New.ReferenceOTU1251
New.CleanUp.ReferenceOTU25670
New.CleanUp.ReferenceOTU154420
New.CleanUp.ReferenceOTU147378
New.CleanUp.ReferenceOTU178244
New.CleanUp.ReferenceOTU85753
New.CleanUp.ReferenceOTU3198
New.CleanUp.ReferenceOTU10973
New.CleanUp.ReferenceOTU7692
New.CleanUp.ReferenceOTU23140
New.CleanUp.ReferenceOTU4178
New.CleanUp.ReferenceOTU8323
New.CleanUp.ReferenceOTU4226

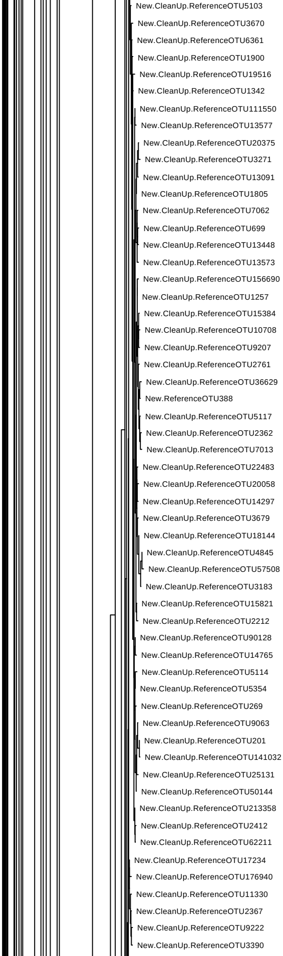
New.CleanUp.ReferenceOTU5103
New.CleanUp.ReferenceOTU3670
New.CleanUp.ReferenceOTU6361
New.CleanUp.ReferenceOTU1900
New.CleanUp.ReferenceOTU19516
New.CleanUp.ReferenceOTU1342
New.CleanUp.ReferenceOTU111550
New.CleanUp.ReferenceOTU13577
New.CleanUp.ReferenceOTU20375
New.CleanUp.ReferenceOTU3271
New.CleanUp.ReferenceOTU13091
New.CleanUp.ReferenceOTU1805
New.CleanUp.ReferenceOTU7062
New.CleanUp.ReferenceOTU699
New.CleanUp.ReferenceOTU13448
New.CleanUp.ReferenceOTU13573
New.CleanUp.ReferenceOTU156690
New.CleanUp.ReferenceOTU1257
New.CleanUp.ReferenceOTU15384
New.CleanUp.ReferenceOTU10708
New.CleanUp.ReferenceOTU9207
New.CleanUp.ReferenceOTU2761
New.CleanUp.ReferenceOTU36629
New.ReferenceOTU388
New.CleanUp.ReferenceOTU5117
New.CleanUp.ReferenceOTU2362
New.CleanUp.ReferenceOTU7013
New.CleanUp.ReferenceOTU22483
New.CleanUp.ReferenceOTU20058
New.CleanUp.ReferenceOTU14297
New.CleanUp.ReferenceOTU3679
New.CleanUp.ReferenceOTU18144
New.CleanUp.ReferenceOTU4845
New.CleanUp.ReferenceOTU57508
New.CleanUp.ReferenceOTU3183
New.CleanUp.ReferenceOTU15821
New.CleanUp.ReferenceOTU2212
New.CleanUp.ReferenceOTU90128
New.CleanUp.ReferenceOTU14765
New.CleanUp.ReferenceOTU5114
New.CleanUp.ReferenceOTU5354
New.CleanUp.ReferenceOTU269
New.CleanUp.ReferenceOTU9063
New.CleanUp.ReferenceOTU201
New.CleanUp.ReferenceOTU141032
New.CleanUp.ReferenceOTU25131
New.CleanUp.ReferenceOTU50144
New.CleanUp.ReferenceOTU213358
New.CleanUp.ReferenceOTU2412
New.CleanUp.ReferenceOTU62211
New.CleanUp.ReferenceOTU17234
New.CleanUp.ReferenceOTU176940
New.CleanUp.ReferenceOTU11330
New.CleanUp.ReferenceOTU2367
New.CleanUp.ReferenceOTU9222
New.CleanUp.ReferenceOTU3390

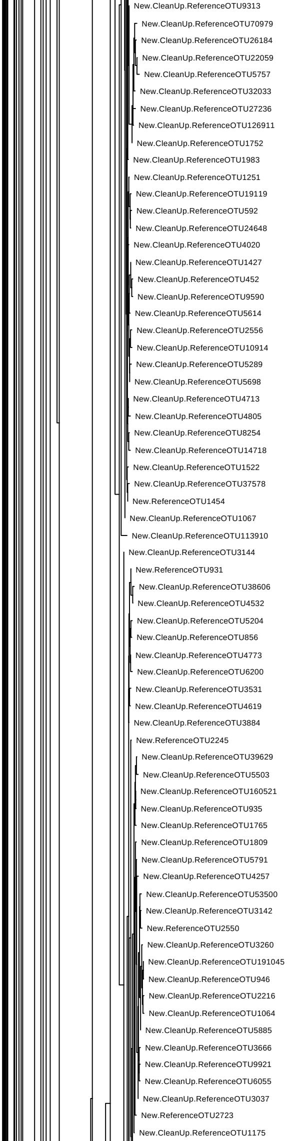
New.CleanUp.ReferenceOTU9313
New.CleanUp.ReferenceOTU70979
New.CleanUp.ReferenceOTU26184
New.CleanUp.ReferenceOTU22059
New.CleanUp.ReferenceOTU5757
New.CleanUp.ReferenceOTU32033
New.CleanUp.ReferenceOTU27236
New.CleanUp.ReferenceOTU126911
New.CleanUp.ReferenceOTU1752
New.CleanUp.ReferenceOTU1983
New.CleanUp.ReferenceOTU1251
New.CleanUp.ReferenceOTU19119
New.CleanUp.ReferenceOTU592
New.CleanUp.ReferenceOTU24648
New.CleanUp.ReferenceOTU4020
New.CleanUp.ReferenceOTU1427
New.CleanUp.ReferenceOTU452
New.CleanUp.ReferenceOTU9590
New.CleanUp.ReferenceOTU5614
New.CleanUp.ReferenceOTU2556
New.CleanUp.ReferenceOTU10914
New.CleanUp.ReferenceOTU5289
New.CleanUp.ReferenceOTU5698
New.CleanUp.ReferenceOTU4713
New.CleanUp.ReferenceOTU4805
New.CleanUp.ReferenceOTU8254
New.CleanUp.ReferenceOTU14718
New.CleanUp.ReferenceOTU1522
New.CleanUp.ReferenceOTU37578
New.ReferenceOTU1454
New.CleanUp.ReferenceOTU1067
New.CleanUp.ReferenceOTU113910
New.CleanUp.ReferenceOTU3144
New.ReferenceOTU931
New.CleanUp.ReferenceOTU38606
New.CleanUp.ReferenceOTU4532
New.CleanUp.ReferenceOTU5204
New.CleanUp.ReferenceOTU856
New.CleanUp.ReferenceOTU4773
New.CleanUp.ReferenceOTU6200
New.CleanUp.ReferenceOTU3531
New.CleanUp.ReferenceOTU4619
New.CleanUp.ReferenceOTU3884
New.ReferenceOTU2245
New.CleanUp.ReferenceOTU39629
New.CleanUp.ReferenceOTU5503
New.CleanUp.ReferenceOTU160521
New.CleanUp.ReferenceOTU935
New.CleanUp.ReferenceOTU1765
New.CleanUp.ReferenceOTU1809
New.CleanUp.ReferenceOTU5791
New.CleanUp.ReferenceOTU4257
New.CleanUp.ReferenceOTU53500
New.CleanUp.ReferenceOTU3142
New.ReferenceOTU2550
New.CleanUp.ReferenceOTU3260
New.CleanUp.ReferenceOTU191045
New.CleanUp.ReferenceOTU946
New.CleanUp.ReferenceOTU2216
New.CleanUp.ReferenceOTU1064
New.CleanUp.ReferenceOTU5885
New.CleanUp.ReferenceOTU3666
New.CleanUp.ReferenceOTU9921
New.CleanUp.ReferenceOTU6055
New.CleanUp.ReferenceOTU3037
New.ReferenceOTU2723
New.CleanUp.ReferenceOTU1175

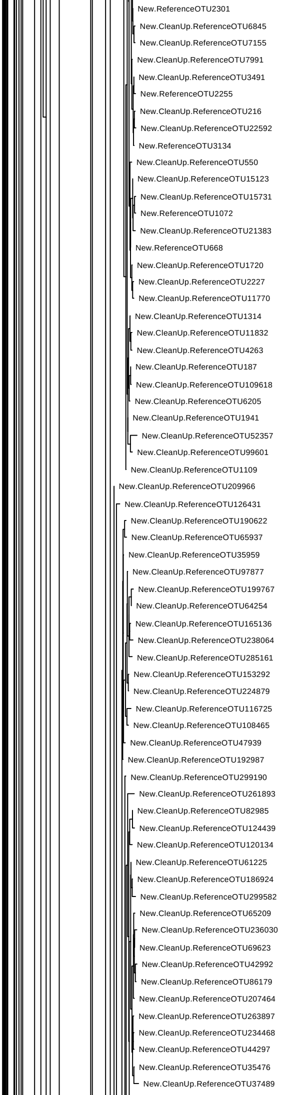

New.ReferenceOTU2301
New.CleanUp.ReferenceOTU6845
New.CleanUp.ReferenceOTU7155
New.CleanUp.ReferenceOTU7991
New.CleanUp.ReferenceOTU3491
New.ReferenceOTU2255
New.CleanUp.ReferenceOTU216
New.CleanUp.ReferenceOTU22592
New.ReferenceOTU3134
New.CleanUp.ReferenceOTU550
New.CleanUp.ReferenceOTU15123
New.CleanUp.ReferenceOTU15731
New.ReferenceOTU1072
New.CleanUp.ReferenceOTU21383
New.ReferenceOTU668
New.CleanUp.ReferenceOTU1720
New.CleanUp.ReferenceOTU2227
New.CleanUp.ReferenceOTU11770
New.CleanUp.ReferenceOTU1314
New.CleanUp.ReferenceOTU11832
New.CleanUp.ReferenceOTU4263
New.CleanUp.ReferenceOTU187
New.CleanUp.ReferenceOTU109618
New.CleanUp.ReferenceOTU6205
New.CleanUp.ReferenceOTU1941
New.CleanUp.ReferenceOTU52357
New.CleanUp.ReferenceOTU99601
New.CleanUp.ReferenceOTU1109
New.CleanUp.ReferenceOTU209966
New.CleanUp.ReferenceOTU126431
New.CleanUp.ReferenceOTU190622
New.CleanUp.ReferenceOTU65937
New.CleanUp.ReferenceOTU35959
New.CleanUp.ReferenceOTU97877
New.CleanUp.ReferenceOTU199767
New.CleanUp.ReferenceOTU64254
New.CleanUp.ReferenceOTU165136
New.CleanUp.ReferenceOTU238064
New.CleanUp.ReferenceOTU285161
New.CleanUp.ReferenceOTU153292
New.CleanUp.ReferenceOTU224879
New.CleanUp.ReferenceOTU116725
New.CleanUp.ReferenceOTU108465
New.CleanUp.ReferenceOTU47939
New.CleanUp.ReferenceOTU192987
New.CleanUp.ReferenceOTU299190
New.CleanUp.ReferenceOTU261893
New.CleanUp.ReferenceOTU82985
New.CleanUp.ReferenceOTU124439
New.CleanUp.ReferenceOTU120134
New.CleanUp.ReferenceOTU61225
New.CleanUp.ReferenceOTU186924
New.CleanUp.ReferenceOTU299582
New.CleanUp.ReferenceOTU65209
New.CleanUp.ReferenceOTU236030
New.CleanUp.ReferenceOTU69623
New.CleanUp.ReferenceOTU42992
New.CleanUp.ReferenceOTU86179
New.CleanUp.ReferenceOTU207464
New.CleanUp.ReferenceOTU263897
New.CleanUp.ReferenceOTU234468
New.CleanUp.ReferenceOTU44297
New.CleanUp.ReferenceOTU35476
New.CleanUp.ReferenceOTU37489

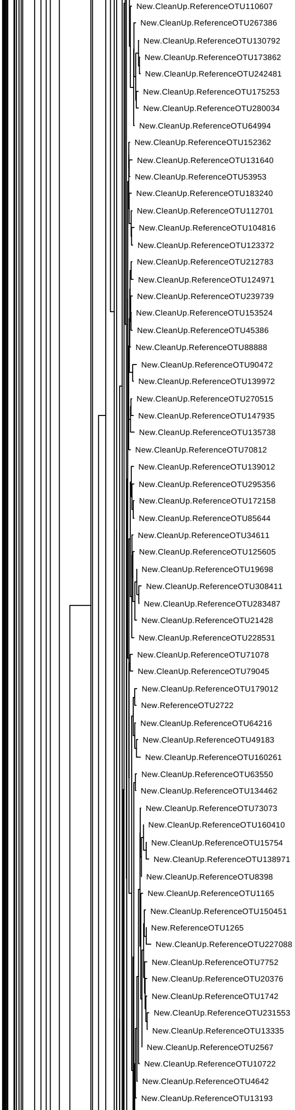
New.CleanUp.ReferenceOTU110607
New.CleanUp.ReferenceOTU267386
New.CleanUp.ReferenceOTU130792
New.CleanUp.ReferenceOTU173862
New.CleanUp.ReferenceOTU242481
New.CleanUp.ReferenceOTU175253
New.CleanUp.ReferenceOTU280034
New.CleanUp.ReferenceOTU64994
New.CleanUp.ReferenceOTU152362
New.CleanUp.ReferenceOTU131640
New.CleanUp.ReferenceOTU53953
New.CleanUp.ReferenceOTU183240
New.CleanUp.ReferenceOTU112701
New.CleanUp.ReferenceOTU104816
New.CleanUp.ReferenceOTU123372
New.CleanUp.ReferenceOTU212783
New.CleanUp.ReferenceOTU124971
New.CleanUp.ReferenceOTU239739
New.CleanUp.ReferenceOTU153524
New.CleanUp.ReferenceOTU45386
New.CleanUp.ReferenceOTU88888
New.CleanUp.ReferenceOTU90472
New.CleanUp.ReferenceOTU139972
New.CleanUp.ReferenceOTU270515
New.CleanUp.ReferenceOTU147935
New.CleanUp.ReferenceOTU135738
New.CleanUp.ReferenceOTU70812
New.CleanUp.ReferenceOTU139012
New.CleanUp.ReferenceOTU295356
New.CleanUp.ReferenceOTU172158
New.CleanUp.ReferenceOTU85644
New.CleanUp.ReferenceOTU34611
New.CleanUp.ReferenceOTU125605
New.CleanUp.ReferenceOTU19698
New.CleanUp.ReferenceOTU308411
New.CleanUp.ReferenceOTU283487
New.CleanUp.ReferenceOTU21428
New.CleanUp.ReferenceOTU228531
New.CleanUp.ReferenceOTU71078
New.CleanUp.ReferenceOTU79045
New.CleanUp.ReferenceOTU179012
New.ReferenceOTU2722
New.CleanUp.ReferenceOTU64216
New.CleanUp.ReferenceOTU49183
New.CleanUp.ReferenceOTU160261
New.CleanUp.ReferenceOTU63550
New.CleanUp.ReferenceOTU134462
New.CleanUp.ReferenceOTU73073
New.CleanUp.ReferenceOTU160410
New.CleanUp.ReferenceOTU15754
New.CleanUp.ReferenceOTU138971
New.CleanUp.ReferenceOTU8398
New.CleanUp.ReferenceOTU1165
New.CleanUp.ReferenceOTU150451
New.ReferenceOTU1265
New.CleanUp.ReferenceOTU227088
New.CleanUp.ReferenceOTU7752
New.CleanUp.ReferenceOTU20376
New.CleanUp.ReferenceOTU1742
New.CleanUp.ReferenceOTU231553
New.CleanUp.ReferenceOTU13335
New.CleanUp.ReferenceOTU2567
New.CleanUp.ReferenceOTU10722
New.CleanUp.ReferenceOTU4642
New.CleanUp.ReferenceOTU13193

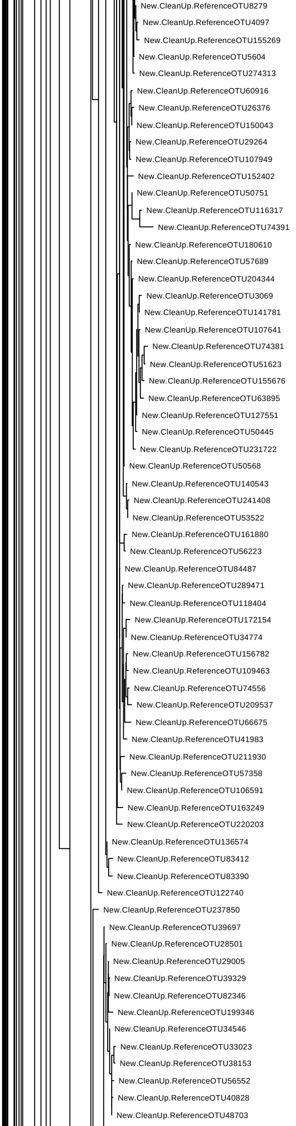
New.CleanUp.ReferenceOTU8279
New.CleanUp.ReferenceOTU4097
New.CleanUp.ReferenceOTU155269
New.CleanUp.ReferenceOTU5604
New.CleanUp.ReferenceOTU274313
New.CleanUp.ReferenceOTU60916
New.CleanUp.ReferenceOTU26376
New.CleanUp.ReferenceOTU150043
New.CleanUp.ReferenceOTU29264
New.CleanUp.ReferenceOTU107949
New.CleanUp.ReferenceOTU152402
New.CleanUp.ReferenceOTU50751
New.CleanUp.ReferenceOTU116317
New.CleanUp.ReferenceOTU74391
New.CleanUp.ReferenceOTU180610
New.CleanUp.ReferenceOTU57689
New.CleanUp.ReferenceOTU204344
New.CleanUp.ReferenceOTU3069
New.CleanUp.ReferenceOTU141781
New.CleanUp.ReferenceOTU107641
New.CleanUp.ReferenceOTU74381
New.CleanUp.ReferenceOTU51623
New.CleanUp.ReferenceOTU155676
New.CleanUp.ReferenceOTU63895
New.CleanUp.ReferenceOTU127551
New.CleanUp.ReferenceOTU50445
New.CleanUp.ReferenceOTU231722
New.CleanUp.ReferenceOTU50568
New.CleanUp.ReferenceOTU140543
New.CleanUp.ReferenceOTU241408
New.CleanUp.ReferenceOTU53522
New.CleanUp.ReferenceOTU161880
New.CleanUp.ReferenceOTU56223
New.CleanUp.ReferenceOTU84487
New.CleanUp.ReferenceOTU289471
New.CleanUp.ReferenceOTU118404
New.CleanUp.ReferenceOTU172154
New.CleanUp.ReferenceOTU34774
New.CleanUp.ReferenceOTU156782
New.CleanUp.ReferenceOTU109463
New.CleanUp.ReferenceOTU74556
New.CleanUp.ReferenceOTU209537
New.CleanUp.ReferenceOTU66675
New.CleanUp.ReferenceOTU41983
New.CleanUp.ReferenceOTU211930
New.CleanUp.ReferenceOTU57358
New.CleanUp.ReferenceOTU106591
New.CleanUp.ReferenceOTU163249
New.CleanUp.ReferenceOTU220203
New.CleanUp.ReferenceOTU136574
New.CleanUp.ReferenceOTU83412
New.CleanUp.ReferenceOTU83390
New.CleanUp.ReferenceOTU122740
New.CleanUp.ReferenceOTU237850
New.CleanUp.ReferenceOTU39697
New.CleanUp.ReferenceOTU28501
New.CleanUp.ReferenceOTU29005
New.CleanUp.ReferenceOTU39329
New.CleanUp.ReferenceOTU82346
New.CleanUp.ReferenceOTU199346
New.CleanUp.ReferenceOTU34546
New.CleanUp.ReferenceOTU33023
New.CleanUp.ReferenceOTU38153
New.CleanUp.ReferenceOTU56552
New.CleanUp.ReferenceOTU40828
New.CleanUp.ReferenceOTU48703

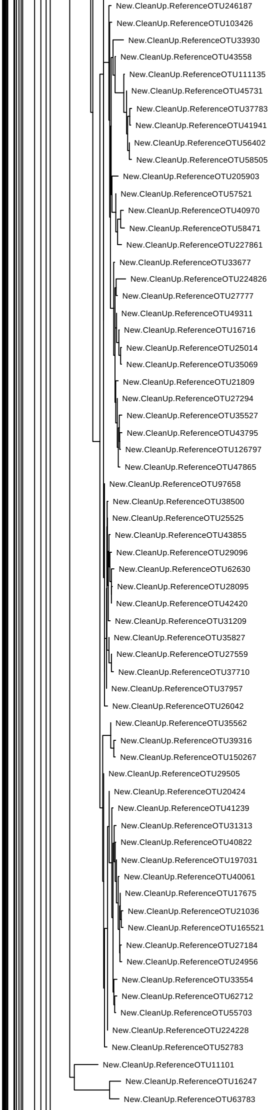
New.CleanUp.ReferenceOTU246187
New.CleanUp.ReferenceOTU103426
New.CleanUp.ReferenceOTU33930
New.CleanUp.ReferenceOTU43558
New.CleanUp.ReferenceOTU111135
New.CleanUp.ReferenceOTU45731
New.CleanUp.ReferenceOTU37783
New.CleanUp.ReferenceOTU41941
New.CleanUp.ReferenceOTU56402
New.CleanUp.ReferenceOTU58505
New.CleanUp.ReferenceOTU205903
New.CleanUp.ReferenceOTU57521
New.CleanUp.ReferenceOTU40970
New.CleanUp.ReferenceOTU58471
New.CleanUp.ReferenceOTU227861
New.CleanUp.ReferenceOTU33677
New.CleanUp.ReferenceOTU224826
New.CleanUp.ReferenceOTU27777
New.CleanUp.ReferenceOTU49311
New.CleanUp.ReferenceOTU16716
New.CleanUp.ReferenceOTU25014
New.CleanUp.ReferenceOTU35069
New.CleanUp.ReferenceOTU21809
New.CleanUp.ReferenceOTU27294
New.CleanUp.ReferenceOTU35527
New.CleanUp.ReferenceOTU43795
New.CleanUp.ReferenceOTU126797
New.CleanUp.ReferenceOTU47865
New.CleanUp.ReferenceOTU97658
New.CleanUp.ReferenceOTU38500
New.CleanUp.ReferenceOTU25525
New.CleanUp.ReferenceOTU43855
New.CleanUp.ReferenceOTU29096
New.CleanUp.ReferenceOTU62630
New.CleanUp.ReferenceOTU28095
New.CleanUp.ReferenceOTU42420
New.CleanUp.ReferenceOTU31209
New.CleanUp.ReferenceOTU35827
New.CleanUp.ReferenceOTU27559
New.CleanUp.ReferenceOTU37710
New.CleanUp.ReferenceOTU37957
New.CleanUp.ReferenceOTU26042
New.CleanUp.ReferenceOTU35562
New.CleanUp.ReferenceOTU39316
New.CleanUp.ReferenceOTU150267
New.CleanUp.ReferenceOTU29505
New.CleanUp.ReferenceOTU20424
New.CleanUp.ReferenceOTU41239
New.CleanUp.ReferenceOTU31313
New.CleanUp.ReferenceOTU40822
New.CleanUp.ReferenceOTU197031
New.CleanUp.ReferenceOTU40061
New.CleanUp.ReferenceOTU17675
New.CleanUp.ReferenceOTU21036
New.CleanUp.ReferenceOTU165521
New.CleanUp.ReferenceOTU27184
New.CleanUp.ReferenceOTU24956
New.CleanUp.ReferenceOTU33554
New.CleanUp.ReferenceOTU62712
New.CleanUp.ReferenceOTU55703
New.CleanUp.ReferenceOTU224228
New.CleanUp.ReferenceOTU52783
New.CleanUp.ReferenceOTU11101
New.CleanUp.ReferenceOTU16247
New.CleanUp.ReferenceOTU63783

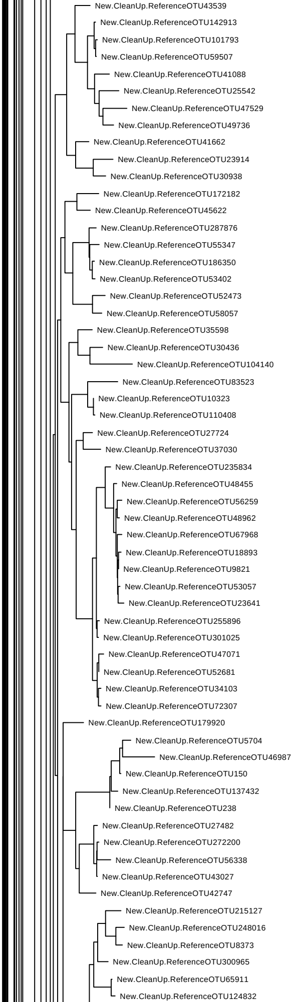

New.CleanUp.ReferenceOTU43539
New.CleanUp.ReferenceOTU142913
New.CleanUp.ReferenceOTU101793
New.CleanUp.ReferenceOTU59507
New.CleanUp.ReferenceOTU41088
New.CleanUp.ReferenceOTU25542
New.CleanUp.ReferenceOTU47529
New.CleanUp.ReferenceOTU49736
New.CleanUp.ReferenceOTU41662
New.CleanUp.ReferenceOTU23914
New.CleanUp.ReferenceOTU30938
New.CleanUp.ReferenceOTU172182
New.CleanUp.ReferenceOTU45622
New.CleanUp.ReferenceOTU287876
New.CleanUp.ReferenceOTU55347
New.CleanUp.ReferenceOTU186350
New.CleanUp.ReferenceOTU53402
New.CleanUp.ReferenceOTU52473
New.CleanUp.ReferenceOTU58057
New.CleanUp.ReferenceOTU35598
New.CleanUp.ReferenceOTU30436
New.CleanUp.ReferenceOTU104140
New.CleanUp.ReferenceOTU83523
New.CleanUp.ReferenceOTU10323
New.CleanUp.ReferenceOTU110408
New.CleanUp.ReferenceOTU27724
New.CleanUp.ReferenceOTU37030
New.CleanUp.ReferenceOTU235834
New.CleanUp.ReferenceOTU48455
New.CleanUp.ReferenceOTU56259
New.CleanUp.ReferenceOTU48962
New.CleanUp.ReferenceOTU67968
New.CleanUp.ReferenceOTU18893
New.CleanUp.ReferenceOTU9821
New.CleanUp.ReferenceOTU53057
New.CleanUp.ReferenceOTU23641
New.CleanUp.ReferenceOTU255896
New.CleanUp.ReferenceOTU301025
New.CleanUp.ReferenceOTU47071
New.CleanUp.ReferenceOTU52681
New.CleanUp.ReferenceOTU34103
New.CleanUp.ReferenceOTU72307
New.CleanUp.ReferenceOTU179920
New.CleanUp.ReferenceOTU5704
New.CleanUp.ReferenceOTU46987
New.CleanUp.ReferenceOTU150
New.CleanUp.ReferenceOTU137432
New.CleanUp.ReferenceOTU238
New.CleanUp.ReferenceOTU27482
New.CleanUp.ReferenceOTU272200
New.CleanUp.ReferenceOTU56338
New.CleanUp.ReferenceOTU43027
New.CleanUp.ReferenceOTU42747
New.CleanUp.ReferenceOTU215127
New.CleanUp.ReferenceOTU248016
New.CleanUp.ReferenceOTU8373
New.CleanUp.ReferenceOTU300965
New.CleanUp.ReferenceOTU65911
New.CleanUp.ReferenceOTU124832

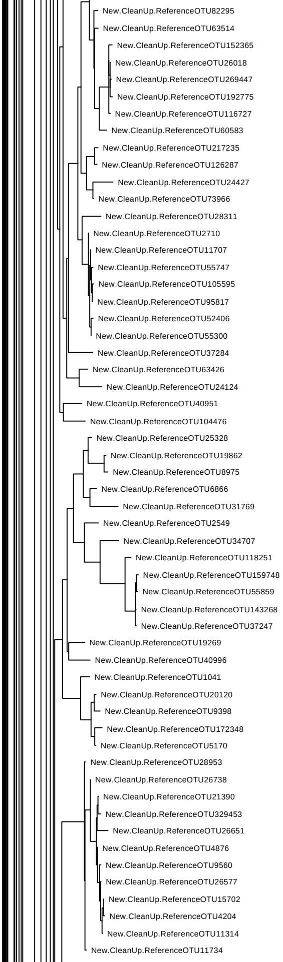
New.CleanUp.ReferenceOTU82295
New.CleanUp.ReferenceOTU63514
New.CleanUp.ReferenceOTU152365
New.CleanUp.ReferenceOTU26018
New.CleanUp.ReferenceOTU269447
New.CleanUp.ReferenceOTU192775
New.CleanUp.ReferenceOTU116727
New.CleanUp.ReferenceOTU60583
New.CleanUp.ReferenceOTU217235
New.CleanUp.ReferenceOTU126287
New.CleanUp.ReferenceOTU24427
New.CleanUp.ReferenceOTU73966
New.CleanUp.ReferenceOTU28311
New.CleanUp.ReferenceOTU2710
New.CleanUp.ReferenceOTU11707
New.CleanUp.ReferenceOTU55747
New.CleanUp.ReferenceOTU105595
New.CleanUp.ReferenceOTU95817
New.CleanUp.ReferenceOTU52406
New.CleanUp.ReferenceOTU55300
New.CleanUp.ReferenceOTU37284
New.CleanUp.ReferenceOTU63426
New.CleanUp.ReferenceOTU24124
New.CleanUp.ReferenceOTU40951
New.CleanUp.ReferenceOTU104476
New.CleanUp.ReferenceOTU25328
New.CleanUp.ReferenceOTU19862
New.CleanUp.ReferenceOTU8975
New.CleanUp.ReferenceOTU6866
New.CleanUp.ReferenceOTU31769
New.CleanUp.ReferenceOTU2549
New.CleanUp.ReferenceOTU34707
New.CleanUp.ReferenceOTU118251
New.CleanUp.ReferenceOTU159748
New.CleanUp.ReferenceOTU55859
New.CleanUp.ReferenceOTU143268
New.CleanUp.ReferenceOTU37247
New.CleanUp.ReferenceOTU19269
New.CleanUp.ReferenceOTU40996
New.CleanUp.ReferenceOTU1041
New.CleanUp.ReferenceOTU20120
New.CleanUp.ReferenceOTU9398
New.CleanUp.ReferenceOTU172348
New.CleanUp.ReferenceOTU5170
New.CleanUp.ReferenceOTU28953
New.CleanUp.ReferenceOTU26738
New.CleanUp.ReferenceOTU21390
New.CleanUp.ReferenceOTU329453
New.CleanUp.ReferenceOTU26651
New.CleanUp.ReferenceOTU4876
New.CleanUp.ReferenceOTU9560
New.CleanUp.ReferenceOTU26577
New.CleanUp.ReferenceOTU15702
New.CleanUp.ReferenceOTU4204
New.CleanUp.ReferenceOTU11314
New.CleanUp.ReferenceOTU11734

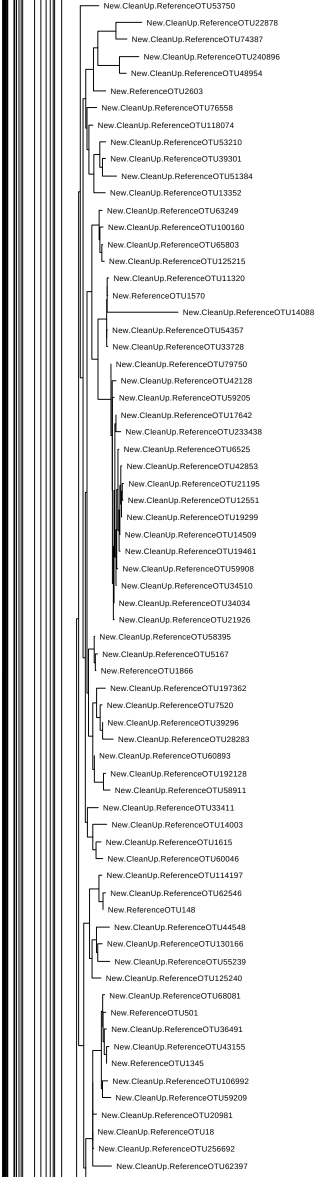
New.CleanUp.ReferenceOTU53750
New.CleanUp.ReferenceOTU22878
New.CleanUp.ReferenceOTU74387
New.CleanUp.ReferenceOTU240896
New.CleanUp.ReferenceOTU48954
New.ReferenceOTU2603
New.CleanUp.ReferenceOTU76558
New.CleanUp.ReferenceOTU118074
New.CleanUp.ReferenceOTU53210
New.CleanUp.ReferenceOTU39301
New.CleanUp.ReferenceOTU51384
New.CleanUp.ReferenceOTU13352
New.CleanUp.ReferenceOTU63249
New.CleanUp.ReferenceOTU100160
New.CleanUp.ReferenceOTU65803
New.CleanUp.ReferenceOTU125215
New.CleanUp.ReferenceOTU11320
New.ReferenceOTU1570
New.CleanUp.ReferenceOTU14088
New.CleanUp.ReferenceOTU54357
New.CleanUp.ReferenceOTU33728
New.CleanUp.ReferenceOTU79750
New.CleanUp.ReferenceOTU42128
New.CleanUp.ReferenceOTU59205
New.CleanUp.ReferenceOTU17642
New.CleanUp.ReferenceOTU233438
New.CleanUp.ReferenceOTU6525
New.CleanUp.ReferenceOTU42853
New.CleanUp.ReferenceOTU21195
New.CleanUp.ReferenceOTU12551
New.CleanUp.ReferenceOTU19299
New.CleanUp.ReferenceOTU14509
New.CleanUp.ReferenceOTU19461
New.CleanUp.ReferenceOTU59908
New.CleanUp.ReferenceOTU34510
New.CleanUp.ReferenceOTU34034
New.CleanUp.ReferenceOTU21926
New.CleanUp.ReferenceOTU58395
New.CleanUp.ReferenceOTU5167
New.ReferenceOTU1866
New.CleanUp.ReferenceOTU197362
New.CleanUp.ReferenceOTU7520
New.CleanUp.ReferenceOTU39296
New.CleanUp.ReferenceOTU28283
New.CleanUp.ReferenceOTU60893
New.CleanUp.ReferenceOTU192128
New.CleanUp.ReferenceOTU58911
New.CleanUp.ReferenceOTU33411
New.CleanUp.ReferenceOTU14003
New.CleanUp.ReferenceOTU1615
New.CleanUp.ReferenceOTU60046
New.CleanUp.ReferenceOTU114197
New.CleanUp.ReferenceOTU62546
New.ReferenceOTU148
New.CleanUp.ReferenceOTU44548
New.CleanUp.ReferenceOTU130166
New.CleanUp.ReferenceOTU55239
New.CleanUp.ReferenceOTU125240
New.CleanUp.ReferenceOTU68081
New.ReferenceOTU501
New.CleanUp.ReferenceOTU36491
New.CleanUp.ReferenceOTU43155
New.ReferenceOTU1345
New.CleanUp.ReferenceOTU106992
New.CleanUp.ReferenceOTU59209
New.CleanUp.ReferenceOTU20981
New.CleanUp.ReferenceOTU18
New.CleanUp.ReferenceOTU256692
New.CleanUp.ReferenceOTU62397

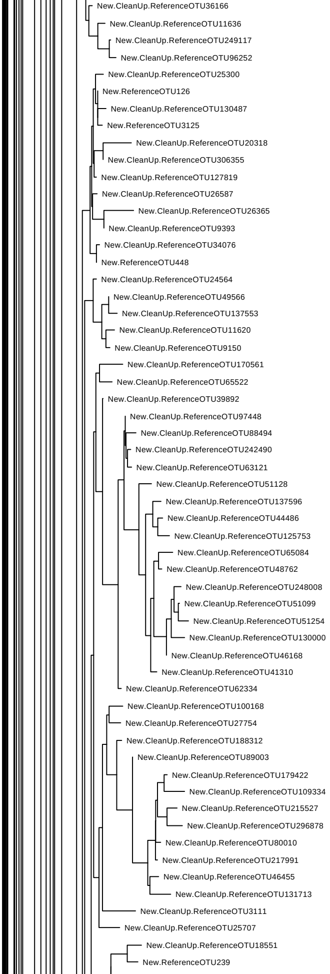
New.CleanUp.ReferenceOTU36166
New.CleanUp.ReferenceOTU11636
New.CleanUp.ReferenceOTU249117
New.CleanUp.ReferenceOTU96252
New.CleanUp.ReferenceOTU25300
New.ReferenceOTU126
New.CleanUp.ReferenceOTU130487
New.ReferenceOTU3125
New.CleanUp.ReferenceOTU20318
New.CleanUp.ReferenceOTU306355
New.CleanUp.ReferenceOTU127819
New.CleanUp.ReferenceOTU26587
New.CleanUp.ReferenceOTU26365
New.CleanUp.ReferenceOTU9393
New.CleanUp.ReferenceOTU34076
New.ReferenceOTU448
New.CleanUp.ReferenceOTU24564
New.CleanUp.ReferenceOTU49566
New.CleanUp.ReferenceOTU137553
New.CleanUp.ReferenceOTU11620
New.CleanUp.ReferenceOTU9150
New.CleanUp.ReferenceOTU170561
New.CleanUp.ReferenceOTU65522
New.CleanUp.ReferenceOTU39892
New.CleanUp.ReferenceOTU97448
New.CleanUp.ReferenceOTU88494
New.CleanUp.ReferenceOTU242490
New.CleanUp.ReferenceOTU63121
New.CleanUp.ReferenceOTU51128
New.CleanUp.ReferenceOTU137596
New.CleanUp.ReferenceOTU44486
New.CleanUp.ReferenceOTU125753
New.CleanUp.ReferenceOTU65084
New.CleanUp.ReferenceOTU48762
New.CleanUp.ReferenceOTU248008
New.CleanUp.ReferenceOTU51099
New.CleanUp.ReferenceOTU51254
New.CleanUp.ReferenceOTU130000
New.CleanUp.ReferenceOTU46168
New.CleanUp.ReferenceOTU41310
New.CleanUp.ReferenceOTU62334
New.CleanUp.ReferenceOTU100168
New.CleanUp.ReferenceOTU27754
New.CleanUp.ReferenceOTU188312
New.CleanUp.ReferenceOTU89003
New.CleanUp.ReferenceOTU179422
New.CleanUp.ReferenceOTU109334
New.CleanUp.ReferenceOTU215527
New.CleanUp.ReferenceOTU296878
New.CleanUp.ReferenceOTU80010
New.CleanUp.ReferenceOTU217991
New.CleanUp.ReferenceOTU46455
New.CleanUp.ReferenceOTU131713
New.CleanUp.ReferenceOTU3111
New.CleanUp.ReferenceOTU25707
New.CleanUp.ReferenceOTU18551
New.ReferenceOTU239

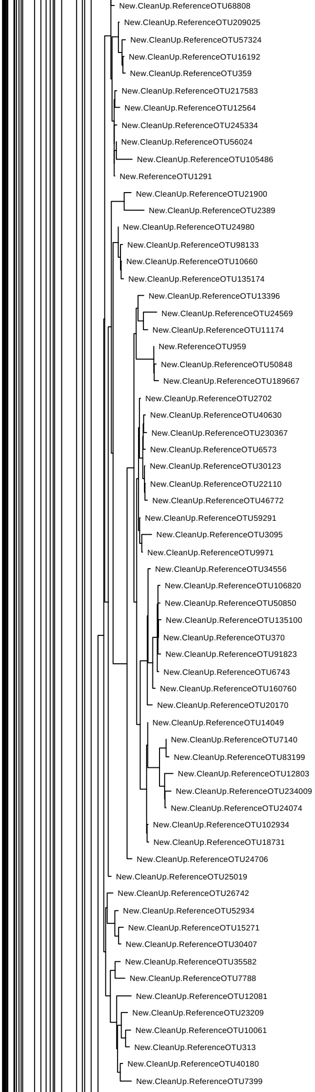
New.CleanUp.ReferenceOTU68808
New.CleanUp.ReferenceOTU209025
New.CleanUp.ReferenceOTU57324
New.CleanUp.ReferenceOTU16192
New.CleanUp.ReferenceOTU359
New.CleanUp.ReferenceOTU217583
New.CleanUp.ReferenceOTU12564
New.CleanUp.ReferenceOTU245334
New.CleanUp.ReferenceOTU56024
New.CleanUp.ReferenceOTU105486
New.ReferenceOTU1291
New.CleanUp.ReferenceOTU21900
New.CleanUp.ReferenceOTU2389
New.CleanUp.ReferenceOTU24980
New.CleanUp.ReferenceOTU98133
New.CleanUp.ReferenceOTU10660
New.CleanUp.ReferenceOTU135174
New.CleanUp.ReferenceOTU13396
New.CleanUp.ReferenceOTU24569
New.CleanUp.ReferenceOTU11174
New.ReferenceOTU959
New.CleanUp.ReferenceOTU50848
New.CleanUp.ReferenceOTU189667
New.CleanUp.ReferenceOTU2702
New.CleanUp.ReferenceOTU40630
New.CleanUp.ReferenceOTU230367
New.CleanUp.ReferenceOTU6573
New.CleanUp.ReferenceOTU30123
New.CleanUp.ReferenceOTU22110
New.CleanUp.ReferenceOTU46772
New.CleanUp.ReferenceOTU59291
New.CleanUp.ReferenceOTU3095
New.CleanUp.ReferenceOTU9971
New.CleanUp.ReferenceOTU34556
New.CleanUp.ReferenceOTU106820
New.CleanUp.ReferenceOTU50850
New.CleanUp.ReferenceOTU135100
New.CleanUp.ReferenceOTU370
New.CleanUp.ReferenceOTU91823
New.CleanUp.ReferenceOTU6743
New.CleanUp.ReferenceOTU160760
New.CleanUp.ReferenceOTU20170
New.CleanUp.ReferenceOTU14049
New.CleanUp.ReferenceOTU7140
New.CleanUp.ReferenceOTU83199
New.CleanUp.ReferenceOTU12803
New.CleanUp.ReferenceOTU234009
New.CleanUp.ReferenceOTU24074
New.CleanUp.ReferenceOTU102934
New.CleanUp.ReferenceOTU18731
New.CleanUp.ReferenceOTU24706
New.CleanUp.ReferenceOTU25019
New.CleanUp.ReferenceOTU26742
New.CleanUp.ReferenceOTU52934
New.CleanUp.ReferenceOTU15271
New.CleanUp.ReferenceOTU30407
New.CleanUp.ReferenceOTU35582
New.CleanUp.ReferenceOTU7788
New.CleanUp.ReferenceOTU12081
New.CleanUp.ReferenceOTU23209
New.CleanUp.ReferenceOTU10061
New.CleanUp.ReferenceOTU313
New.CleanUp.ReferenceOTU40180
New.CleanUp.ReferenceOTU7399

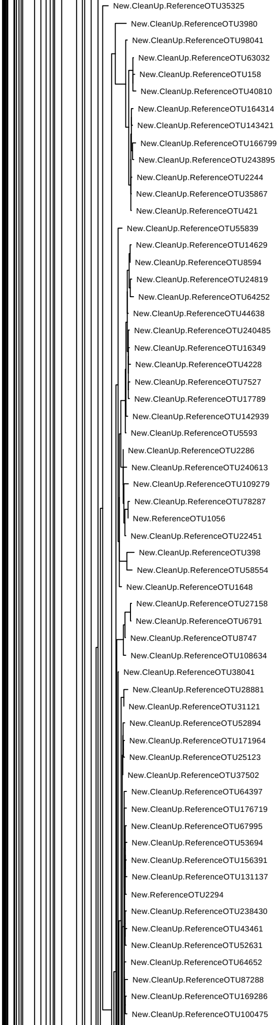

New.CleanUp.ReferenceOTU35325
New.CleanUp.ReferenceOTU3980
New.CleanUp.ReferenceOTU98041
New.CleanUp.ReferenceOTU63032
New.CleanUp.ReferenceOTU158
New.CleanUp.ReferenceOTU40810
New.CleanUp.ReferenceOTU164314
New.CleanUp.ReferenceOTU143421
New.CleanUp.ReferenceOTU166799
New.CleanUp.ReferenceOTU243895
New.CleanUp.ReferenceOTU2244
New.CleanUp.ReferenceOTU35867
New.CleanUp.ReferenceOTU421
New.CleanUp.ReferenceOTU55839
New.CleanUp.ReferenceOTU14629
New.CleanUp.ReferenceOTU8594
New.CleanUp.ReferenceOTU24819
New.CleanUp.ReferenceOTU64252
New.CleanUp.ReferenceOTU44638
New.CleanUp.ReferenceOTU240485
New.CleanUp.ReferenceOTU16349
New.CleanUp.ReferenceOTU4228
New.CleanUp.ReferenceOTU7527
New.CleanUp.ReferenceOTU17789
New.CleanUp.ReferenceOTU142939
New.CleanUp.ReferenceOTU5593
New.CleanUp.ReferenceOTU2286
New.CleanUp.ReferenceOTU240613
New.CleanUp.ReferenceOTU109279
New.CleanUp.ReferenceOTU78287
New.ReferenceOTU1056
New.CleanUp.ReferenceOTU22451
New.CleanUp.ReferenceOTU398
New.CleanUp.ReferenceOTU58554
New.CleanUp.ReferenceOTU1648
New.CleanUp.ReferenceOTU27158
New.CleanUp.ReferenceOTU6791
New.CleanUp.ReferenceOTU8747
New.CleanUp.ReferenceOTU108634
New.CleanUp.ReferenceOTU38041
New.CleanUp.ReferenceOTU28881
New.CleanUp.ReferenceOTU31121
New.CleanUp.ReferenceOTU52894
New.CleanUp.ReferenceOTU171964
New.CleanUp.ReferenceOTU25123
New.CleanUp.ReferenceOTU37502
New.CleanUp.ReferenceOTU64397
New.CleanUp.ReferenceOTU176719
New.CleanUp.ReferenceOTU67995
New.CleanUp.ReferenceOTU53694
New.CleanUp.ReferenceOTU156391
New.CleanUp.ReferenceOTU131137
New.ReferenceOTU2294
New.CleanUp.ReferenceOTU238430
New.CleanUp.ReferenceOTU43461
New.CleanUp.ReferenceOTU52631
New.CleanUp.ReferenceOTU64652
New.CleanUp.ReferenceOTU87288
New.CleanUp.ReferenceOTU169286
New.CleanUp.ReferenceOTU100475

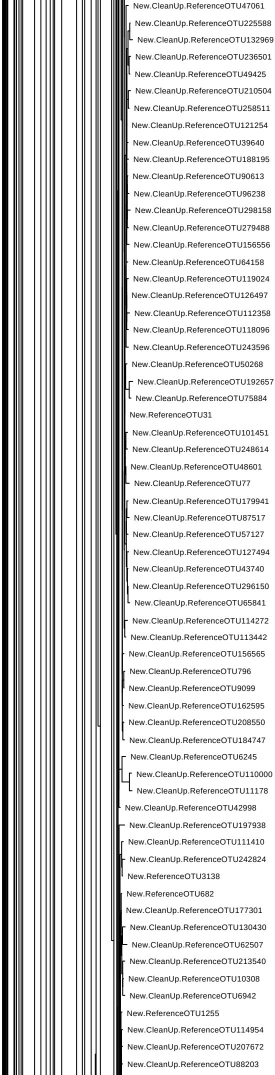
New.CleanUp.ReferenceOTU47061
New.CleanUp.ReferenceOTU225588
New.CleanUp.ReferenceOTU132969
New.CleanUp.ReferenceOTU236501
New.CleanUp.ReferenceOTU49425
New.CleanUp.ReferenceOTU210504
New.CleanUp.ReferenceOTU258511
New.CleanUp.ReferenceOTU121254
New.CleanUp.ReferenceOTU39640
New.CleanUp.ReferenceOTU188195
New.CleanUp.ReferenceOTU90613
New.CleanUp.ReferenceOTU96238
New.CleanUp.ReferenceOTU298158
New.CleanUp.ReferenceOTU279488
New.CleanUp.ReferenceOTU156556
New.CleanUp.ReferenceOTU64158
New.CleanUp.ReferenceOTU119024
New.CleanUp.ReferenceOTU126497
New.CleanUp.ReferenceOTU112358
New.CleanUp.ReferenceOTU118096
New.CleanUp.ReferenceOTU243596
New.CleanUp.ReferenceOTU50268
New.CleanUp.ReferenceOTU192657
New.CleanUp.ReferenceOTU75884
New.ReferenceOTU31
New.CleanUp.ReferenceOTU101451
New.CleanUp.ReferenceOTU248614
New.CleanUp.ReferenceOTU48601
New.CleanUp.ReferenceOTU77
New.CleanUp.ReferenceOTU179941
New.CleanUp.ReferenceOTU87517
New.CleanUp.ReferenceOTU57127
New.CleanUp.ReferenceOTU127494
New.CleanUp.ReferenceOTU43740
New.CleanUp.ReferenceOTU296150
New.CleanUp.ReferenceOTU65841
New.CleanUp.ReferenceOTU114272
New.CleanUp.ReferenceOTU113442
New.CleanUp.ReferenceOTU156565
New.CleanUp.ReferenceOTU796
New.CleanUp.ReferenceOTU9099
New.CleanUp.ReferenceOTU162595
New.CleanUp.ReferenceOTU208550
New.CleanUp.ReferenceOTU184747
New.CleanUp.ReferenceOTU6245
New.CleanUp.ReferenceOTU110000
New.CleanUp.ReferenceOTU11178
New.CleanUp.ReferenceOTU42998
New.CleanUp.ReferenceOTU197938
New.CleanUp.ReferenceOTU111410
New.CleanUp.ReferenceOTU242824
New.ReferenceOTU3138
New.ReferenceOTU682
New.CleanUp.ReferenceOTU177301
New.CleanUp.ReferenceOTU130430
New.CleanUp.ReferenceOTU62507
New.CleanUp.ReferenceOTU213540
New.CleanUp.ReferenceOTU10308
New.CleanUp.ReferenceOTU6942
New.ReferenceOTU1255
New.CleanUp.ReferenceOTU114954
New.CleanUp.ReferenceOTU207672
New.CleanUp.ReferenceOTU88203

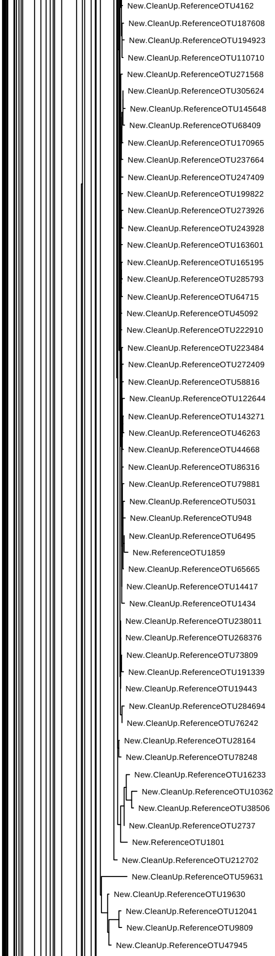
New.CleanUp.ReferenceOTU4162
New.CleanUp.ReferenceOTU187608
New.CleanUp.ReferenceOTU194923
New.CleanUp.ReferenceOTU110710
New.CleanUp.ReferenceOTU271568
New.CleanUp.ReferenceOTU305624
New.CleanUp.ReferenceOTU145648
New.CleanUp.ReferenceOTU68409
New.CleanUp.ReferenceOTU170965
New.CleanUp.ReferenceOTU237664
New.CleanUp.ReferenceOTU247409
New.CleanUp.ReferenceOTU199822
New.CleanUp.ReferenceOTU273926
New.CleanUp.ReferenceOTU243928
New.CleanUp.ReferenceOTU163601
New.CleanUp.ReferenceOTU165195
New.CleanUp.ReferenceOTU285793
New.CleanUp.ReferenceOTU64715
New.CleanUp.ReferenceOTU45092
New.CleanUp.ReferenceOTU222910
New.CleanUp.ReferenceOTU223484
New.CleanUp.ReferenceOTU272409
New.CleanUp.ReferenceOTU58816
New.CleanUp.ReferenceOTU122644
New.CleanUp.ReferenceOTU143271
New.CleanUp.ReferenceOTU46263
New.CleanUp.ReferenceOTU44668
New.CleanUp.ReferenceOTU86316
New.CleanUp.ReferenceOTU79881
New.CleanUp.ReferenceOTU5031
New.CleanUp.ReferenceOTU948
New.CleanUp.ReferenceOTU6495
New.ReferenceOTU1859
New.CleanUp.ReferenceOTU65665
New.CleanUp.ReferenceOTU14417
New.CleanUp.ReferenceOTU1434
New.CleanUp.ReferenceOTU238011
New.CleanUp.ReferenceOTU268376
New.CleanUp.ReferenceOTU73809
New.CleanUp.ReferenceOTU191339
New.CleanUp.ReferenceOTU19443
New.CleanUp.ReferenceOTU284694
New.CleanUp.ReferenceOTU76242
New.CleanUp.ReferenceOTU28164
New.CleanUp.ReferenceOTU78248
New.CleanUp.ReferenceOTU16233
New.CleanUp.ReferenceOTU10362
New.CleanUp.ReferenceOTU38506
New.CleanUp.ReferenceOTU2737
New.ReferenceOTU1801
New.CleanUp.ReferenceOTU212702
New.CleanUp.ReferenceOTU59631
New.CleanUp.ReferenceOTU19630
New.CleanUp.ReferenceOTU12041
New.CleanUp.ReferenceOTU9809
New.CleanUp.ReferenceOTU47945

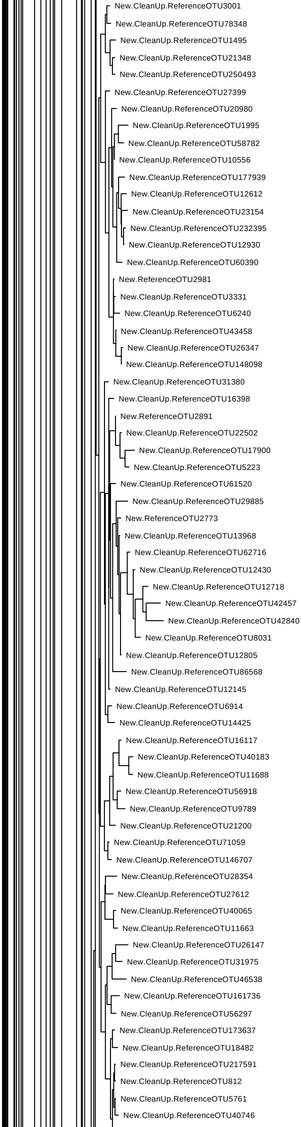
New.CleanUp.ReferenceOTU3001
New.CleanUp.ReferenceOTU78348
New.CleanUp.ReferenceOTU1495
New.CleanUp.ReferenceOTU21348
New.CleanUp.ReferenceOTU250493
New.CleanUp.ReferenceOTU27399
New.CleanUp.ReferenceOTU20980
New.CleanUp.ReferenceOTU1995
New.CleanUp.ReferenceOTU58782
New.CleanUp.ReferenceOTU10556
New.CleanUp.ReferenceOTU177939
New.CleanUp.ReferenceOTU12612
New.CleanUp.ReferenceOTU23154
New.CleanUp.ReferenceOTU232395
New.CleanUp.ReferenceOTU12930
New.CleanUp.ReferenceOTU60390
New.ReferenceOTU2981
New.CleanUp.ReferenceOTU3331
New.CleanUp.ReferenceOTU6240
New.CleanUp.ReferenceOTU43458
New.CleanUp.ReferenceOTU26347
New.CleanUp.ReferenceOTU148098
New.CleanUp.ReferenceOTU31380
New.CleanUp.ReferenceOTU16398
New.ReferenceOTU2891
New.CleanUp.ReferenceOTU22502
New.CleanUp.ReferenceOTU17900
New.CleanUp.ReferenceOTU5223
New.CleanUp.ReferenceOTU61520
New.CleanUp.ReferenceOTU29885
New.ReferenceOTU2773
New.CleanUp.ReferenceOTU13968
New.CleanUp.ReferenceOTU62716
New.CleanUp.ReferenceOTU12430
New.CleanUp.ReferenceOTU12718
New.CleanUp.ReferenceOTU42457
New.CleanUp.ReferenceOTU42840
New.CleanUp.ReferenceOTU8031
New.CleanUp.ReferenceOTU12805
New.CleanUp.ReferenceOTU86568
New.CleanUp.ReferenceOTU12145
New.CleanUp.ReferenceOTU6914
New.CleanUp.ReferenceOTU14425
New.CleanUp.ReferenceOTU16117
New.CleanUp.ReferenceOTU40183
New.CleanUp.ReferenceOTU11688
New.CleanUp.ReferenceOTU56918
New.CleanUp.ReferenceOTU9789
New.CleanUp.ReferenceOTU21200
New.CleanUp.ReferenceOTU71059
New.CleanUp.ReferenceOTU146707
New.CleanUp.ReferenceOTU28354
New.CleanUp.ReferenceOTU27612
New.CleanUp.ReferenceOTU40065
New.CleanUp.ReferenceOTU11663
New.CleanUp.ReferenceOTU26147
New.CleanUp.ReferenceOTU31975
New.CleanUp.ReferenceOTU46538
New.CleanUp.ReferenceOTU161736
New.CleanUp.ReferenceOTU56297
New.CleanUp.ReferenceOTU173637
New.CleanUp.ReferenceOTU18482
New.CleanUp.ReferenceOTU217591
New.CleanUp.ReferenceOTU812
New.CleanUp.ReferenceOTU5761
New.CleanUp.ReferenceOTU40746

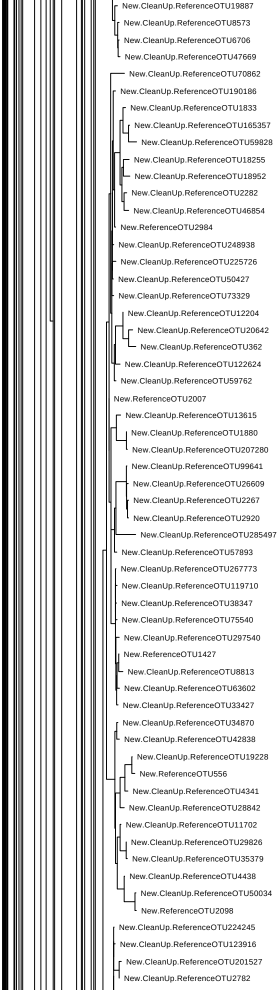

New.CleanUp.ReferenceOTU19887
New.CleanUp.ReferenceOTU8573
New.CleanUp.ReferenceOTU6706
New.CleanUp.ReferenceOTU47669
New.CleanUp.ReferenceOTU70862
New.CleanUp.ReferenceOTU190186
New.CleanUp.ReferenceOTU1833
New.CleanUp.ReferenceOTU165357
New.CleanUp.ReferenceOTU59828
New.CleanUp.ReferenceOTU18255
New.CleanUp.ReferenceOTU18952
New.CleanUp.ReferenceOTU2282
New.CleanUp.ReferenceOTU46854
New.ReferenceOTU2984
New.CleanUp.ReferenceOTU248938
New.CleanUp.ReferenceOTU225726
New.CleanUp.ReferenceOTU50427
New.CleanUp.ReferenceOTU73329
New.CleanUp.ReferenceOTU12204
New.CleanUp.ReferenceOTU20642
New.CleanUp.ReferenceOTU362
New.CleanUp.ReferenceOTU122624
New.CleanUp.ReferenceOTU59762
New.ReferenceOTU2007
New.CleanUp.ReferenceOTU13615
New.CleanUp.ReferenceOTU1880
New.CleanUp.ReferenceOTU207280
New.CleanUp.ReferenceOTU99641
New.CleanUp.ReferenceOTU26609
New.CleanUp.ReferenceOTU2267
New.CleanUp.ReferenceOTU2920
New.CleanUp.ReferenceOTU285497
New.CleanUp.ReferenceOTU57893
New.CleanUp.ReferenceOTU267773
New.CleanUp.ReferenceOTU119710
New.CleanUp.ReferenceOTU38347
New.CleanUp.ReferenceOTU75540
New.CleanUp.ReferenceOTU297540
New.ReferenceOTU1427
New.CleanUp.ReferenceOTU8813
New.CleanUp.ReferenceOTU63602
New.CleanUp.ReferenceOTU33427
New.CleanUp.ReferenceOTU34870
New.CleanUp.ReferenceOTU42838
New.CleanUp.ReferenceOTU19228
New.ReferenceOTU556
New.CleanUp.ReferenceOTU4341
New.CleanUp.ReferenceOTU28842
New.CleanUp.ReferenceOTU11702
New.CleanUp.ReferenceOTU29826
New.CleanUp.ReferenceOTU35379
New.CleanUp.ReferenceOTU4438
New.CleanUp.ReferenceOTU50034
New.ReferenceOTU2098
New.CleanUp.ReferenceOTU224245
New.CleanUp.ReferenceOTU123916
New.CleanUp.ReferenceOTU201527
New.CleanUp.ReferenceOTU2782

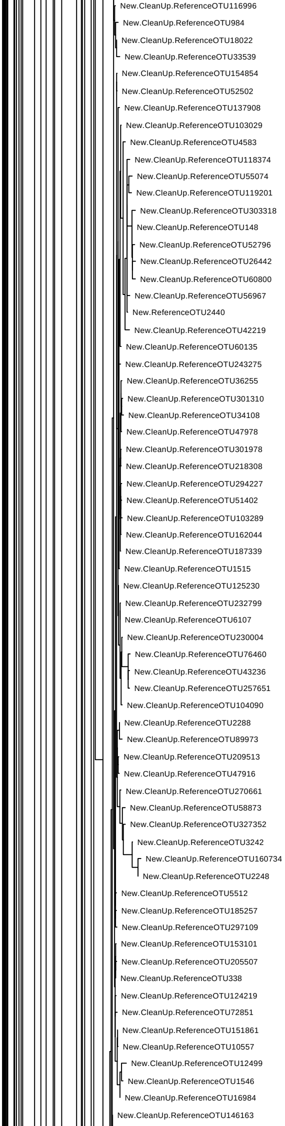
New.CleanUp.ReferenceOTU116996
New.CleanUp.ReferenceOTU984
New.CleanUp.ReferenceOTU18022
New.CleanUp.ReferenceOTU33539
New.CleanUp.ReferenceOTU154854
New.CleanUp.ReferenceOTU52502
New.CleanUp.ReferenceOTU137908
New.CleanUp.ReferenceOTU103029
New.CleanUp.ReferenceOTU4583
New.CleanUp.ReferenceOTU118374
New.CleanUp.ReferenceOTU55074
New.CleanUp.ReferenceOTU119201
New.CleanUp.ReferenceOTU303318
New.CleanUp.ReferenceOTU148
New.CleanUp.ReferenceOTU52796
New.CleanUp.ReferenceOTU26442
New.CleanUp.ReferenceOTU60800
New.CleanUp.ReferenceOTU56967
New.ReferenceOTU2440
New.CleanUp.ReferenceOTU42219
New.CleanUp.ReferenceOTU60135
New.CleanUp.ReferenceOTU243275
New.CleanUp.ReferenceOTU36255
New.CleanUp.ReferenceOTU301310
New.CleanUp.ReferenceOTU34108
New.CleanUp.ReferenceOTU47978
New.CleanUp.ReferenceOTU301978
New.CleanUp.ReferenceOTU218308
New.CleanUp.ReferenceOTU294227
New.CleanUp.ReferenceOTU51402
New.CleanUp.ReferenceOTU103289
New.CleanUp.ReferenceOTU162044
New.CleanUp.ReferenceOTU187339
New.CleanUp.ReferenceOTU1515
New.CleanUp.ReferenceOTU125230
New.CleanUp.ReferenceOTU232799
New.CleanUp.ReferenceOTU6107
New.CleanUp.ReferenceOTU230004
New.CleanUp.ReferenceOTU76460
New.CleanUp.ReferenceOTU43236
New.CleanUp.ReferenceOTU257651
New.CleanUp.ReferenceOTU104090
New.CleanUp.ReferenceOTU2288
New.CleanUp.ReferenceOTU89973
New.CleanUp.ReferenceOTU209513
New.CleanUp.ReferenceOTU47916
New.CleanUp.ReferenceOTU270661
New.CleanUp.ReferenceOTU58873
New.CleanUp.ReferenceOTU327352
New.CleanUp.ReferenceOTU3242
New.CleanUp.ReferenceOTU160734
New.CleanUp.ReferenceOTU2248
New.CleanUp.ReferenceOTU5512
New.CleanUp.ReferenceOTU185257
New.CleanUp.ReferenceOTU297109
New.CleanUp.ReferenceOTU153101
New.CleanUp.ReferenceOTU205507
New.CleanUp.ReferenceOTU338
New.CleanUp.ReferenceOTU124219
New.CleanUp.ReferenceOTU72851
New.CleanUp.ReferenceOTU151861
New.CleanUp.ReferenceOTU10557
New.CleanUp.ReferenceOTU12499
New.CleanUp.ReferenceOTU1546
New.CleanUp.ReferenceOTU16984
New.CleanUp.ReferenceOTU146163

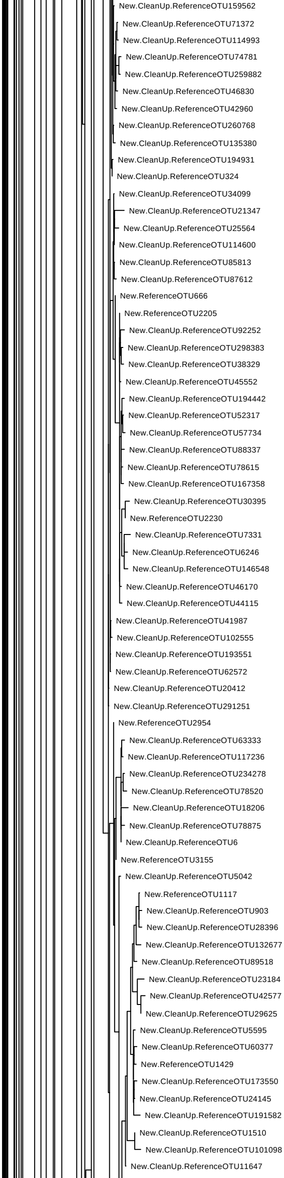
New.CleanUp.ReferenceOTU159562
New.CleanUp.ReferenceOTU71372
New.CleanUp.ReferenceOTU114993
New.CleanUp.ReferenceOTU74781
New.CleanUp.ReferenceOTU259882
New.CleanUp.ReferenceOTU46830
New.CleanUp.ReferenceOTU42960
New.CleanUp.ReferenceOTU260768
New.CleanUp.ReferenceOTU135380
New.CleanUp.ReferenceOTU194931
New.CleanUp.ReferenceOTU324
New.CleanUp.ReferenceOTU34099
New.CleanUp.ReferenceOTU21347
New.CleanUp.ReferenceOTU25564
New.CleanUp.ReferenceOTU114600
New.CleanUp.ReferenceOTU85813
New.CleanUp.ReferenceOTU87612
New.ReferenceOTU666
New.ReferenceOTU2205
New.CleanUp.ReferenceOTU92252
New.CleanUp.ReferenceOTU298383
New.CleanUp.ReferenceOTU38329
New.CleanUp.ReferenceOTU45552
New.CleanUp.ReferenceOTU194442
New.CleanUp.ReferenceOTU52317
New.CleanUp.ReferenceOTU57734
New.CleanUp.ReferenceOTU88337
New.CleanUp.ReferenceOTU78615
New.CleanUp.ReferenceOTU167358
New.CleanUp.ReferenceOTU30395
New.ReferenceOTU2230
New.CleanUp.ReferenceOTU7331
New.CleanUp.ReferenceOTU6246
New.CleanUp.ReferenceOTU146548
New.CleanUp.ReferenceOTU46170
New.CleanUp.ReferenceOTU44115
New.CleanUp.ReferenceOTU41987
New.CleanUp.ReferenceOTU102555
New.CleanUp.ReferenceOTU193551
New.CleanUp.ReferenceOTU62572
New.CleanUp.ReferenceOTU20412
New.CleanUp.ReferenceOTU291251
New.ReferenceOTU2954
New.CleanUp.ReferenceOTU63333
New.CleanUp.ReferenceOTU117236
New.CleanUp.ReferenceOTU234278
New.CleanUp.ReferenceOTU78520
New.CleanUp.ReferenceOTU18206
New.CleanUp.ReferenceOTU78875
New.CleanUp.ReferenceOTU6
New.ReferenceOTU3155
New.CleanUp.ReferenceOTU5042
New.ReferenceOTU1117
New.CleanUp.ReferenceOTU903
New.CleanUp.ReferenceOTU28396
New.CleanUp.ReferenceOTU132677
New.CleanUp.ReferenceOTU89518
New.CleanUp.ReferenceOTU23184
New.CleanUp.ReferenceOTU42577
New.CleanUp.ReferenceOTU29625
New.CleanUp.ReferenceOTU5595
New.CleanUp.ReferenceOTU60377
New.ReferenceOTU1429
New.CleanUp.ReferenceOTU173550
New.CleanUp.ReferenceOTU24145
New.CleanUp.ReferenceOTU191582
New.CleanUp.ReferenceOTU1510
New.CleanUp.ReferenceOTU101098
New.CleanUp.ReferenceOTU11647

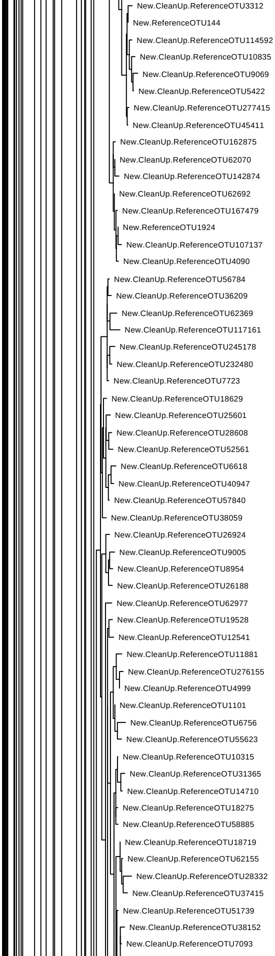
New.CleanUp.ReferenceOTU3312
New.ReferenceOTU144
New.CleanUp.ReferenceOTU114592
New.CleanUp.ReferenceOTU10835
New.CleanUp.ReferenceOTU9069
New.CleanUp.ReferenceOTU5422
New.CleanUp.ReferenceOTU277415
New.CleanUp.ReferenceOTU45411
New.CleanUp.ReferenceOTU162875
New.CleanUp.ReferenceOTU62070
New.CleanUp.ReferenceOTU142874
New.CleanUp.ReferenceOTU62692
New.CleanUp.ReferenceOTU167479
New.ReferenceOTU1924
New.CleanUp.ReferenceOTU107137
New.CleanUp.ReferenceOTU4090
New.CleanUp.ReferenceOTU56784
New.CleanUp.ReferenceOTU36209
New.CleanUp.ReferenceOTU62369
New.CleanUp.ReferenceOTU117161
New.CleanUp.ReferenceOTU245178
New.CleanUp.ReferenceOTU232480
New.CleanUp.ReferenceOTU7723
New.CleanUp.ReferenceOTU18629
New.CleanUp.ReferenceOTU25601
New.CleanUp.ReferenceOTU28608
New.CleanUp.ReferenceOTU52561
New.CleanUp.ReferenceOTU6618
New.CleanUp.ReferenceOTU40947
New.CleanUp.ReferenceOTU57840
New.CleanUp.ReferenceOTU38059
New.CleanUp.ReferenceOTU26924
New.CleanUp.ReferenceOTU9005
New.CleanUp.ReferenceOTU8954
New.CleanUp.ReferenceOTU26188
New.CleanUp.ReferenceOTU62977
New.CleanUp.ReferenceOTU19528
New.CleanUp.ReferenceOTU12541
New.CleanUp.ReferenceOTU11881
New.CleanUp.ReferenceOTU276155
New.CleanUp.ReferenceOTU4999
New.CleanUp.ReferenceOTU1101
New.CleanUp.ReferenceOTU6756
New.CleanUp.ReferenceOTU55623
New.CleanUp.ReferenceOTU10315
New.CleanUp.ReferenceOTU31365
New.CleanUp.ReferenceOTU14710
New.CleanUp.ReferenceOTU18275
New.CleanUp.ReferenceOTU58885
New.CleanUp.ReferenceOTU18719
New.CleanUp.ReferenceOTU62155
New.CleanUp.ReferenceOTU28332
New.CleanUp.ReferenceOTU37415
New.CleanUp.ReferenceOTU51739
New.CleanUp.ReferenceOTU38152
New.CleanUp.ReferenceOTU7093

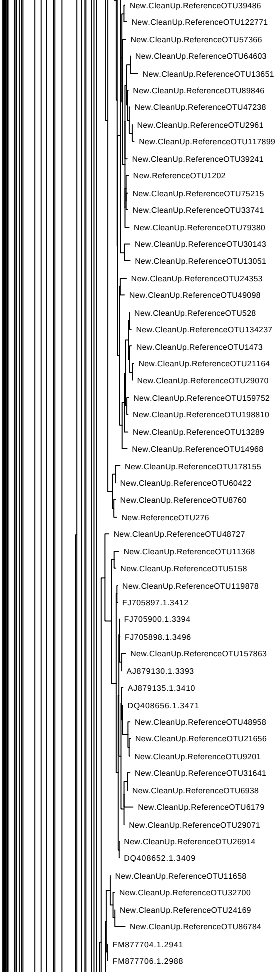
New.CleanUp.ReferenceOTU39486
New.CleanUp.ReferenceOTU122771
New.CleanUp.ReferenceOTU57366
New.CleanUp.ReferenceOTU64603
New.CleanUp.ReferenceOTU13651
New.CleanUp.ReferenceOTU89846
New.CleanUp.ReferenceOTU47238
New.CleanUp.ReferenceOTU2961
New.CleanUp.ReferenceOTU117899
New.CleanUp.ReferenceOTU39241
New.ReferenceOTU1202
New.CleanUp.ReferenceOTU75215
New.CleanUp.ReferenceOTU33741
New.CleanUp.ReferenceOTU79380
New.CleanUp.ReferenceOTU30143
New.CleanUp.ReferenceOTU13051
New.CleanUp.ReferenceOTU24353
New.CleanUp.ReferenceOTU49098
New.CleanUp.ReferenceOTU528
New.CleanUp.ReferenceOTU134237
New.CleanUp.ReferenceOTU1473
New.CleanUp.ReferenceOTU21164
New.CleanUp.ReferenceOTU29070
New.CleanUp.ReferenceOTU159752
New.CleanUp.ReferenceOTU198810
New.CleanUp.ReferenceOTU13289
New.CleanUp.ReferenceOTU14968
New.CleanUp.ReferenceOTU178155
New.CleanUp.ReferenceOTU60422
New.CleanUp.ReferenceOTU8760
New.ReferenceOTU276
New.CleanUp.ReferenceOTU48727
New.CleanUp.ReferenceOTU11368
New.CleanUp.ReferenceOTU5158
New.CleanUp.ReferenceOTU119878
FJ705897.1.3412
FJ705900.1.3394
FJ705898.1.3496
New.CleanUp.ReferenceOTU157863
AJ879130.1.3393
AJ879135.1.3410
DQ408656.1.3471
New.CleanUp.ReferenceOTU48958
New.CleanUp.ReferenceOTU21656
New.CleanUp.ReferenceOTU9201
New.CleanUp.ReferenceOTU31641
New.CleanUp.ReferenceOTU6938
New.CleanUp.ReferenceOTU6179
New.CleanUp.ReferenceOTU29071
New.CleanUp.ReferenceOTU26914
DQ408652.1.3409
New.CleanUp.ReferenceOTU11658
New.CleanUp.ReferenceOTU32700
New.CleanUp.ReferenceOTU24169
New.CleanUp.ReferenceOTU86784
FM877704.1.2941
FM877706.1.2988

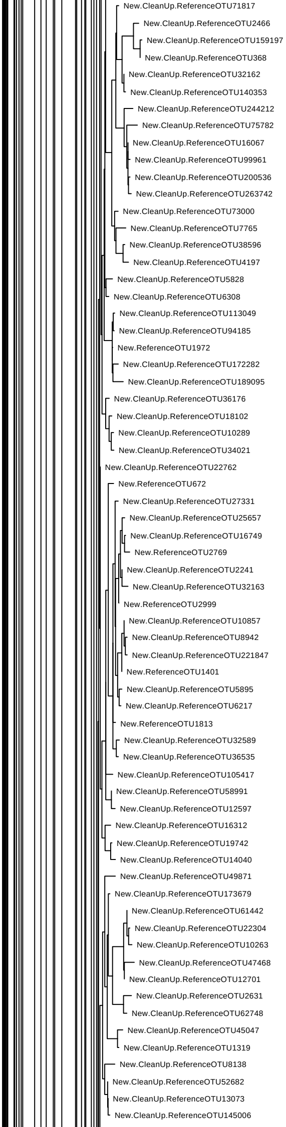
New.CleanUp.ReferenceOTU71817
New.CleanUp.ReferenceOTU2466
New.CleanUp.ReferenceOTU159197
New.CleanUp.ReferenceOTU368
New.CleanUp.ReferenceOTU32162
New.CleanUp.ReferenceOTU140353
New.CleanUp.ReferenceOTU244212
New.CleanUp.ReferenceOTU75782
New.CleanUp.ReferenceOTU16067
New.CleanUp.ReferenceOTU99961
New.CleanUp.ReferenceOTU200536
New.CleanUp.ReferenceOTU263742
New.CleanUp.ReferenceOTU73000
New.CleanUp.ReferenceOTU7765
New.CleanUp.ReferenceOTU38596
New.CleanUp.ReferenceOTU4197
New.CleanUp.ReferenceOTU5828
New.CleanUp.ReferenceOTU6308
New.CleanUp.ReferenceOTU113049
New.CleanUp.ReferenceOTU94185
New.ReferenceOTU1972
New.CleanUp.ReferenceOTU172282
New.CleanUp.ReferenceOTU189095
New.CleanUp.ReferenceOTU36176
New.CleanUp.ReferenceOTU18102
New.CleanUp.ReferenceOTU10289
New.CleanUp.ReferenceOTU34021
New.CleanUp.ReferenceOTU22762
New.ReferenceOTU672
New.CleanUp.ReferenceOTU27331
New.CleanUp.ReferenceOTU25657
New.CleanUp.ReferenceOTU16749
New.ReferenceOTU2769
New.CleanUp.ReferenceOTU2241
New.CleanUp.ReferenceOTU32163
New.ReferenceOTU2999
New.CleanUp.ReferenceOTU10857
New.CleanUp.ReferenceOTU8942
New.CleanUp.ReferenceOTU221847
New.ReferenceOTU1401
New.CleanUp.ReferenceOTU5895
New.CleanUp.ReferenceOTU6217
New.ReferenceOTU1813
New.CleanUp.ReferenceOTU32589
New.CleanUp.ReferenceOTU36535
New.CleanUp.ReferenceOTU105417
New.CleanUp.ReferenceOTU58991
New.CleanUp.ReferenceOTU12597
New.CleanUp.ReferenceOTU16312
New.CleanUp.ReferenceOTU19742
New.CleanUp.ReferenceOTU14040
New.CleanUp.ReferenceOTU49871
New.CleanUp.ReferenceOTU173679
New.CleanUp.ReferenceOTU61442
New.CleanUp.ReferenceOTU22304
New.CleanUp.ReferenceOTU10263
New.CleanUp.ReferenceOTU47468
New.CleanUp.ReferenceOTU12701
New.CleanUp.ReferenceOTU2631
New.CleanUp.ReferenceOTU62748
New.CleanUp.ReferenceOTU45047
New.CleanUp.ReferenceOTU1319
New.CleanUp.ReferenceOTU8138
New.CleanUp.ReferenceOTU52682
New.CleanUp.ReferenceOTU13073
New.CleanUp.ReferenceOTU145006

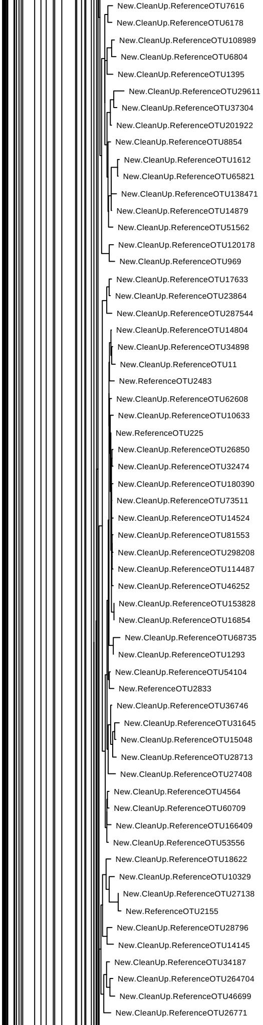
New.CleanUp.ReferenceOTU7616
New.CleanUp.ReferenceOTU6178
New.CleanUp.ReferenceOTU108989
New.CleanUp.ReferenceOTU6804
New.CleanUp.ReferenceOTU1395
New.CleanUp.ReferenceOTU29611
New.CleanUp.ReferenceOTU37304
New.CleanUp.ReferenceOTU201922
New.CleanUp.ReferenceOTU8854
New.CleanUp.ReferenceOTU1612
New.CleanUp.ReferenceOTU65821
New.CleanUp.ReferenceOTU138471
New.CleanUp.ReferenceOTU14879
New.CleanUp.ReferenceOTU51562
New.CleanUp.ReferenceOTU120178
New.CleanUp.ReferenceOTU969
New.CleanUp.ReferenceOTU17633
New.CleanUp.ReferenceOTU23864
New.CleanUp.ReferenceOTU287544
New.CleanUp.ReferenceOTU14804
New.CleanUp.ReferenceOTU34898
New.CleanUp.ReferenceOTU11
New.ReferenceOTU2483
New.CleanUp.ReferenceOTU62608
New.CleanUp.ReferenceOTU10633
New.ReferenceOTU225
New.CleanUp.ReferenceOTU26850
New.CleanUp.ReferenceOTU32474
New.CleanUp.ReferenceOTU180390
New.CleanUp.ReferenceOTU73511
New.CleanUp.ReferenceOTU14524
New.CleanUp.ReferenceOTU81553
New.CleanUp.ReferenceOTU298208
New.CleanUp.ReferenceOTU114487
New.CleanUp.ReferenceOTU46252
New.CleanUp.ReferenceOTU153828
New.CleanUp.ReferenceOTU16854
New.CleanUp.ReferenceOTU68735
New.CleanUp.ReferenceOTU1293
New.CleanUp.ReferenceOTU54104
New.ReferenceOTU2833
New.CleanUp.ReferenceOTU36746
New.CleanUp.ReferenceOTU31645
New.CleanUp.ReferenceOTU15048
New.CleanUp.ReferenceOTU28713
New.CleanUp.ReferenceOTU27408
New.CleanUp.ReferenceOTU4564
New.CleanUp.ReferenceOTU60709
New.CleanUp.ReferenceOTU166409
New.CleanUp.ReferenceOTU53556
New.CleanUp.ReferenceOTU18622
New.CleanUp.ReferenceOTU10329
New.CleanUp.ReferenceOTU27138
New.ReferenceOTU2155
New.CleanUp.ReferenceOTU28796
New.CleanUp.ReferenceOTU14145
New.CleanUp.ReferenceOTU34187
New.CleanUp.ReferenceOTU264704
New.CleanUp.ReferenceOTU46699
New.CleanUp.ReferenceOTU26771

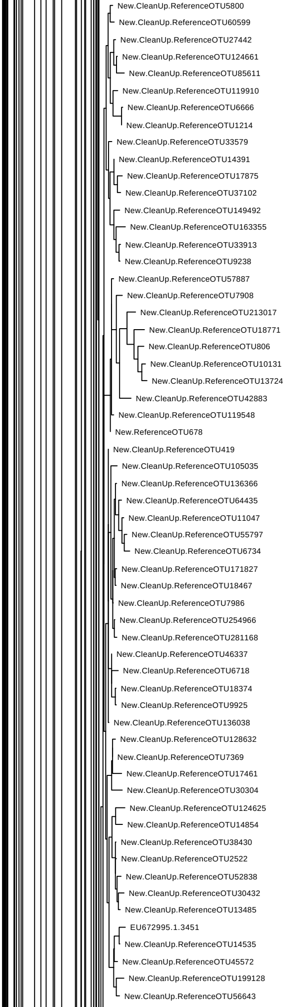
New.CleanUp.ReferenceOTU5800
New.CleanUp.ReferenceOTU60599
New.CleanUp.ReferenceOTU27442
New.CleanUp.ReferenceOTU124661
New.CleanUp.ReferenceOTU85611
New.CleanUp.ReferenceOTU119910
New.CleanUp.ReferenceOTU6666
New.CleanUp.ReferenceOTU1214
New.CleanUp.ReferenceOTU33579
New.CleanUp.ReferenceOTU14391
New.CleanUp.ReferenceOTU17875
New.CleanUp.ReferenceOTU37102
New.CleanUp.ReferenceOTU149492
New.CleanUp.ReferenceOTU163355
New.CleanUp.ReferenceOTU33913
New.CleanUp.ReferenceOTU9238
New.CleanUp.ReferenceOTU57887
New.CleanUp.ReferenceOTU7908
New.CleanUp.ReferenceOTU213017
New.CleanUp.ReferenceOTU18771
New.CleanUp.ReferenceOTU806
New.CleanUp.ReferenceOTU10131
New.CleanUp.ReferenceOTU13724
New.CleanUp.ReferenceOTU42883
New.CleanUp.ReferenceOTU119548
New.ReferenceOTU678
New.CleanUp.ReferenceOTU419
New.CleanUp.ReferenceOTU105035
New.CleanUp.ReferenceOTU136366
New.CleanUp.ReferenceOTU64435
New.CleanUp.ReferenceOTU11047
New.CleanUp.ReferenceOTU55797
New.CleanUp.ReferenceOTU6734
New.CleanUp.ReferenceOTU171827
New.CleanUp.ReferenceOTU18467
New.CleanUp.ReferenceOTU7986
New.CleanUp.ReferenceOTU254966
New.CleanUp.ReferenceOTU281168
New.CleanUp.ReferenceOTU46337
New.CleanUp.ReferenceOTU6718
New.CleanUp.ReferenceOTU18374
New.CleanUp.ReferenceOTU9925
New.CleanUp.ReferenceOTU136038
New.CleanUp.ReferenceOTU128632
New.CleanUp.ReferenceOTU7369
New.CleanUp.ReferenceOTU17461
New.CleanUp.ReferenceOTU30304
New.CleanUp.ReferenceOTU124625
New.CleanUp.ReferenceOTU14854
New.CleanUp.ReferenceOTU38430
New.CleanUp.ReferenceOTU2522
New.CleanUp.ReferenceOTU52838
New.CleanUp.ReferenceOTU30432
New.CleanUp.ReferenceOTU13485
EU672995.1.3451
New.CleanUp.ReferenceOTU14535
New.CleanUp.ReferenceOTU45572
New.CleanUp.ReferenceOTU199128
New.CleanUp.ReferenceOTU56643

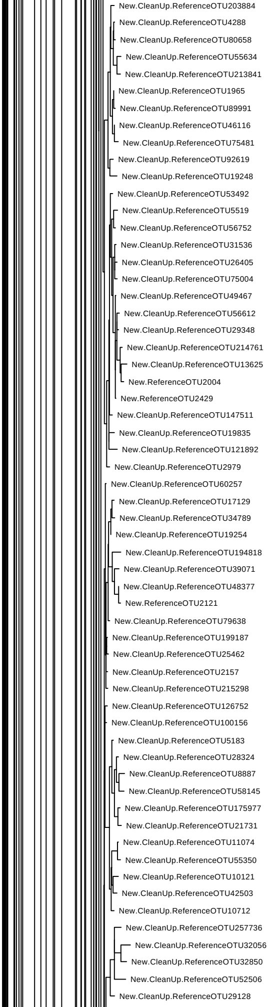
New.CleanUp.ReferenceOTU203884
New.CleanUp.ReferenceOTU4288
New.CleanUp.ReferenceOTU80658
New.CleanUp.ReferenceOTU55634
New.CleanUp.ReferenceOTU213841
New.CleanUp.ReferenceOTU1965
New.CleanUp.ReferenceOTU89991
New.CleanUp.ReferenceOTU46116
New.CleanUp.ReferenceOTU75481
New.CleanUp.ReferenceOTU92619
New.CleanUp.ReferenceOTU19248
New.CleanUp.ReferenceOTU53492
New.CleanUp.ReferenceOTU5519
New.CleanUp.ReferenceOTU56752
New.CleanUp.ReferenceOTU31536
New.CleanUp.ReferenceOTU26405
New.CleanUp.ReferenceOTU75004
New.CleanUp.ReferenceOTU49467
New.CleanUp.ReferenceOTU56612
New.CleanUp.ReferenceOTU29348
New.CleanUp.ReferenceOTU214761
New.CleanUp.ReferenceOTU13625
New.ReferenceOTU2004
New.ReferenceOTU2429
New.CleanUp.ReferenceOTU147511
New.CleanUp.ReferenceOTU19835
New.CleanUp.ReferenceOTU121892
New.CleanUp.ReferenceOTU2979
New.CleanUp.ReferenceOTU60257
New.CleanUp.ReferenceOTU17129
New.CleanUp.ReferenceOTU34789
New.CleanUp.ReferenceOTU19254
New.CleanUp.ReferenceOTU194818
New.CleanUp.ReferenceOTU39071
New.CleanUp.ReferenceOTU48377
New.ReferenceOTU2121
New.CleanUp.ReferenceOTU79638
New.CleanUp.ReferenceOTU199187
New.CleanUp.ReferenceOTU25462
New.CleanUp.ReferenceOTU2157
New.CleanUp.ReferenceOTU215298
New.CleanUp.ReferenceOTU126752
New.CleanUp.ReferenceOTU100156
New.CleanUp.ReferenceOTU5183
New.CleanUp.ReferenceOTU28324
New.CleanUp.ReferenceOTU8887
New.CleanUp.ReferenceOTU58145
New.CleanUp.ReferenceOTU175977
New.CleanUp.ReferenceOTU21731
New.CleanUp.ReferenceOTU11074
New.CleanUp.ReferenceOTU55350
New.CleanUp.ReferenceOTU10121
New.CleanUp.ReferenceOTU42503
New.CleanUp.ReferenceOTU10712
New.CleanUp.ReferenceOTU257736
New.CleanUp.ReferenceOTU32056
New.CleanUp.ReferenceOTU32850
New.CleanUp.ReferenceOTU52506
New.CleanUp.ReferenceOTU29128

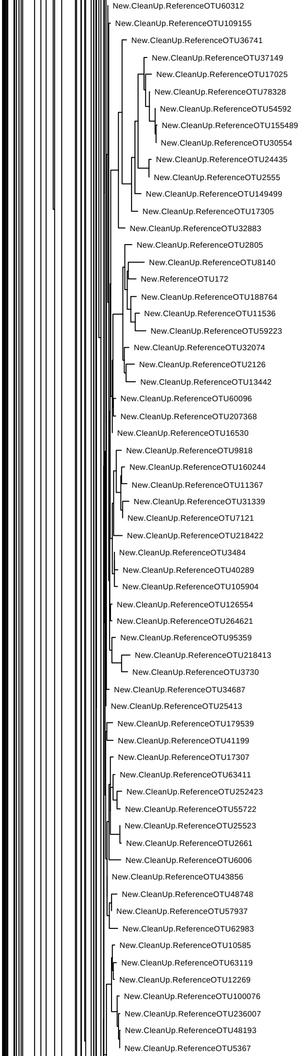
New.CleanUp.ReferenceOTU60312
New.CleanUp.ReferenceOTU109155
New.CleanUp.ReferenceOTU36741
New.CleanUp.ReferenceOTU37149
New.CleanUp.ReferenceOTU17025
New.CleanUp.ReferenceOTU78328
New.CleanUp.ReferenceOTU54592
New.CleanUp.ReferenceOTU155489
New.CleanUp.ReferenceOTU30554
New.CleanUp.ReferenceOTU24435
New.CleanUp.ReferenceOTU2555
New.CleanUp.ReferenceOTU149499
New.CleanUp.ReferenceOTU17305
New.CleanUp.ReferenceOTU32883
New.CleanUp.ReferenceOTU2805
New.CleanUp.ReferenceOTU8140
New.ReferenceOTU172
New.CleanUp.ReferenceOTU188764
New.CleanUp.ReferenceOTU11536
New.CleanUp.ReferenceOTU59223
New.CleanUp.ReferenceOTU32074
New.CleanUp.ReferenceOTU2126
New.CleanUp.ReferenceOTU13442
New.CleanUp.ReferenceOTU60096
New.CleanUp.ReferenceOTU207368
New.CleanUp.ReferenceOTU16530
New.CleanUp.ReferenceOTU9818
New.CleanUp.ReferenceOTU160244
New.CleanUp.ReferenceOTU11367
New.CleanUp.ReferenceOTU31339
New.CleanUp.ReferenceOTU7121
New.CleanUp.ReferenceOTU218422
New.CleanUp.ReferenceOTU3484
New.CleanUp.ReferenceOTU40289
New.CleanUp.ReferenceOTU105904
New.CleanUp.ReferenceOTU126554
New.CleanUp.ReferenceOTU264621
New.CleanUp.ReferenceOTU95359
New.CleanUp.ReferenceOTU218413
New.CleanUp.ReferenceOTU3730
New.CleanUp.ReferenceOTU34687
New.CleanUp.ReferenceOTU25413
New.CleanUp.ReferenceOTU179539
New.CleanUp.ReferenceOTU41199
New.CleanUp.ReferenceOTU17307
New.CleanUp.ReferenceOTU63411
New.CleanUp.ReferenceOTU252423
New.CleanUp.ReferenceOTU55722
New.CleanUp.ReferenceOTU25523
New.CleanUp.ReferenceOTU2661
New.CleanUp.ReferenceOTU6006
New.CleanUp.ReferenceOTU43856
New.CleanUp.ReferenceOTU48748
New.CleanUp.ReferenceOTU57937
New.CleanUp.ReferenceOTU62983
New.CleanUp.ReferenceOTU10585
New.CleanUp.ReferenceOTU63119
New.CleanUp.ReferenceOTU12269
New.CleanUp.ReferenceOTU100076
New.CleanUp.ReferenceOTU236007
New.CleanUp.ReferenceOTU48193
New.CleanUp.ReferenceOTU5367

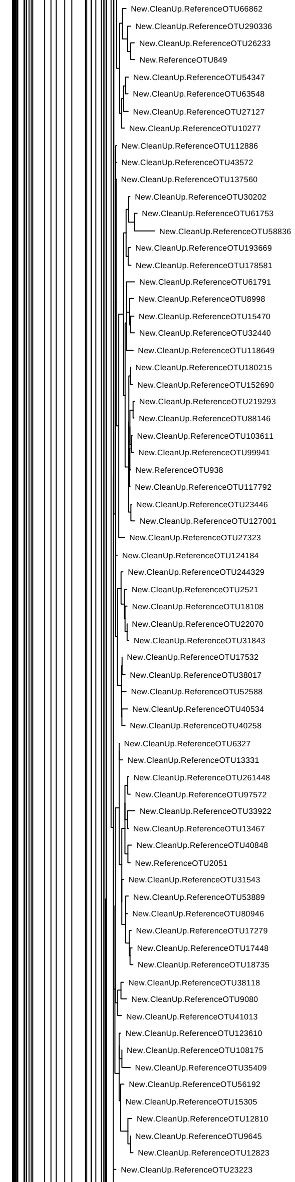
New.CleanUp.ReferenceOTU66862
New.CleanUp.ReferenceOTU290336
New.CleanUp.ReferenceOTU26233
New.ReferenceOTU849
New.CleanUp.ReferenceOTU54347
New.CleanUp.ReferenceOTU63548
New.CleanUp.ReferenceOTU27127
New.CleanUp.ReferenceOTU10277
New.CleanUp.ReferenceOTU112886
New.CleanUp.ReferenceOTU43572
New.CleanUp.ReferenceOTU137560
New.CleanUp.ReferenceOTU30202
New.CleanUp.ReferenceOTU61753
New.CleanUp.ReferenceOTU58836
New.CleanUp.ReferenceOTU193669
New.CleanUp.ReferenceOTU178581
New.CleanUp.ReferenceOTU61791
New.CleanUp.ReferenceOTU8998
New.CleanUp.ReferenceOTU15470
New.CleanUp.ReferenceOTU32440
New.CleanUp.ReferenceOTU118649
New.CleanUp.ReferenceOTU180215
New.CleanUp.ReferenceOTU152690
New.CleanUp.ReferenceOTU219293
New.CleanUp.ReferenceOTU88146
New.CleanUp.ReferenceOTU103611
New.CleanUp.ReferenceOTU99941
New.ReferenceOTU938
New.CleanUp.ReferenceOTU117792
New.CleanUp.ReferenceOTU23446
New.CleanUp.ReferenceOTU127001
New.CleanUp.ReferenceOTU27323
New.CleanUp.ReferenceOTU124184
New.CleanUp.ReferenceOTU244329
New.CleanUp.ReferenceOTU2521
New.CleanUp.ReferenceOTU18108
New.CleanUp.ReferenceOTU22070
New.CleanUp.ReferenceOTU31843
New.CleanUp.ReferenceOTU17532
New.CleanUp.ReferenceOTU38017
New.CleanUp.ReferenceOTU52588
New.CleanUp.ReferenceOTU40534
New.CleanUp.ReferenceOTU40258
New.CleanUp.ReferenceOTU6327
New.CleanUp.ReferenceOTU13331
New.CleanUp.ReferenceOTU261448
New.CleanUp.ReferenceOTU97572
New.CleanUp.ReferenceOTU33922
New.CleanUp.ReferenceOTU13467
New.CleanUp.ReferenceOTU40848
New.ReferenceOTU2051
New.CleanUp.ReferenceOTU31543
New.CleanUp.ReferenceOTU53889
New.CleanUp.ReferenceOTU80946
New.CleanUp.ReferenceOTU17279
New.CleanUp.ReferenceOTU17448
New.CleanUp.ReferenceOTU18735
New.CleanUp.ReferenceOTU38118
New.CleanUp.ReferenceOTU9080
New.CleanUp.ReferenceOTU41013
New.CleanUp.ReferenceOTU123610
New.CleanUp.ReferenceOTU108175
New.CleanUp.ReferenceOTU35409
New.CleanUp.ReferenceOTU56192
New.CleanUp.ReferenceOTU15305
New.CleanUp.ReferenceOTU12810
New.CleanUp.ReferenceOTU9645
New.CleanUp.ReferenceOTU12823
New.CleanUp.ReferenceOTU23223

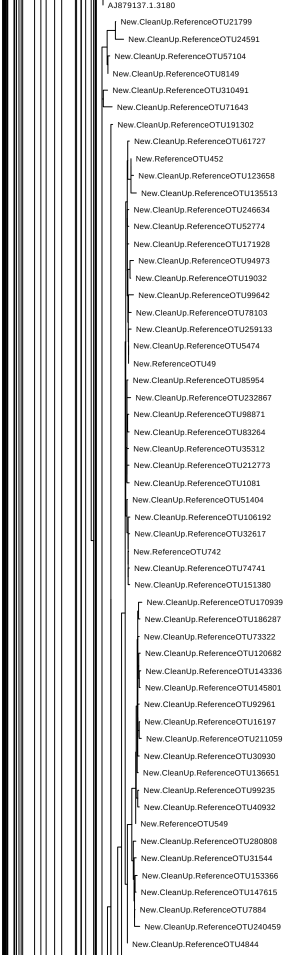
AJ879137.1.3180
New.CleanUp.ReferenceOTU21799
New.CleanUp.ReferenceOTU24591
New.CleanUp.ReferenceOTU57104
New.CleanUp.ReferenceOTU8149
New.CleanUp.ReferenceOTU310491
New.CleanUp.ReferenceOTU71643
New.CleanUp.ReferenceOTU191302
New.CleanUp.ReferenceOTU61727
New.ReferenceOTU452
New.CleanUp.ReferenceOTU123658
New.CleanUp.ReferenceOTU135513
New.CleanUp.ReferenceOTU246634
New.CleanUp.ReferenceOTU52774
New.CleanUp.ReferenceOTU171928
New.CleanUp.ReferenceOTU94973
New.CleanUp.ReferenceOTU19032
New.CleanUp.ReferenceOTU99642
New.CleanUp.ReferenceOTU78103
New.CleanUp.ReferenceOTU259133
New.CleanUp.ReferenceOTU5474
New.ReferenceOTU49
New.CleanUp.ReferenceOTU85954
New.CleanUp.ReferenceOTU232867
New.CleanUp.ReferenceOTU98871
New.CleanUp.ReferenceOTU83264
New.CleanUp.ReferenceOTU35312
New.CleanUp.ReferenceOTU212773
New.CleanUp.ReferenceOTU1081
New.CleanUp.ReferenceOTU51404
New.CleanUp.ReferenceOTU106192
New.CleanUp.ReferenceOTU32617
New.ReferenceOTU742
New.CleanUp.ReferenceOTU74741
New.CleanUp.ReferenceOTU151380
New.CleanUp.ReferenceOTU170939
New.CleanUp.ReferenceOTU186287
New.CleanUp.ReferenceOTU73322
New.CleanUp.ReferenceOTU120682
New.CleanUp.ReferenceOTU143336
New.CleanUp.ReferenceOTU145801
New.CleanUp.ReferenceOTU92961
New.CleanUp.ReferenceOTU16197
New.CleanUp.ReferenceOTU211059
New.CleanUp.ReferenceOTU30930
New.CleanUp.ReferenceOTU136651
New.CleanUp.ReferenceOTU99235
New.CleanUp.ReferenceOTU40932
New.ReferenceOTU549
New.CleanUp.ReferenceOTU280808
New.CleanUp.ReferenceOTU31544
New.CleanUp.ReferenceOTU153366
New.CleanUp.ReferenceOTU147615
New.CleanUp.ReferenceOTU7884
New.CleanUp.ReferenceOTU240459
New.CleanUp.ReferenceOTU4844

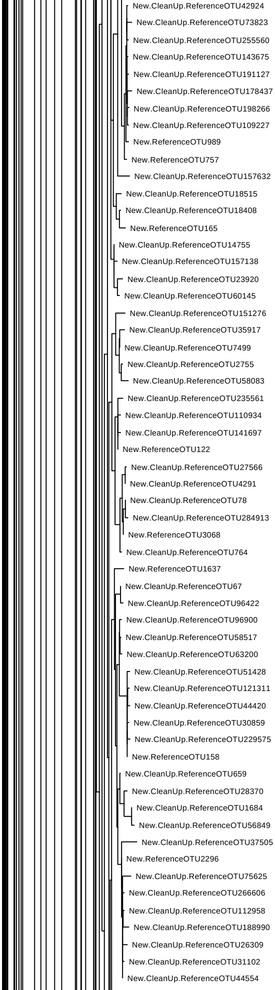
New.CleanUp.ReferenceOTU42924
New.CleanUp.ReferenceOTU73823
New.CleanUp.ReferenceOTU255560
New.CleanUp.ReferenceOTU143675
New.CleanUp.ReferenceOTU191127
New.CleanUp.ReferenceOTU178437
New.CleanUp.ReferenceOTU198266
New.CleanUp.ReferenceOTU109227
New.ReferenceOTU989
New.ReferenceOTU757
New.CleanUp.ReferenceOTU157632
New.CleanUp.ReferenceOTU18515
New.CleanUp.ReferenceOTU18408
New.ReferenceOTU165
New.CleanUp.ReferenceOTU14755
New.CleanUp.ReferenceOTU157138
New.CleanUp.ReferenceOTU23920
New.CleanUp.ReferenceOTU60145
New.CleanUp.ReferenceOTU151276
New.CleanUp.ReferenceOTU35917
New.CleanUp.ReferenceOTU7499
New.CleanUp.ReferenceOTU2755
New.CleanUp.ReferenceOTU58083
New.CleanUp.ReferenceOTU235561
New.CleanUp.ReferenceOTU110934
New.CleanUp.ReferenceOTU141697
New.ReferenceOTU122
New.CleanUp.ReferenceOTU27566
New.CleanUp.ReferenceOTU4291
New.CleanUp.ReferenceOTU78
New.CleanUp.ReferenceOTU284913
New.ReferenceOTU3068
New.CleanUp.ReferenceOTU764
New.ReferenceOTU1637
New.CleanUp.ReferenceOTU67
New.CleanUp.ReferenceOTU96422
New.CleanUp.ReferenceOTU96900
New.CleanUp.ReferenceOTU58517
New.CleanUp.ReferenceOTU63200
New.CleanUp.ReferenceOTU51428
New.CleanUp.ReferenceOTU121311
New.CleanUp.ReferenceOTU44420
New.CleanUp.ReferenceOTU30859
New.CleanUp.ReferenceOTU229575
New.ReferenceOTU158
New.CleanUp.ReferenceOTU659
New.CleanUp.ReferenceOTU28370
New.CleanUp.ReferenceOTU1684
New.CleanUp.ReferenceOTU56849
New.CleanUp.ReferenceOTU37505
New.ReferenceOTU2296
New.CleanUp.ReferenceOTU75625
New.CleanUp.ReferenceOTU266606
New.CleanUp.ReferenceOTU112958
New.CleanUp.ReferenceOTU188990
New.CleanUp.ReferenceOTU26309
New.CleanUp.ReferenceOTU31102
New.CleanUp.ReferenceOTU44554

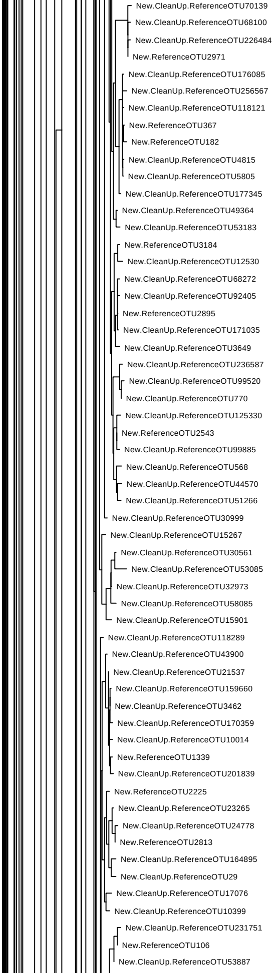
New.CleanUp.ReferenceOTU70139
New.CleanUp.ReferenceOTU68100
New.CleanUp.ReferenceOTU226484
New.ReferenceOTU2971
New.CleanUp.ReferenceOTU176085
New.CleanUp.ReferenceOTU256567
New.CleanUp.ReferenceOTU118121
New.ReferenceOTU367
New.ReferenceOTU182
New.CleanUp.ReferenceOTU4815
New.CleanUp.ReferenceOTU5805
New.CleanUp.ReferenceOTU177345
New.CleanUp.ReferenceOTU49364
New.CleanUp.ReferenceOTU53183
New.ReferenceOTU3184
New.CleanUp.ReferenceOTU12530
New.CleanUp.ReferenceOTU68272
New.CleanUp.ReferenceOTU92405
New.ReferenceOTU2895
New.CleanUp.ReferenceOTU171035
New.CleanUp.ReferenceOTU3649
New.CleanUp.ReferenceOTU236587
New.CleanUp.ReferenceOTU99520
New.CleanUp.ReferenceOTU770
New.CleanUp.ReferenceOTU125330
New.ReferenceOTU2543
New.CleanUp.ReferenceOTU99885
New.CleanUp.ReferenceOTU568
New.CleanUp.ReferenceOTU44570
New.CleanUp.ReferenceOTU51266
New.CleanUp.ReferenceOTU30999
New.CleanUp.ReferenceOTU15267
New.CleanUp.ReferenceOTU30561
New.CleanUp.ReferenceOTU53085
New.CleanUp.ReferenceOTU32973
New.CleanUp.ReferenceOTU58085
New.CleanUp.ReferenceOTU15901
New.CleanUp.ReferenceOTU118289
New.CleanUp.ReferenceOTU43900
New.CleanUp.ReferenceOTU21537
New.CleanUp.ReferenceOTU159660
New.CleanUp.ReferenceOTU3462
New.CleanUp.ReferenceOTU170359
New.CleanUp.ReferenceOTU10014
New.ReferenceOTU1339
New.CleanUp.ReferenceOTU201839
New.ReferenceOTU2225
New.CleanUp.ReferenceOTU23265
New.CleanUp.ReferenceOTU24778
New.ReferenceOTU2813
New.CleanUp.ReferenceOTU164895
New.CleanUp.ReferenceOTU29
New.CleanUp.ReferenceOTU17076
New.CleanUp.ReferenceOTU10399
New.CleanUp.ReferenceOTU231751
New.ReferenceOTU106
New.CleanUp.ReferenceOTU53887

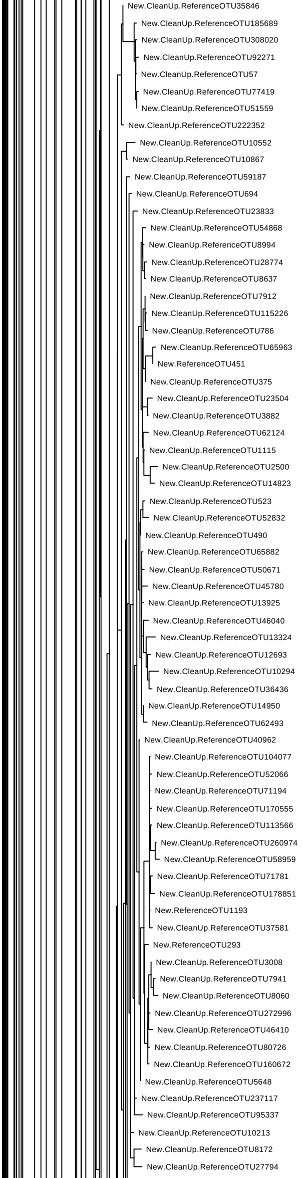
New.CleanUp.ReferenceOTU35846
New.CleanUp.ReferenceOTU185689
New.CleanUp.ReferenceOTU308020
New.CleanUp.ReferenceOTU92271
New.CleanUp.ReferenceOTU57
New.CleanUp.ReferenceOTU77419
New.CleanUp.ReferenceOTU51559
New.CleanUp.ReferenceOTU222352
New.CleanUp.ReferenceOTU10552
New.CleanUp.ReferenceOTU10867
New.CleanUp.ReferenceOTU59187
New.CleanUp.ReferenceOTU694
New.CleanUp.ReferenceOTU23833
New.CleanUp.ReferenceOTU54868
New.CleanUp.ReferenceOTU8994
New.CleanUp.ReferenceOTU28774
New.CleanUp.ReferenceOTU8637
New.CleanUp.ReferenceOTU7912
New.CleanUp.ReferenceOTU115226
New.CleanUp.ReferenceOTU786
New.CleanUp.ReferenceOTU65963
New.ReferenceOTU451
New.CleanUp.ReferenceOTU375
New.CleanUp.ReferenceOTU23504
New.CleanUp.ReferenceOTU3882
New.CleanUp.ReferenceOTU62124
New.CleanUp.ReferenceOTU1115
New.CleanUp.ReferenceOTU2500
New.CleanUp.ReferenceOTU14823
New.CleanUp.ReferenceOTU523
New.CleanUp.ReferenceOTU52832
New.CleanUp.ReferenceOTU490
New.CleanUp.ReferenceOTU65882
New.CleanUp.ReferenceOTU50671
New.CleanUp.ReferenceOTU45780
New.CleanUp.ReferenceOTU13925
New.CleanUp.ReferenceOTU46040
New.CleanUp.ReferenceOTU13324
New.CleanUp.ReferenceOTU12693
New.CleanUp.ReferenceOTU10294
New.CleanUp.ReferenceOTU36436
New.CleanUp.ReferenceOTU14950
New.CleanUp.ReferenceOTU62493
New.CleanUp.ReferenceOTU40962
New.CleanUp.ReferenceOTU104077
New.CleanUp.ReferenceOTU52066
New.CleanUp.ReferenceOTU71194
New.CleanUp.ReferenceOTU170555
New.CleanUp.ReferenceOTU113566
New.CleanUp.ReferenceOTU260974
New.CleanUp.ReferenceOTU58959
New.CleanUp.ReferenceOTU71781
New.CleanUp.ReferenceOTU178851
New.ReferenceOTU1193
New.CleanUp.ReferenceOTU37581
New.ReferenceOTU293
New.CleanUp.ReferenceOTU3008
New.CleanUp.ReferenceOTU7941
New.CleanUp.ReferenceOTU8060
New.CleanUp.ReferenceOTU272996
New.CleanUp.ReferenceOTU46410
New.CleanUp.ReferenceOTU80726
New.CleanUp.ReferenceOTU160672
New.CleanUp.ReferenceOTU5648
New.CleanUp.ReferenceOTU237117
New.CleanUp.ReferenceOTU95337
New.CleanUp.ReferenceOTU10213
New.CleanUp.ReferenceOTU8172
New.CleanUp.ReferenceOTU27794

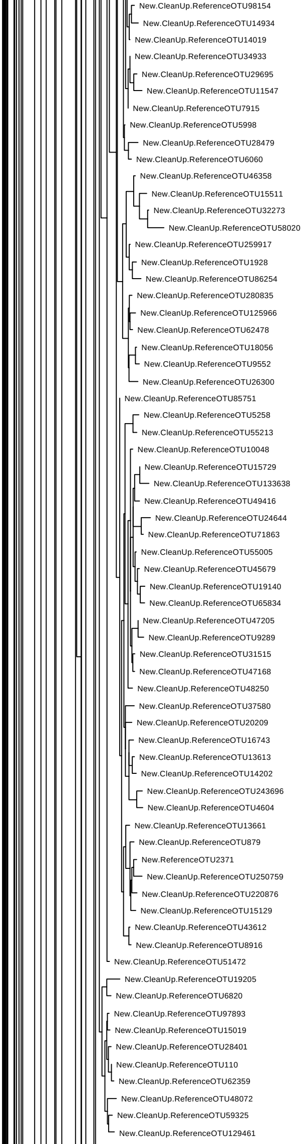
New.CleanUp.ReferenceOTU98154
New.CleanUp.ReferenceOTU14934
New.CleanUp.ReferenceOTU14019
New.CleanUp.ReferenceOTU34933
New.CleanUp.ReferenceOTU29695
New.CleanUp.ReferenceOTU11547
New.CleanUp.ReferenceOTU7915
New.CleanUp.ReferenceOTU5998
New.CleanUp.ReferenceOTU28479
New.CleanUp.ReferenceOTU6060
New.CleanUp.ReferenceOTU46358
New.CleanUp.ReferenceOTU15511
New.CleanUp.ReferenceOTU32273
New.CleanUp.ReferenceOTU58020
New.CleanUp.ReferenceOTU259917
New.CleanUp.ReferenceOTU1928
New.CleanUp.ReferenceOTU86254
New.CleanUp.ReferenceOTU280835
New.CleanUp.ReferenceOTU125966
New.CleanUp.ReferenceOTU62478
New.CleanUp.ReferenceOTU18056
New.CleanUp.ReferenceOTU9552
New.CleanUp.ReferenceOTU26300
New.CleanUp.ReferenceOTU85751
New.CleanUp.ReferenceOTU5258
New.CleanUp.ReferenceOTU55213
New.CleanUp.ReferenceOTU10048
New.CleanUp.ReferenceOTU15729
New.CleanUp.ReferenceOTU133638
New.CleanUp.ReferenceOTU49416
New.CleanUp.ReferenceOTU24644
New.CleanUp.ReferenceOTU71863
New.CleanUp.ReferenceOTU55005
New.CleanUp.ReferenceOTU45679
New.CleanUp.ReferenceOTU19140
New.CleanUp.ReferenceOTU65834
New.CleanUp.ReferenceOTU47205
New.CleanUp.ReferenceOTU9289
New.CleanUp.ReferenceOTU31515
New.CleanUp.ReferenceOTU47168
New.CleanUp.ReferenceOTU48250
New.CleanUp.ReferenceOTU37580
New.CleanUp.ReferenceOTU20209
New.CleanUp.ReferenceOTU16743
New.CleanUp.ReferenceOTU13613
New.CleanUp.ReferenceOTU14202
New.CleanUp.ReferenceOTU243696
New.CleanUp.ReferenceOTU4604
New.CleanUp.ReferenceOTU13661
New.CleanUp.ReferenceOTU879
New.ReferenceOTU2371
New.CleanUp.ReferenceOTU250759
New.CleanUp.ReferenceOTU220876
New.CleanUp.ReferenceOTU15129
New.CleanUp.ReferenceOTU43612
New.CleanUp.ReferenceOTU8916
New.CleanUp.ReferenceOTU51472
New.CleanUp.ReferenceOTU19205
New.CleanUp.ReferenceOTU6820
New.CleanUp.ReferenceOTU97893
New.CleanUp.ReferenceOTU15019
New.CleanUp.ReferenceOTU28401
New.CleanUp.ReferenceOTU110
New.CleanUp.ReferenceOTU62359
New.CleanUp.ReferenceOTU48072
New.CleanUp.ReferenceOTU59325
New.CleanUp.ReferenceOTU129461

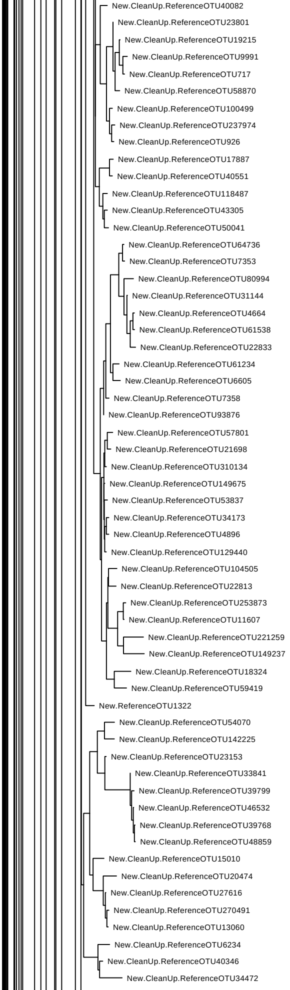
New.CleanUp.ReferenceOTU40082
New.CleanUp.ReferenceOTU23801
New.CleanUp.ReferenceOTU19215
New.CleanUp.ReferenceOTU9991
New.CleanUp.ReferenceOTU717
New.CleanUp.ReferenceOTU58870
New.CleanUp.ReferenceOTU100499
New.CleanUp.ReferenceOTU237974
New.CleanUp.ReferenceOTU926
New.CleanUp.ReferenceOTU17887
New.CleanUp.ReferenceOTU40551
New.CleanUp.ReferenceOTU118487
New.CleanUp.ReferenceOTU43305
New.CleanUp.ReferenceOTU50041
New.CleanUp.ReferenceOTU64736
New.CleanUp.ReferenceOTU7353
New.CleanUp.ReferenceOTU80994
New.CleanUp.ReferenceOTU31144
New.CleanUp.ReferenceOTU4664
New.CleanUp.ReferenceOTU61538
New.CleanUp.ReferenceOTU22833
New.CleanUp.ReferenceOTU61234
New.CleanUp.ReferenceOTU6605
New.CleanUp.ReferenceOTU7358
New.CleanUp.ReferenceOTU93876
New.CleanUp.ReferenceOTU57801
New.CleanUp.ReferenceOTU21698
New.CleanUp.ReferenceOTU310134
New.CleanUp.ReferenceOTU149675
New.CleanUp.ReferenceOTU53837
New.CleanUp.ReferenceOTU34173
New.CleanUp.ReferenceOTU4896
New.CleanUp.ReferenceOTU129440
New.CleanUp.ReferenceOTU104505
New.CleanUp.ReferenceOTU22813
New.CleanUp.ReferenceOTU253873
New.CleanUp.ReferenceOTU11607
New.CleanUp.ReferenceOTU221259
New.CleanUp.ReferenceOTU149237
New.CleanUp.ReferenceOTU18324
New.CleanUp.ReferenceOTU59419
New.ReferenceOTU1322
New.CleanUp.ReferenceOTU54070
New.CleanUp.ReferenceOTU142225
New.CleanUp.ReferenceOTU23153
New.CleanUp.ReferenceOTU33841
New.CleanUp.ReferenceOTU39799
New.CleanUp.ReferenceOTU46532
New.CleanUp.ReferenceOTU39768
New.CleanUp.ReferenceOTU48859
New.CleanUp.ReferenceOTU15010
New.CleanUp.ReferenceOTU20474
New.CleanUp.ReferenceOTU27616
New.CleanUp.ReferenceOTU270491
New.CleanUp.ReferenceOTU13060
New.CleanUp.ReferenceOTU6234
New.CleanUp.ReferenceOTU40346
New.CleanUp.ReferenceOTU34472

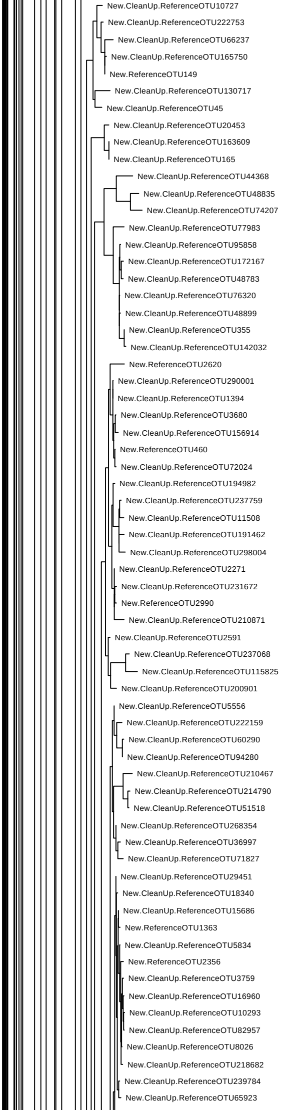
New.CleanUp.ReferenceOTU10727
New.CleanUp.ReferenceOTU222753
New.CleanUp.ReferenceOTU66237
New.CleanUp.ReferenceOTU165750
New.ReferenceOTU149
New.CleanUp.ReferenceOTU130717
New.CleanUp.ReferenceOTU45
New.CleanUp.ReferenceOTU20453
New.CleanUp.ReferenceOTU163609
New.CleanUp.ReferenceOTU165
New.CleanUp.ReferenceOTU44368
New.CleanUp.ReferenceOTU48835
New.CleanUp.ReferenceOTU74207
New.CleanUp.ReferenceOTU77983
New.CleanUp.ReferenceOTU95858
New.CleanUp.ReferenceOTU172167
New.CleanUp.ReferenceOTU48783
New.CleanUp.ReferenceOTU76320
New.CleanUp.ReferenceOTU48899
New.CleanUp.ReferenceOTU355
New.CleanUp.ReferenceOTU142032
New.ReferenceOTU2620
New.CleanUp.ReferenceOTU290001
New.CleanUp.ReferenceOTU1394
New.CleanUp.ReferenceOTU3680
New.CleanUp.ReferenceOTU156914
New.ReferenceOTU460
New.CleanUp.ReferenceOTU72024
New.CleanUp.ReferenceOTU194982
New.CleanUp.ReferenceOTU237759
New.CleanUp.ReferenceOTU11508
New.CleanUp.ReferenceOTU191462
New.CleanUp.ReferenceOTU298004
New.CleanUp.ReferenceOTU2271
New.CleanUp.ReferenceOTU231672
New.ReferenceOTU2990
New.CleanUp.ReferenceOTU210871
New.CleanUp.ReferenceOTU2591
New.CleanUp.ReferenceOTU237068
New.CleanUp.ReferenceOTU115825
New.CleanUp.ReferenceOTU200901
New.CleanUp.ReferenceOTU5556
New.CleanUp.ReferenceOTU222159
New.CleanUp.ReferenceOTU60290
New.CleanUp.ReferenceOTU94280
New.CleanUp.ReferenceOTU210467
New.CleanUp.ReferenceOTU214790
New.CleanUp.ReferenceOTU51518
New.CleanUp.ReferenceOTU268354
New.CleanUp.ReferenceOTU36997
New.CleanUp.ReferenceOTU71827
New.CleanUp.ReferenceOTU29451
New.CleanUp.ReferenceOTU18340
New.CleanUp.ReferenceOTU15686
New.ReferenceOTU1363
New.CleanUp.ReferenceOTU5834
New.ReferenceOTU2356
New.CleanUp.ReferenceOTU3759
New.CleanUp.ReferenceOTU16960
New.CleanUp.ReferenceOTU10293
New.CleanUp.ReferenceOTU82957
New.CleanUp.ReferenceOTU8026
New.CleanUp.ReferenceOTU218682
New.CleanUp.ReferenceOTU239784
New.CleanUp.ReferenceOTU65923

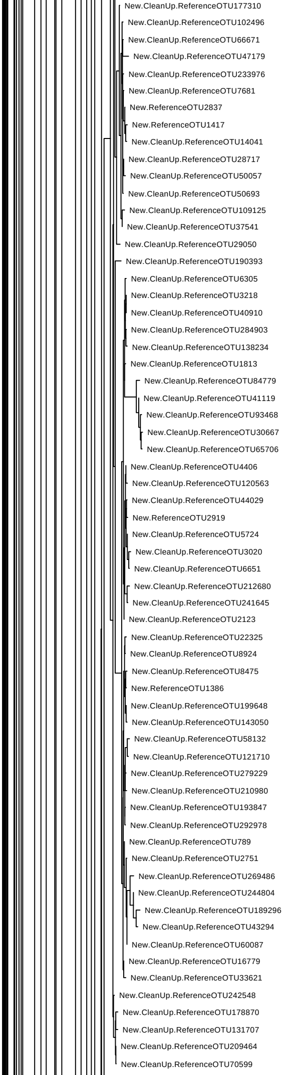

New.CleanUp.ReferenceOTU177310
New.CleanUp.ReferenceOTU102496
New.CleanUp.ReferenceOTU66671
New.CleanUp.ReferenceOTU47179
New.CleanUp.ReferenceOTU233976
New.CleanUp.ReferenceOTU7681
New.ReferenceOTU2837
New.ReferenceOTU1417
New.CleanUp.ReferenceOTU14041
New.CleanUp.ReferenceOTU28717
New.CleanUp.ReferenceOTU50057
New.CleanUp.ReferenceOTU50693
New.CleanUp.ReferenceOTU109125
New.CleanUp.ReferenceOTU37541
New.CleanUp.ReferenceOTU29050
New.CleanUp.ReferenceOTU190393
New.CleanUp.ReferenceOTU6305
New.CleanUp.ReferenceOTU3218
New.CleanUp.ReferenceOTU40910
New.CleanUp.ReferenceOTU284903
New.CleanUp.ReferenceOTU138234
New.CleanUp.ReferenceOTU1813
New.CleanUp.ReferenceOTU84779
New.CleanUp.ReferenceOTU41119
New.CleanUp.ReferenceOTU93468
New.CleanUp.ReferenceOTU30667
New.CleanUp.ReferenceOTU65706
New.CleanUp.ReferenceOTU4406
New.CleanUp.ReferenceOTU120563
New.CleanUp.ReferenceOTU44029
New.ReferenceOTU2919
New.CleanUp.ReferenceOTU5724
New.CleanUp.ReferenceOTU3020
New.CleanUp.ReferenceOTU6651
New.CleanUp.ReferenceOTU212680
New.CleanUp.ReferenceOTU241645
New.CleanUp.ReferenceOTU2123
New.CleanUp.ReferenceOTU22325
New.CleanUp.ReferenceOTU8924
New.CleanUp.ReferenceOTU8475
New.ReferenceOTU1386
New.CleanUp.ReferenceOTU199648
New.CleanUp.ReferenceOTU143050
New.CleanUp.ReferenceOTU58132
New.CleanUp.ReferenceOTU121710
New.CleanUp.ReferenceOTU279229
New.CleanUp.ReferenceOTU210980
New.CleanUp.ReferenceOTU193847
New.CleanUp.ReferenceOTU292978
New.CleanUp.ReferenceOTU789
New.CleanUp.ReferenceOTU2751
New.CleanUp.ReferenceOTU269486
New.CleanUp.ReferenceOTU244804
New.CleanUp.ReferenceOTU189296
New.CleanUp.ReferenceOTU43294
New.CleanUp.ReferenceOTU60087
New.CleanUp.ReferenceOTU16779
New.CleanUp.ReferenceOTU33621
New.CleanUp.ReferenceOTU242548
New.CleanUp.ReferenceOTU178870
New.CleanUp.ReferenceOTU131707
New.CleanUp.ReferenceOTU209464
New.CleanUp.ReferenceOTU70599

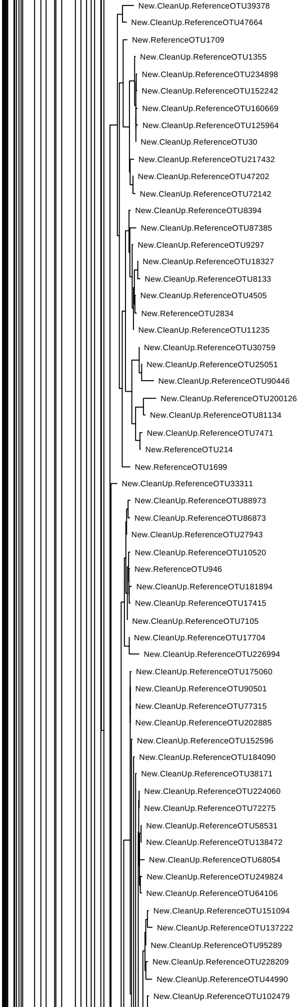
New.CleanUp.ReferenceOTU39378
New.CleanUp.ReferenceOTU47664
New.ReferenceOTU1709
New.CleanUp.ReferenceOTU1355
New.CleanUp.ReferenceOTU234898
New.CleanUp.ReferenceOTU152242
New.CleanUp.ReferenceOTU160669
New.CleanUp.ReferenceOTU125964
New.CleanUp.ReferenceOTU30
New.CleanUp.ReferenceOTU217432
New.CleanUp.ReferenceOTU47202
New.CleanUp.ReferenceOTU72142
New.CleanUp.ReferenceOTU8394
New.CleanUp.ReferenceOTU87385
New.CleanUp.ReferenceOTU9297
New.CleanUp.ReferenceOTU18327
New.CleanUp.ReferenceOTU8133
New.CleanUp.ReferenceOTU4505
New.ReferenceOTU2834
New.CleanUp.ReferenceOTU11235
New.CleanUp.ReferenceOTU30759
New.CleanUp.ReferenceOTU25051
New.CleanUp.ReferenceOTU90446
New.CleanUp.ReferenceOTU200126
New.CleanUp.ReferenceOTU81134
New.CleanUp.ReferenceOTU7471
New.ReferenceOTU214
New.ReferenceOTU1699
New.CleanUp.ReferenceOTU33311
New.CleanUp.ReferenceOTU88973
New.CleanUp.ReferenceOTU86873
New.CleanUp.ReferenceOTU27943
New.CleanUp.ReferenceOTU10520
New.ReferenceOTU946
New.CleanUp.ReferenceOTU181894
New.CleanUp.ReferenceOTU17415
New.CleanUp.ReferenceOTU7105
New.CleanUp.ReferenceOTU17704
New.CleanUp.ReferenceOTU226994
New.CleanUp.ReferenceOTU175060
New.CleanUp.ReferenceOTU90501
New.CleanUp.ReferenceOTU77315
New.CleanUp.ReferenceOTU202885
New.CleanUp.ReferenceOTU152596
New.CleanUp.ReferenceOTU184090
New.CleanUp.ReferenceOTU38171
New.CleanUp.ReferenceOTU224060
New.CleanUp.ReferenceOTU72275
New.CleanUp.ReferenceOTU58531
New.CleanUp.ReferenceOTU138472
New.CleanUp.ReferenceOTU68054
New.CleanUp.ReferenceOTU249824
New.CleanUp.ReferenceOTU64106
New.CleanUp.ReferenceOTU151094
New.CleanUp.ReferenceOTU137222
New.CleanUp.ReferenceOTU95289
New.CleanUp.ReferenceOTU228209
New.CleanUp.ReferenceOTU44990
New.CleanUp.ReferenceOTU102479

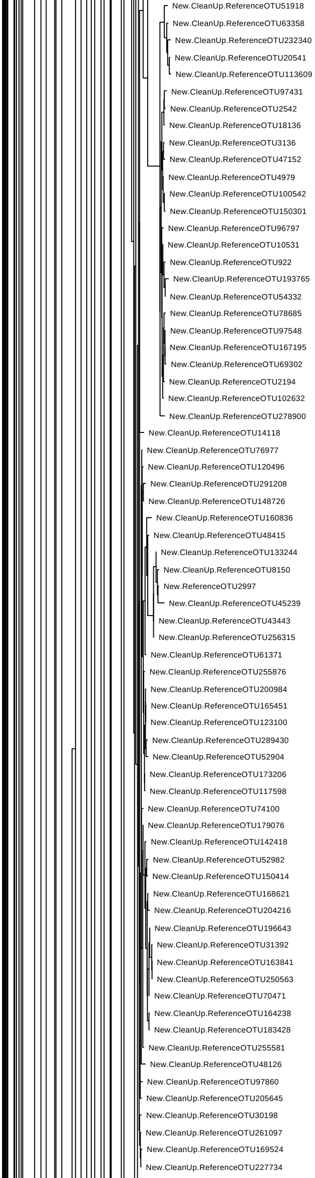
New.CleanUp.ReferenceOTU51918
New.CleanUp.ReferenceOTU63358
New.CleanUp.ReferenceOTU232340
New.CleanUp.ReferenceOTU20541
New.CleanUp.ReferenceOTU113609
New.CleanUp.ReferenceOTU97431
New.CleanUp.ReferenceOTU2542
New.CleanUp.ReferenceOTU18136
New.CleanUp.ReferenceOTU3136
New.CleanUp.ReferenceOTU47152
New.CleanUp.ReferenceOTU4979
New.CleanUp.ReferenceOTU100542
New.CleanUp.ReferenceOTU150301
New.CleanUp.ReferenceOTU96797
New.CleanUp.ReferenceOTU10531
New.CleanUp.ReferenceOTU922
New.CleanUp.ReferenceOTU193765
New.CleanUp.ReferenceOTU54332
New.CleanUp.ReferenceOTU78685
New.CleanUp.ReferenceOTU97548
New.CleanUp.ReferenceOTU167195
New.CleanUp.ReferenceOTU69302
New.CleanUp.ReferenceOTU2194
New.CleanUp.ReferenceOTU102632
New.CleanUp.ReferenceOTU278900
New.CleanUp.ReferenceOTU14118
New.CleanUp.ReferenceOTU76977
New.CleanUp.ReferenceOTU120496
New.CleanUp.ReferenceOTU291208
New.CleanUp.ReferenceOTU148726
New.CleanUp.ReferenceOTU160836
New.CleanUp.ReferenceOTU48415
New.CleanUp.ReferenceOTU133244
New.CleanUp.ReferenceOTU8150
New.ReferenceOTU2997
New.CleanUp.ReferenceOTU45239
New.CleanUp.ReferenceOTU43443
New.CleanUp.ReferenceOTU256315
New.CleanUp.ReferenceOTU61371
New.CleanUp.ReferenceOTU255876
New.CleanUp.ReferenceOTU200984
New.CleanUp.ReferenceOTU165451
New.CleanUp.ReferenceOTU123100
New.CleanUp.ReferenceOTU289430
New.CleanUp.ReferenceOTU52904
New.CleanUp.ReferenceOTU173206
New.CleanUp.ReferenceOTU117598
New.CleanUp.ReferenceOTU74100
New.CleanUp.ReferenceOTU179076
New.CleanUp.ReferenceOTU142418
New.CleanUp.ReferenceOTU52982
New.CleanUp.ReferenceOTU150414
New.CleanUp.ReferenceOTU168621
New.CleanUp.ReferenceOTU204216
New.CleanUp.ReferenceOTU196643
New.CleanUp.ReferenceOTU31392
New.CleanUp.ReferenceOTU163841
New.CleanUp.ReferenceOTU250563
New.CleanUp.ReferenceOTU70471
New.CleanUp.ReferenceOTU164238
New.CleanUp.ReferenceOTU183428
New.CleanUp.ReferenceOTU255581
New.CleanUp.ReferenceOTU48126
New.CleanUp.ReferenceOTU97860
New.CleanUp.ReferenceOTU205645
New.CleanUp.ReferenceOTU30198
New.CleanUp.ReferenceOTU261097
New.CleanUp.ReferenceOTU169524
New.CleanUp.ReferenceOTU227734

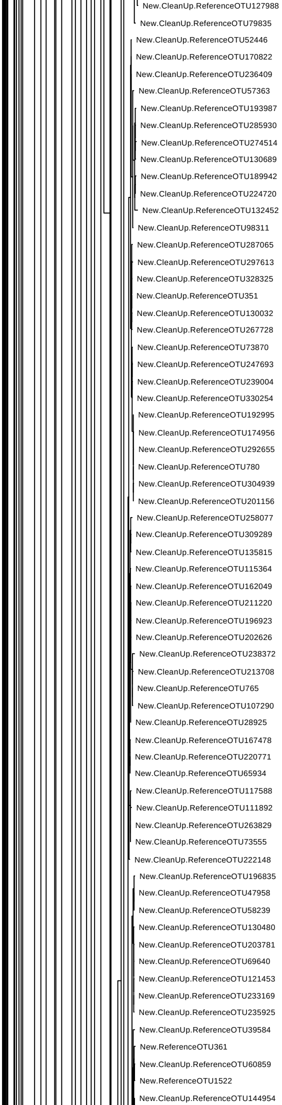
New.CleanUp.ReferenceOTU127988
New.CleanUp.ReferenceOTU79835
New.CleanUp.ReferenceOTU52446
New.CleanUp.ReferenceOTU170822
New.CleanUp.ReferenceOTU236409
New.CleanUp.ReferenceOTU57363
New.CleanUp.ReferenceOTU193987
New.CleanUp.ReferenceOTU285930
New.CleanUp.ReferenceOTU274514
New.CleanUp.ReferenceOTU130689
New.CleanUp.ReferenceOTU189942
New.CleanUp.ReferenceOTU224720
New.CleanUp.ReferenceOTU132452
New.CleanUp.ReferenceOTU98311
New.CleanUp.ReferenceOTU287065
New.CleanUp.ReferenceOTU297613
New.CleanUp.ReferenceOTU328325
New.CleanUp.ReferenceOTU351
New.CleanUp.ReferenceOTU130032
New.CleanUp.ReferenceOTU267728
New.CleanUp.ReferenceOTU73870
New.CleanUp.ReferenceOTU247693
New.CleanUp.ReferenceOTU239004
New.CleanUp.ReferenceOTU330254
New.CleanUp.ReferenceOTU192995
New.CleanUp.ReferenceOTU174956
New.CleanUp.ReferenceOTU292655
New.CleanUp.ReferenceOTU780
New.CleanUp.ReferenceOTU304939
New.CleanUp.ReferenceOTU201156
New.CleanUp.ReferenceOTU258077
New.CleanUp.ReferenceOTU309289
New.CleanUp.ReferenceOTU135815
New.CleanUp.ReferenceOTU115364
New.CleanUp.ReferenceOTU162049
New.CleanUp.ReferenceOTU211220
New.CleanUp.ReferenceOTU196923
New.CleanUp.ReferenceOTU202626
New.CleanUp.ReferenceOTU238372
New.CleanUp.ReferenceOTU213708
New.CleanUp.ReferenceOTU765
New.CleanUp.ReferenceOTU107290
New.CleanUp.ReferenceOTU28925
New.CleanUp.ReferenceOTU167478
New.CleanUp.ReferenceOTU220771
New.CleanUp.ReferenceOTU65934
New.CleanUp.ReferenceOTU117588
New.CleanUp.ReferenceOTU111892
New.CleanUp.ReferenceOTU263829
New.CleanUp.ReferenceOTU73555
New.CleanUp.ReferenceOTU222148
New.CleanUp.ReferenceOTU196835
New.CleanUp.ReferenceOTU47958
New.CleanUp.ReferenceOTU58239
New.CleanUp.ReferenceOTU130480
New.CleanUp.ReferenceOTU203781
New.CleanUp.ReferenceOTU69640
New.CleanUp.ReferenceOTU121453
New.CleanUp.ReferenceOTU233169
New.CleanUp.ReferenceOTU235925
New.CleanUp.ReferenceOTU39584
New.ReferenceOTU361
New.CleanUp.ReferenceOTU60859
New.ReferenceOTU1522
New.CleanUp.ReferenceOTU144954

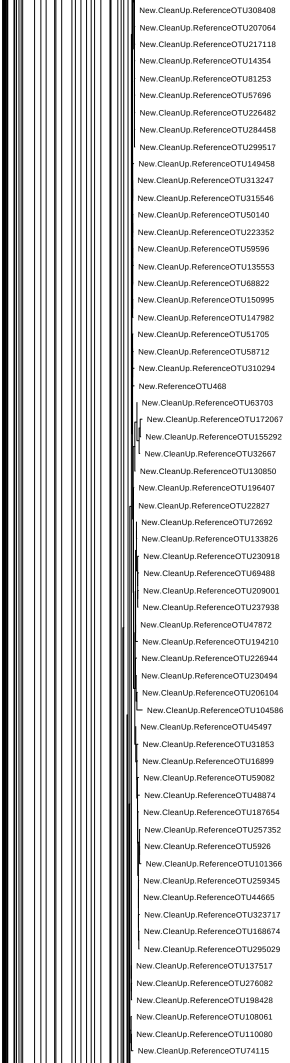
New.CleanUp.ReferenceOTU308408
New.CleanUp.ReferenceOTU207064
New.CleanUp.ReferenceOTU217118
New.CleanUp.ReferenceOTU14354
New.CleanUp.ReferenceOTU81253
New.CleanUp.ReferenceOTU57696
New.CleanUp.ReferenceOTU226482
New.CleanUp.ReferenceOTU284458
New.CleanUp.ReferenceOTU299517
New.CleanUp.ReferenceOTU149458
New.CleanUp.ReferenceOTU313247
New.CleanUp.ReferenceOTU315546
New.CleanUp.ReferenceOTU50140
New.CleanUp.ReferenceOTU223352
New.CleanUp.ReferenceOTU59596
New.CleanUp.ReferenceOTU135553
New.CleanUp.ReferenceOTU68822
New.CleanUp.ReferenceOTU150995
New.CleanUp.ReferenceOTU147982
New.CleanUp.ReferenceOTU51705
New.CleanUp.ReferenceOTU58712
New.CleanUp.ReferenceOTU310294
New.ReferenceOTU468
New.CleanUp.ReferenceOTU63703
New.CleanUp.ReferenceOTU172067
New.CleanUp.ReferenceOTU155292
New.CleanUp.ReferenceOTU32667
New.CleanUp.ReferenceOTU130850
New.CleanUp.ReferenceOTU196407
New.CleanUp.ReferenceOTU22827
New.CleanUp.ReferenceOTU72692
New.CleanUp.ReferenceOTU133826
New.CleanUp.ReferenceOTU230918
New.CleanUp.ReferenceOTU69488
New.CleanUp.ReferenceOTU209001
New.CleanUp.ReferenceOTU237938
New.CleanUp.ReferenceOTU47872
New.CleanUp.ReferenceOTU194210
New.CleanUp.ReferenceOTU226944
New.CleanUp.ReferenceOTU230494
New.CleanUp.ReferenceOTU206104
New.CleanUp.ReferenceOTU104586
New.CleanUp.ReferenceOTU45497
New.CleanUp.ReferenceOTU31853
New.CleanUp.ReferenceOTU16899
New.CleanUp.ReferenceOTU59082
New.CleanUp.ReferenceOTU48874
New.CleanUp.ReferenceOTU187654
New.CleanUp.ReferenceOTU257352
New.CleanUp.ReferenceOTU5926
New.CleanUp.ReferenceOTU101366
New.CleanUp.ReferenceOTU259345
New.CleanUp.ReferenceOTU44665
New.CleanUp.ReferenceOTU323717
New.CleanUp.ReferenceOTU168674
New.CleanUp.ReferenceOTU295029
New.CleanUp.ReferenceOTU137517
New.CleanUp.ReferenceOTU276082
New.CleanUp.ReferenceOTU198428
New.CleanUp.ReferenceOTU108061
New.CleanUp.ReferenceOTU110080
New.CleanUp.ReferenceOTU74115

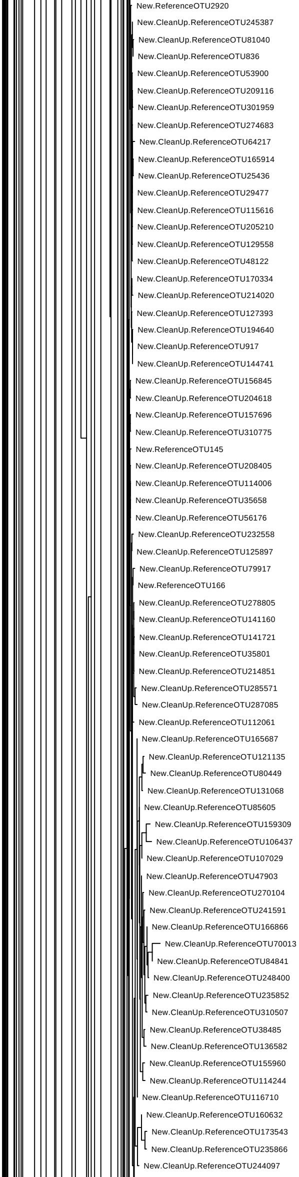
New.ReferenceOTU2920
New.CleanUp.ReferenceOTU245387
New.CleanUp.ReferenceOTU81040
New.CleanUp.ReferenceOTU836
New.CleanUp.ReferenceOTU53900
New.CleanUp.ReferenceOTU209116
New.CleanUp.ReferenceOTU301959
New.CleanUp.ReferenceOTU274683
New.CleanUp.ReferenceOTU64217
New.CleanUp.ReferenceOTU165914
New.CleanUp.ReferenceOTU25436
New.CleanUp.ReferenceOTU29477
New.CleanUp.ReferenceOTU115616
New.CleanUp.ReferenceOTU205210
New.CleanUp.ReferenceOTU129558
New.CleanUp.ReferenceOTU48122
New.CleanUp.ReferenceOTU170334
New.CleanUp.ReferenceOTU214020
New.CleanUp.ReferenceOTU127393
New.CleanUp.ReferenceOTU194640
New.CleanUp.ReferenceOTU917
New.CleanUp.ReferenceOTU144741
New.CleanUp.ReferenceOTU156845
New.CleanUp.ReferenceOTU204618
New.CleanUp.ReferenceOTU157696
New.CleanUp.ReferenceOTU310775
New.ReferenceOTU145
New.CleanUp.ReferenceOTU208405
New.CleanUp.ReferenceOTU114006
New.CleanUp.ReferenceOTU35658
New.CleanUp.ReferenceOTU56176
New.CleanUp.ReferenceOTU232558
New.CleanUp.ReferenceOTU125897
New.CleanUp.ReferenceOTU79917
New.ReferenceOTU166
New.CleanUp.ReferenceOTU278805
New.CleanUp.ReferenceOTU141160
New.CleanUp.ReferenceOTU141721
New.CleanUp.ReferenceOTU35801
New.CleanUp.ReferenceOTU214851
New.CleanUp.ReferenceOTU285571
New.CleanUp.ReferenceOTU287085
New.CleanUp.ReferenceOTU112061
New.CleanUp.ReferenceOTU165687
New.CleanUp.ReferenceOTU121135
New.CleanUp.ReferenceOTU80449
New.CleanUp.ReferenceOTU131068
New.CleanUp.ReferenceOTU85605
New.CleanUp.ReferenceOTU159309
New.CleanUp.ReferenceOTU106437
New.CleanUp.ReferenceOTU107029
New.CleanUp.ReferenceOTU47903
New.CleanUp.ReferenceOTU270104
New.CleanUp.ReferenceOTU241591
New.CleanUp.ReferenceOTU166866
New.CleanUp.ReferenceOTU70013
New.CleanUp.ReferenceOTU84841
New.CleanUp.ReferenceOTU248400
New.CleanUp.ReferenceOTU235852
New.CleanUp.ReferenceOTU310507
New.CleanUp.ReferenceOTU38485
New.CleanUp.ReferenceOTU136582
New.CleanUp.ReferenceOTU155960
New.CleanUp.ReferenceOTU114244
New.CleanUp.ReferenceOTU116710
New.CleanUp.ReferenceOTU160632
New.CleanUp.ReferenceOTU173543
New.CleanUp.ReferenceOTU235866
New.CleanUp.ReferenceOTU244097

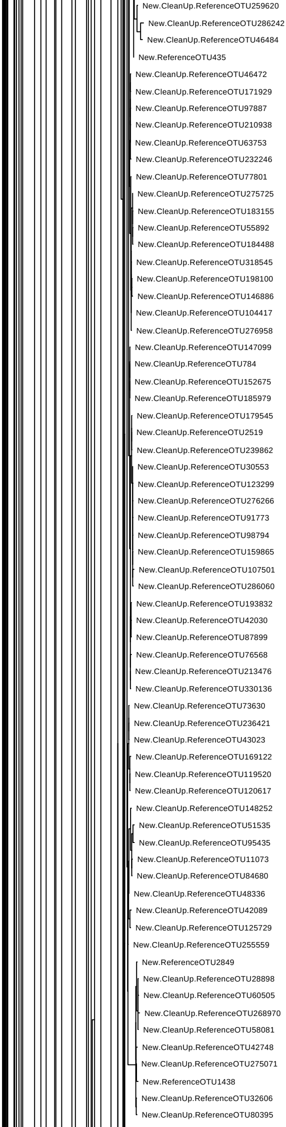
New.CleanUp.ReferenceOTU259620
New.CleanUp.ReferenceOTU286242
New.CleanUp.ReferenceOTU46484
New.ReferenceOTU435
New.CleanUp.ReferenceOTU46472
New.CleanUp.ReferenceOTU171929
New.CleanUp.ReferenceOTU97887
New.CleanUp.ReferenceOTU210938
New.CleanUp.ReferenceOTU63753
New.CleanUp.ReferenceOTU232246
New.CleanUp.ReferenceOTU77801
New.CleanUp.ReferenceOTU275725
New.CleanUp.ReferenceOTU183155
New.CleanUp.ReferenceOTU55892
New.CleanUp.ReferenceOTU184488
New.CleanUp.ReferenceOTU318545
New.CleanUp.ReferenceOTU198100
New.CleanUp.ReferenceOTU146886
New.CleanUp.ReferenceOTU104417
New.CleanUp.ReferenceOTU276958
New.CleanUp.ReferenceOTU147099
New.CleanUp.ReferenceOTU784
New.CleanUp.ReferenceOTU152675
New.CleanUp.ReferenceOTU185979
New.CleanUp.ReferenceOTU179545
New.CleanUp.ReferenceOTU2519
New.CleanUp.ReferenceOTU239862
New.CleanUp.ReferenceOTU30553
New.CleanUp.ReferenceOTU123299
New.CleanUp.ReferenceOTU276266
New.CleanUp.ReferenceOTU91773
New.CleanUp.ReferenceOTU98794
New.CleanUp.ReferenceOTU159865
New.CleanUp.ReferenceOTU107501
New.CleanUp.ReferenceOTU286060
New.CleanUp.ReferenceOTU193832
New.CleanUp.ReferenceOTU42030
New.CleanUp.ReferenceOTU87899
New.CleanUp.ReferenceOTU76568
New.CleanUp.ReferenceOTU213476
New.CleanUp.ReferenceOTU330136
New.CleanUp.ReferenceOTU73630
New.CleanUp.ReferenceOTU236421
New.CleanUp.ReferenceOTU43023
New.CleanUp.ReferenceOTU169122
New.CleanUp.ReferenceOTU119520
New.CleanUp.ReferenceOTU120617
New.CleanUp.ReferenceOTU148252
New.CleanUp.ReferenceOTU51535
New.CleanUp.ReferenceOTU95435
New.CleanUp.ReferenceOTU11073
New.CleanUp.ReferenceOTU84680
New.CleanUp.ReferenceOTU48336
New.CleanUp.ReferenceOTU42089
New.CleanUp.ReferenceOTU125729
New.CleanUp.ReferenceOTU255559
New.ReferenceOTU2849
New.CleanUp.ReferenceOTU28898
New.CleanUp.ReferenceOTU60505
New.CleanUp.ReferenceOTU268970
New.CleanUp.ReferenceOTU58081
New.CleanUp.ReferenceOTU42748
New.CleanUp.ReferenceOTU275071
New.ReferenceOTU1438
New.CleanUp.ReferenceOTU32606
New.CleanUp.ReferenceOTU80395

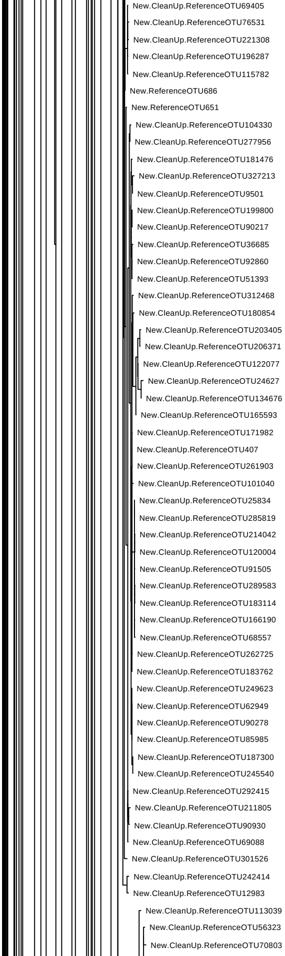
New.CleanUp.ReferenceOTU69405
New.CleanUp.ReferenceOTU76531
New.CleanUp.ReferenceOTU221308
New.CleanUp.ReferenceOTU196287
New.CleanUp.ReferenceOTU115782
New.ReferenceOTU686
New.ReferenceOTU651
New.CleanUp.ReferenceOTU104330
New.CleanUp.ReferenceOTU277956
New.CleanUp.ReferenceOTU181476
New.CleanUp.ReferenceOTU327213
New.CleanUp.ReferenceOTU9501
New.CleanUp.ReferenceOTU199800
New.CleanUp.ReferenceOTU90217
New.CleanUp.ReferenceOTU36685
New.CleanUp.ReferenceOTU92860
New.CleanUp.ReferenceOTU51393
New.CleanUp.ReferenceOTU312468
New.CleanUp.ReferenceOTU180854
New.CleanUp.ReferenceOTU203405
New.CleanUp.ReferenceOTU206371
New.CleanUp.ReferenceOTU122077
New.CleanUp.ReferenceOTU24627
New.CleanUp.ReferenceOTU134676
New.CleanUp.ReferenceOTU165593
New.CleanUp.ReferenceOTU171982
New.CleanUp.ReferenceOTU407
New.CleanUp.ReferenceOTU261903
New.CleanUp.ReferenceOTU101040
New.CleanUp.ReferenceOTU25834
New.CleanUp.ReferenceOTU285819
New.CleanUp.ReferenceOTU214042
New.CleanUp.ReferenceOTU120004
New.CleanUp.ReferenceOTU91505
New.CleanUp.ReferenceOTU289583
New.CleanUp.ReferenceOTU183114
New.CleanUp.ReferenceOTU166190
New.CleanUp.ReferenceOTU68557
New.CleanUp.ReferenceOTU262725
New.CleanUp.ReferenceOTU183762
New.CleanUp.ReferenceOTU249623
New.CleanUp.ReferenceOTU62949
New.CleanUp.ReferenceOTU90278
New.CleanUp.ReferenceOTU85985
New.CleanUp.ReferenceOTU187300
New.CleanUp.ReferenceOTU245540
New.CleanUp.ReferenceOTU292415
New.CleanUp.ReferenceOTU211805
New.CleanUp.ReferenceOTU90930
New.CleanUp.ReferenceOTU69088
New.CleanUp.ReferenceOTU301526
New.CleanUp.ReferenceOTU242414
New.CleanUp.ReferenceOTU12983
New.CleanUp.ReferenceOTU113039
New.CleanUp.ReferenceOTU56323
New.CleanUp.ReferenceOTU70803

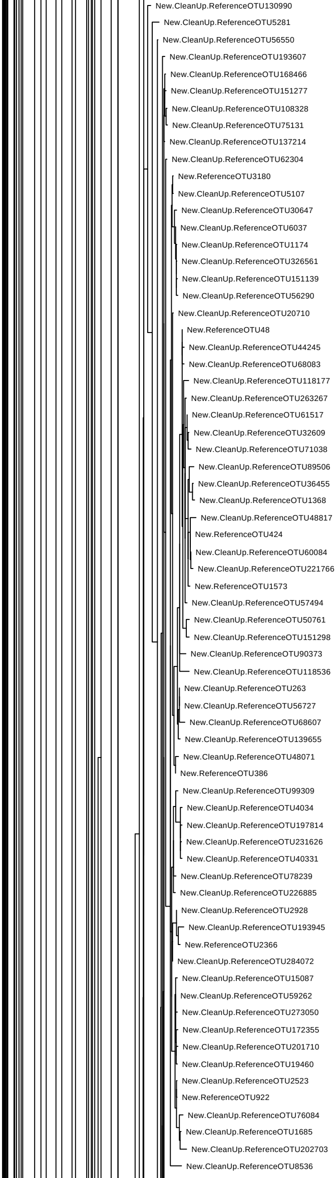
New.CleanUp.ReferenceOTU130990
New.CleanUp.ReferenceOTU5281
New.CleanUp.ReferenceOTU56550
New.CleanUp.ReferenceOTU193607
New.CleanUp.ReferenceOTU168466
New.CleanUp.ReferenceOTU151277
New.CleanUp.ReferenceOTU108328
New.CleanUp.ReferenceOTU75131
New.CleanUp.ReferenceOTU137214
New.CleanUp.ReferenceOTU62304
New.ReferenceOTU3180
New.CleanUp.ReferenceOTU5107
New.CleanUp.ReferenceOTU30647
New.CleanUp.ReferenceOTU6037
New.CleanUp.ReferenceOTU1174
New.CleanUp.ReferenceOTU326561
New.CleanUp.ReferenceOTU151139
New.CleanUp.ReferenceOTU56290
New.CleanUp.ReferenceOTU20710
New.ReferenceOTU48
New.CleanUp.ReferenceOTU44245
New.CleanUp.ReferenceOTU68083
New.CleanUp.ReferenceOTU118177
New.CleanUp.ReferenceOTU263267
New.CleanUp.ReferenceOTU61517
New.CleanUp.ReferenceOTU32609
New.CleanUp.ReferenceOTU71038
New.CleanUp.ReferenceOTU89506
New.CleanUp.ReferenceOTU36455
New.CleanUp.ReferenceOTU1368
New.CleanUp.ReferenceOTU48817
New.ReferenceOTU424
New.CleanUp.ReferenceOTU60084
New.CleanUp.ReferenceOTU221766
New.ReferenceOTU1573
New.CleanUp.ReferenceOTU57494
New.CleanUp.ReferenceOTU50761
New.CleanUp.ReferenceOTU151298
New.CleanUp.ReferenceOTU90373
New.CleanUp.ReferenceOTU118536
New.CleanUp.ReferenceOTU263
New.CleanUp.ReferenceOTU56727
New.CleanUp.ReferenceOTU68607
New.CleanUp.ReferenceOTU139655
New.CleanUp.ReferenceOTU48071
New.ReferenceOTU386
New.CleanUp.ReferenceOTU99309
New.CleanUp.ReferenceOTU4034
New.CleanUp.ReferenceOTU197814
New.CleanUp.ReferenceOTU231626
New.CleanUp.ReferenceOTU40331
New.CleanUp.ReferenceOTU78239
New.CleanUp.ReferenceOTU226885
New.CleanUp.ReferenceOTU2928
New.CleanUp.ReferenceOTU193945
New.ReferenceOTU2366
New.CleanUp.ReferenceOTU284072
New.CleanUp.ReferenceOTU15087
New.CleanUp.ReferenceOTU59262
New.CleanUp.ReferenceOTU273050
New.CleanUp.ReferenceOTU172355
New.CleanUp.ReferenceOTU201710
New.CleanUp.ReferenceOTU19460
New.CleanUp.ReferenceOTU2523
New.ReferenceOTU922
New.CleanUp.ReferenceOTU76084
New.CleanUp.ReferenceOTU1685
New.CleanUp.ReferenceOTU202703
New.CleanUp.ReferenceOTU8536

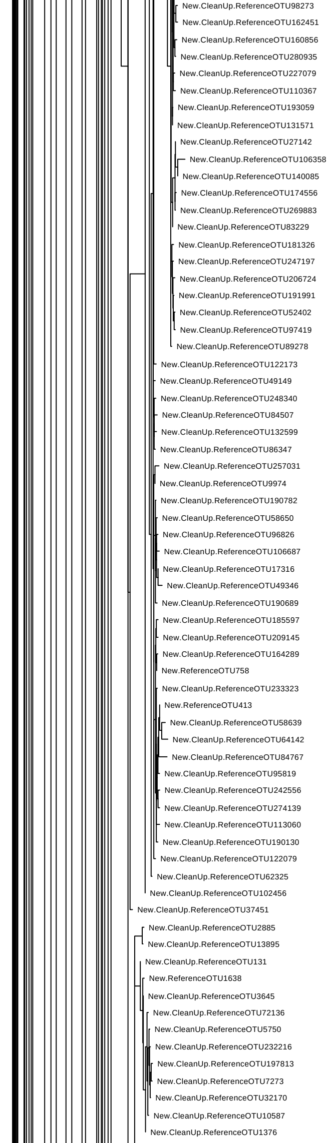
New.CleanUp.ReferenceOTU98273
New.CleanUp.ReferenceOTU162451
New.CleanUp.ReferenceOTU160856
New.CleanUp.ReferenceOTU280935
New.CleanUp.ReferenceOTU227079
New.CleanUp.ReferenceOTU110367
New.CleanUp.ReferenceOTU193059
New.CleanUp.ReferenceOTU131571
New.CleanUp.ReferenceOTU27142
New.CleanUp.ReferenceOTU106358
New.CleanUp.ReferenceOTU140085
New.CleanUp.ReferenceOTU174556
New.CleanUp.ReferenceOTU269883
New.CleanUp.ReferenceOTU83229
New.CleanUp.ReferenceOTU181326
New.CleanUp.ReferenceOTU247197
New.CleanUp.ReferenceOTU206724
New.CleanUp.ReferenceOTU191991
New.CleanUp.ReferenceOTU52402
New.CleanUp.ReferenceOTU97419
New.CleanUp.ReferenceOTU89278
New.CleanUp.ReferenceOTU122173
New.CleanUp.ReferenceOTU49149
New.CleanUp.ReferenceOTU248340
New.CleanUp.ReferenceOTU84507
New.CleanUp.ReferenceOTU132599
New.CleanUp.ReferenceOTU86347
New.CleanUp.ReferenceOTU257031
New.CleanUp.ReferenceOTU9974
New.CleanUp.ReferenceOTU190782
New.CleanUp.ReferenceOTU58650
New.CleanUp.ReferenceOTU96826
New.CleanUp.ReferenceOTU106687
New.CleanUp.ReferenceOTU17316
New.CleanUp.ReferenceOTU49346
New.CleanUp.ReferenceOTU190689
New.CleanUp.ReferenceOTU185597
New.CleanUp.ReferenceOTU209145
New.CleanUp.ReferenceOTU164289
New.ReferenceOTU758
New.CleanUp.ReferenceOTU233323
New.ReferenceOTU413
New.CleanUp.ReferenceOTU58639
New.CleanUp.ReferenceOTU64142
New.CleanUp.ReferenceOTU84767
New.CleanUp.ReferenceOTU95819
New.CleanUp.ReferenceOTU242556
New.CleanUp.ReferenceOTU274139
New.CleanUp.ReferenceOTU113060
New.CleanUp.ReferenceOTU190130
New.CleanUp.ReferenceOTU122079
New.CleanUp.ReferenceOTU62325
New.CleanUp.ReferenceOTU102456
New.CleanUp.ReferenceOTU37451
New.CleanUp.ReferenceOTU2885
New.CleanUp.ReferenceOTU13895
New.CleanUp.ReferenceOTU131
New.ReferenceOTU1638
New.CleanUp.ReferenceOTU3645
New.CleanUp.ReferenceOTU72136
New.CleanUp.ReferenceOTU5750
New.CleanUp.ReferenceOTU232216
New.CleanUp.ReferenceOTU197813
New.CleanUp.ReferenceOTU7273
New.CleanUp.ReferenceOTU32170
New.CleanUp.ReferenceOTU10587
New.CleanUp.ReferenceOTU1376

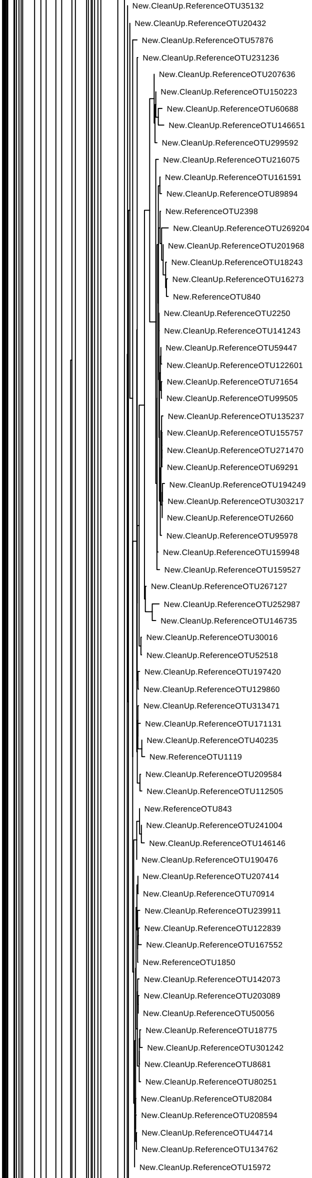
New.CleanUp.ReferenceOTU35132
New.CleanUp.ReferenceOTU20432
New.CleanUp.ReferenceOTU57876
New.CleanUp.ReferenceOTU231236
New.CleanUp.ReferenceOTU207636
New.CleanUp.ReferenceOTU150223
New.CleanUp.ReferenceOTU60688
New.CleanUp.ReferenceOTU146651
New.CleanUp.ReferenceOTU299592
New.CleanUp.ReferenceOTU216075
New.CleanUp.ReferenceOTU161591
New.CleanUp.ReferenceOTU89894
New.ReferenceOTU2398
New.CleanUp.ReferenceOTU269204
New.CleanUp.ReferenceOTU201968
New.CleanUp.ReferenceOTU18243
New.CleanUp.ReferenceOTU16273
New.ReferenceOTU840
New.CleanUp.ReferenceOTU2250
New.CleanUp.ReferenceOTU141243
New.CleanUp.ReferenceOTU59447
New.CleanUp.ReferenceOTU122601
New.CleanUp.ReferenceOTU71654
New.CleanUp.ReferenceOTU99505
New.CleanUp.ReferenceOTU135237
New.CleanUp.ReferenceOTU155757
New.CleanUp.ReferenceOTU271470
New.CleanUp.ReferenceOTU69291
New.CleanUp.ReferenceOTU194249
New.CleanUp.ReferenceOTU303217
New.CleanUp.ReferenceOTU2660
New.CleanUp.ReferenceOTU95978
New.CleanUp.ReferenceOTU159948
New.CleanUp.ReferenceOTU159527
New.CleanUp.ReferenceOTU267127
New.CleanUp.ReferenceOTU252987
New.CleanUp.ReferenceOTU146735
New.CleanUp.ReferenceOTU30016
New.CleanUp.ReferenceOTU52518
New.CleanUp.ReferenceOTU197420
New.CleanUp.ReferenceOTU129860
New.CleanUp.ReferenceOTU313471
New.CleanUp.ReferenceOTU171131
New.CleanUp.ReferenceOTU40235
New.ReferenceOTU1119
New.CleanUp.ReferenceOTU209584
New.CleanUp.ReferenceOTU112505
New.ReferenceOTU843
New.CleanUp.ReferenceOTU241004
New.CleanUp.ReferenceOTU146146
New.CleanUp.ReferenceOTU190476
New.CleanUp.ReferenceOTU207414
New.CleanUp.ReferenceOTU70914
New.CleanUp.ReferenceOTU239911
New.CleanUp.ReferenceOTU122839
New.CleanUp.ReferenceOTU167552
New.ReferenceOTU1850
New.CleanUp.ReferenceOTU142073
New.CleanUp.ReferenceOTU203089
New.CleanUp.ReferenceOTU50056
New.CleanUp.ReferenceOTU18775
New.CleanUp.ReferenceOTU301242
New.CleanUp.ReferenceOTU8681
New.CleanUp.ReferenceOTU80251
New.CleanUp.ReferenceOTU82084
New.CleanUp.ReferenceOTU208594
New.CleanUp.ReferenceOTU44714
New.CleanUp.ReferenceOTU134762
New.CleanUp.ReferenceOTU15972

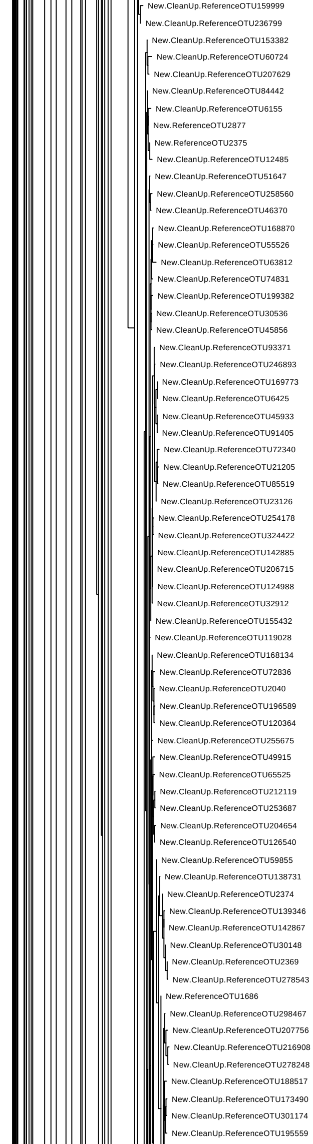
New.CleanUp.ReferenceOTU159999
New.CleanUp.ReferenceOTU236799
New.CleanUp.ReferenceOTU153382
New.CleanUp.ReferenceOTU60724
New.CleanUp.ReferenceOTU207629
New.CleanUp.ReferenceOTU84442
New.CleanUp.ReferenceOTU6155
New.ReferenceOTU2877
New.ReferenceOTU2375
New.CleanUp.ReferenceOTU12485
New.CleanUp.ReferenceOTU51647
New.CleanUp.ReferenceOTU258560
New.CleanUp.ReferenceOTU46370
New.CleanUp.ReferenceOTU168870
New.CleanUp.ReferenceOTU55526
New.CleanUp.ReferenceOTU63812
New.CleanUp.ReferenceOTU74831
New.CleanUp.ReferenceOTU199382
New.CleanUp.ReferenceOTU30536
New.CleanUp.ReferenceOTU45856
New.CleanUp.ReferenceOTU93371
New.CleanUp.ReferenceOTU246893
New.CleanUp.ReferenceOTU169773
New.CleanUp.ReferenceOTU6425
New.CleanUp.ReferenceOTU45933
New.CleanUp.ReferenceOTU91405
New.CleanUp.ReferenceOTU72340
New.CleanUp.ReferenceOTU21205
New.CleanUp.ReferenceOTU85519
New.CleanUp.ReferenceOTU23126
New.CleanUp.ReferenceOTU254178
New.CleanUp.ReferenceOTU324422
New.CleanUp.ReferenceOTU142885
New.CleanUp.ReferenceOTU206715
New.CleanUp.ReferenceOTU124988
New.CleanUp.ReferenceOTU32912
New.CleanUp.ReferenceOTU155432
New.CleanUp.ReferenceOTU119028
New.CleanUp.ReferenceOTU168134
New.CleanUp.ReferenceOTU72836
New.CleanUp.ReferenceOTU2040
New.CleanUp.ReferenceOTU196589
New.CleanUp.ReferenceOTU120364
New.CleanUp.ReferenceOTU255675
New.CleanUp.ReferenceOTU49915
New.CleanUp.ReferenceOTU65525
New.CleanUp.ReferenceOTU212119
New.CleanUp.ReferenceOTU253687
New.CleanUp.ReferenceOTU204654
New.CleanUp.ReferenceOTU126540
New.CleanUp.ReferenceOTU59855
New.CleanUp.ReferenceOTU138731
New.CleanUp.ReferenceOTU2374
New.CleanUp.ReferenceOTU139346
New.CleanUp.ReferenceOTU142867
New.CleanUp.ReferenceOTU30148
New.CleanUp.ReferenceOTU2369
New.CleanUp.ReferenceOTU278543
New.ReferenceOTU1686
New.CleanUp.ReferenceOTU298467
New.CleanUp.ReferenceOTU207756
New.CleanUp.ReferenceOTU216908
New.CleanUp.ReferenceOTU278248
New.CleanUp.ReferenceOTU188517
New.CleanUp.ReferenceOTU173490
New.CleanUp.ReferenceOTU301174
New.CleanUp.ReferenceOTU195559

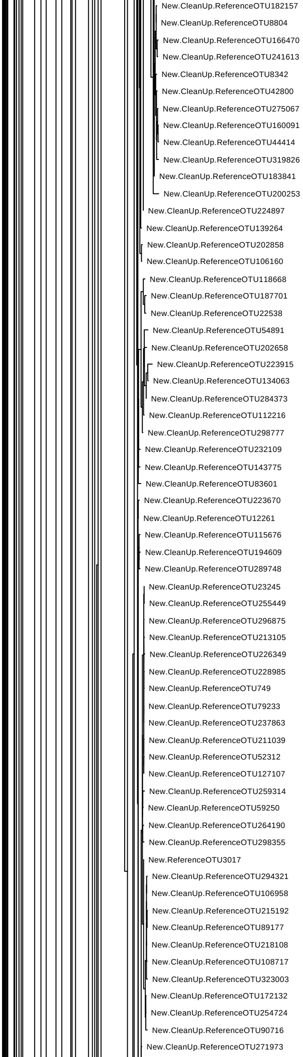
New.CleanUp.ReferenceOTU182157
New.CleanUp.ReferenceOTU8804
New.CleanUp.ReferenceOTU166470
New.CleanUp.ReferenceOTU241613
New.CleanUp.ReferenceOTU8342
New.CleanUp.ReferenceOTU42800
New.CleanUp.ReferenceOTU275067
New.CleanUp.ReferenceOTU160091
New.CleanUp.ReferenceOTU44414
New.CleanUp.ReferenceOTU319826
New.CleanUp.ReferenceOTU183841
New.CleanUp.ReferenceOTU200253
New.CleanUp.ReferenceOTU224897
New.CleanUp.ReferenceOTU139264
New.CleanUp.ReferenceOTU202858
New.CleanUp.ReferenceOTU106160
New.CleanUp.ReferenceOTU118668
New.CleanUp.ReferenceOTU187701
New.CleanUp.ReferenceOTU22538
New.CleanUp.ReferenceOTU54891
New.CleanUp.ReferenceOTU202658
New.CleanUp.ReferenceOTU223915
New.CleanUp.ReferenceOTU134063
New.CleanUp.ReferenceOTU284373
New.CleanUp.ReferenceOTU112216
New.CleanUp.ReferenceOTU298777
New.CleanUp.ReferenceOTU232109
New.CleanUp.ReferenceOTU143775
New.CleanUp.ReferenceOTU83601
New.CleanUp.ReferenceOTU223670
New.CleanUp.ReferenceOTU12261
New.CleanUp.ReferenceOTU115676
New.CleanUp.ReferenceOTU194609
New.CleanUp.ReferenceOTU289748
New.CleanUp.ReferenceOTU23245
New.CleanUp.ReferenceOTU255449
New.CleanUp.ReferenceOTU296875
New.CleanUp.ReferenceOTU213105
New.CleanUp.ReferenceOTU226349
New.CleanUp.ReferenceOTU228985
New.CleanUp.ReferenceOTU749
New.CleanUp.ReferenceOTU79233
New.CleanUp.ReferenceOTU237863
New.CleanUp.ReferenceOTU211039
New.CleanUp.ReferenceOTU52312
New.CleanUp.ReferenceOTU127107
New.CleanUp.ReferenceOTU259314
New.CleanUp.ReferenceOTU59250
New.CleanUp.ReferenceOTU264190
New.CleanUp.ReferenceOTU298355
New.ReferenceOTU3017
New.CleanUp.ReferenceOTU294321
New.CleanUp.ReferenceOTU106958
New.CleanUp.ReferenceOTU215192
New.CleanUp.ReferenceOTU89177
New.CleanUp.ReferenceOTU218108
New.CleanUp.ReferenceOTU108717
New.CleanUp.ReferenceOTU323003
New.CleanUp.ReferenceOTU172132
New.CleanUp.ReferenceOTU254724
New.CleanUp.ReferenceOTU90716
New.CleanUp.ReferenceOTU271973

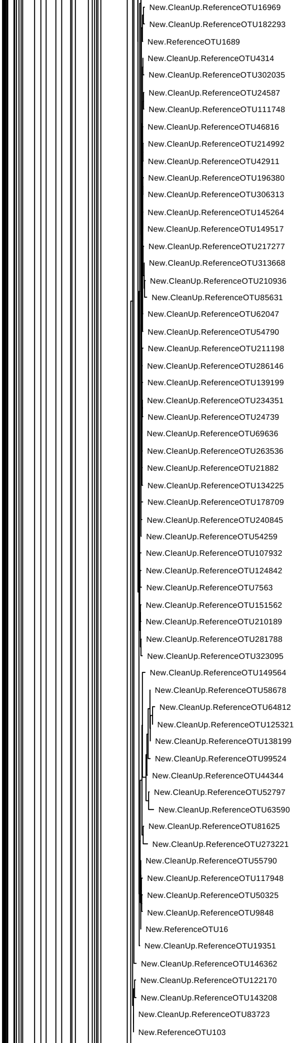
New.CleanUp.ReferenceOTU16969
New.CleanUp.ReferenceOTU182293
New.ReferenceOTU1689
New.CleanUp.ReferenceOTU4314
New.CleanUp.ReferenceOTU302035
New.CleanUp.ReferenceOTU24587
New.CleanUp.ReferenceOTU111748
New.CleanUp.ReferenceOTU46816
New.CleanUp.ReferenceOTU214992
New.CleanUp.ReferenceOTU42911
New.CleanUp.ReferenceOTU196380
New.CleanUp.ReferenceOTU306313
New.CleanUp.ReferenceOTU145264
New.CleanUp.ReferenceOTU149517
New.CleanUp.ReferenceOTU217277
New.CleanUp.ReferenceOTU313668
New.CleanUp.ReferenceOTU210936
New.CleanUp.ReferenceOTU85631
New.CleanUp.ReferenceOTU62047
New.CleanUp.ReferenceOTU54790
New.CleanUp.ReferenceOTU211198
New.CleanUp.ReferenceOTU286146
New.CleanUp.ReferenceOTU139199
New.CleanUp.ReferenceOTU234351
New.CleanUp.ReferenceOTU24739
New.CleanUp.ReferenceOTU69636
New.CleanUp.ReferenceOTU263536
New.CleanUp.ReferenceOTU21882
New.CleanUp.ReferenceOTU134225
New.CleanUp.ReferenceOTU178709
New.CleanUp.ReferenceOTU240845
New.CleanUp.ReferenceOTU54259
New.CleanUp.ReferenceOTU107932
New.CleanUp.ReferenceOTU124842
New.CleanUp.ReferenceOTU7563
New.CleanUp.ReferenceOTU151562
New.CleanUp.ReferenceOTU210189
New.CleanUp.ReferenceOTU281788
New.CleanUp.ReferenceOTU323095
New.CleanUp.ReferenceOTU149564
New.CleanUp.ReferenceOTU58678
New.CleanUp.ReferenceOTU64812
New.CleanUp.ReferenceOTU125321
New.CleanUp.ReferenceOTU138199
New.CleanUp.ReferenceOTU99524
New.CleanUp.ReferenceOTU44344
New.CleanUp.ReferenceOTU52797
New.CleanUp.ReferenceOTU63590
New.CleanUp.ReferenceOTU81625
New.CleanUp.ReferenceOTU273221
New.CleanUp.ReferenceOTU55790
New.CleanUp.ReferenceOTU117948
New.CleanUp.ReferenceOTU50325
New.CleanUp.ReferenceOTU9848
New.ReferenceOTU16
New.CleanUp.ReferenceOTU19351
New.CleanUp.ReferenceOTU146362
New.CleanUp.ReferenceOTU122170
New.CleanUp.ReferenceOTU143208
New.CleanUp.ReferenceOTU83723
New.ReferenceOTU103

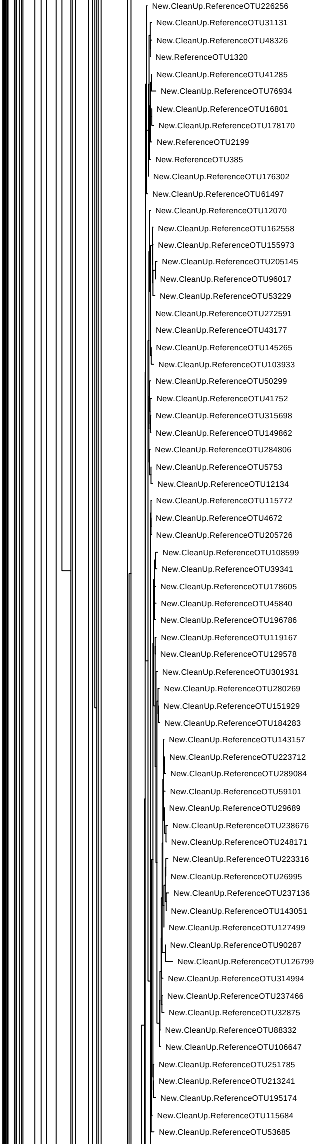
New.CleanUp.ReferenceOTU226256
New.CleanUp.ReferenceOTU31131
New.CleanUp.ReferenceOTU48326
New.ReferenceOTU1320
New.CleanUp.ReferenceOTU41285
New.CleanUp.ReferenceOTU76934
New.CleanUp.ReferenceOTU16801
New.CleanUp.ReferenceOTU178170
New.ReferenceOTU2199
New.ReferenceOTU385
New.CleanUp.ReferenceOTU176302
New.CleanUp.ReferenceOTU61497
New.CleanUp.ReferenceOTU12070
New.CleanUp.ReferenceOTU162558
New.CleanUp.ReferenceOTU155973
New.CleanUp.ReferenceOTU205145
New.CleanUp.ReferenceOTU96017
New.CleanUp.ReferenceOTU53229
New.CleanUp.ReferenceOTU272591
New.CleanUp.ReferenceOTU43177
New.CleanUp.ReferenceOTU145265
New.CleanUp.ReferenceOTU103933
New.CleanUp.ReferenceOTU50299
New.CleanUp.ReferenceOTU41752
New.CleanUp.ReferenceOTU315698
New.CleanUp.ReferenceOTU149862
New.CleanUp.ReferenceOTU284806
New.CleanUp.ReferenceOTU5753
New.CleanUp.ReferenceOTU12134
New.CleanUp.ReferenceOTU115772
New.CleanUp.ReferenceOTU4672
New.CleanUp.ReferenceOTU205726
New.CleanUp.ReferenceOTU108599
New.CleanUp.ReferenceOTU39341
New.CleanUp.ReferenceOTU178605
New.CleanUp.ReferenceOTU45840
New.CleanUp.ReferenceOTU196786
New.CleanUp.ReferenceOTU119167
New.CleanUp.ReferenceOTU129578
New.CleanUp.ReferenceOTU301931
New.CleanUp.ReferenceOTU280269
New.CleanUp.ReferenceOTU151929
New.CleanUp.ReferenceOTU184283
New.CleanUp.ReferenceOTU143157
New.CleanUp.ReferenceOTU223712
New.CleanUp.ReferenceOTU289084
New.CleanUp.ReferenceOTU59101
New.CleanUp.ReferenceOTU29689
New.CleanUp.ReferenceOTU238676
New.CleanUp.ReferenceOTU248171
New.CleanUp.ReferenceOTU223316
New.CleanUp.ReferenceOTU26995
New.CleanUp.ReferenceOTU237136
New.CleanUp.ReferenceOTU143051
New.CleanUp.ReferenceOTU127499
New.CleanUp.ReferenceOTU90287
New.CleanUp.ReferenceOTU126799
New.CleanUp.ReferenceOTU314994
New.CleanUp.ReferenceOTU237466
New.CleanUp.ReferenceOTU32875
New.CleanUp.ReferenceOTU88332
New.CleanUp.ReferenceOTU106647
New.CleanUp.ReferenceOTU251785
New.CleanUp.ReferenceOTU213241
New.CleanUp.ReferenceOTU195174
New.CleanUp.ReferenceOTU115684
New.CleanUp.ReferenceOTU53685

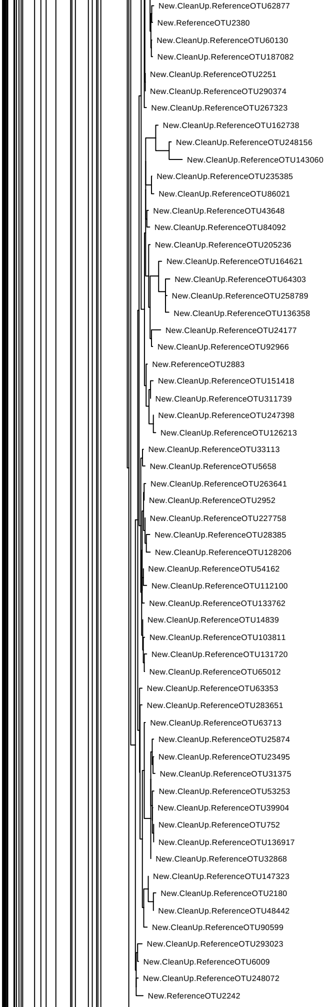
New.CleanUp.ReferenceOTU62877
New.ReferenceOTU2380
New.CleanUp.ReferenceOTU60130
New.CleanUp.ReferenceOTU187082
New.CleanUp.ReferenceOTU2251
New.CleanUp.ReferenceOTU290374
New.CleanUp.ReferenceOTU267323
New.CleanUp.ReferenceOTU162738
New.CleanUp.ReferenceOTU248156
New.CleanUp.ReferenceOTU143060
New.CleanUp.ReferenceOTU235385
New.CleanUp.ReferenceOTU86021
New.CleanUp.ReferenceOTU43648
New.CleanUp.ReferenceOTU84092
New.CleanUp.ReferenceOTU205236
New.CleanUp.ReferenceOTU164621
New.CleanUp.ReferenceOTU64303
New.CleanUp.ReferenceOTU258789
New.CleanUp.ReferenceOTU136358
New.CleanUp.ReferenceOTU24177
New.CleanUp.ReferenceOTU92966
New.ReferenceOTU2883
New.CleanUp.ReferenceOTU151418
New.CleanUp.ReferenceOTU311739
New.CleanUp.ReferenceOTU247398
New.CleanUp.ReferenceOTU126213
New.CleanUp.ReferenceOTU33113
New.CleanUp.ReferenceOTU5658
New.CleanUp.ReferenceOTU263641
New.CleanUp.ReferenceOTU2952
New.CleanUp.ReferenceOTU227758
New.CleanUp.ReferenceOTU28385
New.CleanUp.ReferenceOTU128206
New.CleanUp.ReferenceOTU54162
New.CleanUp.ReferenceOTU112100
New.CleanUp.ReferenceOTU133762
New.CleanUp.ReferenceOTU14839
New.CleanUp.ReferenceOTU103811
New.CleanUp.ReferenceOTU131720
New.CleanUp.ReferenceOTU65012
New.CleanUp.ReferenceOTU63353
New.CleanUp.ReferenceOTU283651
New.CleanUp.ReferenceOTU63713
New.CleanUp.ReferenceOTU25874
New.CleanUp.ReferenceOTU23495
New.CleanUp.ReferenceOTU31375
New.CleanUp.ReferenceOTU53253
New.CleanUp.ReferenceOTU39904
New.CleanUp.ReferenceOTU752
New.CleanUp.ReferenceOTU136917
New.CleanUp.ReferenceOTU32868
New.CleanUp.ReferenceOTU147323
New.CleanUp.ReferenceOTU2180
New.CleanUp.ReferenceOTU48442
New.CleanUp.ReferenceOTU90599
New.CleanUp.ReferenceOTU293023
New.CleanUp.ReferenceOTU6009
New.CleanUp.ReferenceOTU248072
New.ReferenceOTU2242

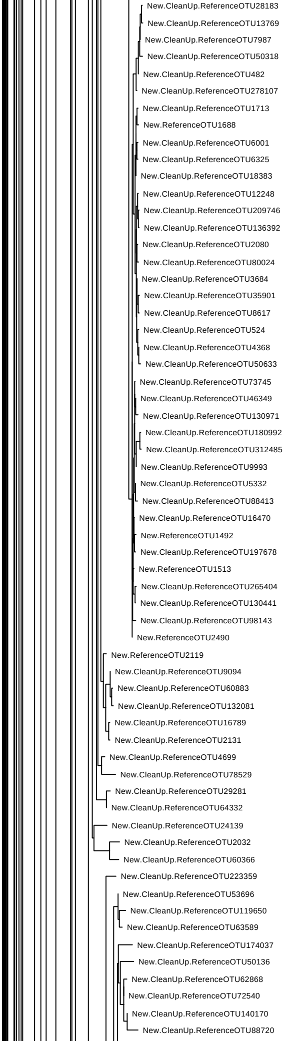
New.CleanUp.ReferenceOTU28183
New.CleanUp.ReferenceOTU13769
New.CleanUp.ReferenceOTU7987
New.CleanUp.ReferenceOTU50318
New.CleanUp.ReferenceOTU482
New.CleanUp.ReferenceOTU278107
New.CleanUp.ReferenceOTU1713
New.ReferenceOTU1688
New.CleanUp.ReferenceOTU6001
New.CleanUp.ReferenceOTU6325
New.CleanUp.ReferenceOTU18383
New.CleanUp.ReferenceOTU12248
New.CleanUp.ReferenceOTU209746
New.CleanUp.ReferenceOTU136392
New.CleanUp.ReferenceOTU2080
New.CleanUp.ReferenceOTU80024
New.CleanUp.ReferenceOTU3684
New.CleanUp.ReferenceOTU35901
New.CleanUp.ReferenceOTU8617
New.CleanUp.ReferenceOTU524
New.CleanUp.ReferenceOTU4368
New.CleanUp.ReferenceOTU50633
New.CleanUp.ReferenceOTU73745
New.CleanUp.ReferenceOTU46349
New.CleanUp.ReferenceOTU130971
New.CleanUp.ReferenceOTU180992
New.CleanUp.ReferenceOTU312485
New.CleanUp.ReferenceOTU9993
New.CleanUp.ReferenceOTU5332
New.CleanUp.ReferenceOTU88413
New.CleanUp.ReferenceOTU16470
New.ReferenceOTU1492
New.CleanUp.ReferenceOTU197678
New.ReferenceOTU1513
New.CleanUp.ReferenceOTU265404
New.CleanUp.ReferenceOTU130441
New.CleanUp.ReferenceOTU98143
New.ReferenceOTU2490
New.ReferenceOTU2119
New.CleanUp.ReferenceOTU9094
New.CleanUp.ReferenceOTU60883
New.CleanUp.ReferenceOTU132081
New.CleanUp.ReferenceOTU16789
New.CleanUp.ReferenceOTU2131
New.CleanUp.ReferenceOTU4699
New.CleanUp.ReferenceOTU78529
New.CleanUp.ReferenceOTU29281
New.CleanUp.ReferenceOTU64332
New.CleanUp.ReferenceOTU24139
New.CleanUp.ReferenceOTU2032
New.CleanUp.ReferenceOTU60366
New.CleanUp.ReferenceOTU223359
New.CleanUp.ReferenceOTU53696
New.CleanUp.ReferenceOTU119650
New.CleanUp.ReferenceOTU63589
New.CleanUp.ReferenceOTU174037
New.CleanUp.ReferenceOTU50136
New.CleanUp.ReferenceOTU62868
New.CleanUp.ReferenceOTU72540
New.CleanUp.ReferenceOTU140170
New.CleanUp.ReferenceOTU88720

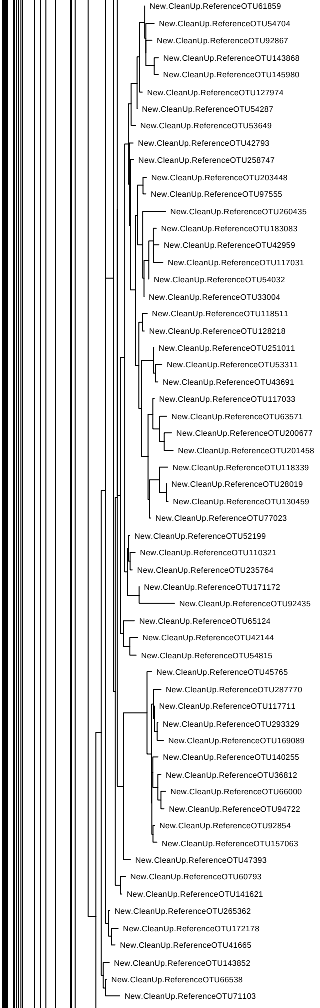
New.CleanUp.ReferenceOTU61859
New.CleanUp.ReferenceOTU54704
New.CleanUp.ReferenceOTU92867
New.CleanUp.ReferenceOTU143868
New.CleanUp.ReferenceOTU145980
New.CleanUp.ReferenceOTU127974
New.CleanUp.ReferenceOTU54287
New.CleanUp.ReferenceOTU53649
New.CleanUp.ReferenceOTU42793
New.CleanUp.ReferenceOTU258747
New.CleanUp.ReferenceOTU203448
New.CleanUp.ReferenceOTU97555
New.CleanUp.ReferenceOTU260435
New.CleanUp.ReferenceOTU183083
New.CleanUp.ReferenceOTU42959
New.CleanUp.ReferenceOTU117031
New.CleanUp.ReferenceOTU54032
New.CleanUp.ReferenceOTU33004
New.CleanUp.ReferenceOTU118511
New.CleanUp.ReferenceOTU128218
New.CleanUp.ReferenceOTU251011
New.CleanUp.ReferenceOTU53311
New.CleanUp.ReferenceOTU43691
New.CleanUp.ReferenceOTU117033
New.CleanUp.ReferenceOTU63571
New.CleanUp.ReferenceOTU200677
New.CleanUp.ReferenceOTU201458
New.CleanUp.ReferenceOTU118339
New.CleanUp.ReferenceOTU28019
New.CleanUp.ReferenceOTU130459
New.CleanUp.ReferenceOTU77023
New.CleanUp.ReferenceOTU52199
New.CleanUp.ReferenceOTU110321
New.CleanUp.ReferenceOTU235764
New.CleanUp.ReferenceOTU171172
New.CleanUp.ReferenceOTU92435
New.CleanUp.ReferenceOTU65124
New.CleanUp.ReferenceOTU42144
New.CleanUp.ReferenceOTU54815
New.CleanUp.ReferenceOTU45765
New.CleanUp.ReferenceOTU287770
New.CleanUp.ReferenceOTU117711
New.CleanUp.ReferenceOTU293329
New.CleanUp.ReferenceOTU169089
New.CleanUp.ReferenceOTU140255
New.CleanUp.ReferenceOTU36812
New.CleanUp.ReferenceOTU66000
New.CleanUp.ReferenceOTU94722
New.CleanUp.ReferenceOTU92854
New.CleanUp.ReferenceOTU157063
New.CleanUp.ReferenceOTU47393
New.CleanUp.ReferenceOTU60793
New.CleanUp.ReferenceOTU141621
New.CleanUp.ReferenceOTU265362
New.CleanUp.ReferenceOTU172178
New.CleanUp.ReferenceOTU41665
New.CleanUp.ReferenceOTU143852
New.CleanUp.ReferenceOTU66538
New.CleanUp.ReferenceOTU71103

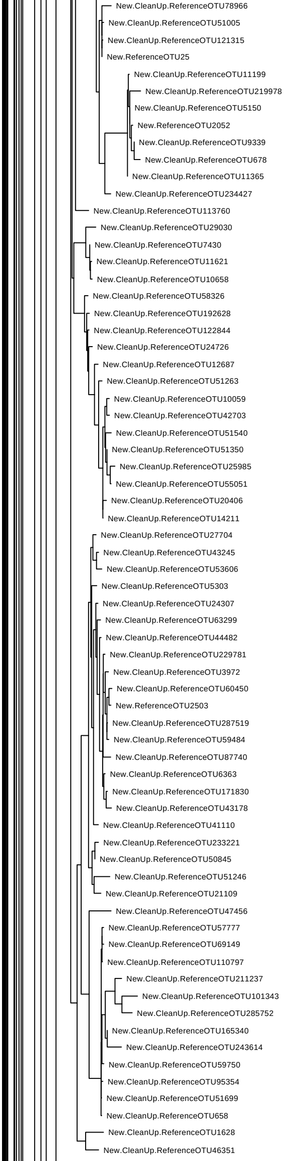
New.CleanUp.ReferenceOTU78966
New.CleanUp.ReferenceOTU51005
New.CleanUp.ReferenceOTU121315
New.ReferenceOTU25
New.CleanUp.ReferenceOTU11199
New.CleanUp.ReferenceOTU219978
New.CleanUp.ReferenceOTU5150
New.ReferenceOTU2052
New.CleanUp.ReferenceOTU9339
New.CleanUp.ReferenceOTU678
New.CleanUp.ReferenceOTU11365
New.CleanUp.ReferenceOTU234427
New.CleanUp.ReferenceOTU113760
New.CleanUp.ReferenceOTU29030
New.CleanUp.ReferenceOTU7430
New.CleanUp.ReferenceOTU11621
New.CleanUp.ReferenceOTU10658
New.CleanUp.ReferenceOTU58326
New.CleanUp.ReferenceOTU192628
New.CleanUp.ReferenceOTU122844
New.CleanUp.ReferenceOTU24726
New.CleanUp.ReferenceOTU12687
New.CleanUp.ReferenceOTU51263
New.CleanUp.ReferenceOTU10059
New.CleanUp.ReferenceOTU42703
New.CleanUp.ReferenceOTU51540
New.CleanUp.ReferenceOTU51350
New.CleanUp.ReferenceOTU25985
New.CleanUp.ReferenceOTU55051
New.CleanUp.ReferenceOTU20406
New.CleanUp.ReferenceOTU14211
New.CleanUp.ReferenceOTU27704
New.CleanUp.ReferenceOTU43245
New.CleanUp.ReferenceOTU53606
New.CleanUp.ReferenceOTU5303
New.CleanUp.ReferenceOTU24307
New.CleanUp.ReferenceOTU63299
New.CleanUp.ReferenceOTU44482
New.CleanUp.ReferenceOTU229781
New.CleanUp.ReferenceOTU3972
New.CleanUp.ReferenceOTU60450
New.ReferenceOTU2503
New.CleanUp.ReferenceOTU287519
New.CleanUp.ReferenceOTU59484
New.CleanUp.ReferenceOTU87740
New.CleanUp.ReferenceOTU6363
New.CleanUp.ReferenceOTU171830
New.CleanUp.ReferenceOTU43178
New.CleanUp.ReferenceOTU41110
New.CleanUp.ReferenceOTU233221
New.CleanUp.ReferenceOTU50845
New.CleanUp.ReferenceOTU51246
New.CleanUp.ReferenceOTU21109
New.CleanUp.ReferenceOTU47456
New.CleanUp.ReferenceOTU57777
New.CleanUp.ReferenceOTU69149
New.CleanUp.ReferenceOTU110797
New.CleanUp.ReferenceOTU211237
New.CleanUp.ReferenceOTU101343
New.CleanUp.ReferenceOTU285752
New.CleanUp.ReferenceOTU165340
New.CleanUp.ReferenceOTU243614
New.CleanUp.ReferenceOTU59750
New.CleanUp.ReferenceOTU95354
New.CleanUp.ReferenceOTU51699
New.CleanUp.ReferenceOTU658
New.CleanUp.ReferenceOTU1628
New.CleanUp.ReferenceOTU46351

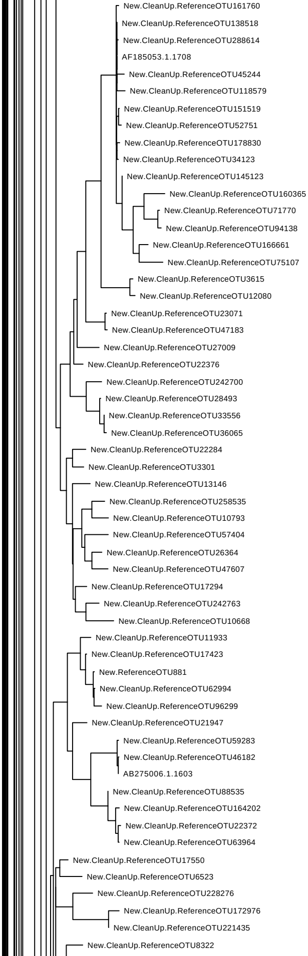
New.CleanUp.ReferenceOTU161760
New.CleanUp.ReferenceOTU138518
New.CleanUp.ReferenceOTU288614
AF185053.1.1708
New.CleanUp.ReferenceOTU45244
New.CleanUp.ReferenceOTU118579
New.CleanUp.ReferenceOTU151519
New.CleanUp.ReferenceOTU52751
New.CleanUp.ReferenceOTU178830
New.CleanUp.ReferenceOTU34123
New.CleanUp.ReferenceOTU145123
New.CleanUp.ReferenceOTU160365
New.CleanUp.ReferenceOTU71770
New.CleanUp.ReferenceOTU94138
New.CleanUp.ReferenceOTU166661
New.CleanUp.ReferenceOTU75107
New.CleanUp.ReferenceOTU3615
New.CleanUp.ReferenceOTU12080
New.CleanUp.ReferenceOTU23071
New.CleanUp.ReferenceOTU47183
New.CleanUp.ReferenceOTU27009
New.CleanUp.ReferenceOTU22376
New.CleanUp.ReferenceOTU242700
New.CleanUp.ReferenceOTU28493
New.CleanUp.ReferenceOTU33556
New.CleanUp.ReferenceOTU36065
New.CleanUp.ReferenceOTU22284
New.CleanUp.ReferenceOTU3301
New.CleanUp.ReferenceOTU13146
New.CleanUp.ReferenceOTU258535
New.CleanUp.ReferenceOTU10793
New.CleanUp.ReferenceOTU57404
New.CleanUp.ReferenceOTU26364
New.CleanUp.ReferenceOTU47607
New.CleanUp.ReferenceOTU17294
New.CleanUp.ReferenceOTU242763
New.CleanUp.ReferenceOTU10668
New.CleanUp.ReferenceOTU11933
New.CleanUp.ReferenceOTU17423
New.ReferenceOTU881
New.CleanUp.ReferenceOTU62994
New.CleanUp.ReferenceOTU96299
New.CleanUp.ReferenceOTU21947
New.CleanUp.ReferenceOTU59283
New.CleanUp.ReferenceOTU46182
AB275006.1.1603
New.CleanUp.ReferenceOTU88535
New.CleanUp.ReferenceOTU164202
New.CleanUp.ReferenceOTU22372
New.CleanUp.ReferenceOTU63964
New.CleanUp.ReferenceOTU17550
New.CleanUp.ReferenceOTU6523
New.CleanUp.ReferenceOTU228276
New.CleanUp.ReferenceOTU172976
New.CleanUp.ReferenceOTU221435
New.CleanUp.ReferenceOTU8322

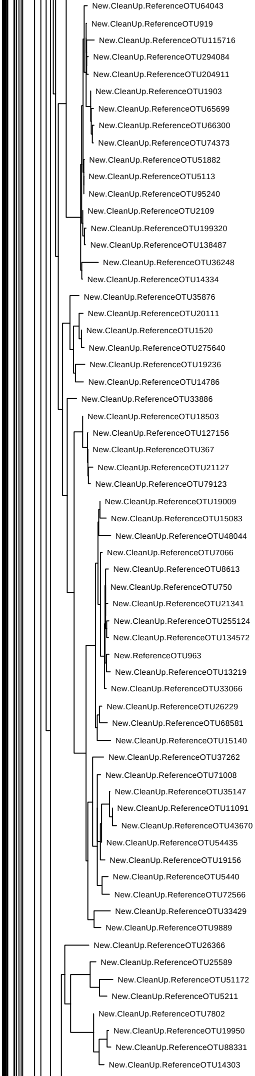
New.CleanUp.ReferenceOTU64043
New.CleanUp.ReferenceOTU919
New.CleanUp.ReferenceOTU115716
New.CleanUp.ReferenceOTU294084
New.CleanUp.ReferenceOTU204911
New.CleanUp.ReferenceOTU1903
New.CleanUp.ReferenceOTU65699
New.CleanUp.ReferenceOTU66300
New.CleanUp.ReferenceOTU74373
New.CleanUp.ReferenceOTU51882
New.CleanUp.ReferenceOTU5113
New.CleanUp.ReferenceOTU95240
New.CleanUp.ReferenceOTU2109
New.CleanUp.ReferenceOTU199320
New.CleanUp.ReferenceOTU138487
New.CleanUp.ReferenceOTU36248
New.CleanUp.ReferenceOTU14334
New.CleanUp.ReferenceOTU35876
New.CleanUp.ReferenceOTU20111
New.CleanUp.ReferenceOTU1520
New.CleanUp.ReferenceOTU275640
New.CleanUp.ReferenceOTU19236
New.CleanUp.ReferenceOTU14786
New.CleanUp.ReferenceOTU33886
New.CleanUp.ReferenceOTU18503
New.CleanUp.ReferenceOTU127156
New.CleanUp.ReferenceOTU367
New.CleanUp.ReferenceOTU21127
New.CleanUp.ReferenceOTU79123
New.CleanUp.ReferenceOTU19009
New.CleanUp.ReferenceOTU15083
New.CleanUp.ReferenceOTU48044
New.CleanUp.ReferenceOTU7066
New.CleanUp.ReferenceOTU8613
New.CleanUp.ReferenceOTU750
New.CleanUp.ReferenceOTU21341
New.CleanUp.ReferenceOTU255124
New.CleanUp.ReferenceOTU134572
New.ReferenceOTU963
New.CleanUp.ReferenceOTU13219
New.CleanUp.ReferenceOTU33066
New.CleanUp.ReferenceOTU26229
New.CleanUp.ReferenceOTU68581
New.CleanUp.ReferenceOTU15140
New.CleanUp.ReferenceOTU37262
New.CleanUp.ReferenceOTU71008
New.CleanUp.ReferenceOTU35147
New.CleanUp.ReferenceOTU11091
New.CleanUp.ReferenceOTU43670
New.CleanUp.ReferenceOTU54435
New.CleanUp.ReferenceOTU19156
New.CleanUp.ReferenceOTU5440
New.CleanUp.ReferenceOTU72566
New.CleanUp.ReferenceOTU33429
New.CleanUp.ReferenceOTU9889
New.CleanUp.ReferenceOTU26366
New.CleanUp.ReferenceOTU25589
New.CleanUp.ReferenceOTU51172
New.CleanUp.ReferenceOTU5211
New.CleanUp.ReferenceOTU7802
New.CleanUp.ReferenceOTU19950
New.CleanUp.ReferenceOTU88331
New.CleanUp.ReferenceOTU14303

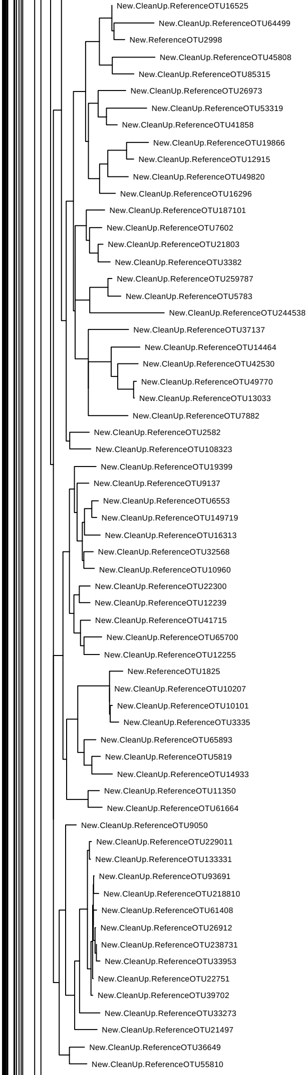
New.CleanUp.ReferenceOTU16525
New.CleanUp.ReferenceOTU64499
New.ReferenceOTU2998
New.CleanUp.ReferenceOTU45808
New.CleanUp.ReferenceOTU85315
New.CleanUp.ReferenceOTU26973
New.CleanUp.ReferenceOTU53319
New.CleanUp.ReferenceOTU41858
New.CleanUp.ReferenceOTU19866
New.CleanUp.ReferenceOTU12915
New.CleanUp.ReferenceOTU49820
New.CleanUp.ReferenceOTU16296
New.CleanUp.ReferenceOTU187101
New.CleanUp.ReferenceOTU7602
New.CleanUp.ReferenceOTU21803
New.CleanUp.ReferenceOTU3382
New.CleanUp.ReferenceOTU259787
New.CleanUp.ReferenceOTU5783
New.CleanUp.ReferenceOTU244538
New.CleanUp.ReferenceOTU37137
New.CleanUp.ReferenceOTU14464
New.CleanUp.ReferenceOTU42530
New.CleanUp.ReferenceOTU49770
New.CleanUp.ReferenceOTU13033
New.CleanUp.ReferenceOTU7882
New.CleanUp.ReferenceOTU2582
New.CleanUp.ReferenceOTU108323
New.CleanUp.ReferenceOTU19399
New.CleanUp.ReferenceOTU9137
New.CleanUp.ReferenceOTU6553
New.CleanUp.ReferenceOTU149719
New.CleanUp.ReferenceOTU16313
New.CleanUp.ReferenceOTU32568
New.CleanUp.ReferenceOTU10960
New.CleanUp.ReferenceOTU22300
New.CleanUp.ReferenceOTU12239
New.CleanUp.ReferenceOTU41715
New.CleanUp.ReferenceOTU65700
New.CleanUp.ReferenceOTU12255
New.ReferenceOTU1825
New.CleanUp.ReferenceOTU10207
New.CleanUp.ReferenceOTU10101
New.CleanUp.ReferenceOTU3335
New.CleanUp.ReferenceOTU65893
New.CleanUp.ReferenceOTU5819
New.CleanUp.ReferenceOTU14933
New.CleanUp.ReferenceOTU11350
New.CleanUp.ReferenceOTU61664
New.CleanUp.ReferenceOTU9050
New.CleanUp.ReferenceOTU229011
New.CleanUp.ReferenceOTU133331
New.CleanUp.ReferenceOTU93691
New.CleanUp.ReferenceOTU218810
New.CleanUp.ReferenceOTU61408
New.CleanUp.ReferenceOTU26912
New.CleanUp.ReferenceOTU238731
New.CleanUp.ReferenceOTU33953
New.CleanUp.ReferenceOTU22751
New.CleanUp.ReferenceOTU39702
New.CleanUp.ReferenceOTU33273
New.CleanUp.ReferenceOTU21497
New.CleanUp.ReferenceOTU36649
New.CleanUp.ReferenceOTU55810

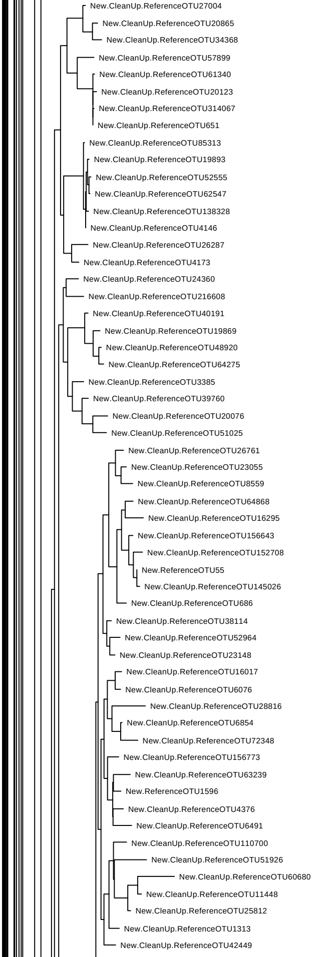
New.CleanUp.ReferenceOTU27004
New.CleanUp.ReferenceOTU20865
New.CleanUp.ReferenceOTU34368
New.CleanUp.ReferenceOTU57899
New.CleanUp.ReferenceOTU61340
New.CleanUp.ReferenceOTU20123
New.CleanUp.ReferenceOTU314067
New.CleanUp.ReferenceOTU651
New.CleanUp.ReferenceOTU85313
New.CleanUp.ReferenceOTU19893
New.CleanUp.ReferenceOTU52555
New.CleanUp.ReferenceOTU62547
New.CleanUp.ReferenceOTU138328
New.CleanUp.ReferenceOTU4146
New.CleanUp.ReferenceOTU26287
New.CleanUp.ReferenceOTU4173
New.CleanUp.ReferenceOTU24360
New.CleanUp.ReferenceOTU216608
New.CleanUp.ReferenceOTU40191
New.CleanUp.ReferenceOTU19869
New.CleanUp.ReferenceOTU48920
New.CleanUp.ReferenceOTU64275
New.CleanUp.ReferenceOTU3385
New.CleanUp.ReferenceOTU39760
New.CleanUp.ReferenceOTU20076
New.CleanUp.ReferenceOTU51025
New.CleanUp.ReferenceOTU26761
New.CleanUp.ReferenceOTU23055
New.CleanUp.ReferenceOTU8559
New.CleanUp.ReferenceOTU64868
New.CleanUp.ReferenceOTU16295
New.CleanUp.ReferenceOTU156643
New.CleanUp.ReferenceOTU152708
New.ReferenceOTU55
New.CleanUp.ReferenceOTU145026
New.CleanUp.ReferenceOTU686
New.CleanUp.ReferenceOTU38114
New.CleanUp.ReferenceOTU52964
New.CleanUp.ReferenceOTU23148
New.CleanUp.ReferenceOTU16017
New.CleanUp.ReferenceOTU6076
New.CleanUp.ReferenceOTU28816
New.CleanUp.ReferenceOTU6854
New.CleanUp.ReferenceOTU72348
New.CleanUp.ReferenceOTU156773
New.CleanUp.ReferenceOTU63239
New.ReferenceOTU1596
New.CleanUp.ReferenceOTU4376
New.CleanUp.ReferenceOTU6491
New.CleanUp.ReferenceOTU110700
New.CleanUp.ReferenceOTU51926
New.CleanUp.ReferenceOTU60680
New.CleanUp.ReferenceOTU11448
New.CleanUp.ReferenceOTU25812
New.CleanUp.ReferenceOTU1313
New.CleanUp.ReferenceOTU42449

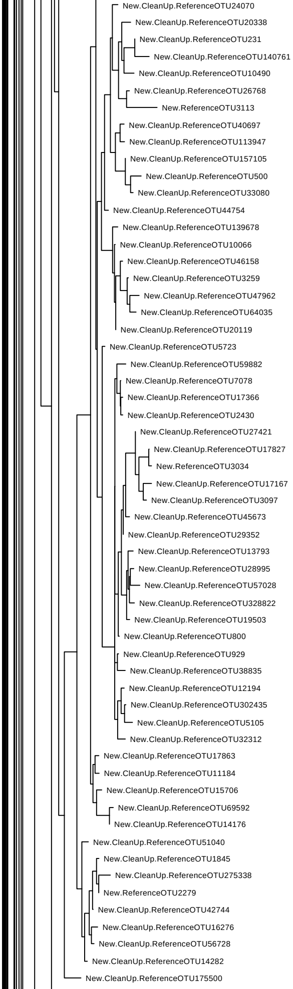
New.CleanUp.ReferenceOTU24070
New.CleanUp.ReferenceOTU20338
New.CleanUp.ReferenceOTU231
New.CleanUp.ReferenceOTU140761
New.CleanUp.ReferenceOTU10490
New.CleanUp.ReferenceOTU26768
New.ReferenceOTU3113
New.CleanUp.ReferenceOTU40697
New.CleanUp.ReferenceOTU113947
New.CleanUp.ReferenceOTU157105
New.CleanUp.ReferenceOTU500
New.CleanUp.ReferenceOTU33080
New.CleanUp.ReferenceOTU44754
New.CleanUp.ReferenceOTU139678
New.CleanUp.ReferenceOTU10066
New.CleanUp.ReferenceOTU46158
New.CleanUp.ReferenceOTU3259
New.CleanUp.ReferenceOTU47962
New.CleanUp.ReferenceOTU64035
New.CleanUp.ReferenceOTU20119
New.CleanUp.ReferenceOTU5723
New.CleanUp.ReferenceOTU59882
New.CleanUp.ReferenceOTU7078
New.CleanUp.ReferenceOTU17366
New.CleanUp.ReferenceOTU2430
New.CleanUp.ReferenceOTU27421
New.CleanUp.ReferenceOTU17827
New.ReferenceOTU3034
New.CleanUp.ReferenceOTU17167
New.CleanUp.ReferenceOTU3097
New.CleanUp.ReferenceOTU45673
New.CleanUp.ReferenceOTU29352
New.CleanUp.ReferenceOTU13793
New.CleanUp.ReferenceOTU28995
New.CleanUp.ReferenceOTU57028
New.CleanUp.ReferenceOTU328822
New.CleanUp.ReferenceOTU19503
New.CleanUp.ReferenceOTU800
New.CleanUp.ReferenceOTU929
New.CleanUp.ReferenceOTU38835
New.CleanUp.ReferenceOTU12194
New.CleanUp.ReferenceOTU302435
New.CleanUp.ReferenceOTU5105
New.CleanUp.ReferenceOTU32312
New.CleanUp.ReferenceOTU17863
New.CleanUp.ReferenceOTU11184
New.CleanUp.ReferenceOTU15706
New.CleanUp.ReferenceOTU69592
New.CleanUp.ReferenceOTU14176
New.CleanUp.ReferenceOTU51040
New.CleanUp.ReferenceOTU1845
New.CleanUp.ReferenceOTU275338
New.ReferenceOTU2279
New.CleanUp.ReferenceOTU42744
New.CleanUp.ReferenceOTU16276
New.CleanUp.ReferenceOTU56728
New.CleanUp.ReferenceOTU14282
New.CleanUp.ReferenceOTU175500

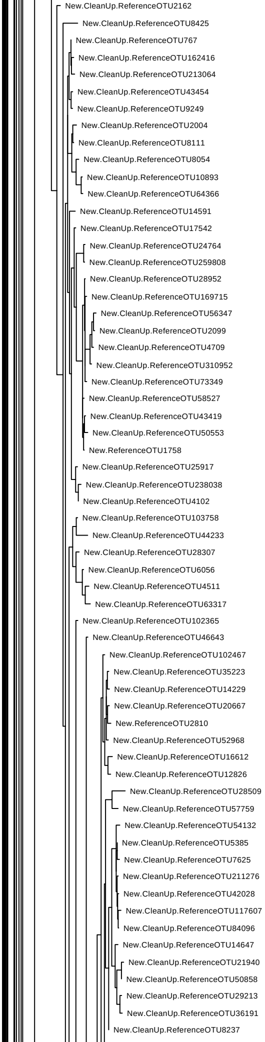
New.CleanUp.ReferenceOTU2162
New.CleanUp.ReferenceOTU8425
New.CleanUp.ReferenceOTU767
New.CleanUp.ReferenceOTU162416
New.CleanUp.ReferenceOTU213064
New.CleanUp.ReferenceOTU43454
New.CleanUp.ReferenceOTU9249
New.CleanUp.ReferenceOTU2004
New.CleanUp.ReferenceOTU8111
New.CleanUp.ReferenceOTU8054
New.CleanUp.ReferenceOTU10893
New.CleanUp.ReferenceOTU64366
New.CleanUp.ReferenceOTU14591
New.CleanUp.ReferenceOTU17542
New.CleanUp.ReferenceOTU24764
New.CleanUp.ReferenceOTU259808
New.CleanUp.ReferenceOTU28952
New.CleanUp.ReferenceOTU169715
New.CleanUp.ReferenceOTU56347
New.CleanUp.ReferenceOTU2099
New.CleanUp.ReferenceOTU4709
New.CleanUp.ReferenceOTU310952
New.CleanUp.ReferenceOTU73349
New.CleanUp.ReferenceOTU58527
New.CleanUp.ReferenceOTU43419
New.CleanUp.ReferenceOTU50553
New.ReferenceOTU1758
New.CleanUp.ReferenceOTU25917
New.CleanUp.ReferenceOTU238038
New.CleanUp.ReferenceOTU4102
New.CleanUp.ReferenceOTU103758
New.CleanUp.ReferenceOTU44233
New.CleanUp.ReferenceOTU28307
New.CleanUp.ReferenceOTU6056
New.CleanUp.ReferenceOTU4511
New.CleanUp.ReferenceOTU63317
New.CleanUp.ReferenceOTU102365
New.CleanUp.ReferenceOTU46643
New.CleanUp.ReferenceOTU102467
New.CleanUp.ReferenceOTU35223
New.CleanUp.ReferenceOTU14229
New.CleanUp.ReferenceOTU20667
New.ReferenceOTU2810
New.CleanUp.ReferenceOTU52968
New.CleanUp.ReferenceOTU16612
New.CleanUp.ReferenceOTU12826
New.CleanUp.ReferenceOTU28509
New.CleanUp.ReferenceOTU57759
New.CleanUp.ReferenceOTU54132
New.CleanUp.ReferenceOTU5385
New.CleanUp.ReferenceOTU7625
New.CleanUp.ReferenceOTU211276
New.CleanUp.ReferenceOTU42028
New.CleanUp.ReferenceOTU117607
New.CleanUp.ReferenceOTU84096
New.CleanUp.ReferenceOTU14647
New.CleanUp.ReferenceOTU21940
New.CleanUp.ReferenceOTU50858
New.CleanUp.ReferenceOTU29213
New.CleanUp.ReferenceOTU36191
New.CleanUp.ReferenceOTU8237

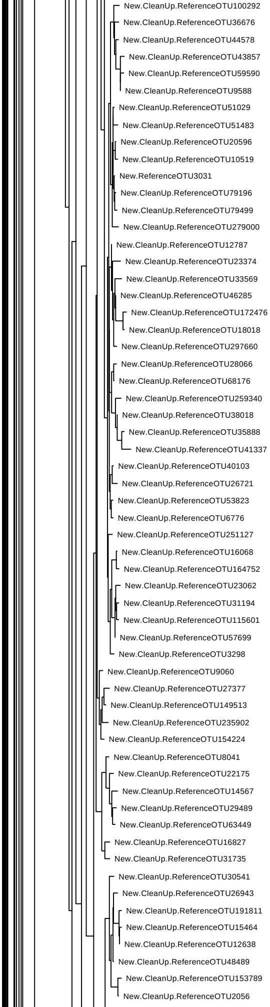
New.CleanUp.ReferenceOTU100292
New.CleanUp.ReferenceOTU36676
New.CleanUp.ReferenceOTU44578
New.CleanUp.ReferenceOTU43857
New.CleanUp.ReferenceOTU59590
New.CleanUp.ReferenceOTU9588
New.CleanUp.ReferenceOTU51029
New.CleanUp.ReferenceOTU51483
New.CleanUp.ReferenceOTU20596
New.CleanUp.ReferenceOTU10519
New.ReferenceOTU3031
New.CleanUp.ReferenceOTU79196
New.CleanUp.ReferenceOTU79499
New.CleanUp.ReferenceOTU279000
New.CleanUp.ReferenceOTU12787
New.CleanUp.ReferenceOTU23374
New.CleanUp.ReferenceOTU33569
New.CleanUp.ReferenceOTU46285
New.CleanUp.ReferenceOTU172476
New.CleanUp.ReferenceOTU18018
New.CleanUp.ReferenceOTU297660
New.CleanUp.ReferenceOTU28066
New.CleanUp.ReferenceOTU68176
New.CleanUp.ReferenceOTU259340
New.CleanUp.ReferenceOTU38018
New.CleanUp.ReferenceOTU35888
New.CleanUp.ReferenceOTU41337
New.CleanUp.ReferenceOTU40103
New.CleanUp.ReferenceOTU26721
New.CleanUp.ReferenceOTU53823
New.CleanUp.ReferenceOTU6776
New.CleanUp.ReferenceOTU251127
New.CleanUp.ReferenceOTU16068
New.CleanUp.ReferenceOTU164752
New.CleanUp.ReferenceOTU23062
New.CleanUp.ReferenceOTU31194
New.CleanUp.ReferenceOTU115601
New.CleanUp.ReferenceOTU57699
New.CleanUp.ReferenceOTU3298
New.CleanUp.ReferenceOTU9060
New.CleanUp.ReferenceOTU27377
New.CleanUp.ReferenceOTU149513
New.CleanUp.ReferenceOTU235902
New.CleanUp.ReferenceOTU154224
New.CleanUp.ReferenceOTU8041
New.CleanUp.ReferenceOTU22175
New.CleanUp.ReferenceOTU14567
New.CleanUp.ReferenceOTU29489
New.CleanUp.ReferenceOTU63449
New.CleanUp.ReferenceOTU16827
New.CleanUp.ReferenceOTU31735
New.CleanUp.ReferenceOTU30541
New.CleanUp.ReferenceOTU26943
New.CleanUp.ReferenceOTU191811
New.CleanUp.ReferenceOTU15464
New.CleanUp.ReferenceOTU12638
New.CleanUp.ReferenceOTU48489
New.CleanUp.ReferenceOTU153789
New.CleanUp.ReferenceOTU2056

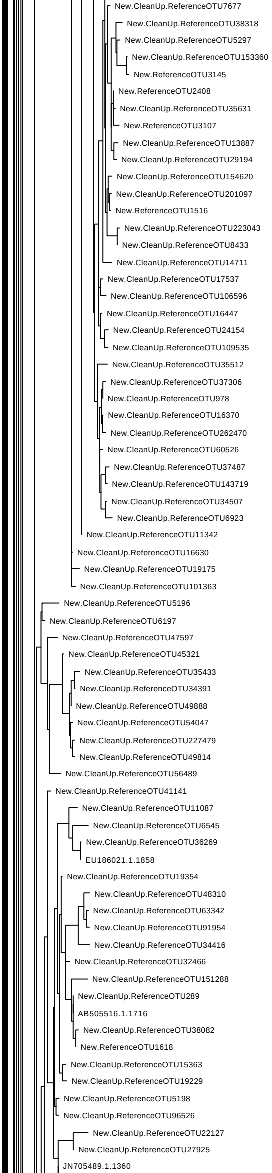
New.CleanUp.ReferenceOTU7677
New.CleanUp.ReferenceOTU38318
New.CleanUp.ReferenceOTU5297
New.CleanUp.ReferenceOTU153360
New.ReferenceOTU3145
New.ReferenceOTU2408
New.CleanUp.ReferenceOTU35631
New.ReferenceOTU3107
New.CleanUp.ReferenceOTU13887
New.CleanUp.ReferenceOTU29194
New.CleanUp.ReferenceOTU154620
New.CleanUp.ReferenceOTU201097
New.ReferenceOTU1516
New.CleanUp.ReferenceOTU223043
New.CleanUp.ReferenceOTU8433
New.CleanUp.ReferenceOTU14711
New.CleanUp.ReferenceOTU17537
New.CleanUp.ReferenceOTU106596
New.CleanUp.ReferenceOTU16447
New.CleanUp.ReferenceOTU24154
New.CleanUp.ReferenceOTU109535
New.CleanUp.ReferenceOTU35512
New.CleanUp.ReferenceOTU37306
New.CleanUp.ReferenceOTU978
New.CleanUp.ReferenceOTU16370
New.CleanUp.ReferenceOTU262470
New.CleanUp.ReferenceOTU60526
New.CleanUp.ReferenceOTU37487
New.CleanUp.ReferenceOTU143719
New.CleanUp.ReferenceOTU34507
New.CleanUp.ReferenceOTU6923
New.CleanUp.ReferenceOTU11342
New.CleanUp.ReferenceOTU16630
New.CleanUp.ReferenceOTU19175
New.CleanUp.ReferenceOTU101363
New.CleanUp.ReferenceOTU5196
New.CleanUp.ReferenceOTU6197
New.CleanUp.ReferenceOTU47597
New.CleanUp.ReferenceOTU45321
New.CleanUp.ReferenceOTU35433
New.CleanUp.ReferenceOTU34391
New.CleanUp.ReferenceOTU49888
New.CleanUp.ReferenceOTU54047
New.CleanUp.ReferenceOTU227479
New.CleanUp.ReferenceOTU49814
New.CleanUp.ReferenceOTU56489
New.CleanUp.ReferenceOTU41141
New.CleanUp.ReferenceOTU11087
New.CleanUp.ReferenceOTU6545
New.CleanUp.ReferenceOTU36269
EU186021.1.1858
New.CleanUp.ReferenceOTU19354
New.CleanUp.ReferenceOTU48310
New.CleanUp.ReferenceOTU63342
New.CleanUp.ReferenceOTU91954
New.CleanUp.ReferenceOTU34416
New.CleanUp.ReferenceOTU32466
New.CleanUp.ReferenceOTU151288
New.CleanUp.ReferenceOTU289
AB505516.1.1716
New.CleanUp.ReferenceOTU38082
New.ReferenceOTU1618
New.CleanUp.ReferenceOTU15363
New.CleanUp.ReferenceOTU19229
New.CleanUp.ReferenceOTU5198
New.CleanUp.ReferenceOTU96526
New.CleanUp.ReferenceOTU22127
New.CleanUp.ReferenceOTU27925
JN705489.1.1360

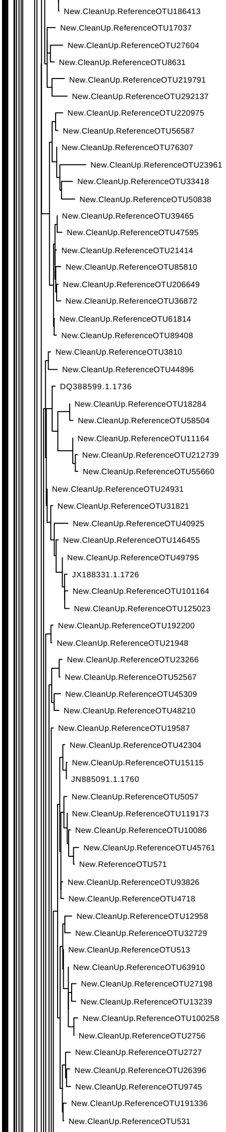
New.CleanUp.ReferenceOTU186413
New.CleanUp.ReferenceOTU17037
New.CleanUp.ReferenceOTU27604
New.CleanUp.ReferenceOTU8631
New.CleanUp.ReferenceOTU219791
New.CleanUp.ReferenceOTU292137
New.CleanUp.ReferenceOTU220975
New.CleanUp.ReferenceOTU56587
New.CleanUp.ReferenceOTU76307
New.CleanUp.ReferenceOTU23961
New.CleanUp.ReferenceOTU33418
New.CleanUp.ReferenceOTU50838
New.CleanUp.ReferenceOTU39465
New.CleanUp.ReferenceOTU47595
New.CleanUp.ReferenceOTU21414
New.CleanUp.ReferenceOTU85810
New.CleanUp.ReferenceOTU206649
New.CleanUp.ReferenceOTU36872
New.CleanUp.ReferenceOTU61814
New.CleanUp.ReferenceOTU89408
New.CleanUp.ReferenceOTU3810
New.CleanUp.ReferenceOTU44896
DQ388599.1.1736
New.CleanUp.ReferenceOTU18284
New.CleanUp.ReferenceOTU58504
New.CleanUp.ReferenceOTU11164
New.CleanUp.ReferenceOTU212739
New.CleanUp.ReferenceOTU55660
New.CleanUp.ReferenceOTU24931
New.CleanUp.ReferenceOTU31821
New.CleanUp.ReferenceOTU40925
New.CleanUp.ReferenceOTU146455
New.CleanUp.ReferenceOTU49795
JX188331.1.1726
New.CleanUp.ReferenceOTU101164
New.CleanUp.ReferenceOTU125023
New.CleanUp.ReferenceOTU192200
New.CleanUp.ReferenceOTU21948
New.CleanUp.ReferenceOTU23266
New.CleanUp.ReferenceOTU52567
New.CleanUp.ReferenceOTU45309
New.CleanUp.ReferenceOTU48210
New.CleanUp.ReferenceOTU19587
New.CleanUp.ReferenceOTU42304
New.CleanUp.ReferenceOTU15115
JN885091.1.1760
New.CleanUp.ReferenceOTU5057
New.CleanUp.ReferenceOTU119173
New.CleanUp.ReferenceOTU10086
New.CleanUp.ReferenceOTU45761
New.ReferenceOTU571
New.CleanUp.ReferenceOTU93826
New.CleanUp.ReferenceOTU4718
New.CleanUp.ReferenceOTU12958
New.CleanUp.ReferenceOTU32729
New.CleanUp.ReferenceOTU513
New.CleanUp.ReferenceOTU63910
New.CleanUp.ReferenceOTU27198
New.CleanUp.ReferenceOTU13239
New.CleanUp.ReferenceOTU100258
New.CleanUp.ReferenceOTU2756
New.CleanUp.ReferenceOTU2727
New.CleanUp.ReferenceOTU26396
New.CleanUp.ReferenceOTU9745
New.CleanUp.ReferenceOTU191336
New.CleanUp.ReferenceOTU531

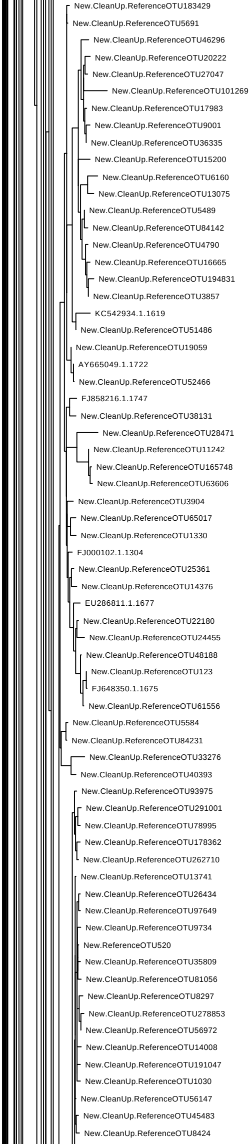
New.CleanUp.ReferenceOTU183429
New.CleanUp.ReferenceOTU5691
New.CleanUp.ReferenceOTU46296
New.CleanUp.ReferenceOTU20222
New.CleanUp.ReferenceOTU27047
New.CleanUp.ReferenceOTU101269
New.CleanUp.ReferenceOTU17983
New.CleanUp.ReferenceOTU9001
New.CleanUp.ReferenceOTU36335
New.CleanUp.ReferenceOTU15200
New.CleanUp.ReferenceOTU6160
New.CleanUp.ReferenceOTU13075
New.CleanUp.ReferenceOTU5489
New.CleanUp.ReferenceOTU84142
New.CleanUp.ReferenceOTU4790
New.CleanUp.ReferenceOTU16665
New.CleanUp.ReferenceOTU194831
New.CleanUp.ReferenceOTU3857
KC542934.1.1619
New.CleanUp.ReferenceOTU51486
New.CleanUp.ReferenceOTU19059
AY665049.1.1722
New.CleanUp.ReferenceOTU52466
FJ858216.1.1747
New.CleanUp.ReferenceOTU38131
New.CleanUp.ReferenceOTU28471
New.CleanUp.ReferenceOTU11242
New.CleanUp.ReferenceOTU165748
New.CleanUp.ReferenceOTU63606
New.CleanUp.ReferenceOTU3904
New.CleanUp.ReferenceOTU65017
New.CleanUp.ReferenceOTU1330
FJ000102.1.1304
New.CleanUp.ReferenceOTU25361
New.CleanUp.ReferenceOTU14376
EU286811.1.1677
New.CleanUp.ReferenceOTU22180
New.CleanUp.ReferenceOTU24455
New.CleanUp.ReferenceOTU48188
New.CleanUp.ReferenceOTU123
FJ648350.1.1675
New.CleanUp.ReferenceOTU61556
New.CleanUp.ReferenceOTU5584
New.CleanUp.ReferenceOTU84231
New.CleanUp.ReferenceOTU33276
New.CleanUp.ReferenceOTU40393
New.CleanUp.ReferenceOTU93975
New.CleanUp.ReferenceOTU291001
New.CleanUp.ReferenceOTU78995
New.CleanUp.ReferenceOTU178362
New.CleanUp.ReferenceOTU262710
New.CleanUp.ReferenceOTU13741
New.CleanUp.ReferenceOTU26434
New.CleanUp.ReferenceOTU97649
New.CleanUp.ReferenceOTU9734
New.ReferenceOTU520
New.CleanUp.ReferenceOTU35809
New.CleanUp.ReferenceOTU81056
New.CleanUp.ReferenceOTU8297
New.CleanUp.ReferenceOTU278853
New.CleanUp.ReferenceOTU56972
New.CleanUp.ReferenceOTU14008
New.CleanUp.ReferenceOTU191047
New.CleanUp.ReferenceOTU1030
New.CleanUp.ReferenceOTU56147
New.CleanUp.ReferenceOTU45483
New.CleanUp.ReferenceOTU8424

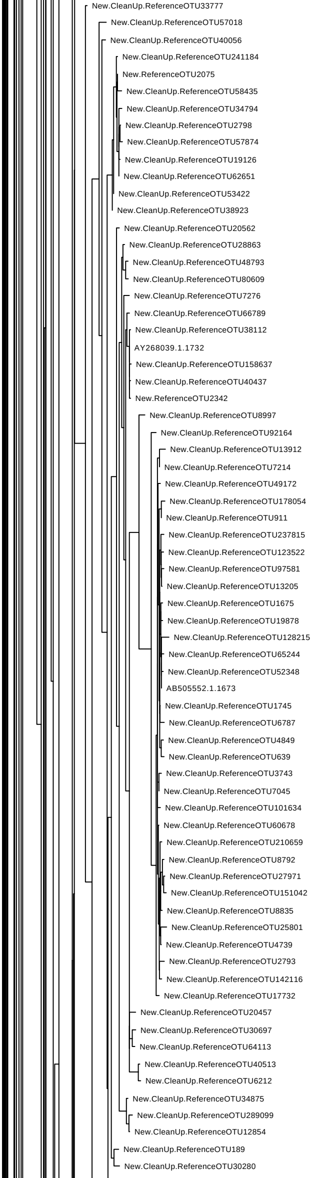
New.CleanUp.ReferenceOTU33777
New.CleanUp.ReferenceOTU57018
New.CleanUp.ReferenceOTU40056
New.CleanUp.ReferenceOTU241184
New.ReferenceOTU2075
New.CleanUp.ReferenceOTU58435
New.CleanUp.ReferenceOTU34794
New.CleanUp.ReferenceOTU2798
New.CleanUp.ReferenceOTU57874
New.CleanUp.ReferenceOTU19126
New.CleanUp.ReferenceOTU62651
New.CleanUp.ReferenceOTU53422
New.CleanUp.ReferenceOTU38923
New.CleanUp.ReferenceOTU20562
New.CleanUp.ReferenceOTU28863
New.CleanUp.ReferenceOTU48793
New.CleanUp.ReferenceOTU80609
New.CleanUp.ReferenceOTU7276
New.CleanUp.ReferenceOTU66789
New.CleanUp.ReferenceOTU38112
AY268039.1.1732
New.CleanUp.ReferenceOTU158637
New.CleanUp.ReferenceOTU40437
New.ReferenceOTU2342
New.CleanUp.ReferenceOTU8997
New.CleanUp.ReferenceOTU92164
New.CleanUp.ReferenceOTU13912
New.CleanUp.ReferenceOTU7214
New.CleanUp.ReferenceOTU49172
New.CleanUp.ReferenceOTU178054
New.CleanUp.ReferenceOTU911
New.CleanUp.ReferenceOTU237815
New.CleanUp.ReferenceOTU123522
New.CleanUp.ReferenceOTU97581
New.CleanUp.ReferenceOTU13205
New.CleanUp.ReferenceOTU1675
New.CleanUp.ReferenceOTU19878
New.CleanUp.ReferenceOTU128215
New.CleanUp.ReferenceOTU65244
New.CleanUp.ReferenceOTU52348
AB505552.1.1673
New.CleanUp.ReferenceOTU1745
New.CleanUp.ReferenceOTU6787
New.CleanUp.ReferenceOTU4849
New.CleanUp.ReferenceOTU639
New.CleanUp.ReferenceOTU3743
New.CleanUp.ReferenceOTU7045
New.CleanUp.ReferenceOTU101634
New.CleanUp.ReferenceOTU60678
New.CleanUp.ReferenceOTU210659
New.CleanUp.ReferenceOTU8792
New.CleanUp.ReferenceOTU27971
New.CleanUp.ReferenceOTU151042
New.CleanUp.ReferenceOTU8835
New.CleanUp.ReferenceOTU25801
New.CleanUp.ReferenceOTU4739
New.CleanUp.ReferenceOTU2793
New.CleanUp.ReferenceOTU142116
New.CleanUp.ReferenceOTU17732
New.CleanUp.ReferenceOTU20457
New.CleanUp.ReferenceOTU30697
New.CleanUp.ReferenceOTU64113
New.CleanUp.ReferenceOTU40513
New.CleanUp.ReferenceOTU6212
New.CleanUp.ReferenceOTU34875
New.CleanUp.ReferenceOTU289099
New.CleanUp.ReferenceOTU12854
New.CleanUp.ReferenceOTU189
New.CleanUp.ReferenceOTU30280

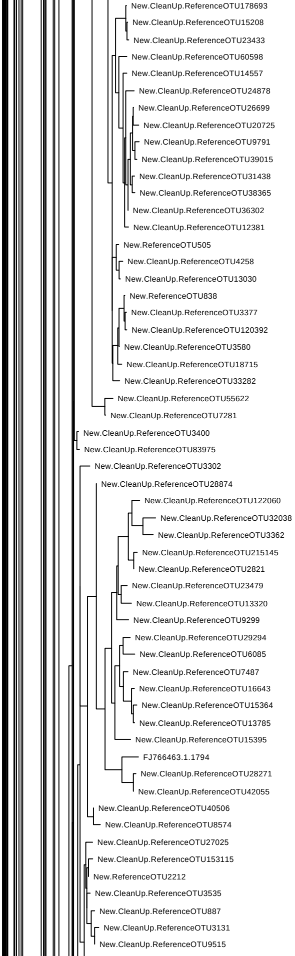
New.CleanUp.ReferenceOTU178693
New.CleanUp.ReferenceOTU15208
New.CleanUp.ReferenceOTU23433
New.CleanUp.ReferenceOTU60598
New.CleanUp.ReferenceOTU14557
New.CleanUp.ReferenceOTU24878
New.CleanUp.ReferenceOTU26699
New.CleanUp.ReferenceOTU20725
New.CleanUp.ReferenceOTU9791
New.CleanUp.ReferenceOTU39015
New.CleanUp.ReferenceOTU31438
New.CleanUp.ReferenceOTU38365
New.CleanUp.ReferenceOTU36302
New.CleanUp.ReferenceOTU12381
New.ReferenceOTU505
New.CleanUp.ReferenceOTU4258
New.CleanUp.ReferenceOTU13030
New.ReferenceOTU838
New.CleanUp.ReferenceOTU3377
New.CleanUp.ReferenceOTU120392
New.CleanUp.ReferenceOTU3580
New.CleanUp.ReferenceOTU18715
New.CleanUp.ReferenceOTU33282
New.CleanUp.ReferenceOTU55622
New.CleanUp.ReferenceOTU7281
New.CleanUp.ReferenceOTU3400
New.CleanUp.ReferenceOTU83975
New.CleanUp.ReferenceOTU3302
New.CleanUp.ReferenceOTU28874
New.CleanUp.ReferenceOTU122060
New.CleanUp.ReferenceOTU32038
New.CleanUp.ReferenceOTU3362
New.CleanUp.ReferenceOTU215145
New.CleanUp.ReferenceOTU2821
New.CleanUp.ReferenceOTU23479
New.CleanUp.ReferenceOTU13320
New.CleanUp.ReferenceOTU9299
New.CleanUp.ReferenceOTU29294
New.CleanUp.ReferenceOTU6085
New.CleanUp.ReferenceOTU7487
New.CleanUp.ReferenceOTU16643
New.CleanUp.ReferenceOTU15364
New.CleanUp.ReferenceOTU13785
New.CleanUp.ReferenceOTU15395
FJ766463.1.1794
New.CleanUp.ReferenceOTU28271
New.CleanUp.ReferenceOTU42055
New.CleanUp.ReferenceOTU40506
New.CleanUp.ReferenceOTU8574
New.CleanUp.ReferenceOTU27025
New.CleanUp.ReferenceOTU153115
New.ReferenceOTU2212
New.CleanUp.ReferenceOTU3535
New.CleanUp.ReferenceOTU887
New.CleanUp.ReferenceOTU3131
New.CleanUp.ReferenceOTU9515

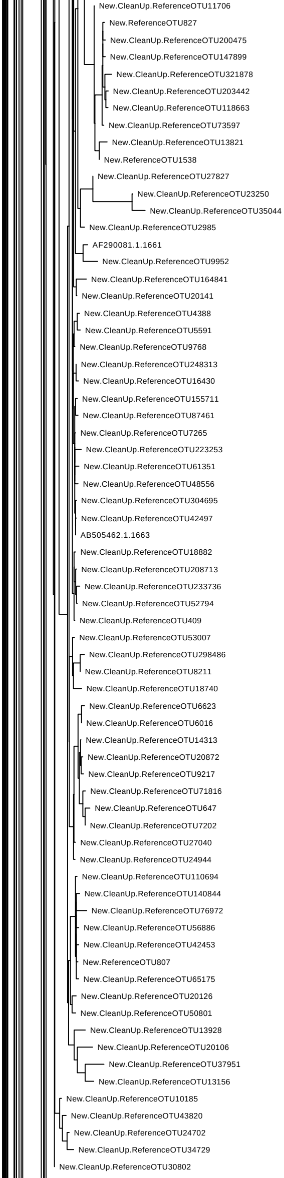
New.CleanUp.ReferenceOTU11706
New.ReferenceOTU827
New.CleanUp.ReferenceOTU200475
New.CleanUp.ReferenceOTU147899
New.CleanUp.ReferenceOTU321878
New.CleanUp.ReferenceOTU203442
New.CleanUp.ReferenceOTU118663
New.CleanUp.ReferenceOTU73597
New.CleanUp.ReferenceOTU13821
New.ReferenceOTU1538
New.CleanUp.ReferenceOTU27827
New.CleanUp.ReferenceOTU23250
New.CleanUp.ReferenceOTU35044
New.CleanUp.ReferenceOTU2985
AF290081.1.1661
New.CleanUp.ReferenceOTU9952
New.CleanUp.ReferenceOTU164841
New.CleanUp.ReferenceOTU20141
New.CleanUp.ReferenceOTU4388
New.CleanUp.ReferenceOTU5591
New.CleanUp.ReferenceOTU9768
New.CleanUp.ReferenceOTU248313
New.CleanUp.ReferenceOTU16430
New.CleanUp.ReferenceOTU155711
New.CleanUp.ReferenceOTU87461
New.CleanUp.ReferenceOTU7265
New.CleanUp.ReferenceOTU223253
New.CleanUp.ReferenceOTU61351
New.CleanUp.ReferenceOTU48556
New.CleanUp.ReferenceOTU304695
New.CleanUp.ReferenceOTU42497
AB505462.1.1663
New.CleanUp.ReferenceOTU18882
New.CleanUp.ReferenceOTU208713
New.CleanUp.ReferenceOTU233736
New.CleanUp.ReferenceOTU52794
New.CleanUp.ReferenceOTU409
New.CleanUp.ReferenceOTU53007
New.CleanUp.ReferenceOTU298486
New.CleanUp.ReferenceOTU8211
New.CleanUp.ReferenceOTU18740
New.CleanUp.ReferenceOTU6623
New.CleanUp.ReferenceOTU6016
New.CleanUp.ReferenceOTU14313
New.CleanUp.ReferenceOTU20872
New.CleanUp.ReferenceOTU9217
New.CleanUp.ReferenceOTU71816
New.CleanUp.ReferenceOTU647
New.CleanUp.ReferenceOTU7202
New.CleanUp.ReferenceOTU27040
New.CleanUp.ReferenceOTU24944
New.CleanUp.ReferenceOTU110694
New.CleanUp.ReferenceOTU140844
New.CleanUp.ReferenceOTU76972
New.CleanUp.ReferenceOTU56886
New.CleanUp.ReferenceOTU42453
New.ReferenceOTU807
New.CleanUp.ReferenceOTU65175
New.CleanUp.ReferenceOTU20126
New.CleanUp.ReferenceOTU50801
New.CleanUp.ReferenceOTU13928
New.CleanUp.ReferenceOTU20106
New.CleanUp.ReferenceOTU37951
New.CleanUp.ReferenceOTU13156
New.CleanUp.ReferenceOTU10185
New.CleanUp.ReferenceOTU43820
New.CleanUp.ReferenceOTU24702
New.CleanUp.ReferenceOTU34729
New.CleanUp.ReferenceOTU30802

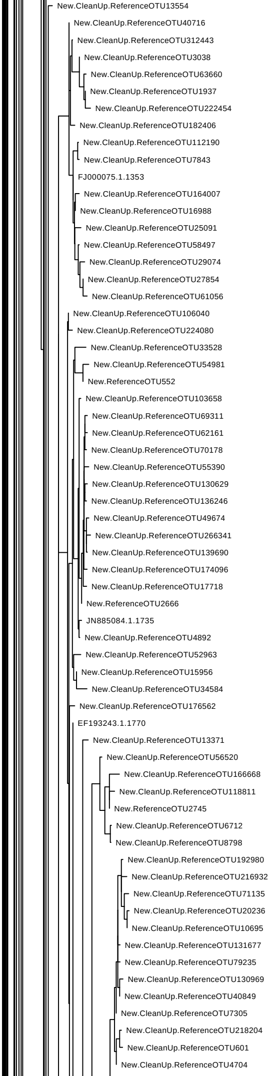
New.CleanUp.ReferenceOTU13554
New.CleanUp.ReferenceOTU40716
New.CleanUp.ReferenceOTU312443
New.CleanUp.ReferenceOTU3038
New.CleanUp.ReferenceOTU63660
New.CleanUp.ReferenceOTU1937
New.CleanUp.ReferenceOTU222454
New.CleanUp.ReferenceOTU182406
New.CleanUp.ReferenceOTU112190
New.CleanUp.ReferenceOTU7843
FJ000075.1.1353
New.CleanUp.ReferenceOTU164007
New.CleanUp.ReferenceOTU16988
New.CleanUp.ReferenceOTU25091
New.CleanUp.ReferenceOTU58497
New.CleanUp.ReferenceOTU29074
New.CleanUp.ReferenceOTU27854
New.CleanUp.ReferenceOTU61056
New.CleanUp.ReferenceOTU106040
New.CleanUp.ReferenceOTU224080
New.CleanUp.ReferenceOTU33528
New.CleanUp.ReferenceOTU54981
New.ReferenceOTU552
New.CleanUp.ReferenceOTU103658
New.CleanUp.ReferenceOTU69311
New.CleanUp.ReferenceOTU62161
New.CleanUp.ReferenceOTU70178
New.CleanUp.ReferenceOTU55390
New.CleanUp.ReferenceOTU130629
New.CleanUp.ReferenceOTU136246
New.CleanUp.ReferenceOTU49674
New.CleanUp.ReferenceOTU266341
New.CleanUp.ReferenceOTU139690
New.CleanUp.ReferenceOTU174096
New.CleanUp.ReferenceOTU17718
New.ReferenceOTU2666
JN885084.1.1735
New.CleanUp.ReferenceOTU4892
New.CleanUp.ReferenceOTU52963
New.CleanUp.ReferenceOTU15956
New.CleanUp.ReferenceOTU34584
New.CleanUp.ReferenceOTU176562
EF193243.1.1770
New.CleanUp.ReferenceOTU13371
New.CleanUp.ReferenceOTU56520
New.CleanUp.ReferenceOTU166668
New.CleanUp.ReferenceOTU118811
New.ReferenceOTU2745
New.CleanUp.ReferenceOTU6712
New.CleanUp.ReferenceOTU8798
New.CleanUp.ReferenceOTU192980
New.CleanUp.ReferenceOTU216932
New.CleanUp.ReferenceOTU71135
New.CleanUp.ReferenceOTU20236
New.CleanUp.ReferenceOTU10695
New.CleanUp.ReferenceOTU131677
New.CleanUp.ReferenceOTU79235
New.CleanUp.ReferenceOTU130969
New.CleanUp.ReferenceOTU40849
New.CleanUp.ReferenceOTU7305
New.CleanUp.ReferenceOTU218204
New.CleanUp.ReferenceOTU601
New.CleanUp.ReferenceOTU4704

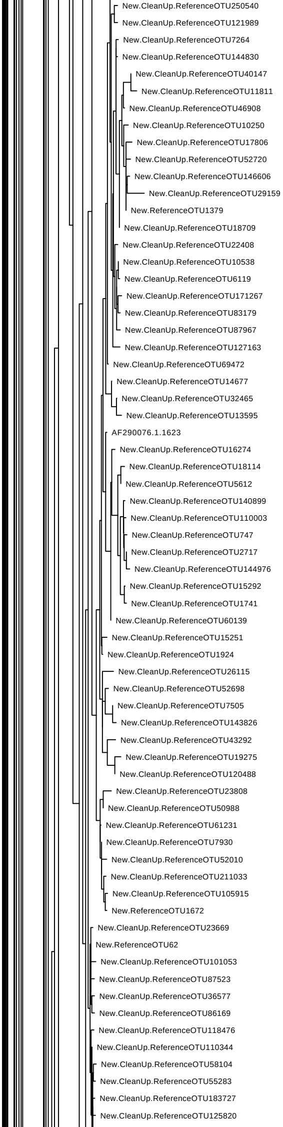
New.CleanUp.ReferenceOTU250540
New.CleanUp.ReferenceOTU121989
New.CleanUp.ReferenceOTU7264
New.CleanUp.ReferenceOTU144830
New.CleanUp.ReferenceOTU40147
New.CleanUp.ReferenceOTU11811
New.CleanUp.ReferenceOTU46908
New.CleanUp.ReferenceOTU10250
New.CleanUp.ReferenceOTU17806
New.CleanUp.ReferenceOTU52720
New.CleanUp.ReferenceOTU146606
New.CleanUp.ReferenceOTU29159
New.ReferenceOTU1379
New.CleanUp.ReferenceOTU18709
New.CleanUp.ReferenceOTU22408
New.CleanUp.ReferenceOTU10538
New.CleanUp.ReferenceOTU6119
New.CleanUp.ReferenceOTU171267
New.CleanUp.ReferenceOTU83179
New.CleanUp.ReferenceOTU87967
New.CleanUp.ReferenceOTU127163
New.CleanUp.ReferenceOTU69472
New.CleanUp.ReferenceOTU14677
New.CleanUp.ReferenceOTU32465
New.CleanUp.ReferenceOTU13595
AF290076.1.1623
New.CleanUp.ReferenceOTU16274
New.CleanUp.ReferenceOTU18114
New.CleanUp.ReferenceOTU5612
New.CleanUp.ReferenceOTU140899
New.CleanUp.ReferenceOTU110003
New.CleanUp.ReferenceOTU747
New.CleanUp.ReferenceOTU2717
New.CleanUp.ReferenceOTU144976
New.CleanUp.ReferenceOTU15292
New.CleanUp.ReferenceOTU1741
New.CleanUp.ReferenceOTU60139
New.CleanUp.ReferenceOTU15251
New.CleanUp.ReferenceOTU1924
New.CleanUp.ReferenceOTU26115
New.CleanUp.ReferenceOTU52698
New.CleanUp.ReferenceOTU7505
New.CleanUp.ReferenceOTU143826
New.CleanUp.ReferenceOTU43292
New.CleanUp.ReferenceOTU19275
New.CleanUp.ReferenceOTU120488
New.CleanUp.ReferenceOTU23808
New.CleanUp.ReferenceOTU50988
New.CleanUp.ReferenceOTU61231
New.CleanUp.ReferenceOTU7930
New.CleanUp.ReferenceOTU52010
New.CleanUp.ReferenceOTU211033
New.CleanUp.ReferenceOTU105915
New.ReferenceOTU1672
New.CleanUp.ReferenceOTU23669
New.ReferenceOTU62
New.CleanUp.ReferenceOTU101053
New.CleanUp.ReferenceOTU87523
New.CleanUp.ReferenceOTU36577
New.CleanUp.ReferenceOTU86169
New.CleanUp.ReferenceOTU118476
New.CleanUp.ReferenceOTU110344
New.CleanUp.ReferenceOTU58104
New.CleanUp.ReferenceOTU55283
New.CleanUp.ReferenceOTU183727
New.CleanUp.ReferenceOTU125820

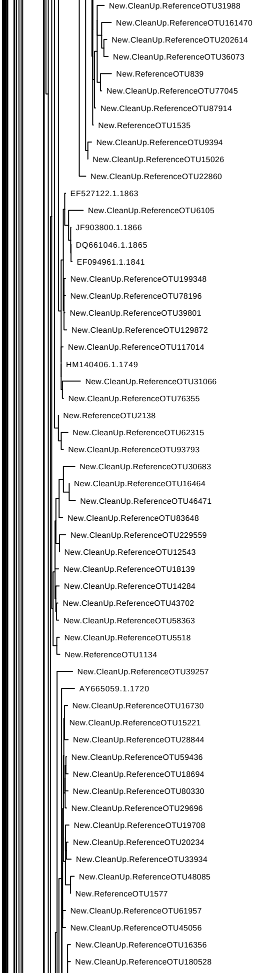
New.CleanUp.ReferenceOTU31988
New.CleanUp.ReferenceOTU161470
New.CleanUp.ReferenceOTU202614
New.CleanUp.ReferenceOTU36073
New.ReferenceOTU839
New.CleanUp.ReferenceOTU77045
New.CleanUp.ReferenceOTU87914
New.ReferenceOTU1535
New.CleanUp.ReferenceOTU9394
New.CleanUp.ReferenceOTU15026
New.CleanUp.ReferenceOTU22860
EF527122.1.1863
New.CleanUp.ReferenceOTU6105
JF903800.1.1866
DQ661046.1.1865
EF094961.1.1841
New.CleanUp.ReferenceOTU199348
New.CleanUp.ReferenceOTU78196
New.CleanUp.ReferenceOTU39801
New.CleanUp.ReferenceOTU129872
New.CleanUp.ReferenceOTU117014
HM140406.1.1749
New.CleanUp.ReferenceOTU31066
New.CleanUp.ReferenceOTU76355
New.ReferenceOTU2138
New.CleanUp.ReferenceOTU62315
New.CleanUp.ReferenceOTU93793
New.CleanUp.ReferenceOTU30683
New.CleanUp.ReferenceOTU16464
New.CleanUp.ReferenceOTU46471
New.CleanUp.ReferenceOTU83648
New.CleanUp.ReferenceOTU229559
New.CleanUp.ReferenceOTU12543
New.CleanUp.ReferenceOTU18139
New.CleanUp.ReferenceOTU14284
New.CleanUp.ReferenceOTU43702
New.CleanUp.ReferenceOTU58363
New.CleanUp.ReferenceOTU5518
New.ReferenceOTU1134
New.CleanUp.ReferenceOTU39257
AY665059.1.1720
New.CleanUp.ReferenceOTU16730
New.CleanUp.ReferenceOTU15221
New.CleanUp.ReferenceOTU28844
New.CleanUp.ReferenceOTU59436
New.CleanUp.ReferenceOTU18694
New.CleanUp.ReferenceOTU80330
New.CleanUp.ReferenceOTU29696
New.CleanUp.ReferenceOTU19708
New.CleanUp.ReferenceOTU20234
New.CleanUp.ReferenceOTU33934
New.CleanUp.ReferenceOTU48085
New.ReferenceOTU1577
New.CleanUp.ReferenceOTU61957
New.CleanUp.ReferenceOTU45056
New.CleanUp.ReferenceOTU16356
New.CleanUp.ReferenceOTU180528

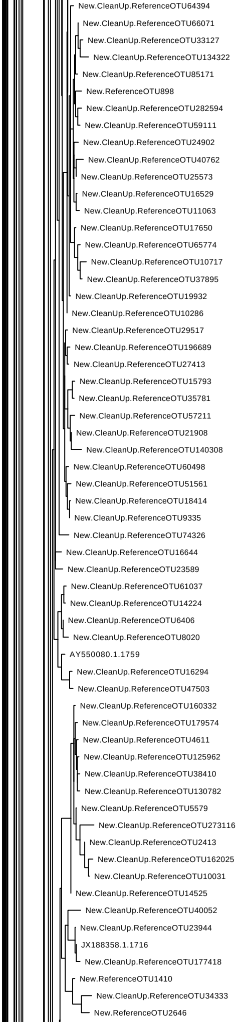
New.CleanUp.ReferenceOTU64394
New.CleanUp.ReferenceOTU66071
New.CleanUp.ReferenceOTU33127
New.CleanUp.ReferenceOTU134322
New.CleanUp.ReferenceOTU85171
New.ReferenceOTU898
New.CleanUp.ReferenceOTU282594
New.CleanUp.ReferenceOTU59111
New.CleanUp.ReferenceOTU24902
New.CleanUp.ReferenceOTU40762
New.CleanUp.ReferenceOTU25573
New.CleanUp.ReferenceOTU16529
New.CleanUp.ReferenceOTU11063
New.CleanUp.ReferenceOTU17650
New.CleanUp.ReferenceOTU65774
New.CleanUp.ReferenceOTU10717
New.CleanUp.ReferenceOTU37895
New.CleanUp.ReferenceOTU19932
New.CleanUp.ReferenceOTU10286
New.CleanUp.ReferenceOTU29517
New.CleanUp.ReferenceOTU196689
New.CleanUp.ReferenceOTU27413
New.CleanUp.ReferenceOTU15793
New.CleanUp.ReferenceOTU35781
New.CleanUp.ReferenceOTU57211
New.CleanUp.ReferenceOTU21908
New.CleanUp.ReferenceOTU140308
New.CleanUp.ReferenceOTU60498
New.CleanUp.ReferenceOTU51561
New.CleanUp.ReferenceOTU18414
New.CleanUp.ReferenceOTU9335
New.CleanUp.ReferenceOTU74326
New.CleanUp.ReferenceOTU16644
New.CleanUp.ReferenceOTU23589
New.CleanUp.ReferenceOTU61037
New.CleanUp.ReferenceOTU14224
New.CleanUp.ReferenceOTU6406
New.CleanUp.ReferenceOTU8020
AY550080.1.1759
New.CleanUp.ReferenceOTU16294
New.CleanUp.ReferenceOTU47503
New.CleanUp.ReferenceOTU160332
New.CleanUp.ReferenceOTU179574
New.CleanUp.ReferenceOTU4611
New.CleanUp.ReferenceOTU125962
New.CleanUp.ReferenceOTU38410
New.CleanUp.ReferenceOTU130782
New.CleanUp.ReferenceOTU5579
New.CleanUp.ReferenceOTU273116
New.CleanUp.ReferenceOTU2413
New.CleanUp.ReferenceOTU162025
New.CleanUp.ReferenceOTU10031
New.CleanUp.ReferenceOTU14525
New.CleanUp.ReferenceOTU40052
New.CleanUp.ReferenceOTU23944
JX188358.1.1716
New.CleanUp.ReferenceOTU177418
New.ReferenceOTU1410
New.CleanUp.ReferenceOTU34333
New.ReferenceOTU2646

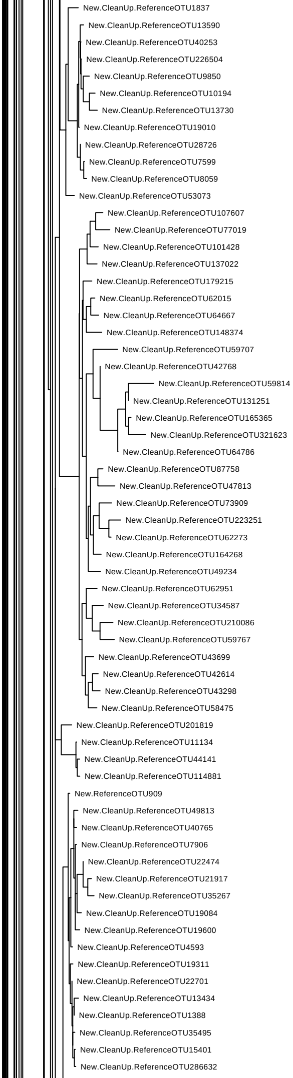
New.CleanUp.ReferenceOTU1837
New.CleanUp.ReferenceOTU13590
New.CleanUp.ReferenceOTU40253
New.CleanUp.ReferenceOTU226504
New.CleanUp.ReferenceOTU9850
New.CleanUp.ReferenceOTU10194
New.CleanUp.ReferenceOTU13730
New.CleanUp.ReferenceOTU19010
New.CleanUp.ReferenceOTU28726
New.CleanUp.ReferenceOTU7599
New.CleanUp.ReferenceOTU8059
New.CleanUp.ReferenceOTU53073
New.CleanUp.ReferenceOTU107607
New.CleanUp.ReferenceOTU77019
New.CleanUp.ReferenceOTU101428
New.CleanUp.ReferenceOTU137022
New.CleanUp.ReferenceOTU179215
New.CleanUp.ReferenceOTU62015
New.CleanUp.ReferenceOTU64667
New.CleanUp.ReferenceOTU148374
New.CleanUp.ReferenceOTU59707
New.CleanUp.ReferenceOTU42768
New.CleanUp.ReferenceOTU59814
New.CleanUp.ReferenceOTU131251
New.CleanUp.ReferenceOTU165365
New.CleanUp.ReferenceOTU321623
New.CleanUp.ReferenceOTU64786
New.CleanUp.ReferenceOTU87758
New.CleanUp.ReferenceOTU47813
New.CleanUp.ReferenceOTU73909
New.CleanUp.ReferenceOTU223251
New.CleanUp.ReferenceOTU62273
New.CleanUp.ReferenceOTU164268
New.CleanUp.ReferenceOTU49234
New.CleanUp.ReferenceOTU62951
New.CleanUp.ReferenceOTU34587
New.CleanUp.ReferenceOTU210086
New.CleanUp.ReferenceOTU59767
New.CleanUp.ReferenceOTU43699
New.CleanUp.ReferenceOTU42614
New.CleanUp.ReferenceOTU43298
New.CleanUp.ReferenceOTU58475
New.CleanUp.ReferenceOTU201819
New.CleanUp.ReferenceOTU11134
New.CleanUp.ReferenceOTU44141
New.CleanUp.ReferenceOTU114881
New.ReferenceOTU909
New.CleanUp.ReferenceOTU49813
New.CleanUp.ReferenceOTU40765
New.CleanUp.ReferenceOTU7906
New.CleanUp.ReferenceOTU22474
New.CleanUp.ReferenceOTU21917
New.CleanUp.ReferenceOTU35267
New.CleanUp.ReferenceOTU19084
New.CleanUp.ReferenceOTU19600
New.CleanUp.ReferenceOTU4593
New.CleanUp.ReferenceOTU19311
New.CleanUp.ReferenceOTU22701
New.CleanUp.ReferenceOTU13434
New.CleanUp.ReferenceOTU1388
New.CleanUp.ReferenceOTU35495
New.CleanUp.ReferenceOTU15401
New.CleanUp.ReferenceOTU286632

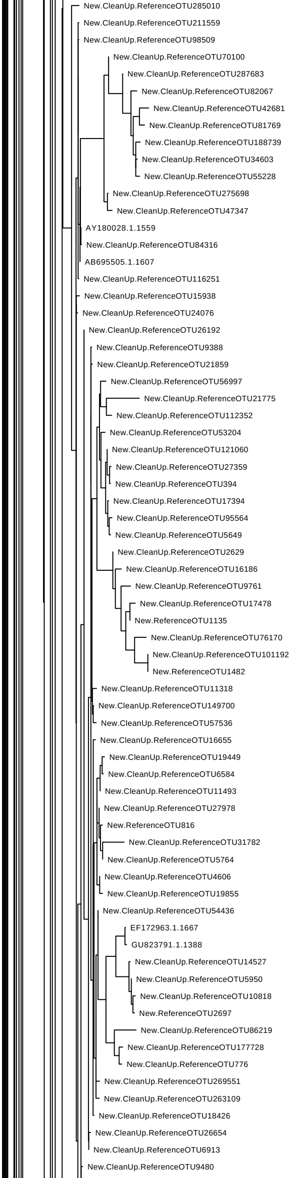
New.CleanUp.ReferenceOTU285010
New.CleanUp.ReferenceOTU211559
New.CleanUp.ReferenceOTU98509
New.CleanUp.ReferenceOTU70100
New.CleanUp.ReferenceOTU287683
New.CleanUp.ReferenceOTU82067
New.CleanUp.ReferenceOTU42681
New.CleanUp.ReferenceOTU81769
New.CleanUp.ReferenceOTU188739
New.CleanUp.ReferenceOTU34603
New.CleanUp.ReferenceOTU55228
New.CleanUp.ReferenceOTU275698
New.CleanUp.ReferenceOTU47347
AY180028.1.1559
New.CleanUp.ReferenceOTU84316
AB695505.1.1607
New.CleanUp.ReferenceOTU116251
New.CleanUp.ReferenceOTU15938
New.CleanUp.ReferenceOTU24076
New.CleanUp.ReferenceOTU26192
New.CleanUp.ReferenceOTU9388
New.CleanUp.ReferenceOTU21859
New.CleanUp.ReferenceOTU56997
New.CleanUp.ReferenceOTU21775
New.CleanUp.ReferenceOTU112352
New.CleanUp.ReferenceOTU53204
New.CleanUp.ReferenceOTU121060
New.CleanUp.ReferenceOTU27359
New.CleanUp.ReferenceOTU394
New.CleanUp.ReferenceOTU17394
New.CleanUp.ReferenceOTU95564
New.CleanUp.ReferenceOTU5649
New.CleanUp.ReferenceOTU2629
New.CleanUp.ReferenceOTU16186
New.CleanUp.ReferenceOTU9761
New.CleanUp.ReferenceOTU17478
New.ReferenceOTU1135
New.CleanUp.ReferenceOTU76170
New.CleanUp.ReferenceOTU101192
New.ReferenceOTU1482
New.CleanUp.ReferenceOTU11318
New.CleanUp.ReferenceOTU149700
New.CleanUp.ReferenceOTU57536
New.CleanUp.ReferenceOTU16655
New.CleanUp.ReferenceOTU19449
New.CleanUp.ReferenceOTU6584
New.CleanUp.ReferenceOTU11493
New.CleanUp.ReferenceOTU27978
New.ReferenceOTU816
New.CleanUp.ReferenceOTU31782
New.CleanUp.ReferenceOTU5764
New.CleanUp.ReferenceOTU4606
New.CleanUp.ReferenceOTU19855
New.CleanUp.ReferenceOTU54436
EF172963.1.1667
GU823791.1.1388
New.CleanUp.ReferenceOTU14527
New.CleanUp.ReferenceOTU5950
New.CleanUp.ReferenceOTU10818
New.ReferenceOTU2697
New.CleanUp.ReferenceOTU86219
New.CleanUp.ReferenceOTU177728
New.CleanUp.ReferenceOTU776
New.CleanUp.ReferenceOTU269551
New.CleanUp.ReferenceOTU263109
New.CleanUp.ReferenceOTU18426
New.CleanUp.ReferenceOTU26654
New.CleanUp.ReferenceOTU6913
New.CleanUp.ReferenceOTU9480

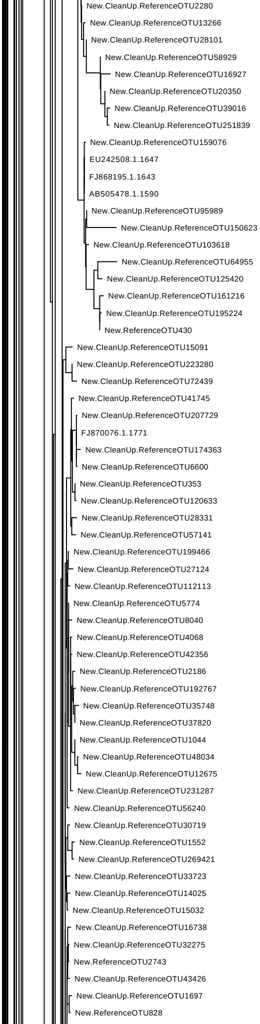
New.CleanUp.ReferenceOTU2280
New.CleanUp.ReferenceOTU13266
New.CleanUp.ReferenceOTU28101
New.CleanUp.ReferenceOTU58929
New.CleanUp.ReferenceOTU16927
New.CleanUp.ReferenceOTU20350
New.CleanUp.ReferenceOTU39016
New.CleanUp.ReferenceOTU251839
New.CleanUp.ReferenceOTU159076
EU242508.1.1647
FJ868195.1.1643
AB505478.1.1590
New.CleanUp.ReferenceOTU95989
New.CleanUp.ReferenceOTU150623
New.CleanUp.ReferenceOTU103618
New.CleanUp.ReferenceOTU64955
New.CleanUp.ReferenceOTU125420
New.CleanUp.ReferenceOTU161216
New.CleanUp.ReferenceOTU195224
New.ReferenceOTU430
New.CleanUp.ReferenceOTU15091
New.CleanUp.ReferenceOTU223280
New.CleanUp.ReferenceOTU72439
New.CleanUp.ReferenceOTU41745
New.CleanUp.ReferenceOTU207729
FJ870076.1.1771
New.CleanUp.ReferenceOTU174363
New.CleanUp.ReferenceOTU6600
New.CleanUp.ReferenceOTU353
New.CleanUp.ReferenceOTU120633
New.CleanUp.ReferenceOTU28331
New.CleanUp.ReferenceOTU57141
New.CleanUp.ReferenceOTU199466
New.CleanUp.ReferenceOTU27124
New.CleanUp.ReferenceOTU112113
New.CleanUp.ReferenceOTU5774
New.CleanUp.ReferenceOTU8040
New.CleanUp.ReferenceOTU4068
New.CleanUp.ReferenceOTU42356
New.CleanUp.ReferenceOTU2186
New.CleanUp.ReferenceOTU192767
New.CleanUp.ReferenceOTU35748
New.CleanUp.ReferenceOTU37820
New.CleanUp.ReferenceOTU1044
New.CleanUp.ReferenceOTU48034
New.CleanUp.ReferenceOTU12675
New.CleanUp.ReferenceOTU231287
New.CleanUp.ReferenceOTU56240
New.CleanUp.ReferenceOTU30719
New.CleanUp.ReferenceOTU1552
New.CleanUp.ReferenceOTU269421
New.CleanUp.ReferenceOTU33723
New.CleanUp.ReferenceOTU14025
New.CleanUp.ReferenceOTU15032
New.CleanUp.ReferenceOTU16738
New.CleanUp.ReferenceOTU32275
New.ReferenceOTU2743
New.CleanUp.ReferenceOTU43426
New.CleanUp.ReferenceOTU1697
New.ReferenceOTU828

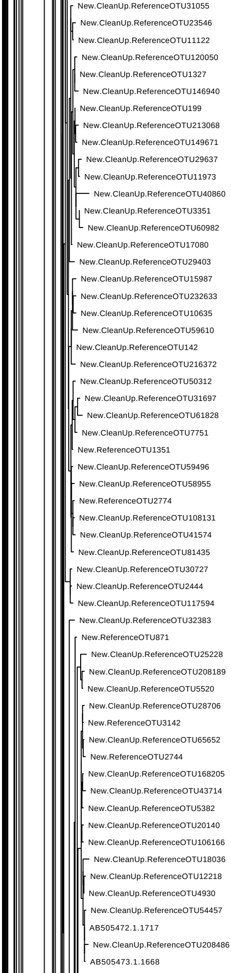
New.CleanUp.ReferenceOTU31055
New.CleanUp.ReferenceOTU23546
New.CleanUp.ReferenceOTU11122
New.CleanUp.ReferenceOTU120050
New.CleanUp.ReferenceOTU1327
New.CleanUp.ReferenceOTU146940
New.CleanUp.ReferenceOTU199
New.CleanUp.ReferenceOTU213068
New.CleanUp.ReferenceOTU149671
New.CleanUp.ReferenceOTU29637
New.CleanUp.ReferenceOTU11973
New.CleanUp.ReferenceOTU40860
New.CleanUp.ReferenceOTU3351
New.CleanUp.ReferenceOTU60982
New.CleanUp.ReferenceOTU17080
New.CleanUp.ReferenceOTU29403
New.CleanUp.ReferenceOTU15987
New.CleanUp.ReferenceOTU232633
New.CleanUp.ReferenceOTU10635
New.CleanUp.ReferenceOTU59610
New.CleanUp.ReferenceOTU142
New.CleanUp.ReferenceOTU216372
New.CleanUp.ReferenceOTU50312
New.CleanUp.ReferenceOTU31697
New.CleanUp.ReferenceOTU61828
New.CleanUp.ReferenceOTU7751
New.ReferenceOTU1351
New.CleanUp.ReferenceOTU59496
New.CleanUp.ReferenceOTU58955
New.ReferenceOTU2774
New.CleanUp.ReferenceOTU108131
New.CleanUp.ReferenceOTU41574
New.CleanUp.ReferenceOTU81435
New.CleanUp.ReferenceOTU30727
New.CleanUp.ReferenceOTU2444
New.CleanUp.ReferenceOTU117594
New.CleanUp.ReferenceOTU32383
New.ReferenceOTU871
New.CleanUp.ReferenceOTU25228
New.CleanUp.ReferenceOTU208189
New.CleanUp.ReferenceOTU5520
New.CleanUp.ReferenceOTU28706
New.ReferenceOTU3142
New.CleanUp.ReferenceOTU65652
New.ReferenceOTU2744
New.CleanUp.ReferenceOTU168205
New.CleanUp.ReferenceOTU43714
New.CleanUp.ReferenceOTU5382
New.CleanUp.ReferenceOTU20140
New.CleanUp.ReferenceOTU106166
New.CleanUp.ReferenceOTU18036
New.CleanUp.ReferenceOTU12218
New.CleanUp.ReferenceOTU4930
New.CleanUp.ReferenceOTU54457
AB505472.1.1717
New.CleanUp.ReferenceOTU208486
AB505473.1.1668

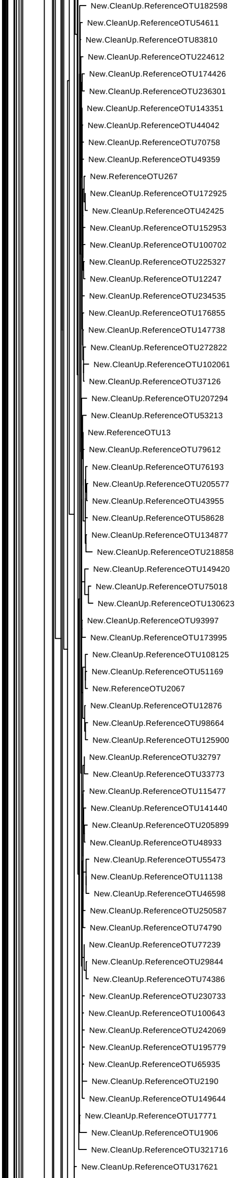
New.CleanUp.ReferenceOTU182598
New.CleanUp.ReferenceOTU54611
New.CleanUp.ReferenceOTU83810
New.CleanUp.ReferenceOTU224612
New.CleanUp.ReferenceOTU174426
New.CleanUp.ReferenceOTU236301
New.CleanUp.ReferenceOTU143351
New.CleanUp.ReferenceOTU44042
New.CleanUp.ReferenceOTU70758
New.CleanUp.ReferenceOTU49359
New.ReferenceOTU267
New.CleanUp.ReferenceOTU172925
New.CleanUp.ReferenceOTU42425
New.CleanUp.ReferenceOTU152953
New.CleanUp.ReferenceOTU100702
New.CleanUp.ReferenceOTU225327
New.CleanUp.ReferenceOTU12247
New.CleanUp.ReferenceOTU234535
New.CleanUp.ReferenceOTU176855
New.CleanUp.ReferenceOTU147738
New.CleanUp.ReferenceOTU272822
New.CleanUp.ReferenceOTU102061
New.CleanUp.ReferenceOTU37126
New.CleanUp.ReferenceOTU207294
New.CleanUp.ReferenceOTU53213
New.ReferenceOTU13
New.CleanUp.ReferenceOTU79612
New.CleanUp.ReferenceOTU76193
New.CleanUp.ReferenceOTU205577
New.CleanUp.ReferenceOTU43955
New.CleanUp.ReferenceOTU58628
New.CleanUp.ReferenceOTU134877
New.CleanUp.ReferenceOTU218858
New.CleanUp.ReferenceOTU149420
New.CleanUp.ReferenceOTU75018
New.CleanUp.ReferenceOTU130623
New.CleanUp.ReferenceOTU93997
New.CleanUp.ReferenceOTU173995
New.CleanUp.ReferenceOTU108125
New.CleanUp.ReferenceOTU51169
New.ReferenceOTU2067
New.CleanUp.ReferenceOTU12876
New.CleanUp.ReferenceOTU98664
New.CleanUp.ReferenceOTU125900
New.CleanUp.ReferenceOTU32797
New.CleanUp.ReferenceOTU33773
New.CleanUp.ReferenceOTU115477
New.CleanUp.ReferenceOTU141440
New.CleanUp.ReferenceOTU205899
New.CleanUp.ReferenceOTU48933
New.CleanUp.ReferenceOTU55473
New.CleanUp.ReferenceOTU11138
New.CleanUp.ReferenceOTU46598
New.CleanUp.ReferenceOTU250587
New.CleanUp.ReferenceOTU74790
New.CleanUp.ReferenceOTU77239
New.CleanUp.ReferenceOTU29844
New.CleanUp.ReferenceOTU74386
New.CleanUp.ReferenceOTU230733
New.CleanUp.ReferenceOTU100643
New.CleanUp.ReferenceOTU242069
New.CleanUp.ReferenceOTU195779
New.CleanUp.ReferenceOTU65935
New.CleanUp.ReferenceOTU2190
New.CleanUp.ReferenceOTU149644
New.CleanUp.ReferenceOTU17771
New.CleanUp.ReferenceOTU1906
New.CleanUp.ReferenceOTU321716
New.CleanUp.ReferenceOTU317621

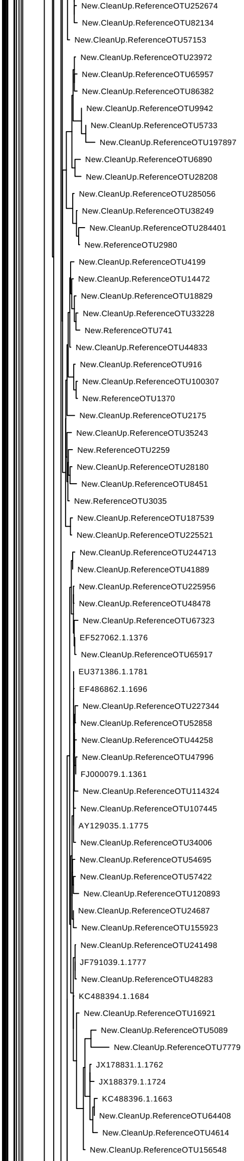
New.CleanUp.ReferenceOTU252674
New.CleanUp.ReferenceOTU82134
New.CleanUp.ReferenceOTU57153
New.CleanUp.ReferenceOTU23972
New.CleanUp.ReferenceOTU65957
New.CleanUp.ReferenceOTU86382
New.CleanUp.ReferenceOTU9942
New.CleanUp.ReferenceOTU5733
New.CleanUp.ReferenceOTU197897
New.CleanUp.ReferenceOTU6890
New.CleanUp.ReferenceOTU28208
New.CleanUp.ReferenceOTU285056
New.CleanUp.ReferenceOTU38249
New.CleanUp.ReferenceOTU284401
New.ReferenceOTU2980
New.CleanUp.ReferenceOTU4199
New.CleanUp.ReferenceOTU14472
New.CleanUp.ReferenceOTU18829
New.CleanUp.ReferenceOTU33228
New.ReferenceOTU741
New.CleanUp.ReferenceOTU44833
New.CleanUp.ReferenceOTU916
New.CleanUp.ReferenceOTU100307
New.ReferenceOTU1370
New.CleanUp.ReferenceOTU2175
New.CleanUp.ReferenceOTU35243
New.ReferenceOTU2259
New.CleanUp.ReferenceOTU28180
New.CleanUp.ReferenceOTU8451
New.ReferenceOTU3035
New.CleanUp.ReferenceOTU187539
New.CleanUp.ReferenceOTU225521
New.CleanUp.ReferenceOTU244713
New.CleanUp.ReferenceOTU41889
New.CleanUp.ReferenceOTU225956
New.CleanUp.ReferenceOTU48478
New.CleanUp.ReferenceOTU67323
EF527062.1.1376
New.CleanUp.ReferenceOTU65917
EU371386.1.1781
EF486862.1.1696
New.CleanUp.ReferenceOTU227344
New.CleanUp.ReferenceOTU52858
New.CleanUp.ReferenceOTU44258
New.CleanUp.ReferenceOTU47996
FJ000079.1.1361
New.CleanUp.ReferenceOTU114324
New.CleanUp.ReferenceOTU107445
AY129035.1.1775
New.CleanUp.ReferenceOTU34006
New.CleanUp.ReferenceOTU54695
New.CleanUp.ReferenceOTU57422
New.CleanUp.ReferenceOTU120893
New.CleanUp.ReferenceOTU24687
New.CleanUp.ReferenceOTU155923
New.CleanUp.ReferenceOTU241498
JF791039.1.1777
New.CleanUp.ReferenceOTU48283
KC488394.1.1684
New.CleanUp.ReferenceOTU16921
New.CleanUp.ReferenceOTU5089
New.CleanUp.ReferenceOTU7779
JX178831.1.1762
JX188379.1.1724
KC488396.1.1663
New.CleanUp.ReferenceOTU64408
New.CleanUp.ReferenceOTU4614
New.CleanUp.ReferenceOTU156548

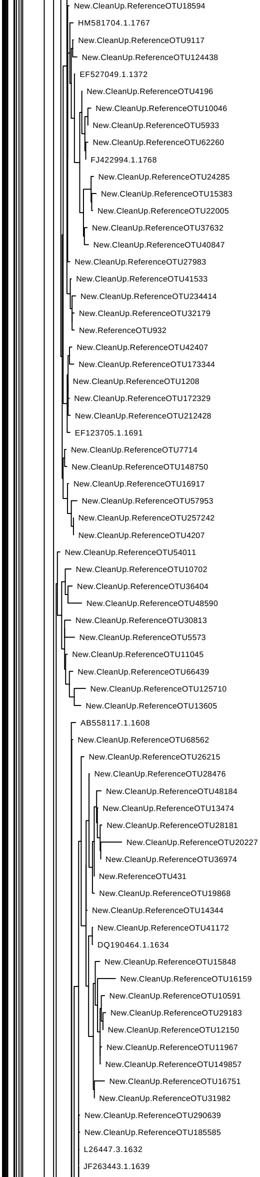
New.CleanUp.ReferenceOTU18594
HM581704.1.1767
New.CleanUp.ReferenceOTU9117
New.CleanUp.ReferenceOTU124438
EF527049.1.1372
New.CleanUp.ReferenceOTU4196
New.CleanUp.ReferenceOTU10046
New.CleanUp.ReferenceOTU5933
New.CleanUp.ReferenceOTU62260
FJ422994.1.1768
New.CleanUp.ReferenceOTU24285
New.CleanUp.ReferenceOTU15383
New.CleanUp.ReferenceOTU22005
New.CleanUp.ReferenceOTU37632
New.CleanUp.ReferenceOTU40847
New.CleanUp.ReferenceOTU27983
New.CleanUp.ReferenceOTU41533
New.CleanUp.ReferenceOTU234414
New.CleanUp.ReferenceOTU32179
New.ReferenceOTU932
New.CleanUp.ReferenceOTU42407
New.CleanUp.ReferenceOTU173344
New.CleanUp.ReferenceOTU1208
New.CleanUp.ReferenceOTU172329
New.CleanUp.ReferenceOTU212428
EF123705.1.1691
New.CleanUp.ReferenceOTU7714
New.CleanUp.ReferenceOTU148750
New.CleanUp.ReferenceOTU16917
New.CleanUp.ReferenceOTU57953
New.CleanUp.ReferenceOTU257242
New.CleanUp.ReferenceOTU4207
New.CleanUp.ReferenceOTU54011
New.CleanUp.ReferenceOTU10702
New.CleanUp.ReferenceOTU36404
New.CleanUp.ReferenceOTU48590
New.CleanUp.ReferenceOTU30813
New.CleanUp.ReferenceOTU5573
New.CleanUp.ReferenceOTU11045
New.CleanUp.ReferenceOTU66439
New.CleanUp.ReferenceOTU125710
New.CleanUp.ReferenceOTU13605
AB558117.1.1608
New.CleanUp.ReferenceOTU68562
New.CleanUp.ReferenceOTU26215
New.CleanUp.ReferenceOTU28476
New.CleanUp.ReferenceOTU48184
New.CleanUp.ReferenceOTU13474
New.CleanUp.ReferenceOTU28181
New.CleanUp.ReferenceOTU20227
New.CleanUp.ReferenceOTU36974
New.ReferenceOTU431
New.CleanUp.ReferenceOTU19868
New.CleanUp.ReferenceOTU14344
New.CleanUp.ReferenceOTU41172
DQ190464.1.1634
New.CleanUp.ReferenceOTU15848
New.CleanUp.ReferenceOTU16159
New.CleanUp.ReferenceOTU10591
New.CleanUp.ReferenceOTU29183
New.CleanUp.ReferenceOTU12150
New.CleanUp.ReferenceOTU11967
New.CleanUp.ReferenceOTU149857
New.CleanUp.ReferenceOTU16751
New.CleanUp.ReferenceOTU31982
New.CleanUp.ReferenceOTU290639
New.CleanUp.ReferenceOTU185585
L26447.3.1632
JF263443.1.1639

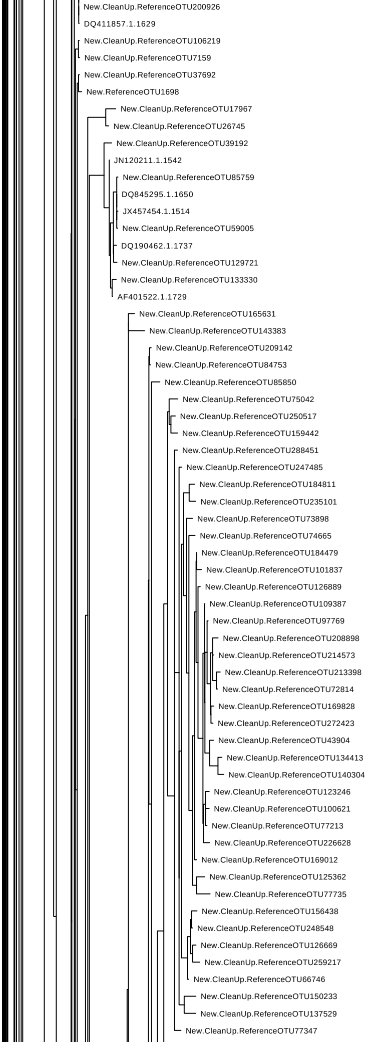
New.CleanUp.ReferenceOTU200926
DQ411857.1.1629
New.CleanUp.ReferenceOTU106219
New.CleanUp.ReferenceOTU7159
New.CleanUp.ReferenceOTU37692
New.ReferenceOTU1698
New.CleanUp.ReferenceOTU17967
New.CleanUp.ReferenceOTU26745
New.CleanUp.ReferenceOTU39192
JN120211.1.1542
New.CleanUp.ReferenceOTU85759
DQ845295.1.1650
JX457454.1.1514
New.CleanUp.ReferenceOTU59005
DQ190462.1.1737
New.CleanUp.ReferenceOTU129721
New.CleanUp.ReferenceOTU133330
AF401522.1.1729
New.CleanUp.ReferenceOTU165631
New.CleanUp.ReferenceOTU143383
New.CleanUp.ReferenceOTU209142
New.CleanUp.ReferenceOTU84753
New.CleanUp.ReferenceOTU85850
New.CleanUp.ReferenceOTU75042
New.CleanUp.ReferenceOTU250517
New.CleanUp.ReferenceOTU159442
New.CleanUp.ReferenceOTU288451
New.CleanUp.ReferenceOTU247485
New.CleanUp.ReferenceOTU184811
New.CleanUp.ReferenceOTU235101
New.CleanUp.ReferenceOTU73898
New.CleanUp.ReferenceOTU74665
New.CleanUp.ReferenceOTU184479
New.CleanUp.ReferenceOTU101837
New.CleanUp.ReferenceOTU126889
New.CleanUp.ReferenceOTU109387
New.CleanUp.ReferenceOTU97769
New.CleanUp.ReferenceOTU208898
New.CleanUp.ReferenceOTU214573
New.CleanUp.ReferenceOTU213398
New.CleanUp.ReferenceOTU72814
New.CleanUp.ReferenceOTU169828
New.CleanUp.ReferenceOTU272423
New.CleanUp.ReferenceOTU43904
New.CleanUp.ReferenceOTU134413
New.CleanUp.ReferenceOTU140304
New.CleanUp.ReferenceOTU123246
New.CleanUp.ReferenceOTU100621
New.CleanUp.ReferenceOTU77213
New.CleanUp.ReferenceOTU226628
New.CleanUp.ReferenceOTU169012
New.CleanUp.ReferenceOTU125362
New.CleanUp.ReferenceOTU77735
New.CleanUp.ReferenceOTU156438
New.CleanUp.ReferenceOTU248548
New.CleanUp.ReferenceOTU126669
New.CleanUp.ReferenceOTU259217
New.CleanUp.ReferenceOTU66746
New.CleanUp.ReferenceOTU150233
New.CleanUp.ReferenceOTU137529
New.CleanUp.ReferenceOTU77347

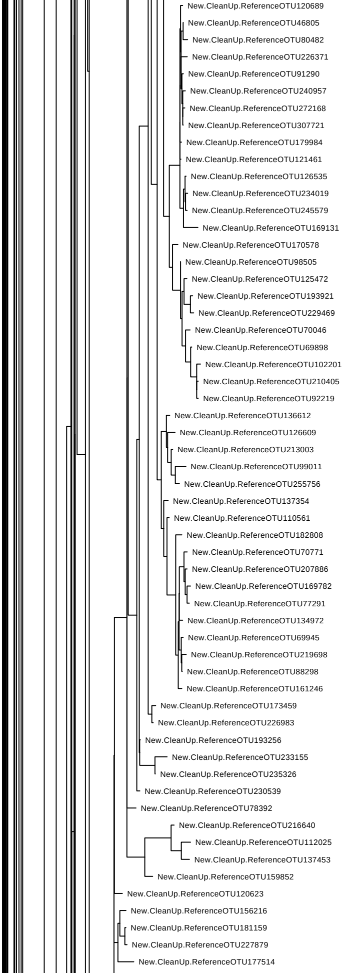
New.CleanUp.ReferenceOTU120689
New.CleanUp.ReferenceOTU46805
New.CleanUp.ReferenceOTU80482
New.CleanUp.ReferenceOTU226371
New.CleanUp.ReferenceOTU91290
New.CleanUp.ReferenceOTU240957
New.CleanUp.ReferenceOTU272168
New.CleanUp.ReferenceOTU307721
New.CleanUp.ReferenceOTU179984
New.CleanUp.ReferenceOTU121461
New.CleanUp.ReferenceOTU126535
New.CleanUp.ReferenceOTU234019
New.CleanUp.ReferenceOTU245579
New.CleanUp.ReferenceOTU169131
New.CleanUp.ReferenceOTU170578
New.CleanUp.ReferenceOTU98505
New.CleanUp.ReferenceOTU125472
New.CleanUp.ReferenceOTU193921
New.CleanUp.ReferenceOTU229469
New.CleanUp.ReferenceOTU70046
New.CleanUp.ReferenceOTU69898
New.CleanUp.ReferenceOTU102201
New.CleanUp.ReferenceOTU210405
New.CleanUp.ReferenceOTU92219
New.CleanUp.ReferenceOTU136612
New.CleanUp.ReferenceOTU126609
New.CleanUp.ReferenceOTU213003
New.CleanUp.ReferenceOTU99011
New.CleanUp.ReferenceOTU255756
New.CleanUp.ReferenceOTU137354
New.CleanUp.ReferenceOTU110561
New.CleanUp.ReferenceOTU182808
New.CleanUp.ReferenceOTU70771
New.CleanUp.ReferenceOTU207886
New.CleanUp.ReferenceOTU169782
New.CleanUp.ReferenceOTU77291
New.CleanUp.ReferenceOTU134972
New.CleanUp.ReferenceOTU69945
New.CleanUp.ReferenceOTU219698
New.CleanUp.ReferenceOTU88298
New.CleanUp.ReferenceOTU161246
New.CleanUp.ReferenceOTU173459
New.CleanUp.ReferenceOTU226983
New.CleanUp.ReferenceOTU193256
New.CleanUp.ReferenceOTU233155
New.CleanUp.ReferenceOTU235326
New.CleanUp.ReferenceOTU230539
New.CleanUp.ReferenceOTU78392
New.CleanUp.ReferenceOTU216640
New.CleanUp.ReferenceOTU112025
New.CleanUp.ReferenceOTU137453
New.CleanUp.ReferenceOTU159852
New.CleanUp.ReferenceOTU120623
New.CleanUp.ReferenceOTU156216
New.CleanUp.ReferenceOTU181159
New.CleanUp.ReferenceOTU227879
New.CleanUp.ReferenceOTU177514

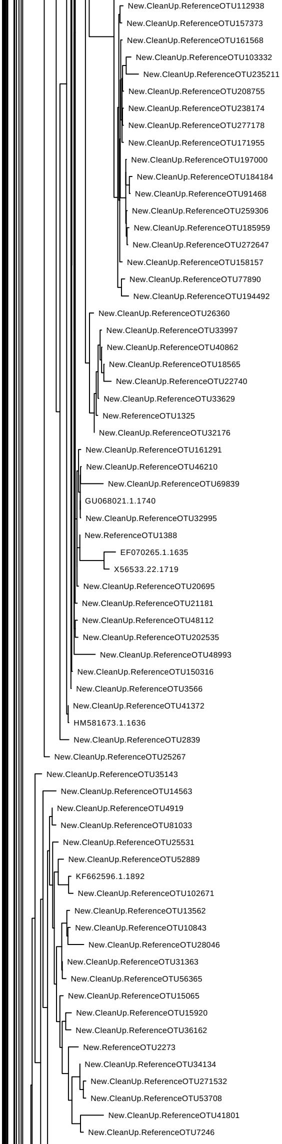
New.CleanUp.ReferenceOTU112938
New.CleanUp.ReferenceOTU157373
New.CleanUp.ReferenceOTU161568
New.CleanUp.ReferenceOTU103332
New.CleanUp.ReferenceOTU235211
New.CleanUp.ReferenceOTU208755
New.CleanUp.ReferenceOTU238174
New.CleanUp.ReferenceOTU277178
New.CleanUp.ReferenceOTU171955
New.CleanUp.ReferenceOTU197000
New.CleanUp.ReferenceOTU184184
New.CleanUp.ReferenceOTU91468
New.CleanUp.ReferenceOTU259306
New.CleanUp.ReferenceOTU185959
New.CleanUp.ReferenceOTU272647
New.CleanUp.ReferenceOTU158157
New.CleanUp.ReferenceOTU77890
New.CleanUp.ReferenceOTU194492
New.CleanUp.ReferenceOTU26360
New.CleanUp.ReferenceOTU33997
New.CleanUp.ReferenceOTU40862
New.CleanUp.ReferenceOTU18565
New.CleanUp.ReferenceOTU22740
New.CleanUp.ReferenceOTU33629
New.ReferenceOTU1325
New.CleanUp.ReferenceOTU32176
New.CleanUp.ReferenceOTU161291
New.CleanUp.ReferenceOTU46210
New.CleanUp.ReferenceOTU69839
GU068021.1.1740
New.CleanUp.ReferenceOTU32995
New.ReferenceOTU1388
EF070265.1.1635
X56533.22.1719
New.CleanUp.ReferenceOTU20695
New.CleanUp.ReferenceOTU21181
New.CleanUp.ReferenceOTU48112
New.CleanUp.ReferenceOTU202535
New.CleanUp.ReferenceOTU48993
New.CleanUp.ReferenceOTU150316
New.CleanUp.ReferenceOTU3566
New.CleanUp.ReferenceOTU41372
HM581673.1.1636
New.CleanUp.ReferenceOTU2839
New.CleanUp.ReferenceOTU25267
New.CleanUp.ReferenceOTU35143
New.CleanUp.ReferenceOTU14563
New.CleanUp.ReferenceOTU4919
New.CleanUp.ReferenceOTU81033
New.CleanUp.ReferenceOTU25531
New.CleanUp.ReferenceOTU52889
KF662596.1.1892
New.CleanUp.ReferenceOTU102671
New.CleanUp.ReferenceOTU13562
New.CleanUp.ReferenceOTU10843
New.CleanUp.ReferenceOTU28046
New.CleanUp.ReferenceOTU31363
New.CleanUp.ReferenceOTU56365
New.CleanUp.ReferenceOTU15065
New.CleanUp.ReferenceOTU15920
New.CleanUp.ReferenceOTU36162
New.ReferenceOTU2273
New.CleanUp.ReferenceOTU34134
New.CleanUp.ReferenceOTU271532
New.CleanUp.ReferenceOTU53708
New.CleanUp.ReferenceOTU41801
New.CleanUp.ReferenceOTU7246

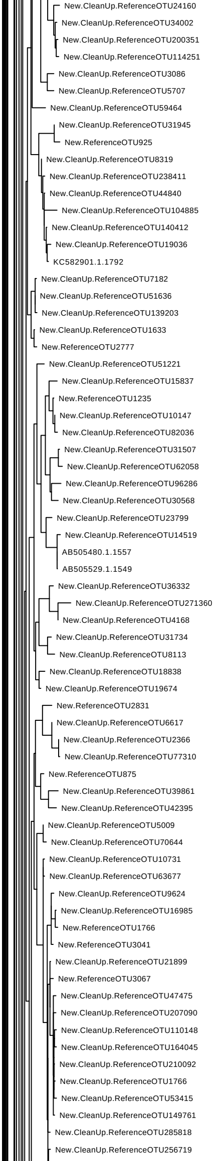
New.CleanUp.ReferenceOTU24160
New.CleanUp.ReferenceOTU34002
New.CleanUp.ReferenceOTU200351
New.CleanUp.ReferenceOTU114251
New.CleanUp.ReferenceOTU3086
New.CleanUp.ReferenceOTU5707
New.CleanUp.ReferenceOTU59464
New.CleanUp.ReferenceOTU31945
New.ReferenceOTU925
New.CleanUp.ReferenceOTU8319
New.CleanUp.ReferenceOTU238411
New.CleanUp.ReferenceOTU44840
New.CleanUp.ReferenceOTU104885
New.CleanUp.ReferenceOTU140412
New.CleanUp.ReferenceOTU19036
KC582901.1.1792
New.CleanUp.ReferenceOTU7182
New.CleanUp.ReferenceOTU51636
New.CleanUp.ReferenceOTU139203
New.CleanUp.ReferenceOTU1633
New.ReferenceOTU2777
New.CleanUp.ReferenceOTU51221
New.CleanUp.ReferenceOTU15837
New.ReferenceOTU1235
New.CleanUp.ReferenceOTU10147
New.CleanUp.ReferenceOTU82036
New.CleanUp.ReferenceOTU31507
New.CleanUp.ReferenceOTU62058
New.CleanUp.ReferenceOTU96286
New.CleanUp.ReferenceOTU30568
New.CleanUp.ReferenceOTU23799
New.CleanUp.ReferenceOTU14519
AB505480.1.1557
AB505529.1.1549
New.CleanUp.ReferenceOTU36332
New.CleanUp.ReferenceOTU271360
New.CleanUp.ReferenceOTU4168
New.CleanUp.ReferenceOTU31734
New.CleanUp.ReferenceOTU8113
New.CleanUp.ReferenceOTU18838
New.CleanUp.ReferenceOTU19674
New.ReferenceOTU2831
New.CleanUp.ReferenceOTU6617
New.CleanUp.ReferenceOTU2366
New.CleanUp.ReferenceOTU77310
New.ReferenceOTU875
New.CleanUp.ReferenceOTU39861
New.CleanUp.ReferenceOTU42395
New.CleanUp.ReferenceOTU5009
New.CleanUp.ReferenceOTU70644
New.CleanUp.ReferenceOTU10731
New.CleanUp.ReferenceOTU63677
New.CleanUp.ReferenceOTU9624
New.CleanUp.ReferenceOTU16985
New.ReferenceOTU1766
New.ReferenceOTU3041
New.CleanUp.ReferenceOTU21899
New.ReferenceOTU3067
New.CleanUp.ReferenceOTU47475
New.CleanUp.ReferenceOTU207090
New.CleanUp.ReferenceOTU110148
New.CleanUp.ReferenceOTU164045
New.CleanUp.ReferenceOTU210092
New.CleanUp.ReferenceOTU1766
New.CleanUp.ReferenceOTU53415
New.CleanUp.ReferenceOTU149761
New.CleanUp.ReferenceOTU285818
New.CleanUp.ReferenceOTU256719

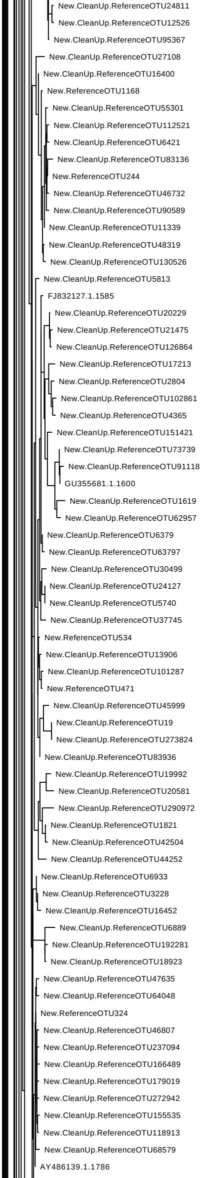

New.CleanUp.ReferenceOTU24811
New.CleanUp.ReferenceOTU12526
New.CleanUp.ReferenceOTU95367
New.CleanUp.ReferenceOTU27108
New.CleanUp.ReferenceOTU16400
New.ReferenceOTU1168
New.CleanUp.ReferenceOTU55301
New.CleanUp.ReferenceOTU112521
New.CleanUp.ReferenceOTU6421
New.CleanUp.ReferenceOTU83136
New.ReferenceOTU244
New.CleanUp.ReferenceOTU46732
New.CleanUp.ReferenceOTU90589
New.CleanUp.ReferenceOTU11339
New.CleanUp.ReferenceOTU48319
New.CleanUp.ReferenceOTU130526
New.CleanUp.ReferenceOTU5813
FJ832127.1.1585
New.CleanUp.ReferenceOTU20229
New.CleanUp.ReferenceOTU21475
New.CleanUp.ReferenceOTU126864
New.CleanUp.ReferenceOTU17213
New.CleanUp.ReferenceOTU2804
New.CleanUp.ReferenceOTU102861
New.CleanUp.ReferenceOTU4365
New.CleanUp.ReferenceOTU151421
New.CleanUp.ReferenceOTU73739
New.CleanUp.ReferenceOTU91118
GU355681.1.1600
New.CleanUp.ReferenceOTU1619
New.CleanUp.ReferenceOTU62957
New.CleanUp.ReferenceOTU6379
New.CleanUp.ReferenceOTU63797
New.CleanUp.ReferenceOTU30499
New.CleanUp.ReferenceOTU24127
New.CleanUp.ReferenceOTU5740
New.CleanUp.ReferenceOTU37745
New.ReferenceOTU534
New.CleanUp.ReferenceOTU13906
New.CleanUp.ReferenceOTU101287
New.ReferenceOTU471
New.CleanUp.ReferenceOTU45999
New.CleanUp.ReferenceOTU19
New.CleanUp.ReferenceOTU273824
New.CleanUp.ReferenceOTU83936
New.CleanUp.ReferenceOTU19992
New.CleanUp.ReferenceOTU20581
New.CleanUp.ReferenceOTU290972
New.CleanUp.ReferenceOTU1821
New.CleanUp.ReferenceOTU42504
New.CleanUp.ReferenceOTU44252
New.CleanUp.ReferenceOTU6933
New.CleanUp.ReferenceOTU3228
New.CleanUp.ReferenceOTU16452
New.CleanUp.ReferenceOTU6889
New.CleanUp.ReferenceOTU192281
New.CleanUp.ReferenceOTU18923
New.CleanUp.ReferenceOTU47635
New.CleanUp.ReferenceOTU64048
New.ReferenceOTU324
New.CleanUp.ReferenceOTU46807
New.CleanUp.ReferenceOTU237094
New.CleanUp.ReferenceOTU166489
New.CleanUp.ReferenceOTU179019
New.CleanUp.ReferenceOTU272942
New.CleanUp.ReferenceOTU155535
New.CleanUp.ReferenceOTU118913
New.CleanUp.ReferenceOTU68579
AY486139.1.1786

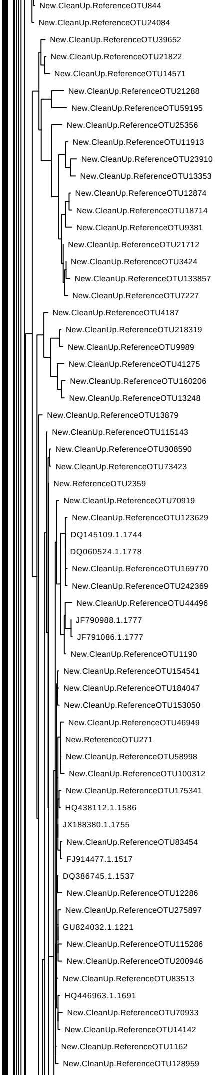

New.CleanUp.ReferenceOTU844
New.CleanUp.ReferenceOTU24084
New.CleanUp.ReferenceOTU39652
New.CleanUp.ReferenceOTU21822
New.CleanUp.ReferenceOTU14571
New.CleanUp.ReferenceOTU21288
New.CleanUp.ReferenceOTU59195
New.CleanUp.ReferenceOTU25356
New.CleanUp.ReferenceOTU11913
New.CleanUp.ReferenceOTU23910
New.CleanUp.ReferenceOTU13353
New.CleanUp.ReferenceOTU12874
New.CleanUp.ReferenceOTU18714
New.CleanUp.ReferenceOTU9381
New.CleanUp.ReferenceOTU21712
New.CleanUp.ReferenceOTU3424
New.CleanUp.ReferenceOTU133857
New.CleanUp.ReferenceOTU7227
New.CleanUp.ReferenceOTU4187
New.CleanUp.ReferenceOTU218319
New.CleanUp.ReferenceOTU9989
New.CleanUp.ReferenceOTU41275
New.CleanUp.ReferenceOTU160206
New.CleanUp.ReferenceOTU13248
New.CleanUp.ReferenceOTU13879
New.CleanUp.ReferenceOTU115143
New.CleanUp.ReferenceOTU308590
New.CleanUp.ReferenceOTU73423
New.ReferenceOTU2359
New.CleanUp.ReferenceOTU70919
New.CleanUp.ReferenceOTU123629
DQ145109.1.1744
DQ060524.1.1778
New.CleanUp.ReferenceOTU169770
New.CleanUp.ReferenceOTU242369
New.CleanUp.ReferenceOTU44496
JF790988.1.1777
JF791086.1.1777
New.CleanUp.ReferenceOTU1190
New.CleanUp.ReferenceOTU154541
New.CleanUp.ReferenceOTU184047
New.CleanUp.ReferenceOTU153050
New.CleanUp.ReferenceOTU46949
New.ReferenceOTU271
New.CleanUp.ReferenceOTU58998
New.CleanUp.ReferenceOTU100312
New.CleanUp.ReferenceOTU175341
HQ438112.1.1586
JX188380.1.1755
New.CleanUp.ReferenceOTU83454
FJ914477.1.1517
DQ386745.1.1537
New.CleanUp.ReferenceOTU12286
New.CleanUp.ReferenceOTU275897
GU824032.1.1221
New.CleanUp.ReferenceOTU115286
New.CleanUp.ReferenceOTU200946
New.CleanUp.ReferenceOTU83513
HQ446963.1.1691
New.CleanUp.ReferenceOTU70933
New.CleanUp.ReferenceOTU14142
New.CleanUp.ReferenceOTU1162
New.CleanUp.ReferenceOTU128959

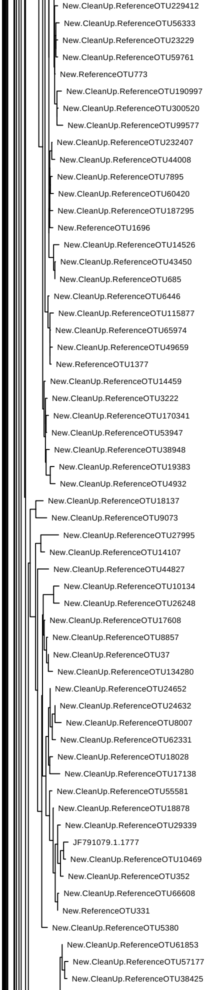
New.CleanUp.ReferenceOTU229412
New.CleanUp.ReferenceOTU56333
New.CleanUp.ReferenceOTU23229
New.CleanUp.ReferenceOTU59761
New.ReferenceOTU773
New.CleanUp.ReferenceOTU190997
New.CleanUp.ReferenceOTU300520
New.CleanUp.ReferenceOTU99577
New.CleanUp.ReferenceOTU232407
New.CleanUp.ReferenceOTU44008
New.CleanUp.ReferenceOTU7895
New.CleanUp.ReferenceOTU60420
New.CleanUp.ReferenceOTU187295
New.ReferenceOTU1696
New.CleanUp.ReferenceOTU14526
New.CleanUp.ReferenceOTU43450
New.CleanUp.ReferenceOTU685
New.CleanUp.ReferenceOTU6446
New.CleanUp.ReferenceOTU115877
New.CleanUp.ReferenceOTU65974
New.CleanUp.ReferenceOTU49659
New.ReferenceOTU1377
New.CleanUp.ReferenceOTU14459
New.CleanUp.ReferenceOTU3222
New.CleanUp.ReferenceOTU170341
New.CleanUp.ReferenceOTU53947
New.CleanUp.ReferenceOTU38948
New.CleanUp.ReferenceOTU19383
New.CleanUp.ReferenceOTU4932
New.CleanUp.ReferenceOTU18137
New.CleanUp.ReferenceOTU9073
New.CleanUp.ReferenceOTU27995
New.CleanUp.ReferenceOTU14107
New.CleanUp.ReferenceOTU44827
New.CleanUp.ReferenceOTU10134
New.CleanUp.ReferenceOTU26248
New.CleanUp.ReferenceOTU17608
New.CleanUp.ReferenceOTU8857
New.CleanUp.ReferenceOTU37
New.CleanUp.ReferenceOTU134280
New.CleanUp.ReferenceOTU24652
New.CleanUp.ReferenceOTU24632
New.CleanUp.ReferenceOTU8007
New.CleanUp.ReferenceOTU62331
New.CleanUp.ReferenceOTU18028
New.CleanUp.ReferenceOTU17138
New.CleanUp.ReferenceOTU55581
New.CleanUp.ReferenceOTU18878
New.CleanUp.ReferenceOTU29339
JF791079.1.1777
New.CleanUp.ReferenceOTU10469
New.CleanUp.ReferenceOTU352
New.CleanUp.ReferenceOTU66608
New.ReferenceOTU331
New.CleanUp.ReferenceOTU5380
New.CleanUp.ReferenceOTU61853
New.CleanUp.ReferenceOTU57177
New.CleanUp.ReferenceOTU38425

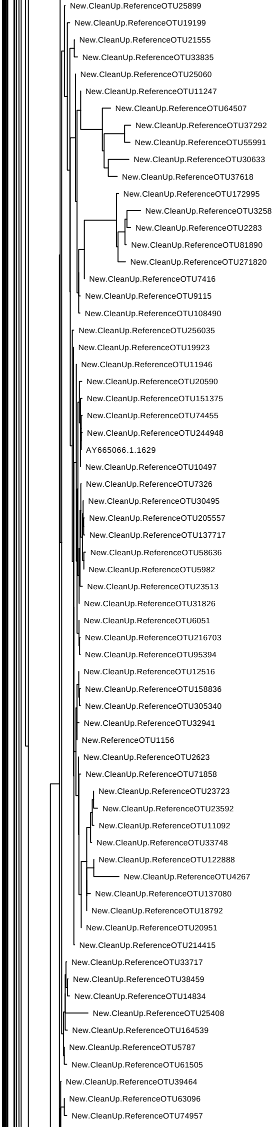
New.CleanUp.ReferenceOTU25899
New.CleanUp.ReferenceOTU19199
New.CleanUp.ReferenceOTU21555
New.CleanUp.ReferenceOTU33835
New.CleanUp.ReferenceOTU25060
New.CleanUp.ReferenceOTU11247
New.CleanUp.ReferenceOTU64507
New.CleanUp.ReferenceOTU37292
New.CleanUp.ReferenceOTU55991
New.CleanUp.ReferenceOTU30633
New.CleanUp.ReferenceOTU37618
New.CleanUp.ReferenceOTU172995
New.CleanUp.ReferenceOTU3258
New.CleanUp.ReferenceOTU2283
New.CleanUp.ReferenceOTU81890
New.CleanUp.ReferenceOTU271820
New.CleanUp.ReferenceOTU7416
New.CleanUp.ReferenceOTU9115
New.CleanUp.ReferenceOTU108490
New.CleanUp.ReferenceOTU256035
New.CleanUp.ReferenceOTU19923
New.CleanUp.ReferenceOTU11946
New.CleanUp.ReferenceOTU20590
New.CleanUp.ReferenceOTU151375
New.CleanUp.ReferenceOTU74455
New.CleanUp.ReferenceOTU244948
AY665066.1.1629
New.CleanUp.ReferenceOTU10497
New.CleanUp.ReferenceOTU7326
New.CleanUp.ReferenceOTU30495
New.CleanUp.ReferenceOTU205557
New.CleanUp.ReferenceOTU137717
New.CleanUp.ReferenceOTU58636
New.CleanUp.ReferenceOTU5982
New.CleanUp.ReferenceOTU23513
New.CleanUp.ReferenceOTU31826
New.CleanUp.ReferenceOTU6051
New.CleanUp.ReferenceOTU216703
New.CleanUp.ReferenceOTU95394
New.CleanUp.ReferenceOTU12516
New.CleanUp.ReferenceOTU158836
New.CleanUp.ReferenceOTU305340
New.CleanUp.ReferenceOTU32941
New.ReferenceOTU1156
New.CleanUp.ReferenceOTU2623
New.CleanUp.ReferenceOTU71858
New.CleanUp.ReferenceOTU23723
New.CleanUp.ReferenceOTU23592
New.CleanUp.ReferenceOTU11092
New.CleanUp.ReferenceOTU33748
New.CleanUp.ReferenceOTU122888
New.CleanUp.ReferenceOTU4267
New.CleanUp.ReferenceOTU137080
New.CleanUp.ReferenceOTU18792
New.CleanUp.ReferenceOTU20951
New.CleanUp.ReferenceOTU214415
New.CleanUp.ReferenceOTU33717
New.CleanUp.ReferenceOTU38459
New.CleanUp.ReferenceOTU14834
New.CleanUp.ReferenceOTU25408
New.CleanUp.ReferenceOTU164539
New.CleanUp.ReferenceOTU5787
New.CleanUp.ReferenceOTU61505
New.CleanUp.ReferenceOTU39464
New.CleanUp.ReferenceOTU63096
New.CleanUp.ReferenceOTU74957

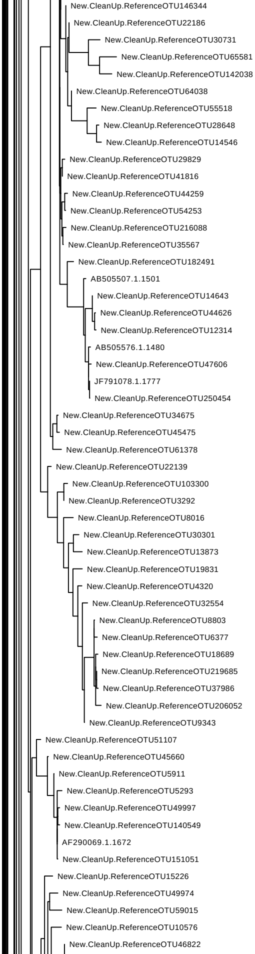
New.CleanUp.ReferenceOTU146344
New.CleanUp.ReferenceOTU22186
New.CleanUp.ReferenceOTU30731
New.CleanUp.ReferenceOTU65581
New.CleanUp.ReferenceOTU142038
New.CleanUp.ReferenceOTU64038
New.CleanUp.ReferenceOTU55518
New.CleanUp.ReferenceOTU28648
New.CleanUp.ReferenceOTU14546
New.CleanUp.ReferenceOTU29829
New.CleanUp.ReferenceOTU41816
New.CleanUp.ReferenceOTU44259
New.CleanUp.ReferenceOTU54253
New.CleanUp.ReferenceOTU216088
New.CleanUp.ReferenceOTU35567
New.CleanUp.ReferenceOTU182491
AB505507.1.1501
New.CleanUp.ReferenceOTU14643
New.CleanUp.ReferenceOTU44626
New.CleanUp.ReferenceOTU12314
AB505576.1.1480
New.CleanUp.ReferenceOTU47606
JF791078.1.1777
New.CleanUp.ReferenceOTU250454
New.CleanUp.ReferenceOTU34675
New.CleanUp.ReferenceOTU45475
New.CleanUp.ReferenceOTU61378
New.CleanUp.ReferenceOTU22139
New.CleanUp.ReferenceOTU103300
New.CleanUp.ReferenceOTU3292
New.CleanUp.ReferenceOTU8016
New.CleanUp.ReferenceOTU30301
New.CleanUp.ReferenceOTU13873
New.CleanUp.ReferenceOTU19831
New.CleanUp.ReferenceOTU4320
New.CleanUp.ReferenceOTU32554
New.CleanUp.ReferenceOTU8803
New.CleanUp.ReferenceOTU6377
New.CleanUp.ReferenceOTU18689
New.CleanUp.ReferenceOTU219685
New.CleanUp.ReferenceOTU37986
New.CleanUp.ReferenceOTU206052
New.CleanUp.ReferenceOTU9343
New.CleanUp.ReferenceOTU51107
New.CleanUp.ReferenceOTU45660
New.CleanUp.ReferenceOTU5911
New.CleanUp.ReferenceOTU5293
New.CleanUp.ReferenceOTU49997
New.CleanUp.ReferenceOTU140549
AF290069.1.1672
New.CleanUp.ReferenceOTU151051
New.CleanUp.ReferenceOTU15226
New.CleanUp.ReferenceOTU49974
New.CleanUp.ReferenceOTU59015
New.CleanUp.ReferenceOTU10576
New.CleanUp.ReferenceOTU46822

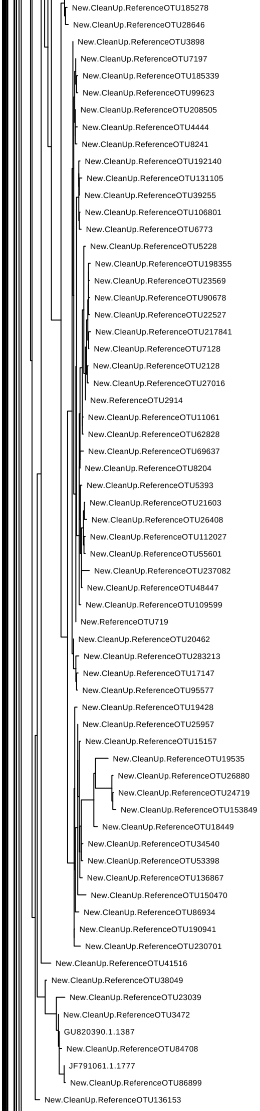
New.CleanUp.ReferenceOTU185278
New.CleanUp.ReferenceOTU28646
New.CleanUp.ReferenceOTU3898
New.CleanUp.ReferenceOTU7197
New.CleanUp.ReferenceOTU185339
New.CleanUp.ReferenceOTU99623
New.CleanUp.ReferenceOTU208505
New.CleanUp.ReferenceOTU4444
New.CleanUp.ReferenceOTU8241
New.CleanUp.ReferenceOTU192140
New.CleanUp.ReferenceOTU131105
New.CleanUp.ReferenceOTU39255
New.CleanUp.ReferenceOTU106801
New.CleanUp.ReferenceOTU6773
New.CleanUp.ReferenceOTU5228
New.CleanUp.ReferenceOTU198355
New.CleanUp.ReferenceOTU23569
New.CleanUp.ReferenceOTU90678
New.CleanUp.ReferenceOTU22527
New.CleanUp.ReferenceOTU217841
New.CleanUp.ReferenceOTU7128
New.CleanUp.ReferenceOTU2128
New.CleanUp.ReferenceOTU27016
New.ReferenceOTU2914
New.CleanUp.ReferenceOTU11061
New.CleanUp.ReferenceOTU62828
New.CleanUp.ReferenceOTU69637
New.CleanUp.ReferenceOTU8204
New.CleanUp.ReferenceOTU5393
New.CleanUp.ReferenceOTU21603
New.CleanUp.ReferenceOTU26408
New.CleanUp.ReferenceOTU112027
New.CleanUp.ReferenceOTU55601
New.CleanUp.ReferenceOTU237082
New.CleanUp.ReferenceOTU48447
New.CleanUp.ReferenceOTU109599
New.ReferenceOTU719
New.CleanUp.ReferenceOTU20462
New.CleanUp.ReferenceOTU283213
New.CleanUp.ReferenceOTU17147
New.CleanUp.ReferenceOTU95577
New.CleanUp.ReferenceOTU19428
New.CleanUp.ReferenceOTU25957
New.CleanUp.ReferenceOTU15157
New.CleanUp.ReferenceOTU19535
New.CleanUp.ReferenceOTU26880
New.CleanUp.ReferenceOTU24719
New.CleanUp.ReferenceOTU153849
New.CleanUp.ReferenceOTU18449
New.CleanUp.ReferenceOTU34540
New.CleanUp.ReferenceOTU53398
New.CleanUp.ReferenceOTU136867
New.CleanUp.ReferenceOTU150470
New.CleanUp.ReferenceOTU86934
New.CleanUp.ReferenceOTU190941
New.CleanUp.ReferenceOTU230701
New.CleanUp.ReferenceOTU41516
New.CleanUp.ReferenceOTU38049
New.CleanUp.ReferenceOTU23039
New.CleanUp.ReferenceOTU3472
GU820390.1.1387
New.CleanUp.ReferenceOTU84708
JF791061.1.1777
New.CleanUp.ReferenceOTU86899
New.CleanUp.ReferenceOTU136153

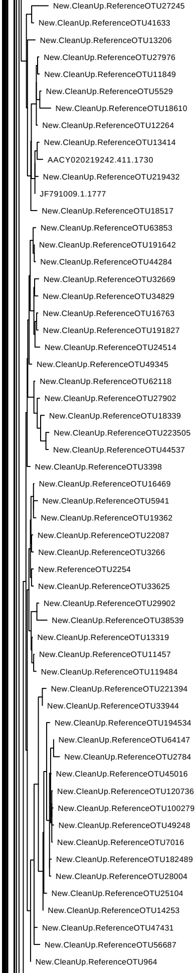
New.CleanUp.ReferenceOTU27245
New.CleanUp.ReferenceOTU41633
New.CleanUp.ReferenceOTU13206
New.CleanUp.ReferenceOTU27976
New.CleanUp.ReferenceOTU11849
New.CleanUp.ReferenceOTU5529
New.CleanUp.ReferenceOTU18610
New.CleanUp.ReferenceOTU12264
New.CleanUp.ReferenceOTU13414
AACY020219242.411.1730
New.CleanUp.ReferenceOTU219432
JF791009.1.1777
New.CleanUp.ReferenceOTU18517
New.CleanUp.ReferenceOTU63853
New.CleanUp.ReferenceOTU191642
New.CleanUp.ReferenceOTU44284
New.CleanUp.ReferenceOTU32669
New.CleanUp.ReferenceOTU34829
New.CleanUp.ReferenceOTU16763
New.CleanUp.ReferenceOTU191827
New.CleanUp.ReferenceOTU24514
New.CleanUp.ReferenceOTU49345
New.CleanUp.ReferenceOTU62118
New.CleanUp.ReferenceOTU27902
New.CleanUp.ReferenceOTU18339
New.CleanUp.ReferenceOTU223505
New.CleanUp.ReferenceOTU44537
New.CleanUp.ReferenceOTU3398
New.CleanUp.ReferenceOTU16469
New.CleanUp.ReferenceOTU5941
New.CleanUp.ReferenceOTU19362
New.CleanUp.ReferenceOTU22087
New.CleanUp.ReferenceOTU3266
New.ReferenceOTU2254
New.CleanUp.ReferenceOTU33625
New.CleanUp.ReferenceOTU29902
New.CleanUp.ReferenceOTU38539
New.CleanUp.ReferenceOTU13319
New.CleanUp.ReferenceOTU11457
New.CleanUp.ReferenceOTU119484
New.CleanUp.ReferenceOTU221394
New.CleanUp.ReferenceOTU33944
New.CleanUp.ReferenceOTU194534
New.CleanUp.ReferenceOTU64147
New.CleanUp.ReferenceOTU2784
New.CleanUp.ReferenceOTU45016
New.CleanUp.ReferenceOTU120736
New.CleanUp.ReferenceOTU100279
New.CleanUp.ReferenceOTU49248
New.CleanUp.ReferenceOTU7016
New.CleanUp.ReferenceOTU182489
New.CleanUp.ReferenceOTU28004
New.CleanUp.ReferenceOTU25104
New.CleanUp.ReferenceOTU14253
New.CleanUp.ReferenceOTU47431
New.CleanUp.ReferenceOTU56687
New.CleanUp.ReferenceOTU964

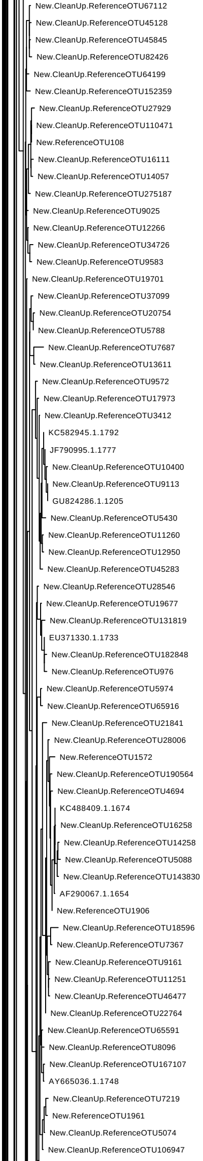
New.CleanUp.ReferenceOTU67112
New.CleanUp.ReferenceOTU45128
New.CleanUp.ReferenceOTU45845
New.CleanUp.ReferenceOTU82426
New.CleanUp.ReferenceOTU64199
New.CleanUp.ReferenceOTU152359
New.CleanUp.ReferenceOTU27929
New.CleanUp.ReferenceOTU110471
New.ReferenceOTU108
New.CleanUp.ReferenceOTU16111
New.CleanUp.ReferenceOTU14057
New.CleanUp.ReferenceOTU275187
New.CleanUp.ReferenceOTU9025
New.CleanUp.ReferenceOTU12266
New.CleanUp.ReferenceOTU34726
New.CleanUp.ReferenceOTU9583
New.CleanUp.ReferenceOTU19701
New.CleanUp.ReferenceOTU37099
New.CleanUp.ReferenceOTU20754
New.CleanUp.ReferenceOTU5788
New.CleanUp.ReferenceOTU7687
New.CleanUp.ReferenceOTU13611
New.CleanUp.ReferenceOTU9572
New.CleanUp.ReferenceOTU17973
New.CleanUp.ReferenceOTU3412
KC582945.1.1792
JF790995.1.1777
New.CleanUp.ReferenceOTU10400
New.CleanUp.ReferenceOTU9113
GU824286.1.1205
New.CleanUp.ReferenceOTU5430
New.CleanUp.ReferenceOTU11260
New.CleanUp.ReferenceOTU12950
New.CleanUp.ReferenceOTU45283
New.CleanUp.ReferenceOTU28546
New.CleanUp.ReferenceOTU19677
New.CleanUp.ReferenceOTU131819
EU371330.1.1733
New.CleanUp.ReferenceOTU182848
New.CleanUp.ReferenceOTU976
New.CleanUp.ReferenceOTU5974
New.CleanUp.ReferenceOTU65916
New.CleanUp.ReferenceOTU21841
New.CleanUp.ReferenceOTU28006
New.ReferenceOTU1572
New.CleanUp.ReferenceOTU190564
New.CleanUp.ReferenceOTU4694
KC488409.1.1674
New.CleanUp.ReferenceOTU16258
New.CleanUp.ReferenceOTU14258
New.CleanUp.ReferenceOTU5088
New.CleanUp.ReferenceOTU143830
AF290067.1.1654
New.ReferenceOTU1906
New.CleanUp.ReferenceOTU18596
New.CleanUp.ReferenceOTU7367
New.CleanUp.ReferenceOTU9161
New.CleanUp.ReferenceOTU11251
New.CleanUp.ReferenceOTU46477
New.CleanUp.ReferenceOTU22764
New.CleanUp.ReferenceOTU65591
New.CleanUp.ReferenceOTU8096
New.CleanUp.ReferenceOTU167107
AY665036.1.1748
New.CleanUp.ReferenceOTU7219
New.ReferenceOTU1961
New.CleanUp.ReferenceOTU5074
New.CleanUp.ReferenceOTU106947

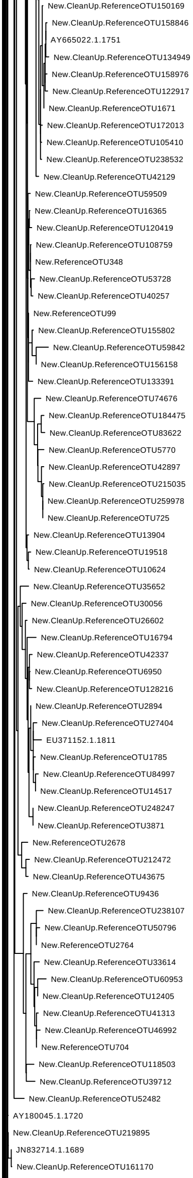
New.CleanUp.ReferenceOTU150169
New.CleanUp.ReferenceOTU158846
AY665022.1.1751
New.CleanUp.ReferenceOTU134949
New.CleanUp.ReferenceOTU158976
New.CleanUp.ReferenceOTU122917
New.CleanUp.ReferenceOTU1671
New.CleanUp.ReferenceOTU172013
New.CleanUp.ReferenceOTU105410
New.CleanUp.ReferenceOTU238532
New.CleanUp.ReferenceOTU42129
New.CleanUp.ReferenceOTU59509
New.CleanUp.ReferenceOTU16365
New.CleanUp.ReferenceOTU120419
New.CleanUp.ReferenceOTU108759
New.ReferenceOTU348
New.CleanUp.ReferenceOTU53728
New.CleanUp.ReferenceOTU40257
New.ReferenceOTU99
New.CleanUp.ReferenceOTU155802
New.CleanUp.ReferenceOTU59842
New.CleanUp.ReferenceOTU156158
New.CleanUp.ReferenceOTU133391
New.CleanUp.ReferenceOTU74676
New.CleanUp.ReferenceOTU184475
New.CleanUp.ReferenceOTU83622
New.CleanUp.ReferenceOTU5770
New.CleanUp.ReferenceOTU42897
New.CleanUp.ReferenceOTU215035
New.CleanUp.ReferenceOTU259978
New.CleanUp.ReferenceOTU725
New.CleanUp.ReferenceOTU13904
New.CleanUp.ReferenceOTU19518
New.CleanUp.ReferenceOTU10624
New.CleanUp.ReferenceOTU35652
New.CleanUp.ReferenceOTU30056
New.CleanUp.ReferenceOTU26602
New.CleanUp.ReferenceOTU16794
New.CleanUp.ReferenceOTU42337
New.CleanUp.ReferenceOTU6950
New.CleanUp.ReferenceOTU128216
New.CleanUp.ReferenceOTU2894
New.CleanUp.ReferenceOTU27404
EU371152.1.1811
New.CleanUp.ReferenceOTU1785
New.CleanUp.ReferenceOTU84997
New.CleanUp.ReferenceOTU14517
New.CleanUp.ReferenceOTU248247
New.CleanUp.ReferenceOTU3871
New.ReferenceOTU2678
New.CleanUp.ReferenceOTU212472
New.CleanUp.ReferenceOTU43675
New.CleanUp.ReferenceOTU9436
New.CleanUp.ReferenceOTU238107
New.CleanUp.ReferenceOTU50796
New.ReferenceOTU2764
New.CleanUp.ReferenceOTU33614
New.CleanUp.ReferenceOTU60953
New.CleanUp.ReferenceOTU12405
New.CleanUp.ReferenceOTU41313
New.CleanUp.ReferenceOTU46992
New.ReferenceOTU704
New.CleanUp.ReferenceOTU118503
New.CleanUp.ReferenceOTU39712
New.CleanUp.ReferenceOTU52482
AY180045.1.1720
New.CleanUp.ReferenceOTU219895
JN832714.1.1689
New.CleanUp.ReferenceOTU161170

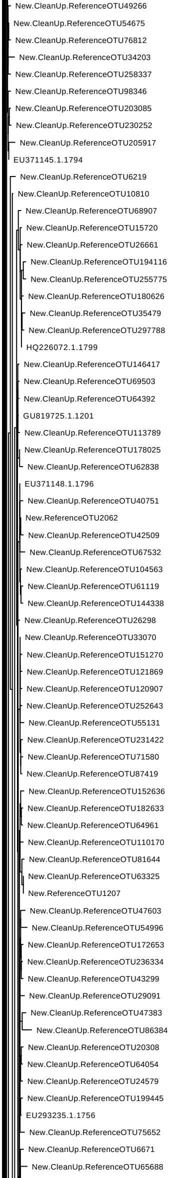
New.CleanUp.ReferenceOTU49266
New.CleanUp.ReferenceOTU54675
New.CleanUp.ReferenceOTU76812
New.CleanUp.ReferenceOTU34203
New.CleanUp.ReferenceOTU258337
New.CleanUp.ReferenceOTU98346
New.CleanUp.ReferenceOTU203085
New.CleanUp.ReferenceOTU230252
New.CleanUp.ReferenceOTU205917
EU371145.1.1794
New.CleanUp.ReferenceOTU6219
New.CleanUp.ReferenceOTU10810
New.CleanUp.ReferenceOTU68907
New.CleanUp.ReferenceOTU15720
New.CleanUp.ReferenceOTU26661
New.CleanUp.ReferenceOTU194116
New.CleanUp.ReferenceOTU255775
New.CleanUp.ReferenceOTU180626
New.CleanUp.ReferenceOTU35479
New.CleanUp.ReferenceOTU297788
HQ226072.1.1799
New.CleanUp.ReferenceOTU146417
New.CleanUp.ReferenceOTU69503
New.CleanUp.ReferenceOTU64392
GU819725.1.1201
New.CleanUp.ReferenceOTU113789
New.CleanUp.ReferenceOTU178025
New.CleanUp.ReferenceOTU62838
EU371148.1.1796
New.CleanUp.ReferenceOTU40751
New.ReferenceOTU2062
New.CleanUp.ReferenceOTU42509
New.CleanUp.ReferenceOTU67532
New.CleanUp.ReferenceOTU104563
New.CleanUp.ReferenceOTU61119
New.CleanUp.ReferenceOTU144338
New.CleanUp.ReferenceOTU26298
New.CleanUp.ReferenceOTU33070
New.CleanUp.ReferenceOTU151270
New.CleanUp.ReferenceOTU121869
New.CleanUp.ReferenceOTU120907
New.CleanUp.ReferenceOTU252643
New.CleanUp.ReferenceOTU55131
New.CleanUp.ReferenceOTU231422
New.CleanUp.ReferenceOTU71580
New.CleanUp.ReferenceOTU87419
New.CleanUp.ReferenceOTU152636
New.CleanUp.ReferenceOTU182633
New.CleanUp.ReferenceOTU64961
New.CleanUp.ReferenceOTU110170
New.CleanUp.ReferenceOTU81644
New.CleanUp.ReferenceOTU63325
New.ReferenceOTU1207
New.CleanUp.ReferenceOTU47603
New.CleanUp.ReferenceOTU54996
New.CleanUp.ReferenceOTU172653
New.CleanUp.ReferenceOTU236334
New.CleanUp.ReferenceOTU43299
New.CleanUp.ReferenceOTU29091
New.CleanUp.ReferenceOTU47383
New.CleanUp.ReferenceOTU86384
New.CleanUp.ReferenceOTU20308
New.CleanUp.ReferenceOTU64054
New.CleanUp.ReferenceOTU24579
New.CleanUp.ReferenceOTU199445
EU293235.1.1756
New.CleanUp.ReferenceOTU75652
New.CleanUp.ReferenceOTU6671
New.CleanUp.ReferenceOTU65688

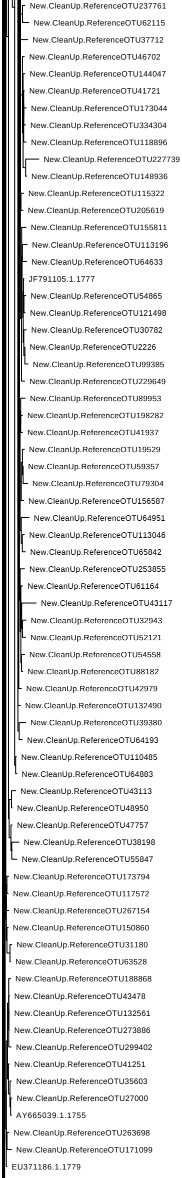

New.CleanUp.ReferenceOTU237761
New.CleanUp.ReferenceOTU62115
New.CleanUp.ReferenceOTU37712
New.CleanUp.ReferenceOTU46702
New.CleanUp.ReferenceOTU144047
New.CleanUp.ReferenceOTU41721
New.CleanUp.ReferenceOTU173044
New.CleanUp.ReferenceOTU334304
New.CleanUp.ReferenceOTU118896
New.CleanUp.ReferenceOTU227739
New.CleanUp.ReferenceOTU148936
New.CleanUp.ReferenceOTU115322
New.CleanUp.ReferenceOTU205619
New.CleanUp.ReferenceOTU155811
New.CleanUp.ReferenceOTU113196
New.CleanUp.ReferenceOTU64633
JF791105.1.1777
New.CleanUp.ReferenceOTU54865
New.CleanUp.ReferenceOTU121498
New.CleanUp.ReferenceOTU30782
New.CleanUp.ReferenceOTU2226
New.CleanUp.ReferenceOTU99385
New.CleanUp.ReferenceOTU229649
New.CleanUp.ReferenceOTU89953
New.CleanUp.ReferenceOTU198282
New.CleanUp.ReferenceOTU41937
New.CleanUp.ReferenceOTU19529
New.CleanUp.ReferenceOTU59357
New.CleanUp.ReferenceOTU79304
New.CleanUp.ReferenceOTU156587
New.CleanUp.ReferenceOTU64951
New.CleanUp.ReferenceOTU113046
New.CleanUp.ReferenceOTU65842
New.CleanUp.ReferenceOTU253855
New.CleanUp.ReferenceOTU61164
New.CleanUp.ReferenceOTU43117
New.CleanUp.ReferenceOTU32943
New.CleanUp.ReferenceOTU52121
New.CleanUp.ReferenceOTU54558
New.CleanUp.ReferenceOTU88182
New.CleanUp.ReferenceOTU42979
New.CleanUp.ReferenceOTU132490
New.CleanUp.ReferenceOTU39380
New.CleanUp.ReferenceOTU64193
New.CleanUp.ReferenceOTU110485
New.CleanUp.ReferenceOTU64883
New.CleanUp.ReferenceOTU43113
New.CleanUp.ReferenceOTU48950
New.CleanUp.ReferenceOTU47757
New.CleanUp.ReferenceOTU38198
New.CleanUp.ReferenceOTU55847
New.CleanUp.ReferenceOTU173794
New.CleanUp.ReferenceOTU117572
New.CleanUp.ReferenceOTU267154
New.CleanUp.ReferenceOTU150860
New.CleanUp.ReferenceOTU31180
New.CleanUp.ReferenceOTU63528
New.CleanUp.ReferenceOTU188868
New.CleanUp.ReferenceOTU43478
New.CleanUp.ReferenceOTU132561
New.CleanUp.ReferenceOTU273886
New.CleanUp.ReferenceOTU299402
New.CleanUp.ReferenceOTU41251
New.CleanUp.ReferenceOTU35603
New.CleanUp.ReferenceOTU27000
AY665039.1.1755
New.CleanUp.ReferenceOTU263698
New.CleanUp.ReferenceOTU171099
EU371186.1.1779

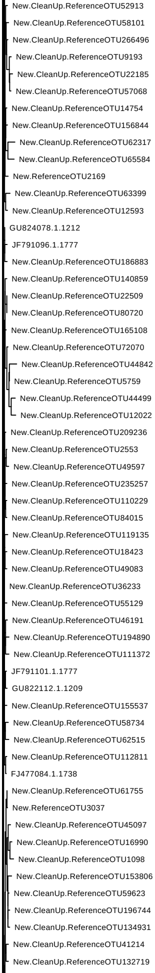
New.CleanUp.ReferenceOTU52913
New.CleanUp.ReferenceOTU58101
New.CleanUp.ReferenceOTU266496
New.CleanUp.ReferenceOTU9193
New.CleanUp.ReferenceOTU22185
New.CleanUp.ReferenceOTU57068
New.CleanUp.ReferenceOTU14754
New.CleanUp.ReferenceOTU156844
New.CleanUp.ReferenceOTU62317
New.CleanUp.ReferenceOTU65584
New.ReferenceOTU2169
New.CleanUp.ReferenceOTU63399
New.CleanUp.ReferenceOTU12593
GU824078.1.1212
JF791096.1.1777
New.CleanUp.ReferenceOTU186883
New.CleanUp.ReferenceOTU140859
New.CleanUp.ReferenceOTU22509
New.CleanUp.ReferenceOTU80720
New.CleanUp.ReferenceOTU165108
New.CleanUp.ReferenceOTU72070
New.CleanUp.ReferenceOTU44842
New.CleanUp.ReferenceOTU5759
New.CleanUp.ReferenceOTU44499
New.CleanUp.ReferenceOTU12022
New.CleanUp.ReferenceOTU209236
New.CleanUp.ReferenceOTU2553
New.CleanUp.ReferenceOTU49597
New.CleanUp.ReferenceOTU235257
New.CleanUp.ReferenceOTU110229
New.CleanUp.ReferenceOTU84015
New.CleanUp.ReferenceOTU119135
New.CleanUp.ReferenceOTU18423
New.CleanUp.ReferenceOTU49083
New.CleanUp.ReferenceOTU36233
New.CleanUp.ReferenceOTU55129
New.CleanUp.ReferenceOTU46191
New.CleanUp.ReferenceOTU194890
New.CleanUp.ReferenceOTU111372
JF791101.1.1777
GU822112.1.1209
New.CleanUp.ReferenceOTU155537
New.CleanUp.ReferenceOTU58734
New.CleanUp.ReferenceOTU62515
New.CleanUp.ReferenceOTU112811
FJ477084.1.1738
New.CleanUp.ReferenceOTU61755
New.ReferenceOTU3037
New.CleanUp.ReferenceOTU45097
New.CleanUp.ReferenceOTU16990
New.CleanUp.ReferenceOTU1098
New.CleanUp.ReferenceOTU153806
New.CleanUp.ReferenceOTU59623
New.CleanUp.ReferenceOTU196744
New.CleanUp.ReferenceOTU134931
New.CleanUp.ReferenceOTU41214
New.CleanUp.ReferenceOTU132719

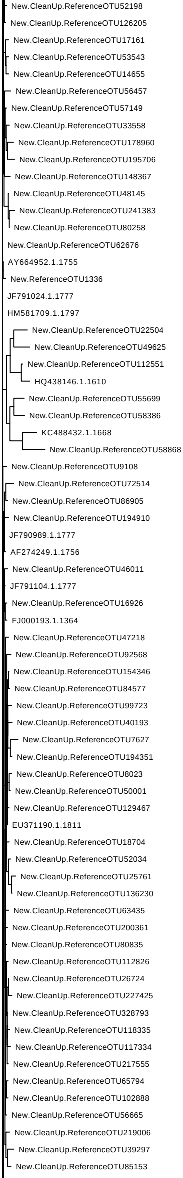
New.CleanUp.ReferenceOTU52198
New.CleanUp.ReferenceOTU126205
New.CleanUp.ReferenceOTU17161
New.CleanUp.ReferenceOTU53543
New.CleanUp.ReferenceOTU14655
New.CleanUp.ReferenceOTU56457
New.CleanUp.ReferenceOTU57149
New.CleanUp.ReferenceOTU33558
New.CleanUp.ReferenceOTU178960
New.CleanUp.ReferenceOTU195706
New.CleanUp.ReferenceOTU148367
New.CleanUp.ReferenceOTU48145
New.CleanUp.ReferenceOTU241383
New.CleanUp.ReferenceOTU80258
New.CleanUp.ReferenceOTU62676
AY664952.1.1755
New.ReferenceOTU1336
JF791024.1.1777
HM581709.1.1797
New.CleanUp.ReferenceOTU22504
New.CleanUp.ReferenceOTU49625
New.CleanUp.ReferenceOTU112551
HQ438146.1.1610
New.CleanUp.ReferenceOTU55699
New.CleanUp.ReferenceOTU58386
KC488432.1.1668
New.CleanUp.ReferenceOTU58868
New.CleanUp.ReferenceOTU9108
New.CleanUp.ReferenceOTU72514
New.CleanUp.ReferenceOTU86905
New.CleanUp.ReferenceOTU194910
JF790989.1.1777
AF274249.1.1756
New.CleanUp.ReferenceOTU46011
JF791104.1.1777
New.CleanUp.ReferenceOTU16926
FJ000193.1.1364
New.CleanUp.ReferenceOTU47218
New.CleanUp.ReferenceOTU92568
New.CleanUp.ReferenceOTU154346
New.CleanUp.ReferenceOTU84577
New.CleanUp.ReferenceOTU99723
New.CleanUp.ReferenceOTU40193
New.CleanUp.ReferenceOTU7627
New.CleanUp.ReferenceOTU194351
New.CleanUp.ReferenceOTU8023
New.CleanUp.ReferenceOTU50001
New.CleanUp.ReferenceOTU129467
EU371190.1.1811
New.CleanUp.ReferenceOTU18704
New.CleanUp.ReferenceOTU52034
New.CleanUp.ReferenceOTU25761
New.CleanUp.ReferenceOTU136230
New.CleanUp.ReferenceOTU63435
New.CleanUp.ReferenceOTU200361
New.CleanUp.ReferenceOTU80835
New.CleanUp.ReferenceOTU112826
New.CleanUp.ReferenceOTU26724
New.CleanUp.ReferenceOTU227425
New.CleanUp.ReferenceOTU328793
New.CleanUp.ReferenceOTU118335
New.CleanUp.ReferenceOTU117334
New.CleanUp.ReferenceOTU217555
New.CleanUp.ReferenceOTU65794
New.CleanUp.ReferenceOTU102888
New.CleanUp.ReferenceOTU56665
New.CleanUp.ReferenceOTU219006
New.CleanUp.ReferenceOTU39297
New.CleanUp.ReferenceOTU85153

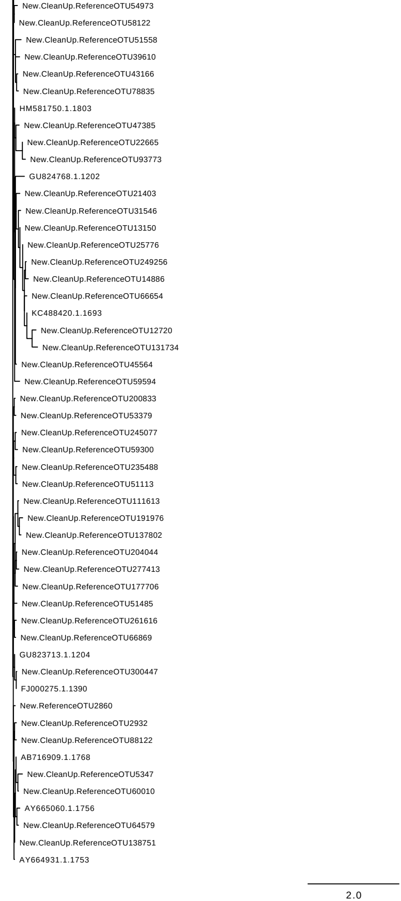
New.CleanUp.ReferenceOTU54973
New.CleanUp.ReferenceOTU58122
New.CleanUp.ReferenceOTU51558
New.CleanUp.ReferenceOTU39610
New.CleanUp.ReferenceOTU43166
New.CleanUp.ReferenceOTU78835
HM581750.1.1803
New.CleanUp.ReferenceOTU47385
New.CleanUp.ReferenceOTU22665
New.CleanUp.ReferenceOTU93773
GU824768.1.1202
New.CleanUp.ReferenceOTU21403
New.CleanUp.ReferenceOTU31546
New.CleanUp.ReferenceOTU13150
New.CleanUp.ReferenceOTU25776
New.CleanUp.ReferenceOTU249256
New.CleanUp.ReferenceOTU14886
New.CleanUp.ReferenceOTU66654
KC488420.1.1693
New.CleanUp.ReferenceOTU12720
New.CleanUp.ReferenceOTU131734
New.CleanUp.ReferenceOTU45564
New.CleanUp.ReferenceOTU59594
New.CleanUp.ReferenceOTU200833
New.CleanUp.ReferenceOTU53379
New.CleanUp.ReferenceOTU245077
New.CleanUp.ReferenceOTU59300
New.CleanUp.ReferenceOTU235488
New.CleanUp.ReferenceOTU51113
New.CleanUp.ReferenceOTU111613
New.CleanUp.ReferenceOTU191976
New.CleanUp.ReferenceOTU137802
New.CleanUp.ReferenceOTU204044
New.CleanUp.ReferenceOTU277413
New.CleanUp.ReferenceOTU177706
New.CleanUp.ReferenceOTU51485
New.CleanUp.ReferenceOTU261616
New.CleanUp.ReferenceOTU66869
GU823713.1.1204
New.CleanUp.ReferenceOTU300447
FJ000275.1.1390
New.ReferenceOTU2860
New.CleanUp.ReferenceOTU2932
New.CleanUp.ReferenceOTU88122
AB716909.1.1768
New.CleanUp.ReferenceOTU5347
New.CleanUp.ReferenceOTU60010
AY665060.1.1756
New.CleanUp.ReferenceOTU64579
New.CleanUp.ReferenceOTU138751
AY664931.1.1753
2.0
